# Weighted Gene Correlation Network Analysis (WGCNA) of *Arabidopsis* Somatic Embryogenesis (SE) and Identification of Key Gene Modules to Uncover SE-Associated Hub Genes

**DOI:** 10.1155/2022/7471063

**Published:** 2022-07-04

**Authors:** Kithmee K. de Silva, Jim M. Dunwell, Anushka M. Wickramasuriya

**Affiliations:** ^1^Department of Plant Sciences, Faculty of Science, University of Colombo, Colombo 03, Sri Lanka; ^2^School of Agriculture, Policy and Development, University of Reading, Reading RG6 6EU, UK

## Abstract

Somatic embryogenesis (SE), which occurs naturally in many plant species, serves as a model to elucidate cellular and molecular mechanisms of embryo patterning in plants. Decoding the regulatory landscape of SE is essential for its further application. Hence, the present study was aimed at employing Weighted Gene Correlation Network Analysis (WGCNA) to construct a gene coexpression network (GCN) for *Arabidopsis* SE and then identifying highly correlated gene modules to uncover the hub genes associated with SE that may serve as potential molecular targets. A total of 17,059 genes were filtered from a microarray dataset comprising four stages of SE, i.e., stage I (zygotic embryos), stage II (proliferating tissues at 7 days of induction), stage III (proliferating tissues at 14 days of induction), and stage IV (mature somatic embryos). This included 1,711 transcription factors and 445 *EMBRYO DEFECTIVE* genes. GCN analysis identified a total of 26 gene modules with the module size ranging from 35 to 3,418 genes using a dynamic cut tree algorithm. The module-trait analysis revealed that four, four, seven, and four modules were associated with stages I, II, III, and IV, respectively. Further, we identified a total of 260 hub genes based on the degree of intramodular connectivity. Validation of the hub genes using publicly available expression datasets demonstrated that at least 78 hub genes are potentially associated with embryogenesis; of these, many genes remain functionally uncharacterized thus far. *In silico* promoter analysis of these genes revealed the presence of *cis*-acting regulatory elements, “soybean embryo factor 4 (SEF4) binding site,” and “E-box” of the napA storage-protein gene of *Brassica napus*; this suggests that these genes may play important roles in plant embryo development. The present study successfully applied WGCNA to construct a GCN for SE in *Arabidopsis* and identified hub genes involved in the development of somatic embryos. These hub genes could be used as molecular targets to further elucidate the molecular mechanisms underlying SE in plants.

## 1. Introduction

The ability to produce embryos from undifferentiated somatic cells *in vitro* is a unique developmental pathway found within the plant kingdom. Since the first report of somatic embryo induction from callus cells of carrot [1, 2], this developmental pathway based on cellular totipotency has been studied extensively due to its biological and scientific significance; it has been recognized as a model system for studying early plant embryogenesis. Until now, most studies have focused on the mechanism of somatic embryo development at the morphological level [2–4] or the development of optimized protocols for the generation of somatic embryos from a range of explants [5–8].

Somatic embryogenesis (SE) involves a complex signaling network [9]; transcriptional regulation of a set of genes in response to stress caused by plant growth regulators, nutrients, certain stress conditions, and other signaling elements triggers cellular reprogramming and transformation of somatic cells into embryos [10, 11]. In 2007, Zeng et al. [12] developed the first draft gene regulatory network for early SE employing a set of transcriptionally regulated SE-related genes in cotton. Although a set of genes have been identified as markers for the initiation phase of SE [13, 14], for example, *SOMATIC EMBRYOGENESIS RECEPTOR-LIKE KINASE1* (*SERK1*) [15, 16], *LEAFY COTYLEDON* (*LEC*) [17–21], *BABY BOOM* (*BBM*) [22], and *WUSCHEL* (*WUS*) [18, 23], the current scientific knowledge on the underlying regulatory landscape of SE is limited. The use of transcriptomics has uncovered a large number of differentially expressed genes (DEGs) during SE in many crops, including *Arabidopsis* [24], rice [25], bread wheat [26], cotton [27], maize [28], and coconut [29]. However, the functions of many of these genes in SE are still not understood.

Gene coexpression networks (GCNs) are increasingly used to understand the interactions among a set of transcriptionally regulated genes. There are many types of coexpression networks: signed/unsigned coexpression networks and weighted/unweighted coexpression networks [30]. In the present study, we have focused on weighted network construction as it is likely to produce more robust findings than unweighted networks [31]. Weighted Correlation Network Analysis (WGCNA) is one of the most popular clustering packages for GCN analysis [31, 32] and the first tool to be employed to construct GCNs from RNA-sequencing (RNA-seq) data. This coexpression tool is easy to use and can be used to find clusters (modules) of highly correlated genes and to identify biologically relevant associations between phenotypes/sample traits and modules from expression data [30]. Recently, WGCNA has been effectively used to identify stage-specific gene expression clusters associated with key stages of *Arabidopsis* zygotic embryo development [33]. In addition, this approach has been successfully used to discover the regulatory landscape of SE in rice [25] and several other biological pathways in plants [34–36]. Here, we have analyzed a transcriptome dataset covering four somatic embryo developmental stages in *Arabidopsis* using WGCNA to understand better the system-level functionality of the transcriptionally regulated genes in dicot SE.

## 2. Materials and Methods

### 2.1. Data Collection and Gene Filtering

The transcriptome data covering somatic embryo developmental stages of wild-type *Arabidopsis* were retrieved from the National Centre for Biotechnology Information (NCBI) GEO database (GEO accession: GSE48915) [37]. The dataset consisted of four developmental stages (zygotic embryos, proliferating tissues at 7 days of induction, proliferating tissues at 14 days of induction, and mature somatic embryos) with two replicates for each stage. Subsequently, the genes with variance greater than the second quartile of variance were filtered to eliminate low-expressed or nonvarying genes, and the remaining genes were used in GCN analysis (https://horvath.genetics.ucla.edu/html/coexpressionnetwork/rpackages/wgcna/faq.html, accessed on 11 May 2022). In addition, DEGs between consecutive embryonic stages were identified by calculating the fold change (FC) in gene expression through a simple *t*-test. Arbitrary FC cut-off of |log_2_ FC| ≥ 2.0 and *p* value of <0.05 were used to reduce false discoveries.

### 2.2. GCN Construction

“WGCNA” package in R software [32] was employed to identify significant gene modules and hub genes in *Arabidopsis* somatic embryo transcriptomes. A gene coexpression similarity matrix was constructed between the expression profiles of the filtered genes using the Pearson correlation. The similarity matrix was then transformed into an adjacency matrix where each entry encodes the connection strength between each pair of genes (“nodes”). The adjacency matrix defines a measure of node dissimilarity from which the nodes (genes) are clustered into network modules. Consequently, the GCN was developed using the automatic one-step network construction and module detection method with the following parameters:
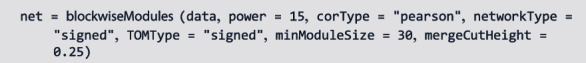


The soft threshold value (power parameter) was decided by the scale-free topology fit index curve.

### 2.3. GCN Visualization

The constructed modular networks were exported to Cytoscape (version 3.7.2) for visualization; gene correlations with *p* value *<0.05* were filtered as significant gene correlations and visualized. The modular networks were analyzed by the “network analyzer” tool in Cytoscape for a concise and informative representation of nodes and edges.

### 2.4. Validation of Network Modules

The robustness of the coexpression modules was assessed through module preservation and quality statistics, which were computed using the modulePreservation function in the WGCNA package [38]. The adjacency matrix of the network was taken as the reference, and the dataset was selected as test data with 200 permutations (nPermutations = 200). The stability of the modules was tested through the statistics median rank and Zsummary.

### 2.5. Inferring Module-Stage Relationships

Module-stage relationships of the GCN were evaluated through module eigengenes (MEs). The correlation relationships between the MEs and different somatic embryo developmental stages were analyzed and visualized through a heatmap. Gene significance was calculated based on the *p* value of the linear regression between the gene expression profile and the associated developmental stage.

### 2.6. Functional Enrichment Analysis

Functional enrichment analysis was performed to detect enriched biological processes in gene modules. Gene Ontology (GO) terms enriched in each module were elucidated using the “singular enrichment analysis” tool provided by agriGO v2.0 [39]. “Arabidopsis genome locus (TAIR10)” was used as the reference, and all other parameters were set as the default for the analysis. Overrepresented GO terms in each network module were identified using the hypergeometric test. To further explore the DEGs mapped to each gene module, the distribution of the following genes across modules was studied: SE-related marker genes [40], plant transcription factors (TFs) (http://planttfdb.cbi.pku.edu.cn/index.php), EMBRYO DEFECTIVE (*EMB*) genes [41], and gene encoding epigenetic regulators [42, 43].

### 2.7. Identification and Validation of Hub Genes

Genes in each module were arranged based on gene connectivity. The top 10 genes of each module were considered as hub genes. The transcriptome dataset published by Wickramasuriya and Dunwell in 2015 was retrieved from the ArrayExpress database (E-MTAB-2403) [24] to study the expression of hub genes during SE.

### 2.8. *In Silico* Analysis of Hub Genes

The promoter sequences of hub genes (1000 bp upstream from the transcription start site) were retrieved from “The Arabidopsis Information Resource” (TAIR) database and analyzed using the Multiple Em for Motif Elicitation (MEME) tool in the MEME Suite 5.3.3 [44]. The following parameters were used in the analysis: number of motifs: 10; motif site distribution: zero or once per occurrence (ZOOPS); minimum width: 6; maximum width: 50; and background model: zero-order model of sequences. Further, the biological significance of the predicted MEME motifs was investigated using the Gene Ontology for MOtifs (GOMo) version 5.3.3 [45] provided in the MEME Suite. Additionally, the retrieved promoter sequences were searched against the Plant *cis*-acting regulatory DNA elements (PLACE) database to identify overrepresented *cis*-acting regulatory elements (CREs; [46]).

## 3. Results

### 3.1. Hierarchical Clustering of Somatic Embryo Transcriptomes

**Figure 1:**
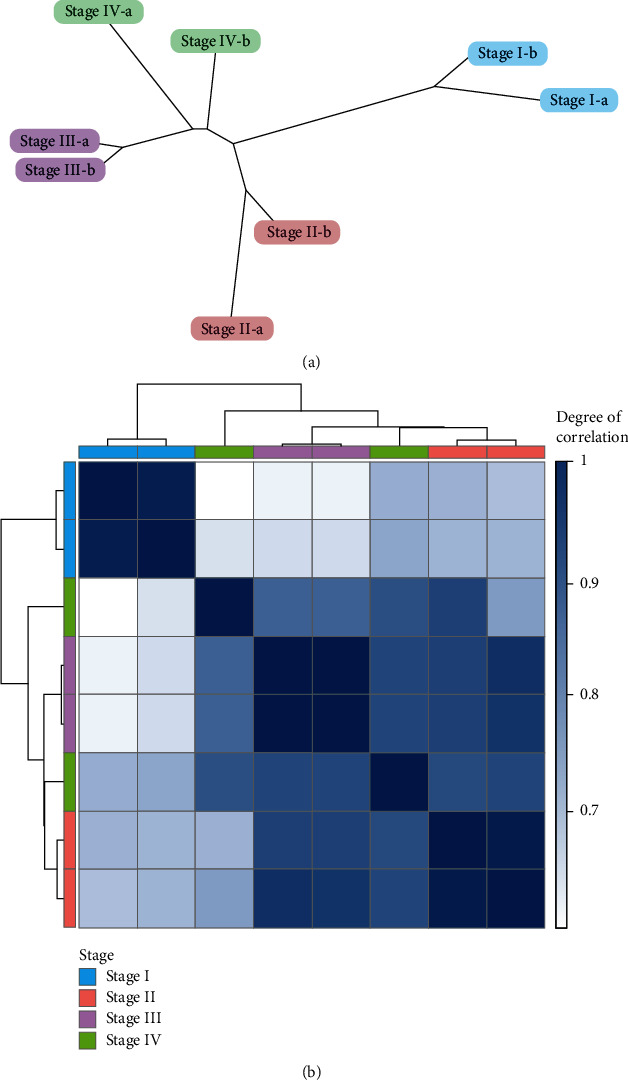
Hierarchical clustering of somatic embryo transcriptomes based on their Euclidean distance using average linkage clustering (replicates of each stage are labeled as “a” and “b”). (a) Unrooted hierarchical clustering dendrogram (the length between nodes corresponds to the distance between samples). (b) Hierarchical clustering heatmap visualizing the correlations between the samples.

In the present study, transcriptome datasets generated through microarray experiments were retrieved from the NCBI covering four somatic embryo developmental stages (with two replicates for each stage), referred to herein as stages I (zygotic embryos), II (proliferating tissues at 7 days of induction), III (proliferating tissues at 14 days of induction), and IV (mature somatic embryos). The hierarchical clustering of samples (Figure 1(a)) confirmed that the sample replicates of each stage have a higher degree of correlation with each other than with other developmental stages; sample outliers were not detected in the dataset. The clustering heatmap clearly distinguished four discrete clusters of related expression patterns corresponding to the stages of somatic embryo development (Figure 1(b)). Further, stage I showed a poor correlation with the other three stages. This suggests that stage I may have a distinct expression profile as compared to other somatic embryo developmental stages.

### 3.2. Filtering of Genes for the GCN Construction and Downstream Analysis

As recommended by Langfelder and Horvath [32], genes were filtered by the variance for the GCN construction; filtering genes for variance greater than 0.25 quantile identified a total of 17,059 genes (see Table S1). This included 445 *EMB* genes [41], 10 SE marker genes [40], and 1,711 *Arabidopsis* TFs (65.3%).

**Figure 2:**
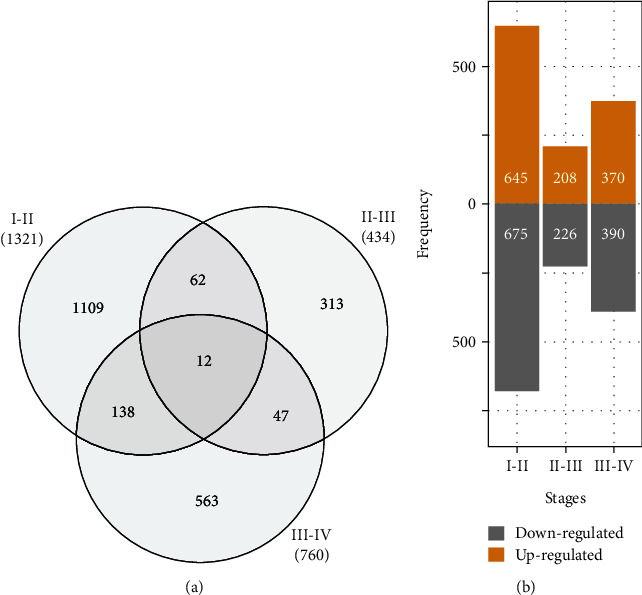
Overview of DEGs. (a**)** Distribution of DEGs between consecutive somatic embryo developmental stages. **(**b**)** Number of up- or downregulated DEGs between stages.

In addition, DEGs were identified by a pairwise ratio of expression between consecutive stages of development. A total of 2,244 genes were identified by threshold filtering based on |log_2_ FC| ≥ 2.0 and *p* value <0.05. 64 *EMB* genes [41], four SE marker genes [40], and 458 TFs were present within the DEGs identified (see Table S2). A total of 12 genes including the genes *STRESS INDUCED FACTOR 2* (AT1G51850), *LIGHT-HARVESTING-LIKE 3 : 1* (AT4G17600), *BETA GLUCOSIDASE 28* (AT2G44460), *FERREDOXIN C 1* (AT4G14890), and *PHOTOSYSTEM II SUBUNIT Q* (AT4G05180) were differentially expressed throughout SE (Figure 2(a)). In addition, a considerable number of genes were up- and downregulated during early embryo developmental stages (Figure 2(b)).

### 3.3. Construction of GCN

**Figure 3:**
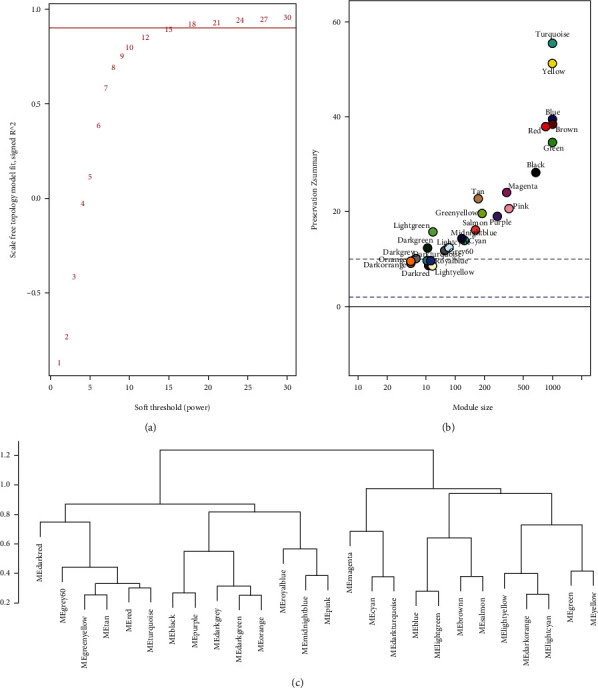
Construction of the draft GCN for SE. (a) Network topology for different soft-thresholding powers. (b) Module preservation statistics. (c) Hierarchical clustering dendrogram of MEs.

The expression profiles of the filtered 17,059 genes were used to construct a scale-free gene expression network with a soft threshold of 15 (Figure 3(a)). The dynamic hierarchical clustering approach integrated with the WGCNA pipeline distinguishes groups of genes with coexpression patterns and clusters them into network modules. In total, 26 distinct coexpression gene modules were detected with the module size ranging from 35 to 3,418 genes (Figures 3(b) and 3(c)); each module was assigned with a unique colour. The module comprising most genes was the turquoise (3,418 genes) followed by the blue (2,973 genes) and brown (2,437 genes) (Figure 3(b)). The expression profiles of coexpressed genes clustered in each module were summarized as MEs. Among the filtered genes, 13 genes that failed to fit within a distinct group were assigned to the grey module and removed from the downstream analysis. Module preservation analysis indicated high module preservation, confirming that the modules generated here can also be found in diverse independent datasets (Figure 3(b)). Each module was exported and visualized using Cytoscape.

### 3.4. Identification of Stage-Related Modules

**Figure 4:**
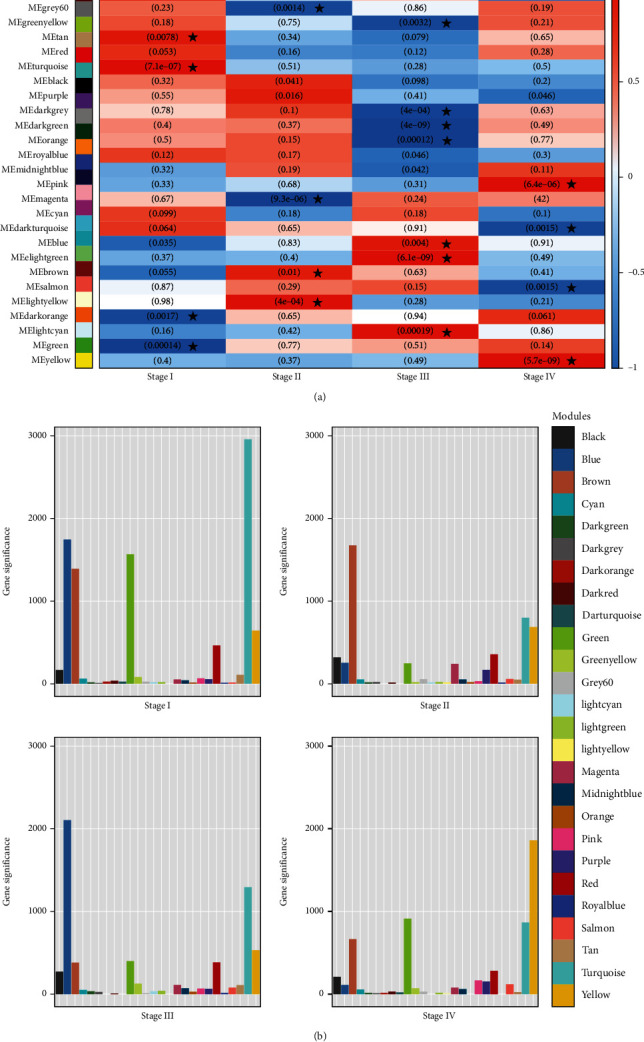
Stage-specific gene modules detected by WGCNA. (a**)** Module-trait relationship heatmap. Each row corresponds to a module, and each column corresponds to a stage. The degree of correlation is illustrated with the colour legend. The numbers in the table correspond to the *p* value. Modules that are significantly associated with each somatic embryo development stage (|*r*| > 0.8 and *p* value ≤0.01) are indicated by an asterisk. (b**)** Gene significance values of coexpression modules related to different somatic embryo developmental stages.

The relationships between the gene modules and different somatic embryo developmental stages were determined by assessing the Pearson correlation coefficient (*r*) between the MEs and developmental stages. Module-trait correlation analyses revealed that multiple modules are related to SE (Figure 4(a)). A total of 18 modules were significantly associated with the somatic embryo developmental stages (|*r*| > 0.8 and *p* value ≤0.01; Figure 4), and these modules were “stage-specific,” i.e., the module was significantly associated with only one particular developmental stage of SE: tan, turquoise, dark-orange, and green to stage I; grey60, magenta, brown, and light-yellow to stage II; green-yellow, dark-gray, dark-green, orange, blue, light-green, and light-cyan to stage III; and pink, dark-turquoise, salmon, and yellow to stage IV. Gene significance, the correlation between modular gene expression and each stage, is shown in Figure 4(b).

### 3.5. Functional Enrichment Analysis of “Stage-Specific” Gene Modules

**Figure 5:**
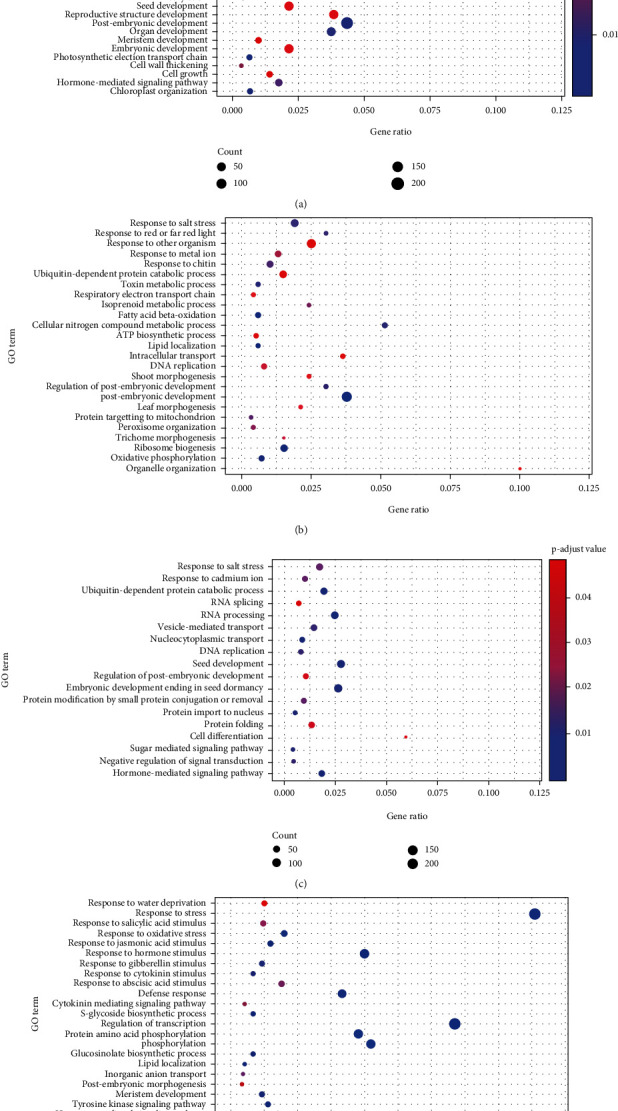
Functional enrichment analysis of the stage-specific modules. (a)–(d) represent the significantly enriched GO terms (*p* value <0.05) in modules specifically associated with stages I–IV, respectively.

GO enrichment analysis performed on “stage-specific” modules showed that the genes in green and turquoise modules which exhibited a significant association with stage I were mainly enriched in the biological processes being involved in postembryonic development, hormone-mediated signaling pathway, biosynthesis pathways (sterol and fatty acids), DNA methylation, and transcription regulation (Figure 5(a)). Genes in brown, light-yellow, and magenta modules, which showed significant association with stage II, were mainly enriched in the biological processes involved in root and shoot development, ATP synthesis, response to the metal ions, and DNA replication (Figure 5(b)), whereas genes in blue and light-cyan modules, which showed significant association with stage III, were enriched for the biological processes involved in transition postembryonic and seed development, hormone- and sugar-mediated signaling pathways, cell differentiation, protein modification, and RNA processing (Figure 5(c)). Moreover, the yellow module, which showed a significant relationship to stage IV, was mainly enriched in biological processes involved in ion transport, postembryonic development, signal transduction, lipid localization, response to oxidative and water stress, as well as response to phytohormones (abscisic acid, gibberellin, cytokinin, and jasmonic acid) (Figure 5(d)).

### 3.6. Analysis of Hub Genes

Hub genes are nodes in a network often hypothesized to be functionally significant due to their high degree of intramodular connectivity. A total of 260 genes (top 10 genes of each module with high connectivity) were identified as potential hub genes; the hub gene with the highest degree of connectivity in each module is given in Table 1 (the complete list of hub genes is given in Table S3). GO enrichment analysis of the hub genes revealed that they are mainly enriched for biological processes such as metabolic processes (mRNA and cellular amino acid), oxidation-reduction, protein folding, and postembryonic development.

Among the hub genes, only 234 genes were functionally annotated; of these, 13 were TFs: *AUXIN RESPONSE FACTOR 9* (*ARF9*), *FLOWERING BHLH 4* (*FBH4*), *BASIC HELIX-LOOP-HELIX 39* (*BHLH39*), *BASIC LEUCINE-ZIPPER 44* (*bZIP44*), *bZIP19*, *ZIM-LIKE 2* (*ZML2*), *AT5G60820*, *AT4G01270*, *KANADI 3* (*KAN3*), *HOMEODOMAIN GLABROUS 4* (*HDG4*), *CELL DIVISION CYCLE 5* (*CDC5*), *NAC DOMAIN CONTAINING PROTEIN 80* (*NAC080*), and *SALT TOLERANCE* (*STO)*). In addition, five genes encoding transposable elements (i.e., *AT2G11560*, *AT3G33066*, *AT5G32430*, *AT3G42820*, and *AT4G28900*) were identified.

*In silico* analysis of the promoter sequences (1000 bp upstream from the transcription start site) of the hub genes using the MEME tool identified four significant motifs ranging in length from 15 to 29 bp (Table 2). Motifs 1, 2, and 3 were detected across 229, 245, and 121 hub genes, respectively. Further analysis of the predicted motifs using the GOMo tool provided in the MEME suite indicated that motifs 1 and 3 may be involved in the DNA endoreduplication, polarity specification of axial/abaxial axis, and hormone-mediated signaling pathways; motifs 1 and 3 seem to function in association to cytokinin and gibberellic acid, respectively.

### 3.7. Validation of Hub Genes

**Figure 6:**
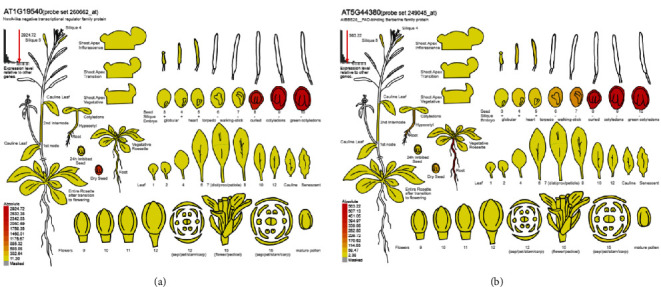
Expression patterns of two hub genes, *AT1G19540* (a) and *AT5G44380* (b), when viewed through the *Arabidopsis* eFP browser. The normalized expression value for each gene is colour-coded as indicated by the legend.

A comparison of hub genes and DEGs showed that 31 hub genes are differentially expressed in SE (the expression values of differentially expressed hub genes are given in Table S4). Further, expression analysis of these genes using the *Arabidopsis* eFP browser demonstrated that two hub genes, *AT1G19540* (Figure 6(a)) and *AT5G44380* (Figure 6(b)), exhibit a seed-specific pattern of expression.

**Figure 7:**
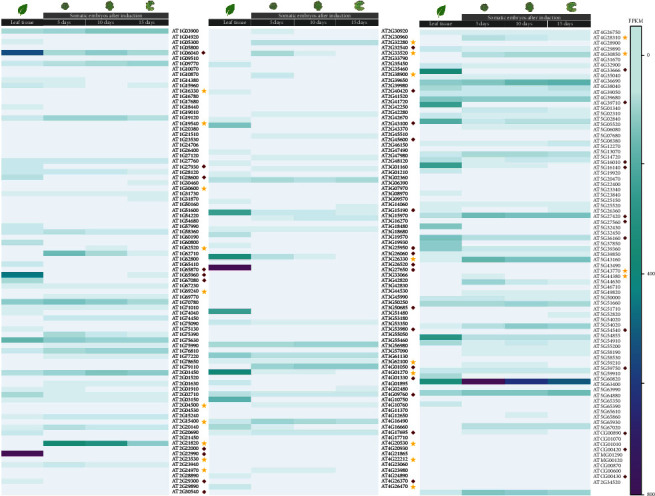
Transcript abundance extracted from the somatic embryo transcriptome dataset, E-MTAB-2465 for the hub genes. The hub genes significantly upregulated (log_2_ FC ≥ 2.0) and downregulated (log_2_ FC ≤ −2.0) in somatic embryonic tissues compared to leaf tissues are indicated with yellow asterisks and red diamonds, respectively. Transcript abundances are shown in fragments per kilobase of transcript per million mapped reads (FPKM).

Moreover, analysis of the expression profiles of hub genes in the *Arabidopsis* somatic embryo transcriptome dataset (E-MTAB-2465) published by Wickramasuriya and Dunwell (2015) revealed that 62 hub genes are differentially expressed in somatic embryonic tissues compared to leaf tissues (|log_2_ FC| ≥ 2.0 and *p* value <0.05; Figure 7). Of these, 15 genes were identified as DEGs in the present analysis. For instance, *CYSTEINE-RICH TRANSMEMBRANE MODULE 7* (*ATHCYSTM7*/AT2G33520), *HEPTAHELICAL TRANSMEMBRANE PROTEIN2* (AT4G30850), *INDOLE-3-ACETIC ACID INDUCIBLE 30* (*IAA30*/AT3G62100), *RPS9C*, *VASCULATURE COMPLEXITY AND CONNECTIVITY* (AT2G32280), *AT2G21820*, *AT2G38900*, and *AT5G43770* showed a marked expression in somatic embryonic tissues as compared to leaf tissues. Expression analysis using the *Arabidopsis* eFP browser further showed that *AT2G29300*, *AT2G21820*, *AT2G38900*, *AT5G43770*, *ATHCYSTM7*, and *AT1G19540* exhibit a seed-specific pattern of gene expression.

As expected, few hub genes highly expressed in leaf tissues were repressed in somatic embryos indicating the importance of gene regulation in SE (Figure 7); for instance, *CELLULOSE SYNTHASE-LIKE B4* (AT2G32540), *CHOLINE/ETHANOLAMINE KINASE 3* (AT4G09760), *GLUTAMATE DECARBOXYLASE 2* (AT1G65960), *ISOPROPYLMALATE ISOMERASE 2* (AT2G43100), *PEROXIREDOXIN Q* (*PRXQ*/AT3G26060), *PHOTOSYNTHETIC NDH SUBCOMPLEX L 4* (*PnsL4/*AT4G39710), *PLASTID RIBOSOMAL PROTEIN S20* (AT3G15190), *STO* (AT1G06040), *SINAPOYLGLUCOSE 1* (*SNG1*/AT2G22990), *THYLAKOID RHODANESE-LIKE* (*TROL*/AT4G01050), *TONOPLAST INTRINSIC PROTEIN 2* (*TIP2*/AT3G26520), *AT3G50685*, *AT4G33666*, *AT5G16010*, and *AT5G54540* genes showed a marked repression in somatic embryos compared to leaf tissues.

**Figure 8:**
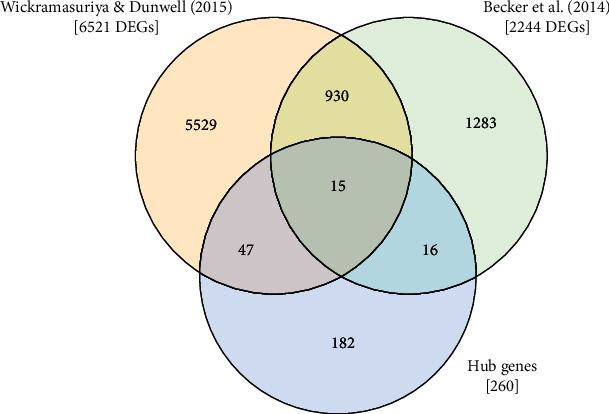
Venn diagram indicating the intersection of hub genes and DEGs (|log_2_  FC| ≥ 2.0 and *p* value <0.05) obtained from E-MTAB-2465 [24] and GSE48915 [37].

**Figure 9:**
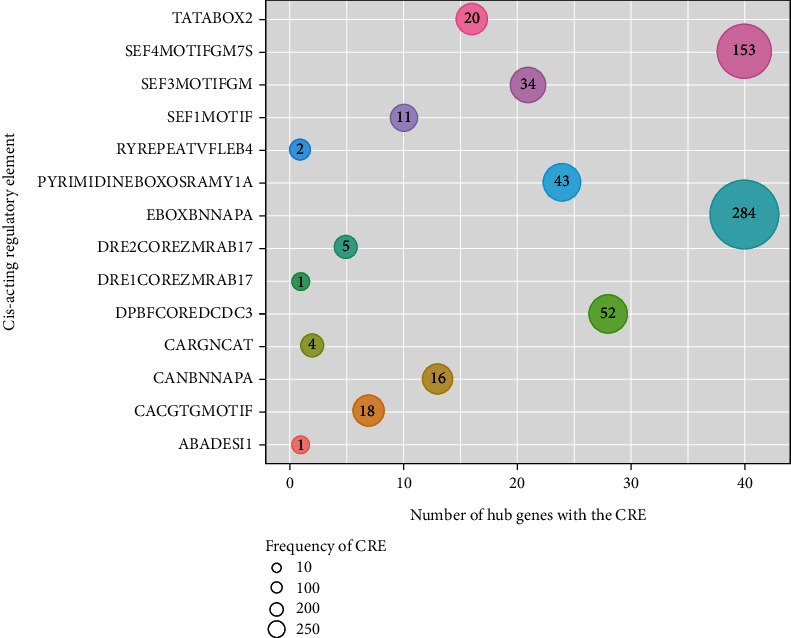
The distribution of several important plant CREs present in the promoter regions of functionally uncharacterized hub genes. The number of hub genes that contain the relevant CRE in their promoter region is indicated by the *x*-axis. The size of the circle depicts the occurrence of the CREs within the promoter regions of the hub genes (as indicated within the circle).

In summary, the present study identified a total of 78 hub genes as potential regulators of SE (Figure 8), including genes showing marked overexpression as well as repression in SE. Of these, 41 genes have not been functionally annotated thus far. The analysis of the promoter sequences of these uncharacterized hub genes using the PLACE database identified a total of 215 different plant CREs; ARR1AT, CAATBOX1, CACTFTPPCA1, DOFCOREZM, GATABOX, GT1CONSENSUS, POLLEN1LELAT52, and WRKY71OS were observed in all 41 functionally uncharacterized potential hub genes. Moreover, several CREs related to embryogenesis were identified (Figure 9). The functions of the predicted CREs are included in Table 3.

### 3.8. Distribution of Embryogenesis-Related Genes across Network Modules

**Figure 10:**
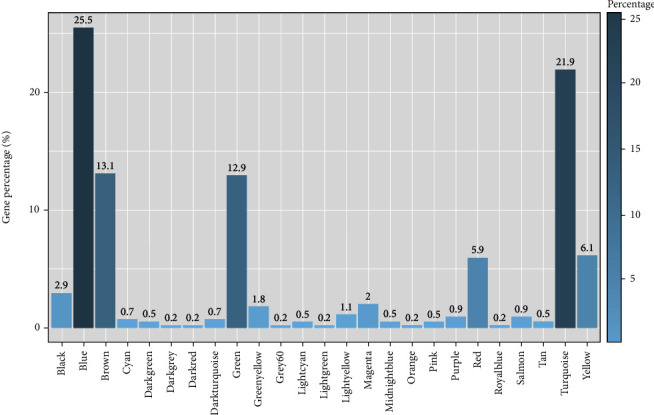
Distribution of *EMB* genes across gene modules. The coloured bars represent the ratio between the number of *EMB* genes in each module and the total number of *EMB* genes in the network.

Further exploration of genes mapped to each network module found that 10 key regulators of SE including *LEC1*, *FUSCA3* (*FUS3*), and *ABSCISIC ACID INSENSITIVE 3* (*ABI3*) are present among the highly connected genes in the network (Table 4); SE-related marker genes, *LEC2*, *SERK1*, *WUS*, *BBM*, and *WUSCHEL RELATED HOMEOBOX 2* (*WOX2*) showed low variance in the present dataset and thus were not included in the GCN analysis. We also observed that the majority of previously published *EMB* genes [41] are localized to the blue and turquoise modules, which showed significant association with stage I and stage III, respectively (Figure 10; see Table S5).

**Figure 11:**
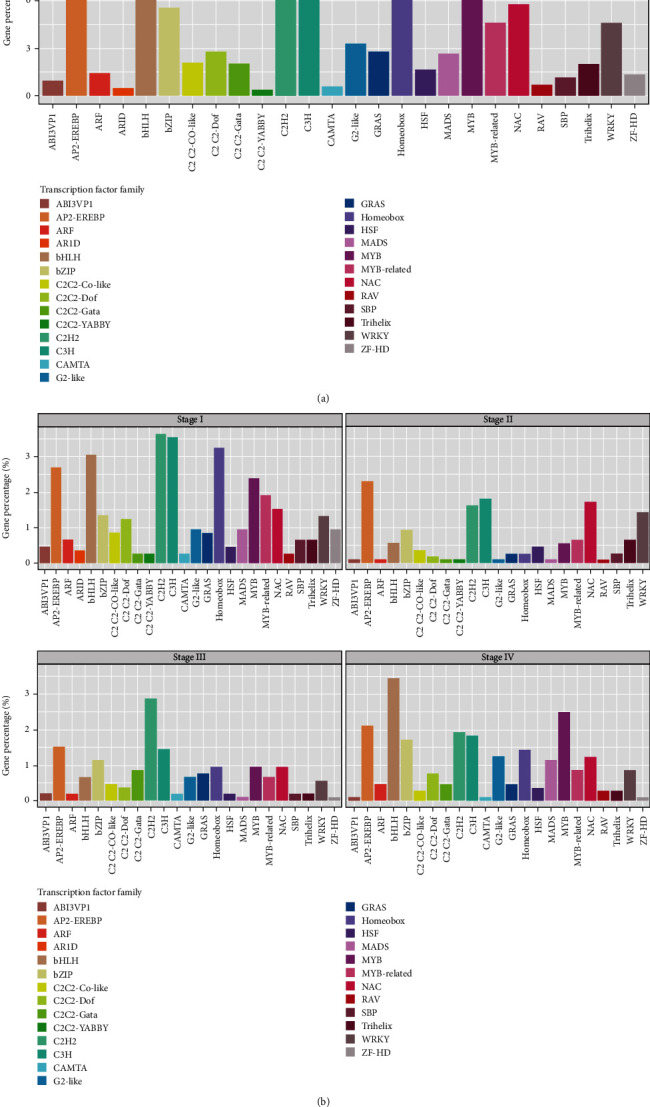
Distribution of TFs in SE. (a) Overall distribution of TFs. The percentage is calculated as the ratio of TFs belonging to each family with respect to the total number of TFs in the network. (b) Distribution of SE-related TFs across different somatic embryo developmental stages. The percentage is calculated for each stage as the ratio of TFs present in each family with respect to the total number of TFs in the network.

In addition, we observed that 1,711 *Arabidopsis* TFs are distributed across all the gene modules except in light-green and royal-blue modules, with the highest number of TFs present in the turquoise module (the complete list of TFs included in the GCN is given in Table S6). Notably, AP2/EREBP (APETALA2/ethylene-responsive element binding proteins), bHLH (basic helix–loop–helix), bZIP, C2H2 (Cys2-His2), HB (homeobox), NAC (NAM, ATAF, and CUC), MYB (MYB-domain), C3H, and WRKY TF families were highly represented (Figure 11(a)). Of these, members of AP2/EREBP, bHLH, C2H2, HB, NAC, MYB, and WRKY TF families were involved in early SE (Figure 11(b)). Interestingly, TFs that are targets of several microRNAs (miRNAs) were also recovered from the GCN (Table S7).

**Figure 12:**
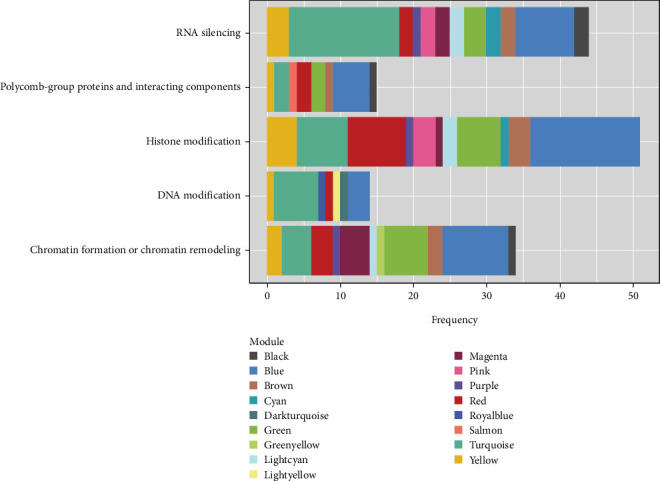
Distribution of genes encoding epigenetic regulators across the network modules.

Notably, several gene encoding epigenetic regulators were localized in network modules (Figure 12). This included 14 genes involved in DNA modification, 51 genes involved in histone modification, 34 genes involved in chromatin remodeling, 15 genes encoding polycomb-group proteins, and 55 genes associated with RNA silencing (see Table S8). Each of these genes directly interacted with numerous modular genes forming a complex network.

## 4. Discussion

Plant embryogenesis is a meticulous developmental process that requires the regulation of multiple genes. A GCN will serve as a map of statistically significant gene interactions that helps in narrowing down the transcriptome to the potential gene interactions involved in biological processes. Recently, Clercq et al. report an integrated gene regulatory network for *Arabidopsis* covering TFs and target genes [47]. In the present study, WGCNA was employed to explore potential clusters of highly coregulated genes and hub genes associated with SE. Although WGCNA has been previously applied to construct a GCN for *Arabidopsis* zygotic embryogenesis (ZE) [33], to the best of our knowledge, this is the first report on the use of WGCNA to construct a GCN for *Arabidopsis* SE and to explore SE-related network modules and hub genes. The findings of this study provide new insights into the molecular mechanism of SE in plants.

The GCN constructed for SE comprised of 26 network modules: black (674 genes), blue (2,973 genes), brown (2,437 genes), cyan (125 genes), dark-green (52 genes), dark-grey (39 genes), dark-orange (35 genes), dark-red (54 genes), dark-turquoise (52 genes), green (2,132 genes), green-yellow (189 genes), grey60 (79 genes), light-cyan (86 genes), light-green (59 genes), light-yellow (58 genes), magenta (338 genes), midnight-blue (117 genes), orange (35 genes), pink (357 genes), purple (271 genes), red (853 genes), royal-blue (56 genes), salmon (162 genes), tan (172 genes), turquoise (3,418 genes), and yellow (2,223 genes) modules. Among them, 18 modules showed strong associations with different stages of SE; module-trait relationship analysis revealed that four, four, seven, and four modules were significantly correlated with stages I, II, III, and IV of SE, respectively. This suggests that SE involves complex genetic networks.

Functional enrichment analysis using GO is one of the most widely used bioinformatic methods to classify genes into functionally related groups [48–50]. GO analysis of the coexpressed gene clusters (or network modules) showed that the initial stages of SE were mainly enriched with biological processes such as hormone-mediated signaling, biosynthesis pathways, ATP synthesis, DNA methylation, and replication. Notably, genes involved in lipid transport, postembryonic development, signal transduction, and seed dormancy were enriched in later stages of SE; this indicates the developmental shift in the maturation phase with the accumulation of embryo-specific food reserves, a process that aids in withstanding dormancy and postembryonic development [2, 10, 51]. Furthermore, genes related to stress responses (e.g., oxidative and water stress), phytohormones (e.g., cytokinin, abscisic acid, gibberellin, and jasmonic acid), and metabolic processes were enriched in all stages of somatic embryo development studied, from the initiation to maturation stage. These findings further confirmed the importance of cell-cell interactions [52], signaling [9, 13, 53], and transcriptional activation of stress responses [54, 55] during plant SE.

High-degree nodes or the genes with high network connectivity in GCN modules (“hub genes”) may have important biological functions [36, 56–58]; often, they may serve as biological markers. Several studies have successfully employed WGCNA to mine hub genes controlling biological processes [34, 59–62]. The present study reports 260 potential hub genes related to SE based on the degree of connectivity. These genes may play pivotal roles in the regulation of SE. Importantly, 13 TFs encoded by hub genes were identified in the coexpression network. They were *ARF9*, *NAC080, ZML2*, *bHLH39*, *KAN3*, *bZIP19*, *bZIP44*, *HDG4*, *FBH4*, *STO*, *CDC5*, *AT5G60820*, and *AT4G01270*; functional roles of many of these genes in the regulation of SE are not reported. Previous studies have reported that ARF9 represses the expression of its target genes such as TOPLESS (TPL) and TPL-related proteins [63, 64]. Wójcikowska and Gaj observed stable expression of *ARF9* during SE [65]. In addition, *KAN3*, a member of GARP TF family, has also exhibited an embryonic expression pattern.

In addition, *ROOT UV-B SENSITIVE 6* (*RUS6*; AT5G49820), which encodes a DUF647 (DOMAIN OF UNKNOWN FUNCTION 647) containing protein, an ankyrin repeat-containing gene designated as *AT5G65860* and a gene that encodes hydroxyproline-*O*-glycosyltransferases (Hyp-*O*-GALT), *GALT4* (*AT1G27120*)), was also identified as hub genes in the coexpression network. The members of the *RUS* gene family play diverse roles in plant development [66]. Interestingly, knockout mutants of *RUS6* have shown a strong embryo-lethal phenotype. In *Arabidopsis*, ankyrin repeat-containing proteins have been classified into 16 groups [67], and of these, proteins with only ankyrin repeats have been associated with disease resistance, antioxidation, embryogenesis, and development [68–70]. For instance, T-DNA mutants of the *EMB* 506 gene, which encodes a protein containing five ankyrin repeats, have shown defective embryo development at the globular-to-heart stage transition [70]. Moreover, Hyp-*O*-GALT enzymes are responsible for hydroxyproline glycosylation of arabinogalactan proteins, which are known to function in various aspects of plant growth and development including SE [71–73]. Although the hub genes identified in the present study are implicated to function in many plant developmental processes, the functions of many of the hub genes in SE remain to be elucidated. Hence, these genes could be potential targets for functional studies in the future.

Promoter analysis of the functionally uncharacterized hub genes using the PLACE database revealed the overrepresentation of two motifs in many of the promoter regions. These were EBOXBNNAPA (consensus sequence: CANNTG) and SEF4MOTIFGM7S (consensus sequence: [A/G]TTTTT[A/G]). Of these, EBOXBNNAPA (“E-box” motif) is a CRE found in the regulatory region of the napin gene, *napA* in *Brassica napus* [74]; this gene encodes a storage protein. Moreover, CANNTG provides the binding site for bHLH TFs [75]. bHLH is one of the most frequently represented gene families in DEGs in ZE [76] and SE and is known to have diverse functions in plants [24] including cell proliferation [75]. The recognition sequence of SEF4MOTIFGM7S motif is known to interact with SEF3, a protein expressed in immature soybean seeds that acts as a transcriptional activator of the *β*-conglycinin *α* subunit gene [77]. Hence, the uncharacterized hub genes that showed considerable expression in embryonic tissues are more likely to play a significant role in plant embryo development.

Differential gene expression analysis of hub genes revealed that 78 genes could be considered as potential regulators of SE; of these, 15 genes were differentially expressed in transcriptome datasets derived from two independent studies related to SE [24, 37]. One of the genes identified was *IAA30*, which is a member of one of the families of auxin signaling proteins (Aux/IAA; [78]). *iaa30* mutants have displayed significantly impaired SE efficiency, producing fewer somatic embryos per explant [76] and suggesting its role in the initiation phase of SE. Moreover, IAA30 is a target of two important SE marker genes, LEC2 and AGL15 [79, 80]. In addition, two hub genes, *AT1G19540* and *AT5G44380*, showed a marked expression in seed development, suggesting their roles in embryogenesis.

To enhance our understanding of the regulatory mechanism of SE, the distribution of embryogenesis-related genes across the gene modules was examined. Horstman et al. report LEC1–LEC2–FUS3–BBM–ABI3 network to induce SE in *Arabidopsis* [81]. Moreover, Zheng et al. suggest a MADS-domain TF encoding gene, and *AGL15* may associate with *LEC2*, *FUS3*, and *ABI3* during SE [82]. However, a recent study has found that *AGL15* is not essential to promote SE [83]. In the present analysis, 10 key regulators of SE including *LEC1*, *ABI3*, *FUS3, AGL15*, and three members of the AINTEGUMENTA-LIKE/PLETHORA (AIL/PLT) subfamily (*ANT*, *AIL5*, and *AIL7*) were identified in the coexpression network. Consistent with previous literature, members of the AP2/EREBP, bHLH, bZIP, MYB, HB, WRKY, NAC, C3H, and C2H2 TF families were overrepresented in the GCN [76, 84]. In addition, members of the TF families (i.e., SPB (SQUAMOSA promoter binding protein-like), GRAS (GRAS-domain), trihelix, G2-like, and CAMTA (CALMODULIN BINDING TRANSCRIPTION ACTIVATOR 3)) that are not or to a lesser extent reported to be involved in SE were identified. The members of GRAS, trihelix, and CAMTA families are known to be involved in the regulation of stress responses [47, 85, 86].

Further, it is reported that miRNAs (e.g., miR156, miR159, miR162, miR164, miR166, miR167, miR169, miR168, miR171, miR319, miR393, and miR396) play an important role in SE [87–91]. Consistent with previous studies, several TFs targeted by miRNAs were recovered from the SE-related GCN. This included seven miR156/157 targeting genes of the SPB TF family, seven miR169 targeting genes of the CCAAT TF family, six miR396 targeting genes of the GRF TF family, five miR166/miR165 targeting genes of the HB TF family, five miR164 targeting genes of the NAC family, and five miR159/miR319 targeting genes of the TCP TF family. These miRNA-targeted TF encoding genes may play a significant role in the regulation of SE responses.

Recent studies have uncovered critical roles of epigenetic modifications in the regulation of SE, in particular, DNA methylation/demethylation [92–94] and histone modifications [91, 95, 96]. Recently, an expression study on *Arabidopsis* embryos at single-cell resolution has provided evidence for distinct expression patterns for many epigenetic regulators across embryonic tissues [97]. Our coexpression network also revealed that many genes encoding epigenetic regulators such as *METHYLTRANSFERASE 1* (*MET1*), *CHROMOMETHYLASE 3* (*CMT3*), *DEMETER* (*DME*), *DEMETER-LIKE* (*DML1,-2*), histone acetyltransferases (*HISTONE ACETYLTRANSFERASE OF THE CBP FAMILY* (*HAC1,-4,-5,-12*), histone deacetylases (i.e., *HISTONE DEACETYLASE* (*HDA1,-2,-3,-5,-6,-8,-9,-14,-15,-17*), and histone demethylases (*JUMONJI DOMAIN-CONTAINING PROTEIN 16* (*JMJ14,-16,-22,-27,-29*) were coexpressed with key genes involved in the regulation of SE.

The present study showed that the WGCNA pipeline could be used to identify biologically relevant modules of SE. However, our analysis has some limitations. The main limitations were the small sample size used in the analysis and the lack of an independent dataset to replicate the findings. Langfelder and Horvth [32] recommend using at least 15 samples to construct robust networks. However, high-quality, clean data could also result in biologically meaningful networks even with <15 samples. Therefore, further experiments are recommended to validate the hub genes discovered in the present study. Furthermore, the GCN built in the present study was based on microarray gene expression data. Although hybridization-based gene expression profiling approaches are high-throughput and relatively inexpensive, they have a number of limitations; most importantly, they provide only an indirect measure of the level of gene expression and can only be used to study the expression levels of genes that the arrays are designed to detect and are subjected to cross-hybridization biases [98]. Given the limitations of this approach, it would be recommended to perform a GCN analysis employing an expression dataset generated through high-throughput transcriptome sequencing (RNA-seq) with an appropriate number of replicates. Unlike microarrays, RNA-seq is not dependent on prior knowledge about the genome sequence and has higher sensitivity to genes expressed either at a low or very high level and also has higher levels of reproducibility than microarrays [99]. Therefore, it could generate a more suitable dataset for GCN analysis.

## 5. Conclusion

In this study, a GCN was successfully constructed for SE employing WGCNA. Gene modules and hub genes related to *Arabidopsis* somatic embryo development were successfully mined based on their statistical significance. The findings reported here provide a unique resource to advance the regulation of SE at the molecular level.

## Figures and Tables

**Table 1:** Top 10 hub genes ordered by the degree of connectivity.

Gene identifier	Degree of connectivity	Gene module	Gene name	Description
AT1G27120	3327	Turquoise	*AT1G27120* (*GALT4*)	Galactosyltransferase family protein
AT5G52820	2853	Blue	*NOTCHLESS* (*NLE*)	WD-40 repeat family protein/notchless protein
AT5G56090	2348	Brown	*CYTOCHROME C OXIDASE 15* (*COX15*)	Encodes a homolog of COX15
AT2G43100	2134	Yellow	*ISOPROPYLMALATE ISOMERASE 2* (*IPMI2*)	Isopropylmalate isomerase 2
AT1G71010	1958	Green	*FORMS APLOID AND BINUCLEATE CELLS 1C* (*FAB1C*)	Encodes a protein that is predicted to act as a phosphatidylinositol-3P 5-kinase but lacks a FYVE domain
AT2G29890	806	Red	*VILLIN 1* (*VLN1*)	Encodes a ubiquitously expressed villin-like protein
AT2G45600	569	Black	*AT2G45600*	Alpha/beta-hydrolases superfamily protein
AT1G72400	321	Magenta	*AT1G72400*	Hypothetical protein
AT3G53980	311	Pink	*AT3G53980*	Bifunctional inhibitor/lipid-transfer protein/seed storage 2S albumin superfamily protein
AT5G65350	243	Purple	*HISTONE 3 11* (*HTR11*)	Histone 3 11
AT5G27560	182	Green-yellow	*AT5G27560*	DUF1995 domain protein, putative (DUF1995)
AT5G54855	170	Tan	*AT5G54855*	Pollen Ole e 1 allergen and extensin family protein
AT1G75630	156	Salmon	*VACUOLAR H+-PUMPING ATPASE 16 KDA PROTEOLIPID SUBUNIT 4* (*AVA-P4*)	Vacuolar H+-pumping ATPase 16 kD proteolipid (ava-p) mRNA
AT1G74450	107	Cyan	*AT1G74450*	BPS1-like protein (DUF793)
AT2G23940	107	Midnight-blue	*AT2G23940*	Transmembrane protein (DUF788)
AT1G30460	82	Light-cyan	*CLEAVAGE AND POLYADENYLATION SPECIFICITY FACTOR 30* (*CPSF30*)	Encodes AtCPSF30, the 30-KDa subunit of cleavage and polyadenylation specificity factor
AT1G06040	74	Grey60	*SALT TOLERANCE* (*STO*)	B-box zinc finger family protein that encodes a salt tolerance protein
AT2G11560	58	Light-green	*AT2G11560*	Mutator-like transposase/similar to MURA transposase of maize
AT3G55050	50	Dark-green	*D-CLADE TYPE 2C PROTEIN PHOSPHATASE 4* (*PP2C.D4*)	Protein phosphatase 2C family protein
ATCG01070	48	Dark-red	*NAD*(*P*)*H-QUINONE OXIDOREDUCTASE SUBUNIT 4*L (*NDHE*)	NADH dehydrogenase ND4L
AT3G25950	48	Royal-blue	*AT3G25950*	TRAM, LAG1, and CLN8 (TLC) lipid-sensing domain containing protein
AT1G65410	39	Dark-turquoise	*ATP-BINDING CASSETTE I13* (*ABCI13*)	Encodes a member of NAP subfamily of transporters
AT5G02310	38	Light-yellow	*PROTEOLYSIS 6* (*PRT6*)	Encodes a component of the N-end rule pathway that targets protein degradation
AT3G61130	37	Dark-grey	*GALACTURONOSYLTRANSFERASE 1* (*GAUT1*)	Encodes a protein with putative galacturonosyltransferase activity
AT3G53350	34	Dark-orange	*ROP INTERACTIVE PARTNER 3* (*RIP3*)	Encodes a microtubule-binding protein
AT5G43490	34	Orange	*AT5G43490*	Myb-like protein X

**Table 2:** Conserved motifs identified in the promoter regions of hub genes using the MEME tool.

	Motif	E-value	Motif width	Sites	Significant GO enriched terms (*q* value<0.05)
1	NAVAAAAAAARAAARARAAARAAAAHMAA Consensus sequence: [AGT]A[AG]AA[AC]AAAA[AG]A[AG][AG][AGC]A[AG]AA[AG][AG]A[AG]AA[ATC][AC]A[AGT]	2.8e-077	29	229	(i) GO:0042023: DNA endoreduplication (ii) GO:0009944: Polarity specification of adaxial/abaxial axis (iii) GO:0009735: Response to cytokinin stimulus (iv) GO:0009744: Response to sucrose stimulus (v) GO:0006468: Protein amino acid phosphorylation (vi) GO:0009965: Leaf morphogenesis
	Consensus logo: 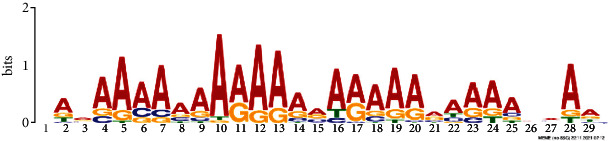				

2	DTTTTTKTTTTKTTY Consensus sequence: [AGT][TG]TTTT[TG]TTTT[TG][TG]T[TCG]	6.0e-020	15	245	
	Consensus logo: 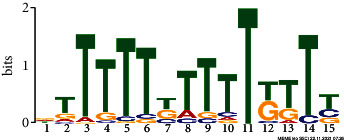				

3	NDRRAGDDDRRWARRRARAGAADRRDAG Consensus sequence: [AGC][GAT][AG][AG][AT][GA][AGT][ATG][AGT][GA][AG][AT]A[GA][AG][GAC]A[AG]A[GA]A[AG][GAT][AG][AG][GTA][AT][GT]	3.6e-025	28	121	(i) GO:0009944: Polarity specification of adaxial/abaxial axis (ii) GO:0048481: Ovule development (iii) GO:0010050: Vegetative phase change (iv) GO:0010051: Xylem and phloem pattern formation (v) GO:0042023: DNA endoreduplication (vi) GO:0035196: Production of miRNAs involved in gene silencing by miRNA (vii) GO:0009740: Gibberellic acid mediated signaling pathway
	Consensus logo: 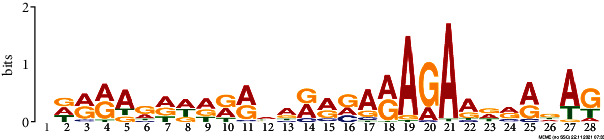				

4	CMAYCTYCTCCDTCHBCATC Consensus sequence: [CT][CA]A[TC]CT[CT]CT[CT]C[TAG][TGC][CT][ATC][GTC]C[AT][TC]C	5.3e-007	20	44	
	Consensus logo: 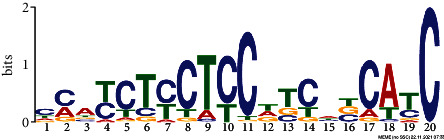				

**Table 3:** Functional roles of several important CREs detected in the functionally uncharacterized hub gene promoter sequences retrieved from the PLACE database [46].

*Cis*-acting regulatory element	Function∗
ABADESI1	“ACGT” motif; transacting factor: TAF-1; responsive to ABA and desiccation. Expressed in seeds late during embryogenesis. Induced by ABA and osmotic stress in vegetative tissues.
CACGTGMOTIF	“CACGTG motif”; essential for expression of beta-phaseolin gene during embryogenesis
CANBNNAPA	Core of “(CA)n element” in storage protein genes; embryo- and endosperm-specific transcription of napin (storage protein) gene
CARGNCAT	Noncanonical CArG motif (CC-Wx8-GG); A relevant *cis*-element for the response to AGL15 (AGAMOUS-like 15) in vivo
DPBFCOREDCDC3	DPBF-1 and 2 (Dc3 promoter-binding factor-1 and 2) binding core sequence; Dc3 expression is embryo-specific and induced by ABA
DRE1COREZMRAB17	“DRE1” core found in maize (Z.M.) rab17 gene promoter; “DRE1” was protected, in in vivo footprinting, by a protein in embryos specifically, but in leaves, was protected when was treated with ABA and drought; rab17 is expressed during late embryogenesis and is induced by ABA
DRE2COREZMRAB17	“DRE2”; core sequence in rab17 gene promoter. rab17 is expressed during late embryogenesis and is induced by ABA
EBOXBNNAPA	“E-box” of napA storage-protein gene
PYRIMIDINEBOXOSRAMY1A	Found in the promoter of alpha-amylase (Amy2/32b) gene which is induced in the aleurone layers in response to GA in embryo
RYREPEATVFLEB4	RY repeat motif; quantitative seed expression; binding site of *Arabidopsis* B3-domain-containing transcription factor FUS3, mediates abscisic acid-induced transcription
SEF1MOTIF	“SEF1 (soybean embryo factor 1)” binding motif; regulates the expression of genes encoding for the beta-conglycinin seed storage proteins
SEF3MOTIFGM	“SEF3 binding site”; regulates the expression of genes encoding for the beta-conglycinin seed storage proteins
SEF4MOTIFGM7S	“SEF4 (soybean embryo factor 4)” binding motif; regulates the expression of genes encoding for the beta-conglycinin seed storage proteins
TATABOX2	“TATA box”; TATA box found in beta-phaseolin promoter which is accurate transcription initiation in the embryo stage

∗Details of PLACE entries were retrieved from the https://www.dna.affrc.go.jp/place/place_seq.shtml (accessed on 19th May 2022).

**Table 4:** Distribution of SE markers across network modules ordered by the number of interactors.

	Gene identifier	Module	Gene name	No. of interactors
1	AT3G26790	Turquoise	*FUSCA3* (*FUS3*)	3346
2	AT5G13790	Turquoise	*AGAMOUS-LIKE 15* (*AGL15*)	3308
3	AT1G21970	Turquoise	*LEAFY COTYLEDON 1* (*LEC1*)	3297
4	AT5G45980	Turquoise	*WUSCHEL RELATED HOMEOBOX 8* (*WOX8*)	3158
5	AT3G24650	Turquoise	*ABSCISIC ACID INSENSITIVE 3* (*ABI3*)	3115
6	AT1G78080	Brown	*WOUND INDUCED DEDIFFERENTIATION 1* (*WIND1*)	2111
7	AT5G57390	Turquoise	*AINTEGUMENTA-LIKE 5* (*AIL5*)	752
8	AT4G37750	Red	*AINTEGUMENTA* (*ANT*)	688
9	AT1G63470	Red	*AT-HOOK MOTIF NUCLEAR LOCALIZED PROTEIN 5* (*AHL5*)	522
10	AT5G65510	Purple	*AINTEGUMENTA-LIKE 7* (*AIL7*)	216

## Data Availability

The datasets used to support the findings of this study are included within the article and within the supplementary information files.

## Abbreviations

*ABI3*: *ABSCISIC ACID INSENSITIVE 3*
AGL: AGAMOUS-LIKE
*AIL*: *AINTEGUMENTA-LIKE*
*BBM*: *BABY BOOM*
bHLH: Basic helix-loop-helix
*bZIP*: *BASIC LEUCINE-*
C2H2: Cys2-His2
CRE: Cis-acting regulatory element
DEG: Differentially expressed gene
*EMB*: *EMBRYO-DEFECTIVE*
FC: Fold change
*FUS3*: *FUSCA3*
GCN: Gene coexpression network
GEO: Gene Expression Omnibus
GO: Gene Ontology
HB: HOMEOBOX
*IAA30*: *INDOLE-3-ACETIC ACID INDUCIBLE 30*
JMJ: JUMONJI DOMAIN-CONTAINING
*KAN3*: *KANADI 3*
LEC: LEAFY COTYLEDON
ME: Module eigengene
MEME: Multiple Em for Motif Elicitation
miRNA: MicroRNA
PLACE: Plant cis-acting regulatory DNA elements
*r*: Pearson correlation coefficient
RNA-Seq: RNA-sequencing
RUS: ROOT UV-B SENSITIVE
SE: Somatic embryogenesis
*STO*: *SALT TOLERANCE*
TF: Transcription factor
WGCNA: Weighted Gene Correlation Network Analysis.

## Supplementary Materials

Supplementary 1
*Table S1*. The list of variance filtered genes (variance >0.25 quantile).

Supplementary 2
*Table S2*. The list of differentially expressed genes.

Supplementary 3
*Table S3*. The top 10 hub genes of each network module.

Supplementary 4
*Table S4*. Important hub genes based on differential gene expression analysis.

Supplementary 5
*Table S5*. *EMB* genes in the coexpression network.

Supplementary 6
*Table S6*. TFs in the coexpression network.

Supplementary 7
*Table S7*. The list of microRNA-targeted TFs.

Supplementary 8
*Table S8*. Distribution of gene encoding epigenetic regulators across network modules.
