# Supplementary material for: Weighted Gene Correlation Network Analysis (WGCNA) of Arabidopsis Somatic Embryogenesis (SE) and Identification of Key Gene Modules to Uncover SE-Associated Hub Genes
Source: Int J Genomics. 2022 Jul 4;2022:7471063. doi: 10.1155/2022/7471063 (PMC9274236; doi:10.1155/2022/7471063)
Supplement: Supplementary 1 — Table S1. The list of variance filtered genes (variance >0.25 quantile). [file 7471063.f1.pdf]

Table S1. List of variance filtered genes (Variance > 0.25 quantile)

Gene identifier	Gene symbol	Gene description
AT1G01010	*NAC DOMAIN CONTAINING PROTEIN 1 (NAC001)*	NAC domain containing protein 1
AT1G01030	*NGATHA3 (NGA3)*	AP2/B3-like transcriptional factor family protein
AT1G01040	*DICER-LIKE 1 (DCL1)*	Encodes a Dicer homolog. Dicer is a RNA helicase involved in microRNA processing. Mutations in this locus can result in embryo lethality. Embryo shape at seed maturity is globular-elongate. Other mutants convert the floral meristems to an indeterminate state, others yet show defects in ovule development. mRNA is expressed in all shoot tissues. DCL1 is able to produce miRNAs and siRNAs. The mRNA is cell-to-cell mobile.
AT1G01050	*PYROPHOSPHORYLASE 1 (PPa1)*	Encodes a soluble protein with inorganic pyrophosphatase activity that is highly specific for Mg-inorganic pyrophosphate.
AT1G01060	*LATE ELONGATED HYPOCOTYL (LHY)*	LHY encodes a myb-related putative transcription factor involved in circadian rhythm along with another myb transcription factor CCA1
AT1G01070	*USUALLY MULTIPLE ACIDS MOVE IN AND OUT TRANSPORTERS 28 (UMAMIT28)*	Encodes a plasma membrane-localized amino acid transporter likely involved in amino acid export in the developing seed.
AT1G01080		RNA-binding (RRM/RBD/RNP motifs) family protein
AT1G01090	*PYRUVATE DEHYDROGENASE E1 ALPHA (PDH-E1 ALPHA)*	pyruvate dehydrogenase E1 alpha subunit
AT1G01110	*IQ-DOMAIN 18 (IQD18)*	Member of IQ67 (CaM binding) domain containing family.
AT1G01120	*3-KETOACYL-COA SYNTHASE 1 (KCS1)*	Encodes a condensing enzyme KCS1 (3-ketoacyl-CoA synthase 1) which is involved in the critical fatty acid elongation process in wax biosynthesis.
AT1G01130		CBL-interacting Serine/Threonine-kinase
AT1G01140	*CBL-INTERACTING PROTEIN KINASE 9 (CIPK9)*	Encodes a CBL-interacting protein kinase with similarity to SOS2
AT1G01150		Homeodomain-like protein with RING/FYVE/PHD-type zinc finger domain-containing protein
AT1G01170		ozone-responsive stress-like protein (DUF1138)
AT1G01180		S-adenosyl-L-methionine-dependent methyltransferases superfamily protein
AT1G01190	*CYTOCHROME P450, FAMILY 78, SUBFAMILY A, POLYPEPTIDE 8 (CYP78A8)*	cytochrome P450, family 78, subfamily A, polypeptide 8
AT1G01200	*RAB GTPASE HOMOLOG A3 (RABA3)*	RAB GTPase homolog A3
AT1G01225		NC domain-containing protein-like protein
AT1G01250		encodes a member of the DREB subfamily A-4 of ERF/AP2 transcription factor family. The protein contains one AP2 domain. There are 17 members in this subfamily including TINY.
AT1G01280	*CYTOCHROME P450, FAMILY 703, SUBFAMILY A, POLYPEPTIDE 2 (CYP703A2)*	member of CYP703A CYP703A2 is expressed specifically in anthers of land plants, catalyzing the in-chain hydroxylation at the C-7 position of medium-chain saturated fatty acids (lauric acid in-chain hydroxylase) which is involved in pollen development (sporopollenin synthesis).
AT1G01290	*COFACTOR OF NITRATE REDUCTASE AND XANTHINE DEHYDROGENASE 3 (CNX3)*	COFACTOR OF NITRATE REDUCTASE AND XANTHINE DEHYDROGENASE 3. Encodes a protein involved in molybdenum cofactor biosynthesis. Homologous to E.coli MoaC. Expression is low in all tissues examined, except in roots. Appears to have targeting signals for chloroplast or mitochondria
AT1G01300		Eukaryotic aspartyl protease family protein
AT1G01320	*REDUCED CHLOROPLAST COVERAGE (REC1)*	Encodes REDUCED CHLOROPLAST COVERAGE 1 (REC1) a protein with similarity to the FLOURY locus in maize. Located in the nucleus and cytosol. Contributes to establishing the size of the chloroplast compartment.
AT1G01330		
AT1G01340	*CYCLIC NUCLEOTIDE GATED CHANNEL 10 (CNGC10)*	member of Cyclic nucleotide gated channel family, regulated by Bacillus amyloliquefaciens under high calcium stress.
AT1G01360	*REGULATORY COMPONENT OF ABA RECEPTOR 1 (RCAR1)*	Encodes RCAR1 (regulatory components of ABA receptor). Interacts with and regulates the type 2C protein phosphatases (PP2Cs) ABI1 and ABI2. Functions as abscisic acid sensor. The mRNA is cell-to-cell mobile.
AT1G01380	*ENHANCER OF TRY AND CPC 1 (ETC1)*	ETC1 is involved in trichome and root hair patterning in Arabidopsis.
AT1G01390		Phosphatidylinositol 4-phosphate 5-kinase (PIP5K) enzyme family member.
AT1G01410	*PUMILIO 22 (PUM22)*	Encodes a member of the Arabidopsis Pumilio (APUM) proteins containing PUF domain (eight repeats of approximately 36 amino acids each). PUF proteins regulate both mRNA stability and translation through sequence-specific binding to the 3' UTR of target mRNA transcripts.
AT1G01420	*UDP-GLUCOSYL TRANSFERASE 72B3 (UGT72B3)*	Phosphatidylinositol 4-phosphate 5-kinase (PIP5K) enzyme family member.
AT1G01430	*TRICHOME BIREFRINGENCE-LIKE 25 (TBL25)*	Encodes a member of the TBL (TRICHOME BIREFRINGENCE-LIKE) gene family containing a plant-specific DUF231 (domain of unknown function) domain. TBL gene family has 46 members, two of which (TBR/AT5G06700 and TBL3/AT5G01360) have been shown to be involved in the synthesis and deposition of secondary wall cellulose, presumably by influencing the esterification state of pectic polymers.Functions as a mannan O-acetyltransferase, catalyzing the 2-O and 3-O-monoacetylation of mannosyl residues A nomenclature for this gene family has been proposed (Volker Bischoff & Wolf Scheible, 2010, personal communication). Phosphatidylinositol 4-phosphate 5-kinase (PIP5K) enzyme family member.
AT1G01450		Protein kinase superfamily protein
AT1G01460	*(PIPK11)*	Type I phosphatidylinositol-4-phosphate 5-kinase, subfamily A.
AT1G01470	*LATE EMBRYOGENESIS ABUNDANT 14 (LEA14)*	Encodes late-embryogenesis abundant protein whose mRNA levels are induced in response to wounding and light stress. Might be involved in protection against desiccation.
AT1G01480	*1-AMINO-CYCLOPROPANE-1-CARBOXYLATE SYNTHASE 2 (ACS2)*	a member of the 1-aminocyclopropane-1-carboxylate (ACC) synthase (S-adenosyl-L-methionine methylthioadenosine-lyase, EC 4.4.1.14) gene family, isolated from a flower-specific cDNA library.
AT1G01490	*HEAVY METAL PROTEIN 1 (ATHMP01)*	Heavy metal transport/detoxification superfamily protein
AT1G01500		Erythronate-4-phosphate dehydrogenase family protein
AT1G01520	*ALTERED SEED GERMINATION 4 (ASG4)*	RVE3 is one of eleven homologous MYB-like transcription factors in Arabidopsis and a member of the RVE8 clade. Plays a minor role in clock regulation.
AT1G01530	*AGAMOUS-LIKE 28 (AGL28)*	AGAMOUS-like 28
AT1G01560	*MAP KINASE 11 (MPK11)*	Member of MAP Kinase family. Flg22-induced activation is blocked by AvrRpt2.
AT1G01570		transferring glycosyl group transferase (DUF604)
AT1G01600	*CYTOCHROME P450, FAMILY 86, SUBFAMILY A, POLYPEPTIDE 4 (CYP86A4)*	Encodes a member of the CYP86A subfamily of cytochrome p450 genes. Expressed significantly at highest level in mature stems and flowers.

AT1G01610	*GLYCEROL-3-PHOSPHATE SN-2-ACYLTRANSFERASE 4 (GPAT4)*	bifunctional sn-glycerol-3-phosphate 2-O-acyltransferase/phosphatase. Involved in cutin assembly and is functionally redundant with GPAT8.
AT1G01620	*PLASMA MEMBRANE INTRINSIC PROTEIN 1C (PIP1C)*	a member of the plasma membrane intrinsic protein subfamily PIP1. localizes to the plasma membrane and exhibits water transport activity in Xenopus oocyte. expressed ubiquitously and protein level decreases slightly during leaf development. Involved redundantly with PIP1;1/2/4/5 in hydraulics and carbon fixation, regulates the expression of related genes that affect plant growth and development.
AT1G01630		Sec14p-like phosphatidylinositol transfer family protein
AT1G01640		Putative role in flower development. Comparison of SALK_011721C to Columbia wild type resulted in a trend toward earlier flowering in the mutant (P=0.1) (Stapleton and Woodruff 2009, personal communication).
AT1G01650	*SIGNAL PEPTIDE PEPTIDASE-LIKE 4 (SPPL4)*	SIGNAL PEPTIDE PEPTIDASE-LIKE 4
AT1G01660	*(PUB55)*	Plant U-box type E3 ubiquitin ligase (PUB).
AT1G01670	*(PUB56)*	Plant U-box type E3 ubiquitin ligase (PUB).
AT1G01690	*PUTATIVE RECOMBINATION INITIATION DEFECTS 3 (PRD3)*	Encodes a novel plant-specific protein that is involved in meiotic double strand break formation.
AT1G01700	*ROP (RHO OF PLANTS) GUANINE NUCLEOTIDE EXCHANGE FACTOR 2 (ROPGEF2)*	Encodes a member of KPP-like gene family, homolog of KPP (kinase partner protein) gene in tomato. Also a member of the RopGEF (guanine nucleotide exchange factor) family, containing the novel PRONE domain (plant-specific Rop nucleotide exchanger), which is exclusively active towards members of the Rop subfamily.
AT1G01720	*(ATAF1)*	Belongs to a large family of putative transcriptional activators with NAC domain. Transcript level increases in response to wounding and abscisic acid. ATAF1 attentuates ABA signaling and sythesis. Mutants are hyposensitive to ABA. The mRNA is cell-to-cell mobile.
AT1G01725		adenylosuccinate synthetase
AT1G01730		hypothetical protein
AT1G01750	*ACTIN DEPOLYMERIZING FACTOR 11 (ADF11)*	actin depolymerizing factor 11
AT1G01760	*ORTHOLOG OF YEAST TAD1 (TAD1)*	denosine deaminases acting on tRNA
AT1G01780	*PLIM2B (PLIM2b)*	Encodes a member of the Arabidopsis LIM proteins: a family of actin bundlers with distinct expression patterns. WLIM1, WLIM2a, and WLIM2b are widely expressed, whereas PLIM2a, PLIM2b, and PLIM2c are predominantly expressed in pollen. Regulates actin cytoskeleton organization. The mRNA is cell-to-cell mobile.
AT1G01790	*K+ EFFLUX ANTIPORTER 1 (KEA1)*	Encodes a member of the cation/proton antiporters-2 antiporter superfamily, the K efflux antiporter KEA1 that is localized to the chloroplast envelope.
AT1G01800		NAD(P)-binding Rossmann-fold superfamily protein
AT1G01830		ARM repeat superfamily protein
AT1G01840		AP2-like ethylene-responsive transcription factor SNZ
AT1G01860	*PALEFACE 1 (PFC1)*	dimethyladenosine transferase
AT1G01900	*(SBTI1.1)*	Encodes AtSBT1.1, a subtilisin-like serine protease. Cleaves the phytosulfokine AtPSK4, a growth promoting peptide.
AT1G01920		SET domain-containing protein
AT1G01940		Cyclophilin-like peptidyl-prolyl cis-trans isomerase family protein
AT1G01980	*(ATBBE1)*	Encodes an oligogalacturonide oxidase that inactivates the elicitor-active oligogalacturonides (OGs).
AT1G02020		nitroreductase family protein
AT1G02050	*LESS ADHESIVE POLLEN 6 (LAP6)*	Chalcone and stilbene synthase family protein
AT1G02060		Tetratricopeptide repeat (TPR)-like superfamily protein
AT1G02070		zinc ion-binding protein
AT1G02100	*SUPPRESSOR OF BRI1 (SBI1)*	Leucine carboxyl methyltransferase
AT1G02120	*VASCULAR ASSOCIATED DEATH1 (VAD1)*	Encodes VAD1 (Vascular Associated Death1), a regulator of cell death and defense responses in vascular tissues. VAD1 is a putative membrane associated protein and contains a GRAM domain. vad1 is a lesion mimic mutant that exhibits light conditional appearance of propagative HR (hypersensitive response)-like lesions along the vascular system. The mRNA is cell-to-cell mobile.
AT1G02180		ferredoxin-like protein
AT1G02190	*ECERIFERUM1-LIKE1 (CER1-L1)*	Fatty acid hydroxylase superfamily
AT1G02200		
AT1G02205	*ECERIFERUM 1 (CER1)*	Expression of the CER1 gene associated with production of stem epicuticular wax and pollen fertility. Biochemical studies showed that cer1 mutants are blocked in the conversion of stem wax C30 aldehydes to C29 alkanes, and they also lack the secondary alcohols and ketones. These suggested the CER1 protein is an aldehyde decarbonylase, but the exact molecular function of this protein remains to be determined.
AT1G02220	*NAC DOMAIN CONTAINING PROTEIN 3 (NAC003)*	NAC domain transcription factor which functions as a negative regulator of the TDIF-PXY module and fine-tunes TDIF signaling in vascular development. Controls the balance of xylem formation and cambial cell divisions.
AT1G02230	*NAC DOMAIN CONTAINING PROTEIN 4 (NAC004)*	NAC domain containing protein 4
AT1G02250	*NAC DOMAIN CONTAINING PROTEIN 5 (NAC005)*	Encodes a member of the NAC family of transcription factors. ANAC005 contains sequences specifying both nuclear and plasma membrane targeting. Overexpression results in increased xylem differentiation suggesting ANAC005 promotes xylem formation.
AT1G02260		Divalent ion symporter
AT1G02280	*TRANSLOCON AT THE OUTER ENVELOPE MEMBRANE OF CHLOROPLASTS 33 (TOC33)*	Encodes a GTP-binding GTP-ase. Component of the chloroplast protein import machinery. Required for import of POR B into plastids. Toc33 phosphorylation may not play an important role in vivo.
AT1G02300	*(ATCATHB1)*	Encodes a capase involved in stress induced cell death.
AT1G02310	*ENDO-BETA-MANNANASE 1 (MAN1)*	Glycosyl hydrolase superfamily protein
AT1G02320		hypothetical protein
AT1G02330	*COP1 SUPPRESSOR 2 (CSU2)*	Encodes a nuclear coiled-coil domain-containing protein that interacts with and negatively regulates COP1's E3 ubiquitin ligase activity, and represses COP1 mediated HY5 degradation in cell-free extracts.

AT1G02340	*LONG HYPOCOTYL IN FAR-RED (HFR1)*	Encodes a light-inducible, nuclear bHLH protein involved in phytochrome signaling. Mutants exhibit a long-hypocotyl phenotype only under far-red light but not under red light and are defective in other phytochrome A-related responses. Mutants also show blue light response defects. HFR1 interacts with COP1, co-localizes to the nuclear specks and is ubiquinated by COP1.
AT1G02360		Chitinase family protein
AT1G02370		Tetratricopeptide repeat (TPR)-like superfamily protein
AT1G02390	*GLYCEROL-3-PHOSPHATE SN-2-ACYLTRANSFERASE 2 (GPAT2)*	putative sn-glycerol-3-phosphate 2-O-acyltransferase
AT1G02400	*GIBBERELLIN 2-OXIDASE 6 (GA2OX6)*	Encodes a gibberellin 2-oxidase that acts on C19 gibberellins but not C20 gibberellins.
AT1G02420		Pentatricopeptide repeat (PPR) superfamily protein
AT1G02430	*ADP-RIBOSYLATION FACTOR D1B (ARFD1B)*	A member of ARF GTPase family. A thaliana has 21 members of this family, known to be essential for vesicle coating and uncoating and functions in GTP-binding. Gene encoding ADP-ribosylation factor and similar to other ARFs and ARF-like proteins.
AT1G02450	*NIM1-INTERACTING 1 (NIMIN1)*	NIMIN1 modulates PR gene expression according the following model: NPR1 forms a ternary complex with NIMIN1 and TGA factors upon SAR induction that binds to a positive regulatory cis-element of the PR-1 promoter, termed LS7. This leads to PR-1 gene induction. NIMIN1 decreases transcriptional activation, possibly through its EAR motif, which results in fine-tuning of PR-1 gene expression.
AT1G02460		Pectin lyase-like superfamily protein
AT1G02470		Polyketide cyclase/dehydrase and lipid transport superfamily protein
AT1G02490		hypothetical protein
AT1G02530	*ATP-BINDING CASSETTE B12 (ABCB12)*	P-glycoprotein 12
AT1G02540		hypothetical protein
AT1G02550		Plant invertase/pectin methylesterase inhibitor superfamily protein
AT1G02560	*NUCLEAR ENCODED CLP PROTEASE 5 (CLPP5)*	One of several nuclear-encoded ClpPs (caseinolytic protease). Contains a highly conserved catalytic triad of Ser-type proteases (Ser-His-Asp). The name reflects nomenclature described in Adam et. al (2001). The mRNA is cell-to-cell mobile.
AT1G02570		transmembrane protein
AT1G02580	*MEDEA (MEA)*	Encodes the imprinted gene MEA that belongs to Polycomb Repressive Complex 2 (PRC2) and has a SET domain for methyltransferase activity and is involved in the stable transcriptional silencing of target genes. It negatively regulates seed development in the absence of fertilization. Mutations in this locus result in embryo lethality. MEA is imprinted in the endosperm. The maternal allele is expressed and the paternal allele is silent. MEA is controlled by DEMETER (DME), a DNA glycosylase required to activate MEA expression, and METHYLTRANSFERASE I (MET1), which maintains CG methylation at the MEA locus. MEA is involved in the negative regulation of its own imprinted gene expression; the effect is not only allele-specific but also dynamically regulated during seed development. In the ovule, the MEA transcripts are accumulated at their highest level before fertilization and gradually decrease after fertilization
AT1G02640	*BETA-XYLOSIDASE 2 (BXL2)*	encodes a protein similar to a beta-xylosidase located in the extracellular matrix. This is a member of glycosyl hydrolase family 3 and has six other closely related members.
AT1G02660	*PLASTID LIPASE2 (PLIP2)*	PLIP2 is a glycerolipid A1 lipase with substrate preference for monogalactosyldiacylglycerol. Expression is induced by ABA.
AT1G02670		P-loop containing nucleoside triphosphate hydrolases superfamily protein
AT1G02680	*TBP-ASSOCIATED FACTOR 13 (TAF13)*	Encodes a TBP-Associated Factor (TAF) that functions together with PRC2 in transcriptional regulation during seed development.
AT1G02700		GATA transcription factor-like protein
AT1G02710		glycine-rich protein
AT1G02720	*GALACTURONOSYLTRANSFERASE-LIKE 5 (GATL5)*	Encodes a protein with putative galacturonosyltransferase activity.
AT1G02730	*CELLULOSE SYNTHASE-LIKE D5 (CSLD5)*	Encodes a gene similar to cellulose synthase. Knock-out mutant has reduced growth, reduced xylan level and reduced xylan synthase activity in stems.It's expression is cell cycle dependent and it appears to function in cell plate formation.
AT1G02740	*MORF RELATED GENE 2 (MRG2)*	MRG1 and MRG2 proteins act as readers of H3K4me3/H3K36me3 marked chromatin. They interact with each other as well as several other protein classes, to modulate the activity of flowering genes.
AT1G02750	*(DIL-2)*	Drought-responsive family protein
AT1G02790	*POLYGALACTURONASE 4 (PGA4)*	encodes a exopolygalacturonase.
AT1G02800	*CELLULASE 2 (CEL2)*	Encodes a protein with similarity to endo-1,4-b-glucanases and is a member of Glycoside Hydrolase Family 9. CEL2 is induced by nemotodes and is expressed in syncitia induced by Heterodera schachtii.May be involved in the development and function of syncitia.
AT1G02810		Plant invertase/pectin methylesterase inhibitor superfamily
AT1G02816		pectinesterase (Protein of unknown function, DUF538)
AT1G02820	*LATE EMBRYOGENESIS ABUNDANT 3 (LEA3)*	Late embryogenesis abundant 3 (LEA3) family protein
AT1G02830		Ribosomal L22e protein family
AT1G02840	*SERINE/ARGININE-RICH PROTEIN SPLICING FACTOR 34 (SR34)*	SR34/SR1 is a plant homologue of the human general/alternative splicing factor SF2/ASF. Barta et al (2010) have proposed a nomenclature for Serine/Arginine-Rich Protein Splicing Factors (SR proteins): Plant Cell. 2010, 22:2926.
AT1G02850	*BETA GLUCOSIDASE 11 (BGLU11)*	beta glucosidase 11
AT1G02860	*NITROGEN LIMITATION ADAPTATION (NLA)*	Encodes a ubiquitin E3 ligase with RING and SPX domains that is involved in mediating immune responses and mediates degradation of PHT1s at plasma membranes. Targeted by MIR827. Ubiquitinates PHT1;3, PHT1;2, PHT1;1/AtPT1 and PHT1;4/AtPT2.
AT1G02870		nucleolar-like protein
AT1G02900	*RAPID ALKALINIZATION FACTOR 1 (RALF1)*	Member of a diversely expressed predicted peptide family showing sequence similarity to tobacco Rapid Alkalinization Factor (RALF), and is believed to play an essential role in the physiology of Arabidopsis. Consists of a single exon and is characterized by a conserved C-terminal motif and N-terminal signal peptide. Mediates Ca2+-dependent signaling. Regulates the splicing of flowering genes and exerts an opposite effect on the flowering time compared with FER.

AT1G02910	*LOW PSII ACCUMULATION1 (LPA1)*	Mutants defective in this gene were shown to have a reduced PSII content (overall reduction in the levels of several PSII subunits) and a disrupted grana stack structure. The N-terminal half of the protein contains two tetratricopeptide repeat (TPR) motifs that are arranged tandemly, each consisting of a 34-residue degenerate consensus sequence. The N-terminal sequence is rich in positive and hydroxylated amino acid residues.
AT1G02930	*GLUTATHIONE S-TRANSFERASE 6 (GSTF6)*	Encodes glutathione transferase belonging to the phi class of GSTs. Naming convention according to Wagner et al. (2002).
AT1G02940	*GLUTATHIONE S-TRANSFERASE (CLASS PHI) 5 (GSTF5)*	Encodes glutathione transferase belonging to the phi class of GSTs. Naming convention according to Wagner et al. (2002).
AT1G02950	*GLUTATHIONE S-TRANSFERASE F4 (GSTF4)*	Encodes glutathione transferase belonging to the phi class of GSTs. Naming convention according to Wagner et al. (2002).
AT1G02960		kinetochore protein
AT1G02980	*CULLIN 2 (CUL2)*	encodes an Arabidopsis cullin
AT1G03010		Phototropic-responsive NPH3 family protein
AT1G03020	*(GRXS1)*	Encodes a member of the CC-type glutaredoxin (ROXY) family that has been shown to interact with the transcription factor TGA2.
AT1G03030		P-loop containing nucleoside triphosphate hydrolases superfamily protein
AT1G03050	*PHOSPHATIDYLINOSITOL BINDING CLATHRIN ASSEMBLY PROTEIN 5A (PICALM5A)*	Phosphatidylinositol binding clathrin assembly protein 5A/B are recent paralogs with overlapping functions in recycling ANXUR proteins to the pollen tube membrane.
AT1G03055	*DWARF27 (D27)*	Encodes the ortholog of rice D27. It is plastid-localized and is required for the inhibition of secondary bud outgrowth and operates on a nonmobile precursor upstream of MAX1 in the SL biosynthesis pathway.
AT1G03060	*SPIRRIG (SPI)*	Encodes a WD/BEACH domain protein involved in cell morphogenesis and ribonucleoprotein particle formation. It interacts with the P-body core component DCP2, associates to mRNA processing bodies (P-bodies), and regulates their assembly upon salt stress. It accumulates at the root hair apex via post-Golgi compartments and positively regulates tip growth by maintaining tip-focused vesicle secretion and filamentous-actin integrity.
AT1G03070	*LIFEGUARD 4 (LFG4)*	Bax inhibitor-1 family protein
AT1G03080	*NETWORKED 1D (NET1D)*	kinase interacting (KIP1-like) family protein
AT1G03100		Pentatricopeptide repeat (PPR) superfamily protein
AT1G03130	*PHOTOSYSTEM I SUBUNIT D-2 (PSAD-2)*	Encodes a protein predicted by sequence similarity with spinach PsaD to be photosystem I reaction center subunit II (PsaD2)
AT1G03140	*PRE-MRNA PROCESSING FACTOR 18A (PRP18A)*	PRP18a is one of two paralogs (the other being PRP18b) which are highly similar to the step II splicing factors in yeast. Loss of function mutations show defects in alternative splicing, mostly intron retention events.
AT1G03170	*FANTASTIC FOUR 2 (FAF2)*	A member of the FAF family proteins encoded by the FANTASTIC FOUR (FAF) genes: AT4G02810 (FAF1), AT1G03170 (FAF2), AT5G19260 (FAF3) and AT3G06020 (FAF4). FAFs have the potential to regulate shoot meristem size in Arabidopsis thaliana. FAFs can repress WUS, which ultimately leads to an arrest of meristem activity in FAF overexpressing lines.
AT1G03180	*(COMET)*	Functions in chromosome axis remodeling and synapsis along with co factors PCH2 and ASY1.Required for chiasma formation.
AT1G03190	*ULTRAVIOLET HYPERSENSITIVE 6 (UVH6)*	UV damage and heat induce a common stress response in plants that leads to tissue death and reduced chloroplast function. The UVH6 product is suggested to be a negative regulator of this response.
AT1G03210		Phenazine biosynthesis PhzC/PhzF protein
AT1G03220	*SECRETED ASPARTIC PROTEASE 2 (SAP2)*	Eukaryotic aspartyl protease family protein
AT1G03240		hypothetical protein
AT1G03270		CBS domain protein with a domain protein (DUF21)
AT1G03290		ELKS/Rab6-interacting/CAST family protein
AT1G03300	*DOMAIN OF UNKNOWN FUNCTION 724 1 (ATDUF1)*	Member of the plant-specific DUF724 protein family. Arabidopsis has 10 DUF724 proteins. Loss of function mutant has a WT phenotype
AT1G03320		hypothetical protein
AT1G03360	*RIBOSOMAL RNA PROCESSING 4 (RRP4)*	Encodes a core subunit of the RNA exosome required for the processing of rRNA, several snoRNA and the degradation of aberrant transcripts.
AT1G03370		C2 calcium/lipid-binding and GRAM domain containing protein
AT1G03390		HXXXD-type acyl-transferase family protein
AT1G03400		A single copy gene that encodes a protein with sequence similarity to tomato E8 (ACC oxidase, the last step in ethylene biosynthesis) involved in ethylene synthesis and fruit ripening in tomato. This gene is not induced by ethylene in siliques. The transcript is found in siliques, etiolated seedlings, leaves, stems and flowers.
AT1G03410	*(2A6)*	2-oxoglutarate (2OG) and Fe(II)-dependent oxygenase superfamily protein
AT1G03420	*SADHU NON-CODING RETROTRANSPOSON 4-2 (Sadhu4-2)*	Member of Sadhu non-coding retrotransposon family
AT1G03430	*HISTIDINE-CONTAINING PHOSPHOTRANSFER FACTOR 5 (AHP5)*	Encodes AHP5, one of the six Arabidopsis thaliana histidine phosphotransfer proteins (AHPs). AHPs function as redundant positive regulators of cytokinin signaling. Members of the AHP gene family include: AT3G21510 (AHP1), AT3G29350 (AHP2), AT5G39340 (AHP3), AT3G16360 (AHP4), AT1G03430 (AHP5) and AT1G80100 (AHP6).
AT1G03440		Leucine-rich repeat (LRR) family protein
AT1G03445	*BRI1 SUPPRESSOR 1 (BSU1)*	encodes a serine?threonine protein phosphatase with an N-terminal Kelch-repeat domain, which is nuclear localized and expressed preferentially in elongating cells. Genetic evidence suggest that this gene plays a redundant role (along with other members of the same gene family) in modulating growth in response to brassinosteroid.
AT1G03457	*BRUNO-LIKE 2 (BRN2)*	RNA-binding (RRM/RBD/RNP motifs) family protein
AT1G03475	*LESION INITIATION 2 (LIN2)*	Encodes coproporphyrinogen III oxidase, a key enzyme in the biosynthetic pathway of chlorophyll and heme, a tetrapyrrole pathway. Mutants express cytological and molecular markers associated with the defense responses, usually activated by pathogen infection.
AT1G03490	*NAC DOMAIN CONTAINING PROTEIN 6 (NAC006)*	NAC domain containing protein 6
AT1G03495		HXXXD-type acyl-transferase family protein
AT1G03530	*NUCLEAR ASSEMBLY FACTOR 1 (NAF1)*	nuclear assembly factor 1
AT1G03540		Pentatricopeptide repeat (PPR-like) superfamily protein
AT1G03550	*SECRETORY CARRIER MEMBRANE PROTEIN 4 (SCAMP4)*	Secretory carrier membrane protein (SCAMP) family protein

AT1G03560		Pentatricopeptide repeat (PPR-like) superfamily protein
AT1G03590		Protein phosphatase 2C family protein
AT1G03600	*(PSB27)*	PSB27 is a chloroplast lumen localized protein that is involved in adaptation to changes in light intensity.
AT1G03610		plant/protein (DUF789)
AT1G03620	*(ELMOD_F)*	Member of the ELMOD family.
AT1G03630	*PROTOCHLOROPHYLLIDE OXIDOREDUCTASE C (POR C)*	Encodes for a protein with protochlorophyllide oxidoreductase activity. The enzyme is NADPH- and light-dependent.
AT1G03660		Ankyrin-repeat containing protein
AT1G03675		
AT1G03687		DTW domain-containing protein
AT1G03700	*CASP-LIKE PROTEIN 1C2 (CASPL1C2)*	Uncharacterized protein family (UPF0497)
AT1G03710		Cystatin/monellin superfamily protein
AT1G03720		Cysteine proteinases superfamily protein
AT1G03760		Prefoldin chaperone subunit family protein
AT1G03770	*RING 1B (RING1B)*	Encodes a nuclear localized protein with similarity to animal polycomb repressive core complex1 (PRC1) core component RING.Appears to function redundantly with ATRING1a, a close paralog. Both interact physically with CLF and LHP1 and appear to function together to repress class I KNOX gene expression.
AT1G03780	*TARGETING PROTEIN FOR XKLP2 (TPX2)*	Homolog of vertebrate TPX2. Protein has three domains involved in nuclear targeting, one in nuclear export and two in microtubule binding. Involved in mitotic spindle assembly during late prophase and early prometaphase.
AT1G03790	*SOMNUS (SOM)*	Encodes SOMNUS (SOM), a nucleus-localized CCCH-type zinc finger protein. SOM negatively regulates light-dependent seed germination downstream of PIL5 (AT2G20180).
AT1G03820		E6-like protein
AT1G03840	*MAGPIE (MGP)*	MGP is a nuclear-localized putative transcription factor with three zinc finger domains. MGP can interact with three proteins implicated in root patterning: SCR, SHR, and JKD in Y2H assays, and these interactions depend on the first zinc finger in MGP. MGP appears to be a direct transcriptional target of SHR and SCR, based on promoter binding assays, though it is not expressed in the QC, based on in situ hybridizations.
AT1G03850	*GLUTAREDOXIN 13 (GRXS13)*	Encodes glutaredoxin ATGRXS13, required to facilitate Botrytis cinerea infection of Arabidopsis thaliana plants. Sylvain La Camera et al (2011, PMID:21756272) reported a third splice variant in addition to the two annotated in TAIR10. It is a member of the CC-type glutaredoxin (ROXY) family that has been shown to interact with the transcription factor TGA2 and suppress ORA59 promoter activity.
AT1G03860	*PROHIBITIN 2 (PHB2)*	prohibitin 2
AT1G03870	*FASCICLIN-LIKE ARABINOOGALACTAN 9 (FLA9)*	fasciclin-like arabinogalactan-protein 9 (Fla9). Possibly involved in embryogenesis and seed development.
AT1G03880	*CRUCIFERIN 2 (CRU2)*	Protein is tyrosine-phosphorylated and its phosphorylation state is modulated in response to ABA in Arabidopsis thaliana seeds.
AT1G03890		RmlC-like cupins superfamily protein
AT1G03900	*ATP-BINDING CASSETTE I18 (ABCI18)*	member of NAP family, an heterogeneous subfamily of the ATP-binding Cassette (ABC) superfamily of membrane transporters. The NAPs proteins are characterized by having only one nucleotide-binding folds (NBFs) domain.
AT1G03920	*NUCLEAR DBF2-RELATED 2 (NDR2)*	Ubiquitously expressed protein kinase.
AT1G03930	*DUAL SPECIFICITY KINASE 1 (ADK1)*	Phosphorylates serine, threonine, and tyrosine
AT1G03950	*VACUOLAR PROTEIN SORTING-ASSOCIATED PROTEIN 2.3 (VPS2.3)*	vacuolar protein sorting-associated protein 2.3
AT1G03960		Calcium-binding EF hand family protein
AT1G03970	*G-BOX BINDING FACTOR 4 (GBF4)*	encodes a basic leucine zipper G-box binding factor that can bind to G-box motifs only as heterodimers with GBF2 or GBF3. A single amino acid change can confer G-box binding as homodimers.
AT1G03980	*PHYTOCHELATIN SYNTHASE 2 (PCS2)*	Encodes a protein with phytochelatin synthase activity which binds Cd2+ and Cd-glutathione complexes with high affinity. The protein has been postulated to be involved in Cd2+ tolerance. AtPCS2 expression appears to be less than that of AtPCS1, explaining the inability of endogenous AtPCS2 to substitute for AtPCS1 in the cad1-3 mutant (AtPCS1 null).
AT1G03990		Long-chain fatty alcohol dehydrogenase family protein
AT1G04000		hypothetical protein
AT1G04020	*BREAST CANCER ASSOCIATED RING 1 (BARD1)*	Encodes a protein containing two tandem BRCA1 C-Terminal (BRCT) domains, which function in phosphorylation-dependent protein-protein interactions. Loss of function mutations cause defects in meristem organization due to failure to repress WUS. BARD1 binds to WUS promoter and over expression of BARD reduces the extent of WUS expression.
AT1G04030		eisosome protein
AT1G04040		HAD superfamily, subfamily IIIB acid phosphatase
AT1G04050	*HOMOLOG OF SU(VAR)3-9 1 (SUVR1)*	Encodes SUVR1, one of the four closely related Arabidopsis SUVR proteins that belong to the SU(VAR)3-9 subgroup of SET-domain proteins. Proteins containing the evolutionarily conserved SET domain are involved in regulation of eukaryotic gene expression and chromatin structure through their histone lysine methyltransferase (HMTase) activity. SUVR1, SUVR2 and SUVR4 proteins contain a novel domain at their N-terminus, and a SUVR specific region preceding the SET domain. Localized to the nucleolus, maybe involved in regulation of rRNA expression.
AT1G04060		
AT1G04090		vacuolar sorting-associated protein (DUF946)
AT1G04110	*STOMATAL DENSITY AND DISTRIBUTION 1 (SDD1)*	Initially identified as a mutation affecting stomatal development and distribution. Encodes a protein similar to serine proteases.
AT1G04120	*ATP-BINDING CASSETTE C5 (ABCC5)*	encodes a high-affinity inositol hexakisphosphate transporter that plays a role in guard cell signaling and phytate storage. It is a member of MRP subfamily / ABC transporter subfamily C.
AT1G04130	*TETRATRICOPEPTIDE REPEAT 2 (TPR2)*	Encodes one of the 36 carboxylate clamp (CC)-tetratricopeptide repeat (TPR) proteins (Prasad 2010, Pubmed ID: 20856808). Interacts with Hsp90/Hsp70 as co-chaperone.

AT1G04150	*MULTIPLE C2 DOMAIN AND TRANSMEMBRANE REGION PROTEIN 10 (MCTP10)*	C2 calcium/lipid-binding plant phosphoribosyltransferase family protein
AT1G04160	*MYOSIN XI B (XIB)*	Encodes a member of the type XI myosin protein family involved in root hair elongation.
AT1G04180	*YUCCA 9 (YUC9)*	YUCCA 9
AT1G04200		dyggve-melchior-clausen syndrome protein
AT1G04220	*3-KETOACYL-COA SYNTHASE 2 (KCS2)*	Encodes KCS2, a member of the 3-ketoacyl-CoA synthase family involved in the biosynthesis of VLCFA (very long chain fatty acids).
AT1G04240	*SHORT HYPOCOTYL 2 (SHY2)*	SHY2/IAA3 regulates multiple auxin responses in roots. It is induced rapidly by IAA, and has been shown to be phosphorylated by oat phytochrome A in vitro.
AT1G04250	*AUXIN RESISTANT 3 (AXR3)*	Transcription regulator acting as repressor of auxin-inducible gene expression. Auxin-inducible AUX/IAA gene. Short-lived nuclear protein with four conserved domains. Domain III has homology to beta alpha alpha dimerization and DNA binding domains. Involved in auxin signaling and is a positive modulator of natural leaf senescence. Auxin induces the degradation of the protein in a dosage-dependent manner in a process mediated by AtRac1. Auxin induced the relocalization of the protein within the nucleus from a diffused nucleoplasmic pattern to a discrete particulated pattern named nuclear protein bodies or NPB in a process also mediated by Rac1. Colocalizes with SCF, CSN and 26S proteasome components.
AT1G04310	*ETHYLENE RESPONSE SENSOR 2 (ERS2)*	encodes an ethylene receptor related to bacterial two-component histidine kinases.
AT1G04330	*(PH2)*	A proline/serine rich protein of unknown function. It interacts with defense related MAP kinase MPK6 and others. May be involved in signaling during defense or stress response.
AT1G04350		encodes a protein whose sequence is similar to 2-oxoglutarate-dependent dioxygenase
AT1G04360	*ARABIDOPSIS TOXICOS EN LEVADURA 1 (ATL1)*	RING/U-box superfamily protein
AT1G04380		encodes a protein similar to a 2-oxoglutarate-dependent dioxygenase
AT1G04390		BTB/POZ domain-containing protein
AT1G04420		NAD(P)-linked oxidoreductase superfamily protein
AT1G04430		S-adenosyl-L-methionine-dependent methyltransferases superfamily protein
AT1G04440	*CASEIN KINASE LIKE 13 (CKL13)*	Member of CKL gene family (CKL-C group).
AT1G04470		hypothetical protein (DUF810)
AT1G04490		hypothetical protein (DUF3527)
AT1G04500		CCT motif family protein
AT1G04510	*MOS4-ASSOCIATED COMPLEX 3A (MAC3A)*	Encodes MAC3A, a U-box proteins with homology to the yeast and human E3 ubiquitin ligase Prp19. Associated with the MOS4-Associated Complex (MAC). Involved in plant innate immunity. Regulator of flowering time.
AT1G04540		Calcium-dependent lipid-binding (CaLB domain) family protein
AT1G04550	*INDOLE-3-ACETIC ACID INDUCIBLE 12 (IAA12)*	IAA12/BDL plays a role in auxin-mediated processes of apical-basal patterning in the embryo. bdl mutants lack a primary root meristem
AT1G04560		AWPM-19-like family protein
AT1G04580	*ALDEHYDE OXIDASE 4 (AO4)*	Encodes aldehyde oxidase AAO4 preferentially expressed in developing seeds.
AT1G04590		PPR containing-like protein
AT1G04600	*MYOSIN XI A (XIA)*	member of Myosin-like proteins
AT1G04610	*YUCCA 3 (YUC3)*	Encodes a member of the YUC family that is expressed in the root apex and is ethylene inducible in the root.
AT1G04620	*7-HYDROXYMETHYL CHLOROPHYLL A (HMCHL) REDUCTASE (HCAR)*	Encodes a 7-hydroxymethyl chlorophyll a reductase, an enzyme of the chlorophyll cycle that converts 7-hydroxymethyl chlorophyll a to chlorophyll a.
AT1G04630	*MATERNAL EFFECT EMBRYO ARREST 4 (MEE4)*	GRIM-19 protein
AT1G04640	*LIPOYLTRANSFERASE 2 (LIP2)*	Lipoyltransferase, located in mitochondria but not found in chloroplasts
AT1G04645		Plant self-incompatibility protein S1 family
AT1G04660		glycine-rich protein
AT1G04670		hypothetical protein
AT1G04680		Pectin lyase-like superfamily protein
AT1G04700		PB1 domain-containing protein tyrosine kinase
AT1G04710	*PEROXISOMAL 3-KETOACYL-COA THIOLASE 4 (PKT4)*	EC2.3.1.16 thiolase. Its transcript levels change after inducing MUTE expression in a mute background.
AT1G04730	*CHROMOSOME TRANSMISSION FIDELITY 18 (CTF18)*	Necessary for sister chromatid cohesion. Acts in synergy with ETG1.
AT1G04760	*VESICLE-ASSOCIATED MEMBRANE PROTEIN 726 (VAMP726)*	member of Synaptobrevin -like protein family
AT1G04770	*SULFUR DEFICIENCY INDUCED 2 (SDI2)*	SDI2 is a member of a small family of TPR proteins in Arabidopsis. Like SDI1 it is induced by low sulfer and appears to play a role in negative regulation of glucosinolate biosynthesis.
AT1G04790		RING/U-box superfamily protein
AT1G04800		glycine-rich protein
AT1G04810		26S proteasome regulatory complex, non-ATPase subcomplex, Rpn2/Psmd1 subunit
AT1G04840		Tetratricopeptide repeat (TPR)-like superfamily protein
AT1G04870	*PROTEIN ARGININE METHYLTRANSFERASE 10 (PRMT10)*	Encodes a type I protein arginine methyltransferase based on the At1g04870.2 gene model. PRMT10 can catalyze the asymmetric dimethylation of arginine 3 on histone 4 and can also methylate myelin basic protein in vitro. Mutants lacking PRMT10 flower late due to defects in the autonomous pathway and they have elevated levels of FLC transcripts.
AT1G04880	*ARID-HMG DNA-BINDING PROTEIN 15 (HMGBD15)*	Encodes a ARID-HMG DNA-binding protein that functions in pollen tube growth through the regulation of gene expression by interacting with the transcription factors AGL66 and AGL104.
AT1G04900		NADH dehydrogenase ubiquinone complex I, assembly factor-like protein (DUF185)
AT1G04910		O-fucosyltransferase family protein
AT1G04920	*SUCROSE PHOSPHATE SYNTHASE 3F (SPS3F)*	Encodes a sucrose-phosphate synthase whose activity is stimulated by Glc-6-P and inhibited by Pi.
AT1G04930	*LIN37 HOMOLOG A (LIN37A)*	Member of the DREAM complex, which represses growth in response to DNA damage, LIN37 type.

AT1G04940	*TRANSLOCON AT THE INNER ENVELOPE MEMBRANE OF CHLOROPLASTS 20-I (Tic20-I)*	Tic20 is believed to function as a component of the protein-conducting channel at the inner envelope membrane. Genes AT1G04940 and AT1G04945 were switched for the TAIR7 genome release to give consistency with MIPs annotation. The Arabidopsis genome encodes four Tic20 homologous proteins, AT1G04940(Tic20-I), AT2G47840(Tic20-II), AT4G03320(Tic20-IV) and AT5G55710(Tic20-V).
AT1G04980	*PDI-LIKE 2-2 (PDIL2-2)*	Encodes a protein disulfide isomerase-like (PDIL) protein, a member of a multigene family within the thioredoxin (TRX) superfamily. Transcript levels for this gene are up-regulated in response to three different chemical inducers of ER stress (dithiothreitol, beta-mercaptoethanol, and tunicamycin). AtIRE1-2 does not appear to be required for this response, but the atbzip60 mutant has a diminished response.
AT1G04990		Zinc finger C-x8-C-x5-C-x3-H type family protein
AT1G05000	*PLANT AND FUNGI ATYPICAL DUAL-SPECIﬁCITY PHOSPHATASE 1 (PFA-DSP1)*	Encodes an atypical dual-speciﬁcity phosphatase.
AT1G05010	*ETHYLENE-FORMING ENZYME (EFE)*	Encodes 1-aminocyclopropane-1-carboxylate oxidase
AT1G05020	*ANTH PROTEIN 180 (AP180)*	ENTH/ANTH/VHS superfamily protein
AT1G05040		UBA-like domain protein
AT1G05060		coiled-coil protein
AT1G05090		dentin sialophosphoprotein-like protein
AT1G05100	*MITOGEN-ACTIVATED PROTEIN KINASE KINASE KINASE 18 (MAPKKK18)*	member of MEKK subfamily. Negatively regulated by RGLG1 and RGLG2; involved in drought stress tolerance.
AT1G05120		Helicase protein with RING/U-box domain-containing protein
AT1G05130		
AT1G05140		Peptidase M50 family protein
AT1G05150		Calcium-binding tetratricopeptide family protein
AT1G05160	*CYTOCHROME P450, FAMILY 88, SUBFAMILY A, POLYPEPTIDE 3 (CYP88A3)*	Encodes an ent-kaurenoic acid hydroxylase, a member of the CYP88A cytochrome p450 family.
AT1G05170		Galactosyltransferase family protein
AT1G05180	*AUXIN RESISTANT 1 (AXR1)*	Encodes a subunit of the RUB1 activating enzyme that regulates the protein degradation activity of Skp1-Cullin-Fbox complexes, primarily, but not exclusively, affecting auxin responses. Acts alongside AS1 to exclude BP expression from leaves. Promotes degradation of the cytokinin response repressor ARR5. Affects expression of key DNA repair and meiotic genes, signifcant role in DNA repair.
AT1G05190	*EMBRYO DEFECTIVE 2394 (emb2394)*	Encodes the plastid 50S ribosomal protein L6.
AT1G05200	*GLUTAMATE RECEPTOR 3.4 (GLR3.4)*	Encodes a putative glutamate receptor GLR3 with dual localization in plastid and plasma membrane.
AT1G05210		Transmembrane protein 97, Putative
AT1G05230	*HOMEODOMAIN GLABROUS 2 (HDG2)*	Encodes a homeobox-leucine zipper family protein belonging to the HD-ZIP IV family. Mutants have trichomes that appear glass-like under a dissecting microscope as compared to the wild-type trichomes. The mutations do not affect trichome growth or branch number.
AT1G05250	*PEROXIDASE 2 (PRX2)*	Encodes a cationic cell-wall-bound peroxidase homolog that is involved in the lignification of cell walls. Regulated by COG1, involved in seed longevity.
AT1G05260	*RARE COLD INDUCIBLE GENE 3 (RCI3)*	Encodes a cold-inducible cationic peroxidase that is involved in the stress response. In response to low temperature, RCI3 transcripts accumulate in the aerial part and in roots of etiolated seedlings but only in roots of light-grown seedlings. The mRNA is cell-to-cell mobile.
AT1G05270		TraB family protein
AT1G05280		ERV-F (C)1 provirus ancestral Env polyprotein, putative (DUF604)
AT1G05290	*ORBITALLY MANIFESTED GENE 1 (OMG1)*	Member of the CONSTANS-Like protein family which is a putative regulator of reactive oxygen species homeostasis.
AT1G05300	*ZINC TRANSPORTER 5 PRECURSOR (ZIP5)*	member of Fe(II) transporter isolog family
AT1G05310		Pectin lyase-like superfamily protein
AT1G05320	*PICC-LIKE (PICL)*	myosin heavy chain, embryonic smooth protein
AT1G05330		hypothetical protein
AT1G05340	*CYSTEINE-RICH TRANSMEMBRANE MODULE 1 (ATHCYSTM1)*	cysteine-rich TM module stress tolerance protein
AT1G05350		NAD(P)-binding Rossmann-fold superfamily protein
AT1G05360	*KILLING ME SLOWLY 2 (KMS2)*	KMS2 encode a endoplasmic reticulum protein involved in the early secretory pathway.
AT1G05370		Sec14p-like phosphatidylinositol transfer family protein
AT1G05385	*LOW PSII ACCUMULATION 19 (LPA19)*	Encodes a Psb27 homolog involved in photosystem II biogenesis.
AT1G05400		hypothetical protein
AT1G05440		C-8 sterol isomerase
AT1G05450		Encodes a Protease inhibitor/seed storage/LTP family protein
AT1G05460	*SILENCING DEFECTIVE (SDE3)*	Encodes a protein with similarity to RNA helicases. Mutants are defective in post-transcriptional gene silencing.
AT1G05470	*COTYLEDON VASCULAR PATTERN 2 (CVP2)*	Encodes an inositol polyphosphate 5' phosphatase (5PTase) that is required for the proper recruitment of cells into developing vascular tissue in leaves and cotyledons. It is most similar to Type I 5PTases that are known to cleave a phosphate from IP3 or IP4. cvp2 mutants have elevated levels of IP3 and are hypersensitive to ABA in seed germination assays.
AT1G05480		
AT1G05490	*CHROMATIN REMODELING 31 (chr31)*	Involved in gene silencing. Locus-specific regulator of 24nt-siRNA expression, works together with CLSY1-4 as the master regulators of essentially all Pol-IV-dependent 24nt-siRNAs.
AT1G05510	*OIL BODY-ASSOCIATED PROTEIN 1A (OBAP1A)*	Protein is tyrosine-phosphorylated and its phosphorylation state is modulated in response to ABA in Arabidopsis thaliana seeds.
AT1G05530	*UDP-GLUCOSYL TRANSFERASE 75B2 (UGT75B2)*	Encodes a protein with glucosyltransferase activity with high sequence homology to UGT1 (AT1G05560). It belongs to an UGT subfamily that binds UDP-glucose but not UDP-glucuronate, UDP-galactose, or UDP-rhamnose as the glycosyl donor. UGT2 was shown to be able to use abscisic acid as glycosylation substrate in the presence of UDP-glucose.
AT1G05550	*DUF295 ORGANELLAR A 2 (ATDOA2)*	DUF295 domain protein of unknown function. Expressed in ovule integuments and nucellus.

AT1G05560	*UDP-GLUCOSYLTRANSFERASE 75B1 (UGT75B1)*	A UDP-glucose transferase localized in the phragmoplast. It has been co-purified with the callose synthase complex and may transfer UDP-glucose from sucrose synthase to the callose synthase and thus help form a substrate channel for the synthesis of callose at the forming cell plate. Induced by salicylic acid. Independent of NPR1 for their induction by salicylic acid. UGT1 encodes a protein with glucosyltransferase activity with high sequence homology to UGT2 (AT1G05530). It belongs to an UGT subfamily that binds UDP-glucose but not UDP-glucuronate, UDP-galactose, or UDP-rhamnose as the glycosyl donor. UGT1 was shown to be able to use abscisic acid as glycosylation substrate in the presence of UDP-glucose. UGT1/UGT75B1 catalyzes the formation of the p aminobenzoate-glucose ester in vitro and in vivo. It appears to be the enzyme predominantly responsible for pABA-Glc formation in Arabidopsis based on assays in leaves, flowers, and siliques.
AT1G05570	*CALLOSE SYNTHASE 1 (CALS1)*	Encodes a callose synthase 1 catalytic subunit . Member of Glycosyltransferase Family- 48.
AT1G05575	*HYPOXIA RESPONSE UNKNOWN PROTEIN 17 (HUP17)*	transmembrane protein
AT1G05580	*CATION/H+ EXCHANGER 23 (CHX23)*	member of Putative Na+/H+ antiporter family
AT1G05610	*ADP-GLUCOSE PYROPHOSPHORYLASE SMALL SUBUNIT 2 (APS2)*	Encodes the small subunit of ADP-glucose pyrophosphorylase. The small subunit is the catalytic isoform responsible for ADP-glucose pyrophosphorylase activity. The presence of the small subunit is required for large subunit stability. Two isoforms of the small subunit (ApS1 and ApS2) have been described. ApS2 is a minor small subunit isoform present in all plant tissues tested.
AT1G05620	*URIDINE-RIBOHYDROLASE 2 (URH2)*	Encodes a cytosolic inosine nucleoside hydrolase. It forms a heterocomplex with NSH1 with almost two orders of magnitude higher catalytic efficiency for xanthosine hydrolysis than observed for NSH1 alone. Transcript levels for this gene are elevated in older leaves suggesting that it may play a role in purine catabolism during senescence.
AT1G05630	*INOSITOL-POLYPHOSPHATE 5-PHOSPHATASE 13 (5PTASE13)*	Encodes an inositol polyphosphate 5-phosphatase with phosphatase activity toward only Ins(1,4,5)P3. Induced in response to ABA and wounding treatments. Expressed in young seedlings and flowers, while no transcripts were detectable in maturated roots, stems, and rosette leaves Modulates the development of cotyledon veins through its regulation of auxin homeostasis. Involved in blue light light?stimulated increase in cytosolic calcium ion.
AT1G05650		Pectin lyase-like superfamily protein
AT1G05670		Pentatricopeptide repeat (PPR-like) superfamily protein
AT1G05680	*URIDINE DIPHOSPHATE GLYCOSYLTRANSFERASE 74E2 (UGT74E2)*	Encodes a UDP-glucosyltransferase, UGT74E2, that acts on IBA (indole-3-butyric acid) and affects auxin homeostasis. The transcript and protein levels of this enzyme are strongly induced by H2O2 and may allow integration of ROS (reactive oxygen species) and auxin signaling. This enzyme can also transfer glycosyl groups to several compounds related to the explosive TNT when this synthetic compound is taken up from the environment.
AT1G05690	*BTB AND TAZ DOMAIN PROTEIN 3 (bt3)*	BTB and TAZ domain protein. Acts redunantly with BT1 and BT2 during female gametophyte development. Acts with BT2 during male gametophyte development.
AT1G05700		Leucine-rich repeat transmembrane protein kinase protein
AT1G05710		basic helix-loop-helix (bHLH) DNA-binding superfamily protein
AT1G05740		FAM136A-like protein (DUF842)
AT1G05760	*RESTRICTED TEV MOVEMENT 1 (RTM1)*	Specifically restrict the long-distance movement of tobacco etch potyvirus (TEV) without involving either hypersensitive cell death or systemic acquired resistance
AT1G05770		Mannose-binding lectin superfamily protein
AT1G05800	*DONGLE (DGL)*	Encodes a galactolipase. Located in the chloroplast. Involved in the initial step of jasmonic acid biosynthesis. Expressed in vegetative tissues and is necessary for the biosynthesis of basal-level JAs in vegetative tissues.
AT1G05805	*ABA-RESPONSIVE KINASE SUBSTRATE 2 (AKS2)*	basic helix-loop-helix (bHLH) DNA-binding superfamily protein
AT1G05810	*RAB GTPASE HOMOLOG A5E (RABA5E)*	Rab GTPase-like A5A protein
AT1G05840		Eukaryotic aspartyl protease family protein
AT1G05850	*POM-POM1 (POM1)*	Encodes an endo chitinase-like protein AtCTL1. Essential for tolerance to heat, salt and drought stresses. Also involved in root hair development, cell expansion and response to cytokinin. Allelic to erh2. 11 alleles described in Hauser (1995). Mutant is defective in acquired thermotolerance, appears semidwarf throughout its life cycle and has extra lateral branches. There are two EMS alleles. Expression of AtHSP101 is not affected in the mutants.
AT1G05880	*ARIADNE 12 (ARI12)*	Encodes ARI12 (ARIADNE 12). ARI12 belongs to a family of `RING between RING fingers' (RBR) domain proteins with E3 ligase activity. Expression of ARI12 is induced by UV-B exposure.
AT1G05890	*ARIADNE 5 (ARI5)*	RING/U-box superfamily protein
AT1G05920		B3 domain protein (DUF313)
AT1G05960	*EFR3 OF PLANT 3 (EFOP3)*	Part of a nanodomain complex that tethers PI4Kα1 to the plasma membrane.
AT1G05990	*ROOT HAIR SPECIFIC 2 (RHS2)*	EF hand calcium-binding protein family
AT1G06000	*(UGT89C1)*	encodes a flavonol-7-O-rhamnosyltransferase involved in the formation of rhamnosylated flavonols
AT1G06010		basic leucine zipper/W2 domain protein
AT1G06020	*FRUCTOKINASE 5 (FRK5)*	Encodes a member of the fructokinase gene family. Nomenclature according to Riggs 2017 has been adopted for the family by the community (personal communication, Boernke, Callis, Granot, Boernke, and Smeekens).
AT1G06040	*SALT TOLERANCE (STO)*	Encodes salt tolerance protein (STO) which confers salt tolerance to yeast cells. Fully complements calcineurin deficient yeast but does not encode a phosphoprotein phosphatase. Sequence has similarities to CONSTANS. STO co-localizes with COP1 and plays a role in light signaling.STO transcript levels are regulated by photoperiod and phtyohormones. STO competes with FLC in the regulation of floral transition genes SOC1 and FT.
AT1G06080	*DELTA 9 DESATURASE 1 (ADS1)*	Encodes a protein homologous to delta 9 acyl-lipid desaturases of cyanobacteria and acyl-CoA desaturases of yeast and mammals. expression down-regulated by cold temperature. It is involved in the desaturation of VLCFAs to make monounsaturated VLCFAs.
AT1G06110	*SKP1/ASK-INTERACTING PROTEIN 16 (SKIP16)*	SKP1/ASK-interacting protein 16
AT1G06120	*ACYL-LIPID DESATURASE1.4 (ADS1.4)*	Membrane bound acyl-lipid desaturases which can perform Δ9 desaturation.

AT1G06140	*MITOCHONDRIAL EDITING FACTOR 3 (MEF3)*	Encodes MEF3 (mitochondrial editing factor 3), a PPR (pentatricopeptide repeat) protein of the E domain subclass. Functions in mitochondrial RNA editing.
AT1G06150	*EMBRYO DEFECTIVE 1444 (EMB1444)*	Encodes a LHW-like protein with 79% amino acid identity to LHW.
AT1G06160	*OCTADECANOID-RESPONSIVE ARABIDOPSIS AP2/ERF 59 (ORA59)*	encodes a member of the ERF (ethylene response factor) subfamily B-3 of ERF/AP2 transcription factor family. The protein contains one AP2 domain. There are 18 members in this subfamily including ATERF-1, ATERF-2, AND ATERF-5.
AT1G06170	*BASIC HELIX LOOP HELIX PROTEIN 89 (BHLH089)*	Encodes a bHLH transcription factor that together with bHLH010 and bHLH091 is important for the normal transcriptome of the developing Arabidopsis anther, possibly by forming a feed-forward loop with DYT1. Recognizes the TCATGTGC box to activate the expression of target genes, including ATA20, EXL4, and MEE48.
AT1G06180	*MYB DOMAIN PROTEIN 13 (MYB13)*	member of MYB3R- and R2R3- type MYB- encoding genes
AT1G06200		Peptidase S24/S26A/S26B/S26C family protein
AT1G06225	*CLAVATA3/ESR-RELATED 3 (CLE3)*	Member of a large family of putative ligands homologous to the Clavata3 gene. Consists of a single exon.
AT1G06230	*GLOBAL TRANSCRIPTION FACTOR GROUP E4 (GTE4)*	This gene is predicted to encode a bromodomain-containing protein. Plant lines expressing RNAi constructs targeted against GTE4 show some resistance to agrobacterium-mediated root transformation.
AT1G06260		Cysteine peptidase,activity detected in leaf and flower.
AT1G06270		Pentatricopeptide repeat (PPR) superfamily protein
AT1G06290	*ACYL-COA OXIDASE 3 (ACX3)*	Encodes an acyl-CoA oxidase with specificity for medium chain fatty acids. The mRNA is cell-to-cell mobile.
AT1G06310	*ACYL-COA OXIDASE 6 (ACX6)*	Encodes a putative acyl-CoA oxidase. However, no transcripts have been detected for this gene and no altered phenotypes have been detected in plants mutant for this gene. This suggests that ACX6 does not significantly contribute to seedling beta-oxidation of fatty acids or indole-3-butyric acid in vivo.
AT1G06320		hypothetical protein
AT1G06340		Plant Tudor-like protein
AT1G06350	*DELTA 9 DESATURASE 4 (ADS4)*	Fatty acid desaturase family protein
AT1G06380		Ribosomal protein L1p/L10e family
AT1G06420		DNA ligase-like protein
AT1G06430	*FTSH PROTEASE 8 (ftsh8)*	encodes a FtsH protease that is localized to the chloroplast
AT1G06440		Ubiquitin carboxyl-terminal hydrolase family protein
AT1G06450	*CCR4-ASSOCIATED FACTOR 1G (CAF1G)*	Deadenylase.
AT1G06460	*ALPHA-CRYSTALLIN DOMAIN 32.1 (ACD32.1)*	ACD32.1 encodes an alpha-crystallin domain containing protein with homology to small heat shock proteins.
AT1G06470		Nucleotide/sugar transporter family protein
AT1G06490	*CALLOSE SYNTHASE 7 (CalS7)*	Encodes Callose Synthase 7 (CalS7), a phloem-specific callose synthase responsible for callose deposition in developing sieve elements during phloem formation and in mature phloem induced by wounding.
AT1G06510		forkhead-associated domain protein
AT1G06520	*GLYCEROL-3-PHOSPHATE SN-2-ACYLTRANSFERASE 1 (GPAT1)*	sn-glycerol-3-phosphate 2-O-acyltransferase. Expressed in flower buds and siliques. Homozygous mutant plants are male sterile.
AT1G06540		hypothetical protein
AT1G06550		ATP-dependent caseinolytic (Clp) protease/crotonase family protein
AT1G06560	*(NOP2C)*	NOL1/NOP2/sun family protein
AT1G06580	*PENTATRICOPEPTIDE REPEAT 1 (PPR1)*	Pentatricopeptide Repeat Protein. During pathogen infection its mRNA is cleaved by MIR400 which down regulates its expression. Mutants are more susceptible to infection ny bacteria.
AT1G06620		encodes a protein whose sequence is similar to a 2-oxoglutarate-dependent dioxygenase The mRNA is cell-to-cell mobile.
AT1G06640		encodes a protein whose sequence is similar to a 2-oxoglutarate-dependent dioxygenase The mRNA is cell-to-cell mobile.
AT1G06650		encodes a protein whose sequence is similar to 2-oxoglutarate-dependent dioxygenase The mRNA is cell-to-cell mobile.
AT1G06680	*PHOTOSYSTEM II SUBUNIT P-1 (PSBP-1)*	Encodes a 23 kD extrinsic protein that is part of photosystem II and participates in the regulation of oxygen evolution. Phosphorylation of this protein is dependent on calcium. In plsp1-1 mutant plastids, the nonmature form of the protein localizes in the stroma. The mRNA is cell-to-cell mobile.
AT1G06690		NAD(P)-linked oxidoreductase superfamily protein
AT1G06710	*MITOCHONDRIAL STABILITY FACTOR 1 (MTSF1)*	Pentatricopeptide repeat protein that stabilizes nad4 mRNA in mitochondria.
AT1G06720		P-loop containing nucleoside triphosphate hydrolases superfamily protein
AT1G06730		pfkB-like carbohydrate kinase family protein
AT1G06750		P-loop containing nucleoside triphosphate hydrolases superfamily protein
AT1G06780	*GALACTURONOSYLTRANSFERASE 6 (GAUT6)*	Encodes a protein with putative galacturonosyltransferase activity. Required for synthesis of native homogalacturonan in growing pollen tubes; critical role in pollen tube growth and male fertility.
AT1G06800	*PHOSPHOLIPASE A I GAMMA 1 (PLA-I{gamma}1)*	Encodes a lipase that hydrolyzes phosphatidylcholine, glycolipids as well as triacylglycerols.
AT1G06810		endonuclease/glycosyl hydrolase
AT1G06830	*CEP DOWNSTREAM 1 (CEPD1)*	Encodes a member of the CC-type glutaredoxin (ROXY) family that has been shown to interact with the transcription factor TGA2. CEPD1 is a non secreted polypeptide that is highly similar to CEPD2 which is another member of a novel family related to glutaredoxins. It is induced by nitrogen starvation. It acts downstream of the CEP1 peptide in systemic N-demand signalling .The RNA is expressed in the phloem of cotelydon and leaf vasculature but the peptide is graft transmissible, traveling from the shoot to the root.
AT1G06840	*HDS-ASSOCIATED RLK1 (HAK1)*	Homomultimers interact with cytoplasmic signaling molecule PBL27, resulting in herbivory resistance, in an ethylene-dependent manner.
AT1G06850	*BASIC LEUCINE-ZIPPER 52 (bZIP52)*	bZIP protein involved in heat stress response. Under heat stress localization moves exclusively to nucleus.
AT1G06870	*PLASTIDIC TYPE I SIGNAL PEPTIDASE 2A (PLSP2A)*	Peptidase S24/S26A/S26B/S26C family protein
AT1G06890	*UDP-XYLOSE TRANSPORTER3 (UXT3)*	UXT3 is a member of the NST-KT subfamily of nucleotide/sugar transporters. It is localized to the golgi and functions as a UDP-Xyl transporter.

AT1G06900		Insulinase (Peptidase family M16) family protein
AT1G06970	*CATION/HYDROGEN EXCHANGER 14 (CHX14)*	member of Putative Na+/H+ antiporter family
AT1G06980		PADRE protein
AT1G06990		GDSL-motif esterase/acyltransferase/lipase. Enzyme group with broad substrate specificity that may catalyze acyltransfer or hydrolase reactions with lipid and non-lipid substrates.
AT1G07000	*EXOCYST SUBUNIT EXO70 FAMILY PROTEIN B2 (EXO70B2)*	A member of EXO70 gene family, putative exocyst subunits, conserved in land plants. Arabidopsis thaliana contains 23 putative EXO70 genes, which can be classified into eight clusters on the phylogenetic tree.
AT1G07010	*SHEWENELLA-LIKE PROTEIN PHOSPHATASE 1 (SLP1)*	Calcineurin-like metallo-phosphoesterase superfamily protein
AT1G07025		Mitochondrial substrate carrier family protein
AT1G07040		plant/protein
AT1G07050	*FITNESS (FITNESS)*	FITNESS encodes a protein with a single CCT domain and belongs to the CCT motif family genes (CMF). FITNESS acts upstream JUB1 thereby controlling H2O2 levels. FITNESS has a role in cellular redox homeostasis controlling H2O2 levels, due to changes in enzymes, metabolites and transcripts related to ROS detoxification.
AT1G07060	*DSB FORMATION (DFO)*	hypothetical protein
AT1G07080		Thioredoxin superfamily protein
AT1G07090	*LIGHT SENSITIVE HYPOCOTYLS 6 (LSH6)*	LIGHT-DEPENDENT SHORT HYPOCOTYLS-like protein (DUF640)
AT1G07120	*IPGA1-LIKE1 (IPGAL1)*	CHUP1-like protein
AT1G07130	*(STN1)*	Encodes a protein with similarity to yeast STN1, an OB fold protein involved in protecting yeast telomeres. In Arabidopsis, loss of STN1 function mutations exhibit gross morphological abnormalities and defects in telomere architecture and maintenance. STN1 likely plays a role in telomere end capping.
AT1G07135		glycine-rich protein
AT1G07140	*(SIRANBP)*	Encodes a putative Ran-binding protein (siRanBP).
AT1G07150	*MITOGEN-ACTIVATED PROTEIN KINASE KINASE KINASE 13 (MAPKKK13)*	Member of MEKK subfamily. Involved in wound induced signaling where it interacts with At5g40440, and activates At1g59580.
AT1G07160		Protein phosphatase 2C family protein
AT1G07180	*ALTERNATIVE NAD(P)H DEHYDROGENASE 1 (NDA1)*	Internal NAD(P)H dehydrogenase in mitochondria. The predicted protein sequence has high homology with other designated NAD(P)H DHs from microorganisms; the capacity for matrix NAD(P)H oxidation via the rotenone-insensitive pathway is significantly reduced in the Atndi1 mutant plant line; the in vitro translation product of AtNDI1 is imported into isolated mitochondria and located on the inside of the inner membrane.
AT1G07190		Lon protease
AT1G07210		Ribosomal protein S18
AT1G07220		O-glucosyltransferase rumi-like protein (DUF821)
AT1G07240	*UDP-GLUCOSYL TRANSFERASE 71C5 (UGT71C5)*	Encodes a UDP-glucosyltransferase that plays a role in abscisic acid (ABA) glucosylation from ABA to ABA-glucose ester and regulates ABA homeostasis, thereby influencing the ABA signal network.
AT1G07250	*UDP-GLUCOSYL TRANSFERASE 71C4 (UGT71C4)*	UDP-glucosyl transferase 71C4
AT1G07260	*UDP-GLUCOSYL TRANSFERASE 71C3 (UGT71C3)*	Encodes a uridine diphosphate-glycosyltransferase that acts on methyl salicylate (MeSA) to form MeSA glucosides in vitro and in vivo and facilitates negative regulation of the SAR response by modulating homeostasis of MeSA and SA.
AT1G07280		Tetratricopeptide repeat (TPR)-like superfamily protein
AT1G07290	*GOLGI NUCLEOTIDE SUGAR TRANSPORTER 2 (GONST2)*	Encodes a GDP-mannose transporter.
AT1G07300		josephin protein-like protein
AT1G07320	*RIBOSOMAL PROTEIN L4 (RPL4)*	encodes a plastid ribosomal protein L4
AT1G07330		dentin sialophosphoprotein
AT1G07340	*SUGAR TRANSPORTER 2 (STP2)*	sugar transporter 2
AT1G07360	*MOS4-ASSOCIATED COMPLEX SUBUNIT 5A (MAC5A)*	Encodes MAC5A, a component of the MOS4-associated complex (MAC) that contributes to snc1- mediated autoimmunity. MAC is a highly conserved nuclear protein complex associated with the spliceosome. Homologues include AT1G07360 (MAC5A), AT2G29580 (MAC5B) and AT5G07060 (MAC5C).
AT1G07380	*NEUTRAL CERAMIDASE 1 (ATNCER1)*	Encodes a neutral ceramidase that is involved in sphingolipid homeostasis and responses to oxidative stress.
AT1G07390	*RECEPTOR LIKE PROTEIN 1 (RLP1)*	receptor like protein 1
AT1G07430	*HIGHLY ABA-INDUCED PP2C GENE 2 (HAI2)*	Encodes a member of the group A protein phosphatase 2C (PP2C) family that is responsible for negatively regulating seed dormancy.
AT1G07440		NAD(P)-binding Rossmann-fold superfamily protein
AT1G07450		NAD(P)-binding Rossmann-fold superfamily protein
AT1G07460		Concanavalin A-like lectin family protein
AT1G07490	*ROTUNDIFOLIA LIKE 3 (RTFL3)*	ROTUNDIFOLIA like 3
AT1G07500	*SIAMESE-RELATED 5 (SMR5)*	SMR5 is a member of the SIAMESE-RELATED Cyclin-Dependent Kinase Inhibitor family. It is induced by ROS/oxidative stress.
AT1G07520		GRAS family transcription factor
AT1G07540	*TRF-LIKE 2 (TRFL2)*	Arabidopsis thaliana telomere-binding protein, putative (At1g07540)
AT1G07550		Leucine-rich repeat protein kinase family protein
AT1G07560		Leucine-rich repeat protein kinase family protein
AT1G07570	*(APK1A)*	Protein kinase capable of phosphorylating tyrosine, serine, and threonine residues
AT1G07590		Tetratricopeptide repeat (TPR)-like superfamily protein
AT1G07600	*METALLOTHIONEIN 1A (MT1A)*	metallothionein, binds to and detoxifies excess copper and other metals, limiting oxidative damage.

AT1G07610	*METALLOTHIONEIN 1C (MT1C)*	one of the five metallothioneins (MTs) genes identified in Arabidopsis. MTs are cysteine-rich proteins required for heavy metal tolerance. The mRNA is cell-to-cell mobile.
AT1G07620	*(ATOBGM)*	GTP-binding protein Obg/CgtA
AT1G07630	*POL-LIKE 5 (PLL5)*	Encodes a protein phosphatase 2C like gene, similar to POL. Involved in leaf development. Knockout mutants have abnormally shaped leaves.
AT1G07640	*(OBP2)*	A member of the DOF transcription factors. Prominently expressed in the phloem of leaves and other organs. Expression is induced by wounding, MeJA and insect feeding. Upregulates glucosinolate biosynthesis. PEAR protein involved in the formation of a short-range concentration gradient that peaks at protophloem sieve elements, and activates gene expression that promotes radial growth. Locally promotes transcription of inhibitory HD-ZIP III genes, and thereby establishes a negative-feedback loop that forms a robust boundary that demarks the zone of cell division.
AT1G07680		transmembrane protein
AT1G07690		transmembrane protein
AT1G07700		Thioredoxin superfamily protein
AT1G07720	*3-KETOACYL-COA SYNTHASE 3 (KCS3)*	Encodes KCS3, a member of the 3-ketoacyl-CoA synthase family involved in the biosynthesis of VLCFA (very long chain fatty acids).
AT1G07730		Disease resistance-responsive (dirigent-like protein) family protein
AT1G07740		Tetratricopeptide repeat (TPR)-like superfamily protein
AT1G07745	*HOMOLOG OF RAD51 D (RAD51D)*	Is a suppressor of SNI1. Encodes a member of the RecA/RAD51 family of DNA recombination and repair proteins. Both RAD51 and SNI1 have a dual role in pathogen-related gene transcription and somatic homologous recombination.
AT1G07750		RmlC-like cupins superfamily protein
AT1G07795		forkhead box protein G1
AT1G07830		ribosomal protein L29 family protein
AT1G07840		Sas10/Utp3/C1D family
AT1G07850		transferring glycosyl group transferase (DUF604)
AT1G07860		Serine/Threonine-kinase RLCKVII protein
AT1G07870		Protein kinase superfamily protein
AT1G07880	*(ATMPK13)*	member of MAP Kinase
AT1G07890	*ASCORBATE PEROXIDASE 1 (APX1)*	Encodes a cytosolic ascorbate peroxidase APX1. Ascorbate peroxidases are enzymes that scavenge hydrogen peroxide in plant cells. Eight types of APX have been described for Arabidopsis: three cytosolic (APX1, APX2, APX6), two chloroplastic types (stromal sAPX, thylakoid tAPX), and three microsomal (APX3, APX4, APX5) isoforms. At least part of the induction of heat shock proteins during light stress in Arabidopsis is mediated by H2O2 that is scavenged by APX1. Expression of the gene is downregulated in the presence of paraquat, an inducer of photoxidative stress. The mRNA is cell-to-cell mobile.
AT1G07910	*RNALIGASE (RNL)*	Encodes a tRNA ligase that resembles the yeast Trl1 RNA ligase in structure and function but very different in sequence. Like Trl1, AtRNL consists of two domains, an N-terminal ligase component and a C-terminal 5'-kinase/2',3'-cyclic phosphodiesterase (CPD) component that can function in tRNA splicing in vivo when expressed as separate polypeptides. Requires a 2'-PO4 end for tRNA splicing in vivo.
AT1G07990		SIT4 phosphatase-associated family protein
AT1G08000	*GATA TRANSCRIPTION FACTOR 10 (GATA10)*	Encodes a member of the GATA factor family of zinc finger transcription factors.
AT1G08020		
AT1G08030	*TYROSYLPROTEIN SULFOTRANSFERASE (TPST)*	Encodes a tyrosylprotein sulfotransferase (TPST). This protein is a 500-aa type I transmembrane protein that shows no sequence similarity to animal TPSTs. Activity confirmed by protein expression in yeast. TPST is expressed throughout the plant body, and the highest levels of expression are in the root apical meristem. TPST acts in the auxin pathway to maintain postembryonic root stem cell niche by defining the expression of the PLETHORA stem cell transcription factor genes. A loss-of-function mutant TPST displayed a marked dwarf phenotype accompanied by stunted roots, pale green leaves, reduction in higher order veins, early senescence, and a reduced number of flowers and siliques. TPST suppresses ethylene production through the action of PSK (phytosulfokine).
AT1G08050		Zinc finger (C3HC4-type RING finger) family protein
AT1G08065	*ALPHA CARBONIC ANHYDRASE 5 (ACA5)*	Mediates stimulation of ATP synthesis by bicarbonate in isolated thylakoids.
AT1G08080	*ALPHA CARBONIC ANHYDRASE 7 (ACA7)*	alpha carbonic anhydrase 7
AT1G08090	*NITRATE TRANSPORTER 2:1 (NRT2:1)*	High-affinity nitrate transporter. Up-regulated by nitrate. Functions as a repressor of lateral root initiation independently of nitrate uptake.
AT1G08100	*NITRATE TRANSPORTER 2.2 (NRT2.2)*	Encodes a high-affinity nitrate transporter.
AT1G08150	*(ATCHX5)*	member of Putative Na+/H+ antiporter family
AT1G08170	*(HTB8)*	Histone superfamily protein
AT1G08180	*SIAMESE-RELATED 2 (SMR2)*	cyclin-dependent kinase inhibitor
AT1G08210		Eukaryotic aspartyl protease family protein
AT1G08220		ATPase complex subunit
AT1G08230		Codes for a H+-driven, high affinity gamma-aminobutyric acid (GABA) transporter. Localized at the plasma membrane. In planta, AtGAT1 expression was highest in flowers and under conditions of elevated GABA concentrations such as wounding or senescence.
AT1G08250	*AROGENATE DEHYDRATASE 6 (ADT6)*	Encodes a plastid-localized arogenate dehydratase involved in phenylalanine biosynthesis. Although this enzyme has sequence similarity to prephenate dehydratases, it is 98 times more active with arogenate than prephenate in enzymatic assays.
AT1G08260	*TILTED 1 (TIL1)*	Similar to POL2A, DNA polymerase epsilon catalytic subunit. Essential for Arabidopsis growth. Null homozygotes are embryo lethal, partial loss of function alleles show embryo patterning defects such as root pole displacement. Delayed progression through cell cycle results in embryos with smaller numbers of larger cells.

AT1G08280	*GLYCOSYLTRANSFERASE 29A (GALT29A)*	Encodes a glycosyltransferase (GT) GALT29A, which belongs to the Carbohydrate Active Enzyme family GT29. GALT29A co-expresses with other arabinogalactan GTs, GALT31A and GLCAT14A. The recombinant GALT29A expressed in Nicotiana benthamiana demonstrated a galactosyltransferase activity, transferring galactose from UDP-galactose to a mixture of various oligosaccharides derived from arabinogalactan proteins.
AT1G08310		alpha/beta-Hydrolases superfamily protein
AT1G08320	*TGACG (TGA) MOTIF-BINDING PROTEIN 9 (TGA9)*	bZIP transcription factor family protein
AT1G08325		
AT1G08340		Rho GTPase activating protein with PAK-box/P21-Rho-binding domain-containing protein
AT1G08380	*PHOTOSYSTEM I SUBUNIT O (PSAO)*	Encodes subunit O of photosystem I.
AT1G08420	*BRI1 SUPPRESSOR 1 (BSU1)-LIKE 2 (BSL2)*	Protein phosphatase which promotes stomatal ACD by establishing kinase-based signalling asymmetry in the two daughter cells.
AT1G08430	*ALUMINUM-ACTIVATED MALATE TRANSPORTER 1 (ALMT1)*	Encodes a Al-activated malate efflux transporter. It is essential for aluminum tolerance but does not represent the major Al tolerance QTL. Staurosporine and calyculin A both block all changes in AtALMT1 gene expression (as a result malate release is totally inhibited). AtALMT1 transcription was clearly induced by indole-3-acetic acid, abscisic acid, low pH, hydrogen peroxide and flg22. STOP1 and CAMTA2 transcription factors are involved in Al-inducible expression of AtALMT1 and both proteins bind to the AtALMT1 promoter.
AT1G08500	*EARLY NODULIN-LIKE PROTEIN 18 (ENODL18)*	early nodulin-like protein 18
AT1G08520	*ALBINA 1 (ALB1)*	Encodes the CHLD subunit of the Mg-chelatase enzyme involved in chlorophyll biosynthesis. Lines carrying recessive mutations of this locus are white and seedling lethal.
AT1G08540	*RNAPOLYMERASE SIGMA SUBUNIT 2 (SIG2)*	Enodes a subunit of chloroplast RNA polymerase, confers the ability to recognize promoter sequences on the core enzyme. SIG1 is induced by red and blue light.
AT1G08550	*NON-PHOTOCHEMICAL QUENCHING 1 (NPQ1)*	Violaxanthin deepoxidase involved in xanthophyll cycle.Two major consequences of the npq1 mutation are the absence of zeaxanthin formation in strong light and the partial inhibition of the quenching of singlet excited chlorophylls in the photosystem II light-harvesting complex
AT1G08560	*SYNTAXIN OF PLANTS 111 (SYP111)*	member of SYP11 syntaxin Gene Family
AT1G08580		hypothetical protein
AT1G08590	*PXY-LIKE1 (PXL1)*	Encodes one of the two putative eLRR kinase closely related to PXY (At1g08590/PXL1 and At4g28650/PXL2). Insertion mutants in either pxl1 or pxl2 do not exhibit an obvious phenotype in the stem; double-mutant combinations of a Col allele, of pxy (pxy-3) with pxl1 and pxl2, generate a more severe vascular phenotype than pxy-3 alone, suggesting that these genes act synergistically with PXY in regulating vascular-tissue development in the stem.
AT1G08600	*(ATRX)*	The Arabidopsis ATRX harbours a N-terminal ADD domain and a C-terminal helicase domain and is devoid of the large central region involved in DAXX interaction in mammals. Arabidopsis ATRX mutant alleles are viable, but with reduced fertility. Their combination with mutants for the H3.3 chaperone HIRA impairs plant survival. ATRX loss affects cellular histone H3.3 pools and modulates the H3.1/H3.3 balance. Notably, at a genome-wide scale, loss of ATRX leads to a reduced H3.3 level at genes characterized by elevated H3.3 occupancy and high expression, including the 45S ribosomal DNA (45S rDNA) loci. Indeed, expression of specific 45S rDNA sequence variants is altered by ATRX loss (DOI:10.1105/tpc.16.00877)
AT1G08610		Pentatricopeptide repeat (PPR) superfamily protein
AT1G08630	*THREONINE ALDOLASE 1 (THA1)*	Encodes a threonine aldolase, involved in threonine degradation to glycine. Primarily expressed in seeds and seedlings.
AT1G08650	*PHOSPHOENOLPYRUVATE CARBOXYLASE KINASE 1 (PPCK1)*	Encodes a phosphoenolpyruvate carboxylase kinase that is expressed at highest levels in leaves. Expression is induced by light. The mRNA is cell-to-cell mobile.
AT1G08660	*MALE GAMETOPHYTE DEFECTIVE 2 (MGP2)*	Encodes a sialyltransferase-like protein that is localized to the Golgi apparatus and is involved in pollen tube growth and pollen germination.
AT1G08670		ENTH/VHS family protein
AT1G08680	*ARF GAP-LIKE ZINC FINGER-CONTAINING PROTEIN ZIGA4 (ZIGA4)*	A member of ARF GAP domain (AGD), A thaliana has 15 members, grouped into four classes. AGD14 belongs to the class 4, together with AGD15.
AT1G08700	*PRESENILIN-1 (PS1)*	Encodes a protein similar to animal presenilin whose expression is increased in response to potassium (K+) deprivation. Located in intracellular membrane compartments.
AT1G08710		F-box protein that is induced in roots by drought stress.
AT1G08720	*ENHANCED DISEASE RESISTANCE 1 (EDR1)*	enhanced disease resistance 1 (EDR1) confers resistance to powdery mildew disease caused by the fungus Erysiphe cichoracearum The mRNA is cell-to-cell mobile.
AT1G08800	*MYOSIN BINDING PROTEIN 1 (MYOB1)*	myosin-binding protein (Protein of unknown function, DUF593)
AT1G08810	*MYB DOMAIN PROTEIN 60 (MYB60)*	putative transcription factor of the R2R3-MYB gene family. Transcript increases under conditions that promote stomatal opening (white and blue light, abi1-1 mutation) and decreases under conditions that trigger stomatal closure (ABA, desiccation, darkness), with the exception of elevated CO2. Expressed exclusively in guard cells of all tissues. It is required for light-induced opening of stomata. Mutant shows reduced stomatal aperture which helps to limit water loss during drought.
AT1G08830	*COPPER/ZINC SUPEROXIDE DISMUTASE 1 (CSD1)*	Encodes a cytosolic copper/zinc superoxide dismutase CSD1 that can detoxify superoxide radicals. Its expression is affected by miR398-directed mRNA cleavage. Regulated by biotic and abiotic stress. Activation of CSD1 in the cytoplasm involves both a CCS-dependent and -independent pathway.
AT1G08860	*BONZAI 3 (BON3)*	Encodes a copine-like protein, which is a member of a newly identified class of calcium-dependent, phospholipid binding proteins that are present in a wide range of organisms. Overexpression of this gene suppresses bon1-1 phenotypes. Double mutant analyses with bon1-1 suggest that BON1 and BON3 have overlapping functions in maintaining cellular homeostasis and inhibiting cell death.
AT1G08900		Major facilitator superfamily protein
AT1G08920	*ERD (EARLY RESPONSE TO DEHYDRATION) SIX-LIKE 1 (ESL1)*	Encodes ESL1, a transporter for monosaccharides.
AT1G08930	*EARLY RESPONSE TO DEHYDRATION 6 (ERD6)*	encodes a putative sucrose transporter whose gene expression is induced by dehydration and cold. The mRNA is cell-to-cell mobile.
AT1G08940		Phosphoglycerate mutase family protein

AT1G08970	*NUCLEAR FACTOR Y, SUBUNIT C9 (NF-YC9)*	Encodes a NUCLEAR FACTOR-Y C (NF-YC) homologue NF-YC9. NF-YC3., NF-YC4 and NF-YC9 redundantly modulate GA- and ABA-mediated seed germination.
AT1G08980	*AMIDASE 1 (AMI1)*	Encodes an enzyme with similarity to bacterial acylamidohydrolases and exhibits indole-3-acetamide amidohydrolase activity in vitro. This enzyme may be involved in the in vivo biosynthesis of indole-acetic acid from indole-3-acetamide, a native metabolite of A. thaliana. It appears to exist as a monomer.
AT1G08990	*PLANT GLYCOGENIN-LIKE STARCH INITIATION PROTEIN 5 (PGSIP5)*	plant glycogenin-like starch initiation protein 5
AT1G09000	*NPK1-RELATED PROTEIN KINASE 1 (NP1)*	NPK1-related protein kinase 1S
AT1G09020	*HOMOLOG OF YEAST SUCROSE NONFERMENTING 4 (SNF4)*	Component of the regulatory subunit of SNF1-related protein kinase. As part of the regulatory complex it binds maltose which promotes kinase activity.
AT1G09030	*NUCLEAR FACTOR Y, SUBUNIT B4 (NF-YB4)*	nuclear factor Y, subunit B4
AT1G09080	*BINDING PROTEIN 3 (BIP3)*	Heat shock protein 70 (Hsp 70) family protein
AT1G09090	*RESPIRATORY BURST OXIDASE HOMOLOG B (RBOHB)*	NADPH-oxidase AtrbohB plays a role in seed after-ripening. Major producer of superoxide in germinating seeds. AtrbohB pre-mRNA is alternatively spliced in seeds in a hormonally and developmentally regulated manner. ABA caused accumulation of AtrbohB-? mRNA and prevented prevented AtrbohB-a mRNA expression in fresh seeds.
AT1G09100	*26S PROTEASOME AAA-ATPASE SUBUNIT RPT5B (RPT5B)*	Encodes RPT5b (Regulatory Particle 5b), one of the six AAA-ATPases of the proteasome regulatory particle. Essential for gametophyte development. In Arabidopsis, the RPT5 subunit is encoded by two highly homologous genes, RPT5a and RPT5b. RPT5a and RPT5b show accession-dependent functional redundancy. In Wassilewskija (Ws) accession: mutant alleles of RPT5a displayed 50% pollen lethality, indicating that RPT5a is essential for male gametophyte development. In the Columbia (Col) accession, a rpt5a mutant allele did not display such a phenotype because the RPT5b Col allele complements the rpt5a defect in the male gametophyte, whereas the RPT5b Ws allele does not. Double rpt5a rpt5b mutants in Col background showed a complete male and female gametophyte lethal phenotype.
AT1G09130		ATP-dependent caseinolytic (Clp) protease/crotonase family protein
AT1G09155	*PHLOEM PROTEIN 2-B15 (PP2-B15)*	phloem protein 2-B15
AT1G09160		Protein phosphatase 2C family protein
AT1G09170		P-loop nucleoside triphosphate hydrolases superfamily protein with CH (Calponin Homology) domain-containing protein
AT1G09180	*SECRETION-ASSOCIATED RAS SUPER FAMILY 1 (SARA1A)*	A member of ARF-like GTPase family. A thaliana has 21 members, in two subfamilies, ARF and ARF-like (ARL) GTPases.
AT1G09190		Tetratricopeptide repeat (TPR)-like superfamily protein
AT1G09200	*HISTONE 3.1 (H3.1)*	Histone superfamily protein
AT1G09220		Pentatricopeptide repeat (PPR) superfamily protein
AT1G09250	*ATBS1 INTERACTING FACTOR 4 (AIF4)*	basic helix-loop-helix (bHLH) DNA-binding superfamily protein
AT1G09280		rhodanese-like domain protein
AT1G09290		hypothetical protein
AT1G09300	*(ATICP55)*	Encodes a mitochondrial protease ICP55. Alters the stability of proteins by removal of a single amino acid from their sequence.
AT1G09310	*(SVB2)*	ABA responsive trichome formation regulator.
AT1G09320	*AGENET DOMAIN (AGD)-CONTAINING P1 (AGDP1)*	Heterochromatin-binding protein which can bind to three H3K9me2 tails. Preferentially binds to long TEs. Required for transcriptional silencing, non-CG DNA methylation, and H3K9 dimethylation at some loci.
AT1G09340	*CHLOROPLAST RNA BINDING (CRB)*	Encodes CHLOROPLAST RNA BINDING (CRB), a putative RNA-binding protein. CRB is important for the proper functioning of the chloroplast. Mutations in CRB also affects the circadian system, altering the expression of both oscillator and output genes. The mRNA is cell-to-cell mobile.
AT1G09350	*GALACTINOL SYNTHASE 3 (GolS3)*	Predicted to encode a galactinol synthase
AT1G09360		Plant invertase/pectin methylesterase inhibitor superfamily protein
AT1G09370		Plant invertase/pectin methylesterase inhibitor superfamily protein
AT1G09380	*USUALLY MULTIPLE ACIDS MOVE IN AND OUT TRANSPORTERS 25 (UMAMIT25)*	nodulin MtN21-like transporter family protein
AT1G09390		GDSL-motif esterase/acyltransferase/lipase. Enzyme group with broad substrate specificity that may catalyze acyltransfer or hydrolase reactions with lipid and non-lipid substrates.
AT1G09400		FMN-linked oxidoreductases superfamily protein
AT1G09410		pentatricopeptide (PPR) repeat-containing protein
AT1G09415	*NIM1-INTERACTING 3 (NIMIN-3)*	encodes a kinase that physically interacts with NPR1/NIM1
AT1G09420	*GLUCOSE-6-PHOSPHATE DEHYDROGENASE 4 (G6PD4)*	Encodes a protein similar to glucose-6-phosphate dehydrogenase but, based on amino acid differences in the active site and lack of activity, does not encode a functional G6PDH. The amino acid sequence for the consensus sequence of the G6PDH active site (DHYLGKE) differs in three places in this protein. gc exon splice site at 20574 is based on protein alignment, and is not confirmed experimentally.
AT1G09430	*ATP-CITRATE LYASE A-3 (ACLA-3)*	Encodes subunit A of the heteromeric enzyme ATP citrate lyase (ACL). In animals, ACL is encoded by a single gene; ACL in Arabidopsis is composed of two polypeptides, ACLA (encoded by 3 genes) and ACLB (encoded by 2 genes). The holoenzyme has an A(4)B(4)stoichiometry. Expression of both ACLA and ACLB but not of either of the subunits alone results in ACL activity.
AT1G09440		Protein kinase superfamily protein
AT1G09450	*HASPIN-RELATED GENE (Haspin)*	Encodes a protein kinase that phosphorylates histone H3 at Thr3 and Thr11 and plays a role in mitotic cell division.
AT1G09460		Carbohydrate-binding X8 domain superfamily protein
AT1G09470	*(ATNEAP3)*	NEAP3 is a member of a small family containing coiled-coil domains, a nuclear localization signal and a C-terminal predicted transmembrane domain. It localizes to the nuclear periphery. Mutants have altered nuclear morphology and chromatin structure.
AT1G09480		similar to Eucalyptus gunnii alcohol dehydrogenase of unknown physiological function (GI:1143445), Vigna unguiculata (gi:1854445), NOT a cinnamyl-alcohol dehydrogenase The mRNA is cell-to-cell mobile.

AT1G09500		similar to Eucalyptus gunnii alcohol dehydrogenase of unknown physiological function (GI:1143445), Vigna unguiculata (gi:1854445), NOT a cinnamyl-alcohol dehydrogenase
AT1G09510		similar to Eucalyptus gunnii alcohol dehydrogenase of unknown physiological function (GI:1143445), Vigna unguiculata (gi:1854445), NOT a cinnamyl-alcohol dehydrogenase
AT1G09530	*PHYTOCHROME INTERACTING FACTOR 3 (PIF3)*	Transcription factor interacting with photoreceptors phyA and phyB. Forms a ternary complex in vitro with G-box element of the promoters of LHY, CCA1. Acts as a negative regulator of phyB signalling. It degrades rapidly after irradiation of dark grown seedlings in a process controlled by phytochromes. Does not play a significant role in controlling light input and function of the circadian clockwork. Binds to G- and E-boxes, but not to other ACEs. Binds to anthocyanin biosynthetic genes in a light- and HY5-independent fashion. PIF3 function as a transcriptional activator can be functionally and mechanistically separated from its role in repression of PhyB mediated processes.
AT1G09540	*MYB DOMAIN PROTEIN 61 (MYB61)*	Encodes putative transcription factor. Mutants lack of mucilage extrusion from the seeds during imbibition. Reduced quantities of mucilage are deposited during the development of the seed coat epidermis in myb61 mutants. Expressed in guard cells,loss of function mutations show an increase in stomatal pore opening suggesting a role in ABA independent regulation of stomatal pore size.
AT1G09550	*PECTIN ACETYLESTERASE 1 (ATPAE1)*	Pectinacetylesterase family protein
AT1G09560	*GERMIN-LIKE PROTEIN 5 (GLP5)*	Encodes a plasodesmata-located protein involved in regulating primary root growth by controlling phloem-mediated allocation of resources between the primary and lateral root meristems. The mRNA is cell-to-cell mobile.
AT1G09570	*PHYTOCHROME A (PHYA)*	Light-labile cytoplasmic red/far-red light photoreceptor involved in the regulation of photomorphogenesis. It exists in two inter-convertible forms: Pr and Pfr (active) and functions as a dimer.The N terminus carries a single tetrapyrrole chromophore, and the C terminus is involved in dimerization. It is the sole photoreceptor mediating the FR high irradiance response (HIR). Major regulator in red-light induction of phototropic enhancement. Involved in the regulation of de-etiolation. Involved in gravitropism and phototropism. Requires FHY1 for nuclear accumulation.
AT1G09600		Protein kinase superfamily protein
AT1G09610	*GLUCURONOXYLAN METHYLTRANSFERASE1 (GXM1)*	glucuronoxylan 4-O-methyltransferase-like protein (DUF579)
AT1G09630	*RAB GTPASE 11C (RAB11c)*	Encodes a putative GTP-binding protein. Associates with organelles on a pathway from the Golgi to the plasma membrane in interphase. In dividing cells acts at the cell plate.
AT1G09660		RNA-binding KH domain-containing protein
AT1G09670		
AT1G09700	*HYPONASTIC LEAVES 1 (HYL1)*	Encodes a nuclear dsRNA binding protein. Involved in mRNA cleavage. The mutant is characterized by shorter stature, delayed flowering, leaf hyponasty, reduced fertility, decreased rate of root growth, and an altered root gravitropic response. It also exhibits less sensitivity to auxin and cytokinin.
AT1G09720	*NETWORKED 2B (NET2B)*	Member of NET domain family of actin binding proteins. Paralog of At3g22790 (NET2A).
AT1G09740		Adenine nucleotide alpha hydrolases-like superfamily protein
AT1G09750		Eukaryotic aspartyl protease family protein
AT1G09780	*2,3-BIPHOSPHOGLYCERATE-INDEPENDENT PHOSPHOGLYCERATE MUTASE 1 (iPGAM1)*	Encodes a 2,3-biphosphoglycerate-independent phosphoglycerate mutase that is involved in pollen development and stomatal movement.
AT1G09790	*COBRA-LIKE PROTEIN 6 PRECURSOR (COBL6)*	COBRA-like protein 6 precursor
AT1G09800		Pseudouridine synthase family protein
AT1G09810	*EVOLUTIONARILY CONSERVED C-TERMINAL REGION 11 (ECT11)*	evolutionarily conserved C-terminal region 11
AT1G09830	*PURINE BIOSYNTHESIS 2 (PUR2)*	glycinamide ribonucleotide synthetase (GAR synthetase) that catalyzes the conversion of phosphoribosyl amine to phosphoribosyl glycineamide
AT1G09840	*SHAGGY-LIKE PROTEIN KINASE 41 (SK41)*	GSK3-like kinase, sub-group IV.
AT1G09850	*XYLEM BARK CYSTEINE PEPTIDASE 3 (XBCP3)*	Arabidopsis thaliana papain-like cysteine peptidase
AT1G09890		Rhamnogalacturonate lyase family protein
AT1G09930	*OLIGOPEPTIDE TRANSPORTER 2 (OPT2)*	oligopeptide transporter
AT1G09940	*(HEMA2)*	Encodes glutamyl-tRNA reductase. Involved in heme biosynthesis in non-photosynthetic tissues and induced by oxidative stress in photosynthetic tissues to supply heme for defensive hemoproteins
AT1G09950	*RESPONSE TO ABA AND SALT 1 (RAS1)*	RESPONSE TO ABA AND SALT 1
AT1G09960	*SUCROSE TRANSPORTER 4 (SUT4)*	low affinity (10mM) sucrose transporter in sieve elements (phloem)
AT1G09970	*(LRR XI-23)*	RLK7 belongs to a leucine-rich repeat class of receptor-likekinase (LRR-RLKs). It is involved in the control of germination speed and the tolerance to oxidant stress. The mRNA is cell-to-cell mobile.
AT1G09990		
AT1G10000		Ribonuclease H-like superfamily protein
AT1G10010	*AMINO ACID PERMEASE 8 (AAP8)*	Encodes a high affinity amino acid transporter that is probably responsible for import of organic nitrogen into developing seeds. One of eight gene family members that encode amino acid permeases. Most closely related to AAP1 (75%) identity.
AT1G10020		formin-like protein (DUF1005)
AT1G10050		Encodes a putative glycosyl hydrolase family 10 protein (xylanase).
AT1G10060	*BRANCHED-CHAIN AMINO ACID TRANSAMINASE 1 (BCAT-1)*	encodes a mitochondrial branched-chain amino acid aminotransferase. Complements the yeast leu/iso-leu/val auxotrophy mutant.
AT1G10070	*BRANCHED-CHAIN AMINO ACID TRANSAMINASE 2 (BCAT-2)*	Encodes a chloroplast branched-chain amino acid aminotransferase. Complements the yeast leu/iso-leu/val auxotrophy mutant. Involved in cell wall development.
AT1G10090		Early-responsive to dehydration stress protein (ERD4)
AT1G10110	*F-BOX/DUF295 BRASSICEAE-SPECIFIC 1 (ATFDB1)*	F-box family protein
AT1G10120	*CRY2-INTERACTING BHLH 4 (CIB4)*	basic helix-loop-helix (bHLH) DNA-binding superfamily protein
AT1G10140		Uncharacterized conserved protein UCP031279
AT1G10150		Carbohydrate-binding protein

AT1G10170	*NF-X-LIKE 1 (NFXL1)*	Encodes AtNFXL1, a homologue of the putative human transcription repressor NF-X1. Functions as a negative regulator of the trichothecene phytotoxin-induced defense response.
AT1G10200	*WLIM1 (WLIM1)*	Encodes a member of the Arabidopsis LIM proteins: a family of actin bundlers with distinct expression patterns. WLIM1, WLIM2a, and WLIM2b are widely expressed, whereas PLIM2a, PLIM2b, and PLIM2c are predominantly expressed in pollen. Regulates actin cytoskeleton organization.
AT1G10210	*MITOGEN-ACTIVATED PROTEIN KINASE 1 (MPK1)*	Encodes ATMPK1. Kinase is activated by wounding.
AT1G10230	*SKP1-LIKE 18 (SK18)*	Involved in protein degradation. One target is PHR1.
AT1G10250		paired amphipathic helix Sin3-like protein
AT1G10260		transposable_element_gene;copia-like retrotransposon family, has a 3.6e-19 P-value blast match to gb|AAG52949.1| gag/pol polyprotein (Endovir1-1) (Arabidopsis thaliana) (Ty1_Copia-family);(source:TAIR10)
AT1G10270	*GLUTAMINE-RICH PROTEIN 23 (GRP23)*	glutamine-rich protein 23
AT1G10300	*(NOG1-2)*	GTPase involved in HA - and ABA-mediated signaling pathways, particularly during defense respnses to pathogens. A truncated version of NOG1-2 has been detected in Col-0, Ler-0, Rsch-4 ecotypes. Functions similarly to the paralogous gene NOG1-1.
AT1G10320		Zinc finger C-x8-C-x5-C-x3-H type family protein
AT1G10330		Tetratricopeptide repeat (TPR)-like superfamily protein
AT1G10340		Ankyrin repeat family protein
AT1G10360	*GLUTATHIONE S-TRANSFERASE TAU 18 (GSTU18)*	Encodes glutathione transferase belonging to the tau class of GSTs. Naming convention according to Wagner et al. (2002).
AT1G10370	*EARLY-RESPONSIVE TO DEHYDRATION 9 (ERD9)*	Encodes GSTU17 (Glutathione S-Transferase U17). Functions as a negative component of stress-mediated signal transduction pathways in drought and salt stress responses.
AT1G10380		Putative membrane lipoprotein
AT1G10390	*DRACULA2 (DRA2)*	DRA2 is a homolog of mammalian nucleoporin 98 and a likely component of the nuclear pore complex in Arabidopsis. It positively participates in the control of the hypocotyl elongation response to plant proximity and control of shade induced gene expression. Nucleoportin which redundantly inhibits flowering together with Nup98b through multiple pathways including clock, photoperiod, and age pathways. Gates flowering in a CONSTANS (CO)-independent mode and bypasses the CO checkpoint in photoperiodic signaling and integrated signals from multiple pathways to directly target FLOWERING LOCUS T (FT) for flowering control.
AT1G10400		UDP-Glycosyltransferase superfamily protein
AT1G10420		
AT1G10460	*GERMIN-LIKE PROTEIN 7 (GLP7)*	germin-like protein (GLP7)
AT1G10470	*RESPONSE REGULATOR 4 (ARR4)*	Encodes a two-component response regulator. Acts redundantly with ARR3 in the control of circadian period in a cytokinin-independent manner.
AT1G10480	*ZINC FINGER PROTEIN 5 (ZFP5)*	Encodes a zinc finger protein containing only a single zinc finger that acts downstream of ZFP6 in regulating trichome development by integrating GA and cytokinin signaling.
AT1G10490		GNAT acetyltransferase (DUF699)
AT1G10522	*PLASTID REDOX INSENSITIVE 2 (PRIN2)*	Encodes PRIN2 (plastid redox insensitive 2). PRIN2 mutants are impaired in PEP (plastid-encoded RNA polymerase) activity and high light-dependent plastid redox signalling to the nucleus.
AT1G10530		PADRE protein
AT1G10540	*NUCLEOBASE-ASCORBATE TRANSPORTER 8 (NAT8)*	nucleobase-ascorbate transporter 8
AT1G10550	*XYLOGLUCAN:XYLOGLUCOSYL TRANSFERASE 33 (XTH33)*	Encodes a membrane-localized protein that is predicted to function during cell wall modification.Overexpression of XTH33 results in abnormal cell morphology. It's expression is under epigenetic control by ATX1.
AT1G10560	*PLANT U-BOX 18 (PUB18)*	Encodes a protein containing a UND, a U-box, and an ARM domain. This protein has E3 ubiquitin ligase activity based on in vitro assays.
AT1G10585		basic helix-loop-helix (bHLH) DNA-binding superfamily protein
AT1G10610		basic helix-loop-helix (bHLH) DNA-binding superfamily protein
AT1G10640		Pectin lyase-like superfamily protein
AT1G10650		SBP (S-ribonuclease binding protein) family protein
AT1G10660		transmembrane protein
AT1G10670	*ATP-CITRATE LYASE A-1 (ACLA-1)*	One of the three genes encoding subunit A of the trimeric protein ATP Citrate Lyase. Antisense ACLA-1 plants cause a reduction in cytosolic acetyl-CoA metabolism and have upregulation of stress-related genes and down-regulation of primary metabolism and growth genes, suggesting the mutation restricts normal growth and developmental processes and puts the plant into a state of stress.
AT1G10690	*(SMR8)*	cyclin-dependent kinase inhibitor
AT1G10710	*POOR HOMOLOGOUS SYNAPSIS 1 (PHS1)*	Computational predictions suggested the presence of a small cysteine-rich protein beginning in intron 9 (Silverstein 2007), but subsequent analysis revealed that this region contains a tenth exon for the At1g10710 gene. PHS1 regulates recombination and pairing of homologous chromosomes during meiotic prophase by controlling transport of RAD50 from cytoplasm to the nucleus.
AT1G10720		BSD domain-containing protein
AT1G10750		carboxyl-terminal peptidase, putative (DUF239)
AT1G10760	*STARCH EXCESS 1 (SEX1)*	Encodes an α-glucan, water dikinase required for starch degradation. Involved in cold-induced freezing tolerance. Mutations that eliminate the GWD protein or affect the dikinase domain of the enzyme dramatically reduce both the amount of phosphate in the amylopectin and the rate of starch degradation. Mature leaves of these mutants accumulate amounts of starch up to seven times greater than those in wild-type leaves. NMR analysis of the mutants, suggests that the gene is specifically involved in the phosphorylation of the glucosyl residues of starch at the C6 position.
AT1G10770		Encodes a putative pectin methylesterase/invertase inhibitor. Anti-sense reduction of this gene's transcript results in pollen tube growth retardation and then partial male sterility and reduced seed set.
AT1G10780		F-box/RNI-like superfamily protein
AT1G10800		voltage-gated hydrogen channel-like protein

AT1G10810		NAD(P)-linked oxidoreductase superfamily protein
AT1G10830	*15-CIS-ZETA-CAROTENE ISOMERASE (Z-ISO)*	Encodes a functional 15-cis-zeta-carotene isomerase (Z-ISO).
AT1G10850		Leucine-rich repeat protein kinase family protein
AT1G10880		Putative role in response to salt stress. Mutants grow larger than the wild type under salt stress condition (Ann Stapleton and Ashley Green, 2009, personal communication).
AT1G10890		arginine/glutamate-rich 1 protein
AT1G10900	*(PIP5K7)*	Phosphatidylinositol-4-phosphate 5-kinase family protein
AT1G10920	*LOCUS ORCHESTRATING VICTORIN EFFECTS1 (LOV1)*	Encodes LOV1, a disease susceptibility gene that, paradoxically, is a member of the NBS-LRR resistance gene family. Conditions susceptibility to the fungus Cochliobolus victoriae and victorin-dependent induction of defense-associated proteins. Saturation mutagenesis identified 59 lov mutations that all display reduced susceptibility to vitorin. Mutations in known defense response pathways do not prevent susceptibility to C. victoriae.
AT1G10940	*SNF1-RELATED PROTEIN KINASE 2.4 (SNRK2.4)*	Encodes a plant protein kinase similar to the calcium/calmodulin-dependent protein kinase subfamily and the SNF1 kinase subfamily (SnRK2) whose activity is activated by ionic (salt) and non-ionic (mannitol) osmotic stress. Kinase activity of its homolog in tobacco is induced by hyperosmotic condition within 1 minute. Phosphatidylinositol 4-phosphate 5-kinase (PIP5K) enzyme family member.
AT1G10960	*FERREDOXIN 1 (FD1)*	Phosphatidylinositol 4-phosphate 5-kinase (PIP5K) enzyme family member.
AT1G10970	*ZINC TRANSPORTER 4 PRECURSOR (ZIP4)*	A member of Zrt- and Irt-related protein (ZIP) family. transcript is induced in response to zinc deficiency in the root and shoot. Expression is regulated by copper, but response to copper deficiency is detected only after three weeks of deficiency. Phosphatidylinositol 4-phosphate 5-kinase (PIP5K) enzyme family member.
AT1G10980		Lung seven transmembrane receptor family protein
AT1G10990		transmembrane protein
AT1G11000	*MILDEW RESISTANCE LOCUS O 4 (MLO4)*	A member of a large family of seven-transmembrane domain proteins specific to plants, homologs of the barley mildew resistance locus o (MLO) protein. The Arabidopsis genome contains 15 genes encoding MLO proteins, with localization in plasma membrane. Phylogenetic analysis revealed four clades of closely-related AtMLO genes. ATMLO4 belongs to the clade I, with AtMLO11 and AtMLO14. The gene is expressed during early seedling growth, in roots and lateral root primordia, in flower and fruit abscission zone, in vascular system of root, cotyledons and young leaves, it was not expressed in mature rosette leaves, as shown by GUS activity patterns. The expression of several phylogenetically closely-related AtMLO genes showed similar or overlapping tissue specificity and analogous responsiveness to external stimuli, suggesting functional redundancy, co-function, or antagonistic function(s).
AT1G11040		HSP40/DnaJ peptide-binding protein
AT1G11070		hydroxyproline-rich glycoprotein family protein
AT1G11080	*SERINE CARBOXYPEPTIDASE-LIKE 31 (scpl31)*	serine carboxypeptidase-like 31
AT1G11120	*(DEG23)*	CTTNBP 2 amino-terminal-like protein
AT1G11130	*STRUBBELIG (SUB)*	Encodes an atypical receptor-like kinase protein with a predicted extracellular domain of six leucine-rich repeats and an intracellular serine-threonine kinase domain expressed throughout the developing root but whose kinase activity is not essential for its function in vivo. Regulates expression of GLABRA2, CAPRICE, WEREWOLF, and ENHANCER OF GLABRA3. Required for floral organ shape, the development of the outer integument of ovules, and stem development. Regulates cell shape and cell division planes in the L2 layer of floral meristems and the L1-derived outer integument of ovules. Controls specification of epidermal root hairs. Participates in the coordination of cell morphogenesis between cell layers during floral development.
AT1G11140		
AT1G11170		lysine ketoglutarate reductase trans-splicing-like protein (DUF707)
AT1G11190	*BIFUNCTIONAL NUCLEASE I (BFN1)*	Encodes a bifunctional nuclease that acts on both RNA and DNA involved in nucleic acid degradation to facilitate nucleotide and phosphate recovery during senescence. It has mismatch-specific endonuclease activity with wide recognition of single base mismatches as well as the ability to cleave indel types of mismatches (heteroduplexes with loops).
AT1G11210		cotton fiber protein, putative (DUF761)
AT1G11220		cotton fiber, putative (DUF761)
AT1G11250	*SYNTAXIN OF PLANTS 125 (SYP125)*	member of SYP12 Gene Family
AT1G11260	*SUGAR TRANSPORTER 1 (STP1)*	Encodes a H+/hexose cotransporter. The mRNA is cell-to-cell mobile.
AT1G11280		S-locus lectin protein kinase family protein
AT1G11290	*CHLORORESPIRATORY REDUCTION22 (CRR22)*	Pentatricopeptide Repeat Protein containing the DYW motif. Required for editing of multiple plastid transcripts. Endonuclease activity.
AT1G11300	*ENHANCED SHOOT GROWTH UNDER MANNITOL STRESS 1 (EGM1)*	The annotation for At1g11300 in TAIR10 is incorrect. This locus has been split into two At1g11300 (symbol: EGM1) and At1g11305 (symbol: EGM2) (Olivier Loudet, personal communication, 2013-04-03). See Comment field for revised annotation.
AT1G11330	*RESISTANT TO DFPM INHIBITION OF ABA SIGNALING 2 (RDA2)*	S-locus lectin protein kinase family protein
AT1G11340		G-type lectin S-receptor-like Serine/Threonine-kinase
AT1G11350	*S-DOMAIN-1 13 (SD1-13)*	S-domain-1 13
AT1G11360		Adenine nucleotide alpha hydrolases-like superfamily protein
AT1G11370		Pectin lyase-like superfamily protein
AT1G11380		PLAC8 family protein
AT1G11390	*ACTIVITY OF BC1 COMPLEX KINASE 10A (ABC1K10A)*	Atypical kinase which functions in plant salt stress tolerance by regulating reactive oxygen species (ROS).
AT1G11410		S-locus lectin protein kinase family protein
AT1G11420	*DOMAIN OF UNKNOWN FUNCTION 724 2 (DUF2)*	Member of the plant-specific DUF724 protein family. Arabidopsis has 10 DUF724 proteins.
AT1G11430	*MULTIPLE ORGANELLAR RNA EDITING FACTOR 9 (MORF9)*	Encodes a protein involved in RNA editing in chloroplasts. The mRNA is cell-to-cell mobile.
AT1G11440		hypothetical protein

AT1G11460	*USUALLY MULTIPLE ACIDS MOVE IN AND OUT TRANSPORTERS 26 (UMAMIT26)*	nodulin MtN21-like transporter family protein
AT1G11470		Pentatricopeptide repeat (PPR) superfamily protein
AT1G11480		eukaryotic translation initiation factor-like protein
AT1G11530	*C-TERMINAL CYSTEINE RESIDUE IS CHANGED TO A SERINE 1 (CXXS1)*	Encodes a monocysteinic thioredoxin, thioredoxin in which the second cysteine of the redox site is replaced by a serine, with low disulfide reductase but efficient disulfide isomerase activity. The mRNA is cell-to-cell mobile.
AT1G11540		Sulfite exporter TauE/SafE family protein
AT1G11545	*XYLOGLUCAN ENDOTRANSGLUCOSYLASE/HYDROLASE 8 (XTH8)*	xyloglucan endotransglucosylase/hydrolase 8
AT1G11560		Oligosaccharyltransferase complex/magnesium transporter family protein
AT1G11570	*NTF2-LIKE (NTL)*	NTF2-like protein
AT1G11580	*METHYLESTERASE PCR A (PMEPCRA)*	methylesterase PCR A
AT1G11590	*(PME19)*	Plant invertase/pectin methylesterase inhibitor superfamily
AT1G11600	*CYTOCHROME P450, FAMILY 77, SUBFAMILY B, POLYPEPTIDE 1 (CYP77B1)*	Plant specific fatty acid epoxygenase.
AT1G11610	*CYTOCHROME P450, FAMILY 71, SUBFAMILY A, POLYPEPTIDE 18 (CYP71A18)*	putative cytochrome P450
AT1G11630	*PENTATRICOPEPTIDE REPEAT 336 (PPR336L)*	Ribosomal pentatricopeptide repeat protein
AT1G11670		MATE efflux family protein
AT1G11680	*CYTOCHROME P450 51G1 (CYP51G1)*	putative obtusifoliol 14-alpha demethylase involved in sterol biosynthesis. The mRNA is cell-to-cell mobile.
AT1G11690		BRANCHLESS TRICHOME-like protein
AT1G11700		senescence regulator (Protein of unknown function, DUF584)
AT1G11710		Pentatricopeptide repeat (PPR) superfamily protein
AT1G11720	*STARCH SYNTHASE 3 (SS3)*	Encodes a starch synthase that in addition to its role in starch biosynthesis also has a negative regulatory function in the biosynthesis of transient starch. The protein apparently contains a starch-binding domain (SBD).
AT1G11730		Galactosyltransferase family protein
AT1G11740		ankyrin repeat family protein
AT1G11760	*MEDIATOR 32 (MED32)*	Required for expression of CBF-controlled cold-responsive genes. Required for recruitment of the Mediator complex and RNA polymerase II to CBF-controlled cold-responsive genes. MED32 has now been annotated as the MED2 subunit of the Mediator complex. Mediator tail subunit, involved in transcriptional regulation. Mediator Complex Subunit, interacts with MED23, MED5, MED16, and MED23 the Regulation of Phenylpropanoid Biosynthesis.
AT1G11770	*(ATBBE2)*	Encodes an oligogalacturonide oxidase that inactivates the elicitor-active oligogalacturonides (OGs).
AT1G11780		oxidoreductase, 2OG-Fe(II) oxygenase family protein
AT1G11790	*AROGENATE DEHYDRATASE 1 (ADT1)*	Encodes a plastid-localized arogenate dehydratase involved in phenylalanine biosynthesis. Not less than six genes encoding ADT were identiﬁed in the Arabidopsis genome: ADT1 [At1g11790]; ADT2 [At3g07630]; ADT3 [At2g27820]; ADT4 [At3g44720]; ADT5 [At5g22630]; and ADT6 [At1g08250].
AT1G11820		O-Glycosyl hydrolases family 17 protein
AT1G11850		transmembrane protein
AT1G11860	*(GLDT)*	T-protein is the aminomethyltransferase of the glycine cleavage multienzyme system GCS.
AT1G11915		wall-associated receptor kinase galacturonan-binding protein
AT1G11920		Pectin lyase-like superfamily protein
AT1G11950	*(JMJ26)*	Transcription factor jumonji (jmjC) domain-containing protein
AT1G11960	*(OSCA1.3)*	Calcium channel that is phosphorylated by BIK1 in the presence of PAMPS and required for stomatal immunity.
AT1G11970		Ubiquitin-like superfamily protein
AT1G11990		O-fucosyltransferase family protein
AT1G12000		Phosphofructokinase family protein
AT1G12010	*1-AMINO-CYCLOPROPANE-1-CARBOXYLIC ACID OXIDASE 3 (ACO3)*	Encodes a protein that appears to have 1-amino-cyclopropane-1-carboxylic acid oxidase activity based on mutant analyses. The mRNA is cell-to-cell mobile.
AT1G12020		hypothetical protein
AT1G12030		phosphoenolpyruvate carboxylase, putative (DUF506)
AT1G12040	*LEUCINE-RICH REPEAT/EXTENSIN 1 (LRX1)*	encodes a a chimeric leucine-rich repeat/extensin protein that regulates root hair morphogenesis and elongation. Null mutants develop root hairs that frequently abort, swell, or branch. Gene is expressed in root hair cells and protein is specifically localized in the wall of the hair proper. The mRNA is cell-to-cell mobile.
AT1G12070		Immunoglobulin E-set superfamily protein
AT1G12080		Vacuolar calcium-binding protein-like protein
AT1G12090	*EXTENSIN-LIKE PROTEIN (ELP)*	extensin-like protein (ELP)
AT1G12100		Bifunctional inhibitor/lipid-transfer protein/seed storage 2S albumin superfamily protein
AT1G12110	*NRT1/ PTR FAMILY 6.3 (NPF6.3)*	Encodes NRT1.1 (CHL1), a dual-affinity nitrate transporter. The protein is expressed in guard cells and function in stomatal opening. Mutants have less transpiration and are more tolerant to drought. Expressed in lateral roots. Involved in nitrate signaling which enables the plant root system to detect and exploit nitrate-rich soil patches. Comparing to the wild type, the mutant displays a strongly decreased lateral root proliferation phenotype in nitrate rich patches on growth medium. Affects flowering time via interaction with the FLC dependent flowering pathway to influence its target gene FT.
AT1G12130	*(FMOGS-OX6)*	Encodes a flavin-containing monooxygenases involved in biosynthesis of aliphatic glucosinolates.
AT1G12150		weak chloroplast movement under blue light protein (DUF827)

AT1G12160	*(FMOGS-OX7)*	Encodes a flavin-containing monooxygenases involved in biosynthesis of aliphatic glucosinolates.
AT1G12180		14.7 kDa heat shock-like protein
AT1G12190		F-box and associated interaction domains-containing protein
AT1G12200	*FLAVIN MONOOXYGENASE (FMO)*	Putative flavin monooxygenase.
AT1G12210	*RPS5-LIKE 1 (RFL1)*	RFL1 has high sequence similarity to the adjacent disease resistance (R) gene RPS5.
AT1G12220	*RESISTANT TO P. SYRINGAE 5 (RPS5)*	Resistance gene, mediates resistance against the bacterial pathogen Pseudomonas syringae. Contains a putative nucleotide binding site composed of kinase-1a (or P-loop), kinase-2a, and putative kinase-3a domains, 13 imperfect leucine-rich repeats, a potential leucine zipper, and two uncharacterized motifs that are well conserved in products of previously isolated R genes. Confers resistance to Pseudomonas syringae strains that express avrPphB.
AT1G12230	*(GSM2-LIKE)*	Aldolase superfamily protein
AT1G12240	*(ATBETAFRUCT4)*	Encodes a vacuolar invertase betaFruct4. betaFruct4 is transported from the endoplasmic reticulum through the intermediate compartments as a membrane protein. The N-terminal cytoplasmic domain contains multiple sequence motifs that are involved at various stages in the trafficking of betaFruct4 from the ER to the central vacuole. The mRNA is cell-to-cell mobile.
AT1G12250	*(TL20.3)*	Pentapeptide repeat-containing protein
AT1G12260	*NAC 007 (NAC007)*	Encodes a NAC-domain transcription factor that is expressed in developing xylem. Over expression of this protein causes ectopic secondary cell wall growth. Complements some of the cell wall defects seen in SND1/NST1 double mutants.
AT1G12270	*HOP1 (Hop1)*	Encodes one of the 36 carboxylate clamp (CC)-tetratricopeptide repeat (TPR) proteins (Prasad 2010, Pubmed ID: 20856808) with potential to interact with Hsp90/Hsp70 as co-chaperones.
AT1G12280	*SUPPRESSOR OF MKK1 MKK2 2 (SUMM2)*	Encodes a NB-LRR protein SUMM2 involved in defense response to bacterium.
AT1G12290	*(L5)*	NLR protein localized to plasma membrane. Overexpression triggers cell death. Has myristolation site at Gly2 which is required for membrane localization.
AT1G12320		ankyrin repeat/KH domain protein (DUF1442)
AT1G12330		cyclin-dependent kinase-like protein
AT1G12370	*PHOTOLYASE 1 (PHR1)*	encodes an amino acid sequence with significant homology to the recently characterized type II photolyases. The uvr2-1 mutant is unable to remove CPDs in vivo, and plant extracts lack detectable photolyase activity , is sensitive to UV-B and is an allele
AT1G12430	*ARMADILLO REPEAT KINESIN 3 (ARK3)*	Encodes the kinesin-like protein PAK has an Armadillo motif tail and is involved in guard cell development in Arabidopsis (from Genbank record AF159052).However, no defect in stomatal complexes has been observed in loss of function mutations. It accumulates at the preprophase band (PPB) in a cell-cycle and microtubule-dependent manner and is most highly expressed in cells where the placement of the division plane (early embryogenesis, stomatal lineages) is critical.
AT1G12450		SNARE associated Golgi protein family
AT1G12480	*OZONE-SENSITIVE 1 (OZS1)*	Encodes a membrane protein with 10 predicted transmembrane helices. SLAC1 is a multispanning membrane protein expressed predominantly in guard cells that plays a role in regulating cellular ion homeostasis and S-type anion currents. SLAC1 is important for normal stomatal closure in response to a variety of signals including elevated CO2, ozone, ABA, darkness, and humidity. SLAC1:GFP localizes to the plasma membrane.
AT1G12490	*ABA-RESPONSIVE FBA DOMAIN-CONTAINING PROTEIN 1 (AFBA1)*	F-box associated ubiquitination effector family protein. Expressed in guard cells and induced by ABA. Mutants are insensitive to ABA and osmotic stressors.
AT1G12500		Nucleotide-sugar transporter family protein
AT1G12520	*COPPER CHAPERONE FOR SOD1 (CCS)*	Copper-zinc superoxide dismutase copper chaperone (delivers copper to the Cu-Zn superoxide dismutase). Localized to the chloroplast. Expressed in roots and shoots. Up-regulated in response to copper and senescence. The AtACC activates all three CuZnSOD activities located in three different subcellular compartments. Contains three domains, central, ATX-1 like and C-terminal. ATX-1 like domain essential for the copper chaperone function of AtCCS in planta.
AT1G12530	*MODIFIER OF SNC1 9 (MOS9)*	Identified in a screen for suppressors of snc1. Regulator of SNC1 and RPP4 mediated immunity. Interacts with methyltransferase ATXR7.
AT1G12550	*HYDROXYPYRUVATE REDUCTASE 3 (HPR3)*	Encodes a hydroxypyruvate reductase that reduces HP to glycerate and shows even more activity with glyoxylate, a more upstream intermediate of the photorespiratory cycle. It is likely targeted to the chloroplast where it could provide a compensatory bypass for the reduction of HP and glyoxylate within this compartment. Together with HPPR2 and TAT1 involved in the biosynthesis of pHPL from tyrosine.
AT1G12560	*EXPANSIN A7 (EXPA7)*	Member of Alpha-Expansin Gene Family. Naming convention from the Expansin Working Group (Kende et al, 2004. Plant Mol Bio). Containing a conserved root hair-specific cis-element RHE. Expressed specifically in root hair cell and involved in root hair elongation.
AT1G12570		Ortholog of maize IPE1 gene which is involved in pollen exine development.
AT1G12600		UDP-N-acetylglucosamine (UAA) transporter family
AT1G12610	*DWARF AND DELAYED FLOWERING 1 (DDF1)*	Encodes a member of the DREB subfamily A-1 of ERF/AP2 transcription factor family (DDF1). The protein contains one AP2 domain. There are six members in this subfamily, including CBF1, CBF2, and CBF3. Overexpression of this gene results in delayed flowering and dwarfism, reduction of gibberellic acid biosynthesis, and increased tolerance to high levels of salt. This gene is expressed in all tissues examined, but most abundantly expressed in upper stems. Overexpression of this gene is also correlated with increased expression of GA biosynthetic genes and RD29A (a cold and drought responsive gene). Under salt stress it induces the expression of GAOX7, which encodes ad C20-GA inhibitor.
AT1G12630		encodes a member of the DREB subfamily A-4 of ERF/AP2 transcription factor family. The protein contains one AP2 domain. There are 17 members in this subfamily including TINY.
AT1G12650		rRNA biogenesis RRP36-like protein
AT1G12660		Predicted to encode a PR (pathogenesis-related) protein. Belongs to the plant thionin (PR-13) family with the following members: At1g66100, At5g36910, At1g72260, At2g15010, At1g12663, At1g12660.

AT1G12700	*RNA PROCESSING FACTOR 1 (RPF1)*	Encodes RNA PROCESSING FACTOR 1 (RPF1), a pentatricopeptide repeat (PPR) protein of the P-class containing canonical PPR-repeats. RPF1 is required for the 5?-end processing of the nad4 mRNA in mitochondria. Ler and other accessions impaired in processing of the nad4 mRNA 5′-end, contain a single nucleotide polymorphism (SNP) 807 nucleotides downstream of the predicted translation start codon (G807A). The resulting premature translation termination codon abolishes the function of the RPF1 gene in Ler. Required for the formation of nad4L-atp4 transcripts with -318 5′ termini.
AT1G12720		transposable_element_gene;Mutator-like transposase family, has a 1.2e-40 P-value blast match to GB:AAA21566 mudrA of transposon=MuDR (MuDr-element) (Zea mays);(source:TAIR10)
AT1G12740	*CYTOCHROME P450, FAMILY 87, SUBFAMILY A, POLYPEPTIDE 2 (CYP87A2)*	encodes a protein with cytochrome P450 domain
AT1G12770	*EMBRYO DEFECTIVE 1586 (EMB1586)*	Encodes a DEAD-box RNA helicase that localizes to mitochondria and is essential for regulating cell-to-cell transport via plasmodesmata. The mRNA is cell-to-cell mobile.
AT1G12790		DNA ligase-like protein
AT1G12800	*S1 DOMAIN-CONTAINING RBP (SDP)*	SDP is a chloroplast localized RNA binding protein that is required for plastid rRNA processing. Plants harboring a mutation in SDP have numerous defects including reduced chlorophyll content, poor growth, yellow leaves and abnormal chloroplasts.
AT1G12810		proline-rich family protein
AT1G12820	*AUXIN SIGNALING F-BOX 3 (AFB3)*	Auxin receptor involved in primary and lateral root growth inhibition in response to nitrate. Target of miR393. Induced by nitrate in primary roots.
AT1G12845		transmembrane protein
AT1G12860	*SCREAM 2 (SCRM2)*	Encodes ICE2 (Inducer of CBF Expression 2), a transcription factor of the bHLH family that participates in the response to deep freezing through the cold acclimation-dependent pathway. Overexpression of ICE2 results in increased tolerance to deep freezing stress after cold acclimation.
AT1G12880	*NUDIX HYDROLASE HOMOLOG 12 (NUDT12)*	nudix hydrolase homolog 12
AT1G12900	*GLYCERALDEHYDE 3-PHOSPHATE DEHYDROGENASE A SUBUNIT 2 (GAPA-2)*	glyceraldehyde 3-phosphate dehydrogenase A subunit 2
AT1G12940	*NITRATE TRANSPORTER2.5 (NRT2.5)*	member of High affinity nitrate transporter family
AT1G12950	*ROOT HAIR SPECIFIC 2 (RHS2)*	root hair specific 2
AT1G12960		Ribosomal protein L18e/L15 superfamily protein
AT1G12990		beta-1,4-N-acetylglucosaminyltransferase family protein
AT1G13000		transmembrane protein, putative (DUF707)
AT1G13030	*COILIN (COILIN)*	Encodes a plant coilin, a protein that in other organisms is a major structural scaffolding protein necessary for Cajal body formation, composition and activity. It has been shown to bind both U1 and U1 snRNAs in vitro.
AT1G13040		Pentatricopeptide repeat (PPR-like) superfamily protein
AT1G13050		proline-rich receptor-like kinase
AT1G13080	*CYTOCHROME P450, FAMILY 71, SUBFAMILY B, POLYPEPTIDE 2 (CYP71B2)*	cytochrome P450 monooxygenase
AT1G13090	*CYTOCHROME P450, FAMILY 71, SUBFAMILY B, POLYPEPTIDE 28 (CYP71B28)*	putative cytochrome P450
AT1G13100	*CYTOCHROME P450, FAMILY 71, SUBFAMILY B, POLYPEPTIDE 29 (CYP71B29)*	putative cytochrome P450
AT1G13110	*CYTOCHROME P450, FAMILY 71 SUBFAMILY B, POLYPEPTIDE 7 (CYP71B7)*	member of CYP71B The mRNA is cell-to-cell mobile.
AT1G13130		Cellulase (glycosyl hydrolase family 5) protein
AT1G13140	*CYTOCHROME P450, FAMILY 86, SUBFAMILY C, POLYPEPTIDE 3 (CYP86C3)*	member of CYP86C
AT1G13150	*CYTOCHROME P450, FAMILY 86, SUBFAMILY C, POLYPEPTIDE 4 (CYP86C4)*	member of CYP86C
AT1G13160		ARM repeat superfamily protein
AT1G13170	*OSBP(OXYSTEROL BINDING PROTEIN)-RELATED PROTEIN 1D (ORP1D)*	OSBP(oxysterol binding protein)-related protein 1D
AT1G13190		RNA-binding (RRM/RBD/RNP motifs) family protein
AT1G13200		F-box and associated interaction domains-containing protein
AT1G13210	*AUTOINHIBITED CA2+/ATPASE II (ACA.l)*	Autoinhibited Ca2+/ATPase II. ALA11 acts redundantly with ALA3, ALA4, ALA5, ALA9, ALA10 in root and shoot development as well as PIN trafficking and polarity .
AT1G13220	*LITTLE NUCLEI2 (LINC2)*	Encodes a nuclear coiled-coil protein related to the carrot peripheral nuclear protein NMCP1 that is involved in the determination of plant nuclear structure. Member of a small gene family in Arabidopsis containing 4 proteins (LNC1-4 or CRWN 1-4) with redundant functions in protection from oxidative damage, control of nuclear morphology and degradation of ABI5.
AT1G13230		Encodes a leucine-rich repeat protein pii-2. Located in the endoplasmic reticulum/plasma membrane continuum in Arabidopsis roots. Required for growth promotion and enhanced seed production mediated by the endophytic fungus Piriformospora indica in Arabidopsis.
AT1G13250	*GALACTURONOSYLTRANSFERASE-LIKE 3 (GATL3)*	Encodes a protein with putative galacturonosyltransferase activity.
AT1G13260	*RELATED TO ABI3/VP1 1 (RAV1)*	Encodes an AP2/B3 domain transcription factor which is upregulated in response to low temperature. It contains a B3 DNA binding domain. It has circadian regulation and may function as a negative growth regulator. The mRNA is cell-to-cell mobile.
AT1G13280	*ALLENE OXIDE CYCLASE 4 (AOC4)*	Encodes allene oxide cyclase. One of four genes in Arabidopsis that encode this enzyme, which catalyzes an essential step in jasmonic acid biosynthesis. Gene expression is reduced during senescence, a process that involves jasmonic acid signalling pathway.
AT1G13290	*DEFECTIVELY ORGANIZED TRIBUTARIES 5 (DOT5)*	Encodes a putative zinc finger protein (C2H2 family, type IIIA, subclass A1d) that has a WIP domain. Seedlings with mutations in DOT5 have a misaligned venation defect in their leaves and cotyledons. Additional developmental abnormalities, such as elongated petioles and aberrant phyllotaxy suggest that DOT5 is required for normal shoot and root development.
AT1G13300	*HYPERSENSITIVITY TO LOW PI-ELICITED PRIMARY ROOT SHORTENING 1 (HRS1)*	Encodes a nuclear localized member of the GARP family of transcription factors. Involved in nitrate/phosphate signaling in roots. It is transcriptionally regulated by nitrate and post transcriptionally by phosphate and functions to integrate these two nutrient signaling pathways in the root. HRS1 and HHO2 are involved in Ni cross regulation of Pi signaling. They function as transcriptional repressors of SPX1, SPX2, and SPX4 as part of a cascade to regulate nitrogen and phosphorus balance.
AT1G13310		Endosomal targeting BRO1-like domain-containing protein

AT1G13330	*ARABIDOPSIS HOP2 HOMOLOG (AHP2)*	Encodes the Arabidopsis Hop2 homologue. In other species, Hop2 is proposed to be involved in inter-homolog bias in double strand break repair.
AT1G13340	*IST1-LIKE 6 (ISTL6)*	Regulator of Vps4 activity in the MVB pathway protein
AT1G13350	*PRP4 KINASE B (PRP4KB)*	Paralog of PRP4KA.
AT1G13370	*(HTR6)*	Histone superfamily protein
AT1G13400	*NUBBIN (NUB)*	Along with JAG, it is involved in stamen and carpel development. Expression is limited to the adaxial side of lateral organs. Activated by AGAMOUS in a cal-1, ap1-1 background.
AT1G13410		Tetratricopeptide repeat (TPR)-like superfamily protein
AT1G13420	*SULFOTRANSFERASE 4B (ST4B)*	Encodes a sulfotransferase. Unlike the related ST4A protein (At2g14920), in vitro experiments show that this enzyme does not act brassinosteroids. ST4B is expressed in the roots and transcript levels rise in response to cytokinin treatment.
AT1G13470		hypothetical protein (DUF1262)
AT1G13540		hypothetical protein (DUF1262)
AT1G13570		F-box/RNI-like superfamily protein
AT1G13580	*LAG1 LONGEVITY ASSURANCE HOMOLOG 3 (LAG13)*	Encodes a ceramide synthase that together with LOH1 is essential for production of ceramides containing Very Long Chain Fatty acid VLCFA-Ceramides(mainly C 22 to 26).
AT1G13590	*PHYTOSULFOKINE 1 PRECURSOR (PSK1)*	Encodes a phytosulfokine-alpha (PSK) precursor, a unique plant peptide growth factor first described in Asparagus.
AT1G13600	*BASIC LEUCINE-ZIPPER 58 (bZIP58)*	basic leucine-zipper 58
AT1G13610		alpha/beta-Hydrolases superfamily protein
AT1G13630		Tetratricopeptide repeat (TPR)-like superfamily protein
AT1G13640		Phosphatidylinositol 3- and 4-kinase family protein
AT1G13650		hypothetical protein
AT1G13670	*BIG GRAIN LIKE 2 (BGL2)*	hypothetical protein
AT1G13680	*PHOSPHOLIPASE C-LIKE 1 (PLCL1)*	Encodes a phospholipase C-like protein that serves as a convergence point for fumonisin B1 and extracellular ATP signalling, and functions in Arabidopsis stress response to fumonisin B1.
AT1G13700	*6-PHOSPHOGLUCONOLACTONASE 1 (PGL1)*	Encodes a cytosolic 6-phosphogluconolactonase (PGL) thought to be involved in the oxidative pentose-phosphate pathway (OPPP).
AT1G13710	*CYTOCHROME P450, FAMILY 78, SUBFAMILY A, POLYPEPTIDE 5 (CYP78A5)*	Encodes the cytochrome P450 CYP78A5 monooxygenase. Contributes to the generation of a growth-stimulating signal distinct from the classical phytohormones that prevents proliferation arrest, promoting organ growth. In ovules it is required for megagametogenesis, maternal control of seed size and restricting megaspore mother cell fate to a single cell.
AT1G13740	*ABI FIVE BINDING PROTEIN 2 (AFP2)*	Encodes a member of a small plant-specific gene family whose members interact with ABI5 and appear to be involved in mediating stress responses. AFP2 mutants affect a number of ABA mediated processes such as germination and response to osmotic and sugar stress. AFP2 nuclear localization is stress dependent.
AT1G13750	*PURPLE ACID PHOSPHATASE 1 (PAP1)*	Encodes a purple acid phosphatase whose expression is responsive to both phosphate (Pi) and phosphite (Phi) in roots.
AT1G13760		hypothetical protein
AT1G13790	*FACTOR OF DNA METHYLATION 4 (FDM4)*	Belongs to a subgroup of SGS3-like proteins that act redundantly in RNA-directed DNA methylation: AT1G15910 (FDM1), AT4G00380 (FDM2), AT3G12550 (FDM3), AT1G13790 (FDM4), AT1G80790 (FDM5).
AT1G13800	*EMBRYONIC FACTOR 19 (FAC19)*	Encodes a PPR (pentatricopeptide repeat motif) protein that is essential for the initiation of zygotic embryogenesis.
AT1G13810		Restriction endonuclease, type II-like superfamily protein
AT1G13830		Carbohydrate-binding X8 domain superfamily protein
AT1G13870	*DEFORMED ROOTS AND LEAVES 1 (DRL1)*	Encodes a homolog of the yeast TOT4/KTI12 protein. Yeast TOT4/KTI12 associates with Elongator, a multisubunit complex that binds the RNA polymerase II transcription elongation complex. Ds insertion mutant has enlarged shoot apical region, 4 to 6 long slender leaves followed by spike-like structures, short roots. Mutants also have no ncm5U (5-carbamoylmethyluridine).
AT1G13890	*SOLUBLE N-ETHYLMALEIMIDE-SENSITIVE FACTOR ADAPTOR PROTEIN 30 (SNAP30)*	Encodes a member of a gene family homologous to mammalian SNAP25, a type of SNARE proteins with two chains. There are three members in Arabidopsis: SNAP30, SNAP29, and SNAP33.
AT1G13910		Leucine-rich repeat (LRR) family protein
AT1G13920		Remorin family protein
AT1G13930		Involved in response to salt stress. Knockout mutants are hypersensitive to salt stress. The mRNA is cell-to-cell mobile.
AT1G13950	*EUKARYOTIC ELONGATION FACTOR 5A-1 (ELF5A-1)*	Encodes eukaryotic translation initiation factor 5A (EIF-5A).In mammalian cells it functions as a shuttle protein that translocates mRNA from the nucleus to cytoplasmic ribosomes. Overexpression results in an increase in both primary and secondary xylem formation. In RNAi suppressed lines, xylem formation is reduced.
AT1G13960	*WRKY DNA-BINDING PROTEIN 4 (WRKY4)*	Encodes WRKY DNA-binding protein 4 (WRKY4).
AT1G13980	*GNOM (GN)*	Encodes a GDP/GTP exchange factor for small G-proteins of the ADP ribosylation factor (RAF) class, and as regulator of intracellular trafficking. Homologous to Sec7p and YEC2 from yeast. Involved in the specification of apical-basal pattern formation. Essential for cell division, expansion and adhesion. It appears that heteotypic binding between the DCB and C-terminal domains of two GNOM proteins is required for membrane association, however, GNOM appears to exist predominantly as a heterodimer formed through DCB-DCB interactions. BFA inhibits GNOM trafficking and BFA resistant lines are more resistant to cold stress.
AT1G13990		plant/protein
AT1G14000	*VH1-INTERACTING KINASE (VIK)*	Encodes a protein with similarity to members of the C1 subgroup of MAP kinase kinase kinases. Interacts physically with the receptor kinase BRL2/VH1 and appears to be involved in auxin and brassinosteriod signaling. The mRNA is cell-to-cell mobile.
AT1G14030	*LYSINE METHYLTRANSFERASE (LSMT)-LIKE (LSMT-L)*	Encodes a lysine methyltransferase whose main soluble physiological substrates are chloroplastic fructose 1,6-bisphosphate aldolases, FBA1, FBA2, and FBA3. Lysines near the C-terminal end of the target proteins are trimethylated.

AT1G14040	*PHO1 HOMOLOG 3 (PHO1;H3)*	Encodes a PHO1 homologue that is upregulated in response to Zn deficiency and is involved in Pi homeostasis in response to Zn deficiency. The mRNA is cell-to-cell mobile.
AT1G14050		
AT1G14070	*FUCOSYLTRANSFERASE 7 (FUT7)*	member of Xyloglucan fucosyltransferase family
AT1G14080	*FUCOSYLTRANSFERASE 6 (FUT6)*	Encodes an alpha-(1,2)-fucosyltransferase.
AT1G14090		pseudogene of Pentatricopeptide repeat (PPR) superfamily protein
AT1G14100	*FUCOSYLTRANSFERASE 8 (FUT8)*	member of Glycosyltransferase Family- 37. FUT8 was previously associated to AT1G14110
AT1G14110	*FUCOSYLTRANSFERASE 9 (FUT9)*	member of xyloglucan fucosyltransferase family
AT1G14120	*AUXIN OXIDASE (ATDAO2)*	DAO2 is an IAA oxidase expressed in root caps. it is a member of a family of dioxygenase and 2OG Fe(II) oxygenase domain and DAO domain containing proteins. It is expressed specifically in root cap cells and does not appear to be the major IAA oxidase in planta.
AT1G14150	*PHOTOSYNTHETIC NDH SUBCOMPLEX L 2 (PnsL2)*	Encodes a subunit of the NAD(P)H dehydrogenase complex located in the chloroplast thylakoid lumen.
AT1G14190		Glucose-methanol-choline (GMC) oxidoreductase family protein
AT1G14200	*(SNIPER1)*	E3 ligase involved in the regulation of the homeostasis of sensor NLR immune receptors.
AT1G14210		Ribonuclease T2 family protein
AT1G14220		Ribonuclease T2 family protein
AT1G14240	*APYRASE3 (APY3)*	GDA1/CD39 nucleoside phosphatase family protein
AT1G14250		GDA1/CD39 nucleoside phosphatase family protein
AT1G14260		RING/FYVE/PHD zinc finger superfamily protein
AT1G14270		CAAX amino terminal protease family protein
AT1G14280	*PHYTOCHROME KINASE SUBSTRATE 2 (PKS2)*	Encodes phytochrome kinase substrate 2. PKS proteins are critical for hypocotyl phototropism. Forms a complex with Phot1, Phot2 and NPH3.
AT1G14290	*SPHINGOID BASE HYDROXYLASE 2 (SBH2)*	Encodes one of the two redundant sphingoid base hydroxylases (SBH). Involved in sphingolipid trihydroxy long-chain base (4-hydroxysphinganine) biosynthesis. Double mutants of SBHs were dwarfed and not able to progress from vegetative to reproductive growth.
AT1G14300		ARM repeat superfamily protein
AT1G14330		Galactose oxidase/kelch repeat superfamily protein
AT1G14340	*BPA1-LIKE 3 (BPL3)*	ACD11 binding partner, negatively regulates ROS-mediated defense response.
AT1G14345		NAD(P)-linked oxidoreductase superfamily protein
AT1G14350	*FOUR LIPS (FLP)*	Encodes a putative MYB transcription factor involved in stomata development, loss of FLP activity results in a failure of guard mother cells (GMCs) to adopt the guard cell fate, thus they continue to divide resulting in abnormal stomata consisting of clusters of numerous guard cell-like cells. This phenotype is enhanced in double mutants with MYB88. Its transcript levels change after inducing MUTE expression in a mute background. Also regulates female reproductive development.
AT1G14370	*PROTEIN KINASE 2A (APK2A)*	Encodes protein kinase APK2a. Protein is N-myristoylated.
AT1G14390		Leucine-rich repeat protein kinase family protein
AT1G14410	*WHIRLY 1 (WHY1)*	Encodes a homolog of the potato p24 protein. Binds single strand telomeric repeats. Negatively regulates telomerase activity and telomere length.
AT1G14420	*(AT59)*	Pectate lyase family protein
AT1G14430	*GALACTOSE OXIDASE-LIKE 5 (GOXL5)*	Galactose oxydase; may function in tissues that require mechanical reinforcements in the absence of lignification.
AT1G14440	*HOMEOBOX PROTEIN 31 (HB31)*	homeobox protein 31
AT1G14450		NADH dehydrogenase (ubiquinone)s
AT1G14460		AAA-type ATPase family protein
AT1G14470		Pentatricopeptide repeat (PPR) superfamily protein
AT1G14480		Ankyrin repeat family protein
AT1G14490	*AT-HOOK MOTIF NUCLEAR LOCALIZED PROTEIN 28 (AHL28)*	Putative AT-hook DNA-binding family protein
AT1G14520	*MYO-INOSITOL OXYGENASE 1 (MIOX1)*	Encodes MIOX1. Belongs to myo-inositol oxygenase gene family.
AT1G14530	*TOM THREE HOMOLOG 1 (THH1)*	tobamovirus multiplication-like protein (DUF1084)
AT1G14540	*PEROXIDASE 4 (PER4)*	Class III peroxidase cell wall-targeted protein localized to the micropylar endosperm facing the radicle. Involved in seed germination.
AT1G14550		Peroxidase superfamily protein
AT1G14580	*BLUEJAY (BLJ)*	C2H2-like zinc finger protein
AT1G14620	*DECOY (DECOY)*	decoy
AT1G14660	*NA+/H+ EXCHANGER 8 (NHX8)*	member of putative Na+/H+ antiporter (AtNHX) family. Functions as a plasma membrane Li+/H+ antiporter. Involved in Li+ efflux and detoxification.
AT1G14670		Endomembrane protein 70 protein family
AT1G14680		early endosome antigen
AT1G14685	*BASIC PENTACYSTEINE 2 (BPC2)*	Encodes a member of the BASIC PENTACYSTEINE (BPC) proteins. BPC proteins are plant-specific transcription factors present throughout land plants. BPC transcription factor family is integral for a wide range of processes that support normal growth and development.Along with BPC1, BPC2 binds to the promoter of and represses GALS1 thereby reducing beta 1,4- galactan accumulation.
AT1G14687	*HOMEOBOX PROTEIN 32 (HB32)*	homeobox protein 32
AT1G14690	*MICROTUBULE-ASSOCIATED PROTEIN 65-7 (MAP65-7)*	microtubule-associated protein 65-7
AT1G14700	*PURPLE ACID PHOSPHATASE 3 (PAP3)*	purple acid phosphatase 3
AT1G14710		hydroxyproline-rich glycoprotein family protein
AT1G14720	*XYLOGLUCAN ENDOTRANSGLUCOSYLASE/HYDROLASE 28 (XTH28)*	member of Glycoside Hydrolase Family 16
AT1G14730		Cytochrome b561/ferric reductase transmembrane protein family

AT1G14740	*TITANIA 1 (TTA1)*	Encodes a PHD-finger protein that, with TTA1, is redundantly required for MP-dependent embryonic root meristem initiation.
AT1G14760	*KNOX ARABIDOPSIS THALIANA MEINOX (KNATM)*	Encodes a novel Arabidopsis KNOX gene that encodes a MEINOX domain but lacks the homeodomain and interacts with TALE-class homeodomain proteins to modulate their activities
AT1G14770		RING/FYVE/PHD zinc finger superfamily protein
AT1G14780		MAC/Perforin domain-containing protein
AT1G14790	*RNA-DEPENDENT RNA POLYMERASE 1 (RDR1)*	Encodes RNA-dependent RNA polymerase. While not required for virus-induced post-transcriptional gene silencing (PTGS), it can promote turnover of viral RNAs in infected plants. Nomenclature according to Xie, et al. (2004). Involved in the production of Cucumber Mosaic Virus siRNAs.
AT1G14800		Nucleic acid-binding, OB-fold-like protein
AT1G14810		encodes an aspartate semialdehyde dehydrogenase, which produces the branch point intermediate for lysine and threonine/methionine biosynthesis
AT1G14820		Sec14p-like phosphatidylinositol transfer family protein
AT1G14830	*DYNAMIN-LIKE 1C (DL1C)*	Encodes a dynamin-like protein that is involved in mitochondrial morphogenesis and pollen development. Protein is localized as speckles in the cytoplasm, partially co-localizes with mitochondrial markers, cell plate of dividing cells, and the tip of root hairs, root cap cells, and expanding part of trichoblasts.
AT1G14840	*MICROTUBULE-ASSOCIATED PROTEINS 70-4 (MAP70-4)*	Encodes a microtubule associated protein (MAP70-4). Expressed in all tissues.
AT1G14850	*NUCLEOPORIN 155 (NUP155)*	Encodes a protein similar to nucleoporin, a a major component of the nuclear pore complex (NPC) involved in cellular nucleo-cytoplasmic transport
AT1G14870	*PLANT CADMIUM RESISTANCE 2 (PCR2)*	PCR2 encodes a membrane protein involved in zinc transport and detoxification.
AT1G14890		Plant invertase/pectin methylesterase inhibitor superfamily protein
AT1G14910	*(PICALM1B)*	ANTH domain-containing protein which functions as adaptor protein for clathrin-mediated endocytosis (CME) of the secretory vesicle-associated longintype R-SNARE VAMP72 group. Interacts with the SNARE domain of VAMP72 and clathrin at the plasma membrane. Required for recycling of R-SNARE proteins.
AT1G14930		Polyketide cyclase/dehydrase and lipid transport superfamily protein
AT1G14940		Polyketide cyclase/dehydrase and lipid transport superfamily protein
AT1G14950		Polyketide cyclase/dehydrase and lipid transport superfamily protein
AT1G14960		Polyketide cyclase/dehydrase and lipid transport superfamily protein
AT1G14980	*CHAPERONIN 10 (CPN10)*	Encodes mitochondrial-localized chaperonin 10 that complements the E.coli groES mutant. Its mRNA is upregulated in response to heat shock treatment and is expressed uniformly in various organs.
AT1G14990		transmembrane protein
AT1G15000	*SERINE CARBOXYPEPTIDASE-LIKE 50 (scpl50)*	serine carboxypeptidase-like 50
AT1G15010		mediator of RNA polymerase II transcription subunit
AT1G15020	*QUIESCIN-SULFHYDRYL OXIDASE 1 (QSOX1)*	Encodes a protein disulfide isomerase-like (PDIL) protein, a member of a multigene family within the thioredoxin (TRX) superfamily. This protein also belongs to the quiescin-sulfhydryl oxidase (QSOX) family, which possess an Erv1-like domain at the COOH terminus in addition to a TRX domain.
AT1G15040	*GLUTAMINE AMIDOTRANSFERASE 1_2.1 (GAT1_2.1)*	Encodes a nitrogen regulated putative glutamine amidotransferase that represses shoot branching.
AT1G15045		
AT1G15050	*INDOLE-3-ACETIC ACID INDUCIBLE 34 (IAA34)*	Belongs to auxin inducible gene family.
AT1G15080	*LIPID PHOSPHATE PHOSPHATASE 2 (LPP2)*	Encodes phosphatidic acid phosphatase. Involved in ABA signaling. Functions as a negative regulator upstream of ABI4. Expressed during germination and seed development. Expressed overall in young seedlings, in roots, hypocotyls, and vascular cells of cotyledons and leaves of 10 day-old seedlings, in flower filaments and stem elongation zones. Not expressed in anthers, pollen nor petals.
AT1G15100	*RING-H2 FINGER A2A (RHA2A)*	Encodes a putative RING-H2 finger protein RHA2a.
AT1G15140	*FERREDOXIN-NADP(+) OXIDOREDUCTASE -LIKE (FNRL)*	FAD/NAD(P)-binding oxidoreductase
AT1G15150		MATE efflux family protein
AT1G15170		MATE efflux family protein
AT1G15180		MATE efflux family protein
AT1G15190	*FASCICLIN-LIKE ARABINOGALACTAN PROTEIN 19 (FLA19)*	Fasciclin-like arabinogalactan protein. Possibly involved in embryogenesis and seed development.
AT1G15210	*ATP-BINDING CASSETTE G35 (ABCG35)*	pleiotropic drug resistance 7
AT1G15220	*(CCMH)*	Encodes a protein with oxidoreductase activity present in the inner membrane of mitochondria. CCMH is postulated to play a central role in mitochondrial cytochrome c maturation, probably as part of a heme lyase complex that also holds activity of reducing apocytochrome c. CCMH interacts with apocytochrome AtCYTc-a and is shown to be present in a 500 kDa-complex along with CcmFN2.
AT1G15230		hypothetical protein
AT1G15250	*(RPL37A)*	cytosolic ribosomal protein gene, part of eL20 family
AT1G15260		LOW protein: ATP-dependent RNA helicase-like protein
AT1G15290	*REDUCED CHLOROPLAST COVERAGE 3 (REC3)*	Encodes REDUCED CHLOROPLAST COVERAGE 3 (REC3). Contributes to establishing the size of the chloroplast compartment.
AT1G15300	*CYTOSLEEPER (CYTOSLEEPER)*	transposable_element_gene;hAT-like transposase family (hobo/Ac/Tam3), has a 5.1e-59 P-value blast match to GB:CAA29005 ORFa of Maize Ac (hAT-element) (Zea mays);(source:TAIR10)
AT1G15310	*SIGNAL RECOGNITION PARTICLE 54 KDA SUBUNIT (ATHSRP54A)*	54 kDa protein subunit of SRP that interacts with the signal peptide of secreted proteins
AT1G15320	*(INP2)*	seed dormancy control protein
AT1G15330		Cystathionine beta-synthase (CBS) protein
AT1G15340	*METHYL-CPG-BINDING DOMAIN 10 (MBD10)*	Protein containing methyl-CpG-binding domain.Has sequence similarity to human MBD proteins.
AT1G15350		DUF4050 family protein

AT1G15360	*SHINE 1 (SHN1)*	Encodes a member of the ERF (ethylene response factor) subfamily B-6 of ERF/AP2 transcription factor family. The protein contains one AP2 domain. There are 12 members in this subfamily including RAP2.11. This gene is involved in wax biosynthesis. Over-expression of the gene results in glossy leaf phenotype and increased drought tolerance. Two closely related genes, AT5G25390 and AT5G11190 have similar phenotypes when over-expressed. Strong expression levels in flowers. Binds to the promoter of LACS2.
AT1G15380	*GLYOXYLASE I 4 (GLYI4)*	Glyoxalase which affects ABA?JA crosstalk. Member of the GLYI family. glyI4 mutant plants show a general stress phenotype characterized by compromised MG scavenging, accumulation of reactive oxygen species (ROS), stomatal closure, andreduced fitness was observed.
AT1G15390	*PEPTIDE DEFORMYLASE 1A (PDF1A)*	encodes a peptide deformylase-like protein. Removes N-formyl groups, a prerequisite for the action of methionine aminopeptidase during protein synthesis. Targeted to mitochondria. Requires Zn for catalysis.
AT1G15400	*MAPK SUBSTRATES IN THE STOMATAL LINEAGE 2 (MASS2)*	Tightly connected with MAPK signaling to fine-tune stomatal production and patterning.
AT1G15410		aspartate-glutamate racemase family
AT1G15425		
AT1G15430		hypothetical protein (DUF1644)
AT1G15440	*PERIODIC TRYPTOPHAN PROTEIN 2 (PWP2)*	Encodes a nucleolar protein that is a ribosome biogenesis co-factor. Mutants display aberrant RNA processing and female gametophyte development.
AT1G15460	*REQUIRES HIGH BORON 4 (BOR4)*	Encodes a efflux-type boron transporter. Over-expression improved plant growth under B toxic conditions.
AT1G15480	*PRECOCIOUS1 (POCO1)*	PPR motif containing protein. Found in mitochondria. Mutants flower early and have reduced levels of the ABI5, a regulator of FLC expression.
AT1G15510	*EARLY CHLOROPLAST BIOGENESIS2 (ECB2)*	Encodes a pentatricopeptide repeat protein required for chloroplast transcript accD RNA editing and early chloroplast biogenesis.
AT1G15520	*ATP-BINDING CASSETTE G40 (ABCG40)*	ABC transporter family involved in ABA transport and resistance to lead. Localizes to plasma membrane. Upregulated by lead. Expressed in leaves, flowers, stomata and roots.
AT1G15530	*L-TYPE LECTIN RECEPTOR KINASE S.1 (LECRK-S.1)*	Concanavalin A-like lectin protein kinase family protein
AT1G15550	*GIBBERELLIN 3-OXIDASE 1 (GA3OX1)*	Involved in later steps of the gibberellic acid biosynthetic pathway. Activated by AGAMOUS in a cal-1, ap1-1 background. Deletion of 208 bp from -1016 to -809 (Δ-808) resulted in loss of GA-negative feedback (this sequence, which contains a 43-bp sequence GNFEI, was shown to be sufficient for GA-negative feedback).
AT1G15580	*INDOLE-3-ACETIC ACID INDUCIBLE 5 (IAA5)*	auxin induced protein
AT1G15590		E3 ubiquitin-protein ligase
AT1G15600		transmembrane protein
AT1G15640		transmembrane protein
AT1G15670	*KISS ME DEADLY 2 (KMD2)*	Encodes a member of a family of F-box proteins, called the KISS ME DEADLY (KMD) family, that targets type-B ARR proteins for degradation and is involved in the negative regulation of the cytokinin response. Also named as KFB1, a member of a group of Kelch repeat F-box proteins that negatively regulate phenylpropanoid biosynthesis by targeting the phenypropanoid biosynthesis enzyme phenylalanine ammonia-lyase.
AT1G15700	*(ATPC2)*	One of two genes that encode the gamma subunit of Arabidopsis chloroplast ATP synthase. It is thought to be involved in the regulation of the ATP synthase activity.
AT1G15710	*TYRA AROGENATE DEHYDROGENASE 2 (TYRA2)*	prephenate dehydrogenase family protein
AT1G15730		Cobalamin biosynthesis CobW-like protein
AT1G15780	*NON-RECOGNITION-OF-BTH 4 (NRB4)*	mediator of RNA polymerase II transcription subunit 15a-like protein
AT1G15790		mediator of RNA polymerase II transcription subunit 15a-like protein
AT1G15800		hypothetical protein
AT1G15810		S15/NS1, RNA-binding protein
AT1G15820	*LIGHT HARVESTING COMPLEX PHOTOSYSTEM II SUBUNIT 6 (LHCB6)*	Lhcb6 protein (Lhcb6), light harvesting complex of photosystem II.
AT1G15830		hypothetical protein
AT1G15840		hypothetical protein
AT1G15850		Transducin/WD40 repeat-like superfamily protein
AT1G15870		Mitochondrial glycoprotein family protein
AT1G15880	*GOLGI SNARE 11 (GOS11)*	Golgi SNARE 11 protein (GOS11)
AT1G15890		Disease resistance protein (CC-NBS-LRR class) family
AT1G15900		transmembrane protein
AT1G15910	*FACTOR OF DNA METHYLATION 1 (FDM1)*	Belongs to a subgroup of SGS3-like proteins that act redundantly in RNA-directed DNA methylation: AT1G15910 (FDM1), AT4G00380 (FDM2), AT3G12550 (FDM3), AT1G13790 (FDM4), AT1G80790 (FDM5).
AT1G15940	*(PDS5E)*	One of 5 PO76/PDS5 cohesion cofactor orthologs of Arabidopsis.
AT1G15960	*NRAMP METAL ION TRANSPORTER 6 (NRAMP6)*	member of Nramp2 family
AT1G15980	*PHOTOSYNTHETIC NDH SUBCOMPLEX B 1 (PnsB1)*	encodes a novel subunit of the chloroplast NAD(P)H dehydrogenase complex, involved in cyclic electron flow around photosystem I to produce ATP.
AT1G15990	*CYCLIC NUCLEOTIDE GATED CHANNEL 7 (CNGC7)*	Encodes a plasma membrane localized member of the cyclic nucleotide gated channel (CNGC) family that is essential for male reproductive fertility.
AT1G16000	*OUTER ENVELOPE PROTEIN 9.1 (OEP9.1)*	Member of the Arabidopsis 7-kDa OEP family. Tail-anchored (TA) membrane protein which possesses a single C-terminal transmembrane domain targeting post-translationally to plastids.
AT1G16030	*HEAT SHOCK PROTEIN 70B (Hsp70b)*	heat shock protein 70B
AT1G16040		phosphatidylinositol-glycan biosynthesis class F-like protein
AT1G16060	*ARIA-INTERACTING DOUBLE AP2 DOMAIN PROTEIN (ADAP)*	Encodes ADAP, an AP2-domain protein that interacts with ARIA. ADAP is a positive regulator of the ABA response and is also involved in regulating seedling growth.
AT1G16130	*WALL ASSOCIATED KINASE-LIKE 2 (WAKL2)*	Encodes a WAK-like receptor-like kinase with a cytoplasmic Ser/Thr protein kinase domain and an extracellular domain with EGF-like repeats.
AT1G16150	*WALL ASSOCIATED KINASE-LIKE 4 (WAKL4)*	Encodes a WAK-like receptor-like kinase with a cytoplasmic Ser/Thr protein kinase domain and an extracellular domain with EGF-like repeats. Likely involved in Arabidopsis root mineral responses to Zn2+, Cu2+, K+, Na+ and Ni+. The mRNA is cell-to-cell mobile.

AT1G16160	*WALL ASSOCIATED KINASE-LIKE 5 (WAKL5)*	WAK-like kinase The mRNA is cell-to-cell mobile.
AT1G16190	*RADIATION SENSITIVE23A (RAD23A)*	Encodes a member of the RADIATION SENSITIVE23 (RAD23) family: AT1G16190(RAD23A), AT1G79650(RAD23B), AT3G02540(RAD23C), AT5G38470(RAD23D). RAD23 proteins play an essential role in the cell cycle, morphology, and fertility of plants through their delivery of UPS (ubiquitin/26S proteasome system) substrates to the 26S proteasome.
AT1G16260	*WALL ASSOCIATED KINASE LIKE 8 (WAKL8)*	Wall-associated kinase family protein
AT1G16310	*METAL-TOLERANCE PROTEIN 10 (MTP10)*	Cation efflux family protein which affects ABA-JA crosstalk and susceptibility to Mamestra brassicae herbivory.
AT1G16330	*CYCLIN B3;1 (CYCB3;1)*	core cell cycle genes
AT1G16350		Aldolase-type TIM barrel family protein
AT1G16360		LEM3 (ligand-effect modulator 3) family protein / CDC50 family protein
AT1G16370	*ORGANIC CATION/CARNITINE TRANSPORTER 6 (OCT6)*	organic cation/carnitine transporter 6
AT1G16380	*(ATCHX1)*	member of Putative Na+/H+ antiporter family
AT1G16390	*ORGANIC CATION/CARNITINE TRANSPORTER 3 (OCT3)*	organic cation/carnitine transporter 3
AT1G16410	*CYTOCHROME P450 79F1 (CYP79F1)*	member of CYP79F The mRNA is cell-to-cell mobile.
AT1G16420	*METACASPASE 8 (MC8)*	Encodes a metacaspase (cysteine-type endopeptidase) that is involved in promoting programmed cell death in response to hydrogen peroxide (H2O2), UV light, and methyl viologen (MV). Transcript levels rise in response to UV-C, H2O2, and MV. In vitro assays demonstrate that this enzyme has a preference for cleaving after an arginine residue, and it has a pH optimum of 8.0.
AT1G16440	*ROOT HAIR SPECIFIC 3 (RSH3)*	Member of AGC VIIIa Kinase gene family. Invovled in the maintenance of (p)ppGpp level to accustom plastidial gene expression to darkness.
AT1G16460	*RHODANESE HOMOLOGUE 2 (RDH2)*	encodes a cytoplasmic thiosulfate:cyanide sulfurtransferase, activity of which increased the rhodanese activity of transgenic yeast. Can also act as a mercaptopyruvate sulfurtransferase.
AT1G16490	*MYB DOMAIN PROTEIN 58 (MYB58)*	Member of the R2R3 factor gene family.
AT1G16500		filamentous hemagglutinin transporter
AT1G16510	*SMALL AUXIN UPREGULATED 41 (SAUR41)*	Encodes a clade III SAUR gene with a distinctive expression pattern in root meristems. It is normally expressed in the quiescent center and cortex/endodermis initials and upon auxin stimulation, the expression is found in the endodermal layer. Overexpression studies suggest roles in cell expansion and auxin transport.
AT1G16530	*ASYMMETRIC LEAVES 2-LIKE 9 (ASL9)*	ASYMMETRIC LEAVES 2-like 9
AT1G16540	*ABA DEFICIENT 3 (ABA3)*	Encodes molybdenum cofactor sulfurase. Involved in Moco biosynthesis. Involved in the conversion of ABA-aldehyde to ABA, the last step of abscisic acid (ABA) biosynthesis. <i>sir</i> loss-of-function mutants are resistant to sirtinol, a modulator of auxin signaling.N terminal domain is similar to bacterial NifS suggesting a common mechanism for sulphur mobilization and transfer. Also involved in protein import into chloroplasts.
AT1G16590	*(REV7)*	putative translesion synthesis polymerase zeta subunit, homologous to Y-family DNA polymerases, contains BRCT domain. Mutants are sensitive to UV-B radiation. Gene is involved in damage-tolerance mechanisms through translesion synthesis(TLS).
AT1G16610	*ARGININE/SERINE-RICH 45 (SR45)*	Encodes SR45, a member of the highly conserved family of serine/arginine-rich (SR) proteins, which play key roles in pre-mRNA splicing and other aspects of RNA metabolism. SR45 is a spliceosome protein, interacts with SR33 and the U1-70K protein of the U1 snRNP. Also involved in plant sugar response. sr45-1 mutation confers hypersensitivity to glucose during early seedling growth.
AT1G16640		AP2/B3-like transcriptional factor family protein
AT1G16670	*COLD-RESPONSIVE PROTEIN KINASE 1 (CRPK1)*	Encodes a cold-activated plasma membrane protein cold-responsive protein kinase that phosphorylates 14-3-3 proteins. The phosphorylated 14-3-3 proteins shuttle from the cytosol to the nucleus, where they interact with and destabilize the key cold-responsive C-repeat-binding factor (CBF) proteins, modulate CBF stability and the response to cold stress.
AT1G16705		p300/CBP acetyltransferase-related protein-like protein
AT1G16710	*HISTONE ACETYLTRANSFERASE OF THE CBP FAMILY 12 (HAC12)*	Encodes an enzyme with histone acetyltransferase activity that can use both H3 and H4 histones as substrates. No single prior lysine acetylation is sufficient to block HAC12 acetylation of the H3 or H4 peptides, suggesting that HAC12 can acetylate any of several lysines present in the peptides.
AT1G16720	*HIGH CHLOROPHYLL FLUORESCENCE PHENOTYPE 173 (HCF173)*	Encodes HCF173, a protein with weak similarities to the superfamily of the short-chain dehydrogenases/reductases. HCF173 is involved in the initiation of translation of the psbA mRNA and binds a specific site in the 5' UTR of psbA mRNA. Mutants shows a high chlorophyll fluorescence phenotype (hcf) and are severely affected in the accumulation of PSII subunits. The protein HCF173 is localized in the chloroplast, where it is mainly associated with the membrane system and is part of a higher molecular weight complex with psbA mRNA as a component of this complex.
AT1G16730	*UNKNOWN PROTEIN 6 (UP6)*	hypothetical protein
AT1G16740		Ribosomal protein L20
AT1G16750		GPI-anchored adhesin-like protein, putative (Protein of unknown function, DUF547)
AT1G16760		kinase with adenine nucleotide alpha hydrolases-like domain-containing protein
AT1G16770		hypothetical protein
AT1G16820		vacuolar ATP synthase catalytic subunit-related / V-ATPase-related / vacuolar proton pump-like protein
AT1G16830		Pentatricopeptide repeat (PPR) superfamily protein
AT1G16840		hypothetical protein
AT1G16850		transmembrane protein
AT1G16860	*(SHOU4L)*	Plasma membrane-localized proteins that negatively regulate cellulose synthesis by inhibiting the exocytosis of CESAs.
AT1G16870		mitochondrial 28S ribosomal protein S29-like protein
AT1G16880	*ACT DOMAIN REPEATS 11 (ACR11)*	Encodes a ACT domain-containing protein. The ACT domain, named after bacterial aspartate kinase, chorismate mutase and TyrA (prephenate dehydrogenase), is a regulatory domain that serves as an amino acid-binding site in feedback-regulated amino acid metabolic enzymes. The mRNA is cell-to-cell mobile.
AT1G16940		F-box/RNI-like/FBD-like domains-containing protein
AT1G16950		transmembrane protein

AT1G16980	*TREHALOSE-PHOSPHATASE/SYNTHASE 2 (TPS2)*	Encodes an enzyme putatively involved in trehalose biosynthesis. The protein has a trehalose synthase (TPS)-like domain that may or may not be active but no trehalose phosphatase (TPP)-like domain.
AT1G17000	*TREHALOSE-PHOSPHATASE/SYNTHASE 3 (TPS3)*	Encodes an enzyme putatively involved in trehalose biosynthesis. The protein has a trehalose synthase (TPS)-like domain that may or may not be active but no trehalose phosphatase (TPP)-like domain.
AT1G17010		2-oxoglutarate (2OG) and Fe(II)-dependent oxygenase superfamily protein
AT1G17020	*SENESCENCE-RELATED GENE 1 (SRG1)*	Encodes a novel member of the Fe(II)/ascorbate oxidase gene family; senescence-related gene.
AT1G17040	*SH2 DOMAIN PROTEIN A (SHA)*	Encodes a protein that contains an SH2 domain. It can pull down a 120-kD tyrosine-phosphorylated protein in vitro. It is predicted to act as a transcription factor.
AT1G17060	*CYTOCHROME P450 72C1 (CYP72C1)*	Encodes a protein with similarity to other cytochrome P450's and is a homolog of BAS1. Over expression causes a dwarf phenotype resembling brassinolide resistant mutants. Double mutant analysis of sob7/bas1 loss of function mutants suggests these genes have redundant functions in light responsiveness. SOB7 may function in metabolizing brassinolides. Expressed in leaf, root, stem and silique but expression highest in flower and cauline leaves. Dominant overexpressing plants have dwarf phenotype, short siliques/seeds, rounded dark green leaves and short hypocotyls in light and dark. Loss of function alleles result in plants with long hypocotyls.
AT1G17090		transmembrane protein
AT1G17100	*HAEM-BINDING PROTEIN 1 (HBP1)*	Encodes a cytosolic heme binding protein(cHBP)that can reversibly bind tetrapyrroles including heme, protoporphyrin IX and Mg-protoporphyrin IX dimethyl ester with distinct binding afﬁnities.
AT1G17130	*(CWC16B)*	DUF572 domain protein involved in alternative splicing.
AT1G17140	*INTERACTOR OF CONSTITUTIVELY ACTIVE ROP 1 (ICR1)*	Encodes a ROP/RAC effector, designated interactor of constitutive active ROPs 1 (ICR1), that interacts with GTP-bound ROPs. ICR1 is a scaffold mediating formation of protein complexes that are required for cell polarity. ICR1 is comprised of coiled-coil domains and forms complexes with itself and the exocyst vesicle-tethering complex subunit SEC3.
AT1G17150		Pectin lyase-like superfamily protein
AT1G17170	*GLUTATHIONE S-TRANSFERASE TAU 24 (GSTU24)*	Encodes glutathione transferase belonging to the tau class of GSTs. Naming convention according to Wagner et al. (2002). It is involved in the detoxification of the environmental pollutant 2,4,6-trinitrotoluene. Arabidopsis plants over-expressing At1g17170 were more resistant to TNT, removed more TNT from sterile and soil-based media, and had reduced levels of glutathione when grown in the presence of TNT.
AT1G17180	*GLUTATHIONE S-TRANSFERASE TAU 25 (GSTU25)*	Encodes glutathione transferase belonging to the tau class of GSTs. Naming convention according to Wagner et al. (2002). Detoxification of the environmental pollutant 2,4,6-trinitrotoluene. Arabidopsis plant over-expressing At1g17180 were more resistant to TNT, removed more TNT from sterile and soil-based media, and had reduced levels of glutathione when grown in the presence of TNT.
AT1G17190	*GLUTATHIONE S-TRANSFERASE TAU 26 (GSTU26)*	Encodes glutathione transferase belonging to the tau class of GSTs. Naming convention according to Wagner et al. (2002).
AT1G17200	*CASP-LIKE PROTEIN 2A1 (CASPL2A1)*	Uncharacterized protein family (UPF0497)
AT1G17220	*FU-GAERI1 (FUG1)*	Encodes a chloroplast localized protein with similarity to translation initiation factor 2. Can complement loss of INFB in E.coli suggesting FUG1 does function as a translation initiation factor in vivo. Identified as a suppressor of the leaf variegation mutant var2-6. Suppression is only seen in hypomorphs as complete loss of function alleles are embryo lethal. The mRNA is cell-to-cell mobile.
AT1G17230		Leucine-rich receptor-like protein kinase family protein
AT1G17240	*RECEPTOR LIKE PROTEIN 2 (RLP2)*	Encodes a CLAVATA2 (CLV2)-related gene. Complements the clv2 mutant when expressed under the control of the CLV2 promoter.
AT1G17290	*ALANINE AMINOTRANSFERASE (AlaAT1)*	Encodes for alanine aminotransferase (ALAAT1), involved in alanine catabolism during plants recovery from hypoxia The mRNA is cell-to-cell mobile.
AT1G17300		hypothetical protein
AT1G17310	*AGAMOUS-LIKE 100 (AGL100)*	MADS-box transcription factor family protein
AT1G17330	*CYCLIC NUCLEOTIDEDEPENDENT PHOSPHODIESTERASE 1 (CN-PDE1)*	cGMP-activated phosphodiesterase responsible for UVA induced decrease in cGMP.
AT1G17360		LOW protein: protein phosphatase 1 regulatory subunit-like protein
AT1G17380	*JASMONATE-ZIM-DOMAIN PROTEIN 5 (JAZ5)*	jasmonate-zim-domain protein 5
AT1G17390		transposable_element_gene;similar to RNase H domain-containing protein [Arabidopsis thaliana] (TAIR:AT5G36905.1);(source:TAIR10)
AT1G17420	*LIPOXYGENASE 3 (LOX3)*	LOX3 encode a Lipoxygenase. Lipoxygenases (LOXs) catalyze the oxygenation of fatty acids (FAs).
AT1G17430		alpha/beta-Hydrolases superfamily protein
AT1G17460	*TRF-LIKE 3 (TRFL3)*	Arabidopsis thaliana myb family transcription factor (At1g17460)
AT1G17480	*IQ-DOMAIN 7 (IQD7)*	Transient expression of Pro35S:GFP-IQD7 in leaves of N. benthamiana alters microtubule organization, in patterns similar to Pro35S:GFP-IQD8 and Pro35S:GFP-IQD6.Member of IQ67 (CaM binding) domain containing family.
AT1G17500	*AMINOPHOSPHOLIPID ATPASE 4 (ALA4)*	ATPase E1-E2 type family protein / haloacid dehalogenase-like hydrolase family protein. ALA4 acts redundantly with ALA3, ALA5, ALA9, ALA10 and ALA11 in root and shoot development
AT1G17510		hypothetical protein
AT1G17530	*TRANSLOCASE OF INNER MITOCHONDRIAL MEMBRANE 23 (TIM23-1)*	Encodes a translocase of inner mitochondrial membrane.
AT1G17540		kinase with adenine nucleotide alpha hydrolases-like domain-containing protein
AT1G17545		Protein phosphatase 2C family protein
AT1G17560	*HUELLENLOS (HLL)*	Encodes HUELLENLOS (HLL), a mitochondrial ribosome protein, similar to L14 ribosomal protein of eubacteria. HLL is essential for normal ovule development.
AT1G17590	*NUCLEAR FACTOR Y, SUBUNIT A8 (NF-YA8)*	Binds directly to CCAAT cis-elements in the promoters of multiple MIR156 genes and inhibits the juvenile-to adult transition by activating transcription of these MIR156s.
AT1G17600	*(SOC3)*	SOC3 is a TIR-NB-leucine-rich repeat (TNL) protein.Mutants suppress loss of chs2 phenotype of auto-activation of immunity. When the TIR domain of SOC3 interacts with CHS2 the binding results in temperature activation of cell death, the suppressors inhibit this interaction.

AT1G17610	*CHILLING SENSITIVE 1 (CHS1)*	TN-type protein that controls temperature-dependent growth and defense responses .Mutant accumulates steryl-esters at low temperatures and shows temperature dependent activation of defense responses..
AT1G17615	*TIR-NBS2 (TN2)*	TN2 is an atypical TIR-NBS protein that lacks the LRR domain common in typical NLR receptors. It interacts with EXO70B1, a subunit of the exocyst complex. Loss of function mutants in TN2 can suppress EXO70B1 mutants suggesting that EXO70B1 acts through TN2.
AT1G17620		Late embryogenesis abundant (LEA) hydroxyproline-rich glycoprotein family
AT1G17630	*CELL WALL MAINTAINER 1 (CWM1)*	Encodes a PPR protein involved in mitochondrial functioning. Mutants suppress cell wall defects caused by C17 chemical inhibitor. Mutants are defective in cytochrome c maturation and activation of mitochondrial retrograde signalling.
AT1G17640	*RNA-BINDING GLYCINE-RICH PROTEIN D1 (RBGD1)*	Belongs to a member of the RNA-binding glycine-rich (RBG) gene superfamily.
AT1G17650	*GLYOXYLATE REDUCTASE 2 (GLYR2)*	Glyoxylate reductase located in chloroplasts.
AT1G17665		CA-responsive protein
AT1G17710	*PHOSPHOETHANOLAMINE/PHOSPHOCHOLINE PHOSPHATASE 1 (PEPC1)*	Encodes a phosphoethanolamine/phosphocholine phosphatase. It is likely to be involved in the liberation of inorganic phosphate from intracellular sources. Expression is upregulated in the shoot of cax1/cax3 mutant.
AT1G17730	*VACUOLAR PROTEIN SORTING 46.1 (VPS46.1)*	Encodes an ESCRT-related protein: CHMP1A/AT1G73030; CHMP1B/AT1G17730. CHMP1A and B mediate multivesicular body sorting of auxin carriers and are required for plant development. ESCRT: Endosomal Sorting Complexes Required For Transport machinery; CHMP: Charged Multivesicular Body Protein/Chromatin Modifying Protein.
AT1G17740		
AT1G17745	*3-PHOSPHOGLYCERATE DEHYDROGENASE (PGDH)*	encodes a 3-Phosphoglycerate dehydrogenase The mRNA is cell-to-cell mobile.
AT1G17750	*PEP1 RECEPTOR 2 (PEPR2)*	Encodes PEPR2, a plasma membrane leucine-rich repeat receptor kinase functioning as a receptor for the Pep1 and Pep2 peptides. Pep1 and Pep2 are amino acids that induce the transcription of defense-related genes.
AT1G17810	*BETA-TONOPLAST INTRINSIC PROTEIN (BETA-TIP)*	beta-tonoplast intrinsic protein (beta-TIP) mRNA, complete
AT1G17830		hypothetical protein (DUF789)
AT1G17840	*ATP-BINDING CASSETTE G11 (ABCG11)*	Encodes a plasma membrane-localized ATP-binding cassette transporter, that is required for cutin transport to the extracellular matrix. The mRNA is cell-to-cell mobile.
AT1G17860	*ARABIDOPSIS THALIANA KUNITZ TRYPSIN INHIBITOR 5 (ATKTI5)*	Member of Kunitz trypsin inhibitor (KTI) family involved in plant defense response against spider mites.
AT1G17870	*ETHYLENE-DEPENDENT GRAVITROPISM-DEFICIENT AND YELLOW-GREEN-LIKE 3 (EGY3)*	S2P-like putative metalloprotease, also contain transmembrane helices near their C-termini and many of them, five of seven, contain a conserved zinc-binding motif HEXXH. Homolog of EGY1. Each of the EGY1 and EGY-like proteins share two additional highly conserved motifs, the previously reported NPDG motif (aa 442?454 in EGY1, Rudner et al., 1999) and a newly defined GNLR motif (aa 171?179 in EGY1). The GNLR motif is a novel signature motif unique to EGY1 and EGY-like proteins as well as other EGY1 orthologs found in cyanobacteria. Mediates chloroplastic ROS homeostasis and promotes retrograde signaling in response to salt stress.
AT1G17890	*(GER2)*	NAD(P)-binding Rossmann-fold superfamily protein
AT1G17900		transposable_element_gene;similar to unknown protein [Arabidopsis thaliana] (TAIR:AT5G27590.1);(source:TAIR10)
AT1G17910		Wall-associated kinase family protein
AT1G17920	*HOMEODOMAIN GLABROUS 12 (HDG12)*	Encodes a homeobox-leucine zipper family protein belonging to the HD-ZIP IV family. Together with HDG11, it is involved in trichome branching.
AT1G17950	*MYB DOMAIN PROTEIN 52 (MYB52)*	R2R3-MYB transcription family. Absence of the MYB52 transcription factor is correlated with an increase in PME activity and a decrease in the degree of pectin methylesterification. Transcriptionally activates PECTIN METHYLESTERASE INHIBITOR6 (PMEI6), PMEI14, and SUBTILISIN-LIKE SER PROTEASE1.7 (SBT1.7) by binding to their promoters.
AT1G17960		Threonyl-tRNA synthetase
AT1G17970	*(CTL15)*	RING/U-box superfamily protein
AT1G17990		FMN-linked oxidoreductases superfamily protein
AT1G18010		Major facilitator superfamily protein
AT1G18030		Protein phosphatase 2C family protein
AT1G18060	*(FBN-LIKE)*	localized to chloroplasts
AT1G18090		5-3 exonuclease family protein
AT1G18100	*(E12A11)*	Encodes a member of the FT and TFL1 family of phosphatidylethanolamine-binding proteins. It is expressed in seeds and up-regulated in response to ABA. Loss of function mutants show decreased rate of germination in the presence of ABA. ABA dependent regulation is mediated by both ABI3 and ABI5. ABI5 promotes MFT expression, primarily in the radicle-hypocotyl transition zone and ABI3 suppresses it in the seed.
AT1G18130		Class II aaRS and biotin synthetases superfamily protein
AT1G18140	*LACCASE 1 (LAC1)*	putative laccase, a member of laccase family of genes (with 17 members in Arabidopsis).
AT1G18150	*(ATMPK8)*	Encodes mitogen-activated protein kinase 8 (MPK8). MPK8 connects protein phosphorylation, Ca2+, and ROS in the wound-signaling pathway.
AT1G18170		FKBP-like peptidyl-prolyl cis-trans isomerase family protein
AT1G18180		3-oxo-5-alpha-steroid 4-dehydrogenase (DUF1295)
AT1G18200	*RAB GTPASE HOMOLOG A6B (RABA6b)*	Rab GTPase-like A1I protein
AT1G18220	*PURINE PERMEASE 9 (PUP9)*	Member of a family of proteins related to PUP1, a purine transporter. May be involved in the transport of purine and purine derivatives such as cytokinins, across the plasma membrane.
AT1G18280	*GLYCOSYLPHOSPHATIDYLINOSITOL-ANCHORED LIPID PROTEIN TRANSFER 3 (LTPG3)*	Bifunctional inhibitor/lipid-transfer protein/seed storage 2S albumin superfamily protein
AT1G18290		PADRE protein up-regulated after infection by S. sclerotiorum.
AT1G18310		glycosyl hydrolase family 81 protein
AT1G18320	*(ATTIM22)*	Mitochondrial import inner membrane translocase subunit Tim17/Tim22/Tim23 family protein
AT1G18330	*EARLY-PHYTOCHROME-RESPONSIVE1 (EPR1)*	EARLY-PHYTOCHROME-RESPONSIVE1

AT1G18350	*MAP KINASE KINASE 7 (MKK7)*	MAP kinase kinase7. Member of plant mitogen-activated protein kinase kinase group D. Negative regulator of polar auxin transport. Overexpression leads to activation of basal and systemic acquired resistance.
AT1G18360	*(MAGL2)*	alpha/beta-Hydrolases superfamily protein
AT1G18370	*HINKEL (HIK)*	Encodes a kinesin HINKEL. Required for cytokinesis in pollen. Mutant has cytokinesis defects; seedling lethal.
AT1G18380		
AT1G18390	*LEAF RUST 10 DISEASE-RESISTANCE LOCUS RECEPTOR- LIKE PROTEIN KINASE-LIKE 1.2 (LRK10L1.2)*	Serine/Threonine kinase family catalytic domain protein
AT1G18400	*BR ENHANCED EXPRESSION 1 (BEE1)*	Encodes the brassinosteroid signaling component BEE1 (BR-ENHANCED EXPRESSION 1). Positively modulates the shade avoidance syndrome in Arabidopsis seedlings.
AT1G18410		P-loop containing nucleoside triphosphate hydrolases superfamily protein
AT1G18460	*ALPHA/BETA-HYDROLASE (alpha/beta-HYD)*	Alpha/beta hydrolase
AT1G18480	*SHEWENELLA-LIKE PROTEIN PHOSPHATASE 2 (SLP2)*	Calcineurin-like metallo-phosphoesterase superfamily protein
AT1G18485		Pentatricopeptide repeat (PPR) superfamily protein
AT1G18500	*METHYLTHIOALKYLMALATE SYNTHASE-LIKE 4 (MAML-4)*	Encodes an active Arabidopsis isopropylmalate synthase IPMS1. Involved in leucine biosynthesis. Do not participate in the chain elongation of glucosinolates. Expressed constitutively throughout the plant. Loss of IPMS1 can be compensated by a second isopropylmalate synthase gene IPMS2 (At1g74040). The mRNA is cell-to-cell mobile.
AT1G18510	*TETRASPANIN 16 (TET16)*	Member of TETRASPANIN family
AT1G18520	*TETRASPANIN11 (TET11)*	Member of TETRASPANIN family
AT1G18530	*CALMODULIN LIKE 15 (CML15)*	Calmodulin like protein. Paralog of CML16. Expression in flowers is restricted to anthers and mature pollen.
AT1G18550		ATP binding microtubule motor family protein
AT1G18570	*MYB DOMAIN PROTEIN 51 (MYB51)*	Encodes a member of the R2R3-MYB transcription family. Involved in indole glucosinolate biosynthesis. The mRNA is cell-to-cell mobile.
AT1G18580	*GALACTURONOSYLTRANSFERASE 11 (GAUT11)*	Encodes a protein with putative galacturonosyltransferase activity.GAUT11 is required for pectin synthesis in seed coat epidermal cells and normal mucilage release.
AT1G18590	*SULFOTRANSFERASE 17 (SOT17)*	encodes a desulfoglucosinolate sulfotransferase, involved in the final step of glucosinolate core structure biosynthesis. Has a broad-substrate specificity with preference with methionine-derived desulfoglucosinolates.
AT1G18620	*TON1 RECRUITING MOTIF 3 (TRM3)*	Member of a small gene family in Arabidopsis. Quadruple mutants in this family display defects in cell elongation.
AT1G18630	*RNA-BINDING GLYCINE-RICH PROTEIN A1 (RBGA1)*	encodes a glycine-rich RNA binding protein.
AT1G18640	*3-PHOSPHOSERINE PHOSPHATASE (PSP)*	Encodes a 3-phosphoserine phosphatase acting in the last step of serine biosynthesis within the chloroplast. Essential for embryo, pollen and root development.
AT1G18650	*PLASMODESMATA CALLOSE-BINDING PROTEIN 3 (PDCB3)*	Encodes a member of the X8-GPI family of proteins. It localizes to the plasmodesmata and is predicted to bind callose.
AT1G18660	*IAN9-ASSOCIATED PROTEIN1 (IAP1)*	Membrane localized protein of unknown function. Involved in negative regulation of immune response. Mutants have increased resistance to pathogens.
AT1G18670	*IMPAIRED IN BABA-INDUCED STERILITY 1 (IBS1)*	Encodes a cyclin-dependent kinase-like protein with a ser/thr protein kinase domain and an N-terminal myristoylation sequence. Mutants in this gene are unable to express female sterility in response to beta-aminobutyric acid, as wild type plants do.
AT1G18690	*XYLOGLUCAN XYLOSYLTRANSFERASE 4 (XXT4)*	Galactosyl transferase GMA12/MNN10 family protein
AT1G18710	*MYB DOMAIN PROTEIN 47 (MYB47)*	Member of the R2R3 factor gene family. Promotes seed longevity (viability of seed over time.) Expressed in the chalazal seed coat. Overexpresion enhances resistance of seed to deterioration (PMID:32519347).
AT1G18730	*PHOTOSYNTHETIC NDH SUBCOMPLEX B 4 (PnsB4)*	likely a subunit of the chloroplast NAD(P)H dehydrogenase complex, involved in PSI cyclic electron transport. Located on the thylakoid membrane. Mutant has impaired NAD(P)H dehydrogenase activity. The mRNA is cell-to-cell mobile.
AT1G18740	*BYPASS1-LIKE (B1L)*	DUF793 domain containing protein. Expression is induced by cold. Loss of function mutations are more sensitive to freezing and have reduced levels of CBFs. May act by preventing degradation of CBFs.
AT1G18800	*NAP1-RELATED PROTEIN 2 (NRP2)*	Double nrp1-1 nrp2-1 mutants show arrest of cell cycle progression at G2/M and disordered cellular organization occurred in root tips. Localize in the nucleus and can form homomeric and heteromeric protein complexes with NRP1. Bind histones Histone2A and Histone2B and associate with chromatin in vivo. Plant mutated in both NRP1 and NRP2 genes show hypersensitivity to genotoxic stresses including UV and DSB-inducing agent Bleomycin. NRP genes act synergistically with NAP1 genes in promoting somatic homologous recombination.
AT1G18810		phytochrome kinase substrate-like protein
AT1G18830	*(SEC31A)*	Together with SEC31B a component of the coat protein complex II (COPII) which promotes the formation of transport vesicles from the endoplasmic reticulum (ER).
AT1G18840	*IQ-DOMAIN 30 (IQD30)*	Member of IQ67 (CaM binding) domain containing family.
AT1G18850	*LANT-SPECIFIC COMPONENT OF THE PRE- RRNA PROCESSING COMPLEX2 (PCP2)*	PCP2 encodes a novel plant specific protein that is co-expressed with components of pre-rRNA processing complex. Co-localizes with NuGWD1 and SWA1.
AT1G18860	*WRKY DNA-BINDING PROTEIN 61 (WRKY61)*	member of WRKY Transcription Factor; Group II-b
AT1G18870	*ISOCHORISMATE SYNTHASE 2 (ICS2)*	Encodes a protein with isochorismate synthase activity involved in phylloquinone biosynthesis. Mutant studies of this gene's function suggest that its function is redundant with that of ICS1 (AT1G7410).
AT1G18880	*NRT1/ PTR FAMILY 2.9 (NPF2.9)*	Encodes a low-afﬁnity plasma membrane nitrate transporter expressed in the companion cells of root phloem.
AT1G18890	*CALCIUM-DEPENDENT PROTEIN KINASE 1 (CDPK1)*	encodes a calcium-dependent protein kinase whose gene expression is induced by dehydration and high salt. Kinase activity could not be detected in vitro.
AT1G18900		Pentatricopeptide repeat (PPR) superfamily protein
AT1G18910	*BRUTUS LIKE 2 (BTSL2)*	E3 ubiquitin ligase that functions redundantly in the root with BTSL1 to negatively regulate iron uptake.
AT1G18940		Nodulin-like / Major Facilitator Superfamily protein

AT1G18950	*DDT-RELATED PROTEIN4 (DDR4)*	DDT domain superfamily
AT1G18970	*GERMIN-LIKE PROTEIN 4 (GLP4)*	Encodes a germin-like protein with possible oxalate oxidase activity (based on GenBank record).
AT1G18980		RmlC-like cupins superfamily protein
AT1G18990		myosin-binding protein, putative (Protein of unknown function, DUF593)
AT1G19010		hypothetical protein
AT1G19020	*SMALL DEFENSE-ASSOCIATED PROTEIN 1 (SDA1)*	Modulates defense against bacterial pathogens and tolerance to oxidative stress.
AT1G19030		transposable_element_gene;similar to unknown protein [Arabidopsis thaliana] (TAIR:AT5G13655.1);(source:TAIR10)
AT1G19040		NAC (No Apical Meristem) domain transcriptional regulator superfamily protein
AT1G19060		hypothetical protein (DUF626)
AT1G19090	*RECEPTOR-LIKE SERINE/THREONINE KINASE 2 (RKF2)*	receptor-like serine/threonine kinase (RKF2)
AT1G19100	*DEFECTIVE IN MERISTEM SILENCING 11 (DMS11)*	Encodes a member of the conserved Microrchidia (MORC) adenosine triphosphatase (ATPase) family, predicted to catalyze alterations in chromosome superstructure. Required for heterochromatin condensation and gene silencing.
AT1G19130		RmlC-like jelly roll fold protein
AT1G19150	*PHOTOSYSTEM I LIGHT HARVESTING COMPLEX GENE 6 (LHCA6)*	PSI type II chlorophyll a/b-binding protein (Lhca2*1) mRNA, The mRNA is cell-to-cell mobile.
AT1G19160		F-box family protein
AT1G19180	*JASMONATE-ZIM-DOMAIN PROTEIN 1 (JAZ1)*	JAZ1 is a nuclear-localized protein involved in jasmonate signaling. JAZ1 transcript levels rise in response to a jasmonate stimulus. JAZ1 can interact with the COI1 F-box subunit of an SCF E3 ubiquitin ligase in a yeast-two-hybrid assay only in the presence of jasmonate-isoleucine (JA-ILE) or coronatine. Application of jasmonate methyl ester to Arabidopsis roots reduces the levels of a JAZ1:GUS fusion protein, presumably by stimulating ubiquitin-proteasome-mediated degradation. The Jas domain appears to be important for JAZ1-COI1 interactions in the presence of coronatine. Two positive residues (R205 and R206) in the Jas domain shown to be important for coronatine -dependent COI1 binding are not required for binding AtMYC2. The mRNA is cell-to-cell mobile.
AT1G19190	*(NPF5.1)*	Controls leaf stomatal aperture by regulating abscisic acid transport.
AT1G19200		cyclin-dependent kinase, putative (DUF581)
AT1G19210	*(ERF017)*	encodes a member of the DREB subfamily A-5 of ERF/AP2 transcription factor family. The protein contains one AP2 domain. There are 15 members in this subfamily including RAP2.1, RAP2.9 and RAP2.10.
AT1G19220	*AUXIN RESPONSE FACTOR 19 (ARF19)*	Encodes an auxin response factor that contains the conserved VP1-B3 DNA-binding domain at its N-terminus and the Aux/IAA-like domains III and IV present in most ARFs at its C-terminus. The protein interacts with IAA1 (yeast two hybrid) and other auxin response elements such as ER7 and ER9 (yeast one hybrid). ARF19 protein can complement many aspects of the arf7 mutant phenotype and , together with ARF7, is involved in the response to ethylene. In the arf7 arf19 double mutant, several auxin-responsive genes (e.g. IAA5, LBD16, LBD29 and LBD33) are no longer upregulated by auxin.
AT1G19230	*(ATRBOHE)*	Riboflavin synthase-like superfamily protein
AT1G19250	*FLAVIN-DEPENDENT MONOOXYGENASE 1 (FMO1)*	FMO1 is required for full expression of TIR-NB-LRR conditioned resistance to avirulent pathogens and for basal resistance to invasive virulent pathogens. Functions in an EDS1-regulated but SA-independent mechanism that promotes resistance and cell death at pathogen infection sites. FMO1 functions as a pipecolate N-hydroxylase and catalyzes the biochemical conversion of pipecolic acid to N-hydroxypipecolic acid (NHP). NHP systemically accumulates in the plant foliage and induces systemic acquired resistance to pathogen infection.
AT1G19290	*TANG 2 (TANG2)*	Pentatricopeptide repeat protein involved in mitochondrial RNA processing.
AT1G19300	*PARVUS (PARVUS)*	The PARVUS/GLZ1 gene encodes a putative family 8 glycosyl transferase that contributes to xylan biosynthesis. Its gene expression shows good co-variance with the IRX3 gene involved in secondary cell wall synthesis. PARVUS/GLZ1 is predicted to have galacturonosyltransferase activity and may be involved in the formation of the complex oligosaccharide sequence present at the reducing end of xylan. PARVUS is expressed in cells undergoing secondary wall thickening, and parvus mutants have thinner cell walls.
AT1G19320		Pathogenesis-related thaumatin superfamily protein
AT1G19330	*SAP30 FUNCTION-RELATED 2 (AFR2)*	Evening-expressed key component of Sin3-HDAC complex, which bind directly to the CIRCADIAN CLOCK ASSOCIATED 1 (CCA1) and PSEUDO-RESPONSE REGULATOR 9 (PRR9) promoters and catalyze histone 3 (H3) deacetylation at the cognate regions to repress expression, allowing the declining phase of their expression at dusk.
AT1G19340		Methyltransferase MT-A70 family protein
AT1G19370		membrane protein
AT1G19380		sugar, putative (DUF1195)
AT1G19390		Wall-associated kinase family protein
AT1G19410		FBD / Leucine Rich Repeat domains containing protein
AT1G19430		S-adenosyl-L-methionine-dependent methyltransferases superfamily protein
AT1G19450		Major facilitator superfamily protein
AT1G19470		Galactose oxidase/kelch repeat superfamily protein
AT1G19500		hypothetical protein
AT1G19510	*RAD-LIKE 5 (RL5)*	RAD-like 5
AT1G19520	*NUCLEAR FUSION DEFECTIVE 5 (NFD5)*	Ribosomal pentatricopeptide repeat protein
AT1G19530	*RGA TARGET 1 (RGAT1)*	Direct target of RGA, plays an essential role in GA-mediated tapetum and pollen development.
AT1G19540		NmrA-like negative transcriptional regulator family protein

AT1G19570	*(PMID:28381499).*	Encodes a member of the dehydroascorbate reductase gene family. Critical for a mutualistic symbiosis between the host Arabidopsis and the root colonizing fungus Piriformospora indica.Encodes about 50-60% of extractable leaf GSH-dependent DHAR activity, but single knockout mutants show unaltered ascorbate and glutathione status in optimal and oxidative stress conditions (PMID:28381499). Acts redundantly with DHAR2 to oxidize glutathione in response to increased intracelullar hydrogen peroxide (catalase deficiency) . Complementation of a cat2 dhar1 dhar2 dhar3 quadruple mutant with DHAR1 fully restores cat2 phenotype and pathogenesis-related responses
AT1G19600		pfkB-like carbohydrate kinase family protein
AT1G19610	*(PDF1.4)*	Predicted to encode a PR (pathogenesis-related) protein. Belongs to the plant defensin (PDF) family with the following members: At1g75830/PDF1.1, At5g44420/PDF1.2a, At2g26020/PDF1.2b, At5g44430/PDF1.2c, At2g26010/PDF1.3, At1g19610/PDF1.4, At1g55010/PDF1.5, At2g02120/PDF2.1, At2g02100/PDF2.2, At2g02130/PDF2.3, At1g61070/PDF2.4, At5g63660/PDF2.5, At2g02140/PDF2.6, At5g38330/PDF3.1 and At4g30070/PDF3.2.
AT1G19620		transmembrane protein
AT1G19630	*CYTOCHROME P450, FAMILY 722, SUBFAMILY A, POLYPEPTIDE 1 (CYP722A1)*	cytochrome P450, family 722, subfamily A, polypeptide 1
AT1G19640	*JASMONIC ACID CARBOXYL METHYLTRANSFERASE (JMT)*	Encodes a S-adenosyl-L-methionine:jasmonic acid carboxyl methyltransferase that catalyzes the formation of methyljasmonate from jasmonic acid. Its expression is induced in response to wounding or methyljasmonate treatment.
AT1G19660	*BIFUNCTIONAL NUCLEASE IN BASAL DEFENSE RESPONSE 2 (BBD2)*	Wound-responsive family protein
AT1G19670	*CHLOROPHYLLASE 1 (CLH1)*	Chlorophyllase is the first enzyme involved in chlorophyll degradation. It catalyzes the hydrolysis of the ester bond to yield chlorophyllide and phytol. AtCLH1 lacks a typical signal sequence for the chloroplast. Its expression is induced rapidly by methyljasmonate, a known promoter of senescence and chlorophyll degradation.
AT1G19680		RING/U-box superfamily protein
AT1G19700	*BEL1-LIKE HOMEODOMAIN 10 (BEL10)*	Encodes a member of the BEL family of homeodomain proteins. Its interaction with PLP (PAS/LOV PROTEIN) is diminished by blue light.
AT1G19710		UDP-Glycosyltransferase superfamily protein
AT1G19720		Pentatricopeptide repeat (PPR-like) superfamily protein
AT1G19730	*(ATTRX4)*	encodes a cytosolic thioredoxin that reduces disulfide bridges of target proteins by the reversible formation of a disulfide bridge between two neighboring Cys residues present in the active site. Thioredoxins have been found to regulate a variety of biological reactions in prokaryotic and eukaryotic cells.
AT1G19740	*LON DOMAIN-CONTAINING PROTEIN 1 (LCP1)*	Chloroplast localized ATP-dependent protease La (LON) domain protein. Mutants are defective in anther dehiscence due to defects in lignification that may result from mis regulation of ROS scavenging enzymes.
AT1G19780	*CYCLIC NUCLEOTIDE GATED CHANNEL 8 (CNGC8)*	Encodes a member of the cyclic nucleotide gated channel (CNGC) family that is essential for male reproductive fertility.
AT1G19830	*SMALL AUXIN UPREGULATED RNA 54 (SAUR54)*	SAUR-like auxin-responsive protein family
AT1G19835	*TRICHOME CELL SHAPE 1 (TCS1)*	TCS1 encodes a coiled-coil domain protein that binds to microtubules and co-localizes with the cortical microtubules. Mutants have defects in trichome branching and hypocotyl elongation. TCS1 interacts with ZWI and appears to be involved in microtubule assembly.
AT1G19850	*MONOPTEROS (MP)*	Encodes a transcription factor (IAA24) mediating embryo axis formation and vascular development. Similar to AUXIN RESPONSIVE FACTOR 1 (ARF1) shown to bind to auxin responsive elements (AREs), and to the maize transcriptional activator VIVIPAROUS 1(VP1). In situ hybridization shows expression in provascular tissue of embryos, the emerging shoot primordia, then is restricted to provascular tissue, and in the root central vascular cylinder.
AT1G19880	*PLASTICITY OF ROSETTE TO NITROGEN 1 (PROTON1)*	Encodes a regulator of chromatin condensation 1 (RCC1) family protein; confers plasticity of rosette diameter in response to changes in N availability.
AT1G19890	*MALE-GAMETE-SPECIFIC HISTONE H3 (MGH3)*	histone 3.3, male-gamete-specific expression. Direct target promoter of the male germline-specific transcription factor DUO1.
AT1G19900	*RUBY PARTICLES IN MUCILAGE (RUBY)*	RUBY encodes a secreted galactose oxidase involved in cell wall modification.
AT1G19920	*(APS2)*	encodes a chloroplast form of ATP sulfurylase.
AT1G19930		Galactose oxidase/kelch repeat superfamily protein
AT1G19940	*GLYCOSYL HYDROLASE 9B5 (GH9B5)*	glycosyl hydrolase 9B5
AT1G19950	*HVA22-LIKE PROTEIN H (ATHVA22H) (HVA22H)*	HVA22-like protein H (ATHVA22H)
AT1G19960		Unknown gene, expression decreased in response to Mn and increased by cytokinin.
AT1G19970		ER lumen protein retaining receptor family protein
AT1G20010	*TUBULIN BETA-5 CHAIN (TUB5)*	beta tubulin
AT1G20020	*FERREDOXIN-NADP(+)-OXIDOREDUCTASE 2 (FNR2)*	Encodes a leaf-type ferredoxin:NADP(H) oxidoreductase. It is present in both chloroplast stroma and thylakoid membranes but is more abundant in the stroma The mRNA is cell-to-cell mobile.
AT1G20030		Pathogenesis-related thaumatin superfamily protein
AT1G20070		hypothetical protein
AT1G20080	*(SYTB)*	Encodes a synaptotagmin localized on the Golgi apparatus and that regulates protein secretion via the unconventional protein transport from the cytosol to the extracellular matrix in plant cells.
AT1G20090	*RHO-RELATED PROTEIN FROM PLANTS 2 (ROP2)*	Member of the Rho GTPase family. Functions to organize the microtubular cytoskeleton in combination with RIC1 and RIC4. These interactions affect pavement cell morphogenesis and pollen tube growth. ROP2 expression is stimulated by brassinosteroid treatment. Inhibit light-induced stomatal opening. The mRNA is cell-to-cell mobile.
AT1G20100		DNA ligase-like protein
AT1G20120		GDSL-motif esterase/acyltransferase/lipase. Enzyme group with broad substrate specificity that may catalyze acyltransfer or hydrolase reactions with lipid and non-lipid substrates.
AT1G20130		GDSL-motif esterase/acyltransferase/lipase. Enzyme group with broad substrate specificity that may catalyze acyltransfer or hydrolase reactions with lipid and non-lipid substrates.

AT1G20150		Subtilisin-like serine endopeptidase family protein
AT1G20160	*(ATSBT5.2)*	Encodes two isoforms. One (SBT5.2(a)) is a secreted, cell wall localized subtilisin-like serine protease that is involved in regulation of stomatal development. The second isoform (SBT5.2(b) is localized to endosomes.
AT1G20180		transmembrane protein (DUF677)
AT1G20190	*EXPANSIN 11 (EXPA11)*	member of Alpha-Expansin Gene Family. Naming convention from the Expansin Working Group (Kende et al, 2004. Plant Mol Bio)
AT1G20280		homeobox-leucine zipper protein-like protein
AT1G20290		SWI-SNF-related chromatin binding protein
AT1G20300		Pentatricopeptide repeat (PPR) superfamily protein
AT1G20310		syringolide-induced protein
AT1G20330	*STEROL METHYLTRANSFERASE 2 (SMT2)*	Encodes a sterol-C24-methyltransferases involved in sterol biosynthesis. Mutants display altered sterol composition, serrated petals and sepals and altered cotyledon vascular patterning as well as ectopic endoreduplication. This suggests that suppression of endoreduplication is important for petal morphogenesis and that normal sterol composition is required for this suppression.
AT1G20340	*DNA-DAMAGE-REPAIR/TOLERATION PROTEIN 112 (DRT112)*	recombination and DNA-damage resistance protein (DRT112) One of two Arabidopsis plastocyanin genes. Predominant form, expressed 10x higher than PETE1. PETE2 is thought to be post-transcriptionally regulated via copper accumulation and is involved in copper homeostasis. Mutation of this gene does not have obvious effect on photosynthesis. In plsp1-1 mutant plastids, the nonmature form of the protein localizes in the membrane.
AT1G20350	*TRANSLOCASE INNER MEMBRANE SUBUNIT 17-1 (TIM17-1)*	mitochondrial inner membrane translocase
AT1G20370		Pseudouridine synthase family protein
AT1G20390		transposable_element_gene;gypsy-like retrotransposon family, has a 1.5e-251 P-value blast match to GB:AAD19359 polyprotein (gypsy_Ty3-element) (Sorghum bicolor);(source:TAIR10)
AT1G20400		hypothetical protein (DUF1204)
AT1G20410		Pseudouridine synthase family protein
AT1G20440	*COLD-REGULATED 47 (COR47)*	Belongs to the dehydrin protein family, which contains highly conserved stretches of 7-17 residues that are repetitively scattered in their sequences, the K-, S-, Y- and lysine rich segments. Cold regulated gene, amino acid sequence homology with Group II LEA (late embryogenesis abundant) proteins. Also responds to osmotic stress, ABA, dehydration and inhibits e.coli growth while overexpressed. COR47 and RAB18 double overexpressor plants are cold tolerant. Regulated by heat shock.
AT1G20450	*EARLY RESPONSIVE TO DEHYDRATION 10 (ERD10)*	Encodes a gene induced by low temperature and dehydration. Inhibits e.coli growth while overexpressed. Belongs to the dehydrin protein family, which contains highly conserved stretches of 7-17 residues that are repetitively scattered in their sequences, the K-, S-, Y- and lysine rich segments. LTI29 and LTI30 double overexpressors confer cold tolerance. Localized to membranes and cytoplasm.
AT1G20480		AMP-dependent synthetase and ligase family protein
AT1G20490		AMP-dependent synthetase and ligase family protein
AT1G20500		AMP-dependent synthetase and ligase family protein
AT1G20510	*OPC-8:0 COA LIGASE1 (OPCL1)*	OPC-8:0 CoA ligase1
AT1G20520		DUF241 domain protein, putative (DUF241)
AT1G20530		girdin (DUF630 and DUF632)
AT1G20580	*(SMD3B)*	Core component of spliceosomal snRNP.
AT1G20590		cyclin
AT1G20620	*CATALASE 3 (CAT3)*	Catalase, catalyzes the breakdown of hydrogen peroxide (H2O2) into water and oxygen. The mRNA is cell-to-cell mobile.
AT1G20630	*CATALASE 1 (CAT1)*	Catalyzes the reduction of hydrogen peroxide using heme group as cofactor. Protects cells from toxicity by H2O2.
AT1G20650	*ALTERED SEED GERMINATION 5 (ASG5)*	Protein kinase superfamily protein
AT1G20680		transport/golgi organization-like protein (DUF833)
AT1G20690		SWI-SNF-related chromatin binding protein
AT1G20693	*HIGH MOBILITY GROUP B2 (HMGB2)*	Encodes a protein belonging to the subgroup of HMGB (high mobility group B) proteins that have a distinctive DNA-binding motif, the HMG-box domain. The motif confers non-sequence specific interaction with linear DNA and structure-specific binding to distorted DNA sites. The HMGB proteins are involved in the assembly of nucleoprotein complexes. Can be phosphorylated by CK2alpha. The mRNA is cell-to-cell mobile.
AT1G20700	*WUSCHEL RELATED HOMEOBOX 14 (WOX14)*	Encodes WOX14, a WUSCHEL-related homeobox gene family member with 65 amino acids in its homeodomain. Proteins in this family contain a sequence of eight residues (TLPLFPMH) downstream of the homeodomain called the WUS box. Functions in the shoot meristem organizing center to maintain the stem cells in an undifferentiated state. WOX4 and WOX14 act downstream of the PXY receptor kinase to regulate plant vascular proliferation independently of any role in vascular organisation.
AT1G20720		RAD3-like DNA-binding helicase protein
AT1G20750		RAD3-like DNA-binding helicase protein
AT1G20770		coiled-coil protein
AT1G20780	*SENESCENCE-ASSOCIATED E3 UBIQUITIN LIGASE 1 (SAUL1)*	Encodes a protein containing a U-box and an ARM domain. Homozygous mutant seedlings have a seedling lethal phenotype with widespread cell death lesions throughout the cotyledons and roots.
AT1G20800		F-box family protein
AT1G20810		FKBP-like peptidyl-prolyl cis-trans isomerase family protein
AT1G20823	*(ATATL80)*	Encodes a RING E3 ubiquitin ligase ATL80. Involved in phosphate mobilization and cold stress response in sufficient phosphate growth conditions. The mRNA is cell-to-cell mobile.
AT1G20830	*MULTIPLE CHLOROPLAST DIVISION SITE 1 (MCD1)*	Encodes MCD1 (MULTIPLE CHLOROPLAST DIVISION SITE 1). Determines the site of chloroplast division in concert with MinD (AT5G24020).
AT1G20840	*TONOPLAST MONOSACCHARIDE TRANSPORTER1 (TMT1)*	The protein encoded by this gene is found in the tonoplast (vacuole membrane) of Arabidopsis cells. The gene is expressed at highest levels in juvenile (sink) and adult (source) leaves, followed by flower tissues.

AT1G20850	*XYLEM CYSTEINE PEPTIDASE 2 (XCP2)*	Cysteine peptidase. Enzyme activity detected in leaf.
AT1G20860	*PHOSPHATE TRANSPORTER 1;8 (PHT1;8)*	Encodes Pht1;8, a member of the Pht1 family of phosphate transporters which include: Pht1;1/At5g43350, Pht1;2/At5g43370, Pht1;3/At5g43360, Pht1;4/At2g38940, Pht1;5/At2g32830, Pht1;6/At5g43340, Pht1;7/At3g54700, Pht1;8/At1g20860, Pht1;9/At1g76430 (Plant Journal 2002, 31:341).
AT1G20870	*INCREASED DNA METHYLATION 3 (IDM3)*	Encodes an anti-silencing factor that prevents gene repression and DNA hypermethylation.
AT1G20900	*AT-HOOK MOTIF NUCLEAR-LOCALIZED PROTEIN 27 (AHL27)*	Encodes an AT hook domain containing protein that acts redundantly with SOB3 to modulate hypocotyl growth inhibition in response to light.
AT1G20920	*REGULATOR OF CBF GENE EXPRESSION 1 (RCF1)*	P-loop containing nucleoside triphosphate hydrolases superfamily protein
AT1G20940		F-box family protein
AT1G20970		calponin-like domain protein
AT1G21000	*(PLATZ1)*	PLATZ transcription factor family protein
AT1G21010		PADRE proteinup-regulated after infection by S. sclerotiorun.
AT1G21020		transposable_element_gene;similar to Ulp1 protease family protein [Arabidopsis thaliana] (TAIR:AT1G08740.1);(source:TAIR10)
AT1G21050		MIZU-KUSSEI-like protein (Protein of unknown function, DUF617)
AT1G21060		Serine/Threonine-kinase, putative (Protein of unknown function, DUF547)
AT1G21070	*UDP-RHA/UDP-GAL TRANSPORTER 2 (URGT2)*	Nucleotide-sugar transporter family protein
AT1G21090		Cupredoxin superfamily protein
AT1G21100	*INDOLE GLUCOSINOLATE O-METHYLTRANSFERASE 1 (IGMT1)*	O-methyltransferase family protein
AT1G21110	*INDOLE GLUCOSINOLATE O-METHYLTRANSFERASE 3 (IGMT3)*	O-methyltransferase family protein
AT1G21120	*INDOLE GLUCOSINOLATE O-METHYLTRANSFERASE 2 (IGMT2)*	O-methyltransferase family protein
AT1G21130	*INDOLE GLUCOSINOLATE O-METHYLTRANSFERASE 4 (IGMT4)*	O-methyltransferase family protein
AT1G21140	*VACUOLAR IRON TRANSPORTER-LIKE 1 (VTL1)*	The gene encodes nodulin-like1 whose transcript abundance was repressed under conditions of Fe-deficient growth.
AT1G21160		eukaryotic translation initiation factor 2 (eIF-2) family protein
AT1G21200		sequence-specific DNA binding transcription factor
AT1G21210	*WALL ASSOCIATED KINASE 4 (WAK4)*	cell wall-associated ser/thr kinase involved in cell elongation and lateral root development
AT1G21220		transposable_element_gene;copia-like retrotransposon family, has a 3.9e-26 P-value blast match to GB:CAA72989 open reading frame 1 (Ty1_Copia-element) (Brassica oleracea);(source:TAIR10)
AT1G21230	*WALL ASSOCIATED KINASE 5 (WAK5)*	encodes a wall-associated kinase The mRNA is cell-to-cell mobile.
AT1G21240	*WALL ASSOCIATED KINASE 3 (WAK3)*	encodes a wall-associated kinase The mRNA is cell-to-cell mobile.
AT1G21250	*CELL WALL-ASSOCIATED KINASE 1 (WAK1)*	Encodes a cell wall-associated kinase that interacts with AtGRP3 and may function as a signaling receptor of extracellular matrix component such as oligogalacturonides. The mRNA is cell-to-cell mobile.
AT1G21290		transposable_element_gene;copia-like retrotransposon family, has a 3.7e-25 P-value blast match to GB:CAA72989 open reading frame 1 (Ty1_Copia-element) (Brassica oleracea);(source:TAIR10)
AT1G21300		transposable_element_gene;copia-like retrotransposon family, has a 3.9e-15 P-value blast match to GB:CAA72989 open reading frame 1 (Ty1_Copia-element) (Brassica oleracea);(source:TAIR10)
AT1G21310	*EXTENSIN 3 (EXT3)*	Encodes extensin 3.
AT1G21320	*(NSRB)*	nucleic acid/nucleotide binding protein
AT1G21340		Dof-type zinc finger DNA-binding family protein
AT1G21360	*GLYCOLIPID TRANSFER PROTEIN 2 (GLTP2)*	glycolipid transfer protein 2
AT1G21390	*EMBRYO DEFECTIVE 2170 (emb2170)*	embryo defective 2170
AT1G21400	*(E1A1)*	Branched chain alpha-keto acid dehydrogenase E1 alpha.
AT1G21430	*(YUC11)*	Flavin-binding monooxygenase family protein
AT1G21440		Phosphoenolpyruvate carboxylase family protein
AT1G21460	*(SWEET1)*	Nodulin MtN3 family protein
AT1G21500		hypothetical protein
AT1G21510		TPRXL
AT1G21520		hypothetical protein
AT1G21530		AMP-dependent synthetase and ligase family protein
AT1G21540	*(AAE9)*	Isobutyl-CoA synthetase involved in iso-branched wax biosynthesis.
AT1G21550		Calcium-binding EF-hand family protein
AT1G21560		hypothetical protein
AT1G21590		kinase with adenine nucleotide alpha hydrolases-like domain-containing protein
AT1G21600	*PLASTID TRANSCRIPTIONALLY ACTIVE 6 (PTAC6)*	Present in transcriptionally active plastid chromosomes. Involved in plastid gene expression. essential subunit of the plastid-encoded RNA polymerase (PEP). Mediates phytochrome signaling.
AT1G21640	*NAD KINASE 2 (NADK2)*	Encodes a protein with NAD kinase activity. The protein was also shown to bind calmodulin.
AT1G21650	*(SECA2)*	Encodes a component of the thylakoid-localized Sec system involved in the translocation of cytoplasmic proteins into plastid. Loss-of-function mutations result in arrest at the globular stage and embryo lethality.
AT1G21670		DPP6 amino-terminal domain protein
AT1G21680		DPP6 N-terminal domain-like protein
AT1G21710	*8-OXOGUANINE-DNA GLYCOSYLASE 1 (OGG1)*	Encodes 8-oxoguanine-DNA glycosylase. DNA repair enzyme.
AT1G21740		DUF630 family protein, putative (DUF630 and DUF632)

AT1G21750	*PDI-LIKE 1-1 (PDIL1-1)*	Encodes a protein disulfide isomerase-like (PDIL) protein, a member of a multigene family within the thioredoxin (TRX) superfamily; isoform contains non-consensus GA donor splice site at intron 9. Transcript levels for this gene are up-regulated in response to three different chemical inducers of ER stress (dithiothreitol, beta-mercaptoethanol, and tunicamycin). Neither AtIRE1-2 nor AtbZIP60 appear to be required for this response. The mRNA is cell-to-cell mobile.
AT1G21760	*F-BOX PROTEIN 7 (FBP7)*	This gene is predicted to encode an F-box protein that is evolutionarily conserved between Arabidopsis and other eukaryotes including S.cerevisiae and humans. It may play a role in regulating translation under conditions of temperature stress. FBP7 transcript levels are increased at high and low temperatures. The mRNA is cell-to-cell mobile.
AT1G21790		TRAM, LAG1 and CLN8 (TLC) lipid-sensing domain containing protein
AT1G21810	*VESICLE TETHERING 2 (VETH2)*	Encodes a protein that localizes at motile vesicle-like small compartments in differentiating xylem cells that is associated with microtubule plus-ends. VETH-positive compartments are unlikely to be elements in conventional endomembrane trafficking pathways. It can associate with COG2, and together these two proteins co-localize with the EXO70A1 exocyst subunit, tethering EXO70A1 to compartments associated with cortical microtubules.
AT1G21820		
AT1G21830		hypothetical protein
AT1G21850	*SKU5 SIMILAR 8 (sks8)*	SKU5 similar 8
AT1G21860	*SKU5 SIMILAR 7 (sks7)*	SKU5 similar 7
AT1G21870	*GOLGI NUCLEOTIDE SUGAR TRANSPORTER 5 (GONST5)*	Encodes a Golgi-localized nucleotide-sugar transporter.
AT1G21890	*USUALLY MULTIPLE ACIDS MOVE IN AND OUT TRANSPORTERS 19 (UMAMIT19)*	nodulin MtN21-like transporter family protein
AT1G21910	*DEHYDRATION RESPONSE ELEMENT-BINDING PROTEIN 26 (DREB26)*	encodes a member of the DREB subfamily A-5 of ERF/AP2 transcription factor family. The protein contains one AP2 domain. There are 15 members in this subfamily including RAP2.1, RAP2.9 and RAP2.10.
AT1G21920	*MORN-MOTIF REPEAT PROTEIN REGULATING FLOWERING 1 ((MRF1)*	MRF1 is related to SET7/9 proteins but contains an atypical SET domain. It is expressed in phloem and mutants have a weak late flowering phenotype. Phosphatidylinositol 4-phosphate 5-kinase (PIP5K) enzyme family member.
AT1G21950		transmembrane protein
AT1G21970	*LEAFY COTYLEDON 1 (LEC1)*	Transcriptional activator of genes required for both embryo maturation and cellular differentiation. Sequence is similar to HAP3 subunit of the CCAAT-box binding factor. HAP3 subunit is divided into three domains: an amino-terminal A domain, a central B domain, and a carboxyl-terminal C domain. LEC1 shared high similarity with other HAP3 homologs only in central, B domain. LEC1 is required for the specification of cotyledon identity and the completion of embryo maturation. It was sufficient to induce embryogenic programs in vegetative cells, suggesting that LEC1 is a major embryonic regulator that mediates the switch between embryo and vegetative development. Mutants are desiccation intolerant, have trichomes on cotyledons and exhibit precocious meristem activation. Levels of the ABI3 and FUS3 transcripts were significantly reduced in developing siliques of the lec1-1 mutants, indicating that LEC1 down-regulates FUS3 and ABI3.When LEC1 is overexpressed from an inducible promoter, the expression of numerous genes involved in fatty acid biosynthesis is increased suggesting a role in positive regulation of FA biosynthesis. LEC is required for FLC activation in embryos following vernalization.
AT1G21980	*PHOSPHATIDYLINOSITOL-4-PHOSPHATE 5-KINASE 1 (PIP5K1)*	Type I phosphatidylinositol-4-phosphate 5-kinase. Preferentially phosphorylates PtdIns4P. Induced by water stress and abscisic acid in Arabidopsis thaliana. Expressed in procambial cells of leaves, flowers and roots. A N-terminal Membrane Occupation and Recognition Nexus (MORN)affects enzyme activity and distribution.
AT1G22010		hypothetical protein
AT1G22015	*(DD46)*	Glycosyltransferase-31 (GT31) family member; β-(1,3)-galactosyltransferase (GalT) that catalyzes the synthesis of a β-(1,3)-galactan. GT31 β-(1,3)-GalTs play a role in elaborating type II AGs that decorate AGPs and pectins, thereby imparting functional consequences on plant growth and development.
AT1G22030		Phosphatidylinositol 4-phosphate 5-kinase (PIP5K) enzyme family member.
AT1G22040		Galactose oxidase/kelch repeat superfamily protein
AT1G22060		sporulation-specific protein
AT1G22070	*TGA1A-RELATED GENE 3 (TGA3)*	Encodes a transcription factor. Like other TGAla-related factors, TGA3 has a highly conserved bZIP region and exhibits similar DNA-binding properties.
AT1G22080		Cysteine proteinases superfamily protein
AT1G22090	*EMBRYO DEFECTIVE 2204 (emb2204)*	hypothetical protein
AT1G22110		structural constituent of ribosome
AT1G22130	*AGAMOUS-LIKE 104 (AGL104)*	Encodes a member of the MIKC (MADS box, Keratin binding domain, and C terminal domain containing)family of transcriptional regulators. AGL104 is expressed in pollen.It forms heterodimers with other MICK family members (AGL65 and AGL30). Involved in late stages of pollen development and pollen tube growth.
AT1G22140		zinc finger CCCH domain protein
AT1G22150	*SULFATE TRANSPORTER 1;3 (SULTR1;3)*	sulfate transporter Sultr1;3
AT1G22160		senescence-associated family protein (DUF581)
AT1G22170		Phosphoglycerate mutase family protein
AT1G22180		Sec14p-like phosphatidylinositol transfer family protein
AT1G22210	*TREHALOSE-6-PHOSPHATE PHOSPHATASE C (TPPC)*	Haloacid dehalogenase-like hydrolase (HAD) superfamily protein
AT1G22240	*PUMILIO 8 (PUM8)*	Encodes a member of the Arabidopsis Pumilio (APUM) proteins containing PUF domain (eight repeats of approximately 36 amino acids each). PUF proteins regulate both mRNA stability and translation through sequence-specific binding to the 3' UTR of target mRNA transcripts.

AT1G22275	*(ZYP1b)*	One of two nearly identical proteins (ZYP1a) identified by similarity to transverse filament (TF) proteins. These proteins are involved in chromosome synapsis during meiosis I and localize to the synaptonemal complex (SC). Single mutants have reduced fertility and double mutants (induced by RNAi) have severely reduced fertility.
AT1G22280	*PHYTOCHROME-ASSOCIATED PROTEIN PHOSPHATASE TYPE 2C (PAPP2C)*	Encodes a phytochrome-associated protein, PAPP2C (phytochrome-associated protein phosphatase type 2C). PAPP2C interacts in the nucleus with phyA (phytochrome A) and phyB. Functions as a regulator of phytochrome-interacting factor PIF3 by dephosphorylating phytochromes in the nucleus.
AT1G22290		14-3-3 family protein
AT1G22330		RNA-binding (RRM/RBD/RNP motifs) family protein
AT1G22335		
AT1G22340	*UDP-GLUCOSYL TRANSFERASE 85A7 (UGT85A7)*	UDP-glucosyl transferase 85A7
AT1G22360	*UDP-GLUCOSYL TRANSFERASE 85A2 (UGT85A2)*	UDP-glucosyl transferase 85A2
AT1G22370	*UDP-GLUCOSYL TRANSFERASE 85A5 (UGT85A5)*	UDP-glucosyl transferase 85A5
AT1G22380	*UDP-GLUCOSYL TRANSFERASE 85A3 (UGT85A3)*	Encodes a putative UDP-glucosyl transferase. At1g22380 was initially identified as encoding the protein AAF87154, which has been classified as a bHLH protein (AtbHLH152). Subsequently it has been found that the AAF87154 protein appears to be encoded by the AT1G23970 genomic locus.
AT1G22400	*(UGT85A1)*	UDP-Glycosyltransferase superfamily protein
AT1G22420		hydroxyproline-rich glycoprotein family protein
AT1G22430		GroES-like zinc-binding dehydrogenase family protein
AT1G22440		Zinc-binding alcohol dehydrogenase family protein
AT1G22460		O-fucosyltransferase family protein
AT1G22480		Cupredoxin superfamily protein
AT1G22490	*(BHLH094)*	basic helix-loop-helix (bHLH) DNA-binding superfamily protein
AT1G22500	*ARABIDOPSIS TOXICOS EN LEVADURA 15 (ATL15)*	Gene encodes a putative C3HC4-type RING zinc finger factor. it is induced in response to light and ascorbate stimulus.
AT1G22530	*PATELLIN 2 (PATL2)*	PATLs belong to a family of proteins having a Golgi dynamics (GOLD) domain in tandem with the Sec14p-like domain. PATLs are auxin regulated. Quadruple mutants (patl2456) show altered PIN1 lateralization in root endodermis cells.
AT1G22550	*(NPF5.16)*	Tonoplast localized pH dependent, low affinity nitrogen transporter.In shoots, expressed in leaf veins and mesophyll. In roots, GUS activity was detected in root vascular stele. More highly expressed in roots.
AT1G22570		Major facilitator superfamily protein
AT1G22590	*AGAMOUS-LIKE 87 (AGL87)*	AGAMOUS-like 87
AT1G22600		Late embryogenesis abundant protein (LEA) family protein
AT1G22630		SSUH2-like protein
AT1G22640	*MYB DOMAIN PROTEIN 3 (MYB3)*	MYB-type transcription factor (MYB3) that represses phenylpropanoid biosynthesis gene expression
AT1G22650	*ALKALINE/NEUTRAL INVERTASE D (A/N-InvD)*	Plant neutral invertase family protein
AT1G22690	*(GASA9)*	Gibberellin-regulated family protein
AT1G22700	*(PYG7)*	Encodes a TPR protein with homology to Ycf37 from Synechocystis that is localized to the thylakoid membrane and is involved in photosystem I biogenesis.
AT1G22710	*SUCROSE-PROTON SYMPORTER 2 (SUC2)*	Encodes for a high-affinity transporter essential for phloem loading and long-distance transport. A major sucrose transporter, AtSUC2 can also transport a wide range of physiological and synthetic glucose conjugates with both α- or β-linkage.
AT1G22720		Protein kinase superfamily protein
AT1G22740	*RAB GTPASE HOMOLOG G3B (RABG3B)*	GTP-binding protein Rab7
AT1G22760	*POLY(A) BINDING PROTEIN 3 (PAB3)*	Putative poly(A) binding protein May there fore function in posttranscriptional regulation, including mRNA turnover and translational initiation. Expression detected only in floral organs.
AT1G22770	*GIGANTEA (GI)*	Together with CONSTANTS (CO) and FLOWERING LOCUS T (FT), GIGANTEA promotes flowering under long days in a circadian clock-controlled flowering pathway. GI acts earlier than CO and FT in the pathway by increasing CO and FT mRNA abundance. Located in the nucleus. Regulates several developmental processes, including photoperiod-mediated flowering, phytochrome B signaling, circadian clock, carbohydrate metabolism, and cold stress response. The gene's transcription is controlled by the circadian clock and it is post-transcriptionally regulated by light and dark. Forms a complex with FKF1 on the CO promoter to regulate CO expression. The mRNA is cell-to-cell mobile.
AT1G22810	*(ATERF019)*	encodes a member of the DREB subfamily A-5 of ERF/AP2 transcription factor family. The protein contains one AP2 domain. There are 16 members in this subfamily including RAP2.1, RAP2.9 and RAP2.10. Overexpression leands to delayed senescence and delayed flowering. Negatively regulates plant resistance to P. parasitica by suppressing PAMP-triggered immunity.
AT1G22830		Tetratricopeptide repeat (TPR)-like superfamily protein
AT1G22840	*CYTOCHROME C-1 (CYTC-1)*	Encodes cytochrome c. Contains two site II (TGGGCC/T) elements, which interact with a TCP-domain transcription factor, and a downstream internal telomeric repeat, and are required for expression of the Cytc-1 gene. Promoter directs preferential expression in root and shoot meristems and in anthers. Double mutants with CYTC-2 accumulate starch during the day, have delayed growth and development and reduced GA and DELLA proteins linking cellular metabolism and GA homeostasis.
AT1G22850		SNARE associated Golgi protein family
AT1G22870	*(SCYL2A)*	One of two paralogs in Arabidopsis.Loss of both SCYL2B and SCYL2A results in severe growth defects.
AT1G22880	*CELLULASE 5 (CEL5)*	cellulase 5
AT1G22885	*(PROSCOOP13)*	transmembrane protein
AT1G22890	*(STMP2)*	Secreted peptide which functions in plant growth and pathogen defense.
AT1G22900		Disease resistance-responsive (dirigent-like protein) family protein
AT1G22910		RNA-binding (RRM/RBD/RNP motifs) family protein

AT1G22930		T-complex protein 11
AT1G22960		Pentatricopeptide repeat (PPR) superfamily protein
AT1G22980		Grap2/cyclin-D-interacting protein
AT1G22985	*CYTOKININ RESPONSE FACTOR 7 (CRF7)*	encodes a member of the ERF (ethylene response factor) subfamily B-5 of ERF/AP2 transcription factor family. The protein contains one AP2 domain. There are 7 members in this subfamily.
AT1G22990	*HEAVY METAL ASSOCIATED ISOPRENYLATED PLANT PROTEIN 22 (HIPP22)*	Heavy metal transport/detoxification superfamily protein
AT1G23000	*HEAVY METAL ASSOCIATED PROTEIN 5 (ATHMP05)*	Heavy metal transport/detoxification superfamily protein
AT1G23010	*LOW PHOSPHATE ROOT1 (LPR1)*	Encodes a protein with multicopper oxidase activity. Located in ER. Function together with LPR2 (AT1G71040) and a P5-type ATPase (At5g23630/PDR2) in a common pathway that adjusts root meristem activity to Pi (inorganic phosphate) availability.
AT1G23020	*FERRIC REDUCTION OXIDASE 3 (FRO3)*	Encodes a ferric chelate reductase whose transcription is regulated by FIT1. Expressed in the root, shoot, flower and cotyledon.
AT1G23030	*PLANT U-BOX PROTEIN 11 (PUB11)*	Encodes a plant U-Box protein that is capable of binding and ubiquitinating a variety of targets including MYC2,LRR1,KIN and acting as an E3 ligase. Regulates a number of physiological hormonal and environment al responses via selective degradation of targets.Unlike PUB10, its closest homolog in Arabidopsis, it does not appear to play a major role in the MeJA-mediated response.
AT1G23040		hydroxyproline-rich glycoprotein family protein
AT1G23050		hydroxyproline-rich glycoprotein family protein
AT1G23060	*MICROTUBULE DESTABILIZING PROTEIN 40 (MDP40)*	hypothetical protein
AT1G23070		organic solute transporter ostalpha protein (DUF300)
AT1G23080	*PIN-FORMED 7 (PIN7)*	Encodes a novel component of auxin efflux that is located apically in the basal cell and is involved during embryogenesis in setting up the apical-basal axis in the embryo. It is also involved in pattern specification during root development. In roots, it is expressed at lateral and basal membranes of provascular cells in the meristem and elongation zone, whereas in the columella cells it coincides with the PIN3 domain. Plasma membrane-localized PIN proteins mediate a saturable efflux of auxin. PINs mediate auxin efflux from mammalian and yeast cells without needing additional plant-specific factors. The action of PINs in auxin efflux is distinct from PGPs, rate-limiting, specific to auxins and sensitive to auxin transport inhibitors. PINs are directly involved of in catalyzing cellular auxin efflux.
AT1G23090	*(SULTR3;3)*	Encodes AST91 mRNA for sulfate transporter.
AT1G23100		GroES-like family protein
AT1G23120		Polyketide cyclase/dehydrase and lipid transport superfamily protein
AT1G23130		Polyketide cyclase/dehydrase and lipid transport superfamily protein
AT1G23140	*C2-DOMAIN ABA-RELATED8 (CAR8)*	Expression is upregulated in the shoot of cax1/cax3 mutant.
AT1G23160	*GRETCHEN HAGEN 3.7 (GH3.7)*	Acyl acid amido synthetase involved in conjugation of an amino acid to a plant hormone, a reaction that regulates free hormone concentration and downstream responses. Can also conjugate chorismate, a precursor of aromatic amino acids and salicylic acid, to cysteine and glutamate, respectively.
AT1G23180		ARM repeat superfamily protein
AT1G23190	*PHOSPHOGLUCOMUTASE 3 (PGM3)*	Encodes a cytosolic phosphoglucomutase (PGM). Two Arabidopsis PGM proteins (AT1G70730/PGM2 and AT1G23190/PGM3) have high sequence similarities and redundant functions. Mature plants possessing a single cPGM allele had a major reduction in cPGM activity. Whereas pgm2 and pgm3 single mutants are undistinguishable from the wild type, loss of both PGM2 and PGM3 severely impairs male and female gametophyte development. The mRNA is cell-to-cell mobile.
AT1G23200	*HIGHLY METHYL ESTERIFIED SEEDS (HMS)*	ProPME pectin methyl esterase involved in embryo development.
AT1G23205		Plant invertase/pectin methylesterase inhibitor superfamily protein
AT1G23210	*GLYCOSYL HYDROLASE 9B6 (GH9B6)*	glycosyl hydrolase 9B6
AT1G23240		Caleosin-related family protein
AT1G23250		Caleosin-related family protein
AT1G23270		hypothetical protein
AT1G23280		MAK16 protein-like protein
AT1G23300		MATE efflux family protein
AT1G23310	*GLUTAMATE:GLYOXYLATE AMINOTRANSFERASE (GGT1)*	Identified by cloning the gene that corresponded to a purified protein having glyoxylate aminotransferase activity. Localized to the peroxisome and thought to be involved in photorespiration/ metabolic salvage pathway.
AT1G23320	*TRYPTOPHAN AMINOTRANSFERASE RELATED 1 (TAR1)*	Encodes a protein with similarity to the TAA1 trytophan aminotransferase involved in IAA biosynthesis. This gene appears to be expressed at a very low level during seedling development. Triple mutant analyses implicate this gene in embryonic development.
AT1G23330		alpha/beta-Hydrolases superfamily protein
AT1G23340		carboxyl-terminal proteinase, putative (DUF239)
AT1G23350		Plant invertase/pectin methylesterase inhibitor superfamily protein
AT1G23370		
AT1G23390	*KELCH DOMAIN-CONTAINING F-BOX PROTEIN (KFB)*	A kelch domain-containing F-box protein. Its N terminus contains a typical F-box motif but its C-terminal domain only consists of one predicted kelch motif. Predicted to be stu Interacts with chalcone synthase CHS to mediate CHS ubiquitination and degradation.
AT1G23400	*CHLOROPLAST RNA SPLICING2-ASSOCIATED FACTOR 2 (CAF2)*	Promotes the splicing of chloroplast group II introns.
AT1G23410	*(RPS27AA)*	cytosolic ribosomal protein gene, part of eS31 family
AT1G23420	*INNER NO OUTER (INO)*	Essential for formation and asymmetric growth of the ovule outer integument. Member of the YABBY protein family of putative transcription factors that contain apparent Cys(2)-Cys(2) zinc-finger domains and regions of similarity to the high mobility group (HMG) transcription factors. INO may be required for polarity determination in the central part of the ovule.
AT1G23430		

AT1G23440		Peptidase C15, pyroglutamyl peptidase I-like protein
AT1G23450		pentatricopeptide (PPR) repeat-containing protein
AT1G23460		Pectin lyase-like superfamily protein
AT1G23480	*CELLULOSE SYNTHASE-LIKE A3 (CSLA03)*	encodes a gene similar to cellulose synthase
AT1G23510		OBP32pep protein
AT1G23520		hypothetical protein (DUF220)
AT1G23530		transmembrane protein
AT1G23540	*PROLINE-RICH EXTENSIN-LIKE RECEPTOR KINASE 12 (PERK12)*	Encodes a member of the PERK family of putative receptor kinases. Overexpression leads to morphological defects and reduced fertility and increased expression of MAX genes.
AT1G23550	*SIMILAR TO RCD ONE 2 (SRO2)*	Encodes a protein with similarity to RCD1 but without the WWE domain. The protein does have a PARP signature upstream of the C-terminal protein interaction domain. The PARP signature may bind NAD+ and attach the ADP-ribose-moiety from NAD+ to the target molecule. Its presence suggests a role for the protein in ADP ribosylation. Its transcript level is up-regulated by tunicamycin (N-linked glycosylation inhibitor causing ER stress).
AT1G23560		OBP32pep, putative (DUF220)
AT1G23570		hypothetical protein (DUF220)
AT1G23580		transmembrane protein, putative (Domain of unknown function DUF220)
AT1G23590		OBP32pep protein, putative (Domain of unknown function DUF220)
AT1G23600		OBP32pep protein, putative (Domain of unknown function DUF220)
AT1G23610		hypothetical protein
AT1G23630		
AT1G23640		OBP32pep protein
AT1G23650		hypothetical protein
AT1G23670		OBP32pep protein, putative (DUF220)
AT1G23680		hypothetical protein (DUF220)
AT1G23690		hypothetical protein (Domain of unknown function DUF220)
AT1G23700		Protein kinase superfamily protein
AT1G23710		hypothetical protein (DUF1645)
AT1G23720	*EXTENSIN 15 (EXT15)*	Proline-rich extensin-like family protein
AT1G23730	*BETA CARBONIC ANHYDRASE 3 (BCA3)*	beta carbonic anhydrase 3
AT1G23740	*ALKENAL/ONE OXIDOREDUCTASE (AOR)*	AOR is an alkenal/one oxidoreductase that acts on compounds with unsaturated alpha,beta-carbonyls. The activity of this enzyme with a number of substrates, including acrolein and 3-buten-2-one, was demonstrated in vitro using a truncated form of the protein that lacked approximately 80 of the first amino acids. This protein appears to localize to the chloroplast where it likely helps to maintain the photosynthetic process by detoxifying reactive carbonyls formed during lipid peroxidation.
AT1G23750		Nucleic acid-binding, OB-fold-like protein
AT1G23760	*(JP630)*	Encodes aromatic rich glycoprotein JP630.
AT1G23780		F-box family protein
AT1G23790	*CORTICAL MICROTUBULE DISORDERING4 (CORD4)*	dicer-like protein (DUF936)
AT1G23800	*ALDEHYDE DEHYDROGENASE 2B7 (ALDH2B7)*	Encodes a mitochondrial aldehyde dehydrogenase; nuclear gene for mitochondrial product.
AT1G23810		Paired amphipathic helix (PAH2) superfamily protein
AT1G23820	*SPERMIDINE SYNTHASE 1 (SPDS1)*	Spermidine synthase.
AT1G23830		transmembrane protein
AT1G23850		transmembrane protein
AT1G23870	*TREHALOSE-PHOSPHATASE/SYNTHASE 9 (TPS9)*	Encodes an enzyme putatively involved in trehalose biosynthesis. The protein has a trehalose synthase (TPS)-like domain that may or may not be active as well as a trehalose phosphatase (TPP)-like domain. The mRNA is cell-to-cell mobile.
AT1G23910		Polyketide cyclase/dehydrase and lipid transport superfamily protein
AT1G23940		
AT1G23950		hypothetical protein (DUF626)
AT1G23960		hypothetical protein (DUF626)
AT1G23990		transposable_element_gene;non-LTR retrotransposon family (LINE), has a 2.0e-30 P-value blast match to GB:NP_038603 L1 repeat, Tf subfamily, member 23 (LINE-element) (Mus musculus);(source:TAIR10)
AT1G24000		Polyketide cyclase/dehydrase and lipid transport superfamily protein
AT1G24010		Polyketide cyclase/dehydrase and lipid transport superfamily protein
AT1G24020	*MLP-LIKE PROTEIN 423 (MLP423)*	MLP-like protein 423
AT1G24030	*PBS1-LIKE 28 (PBL28)*	Protein kinase superfamily protein
AT1G24060		hypothetical protein
AT1G24090	*(RNH1C)*	RNase H family protein
AT1G24100	*UDP-GLUCOSYL TRANSFERASE 74B1 (UGT74B1)*	Encodes a UDP-glucose:thiohydroximate S-glucosyltransferase, involved in glucosinolate biosynthesis
AT1G24110		Peroxidase superfamily protein
AT1G24140	*(AT3-MMP)*	Expression induced by fungal and bacterial pathogens.

AT1G24180	*IAA-CONJUGATE-RESISTANT 4 (IAR4)*	Arabidopsis thaliana pyruvate dehydrogenase E1a-like subunit. 81% identical to a previously characterized Arabidopsis mitochondrial PDH E1a-subunit, At1g59900. Serine 296 phosphorylation of IAR4 has critical function in root hair formation and root development. Changing Ser296 in IAR4 to Ala resulted in a phenotype intermediate between mutant and wild-type, while substitution to Asp was either lethal or caused an extreme dwarf phenotype.
AT1G24210		Paired amphipathic helix (PAH2) superfamily protein
AT1G24260	*SEPALLATA3 (SEP3)*	Member of the MADs box transcription factor family. SEP3 is redundant with SEP1 and 2. Flowers of SEP1/2/3 triple mutants show a conversion of petals and stamens to sepals.SEP3 forms heterotetrameric complexes with other MADS box family members and binds to the CArG box motif.
AT1G24320		Six-hairpin glycosidases superfamily protein
AT1G24330	*(PUB6)*	Plant U-box type E3 ubiquitin ligase (PUB).
AT1G24340	*EMBRYO DEFECTIVE 2421 (emb2421)*	A locus involved in embryogenesis. Mutations in this locus result in embryo lethality. The mRNA is cell-to-cell mobile.
AT1G24360	*(KR)*	Dual mitochondrial and plastid localized b-ketoacyl- ACP reductase, a component of the fatty acid synthase complex.
AT1G24390		hypothetical protein
AT1G24400	*LYSINE HISTIDINE TRANSPORTER 2 (LHT2)*	High-affinity transporter for neutral and acidic amino acids, expressed in tapetum tissue of anthers. Transport of 1-Aminocyclopropane-1-carboxylic acid (ACC).
AT1G24420		HXXXD-type acyl-transferase family protein
AT1G24430		HXXXD-type acyl-transferase family protein
AT1G24440		RING/U-box superfamily protein
AT1G24460	*TGN-LOCALIZED SYP41 INTERACTING PROTEIN (TNO1)*	Encodes a novel protein that co-immunoprecipitates with SYP41. It is involved in vacuolar trafficking and salt tolerance, potentially via a role in vesicle fusion and in maintaining TGN structure or identity. Mutants display delayed formation of the Brefeldin A (BFA) compartment in cotyledons upon application of BFA.
AT1G24470	*BETA-KETOACYL REDUCTASE 2 (KCR2)*	Encodes one of the two Arabidopsis homologues to YBR159w encoding a S. cerevisiae beta-ketoacyl reductase (KCR), which catalyzes the first reduction during VLCFA (very long chain fatty acids, >18 carbon) elongation: KCR1 (At1g67730), KCR2 (At1g24470). Complementation of the yeast ybr159Delta mutant demonstrated that the two KCR proteins are divergent and that only AtKCR1 can restore heterologous elongase activity similar to the native yeast KCR gene.
AT1G24490	*ALBINA 4 (ALB4)*	Homologue of the Alb3/Oxa1/YidC family. ALB4 is almost identical to the Alb3/Oxa1/YidC domain of the previously described 110 kDa inner envelope protein ARTEMIS. However, ALB4 is expressed as a separate 55 kDa protein and is located in the thylakoid membrane of chloroplasts. Analysis of a T-DNA insertion line with a reduced level of Alb4 revealed chloroplasts with an altered ultrastructure. Mutant plastids are larger, more spherical in appearance and the grana stacks within the mutant lines are less appressed than in the wild-type chloroplasts. ALB4 is required for proper chloroplast biogenesis. The mRNA is cell-to-cell mobile.
AT1G24520	*HOMOLOG OF BRASSICA CAMPESTRIS POLLEN PROTEIN 1 (BCP1)*	Male fertility gene acting on tapetum and microspore
AT1G24530		Transducin/WD40 repeat-like superfamily protein
AT1G24540	*CYTOCHROME P450, FAMILY 86, SUBFAMILY C, POLYPEPTIDE 1 (CYP86C1)*	member of CYP86C
AT1G24560	*PLANT-UNIQUE RAB5 EFFECTOR 2 (PUF2)*	Effector molecule of the plant-unique RAB5, ARA6. Acts in the vacuolar/endocytic trafficking pathway with canonical RAB5 and SYP. Promotes recruitment of VSP9a onto the endosome, which is required for efficient RAB5 activation. Colocalizes with RAB5 on endosomes, where it coordinates transport with canonical RAB5.
AT1G24575		DEAD-box ATP-dependent RNA helicase-like protein
AT1G24600		hypothetical protein
AT1G24620	*CALMODULIN LIKE 25 (CML25)*	Encodes a EF-hand calcium-binding protein family member. Mutants exhibit longer root hairs under phosphate-deficient conditions.
AT1G24625	*ZINC FINGER PROTEIN 7 (ZFP7)*	Encodes a zinc finger protein containing only a single zinc finger.
AT1G24640		transposable_element_gene;non-LTR retrotransposon family (LINE), has a 4.3e-37 P-value blast match to GB:NP_038602 L1 repeat, Tf subfamily, member 18 (LINE-element) (Mus musculus);(source:TAIR10)
AT1G24650		Leucine-rich repeat protein kinase family protein
AT1G24678		
AT1G24735		S-adenosyl-L-methionine-dependent methyltransferases superfamily protein
AT1G24764	*MICROTUBULE-ASSOCIATED PROTEINS 70-2 (MAP70-2)*	Member of the MAP70 protein family.
AT1G24938		transposable_element_gene;similar to unknown protein [Arabidopsis thaliana] (TAIR:AT1G35100.1);(source:TAIR10)
AT1G24996		hypothetical protein
AT1G25025		hypothetical protein
AT1G25230		Calcineurin-like metallo-phosphoesterase superfamily protein
AT1G25240	*(PICALM9A)*	ENTH/VHS/GAT family protein
AT1G25250	*INDETERMINATE(ID)-DOMAIN 16 (IDD16)*	Encodes a transcription factor that, together with IDD14 and IDD15, regulates auxin biosynthesis and transport and thus aerial organ morphogenesis and gravitropic responses . IDD16 also binds to the SPCH promoter and regulates stomata initiation.
AT1G25260	*REDUCED POLLEN NUMBER (RDP)*	Involved in male gamete development. Trans-acting factor in the assembly of the pre-60S particle.
AT1G25270	*USUALLY MULTIPLE ACIDS MOVE IN AND OUT TRANSPORTERS 24 (UMAMIT24)*	nodulin MtN21-like transporter family protein
AT1G25275		Thionin-like gene involved in resistance against the beet cyst nematode (Heterodera schachtii).
AT1G25330	*CESTA (CES)*	Encodes CESTA, a positive regulator of brassinosteroid biosynthesis.
AT1G25340	*MYB DOMAIN PROTEIN 116 (MYB116)*	putative transcription factor (MYB116)
AT1G25350	*OVULE ABORTION 9 (OVA9)*	glutamine-tRNA ligase, putative / glutaminyl-tRNA synthetase, putative / GlnRS
AT1G25360	*D ORGANELLAR TRANSCRIPT PROCESSING 90 (OTP90)*	DYW-type pentatricopeptide repeat protein involved in C to U editing in mitochondria and chloroplasts.

AT1G25390	*LEAF RUST 10 DISEASE-RESISTANCE LOCUS RECEPTOR- LIKE PROTEIN KINASE-LIKE 4 (LRK10L4)*	Protein kinase superfamily protein
AT1G25400		transmembrane protein
AT1G25410	*ISOPENTENYLTRANSFERASE 6 (IPT6)*	AB061404 Arabidopsis thaliana AtIPT6 mRNA for cytokinin synthase, complete cds
AT1G25450	*3-KETOACYL-COA SYNTHASE 5 (KCS5)*	Encodes KCS5, a member of the 3-ketoacyl-CoA synthase family involved in the biosynthesis of VLCFA (very long chain fatty acids).
AT1G25460		NAD(P)-binding Rossmann-fold superfamily protein
AT1G25490	*ROOTS CURL IN NPA (RCN1)*	One of three genes encoding phosphoprotein phosphatase 2A regulatory subunit A; Recessive ethylene-response mutant EER1 displays increased ethylene sensitivity in the hypocotyl and stem
AT1G25500		Plasma-membrane choline transporter family protein
AT1G25510		Eukaryotic aspartyl protease family protein
AT1G25530		Transmembrane amino acid transporter family protein
AT1G25550	*HRS1 HOMOLOG3 (HHO3)*	Member of HHO/HRS GARP type transcriptional repressor family. Involved in Pi uptake and Pi starvation signaling. Transcriptional repressors that functions with other NIGT genes as an important hub in the nutrient signaling network associated with the acquisition and use of nitrogen and phosphorus.
AT1G25560	*TEMPRANILLO 1 (TEM1)*	Encodes a member of the RAV transcription factor family that contains AP2 and B3 binding domains. Involved in the regulation of flowering under long days. Loss of function results in early flowering. Overexpression causes late flowering and repression of expression of FT. Novel transcriptional regulator involved in ethylene signaling. Promoter bound by EIN3. EDF1 in turn, binds to promoter elements in ethylene responsive genes.
AT1G25570	*(LLR1)*	Di-glucose binding protein with Leucine-rich repeat domain-containing protein
AT1G25580	*SUPPRESSOR OF GAMMA RADIATION 1 (SOG1)*	Encodes suppressor of gamma response 1 (SOG1), a putative transcription factor governing multiple responses to DNA damage.
AT1G26150	*PROLINE-RICH EXTENSIN-LIKE RECEPTOR KINASE 10 (PERK10)*	Encodes a member of the proline-rich extensin-like receptor kinase (PERK) family. This family consists of 15 predicted receptor kinases (PMID: 15653807).
AT1G26180		membrane protein
AT1G26190	*TRIPHOSPHATE TUNNEL METALLOENZYME 2 (TTM2)*	TTM2 is a triphosphate tunnel metalloenzyme that displays pyrophosphatase activity.It contains both a uridine kinase (UK) domain and CYTH domain. TTM2 is involved in negative regulation defense response to pathogens (PMID:28733390).
AT1G26210	*SOB FIVE-LIKE 1 (SOFL1)*	AtSOFL1 acts redundantly with AtSOFL2 as positive regulator of cytokinin levels.
AT1G26220	*SEROTONIN N-ACETYLTRANSFERASE 2 (SNAT2)*	Catalyzes formation of N-acetylserotonin (NAS) from serotonin. Involved in the GA-related flowering pathway.
AT1G26230	*CHAPERONIN-60BETA4 (CPN60BETA4)*	Encodes a subunit of chloroplasts chaperonins that are involved in mediating the folding of newly synthesized, translocated, or stress-denatured proteins. Cpn60 subunits are: Cpn60alpha1 (At2g28000), AtCpn60alpha2 (At5g18820), AtCpn60beta1 (At1g55490), AtCpn60beta2 (At3g13470), AtCpn60beta3 (At5g56500), AtCpn60beta4 (At1g26230).
AT1G26240	*EXTENSIN 19 (EXT19)*	Proline-rich extensin-like family protein
AT1G26250	*EXTENSIN 18 (EXT18)*	Proline-rich extensin-like family protein
AT1G26260	*CRYPTOCHROME-INTERACTING BASIC-HELIX-LOOP-HELIX 5 (CIB5)*	Encodes CIB5 (cryptochrome-interacting basic-helix-loop-helix). Related to CIB1 (AT4G34530). CIB5 interacts with CRY2 and forms heterodimer with CIB1 in vitro. Regulates flowering time redundantly with CIB1.
AT1G26270		Phosphatidylinositol 3- and 4-kinase family protein
AT1G26290		hypothetical protein
AT1G26310	*CAULIFLOWER (CAL)*	Floral homeotic gene encoding a MADS domain protein homologous to AP1. Enhances the flower to shoot transformation in ap1 mutants.
AT1G26320		Zinc-binding dehydrogenase family protein
AT1G26340	*CYTOCHROME B5 ISOFORM A (CB5-A)*	encodes a member of the cytochromes b5 family of proteins that localizes to the outer envelope of the chloroplast. The C-terminal portion of the protein appears to be capable of inserting into a plant microsomal membrane in vitro.
AT1G26350		hypothetical protein
AT1G26360	*METHYL ESTERASE 13 (MES13)*	Encodes a protein predicted to act as a carboxylesterase. It has similarity to the SABP2 methyl salicylate esterase from tobacco but no enzymatic activity has been identified for this protein.
AT1G26380	*FAD-LINKED OXIDOREDUCTASE 1 (FOX1)*	Functions in the biosynthesis of 4-hydroxy indole-3-carbonyl nitrile (4-OH-ICN), a cyanogenic phytoalexin in Arabidopsis. FOX1 acts as a dehydrogenase on indole cyanohydrin to form indole carbonyl nitrile.
AT1G26390	*(ATBBE4)*	FAD-binding Berberine family protein
AT1G26400	*(ATBBE5)*	FAD-binding Berberine family protein
AT1G26410	*(ATBBE6)*	FAD-binding Berberine family protein
AT1G26420	*(ATBBE7)*	FAD-binding Berberine family protein
AT1G26440	*UREIDE PERMEASE 5 (UPS5)*	Encodes a ureide permease, uptake assays in yeast mutants indicated the longer splice variant is a cellular importer for allantoin, uracil and xanthine. Encodes 2 splice variants, UPS5L and UPS5S, which under nonstress conditions may function in allantoin degradation for nutrient recycling, whereas under stress, both genes may be involved in vesicular export allowing allantoin translocation from roots to shoots.
AT1G26450		Carbohydrate-binding X8 domain superfamily protein
AT1G26460		Tetratricopeptide repeat (TPR)-like superfamily protein
AT1G26470	*SNRK2-SUBSTRATE 1 (SNS1)*	chromatin modification-like protein
AT1G26480	*GENERAL REGULATORY FACTOR 12 (GRF12)*	14-3-3 protein GF14iota (grf12)
AT1G26500		Pentatricopeptide repeat (PPR) superfamily protein
AT1G26530		PIN domain-like family protein
AT1G26540		Agenet domain-containing protein
AT1G26560	*BETA GLUCOSIDASE 40 (BGLU40)*	beta glucosidase 40

AT1G26570	*UDP-GLUCOSE DEHYDROGENASE 1 (UGD1)*	UDP-glucose dehydrogenase 1
AT1G26590		C2H2-like zinc finger protein
AT1G26600	*CLAVATA3/ESR-RELATED 9 (CLE9)*	Member of a large family of putative ligands homologous to the Clavata3 gene. Consists of a single exon. Can partially replace CLV3 function in vivo.
AT1G26610		C2H2-like zinc finger protein
AT1G26620		T-box transcription factor, putative (DUF863)
AT1G26640	*ISOPENTENYL PHOSPHATE KINASE (IPK)*	Encodes a cytosolic isopentenyl phosphate kinase that plays an important role in regulating the formation of both MVA (mevalonic acid) and MEP (methylerythritol phosphate) pathway-derived terpenoid compounds by controlling the ratio of IP/DMAP to IPP/DMAPP. IPP and DMAPP are the universal C5 building blocks of all natural terpenoids. IPK enhances terpenoid formation by returning IP/DMAP to the terpenoid biosynthetic network.
AT1G26660		Prefoldin chaperone subunit family protein
AT1G26665	*MEDIATOR 10B (MED10B)*	Mediator complex, subunit Med10
AT1G26680		transcriptional factor B3 family protein
AT1G26700	*MILDEW RESISTANCE LOCUS O 14 (MLO14)*	A member of a large family of seven-transmembrane domain proteins specific to plants, homologs of the barley mildew resistance locus o (MLO) protein. The Arabidopsis genome contains 15 genes encoding MLO proteins, with localization in plasma membrane. Phylogenetic analysis revealed four clades of closely-related AtMLO genes. ATMLO14 belongs to the clade I, with AtMLO4 and AtMLO11. The gene is expressed during early seedling growth, in developing primary root, and particularly in root tips of 10-day old seedlings; it was not expressed in leaves or flowers, as shown by GUS activity patterns. The expression of several phylogenetically closely-related AtMLO genes showed similar or overlapping tissue specificity and analogous responsiveness to external stimuli, suggesting functional redundancy, co-function, or antagonistic function(s).
AT1G26710		transmembrane protein
AT1G26730		EXS (ERD1/XPR1/SYG1) family protein
AT1G26740		Ribosomal L32p protein family
AT1G26760	*SET DOMAIN PROTEIN 35 (SDG35)*	SET domain protein 35
AT1G26770	*EXPANSIN A10 (EXPA10)*	Encodes an expansin. Naming convention from the Expansin Working Group (Kende et al, Plant Mol Bio). Involved in the formation of nematode-induced syncytia in roots of Arabidopsis thaliana.
AT1G26780	*MYB DOMAIN PROTEIN 117 (MYB117)*	Encodes LOF1 (LATERAL ORGAN FUSION1), a MYB-domain transcription factor expressed in organ boundaries. Functions in boundary specification, meristem initiation and maintenance, and organ patterning. Also see LOF2 (At1g69560).
AT1G26790	*CYCLING DOF FACTOR 6 (CDF6)*	Dof-type zinc finger DNA-binding family protein
AT1G26795		Plant self-incompatibility protein S1 family
AT1G26800	*MISFOLDED PROTEIN SENSING RING E3 LIGASE 1 (MPSR1)*	MPSR1 is cytoplasmic E3 ligase that senses misfolded proteins independently of chaperones and targets those proteins for degradation via the 26S proteasome. Involved in the regulation of the homeostasis of sensor NLR immune receptors.
AT1G26820	*RIBONUCLEASE 3 (RNS3)*	Encodes ribonuclease RNS3.
AT1G26840	*ORIGIN RECOGNITION COMPLEX PROTEIN 6 (ORC6)*	Origin Recognition Complex subunit 6. Involved in the initiation of DNA replication. Regulated transcriptionally during cell cycle, peaking at G1/S-phase. Target of E2F/DF family of transcription factors. It acts downstream of BRP4, and is, at least in part, involved in the BRP4-mediated mitotic cell-cycle progression.
AT1G26850		S-adenosyl-L-methionine-dependent methyltransferases superfamily protein
AT1G26860		transposable_element_gene;similar to unknown protein [Arabidopsis thaliana] (TAIR:AT2G01700.1);(source:TAIR10)
AT1G26870	*FEZ (FEZ)*	NAC-domain protein. Expressed in root cap stem cells, where it promotes periclinal root cap-forming divisions. Involved in a regulatory feedback loop with SMB. FEZ activates SMB in hte root cap daughter cells soon after division, and SMB in turn represses FEZ expression in these cells, thereby preventing further stem cell divisions.
AT1G26920		zinc finger CCHC domain protein
AT1G26930		Galactose oxidase/kelch repeat superfamily protein
AT1G26960	*HOMEOBOX PROTEIN 23 (AtHB23)*	Encodes a homeodomain leucine zipper class I (HD-Zip I) protein. Participates in the gene regulatory network controlling root branching by mediating the regulation of LAX3 by ARF7/19.
AT1G26970	*PBS1-LIKE 4 (PBL4)*	Protein kinase superfamily protein
AT1G26990		transposable_element_gene;copia-like retrotransposon family, has a 2.0e-294 P-value blast match to GB:CAA72989 open reading frame 1 (Ty1_Copia-element) (Brassica oleracea);(source:TAIR10)
AT1G27000		GRIP/coiled-coil protein, putative (DUF1664)
AT1G27030		hypothetical protein
AT1G27040	*NRT1/ PTR FAMILY 4.5 (NPF4.5)*	Major facilitator superfamily protein
AT1G27050		Encodes a protein with a RNA recognition motif. Previously annotated as ATHB54, a homeodomain leucine zipper (HD-Zip) family protein. In the TAIR10 genome release (2010), this locus was split into two loci: AT1G27045 (containing homeodomain and leucine zipper domains) and AT1G27050 (containing a RNA recognition motif). AT1G27045 is now named ATHB54. Note that Affymetrix ATH1 Probe Set linked to symbol ATHB54 is in fact directed against the product of the AT1G27050 locus (the mRNA coding for the RNA-recognition-motif protein).
AT1G27060	*SENSITIVE TO ABA 1 (SAB1)*	Regulator of chromosome condensation (RCC1) family protein
AT1G27080	*NRT1/ PTR FAMILY 2.12 (NPF2.12)*	Encodes a protein with low-affinity nitrate transporter activity that is expressed in the vascular tissue of the funiculus and the silique. This plasma membrane-localized enzyme is predicted to have 12 transmembrane domains. Plants lacking NRT1.6 have reduced levels of nitrate in their seeds and have increased levels of early embryonic developmental defects and seed abortion.
AT1G27090		glycine-rich protein
AT1G27100		Actin cross-linking protein
AT1G27120	*(GALT4)*	Encodes a Golgi-localized hydroxyproline-O-galactosyltransferase.

AT1G27130	*GLUTATHIONE S-TRANSFERASE TAU 13 (GSTU13)*	Encodes glutathione transferase belonging to the tau class of GSTs. Naming convention according to Wagner et al. (2002). GSTU13 acts in the pathogen triggered pathway for indole glucosinolate metabolisms that involves also PENETRATION2 myrosinase. It is likely the enzyme that conjugates GSH with unstable indol-3-ylmethyl-ITCs formed upon PEN2-mediated IG hydrolysis, particularly in the branch of this pathway in which 4-substituted IGs are processed.
AT1G27140	*GLUTATHIONE S-TRANSFERASE TAU 14 (GSTU14)*	Encodes glutathione transferase belonging to the tau class of GSTs. Naming convention according to Wagner et al. (2002).
AT1G27160		valyl-tRNA synthetase / valine-tRNA ligase-like protein
AT1G27170		transmembrane receptors / ATP binding protein
AT1G27190	*BAK1-INTERACTING RECEPTOR-LIKE KINASE 3 (BIR3)*	Activated by TCP8/14/15/22, involved in modulation of GA-dependent stamen filament elongation.
AT1G27200		glycosyltransferase family protein (DUF23)
AT1G27220		paired amphipathic helix repeat-containing protein
AT1G27240		Paired amphipathic helix (PAH2) superfamily protein
AT1G27285		transposable_element_gene;copia-like retrotransposon family, has a 0. P-value blast match to GB:AAC02666 polyprotein (Ty1_Copia-element) (Arabidopsis thaliana);(source:TAIR10)
AT1G27290		transmembrane protein
AT1G27300		transmembrane protein
AT1G27320	*HISTIDINE KINASE 3 (HK3)*	Encodes a histidine kinases, a cytokinin receptor that controls cytokinin-mediated leaf longevity through a specific phosphorylation of the response regulator, ARR2. The mRNA is cell-to-cell mobile.
AT1G27330		Ribosome associated membrane protein RAMP4
AT1G27340	*LEAF CURLING RESPONSIVENESS (LCR)*	Encodes a putative F-box protein that is involved in the regulation of leaf morphology.
AT1G27360	*SQUAMOSA PROMOTER-LIKE 11 (SPL11)*	In conjunction with SPL10 and SPL2, SPL11 redundantly controls proper development of lateral organs in association with shoot maturation in the reproductive phase. SPL2, SPL10, and SPL11, suppress root regeneration with age by inhibiting wound-induced auxin biosynthesis.
AT1G27370	*SQUAMOSA PROMOTER BINDING PROTEIN-LIKE 10 (SPL10)*	In conjunction with SPL11 and SPL2, SPL10 redundantly controls proper development of lateral organs in association with shoot maturation in the reproductive phase. SPL2, SPL10, and SPL11, suppress root regeneration with age by inhibiting wound-induced auxin biosynthesis. SPL10 also controls lamina shape during vegetative development.
AT1G27380	*ROP-INTERACTIVE CRIB MOTIF-CONTAINING PROTEIN 2 (RIC2)*	encodes a member of a novel protein family that contains contain a CRIB (for Cdc42/Rac-interactive binding) motif required for their specific interaction with GTP-bound Rop1 (plant-specific Rho GTPase). Interacts with Rop1 and is involved in pollen tube growth and function. Protein most similar to RIC4 (family subgroup V). Gene is expressed in all tissues examined.
AT1G27390	*TRANSLOCASE OUTER MEMBRANE 20-2 (TOM20-2)*	Form of TOM20, which is a component of the TOM complex, involved in transport of nuclear-encoded mitochondrial proteins
AT1G27435		hypothetical protein
AT1G27460	*NO POLLEN GERMINATION RELATED 1 (NPGR1)*	encodes a calmodulin-binding protein that is expressed in pollen, suspension culture cells, flowers, and fruits. The mRNA is cell-to-cell mobile.
AT1G27470		transducin family protein / WD-40 repeat family protein
AT1G27480		alpha/beta-Hydrolases superfamily protein
AT1G27520	*MANNOSIDASE 5 (MNS5)*	Glycosyl hydrolase family 47 protein
AT1G27530		ubiquitin-fold modifier-conjugating enzyme
AT1G27540	*F-BOX/DUF295 BRASSICEAE-SPECIFIC 2 (ATFDB2)*	F-box protein (DUF295)
AT1G27550	*F-BOX/DUF295 BRASSICEAE-SPECIFIC 3 (ATFDB3)*	F-box family protein
AT1G27570		phosphatidylinositol 3- and 4-kinase family protein
AT1G27590		
AT1G27610		hypothetical protein
AT1G27620		HXXXD-type acyl-transferase family protein
AT1G27630	*CYCLIN T 1;3 (CYCT1;3)*	cyclin T 1
AT1G27640		Putative role in leaf development. Comparison of SALK_123839C to Columbia under normal growth conditions resulted in a trend toward increased leaf length in the mutant (P=0.13; median 22 for mutant,17 for Columbia) (Ann Stapleton and Delita Pardue, 2009, personal communication).
AT1G27650	*(ATU2AF35A)*	U2 auxiliary factor small subunit. The atU2AF35a protein and its homolog, atU2AF35b, contain most of the conserved domains of hsU2AF35, including the psiRRM, one RS domain, two zinc fingers, and the two regions for interacting with U2AF large subunit. Both proteins lack the stretch of glycines present in human U2AF35. The sequences are overall 83% identical, and each Arabidopsis homolog shows approximately 70% similarity to hsU2AF35. U2AF(35) homologs were also identified from maize, rice and other plants with large-scale EST projects. Both genes are expressed in all major tissues, with atU2AF(35)a expressed at a higher level than atU2AF(35)b in most tissues. The expression patterns were different in roots: atU2AF(35)b expressed strongly in whole young roots and root tips and atU2AF(35)a limited to root vascular regions.
AT1G27670		transmembrane protein
AT1G27690	*BOUNDARY OF ROP DOMAIN3 (BDR3)*	lipase, putative (DUF620)
AT1G27700		Syntaxin/t-SNARE family protein
AT1G27720	*TBP-ASSOCIATED FACTOR 4B (TAF4b)*	TBP-associated factor 4B
AT1G27730	*SALT TOLERANCE ZINC FINGER (STZ)*	Related to Cys2/His2-type zinc-finger proteins found in higher plants. Compensated for a subset of calcineurin deficiency in yeast. Salt tolerance produced by ZAT10 appeared to be partially dependent on ENA1/PMR2, a P-type ATPase required for Li+ and Na+ efflux in yeast. The protein is localized to the nucleus, acts as a transcriptional repressor and is responsive to chitin oligomers. Also involved in response to photooxidative stress.
AT1G27740	*ROOT HAIR DEFECTIVE 6-LIKE 4 (RSL4)*	Basic helix-loop-helix (bHLH) transcription factor that is sufficient to promote postmitotic cell growth in root-hair cells. RSL4 is a direct transcriptional target of RHD6
AT1G27790		transposable_element_gene;similar to unknown protein [Arabidopsis thaliana] (TAIR:AT3G24380.1);(source:TAIR10)
AT1G27820	*CCR4-ASSOCIATED FACTOR 1C (CAF1C)*	Deadenylase.

AT1G27840	*(ATCSA-1)*	Encodes a DDB1a interacting protein ATCSA-1 required for UV-B tolerance and genomic integrity. Involved in dictating the efficiency and the strand preference of repair of transcribed genes, works as the dominant protein in coupling repair to transcription with minor contribution from CSA2.
AT1G27850	*BASIC PROLINE-RICH PROTEIN5 (BPP5)*	Encodes a microtubule-associated protein involved in cortical microtubule organization during leaf development.
AT1G27880		DEAD/DEAH box RNA helicase family protein
AT1G27900		RNA helicase family protein
AT1G27920	*MICROTUBULE-ASSOCIATED PROTEIN 65-8 (MAP65-8)*	microtubule-associated protein 65-8
AT1G27930	*(AGM1)*	Arabinogalactan methylesterase,involved in arabinogalactan glucuronic acid methylation. Interacts with eIF3.
AT1G27950	*GLYCOSYLPHOSPHATIDYLINOSITOL-ANCHORED LIPID PROTEIN TRANSFER 1 (LTPG1)*	Encodes LTPG1, a lipid transfer protein with a predicted GPI (glycosylphosphatidylinositol)-anchor domain. Localized in the plasma membrane. Disruption of the LTPG1 gene causes alterations of cuticular lipid composition, but no significant changes on total wax and cutin monomer loads are seen. The mRNA is cell-to-cell mobile.
AT1G27990		transmembrane protein
AT1G28010	*ATP-BINDING CASSETTE B14 (ABCB14)*	Encodes an ATP-binding cassette (ABC) transporter. Expressed in the vascular tissue of primary stem.
AT1G28030		2-oxoglutarate (2OG) and Fe(II)-dependent oxygenase superfamily protein
AT1G28040	*ARABIDOPSIS T??XICOS EN LEVADURA 20 (ATL20)*	RING/U-box superfamily protein
AT1G28050	*B-BOX DOMAIN PROTEIN 13 (BBX13)*	B-box type zinc finger protein with CCT domain-containing protein
AT1G28060	*RNA-DIRECTED DNA METHYLATION 16 (RDM16)*	Pre-mRNA-splicing factor 3
AT1G28070		ATP-dependent RNA helicase
AT1G28080		RING finger protein
AT1G28100		hypothetical protein
AT1G28110	*SERINE CARBOXYPEPTIDASE-LIKE 45 (SCPL45)*	serine carboxypeptidase-like 45
AT1G28130	*GRETCHEN HAGEN 3.17 (GH3.17)*	Encodes an IAA-amido synthase that conjugates Asp and other amino acids to auxin in vitro. Lines carrying insertions in this gene are hypersensitive to auxin.
AT1G28135		hypothetical protein
AT1G28140		integral membrane family protein
AT1G28150		hypothetical protein
AT1G28160		encodes a member of the ERF (ethylene response factor) subfamily B-1 of ERF/AP2 transcription factor family. The protein contains one AP2 domain. There are 15 members in this subfamily including ATERF-3, ATERF-4, ATERF-7, and leafy petiole.
AT1G28170	*SULPHOTRANSFERASE 7 (SOT7)*	sulfotransferase 7
AT1G28190		PADRE protein up-regulated after infection by S. sclerotiorum.
AT1G28260		Telomerase activating protein Est1
AT1G28270	*RALF-LIKE 4 (RALFL4)*	Member of a diversely expressed predicted peptide family showing sequence similarity to tobacco Rapid Alkalinization Factor (RALF), and is believed to play an essential role in the physiology of Arabidopsis. Consists of a single exon and is characterized by a conserved C-terminal motif and N-terminal signal peptide. RALF4 and RALF19 act redundantly in the pollen tube to regulate pollen tube growth.
AT1G28280	*MPK3/6-TARGETED VQP 1 (MVQ1)*	VQ motif-containing protein
AT1G28300	*LEAFY COTYLEDON 2 (LEC2)*	Transcription factor that contains a B3 domain, a DNA-binding motif unique to plants and characteristic of several transcription factors. Plays critical roles both early and late during embryo development. LEC2 RNA accumulates primarily during seed development. LEC2 is required for the maintenance of suspensor morphology, specification of cotyledon identity, progression through the maturation phase, and suppression of premature germination. It establishes a cellular environment sufficient to initiate embryo development - ectopic, postembryonic expression of LEC2 in transgenic plants induces the formation of somatic embryos and other organ-like structures and often confers embryonic characteristics to seedlings and to reproductive and vegetative organs of mature plants.
AT1G28327		E3 ubiquitin-protein ligase
AT1G28330	*DORMANCY-ASSOCIATED PROTEIN-LIKE 1 (DYL1)*	dormancy-associated protein (DRM1)
AT1G28360	*ERF DOMAIN PROTEIN 12 (ERF12)*	encodes a member of the ERF (ethylene response factor) subfamily B-1 of ERF/AP2 transcription factor family (ERF12). The protein contains one AP2 domain. There are 15 members in this subfamily including ATERF-3, ATERF-4, ATERF-7, and leafy petiole. Regulates floral development.
AT1G28375		transmembrane protein
AT1G28390		Protein kinase superfamily protein
AT1G28395		hypothetical protein
AT1G28400		GATA zinc finger protein
AT1G28420	*HOMEOBOX-1 (HB-1)*	homeobox-1
AT1G28430	*CYTOCHROME P450, FAMILY 705, SUBFAMILY A, POLYPEPTIDE 24 (CYP705A24)*	member of CYP705A
AT1G28440	*HAESA-LIKE 1 (HSL1)*	HAESA-like 1
AT1G28460	*AGAMOUS-LIKE 59 (AGL59)*	AGAMOUS-like 59
AT1G28470	*NAC DOMAIN CONTAINING PROTEIN 10 (NAC010)*	NAC domain containing protein 10
AT1G28480	*(GRX480)*	Encodes GRX480, a member of the glutaredoxin family that regulates protein redox state. GRX480 interacts with TGA factors and suppresses JA-responsive PDF1.2 transcription. GRX480 transcription is SA-inducible and requires NPR1. Maybe involved in SA/JA cross-talk. It has also been shown to interact with the transcription factor TGA2 and suppress ORA59 promoter activity.
AT1G28500		hypothetical protein (DUF626)
AT1G28510		Optic atrophy 3 protein (OPA3)
AT1G28520	*VASCULAR PLANT ONE ZINC FINGER PROTEIN (VOZ1)*	VOZ transcription factor which acts as positive regulator of several salt-responsive genes. Functionally redundant in salt stress with VOZ2.
AT1G28540		Tail-anchored (TA) OEP membrane protein which possesses a single C-terminal transmembrane domain targeting post-translationally to plastids.

AT1G28550	*RAB GTPASE HOMOLOG A1I (RABA1i)*	RAB GTPase homolog A1I
AT1G28570		SGNH hydrolase-type esterase superfamily protein
AT1G28580		GDSL-motif esterase/acyltransferase/lipase. Enzyme group with broad substrate specificity that may catalyze acyltransfer or hydrolase reactions with lipid and non-lipid substrates.
AT1G28590		GDSL-motif esterase/acyltransferase/lipase. Enzyme group with broad substrate specificity that may catalyze acyltransfer or hydrolase reactions with lipid and non-lipid substrates.
AT1G28600	*GUARD-CELL-ENRICHED GDSL LIPASE 2 (GGL2)*	GDSL-motif esterase/acyltransferase/lipase. Enzyme group with broad substrate specificity that may catalyze acyltransfer or hydrolase reactions with lipid and non-lipid substrates.
AT1G28610	*GUARD-CELL-ENRICHED GDSL LIPASE 3 (GGL3)*	GDSL-motif esterase/acyltransferase/lipase. Enzyme group with broad substrate specificity that may catalyze acyltransfer or hydrolase reactions with lipid and non-lipid substrates.
AT1G28630		transcriptional regulator EFH1-like protein
AT1G28640		GDSL-motif esterase/acyltransferase/lipase. Enzyme group with broad substrate specificity that may catalyze acyltransfer or hydrolase reactions with lipid and non-lipid substrates.
AT1G28650		GDSL-motif esterase/acyltransferase/lipase. Enzyme group with broad substrate specificity that may catalyze acyltransfer or hydrolase reactions with lipid and non-lipid substrates.
AT1G28670	*(ARAB-1)*	Arabidopsis thaliana lipase
AT1G28680	*COUMARIN SYNTHASE (COSY)*	Catalyses trans-cis isomerization and lactonization in the biosynthesis of coumarins in roots.
AT1G28690		Tetratricopeptide repeat (TPR)-like superfamily protein
AT1G28710		Nucleotide-diphospho-sugar transferase family protein
AT1G28960	*NUDIX HYDROLASE HOMOLOG 15 (NUDX15)*	Encodes a ppGpp pyrophosphohydrolase.
AT1G29020		Calcium-binding EF-hand family protein
AT1G29040		50S ribosomal protein L34
AT1G29050	*TRICHOME BIREFRINGENCE-LIKE 38 (TBL38)*	Encodes a member of the TBL (TRICHOME BIREFRINGENCE-LIKE) gene family containing a plant-specific DUF231 (domain of unknown function) domain. TBL gene family has 46 members, two of which (TBR/AT5G06700 and TBL3/AT5G01360) have been shown to be involved in the synthesis and deposition of secondary wall cellulose, presumably by influencing the esterification state of pectic polymers. A nomenclature for this gene family has been proposed (Volker Bischoff & Wolf Scheible, 2010, personal communication).
AT1G29070	*PLASTID RIBOSOMAL PROTEINS OF THE 50S SUBUNIT 34 (PRPL34)*	Ribosomal protein L34
AT1G29080		Papain family cysteine protease
AT1G29090		Cysteine proteinases superfamily protein
AT1G29110		Cysteine proteinases superfamily protein
AT1G29130		
AT1G29140		Pollen Ole e 1 allergen and extensin family protein
AT1G29160	*COGWHEEL1 (COG1)*	Encodes a DOF transcription factor involved in seed coat development. Regulates PRX2 and PRX25, involved in seed longevity.
AT1G29180		Cysteine/Histidine-rich C1 domain family protein
AT1G29195		PADRE protein up-regulated after infection by S. sclerotiorum.
AT1G29200		O-fucosyltransferase family protein
AT1G29230	*CBL-INTERACTING PROTEIN KINASE 18 (CIPK18)*	Encodes a member of the SNF1-related kinase (SnRK) gene family (SnRK3.20), which has also been reported as a member of the CBL-interacting protein kinases (CIPK18).
AT1G29240		transcription initiation factor TFIID subunit, putative (DUF688)
AT1G29270		transcription factor bHLH35-like protein
AT1G29280	*WRKY DNA-BINDING PROTEIN 65 (WRKY65)*	member of WRKY Transcription Factor; Group II-e The mRNA is cell-to-cell mobile.
AT1G29330	*ENDOPLASMIC RETICULUM RETENTION DEFECTIVE 2 (ERD2)*	Encodes a protein similar in sequence to animal and yeast endoplasmic reticulum retention signal receptor. This protein can functionally complement the yeast homologue. Transcript is detected in flower buds, stems, root, and leaves.
AT1G29340	*PLANT U-BOX 17 (PUB17)*	Encodes a protein containing a UND, a U-box, and an ARM domain. This protein has E3 ubiquitin ligase activity. It is required for cell death and full resistance specified by Arabidopsis RPM1 and RPS4 resistance proteins against Pseudomonas syringae pv tomato. The mRNA is cell-to-cell mobile.
AT1G29390	*COLD REGULATED 314 THYLAKOID MEMBRANE 2 (COR314-TM2)*	Integral membrane protein in the inner envelope of chloroplasts. Provide freezing tolerance.
AT1G29395	*COLD REGULATED 314 INNER MEMBRANE 1 (COR413IM1)*	Integral membrane protein in the inner envelope of chloroplasts. Provide freezing tolerance. Expression is induced by short-term cold-treatment, water deprivation, and abscisic acid treatment. involved in response to salt tolerance
AT1G29400	*MEI2-LIKE PROTEIN 5 (ML5)*	A member of mei2-like gene family, predominantly plant-based family of genes encoding RNA binding proteins with characteristic presence of a highly conserved RNA binding motif first described in the mei2 gene of the fission yeast S. pombe. In silico analyses reveal nine mei2 -like genes in A. thaliana. They were grouped into four distinct clades, based on overall sequence similarity and subfamily-specific sequence elements. AML5 is a member of two sister clades of mei2-like gene family, AML1 through AML5, and belongs to the clade named ALM235. Among mei2-like genes, AML5 is the transcript with highest frequency of alternative splicing. Expression was detected during embryo development (heart and torpedo stage) and in vegetative and floral apices.
AT1G29430	*SMALL AUXIN UPREGULATED RNA 62 (SAUR62)*	SAUR762 expression is induced during pollination and expressed in pollen tubes. SAUR62 likely functions in translation of proteins required for pollen tube development/function.
AT1G29440	*SMALL AUXIN UP RNA 63 (SAUR63)*	SAUR-like auxin-responsive protein family
AT1G29450	*SMALL AUXIN UPREGULATED RNA 64 (SAUR64)*	SAUR-like auxin-responsive protein family
AT1G29460	*SMALL AUXIN UPREGULATED RNA 65 (SAUR65)*	SAUR-like auxin-responsive protein family
AT1G29480		hypothetical protein

AT1G29490	*SMALL AUXIN UPREGULATED 68 (SAUR68)*	SAUR-like auxin-responsive protein family
AT1G29500	*SMALL AUXIN UPREGULATED RNA 66 (SAUR66)*	SAUR-like auxin-responsive protein family
AT1G29510	*SMALL AUXIN UPREGULATED RNA 67 (SAUR67)*	This locus was referred to as SAUR68 in PMID:17948056 but the nomenclature should be SAUR67.
AT1G29520		AWPM-19-like family protein
AT1G29530		hypothetical protein
AT1G29570		Zinc finger C-x8-C-x5-C-x3-H type family protein
AT1G29600		Zinc finger C-x8-C-x5-C-x3-H type family protein
AT1G29610		
AT1G29620		Cytochrome C oxidase polypeptide VIB family protein
AT1G29630		5-3 exonuclease family protein
AT1G29650		transposable_element_gene;non-LTR retrotransposon family (LINE), has a 1.5e-28 P-value blast match to GB:NP_038603 L1 repeat, Tf subfamily, member 23 (LINE-element) (Mus musculus);(source:TAIR10)
AT1G29660	*(GGL5)*	GDSL-motif esterase/acyltransferase/lipase. Enzyme group with broad substrate specificity that may catalyze acyltransfer or hydrolase reactions with lipid and non-lipid substrates.
AT1G29670	*(GDSL1)*	GDSL-motif esterase/acyltransferase/lipase. Enzyme group with broad substrate specificity that may catalyze acyltransfer or hydrolase reactions with lipid and non-lipid substrates. The mRNA is cell-to-cell mobile.
AT1G29680		histone acetyltransferase (DUF1264)
AT1G29690	*CONSTITUTIVELY ACTIVATED CELL DEATH 1 (CAD1)*	Encodes a protein containing a domain with significant homology to the MACPF (membrane attack complex and perforin) domain of complements and perforin proteins that are involved in innate immunity in animals. Transgenic <i>cad1-1</i> mutant plants show lesions seen in the hypersensitive response, as well as a spontaneous activation of expression of pathogenesis-related genes and leading to a 32-fold increase in salicylic acid (SA). CAD1 is postulated to act as a negative regulator controlling SA-mediated pathway of programmed cell death in plant immunity.
AT1G29700		Metallo-hydrolase/oxidoreductase superfamily protein
AT1G29720	*RKF-LIKE 1 (RKFL1)*	Encodes one of three RECEPTOR-LIKE KINASE IN FLOWERS 1 (RKF1) paralogues that is required in the stigmatic papillae and the female reproductive tract to promote compatible pollen grain hydration and pollen tube growth.
AT1G29730	*RKF-LIKE 2 (RKFL2)*	Encodes one of three RECEPTOR-LIKE KINASE IN FLOWERS 1 (RKF1) paralogues that is required in the stigmatic papillae and the female reproductive tract to promote compatible pollen grain hydration and pollen tube growth.
AT1G29770		Haloacid dehalogenase-like hydrolase (HAD) superfamily protein
AT1G29780		Haloacid dehalogenase-like hydrolase (HAD) superfamily protein
AT1G29790		S-adenosyl-L-methionine-dependent methyltransferases superfamily protein
AT1G29820		Magnesium transporter CorA-like family protein
AT1G29860	*WRKY DNA-BINDING PROTEIN 71 (WRKY71)*	member of WRKY Transcription Factor; Group II-c
AT1G29870		tRNA synthetase class II (G, H, P and S) family protein
AT1G29910	*CHLOROPHYLL A/B BINDING PROTEIN 3 (CAB3)*	member of Chlorophyll a/b-binding protein family
AT1G29940	*NUCLEAR RNA POLYMERASE A2 (NRPA2)*	Encodes a subunit of RNA polymerase 1 (aka RNA polymerase A).
AT1G29950	*(SACL3)*	basic helix-loop-helix (bHLH) DNA-binding superfamily protein
AT1G29960		Peptidase S24/S26A/S26B/S26C family protein
AT1G30000	*ALPHA-MANNOSIDASE 3 (MNS3)*	alpha-mannosidase 3
AT1G30010	*NUCLEAR MATURASE 1 (NMAT1)*	Analysis of the RNA profiles of wild-type and mutant plants establishes a role for nMAT1 in the trans-splicing of nad1 intron 1 and in the cis-splicing of nad2 intron 1 and nad4 intron 2.
AT1G30020	*(SVB4)*	SVB family gene.
AT1G30040	*GIBBERELLIN 2-OXIDASE (GA2OX2)*	Encodes a gibberellin 2-oxidase that acts on C-19 gibberellins. AtGA2OX2 expression is responsive to cytokinin and KNOX activities.
AT1G30070		SGS domain-containing protein
AT1G30080		Glycosyl hydrolase superfamily protein
AT1G30100	*NINE-CIS-EPOXYCAROTENOID DIOXYGENASE 5 (NCED5)*	Encodes 9-cis-epoxycarotenoid dioxygenase, a key enzyme in the biosynthesis of abscisic acid. The expression of this gene increases during the first 6h of imbibition.
AT1G30110	*NUDIX HYDROLASE HOMOLOG 25 (NUDX25)*	Encodes a ppGpp pyrophosphohydrolase.
AT1G30120	*PYRUVATE DEHYDROGENASE E1 BETA (PDH-E1 BETA)*	Encodes a putative plastid pyruvate dehydrogenase E1 beta subunit that is distinct from the mitochondrial pyruvate dehydrogenase E1 beta subunit.
AT1G30140		Myb/SANT-like DNA-binding domain protein
AT1G30150		transposable_element_gene;CACTA-like transposase family (Tnp1/En/Spm), has a 2.2e-24 P-value blast match to ref|NP_189784.1| TNP1-related protein (Arabidopsis thaliana) (CACTA-element);(source:TAIR10)
AT1G30160	*DUF295 ORGANELLAR A 3 (ATDOA3)*	hypothetical protein (DUF295)
AT1G30200		F-box family protein
AT1G30210	*TEOSINTE BRANCHED 1, CYCLOIDEA, AND PCF FAMILY 24 (TCP24)*	TCP family protein involved in heterochronic regulation of leaf differentiation.
AT1G30220	*INOSITOL TRANSPORTER 2 (INT2)*	Inositol transporter presenting conserved extracellular loop domains homologs of plexins/semaphorin/integrin (PSI) domains from animal type I receptors.
AT1G30240		proline-, glutamic acid/leucine-rich protein
AT1G30250		hypothetical protein
AT1G30260		galactosyltransferase family protein
AT1G30270	*CBL-INTERACTING PROTEIN KINASE 23 (CIPK23)*	Arabidopsis thaliana CBL-interacting protein kinase 23. CIPK23 serves as a positive regulator of the potassium transporter AKT1 by directly phosphorylating AKT1. CIPK23 is activated by the binding of two calcineurin B-like proteins, CBL1 and CBL9. The mRNA is cell-to-cell mobile.

AT1G30300		Metallo-hydrolase/oxidoreductase superfamily protein
AT1G30350		Pectin lyase-like superfamily protein
AT1G30380	*PHOTOSYSTEM I SUBUNIT K (PSAK)*	Encodes subunit K of photosystem I reaction center. The mRNA is cell-to-cell mobile.
AT1G30410	*ATP-BINDING CASSETTE C12 (ABCC12)*	member of MRP subfamily
AT1G30440		Phototropic-responsive NPH3 family protein
AT1G30450	*CATION-CHLORIDE CO-TRANSPORTER 1 (CCC1)*	member of Cation-chloride co-transporter family
AT1G30460	*CLEAVAGE AND POLYADENYLATION SPECIFICITY FACTOR 30 (CPSF30)*	Encodes AtCPSF30, the 30-KDa subunit of cleavage and polyadenylation specificity factor. AtCPSF30 is a probable processing endonuclease. Nucleus-localized RNA binding protein capable of interacting with itself and with calmodulin. Its RNA-binding activity is inhibited by calmodulin in a calcium-dependent fashion.
AT1G30470		SIT4 phosphatase-associated family protein
AT1G30490	*PHAVOLUTA (PHV)*	Dominant PHV mutations cause transformation of abaxial leaf fates into adaxial leaf fates. Has overlapping functions with PHABULOSA, REVOLUTA and CORONA/ATHB15 in patterning the apical portion of the embryo. Encodes a member of HD-Zip family which contains homeodomain-leucine zipper domains and domain similar to a mammalian sterol binding domain.
AT1G30500	*NUCLEAR FACTOR Y, SUBUNIT A7 (NF-YA7)*	nuclear factor Y, subunit A7
AT1G30510	*ROOT FNR 2 (RFNR2)*	Encodes a root-type ferredoxin:NADP(H) oxidoreductase.
AT1G30515		GATA zinc finger protein
AT1G30520	*ACYL-ACTIVATING ENZYME 14 (AAE14)*	Encodes a chloroplast O-succinylbenzoyl-CoA ligase. Involved in phylloquinone biosynthesis. Knock mutant is seedling lethal.
AT1G30530	*UDP-GLUCOSYL TRANSFERASE 78D1 (UGT78D1)*	The At1g30530 gene encodes a UDP-rhamnose:flavonol-3-O-rhamnosyltransferase (UGT78D1) attaching a rhamnosyl residue to the 3-O-position of the flavonols kaempferol and quercetin
AT1G30550		S-adenosyl-L-methionine-dependent methyltransferase superfamily protein
AT1G30560	*GLYCEROL-3-PHOSPHATE PERMEASE 3 (G3Pp3)*	Encodes a member of the phosphate starvation-induced glycerol-3-phosphate permease gene family: AT3G47420(G3Pp1), AT4G25220(G3Pp2), AT1G30560(G3Pp3), AT4G17550(G3Pp4) and AT2G13100(G3Pp5).
AT1G30570	*HERCULES RECEPTOR KINASE 2 (HERK2)*	Encodes HERCULES2 (HERK2), a receptor kinase regulated by Brassinosteroids and required for cell elongation during vegetative growth.
AT1G30610	*EMBRYO DEFECTIVE 2279 (emb2279)*	Pentatricopeptide repeat protein .Mutations in this locus result in embryo lethality due to defects in chloroplast development. Embryo shape at seed maturity is globular.
AT1G30620	*MURUS 4 (MUR4)*	encodes a type-II membrane protein that catalyzes 4-epimerization of UDP-D-Xylose to UDP-L-Arabinose in vitro, the nucleotide sugar used by glycosyltransferases in the arabinosylation of cell wall polysaccharides and wall-resident proteoglycans.
AT1G30640	*NUCLEAR DBF2-RELATED 7 (NDR7)*	Ubiquitously expressed protein kinase.
AT1G30650	*WRKY DNA-BINDING PROTEIN 14 (WRKY14)*	member of WRKY Transcription Factor; Group II-e
AT1G30660	*(TWINKY)*	A truncated version of Twinkle that retains only the DNA primase domain.
AT1G30690	*(PATL4)*	Sec14p-like phosphatidylinositol transfer family protein
AT1G30700	*(ATBBE8)*	FAD-binding Berberine family protein
AT1G30710	*(ATBBE9)*	FAD-binding Berberine family protein
AT1G30720	*(ATBBE10)*	FAD-binding Berberine family protein
AT1G30740	*(ATBBE12)*	FAD-binding Berberine family protein
AT1G30750		TPRXL
AT1G30760	*BERBERINE BRIDGE ENZYME-LIKE 13 (ATBBE-LIKE 13)*	Encodes a BBE-like enzyme that acts in monolignol metabolism by catalyzing the oxidation of aromatic allylic alcohols, such as coumaryl-, sinapyl-, and coniferyl alcohol, to the corresponding aldehydes. The mRNA is cell-to-cell mobile.
AT1G30780		F-box associated ubiquitination effector family protein
AT1G30790		F-box and associated interaction domains-containing protein
AT1G30795		Glycine-rich protein family
AT1G30800		Fasciclin-like arabinogalactan family protein
AT1G30810	*JUMONJI DOMAIN-CONTAINING PROTEIN 18 (JMJ18)*	JMJ18 encodes a novel JmjC domain- containing histone H3K4 demethylase. PHD finger-containing protein.
AT1G30840	*PURINE PERMEASE 4 (PUP4)*	Member of a family of proteins related to PUP1, a purine transporter. May be involved in the transport of purine and purine derivatives such as cytokinins, across the plasma membrane.
AT1G30850	*ROOT HAIR SPECIFIC 4 (RSH4)*	root hair specific 4
AT1G30860		RING/U-box superfamily protein
AT1G30870	*PEROXIDASE7 (PER7)*	Peroxidase superfamily protein
AT1G30880		hypothetical protein
AT1G30900	*VACUOLAR SORTING RECEPTOR 6 (VSR6)*	VACUOLAR SORTING RECEPTOR 6
AT1G30940		pseudogene of F-box family protein
AT1G30950	*UNUSUAL FLORAL ORGANS (UFO)*	Required for the proper identity of the floral meristem. Involved in establishing the whorled pattern of floral organs, in the control of specification of the floral meristem, and in the activation of APETALA3 and PISTILLATA. UFO is found at the AP3 promoter in a LFY-dependent manner, suggesting that it works with LFY to regulate AP3 expression. UFO may also promote the ubiquitylation of LFY.
AT1G30960	*(ERA-2)*	Ortholog of ERA (E. coli RAS-like protein)-related GTPase (ERG). Mitochondrial protein that associates with 18sRNA. Heterozygous mutants segregate for embryo lethality inherited as a sporphytic maternal effect. Increased ROS in the mutant ovule suggests a heritable mitochondrial defect results in lethality.
AT1G30990		Polyketide cyclase/dehydrase and lipid transport superfamily protein
AT1G31000		F-box/associated interaction domain protein

AT1G31030		transposable_element_gene;non-LTR retrotransposon family (LINE), has a 1.0e-41 P-value blast match to GB:NP_038603 L1 repeat, Tf subfamily, member 23 (LINE-element) (Mus musculus);(source:TAIR10)
AT1G31050	*PERICYCLE FACTOR TYPE-A 1 (PFA1)*	Together with PFA2 and PFA3 governs the competence of pericycle cells to initiate lateral root primordium formation.
AT1G31060		
AT1G31070	*N-ACETYLGLUCOSAMINE-1-PHOSPHATE URIDYLYLTRANSFERASE 1 (GlcNAc1pUT1)*	Encodes a protein that functions as an N-acetylglucosamine-1-phosphate uridylyltransferase that catalyzes the formation of UDP-N-acetylglucosamine (UDP-GlcNAc). This is an essential precursor for glycolipid and glycoprotein synthesis and is also used for regulatory protein modification in signaling pathways. The enzyme can also catalyze the reverse reaction using both UDP-GlcNAc and the less common UDP-N-acetylgalactosamine as substrates.
AT1G31080		F-box family protein
AT1G31120	*K+ UPTAKE PERMEASE 10 (KUP10)*	potassium transporter
AT1G31150		K-box region protein (DUF1985)
AT1G31170	*SULFIREDOXIN (SRX)*	encodes a cysteine-sulfinic acid reductase (sulfiredoxin - EC 1.8.98.2) capable of reducing overoxidized plastidic 2-Cys-Prx involved in peroxide detoxification and response to oxidative stress
AT1G31180	*ISOPROPYLMALATE DEHYDROGENASE 3 (IMD3)*	The AtIMD3 is one out of 3 genes encoding the enzyme 3-isopropylmalate dehydrogenase involved in leucine biosynthesis in Arabidopsis. Its subcellular location has been targeted to plastids.
AT1G31200	*PHLOEM PROTEIN 2-A9 (PP2-A9)*	phloem protein 2-A9
AT1G31210		transposable_element_gene;copia-like retrotransposon family, has a 2.3e-253 P-value blast match to dbj|BAA78425.1| polyprotein (Arabidopsis thaliana) (AtRE1) (Ty1_Copia-element);(source:TAIR10)
AT1G31230	*ASPARTATE KINASE-HOMOSERINE DEHYDROGENASE I (AK-HSDH I)*	Encodes a bifunctional aspartate kinase/homoserine dehydrogenase. These two activities catalyze the first and the third steps toward the synthesis of the essential amino acids threonine, isoleucine and methionine.
AT1G31250		proline-rich family protein
AT1G31260	*ZINC TRANSPORTER 10 PRECURSOR (ZIP10)*	member of Fe(II) transporter isolog family
AT1G31270		hypothetical protein
AT1G31280	*ARGONAUTE 2 (AGO2)*	Encodes Argonaute gene that binds viral siRNAs and is involved in antiviral defense response. Regulates innate immunity.Mutants have increased susceptibility to Potato virus X and tobacco rattle virus.
AT1G31290	*ARGONAUTE 3 (AGO3)*	ARGONAUTE 3
AT1G31310		hydroxyproline-rich glycoprotein family protein
AT1G31320	*LOB DOMAIN-CONTAINING PROTEIN 4 (LBD4)*	LOB domain-containing protein 4
AT1G31330	*PHOTOSYSTEM I SUBUNIT F (PSAF)*	Encodes subunit F of photosystem I.
AT1G31335		transmembrane protein
AT1G31350	*KAR-UP F-BOX 1 (KUF1)*	KAR-UP F-box 1
AT1G31360	*RECQ HELICASE L2 (RECQL2)*	Encodes a (d)NTP-dependent 3'->5' DNA helicase. This protein can also disrupt D loop structures and may mediate branch migration of Holliday junctions when tested in vitro. The unwinding activity of the enzyme depends on the presence of divalent cations (Mg2+, Mn2+, or Ca2+, but not Zn2+).(d)NTPs are also required with ATP and dATP supporting the greatest amount of DNA unwinding in vitro.
AT1G31370		Ubiquitin-specific protease family C19-related protein
AT1G31380		TRAF-like family protein
AT1G31400		TRAF-like family protein
AT1G31450	*(ECS1)*	Encodes an egg cell specific endopeptidase that is secreted upon successful fertilization (gamete fusion). The secreted protein targets the pollen attractant, LURE1.2 to effectively block polytuby.
AT1G31490		HXXXD-type acyl-transferase family protein
AT1G31510		F-box associated ubiquitination effector family protein
AT1G31520		hypothetical protein
AT1G31530	*CATABOLITE REPRESSOR 4G (CCR4G)*	Deadenylase.
AT1G31540		Disease resistance protein (TIR-NBS-LRR class) family
AT1G31550		GDSL-motif esterase/acyltransferase/lipase. Enzyme group with broad substrate specificity that may catalyze acyltransfer or hydrolase reactions with lipid and non-lipid substrates.
AT1G31580	*(ECS1)*	Encodes cell wall protein. ECS1 is not a Xcc750 resistance gene, but the genetic data indicate that ECS1 is linked to a locus influencing resistance to Xcc750. The mRNA is cell-to-cell mobile.
AT1G31600	*TRNA METHYLTRANSFERASE 9 (TRM9)*	RNA-binding (RRM/RBD/RNP motifs) family protein
AT1G31620		hypothetical protein
AT1G31630	*AGAMOUS-LIKE 86 (AGL86)*	AGAMOUS-like 86
AT1G31640	*AGAMOUS-LIKE 92 (AGL92)*	A paternally expressed imprinted gene.
AT1G31650	*ROP (RHO OF PLANTS) GUANINE NUCLEOTIDE EXCHANGE FACTOR 14 (ROPGEF14)*	Encodes a member of KPP-like gene family, homolog of KPP (kinase partner protein) gene in tomato. Also a member of the RopGEF (guanine nucleotide exchange factor) family, containing the novel PRONE domain (plant-specific Rop nucleotide exchanger), which is exclusively active towards members of the Rop subfamily.
AT1G31660	*ESSENTIAL NUCLEAR PROTEIN 1 (ENP1)*	Encodes a protein that is a ribosome biogenesis co-factor. Mutants display aberrant RNA processing and male and female gametophyte development.
AT1G31670		Copper amine oxidase family protein
AT1G31680		
AT1G31690	*COPPER AMINE OXIDASE ALPHA 2 (CuAOalpha2)*	Copper amine oxidase family protein
AT1G31700		

AT1G31710	*COPPER AMINE OXIDASE ALPHA 3 (CuAOalpha3)*	Copper amine oxidase family protein
AT1G31720	*MODIFYING WALL LIGNIN-1 (MWL1)*	chitin synthase, putative (DUF1218)
AT1G31750	*GLYCINE AND PROLINE RICH PROTEIN 1 (GPRP1)*	proline-rich family protein
AT1G31770	*ATP-BINDING CASSETTE G14 (ABCG14)*	ATP-binding cassette 14
AT1G31790		Tetratricopeptide repeat (TPR)-like superfamily protein
AT1G31812	*ACYL-COA-BINDING PROTEIN 6 (ACBP6)*	Acyl-CoA-binding protein. Bind acyl-CoA esters and protect acyl-CoAs from degradation by microsomal acyl-hydrolases. Plays a role in determining seed oil content.
AT1G31817	*NUCLEAR FUSION DEFECTIVE 3 (NFD3)*	Ribosomal L18p/L5e family protein
AT1G31820	*POLYAMINE UPTAKE TRANSPORTER 1 (PUT1)*	Encodes POLYAMINE UPTAKE TRANSPORTER 1, an amino acid permease family protein.
AT1G31830	*POLYAMINE UPTAKE TRANSPORTER 2 (PUT2)*	Encodes POLYAMINE UPTAKE TRANSPORTER 2, an amino acid permease family protein.
AT1G31840		Tetratricopeptide repeat (TPR)-like superfamily protein
AT1G31850		S-adenosyl-L-methionine-dependent methyltransferases superfamily protein
AT1G31910		GHMP kinase family protein
AT1G31920		Tetratricopeptide repeat (TPR)-like superfamily protein
AT1G31930	*EXTRA-LARGE GTP-BINDING PROTEIN 3 (XLG3)*	Encodes XLG3 (extra-large G protein 3) that shows significant similarity to the G protein alpha subunit in its C terminal region. Involved in the regulation of root morphological and growth responses.
AT1G31940		cystic fibrosis transmembrane conductance regulator
AT1G31950	*TERPENE SYNTHASE 29 (TPS29)*	Sesterterpene synthase which produces various sesterpne backbones bia type-A cyclization mechanism.
AT1G31960		hypothetical protein
AT1G31970	*STRESS RESPONSE SUPPRESSOR 1 (STRS1)*	DEA(D/H)-box RNA helicase family protein
AT1G32000		hypothetical protein
AT1G32010		myosin heavy chain-like protein
AT1G32020		F-box family protein
AT1G32030		plant-specific B3-DNA-binding domain protein (DUF313)
AT1G32040		transposable_element_gene;similar to unknown protein [Arabidopsis thaliana] (TAIR:AT4G06603.1);(source:TAIR10)
AT1G32060	*PHOSPHORIBULOKINASE (PRK)*	phosphoribulokinase
AT1G32070	*NUCLEAR SHUTTLE INTERACTING (NSI)*	Encodes a acetyltransferase (NSI) that is localized in the nucleus and chloroplast. It interacts with the geminivirus movement protein NSP. This interaction is required for viral infection and systemic spread. Acetylates the viral coat protein (CP) in vitro, but not NSP. NSP inhibits NSI activity in vitro. In the chloroplast NSI functions in the dynamic reorganization thylakoid membrane complexes. NSI is highly transcribed in phloem and in xylem parenchyma cells, and in the apical meristem and guard cells, within young tissues in Arabidopsis, and its expression is turned off as tissues mature.Mutants have reduced melatonin and anthocyanin levels and do not accumulate the PSI-LHCII state transition complex.The protein has distinct lysine acetylation and relaxed N-terminal acetylation specificities on chloroplast proteins as determined by in vitro as well as in vivo analyses using quantitative protein mass spectrometry (PMID:32633465).
AT1G32080	*(LrgB)*	Encodes a plant LrgAB/CidAB protein localized to the chloroplast envelope that is involved in chloroplast development, carbon partitioning, ABA/drought response, and leaf senescence. The gene may have evolved from gene fusion of bacterial lrgA and lrgB.
AT1G32100	*PINORESINOL REDUCTASE 1 (PRR1)*	Encodes a pinoresinol reductase involved in lignan biosynthesis. Expressed strongly in roots and less strongly in stems. Shows specificity for pinoresinol and not lariciresinol.
AT1G32120		serine/threonine-protein phosphatase 7 long form-like protein
AT1G32170	*XYLOGLUCAN ENDOTRANSGLUCOSYLASE/HYDROLASE 30 (XTH30)*	xyloglucan endotransglycosylase-related protein (XTR4) The mRNA is cell-to-cell mobile.
AT1G32180	*CELLULOSE SYNTHASE-LIKE D6 (CSLD6)*	encodes a gene similar to cellulose synthase
AT1G32190		alpha/beta-Hydrolases superfamily protein
AT1G32200	*(ATS1)*	Encodes a chloroplast glycerol-3-phosphate acyltransferase.Involved in the biosynthesis of chloroplast phosphatidylglycerol.
AT1G32220		NAD(P)-binding Rossmann-fold superfamily protein
AT1G32230	*RADICAL-INDUCED CELL DEATH1 (RCD1)*	Encodes a protein belonging to the (ADP-ribosyl)transferase domain-containing subfamily of WWE protein-protein interaction domain protein family. Superoxide radicals are necessary and sufficient to propagate cell death or lesion formation in rcd1 mutants. Without stress treatment, RCD1 is localized in the nucleus. Under high salt or oxidative stress, RCD1 is found not only in the nucleus but also in the cytoplasm. The mRNA is cell-to-cell mobile.
AT1G32240	*KANADI 2 (KAN2)*	Encodes a member of the KANADI family of putative transcription factors. Together with KAN1, this gene appears to be involved in the development of the carpel and the outer integument of the ovule.Along with KAN1 and KAN4 appears to regulate the proper localization of PIN1 in early embryogenesis.
AT1G32250		Calcium-binding EF-hand family protein
AT1G32270	*(ATSYP24)*	member of SYP2 Gene Family
AT1G32290		
AT1G32300	*L -GULONO-1,4-LACTONE (L -GULL) OXIDASE 1 (GULLO1)*	D-arabinono-1,4-lactone oxidase family protein
AT1G32330	*HEAT SHOCK TRANSCRIPTION FACTOR A1D (HSFA1D)*	Member of Heat Stress Transcription Factor (Hsf) family. Negatively regulated by HSP90.2.
AT1G32350	*ALTERNATIVE OXIDASE 1D (AOX1D)*	Alternative oxidase (AOX); terminal oxidases of electron transfer in mitochondria.
AT1G32360		Zinc finger (CCCH-type) family protein
AT1G32375		F-box/RNI-like/FBD-like domains-containing protein
AT1G32390		transposable_element_gene;similar to unknown protein [Arabidopsis thaliana] (TAIR:AT4G11710.1);(source:TAIR10)

AT1G32400	*TOBAMOVIRUS MULTIPLICATION 2A (TOM2A)*	TOM2A encodes a 280 amino acid putative four-pass transmembrane protein with a C-terminal farnesylation signal, essential for efficient multiplication of tobacco mosaic viruses.
AT1G32430		F-box and associated interaction domains-containing protein
AT1G32450	*NRT1/ PTR FAMILY 7.3 (NPF7.3)*	Transmembrane nitrate transporter. Involved in xylem transport of nitrate from root to shoot. Induced in response to high and low concentrations of nitrate. Not involved in nitrate uptake. Expressed in root pericycle cells under the control of MYB59. Also functions as a proton-coupled H+/K+ antiporter for K+ loading into the xylem.
AT1G32470	*(GDC-H1)*	Single hybrid motif superfamily protein
AT1G32480	*ISOCITRATE DEHYDROGENASE IV (IDH-IV)*	Predicted to encode a protein related isocitrate dehydrogenases, but it appears to be missing the sequences encoding the N-terminal portion of the protein.
AT1G32520	*OXIDATION RESISTANCE 4 (OXR4)*	TLDc domain protein
AT1G32530	*PP2CA INTERACTING RING FINGER PROTEIN 2 (PIR2)*	RING/U-box superfamily protein
AT1G32540	*LSD ONE LIKE 1 (LOL1)*	Encodes a protein with 3 plant-specific zinc finger domains that acts as a positive regulator of cell death.
AT1G32550	*FERREDOXIN C 2 (FdC2)*	Encodes FdC2, a ferredoxin protein capable of alternative electron partitioning. FdC1 level increases in conditions of acceptor limitation at PSI.
AT1G32560	*LATE EMBRYOGENESIS ABUNDANT 4-1 (AtLEA4-1)*	Encodes LEA4-1, a member of the Late Embryogenesis Abundant (LEA) proteins which typically accumulate in response to low water availability conditions imposed during development or by the environment.
AT1G32570		hypothetical protein
AT1G32580	*MULTIPLE ORGANELLAR RNA EDITING FACTOR 5 (MORF5)*	Member of MORF family consisting of of nine full-length proteins encoded in the nuclear genome. MORF proteins are required for all RNA editing events in plastids and for many, possibly also all, sites in mitochondria. Potential link between the RNA binding PPR protein and the protein contributing the enzymatic activity in RNA editing.
AT1G32590		transposable_element_gene;copia-like retrotransposon family, has a 1.2e-230 P-value blast match to gb|AAO73527.1| gag-pol polyprotein (Glycine max) (SIRE1) (Ty1_Copia-family);(source:TAIR10)
AT1G32600		F-box associated ubiquitination effector family protein
AT1G32640	*(MYC2)*	Encodes a MYC-related transcriptional activator with a typical DNA binding domain of a basic helix-loop-helix leucine zipper motif. Binds to an extended G-Box promoter motif and interacts with Jasmonate ZIM-domain proteins. MYC2 interacts with EIN3 and EIL1 to repress hook curvature and resistance to Botrytis cinera.Its transcription is induced by dehydration stress, ABA treatment and blue light via CRY1. Negative regulator of blue light-mediated photomorphogenic growth and blue and far-red-light-regulated gene expression. Positive regulator of lateral root formation. Regulates diverse JA-dependent functions. Negatively regulates Trp metabolism and biosynthesis of Trp-derived secondary metabolites. Positively regulates flavonoid biosynthesis, resistance to insects, and response to oxidative stress. Regulates other transcription factors, and negatively regulates its own expression. For example it binds to and regulates the expression of NST1. Its stability is modulated by PUB10 through polyubiquitination.
AT1G32650		hypothetical protein
AT1G32660		F-box and associated interaction domains-containing protein
AT1G32670		
AT1G32680		transposable_element_gene;similar to unknown protein [Arabidopsis thaliana] (TAIR:AT5G35880.1);(source:TAIR10)
AT1G32690		DUF740 family protein
AT1G32700		PLATZ transcription factor family protein
AT1G32740		SBP (S-ribonuclease binding protein) family protein
AT1G32750	*(HAF01)*	This gene is predicted to encode a histone acetyltransferase. Five lines with RNAi constructs directed against HAF1 grow normally and can produce root calli, but have defects in agrobacterium-mediated transformation.
AT1G32760		Glutaredoxin family protein
AT1G32770	*NAC DOMAIN CONTAINING PROTEIN 12 (NAC012)*	Encodes SND1, a NAC Domain transcription factor involved in secondary wall biosynthesis in fibers. Expressed specifically in interfascicular fibers and xylary fibers in stems. Expressed in the procambium of stem inflorescences and root. May act as a negative regulator of secondary wall thickening in xylary fibers. Acts redundantly with NST1 to control development of secondary walls in siliques.
AT1G32780		GroES-like zinc-binding dehydrogenase family protein
AT1G32790	*CTC-INTERACTING DOMAIN 11 (CID11)*	RNA-binding protein, putative, similar to RNA-binding protein GB:CAB40027 GI:4539439 from (Arabidopsis thaliana).Member of a family of PAB2 binding domain proteins.
AT1G32800		
AT1G32830		transposable_element_gene;similar to heat shock protein binding [Arabidopsis thaliana] (TAIR:AT3G30450.1);(source:TAIR10)
AT1G32840		transposable_element_gene;similar to Ulp1 protease family protein [Arabidopsis thaliana] (TAIR:AT3G47260.1);(source:TAIR10)
AT1G32850	*UBIQUITIN-SPECIFIC PROTEASE 11 (UBP11)*	ubiquitin-specific protease 11
AT1G32860		Glycosyl hydrolase superfamily protein
AT1G32870	*NAC DOMAIN PROTEIN 13 (NAC13)*	Expression in rosette leaves is activated by high concentration of boron.
AT1G32880		ARM repeat superfamily protein
AT1G32900	*GRANULE BOUND STARCH SYNTHASE 1 (GBSS1)*	UDP-Glycosyltransferase superfamily protein
AT1G32920		hypothetical protein
AT1G32940	*(SBT3.5)*	Subtilase family protein
AT1G32950		Subtilase family protein
AT1G32960	*(SBT3.3)*	Subtilase family protein
AT1G32970		Subtilisin-like serine endopeptidase family protein
AT1G32980		Subtilisin-like serine endopeptidase family protein

AT1G32990	*PLASTID RIBOSOMAL PROTEIN L11 (PRPL11)*	mutant has Decreased effective quantum yield of photosystem II; Pale green plants; Reduced growth rate; Plastid Ribosomal Protein L11 The mRNA is cell-to-cell mobile.
AT1G33000		transposable_element_gene;similar to nucleic acid binding / ribonuclease H [Arabidopsis thaliana] (TAIR:AT4G08860.1);(source:TAIR10)
AT1G33030		O-methyltransferase family protein
AT1G33050		hypothetical protein
AT1G33055	*HYPOXIA RESPONSE UNKNOWN PROTEIN 32 (HUP32)*	hypothetical protein
AT1G33070		MADS-box family protein
AT1G33080		MATE efflux family protein
AT1G33090		MATE efflux family protein
AT1G33110		MATE efflux family protein
AT1G33130		transposable_element_gene;CACTA-like transposase family (Ptta/En/Spm), has a 1.3e-125 P-value blast match to At5g36655.1/81-333 CACTA-like transposase family (Ptta/En/Spm) (CACTA-element) (Arabidopsis thaliana);(source:TAIR10)
AT1G33170		S-adenosyl-L-methionine-dependent methyltransferases superfamily protein
AT1G33220		Glycosyl hydrolase superfamily protein
AT1G33260		Protein kinase superfamily protein
AT1G33265	*FATTY ACID EXPORT 4 (FAX4)*	Encodes a chloroplast membrane-localized fatty acid exporter that plays a critical roles in transporting plastid fatty acids for TAG biosynthesis during seed and embryo development.
AT1G33270		Acyl transferase/acyl hydrolase/lysophospholipase superfamily protein
AT1G33280	*NAC DOMAIN CONTAINING PROTEIN 15 (NAC015)*	NAC domain protein. SMB, BRN1, and BRN2 act to regulate root cap maturation, in a partially redundant fashion.BRN1 and BRN2, control the cell wall maturation processes that are required to detach root cap layers from the root.
AT1G33320		Pyridoxal phosphate (PLP)-dependent transferases superfamily protein
AT1G33340	*(PICALM8)*	ENTH/ANTH/VHS superfamily protein
AT1G33350		Pentatricopeptide repeat (PPR) superfamily protein
AT1G33410	*SUPPRESSOR OF AUXIN RESISTANCE1 (SAR1)*	Encodes a nucleoporin that regulates CONSTANS (CO) protein stability through affecting nuclear pore complex localization of an E3-ubiquitin ligase, HIGH EXPRESSION OF OSMOTICALLY RESPONSIVE GENES1 (HOS1), which destabilizes CO protein in the morning period.
AT1G33440	*NRT1/ PTR FAMILY 4.4 (NPF4.4)*	Major facilitator superfamily protein
AT1G33450		transposable_element_gene;similar to unknown protein [Arabidopsis thaliana] (TAIR:AT4G04780.2);(source:TAIR10)
AT1G33460		transposable_element_gene;Mutator-like transposase family, has a 3.6e-40 P-value blast match to GB:AAA21566 mudrA of transposon=MuDR (MuDr-element) (Zea mays);(source:TAIR10)
AT1G33470		RNA-binding (RRM/RBD/RNP motifs) family protein
AT1G33480	*ARABIDOPSIS T??XICOS EN LEVADURA 58 (ATL58)*	RING/U-box superfamily protein
AT1G33490		E3 ubiquitin-protein ligase
AT1G33500		tropomyosin
AT1G33540	*SERINE CARBOXYPEPTIDASE-LIKE 18 (scpl18)*	serine carboxypeptidase-like 18
AT1G33560	*ACTIVATED DISEASE RESISTANCE 1 (ADR1)*	Encodes a NBS-LRR disease resistance protein that possesses N-terminal kinase subdomains. Activation tagged mutant of ADR1 showed elevated levels of SA and reactive oxygen species in addition to number of defense gene transcripts. Exhibits resistance to number of microbial pathogens.
AT1G33580		transposable_element_gene;CACTA-like transposase family (Tnp2/En/Spm), has a 5.5e-96 P-value blast match to gb|AAG52024.1|AC022456_5 Tam1-homologous transposon protein TNP2, putative;(source:TAIR10)
AT1G33590		Leucine-rich repeat (LRR) family protein
AT1G33600		Leucine-rich repeat (LRR) family protein
AT1G33610		Leucine-rich repeat (LRR) family protein
AT1G33640		hypothetical protein
AT1G33660		Pseudogene of AT1G33660; peroxidase family protein
AT1G33680		KH domain-containing protein
AT1G33690		
AT1G33700		Beta-glucosidase, GBA2 type family protein
AT1G33710		RNA-directed DNA polymerase (reverse transcriptase)-related family protein
AT1G33720	*CYTOCHROME P450, FAMILY 76, SUBFAMILY C, POLYPEPTIDE 6 (CYP76C6)*	cytochrome P450, family 76, subfamily C, polypeptide 6
AT1G33750	*TERPENE SYNTHASE 22 (TPS22)*	Sesterterpene synthase which produces various sesterpne backbones bia type-A cyclization mechanism.
AT1G33760	*ETHYLENE RESPONSE FACTOR022 (ERF022)*	encodes a member of the DREB subfamily A-4 of ERF/AP2 transcription factor family. The protein contains one AP2 domain. There are 17 members in this subfamily including TINY.
AT1G33770		Protein kinase superfamily protein
AT1G33790		jacalin lectin family protein
AT1G33800	*GLUCURONOXYLAN METHYLTRANSFERASE 1 (GXMT1)*	Encodes a glucuronoxylan(GX)-speciﬁc 4-O-methyltransferase responsible for methylating GlcA residues in GX. Reduced methylation of GX ingxmt1-1 plants is correlated with altered lignin composition. The mRNA is cell-to-cell mobile.
AT1G33811	*(GGL7)*	GDSL-motif esterase/acyltransferase/lipase. Enzyme group with broad substrate specificity that may catalyze acyltransfer or hydrolase reactions with lipid and non-lipid substrates.
AT1G33813		transposable_element_gene;copia-like retrotransposon family, has a 5.8e-39 P-value blast match to gb|AAO73529.1| gag-pol polyprotein (Glycine max) (SIRE1) (Ty1_Copia-family);(source:TAIR10)

AT1G33817		transposable_element_gene;copia-like retrotransposon family, has a 8.1e-100 P-value blast match to gb|AAO73521.1| gag-pol polyprotein (Glycine max) (SIRE1) (Ty1_Copia-family);(source:TAIR10)
AT1G33820		hypothetical protein
AT1G33830	*IMMUNE ASSOCIATED NUCLEOTIDE BINDING 1 (IAN1)*	P-loop containing nucleoside triphosphate hydrolases superfamily protein
AT1G33840		LURP-one-like protein (DUF567)
AT1G33850		Ribosomal protein S19 family protein
AT1G33860		hypothetical protein
AT1G33870		P-loop containing nucleoside triphosphate hydrolases superfamily protein
AT1G33880	*IMMUNE ASSOCIATED NUCLEOTIDE BINDING 2 (IAN2)*	One of a cluster of paralogs (IAN2-6) that are associated with variation in heat tolerance.
AT1G33890	*IMMUNE ASSOCIATED NUCLEOTIDE BINDING 3 (IAN3)*	One of a cluster of paralogs (IAN2-6) that are associated with variation in heat tolerance.
AT1G33900	*IMMUNE ASSOCIATED NUCLEOTIDE BINDING 4 (IAN4)*	One of a cluster of paralogs (IAN2-6) that are associated with variation in heat tolerance.
AT1G33910	*IMMUNE ASSOCIATED NUCLEOTIDE BINDING 5 (IAN5)*	One of a cluster of paralogs (IAN2-6) that are associated with variation in heat tolerance.
AT1G33920	*PHLOEM PROTEIN 2-A4 (PP2-A4)*	phloem protein 2-A4
AT1G33930	*IMMUNE ASSOCIATED NUCLEOTIDE BINDING 6 (IAN6)*	One of a cluster of paralogs (IAN2-6) that are associated with variation in heat tolerance.
AT1G33940		Serine/Threonine-kinase ULK4-like protein
AT1G33950	*IMMUNE ASSOCIATED NUCLEOTIDE BINDING 7 (IAN7)*	Avirulence induced gene (AIG1) family protein
AT1G33960	*AVRRPT2-INDUCED GENE 1 (AIG1)*	Identified as a gene that is induced by avirulence gene avrRpt2 and RPS2 after infection with Pseudomonas syringae pv maculicola strain ES4326 carrying avrRpt2
AT1G33970	*IMMUNE ASSOCIATED NUCLEOTIDE BINDING 9 (IAN9)*	IAN9 is a member of a small family of proteins. It's expression is repressed upon pathogen infection and loss of function mutants show increased resistance to bacterial pathogens.
AT1G33980	*(UPF3)*	Involved in mRNA surveillance, detects exported mRNAs with truncated open reading frames and initiates nonsense-mediated mRNA decay (NMD). Regulates AT1G72910, AT1G72940, and ADR1-LIKE 2 in a temperature dependent manner.
AT1G34000	*ONE-HELIX PROTEIN 2 (OHP2)*	Encodes a novel member of the Lhc family from Arabidopsis with one predicted transmembrane alpha-helix closely related to helix I of Lhc protein from PSI (Lhca4). Gene expression is triggered by light stress and both transcript and protein accumulate in a light intensity-dependent manner. Ohp2 is associated with PSI under low- or high-light conditions. Together with OHP1, OHP2 is essential for the formation of photosystem II reaction center, even though neither is a part of the final PSII RC. It forms a complex with OHP1 and HCF244, D1, D2, PsbI, and cytochrome b559 at an early stage of PSII de novo assembly and of PSII repair under high-light conditions.
AT1G34010		hypothetical protein
AT1G34040	*TRYPTOPHAN AMINOTRANSFERASE RELATED 3 (TAR3)*	Pyridoxal phosphate (PLP)-dependent transferases superfamily protein
AT1G34065	*S-ADENOSYLMETHIONINE CARRIER 2 (SAMC2)*	S-adenosylmethionine carrier 2
AT1G34070		Copia-like polyprotein/retrotransposon
AT1G34080		transposable_element_gene;gypsy-like retrotransposon family, has a 4.0e-88 P-value blast match to GB:AAD19359 polyprotein (gypsy_Ty3-element) (Sorghum bicolor);(source:TAIR10)
AT1G34090		transposable_element_gene;copia-like retrotransposon family, has a 8.8e-131 P-value blast match to GB:BAA78423 polyprotein (Ty1_Copia-element) (Arabidopsis thaliana)GB:BAA78423 polyprotein (Ty1_Copia-element) (Arabidopsis thaliana)GB:BAA78423 polyprotein (Ty1_Copia-element) (Arabidopsis thaliana)gi|4996361|dbj|BAA78423.1| polyprotein (Arabidopsis thaliana) (Ty1_Copia-element);(source:TAIR10)
AT1G34100		pseudogene of Protein kinase superfamily protein
AT1G34110	*RGF1 INSENSITIVE 5 (RGI5)*	Leucine-rich receptor-like protein kinase family protein
AT1G34130	*STAUROSPORIN AND TEMPERATURE SENSITIVE 3-LIKE B (STT3B)*	Encodes homolog of yeast STT3, a subunit of oligosaccharyltransferase.
AT1G34140	*POLY(A) BINDING PROTEIN 1 (PAB1)*	polyadenylate-binding protein, putative / PABP, putative, non-consensus splice donor TA at exon 1; similar to polyadenylate-binding protein (poly(A)-binding protein) from (Triticum aestivum) GI:1737492, (Nicotiana tabacum) GI:7673355, {Arabidopsis thaliana} SP:P42731; contains InterPro entry IPR000504: RNA-binding region RNP-1 (RNA recognition motif) (RRM). Only member of the class IV PABP family.
AT1G34150		Pseudouridine synthase family protein
AT1G34170	*AUXIN RESPONSE FACTOR 13 (ARF13)*	AUXIN RESPONSE FACTOR 13
AT1G34180	*NAC DOMAIN CONTAINING PROTEIN 16 (NAC016)*	NAC domain containing protein 16
AT1G34200		Glyceraldehyde-3-phosphate dehydrogenase-like family protein
AT1G34240		transposable_element_gene;hAT-like transposase family (hobo/Ac/Tam3), has a 5.6e-48 P-value blast match to GB:AAD24567 transposase Tag2 (hAT-element) (Arabidopsis thaliana);(source:TAIR10)
AT1G34245	*EPIDERMAL PATTERNING FACTOR 2 (EPF2)*	Encodes a secretory peptide EPF2 expressed in proliferating cells of the stomatal lineage, known as meristemoids, and in guard mother cells, the progenitors of stomata. Controls asymmetric cell divisions during stomatal development. EPF2 is related to EPF1, also involved in stomatal development. Its transcript levels change after inducing MUTE expression in a mute background. EPF2 binds to the ER receptor triggering MAPK activation that in turn inhibits stomatal development. EPF2 competes with STOMAGEN for binding to receptor protein kinases ER, and TMM.
AT1G34260	*FORMS APLOID AND BINUCLEATE CELLS 1A (FAB1D)*	Encodes a protein that is predicted to act as a phosphatidylinositol-3P 5-kinase, but, because it lacks a FYVE domain, it is unlikely to be efficiently targeted to membranes containing the porposed phosphatidylinositol-3P substrate. Therefore, its molecular function remains unknown. The mRNA is cell-to-cell mobile.
AT1G34280		
AT1G34290	*RECEPTOR LIKE PROTEIN 5 (RLP5)*	receptor like protein 5
AT1G34300		lectin protein kinase family protein
AT1G34310	*AUXIN RESPONSE FACTOR 12 (ARF12)*	auxin response factor 12
AT1G34330		Possibly not a pseudogene based on evidence for transcription (RNA-seq) and translation (Ribo-seq) described in PMID:27791167

AT1G34340		alpha/beta-Hydrolases superfamily protein
AT1G34355	*PARALLEL SPINDLE 1 (PS1)*	Encodes PS1 (Parallel Spindle 1). Mutations in PS1 lead to diploid male spores, diploid pollen grains, and spontaneous triploid plants in the next generation. Female meiosis is not affected in the mutants.
AT1G34360	*(ATINFC-1)*	translation initiation factor 3 (IF-3) family protein
AT1G34400		hypothetical protein
AT1G34410	*AUXIN RESPONSE FACTOR 21 (ARF21)*	auxin response factor 21
AT1G34420		leucine-rich repeat transmembrane protein kinase family protein
AT1G34430	*EMBRYO DEFECTIVE 3003 (EMB3003)*	2-oxoacid dehydrogenases acyltransferase family protein
AT1G34440		transmembrane protein
AT1G34460	*CYCLIN B1;5 (CYCB1;5)*	B1 type cyclin
AT1G34480		Cysteine/Histidine-rich C1 domain family protein
AT1G34500		MBOAT (membrane bound O-acyl transferase) family protein
AT1G34510		Peroxidase superfamily protein
AT1G34520		MBOAT (membrane bound O-acyl transferase) family protein
AT1G34530		transposable_element_gene;CACTA-like transposase family (Ptta/En/Spm), has a 7.8e-116 P-value blast match to At5g36655.1/81-333 CACTA-like transposase family (Ptta/En/Spm) (CACTA-element) (Arabidopsis thaliana);(source:TAIR10)
AT1G34540	*CYTOCHROME P450, FAMILY 94, SUBFAMILY D, POLYPEPTIDE 1 (CYP94D1)*	member of CYP94D
AT1G34545		transposable_element_gene;copia-like retrotransposon family, has a 3.0e-112 P-value blast match to GB:CAA72990 open reading frame 2 (Ty1_Copia-element) (Brassica oleracea);(source:TAIR10)
AT1G34550	*EMBRYO DEFECTIVE 2756 (EMB2756)*	transmembrane protein (DUF616)
AT1G34575	*(ATBBE14)*	FAD-binding Berberine family protein
AT1G34580		Major facilitator superfamily protein
AT1G34590		transposable_element_gene;similar to unknown protein [Arabidopsis thaliana] (TAIR:AT4G06603.1);(source:TAIR10)
AT1G34620		transposable_element_gene;Mutator-like transposase family, has a 8.5e-75 P-value blast match to Q9SJR8 /172-333 Pfam PF03108 MuDR family transposase (MuDr-element domain);(source:TAIR10)
AT1G34640		peptidase
AT1G34650	*HOMEODOMAIN GLABROUS 10 (HDG10)*	Encodes a homeobox-leucine zipper family protein belonging to the HD-ZIP IV family.
AT1G34660		transposable_element_gene;CACTA-like transposase family (En/Spm), has a 1.1e-114 P-value blast match to GB:BAA20532 ORF of transposon Tdc1 (CACTA-element) (Daucus carota);(source:TAIR10)
AT1G34670	*MYB DOMAIN PROTEIN 93 (MYB93)*	Encodes a member of the R2R3 transcription factor gene family that is a negative regulator of lateral root (LR) development. It has been proposed that this transcription factor is part of a novel negative feedback loop stimulated specifically in the endodermis upon LR initiation to ensure that LRs are formed only in the correct place.
AT1G34740		transposable_element_gene;similar to Ulp1 protease family protein [Arabidopsis thaliana] (TAIR:AT4G05280.1);(source:TAIR10)
AT1G34750	*CERK-1 INTERACTING PROTEIN PHOSPHATASE 1 (CIPP1)*	Protein phosphatase 2C family protein
AT1G34760	*GENERAL REGULATORY FACTOR 11 (GRF11)*	Encodes a 14-3-3 protein. Binds H+-ATPase in response to blue light.
AT1G34842		transposable_element_gene;non-LTR retroelement reverse transcriptase;(source:TAIR10)
AT1G34967		transposable_element_gene;copia-like retrotransposon family, has a 8.6e-83 P-value blast match to GB:CAA72989 open reading frame 1 (Ty1_Copia-element) (Brassica oleracea);(source:TAIR10)
AT1G35030		hypothetical protein
AT1G35040		hypothetical protein
AT1G35050		transposable_element_gene;copia-like retrotransposon family, has a 1.8e-61 P-value blast match to GB:CAA32025 ORF (Ty1_Copia-element) (Nicotiana tabacum)GB:CAA32025 ORF (Ty1_Copia-element) (Nicotiana tabacum);(source:TAIR10)
AT1G35140	*PHOSPHATE-INDUCED 1 (PHI-1)*	EXL1 is involved in the C-starvation response. Phenotypic changes of an exl1 loss of function mutant became evident only under corresponding experimental conditions. For example, the mutant showed diminished biomass production in a short-day/low light growth regime, impaired survival during extended night, and impaired survival of anoxia stress.
AT1G35143		transposable_element_gene;similar to replication protein-related [Arabidopsis thaliana] (TAIR:AT1G52950.1);(source:TAIR10)
AT1G35150		General transcription factor 2-related zinc finger protein
AT1G35180		TRAM, LAG1 and CLN8 (TLC) lipid-sensing domain containing protein
AT1G35183		zinc finger, C3HC4 type (RING finger) protein
AT1G35190		2-oxoglutarate (2OG) and Fe(II)-dependent oxygenase superfamily protein
AT1G35200		pseudogene of Ribosomal protein L4/L1 family
AT1G35210		hypothetical protein
AT1G35230	*ARABINOGALACTAN PROTEIN 5 (AGP5)*	Encodes arabinogalactan-protein (AGP5). The mRNA is cell-to-cell mobile.
AT1G35250	*ACYL-LIPID THIOESTERASE 2 (ALT2)*	Thioesterase superfamily protein
AT1G35260	*MLP-LIKE PROTEIN 165 (MLP165)*	MLP-like protein 165
AT1G35280		transposable_element_gene;CACTA-like transposase family (Tnp2/En/Spm), has a 2.5e-207 P-value blast match to gb|AAG52024.1|AC022456_5 Tam1-homologous transposon protein TNP2, putative;(source:TAIR10)
AT1G35290	*ACYL-LIPID THIOESTERASE 1 (ALT1)*	Thioesterase superfamily protein
AT1G35310	*MLP-LIKE PROTEIN 168 (MLP168)*	MLP-like protein 168
AT1G35320		transmembrane protein

AT1G35330	*ARABIDOPSIS T??XICOS EN LEVADURA 34 (ATL34)*	RING/U-box superfamily protein
AT1G35340		ATP-dependent protease La (LON) domain protein
AT1G35350		EXS (ERD1/XPR1/SYG1) family protein
AT1G35370		transposable_element_gene;gypsy-like retrotransposon family, has a 8.7e-191 P-value blast match to GB:AAD22153 polyprotein (Gypsy_Ty3-element) (Sorghum bicolor);(source:TAIR10)
AT1G35380		transposable_element_gene;retrotransposon family;(source:TAIR10)
AT1G35410		hypothetical protein (DUF1184)
AT1G35420		alpha/beta-Hydrolases superfamily protein
AT1G35430		transmembrane protein
AT1G35480		transposable_element_gene;hAT-like transposase family (hobo/Ac/Tam3), has a 8.9e-45 P-value blast match to GB:AAD24567 transposase Tag2 (hAT-element) (Arabidopsis thaliana);(source:TAIR10)
AT1G35490		bZIP family transcription factor
AT1G35500		hypothetical protein
AT1G35513		pseudogene of isochorismate synthase-related / isochorismate mutase-related
AT1G35516		myb-like transcription factor family protein
AT1G35530	*FANCONI ANEMIA COMPLEMENTATION GROUP M (FANCM)*	Encodes FANCM, a highly conserved helicase that functions as a major factor limiting meiotic crossover formation. It is not directly involved in the repair of DNA lesions but suppresses spontaneous somatic homologous recombination via a RecQ helicase (At-RECQ4A)-independent pathway.
AT1G35555		
AT1G35560	*TCP DOMAIN PROTEIN 23 (TCP23)*	Encodes a member of the TCP-P subfamily that is involved in flowering time control and plant development. Mutants present an early flowering phenotype.
AT1G35570		transposable_element_gene;similar to unknown protein [Arabidopsis thaliana] (TAIR:AT4G11710.1);(source:TAIR10)
AT1G35580	*CYTOSOLIC INVERTASE 1 (CINV1)*	CINV1 / A/N-InvG is an alkaline/neutral invertase that breaks sucrose down into fructose and glucose (GH100). The exact localization of CINV1 remains under investigation but there is evidence that fluorescently-tagged CINV1 localizes to the cytoplasm. atinvg mutants have reduced root growth, reduced invertase activity, and increased expression of antioxidant genes under basal conditions. The levels of CINV1 / A/N-InvG transcripts rise in response to a hydrogen peroxide treatment. The protein has been shown to interact with PIP5K9.
AT1G35590		transposable_element_gene;CACTA-like transposase family (Tnp2/En/Spm), has a 1.2e-150 P-value blast match to GB:CAA40555 TNP2 (CACTA-element) (Antirrhinum majus);(source:TAIR10)
AT1G35610		Cysteine/Histidine-rich C1 domain family protein
AT1G35612		pseudogene of Ulp1 protease family protein
AT1G35614		hypothetical protein
AT1G35617		hypothetical protein
AT1G35625		RING/U-box superfamily protein
AT1G35640		transposable_element_gene
AT1G35647		transposable_element_gene;gypsy-like retrotransposon family, has a 3.6e-209 P-value blast match to GB:AAD27547 polyprotein (Gypsy_Ty3-element) (Oryza sativa subsp. indica);(source:TAIR10)
AT1G35650		transposable_element_gene;similar to Ulp1 protease family protein [Arabidopsis thaliana] (TAIR:AT2G29240.1);(source:TAIR10)
AT1G35660		erythroid differentiation factor-like protein
AT1G35663		transposable_element_gene;similar to DNA binding / transposase [Arabidopsis thaliana] (TAIR:AT4G04635.1);(source:TAIR10)
AT1G35670	*CALCIUM-DEPENDENT PROTEIN KINASE 2 (CDPK2)*	Encodes a Ca(2+)-dependent, calmodulin-independent protein kinase that is rapidly induced by drought and high-salt stress but not by low-temperature stress or heat stress. Positive regulator of ABA signaling. Phosphorylates ABA responsive transcription factors ABF1 and ABF4.
AT1G35680	*CHLOROPLAST RIBOSOMAL PROTEIN L21 (RPL21C)*	Encodes a chloroplast ribosomal protein L21 that is required for chloroplast development and embryogenesis. The mRNA is cell-to-cell mobile.
AT1G35710		kinase family with leucine-rich repeat domain-containing protein
AT1G35720	*ANNEXIN 1 (ANNAT1)*	Encodes a member of the annexin gene family, a diverse, multigene family of calcium-dependent, membrane-binding proteins. The protein was determined to have peroxidase activity. This activity is thought to be dependent on the presence of post-translational modifications (most likely phosphorylation). The protein was shown to be present as a mixture of monomer and homodimer. The homodimerization seems to be dependent on the presence of Ca2+ or H2O2. The dimerization was prevented by the addition of DTT, β-mercaptoethanol and TCEP. Annat1 mRNA is expressed in flowers, roots,leaves and stems and is most abundant in stems. mRNA levels are increased in response to oxidative stress. Developmental expression patterns suggest a role in Golgi-mediated polysaccharide secretion. It is a Ca 2+-permeable transporter providing a molecular link between reactive oxygen species and cytosolic Ca 2+ in plants. The mRNA is cell-to-cell mobile.
AT1G35730	*PUMILIO 9 (PUM9)*	Encodes a member of the Arabidopsis Pumilio (APUM) proteins containing PUF domain (eight repeats of approximately 36 amino acids each). PUF proteins regulate both mRNA stability and translation through sequence-specific binding to the 3' UTR of target mRNA transcripts.
AT1G35740		pseudogene of glucan synthase-like 9
AT1G35750	*PUMILIO 10 (PUM10)*	Encodes a member of the Arabidopsis Pumilio (APUM) proteins containing PUF domain (eight repeats of approximately 36 amino acids each). PUF proteins regulate both mRNA stability and translation through sequence-specific binding to the 3' UTR of target mRNA transcripts.
AT1G35770		transposable_element_gene;similar to Ulp1 protease family protein [Arabidopsis thaliana] (TAIR:AT2G06860.1);(source:TAIR10)
AT1G35780		N-lysine methyltransferase
AT1G35790		transposable_element_gene;gypsy-like retrotransposon family (Athila), has a 1.3e-42 P-value blast match to GB:CAA57397 Athila ORF 1 (Arabidopsis thaliana);(source:TAIR10)
AT1G35820		heat shock protein

AT1G35850	*PUMILIO 17 (PUM17)*	Encodes a member of the Arabidopsis Pumilio (APUM) proteins containing PUF domain (eight repeats of approximately 36 amino acids each). PUF proteins regulate both mRNA stability and translation through sequence-specific binding to the 3' UTR of target mRNA transcripts.
AT1G35860	*TRANSLOCON OUTER MEMBRANE COMPLEX 75-I (TOC75-I)*	TOC75 pseudogene due to a 5.4-kb gypsy/Ty3-related retrotransposon inserted at the 5' end of the gene
AT1G35880		hypothetical protein
AT1G35890		NAC (No Apical Meristem) domain transcriptional regulator superfamily protein
AT1G35900		
AT1G35910	*TREHALOSE-6-PHOSPHATE PHOSPHATASE D (TPPD)*	Haloacid dehalogenase-like hydrolase (HAD) superfamily protein
AT1G35940		transposable_element_gene;similar to unknown protein [Arabidopsis thaliana] (TAIR:AT3G13250.1);(source:TAIR10)
AT1G35970		transposable_element_gene;gypsy-like retrotransposon family (Athila), has a 3.8e-37 P-value blast match to GB:CAA57397 Athila ORF 1 (Arabidopsis thaliana);(source:TAIR10)
AT1G35995		transposable_element_gene;Mutator-like transposase family, has a 4.4e-38 P-value blast match to GB:AAA21566 mudrA of transposon=MuDR (MuDr-element) (Zea mays);(source:TAIR10)
AT1G36000	*LOB DOMAIN-CONTAINING PROTEIN 5 (LBD5)*	LOB domain-containing protein 5
AT1G36020		DEAD/DEAH-box RNA helicase family protein
AT1G36030		Encodes a member of the F-box family, whose members involved in regulating diverse cellular processes including cell cycle transition, transcriptional regulation and signal transduction.
AT1G36050		Endoplasmic reticulum vesicle transporter protein
AT1G36060	*TRANSLUCENT GREEN (TG)*	encodes a member of the DREB subfamily A-6 of ERF/AP2 transcription factor family. The protein contains one AP2 domain. There are 8 members in this subfamily including RAP2.4.Overexpression results in increased drought tolerance and vitrified leaves. Binds to DRE/GCC promoter elements and activates expression of aquaporin genes AtTIP1;1, AtTIP2;3, and AtPIP2;2.
AT1G36070		Transducin/WD40 repeat-like superfamily protein
AT1G36095		DNA binding protein
AT1G36100		myosin heavy chain-like protein
AT1G36105		transposable_element_gene;Mutator-like transposase family, has a 1.7e-33 P-value blast match to GB:AAA21566 mudrA of transposon=MuDR (MuDr-element) (Zea mays);(source:TAIR10)
AT1G36110		transposable_element_gene;copia-like retrotransposon family, has a 2.2e-59 P-value blast match to gb|AAO73521.1| gag-pol polyprotein (Glycine max) (SIRE1) (Ty1_Copia-family);(source:TAIR10)
AT1G36120		transposable_element_gene;gypsy-like retrotransposon family, has a 9.8e-283 P-value blast match to GB:AAD27547 polyprotein (Gypsy_Ty3-element) (Oryza sativa subsp. indica);(source:TAIR10)
AT1G36130		transposable_element_gene;gypsy-like retrotransposon family (Athila), has a 4.2e-84 P-value blast match to GB:CAA57397 Athila ORF 1 (Arabidopsis thaliana);(source:TAIR10)
AT1G36150	*GLYCOSYLPHOSPHATIDYLINOSITOL-ANCHORED LIPID PROTEIN TRANSFER 33 (LTPG33)*	Bifunctional inhibitor/lipid-transfer protein/seed storage 2S albumin superfamily protein
AT1G36160	*ACETYL-COA CARBOXYLASE 1 (ACC1)*	Encodes acetyl-CoA carboxylase. Mutant displays uncoordinated cell divisions which are enhanced by cytokinins. Mutant also has aberrant organization of the apical region in the embryo and abnormal root and shoot development and is deficient in freezing tolerance after cold acclimation. Essential for very long chain fatty acid elongation. The mRNA is cell-to-cell mobile.
AT1G36180	*ACETYL-COA CARBOXYLASE 2 (ACC2)*	acetyl-CoA carboxylase 2 (ACC2) The mRNA is cell-to-cell mobile.
AT1G36190		transposable_element_gene;CACTA-like transposase family (Ptta/En/Spm), has a 8.5e-119 P-value blast match to At1g36190.1/92-340 CACTA-like transposase family (Ptta/En/Spm) (CACTA-element) (Arabidopsis thaliana);(source:TAIR10)
AT1G36230		hypothetical protein
AT1G36310	*TRNA METHYLTRANSFERASE 9 (TRM9)*	S-adenosyl-L-methionine-dependent methyltransferases superfamily protein
AT1G36340	*UBIQUITIN-CONJUGATING ENZYME 31 (UBC31)*	ubiquitin-conjugating enzyme 31
AT1G36370	*MORE SULPHUR ACCUMULATION1 (MSA1)*	Encodes a nuclear localised protein MSA1 (MORE SULPHUR ACCUMULATION1). Epigenetically regulates sulphur homeostasis. Has sequence similarity to SHM (serine hydroxymethyltransferase) but lacks SHM activity in vitro.
AT1G36380		transmembrane protein
AT1G36390	*CHLOROPLAST GRPE 2 (CGE2)*	Chloroplast GrpE protein.
AT1G36430		transposable_element_gene;retrotransposon family;(source:TAIR10)
AT1G36440		transposable_element_gene;pseudogene, hypothetical protein, contains Pfam profile PF03384: Drosophila protein of unknown function, DUF287;(source:TAIR10)
AT1G36470		transposable_element_gene;CACTA-like transposase family (Tnp2/En/Spm), has a 4.8e-152 P-value blast match to GB:CAA40555 TNP2 (CACTA-element) (Antirrhinum majus);(source:TAIR10)
AT1G36480		transposable_element_gene;CACTA-like transposase family (En/Spm), has a 1.6e-35 P-value blast match to dbj|BAB64937.1| TdcA1-ORF1-ORF2 (Daucus carota) Spm/En-like (CACTA-like);(source:TAIR10)
AT1G36510		Nucleic acid-binding proteins superfamily
AT1G36550		transposable_element_gene;similar to retrotransposon protein, putative, Ty1-copia subclass [Oryza sativa (japonica cultivar-group)] (GB:ABA98367.2);(source:TAIR10)
AT1G36560		transposable_element_gene;gypsy-like retrotransposon family (Athila), has a 3.1e-108 P-value blast match to GB:CAA57397 Athila ORF 1 (Arabidopsis thaliana);(source:TAIR10)
AT1G36590		transposable_element_gene;gypsy-like retrotransposon family, has a 2.7e-200 P-value blast match to GB:AAD27547 polyprotein (Gypsy_Ty3-element) (Oryza sativa subsp. indica);(source:TAIR10)

AT1G36600		transposable_element_gene;copia-like retrotransposon family, has a 8.9e-21 P-value blast match to gb|AAG52950.1| putative envelope protein (Endovir1-1) (Arabidopsis thaliana) (Ty1_Copia-family);(source:TAIR10)
AT1G36640		transmembrane protein
AT1G36670		transposable_element_gene;similar to unknown protein [Arabidopsis thaliana] (TAIR:AT1G37050.1);(source:TAIR10)
AT1G36756		hypothetical protein
AT1G36810		transposable_element_gene;pseudogene, hypothetical protein;(source:TAIR10)
AT1G36820		
AT1G36830		transposable_element_gene;gypsy-like retrotransposon family, has a 5.9e-38 P-value blast match to GB:BAA84458 GAG-POL precursor (gypsy_Ty3-element) (Oryza sativa)gi|5902445|dbj|BAA84458.1| GAG-POL precursor (Oryza sativa (japonica cultivar-group)) (RIRE2) (Gypsy_Ty3-family);(source:TAIR10)
AT1G36900		
AT1G36910		transposable_element_gene;CACTA-like transposase family (Ptta/En/Spm), has a 4.0e-81 P-value blast match to At5g29026.1/8-244 CACTA-like transposase family (Ptta/En/Spm) (CACTA-element) (Arabidopsis thaliana);(source:TAIR10)
AT1G36920		hypothetical protein
AT1G36940		myotubularin-like protein
AT1G36970		transmembrane protein, putative (DUF1985)
AT1G37000		Beta-galactosidase related protein
AT1G37010		spindle pole body component
AT1G37020		Cysteine proteinases superfamily protein
AT1G37057		transposable_element_gene;hAT-like transposase family (hobo/Ac/Tam3), has a 7.8e-25 P-value blast match to GB:AAD24567 transposase Tag2 (hAT-element) (Arabidopsis thaliana);(source:TAIR10)
AT1G37060		transposable_element_gene;gypsy-like retrotransposon family (Athila), has a 1.4e-248 P-value blast match to GB:CAA57397 Athila ORF 1 (Arabidopsis thaliana);(source:TAIR10)
AT1G37063		transposable_element_gene;transposase-related, weak similarity to Tam3-transposase (Antirrhinum majus) GI:16064, PIR|A39652 Hobo element transposase HFL1 (Drosophila melanogaster);(source:TAIR10)
AT1G37080		transposable_element_gene;similar to DNA binding [Arabidopsis thaliana] (TAIR:AT4G01980.1);(source:TAIR10)
AT1G37110		transposable_element_gene;copia-like retrotransposon family, has a 0. P-value blast match to GB:CAA31653 polyprotein (Ty1_Copia-element) (Arabidopsis thaliana);(source:TAIR10)
AT1G37130	*NITRATE REDUCTASE 2 (NIA2)*	Identified as a mutant resistant to chlorate. Encodes nitrate reductase structural gene. Involved in nitrate assimilation. Has nitrate reductase activity. Up-regulated by the fungus P. indica. Binds transcription factor At2g35940. The mRNA is cell-to-cell mobile.
AT1G37140	*MEI2 C-TERMINAL RRM ONLY LIKE 1 (MCT1)*	Amember of mei2-like gene family; phylogenetic analysis revealed that it belongs to the fourth clade of mei2-like proteins, with conserved C-terminal RNA recognition motif (RRM) only. MCT1 expression is increased in the presence of ABA and RNAi suppression showed increased germination rates in the presence of ABA.
AT1G37150	*HOLOCARBOXYLASE SYNTHETASE 2 (HCS2)*	Although HCS2 is predicted to encode a biotin protein ligase / holocarboxylase synthetase (HCS), hcs2 mutants do not show a decrease in HCS activity. A dual-targeted HCS1 (At2g25710) might account for the HCS activity observed in multiple subcellular compartments in Arabidopsis.
AT1G37160		transposable_element_gene;gypsy-like retrotransposon family (Athila), similar to putative Athila retroelement ORF1 protein GI:4567296 from (Arabidopsis thaliana);(source:TAIR10)
AT1G37170		transposable_element_gene;Mutator-like transposase family, has a 1.1e-84 P-value blast match to O22278 /203-375 Pfam PF03108 MuDR family transposase (MuDr-element domain);(source:TAIR10)
AT1G37826		
AT1G37900		
AT1G38340		transposable_element_gene;gypsy-like retrotransposon family (Athila), has a 3.7e-36 P-value blast match to GB:CAA57397 Athila ORF 1 (Arabidopsis thaliana);(source:TAIR10)
AT1G38470		
AT1G38950		
AT1G39190		transposable_element_gene;CACTA-like transposase family (Tnp2/En/Spm), has a 6.1e-139 P-value blast match to GB:CAA40555 TNP2 (CACTA-element) (Antirrhinum majus);(source:TAIR10)
AT1G39270		transposable_element_gene;gypsy-like retrotransposon family (Athila), has a 6.7e-105 P-value blast match to gb|AAL06422.1|AF378081_1 reverse transcriptase (Athila4) (Arabidopsis thaliana) (Gypsy_Ty3-family);(source:TAIR10)
AT1G39350		transposable_element_gene
AT1G39430		transposable_element_gene;similar to unknown protein [Arabidopsis thaliana] (TAIR:AT3G43100.1);(source:TAIR10)
AT1G39750		transposable_element_gene;gypsy-like retrotransposon family (Athila), has a 3.8e-35 P-value blast match to GB:CAA57397 Athila ORF 1 (Arabidopsis thaliana);(source:TAIR10)
AT1G40150		transposable_element_gene;gypsy-like retrotransposon family (Athila), has a 3.7e-38 P-value blast match to GB:CAA57397 Athila ORF 1 (Arabidopsis thaliana);(source:TAIR10)
AT1G40230		transposable_element_gene
AT1G40390		DNAse I-like superfamily protein
AT1G40550		
AT1G40710		
AT1G40871		

AT1G40952		
AT1G41114		
AT1G41276		
AT1G41750		transposable_element_gene;similar to unknown protein [Arabidopsis thaliana] (TAIR:AT3G32903.1);(source:TAIR10)
AT1G41770		transposable_element_gene;similar to unknown protein [Arabidopsis thaliana] (TAIR:AT3G33064.1);(source:TAIR10)
AT1G41795		transposable_element_gene;gypsy-like retrotransposon family (Athila), has a 3.6e-264 P-value blast match to GB:CAA57397 Athila ORF 1 (Arabidopsis thaliana);(source:TAIR10)
AT1G41797		transposable_element_gene;gypsy-like retrotransposon family (Athila), has a 4.2e-119 P-value blast match to GB:CAA57397 Athila ORF 1 (Arabidopsis thaliana);(source:TAIR10)
AT1G41810		transposable_element_gene;similar to unknown protein [Arabidopsis thaliana] (TAIR:AT1G34590.1);(source:TAIR10)
AT1G41820		hypothetical protein
AT1G41825		transposable_element_gene;gypsy-like retrotransposon family (Athila), has a 1.9e-98 P-value blast match to gb|AAL06422.1|AF378081_1 reverse transcriptase (Athila4) (Arabidopsis thaliana) (Gypsy_Ty3-family);(source:TAIR10)
AT1G41830	*SKU5-SIMILAR 6 (SKS6)*	SKU5-similar 6
AT1G41860		transposable_element_gene;contains InterPro domain Retrotransposon gag protein;(source:TAIR10)
AT1G41870		transposable_element_gene;similar to unknown protein [Arabidopsis thaliana] (TAIR:AT3G42120.1);(source:TAIR10)
AT1G41900		pseudogene of myosin heavy chain-related
AT1G41920		General transcription factor 2-related zinc finger protein
AT1G41930		transposable_element_gene;CACTA-like transposase family (Tnp1/En/Spm), has a 3.8e-118 P-value blast match to ref|NP_189784.1| TNP1-related protein (Arabidopsis thaliana) (CACTA-element);(source:TAIR10)
AT1G42190		GAG/POL/ENV polyprotein
AT1G42200		transposable_element_gene;CACTA-like transposase family (Ptta/En/Spm), has a 2.3e-27 P-value blast match to At5g36655.1/81-333 CACTA-like transposase family (Ptta/En/Spm) (CACTA-element) (Arabidopsis thaliana);(source:TAIR10)
AT1G42220		transposable_element_gene;CACTA-like transposase family (Ptta/En/Spm), has a 3.7e-14 P-value blast match to At5g36655.1/81-333 CACTA-like transposase family (Ptta/En/Spm) (CACTA-element) (Arabidopsis thaliana);(source:TAIR10)
AT1G42250		transposable_element_gene;similar to unknown protein [Arabidopsis thaliana] (TAIR:AT2G05087.1);(source:TAIR10)
AT1G42360		transposable_element_gene;pseudogene, hypothetical protein;(source:TAIR10)
AT1G42375		transposable_element_gene;gypsy-like retrotransposon family (Athila), has a 3.3e-236 P-value blast match to GB:CAA57397 Athila ORF 1 (Arabidopsis thaliana);(source:TAIR10)
AT1G42400		transposable_element_gene;similar to unknown protein [Arabidopsis thaliana] (TAIR:AT1G35090.1);(source:TAIR10)
AT1G42410		transposable_element_gene;CACTA-like transposase family (Ptta/En/Spm), has a 1.0e-124 P-value blast match to At5g36655.1/81-333 CACTA-like transposase family (Ptta/En/Spm) (CACTA-element) (Arabidopsis thaliana);(source:TAIR10)
AT1G42420		transposable_element_gene;pseudogene, hypothetical protein;(source:TAIR10)
AT1G42440		pre-rRNA-processing TSR1-like protein
AT1G42450		transposable_element_gene;Mutator-like transposase family, has a 4.3e-96 P-value blast match to Q9SHN7 /450-633 Pfam PF03108 MuDR family transposase (MuDr-element domain);(source:TAIR10)
AT1G42460		transposable_element_gene;similar to Ulp1 protease family protein [Arabidopsis thaliana] (TAIR:AT2G07240.1);(source:TAIR10)
AT1G42470	*NIEMANN-PICK DISEASE TYPE C1-1 (ATNPC1 - 1)*	Patched family protein
AT1G42480		TLR4 regulator/MIR-interacting MSAP protein
AT1G42540	*GLUTAMATE RECEPTOR 3.3 (GLR3.3)*	member of Putative ligand-gated ion channel subunit family
AT1G42550	*PLASTID MOVEMENT IMPAIRED1 (PMI1)*	Encodes a plant-specific protein of unknown function that appears to be conserved among angiosperms. The mRNA is cell-to-cell mobile.
AT1G42560	*MILDEW RESISTANCE LOCUS O 9 (MLO9)*	A member of a large family of seven-transmembrane domain proteins specific to plants, homologs of the barley mildew resistance locus o (MLO) protein. The Arabidopsis genome contains 15 genes encoding MLO proteins, with localization in plasma membrane. Phylogenetic analysis revealed four clades of closely-related AtMLO genes. ATMLO9 belongs to the clade III, with AtMLO5, AtMLO7, AtMLO8, and AtMLO10. The gene is expressed during early seedling growth, in cotyledon vascular system, in flowers (with strong expression in anthers) in siliques and fruit abscission zone; not expressed in roots, or in mature rosette leaves, as shown by GUS activity patterns. The expression of several phylogenetically closely-related AtMLO genes showed similar or overlapping tissue specificity and analogous responsiveness to external stimuli, suggesting functional redundancy, co-function, or antagonistic function(s).Acts redundantly with MLO5 to tether Ca2+ channels to the pollen tube plasma membrane to effect pollen tube guidance.
AT1G42570		pseudogene of FAR1-related sequence 5
AT1G42580		transposable_element_gene;similar to DNA binding [Arabidopsis thaliana] (TAIR:AT3G47680.1);(source:TAIR10)
AT1G42610		transposable_element_gene;pseudogene, hypothetical protein;(source:TAIR10)
AT1G42630		transposable_element_gene;similar to nucleic acid binding / zinc ion binding [Arabidopsis thaliana] (TAIR:AT1G40080.1);(source:TAIR10)
AT1G42640		
AT1G42650		transposable_element_gene;pseudogene, hypothetical protein;(source:TAIR10)
AT1G42680		P-loop containing nucleoside triphosphate hydrolases superfamily protein
AT1G42690		transposable_element_gene;pseudogene, similar to B, blastp match of 48%25 identity and 4.4e-72 P-value to GP|22830897|dbj|BAC15771.1||AB087616 B {Oryza sativa (japonica cultivar-group)};(source:TAIR10)
AT1G42697		transposable_element_gene;CACTA-like transposase family (Tnp2/En/Spm), has a 1.5e-82 P-value blast match to gb|AAG52024.1|AC022456_5 Tam1-homologous transposon protein TNP2, putative;(source:TAIR10)

AT1G42700		hypothetical protein
AT1G42705		transposable_element_gene;hAT-like transposase family (hobo/Ac/Tam3), has a 2.3e-66 P-value blast match to GB:AAD24567 transposase Tag2 (hAT-element) (Arabidopsis thaliana);(source:TAIR10)
AT1G42710		General transcription factor 2-related zinc finger protein
AT1G42960	*CARBOXYLTRANSFERASE INTERACTOR1 (CTI1)*	expressed protein localized to the inner membrane of the chloroplast.
AT1G42970	*GLYCERALDEHYDE-3-PHOSPHATE DEHYDROGENASE B SUBUNIT (GAPB)*	Encodes chloroplast localized glyceraldehyde-3-phosphate dehydrogenase that can use both NADH and NADPH to reduce 1,3-diphosphate glycerate. It forms A2B2 heterotetramers with GapA forms of the GADPH enzyme. These complexes are active in the light under reducing conditions, but show reduced NADPH-dependent activity in response to oxidized thioredoxins and increased NAD(H)/NADP(H) ratios due to the formation of inactive A8B8 hexadecamers. The mRNA is cell-to-cell mobile.
AT1G42980		Actin-binding FH2 (formin homology 2) family protein
AT1G42990	*BASIC REGION/LEUCINE ZIPPER MOTIF 60 (BZIP60)*	bZIP60 consists of a bZIP DNA binding domain followed by a putative transmembrane domain. bZIP60 mRNA is upregulated by the addition of ER stress inducers, tunicamycin (inhibitor of N-linked glycosylation), DTT (inhibitor of disulfide bond formation) and azetin-2-carboxylate (proline analog perturbing protein structure). Upon ER stress, bZIP60 mRNA is spliced by IRE1A and IRE1B to produce bZIP60-S, an active transcription factor without the transmembrane domain. bZIP60-U, a product of unspliced form of bZIP60 mRNA, is localized at the ER membrane and bZIP60-S is localized in the nucleus.
AT1G43000		PLATZ transcription factor family protein
AT1G43010		Pentatricopeptide repeat (PPR) superfamily protein
AT1G43020		electron protein, putative (Protein of unknown function, DUF547)
AT1G43040	*SMALL AUXIN UPREGULATED RNA 58 (SAUR58)*	SAUR-like auxin-responsive protein family
AT1G43080		Pectin lyase-like superfamily protein
AT1G43140		Cullin family protein
AT1G43160	*RELATED TO AP2 6 (RAP2.6)*	encodes a member of the ERF (ethylene response factor) subfamily B-4 of ERF/AP2 transcription factor family (RAP2.6). The protein contains one AP2 domain. There are 7 members in this subfamily.
AT1G43200		transposable_element_gene;pseudogene, hypothetical protein, contains Pfam profile PF03384: Drosophila protein of unknown function, DUF287;(source:TAIR10)
AT1G43220		transposable_element_gene;pseudogene, hypothetical protein;(source:TAIR10)
AT1G43230		transposable_element_gene;similar to unknown protein [Arabidopsis thaliana] (TAIR:AT4G07523.1);(source:TAIR10)
AT1G43260		hAT transposon superfamily protein
AT1G43290		transposable_element_gene;pseudogene, hypothetical protein;(source:TAIR10)
AT1G43310		Nucleotide-sugar transporter family protein
AT1G43320		hypothetical protein
AT1G43330		Homeodomain-like superfamily protein
AT1G43560	*THIOREDOXIN Y2 (ty2)*	thioredoxin Y2
AT1G43570		transposable_element_gene;similar to unknown protein [Arabidopsis thaliana] (TAIR:AT5G52065.1);(source:TAIR10)
AT1G43590		transposable_element_gene;similar to unknown protein [Arabidopsis thaliana] (TAIR:AT5G34838.1);(source:TAIR10)
AT1G43610		Haloacid dehalogenase-like hydrolase (HAD) superfamily protein
AT1G43630		plant/protein (DUF793)
AT1G43640	*TUBBY LIKE PROTEIN 5 (TLP5)*	Member of TLP family of tubby like proteins that also contain an F-Box.
AT1G43650	*USUALLY MULTIPLE ACIDS MOVE IN AND OUT TRANSPORTERS 22 (UMAMIT22)*	nodulin MtN21-like transporter family protein
AT1G43660		transposable_element_gene;similar to unknown protein [Arabidopsis thaliana] (TAIR:AT1G48290.1);(source:TAIR10)
AT1G43680		nucleic acid-binding/zinc ion-binding protein
AT1G43710	*EMBRYO DEFECTIVE 1075 (emb1075)*	Encodes a serine decarboxylase that is involved in ethanolamine metabolism and is crucial for plant growth.
AT1G43715		transposable_element_gene;copia-like retrotransposon family, has a 2.6e-130 P-value blast match to GB:BAA78424 polyprotein (Ty1_Copia-element) (Arabidopsis thaliana)gi|4996363|dbj|BAA78424.1| polyprotein (AtRE2) (Arabidopsis thaliana) (Ty1_Copia-element);(source:TAIR10)
AT1G43730		RNA-directed DNA polymerase (reverse transcriptase)-related family protein
AT1G43740		transposable_element_gene;gypsy-like retrotransposon family (Athila);(source:TAIR10)
AT1G43745		transposable_element_gene;gypsy-like retrotransposon family (Athila), has a 1.4e-218 P-value blast match to GB:CAA57397 Athila ORF 1 (Arabidopsis thaliana);(source:TAIR10)
AT1G43760		DNAse I-like superfamily protein
AT1G43780	*SERINE CARBOXYPEPTIDASE-LIKE 44 (scpl44)*	serine carboxypeptidase-like 44
AT1G43790	*TRACHEARY ELEMENT DIFFERENTIATION-RELATED 6 (TED6)*	tracheary element differentiation-related 6
AT1G43800	*FLORAL TRANSITION AT THE MERISTEM1 (FTM1)*	Δ9 stearoyl-ACP desaturase which together with FAB2, AAD1, and AAD5 redundantly participates in oil storage during the maturation phase.
AT1G43810		hypothetical protein
AT1G43840		transposable_element_gene;CACTA-like transposase family (Ptta/En/Spm), has a 5.2e-121 P-value blast match to At5g36655.1/81-333 CACTA-like transposase family (Ptta/En/Spm) (CACTA-element) (Arabidopsis thaliana);(source:TAIR10)
AT1G43870		transposable_element_gene;similar to myosin heavy chain-related [Arabidopsis thaliana] (TAIR:AT4G08113.1);(source:TAIR10)
AT1G43880		transposable_element_gene;similar to unknown protein [Arabidopsis thaliana] (TAIR:AT3G30810.1);(source:TAIR10)
AT1G43886		transposable_element_gene;copia-like retrotransposon family, has a 5.3e-165 P-value blast match to GB:AAA57005 Hopscotch polyprotein (Ty1_Copia-element) (Zea mays);(source:TAIR10)
AT1G43910		P-loop containing nucleoside triphosphate hydrolases superfamily protein

AT1G43920		transposable_element_gene;similar to unknown protein [Arabidopsis thaliana] (TAIR:AT2G07090.1);(source:TAIR10)
AT1G43930		transposable_element_gene;Mutator-like transposase family, has a 1.2e-56 P-value blast match to GB:AAA21566 mudrA of transposon=MuDR (MuDr-element) (Zea mays);(source:TAIR10)
AT1G43940		transposable_element_gene;similar to unknown protein [Arabidopsis thaliana] (TAIR:AT3G42540.1);(source:TAIR10)
AT1G43950	*AUXIN RESPONSE FACTOR 23 (ARF23)*	auxin response factor 23
AT1G43980		Tetratricopeptide repeat (TPR)-like superfamily protein
AT1G43995		transposable_element_gene;similar to RNase H domain-containing protein [Arabidopsis thaliana] (TAIR:AT2G22350.1);(source:TAIR10)
AT1G44000	*STAY-GREEN LIKE (SGRL)*	STAY-GREEN-like protein
AT1G44030		Cysteine/Histidine-rich C1 domain family protein
AT1G44050		Cysteine/Histidine-rich C1 domain family protein
AT1G44060		transposable_element_gene;CACTA-like transposase family (Ptta/En/Spm), has a 2.0e-22 P-value blast match to At5g29026.1/8-244 CACTA-like transposase family (Ptta/En/Spm) (CACTA-element) (Arabidopsis thaliana);(source:TAIR10)
AT1G44070		transposable_element_gene;CACTA-like transposase family (Tnp2/En/Spm), has a 2.6e-142 P-value blast match to GB:CAA40555 TNP2 (CACTA-element) (Antirrhinum majus);(source:TAIR10)
AT1G44090	*GIBBERELLIN 20-OXIDASE 5 (GA20OX5)*	Encodes a gibberellin 20-oxidase.
AT1G44100	*AMINO ACID PERMEASE 5 (AAP5)*	amino acid permease 5
AT1G44110	*CYCLIN A1;1 (CYCA1;1)*	Cyclin A1
AT1G44120	*CELLULOSE SYNTHASE INTERACTIVE 2 (CSI2)*	CELLULOSE SYNTHASE INTERACTIVE 2
AT1G44130		Eukaryotic aspartyl protease family protein
AT1G44160		HSP40/DnaJ peptide-binding protein
AT1G44170	*ALDEHYDE DEHYDROGENASE 3H1 (ALDH3H1)*	Encodes an aldehyde dehydrogenase induced by ABA and dehydration that can oxidize saturated aliphatic aldehydes. It is also able to oxidize beta-unsaturated aldehydes, but not aromatic aldehydes. Activity of ALDH3H1 is NAD +-dependent.
AT1G44222		
AT1G44254		
AT1G44318	*(hemb2)*	Aldolase superfamily protein
AT1G44350	*IAA-LEUCINE RESISTANT (ILR)-LIKE GENE 6 (ILL6)*	encodes a protein similar to IAA amino acid conjugate hydrolase. NO-induced, involved in growth and disease resistance. Plays a positive role in the activation of SAR when challenged with the avirulent pathogen Pst DC3000 (avrB) at 5 ? 106 CFU.
AT1G44414		zinc-ribbon domain protein
AT1G44446	*CHLORINA 1 (CH1)*	Encodes chlorophyllide <i>a</i> oxygenase which converts chlorophyllide <i>a</i> to chlorophyllide <i>b</i> by catalyzing two successive hydroxylations at the 7-methyl group of chlorophyllide <i>a</i> . Mutants are deficient in pigments that associate with thylakoid membrane proteins, lacking chlorophyll <i>b</i> and light-harvesting proteins of photosystem II. The protein was shown through cross-linking experiments to interact with Toc75, Toc34, Tic40, Tic20 and Tic22.
AT1G44478		Cyclophilin
AT1G44510		transposable_element_gene;copia-like retrotransposon family, has a 0. P-value blast match to dbj|BAA78426.1| polyprotein (AtRE2-1) (Arabidopsis thaliana) (Ty1_Copia-element);(source:TAIR10)
AT1G44575	*NONPHOTOCHEMICAL QUENCHING 4 (NPQ4)*	Encoding PSII-S (CP22), a ubiquitous pigment-binding protein associated with photosystem II (PSII) of higher plants. Involved in nonphotochemical quenching rather than in photosynthesis. Mutant has a normal violaxanthin cycle but has a limited capacity of quenching singlet excited chlorophylls and is tolerant to lipid peroxidation.
AT1G44740		hypothetical protein
AT1G44760		Adenine nucleotide alpha hydrolases-like superfamily protein
AT1G44770		elongation factor
AT1G44780		translation initiation factor
AT1G44790		ChaC-like family protein
AT1G44800	*SILIQUES ARE RED 1 (SIAR1)*	Encodes Siliques Are Red 1 (SIAR1). Functions as a bidirectional amino acid transporter that is crucial for the amino acid homeostasis of siliques. Member of nodulin MtN21-like transporter family.
AT1G44810	*GEBP-LIKE 4 (GPL4)*	GeBP transcription factor required for Cd‐induced growth inhibition.
AT1G44830	*ERF TRANSCRIPTION FACTOR 14 (ERF014)*	Encodes a nuclear-localized member of the DREB subfamily A-5 of ERF/AP2 transcription factor family. The protein contains one AP2 domain. There are 15 members in this subfamily including RAP2.1, RAP2.9 and RAP2.10. Overexpression in cultured cells results in an increase in pectin deposition.ERF014 differentially regulates responses to bacterial and fungal pathogens.
AT1G44840		transposable_element_gene;Mutator-like transposase family, has a 1.2e-64 P-value blast match to Q9S9L6 /322-461 Pfam PF03108 MuDR family transposase (MuDr-element domain);(source:TAIR10)
AT1G44890		inner membrane OXA1-like protein
AT1G44900	*MINICHROMOSOME MAINTENANCE 2 (MCM2)*	Encodes MCM2 (MINICHROMOSOME MAINTENANCE 2), a protein essential to embryo development. Overexpression results in altered root meristem function.
AT1G44910	*PRE-MRNA-PROCESSING PROTEIN 40A (PRP40A)*	Binds the carboxyl-terminal domain (CTD) of the largest subunit of RNA polymerase II and functions as a scaffold for RNA processing machineries.
AT1G44920		transmembrane protein
AT1G44930		
AT1G44960		SNARE associated Golgi protein family
AT1G44970	*PEROXIDASE9 (PRX9)*	Encodes a class III peroxidase that is genetically redundant with PRX40, expressed in the tapetum, and essential for proper anther and pollen development. Peroxidase required for casparian strip lignification as well as partially required for SGN-dependent compensatory lignification.

AT1G44980	*PECTIN METHYLESTERASE 7 (PME7)*	Type II pectin methylesterase
AT1G44990		transmembrane protein
AT1G45070		transposable_element_gene;Mutator-like transposase family, has a 7.5e-84 P-value blast match to Q9SL18 /349-510 Pfam PF03108 MuDR family transposase (MuDr-element domain);(source:TAIR10)
AT1G45090		transposable_element_gene;similar to unknown protein [Arabidopsis thaliana] (TAIR:AT5G28480.1);(source:TAIR10)
AT1G45100		RNA-binding (RRM/RBD/RNP motifs) family protein
AT1G45110		Tetrapyrrole (Corrin/Porphyrin) Methylase
AT1G45130	*BETA-GALACTOSIDASE 5 (BGAL5)*	beta-galactosidase 5
AT1G45145	*THIOREDOXIN H-TYPE 5 (TRX5)*	encodes a cytosolic thioredoxin that reduces disulfide bridges of target proteins by the reversible formation of a disulfide bridge between two neighboring Cys residues present in the active site. Thioredoxins have been found to regulate a variety of biological reactions in prokaryotic and eukaryotic cells.
AT1G45180	*(CTL05)*	RING/U-box superfamily protein
AT1G45190	*DOWNREGULATED IN DIF1 18 (DD18)*	downregulated in DIF1 18
AT1G45261		
AT1G45474	*PHOTOSYSTEM I LIGHT HARVESTING COMPLEX GENE 5 (LHCA5)*	Encodes a component of the light harvesting complex of photosystem I.
AT1G45545		WEAK CHLOROPLAST MOVEMENT UNDER BLUE LIGHT-like protein (DUF827)
AT1G45616	*RECEPTOR LIKE PROTEIN 6 (RLP6)*	receptor like protein 6
AT1G46264	*HEAT SHOCK TRANSCRIPTION FACTOR B4 (HSFB4)*	Encodes SCHIZORIZA, a member of Heat Shock Transcription Factor (Hsf) family. Functions as a nuclear factor regulating asymmetry of stem cell divisions.
AT1G46336		transmembrane protein
AT1G46696		hypothetical protein (Protein of unknown function, DUF601)
AT1G46768	*RELATED TO AP2 1 (RAP2.1)*	encodes a member of the DREB subfamily A-5 of ERF/AP2 transcription factor family (RAP2.1). The protein contains one AP2 domain. There are 16 members in this subfamily including RAP2.9 and RAP2.10.
AT1G46840		F-box family protein
AT1G46912		F-box associated ubiquitination effector family protein
AT1G47128	*RESPONSIVE TO DEHYDRATION 21A (RD21A)*	Cysteine proteinase precursor-like protein/ dehydration stress-responsive gene (RD21). Has been shown to have peptide ligase activity and protease activity in vitro. RD21 is involved in immunity to the necrotrophic fungal pathogen Botrytis cinerea.Activity detected in root, leaf, flower and cell culture.
AT1G47210	*CYCLIN-DEPENDENT PROTEIN KINASE 3;2 (CYCA3;2)*	cyclin-dependent protein kinase 3
AT1G47220	*CYCLIN A3;3 (CYCA3;3)*	Cyclin A3
AT1G47270	*TUBBY LIKE PROTEIN 6 (TLP6)*	Member of TLP family of tubby like proteins that also contain an F-Box. Localized to the plasma membrane.
AT1G47280		hypothetical protein
AT1G47290	*3BETA-HYDROXYSTEROID-DEHYDROGENASE/DECARBOXYLASE ISOFORM 1 (3BETAHSD/D1)*	Encodes an enzyme with 3β-hydroxysteroid dehydrogenase/C4-decarboxylase activity <i>in vitro</i>. The activity of the enzyme was determined using microsomal extracts of yeast overexpressing the <i>Arabidopsis</i> gene. Cytosolic fractions failed to be associated to the activity, leading to the speculation that the enzyme is membrane-bound.
AT1G47300		F-box family protein
AT1G47350		F-box associated ubiquitination effector family protein
AT1G47360		transposable_element_gene;copia-like retrotransposon family, has a 1.2e-307 P-value blast match to GB:CAA31653 polyprotein (Ty1_Copia-element) (Arabidopsis thaliana);(source:TAIR10)
AT1G47370	*ESPONSE TO THE BACTERIAL TYPE III EFFECTOR PROTEIN HOPBA1 (RBA1)*	RBA1 variant in Ag0 background is a TIR-only receptor protein that binds to the bacterial type III effector protein HopBA. The Col-0 variant, which is not expressed, is likely a psuedogene and more highly methylated than the Ag0 variant which is expressed.
AT1G47380		Protein phosphatase 2C family protein
AT1G47400	*FE-UPTAKE-INDUCING PEPTIDE3 (FEP3)*	Involved in regulation of iron deficiency response genes. Overexpression results in hyperaccumulation of Fe and Mn.
AT1G47410		hypothetical protein
AT1G47465		transposable_element_gene;gypsy-like retrotransposon family (Athila), has a 9.7e-17 P-value blast match to GB:CAA57397 Athila ORF 1 (Arabidopsis thaliana);(source:TAIR10)
AT1G47470		ECA1 gametogenesis family protein (DUF784)
AT1G47480		alpha/beta-Hydrolases superfamily protein
AT1G47485	*C-TERMINALLY ENCODED PEPTIDE 1 (CEP1)*	Encodes CEP1, a 15-amino-acid peptide, which is mainly expressed in the lateral root primordia. When overexpressed or externally applied, CEP1 arrests root growth. CEP1 is a candidate for a novel peptide plant hormone.
AT1G47500	*RNA-BINDING PROTEIN 47C' (RBP47C')*	RNA-binding protein 47C
AT1G47520		transposable_element_gene;copia-like retrotransposon family, has a 3.3e-256 P-value blast match to GB:CAA72989 open reading frame 1 (Ty1_Copia-element) (Brassica oleracea);(source:TAIR10)
AT1G47540		Scorpion toxin-like knottin superfamily protein
AT1G47550	*EXOCYST COMPLEX COMPONENT SEC3A (SEC3A)*	Encodes a member of the exocyst complex gene family. The exocyst is a protein complex involved in tethering vesicles to the plasma membrane during regulated or polarized secretion. It binds phosphoinositide lipids.
AT1G47600	*BETA GLUCOSIDASE 34 (BGLU34)*	Encodes a myrosinase. Over-expression led to a glucosinolate profile change.
AT1G47610		Transducin/WD40 repeat-like superfamily protein
AT1G47620	*CYTOCHROME P450, FAMILY 96, SUBFAMILY A, POLYPEPTIDE 8 (CYP96A8)*	member of CYP96A
AT1G47655		Dof-type zinc finger DNA-binding family protein

AT1G47660		hypothetical protein
AT1G47670		Transmembrane amino acid transporter family protein
AT1G47680		hypothetical protein
AT1G47710	*(SERPIN1)*	Inhibitor of pro-apoptotic proteases, which is involved in the regulation of the programmed cell death induction.
AT1G47720	*ORGANELLAR SINGLE-STRANDED (OSB1)*	Encodes an organellar single-strand DNA binding protein, located in mitochondria, controls the stoichiometry of alternative mitochondrial DNA forms generated by homologous recombination.
AT1G47730		F-box and associated interaction domains-containing protein
AT1G47750	*PEROXIN 11A (PEX11A)*	member of the peroxin11 (PEX11) gene family, integral to peroxisome membrane, controls peroxisome proliferation.
AT1G47765		F-box and associated interaction domains-containing protein
AT1G47780		alpha/beta-Hydrolases superfamily protein
AT1G47790		F-box and associated interaction domains-containing protein
AT1G47810		F-box and associated interaction domains-containing protein
AT1G47816		transposable_element_gene;Mutator-like transposase family, has a 2.5e-26 P-value blast match to Q9SLM0 /314-478 Pfam PF03108 MuDR family transposase (MuDr-element domain);(source:TAIR10)
AT1G47820		hypothetical protein
AT1G47840	*HEXOKINASE 3 (HXK3)*	Encodes a putative hexokinase.
AT1G47870	*(ATE2F2)*	Member of the E2F transcription factors, (cell cycle genes), key components of the cyclin D/retinoblastoma/E2F pathway. AtE2Fc is regulated by a balance between gene expression and ubiquitin-proteasome proteolysis. AtE2Fc might play a role in cell division and during the transition from skotomorphogenesis to photomorphogenesis. E2Fc has been shown to interact with DPB in its nonphosphorylated form; when E2Fc is phosphorylated, the formation of the E2Fc/DPB heterodimer is lost. E2Fc is required for miR396 activity on cell proliferation under UV-B. Its role is independent of E2Fe, probably modulating DNA damage responses through the regulation of SOG1 and ATR transcript levels.
AT1G47885		Ribonuclease inhibitor
AT1G47890	*RECEPTOR LIKE PROTEIN 7 (RLP7)*	receptor like protein 7
AT1G47900		filament-like protein (DUF869)
AT1G47920	*(SYP81)*	RNI-like superfamily protein
AT1G47940		Pentatricopeptide repeat (PPR) superfamily protein
AT1G47960	*CELL WALL / VACUOLAR INHIBITOR OF FRUCTOSIDASE 1 (C/VIF1)*	Plant cell wall (CWI) and vacuolar invertases (VI) play important roles in carbohydrate metabolism, stress responses and sugar signaling. This protein may inhibit their activity.
AT1G47980		desiccation-like protein
AT1G47990	*GIBBERELLIN 2-OXIDASE 4 (GA2OX4)*	Encodes a gibberellin 2-oxidase that acts on C19 gibberellins. AtGA2OX4 expression is responsive to cytokinin and KNOX activities.
AT1G48000	*MYB DOMAIN PROTEIN 112 (MYB112)*	Encodes a putative transcription factor (MYB112).
AT1G48010		Plant invertase/pectin methylesterase inhibitor superfamily protein
AT1G48050	*(KU80)*	Ku80 and ku70 form the heterodimer complex Ku, required for proper maintenance of the telomeric C strand. Ku regulates the extension of the telomeric G strand. Interacts with WEX, and this interaction stimulates the WEX exonuclease activity. Binds double stranded DNA breaks as a heterodimer with Ku70, involved in non-homologous end joining repair. Mutants are defective in T-DNA integration. Over expression confers increased resistance to DNA damage agents and increased susceptibility to T-DNA transformation.
AT1G48060		F-box/associated interaction domain protein
AT1G48070		Thioredoxin superfamily protein
AT1G48095		nucleic acid-binding/zinc ion-binding protein
AT1G48100	*POLYGALACTURONASE INVOLVED IN EXPANSION3 (PGX3)*	Pectin lyase-like superfamily protein
AT1G48120	*MAIN-LIKE 3 (MAIL3)*	Encodes a nuclear localized aminotransferase-like protein containing a plant mobile domain.
AT1G48130	*1-CYSTEINE PEROXIREDOXIN 1 (PER1)*	encodes a protein similar to the 1-cysteine (1-Cys) peroxiredoxin family of antioxidants. Expression is limited to seed (aleurone and embryo) and is not induced by ABA or drought.
AT1G48140	*DOLICHOL PHOSPHATE MANNOSE SYNTHASE 3 (DPMS3)*	Encodes a subunit of the dolichol phosphate mannase synthase (DPMS) complex that may serve as membrane anchors for the catalytic core, DPMS1, or provide catalytic assistance. It is localized in the ER and mediates isoprenyl-linked glycan biogenesis.
AT1G48160		signal recognition particle 19 kDa protein, putative / SRP19
AT1G48180		target of trans acting-siR480/255 protein
AT1G48210		Protein kinase superfamily protein
AT1G48260	*CBL-INTERACTING PROTEIN KINASE 17 (CIPK17)*	Encodes a member of the SNF1-related kinase (SnRK) gene family (SnRK3.21), which has also been reported as a member of the CBL-interacting protein kinases (CIPK17).
AT1G48280	*IPGA1-LIKE2 (IPGAL2)*	hydroxyproline-rich glycoprotein family protein
AT1G48290		transposable_element_gene;similar to unknown protein [Arabidopsis thaliana] (TAIR:AT1G43660.1);(source:TAIR10)
AT1G48300	*DIACYLGLYCEROL ACYLTRANSFERASE 3 (DGAT3)*	Cytosolic iron-sulfur protein with a [2Fe-2S] cluster which synthesizes triacylglycerol (DGAT activity).
AT1G48320	*DHNA-COA THIOESTERASE 1 (DHNAT1)*	Encodes one of the two functional DHNA-CoA (1,4-dihydroxy-2-naphthoyl-CoA) thioesterases found in Arabidopsis.
AT1G48330		SsrA-binding protein
AT1G48350	*EMBRYO DEFECTIVE 3105 (EMB3105)*	Member of the uL18 RNA-binding protein family. uL18 proteins share a short structurally conserved domain that binds the 5S rRNA and allow its incorporation into ribosomes.
AT1G48370	*YELLOW STRIPE LIKE 8 (YSL8)*	Arabidopsis thaliana metal-nicotianamine transporter YSL4
AT1G48390		RNI-like superfamily protein
AT1G48430		Dihydroxyacetone kinase

AT1G48450		alanine-tRNA ligase, putative (DUF760)
AT1G48460		tRNA-processing ribonuclease BN
AT1G48470	*GLUTAMINE SYNTHETASE 1;5 (GLN1;5)*	Encodes cytosolic glutamine synthase isozyme. Expression of mRNA is not detectable in roots.
AT1G48480	*RECEPTOR-LIKE KINASE 1 (RKL1)*	Arabidopsis thaliana receptor-like protein kinase (RKL1) gene
AT1G48500	*JASMONATE-ZIM-DOMAIN PROTEIN 4 (JAZ4)*	Jasmonate zim domain transcription factor family protein.Involved in freezing tolerance and JA iduceed leaf senesence.
AT1G48510	*SURFEIT 1B (SURF1B)*	Encodes one of two Arabidopsis mitochondrial proteins similar to human SURF1 which is known to be involved in cytochrome c oxidase assembly. Mutations result in defects in hypocotyl elongation and changes in GA homeostasis.
AT1G48520	*GLU-ADT SUBUNIT B (GATB)*	Encodes Glu-tRNA(Gln) amidotransferase subunit B (from Genbank record AF239836).
AT1G48550	*VACUOLAR PROTEIN SORTING (VPS26C)*	VPS26C is a component of a retromer complex, it is involved in endosome to lysosome protein transport and root hair growth.
AT1G48560		hypothetical protein
AT1G48570		zinc finger (Ran-binding) family protein
AT1G48580		nuclear localized protein
AT1G48600	*PHOSPHOETHANOLAMINE N-METHYLTRANSFERASE (PMEAMT)*	Encodes a phosphoethanolamine N-methyltransferase that catalyses the last two methylation steps of the three sequential methylations of phosphoethanolamine (PEA) that are required for the synthesis of phosphocholine (PCho) in plants.
AT1G48610		AT hook motif-containing protein
AT1G48620	*HIGH MOBILITY GROUP A5 (HON5)*	This gene is predicted to encodes a histone H1/H5 family member. A plant line expressing an RNAi construct targeted against HON5 shows a reduced level of agrobacterium-mediated root transformation.
AT1G48640		Transmembrane amino acid transporter family protein
AT1G48650		DEA(D/H)-box RNA helicase family protein
AT1G48660		Auxin-responsive GH3 family protein
AT1G48670		auxin-responsive GH3 family protein
AT1G48690		Auxin-responsive GH3 family protein
AT1G48720		Copia-like polyprotein/retrotransposon
AT1G48730		hypothetical protein
AT1G48750		Bifunctional inhibitor/lipid-transfer protein/seed storage 2S albumin superfamily protein
AT1G48760	*DELTA-ADAPTIN (delta-ADR)*	Encodes the putative delta subunit of the AP(adaptor protein)-3 complex and plays a role in vacuolar function.
AT1G48800		Terpenoid cyclases/Protein prenyltransferases superfamily protein
AT1G48820		Terpenoid cyclases/Protein prenyltransferases superfamily protein
AT1G48840		Plant protein of unknown function (DUF639);(source:TAIR10)
AT1G48870		Transducin/WD40 repeat-like superfamily protein
AT1G48910	*YUCCA 10 (YUC10)*	A paternally expressed imprinted gene.
AT1G48920	*NUCLEOLIN LIKE 1 (NUC-L1)*	Encodes ATNUC-L1 (NUCLEOLIN LIKE 1), the predominant form of the two nucleolin proteins found in Arabidopsis. This protein is involved in rRNA processing, ribosome biosynthesis, and vascular pattern formation. PARL1 localizes to the nucleolus and parl1 mutants accumulate elevated levels of the unspliced 35S pre-rRNA. parl1 mutants also have defects in cotyledon, leaf, sepal, and petal vein patterning and have reduced stature, reduced fertility, increased bushiness, and reduced root length. The sugar-induced expression of ribosome proteins is also reduced in parl1 mutants. The mRNA is cell-to-cell mobile.
AT1G48930	*GLYCOSYL HYDROLASE 9C1 (GH9C1)*	glycosyl hydrolase 9C1
AT1G48940	*EARLY NODULIN-LIKE PROTEIN 6 (ENODL6)*	early nodulin-like protein 6
AT1G48960		Adenine nucleotide alpha hydrolases-like superfamily protein
AT1G48980	*ATALKBH9A (ALKBH9A)*	2-oxoglutarate (2OG) and Fe(II)-dependent oxygenase superfamily protein
AT1G49000		transmembrane protein
AT1G49010	*(MYBS1)*	Duplicated homeodomain-like superfamily protein
AT1G49015		DPP6 N-terminal domain-like protein
AT1G49030		PLAC8 family protein
AT1G49050	*(APCB1)*	Encodes a member of the aspartyl protease family. Interacts with BAGP1 and BAG6 and appears to be required for cleavage of BAG6 as BAG6 is not cleaved in APCB1 mutant backgrounds.
AT1G49100		Leucine-rich repeat protein kinase family protein
AT1G49110		hypothetical protein
AT1G49150		transmembrane protein
AT1G49160	*(WNK7)*	Encodes a member of the WNK family (9 members in all) of protein kinases, the structural design of which is clearly distinct from those of other known protein kinases, such as receptor-like kinases and mitogen-activated protein kinases. Its
AT1G49170		hypothetical protein
AT1G49180		protein kinase family protein
AT1G49190	*RESPONSE REGULATOR 19 (RR19)*	member of Response Regulator: B- Type
AT1G49200	*ARABIDOPSIS T??XICOS EN LEVADURA 75 (ATL75)*	RING/U-box superfamily protein
AT1G49210	*ARABIDOPSIS T??XICOS EN LEVADURA 76 (ATL76)*	RING/U-box superfamily protein
AT1G49220	*ARABIDOPSIS T??XICOS EN LEVADURA 10 (ATL10)*	RING/U-box superfamily protein
AT1G49230	*ARABIDOPSIS TOXICOS EN LEVADURA 78 (ATL78)*	RING/U-box superfamily protein
AT1G49240	*ACTIN 8 (ACT8)*	Member of a subclass of actins composed of ACT2 and ACT8. Its mRNA is strongly expressed in strongly expressed in leaves, roots, stems, flowers, pollen, and siliques. However, protein expression, assayed by a ACT8:GUS fusion reporter, is very low in pollen.

AT1G49245		Prefoldin chaperone subunit family protein
AT1G49250		ATP-dependent DNA ligase
AT1G49260		mechanosensitive ion channel-like protein
AT1G49290	*(PEG2)*	Paternally expressed gene that is localized around the sperm nuclei of pollen. PEG2 acts as a sponge for siRNA854 during endosperm development, this action is necessary to induce triploid seed abortion.
AT1G49310		transmembrane protein
AT1G49320	*UNKNOWN SEED PROTEIN LIKE 1 (USPL1)*	Encodes USPL1, a BURP domain protein targeted to the protein storage vacuoles. Overexpression of USPL1 affects seed development, protein storage vacuoles and lipid vesicles morphology and function.
AT1G49370		pseudogene of Transmembrane amino acid transporter family protein
AT1G49390		2-oxoglutarate (2OG) and Fe(II)-dependent oxygenase superfamily protein
AT1G49400	*EMBRYO DEFECTIVE 1129 (emb1129)*	Nucleic acid-binding, OB-fold-like protein
AT1G49410	*TRANSLOCASE OF THE OUTER MITOCHONDRIAL MEMBRANE 6 (TOM6)*	translocase of the outer mitochondrial membrane 6
AT1G49430	*LONG-CHAIN ACYL-COA SYNTHETASE 2 (LACS2)*	Encodes a long chain acyl-CoA synthetase that catalyzes the synthesis of omega-hydroxy fatty acyl-CoA intermediates in the pathway to cutin synthesis. Required for repression of lateral root formation.
AT1G49450	*ABA-INDUCED WD40 REPEAT 2 (AIW2)*	Transducin/WD40 repeat-like superfamily protein
AT1G49470		transmembrane epididymal protein (DUF716)
AT1G49480	*RELATED TO VERNALIZATION1 1 (RTV1)*	Encodes a nuclear-localized DNA-binding protein that interacts with ITN1 at the PM and nuclei in vivo and may regulate ITN's subcellular localization.
AT1G49490	*LEUCINE-RICH REPEAT/EXTENSIN 9 (LRX9)*	Pollen expressed protein required for pollen tube growth.Along with other members of the LRX family, itnteracts with RALF4 to control pollen tube growth and integrity. Loss of function results in premature pollen tube rupture and reduced fertility.
AT1G49500		transcription initiation factor TFIID subunit 1b-like protein
AT1G49520		SWIB complex BAF60b domain-containing protein
AT1G49540	*ELONGATOR PROTEIN 2 (ELP2)*	elongator protein 2
AT1G49560	*HRS1 HOMOLOG6 (HHO6)*	Homeodomain-like superfamily protein
AT1G49570		Peroxidase superfamily protein
AT1G49580		Calcium-dependent protein kinase (CDPK) family protein
AT1G49600	*RNA-BINDING PROTEIN 47A (RBP47A)*	RNA-binding protein 47A
AT1G49620	*(ICK5)*	Kip-related protein (KRP) gene, encodes CDK (cyclin-dependent kinase) inhibitor (CKI), negative regulator of cell division. A member of seven KRP genes found in Arabidopsis thaliana. Differential expression patterns for distinct KRPs were revealed by in situ hybridization. Binds to D type cyclins and may inhibit cell cycle.
AT1G49650		alpha/beta-Hydrolases superfamily protein
AT1G49660	*CARBOXYESTERASE 5 (CXE5)*	Encodes a protein with carboxylesterase whose activity was tested using pNA.
AT1G49680		mutator transposase MUDRA protein
AT1G49700		Plant protein 1589 of unknown function
AT1G49720	*ABSCISIC ACID RESPONSIVE ELEMENT-BINDING FACTOR 1 (ABF1)*	Identified as a protein that binds to abscisic acid response elements. May mediate transcriptional regulation of ABA responses.
AT1G49730		Protein kinase superfamily protein
AT1G49740		PLC-like phosphodiesterases superfamily protein
AT1G49750		Leucine-rich repeat (LRR) family protein
AT1G49780	*PLANT U-BOX 26 (PUB26)*	PUB25 and PUB26 are closely related paralogs that encode functional E3 ligases. They function in immune response pathway by targeting BIK1 for degradation.
AT1G49800	*PRECURSOR OF PAMP-INDUCED PEPTIDE-LIKE 1 (PREPIPL1)*	Homolog of PIP1.
AT1G49810	*NA+/H+ ANTIPORTER 2 (NHD2)*	member of Na+/H+ antiporter-Putative family
AT1G49830		basic helix-loop-helix (bHLH) DNA-binding superfamily protein
AT1G49840	*BOUNDARY OF ROP DOMAINS5 (BDR5)*	glutamyl-tRNA (Gln) amidotransferase subunit A (DUF620)
AT1G49860	*GLUTATHIONE S-TRANSFERASE (CLASS PHI) 14 (GSTF14)*	Encodes glutathione transferase belonging to the phi class of GSTs. Naming convention according to Wagner et al. (2002). The mRNA is cell-to-cell mobile.
AT1G49870		myosin-2 heavy chain-like protein
AT1G49890	*QWRF DOMAIN CONTAINING 2 (QWRF2)*	Together with QWRF1 redundantly modulates cortical microtubule arrangement in floral organ growth and fertility.
AT1G49900		C2H2 type zinc finger transcription factor family
AT1G49920		MuDR family transposase
AT1G49930		alanine-tRNA ligase
AT1G49940		hypothetical protein
AT1G49960		Xanthine/uracil permease family protein
AT1G49970	*CLP PROTEASE PROTEOLYTIC SUBUNIT 1 (CLPR1)*	Encodes a ClpP-related sequence. Though similar to ClpP proteins, this does not contains the highly conserved catalytic triad of Ser-type proteases (Ser-His-Asp). The name reflects nomenclature described in Adam et. al (2001).
AT1G49975		photosystem I reaction center subunit N
AT1G50010	*TUBULIN ALPHA-2 CHAIN (TUA2)*	Encodes alpha-2,4 tubulin. TUA2 and TUA4 encode identical proteins. The mRNA is cell-to-cell mobile.
AT1G50040		formin-like protein, putative (DUF1005)
AT1G50050		CAP (Cysteine-rich secretory proteins, Antigen 5, and Pathogenesis-related 1 protein) superfamily protein
AT1G50060		CAP (Cysteine-rich secretory proteins, Antigen 5, and Pathogenesis-related 1 protein) superfamily protein

AT1G50080		ribonuclease
AT1G50110	*BRANCHED-CHAIN AMINOTRANSFERASE 6 (BCAT6)*	Encodes a cytosolic branched-chain aminotransferase that acts on Leu, Ile, Val and also on Met. Together with BCAT4 and BCAT3, it is involved in methionine salvage and glucosinolate biosynthesis.
AT1G50140		P-loop containing nucleoside triphosphate hydrolases superfamily protein
AT1G50150		
AT1G50160		ribonuclease H superfamily polynucleotidyl transferase
AT1G50180	*CEL-ACTIVATED RESISTANCE 1 (CAR1)*	Host immune receptor which recognizes the conserved effectors AvrE and HopAA1.
AT1G50190		Cysteine/Histidine-rich C1 domain family protein
AT1G50200	*ALANYL-TRNA SYNTHETASE (ALATS)*	Alanyl-tRNA synthetase
AT1G50230		
AT1G50240	*FUSED (FU)*	The FUSED (FU) gene belongs to Ser/Thr protein kinase family and has a key role in the hedgehog signaling pathway known to control cell proliferation and patterning in fruit flies and humans . Arabidopsis thaliana genome has a single Fu gene that is involved in male meiosis cytokinesis. Cytokinesis-defective mutants, named two-in-one (tio), result from mutations in Arabidopsis Fu. Phenotypic analysis of tio mutants reveals an essential role for TIO in conventional modes of cytokinesis in plant meristems and during male gametogenesis. TIO is tightly localized to the midline of the nascent phragmoplast and remains associated with the expanding phragmoplast ring. This gene was previously annotated as two gene models, AT1G50230.1 and AT1G50240.1, however the experimental evidence exists (Oh et al, Current Biology, 2005) showing that these two models are in fact single gene, named FUSED.
AT1G50250	*FTSH PROTEASE 1 (FTSH1)*	encodes an FTSH protease that is localized to the chloroplast. Involved in the D1 repair cycle of Photosystem II. FtsH1 and FtsH5 are interchangeable in thylakoid membranes.
AT1G50280	*BTB/POZ PROTEIN HYPERSENSITIVE TO ABA 1 (BPH1)*	BTB/POZ protein that forms a complex with CUL3a. Involved in repression of ABA responses.
AT1G50290		hypothetical protein
AT1G50300	*TBP-ASSOCIATED FACTOR 15 (TAF15)*	TBP-associated factor 15
AT1G50310	*SUGAR TRANSPORTER 9 (STP9)*	Sucrose transporter, expressed in pollen tubes.
AT1G50320	*THIOREDOXIN X (THX)*	encodes a prokaryotic thioredoxin
AT1G50340		Plant invertase/pectin methylesterase inhibitor superfamily protein
AT1G50350		E3 ubiquitin-protein ligase RING1-like protein
AT1G50390		pfkB-like carbohydrate kinase family protein
AT1G50400		Eukaryotic porin family protein
AT1G50420	*SCARECROW-LIKE 3 (SCL3)*	Encodes a scarecrow-like protein (SCL3) Putative transcription factors interacting with the gene product of VHA-B1 (vacuolar ATPase subunit B1; as shown through yeast two-hybrid assay).
AT1G50430	*DWARF 5 (DWF5)*	Mutants are defective in Brassinosteroid biosynthesis (delta7-sterol-C7 reduction step) and have a dwarf phenotype. EXO70 interactor and presumed negative secretion regulator.
AT1G50460	*HEXOKINASE-LIKE 1 (HKL1)*	Involved in glucose-ethylene crosstalk.
AT1G50470		F-box associated ubiquitination effector family protein
AT1G50480	*10-FORMYLTETRAHYDROFOLATE SYNTHETASE (THFS)*	10-formyltetrahydrofolate synthetase (THFS) mRNA, complete The mRNA is cell-to-cell mobile.
AT1G50520	*CYTOCHROME P450, FAMILY 705, SUBFAMILY A, POLYPEPTIDE 27 (CYP705A27)*	member of CYP705A The mRNA is cell-to-cell mobile.
AT1G50530		hypothetical protein
AT1G50560	*CYTOCHROME P450, FAMILY 705, SUBFAMILY A, POLYPEPTIDE 25 (CYP705A25)*	member of CYP705A
AT1G50570		Calcium-dependent lipid-binding (CaLB domain) family protein
AT1G50575		Putative lysine decarboxylase family protein
AT1G50580		UDP-Glycosyltransferase superfamily protein
AT1G50590		RmlC-like cupins superfamily protein
AT1G50600	*SCARECROW-LIKE 5 (SCL5)*	Encodes a scarecrow-like protein (SCL5). Member of GRAS gene family.
AT1G50610	*POLLEN RECEPTOR LIKE KINASE 5 (PRK5)*	Leucine-rich repeat protein kinase family protein
AT1G50630		extracellular ligand-gated ion channel protein (DUF3537)
AT1G50640	*ETHYLENE RESPONSIVE ELEMENT BINDING FACTOR 3 (ERF3)*	encodes a member of the ERF (ethylene response factor) subfamily B-1 of ERF/AP2 transcription factor family (ATERF-3). The protein contains one AP2 domain. There are 15 members in this subfamily including ATERF-3, ATERF-4, ATERF-7, and leafy petiole.
AT1G50650	*KERBEROS (KRS)*	KRS is a member of the STIG1 family of peptides. Its expression in embryos appears to be dependent upon ZOU.Loss of function results in a reduction of α-JIM12 labelled 'sheath' around the developing embryo.
AT1G50660		actin cytoskeleton-regulatory complex pan-like protein
AT1G50680		AP2/B3 transcription factor family protein
AT1G50700	*CALCIUM-DEPENDENT PROTEIN KINASE 33 (CPK33)*	Member of Calcium Dependent Protein Kinase. Mediates Strigolactone-Induced Stomatal Closure
AT1G50720		Stigma-specific Stig1 family protein
AT1G50730		hypothetical protein
AT1G50740		Transmembrane proteins 14C
AT1G50750		aminotransferase-like, mobile domain protein
AT1G50760		Aminotransferase-like, plant mobile domain family protein
AT1G50780		2Fe-2S ferredoxin-like superfamily protein
AT1G50800		
AT1G50890		ARM repeat superfamily protein

AT1G50900	*GRANA DEFICIENT CHLOROPLAST 1 (GDC1)*	Encodes GDC1 (Grana Deficient Chloroplast 1), an ankyrin domain containing protein required fro chloroplast thylakoid grana formation. The mRNA is cell-to-cell mobile.
AT1G50920	*(NOG1-1)*	Putative GTPase involved in HA - and ABA-mediated signaling pathways, particularly during defense respnses to pathogens. Has paralog NOG1-2.
AT1G50930	*VASCULAR-RELATED UNKNOWN PROTEIN 2 (VUP2)*	Serine/Threonine-kinase
AT1G50960	*GIBBERELLIN 2-OXIDASE 7 (GA2OX7)*	Encodes a protein with gibberellin 2-oxidase activity which acts specifically on C-20 gibberellins. DDF1 binds to GA2OX7 and regulates its expression in response to salt stress.
AT1G50970		Membrane trafficking VPS53 family protein
AT1G50990	*BRASSINOSTEROID-SIGNALING KINASE 11 (BSK11)*	kinase with tetratricopeptide repeat domain-containing protein
AT1G51000		hypothetical protein
AT1G51060	*HISTONE H2A 10 (HTA10)*	Encodes HTA10, a histone H2A protein. The mRNA is cell-to-cell mobile.
AT1G51070	*BASIC HELIX-LOOP-HELIX 115 (bHLH115)*	bHLH115 is a basic helix loop helix protein of the IVc subgroup that plays a role in iron homeostasis. It interacts with related family members and targets PYE and other genes involved in response to Fe.
AT1G51080		golgin family A protein
AT1G51090	*(ATHMAD1)*	Heavy metal associated domain containing protein involved in plant immunity.Mutants show an increase in root length under NO stress and reduction in root length under H2O2 stress conditions. Mutants show increases in defense responses to pathogens including hypersensitive lesions, increased resistance and induction of SAR genes.
AT1G51100	*CHLORORESPIRATORY REDUCTION 41 (CRR41)*	Chloroplast NADH dehydrogenase assembly protein. Mutants are defective in the accumulation of subcomplex A.
AT1G51110	*FIBRILLIN10 (FBN10)*	localized to chloroplasts
AT1G51115		
AT1G51140	*FLOWERING BHLH 3 (FBH3)*	Encodes a basic helix-loop-helix-type transcription factor involved in photoperiodism flowering. Binds to the E-box cis-element in the CONSTANS (CO) promoter to regulate flowering. Interacts with CFL1 and along with CFLAP2 negatively regulates cuticle development. Binds to the potassium channel gene KAT1 as a dimer. The DNA-binding capacity is inhibited in response to ABA through phosphorylation-dependent monomerization.
AT1G51190	*PLETHORA 2 (PLT2)*	Encodes a member of the AINTEGUMENTA-like (AIL) subclass of the AP2/EREBP family of transcription factors and is essential for quiescent center (QC) specification and stem cell activity. It is a key effector for establishment of the stem cell niche during embryonic pattern formation. It is transcribed in response to auxin accumulation and is dependent on auxin response transcription factors.
AT1G51210		UDP-Glycosyltransferase superfamily protein
AT1G51250		Plant self-incompatibility protein S1 family
AT1G51260	*LYSOPHOSPHATIDYL ACYLTRANSFERASE 3 (LPAT3)*	ACYL-COA:1-ACYLGLYCEROL-3-PHOSPHATE ACYLTRANSFERASE, PUTATIVE SIMILAR TO ACYL-COA:1-ACYLGLYCEROL-3-PHOSPHATE ACYLTRANSFERASE GI:4583544 FROM [BRASSICA NAPUS]
AT1G51270		vesicle-associated protein 1-4
AT1G51280		
AT1G51290		F-box and associated interaction domains-containing protein
AT1G51300		alpha/beta-Hydrolases superfamily protein
AT1G51310		tRNA (5-methylaminomethyl-2-thiouridylate)-methyltransferase
AT1G51320		F-box and associated interaction domains-containing protein
AT1G51340	*MULTI-DRUG AND TOXIC COMPOUND EXTRUSION (MATE)*	Encodes a root citrate transporter which together with the root malate transporter ALMT1 are the primary mechanism of aluminum tolerance.
AT1G51360	*DIMERIC A/B BARREL DOMAINS-PROTEIN 1 (DABB1)*	Involved in defense against fungal pathogens and located in cytosol.
AT1G51380		DEA(D/H)-box RNA helicase family protein
AT1G51390	*NFU DOMAIN PROTEIN 5 (NFU5)*	Encodes a protein containing the NFU domain that may be involved in iron-sulfur cluster assembly. Part of a five member gene family, more closely related to NFU4 than to NFU1,2, and 3. Targeted to the mitochondrion. The mRNA is cell-to-cell mobile.
AT1G51400		Photosystem II 5 kD protein
AT1G51410		similar to Eucalyptus gunnii alcohol dehydrogenase of unknown physiological function (GI:1143445), apple tree, PIR:T16995; NOT a cinnamyl-alcohol dehydrogenase
AT1G51420	*SUCROSE-PHOSPHATASE 1 (SPP1)*	sucrose-phosphatase 1
AT1G51440	*DAD1-LIKE LIPASE 2 (DALL2)*	Encodes a lipase that hydrolyzes phosphatidylcholine, glycolipids as well as triacylglycerols.
AT1G51450	*TRAUCO (TRO)*	Nuclear protein required for early embryogenesis. Encodes a structural core component of a COMPASS-like H3K4 histone methylation complex that is also involved in the timing of the floral transition and in leaf morphogenesis. Mutants are decreased in H3K4 histone trimethylation.
AT1G51460	*ATP-BINDING CASSETTE G13 (ABCG13)*	ABCG13 encodes a member of the ATP-binding cassette (ABC) transporter family protein. Mutants show defects in petal elongation resulting in a folded petal phenotype.
AT1G51480	*RESISTANCE SILENCED GENE 1 (RSG1)*	disease resistance protein (CC-NBS-LRR class) family protein
AT1G51490	*BETA GLUCOSIDASE 36 (BGLU36)*	This locus encodes a myrosinase. Arabidopsis thaliana ecotype Col-0 contains a non-functional copy but some other (not all) ecotypes contain a functional copy (reference: PubMed ID 26907263).
AT1G51500	*ATP-BINDING CASSETTE G12 (ABCG12)*	Encodes an ABC transporter involved in cuticular wax biosynthesis. Lines carrying recessive mutations in this locus have weakly glaucous stem surface, and relative elevated secondary alcohols and ketones.
AT1G51520		RNA-binding (RRM/RBD/RNP motifs) family protein
AT1G51550		Kelch repeat-containing F-box family protein
AT1G51570	*MULTIPLE C2 DOMAIN AND TRANSMEMBRANE REGION PROTEIN 4 (MCTP4)*	Required for maintenance of inflorescence and shoot SAMs and normal development of the derived vascular cambium, functions in the SAM to promote continuous organogenesis, affects SAM development through STM, where it affects intracellular localization of STM in SAM cells in the peripheral region and prevents STM localization toward the cell wall of SAM cells in the peripheral region.
AT1G51590	*ALPHA-MANNOSIDASE 1 (MNS1)*	Encodes an alpha-mannosidase I enzyme responsible for N-glycan maturation.

AT1G51600	*ZIM-LIKE 2 (ZML2)*	member of a novel family of plant-specific GATA-type transcription factors.
AT1G51610		Cation efflux family protein
AT1G51620		Protein kinase superfamily protein
AT1G51640	*EXOCYST SUBUNIT EXO70 FAMILY PROTEIN G2 (EXO70G2)*	A member of EXO70 gene family, putative exocyst subunits, conserved in land plants. Arabidopsis thaliana contains 23 putative EXO70 genes, which can be classified into eight clusters on the phylogenetic tree.
AT1G51660	*MITOGEN-ACTIVATED PROTEIN KINASE KINASE 4 (MKK4)*	Encodes a mitogen-activated map kinase kinase (there are nine in Arabidopsis) involved in innate immunity. This protein activates MPK3/MPK6 and early-defense genes redundantly with MKK5. In plants with both MKK5 and MKK4 levels reduced by RNAi plants, floral organs do not abscise suggestion a role for both proteins in mediating floral organ abscission. The mRNA is cell-to-cell mobile.
AT1G51670	*HEAT-INDUCED TAS1 TARGET 5 (HTT5)*	hypothetical protein
AT1G51680	*4-COUMARATE:COA LIGASE 1 (4CL1)*	encodes an isoform of 4-coumarate:CoA ligase (4CL), which is involved in the last step of the general phenylpropanoid pathway. In addition to 4-coumarate, it also converts ferulate. The catalytic efficiency was in the following (descending) order: p-coumaric acid, ferulic acid, caffeic acid and 5-OH-ferulic acid. At4CL1 was unable to use sinapic acid as substrate.
AT1G51690	*PROTEIN PHOSPHATASE 2A 55 KDA REGULATORY SUBUNIT B ALPHA ISOFORM (B ALPHA)*	55 kDa B regulatory subunit of phosphatase 2A mRNA,
AT1G51700	*DOF ZINC FINGER PROTEIN 1 (DOF1)*	Encodes dof zinc finger protein (adof1). The mRNA is cell-to-cell mobile.
AT1G51730		Ubiquitin-conjugating enzyme family protein
AT1G51745	*(SL1)*	Tudor/PWWP/MBT superfamily protein
AT1G51750		transposable_element_gene;non-LTR retrotransposon family (LINE), has a 1.6e-20 P-value blast match to GB:NP_038605 L1 repeat, Tf subfamily, member 30 (LINE-element) (Mus musculus);(source:TAIR10)
AT1G51780	*IAA-LEUCINE RESISTANT (ILR)-LIKE GENE 5 (ILL5)*	encodes a member of the six Arabidopsis IAA-amino acid conjugate hydrolase subfamily and conjugates and is very similar to IAR3.
AT1G51790		Leucine-rich repeat protein kinase family protein
AT1G51800	*IMPAIRED OOMYCETE SUSCEPTIBILITY 1 (IOS1)*	The gene encodes a putative member of the LRR-RLK protein family. Expressin and mutant analysis revealed that it contributes to the interaction between Arabidopsis and Hyaloperonospora arabidopsidis. and The mRNA is cell-to-cell mobile.
AT1G51805	*STRESS INDUCED FACTOR 3 (SIF3)*	Leucine-rich repeat protein kinase family protein
AT1G51810		Leucine-rich repeat protein kinase family protein
AT1G51820	*STRESS INDUCED FACTOR 4 (SIF4)*	Leucine-rich repeat protein kinase family protein
AT1G51830	*STRESS INDUCED FACTOR 1 (SIF1)*	Leucine-rich repeat protein kinase family protein
AT1G51840	*STRESS INDUCED FACTOR 1 (SIF1)*	kinase-like protein
AT1G51850	*STRESS INDUCED FACTOR 2 (SIF2)*	Malectin-like receptor-like kinase involved in MAMP mediated stomatal immunity. Interacts with BAK1/FLS2 signaling complex and subsequently phosphorylates and activates SLAC1.
AT1G51860		Leucine-rich repeat protein kinase family protein
AT1G51880	*ROOT HAIR SPECIFIC 6 (RHS6)*	root hair specific 6
AT1G51890		Leucine-rich repeat protein kinase family protein
AT1G51900	*IST1-LIKE 12 (ISTL12)*	Regulator of Vps4 activity in the MVB pathway protein
AT1G51910		Leucine-rich repeat protein kinase family protein
AT1G51915		cryptdin protein-like protein
AT1G51920		transmembrane protein
AT1G51960	*IQ-DOMAIN 27 (IQD27)*	Member of IQ67 (CaM binding) domain containing family.
AT1G51980		Insulinase (Peptidase family M16) protein
AT1G51990		O-methyltransferase family protein
AT1G52000		Mannose-binding lectin superfamily protein
AT1G52010		transposable_element_gene;Mutator-like transposase family, has a 2.8e-81 P-value blast match to O65231 /281-442 Pfam PF03108 MuDR family transposase (MuDr-element domain);(source:TAIR10)
AT1G52020		transposable_element_gene;pseudogene, Ulp1 protease family, contains Pfam profile PF02902: Ulp1 protease family, C-terminal catalytic domain;(source:TAIR10)
AT1G52040	*MYROSINASE-BINDING PROTEIN 1 (MBP1)*	Encodes myrosinase-binding protein expressed in flowers.
AT1G52050		Mannose-binding lectin superfamily protein
AT1G52060		Mannose-binding lectin superfamily protein
AT1G52070		Mannose-binding lectin superfamily protein
AT1G52080	*(AR791)*	actin binding protein family
AT1G52090		transposable_element_gene;similar to unknown protein [Arabidopsis thaliana] (TAIR:AT5G27590.1);(source:TAIR10)
AT1G52100		Mannose-binding lectin superfamily protein
AT1G52140		Avr9/Cf-9 rapidly elicited protein
AT1G52150	*(ATHB-15)*	Member of the class III HD-ZIP protein family. Contains homeodomain and leucine zipper domain. Critical for vascular development and negatively regulates vascular cell differentiation.
AT1G52160	*TRNASE Z3 (TRZ3)*	Encodes a tRNase Z.
AT1G52190	*NRT1/ PTR FAMILY 1.2 (NPF1.2)*	Encodes a low affinity nitrate transporter that is expressed in the plasma membrane and found in the phloem of the major veins of leaves. It is responsible for nitrate redistribution to young leaves.
AT1G52200		PLAC8 family protein
AT1G52220	*CURVATURE THYLAKOID 1C (CURT1C)*	Thylakoid membrane localized protein that interacts with other CURT family proteins. Oligomerization is associated with grana thylakoid curavature.
AT1G52230	*PHOTOSYSTEM I SUBUNIT H2 (PSAH2)*	Phosphorylation of this protein is dependent on calcium. The mRNA is cell-to-cell mobile.

AT1G52240	*ROP (RHO OF PLANTS) GUANINE NUCLEOTIDE EXCHANGE FACTOR 11 (ROPGEF11)*	Encodes a member of KPP-like gene family, homolog of KPP (kinase partner protein) gene in tomato. Also a member of the RopGEF (guanine nucleotide exchange factor) family, containing the novel PRONE domain (plant-specific Rop nucleotide exchanger), which is exclusively active towards members of the Rop subfamily .
AT1G52250		
AT1G52260	*PDI-LIKE 1-5 (PDIL1-5)*	Encodes a protein disulfide isomerase-like (PDIL) protein, a member of a multigene family within the thioredoxin (TRX) superfamily. Unlike several other PDI family members, transcript levels for this gene are not up-regulated in response to three different chemical inducers of ER stress (dithiothreitol, beta-mercaptoethanol, and tunicamycin).
AT1G52280	*RAB GTPASE HOMOLOG G3D (RABG3d)*	RAB GTPase homolog G3D
AT1G52340	*ABA DEFICIENT 2 (ABA2)*	Encodes a cytosolic short-chain dehydrogenase/reductase involved in the conversion of xanthoxin to ABA-aldehyde during ABA biosynthesis. Mutants are insensitive to sucrose and glucose.
AT1G52360		Coatomer, beta subunit
AT1G52390		hypothetical protein
AT1G52400	*BETA GLUCOSIDASE 18 (BGLU18)*	encodes a member of glycosyl hydrolase family 1, located in inducible ER bodies which were formed after wounding, required in inducible ER body formation The mRNA is cell-to-cell mobile.
AT1G52410	*TSK-ASSOCIATING PROTEIN 1 (TSA1)*	Contains a novel calcium-binding repeat sequence. Binds TSK in vitro. Localizes to small cytoplasmic vesicles in interphase cells. In cells synchronized for cell division, TSA1 and TSK relocalize to ends of spindle microtubules that are ahead of separating chromatids during metaphase and anaphase of mitosis. May be involved in mitosis together with TSK. Expressed preferentially in the flower and shoot apex. Can form multimers. The mRNA is cell-to-cell mobile.
AT1G52450		Ubiquitin carboxyl-terminal hydrolase-related protein
AT1G52460		α/β‐hydrolase involved in dolichol accumulation.
AT1G52470		alpha/beta-Hydrolases superfamily protein
AT1G52500	*MUTM HOMOLOG-1 (MMH-1)*	Encodes one of the splice variants of Arabidopsis thaliana MutM homolog. Encodes a formamidopyrimidine-DNA glycosylase that has abasic lyase activity and is able to nick double- stranded oligonucleotides containing 8-oxo-7,8-dihydroguanine (8-oxoG) in vitro.
AT1G52510		alpha/beta-Hydrolases superfamily protein
AT1G52520	*FAR1-RELATED SEQUENCE 6 (FRS6)*	FAR1-related sequence 6
AT1G52540		Protein kinase superfamily protein
AT1G52560	*(HSP26.5)*	HSP20-like chaperones superfamily protein
AT1G52570	*PHOSPHOLIPASE D ALPHA 2 (PLDALPHA2)*	member of C2-PLD subfamily
AT1G52580	*RHOMBOID-LIKE PROTEIN 5 (RBL5)*	RHOMBOID-like protein 5
AT1G52590		Putative thiol-disulfide oxidoreductase DCC
AT1G52610		transposable_element_gene;Mutator-like transposase family, has a 2.0e-18 P-value blast match to GB:AAA21566 mudrA of transposon=MuDR (MuDr-element) (Zea mays);(source:TAIR10)
AT1G52630		O-fucosyltransferase family protein
AT1G52640		Pentatricopeptide repeat (PPR) superfamily protein
AT1G52660		P-loop containing nucleoside triphosphate hydrolases superfamily protein
AT1G52670	*BCCP-LIKE PROTEIN 1 (BLP1)*	Single hybrid motif superfamily protein
AT1G52680		late embryogenesis abundant protein-related / LEA protein-like protein
AT1G52690	*LATE EMBRYOGENESIS ABUNDANT 7 (LEA7)*	Late embryogenesis abundant protein (LEA) family protein
AT1G52700		alpha/beta-Hydrolases superfamily protein
AT1G52710		Rubredoxin-like superfamily protein
AT1G52720		hypothetical protein
AT1G52760	*LYSOPHOSPHOLIPASE 2 (LysoPL2)*	Encodes caffeoyl shikimate esterase and is involved in lignin biosynthesis. CSE converts caffeoyl shikimate to caffiate. Loss of function mutations have reduced lignin content and collapsed vessel elements. It is also reported to function as a lysophospholipase 2 (LysoPL2) involved in tolerance to cadmium-induced oxidative stress. Binds Acyl-CoA-binding protein 2 (ACBP2).
AT1G52770		Phototropic-responsive NPH3 family protein
AT1G52790		encodes a putative oxidoreductase, 2OG-Fe(II) oxygenase family protein, similar to GS-AOP loci (GI:16118889, GI:16118887, GI:16118891, GI:16118893); contains PF03171 2OG-Fe(II) oxygenase superfamily domain
AT1G52800		2-oxoglutarate (2OG) and Fe(II)-dependent oxygenase superfamily protein
AT1G52810		2-oxoglutarate (2OG) and Fe(II)-dependent oxygenase superfamily protein
AT1G52820		2-oxoglutarate (2OG) and Fe(II)-dependent oxygenase superfamily protein
AT1G52830	*INDOLE-3-ACETIC ACID 6 (IAA6)*	An extragenic dominant suppressor of the hy2 mutant phenotype. Also exhibits aspects of constitutive photomorphogenetic phenotype in the absence of hy2. Mutants have dominant leaf curling phenotype shortened hypocotyls and reduced apical hook. Induced by indole-3-acetic acid.
AT1G52840		transposable_element_gene;similar to unknown protein [Arabidopsis thaliana] (TAIR:AT2G13865.1);(source:TAIR10)
AT1G52870		Peroxisomal membrane 22 kDa (Mpv17/PMP22) family protein
AT1G52880	*NO APICAL MERISTEM (NAM)*	Transcription factor with a NAC domain. Homologous to the petunia gene NAM which is required for the development of the shoot. Expressed in the embryo.
AT1G52890	*NAC DOMAIN CONTAINING PROTEIN 19 (NAC019)*	encodes a NAC transcription factor whose expression is induced by drought, high salt, and abscisic acid. This gene binds to ERD1 promoter in vitro.
AT1G52900		Toll-Interleukin-Resistance (TIR) domain family protein
AT1G52910		fiber (DUF1218)

AT1G52920	*G PROTEIN COUPLED RECEPTOR (GPCR)*	Encodes a plasma membrane?localized ABA receptor, which interacts with the Gαβγ complex. It has been postulated that the binding of ABA to GCR2 results in the release of the G protein and dissociation of the heterotrimeric complex into Gα and the Gβγ dimer to activate downstream ABA effectors and to trigger the ABA responses.
AT1G52930	*ARABIDOPSIS HOMOLOGUE OF YEAST BRX1 2 (ATBRX1-2)*	Encodes one of two Arabidopsis orthologs of yeast BRX1, a protein involved in maturation of the large ribosomal subunit. The proteins are mainly localized in nucleolus. Mutant plants are affected in pre-rRNA processing.
AT1G52940	*PURPLE ACID PHOSPHATASE 5 (PAP5)*	Encodes a purple acid phosphatase that is induced under prolonged phosphate (Pi) starvation and is required for maintaining basal resistance against Pseudomonas syringae and Botrytis cinerea.
AT1G52950		Nucleic acid-binding, OB-fold-like protein
AT1G52970	*DOWNREGULATED IN DIF1 11 (DD11)*	downregulated in DIF1 11
AT1G52980	*NUCLEAR/NUCLEOLAR GTPASE 2 (AtNug2)*	Encodes a GTPase that belongs to the subfamily of YlqF/YawG GTPases. Functions in Pre-60S ribosomal subunit maturation. The mRNA is cell-to-cell mobile.
AT1G52990		thioredoxin family protein
AT1G53010	*ARABIDOPSIS T??XICOS EN LEVADURA 19 (ATL19)*	RING/U-box superfamily protein
AT1G53020	*UBIQUITIN-CONJUGATING ENZYME 26 (UBC26)*	Cognate nuclear E2 enzyme that interacts with the RFA4 E3 ligase and forms UBC26-RFA4-Receptor complexes in nuclear speckles.
AT1G53030		encodes a copper chaperone, can functional complements the yeast COX17 null mutant. May play a role in the delivery of copper to mitochondria. Expressed in roots and thus may also play a role in copper transport in the roots.
AT1G53035		transmembrane protein
AT1G53060		Legume lectin family protein
AT1G53070		Legume lectin family protein
AT1G53080		Legume lectin family protein
AT1G53090	*SPA1-RELATED 4 (SPA4)*	Encodes a member of the SPA (suppressor of phyA-105) protein family (SPA1-SPA4). SPA proteins contain an N-terminal serine/threonine kinase-like motif followed by a coiled-coil structure and a C-terminal WD-repeat domain. SPA proteins function redundantly in suppressing photomorphogenesis in dark- and light-grown seedlings. SPA4 (and SPA3) predominantly regulates elongation growth in adult plants.
AT1G53100		Core-2/I-branching beta-1,6-N-acetylglucosaminyltransferase family protein
AT1G53140	*DYNAMIN RELATED PROTEIN 5A (DRP5A)*	Encodes DRP5A, a dynamin protein involved in cytokinesis in Arabidopsis.
AT1G53165	*(ATMAP4K ALPHA1)*	Protein kinase superfamily protein
AT1G53170	*ETHYLENE RESPONSE FACTOR 8 (ERF8)*	encodes a member of the ERF (ethylene response factor) subfamily B-1 of ERF/AP2 transcription factor family (ATERF-8). The protein contains one AP2 domain. There are 15 members in this subfamily including ATERF-3, ATERF-4, ATERF-7, and leafy petiole.
AT1G53180		hypothetical protein
AT1G53200		TAF RNA polymerase I subunit A
AT1G53230	*TEOSINTE BRANCHED 1, CYCLOIDEA AND PCF TRANSCRIPTION FACTOR 3 (TCP3)*	Encodes a member of a recently identified plant transcription factor family that includes Teosinte branched 1, Cycloidea 1, and proliferating cell nuclear antigen (PCNA) factors, PCF1 and 2. Regulated by miR319. Involved in heterochronic regulation of leaf differentiation.
AT1G53260		hypothetical protein
AT1G53270	*ATP-BINDING CASSETTE G10 (ABCG10)*	ABC-2 type transporter family protein
AT1G53280	*DJ-1 HOMOLOG B (DJ1B)*	Encodes a homolog of animal DJ-1 superfamily protein. In the A. thaliana genome, three genes encoding close homologs of human DJ-1 were identified AT3G14990 (DJ1A), AT1G53280 (DJ1B) and AT4G34020 (DJ1C). Among the three homologs, DJ1C is essential for chloroplast development and viability. It exhibits glyoxalase activity towards glyoxal and methylglyoxal.
AT1G53300	*TETRATRICOPEPTIDE-REPEAT THIOREDOXIN-LIKE 1 (TTL1)*	Encodes one of the 36 carboxylate clamp (CC)-tetratricopeptide repeat (TPR) proteins (Prasad 2010, Pubmed ID: 20856808) with potential to interact with Hsp90/Hsp70 as co-chaperones. The TTL family is required for osmotic stress tolerance and male sporogenesis. The mRNA is cell-to-cell mobile.
AT1G53310	*PHOSPHOENOLPYRUVATE CARBOXYLASE 1 (PPC1)*	Encodes one of four Arabidopsis phosphoenolpyruvate carboxylase proteins.Plays an important role in carbon and nitrogen metabolism.
AT1G53320	*TUBBY LIKE PROTEIN 7 (TLP7)*	Member of plant TLP family. TLP7 is tethered to the PM but detaches upon stimulus and translocates to the nucleus. Has DNA binding activity but lacks conservation of the transcription activation domain.
AT1G53325		F-box associated ubiquitination effector family protein
AT1G53340		Cysteine/Histidine-rich C1 domain family protein
AT1G53345		DHHA1 domain protein
AT1G53350		Disease resistance protein (CC-NBS-LRR class) family
AT1G53360		F-box/associated interaction domain protein
AT1G53390	*ATP-BINDING CASSETTE G24 (ABCG24)*	P-loop containing nucleoside triphosphate hydrolases superfamily protein
AT1G53420		Leucine-rich repeat transmembrane protein kinase
AT1G53430	*NEMATODE-INDUCED LRR-RLK 2 (NILR2)*	Probable LRR receptor-like ser/thr-protein kinase; Commonly-enriched candidate LPS-interacting PM-associated proteins for both LPS chemotypes subsequent to the polymyxin B affinity chromatography strategy.
AT1G53440		Leucine-rich repeat transmembrane protein kinase
AT1G53450		epstein-barr nuclear antigen
AT1G53500	*MUCILAGE-MODIFIED 4 (MUM4)*	encodes a putative NDP-L-rhamnose synthase, an enzyme required for the synthesis of the pectin rhamnogalacturonan I, the major component of Arabidopsis mucilage. Gene is involved in seed coat mucilage cell development. Mutant analyses suggest that MUM4 is required for complete mucilage synthesis, cytoplasmic rearrangement and seed coat development.
AT1G53520	*FATTY-ACID-BINDING PROTEIN 3 (FAP3)*	Encodes a plastid stroma localized fatty acid binding protein involved in fatty acid metabolism.
AT1G53530	*(ATIMP1A)*	Mitochondrial ATP-independent protease
AT1G53540	*(HSP17.6C)*	Member of the class I small heat-shock protein (sHSP) family, which accounts for the majority of sHSPs in maturing seeds

AT1G53560		Ribosomal protein L18ae family
AT1G53580	*GLYOXALASE II 3 (GLY3)*	Mononuclear Fe(II)-containing member of the b-lactamase fold superfamily. ETHE1 is homodimeric in solution, exhibits low-level esterase activity, and specifically binds a single Fe(II) atom in the active site.
AT1G53600		Tetratricopeptide repeat (TPR)-like superfamily protein
AT1G53620		transmembrane protein
AT1G53640		transmembrane protein
AT1G53645		hydroxyproline-rich glycoprotein family protein
AT1G53650	*CTC-INTERACTING DOMAIN 8 (CID8)*	RNA-binding protein, putative, similar to RNA-binding protein GB:AAA86641 GI:1174153 from (Arabidopsis thaliana).Contains PAB2 domain which facilitates binding to PABC proteins.
AT1G53660		Nucleotide/sugar transporter family protein
AT1G53670	*METHIONINE SULFOXIDE REDUCTASE B 1 (MSRB1)*	1-Cys methionine sulfoxide reductase.
AT1G53680	*GLUTATHIONE S-TRANSFERASE TAU 28 (GSTU28)*	Encodes glutathione transferase belonging to the tau class of GSTs. Naming convention according to Wagner et al. (2002).
AT1G53690		Protein of unknown function that is homologous to At5g41010, which encodes a non-catalytic subunit common to nuclear DNA-dependent RNA polymerases II, IV and V; homologous to budding yeast RPB12.
AT1G53700	*WAG 1 (WAG1)*	The WAG1 and its homolog, WAG2 each encodes a protein-serine/threonine kinase that are nearly 70% identical to PsPK3 protein. All three together with CsPK3 belong to PsPK3-type kinases. At the N-terminus, all four possess a serine/threonine-rich domain. They are closely related to Arabidopsis kinases PINOID. wag1/wag2 double mutants exhibit a pronounced wavy root phenotype when grown vertically on agar plates (while wild-type plants develop wavy roots only on plates inclined to angles less than 90 degrees), indicating an overlapping role for WAG1 and WAG2 as suppressors of root waving. Simultaneous disruption of PID(AT2G34650) and its 3 closest homologs (PID2/AT2G26700, WAG1/AT1G53700, and WAG2/AT3G14370) abolishes the formation of cotyledons.
AT1G53710		Calcineurin-like metallo-phosphoesterase superfamily protein
AT1G53720	*CYCLOPHILIN 59 (CYP59)*	Encodes a cyclophilin, member of a family modular proteins consisting of a peptidyl-prolyl cis? trans isomerase (PPIase) domain, followed by an RNA recognition motif (RRM), and a C-terminal domain enriched in charged amino acids. Interacts with with SCL33/SR33 and with a majority of Arabidopsis SR proteins and the largest subunit of RNA polymerase II. Localizes to the nucleus, but it does not significantly colocalize with SR proteins in nuclear speckles.
AT1G53750	*REGULATORY PARTICLE TRIPLE-A 1A (RPT1A)*	26S proteasome AAA-ATPase subunit RPT1a (RPT1a) mRNA,
AT1G53760		K+-H+ exchange-like protein
AT1G53790		F-box and associated interaction domains-containing protein
AT1G53800		muscle M-line assembly protein
AT1G53810		transposable_element_gene;copia-like retrotransposon family, has a 8.0e-248 P-value blast match to GB:AAA57005 Hopscotch polyprotein (Ty1_Copia-element) (Zea mays);(source:TAIR10)
AT1G53820	*ARABIDOPSIS T??XICOS EN LEVADURA 60 (ATL60)*	RING/U-box superfamily protein
AT1G53830	*PECTIN METHYLESTERASE 2 (PME2)*	encodes a pectin methylesterase
AT1G53885		linoleate 9S-lipoxygenase-4 protein, putative (DUF581). Senescence associated protein, expression regulated by cytokinin.
AT1G53910	*RELATED TO AP2 12 (RAP2.12)*	Encodes a member of the ERF (ethylene response factor) subfamily B-2 of ERF/AP2 transcription factor family (RAP2.12). The protein contains one AP2 domain. There are 5 members in this subfamily including RAP2.2 AND RAP2.12. Involved in oxygen sensing. It plays a key role in controlling root bending in response to hypoxia.
AT1G53920	*GDSL-MOTIF LIPASE 5 (GLIP5)*	Contains lipase signature motif and GDSL domain.
AT1G53930		Ubiquitin-like superfamily protein
AT1G53940	*GDSL-MOTIF LIPASE 2 (GLIP2)*	Encodes a lipase, has in vitro lipase activity with p-nitrophenyl acetate and p-nitrophenyl butyrate, gene expression induced by hormones, negatively regulates auxin signaling, involved in disease resistance
AT1G53950		ubiquitin-40S ribosomal S27a-like protein
AT1G53970		GDSL esterase/lipase-like protein
AT1G53980		Ubiquitin-like superfamily protein
AT1G53990	*GDSL-MOTIF LIPASE 3 (GLIP3)*	Contains lipase signature motif and GDSL domain. The mRNA is cell-to-cell mobile.
AT1G54010	*GDSL-LIKE LIPASE 23 (GLL23)*	GDSL-motif esterase/acyltransferase/lipase. Enzyme group with broad substrate specificity that may catalyze acyltransfer or hydrolase reactions with lipid and non-lipid substrates.
AT1G54020		GDSL-motif esterase/acyltransferase/lipase. Enzyme group with broad substrate specificity that may catalyze acyltransfer or hydrolase reactions with lipid and non-lipid substrates.
AT1G54030	*MODIFIED VACUOLE PHENOTYPE 1 (MVP1)*	Encodes a vacuolar protein. Mutation causes organizational defects in the endoplasmic reticulum and aberrant protein trafficking in the plant secretory pathway. The mRNA is cell-to-cell mobile.
AT1G54040	*EPITHIOSPECIFIER PROTEIN (ESP)*	Epithiospecifier protein, interacts with WRKY53. Involved in pathogen resistance and leaf senescence.
AT1G54050	*(HSP17.4B)*	HSP20-like chaperones superfamily protein
AT1G54060	*6B-INTERACTING PROTEIN 1-LIKE 1 (ASIL1)*	Member of the trihelix DNA binding protein family. Nuclear localized. Involved in repressing seed maturation genes during seed germination and seedling development.
AT1G54070		Dormancy/auxin associated family protein
AT1G54100	*ALDEHYDE DEHYDROGENASE 7B4 (ALDH7B4)*	Aldehyde dehydrogenase
AT1G54110		Membrane fusion protein Use1
AT1G54120		hypothetical protein

AT1G54130	*RELA/SPOT HOMOLOG 3 (RSH3)*	This gene appears to be at least partially redundant with RSH2 (At3g14050). Guanosine tetraphosphate synthesized by RSH2/RSH3 (and CRSH At3g17470) to an unknown extent can repress chloroplast gene expression, and also reduce chloroplast size. Involved in the maintenance of the (p)ppGp level to accustom plastidial gene expression to darkness.
AT1G54160	*NUCLEAR FACTOR Y, SUBUNIT A5 (NF-YA5)*	Encodes a member of the CCAAT-binding transcription factor (CBF-B/NF-YA) family. Expression is upregulated in response to ABA and drought. This regulation appears to be mediated by MIR169A which is downregulated in response to drought. NFYA5 is a target of MIR169A. Loss of function mutations are hypersensitive to drought.
AT1G54180	*BREVIS RADIX-LIKE 3 (BRX-LIKE3)*	Belongs to five-member BRX gene family. Arabidopsis BRX genes share high levels of similarity among each others, with several conserved domains. The most distinct is BRX domain - highly conserved in all BRX genes among distantly related species. This protein-protein interaction domain is required and sufficient for BRX activity.
AT1G54190		
AT1G54200	*BIG GRAIN 3 (BG3)*	DNA mismatch repair Msh6-like protein
AT1G54220	*MITOCHONDRIAL PYRUVATE DEHYDROGENASE SUBUNIT 2-3 (MTE2-3)*	Encodes a subunit of the mitochondrial pyruvate dehydrogenase complex.
AT1G54230		Winged helix-turn-helix transcription repressor DNA-binding protein
AT1G54240		winged-helix DNA-binding transcription factor family protein
AT1G54250	*(NRPB8A)*	One of two highly similar proteins that can serve as non-catalytic subunits of Nuclear RNA polymerases II and V; homologous to budding yeast RPB8. Probably redundant with At3g59600.
AT1G54260		winged-helix DNA-binding transcription factor family protein
AT1G54270	*EIF4A-2 (EIF4A-2)*	member of eIF4A - eukaryotic initiation factor 4A
AT1G54280	*AMINOPHOSPHOLIPID ATPASE 6 (ALA6)*	Encodes a member of the P4 subfamily of P-type ATPases expressed in the pollen plasma membrane. Double mutants with ALA7 display pollen and pollen tube defects.
AT1G54310		S-adenosyl-L-methionine-dependent methyltransferases superfamily protein
AT1G54330	*NAC DOMAIN CONTAINING PROTEIN 20 (NAC020)*	NAC domain containing protein 20
AT1G54340	*ISOCITRATE DEHYDROGENASE (ICDH)*	NADP-specific isocitrate dehydrogenase (ICDH) The mRNA is cell-to-cell mobile.
AT1G54360	*TBP-ASSOCIATED FACTOR 6B (TAF6B)*	Encodes one of two Arabidopsis proteins with significant similarity to the histone fold TBP-associated factor TAF6.
AT1G54410	*DEHYDRIN 11KDA (HIRD11)*	Encodes a KS-type dehydrin can reduce the formation of reactive oxygen species (ROS) from Cu.
AT1G54420		hypothetical protein
AT1G54430		transposable_element_gene;similar to unknown protein [Arabidopsis thaliana] (TAIR:AT3G13250.1);(source:TAIR10)
AT1G54450		Calcium-binding EF-hand family protein
AT1G54460		TPX2 (targeting protein for Xklp2) protein family
AT1G54470	*RESISTANCE TO PERONOSPORA PARASITICA 27 (RPP27)*	Encodes a Cf-like gene in Arabidopsis that confers downy mildew resistance to several isolates of Peronospora parasitica.
AT1G54480		
AT1G54490	*EXORIBONUCLEASE 4 (XRN4)*	Involved in the ethylene response. XRN4 does not appear to regulate ethylene signaling via an RNA-INDUCED SILENCING COMPLEX-based RNA silencing mechanism but acts by independent means. Endogenous suppressor of posttranscriptional gene silencing. The mRNA is cell-to-cell mobile.
AT1G54500		RBD1 is a thylakoid membrane-bound iron-binding protein that is required for the proper assembly of photosystem II in Arabidopsis. It is found in all oxygenic photoautotrophic organisms (plants, algae and cyanobacteria).
AT1G54510	*NIMA-RELATED SERINE/THREONINE KINASE 1 (NEK1)*	Encodes AtNEK1, a member of the NIMA-related serine/threonine kinases (Neks) that have been linked to cell-cycle regulation in fungi and mammals. Plant Neks might be involved in plant development processes.
AT1G54540	*(NHL4)*	Late embryogenesis abundant (LEA) hydroxyproline-rich glycoprotein family
AT1G54560	*(XIE)*	Encodes a class XI myosin that is involved in organelle motility, actin organization, and optimal growth of pollen tubes.
AT1G54570	*PHYTYL ESTER SYNTHASE 1 (PES1)*	Encodes a protein with phytyl ester synthesis and diacylglycerol acyltransferase activities that is involved in the deposition of free phytol and free fatty acids in the form of phytyl esters in chloroplasts, a process involved in maintaining the integrity of the photosynthetic membrane during abiotic stress and senescence.
AT1G54630	*ACYL CARRIER PROTEIN 3 (ACP3)*	encodes an acyl carrier protein expressed in leaves, roots, and dry seeds. Gene expression is not regulated by light.
AT1G54640		F-box family protein-like protein
AT1G54650	*TRNA METHYLTRANSFERASE 140B (TRM140B)*	Methyltransferase family protein
AT1G54690	*GAMMA HISTONE VARIANT H2AX (GAMMA-H2AX)*	Encodes HTA3, a histone H2A protein. H2AX is a meiosis-specific isoform of histone H2A. Upon DSB formation, rapid accumulation of phosphorylated H2AX (γ-H2AX) occurs around the break site. H2AX foci accumulate in early G2. Immunolocalization studies in spread preparations of wild-type meiocytes at G2/early leptotene revealed the accumulation of numerous rather diffuse γ-H2AX foci throughout the chromatin. However, their accumulation is not contemporaneous with that of AtSPO11-1. At 3 h post-S, no γ-H2AX foci are detected. During the 3- to 5-h window when AtSPO11-1 foci rapidly disappear, there is an equally swift accumulation of γ-H2AX to a maximum of >50 diffuse foci. The level of γH2AX then remains constant for a further 13 h before undergoing a gradual decrease to 10?20 foci in the 18- to 24-h post-S period. By 30 h the foci have disappeared from the chromatin.
AT1G54700		hypothetical protein
AT1G54720		early-responsive to dehydration protein-related / ERD protein-like protein
AT1G54730		Major facilitator superfamily protein
AT1G54740		FANTASTIC four-like protein (DUF3049)
AT1G54780	*THYLAKOID LUMEN PROTEIN 18.3 (TLP18.3)*	Encodes a thylakoid lumen protein regulating photosystem II repair cycle. Has acid phosphatase activity. The mRNA is cell-to-cell mobile.
AT1G54790		GDSL-motif esterase/acyltransferase/lipase. Enzyme group with broad substrate specificity that may catalyze acyltransfer or hydrolase reactions with lipid and non-lipid substrates.
AT1G54820		Protein kinase superfamily protein

AT1G54830	*NUCLEAR FACTOR Y, SUBUNIT C3 (NF-YC3)*	Encodes a NUCLEAR FACTOR-Y C (NF-YC) homologue NF-YC3. NF-YC3., NF-YC4 and NF-YC9 redundantly modulate GA- and ABA-mediated seed germination.
AT1G54840	*INCREASED DNA METHYLATION 2 (IDM2)*	Encodes an atypical member of the sHSP20 family that is involved in histone demethylation. Loss of function mutations show increased methylation. IMD2 co-localizes to the nucleus with, and physically interacts with, IMD1, a protein involved in RNA directed DNA methylation. IMD2 contains an alpha crystallin domain , that is required for its function.
AT1G54860		Glycoprotein membrane precursor GPI-anchored
AT1G54870	*CHLOROPLAST ALDEHYDE REDUCTASE (CHLADR)*	ChlADR is an aldehyde reductase that catalyzes the reduction of the aldehyde carbonyl groups on saturated and alpha,beta-unsaturated aldehydes with more than 5 carbons in vitro. The N-terminal region of this protein directs GFP to the chloroplast where where ChlADR likely helps to maintain the photosynthetic process by detoxifying reactive carbonyls formed during lipid peroxidation. In addition, this enzyme can also reduce cis-3-hexenal, a major plant volatile compound that contributes to green leaf odor, as well as methylglyoxal in vitro.
AT1G54880		
AT1G54890		Late embryogenesis abundant (LEA) protein-like protein
AT1G54920		hypothetical protein
AT1G54923		transposable_element_gene;similar to unknown protein [Arabidopsis thaliana] (TAIR:AT3G30816.1);(source:TAIR10)
AT1G54926		transposable_element_gene;similar to unknown protein [Arabidopsis thaliana] (TAIR:AT3G47240.1);(source:TAIR10)
AT1G54940	*PLANT GLYCOGENIN-LIKE STARCH INITIATION PROTEIN 4 (PGSIP4)*	Encodes a xylan glucuronosyltransferase.
AT1G54955		transposable_element_gene;similar to unknown protein [Arabidopsis thaliana] (TAIR:AT4G05145.1);(source:TAIR10)
AT1G54990	*AUXIN RESISTANT 4 (AXR4)*	auxin response mutant (AXR4) The mRNA is cell-to-cell mobile.
AT1G55020	*LIPOXYGENASE 1 (LOX1)*	lipoxygenase, a defense gene conferring resistance Xanthomonas campestris The mRNA is cell-to-cell mobile.
AT1G55030		RNI-like superfamily protein
AT1G55040	*SLOW EMBRYO DEVELOPMENT1 (SED1)*	SED1 is a protein of unknown function that is located in the mitochondrion. sed1 mutants are embryo lethal.
AT1G55050	*(SANT4)*	hypothetical protein
AT1G55060	*UBIQUITIN 12 (UBQ12)*	Ubiquitin-like gene, believed to be a pseudogene because of amino acid substitutions in 3 of the 5 ubiquitin repeats found in the UBQ12 gene product
AT1G55080	*(MED9)*	mediator of RNA polymerase II transcription subunit-like protein
AT1G55090		carbon-nitrogen hydrolase family protein
AT1G55110	*INDETERMINATE(ID)-DOMAIN 7 (IDD7)*	indeterminate(ID)-domain 7
AT1G55120	*BETA-FRUCTOFURANOSIDASE 5 (FRUCT5)*	Encodes a protein with fructan exohydrolase (FEH) activity acting on levan-type fructans (6-FEH, levanase). The enzyme does not have invertase activity.
AT1G55180	*PHOSPHOLIPASE D ALPHA 4 (PLDEPSILON)*	member of C2-PLD. subfamily Represents a phospholipase D (PLD) gene with four exons, hence it is a member of the alpha class. Its amino acid sequence is quite different from other PLDs, therefore it might possess unique structural and/or catalytic properties.
AT1G55200		kinase with adenine nucleotide alpha hydrolases-like domain-containing protein
AT1G55210		Disease resistance-responsive (dirigent-like protein) family protein
AT1G55220		hypothetical protein
AT1G55240		proteinase inhibitor I4, serpin (DUF716)
AT1G55250	*HISTONE MONO-UBIQUITINATION 2 (HUB2)*	Encodes one of two orthologous E3 ubiquitin ligases in Arabidopsis that are involved in monoubiquitination of histone H2B.
AT1G55260	*GLYCOSYLPHOSPHATIDYLINOSITOL-ANCHORED LIPID PROTEIN TRANSFER 6 (LTPG6)*	Bifunctional inhibitor/lipid-transfer protein/seed storage 2S albumin superfamily protein
AT1G55265		DUF538 family protein, putative (Protein of unknown function, DUF538)
AT1G55290	*(F6'H2)*	encodes a protein whose sequence is similar to oxidoreductase, 2OG-Fe(II) oxygenase
AT1G55315		
AT1G55330	*ARABINOGALACTAN PROTEIN 21 (AGP21)*	Encodes a putative arabinogalactan-protein (AGP21).
AT1G55360		tRNA-splicing ligase (DUF239)
AT1G55370	*NDH-DEPENDENT CYCLIC ELECTRON FLOW 5 (NDF5)*	NDH-dependent cyclic electron flow 5
AT1G55380		Cysteine/Histidine-rich C1 domain family protein
AT1G55390		Cysteine/Histidine-rich C1 domain family protein
AT1G55410		pseudogene of Cysteine/Histidine-rich C1 domain family protein
AT1G55430		Cysteine/Histidine-rich C1 domain family protein
AT1G55440		Cysteine/Histidine-rich C1 domain family protein
AT1G55450		S-adenosyl-L-methionine-dependent methyltransferases superfamily protein
AT1G55480	*PROTEIN CONTAINING PDZ DOMAIN, A K-BOX DOMAIN, AND A TPR REGION (ZKT)*	Encodes a member of a novel plant protein family containing a PDZ, a K-box, and a TPR motif. mRNA but not protein levels decrease after wounding. ZKT is phosphorylated at Thr and Ser residues after wounding. The mRNA is cell-to-cell mobile.
AT1G55490	*CHAPERONIN 60 BETA (CPN60B)*	encodes the beta subunit of the chloroplast chaperonin 60, a homologue of bacterial GroEL. Mutants in this gene develops lesions on its leaves, expresses systemic acquired resistance (SAR) and develops accelerated cell death to heat shock stress. The protein has molecular chaperone activity for suppressing protein aggregation in vitro.
AT1G55500	*EVOLUTIONARILY CONSERVED C-TERMINAL REGION 4 (ECT4)*	YTHDF protein which togeteher with ECT2 and ECT3 is involved in cell proliferation during plant organogenesis.
AT1G55530	*BCA2A ZINC FINGER ATL 6 (BTL06)*	RING/U-box superfamily protein
AT1G55545		
AT1G55550		P-loop containing nucleoside triphosphate hydrolases superfamily protein
AT1G55560	*SKU5 SIMILAR 14 (sks14)*	SKU5 similar 14
AT1G55570	*SKU5 SIMILAR 12 (sks12)*	SKU5 similar 12

AT1G55580	*LATERAL SUPPRESSOR (LAS)*	Encodes a member of the GRAS family of putative transcriptional regulators. It is involved in the initiation of axillary meristems during both the vegetative and reproductive growth phases and functions upstream of REV and AXR1 in the regulation of shoot branching.
AT1G55600	*WRKY DNA-BINDING PROTEIN 10 (WRKY10)*	member of WRKY Transcription Factor; Group I. It has WRKY domain at its N terminal end and zinc-finger like motif.
AT1G55610	*BRI1 LIKE (BRL1)*	mutant has Altered vascular cell differentiation; LRR Receptor Kinase
AT1G55640	*PRENYLATED RAB ACCEPTOR 1.G1 (PRA1.G1)*	prenylated RAB acceptor 1.G1
AT1G55660	*F-BOX OF FLOWERING 2 (FOF2)*	FOF2, is the F-box protein family. Overexpression of FOF2 results in delayed transitions to flowering under both LD and SD conditions.FOF2 expression is induced by ABA during seed germination where it acts through ABI3 and ABI5 to modulate germination.
AT1G55670	*PHOTOSYSTEM I SUBUNIT G (PSAG)*	Encodes subunit G of photosystem I, an 11-kDa membrane protein that plays an important role in electron transport between plastocyanin and PSI and is involved in the stability of the PSI complex. PSI-G subunit is bound to PSI-B and is in contact with Lhca1. The protein inserts into thylakoids by a direct or "spontaneous" pathway that does not involve the activities of any known chloroplast protein-targeting machinery. PSI-G appears to be directly or indirectly involved in the interaction between Photosystem I and plastocyanin.
AT1G55700		Cysteine/Histidine-rich C1 domain family protein
AT1G55730	*CATION EXCHANGER 5 (CAX5)*	member of Low affinity calcium antiporter CAX2 family
AT1G55740	*SEED IMBIBITION 1 (SIP1)*	seed imbibition 1
AT1G55760	*STRESS-INDUCED BTB PROTEIN1 (SIBP1)*	Expression induced under NaCl, mannitol, ABA and indole-3-acetic acid (IAA) treatment.
AT1G55780		
AT1G55800		hypothetical protein
AT1G55810	*URIDINE KINASE-LIKE 3 (UKL3)*	One of the homologous genes predicted to encode proteins with UPRT domains (Uracil phosphoribosyltransferase). Five of these genes (At5g40870, At3g27190, At1g55810, At4g26510 and At3g27440) show a high level of identity, and are annotated as also containing a N-terminal uracil kinase (UK) domain. These genes are referred to as UKL1 (UK-like 1), UKL2, UKL3, UKL4 and UKL5, respectively.
AT1G55820		lysine-specific demethylase, putative (DUF1296)
AT1G55830		coiled-coil protein
AT1G55840		Sec14p-like phosphatidylinositol transfer family protein
AT1G55850	*CELLULOSE SYNTHASE LIKE E1 (CSLE1)*	encodes a protein similar to cellulose synthase The mRNA is cell-to-cell mobile.
AT1G55870	*ABA-HYPERSENSITIVE GERMINATION 2 (AHG2)*	Encodes a poly(A)-specific ribonuclease, AtPARN. Expression of AtPARN is upregulated by ABA or stress treatment. Mutant is hypersensitivity to salicylic acid as well as ABA. Functions with AGS1 to regulate the poly(A) status of mitochondrial mRNA.
AT1G55880		Pyridoxal-5-phosphate-dependent enzyme family protein
AT1G55900	*(TIM50)*	component of a translocase in the mitochondrial inner membrane
AT1G55910	*ZINC TRANSPORTER 11 PRECURSOR (ZIP11)*	member of Putative zinc transporter ZIP2 - like family
AT1G55915	*WSS1/SPRTN TYPE REPAIR PROTEASE A (WSS1A)*	DNA-protein crosslink repair protease. WSS1A is involved in DNA-protein crosslink (DPC) repair and acts in independent pathways of the nuclease MUS81 and the phosphodiesterase TDP1. WSS1A is involved in the repair of replicative DNA damage (determination of dead cells in the root meristem using propidium Iodide staining). CRISPR wss1A mutants are camptothecin (CPT) and cis-platin hypersensitive and exhibit a fasciated growth phenotype and shortened roots
AT1G55920	*SERINE ACETYLTRANSFERASE 2;1 (SERAT2;1)*	Encodes a chloroplast/cytosol localized serine O-acetyltransferase involved in sulfur assimilation and cysteine biosynthesis. Expressed in the vascular system. The mRNA is cell-to-cell mobile.
AT1G55940	*CYTOCHROME P450, FAMILY 708, SUBFAMILY A, POLYPEPTIDE 1 (CYP708A1)*	cytochrome P450 family protein
AT1G55950		DNA-binding storekeeper protein-related transcriptional regulator
AT1G55970	*HISTONE ACETYLTRANSFERASE OF THE CBP FAMILY 4 (HAC4)*	HAC4 is most likely to be an expressed pseudogene that lacks HAT function. there is a single nucleotide deletion in both the HAC4 genomic and cDNA sequences relative to its homologs. The resulting frameshift within the open reading frame causes a stop codon to occur within the predicted acetyltransferase catalytic domain.
AT1G55980		FAD/NAD(P)-binding oxidoreductase family protein
AT1G55990		glycine-rich protein
AT1G56010	*NAC DOMAIN CONTAINING PROTEIN 1 (NAC1)*	Encodes a transcription factor involved auxin-mediated lateral root formation. Acts downstream of TIR1 and is regulated post-transcriptionally by miRNA164 and by SINAT5-dependent ubiquitination.
AT1G56020		serine/arginine repetitive matrix-like protein
AT1G56030	*(PUB57)*	RING/U-box protein
AT1G56040	*(PUB58)*	HEAT/U-box protein
AT1G56060	*CYSTEINE-RICH TRANSMEMBRANE MODULE 3 (ATHCYSTM3)*	CYSTM3 is a mitochondrial protein that is induced by salt stress and is a negative regulator of salt stress.
AT1G56080	*NAI2-INTERACTING PROTEIN 3 (NAIP3)*	NAI1 interacting protein, involved in ER body formation.
AT1G56090		Tetratricopeptide repeat (TPR)-like superfamily protein
AT1G56100	*(PMEI14)*	Plant invertase/pectin methylesterase inhibitor superfamily protein
AT1G56110	*HOMOLOG OF NUCLEOLAR PROTEIN NOP56 (NOP56)*	NOP56-like protein
AT1G56120		Leucine-rich repeat transmembrane protein kinase
AT1G56150	*SMALL AUXIN UPREGULATED 71 (SAUR71)*	SAUR-like auxin-responsive protein family
AT1G56160	*MYB DOMAIN PROTEIN 72 (MYB72)*	Encodes a member of the R2R3 transcription factor gene family that is involved in mediating induced systemic resistance. Genetic analysis of loss of function mutants and overexpressor lines indicates MYB72 is necessary but not sufficient for ISR.Interacts in vivo with EIL3.
AT1G56170	*NUCLEAR FACTOR Y, SUBUNIT C2 (NF-YC2)*	Encodes a protein with similarity to a subunit of the CCAAT promoter motif binding complex of yeast.One of two members of this class (HAP5B) and expressed in vegetative and reproductive tissues. Involved in the regulation of response to nutrient levels.

AT1G56190	*(CPGK2)*	One of a pair of plastid localized phosphoglycerate kinases involved in galactolipid biosynthesis. Functions redundantly with AT3g12780 (PGK1) in the chloroplast in the biosynthesis of thylakoid membrane galactolipids. Double mutants are photosynthetically incompetent, plants are albino and seedling lethal.
AT1G56200	*EMBRYO DEFECTIVE 1303 (emb1303)*	Encodes a chloroplast localized protein that is essential for chloroplast development.
AT1G56210	*HEAVY METAL ASSOCIATED PROTEIN 10 (ATHMP10)*	Heavy metal transport/detoxification superfamily protein
AT1G56220		Dormancy/auxin associated family protein
AT1G56230		enolase (DUF1399)
AT1G56270		RPB1a
AT1G56300	*(DJC53)*	Chaperone DnaJ-domain superfamily protein
AT1G56310	*ATRIMMER2 (ATRM2)*	DEDDy-type 3′ -> 5′ exoribonuclease involved in miRNA degradation.
AT1G56320		hypothetical protein
AT1G56340	*CALRETICULIN 1A (CRT1a)*	Encodes one of three Arabidopsis calreticulins. In CRT-deficient mouse fibroblasts, this protein restores ER Ca2+ levels. Post-transcriptionally regulates together with CRT2 VAMP721/722 levels under ER stress. Loss-of-function results in activation of the ethylene signaling pathway, reduced susceptibility to Verticillium longisporum.
AT1G56345		Pseudouridine synthase family protein
AT1G56360	*PURPLE ACID PHOSPHATASE 6 (PAP6)*	purple acid phosphatase 6
AT1G56380	*(MTERF23)*	Mitochondrial transcription termination factor family protein
AT1G56410	*EARLY-RESPONSIVE TO DEHYDRATION 2 (ERD2)*	encodes a heat shock protein whose gene expression is induced by heat and dehydration.
AT1G56430	*NICOTIANAMINE SYNTHASE 4 (NAS4)*	Encodes a protein with nicotianamine synthase activity.
AT1G56440	*TETRATRICOPEPTIDE REPEAT 5 (TPR5)*	Encodes one of the 36 carboxylate clamp (CC)-tetratricopeptide repeat (TPR) proteins (Prasad 2010, Pubmed ID: 20856808) with potential to interact with Hsp90/Hsp70 as co-chaperones.
AT1G56500	*SUPPRESSOR OF QUENCHING 1 (SOQ1)*	Encodes a thylakoid membrane protein with thioredoxin-like and beta-propeller domains located in the lumen and a haloacid-dehalogenase domain exposed to the chloroplast stroma. The protein's role may be to prevent formation of a slowly reversible form of antenna quenching, thereby maintaining the efficiency of light harvesting. The mRNA is cell-to-cell mobile.
AT1G56505		
AT1G56510	*WHITE RUST RESISTANCE 4 (WRR4)*	TIR-NB-LRR protein that confers resistance to four races of Albugo candida. The mRNA is cell-to-cell mobile.
AT1G56520		Disease resistance protein (TIR-NBS-LRR class) family
AT1G56530		Predicted as a potential part of a trans-NAT (natural antisense transcript) pair (At4g19270::At1g56530).
AT1G56540		Disease resistance protein (TIR-NBS-LRR class) family
AT1G56560	*ALKALINE/NEUTRAL INVERTASE A (A/N-InvA)*	A/N-InvA is a neutral invertase that breaks sucrose down into fructose and glucose. It is member of the larger family of alkaline/neutral invertases (GH100). GFP-tagged A/N-InvA localizes to the mitochondria. atinva mutants have reduced root growth, reduced invertase activity, and increased expression of antioxidant genes under basal conditions. The levels of A/N-InvA transcripts rise in response to a hydrogen peroxide treatment.
AT1G56580	*SMALLER WITH VARIABLE BRANCHES (SVB)*	Encodes SMALLER WITH VARIABLE BRANCHES (SVB), a protein with a conserved domain of unknown function (DUF538). The trichomes of the SVB mutants are smaller and exhibit branches of variable length and number. ABA responsive trichome formation regulator.
AT1G56600	*GALACTINOL SYNTHASE 2 (GolS2)*	GolS2 is a galactinol synthase that catalyzes the formation of galactinol from UDP-galactose and myo-inositol. GolS2 transcript levels rise in response to methyl viologen, an oxidative damage-inducing agent. Plants over-expressing GolS2 have increased tolerance to salt, chilling, and high-light stress.
AT1G56620		Plant invertase/pectin methylesterase inhibitor superfamily protein
AT1G56650	*PRODUCTION OF ANTHOCYANIN PIGMENT 1 (PAP1)*	Encodes a putative MYB domain containing transcription factor involved in anthocyanin metabolism and radical scavenging. Essential for the sucrose-mediated expression of the dihydroflavonol reductase gene. Auxin and ethylene responsiveness of PAP1 transcription is lost in myb12 mutants. Interacts with JAZ proteins to regulate anthocyanin accumulation.
AT1G56660		MAEBL domain protein
AT1G56670		GDSL-motif esterase/acyltransferase/lipase. Enzyme group with broad substrate specificity that may catalyze acyltransfer or hydrolase reactions with lipid and non-lipid substrates.
AT1G56675		transposable_element_gene;copia-like retrotransposon family, has a 1.7e-196 P-value blast match to GB:AAC02666 polyprotein (Ty1_Copia-element) (Arabidopsis thaliana);(source:TAIR10)
AT1G56680		Chitinase family protein
AT1G56690		Pentatricopeptide repeat (PPR) superfamily protein
AT1G56700		Peptidase C15, pyroglutamyl peptidase I-like protein
AT1G56710	*POLYGALACTURONASE LIKE 1 (PGL1)*	Pectin lyase-like superfamily protein
AT1G56720		Protein kinase superfamily protein
AT1G57550		Low temperature and salt responsive protein family
AT1G57570		Mannose-binding lectin superfamily protein
AT1G57590	*PECTIN ACETYESTERASE 2 (PAE2)*	Pectinacetylesterase family protein
AT1G57630		Toll-Interleukin-Resistance (TIR) domain family protein
AT1G57650		ATP binding protein
AT1G57670		Toll-Interleukin-Resistance (TIR) domain family protein
AT1G57750	*CYTOCHROME P450, FAMILY 96, SUBFAMILY A, POLYPEPTIDE 15 (CYP96A15)*	Encodes a CYP96A15, midchain alkane hydroxylase, involved in cuticular wax biosynthesis.
AT1G57760		Encodes a ECA1 gametogenesis related family protein
AT1G57770		FAD/NAD(P)-binding oxidoreductase family protein

AT1G57780	*HEAVY METAL ASSOCIATED PROTEIN 11 (ATHMP11)*	heavy-metal-associated domain-containing protein
AT1G57790	*FBOX/DUF295-RELATED 1 (ATFDR1)*	F-box family protein
AT1G57830		Toll-Interleukin-Resistance (TIR) domain family protein
AT1G57850		Toll-Interleukin-Resistance (TIR) domain family protein
AT1G57906	*F-BOX/DUF295 ANCESTRAL 2 (ATFDA2)*	F-box protein
AT1G57943	*PURINE PERMEASE 17 (PUP17)*	Member of a family of proteins related to PUP1, a purine transporter. May be involved in the transport of purine and purine derivatives such as cytokinins, across the plasma membrane.
AT1G57990	*PURINE PERMEASE 18 (PUP18)*	Member of a family of proteins related to PUP1, a purine transporter. May be involved in the transport of purine and purine derivatives such as cytokinins, across the plasma membrane.
AT1G58025		DNA-binding bromodomain-containing protein
AT1G58032		
AT1G58070	*WALLIN (WAL)*	WALLIN is an actin binding protein involved in ROP11 mediated xylem pit patterning.
AT1G58080	*ATP PHOSPHORIBOSYL TRANSFERASE 1 (ATP-PRT1)*	ATP phosphoribosyl transferase, catalyses first step of histidine biosynthesis
AT1G58110		Basic-leucine zipper (bZIP) transcription factor family protein
AT1G58120		hypothetical protein
AT1G58150		phosphoglycerate kinase
AT1G58160	*JACALIN-TYPE LECTIN REQUIRED FOR POTEXVIRUS RESISTANCE 1 (JAX1)*	At1g58160 in Col-0 has been shown to be a pseudogene due to a stop codon in the first exon (PMID:22307853). Its functional copy in other ecotypes (Bay-0) encodes JAX1, a jacalin-type lectin gene that confers resistance against potexviruses.
AT1G58170		Disease resistance-responsive (dirigent-like protein) family protein
AT1G58180	*BETA CARBONIC ANHYDRASE 6 (BCA6)*	beta carbonic anhydrase 6
AT1G58190	*RECEPTOR LIKE PROTEIN 9 (RLP9)*	receptor like protein 9
AT1G58200	*MSCS-LIKE 3 (MSL3)*	A member of MscS-like gene family, structurally very similar to MSL2, comprising of an N-terminal chloroplast transit peptide, five trans-membrane helices and a C-terminal cytoplasmic domain. Mutant plants showed abnormalities in the size and shape of plastids. MSL3-GFP was localized to discrete foci on the plastid envelope and co-localize with the plastid division protein AtMinE. MSL3 was capable of increasing the osmotic-shock survival of a mutant bacterial strain lacking MS-ion-channel activity.
AT1G58210	*EMBRYO DEFECTIVE 1674 (EMB1674)*	Encodes a member of the NET superfamily of proteins that potentially couples different membranes to the actin cytoskeleton in plant cells. It colocalizes with filamentous actin and is localized to the plasma membrane.
AT1G58250	*SABRE (SAB)*	SABRE, putative gene of unknown function, homologous to maize apt1 gene. Required for normal cell expansion in the root cortex. The sabre mutation results in abnormal cell expansion. Encodes a rare message; very low level of expression was detected in roots and shoots.
AT1G58270	*(ZW9)*	ZW9 mRNA, complete cds The mRNA is cell-to-cell mobile.
AT1G58290	*(HEMA1)*	Encodes a protein with glutamyl-tRNA reductase (GluTR) activity, catalyzing the NADPH-dependent reduction of Glu-tRNA(Glu) to glutamate 1-semialdehyde (GSA) with the release of free tRNA(Glu). It is involved in the early steps of chlorophyll biosynthesis.
AT1G58300	*HEME OXYGENASE 4 (ho4)*	Encodes a member (HO4) of the heme oxygenase family.
AT1G58330	*(ZW2)*	transcription factor-like protein
AT1G58340	*(ZF14)*	Encodes a plant MATE (multidrug and toxic compound extrusion) transporter that is localized to the Golgi complex and small organelles and is involved in determining the rate of organ initiation. It is also involved in iron homeostasis when plants are under osmotic stress.
AT1G58360	*AMINO ACID PERMEASE 1 (AAP1)*	Encodes AAP1 (amino acid permease 1), a neutral amino acid transporter expressed in seeds. Functions in amino acid uptake into embryos. The transporter also functions in acquisition of glutamate and neutral amino acids by the root.
AT1G58390		Disease resistance protein (CC-NBS-LRR class) family
AT1G58410		Disease resistance protein (CC-NBS-LRR class) family
AT1G58420		Uncharacterized conserved protein UCP031279
AT1G58430	*(RXF26)*	Encodes an anther-specific proline-rich protein.
AT1G58440	*(XF1)*	Encodes a putative protein that has been speculated, based on sequence similarities, to have squalene monooxygenase activity.
AT1G58450	*TETRATRICOPEPTIDE REPEAT 6 (TPR6)*	Encodes one of the 36 carboxylate clamp (CC)-tetratricopeptide repeat (TPR) proteins (Prasad 2010, Pubmed ID: 20856808) with potential to interact with Hsp90/Hsp70 as co-chaperones.
AT1G58842		
AT1G59124		Disease resistance protein (CC-NBS-LRR class) family
AT1G59171		Inositol-pentakisphosphate 2-kinase family protein
AT1G59265		transposable_element_gene;copia-like retrotransposon family, has a 0. P-value blast match to dbj|BAA78425.1| polyprotein (Arabidopsis thaliana) (AtRE1) (Ty1_Copia-element);(source:TAIR10)
AT1G59500	*(GH3.4)*	encodes an IAA-amido synthase that conjugates Asp and other amino acids to auxin in vitro.
AT1G59520	*(CW7)*	Encodes CW7.
AT1G59530	*BASIC LEUCINE-ZIPPER 4 (bZIP4)*	basic leucine-zipper 4
AT1G59535		
AT1G59540	*(ZCF125)*	Encodes a kinesin-like protein.
AT1G59550		This locus is annotated as a protein-coding gene in TAIR10. Based on communication with Jean-Luc GALLOIS (April 2013), this gene is re-annotated as a UBX domain-containing pseudogene. Note that the Map Detail Image on the locus detial page and in GBrowse will not be updated until after the next genome release.
AT1G59590	*(ZCF37)*	ZCF37 mRNA, complete cds The mRNA is cell-to-cell mobile.
AT1G59620	*(CW9)*	Encodes CW9.

AT1G59630		F-box associated ubiquitination effector family protein
AT1G59640	*BIG PETAL P (BPEP)*	A basic helix-loop-helix encoding gene (BIGPETAL, BPE) involved in the control of petal size. BPE is expressed via two mRNAs derived from an alternative splicing event. The BPEub (AT1G59640.1)transcript is expressed ubiquitously, whereas the BPEp (AT1G59640.2) transcript is preferentially expressed in petals. Plants that lack the petal-expressed variant BPEp have larger petals as a result of increased cell size. BPEp is positively regulated downstream of APETALA3, PISTILLATA, APETALA1 and PISTILLATA3 and is negatively regulated downstream of AGAMOUS.
AT1G59660	*DRA2-LIKE (DRAL)*	Encodes a protein with similarity to mammalian nucleoporin Nup98.Its expression is upregulated in mutants that are NUP deficient. Nucleoportin which redundantly inhibits flowering together with Nup98a through multiple pathways including clock, photoperiod, and age pathways. Gates flowering in a CONSTANS (CO)-independent mode and bypasses the CO checkpoint in photoperiodic signaling and integrated signals from multiple pathways to directly target FLOWERING LOCUS T (FT) for flowering control.
AT1G59670	*GLUTATHIONE S-TRANSFERASE TAU 15 (GSTU15)*	Encodes glutathione transferase belonging to the tau class of GSTs. Naming convention according to Wagner et al. (2002).
AT1G59680	*EMBRYO SAC DEVELOPMENT ARREST 1 (EDA1)*	F-box associated ubiquitination effector family protein
AT1G59700	*GLUTATHIONE S-TRANSFERASE TAU 16 (GSTU16)*	Encodes glutathione transferase belonging to the tau class of GSTs. Naming convention according to Wagner et al. (2002). The mRNA is cell-to-cell mobile.
AT1G59710		actin cross-linking protein (DUF569)
AT1G59720	*CHLORORESPIRATORY REDUCTION28 (CRR28)*	Pentatricopeptide Repeat Protein containing the DYW motif. Required for editing of multiple plastid transcripts. Endonuclease activity.
AT1G59725		DNAJ heat shock family protein
AT1G59730	*THIOREDOXIN H-TYPE 7 (TH7)*	Thioredoxin H-type 7 , oxidoreductase located in cytosol and ER. Interacts with GPT1.
AT1G59740	*NRT1/ PTR FAMILY 4.3 (NPF4.3)*	Major facilitator superfamily protein
AT1G59750	*AUXIN RESPONSE FACTOR 1 (ARF1)*	Encodes a member of the auxin response factor family. ARFs bind to the cis element 5'-TGTCTC-3' ARFs mediate changes in gene expression in response to auxin. ARF's form heterodimers with IAA/AUX genes. ARF1 enhances mutant phenotypes of ARF2 and may act with ARF2 to control aspects of maturation and senescence.ARF1:LUC and 3xHA:ARF1 proteins have a half-life of ~3-4 hours and their degradation is reduced by proteasome inhibitors. 3xHA:ARF1 degradation is not affected by a pre-treatment with IAA. A nuclear-targeted fusion protein containing the middle region of ARF1 linked to LUC:NLS has a similar half-life to the full-length ARF1:LUC construct. The degradation of 3xHA:ARF1 is not affected in an axr6-3 mutant grown at room temperature, although the degradation of AXR2/IAA7 is slowed under these conditions.
AT1G59760	*HOMOLOG OF YEAST MTR4 (MTR4)*	Encodes MTR4, a putative RNA helicase and exosome co-factor. Required for proper rRNA biogenesis and development.
AT1G59790		Cullin family protein
AT1G59800		Cullin family protein
AT1G59840	*COFACTOR ASSEMBLY OF COMPLEX C (CCB4)*	cofactor assembly of complex C
AT1G59850		ARM repeat superfamily protein
AT1G59860	*(HSP17.6A)*	HSP20-like chaperones superfamily protein
AT1G59870	*PENETRATION 3 (PEN3)*	ATP binding cassette transporter. Localized to the plasma membrane in uninfected cells. In infected leaves, the protein concentrated at infection sites. Contributes to nonhost resistance to inappropriate pathogens that enter by direct penetration in a salicylic acid?dependent manner. Required for mlo resistance. Has Cd transporter activity (Cd2+ extrusion pump) and contributes to heavy metal resistance. The mRNA is cell-to-cell mobile.
AT1G59890	*SIN3-LIKE 5 (SNL5)*	SIN3-like 5
AT1G59900	*PYRUVATE DEHYDROGENASE COMPLEX E1 ALPHA SUBUNIT (E1 ALPHA)*	encodes the e1 alpha subunit of the pyruvate dehydrogenase complex (PDC) The mRNA is cell-to-cell mobile.
AT1G59910	*FORMIN HOMOLOGUE 7 (FORMIN7)*	Member of family of cytoskeletal-interacting proteins which have the ability to stimulate actin nucleation and barbed-end capping through the combined activity of conserved formin-homology 1 (FH1) and formin-homology 2 (FH2) domains.
AT1G59930		Encodes a maternally expressed imprinted gene.
AT1G59940	*RESPONSE REGULATOR 3 (ARR3)*	Type A response regulator highly similar to bacterial two-component response regulators. Rapidly induced by cytokinin. Involved in red-light signaling. Acts redundantly with ARR3 in the control of circadian period in a cytokinin-independent manner.
AT1G59950		NAD(P)-linked oxidoreductase superfamily protein
AT1G59960		NAD(P)-linked oxidoreductase superfamily protein
AT1G59970	*(AT5-MMP)*	Matrix metalloproteinase important for root development and root bacterial communities. Modulates auxin/ABA signaling rendering the plant sensitive to drought stress and recruiting differential root bacterial communities.
AT1G59980	*ARG1-LIKE 2 (ARL2)*	ARG1-like 2
AT1G59990	*EMBRYO DEFECTIVE 3108 (EMB3108)*	DEA(D/H)-box RNA helicase family protein
AT1G60000		RNA-binding (RRM/RBD/RNP motifs) family protein
AT1G60010		PADRE protein down-regulated after infection by S. sclerotiorun.
AT1G60020		transposable_element_gene;copia-like retrotransposon family, has a 0. P-value blast match to GB:AAC02672 polyprotein (Ty1_Copia-element) (Arabidopsis arenosa);(source:TAIR10)
AT1G60030	*NUCLEOBASE-ASCORBATE TRANSPORTER 7 (NAT7)*	nucleobase-ascorbate transporter 7
AT1G60040	*AGAMOUS-LIKE 49 (AGL49)*	AGAMOUS-like 49
AT1G60060		Serine/threonine-protein kinase WNK (With No Lysine)-like protein
AT1G60080		3-5-exoribonuclease family protein
AT1G60090	*BETA GLUCOSIDASE 4 (BGLU4)*	beta glucosidase 4
AT1G60130		Mannose-binding lectin superfamily protein
AT1G60140	*TREHALOSE PHOSPHATE SYNTHASE (TPS10)*	Encodes an enzyme putatively involved in trehalose biosynthesis. The protein has a trehalose synthase (TPS)-like domain that may or may not be active as well as a trehalose phosphatase (TPP)-like domain.
AT1G60160	*K TRANSPORTER12 (KT12)*	Member of the KT/KUP/HAK family of proton-coupled potassium transporters which have potential effect on cellular expansion.

AT1G60180		pseudogene of F-box family protein
AT1G60190	*PLANT U-BOX 19 (PUB19)*	Encodes PUB19, a plant U-box armadillo repeat protein. Involved in salt inhibition of germination together with PUB18. The mRNA is cell-to-cell mobile.
AT1G60200	*RNA-BINDING PROTEIN 25 (RBM25)*	RBM25 is an alternative splicing factor involved in mediation of abiotic stress response and ABA response. Its expression is modulated by a variety of stressors and it in turn appears to affect the ratio of splice variants of stress responsive genes such as HAB1.2/HAB1.1.
AT1G60210		
AT1G60220	*UB-LIKE PROTEASE 1D (ULP1D)*	Encodes a deSUMOylating enzyme. In vitro it has both peptidase activity and isopeptidase activity: it can cleave C-terminal residues from SUMO to activate it for attachment to a target protein and it can also act on the isopeptide bond between SUMO and another protein. sGFP:OTS1 protein accumulates in the nucleus. Double mutant analysis with ULP1C/OTS2 indicates that these genes are involved in salt stress responses and flowering time regulation. Over-expression of 35S:OTS1 increases salt tolerance and reduces the level of SUMO-conjugated proteins. OTS1 transcript levels do not appear to change in response to salt, but, salt stress reduces the level of OTS1 protein in a proteasome-dependent manner.
AT1G60260	*BETA GLUCOSIDASE 5 (BGLU5)*	beta glucosidase 5
AT1G60270	*BETA GLUCOSIDASE 6 (BGLU6)*	beta glucosidase 6
AT1G60280	*NAC DOMAIN CONTAINING PROTEIN 23 (NAC023)*	NAC domain containing protein 23
AT1G60300		NAC (No Apical Meristem) domain transcriptional regulator superfamily protein
AT1G60320		Toll-Interleukin-Resistance (TIR) domain family protein
AT1G60350	*NAC DOMAIN CONTAINING PROTEIN 24 (NAC024)*	NAC domain containing protein 24
AT1G60380		NAC (No Apical Meristem) domain transcriptional regulator superfamily protein
AT1G60390	*POLYGALACTURONASE 1 (PG1)*	polygalacturonase 1
AT1G60400	*(PEG3)*	F-box/RNI-like superfamily protein
AT1G60440	*PANTOTHENATE KINASE 1 (PANK1)*	The gene AT1G60440 encodes pantothenate kinase 1. Its molecular function was shown to phosphorylate pantothenate to form 4?-phosphopantothenate.
AT1G60450	*GALACTINOL SYNTHASE 7 (GolS7)*	Predicted to encode a galactinol synthase.
AT1G60470	*GALACTINOL SYNTHASE 4 (GolS4)*	Predicted to encode a galactinol synthase.
AT1G60500	*DYNAMIN RELATED PROTEIN 4C (DRP4C)*	Dynamin related protein 4C
AT1G60530	*DYNAMIN RELATED PROTEIN 4A (DRP4A)*	Dynamin related protein 4A
AT1G60540	*DYNAMIN RELATED PROTEIN 4B (DRP4B)*	Annotated as pseudogene of the dynamin family.Possibly not a pseudogene based on evidence for transcription (RNA-seq) and translation (Ribo-seq) described in PMID:27791167
AT1G60550	*ENOYL-COA HYDRATASE/ISOMERASE D (ECHID)*	enoyl-CoA hydratase/isomerase D
AT1G60570		Galactose oxidase/kelch repeat superfamily protein
AT1G60590		Pectin lyase-like superfamily protein
AT1G60600	*ABERRANT CHLOROPLAST DEVELOPMENT 4 (ABC4)*	Encodes a protein similar to 1,4-dihydroxy-2-naphthoic acid phytyltransferase involved in phylloquinone and plastoquinone biosynthesis. Mutants are pale green and heterotrophic with defects in photosynthetic electron transport.
AT1G60630		Leucine-rich repeat protein kinase family protein
AT1G60650	*RNA-BINDING GLYCINE-RICH PROTEIN B1 (RBGB1)*	Encodes one of the zinc finger-containing glycine-rich RNA-binding proteins involved in cold tolerance: AT3G26420 (ATRZ-1A), AT1G60650 (AtRZ-1b), AT5G04280 (AtRZ-1c). It also, along with AtRZ-1c, plays important roles in plant development, pre- mRNA splicing, and general gene expression.
AT1G60680		NAD(P)-linked oxidoreductase superfamily protein
AT1G60690		NAD(P)-linked oxidoreductase superfamily protein
AT1G60710	*(ATB2)*	Encodes ATB2.
AT1G60730		NAD(P)-linked oxidoreductase superfamily protein
AT1G60740	*PEROXIREDOXIN IID (PRXIID)*	Thioredoxin superfamily protein
AT1G60750		NAD(P)-linked oxidoreductase superfamily protein
AT1G60770	*RIBOSOMAL PENTATRICOPEPTIDE REPEAT PROTEIN 4 (RPPR4)*	Ribosomal pentatricopeptide repeat protein
AT1G60790	*TRICHOME BIREFRINGENCE-LIKE 2 (TBL2)*	Encodes a member of the TBL (TRICHOME BIREFRINGENCE-LIKE) gene family containing a plant-specific DUF231 (domain of unknown function) domain. TBL gene family has 46 members, two of which (TBR/AT5G06700 and TBL3/AT5G01360) have been shown to be involved in the synthesis and deposition of secondary wall cellulose, presumably by influencing the esterification state of pectic polymers. A nomenclature for this gene family has been proposed (Volker Bischoff & Wolf Scheible, 2010, personal communication).
AT1G60800	*NSP-INTERACTING KINASE 3 (NIK3)*	Encodes one of a group of LRR-RLKs, designated as CLAVATA3 INSENSITIVE RECEPTOR KINASES (CIKs), that act as co-receptors and have essential roles in regulating CLV3-mediated stem cell homeostasis.
AT1G60810	*ATP-CITRATE LYASE A-2 (ACLA-2)*	One of the three genes encoding subunit A of the trimeric enzyme ATP Citrate lyase
AT1G60850	*(ATRPAC42)*	DNA-directed RNA polymerase family protein
AT1G60870	*MATERNAL EFFECT EMBRYO ARREST 9 (MEE9)*	maternal effect embryo arrest 9
AT1G60940	*SNF1-RELATED PROTEIN KINASE 2.10 (SNRK2.10)*	encodes a member of SNF1-related protein kinases (SnRK2) whose activity is activated by ionic (salt) and non-ionic (mannitol) osmotic stress.
AT1G60950	*(FED A)*	encodes a major leaf ferredoxin
AT1G60960	*IRON REGULATED TRANSPORTER 3 (IRT3)*	Encodes a plasma membrane localized zinc/iron transporter.
AT1G60970		SNARE-like superfamily protein
AT1G60980	*GIBBERELLIN 20-OXIDASE 4 (GA20OX4)*	gibberellin 20-oxidase 4
AT1G60985	*SCR-LIKE 6 (SCRL6)*	Encodes a member of a family of small, secreted, cysteine rich proteins with sequence similarity to SCR (S locus cysteine-rich protein).
AT1G60990	*IBA57.2 (IBA57.2)*	Encodes a chloroplast-localized COG0354 protein that requires folate for its function in Fe/S cluster biogenesis.

AT1G61065		1,3-beta-glucan synthase component (DUF1218)
AT1G61070	*LOW-MOLECULAR-WEIGHT CYSTEINE-RICH 66 (LCR66)*	Predicted to encode a PR (pathogenesis-related) protein. Belongs to the plant defensin (PDF) family with the following members: At1g75830/PDF1.1, At5g44420/PDF1.2a, At2g26020/PDF1.2b, At5g44430/PDF1.2c, At2g26010/PDF1.3, At1g19610/PDF1.4, At1g55010/PDF1.5, At2g02120/PDF2.1, At2g02100/PDF2.2, At2g02130/PDF2.3, At1g61070/PDF2.4, At5g63660/PDF2.5, At2g02140/PDF2.6, At5g38330/PDF3.1 and At4g30070/PDF3.2.
AT1G61080		Hydroxyproline-rich glycoprotein family protein
AT1G61090		hypothetical protein
AT1G61100		disease resistance protein (TIR class)
AT1G61110	*NAC DOMAIN CONTAINING PROTEIN 25 (NAC025)*	NAC transcription regulator. Regulates endosperm cell expansion during germination.
AT1G61120	*TERPENE SYNTHASE 04 (TPS04)*	Encodes a geranyllinalool synthase that produces a precursor to TMTT, a volatile plant defense C16-homoterpene. GES transcript levels rise in response to alamethicin, a fungal peptide mixture that damages membranes. This transcriptional response is blocked in JA biosynthetic and JA signaling mutants, but GES transcript levels still rise in response to alamethicin in mutants with salicylic acid and ethylene biosynthetic and/or signaling defects. GES transcripts also accumulate in response to a larval infestation. This enzyme does not localize to the plastids, and it may be present in the cytosol or endoplasmic reticulum. The mRNA is cell-to-cell mobile.
AT1G61130	*SERINE CARBOXYPEPTIDASE-LIKE 32 (SCPL32)*	serine carboxypeptidase-like 32
AT1G61160		retrotransposon gag
AT1G61170		hypothetical protein
AT1G61210	*DWD HYPERSENSITIVE TO ABA 3 (DWA3)*	DWA3 encodes a DWD(DDB1 binding WD40) protein. Invitro analyses suggest its involvement in the negative regulation of ABA responses.One of four katanin p80 subunits. Involved in targeting of katanin complex to crossover and branch points to properly sever microtubules.
AT1G61240		lysine ketoglutarate reductase trans-splicing-like protein (DUF707)
AT1G61260		cotton fiber (DUF761)
AT1G61270	*HISTIDINE TRANSPORTER 3 (LHT3)*	Involved in transport of 1-Aminocyclopropane-1-carboxylic acid (ACC).
AT1G61280		Phosphatidylinositol N-acetylglucosaminyltransferase, GPI19/PIG-P subunit
AT1G61290	*SYNTAXIN OF PLANTS 124 (SYP124)*	member of SYP12 Gene Family
AT1G61320		FBD / Leucine Rich Repeat domains containing protein
AT1G61340	*F-BOX STRESS INDUCED 1 (FBS1)*	Encodes a F-box protein induced by various biotic or abiotic stress.
AT1G61360		S-locus lectin protein kinase family protein
AT1G61370		S-locus lectin protein kinase family protein
AT1G61380	*S-DOMAIN-1 29 (SD1-29)*	Encodes a membrane localized S-domain receptor kinase that is involved in lipopolysaccharide (LPS) sensing. SD1-29 detected LPS of Pseudomonas and Xanthomonas species for which it serves as a microbe associated molecular pattern triggering innate immunity. Loses of function mutants are hyper susceptible to P.syringae.
AT1G61390		S-locus lectin protein kinase family protein
AT1G61400		S-locus lectin protein kinase family protein
AT1G61410		DNA double-strand break repair and VJ recombination XRCC4
AT1G61420		S-locus lectin protein kinase family protein
AT1G61430		S-locus lectin protein kinase family protein
AT1G61440		S-locus lectin protein kinase family protein
AT1G61450		CAP-gly domain linker
AT1G61490		S-locus lectin protein kinase family protein
AT1G61500		S-locus lectin protein kinase family protein
AT1G61520	*PHOTOSYSTEM I LIGHT HARVESTING COMPLEX GENE 3 (LHCA3)*	PSI type III chlorophyll a/b-binding protein (Lhca3*1) The mRNA is cell-to-cell mobile.
AT1G61550		S-locus lectin protein kinase family protein
AT1G61560	*MILDEW RESISTANCE LOCUS O 6 (MLO6)*	A member of a large family of seven-transmembrane domain proteins specific to plants, homologs of the barley mildew resistance locus o (MLO) protein. The Arabidopsis genome contains 15 genes encoding MLO proteins, with localization in plasma membrane. Phylogenetic analysis revealed four clades of closely-related AtMLO genes. ATMLO6 belongs to the clade IV, with AtMLO2, AtMLO3 and AtMLO12. The gene is expressed during early seedling growth, in roots and lateral root primordia, in flower and fruit abscission zone, in vascular system of cotyledons, young leaves and petals, in mature rosette leaves, in anthers, as shown by GUS activity patterns. The expression of several phylogenetically closely-related AtMLO genes showed similar or overlapping tissue specificity and analogous responsiveness to external stimuli, suggesting functional redundancy, co-function, or antagonistic function(s).
AT1G61566	*RALF-LIKE 9 (RALFL9)*	Member of a diversely expressed predicted peptide family showing sequence similarity to tobacco Rapid Alkalinization Factor (RALF), and is believed to play an essential role in the physiology of Arabidopsis. Consists of a single exon and is characterized by a conserved C-terminal motif and N-terminal signal peptide.
AT1G61570	*TRANSLOCASE OF THE INNER MITOCHONDRIAL MEMBRANE 13 (TIM13)*	Encodes a putative small zinc finger-like protein (TIM13); nucleus-encoded gene whose product is found in the mitochondrial inner membrane space.
AT1G61580	*R-PROTEIN L3 B (RPL3B)*	R-protein L3 B
AT1G61590	*PBS1-LIKE 15 (PBL15)*	Protein kinase superfamily protein
AT1G61610		S-locus lectin protein kinase family protein
AT1G61620	*COP1 SUPPRESSOR 1 (CSU1)*	Encodes a RING-finger E3 ubiquitin ligase that plays a major role in maintaining COP1 homeostasis by targeting COP1 for ubiquitination and degradation in dark-grown seedlings. The mRNA is cell-to-cell mobile.
AT1G61630	*EQUILIBRATIVE NUCLEOSIDE TRANSPORTER 7 (ENT7)*	equilibrative nucleoside transporter 7
AT1G61640		Protein kinase superfamily protein

AT1G61660	*(ATBHLH112)*	Encodes a transcriptional activator that regulates the expression of genes by binding to their GCG- or E-boxes to mediate physiological responses, including proline biosynthesis and ROS scavenging pathways, to enhance stress tolerance. Governs the competence of pericycle cells to initiate lateral root primordium formation.
AT1G61667		serine protease, putative (Protein of unknown function, DUF538)
AT1G61680	*TERPENE SYNTHASE 14 (TPS14)*	terpene synthase 14
AT1G61700		Protein of unknown function that is homologous to the At1g11475 locus that encodes a non-catalytic subunit common to nuclear DNA-dependent RNA polymerases II, IV and V. Homologous to budding yeast RPB10.
AT1G61710		Cysteine/Histidine-rich C1 domain family protein
AT1G61720	*BANYULS (BAN)*	Negative regulator of flavonoid biosynthesis, mutants accumulate flavonoid pigments in their seed coat, putative oxidoreductase. It is thought that a ternary complex composed of TT2, TT8 and TTG1 is necessary for correct expression of BAN in seed endothelium.
AT1G61740		Sulfite exporter TauE/SafE family protein
AT1G61750		Receptor-like protein kinase-related family protein
AT1G61760		Late embryogenesis abundant (LEA) hydroxyproline-rich glycoprotein family
AT1G61800	*GLUCOSE-6-PHOSPHATE/PHOSPHATE TRANSLOCATOR 2 (GPT2)*	glucose6-Phosphate/phosphate transporter 2. Expression is upregulated in the shoot of cax1/cax3 mutant. The mRNA is cell-to-cell mobile.
AT1G61810	*BETA-GLUCOSIDASE 45 (BGLU45)*	beta-glucosidase 45
AT1G61820	*BETA GLUCOSIDASE 46 (BGLU46)*	beta glucosidase 46
AT1G61830		pseudogene of Cysteine/Histidine-rich C1 domain family protein
AT1G61840		Cysteine/Histidine-rich C1 domain family protein
AT1G61860	*PBS1-LIKE 41 (PBL41)*	Protein kinase superfamily protein
AT1G61870	*PENTATRICOPEPTIDE REPEAT 336 (PPR336)*	Generic translation factor involved in mitochondrial translation.
AT1G61890		MATE efflux family protein
AT1G61920		transmembrane protein
AT1G61930	*(S40-6)*	senescence regulator (Protein of unknown function, DUF584)
AT1G61940	*TUBBY LIKE PROTEIN 4 (TLP4)*	Member of TLP family
AT1G61950	*CALCIUM-DEPENDENT PROTEIN KINASE 19 (CPK19)*	Member of Calcium Dependent Protein Kinase, regulated by Bacillus amyloliquefaciens under high calcium stress.
AT1G62010	*(MTERF14)*	Mitochondrial transcription termination factor family protein
AT1G62030		Cysteine/Histidine-rich C1 domain family protein
AT1G62040	*AUTOPHAGY 8C (ATG8C)*	Autophagy protein.
AT1G62050		Ankyrin repeat family protein
AT1G62060	*TESTA ABUNDANT 2 (TBA2)*	Encodes a member of a mucilage protein family. Predicted in silico to be glycosylated.
AT1G62070		hypothetical protein
AT1G62100		
AT1G62110	*(MTERF31)*	Mitochondrial transcription termination factor family protein
AT1G62150	*(MTERF20)*	Mitochondrial transcription termination factor family protein
AT1G62160		Serine protease inhibitor (SERPIN) family protein
AT1G62170		Serine protease inhibitor (SERPIN) family protein
AT1G62180	*5'ADENYLYLPHOSPHOSULFATE REDUCTASE 2 (APR2)*	encodes a adenosine 5'-phosphosulfate reductase, involved in sulfate assimilation. Is a major effect locus for natural variation of shoot sulfate content in Arabidopsis.
AT1G62200	*NRT1/ PTR FAMILY 8.5 (NPF8.5)*	Major facilitator superfamily protein
AT1G62210		hypothetical protein
AT1G62250		orotidine 5-phosphate decarboxylase
AT1G62280	*SLAC1 HOMOLOGUE 1 (SLAH1)*	Encodes a protein with ten predicted transmembrane helices. The SLAH1 protein has similarity to the SLAC1 protein involved in ion homeostasis in guard cells. Although it is not expressed in guard cells, it can complement a slac1-2 mutant suggesting that it performs a similar function. SLAH1:GFP localizes to the plasma membrane.
AT1G62290	*PUTATIVE ASPARTIC PROTEINASE A2 (PASPA2)*	Saposin-like aspartyl protease family protein
AT1G62300	*(WRKY6)*	Encodes a transcription factor WRKY6. Regulates Phosphate1 (Pho1) expression in response to low phosphate (Pi) stress.
AT1G62310	*(JMJ29)*	Encodes a probable H3K9me2 demethylase. Functions in trichome morphogenesis via regulation of GL3.
AT1G62320		ERD (early-responsive to dehydration stress) family protein
AT1G62340	*ABNORMAL LEAF-SHAPE 1 (ALE1)*	Subtilisin-like serine protease required for epidermal surface formation in embryos and juvenile plants
AT1G62350		Pentatricopeptide repeat (PPR) superfamily protein
AT1G62360	*SHOOT MERISTEMLESS (STM)*	Class I knotted-like homeodomain protein that is required for shoot apical meristem (SAM) formation during embryogenesis and for SAM function throughout the lifetime of the plant. Functions by preventing incorporation of cells in the meristem center into differentiating organ primordia. It has also been shown to have a role in the specification of flower meristem identity.
AT1G62380	*ACC OXIDASE 2 (ACO2)*	Encodes a protein similar to 1-aminocyclopropane-1-carboxylic oxidase (ACC oxidase). Expression of the AtACO2 transcripts is affected by ethylene.
AT1G62390	*PHOX2 (Phox2)*	Encodes one of the 36 carboxylate clamp (CC)-tetratricopeptide repeat (TPR) proteins (Prasad 2010, Pubmed ID: 20856808) with potential to interact with Hsp90/Hsp70 as co-chaperones.
AT1G62410		MIF4G domain-containing protein
AT1G62420		DUF506 family protein (DUF506)
AT1G62440	*LEUCINE-RICH REPEAT/EXTENSIN 2 (LRX2)*	encodes a paralog of LRX1 (LEUCINE-RICH REPEAT/EXTENSIN 1) which acts synergistically with LRX1 in root hair cell morphogenesis.

AT1G62450		Immunoglobulin E-set superfamily protein
AT1G62480		Vacuolar calcium-binding protein-like protein
AT1G62490	*(MTERF25)*	Mitochondrial transcription termination factor family protein
AT1G62500	*(DEG27)*	Bifunctional inhibitor/lipid-transfer protein/seed storage 2S albumin superfamily protein
AT1G62510		Expressed in the root cortex.
AT1G62520		sulfated surface-like glycoprotein
AT1G62530		hypothetical protein (DUF863)
AT1G62540	*FLAVIN-MONOOXYGENASE GLUCOSINOLATE S-OXYGENASE 2 (FMO GS-OX2)*	belongs to the flavin-monooxygenase (FMO) family, encodes a glucosinolate S-oxygenase that catalyzes the conversion of methylthioalkyl glucosinolates to methylsulfinylalkyl glucosinolates The mRNA is cell-to-cell mobile.
AT1G62560	*FLAVIN-MONOOXYGENASE GLUCOSINOLATE S-OXYGENASE 3 (FMO GS-OX3)*	belongs to the flavin-monooxygenase (FMO) family, encodes a glucosinolate S-oxygenase that catalyzes the conversion of methylthioalkyl glucosinolates to methylsulfinylalkyl glucosinolates The mRNA is cell-to-cell mobile.
AT1G62570	*FLAVIN-MONOOXYGENASE GLUCOSINOLATE S-OXYGENASE 4 (FMO GS-OX4)*	belongs to the flavin-monooxygenase (FMO) family, encodes a glucosinolate S-oxygenase that catalyzes the conversion of methylthioalkyl glucosinolates to methylsulfinylalkyl glucosinolates The mRNA is cell-to-cell mobile.
AT1G62590	*PENTATRICOPEPTIDE (PPR) ADENYLATE CYCLASE (PPR-AC)*	Encodes a functional adenylate cyclase.
AT1G62610		NAD(P)-binding Rossmann-fold superfamily protein
AT1G62620		Flavin-binding monooxygenase family protein
AT1G62630		Disease resistance protein (CC-NBS-LRR class) family
AT1G62640	*3-KETOACYL-ACYL CARRIER PROTEIN SYNTHASE III (KAS III)*	3-ketoacyl-acyl carrier protein synthase III (KAS III)
AT1G62660	*VACUOLAR INVERTASE 1 (VI1)*	Glycosyl hydrolases family 32 protein
AT1G62670	*RNA PROCESSING FACTOR 2 (RPF2)*	Encodes a pentatricopeptide repeat protein required for 5' end processing of nad9 and cox3 mRNAs in mitochondria.
AT1G62680		Pentatricopeptide repeat (PPR) superfamily protein
AT1G62690		hypothetical protein
AT1G62700	*ARABIDOPSIS NAC DOMAIN CONTAINING PROTEIN 26 (ANAC026)*	Encodes a NAC-domain transcription factor. Expressed in the vascular tissue.
AT1G62710	*BETA VACUOLAR PROCESSING ENZYME (BETA-VPE)*	Encodes a vacuolar processing enzyme belonging to a novel group of cysteine proteases that is expressed specifically in seeds and is essential for the proper processing of storage proteins.
AT1G62720	*NOVEL GENE 1 (NG1)*	Encodes a PPR protein gene that localizes to the mitochondrion and is required for seed germination.
AT1G62730		Terpenoid synthases superfamily protein
AT1G62750	*SNOWY COTYLEDON 1 (SCO1)*	Nuclear encoded protein consists of the five domains conserved in EF-G proteins, with two GTP-binding sites in the first domain, and an additional transit peptide at the N-terminus. Localized in chloroplasts. Point mutation results in a delay in the onset of germination. At early developmental stage embryos still contain undifferentiated proplastids. The greening of cotyledons is severely impaired in light-grown mutant sco1 seedlings, whereas the following true leaves develop normally as in wild-type plants.
AT1G62760	*(ATPMEI10)*	Pectin methylesterase inhibitor that controls PME activity and pectin methylesterification during Botrytis infection.
AT1G62763		
AT1G62770	*(PMEI9)*	PMEI9 pectin methyleseterase inhibitor. Expressed in many plant tissues.
AT1G62780		dimethylallyl, adenosine tRNA methylthiotransferase
AT1G62800	*ASPARTATE AMINOTRANSFERASE 4 (ASP4)*	Encodes aspartate aminotransferase (Asp4).
AT1G62810	*COPPER AMINE OXIDASE1 (CUAO1)*	Encodes COPPER AMINE OXIDASE1 (CuAO1). Contributes to abscisic acid- and polyamine-induced nitric oxide biosynthesis and abscisic acid signal transduction.
AT1G62820	*(CML14)*	Calcium-binding EF-hand family protein
AT1G62830	*LSD1-LIKE 1 (LDL1)*	Encodes a homolog of human Lysine-Specific Demethylase1. Involved in H3K4 methylation of target genes including the flowering time loci FLC and FWA. Located in nucleus. Negatively regulates root elongation. Involved in repression of LRP1 via histone deacetylation.
AT1G62840		ankyrin repeat/KH domain protein (DUF1442)
AT1G62850		Class I peptide chain release factor
AT1G62860		pseudogene of pentatricopeptide (PPR) repeat-containing protein
AT1G62870		hypothetical protein
AT1G62890		transposable_element_gene;similar to unknown protein [Arabidopsis thaliana] (TAIR:AT3G17200.1);(source:TAIR10)
AT1G62900		S-adenosyl-L-methionine-dependent methyltransferases superfamily protein
AT1G62910	*RF-LIKE 9 (RFL9)*	Encodes PPR protein involved in mitochondrial 5' end processing. Ecotype variants show differences in processing.
AT1G62930	*RNA PROCESSING FACTOR 3 (RPF3)*	RPF3 encodes a pentatricopeptide repeat (PPR) protein involved in 5' processing of different mitochondrial mRNAs.
AT1G62940	*ACYL-COA SYNTHETASE 5 (ACOS5)*	encodes an acyl-CoA synthetase, has in vitro activity towards medium- to long-chain fatty acids and their hydroxylated derivatives. Expressed in the tapetum. Involved in pollen wall exine formation. Null mutants were devoid of pollen grains at anther maturity and were completely male sterile.
AT1G62950		leucine-rich repeat transmembrane protein kinase family protein
AT1G62970	*SUVH1/3-INTERACTING DNAJ DOMAIN-CONTAINING PROTEIN 3 (SDJ3)*	SDJ3 functions partially redundantly with SDJ1 and SDJ2 and interacts with SUVH1 and SUVH3 to form a SUVH-SDJ complex. The complex binds promoters with DNA methylation and mediates transcriptional activation of promoter methylated genes.
AT1G62980	*EXPANSIN A18 (EXPA18)*	Alpha-expansin 18. Naming convention from the Expansin Working Group (Kende et al, 2004. Plant Mol Bio)
AT1G62990	*KNOTTED-LIKE HOMEOBOX OF ARABIDOPSIS THALIANA 7 (KNAT7)*	Encodes a homeodomain transcription factor of the Knotted family. May be involved in secondary cell wall biosynthesis. Mutants have moderately irregular xylem development. Expression of this gene is upregulated by SND1 and MYB46.
AT1G63010	*VACUOLAR PHOSPHATE TRANSPORTER 1 (VPT1)*	Encodes an SPX domain protein that transports Pi into the vacuole and is essential for phosphate homeostasis.
AT1G63020	*NUCLEAR RNA POLYMERASE D1A (NRPD1A)*	Encodes one of two alternative largest subunits of a putative plant-specific RNA polymerase IV (aka RNA polymerase D). Required for posttranscriptional gene silencing.

AT1G63030	*DWARF AND DELAYED FLOWERING 2 (ddf2)*	encodes a member of the DREB subfamily A-1 of ERF/AP2 transcription factor family (DDF2). The protein contains one AP2 domain. There are six members in this subfamily, including CBF1, CBF2, and CBF3. Overexpression of this gene results in the reduction of gibberellic acid biosynthesis. This gene is expressed in all tissues examined, but most abundantly expressed in rosette leaves and stems. Overexpression of DDF1, a putative paralog of this gene, also reduces gibberellic acid biosynthesis and makes the plants more tolerant to high-salinity levels.
AT1G63040		a pseudogene member of the DREB subfamily A-4 of ERF/AP2 transcription factor family. The translated product contains one AP2 domain. There are 17 members in this subfamily including TINY.
AT1G63050	*LYSOPHOSPHOLIPID ACYLTRANSFERASE 2 (LPLAT2)*	Encodes a lysophosphatidylcholine acyltransferase (LPCAT). Participates in the Lands cycle in developing seeds. Involved in triacylglycerol biosynthesis.
AT1G63060		ribosome biogenesis NEP1-like protein
AT1G63070		pentatricopeptide (PPR) repeat-containing protein
AT1G63090	*PHLOEM PROTEIN 2-A11 (PP2-A11)*	phloem protein 2-A11
AT1G63100	*SCARECROW-LIKE 28 (SCL28)*	Transcription factor belonging to the GRAS family which controls the mitotic cell cycle and division plane orientation.
AT1G63120	*RHOMBOID-LIKE 2 (RBL2)*	AtRBL2 has been identified as a rhomboid protein involved in regulated intramembrane proteolysis (RIP). The enzyme has the proteolytic activity and substrate specificity comparable to the Drosophila Rho-1 protein.
AT1G63150		Transacting siRNA generating locus.
AT1G63160	*REPLICATION FACTOR C 2 (RFC2)*	replication factor C 2
AT1G63170		Zinc finger, C3HC4 type (RING finger) family protein
AT1G63180	*UDP-D-GLUCOSE/UDP-D-GALACTOSE 4-EPIMERASE 3 (UGE3)*	Encodes a protein with UDP-D-glucose 4-epimerase activity. Involved in pollen development.
AT1G63190		Cystatin/monellin superfamily protein
AT1G63200		Cystatin/monellin superfamily protein
AT1G63210	*(SPT6)*	SPT6L encodes a putative WG/GW-repeat protein involved in the regulation of apical-basal polarity of embryo
AT1G63220		Calcium-dependent lipid-binding (CaLB domain) family protein
AT1G63240	*ROS1-ASSOCIATED METHYL-DNA BINDING PROTEIN 1 (RMB1)*	Methyl-DNA binding protein which interacts with RMB1 and ROS1 acting in the base excision repair pathway through DNA methylation.
AT1G63280		Serine protease inhibitor (SERPIN) family protein
AT1G63300		Myosin heavy chain-related protein
AT1G63310		hypothetical protein
AT1G63320		Pentatricopeptide repeat (PPR) superfamily protein
AT1G63340		Flavin-containing monooxygenase family protein
AT1G63350		Disease resistance protein (CC-NBS-LRR class) family
AT1G63390		flavin containing monooxygenase FMO GS-OX-like protein
AT1G63400		Pentatricopeptide repeat (PPR) superfamily protein
AT1G63430		Leucine-rich repeat protein kinase family protein
AT1G63440	*HEAVY METAL ATPASE 5 (HMA5)*	The Arabidopsis P-type ATPase HMA5 is involved in Cu detoxification. hma5 mutant plants exhibit Cu hypersensitivity, which is especially dramatic in roots where HMA5 is mostly expressed.
AT1G63450	*ROOT HAIR SPECIFIC 8 (RHS8)*	Encodes a xyloglucan-specific galacturonosyltransferase (XUT1) that forms the beta-d-galactosyluronic acid-(1->2)-alpha-d-xylosyl linkage.
AT1G63490	*JUMONJI DOMAIN-CONTAINING PROTEIN 17 (JMJ17)*	Histone demethylase belonging to the KDM5/JARID1 family which plays crucial roles in response to dehydration stress and abscisic acid (ABA). Directly binds the chromatin of OPEN STOMATA 1 (OST1) and demethylated H3K4me3 for the regulation of OST1 mRNA abundance, thereby modulating the dehydration stress response.
AT1G63500	*BRASSINOSTEROID-SIGNALING KINASE 7 (BSK7)*	kinase with tetratricopeptide repeat domain-containing protein
AT1G63520		hypothetical protein (DUF3527)
AT1G63530		hypothetical protein
AT1G63540		hydroxyproline-rich glycoprotein family protein
AT1G63550		Receptor-like protein kinase-related family protein
AT1G63560		Receptor-like protein kinase-related family protein; CONTAINS InterPro DOMAIN/s: Protein of unknown function DUF26 (InterPro:IPR002902); BEST Arabidopsis thaliana protein match is: Receptor-like protein kinase-related family protein (TAIR:AT1G63600.1)
AT1G63580		Encodes a plasma membrane-localized protein with two DUF26 domains and a GPI anchor domain.
AT1G63590		Receptor-like protein kinase-related family protein
AT1G63600		Receptor-like protein kinase-related family protein
AT1G63610		hypothetical protein
AT1G63630		Tetratricopeptide repeat (TPR)-like superfamily protein
AT1G63650	*ENHANCER OF GLABRA 3 (EGL3)*	Mutant has reduced trichomes, anthocyanin, and seed coat mucilage and abnormally patterned stomates. Mutants are defective in jasmonate-induced anthocyanin accumulation. Encodes a bHLH Transcription Factor 1. The protein is functionally redundant with GL3 and TT8 and interacts with TTG1, the myb proteins GL1, PAP1 and 2, CPC and TRY, and it will form heterodimers with GL3. Expression in N (non-hair cell forming) cell layers is negatively regulated by WER. Expression in H cells (hair cell forming) is promoted by CPC/TRY.
AT1G63660		GMP synthase (glutamine-hydrolyzing), putative / glutamine amidotransferase
AT1G63670	*TON1 RECRUITING MOTIF 12 (TRM12)*	hypothetical protein (DUF3741)
AT1G63690	*SIGNAL PEPTIDE PEPTIDASE-LIKE 2 (SPPL2)*	SIGNAL PEPTIDE PEPTIDASE-LIKE 2
AT1G63710	*CYTOCHROME P450, FAMILY 86, SUBFAMILY A, POLYPEPTIDE 7 (CYP86A7)*	Encodes a member of the CYP86A subfamily of cytochrome p450 genes. Expressed at highest level in mature stems and flowers.
AT1G63720		hydroxyproline-rich glycoprotein family protein
AT1G63740		Disease resistance protein (TIR-NBS-LRR class) family

AT1G63750		miR825-5p target proposed as a phasiRNA producing locus.
AT1G63770		Peptidase M1 family protein
AT1G63780	*(IMP4)*	Small nucleolar ribonucleoprotein protein involved in ribosomal RNA processing. Located in nucleolus and cajal bodies.
AT1G63800	*UBIQUITIN-CONJUGATING ENZYME 5 (UBC5)*	ubiquitin-conjugating enzyme 5
AT1G63810		nucleolar protein
AT1G63820		CCT motif family protein
AT1G63840		RING/U-box superfamily protein
AT1G63850		BTB/POZ domain-containing protein
AT1G63870		Disease resistance protein (TIR-NBS-LRR class) family
AT1G63880		Encodes a TIR-NBS-LRR class of disease resistance protein effective against Leptosphaeria maculans. The mRNA is cell-to-cell mobile.
AT1G63910	*MYB DOMAIN PROTEIN 103 (AtMYB103)*	member of MYB3R- and R2R3- type MYB- encoding genes
AT1G63930	*FROM THE CZECH 'ROH' MEANING 'CORNER' (ROH1)*	EXO70 interactor and presumed negative secretion regulator.
AT1G63940	*MONODEHYDROASCORBATE REDUCTASE 6 (MDAR6)*	monodehydroascorbate reductase 6
AT1G63950	*HEAVY METAL ASSOCIATED PROTEIN 13 (ATHMP13)*	Heavy metal transport/detoxification superfamily protein
AT1G63960		Copper transport protein family
AT1G64000	*WRKY DNA-BINDING PROTEIN 56 (WRKY56)*	member of WRKY Transcription Factor; Group II-c
AT1G64010		Serine protease inhibitor (SERPIN) family protein
AT1G64020		Serine protease inhibitor (SERPIN) family protein
AT1G64030	*SERPIN 3 (SRP3)*	serpin 3
AT1G64040	*TYPE ONE SERINE/THREONINE PROTEIN PHOSPHATASE 3 (TOPP3)*	Encodes the catalytic subunit of a Type 1 phosphoprotein Ser/Thr phosphatase, expressed in roots, shoots and flowers.
AT1G64050		hypothetical protein
AT1G64060	*RESPIRATORY BURST OXIDASE PROTEIN F (RBOH F)*	Interacts with AtrbohD gene to fine tune the spatial control of ROI production and hypersensitive response to cell in and around infection site.
AT1G64100		pentatricopeptide (PPR) repeat-containing protein
AT1G64110	*DUO1-ACTIVATED ATPASE 1 (DAA1)*	Target promoter of the male germline-specific transcription factor DUO1.
AT1G64130		Polyketide cyclase/dehydrase and lipid transport superfamily protein
AT1G64140		WRKY transcription factor
AT1G64150	*BIVALENT CATION TRANSPORTER 1 (BICAT1)*	Encodes an integral thylakoid membrane protein that is required for normal operation of oxygen-evolving complex (as evidenced by oxygen evolution rates) and for manganese incorporation. PAM71 belongs to a small gene family in Arabidopsis comprising five members. PAM71 is well conserved in the green lineage and shares homology with putative Ca2+/H+ exchangers from yeast (Saccharomyces cerevisiae) (GDT1) and human (Homo sapiens) (TMEM165).
AT1G64160	*DIRIGENT PROTEIN 5 (DIR5)*	Encodes a dirigent protein involved in the synthesis of (-)pinoresinol. Dirigent proteins impart stereoselectivity on the phenoxy radical coupling reaction yielding optically active lignans from two molecules of coniferyl alcohol.
AT1G64170	*CATION/H+ EXCHANGER 16 (CHX16)*	member of Putative Na+/H+ antiporter family
AT1G64180		intracellular protein transport protein USO1-like protein
AT1G64200	*VACUOLAR H+-ATPASE SUBUNIT E ISOFORM 3 (VHA-E3)*	vacuolar H+-ATPase subunit E isoform 3
AT1G64210		Leucine-rich repeat protein kinase family protein
AT1G64220	*TRANSLOCASE OF OUTER MEMBRANE 7 KDA SUBUNIT 2 (TOM7-2)*	translocase of outer membrane 7 kDa subunit 2
AT1G64230	*UBIQUITIN-CONJUGATING ENZYME 28 (UBC28)*	ubiquitin-conjugating enzyme 28
AT1G64250		transposable_element_gene;Mutator-like transposase family, has a 2.2e-20 P-value blast match to GB:AAA21566 mudrA of transposon=MuDR (MuDr-element) (Zea mays);(source:TAIR10)
AT1G64260		MuDR family transposase
AT1G64270		transposable_element_gene;Mutator-like transposase family, has a 1.0e-06 P-value blast match to GB:AAA21566 mudrA of transposon=MuDR (MuDr-element) (Zea mays);(source:TAIR10)
AT1G64290		F-box protein-like protein
AT1G64300		Protein kinase family protein
AT1G64310	*ORGANELLE TRANSCRIPT PROCESSING 71 (OTP71)*	Encodes a pentatricopeptide repeat (PPR) protein involved in RNA editing in mitochondria.
AT1G64320		myosin heavy chain-like protein
AT1G64330		myosin heavy chain-like protein
AT1G64355		1-acyl-sn-glycerol-3-phosphate acyltransferase
AT1G64360	*ENESCENCE-ASSOCIATED AND QQS-RELATED (SAQR)*	SAQR is a clade specific protein present in single copy in Arabidopsis.It's expression is increased during light induced oxidative stress ,drought stress and also during senescence. Promoter contains two AGL15 binding sites.
AT1G64370	*PHLOEM ASSOCIATED RNA CHAPERONE-LIKE (PARCL)*	filaggrin-like protein
AT1G64380		encodes a member of the DREB subfamily A-6 of ERF/AP2 transcription factor family. The protein contains one AP2 domain. There are 8 members in this subfamily including RAP2.4.
AT1G64390	*GLYCOSYL HYDROLASE 9C2 (GH9C2)*	glycosyl hydrolase 9C2
AT1G64400	*LONG-CHAIN ACYL-COA SYNTHETASE 3 (LACS3)*	AMP-dependent synthetase and ligase family protein
AT1G64405		hypothetical protein
AT1G64410		transposable_element_gene;similar to unknown protein [Arabidopsis thaliana] (TAIR:AT1G52960.1);(source:TAIR10)
AT1G64430		Pentatricopeptide repeat (PPR) superfamily protein

AT1G64440	*ROOT HAIR DEFECTIVE 1 (RHD1)*	Encodes a protein with UDP-D-glucose 4-epimerase activity. Mutants in RHD1 have abnormally shaped root hairs with a bulbous region at the base. Allelic to REB1 encoding a UDP-D-glucose 4-epimerase involved in cell wall biosynthesis.Involved in growth and cell wall carbohydrate biosynthesis.
AT1G64450		Glycine-rich protein family
AT1G64460	*(PI4Kgamma2)*	Phosphatidylinositol 4-kinase involved in root elongation and hypersensitive response.Affects stability of MIEL1 which in turn affects MYB30 turnover.
AT1G64470		
AT1G64500	*(THRUMIN1)*	A member of a protein family found in plants and animals that contain conserved C-terminal glutaredoxin-like and putative zinc-binding cysteine-rich domains. It is involved in light stimulated actin bundling and chloroplast movement. The mRNA is cell-to-cell mobile.
AT1G64510	*PLASTID RIBOSOMAL PROTEIN OF THE 30S SUBUNIT 6 (PRPS6)*	Translation elongation factor EF1B/ribosomal protein S6 family protein
AT1G64530	*NIN-LIKE PROTEIN 6 (NLP6)*	Plant regulator RWP-RK family protein
AT1G64550	*ATP-BINDING CASSETTE F3 (ABCF3)*	Encodes a member of GCN subfamily. Predicted to be involved in stress-associated protein translation control. The mutant is affected in MAMP ((microbe-associated molecular patterns)-induced stomatal closure, but not other MAMP-induced responses in the leaves. Arabidopsis has five ABCF proteins, which are all closely related by sequence to yeast GCN20. None of these five are individually required for GCN2 kinase activity.
AT1G64560		pseudogene of S-adenosylmethionine-dependent methyltransferase/rRNA (adenine-N6,N6-)-dimethyltransferase/rRNA methyltransferase
AT1G64590		NAD(P)-binding Rossmann-fold superfamily protein
AT1G64600		copper ion binding / methyltransferase
AT1G64610		Transducin/WD40 repeat-like superfamily protein
AT1G64620	*(DOF1.8)*	Plant-specific Dof transcription factor which regulates vascular cell di#erentiation and lignin biosynthesis.
AT1G64670	*BODYGUARD1 (BDG1)*	Encodes a epidermally expressed extracellular protein that likely functions as an alpha-beta hydrolase and is required for normal cuticle formation. Homozygous mutant plants are dwarfed and have abnormal leaves, collapsed cells, reduced numbers of trichomes. The specific role of BDG is unclear: it may function in cutin biosynthesis or as a cross-linking enzyme in the cell wall itself.
AT1G64680		beta-carotene isomerase D27
AT1G64690	*BRANCHLESS TRICHOMES (BLT)*	Encodes BRANCHLESS TRICHOME (BLT) involved in trichome development. A large portion of the internal amino acid sequence of BLT is predicted to form a coiled-coil domain. BLT mutants form branchless trichomes with blunt tips.
AT1G64700		PADRE protein up-regulated after infection by S. sclerotiorun.
AT1G64710		GroES-like zinc-binding alcohol dehydrogenase family protein
AT1G64720	*MEMBRANE RELATED PROTEIN CP5 (CP5)*	membrane related protein CP5
AT1G64740	*ALPHA-1 TUBULIN (TUA1)*	alpha-tubulin expressed primarily in stamens and mature pollen
AT1G64770	*PHOTOSYNTHETIC NDH SUBCOMPLEX B 2 (PnsB2)*	encodes a novel subunit of the chloroplast NAD(P)H dehydrogenase complex, involved in cyclic electron flow around photosystem I to produce ATP.
AT1G64780	*AMMONIUM TRANSPORTER 1;2 (AMT1;2)*	encodes an ammonium transporter protein believed to act as a high affinity transporter. It is expressed in the root, primarily in endodermal and cortical cells, and contributes to ammonium uptake in the root.
AT1G64790	*ILITHYIA (ILA)*	ILITHYIA (ILA) is a HEAT repeat protein involved in plant immunity. The gene is also involved in systemic acquired resistance induced by P. syringae expressing avrRps4. Loss-of-function mutants of ILA caused pleiotropic defects in the mutant plants. The mutant plants are smaller in size and the leaves are serrated and yellow to light green in color. Required for bacterium-triggered stomatal closure.
AT1G64800		DNA binding / transcription factor
AT1G64820		MATE efflux family protein
AT1G64830		Eukaryotic aspartyl protease family protein
AT1G64860	*SIGMA FACTOR A (SIGA)*	Subunit of chloroplast RNA polymerase, confers the ability to recognize promoter sequences on the core enzyme
AT1G64880		Ribosomal protein S5 family protein
AT1G64900	*CYTOCHROME P450, FAMILY 89, SUBFAMILY A, POLYPEPTIDE 2 (CYP89A2)*	Encodes cytochrome P450 (CYP89A2). The mRNA is cell-to-cell mobile.
AT1G64910		UDP-Glycosyltransferase superfamily protein
AT1G64920		UDP-Glycosyltransferase superfamily protein
AT1G64930	*CYTOCHROME P450, FAMILY 87, SUBFAMILY A, POLYPEPTIDE 7 (CYP89A7)*	member of CYP89A
AT1G64940	*CYTOCHROME P450, FAMILY 87, SUBFAMILY A, POLYPEPTIDE 6 (CYP89A6)*	member of CYP89A
AT1G64950	*CYTOCHROME P450, FAMILY 89, SUBFAMILY A, POLYPEPTIDE 5 (CYP89A5)*	member of CYP89A The mRNA is cell-to-cell mobile.
AT1G64960	*HYPERSENSITIVE TO EXCESS BORON 1 (HEB1)*	ARM repeat superfamily protein
AT1G64970	*GAMMA-TOCOPHEROL METHYLTRANSFERASE (G-TMT)*	gamma-tocopherol methyltransferase (g-TMT) mRNA, nuclear; mutant has Deficient in alpha and beta tocopherol; Accumulates gamma tocopherol in leaves
AT1G64980	*CADMIUM 2+ INDUCED (CDI)*	Encodes a putative nucleotide-diphospho-sugar transferase required for pollen germination and tube growth.
AT1G65000		F-box only protein
AT1G65010		Encodes a microtubule-associated protein. Putative role in flower development. Comparison of SALK_061426C to Columbia wild type in normal lighting and under low light of 33 micromoles per meter-squared per second resulted in a trend toward earlier bolting in the mutant under low light (P=0.055) (Ann Stapleton and Patrick Pridgen, 2009, personal communication).
AT1G65040	*HOMOLOG OF YEAST HRD1 (Hrd1B)*	Encodes one of the Arabidopsis homologs of the yeast/human Hrd1 protein: AT3G16090 (Hrd1A), AT1G65040 (Hrd1B). Involved in ERAD (Endoplasmic reticulum-associated degradation).
AT1G65070		DNA mismatch repair protein MutS, type 2
AT1G65080	*(OXA2A)*	Structurally distinct member of Oxa1 superfamily , has tetratricopeptide repeat (TPR) domain at the C terminus. Paralog of OXA2b.Involved in maturation of mitochondrial cytochrome c.
AT1G65090		nucleolin

AT1G65110		Ubiquitin carboxyl-terminal hydrolase-related protein
AT1G65120		Ubiquitin carboxyl-terminal hydrolase-related protein
AT1G65130		Ubiquitin carboxyl-terminal hydrolase-related protein
AT1G65170		Ubiquitin carboxyl-terminal hydrolase family protein
AT1G65180		Cysteine/Histidine-rich C1 domain family protein
AT1G65190	*(ZRK13)*	Protein kinase superfamily protein
AT1G65210		Galactose-binding protein
AT1G65220		ARM repeat superfamily protein
AT1G65230		transmembrane protein, putative (DUF2358)
AT1G65240	*(A39)*	Eukaryotic aspartyl protease family protein
AT1G65270		ER membrane protein complex subunit-like protein
AT1G65280		DNAJ heat shock N-terminal domain-containing protein
AT1G65290	*MITOCHONDRIAL ACYL CARRIER PROTEIN 2 (mtACP2)*	Encodes a member of the mitochondrial acyl carrier protein (ACP) family that forms part of the membrane arm of mitochondrial complex and contributes to the mitochondrial respiratory chain. The mRNA is cell-to-cell mobile. The designations of mtACP-1 and mtACP-2 in Klusch et al. 2021 (DOI:10.1093/plcell/koab092)are flipped with respect to the nomenclature published by Meyer et al. 2007 (DOI:10.1007/s11103-007-9156-9).
AT1G65310	*XYLOGLUCAN ENDOTRANSGLUCOSYLASE/HYDROLASE 17 (XTH17)*	Encodes a xyloglucan endotransglucosylase/hydrolase with only only the endotransglucosylase (XET; EC 2.4.1.207) activity towards xyloglucan and non-detectable endohydrolytic (XEH; EC 3.2.1.151) activity. Expressed in the mature or basal regions of both the main and lateral roots, but not in the tip of these roots where cell division occurs.
AT1G65330	*PHERES1 (PHE1)*	Type I MADS-box protein, regulated by MEA and FIE, expressed transiently after fertilization in embryo and endosperm.
AT1G65340	*CYTOCHROME P450, FAMILY 96, SUBFAMILY A, POLYPEPTIDE 3 (CYP96A3)*	member of CYP96A
AT1G65350	*UBIQUITIN 13 (UBQ13)*	polyubiquitin gene,Columbia ecotype revealed that the gene contained a 3.9-kb insertion in the coding region from mitochondrial DNA.
AT1G65360	*AGAMOUS-LIKE 23 (AGL23)*	Encodes AGL23, a Type I MADS-box gene that controls female gametophyte development and the biogenesis of organelles during embryo development.
AT1G65370		TRAF-like family protein
AT1G65390	*PHLOEM PROTEIN 2 A5 (PP2-A5)*	Phloem Protein2 family gene encoding a two-domain protein containing predicted lectin and Toll/Interleukin-1 receptor domains, which is induced upon spider mite attack and improves the ability to defend against T. urticae by participating in the tight regulation of hormonal cross talk upon mite feeding.
AT1G65400		
AT1G65440	*GLOBAL TRANSCRIPTION FACTOR GROUP B1 (GTB1)*	Related to yeast Spt6 protein, which functions as part of a protein complex in transcription initiation and also plays a role in chromatin structure / assembly. It encodes a putative WG/GW-repeat protein involved in the regulation of apical-basal polarity of embryo
AT1G65445		
AT1G65450	*GLAUCE (GLC)*	Contains dual transcription units and alternative splicing that could rescue the sterility defect of glc mutants. Shares homology to BAHD (for BEAT, AHCT, HCBT, and DAT) acyl-transferases. Functions in double fertilization.
AT1G65470	*FASCIATA 1 (FAS1)*	Chromatin Assembly Factor-1 (CAF-1) p150 subunit. Mutants have reduced heterochromatin content. In Arabidopsis, the three CAF-1 subunits are encoded by FAS1, FAS2 and, most likely, MSI1, respectively. Mutations in FAS1 or FAS2 lead to increased frequency of homologous recombination and T-DNA integration in Arabidopsis.
AT1G65480	*FLOWERING LOCUS T (FT)*	FT, together with LFY, promotes flowering and is antagonistic with its homologous gene, TERMINAL FLOWER1 (TFL1). Together with TSF, it plays an antagonistic role to TFL1 in the determination of inflorescence meristem identity. FT is expressed in leaves and is induced by long day treatment. Either the FT mRNA or protein is translocated to the shoot apex where it induces its own expression. Recent data suggests that FT protein acts as a long-range signal. FT is a target of CO and acts upstream of SOC1.
AT1G65490	*(STMP5)*	Secreted peptide which functions in plant growth and pathogen defense.
AT1G65500	*(STMP6)*	Secreted peptide which functions in plant growth and pathogen defense.
AT1G65510	*(STMP7)*	Secreted peptide which functions in plant growth and pathogen defense.
AT1G65560		Zinc-binding dehydrogenase family protein
AT1G65570	*ROOT CAP POLYGALACTURONASE (RCPG)*	Encodes a glycosyl hydrolase 28 (GH28) family polygalacturonase (PG) protein. Involved in root cap development.
AT1G65590	*BETA-HEXOSAMINIDASE 3 (HEXO3)*	Encodes a protein with beta-hexosaminidase activity. Located on the plasma membrane.
AT1G65610	*KORRIGAN 2 (KOR2)*	Six-hairpin glycosidases superfamily protein
AT1G65620	*ASYMMETRIC LEAVES 2 (AS2)*	required for formation of a symmetric flat leaf lamina, encodes a member of a family of proteins characterized by cysteine repeats and a leucine zipper; involved in KNOX gene regulation. Acts together with ASL1 in proximal-distal symmetry determination. Forms a complex with AS1 that binds to the BP promoter and leads to silencing of BP.
AT1G65630	*DEGRADATION OF PERIPLASMIC PROTEINS 3 (DEG3)*	Encodes a putative DegP protease.
AT1G65640	*DEGRADATION OF PERIPLASMIC PROTEINS 4 (DEG4)*	Encodes a putative DegP protease.
AT1G65660	*SWELLMAP 1 (SMP1)*	Encodes a CCHC zinc finger protein that may function as a step II splicing factor. In an epigenetic allele of SMP1 (in which SMP1 and SMP2 mRNA is reduced) organs are smaller and contain fewer cells.
AT1G65670	*CYTOCHROME P450, FAMILY 702, SUBFAMILY A, POLYPEPTIDE 1 (CYP702A1)*	a member of the cytochrome P450 gene family. molecular function unknown.
AT1G65690	*NDR1/HIN1-LIKE 6 (NHL6)*	Encodes NHL6 (NDR1/HIN1-like 6). Plays an important role in the abiotic stresses-induced ABA signaling and biosynthesis, particularly during seed germination and early seedling development.
AT1G65730	*YELLOW STRIPE LIKE 7 (YSL7)*	Arabidopsis thaliana metal-nicotianamine transporter YSL4
AT1G65740	*UPWARD CURLY LEAF1 (UCL1)*	ascorbic acid mannose pathway regulator (DUF295)
AT1G65760	*F-BOX/DUF295 ANCESTRAL 6 (ATFDA6)*	ascorbic acid mannose pathway regulator (DUF295)

AT1G65790	*RECEPTOR KINASE 1 (RK1)*	An alternatively spliced gene that encodes a functional transmembrane receptor serine/threonine kinase, alternate form may not have transmembrane domain.
AT1G65810		P-loop containing nucleoside triphosphate hydrolases superfamily protein
AT1G65820		microsomal glutathione s-transferase
AT1G65850		Disease resistance protein (TIR-NBS-LRR class) family
AT1G65860	*FLAVIN-MONOOXYGENASE GLUCOSINOLATE S-OXYGENASE 1 (FMO GS-OX1)*	belongs to the flavin-monooxygenase (FMO) family, encodes a glucosinolate S-oxygenase that catalyzes the conversion of methylthioalkyl glucosinolates to methylsulfinylalkyl glucosinolates
AT1G65870		Disease resistance-responsive (dirigent-like protein) family protein
AT1G65880	*BENZOYLOXYGLUCOSINOLATE 1 (BZO1)*	Encodes a benzoate-CoA ligase. Involved in the biosynthesis of benzoyloxyglucosinolate in Arabidopsis seeds.
AT1G65890	*ACYL ACTIVATING ENZYME 12 (AAE12)*	acyl activating enzyme 12
AT1G65900		plant/protein
AT1G65910	*NAC DOMAIN CONTAINING PROTEIN 28 (NAC028)*	NAC domain containing protein 28
AT1G65920		Regulator of chromosome condensation (RCC1) family with FYVE zinc finger domain-containing protein
AT1G65940		pseudogene of Dof-type zinc finger domain-containing protein
AT1G65960	*GLUTAMATE DECARBOXYLASE 2 (GAD2)*	glutamate decarboxylase (GAD2) The mRNA is cell-to-cell mobile.
AT1G65990	*PEROXIREDOXIN IIA (PRXIIA)*	type 2 peroxiredoxin-related / thiol specific antioxidant / mal allergen family protein
AT1G66000		hypothetical protein (DUF577)
AT1G66020	*TERPENE SYNTHASE 26 (TPS26)*	Terpenoid cyclases/Protein prenyltransferases superfamily protein
AT1G66030	*CYTOCHROME P450, FAMILY 96, SUBFAMILY A, POLYPEPTIDE 14 PSEUDOGENE (CYP96A14P)*	Encodes a protein with cytochrome P450 domain. Probable psuedogene.
AT1G66080	*HIKESHI-LIKE PROTEIN1 (HLP1)*	Encodes a glucose-regulated protein that binds to the promoters of glucose-regulated heat shock responsive genes and promotes chromatin acetylation. HLP1 is required in maintaining histone H3K acetylation and H3K4 methylation marks at the promoters of heat shock protein genes in providing thermotolerance/thermomemory response.
AT1G66090	*TIR-NBS3 (TN3)*	Disease resistance protein (TIR-NBS class)
AT1G66100		Predicted to encode a PR (pathogenesis-related) protein. Belongs to the plant thionin (PR-13) family with the following members: At1g66100, At5g36910, At1g72260, At2g15010, At1g12663, At1g12660.
AT1G66110		hypothetical protein (DUF577)
AT1G66120	*ACYL-ACTIVATING ENZYME 11 (AAE11)*	AMP-dependent synthetase and ligase family protein
AT1G66130		NAD(P)-binding Rossmann-fold superfamily protein
AT1G66140	*ZINC FINGER PROTEIN 4 (ZFP4)*	Encodes a zinc finger protein containing only a single zinc finger.
AT1G66150	*TRANSMEMBRANE KINASE 1 (TMK1)*	Receptor-like transmembrane kinase I (TMK1); key regulator in auxin signaling. High auxin and TMK1 play essential and positive roles in ABA signaling through regulating ABA INSENSITIVE 1 and 2 (ABI1/2). Inhibits the phosphatase activity of ABI2 by direct phosphorylation of threonine 321 (T321), a conserved phosphorylation site in ABI2 proteins, whose phosphorylation status is important for both auxin and ABA responses.
AT1G66160	*CYS, MET, PRO, AND GLY PROTEIN 1 (CMPG1)*	CYS, MET, PRO, and GLY protein 1
AT1G66170	*MALE MEIOCYTE DEATH 1 (MMD1)*	Encodes a PHD-domain containing protein required for male meiosis. Gene is expressed in developing male meiocytes and protein is localized to nuclear euchromatin specifically during diplotene. Required to regulate microtubule organization and cell cycle transitions during male meiosis, and functions as a direct transcription activator of the meiotic gene TDM1.
AT1G66180		The gene encodes a putative aspartyl protease (ASP). Its expression is induced in response to light and ascorbate. The mRNA is cell-to-cell mobile.
AT1G66210		Subtilisin-like serine endopeptidase family protein
AT1G66220		Subtilase family protein
AT1G66230	*MYB DOMAIN PROTEIN 20 (MYB20)*	Encodes a transcriptional regulator that directly activates lignin biosynthesis genes and phenylalanine biosynthesis genes during secondary wall formation.
AT1G66250		O-Glycosyl hydrolases family 17 protein
AT1G66260	*(ALY3)*	RNA-binding (RRM/RBD/RNP motifs) family protein
AT1G66280	*(BGLU22)*	Glycosyl hydrolase superfamily protein
AT1G66310		F-box/RNI-like/FBD-like domains-containing protein
AT1G66350	*RGA-LIKE 1 (RGL1)*	Negative regulator of GA responses, member of GRAS family of transcription factors. Also belongs to the DELLA proteins that restrain the cell proliferation and expansion that drives plant growth. RGL1 may be involved in reducing ROS accumulation in response to stress by up-regulating the transcription of superoxide dismutases. Rapidly degraded in response to GA. Involved in flower and fruit development.
AT1G66360	*C2-DOMAIN ABA-RELATED2 (CAR2)*	Calcium-dependent lipid-binding (CaLB domain) family protein
AT1G66370	*MYB DOMAIN PROTEIN 113 (MYB113)*	Encodes a member of the MYB family of transcription factors. Involved in regulation of anthocyanin biosynthesis. Affects the expression of enzymes involved in later steps of anthocyanin biosynthesis.
AT1G66380	*MYB DOMAIN PROTEIN 114 (MYB114)*	Encodes a member of the MYB family of transcription factors. Involved in regulation of anthocyanin biosynthesis. Affects the expression of enzymes involved in later steps of anthocyanin biosynthesis
AT1G66390	*MYB DOMAIN PROTEIN 90 (MYB90)*	Production of anthocyanin pigment 2 protein (PAP2).
AT1G66400	*CALMODULIN LIKE 23 (CML23)*	Encodes a calmodulin-like protein. Regulates nitric oxide levels and transition to flowering.
AT1G66430	*FRUCTOKINASE 3 (FRK3)*	Encodes one of the several Arabidopsis fructokinases. Nomenclature according to Riggs 2017 has been adopted for the family by the community (personal communication, Boernke, Callis, Granot, Boernke, and Smeekens). Important for seed oil accumulation and vascular development.
AT1G66440		Cysteine/Histidine-rich C1 domain family protein
AT1G66450		Cysteine/Histidine-rich C1 domain family protein
AT1G66460		Protein kinase superfamily protein

AT1G66470	*ROOT HAIR DEFECTIVE6 (RHD6)*	ROOT HAIR DEFECTIVE6
AT1G66480		Involved in chloroplast avoidance movement under intermediate and high light intensities; PADRE protein up-regulated after infection by S. sclerotiorun.
AT1G66490		F-box and associated interaction domains-containing protein
AT1G66500		Pre-mRNA cleavage complex II
AT1G66540		Cytochrome P450 superfamily protein
AT1G66550	*WRKY DNA-BINDING PROTEIN 67 (WRKY67)*	member of WRKY Transcription Factor; Group III
AT1G66570	*SUCROSE-PROTON SYMPORTER 7 (SUC7)*	sucrose-proton symporter 7
AT1G66610		TRAF-like superfamily protein
AT1G66620		Protein with RING/U-box and TRAF-like domain
AT1G66630	*(PEG4)*	Protein with RING/U-box and TRAF-like domain
AT1G66640		RNI-like superfamily protein
AT1G66645		
AT1G66660		Protein with RING/U-box and TRAF-like domain
AT1G66670	*CLP PROTEASE PROTEOLYTIC SUBUNIT 3 (CLPP3)*	One of several nuclear-encoded ClpPs (caseinolytic protease). Contains a highly conserved catalytic triad of Ser-type proteases (Ser-His-Asp). The name reflects nomenclature described in Adam et. al (2001).
AT1G66690		SABATH family methyltransferase.
AT1G66720		S-adenosyl-L-methionine-dependent methyltransferases superfamily protein
AT1G66750	*CDK-ACTIVATING KINASE 4 (CAK4)*	Encodes a CDK-activating kinase that interacts with SPT5, a regulator of transcription and histone methylation.
AT1G66760		MATE efflux family protein
AT1G66770	*(SWEET6)*	Nodulin MtN3 family protein
AT1G66780		MATE efflux family protein
AT1G66790		
AT1G66800		Its expression is enriched in non-root hair cells (compared to root hair cells) and this enrichment is associated with increase in the transcription-associated mark trimethylation of H3 lysine 4 (H3K4me3) and decrease in the Polycomb silencing-associated mark trimethylation of H3 lysine 27 (H3K27me3) in non-root hair cells relative to root-hair cells. Protein sequence is similar to Eucalyptus gunnii alcohol dehydrogenase of unknown physiological function (GI:1143445), apple tree, PIR:T16995; NOT a cinnamyl-alcohol dehydrogenase. The mRNA is cell-to-cell mobile.
AT1G66810	*(ATC3H14)*	Encodes a tandem CCCH zinc finger (TZF) protein that can bind DNA and RNA, function as a transcriptional activator, and is involved in secondary wall biosynthesis.
AT1G66830		Leucine-rich repeat protein kinase family protein
AT1G66840	*PLASTID MOVEMENT IMPAIRED 2 (PMI2)*	Encodes a coiled-coil protein WEB2 (weak chloroplast movement under blue light 2, also named PMI2/plastid movement impaired 2). Involved in chloroplast avoidance movement under intermediate and high light intensities. WEB2, together with another coiled-coil protein WEB1 (AT2G26570), maintains the chloroplast photorelocation movement velocity.
AT1G66850		Bifunctional inhibitor/lipid-transfer protein/seed storage 2S albumin superfamily protein
AT1G66860		Class I glutamine amidotransferase-like superfamily protein
AT1G66880		Protein kinase superfamily protein
AT1G66890		50S ribosomal-like protein
AT1G66920		Protein kinase superfamily protein
AT1G66930	*LEAF RUST 10 DISEASE-RESISTANCE LOCUS RECEPTOR- LIKE PROTEIN KINASE-LIKE2 (LRK10L2)*	Protein kinase superfamily protein
AT1G66940		kinase-like protein
AT1G66950	*ATP-BINDING CASSETTE G39 (ABCG39)*	Encodes a plasma membrane-localized ABC transporter. Confers tolerance to herbicide paraquat.
AT1G66960	*(LUP5)*	Terpenoid cyclases family protein
AT1G66970	*SHV3-LIKE 2 (SVL2)*	Encodes a member of the glycerophosphodiester phosphodiesterase like (GDPD-like) family.
AT1G66980	*SUPPRESSOR OF NPR1-1 CONSTITUTIVE 4 (SNC4)*	Encodes SNC4 (suppressor of npr1-1, constitutive 4), an atypical receptor-like kinase with two predicted extracellular glycerophosphoryl diester phosphodiesterase domains.
AT1G67000		Protein kinase superfamily protein
AT1G67030	*ZINC FINGER PROTEIN 6 (ZFP6)*	Encodes a novel C2H2 zinc finger protein containing only a single zinc finger which plays a key role in regulating trichome development by integrating GA and cytokinin signaling. The mRNA is cell-to-cell mobile.
AT1G67040	*TON1 RECRUITING MOTIF 22 (TRM22)*	DnaA initiator-associating protein
AT1G67050		membrane-associated kinase regulator
AT1G67060		peptidase M50B-like protein
AT1G67070	*DARK INDUCIBLE 9 (DIN9)*	Encodes a protein with phosphomannose isomerase activity that is involved in synthesis of ascorbic acid. Expression is induced after 24 hours of dark treatment, in senescing leaves and treatment with exogenous photosynthesis inhibitor. Induction of gene expression was suppressed in excised leaves supplied with sugar. The authors suggest that the gene's expression pattern is responding to the level of sugar in the cell.
AT1G67080	*ABSCISIC ACID (ABA)-DEFICIENT 4 (ABA4)*	Encodes a protein involved in the photoprotection of PSII. An aba4-1 mutant completely lacks neoxanthin,a component of the chromophore of the peripheral antenna system in PSII. ABA4 is required for neoxanthin biosynthesis, an intermediary step in abscisic acid biosynthesis, but no catalytic activity has been detected for the ABA4 protein.
AT1G67090	*RIBULOSE BISPHOSPHATE CARBOXYLASE SMALL CHAIN 1A (RBCS1A)*	Encodes a member of the Rubisco small subunit (RBCS) multigene family: RBCS1A (At1g67090), RBCS1B (At5g38430), RBCS2B (At5g38420), and RBCS3B (At5g38410). Functions to yield sufficient Rubisco content for leaf photosynthetic capacity.

AT1G67100	*LOB DOMAIN-CONTAINING PROTEIN 40 (LBD40)*	LOB domain-containing protein 40
AT1G67110	*CYTOCHROME P450, FAMILY 735, SUBFAMILY A, POLYPEPTIDE 2 (CYP735A2)*	cytochrome P450, family 735, subfamily A, polypeptide 2
AT1G67120	*MIDASIN 1 (MDN1)*	Represents a homolog of the yeast MDN gene, which encodes a non-ribosomal protein involved in the maturation and assembly of the 60S ribosomal subunit. In Arabidopsis, it is essential for female gametogenesis progression.
AT1G67210	*(ZCCHC8B)*	Proline-rich spliceosome-associated (PSP) family protein / zinc knuckle (CCHC-type) family protein
AT1G67230	*LITTLE NUCLEI1 (LINC1)*	Encodes a nuclear coiled-coil protein related to the carrot peripheral nuclear protein NMCP1 that is involved in the determination of plant nuclear structure. Member of a small gene family in Arabidopsis containing 4 proteins (LNC1-4 or CRWN 1-4) with redundant functions in protection from oxidative damage, control of nuclear morphology and degradation of ABI5.
AT1G67240		transposable_element_gene;Mutator-like transposase family, has a 4.5e-23 P-value blast match to GB:AAA21566 mudrA of transposon=MuDR (MuDr-element) (Zea mays);(source:TAIR10)
AT1G67270		Zinc-finger domain of monoamine-oxidase A repressor R1 protein
AT1G67320	*EMBRYO DEFECTIVE 2813 (EMB2813)*	DNA primase, large subunit family
AT1G67330	*(AGM2)*	Arabinogalactan methylesterase,involved in arabinogalactan glucuronic acid methylation.
AT1G67360	*LD-ASSOCIATED PROTEIN 1 (LDAP1)*	Encodes a small rubber particle protein homolog. Plays dual roles as positive factors for tissue growth and development and in drought stress responses.
AT1G67370	*ASYNAPTIC 1 (ASY1)*	Meiotic asynaptic mutant 1 (ASY1). ASY1 protein is initially distributed as numerous foci throughout the chromatin. During early G2, the foci are juxtaposed to the nascent chromosome axes to form a continuous axis associated signal.
AT1G67440	*EMBRYO DEFECTIVE 1688 (emb1688)*	Homolog of bacterial rsgA, functions in chloroplast ribosome biogenesis.
AT1G67450		F-box and associated interaction domains-containing protein
AT1G67460		Minichromosome maintenance (MCM2/3/5) family protein
AT1G67470	*(ZRK12)*	Protein kinase superfamily protein
AT1G67480		Galactose oxidase/kelch repeat superfamily protein
AT1G67490	*GLUCOSIDASE 1 (GCS1)*	Encodes an alpha-glucosidase I enzyme that catalyzes the first step in N-linked glycan processing. Localized to the endoplasmic reticulum (ER).
AT1G67500	*RECOVERY PROTEIN 3 (REV3)*	Encodes the catalytic subunit of DNA polymerase zeta.Mutants are sensitive to UV-B radiation. Gene is involved in damage-tolerance mechanisms through translesion synthesis(TLS).
AT1G67510		Leucine-rich repeat protein kinase family protein
AT1G67520		lectin protein kinase family protein
AT1G67540		transmembrane protein
AT1G67550	*UREASE (URE)*	Encodes a nickel-containing urea hydrolase involved in nitrogen recycling. It requires three urease accessory proteins for its activation. The mRNA is cell-to-cell mobile.
AT1G67560	*LIPOXYGENASE 6 (LOX6)*	PLAT/LH2 domain-containing lipoxygenase family protein
AT1G67570		zinc finger CONSTANS-like protein (DUF3537)
AT1G67580	*CYCLIN-DEPENDENT KINASE G2 (CDKG2)*	Protein kinase superfamily protein
AT1G67590		Remorin family protein
AT1G67600		Acid phosphatase/vanadium-dependent haloperoxidase-related protein
AT1G67620		Lojap-related protein
AT1G67623		F-box family protein
AT1G67630	*DNA POLYMERASE ALPHA 2 (POLA2)*	DNA polymerase alpha 2
AT1G67635		phosphatidylinositol 4-kinase gamma-like protein
AT1G67640		Transmembrane amino acid transporter family protein
AT1G67670		hypothetical protein
AT1G67700	*HYPERSENSITIVE TO HIGH LIGHT 1 (HHL1)*	multidrug resistance protein
AT1G67710	*RESPONSE REGULATOR 11 (ARR11)*	Encodes an Arabidopsis response regulator (ARR) protein that acts in concert with other type-B ARRs in the cytokinin signaling pathway. Affects ABA-JA crosstalk.
AT1G67720		Leucine-rich repeat protein kinase family protein
AT1G67730	*BETA-KETOACYL REDUCTASE 1 (KCR1)*	Encodes one of the two Arabidopsis homologues to YBR159w encoding a S. cerevisiae beta-ketoacyl reductase (KCR), which catalyzes the first reduction during VLCFA (very long chain fatty acids, >18 carbon) elongation: KCR1 (At1g67730), KCR2 (At1g24470). Complementation of the yeast ybr159Delta mutant demonstrated that the two KCR proteins are divergent and that only AtKCR1 can restore heterologous elongase activity similar to the native yeast KCR gene. The mRNA is cell-to-cell mobile.
AT1G67740	*PHOTOSYSTEM II BY (PSBY)*	PsbY precursor (psbY) mRNA. This single nuclear gene is imported into the chloroplasts where it is processed into two integral membrane proteins with identical topology (PsbY-1 and PsbY-2). The protein appears to bind manganese. Important for the redox control of cytochrome b559.
AT1G67750		Pectate lyase family protein
AT1G67770	*TERMINAL EAR1-LIKE 2 (TEL2)*	Similar to terminal ear1 in Zea mays. A member of mei2-like gene family; phylogenetic analysis revealed that TEL2 belongs to the third clade of mei2-like proteins (TEL clade), with conserved two N-terminal RNA recognition motifs (RRM), in addition to the C-terminal RRM, shared among all mei2-like proteins. Expression patterns were similar to TEL1, with lower expression levels in most tissues examined.
AT1G67780	*DDT-RELATED PROTEIN3 (DDR3)*	Zinc-finger domain of monoamine-oxidase A repressor R1 protein
AT1G67790		sieve element occlusion protein
AT1G67810	*SULFUR E2 (SUFE2)*	Encodes a protein capable of stimulating the cysteine desulfurase activity of CpNifS (AT1G08490) in vitro. SufE2:GFP localizes to the chloroplasts where it is likely to play a role in iron-sulfur cluster assembly. Transcript levels for this gene are high in the pollen relative to other organs based on RT-PCR analysis. The mRNA is cell-to-cell mobile.

AT1G67830	*ALPHA-FUCOSIDASE 1 (FXG1)*	Encodes a protein with α-fucosidase activity. The activity was assessed on 2'-fucosyl-lactitol. AtFXG1 was able to remove the t-fucosyl residues of XXFG xyloglucan oligosaccharides.
AT1G67850		lysine ketoglutarate reductase trans-splicing protein (DUF707)
AT1G67855		hypothetical protein
AT1G67860		transmembrane protein
AT1G67865		hypothetical protein
AT1G67870		glycine-rich protein
AT1G67910		hypothetical protein
AT1G67920		hypothetical protein
AT1G67940	*ATP-BINDING CASSETTE I17 (ABCI17)*	member of NAP subfamily The mRNA is cell-to-cell mobile.
AT1G67980	*CAFFEOYL-COA 3-O-METHYLTRANSFERASE (CCOAMT)*	Encodes S-adenosyl-L-methionine: transcaffeoyl Coenzyme A 3-O-methyltransferase. Methyltransferase in the lignin biosynthetic pathway.
AT1G67990	*(TSM1)*	Encodes a tapetum-specific O-methyltransferase. In vitro enzyme assay indicated activity with caffeoyl-CoA, caffeoyl glucose, chlorogenic acid and polyamine conjugates. RNAi mutants had impaired silique development and seed setting.
AT1G68010	*HYDROXYPYRUVATE REDUCTASE (HPR)*	Encodes hydroxypyruvate reductase.
AT1G68020	*(ATTPS6)*	Encodes an enzyme putatively involved in trehalose biosynthesis. The protein has a trehalose synthase (TPS)-like domain and a trehalose phosphatase (TPP)-like domain. It can complement a yeast mutant lacking both of these activities suggesting that this is a bifunctional enzyme.
AT1G68040		S-adenosyl-L-methionine-dependent methyltransferases superfamily protein
AT1G68060	*MICROTUBULE-ASSOCIATED PROTEINS 70-1 (MAP70-1)*	Encodes a microtubule associated protein (MAP70-1). Expressed in all tissues.
AT1G68090	*ANNEXIN 5 (ANN5)*	Encodes a calcium-binding protein annexin (AnnAt5). Plays a vital role in pollen development via Ca2+ dependent membrane trafficking.
AT1G68110	*CLATHRIN ASSEMBLY PROTEIN (CLAP)*	An ENTH (Epsin NH2 terminal homology)/ANTH/VHS superfamily protein with adenylate cyclase activity and a role in clathrin assembly and endocytosis.
AT1G68120	*BASIC PENTACYSTEINE 3 (BPC3)*	BASIC PENTACYSTEINE protein
AT1G68130	*INDETERMINATE(ID)-DOMAIN 14 (IDD14)*	Encodes the longer of two splice variants of a transcription factor involved in regulating starch metabolism in response to cold.
AT1G68140		zinc finger/BTB domain protein, putative (DUF1644)
AT1G68150	*WRKY DNA-BINDING PROTEIN 9 (WRKY9)*	member of WRKY Transcription Factor; Group II-b The mRNA is cell-to-cell mobile.
AT1G68160		ZZ-type zinc finger protein, putative (DUF3755)
AT1G68170	*USUALLY MULTIPLE ACIDS MOVE IN AND OUT TRANSPORTERS 23 (UMAMIT23)*	nodulin MtN21-like transporter family protein
AT1G68190	*B-BOX DOMAIN PROTEIN 27 (BBX27)*	B-box zinc finger family protein
AT1G68200	*CALLOSE DEFECTIVE MICROSPORE1 (CDM1)*	Zinc finger C-x8-C-x5-C-x3-H type family protein
AT1G68230		Reticulon family protein
AT1G68240		basic helix-loop-helix (bHLH) DNA-binding superfamily protein
AT1G68250		hypothetical protein
AT1G68260	*ACYL-LIPID THIOESTERASE 3 (ALT3)*	Thioesterase superfamily protein
AT1G68270		AMP-dependent synthetase and ligase family protein
AT1G68290	*ENDONUCLEASE 2 (ENDO2)*	Encodes an endonuclease ENDO2. ENDO2 purified from transgenic Arabidopsis digests RNA, ssDNA, and dsDNA, with a substrate preference for ssDNA and RNA. ENDO2 produced and purified from Nicotiana benthamiana expression showed no demonstrable endonuclease activity, either towards single stranded DNA or mismatches, in vitro.
AT1G68320	*MYB DOMAIN PROTEIN 62 (MYB62)*	putative transcription factor: R2R3-MYB transcription family. Involved in regulation of phosphate starvation responses and gibberellic acid biosynthesis.
AT1G68330		membrane-associated kinase regulator
AT1G68340		hypothetical protein (DUF1639)
AT1G68360	*GLABROUS INFLORESCENCE STEMS 3 (GIS3)*	Encodes a nuclear localized member of the C2H2 family of TFIIIA transcription factors.GIS3 is involved in trichome initiation and development downstream of GA and cytokinin signaling. GIS regulates the expression GIS and GIS2.
AT1G68370	*ALTERED RESPONSE TO GRAVITY 1 (ARG1)*	DnaJ-like protein with homology to coiled coils found in cytoskeleton-interacting proteins.
AT1G68380		Core-2/I-branching beta-1,6-N-acetylglucosaminyltransferase family protein
AT1G68400		leucine-rich repeat transmembrane protein kinase family protein
AT1G68410		Protein phosphatase 2C family protein
AT1G68420		Class II aaRS and biotin synthetases superfamily protein
AT1G68440		Transmembrane protein. Expression induced by abiotic stressors such as ABA, drought, heat, light, NaCl, osmotic stress and wounding.
AT1G68450	*PIGMENT DEFECTIVE 337 (PDE337)*	VQ motif-containing protein
AT1G68470		Exostosin family protein
AT1G68480	*JAGGED (JAG)*	Encodes a putative zinc finger transcription factor that is necessary for proper lateral organ shape and is sufficient to induce the proliferation of lateral organ tissue. Together with NUB, it is involved in stamen and carpel development.
AT1G68500		hypothetical protein
AT1G68510	*LOB DOMAIN-CONTAINING PROTEIN 42 (LBD42)*	LOB domain protein.
AT1G68520	*B-BOX DOMAIN PROTEIN 14 (BBX14)*	B-box type zinc finger protein with CCT domain-containing protein
AT1G68530	*3-KETOACYL-COA SYNTHASE 6 (KCS6)*	Encodes KCS6, a member of the 3-ketoacyl-CoA synthase family involved in the biosynthesis of VLCFA (very long chain fatty acids).
AT1G68540	*TETRAKETIDE ALPHA-PYRONE REDUCTASE 2 (TKPR2)*	NAD(P)-binding Rossmann-fold superfamily protein
AT1G68560	*ALPHA-XYLOSIDASE 1 (XYL1)*	Encodes a bifunctional alpha-l-arabinofuranosidase/beta-d-xylosidase that belongs to family 3 of glycoside hydrolases.
AT1G68570	*NRT1/ PTR FAMILY 3.1 (NPF3.1)*	NPF3.1 is a membrane localized GA transporter that is expressed in the root endodermis.

AT1G68580		Agenet and bromo-adjacent homology (BAH) domain-containing protein
AT1G68585		hypothetical protein
AT1G68590	*PLASTID-SPECIﬁC RIBOSOMAL PROTEIN 3/1 (PSRP3/1)*	Ribosomal protein PSRP-3/Ycf65
AT1G68600		aluminum activated malate transporter family protein
AT1G68610	*PLANT CADMIUM RESISTANCE 11 (PCR11)*	Target promoter of the male germline-specific transcription factor DUO1.
AT1G68620		alpha/beta-Hydrolases superfamily protein
AT1G68630		PLAC8 family protein
AT1G68640	*PERIANTHIA (PAN)*	Encodes bZIP-transcription factor. Mutant plants have extra floral organs. PAN is essential for AG activation in early flowers of short-day-grown plants. Binds directly to 5'-AAGAAT-3'regulatory sequence in AG promoter.
AT1G68650	*PHOTOSYNTHESIS-AFFECTED MUTANT 71 LIKE 5 (PML5)*	Member of the UPF0016 family of membrane proteins, belongs to the conserved group of Mn/Ca transporters. Might act to fine tune Mn allocation into the endoplasmic reticulum of specific cell types.
AT1G68660	*CASEINOLYTIC PROTEASE S 1 (CLPS1)*	ClpS1 is a member of the caseionolytic proteinase S family of N-recognins. It is involved in proteolysis in the chloroplast stroma. An arginine residue (Arg50) controls low-affinity substrate binding.
AT1G68670	*(HHO2)*	HHO2 is a HRS1 homolog. Nitrate-inducible expression. Also induced in roots by low Pi and is likely involved in maintaining phosphate homeostasis. It is target of PHR1.Both HHO2 and HRS1 are involved in Ni cross regulation of Pi signaling. They function as transcriptional repressors of SPX1, SPX2, and SPX4 as part of a cascade to regulate nitrogen and phosphorus balance. Transcriptional repressors that functions with other NIGT genes as an important hub in the nutrient signaling network associated with the acquisition and use of nitrogen and phosphorus.
AT1G68680		SH3/FCH domain protein
AT1G68690	*PROLINE-RICH EXTENSIN-LIKE RECEPTOR KINASE 9 (PERK9)*	Encodes a member of the proline-rich extensin-like receptor kinase (PERK) family. This family consists of 15 predicted receptor kinases (PMID: 15653807).
AT1G68700		transmembrane protein
AT1G68710	*AMINOPHOSPHOLIPID ATPASE 9 (ALA9)*	ATPase E1-E2 type family protein / haloacid dehalogenase-like hydrolase family protein
AT1G68720	*TRNA ARGININE ADENOSINE DEAMINASE (TADA)*	Encodes the chloroplastic A-to-I tRNA editing enzyme.
AT1G68740	*(PHO1;H1)*	Encodes PHO1;H1, a member of the PHO1 family. Involved in inorganic phosphate (Pi) transport and homeostasis. Complements pho1 mutation. Its expression is responsive to both phosphate (Pi) and phosphite (Phi) in shoots.
AT1G68750	*PHOSPHOENOLPYRUVATE CARBOXYLASE 4 (PPC4)*	Encodes one of four Arabidopsis phosphoenolpyruvate (PEP) carboxylase proteins. But, it is more similar to bacterial PEP carboxylase than plant PEP carboxylase. Efforts to express this enzyme and to demonstrate its enzymatic activity in E.coli failed.
AT1G68765	*INFLORESCENCE DEFICIENT IN ABSCISSION (IDA)*	Encodes a small protein of 77 amino acids. Loss of function mutations are defective in the process of ethylene independent floral organ abscission. Although the mutants have a normal appearing abscission zone, the floral organs do not abscise. The peptide appears to be secreted and may function as a ligand. Arabidopsis 35S:IDA lines constitutively overexpressing IDA exhibit earlier abscission of floral organs, showing that the abscission zones are responsive to IDA soon after the opening of the flowers. In addition, ectopic abscission was observed at the bases of the pedicel, branches of the inflorescence, and cauline leaves. The silique valves also dehisced prematurely.
AT1G68780		RNI-like superfamily protein
AT1G68790	*LITTLE NUCLEI3 (LINC3)*	Member of small gene family in Arabidopsis containing 4 members (LNC1-4 or CRWN 1-4) with redundant functions in protection from oxidative damage, control of nuclear morphology and degradation of ABI5.
AT1G68795	*CLAVATA3/ESR-RELATED 12 (CLE12)*	Member of a large family of putative ligands homologous to the Clavata3 gene. Consists of a single exon.
AT1G68810	*ABNORMAL SHOOT 5 (ABS5)*	basic helix-loop-helix (bHLH) DNA-binding superfamily protein
AT1G68830	*STT7 HOMOLOG STN7 (STN7)*	STN7 protein kinase; required for state transitions, phosphorylation of the major antenna complex (LHCII) between PSII and PSI, and light adaptation. STN7 is involved in state transitions.
AT1G68840	*RELATED TO ABI3/VP1 2 (RAV2)*	Rav2 is part of a complex that has been named `regulator of the (H+)-ATPase of the vacuolar and endosomal membranes' (RAVE) The mRNA is cell-to-cell mobile.
AT1G68850		Peroxidase superfamily protein
AT1G68875		hypothetical protein
AT1G68880	*BASIC LEUCINE-ZIPPER 8 (bZIP)*	basic leucine-zipper 8
AT1G68890	*PHYLLO (PHYLLO)*	Homologous to the four eubacterial men genes involved in menanoquinone biosynthesis. Studies of mutants defective in this gene demonstrated its involvement in phylloquinone biosynthesis in Arabidopsis. The mRNA is cell-to-cell mobile.
AT1G68910	*WPP DOMAIN-INTERACTING PROTEIN 2 (WIT2)*	Encodes one of the WPP domain-interacting proteins (WIT1/AT5G11390, WIT2/AT1G68910) required for RanGAP nuclear envelope association in root tip cells. Ran GTPase plays essential roles in multiple cellular processes, including nucleocytoplasmic transport, spindle formation, and postmitotic nuclear envelope reassembly. The cytoplasmic Ran GTPase activating protein RanGAP is critical to establish a functional RanGTP/RanGDP gradient across the nuclear envelope and is associated with the outer surface of the nuclear envelope in metazoan and higher plant cells. Arabidopsis thaliana RanGAP association with the root tip nuclear envelope requires a family of likely plant-specific nucleoporins combining coiled-coil and transmembrane domains (CC-TMD) and WPP domain-interacting proteins (WIPs). WIT1 and WIT2 have been identified as a second family of CC-TMD proteins, structurally similar, yet clearly distinct from the WIP family, that is required for RanGAP nuclear envelop association in root tip cells.
AT1G68940	*(PUB42)*	Plant U-box type E3 ubiquitin ligase (PUB).
AT1G68990	*MALE GAMETOPHYTE DEFECTIVE 3 (MGP3)*	MGP3 (male gametophyte-defective 3) belongs to a small family of nuclear-encoded Phage type RNA polymerases (RPOTs) involved in the transcription of mitochondrial genes in Arabidopsis thaliana. Mutation in MGP 3 significantly retarded pollen tube growth and caused defective embryo development.
AT1G69040	*ACT DOMAIN REPEAT 4 (ACR4)*	ACT-domain containing protein involved in feedback regulation of amino acid metabolism
AT1G69050		hypothetical protein

AT1G69070		nucleolar-like protein
AT1G69080		Adenine nucleotide alpha hydrolases-like superfamily protein
AT1G69100		Eukaryotic aspartyl protease family protein
AT1G69120	*APETALA1 (AP1)*	Floral homeotic gene encoding a MADS domain protein homologous to SRF transcription factors. Specifies floral meristem and sepal identity. Required for the transcriptional activation of AGAMOUS. Interacts with LEAFY.Binds to promoter and regulates the expression of flowering time genes SVP, SOC1 and AGL24.
AT1G69150		Cysteine/Histidine-rich C1 domain family protein
AT1G69160	*BIG GRAIN LIKE 1 (BGL1)*	suppressor
AT1G69170	*SQUAMOSA PROMOTER BINDING PROTEIN (SBP)-DOMAIN TRANSCRIPTION FACTOR 6 (SPL6)*	Encodes SPL6. Required for the resistance mediated by the TIR-NB-LRR RPS4 against Pseudomonas syringae carrying the avrRps4 effector. Transcriptome analysis indicates that SPL6 positively regulates a subset of defense genes.
AT1G69180	*CRABS CLAW (CRC)*	Putative transcription factor with zinc finger and helix-loop-helix domains, the later similar to HMG boxes. Involved in specifying abaxial cell fate in the carpel. Four putative LFY binding sites (CCANTG) and two potential binding sites for MADS box proteins known as CArG boxes (CC(A/T)6GG) were found in the region spanning 3.8 Kb upstream of the CRC coding region. CRC targets YABBY genes such as YUC4 in gynoecium development.
AT1G69190		encodes a bifunctional cytosolic hydroxymethyldihydropterin pyrophosphokinase/ dihydropteroate synthase (HPPK/DHPS)that is involved in tetrahydrofolate biosynthesis and is responsive to oxidative stress.
AT1G69210		Uncharacterized protein family UPF0090
AT1G69230	*SPIRAL1-LIKE2 (SP1L2)*	SPIRAL1-LIKE2 belongs to a six-member gene family in Arabidopsis; all members share a high sequence similarity in amino- and carboxy-terminal regions. Regulates cortical microtubule organization. Mutant plants exhibit altered patterns of root and organ growth as a result of defective anisotropic cell expansion.
AT1G69240	*METHYL ESTERASE 15 (MES15)*	Encodes a protein predicted to act as a carboxylesterase. It has similarity to the SABP2 methyl salicylate esterase from tobacco but no enzymatic activity has been identified for this protein.
AT1G69250		Nuclear transport factor 2 (NTF2) family protein with RNA binding (RRM-RBD-RNP motifs) domain-containing protein
AT1G69260	*ABI FIVE BINDING PROTEIN (AFP1)*	ABI five binding protein
AT1G69295	*PLASMODESMATA CALLOSE-BINDING PROTEIN 4 (PDCB4)*	Encodes a member of the X8-GPI family of proteins. It localizes to the plasmodesmata and is predicted to bind callose.
AT1G69310	*WRKY DNA-BINDING PROTEIN 57 (WRKY57)*	Encodes WRKY57, a member of the WRKY Transcription Factor. Activation of WRKY57 confers drought tolerance.
AT1G69320	*CLAVATA3/ESR-RELATED 10 (CLE10)*	Member of a large family of putative ligands homologous to the Clavata3 gene. Consists of a single exon.
AT1G69330		RING/U-box superfamily protein
AT1G69350		Tetratricopeptide repeat (TPR)-like superfamily protein
AT1G69360		T-box transcription factor, putative (DUF863)
AT1G69380	*RETARDED ROOT GROWTH (RRG)*	Encodes a mitochondria-localized protein that is required for cell division in the root meristem.
AT1G69390	*HOMOLOGUE OF BACTERIAL MINE 1 (MINE1)*	Encodes an Arabidopsis homologue of the bacterial MinE topological specificity factor ensuring correct division site placement. It is an essential integral component of the plastid division machinery.
AT1G69430		Son of sevenless protein
AT1G69440	*ARGONAUTE7 (AGO7)*	Encodes ARGONAUTE7, a member of the ARGONAUTE family, characterised by the presence of PAZ and PIWI domains. Involved in the regulation of developmental timing. Required for the accumulation of TAS3 ta-siRNAs but not for accumulation of miR171, miR173, miR390 or mi391. Localized in mature rosette leaves and floral buds.
AT1G69450		Early-responsive to dehydration stress protein (ERD4)
AT1G69470		heat shock protein-binding protein
AT1G69480		EXS (ERD1/XPR1/SYG1) family protein
AT1G69490	*NAC-LIKE, ACTIVATED BY AP3/PI (NAP)*	Encodes a member of the NAC transcription factor gene family. It is expressed in floral primordia and upregulated by AP3 and PI. Its expression is associated with leaf senescence. The mRNA is cell-to-cell mobile.
AT1G69500	*CYTOCHROME P450, FAMILY 704, SUBFAMILY B, POLYPEPTIDE 1 (CYP704B1)*	Encodes a cytochrome P450, designated CYP704B1. Expressed in the developing anthers. Essential for pollen exine development. Mutations in CYP704B1 result in impaired pollen walls that lack a normal exine layer and exhibit a characteristic striped surface, termed zebra phenotype. Heterologous expression of CYP704B1 in yeast cells demonstrated that it catalyzes omega-hydroxylation of long-chain fatty acids, implicating these molecules in sporopollenin synthesis.
AT1G69510		cAMP-regulated phosphoprotein 19-related protein
AT1G69523		S-adenosyl-L-methionine-dependent methyltransferases superfamily protein
AT1G69526		S-adenosyl-L-methionine-dependent methyltransferases superfamily protein
AT1G69540	*AGAMOUS-LIKE 94 (AGL94)*	Encodes a member of the MIKC (MADS box, Keratin binding domain, and C terminal domain containing)family of transcriptional regulators.
AT1G69550		disease resistance protein (TIR-NBS-LRR class)
AT1G69560	*MYB DOMAIN PROTEIN 105 (MYB105)*	Encodes LOF2 (LATERAL ORGAN FUSION2), a MYB-domain transcription factor expressed in organ boundaries. Functions in boundary specification, meristem initiation and maintenance, and organ patterning. Also see LOF1 (At1g26780).
AT1G69570	*CYCLING DOF FACTOR 5 (CDF5)*	CDF5 is a circadian regulated transcript that is antiphasic with respect to its natural antisense transcript (NAT) FLORE (AT1G69572).CDF5 transcript accumulation delays flowering. CDF5 links circadian oscillation and photoperiodism.
AT1G69580		Homeodomain-like superfamily protein
AT1G69600	*ZINC FINGER HOMEODOMAIN 1 (ZFHD1)*	Encodes ZFHD1, a member of the zinc finger homeodomain transcriptional factor family. Binds to the 62 bp promoter region of ERD1 (early responsive to dehydration stress 1). Expression of ZFHD1 is induced by drought, high salinity and abscisic acid.
AT1G69610		zinc finger FYVE domain protein, putative (DUF1666)
AT1G69630		F-box/RNI-like superfamily protein

AT1G69660		TRAF-like family protein
AT1G69700	*HVA22 HOMOLOGUE C (HVA22C)*	Part of the AtHVA22 family. Protein expression is ABA- and stress-inducible. The mRNA is cell-to-cell mobile.
AT1G69720	*HEME OXYGENASE 3 (ho3)*	Encodes a member (HO3) of the heme oxygenase family.
AT1G69730		Wall-associated kinase family protein
AT1G69740	*(HEMB1)*	Encodes a putative 5-aminolevulinate dehydratase involved in chlorophyll biosynthesis.
AT1G69750	*CYTOCHROME C OXIDASE 19-2 (COX19-2)*	cytochrome c oxidase 19-2
AT1G69770	*CHROMOMETHYLASE 3 (CMT3)*	Encodes a chromomethylase involved in methylating cytosine residues at non-CG sites. Involved in preferentially methylating transposon-related sequences, reducing their mobility. CMT3 interacts with an Arabidopsis homologue of HP1 (heterochromatin protein 1), which in turn interacts with methylated histones. Involved in gene silencing.
AT1G69780	*(ATHB13)*	Encodes a homeodomain leucine zipper class I (HD-Zip I) protein which is expressed during the seed-to-seedling transition, regulates some of the network nodes, and affects late seedling establishment. Knock-out mutants for athb13 showed increased primary root length as compared with wild type (Col-0) seedlings, suggesting that this transcription factor is a negative regulator of early root growth, possibly repressing cell division and/or cell elongation or the length of time cells elongate.
AT1G69790	*PBS1-LIKE 18 (PBL18)*	Protein kinase superfamily protein
AT1G69800		Cystathionine beta-synthase (CBS) protein
AT1G69810	*WRKY DNA-BINDING PROTEIN 36 (WRKY36)*	member of WRKY Transcription Factor; Group II-b
AT1G69820	*GAMMA-GLUTAMYL TRANSPEPTIDASE 3 (GGT3)*	Note that conflicting nomenclature exists in the literature: At1g69820 is named as GGT4 in Plant J. 2007 Mar 49(5):878-88; and as GGT3 in Plant Physiol. 2007 Aug 144(4):1715-32.
AT1G69850	*NRT1/ PTR FAMILY 4.6 (NPF4.6)*	Encodes an inducible component of low-affinity nitrate uptake. mRNA found primarily in root hairs and the epidermis of roots. It also acts as an ABA importer at the site of ABA biosynthesis and is important for the regulation of stomatal aperture in inflorescence stems.
AT1G69860		Major facilitator superfamily protein
AT1G69870	*NRT1/ PTR FAMILY 2.13 (NPF2.13)*	Encodes a low affinity nitrate transporter NRT1.7. Expressed in phloem. Responsible for source-to-sink remobilization of nitrate. The mRNA is cell-to-cell mobile.
AT1G69880	*THIOREDOXIN H-TYPE 8 (TH8)*	thioredoxin H-type 8
AT1G69910		Protein kinase superfamily protein
AT1G69920	*GLUTATHIONE S-TRANSFERASE TAU 12 (GSTU12)*	Encodes glutathione transferase belonging to the tau class of GSTs. Naming convention according to Wagner et al. (2002).
AT1G69930	*GLUTATHIONE S-TRANSFERASE TAU 11 (GSTU11)*	Encodes glutathione transferase belonging to the tau class of GSTs. Naming convention according to Wagner et al. (2002).
AT1G69970	*CLAVATA3/ESR-RELATED 26 (CLE26)*	Member of a large family of putative ligands homologous to the Clavata3 gene. Consists of a single exon. Can not replace CLV3 function in vivo.
AT1G69990	*BAK1-INTERACTING RECEPTOR-LIKE KINASE 4 (BIR4)*	Leucine-rich repeat protein kinase family protein
AT1G70000	*(MYBD)*	Encodes a MYB-like Domain transcription factor that plays a positive role in anthocyanin accumulation in response to light and cytokinin via repression of MYBL2.MYBD expression increased in response to light or cytokinin, and MYBD enhanced anthocyanin biosynthesis via the repression of MYBL2 encoding for a transcription factor that had a negative effect on this process. In addition, MYBD can bind in vivo to the MYBL2 promoter and a lower level of histone H3K9 acetylation (H3K9ac) at upstream region of MYBL2 in MYBD-OX in comparison to wild-type plants, implies that MYBD represses MYBL2 expression via an epigenetic mechanism.
AT1G70040		transmembrane protein, putative (DUF1163)
AT1G70080	*TERPENE SYNTHASE 6 (TPS6)*	Terpene synthase. Expressed in roots and has low enzyme activity in vitro. Products include dolabellane type diterpenes. Sesterterpene synthase which produces various sesterpne backbones via type-B cyclization mechanism.
AT1G70090	*GLUCOSYL TRANSFERASE FAMILY 8 (LGT8)*	Encodes a protein with putative galacturonosyltransferase activity.
AT1G70100		neurofilament heavy protein
AT1G70110	*L-TYPE LECTIN RECEPTOR KINASE V.1 (LECRK-V.1)*	Concanavalin A-like lectin protein kinase family protein
AT1G70130	*L-TYPE LECTIN RECEPTOR KINASE V.2 (LECRK-V.2)*	Concanavalin A-like lectin protein kinase family protein
AT1G70140	*FORMIN 8 (FH8)*	Encodes a group I formin. Binds to F-actin barbed ends. Has severing actin filaments activity. Binds profilin. Involved in the initiation and tip growth of root hairs through regulation of actin cytoskeleton.
AT1G70150		zinc ion binding protein
AT1G70170	*MATRIX METALLOPROTEINASE (MMP)*	Matrix metalloprotease. Expression induced by fungal and bacterial pathogens. Mutants are late flowering with early senescence.
AT1G70180		Sterile alpha motif (SAM) domain-containing protein
AT1G70190		ribosomal protein L7/L12 domain-containing protein
AT1G70200	*(RBD1)*	Encodes a RNA-Binding Protein RBD1. Promotes chilling tolerance through 23S rRNA processing.
AT1G70210	*CYCLIN D1;1 (CYCD1;1)*	Encodes a D-type cyclin that physically interacts with CDC2A. Its expression is upregulated early during germination.
AT1G70230	*TRICHOME BIREFRINGENCE-LIKE 27 (TBL27)*	Encodes a member of the TBL (TRICHOME BIREFRINGENCE-LIKE) gene family containing a plant-specific DUF231 (domain of unknown function) domain. A putative xyloglucan O-acetyltransferase. TBL gene family has 46 members, two of which (TBR/AT5G06700 and TBL3/AT5G01360) have been shown to be involved in the synthesis and deposition of secondary wall cellulose, presumably by influencing the esterification state of pectic polymers. A nomenclature for this gene family has been proposed (Volker Bischoff & Wolf Scheible, 2010, personal communication).
AT1G70240		
AT1G70250	*GLYCOSYLPHOSPHATIDYLINOSITOL-ANCHORED LIPID PROTEIN TRANSFER 32 (LTPG32)*	Encodes a Protease inhibitor/seed storage/LTP family protein.
AT1G70270		transcription factor
AT1G70290	*TREHALOSE-6-PHOSPHATASE SYNTHASE S8 (TPS8)*	Encodes an enzyme putatively involved in trehalose biosynthesis. Though the protein has both trehalose-6-phosphate synthase (TPS)-like and trehalose-6-phosphate phosphatase (TPP)-like domains, neither activity has been detected in enzymatic assays nor has the protein been able to complement yeast TPS or TPP mutants.

AT1G70300	*K+ UPTAKE PERMEASE 6 (KUP6)*	potassium transporter
AT1G70310	*SPERMIDINE SYNTHASE 2 (SPDS2)*	Spermidine synthase.
AT1G70330	*EQUILIBRATIVE NUCLEOTIDE TRANSPORTER 1 (ENT1)*	encodes an adenosine transporter that catalyze a proton-dependent adenosine transport. The mRNA is cell-to-cell mobile.
AT1G70350		hypothetical protein
AT1G70370	*POLYGALACTURONASE 2 (PG2)*	Polygalacturonase involved in cell wall modification.
AT1G70400		NOSIC domain protein
AT1G70410	*BETA CARBONIC ANHYDRASE 4 (BCA4)*	Encodes a putative beta-carbonic anhydrase betaCA4. Together with betaCA1 (At3g01500) regulates CO2-controlled stomatal movements in guard cells, as well as attenuates immunity. Differential CA gene expression in response to changing atmospheric CO2 conditions contribute to altered disease resistance levels.
AT1G70420		DNA ligase-like protein, putative (DUF1645)
AT1G70430		Protein kinase superfamily protein
AT1G70450		Its expression is enriched in root hair cells (compared to non-root hair cells) and this enrichment is associated with increase in the transcription-associated mark trimethylation of H3 lysine 4 (H3K4me3) and decrease in the Polycomb silencing-associated mark trimethylation of H3 lysine 27 (H3K27me3) in root hair cells relative to non-root hair cells.
AT1G70460	*PROLINE-RICH EXTENSIN-LIKE RECEPTOR KINASE 13 (PERK13)*	Encodes a member of the proline-rich extensin-like receptor kinase (PERK) family. This family consists of 15 predicted receptor kinases (PMID: 15653807).
AT1G70470		transmembrane protein
AT1G70480	*(JASSY)*	Protein residing in the chloroplast outer membrane, has channel-like properties facilitating the export of the jasmonate precursor 12-oxophytodienoic acid (OPDA) from the chloroplast.
AT1G70500		Pectin lyase-like superfamily protein
AT1G70510	*KNOTTED-LIKE FROM ARABIDOPSIS THALIANA 2 (KNAT2)*	A member of class I knotted1-like homeobox gene family (together with KNAT1). Similar to the knotted1 (kn1) homeobox gene of maize. KNAT2 acts synergistically with cytokinins and antagonistically with ethylene based on ectopic expression studies in different mutant backgrounds and hormone treatments. In addition, KNAT2 is negatively regulated by AS and YABBY genes. KNAT2 is strongly expressed in the shoot apex of seedlings, while in mature plants the gene is primarily expressed in flowers and inflorescence stems.
AT1G70520	*CYSTEINE-RICH RLK2 (CRK2)*	Encodes a cysteine-rich receptor-like protein kinase located to the plasma membrane. Involved in regulating microbe-associated molecular pattern-triggered ROS production and stress induced callose deposition at the plasmodesmata in roots. Required for MAMP-triggered responses and resistance to Pseudomonas syringae pv. tomato 118 DC3000 .
AT1G70530	*CYSTEINE-RICH RLK (RECEPTOR-LIKE PROTEIN KINASE) 3 (CRK3)*	Encodes a cysteine-rich receptor-like protein kinase.
AT1G70540	*EMBRYO SAC DEVELOPMENT ARREST 24 (EDA24)*	Plant invertase/pectin methylesterase inhibitor superfamily protein
AT1G70550		NEP-interacting protein, putative (DUF239)
AT1G70560	*TRYPTOPHAN AMINOTRANSFERASE OF ARABIDOPSIS 1 (TAA1)*	TAA1 is involved in the shade-induced production of indole-3-pyruvate (IPA), a precursor to IAA, a biologically active auxin. It is also involved in regulating many aspects of plant growth and development from embryogenesis to flower formation and plays a role in ethylene-mediated signaling. This enzyme can catalyze the formation of IPA from L-tryptophan. Though L-Trp is expected to be the preferred substrate in vivo, TAA1 also acts as an aminotransferase using L-Phe, L-Tyr, L-Leu, L-Ala, L-Met, and L-Gln. Lines carrying mutations in this gene are unaffected by auxin transporter inhibitor NPA. Double mutant analysis and exogenous auxin treatment suggest that this gene is required for auxin signaling during lateral root and root meristem development. The activity of TAA1 can be controlled by phosphorylation of residue T101, which, when phosphorylated results in loss of activity. TAA1 is a target of TMK4.
AT1G70580	*ALANINE-2-OXOGLUTARATE AMINOTRANSFERASE 2 (AOAT2)*	Encodes a protein with glyoxylate aminotransferase activity. It can act on a number of different small substrates and amino acids in vitro.
AT1G70610	*ATP-BINDING CASSETTE B26 (ABCB26)*	member of TAP subfamily
AT1G70620		cyclin-like protein
AT1G70630	*REDUCED ARABINOSE YARIV 1 (RAY1)*	Nucleotide-diphospho-sugar transferase family protein
AT1G70650		Ran BP2/NZF zinc finger-like superfamily protein
AT1G70660	*MMS ZWEI HOMOLOGUE 2 (MMZ2)*	MMZ2/UEV1B encodes a protein that may play a role in DNA damage responses and error-free post-replicative DNA repair by participating in lysine-63-based polyubiquitination reactions. UEV1A can form diubiquitin and triubiquitin chains in combination with UBC13A/UBC35 in vitro. It can also functionally complement an mms2 mutation in budding yeast, both by increasing mms2 mutant viability in the presence of the DNA damaging agent MMS, and by reducing the rate of spontaneous DNA mutation. However, a combination of MMZ2/UEV1B and UBC13A do not do a good job of rescuing an mms2 ubc13 double mutant in yeast. MMZ2/UEV1B transcripts are found in most plant organs, but not in the pollen or in seedlings 6 hours or 2 days post-germination. The transcript levels do not appear to be stress-inducible. The mRNA is cell-to-cell mobile.
AT1G70670	*CALEOSIN 4 (CLO4)*	The gene encodes a stress-responsive and OB-associated non-seed caleosin-like protein. It plays a negative regulator role in ABA signaling.
AT1G70690	*HOPW1-1-INDUCED GENE1 (HWI1)*	Encodes a plasmodesmal protein that may be involved in the intercellular movement of molecules through the plasmodesmata. The protein has two DUF26 domains and a single transmembrane domain.
AT1G70700	*(TIFY7)*	JAZ9 is a protein presumed to be involved in jasmonate signaling. JAZ9 transcript levels rise in response to a jasmonate stimulus. JAZ9 can interact with the COI1 F-box subunit of an SCF E3 ubiquitin ligase in a yeast-two-hybrid assay only in the presence of jasmonate-isoleucine (JA-ILE) or coronatine. The Jas domain appears to be important for JAZ9-COI1 interactions in the presence of coronatine. Two positive residues (R205 and R206) in the Jas domain shown to be important for coronatine -dependent COI1 binding are not required for binding AtMYC2. The mRNA is cell-to-cell mobile.
AT1G70710	*GLYCOSYL HYDROLASE 9B1 (GH9B1)*	endo-1,4-beta-glucanase. Involved in cell elongation.
AT1G70720		Plant invertase/pectin methylesterase inhibitor superfamily protein
AT1G70750	*MYOSIN BINDING PROTEIN 2 (MYOB2)*	myosin-binding protein (Protein of unknown function, DUF593)

AT1G70760	*NADH DEHYDROGENASE-LIKE COMPLEX L (NdhL)*	a subunit of the chloroplast NAD(P)H dehydrogenase complex, involved in PSI cyclic electron transport. Located on the thylakoid membrane. Mutant has impaired NAD(P)H dehydrogenase activity. The mRNA is cell-to-cell mobile.
AT1G70810	*C2-DOMAIN ABA-RELATED7 (CAR7)*	Calcium-dependent lipid-binding (CaLB domain) family protein
AT1G70820		phosphoglucomutase, putative / glucose phosphomutase
AT1G70830	*MLP-LIKE PROTEIN 28 (MLP28)*	MLP-like protein 28
AT1G70840	*MLP-LIKE PROTEIN 31 (MLP31)*	MLP-like protein 31
AT1G70850	*MLP-LIKE PROTEIN 34 (MLP34)*	MLP-like protein 34
AT1G70870		Polyketide cyclase/dehydrase and lipid transport superfamily protein
AT1G70880		Polyketide cyclase/dehydrase and lipid transport superfamily protein
AT1G70890	*MLP-LIKE PROTEIN 43 (MLP43)*	MLP-like protein 43
AT1G70895	*CLAVATA3/ESR-RELATED 17 (CLE17)*	Member of a large family of putative ligands homologous to the Clavata3 gene. Consists of a single exon.
AT1G70900		hypothetical protein
AT1G70910	*DESPIERTO (DEP)*	Encodes DESPIERTO (DEP), a RING finger protein involved in ABA sensitivity during seed development. Regulates the expression of ABI3, and produces a complete loss of dormancy when mutated.
AT1G70920	*HOMEOBOX-LEUCINE ZIPPER PROTEIN 18 (HB18)*	homeobox-leucine zipper protein 18
AT1G70930		
AT1G70940	*PIN-FORMED 3 (PIN3)*	A regulator of auxin efflux and involved in differential growth. PIN3 is expressed in gravity-sensing tissues, with PIN3 protein accumulating predominantly at the lateral cell surface. PIN3 localizes to the plasma membrane and to vesicles. In roots, PIN3 is expressed without pronounced polarity in tiers two and three of the columella cells, at the basal side of vascular cells, and to the lateral side of pericycle cells of the elongation zone. PIN3 overexpression inhibits root cell growth. Protein phosphorylation plays a role in regulating PIN3 trafficking to the plasma membrane. The mRNA is cell-to-cell mobile.
AT1G70950	*WAVE DAMPENED 7 (WDL7)*	Microtubule-stabilizing protein. Module with MREL57 regulates microtubule disassembly to mediate stomatal closure in response to drought stress and ABA treatment. MREL57 interacts with, ubiquitinates and degrades WDL7, effect is enhanced by ABA.
AT1G70980	*(SYNC3)*	Class II aminoacyl-tRNA and biotin synthetases superfamily protein
AT1G70985		hydroxyproline-rich glycoprotein family protein
AT1G70990	*EXTENSIN 33 (EXT33)*	Short extensin family protein required during the first phase of dark-grown hypocotyl elongation, regulates the moment and extent of the growth acceleration by modulating cell wall extensibility.
AT1G71000		Chaperone DnaJ-domain superfamily protein
AT1G71010	*FORMS APLOID AND BINUCLEATE CELLS 1C (FAB1C)*	Encodes a protein that is predicted to act as a phosphatidylinositol-3P 5-kinase, but, because it lacks a FYVE domain, it is unlikely to be efficiently targeted to membranes containing the proposed phosphatidylinositol-3P substrate. Therefore, its molecular function remains unknown. The mRNA is cell-to-cell mobile.
AT1G71030	*MYB-LIKE 2 (MYBL2)*	Encodes a putative myb family transcription factor. In contrast to most other myb-like proteins its myb domain consists of a single repeat. A proline-rich region potentially involved in transactivation is found in the C-terminal part of the protein. Its transcript accumulates mainly in leaves.
AT1G71040	*LOW PHOSPHATE ROOT2 (LPR2)*	Encodes LPR2. Function together with LPR1 (AT1G23010) and a P5-type ATPase (At5g23630/PDR2) in a common pathway that adjusts root meristem activity to Pi (inorganic phosphate) availability.
AT1G71050	*HEAVY METAL ASSOCIATED ISOPRENYLATED PLANT PROTEIN 20 (HIPP20)*	Heavy metal transport/detoxification superfamily protein
AT1G71080		RNA polymerase II transcription elongation factor
AT1G71090	*PIN-LIKES 2 (PILS2)*	Auxin efflux carrier family protein
AT1G71100	*RADIAL SWELLING 10 (RSW10)*	Encodes a ribose 5-phosphate isomerase involved in the formation of uridine used for the synthesis of UDP-sugars. Mutants of this gene are affected in cellulose biosynthesis.
AT1G71120	*GDSL-MOTIF LIPASE/HYDROLASE 6 (GLIP6)*	Contains lipase signature motif and GDSL domain.
AT1G71130	*CYTOKININ RESPONSE FACTOR 8 (CRF8)*	encodes a member of the ERF (ethylene response factor) subfamily B-5 of ERF/AP2 transcription factor family. The protein contains one AP2 domain. There are 7 members in this subfamily.
AT1G71140	*PROTEIN DETOXIFICATION 14 (DTX14)*	MATE transporter that can export the antibiotic norfloxacin.
AT1G71160	*3-KETOACYL-COA SYNTHASE 7 (KCS7)*	Encodes KCS7, a member of the 3-ketoacyl-CoA synthase family involved in the biosynthesis of VLCFA (very long chain fatty acids).
AT1G71170		6-phosphogluconate dehydrogenase family protein
AT1G71200	*CU-DEFICIENCY INDUCED TRANSCRIPTION FACTOR 1 (CITF1)*	bHLH160 transcription factor. Induced by copper deficiency and seems to mediate copper uptake along with SPL7. Alternative splicing variant in response to MeJa treatment has potential novel function where it can dimerize but not bind DNA, resulting in a function opposite of the primary isoform.
AT1G71210		Pentatricopeptide repeat (PPR) superfamily protein
AT1G71220	*EMS-MUTAGENIZED BRI1 SUPPRESSOR 1 (EBS1)*	Encodes UDP-glucose:glycoprotein glucosyltransferase. Non-receptor component required for EFR-mediated immunity. Mutants show de-repressed anthocyanin accumulation in the presence of elf18, and EFR accumulation and signalling.
AT1G71250		GDSL-motif esterase/acyltransferase/lipase. Enzyme group with broad substrate specificity that may catalyze acyltransfer or hydrolase reactions with lipid and non-lipid substrates.
AT1G71260	*WHIRLY 2 (ATWHY2)*	Encodes WHY2, a homolog of the potato p24 protein. It shares the conserved KGKAAL domain, a putative DNA-binding domain, with potato p24 and is localized to mitochondria and not the nucleus. WHY2 is a member of the Whirly family proteins present mainly in the plant kingdom performing various activities related to DNA metabolism. Crystal structure of Solanum tuberosum WHY2, a close homolog of Arabidopsis WHY2, reveal that Whirly proteins bind to single strand DNA to promote accurate repair of DNA double-strand breaks over an error-prone repair pathway.
AT1G71280		DEA(D/H)-box RNA helicase family protein
AT1G71290		F-box associated ubiquitination effector family protein

AT1G71330	*NON-INTRINSIC ABC PROTEIN 5 (NAP5)*	member of NAP subfamily
AT1G71340	*GLYCEROPHOSPHODIESTER PHOSPHODIESTERASE 4 (GDPD4)*	Encodes a member of the glycerophosphodiester phosphodiesterase (GDPD) family.
AT1G71380	*CELLULASE 3 (CEL3)*	cellulase 3
AT1G71390	*RECEPTOR LIKE PROTEIN 11 (RLP11)*	receptor like protein 11
AT1G71400	*RECEPTOR LIKE PROTEIN 12 (RLP12)*	Encodes a CLAVATA2 (CLV2)-related gene. Complements the clv2 mutant when expressed under the control of the CLV2 promoter. The mRNA is cell-to-cell mobile.
AT1G71410	*(SCYL2B)*	One of two paralogs in Arabidopsis.SCYL2B is a membrane localized protein that interacts with components of clathrin-mediated vesicle trafficking pathways. Loss of both SCYL2B and .SCYL2A results in severe growth defects.
AT1G71420		Tetratricopeptide repeat (TPR)-like superfamily protein
AT1G71450	*FYF UP-REGULATING 321 FACTOR 1 (FUF1)*	Encodes a member of the DREB subfamily A-4 of ERF/AP2 transcription factor family. The protein contains one AP2 domain. There are 17 members in this subfamily including TINY. FUF1 appears to negatively regulate certain ethylene responsive EDF genes thereby negatively regulating flower senescence.
AT1G71470		hypothetical protein
AT1G71480		Nuclear transport factor 2 (NTF2) family protein
AT1G71490		Tetratricopeptide repeat (TPR)-like superfamily protein
AT1G71500	*PHOTOSYSTEM B PROTEIN 33 (PSB33)*	Encodes PSB33, a protein conserved in the plastid lineage. PSB33 is associated with the chloroplast thylakoid membrane and provides stability to Photosystem II. The mRNA is cell-to-cell mobile.
AT1G71530		Protein kinase superfamily protein
AT1G71680		Transmembrane amino acid transporter family protein
AT1G71690		glucuronoxylan 4-O-methyltransferase-like protein (DUF579)
AT1G71691		GDSL-motif esterase/acyltransferase/lipase. Enzyme group with broad substrate specificity that may catalyze acyltransfer or hydrolase reactions with lipid and non-lipid substrates.
AT1G71692	*AGAMOUS-LIKE 12 (AGL12)*	Encodes a member of the MADS box family of transcription factors. Involved in root cell differentiation and flowering time. Loss of function mutations have abnormal cellular differentiation in the roots and are late flowering. AGL12 along with AGL14, and AGL17 is preferentially expressed in root tissues and represent the only characterized MADS box genes expressed in roots.
AT1G71695		Peroxidase superfamily protein
AT1G71710		DNAse I-like superfamily protein
AT1G71720	*PIGMENT DEFECTIVE 338 (PDE338)*	Encodes a chloroplast localized protein that regulates the translation of Ycf1 by binding to its mRNA. It is involved in the biogenesis of photosynthetic complexes.
AT1G71730		hypothetical protein
AT1G71740		nucleolar protein
AT1G71760		hypothetical protein
AT1G71770	*POLY(A)-BINDING PROTEIN 5 (PAB5)*	Encodes a Class I polyA-binding protein. Expressed in floral organs. Binds polyA sepharose in vitro.
AT1G71780		WD repeat protein
AT1G71790	*CAPPING PROTEIN B (CPB)*	Encodes a heterodimeric actin binding protein composed of an alpha and a beta sumunit. Stabilizes actin filament cytoskeleton by capping.
AT1G71810		Protein kinase superfamily protein
AT1G71830	*SOMATIC EMBRYOGENESIS RECEPTOR-LIKE KINASE 1 (SERK1)*	Plasma membrane LRR receptor-like serine threonine kinase expressed during embryogenesis in locules until stage 6 anthers, with higher expression in the tapetal cell layer. SERK1 and SERK2 receptor kinases function redundantly as an important control point for sporophytic development controlling male gametophyte production. SERK1 interacts with and transphosphorylates EMS1
AT1G71840		transducin family protein / WD-40 repeat family protein
AT1G71850		Ubiquitin carboxyl-terminal hydrolase family protein
AT1G71860	*PROTEIN TYROSINE PHOSPHATASE 1 (PTP1)*	Encodes a protein with tyrosine phosphatase activity that is downregulated in response to cold and upregulated in response to salt stress.
AT1G71870	*(BIGE1A)*	Metabolite transporter involved in the anthocyanin response to anthocyanin induction conditions. Affects ABA signaling and localization.
AT1G71880	*SUCROSE-PROTON SYMPORTER 1 (SUC1)*	Sucrose transporter gene induced in response to nematodes; member of Sucrose-proton symporter family. The mRNA is cell-to-cell mobile.
AT1G71890	*(SUC5)*	Encodes a sucrose transporter that is expressed in the endosperm. Mutants have delayed accumulation of fatty acids and embryo maturation.
AT1G71900	*(ENOR3L4)*	magnesium transporter, putative (DUF803)
AT1G71910		hypothetical protein
AT1G71930	*VASCULAR RELATED NAC-DOMAIN PROTEIN 7 (VND7)*	Encodes a NAC-domain transcription factor with transcriptional activation activity that is involved in xylem formation. Induces transdifferentiation of various cells into protoxylem vessel elements. Located in the nucleus. Expression induced in the presence of auxin, cytokinin and brassinosteroids.
AT1G71950	*SUBTILISIN PROPEPTIDE-LIKE INHIBITOR 1 (SPI-1)*	SPI-1 is a member of the I9 inhibitor family. It is an inhibitor of SBT4.13 subtilase.
AT1G71960	*ATP-BINDING CASETTE G25 (ABCG25)*	Encodes a plasma membrane localized ABC transporter involved in abscisic acid transport and responses.
AT1G71970		hypothetical protein
AT1G72000	*ALKALINE/NEUTRAL INVERTASE F (A/N-InvF)*	Plant neutral invertase family protein
AT1G72030	*GCN5‐RELATED N‐ACETYLTRANSFERASE 10 (GNAT10)*	Acyl-CoA N-acyltransferases (NAT) superfamily protein
AT1G72060		serine-type endopeptidase inhibitor
AT1G72070		Chaperone DnaJ-domain superfamily protein
AT1G72080		hypothetical protein
AT1G72100		late embryogenesis abundant domain-containing protein / LEA domain-containing protein
AT1G72110		O-acyltransferase (WSD1-like) family protein
AT1G72120	*NRT1/ PTR FAMILY 5.14 (NPF5.14)*	Major facilitator superfamily protein

AT1G72130	*(NPF5.11)*	Tonoplast localized pH dependent, low affinity nitrogen transporter.In shoots, expressed in leaf veins and mesophyll. In roots, GUS activity was detected in root vascular stele. More highly expressed in roots.
AT1G72140	*(NPF5.12)*	Tonoplast localized pH dependent, low affinity nitrogen transporter.In shoots, expressed in leaf veins and mesophyll. In roots, GUS activity was detected in root vascular stele. More highly expressed in roots.
AT1G72150	*PATELLIN 1 (PATL1)*	novel cell-plate-associated protein that is related in sequence to proteins involved in membrane trafficking in other eukaryotes The mRNA is cell-to-cell mobile.
AT1G72160	*(PATL3)*	Sec14p-like phosphatidylinositol transfer family protein
AT1G72180	*C-TERMINALLY ENCODED PEPTIDE RECEPTOR 2 (CEPR2)*	Encodes a leucine-rich repeat receptor kinase that functions as a receptor for CEP1 peptide. Mediates nitrate uptake signaling.
AT1G72190		D-isomer specific 2-hydroxyacid dehydrogenase family protein
AT1G72200	*ARABIDOPSIS T??XICOS EN LEVADURA 11 (ATL11)*	RING/U-box superfamily protein
AT1G72220	*ARABIDOPSIS T??XICOS EN LEVADURA 54 (ATL54)*	RING/U-box superfamily protein
AT1G72230		Cupredoxin superfamily protein
AT1G72240		hypothetical protein
AT1G72250	*MALECTIN DOMAIN KINESIN 1 (MDKIN1)*	Malectin domain kinesin.
AT1G72260	*THIONIN 2.1 (THI2.1)*	Encodes a thionin which is a cysteine rich protein having antimicrobial properties. Thi2.1 is expressed in response to a variety of pathogens and induced by ethylene and jasmonic acid. Belongs to the plant thionin (PR-13) family with the following members: At1g66100, At5g36910, At1g72260, At2g15010, At1g12663, At1g12660.
AT1G72270	*IDM1-ASSOCIATED PROTEIN 1 (IDAP1)*	Encodes IDAP1. Acts together with IDAP2 and IDM1 to regulate active DNA demethylation.
AT1G72280	*ENDOPLASMIC RETICULUM OXIDOREDUCTINS 1 (ERO1)*	Encodes an oxidoreductin required for oxidative protein folding in the ER and exists in two distinct oxidized isoforms (Ox1 and Ox2), which are determined by the formation or breakage of the putative regulatory disulfide. AtERO1 is mainly present in the Ox1 redox state.
AT1G72290	*WATER-SOLUBLE CHLOROPHYLL PROTEIN (ATWSCP)*	Encodes a Kunitz-protease inhibitor, a water-soluble chlorophyll protein involved in herbivore resistance activation.
AT1G72300	*PSY1 RECEPTOR (PSY1R)*	Encodes a leucine-rich repeat receptor kinase (LRR-RK) involved in the perception of PSY1. PSY1 is an 18-aa tyrosine-sulfated glycopeptide encoded by AT5G58650 that promotes cellular proliferation and expansion.
AT1G72320	*PUMILIO 23 (PUM23)*	Encodes a member of the Arabidopsis Pumilio (APUM) proteins containing PUF domain (eight repeats of approximately 36 amino acids each). PUF proteins regulate both mRNA stability and translation through sequence-specific binding to the 3' UTR of target mRNA transcripts.
AT1G72350	*AGAMOUS-LIKE 60 (AGL60)*	MADS-box transcription factor family protein
AT1G72360	*ETHYLENE RESPONSE FACTOR 73 (ERF73)*	Encodes a member of the ERF (ethylene response factor) subfamily B-2 of ERF/AP2 transcription factor family. The protein contains one AP2 domain. There are 5 members in this subfamily including RAP2.2 AND RAP2.12.
AT1G72380		
AT1G72400		
AT1G72410		COP1-interacting protein-like protein
AT1G72420		NADH:ubiquinone oxidoreductase intermediate-associated protein 30
AT1G72430	*SMALL AUXIN UPREGULATED RNA 78 (SAUR78)*	SAUR-like auxin-responsive protein family
AT1G72450	*JASMONATE-ZIM-DOMAIN PROTEIN 6 (JAZ6)*	JAZ6 transcript levels rise in response to a jasmonate stimulus and a GFP:JAZ6 fusion protein localizes to the nucleus. Application of jasmonate methyl ester to Arabidopsis roots reduces the levels of a JAZ6:GUS fusion protein, presumably by stimulating ubiquitin-proteasome-mediated degradation.
AT1G72470	*EXOCYST SUBUNIT EXO70 FAMILY PROTEIN D1 (EXO70D1)*	A member of EXO70 gene family, putative exocyst subunits, conserved in land plants. Arabidopsis thaliana contains 23 putative EXO70 genes, which can be classified into eight clusters on the phylogenetic tree.
AT1G72480		Lung seven transmembrane receptor family protein
AT1G72490	*DEEPER ROOTING 1 (DRO1)*	DRO1 is a member of the IGT gene family and has a unknown function . It is expressed in roots and involved in leaf root architecture, specifically the orientation of lateral root angles. Involved in determining lateral root branch angle.
AT1G72500		inter alpha-trypsin inhibitor, heavy chain-like protein
AT1G72510		DUF1677 family protein (DUF1677)
AT1G72520	*LIPOXYGENASE 4 (LOX4)*	PLAT/LH2 domain-containing lipoxygenase family protein
AT1G72530	*RNA EDITING-INTERACTING PROTEIN (RIP7)*	Member of MORF family consisting of of nine full-length proteins encoded in the nuclear genome. MORF proteins are required for all RNA editing events in plastids and for many, possibly also all, sites in mitochondria. Potential link between the RNA binding PPR protein and the protein contributing the enzymatic activity in RNA editing.
AT1G72540	*PBS1-LIKE 33 (PBL33)*	Protein kinase superfamily protein
AT1G72560	*PAUSED (PSD)*	Encodes a karyopherin, specifically the Arabidopsis ortholog of LOS1/XPOT, a protein that mediates nuclear export of tRNAs in yeast and mammals. PSD is capable of rescuing the tRNA export defect of los1 in S. cerevisiae. psd mutants display disrupted initiation of the shoot apical meristem and delay leaf initiation after germination; they also display delayed transition from vegetative to reproductive development.
AT1G72570	*AP2-LIKE ETHYLENE-RESPONSIVE TRANSCRIPTION FACTOR (AIL1)*	Integrase-type DNA-binding superfamily protein
AT1G72580		hypothetical protein
AT1G72600		hydroxyproline-rich glycoprotein family protein
AT1G72610	*GERMIN-LIKE PROTEIN 1 (GER1)*	germin-like protein (GLP1)
AT1G72630	*ELF4-LIKE 2 (ELF4-L2)*	ELF4-like 2
AT1G72640		NAD(P)-binding Rossmann-fold superfamily protein
AT1G72660	*(DRG1-3)*	P-loop containing nucleoside triphosphate hydrolases superfamily protein
AT1G72680	*CINNAMYL-ALCOHOL DEHYDROGENASE (CAD1)*	cinnamyl-alcohol dehydrogenase
AT1G72730		DEA(D/H)-box RNA helicase family protein

AT1G72740		Single Myb Histone (SMH) gene family member. Contains terminal acidic SANT domain.
AT1G72750	*TRANSLOCASE INNER MEMBRANE SUBUNIT 23-2 (TIM23-2)*	translocase inner membrane subunit 23-2
AT1G72760		Protein kinase superfamily protein
AT1G72770	*HYPERSENSITIVE TO ABA1 (HAB1)*	mutant has ABA hypersensitive inhibition of seed germination; Protein Phosphatase 2C; regulates the activation of the Snf1-related kinase OST1 by abscisic acid. The mRNA is cell-to-cell mobile.
AT1G72790		hydroxyproline-rich glycoprotein family protein
AT1G72800		RNA-binding (RRM/RBD/RNP motifs) family protein
AT1G72810	*THREONINE SYNTHASE 2 (TSY)*	Pyridoxal-5-phosphate-dependent enzyme family protein
AT1G72820		Mitochondrial substrate carrier family protein
AT1G72830	*NUCLEAR FACTOR Y, SUBUNIT A3 (NF-YA3)*	Encodes a subunit of CCAAT-binding complex, binds to CCAAT box motif present in some plant promoter sequences. One of three members of this class (HAP2A, HAP2B, HAP2C), it is expressed in vegetative and reproductive tissues. Expression is upregulated in the shoot of cax1/cax3 mutant.
AT1G72840	*(TNL40)*	NBS TIR LRR protein. It is induced in response to bacterial pathogens and overexpression results in cell death in leaves.
AT1G72850	*TIR-NBS4 (TN4)*	Disease resistance protein (TIR-NBS class)
AT1G72860	*(TNL60)*	NBS TIR LRR protein. It is induced in response to bacterial pathogens and overexpression results in cell death in leaves.
AT1G72870	*TIR-NBS5 (TN5)*	TIR-NBS gene.
AT1G72890	*TIR-NBS6 (TN6)*	NBS TIR protein.
AT1G72900	*TIR-NBS7 (TN7)*	Toll-Interleukin-Resistance (TIR) domain-containing protein
AT1G72920	*TIR-NBS9 (TN9)*	Toll-Interleukin-Resistance (TIR) domain family protein
AT1G72930	*TOLL/INTERLEUKIN-1 RECEPTOR-LIKE (TIR)*	Toll/interleukin-1 receptor-like protein (TIR) protein. It is induced in response to bacterial pathogens and overexpression results in cell death in leaves.
AT1G72940	*TIR-NBS11 (TN11)*	Nucleotide-binding, leucine-rich repeat (NLR) gene regulated by nonsense-mediated mRNA decay (NMD) genes UPF1 and UPF3.
AT1G72950	*TIR-NBS12 (TN12)*	Disease resistance protein (TIR-NBS class)
AT1G72960		Root hair defective 3 GTP-binding protein (RHD3)
AT1G72970	*HOTHEAD (HTH)*	Originally identified as a mutation that causes floral organs to fuse together. About 10-20% of mutants also have defects in ovules. Mutants have reduced fertility most likely as because of fusions that pistil emergence. The protein has similarity to the mandelonitrile lyase family of FAD containing oxidoreductases and is predicted to be secreted (SignalP).It is expressed in all tissue layers of roots, inflorescences, stems, leaves, and flowers and is also expressed in siliques. Expression is highest in inflorescence and flower tissue.Transmission of mutant alleles to the progeny shows non mendelian segregation- a percentage of mutant alleles revert back to a previous parental (e.g. grandparental) wild type allele. It has been suggested that an RNA template driven or other extra-DNA genomic mechanism may be responsible for the non-mendelian inheritance of HTH. Reversion events in alleles at other loci have also been observed to occur in plants with an hth mutant background indicating a genome wide effect.
AT1G73000	*PYR1-LIKE 3 (PYL3)*	Encodes a member of the PYR (pyrabactin resistance)/PYL(PYR1-like)/RCAR (regulatory components of ABA receptor) family proteins with 14 members. PYR/PYL/RCAR family proteins function as abscisic acid sensors. Mediate ABA-dependent regulation of protein phosphatase 2Cs ABI1 and ABI2.
AT1G73010	*PHOSPHATE STARVATION-INDUCED GENE 2 (PS2)*	Encodes PPsPase1, a pyrophosphate-specific phosphatase catalyzing the specific cleavage of pyrophosphate (Km 38.8 uM) with an alkaline catalytic pH optimum. Expression is upregulated in the shoot of cax1/cax3 mutant.
AT1G73020	*TRANS MEMBRANE PROTEIN 16 (TMEM16)*	anoctamin-like protein
AT1G73040		Mannose-binding lectin superfamily protein
AT1G73050		Glucose-methanol-choline (GMC) oxidoreductase family protein
AT1G73060	*LOW PSII ACCUMULATION 3 (LPA3)*	Low PSII Accumulation 3
AT1G73070		
AT1G73110		P-loop containing nucleoside triphosphate hydrolases superfamily protein
AT1G73120		F-box/RNI superfamily protein
AT1G73160		UDP-Glycosyltransferase superfamily protein
AT1G73170		P-loop containing nucleoside triphosphate hydrolases superfamily protein
AT1G73180		Eukaryotic translation initiation factor eIF2A family protein
AT1G73190	*(TIP3;1)*	Moves to the Protein Storage Vacuole in a Golgi independent manner
AT1G73220	*ORGANIC CATION/CARNITINE TRANSPORTER1 (OCT1)*	Encodes Organic Cation Transporter 1 (OCT1), likely to be involved in polyamine transport.
AT1G73240		nucleoporin protein Ndc1-Nup protein
AT1G73250	*GDP-4-KETO-6-DEOXYMANNOSE-3,5-EPIMERASE-4-REDUCTASE 1 (GER1)*	encodes a bifunctional 3, 5-epimerase-4-reductase in L-fucose synthesis and converts GDP-D-mannose to GDP-L-fucose in vitro along with MUR1 (GDP-D-mannose 4,6-dehydratase). It is expressed in all tissues examined, but most abundantly in roots and flowers.
AT1G73260	*KUNITZ TRYPSIN INHIBITOR 1 (KTI1)*	Encodes a trypsin inhibitor involved in modulating programmed cell death in plant-pathogen interactions.
AT1G73270	*SERINE CARBOXYPEPTIDASE-LIKE 6 (scpl6)*	serine carboxypeptidase-like 6
AT1G73280	*SERINE CARBOXYPEPTIDASE-LIKE 3 (scpl3)*	serine carboxypeptidase-like 3
AT1G73290	*SERINE CARBOXYPEPTIDASE-LIKE 5 (scpl5)*	serine carboxypeptidase-like 5
AT1G73300	*SERINE CARBOXYPEPTIDASE-LIKE 2 (scpl2)*	serine carboxypeptidase-like 2
AT1G73320		S-adenosyl-L-methionine-dependent methyltransferases superfamily protein
AT1G73330	*DROUGHT-REPRESSED 4 (DR4)*	encodes a plant-specific protease inhibitor-like protein whose transcript level in root disappears in response to progressive drought stress. The decrease in transcript level is independent from abscisic acid level.
AT1G73340	*ABIETANE DITERPENE OXIDASE 1 (ADTO1)*	ADTO1 is required for the activation of systemic acquired resistance.

AT1G73360	*HOMEODOMAIN GLABROUS 11 (HDG11)*	Encodes a homeobox-leucine zipper family protein belonging to the HD-ZIP IV family. It is involved in trichome branching. The transcription factor directly upregulates the expression of several cell-wall-loosening protein genes and reveals the important role that these target genes play in coordinating cell-wall extensibility with root development.
AT1G73370	*SUCROSE SYNTHASE 6 (SUS6)*	Encodes a protein with sucrose synthase activity (SUS6).
AT1G73410	*MYB DOMAIN PROTEIN 54 (MYB54)*	Encodes a putative transcription factor that is a member of the R2R3-MYB family.
AT1G73430	*CONSERVED OLIGOMERIC GOLGI COMPLEX 3 (COG3)*	COG3 is a component of a putative conserved oligomeric Golgi (COG) complex that is thought to be involved in tethering of retrograde intra Golgi vesicles. In mutant pollen,golgi appear abnormal. It is required for proper deposition of cell wall materials in pollen tube growth. When homozygotes can be produced (by complementing the defect in pollen), the plants are embryo lethal suggesting an essential function. COG3 interacts with several other putative COG components.
AT1G73470		hypothetical protein
AT1G73510		hypothetical protein
AT1G73530	*ORGANELLE RNA RECOGNITION MOTIF PROTEIN 6 (ORRM6)*	RNA-binding (RRM/RBD/RNP motifs) family protein
AT1G73540	*NUDIX HYDROLASE HOMOLOG 21 (NUDT21)*	nudix hydrolase homolog 21
AT1G73560	*GLYCOSYLPHOSPHATIDYLINOSITOL-ANCHORED LIPID PROTEIN TRANSFER 9 (LTPG9)*	Bifunctional inhibitor/lipid-transfer protein/seed storage 2S albumin superfamily protein
AT1G73590	*PIN-FORMED 1 (PIN1)*	Encodes an auxin efflux carrier involved in shoot and root development. It is involved in the maintenance of embryonic auxin gradients. Loss of function severely affects organ initiation, pin1 mutants are characterised by an inflorescence meristem that does not initiate any flowers, resulting in the formation of a naked inflorescence stem. PIN1 is involved in the determination of leaf shape by actively promoting development of leaf margin serrations. In roots, the protein mainly resides at the basal end of the vascular cells, but weak signals can be detected in the epidermis and the cortex. Expression levels and polarity of this auxin efflux carrier change during primordium development suggesting that cycles of auxin build-up and depletion accompany, and may direct, different stages of primordium development. PIN1 action on plant development does not strictly require function of PGP1 and PGP19 proteins.
AT1G73600	*(NMT3)*	Encodes a S-adenosyl-L-methionine-dependent phosphoethanolamine N-methyltransferase whose expression is responsive to both phosphate (Pi) and phosphite (Phi) in roots. It catalyzes the three sequential P-base methylation of phosphoethanolamine to phosphocholine. Homologous biochemical function to NMT1 (At3g18000). Double mutants of NMT1 and NMT3 are defective in leaf, root, flower, seed, and pollen development.
AT1G73610		GDSL-motif esterase/acyltransferase/lipase. Enzyme group with broad substrate specificity that may catalyze acyltransfer or hydrolase reactions with lipid and non-lipid substrates.
AT1G73620		Pathogenesis-related thaumatin superfamily protein
AT1G73630		EF hand calcium-binding protein family
AT1G73640	*RAB GTPASE HOMOLOG A6A (RABA6a)*	RAB GTPase homolog A6A
AT1G73650		3-oxo-5-alpha-steroid 4-dehydrogenase (DUF1295)
AT1G73655		Peptidyl-prolyl cis-trans isomerase family protein; protein interaction with begomovirus movement proteins.
AT1G73660	*SUGAR INSENSITIVE 8 (SIS8)*	Encodes a protein with similarity to MAPKKKs. May function as a negative regulator of salt tolerance.
AT1G73670	*MAP KINASE 15 (MPK15)*	member of MAP Kinase The mRNA is cell-to-cell mobile.
AT1G73680	*ALPHA DIOXYGENASE (ALPHA DOX2)*	Encodes an alpha dioxygenase. Recombinant protein catalyzes the conversion of a wide range of fatty acids into 2(R)-hydroperoxy derivatives.
AT1G73700		MATE efflux family protein
AT1G73710		Pentatricopeptide repeat (PPR) superfamily protein
AT1G73750		alpha/beta hydrolase family protein
AT1G73760	*(CTL14)*	RING/U-box superfamily protein
AT1G73780		Bifunctional inhibitor/lipid-transfer protein/seed storage 2S albumin superfamily protein
AT1G73805	*SAR DEFICIENT 1 (SARD1)*	Encodes SAR Deficient 1 (SARD1), a key regulator for ICS1 (Isochorismate Synthase 1) induction and salicylic acid (SA) synthesis.
AT1G73830	*BR ENHANCED EXPRESSION 3 (BEE3)*	Encodes the brassinosteroid signaling component BEE3 (BR-ENHANCED EXPRESSION 3). Positively modulates the shade avoidance syndrome in Arabidopsis seedlings.
AT1G73850		DNA ligase (DUF1666)
AT1G73860		P-loop containing nucleoside triphosphate hydrolases superfamily protein
AT1G73870	*B-BOX DOMAIN PROTEIN 16 (BBX16)*	B-box type zinc finger protein with CCT domain-containing protein
AT1G73910	*ACTIN-RELATED PROTEINS 4A (ARP4A)*	Encodes a gene similar to actin-related proteins in other organisms. Member of nuclear ARP family of genes. Component of chromatin remodeling complexes, involved in chromatin-mediated gene regulation.
AT1G73920		alpha/beta-Hydrolases superfamily protein
AT1G73940		tumor necrosis factor receptor family protein
AT1G73950		Transmembrane Fragile-X-F-associated protein
AT1G73960	*TBP-ASSOCIATED FACTOR 2 (TAF2)*	Member of TFIID complex.
AT1G73970		obscurin-like protein
AT1G73980	*TRIPHOSPHATE TUNNEL METALLOENZYME 1 (TTM1)*	TTM1 is a triphosphate tunnel metalloenzyme that displays pyrophosphatase activity. It contains both a uridine kinase (UK) domain,CYTH domain, a coiled-coil domain and a transmembrane domain at the C-terminal Mutants show a delay in leaf senescence. Can functionally complement TTM1 and vise versa. (PMID:28733390)
AT1G74000	*STRICTOSIDINE SYNTHASE 3 (SS3)*	encodes a protein similar to strictosidine synthase, which is involved in the production of monoterpene indole alkaloids. This gene belongs to a family of 13 members in Arabidopsis.
AT1G74010		Calcium-dependent phosphotriesterase superfamily protein
AT1G74020	*STRICTOSIDINE SYNTHASE 2 (SS2)*	Encodes AtSS-2 strictosidine synthase.
AT1G74030	*ENOLASE 1 (ENO1)*	Encodes the plastid-localized phosphoenolpyruvate enolase. Mutant plants have abnormal trichomes, defects in fatty acid metabolism.

AT1G74055		transmembrane protein
AT1G74070		Cyclophilin-like peptidyl-prolyl cis-trans isomerase family protein
AT1G74080	*MYB DOMAIN PROTEIN 122 (MYB122)*	Encodes a putative transcription factor, member of the R2R3 factor gene family (MYB122).
AT1G74090	*DESULFO-GLUCOSINOLATE SULFOTRANSFERASE 18 (SOT18)*	encodes a desulfoglucosinolate sulfotransferase, involved in the final step of glucosinolate core structure biosynthesis. Has a broad-substrate specificity with preference with methionine-derived desulfoglucosinolates.
AT1G74100	*SULFOTRANSFERASE 16 (SOT16)*	encodes a desulfoglucosinolate sulfotransferase, involved in the final step of glucosinolate core structure biosynthesis. Has a broad-substrate specificity with different desulfoglucosinolates, the best substrate is indole-3-methyl-dsGS, followed by benzyl-dsGS. Expression was induced by wounding, jasmonate and ethylene stimulates.
AT1G74110	*CYTOCHROME P450, FAMILY 78, SUBFAMILY A, POLYPEPTIDE 10 (CYP78A10)*	member of CYP78A
AT1G74120	*MITOCHONDRIAL TRANSCRIPTION TERMINATION FACTOR 15 (MTERF15)*	Encodes a mitochondrial transcription termination factor mTERF15. Required for mitochondrial nad2 intron 3 splicing and functional complex I activity.
AT1G74140		Rhomboid-related intramembrane serine protease family protein
AT1G74150		Galactose oxidase/kelch repeat superfamily protein
AT1G74170	*RECEPTOR LIKE PROTEIN 13 (RLP13)*	receptor like protein 13
AT1G74180	*RECEPTOR LIKE PROTEIN 14 (RLP14)*	receptor like protein 14
AT1G74190	*RECEPTOR LIKE PROTEIN 15 (RLP15)*	receptor like protein 15
AT1G74200	*RECEPTOR LIKE PROTEIN 16 (RLP16)*	receptor like protein 16
AT1G74220		homeobox-like protein
AT1G74240		Mitochondrial substrate carrier family protein
AT1G74250		DNAJ heat shock N-terminal domain-containing protein
AT1G74290		alpha/beta-Hydrolases superfamily protein
AT1G74300		alpha/beta-Hydrolases superfamily protein
AT1G74310	*HEAT SHOCK PROTEIN 101 (HSP101)*	Encodes ClpB1, which belongs to the Casein lytic proteinase/heat shock protein 100 (Clp/Hsp100) family. Involved in refolding of proteins which form aggregates under heat stress. Also known as AtHsp101. AtHsp101 is a cytosolic heat shock protein required for acclimation to high temperature.
AT1G74350	*NUCLEAR-ENCODED MATURASE-RELATED 4 (NMAT4)*	Encodes nMAT4, a maturase factor required for nad1 pre-mRNA processing and maturation. Essential for holocomplex I biogenesis in Arabidopsis mitochondria.
AT1G74360	*NEMATODE-INDUCED LRR-RLK 1 (NILR1)*	NILR1 encodes a serine/threonine kinase involved in defense response to nematodes.
AT1G74370		RING/U-box superfamily protein
AT1G74400		Tetratricopeptide repeat (TPR)-like superfamily protein
AT1G74410	*ARABIDOPSIS T??XICOS EN LEVADURA 24 (ATL24)*	RING/U-box superfamily protein
AT1G74420	*FUCOSYLTRANSFERASE 3 (FUT3)*	Predicted fucosyltransferase, based on similarity to FUT1, but not functionally redundantwith FUT1.
AT1G74430	*MYB DOMAIN PROTEIN 95 (MYB95)*	Encodes a putative transcription factor (MYB95). The mRNA is cell-to-cell mobile.
AT1G74440	*(MHL)*	Similar to MPH1, can complement mph1-1 salt sensitivity phenotype.
AT1G74450	*(ROH1D)*	Plants overexpressing At1g74450 are stunted in height and have reduced male fertility.
AT1G74460		GDSL-motif esterase/acyltransferase/lipase. Enzyme group with broad substrate specificity that may catalyze acyltransfer or hydrolase reactions with lipid and non-lipid substrates.
AT1G74470		Encodes for a multifunctional protein with geranylgeranyl reductase activity shown to catalyze the reduction of prenylated geranylgeranyl-chlorophyll a to phytyl-chlorophyll a (chlorophyll a) and free geranylgeranyl pyrophosphate to phytyl pyrophosphate. The mRNA is cell-to-cell mobile.
AT1G74480	*RWP-RK DOMAIN CONTAINING 2 (RKD2)*	RWP-RK domain-containing protein
AT1G74490	*PBS1-LIKE 29 (PBL29)*	Protein kinase superfamily protein
AT1G74500	*ACTIVATION-TAGGED BRI1(BRASSINOSTEROID-INSENSITIVE 1)-SUPPRESSOR 1 (BS1)*	Encodes a basic helix/loop/helix transcription factor that acts downstream of MP in root initiation. TMO7 protein moves to the hypophysis and to vascular cells, contributing to MP-dependent root formation. Promotes the correct definition of the hypophysis cell division plane.
AT1G74510		Galactose oxidase/kelch repeat superfamily protein
AT1G74540	*CYTOCHROME P450, FAMILY 98, SUBFAMILY A, POLYPEPTIDE 8 (CYP98A8)*	Encodes a tricoumaroylspermidine / triferuloylspermidine meta-hydroxylase that participates in the formation of N¹,N⁵-di(hydroxyferuloyol)- N¹⁰-sinapoyl spermidine, an important constituent of pollen. This gene appears to be expressed in young flower buds and inflorescence tips with notably high levels of expression in the tapetum and pollen.
AT1G74550	*CYTOCHROME P450, FAMILY 98, SUBFAMILY A, POLYPEPTIDE 9 (CYP98A9)*	Encodes a tricoumaroylspermidine meta-hydroxylase that participates in the formation of N¹,N⁵-di(hydroxyferuloyol)-N¹⁰-sinapoylspermidine, an important constituent of pollen. This gene appears to be expressed in young flower buds and inflorescence tips with notably high levels of expression in the tapetum and pollen. It is also expressed in root tips.
AT1G74560	*NAP1-RELATED PROTEIN 1 (NRP1)*	Double nrp1-1 nrp2-1 mutants show arrest of cell cycle progression at G2/M and disordered cellular organization occurred in root tips. Localize in the nucleus and can form homomeric and heteromeric protein complexes with NRP2. Bind histones Histone2A and Histone2B and associate with chromatin in vivo. Plant mutated in both NRP1 and NRP2 genes show hypersensitivity to genotoxic stresses including UV and DSB-inducing agent Bleomycin. NRP genes act synergistically with NAP1 genes in promoting somatic homologous recombination.
AT1G74580		Pentatricopeptide repeat (PPR) superfamily protein
AT1G74590	*GLUTATHIONE S-TRANSFERASE TAU 10 (GSTU10)*	Encodes glutathione transferase belonging to the tau class of GSTs. Naming convention according to Wagner et al. (2002).
AT1G74600	*ORGANELLE TRANSCRIPT PROCESSING 87 (OTP87)*	OTP87 appears to act as a trans-factor involved in the recognition of the two editing sites in mitochondrial genes nad7-C24 and atp1-C1178.
AT1G74620		RING/U-box superfamily protein
AT1G74640		alpha/beta-Hydrolases superfamily protein
AT1G74650	*MYB DOMAIN PROTEIN 31 (MYB31)*	Member of the R2R3 factor gene family.

AT1G74660	*MINI ZINC FINGER 1 (MIF1)*	Encodes MINI ZINC FINGER 1 (MIF1) which has a zinc finger domain but lacks other protein motifs normally present in transcription factors. MIF1 physically interact with a group of zinc finger-homeodomain (ZHD) transcription factors, such as ZHD5 (AT1G75240), that regulate floral architecture and leaf development. Gel mobility shift assays revealed that MIF1 blocks the DNA binding activity of ZHD5 homodimers by competitively forming MIF1-ZHD5 heterodimers. Constitutive overexpression of MIF1 caused dramatic developmental defects, seedlings were non-responsive to gibberellin (GA) for cell elongation, hypersensitive to the GA synthesis inhibitor paclobutrazol (PAC) and abscisic acid (ABA), and hyposensitive to auxin, brassinosteroid and cytokinin, but normally responsive to ethylene.
AT1G74670	*GA-STIMULATED ARABIDOPSIS 6 (GASA6)*	Gibberellin-regulated family protein
AT1G74680		Exostosin family protein
AT1G74700	*TRNASE Z1 (TRZ1)*	Encodes a protein with RNAse Z activity suggesting a role in tRNA processing.
AT1G74710	*ENHANCED DISEASE SUSCEPTIBILITY TO ERYSIPHE ORONTII 16 (EDS16)*	Encodes a protein with isochorismate synthase activity. Mutants fail to accumulate salicylic acid. Its function may be redundant with that of ICS2 (AT1G18870).
AT1G74730	*(RIQ2)*	Encodes a grana core localized protein, is homologous to RIG1. Mutant plants have reduced NPQ, affected organization of light-havesting complex II and an enhanced grana stacking.
AT1G74740	*CALCIUM-DEPENDENT PROTEIN KINASE 30 (CPK30)*	member of Calcium Dependent Protein Kinase
AT1G74750		Pentatricopeptide repeat (PPR) superfamily protein
AT1G74760		
AT1G74770	*BRUTUS LIKE 1 (BTSL1)*	zinc ion binding protein
AT1G74780		Nodulin-like / Major Facilitator Superfamily protein
AT1G74790		catalytics
AT1G74810	*REQUIRES HIGH BORON 5 (BOR5)*	HCO3- transporter family
AT1G74840		Homeodomain-like superfamily protein
AT1G74850	*PLASTID TRANSCRIPTIONALLY ACTIVE 2 (PTAC2)*	Present in transcriptionally active plastid chromosomes. Involved in plastid gene expression. PEP complex component.
AT1G74870		RING/U-box superfamily protein
AT1G74880	*NADH DEHYDROGENASE-LIKE COMPLEX) (NdhO)*	Encodes subunit NDH-O of NAD(P)H:plastoquinone dehydrogenase complex (Ndh complex) present in the thylakoid membrane of chloroplasts. This subunit is thought to be required for Ndh complex assembly.
AT1G74890	*RESPONSE REGULATOR 15 (ARR15)*	Encodes a nuclear response regulator that acts as a negative regulator in cytokinin-mediated signal transduction. Transcript accumulates in leaves and roots in response to cytokinin treatment.
AT1G74900	*ORGANELLE TRANSCRIPT PROCESSING DEFECT 43 (OTP43)*	Pentatricopeptide repeat (PPR) superfamily protein
AT1G74930	*(ORA47)*	encodes a member of the DREB subfamily A-5 of ERF/AP2 transcription factor family. The protein contains one AP2 domain. There are 15 members in this subfamily including RAP2.1, RAP2.9 and RAP2.10. The mRNA is cell-to-cell mobile.
AT1G74940		cyclin-dependent kinase, putative (DUF581)
AT1G74950	*(TIFY10B)*	Key regulator in alternative splicing in the jasmonate signaling pathway, alone and in collaboration with other regulators.
AT1G74960	*FATTY ACID BIOSYNTHESIS 1 (FAB1)*	Encodes a plastidic beta-ketoacyl-ACP synthase II, involved in fatty acid elongation from 16:0-ACP to 18:0-ACP. Homozygous knock-out mutants are embryo lethal, indicating early embryo development is sensitive to elevated 16:0.
AT1G74970	*RIBOSOMAL PROTEIN S9 (RPS9)*	Ribosomal protein S9, nuclear encoded component of the chloroplast ribosome
AT1G74990		RING/U-box superfamily protein
AT1G75020	*LYSOPHOSPHATIDYL ACYLTRANSFERASE 4 (LPAT4)*	lysophosphatidyl acyltransferase 4
AT1G75030	*THAUMATIN-LIKE PROTEIN 3 (TLP-3)*	encodes a PR5-like protein
AT1G75040	*PATHOGENESIS-RELATED GENE 5 (PR5)*	Thaumatin-like protein involved in response to pathogens. mRNA level of the PR-5 gene (At1g75040)is significantly changed after cutting the inflorescence stem indicating the existence of a network of signal transducing pathways as other stress-regulated genes (At5g01410, At3g17800, At1g29930)do not response to the treatment. The mRNA is cell-to-cell mobile.
AT1G75050		Pathogenesis-related thaumatin superfamily protein
AT1G75080	*BRASSINAZOLE-RESISTANT 1 (BZR1)*	Encodes a positive regulator of the brassinosteroid (BR) signalling pathway that mediates both downstream BR responses and negative feedback regulation of BR biosynthesis. There is evidence for phosphorylation-dependent nucleocytoplasmic shuttling of BZR1. GSK3-like kinases (including BIN2), 14-3-3 proteins, and the phosphatase BSU1 seem to participate in this process. Phosphorylation also appears to affect BZR1's transcriptional activities.
AT1G75100	*J-DOMAIN PROTEIN REQUIRED FOR CHLOROPLAST ACCUMULATION RESPONSE 1 (JAC1)*	Contains a J-domain at the C-terminus which is similar to the J-domain of auxilin, a clathrin-uncoating factor in cow, yeast and worm. Arabidopsis contains 6 other proteins similar to auxilin. Expressed in leaves and stems, but not in roots. Localized in the cytoplasm. Required for the chloroplast accumulation response, but not for the avoidance response. No molecular function known. Influences the composition of photosynthetic pigments, the efficiency of photosynthesis, and the CO2 uptake rate. Positive effect on water use efficiency (WUE) by reducing stomatal aperture and water vapor conductance; involved in the fine-tuning of H2O2 foliar levels, antioxidant enzymes activities and cell death after UV-C photooxidative stress.
AT1G75110	*REDUCED RESIDUAL ARABINOSE 2 (RRA2)*	Encodes an arabinosyltransferase that modifies extensin proteins in root hair cells.
AT1G75150		DNA ligase-like protein
AT1G75160	*BOUNDARY OF ROP DOMAIN9 (BDR9)*	DUF620 family protein (DUF620)
AT1G75170		Sec14p-like phosphatidylinositol transfer family protein
AT1G75180		Erythronate-4-phosphate dehydrogenase family protein
AT1G75190		hypothetical protein
AT1G75200		flavodoxin family protein / radical SAM domain-containing protein
AT1G75220	*ERD6-LIKE 6 (ERDL6)*	Encodes a vacuolar glucose exporter that is induced in response to factors that activate vacuolar glucose pools like darkness, heat stress and wounding and repressed during conditions that trigger glucose accumulation in the vacuole like cold stress and external sugar supply.

AT1G75240	*HOMEOBOX PROTEIN 33 (HB33)*	Encodes a zinc finger-homeodomain transcription factor ZHD5. Nuclear import and DNA binding of the ZHD5 transcription factor is modulated by a competitive peptide inhibitor MIF1 (AT1G74660).
AT1G75250	*RAD-LIKE 6 (RL6)*	RAD-like 6
AT1G75270	*DEHYDROASCORBATE REDUCTASE 2 (DHAR2)*	dehydroascorbate reductase 2
AT1G75280		isoflavone reductase, putative, identical to SP:P52577 Isoflavone reductase homolog P3 (EC 1.3.1.-) {Arabidopsis thaliana}; contains Pfam profile PF02716: isoflavone reductase. Involved in response to oxidative stress. The mRNA is cell-to-cell mobile.
AT1G75290		encodes a protein whose sequence is similar to an isoflavone reductase
AT1G75300		encodes a protein whose sequence is similar to an isoflavone reductase
AT1G75310	*AUXILIN-LIKE 1 (AUL1)*	auxin-like 1 protein
AT1G75340		Zinc finger C-x8-C-x5-C-x3-H type family protein
AT1G75350	*EMBRYO DEFECTIVE 2184 (emb2184)*	Ribosomal protein L31
AT1G75410	*BEL1-LIKE HOMEODOMAIN 3 (BLH3)*	BEL1-like homeodomain 3 (BLH3)
AT1G75430	*BEL1-LIKE HOMEODOMAIN 11 (BLH11)*	BEL1-like homeodomain 11
AT1G75440	*UBIQUITIN-CONJUGATING ENZYME 16 (UBC16)*	ubiquitin-conjugating enzyme 16
AT1G75450	*CYTOKININ OXIDASE 5 (CKX5)*	This gene used to be called AtCKX6. It encodes a protein whose sequence is similar to cytokinin oxidase/dehydrogenase, which catalyzes the degradation of cytokinins.
AT1G75460		ATP-dependent protease La (LON) domain protein
AT1G75470	*PURINE PERMEASE 15 (PUP15)*	Member of a family of proteins related to PUP1, a purine transporter. May be involved in the transport of purine and purine derivatives such as cytokinins, across the plasma membrane.
AT1G75490		encodes a member of the DREB subfamily A-2 of ERF/AP2 transcription factor family. The protein contains one AP2 domain. There are eight members in this subfamily including DREB2A AND DREB2B that are involved in response to drought.
AT1G75500	*WALLS ARE THIN 1 (WAT1)*	An Arabidopsis thaliana homolog of Medicago truncatula NODULIN21 (MtN21). The gene encodes a plant-specific, predicted integral membrane protein and is a member of the Plant-Drug/Metabolite Exporter (P-DME) family (Transporter Classification number: TC 2.A.7.3) and the nodulin MtN21-like transporter family.
AT1G75520	*SHI-RELATED SEQUENCE 5 (SRS5)*	A member of SHI gene family. Arabidopsis thaliana has ten members that encode proteins with a RING finger-like zinc finger motif. Despite being highly divergent in sequence, many of the SHI-related genes are partially redundant in function and synergistically promote gynoecium, stamen and leaf development in Arabidopsis.SRS5 is a positive regulator of photomorphogenesis.
AT1G75540	*B-BOX DOMAIN PROTEIN 21 (BBX21)*	Encodes a B-box zinc finger transcription factor BBX21 (also named STH2/salt tolerance homolog2 and LHUS/long hypocotyl under shade). Interacts with COP1 to control de-etiolation. Also genetically interacts with COP1 to regulate shade avoidance. The mRNA is cell-to-cell mobile.
AT1G75550		glycine-rich protein
AT1G75580	*SMALL AUXIN UPREGULATED RNA 51 (SAUR51)*	SAUR-like auxin-responsive protein family
AT1G75590	*SMALL AUXIN UPREGULATED RNA 52 (SAUR52)*	SAUR-like auxin-responsive protein family
AT1G75600	*(HTR14)*	Histone superfamily protein
AT1G75610		pseudogene of Histone superfamily protein
AT1G75620	*GALACTOSE OXIDASE-LIKE 3 (GOXL3)*	Galactose oxydase; may function in tissues that require mechanical reinforcements in the absence of lignification.
AT1G75640	*MUSTACHES (MUS)*	Encodes a Leucine-Rich Repeat Receptor-Like Kinase MUSTACHES (MUS). Regulates stomatal bilateral symmetry. Mutants have abnormally shaped guard cells, absent or skewed stomatal pores.
AT1G75660	*5'-3' EXORIBONUCLEASE 3 (XRN3)*	Encodes a protein with similarity to yeast 5'-3'exonucleases and can functionally complement the yeast mutations. In Arabidopsis XRN3 acts as a suppressor of posttranscriptional gene silencing. Mutants accumulate excised miRNA products suggesting that XRN3 is involved in degradation of these products.
AT1G75670		DNA-directed RNA polymerase
AT1G75680	*GLYCOSYL HYDROLASE 9B7 (GH9B7)*	glycosyl hydrolase 9B7
AT1G75690	*LOW QUANTUM YIELD OF PHOTOSYSTEM II 1 (LQY1)*	Thylakoid Thiol/Disulfide-Modulating Protein.
AT1G75710		C2H2-like zinc finger protein
AT1G75720		WEB family protein (DUF827)
AT1G75730		hypothetical protein
AT1G75750	*GAST1 PROTEIN HOMOLOG 1 (GASA1)*	GA-responsive GAST1 protein homolog regulated by BR and GA antagonistically. Possibly involved in cell elongation based on expression data The mRNA is cell-to-cell mobile.
AT1G75770		hypothetical protein
AT1G75780	*TUBULIN BETA-1 CHAIN (TUB1)*	beta tubulin gene downregulated by phytochrome A (phyA)-mediated far-red light high-irradiance and the phytochrome B (phyB)-mediated red light high-irradiance responses
AT1G75790	*SKU5 SIMILAR 18 (sks18)*	SKU5 similar 18
AT1G75800		Pathogenesis-related thaumatin superfamily protein
AT1G75820	*CLAVATA 1 (CLV1)*	Putative receptor kinase with an extracellular leucine-rich domain. Controls shoot and floral meristem size, and contributes to establish and maintain floral meristem identity. Negatively regulated by KAPP (kinase-associated protein phosphatase). CLV3 peptide binds directly CLV1 ectodomain.
AT1G75830	*LOW-MOLECULAR-WEIGHT CYSTEINE-RICH 67 (LCR67)*	Predicted to encode a PR (pathogenesis-related) protein. Belongs to the plant defensin (PDF) family with the following members: At1g75830/PDF1.1, At5g44420/PDF1.2a, At2g26020/PDF1.2b, At5g44430/PDF1.2c, At2g26010/PDF1.3, At1g19610/PDF1.4, At1g55010/PDF1.5, At2g02120/PDF2.1, At2g02100/PDF2.2, At2g02130/PDF2.3, At1g61070/PDF2.4, At5g63660/PDF2.5, At2g02140/PDF2.6, At5g38330/PDF3.1 and At4g30070/PDF3.2.
AT1G75860		DNA ligase

AT1G75870		hypothetical protein
AT1G75880	*(GGL12)*	Guard-cell-enriched GDSL Lipase family member.
AT1G75890		GDSL-motif esterase/acyltransferase/lipase. Enzyme group with broad substrate specificity that may catalyze acyltransfer or hydrolase reactions with lipid and non-lipid substrates.
AT1G75900	*(GGL13)*	GDSL-motif esterase/acyltransferase/lipase. Enzyme group with broad substrate specificity that may catalyze acyltransfer or hydrolase reactions with lipid and non-lipid substrates.
AT1G75910	*EXTRACELLULAR LIPASE 4 (EXL4)*	Member of Lipase proteins. Involved in lipid metabolism and pollen wall formation. DYT1 and bHLH089 specifically recognize the TCATGTGC box to activate expression.
AT1G75920	*(EXL5)*	GDSL-motif esterase/acyltransferase/lipase. Enzyme group with broad substrate specificity that may catalyze acyltransfer or hydrolase reactions with lipid and non-lipid substrates. The mRNA is cell-to-cell mobile.
AT1G75930	*EXTRACELLULAR LIPASE 6 (EXL6)*	member of Lipase proteins
AT1G75940	*(ATA27)*	encodes a protein similar to the BGL4 beta-glucosidase from Brassica napus. The ATA27 protein is predicted to have an ER retention signal and an acidic isoelectric point, suggesting that it may be localized to the ER lumen.
AT1G75960		AMP-dependent synthetase and ligase family protein
AT1G75980		Single hybrid motif superfamily protein
AT1G76010	*(ALBA1)*	Alba DNA/RNA-binding protein
AT1G76020		Thioredoxin superfamily protein
AT1G76035		
AT1G76040	*CALCIUM-DEPENDENT PROTEIN KINASE 29 (CPK29)*	member of Calcium Dependent Protein Kinase
AT1G76060	*EMBRYO DEFECTIVE 1793 (EMB1793)*	CIAF1 mitochondrial protein required for assembly of the 1000-kD complex I holoenzyme.
AT1G76070		hypothetical protein
AT1G76080	*CHLOROPLASTIC DROUGHT-INDUCED STRESS PROTEIN OF 32 KD (CDSP32)*	Encodes a thioredoxin like protein. Localizes to the chloroplast and is redistributed to the chloroplast envelope under heat stress. It is involved in non host resistance and thermotolerance.
AT1G76090	*STEROL METHYLTRANSFERASE 3 (SMT3)*	Encodes S-adenosyl-methionine-sterol-C-methyltransferase, an enzyme in the sterol biosynthetic pathway.
AT1G76100	*PLASTOCYANIN 1 (PETE1)*	One of two Arabidopsis plastocyanin genes. Expressed at 1/10th level of PETE2. Does not respond to increased copper levels and is thought to be the isoform that participates in electron transport under copper-limiting conditions. Mutation of this gene does not have obvious effect on photosynthesis.
AT1G76110		HMG (high mobility group) box protein with ARID/BRIGHT DNA-binding domain-containing protein
AT1G76130	*ALPHA-AMYLASE-LIKE 2 (AMY2)*	alpha-amylase, putative / 1,4-alpha-D-glucan glucanohydrolase, putative, strong similarity to alpha-amylase GI:7532799 from (Malus x domestica);contains Pfam profile PF00128: Alpha amylase, catalytic domain. Predicted to be secreted based on SignalP analysis.
AT1G76150	*ENOYL-COA HYDRATASE 2 (ECH2)*	Encodes a monofunctional enoyl-CoA hydratase 2, involved in the degradation of even cis-unsaturated fatty acids, gene expression is enhanced during the first 2 days of germination, as well as in senescent leaves.
AT1G76160	*SKU5 SIMILAR 5 (sks5)*	SKU5 similar 5
AT1G76170		2-thiocytidine tRNA biosynthesis protein, TtcA
AT1G76180	*EARLY RESPONSE TO DEHYDRATION 14 (ERD14)*	Encodes a dehydrin protein whose expression is induced early on in response to dehydration stress. This gene's expression to cold occurs in two waves, with early induction occurring within 1 h and secondary induction occurring 5 h after the beginning of cold stress. Expression is also induced in response to ABA but not in response to 2,4-D, BA, and GA3. ERD14 protein is capable of binding Ca2+, especially when the protein is phosphorylated.
AT1G76190	*SMALL AUXIN UPREGULATED RNA 56 (SAUR56)*	SAUR-like auxin-responsive protein family
AT1G76210		DUF241 domain protein, putative (DUF241)
AT1G76220		hypothetical protein (DUF241)
AT1G76230		hypothetical protein
AT1G76240		DUF241 domain protein (DUF241)
AT1G76250		transmembrane protein
AT1G76260	*DWD (DDB1-BINDING WD40 PROTEIN) HYPERSENSITIVE TO ABA 2 (DWA2)*	DWD (DDB1-binding WD40 protein) hypersensitive to ABA 2
AT1G76280		Tetratricopeptide repeat (TPR)-like superfamily protein
AT1G76290		AMP-dependent synthetase and ligase family protein
AT1G76300	*SNRNP CORE PROTEIN SMD3 (SMD3A)*	snRNP core protein SMD3
AT1G76310	*CYCLIN B2;4 (CYCB2;4)*	core cell cycle genes
AT1G76340	*GOLGI NUCLEOTIDE SUGAR TRANSPORTER 3 (GONST3)*	Encodes a nucleotide-sugar transporter. It is is likely the primary Golgi GDP-L-galactose transporter, and provides GDP-L-galactose for RG-II biosynthesis. Knockout lines are lethal. RNAi suppressor lines were used for analysis. GDP-L-Galactose transport was affected. This process was required for pectic RG-II biosynthesis.
AT1G76360	*(PBL31)*	Protein kinase superfamily protein
AT1G76370	*PBS1-LIKE 22 (PBL22)*	Protein kinase superfamily protein
AT1G76405	*(OEP2)*	outer envelope pore 21B-like protein
AT1G76410	*(ATL8)*	RING/U-box superfamily protein
AT1G76420	*CUP SHAPED COTYLEDON3 (CUC3)*	Identified in an enhancer trap line; member of the NAC family of proteins. Expressed at the boundary between the shoot meristem and lateral organs and the polar nuclei in the embryo sac. Together with CUC2-DA1-UBP15 part of a regulatory module which controls the initiation of axillary meristems, thereby determining plant architecture. Regulates axillary meristem initiation by directly binding to the DA1 promoter.
AT1G76430	*PHOSPHATE TRANSPORTER 1;9 (PHT1;9)*	Encodes Pht1;9, a member of the Pht1 family of phosphate transporters which include: Pht1;1/At5g43350, Pht1;2/At5g43370, Pht1;3/At5g43360, Pht1;4/At2g38940, Pht1;5/At2g32830, Pht1;6/At5g43340, Pht1;7/At3g54700, Pht1;8/At1g20860, Pht1;9/At1g76430 (Plant Journal 2002, 31:341).

AT1G76450		Photosystem II reaction center PsbP family protein
AT1G76470		NAD(P)-binding Rossmann-fold superfamily protein
AT1G76490	*HYDROXY METHYLGLUTARYL COA REDUCTASE 1 (HMG1)*	Encodes a 3-hydroxy-3-methylglutaryl coenzyme A reductase, which is involved in melavonate biosynthesis and performs the first committed step in isoprenoid biosynthesis. Expression is activated in dark in leaf tissue but not controlled by light in the root (confine The mRNA is cell-to-cell mobile.
AT1G76510	*AT RICH INTERACTION DOMAIN 4 (ARID4)*	ARID/BRIGHT DNA-binding domain-containing protein
AT1G76520	*PIN-LIKES 3 (PILS3)*	Auxin efflux carrier family protein
AT1G76530	*PIN-LIKES 4 (PILS4)*	Auxin efflux carrier family protein
AT1G76540	*CYCLIN-DEPENDENT KINASE B2;1 (CDKB2;1)*	Encodes a cyclin-dependent protein kinase involved in regulation of the G2/M transition of the mitotic cell cycle. Specifically binds to the cyclin CYCD4;1, expressed in shoot meristem, young leaves and vascular tissue during the G2/M phase. Required for proper organization of the shoot apical meristem and for hormone signaling.
AT1G76550		Phosphofructokinase family protein. Target of miRNA sRNA6.
AT1G76560	*CP12 DOMAIN-CONTAINING PROTEIN 3 (CP12-3)*	CP12 domain-containing protein 3
AT1G76570	*LIGHT-HARVESTING COMPLEX B7 (LHCB7)*	Chlorophyll A-B binding family protein
AT1G76590	*(PLATZ2)*	PLATZ transcription factor family protein
AT1G76600		PADRE protein up-regulated after infection by S. sclerotiorun.
AT1G76620	*PIGMENT DEFECTIVE EMBRYO 339 (PDE339)*	Serine/Threonine-kinase, putative (Protein of unknown function, DUF547)
AT1G76630	*(SKI3)*	SKI3 encodes a cytplasmically localized component of the SKI complex which is involved in exosome mediated RNA decay.
AT1G76640	*CALMODULIN LIKE 39 (CML39)*	Calcium-binding EF-hand family protein
AT1G76650	*CALMODULIN-LIKE 38 (CML38)*	calmodulin-like 38
AT1G76660		hydroxyproline-rich glycoprotein family protein
AT1G76690	*12-OXOPHYTODIENOATE REDUCTASE 2 (OPR2)*	Encodes one of the closely related 12-oxophytodienoic acid reductases. This enzyme is not expected to participate in jasmonic acid biosynthesis because during in vitro assays, it shows very little activity with the naturally occurring OPDA isomer. Shows activity towards 2,4,6-trinitrotoluene. Expressed predominately in root. Predicted to be a cytosolic protein.
AT1G76700		DNAJ heat shock N-terminal domain-containing protein
AT1G76720		eukaryotic translation initiation factor 2 (eIF-2) family protein
AT1G76730	*CLUSTERS OF ORTHOLOGOUS GROUP 212 (COG0212)*	Encodes a paralog of ATP-dependent folate salvage enzyme 5-formyltetrahydrofolate cycloligase (5-FCL) that is targeted to chloroplasts and to be required for embryo viability and lacks 5-FCL activity.
AT1G76740		hypothetical protein
AT1G76770		HSP20-like chaperone
AT1G76780		HSP20-like chaperones superfamily protein
AT1G76790	*INDOLE GLUCOSINOLATE O-METHYLTRANSFERASE 5 (IGMT5)*	Encodes a protein with similarity to N-acetylserotonin O-methyltransferase (ASMT) but it does not have ASMT activity in vitro.
AT1G76800	*VACUOLAR IRON TRANSPORTER-LIKE 2 (VTL2)*	The gene encodes nodulin-like2 whose transcript abundance was repressed under conditions of Fe-deficient growth.
AT1G76810	*(EIF5B1)*	eukaryotic translation initiation factor 2 (eIF-2) family protein
AT1G76820		eukaryotic translation initiation factor 2 (eIF-2) family protein
AT1G76870		transcription factor
AT1G76880	*(DF1)*	DF1 is a putative transcription factor required for the synthesis of seed mucilage polysaccharides. The df1 seeds produce almost 50% less mucilage than wild-type, but show less severe defects than the myb5 and ttg2 mutants.
AT1G76890	*(GT2)*	encodes a plant trihelix DNA-binding protein
AT1G76900	*TUBBY LIKE PROTEIN 1 (TLP1)*	Member of plant TLP family. Contains terminal F-box domain, interacts with ASK proteins. Tethered to the PM.
AT1G76910		hypothetical protein
AT1G76920	*(BAF1)*	F-box family E3 ubiquitin ligase. Involved in BES1 degradation via selective autophagy in a DSK2 dependent manner.
AT1G76930	*EXTENSIN 4 (EXT4)*	Encodes an Arabidopsis extensin gene that belongs to cell-wall hydroxyproline-rich glycoproteins. The cross-link of extensins enforces cell wall strength. Transgenic plants overexpressing this gene show an increase in stem thickness.
AT1G76960		Unknown protein, contains WRKY40 binding motifs.
AT1G76990	*ACT DOMAIN REPEAT 3 (ACR3)*	ACT domain repeat 3
AT1G77000	*(SKP2B)*	AtSKP2;2 is a homolog of human SKP2, the human F-box protein that recruits E2F1. Contains an F-box motif at the N-terminal region and a C-terminal Leu-rich repeat domain. Forms part of an E3-ubiquitin-ligase SCF (Skp1, cullin, F-box) complex and recruits phosphorylated AtE2Fc, a transcriptional factor that might play a role in cell division and during the transition from skotomorphogenesis to photomorphogenesis. AtSKP2;1 (At1g21410) and AtSKP2;2 (At1g77000) may be duplicated genes. AtSKP2b may also be involved in the degradation of KRP1/ICK1, a CDK inhibitor.
AT1G77020		DNAJ heat shock N-terminal domain-containing protein
AT1G77050		
AT1G77060		Phosphoenolpyruvate carboxylase family protein
AT1G77070		
AT1G77090		thylakoid lumenal protein (Mog1/PsbP/DUF1795-like photosystem II reaction center PsbP family protein)
AT1G77100		peroxidase superfamily protein
AT1G77110	*PIN-FORMED 6 (PIN6)*	Rate-limiting factor in saturable efflux of auxins. PINs are directly involved of in catalyzing cellular auxin efflux.
AT1G77120	*ALCOHOL DEHYDROGENASE 1 (ADH1)*	Catalyzes the reduction of acetaldehyde using NADH as reductant. Requires zinc for activity. Dimer. Anaerobic response polypeptide (ANP). Fermentation. The protein undergoes thiolation following treatment with the oxidant tert-butylhydroperoxide. The mRNA is cell-to-cell mobile.
AT1G77130	*PLANT GLYCOGENIN-LIKE STARCH INITIATION PROTEIN 2 (PGSIP2)*	Encodes a glucuronyltransferase responsible for the addition of GlcA residues onto xylan and for secondary wall deposition.

AT1G77170		Tetratricopeptide repeat (TPR)-like superfamily protein
AT1G77180	*SNW/SKI-INTERACTING PROTEIN (SKIP)*	Encodes a putative transcriptional factor. Shows transcriptional activator activity in yeast. Involved in response to abscisic acid, salt and osmotic stress. SKIP lengthens period of the circadian clock by impairing the alternative splicing of PRR7 and PRR9. SKIP regulates the splicing of SEF pre-mRNA and suppresses flowering by activation of FLC.
AT1G77200		encodes a member of the DREB subfamily A-4 of ERF/AP2 transcription factor family. The protein contains one AP2 domain. There are 17 members in this subfamily including TINY.
AT1G77210	*SUGAR TRANSPORT PROTEIN 14 (STP14)*	AtSTP14 belongs to the family of sugar transport proteins (AtSTPs)in volved in monosaccharide transport. Heterologous expression in yeast revealed that AtSTP14 is the transporter specifc for galactose and does not transport other monosaccharides such as glucose or fructose.
AT1G77220	*LAZ1 HOMOLOG1 (LAZ1H1)*	LAZ1H1 is a DUF300 that is localized to the tonoplast. Along with LAZ1 it appears to play a role in maintaining the structural integrity of vacuoles and regulating BR signaling by modulating downstream subcellular distribution of BAK1.
AT1G77230		Tetratricopeptide repeat (TPR)-like superfamily protein
AT1G77240	*ACYL ACTIVATING ENZYME 2 (AAE2)*	AMP-dependent synthetase and ligase family protein
AT1G77250		RING/FYVE/PHD-type zinc finger family protein
AT1G77260		S-adenosyl-L-methionine-dependent methyltransferases superfamily protein
AT1G77270		hypothetical protein
AT1G77280		kinase with adenine nucleotide alpha hydrolases-like domain-containing protein
AT1G77330	*ACC OXIDASE 5 (ACO5)*	similar to 1-aminocyclopropane-1-carboxylate oxidase GI:3386565 from (Sorghum bicolor)
AT1G77340		Pentatricopeptide repeat (PPR) superfamily protein
AT1G77380	*AMINO ACID PERMEASE 3 (AAP3)*	Amino acid permease which transports basic amino acids.
AT1G77400		extensin-like protein
AT1G77405		Pentatricopeptide repeat (PPR) superfamily protein
AT1G77450	*NAC DOMAIN CONTAINING PROTEIN 32 (NAC032)*	NAC domain transcriptional regulator that is induced by ROS in roots where it regulates the expression of downstream genes such as MYB30.
AT1G77460	*CELLULOSE SYNTHASE INTERACTIVE 3 (CSI3)*	Encodes a plasma membrane, microtubule associated protein with sequence similarity to CSI1 that is involved in cellulose biosynthesis and cell elongation. A mutation in CSI3 alone do not appear to affect growth but enhances the cell elongation phenotype of CSI1 mutants. CSI3 co localizes with CSI1 and CESA3 and CESA6.
AT1G77490	*THYLAKOIDAL ASCORBATE PEROXIDASE (TAPX)*	Encodes a chloroplastic thylakoid ascorbate peroxidase tAPX. Ascorbate peroxidases are enzymes that scavenge hydrogen peroxide in plant cells. Eight types of APX have been described for Arabidopsis: three cytosolic (APX1, APX2, APX6), two chloroplastic types (stromal sAPX, thylakoid tAPX), and three microsomal (APX3, APX4, APX5) isoforms.
AT1G77510	*PDI-LIKE 1-2 (PDIL1-2)*	Encodes a protein disulfide isomerase-like (PDIL) protein, a member of a multigene family within the thioredoxin (TRX) superfamily. Transcript levels for this gene are up-regulated in response to three different chemical inducers of ER stress (dithiothreitol, beta-mercaptoethanol, and tunicamycin). AtIRE1-2 does not appear to be required for this response, but the atbzip60 mutant has a diminished response. This protein has been shown to be an attenuator of D1 synthesis, modulating photoinhibition in a light-regulated manner.
AT1G77530		O-methyltransferase family protein
AT1G77540		Encodes a H3/H4 histone acetyltransferase. Belongs to the GNAT family, whose many members are involved in histone acetylation and chromatin remodeling, and are important for the regulation of cell growth and development.
AT1G77550		tubulin-tyrosine ligase
AT1G77570	*RESTRICTED TO NUCLEOLUS 1 (REN1)*	Winged helix-turn-helix transcription repressor DNA-binding. Expressed in pollen and mutants show enlarged pollen grain nucleoli.
AT1G77580	*VESICLE TETHERING 3 (VETH3)*	filament-like protein (DUF869)
AT1G77590	*LONG CHAIN ACYL-COA SYNTHETASE 9 (LACS9)*	Encodes major plastidic long chain acyl-CoA synthetase with a slight substrate preference of oleic acid over any of the other fatty acids.
AT1G77610		EamA-like transporter family protein
AT1G77640		encodes a member of the DREB subfamily A-5 of ERF/AP2 transcription factor family. The protein contains one AP2 domain. There are 15 members in this subfamily including RAP2.1, RAP2.9 and RAP2.10.
AT1G77655		hypothetical protein
AT1G77670		Pyridoxal phosphate (PLP)-dependent transferases superfamily protein
AT1G77680	*RRP44 HOMOLOG B (RRP44B)*	Ribonuclease II/R family protein
AT1G77690	*LIKE AUX1 3 (LAX3)*	Encodes an auxin influx carrier LAX3 (Like Aux1) that promotes lateral root emergence. Auxin-induced expression of LAX3 in turn induces a selection of cell-wall-remodelling enzymes, which are likely to promote cell separation in advance of developing lateral root primordia.
AT1G77700	*UBIQUITIN FOLD MODIFIER 1 (UFM1)*	Osmotin-like protein.
AT1G77710	*CONSERVED IN CILIATED SPECIES AND IN THE LAND PLANTS 2 (CCP2)*	ubiquitin-fold modifier
AT1G77730		Pleckstrin homology (PH) domain superfamily protein
AT1G77750		Ribosomal protein S13/S18 family
AT1G77760	*NITRATE REDUCTASE 1 (NIA1)*	Encodes the cytosolic minor isoform of nitrate reductase (NR). Involved in the first step of nitrate assimilation, it contributes about 15% of the nitrate reductase activity in shoots. Similar to molybdopterin oxidoreductases at the N-terminus, and to FAD/NAD-binding cytochrome reductases at the C-terminus. Cofactors: FAD, heme iron (cytochrome B-557), and molybdenum-pterin.
AT1G77780		Glycosyl hydrolase superfamily protein
AT1G77790		Glycosyl hydrolase superfamily protein
AT1G77820		transposable_element_gene;pseudogene, endonuclease/exonuclease/phosphatase family protein, contains similarity to reverse transcriptase GI:976278 from (Arabidopsis thaliana);(source:TAIR10)
AT1G77830		RING/U-box superfamily protein
AT1G77840		Translation initiation factor IF2/IF5

AT1G77850	*AUXIN RESPONSE FACTOR 17 (ARF17)*	Encodes a transcriptional regulator that directly binds to the promoter of MYB108 and plays a crucial role in anther dehiscence, pollen wall pattern formation, tapetum development, and auxin signal transduction in anthers. It is post-transcriptionally regulated by miR160 and regulates early auxin response genes.
AT1G77860	*KOMPEITO (KOM)*	Mutant has Altered morphology of pollen exine wall; Seven-Path Transmembrane Protein
AT1G77870	*MEMBRANE-ANCHORED UBIQUITIN-FOLD PROTEIN 5 PRECURSOR (MUB5)*	membrane-anchored ubiquitin-fold protein 5 precursor
AT1G77890	*BECLIN 1-ASSOCIATED AUTOPHAGY-RELATED KEY REGULATOR 14A (ATG14A)*	One of a pair of paralogs (the other is AT4G08540)that is a subunit of the lass III phosphatidylinositol 3-kinase (PI3K) complex but is not essential for PI3P biosynthesis.
AT1G77900		
AT1G77910		transmembrane protein
AT1G77920	*TGACG SEQUENCE-SPECIFIC BINDING PROTEIN 7 (TGA7)*	bZIP transcription factor family protein
AT1G77950	*AGAMOUS-LIKE 67 (AGL67)*	Cooperates with the histone mark reader EBS to modulate seed germination under high temperature.
AT1G77960		repressor ROX1-like protein
AT1G77970		
AT1G77980	*AGAMOUS-LIKE 66 (AGL66)*	Encodes a member of the MIKC (MADS box, Keratin binding domain, and C terminal domain containing)family of transcriptional regulators. AGL66 is expressed in pollen.It forms heterodimers with other MICK family members (AGL104). Involved in late stages of pollen development and pollen tube growth.
AT1G77990	*SULPHATE TRANSPORTER 2;2 (SULTR2;2)*	Encodes a low-affinity sulfate transporter.
AT1G78000	*SULFATE TRANSPORTER 1;2 (SULTR1;2)*	Encodes a sulfate transporter that can restore sulfate uptake capacity of a yeast mutant lacking sulfate transporter genes.
AT1G78010	*(TRME)*	tRNA modification GTPase
AT1G78020	*FCS LIKE ZINC FINGER 6 (FLZ6)*	FCS like zinc finger 6 is induced during energy starvation through SnRK1 signaling. Mutants accumulate more SnRK1alpha1 which results in the inhibition of seedling growth under favorable growth conditions. Increased SnRK1 activity in the mutant also results in the downregulation of TOR signaling (DOI:10.1111/tpj.13854).
AT1G78030		hypothetical protein
AT1G78050	*PHOSPHOGLYCERATE/BISPHOSPHOGLYCERATE MUTASE (PGM)*	phosphoglycerate/bisphosphoglycerate mutase
AT1G78060		Glycosyl hydrolase family protein
AT1G78070		Transducin/WD40 repeat-like superfamily protein
AT1G78080	*RELATED TO AP2 4 (RAP2.4)*	Encodes a member of the DREB subfamily A-6 of ERF/AP2 transcription factor family (RAP2.4). The protein contains one AP2 domain. Role in mediating light and ethylene signaling. The mRNA is cell-to-cell mobile.
AT1G78090	*TREHALOSE-6-PHOSPHATE PHOSPHATASE B (TPPB)*	homologous to the C-terminal part of microbial trehalose-6-phosphate phosphatases
AT1G78100	*AUXIN UP-REGULATED F-BOX PROTEIN 1 (AUF1)*	F-box family protein
AT1G78110		nucleolar GTP-binding protein
AT1G78120	*TETRATRICOPEPTIDE REPEAT 12 (TPR12)*	Encodes one of the 36 carboxylate clamp (CC)-tetratricopeptide repeat (TPR) proteins (Prasad 2010, Pubmed ID: 20856808) with potential to interact with Hsp90/Hsp70 as co-chaperones.
AT1G78130	*UNFERTILIZED EMBRYO SAC 2 (UNE2)*	Major facilitator superfamily protein
AT1G78150		N-lysine methyltransferase
AT1G78160	*PUMILIO 7 (PUM7)*	Encodes a member of the Arabidopsis Pumilio (APUM) proteins containing PUF domain (eight repeats of approximately 36 amino acids each). PUF proteins regulate both mRNA stability and translation through sequence-specific binding to the 3' UTR of target mRNA transcripts.
AT1G78170		E3 ubiquitin-protein ligase
AT1G78180		Mitochondrial substrate carrier family protein
AT1G78200		Protein phosphatase 2C family protein
AT1G78210		alpha/beta-Hydrolases superfamily protein
AT1G78220	*GENERAL REGULATORY FACTOR 13 (GRF13)*	14-3-3 protein GF14 pi
AT1G78270	*UDP-GLUCOSYL TRANSFERASE 85A4 (UGT85A4)*	UDP-glucosyl transferase 85A4
AT1G78290	*SNF1-RELATED PROTEIN KINASE 2-8 (SNRK2-8)*	encodes a member of SNF1-related protein kinase (SnRK2) family whose activity is activated by ionic (salt) and non-ionic (mannitol) osmotic stress and dehydration.
AT1G78300	*GENERAL REGULATORY FACTOR 2 (GRF2)*	G-box binding factor GF14 omega encoding a 14-3-3 protein The mRNA is cell-to-cell mobile.
AT1G78320	*GLUTATHIONE S-TRANSFERASE TAU 23 (GSTU23)*	Encodes glutathione transferase belonging to the tau class of GSTs. Naming convention according to Wagner et al. (2002).
AT1G78340	*GLUTATHIONE S-TRANSFERASE TAU 22 (GSTU22)*	Encodes glutathione transferase belonging to the tau class of GSTs. Naming convention according to Wagner et al. (2002).
AT1G78360	*GLUTATHIONE S-TRANSFERASE TAU 21 (GSTU21)*	Encodes glutathione transferase belonging to the tau class of GSTs. Naming convention according to Wagner et al. (2002).
AT1G78370	*GLUTATHIONE S-TRANSFERASE TAU 20 (GSTU20)*	Encodes glutathione transferase belonging to the tau class of GSTs. Naming convention according to Wagner et al. (2002).
AT1G78390	*NINE-CIS-EPOXYCAROTENOID DIOXYGENASE 9 (NCED9)*	Encodes 9-cis-epoxycarotenoid dioxygenase, a key enzyme in the biosynthesis of abscisic acid. The expression of this gene increases during the first 6h of imbibition.
AT1G78400	*POLYGALACTURONASE INVOLVED IN EXPANSION2 (PGX2)*	PGX2 is a cell wall protein that codes for a polygalacturonase.
AT1G78410		VQ motif-containing protein
AT1G78430	*ROP INTERACTIVE PARTNER 2 (RIP2)*	Encodes RIP2 (ROP interactive partner 2), a putative Rho protein effector, interacting specifically with the active form of ROPs (Rho proteins of plants).
AT1G78440	*ARABIDOPSIS THALIANA GIBBERELLIN 2-OXIDASE 1 (ATGA2OX1)*	Encodes a gibberellin 2-oxidase that acts on C19 gibberellins.
AT1G78450		SOUL heme-binding family protein
AT1G78460	*CYTOSOLIC HEME (TETRAPYRROLE) BINDING PROTEIN 3 (CHBP3)*	SOUL heme-binding family protein
AT1G78470		F-box/LRR protein

AT1G78480		Prenyltransferase family protein
AT1G78490	*CYTOCHROME P450, FAMILY 708, SUBFAMILY A, POLYPEPTIDE 3 (CYP708A3)*	member of CYP708A family. The mRNA is cell-to-cell mobile.
AT1G78500	*PENTACYCLIC TRITERPENE SYNTHASE 6 (PEN6)*	Encodes a protein with pentacyclic triterpene synthase activity. In addition to the compounds lupeol, α-amyrin and bauerenol, this enzyme was also shown to produce two <i>seco</i>-triterpenes: α- and β-<i>seco</i>-amyrin.
AT1G78530		Protein kinase superfamily protein
AT1G78550		2-oxoglutarate (2OG) and Fe(II)-dependent oxygenase superfamily protein
AT1G78580	*TREHALOSE-6-PHOSPHATE SYNTHASE (TPS1)*	Encodes an enzyme putatively involved in trehalose biosynthesis. The protein has a trehalose synthase (TPS)-like domain but no trehalose phosphatase (TPP)-like domain. ATTPS1 is able to complement yeast tps1 mutants in vivo. The gene product modulates cell growth but not cell differentiation by determining cell wall deposition and cell division. The N-terminal domain of TPS1 has a nuclear localization signal and an autoinhibitory function. The C-terminal domain is important for catalytic fidality of TPS1 and for appropriate signaling of the sucrose status by trehalose 6-phosphate levels in the plant (10.1105/tpc.19.00837).
AT1G78600	*LIGHT-REGULATED ZINC FINGER PROTEIN 1 (LZF1)*	light-regulated zinc finger protein 1
AT1G78630	*EMBRYO DEFECTIVE 1473 (emb1473)*	Ribosomal protein L13 family protein
AT1G78650	*(POLD3)*	Similar to DNA polymerase delta (POLD3), which in other organism was shown to be involved in the elongation of DNA replication.
AT1G78660	*GAMMA-GLUTAMYL HYDROLASE 1 (GGH1)*	The Arabidopsis protein AtGGH1 is a gamma-glutamyl hydrolase cleaving pentaglutamates to yield di- and triglutamates. The enzyme is involved in the tetrahydrofolate metabolism and located to the vacuole.
AT1G78670	*GAMMA-GLUTAMYL HYDROLASE 3 (GGH3)*	gamma-glutamyl hydrolase 3
AT1G78680	*GAMMA-GLUTAMYL HYDROLASE 2 (GGH2)*	The Arabidopsis protein AtGGH2 is a gamma-glutamyl hydrolase acting specifically on monoglutamates. The enzyme is involved in the tetrahydrofolate metabolism and located to the vacuole.
AT1G78690	*(At1g78690p)*	Encodes a lysoglycerophospholipid O-acyltransferase that acylates 1-acyl lyso PE and 1-acyl lyso PG but not PE or PG.
AT1G78710	*TRICHOME BIREFRINGENCE-LIKE 42 (TBL42)*	Encodes a member of the TBL (TRICHOME BIREFRINGENCE-LIKE) gene family containing a plant-specific DUF231 (domain of unknown function) domain. TBL gene family has 46 members, two of which (TBR/AT5G06700 and TBL3/AT5G01360) have been shown to be involved in the synthesis and deposition of secondary wall cellulose, presumably by influencing the esterification state of pectic polymers. A nomenclature for this gene family has been proposed (Volker Bischoff & Wolf Scheible, 2010, personal communication).
AT1G78720		SecY protein transport family protein
AT1G78740		RNI-like superfamily protein
AT1G78750		F-box/RNI-like superfamily protein
AT1G78770	*ANAPHASE PROMOTING COMPLEX 6 (APC6)*	anaphase promoting complex 6
AT1G78780		pathogenesis-related family protein
AT1G78800		UDP-Glycosyltransferase superfamily protein
AT1G78815	*LIGHT SENSITIVE HYPOCOTYLS 7 (LSH7)*	LIGHT-DEPENDENT SHORT HYPOCOTYLS-like protein (DUF640)
AT1G78820		D-mannose binding lectin protein with Apple-like carbohydrate-binding domain-containing protein
AT1G78840		F-box/RNI-like/FBD-like domains-containing protein
AT1G78860	*APPLE DOMAIN LECTIN-2 (GAL2)*	curculin-like (mannose-binding) lectin family protein, low similarity to Ser/Thr protein kinase (Zea mays) GI:2598067; contains Pfam profile PF01453: Lectin (probable mannose binding) but not the protein kinase domain of the Z. mays protein
AT1G78930	*(MTERF16)*	Mitochondrial transcription termination factor family protein
AT1G78940		kinase with adenine nucleotide alpha hydrolases-like domain-containing protein
AT1G78950	*BETA-AMYRIN SYNTHASE (BAS)*	Terpenoid cyclases family protein
AT1G78960	*LUPEOL SYNTHASE 2 (LUP2)*	Encodes a multifunctional 2-3-oxidosqualene (OS)-triterpene cyclase that can cyclize OS into lupeol, alpha- and beta-amyrin.
AT1G78970	*LUPEOL SYNTHASE 1 (LUP1)*	Lupeol synthase. Converts oxidosqualene to multiple triterpene alcohols and a triterpene diols. This conversion proceeds through the formation of a 17β-dammarenyl cation.
AT1G78980	*STRUBBELIG-RECEPTOR FAMILY 5 (SRF5)*	STRUBBELIG-receptor family 5
AT1G78990		HXXXD-type acyl-transferase family protein
AT1G78995		hypothetical protein
AT1G79000	*HISTONE ACETYLTRANSFERASE OF THE CBP FAMILY 1 (HAC1)*	Homologous to CREB-binding protein, a co-activator of transcription with histone acetyl-transferase activity. No single prior lysine acetylation is sufficient to block HAC1 acetylation of the H3 or H4 peptides, suggesting that HAC1, HAC5, and HAC12 can acetylate any of several lysines present in the peptides. HAM2 acetylates histone H4 lysine 5. A plant line expressing an RNAi construct targeted against HAC1 has reduced rates of agrobacterium-mediated root transformation.
AT1G79040	*PHOTOSYSTEM II SUBUNIT R (PSBR)*	Encodes for the 10 kDa PsbR subunit of photosystem II (PSII). This subunit appears to be involved in the stable assembly of PSII, particularly that of the oxygen-evolving complex subunit PsbP. Mutants defective in this gene have reduced amounts of subunits PsbP and PsbQ in PSII. In turn, assembly of PsbR is dependent on the presence of PsbJ.
AT1G79060		TPRXL
AT1G79080		Pentatricopeptide repeat (PPR) superfamily protein
AT1G79100		arginine/serine-rich protein-like protein
AT1G79110	*BOI-RELATED GENE 2 (BRG2)*	Encodes one of the BRGs (BOI-related gene) involved in resistance to Botrytis cinerea.
AT1G79140		
AT1G79150	*NUCLEOLAR COMPLEX ASSOCIATED 3 (NOC3)*	binding protein
AT1G79160		filamentous hemagglutinin transporter
AT1G79170		transmembrane protein
AT1G79180	*MYB DOMAIN PROTEIN 63 (MYB63)*	Member of the R2R3 factor gene family.

AT1G79190		ARM repeat superfamily protein
AT1G79230	*MERCAPTOPYRUVATE SULFURTRANSFERASE 1 (MST1)*	encodes a sulfurtransferase/rhodaneses, which belongs to a group of enzymes widely distributed in all three phyla that catalyze the transfer of sulfur from a donor to a thiophilic acceptor substrate. The protein and transcript levels are NOT affected by senescence or exogenous cyanide, suggesting that sulfurtransferases are involved in cyanide detoxification.
AT1G79250	*AGC KINASE 1.7 (AGC1.7)*	AGC kinase 1.7
AT1G79260		nitrobindin heme-binding domain protein
AT1G79270	*EVOLUTIONARILY CONSERVED C-TERMINAL REGION 8 (ECT8)*	evolutionarily conserved C-terminal region 8
AT1G79310	*METACASPASE 7 (MC7)*	Encodes a putative metacaspase. Arabidopsis contains three type I MCP genes (MCP1a-c) and six type II MCP genes (MCP2a?f): AtMCP1a/At5g64240, AtMCP1b/At1g02170, AtMCP1c/At4g25110, AtMCP2a/At1g79310, AtMCP2b/At1g79330, AtMCP2c/At1g79320, AtMCP2d/At1g79340, AtMCP2e/At1g16420, AtMCP2f/At5g04200.
AT1G79320	*METACASPASE 6 (MC6)*	Encodes a putative metacaspase. Arabidopsis contains three type I MCP genes (MCP1a-c) and six type II MCP genes (MCP2a?f): AtMCP1a/At5g64240, AtMCP1b/At1g02170, AtMCP1c/At4g25110, AtMCP2a/At1g79310, AtMCP2b/At1g79330, AtMCP2c/At1g79320, AtMCP2d/At1g79340, AtMCP2e/At1g16420, AtMCP2f/At5g04200.
AT1G79330	*METACASPASE 5 (MC5)*	Metacaspase AtMCPb2/AMC6. Caspase family protein. Arginine/lysine-specific cysteine protease activity. Induces apoptosis in yeast. Contains Pfam domain, PF00656: ICE-like protease (caspase) p20 domain. Arabidopsis contains three type I MCP genes (MCP1a-c) and six type II MCP genes (MCP2a?f): AtMCP1a/At5g64240, AtMCP1b/At1g02170, AtMCP1c/At4g25110, AtMCP2a/At1g79310, AtMCP2b/At1g79330, AtMCP2c/At1g79320, AtMCP2d/At1g79340, AtMCP2e/At1g16420, AtMCP2f/At5g04200.
AT1G79340	*METACASPASE 4 (MC4)*	Encodes MCP2d, the predominant and constitutively expressed member of type II metacaspases (MCPs). MCP2d plays a positive regulatory role in biotic and abiotic stress-induced programmed cell death (PCD). Arabidopsis contains three type I MCP genes (MCP1a-c) and six type II MCP genes (MCP2a?f): AtMCP1a/At5g64240, AtMCP1b/At1g02170, AtMCP1c/At4g25110, AtMCP2a/At1g79310, AtMCP2b/At1g79330, AtMCP2c/At1g79320, AtMCP2d/At1g79340, AtMCP2e/At1g16420, AtMCP2f/At5g04200. The mRNA is cell-to-cell mobile.
AT1G79360	*ORGANIC CATION/CARNITINE TRANSPORTER 2 (OCT2)*	organic cation/carnitine transporter 2
AT1G79370	*CYTOCHROME P450, FAMILY 79, SUBFAMILY C, POLYPEPTIDE 1 (CYP79C1)*	member of CYP79C
AT1G79380	*RING DOMAIN LIGASE 4 (RGLG4)*	Encodes a ubiquitin ligase that is an essential upstream modulator of JA signaling in response to various stimuli.
AT1G79390		centrosomal protein
AT1G79400	*CATION/H+ EXCHANGER 2 (CHX2)*	member of Putative Na+/H+ antiporter family
AT1G79410	*ORGANIC CATION/CARNITINE TRANSPORTER5 (OCT5)*	organic cation/carnitine transporter5
AT1G79420	*BOUNDARY OF ROP DOMAIN2 (BDR2)*	C-type mannose receptor (DUF620)
AT1G79430	*ALTERED PHLOEM DEVELOPMENT (APL)*	Encodes gene product that is required for several aspects of phloem development in the root: (1) the specific divisions organizing the phloem pole, (2) sieve element differentiation and (3) the expression of a companion-specific gene. Mutant has a defect in the organization of phloem poles in the root. apl seedlings have a short, determinate root with only occasional lateral branches.
AT1G79440	*ALDEHYDE DEHYDROGENASE 5F1 (ALDH5F1)*	Encodes a mitochondrial succinic semialdehyde dehydrogenase (SSADH). Nomenclature according to Kirch, et al (2004).
AT1G79450	*ALA-INTERACTING SUBUNIT 5 (ALIS5)*	ALA-interacting subunit 5
AT1G79460	*GA REQUIRING 2 (GA2)*	Encodes for a protein with ent-kaurene synthase B activity which catalyzes the second step in the cyclization of GGPP to ent-kaurene in the gibberellins biosynthetic pathway.
AT1G79470		Aldolase-type TIM barrel family protein
AT1G79490	*EMBRYO DEFECTIVE 2217 (EMB2217)*	Pentatricopeptide repeat (PPR) superfamily protein
AT1G79510		hypothetical protein (DUF2358)
AT1G79520		Cation efflux family protein
AT1G79530	*GLYCERALDEHYDE-3-PHOSPHATE DEHYDROGENASE OF PLASTID 1 (GAPCP-1)*	Encodes one of the chloroplast/plastid localized GAPDH isoforms (GAPCp1/At1g79530 and GAPCp2/At1g16300). gapcp double mutants display a drastic phenotype of arrested root development, dwarfism and sterility. GAPCps are important for the synthesis of serine in roots.
AT1G79540		Pentatricopeptide repeat (PPR) superfamily protein
AT1G79580	*SOMBRERO (SMB)*	NAC-domain protein. Involved in root cap development. Involved in a regulatory feedback loop with FEZ. FEZ activates SMB in hte root cap daughter cells soon after division, and SMB in turn represses FEZ expression in these cells, thereby preventing further stem cell divisions.
AT1G79610	*NA+/H+ ANTIPORTER 6 (NHX6)*	Encodes an endosomal Na(+)/H(+) antiporter: AT1G54370 (NHX5), AT1G79610 (NHX6). Double knockout nhx5 nhx6 showed reduced growth, with smaller and fewer cells and increased sensitivity to salinity.
AT1G79615		
AT1G79620	*VASCULAR-RELATED RLK 1 (VRLK1)*	VRLK1 is a LRR kinase involved in switching between cell elongation and secondary cell wall thickening.VRLK1 is a member of a gene family that includes a small number of recently duplicated paralogs.
AT1G79640		Protein kinase superfamily protein
AT1G79660		ephrin-A3 protein
AT1G79670	*RESISTANCE TO FUSARIUM OXYSPORUM 1 (RFO1)*	Encodes a receptor-like kinase that does not contain an extracellular leucine-rich repeat domain. A novel type of dominant disease-resistance protein that confers resistance to a broad spectrum of Fusarium races.
AT1G79690	*NUDIX HYDROLASE HOMOLOG 3 (NUDT3)*	Encodes a dual activity enzyme which catalyses the hydrolysis of a peptide bond and of a phosphate bond, acting both as a dipeptidyl peptidase III and an atypical Nudix hydrolase.
AT1G79700	*WRINKLED 4 (WRI4)*	WRI4 encodes an AP2/ERF-type transcriptional activator that specifically controls cuticular wax biosynthesis in Arabidopsis stems. It also functions to activate transcription of genes involved fatty acid biosynthesis during seed and flower development as well as stem wax biosynthesis. Targets identified by ChIP-seq include: LACS1, KCR1, PAS2, ECR, and WSD1.
AT1G79710		Major facilitator superfamily protein
AT1G79720		Eukaryotic aspartyl protease family protein

AT1G79750	*NADP-MALIC ENZYME 4 (NADP-ME4)*	The malic enzyme (EC 1.1.1.40) encoded by AtNADP-ME4 is localized to chloroplasts. The gene is expressed throughout the whole plant and during embryogenesis and germination. A possible involvement in the fatty acid biosynthesis has been proposed.
AT1G79760	*DOWNSTREAM TARGET OF AGL15-4 (DTA4)*	Identified as target of the AGL15 binding motif CArG.
AT1G79770		CASP-like protein (DUF1677)
AT1G79790	*FLAVIN MONONUCLEOTIDE HYDROLASE 1 (FHY1)*	Encodes a chloroplast-localized FMN hydrolase that whose phosphatase activity is FMN-specific.
AT1G79800	*EARLY NODULIN-LIKE PROTEIN 7 (ENODL7)*	early nodulin-like protein 7
AT1G79810	*REVERSAL OF THE DET PHENOTYPE 3 (TED3)*	Dominant suppressor of det1 phenotypes. Encodes a peroxisomal protein essential for Arabidopsis growth. Inserted directly from the cytosol into peroxisomes.
AT1G79830	*GOLGIN CANDIDATE 5 (GC5)*	This gene is predicted to encode a protein that functions as a Golgi apparatus structural component known as a golgin in mammals and yeast. A fluorescently-tagged version of GC5 co-localizes with Golgi markers, and this localization appears to be replicated using the C-terminal (139 aa) portion of the protein. The C-terminal portion of the protein can also specifically interact with two members of the Rab family of GTPases (RabH1b and RabH1c).
AT1G79840	*GLABRA 2 (GL2)*	Glabra 2, a homeodomain protein affects epidermal cell identity including trichomes, root hairs, and seed coat. It also down-regulates seed oil content. Expressed in atrichoblasts and required to suppress root hair development. Also expressed abundantly during early seed development. Directly regulated by WER.
AT1G79850	*RIBOSOMAL PROTEIN S17 (RPS17)*	nuclear-encoded 30S chloroplast ribosomal protein S17
AT1G79860	*ROP (RHO OF PLANTS) GUANINE NUCLEOTIDE EXCHANGE FACTOR 12 (ROPGEF12)*	Encodes a member of KPP-like gene family, homolog of KPP (kinase partner protein) gene in tomato. Also a member of the RopGEF (guanine nucleotide exchange factor) family, containing the novel PRONE domain (plant-specific Rop nucleotide exchanger), which is exclusively active towards members of the Rop subfamily. Coexpression of AtPRK2a with AtRopGEF12 resulted in isotropic pollen tube growth Growth.
AT1G79870	*HYDROXYPHENYLPYRUVATE REDUCTASE 2 (HPPR2)*	Hydroxyphenylpyruvate reductase (HPPR), which catalyzes the reduction of 4-hydroxyphenylpyruvic acid (pHPP) to 4-hydroxyphenyllactic acid (pHPL). Together with HPPR3 and TAT1 involved in the biosynthesis of pHPL from tyrosine.
AT1G79880	*LA PROTEIN 2 (LA2)*	RNA recognition motif (RRM)-containing protein
AT1G79890		RAD3-like DNA-binding helicase protein
AT1G79910	*IST1-LIKE 8 (ISTL8)*	Regulator of Vps4 activity in the MVB pathway protein
AT1G79920	*HEAT SHOCK PROTEIN 70-15 (Hsp70-15)*	Heat shock protein 70 (Hsp 70) family protein
AT1G79940	*(ATERDJ2A)*	J domain protein localized in ER membrane. Mutants have defective pollen germination.
AT1G79960	*OVATE FAMILY PROTEIN 14 (OFP14)*	ovate family protein 14
AT1G79970		hypothetical protein
AT1G79990		coatomer subunit beta-2
AT1G80000	*CASC3/BARENTSZ ELF4AIII-BINDING PROTEIN (BTZ1)*	CASC3/Barentsz eIF4AIII binding protein
AT1G80030	*DNA J PROTEIN A7 (DJA7)*	Molecular chaperone Hsp40/DnaJ family protein
AT1G80050	*ADENINE PHOSPHORIBOSYL TRANSFERASE 2 (APT2)*	Encodes an adenosine phosphoribosyl transferase(E.C:2.4.2.7), a constitutively expressed enzyme involved in the one-step salvage of adenine to AMP. This isozyme has high affinity for cytokinins and is likely to be localized to the cytosol.
AT1G80060		Ubiquitin-like superfamily protein
AT1G80080	*TOO MANY MOUTHS (TMM)*	Encodes a transmembrane leucine-repeat containing receptor-like protein that is expressed in proliferative postprotodermal cells. Recessive mutation leads to disruption of asymmetric cell division during stomata development. Its transcript levels change after inducing MUTE expression in a mute background.
AT1G80090	*CBS DOMAIN CONTAINING PROTEIN 4 (CBSX4)*	Cystathionine beta-synthase (CBS) family protein
AT1G80100	*HISTIDINE PHOSPHOTRANSFER PROTEIN 6 (HP6)*	AHP6 lacks the conserved histidine residue (Asn83 in AHP6b), which is required for phosphotransfer, present in the other AHPs. AHP6 does not appear to have phosphotransfer activity. Acts as an inhibitor of cytokinin signaling by interacting with the phosphorelay machinery. Expressed in developing protoxylem and associated pericycle cell files. Negative regulator of cytokinin signaling. Expression is down-regulated by cytokinins. There are two alternatively spliced genes for this locus, AHP6a and AHP6b, differing in the length of the first exon. In ahp6-2 seedlings, only the AHP6a transcript is present. Members of the AHP gene family include: AT3G21510 (AHP1), AT3G29350 (AHP2), AT5G39340 (AHP3), AT3G16360 (AHP4), AT1G03430 (AHP5) and AT1G80100 (AHP6).
AT1G80110	*PHLOEM PROTEIN 2-B11 (PP2-B11)*	phloem protein 2-B11
AT1G80130		Tetratricopeptide repeat (TPR)-like superfamily protein
AT1G80140		Pectin lyase-like superfamily protein
AT1G80160	*GLYOXYLASE I 7 (GLYI7)*	Vicinal oxygen chelate (VOC) superfamily member.
AT1G80170		Pectin lyase-like superfamily protein
AT1G80180	*MAPK SUBSTRATES IN THE STOMATAL LINEAGE 1 (MASS1)*	Encodes a substrate of the MAPK kinases. Phenotypic analyses of Arabidopsis expressing phosphorylation site mutant forms of At1g80180.1 showed clustered stomata and higher stomatal index in cotyledons expressing the phosphomimetic form of At1g80180.1. Tightly connected with MAPK signaling to fine-tune stomatal production and patterning.
AT1G80190	*PARTNER OF SLD FIVE 1 (PSF1)*	Similar to the PSF1 component of GINS complex, which in other organism was shown to be involved in the initiation of DNA replication.
AT1G80230		Rubredoxin-like superfamily protein
AT1G80240	*DUF642 L-GALL RESPONSIVE GENE 1 (DGR1)*	DUF642 gene
AT1G80245		Spc97 / Spc98 family of spindle pole body (SBP) component
AT1G80270	*PENTATRICOPEPTIDE REPEAT 596 (PPR596)*	PENTATRICOPEPTIDE REPEAT 596
AT1G80280		alpha/beta-Hydrolases superfamily protein
AT1G80290		a member of the Glycosyltransferase Family 64 (according to CAZy Database)

AT1G80310	*MOLYBDATE TRANSPORTER 2 (MOT2)*	MOT2 encodes a molybdate transporter which locates to the vacuolar membrane. Loss-of-function (knock out) mutants show elevated molydate levels in rosette leaves and in fully senescent leaves, but decreased MoO4 levels in seeds. Under conditions of molybdate deficiency leaves from mot2::tDNA mutants show strongly reduced nitrate reductase activity. The mot2 gene is slightly expressed in young and mature leaves, but strongly in senescing leaves. This observation points to a function of MOT2 in molybdate transfer from leaves to seeds during plant senescence.
AT1G80320		2-oxoglutarate (2OG) and Fe(II)-dependent oxygenase superfamily protein
AT1G80330	*GIBBERELLIN 3-OXIDASE 4 (GA3OX4)*	Encodes a protein with gibberellin 3-oxidase activity. The enzyme, expressed and purified in E.coli, was shown to catalyze the 3β-hydroxylation of GA20 into GA29.
AT1G80340	*GIBBERELLIN 3-OXIDASE 2 (GA3OX2)*	Encodes a protein with gibberellin 3 β-hydroxylase activity. The protein was heterologously expressed in E. coli and shown to catalyze the hydroxylation of both GA9 and GA20.
AT1G80350	*ECTOPIC ROOT HAIR 3 (ERH3)*	encodes a p60 katanin protein that is expressed throughout the plant. Required for the specification of cell fates from early in development (in the meristem) through differentiation and for normal postmitotic organization of cortical microtubules into transverse arrays in root epidermis cells. Mutants display cytoskeletal defects.
AT1G80360	*REVERSAL OF SAV3 PHENOTYPE 1 (VAS1)*	Encodes a methionine-specific aminotransferase that uses the ethylene biosynthetic intermediate methionine as an amino donor and the auxin biosynthetic intermediate indole-3-pyruvic acid as an amino acceptor to produce L-tryptophan and 2-oxo-4-methylthiobutyric acid. These actions allow VAS1 to coordinate both auxin and ethylene biosynthesis. It functions downstream of TAA1/SAV3 but upstream of YUCs to negatively modulate IAA biosynthesis directly by altering the 3-IPA pool.
AT1G80380	*GLYCERATE KINASE (GLYK)*	encodes a glycerate kinase which catalyzes the last step of photorespiration C2 cycle.
AT1G80410	*NAA15 (NAA15)*	Encodes the catalytic subunit of a N-terminal acetyltransferase.
AT1G80440	*KISS ME DEADLY 1 (KMD1)*	Encodes a member of a family of F-box proteins, called the KISS ME DEADLY (KMD) family, that targets type-B ARR proteins for degradation and is involved in the negative regulation of the cytokinin response. Also named as KFB20, a member of a group of Kelch repeat F-box proteins that negatively regulate phenylpropanoid biosynthesis by targeting the phenypropanoid biosynthesis enzyme phenylalanine ammonia-lyase. The mRNA is cell-to-cell mobile.
AT1G80450		VQ motif-containing protein
AT1G80520		Sterile alpha motif (SAM) domain-containing protein
AT1G80550		Pentatricopeptide repeat (PPR) superfamily protein
AT1G80560	*ISOPROPYLMALATE DEHYDROGENASE 2 (IMD2)*	The AtIMD2 is one out of 3 genes encoding the enzyme 3-isopropylmalate dehydrogenase involved in leucine biosynthesis in Arabidopsis. Its subcellular location has been targeted to plastids. The mRNA is cell-to-cell mobile.
AT1G80570		RNI-like superfamily protein
AT1G80580		encodes a member of the ERF (ethylene response factor) subfamily B-1 of ERF/AP2 transcription factor family. The protein contains one AP2 domain. There are 15 members in this subfamily including ATERF-3, ATERF-4, ATERF-7, and leafy petiole.
AT1G80615		
AT1G80660	*H(+)-ATPASE 9 (HA9)*	H[+]-ATPase 9
AT1G80680	*SUPPRESSOR OF AUXIN RESISTANCE 3 (SAR3)*	Mutant has early-flowering phenotype, encodes a putative nucleoporin. Required for the activation of downstream defense pathways by the snc1 mutation. Involved in basal resistance against bacterial pathogens.
AT1G80690		PPPDE putative thiol peptidase family protein
AT1G80700		stress response NST1-like protein
AT1G80720		Mitochondrial glycoprotein family protein
AT1G80730	*ZINC-FINGER PROTEIN 1 (ZFP1)*	Encodes a zinc finger protein and is expressed at high levels in the shoot apex, including the apical meristem, developing leaves and the developing vascular system. expression induced three days post germination. T-DNA insertion mutant has a dominant phenotype in leaf initiation.
AT1G80750	*(RPL7A)*	Cytosolic ribosomal 60S subunit protein.
AT1G80760	*NOD26-LIKE INTRINSIC PROTEIN 6;1 (NIP6;1)*	Encodes a protein with boron transporter activity. It helps to preferentially direct boron to young developing tissues in the shoot, such as immature leaves, under low boron conditions. This boron channel appears to be impermeable to water, unlike the closely related NIP5;1 boron transporter. This protein also allows the transport of glycerol, urea, and formimide but not larger uncharged solutes such as arabitol and sucrose when it is expressed heterologously.
AT1G80780	*CCR4-ASSOCIATED FACTOR 1J (CAF1J)*	Deadenylase.
AT1G80790	*FACTOR OF DNA METHYLATION 5 (FDM5)*	Belongs to a subgroup of SGS3-like proteins that act redundantly in RNA-directed DNA methylation: AT1G15910 (FDM1), AT4G00380 (FDM2), AT3G12550 (FDM3), AT1G13790 (FDM4), AT1G80790 (FDM5). The mRNA is cell-to-cell mobile.
AT1G80800		pseudogene of Ribosomal protein L7Ae/L30e/S12e/Gadd45 family protein
AT1G80810	*(PDS5D)*	One of 5 PO76/PDS5 cohesion cofactor orthologs of Arabidopsis.
AT1G80820	*CINNAMOYL COA REDUCTASE (CCR2)*	Encodes an cinnamoyl CoA reductase isoform. Involved in lignin biosynthesis.
AT1G80830	*NATURAL RESISTANCE-ASSOCIATED MACROPHAGE PROTEIN 1 (NRAMP1)*	Thought to be involved in iron homeostasis. Induced in leaves in response to iron deficiency. Transgenic plants accumulate toxic levels of iron. Gene complements yeast iron uptake mutants.
AT1G80840	*WRKY DNA-BINDING PROTEIN 40 (WRKY40)*	Pathogen-induced transcription factor. Binds W-box sequences in vitro. Forms protein complexes with itself and with WRKY40 and WRKY60. Coexpression with WRKY18 or WRKY60 made plants more susceptible to both P. syringae and B. cinerea. WRKY18, WRKY40, and WRKY60 have partially redundant roles in response to the hemibiotrophic bacterial pathogen Pseudomonas syringae and the necrotrophic fungal pathogen Botrytis cinerea, with WRKY18 playing a more important role than the other two. The mRNA is cell-to-cell mobile.
AT1G80850		DNA glycosylase superfamily protein
AT1G80870		Protein kinase superfamily protein
AT1G80910	*CALCIUM CAFFEINE ZINC SENSITIVITY 1B (CCZ1B)*	vacuolar fusion CCZ1-like protein (DUF1712)
AT1G80920	*(J8)*	A nuclear encoded soluble protein found in the chloroplast stroma. Negatively regulated by light and has rapid turnover in darkness.

AT1G80930		MIF4G domain-containing protein / MA3 domain-containing protein
AT1G80940		Snf1 kinase interactor-like protein
AT1G80960		F-box and Leucine Rich Repeat domains containing protein
AT1G80970		XH domain-containing protein
AT2G01100		FAM133-like protein
AT2G01110	*ALBINO AND PALE GREEN 2 (APG2)*	mutant is Albino and pale green; Chloroplast Protein Translocation (tatC). Core subunit of the chloroplast Tat translocase. Integral chloroplast thylakoid membrane protein.
AT2G01120	*ORIGIN RECOGNITION COMPLEX SUBUNIT 4 (ORC4)*	Origin Recognition Complex subunit 4. Involved in the initiation of DNA replication. Regulated transcriptionally during cell cycle, peaking at G1/S-phase. Target of E2F/DF family of transcription factors. Interacts with all ORC subunits except ORC1b.
AT2G01130		DEA(D/H)-box RNA helicase family protein
AT2G01180	*PHOSPHATIDIC ACID PHOSPHATASE 1 (PAP1)*	Encodes phosphatidate phosphatase. Up-regulated by genotoxic stress (gamma ray or UV-B) and elicitor treatments with mastoparan and harpin. Expressed in roots and leaves.
AT2G01190	*PIGMENT DEFECTIVE 331 (PDE331)*	Octicosapeptide/Phox/Bem1p family protein
AT2G01200	*INDOLE-3-ACETIC ACID INDUCIBLE 32 (IAA32)*	Belongs to auxin inducible gene family.
AT2G01210	*ZYGOTIC ARREST 1 (ZAR1)*	ZAR1 encodes a plasma membrane localized leucine-rich repeat receptor-like kinase (LRR-RLK) that contains a putative CaM-binding domain and a Gβ-binding motif within its intracellular kinase region. Homozygous of function mutations are embryo-lethal and fail to properly make the first asymmetric division of the zygote. ZAR1 interacts with both CaM and Gβ in vivo and that interaction activates ZAR1 kinase activity.
AT2G01240		reticulon-like protein B15
AT2G01260		hypothetical protein (DUF789)
AT2G01270	*QUIESCIN-SULFHYDRYL OXIDASE 2 (QSOX2)*	Encodes a protein disulfide isomerase-like (PDIL) protein, a member of a multigene family within the thioredoxin (TRX) superfamily. This protein also belongs to the quiescin-sulfhydryl oxidase (QSOX) family, which possess an Erv1-like domain at the COOH terminus in addition to a TRX domain.
AT2G01280	*MATERNAL EFFECT EMBRYO ARREST 65 (MEE65)*	Involved in regulation of thermo tolerance.
AT2G01290	*RIBOSE-5-PHOSPHATE ISOMERASE 2 (RPI2)*	Cytosolic ribose-5-phosphate isomerase. Knockout mutation causes chloroplast dysfunction, late flowering and premature cell death.
AT2G01300		mediator of RNA polymerase II transcription subunit
AT2G01310		hypothetical protein
AT2G01320	*ATP-BINDING CASSETTE G7 (ABCG7)*	ABC-2 type transporter family protein
AT2G01330	*ACTIN-INTERACTING PROTEIN 1-1 (AIP1-1)*	nucleotide binding protein
AT2G01380		
AT2G01390	*EMBRYO DEFECTIVE 3111 (EMB3111)*	Tetratricopeptide repeat (TPR)-like superfamily protein
AT2G01400		hypothetical protein
AT2G01410		NHL domain-containing protein
AT2G01420	*PIN-FORMED 4 (PIN4)*	Encodes a putative auxin efflux carrier that is localized in developing and mature root meristems. It is involved in the maintenance of embryonic auxin gradients. A role for AtPIN4 in generating a sink for auxin below the quiescent center of the root meristem that is essential for auxin distribution and patterning is proposed. In the root, PIN4 is detected around the quiescent center and cells surrounding it, and localizes basally in provascular cells. PIN4 expression is upregulated in brassinosteroid-insensitive mutant (PMID 16141452).
AT2G01430	*HOMEOBOX-LEUCINE ZIPPER PROTEIN 17 (HB17)*	ATHB17 is a member of the HD-Zip transcription factor family. It is expressed most strongly in roots at different stages of development and induced by ABA, paraquat, drought, and NaCl treatments. Loss of function mutants are more sensitive to salt and drought stress.The protein is nuclear localized and has been shown to bind to the promoter of SIG5 and other genes.
AT2G01460		P-loop containing nucleoside triphosphate hydrolases superfamily protein
AT2G01480	*ESMERALDA1 (ESMD1)*	ESMD1 is a golgi localized putative O-fucosyltransferase.
AT2G01490	*PHYTANOYL-COA 2-HYDROXYLASE (PAHX)*	Encodes a phytanoyl-CoA 2-hydroxylase (PAHX). The mRNA is cell-to-cell mobile.
AT2G01500	*PRETTY FEW SEEDS 2 (PFS2)*	PFS2 encodes a homeodomain gene that is a member of the WUS clade of transcription factors. It delays differentiation and maturation of primordia and regulates ovule patterning. The pfs2 mutant exhibits developmental defects in the maternal integuments and gametophyte, specifically, the boundary between the chalaza and the nucellus shifted towards the distal end of pfs2 ovule primordia. In addition, leaves displayed curling and petals were wavy and crenulated. Overexpression of PFS2 affects floral organ and leaf development. Single- and double-mutant analyses reveal that PFS2 activity represses AGAMOUS expression in young floral primordia. Also involved in regulation of response to low temperature.
AT2G01510		Tetratricopeptide repeat (TPR)-like superfamily protein
AT2G01520	*MLP-LIKE PROTEIN 328 (MLP328)*	Encodes a cis-cinnamic acid responsive gene that is a member of the major latex protein-like gene family and plays a role in promoting vegetative growth and delaying ﬂowering. The mRNA is cell-to-cell mobile.
AT2G01530	*MLP-LIKE PROTEIN 329 (MLP329)*	MLP-like protein 329
AT2G01540	*C2-DOMAIN ABA-RELATED 10 (CAR10)*	Calcium-dependent lipid-binding (CaLB domain) family protein
AT2G01550		transposable_element_gene;non-LTR retrotransposon family (LINE), has a 4.9e-48 P-value blast match to GB:AAA39398 ORF2 (Mus musculus) (LINE-element);(source:TAIR10)
AT2G01570	*REPRESSOR OF GA1-3 1 (RGA1)*	Member of the VHIID/DELLA regulatory family. Contains homopolymeric serine and threonine residues, a putative nuclear localization signal, leucine heptad repeats, and an LXXLL motif. Putative transcriptional regulator repressing the gibberellin response and integration of phytohormone signalling. DELLAs repress cell proliferation and expansion that drives plant growth. The protein undergoes degradation in response to GA via the 26S proteasome. RGA1 binds to PIF3 and inhibits its DNA binding activity and thus affects the expression of PIF3 regulated genes. RGA may be involved in reducing ROS accumulation in response to stress by up-regulating the transcription of superoxide dismutases. Represses GA-induced vegetative growth and floral initiation. Rapidly degraded in response to GA. Involved in fruit and flower development.

AT2G01580		transmembrane protein
AT2G01590	*CHLORORESPIRATORY REDUCTION 3 (CRR3)*	Likely a subunit of the chloroplast NAD(P)H dehydrogenase complex, involved in PSI cyclic electron transport. Located in the membrane fraction of chloroplast. Mutant has impaired NAD(P)H dehydrogenase activity.
AT2G01610		Plant invertase/pectin methylesterase inhibitor superfamily protein
AT2G01650	*PLANT UBX DOMAIN-CONTAINING PROTEIN 2 (PUX2)*	encodes a peripheral membrane protein that contains UBX domain and interacts with AtCDC48 in vitro and co-fractionates with membrane-associated but not soluble AtCDC48 in vivo.
AT2G01660	*PLASMODESMATA-LOCATED PROTEIN 6 (PDLP6)*	Encodes a plasmodesmal protein that may be involved in the intercellular movement of molecules through the plasmodesmata. The protein has two DUF26 domains and a single transmembrane domain.
AT2G01670	*NUDIX HYDROLASE HOMOLOG 17 (NUDT17)*	nudix hydrolase homolog 17
AT2G01680		Ankyrin repeat family protein
AT2G01700		transposable_element_gene;similar to unknown protein [Arabidopsis thaliana] (TAIR:AT1G26860.1);(source:TAIR10)
AT2G01710	*SUVH1/3-INTERACTING DNAJ DOMAIN-CONTAINING PROTEIN 2 (SDJ2)*	SDJ2 functions partially redundantly with SDJ1 and SDJ3 and interacts with SUVH1 and SUVH3 to form a SUVH-SDJ complex. The complex binds promoters with DNA methylation and mediates transcriptional activation of promoter methylated genes.
AT2G01760	*RESPONSE REGULATOR 14 (RR14)*	member of Response Regulator: B- Type
AT2G01770	*VACUOLAR IRON TRANSPORTER 1 (VIT1)*	Encodes an iron transporter required for iron sequestration into vacuoles. Expressed in developing embryo and seed. Localized in the vacuolar membrane.
AT2G01780		Curculin-like (mannose-binding) lectin family protein
AT2G01790		TRAF-like family protein
AT2G01800		COP1-interacting protein-like protein
AT2G01810		RING/FYVE/PHD zinc finger superfamily protein
AT2G01830	*WOODEN LEG (WOL)*	Histidine kinase: cytokinin-binding receptor that transduces cytokinin signals across the plasma membrane
AT2G01840		transposable_element_gene;non-LTR retrotransposon family (LINE), has a 9.6e-34 P-value blast match to GB:NP_038607 L1 repeat, Tf subfamily, member 9 (LINE-element) (Mus musculus);(source:TAIR10)
AT2G01850	*ENDOXYLOGLUCAN TRANSFERASE A3 (EXGT-A3)*	EXGT-A3 has homology to xyloglucan endotransglucosylases/hydrolases (XTHs). Mutants in this gene show a lesion mimic phenotype associated with leaf maturation and a reduction in the number of tertiary veins. Individual tracheary elements in the mutants are shorter, but phloem transport activity is not severely affected. EXGT-A3 plays a role in xyloglucan degradation in the differentiating tracheary elements of rosette leaves. The mRNA is cell-to-cell mobile.
AT2G01870		transmembrane protein
AT2G01880	*PURPLE ACID PHOSPHATASE 7 (PAP7)*	PEP complex component.
AT2G01890	*PURPLE ACID PHOSPHATASE 8 (PAP8)*	Encodes a purple acid phosphatase (PAP) belonging to the low molecular weight plant PAP group.
AT2G01900	*INOSITOL POLYPHOSPHATE PHOSPHATIDYLINOSITOL 5-PHOSPHATASE9 (T5PTASE9)*	Encodes an inositol polyphosphate phosphatidylinositol 5-phosphatase that is expressed in roots and is involved in mediating salt tolerance through endocytosis.
AT2G01910	*(ATMAP65-6)*	Binds microtubules. Induces a crisscross mesh of microtubules, not bundles. Not involved in microtubule polymerization nor nucleation. Localizes to mitochondria. The mRNA is cell-to-cell mobile.
AT2G01920	*(PICALM9C)*	ENTH/VHS/GAT family protein
AT2G01930	*BASIC PENTACYSTEINE1 (BPC1)*	BASIC PENTACYSTEINE1 (BPC1) is a regulator of the homeotic Arabidopsis thaliana gene SEEDSTICK (STK), which controls ovule identity. BPC1 induces conformational changes by cooperative binding to purine-rich elements present in the STK regulatory sequence. STK is upregulated in bpc1 mutant.Along with BPC2, BPC1 binds to the promoter of and represses GALS1 thereby reducing beta 1,4- galactan accumulation.
AT2G01940	*SHOOT GRAVITROPISM 5 (SGR5)*	Encodes a transcription factor that, together with IDD14 and IDD16, regulates auxin biosynthesis and transport and thus aerial organ morphogenesis and gravitropic responses. May be involved in an early event in shoot gravitropism such as gravity perception and/or a signaling process subsequent to amyloplast sedimentation as a putative transcription factor in gravity-perceptive cells.
AT2G01950	*BRI1-LIKE 2 (BRL2)*	Encodes a leucine rich repeat receptor kinase and associated with provascular/procambial cells. Similar to BRI, brassinosteroid receptor protein.
AT2G01960	*TETRASPANIN14 (TET14)*	Member of TETRASPANIN family
AT2G01990		XRI1-like protein
AT2G02010	*GLUTAMATE DECARBOXYLASE 4 (GAD4)*	glutamate decarboxylase 4
AT2G02020	*NRT1/ PTR FAMILY 8.4 (NPF8.4)*	Major facilitator superfamily protein
AT2G02050		NADH-ubiquinone oxidoreductase B18 subunit
AT2G02060		Homeodomain-like superfamily protein
AT2G02070	*INDETERMINATE(ID)-DOMAIN 5 (IDD5)*	RAVEN is part of the network regulated by BLJUEJAY, JACKDAW, SACRECROW and SHORT-ROOT to regulate root tissue patterning through cell lineage specification and asymmetric cell division. RAVEN is directly activated by SHORT-ROOT and directly repressed by JACKDAW.
AT2G02080	*INDETERMINATE(ID)-DOMAIN 4 (IDD4)*	C2H2 BIRD transcription factor family.
AT2G02100	*LOW-MOLECULAR-WEIGHT CYSTEINE-RICH 69 (LCR69)*	Predicted to encode a PR (pathogenesis-related) protein. Belongs to the plant defensin (PDF) family with the following members: At1g75830/PDF1.1, At5g44420/PDF1.2a, At2g26020/PDF1.2b, At5g44430/PDF1.2c, At2g26010/PDF1.3, At1g19610/PDF1.4, At1g55010/PDF1.5, At2g02120/PDF2.1, At2g02100/PDF2.2, At2g02130/PDF2.3, At1g61070/PDF2.4, At5g63660/PDF2.5, At2g02140/PDF2.6, At5g38330/PDF3.1 and At4g30070/PDF3.2. The mRNA is cell-to-cell mobile.
AT2G02120	*(PDF2.1)*	Predicted to encode a PR (pathogenesis-related) protein. Belongs to the plant defensin (PDF) family with the following members: At1g75830/PDF1.1, At5g44420/PDF1.2a, At2g26020/PDF1.2b, At5g44430/PDF1.2c, At2g26010/PDF1.3, At1g19610/PDF1.4, At1g55010/PDF1.5, At2g02120/PDF2.1, At2g02100/PDF2.2, At2g02130/PDF2.3, At1g61070/PDF2.4, At5g63660/PDF2.5, At2g02140/PDF2.6, At5g38330/PDF3.1 and At4g30070/PDF3.2. Mediates ammonium metabolism by regulatingglutamine synthetase activity.

AT2G02140	*LOW-MOLECULAR-WEIGHT CYSTEINE-RICH 72 (LCR72)*	Predicted to encode a PR (pathogenesis-related) protein. Belongs to the plant defensin (PDF) family with the following members: At1g75830/PDF1.1, At5g44420/PDF1.2a, At2g26020/PDF1.2b, At5g44430/PDF1.2c, At2g26010/PDF1.3, At1g19610/PDF1.4, At1g55010/PDF1.5, At2g02120/PDF2.1, At2g02100/PDF2.2, At2g02130/PDF2.3, At1g61070/PDF2.4, At5g63660/PDF2.5, At2g02140/PDF2.6, At5g38330/PDF3.1 and At4g30070/PDF3.2.
AT2G02160	*(ATC3H17)*	Non- tandem CCCH zinc finger protein.
AT2G02200		transposable_element_gene;pseudogene, hypothetical protein, similar to At2g04970, At2g15200, At1g32830, At2g14140, At3g30450, At4g03990, At5g34895, At3g47270;(source:TAIR10)
AT2G02210		transposable_element_gene;pseudogene, Ulp1 protease family, contains Pfam profile PF02902: Ulp1 protease family, C-terminal catalytic domain;(source:TAIR10)
AT2G02220	*PHYTOSULFOKIN RECEPTOR 1 (PSKR1)*	Encodes a protein interacting with phytosulfokine, a five amino acid sulfated peptide (YIYTQ). Contains dual guanylate cyclase and kinase catalytic activities that operate in vivo.
AT2G02250	*PHLOEM PROTEIN 2-B2 (PP2-B2)*	phloem protein 2-B2
AT2G02280	*PHLOEM PROTEIN 2-B4 (PP2-B4)*	phloem protein 2-B4
AT2G02290		Haloacid dehalogenase-like hydrolase (HAD) superfamily protein
AT2G02300	*PHLOEM PROTEIN 2-B5 (PP2-B5)*	phloem protein 2-B5
AT2G02340	*PHLOEM PROTEIN 2-B8 (PP2-B8)*	phloem protein 2-B8
AT2G02360	*PHLOEM PROTEIN 2-B10 (PP2-B10)*	Encodes an F-box protein containing a Nictaba-related lectin domain that can act as a carbohydrate-binding protein.Expression is induced by SA and pathogenic bacteria.
AT2G02380	*GLUTATHIONE S-TRANSFERASE (CLASS ZETA) 2 (GSTZ2)*	Encodes glutathione transferase belonging to the zeta class of GSTs. Naming convention according to Wagner et al. (2002).
AT2G02390	*GLUTATHIONE S-TRANSFERASE ZETA 1 (GSTZ1)*	Encodes glutathione transferase belonging to the zeta class of GSTs. Naming convention according to Wagner et al. (2002). The protein undergoes spontaneous thiolation following treatment with the oxidant tert-butylhydroperoxide. It functions in vitro as a maleylacetoacetate isomerase and is likely to be involved in tyrosine catabolism.
AT2G02400		NAD(P)-binding Rossmann-fold superfamily protein
AT2G02410		yacP-like NYN domain protein
AT2G02420		
AT2G02440		transmembrane protein
AT2G02450	*LONG VEGETATIVE PHASE 1 (LOV1)*	NAC domain containing protein 35
AT2G02470	*ALFIN-LIKE 6 (AL6)*	Encodes a member of the Alfin1-like family of nuclear-localized PHD (plant homeodomain) domain containing proteins that acts as a novel upstream regulator of root hair formation during Pi starvation. All AL proteins except AL3 bind to di- or trimethylated histone H3 (H3K4me3/2). Members of this family include: AT5G05610 (AL1), AT3G11200 (AL2), AT3G42790 (AL3), AT5G26210 (AL4), AT5G20510 (AL5), AT2G02470 (AL6), AT1G14510 (AL7).
AT2G02490		transmembrane protein
AT2G02500	*(ISPD)*	Encodes a protein with 4-Diphosphocytidyl-2C-methyl-D-erythritol synthase activity. The enzyme has an absolute requirement for divalent cations (Mg2+ reaches the highest catalytic activity).
AT2G02515		hypothetical protein
AT2G02520		RNA-directed DNA polymerase (reverse transcriptase)-related family protein
AT2G02530		
AT2G02540	*HOMEOBOX PROTEIN 21 (HB21)*	Zinc finger homeobox protein. Expressed in vascular tissue. In a yeast one hybrid system was not able to transactivate a reporter gene.
AT2G02550		PIN domain-like family protein
AT2G02580	*CYTOCHROME P450, FAMILY 71, SUBFAMILY B, POLYPEPTIDE 9 (CYP71B9)*	member of CYP71B
AT2G02590		small multi-drug export protein
AT2G02610		Cysteine/Histidine-rich C1 domain family protein
AT2G02620		Cysteine/Histidine-rich C1 domain family protein
AT2G02630		Cysteine/Histidine-rich C1 domain family protein
AT2G02640		Cysteine/Histidine-rich C1 domain family protein
AT2G02650		Ribonuclease H-like superfamily protein
AT2G02660		F-box associated ubiquitination effector family protein
AT2G02670		pseudogene of Cysteine/Histidine-rich C1 domain family protein
AT2G02680		Cysteine/Histidine-rich C1 domain family protein
AT2G02690		Cysteine/Histidine-rich C1 domain family protein
AT2G02700		Cysteine/Histidine-rich C1 domain family protein
AT2G02720		Pectate lyase family protein
AT2G02730		GRIP/coiled-coil protein, putative (DUF1664)
AT2G02740	*WHIRLY 3 (WHY3)*	Encodes a homolog of the potato p24 protein. It shares the conserved KGKAAL domain, a putative DNA-binding domain, with potato p24 and is localized to the plastid and not the nucleus.
AT2G02750		Pentatricopeptide repeat (PPR) superfamily protein
AT2G02770		4-phosphopantetheinyl transferase domain protein
AT2G02780		Leucine-rich repeat protein kinase family protein
AT2G02830		transposable_element_gene;copia-like retrotransposon family, has a 1.3e-37 P-value blast match to GB:CAA72990 open reading frame 2 (Ty1_Copia-element) (Brassica oleracea);(source:TAIR10)

AT2G02850	*PLANTACYANIN (ARPN)*	Encodes plantacyanin one of blue copper proteins. Involved in anther development and pollination. Expressed in the transmitting tract of the pistil.
AT2G02860	*SUCROSE TRANSPORTER 2 (SUT2)*	encodes a sucrose transporter in sieve elements and a number of sink tissues and cell types. Gene expression is induced by wounding.
AT2G02930	*GLUTATHIONE S-TRANSFERASE F3 (GSTF3)*	Encodes glutathione transferase belonging to the phi class of GSTs. Naming convention according to Wagner et al. (2002).
AT2G02950	*PHYTOCHROME KINASE SUBSTRATE 1 (PKS1)*	Encodes a basic soluble protein which can independently bind to either PHYA or PHYB, regardless of whether the phytochromes are in the Pr or Pfr state. PKS1 can be phosphorylated by oat phyA in vitro in a light regulated manner. It is postulated to be a negative regulator of phyB signalling.
AT2G02970	*APYRASE 6 (APY6)*	Encodes a putative apyrase involved in pollen exine pattern formation and anther dehiscence.
AT2G02990	*RIBONUCLEASE 1 (RNS1)*	Encodes a member of the ribonuclease T2 family that responds to inorganic phosphate starvation, and inhibits production of anthocyanin. Also involved in wound-induced signaling independent of jasmonic acid. Its expression is responsive to both phosphate (Pi) and phosphite (Phi) in roots.
AT2G03020		Heat shock protein HSP20/alpha crystallin family
AT2G03040		emp24/gp25L/p24 family/GOLD family protein
AT2G03060	*AGAMOUS-LIKE 30 (AGL30)*	Encodes a member of the MIKC (MADS box, Keratin binding domain, and C terminal domain containing)family of transcriptional regulators. AGL30 is expressed in pollen.It forms heterodimers with other MICK family members.
AT2G03080		transposable_element_gene;copia-like retrotransposon family, has a 1.4e-10 P-value blast match to GB:AAC24836 pol polyprotein (Ty1_Copia-element) (Candida albicans);(source:TAIR10)
AT2G03090	*EXPANSIN A15 (EXPA15)*	member of Alpha-Expansin Gene Family. Naming convention from the Expansin Working Group (Kende et al, 2004. Plant Mol Bio). Involved in the formation of nematode-induced syncytia in roots of Arabidopsis thaliana.
AT2G03110		putative RNA-binding protein
AT2G03130		Ribosomal protein L12/ ATP-dependent Clp protease adaptor protein ClpS family protein
AT2G03150	*SHORT ROOT IN SALT MEDIUM 1 (RSA1)*	Encodes a nuclear-localized calcium-binding protein RSA1 (SHORT ROOT IN SALT MEDIUM 1), which is required for salt tolerance.
AT2G03160	*SKP1-LIKE 19 (SK19)*	SKP1-like 19
AT2G03170	*SKP1-LIKE 14 (SK14)*	SKP1-like 14
AT2G03180		hypothetical protein
AT2G03190	*SKP1-LIKE 16 (SK16)*	one of SKP1 homologs. Gene is expressed specifically in the silique.
AT2G03200	*ATYPICAL ASPARTIC PROTEASE IN ROOTS 1 (ASPR1)*	Atypical aspartic protease which modulates lateral root development.
AT2G03210	*FUCOSYLTRANSFERASE 2 (FUT2)*	member of Glycosyltransferase Family- 37
AT2G03220	*FUCOSYLTRANSFERASE 1 (FT1)*	member of Glycosyltransferase Family- 37
AT2G03240		EXS (ERD1/XPR1/SYG1) family protein
AT2G03260		EXS (ERD1/XPR1/SYG1) family protein
AT2G03300	*(ATTX12)*	TX12 is a Toll/Interleukin-1 receptor domain containing protein. Misexpression results in ectopic activation of defense response genes.
AT2G03310		transmembrane protein
AT2G03320		hypothetical protein
AT2G03330		transmembrane protein
AT2G03360		Glycosyltransferase family 61 protein
AT2G03370		O-linked-mannose beta-1,4-N-acetylglucosaminyltransferase-like protein
AT2G03380	*PENTATRICOPEPTIDE REPEAT PROTEIN 96 (PPR96)*	Pentatricopeptide repeat (PPR) superfamily protein
AT2G03400		
AT2G03410		Mo25 family protein
AT2G03420		hypothetical protein
AT2G03460		Galactose oxidase/kelch repeat superfamily protein
AT2G03480	*QUASIMODO2 LIKE 2 (QUL2)*	Encodes QUASIMODO2 LIKE2 (QUL2), a paralog of QUASIMODO2 (QUA2). AT1G78240 (QUA2), AT1G13860 (QUL1) and AT2G03480 (QUL2) form a clade with a possible role in plant vasculature development.
AT2G03500	*EARLY FLOWERING MYB PROTEIN (EFM)*	Encodes a nuclear localized member of the MYB family of transcriptional regulators that is involved in negative regulation of flowering. It is expressed in vascular tissues and at low levels in the shoot apex during the transition to flowering. Loss of function mutations are early flowering.EFM is involved in the autonomous, thermosensory and GA pathways and expression is directly regulated by SVP. EFM interacts with JMJD5 to repress FT expression.
AT2G03520	*UREIDE PERMEASE 4 (UPS4)*	Encodes AtUPS4, a member of the Arabidopsis ureide permease family.
AT2G03530	*UREIDE PERMEASE 2 (UPS2)*	Mediate high-affinity uracil and 5-FU (a toxic uracil analogue) transport when expressed in yeast and Xenopus oocytes.Involved in allantoin transport.
AT2G03540		transposable_element_gene;similar to unknown protein [Arabidopsis thaliana] (TAIR:AT4G11710.1);(source:TAIR10)
AT2G03550		alpha/beta-Hydrolases superfamily protein
AT2G03560	*F-BOX/DUF295 BRASSICEAE-SPECIFIC 7 (ATFDB7)*	F-box only protein (DUF295)
AT2G03600	*UREIDE PERMEASE 3 (UPS3)*	Encodes UPS3 (ureide permease 3), similar to UPS1, an allantoin transporter.
AT2G03630		suppressor SRP40-like protein
AT2G03670	*CELL DIVISION CYCLE 48B (CDC48B)*	CDC48 - like protein AAA-type ATPaseCell. division control protein 48 homolog B
AT2G03710	*SEPALLATA 4 (SEP4)*	This gene belongs to the family of SEP genes. It is involved in the development of sepals, petals, stamens and carpels. Additionally, it plays a central role in the determination of flower meristem and organ identity.
AT2G03720	*MORPHOGENESIS OF ROOT HAIR 6 (MRH6)*	Involved in root hair development
AT2G03730	*ACT DOMAIN REPEAT 5 (ACR5)*	Member of a small family of ACT domain containing proteins. ACT domains are thought to be involved in amino acid binding.
AT2G03740	*LATE EMBRYOGENESIS ABUNDANT 11 (LEA11)*	Late embryogenesis abundant protein. Associates with and stabilizes membranes as part of cryoprotective response.
AT2G03750		P-loop containing nucleoside triphosphate hydrolases superfamily protein

AT2G03760	*SULPHOTRANSFERASE 12 (SOT12)*	Encodes a brassinosteroid sulfotransferase. In vitro experiements show that this enzyme has a preference for 24-epibrassinosteroids, particularly 24-epicathasterone, but does not act on castasterone and brassinolide. It also shows sulfating activity toward flavonoids. It is differentially expressed during development, being more abundant in young seedlings and actively growing cell cultures. Expression is induced in response to salicylic acid and methyl jasmonate and bacterial pathogens.
AT2G03770	*SULFOTRANSFERASE 202E1 (SULT202E1)*	Encodes a sulfotransferase with sulfating activity toward flavonoids, specifically kaempferol.
AT2G03800	*GEKO1 (GEK1)*	encodes a D-aminoacyl-tRNA deacylase. Involved in detoxification of D-aminoacyl-tRNA. Mutants also show ethanol-hypersensitive phenotype.
AT2G03820	*NONSENSE-MEDIATED MRNA DECAY 3 (NMD3)*	Encodes a protein involved in the nuclear export of the 60S ribosomal subunit and formation of the secondary cell wall.
AT2G03830	*ROOT MERISTEM GROWTH FACTOR 8 (RGF8)*	Encodes a root meristem growth factor (RGF). Belongs to a family of functionally redundant homologous peptides that are secreted, tyrosine-sulfated, and expressed mainly in the stem cell area and the innermost layer of central columella cells. RGFs are required for maintenance of the root stem cell niche and transit amplifying cell proliferation. Members of this family include: At5g60810 (RGF1), At1g13620 (RGF2), At2g04025 (RGF3), At3g30350 (RGF4), At5g51451 (RGF5), At4g16515 (RGF6), At3g02240 (RGF7), At2g03830 (RGF8) and At5g64770 (RGF9).
AT2G03840	*TETRASPANIN13 (TET13)*	TET13 encodes a member of the TETRASPANIN gene family that is expressed in the hypophysis, QC, root stem cells, lateral root primordia and is involved in primary root growth and lateral root development.
AT2G03850		Late embryogenesis abundant protein (LEA) family protein
AT2G03880	*REQUIRED FOR EFFICIENCY OF MITOCHONDRIAL EDITING 1 (REME1)*	Pentatricopeptide repeat (PPR) superfamily protein
AT2G03890	*PHOSPHOINOSITIDE 4-KINASE GAMMA 7 (PI4K GAMMA 7)*	Phosphoinositide kinase which undergo autophosphorylation and phosphorylate serine/threonine residues of protein substrates. Contains phosphoinositide 3/4-kinase and ubiquitin-like domains. The mRNA is cell-to-cell mobile.
AT2G03980		GDSL-motif esterase/acyltransferase/lipase. Enzyme group with broad substrate specificity that may catalyze acyltransfer or hydrolase reactions with lipid and non-lipid substrates.
AT2G04000		transposable_element_gene;similar to unknown protein [Arabidopsis thaliana] (TAIR:AT3G29700.1);(source:TAIR10)
AT2G04020		GDSL-motif esterase/acyltransferase/lipase. Enzyme group with broad substrate specificity that may catalyze acyltransfer or hydrolase reactions with lipid and non-lipid substrates.
AT2G04025	*ROOT MERISTEM GROWTH FACTOR 3 (RGF3)*	Encodes a root meristem growth factor (RGF). Belongs to a family of functionally redundant homologous peptides that are secreted, tyrosine-sulfated, and expressed mainly in the stem cell area and the innermost layer of central columella cells. RGFs are required for maintenance of the root stem cell niche and transit amplifying cell proliferation. Members of this family include: At5g60810 (RGF1), At1g13620 (RGF2), At2g04025 (RGF3), At3g30350 (RGF4), At5g51451 (RGF5), At4g16515 (RGF6), At3g02240 (RGF7), At2g03830 (RGF8) and At5g64770 (RGF9).
AT2G04032	*ZINC TRANSPORTER 7 PRECURSOR (ZIP7)*	zinc transporter 7 precursor
AT2G04038	*BASIC LEUCINE-ZIPPER 48 (bZIP48)*	basic leucine-zipper 48
AT2G04039	*(NDHV)*	NdhV is loosely associated with the NDH complex and is required for stabilizing NDH subcomplexes A and E.
AT2G04040	*DETOXIFICATION 1 (DTX1)*	AtDTX1 (At2g04040) has been identified as a detoxifying efflux carrier for plant-derived antibiotics and other toxic compounds, including Cd^{2+}. Expression in rosette leaves is activated by high concentration of boron.Mistakenly referred to as At2g04070 in PMID:11739388.
AT2G04050		MATE efflux family protein
AT2G04060		glycosyl hydrolase family 35 protein
AT2G04063	*GLYCINE AND PROLINE RICH PROTEIN 5 (GPRP5)*	glycine-rich protein
AT2G04070		Expression in rosette leaves is activated by high concentration of boron.
AT2G04080		MATE efflux family protein
AT2G04100		MATE efflux family protein
AT2G04160	*AUXIN-INDUCED IN ROOT CULTURES 3 (AIR3)*	isolated from differential screening of a cDNA library from auxin-treated root culture. encodes a protein similar to subtilisin-like serine protease which is believed to be active outside the plant cell.
AT2G04210		transposable_element_gene;similar to unknown protein [Arabidopsis thaliana] (TAIR:AT2G10070.2);(source:TAIR10)
AT2G04220		DUF868 family protein (DUF868)
AT2G04230		FBD, F-box and Leucine Rich Repeat domains containing protein
AT2G04270	*RNASE E/G-LIKE (RNEE/G)*	Similar to E.coli endoribonuclease E. Functions as a ribonuclease, is located in the chloroplast, and is involved in chloroplast development. Loss of function mutants are white and arrest at the cotyledon stage. The phenotype is rescued by providing sucrose.
AT2G04300		Leucine-rich repeat protein kinase family protein
AT2G04305		Magnesium transporter CorA-like family protein
AT2G04330		transposable_element_gene;similar to unknown protein [Arabidopsis thaliana] (TAIR:AT2G23480.1);(source:TAIR10)
AT2G04350	*LONG-CHAIN ACYL-COA SYNTHETASE 8 (LACS8)*	AMP-dependent synthetase and ligase family protein
AT2G04370		
AT2G04380		hypothetical protein
AT2G04400	*INDOLE-3-GLYCEROL PHOSPHATE SYNTHASE (IGPS)*	Acts during tryptophan biosynthesis controlled by ERF109.
AT2G04420		Polynucleotidyl transferase, ribonuclease H-like superfamily protein
AT2G04440		MutT/nudix family protein
AT2G04450	*NUDIX HYDROLASE HOMOLOG 6 (NUDT6)*	Encodes a protein with NADH pyrophosphatase activity. Although this protein was also shown to have ADP-ribose diphosphatase activity in vitro, mutant analyses suggest that NUDX6 is involved in NADH metabolism in vivo.
AT2G04460		transposable_element_gene;similar to unknown protein [Arabidopsis thaliana] (TAIR:AT2G10090.1);(source:TAIR10)
AT2G04480		hypothetical protein
AT2G04490		transposable_element_gene;copia-like retrotransposon family, has a 0. P-value blast match to GB:CAA72989 open reading frame 1 (Ty1_Copia-element) (Brassica oleracea);(source:TAIR10)
AT2G04500		Cysteine/Histidine-rich C1 domain family protein

AT2G04530	*(CPZ)*	Encodes a protein with RNAse Z activity suggesting a role in tRNA processing. Protein contains a signal sequence for import into the chloroplast.
AT2G04560	*LIPID X B (LPXB)*	transferases, transferring glycosyl groups
AT2G04570	*OCCLUDED STOMATAL PORES 1 (OSP1)*	GDSL-motif esterase/acyltransferase/lipase. Enzyme group with broad substrate specificity that may catalyze acyltransfer or hydrolase reactions with lipid and non-lipid substrates. The mRNA is cell-to-cell mobile.
AT2G04580		transposable_element_gene;similar to unknown protein [Arabidopsis thaliana] (TAIR:AT2G22440.1);(source:TAIR10)
AT2G04600		transposable_element_gene;similar to unknown protein [Arabidopsis thaliana] (TAIR:AT2G13865.1);(source:TAIR10)
AT2G04640		
AT2G04650	*KONJAC 2 (KJC2)*	KONJAC2 is imilar to sugar pyrophosphorylases but has an insertion of 2 AA in the pyrophosphorylase consensus motif that is highly conserved in GMPPs. KONJAC2 does not display GDP-mannose pyrophosphorylase activity. It can simulate GDP-mannose pyrophosphorylase activity of VTC1.
AT2G04670		transposable_element_gene;gypsy-like retrotransposon family, has a 1.2e-313 P-value blast match to GB:AAD27547 polyprotein (Gypsy_Ty3-element) (Oryza sativa subsp. indica);(source:TAIR10)
AT2G04675		hypothetical protein
AT2G04680		Cysteine/Histidine-rich C1 domain family protein
AT2G04690		Pyridoxamine 5-phosphate oxidase family protein
AT2G04710		transposable_element_gene;hAT-like transposase family (hobo/Ac/Tam3), has a 1.6e-29 P-value blast match to GB:CAA29005 ORFa of Maize Ac (hAT-element) (Zea mays);(source:TAIR10)
AT2G04720		pseudogene of GTP-binding 2
AT2G04730		pseudogene of F-box and associated interaction domains-containing protein
AT2G04740		ankyrin repeat family protein
AT2G04750	*(ATFIM3)*	Encodes a member of the fimbrin family. Different members of the fimbrin/plastin family have diverged biochemically during evolution to generate either tight actin bundles or loose networks with distinct biochemical and biophysical properties. FIM4 generates both actin bundles and branched actin filaments whereas FIM5 only generates actin bundles.
AT2G04760		transposable_element_gene;non-LTR retrotransposon family (LINE), has a 1.8e-38 P-value blast match to GB:NP_038605 L1 repeat, Tf subfamily, member 30 (LINE-element) (Mus musculus);(source:TAIR10)
AT2G04770		transposable_element_gene;CACTA-like transposase family (Ptta/En/Spm), has a 6.0e-20 P-value blast match to At5g29026.1/8-244 CACTA-like transposase family (Ptta/En/Spm) (CACTA-element) (Arabidopsis thaliana);(source:TAIR10)
AT2G04780	*FASCICLIN-LIKE ARABINOOGALACTAN 7 (FLA7)*	fasciclin-like arabinogalactan-protein 7 (Fla7). Possibly involved in embryogenesis and seed development.
AT2G04790		PTB domain engulfment adapter
AT2G04795		hypothetical protein
AT2G04800		hypothetical protein
AT2G04820		transposable_element_gene;copia-like retrotransposon family, has a 2.8e-214 P-value blast match to GB:AAC02666 polyprotein (Ty1_Copia-element) (Arabidopsis thaliana);(source:TAIR10)
AT2G04830	*F-BOX/DUF295 BRASSICEAE-SPECIFIC 10 (ATFDB10)*	F-box only protein (DUF295)
AT2G04840	*F-BOX/DUF295 BRASSICEAE-SPECIFIC 11 (ATFDB11)*	F-box only protein (DUF295)
AT2G04850		Auxin-responsive family protein
AT2G04860		Tetratricopeptide repeat (TPR)-like superfamily protein
AT2G04870		hypothetical protein
AT2G04880	*ZINC-DEPENDENT ACTIVATOR PROTEIN-1 (ZAP1)*	Encodes WRKY1, a member of the WRKY transcription factors in plants involved in disease resistance, abiotic stress, senescence as well as in some developmental processes. WRKY1 is involved in the salicylic acid signaling pathway. The crystal structure of the WRKY1 C-terminal domain revealed a zinc-binding site and identified the DNA-binding residues of WRKY1. The mRNA is cell-to-cell mobile.
AT2G04890	*SCARECROW-LIKE 21 (SCL21)*	Encodes a scarecrow-like protein (SCL21). Member of GRAS gene family.
AT2G04920		F-box and associated interaction domains-containing protein
AT2G04940		scramblase-like protein
AT2G04970		transposable_element_gene;similar to heat shock protein binding [Arabidopsis thaliana] (TAIR:AT1G32830.1);(source:TAIR10)
AT2G04980		transposable_element_gene;pseudogene, Ulp1 protease family, similar to At1g32840, At4g04010, At2g06430, At2g15140, At2g14130, At3g44500, At2g15190, At3g47260, At5g34900, At3g29210, At2g02210, At3g32900;(source:TAIR10)
AT2G04990		transposable_element_gene;pseudogene, hypothetical protein, contains Pfam profile PF03384: Drosophila protein of unknown function, DUF287;(source:TAIR10)
AT2G05000		transposable_element_gene;similar to unknown protein [Arabidopsis thaliana] (TAIR:AT3G47320.1);(source:TAIR10)
AT2G05010		transposable_element_gene;Mutator-like transposase family, has a 3.6e-93 P-value blast match to Q9SI25 /181-349 Pfam PF03108 MuDR family transposase (MuDr-element domain);(source:TAIR10)
AT2G05020		transposable_element_gene;pseudogene, similar to putative transposable element, blastp match of 61%25 identity and 9.4e-140 P-value to GP|13122426|dbj|BAB32907.1||AP003047 putative transposable element {Oryza sativa (japonica cultivar-group)};(source:TAIR10)
AT2G05030		transposable_element_gene;contains domain GAG/POL/ENV POLYPROTEIN (PTHR10178);(source:TAIR10)
AT2G05070	*PHOTOSYSTEM II LIGHT HARVESTING COMPLEX GENE 2.2 (LHCB2.2)*	Encodes Lhcb2.2. Belongs to the Lhc super-gene family encodes the light-harvesting chlorophyll a/b-binding (LHC) proteins that constitute the antenna system of the photosynthetic apparatus.
AT2G05080		transposable_element_gene;similar to unknown protein [Arabidopsis thaliana] (TAIR:AT3G13250.1);(source:TAIR10)
AT2G05090		transposable_element_gene;similar to unknown protein [Arabidopsis thaliana] (TAIR:AT5G37080.1);(source:TAIR10)
AT2G05120	*(NUP133)*	Nucleoporin, Nup133/Nup155-like protein
AT2G05160		CCCH-type zinc fingerfamily protein with RNA-binding domain-containing protein

AT2G05200		transposable_element_gene;non-LTR retrotransposon family (LINE), has a 8.3e-42 P-value blast match to GB:NP_038605 L1 repeat, Tf subfamily, member 30 (LINE-element) (Mus musculus);(source:TAIR10)
AT2G05270		hypothetical protein
AT2G05290		transposable_element_gene;similar to unknown protein [Arabidopsis thaliana] (TAIR:AT2G41570.1);(source:TAIR10)
AT2G05310		transmembrane protein
AT2G05320	*N-ACETYLGLUCOSAMINYLTRANSFERASE II (GNT-II)*	beta-1,2-N-acetylglucosaminyltransferase II
AT2G05350		hypothetical protein
AT2G05370		RNA-dependent RNA polymerase-like protein
AT2G05380	*GLYCINE-RICH PROTEIN 3 SHORT ISOFORM (GRP3S)*	glycine-rich protein 3 short isoform (GRP3S) mRNA, complete The mRNA is cell-to-cell mobile.
AT2G05390		transposable_element_gene;copia-like retrotransposon family, has a 7.2e-200 P-value blast match to gb|AAG52949.1| gag/pol polyprotein (Endovir1-1) (Arabidopsis thaliana) (Ty1_Copia-family);(source:TAIR10)
AT2G05400		Ubiquitin-specific protease family C19-related protein
AT2G05430		Ubiquitin-specific protease family C19-related protein
AT2G05450		transposable_element_gene;pseudogene, Ulp1 protease family, contains Pfam profile PF02902: Ulp1 protease family, C-terminal catalytic domain;(source:TAIR10)
AT2G05460		pseudogene of Ulp1 protease family protein
AT2G05470		transposable_element_gene;pseudogene, hypothetical protein, similar to At2g06630, At2g12120, At1g45090, At5g28482;(source:TAIR10)
AT2G05500		
AT2G05510		Glycine-rich protein family
AT2G05520	*GLYCINE-RICH PROTEIN 3 (GRP-3)*	Encodes a glycine-rich protein that is expressed mainly in stems and leaves. AtGRP3 functions in root size determination during development and in Al stress. mRNA levels are upregulated in response to ABA, salicylic acid and ethylene but downregulated in response to desiccation. The mRNA is cell-to-cell mobile.
AT2G05530		Glycine-rich protein family
AT2G05540		Glycine-rich protein family
AT2G05550		transposable_element_gene;non-LTR retrotransposon family (LINE), has a 6.6e-37 P-value blast match to GB:NP_038605 L1 repeat, Tf subfamily, member 30 (LINE-element) (Mus musculus);(source:TAIR10)
AT2G05560		transposable_element_gene;pseudogene, Ulp1 protease family, contains Pfam profile PF02902: Ulp1 protease family, C-terminal catalytic domain;(source:TAIR10)
AT2G05580		Glycine-rich protein family
AT2G05600		F-box associated ubiquitination effector family protein
AT2G05610		transposable_element_gene;gypsy-like retrotransposon family, has a 1.2e-179 P-value blast match to GB:AAD27547 polyprotein (Gypsy_Ty3-element) (Oryza sativa subsp. indica);(source:TAIR10)
AT2G05620	*PROTON GRADIENT REGULATION 5 (PGR5)*	Involved in electron flow in Photosystem I. Essential for photoprotection.
AT2G05630	*(ATG8D)*	in the Arabidopsis autophagy pathway
AT2G05640		transposable_element_gene;pseudogene, similar to putative helicase, low similarity to SP|Q9UUA2 DNA repair and recombination protein pif1, mitochondrial precursor {Schizosaccharomyces pombe};(source:TAIR10)
AT2G05650		
AT2G05710	*ACONITASE 3 (ACO3)*	Encodes an aconitase that can catalyze the conversion of citrate to isocitrate through a cis-aconitate intermediate, indicating that it may participate in the TCA cycle and other primary metabolic pathways. The protein is believed to accumulate in the mitochondria and the cytosol. It affects CSD2 (At2g28190 - a superoxide dismutase) transcript levels and may play a role in the response to oxidative stress. One member of the family (ACO1 - At35830) was shown to specifically bind to the 5' UTR of CSD2 in vitro. ACO3 is tyrosine-phosphorylated and its phosphorylation state is modulated in response to ABA in Arabidopsis thaliana seeds. The mRNA is cell-to-cell mobile.
AT2G05720		Transducin/WD40 repeat-like superfamily protein
AT2G05760	*NUCLEOBASE ASCORBATE TRANSPORTER 1 (NAT1)*	Xanthine/uracil permease family protein
AT2G05790		O-Glycosyl hydrolases family 17 protein
AT2G05810		ARM repeat superfamily protein
AT2G05820		transposable_element_gene;CACTA-like transposase family (Tnp2/En/Spm), has a 6.4e-71 P-value blast match to gb|AAG52024.1|AC022456_5 Tam1-homologous transposon protein TNP2, putative;(source:TAIR10)
AT2G05830	*5-METHYLTHIORIBOSE KINASE 1 (MTI1)*	Encodes a 5-methylthioribose-1-phosphate isomerase.
AT2G05840	*20S PROTEASOME SUBUNIT PAA2 (PAA2)*	Encodes 20S proteasome subunit PAA2 (PAA2).
AT2G05850	*SERINE CARBOXYPEPTIDASE-LIKE 38 (scpl38)*	serine carboxypeptidase-like 38
AT2G05870		transposable_element_gene;similar to cytochrome P-450 aromatase-related [Arabidopsis thaliana] (TAIR:AT4G07435.1);(source:TAIR10)
AT2G05880		transposable_element_gene;similar to replication protein-related [Arabidopsis thaliana] (TAIR:AT5G35260.1);(source:TAIR10)
AT2G05890		transposable_element_gene;similar to unknown protein [Arabidopsis thaliana] (TAIR:AT3G30550.1);(source:TAIR10)
AT2G05900	*SET DOMAIN PROTEIN 11 (SDG11)*	Predicted to encodes a SU(VAR)3-9 homolog, a SET domain protein. Known SET domain proteins are involved in epigenetic control of gene expression and act as histone methyltransferases. There are 10 SUVH genes in Arabidopsis and members of this subfamily of the SET proteins have an additional conserved SRA domain.
AT2G05910		LURP-one-like protein (DUF567)
AT2G05930		transposable_element_gene;copia-like retrotransposon family, has a 0. P-value blast match to gb|AAG52949.1| gag/pol polyprotein (Endovir1-1) (Arabidopsis thaliana) (Ty1_Copia-family);(source:TAIR10)

AT2G05940	*RPM1-INDUCED PROTEIN KINASE (RIPK)*	Encodes a receptor-like cytoplasmic kinase that phosphorylates the host target RIN4, leading to the activation of a plant innate immune receptor RPM1.
AT2G05950		transposable_element_gene;similar to unknown protein [Arabidopsis thaliana] (TAIR:AT3G32050.1);(source:TAIR10)
AT2G05960		transposable_element_gene;copia-like retrotransposon family, has a 6.2e-200 P-value blast match to gb|AAG52949.1| gag/pol polyprotein (Endovir1-1) (Arabidopsis thaliana) (Ty1_Copia-family);(source:TAIR10)
AT2G05980		transposable_element_gene;non-LTR retrotransposon family (LINE), has a 1.0e-42 P-value blast match to GB:NP_038607 L1 repeat, Tf subfamily, member 9 (LINE-element) (Mus musculus);(source:TAIR10)
AT2G05990	*MOSAIC DEATH 1 (MOD1)*	Encodes enoyl-ACP reductase a component of the fatty acid synthase complex. A reduced function mutation in this gene, mod1, was found in a screen for premature cell death mutants. Mutant plants have reduced lipid level and pleiotropic morphological defects, including chlorotic and abnormally shaped leaves. The mRNA is cell-to-cell mobile.
AT2G06000		Pentatricopeptide repeat (PPR) superfamily protein
AT2G06005	*FRIGIDA INTERACTING PROTEIN 1 (FIP1)*	Encodes one of the FRI interacting proteins: FRIGIDA INTERACTING PROTEIN 1 (FIP1)/At2g06005, FIP2/ At4g17060. FRI (At4G00650) is a major determinant of natural variation in Arabidopsis flowering time.
AT2G06010	*OBP3-RESPONSIVE GENE 4 (ORG4)*	encodes a novel protein whose expression level is induced in lines overexpressing salicylic-acid (SA)-inducible Arabidopsis DNA binding with one finger (Dof) transcription factor, called OBF-binding protein 3.
AT2G06020		Homeodomain-like superfamily protein
AT2G06030		
AT2G06040	*RADIATION SENSITIVE 7A (RAD7A)*	Contributes to UV tolerance through nucleotide excision repair.
AT2G06050	*OXOPHYTODIENOATE-REDUCTASE 3 (OPR3)*	Encodes a 12-oxophytodienoate reductase that is required for jasmonate biosynthesis. Mutants are male sterile and defective in pollen dehiscence. Shows activity towards 2,4,6-trinitrotoluene. CFA-Ile, CFA-Leu, CFA-Val, CFA-Met and CFA-Ala can restore the fertility of opr3 plants by inducing filament elongation and anther dehiscence.
AT2G06090		Plant self-incompatibility protein S1 family
AT2G06110		transposable_element_gene;gypsy-like retrotransposon family (Athila), has a 6.4e-72 P-value blast match to GB:CAA57397 Athila ORF 1 (Arabidopsis thaliana);(source:TAIR10)
AT2G06120		transposable_element_gene;similar to unknown protein [Arabidopsis thaliana] (TAIR:AT3G15600.1);(source:TAIR10)
AT2G06130		transposable_element_gene;Mutator-like transposase family, has a 2.7e-30 P-value blast match to O80466 /172-336 Pfam PF03108 MuDR family transposase (MuDr-element domain);(source:TAIR10)
AT2G06140		transposable_element_gene;similar to unknown protein [Arabidopsis thaliana] (TAIR:AT5G32605.1);(source:TAIR10)
AT2G06150		transposable_element_gene;pseudogene, hypothetical protein;(source:TAIR10)
AT2G06160		transposable_element_gene;gypsy-like retrotransposon family (Athila), has a 0. P-value blast match to GB:CAA57397 Athila ORF 1 (Arabidopsis thaliana);(source:TAIR10)
AT2G06170		transposable_element_gene;gypsy-like retrotransposon family, has a 1.7e-91 P-value blast match to GB:AAD22153 polyprotein (Gypsy_Ty3-element) (Sorghum bicolor);(source:TAIR10)
AT2G06180		transposable_element_gene;pseudogene, hypothetical protein;(source:TAIR10)
AT2G06190		transposable_element_gene;gypsy-like retrotransposon family, has a 5.2e-36 P-value blast match to GB:CAA73042 polyprotein (Gypsy_Ty3-element) (Ananas comosus);(source:TAIR10)
AT2G06200	*GROWTH-REGULATING FACTOR 6 (GRF6)*	Growth regulating factor encoding transcription activator. One of the nine members of a GRF gene family, containing nuclear targeting domain. Involved in leaf development and expressed in root, shoot and flower
AT2G06210	*EARLY FLOWERING 8 (ELF8)*	Encodes a yeast CTR9 homolog that is involved in the control of flowering time by elevating FLC expression to a level that creates the vernalization-responsive, winter-annual habit. Yeast CTR9 is a component of a five-member PAF1 complex that associates with RNA pol II and is thought to regulate gene expression by recruiting SET1 (a histone 3 Lys 4 [H3-K4] methyl transferase) to the initially transcribed [5'] regions of target chromatin. Mutants display reduced H3-K4 methylation in both FLC and FLM chromatin. Member of PAF-C complex.
AT2G06220		transposable_element_gene;pseudogene, expressed protein, contains Pfam profile PF03384: Drosophila protein of unknown function, DUF287;(source:TAIR10)
AT2G06230		transposable_element_gene;similar to unknown protein [Arabidopsis thaliana] (TAIR:AT3G33230.1);(source:TAIR10)
AT2G06255	*ELF4-LIKE 3 (ELF4-L3)*	DUF1313 domain containing protein.
AT2G06260		transposable_element_gene;gypsy-like retrotransposon family (Athila), has a 3.0e-28 P-value blast match to GB:CAA57397 Athila ORF 1 (Arabidopsis thaliana);(source:TAIR10)
AT2G06310		transposable_element_gene;gypsy-like retrotransposon family (Athila), has a 2.2e-219 P-value blast match to GB:CAA57397 Athila ORF 1 (Arabidopsis thaliana);(source:TAIR10)
AT2G06320		
AT2G06330		transposable_element_gene;pseudogene, hypothetical protein;(source:TAIR10)
AT2G06390		transposable_element_gene
AT2G06410		transposable_element_gene;Mutator-like transposase family, has a 7.0e-85 P-value blast match to Q9SJR8 /172-333 Pfam PF03108 MuDR family transposase (MuDr-element domain);(source:TAIR10)
AT2G06430		transposable_element_gene;pseudogene, Ulp1 protease family, contains Pfam profile PF02902: Ulp1 protease family, C-terminal catalytic domain;(source:TAIR10)
AT2G06470		transposable_element_gene;gypsy-like retrotransposon family, has a 0. P-value blast match to GB:AAD27547 polyprotein (Gypsy_Ty3-element) (Oryza sativa subsp. indica);(source:TAIR10)
AT2G06480		transposable_element_gene

AT2G06490		transposable_element_gene;CACTA-like transposase family (Ptta/En/Spm), has a 7.5e-83 P-value blast match to At5g29026.1/8-244 CACTA-like transposase family (Ptta/En/Spm) (CACTA-element) (Arabidopsis thaliana);(source:TAIR10)
AT2G06500		hAT family dimerization domain-containing protein
AT2G06520	*PHOTOSYSTEM II SUBUNIT X (PSBX)*	Encodes a protein with sequence similarity to the spinach photosystem II subunit PsbX.
AT2G06630		transposable_element_gene;similar to unknown protein [Arabidopsis thaliana] (TAIR:AT2G12120.1);(source:TAIR10)
AT2G06640		transposable_element_gene;pseudogene, hypothetical protein;(source:TAIR10)
AT2G06660		transposable_element_gene;Mariner-like transposase family, has a 2.3e-43 P-value blast match to GB:S20478 hypothetical protein (Mariner_Tc1-element) (Drosophila melanogaster);(source:TAIR10)
AT2G06700		transposable_element_gene;similar to unknown protein [Arabidopsis thaliana] (TAIR:AT2G12130.1);(source:TAIR10)
AT2G06730		
AT2G06820		transposable_element_gene;similar to unknown protein [Arabidopsis thaliana] (TAIR:AT4G09700.1);(source:TAIR10)
AT2G06840		transposable_element_gene;copia-like retrotransposon family, has a 3.5e-184 P-value blast match to GB:AAC02666 polyprotein (Ty1_Copia-element) (Arabidopsis thaliana);(source:TAIR10)
AT2G06850	*XYLOGLUCAN ENDOTRANSGLUCOSYLASE/HYDROLASE 4 (XTH4)*	endoxyloglucan transferase (EXGT-A1) gene
AT2G06870		transposable_element_gene;Mutator-like transposase family, has a 2.3e-49 P-value blast match to Q9SHN7 /450-633 Pfam PF03108 MuDR family transposase (MuDr-element domain);(source:TAIR10)
AT2G06890	*(NTP10)*	transposable_element_gene;gypsy-like retrotransposon family, has a 7.3e-184 P-value blast match to GB:AAD22153 polyprotein (Gypsy_Ty3-element) (Sorghum bicolor);(source:TAIR10)
AT2G06925	*(PLA2-ALPHA)*	Encodes a secretory phospholipase A2 enzyme, which specifically hydrolyzes the sn-2 position of phospholipids. The enzyme has a preference towards linoleoyl acyl chain over palmitoyl acyl chain. It also has a slight preference for phosphatidylcholine over phosphatidylethanolamine.
AT2G06950		transposable_element_gene;copia-like retrotransposon family, has a 2.7e-243 P-value blast match to dbj|BAA78426.1| polyprotein (AtRE2-1) (Arabidopsis thaliana) (Ty1_Copia-element);(source:TAIR10)
AT2G06960		2-oxoglutarate (2OG) and Fe(II)-dependent oxygenase superfamily protein
AT2G06970		
AT2G07000		hypothetical protein
AT2G07010		transposable_element_gene;copia-like retrotransposon family, has a 2.9e-175 P-value blast match to GB:CAA72989 open reading frame 1 (Ty1_Copia-element) (Brassica oleracea);(source:TAIR10)
AT2G07020		kinase with adenine nucleotide alpha hydrolases-like domain-containing protein
AT2G07030		transposable_element_gene;Mutator-like transposase family, has a 2.9e-11 P-value blast match to Q9XE24 /118-277 Pfam PF03108 MuDR family transposase (MuDr-element domain);(source:TAIR10)
AT2G07040	*(PRK2A)*	Pollen receptor kinase. Coexpression of AtPRK2a with AtRopGEF12 resulted in isotropic pollen tube growth.
AT2G07050	*CYCLOARTENOL SYNTHASE 1 (CAS1)*	Involved in the biosynthesis of brassinosteroids. Catalyzes the reaction from epoxysqualene to cycloartenol.
AT2G07070		transposable_element_gene
AT2G07080		transposable_element_gene;copia-like retrotransposon family, has a 5.0e-74 P-value blast match to gb|AAG52949.1| gag/pol polyprotein (Endovir1-1) (Arabidopsis thaliana) (Ty1_Copia-family);(source:TAIR10)
AT2G07110		exosome complex exonuclease RRP46-like protein
AT2G07120		F-box associated ubiquitination effector family protein
AT2G07130		pseudogene of F-box family protein
AT2G07170		ARM repeat superfamily protein
AT2G07200		Cysteine proteinases superfamily protein
AT2G07230		transposable_element_gene;Mutator-like transposase family, has a 8.1e-59 P-value blast match to Q9SHN7 /450-633 Pfam PF03108 MuDR family transposase (MuDr-element domain);(source:TAIR10)
AT2G07240		cysteine-type peptidase
AT2G07280		hypothetical protein
AT2G07290		hypothetical protein
AT2G07300		transposable_element_gene;similar to unknown protein [Arabidopsis thaliana] (TAIR:AT1G35090.1);(source:TAIR10)
AT2G07320		transposable_element_gene;similar to unknown protein [Arabidopsis thaliana] (TAIR:AT5G37880.1);(source:TAIR10)
AT2G07330		transposable_element_gene;Mutator-like transposase family, has a 1.9e-87 P-value blast match to Q9ZQK9 /304-464 Pfam PF03108 MuDR family transposase (MuDr-element domain);(source:TAIR10)
AT2G07350		
AT2G07360	*TPLATE-ASSOCIATED SH3 DOMAIN CONTAINING PROTEIN (TASH3)*	TPLATE-associated SH3 domain containing protein.
AT2G07370		
AT2G07380		transposable_element_gene;Mutator-like transposase family, has a 5.0e-62 P-value blast match to O22273 /233-373 Pfam PF03108 MuDR family transposase (MuDr-element domain);(source:TAIR10)
AT2G07430		transposable_element_gene;gypsy-like retrotransposon family (Athila), has a 4.0e-125 P-value blast match to GB:CAA57397 Athila ORF 1 (Arabidopsis thaliana);(source:TAIR10)
AT2G07440		two-component responsive regulator-related / response regulator protein-like protein
AT2G07450		transposable_element_gene;gypsy-like retrotransposon family (Athila), has a 3.1e-223 P-value blast match to GB:CAA57397 Athila ORF 1 (Arabidopsis thaliana);(source:TAIR10)

AT2G07490		transposable_element_gene;gypsy-like retrotransposon family (Athila), has a 4.1e-27 P-value blast match to GB:CAA57397 Athila ORF 1 (Arabidopsis thaliana);(source:TAIR10)
AT2G07500		transposable_element_gene;Mutator-like transposase family, has a 1.9e-70 P-value blast match to Q9SJR8 /172-333 Pfam PF03108 MuDR family transposase (MuDr-element domain);(source:TAIR10)
AT2G07505		zinc ion binding protein
AT2G07510		pseudogene of Ulp1 protease family protein
AT2G07520		transposable_element_gene;similar to unknown protein [Arabidopsis thaliana] (TAIR:AT3G15310.1);(source:TAIR10)
AT2G07550		transposable_element_gene;copia-like retrotransposon family, has a 4.7e-313 P-value blast match to GB:CAA31653 polyprotein (Ty1_Copia-element) (Arabidopsis thaliana);(source:TAIR10)
AT2G07560	*H(+)-ATPASE 6 (HA6)*	H[+]-ATPase 6
AT2G07620		transposable_element_gene;pseudogene, similar to putative helicase, very low similarity to SP|Q9UUA2 DNA repair and recombination protein pif1, mitochondrial precursor {Schizosaccharomyces pombe};(source:TAIR10)
AT2G07630		transposable_element_gene;similar to unknown protein [Arabidopsis thaliana] (TAIR:AT3G31990.1);(source:TAIR10)
AT2G07650		transposable_element_gene;non-LTR retrotransposon family (LINE), has a 3.8e-41 P-value blast match to GB:AAA39398 ORF2 (Mus musculus) (LINE-element);(source:TAIR10)
AT2G07670		Pseudogene of AT2G07702
AT2G07672		hypothetical protein
AT2G07673		hypothetical protein
AT2G07675		Ribosomal protein S12/S23 family protein
AT2G07676		hypothetical protein
AT2G07677		pseudogene of DNA binding
AT2G07680	*ATP-BINDING CASSETTE C13 (ABCC13)*	Encodes ABCC13/MRP11, a member of the multidrug resistance associated protein MRP/ABCC subfamily. Its expression is induced by gibberellic acid and downregulated by naphthalene acetic acid, abscisic acid, and zeatin.
AT2G07681	*ATP-BINDING CASSETTE I4 (ABCI4)*	Cytochrome C assembly protein
AT2G07683		transposable_element_gene;copia-like retrotransposon family, has a 2.2e-110 P-value blast match to GB:AAA57005 Hopscotch polyprotein (Ty1_Copia-element) (Zea mays);(source:TAIR10)
AT2G07684		hypothetical protein
AT2G07685		transposable_element_gene;gypsy-like retrotransposon family (Athila), has a 8.2e-13 P-value blast match to GB:CAA57397 Athila ORF 1 (Arabidopsis thaliana);(source:TAIR10)
AT2G07687		Cytochrome c oxidase, subunit III
AT2G07690	*MINICHROMOSOME MAINTENANCE 5 (MCM5)*	Member of the minichromosome maintenance complex, involved in DNA replication initiation. Abundant in proliferating and endocycling tissues. Localized in the nucleus during G1, S and G2 phases of the cell cycle, and are released into the cytoplasmic compartment during mitosis. Binds chromatin.
AT2G07691		hypothetical protein
AT2G07692		hypothetical protein
AT2G07693		transposable_element_gene;copia-like retrotransposon family, has a 6.2e-16 P-value blast match to GB:CAA37924 orf 2 (Ty1_Copia-element) (Arabidopsis thaliana);(source:TAIR10)
AT2G07695		Cytochrome C oxidase subunit II-like, transmembrane domain-containing protein
AT2G07698		ATPase, F1 complex, alpha subunit protein
AT2G07700		transposable_element_gene;gypsy-like retrotransposon family, has a 4.7e-21 P-value blast match to GB:226407 retrotransposon del1-46 (Gypsy_Ty3-element) (Lilium henryi);(source:TAIR10)
AT2G07701		hypothetical protein
AT2G07702		hypothetical protein
AT2G07705		hypothetical protein
AT2G07706		hypothetical protein
AT2G07707		Plant mitochondrial ATPase, F0 complex, subunit 8 protein
AT2G07713		hypothetical protein
AT2G07714		transcription factor-like protein
AT2G07715		Nucleic acid-binding, OB-fold-like protein
AT2G07719		Putative membrane lipoprotein
AT2G07720		transposable_element_gene;gypsy-like retrotransposon family (Athila), has a 1.1e-59 P-value blast match to GB:CAA57397 Athila ORF 1 (Arabidopsis thaliana);(source:TAIR10)
AT2G07721		hypothetical protein
AT2G07722		transmembrane protein
AT2G07724		hypothetical protein
AT2G07725		Ribosomal L5P family protein
AT2G07727		cytochrome b
AT2G07728		hypothetical protein
AT2G07730		transposable_element_gene;non-LTR retrotransposon family (LINE), has a 5.0e-32 P-value blast match to GB:NP_038603 L1 repeat, Tf subfamily, member 23 (LINE-element) (Mus musculus);(source:TAIR10)

AT2G07734		Alpha-L RNA-binding motif/Ribosomal protein S4 family protein
AT2G07736		transposable_element_gene;copia-like retrotransposon family, has a 4.9e-29 P-value blast match to GB:CAA37924 orf 2 (Ty1_Copia-element) (Arabidopsis thaliana);(source:TAIR10)
AT2G07738		hypothetical protein
AT2G07739		Ycf1 protein
AT2G07740		transposable_element_gene;similar to nucleic acid binding / zinc ion binding [Arabidopsis thaliana] (TAIR:AT2G01050.1);(source:TAIR10)
AT2G07741		ATPase, F0 complex, subunit A protein
AT2G07750		DEA(D/H)-box RNA helicase family protein
AT2G07760		Zinc knuckle (CCHC-type) family protein
AT2G09840		nucleic acid/zinc ion-binding protein
AT2G09910		transposable_element_gene;similar to ASY2, DNA binding [Arabidopsis thaliana] (TAIR:AT4G32200.1);(source:TAIR10)
AT2G09960		transposable_element_gene;expressed protein, includes At2g05890, At4g07450, At3g30630, At3g43100, At2g09960, At3g30550, At1g39430, At2g10460, At4g03640, At5g35250;(source:TAIR10)
AT2G10000		transposable_element_gene;CACTA-like transposase family (Tnp1/En/Spm), has a 3.1e-154 P-value blast match to ref|NP_189784.1| TNP1-related protein (Arabidopsis thaliana) (CACTA-element);(source:TAIR10)
AT2G10010		transposable_element_gene;CACTA-like transposase family (Tnp2/En/Spm), has a 0. P-value blast match to gb|AAG52024.1|AC022456_5 Tam1-homologous transposon protein TNP2, putative;(source:TAIR10)
AT2G10020		hypothetical protein
AT2G10050		transposable_element_gene;similar to pol polyprotein-like [Solanum tuberosum] (GB:AAU89775.1);(source:TAIR10)
AT2G10070		transposable_element_gene;similar to unknown protein [Arabidopsis thaliana] (TAIR:AT1G40125.1);(source:TAIR10)
AT2G10090		transposable_element_gene;similar to unknown protein [Arabidopsis thaliana] (TAIR:AT2G04460.1);(source:TAIR10)
AT2G10140		transposable_element_gene;CACTA-like transposase family (Tnp2/En/Spm), has a 1.7e-140 P-value blast match to GB:CAA40555 TNP2 (CACTA-element) (Antirrhinum majus);(source:TAIR10)
AT2G10340		
AT2G10370		transposable_element_gene;similar to unknown protein [Arabidopsis thaliana] (TAIR:AT1G44860.1);(source:TAIR10)
AT2G10380		transposable_element_gene;similar to unknown protein [Arabidopsis thaliana] (TAIR:AT5G36070.1);(source:TAIR10)
AT2G10390		transposable_element_gene;similar to unknown protein [Arabidopsis thaliana] (TAIR:AT1G35100.1);(source:TAIR10)
AT2G10400		transposable_element_gene;Mutator-like transposase family, has a 3.7e-61 P-value blast match to Q9S9L6 /322-461 Pfam PF03108 MuDR family transposase (MuDr-element domain);(source:TAIR10)
AT2G10440	*MEDIATOR 15_2 (MED15_2)*	mediator of RNA polymerase II transcription subunit
AT2G10450		14-3-3 family protein
AT2G10460		transposable_element_gene;similar to unknown protein [Arabidopsis thaliana] (TAIR:AT4G07450.1);(source:TAIR10)
AT2G10465		transposable_element_gene;similar to replication protein-related [Arabidopsis thaliana] (TAIR:AT5G35260.1);(source:TAIR10)
AT2G10490		transposable_element_gene;CACTA-like transposase family (Ptta/En/Spm), has a 2.0e-43 P-value blast match to At5g29026.1/8-244 CACTA-like transposase family (Ptta/En/Spm) (CACTA-element) (Arabidopsis thaliana);(source:TAIR10)
AT2G10500		transposable_element_gene;similar to unknown protein [Arabidopsis thaliana] (TAIR:AT4G07430.1);(source:TAIR10)
AT2G10610		transposable_element_gene;copia-like retrotransposon family, has a 2.6e-162 P-value blast match to gb|AAG52949.1| gag/pol polyprotein (Endovir1-1) (Arabidopsis thaliana) (Ty1_Copia-family);(source:TAIR10)
AT2G10620		transposable_element_gene;gypsy-like retrotransposon family (Athila), has a 5.5e-59 P-value blast match to GB:CAA57397 Athila ORF 1 (Arabidopsis thaliana);(source:TAIR10)
AT2G10630		transposable_element_gene;CACTA-like transposase family (En/Spm), has a 2.7e-97 P-value blast match to GB:BAA20532 ORF of transposon Tdc1 (CACTA-element) (Daucus carota);(source:TAIR10)
AT2G10640		transposable_element_gene;CACTA-like transposase family (Ptta/En/Spm), has a 7.4e-42 P-value blast match to At5g29026.1/8-244 CACTA-like transposase family (Ptta/En/Spm) (CACTA-element) (Arabidopsis thaliana);(source:TAIR10)
AT2G10650		transposable_element_gene;gypsy-like retrotransposon family (Athila), has a 3.5e-43 P-value blast match to GB:CAA57397 Athila ORF 1 (Arabidopsis thaliana);(source:TAIR10)
AT2G10660		transposable_element_gene;gypsy-like retrotransposon family (Athila), has a 4.3e-277 P-value blast match to GB:CAA57397 Athila ORF 1 (Arabidopsis thaliana);(source:TAIR10)
AT2G10690		transposable_element_gene;gypsy-like retrotransposon family (Athila), has a 9.8e-118 P-value blast match to gb|AAL06421.1|AF378079_1 reverse transcriptase (Athila4) (Arabidopsis thaliana) (Gypsy_Ty3-family);(source:TAIR10)
AT2G10740		transposable_element_gene;gypsy-like retrotransposon family (Athila), has a 2.7e-102 P-value blast match to GB:CAA57397 Athila ORF 1 (Arabidopsis thaliana);(source:TAIR10)
AT2G10750		
AT2G10760		transposable_element_gene;copia-like retrotransposon family, has a 6.5e-285 P-value blast match to GB:CAA31653 polyprotein (Ty1_Copia-element) (Arabidopsis thaliana);(source:TAIR10)
AT2G10780		transposable_element_gene;gypsy-like retrotransposon family, has a 0. P-value blast match to GB:AAD27547 polyprotein (Gypsy_Ty3-element) (Oryza sativa subsp. indica);(source:TAIR10)
AT2G10840		transposable_element_gene;pseudogene, hypothetical protein, similar to At5g35280, At2g10500, At2g05860, At4g07310, At4g07430, At1g39270;(source:TAIR10)
AT2G10850		transposable_element_gene;similar to unknown protein [Arabidopsis thaliana] (TAIR:AT1G43970.1);(source:TAIR10)

AT2G10870		transposable_element_gene;similar to unknown protein [Arabidopsis thaliana] (TAIR:AT1G48250.1);(source:TAIR10)
AT2G10880		transposable_element_gene;hypothetical protein, similar to hypothetical protein GB:AAC26673;(source:TAIR10)
AT2G10900		similarity to non-LTR retroelement protein
AT2G10920		hypothetical protein
AT2G10940		Bifunctional inhibitor/lipid-transfer protein/seed storage 2S albumin superfamily protein
AT2G10970		Plant invertase/pectin methylesterase inhibitor superfamily protein
AT2G11010		hypothetical protein
AT2G11090		transposable_element_gene;similar to unknown protein [Arabidopsis thaliana] (TAIR:AT2G07880.1);(source:TAIR10)
AT2G11110		transposable_element_gene;CACTA-like transposase family (Ptta/En/Spm), has a 6.4e-110 P-value blast match to At5g36655.1/81-333 CACTA-like transposase family (Ptta/En/Spm) (CACTA-element) (Arabidopsis thaliana);(source:TAIR10)
AT2G11120		similarity to putative Athila retroelement ORF1 protein
AT2G11130		transposable_element_gene;gypsy-like retrotransposon family, has a 1.0e-35 P-value blast match to GB:BAA84458 GAG-POL precursor (gypsy_Ty3-element) (Oryza sativa)gi|5902445|dbj|BAA84458.1| GAG-POL precursor (Oryza sativa (japonica cultivar-group)) (RIRE2) (Gypsy_Ty3-family);(source:TAIR10)
AT2G11140		transposable_element_gene;copia-like retrotransposon family, has a 1.0e-71 P-value blast match to GB:CAA72989 open reading frame 1 (Ty1_Copia-element) (Brassica oleracea);(source:TAIR10)
AT2G11150		pseudogene of putative replication protein A1
AT2G11160		
AT2G11170		transposable_element_gene;pseudogene, hypothetical protein;(source:TAIR10)
AT2G11190		transposable_element_gene;copia-like retrotransposon family, has a 3.5e-90 P-value blast match to GB:CAA32025 ORF (Ty1_Copia-element) (Nicotiana tabacum)GB:CAA32025 ORF (Ty1_Copia-element) (Nicotiana tabacum);(source:TAIR10)
AT2G11210		transposable_element_gene;Mutator-like transposase family, has a 1.1e-93 P-value blast match to Q9ZQM3 /24-192 Pfam PF03108 MuDR family transposase (MuDr-element domain);(source:TAIR10)
AT2G11220		transposable_element_gene;copia-like retrotransposon family, has a 3.5e-16 P-value blast match to gb|AAO73521.1| gag-pol polyprotein (Glycine max) (SIRE1) (Ty1_Copia-family);(source:TAIR10)
AT2G11230		transposable_element_gene;gypsy-like retrotransposon family (Athila), has a 8.9e-61 P-value blast match to GB:CAA57397 Athila ORF 1 (Arabidopsis thaliana);(source:TAIR10)
AT2G11240		transposable_element_gene;non-LTR retrotransposon family (LINE), has a 1.1e-38 P-value blast match to GB:NP_038603 L1 repeat, Tf subfamily, member 23 (LINE-element) (Mus musculus);(source:TAIR10)
AT2G11260		
AT2G11270		citrate synthase-like protein
AT2G11360		transposable_element_gene;similar to unknown protein [Arabidopsis thaliana] (TAIR:AT2G24900.1);(source:TAIR10)
AT2G11370		transposable_element_gene;similar to unknown protein [Arabidopsis thaliana] (TAIR:AT3G30590.1);(source:TAIR10)
AT2G11390		transposable_element_gene;CACTA-like transposase family (Ptta/En/Spm), has a 1.2e-21 P-value blast match to At5g59620.1/14-257 CACTA-like transposase family (Ptta/En/Spm) (CACTA-element) (Arabidopsis thaliana);(source:TAIR10)
AT2G11480		transposable_element_gene;pseudogene, hypothetical protein;(source:TAIR10)
AT2G11490		transposable_element_gene;similar to unknown protein [Arabidopsis thaliana] (TAIR:AT5G28980.1);(source:TAIR10)
AT2G11540		
AT2G11560		
AT2G11580		
AT2G11600		transposable_element_gene;pseudogene, hypothetical protein, and genefinder;(source:TAIR10)
AT2G11650		transposable_element_gene;pseudogene, hypothetical protein, and genefinder;(source:TAIR10)
AT2G11680		transposable_element_gene;gypsy-like retrotransposon family, has a 3.4e-54 P-value blast match to GB:CAB39733 rotease, reverse transcriptase, ribonuclease H, integrase (Gypsy_Ty3-element) (Drosophila buzzatii);(source:TAIR10)
AT2G11690		transposable_element_gene;CACTA-like transposase family (Tnp1/En/Spm), has a 4.8e-34 P-value blast match to ref|NP_189784.1| TNP1-related protein (Arabidopsis thaliana) (CACTA-element);(source:TAIR10)
AT2G11810	*MONOGALACTOSYLDIACYLGLYCEROL SYNTHASE TYPE C (MGDC)*	MGD3 is the major enzyme for galactolipid metabolism during phosphate starvation. Does not contribute to galactolipid synthesis under P1-sufficient conditions.
AT2G11830		transposable_element_gene;similar to unknown protein [Arabidopsis thaliana] (TAIR:AT5G47225.1);(source:TAIR10)
AT2G11890	*TRIPHOSPHATE TUNNEL METALLOENZYME 3 (TTM3)*	Encodes a tripolyphosphatase that is involved in root development.
AT2G11910		hypothetical protein
AT2G11920		
AT2G11940		transposable_element_gene;gypsy-like retrotransposon family, has a 7.0e-189 P-value blast match to GB:AAD19359 polyprotein (gypsy_Ty3-element) (Sorghum bicolor);(source:TAIR10)
AT2G12050		pseudogene of Reticulon family protein
AT2G12150		transposable_element_gene;Mutator-like transposase family, has a 1.1e-84 P-value blast match to Q9SL18 /349-510 Pfam PF03108 MuDR family transposase (MuDr-element domain);(source:TAIR10)
AT2G12170		hypothetical protein
AT2G12190		Cytochrome P450 superfamily protein

AT2G12210		transposable_element_gene;CACTA-like transposase family (Tnp2/En/Spm), has a 0. P-value blast match to gb|AAG52024.1|AC022456_5 Tam1-homologous transposon protein TNP2, putative;(source:TAIR10)
AT2G12220		
AT2G12230		pseudogene of P-loop containing nucleoside triphosphate hydrolases superfamily protein
AT2G12240		transposable_element_gene;CACTA-like transposase family (Ptta/En/Spm), has a 6.6e-45 P-value blast match to At5g29026.1/8-244 CACTA-like transposase family (Ptta/En/Spm) (CACTA-element) (Arabidopsis thaliana);(source:TAIR10)
AT2G12250		similarity to En/Spm-like transposon protein
AT2G12290		hypothetical protein
AT2G12300		transposable_element_gene;CACTA-like transposase family (Ptta/En/Spm), has a 3.8e-41 P-value blast match to At5g29026.1/8-244 CACTA-like transposase family (Ptta/En/Spm) (CACTA-element) (Arabidopsis thaliana);(source:TAIR10)
AT2G12320		transposable_element_gene;similar to unknown protein [Arabidopsis thaliana] (TAIR:AT5G32169.1);(source:TAIR10)
AT2G12350		
AT2G12420		transposable_element_gene;CACTA-like transposase family (Ptta/En/Spm), has a 3.9e-19 P-value blast match to At5g29026.1/8-244 CACTA-like transposase family (Ptta/En/Spm) (CACTA-element) (Arabidopsis thaliana);(source:TAIR10)
AT2G12480	*SERINE CARBOXYPEPTIDASE-LIKE 43 (SCPL43)*	serine carboxypeptidase-like 43
AT2G12520		transposable_element_gene;similar to unknown protein [Arabidopsis thaliana] (TAIR:AT4G04070.1);(source:TAIR10)
AT2G12610		transposable_element_gene;similar to unknown protein [Arabidopsis thaliana] (TAIR:AT3G42120.1);(source:TAIR10)
AT2G12720		transposable_element_gene;Mutator-like transposase family, has a 7.6e-66 P-value blast match to O22273 /233-373 Pfam PF03108 MuDR family transposase (MuDr-element domain);(source:TAIR10)
AT2G12870		transposable_element_gene;gypsy-like retrotransposon family, has a 3.8e-07 P-value blast match to GB:AAD19359 polyprotein (gypsy_Ty3-element) (Sorghum bicolor);(source:TAIR10)
AT2G12880		Zinc knuckle (CCHC-type) family protein
AT2G12900	*BASIC LEUCINE-ZIPPER 33 (BZIP33)*	Basic-leucine zipper (bZIP) transcription factor family protein
AT2G12910		transposable_element_gene;pseudogene, hypothetical protein;(source:TAIR10)
AT2G12920		transposable_element_gene;gypsy-like retrotransposon family, has a 1.2e-128 P-value blast match to GB:AAD19359 polyprotein (gypsy_Ty3-element) (Sorghum bicolor);(source:TAIR10)
AT2G12940	*UNFERTILIZED EMBRYO SAC 4 (UNE4)*	Basic-leucine zipper (bZIP) transcription factor family protein
AT2G12980		transposable_element_gene;CACTA-like transposase family (Ptta/En/Spm), has a 6.5e-99 P-value blast match to At5g36655.1/81-333 CACTA-like transposase family (Ptta/En/Spm) (CACTA-element) (Arabidopsis thaliana);(source:TAIR10)
AT2G12990		transposable_element_gene;CACTA-like transposase family (En/Spm), has a 9.4e-25 P-value blast match to dbj|BAB64937.1| TdcA1-ORF1-ORF2 (Daucus carota) Spm/En-like (CACTA-like);(source:TAIR10)
AT2G13000		transposable_element_gene;CACTA-like transposase family (Tnp2/En/Spm), has a 9.0e-151 P-value blast match to GB:CAA40555 TNP2 (CACTA-element) (Antirrhinum majus);(source:TAIR10)
AT2G13020		transposable_element_gene;gypsy-like retrotransposon family (Athila), has a 7.3e-101 P-value blast match to GB:CAA57397 Athila ORF 1 (Arabidopsis thaliana);(source:TAIR10)
AT2G13070		transposable_element_gene;similar to unknown protein [Arabidopsis thaliana] (TAIR:AT2G12520.1);(source:TAIR10)
AT2G13100	*GLYCEROL-3-PHOSPHATE PERMEASE 5 (G3Pp5)*	Encodes a member of the phosphate starvation-induced glycerol-3-phosphate permease gene family: AT3G47420(G3Pp1), AT4G25220(G3Pp2), AT1G30560(G3Pp3), AT4G17550(G3Pp4) and AT2G13100(G3Pp5). The mRNA is cell-to-cell mobile.
AT2G13150	*BASIC LEUCINE-ZIPPER 31 (BZIP31)*	Basic-leucine zipper (bZIP) transcription factor family protein
AT2G13160		transposable_element_gene;CACTA-like transposase family (Ptta/En/Spm), has a 1.1e-126 P-value blast match to At5g36655.1/81-333 CACTA-like transposase family (Ptta/En/Spm) (CACTA-element) (Arabidopsis thaliana);(source:TAIR10)
AT2G13210		
AT2G13230		transposable_element_gene;gypsy-like retrotransposon family, has a 8.2e-157 P-value blast match to GB:BAA84458 GAG-POL precursor (gypsy_Ty3-element) (Oryza sativa)gi|5902445|dbj|BAA84458.1| GAG-POL precursor (Oryza sativa (japonica cultivar-group)) (RIRE2) (Gypsy_Ty3-family);(source:TAIR10)
AT2G13250		transposable_element_gene;gypsy-like retrotransposon family (Athila), has a 4.1e-91 P-value blast match to GB:CAA57397 Athila ORF 1 (Arabidopsis thaliana);(source:TAIR10)
AT2G13270		transposable_element_gene;similar to unknown protein [Arabidopsis thaliana] (TAIR:AT3G42120.1);(source:TAIR10)
AT2G13310		transposable_element_gene;CACTA-like transposase family (Ptta/En/Spm), has a 1.3e-123 P-value blast match to At5g36655.1/81-333 CACTA-like transposase family (Ptta/En/Spm) (CACTA-element) (Arabidopsis thaliana);(source:TAIR10)
AT2G13330		transposable_element_gene;gypsy-like retrotransposon family, has a 2.0e-156 P-value blast match to GB:AAD11615 prpol (gypsy_Ty3-element) (Zea mays);(source:TAIR10)
AT2G13335		transposable_element_gene;gypsy-like retrotransposon family, has a 9.7e-06 P-value blast match to GB:BAA84457 GAG-POL precursor (gypsy_Ty3-element) (Oryza sativa)gi|5902444|dbj|BAA84457.1| GAG-POL precursor (Oryza sativa (japonica cultivar-group)) (RIRE2) (Gypsy_Ty3-family);(source:TAIR10)
AT2G13350		Calcium-dependent lipid-binding (CaLB domain) family protein
AT2G13360	*ALANINE:GLYOXYLATE AMINOTRANSFERASE (AGT)*	Encodes a peroxisomal photorespiratory enzyme that catalyzes transamination reactions with multiple substrates. It is involved in photorespiration.
AT2G13430		hypothetical protein
AT2G13500		Ta11-like non-LTR retrotransposon
AT2G13510		Ta11-like non-LTR retrotransposon

AT2G13550		hypothetical protein
AT2G13570	*NUCLEAR FACTOR Y, SUBUNIT B7 (NF-YB7)*	nuclear factor Y, subunit B7
AT2G13610	*ATP-BINDING CASSETTE G5 (ABCG5)*	ABC-2 type transporter family protein
AT2G13620	*CATION/HYDROGEN EXCHANGER 15 (CHX15)*	member of Putative Na+/H+ antiporter family
AT2G13630		F-box associated ubiquitination effector family protein
AT2G13660		hypothetical protein
AT2G13670		
AT2G13675		
AT2G13690		PRLI-interacting factor
AT2G13720		putative DNA topoisomerase
AT2G13730		transposable_element_gene
AT2G13750		transposable_element_gene;CACTA-like transposase family (Ptta/En/Spm), has a 2.5e-17 P-value blast match to At5g29026.1/8-244 CACTA-like transposase family (Ptta/En/Spm) (CACTA-element) (Arabidopsis thaliana);(source:TAIR10)
AT2G13760		no-apical-meristem-associated carboxy-terminal domain protein
AT2G13770		nuclease
AT2G13780		
AT2G13790	*SOMATIC EMBRYOGENESIS RECEPTOR-LIKE KINASE 4 (SERK4)*	somatic embryogenesis receptor-like kinase 4
AT2G13810	*AGD2-LIKE DEFENSE RESPONSE PROTEIN 1 (ALD1)*	ALD1 is a L-lysine alpha-aminotransferase. It is part of the pipecolic acid biosynthetic pathway, where it catalyzes the biochemical conversion of lysine to epsilon-amino-alpha-ketocaproic acid (KAC) which is subject to subsequent transamination, cyclization and isomerization to form 2,3-dehydropipecolic acid.
AT2G13820	*XYLOGEN PROTEIN 2 (XYP2)*	Bifunctional inhibitor/lipid-transfer protein/seed storage 2S albumin superfamily protein
AT2G13840		Polymerase/histidinol phosphatase-like protein
AT2G13870		transposable_element_gene;CACTA-like transposase family (Ptta/En/Spm), has a 1.4e-103 P-value blast match to At5g36655.1/81-333 CACTA-like transposase family (Ptta/En/Spm) (CACTA-element) (Arabidopsis thaliana);(source:TAIR10)
AT2G13880		
AT2G13890		transposable_element_gene;pseudogene, zinc knuckle (CCHC type) protein family, contains Pfam domain, PF00098: Zinc knuckle;(source:TAIR10)
AT2G13900		Cysteine/Histidine-rich C1 domain family protein
AT2G13920		pseudogene of Cysteine/Histidine-rich C1 domain family protein
AT2G13930		transposable_element_gene;copia-like retrotransposon family, has a 2.8e-77 P-value blast match to reverse transcriptase (Ty1_Copia-element) (Arabidopsis thaliana);(source:TAIR10)
AT2G13940		transposable_element_gene;copia-like retrotransposon family, has a 4.4e-197 P-value blast match to GB:AAA57005 Hopscotch polyprotein (Ty1_Copia-element) (Zea mays);(source:TAIR10)
AT2G13950		Cysteine/Histidine-rich C1 domain family protein
AT2G13960		Homeodomain-like superfamily protein
AT2G13970		transposable_element_gene;Mutator-like transposase family, has a 4.1e-39 P-value blast match to GB:AAA21566 mudrA of transposon=MuDR (MuDr-element) (Zea mays);(source:TAIR10)
AT2G14010		transposable_element_gene;similar to Ulp1 protease family protein [Arabidopsis thaliana] (TAIR:AT2G29240.1);(source:TAIR10)
AT2G14020		transposable_element_gene;similar to unknown protein [Arabidopsis thaliana] (TAIR:AT5G44880.1);(source:TAIR10)
AT2G14030		transposable_element_gene;Mutator-like transposase family, has a 3.1e-78 P-value blast match to Q9SLM0 /314-478 Pfam PF03108 MuDR family transposase (MuDr-element domain);(source:TAIR10)
AT2G14040		transposable_element_gene;gypsy-like retrotransposon family (Athila), has a 1.0e-99 P-value blast match to gb|AAL06421.1|AF378079_1 reverse transcriptase (Athila4) (Arabidopsis thaliana) (Gypsy_Ty3-family);(source:TAIR10)
AT2G14060		encodes a protein whose sequence is similar to SAM:salicylic acid carboxyl methyltransferase (SAMT) (GI:6002712)(Clarkia breweri) and to SAM:benzoic acid carboxyl methyltransferase (BAMT)(GI:9789277)(Antirrhinum majus)
AT2G14080		Disease resistance protein (TIR-NBS-LRR class) family
AT2G14095		hypothetical protein
AT2G14100	*CYTOCHROME P450, FAMILY 705, SUBFAMILY A, POLYPEPTIDE 13 (CYP705A13)*	a member of the cytochrome P450 family
AT2G14110		Haloacid dehalogenase-like hydrolase (HAD) superfamily protein
AT2G14120	*DYNAMIN RELATED PROTEIN (DRP3B)*	Encodes a dynamin related protein. DRPs are self-assembling GTPasse involved in fission and fusion of membranes. DRP3B functions in mitochondrion and peroxisome fission in combination with DRP3A.
AT2G14130		transposable_element_gene;similar to Ulp1 protease family protein [Arabidopsis thaliana] (TAIR:AT3G47260.1);(source:TAIR10)
AT2G14140		transposable_element_gene;similar to heat shock protein binding [Arabidopsis thaliana] (TAIR:AT3G47270.1);(source:TAIR10)
AT2G14160		RNA-binding (RRM/RBD/RNP motifs) family protein
AT2G14210	*AGAMOUS-LIKE 44 (AGL44)*	MADS box gene, transcription factor
AT2G14230		transposable_element_gene;CACTA-like transposase family (Ptta/En/Spm), has a 2.0e-119 P-value blast match to At5g36655.1/81-333 CACTA-like transposase family (Ptta/En/Spm) (CACTA-element) (Arabidopsis thaliana);(source:TAIR10)
AT2G14250		
AT2G14270		Cytochrome C oxidase polypeptide VIB family protein
AT2G14290	*F-BOX/DUF295 BRASSICEAE-SPECIFIC 13 (ATFDB13)*	LL-diaminopimelate protein (DUF295)

AT2G14300		transposable_element_gene;pseudogene, similar to putative helicase, blastp match of 40%25 identity and 2.5e-288 P-value to GP|14140296|gb|AAK54302.1|AC034258_20|AC034258 putative helicase {Oryza sativa (japonica cultivar-group)};(source:TAIR10)
AT2G14330		transposable_element_gene;similar to unknown protein [Arabidopsis thaliana] (TAIR:AT3G45350.1);(source:TAIR10)
AT2G14350		transposable_element_gene;expressed protein, contains Pfam profile PF03384: Drosophila protein of unknown function, DUF287;(source:TAIR10)
AT2G14360		
AT2G14380		transposable_element_gene;gypsy-like retrotransposon family, has a 1.6e-123 P-value blast match to GB:AAD19359 polyprotein (gypsy_Ty3-element) (Sorghum bicolor);(source:TAIR10)
AT2G14390		hypothetical protein
AT2G14400		transposable_element_gene;gypsy-like retrotransposon family, has a 5.4e-191 P-value blast match to GB:BAA84458 GAG-POL precursor (gypsy_Ty3-element) (Oryza sativa)gi|5902445|dbj|BAA84458.1| GAG-POL precursor (Oryza sativa (japonica cultivar-group)) (RIRE2) (Gypsy_Ty3-family);(source:TAIR10)
AT2G14410		pseudogene of myosin heavy chain-like protein
AT2G14420		transposable_element_gene;Mutator-like transposase family, has a 8.4e-10 P-value blast match to GB:AAA21566 mudrA of transposon=MuDR (MuDr-element) (Zea mays);(source:TAIR10)
AT2G14430		transposable_element_gene;non-LTR retrotransposon family (LINE), has a 2.5e-45 P-value blast match to GB:BAA20419 reverse transcriptase (LINE-element) (Mus musculus);(source:TAIR10)
AT2G14450		transposable_element_gene;similar to unknown protein [Arabidopsis thaliana] (TAIR:AT1G35920.1);(source:TAIR10)
AT2G14460		hypothetical protein
AT2G14470		transposable_element_gene;pseudogene, similar to putative helicase, low similarity to SP|Q9UUA2 DNA repair and recombination protein pif1, mitochondrial precursor {Schizosaccharomyces pombe};(source:TAIR10)
AT2G14490		pseudogene of Calcium-dependent ARF-type GTPase activating protein family
AT2G14510		Leucine-rich repeat protein kinase family protein
AT2G14520		CBS domain protein (DUF21)
AT2G14540	*SERPIN 2 (SRP2)*	serpin 2
AT2G14550		pseudogene of RNA-binding (RRM/RBD/RNP motifs) family protein
AT2G14560	*LATE UPREGULATED IN RESPONSE TO HYALOPERONOSPORA PARASITICA (LURP1)*	Encodes LURP1, a member of the LURP cluster (late upregulated in response to Hyaloperonospora parasitica) which exhibits a pronounced upregulation after recognition of the pathogenic oomycte H. parasitica. LURP1 is required for full basal defense to H. parasitica and resistance to this pathogen mediated by the R-proteins RPP4 and RPP5. The mRNA is cell-to-cell mobile.
AT2G14570		transposable_element_gene;similar to unknown protein [Arabidopsis thaliana] (TAIR:AT1G48290.1);(source:TAIR10)
AT2G14580	*BASIC PATHOGENESIS-RELATED PROTEIN 1 (PRB1)*	pathogenesis related protein, encodes a basic PR1-like protein. Expresses in flowers, roots, and not in leaves and responses to ethylene and methyl jasmonate. Salicylic acid represses gene expression.
AT2G14595		transposable_element_gene;Mutator-like transposase family, has a 4.1e-55 P-value blast match to Q9SHN7 /450-633 Pfam PF03108 MuDR family transposase (MuDr-element domain);(source:TAIR10)
AT2G14610	*PATHOGENESIS-RELATED GENE 1 (PR1)*	PR1 gene expression is induced in response to a variety of pathogens. It is a useful molecular marker for the SAR response. Though the Genbank record for the cDNA associated to this gene is called 'PR-1-like', the sequence actually corresponds to PR1. Expression of this gene is salicylic-acid responsive.
AT2G14620	*XYLOGLUCAN ENDOTRANSGLUCOSYLASE/HYDROLASE 10 (XTH10)*	xyloglucan endotransglucosylase/hydrolase 10
AT2G14630		transposable_element_gene;similar to unknown protein [Arabidopsis thaliana] (TAIR:AT5G28785.1);(source:TAIR10)
AT2G14640		transposable_element_gene;gypsy-like retrotransposon family, has a 2.5e-119 P-value blast match to GB:AAD27547 polyprotein (Gypsy_Ty3-element) (Oryza sativa subsp. indica);(source:TAIR10)
AT2G14650		transposable_element_gene;gypsy-like retrotransposon family, has a 1.6e-311 P-value blast match to GB:AAD27547 polyprotein (Gypsy_Ty3-element) (Oryza sativa subsp. indica);(source:TAIR10)
AT2G14660		thymocyte nuclear-like protein
AT2G14670	*SUCROSE-PROTON SYMPORTER 8 (SUC8)*	sucrose-proton symporter 8
AT2G14680	*MATERNAL EFFECT EMBRYO ARREST 13 (MEE13)*	myosin heavy chain-like protein
AT2G14690		Encodes a putative glycosyl hydrolase family 10 protein (xylanase).
AT2G14700		hypothetical protein
AT2G14730		transposable_element_gene;similar to unknown protein [Arabidopsis thaliana] (TAIR:AT5G32621.1);(source:TAIR10)
AT2G14750	*APS KINASE (APK)*	Encodes adenosine-5'-phosphosulfate kinase. Provides activated sulfate for sulfation of secondary metabolites, including the glucosinolates. Essential for pollen viability. The mRNA is cell-to-cell mobile.
AT2G14760		basic helix-loop-helix (bHLH) DNA-binding superfamily protein
AT2G14770		transposable_element_gene;similar to Ulp1 protease family protein [Arabidopsis thaliana] (TAIR:AT1G27780.1);(source:TAIR10)
AT2G14800		hypothetical protein
AT2G14810		hypothetical protein
AT2G14830	*IST1-LIKE 10 (ISTL10)*	Ist1p
AT2G14880	*(SWIB2)*	SWIB/MDM2 domain superfamily protein
AT2G14900	*(GASA7)*	Gibberellin-regulated family protein
AT2G14930		transposable_element_gene;copia-like retrotransposon family, has a 9.9e-255 P-value blast match to dbj|BAA78426.1| polyprotein (AtRE2-1) (Arabidopsis thaliana) (Ty1_Copia-element);(source:TAIR10)

AT2G14950		transposable_element_gene;hAT-like transposase family (hobo/Ac/Tam3), has a 1.5e-57 P-value blast match to GB:AAD24567 transposase Tag2 (hAT-element) (Arabidopsis thaliana);(source:TAIR10)
AT2G14960	*(GH3.1)*	encodes a protein similar to IAA-amido synthases. Lines carrying an insertion in this gene are hypersensitive to auxin.
AT2G14980		transposable_element_gene;CACTA-like transposase family (Tnp2/En/Spm), has a 1.2e-247 P-value blast match to gb|AAG52024.1|AC022456_5 Tam1-homologous transposon protein TNP2, putative;(source:TAIR10)
AT2G15000		caspase-6 protein
AT2G15010		Predicted to encode a PR (pathogenesis-related) protein. Belongs to the plant thionin (PR-13) family with the following members: At1g66100, At5g36910, At1g72260, At2g15010, At1g12663, At1g12660.
AT2G15020		hypothetical protein
AT2G15040	*RECEPTOR LIKE PROTEIN 18 (RLP18)*	pseudogene of receptor like protein 53
AT2G15050	*LIPID TRANSFER PROTEIN (LTP)*	Predicted to encode a PR (pathogenesis-related) protein. Belongs to the lipid transfer protein (PR-14) family with the following members: At2g38540/LTP1, At2g38530/LTP2, At5g59320/LTP3, At5g59310/LTP4, At3g51600/LTP5, At3g08770/LTP6, At2g15050/LTP7, At2g18370/LTP8, At2g15325/LTP9, At5g01870/LTP10, At4g33355/LTP11, At3g51590/LTP12, At5g44265/LTP13, At5g62065/LTP14, At4g08530/LTP15.
AT2G15060		transposable_element_gene;CACTA-like transposase family (Tnp1/En/Spm), has a 1.8e-63 P-value blast match to ref|NP_189784.1| TNP1-related protein (Arabidopsis thaliana) (CACTA-element);(source:TAIR10)
AT2G15070		transposable_element_gene;CACTA-like transposase family (Tnp2/En/Spm), has a 8.4e-192 P-value blast match to gb|AAG52024.1|AC022456_5 Tam1-homologous transposon protein TNP2, putative;(source:TAIR10)
AT2G15080	*RECEPTOR LIKE PROTEIN 19 (RLP19)*	receptor like protein 19
AT2G15090	*3-KETOACYL-COA SYNTHASE 8 (KCS8)*	Encodes KCS8, a member of the 3-ketoacyl-CoA synthase family involved in the biosynthesis of VLCFA (very long chain fatty acids). The mRNA is cell-to-cell mobile.
AT2G15100		transposable_element_gene;gypsy-like retrotransposon family, has a 4.0e-216 P-value blast match to GB:AAD19359 polyprotein (gypsy_Ty3-element) (Sorghum bicolor);(source:TAIR10)
AT2G15110		hypothetical protein (Protein of unknown function, DUF601)
AT2G15120		pseudogene of Plant basic secretory protein (BSP) family protein
AT2G15130		Plant basic secretory protein (BSP) family protein
AT2G15140		transposable_element_gene;pseudogene, Ulp1 protease family, contains Pfam profile PF02902: Ulp1 protease family, C-terminal catalytic domain;(source:TAIR10)
AT2G15150		transposable_element_gene;Mutator-like transposase family, has a 1.8e-84 P-value blast match to Q9SKL7 /23-182 Pfam PF03108 MuDR family transposase (MuDr-element domain);(source:TAIR10)
AT2G15190		transposable_element_gene;pseudogene, Ulp1 protease family, contains Pfam profile PF02902: Ulp1 protease family, C-terminal catalytic domain;(source:TAIR10)
AT2G15200		transposable_element_gene;pseudogene, hypothetical protein, similar to At2g04970, At1g32830, At2g14140, At3g30450, At4g03990, At5g34895, At3g47270, At2g02200;(source:TAIR10)
AT2G15240		UNC-50 family protein
AT2G15260		RING/U-box superfamily protein
AT2G15300		Leucine-rich repeat protein kinase family protein
AT2G15310	*ADP-RIBOSYLATION FACTOR B1A (ARFB1A)*	A member of ARF GTPase family. A thaliana has 21 members of this family, known to be essential for vesicle coating and uncoating and functions in GTP-binding. Gene encoding ADP-ribosylation factor and similar to ADP-ribosylation factor (GI:861205) (Chlamydomonas reinhardtii), other ARFs and ARF-like proteins.
AT2G15320		Leucine-rich repeat (LRR) family protein
AT2G15325		Predicted to encode a PR (pathogenesis-related) protein. Belongs to the lipid transfer protein (PR-14) family with the following members: At2g38540/LTP1, At2g38530/LTP2, At5g59320/LTP3, At5g59310/LTP4, At3g51600/LTP5, At3g08770/LTP6, At2g15050/LTP7, At2g18370/LTP8, At2g15325/LTP9, At5g01870/LTP10, At4g33355/LTP11, At3g51590/LTP12, At5g44265/LTP13, At5g62065/LTP14, At4g08530/LTP15.
AT2G15340		glycine-rich protein
AT2G15360		fucosyltransferase
AT2G15370	*FUCOSYLTRANSFERASE 5 (FUT5)*	Predicted fucosyltransferase, based on similarity to FUT1, but not functionally redundant with FUT1.
AT2G15380		transposable_element_gene;non-LTR retrotransposon family (LINE), has a 1.1e-29 P-value blast match to GB:NP_038603 L1 repeat, Tf subfamily, member 23 (LINE-element) (Mus musculus);(source:TAIR10)
AT2G15390	*FUCOSYLTRANSFERASE 4 (FUT4)*	Encodes an alpha-(1,2)-fucosyltransferase.
AT2G15410		transposable_element_gene;gypsy-like retrotransposon family, has a 4.1e-217 P-value blast match to GB:AAD19359 polyprotein (gypsy_Ty3-element) (Sorghum bicolor);(source:TAIR10)
AT2G15420		myosin heavy chain-like protein
AT2G15440		polysaccharide biosynthesis protein (DUF579)
AT2G15470		Pectin lyase-like superfamily protein
AT2G15480	*UDP-GLUCOSYL TRANSFERASE 73B5 (UGT73B5)*	UDP-glucosyl transferase 73B5
AT2G15490	*UDP-GLYCOSYLTRANSFERASE 73B4 (UGT73B4)*	UDP-glycosyltransferase 73B4
AT2G15500		RNA-binding protein
AT2G15510		transposable_element_gene;non-LTR retrotransposon family (LINE), has a 3.0e-20 P-value blast match to GB:NP_038602 L1 repeat, Tf subfamily, member 18 (LINE-element) (Mus musculus);(source:TAIR10)
AT2G15520		transposable_element_gene;similar to zinc finger protein, putative [Arabidopsis thaliana] (TAIR:AT3G24680.1);(source:TAIR10)

AT2G15530	*MED25 BINDING RING-H2 PROTEIN 1 (MBR1)*	RING/U-box superfamily protein
AT2G15535	*LOW-MOLECULAR-WEIGHT CYSTEINE-RICH 10 (LCR10)*	low-molecular-weight cysteine-rich 10
AT2G15540		transposable_element_gene;non-LTR retrotransposon family (LINE), has a 2.7e-33 P-value blast match to GB:NP_038607 L1 repeat, Tf subfamily, member 9 (LINE-element) (Mus musculus);(source:TAIR10)
AT2G15550		transposable_element_gene;similar to unknown protein [Arabidopsis thaliana] (TAIR:AT2G16410.1);(source:TAIR10)
AT2G15560		Putative endonuclease
AT2G15570	*(ATHM3)*	chloroplast protein similar to prokaryotic thioredoxin.
AT2G15580		RING/U-box superfamily protein
AT2G15600		transposable_element_gene;similar to unknown protein [Arabidopsis thaliana] (TAIR:AT5G41505.1);(source:TAIR10)
AT2G15610		hypothetical protein (DUF1685)
AT2G15620	*NITRITE REDUCTASE 1 (NIR1)*	Involved in the second step of nitrate assimilation. Its expression is induced by nitrate. The mRNA is cell-to-cell mobile.
AT2G15630		Pentatricopeptide repeat (PPR) superfamily protein
AT2G15640		F-box family protein
AT2G15650		transposable_element_gene;copia-like retrotransposon family, has a 5.0e-227 P-value blast match to gb|AAO73527.1| gag-pol polyprotein (Glycine max) (SIRE1) (Ty1_Copia-family);(source:TAIR10)
AT2G15660	*AGAMOUS-LIKE 95 (AGL95)*	AGAMOUS-like 95
AT2G15670		transmembrane protein
AT2G15680	*CALMODULIN-LIKE 30 (CML30)*	Encodes a calmodulin-like protein.
AT2G15690	*DYW DOMAIN PROTEIN 2 (DYW2)*	Encodes an atypical PPR-DYW protein containing five predicted PPR domains and a C-terminal DYW domain separated by an amino acid sequence that do not clearly correspond to an E domain. It is expressed in both the mitochondrion and chloroplast and is also involved in RNA editing in the mitochondrion and chloroplast as a core member of E+-type PPR editosomes.
AT2G15700		transposable_element_gene;copia-like retrotransposon family, has a 2.4e-308 P-value blast match to GB:CAA31653 polyprotein (Ty1_Copia-element) (Arabidopsis thaliana);(source:TAIR10)
AT2G15720		transposable_element_gene;non-LTR retrotransposon family (LINE), has a 5.4e-35 P-value blast match to GB:AAB41224 ORF2 (LINE-element) (Rattus norvegicus);(source:TAIR10)
AT2G15740	*GAZ-LIKE 1 (GAL1)*	Member of a small family of zinc finger containing putative transcription factors.Similar to GAZ.
AT2G15750		transposable_element_gene;similar to unknown protein [Arabidopsis thaliana] (TAIR:AT2G06845.1);(source:TAIR10)
AT2G15760		calmodulin-binding protein (DUF1645)
AT2G15780		Cupredoxin superfamily protein
AT2G15790	*SQUINT (SQN)*	SQN encodes the Arabidopsis homolog of cyclophilin 40 (CyP40). It is specifically required for the vegetative but not the reproductive maturation of the shoot. Belongs to one of the 36 carboxylate clamp (CC)-tetratricopeptide repeat (TPR) proteins (Prasad 2010, Pubmed ID: 20856808) with potential to interact with Hsp90/Hsp70 as co-chaperones.
AT2G15800		transposable_element_gene
AT2G15810		transposable_element_gene;Mutator-like transposase family, has a 1.5e-12 P-value blast match to GB:AAA21566 mudrA of transposon=MuDR (MuDr-element) (Zea mays);(source:TAIR10)
AT2G15830		hypothetical protein
AT2G15860		BAT2 domain protein
AT2G15870		transposable_element_gene;copia-like retrotransposon family, has a 0. P-value blast match to GB:CAA72989 open reading frame 1 (Ty1_Copia-element) (Brassica oleracea);(source:TAIR10)
AT2G15880	*LEUCINE-RICH REPEAT/EXTENSIN 10 (LRX10)*	Pollen expressed protein required for pollen tube growth.Along with other members of the LRX family, itnteracts with RALF4 to control pollen tube growth and integrity. Loss of function results in premature pollen tube rupture and reduced fertility.
AT2G15890	*MATERNAL EFFECT EMBRYO ARREST 14 (MEE14)*	Encodes CBP1, a regulator of transcription initiation in central cell-mediated pollen tube guidance.
AT2G15920		transposable_element_gene;copia-like retrotransposon family, has a 0. P-value blast match to GB:CAA72989 open reading frame 1 (Ty1_Copia-element) (Brassica oleracea);(source:TAIR10)
AT2G15930		
AT2G15940		transposable_element_gene;hAT-like transposase family (hobo/Ac/Tam3), has a 1.1e-114 P-value blast match to GB:AAD24567 transposase Tag2 (hAT-element) (Arabidopsis thaliana);(source:TAIR10)
AT2G15960		Unknown protein. Expression decreased in response to proline.
AT2G15970	*COLD REGULATED 413 PLASMA MEMBRANE 1 (COR413-PM1)*	encodes an alpha form of a protein similar to the cold acclimation protein WCOR413 in wheat. Expression is induced by short-term cold-treatment, water deprivation, and abscisic acid treatment. The mRNA is cell-to-cell mobile.
AT2G16000		transposable_element_gene;copia-like retrotransposon family, has a 2.6e-248 P-value blast match to GB:CAA72989 open reading frame 1 (Ty1_Copia-element) (Brassica oleracea);(source:TAIR10)
AT2G16005	*INTERACTOR OF SYNAPTOTAGMIN1 (ROSY1)*	ROSY1 protein contains a MD-2-related lipid-recognition domain. It is rapidly upregulated in response to gravistimulation.
AT2G16010		transposable_element_gene;similar to unknown protein [Arabidopsis thaliana] (TAIR:AT2G07630.1);(source:TAIR10)
AT2G16020		hypothetical protein
AT2G16040		hAT dimerization domain-containing protein / transposase-like protein
AT2G16060	*HEMOGLOBIN 1 (HB1)*	Encodes a class 1 nonsymbiotic hemoglobin induced by low oxygen levels with very high oxygen affinity. It is not likely to be a hemoglobin transporter because of its extremely high affinity for oxygen. Overexpression impairs cold stress-induced nitric oxide (NO) production.

AT2G16070	*PLASTID DIVISION2 (PDV2)*	An integral outer envelope membrane protein (its homolog in A thaliana PDV1), component of the plastid division machinery. Similar to ARC6, PDV2 localizes to a continuous ring at the division site in wild-type plants. PDV1 and PDV2 are required for localization of ARC5 at the chloroplast division site.
AT2G16100		pseudogene hypothetical protein
AT2G16110		pseudogene of RNA-directed DNA polymerase (reverse transcriptase)-related family protein
AT2G16160		transposable_element_gene;similar to unknown protein [Arabidopsis thaliana] (TAIR:AT1G45080.1);(source:TAIR10)
AT2G16190		hypothetical protein
AT2G16200	*GAMMA1 COAT PROTEIN (gamma1-COP)*	Member of the Coat Protein I (COPI) complex is a seven-subunit coatomer complex consisting of the α, β, β′, γ, δ, ε, and ζ proteins. COPI is required for retrograde transport from the Golgi to the endoplasmic reticulum, Golgi maintenance, and cell plate formation.
AT2G16210	*REPRODUCTIVE MERISTEM 24 (REM24)*	Encodes a member of the REM (Reproductive Meristem) gene family, a part of the B3 DNA-binding domain superfamily.
AT2G16230		O-Glycosyl hydrolases family 17 protein
AT2G16270		transmembrane protein
AT2G16290	*F-BOX/DUF295 ANCESTRAL 8 (ATFDA8)*	F-box SKIP23-like protein (DUF295)
AT2G16330		transposable_element_gene;similar to unknown protein [Arabidopsis thaliana] (TAIR:AT1G45080.1);(source:TAIR10)
AT2G16340		hypothetical protein
AT2G16350		
AT2G16370	*DIHYDROFOLATE REDUCTASE THYMIDYLATE SYNTHASE 1 (DHFR-TS-1)*	Encodes a bifunctional dihydrofolate reductase - thymidylate synthase gene. This is unique in Arabidopsis and protozoa. Other organisms have independent genes for this function. This alternative transcript is produced in tissues other than leaves.
AT2G16380		Sec14p-like phosphatidylinositol transfer family protein
AT2G16390	*DEFECTIVE IN RNA-DIRECTED DNA METHYLATION 1 (DRD1)*	Putative chromatin remodeling protein, member of a plant-specific subfamily of SWI2/SNF2-like proteins. Mutations nearly eliminate non-CpG methylation at a target promoter but do not affect rDNA or centromere methylation. Cooperates with PolIVb to facilitate RNA-directed de novo methylation and silencing of homologous DNA. Endogenous targets include intergenic regions near retrotransposon LTRs or short RNA encoding sequences that might epigenetically regulate adjacent genes. May be used to establish a basal yet reversible level of silencing in euchromatin.
AT2G16400	*BEL1-LIKE HOMEODOMAIN 7 (BLH7)*	BEL1-like homeodomain 7
AT2G16410		transposable_element_gene;similar to unknown protein [Arabidopsis thaliana] (TAIR:AT2G15550.1);(source:TAIR10)
AT2G16430	*PURPLE ACID PHOSPHATASE 10 (PAP10)*	Encodes an acid phosphatase involved plant acclimation to Pi deprivation.
AT2G16440	*MINICHROMOSOME MAINTENANCE 4 (MCM4)*	Regulates DNA replication via interaction with BICE1 and MCM7.
AT2G16450		F-box and associated interaction domains-containing protein
AT2G16480		
AT2G16510	*(VHA-C5)*	Member of V-ATPase family. Vacuolar-type H + -ATPase (V-ATPase) is a multisubunit proton pump located on the endomembranes.
AT2G16520		RING/U-box protein with C6HC-type zinc finger protein
AT2G16570	*GLN PHOSPHORIBOSYL PYROPHOSPHATE AMIDOTRANSFERASE 1 (ASE1)*	Amidophosphoribosyltransferase (ATase: EC 2.4.2.14) is a key enzyme in the pathway of purine nucleotide biosynthesis
AT2G16580	*SMALL AUXIN UPREGULATED RNA 8 (SAUR8)*	SAUR-like auxin-responsive protein family
AT2G16590		
AT2G16595		Translocon-associated protein (TRAP), alpha subunit
AT2G16610		transposable_element_gene;CACTA-like transposase family (En/Spm), has a 6.1e-89 P-value blast match to GB:BAA20532 ORF of transposon Tdc1 (CACTA-element) (Daucus carota);(source:TAIR10)
AT2G16620		Protein kinase superfamily protein
AT2G16630	*FUSED OUTER CUTICULAR LEDGE1 (FOCL1)*	Pollen Ole e 1 allergen and extensin family protein
AT2G16650	*PROTEINACEOUS RNASE P 2 (PRORP2)*	Encodes a proteinaceous RNase P that supports RNase P activity in vivo in both organelles and the nucleus. It is also involved in the maturation of small nucleolar RNA (snoRNA) and mRNA.
AT2G16670		transposable_element_gene;copia-like retrotransposon family, has a 1.2e-190 P-value blast match to GB:AAB82754 retrofit (TY1_Copia-element) (Oryza longistaminata);(source:TAIR10)
AT2G16680		transposable_element_gene;non-LTR retrotransposon family (LINE), has a 5.0e-35 P-value blast match to GB:NP_038605 L1 repeat, Tf subfamily, member 30 (LINE-element) (Mus musculus);(source:TAIR10)
AT2G16700	*ACTIN DEPOLYMERIZING FACTOR 5 (ADF5)*	Encodes actin depolymerizing factor 5 (ADF5).
AT2G16720	*MYB DOMAIN PROTEIN 7 (MYB7)*	Encodes a member of MYB3R- and R2R3- type MYB- encoding gene family that acts as a repressor of flavonol biosynthesis. AtMYB7 gene expression is induced by salt treatment.
AT2G16730	*BETA-GALACTOSIDASE 13 (BGAL13)*	putative beta-galactosidase (BGAL13 gene)
AT2G16740	*UBIQUITIN-CONJUGATING ENZYME 29 (UBC29)*	ubiquitin-conjugating enzyme 29
AT2G16750		kinase with adenine nucleotide alpha hydrolases-like domain-containing protein
AT2G16760		Calcium-dependent phosphotriesterase superfamily protein
AT2G16780	*MULTICOPY SUPPRESSOR OF IRA1 2 (MSI2)*	Encodes a WD-40 repeat protein similar to yeast MSI1.
AT2G16790		P-loop containing nucleoside triphosphate hydrolases superfamily protein
AT2G16810		F-box and associated interaction domains-containing protein
AT2G16820		transposable_element_gene;similar to unknown protein [Arabidopsis thaliana] (TAIR:AT5G27590.1);(source:TAIR10)
AT2G16830		a pseudogene whose sequence is similar to plasma membrane intrinsic protein 3 (PIP3)
AT2G16835		Aquaporin-like superfamily protein

AT2G16840		transposable_element_gene;copia-like retrotransposon family, has a 7.7e-06 P-value blast match to GB:AAC64917 gag-pol polyprotein (Ty1_Copia-element) (Glycine max);(source:TAIR10)
AT2G16870		Disease resistance protein (TIR-NBS-LRR class) family
AT2G16890		UDP-Glycosyltransferase superfamily protein
AT2G16900		phospholipase-like protein (PEARLI 4) family protein
AT2G16910	*ABORTED MICROSPORES (AMS)*	Encodes a basic helix-loop helix transcription factor involved in tapetal cell development, that directly regulates MGT5 expression in tapetum cells. Loss of function mutations are male sterile. AMS binds to a region termed the E box of target gene promoters.
AT2G16950	*TRANSPORTIN 1 (TRN1)*	TRN1 is an importin beta protein that functions as a nuclear import receptor for AtGRP7 and in interacts with AGO1 to affect miRNA loading.
AT2G16970	*MATERNAL EFFECT EMBRYO ARREST 15 (MEE15)*	Major facilitator superfamily protein
AT2G16980		Major facilitator superfamily protein
AT2G16990		Major facilitator superfamily protein
AT2G17000		Mechanosensitive ion channel family protein
AT2G17020		F-box/RNI-like superfamily protein
AT2G17040	*NAC DOMAIN CONTAINING PROTEIN 36 (NAC036)*	Member of the NAC transcription factor family and more specifically, the ONAC022 subfamily. Involved in leaf and inflorescence stem morphogenesis. The mRNA is cell-to-cell mobile.
AT2G17050		disease resistance protein (TIR-NBS-LRR class)
AT2G17060		Disease resistance protein (TIR-NBS-LRR class) family
AT2G17070		hypothetical protein (DUF241)
AT2G17080		hypothetical protein (DUF241)
AT2G17090	*SHORT SUSPENSOR (SSP)*	Encodes a N-myrystolylated plasma membrane associated member of the RLCK II family of IRAK/Pelle-like kinases that regulates the MAPK pathway that promotes the elongation of the Arabidopsis zygote and the development of its basal daughter cell into the extra-embryonic suspensor. SSP transcripts are produced in mature pollen but are not translated until delivery to the zygote and the endosperm after fertilization, exerting a paternal effect on embryonic development. The primary role of its kinase domain may lie in protein binding rather than in catalysis as key residues of the active site are absent.
AT2G17120	*LYSM-CONTAINING RECEPTOR PROTEIN 1 (LYP1)*	Induction of chitin-responsive genes by chitin treatment is not blocked in the mutant. It contains a C-terminal GPI anchor signal and is an ortholog of OsCEBiP.
AT2G17130	*ISOCITRATE DEHYDROGENASE SUBUNIT 2 (IDH2)*	Encodes a regulatory subunit of the mitochondrially-localized NAD+- dependent isocitrate dehydrogenase.
AT2G17140		Pentatricopeptide repeat (PPR) superfamily protein
AT2G17150	*NIN-LIKE PROTEIN 1 (NLP1)*	Plant regulator RWP-RK family protein
AT2G17160		Interleukin-1 receptor-associated kinase 4 protein
AT2G17170		Protein kinase superfamily protein
AT2G17180	*DUO1-ACTIVATED ZINC FINGER 1 (DAZ1)*	Target promoter of the male germline-specific transcription factor DUO1.
AT2G17210		Tetratricopeptide repeat (TPR)-like superfamily protein
AT2G17230	*EXORDIUM LIKE 5 (EXL5)*	EXORDIUM like 5
AT2G17250	*EMBRYO DEFECTIVE 2762 (EMB2762)*	Encodes a nucleolar protein that is a ribosome biogenesis co-factor. Mutants display aberrant RNA processing and homozygous embryos arrest in the globular stage of development.
AT2G17260	*GLUTAMATE RECEPTOR 2 (GLR2)*	Encodes a glutamate receptor. Involved in calcium-programmed stomatal closure.
AT2G17280		Phosphoglycerate mutase family protein
AT2G17290	*CALCIUM DEPENDENT PROTEIN KINASE 6 (CPK6)*	Encodes calcium dependent protein kinase 6 (CPK6), a member of the Arabidopsis CDPK gene family. CDPKs contain an intrinsic Ca2+-activation domain with four EF hand Ca2+-binding sites. CDPKs protein kinases have been proposed to function in multiple plant signal transduction pathways downstream of [Ca2+]cyt elevations, thus transducing various physiological responses. CPK6 is expressed in both guard cells and mesophyll cells. Functions in guard cell ion channel regulation. ABA and Ca(2+) activation of slow-type anion channels and, interestingly, ABA activation of plasma membrane Ca(2+)-permeable channels were impaired in independent alleles of single and double cpk3cpk6 mutant guard cells. Furthermore, ABA- and Ca(2+)-induced stomatal closing were partially impaired in these cpk3cpk6 mutant alleles. The protein kinase CPK6 is shown in biochemical assays to be directly activated by elevations in calcium concentrations in the physiological range (Laanements et al., 2013 PlantPhys.; PMID: 23766366). These data correlate with the in vivo function of CPK6 in Ca2+ and ABA activation of S-type anion channels (Mori et al., 2006 PLoS Biol.; PMID: 17032064) and the ability of CPK6 to mediate ABA activation of SLAC1 (Brandt et al., 2012 PNAS; PMID: 22689970). The mRNA is cell-to-cell mobile.
AT2G17310	*SUPPRESSOR OF NIM1-1 1 (SON1)*	Encodes an F-Box protein that regulates a novel induced defense response independent of both salicylic acid and systemic acquired resistance
AT2G17330	*CYTOCHROME P450 51G2 (CYP51G2)*	putative obtusifoliol 14-alpha demethylase. Expressed pseudogene.
AT2G17340		pantothenate kinase
AT2G17350		beta-mannosyltransferase-like protein
AT2G17370	*3-HYDROXY-3-METHYLGLUTARYL-COA REDUCTASE 2 (HMG2)*	Encodes a 3-hydroxy-3-methylglutaryl-CoA reductase (HMGR) that is involved in the synthesis of sterol and triterpenoid compounds.
AT2G17400		
AT2G17420	*NADPH-DEPENDENT THIOREDOXIN REDUCTASE A (NTRA)*	NADPH-dependent thioredoxin reductase, major cytosolic isoform The mRNA is cell-to-cell mobile.

AT2G17430	*MILDEW RESISTANCE LOCUS O 7 (MLO7)*	A member of a large family of seven-transmembrane domain proteins specific to plants, homologs of the barley mildew resistance locus o (MLO) protein. Controls pollen tube reception in synergids. The Arabidopsis genome contains 15 genes encoding MLO proteins, with localization in plasma membrane. Phylogenetic analysis revealed four clades of closely-related AtMLO genes. ATMLO7 belongs to the clade III, with AtMLO5, AtMLO8, AtMLO9, and AtMLO10. The gene is expressed in vegetative organs (RT-PCR experiments)and in pollen grains, as shown by GUS activity patterns. The expression of several phylogenetically closely-related AtMLO genes showed similar or overlapping tissue specificity and analogous responsiveness to external stimuli, suggesting functional redundancy, co-function, or antagonistic function(s).
AT2G17440	*PLANT INTRACELLULAR RAS GROUP-RELATED LRR 5 (PIRL5)*	Encodes PIRL5, a member of the Plant Intracellular Ras-group-related LRRs (Leucine rich repeat proteins). PIRLs are a distinct, plant-specific class of intracellular LRRs that likely mediate protein interactions, possibly in the context of signal transduction. The mRNA is cell-to-cell mobile.
AT2G17470	*ALUMINIUM ACTIVATED MALATE TRANSPORTER 6 (ALMT6)*	Encodes ALMT6, a member of the aluminum-activated malate transporter family.
AT2G17490		transposable_element_gene;copia-like retrotransposon family, has a 9.3e-199 P-value blast match to gb|AAO73521.1| gag-pol polyprotein (Glycine max) (SIRE1) (Ty1_Copia-family);(source:TAIR10)
AT2G17500	*PIN-LIKES 5 (PILS5)*	Auxin efflux carrier family protein
AT2G17510	*EMBRYO DEFECTIVE 2763 (EMB2763)*	ribonuclease II family protein
AT2G17530		Protein kinase superfamily protein
AT2G17540		hypothetical protein
AT2G17550	*TON1 RECRUITING MOTIF 26 (TRM26)*	RB1-inducible coiled-coil protein
AT2G17570	*CIS-PRENYLTRANSFERASE 1 (CPT1)*	Cis‐prenyltransferase involved in dolichol accumulation.
AT2G17580	*AHG2-1 SUPPRESSOR 1 (AGS1)*	Encodes a bacterial-type poly(A) polymerase, AGS1.
AT2G17600		Cysteine/Histidine-rich C1 domain family protein
AT2G17610		transposable_element_gene;non-LTR retrotransposon family (LINE), has a 3.6e-21 P-value blast match to GB:AAA39398 ORF2 (Mus musculus) (LINE-element);(source:TAIR10)
AT2G17620	*CYCLIN B2;1 (CYCB2;1)*	Cyclin B2
AT2G17630	*PHOSPHOSERINE AMINOTRANSFERASE 2 (PSAT2)*	Pyridoxal phosphate (PLP)-dependent transferases superfamily protein
AT2G17640	*(ATSERAT3;1)*	Encodes a cytosolic serine O-acetyltransferase involved in sulfur assimilation and cysteine biosynthesis. Expressed in the vascular system. Expression is induced after long-term sulfur starvation.
AT2G17650		AMP-dependent synthetase and ligase family protein
AT2G17660		RPM1-interacting protein 4 (RIN4) family protein
AT2G17670		Tetratricopeptide repeat (TPR)-like superfamily protein
AT2G17680		DUF241 domain protein, putative (DUF241)
AT2G17710		Big1
AT2G17730	*NEP-INTERACTING PROTEIN 2 (NIP2)*	Intrinsic thylakoid membrane protein that fixes RPOTmp on the stromal side of the thylakoid membrane.
AT2G17740	*VACUOLELESS GAMETOPHYTES (VLG)*	VACUOLELESS GAMETOPHYTES (VLG) as a DC1 domain containing protein that is found in the endomembrane system. It is essential for both female and male gametophyte development.
AT2G17750	*NEP-INTERACTING PROTEIN 1 (NIP1)*	Intrinsic thylakoid membrane protein that fixes RPOTmp on the stromal side of the thylakoid membrane.
AT2G17760		Eukaryotic aspartyl protease family protein
AT2G17770	*BASIC REGION/LEUCINE ZIPPER MOTIF 27 (BZIP27)*	Encodes a paralog of bZIP transcription factor FD. This protein interacts with FD and FT.
AT2G17790	*VPS35 HOMOLOG A (VPS35A)*	Encodes a protein with similarity to yeast VPS35 which encodes a component of the retromer involved in retrograde endosomal transport. Mutants partially suppress the loss of VTI11 function in Arabidopsis and restores gravitropism in the double mutant. The mRNA is cell-to-cell mobile.
AT2G17820	*HISTIDINE KINASE 1 (HK1)*	Encodes a member of the histidine kinase family.
AT2G17845		NAD(P)-binding Rossmann-fold superfamily protein
AT2G17850		Rhodanese/Cell cycle control phosphatase superfamily protein
AT2G17860		Pathogenesis-related thaumatin superfamily protein
AT2G17870	*COLD SHOCK DOMAIN PROTEIN 3 (CSP3)*	Encodes COLD SHOCK DOMAIN PROTEIN 3 (CSP3), involved in the acquisition of freezing tolerance.
AT2G17880	*DNA J PROTEIN C24 (DJC24)*	Chaperone DnaJ-domain superfamily protein
AT2G17890	*CALCIUM-DEPENDENT PROTEIN KINASE 16 (CPK16)*	Encodes a member of Calcium Dependent Protein Kinase. Protein is N-myristoylated. Localizes to the plasma membrane. Localizes to the chloroplast when the myristoylation motif is mutated.
AT2G17910		transposable_element_gene;non-LTR retrotransposon family (LINE), has a 1.8e-42 P-value blast match to GB:AAB41224 ORF2 (LINE-element) (Rattus norvegicus);(source:TAIR10)
AT2G17920		nucleic acid binding / zinc ion binding protein
AT2G17940		WEB family protein (DUF827)
AT2G17950	*WUSCHEL (WUS)*	Homeobox gene controlling the stem cell pool. Expressed in the stem cell organizing center of meristems. Required to keep the stem cells in an undifferentiated state. Regulation of WUS transcription is a central checkpoint in stem cell control. The size of the WUS expression domain controls the size of the stem cell population through WUS indirectly activating the expression of CLAVATA3 (CLV3) in the stem cells and CLV3 repressing WUS transcription through the CLV1 receptor kinase signaling pathway. Repression of WUS transcription through AGAMOUS (AG) activity controls stem cell activity in the determinate floral meristem. Binds to TAAT element core motif. WUS is also involved in cell differentiation during anther development. Responds to CMV infection and represses virus accumulation in the meristem central and peripheral zones; inhibits viral protein synthesis by repressing the expression of plant S-adenosyl-L-methionine?dependent methyltransferases, which are involved in ribosomal RNA processing and ribosome stability.
AT2G17960		hypothetical protein
AT2G17970	*(ALKBH9B)*	2-oxoglutarate (2OG) and Fe(II)-dependent oxygenase superfamily protein

AT2G17972		transmembrane protein
AT2G17990	*CALCIUM-DEPENDENT PROTEIN KINASE 1 ADAPTOR PROTEIN 2 (AtCAP2)*	Calcium-dependent protein kinase 1 adaptor protein involved in vacuolar transport and lytic vacuole biogenesis.
AT2G18010	*SMALL AUXIN UPREGULATED RNA 10 (SAUR10)*	SAUR-like auxin-responsive protein family
AT2G18030	*METHIONINE SULFOXIDE REDUCTASE A5 (MSRA5)*	Peptide methionine sulfoxide reductase family protein
AT2G18050	*HISTONE H1-3 (HIS1-3)*	encodes a structurally divergent linker histone whose gene expression is induced by dehydration and ABA. The mRNA is cell-to-cell mobile.
AT2G18060	*VASCULAR RELATED NAC-DOMAIN PROTEIN 1 (VND1)*	Encodes a NAC-domain transcription factor that is expressed in developing vessels and protoxylem. Along with other members of this family, VND1 appears to regulate the development of genes required for secondary cell wall biosynthesis.
AT2G18070		hypothetical protein
AT2G18080	*EMBRYO SAC DEVELOPMENT ARREST 2 (EDA2)*	Serine carboxypeptidase S28 family protein
AT2G18090		PHD finger family protein / SWIB complex BAF60b domain-containing protein / GYF domain-containing protein
AT2G18115		pseudogene of glycine-rich protein
AT2G18120	*SHI-RELATED SEQUENCE 4 (SRS4)*	A member of SHI gene family. Arabidopsis thaliana has ten members that encode proteins with a RING finger-like zinc finger motif. Despite being highly divergent in sequence, many of the SHI-related genes are partially redundant in function and synergistically promote gynoecium, stamen and leaf development in Arabidopsis.
AT2G18140		Peroxidase superfamily protein
AT2G18160	*BASIC LEUCINE-ZIPPER 2 (bZIP2)*	Encodes a b-ZIP transcription factor.
AT2G18180		Sec14p-like phosphatidylinositol transfer family protein
AT2G18190		P-loop containing nucleoside triphosphate hydrolases superfamily protein
AT2G18200		transmembrane protein
AT2G18210		hypothetical protein
AT2G18220	*NUCLEOLAR COMPLEX ASSOCIATED 2 (NOC2)*	Noc2p family
AT2G18230	*PYROPHOSPHORYLASE 2 (PPa2)*	Encodes a protein that might have inorganic pyrophosphatase activity.
AT2G18240		Rer1 family protein
AT2G18260	*SYNTAXIN OF PLANTS 112 (SYP112)*	member of SYP11 Gene Family
AT2G18270		hypothetical protein
AT2G18280	*TUBBY LIKE PROTEIN 2 (TLP2)*	Member of TLP family The mRNA is cell-to-cell mobile.
AT2G18300	*HOMOLOG OF BEE2 INTERACTING WITH IBH 1 (HBI1)*	DNA-binding bHLH protein involved in positive regulation of cell elongation and proliferation and, negative control of plant immunity.One component of PRE-IBH1-HBI1 tripartite module.
AT2G18320		F-box associated ubiquitination effector family protein
AT2G18330	*SHOT1 BINDING ATPASE 3 (SBA3)*	Homologue of animal ATPase Family AAA Domain-Containing Protein 3 (ATAD3), which is involved in mitochondrial nucleoid organization; interacts with SHOT1.
AT2G18340	*(LEA13)*	Late embryogenesis abundant (LEA) protein involved in drought tolerance and participate in stomatal density.
AT2G18350	*HOMEOBOX PROTEIN 24 (HB24)*	homeobox protein 24
AT2G18360		alpha/beta-Hydrolases superfamily protein
AT2G18370		Predicted to encode a PR (pathogenesis-related) protein. Belongs to the lipid transfer protein (PR-14) family with the following members: At2g38540/LTP1, At2g38530/LTP2, At5g59320/LTP3, At5g59310/LTP4, At3g51600/LTP5, At3g08770/LTP6, At2g15050/LTP7, At2g18370/LTP8, At2g15325/LTP9, At5g01870/LTP10, At4g33355/LTP11, At3g51590/LTP12, At5g44265/LTP13, At5g62065/LTP14, At4g08530/LTP15.
AT2G18380	*GATA TRANSCRIPTION FACTOR 20 (GATA20)*	Encodes a member of the GATA factor family of zinc finger transcription factors.
AT2G18390	*TITAN 5 (TTN5)*	Encodes a member of ARF-like GTPase family. A thaliana has 21 members, in two subfamilies, ARF and ARF-like (ARL) GTPases. Mutant has abnormal mitosis and cell cycle control during seed development.
AT2G18410	*ELONGATOR COMPLEX PROTEIN 5 (ELP5)*	elongator complex protein
AT2G18420	*(GASA11)*	Encodes a Gibberellin-regulated GASA/GAST/Snakin family protein
AT2G18440	*GENE WITH UNSTABLE TRANSCRIPT 15 (GUT15)*	Encodes a noncoding RNA, a member of an emerging class of transcripts that lack significant open reading frames and encode RNA as their final product. Has been identified as a translated small open reading frame by ribosome profiling.
AT2G18450	*SUCCINATE DEHYDROGENASE 1-2 (SDH1-2)*	Nuclear encoded mitochondrial flavoprotein subunit of succinate dehydrogenase complex .
AT2G18470	*PROLINE-RICH EXTENSIN-LIKE RECEPTOR KINASE 4 (PERK4)*	Proline-rich extensin-like receptor kinase 4. Functions at an early stage of ABA signalling inhibiting primary root cell elongation by perturbing Ca2+ homeostasis.
AT2G18480		Major facilitator superfamily protein
AT2G18490	*GA- AND ABA-RESPONSIVE ZINC FINGER (GAZ)*	GAZ is a nuclear localized transcriptional activator that is regulated (decreased) by GA and ABA levels. GAZ is expressed in the root stele and may function non-cell autonomously to effect hormone mediated control of ground tissue maturation.
AT2G18500	*OVATE FAMILY PROTEIN 7 (OFP7)*	ovate family protein 7
AT2G18510	*EMBRYO DEFECTIVE 2444 (emb2444)*	Essential gene (embryo lethal) that is similar to component of splicosome. Regulates embryonic pattern formation through Pol II-Mediated transcription of WOX2 an PIN7 (DOI:10.1016/j.isci.2019.09.004). JANUS positively regulates PLT1 expression in the root meristem by recruiting RNA polymerase II (Pol II) to PLT1 and by interacting with PLT1. Nuclear accumulation of JANUS in root meristem depends on IMB4. (DOI:10.1105/tpc.20.00108)
AT2G18530		Protein kinase superfamily protein
AT2G18540		RmlC-like cupins superfamily protein
AT2G18550	*HOMEOBOX PROTEIN 21 (HB21)*	Encodes a homeodomain leucine zipper class I (HD-Zip I) protein.
AT2G18570		UDP-Glycosyltransferase superfamily protein

AT2G18580		transposable_element_gene;hAT-like transposase family (hobo/Ac/Tam3), has a 3.7e-98 P-value blast match to GB:AAD24567 transposase Tag2 (hAT-element) (Arabidopsis thaliana);(source:TAIR10)
AT2G18590		Major facilitator superfamily protein
AT2G18600		Ubiquitin-conjugating enzyme family protein
AT2G18610		
AT2G18620	*GERANYLGERANYL PYROPHOSPHATE SYNTHASE 2 (GGPPS2)*	Terpenoid synthases superfamily protein
AT2G18630		transmembrane protein, putative (DUF677)
AT2G18640	*GERANYLGERANYL PYROPHOSPHATE SYNTHASE 4 (GGPS4)*	Encodes an endoplasmic reticulum-targeted geranylgeranyl pyrophosphate synthase
AT2G18650	*MATERNAL EFFECT EMBRYO ARREST 16 (MEE16)*	RING/U-box superfamily protein
AT2G18660	*PLANT NATRIURETIC PEPTIDE A (PNP-A)*	Encodes PNP-A (Plant Natriuretic Peptide A). PNPs are a class of systemically mobile molecules distantly related to expansins; their biological role has remained elusive. PNP-A contains a signal peptide domain and is secreted into the extracellular space. Co-expression analyses using microarray data suggest that PNP-A may function as a component of plant defence response and SAR in particular, and could be classified as a newly identified PR protein. It is stress responsive and can enhance its own expression.
AT2G18670	*ARABIDOPSIS T??XICOS EN LEVADURA 56 (ATL56)*	RING/U-box superfamily protein
AT2G18680		transmembrane protein
AT2G18690		transmembrane protein
AT2G18720		Translation elongation factor EF1A/initiation factor IF2gamma family protein
AT2G18730	*DIACYLGLYCEROL KINASE 3 (DGK3)*	diacylglycerol kinase 3
AT2G18750	*(CBP60C)*	Calmodulin-binding protein
AT2G18760	*CHROMATIN REMODELING 8 (CHR8)*	chromatin remodeling 8
AT2G18800	*XYLOGLUCAN ENDOTRANSGLUCOSYLASE/HYDROLASE 21 (XTH21)*	xyloglucan endotransglucosylase/hydrolase 21
AT2G18810		B3 domain protein (DUF313)
AT2G18830		RNA-binding (RRM/RBD/RNP motif) family protein
AT2G18880	*VERNALIZATION5/VIN3-LIKE 2 (VEL2)*	vernalization5/VIN3-like protein
AT2G18890	*RECEPTOR-LIKE CYTOPLASMIC KINASE VI_A2 (RLCK VI_A2)*	RLCK VI_A class kinase which activity is regulated by Rho-of-plants (ROP) GTPases. Controls seedling and plant growth in parallel with gibberrellin.
AT2G18900		Transducin/WD40 repeat-like superfamily protein
AT2G18915	*LOV KELCH PROTEIN 2 (LKP2)*	encodes a member of F-box proteins that includes two other proteins in Arabidopsis (ZTL and FKF1). These proteins contain a unique structure containing a PAS domain at their N-terminus, an F-box motif, and 6 kelch repeats at their C-terminus. Overexpression results in arrhythmic phenotypes for a number of circadian clock outputs in both constant light and constant darkness, long hypocotyls under multiple fluences of both red and blue light, and a loss of photoperiodic control of flowering time. Although this the expression of this gene itself is not regulated by circadian clock, it physically interacts with Dof transcription factors that are transcriptionally regulated by circadian rhythm. LKP2 interacts with Di19, CO/COL family proteins.
AT2G18920		hypothetical protein
AT2G18930		
AT2G18940		Tetratricopeptide repeat (TPR)-like superfamily protein
AT2G18950	*HOMOGENTISATE PHYTYLTRANSFERASE 1 (HPT1)*	Encodes homogentisate phytyltransferase involved in tocopherol biosynthesis. Has impact on seed longevity and plays a role in the adaptation to low temperature stress, notably phloem loading.
AT2G18970		hypothetical protein
AT2G18980	*(PRX16)*	Class III peroxidase cell wall-targeted protein localized to the micropylar endosperm facing the radicle. Involved in seed germination.
AT2G19000		hypothetical protein
AT2G19040	*RALF-LIKE 12 (RALFL12)*	Member of a diversely expressed predicted peptide family showing sequence similarity to tobacco Rapid Alkalinization Factor (RALF), and is believed to play an essential role in the physiology of Arabidopsis. Consists of a single exon and is characterized by a conserved C-terminal motif and N-terminal signal peptide.
AT2G19050		GDSL-motif esterase/acyltransferase/lipase. Enzyme group with broad substrate specificity that may catalyze acyltransfer or hydrolase reactions with lipid and non-lipid substrates.
AT2G19060		SGNH hydrolase-type esterase superfamily protein
AT2G19070	*SPERMIDINE HYDROXYCINNAMOYL TRANSFERASE (SHT)*	encodes a protein whose sequence is similar to anthranilate N-hydroxycinnamoyl/benzoyltransferase from Dianthus caryophyllus (gi:2239091). BAHD acyltransferase. Uses hydroxycinnamoyl CoAs, including caffeoyl/feruoyl/p-coumaroyl/sinapoyl-CoA as acyl donors to fully substitute the N1, N5, and N10 positions of spermidine.
AT2G19080		metaxin-like protein
AT2G19100		transposable_element_gene;non-LTR retrotransposon family (LINE), has a 3.2e-33 P-value blast match to GB:AAB41224 ORF2 (LINE-element) (Rattus norvegicus);(source:TAIR10)
AT2G19110	*HEAVY METAL ATPASE 4 (HMA4)*	Encodes a protein with similarity to Zn ATPase. Can rescue Zn deficiency in yeast and Cd resistance, suggesting a role in Zn and Cd transport. The mRNA is cell-to-cell mobile.
AT2G19120		P-loop containing nucleoside triphosphate hydrolases superfamily protein
AT2G19130		S-locus lectin protein kinase family protein
AT2G19140		transposable_element_gene;copia-like retrotransposon family, has a 2.0e-09 P-value blast match to GB:CAA72989 open reading frame 1 (Ty1_Copia-element) (Brassica oleracea);(source:TAIR10)
AT2G19150		Pectin lyase-like superfamily protein
AT2G19180		hypothetical protein

AT2G19190	*FLG22-INDUCED RECEPTOR-LIKE KINASE 1 (FRK1)*	Encodes a receptor-like protein kinase that is involved in early defense signaling. Expression of this gene is strongly induced during leaf senescence. It is a target of the transcription factor AtWRKY6.
AT2G19200		pseudogene of hypothetical protein (DUF626)
AT2G19210		Leucine-rich repeat transmembrane protein kinase protein
AT2G19230		Leucine-rich repeat transmembrane protein kinase protein
AT2G19250		pseudogene of ALBINA 1
AT2G19290		hypothetical protein
AT2G19300		hypothetical protein
AT2G19320		hypothetical protein
AT2G19330	*PLANT INTRACELLULAR RAS GROUP-RELATED LRR 6 (PIRL6)*	Encodes PIRL6, a member of the Plant Intracellular Ras-group-related LRRs (Leucine rich repeat proteins). PIRLs are a distinct, plant-specific class of intracellular LRRs that likely mediate protein interactions, possibly in the context of signal transduction.
AT2G19360		tRNA-splicing ligase, putative (DUF239)
AT2G19370		
AT2G19385		zinc ion binding protein
AT2G19390	*SAGA COMPLEX SUBUNIT 2A (SCS2A)*	SAGA complex subunit. Regulates gene expression by affecting histone H3 acetylation.
AT2G19400	*NUCLEAR DBF2-RELATED 4 (NDR4)*	AGC (cAMP-dependent, cGMP-dependent and protein kinase C) kinase family protein
AT2G19410	*(PUB34)*	Plant U-box type E3 ubiquitin ligase (PUB).
AT2G19420		hypothetical protein
AT2G19440	*ZERZAUST HOMOLOG (ZETH)*	Homolog of ZET, an atypical β-1,3 glucanase. Differentially expressed in Ler (very low) vs Col (very high) backgrounds.
AT2G19450	*TRIACYLGLYCEROL BIOSYNTHESIS DEFECT 1 (TAG1)*	Encodes Acyl-CoA:diacylglycerol acyltransferase (DGAT) catalyzes the final step of the triacylglycerol synthesis pathway. An insertion mutation in the TAG1 gene results in altered lipid phenotype. Role in senescence and seed development. Its preferred substrate is linolenoyl-CoA (C18:3-CoA).
AT2G19460		DUF3511 domain protein (DUF3511)
AT2G19470	*CASEIN KINASE I-LIKE 5 (ckl5)*	Member of CKL gene family (CKL-B group)
AT2G19480	*NUCLEOSOME ASSEMBLY PROTEIN 1;2 (NAP1;2)*	This gene is predicted to encode a nucleosome assembly protein. Plant lines expressing an RNAi construct directed against this gene show a reduction in agrobacterium-mediated root transformation. The mRNA is cell-to-cell mobile. Plants mutated in three ubiquitously expressed NAP1 genes (NAP1;1~NAP1;3) and organ-specifically expressed NAP1;4 gene show hypersensitivity to genotoxic stresses including UV and DSB-inducing agent Bleomycin. The NAP1 genes act synergistically with NRP genes in promoting somatic homologous recombination.
AT2G19500	*CYTOKININ OXIDASE 2 (CKX2)*	It encodes a protein whose sequence is similar to cytokinin oxidase/dehydrogenase, which catalyzes the degradation of cytokinins.
AT2G19510	*LOB DOMAIN-CONTAINING PROTEIN 8 (LBD8)*	LOB domain-containing protein 8
AT2G19540	*HEAT STRESS TOLERANT DWD 1 (HTD1)*	Transducin family protein / WD-40 repeat family protein
AT2G19550		alpha/beta-Hydrolases superfamily protein
AT2G19560	*ENHANCED ETHYLENE RESPONSE 5 (EER5)*	encodes a protein with a PAM domain involved in ethylene signaling. eer5 mutants show ethylene hypersensitivity in relation to hypocotyl elongation. EER5 interacts with EIN2 and with COP9 in Y2H assays. EIN3 protein levels are the same in WT and eer5-1 mutants. EER5 may be involved in promoting a dampening of the ethylene response.
AT2G19570	*CYTIDINE DEAMINASE 1 (CDA1)*	Encodes a cytidine deaminase that deaminates cytidine and deoxycytidine and is competitively inhibited by cytosine-containing compounds.
AT2G19580	*TETRASPANIN2 (TET2)*	Member of TETRASPANIN family
AT2G19590	*ACC OXIDASE 1 (ACO1)*	encodes a protein whose sequence is similar to 1-aminocyclopropane-1-carboxylate oxidase
AT2G19600	*K+ EFFLUX ANTIPORTER 4 (KEA4)*	member of Putative potassium proton antiporter family
AT2G19610		RING/U-box superfamily protein
AT2G19620	*N-MYC DOWNREGULATED-LIKE 3 (NDL3)*	Plays a role in dehydration stress response.
AT2G19630		F-box and associated interaction domains-containing protein
AT2G19640	*ASH1-RELATED 2 (ASHR2)*	ASH1-related protein 2
AT2G19650		Cysteine/Histidine-rich C1 domain family protein
AT2G19660		Cysteine/Histidine-rich C1 domain family protein
AT2G19670	*PROTEIN ARGININE METHYLTRANSFERASE 1A (PRMT1A)*	protein arginine methyltransferase 1A
AT2G19700		hypothetical protein
AT2G19710	*IST1-LIKE 5 (ISTL5)*	Regulator of Vps4 activity in the MVB pathway protein
AT2G19770	*PROFILIN 5 (PRF5)*	Encodes profilin 5, originally named profilin 4 (PRO4/PFN4). Low-molecular weight, actin monomer-binding protein that regulates the organization of actin cytoskeleton. Pollen-specific plant profilin present predominantly in mature pollen and growing pollen tubes.
AT2G19780		Leucine-rich repeat (LRR) family protein
AT2G19800	*MYO-INOSITOL OXYGENASE 2 (MIOX2)*	Encodes a myo-inositol oxygenase family gene.
AT2G19810	*OXIDATION-RELATED ZINC FINGER 1 (OZF1)*	Encodes Oxidation-related Zinc Finger 1 (OZF1), a plasma membrane protein involved in oxidative stress.
AT2G19840		transposable_element_gene;copia-like retrotransposon family, has a 3.5e-301 P-value blast match to GB:CAA31653 polyprotein (Ty1_Copia-element) (Arabidopsis thaliana);(source:TAIR10)
AT2G19850		transcription repressor
AT2G19860	*HEXOKINASE 2 (HXK2)*	Encodes a protein with hexokinase activity (AtHXK2) and acts as a sensor for plant sugar responses.
AT2G19880	*GLCCER SYNTHASE (GCS)*	Encodes Glucosylceramide synthase (GCS) which catalyzes the final step in glucosylceramide (GlcCer) synthesis by transferring a glucosyl residue from UDP-Glc to the ceramide backbone.
AT2G19890		hypothetical protein

AT2G19900	*NADP-MALIC ENZYME 1 (NADP-ME1)*	The malic enzyme (EC 1.1.1.40) encoded by AtNADP-ME1 is expressed in response to developmental and cell-specific signals. The enzyme is active in vitro and appears to function as a homohexamer or homooctamer. It is believed to be a cytosolic protein.
AT2G19930		RNA-dependent RNA polymerase family protein
AT2G19960		hAT family dimerization domain-containing protein
AT2G19970		CAP (Cysteine-rich secretory proteins, Antigen 5, and Pathogenesis-related 1 protein) superfamily protein
AT2G19980		CAP (Cysteine-rich secretory proteins, Antigen 5, and Pathogenesis-related 1 protein) superfamily protein
AT2G19990	*PATHOGENESIS-RELATED PROTEIN-1-LIKE (PR-1-LIKE)*	Encodes a PR-1-like protein homolog that is differentially expressed in resistant compared to susceptible cultivars by powdery mildew infection. The deduced amino acid sequence has 24 amino acids comprising the signal peptide and 140 amino acids of the mature peptide (15 kDa). Northern blot analysis showed accumulation of the corresponding mRNA 12 h after inoculation of resistant barley cultivars with Erysiphe graminis. Though the Genbank record for the cDNA associated to this gene model is called 'PR-1', the sequence actually corresponds to PR-1-like. Expression of this gene is not salicylic-acid responsive.
AT2G20030	*ARABIDOPSIS T??XICOS EN LEVADURA 12 (ATL12)*	RING/U-box superfamily protein
AT2G20040		
AT2G20050		protein phosphatase 2C and cyclic nucleotide-binding/kinase domain-containing protein
AT2G20060		Ribosomal protein L4/L1 family
AT2G20070		defensin-like protein
AT2G20080	*TCP INTERACTOR CONTAINING EAR MOTIF PROTEIN 2 (TIE2)*	hypothetical protein
AT2G20090		
AT2G20100	*UNKNOWN TRANSCRIPTION FACTOR 1 (UKTF1)*	Together with PFA1 and PFA3 governs the competence of pericycle cells to initiate lateral root primordium formation.
AT2G20145		
AT2G20150		hypothetical protein
AT2G20160	*MEIDOS (MEO)*	E3 ubiquitin ligase SCF complex subunit SKP1/ASK1 family protein
AT2G20170		NEP-interacting protein, putative (DUF239)
AT2G20180	*PHYTOCHROME INTERACTING FACTOR 3-LIKE 5 (PIL5)*	Encodes a novel Myc-related bHLH transcription factor that has transcriptional activation activity in the dark. It is a key negative regulator of phytochrome-mediated seed germination and acts by inhibiting chlorophyll biosynthesis, light-mediated suppression of hypocotyl elongation and far-red light-mediated suppression of seed germination, and promoting negative gravitropism in hypocotyls. Light reduces this activity in a phy-dependent manner. The protein preferentially interacts with the Pfr forms of Phytochrome A (PhyA) and Phytochrome B (PhyB), is physically associated with APRR1/TOC1 and is degraded in red (R) and far-red (FR) light through the ubiquitin (ub)-26S proteasome pathway to optimize photomorphogenic development in Arabidopsis. It also negatively regulates GA3 oxidase expression.
AT2G20200		
AT2G20210		RNI-like superfamily protein
AT2G20220		
AT2G20250		hypothetical protein
AT2G20260	*PHOTOSYSTEM I SUBUNIT E-2 (PSAE-2)*	Encodes subunit E of photosystem I. The mRNA is cell-to-cell mobile.
AT2G20280		Zinc finger C-x8-C-x5-C-x3-H type family protein
AT2G20290	*MYOSIN-LIKE PROTEIN XIG (XIG)*	member of Myosin-like proteins
AT2G20320		DENN (AEX-3) domain-containing protein
AT2G20350		encodes a member of the ERF (ethylene response factor) subfamily B-6 of ERF/AP2 transcription factor family. The protein contains one AP2 domain. There are 12 members in this subfamily including RAP2.11.
AT2G20370	*MURUS 3 (MUR3)*	Encodes a xyloglucan galactosyltransferase located in the membrane of Golgi stacks that is involved in the biosynthesis of fucose. It is also involved in endomembrane organization. It is suggested that it is a dual-function protein that is responsible for actin organization and the synthesis of cell wall materials. The mRNA is cell-to-cell mobile.
AT2G20380		Galactose oxidase/kelch repeat superfamily protein
AT2G20390		cytochrome oxidase complex assembly protein
AT2G20400	*PHR1-LIKE 4 (PHL4)*	MYB-CC transcription factor. PHL4 is related to PHR1 (which regulates plant Pi starvation response) but it does not seem to have a significant role in Pi starvation.
AT2G20410		RNA-binding ASCH domain protein
AT2G20420		ATP citrate lyase (ACL) family protein
AT2G20430	*ROP-INTERACTIVE CRIB MOTIF-CONTAINING PROTEIN 6 (RIC6)*	encodes a member of a novel protein family that contains contain a CRIB (for Cdc42/Rac-interactive binding) motif required for their specific interaction with GTP-bound Rop1 (plant-specific Rho GTPase). Most similar to RIC7 and RIC8 (subfamily group II). Gene is expressed predominantly in inflorescence and flower tissue.
AT2G20440		Ypt/Rab-GAP domain of gyp1p superfamily protein
AT2G20450		Ribosomal protein L14
AT2G20470	*NUCLEAR DBF2-RELATED 5 (NDR5)*	AGC (cAMP-dependent, cGMP-dependent and protein kinase C) kinase family protein
AT2G20480		hypothetical protein
AT2G20490	*(NOP10)*	nucleolar RNA-binding Nop10p family protein
AT2G20500		hypothetical protein
AT2G20510	*TRANSLOCASE INNER MEMBRANE SUBUNIT 44-1 (TIM44-1)*	One of two loci encoding the TIM44 subunit of the mitochondrial inner membrane translocase complex. TIM44 subunit is the part of the complex that hydrolyzes ATP to provide energy for protein translocation to the inner membrane.
AT2G20515		pollen Ole e I family allergen protein

AT2G20520	*FASCICLIN-LIKE ARABINOGALACTAN 6 (FLA6)*	fasciclin-like arabinogalactan-protein 6 (Fla6). Possibly involved in embryogenesis and seed development.
AT2G20530	*PROHIBITIN 6 (PHB6)*	prohibitin 6
AT2G20540	*MITOCHONDRIAL EDITING FACTOR 21 (MEF21)*	Encodes a pentatricopeptide repeat protein (PPR) protein involved in mitochondrial mRNA editing.
AT2G20550		HSP40/DnaJ peptide-binding protein
AT2G20560	*DNAJ PROTEIN (DNAJ)*	DNAJ heat shock family protein
AT2G20570	*GBF'S PRO-RICH REGION-INTERACTING FACTOR 1 (GPRI1)*	Encodes GLK1, Golden2-like 1, one of a pair of partially redundant nuclear transcription factors that regulate chloroplast development in a cell-autonomous manner. GLK2, Golden2-like 2, is encoded by At5g44190. GLK1 and GLK2 regulate the expression of the photosynthetic apparatus. GLK1 is also a member of the GARP transcription factor family.
AT2G20585	*NUCLEAR FUSION DEFECTIVE 6 (NFD6)*	nuclear fusion defective 6
AT2G20595		highly expressed in the female gametophyte central cell and expressed at low levels in the egg and synergids
AT2G20610	*SUPERROOT 1 (SUR1)*	Confers auxin overproduction. Mutants have an over-proliferation of lateral roots. Encodes a C-S lyase involved in converting S-alkylthiohydroximate to thiohydroximate in glucosinolate biosynthesis. Induced in epidermal cells attacked by powdery mildew. The RTY enzyme is expected to function as a dimer (or a higher order multimeric complex), as all RTY-related enzymes with a defined crystal structure are known to form dimers or tetramers.
AT2G20616		QWRF motif protein (DUF566)
AT2G20620		hypothetical protein (DUF626)
AT2G20625		hypothetical protein (DUF626)
AT2G20630	*PP2C INDUCED BY AVRRPM1 (PIA1)*	PP2C induced by AVRRPM1
AT2G20660	*RALF-LIKE 14 (RALFL14)*	Member of a diversely expressed predicted peptide family showing sequence similarity to tobacco Rapid Alkalinization Factor (RALF), and is believed to play an essential role in the physiology of Arabidopsis. Consists of a single exon and is characterized by a conserved C-terminal motif and N-terminal signal peptide.
AT2G20670		sugar phosphate exchanger, putative (DUF506)
AT2G20690		A synthetic gene encoding the catalytic domain of the Arabidopsis thaliana gene At2g20690 was recombinant expressed in E. coli demonstrating the molecular function of riboflavin synthase. The mRNA is cell-to-cell mobile.
AT2G20700	*LORELEI-LIKE-GPI ANCHORED PROTEIN 2 (LLG2)*	LLG2/3 are involved in regulation of pollen tube growth by trafficking of ANX/BUPS to the apical PM of pollen tubes. In the PM they function as co-receptors with ANX/BUPS to regulate ROS production.
AT2G20710		Tetratricopeptide repeat (TPR)-like superfamily protein
AT2G20720		Pentatricopeptide repeat (PPR) superfamily protein
AT2G20740		Tetraspanin family protein
AT2G20750	*EXPANSIN B1 (EXPB1)*	member of BETA-EXPANSINS. Naming convention from the Expansin Working Group (Kende et al, 2004. Plant Mol Bio)
AT2G20770	*GCR2-LIKE 2 (GCL2)*	Encodes a protein with reported similarity to GCR2 a putative G protein coupled receptor thought to be an ABA receptor.GCL2 also has similarity to LANCL1 and LANCL2, human homologs of bacterial lanthionine synthetase.
AT2G20780		Major facilitator superfamily protein
AT2G20800	*NAD(P)H DEHYDROGENASE B4 (NDB4)*	NAD(P)H dehydrogenase B4
AT2G20805		DNA-binding storekeeper protein transcriptional regulator-like protein
AT2G20810	*GALACTURONOSYLTRANSFERASE 10 (GAUT10)*	Galacturonosyltransferase,regulator of root meristem maintenance.
AT2G20830		folic acid binding / transferase
AT2G20850	*STRUBBELIG-RECEPTOR FAMILY 1 (SRF1)*	STRUBBELIG-receptor family 1
AT2G20870		cell wall protein precursor
AT2G20875	*EPIDERMAL PATTERNING FACTOR 1 (EPF1)*	Encodes a secretory peptide EPF1 involved in stomatal development. EPF1 is related to EPF2 which controls asymmetric cell divisions during stomatal devlopment. Its transcript levels change after inducing MUTE expression in a mute background.
AT2G20880	*ERF DOMAIN 53 (ERF53)*	Encodes ERF53, a drought-induced transcription factor. Belongs to the AP2/ERF superfamily, and has a highly conserved AP2 domain. Regulates drought-responsive gene expressions by binding to the GCC box and/or dehydration-responsive element (DRE) in the promoter of downstream genes. Overexpression of AtERF53 driven by the CaMV35S promoter resulted in an unstable drought-tolerant phenotype in T2 transgenic plants. Involved in heat shock response.
AT2G20890	*PHOTOSYSTEM II REACTION CENTER PSB29 PROTEIN (PSB29)*	Chloroplast-localized Thylakoid formation1 gene product involved in vesicle-mediated formation of thylakoid membranes. Thf1 antisense lines contain abnormal chloroplasts early in leaf development (chloroplasts have loosely stacked thylakoid membranes). Expression was induced in the light and decreased under dark conditions. G-alpha interaction partner that functions downstream of the plasma membrane?delimited heterotrimeric G-protein (GPA1) in a D-glucose signaling pathway. Localized to both the outer plastid membrane and the stroma. Probably involved in the metabolic pathway that controls the assembly of the PS II complex. The mRNA is cell-to-cell mobile.
AT2G20940		transmembrane protein, putative (DUF1279)
AT2G20950		phospholipase-like protein (PEARLI 4) family protein
AT2G20960	*(pEARLI4)*	phospholipase-like protein (PEARLI 4) family protein
AT2G20970		lipid-binding protein
AT2G20980	*MINICHROMOSOME MAINTENANCE 10 (MCM10)*	Similar to MCM10, which in other organism was shown to be involved in the initiation of DNA replication.
AT2G21000		transposable_element_gene;copia-like retrotransposon family, has a 1.8e-22 P-value blast match to GB:CAA72989 open reading frame 1 (Ty1_Copia-element) (Brassica oleracea);(source:TAIR10)
AT2G21020		pseudogene of NOD26-like intrinsic protein 3
AT2G21030		BREVIS RADIX-like protein
AT2G21040		C2 domain-containing protein. Possible pseudogene of AT2G20990.
AT2G21045	*HIGH ARSENIC CONTENT 1 (HAC1)*	Arsenate reductase. Contributes to QTL for arsenate tolerance. Col is resistant and Kas-1 represents sensitive strain.

AT2G21050	*LIKE AUXIN RESISTANT 2 (LAX2)*	Encodes LAX2 (LIKE AUXIN RESISTANT), a member of the AUX1 LAX family of auxin influx carriers. Required for the establishment of embryonic root cell organization.
AT2G21060	*GLYCINE-RICH PROTEIN 2B (GRP2B)*	Glycine-rich protein (AtGRP2b). Also named as CSP4 (cold shock domain protein 4) containing a well conserved cold shock domain (CSD) and glycine-rich motifs interspersed by two retroviral-like CCHC zinc fingers. AtCSP4 is expressed in all tissues but accumulates in reproductive tissues and those undergoing cell divisions. Overexpression of AtCSP4 reduces silique length and induces embryo lethality.
AT2G21070	*FIONA1 (FIO1)*	This gene is predicted to an encode a nuclear-localized protein that is involved in regulating the period of circadian rhythms without affecting their amplitude or robustness. FIONA1 seems to act as a central oscillator-associated component, but its transcript levels are not regulated in a circadian or light-dependent manner. FIONA1 also appears to be involved in photoperiod-dependent flowering.
AT2G21080		Ras guanine nucleotide exchange factor K
AT2G21090		Pentatricopeptide repeat (PPR-like) superfamily protein
AT2G21100		Disease resistance-responsive (dirigent-like protein) family protein
AT2G21110		Disease resistance-responsive (dirigent-like protein) family protein
AT2G21130		Cyclophilin-like peptidyl-prolyl cis-trans isomerase family protein
AT2G21140	*PROLINE-RICH PROTEIN 2 (PRP2)*	Proline-rich protein expressed in expanding leaves, stems, flowers, and siliques.
AT2G21150	*XAP5 CIRCADIAN TIMEKEEPER (XCT)*	Encodes a nuclear localized XAP5 family protein involved in light regulation of the circadian clock and photomorphogenesis. XCT loss of function mutations also show decreased levels of DCL1, 3 and 4 and correspondingly lower levels of certain small RNAs suggesting a role in sRNA biogenesis.
AT2G21160		Translocon-associated protein (TRAP), alpha subunit
AT2G21180		transmembrane protein
AT2G21185		transmembrane protein
AT2G21190		ER lumen protein retaining receptor family protein
AT2G21200	*SMALL AUXIN UPREGULATED RNA 7 (SAUR7)*	SAUR-like auxin-responsive protein family
AT2G21210	*SMALL AUXIN UPREGULATED RNA 6 (SAUR6)*	Putative auxin-regulated protein whose expression is downregulated in response to chitin oligomers.
AT2G21220	*SMALL AUXIN UPREGULATED RNA 12 (SAUR12)*	SAUR-like auxin-responsive protein family
AT2G21260		NAD(P)-linked oxidoreductase superfamily protein
AT2G21280	*(SULA)*	A nuclear-encoded, plastid-targeted protein (AtSulA) whose overexpression causes severe yet stochastic plastid (shown in chloroplasts and leucoplasts) division defects. The protein does not appear to interact with either AtFtsZ proteins when studied in a yeast two-hybrid system.
AT2G21300		ATP binding microtubule motor family protein
AT2G21310		transposable_element_gene;copia-like retrotransposon family, has a 1.8e-151 P-value blast match to GB:CAA31653 polyprotein (Ty1_Copia-element) (Arabidopsis thaliana);(source:TAIR10)
AT2G21320	*B-BOX DOMAIN PROTEIN 18 (BBX18)*	B-box zinc finger family protein
AT2G21330	*FRUCTOSE-BISPHOSPHATE ALDOLASE 1 (FBA1)*	fructose-bisphosphate aldolase 1
AT2G21370	*XYLULOSE KINASE-1 (XK-1)*	Although this gene has a sequence similar to xylulose kinases, several lines of experimental evidence suggest that it does not act on xylulose or deoxy-xylulose.
AT2G21385	*CONSERVED IN THE GREEN LINEAGE AND DIATOMS 11 (CGLD11)*	Encodes a chloroplast stroma localized protein that is found only in the green plant lineage. It is involved in assembly of the chloroplast ATP synthase complex.
AT2G21420	*RING FINGER ABA-RELATED 4 (RFA4)*	E3 ubiquitin ligases, member of the RING between RING fingers (RBR)-type RSL1/RFA family, key regulator of ABA receptor stability in root and leaf tissues, targeting ABA receptors for degradation in different subcellular locations.
AT2G21450	*CHROMATIN REMODELING 34 (CHR34)*	chromatin remodeling 34
AT2G21460		transposable_element_gene;copia-like retrotransposon family, has a 1.3e-303 P-value blast match to GB:CAA31653 polyprotein (Ty1_Copia-element) (Arabidopsis thaliana);(source:TAIR10)
AT2G21470	*SUMO-ACTIVATING ENZYME 2 (SAE2)*	Encodes one of the two subunits of the SUMO activation enzyme required during sumolation. Sumolation is a post-translational protein modification process similar to ubiquitination during which a polypeptide (SUMO) is covalently attached to a target protein.
AT2G21480	*BUDDHAS PAPER SEAL 2 (BUPS2)*	BUSP2 plays a smaller role than BUSP1 in pollen tube growth. bups1/2 double mutants have reduced feritlity due to premature rupture of pollen tubes before they reach the ovule but single busp2 mutants are fertile. BUSP2 interacts with RALF4/19 peptide ligands and ANX1/2 receptors. BUPS/ANX signaling may regulate and promote pollen tube growth.
AT2G21490	*DEHYDRIN LEA (LEA)*	dehydrin LEA
AT2G21510		DNAJ heat shock N-terminal domain-containing protein
AT2G21530		SMAD/FHA domain-containing protein
AT2G21540	*SEC14-LIKE 3 (SFH3)*	SEC14-like 3
AT2G21550	*(DRTS3)*	One of three DRTS genes, this is the most divergent one.THY3/DRTS3 is preferentially expressed in the shoot apex, stipules and root caps.
AT2G21560		nucleolar-like protein
AT2G21590	*(APL4)*	Encodes the large subunit of ADP-glucose pyrophosphorylase, the enzyme which catalyzes the first and limiting step in starch biosynthesis. The large subunit plays a regulatory role whereas the small subunit (ApS) is the catalytic isoform. Four isoforms of the large subunit (ApL1-4) have been described.
AT2G21610	*PECTINESTERASE 11 (PE11)*	pectinesterase 11
AT2G21640		Encodes a protein of unknown function that is a marker for oxidative stress response.Expression in rosette leaves is activated by high concentration of boron.
AT2G21650	*MATERNAL EFFECT EMBRYO ARREST 3 (MEE3)*	RSM1 is a member of a small sub-family of single MYB transcription factors. Analysis of overexpressin lines indicate its involvement during early morphogenesis.

AT2G21655		ECA1 gametogenesis family protein (DUF784)
AT2G21660	*COLD, CIRCADIAN RHYTHM, AND RNA BINDING 2 (CCR2)*	Encodes a small glycine-rich RNA binding protein that is part of a negative-feedback loop through which AtGRP7 regulates the circadian oscillations of its own transcript. Gene expression is induced by cold. GRP7 appears to promote stomatal opening and reduce tolerance under salt and dehydration stress conditions, but, promotes stomatal closing and thereby increases stress tolerance under conditions of cold tolerance. Loss of function mutations have increased susceptibility to pathogens suggesting a role in mediating innate immune response. Mutants are also late flowering in a non-photoperiodic manner and are responsive to vernalization suggesting an interaction with the autonomous flowering pathway. There is a reduction of mRNA export from the nucleus in grp7 mutants. GRP7:GFP fusion proteins can be found in the cytosol and nucleus. A substrate of the type III effector HopU1 (mono-ADP-ribosyltransferase).
AT2G21680		Galactose oxidase/kelch repeat superfamily protein
AT2G21690		RNA-binding (RRM/RBD/RNP motifs) family protein
AT2G21710	*EMBRYO DEFECTIVE 2219 (EMB2219)*	Mitochondrial transcription termination factor family protein
AT2G21740	*EGG CELL 1.2 (EC1.2)*	Encodes a small cysteine-rich protein that is secreted by the egg cell during gamete interactions. The regulated secretion of EC1 by the egg cell upon sperm-egg interaction is proposed to ensure the appropriate localization of the cell-fusion machinery in distinct sperm membrane domains to accomplish gamete fusion.
AT2G21770	*CELLULOSE SYNTHASE A9 (CESA9)*	cellulose synthase, related to CESA6.
AT2G21780		hypothetical protein
AT2G21790	*RIBONUCLEOTIDE REDUCTASE 1 (RNR1)*	encodes large subunit of ribonucleotide reductase involved in the production of deoxyribonucleoside triphosphates (dNTPs) for DNA replication and repair
AT2G21820		seed maturation protein
AT2G21830		Cysteine/Histidine-rich C1 domain family protein
AT2G21850		Cysteine/Histidine-rich C1 domain family protein
AT2G21860		violaxanthin de-epoxidase-like protein
AT2G21880	*RAB GTPASE HOMOLOG 7A (RAB7A)*	RAB GTPase homolog 7A
AT2G21890	*CINNAMYL ALCOHOL DEHYDROGENASE HOMOLOG 3 (CAD3)*	cinnamyl alcohol dehydrogenase homolog 3
AT2G21900	*WRKY DNA-BINDING PROTEIN 59 (WRKY59)*	member of WRKY Transcription Factor; Group II-c
AT2G21910	*CYTOCHROME P450, FAMILY 96, SUBFAMILY A, POLYPEPTIDE 5 (CYP96A5)*	member of CYP96A
AT2G21920		F-box associated ubiquitination effector family protein
AT2G21930		A paternally expressed imprinted gene.
AT2G21950	*SKP1 INTERACTING PARTNER 6 (SKIP6)*	Encodes an SKP1 interacting partner (SKIP6).
AT2G21960		transmembrane protein
AT2G21990		MIZU-KUSSEI-like protein (Protein of unknown function, DUF617)
AT2G22000	*ELICITOR PEPTIDE 6 PRECURSOR (PROPEP6)*	elicitor peptide 6 precursor
AT2G22010	*RELATED TO KPC1 (RKP)*	Encodes a protein predicted to act as a RING E3 ubiquitin ligase. It appears to regulate the stability of the KRP1/ICK1 cyclin dependent kinase inhibitor. Induced by beet severe curly virus (BSCTV) C4 protein.
AT2G22020		
AT2G22030		Galactose oxidase/kelch repeat superfamily protein
AT2G22040	*LETHAL WITH SEC THIRTEEN 8-2 (LST8-2)*	Encodes a homolog of LST8 (Lethal with Sec Thirteen 8/G protein b subunit-like (LST8/GbL).
AT2G22050		Galactose oxidase/kelch repeat superfamily protein
AT2G22060		galactose oxidase/kelch repeat protein
AT2G22070		pentatricopeptide (PPR) repeat-containing protein
AT2G22080		transmembrane protein
AT2G22100		RNA-binding (RRM/RBD/RNP motifs) family protein
AT2G22160		Cysteine proteinases superfamily protein
AT2G22170	*PLAT DOMAIN PROTEIN 2 (PLAT2)*	Lipase/lipooxygenase, PLAT/LH2 family protein
AT2G22180		hydroxyproline-rich glycoprotein family protein
AT2G22190	*TREHALOSE-6-PHOSPHATE PHOSPHATASE E (TPPE)*	Haloacid dehalogenase-like hydrolase (HAD) superfamily protein
AT2G22200		encodes a member of the DREB subfamily A-6 of ERF/AP2 transcription factor family. The protein contains one AP2 domain. There are 8 members in this subfamily including RAP2.4.
AT2G22230		Thioesterase superfamily protein
AT2G22240	*MYO-INOSITOL-1-PHOSPHATE SYNTHASE 2 (MIPS2)*	** Referred to as MIPS1 in Mitsuhashi et al 2008. Myo-inositol-1-phosphate synthase isoform 2. Expressed in leaf, root and silique. Immunolocalization experiments with an antibody recognizing MIPS1, MIPS2, and MIPS3 showed endosperm localization.
AT2G22270		hematological/neurological-like protein
AT2G22290	*RAB GTPASE HOMOLOG H1D (RABH1d)*	RAB GTPase homolog H1D
AT2G22300	*SIGNAL RESPONSIVE 1 (SR1)*	Encodes a putative CAM binding transcription factor. Loss of function mutations show enhanced resistance to fungal and bacterial pathogens suggesting that CAMTA functions to suppress defense responses.It acts in the cold response pathway, it can bind to and activate the expression of DREB1 genes.
AT2G22320		hypothetical protein
AT2G22330	*CYTOCHROME P450, FAMILY 79, SUBFAMILY B, POLYPEPTIDE 3 (CYP79B3)*	Encodes a cytochrome P450. Involved in tryptophan metabolism. Converts Trp to indole-3-acetaldoxime (IAOx), a precursor to IAA and indole glucosinolates. The mRNA is cell-to-cell mobile.
AT2G22340		transmembrane protein

AT2G22350		transposable_element_gene;similar to RNase H domain-containing protein [Arabidopsis thaliana] (TAIR:AT5G36905.1);(source:TAIR10)
AT2G22400	*(TRM4B)*	TRM4B is a cytosine-5--methyltransferase. Mutants have decreased methyl cytosine and defects in root development.
AT2G22420	*(PRX17)*	Encodes a cell wall-localized class III peroxidase that is directly regulated by the MADS-box transcription factor AGL15 and is involved in lignified tissue formation.
AT2G22430	*HOMEOBOX PROTEIN 6 (HB6)*	Encodes a homeodomain leucine zipper class I (HD-Zip I) protein that is a target of the protein phosphatase ABI1 and regulates hormone responses in Arabidopsis.
AT2G22440		Frigida like gene.
AT2G22470	*ARABINOGALACTAN PROTEIN 2 (AGP2)*	Encodes arabinogalactan-protein (AGP2).
AT2G22480	*PHOSPHOFRUCTOKINASE 5 (PFK5)*	Phosphofructokinase isoform; target of plastidic thioredoxin-f-dependent redox regulation.
AT2G22490	*CYCLIN D2;1 (CYCD2;1)*	encodes a D-type cyclin whose transcription level is regulated by sucrose but not phytohormones or nitrate. Protein physically interacts with CDC2A. CycD2 kinase activity is regulated by sequestration of CycD2 protein in a form inaccessible to immunoprecipitation and probably not complexed to CDC2A.
AT2G22500	*UNCOUPLING PROTEIN 5 (UCP5)*	Encodes one of the mitochondrial dicarboxylate carriers (DIC): DIC1 (AT2G22500), DIC2 (AT4G24570), DIC3 (AT5G09470).
AT2G22510		hydroxyproline-rich glycoprotein family protein
AT2G22520		hypothetical protein
AT2G22540	*SHORT VEGETATIVE PHASE (SVP)*	Encodes a nuclear protein that acts as a floral repressor and that functions within the thermosensory pathway. SVP represses FT expression via direct binding to the vCArG III motif in the FT promoter.
AT2G22550		
AT2G22560	*NETWORKED 2D (NET2D)*	Kinase interacting (KIP1-like) family protein
AT2G22590		UDP-Glycosyltransferase superfamily protein
AT2G22600		RNA-binding KH domain-containing protein
AT2G22610	*MALECTIN DOMAIN KINESIN 2 (MDKIN2)*	Malectin domain kinesin. Possible role in cell division, with a possible secondary function in the nuclei.
AT2G22620	*RHAMNOGALACTURONAN LYASE6 (RGIL6)*	Rhamnogalacturonate lyase family protein
AT2G22630	*AGAMOUS-LIKE 17 (AGL17)*	Encodes a MADs domain containing protein involved in promoting flowering. Loss of function mutations show delayed flowering in long days and reduced levels of LFY and AP1 expression.
AT2G22650		FAD-dependent oxidoreductase family protein
AT2G22660	*GLYCINE-RICH DOMAIN PROTEIN1 (ATGRDP1)*	Encodes a member of a family of DUF1399 domain containing proteins. GRDP1 is involved in germination and response to ABA. Loss of function mutants have reduced germination in the presence of osmotic stressors.
AT2G22670	*INDOLEACETIC ACID-INDUCED PROTEIN 8 (IAA8)*	Encodes a transcriptional repressor of the auxin response that is auxin inducible and is involved in lateral root formation. The mRNA is cell-to-cell mobile.
AT2G22680	*WAV3 HOMOLOG 1 (WAVH1)*	Zinc finger (C3HC4-type RING finger) family protein
AT2G22700		
AT2G22740	*SU(VAR)3-9 HOMOLOG 6 (SUVH6)*	Encodes a SU(VAR)3-9 homolog, a methyltransferase involved in histone methylation. The protein was shown to bind to methylated cytosines of CG, CNG and CNN motifs but has a preference for the latter two. This is a member of a subfamily of SET proteins that shares a conserved SRA domain.
AT2G22750		basic helix-loop-helix (bHLH) DNA-binding superfamily protein
AT2G22760		basic helix-loop-helix (bHLH) DNA-binding superfamily protein
AT2G22770	*(NAI1)*	Regulates the development of ER bodies. also involves in response to the endophytic fungus Piriformospora indica.
AT2G22780	*PEROXISOMAL NAD-MALATE DEHYDROGENASE 1 (PMDH1)*	encodes an peroxisomal NAD-malate dehydrogenase that is involved in fatty acid beta-oxidation through providing NAD to the process of converting fatty acyl CoA to acetyl CoA.
AT2G22800	*(HAT9)*	Encodes homeobox protein HAT9.
AT2G22810	*1-AMINOCYCLOPROPANE-1-CARBOXYLATE SYNTHASE 4 (ACS4)*	key regulatory enzyme in the biosynthesis of the plant hormone ethylene. ACS4 is specifically induced by indoleacetic acid (IAA).
AT2G22840	*GROWTH-REGULATING FACTOR 1 (GRF1)*	Growth regulating factor encoding transcription activator. One of the nine members of a GRF gene family, containing nuclear targeting domain. Mutants result in smaller leaves indicating the role of the gene in leaf development. Expressed in root, shoot and flower
AT2G22850	*BASIC LEUCINE-ZIPPER 6 (bZIP6)*	basic leucine-zipper 6
AT2G22860	*PHYTOSULFOKINE 2 PRECURSOR (PSK2)*	Phytosulfokine 2 precursor, coding for a unique plant peptide growth factor. The mRNA is cell-to-cell mobile.
AT2G22870	*EMBRYO DEFECTIVE 2001 (EMB2001)*	P-loop containing nucleoside triphosphate hydrolases superfamily protein
AT2G22880	*(VQ12)*	VQ motif-containing protein
AT2G22890		Kua-ubiquitin conjugating enzyme hybrid localization domain-containing protein
AT2G22900	*MUCILAGE-RELATED10 (MUCI10)*	Encodes MUCI10, a galactomannan-1,6-galactosyltransferase. MUCI10 likely decorates glucomannan, synthesized by CSLA2, with galactose residues in vivo. The degree of galactosylation is essential for the synthesis of the GGM backbone, the structure of cellulose, mucilage density, as well as the adherence of pectin.
AT2G22920	*SERINE CARBOXYPEPTIDASE-LIKE 12 (SCPL12)*	serine carboxypeptidase-like 12
AT2G22930		UDP-Glycosyltransferase superfamily protein
AT2G22940		hypothetical protein
AT2G22950	*AUTO-REGULATED CA2+-ATPASE 7 (ACA7)*	Encodes a putative auto-regulated Ca2+-ATPase located in the plasma membrane involved in transporting Ca2+ outside developing pollen grains. This activity is important to support normal pollen development, particularly the progression to uninucleated microspores to bicellular pollen grains.
AT2G22960	*LAVONOL-PHENYLACYLTRANSFERASE 2 (FPT2)*	Encodes a putative flavonol-phenylacyltransferase. Some accessions (e.g. C24) contain a full length protein that produces high levels of saiginols compared to Col which is non producing. The producer strains also appear to be more resistant to UV-B irradiation.
AT2G22970	*SERINE CARBOXYPEPTIDASE-LIKE 11 (SCPL11)*	serine carboxypeptidase-like 11
AT2G22980	*SERINE CARBOXYPEPTIDASE-LIKE 13 (SCPL13)*	serine carboxypeptidase-like 13

AT2G22990	*SINAPOYLGLUCOSE 1 (SNG1)*	sinapoylglucose:malate sinapoyltransferase. Catalyzes the formation of sinapoylmalate from sinapoylglucose. Mutants accumulate excess sinapoylglucose.
AT2G23000	*SERINE CARBOXYPEPTIDASE-LIKE 10 (scpl10)*	serine carboxypeptidase-like 10
AT2G23030	*SNF1-RELATED PROTEIN KINASE 2.9 (SNRK2.9)*	encodes a member of SNF1-related protein kinases (SnRK2)
AT2G23040		hypothetical protein
AT2G23050	*NAKED PINS IN YUC MUTANTS 4 (NPY4)*	A member of the NPY gene family (NPY1/AT4G31820, NPY2/AT2G14820, NPY3/AT5G67440, NPY4/AT2G23050, NPY5/AT4G37590). Involved in auxin-mediated organogenesis.
AT2G23060		Acyl-CoA N-acyltransferases (NAT) superfamily protein
AT2G23070	*CK2 ALPHA CP (CKA4)*	Encodes a chloroplast localized subunit of casien kinase4. Functions reduntantly with other alpha subunits (1, 2,and 3) in ABA mediated suppression of seed germination, lateral root development and flowering time.
AT2G23100		Cysteine/Histidine-rich C1 domain family protein
AT2G23110		Late embryogenesis abundant protein, group 6
AT2G23130	*ARABINOGALACTAN PROTEIN 17 (AGP17)*	AGP17 is a lysine-rich arabinogalactan-protein (AGP) and part of a multi-gene family of glycoproteins with approx. 50 members. It falls into one subclass with AGP18 and AGP19, other lysine-rich AGPs. 84% of its proline residues are hydroxylated to hydroproline and its heavy glycosylation accounts for appr. 69% of the molecular weight. The main glycosyl residues are arabinose (30.1%) and galactose (55.1%). Glycosyl linkages are consistent with type II arabinogalactans. AGP17 is predicted to have a glycosylphosphatidylinositol (GPI)anchor and is localized to the plasma membrane and Hechtian strands. It is expressed in young/old leaves, shoots, suspension cultures and flowers.
AT2G23150	*NATURAL RESISTANCE-ASSOCIATED MACROPHAGE PROTEIN 3 (NRAMP3)*	Encodes a member of the Nramp2 metal transporter family; like its homolog Atnramp4, localized in vacuolar membrane. Seedlings of double mutant, atnramp3-1 atnramp4-1, were arrested at early germination.
AT2G23160		F-box family protein
AT2G23170	*(GH3.3)*	encodes an IAA-amido synthase that conjugates Asp and other amino acids to auxin in vitro.
AT2G23180	*CYTOCHROME P450, FAMILY 96, SUBFAMILY A, POLYPEPTIDE 1 (CYP96A1)*	member of CYP96A
AT2G23200		Protein kinase superfamily protein
AT2G23210		UDP-Glycosyltransferase superfamily protein
AT2G23220	*CYTOCHROME P450, FAMILY 81, SUBFAMILY D, POLYPEPTIDE 6 (CYP81D6)*	member of CYP81D
AT2G23230		Terpenoid cyclases/Protein prenyltransferases superfamily protein
AT2G23240	*ARABIDOPSIS THALIANA METALLOTHIONEIN 4B (ATMT4B)*	AtMT4b is a member of Type 4 metallothionein (MT) genes. It is involved in the early develoment of the embryo and in the accumulation of metal ions especially Zn in the seeds.
AT2G23250	*UDP-GLUCOSYL TRANSFERASE 84B2 (UGT84B2)*	UDP-glucosyl transferase 84B2
AT2G23260	*UDP-GLUCOSYL TRANSFERASE 84B1 (UGT84B1)*	UDP-glucosyltransferase that acts on a number of substrates including IAA and PAA.
AT2G23270	*PRECURSOR OF PAMP-INDUCED PEPTIDE 3 (PREPIP3)*	Encoding a precursor protein of a secreted peptide that is responsive to flg22 stimulus. Finetuning role in modulation of immunity through the regulation of SA and JA biosynthesis and signalling pathways.
AT2G23280		
AT2G23290	*MYB DOMAIN PROTEIN 70 (MYB70)*	Member of the R2R3 factor gene family. Modulates seed germination and root system development.
AT2G23310	*(ATRER1C1)*	Encodes AtRER1C1, a Golgi membrane protein involved in returning the molecules that are exported from the endoplasmic reticulum (ER) to the Golgi apparatus back to the ER (a mechanism known as retrieval). There are two Arabidopsis homologues of AtRERC1: AtRER1A and AtRER1B.
AT2G23320	*WRKY DNA-BINDING PROTEIN 15 (WRKY15)*	Encodes WRKY DNA-binding protein 15 (WRKY15).
AT2G23330		transposable_element_gene;copia-like retrotransposon family, has a 3.9e-195 P-value blast match to GB:AAA57005 Hopscotch polyprotein (Ty1_Copia-element) (Zea mays);(source:TAIR10)
AT2G23340	*DREB AND EAR MOTIF PROTEIN 3 (DEAR3)*	encodes a member of the DREB subfamily A-5 of ERF/AP2 transcription factor family. The protein contains one AP2 domain. There are 16 members in this subfamily including RAP2.1, RAP2.9 and RAP2.10.
AT2G23360		filament-like protein (DUF869)
AT2G23380	*CURLY LEAF (CLF)*	Similar to the product of the Polycomb-group gene Enhancer of zeste. Catalytic component of the PRC2 complex.Required for stable repression of AG and AP3. Putative role in cell fate determination. Involved in the control of leaf morphogenesis. mutants exhibit curled, involute leaves. AGAMOUS and APETALA3 are ectopically expressed in the mutant.
AT2G23400	*CIS-PRENYLTRANSFERASE 2 (CPT2)*	Undecaprenyl pyrophosphate synthetase family protein
AT2G23410	*CIS-PRENYLTRANSFERASE (CPT)*	Encodes cis-prenyltransferase involved in dolichol biosynthesis.
AT2G23420	*NICOTINATE PHOSPHORIBOSYLTRANSFERASE 2 (NAPRT2)*	nicotinate phosphoribosyltransferase 2
AT2G23430	*(ICK1)*	Encodes a cyclin-dependent kinase inhibitor protein that functions as a negative regulator of cell division and promoter of endoreduplication. A member of seven KRP genes found in Arabidopsis thaliana. Differential expression patterns for distinct KRPs were revealed by in situ hybridization. Both SKP2b and RKP appear to be involved in the degradation of KRP1.
AT2G23440	*C-TERMINALLY ENCODED PEPTIDE 3 (CEP3)*	transmembrane protein
AT2G23470	*ROOT UV-B SENSITIVE 4 (RUS4)*	DUF647 domain containing protein. Mutants are male sterile with defects in endothecium, tapetum and stamen maturation.
AT2G23480		transposable_element_gene;similar to unknown protein [Arabidopsis thaliana] (TAIR:AT5G35794.1);(source:TAIR10)
AT2G23500		transposable_element_gene;Mutator-like transposase family, has a 2.4e-80 P-value blast match to O80466 /172-336 Pfam PF03108 MuDR family transposase (MuDr-element domain);(source:TAIR10)
AT2G23510	*SPERMIDINE DISINAPOYL ACYLTRANSFERASE (SDT)*	BAHD acyltransferase which transfers acyl-groups from different acyl-donors specifically to amines. Catalyzes the multisite acylation of spermidine. Uses caffeoyl/feruoyl/sinapoyl-CoA with the N1 and N10 positions of spermidine.
AT2G23530		Zinc-finger domain of monoamine-oxidase A repressor R1

AT2G23540		GDSL-motif esterase/acyltransferase/lipase. Enzyme group with broad substrate specificity that may catalyze acyltransfer or hydrolase reactions with lipid and non-lipid substrates.
AT2G23550	*METHYL ESTERASE 6 (MES6)*	Encodes a protein predicted to act as a carboxylesterase. It has similarity to the SABP2 methyl salicylate esterase from tobacco but no enzymatic activity has been identified for this protein.
AT2G23560	*METHYL ESTERASE 7 (MES7)*	Encodes a protein shown to have carboxylesterase activity, methyl salicylate esterase activity, and methyl IAA esterase activity in vitro. This protein does not act on methyl JA, MeGA4, or MEGA9 in vitro. MES7 appears to be involved in MeSA hydrolysis in planta. Expression of MES7 can restore systemic acquired resistance in SAR-deficient tobacco plants.
AT2G23570	*METHYL ESTERASE 19 (MES19)*	Encodes a protein with similarity to SABP2, a methyl salicylate esterase from tobacco. However, this protein is truncated and lacks one of the residues of the predicted catalytic triad, suggesting that it does not have this enzymatic activity.
AT2G23580	*METHYL ESTERASE 4 (MES4)*	Encodes a protein shown to have carboxylesterase activity and methyl salicylate esterase activity in vitro. This protein does not act on methyl IAA, methyl JA, MeGA4, or MEGA9 in vitro.
AT2G23590	*METHYL ESTERASE 8 (MES8)*	Encodes a protein shown to have carboxylesterase activity in vitro. It has similarity to the SABP2 methyl salicylate esterase from tobacco. This protein does not act on methyl IAA, methyl JA, MeSA, MeGA4, or MEGA9 in vitro.
AT2G23610	*METHYL ESTERASE 3 (MES3)*	Encodes a protein shown to have carboxylesterase activity, methyl IAA esterase activity, and methyl jasmonate esterase activity in vitro. This protein does not act on methyl salicylate, MeGA4, or MEGA9 in vitro.
AT2G23620	*METHYL ESTERASE 1 (MES1)*	Encodes a protein shown to have carboxylesterase activity, methyl salicylate esterase activity, methyl jasmonate esterase activity, and methyl IAA esterase activity in vitro. MES1 appears to be involved in MeSA hydrolysis in planta. Expression of MES1 can restore systemic acquired resistance in SAR-deficient tobacco plants. This protein does not act on MeGA4, or MEGA9 in vitro.
AT2G23630	*SKU5 SIMILAR 16 (sks16)*	SKU5 similar 16
AT2G23640	*RETICULAN LIKE PROTEIN B13 (RTNLB13)*	Encodes RTNLB13, a reticulon protein integral to the endoplasmic reticulum (ER) membrane that have the ability to shape the ER into tubules.
AT2G23660	*LOB DOMAIN-CONTAINING PROTEIN 10 (LBD10)*	LOB domain-containing protein 10
AT2G23670	*HOMOLOG OF SYNECHOCYSTIS YCF37 (YCF37)*	Phosphorylated protein in the thylakoid lumen.
AT2G23680		Cold acclimation protein WCOR413 family
AT2G23690		PADRE protein.
AT2G23700		Itga6 (Protein of unknown function, DUF547)
AT2G23720		transposable_element_gene;Mutator-like transposase family, has a 6.7e-76 P-value blast match to Q9SI25 /181-349 Pfam PF03108 MuDR family transposase (MuDr-element domain);(source:TAIR10)
AT2G23730		
AT2G23740	*SU(VAR)3-9-RELATED PROTEIN 5 (SUVR5)*	Encodes a SET-domain protein SUVR5 that mediates H3K9me2 deposition and silencing at stimulus response genes in a DNA methylation-independent manner.
AT2G23760	*BEL1-LIKE HOMEODOMAIN 4 (BLH4)*	Encodes a member of the BEL family of homeodomain proteins. Plants doubly mutant for saw1/saw2 (blh2/blh4) have serrated leaves. BP is expressed in the serrated leaves, therefore saw2 and saw1 may act redundantly to repress BP in leaves. Regulates together with BLH2 demethylesterification of homogalacturonan in seed mucilage.
AT2G23770	*LYSM-CONTAINING RECEPTOR-LIKE KINASE 4 (LYK4)*	Encodes a putative LysM-containing receptor-like kinase LYK4. Shares overlapping function with LYK5 in mediating chitin-triggered immune responses. Based on protein sequence alignment analysis, it was determined as a pseudo kinase due to a lack of the ATP-binding P-loop in the kinase domain.
AT2G23800	*GERANYLGERANYL PYROPHOSPHATE SYNTHASE 2 (GGPS2)*	encodes an endoplasmic reticulum-targeted geranylgeranyl pyrophosphate synthase
AT2G23810	*TETRASPANIN8 (TET8)*	Member of TETRASPANIN family
AT2G23830	*(PVA31)*	PapD-like superfamily protein
AT2G23840		HNH endonuclease
AT2G23860		pseudogene of PapD-like superfamily protein
AT2G23890		HAD-superfamily hydrolase, subfamily IG, 5-nucleotidase
AT2G23900	*POLYGALACTURONASE CLADE F 3 (PGF3)*	Pectin lyase-like superfamily protein
AT2G23910		NAD(P)-binding Rossmann-fold superfamily protein
AT2G23920		
AT2G23930	*PROBABLE SMALL NUCLEAR RIBONUCLEOPROTEIN G (SNRNP-G)*	Putative small nuclear ribonucleoprotein G
AT2G23945		Eukaryotic aspartyl protease family protein
AT2G23950	*CLE-RESISTANT RECEPTOR KINASE (CLERK)*	Encodes an LLR receptor kinase that is expressed in protophloem and is required for CLE peptide sensing in roots. One of a group of LRR-RLKs, designated as CLAVATA3 INSENSITIVE RECEPTOR KINASES (CIKs), that acts as a co-regulator and has essential roles in regulating CLV3-mediated stem cell homeostasis.
AT2G23970		Class I glutamine amidotransferase-like superfamily protein
AT2G23980	*CYCLIC NUCLEOTIDE-GATED CHANNEL 6 (CNGC6)*	Encodes a cyclic GMP-activated non-selective cation channel in the plasma membrane of guard cells. Required for constitutive growth of root hairs as Ca2+-permeable channels. Regulates Ca2+ infux during heat shock.
AT2G23990	*EARLY NODULIN-LIKE PROTEIN 11 (ENODL11)*	early nodulin-like protein 11
AT2G24000	*SERINE CARBOXYPEPTIDASE-LIKE 22 (scpl22)*	serine carboxypeptidase-like 22
AT2G24010	*SERINE CARBOXYPEPTIDASE-LIKE 23 (scpl23)*	serine carboxypeptidase-like 23
AT2G24020	*SUPPRESSOR OF TIC40 2 (STIC2)*	STIC2 was identified in a screen for suppressors of chloroplast protein import defect in tic40.
AT2G24050	*EUKARYOTIC TRANSLATION INITIATION FACTOR ISOFORM 4G2 (eIFiso4G2)*	Encodes a putative eukaryotic translation initiation factor.

AT2G24060	*(ATINFC-2)*	SUPPRESSOR OF VARIEGATION9 (SVR9),encodes a chloroplast-localized prokaryotic type translation initiation factor 3. Mutant plants shows both chloroplast development defect, and a series of leaf developmental abnormalities including more serrated leaf margin, disorganized mesophyll cells, and altered cotyledon venation patterns.
AT2G24070	*QWRF DOMAIN CONTAINING 4 (QWRF4)*	QWRF motif protein (DUF566)
AT2G24080	*F-BOX/DUF295 BRASSICEAE-SPECIFIC 15 (ATFDB15)*	F-box protein (DUF295)
AT2G24090	*PLASTID RIBOSOMAL PROTEIN L35 (PRPL35)*	Ribosomal protein L35
AT2G24120	*SCABRA 3 (SCA3)*	DNA/RNA polymerases superfamily protein
AT2G24130		Leucine-rich receptor-like protein kinase family protein
AT2G24140	*(MRF7)*	MyoB myosin receptor which specifically localises to the Golgi membrane and affects its movement.
AT2G24160		pseudogene of receptor like protein 37
AT2G24170		Endomembrane protein 70 protein family
AT2G24180	*CYTOCHROME P450 71B6 (CYP71B6)*	Encodes a cytochrome P450 monooxygenase that converts indole-3-acetonitrile to indole-3-aldehyde / indole-3-carboxylic acid and cyanide. The mRNA is cell-to-cell mobile.
AT2G24190	*SHORT-CHAIN DEHYDROGENASE/REDUCTASE 2 (SDR2)*	Encodes an aldehyde reductase that catalyzes the reduction of the aldehyde carbonyl groups on saturated and alpha,beta-unsaturated aldehydes with more than 5 carbons in vitro. In addition, this enzyme can reduce methylglyoxal in vitro. It is believed that this enzyme localizes to the cytosol like the closely related protein encoded by AT3G61220.
AT2G24200	*LEUCYL AMINOPEPTIDASE 1 (LAP1)*	Cytosol aminopeptidase family protein
AT2G24210	*TERPENE SYNTHASE 10 (TPS10)*	terpene synthase 10
AT2G24220	*PURINE PERMEASE 5 (PUP5)*	Member of a family of proteins related to PUP1, a purine transporter. May be involved in the transport of purine and purine derivatives such as cytokinins, across the plasma membrane.
AT2G24230		Leucine-rich repeat protein kinase family protein
AT2G24240		BTB/POZ domain with WD40/YVTN repeat-like protein
AT2G24250	*F-BOX/DUF295 ANCESTRAL 14 (ATFDA14)*	LOW protein: F-box/kelch-repeat protein (DUF295)
AT2G24255	*F-BOX/DUF295 ANCESTRAL 15 (ATFDA15)*	LOW protein: F-box/kelch-repeat protein
AT2G24260	*LJRHL1-LIKE 1 (LRL1)*	Encodes a basic helix-loop-helix (bHLH) protein that regulates root hair and sperm cell development. One of the three Arabidopsis homologs of the Lotus japonicus ROOTHAIRLESS1 (LjRHL1) gene: At2g24260 (AtLRL1), At4g30980 (AtLRL2), and At5g58010 (AtLRL3).
AT2G24270	*ALDEHYDE DEHYDROGENASE 11A3 (ALDH11A3)*	Encodes a protein with non-phosphorylating NADP-dependent glyceraldehyde-3-phosphate dehydrogenase activity. The activity of the enzyme was determined from leaf extracts; the enzyme has not been purified to confirm activity.
AT2G24280		alpha/beta-Hydrolases superfamily protein
AT2G24290	*(ELMO5)*	ubiquitin-associated protein (DUF1068)
AT2G24330	*LUNAPARK1 (LNP1)*	Encodes one of two LUNAPARK proteins in Arabidopsis. Both LNPA and LNPB are predominantly distributed throughout the ER, but not preferentially localized at the three-way junctions. Mutation of both LNPA and LNPB together caused the cortical ER to develop poor ER cisternae and a less dense tubular network. E3 ligase involved in degradation of RHD3 to maintain a tubular ER network.
AT2G24340		sequence-specific DNA binding transcription factor
AT2G24350		RNA binding (RRM/RBD/RNP motifs) family protein
AT2G24370		kinase with adenine nucleotide alpha hydrolases-like domain-containing protein
AT2G24390		AIG2-like (avirulence induced gene) family protein
AT2G24400	*SMALL AUXIN UPREGULATED RNA 38 (SAUR38)*	SAUR-like auxin-responsive protein family
AT2G24410		SMAD/FHA domain protein
AT2G24420		DNA repair ATPase-like protein
AT2G24430	*NAC DOMAIN CONTAINING PROTEIN 38 (NAC038)*	NAC domain containing protein 38
AT2G24440		selenium binding protein
AT2G24450	*FASCICLIN-LIKE ARABINOGALACTAN PROTEIN 3 PRECURSOR (FLA3)*	Fasciclin-like arabinogalactan protein. Possibly involved in embryogenesis and seed development.
AT2G24460		C3HC4-type RING finger protein
AT2G24470		filament-like protein (DUF869)
AT2G24490	*REPLICON PROTEIN A2 (RPA2)*	Encodes a component of Replication Protein A. Component of transcriptional gene silencing which does not affect endogenous small RNA accumulation nor DNA methylation. Localized in the nucleus. Involved in DNA repair. Interacts physically with ROS1.
AT2G24500	*(FZF)*	Encodes a C2H2 zinc finger protein FZF. Involved in cold regulated rRNA processing and the accumulation of CBF protein. Cytosolic ribosomal 60S-biogenesis factor.
AT2G24520	*H(+)-ATPASE 5 (HA5)*	plasma membrane H+-ATPase
AT2G24530	*(ADA1E)*	Member of SAGA complex, SPT modulu subunit, interacts with HAG1.
AT2G24540	*ATTENUATED FAR-RED RESPONSE (AFR)*	Galactose oxidase/kelch repeat superfamily protein
AT2G24550		major centromere autoantigen B-like protein
AT2G24560	*GUARD-CELL-ENRICHED GDSL LIPASE 15 (GGL15)*	GDSL-motif esterase/acyltransferase/lipase. Enzyme group with broad substrate specificity that may catalyze acyltransfer or hydrolase reactions with lipid and non-lipid substrates.
AT2G24570	*WRKY DNA-BINDING PROTEIN 17 (WRKY17)*	member of WRKY Transcription Factor; Group II-d; negative regulator of basal resistance to Pseudomonas syringae.
AT2G24580		FAD-dependent oxidoreductase family protein
AT2G24600		Ankyrin repeat family protein
AT2G24610	*CYCLIC NUCLEOTIDE-GATED CHANNEL 14 (CNGC14)*	Member of Cyclic nucleotide gated channel family; a plasma membrane localized Ca2+ chan nel, which is required for the normal growth of Arabidopsis root hair tip.

AT2G24620		S-locus glycoprotein family protein
AT2G24650		B3 domain-containing protein REM13
AT2G24660		transposable_element_gene;copia-like retrotransposon family, has a 1.7e-166 P-value blast match to GB:BAA78424 polyprotein (Ty1_Copia-element) (Arabidopsis thaliana)gi|4996363|dbj|BAA78424.1| polyprotein (AtRE2) (Arabidopsis thaliana) (Ty1_Copia-element);(source:TAIR10)
AT2G24710	*GLUTAMATE RECEPTOR 2.3 (GLR2.3)*	member of Putative ligand-gated ion channel subunit family
AT2G24740	*SET DOMAIN GROUP 21 (SDG21)*	Encodes a SU(VAR)3-9 homolog, a SET domain protein (Homology Subgroup V; Orthology Group 1). Known SET domain proteins are involved in epigenetic control of gene expression. There are 10 SUVH genes in Arabidopsis and members of this subfamily of the SET proteins have an additional conserved SRA domain. This protein is a putative histone methyltransferase (predicted to methylate H3K9/20) related to the the Drosophila Su(var)3-9 and mammalian G9a proteins.
AT2G24762	*GLUTAMINE DUMPER 4 (GDU4)*	Encodes a member of the GDU (glutamine dumper) family proteins involved in amino acid export: At4g31730 (GDU1), At4g25760 (GDU2), At5g57685 (GDU3), At2g24762 (GDU4), At5g24920 (GDU5), At3g30725 (GDU6) and At5g38770 (GDU7).
AT2G24780		hypothetical protein
AT2G24790	*CONSTANS-LIKE 3 (COL3)*	Positive regulator of photomorphogenesis that acts downstream of COP1 but can promote lateral root development independently of COP1 and also function as a daylength-sensitive regulator of shoot branching. The mRNA is cell-to-cell mobile.
AT2G24800		Peroxidase superfamily protein
AT2G24810		Pathogenesis-related thaumatin superfamily protein
AT2G24820	*TRANSLOCON AT THE INNER ENVELOPE MEMBRANE OF CHLOROPLASTS 55-II (TIC55-II)*	translocon at the inner envelope membrane of chloroplasts 55-II
AT2G24850	*TYROSINE AMINOTRANSFERASE 3 (TAT3)*	Encodes a tyrosine aminotransferase that is responsive to treatment with jasmonic acid.
AT2G24860	*EDS1 -INTERACTING J PROTEIN 1 (EIJ1)*	Loss-of-function mutant of EIJ1 presents normal growth, but a stronger resistance to Pst DC3000 compared with the wild type.
AT2G24880		Plant self-incompatibility protein S1 family
AT2G24920		transposable_element_gene;similar to unknown protein [Arabidopsis thaliana] (TAIR:AT3G30470.1);(source:TAIR10)
AT2G24930		transposable_element_gene;similar to Ulp1 protease family protein [Arabidopsis thaliana] (TAIR:AT3G30640.1);(source:TAIR10)
AT2G24940	*MEMBRANE-ASSOCIATED PROGESTERONE BINDING PROTEIN 2 (MAPR2)*	membrane-associated progesterone binding protein 2
AT2G24950		transmembrane protein, putative (DUF239)
AT2G24960		Myb/SANT-like DNA-binding domain protein
AT2G24970		spindle/kinetochore-associated protein
AT2G24980	*EXTENSIN 6 (EXT6)*	Proline-rich extensin-like family protein
AT2G25000	*WRKY DNA-BINDING PROTEIN 60 (WRKY60)*	Pathogen-induced transcription factor. Forms protein complexes with itself and with WRKY40. Coexpression with WRKY18 or WRKY40 made plants more susceptible to both P. syringae and B. cinerea. WRKY18, WRKY40, and WRKY60 have partially redundant roles in response to the hemibiotrophic bacterial pathogen Pseudomonas syringae and the necrotrophic fungal pathogen Botrytis cinerea, with WRKY18 playing a more important role than the other two.
AT2G25030		pseudogene of casein lytic proteinase B4
AT2G25060	*EARLY NODULIN-LIKE PROTEIN 14 (ENODL14)*	early nodulin-like protein 14
AT2G25070		Protein phosphatase 2C family protein
AT2G25090	*CBL-INTERACTING PROTEIN KINASE 16 (CIPK16)*	Encodes a member of the SNF1-related kinase (SnRK) gene family (SnRK3.18), which has also been reported as a member of the CBL-interacting protein kinases (CIPK16) and is involved in salinity tolerance.
AT2G25100		Polynucleotidyl transferase, ribonuclease H-like superfamily protein
AT2G25140	*CASEIN LYTIC PROTEINASE B4 (CLPB4)*	Encodes ClpB4, which belongs to the Casein lytic proteinase/heat shock protein 100 (Clp/Hsp100) family. Targeted to the mitochondrion, also referred to as ClpB-m. Transcripts of ClpB4 accumulate dramatically at high temperatures, suggesting that it may be involved in response to heat stress.
AT2G25150		HXXXD-type acyl-transferase family protein
AT2G25160	*CYTOCHROME P450, FAMILY 82, SUBFAMILY F, POLYPEPTIDE 1 (CYP82F1)*	cytochrome P450, family 82, subfamily F, polypeptide 1
AT2G25190		PPPDE putative thiol peptidase family protein
AT2G25200		hypothetical protein (DUF868)
AT2G25220		Protein kinase superfamily protein
AT2G25230	*MYB DOMAIN PROTEIN 100 (MYB100)*	Encodes a putative transcription factor (MYB100).
AT2G25240	*CONSERVED IN CILIATED SPECIES AND IN THE LAND PLANTS 3 (CCP3)*	Serine protease inhibitor (SERPIN) family protein. Involved in stress response regulated cell death.
AT2G25250		serine/arginine repetitive matrix-like protein
AT2G25300	*HYDROXYPROLINE O-GALACTOSYLTRANSFERASE 3 (HPGT3)*	Encodes a hydroxyproline O-galactosyltransferase.
AT2G25310		ER membrane protein complex subunit-like protein (DUF2012)
AT2G25330		TRAF-like family protein
AT2G25340	*VESICLE-ASSOCIATED MEMBRANE PROTEIN 712 (VAMP712)*	Member of Synaptobrevin-like AtVAMP7C, v-SNARE protein family.
AT2G25355		PNAS-3-like protein
AT2G25360		RING/U-box superfamily protein
AT2G25370		RING/U-box superfamily protein
AT2G25380		pseudogene of zinc finger protein-related
AT2G25390		pseudogene of F-box and associated interaction domains-containing protein
AT2G25420	*(RWD40)*	WD40 domain protein which interacts with ROS1 in the base excision repair pathway through DNA methylation.
AT2G25430	*(PICALM4A)*	AP180 N-terminal homology domain, TPLATE complex protein involved in clathrin-mediated endocytosis.
AT2G25440	*RECEPTOR LIKE PROTEIN 20 (RLP20)*	receptor like protein 20

AT2G25450	*GLUCOSINOLATE HYDROXYLASE (GSL-OH)*	Encodes a 2-oxoacid-dependent dioxygenase involved in the production of 2-hydroxybut-3-enyl glucosinolate.
AT2G25460		EEIG1/EHBP1 protein amino-terminal domain protein
AT2G25470	*RECEPTOR LIKE PROTEIN 21 (RLP21)*	receptor like protein 21
AT2G25490	*EIN3-BINDING F BOX PROTEIN 1 (EBF1)*	Encodes an F-box protein involved in the ubiquitin/proteasome-dependent proteolysis of EIN3. The mRNA is cell-to-cell mobile.
AT2G25500		Inosine triphosphate pyrophosphatase family protein
AT2G25510		transmembrane protein
AT2G25540	*CELLULOSE SYNTHASE 10 (CESA10)*	cellulose synthase
AT2G25550		transposable_element_gene;non-LTR retrotransposon family (LINE), has a 3.1e-44 P-value blast match to GB:AAA39398 ORF2 (Mus musculus) (LINE-element);(source:TAIR10)
AT2G25565		C3HC4-type RING finger protein
AT2G25590		Plant Tudor-like protein
AT2G25600	*SHAKER POLLEN INWARD K+ CHANNEL (SPIK)*	Encodes SPIK, a member of the Shaker family potassium ion (K+) channel. This family includes five groups based on phylogenetic analysis (FEBS Letters (2007) 581: 2357): I (inward rectifying channel): AKT1 (AT2G26650), AKT5 (AT4G32500) and SPIK (also known as AKT6, AT2G25600); II (inward rectifying channel): KAT1 (AT5G46240) and KAT2 (AT4G18290); III (weakly inward rectifying channel): AKT2 (AT4G22200); IV (regulatory subunit involved in inwardly rectifying conductance formation): KAT3 (also known as AtKC1, AT4G32650); V (outward rectifying channel): SKOR (AT3G02850) and GORK (AT5G37500). Mutant plants have impaired pollen-tube growth.
AT2G25625	*CHLOROPLAST VESICULATION (CV)*	Histone deacetylase-like protein. Induced by senescence and abiotic stresses.
AT2G25630	*BETA GLUCOSIDASE 14 (BGLU14)*	beta glucosidase 14
AT2G25640		SPOC domain / Transcription elongation factor S-II protein
AT2G25650	*GEBP-LIKE PROTEIN 1 (GPL1)*	DNA-binding storekeeper protein-related transcriptional regulator
AT2G25680	*MOLYBDATE TRANSPORTER 1 (MOT1)*	Encodes a high-affinity molybdate transporter. Mutant has reduced concentrations of molybdate in roots and shoots, and reduced shoot and root length when growing on Mo-limited medium.
AT2G25700	*SKP1-LIKE 3 (SK3)*	E3 ubiquitin ligase SCF complex subunit SKP1/ASK1 (At3), putative, E3 ubiquitin ligase; similar to fimbriata-associated protein fap1 GI:2673868 from (Antirrhinum majus. Interacts with F-box proteins.
AT2G25710	*HOLOCARBOXYLASE SYNTHASE 1 (HCS1)*	Encodes a dual-targeted biotin holocarboxylase synthetase that can localize to the chloroplast and the cytosol. In vitro, it has been shown to catalyze the addition of biotin to the BCCP subunit of acetyl-CoA carboxylase and it can also biotinylate methylcrotonyl-CoA carboxylase. A small upstream ORF in the 5'UTR (uORF24) regulates the differential targeting of this enzyme.
AT2G25730		zinc finger FYVE domain protein
AT2G25735		hypothetical protein
AT2G25740		ATP-dependent protease La (LON) domain protein
AT2G25760	*PHOTOREGULATORY PROTEIN KINASE 4 (PPK4)*	Casein kinase involved in phosphorylation and ubiquination of RYR/PYLs, resulting in negative regulation of ABA response. Plays a role in repressing the transition from vegetative to reproductive phase.
AT2G25780		hypothetical protein (DUF1677)
AT2G25790	*STERILITY-REGULATING KINASE MEMBER 1 (SKM1)*	Leucine-rich receptor-like protein kinase family protein
AT2G25810	*TONOPLAST INTRINSIC PROTEIN 4;1 (TIP4;1)*	tonoplast intrinsic protein 4
AT2G25820	*ETHYLENE AND SALT INDUCIBLE 2 (ESE2)*	encodes a member of the DREB subfamily A-4 of ERF/AP2 transcription factor family. The protein contains one AP2 domain. There are 17 members in this subfamily including TINY.
AT2G25830		YebC-like protein
AT2G25850	*POLY(A) POLYMERASE 2 (PAPS2)*	Encodes a poly(A) polymerase. Located in the nucleus.
AT2G25860		
AT2G25880	*ATAURORA2 (AUR2)*	Encodes a member of a family of Ser/Thr kinases whose activities peak during cell division. Transcripts are abundant in tissues rich in dividing cells like roots and flowers but are low or absent in fully expanded leaves and stems. In interphase cells, the protein is predominantly nuclear. During mitosis, the protein associates with plant-specific cytoskeletal structures (preprophase band, phragmoplast, nascent cell plate) that are necessary for cytokinesis as well as with the microtubule spindle.
AT2G25890		Oleosin family protein
AT2G25900	*(ATCTH)*	Encodes a protein with two tandem-arrayed CCCH-type zinc fingers that binds RNA and is involved in RNA turnover. The mRNA is cell-to-cell mobile.
AT2G25930	*EARLY FLOWERING 3 (ELF3)*	Encodes a nuclear protein that is expressed rhythmically and interacts with phytochrome B to control plant development and flowering through a signal transduction pathway. Required component of the core circadian clock regardless of light conditions.
AT2G25940	*ALPHA-VACUOLAR PROCESSING ENZYME (ALPHA-VPE)*	Encodes a vacuolar processing enzyme belonging to a novel group of cysteine proteinases that is expressed in vegetative organs and is upregulated in association with various types of cell death and under stressed conditions.
AT2G25980		Mannose-binding lectin superfamily protein
AT2G25990		hypothetical protein
AT2G26000	*BRAP2 RING ZNF UBP DOMAIN-CONTAINING PROTEIN 2 (BRIZ2)*	Encodes a RING domain containing protein BRIZ2. BRIZ1 (At2g42160) and BRIZ2 (At2g26000) proteins form a heteromeric E3 ligase complex required for seed germination and post-germination growth.
AT2G26020	*PLANT DEFENSIN 1.2B (PDF1.2b)*	Predicted to encode a PR (pathogenesis-related) protein. Belongs to the plant defensin (PDF) family with the following members: At1g75830/PDF1.1, At5g44420/PDF1.2a, At2g26020/PDF1.2b, At5g44430/PDF1.2c, At2g26010/PDF1.3, At1g19610/PDF1.4, At1g55010/PDF1.5, At2g02120/PDF2.1, At2g02100/PDF2.2, At2g02130/PDF2.3, At1g61070/PDF2.4, At5g63660/PDF2.5, At2g02140/PDF2.6, At5g38330/PDF3.1 and At4g30070/PDF3.2.

AT2G26040	*PYR1-LIKE 2 (PYL2)*	Encodes a member of the PYR (pyrabactin resistance)/PYL(PYR1-like)/RCAR (regulatory components of ABA receptor) family proteins with 14 members. PYR/PYL/RCAR family proteins function as abscisic acid sensors. Mediate ABA-dependent regulation of protein phosphatase 2Cs ABI1 and ABI2.
AT2G26050		hypothetical protein (DUF1644)
AT2G26070	*REVERSION-TO-ETHYLENE SENSITIVITY1 (RTE1)*	Encodes a predicted membrane protein. Similar sequences are widely distributed and conserved in plants, animals and protists but absent in fungi and prokaryotes. The sequence has no known motifs and no biological function has been assigned in any species. In Arabidopsis, it appears to be involved in the negative regulation of the response to ethylene, is localized to the Golgi and is a positive regulator of ETR1.
AT2G26110		bromodomain protein (DUF761)
AT2G26120		glycine-rich protein
AT2G26130	*RING FINGER OF SEED LONGEVITY 1 (RSL1)*	Encodes a RING-type zinc finger ubiquitin ligase involved in seed longevity.Gain of function (35S promoter) increases, and loss of function decreases, seed longevity.
AT2G26150	*HEAT SHOCK TRANSCRIPTION FACTOR A2 (HSFA2)*	member of Heat Stress Transcription Factor (Hsf) family. Involved in response to misfolded protein accumulation in the cytosol. Regulated by alternative splicing and non-sense-mediated decay.
AT2G26160	*F-BOX/DUF295 ANCESTRAL 16 (ATFDA16)*	F-box SKIP23-like protein (DUF295)
AT2G26170	*CYTOCHROME P450, FAMILY 711, SUBFAMILY A, POLYPEPTIDE 1 (CYP711A1)*	Encodes a protein with similarity to thromboxane-A synthase, member of the CYP711A cytochrome P450 family. MAX1 is a specific repressor of vegetative axillary buds generated by the axillary meristem. Expressed in vascular traces in the rosette stem and axillary buds throughout plant development. Mutants have increased axillary branches. Along with MAX3,4 thought to mediate control of shoot branching via synthesis of a signal molecule which is transported over long distance mediated by MAX2. cDNA supports the existence of the longer transcript predicted for this locus, no cDNA isolated for shorter transcript. MAX1 downregulates 11 genes involved in flavonoid pathway (CHS, CHI, F3H, F3'H, FLS, DFR, ANS, UFGT, RT, AAC and GST).
AT2G26180	*IQ-DOMAIN 6 (IQD6)*	Transient Expression of Pro35S:YFP-IQD5 in leaves of N. benthamiana alters microtubule organization.Member of IQ67 (CaM binding) domain containing family.
AT2G26200	*TRNA METHYLTRANSFERASE 140A (TRM140A)*	S-adenosyl-L-methionine-dependent methyltransferases superfamily protein
AT2G26210		Ankyrin repeat family protein
AT2G26230	*URATE OXIDASE (UOX)*	Encodes a urate oxidase that is involved in peroxisome maintenance.
AT2G26240		Transmembrane proteins 14C
AT2G26250	*3-KETOACYL-COA SYNTHASE 10 (KCS10)*	epidermis-specific, encodes KCS10, a putative 3-ketoacyl-CoA synthase. probably involved in the synthesis of long-chain lipids found in the cuticle.
AT2G26260	*3BETA-HYDROXYSTEROID-DEHYDROGENASE/DECARBOXYLASE ISOFORM 2 (3BETAHSD/D2)*	Encodes an enzyme with 3β-hydroxysteroid dehydrogenase/C4-decarboxylase activity <i>in vitro</i>. The activity of the enzyme was determined using microsomal extracts of yeast overexpressing the <i>Arabidopsis</i> gene. Cytosolic fractions failed to be associated to the activity, leading to the speculation that the enzyme is membrane-bound.
AT2G26290	*ROOT-SPECIFIC KINASE 1 (ARSK1)*	root-specific kinase 1
AT2G26300	*G PROTEIN ALPHA SUBUNIT 1 (GP ALPHA 1)*	Encodes an alpha subunit of a heterotrimeric GTP-binding protein. The active GTP-bound form of GPA1 binds to the GTG1 and GTG2 abscisic acid (ABA) receptors and appears to affect their GTPase and GTP-binding activity, and hence, ABA binding abilities. GPA1 is a positive regulator in ABA-mediated inhibition of stomatal opening. Plants with recessive mutant alleles have complex phenotypes including: reduced brassinolide response, reduced cell divisions, round leaves, short hypocotyls. It is likely to be involved in the signaling events that trigger unfolded protein response-associated cell death. GPA1 is also involved in sugar signaling. The mRNA is cell-to-cell mobile.
AT2G26320	*AGAMOUS-LIKE 33 (AGL33)*	AGAMOUS-like 33
AT2G26330	*ERECTA (ER)*	Homologous to receptor protein kinases. Involved in specification of organs originating from the shoot apical meristem. Contains a cytoplasmic protein kinase catalytic domain, a transmembrane region, and an extracellular leucine-rich repeat. ER has been identified as a quantitative trait locus for transpiration efficiency by influencing epidermal and mesophyll development, stomatal density and porosity of leaves. It has been implicated in resistance to the bacterium Ralstonia solanacearum and to the necrotrophic fungus Plectosphaerella cucumerina. Together with ERL1 and ERL2, ER governs the initial decision of protodermal cells to either divide proliferatively to produce pavement cells or divide asymmetrically to generate stomatal complexes. ER binds to the peptides STOMAGEN and EPF2 which compete for the same binding site. The ER-EFP2 complex activates MAPK signaling that inhibits stomatal development. ER-STOMAGEN does not activate MAPK signaling. Plants harboring loss of function alleles of er are more susceptible to heat stress than wild type. In Arabidopsis and other organisms, overexpression of ER confers thermotolerance via as yet undefined mechanisms.
AT2G26340		hypothetical protein
AT2G26360		Mitochondrial substrate carrier family protein
AT2G26380		Leucine-rich repeat (LRR) family protein
AT2G26390	*SERPIN4 (SERP4)*	Serine protease inhibitor (SERPIN) family protein. Involved in stress response regulated cell death.
AT2G26400	*ACIREDUCTONE DIOXYGENASE 3 (ARD3)*	Encodes a protein predicted to belong to the acireductone dioxygenase (ARD/ARD?)family.
AT2G26410	*IQ-DOMAIN 4 (Iqd4)*	Member of IQ67 (CaM binding) domain containing family.
AT2G26430	*ARGININE-RICH CYCLIN 1 (RCY1)*	Encodes an ania-6a type arginine-rich cyclin which confers tolerance to LiCl and NaCl when expressed in yeast.
AT2G26440	*PECTIN METHYLESTERASE 12 (PME12)*	Plant invertase/pectin methylesterase inhibitor superfamily
AT2G26450		Plant invertase/pectin methylesterase inhibitor superfamily
AT2G26470		embryonic stem cell-specific 5-hydroxymethylcytosine-binding protein
AT2G26490	*JINGUBANG (JGB)*	JGB contains seven WD40 repeats and is highly conserved in flowering plants. Overexpression inhibits pollen germination. suggesting JGB is a negative regulator of pollen germination
AT2G26500	*(PETM)*	Essential for the stabilization and function of the cytochrome b6f complex.
AT2G26520		transmembrane protein

AT2G26530	*(AR781)*	Pheromone receptor-like protein involved in the early elicitor signaling events which occur within minutes and include ion fluxes across the plasma membrane, activation of MPKs and the formation of ROS related to PGPS1 and WRKY33.
AT2G26540	*(HEMD)*	Encodes a uroporphyrinogen-III synthase involved in tetrapyrrole biosynthesis. The protein localizes to the chloroplast. Member of the plant-specific DUF724 protein family. Arabidopsis has 10 DUF724 proteins. Loss of function mutant has a WT phenotype
AT2G26560	*PHOSPHOLIPASE A 2A (PLA2A)*	Encodes a lipid acyl hydrolase with wide substrate specificity that accumulates upon infection by fungal and bacterial pathogens. Protein is localized in the cytoplasm in healthy leaves, and in membranes in infected cells. Plays a role in cell death and differentially affects the accumulation of oxylipins. Contributes to resistance to virus.
AT2G26570	*WEAK CHLOROPLAST MOVEMENT UNDER BLUE LIGHT 1 (WEB1)*	Encodes a coiled-coil protein WEB1 (weak chloroplast movement under blue light 1). WEB1, together with another coiled-coil protein WEB2/PMI2 (At1g66840), maintains the chloroplast photorelocation movement velocity.
AT2G26580	*YABBY5 (YAB5)*	plant-specific transcription factor YABBY family protein
AT2G26620		Pectin lyase-like superfamily protein
AT2G26630		transposable_element_gene;transposase IS4 family protein, contains Pfam profile: PF01609 transposase DDE domain;(source:TAIR10)
AT2G26640	*3-KETOACYL-COA SYNTHASE 11 (KCS11)*	Encodes KCS11, a member of the 3-ketoacyl-CoA synthase family involved in the biosynthesis of VLCFA (very long chain fatty acids).
AT2G26650	*K+ TRANSPORTER 1 (KT1)*	Encodes AKT1, a member of the Shaker family inward rectifying potassium channel predominantly expressed in predominantly in root hairs and root endodermis. This family includes five groups based on phylogenetic analysis (FEBS Letters (2007) 581: 2357): I (inward rectifying channel): AKT1 (AT2G26650), AKT5 (AT4G32500) and SPIK (also known as AKT6, AT2G25600); II (inward rectifying channel): KAT1 (AT5G46240) and KAT2 (AT4G18290); III (weakly inward rectifying channel): AKT2 (AT4G22200); IV (regulatory subunit involved in inwardly rectifying conductance formation): KAT3 (also known as AtKC1, AT4G32650); V (outward rectifying channel): SKOR (AT3G02850) and GORK (AT5G37500).
AT2G26660	*SPX DOMAIN GENE 2 (SPX2)*	SPX domain-containing protein 2 (SPX2)
AT2G26670	*REVERSAL OF THE DET PHENOTYPE 4 (TED4)*	Encodes a plastid heme oxygenase necessary for phytochrome chromophore biosynthesis and for coupling the expression of some nuclear genes to the functional state of the chloroplast.
AT2G26680		FkbM family methyltransferase
AT2G26690	*NRT1/ PTR FAMILY 6.2 (NPF6.2)*	Major facilitator superfamily protein
AT2G26700	*PINOID2 (PID2)*	Member of AGC VIIIa Kinase gene family. Encodes PID2, a homolog of PID. Simultaneous disruption of PID(AT2G34650) and its 3 closest homologs (PID2/AT2G26700, WAG1/AT1G53700, and WAG2/AT3G14370) abolishes the formation of cotyledons.
AT2G26710	*PHYB ACTIVATION TAGGED SUPPRESSOR 1 (BAS1)*	Encodes a member of the cytochrome p450 family that serves as a control point between multiple photoreceptor systems and brassinosteroid signal transduction. Involved in brassinolide metabolism. Mediates response to a variety of light signals including hypocotyl elongation and cotyledon expansion.
AT2G26720		Cupredoxin superfamily protein
AT2G26730		Leucine-rich repeat protein kinase family protein
AT2G26740	*SOLUBLE EPOXIDE HYDROLASE (SEH)*	Encodes a soluble epoxide hydrolase whose expression is induced by auxin and water stress.
AT2G26760	*CYCLIN B1;4 (CYCB1;4)*	Cyclin B1
AT2G26770	*STOMATAL CLOSURE-RELATED ACTIN BINDING PROTEIN 1 (SCAB1)*	Encodes a plant-specific actin binding protein SCAB1 (STOMATAL CLOSURE-RELATED ACTIN BINDING PROTEIN1). SCAB1 stabilizes actin filaments and regulates stomatal movement.
AT2G26780		ARM repeat superfamily protein
AT2G26790		Pentatricopeptide repeat (PPR) superfamily protein
AT2G26810		Putative methyltransferase family protein
AT2G26820	*PHLOEM PROTEIN 2-A3 (PP2-A3)*	phloem protein 2-A3
AT2G26850		F-box family protein
AT2G26870	*NON-SPECIFIC PHOSPHOLIPASE C2 (NPC2)*	Non-specific phospholipase C2 involved in gametophyte development.
AT2G26880	*AGAMOUS-LIKE 41 (AGL41)*	AGAMOUS-like 41
AT2G26900	*BILE ACID:SODIUM SYMPORTER FAMILY PROTEIN 2 (BASS2)*	Sodium Bile acid symporter family
AT2G26910	*ATP-BINDING CASSETTE G32 (ABCG32)*	Encodes a member of the PLEIOTROPIC DRUG RESISTANCE family of ATP binding cassette transporters. Required for the formation of a functional cuticle.
AT2G26940		C2H2-type zinc finger family protein
AT2G26950	*MYB DOMAIN PROTEIN 104 (MYB104)*	Member of the R2R3 factor gene family.
AT2G26960	*MYB DOMAIN PROTEIN 81 (MYB81)*	Member of the R2R3 factor gene family.Expressed in microspores and required for progression into pollen mitosis I.
AT2G27000	*CYTOCHROME P450, FAMILY 705, SUBFAMILY A, POLYPEPTIDE 8 (CYP705A8)*	member of CYP705A
AT2G27010	*CYTOCHROME P450, FAMILY 705, SUBFAMILY A, POLYPEPTIDE 9 (CYP705A9)*	member of CYP705A
AT2G27040	*ARGONAUTE 4 (AGO4)*	AGO4 is a member of a class of PAZ/PIWI domain containing proteins involved in siRNA mediated gene silencing.Loss of function mutations have reduced site specific CpNpG and CpHpH methylation, abnormal ovule/megagametophyte develoment and increased susceptibility to bacterial pathogens including Tobacco rattle virus.
AT2G27050	*ETHYLENE-INSENSITIVE3-LIKE 1 (EIL1)*	ethylene-insensitive3-like1 (EIL1) The mRNA is cell-to-cell mobile.
AT2G27080	*NDR/HIN1-LIKE 13 (NHL13)*	Late embryogenesis abundant (LEA) hydroxyproline-rich glycoprotein family
AT2G27090		bZIP transcription factor (DUF630 and DUF632)
AT2G27120	*TILTED 2 (TIL2)*	Encodes a protein with similarity to DNA polymerase epsilon catalytic subunit. Based on yeast two hybrid analysis, not predicted to be a subunit of the DNA polymerase epsilon complex. No phenotype observed in homozygous mutant embryos or plants but in combination with TIL1-1/til1-1 heterozygotes arrest earlier than til1 homozygotes suggesting TIL2 functions redundantly with TIL1.
AT2G27140		HSP20-like chaperones superfamily protein
AT2G27150	*ABSCISIC ALDEHYDE OXIDASE 3 (AAO3)*	Encodes the aldehyde oxidase delta isoform catalyzing the final step in abscisic acid biosynthesis.

AT2G27160		hypothetical protein
AT2G27180		hypothetical protein
AT2G27190	*PURPLE ACID PHOSPHATASE 12 (PAP12)*	Encodes a root-secreted purple acid phosphatase involved in extracellular phosphate-scavenging. PEP complex component.
AT2G27200	*YEAST LSG1 ORTHOLOGUE 1 (LSG1-1)*	Encodes a cytosolic protein that shares 77.3% identity with AtLSG1-2 at the protein sequence level. The mRNA is cell-to-cell mobile.
AT2G27220	*BEL1-LIKE HOMEODOMAIN 5 (BLH5)*	BEL1-like homeodomain 5
AT2G27230	*LONESOME HIGHWAY (LHW)*	Encodes a nuclear-localized transcriptional activator with weak sequence similarity to basic helix-loop-helix(bHLH)-domain proteins. It promotes the production of stele cells in root meristems and is required to establish and maintain the normal vascular cell number and pattern in primary and lateral roots.
AT2G27240		aluminum activated malate transporter family protein
AT2G27250	*CLAVATA3 (CLV3)*	One of the three CLAVATA genes controlling the size of the shoot apical meristem (SAM) in Arabidopsis. Belongs to a large gene family called CLE for CLAVATA3/ESR-related. Encodes a stem cell-specific protein CLV3 presumed to be a precursor of a secreted peptide hormone. The deduced ORF encodes a 96-amino acid protein with an 18-amino acid N-terminal signal peptide. The functional form of CLV3 (MCLV3) was first reported to be a posttranscriptionally modified 12-amino acid peptide, in which two of the three prolines were modified to hydroxyproline (Ito et al., Science 2006, 313:842; Kondo et al., Science 2006, 313:845). Ohyama et al. (2009) later reported that the active mature CLV3 is a 13-amino-acid arabinosylated glycopeptide (Nature Chemical Biology, 5:578). CLV3 binds the ectodomain of the CLAVATA1 (CLV1) receptor-kinase. Regulates shoot and floral meristem development. Required for CLAVATA1 receptor-like kinase assembly into a signaling complex that includes KAPP and a Rho-related protein. It restricts its own domain of expression, the central zone (CZ) of the shoot apical meristem (SAM), by preventing differentiation of peripheral zone cells, which surround the CZ, into CZ cells and restricts overall SAM size by a separate, long-range effect on cell division rate. CLE domain of CLV3 is sufficient for function. Results obtained from whole seedlings challenge the concept that the immune receptor FLS2 perceives the meristematic regulatory peptide CLV3p in mesophyll, seedlings, and SAM cells and that CLV3p contributes to SAM immunity against bacterial infection (PMID:22923673).
AT2G27270		transmembrane protein
AT2G27280		coiled-coil protein (DUF2040)
AT2G27290		FAM210B-like protein, putative (DUF1279)
AT2G27300	*NTM1-LIKE 8 (NTL8)*	NTL8 is a membrane-associated NAC transcription factor that binds both TRY and TCL1. Overexpression results in fewer trichomes.
AT2G27310		F-box family protein
AT2G27360		GDSL-motif esterase/acyltransferase/lipase. Enzyme group with broad substrate specificity that may catalyze acyltransfer or hydrolase reactions with lipid and non-lipid substrates.
AT2G27370	*CASPARIAN STRIP MEMBRANE DOMAIN PROTEIN 3 (CASP3)*	Uncharacterized protein family (UPF0497)
AT2G27375		transposable_element_gene;similar to RNase H domain-containing protein [Arabidopsis thaliana] (TAIR:AT4G09490.1);(source:TAIR10)
AT2G27380	*EXTENSIN PROLINE-RICH 1 (EPR1)*	Encodes an extensin like gene involved in seed germination.
AT2G27390		proline-rich family protein
AT2G27395		pseudogene of cysteine protease-related
AT2G27410		B3 domain protein, putative (DUF313)
AT2G27420		Cysteine proteinases superfamily protein
AT2G27440		pseudogene of rac GTPase activating protein
AT2G27450	*NITRILASE-LIKE PROTEIN 1 (NLP1)*	Encodes N-carbamoylputrescine amidohydrolase that is involved in putrescine and polyamine biosynthesis.
AT2G27470	*NUCLEAR FACTOR Y, SUBUNIT B11 (NF-YB11)*	nuclear factor Y, subunit B11
AT2G27480		Calcium-binding EF-hand family protein
AT2G27500		Glycosyl hydrolase superfamily protein
AT2G27540		F-box associated ubiquitination effector family protein
AT2G27550	*CENTRORADIALIS (ATC)*	encodes a protein similar to TFL1. overexpression leads to similar phenotype as TFL1 overexpression. expressed specifically in the hypocotyl and null mutation does not result in phenotypes exhibited by TFL1 null mutations. It acts non-cell autonomously to inhibit floral initiation.
AT2G27570		P-loop containing nucleoside triphosphate hydrolases superfamily protein
AT2G27590		S-adenosyl-L-methionine-dependent methyltransferases superfamily protein
AT2G27620		
AT2G27650		Ubiquitin carboxyl-terminal hydrolase-related protein
AT2G27660		Cysteine/Histidine-rich C1 domain family protein
AT2G27680		NAD(P)-linked oxidoreductase superfamily protein
AT2G27690	*CYTOCHROME P450, FAMILY 94, SUBFAMILY C, POLYPEPTIDE 1 (CYP94C1)*	Encodes a CYP94C1. Has highest omega-hydroxylase activity with 9,10-epoxystearic acid, while also metabolized lauric acid (C12:0) and C18 unsaturated fatty acids. Gene expression is induced in response to wounding and jasmonic acid treatment.
AT2G27700		eukaryotic translation initiation factor 2 family protein / eIF-2 family protein
AT2G27740		RAB6-interacting golgin (DUF662)
AT2G27750		Surfeit locus protein 6
AT2G27775		PPR containing protein
AT2G27800		Tetratricopeptide repeat (TPR)-like superfamily protein
AT2G27810	*NUCLEOBASE-ASCORBATE TRANSPORTER 12 (NAT12)*	Encodes a plasma-membrane localized nucleobase transporter capable of transporting adenine, guanine, uracil and hypoxanthine. Likely to be a proton-nucleobase symporter.
AT2G27830		hypothetical protein
AT2G27840	*(HDT4)*	Belongs to the plant specific HD2 type proteins; similar to nucleolar Zea mays histone deacetylase; HD2-p39

AT2G27860	*UDP-D-APIOSE/UDP-D-XYLOSE SYNTHASE 1 (AXS1)*	Encodes UDP-d-apiose/UDP-d-xylose synthase that requires NAD+ for enzymatic activity and is strongly inhibited by UDP-d-galacturonate.
AT2G27870		transposable_element_gene;similar to RNase H domain-containing protein [Arabidopsis thaliana] (TAIR:AT2G22350.1);(source:TAIR10)
AT2G27880	*ARGONAUTE 5 (AGO5)*	AGO5.Required for antiviral RNA silencing.Confers resistance to Potato virus X.
AT2G27890		
AT2G27920	*SERINE CARBOXYPEPTIDASE-LIKE 51 (SCPL51)*	serine carboxypeptidase-like 51
AT2G27940	*ARABIDOPSIS T??XICOS EN LEVADURA 57 (ATL57)*	RING/U-box superfamily protein
AT2G27950		Ring/U-Box superfamily protein
AT2G27980		Acyl-CoA N-acyltransferase with RING/FYVE/PHD-type zinc finger domain-containing protein
AT2G27990	*BEL1-LIKE HOMEODOMAIN 8 (BLH8)*	Encodes a BEL1-like homeobox gene that functions together with PNY in meristem maintenance by regulating the allocation process during vegetative and reproductive development. Both gene products are required for the competence of the SAM to respond properly to floral inductive signals.
AT2G28010		Eukaryotic aspartyl protease family protein
AT2G28030		Eukaryotic aspartyl protease family protein
AT2G28050	*RNA PROCESSING FACTOR 7 (RPF7)*	Encodes a relatively short regular p-class pentatricopeptide repeat protein comprising seven canonical p repeats and a single short s repeat that is necessary for the 5'-processing and stability of nad2 mRNA in mitochondria.
AT2G28080		UDP-Glycosyltransferase superfamily protein
AT2G28085	*SMALL AUXIN UPREGULATED RNA 42 (SAUR42)*	SAUR-like auxin-responsive protein family
AT2G28090	*HEAVY METAL ASSOCIATED PROTEIN 17 (ATHMP17)*	Heavy metal transport/detoxification superfamily protein
AT2G28100	*ALPHA-L-FUCOSIDASE 1 (FUC1)*	Encodes a protein with α-fucosidase activity. The activity was assessed on 2'-fucosyl-lactitol. AtFUC1 was not able to act on XXFG substrates, at least when heterologously expressed in <i>Pichia pastoris</i>. The enzyme has been postulated to act on fucosylated substrates other than xyloglucan oligosaccharides. was shown (<i>Pichia pastoris</i>) to hydrolyze fucose in 3- and 4-linkage , hence was characterized as alpha-L-3,4-fucosidase The mRNA is cell-to-cell mobile.
AT2G28110	*FRAGILE FIBER 8 (FRA8)*	Homolog to AT5G22940, a member of glycosyltransferase family 47 that is involved in secondary cell wall biosynthesis. It exhibits high sequence similarity to tobacco (Nicotiana plumbaginifolia) pectin glucuronyltransferase. Protein has a domain that shares significant similarity with the pfam03016 domain. It is expressed specifically in developing vessels and fiber cells, and FRA8 is targeted to Golgi. Mutants have irregular xylem formation, reduced cellulose levels and plants are smaller than normal siblings.
AT2G28120	*OVARIAN TUMOR DOMAIN (OTU)-CONTAINING DUB (DEUBIQUITILATING ENZYME) 1 (OTU1)*	Major facilitator superfamily protein
AT2G28160	*FER-LIKE IRON DEFICIENCY INDUCED TRANSCRIPTION FACTOR (FIT)*	Encodes a putative transcription factor that regulates iron uptake responses. mRNA is detected in the outer cell layers of the root and accumulates in response to iron deficiency. The expression of many iron-regulated genes is dependent on FIT1. It specifically regulates FRO2 at the level of mRNA accumulation and IRT1 at the level of protein accumulation.Similar to FER in tomato and is a regulator of iron uptake. It is post-transcriptionally controlled.
AT2G28170	*(ATCHX7)*	member of Putative Na+/H+ antiporter family
AT2G28180	*(ATCHX8)*	member of Putative Na+/H+ antiporter family
AT2G28200		C2H2-type zinc finger family protein
AT2G28210	*ALPHA CARBONIC ANHYDRASE 2 (ACA2)*	alpha carbonic anhydrase 2
AT2G28250	*(NCRK)*	Protein kinase superfamily protein
AT2G28260	*CYCLIC NUCLEOTIDE-GATED CHANNEL 15 (CNGC15)*	member of Cyclic nucleotide gated channel family, involved in response to calcium.
AT2G28270		Cysteine/Histidine-rich C1 domain family protein
AT2G28305	*LONELY GUY 1 (LOG1)*	Putative lysine decarboxylase family protein
AT2G28310		trimethylguanosine synthase (DUF707)
AT2G28320		Pleckstrin homology (PH) and lipid-binding START domains-containing protein
AT2G28350	*AUXIN RESPONSE FACTOR 10 (ARF10)*	Involved in root cap cell differentiation.
AT2G28355	*LOW-MOLECULAR-WEIGHT CYSTEINE-RICH 5 (LCR5)*	low-molecular-weight cysteine-rich 5
AT2G28380	*DSRNA-BINDING PROTEIN 2 (DRB2)*	Encodes a cytoplasmic dsRNA-binding protein DRB2. A maternally expressed imprinted gene. DRB2 and DRB4 have an antagonistic impact on polymerase IV-dependent siRNA levels.
AT2G28400		senescence regulator (Protein of unknown function, DUF584)
AT2G28410		transmembrane protein
AT2G28420	*GLYOXYLASE I 8 (GLYI8)*	Vicinal oxygen chelate (VOC) superfamily member.
AT2G28440		proline-rich family protein
AT2G28450	*TRNA METHYLTRANSFERASE 2B (TRM2B)*	zinc finger (CCCH-type) family protein
AT2G28460		Cysteine/Histidine-rich C1 domain family protein
AT2G28470	*BETA-GALACTOSIDASE 8 (BGAL8)*	putative beta-galactosidase (BGAL8 gene)
AT2G28490		RmlC-like cupins superfamily protein
AT2G28510	*DOF PROTEIN 2.1 (DOF2.1)*	DOF transcription factor with a conserved zinc finger (ZF) DNA-binding domain.
AT2G28530		
AT2G28550	*RELATED TO AP2.7 (RAP2.7)*	AP2 family transcription factor that is involved in regulation of flowering and innate immunity.Interacts with CRY2 to regulate CO and FT. TOE1 binds to activation domain of CO and binds CORE sequences of the FT promoter.TOE1/TOE2 are also targets of MiR172b and function in regulation of innate immunity.
AT2G28560	*(RAD51B)*	Encodes a protein of the RAD51B family involved in double stranded DNA repair. Homozygous mutant plants show increased sensitivity to mitomycin which induces DS breaks.
AT2G28570		hypothetical protein

AT2G28580		transmembrane protein, putative (DUF247)
AT2G28590	*PBS1-LIKE 6 (PBL6)*	Protein kinase superfamily protein
AT2G28600		P-loop containing nucleoside triphosphate hydrolases superfamily protein
AT2G28605		Encodes a PsbP domain-OEC23 like protein localized in thylakoid (peripheral-lumenal side).
AT2G28610	*PRESSED FLOWER (PRS)*	Encodes a homeodomain containing protein that regulates lateral axis-dependent development of Arabidopsis flowers and is required for cell proliferation. It is expressed in a restricted number of L1 cells at the lateral regions of flower primordia, floral organ primordia, and young leaf primordia.
AT2G28620	*(RSW7)*	Mutants have radially swollen roots but do not exhibit defects in abundance or orientation of cortical microtubules, nor are microfibrils reduced. Cellulose synthesis is also unchanged with respect to wild type. There is a disruption in the normal pattern of cell wall placement.
AT2G28630	*3-KETOACYL-COA SYNTHASE 12 (KCS12)*	Encodes KCS12, a member of the 3-ketoacyl-CoA synthase family involved in the biosynthesis of VLCFA (very long chain fatty acids).
AT2G28640	*EXOCYST SUBUNIT EXO70 FAMILY PROTEIN H5 (EXO70H5)*	A member of EXO70 gene family, putative exocyst subunits, conserved in land plants. Arabidopsis thaliana contains 23 putative EXO70 genes, which can be classified into eight clusters on the phylogenetic tree.
AT2G28650	*EXOCYST SUBUNIT EXO70 FAMILY PROTEIN H8 (EXO70H8)*	A member of EXO70 gene family, putative exocyst subunits, conserved in land plants. Arabidopsis thaliana contains 23 putative EXO70 genes, which can be classified into eight clusters on the phylogenetic tree.
AT2G28660	*HEAVY METAL ASSOCIATED PROTEIN 18 (ATHMP18)*	Chloroplast-targeted copper chaperone protein
AT2G28670	*ENHANCED SUBERIN 1 (ESB1)*	Encodes a protein of unknown function that is involved in formation of the casparian strip. ESB1 is localized to the casparian strip and this localization is depended on CASP1 and 2. esb1 mutants have increased levels of suberin, disordered casparian strip and altered levels of several ions in their leaves.
AT2G28680		RmlC-like cupins superfamily protein
AT2G28690		TOX high mobility group box protein, putative (DUF1635)
AT2G28700	*AGAMOUS-LIKE 46 (AGL46)*	AGAMOUS-like 46
AT2G28710		C2H2-type zinc finger family protein
AT2G28720	*(HTB3)*	Histone superfamily protein
AT2G28755		UDP-D-glucuronate carboxy-lyase-like protein
AT2G28760	*UDP-XYL SYNTHASE 6 (UXS6)*	Encodes a cytosolic isoform of UDP-glucuronic acid decarboxylase.
AT2G28780		P-hydroxybenzoic acid efflux pump subunit
AT2G28810		Dof-type zinc finger DNA-binding family protein
AT2G28820		
AT2G28840	*XB3 ORTHOLOG 1 IN ARABIDOPSIS THALIANA (XBAT31)*	Putative E3 Ub protein ligase; regulates thermoresponsive hypocotyl growth through mediating degradation of the thermosensor ELF3.
AT2G28850	*CYTOCHROME P450, FAMILY 710, SUBFAMILY A, POLYPEPTIDE 3 (CYP710A3)*	member of CYP710A
AT2G28870	*(SMR10)*	cyclin-dependent kinase inhibitor SMR1-like protein
AT2G28900	*OUTER PLASTID ENVELOPE PROTEIN 16-1 (OEP16-1)*	Encodes AtOEP16, a 16-KDa plastid outer membrane protein involved in plastid import of protochlorophyllide oxidoreductase A. Predominantly expressed in leaves and is also inducible by cold treatment.
AT2G28930	*PROTEIN KINASE 1B (PK1B)*	protein kinase 1B
AT2G28940	*PBS1-LIKE 37 (PBL37)*	Protein kinase superfamily protein
AT2G28950	*EXPANSIN A6 (EXPA6)*	Encodes an expansin. Naming convention from the Expansin Working Group (Kende et al, 2004. Plant Mol Bio). Involved in the formation of nematode-induced syncytia in roots of Arabidopsis thaliana.
AT2G28960		Leucine-rich repeat protein kinase family protein
AT2G28970		Leucine-rich repeat protein kinase family protein
AT2G28990		Leucine-rich repeat protein kinase family protein
AT2G29000		Leucine-rich repeat protein kinase family protein
AT2G29040	*(GT11)*	A MUR3 xyloglucan galactosyltransferase homolog which is highly expressed in pollen and which regulates the cell wall stability of pollen tubes.
AT2G29070		Ubiquitin fusion degradation UFD1 family protein
AT2G29090	*CYTOCHROME P450, FAMILY 707, SUBFAMILY A, POLYPEPTIDE 2 (CYP707A2)*	Encodes a protein with ABA 8'-hydroxylase activity, involved in ABA catabolism. Member of the CYP707A gene family. This gene predominantly accumulates in dry seeds and is up-regulated immediately following imbibition. CYP707A2 appears to play a major role in the rapid decrease in ABA levels during early seed imbibition.
AT2G29100	*GLUTAMATE RECEPTOR 2.9 (GLR2.9)*	member of Putative ligand-gated ion channel subunit family
AT2G29110	*GLUTAMATE RECEPTOR 2.8 (GLR2.8)*	member of Putative ligand-gated ion channel subunit family
AT2G29120	*GLUTAMATE RECEPTOR 2.7 (GLR2.7)*	member of Putative ligand-gated ion channel subunit family
AT2G29140	*PUMILIO 3 (PUM3)*	Encodes a member of the Arabidopsis Pumilio (APUM) proteins containing PUF domain (eight repeats of approximately 36 amino acids each). PUF proteins regulate both mRNA stability and translation through sequence-specific binding to the 3' UTR of target mRNA transcripts. The mRNA is cell-to-cell mobile.
AT2G29150		NAD(P)-binding Rossmann-fold superfamily protein
AT2G29170		NAD(P)-binding Rossmann-fold superfamily protein
AT2G29180		transmembrane protein
AT2G29190	*PUMILIO 2 (PUM2)*	Encodes a member of the Arabidopsis Pumilio (APUM) proteins containing PUF domain (eight repeats of approximately 36 amino acids each). PUF proteins regulate both mRNA stability and translation through sequence-specific binding to the 3' UTR of target mRNA transcripts.
AT2G29220	*L-TYPE LECTIN RECEPTOR KINASE III.1 (LECRK-III.1)*	Concanavalin A-like lectin protein kinase family protein
AT2G29250	*L-TYPE LECTIN RECEPTOR KINASE III.2 (LECRK-III.2)*	Concanavalin A-like lectin protein kinase family protein
AT2G29260		NAD(P)-binding Rossmann-fold superfamily protein

AT2G29290	*(DEG14)*	NAD(P)-binding Rossmann-fold superfamily protein
AT2G29300		NAD(P)-binding Rossmann-fold superfamily protein
AT2G29310		NAD(P)-binding Rossmann-fold superfamily protein
AT2G29320		NAD(P)-binding Rossmann-fold superfamily protein
AT2G29330	*TROPINONE REDUCTASE (TRI)*	tropinone reductase
AT2G29340		NAD-dependent epimerase/dehydratase family protein
AT2G29350	*SENESCENCE-ASSOCIATED GENE 13 (SAG13)*	Encodes a senescence associated protein required for resistance against fungal pathogens. Negative regulator of defense against bacterial pathogens. Induced by ROS. Required for defense against ROS and fungal pathogens most likely by activating anthocyanin biosynthesis.
AT2G29360		NAD(P)-binding Rossmann-fold superfamily protein
AT2G29370		NAD(P)-binding Rossmann-fold superfamily protein
AT2G29380	*HIGHLY ABA-INDUCED PP2C GENE 3 (HAI3)*	highly ABA-induced PP2C protein 3
AT2G29400	*TYPE ONE PROTEIN PHOSPHATASE 1 (TOPP1)*	Type 1 protein phosphatase, expressed in roots, rosettes and flowers
AT2G29410	*METAL TOLERANCE PROTEIN B1 (MTPB1)*	member of Zinc transporter (ZAT) family
AT2G29420	*GLUTATHIONE S-TRANSFERASE TAU 7 (GSTU7)*	Encodes glutathione transferase belonging to the tau class of GSTs. Naming convention according to Wagner et al. (2002). Induced by Salicylic acid. Independent of NPR1 for their induction by salicylic acid.
AT2G29440	*GLUTATHIONE S-TRANSFERASE TAU 6 (GSTU6)*	Encodes glutathione transferase belonging to the tau class of GSTs. Naming convention according to Wagner et al. (2002).
AT2G29450	*GLUTATHIONE S-TRANSFERASE TAU 5 (GSTU5)*	Encodes a member of the TAU glutathione S-transferase gene family. Gene expression is induced by exposure to auxin, pathogen and herbicides. Naming convention according to Wagner et al. (2002)
AT2G29460	*GLUTATHIONE S-TRANSFERASE TAU 4 (GSTU4)*	Encodes glutathione transferase belonging to the tau class of GSTs. Naming convention according to Wagner et al. (2002). Role in the degradation of H2O2 to water using glutathione as electron donor
AT2G29470	*GLUTATHIONE S-TRANSFERASE TAU 3 (GSTU3)*	Encodes glutathione transferase belonging to the tau class of GSTs. Naming convention according to Wagner et al. (2002).
AT2G29480	*GLUTATHIONE S-TRANSFERASE TAU 2 (GSTU2)*	Encodes glutathione transferase belonging to the tau class of GSTs. Naming convention according to Wagner et al. (2002).
AT2G29490	*GLUTATHIONE S-TRANSFERASE TAU 1 (GSTU1)*	Encodes glutathione transferase belonging to the tau class of GSTs. Naming convention according to Wagner et al. (2002).
AT2G29500	*(HSP17.6B)*	HSP20-like chaperones superfamily protein
AT2G29510		hypothetical protein (DUF3527)
AT2G29530	*TRANSLOCASE OF THE INNER MEMBRANE 10 (TIM10)*	Encodes a small zinc finger-like protein that is a component of the mitochondrial protein import apparatus. Together with AtTIM9, AtTIM10 is non-redundantly essential for maintaining mitochondrial function of early embryo proper cells and endosperm free-nuclei.
AT2G29540	*RNAPOLYMERASE 14 KDA SUBUNIT (RPC14)*	RNA polymerase I(A) and III(C) 14 kDa subunit
AT2G29590		Thioesterase superfamily protein
AT2G29600		Galactose oxidase/kelch repeat superfamily protein
AT2G29605		Plant protein 1589 of unknown function
AT2G29610		pseudogene of the F-box protein family, contains Pfam profile PF00646: F-box domain
AT2G29620		dentin sialophosphoprotein
AT2G29630	*THIAMINC (THIC)*	Encodes a protein involved in thiamin biosynthesis. The protein is an iron-sulfur cluster protein predicted to catalyze the conversion of 5-aminoimidazole ribonucleotide (AIR) to hydroxymethylpyrimidine (HMP) or hydroxymethylpyrimidine phosphate (HMP-P). A severe reduction of THIC levels in plants decreases vitamin B1 (thiamin diphosphate (TPP)) levels and also leads to changes in the levels of numerous other metabolites since so many primary metabolic enzymes require a TPP co-factor. thiC mutants are chlorotic and arrest in their development at the cotyledon stage. A N-terminal targeting sequence directs the THIC protein to the chloroplast stroma. A conserved TPP-binding site is located in the 3' UTR of the At2g29630.2 gene model, and is predicted to function as a riboswitch. The riboswitch controls the formation of transcripts with alternative 3' UTR lengths, which affect mRNA accumulation and protein production. THIC transcripts are observed in seedlings 5 or more days after germination, and light promotes the expression of this gene. Recessive mutant isolated by Redei. Leaves but not cotyledons white, lethal; restored to normal by thiamine or 2,5-dimethyl-4-aminopyrimidine.
AT2G29650	*PHOSPHATE TRANSPORTER 4;1 (PHT4;1)*	Encodes an inorganic phosphate transporter (PHT4;1) that is localized to the thylakoid membrane.
AT2G29660		zinc finger (C2H2 type) family protein
AT2G29670		Tetratricopeptide repeat (TPR)-like superfamily protein
AT2G29720	*(CTF2B)*	Encodes CTF2B.
AT2G29730	*UDP-GLUCOSYL TRANSFERASE 71D1 (UGT71D1)*	UDP-glucosyl transferase 71D1
AT2G29740	*UDP-GLUCOSYL TRANSFERASE 71C2 (UGT71C2)*	UDP-glucosyl transferase 71C2
AT2G29750	*UDP-GLUCOSYL TRANSFERASE 71C1 (UGT71C1)*	UDP-glucosyl transferase 71C1
AT2G29760	*ORGANELLE TRANSCRIPT PROCESSING 81 (OTP81)*	Encodes a chloroplast RNA editing factor.
AT2G29770		Galactose oxidase/kelch repeat superfamily protein
AT2G29780		Galactose oxidase/kelch repeat superfamily protein
AT2G29790	*POLLEN COAT PROTEIN B CLASS BETA (PCP-BBETA)*	Encodes a Maternally expressed gene (MEG) family protein [pseudogene]
AT2G29820		Galactose oxidase/kelch repeat superfamily protein
AT2G29860		Galactose oxidase/kelch repeat superfamily protein
AT2G29880		Myb/SANT-like DNA-binding domain protein
AT2G29890	*VILLIN 1 (VLN1)*	Encodes a ubiquitously expressed villin-like protein, whose mRNA may be alternatively processed. Villin belongs to a superfamily of actin binding proteins called the villin/gelsolin family. Animal villins are involved in actin binding. VLN1 protein co-localizes with actin filaments in several assays. VLN1 binds and bundles F-actin in a calcium-independent manner. It does not nucleate, cap or sever actin filaments and it stabilizes actin filaments, protecting them from ADF-mediated depolymerization.

AT2G29900	*PRESENILIN-2 (PS2)*	Encodes a gamma-secretase subunit. Associates with other subunits in intracellular membrane compartments.
AT2G29920		hypothetical protein
AT2G29940	*ATP-BINDING CASSETTE G31 (ABCG31)*	pleiotropic drug resistance 3
AT2G29970	*SMAX1-LIKE 7 (SMXL7)*	Encodes a member of an eight-gene family (SMAX1 and SMAX1-like) that has weak similarity to AtHSP101, a ClpB chaperonin required for thermotolerance. The mRNA is cell-to-cell mobile.
AT2G29980	*FATTY ACID DESATURASE 3 (FAD3)*	Endoplasmic reticulum enzyme responsible for the synthesis of 18:3 fatty acids from phospholipids. Uses cytochrome b5 as electron donor.
AT2G29995		PSY3-like protein
AT2G30000		PHF5-like protein
AT2G30010	*TRICHOME BIREFRINGENCE-LIKE 45 (TBL45)*	Encodes a member of the TBL (TRICHOME BIREFRINGENCE-LIKE) gene family containing a plant-specific DUF231 (domain of unknown function) domain. TBL gene family has 46 members, two of which (TBR/AT5G06700 and TBL3/AT5G01360) have been shown to be involved in the synthesis and deposition of secondary wall cellulose, presumably by influencing the esterification state of pectic polymers. A nomenclature for this gene family has been proposed (Volker Bischoff & Wolf Scheible, 2010, personal communication).
AT2G30020	*(AP2C1)*	Encodes AP2C1. Belongs to the clade B of the PP2C-superfamily. Acts as a MAPK phosphatase that negatively regulates MPK4 and MPK6.
AT2G30030		
AT2G30040	*MITOGEN-ACTIVATED PROTEIN KINASE KINASE KINASE 14 (MAPKKK14)*	Member of MEKK subfamily. Induced by jasmonic acid and wounding in involved in insectivory response signaling. Iinteracts with At5g40440, and activates At1g59580.
AT2G30060		Pleckstrin homology (PH) domain superfamily protein
AT2G30070	*POTASSIUM TRANSPORTER 1 (KT1)*	Encodes a high affinity potassium transporter.
AT2G30080	*(ZIP6)*	member of Fe(II) transporter isolog family. Gene expression is not regulated by iron, copper, or zinc deficiency or excess.
AT2G30090		Acyl-CoA N-acyltransferases (NAT) superfamily protein
AT2G30130	*(ASL5)*	Overexpression/activation tagged allele has epinastic leaves, reduced apical dominance and is sterile. Gene is similar to asymmetric leaves (AS)/lateral organ boundary (LOB) genes which repress KNOX gene expression.
AT2G30140	*UDP-GLUCOSYL TRANSFERASE 87A2 (UGT87A2)*	Encodes a putative glycosyltransferase. Regulates flowering time via FLOWERING LOCUS C.
AT2G30150		UDP-Glycosyltransferase superfamily protein
AT2G30160	*MITOCHONDRIAL IRON TRANSPORTER 1 (MIT1)*	Mitochondrial iron transport protein. Member of the substrate carrier family (MCF) of protein transporters.
AT2G30200	*EMBRYO DEFECTIVE 3147 (EMB3147)*	Malonyl-ACP expressed in developing seeds. Loss of function mutants are embryo lethal and over expression in seeds leads to increased seed oil content.
AT2G30210	*LACCASE 3 (LAC3)*	putative laccase, a member of laccase family of genes (17 members in Arabidopsis).
AT2G30240	*(ATCHX13)*	Encodes a plasma membrane localized potassium transporter.
AT2G30250	*WRKY DNA-BINDING PROTEIN 25 (WRKY25)*	member of WRKY Transcription Factor; Group I. Located in nucleus. Involved in response to various abiotic stresses - especially salt stress.
AT2G30270		LURP-one-like protein (DUF567)
AT2G30280	*RNA-DIRECTED DNA METHYLATION 4 (RDM4)*	Encodes RDM4, a transcriptional regulator functioning in RNA-directed DNA methylation and plant development.
AT2G30290	*VACUOLAR SORTING RECEPTOR 2 (VSR2)*	VACUOLAR SORTING RECEPTOR 2
AT2G30300		Major facilitator superfamily protein
AT2G30310		GDSL-motif esterase/acyltransferase/lipase. Enzyme group with broad substrate specificity that may catalyze acyltransfer or hydrolase reactions with lipid and non-lipid substrates.
AT2G30320		Pseudouridine synthase family protein
AT2G30340	*LOB DOMAIN-CONTAINING PROTEIN 13 (LBD13)*	Lateral Organ Boundaries domain protein. LOB13 promotes lateral root formation.
AT2G30360	*SOS3-INTERACTING PROTEIN 4 (SIP4)*	Encodes a SOS2-like protein kinase that is a member of the CBL-interacting protein kinase family.Loss of function mutants show a decrease in sensitivity to high pH.Phosphorylates AHA2, a plasma membrane H+ ATPase.This phosphorylation appears to regulate the activity of the proton transporter.
AT2G30370	*CHALLAH (CHAL)*	Encodes a small, potentially secreted protein that acts as an inhibitor of stomatal production though likely not through direct interaction with the TMM receptor. It is homologous to known stomatal regulators EPF1 and EPF2. Memmber of the EPF/EPFL (epidermal patterning factor/EPF-like) gene family, which genes encode plant-specific secretory peptides, several of which play a role in controlling stomatal density and patterning in the plant epidermis.
AT2G30380	*CHIQUITA1-LIKE 8 (CHIQL8)*	MYB family transcription factor
AT2G30390	*FERROCHELATASE 2 (FC2)*	Encodes one of two ferrochelatase genes in Arabidopsis. Ferrochelatase is the terminal enzyme of heme biosynthesis. FC-II is speculated to operate in photosynthetic cytochromes.
AT2G30400	*OVATE FAMILY PROTEIN 2 (OFP2)*	ovate family protein 2
AT2G30420	*ENHANCER OF TRY AND CPC 2 (ETC2)*	In a tandem repeat with AT2G30424 and AT2G30432
AT2G30430		hypothetical protein
AT2G30460	*UDP-XYLOSE TRANSPORTER2 (UXT2)*	UXT2 is a member of the NST-KT subfamily of nucleotide/sugar transporters. It is localized to the golgi and functions as a UDP-Xyl transporter.

AT2G30470	*HIGH-LEVEL EXPRESSION OF SUGAR-INDUCIBLE GENE 2 (HSI2)*	HSI2 is a member of the ABI3 family of B3 domain proteins and functions as an active repressor of the Spo minimal promoter through the EAR motif. It contains a plant-specific B3 DNA-binding domain. It is expressed at similar levels in all organs. Treatment with 6% sucrose showed a slight increase in transcript levels after 24 h. No changes were observed after treatment with 50?M ABA. It is localized in the nucleus via a nuclear localization sequence located in the fourth conserved region of the C-terminal B3 domain. HSI2 is also an epigenetic repressor as it also contains functional plant homeodomain-like (PHD-L) and zinc-finger Cys- and Trp-containing (CW) domains associated with epigenetic regulation. The PHD-L domain of HSI2 is connected to promoting trimethylation of Lys-27 on histone 3 (H3K27me3), while the CW domain can bind directly to H3K4me3. Through these domains, HSI2 represses the seed maturation program during seed germination by repressing transcription of the core LAFL (LEC1, ABI3, FUS3, and LEC2) seed developmental transcriptional regulators. In developing A. thaliana embryos, HSI2 suppresses expression of a large number of genes, many identified as targets of FUS3. However, the absence of HSI2 had no effect on transcript levels of the LAFL regulators and the levels of measured metabolites and phytohormones (ABA, auxin, and JA derivatives) in developing Arabidopsis embryos. HSI2 likely fine-tunes seed maturation by repressing genes involved in early embryogenesis that are not required later for seed maturation and desiccation.
AT2G30480		hypothetical protein
AT2G30500	*NETWORKED 4B (NET4B)*	Kinase interacting (KIP1-like) family protein
AT2G30510		
AT2G30520	*ROOT PHOTOTROPISM 2 (RPT2)*	Encodes a phototropin-interacting NRL protein that is an early signaling component in the phototrophic response and is essential for the phototropin-mediated chloroplast accumulation response but is not involved in the chloroplast avoidance response or stomatal opening.
AT2G30540	*(ROXY7)*	Encodes a member of the CC-type glutaredoxin (ROXY) family that has been shown to interact with the transcription factor TGA2.
AT2G30550	*DAD1-LIKE LIPASE 3 (DALL3)*	Encodes a lipase that hydrolyzes phosphatidylcholine, glycolipids as well as triacylglycerols.
AT2G30560		Needs to be reannotated and split into two genes, AtEAL2 and AtEAL3, both encoding maize Ebb apparatus 1-like proteins. The current predicted structure is not well supported (T8, one *). The predicted proteins can be found in doi.org/10.1007/s00425-005-0174-z
AT2G30570	*PHOTOSYSTEM II REACTION CENTER W (PSBW)*	Encodes PsbW, a protein similar to photosystem II reaction center subunit W. Loss of PsbW destabilizes the supramolecular organization of PSII.
AT2G30580	*DREB2A-INTERACTING PROTEIN 2 (DRIP2)*	Encodes a C3HC4 RING-domain-containing ubiquitin E3 ligase capable of interacting with DREB2A. DRIP2 seems to be involved in regulating stress-related transcriptional changes and drought tolerance.
AT2G30600		BTB/POZ domain-containing protein
AT2G30610		
AT2G30630		P-loop containing nucleoside triphosphate hydrolases superfamily protein
AT2G30650		ATP-dependent caseinolytic (Clp) protease/crotonase family protein
AT2G30660		ATP-dependent caseinolytic (Clp) protease/crotonase family protein
AT2G30670		NAD(P)-binding Rossmann-fold superfamily protein
AT2G30680		callose synthase-like protein
AT2G30690	*(MYOB4)*	lateral signaling target-like protein (Protein of unknown function, DUF593)
AT2G30695		bacterial trigger factor
AT2G30730		Protein kinase superfamily protein
AT2G30740		Protein kinase superfamily protein
AT2G30750	*CYTOCHROME P450, FAMILY 71, SUBFAMILY A, POLYPEPTIDE 12 (CYP71A12)*	Putative cytochrome P450; together with CYP71A13 produces dihydrocamalexic acid (DHCA), the precursor to the defense-related compound camalexin, which accumulates in the intercellular space and contributes to the resistance of mature Arabidopsis to P. syringae without directly inhibiting bacterial growth.
AT2G30760		hypothetical protein
AT2G30770	*CYTOCHROME P450, FAMILY 71, SUBFAMILY A, POLYPEPTIDE 13 (CYP71A13)*	Putative cytochrome P450; together with CYP71A12 produces dihydrocamalexic acid (DHCA), the precursor to the defense-related compound camalexin, which accumulates in the intercellular space and contributes to the resistance of mature Arabidopsis to P. syringae without directly inhibiting bacterial growth.
AT2G30780		Tetratricopeptide repeat (TPR)-like superfamily protein
AT2G30790	*PHOTOSYSTEM II SUBUNIT P-2 (PSBP-2)*	Encodes a 23 kD extrinsic protein that is part of photosystem II and participates in the regulation of oxygen evolution.
AT2G30810	*(GASA12)*	Gibberellin-regulated family protein
AT2G30820		aspartyl/glutamyl-tRNA(Asn/Gln) amidotransferase subunit
AT2G30830		encodes a protein whose sequence is similar to 2-oxoglutarate-dependent dioxygenase
AT2G30840		encodes a protein whose sequence is similar to 2-oxoglutarate-dependent dioxygenase
AT2G30860	*GLUTATHIONE S-TRANSFERASE PHI 9 (GSTF9)*	Encodes glutathione transferase belonging to the phi class of GSTs. Naming convention according to Wagner et al. (2002).
AT2G30870	*GLUTATHIONE S-TRANSFERASE PHI 10 (GSTF10)*	early dehydration-induced gene ERD13 homologous to tobacco and maize glutathione S-transferases. Encodes glutathione transferase belonging to the phi class of GSTs. Naming convention according to Wagner et al. (2002)
AT2G30890		Cytochrome b561/ferric reductase transmembrane protein family
AT2G30900	*TRICHOME BIREFRINGENCE-LIKE 43 (TBL43)*	Encodes a member of the TBL (TRICHOME BIREFRINGENCE-LIKE) gene family containing a plant-specific DUF231 (domain of unknown function) domain. TBL gene family has 46 members, two of which (TBR/AT5G06700 and TBL3/AT5G01360) have been shown to be involved in the synthesis and deposition of secondary wall cellulose, presumably by influencing the esterification state of pectic polymers. A nomenclature for this gene family has been proposed (Volker Bischoff & Wolf Scheible, 2010, personal communication).
AT2G30925		transmembrane protein
AT2G30930		hypothetical protein
AT2G30940		Protein kinase superfamily protein

AT2G30950	*VARIEGATED 2 (VAR2)*	Metalloprotease that functions in thylakoid membrane biogenesis. Involved in the repair of PSII following damaged incurred during photoinhibition. Forms a complex with VAR1. Mutants show a variegated phenotype, which decreases during development. Transcript and protein levels increase with light intensity. In plsp1-1 mutant plastids, the nonmature form of the protein localizes in the membrane.
AT2G30970	*ASPARTATE AMINOTRANSFERASE 1 (ASP1)*	ASPARTATE AMINOTRANSFERASE 1
AT2G31000		
AT2G31030	*OSBP(OXYSTEROL BINDING PROTEIN)-RELATED PROTEIN 1B (ORP1B)*	OSBP(oxysterol binding protein)-related protein 1B
AT2G31035		oxysterol-binding-like protein
AT2G31040	*CONSERVED ONLY IN THE GREEN LINEAGE 160 (CGL160)*	Encodes an integral thylakoid protein that facilitates assembly of the membranous part of the chloroplast ATPase.
AT2G31060	*EMBRYO DEFECTIVE 2785 (EMB2785)*	elongation factor family protein
AT2G31070	*TCP DOMAIN PROTEIN 10 (tcp10)*	TCP family protein involved in heterchronic regulation of leaf differentiation.
AT2G31080		transposable_element_gene;non-LTR retrotransposon family (LINE), has a 1.3e-49 P-value blast match to GB:NP_038603 L1 repeat, Tf subfamily, member 23 (LINE-element) (Mus musculus);(source:TAIR10)
AT2G31085	*CLAVATA3/ESR-RELATED 6 (CLE6)*	Member of a large family of putative ligands homologous to the Clavata3 gene. Consists of a single exon. Can replace CLV3 function in vivo.
AT2G31090	*TAXIMIN 1 (TAX1)*	Encodes a signalling peptide influencing lateral organ separation.
AT2G31100		alpha/beta-Hydrolases superfamily protein
AT2G31110		Encodes a member of the TBL (TRICHOME BIREFRINGENCE-LIKE) gene family containing a plant-specific DUF231 (domain of unknown function) domain. TBL gene family has 46 members, two of which (TBR/AT5G06700 and TBL3/AT5G01360) have been shown to be involved in the synthesis and deposition of secondary wall cellulose, presumably by influencing the esterification state of pectic polymers. A nomenclature for this gene family has been proposed (Volker Bischoff & Wolf Scheible, 2010, personal communication).
AT2G31120		
AT2G31150		ATP binding / ATPase
AT2G31160	*LIGHT SENSITIVE HYPOCOTYLS 3 (LSH3)*	LIGHT-DEPENDENT SHORT HYPOCOTYLS-like protein (DUF640)
AT2G31180	*MYB DOMAIN PROTEIN 14 (MYB14)*	Member of the R2R3 factor gene family.
AT2G31210	*BASIC HELIX LOOP HELIX PROTEIN 91 (BHLH091)*	Encodes a bHLH transcription factor that together with bHLH089 and bHLH010 is important for the normal transcriptome of the developing Arabidopsis anther, possibly by forming a feed-forward loop with DYT1.
AT2G31230	*ETHYLENE-RESPONSIVE ELEMENT BINDING FACTOR 15 (ERF15)*	encodes a member of the ERF (ethylene response factor) subfamily B-3 of ERF/AP2 transcription factor family. The protein contains one AP2 domain. There are 18 members in this subfamily including ATERF-1, ATERF-2, AND ATERF-5.
AT2G31240		Tetratricopeptide repeat (TPR)-like superfamily protein
AT2G31250	*(HEMA3)*	Glutamyl-tRNA reductase family protein
AT2G31290		Ubiquitin carboxyl-terminal hydrolase family protein
AT2G31320	*POLY(ADP-RIBOSE) POLYMERASE 1 (PARP1)*	Encodes a poly(ADP-ribose) polymerase.
AT2G31360	*16:0DELTA9 DESATURASE 2 (ADS2)*	Encodes a protein homologous to delta 9 acyl-lipid desaturases of cyanobacteria and acyl-CoA desaturases of yeast and mammals. expression up-regulated by cold temperature. It is involved in the synthesis of the 24:1n-9 and 26:1n-9 components of seed lipids, sphingolipids and the membrane phospholipids phosphatidylserine (PS), and phosphatidylethanolamine (PE).
AT2G31370	*POSF21 (POSF21)*	Basic-leucine zipper (bZIP) transcription factor family protein
AT2G31380	*SALT TOLERANCE HOMOLOGUE (STH)*	a B-box zinc finger protein that interacts with COP1. contains a novel 11 amino acid motif at the C-terminus (also found at the N-terminus of HY5) that is involved in the COP1 interaction.
AT2G31390	*FRUCTOKINASE 2 (FRK2)*	Encodes a member of the fructokinase gene family. Nomenclature according to Riggs 2017 has been adopted for the family by the community (personal communication, Boernke, Callis, Granot, Boernke, and Smeekens).
AT2G31410		coiled-coil protein
AT2G31420		B3 domain protein (DUF313)
AT2G31440	*APH-1 (APH-1)*	Encodes a gamma-secretase subunit. Associates with other subunits in intracellular membrane compartments.
AT2G31460		B3 domain protein, putative (DUF313)
AT2G31470	*DROUGHT TOLERANCE REPRESSOR (DOR)*	Encodes a F-Box protein DOR (Drought tolerance Repressor) functionally as an inhibitory factor for abscisic acid-induced stomatal closure under drought stress.
AT2G31480		hypothetical protein
AT2G31490		neuronal acetylcholine receptor subunit alpha-5
AT2G31500	*CALCIUM-DEPENDENT PROTEIN KINASE 24 (CPK24)*	member of Calcium Dependent Protein Kinase
AT2G31550		SGNH hydrolase-type esterase superfamily protein
AT2G31570	*GLUTATHIONE PEROXIDASE 2 (GPX2)*	glutathione peroxidase GPx
AT2G31590		hypothetical protein
AT2G31640		
AT2G31660	*SUPER SENSITIVE TO ABA AND DROUGHT2 (SAD2)*	SAD2 (super sensitive to ABA and drought 2) encodes an importin beta-domain family protein likely to be involved in nuclear transport in ABA signaling. Subcellular localization of GFP-tagged SAD2 showed a predominantly nuclear localization, consistent with a role for SAD2 in nuclear transport. Mutation of SAD2 in Arabidopsis alters abscisic acid sensitivity. SAD2 was ubiquitously expressed at low levels in all tissues except flowers. SAD2 expression was not induced by ABA or stress. Loss of function mutations in SAD2 exhibit increased tolerance for UV stress, increased production of UV protective secondary metabolites and suppression of nuclear localization of MYB4 (a repressor of UV stress response genes). Regulates microRNA activity. Defective trichome activity.
AT2G31670	*UP3 (UP3)*	Stress responsive alpha-beta barrel domain protein
AT2G31680	*RAB GTPASE HOMOLOG A5D (RABA5d)*	RAB GTPase homolog A5D

AT2G31690	*DAD1-LIKE LIPASE 5 (DALL5)*	encodes a triacylglycerol lipase located in plastoglobuli and involved in the degradation of triacylglycerol. It also has impact on leaf senescence and maintaining the structural integrity of thylakoids.
AT2G31700		transmembrane protein
AT2G31725		FAM136A-like protein (DUF842)
AT2G31730		basic helix-loop-helix (bHLH) DNA-binding superfamily protein
AT2G31750	*UDP-GLUCOSYL TRANSFERASE 74D1 (UGT74D1)*	Encodes an auxin glycosyltransferase that is likely to be involved in regulation of auxin by glycosylation.
AT2G31760	*ARIADNE 10 (ARI10)*	RING/U-box superfamily protein
AT2G31770	*ARIADNE 9 (ARI9)*	RING/U-box superfamily protein
AT2G31780	*ARIADNE 11 (ARI11)*	RING/U-box superfamily protein
AT2G31790		UDP-Glycosyltransferase superfamily protein
AT2G31800		Integrin-linked protein kinase family
AT2G31820		Ankyrin repeat family protein
AT2G31850		hypothetical protein
AT2G31860		pseudogene of poly(ADP-ribose) glycohydrolase 2
AT2G31880	*SUPPRESSOR OF BIR1 1 (SOBIR1)*	Encodes a putative leucine rich repeat transmembrane protein that is expressed in response to Pseudomonas syringae. Expression of SRRLK may be required for silencing via lsiRNAs. Regulates cell death and innate immunity.
AT2G31900	*MYOSIN-LIKE PROTEIN XIF (XIF)*	Encodes an novel myosin isoform.
AT2G31910	*CATION/H+ EXCHANGER 21 (CHX21)*	member of Putative Na+/H+ antiporter family
AT2G31930		protein of unknown function
AT2G31940		oxidoreductase/transition metal ion-binding protein
AT2G31945		transmembrane protein
AT2G31950		
AT2G31955	*COFACTOR OF NITRATE REDUCTASE AND XANTHINE DEHYDROGENASE 2 (CNX2)*	COFACTOR OF NITRATE REDUCTASE AND XANTHINE DEHYDROGENASE 2. Encodes a protein involved in molybdenum cofactor biosynthesis. Homologous to E.coli moaA. Expression is abundant in all tissues examined, particularly in roots. Appears to have targeting signals for chloroplast or mitochondria.
AT2G31980	*PHYTOCYSTATIN 2 (CYS2)*	Cystatins are inhibitors of cysteine proteinases.
AT2G31990		Exostosin family protein
AT2G32010	*CVP2 LIKE 1 (CVL1)*	Encodes an inositol polyphosphate 5?-phosphatase (5PTase). Mediating phosphoinositide signaling. Involved in establishment of foliar vein patterns.
AT2G32020		Acyl-CoA N-acyltransferases (NAT) superfamily protein
AT2G32030		Acyl-CoA N-acyltransferases (NAT) superfamily protein
AT2G32050		cell cycle control-like protein (DUF572)
AT2G32070	*CCR4-ASSOCIATED FACTOR 1K (CAF1K)*	Deadenylase.
AT2G32090	*GLYOXALASE I-LIKE;9 (GLXI-LIKE;9)*	Lactoylglutathione lyase / glyoxalase I family protein
AT2G32100	*OVATE FAMILY PROTEIN 16 (OFP16)*	ovate family protein 16
AT2G32140		transmembrane receptor
AT2G32150	*XMP-SPECIFIC PHOSPHATASE (XMPP)*	Encodes a xanthosine monophosphate (XMP)phosphatase. Dephosphorylates XMP to xanthosine as an initial step in purine nucleotide catabolism.
AT2G32160		S-adenosyl-L-methionine-dependent methyltransferases superfamily protein
AT2G32190	*CYSTEINE-RICH TRANSMEMBRANE MODULE 4 (ATHCYSTM4)*	cysteine-rich/transmembrane domain A-like protein
AT2G32210	*CYSTEINE-RICH TRANSMEMBRANE MODULE 6 (ATHCYSTM6)*	cysteine-rich/transmembrane domain A-like protein
AT2G32220		Ribosomal L27e protein family
AT2G32240	*PAMP-INDUCED COILED COIL (PICC)*	PAMP induced protein involved in defense response. Interaction with UBAC2 proteins in the ER, is necessary for PAMP mediated accumulation of the callose synthase PMR4.
AT2G32250	*FAR1-RELATED SEQUENCE 2 (FRS2)*	FAR1-related sequence 2
AT2G32270	*ZINC TRANSPORTER 3 PRECURSOR (ZIP3)*	A member of Zrt- and Irt-related protein (ZIP) family. transcript is induced in response to zinc deficiency in the root. also response to iron deficiency.
AT2G32280	*VASCULATURE COMPLEXITY AND CONNECTIVITY (VCC)*	Encodes a member of a plant-specific gene family that is required for embryo provasculature development. The gene product regulates vascular network complexity and connectivity in cotyledons.
AT2G32290	*BETA-AMYLASE 6 (BAM6)*	beta-amylase 6
AT2G32300	*UCLACYANIN 1 (UCC1)*	Encodes a uclacyanin, a protein precursor that is closely related to precursors of stellacyanins and a blue copper protein from pea pods.
AT2G32330		
AT2G32340		TraB family protein
AT2G32360		Ubiquitin-like superfamily protein
AT2G32370	*HOMEODOMAIN GLABROUS 3 (HDG3)*	Encodes a homeobox-leucine zipper family protein belonging to the HD-ZIP IV family. Together with ATML1 and PDF2, it is involved in cotyledon development.
AT2G32380		Transmembrane protein 97, predicted
AT2G32420		
AT2G32430		Galactosyltransferase family protein
AT2G32440	*ENT-KAURENOIC ACID HYDROXYLASE 2 (KAO2)*	ent-kaurenoic acid hydroxylase (KAO2)
AT2G32460	*MYB DOMAIN PROTEIN 101 (MYB101)*	Member of the R2R3 factor gene family.
AT2G32470		F-box associated ubiquitination effector family protein

AT2G32490		pseudogene of 3'-5' exonuclease domain-containing protein
AT2G32500		Stress responsive alpha-beta barrel domain protein
AT2G32510	*MITOGEN-ACTIVATED PROTEIN KINASE KINASE KINASE 17 (MAPKKK17)*	Member of MEKK subfamily involved in wound and JA induced signaling.Interacts with At5g40440, and activates At1g59580.
AT2G32540	*CELLULOSE SYNTHASE-LIKE B4 (CSLB04)*	encodes a gene similar to cellulose synthase The mRNA is cell-to-cell mobile.
AT2G32560		F-box family protein
AT2G32570		
AT2G32600		hydroxyproline-rich glycoprotein family protein
AT2G32610	*CELLULOSE SYNTHASE-LIKE B1 (CSLB01)*	encodes a gene similar to cellulose synthase
AT2G32620	*CELLULOSE SYNTHASE-LIKE B (CSLB02)*	encodes a gene similar to cellulose synthase
AT2G32630		Pentatricopeptide repeat (PPR-like) superfamily protein
AT2G32640	*LYCOPENE BETA-CYLCASE (LCYB)*	Encodes a lycopene beta cyclase that catalyzes the addition of beta-ionone end groups to the end of lycopene molecules.
AT2G32650		RmlC-like cupins superfamily protein
AT2G32660	*RECEPTOR LIKE PROTEIN 22 (RLP22)*	receptor like protein 22
AT2G32670	*VESICLE-ASSOCIATED MEMBRANE PROTEIN 725 (VAMP725)*	member of Synaptobrevin -like protein family
AT2G32680	*RECEPTOR LIKE PROTEIN 23 (RLP23)*	NLP20 LRR receptor protein involved in PAMP mediated immunity.
AT2G32690	*GLYCINE-RICH PROTEIN 23 (GRP23)*	Glycine-rich protein similar in structure to GRP5. The expression of GRP23 is induced by HPA (cutin monomer, salicylic acid, and abscisic acid.
AT2G32710	*KIP-RELATED PROTEIN 4 (KRP4)*	Kip-related protein (KRP) gene, encodes CDK (cyclin-dependent kinase) inhibitor (CKI). A member of seven KRP genes found in Arabidopsis thaliana. Negative regulator of cell division. Expressed in actively dividing cells.
AT2G32720	*CYTOCHROME B5 ISOFORM B (CB5-B)*	Participates with ELO2 in VLCFA synthesis.
AT2G32740	*GALACTOSYLTRANSFERASE 13 (GT13)*	galactosyltransferase 13
AT2G32750		Exostosin family protein
AT2G32765	*SMALL UBIQUITINRELATED MODIFIER 5 (SUMO5)*	Encodes a small ubiquitin-like modifier (SUMO) protein that becomes covalently attached to various intracellular protein targets through an isopeptide bond. SUMOylation typically has a post-translational effect on the behavior of the target protein.
AT2G32770	*PURPLE ACID PHOSPHATASE 13 (PAP13)*	purple acid phosphatase 13
AT2G32780	*UBIQUITIN-SPECIFIC PROTEASE 1 (UBP1)*	ubiquitin-specific protease 1
AT2G32790		Ubiquitin-conjugating enzyme family protein
AT2G32800	*L-TYPE LECTIN RECEPTOR KINASE S.2 (LECRK-S.2)*	protein kinase family protein
AT2G32830	*PHOSPHATE TRANSPORTER 1;5 (PHT1;5)*	Encodes Pht1;5, a member of the Pht1 family of phosphate transporters which include: Pht1;1/At5g43350, Pht1;2/At5g43370, Pht1;3/At5g43360, Pht1;4/At2g38940, Pht1;5/At2g32830, Pht1;6/At5g43340, Pht1;7/At3g54700, Pht1;8/At1g20860, Pht1;9/At1g76430 (Plant Journal 2002, 31:341).
AT2G32850		Protein kinase superfamily protein
AT2G32860	*BETA GLUCOSIDASE 33 (BGLU33)*	beta glucosidase 33
AT2G32880		TRAF-like family protein
AT2G32890	*RALF-LIKE 17 (RALFL17)*	Member of a diversely expressed predicted peptide family showing sequence similarity to tobacco Rapid Alkalinization Factor (RALF), and is believed to play an essential role in the physiology of Arabidopsis. Consists of a single exon and is characterized by a conserved C-terminal motif and N-terminal signal peptide. This gene is contained within a highly AT-rich repetitive sequence region.
AT2G32920	*PDI-LIKE 2-3 (PDIL2-3)*	Encodes a protein disulfide isomerase-like (PDIL) protein, a member of a multigene family within the thioredoxin (TRX) superfamily. Transcript levels for this gene are up-regulated in response to three different chemical inducers of ER stress (dithiothreitol, beta-mercaptoethanol, and tunicamycin). AtIRE1-2 does not appear to be required for this response, but the atbzip60 mutant has a diminished response.
AT2G32930	*ZINC FINGER NUCLEASE 2 (ZFN2)*	Encodes a zinc finger protein.
AT2G32940	*ARGONAUTE 6 (AGO6)*	Encodes a nuclear localized 879-amino-acid protein that contains conserved PAZ and PIWI domains that is important for the accumulation of specific heterochromatin-related siRNAs, and for DNA methylation and transcriptional gene silencing.
AT2G32990	*GLYCOSYL HYDROLASE 9B8 (GH9B8)*	glycosyl hydrolase 9B8
AT2G33010		Ubiquitin-associated (UBA) protein
AT2G33020	*RECEPTOR LIKE PROTEIN 24 (RLP24)*	receptor like protein 24
AT2G33030	*RECEPTOR LIKE PROTEIN 25 (RLP25)*	receptor like protein 25
AT2G33070	*NITRILE SPECIFIER PROTEIN 2 (NSP2)*	Encodes a nitrile-specifier protein NSP2. NSP2 is one out of five (At3g16400/NSP1, At2g33070/NSP2, At3g16390/NSP3, At3g16410/NSP4 and At5g48180/NSP5) A. thaliana epithiospecifier protein (ESP) homologues that promote simple nitrile, but not epithionitrile or thiocyanate formation.
AT2G33080	*RECEPTOR LIKE PROTEIN 28 (RLP28)*	receptor like protein 28
AT2G33100	*CELLULOSE SYNTHASE-LIKE D1 (CSLD1)*	encodes a gene similar to cellulose synthase
AT2G33130	*RALF-LIKE 18 (RALFL18)*	Member of a diversely expressed predicted peptide family showing sequence similarity to tobacco Rapid Alkalinization Factor (RALF), and is believed to play an essential role in the physiology of Arabidopsis. Consists of a single exon and is characterized by a conserved C-terminal motif and N-terminal signal peptide.
AT2G33150	*PEROXISOMAL 3-KETOACYL-COA THIOLASE 3 (PKT3)*	Encodes an organellar (peroxisome, glyoxysome) 3-ketoacyl-CoA thiolase, involved in fatty acid b-oxidation during germination and subsequent seedling growth. Mutants have defects in glyoxysomal fatty acid beta-oxidation. EC2.3.1.16 thiolase.
AT2G33160	*NIMNA (SANSKRIT FOR '"SUNKEN" OR "LOW") (NMA)*	Gene structure annotation for AT2G33160.1 is inaccurate in TAIR10, see PMID:23709666 and Comments field on the locus page for updated annotation.
AT2G33170		Leucine-rich repeat receptor-like protein kinase family protein
AT2G33180		hypothetical protein
AT2G33210	*HEAT SHOCK PROTEIN 60-2 (HSP60-2)*	Involved in the RNA splicing of rpl2 and ccmFC introns in mitochondria.
AT2G33230	*YUCCA 7 (YUC7)*	Encodes a flavin monooxygenase gene which belongs to the tryptophan-dependent auxin biosynthetic pathway and enhances drought resistance.

AT2G33240	*MYOSIN XI D (XID)*	member of Myosin-like proteins
AT2G33255		Haloacid dehalogenase-like hydrolase (HAD) superfamily protein
AT2G33260		Tryptophan/tyrosine permease
AT2G33270	*ATYPICAL CYS HIS RICH THIOREDOXIN 3 (ACHT3)*	Encodes a member of the thioredoxin family protein. Located in the chloroplast.
AT2G33310	*AUXIN-INDUCED PROTEIN 13 (IAA13)*	Auxin induced gene, IAA13 (IAA13).
AT2G33320		Calcium-dependent lipid-binding (CaLB domain) family protein
AT2G33330	*PLASMODESMATA-LOCATED PROTEIN 3 (PDLP3)*	Encodes a plasmodesmal protein that affects the intercellular movement of molecules through the plasmodesmata. The protein has two DUF26 domains and a single transmembrane domain.
AT2G33350		CCT motif family protein
AT2G33380	*RESPONSIVE TO DESICCATION 20 (RD20)*	Encodes a calcium binding protein whose mRNA is induced upon treatment with NaCl, ABA and in response to desiccation. mRNA expression under drought conditions is apparent particularly in leaves and flowers. Isoform of caleosin with a role as a peroxygenase involved in oxylipin metabolism during biotic and abiotic stress. Involved in the production of 2-hydroxy-octadecatrienoic acid. The peroxygenase has a narrow substrate specificity thus acting as a fatty acid hydroperoxide reductase in vivo.
AT2G33410	*RNA-BINDING GLYCINE-RICH PROTEIN D2 (RBGD2)*	Belongs to a member of the RNA-binding glycine-rich (RBG) gene superfamily. The mRNA is cell-to-cell mobile.
AT2G33420	*CELLULOSE-RELATED DUF810 (CRD1)*	hypothetical protein (DUF810)
AT2G33440		
AT2G33450	*PLASTID RIBOSOMAL PROTEIN L28 (PRPL28)*	Ribosomal L28 family
AT2G33460	*ROP-INTERACTIVE CRIB MOTIF-CONTAINING PROTEIN 1 (RIC1)*	encodes a member of a novel protein family that contains contain a CRIB (for Cdc42/Rac-interactive binding) motif required for their specific interaction with GTP-bound Rop1 (plant-specific Rho GTPase). Interacts with Rop1 and is involved in pollen tube growth and function. Protein most similar to RIC3(family subgroup III). RIC1 is localized to the apical region of the plasma membrane in pollen tube and mutation analyses indicate that this localization is dependent on ROP1 binding. Gene is expressed in all tissues examined.Analysis of overexpression and loss of function mutants indicates a role in cortical microtubule organization during pavement cell morphogenesis.
AT2G33480	*NAC DOMAIN CONTAINING PROTEIN 41 (NAC041)*	NAC domain containing protein 41
AT2G33520	*CYSTEINE-RICH TRANSMEMBRANE MODULE 7 (ATHCYSTM7)*	cysteine-rich/transmembrane domain protein A
AT2G33530	*SERINE CARBOXYPEPTIDASE-LIKE 46 (scpl46)*	serine carboxypeptidase-like 46
AT2G33540	*C-TERMINAL DOMAIN PHOSPHATASE-LIKE 3 (CPL3)*	C-terminal domain phosphatase-like 3
AT2G33560	*BUB1-RELATED (BUB1: BUDDING UNINHIBITED BY BENZYMIDAZOL 1) (BUBR1)*	Encodes BUBR1. May have the spindle assembly checkpoint protein functions conserved from yeast to humans.
AT2G33570	*GALACTAN SYNTHASE 1 (GALS1)*	glycosyltransferase family protein (DUF23)
AT2G33580	*LYSM-CONTAINING RECEPTOR-LIKE KINASE 5 (LYK5)*	Encodes a putative LysM-containing receptor-like kinase. LYK5 is a major chitin receptor and forms a chitin-induced complex with related kinase CERK1. Based on protein sequence alignment analysis, it was determined as a pseudo kinase due to a lack of the ATP-binding P-loop in the kinase domain.
AT2G33590	*CCR(CINNAMOYL COA:NADP OXIDOREDUCTASE)-LIKE 1 (CRL1)*	Encodes a protein with homology to members of the dihydroflavonol-4-reductase (DFR) superfamily. The expression pattern of AtCRL1 indicates that CRL1 has a role in embryogenesis and seed germination. AtCRL1 is induced by ABA, drought and heat, and is highly expressed in seeds. The mRNA is cell-to-cell mobile.
AT2G33610	*SWITCH SUBUNIT 3 (SWI3B)*	Homologous to yeast SWI3 & RSC8, components of the SWI/SNF and RSC chromatin remodeling complexes. Interacts with BSH, AtSWI3A, SWI3C and FCA. Expressed ubiquitously.
AT2G33630		NAD(P)-binding Rossmann-fold superfamily protein
AT2G33640	*PROTEIN S-ACYL TRANSFERASE 21 (PAT21)*	DHHC-type zinc finger family protein that encodes a functional s-acyl transferase.
AT2G33670	*MILDEW RESISTANCE LOCUS O 5 (MLO5)*	A member of a large family of seven-transmembrane domain proteins specific to plants, homologs of the barley mildew resistance locus o (MLO) protein. The Arabidopsis genome contains 15 genes encoding MLO proteins, with localization in plasma membrane. Phylogenetic analysis revealed four clades of closely-related AtMLO genes. ATMLO5 belongs to the clade III, with AtMLO7, AtMLO8, AtMLO9, and AtMLO10. The gene is expressed during seedling growth, in cotyledon vascular system, and in stigma, anther and pollen grains; it was not expressed in rosette leaves, as shown by GUS activity patterns. The expression of several phylogenetically closely-related AtMLO genes showed similar or overlapping tissue specificity and analogous responsiveness to external stimuli, suggesting functional redundancy, co-function, or antagonistic function(s). Acts redundantly with MLO9 to tether Ca2+ channels to the pollen tube plasma membrane to effect pollen tube guidance.
AT2G33680	*(RID4)*	The pentatricopeptide repeat protein required for root development and high temperature tolerance.
AT2G33690		Late embryogenesis abundant protein, group 6
AT2G33710		encodes a member of the ERF (ethylene response factor) subfamily B-4 of ERF/AP2 transcription factor family. The protein contains one AP2 domain. There are 7 members in this subfamily.
AT2G33720		AP2/B3-like transcriptional factor family protein
AT2G33730	*SMALL1 (SMA1)*	Homolog of the DEADbox pre-mRNA splicing factor Prp28 which regulates abundance of miRNA. It plays essential roles in miRNA biogenesis and interacts with the DCL1 complex and positively influences pri-miRNA processing. SMA1 binds the promoter region of genes encoding pri-miRNAs (MIRs) and is required for MIR transcription. It enhances the abundance of the DCL1 protein levels through promoting the splicing of the DCL1 pre-mRNAs.
AT2G33770	*PHOSPHATE 2 (PHO2)*	Encodes a ubiquitin-conjugating E2 enzyme. UBC24 mRNA accumulation is suppressed by miR399f, miR399b and miR399c. Involved in phosphate starvation response and mediates degradation of PHO1 and PHT1s at endomembrane. Its expression is responsive to phosphate (Pi) and not phosphite (Phi) in roots and shoots. The mRNA is cell-to-cell mobile.
AT2G33790	*ARABINOGALACTAN PROTEIN 30 (AGP30)*	pollen Ole e 1 allergen protein containing 14.6% proline residues, similar to arabinogalactan protein (Daucus carota) GI:11322245, SP:Q03211 Pistil-specific extensin-like protein precursor (PELP) {Nicotiana tabacum}; contains Pfam profile PF01190: Pollen proteins Ole e I family

AT2G33800	*SCABRA 1 (SCA1)*	Encodes SCABRA1 (SCA1), a nuclear gene encoding a plastid-type ribosomal protein that functions  as a structural component of the 70S plastid ribosome. The sca1-rps5 allele exhibits defects in plastid 16SrRNA processing and a resulting decrease in accumulation of photosynthetic proteins. Loss-of-function mutations enhance the polarity defects of the as2 mutants.
AT2G33810	*SQUAMOSA PROMOTER BINDING PROTEIN-LIKE 3 (SPL3)*	Encodes a member of the SPL (squamosa-promoter binding protein-like)gene family, a novel gene family encoding DNA binding proteins and putative transcription factors. Contains the SBP-box, which encodes the SBP-domain, required and sufficient for interaction with DNA. It binds DNA, may directly regulate AP1, and is involved in regulation of flowering and vegetative phase change. Its temporal expression is regulated by the microRNA miR156. The target site for the microRNA is in the 3'UTR.
AT2G33830	*DORMANCY ASSOCIATED GENE 2 (DRM2)*	Negative regulator of local and systemic acquired resistance; target of FLD for activation of SAR.
AT2G33840		Tyrosyl-tRNA synthetase, class Ib, bacterial/mitochondrial
AT2G33845		Nucleic acid-binding, OB-fold-like protein
AT2G33850	*E6-LIKE 1 (E6L1)*	Stigmatic factor that plays a role during the early post-pollination stages.
AT2G33860	*ETTIN (ETT)*	ettin (ett) mutations have pleiotropic effects on Arabidopsis flower development, causing increases in perianth organ number, decreases in stamen number and anther formation, and apical-basal patterning defects in the gynoecium. The ETTIN gene encodes a protein with homology to DNA binding proteins which bind to auxin response elements. ETT transcript is expressed throughout stage 1 floral meristems and subsequently resolves to a complex pattern within petal, stamen and carpel primordia. ETT probably functions to impart regional identity in floral meristems that affects perianth organ number spacing, stamen formation, and regional differentiation in stamens and the gynoecium. During stage 5, ETT expression appears in a ring at the top of the floral meristem before morphological appearance of the gynoecium, consistent with the proposal that ETT is involved in prepatterning apical and basal boundaries in the gynoecium primordium. It is a target of the ta-siRNA tasiR-ARF. ETT is also a target of AP2; integrateing the functions of AGAMOUS and APETALA2 in floral meristem determinacy. Positive regulation of drought stress response genes.
AT2G33870	*RAB GTPASE HOMOLOG A1H (ArRABA1h)*	RAB GTPase homolog A1H
AT2G33880	*HOMEOBOX-3 (HB-3)*	Encodes a protein with similarity to WUS type homeodomain protein. Required for meristem growth and development and acts through positive regulation of WUS. Loss of function phenotypes include embryo lethality, hyponastic cotyledons, reduced root development and smaller meristems. Phenotypes can be rescued by addition of sucrose in the growth media. Overexpression can partially rescue the triple mutant cytokinin receptor phenotype suggesting HB-3 is a downstream effector of cytokinin signaling.
AT2G33980	*NUDIX HYDROLASE HOMOLOG 22 (NUDT22)*	nudix hydrolase homolog 22
AT2G34020		Calcium-binding EF-hand family protein
AT2G34060		Peroxidase superfamily protein
AT2G34080		Cysteine proteinases superfamily protein
AT2G34100		nonsense-mediated mRNA decay-like protein
AT2G34110		hypothetical protein
AT2G34120		Cytochrome C oxidase polypeptide VIB family protein
AT2G34130	*MATERNAL EFFECT EMBRYO ARREST 19 (MEE19)*	transposable_element_gene;CACTA-like transposase family (Ptta/En/Spm), has a 5.8e-123 P-value blast match to At5g36655.1/81-333 CACTA-like transposase family (Ptta/En/Spm) (CACTA-element) (Arabidopsis thaliana);(source:TAIR10)
AT2G34140	*CYCLING DOF FACTOR 4 (CDF4)*	CDF4 is member of the group II DOF transcription factor family is involved in regulation of differentiation root columella cells. It is a direct target of the transcriptional repressor WOX5. CDF4 itself is a transcriptional repressor that appears to repress root columella stem cell identity. Ectopic expression of CDF leads to premature differentiation of root columella cells.
AT2G34150	*WISKOTT-ALDRICH SYNDROME PROTEIN FAMILY VERPROLIN HOMOLOGOUS PROTEIN 1 (WAVE1)*	Encodes a member of the SCAR family.These proteins are part of a complex (WAVE) complex.The SCAR subunit activates the ARP2/3 complex which in turn act as a nucleator for actin filaments.
AT2G34160	*NVOLVED IN RRNA PROCESSING 7 (IRP7)*	Alba DNA/RNA-binding protein
AT2G34180	*CBL-INTERACTING PROTEIN KINASE 13 (CIPK13)*	Encodes CBL-interacting protein kinase 13 (CIPK13).
AT2G34190		Xanthine/uracil permease family protein
AT2G34210	*(SPT5-1)*	Transcription elongation factor Spt5
AT2G34230		ubiquitin carboxyl-terminal hydrolase-like protein, putative (DUF627 and DUF629)
AT2G34260	*HUMAN WDR55 (WD40 REPEAT) HOMOLOG (WDR55)*	Encodes a WDxR motif-containing protein that is required for gametogenesis, seed and endosperm development.
AT2G34270		hypothetical protein
AT2G34290		Protein kinase superfamily protein
AT2G34300		S-adenosyl-L-methionine-dependent methyltransferases superfamily protein
AT2G34315		avirulence induced family protein
AT2G34320		Polynucleotidyl transferase, ribonuclease H-like superfamily protein
AT2G34330		LOW protein: protein BOBBER-like protein
AT2G34340		senescence regulator (Protein of unknown function, DUF584)
AT2G34350		Nodulin-like / Major Facilitator Superfamily protein
AT2G34360		MATE efflux family protein
AT2G34370	*DYW DOMAIN PROTEIN 3 (DYW3)*	Pentatricopeptide repeat (PPR) superfamily protein
AT2G34390	*NOD26-LIKE INTRINSIC PROTEIN 2;1 (NIP2;1)*	aquaporin NIP2.1; encodes a member of the "Nodulin 26-like Intrinsic Protein" (NIP) subgroup of the aquaporin superfamily of membrane channel proteins. Expression is limited to the "anoxia core" region of the root stele under normal growth conditions, but shows substantial induction (up to 1,000-fold by 2-4?h of hypoxia) by low oxygen stress, and accumulation in all root tissues.
AT2G34400		Pentatricopeptide repeat (PPR-like) superfamily protein

AT2G34410	*REDUCED WALL ACETYLATION 3 (RWA3)*	Encodes a homolog of the protein Cas1p known to be involved in polysaccharide O-acetylation in Cryptococcus neoformans. Has high similarity to RWA2 whose mutant displays reduced acetylation. The protein is expressed in the Golgi and is involved in the acetylation of xylan during secondary wall biosynthesis.
AT2G34430	*LIGHT-HARVESTING CHLOROPHYLL-PROTEIN COMPLEX II SUBUNIT B1 (LHB1B1)*	Photosystem II type I chlorophyll a/b-binding protein The mRNA is cell-to-cell mobile.
AT2G34440	*AGAMOUS-LIKE 29 (AGL29)*	AGL29 MADS box gene.
AT2G34460		NAD(P)-binding Rossmann-fold superfamily protein
AT2G34490	*CYTOCHROME P450, FAMILY 710, SUBFAMILY A, POLYPEPTIDE 2 (CYP710A2)*	Encodes a protein with C22-sterol desaturase activity. The enzyme was shown to catalyze the conversion of both 24-<i>epi</i>-campesterol and β-sitosterol to brassicasterol and stigmasterol, respectively, in the presence of NADPH.
AT2G34500	*CYTOCHROME P450, FAMILY 710, SUBFAMILY A, POLYPEPTIDE 1 (CYP710A1)*	Encodes a protein with C22-sterol desaturase activity. The enzyme was shown to catalyze in the presence of NADPH the conversion of β-sitosterol to stigmasterol, but not that of 24-<i>epi</i>-campesterol to brassicasterol (unlike CYP710A2).
AT2G34510	*(ATHB-1)*	Protein of unknown function, DUF642. Found in cellulose enriched cell wall fractions.
AT2G34520	*MITOCHONDRIAL RIBOSOMAL PROTEIN S14 (RPS14)*	nuclear-encoded mitochondrial ribosomal protein S14
AT2G34530		transmembrane protein
AT2G34540		hypothetical protein
AT2G34550		
AT2G34555	*GIBBERELLIN 2-OXIDASE 3 (ATGA2OX3)*	Encodes a gibberellin 2-oxidase that acts on C19 gibberellins to deactivate them.
AT2G34560	*CONSERVED IN CILIATED SPECIES AND IN THE LAND PLANTS 1 (CCP1)*	P-loop containing nucleoside triphosphate hydrolases superfamily protein
AT2G34570	*MATERNAL EFFECT EMBRYO ARREST 21 (MEE21)*	PIN domain-like family protein
AT2G34580		cytomegalovirus UL139 protein
AT2G34585		transmembrane protein
AT2G34590		Transketolase family protein
AT2G34600	*JASMONATE-ZIM-DOMAIN PROTEIN 7 (JAZ7)*	Key regulator in alternative splicing in the jasmonate signaling pathway, alone and in collaboration with other regulators.
AT2G34610		cotton fiber protein
AT2G34620	*(MTERF10)*	Mitochondrial transcription termination factor family member.
AT2G34630	*GERANYL DIPHOSPHATE SYNTHASE 1 (GPS1)*	Encodes a geranyl diphosphate synthase. RNAi lines are dwarf. T-DNA knock-out lines are embryo lethal.
AT2G34640	*PLASTID TRANSCRIPTIONALLY ACTIVE 12 (PTAC12)*	Present in transcriptionally active plastid chromosomes. Involved in plastid gene expression.
AT2G34650	*PINOID (PID)*	Encodes a protein serine/threonine kinase that may act as a positive regulator of cellular auxin efflux, as a a binary switch for PIN polarity, and as a negative regulator of auxin signaling. Recessive mutants exhibit similar phenotypes as pin-formed mutants in flowers and inflorescence but distinct phenotypes in cotyledons and leaves. Expressed in the vascular tissue proximal to root and shoot meristems, shoot apex, and embryos. Expression is induced by auxin. Overexpression of the gene results in phenotypes in the root and shoot similar to those found in auxin-insensitive mutants. The protein physically interacts with TCH3 (TOUCH3) and PID-BINDING PROTEIN 1 (PBP1), a previously uncharacterized protein containing putative EF-hand calcium-binding motifs. Acts together with ENP (ENHANCER OF PINOID) to instruct precursor cells to elaborate cotyledons in the transition stage embryo. Interacts with PDK1. PID autophosphorylation is required for the ability of PID to phosphorylate an exogenous substrate. PID activation loop is required for PDK1-dependent PID phosphorylation and requires the PIF domain. Negative regulator of root hair growth. PID kinase activity is critical for the inhibition of root hair growth and for maintaining the proper subcellular localization of PID.
AT2G34660	*ATP-BINDING CASSETTE C2 (ABCC2)*	encodes a multidrug resistance-associated protein that is MgATP-energized glutathione S-conjugate pump. An ABCC-type arsenite-phytochelatin transporter. The expression of this gene is upregulated by herbicide safeners such as benoxacor and fenclorim. The mRNA is cell-to-cell mobile.
AT2G34680	*AUXIN-INDUCED IN ROOT CULTURES 9 (AIR9)*	isolated from differential screening of a cDNA library from auxin-treated root culture. sequence does not show homology to any known proteins and is predicted to be extracellular.
AT2G34690	*ACCELERATED CELL DEATH 11 (ACD11)*	Gene product transports the glycolipid precursor sphingosine between membranes in vitro. Mutant constitutively expresses defense-related genes that accompany the hypersensitive response normally triggered by avirulent pathogens. The mRNA is cell-to-cell mobile.
AT2G34700		Pollen Ole e 1 allergen and extensin family protein
AT2G34710	*PHABULOSA (PHB)*	Dominant PHB mutations cause transformation of abaxial leaf fates into adaxial leaf fates. Encodes a member of HD-Zip family which contains homeodomain-leucine zipper domains and domain similar to a mammalian sterol binding domain. Has overlapping functions with PHAVOLUTA, REVOLUTA and CORONA.
AT2G34720	*NUCLEAR FACTOR Y, SUBUNIT A4 (NF-YA4)*	nuclear factor Y, subunit A4
AT2G34740		protein phosphatase 2C family protein
AT2G34750		RNA polymerase I specific transcription initiation factor RRN3 protein
AT2G34770	*FATTY ACID HYDROXYLASE 1 (FAH1)*	encodes a fatty acid hydroxylase, required for the AtBI-1-mediated suppression of programmed cell death.
AT2G34790	*MATERNAL EFFECT EMBRYO ARREST 23 (MEE23)*	Encodes a BBE-like enzyme that acts in monolignol metabolism by catalyzing the oxidation of aromatic allylic alcohols, such as coumaryl-, sinapyl-, and coniferyl alcohol, to the corresponding aldehydes.
AT2G34800		
AT2G34810	*(ATBBE16)*	FAD-binding Berberine family protein
AT2G34820		basic helix-loop-helix (bHLH) DNA-binding superfamily protein
AT2G34830	*WRKY DNA-BINDING PROTEIN 35 (WRKY35)*	member of WRKY Transcription Factor; Group II-e
AT2G34850	*MATERNAL EFFECT EMBRYO ARREST 25 (MEE25)*	NAD(P)-binding Rossmann-fold superfamily protein
AT2G34860	*EMBRYO SAC DEVELOPMENT ARREST 3 (EDA3)*	DnaJ-like zinc finger domain-containing protein which regulates the assembly of photosystem I (PSI) and seed development.
AT2G34870	*MATERNAL EFFECT EMBRYO ARREST 26 (MEE26)*	hydroxyproline-rich glycoprotein family protein
AT2G34880	*MATERNAL EFFECT EMBRYO ARREST 27 (MEE27)*	JMJ15 is a novel H3K4 demethylase that regulates genes involved in flowering and response to stress. It is also a maternally expressed, imprinted gene.

AT2G34890	*CTP SYNTHASE 5 (CTPS5)*	Cytidine triphosphate synthase.
AT2G34900	*IMBIBITION-INDUCIBLE 1 (IMB1)*	Encodes a member of the BET subgroup of bromodomain proteins, a novel class of putative transcription factors. Its expression is induced during seed imbibition and downregulated during germination. Seeds of a loss-of-function mutant allele, imb1, show impaired cotyledon greening during germination in abscisic acid (ABA) and express higher levels of ABI5 protein than the wild type. Moreover, imb1 seeds are deficient in the phytochrome A (phyA)-mediated very-low-fluence response of germination.
AT2G34910		root hair specific protein
AT2G34920	*EMBRYO SAC DEVELOPMENT ARREST 18 (EDA18)*	RING/U-box superfamily protein
AT2G34930		disease resistance family protein / LRR family protein
AT2G34960	*CATIONIC AMINO ACID TRANSPORTER 5 (CAT5)*	Encodes a member of the cationic amino acid transporter (CAT) subfamily of amino acid polyamine choline transporters. Mediates efficient uptake of Lys, Arg and Glu in a yeast system. Localized to the plasma membrane.
AT2G34970		Trimeric LpxA-like enzyme
AT2G34990	*ARABIDOPSIS T??XICOS EN LEVADURA 38 (ATL38)*	RING/U-box superfamily protein
AT2G35000	*ARABIDOPSIS TOXICOS EN LEVADURA 9 (ATL9)*	ATL9 is an E3 ligase-like protein that is induced by chitin oligomers and contributes to fungal resistance.It differs from other members of the ATL family in that it has a PEST domain. It is a short lived protein that is subject to proteosome mediated degradation. It is expressed in many aerial tissues in a pattern that varies with developmental stage.
AT2G35020	*N-ACETYLGLUCOSAMINE-1-PHOSPHATE URIDYLYLTRANSFERASE 2 (GlcNAc1pUT2)*	Encodes a protein that functions as an N-acetylglucosamine-1-phosphate uridylyltransferase that catalyzes the formation of UDP-N-acetylglucosamine (UDP-GlcNAc). This is an essential precursor for glycolipid and glycoprotein synthesis and is also used for regulatory protein modification in signaling pathways. The enzyme can also catalyze the reverse reaction using both UDP-GlcNAc and the less common UDP-N-acetylgalactosamine as substrates. This enzyme can also act on glucose-1-phosphate to produce UDP-glucose. The mRNA is cell-to-cell mobile.
AT2G35030	*CYTOCHROME C OXIDASE DEFICIENT 1 (COD1)*	Pentatricopeptide repeat (PPR) superfamily protein
AT2G35040		AICARFT/IMPCHase bienzyme family protein
AT2G35070		transmembrane protein
AT2G35075		hypothetical protein
AT2G35150	*EXORDIUM LIKE 7 (EXL7)*	Encodes EXORDIUM LIKE 7.
AT2G35155		Trypsin family protein
AT2G35160	*SU(VAR)3-9 HOMOLOG 5 (SUVH5)*	Encodes SU(var)3-9 homologue 5 (SUVH5). SUVH5 has histone methyltransferase (MTase) activity in vitro and contributes to the maintenance of H3 mK9 (methylation of histone H3 at Lys-9) and CMT3-mediated non-CG methylation in vivo. This is a member of a subfamily of SET proteins that shares a conserved SRA domain.
AT2G35170		Histone H3 K4-specific methyltransferase SET7/9 family protein
AT2G35210	*ROOT AND POLLEN ARFGAP (RPA)*	A member of ARF GAP domain (AGD), A thaliana has 15 members, grouped into four classes.
AT2G35260	*BALANCE OF CHLOROPHYLL METABOLISM 1 (BCM1)*	CAAX protease self-immunity protein
AT2G35270	*AT-HOOK MOTIF NUCLEAR LOCALIZED PROTEIN 21 (AHL21)*	Direct target of AGAMOUS. Regulates patterning and differentiation of reproductive organs.
AT2G35290	*SMALL AUXIN UPREGULATED RNA 79 (SAUR79)*	hypothetical protein
AT2G35300	*LATE EMBRYOGENESIS ABUNDANT 18 (LEA18)*	Encodes LEA4-2/LEA18, a member of the Late Embryogenesis Abundant (LEA) proteins which typically accumulate in response to low water availability conditions imposed during development or by the environment.
AT2G35310	*REPRODUCTIVE MERISTEM 23 (REM23)*	Encodes a member of the REM (Reproductive Meristem) gene family, a part of the B3 DNA-binding domain superfamily.
AT2G35340	*MATERNAL EFFECT EMBRYO ARREST 29 (MEE29)*	helicase domain-containing protein
AT2G35370	*GLYCINE DECARBOXYLASE COMPLEX H (GDCH)*	Encodes glycine decarboxylase complex H protein. Involved in photorespiration. The mRNA is cell-to-cell mobile.
AT2G35380		Peroxidase superfamily protein
AT2G35400		
AT2G35410		RNA-binding (RRM/RBD/RNP motifs) family protein
AT2G35430		Zinc finger C-x8-C-x5-C-x3-H type family protein
AT2G35440		
AT2G35450		catalytic/ hydrolase
AT2G35460		Late embryogenesis abundant (LEA) hydroxyproline-rich glycoprotein family
AT2G35470		ribosome maturation factor
AT2G35480		envelope glycoprotein
AT2G35490	*FIBRILLIN2 (FBN2)*	Involved in photoprotection of photosystem II.
AT2G35500	*SHIKIMATE KINASE-LIKE 2 (SKL2)*	Encodes a protein with some sequence similarity to shikimate kinases, but a truncated form of this protein (lacking a putative N-terminal chloroplast transit peptide) does not have shikimate kinase activity in vitro.
AT2G35530	*BASIC REGION/LEUCINE ZIPPER TRANSCRIPTION FACTOR 16 (bZIP16)*	Encodes a G group bZIP transcription factor family member that can bind cis elements with an ACGT core, such as G-box, Hex, C-box and As-1. The protein is localized in the nucleus and can homodimerize and can heterodimerize with other G group members.
AT2G35550	*BASIC PENTACYSTEINE 7 (BPC7)*	basic pentacysteine 7
AT2G35560		
AT2G35570		pseudogene of Serine protease inhibitor (SERPIN) family protein
AT2G35580		Serine protease inhibitor (SERPIN) family protein
AT2G35585		cystic fibrosis transmembrane conductance regulator
AT2G35590		pseudogene of Serine protease inhibitor (SERPIN) family protein

AT2G35600	*BREVIS RADIX-LIKE 1 (BRXL1)*	Belongs to five-member BRX gene family. Arabidopsis BRX genes share high levels of similarity among each others, with several conserved domains. The most distinct is BRX domain - highly conserved in all BRX genes among distantly related species. This protein-protein interaction domain is required and sufficient for BRX activity.
AT2G35650	*CELLULOSE SYNTHASE LIKE (CSLA07)*	a member of Glycosyltransferase- Family 2 and encodes a beta-mannan synthase based on in vitro enzyme assays from heterologously expressed protein. Mutants exhibit defects in pollen tube growth and embryo development. The defective embryonic development was associated with reduced proliferation and failed cellularization of the endosperm.
AT2G35670	*FERTILIZATION INDEPENDENT SEED 2 (FIS2)*	Encodes a negative regulator of seed development in the absence of pollination. In the ovule, the FIS2 transcripts are accumulated at their highest level before fertilization and gradually decrease after fertilization.
AT2G35680	*PROTEIN TYROSINE PHOSPHATASE LOCALIZED TO MITOCHONDRION 1 (PTPMT1)*	Encodes a phosphatidylglycerophosphate (PGP) phosphatase involved in the synthesis of plastidial Phosphatidylglycerol (PG) in conjunction with PGPP1 and PTPMT2 in root. PTPMT1 levels were higher in node, cauline leaf, and flower than in root, leaf, and stem.
AT2G35690	*ACYL-COA OXIDASE 5 (ACX5)*	Encodes an acyl-CoA oxidase. Involved in jasmonate biosynthesis. Expressed uniformly in seedlings and throughout development.
AT2G35700	*ERF FAMILY PROTEIN 38 (ERF38)*	encodes a member of the DREB subfamily A-4 of ERF/AP2 transcription factor family. The protein contains one AP2 domain. There are 17 members in this subfamily including TINY. Thought to be involved in secondary cell wall metabolism.
AT2G35710	*PLANT GLYCOGENIN-LIKE STARCH INITIATION PROTEIN 7 (PGSIP7)*	Nucleotide-diphospho-sugar transferases superfamily protein
AT2G35715		
AT2G35720	*ORIENTATION UNDER VERY LOW FLUENCES OF LIGHT 1 (OWL1)*	Encodes OWL1, a J-domain protein involved in perception of very low light fluences.
AT2G35730	*HEAVY METAL ASSOCIATED PROTEIN 19 (ATHMP19)*	Heavy metal transport/detoxification superfamily protein
AT2G35733		hypothetical protein
AT2G35736		hypothetical protein
AT2G35740	*NOSITOL TRANSPORTER 3 (INT3)*	nositol transporter 3
AT2G35750		transmembrane protein
AT2G35760	*CASP-LIKE PROTEIN 2B2 (CASPL2B2)*	Uncharacterized protein family (UPF0497)
AT2G35770	*SERINE CARBOXYPEPTIDASE-LIKE 28 (scpl28)*	serine carboxypeptidase-like 28
AT2G35780	*SERINE CARBOXYPEPTIDASE-LIKE 26 (scpl26)*	serine carboxypeptidase-like 26
AT2G35790		transmembrane protein
AT2G35795	*(PAM18-1)*	Chaperone DnaJ-domain superfamily protein
AT2G35810		ureidoglycolate hydrolase
AT2G35820		ureidoglycolate hydrolase
AT2G35840		Sucrose-6F-phosphate phosphohydrolase family protein
AT2G35860	*FASCICLIN-LIKE ARABINOGALACTAN PROTEIN 16 PRECURSOR (FLA16)*	Fasciclin-like arabinogalactan protein. Possibly involved in embryogenesis and seed development.
AT2G35870		
AT2G35880	*WAVE-DAMPENED2-LIKE4 (WDL4)*	Microtubule-stabilizing protein. Expression is regulated by ethylene and it is a direct target of EIN3.During apical hook formation, it acts to modulate auxin maxima by regulating PIN traffiking.
AT2G35890	*CALCIUM-DEPENDENT PROTEIN KINASE 25 (CPK25)*	member of Calcium Dependent Protein Kinase
AT2G35900		Mal d 1-associated protein
AT2G35910	*ARABIDOPSIS T??XICOS EN LEVADURA 70 (ATL70)*	RING/U-box superfamily protein
AT2G35920		RNA helicase family protein
AT2G35930	*PLANT U-BOX 23 (PUB23)*	Encodes a cytoplasmically localized U-box domain containing E3 ubiquitin ligase that is involved in the response to water stress and acts as a negative regulator of PAMP-triggered immunity.
AT2G35940	*BEL1-LIKE HOMEODOMAIN 1 (BLH1)*	Encodes a member of the BEL-like homeodomain protein family. Ecotopic expression in the embryo sac leads to defects in nuclear migration and cellularization and embryo sacs with multiple egg cells. Loss of function alleles have no female gametophyte defects. The ecotopic expression phenotype requires KNAT3 because it can be suppressed by loss of KNAT3 function alleles. Localized to the nucleus but interaction with OFP1 relocates it to the cytoplasm.
AT2G35950	*EMBRYO SAC DEVELOPMENT ARREST 12 (EDA12)*	embryo sac development arrest 12
AT2G35960	*NDR1/HIN1-LIKE 12 (NHL12)*	Encodes a protein whose sequence is similar to tobacco hairpin-induced gene (HIN1) and Arabidopsis non-race specific disease resistance gene (NDR1). Expression is not altered in response to cucumber mosaic virus or spermine.
AT2G35970		Late embryogenesis abundant (LEA) hydroxyproline-rich glycoprotein family
AT2G35980	*YELLOW-LEAF-SPECIFIC GENE 9 (YLS9)*	Encodes a protein whose sequence is similar to tobacco hairpin-induced gene (HIN1) and Arabidopsis non-race specific disease resistance gene (NDR1). Expression of this gene is induced by cucumber mosaic virus, spermine and during senescence. The gene product is localized to the chloroplast. The mRNA is cell-to-cell mobile.
AT2G35990	*LONELY GUY 2 (LOG2)*	Putative lysine decarboxylase family protein
AT2G36000	*EMBRYO DEFECTIVE 3114 (EMB3114)*	Encodes an mTERF protein localized in the chloroplast stroma.
AT2G36010	*E2F TRANSCRIPTION FACTOR 3 (E2F3)*	Member of the E2F transcription factors, (cell cycle genes), key components of the cyclin D/retinoblastoma/E2F pathway.
AT2G36020	*HVA22-LIKE PROTEIN J (HVA22J)*	HVA22-like protein J
AT2G36030		hypothetical protein
AT2G36040		transposable_element_gene;similar to unknown protein [Arabidopsis thaliana] (TAIR:AT3G30610.1);(source:TAIR10)
AT2G36050	*OVATE FAMILY PROTEIN 15 (OFP15)*	ovate family protein 15
AT2G36070	*TRANSLOCASE INNER MEMBRANE SUBUNIT 44-2 (TIM44-2)*	One of two genes in Arabidopsis that encode a putative subunit of the mitochondrial inner membrane translocase complex. TIM44 subunit is thought to provide the energy for translocation via hydrolysis of ATP.
AT2G36080	*ABNORMAL SHOOT 2 (ABS2)*	Encodes a plant-specific B3 DNA-binding domain transcription factor. Has transcription repressor activity.

AT2G36090		F-box family protein
AT2G36100	*CASPARIAN STRIP MEMBRANE DOMAIN PROTEIN 1 (CASP1)*	Encodes a membrane bound protein involved in formation of the casparian strip. Along with CASP 2 it is required for the localization of ESB1.
AT2G36120	*DEFECTIVELY ORGANIZED TRIBUTARIES 1 (DOT1)*	Encodes a glycine rich protein that is involved in leaf vascular patterning. dot1 mutants have an aberrant open-class venation pattern in leaves and cotyledons, as well as several other leaf development defects. The mRNA is cell-to-cell mobile.
AT2G36145		hypothetical protein
AT2G36180		EF hand calcium-binding protein family
AT2G36190	*CELL WALL INVERTASE 4 (cwINV4)*	cwINV4 appears to function as a cell wall-localized invertase (that can catalyze the hydrolysis of sucrose into fructose and glucose) based on the phenotype of cwinv4 mutants. cwINV4 transcripts are expressed at high levels in lateral and median nectaries and this enzyme plays an important role in nectar production. Also expressed in ovary placenta and appears to play a role linking sugar sensing to ovule intitiation.
AT2G36200		P-loop containing nucleoside triphosphate hydrolases superfamily protein
AT2G36210	*SMALL AUXIN UPREGULATED RNA 45 (SAUR45)*	SAUR-like auxin-responsive protein family
AT2G36220		hypothetical protein
AT2G36270	*ABA INSENSITIVE 5 (ABI5)*	Encodes a member of the basic leucine zipper transcription factor family, involved in ABA signalling during seed maturation and germination. The Arabidopsis abscisic acid (ABA)-insensitive abi5 mutants have pleiotropic defects in ABA response, including decreased sensitivity to ABA inhibition of germination and altered expression of some ABA-regulated genes. Comparison of seed and ABA-inducible vegetative gene expression in wild-type and abi5-1 plants indicates that ABI5 regulates a subset of late embryogenesis-abundant genes during both developmental stages. Responsible for reducing cadmium uptake, mediated by interaction with MYB49 .
AT2G36290		alpha/beta-Hydrolases superfamily protein
AT2G36310	*URIDINE-RIBOHYDROLASE 1 (URH1)*	Encodes a cytoplasmic nucleoside hydrolase. It has the highest levels of activity with uridine followed by xanthosine. It shows little activity with inosine and none with cytidine. Mutant analyses indicate that it plays a role in purine and pyrimidine catabolism.
AT2G36320		A20/AN1-like zinc finger family protein
AT2G36330	*CASP-LIKE PROTEIN 4A3 (CASPL4A3)*	Uncharacterized protein family (UPF0497)
AT2G36360		Galactose oxidase/kelch repeat superfamily protein
AT2G36380	*ATP-BINDING CASSETTE G34 (ABCG34)*	pleiotropic drug resistance 6
AT2G36400	*GROWTH-REGULATING FACTOR 3 (GRF3)*	Growth regulating factor encoding transcription activator. One of the nine members of a GRF gene family, containing nuclear targeting domain. Mutants result in smaller leaves indicating the role of the gene in leaf development. Expressed in root, shoot and flower.
AT2G36410		transcriptional activator (DUF662)
AT2G36430		transmembrane protein, putative (DUF247)
AT2G36440		hypothetical protein
AT2G36450	*HARDY (HRD)*	encodes a member of the DREB subfamily A-4 of ERF/AP2 transcription factor family. The protein contains one AP2 domain. There are 17 members in this subfamily including TINY. Ectopic overexpression of HRD increases the density of the root network and improves water and salt stress tolerance in Arabidopsis. Overexpression of HRD in rice causes an increase in plant biomass and drought resistance.
AT2G36460	*FRUCTOSE-BISPHOSPHATE ALDOLASE 6 (FBA6)*	Aldolase superfamily protein
AT2G36470		DUF868 family protein, putative (DUF868)
AT2G36490	*DEMETER-LIKE 1 (DML1)*	A repressor of transcriptional gene silencing. Functions by demethylating the target promoter DNA. Interacts physically with RPA2/ROR1. In the ros1 mutants, an increase in methylation is observed in a number of gene promoters. Among the loci affected by ros1, a few (RD29A and At1g76930) are affected in cytosine methylation in all sequence contexts (CpG, CpNpG or CpNpN), although many others are affected primarily in non-CpG contexts. The ros1 mutant is more susceptible to biotrophic pathogens and is repressed in its responsiveness of salyclic acid-dependent defence genes.
AT2G36530	*LOW EXPRESSION OF OSMOTICALLY RESPONSIVE GENES 2 (LOS2)*	Involved in light-dependent cold tolerance and encodes an enolase. Protein is tyrosine-phosphorylated and its phosphorylation state is modulated in response to ABA in Arabidopsis thaliana seeds. Affects seed size and weight by adjusting cytokinin content and forming ENO2-bZIP75 complex. The los2 mutant has an autoimmune response through a perturbation of glycolysis from the reduction of the enolase activity results in activation of NLR-involved immune responses which further influences primary metabolism and plant growth.
AT2G36550		haloacid dehalogenase-like hydrolase family protein
AT2G36560	*(PEG5)*	A paternally expressed imprinted gene.
AT2G36570	*PXY/TDR-CORRELATED 1 (PXC1)*	Leucine-rich repeat protein kinase family protein
AT2G36580		Pyruvate kinase family protein
AT2G36590	*PROLINE TRANSPORTER 3 (ProT3)*	Encodes a proline transporter with affinity for gly betaine, proline, and GABA. Protein is expressed in leaves, flowers and siliques but to a much lesser extent in roots.
AT2G36610	*HOMEOBOX PROTEIN 22 (HB22)*	Encodes a homeodomain leucine zipper class I (HD-Zip I) protein.
AT2G36630		Sulfite exporter TauE/SafE family protein
AT2G36640	*EMBRYONIC CELL PROTEIN 63 (ECP63)*	Encodes putative phosphotyrosine protein belonging to late embryogenesis abundant (LEA) protein in group 3 that might be involved in maturation and desiccation tolerance of seeds. RFLP and CAPS mapping place it on chromosome 4 but the nucleotide sequence maps it to chromosome 2.
AT2G36650		CHUP1-like protein
AT2G36660	*POLY(A) BINDING PROTEIN 7 (PAB7)*	polyadenylate-binding protein, putative / PABP, putative. Member of the class III family of PABP proteins.
AT2G36690	*GERMINATION INSENSITIVE TO ABA MUTANT 2 (GIM2)*	Protein belonging to the Fe-dependent 2-oxoglutarate dioxygenase superfamily, catalyzes the stereospecific hydration of GA12 to produce DHGA12, negatively regulates ABA sensitivity during germination, phototrophic establishment and seedling development.
AT2G36700		Pectin lyase-like superfamily protein
AT2G36710		Pectin lyase-like superfamily protein
AT2G36720		Acyl-CoA N-acyltransferase with RING/FYVE/PHD-type zinc finger domain-containing protein
AT2G36730		Pentatricopeptide repeat (PPR) superfamily protein

AT2G36750	*UDP-GLUCOSYL TRANSFERASE 73C1 (UGT73C1)*	UDP-glucosyl transferase 73C1
AT2G36760	*UDP-GLUCOSYL TRANSFERASE 73C2 (UGT73C2)*	UDP-glucosyl transferase 73C2
AT2G36770		UDP-Glycosyltransferase superfamily protein
AT2G36790	*UDP-GLUCOSYL TRANSFERASE 73C6 (UGT73C6)*	The At2g36790 gene encodes a UDP-glucose:flavonol-3-O-glycoside-7-O-glucosyltransferase (UGT73C6)attaching a glucosyl residue to the 7-O-position of the flavonols kaempferol, quercetin and their 3-O-glycoside derivatives.
AT2G36810	*SHOOT GRAVITROPISM 6 (SGR6)*	Specifically involved in gravity perception and/or gravity signal transduction for the shoot gravitropic response. Effects gravitropism only in inflorescence stems but normal in both hypocotyls and roots.
AT2G36820		
AT2G36830	*GAMMA TONOPLAST INTRINSIC PROTEIN (GAMMA-TIP)*	Encodes a tonoplast intrinsic protein, which functions as water channel. It has also been shown to be able to facilitate the transport of urea and hydrogen peroxide. Highly expressed in vascular tissues of the root, stem, cauline leaves and flowers but not in the apical meristems. The mRNA is cell-to-cell mobile.
AT2G36840	*ACT DOMAIN REPEATS 10 (ACR10)*	Encodes a ACT domain-containing protein. The ACT domain, named after bacterial aspartate kinase, chorismate mutase and TyrA (prephenate dehydrogenase), is a regulatory domain that serves as an amino acid-binding site in feedback-regulated amino acid metabolic enzymes.
AT2G36870	*XYLOGLUCAN ENDOTRANSGLUCOSYLASE/HYDROLASE 32 (XTH32)*	Encodes a xyloglucan endotransglycosylase/hydrolase. Protein sequence and phylogenetic analysis indicates that this enzyme resides in Group III-A of the XTH family, with high similarity to Tropaeolum majus (nasturtium) xyloglucanase 1 (TmNXG1). By sequence similarity to XTH31 (At3g44990) and in vivo analysis, likely to exhibit predominant xyloglucan endo-hydrolase activity (EC 3.2.1.151) with only limited potential to act as a xyloglucan endo-transglycosylase (EC 2.4.1.207).
AT2G36885		translation initiation factor
AT2G36890	*REGULATOR OF AXILLARY MERISTEMS 2 (RAX2)*	Putative homolog of the Blind gene in tomato. Together with RAX1 and RAX3 belong to the class R2R3 MYB genes; encoded by the Myb-like transcription factor MYB38, regulates axillary meristem formation. The mRNA is cell-to-cell mobile.
AT2G36900	*MEMBRIN 11 (MEMB11)*	member of Membrin Gene Family
AT2G36910	*ATP-BINDING CASSETTE B1 (ABCB1)*	Belongs to the family of ATP-binding cassette (ABC) transporters. Also known as AtMDR1.Possibly regulates auxin-dependent responses by influencing basipetal auxin transport in the root. Exerts nonredundant, partially overlapping functions with the ABC transporter encoded by AT3G28860. PGP1 mediates cellular efflux of IAA and interacts with PIN genes that may confer an accelerated vectoral component to PGP-mediated transport. The non-polar localization of PGP1 at root and shoot apices, where IAA gradient-driven transport is impaired, may be required to confer directionality to auxin transport in those tissues. The mRNA is cell-to-cell mobile.
AT2G36920		B3 domain protein
AT2G36930		zinc finger (C2H2 type) family protein
AT2G36940		
AT2G36970		UDP-Glycosyltransferase superfamily protein
AT2G36990	*RNAPOLYMERASE SIGMA-SUBUNIT F (SIGF)*	Encodes a general sigma factor in chloroplasts and is probably responsible for the recognition of sigma 70 type standard bacteria-type multi-subunit RNA polymerase (PEP) promoters in young cotyledons. It is a substrate for regulatory phosphorylation by cpCK2, a nuclear-coded plastid-targeted casein kinase 2, that has been implicated as a key component in plant sigma factor phosphorylation and transcriptional regulation.
AT2G37000		TCP family transcription factor
AT2G37010	*NON-INTRINSIC ABC PROTEIN 12 (NAP12)*	member of NAP subfamily
AT2G37020		Translin family protein
AT2G37030	*SMALL AUXIN UPREGULATED RNA 46 (SAUR46)*	SAUR-like auxin-responsive protein family
AT2G37040	*PHE AMMONIA LYASE 1 (pal1)*	Encodes PAL1, a phenylalanine ammonia-lyase. Arabidopsis has four PALs: AT2G37040 (PAL1), AT3G53260 (PAL2), AT5G04230 (PAL3) and AT3G10340 (PAL4).
AT2G37050	*SALT INDUCED MALECTIN-LIKE DOMAIN-CONTAINING PROTEIN1 (SIMP1)*	Leucine-rich repeat protein kinase family protein
AT2G37060	*NUCLEAR FACTOR Y, SUBUNIT B8 (NF-YB8)*	nuclear factor Y, subunit B8
AT2G37070	*GROWING PLUS-END TRACKING 1 (GPT1)*	Encodes a microtubule-associated protein track growing microtubule plus ends.
AT2G37080	*ROP INTERACTIVE PARTNER 2 (RIP2)*	Encodes RIP2 (ROP interactive partner 2), a putative Rho protein effector, interacting specifically with the active form of ROPs (Rho proteins of plants).
AT2G37090	*IRREGULAR XYLEM 9 (IRX9)*	The IRX9 gene encodes a putative family 43 glycosyl transferase. It was coordinately expressed with the cellulose synthase subunits during secondary cell wall formation. Cell wall analysis revealed a decrease in the abundance of xylan in the irx9 mutant, indicating that IRX9 is required for xylan synthesis. Mutants have irregular xylem phenotype suggesting a role in secondary cell wall biosynthesis.IRX9 was identified as MUCI65 in a reverse genetic screen for MUCILAGE-RELATED genes. Despite producing only a few seeds, the irx9-1 mutant displays normal mucilage properties.
AT2G37110		PLAC8 family protein
AT2G37120		S1FA-like DNA-binding protein
AT2G37130		Peroxidase superfamily protein
AT2G37140		Terpenoid synthases superfamily protein
AT2G37150	*(CTL10)*	RING/U-box superfamily protein
AT2G37180	*RESPONSIVE TO DESICCATION 28 (RD28)*	a member of the plasma membrane intrinsic protein PIP2. functions as aquaporin and is involved in desiccation.
AT2G37210	*LONELY GUY 3 (LOG3)*	Encodes a protein of unknown function. It has been crystallized and shown to be structurally almost identical to the protein encoded by At5g11950.
AT2G37220		Encodes a chloroplast RNA binding protein. A substrate of the type III effector HopU1 (mono-ADP-ribosyltransferase). Protein is tyrosine-phosphorylated and its phosphorylation state is modulated in response to ABA in Arabidopsis thaliana seeds.
AT2G37250	*ADENOSINE KINASE (ADK)*	encodes adenylate kinase that is located in the chloroplast involved in the coordination of metabolism and growth

AT2G37260	*TRANSPARENT TESTA GLABRA 2 (TTG2)*	Encodes a protein similar to WRKY transcription factors that is expressed in the seed integument and endosperm. Mutants are defective in proanthocyanidin synthesis and seed mucilate deposition. Seeds are yellow colored. Seed size is also affected; seeds are reduced in size but only when the mutant allele is transmitted through the female parent.Loss of function alleles are associated with a reduction in interploidy lethality.
AT2G37280	*ATP-BINDING CASSETTE G33 (ABCG33)*	Encodes an ATP-binding cassette (ABC) transporter. Expressed in the vascular tissue of primary stem.
AT2G37290		Ypt/Rab-GAP domain of gyp1p superfamily protein
AT2G37300	*ATP-BINDING CASSETTE I16 (ABCI16)*	transmembrane protein
AT2G37310		Pentatricopeptide repeat (PPR) superfamily protein
AT2G37320		Tetratricopeptide repeat (TPR)-like superfamily protein
AT2G37330	*ALUMINUM SENSITIVE 3 (ALS3)*	Encodes an ABC transporter-like protein, without an ATPase domain, required for aluminum (Al) resistance/tolerance and may function to redistribute accumulated Al away from sensitive tissues in order to protect the growing root from the toxic effects of Al.
AT2G37340	*ARGININE/SERINE-RICH ZINC KNUCKLE-CONTAINING PROTEIN 33 (RS2Z33)*	encodes an RS-containing Zinc knuckle protein with molecular mass of 33kDa that is localized to nuclear specks. Barta et al (2010) have proposed a nomenclature for Serine/Arginine-Rich Protein Splicing Factors (SR proteins): Plant Cell. 2010, 22:2926.
AT2G37360	*ATP-BINDING CASSETTE G2 (ABCG2)*	Belongs to a clade of five Arabidopsis thaliana ABCG half-transporters that are required for synthesis of an effective suberin barrier in roots and seed coats (ABCG2, ABCG6, and ABCG20) and for synthesis of an intact pollen wall (ABCG1 and ABCG16).
AT2G37380	*MEMBRANE-ASSOCIATED KINASE REGULATOR 3 (MAKR3)*	Encodes a member of the MAKR (MEMBRANE-ASSOCIATED KINASE REGULATOR) gene family. MAKRs have putative kinase interacting motifs and membrane localization signals. Known members include: AT5G26230 (MAKR1), AT1G64080 (MAKR2), AT2G37380 (MAKR3), AT2G39370 (MAKR4), AT5G52870 (MAKR5) and AT5G52900 (MAKR6).
AT2G37390	*SODIUM POTASSIUM ROOT DEFECTIVE 2 (NAKR2)*	Chloroplast-targeted copper chaperone protein
AT2G37400		Tetratricopeptide repeat (TPR)-like superfamily protein
AT2G37410	*TRANSLOCASE INNER MEMBRANE SUBUNIT 17-2 (TIM17-2)*	Mitochondrial inner membrane translocase. Together with AtTIM17-1, TIM17-2 has a long C-terminal extension not present in other TIMs. The extension is located in the outer membrane and so TIM17-2 links the inner and outer mitochondrial membranes. The C-terminal region is essential for protein import into mitochondria via the general import pathway but is not necessary for import via the carrier pathway.
AT2G37420		ATP binding microtubule motor family protein
AT2G37430	*ZINC FINGER OF ARABIDOPSIS THALIANA 11 (ZAT11)*	Encodes a member of the zinc finger family of transcriptional regulators. It is expressed in many root tips, primary roots, cotyledons and hypocotyl. The protein is localized to the nucleus. Overexpression of ZAT11 causes increased root growth and increased sensitivity to nickel ions. The mRNA is cell-to-cell mobile.
AT2G37440		DNAse I-like superfamily protein
AT2G37450	*USUALLY MULTIPLE ACIDS MOVE IN AND OUT TRANSPORTERS 13 (UMAMIT13)*	nodulin MtN21-like transporter family protein
AT2G37460	*USUALLY MULTIPLE ACIDS MOVE IN AND OUT TRANSPORTERS 12 (UMAMIT12)*	nodulin MtN21-like transporter family protein
AT2G37470	*(HTB5)*	Histone superfamily protein
AT2G37480		hypothetical protein
AT2G37510		RNA-binding (RRM/RBD/RNP motifs) family protein
AT2G37520		Acyl-CoA N-acyltransferase with RING/FYVE/PHD-type zinc finger domain-containing protein
AT2G37540		NAD(P)-binding Rossmann-fold superfamily protein
AT2G37560	*ORIGIN RECOGNITION COMPLEX SECOND LARGEST SUBUNIT 2 (ORC2)*	Origin Recognition Complex subunit 2. Involved in the initiation of DNA replication. Regulated transcriptionally during cell cycle, peaking at G1/S-phase. Target of E2F/DF family of transcription factors. Interacts strongly with all ORC subunits.
AT2G37580	*ARABIDOPSIS T??XICOS EN LEVADURA 33 (ATL33)*	RING/U-box superfamily protein
AT2G37610	*(SMR12)*	hypothetical protein
AT2G37630	*ASYMMETRIC LEAVES 1 (AS1)*	Encodes a MYB-domain protein involved in specification of the leaf proximodistal axis. Mutation results in lobed and dissected leaves with a characteristic asymmetry. Homologous to the Antirrhinum PHANTASTICA (PHAN) and maize ROUGH SHEATH2 (RS2) genes Asymmetric placement of auxin response at the distal leaf tip precedes visible asymmetric leaf growth. Acts alongside AXR1 to exclude BP expression in leaves and with PIN1 to repress BP and promote lateral organ growth. Interacts physically with AS2 to form a complex that binds to the BP promoter and silences BP. Also functions as a regulator of the plant immune response.
AT2G37640	*(EXP3)*	member of Alpha-Expansin Gene Family. Naming convention from the Expansin Working Group (Kende et al, 2004. Plant Mol Bio). Involved in the formation of nematode-induced syncytia in roots of Arabidopsis thaliana.
AT2G37650		GRAS family transcription factor
AT2G37660		NAD(P)-binding Rossmann-fold superfamily protein
AT2G37670		Transducin/WD40 repeat-like superfamily protein
AT2G37680		glucose-induced degradation-like protein
AT2G37690		phosphoribosylaminoimidazole carboxylase, putative / AIR carboxylase
AT2G37700		Fatty acid hydroxylase superfamily
AT2G37710	*L-TYPE LECTIN RECEPTOR KINASE IV.1 (LECRK-IV.1)*	Induced in response to Salicylic acid. The mRNA is cell-to-cell mobile.
AT2G37720	*TRICHOME BIREFRINGENCE-LIKE 15 (TBL15)*	Encodes a member of the TBL (TRICHOME BIREFRINGENCE-LIKE) gene family containing a plant-specific DUF231 (domain of unknown function) domain. TBL gene family has 46 members, two of which (TBR/AT5G06700 and TBL3/AT5G01360) have been shown to be involved in the synthesis and deposition of secondary wall cellulose, presumably by influencing the esterification state of pectic polymers. A nomenclature for this gene family has been proposed (Volker Bischoff & Wolf Scheible, 2010, personal communication).
AT2G37730		glycosyltransferase (DUF604)
AT2G37740	*ZINC-FINGER PROTEIN 10 (ZFP10)*	zinc-finger protein 10
AT2G37750		hypothetical protein

AT2G37770	*CHLOROPLASTIC ALDO-KETO REDUCTASE (ChlAKR)*	Encodes an NADPH-dependent aldo-keto reductase that can act on a wide variety of substrates in vitro including saturated and unsaturated aldehydes, steroids, and sugars. GFP-tagged AKR4C9 localizes to the chloroplast where it may play a role in detoxifying reactive carbonyl compounds that threaten to impair the photosynthetic process. Transcript levels for this gene are up-regulated in response to cold, salt, and drought stress.
AT2G37780		Cysteine/Histidine-rich C1 domain family protein
AT2G37790	*ALDO-KETO REDUCTASE FAMILY 4 MEMBER C10 (AKR4C10)*	NAD(P)-linked oxidoreductase superfamily protein
AT2G37800		cysteine/histidine-rich C1 domain protein
AT2G37810		Cysteine/Histidine-rich C1 domain family protein
AT2G37820		Cysteine/Histidine-rich C1 domain family protein
AT2G37830		Probably not a pseudogene based on evidence for transcription (RNA-seq) and translation (Ribo-seq) described in PMID:27791167.
AT2G37850		
AT2G37860	*LOWER CELL DENSITY 1 (LCD1)*	Encodes a protein involved in differential development of bundle sheath and mesophyll cell chloroplasts.
AT2G37870		Bifunctional inhibitor/lipid-transfer protein/seed storage 2S albumin superfamily protein
AT2G37880		MIZU-KUSSEI-like protein (Protein of unknown function, DUF617)
AT2G37890		Mitochondrial substrate carrier family protein
AT2G37900		Major facilitator superfamily protein
AT2G37910		cation/hydrogen exchanger, putative (CHX21)
AT2G37920	*EMBRYO DEFECTIVE 1513 (emb1513)*	copper ion transmembrane transporter
AT2G37940	*ARABIDOPSIS INOSITOL PHOSPHORYLCERAMIDE SYNTHASE 2 (AtIPCS2)*	Inositol phosphorylceramide synthase 2
AT2G37950		RING/FYVE/PHD zinc finger superfamily protein
AT2G37970	*(SOUL-1)*	Encodes a cytosolic heme binding protein(cHBP)that can reversibly bind tetrapyrroles including heme, protoporphyrin IX and Mg-protoporphyrin IX dimethyl ester with distinct binding afﬁnities.
AT2G37975		Yos1-like protein
AT2G37990	*ARABIDOPSIS HOMOLOG OF YEAST RRS1 (ARRS1)*	ribosome biogenesis regulatory protein (RRS1) family protein
AT2G38010	*NEUTRAL CERAMIDASE 2 (ATNCER2)*	Neutral/alkaline non-lysosomal ceramidase
AT2G38040	*ACETYL CO-ENZYME A CARBOXYLASE CARBOXYLTRANSFERASE ALPHA SUBUNIT (CAC3)*	encodes the carboxyltransferase alpha subunit of acetyl-CoA carboxylase, involved in de novo fatty acid biosynthesis
AT2G38080	*IRREGULAR XYLEM 12 (IRX12)*	LAC4 appears to have laccase activity based on enzyme assays performed using lac4 mutants. These mutants also have reduced levels of lignin. LAC4 is expressed in vascular bundles and fibers and likely contributes to lignin biosynthesis, and hence cell wall biosynthesis, there. lac4/irx12 mutants have a mild irregular xylem phenotype.
AT2G38090		Duplicated homeodomain-like superfamily protein
AT2G38100	*(NPF5.5)*	Encodes a nitrate transporter that is involved in nitrogen accumulation in embryos.
AT2G38110	*GLYCEROL-3-PHOSPHATE SN-2-ACYLTRANSFERASE 6 (GPAT6)*	bifunctional sn-glycerol-3-phosphate 2-O-acyltransferase/phosphatase. Involved in cutin assembly.
AT2G38120	*AUXIN RESISTANT 1 (AUX1)*	Encodes an auxin influx transporter. AUX1 resides at the apical plasma membrane of protophloem cells and at highly dynamic subpopulations of Golgi apparatus and endosomes in all cell types. AUX1 action in the lateral root cap and/or epidermal cells influences lateral root initiation and positioning. Shoot supplied ammonium targets AUX1 and inhibits lateral root emergence. The mRNA is cell-to-cell mobile.
AT2G38140	*PLASTID-SPECIFIC RIBOSOMAL PROTEIN 4 (PSRP4)*	plastid-specific ribosomal protein 4 (PSRP4) mRNA, complete The mRNA is cell-to-cell mobile.
AT2G38150		alpha 1,4-glycosyltransferase family protein
AT2G38160	*BASIC PROLINE-RICH PROTEIN4 (BPP4)*	hypothetical protein
AT2G38170	*CATION EXCHANGER 1 (CAX1)*	Encodes a high affinity vacuolar calcium antiporter. The residue His 338 is critical to Ca^{2+} transport activity. Disruption of CAX1 reduces manganese and zinc of shoot tissue and results in a decrease in the activity of vacuolar V-type proton ATPase.
AT2G38180		SGNH hydrolase-type esterase superfamily protein
AT2G38210	*PUTATIVE PDX1-LIKE PROTEIN 4 (PDX1L4)*	putative PDX1-like protein 4
AT2G38220	*ABERRANT POLLEN DEVELOPMENT 3 (APD3)*	RING/U-box superfamily protein
AT2G38240	*JASMONATE-INDUCED OXYGENASE4 (JOX4)*	One of 4 paralogs encoding a 2-oxoglutarate/Fe(II)-dependent oxygenases that hydroxylates JA to 12-OH-JA.
AT2G38250		Homeodomain-like superfamily protein
AT2G38270	*CAX-INTERACTING PROTEIN 2 (CXIP2)*	Encodes protein homologous to CXIP1. CXIP1 is a PICOT domain containing protein interacts with CAX1, a high capacity calcium transporter. However, CXP2 does not interact with CAX1 and only moderately activates another calcium transporter CAX4.
AT2G38280	*EMBRYONIC FACTOR1 (FAC1)*	Encodes a protein with in vitro AMP deaminase activity that is involved in embryogenesis. Homozygous mutant embryos fail to develop past the zygote stage.
AT2G38290	*AMMONIUM TRANSPORTER 2 (AMT2)*	encodes a high-affinity ammonium transporter, which is expressed in shoot and root. Expression in root and shoot is under nitrogen and carbon dioxide regulation, respectively.
AT2G38300		myb-like HTH transcriptional regulator family protein
AT2G38310	*PYR1-LIKE 4 (PYL4)*	Encodes a member of the PYR (pyrabactin resistance)/PYL(PYR1-like)/RCAR (regulatory components of ABA receptor) family proteins with 14 members. PYR/PYL/RCAR family proteins function as abscisic acid sensors. Mediate ABA-dependent regulation of protein phosphatase 2Cs ABI1 and ABI2. The mRNA is cell-to-cell mobile.
AT2G38320	*TRICHOME BIREFRINGENCE-LIKE 34 (TBL34)*	Encodes a member of the TBL (TRICHOME BIREFRINGENCE-LIKE) gene family containing a plant-specific DUF231 (domain of unknown function) domain. TBL gene family has 46 members, two of which (TBR/AT5G06700 and TBL3/AT5G01360) have been shown to be involved in the synthesis and deposition of secondary wall cellulose, presumably by influencing the esterification state of pectic polymers. A nomenclature for this gene family has been proposed (Volker Bischoff & Wolf Scheible, 2010, personal communication).TBL34 are required only for xylan 3-O-monoacetylation and 2,3-di-O-acetylation. This biochemical phenotype can be observed in tbl34 esk1, double mutant and tbl34 tbl35 esk1 triple mutants.

AT2G38330		MATE efflux family protein
AT2G38340	*DEHYDRATION RESPONSE ELEMENT-BINDING PROTEIN 19 (DREB19)*	encodes a member of the DREB subfamily A-2 of ERF/AP2 transcription factor family. The protein contains one AP2 domain. There are eight members in this subfamily including DREB2A AND DREB2B that are involved in response to drought.
AT2G38350		hypothetical protein
AT2G38360	*PRENYLATED RAB ACCEPTOR 1.B4 (PRA1.B4)*	prenylated RAB acceptor 1.B4
AT2G38370		weak chloroplast movement under blue light protein (DUF827)
AT2G38390		Peroxidase superfamily protein
AT2G38400	*ALANINE:GLYOXYLATE AMINOTRANSFERASE 3 (AGT3)*	alanine:glyoxylate aminotransferase 2 homolog (AGT3) mRNA,
AT2G38450		Sel1 repeat protein
AT2G38460	*IRON REGULATED 1 (IREG1)*	Encodes IRON REGULATED1 (IREG1/FPN1), one of the Arabidopsis orthologs (AT2G38460/IREG1/FPN1 and AT5G03570/IREG2/FPN2) the iron efflux transporter ferroportin (FPN) identified in animals.
AT2G38465		hypothetical protein
AT2G38470	*WRKY DNA-BINDING PROTEIN 33 (WRKY33)*	Member of the plant WRKY transcription factor family. Regulates the antagonistic relationship between defense pathways mediating responses to P. syringae and necrotrophic fungal pathogens. Located in nucleus. Involved in response to various abiotic stresses - especially salt stress. Regulates cytochrome P450 gene CYP94B1 to control apoplastic barrier formation in roots to confer salt tolerance.
AT2G38480	*CASP-LIKE PROTEIN 4B1 (CASPL4B1)*	Uncharacterized protein family (UPF0497)
AT2G38490	*CBL-INTERACTING PROTEIN KINASE 22 (CIPK22)*	member of AtCIPKs
AT2G38500		2-oxoglutarate (2OG) and Fe(II)-dependent oxygenase superfamily protein
AT2G38510		MATE efflux family protein
AT2G38520		transposable_element_gene;copia-like retrotransposon family, has a 8.4e-60 P-value blast match to dbj|BAA78427.1| polyprotein (AtRE2-2) (Arabidopsis thaliana) (Ty1_Copia-element);(source:TAIR10)
AT2G38530	*LIPID TRANSFER PROTEIN 2 (LTP2)*	Involved in lipid transfer between membranes and plays a role in maintaining the integrity of the cuticle-cell wall interface. Belongs to a family of Lipid transfer proteins. Sequence similarity to other plant/Arabidopsis LPT genes but highest similarity to LPT1. Stress and pathogen-inducible motifs found in the upstream region. Expressed in flower, leaves and siliques but absent in roots. Predicted to be a member of PR-14 pathogenesis-related protein family with the following members: At2g38540/LTP1, At2g38530/LTP2, At5g59320/LTP3, At5g59310/LTP4, At3g51600/LTP5, At3g08770/LTP6, At2g15050/LTP7, At2g18370/LTP8, At2g15325/LTP9, At5g01870/LTP10, At4g33355/LTP11, At3g51590/LTP12, At5g44265/LTP13, At5g62065/LTP14, At4g08530/LTP15.
AT2G38540	*LIPID TRANSFER PROTEIN 1 (LP1)*	Non-specific lipid transfer protein. Binds calmodulin in a Ca2+-independent manner. Localized to the cell wall. Specifically expressed in L1 epidermal layer. Predicted to be a member of PR-14 pathogenesis-related protein family with the following members: At2g38540/LTP1, At2g38530/LTP2, At5g59320/LTP3, At5g59310/LTP4, At3g51600/LTP5, At3g08770/LTP6, At2g15050/LTP7, At2g18370/LTP8, At2g15325/LTP9, At5g01870/LTP10, At4g33355/LTP11, At3g51590/LTP12, At5g44265/LTP13, At5g62065/LTP14, At4g08530/LTP15. The mRNA is cell-to-cell mobile.
AT2G38560	*TRANSCRIPT ELONGATION FACTOR IIS (TFIIS)*	Encodes RNA polymerase II transcript elongation factor TFIIS. Complements yeast TFIIS mutation. Mutant plants display essentially normal development, but they flower slightly earlier than the wild type and show clearly reduced seed dormancy.
AT2G38580		Mitochondrial ATP synthase D chain-related protein
AT2G38590		F-box and associated interaction domains-containing protein
AT2G38640		LURP-one-like protein (DUF567)
AT2G38700	*MEVALONATE DIPHOSPHATE DECARBOXYLASE 1 (MVD1)*	Encodes mevalonate diphosphate decarboxylase, the enzyme that catalyzes the synthesis of isopentenyl diphosphate, used in sterol and isoprenoid biosynthesis. The protein appears to form a homodimeric complex. Incidentally, it was shown that the Arabidopsis MVD protein could also interact with its yeast homolog to form a heterodimer.
AT2G38740	*BROAD-RANGE SUGAR PHOSPHATE PHOSPHATASE (SGPP)*	HAD-type phosphosugar phosphatase.
AT2G38750	*ANNEXIN 4 (ANNAT4)*	Annexins are a family of calcium dependent membrane binding proteins though to be involved in Golgi mediated secretion. This is one of four annexins identified in Arabidopsis.
AT2G38760	*ANNEXIN 3 (ANNAT3)*	Annexins are calcium binding proteins that are localized in the cytoplasm. When cytosolic Ca2+ increases, they relocate to the plasma membrane. The mRNA is cell-to-cell mobile.
AT2G38770	*EMBRYO DEFECTIVE 2765 (EMB2765)*	P-loop containing nucleoside triphosphate hydrolases superfamily protein
AT2G38780		cytochrome C oxidase subunit
AT2G38790		hypothetical protein
AT2G38800		Plant calmodulin-binding protein-like protein
AT2G38820		DNA-directed RNA polymerase subunit beta-beta protein, putative (DUF506)
AT2G38830		Ubiquitin-conjugating enzyme/RWD-like protein
AT2G38840	*GUANYLATE-BINDING PROTEIN-LIKE 2 (GBPL2)*	Guanylate-binding family protein
AT2G38860	*(YLS5)*	Encodes protease I (pfpI)-like protein YLS5.
AT2G38870		Predicted to encode a PR (pathogenesis-related) peptide that belongs to the PR-6 proteinase inhibitor family. Six putative PR-6-type protein encoding genes are found in Arabidopsis: At2g38900, At2g38870, At5g43570, At5g43580, At3g50020 and At3g46860.
AT2G38890		hypothetical protein
AT2G38900		Predicted to encode a PR (pathogenesis-related) peptide that belongs to the PR-6 proteinase inhibitor family. Six putative PR-6-type protein encoding genes are found in Arabidopsis: At2g38900, At2g38870, At5g43570, At5g43580, At3g50020 and At3g46860.
AT2G38905		Low temperature and salt responsive protein family
AT2G38910	*CALCIUM-DEPENDENT PROTEIN KINASE 20 (CPK20)*	member of Calcium Dependent Protein Kinase

AT2G38920		SPX (SYG1/Pho81/XPR1) domain-containing protein / zinc finger (C3HC4-type RING finger) protein-like protein
AT2G38930		
AT2G38940	*PHOSPHATE TRANSPORTER 1;4 (PHT1;4)*	Encodes Pht1;4, a member of the Pht1 family of phosphate transporters which include: Pht1;1/At5g43350, Pht1;2/At5g43370, Pht1;3/At5g43360, Pht1;4/At2g38940, Pht1;5/At2g32830, Pht1;6/At5g43340, Pht1;7/At3g54700, Pht1;8/At1g20860, Pht1;9/At1g76430 (Plant Journal 2002, 31:341). Expression is upregulated in the shoot of cax1/cax3 mutant and is responsive to phosphate (Pi) and not phosphite (Phi) in roots and shoots. The mRNA is cell-to-cell mobile.
AT2G38970		Zinc finger (C3HC4-type RING finger) family protein
AT2G39000	*(ATNAA70)*	Encodes a chloroplast localized n-acetyltransfefase involved in N-terminal protein amino acid acetylation.
AT2G39010	*PLASMA MEMBRANE INTRINSIC PROTEIN 2E (PIP2E)*	plasma membrane intrinsic protein 2E
AT2G39020	*(NATA2)*	Although this locus shares considerable sequence similarity with the adjacent NATA1 gene (At2g39030), they appear to encode genes with different functions. NATA1 is involved in the production of N-delta-acetylornithine, but, overexpression of At2g39020 in tobacco does not lead to the formation of this defense compound. The mRNA is cell-to-cell mobile.
AT2G39030	*N-ACETYLTRANSFERASE ACTIVITY 1 (NATA1)*	Encodes a protein that acts as an ornithine N-delta-acetyltransferase, leading to the formation of N-delta-actetylornithine. This compound is likely used in plant defense and levels of it are increased in Arabidopsis plants in response to MeJA and ABA. The mRNA is cell-to-cell mobile.
AT2G39040		Peroxidase superfamily protein
AT2G39050	*EUONYMUS LECTIN S3 (EULS3)*	Encodes a nucleocytoplasmic lectin that is capable of binding carbohydrates. It is involved in ABA mediated stomatal movement and increased expression is correlated with increased resistance to Pseudomonas syringae.
AT2G39060	*(SWEET9)*	Encodes a sucrose transporter that is expressed in nectaries and is involved in nectar secretion.
AT2G39080	*EMBRYO DEFECTIVE 2799 (EMB2799)*	NAD(P)-binding Rossmann-fold superfamily protein
AT2G39100		RING/U-box superfamily protein
AT2G39110	*PBS1-LIKE 38 (PBL38)*	Protein kinase superfamily protein
AT2G39130		Transmembrane amino acid transporter family protein
AT2G39140	*SUPPRESSOR OF VARIEGATION 1 (SVR1)*	Suppressor of var2 variegation phenotype. Chloroplast localized. Loss of function mutant has defects in chloroplast protein translation and rRNA processing. Similar in sequence to pseudouridine synthase proteins.
AT2G39160		hypothetical protein
AT2G39170		MEF2BNB-like protein
AT2G39180	*CRINKLY4 RELATED 2 (CCR2)*	CRINKLY4 related 2
AT2G39190	*(ATATH8)*	member of ATH subfamily
AT2G39200	*MILDEW RESISTANCE LOCUS O 12 (MLO12)*	A member of a large family of seven-transmembrane domain proteins specific to plants, homologs of the barley mildew resistance locus o (MLO) protein. The Arabidopsis genome contains 15 genes encoding MLO proteins, with localization in plasma membrane. Phylogenetic analysis revealed four clades of closely-related AtMLO genes. ATMLO6 belongs to the clade IV, with AtMLO2, AtMLO3 and AtMLO12. The gene is expressed during early seedling growth, in root tips and cotyledon vascular system, in floral organs (anthers and stigma), and in fruit abscission zone, as shown by GUS activity patterns. The expression of several phylogenetically closely-related AtMLO genes showed similar or overlapping tissue specificity and analogous responsiveness to external stimuli, suggesting functional redundancy, co-function, or antagonistic function(s).
AT2G39210	*PICLORAM RESISTANT30 (PIC30)*	Major facilitator superfamily transmembrane transporter responsible for the uptake of picolinate herbicides.
AT2G39220	*PATATIN-LIKE PROTEIN 6 (PLP6)*	Phospholipase pPLAIIIa involved in seed germination and resistance to Turnip Crinkle Virus.
AT2G39230	*LATERAL ORGAN JUNCTION (LOJ)*	Encodes a pentatricopeptide protein (LOJ) that is specifically expressed in lateral organ junctions.
AT2G39240		RNA polymerase I specific transcription initiation factor RRN3 protein
AT2G39250	*SCHNARCHZAPFEN (SNZ)*	Encodes a AP2 domain transcription factor that can repress flowering. SNZ and its paralogous gene, SCHLAFMUTZE (SMZ), share a signature with partial complementarity to the miR172 microRNA, whose precursor is induced upon flowering.
AT2G39260	*(UPF2)*	Nonsense mediated decay (NMD)factor.
AT2G39280		Ypt/Rab-GAP domain of gyp1p superfamily protein
AT2G39300		CAP-gly domain linker
AT2G39310	*JACALIN-RELATED LECTIN 22 (JAL22)*	jacalin-related lectin 22
AT2G39320		Cysteine proteinases superfamily protein
AT2G39330	*JACALIN-RELATED LECTIN 23 (JAL23)*	jacalin-related lectin 23
AT2G39340	*YEAST SAC3 HOMOLOG A (SAC3A)*	Putative mRNA export factor that is highly co-expressed with PRP4KA.
AT2G39350	*ATP-BINDING CASSETTE G1 (ABCG1)*	Belongs to a clade of five Arabidopsis thaliana ABCG half-transporters that are required for synthesis of an effective suberin barrier in roots and seed coats (ABCG2, ABCG6, and ABCG20) and for synthesis of an intact pollen wall (ABCG1 and ABCG16).
AT2G39370	*MEMBRANE-ASSOCIATED KINASE REGULATOR 4 (MAKR4)*	Encodes a member of the MAKR (MEMBRANE-ASSOCIATED KINASE REGULATOR) gene family. MAKRs have putative kinase interacting motifs and membrane localization signals. Known members include: AT5G26230 (MAKR1), AT1G64080 (MAKR2), AT2G37380 (MAKR3), AT2G39370 (MAKR4), AT5G52870 (MAKR5) and AT5G52900 (MAKR6).
AT2G39400	*(MAGL6)*	alpha/beta-Hydrolases superfamily protein
AT2G39410	*(MAGL7)*	alpha/beta-Hydrolases superfamily protein
AT2G39420	*(MAGL8)*	alpha/beta-Hydrolases superfamily protein
AT2G39430		Disease resistance-responsive (dirigent-like protein) family protein
AT2G39440		ribonuclease H2 subunit C-like protein
AT2G39470	*PHOTOSYNTHETIC NDH SUBCOMPLEX L 1 (PnsL1)*	PsbP-like protein 2
AT2G39510	*USUALLY MULTIPLE ACIDS MOVE IN AND OUT TRANSPORTERS 14 (UMAMIT14)*	Encodes a plasma membrane-localized amino acid transporter likely involved in amino acid export in the developing seed.
AT2G39520		hypothetical protein

AT2G39530	*CASP-LIKE PROTEIN 4D1 (CASPL4D1)*	Uncharacterized protein family (UPF0497)
AT2G39540	*(GASA8)*	Gibberellin-regulated family protein
AT2G39550	*(PGGT-I)*	encodes the beta subunit of geranylgeranyl transferase (GGT-IB), involved in both ABA-mediated and auxin signaling pathways.
AT2G39560		Putative membrane lipoprotein
AT2G39570	*ACT DOMAIN REPEATS 9 (ACR9)*	Encodes a ACT domain-containing protein. The ACT domain, named after bacterial aspartate kinase, chorismate mutase and TyrA (prephenate dehydrogenase), is a regulatory domain that serves as an amino acid-binding site in feedback-regulated amino acid metabolic enzymes.
AT2G39590		40S ribosomal protein S15a
AT2G39620		Pentatricopeptide repeat (PPR) superfamily protein
AT2G39640		glycosyl hydrolase family 17 protein
AT2G39650		cruciferin (DUF506)
AT2G39660	*BOTRYTIS-INDUCED KINASE1 (BIK1)*	Encodes a plasma membrane-localized ser/thr protein kinase that is a crucial component of host response signaling required to activate the resistance responses to Botrytis and A. brassicicola infection. It is likely a negative regulator of salicylic acid accumulation and basal defense against virulent bacterial pathogens. Together with ER plays opposing roles in leaf morphogenesis and inflorescence architecture. Required to maintain appropriate auxin response during leaf margin morphogenesis. Interacts with ER-family proteins and directly phosphorylates ER.
AT2G39680		
AT2G39690		ternary complex factor MIP1 leucine-zipper protein (Protein of unknown function, DUF547)
AT2G39700	*EXPANSIN A4 (EXPA4)*	putative expansin. Naming convention from the Expansin Working Group (Kende et al, 2004. Plant Mol Bio). Involved in the formation of nematode-induced syncytia in roots of Arabidopsis thaliana.
AT2G39705	*ROTUNDIFOLIA LIKE 8 (RTFL8)*	ROTUNDIFOLIA like 8
AT2G39710		Encodes a Cysteine-rich peptide (CRP) family protein
AT2G39720	*RING-H2 FINGER C2A (RHC2A)*	Encodes a putative RING-H2 finger protein RHC2a.
AT2G39725	*(SDHAF1)*	LYR family of Fe/S cluster biogenesis protein
AT2G39730	*RUBISCO ACTIVASE (RCA)*	Rubisco activase, a nuclear-encoded chloroplast protein that consists of two isoforms arising from alternative splicing in most plants. Required for the light activation of rubisco. Involved in jasmonate-induced leaf senescence.
AT2G39770	*CYTOKINESIS DEFECTIVE 1 (CYT1)*	Encodes a GDP-mannose pyrophosphorylase/ mannose-1-pyrophosphatase. This enzyme provides GDP-mannose, which is used for cell wall carbohydrate biosynthesis and protein glycosylation as well as for ascorbate (vitamin C) biosynthesis. Mutations in this gene confer hypersensitivity to NH4+.
AT2G39790		Mitochondrial glycoprotein family protein
AT2G39795		Mitochondrial glycoprotein family protein
AT2G39820	*EUKARYOTIC INITIATION FACTOR 6B (eIF6B)*	Translation initiation factor IF6
AT2G39830	*DA1-RELATED PROTEIN 2 (DAR2)*	Essential for early phloem development and function, and for root system development.DAR2 is a member of a small (7 member) ubiquitin binding protein family. It appears to play a role in regulation of endoreduplication in leaf epidermal tissue.
AT2G39850		Subtilisin-like serine endopeptidase family protein
AT2G39870		hypothetical protein
AT2G39890	*PROLINE TRANSPORTER 1 (PROT1)*	Encodes a proline transporter with affinity for gly betaine, proline and GABA. Protein is expressed in the vascular tissue, specifically the phloem.
AT2G39900	*WLIM2A (WLIM2a)*	Encodes a member of the Arabidopsis LIM proteins: a family of actin bundlers with distinct expression patterns. WLIM1, WLIM2a, and WLIM2b are widely expressed, whereas PLIM2a, PLIM2b, and PLIM2c are predominantly expressed in pollen. Regulates actin cytoskeleton organization.
AT2G39920		HAD superfamily, subfamily IIIB acid phosphatase
AT2G39940	*CORONATINE INSENSITIVE 1 (COI1)*	Encodes a protein containing Leu-rich repeats and a degenerate F-box motif. Associates with AtCUL1, AtRbx1, and the Skp1-like proteins ASK1 and ASK2 to assemble SCF COI1 ubiquitin-ligase complexes in planta. A single amino acid substitution in the F-box motif of COI1 abolishes the formation of the SCF(COI1) complexes and results in loss of the JA response. Required for wound- and jasmonates-induced transcriptional regulation. Amino acid mutations in COI1 distinctively affect jasmonate-regulated male fertility.CFA-Ile, CFA-Leu, CFA-Val, CFA-Met and CFA-Ala could not inhibit the root length and restoration of fertility in coi1-1 mutants.
AT2G39950		flocculation protein
AT2G39960		Microsomal signal peptidase 25 kDa subunit (SPC25)
AT2G39970	*PEROXISOMAL NAD CARRIER (PXN)*	Encodes peroxisomal membrane protein 38 (PMP38). Mutation in this protein results in enlargement of peroxisomes. Delivers NAD+ for optimal fatty acid degradation during storage oil mobilization.
AT2G39980		HXXXD-type acyl-transferase family protein
AT2G39990	*EUKARYOTIC TRANSLATION INITIATION FACTOR 2 (EIF2)*	translation initiation factor eIF2 p47 subunit homolog
AT2G40000	*ORTHOLOG OF SUGAR BEET HS1 PRO-1 2 (HSPRO2)*	ortholog of sugar beet HS1 PRO-1 2
AT2G40010		Ribosomal protein L10 family protein
AT2G40020		Nucleolar histone methyltransferase-related protein
AT2G40030	*NUCLEAR RNA POLYMERASE D1B (NRPD1B)*	Encodes the unique largest subunit of nuclear DNA-dependent RNA polymerase V; homologous to budding yeast RPB1 and the E. coli RNA polymerase beta prime subunit. Required for normal RNA-directed DNA methylation at non-CG methylation sites and transgene silencing. The nrpe1 mutant is more resistant to biotrophic pathogens and is primed to activate salicylic acid-dependent defence genes.
AT2G40040		
AT2G40050		Cysteine/Histidine-rich C1 domain family protein

AT2G40080	*EARLY FLOWERING 4 (ELF4)*	Encodes a novel nuclear 111 amino-acid phytochrome-regulated component of a negative feedback loop involving the circadian clock central oscillator components CCA1 and LHY. ELF4 is necessary for light-induced expression of both CCA1 and LHY, and conversely, CCA1 and LHY act negatively on light-induced ELF4 expression. ELF4 promotes clock accuracy and is required for sustained rhythms in the absence of daily light/dark cycles. It is involved in the phyB-mediated constant red light induced seedling de-etiolation process and may function to coregulate the expression of a subset of phyB-regulated genes.
AT2G40090	*ABC2 HOMOLOG 9 (ATH9)*	member of ATH subfamily
AT2G40100	*LIGHT HARVESTING COMPLEX PHOTOSYSTEM II (LHCB4.3)*	Lhcb4:3 protein (Lhcb4.3, light harvesting complex of photosystem II The mRNA is cell-to-cell mobile.
AT2G40110		Yippee family putative zinc-binding protein
AT2G40130	*SMAX1-LIKE 8 (SMXL8)*	Encodes a member of an eight-gene family (SMAX1 and SMAX1-like) that has weak similarity to AtHSP101, a ClpB chaperonin required for thermotolerance.
AT2G40150	*TRICHOME BIREFRINGENCE-LIKE 28 (TBL28)*	Encodes a member of the TBL (TRICHOME BIREFRINGENCE-LIKE) gene family containing a plant-specific DUF231 (domain of unknown function) domain. TBL gene family has 46 members, two of which (TBR/AT5G06700 and TBL3/AT5G01360) have been shown to be involved in the synthesis and deposition of secondary wall cellulose, presumably by influencing the esterification state of pectic polymers. A nomenclature for this gene family has been proposed (Volker Bischoff & Wolf Scheible, 2010, personal communication).
AT2G40160	*TRICHOME BIREFRINGENCE-LIKE (TBL30)*	Encodes a member of the TBL (TRICHOME BIREFRINGENCE-LIKE) gene family containing a plant-specific DUF231 (domain of unknown function) domain. TBL gene family has 46 members, two of which (TBR/AT5G06700 and TBL3/AT5G01360) have been shown to be involved in the synthesis and deposition of secondary wall cellulose, presumably by influencing the esterification state of pectic polymers. A nomenclature for this gene family has been proposed (Volker Bischoff & Wolf Scheible, 2010, personal communication).
AT2G40170	*LATE EMBRYOGENESIS ABUNDANT 6 (GEA6)*	Encodes a group 1 LEA gene that is activated by direct binding of ABI5 to its promoter and is involved in response to ABA. Is required for normal seed development. Involved in regulating the timing of desiccation tolerance and rate of water loss during seed maturation.
AT2G40180	*PHOSPHATASE 2C5 (PP2C5)*	Encodes PP2C5, a member of the PP2C family phosphatases. PP2C5 acts as a MAPK phosphatase that positively regulates seed germination, stomatal closure and ABA-inducible gene expression.
AT2G40200		basic helix-loop-helix (bHLH) DNA-binding superfamily protein
AT2G40210	*AGAMOUS-LIKE 48 (AGL48)*	AGAMOUS-like 48
AT2G40220	*ABA INSENSITIVE 4 (ABI4)*	Encodes a member of the DREB subfamily A-3 of ERF/AP2 transcription factor family (ABI4). The protein contains one AP2 domain. There is only one member in this family. Involved in abscisic acid (ABA) signal transduction, ABA-mediated glucose response, and hexokinase-dependent sugar responses. Acts downstream of GUN1 in retrograde signaling. Expressed most abundantly in developing siliques and to a lesser degree in seedlings.
AT2G40230		HXXXD-type acyl-transferase family protein
AT2G40240		Tetratricopeptide repeat (TPR)-like superfamily protein
AT2G40250		SGNH hydrolase-type esterase superfamily protein
AT2G40260		Homeodomain-like superfamily protein
AT2G40270		Protein kinase family protein
AT2G40280		S-adenosyl-L-methionine-dependent methyltransferases superfamily protein
AT2G40290		Encodes an eIF2alpha homolog that can be phosphorylated by GCN2 in vitro.
AT2G40300	*FERRITIN 4 (FER4)*	Encodes FERRITIN 4, AtFER4. Ferritins are a class of 24-mer multi-meric proteins found in all kingdoms of life. Function as the main iron store in mammals. Evidence suggests that Arabidopsis ferritins are essential to protect cells against oxidative damage, but they do not constitute the major iron pool. Localize to mitochondria. Knock out mutants are not sensitive to abiotic stress.
AT2G40310		Pectin lyase-like superfamily protein
AT2G40316		autophagy-like protein
AT2G40330	*PYR1-LIKE 6 (PYL6)*	Encodes a member of the PYR (pyrabactin resistance)/PYL(PYR1-like)/RCAR (regulatory components of ABA receptor) family proteins with 14 members. PYR/PYL/RCAR family proteins function as abscisic acid sensors. Mediate ABA-dependent regulation of protein phosphatase 2Cs ABI1 and ABI2.
AT2G40350		encodes a member of the DREB subfamily A-2 of ERF/AP2 transcription factor family. The protein contains one AP2 domain. There are eight members in this subfamily including DREB2A AND DREB2B that are involved in response to drought.
AT2G40360	*ARABIDOPSIS THALIANA PESCADILLO ORTHOLOG1 (ATPEP1)*	Encodes BOP1, an ortholog of Block of cell proliferation (BOP) protein. A T-DNA null allele of the BOP1 gene is lethal, and a 50% decrease in transcript accumulation is sufficient to cause severe developmental defects linked to defective cell division.
AT2G40370	*LACCASE 5 (LAC5)*	putative laccase, a member of laccase family of genes (17 members in Arabidopsis). Together with DP1/DIR12 involved in neolignan biosynthesis via sinapoylcholine/feruloylcholine.
AT2G40400	*BRZ-INSENSITIVE-PALE GREEN 3 (BPG3)*	Encodes a chloroplast localized protein of unknown function that is involved in regulation of chloroplast development.
AT2G40410	*CA2+-DEPENDENT NUCLEASE (ATCAN2)*	Encodes a Ca(2+)-dependent nuclease that can degrade both DNA and RNA.
AT2G40420		Encodes a putative amino acid transporter.
AT2G40435		transcription factor SCREAM-like protein
AT2G40440		BTB/POZ domain protein
AT2G40460		Major facilitator superfamily protein
AT2G40470	*LOB DOMAIN-CONTAINING PROTEIN 15 (LBD15)*	LOB-domain containing protein. Involved in regulation of xylem differentiation- acts as a regulator of VND7 which is a master regulator of xylem cell differentiation.
AT2G40475	*ALTERED SEED GERMINATION 8 (ASG8)*	hypothetical protein
AT2G40480		WEB family protein (DUF827)
AT2G40490	*(HEME2)*	Uroporphyrinogen decarboxylase
AT2G40500		Protein kinase superfamily protein

AT2G40530		transmembrane protein
AT2G40550	*E2F TARGET GENE 1 (ETG1)*	Encodes a nuclear localized target of E2Fa-DPa, transcription factors controlling cell cycle progression. Required for sister chromatid cohesion and DNA repair.
AT2G40570		initiator tRNA phosphoribosyl transferase family protein
AT2G40580		Protein kinase superfamily protein
AT2G40610	*EXPANSIN A8 (EXPA8)*	member of Alpha-Expansin Gene Family. Naming convention from the Expansin Working Group (Kende et al, 2004. Plant Mol Bio). Involved in the formation of nematode-induced syncytia in roots of Arabidopsis thaliana.
AT2G40640	*(PUB62)*	Plant U-box type E3 ubiquitin ligase (PUB).
AT2G40670	*RESPONSE REGULATOR 16 (RR16)*	response regulator 16
AT2G40680		transposable_element_gene;similar to unknown protein [Arabidopsis thaliana] (TAIR:AT5G52065.1);(source:TAIR10)
AT2G40690	*(GLY1)*	Encodes a putative dihydroxyacetone phosphate (DHAP) reductase involved in glycerol-3-phosphate supply within the chloroplast for synthesis of glycerolipids. Mutants have reduced levels of hexadecatrienoic acid, which is rescued by exogenous glycerol-3-phosphate. This gene appears to be involved in the flux of fatty acids in the prokaryotic glyerolipid biosynthesis pathway.
AT2G40700	*RNA HELICASE 17 (RH17)*	DEAD-box helicase family protein. Overexpression confers tolerance to salt stress.
AT2G40720		Tetratricopeptide repeat (TPR)-like superfamily protein
AT2G40740	*WRKY DNA-BINDING PROTEIN 55 (WRKY55)*	member of WRKY Transcription Factor; Group III
AT2G40750	*WRKY DNA-BINDING PROTEIN 54 (WRKY54)*	member of WRKY Transcription Factor; Group III. Together with WRKY70 positively regulates SARD1 and CBP60g expression in plant immunity.
AT2G40760		Rhodanese/Cell cycle control phosphatase superfamily protein
AT2G40765		transmembrane protein
AT2G40790	*C-TERMINAL CYSTEINE RESIDUE IS CHANGED TO A SERINE 2 (CXXS2)*	Encodes a monocysteinic thioredoxin, thioredoxin in which the second cysteine of the redox site is replaced by a serine, with low disulfide reductase but efficient disulfide isomerase activity. It contains a large N-terminal extension with respect to the dicysteinic thioredoxins that is enriched with 8 cysteines and positively charged residues.
AT2G40800	*ATTIM21-LIKE 1 (ATTIM21L-1)*	TIM domain protein. Associates with components of mitochondrial complex I and III. May be involved in biogenesis of respiratory chain components.
AT2G40830	*RING-H2 FINGER C1A (RHC1A)*	Encodes an E3 ubiquitin ligase for the GA-receptor GID1 that functions as a negative regulator of GA signaling in seedlings and seeds by inducing ubiquitin-dependent proteolysis of GID1s. Tyr321 phosphorylation of GARU by TAGK2 inactivates GARU.
AT2G40840	*DISPROPORTIONATING ENZYME 2 (DPE2)*	Encodes a cytosolic protein with transglucosidase and amylomaltase activity. It is an essential component of the pathway from starch to sucrose and cellular metabolism in leaves at night. The protein binds to heteroglycans and utilizes glucose, mannose and xylose as acceptors. Fucose and galactose can also act as acceptors but less efficiently than the previous three. It was also was also recently reported to act on maltodextrins. On the other hand, arabinose and fructose were not efficiently used. Its role probably includes metabolizing maltose exported from the chloroplast. Studies using maltose extracted from the double mutant be2-1 be3-2 showed that this enzyme is preferentially active of β-maltose. The mRNA is cell-to-cell mobile.
AT2G40870		
AT2G40880	*CYSTATIN A (CYSA)*	Encodes a protein with cysteine proteinase inhibitor activity. Overexpression increases tolerance to abiotic stressors (i.e.salt,osmotic, cold stress). The mRNA is cell-to-cell mobile.
AT2G40890	*CYTOCHROME P450, FAMILY 98, SUBFAMILY A, POLYPEPTIDE 3 (CYP98A3)*	encodes coumarate 3-hydroxylase (C3H), a P450-dependent monooxygenase. Involved in lignin biosynthesis and flavonoid biosynthesis. Also affects the biosynthesis of coumarins such as scopoletin and scopolin as a branching-out-pathway from the phenylpropanoid acid level.
AT2G40900	*USUALLY MULTIPLE ACIDS MOVE IN AND OUT TRANSPORTERS 11 (UMAMIT11)*	Encodes a plasma membrane-localized amino acid transporter likely involved in amino acid export in the developing seed.
AT2G40910		F-box and associated interaction domains-containing protein
AT2G40940	*ETHYLENE RESPONSE SENSOR 1 (ERS1)*	Ethylene receptor, subfamily 1. Has histidine kinase activity.
AT2G40955		hypothetical protein
AT2G40970	*(MYBC1)*	Homeodomain-like superfamily protein
AT2G40990		DHHC-type zinc finger family protein
AT2G41000		Chaperone DnaJ-domain superfamily protein
AT2G41010	*CALMODULIN (CAM)-BINDING PROTEIN OF 25 KDA (CAMBP25)*	Encodes a novel calmodulin binding protein whose gene expression is induced by dehydration and ionic (salt) and non-ionic (mannitol) osmotic stress. Lines over-expressing this gene are more sensitive and anti-sense lines are more tolerant to osmotic stress, suggesting this gene may be a negative regulator of response to osmotic stress.
AT2G41040		S-adenosyl-L-methionine-dependent methyltransferases superfamily protein
AT2G41050		PQ-loop repeat family protein / transmembrane family protein
AT2G41060		RNA-binding (RRM/RBD/RNP motifs) family protein
AT2G41070	*ENHANCED EM LEVEL (EEL)*	Transcription factor homologous to ABI5. Regulates AtEm1 expression by binding directly at the AtEm1 promoter. Located in the nucleus and expressed during seed maturation in the cotyledons and later in the whole embryo.
AT2G41080		pentatricopeptide (PPR) repeat protein
AT2G41090	*CALMODULIN LIKE 10 (CML10)*	Encodes a cytoplasmic, calcium binding calmodulin variant. CML10 interacts with phosphomannomutase (PMM)in vivo and increases its activity thereby affecting ascorbic acid biosynthesis. Its expression is induced by oxidative and other stress. The mRNA is cell-to-cell mobile.
AT2G41100	*TOUCH 3 (TCH3)*	encodes a calmodulin-like protein, with six potential calcium binding domains. Calcium binding shown by Ca(2+)-specific shift in electrophoretic mobility. Expression induced by touch and darkness. Expression may also be developmentally controlled. Expression in growing regions of roots, vascular tissue, root/shoot junctions, trichomes, branch points of the shoot, and regions of siliques and flowers. The mRNA is cell-to-cell mobile.
AT2G41120		DUF309 domain protein
AT2G41140	*CDPK-RELATED KINASE 1 (CRK1)*	Encodes CDPK-related kinase 1 (CRK1).
AT2G41180	*SIGMA FACTOR BINDING PROTEIN 2 (SIB2)*	VQ motif-containing protein
AT2G41190		Transmembrane amino acid transporter family protein

AT2G41200		transmembrane protein
AT2G41210	*PHOSPHATIDYLINOSITOL- 4-PHOSPHATE 5-KINASE 5 (PIP5K5)*	Encodes a protein with phosphatidylinositol-4-phosphate 5-kinase activity that plays a role in pollen tip growth. The enzyme localizes to the apical plasma membrane and adjacent cytosolic region of pollen tubes. Overexpression of this gene leads to increased deposition of pectin in the cell wall at the tip of the pollen tube and causes altered pollen tube morphology.
AT2G41230	*ORGAN SIZE RELATED 1 (OSR1)*	Encodes an ER-localized plant hormone-responsive gene and appears to act redundantly with ARGOS and ARL during organ growth. Over-expression modifies plant sensitivity to ethylene, leading to improved drought tolerance.
AT2G41250		Haloacid dehalogenase-like hydrolase (HAD) superfamily protein
AT2G41260	*(M17)*	Late-embryogenesis-abundant gene. Involved in the acquisition of desiccation tolerance during late phase of embryogenesis.
AT2G41280	*(M10)*	Encodes a hydrophilic protein similar to Late Embryogenesis Activated (LEA) proteins expressed during embryogenesis, which are thought to be involved in the acquisition of desiccation tolerance.
AT2G41290	*STRICTOSIDINE SYNTHASE-LIKE 2 (SSL2)*	Although this enzyme is predicted to encode a strictosidine synthase (SS), it lacks a conserved catalytic glutamate residue found in active SS enzymes and it is not expected to have SS activity.
AT2G41300	*STRICTOSIDINE SYNTHASE-LIKE 1 (SSL1)*	Although this enzyme is predicted to encode a strictosidine synthase (SS), it lacks a conserved catalytic glutamate residue found in active SS enzymes and it is not expected to have SS activity.
AT2G41310	*RESPONSE REGULATOR 3 (RR3)*	Encodes an A- type response Regulator that is primarily expressed in the root and is involved in cytokinin-mediated signalling. Phosphatidylinositol 4-phosphate 5-kinase (PIP5K) enzyme family member.
AT2G41330		Glutaredoxin family protein
AT2G41340	*RNA POLYMERASE II FIFTH LARGEST SUBUNIT, D (RPB5D)*	NRPE5-like protein of unknown function; homologous to budding yeast RPB5
AT2G41370	*BLADE ON PETIOLE2 (BOP2)*	Encodes BOP2, a cytoplasmic and nuclear-localized NPR1 like protein with BTB/POZ domain and ankyrin repeats. Interacts with BOP1 and appears to be genetically redundant with BOP1.bop1/bop2 double mutants have longer leaves, often with leaflets on the petiole, asymmetric flowers with extra organs and no nectaries. Also defective in floral organ abscission. BOP1/2 promotes floral meristem fate and determinacy in a pathway targetting APETALA1 and AGAMOUS-LIKE24. PUCHI, BOP1 and BOP2 are redundantly required for expression of LFY and AP1. BOP2 is expressed in valve margin. Misexpression in stems causes short internodes and ectopic biosynthesis of lignin. BOP2 activity is antagonistic to BP (At4g08150) and PNY (At5g02030). BOP3 expression is restricted to pedicel axils by BP and PNY; promotes KNAT6 (At1g23380) expression.
AT2G41380		S-adenosyl-L-methionine-dependent methyltransferases superfamily protein
AT2G41390		Pollen Ole e 1 allergen and extensin family protein
AT2G41410		Calcium-binding EF-hand family protein
AT2G41420	*WINDHOSE 2 (WIH2)*	proline-rich family protein
AT2G41440		agamous-like MADS-box protein
AT2G41450		N-acetyltransferase
AT2G41470		agamous-like MADS-box protein
AT2G41480	*PEROXIDASE 25 (PRX25)*	Encodes a cationic cell-wall-bound peroxidase homolog that is involved in the lignification of cell walls. Regulated by COG1, involved in seed longevity.
AT2G41490	*UDP-GLCNAC-ADOLICHOL PHOSPHATE GLCNAC-1-P-TRANSFERASE (GPT)*	UDP-GlcNAc:dolichol phosphate N-acetylglucosamine-1-phosphate transferase
AT2G41500	*LACHESIS (LIS)*	Encodes LACHESIS (LIS), a protein with seven WD40 repeats. LIS is homologous to the yeast splicing factor PRP4 which is associated with the U4/U6 complex of the spliceosome. LIS is involved in a mechanism that prevents accessory cells from adopting gametic cell fate: lis mutant forms supernumerary egg cells.
AT2G41510	*CYTOKININ OXIDASE/DEHYDROGENASE 1 (CKX1)*	It encodes a protein whose sequence is similar to cytokinin oxidase/dehydrogenase, which catalyzes the degradation of cytokinins.Acts on zeatin 9-riboside-50-triphosphate substrate.
AT2G41540	*(GPDHC1)*	Encodes a protein with NAD-dependent glycerol-3-phosphate (G3P) dehydrogenase which was shown to complement an Escherichia coli strain: BB20-14, auxotrophic for glycerol/G3P due to a loss-of-function mutation in the gpsA gene.
AT2G41550		Rho termination factor
AT2G41560	*AUTOINHIBITED CA(2+)-ATPASE, ISOFORM 4 (ACA4)*	Encodes a calmodulin-regulated Ca(2+)-ATPase that improves salt tolerance in yeast. Localized to the vacuole. Lesion mimic phenotype when mutation in the gene is combined with a mutation in ACA11. Lesion mimic phenotype of double knockout can be suppressed by nutritional supplements that increase anion levels (e.g. 15 mM Nitrate, Chloride, or Phosphate).
AT2G41570		transposable_element_gene;similar to unknown protein [Arabidopsis thaliana] (TAIR:AT2G16690.1);(source:TAIR10)
AT2G41580		transposable_element_gene;non-LTR retrotransposon family (LINE), has a 4.7e-42 P-value blast match to GB:NP_038602 L1 repeat, Tf subfamily, member 18 (LINE-element) (Mus musculus);(source:TAIR10)
AT2G41590		Ta11-like non-LTR retrotransposon
AT2G41610	*DEFECT IN CELL ELONGATION1 (DICE1)*	Transmembrane protein from a plant specific gene family. Overexpression causes abnormal cell wall composition and defects in cell growth.
AT2G41620		Nucleoporin interacting component (Nup93/Nic96-like) family protein
AT2G41630	*TRANSCRIPTION FACTOR IIB 1 (TFIIB1)*	Encodes a transcription factor, TFIIB1, that plays important roles in pollen tube growth, guidance, and reception as well as endosperm development and is partially functionally different from AtTFIIB2 and AtTFIIB3/AtpBRP2.
AT2G41640		Glycosyltransferase family 61 protein
AT2G41650		hypothetical protein
AT2G41660	*MIZU-KUSSEI 1 (MIZ1)*	Essential for hydrotropism in roots. Mutant roots are defective in hydrotropism, and have slightly reduced phototropism and modified wavy growth response. Has normal gravitropism and root elongation.
AT2G41680	*NADPH-DEPENDENT THIOREDOXIN REDUCTASE C (NTRC)*	Encodes a NADPH thioredoxin reductase involved in chloroplast protection against oxidative damage.
AT2G41690	*HEAT SHOCK TRANSCRIPTION FACTOR B3 (HSFB3)*	member of Heat Stress Transcription Factor (Hsf) family
AT2G41700	*ATP-BINDING CASSETTE A1 (ABCA1)*	ATP-binding cassette A1

AT2G41705	*FLUORIDE EXPORT PROTEIN (FEX)*	Encodes a fluoride export protein.
AT2G41730	*(HRG1)*	Expression in rosette leaves is activated by high concentration of boron.
AT2G41780		hypothetical protein
AT2G41800	*TEEBE, LONG IN THE MAYO-YOREME LANGUAGE (TEB)*	Encodes a DUF642 cell wall protein that is highly induced during the M/G1 phases of the cell cycle and is involved in hypocotyl cell elongation.
AT2G41810		imidazolonepropionase (Protein of unknown function, DUF642)
AT2G41820	*PXY/TDR-CORRELATED 3 (PXC3)*	Leucine-rich repeat protein kinase family protein
AT2G41850	*POLYGALACTURONASE ABSCISSION ZONE A. THALIANA (PGAZAT)*	ADPG2.
AT2G41860	*CALCIUM-DEPENDENT PROTEIN KINASE 14 (CPK14)*	member of Calcium Dependent Protein Kinase
AT2G41870	*REMORIN GROUP 4 2 (REM4.2)*	Remorin family protein
AT2G41880	*GUANYLATE KINASE 1 (GK-1)*	Guanylate kinase. Involved in nucleotide metabolism.
AT2G41900	*OXIDATIVE STRESS 2 (OXS2)*	AtOXS2 specifcally entered the nuclear under salt stress. Te specifc nuclear localization of AtOXS2 could play a role in salt tolerance at the molecular level. Tese results implied that AtOXS2 might target some downstream cis-elements which are required for salt stress responses
AT2G41930	*(NIT1)*	Protein kinase superfamily protein
AT2G41940	*ZINC FINGER PROTEIN 8 (ZFP8)*	Encodes a zinc finger protein containing only a single zinc finger.
AT2G41970	*MARIS (MRI)*	Encodes MRI, a plasma membrane-localized member of the RLCK-VIII subfamily. Preferentially expressed in both pollen tubes and root hairs. mri-knockout mutants display spontaneous pollen tube and root-hair bursting.
AT2G41990	*COMPANION OF CELLULOSE SYNTHASE 3 (CC3)*	late embryogenesis abundant protein
AT2G42000	*ARABIDOPSIS THALIANA METALLOTHIONEIN 4A (ATMT4A)*	AtMT4a is a member of Type 4 metallothionein (MT) genes. It is involved in the early develoment of the embryo and in the accumulation of metal ions especially Zn in the seeds.
AT2G42040		WRC protein
AT2G42050		transposable_element_gene;similar to unknown protein [Arabidopsis thaliana] (TAIR:AT5G35860.1);(source:TAIR10)
AT2G42060		Cysteine/Histidine-rich C1 domain family protein
AT2G42070	*NUDIX HYDROLASE HOMOLOG 23 (NUDX23)*	Encodes a plastid-localized Nudix hydrolase that has FAD pyrophosphohydrolase activity. Negative feedback regulation of the metabolism of flavins through the hydrolysis of FAD by AtNUDX23 in plastids is involved in the flavin homeostasis in plant cells.
AT2G42090	*ACTIN 9 (ACT9)*	actin related gene or pseudogene, based on sequence divergence and lack of expression
AT2G42100		Actin-like ATPase superfamily protein
AT2G42110		hypothetical protein
AT2G42130		Plastid-lipid associated protein PAP / fibrillin family protein
AT2G42140		VQ motif-containing protein
AT2G42150		DNA-binding bromodomain-containing protein
AT2G42160	*BRAP2 RING ZNF UBP DOMAIN-CONTAINING PROTEIN 1 (BRIZ1)*	Encodes a RING domain containing protein BRIZ1. BRIZ1 (At2g42160) and BRIZ2 (At2g26000) proteins form a heteromeric E3 ligase complex required for seed germination and post-germination growth.
AT2G42180		cotton fiber protein
AT2G42190		rho GTPase-activating gacO-like protein
AT2G42200	*SQUAMOSA PROMOTER BINDING PROTEIN-LIKE 9 (SPL9)*	Encodes a putative transcriptional regulator that is involved in the vegetative to reproductive phase transition. Expression is regulated by MIR156b. SPL activity nonautonomously inhibits initiation of new leaves at the shoot apical meristem.
AT2G42220		Rhodanese/Cell cycle control phosphatase superfamily protein
AT2G42240		RNA-binding (RRM/RBD/RNP motifs) family protein
AT2G42250	*CYTOCHROME P450, FAMILY 712, SUBFAMILY A, POLYPEPTIDE 1 (CYP712A1)*	member of CYP712A
AT2G42270	*(BRR2B)*	Similar to yeast Brr2p DEAD/DExH box ATP-dependent RNA helicase.
AT2G42280	*FLOWERING BHLH 4 (FBH4)*	basic helix-loop-helix (bHLH) DNA-binding superfamily protein
AT2G42290		Leucine-rich repeat protein kinase family protein
AT2G42300	*(BHLH48)*	Together with bHLH60 associates with phytochrome interacting factor 7 to regulate hypocotyl elongation.
AT2G42320		nucleolar protein gar2-like protein
AT2G42330	*TRANS-MEMBRANE KINASE 4 (TMK4)*	Transmembrane kinase involved in auxin signaling. Phosphorylates T101 of TAA1 to inactive the transaminase and block auxin biosynthesis.
AT2G42340		hypothetical protein
AT2G42350	*ARABIDOPSIS T??XICOS EN LEVADURA 40 (ATL40)*	RING/U-box superfamily protein
AT2G42360	*ARABIDOPSIS T??XICOS EN LEVADURA 41 (ATL41)*	RING/U-box superfamily protein
AT2G42370		hypothetical protein
AT2G42380	*(BZIP34)*	Encodes a member of the BZIP family of transcription factors. Forms heterodimers with the related protein AtbZIP61. Binds to G-boxes in vitro and is localized to the nucleus in onion epidermal cells.
AT2G42390		kinase C substrate, heavy chain-like protein
AT2G42410	*ZINC FINGER PROTEIN 11 (ZFP11)*	Encodes a zinc finger protein ZFP11. Overexpression of ZFP11 causes mortality and a deformed phenotype.
AT2G42430	*LATERAL ORGAN BOUNDARIES-DOMAIN 16 (LBD16)*	LOB-domain protein gene LBD16. This gene contains one auxin-responsive element (AuxRE). Regluates lateral root formation.
AT2G42440	*ASYMMETRIC LEAVES 2-LIKE 15 (ASL15)*	Lateral organ boundaries (LOB) domain family protein
AT2G42480		MATH domain/coiled-coil protein
AT2G42490	*COPPER AMINE OXIDASE ZETA (CuAO-zeta)*	Peroxisome-localized copper amine oxidase involved in lateral root formation.
AT2G42520	*RNA HELICASE 37 (RH37)*	P-loop containing nucleoside triphosphate hydrolases superfamily protein
AT2G42530	*COLD REGULATED 15B (COR15B)*	Encodes COR15B, a protein that protects chloroplast membranes during freezing.

AT2G42540	*COLD-REGULATED 15A (COR15A)*	A cold-regulated gene whose product is targeted to the chloroplast. Cor15am protects stromal proteins from aggregation under various stress conditions. Constitutive expression increases freezing tolerance in protoplasts in vitro and chloroplasts in vivo. NMR and x-ray diffraction studies suggest that COR15a alters the intrinsic curvature of the inner membrane of chloroplast envelope. Late Embryogenesis abundant protein (LEA). Protects chloroplast membranes during freezing.
AT2G42560	*LATE EMBRYOGENESIS ABUNDANT 25 (LEA25)*	Late embryogenesis protein. Based on in vitro studies, likely plays a role in stablizing membranes in response to freezing stress.
AT2G42570	*TRICHOME BIREFRINGENCE-LIKE 39 (TBL39)*	Encodes a member of the TBL (TRICHOME BIREFRINGENCE-LIKE) gene family containing a plant-specific DUF231 (domain of unknown function) domain. TBL gene family has 46 members, two of which (TBR/AT5G06700 and TBL3/AT5G01360) have been shown to be involved in the synthesis and deposition of secondary wall cellulose, presumably by influencing the esterification state of pectic polymers. A nomenclature for this gene family has been proposed (Volker Bischoff & Wolf Scheible, 2010, personal communication).
AT2G42590	*GENERAL REGULATORY FACTOR 9 (GRF9)*	14-3-3 gene. Binds calcium and displays induced structural changes.
AT2G42600	*PHOSPHOENOLPYRUVATE CARBOXYLASE 2 (PPC2)*	Encodes one of four Arabidopsis phosphoenolpyruvate carboxylase proteins.PPC1 and PPC2 are crucial for balancing carbon and nitrogen metabolism.
AT2G42610	*LIGHT SENSITIVE HYPOCOTYLS 10 (LSH10)*	LIGHT-DEPENDENT SHORT HYPOCOTYLS-like protein (DUF640)
AT2G42620	*MORE AXILLARY BRANCHES 2 (MAX2)*	The mutations at MAX2 cause increased hypocotyl and petiole elongation in light-grown seedlings. Positional cloning identifies MAX2 as a member of the F-box leucine-rich repeat family of proteins. MAX2 is identical to ORE9, a proposed regulator of leaf senescence. Involved in positive regulation of light responses. The mRNA is cell-to-cell mobile.
AT2G42630		
AT2G42660		Homeodomain-like superfamily protein
AT2G42690	*ACYLATED GALACTOLIPID- ASSOCIATED PHOSPHOLIPASE 1 (AGAP1)*	alpha/beta-Hydrolases superfamily protein
AT2G42710		Ribosomal protein L1p/L10e family
AT2G42720		FBD, F-box, Skp2-like and Leucine Rich Repeat domains containing protein
AT2G42750	*DNA J PROTEIN C77 (DJC77)*	DNAJ heat shock N-terminal domain-containing protein
AT2G42760		DUF1685 family protein
AT2G42790	*CITRATE SYNTHASE 3 (CSY3)*	Encodes a peroxisomal citrate synthase that is expressed throughout seedling and shoot development.
AT2G42800	*RECEPTOR LIKE PROTEIN 29 (RLP29)*	receptor like protein 29
AT2G42810	*PROTEIN PHOSPHATASE 5 (PP5)*	Encodes a phytochrome-specific type 5 serine/threonine protein phosphatase. It dephosphorylates active Pfr-phytochromes. Controls light signal flux by enhancing phytochrome stability and affinity for a signal transducer. The gene is alternately spliced. This variant is an integral membrane protein localized to the ER and nuclear envelope. Belongs to one of the 36 carboxylate clamp (CC)-tetratricopeptide repeat (TPR) proteins (Prasad 2010, Pubmed ID: 20856808) with potential to interact with Hsp90/Hsp70 as co-chaperones. It also regulates tetrapyrrole biosynthesis through the accumulation of Mg-ProtoIX and acts as a negative regulator of photosynthesis associated nuclear gene expression during chloroplast biogenesis and development.
AT2G42830	*SHATTERPROOF 2 (SHP2)*	AGAMOUS [AG]-like MADS box protein (AGL5) involved in fruit development (valve margin and dehiscence zone differentiation). A putative direct target of AG. SHP2 has been shown to be a downstream gene of the complex formed by AG and SEP proteins (SEP4 alone does not form a functional complex with AG).
AT2G42840	*PROTODERMAL FACTOR 1 (PDF1)*	Encodes a putative extracellular proline-rich protein is exclusively expressed in the L1 layer of vegetative, inflorescence and floral meristems and the protoderm of organ primordia.
AT2G42850	*CYTOCHROME P450, FAMILY 718 (CYP718)*	cytochrome P450, family 718
AT2G42860		hypothetical protein
AT2G42870	*PHY RAPIDLY REGULATED 1 (PAR1)*	Encodes PHYTOCHROME RAPIDLY REGULATED1 (PAR1), an atypical basic helix-loop-helix (bHLP) protein. Closely related to PAR2 (At3g58850). Up regulated after simulated shade perception. Acts in the nucleus to control plant development and as a negative regulator of shade avoidance response. Functions as transcriptional repressor of auxin-responsive genes SAUR15 (AT4G38850) and SAUR68 (AT1G29510).
AT2G42900		Plant basic secretory protein (BSP) family protein
AT2G42910	*PHOSPHORIBOSYL DIPHOSPHATE SYNTHASE 4 (PRS4)*	Phosphoribosyltransferase family protein
AT2G42920		Pentatricopeptide repeat (PPR-like) superfamily protein
AT2G42930		Carbohydrate-binding X8 domain superfamily protein
AT2G42940	*AT-HOOK MOTIF NUCLEAR-LOCALIZED PROTEIN 16 (AHL16)*	Encodes a nuclear matrix protein with AT-hook DNA binding motifs that acts in the maintenance of genomic integrity by silencing TEs and repeat-containing genes through epigenetic machinery. It interacts with FVE and MSI5 which are components of HDAC corepressor complexes. It is expressed in tapetum during the tetrad stage.
AT2G42950		Magnesium transporter CorA-like family protein
AT2G42960		Protein kinase superfamily protein
AT2G42980		Eukaryotic aspartyl protease family protein
AT2G42990		GDSL-motif esterase/acyltransferase/lipase. Enzyme group with broad substrate specificity that may catalyze acyltransfer or hydrolase reactions with lipid and non-lipid substrates.
AT2G43000	*NAC DOMAIN CONTAINING PROTEIN 42 (NAC042)*	Encodes a NAC transcription factor induced by hydrogen peroxide (H2O2). Involved in senescence. Over expression of the gene strongly delays senescence and enhances tolerance to various abiotic stresses.
AT2G43010	*PHYTOCHROME INTERACTING FACTOR 4 (PIF4)*	Isolated as a semidominant mutation defective in red -light responses. Encodes a nuclear localized bHLH protein that interacts with active PhyB protein. Negatively regulates phyB mediated red light responses. Involved in shade avoidance response. Protein abundance is negatively regulated by PhyB.Involved in the regulation of response to nutrient levels. Controls the resistance to B. cinerea in a COI1- and EIN2-dependent manner.
AT2G43030	*PLASTID RIBOSOMAL PROTEINS OF THE 50S SUBUNIT (PRPL3)*	Ribosomal protein L3 family protein
AT2G43040	*NO POLLEN GERMINATION 1 (NPG1)*	encodes a calmodulin-binding protein that is expressed specifically in pollen and is required for pollen development.

AT2G43050	*(ATPMEPCRD)*	Plant invertase/pectin methylesterase inhibitor superfamily
AT2G43060	*ILI1 BINDING BHLH 1 (IBH1)*	ILI1 binding bHLH 1
AT2G43080	*P4H ISOFORM 1 (AT-P4H-1)*	Encodes a prolyl-4 hydroxylase that can hydroxylate poly(L-proline),the collagen model peptide (Pro-Pro-Gly)10 and other proline rich peptides.
AT2G43100	*ISOPROPYLMALATE ISOMERASE 2 (IPMI2)*	Small subunit, which together with IPMI SSU2, IPMI SSU3 and IPMI LSU1, is a member of heterodimeric isopropylmalate isomerase (IPMI). Together with IPMI SSU3 participates in the Met chain elongation pathway.
AT2G43140	*(BHLH129)*	bHLH129 is a nuclear localized basic helix loop helix protein. It has been shown to function as a transcriptional repressor. Overexpression of bHLH129 regulates root elongation and ABA response.
AT2G43150	*EXTENSIN 21 (EXT21)*	Proline-rich extensin-like family protein
AT2G43160	*EPSIN2 (EPS2)*	Involved in plant trans-Golgi network (TGN) transport.
AT2G43170		
AT2G43180		Phosphoenolpyruvate carboxylase family protein
AT2G43220		Cysteine/Histidine-rich C1 domain family protein
AT2G43230	*CYTOSOLIC ABA RECEPTOR KINASE 6 (CARK6)*	Member of cytosolic ABA receptor kinases; interacts with ABA receptors RCAR11-14. Positively regulates germination, seedling architecture and root growth in response to ABA.
AT2G43260		F-box and associated interaction domains-containing protein
AT2G43270		F-box and associated interaction domains-containing protein
AT2G43280	*FAR1-RELATED SEQUENCES-RELATED FACTOR4 (FRF4)*	Encodes one of four FRS (FAR1-RELATED SEQUENCE) factor-like genes in Arabidopsis. FRS factors are characterized by having an N-terminal C2H2-type chelating motif of the WRKY- Glial Cell Missing1 family, a central core transposase domain of Mutator-like element transposases, and a C-terminal SWIM domain. The four FRF-like genes in Arabidopsis share only the N-terminal motif with FRS proteins.
AT2G43310	*(UL18-L6)*	Member of the uL18 RNA-binding protein family. uL18 proteins share a short structurally conserved domain that binds the 5S rRNA and allow its incorporation into ribosomes.
AT2G43350	*GLUTATHIONE PEROXIDASE 3 (GPX3)*	Glutathione peroxidase. Functions as both a redox transducer and a scavenger in abscisic acid and drought stress responses. Interacts with ABI2 and ABI1.
AT2G43360	*BIOTIN AUXOTROPH 2 (BIO2)*	Catalyzes the conversion of dethiobiotin to biotin.
AT2G43370		RNA-binding (RRM/RBD/RNP motifs) family protein
AT2G43380		
AT2G43390		hypothetical protein
AT2G43420	*RETICULON 20 (RTN20)*	3-beta hydroxysteroid dehydrogenase/isomerase family protein
AT2G43450		hypothetical protein
AT2G43480		Peroxidase superfamily protein
AT2G43500	*NIN-LIKE PROTEIN 8 (NLP8)*	Plant regulator RWP-RK family protein
AT2G43510	*TRYPSIN INHIBITOR PROTEIN 1 (TI1)*	Member of the defensin-like (DEFL) family. Encodes putative trypsin inhibitor protein which may function in defense against herbivory.
AT2G43520	*TRYPSIN INHIBITOR PROTEIN 2 (TI2)*	Encodes putative trypsin inhibitor protein which may function in defense against herbivory. Member of the defensin-like (DEFL) family.
AT2G43530		Encodes a defensin-like (DEFL) family protein. The mRNA is cell-to-cell mobile.
AT2G43535		Encodes a defensin-like (DEFL) family protein.
AT2G43540		transmembrane protein
AT2G43550		Encodes a defensin-like (DEFL) family protein.
AT2G43560		FKBP-like peptidyl-prolyl cis-trans isomerase family protein
AT2G43570	*CHITINASE, PUTATIVE (CHI)*	chitinase
AT2G43580		Chitinase family protein
AT2G43590		Chitinase family protein
AT2G43600		Chitinase family protein
AT2G43610		Chitinase family protein
AT2G43620		Chitinase family protein
AT2G43650	*EMBRYO DEFECTIVE 2777 (EMB2777)*	SAS10/C1D family protein. Loss of function mutants are embryo lethal.Ubiquitously expressed, with preference for tissues undergoing rapid cellular growth and differentiation.
AT2G43660		Carbohydrate-binding X8 domain superfamily protein
AT2G43670		Carbohydrate-binding X8 domain superfamily protein
AT2G43680	*IQ-DOMAIN 14 (IQD14)*	Member of IQ67 (CaM binding) domain containing family.
AT2G43690	*L-TYPE LECTIN RECEPTOR KINASE V.3 (LECRK-V.3)*	Concanavalin A-like lectin protein kinase family protein
AT2G43710	*SUPPRESSOR OF SA INSENSITIVE 2 (SSI2)*	Encodes a stearoyl-ACP desaturase, involved in fatty acid desaturation. The ssi2 mutants have increased 18:0 and reduced 18:1 fatty acids. Exogenous application of glycerol to wild type plants mimics the ssi2 mutant phenotype. The altered 18:1 fatty acid content in the ssi2 mutants has an impact on SA- and JA-mediated defense signaling. ssi2 mutants resulted in hyper-resistance to green peach aphid and antibiosis activity in petiole exudates. Redundant Δ9 stearoyl-ACP desaturase gene which together with AAD1 and AAD5 during embryo development provide precursors for the elaboration of embryo cuticle and therefore plays a specific role during the phase of invasive embryo growth through the endosperm. Together with AAD1, AAD5, and AAD6 redundantly participates in oil storage during the maturation phase.
AT2G43730		Mannose-binding lectin superfamily protein
AT2G43750	*O-ACETYLSERINE (THIOL) LYASE B (OASB)*	Arabidopsis thaliana O-acetylserine (thiol) lyase (OAS-TL) isoform oasB, the key enzyme for fixation of inorganic sulfide. It catalyzes the formation of cysteine from O-acetylserine and inorganic sulfide. Required for pollen tube growth and/or fertilization.
AT2G43760	*COFACTOR OF NITRATE REDUCTASE AND XANTHINE DEHYDROGENASE 6 (CNX6)*	molybdopterin biosynthesis MoaE family protein

AT2G43780		cytochrome oxidase assembly protein
AT2G43800	*FORMIN 2 (FH2)*	Localizes to plasmodesmata (PD) through its transmembrane domain and is required for normal intercellular trafficking. Functions in a partially redundant manner with its closest homolog AtFH1. Regulates PD's permeability by anchoring actin filaments to PD. Caps the barbed end of actin filaments and stabilizes them in vitro.
AT2G43820	*UDP-GLUCOSYLTRANSFERASE 74F2 (UGT74F2)*	Encodes a nicotinate-O-glycosyltransferase. Induced by Salicylic acid, virus, fungus and bacteria. Also involved in the tryptophan synthesis pathway. Independent of NPR1 for their induction by salicylic acid. UGT74F1 transfers UDP:glucose to salicylic acid (forming a glucoside (SAG) and a glucose ester (SGE)), benzoic acid, and anthranilate in vitro. UGT74F2 shows a weak ability to catalyze the formation of the p-aminobenzoate-glucose ester in vitro. But, UGT75B1 appears to be the dominant pABA acylglucosyltransferase in vivo based on assays in leaves, flowers, and siliques.
AT2G43840	*UDP-GLYCOSYLTRANSFERASE 74 F1 (UGT74F1)*	UGT74F1 transfers UDP:glucose to salicylic acid (forming a glucoside), benzoic acid, quercetin, and athranilate in vitro. UGT74F1 shows a weak ability to catalyze the formation of the p-aminobenzoate-glucose ester in vitro. But, UGT75B1 appears to be the dominant pABA acylglucosyltransferase in vivo based on assays in leaves, flowers, and siliques. The true biological substrate(s) of UGT74F1 are not known, but mutant plants lacking UGT74F1 have a decreased level of salicylate glucoside.
AT2G43850	*INTEGRIN-LINKED KINASE1 (ILK1)*	Integrin-linked protein kinase family
AT2G43860		Pectin lyase-like superfamily protein
AT2G43870		Pectin lyase-like superfamily protein
AT2G43880		Pectin lyase-like superfamily protein
AT2G43890		Pectin lyase-like superfamily protein
AT2G43920	*HARMLESS TO OZONE LAYER 2 (HOL2)*	S-adenosyl-L-methionine-dependent methyltransferases superfamily protein
AT2G43930		Protein kinase superfamily protein
AT2G43950	*CHLOROPLAST OUTER ENVELOPE PROTEIN 37 (OEP37)*	Constitutes a peptide sensitive ion channel in chloroplast outer membranes. Accumulates in germinating seeds and developing embryos.
AT2G43970	*LA RELATED PROTEIN 6B (LARP6B)*	RNA-binding protein
AT2G44000		Late embryogenesis abundant (LEA) hydroxyproline-rich glycoprotein family
AT2G44010		hypothetical protein
AT2G44040		Dihydrodipicolinate reductase, bacterial/plant
AT2G44060	*LATE EMBRYOGENESIS ABUNDANT 26 (LEA26)*	Late embryogenesis abundant protein, group 2
AT2G44065		Ribosomal protein L2 family
AT2G44070		NagB/RpiA/CoA transferase-like superfamily protein
AT2G44080	*ARGOS-LIKE (ARL)*	Encodes ARL, a gene similar to ARGOS involved in cell expansion-dependent organ growth. Upregulated by brassinosteroid. Acts downstream of BRI1. The mRNA is cell-to-cell mobile.
AT2G44110	*MILDEW RESISTANCE LOCUS O 15 (MLO15)*	A member of a large family of seven-transmembrane domain proteins specific to plants, homologs of the barley mildew resistance locus o (MLO) protein. The Arabidopsis genome contains 15 genes encoding MLO proteins, with localization in plasma membrane. Phylogenetic analysis revealed four clades of closely-related AtMLO genes. ATMLO15 belongs to the clade II, with ATMLO13 and ATMLO15. The gene is expressed during early seedling growth, in root tips and flower (papillae, anthers and pollen grains), as shown by GUS activity patterns. The expression of several phylogenetically closely-related AtMLO genes showed similar or overlapping tissue specificity and analogous responsiveness to external stimuli, suggesting functional redundancy, co-function, or antagonistic function(s).
AT2G44130	*KISS ME DEADLY 3 (KMD3)*	Encodes a member of a family of F-box proteins, called the KISS ME DEADLY (KMD) family. Component of SCF ubiquitin protein ligase, interacts with phenylalanine ammonia-lyase. AtKFB39 is a homolog of previously identified AtKFB50 (At3g59940) and specifically interacts with Arabidopsis PAL3 and PAL4 in vitro. In planta, together with AtKFB01, KFB20 and KFB50, it regulates PAL protein stability thus controlling phenylpropanoid biosynthesis .
AT2G44150	*HISTONE-LYSINE N-METHYLTRANSFERASE ASHH3 (ASHH3)*	Encodes a protein-lysine N-methyltransferase. Located in ER.
AT2G44160	*METHYLENETETRAHYDROFOLATE REDUCTASE 2 (MTHFR2)*	methylenetetrahydrofolate reductase MTHFR2 mRNA, complete The mRNA is cell-to-cell mobile.
AT2G44170	*N-MYRISTOYLTRANSFERASE 2 (NMT2)*	pseudogene of myristoyl-CoA:protein N-myristoyltransferase
AT2G44180	*METHIONINE AMINOPEPTIDASE 2A (MAP2A)*	Encodes a MAP2 like methionine aminopeptidase. In MAP1A mutant background plants show an increased sensitivity to fumagillin resulting in defects in development. Phenotype is similar to RNAi lines which knock out all MAP2/MAP1 loci.
AT2G44195		pre-mRNA splicing factor domain-containing protein
AT2G44200		pre-mRNA splicing factor domain-containing protein
AT2G44210		carboxyl-terminal peptidase (DUF239)
AT2G44230		hypothetical protein (DUF946)
AT2G44240		NEP-interacting protein (DUF239)
AT2G44250		tRNA-splicing ligase, putative (DUF239)
AT2G44260		DUF946 family protein (DUF946)
AT2G44270	*REPRESSOR OF LRX1 (ROL5)*	Encodes ROL5, a repressor of lrx1 mutants that develop aberrant root hairs. ROL5 is a homolog of yeast Ncs6p that affects TOR signaling. The target of rapamycin (TOR) pathway is a major regulator of cell growth in eukaryotes, and inhibition of this pathway by rapamycin reduces cell growth. ROL5 might function as a mitochondrial component of the TOR pathway that influences the plant's response to ROS (reactive oxygen species).
AT2G44300	*GLYCOSYLPHOSPHATIDYLINOSITOL-ANCHORED LIPID PROTEIN TRANSFER 14 (LTPG14)*	Bifunctional inhibitor/lipid-transfer protein/seed storage 2S albumin superfamily protein
AT2G44350	*(ATCS)*	encodes a mitochrondrion targeted citrate synthase, the first enzyme of the tricarboxylic acid cycle, catalyzing the condensation of acetyl-CoA and oxaloacetate, finally yielding citrate and CoA.
AT2G44360		ecotropic viral integration site protein
AT2G44370		Cysteine/Histidine-rich C1 domain family protein

AT2G44380		Cysteine/Histidine-rich C1 domain family protein
AT2G44430		DNA-binding bromodomain-containing protein
AT2G44450	*BETA GLUCOSIDASE 15 (BGLU15)*	beta glucosidase 15
AT2G44460	*BETA GLUCOSIDASE 28 (BGLU28)*	Beta-glucosidase, major myrosinase which initiates sulfur reallocation by hydrolyzing particular GL species, conferring sulfur deficiency tolerance, especially during early development.
AT2G44470	*BETA GLUCOSIDASE 29 (BGLU29)*	beta glucosidase 29
AT2G44480	*BETA GLUCOSIDASE 17 (BGLU17)*	beta glucosidase 17
AT2G44490	*PENETRATION 2 (PEN2)*	Encodes a glycosyl hydrolase that localizes to peroxisomes and acts as a component of an inducible preinvasion resistance mechanism. Required for mlo resistance. The mRNA is cell-to-cell mobile.
AT2G44510		CDK inhibitor P21 binding protein
AT2G44520	*CYTOCHROME C OXIDASE 10 (COX10)*	cytochrome c oxidase 10
AT2G44540	*GLYCOSYL HYDROLASE 9B9 (GH9B9)*	glycosyl hydrolase 9B9
AT2G44560	*GLYCOSYL HYDROLASE 9B11 (GH9B11)*	glycosyl hydrolase 9B11
AT2G44570	*GLYCOSYL HYDROLASE 9B12 (GH9B12)*	glycosyl hydrolase 9B12
AT2G44580		zinc ion binding protein
AT2G44590	*DYNAMIN-LIKE 1D (DL1D)*	DYNAMIN-like 1D
AT2G44650	*CHLOROPLAST CHAPERONIN 10 (CHL-CPN10)*	Encodes a chloroplast-localized chaperonin 10 whose mRNA is expressed in leaves and stems but not roots.
AT2G44660		ALG6, ALG8 glycosyltransferase family
AT2G44690	*ARABIDOPSIS RAC-LIKE 9 (ARAC9)*	A member of ROP GTPase gene family; promotes autophagy.
AT2G44710		RNA-binding (RRM/RBD/RNP motifs) family protein
AT2G44720		
AT2G44730		Alcohol dehydrogenase transcription factor Myb/SANT-like family protein
AT2G44740	*CYCLIN P4;1 (CYCP4;1)*	cyclin p4
AT2G44745	*WRKY DNA-BINDING PROTEIN 12 (WRKY12)*	WRKY gene family member involved in vascular/pith development.
AT2G44750	*THIAMIN PYROPHOSPHOKINASE 2 (TPK2)*	Encodes a thiamine pyrophosphokinase capable of producing thiamine pyrophosphate from free thiamine.
AT2G44760		dihydroorotate dehydrogenase (DUF3598)
AT2G44770	*(ELMOD_B)*	Acts upstream of aperture proteins, their expression levels influence the number of aperture domains that form on the surface of developing pollen grains.
AT2G44790	*UCLACYANIN 2 (UCC2)*	Encodes a uclacyanin, a protein precursor that is closely related to precursors of stellacyanins and a blue copper protein from pea pods.
AT2G44800		2-oxoglutarate (2OG) and Fe(II)-dependent oxygenase superfamily protein
AT2G44810	*DEFECTIVE ANTHER DEHISCENCE 1 (DAD1)*	Mutant has defects in anther dehiscence, pollen maturation, and flower opening. The DAD1 protein is a chloroplastic phospholipase A1 that catalyzes the initial step of jasmonic acid biosynthesis.
AT2G44820		axoneme-associated protein MST101(2) protein
AT2G44830	*PROTEIN KINASE ASSOCIATED WITH BRX (PAX)*	AGCVIII kinase involved in the pulse-induced first positive phototropism. Plasma-membrane-associated element of a molecular rheostat that modulates auxin flux through developing protophloem sieve elements (PPSEs) while interacting with BRX, thereby timing PPSE differentiation. Activates PIN-mediated auxin efflux.
AT2G44840	*ETHYLENE-RESPONSIVE ELEMENT BINDING FACTOR 13 (ERF13)*	encodes a member of the ERF (ethylene response factor) subfamily B-3 of ERF/AP2 transcription factor family. The protein contains one AP2 domain. There are 18 members in this subfamily including ATERF-1, ATERF-2, AND ATERF-5. The mRNA is cell-to-cell mobile.
AT2G44850		hypothetical protein
AT2G44860	*RIBOSOMAL PROTEIN L24C (RPL24C)*	cytosolic ribosomal protein gene, part of eL24 family
AT2G44880	*ABA HYPERSENSITIVE GERMINATION 11 (AHG11)*	Pentatricopeptide repeat (PPR-like) superfamily protein
AT2G44910	*HOMEOBOX-LEUCINE ZIPPER PROTEIN 4 (HB4)*	Encodes a homeodomain protein whose expression displays a dependence on phyB for both red and far-red light response. Also involved in the shade avoidance syndrome.
AT2G44920		Encodes a pentapeptide-repeat protein (PRP) composed of 25 repeats capped by N- and C-terminal a-helices. Unlike other PRPs, At2g44920 consists exclusively of type II b-turns
AT2G44930		transmembrane protein, putative (DUF247)
AT2G44940	*ETHYLENE-RESPONSIVE TRANSCRIPTION FACTOR 34 (ERF34)*	encodes a member of the DREB subfamily A-4 of ERF/AP2 transcription factor family. The protein contains one AP2 domain. There are 17 members in this subfamily including TINY.
AT2G44960		
AT2G44980	*ALTERED SEED GERMINATION 3 (ASG3)*	SNF2 domain-containing protein / helicase domain-containing protein
AT2G44990	*CAROTENOID CLEAVAGE DIOXYGENASE 7 (CCD7)*	More Axillary Branching; carotenoid cleavage dioxygenases.
AT2G45000	*EMBRYO DEFECTIVE 2766 (EMB2766)*	Encodes a nucleoporin, a component of the nuclear pore complex, that appears to be a major negative regulator of auxin signalling. Loss of function mutants are embryo lethal.
AT2G45010		PLAC8 family protein
AT2G45030		Translation elongation factor EFG/EF2 protein
AT2G45040		Matrixin family protein
AT2G45050	*GATA TRANSCRIPTION FACTOR 2 (GATA2)*	Encodes a member of the GATA factor family of zinc finger transcription factors. A positive regulator of photomorphogenesis.
AT2G45070	*SUPPRESSORS OF SECRETION-DEFECTIVE 61 BETA (SEC61 BETA)*	Sec61 Beta Subunit
AT2G45080	*CYCLIN P3;1 (cycp3;1)*	cyclin p3
AT2G45110	*EXPANSIN B4 (EXPB4)*	member of BETA-EXPANSINS. Naming convention from the Expansin Working Group (Kende et al, 2004. Plant Mol Bio)

AT2G45120	*(ZAT4)*	C2H2-like zinc finger protein
AT2G45130	*SPX DOMAIN GENE 3 (SPX3)*	Expression is upregulated in the shoot of cax1/cax3 mutant.
AT2G45170	*AUTOPHAGY 8E (ATG8E)*	Involved in autophagy. Under nutrient starvation the protein localizes to autophagosomes. Involved in submergence (hypoxia) tolerance; ethanol induces autophagy.
AT2G45180	*DISEASE RELATED NONSPECIﬁC LIPID TRANSFER PROTEIN 1 (DRN1)*	nsLTP family-related gene. Expression is strongly suppressed by bacterial pathogens. Mutants are more susceptible to pathogens and abiotic stressors suggesting a function in basal stress response.
AT2G45190	*ABNORMAL FLORAL ORGANS (AFO)*	Encodes a member of the YABBY family of transcriptional regulators that is involved in abaxial cell type specification in leaves and fruits. YAB1 acts in a non-cell autonomous fashion within the meristem to affect phyllotactic patterning. The non-autonomous effect on the central region of the meristem is mediated through the activity if Lateral Suppressor (LAS).
AT2G45210	*SMALL AUXIN UPREGULATED 36 (SAUR36)*	SAUR-like auxin-responsive protein family
AT2G45220	*PECTIN METHYLESTERASE 17 (PME17)*	Pectin methylesterase involved in pectin remodelling. Regulated by its PRO region that triggers PME activity in the resistance to Botrytis cinerea.
AT2G45230		transposable_element_gene;non-LTR retrotransposon family (LINE), has a 3.9e-46 P-value blast match to GB:AAB41224 ORF2 (LINE-element) (Rattus norvegicus);(source:TAIR10)
AT2G45250	*(LIN52A)*	Member of the DREAM complex, which represses growth in response to DNA damage, LIN52 type.
AT2G45280	*RAS ASSOCIATED WITH DIABETES PROTEIN 51C (RAD51C)*	Encodes a protein similar to RAD51C involved in double stranded break repair via homologous recombination. Sensitive to DSB induced by Mitomycin C and gamma irradiation, interacts with Atxrcc3 in yeast two-hybrid assay. Required for female meiosis but not critical for mitosis under normal conditions.
AT2G45290	*TRANSKETOLASE 2 (TKL2)*	Transketolase
AT2G45310	*UDP-D-GLUCURONATE 4-EPIMERASE 4 (GAE4)*	UDP-D-glucuronate 4-epimerase
AT2G45320		polyphosphatidylinositol phosphatase
AT2G45340		Leucine-rich repeat protein kinase family protein
AT2G45350	*CHLORORESPIRATORY REDUCTION 4 (CRR4)*	Encodes a member of a PCMP (plant combinatorial and modular protein) family (PCMP-E subfamily) with 11 pentatricopeptide (PPR) repeats. The protein is involved in RNA editing of the initiation codon of ndhD in the chloroplast.
AT2G45380		myeloid leukemia factor
AT2G45400	*BRI1-5 ENHANCED 1 (BEN1)*	involved in the regulation of brassinosteroid metabolic pathway
AT2G45420	*LOB DOMAIN-CONTAINING PROTEIN 18 (LBD18)*	LOB domain-containing protein 18
AT2G45430	*AT-HOOK MOTIF NUCLEAR-LOCALIZED PROTEIN 22 (AHL22)*	Encodes a nuclear localized AT hook domain containing protein that can bind AT rich DNA in vitro. Overexpression of the gene results in delayed flowering. Is likely to act redundantly with AHL18, AHL27 and AHL29 in the regulation of flowering. It is also involved in both photo- and skotomorphogenesis.
AT2G45450	*LITTLE ZIPPER 1 (ZPR1)*	ZPR1, a small leucine zipper-containing protein that interacts with REV HD-ZIPIII and is involved in the establishment of leaf polarity.
AT2G45470	*FASCICLIN-LIKE ARABINOGALACTAN PROTEIN 8 (FLA8)*	Fasciclin-like arabinogalactan protein. Possibly involved in embryogenesis and seed development.
AT2G45490	*ATAURORA3 (AUR3)*	Encodes a member of a family of Ser/Thr kinases whose activities peak during cell division. Transcripts are abundant in tissues rich in dividing cells like roots and flowers but are low or absent in fully expanded leaves and stems. In interphase cells, the protein is predominantly nuclear. During mitosis, the protein associates with plant-specific cytoskeletal structures (preprophase band, phragmoplast, nascent cell plate) that are necessary for cytokinesis as well as with the microtubule spindle. The protein is concentrated in nuclear dots arranged around the nucleolus and the nuclear periphery in early prophase cells.
AT2G45500		AAA-type ATPase family protein
AT2G45510	*CYTOCHROME P450, FAMILY 704, SUBFAMILY A, POLYPEPTIDE 2 (CYP704A2)*	member of CYP704A
AT2G45530		RING/U-box superfamily protein
AT2G45550	*CYTOCHROME P450, FAMILY 76, SUBFAMILY C, POLYPEPTIDE 4 (CYP76C4)*	Member of CYP76C family of cytochrome P450 enzymes.Has geraniol 9- or 8-hydroxylase activity.
AT2G45560	*CYTOCHROME P450, FAMILY 76, SUBFAMILY C, POLYPEPTIDE 1 (CYP76C1)*	cytochrome P450 monooxygenase
AT2G45570	*CYTOCHROME P450, FAMILY 76, SUBFAMILY C, POLYPEPTIDE 2 (CYP76C2)*	member of CYP76C
AT2G45590		Protein kinase superfamily protein
AT2G45600		alpha/beta-Hydrolases superfamily protein
AT2G45610		alpha/beta-Hydrolases superfamily protein
AT2G45620	*UTP:RNA URIDYLYLTRANSFERASE 1 (URT1)*	Nucleotidyltransferase family protein involved in transcript polyadenylation. TUTase which connects decapping activators and prevents the accumulation of excessively deadenylated mRNAs to avoid siRNA biogenesis.
AT2G45630	*HYDROXYPHENYLPYRUVATE REDUCTASE 4 (HPPR4)*	Hydroxyphenylpyruvate reductase (HPPR) family member with low activity.
AT2G45650	*AGAMOUS-LIKE 6 (AGL6)*	Sequence suggests this encodes a MADS-box transcription factor. Negatively regulates the FLC/MAF clade genes and positively regulates FT in Arabidopsis.
AT2G45660	*AGAMOUS-LIKE 20 (AGL20)*	Controls flowering and is required for CO to promote flowering. It acts downstream of FT. Overexpression of (SOC1) AGL20 suppresses not only the late flowering of plants that have functional FRI and FLC alleles but also the delayed phase transitions during the vegetative stages of development. AGL20/SOC1 acts with AGL24 to promote flowering and inflorescence meristem identity.AGL20 upregulates expression of AGL24 in response to GA.
AT2G45670	*LYSOPHOSPHATIDYLETHANOLAMINE ACYLTRANSFERASE2 (LPEAT2)*	Encodes an acyl-CoA: lysophosphatidylethanolamine acyltransferase with 20:0-CoA being the best acyl donor. Mutations adversely affect the growth of plants and result in decreased lipid content in roots and seeds.
AT2G45680	*TCP DOMAIN PROTEIN 9 (TCP9)*	TCP family transcription factor

AT2G45690	*SHRUNKEN SEED 1 (SSE1)*	Encodes a protein with similarity to yeast Pep16p, a membrane localized protein involved in peroxisome assembly and protein-trafficking. SSE1 mutant seeds do not accumulate oils and dessicated seeds have a shrunken appearance. Involved in protein and oil body biogenesis. SSE is expressed during seed development, reaching the highest peak in mature siliques. Expression in leaves and roots is low compared to cotyledons and flowers. Located in peroxisomes and endoplasmic reticulum. Homologous to the peroxin PEX16 and complements the pex16 mutants of the yeast Yarrowia lipolytica.
AT2G45720		ARM repeat superfamily protein
AT2G45730	*TRNA METHYLTRANSFERASE 6 (TRM6)*	Part of complex with TRM61, plays a critical role in maintaining the stability of initiator methionyl-tRNA, embryo and endosperm development. In the complex TRM61 functions as the catalytic subunit, and TRM6 performs the role of the binding subunit.
AT2G45740	*PEROXIN 11D (PEX11D)*	member of the peroxin11 (PEX11) gene family, integral to peroxisome membrane, controls peroxisome proliferation. The mRNA is cell-to-cell mobile.
AT2G45750		S-adenosyl-L-methionine-dependent methyltransferases superfamily protein
AT2G45760	*BON ASSOCIATION PROTEIN 2 (BAP2)*	encodes a protein that is similar to BONZAI1-binding protein BAP1.
AT2G45770	*(CPFTSY)*	chloroplast SRP receptor homolog, alpha subunit CPFTSY. Required for LHCP integration into isolated thylakoids.
AT2G45780		other_RNA
AT2G45790	*PHOSPHOMANNOMUTASE (PMM)*	Encodes a cytoplasmic phosphomannomutase, involved in ascorbate biosynthesis
AT2G45800	*PLIM2A (PLIM2a)*	Encodes a member of the Arabidopsis LIM proteins: a family of actin bundlers with distinct expression patterns. WLIM1, WLIM2a, and WLIM2b are widely expressed, whereas PLIM2a, PLIM2b, and PLIM2c are predominantly expressed in pollen. Regulates actin cytoskeleton organization.
AT2G45820	*REMORIN 1.3 (Rem1.3)*	Lipid raft regulatory protein, crucial for plasma membrane nanodomain assembly to control plasmodesmata aperture and functionality.
AT2G45830	*DOWNSTREAM TARGET OF AGL15 2 (DTA2)*	downstream target of AGL15 2
AT2G45840		O-glucosyltransferase rumi-like protein (DUF821)
AT2G45850	*AT-HOOK MOTIF NUCLEAR LOCALIZED PROTEIN 9 (AHL9)*	AT hook motif DNA-binding family protein
AT2G45860		hypothetical protein
AT2G45870	*VCCN2 (VCCN2)*	Encodes a bestrophin-like protein (Best2). Minor isoform (10% transcript of AtBest1). Putative chloride ion channel. Proposed to modulate proton motive force partitioning by mediating chloride ion influx in the thylakoid lumen.
AT2G45880	*BETA-AMYLASE 7 (BAM7)*	Encodes a beta-amylase-like protein present in the nucleus rather than targeted to the chloroplast. Contains BRASSINAZOLE RESISTANT1 (BZR1)-type DNA binding domains. Activates gene expression in protoplast transactivation assays.
AT2G45890	*ROP (RHO OF PLANTS) GUANINE NUCLEOTIDE EXCHANGE FACTOR 4 (ROPGEF4)*	Encodes a member of KPP-like gene family, homolog of KPP (kinase partner protein) gene in tomato. Also a member of the RopGEF (guanine nucleotide exchange factor) family, containing the novel PRONE domain (plant-specific Rop nucleotide exchanger), which is exclusively active towards members of the Rop subfamily. Mutants exhibit longer root hairs under phosphate-deficient conditions. Involved in cell wall patterning. Encodes ROP activator, regulates the formation of ROP-activated domains; these in turn determine the pattern of cell wall pits. Forms a dimer that interacts with activated ROP11 in vivo, which could provide positive feedback for ROP activation. Required for periodic formation of secondary cell wall pits
AT2G45900	*TON1 RECRUITING MOTIF 13 (TRM13)*	Phosphatidylinositol N-acetylglucosaminlytransferase subunit P-like protein
AT2G45920		U-box domain-containing protein
AT2G45930		hypothetical protein
AT2G45940	*DUF295 ORGANELLAR A 6 (ATDOA6)*	hypothetical protein (DUF295)
AT2G45950	*SKP1-LIKE 20 (SK20)*	SKP1-like 20
AT2G45960	*PLASMA MEMBRANE INTRINSIC PROTEIN 1B (PIP1B)*	a member of the plasma membrane intrinsic protein subfamily PIP1. localizes to the plasma membrane and exhibits water transport activity in Xenopus oocyte. expressed ubiquitously and protein level decreases slightly during leaf development. Involved redundantly with PIP1;1/3/4/5 in hydraulics and carbon fixation, regulates the expression of related genes that affect plant growth and development.
AT2G45970	*CYTOCHROME P450, FAMILY 86, SUBFAMILY A, POLYPEPTIDE 8 (CYP86A8)*	Encodes a member of the CYP86A subfamily of cytochrome p450 genes. Expressed at moderate levels in flowers, leaves, roots and stems.Mutant seeds have reduced seed longevity, higher tetrazolium salt uptake and reduction, and reduced lipid polyester barriers (PMID:32519347).
AT2G45980	*ATG8-INTERACTING PROTEIN 1 (ATI1)*	Encodes an Atg8-interacting protein that is partially associated with the ER during favorable growth conditions and becomes mainly associated with a spherical compartment that dynamically moves along the ER network. In stress induced plants, ATI1 is localized to a novel plastid associated bodies that are transported to vesicles, in what appears to be an autophagy dependent process. ATI1 interacts with number of other plastid proteins such as NPQ4 and APE1.
AT2G46010		
AT2G46030	*UBIQUITIN-CONJUGATING ENZYME 6 (UBC6)*	Ubiquitin conjugating enzyme E2
AT2G46040	*AT-RICH INTERACTING DOMAIN 1 (ARID1)*	Encodes a transcriptional activator that is involved in pollen development. ARID1 is expressed in nuclear bodies of microspore, vegetative and generative cells, and binds to and activates DUO during microgametogenesis.
AT2G46050	*MITOCHONDRIAL EDITING FACTOR 31 (MEF31)*	E-PPR protein involved in mitochondrial RNA editing.It is involved in editing of the mitochondrial tatC transcript at site 581.
AT2G46070	*MITOGEN-ACTIVATED PROTEIN KINASE 12 (MPK12)*	Encodes a MAP kinase protein. MPK12 interacts with the IBR5 protein phosphatase in vitro and in vivo, and it can be dephosphorylated and inactivated by IBR5. MPK12 appears to be a negative regulator of auxin signlaing. MPK12 RNAi lines are hypersensitive to auxin in root elongation and transcriptional response assays, but they appear to have normal sensitivity to ABA. MPK12 is a nuclear protein and its kinase activity is increased following auxin treatment. MPK12 transcripts are widely expressed in seedlings, but MPK12 expression is stronger in guard cells than in other cell types in mature plants.
AT2G46100		Nuclear transport factor 2 (NTF2) family protein
AT2G46110	*KETOPANTOATE HYDROXYMETHYLTRANSFERASE 1 (KPHMT1)*	Encodes a ketopentoate hydroxymethyltransferase that appears to localize to the mitochondria. This protein is expected to play a role in pantothenate (vitamin B5) biosynthesis.
AT2G46130	*WRKY DNA-BINDING PROTEIN 43 (WRKY43)*	member of WRKY Transcription Factor; Group II-c

AT2G46140	*LATE EMBRYOGENESIS ABUNDANT 27 (LEA27)*	Late embryogenesis abundant protein
AT2G46150		Late embryogenesis abundant (LEA) hydroxyproline-rich glycoprotein family
AT2G46180	*GOLGIN CANDIDATE 4 (GC4)*	This gene is predicted to encode a protein that functions as a Golgi apparatus structural component known as a golgin in mammals and yeast. A fluorescently-tagged version of GC4 co-localizes with Golgi markers, and this localization appears to be replicated using the C-terminal (169 aa) portion of the protein.
AT2G46190		Mitochondrial glycoprotein family protein
AT2G46200		U11/U12 small nuclear ribonucleoprotein
AT2G46210	*SPHINGOID LCB DESATURASE 2 (SLD2)*	Fatty acid/sphingolipid desaturase
AT2G46220		DUF2358 family protein (DUF2358)
AT2G46240	*BCL-2-ASSOCIATED ATHANOGENE 6 (BAG6)*	A member of Arabidopsis BAG (Bcl-2-associated athanogene) proteins, plant homologs of mammalian regulators of apoptosis. Expression of BAG6 in leaves was strongly induced by heat stress. Knockout mutants exhibited enhanced susceptibility to fungal pathogen Botrytis cinerea. Plant BAG proteins are multi-functional and remarkably similar to their animal counterparts, as they regulate apoptotic-like processes ranging from pathogen attack, to abiotic stress, to plant development. The mRNA is cell-to-cell mobile.
AT2G46250		myosin heavy chain-like protein
AT2G46260	*LIGHT-RESPONSE BTB 1 (LRB1)*	Involvement in protein ubiquitylation is predicted based on physical interaction with CULLIN 3 proteins. LRBs physically interact with photoexcited and phosphorylated CRY2, at the CCE domain of CRY2, to facilitate polyubiquitination and degradation of CRY2 in response to blue light.
AT2G46270	*G-BOX BINDING FACTOR 3 (GBF3)*	encodes a bZIP G-box binding protein whose expression is induced by ABA. It has been shown to bind to Adh that contains the G-box and is induced by cold and water deprivation. GBF3 has been shown to be expressed mostly in the root and dark-grown leaves. GBF3 can act as homodimers and as heterodimers with GFB1, GBF2 and GBF4. In addition, GBF3!?s DNA binding activity is enhanced by GIP1, GPRI1 and GPRI2.
AT2G46280	*TGF-BETA RECEPTOR INTERACTING PROTEIN 1 (TRIP-1)*	Encodes a homolog of mammalian TGF-beta receptor interacting protein. Co-immunoprecipitates with BRI1 and can be phosphorylated in vitro by BRI1 at specific sites (Thr-14, Thr-89, and either Thr-197 or Ser-198). May therefore be a cytoplasmic BRI1 substrate and involved in brassinosteroid regulated plant growth and development.The encoded protein has two DWD motifs. It can bind to DDB1a in Y2H assays, and DDB1b in co-IP assays, and may be involved in the formation of a CUL4-based E3 ubiquitin ligase
AT2G46300		Late embryogenesis abundant (LEA) hydroxyproline-rich glycoprotein family
AT2G46310	*CYTOKININ RESPONSE FACTOR 5 (CRF5)*	CRF5 encodes one of the six cytokinin response factors. It is transcriptionally upregulated in response to cytokinin. CRF5 belongs to the AP2/ERF superfamily of the transcriptional factors. CRF proteins rapidly relocalize to the nucleus in response to cytokinin. Analysis of loos-of-function mutants revealed that the CRFs function redundantly to regulate the development of embryos, cotyledons and leaves.
AT2G46340	*SUPPRESSOR OF PHYA-105 1 (SPA1)*	Encodes a member of the SPA (suppressor of phyA-105) protein family (SPA1-SPA4). SPA proteins contain an N-terminal serine/threonine kinase-like motif followed by a coiled-coil structure and a C-terminal WD-repeat domain. SPA1 is a PHYA signaling intermediate, putative regulator of PHYA signaling pathway. Light responsive repressor of photomorphogenesis. Involved in regulating circadian rhythms and flowering time in plants. Under constant light, the abundance of SPA1 protein exhibited circadian regulation, whereas under constant darkness, SPA1 protein levels remained unchanged. In addition, the spa1-3 mutation slightly shortened circadian period of CCA1, TOC1/PRR1 and SPA1 transcript accumulation under constant light.
AT2G46360		hypothetical protein
AT2G46370	*JASMONATE RESISTANT 1 (JAR1)*	Encodes a jasmonate-amido synthetase that is a member of the GH3 family of proteins. JAR1 catalyzes the formation of a biologically active jasmonyl-isoleucine (JA-Ile) conjugate. JA-Ile promotes the interaction between JAZ1 and COI1 in the jasmonate signaling pathway. JAR1 localizes to the cytoplasm and is also a phytochrome A signaling component. JAR1 is an auxin-induced gene. Loss of function mutants are defective in a variety of responses to jasmonic acid. JAR1 has additional enzymatic activities in vitro, (e.g. the ability to synthesize adenosine 5'-tetraphosphate and other JA conjugates), but there are no data to show whether JAR1 catalyzes many of these reactions in vivo. JAR1 is involved in pathogen defense, sensitivity to ozone, and wound responses.
AT2G46375		hypothetical protein
AT2G46390	*SUCCINATE DEHYDROGENASE 8 (SDH8)*	predicted to encode subunit 8 of mitochondrial complex II and to participate in the respiratory chain
AT2G46400	*WRKY DNA-BINDING PROTEIN 46 (WRKY46)*	Encodes a WRKY transcription factor that contributes to the feedforward inhibition of osmotic/salt stress-dependent LR inhibition via regulation of ABA signaling and auxin homeostasis.
AT2G46410	*CAPRICE (CPC)*	Nuclear-localized R3-type MYB transcription factor. Positive regulator of hair-cell differentiation. Preferentially transcribed in hairless cells. Moves from atrichoblasts into trichoblast via plasmodesmata in a tissue-specific mode. N-terminus and part of the Myb domain are required for this movement, with W76 playing a crucial role. Capability to increase the size-exclusion limit of plasmodesmata. Regulated by WEREWOLF.
AT2G46420		helicase with zinc finger protein
AT2G46440	*CYCLIC NUCLEOTIDE-GATED CHANNEL 11 (CNGC11)*	Member of Cyclic nucleotide gated channel family. Positive regulator of resistance against avirulent fungal pathogen. The mRNA is cell-to-cell mobile.
AT2G46450	*CYCLIC NUCLEOTIDE-GATED CHANNEL 12 (CNGC12)*	Member of Cyclic nucleotide gated channel family.Positive regulator of resistance against avirulent fungal pathogen.Suppresses the phenotype conferred by cpr22 in a dosage-dependent manner.
AT2G46480	*GALACTURONOSYLTRANSFERASE 2 (GAUT2)*	Encodes a protein with putative galacturonosyltransferase activity.
AT2G46505	*SUCCINATE DEHYDROGENASE SUBUNIT 4 (SDH4)*	Encodes succinate dehydrogenase ,a component of mitochondrial respiratory complex II. Nuclear encoded gene which is imported into the mitochondrion.
AT2G46520	*(XPO2)*	cellular apoptosis susceptibility protein, putative / importin-alpha re-exporter
AT2G46530	*AUXIN RESPONSE FACTOR 11 (ARF11)*	auxin response factor 11
AT2G46550		transmembrane protein
AT2G46570	*LACCASE 6 (LAC6)*	putative laccase, a member of laccase family of genes (with 17 members in Arabidopsis).

AT2G46590	*DOF AFFECTING GERMINATION 2 (DAG2)*	Encodes a protein containing Dof zinc finger motifs that is a positive regulator of light-mediated seed germination. Its expression is limited to vascular system of the mother plant. A recessive mutation is inherited as maternal-effect and expression is not detected in the embryo. Mutants are defective in seed germination and are more dependent on light and cold treatment and less sensitive to gibberellin during seed germination. It plays its main role downstream of PIL5 and DAG1 in the phytochrome B (phyB)-mediated pathway.
AT2G46600		Calcium-binding EF-hand family protein
AT2G46610	*ARGININE/SERINE-RICH SPLICING FACTOR 31A (RS31a)*	Barta et al (2010) have proposed a nomenclature for Serine/Arginine-Rich Protein Splicing Factors (SR proteins): Plant Cell. 2010, 22:2926.
AT2G46620		P-loop containing nucleoside triphosphate hydrolases superfamily protein
AT2G46630		serine/arginine repetitive matrix protein
AT2G46640	*TILLER ANGLE CONTROL 1 (TAC1)*	Encodes TAC1 (Tiller Angle Control 1). Influences axillary branch growth angle. Inflorescence stems of TAC1 mutants are vertically oriented and have axillary shoots with narrow branch angles.
AT2G46650	*CYTOCHROME B5 ISOFORM C (CB5-C)*	member of Cytochromes b5 The mRNA is cell-to-cell mobile.
AT2G46660	*CYTOCHROME P450, FAMILY 78, SUBFAMILY A, POLYPEPTIDE 6 (CYP78A6)*	Encodes a member of CYP78A cytochrome P450 monooxygenase protein family that is required in the sporophytic tissue of the mother plant to promote seed growth.
AT2G46680	*HOMEOBOX 7 (HB-7)*	encodes a putative transcription factor that contains a homeodomain closely linked to a leucine zipper motif. Transcript is detected in all tissues examined. Is transcriptionally regulated in an ABA-dependent manner and may act in a signal transduction pathway which mediates a drought response.
AT2G46690	*SMALL AUXIN UPREGULATED RNA 32 (SAUR32)*	Regulates ABA-mediated responses to drought stress.
AT2G46710	*ROP GUANOSINE TRIPHOSPHATASE (GTPASE)-ACTIVATING PROTEIN 3 (ROPGAP3)*	ROP (Rho of plant GTPases) family member Involved in cell wall patterning. Encodes ROP inactivator, regulates the formation of ROP-activated domains; these in turn determined the pattern of cell wall pits. Positively regulates pit formation, but negatively regulates pit size, required for periodic formation of secondary cell wall pits.
AT2G46720	*3-KETOACYL-COA SYNTHASE 13 (KCS13)*	Encodes KCS13, a member of the 3-ketoacyl-CoA synthase family involved in the biosynthesis of VLCFA (very long chain fatty acids).
AT2G46740	*L -GULONO-1,4-LACTONE (L -GULL) OXIDASE 5 (GULLO5)*	Encodes a homolog of rat L-gulono-1,4-lactone (L-GulL) oxidase that is involved in the biosynthesis of L-ascorbic acid.
AT2G46750	*L -GULONO-1,4-LACTONE (L -GULL) OXIDASE 2 (GULLO2)*	Encodes a homolog of rat L-gulono-1,4-lactone (L-GulL) oxidase that is involved in the biosynthesis of L-ascorbic acid.
AT2G46760	*L -GULONO-1,4-LACTONE (L -GULL) OXIDASE 6 (GULLO6)*	D-arabinono-1,4-lactone oxidase family protein
AT2G46770	*NAC SECONDARY WALL THICKENING PROMOTING FACTOR1 (NST1)*	NAC transcription factor NST1. NST1 and NST2 are redundant in regulating secondary wall thickening in anther walls and siliques. An NST1 promoter fusion was detected in various tissues in which lignified secondary walls develop. Both MYC2 and MYC4 bind to the NST1 promoter and appear to regulate its expression in response to blue light.
AT2G46780		RNA-binding (RRM/RBD/RNP motifs) family protein
AT2G46790	*PSEUDO-RESPONSE REGULATOR 9 (PRR9)*	Pseudo-response regulator PRR9. Involved in clock function. PRR7 and PRR9 are partially redundant essential components of a temperature-sensitive circadian system. CCA1 and LHY had a positive effect on PRR9. Interact with TOC1 in a yeast two-hybrid assay. Acts as transcriptional repressor of CCA1 and LHY. Acts additively with EC, PRR5 and PRR7 to regulate hypocotyl growth under photoperiodic conditions.
AT2G46820	*PHOTOSYSTEM I P SUBUNIT (PSI-P)*	Encodes the P subunit of Photosystem I. About 25% of the TMP14 pool appeared to be phosphorylated, and this ratio is not affected by light. Contains seven phosphorylation sites on threonine residue and chloroplast targeting signal. Located in the proximity of PSI-L, -H and -O subunits. Forms oligomers with other members of CURT1 family to modulate grana structure.
AT2G46830	*CIRCADIAN CLOCK ASSOCIATED 1 (CCA1)*	Encodes a transcriptional repressor that performs overlapping functions with LHY in a regulatory feedback loop that is closely associated with the circadian oscillator of Arabidopsis. Binds to the evening element in the promoter of TOC1 and represses TOC1 transcription. CCA1 and LHY colocalize in the nucleus and form heterodimers in vivo. CCA1 and LHY function synergistically in regulating circadian rhythms of Arabidopsis. CCA1 binds the GI promoter.
AT2G46840	*DOMAIN OF UNKNOWN FUNCTION 724 4 (DUF4)*	Member of the plant-specific DUF724 protein family. Arabidopsis has 10 DUF724 proteins. Loss of function mutant has a WT phenotype. Overexpression increases plant organ size, possibly by influencing the expression of the cell wall formation and auxin transporter genes that regulate cell size.
AT2G46850		Protein kinase superfamily protein
AT2G46860	*PYROPHOSPHORYLASE 3 (PPa3)*	Encodes a protein that might have inorganic pyrophosphatase activity.
AT2G46880	*PURPLE ACID PHOSPHATASE 14 (PAP14)*	purple acid phosphatase 14
AT2G46900		transcription factor-like protein
AT2G46910		Plastid-lipid associated protein PAP / fibrillin family protein
AT2G46915		DUF3754 family protein, putative (DUF3754)
AT2G46940		fold protein
AT2G46950	*CYTOCHROME P450, FAMILY 709, SUBFAMILY B, POLYPEPTIDE 2 (CYP709B2)*	cytochrome P450, family 709, subfamily B, polypeptide 2
AT2G46960	*CYTOCHROME P450, FAMILY 709, SUBFAMILY B, POLYPEPTIDE 1 (CYP709B1)*	member of CYP709B
AT2G46980	*ASYNAPTIC 3 (ASY3)*	Encodes ASY3, a coiled-coil domain protein that is required for normal meiosis.
AT2G46990	*INDOLE-3-ACETIC ACID INDUCIBLE 20 (IAA20)*	Encodes a member of the Aux/IAA family of proteins implicated in auxin signaling. IAA20 lacks the conserved degron (domain II) found in many family members, and IAA20 fusion proteins are stable in Arabidopsis seedlings. IAA20 transcripts are induced by auxin treatment, and overexpression of IAA20 leads to defects in gravitropism, root development, root meristem maintenance, etiolation, and cotyledon vascular development.
AT2G47000	*ATP-BINDING CASSETTE B4 (ABCB4)*	Encodes an auxin efflux transmembrane transporter that is a member of the multidrug resistance P-glycoprotein (MDR/PGP) subfamily of ABC transporters. Functions in the basipetal redirection of auxin from the root tip. Exhibits apolar plasma membrane localization in the root cap and polar localization in tissues above and is involved in root hair elongation.
AT2G47010		calcium/calcium/calmodulin-dependent Serine/Threonine-kinase
AT2G47020		Peptide chain release factor 1

AT2G47040	*VANGUARD1 (VGD1)*	Share high homologies with a group of pectin methylesterases (PME), pollen specific, and is required for enhancing the growth of pollen tube in style and transmitting tract tissues.
AT2G47050	*(PMEIL)*	Encodes a pollen-expressed pectin methylesterase inhibitor that affects male fertility by regulating pollen viability and pollen tube growth.
AT2G47070	*SQUAMOSA PROMOTER BINDING PROTEIN-LIKE 1 (SPL1)*	member of SPL gene family, encodes DNA binding proteins and putative transcription factors. All have the SBP-box, which encodes the SBP-domain, required for and sufficient for interaction with DNA.
AT2G47120		NAD(P)-binding Rossmann-fold superfamily protein
AT2G47130	*SHORT-CHAIN DEHYDROGENASE/REDUCTASE 3 (SDR3)*	Encodes a short-chain dehydrogenase/reductase that is not involved in ABA biosynthesis but plays an important role in plant defense response to bacteria.
AT2G47140	*SHORT-CHAIN DEHYDROGENASE REDUCTASE 5 (SDR5)*	NAD(P)-binding Rossmann-fold superfamily protein
AT2G47150		NAD(P)-binding Rossmann-fold superfamily protein
AT2G47160	*REQUIRES HIGH BORON 1 (BOR1)*	Encodes a key transporter under boron (B) limitation in the soil. Protein accumulates in shoots and roots under conditions of boron deficiency and is degraded within several hours of restoring boron supply. Localized to the plasma membrane under B limitation, and to the cytoplasm after B application before degradation. Protein is transferred via the endosomes to the vacuole for degradation. Localized to the inner plasma membrane domain in the columella, lateral root cap, epidermis, and endodermis in the root tip region, and in the epidermis and endodermis in the elongation zone. Under high-boron is transported to the vacuole for degradation. Thought to be a B transceptor, directly senses the B concentration and promotes its own polyubiquitination and vacuolar sorting for quick and precise maintenance of B homeostasis.
AT2G47180	*GALACTINOL SYNTHASE 1 (GolS1)*	GolS1 is a galactinol synthase that catalyzes the formation of galactinol from UDP-galactose and myo-inositol. GolS1 transcript levels rise in response to methyl viologen, an oxidative damage-inducing agent. Plants over-expressing GolS1 have increased tolerance to salt, chilling, and high-light stress.
AT2G47190	*MYB DOMAIN PROTEIN 2 (MYB2)*	Encodes a MYB transcription factor that possesses an R2R3 MYB DNA binding domain and is known to regulate the expression of salt- and dehydration-responsive genes. Has been shown to bind calmodulin.
AT2G47200		hypothetical protein
AT2G47230	*DOMAIN OF UNKNOWN FUNCTION 724 6 (DUF6)*	Member of the plant-specific DUF724 protein family. Arabidopsis has 10 DUF724 proteins.
AT2G47240	*LONG-CHAIN ACYL-COA SYNTHASE 1 (LACS1)*	Encodes an acyl-CoA synthetase that acts on long-chain and very-long-chain fatty acids, involved in cuticular wax and cutin biosynthesis The mRNA is cell-to-cell mobile.
AT2G47250		RNA helicase family protein
AT2G47260	*WRKY DNA-BINDING PROTEIN 23 (WRKY23)*	Encodes a member of WRKY Transcription Factor; Group I. Involved in nematode feeding site establishment and auxin mediated PIN polar localization in roots. Expression is induced by auxin.
AT2G47270	*UPBEAT1 (UPB1)*	Encodes UPBEAT1 (UPB1), a transcription factor with a bHLH domain. Regulates the expression of a set of peroxidases that modulate the balance of reactive oxygen species (ROS) between the zones of cell proliferation and the zone of cell elongation where differentiation begins. Disruption of UPB1 activity alters this ROS balance, leading to a delay in the onset of differentiation. Regulates growth by mediating cell cycle progression.
AT2G47280		Pectin lyase-like superfamily protein
AT2G47290		
AT2G47300	*SIMILAR TO YEAST POP1 (POP1)*	Encodes a protein involved in rRNA but not tRNA maturation.
AT2G47310	*SISTER OF FCA (SSF)*	Functions in an antagonistic manner to its close homolog FCA. The SSF414N protein variant interacts more strongly with CUL1, a component of the E3 ubiquitination complex, than the SSF414D form, mediating differences in SSF protein degradation and FLC expression.
AT2G47340		Plant invertase/pectin methylesterase inhibitor superfamily protein
AT2G47380		Cytochrome c oxidase subunit Vc family protein
AT2G47390	*CHLOROPLAST GLUTAMYL PEPTIDASE (CGEP)*	Chloroplast stroma localized glutamyl peptidase.
AT2G47400	*CP12 DOMAIN-CONTAINING PROTEIN 1 (CP12-1)*	CP12-1 encodes a small peptide found in the chloroplast stroma. It belongs to the CP12 gene family thought to be involved in the formation of a supramolecular complex with glyceraldehyde-3-phosphate dehydrogenase (GAPDH) and phosphoribulokinase (PRK) embedded in the Calvin cycle. The mRNA is cell-to-cell mobile.
AT2G47420	*ADENOSINE DIMETHYL TRANSFERASE 1A (DIM1A)*	Encodes a putative rRNA dimethyltransferase.
AT2G47430	*CYTOKININ-INDEPENDENT 1 (CKI1)*	Encodes a putative plasma membrane-bound hybrid histidine kinase and cytokinin sensor that is expressed within the female gametophyte.
AT2G47440		Tetratricopeptide repeat (TPR)-like superfamily protein
AT2G47450	*CHAOS (CAO)*	A component of the chloroplast signal recognition particle pathway that is involved in LHCP targeting. It is downregulated in response to high light. It recognizes the DPLG motif in Lhcb1. The mRNA is cell-to-cell mobile.
AT2G47460	*MYB DOMAIN PROTEIN 12 (MYB12)*	MYB12 belongs to subgroup 7 of the R2R3-MYB family. It strongly activates the promoters of chalcone synthase (CHS), flavanone 3-hydroxylase (F3H), flavonol synthase (FLS) and - to a lesser extent - chalcone flavanone isomerase (CHI), but cannot activate the promoters of flavonoid-3'hydroxylase (F3'H) and dihydroflavonol 4-reductase (DF). The activation requires a functional MYB recognition element (MRE). Results from the myb12-1f allele indicate that an activation domain might be present in the C-terminus. Overexpression or knock-out plants do not show any obvious phenotype under greenhouse conditions. Young myb12-ko seedlings contain reduced amounts of flavonoids (quercetin and kaempferol), while seedlings as well as leaves of MYB12-OX plants displayed an increased flavonoid content. They did not show any significant difference in anthocyanin content. Expression of CHS and FLS shows a clear correlation to MYB12 expression levels. CHI and F3H show increased transcript levels in the MYB12-OX lines, but no differences in the knock-out. Even in the absence of functional MYB12, flavonol biosynthesis is not completely absent, suggesting functional redundancy. The redundant factors are MYB11 and MYB111 although MYB12 is primarily required for flavonol biosynthesis in roots. Mutations in MYB12 block both auxin and ethylene stimulation of flavonoid synthesis.
AT2G47490	*NAD+ TRANSPORTER 1 (NDT1)*	Encodes a chloroplast-localized NAD+ transporter that transports NAD+ in a counter exchange mode with ADP and AMP in vitro.
AT2G47500		P-loop nucleoside triphosphate hydrolases superfamily protein with CH (Calponin Homology) domain-containing protein

AT2G47520	*ETHYLENE RESPONSE FACTOR 71 (ERF71)*	encodes a member of the ERF (ethylene response factor) subfamily B-2 of ERF/AP2 transcription factor family. The protein contains one AP2 domain. There are 5 members in this subfamily including RAP2.2 AND RAP2.12. It plays a role in hypoxia-induced root slanting.
AT2G47540		Pollen Ole e 1 allergen and extensin family protein
AT2G47550		Plant invertase/pectin methylesterase inhibitor superfamily
AT2G47560	*ARABIDOPSIS T??XICOS EN LEVADURA 64 (ATL64)*	RING/U-box superfamily protein
AT2G47570		60S ribosomal protein L18
AT2G47600	*MAGNESIUM/PROTON EXCHANGER (MHX)*	Encodes a magnesium/proton exchanger, member of putative Na+/Ca2+ antiporter gene family
AT2G47660		
AT2G47670	*PECTIN METHYLESTERASE INHIBITOR 6 (PMEI6)*	PMEI6 pectin methylesterase inhibitor functions in establishing a patter of homogalacturonan methylesterification of seed coat cell wall proteins .
AT2G47720		hypothetical protein
AT2G47750	*PUTATIVE INDOLE-3-ACETIC ACID-AMIDO SYNTHETASE GH3.9 (GH3.9)*	Encodes GH3.9, a member of the GH3 family auxin-responsive genes. gh3.9-1 mutants had greater primary root length, increased sensitivity to indole-3-acetic acid (IAA)-mediated root growth inhibition, but no obvious effects on apical dominance or leaf morphology.
AT2G47770	*TSPO(OUTER MEMBRANE TRYPTOPHAN-RICH SENSORY PROTEIN)-RELATED (TSPO)*	Encodes a membrane-bound protein designated AtTSPO (Arabidopsis thaliana TSPO-related). AtTSPO is related to the bacterial outer membrane tryptophan-rich sensory protein (TspO) and the mammalian mitochondrial 18 kDa Translocator Protein (18 kDa TSPO), members of the TspO/MBR domain-containing membrane proteins. Mainly detected in dry seeds, but can be induced in vegetative tissues by osmotic or salt stress or abscisic acid treatment. Located in endoplasmic reticulum and the Golgi stacks. It is degraded through the autophagy pathway.
AT2G47780	*LD-ASSOCIATED PROTEIN 2 (LDAP2)*	Encodes a small rubber particle protein homolog. Plays dual roles as positive factors for tissue growth and development and in drought stress responses.
AT2G47790	*GIGANTUS 1 (GTS1)*	Encodes GIGANTUS1 (GTS1), a member of Transducin/WD40 protein superfamily. Controls seed germination, growth and biomass accumulation.
AT2G47800	*ATP-BINDING CASSETTE C4 (ABCC4)*	Encodes a plasma membrane localized ATPase transporter involved in multidrug transport. The expression of this gene is upregulated by herbicide safeners such as benoxacor, fluxofenim and fenclorim. The mRNA is cell-to-cell mobile.
AT2G47810	*NUCLEAR FACTOR Y, SUBUNIT B5 (NF-YB5)*	nuclear factor Y, subunit B5
AT2G47830		Cation efflux family protein
AT2G47860	*(SETH6)*	Phototropic-responsive NPH3 family protein
AT2G47880	*CEP DOWNSTREAM 2 (CEPD2)*	Encodes a member of the CC-type glutaredoxin (ROXY) family. CEPD2 is a non secreted polypeptide that is highly similar to CEPD1 which is another member of a novel family related to glutaredoxins. It is induced by nitrogen starvation. It acts downstream of the CEP1 peptide in systemic N-demand signalling .The RNA is expressed in the phloem of cotelydon and leaf vasculature but the peptide is graft transmissible, traveling from the shoot to the root.
AT2G47890	*B-BOX PROTEIN 11 (BBX11)*	Acts as a positive regulator of red light signaling; overexpression causes markedly shortened hypocotyls under various light states. Binds to the HY5 promoter to activate its transcription, while both BBX21 and HY5 associate with its promoter to positively regulate its expression. T
AT2G47910	*CHLORORESPIRATORY REDUCTION 6 (CRR6)*	Encodes a chloroplast thylakoid membrane protein. Required for the assembly/accumulation of the NAD(P)H dehydrogenase complex of the photosynthetic electron transport chain.
AT2G47920	*NETWORKED 3C (NET3C)*	Kinase interacting (KIP1-like) family protein
AT2G47930	*ARABINOGALACTAN PROTEIN 26 (AGP26)*	arabinogalactan protein 26
AT2G47940	*DEGRADATION OF PERIPLASMIC PROTEINS 2 (DEG2)*	Encodes DegP2 protease (DEGP2); nuclear gene for chloroplast product.
AT2G47950		myelin transcription factor-like protein
AT2G47960	*(TRAPPC13)*	Part of multi-protein complex, acting as guanine nucleotide exchange factors (GEFs) and possibly as tethers, regulating intracellular trafficking.
AT2G47970		Nuclear pore localization protein NPL4
AT2G47990	*SLOW WALKER1 (SWA1)*	Encodes a transducin family nucleolar protein with six WD40 repeats that is most likely involved in 18S rRNA biogenesis. The slow progression of the gametophytic division cycles in swa1 suggested that the SWA1 protein is required for the normal progression of mitotic division cycles through the regulation of cell metabolism. Ubiquitously expressed throughout the plant.
AT2G48020	*ZINC-INDUCED FACILITATOR 2 (ZIF2)*	Encodes a zinc transporter ZIF2. Expression of ZIF2 is regulated by alternative splicing.
AT2G48040		
AT2G48050		
AT2G48060	*(PZO1)*	Similar to mechanically sensitive ion channel identified in mouse. Mutants display root helical growth phenotype in agar media suggesting a role in mechanoperception at the root cap.
AT2G48080	*ATALKBH10A (ALKBH10A)*	oxidoreductase, 2OG-Fe(II) oxygenase family protein
AT2G48090		hypothetical protein
AT2G48110	*REDUCED EPIDERMAL FLUORESCENCE 4 (REF4)*	Encodes a novel protein of unknown function with homologs in non-seed plants. Sequence analysis predicts membrane spanning domains and a putative protein-protein interaction domain. Semi-dominant mutations display defects in phenylpropanoid accumulation suggesting a role in phenylpropanoid metabolism. It has been shown to physically associate with the conserved transcriptional coregulatory complex, Mediator, and is involved in the regulation of phenylpropanoid homeostasis. Required for expression of some dark-upregulated genes.
AT2G48130	*GLYCOSYLPHOSPHATIDYLINOSITOL-ANCHORED LIPID PROTEIN TRANSFER 15 (LTPG15)*	Encodes a plasma membrane-localized glycosylphosphatidylinositol-anchored lipid transfer protein expressed in root endodermis and seed coats that is involved in very long chain fatty acid (and their derivatives) transport.
AT2G48140	*EMBRYO SAC DEVELOPMENT ARREST 4 (EDA4)*	Bifunctional inhibitor/lipid-transfer protein/seed storage 2S albumin superfamily protein
AT2G48150	*GLUTATHIONE PEROXIDASE 4 (GPX4)*	Encodes glutathione peroxidase.
AT2G48160	*HUA2 LIKE 2 (HULK2)*	HUA and HUA-LIKE (HULK) genes act redundantly to regulate a subset of essential genes, with some (or all) family members also having specific functions.
AT3G01010		UDP-glucose/GDP-mannose dehydrogenase family protein

AT3G01015	*MICROTUBULE DESTABILIZING PROTEIN60 (MD60)*	MDP60 is a member of the TPX2 protein family. It co-localizes with microtubules and appears to function to destabilize them during light mediated hypocotyl growth.
AT3G01020	*ISCU-LIKE 2 (ISU2)*	Encodes a mitochondrial protein similar to E.coli IscU. In bacteria, IscU is a scaffold protein accepting sulfur and iron to build a transient Fe-S cluster,which is subsequently transferred to a target apoprotein.
AT3G01060		lysine-tRNA ligase
AT3G01070	*EARLY NODULIN-LIKE PROTEIN 16 (ENODL16)*	early nodulin-like protein 16
AT3G01080	*WRKY DNA-BINDING PROTEIN 58 (WRKY58)*	member of WRKY Transcription Factor; Group I
AT3G01100	*HYPOTHETICAL PROTEIN 1 (HYP1)*	unknown protein, has cDNAs and ESTs associated to it
AT3G01120	*METHIONINE OVERACCUMULATION 1 (MTO1)*	encodes a cystathionine gamma-synthase, which performs the first committed step in methionine biosynthesis. A conserved motif of 13 amino acids in the first exon is required for posttranscriptional autoregulation. This enzyme shares the same substrate as threonine synthase (TS) and its absence transcriptionally affects 8 genes in the genome.
AT3G01140	*MYB DOMAIN PROTEIN 106 (MYB106)*	Encodes a MIXTA-like MYB gene NOECK (NOK). Loss of function mutations show an increased number of branchpoints in leaf trichomes suggesting a role in negative regulation of trichome branching.
AT3G01160		pre-rRNA-processing ESF1-like protein
AT3G01170		Ribosomal protein L34e superfamily protein
AT3G01175		transmembrane protein
AT3G01180	*STARCH SYNTHASE 2 (SS2)*	Starch synthase 2 involved in amylopectin metabolism.
AT3G01190		Peroxidase superfamily protein
AT3G01210	*BPA1 LIKE 6 (BPL6)*	ACD11 binding partner, may be involved in negative regulation of ROS-mediated defense response.
AT3G01220	*HOMEOBOX PROTEIN 20 (HB20)*	Encodes a homeodomain leucine zipper class I (HD-Zip I) protein. Expressed during seed germination in the micropylar endosperm and in the root cap, and increases ABA sensitivity and seed dormancy when mutated. The mRNA is cell-to-cell mobile.
AT3G01240		splicing regulatory glutamine/lysine-rich-like protein
AT3G01250		transmembrane protein
AT3G01260		Galactose mutarotase-like superfamily protein
AT3G01270		Pectate lyase family protein
AT3G01290	*HYPERSENSITIVE INDUCED REACTION 2 (HIR2)*	SPFH/Band 7/PHB domain-containing membrane-associated protein family
AT3G01310	*ARABIDOPSIS HOMOLOG OF YEAST VIP1 1 (ATVIP1)*	Encodes a functional VIP1/PPIP5K-type ATP-grasp kinase that is involved in both InsP6 to InsP7 conversion and InsP7 to InsP8 conversion, producing the InsP8 cofactor of the ASK1-COI1-JAZ-jasmonate co-receptor complex. It is the major isoform in plants, is required for jasmonate-dependent defenses, and plays an important role in plant defenses against necrotrophic fungi and insect herbivores.
AT3G01350		Major facilitator superfamily protein
AT3G01360		plant viral-response family protein (DUF716)
AT3G01370	*CRM FAMILY MEMBER 2 (CFM2)*	Encodes a protein containing a CRM domain that is involved in group I and group II intron splicing.
AT3G01390	*VACUOLAR MEMBRANE ATPASE 10 (VMA10)*	Subunit G of the vacuolar membrane ATPAse complex
AT3G01420	*(DOX1)*	Encodes an alpha-dioxygenase involved in protection against oxidative stress and cell death. Induced in response to Salicylic acid and oxidative stress. Independent of NPR1 in induction by salicylic acid. The mRNA is cell-to-cell mobile.
AT3G01430		NHL domain protein
AT3G01440	*PHOTOSYNTHETIC NDH SUBCOMPLEX L 3 (PnsL3)*	Encodes a subunit of the NAD(P)H complex located in the chloroplast thylakoid lumen.
AT3G01450		ARM repeat superfamily protein
AT3G01470	*HOMEOBOX 1 (HB-1)*	Encodes a homeodomain leucine zipper class I (HD-Zip I) transcriptional activator involved in leaf and hypocotyl development. Its promoter is bound by PIF1 which likely regulates its expression. Its translation is regulated by a conserved upstream ORF (CPuORF33).
AT3G01480	*CYCLOPHILIN 38 (CYP38)*	Encodes a chloroplast cyclophilin functioning in the assembly and maintenance of photosystem II (PSII) supercomplexes. The mRNA is cell-to-cell mobile.
AT3G01490	*CONVERGENCE OF BLUE LIGHT AND CO2 (CBC1)*	Belongs to the Raf-like kinase subfamily of the mitogen-activated protein kinase kinase kinase (MAPKKK) family. Negatively regulates stomatal opening by negatively regulating plasma membrane H+-ATPase phosphorylation.
AT3G01500	*CARBONIC ANHYDRASE 1 (CA1)*	Encodes a putative beta-carbonic anhydrase betaCA1. Together with betaCA4 (At1g70410) regulates CO2-controlled stomatal movements in guard cells, as well as attenuates immunity. Differential CA gene expression in response to changing atmospheric CO2 conditions contribute to altered disease resistance levels. Activated by OXS2 under the treatment of salt.
AT3G01510	*LIKE SEX4 1 (LSF1)*	Encodes a putative phosphatase, LSF1, required for normal starch turnover in leaves.
AT3G01520		Encodes a universal stress protein (USP)-like protein that has been crystallized in complex with AMP, suggesting that it belongs to the ATP-binding USP subfamily. The mRNA is cell-to-cell mobile.
AT3G01530	*MYB DOMAIN PROTEIN 57 (MYB57)*	Member of the R2R3 factor gene family.MYB57 interacts with JAZ proteins, and functions redundantly with MYB21 and MYB24 to regulate stamen development. Promote flavonol biosynthesis through regulation of FLS1 gene expression.
AT3G01540	*DEAD BOX RNA HELICASE 1 (DRH1)*	RNA HELICASE DRH1
AT3G01550	*PHOSPHOENOLPYRUVATE (PEP)/PHOSPHATE TRANSLOCATOR 2 (PPT2)*	phosphoenolpyruvate (pep)/phosphate translocator 2
AT3G01570		Oleosin family protein
AT3G01580		Tetratricopeptide repeat (TPR)-like superfamily protein
AT3G01620		beta-1,4-N-acetylglucosaminyltransferase family protein
AT3G01630		Major facilitator superfamily protein
AT3G01640	*GLUCURONOKINASE G (GLCAK)*	AtGlcAK is a sugar kinase able to phosphorylate D-GlcA to D-GlcA-1-phosphate in the presence of ATP.

AT3G01650	*RING DOMAIN LIGASE1 (RGLG1)*	Encodes RGLG1 (RING domain ligase 1), a RING domain ubiquitin E3 ligase that negatively regulates the drought stress response by mediating ERF53 transcriptional activity. ABA inhibits myristoylation and induces shuttling of the RGLG1 to promote nuclear degradation of PP2CA.
AT3G01660		S-adenosyl-L-methionine-dependent methyltransferases superfamily protein
AT3G01670	*SIEVE ELEMENT OCCLUSION A (SEOA)*	Encodes a protein localized to phloem filaments that is required for phloem filament formation. The mRNA is cell-to-cell mobile.
AT3G01680	*SIEVE-ELEMENT-OCCLUSION-RELATED 1 (SEOR1)*	Encodes a protein localized to phloem filaments that is required for phloem filament formation. The mRNA is cell-to-cell mobile.
AT3G01690		alpha/beta-Hydrolases superfamily protein
AT3G01700	*ARABINOGALACTAN PROTEIN 11 (AGP11)*	Encodes an arabinogalactan protein that is expressed in pollen, pollen sac and pollen tube. Loss of AGP11 function results in decreased fertility due to defects in pollen tube growth.
AT3G01730		Mutants exhibit shorter root hairs under phosphate-deficient conditions.
AT3G01750		Ankyrin repeat family protein
AT3G01780	*(TPLATE)*	Encodes TPLATE, a cytokinesis protein targeted to the cell plate. Functions in vesicle-trafficking events required for site-specific cell wall modifications during pollen germination and for anchoring of the cell plate to the mother wall at the correct cortical position.
AT3G01790		Ribosomal protein L13 family protein
AT3G01800		Ribosome recycling factor
AT3G01810		EEIG1/EHBP1 protein amino-terminal domain protein
AT3G01820		P-loop containing nucleoside triphosphate hydrolases superfamily protein
AT3G01830		Calcium-binding EF-hand family protein
AT3G01840	*LYSM-CONTAINING RECEPTOR-LIKE KINASE 2 (LYK2)*	Encodes a putative LysM-containing receptor-like kinase. Induction of chitin-responsive genes by chitin treatment is not blocked in the mutant. Based on protein sequence alignment analysis, it was determined to be a pseudo kinase since lack of the ATP-binding P-loop in the kinase domain.
AT3G01860		hypothetical protein
AT3G01880		vacuolar sorting-associated protein (DUF946)
AT3G01890	*SWP73A (SWP73A)*	Encodes SWP73A, a subunit of the SWI/SNF chromatin remodeling complex. While undergoing normal vegetative development, swp73a mutants display reduced expression of FLOWERING LOCUS C and early flowering in short days.
AT3G01900	*CYTOCHROME P450, FAMILY 94, SUBFAMILY B, POLYPEPTIDE 2 (CYP94B2)*	member of CYP94B
AT3G01910	*SULFITE OXIDASE (SOX)*	Encodes a homodimeric Mo-enzyme with molybdopterin as organic component of the molybdenum cofactor. It lacks the heme domain that other eukaryotic Mo-enzymes possess and has no redox-active centers other than the molybdenum. SO protein has been found in all parts of the plant. The plant SO combines its enzymatic sulfite oxidation with a subsequent nonenzymatic step using its reaction product H2O2 as intermediate for oxidizing another molecule of sulfite.
AT3G01920		DHBP synthase RibB-like alpha/beta domain-containing protein
AT3G01930		Major facilitator superfamily protein
AT3G01960		hypothetical protein
AT3G01970	*WRKY DNA-BINDING PROTEIN 45 (WRKY45)*	member of WRKY Transcription Factor; Group I
AT3G01990	*ACT DOMAIN REPEAT 6 (ACR6)*	Member of a small family of ACT domain containing proteins in Arabidopsis. ACT domains are involved in amino acid binding.
AT3G02000	*(ROXY1)*	Encodes a member of the CC-type glutaredoxin (ROXY) family that has been shown to interact with the transcription factor TGA2 and suppress ORA59 promoter activity. It is required for proper petal initiation and organogenesis. It is likely to function in the temporal and spatial expression regulation of AGAMOUS in the first and second whorl. Its function is dependent on the Cysteine 49 residue and its nuclear localization. ROXY1 interacts in vitro and in vivo with members of the TGA family of transcription factors (e.g. TGA2, TGA3, TGA7 and PAN).
AT3G02010		Pentatricopeptide repeat (PPR) superfamily protein
AT3G02020	*ASPARTATE KINASE 3 (AK3)*	encodes a monofunctional aspartate kinase
AT3G02040	*SENESCENCE-RELATED GENE 3 (SRG3)*	Encodes a member of the glycerophosphodiester phosphodiesterase (GDPD) family. Has glycerophosphodiester phosphodiesterase activity. Functions in maintaining cellular phosphate homeostasis under phosphate starvation. The mRNA is cell-to-cell mobile.
AT3G02060		DEAD/DEAH box helicase
AT3G02090	*(MPPBETA)*	Insulinase (Peptidase family M16) protein
AT3G02100		UDP-Glycosyltransferase superfamily protein
AT3G02110	*SERINE CARBOXYPEPTIDASE-LIKE 25 (scpl25)*	serine carboxypeptidase-like 25
AT3G02120		hydroxyproline-rich glycoprotein family protein
AT3G02125		pinin-like protein
AT3G02140	*TWO OR MORE ABRES-CONTAINING GENE 2 (TMAC2)*	Encodes a protein that acts in the nucleus and is an important negative regulator of ABA and salt stress responses, and could play a critical role in controlling root elongation, floral initiation and starch degradation.
AT3G02150	*PLASTID TRANSCRIPTION FACTOR 1 (PTF1)*	a chloroplast trans-acting factor of the psbD light-responsive promoter.TCP gene involved in heterochronic control of leaf differentiation.
AT3G02170	*LONGIFOLIA2 (LNG2)*	Encodes LONGIFOLIA2 (LNG2). Regulates leaf morphology by promoting cell expansion in the leaf-length direction. The LNG2 homologue LNG1 (At5g15580) has similar function.
AT3G02190		Ribosomal protein L39 family protein
AT3G02200		Proteasome component (PCI) domain protein
AT3G02210	*COBRA-LIKE PROTEIN 1 PRECURSOR (COBL1)*	COBRA-like protein 1 precursor
AT3G02240	*ROOT MERISTEM GROWTH FACTOR 7 (RGF7)*	Encodes a root meristem growth factor (RGF). Belongs to a family of functionally redundant homologous peptides that are secreted, tyrosine-sulfated, and expressed mainly in the stem cell area and the innermost layer of central columella cells. RGFs are required for maintenance of the root stem cell niche and transit amplifying cell proliferation. Members of this family include: At5g60810 (RGF1), At1g13620 (RGF2), At2g04025 (RGF3), At3g30350 (RGF4), At5g51451 (RGF5), At4g16515 (RGF6), At3g02240 (RGF7), At2g03830 (RGF8) and At5g64770 (RGF9).
AT3G02250	*(RRT2)*	O-fucosyltransferase family protein

AT3G02270		Trimeric LpxA-like enzyme
AT3G02310	*SEPALLATA 2 (SEP2)*	MADS-box protein, binds K domain of AG in vivo
AT3G02320	*TRNA METHYLTRANSFERASE 1A (TRM1A)*	Involved in posttranscriptional modification of tRNA.
AT3G02330	*MITOCHONDRIAL EDITING FACTOR 13 (MEF13)*	Involved in cytidine to uridine editing of the mitochondrial mRNA AtMg00510.
AT3G02340		RING/U-box superfamily protein
AT3G02380	*CONSTANS-LIKE 2 (COL2)*	homologous to the flowering-time gene CONSTANS (CO) encoding zinc-finger proteins
AT3G02390		hypothetical protein
AT3G02400	*FORKHEADASSOCIATED DOMAIN 2 (FHA3)*	Contains a single exon and encodes a ~66-kD protein with a Forkhead- Associated domain. Binds the promoter of PEX11b and expression is correlated with negative regulation of PEX11b.
AT3G02420		dihydroflavonol 4-reductase/flavanone protein
AT3G02440	*TRICHOME BIREFRINGENCE-LIKE 20 (TBL20)*	Encodes a member of the TBL (TRICHOME BIREFRINGENCE-LIKE) gene family containing a plant-specific DUF231 (domain of unknown function) domain. TBL gene family has 46 members, two of which (TBR/AT5G06700 and TBL3/AT5G01360) have been shown to be involved in the synthesis and deposition of secondary wall cellulose, presumably by influencing the esterification state of pectic polymers. A nomenclature for this gene family has been proposed (Volker Bischoff & Wolf Scheible, 2010, personal communication).
AT3G02480	*ABA-RESPONSE PROTEIN (ABR)*	Late embryogenesis abundant protein (LEA) family protein
AT3G02500		mental retardation GTPase activating protein
AT3G02540	*RADIATION SENSITIVE23C (RAD23C)*	Encodes a member of the RADIATION SENSITIVE23 (RAD23) family: AT1G16190(RAD23A), AT1G79650(RAD23B), AT3G02540(RAD23C), AT5G38470(RAD23D). RAD23 proteins play an essential role in the cell cycle, morphology, and fertility of plants through their delivery of UPS (ubiquitin/26S proteasome system) substrates to the 26S proteasome.
AT3G02550	*LOB DOMAIN-CONTAINING PROTEIN 41 (LBD41)*	LOB domain-containing protein 41
AT3G02555		hypothetical protein
AT3G02570	*MATERNAL EFFECT EMBRYO ARREST 31 (MEE31)*	Encodes a protein with phosphomannose isomerase activity.
AT3G02590		Fatty acid hydroxylase superfamily protein
AT3G02610	*ACYL-ACYL CARRIER PROTEIN DESATURASE2 (AAD2)*	Encodes one of two ∆9 palmitoyl-ACP desaturases responsible for the biosynthesis of ω-7 fatty acids in the maturing endosperm.
AT3G02630	*ACYL?ACYL CARRIER PROTEIN (ACP) DESATURASE 5 (AAD5)*	One of seven acyl acyl carrier proteins. Expressed primarily in developing seeds.Involved in fatty acid metabolism. Redundant Δ9 stearoyl-ACP desaturase gene which together with FAB2 and AAD1 during embryo development provide precursors for the elaboration of embryo cuticle and therefore plays a specific role during the phase of invasive embryo growth through the endosperm. Together with FAB2, AAD5, and AAD6 redundantly participates in oil storage during the maturation phase.
AT3G02640		transmembrane protein
AT3G02650		Tetratricopeptide repeat (TPR)-like superfamily protein
AT3G02670		Glycine-rich protein family
AT3G02680	*NIJMEGEN BREAKAGE SYNDROME 1 (NBS1)*	DNA repair and meiotic recombination protein, component of MRE11 complex with RAD50 and MRE11
AT3G02690		Nucleotide/sugar transporter family protein
AT3G02700		NC domain-containing protein-like protein
AT3G02710		Encodes a protein with a putative role in mRNA splicing.
AT3G02730	*THIOREDOXIN F-TYPE 1 (TRXF1)*	Encodes a type-f thioredoxin. Has a role in the short-term activation of carbon metabolism. Loss affects growth under short-day conditions.
AT3G02750		Protein phosphatase 2C family protein
AT3G02760		Class II aaRS and biotin synthetases superfamily protein
AT3G02770		Ribonuclease E inhibitor RraA/Dimethylmenaquinone methyltransferase
AT3G02800	*PLANT AND FUNGI ATYPICAL DUAL-SPECIﬁCITY PHOSPHATASE 3 (PFA-DSP3)*	Encodes an atypical dual-speciﬁcity phosphatase.
AT3G02810	*LOST IN POLLEN TUBE GUIDANCE 2 (LIP2)*	Encodes a receptor-like cytoplasmic kinase localized in the membrane of pollen tube tip regions that controls micropylar pollen tube guidance in Arabidopsis.
AT3G02820		zinc knuckle (CCHC-type) family protein
AT3G02830	*ZINC FINGER PROTEIN 1 (ZFN1)*	Encodes a zinc finger protein that binds to PORA mRNA in vivo and recruits the Pfr form of phytochrome to the 5′-UTR of PORA mRNA to regulate translation of the mRNA.
AT3G02840		ARM repeat superfamily protein
AT3G02850	*STELAR K+ OUTWARD RECTIFIER (SKOR)*	Encodes SKOR, a member of Shaker family potassium ion (K+) channel. This family includes five groups based on phylogenetic analysis (FEBS Letters (2007) 581: 2357): I (inward rectifying channel): AKT1 (AT2G26650), AKT5 (AT4G32500) and SPIK (also known as AKT6, AT2G25600); II (inward rectifying channel): KAT1 (AT5G46240) and KAT2 (AT4G18290); III (weakly inward rectifying channel): AKT2 (AT4G22200); IV (regulatory subunit involved in inwardly rectifying conductance formation): KAT3 (also known as AtKC1, AT4G32650); V (outward rectifying channel): SKOR (AT3G02850) and GORK (AT5G37500). Mediates the delivery of K+ from stelar cells to the xylem in the roots towards the shoot. mRNA accumulation is modulated by abscisic acid. K+ gating activity is modulated by external and internal K+. Involved in response to low potassium.
AT3G02870	*(VTC4)*	Encodes a L-galactose-1-phosphate phosphatase, involved in ascorbate biosynthesis.
AT3G02875	*IAA-LEUCINE RESISTANT 1 (ILR1)*	Hydrolyzes amino acid conjugates of the plant growth regulator indole-3-acetic acid (IAA), including IAA-Leu and IAA-Phe. Uses Mg and Co ions as cofactors.
AT3G02885	*GAST1 PROTEIN HOMOLOG 5 (GASA5)*	GASA5, is involved in the regulation of seedling thermotolerance.
AT3G02890	*ASI1-IMMUNOPRECIPITATED PROTEIN 2 (AIPP2)*	PHD protein which cooperates with PAIPP2 and BAH domain protein AIPP3 to read H3K4 histone marks. The BAH-PHD bivalent histone reader complex silences a substantial subset of H3K27me3-enriched loci, including development and stress response-related genes. Interacts with BDT1, acts with other PHD proteins to associate with flowering genes and thereby suppress their transcription.

AT3G02900		Low-density receptor-like protein
AT3G02910		AIG2-like (avirulence induced gene) family protein
AT3G02920	*(RPA32B)*	Replication protein A, subunit RPA32
AT3G02930		Encodes a microtubule-associated protein.
AT3G02940	*MYB DOMAIN PROTEIN 107 (MYB107)*	Encodes a putative transcription factor (MYB107).
AT3G02950	*(THO7)*	Encodes a component of the putative Arabidopsis THO/TREX complex: THO1 or HPR1 (At5g09860), THO2 (At1g24706), THO3 or TEX1 (At5g56130), THO5 (At5g42920, At1g45233), THO6 (At2g19430), and THO7 (At5g16790, At3g02950). THO/TREX complexes in animals have been implicated in the transport of mRNA precursors. Mutants of THO3/TEX1, THO1, THO6 accumulate reduced amount of small interfering (si)RNA, suggesting a role of the putative Arabidopsis THO/TREX in siRNA biosynthesis.
AT3G02970	*EXORDIUM LIKE 6 (EXL6)*	EXORDIUM like 6
AT3G02980	*MEIOTIC CONTROL OF CROSSOVERS1 (MCC1)*	Encodes MEIOTIC CONTROL OF CROSSOVERS1 (MCC1), a GCN5-related histone N-acetyltransferase. MCC1 appeared to be required in meiosis for normal chiasma number and distribution and for chromosome segregation. Activation tagging line has increased level of histone H3 acetylation.
AT3G03000		Calmodulin like protein localized in the plant vacuolar compartment with a function of binding and modifying the activity of a tonoplast transporter (AtNHX1) from within the vacuole in a Ca+2- and pH-dependent manner
AT3G03020		hypothetical protein
AT3G03040		F-box/RNI-like superfamily protein. Idenfitied in GWAS as locus involved in response to the defense molecule, allyl glucosinolate.
AT3G03060	*SHOT1 BINDING ATPASE 1 (SBA1)*	Homologue of animal ATPase Family AAA Domain-Containing Protein 3 (ATAD3), which is involved in mitochondrial nucleoid organization; interacts with SHOT1.
AT3G03080		Zinc-binding dehydrogenase family protein
AT3G03090	*VACUOLAR GLUCOSE TRANSPORTER 1 (VGT1)*	Encodes a vacuolar membrane-localized glucose transporter that can also transport fructose. Mutations in these gene have effects on seed germination and time to flowering.
AT3G03120	*ADP-RIBOSYLATION FACTOR B1C (ARFB1C)*	A member of ARF GTPase family. A thaliana has 21 members of this family, known to be essential for vesicle coating and uncoating and functions in GTP-binding. Gene encoding ADP-ribosylation factor and similar to ADP-ribosylation factor 1; ARF 1 (GP:385340) {Drosophila melanogaster}, other ARFs and ARF-like proteins.
AT3G03130		lisH domain-like protein
AT3G03150		hypothetical protein
AT3G03170		hypothetical protein
AT3G03180	*GOLGI TRANSPORT 1 (GOT1)*	Got1/Sft2-like vescicle transport protein family
AT3G03190	*GLUTATHIONE S-TRANSFERASE F11 (GSTF11)*	Encodes glutathione transferase belonging to the phi class of GSTs. Naming convention according to Wagner et al. (2002).
AT3G03200	*NAC DOMAIN CONTAINING PROTEIN 45 (NAC045)*	NAC domain containing protein 45
AT3G03220	*EXPANSIN A13 (EXPA13)*	member of Alpha-Expansin Gene Family. Naming convention from the Expansin Working Group (Kende et al, 2004. Plant Mol Bio)
AT3G03230		alpha/beta-Hydrolases superfamily protein
AT3G03240		alpha/beta-Hydrolases superfamily protein
AT3G03250	*UDP-GLUCOSE PYROPHOSPHORYLASE 1 (UGP1)*	Is thought to encode a cytosolic UDP-glucose pyrophosphorylase with strong similarity to potato UTP--glucose-1-phosphate uridylyltransferase. Downregulated by flooding.
AT3G03260	*HOMEODOMAIN GLABROUS 8 (HDG8)*	Encodes a homeobox-leucine zipper family protein belonging to the HD-ZIP IV family.
AT3G03270	*HYPOXIA RESPONSIVE UNIVERSAL STRESS PROTEIN 1 (HRU1)*	HRU1 is a hypoxia induced universal stress protein. It exists as two splice variants with AT3G03270.2 , which contains a putative dimerization domain, the predominant transcript found under anoxia. It is induced by RAP2.12. Subcellular localization is dynamic; under anoxia the localization of HRU1 shifts from cytoplasm to the plasma membrane.
AT3G03290		Adenine nucleotide alpha hydrolases-like superfamily protein
AT3G03300	*DICER-LIKE 2 (DCL2)*	Encodes a Dicer-like protein that functions in the antiviral silencing response in turnip-crinkle virus-infected plants but not in TMV or CMV-strain-Y-infected plants. Involved in the production of ta-siRNAs. Partially antagonizes the production of miRNAs by DCL1. Substitutes for DCL4 to produce viral siRNA when DCL4 is missing or inhibited. Able to produce siRNAs but not miRNAs.
AT3G03305		Calcineurin-like metallo-phosphoesterase superfamily protein
AT3G03310	*LECITHIN:CHOLESTEROL ACYLTRANSFERASE 3 (LCAT3)*	lecithin:cholesterol acyltransferase 3
AT3G03370		hypothetical protein
AT3G03380	*DEGRADATION OF PERIPLASMIC PROTEINS 7 (DEG7)*	Encodes a putative DegP protease. The mRNA is cell-to-cell mobile.
AT3G03390		
AT3G03400		EF hand calcium-binding protein family
AT3G03410		EF hand calcium-binding protein family
AT3G03420	*(ATATP23)*	Ku70-binding family protein
AT3G03430		Calcium-binding EF-hand family protein
AT3G03450	*RGA-LIKE 2 (RGL2)*	Encodes a DELLA protein, a member of the GRAS superfamily of putative transcription factors. DELLA proteins restrain the cell proliferation and expansion that drives plant growth. Negative regulator of the response to GA in controlling seed germination. GA triggers the degradation of RGL2 protein in a process blocked by both proteasome inhibitors and serine/threonine phosphatase inhibitors. The protein undergoes degradation in response to GA via the 26S proteasome. RGL2 may be involved in reducing ROS accumulation in response to stress by up-regulating the transcription of superoxide dismutases. Rapidly degraded in response to GA. Regulates GA-promoted seed germination. Involved in flower and fruit development.
AT3G03470	*CYTOCHROME P450, FAMILY 87, SUBFAMILY A, POLYPEPTIDE 9 (CYP89A9)*	P450 monooxygenase CYP89A9. Involved in NDCC accumulation during Arabidopsis leaf senescence.
AT3G03480	*ACETYL COA:(Z)-3-HEXEN-1-OL ACETYLTRANSFERASE (CHAT)*	acetyl CoA:(Z)-3-hexen-1-ol acetyltransferase

AT3G03490	*PEROXIN 19-1 (PEX19-1)*	Encodes one of two PEX19 peroxin isoforms, a cytosolic chaperone for peroxisome membrane proteins (PMPs) that delivers PMPs to the endoplasmic reticulum or peroxisomal membrane. It is predominantly cytosolic, forms dimers, promotes peroxisome function and is essential for viability. The protein is farnesylated in vivo through the actions of ERA1 and PLP.
AT3G03510		Phototropic-responsive NPH3 family protein
AT3G03520	*NON-SPECIFIC PHOSPHOLIPASE C3 (NPC3)*	Lysophosphatidic acid phosphatase highly expressed during phosphate starvation and abiotic stresses. Role in lipid synthesis.
AT3G03540	*NON-SPECIFIC PHOSPHOLIPASE C5 (NPC5)*	Encodes a nonspecific phospholipase C. Located in the cytosol. Involved in the conversion of phospholipids to glycolipids under phosphate deprivation conditions.
AT3G03550	*ARABIDOPSIS T??XICOS EN LEVADURA 51 (ATL51)*	RING/U-box superfamily protein
AT3G03560		hypothetical protein
AT3G03570		signal transducer, putative (DUF3550/UPF0682)
AT3G03590	*(SWIB4)*	SWIB/MDM2 domain superfamily protein
AT3G03600	*RIBOSOMAL PROTEIN S2 (RPS2)*	Structural component of the mitochondrial ribosome small subunit
AT3G03600	*RIBOSOMAL PROTEIN S2 (RPS2)*	Structural component of the mitochondrial ribosome small subunit
AT3G03620		MATE efflux family protein
AT3G03640	*BETA GLUCOSIDASE 25 (BGLU25)*	Encodes beta-glucosidase (GLUC).
AT3G03660	*WUSCHEL RELATED HOMEOBOX 11 (WOX11)*	Encodes a WUSCHEL-related homeobox gene family member with 65 amino acids in its homeodomain. Proteins in this family contain a sequence of eight residues (TLPLFPMH) downstream of the homeodomain called the WUS box.
AT3G03670		Peroxidase superfamily protein
AT3G03680	*MULTIPLE C2 DOMAIN AND TRANSMEMBRANE REGION PROTEIN 14 (MCTP14)*	Member of a family of Multiple C2 Domain and Transmembrane Region Proteins.
AT3G03700		Plasma-membrane choline transporter family protein
AT3G03710	*RESISTANT TO INHIBITION WITH FSM 10 (RIF10)*	Encodes a chloroplast polynucleotide phosphorylase (PNPase). Involved in response to phosphorus (P) starvation. Mutants impaired in the expression of this gene have been selected through their resistance to fosmidomycin, a strong inhibitor of DXR, an enzyme of the methylerythritol-dependent IPP biosynthesis pathway. The pathway enzymes were upregulated in the mutant seedlings.
AT3G03720	*CATIONIC AMINO ACID TRANSPORTER 4 (CAT4)*	Encodes a member of the cationic amino acid transporter (CAT) subfamily of amino acid polyamine choline transporters.
AT3G03740	*BTB-POZ AND MATH DOMAIN 4 (BPM4)*	Encodes a member of the MATH-BTB domain proteins (BPMs) that directly interact with and target for proteasomal degradation the class I homeobox-leucine zipper (HD-ZIP) transcription factor ATHB6. Known members include AT5G19000 (BPM1), AT3G06190 (BPM2), AT2G39760 (BPM3), AT3G03740 (BPM4), AT5G21010 (BPM5) and AT3G43700 (BPM6).
AT3G03770		Leucine-rich repeat protein kinase family protein
AT3G03790		ankyrin repeat family protein / regulator of chromosome condensation (RCC1) family protein
AT3G03800	*SYNTAXIN OF PLANTS 131 (SYP131)*	member of SYP13 Gene Family
AT3G03830	*SMALL AUXIN UP RNA 28 (SAUR28)*	SAUR-like auxin-responsive protein family
AT3G03840	*SMALL AUXIN UP RNA 27 (SAUR27)*	SAUR-like auxin-responsive protein family
AT3G03870		transmembrane protein
AT3G03880		sterol O-acyltransferase, putative (DUF1639)
AT3G03890	*(HOZ)*	Dimeric β-barrel protein that is structurally related to the putative non-canonical heme oxygenase (HO) and is located in chloroplasts. May function additionally in the tetrapyrrole biosynthetic pathway.
AT3G03900	*ADENOSINE-5'-PHOSPHOSULFATE (APS) KINASE 3 (APK3)*	Provides activated sulfate for the sulfation of secondary metabolites, including the glucosinolates. Redundant with APK4.
AT3G03910	*GLUTAMATE DEHYDROGENASE 3 (GDH3)*	GDH3 encodes a member of the glutamate dehydrogenease family. Its expression is upregulated in response to cytokinin and it may play a role in the control of nitrogen metabolism in leaf development.
AT3G03920		H/ACA ribonucleoprotein complex, subunit Gar1/Naf1 protein
AT3G03950	*EVOLUTIONARILY CONSERVED C-TERMINAL REGION 1 (ECT1)*	Physically interacts with CIPK1. Located in the nucleus.
AT3G03980		NAD(P)-binding Rossmann-fold superfamily protein
AT3G03990	*DWARF 14 (D14)*	Encodes an alpha/beta hydrolase essential for strigolactone signaling. Degradation of the protein is promoted by strigolactone. The mRNA is cell-to-cell mobile.
AT3G04000	*CHLOROPLAST ALDEHYDE REDUCTASE (CHLADR)*	ChlADR is an aldehyde reductase that catalyzes the reduction of the aldehyde carbonyl groups on saturated and alpha,beta-unsaturated aldehydes with more than 5 carbons in vitro. The N-terminal region of this protein directs GFP to the chloroplast where where ChlADR likely helps to maintain the photosynthetic process by detoxifying reactive carbonyls formed during lipid peroxidation. In addition, this enzyme can also reduce cis-3-hexenal, a major plant volatile compound that contributes to green leaf odor, as well as methylglyoxal in vitro.
AT3G04010		O-Glycosyl hydrolases family 17 protein
AT3G04020		hypothetical protein
AT3G04030	*(MYR2)*	Homeodomain-like superfamily protein
AT3G04050		Pyruvate kinase family protein
AT3G04060	*NAC DOMAIN CONTAINING PROTEIN 46 (NAC046)*	NAC046 is a member of the NAC domain containing family of transcription factors. It was identified in a screen for regulators of chlorophyll protein gene expression. Mutants in NAC046 have delayed senescence and increased CHL content suggesting a role in regulation of senescence and chlorophyll degradation.
AT3G04070	*NAC DOMAIN CONTAINING PROTEIN 47 (NAC047)*	NAC domain containing protein 47
AT3G04100	*AGAMOUS-LIKE 57 (AGL57)*	AGAMOUS-like 57
AT3G04110	*GLUTAMATE RECEPTOR 1.1 (GLR1.1)*	putative glutamate receptor (GLR1.1). Contains a functional cation - permeable pore domain. Involved in cellular cation homeostasis.
AT3G04120	*GLYCERALDEHYDE-3-PHOSPHATE DEHYDROGENASE C SUBUNIT 1 (GAPC1)*	encodes cytosolic GADPH (C subunit) involved in the glycolytic pathway but also interacts with H2O2 potentially placing it in a signalling cascade induced by ROS. The mRNA is cell-to-cell mobile.

AT3G04130		Tetratricopeptide repeat (TPR)-like superfamily protein
AT3G04150		RmlC-like cupins superfamily protein
AT3G04160		U11/U12 small nuclear ribonucleoprotein
AT3G04170		RmlC-like cupins superfamily protein
AT3G04190		RmlC-like cupins superfamily protein
AT3G04200		RmlC-like cupins superfamily protein
AT3G04210	*(TN13)*	TN13 is a TIR-NBS protein involved in immune response. It co localizes with the ER and perinuclear membranes and interacts with MOS6. It also associates with the CC-NBS-LRR resistance protein RPS5 and contributes to RPS5-triggered immunity.
AT3G04220		Target of miR825/825. Mutants have decreased resistance to fungal pathogens.
AT3G04230		Ribosomal protein S5 domain 2-like superfamily protein
AT3G04250		F-box associated ubiquitination effector family protein
AT3G04260	*PLASTID TRANSCRIPTIONALLY ACTIVE 3 (PTAC3)*	PEP complex component.
AT3G04270		two-component response regulator ARR22-like protein
AT3G04280	*RESPONSE REGULATOR 22 (RR22)*	Encodes an atypical subtype of the ARR (Arabidopsis response regulator) protein family. ARR22 is more similar to the receiver domains of hybrid kinases than other response regulators. It acts as a phosphohistidine phosphatase when tested with phospho-AHP5 in vitro suggesting that it might be involved in a two-component phosphorelay. Expression of ARR22 transcripts appears to be localized to the chalaza and to be induced by wounding. Ectopic expression of ARR in other parts of the plant leads to reduced cytokinin-related responses and impaired root, shoot, and flower development. Overexpression of wild-type ARR22 in an arr22 mutant background causes variable defects in plant growth and fertility. But, in the same ar22 background, over-expression of versions of ARR22 that should act as dominant-negative or constitutively active proteins, based on mutations to the conserved Asp residue, do not show any phenotypic abnormalities, raising the possibility that these may not act as canonical response regulators.
AT3G04290	*LI-TOLERANT LIPASE 1 (LTL1)*	Li-tolerant lipase 1
AT3G04310		transmembrane protein
AT3G04320		Kunitz family trypsin and protease inhibitor protein
AT3G04350		vacuolar sorting-associated protein (DUF946)
AT3G04360		Calcium-dependent lipid-binding (CaLB domain) family protein
AT3G04370	*PLASMODESMATA-LOCATED PROTEIN 4 (PDLP4)*	Encodes a plasmodesmal protein that may be involved in the intercellular movement of molecules through the plasmodesmata. The protein has two DUF26 domains and a single transmembrane domain.
AT3G04390		Aldehyde oxidase/xanthine dehydrogenase, molybdopterin binding protein
AT3G04410		NAC (No Apical Meristem) domain transcriptional regulator superfamily protein
AT3G04420	*NAC DOMAIN CONTAINING PROTEIN 48 (NAC048)*	NAC domain containing protein 48
AT3G04480		endoribonuclease
AT3G04510	*LIGHT SENSITIVE HYPOCOTYLS 2 (LSH2)*	LIGHT-DEPENDENT SHORT HYPOCOTYLS-like protein (DUF640)
AT3G04520	*THREONINE ALDOLASE 2 (THA2)*	Encodes a threonine aldolase, involved in threonine degradation to glycine. Expressed in vascular tissue through out the plant.
AT3G04530	*PHOSPHOENOLPYRUVATE CARBOXYLASE KINASE 2 (PPCK2)*	Encodes a second Arabidopsis phosphoenolpyruvate carboxylase kinase gene product with a different expression pattern from PPCK1. Expression of the gene is upregulated by exposure of the plant to light and is responsive to both phosphate (Pi) and phosphite (Phi) in shoots.
AT3G04540		Encodes a defensin-like (DEFL) family protein.
AT3G04550	*RUBISCO ACCUMULATION FACTOR 1 (RAF1)*	Encodes an ancillary chaperone protein that functions in Rubisco biogenesis. RAF1 dimers function in the assembly of the large subunit of Rubisco. Co-expression of RAF1 and rbcL in tobacco cells results in increased photosynthesis and plant growth. The mRNA is cell-to-cell mobile.
AT3G04570	*AT-HOOK MOTIF NUCLEAR-LOCALIZED PROTEIN 19 (AHL19)*	AT-hook motif nuclear-localized protein 19
AT3G04580	*ETHYLENE INSENSITIVE 4 (EIN4)*	Ethylene receptor, subfamily 2. Has serine kinase activity.
AT3G04600		Nucleotidylyl transferase superfamily protein
AT3G04630	*WVD2-LIKE 1 (WDL1)*	Member of a small gene family which have a KLEEK domain which may be involved in protein- protein interactions. Over expression of WDL1 results in abnormal root development.
AT3G04640		glycine-rich protein
AT3G04660		F-box and associated interaction domains-containing protein
AT3G04690	*ANXUR1 (ANX1)*	Receptor-like kinase required for maintenance of pollen tube growth. Display polar localization at the plasma membrane of the pollen tube tip.
AT3G04700		carboxylate clamp-TPR protein (DUF1685)
AT3G04710	*TETRATRICOPEPTIDE REPEAT 10 (TPR10)*	Encodes one of the 36 carboxylate clamp (CC)-tetratricopeptide repeat (TPR) proteins (Prasad 2010, Pubmed ID: 20856808) with potential to interact with Hsp90/Hsp70 as co-chaperones.
AT3G04720	*PATHOGENESIS-RELATED 4 (PR4)*	Encodes a protein similar to the antifungal chitin-binding protein hevein from rubber tree latex. mRNA levels increase in response to ethylene and turnip crinkle virus infection. The mRNA is cell-to-cell mobile.
AT3G04730	*INDOLEACETIC ACID-INDUCED PROTEIN 16 (IAA16)*	early auxin-induced (IAA16)
AT3G04750		Tetratricopeptide repeat (TPR)-like superfamily protein
AT3G04760		Pentatricopeptide repeat (PPR-like) superfamily protein
AT3G04770	*40S RIBOSOMAL PROTEIN SA B (RPSAb)*	40s ribosomal protein SA B
AT3G04790	*EMBRYO DEFECTIVE 3119 (EMB3119)*	Ribose 5-phosphate isomerase, type A protein
AT3G04800	*TRANSLOCASE INNER MEMBRANE SUBUNIT 23-3 (TIM23-3)*	translocase inner membrane subunit 23-3
AT3G04820		Pseudouridine synthase family protein
AT3G04880	*DNA-DAMAGE-REPAIR/TOLERATION 2 (DRT102)*	encodes a novel protein involved in DNA repair from UV damage. Isolated by functional complementation of E. coli UV-sensitive mutants (UVR genes).

AT3G04890		adenine phosphoribosyltransferase-like protein, putative (DUF2358)
AT3G04900		Heavy metal transport/detoxification superfamily protein
AT3G04910	*WITH NO LYSINE (K) KINASE 1 (WNK1)*	Serine/threonine protein kinase, whose transcription is regulated by circadian rhythm.
AT3G04940	*CYSTEINE SYNTHASE D1 (CYSD1)*	Encodes cysteine synthase CysD1.
AT3G04950		SEC-C motif protein
AT3G04960		trichohyalin, putative (DUF3444)
AT3G04970		DHHC-type zinc finger family protein
AT3G04980		DNAJ heat shock N-terminal domain-containing protein
AT3G04990		intracellular protein transporter
AT3G05020	*ACYL CARRIER PROTEIN 1 (ACP1)*	encodes an acyl carrier protein expressed in leaves, roots, and dry seeds. Protein is not regulated by light.
AT3G05040	*HASTY (HST)*	Encodes member of importin/exportin family. Involved in timing of shoot maturation. Involved in miRNA transport. Mutants flower early and have small, curled leaves and reduced abundance of certain miRNA species.
AT3G05050		Protein kinase superfamily protein
AT3G05060		SAR DNA-binding protein, putative, strong similarity to SAR DNA-binding protein-1 (Pisum sativum) GI:3132696; contains Pfam profile PF01798: Putative snoRNA binding domain; encodes NOP58-like protein
AT3G05080		hypothetical protein
AT3G05110		early endosome antigen-like protein, putative (DUF3444)
AT3G05120	*GA INSENSITIVE DWARF1A (GID1A)*	Encodes a gibberellin (GA) receptor ortholog of the rice GA receptor gene (OsGID1). Has GA-binding activity, showing higher affinity to GA4. Interacts with DELLA proteins in vivo in the presence of GA4. The DELLA region alone can interact with GID1A in GA-dependent manner in a Y2H assay.
AT3G05140	*ROP BINDING PROTEIN KINASES 2 (RBK2)*	ROP binding protein kinases 2
AT3G05150		Major facilitator superfamily protein
AT3G05165		Major facilitator superfamily protein
AT3G05180	*(GGL16)*	GDSL-motif esterase/acyltransferase/lipase. Enzyme group with broad substrate specificity that may catalyze acyltransfer or hydrolase reactions with lipid and non-lipid substrates.
AT3G05190		D-aminoacid aminotransferase-like PLP-dependent enzymes superfamily protein
AT3G05200	*ARABIDOPSIS TOXICOS EN LEVADURA 6 (ATL6)*	Encodes a putative RING-H2 zinc finger protein ATL6 (ATL6). The mRNA is cell-to-cell mobile.
AT3G05210	*(ERCC1)*	encodes a homolog of human ERCC1 protein (yeast RAD10), which is a DNA repair endonuclease. Mutants are sensitive to UV-B and gamma radiation (G2 cell cycle phase arrest) and are defective in dark-repair of pyrimidine pyrimidone dimers. This protein incises the 5' end of damaged DNA, similar to ERCC1/RAD10.
AT3G05260		NAD(P)-binding Rossmann-fold superfamily protein
AT3G05270	*VESICLE TETHERING 1 (VETH1)*	Encodes a protein that localizes at motile vesicle-like small compartments in differentiating xylem cells that is associated with microtubule plus-ends. VETH-positive compartments are unlikely to be elements in conventional endomembrane trafficking pathways. It can associate with COG2, and together these two proteins co-localize with the EXO70A1 exocyst subunit, tethering EXO70A1 to compartments associated with cortical microtubules.
AT3G05310	*MIRO-RELATED GTP-ASE 3 (MIRO3)*	Encodes a protein with similarity to MIRO GTPases.
AT3G05320	*O-FUCOSYL TRANSFERASE 1 (OFT1)*	Golgi localized protein with similarity to protein O-fucosyltransferases. Mutants show lower seed set/reduced fertility. Mutant pollen fails to compete with wild type due to the inability to penetrate the stigma-style boundary.
AT3G05360	*RECEPTOR LIKE PROTEIN 30 (RLP30)*	receptor like protein 30
AT3G05390		S-adenosyl-L-methionine-dependent methyltransferase
AT3G05400		Major facilitator superfamily protein
AT3G05440		C2 domain-containing protein
AT3G05460		sporozoite surface protein-like protein
AT3G05470	*FORMIN 11 (FH11)*	Actin-binding FH2 (formin homology 2) family protein
AT3G05490	*RALF-LIKE 22 (RALFL22)*	Member of a diversely expressed predicted peptide family showing sequence similarity to tobacco Rapid Alkalinization Factor (RALF), and is believed to play an essential role in the physiology of Arabidopsis. Consists of a single exon and is characterized by a conserved C-terminal motif and N-terminal signal peptide.
AT3G05500	*LD-ASSOCIATED PROTEIN 3 (LDAP3)*	Encodes a protein that associates with lipid droplet surfaces and shares sequence homology with family of small rubber particle proteins. The mRNA is cell-to-cell mobile.
AT3G05510		Phospholipid/glycerol acyltransferase family protein
AT3G05540	*TRANSLATIONALLY CONTROLLED TUMOR PROTEIN 2 (TCTP2)*	Encodes TCTP2, a homolog of Translationally Controlled Tumor Protein. Enhances in vitro plant regeneration.
AT3G05580	*TYPE ONE PROTEIN PHOSPHATASE 9 (TOPP9)*	Encodes a Type One Protein Phosphatase that acts as a nucleocytoplasmic negative regulator of tip growth. Mutants affect pollen germination, pollen tube growth, and root hair growth. It acts genetically downstream of ANX1 (AT3G04690) and ANX2 (AT5G28680) and is functionally redundant with TOPP8 (AT5G27840).
AT3G05600	*EPOXIDE HYDROLASE 1 (ATEH1)*	Encodes a cytosolic epoxide hydrolase capable of acting on 9,10-epoxystearic acid and on 12,13- epoxyoctadec-9-enoic acid that is involved in the synthesis of poly-hydroxylated cutin monomers.
AT3G05610		Plant invertase/pectin methylesterase inhibitor superfamily
AT3G05620		Plant invertase/pectin methylesterase inhibitor superfamily

AT3G05630	*PHOSPHOLIPASE D P2 (PLDP2)*	Encodes a member of the PXPH-PLD subfamily of phospholipase D proteins. Regulates vesicle trafficking. Required for auxin transport and distribution and hence auxin responses. This subfamily is novel structurally different from the majority of plant PLDs by having phox homology (PX) and pleckstrin homology (PH) domains. Involved regulating root development in response to nutrient limitation. Plays a major role in phosphatidic acid production during phosphate deprivation. Induced upon Pi starvation in both shoots and roots. Involved in hydrolyzing phosphatidylcholine and phosphatidylethanolamine to produce diacylglycerol for digalactosyldiacylglycerol synthesis and free Pi to sustain other Pi-requiring processes. Does not appear to be involved in root hair patterning. Expression is upregulated in the shoot of cax1/cax3 mutant and is responsive to phosphate (Pi) and not phosphite (Phi) in roots and shoots.
AT3G05640	*E GROWTH-REGULATING 1 (EGR1)*	EGR1 functions as a negative regulator of plant growth with prominent effect on plant growth during drought stress.EGR1 regulates microtubule organization and likely affects additional cytoskeleton and trafficking processes along the plasma membrane.
AT3G05650	*RECEPTOR LIKE PROTEIN 32 (RLP32)*	receptor like protein 32
AT3G05660	*RECEPTOR LIKE PROTEIN 33 (RLP33)*	receptor like protein 33
AT3G05670		RING/U-box protein
AT3G05675		BTB/POZ domain-containing protein
AT3G05690	*NUCLEAR FACTOR Y, SUBUNIT A2 (NF-YA2)*	Encodes a subunit of CCAAT-binding complex, binds to CCAAT box motif present in some plant promoter sequences. One of three members of this class (HAP2A, HAP2B, HAP2C), it is expressed in vegetative and reproductive tissues.
AT3G05720	*IMPORTIN ALPHA ISOFORM 7 (IMPA-7)*	Putative importin alpha isoform. When overexpressed can rescue the impa-4 decreased transformation susceptibility phenotype.
AT3G05730		Encodes a defensin-like (DEFL) family protein. The mRNA is cell-to-cell mobile.
AT3G05760		C2H2 and C2HC zinc fingers superfamily protein
AT3G05770		hypothetical protein
AT3G05780	*LON PROTEASE 3 (LON3)*	Encodes a member of the Lon protease-like proteins (Lon1/At5g26860, Lon2/At5g47040, Lon3/At3g05780, Lon4/At3g05790). Lon is a multifunctional ATP-dependent protease which exists in bacteria, archaea and within organelles in eukaryotic cells. Lon proteases are responsible for the degradation of abnormal, damaged and unstable proteins.
AT3G05790	*LON PROTEASE 4 (LON4)*	Encodes a member of the Lon protease-like proteins (Lon1/At5g26860, Lon2/At5g47040, Lon3/At3g05780, Lon4/At3g05790). Lon is a multifunctional ATP-dependent protease which exists in bacteria, archaea and within organelles in eukaryotic cells. Lon proteases are responsible for the degradation of abnormal, damaged and unstable proteins.
AT3G05800	*ATBS1(ACTIVATION-TAGGED BRI1 SUPPRESSOR 1)-INTERACTING FACTOR 1 (AIF1)*	AtBS1(activation-tagged BRI1 suppressor 1)-interacting factor 1
AT3G05810		IGR motif protein
AT3G05820	*INVERTASE H (INVH)*	Encodes a putative plastid-targeted alkaline/neutral invertase.Expression is induced by salt, osmotic and ABA treatments. Loss of function affects mitochondrial functioning and ROS production.
AT3G05830	*(ATNEAP1)*	Encodes alpha-helical IF (intermediate filament)-like protein.NEAP1 is a member of a small family containing coiled-coil domains, a nuclear localization signal and a C-terminal predicted transmembrane domain. It localizes to the nuclear periphery. Mutants have altered nuclear morphology and chromatin structure.
AT3G05860	*AGAMOUS-LIKE 45 (AGL45)*	MADS-box transcription factor family protein
AT3G05880	*RARE-COLD-INDUCIBLE 2A (RCI2A)*	Induced by low temperatures, dehydration and salt stress and ABA. Encodes a small (54 amino acids), highly hydrophobic protein that bears two potential transmembrane domains.
AT3G05890	*RARE-COLD-INDUCIBLE 2B (RCI2B)*	Low temperature and salt responsive protein family
AT3G05900		neurofilament protein-like protein
AT3G05920	*HEAVY METAL ASSOCIATED PROTEIN 24 (ATHMP24)*	Heavy metal transport/detoxification superfamily protein
AT3G05930	*GERMIN-LIKE PROTEIN 8 (GLP8)*	germin-like protein (GLP8)
AT3G05950		RmlC-like cupins superfamily protein
AT3G05970	*LONG-CHAIN ACYL-COA SYNTHETASE 6 (LACS6)*	encode peroxisomal long-chain acyl-CoA synthetase (LACS) isozymes
AT3G05980		hypothetical protein
AT3G06000		RNI-like superfamily protein
AT3G06010	*(ATCHR12)*	Encodes AtCHR12, a SNF2/Brahma-type chromatin-remodeling protein. AtCHR12 mediates temporary growth arrest in Arabidopsis upon perceiving environmental stress.
AT3G06020	*FANTASTIC FOUR 4 (FAF4)*	A member of the FAF family proteins encoded by the FANTASTIC FOUR (FAF) genes: AT4G02810 (FAF1), AT1G03170 (FAF2), AT5G19260 (FAF3) and AT3G06020 (FAF4). FAFs have the potential to regulate shoot meristem size in Arabidopsis thaliana. FAFs can repress WUS, which ultimately leads to an arrest of meristem activity in FAF overexpressing lines.
AT3G06030	*NPK1-RELATED PROTEIN KINASE 3 (NP3)*	NPK1-related protein kinase 3
AT3G06035		Glycoprotein membrane precursor GPI-anchored
AT3G06070		hypothetical protein
AT3G06080	*TRICHOME BIREFRINGENCE-LIKE 10 (TBL10)*	Encodes a member of the TBL (TRICHOME BIREFRINGENCE-LIKE) gene family containing a plant-specific DUF231 (domain of unknown function) domain. TBL gene family has 46 members, two of which (TBR/AT5G06700 and TBL3/AT5G01360) have been shown to be involved in the synthesis and deposition of secondary wall cellulose, presumably by influencing the esterification state of pectic polymers. A nomenclature for this gene family has been proposed (Volker Bischoff & Wolf Scheible, 2010, personal communication).
AT3G06090	*PRECURSOR OF PAMP-INDUCED PEPTIDE-LIKE 2 (PREPIPL2)*	homolog of prePIP1
AT3G06100	*NOD26-LIKE INTRINSIC PROTEIN 7;1 (NIP7;1)*	Encodes NIP7;1, an anther-specific boric acid transporter of the aquaporin superfamily regulated by an unusual tyrosine in helix 2 of the transport pore.
AT3G06110	*MAPK PHOSPHATASE 2 (MKP2)*	Encodes a nuclear-localized MAP kinase phosphatase. Plants with reduced levels of MKP2 transcripts are hypersensitive to ozone and ozone-mediated activation of MPK3 and MPK6 is prolonged in these plants.

AT3G06120	*MUTE (MUTE)*	Encodes a basic helix-loop-helix (bHLH) protein that controls meristemoid differentiation during stomatal development. In the absence of MUTE, meristemoids abort after excessive asymmetric divisions and fail to differentiate stomata. MUTE expression in the meristemoid is required for SLGCs differentiation as pavement cells. Epidermal cells lose their competence to respond to MUTE overexpression during cotyledon development.
AT3G06130	*HEAVY METAL ASSOCIATED PROTEIN 25 (ATHMP25)*	Heavy metal transport/detoxification superfamily protein
AT3G06160	*(REM18)*	AP2/B3-like transcriptional factor family protein
AT3G06170		Serinc-domain containing serine and sphingolipid biosynthesis protein
AT3G06200		P-loop containing nucleoside triphosphate hydrolases superfamily protein
AT3G06210		ARM repeat superfamily protein
AT3G06220	*(REM19)*	AP2/B3-like transcriptional factor family protein
AT3G06230	*MAP KINASE KINASE 8 (MKK8)*	member of MAP Kinase Kinase
AT3G06260	*GALACTURONOSYLTRANSFERASE-LIKE 4 (GATL4)*	Encodes a protein with putative galacturonosyltransferase activity.
AT3G06280		F-box associated ubiquitination effector family protein
AT3G06300	*PROLYL 4-HYDROXYLASE 2 (P4H2)*	Encodes a prolyl-4 hydroxylase that can hydroxylate poly(L-proline)and other proline rich peptides, including those with sequences corresponding to those in arabinogalactan proteins and extensins. The mRNA is cell-to-cell mobile.
AT3G06360	*ARABINOGALACTAN PROTEIN 27 (AGP27)*	Encodes an arabinogalactan-protein (AGP27).
AT3G06380	*TUBBY-LIKE PROTEIN 9 (TLP9)*	Member of plant TLP family which differs in having an F box domain. Interacts with ASK proteins.Plasma membrane tethering is mediated by PIP2 binding domain. Mutants are insensitive to ABA. May act redundantly with its paralog TPL3.
AT3G06390	*CASP-LIKE PROTEIN 1D2 (CASPL1D2)*	Uncharacterized protein family (UPF0497)
AT3G06420	*AUTOPHAGY 8H (ATG8H)*	Autophagy protein.
AT3G06440	*(GALT3)*	Encodes a Golgi-localized hydroxyproline-O-galactosyltransferase. Mutants display multiple phenotypes including reduced root hair growth and reduced seed coat mucilage.
AT3G06460	*ELO HOMOLOG 1 (ELO1)*	ELO family protein containing a characteristic histidine motif which binds to AtCb5-B, interacts with AtBI-1
AT3G06470	*ELO HOMOLOG 2 (ELO2)*	ELO family protein containing a characteristic histidine motif which binds to AtCb5-B, interacts with AtBI-1. Together with AtCb5-B interacts with KCR1, PAS2, and CER10, which are essential for the synthesis of VLCFAs.
AT3G06483	*PYRUVATE DEHYDROGENASE KINASE (PDK)*	Pyruvate dehydrogenase kinase (PDK) specifically phosphorylates the E1α subunit of the pyruvate dehydrogenase complex (PDC) on a Ser residue using ATP as a phosphate donor. PDK is a unique type of protein kinase having a His-kinase-like sequence but Ser-kinase activity. Site-directed mutagenesis and structural analysis indicate that PDK belongs to the GHKL superfamily.
AT3G06490	*MYB DOMAIN PROTEIN 108 (MYB108)*	Encodes a MYB transcription factor involved in regulating anther dehiscence as well as regulating cell death, and cuticle-related Botrytis immunity.
AT3G06510	*SENSITIVE TO FREEZING 2 (SFR2)*	Encodes a protein with beta-glucosidase and galactosyltransferase activity, mutants show increased sensitivity to freezing. Though it is classified as a family I glycosyl hydrolase, it has no hydrolase activity in vitro.
AT3G06530		ARM repeat superfamily protein
AT3G06540	*RAB ESCORT PROTEIN (REP)*	Encodes a cytoplasmic Rab escort protein that preferentially binds the GDP-bound form of Rab and stimulates geranylgeranylation of various Rab GTPases in Arabidopsis extracts in vitro.
AT3G06560	*POLY(A) POLYMERASE 3 (PAPS3)*	Encodes a poly(A) polymerase. Located in the cytoplasm.
AT3G06590	*ATBS1 INTERACTING FACTOR 2 (AIF2)*	Encodes RITF1, a bHLH transcription factor that regulates the transcription of several genes involved in the detoxification of reactive oxygen species generated by salt stress.
AT3G06600	*(SINE3)*	At3G06600 encodes the plant KASH protein SINE3; SINE3 interacts with SUN1 and SUN2 and is localized at the nuclear envelope.
AT3G06630		protein kinase family protein
AT3G06640		PAS domain-containing protein tyrosine kinase family protein
AT3G06650	*ATP-CITRATE LYASE B-1 (ACLB-1)*	One of the two genes encoding subunit B of the trimeric enzyme ATP Citrate lyase
AT3G06710		E3 ubiquitin ligase
AT3G06740	*GATA TRANSCRIPTION FACTOR 15 (GATA15)*	Encodes a member of the GATA factor family of zinc finger transcription factors.
AT3G06760	*DROUGHT INDUCED 9-4 (ATDIL9-4)*	Drought-responsive family protein
AT3G06780		glycine-rich protein
AT3G06800		
AT3G06810	*IBA-RESPONSE 3 (IBR3)*	Encodes a protein with similarity to acyl-CoA dehydrogenases. Mutations in IBR3 render plants resistant to indole-3-butryic acid, a putative storage form of the biologically active auxin IAA (indole-3-acetic acid). IBR3 is hypothesized to carry out the second step in a β-oxidation-like process of IBA metabolism in Arabidopsis. Though its subcellular location has not been determined, IBR3 has a peroxisomal targeting sequence and two other putative IBA metabolic enzymes (IBR1 and IBR10) can be found in this organelle. No specific enzymatic activity has been documented for IBR3, but double mutant analyses with CHY1 argue against a role for IBR3 in general fatty acid β-oxidation. The mRNA is cell-to-cell mobile.
AT3G06830		Plant invertase/pectin methylesterase inhibitor superfamily
AT3G06840		hypothetical protein
AT3G06850	*(BCE2)*	dihydrolipoamide branched chain acyltransferase
AT3G06860	*MULTIFUNCTIONAL PROTEIN 2 (MFP2)*	Encodes a multifunctional protein. Involved in peroxisomal fatty acid beta oxidation. Loss-of-function mutant lacks hydroxyacyl-CoA dehydrogenase activity and have reduced levels of long-chain enoyl-CoA hydratase activity. The mutant has fewer but larger peroxisomes. The mRNA is cell-to-cell mobile.
AT3G06870		proline-rich family protein
AT3G06890		transmembrane protein
AT3G06895		syntaxin KNOLLE-like protein

AT3G06930	*PROTEIN ARGININE METHYLTRANSFERASE 4B (PRMT4B)*	Encodes an type I protein arginine methyltransferase. PRMT4b can catalyze the asymmetric dimethylation of arginines 2,17, and 26 on histone 3 and can also methylate myelin basic protein in vitro. Double mutants lacking PRMT4a and 4b have reduced levels of histone 3 methylated at R17. These double mutants flower late due to defects in the autonomous pathway and they have elevated levels of FLC transcripts.
AT3G06970		RNA-binding (RRM/RBD/RNP motifs) family protein
AT3G06990		Cysteine/Histidine-rich C1 domain family protein
AT3G07000		Cysteine/Histidine-rich C1 domain family protein
AT3G07010		Pectin lyase-like superfamily protein
AT3G07030	*(ALBA6)*	Alba DNA/RNA-binding protein
AT3G07040	*RESISTANCE TO P. SYRINGAE PV MACULICOLA 1 (RPM1)*	Contains an N-terminal tripartite nucleotide binding site and a C-terminal tandem array of leucine-rich repeats. Confers resistance to Pseudomonas syringae strains that carry the avirulence genes avrB and avrRpm1.
AT3G07050	*NUCLEOSTEMIN-LIKE 1 (NSN1)*	Arabidopsis NSN1 encodes a nucleolar GTP- binding protein and is required for maintenance of inflorescence meristem identity and floral organ development.
AT3G07070	*PBS1-LIKE 26 (PBL26)*	Protein kinase superfamily protein
AT3G07130	*PURPLE ACID PHOSPHATASE 15 (PAP15)*	Encodes PAP15, a purple acid phosphatase with phytase activity. Expression of PAP15 is developmentally and temporally regulated, with strong expression at the early stages of seedling growth and pollen germination. The expression is also organ/tissue-specific, with strongest expression in the vasculature, pollen grains, and roots. Recombinant PAP protein exhibits broad substrate specificity with moderate phytase activity. PAP15 likely mobilizes phosphorus reserves in plants, particularly during seed and pollen germination.
AT3G07140		GPI transamidase component Gpi16 subunit family protein
AT3G07150		amino acid-ligase
AT3G07200	*SUMO-TARGETED UBIQUITIN E3 LIGASE 3 (STUBL3)*	RING/U-box superfamily protein. SUMO- targeted ubiquitin ligase.
AT3G07240		
AT3G07250		RNA-binding (RRM-RBD-RNP motif) domain nuclear transport factor 2 family protein
AT3G07260		SMAD/FHA domain-containing protein
AT3G07270		GTP cyclohydrolase I
AT3G07300		NagB/RpiA/CoA transferase-like superfamily protein
AT3G07310		phosphoserine aminotransferase, putative (DUF760)
AT3G07320		O-Glycosyl hydrolases family 17 protein
AT3G07340	*CRY2-INTERACTING BHLH 3 (CIB3)*	basic helix-loop-helix (bHLH) DNA-binding superfamily protein
AT3G07350		sulfate/thiosulfate import ATP-binding protein, putative (DUF506)
AT3G07360	*PLANT U-BOX 9 (PUB9)*	Encodes a protein containing a U-box and an ARM domain. This protein has E3 ubiquitin ligase activity based on in vitro assays.
AT3G07370	*CARBOXYL TERMINUS OF HSC70-INTERACTING PROTEIN (CHIP)*	Encodes AtCHIP, a new class of E3 ubiquitin ligases with three tetratricopeptide repeats and a U-box domain, structurally similar to the animal CHIP proteins. Plays an important role in plant cellular metabolism under temperature stress conditions. Functions as an E3 ubiquitin ligase of protein phosphatase 2A subunits and alters plant response to abscisic acid treatment. Belongs to one of the 36 carboxylate clamp (CC)-tetratricopeptide repeat (TPR) proteins (Prasad 2010, Pubmed ID: 20856808) with potential to interact with Hsp90/Hsp70 as co-chaperones.
AT3G07380		glycosyltransferase family protein (DUF23)
AT3G07390	*AUXIN-INDUCED IN ROOT CULTURES 12 (AIR12)*	isolated from differential screening of a cDNA library from auxin-treated root culture. sequence does not show homology to any known proteins and is predicted to be extracellular. The mRNA is cell-to-cell mobile.
AT3G07410	*RAB GTPASE HOMOLOG A5B (RABA5b)*	RAB GTPase homolog A5B
AT3G07420	*ASPARAGINYL-TRNA SYNTHETASE 2 (NS2)*	Encodes an asparaginyl-tRNA synthetase.
AT3G07450		Bifunctional inhibitor/lipid-transfer protein/seed storage 2S albumin superfamily protein
AT3G07470		DUF538 protein
AT3G07490	*ARF-GAP DOMAIN 11 (AGD11)*	A member of ARF GAP domain (AGD), A thaliana has 15 members, grouped into four classes.
AT3G07540	*FORMIN 10 (FH10)*	Actin-binding FH2 (formin homology 2) family protein
AT3G07550		RNI-like superfamily protein
AT3G07560	*PEROXIN 13 (PEX13)*	Encodes peroxin 13 (PEX13) involved in protein transport into peroxisomes, for example, peroxisomal import of nitric oxide synthase.
AT3G07570		Cytochrome b561/ferric reductase transmembrane with DOMON related domain-containing protein
AT3G07580		hypothetical protein
AT3G07590	*(SMD1A)*	SmD1a is one of two Yeast SmD1 orthologs, lower levels than SmD1b.It is localized to the nucleus and may play a minor role in RNA splicing and indirectly facilitating PTGS.
AT3G07600		Heavy metal transport/detoxification superfamily protein
AT3G07610	*INCREASE IN BONSAI METHYLATION 1 (IBM1)*	IBM1 likely encodes a protein with histone H3mK9 demethylation activity. It may preferentially demethylate H3mK9 at low-copy loci to protect them from silencing by nearby heterochromatin by preventing the spread of cytosine methylation. BONSAI (At1g73177) is hypermethylated in ibm1 mutants. ibm1 mutants have morphological defects that become apparent at the F3 generation, including small narrow leaves, arrested flower development, and faulty pollen development. These phenotypes cannot result solely from the BONSAI hypermethylation. Aberrant phenotypes in ibm1 mutants in both DNA methylation and plant development can be suppressed by mutations in the KYP H3K9 methyltransferase or the CMT3 non CG-cytosine methylase.
AT3G07630	*AROGENATE DEHYDRATASE 2 (ADT2)*	Encodes a plastid-localized arogenate dehydratase involved in phenylalanine biosynthesis. Not less than six genes encoding ADT were identiﬁed in the Arabidopsis genome: ADT1 [At1g11790]; ADT2 [At3g07630]; ADT3 [At2g27820]; ADT4 [At3g44720]; ADT5 [At5g22630]; and ADT6 [At1g08250].
AT3G07640	*(PBAC5)*	period circadian protein

AT3G07650	*CONSTANS-LIKE 9 (COL9)*	This gene belongs to the CO (CONSTANS) gene family. This gene family is divided in three subgroups: groups III, to which COL9 belongs, is characterised by one B-box (supposed to regulate protein-protein interactions) and a second diverged zinc finger. COL9 downregulates expression of CO (CONSTANS) as well as FT and SOC1 which are known regulatory targets of CO. The mRNA is cell-to-cell mobile.
AT3G07670		Rubisco methyltransferase family protein
AT3G07680	*P24 SUBFAMILY BETA 2 (P24BETA2)*	Encodes an Golgi-localized p24 protein. Interacts with p24delta5 at ER export sites for ER exit and coupled transport to the Golgi apparatus. The mRNA is cell-to-cell mobile.
AT3G07690		6-phosphogluconate dehydrogenase family protein
AT3G07700	*SALT-INDUCED ABC1 KINASE 1 (SIA1)*	ABC1K7 is a member of an atypical protein kinase family that is induced by salt stress. Loss of function mutations affect the metabolic profile of chloroplast lipids. It appears to function along with ABC1K8 in mediating lipid membrane changes in response to stress.
AT3G07710		hypothetical protein
AT3G07730		hypothetical protein
AT3G07750	*RIBOSOMAL RNA PROCESSING 42 (RRP42)*	3-5-exoribonuclease family protein
AT3G07770	*HEAT SHOCK PROTEIN 89.1 (Hsp89.1)*	HEAT SHOCK PROTEIN 89.1
AT3G07820		Pectin lyase-like superfamily protein
AT3G07830	*POLYGALACTURONASE 3 (PGA3)*	Pectin lyase-like superfamily protein
AT3G07840		Pectin lyase-like superfamily protein
AT3G07850		Pectin lyase-like superfamily protein
AT3G07860		Ubiquitin-like superfamily protein
AT3G07870	*F-BOX PROTEIN92 (FBX92)*	FBX92 is an F-box containing protein. Overexpression produces plants with smaller leaves while reduced expression is correlated with increased leaf size and increased rates of cell proliferation.
AT3G07880	*SUPERCENTIPEDE1 (SCN1)*	RhoGTPase GDP dissociation inhibitor (RhoGDI) that spatially restricts the sites of growth to a single point on the trichoblast. It regulates the NADPH oxidase RHD2/AtrbohC, which is required for hair growth.
AT3G07900		O-fucosyltransferase family protein
AT3G07920		Translation initiation factor IF2/IF5
AT3G07940		Calcium-dependent ARF-type GTPase activating protein family
AT3G07960	*PHOSPHATIDYLINOSITOL-4-PHOSPHATE 5-KINASE 6 (PIP5K6)*	Encodes phosphatidylinositol-4-phosphate 5-kinase 6 (PIP5K6). Regulates clathrin-dependent endocytosis in pollen tubes.
AT3G07970	*QUARTET 2 (QRT2)*	Required for pollen separation during normal development. In qrt mutants, the outer walls of the four meiotic products of the pollen mother cell are fused, and pollen grains are released in tetrads.May be required for cell type-specific pectin degradation.
AT3G07990	*SERINE CARBOXYPEPTIDASE-LIKE 27 (SCPL27)*	serine carboxypeptidase-like 27
AT3G08010	*(ATAB2)*	Encodes a chloroplast-localized protein ATAB2. ATAB2 is involved in the biogenesis of Photosystem I and II. ATAB2 has A/U-rich RNA-binding activity and presumably functions as an activator of translation with targets at PS I and PS II.
AT3G08030	*(ATHA2-1)*	The mRNA of this gene is expressed in viable seeds. Its detection in a dry seed lot has potential for use as a molecular marker for germination performance as absence of expression correlates with decreased germination. Encodes DUF642 cell wall protein.
AT3G08040	*FERRIC REDUCTASE DEFECTIVE 3 (FRD3)*	Encodes a member of the MATE (multidrug and toxin efflux family), expressed in roots but not shoots. Mutants accumulate excess iron, manganese and zinc, and express root Fe(III) chelatase activity even under iron sufficiency conditions. FRD3 is likely to function in root xylem loading of an iron chelator or other factor necessary for efficient iron uptake out of the xylem or apoplastic space and into leaf cells.
AT3G08490		delta-latroinsectotoxin-Lt1a protein
AT3G08500	*MYB DOMAIN PROTEIN 83 (MYB83)*	Encodes a putative R2R3-type MYB transcription factor (MYB83).
AT3G08510	*PHOSPHOLIPASE C 2 (PLC2)*	Phosphoinositide-specific phospholipase C (PI-PLC), catalyzes hydrolysis of phosphatidylinositol 4,5-bisphosphate into inositol 1,4,5-trisphosphate and diacylglycerol. It is involved in auxin biosynthesis and signaling, modulating development of both male and female gametophytes. It also regulates MAMP-triggered immunity by modulating ROS production.
AT3G08560	*VACUOLAR H+-ATPASE SUBUNIT E ISOFORM 2 (VHA-E2)*	vacuolar H+-ATPase subunit E isoform 2
AT3G08570		Phototropic-responsive NPH3 family protein
AT3G08590	*2,3-BIPHOSPHOGLYCERATE-INDEPENDENT PHOSPHOGLYCERATE MUTASE 2 (iPGAM2)*	Encodes a 2,3-biphosphoglycerate-independent phosphoglycerate mutase that is involved in pollen development and stomatal movement.
AT3G08600		transmembrane protein, putative (DUF1191)
AT3G08610		NADH dehydrogenase ubiquinone 1 alpha subcomplex subunit
AT3G08630	*RETICULATA-RELATED 2 (RER2)*	alphavirus core family protein (DUF3411)
AT3G08660		Phototropic-responsive NPH3 family protein
AT3G08690	*UBIQUITIN-CONJUGATING ENZYME 11 (UBC11)*	ubiquitin-conjugating enzyme 11
AT3G08700	*UBIQUITIN-CONJUGATING ENZYME 12 (UBC12)*	ubiquitin-conjugating enzyme 12
AT3G08730	*PROTEIN-SERINE KINASE 1 (PK1)*	Encodes a protein-serine kinase that phosphorylates ribosomal protein in vitro. Activation of AtS6k is regulated by 1-naphthylacetic acid and kinetin, at least in part, via a lipid kinase-dependent pathway. Involved in translational up-regulation of ribosomal proteins. Phosphorylated by PDK1. Interacts with RAPTOR1, which in turn interacts with TOR. SPK6 activity is affected by osmotic stress, and plants overexpressing S6k1 are hypersensitive to osmotic stress. The gene is expressed in all tissues examined, with highest expression level detected in metabolically active tissues.
AT3G08740		elongation factor P (EF-P) family protein
AT3G08750		F-box and associated interaction domains-containing protein
AT3G08760	*(ATSIK)*	Encodes an osmotic stress-inducible kinase that functions as a negative regulator of osmotic stress signaling in plants.
AT3G08770	*LIPID TRANSFER PROTEIN 6 (LTP6)*	Predicted to encode a PR (pathogenesis-related) protein. Belongs to the lipid transfer protein (PR-14) family with the following members: At2g38540/LTP1, At2g38530/LTP2, At5g59320/LTP3, At5g59310/LTP4, At3g51600/LTP5, At3g08770/LTP6, At2g15050/LTP7, At2g18370/LTP8, At2g15325/LTP9, At5g01870/LTP10, At4g33355/LTP11, At3g51590/LTP12, At5g44265/LTP13, At5g62065/LTP14, At4g08530/LTP15.

AT3G08780		BRISC complex subunit Abro1-like protein
AT3G08810		Galactose oxidase/kelch repeat superfamily protein
AT3G08820		Pentatricopeptide repeat (PPR) superfamily protein
AT3G08840		D-alanine-D-alanine ligase family
AT3G08850	*(RAPTOR1)*	Encodes one of two Arabidopsis RAPTOR/KOG1 homologs. RAPTOR proteins are binding partners of the target of rapamycin kinase that is present in all eukaryotes and play a central role in the stimulation of cell growth and metabolism in response to nutrients. Mutants show embryo lethal phenotype which occurs at pre-globular stage. May interact with TOR kinase in a rapamycin like signaling pathway. Interacts with TOR and S6K1 in vivo. Overexpression of RAPTOR1 rendered the S6K1 osmotic stress insensitive.
AT3G08860	*PYRIMIDINE 4 (PYD4)*	Encodes a protein that is predicted to have beta-alanine aminotransferase activity.
AT3G08870	*L-TYPE LECTIN RECEPTOR KINASE VI.1 (LECRK-VI.1)*	Concanavalin A-like lectin protein kinase family protein
AT3G08880	*MERISTEMS UNSTRUCTURED (MUN)*	Encodes a kinetochore hub-protein that is required for chromosome segregation to ensure proper cell division and the maintenance of plant architecture.
AT3G08890		hypothetical protein (Protein of unknown function, DUF538)
AT3G08900	*REVERSIBLY GLYCOSYLATED POLYPEPTIDE 3 (RGP3)*	RGP3 is a UDP-arabinose mutase that catalyzes the interconversion between the pyranose and furanose forms of UDP-L-arabinose. It is a reversibly autoglycosylated protein. Fluorescently-tagged RGP3 is found in the cytosol and associated with Golgi-like particles when expressed in tobacco leaves. An RGP3-YFP fusion protein under the control a native promoter can be found in the endosperm of Arabidopsis embryos during the linear and bent cotyledon stages of development.
AT3G08920	*RHODANESE-LIKE PROTEIN (STR10)*	Rhodanese/Cell cycle control phosphatase superfamily protein
AT3G08940	*LIGHT HARVESTING COMPLEX PHOTOSYSTEM II (LHCB4.2)*	Lhcb4.2 protein (Lhcb4.2, protein involved in the light harvesting complex of photosystem II The mRNA is cell-to-cell mobile.
AT3G08950	*HOMOLOGUE OF THE COPPER CHAPERONE SCO1 (HCC1)*	Encodes HCC1, homologue of the copper chaperone SCO1 (synthesis of cytochrome c oxidase 1) from the yeast Saccharomyces cerevisiae. SCO1 encodes a mitochondrial protein that is essential for the correct assembly of complex IV in the respiratory chain. HCC1 is localized in the mitochondrion. A chimeric yeast Sco1-Arabidopsis HCC1 protein complements yeast Sco1 activity. Embryos of hcc1 mutants became arrested at various developmental stages, mostly at the heart stage.
AT3G08960	*(KA120)*	Ran effector.
AT3G08970	*(ATERDJ3A)*	J domain protein localized in ER lumen. Can compensate for the growth defect in jem1 scj1 mutant yeast. Also shows similarity to HSP40 proteins and is induced by heat stress. At high temperatures, mutant alleles are not transmitted through the pollen due to defects in pollen tube growth.
AT3G08980		Peptidase S24/S26A/S26B/S26C family protein
AT3G08990		Yippee family putative zinc-binding protein
AT3G09010		Protein kinase superfamily protein
AT3G09020		alpha 1,4-glycosyltransferase family protein
AT3G09030	*ENDOPLASMIC RETICULUM-ARRESTED PEN3 (EAP3)*	EAP3 is a cytolsolic BTB/POZ-domain protein involved in trafficking of PEN3.
AT3G09040	*MITOCHONDRIAL RNA EDITING FACTOR 12 (MEF12)*	Pentatricopeptide repeat (PPR) superfamily protein
AT3G09050		8-amino-7-oxononanoate synthase
AT3G09060		Pentatricopeptide repeat (PPR) superfamily protein
AT3G09070	*OCTOPUS (OPS)*	Encodes a polarly localised membrane-associated protein that regulates phloem differentiation entry.
AT3G09080		Transducin/WD40 repeat-like superfamily protein
AT3G09085		transmembrane protein (DUF962)
AT3G09100		mRNA capping enzyme family protein
AT3G09130		hypothetical protein
AT3G09140		hypothetical protein (DUF674)
AT3G09160		RNA-binding (RRM/RBD/RNP motifs) family protein
AT3G09170		transposable_element_gene;similar to Ulp1 protease family protein [Arabidopsis thaliana] (TAIR:AT4G04545.1);(source:TAIR10)
AT3G09210	*PLASTID TRANSCRIPTIONALLY ACTIVE 13 (PTAC13)*	plastid transcriptionally active 13
AT3G09220	*LACCASE 7 (LAC7)*	putative laccase, a member of laccase family of genes (17 members in Arabidopsis).
AT3G09240	*BRASSINOSTEROID-SIGNALING KINASE 9 (BSK9)*	kinase with tetratricopeptide repeat domain-containing protein
AT3G09260	*(PYK10)*	Encodes beta-glucosidase.The major constituent of ER bodies. One of the most abundant proteins in Arabidopsis seedlings. Exist in an soluble (inactive) and non-soluble (active) form, most probably formed in a polymerization process. Involved in the mutualistic interaction between Arabidopsis and the endophytic fungus Piriformospora indica.
AT3G09270	*GLUTATHIONE S-TRANSFERASE TAU 8 (GSTU8)*	Encodes glutathione transferase belonging to the tau class of GSTs. Naming convention according to Wagner et al. (2002).
AT3G09280		transmembrane protein
AT3G09290	*TELOMERASE ACTIVATOR1 (TAC1)*	encodes activation factor TAC1 which mediates telomerase activity
AT3G09310		membrane protein insertion efficiency factor
AT3G09320		DHHC-type zinc finger family protein
AT3G09330		Transmembrane amino acid transporter family protein
AT3G09360	*(BRF2)*	Cyclin/Brf1-like TBP-binding protein. Double mutants with BRF1 show defects in pollen development. Controls FES1A regulated thermosensitivity.
AT3G09380		MIP18 family protein
AT3G09390	*METALLOTHIONEIN 2A (MT2A)*	metallothionein, binds to and detoxifies excess copper and other metals, limiting oxidative damage
AT3G09410	*PECTIN ACETYESTERASE 5 (ATPAE5)*	Pectinacetylesterase family protein
AT3G09420		
AT3G09430		peptide transporter family protein

AT3G09440		Heat shock protein 70 (Hsp 70) family protein
AT3G09470	*AT UNCOORDINATED PROTEIN 93 (ATUNC93)*	Protein similar to UNC93 of C.elegans. Mutants are hypersensitive to ABA treatment and salt sensitive and have disregulated K+ accumulation.
AT3G09480	*(HTB7)*	Histone superfamily protein
AT3G09490		Tetratricopeptide repeat (TPR)-like superfamily protein
AT3G09520	*EXOCYST SUBUNIT EXO70 FAMILY PROTEIN H4 (EXO70H4)*	A member of EXO70 gene family, putative exocyst subunits, conserved in land plants. Arabidopsis thaliana contains 23 putative EXO70 genes, which can be classified into eight clusters on the phylogenetic tree.
AT3G09530	*EXOCYST SUBUNIT EXO70 FAMILY PROTEIN H3 (EXO70H3)*	A member of EXO70 gene family, putative exocyst subunits, conserved in land plants. Arabidopsis thaliana contains 23 putative EXO70 genes, which can be classified into eight clusters on the phylogenetic tree.
AT3G09560	*PHOSPHATIDIC ACID PHOSPHOHYDROLASE 1 (PAH1)*	The PAH1 gene encodes a phosphatidate phosphohydrolase. Mutant analysis revealed its involvement in galactolipid synthesis pathway, and the membrane lipid remodeling. The pah1pah2 double-mutant showed enhanced Al-susceptibility under low-P conditions, but there was no significant differences in Al tolerance between pah1pah2 and wild type when they were grown in a solution containing 35 μM Pi.
AT3G09580		FAD/NAD(P)-binding oxidoreductase family protein
AT3G09590		CAP (Cysteine-rich secretory proteins, Antigen 5, and Pathogenesis-related 1 protein) superfamily protein
AT3G09600	*REVEILLE 8 (RVE8)*	Encodes a MYB-like transcription factor similar to CIRCADIAN CLOCK-ASSOCIATED1 (CCA1) and ELONGATED HYPOCOTYL (LHY). Involved in the regulation of circadian clock by modulating the pattern of histone 3 (H3) acetylation. Functions as a transcriptional activator of evening element containing clock genes. Involved in heat shock response.
AT3G09610		
AT3G09620		P-loop containing nucleoside triphosphate hydrolases superfamily protein
AT3G09640	*ASCORBATE PEROXIDASE 2 (APX2)*	Encodes a cytosolic ascorbate peroxidase APX2. Ascorbate peroxidases are enzymes that scavenge hydrogen peroxide in plant cells. Eight types of APX have been described for Arabidopsis: three cytosolic (APX1, APX2, APX6), two chloroplastic types (stromal sAPX, thylakoid tAPX), and three microsomal (APX3, APX4, APX5) isoforms.
AT3G09650	*HIGH CHLOROPHYLL FLUORESCENCE 152 (HCF152)*	RNA binding protein involved in the processing of chloroplast psbB-psbT-psbH-petB-petD transcript unit.
AT3G09660	*MINICHROMOSOME MAINTENANCE 8 (MCM8)*	Encodes a minichromosome maintenance protein that is involved with RAD51 in a backup pathway that repairs meiotic double strand breaks without giving meiotic crossovers when the major pathway, which relies on DMC1, fails.
AT3G09700	*(PAM18-2)*	Chaperone DnaJ-domain superfamily protein
AT3G09710	*IQ-DOMAIN 1 (IQD1)*	Ca(2+)-dependent calmodulin-binding protein. Targeted to the nucleus. Involved in glucosinolate metabolism in response to biotic challenge. Expressed in vascular tissue.Member of IQ67 (CaM binding) domain containing family.
AT3G09720	*RNA HELICASE 57 (RH57)*	P-loop containing nucleoside triphosphate hydrolases superfamily protein
AT3G09750		Galactose oxidase/kelch repeat superfamily protein
AT3G09760		RING/U-box superfamily protein
AT3G09770	*LOSS OF GDU 2 (LOG2)*	Encodes a ubiquitin E3 ligase LOG2 (LOSS OF GDU2). Required for GLUTAMINE DUMPER1(GDU1)-induced amino secretion.
AT3G09780	*CRINKLY4 RELATED 1 (CCR1)*	CRINKLY4 related 1
AT3G09790	*UBIQUITIN 8 (UBQ8)*	encodes a ubiquitin-like protein that contains tandem repeats of the ubiquitin coding region, but at least one repeat per gene encodes a protein with amino acid substitutions.
AT3G09805		
AT3G09830	*PATTERN-TRIGGERED IMMUNITY (PTI) COMPROMISED RECEPTOR-LIKE CYTOPLASMIC KINASE 1 (PCRK1)*	Encodes a member of subfamily VIIa of the receptor-like cytoplasmic kinases (RLCKs). It contributes to pattern-triggered immunity in response to P. syringae.
AT3G09860		actin T1-like protein
AT3G09870	*SMALL AUXIN UPREGULATED RNA 48 (SAUR48)*	SAUR-like auxin-responsive protein family
AT3G09880	*(ATB' BETA)*	Encodes B' regulatory subunit of PP2A (AtB'beta). Functions redundantly with the alpha subunit do maintain sister chromatid cohesion during meiosis.
AT3G09900	*RAB GTPASE HOMOLOG E1E (RABE1e)*	RAB GTPase homolog E1E
AT3G09910	*RAB GTPASE HOMOLOG C2B (RABC2b)*	RAB GTPase homolog C2B
AT3G09930		GDSL-motif esterase/acyltransferase/lipase. Enzyme group with broad substrate specificity that may catalyze acyltransfer or hydrolase reactions with lipid and non-lipid substrates.
AT3G09940	*MONODEHYDROASCORBATE REDUCTASE 3 (MDAR3)*	Encodes a member of the monodehydroascorbate reductase gene family. Critical for a mutualistic symbiosis between the host Arabidopsis and the root colonizing fungus Piriformospora indica.
AT3G09960		Calcineurin-like metallo-phosphoesterase superfamily protein
AT3G09980	*ACETYLATED INTERACTING PROTEIN 1 (ACIP1)*	Encodes ACIP1, a microtubules-associated protein required for bacterial immunity. The mRNA is cell-to-cell mobile.
AT3G10010	*DEMETER-LIKE 2 (DML2)*	Encodes a protein with DNA glycosylase activity that is involved in maintaining methylation marks.
AT3G10020	*HYPOXIA RESPONSE UNKNOWN PROTEIN 26 (HUP26)*	plant/protein
AT3G10030		aspartate/glutamate/uridylate kinase family protein
AT3G10040	*HYPOXIA RESPONSE ATTENUATOR1 (HRA1)*	Encodes HRA1 (HYPOXIA RESPONSE ATTENUATOR1), a low oxygen-inducible transcription factor.
AT3G10050	*L-O-METHYLTHREONINE RESISTANT 1 (OMR1)*	first enzyme in the biosynthetic pathway of isoleucine
AT3G10060		FKBP-like peptidyl-prolyl cis-trans isomerase family protein
AT3G10080		RmlC-like cupins superfamily protein
AT3G10090		Nucleic acid-binding, OB-fold-like protein
AT3G10100		transposable_element_gene;similar to ASY2, DNA binding [Arabidopsis thaliana] (TAIR:AT4G32200.1);(source:TAIR10)
AT3G10120		PADRE protein down-regulated after infection by S. sclerotiorum.
AT3G10150	*PURPLE ACID PHOSPHATASE 16 (PAP16)*	purple acid phosphatase 16

AT3G10160	*DHFS-FPGS HOMOLOG C (DFC)*	Encodes a protein with tetrahydrofolylpolyglutamate synthase activity that is located in the mitochondrial matrix. One of the three folylpolyglutamate synthetase isoforms (FPGSs): FPGS1 (At5g05980, plastidic), FPGS2 (At3g10160, mitochondrial) and FPGS3 (At3g55630, cytosolic).
AT3G10170		
AT3G10180		P-loop containing nucleoside triphosphate hydrolases superfamily protein
AT3G10190	*CALMODULIN-LIKE 36 (CML36)*	Encodes a protein with sequence similarity to calmodulins. Loss of function mutations have decreased response to chitin elicitors suggesting a role in plant response to fungal pathogens.
AT3G10200		S-adenosyl-L-methionine-dependent methyltransferases superfamily protein
AT3G10220	*EMBRYO DEFECTIVE 2804 (EMB2804)*	Encodes a tubulin-binding cofactor. Homozygous mutant plants are embryo lethal. Heterozygous mutant plants showed increased ploidy and higher numbers of spindles and phragmoplasts, suggesting a role in cell division. The mRNA is cell-to-cell mobile.
AT3G10240		F-box and associated interaction domains-containing protein
AT3G10260	*RETICULON-LIKE B 8 (RTNLB8)*	Reticulon family protein
AT3G10270	*DNA GYRASE B1 (GYRB1)*	Protein targeting to mitochondria is influenced by UTR sequences.
AT3G10300		Calcium-binding EF-hand family protein
AT3G10310		P-loop nucleoside triphosphate hydrolases superfamily protein with CH (Calponin Homology) domain-containing protein
AT3G10320	*MUCILAGE-RELATED 21 (MUCI21)*	MUCI21 is a GT61 protein required for the production of highly branched xylan in seed coat mucilage. MUCI21 likely decorates xylan with xylose side chains that seem to be necessary for pectin attachment to the seed surface.
AT3G10340	*PHENYLALANINE AMMONIA-LYASE 4 (PAL4)*	Encodes PAL4, a putative a phenylalanine ammonia-lyase. Arabidopsis has four PALs: AT2G37040 (PAL1), AT3G53260 (PAL2), AT5G04230 (PAL3) and AT3G10340 (PAL4).
AT3G10370	*SUGAR-DEPENDENT 6 (SDP6)*	mitochondrial FAD-dependent glycerol-3-phosphate dehydrogenase. possibly involved in storage lipid catabolism and glycerol assimilation, and in glycerol-3-phosphate shuttle which transports reducing power from cytosol to mitochondrion.
AT3G10380	*SUBUNIT OF EXOCYST COMPLEX 8 (SEC8)*	Subunit of the Putative Arabidopsis Exocyst Complex
AT3G10420	*SEEDLING PLASTID DEVELOPMENT 1 (SPD1)*	P-loop containing nucleoside triphosphate hydrolases superfamily protein
AT3G10450	*SERINE CARBOXYPEPTIDASE-LIKE 7 (SCPL7)*	serine carboxypeptidase-like 7
AT3G10460		Plant self-incompatibility protein S1 family
AT3G10470		C2H2-type zinc finger family protein
AT3G10490	*NAC DOMAIN CONTAINING PROTEIN 52 (ANAC052)*	Encodes a NAC transcription factor that physically associates with the histone H3K4 demethylase JMJ14 and through that association is involved in transcriptional repression and flowering time control.
AT3G10500	*NAC DOMAIN CONTAINING PROTEIN 53 (NAC053)*	Encodes a transcriptional activator that is associated with the plasma membrane in a dormant form and is proteolytically cleaved to create a form that can enter the nucleus. It is thought to promote ROS production by binding directly to the promoters of genes encoding ROS biosynthetic enzymes during drought-induced leaf senescence. The mRNA is cell-to-cell mobile.
AT3G10520	*HAEMOGLOBIN 2 (HB2)*	Encodes a class 2 non-symbiotic hemoglobin. Over-expression of AHb2 in seeds led to a 40% increase in the total fatty acid content of developing and mature seeds in three subsequent generations. This was mainly due to an increase in the poly-unsaturated C18:2 (omega-6) linoleic and C18:3 (omega-3) alpha-linolenic acids.
AT3G10525	*LOSS OF GIANT CELLS FROM ORGANS (LGO)*	Encodes LGO (loss of giant cells from organs) required for endoreduplication in sepal giant cell formation. Giant cells in both leaves and sepals are absent in lgo mutants. LGO is a member of a plant specific cell cycle inhibitor family SIAMESE and was originally named as SMR1(SIAMESE RELATED 1).
AT3G10530		Transducin/WD40 repeat-like superfamily protein
AT3G10560	*UNFERTILIZED EMBRYO SAC 9 (UNE9)*	member of CYP77A
AT3G10570	*CYTOCHROME P450, FAMILY 77, SUBFAMILY A, POLYPEPTIDE 6 (CYP77A6)*	member of CYP77A
AT3G10580		Homeodomain-like superfamily protein
AT3G10590		Duplicated homeodomain-like superfamily protein
AT3G10600	*CATIONIC AMINO ACID TRANSPORTER 7 (CAT7)*	Encodes a member of the cationic amino acid transporter (CAT) subfamily of amino acid polyamine choline transporters.
AT3G10630		UDP-Glycosyltransferase superfamily protein
AT3G10660	*CALMODULIN-DOMAIN PROTEIN KINASE CDPK ISOFORM 2 (CPK2)*	predicted to encode calcium-dependent protein kinase and is localized to the ER. Protein is myristoylated in a cell-free extract. Changing the proposed myristoylated site, G residue in the amino terminal, to A prevented the meristoylation . The G to A mutation decreased AtCPK2 membrane association by approximately 50%.
AT3G10680	*SIEVE ELEMENT-LINING CHAPERONE 1 (SLI1)*	SLI1 is a heat shock like protein that is found in sieve elements, sieve plates and spherical bodies peripheral to the mitochondria. Mutants show increased phloem feeding by aphids and decreased heat tolerance.
AT3G10700	*GALACTURONIC ACID KINASE (GalAK)*	Encodes a GHMP kinase family protein that acts as a galacturonic acid-1-phosphate kinase that catalyzes the production of galacturonic acid-1-phosphate. This is a precursor of the important cell wall building block UDP-galacturonic acid. Based on gene trap line GT8007, the gene appears to be expressed in a petal and stamen-specific manner, between flower stages 8 to 11, however, later RT-qPCR analysis demonstrates that the transcript is present throughout the plant in all tissues tested.
AT3G10720		Plant invertase/pectin methylesterase inhibitor superfamily
AT3G10740	*ALPHA-L-ARABINOFURANOSIDASE 1 (ASD1)*	Encodes a bifunctional alpha-l-arabinofuranosidase/beta-d-xylosidase that belongs to family 51 of glycoside hydrolases. It may be involved in cell wall modification.
AT3G10750		FBD domain family
AT3G10760		Homeodomain-like superfamily protein
AT3G10770		Single-stranded nucleic acid binding R3H protein
AT3G10780		emp24/gp25L/p24 family/GOLD family protein
AT3G10830		

AT3G10840		alpha/beta-Hydrolases superfamily protein
AT3G10870	*METHYL ESTERASE 17 (MES17)*	Encodes a methyl IAA esterase. Methyl IAA is believed to be an inactive form of auxin that needs to be demethylated to exert a biological effect. MES17 does not act on methyl JA, MeSA, MeGA4, or MEGA9 in vitro. This gene is expressed in several tissues of seedlings and adult plants, with a higher relative level of expression in the seedling shoot apex and the adult stem.
AT3G10880		tropomyosin
AT3G10890	*ENDO-BETA-MANNANASE 3 (MAN3)*	Encodes an endo beta mannanase that is localized to the apoplast and involved in glutathione mediated cadmium tolerance.
AT3G10910	*DAF-LIKE GENE 1 (DAFL1)*	RING/U-box superfamily protein
AT3G10930	*IDA-LIKE7 (IDL7)*	Encodes a small secreted signaling peptide that processed both N- and C-terminally after translation and is rapidly induced in response to ROS and flg22-induced stress and may act as a negative modulator of stress-induced ROS signalling.
AT3G10950		Zinc-binding ribosomal protein family protein
AT3G10960	*AZA-GUANINE RESISTANT1 (AZG1)*	Encodes a homolog of the adenine-guanine-hypoxanthine transporter AzgA of Aspergillus nidulans. Function as a plant adenine-guanine transporter. Two closely related genes exist in Arabidopsis: AT3G10960 (Azg1) and AT5G50300 (Azg2).
AT3G10980		PLAC8 family protein
AT3G10990		F-box associated ubiquitination effector family protein
AT3G11000		DCD (Development and Cell Death) domain protein
AT3G11010	*RECEPTOR LIKE PROTEIN 34 (RLP34)*	receptor like protein 34
AT3G11020	*DRE/CRT-BINDING PROTEIN 2B (DREB2B)*	encodes a member of the DREB subfamily A-2 of ERF/AP2 transcription factor family (DREB2B). The protein contains one AP2 domain. There are eight members in this subfamily including DREB2A.
AT3G11040	*ENDO-BETA-N-ACETYGLUCOSAMINIDASE 85B (ENGase85B)*	Encodes a cytosolic beta-endo-N-acetyglucosaminidase (ENGase). ENGases N-glycans cleave the O-glycosidic linkage between the two GlcNAc residues of the N-glycan core structure and thus generate a protein with a single GlcNAc attached to asparagine.
AT3G11050	*FERRITIN 2 (FER2)*	ferritin 2
AT3G11060		transmembrane protein
AT3G11080	*RECEPTOR LIKE PROTEIN 35 (RLP35)*	receptor like protein 35
AT3G11090	*LOB DOMAIN-CONTAINING PROTEIN 21 (LBD21)*	LOB domain-containing protein 21
AT3G11100	*VFP3 (VFP3)*	SANT and trihelix-domain protein. Interacts with agrobacterium virulence protein VirF. It also plays a role in regulation of histone acetylation during ethylene signaling. It binds EIN2 and EIN3 and histone H3.
AT3G11110	*ARABIDOPSIS T??XICOS EN LEVADURA 66 (ATL66)*	RING/U-box superfamily protein
AT3G11120		Ribosomal protein L41 family
AT3G11150		2-oxoglutarate (2OG) and Fe(II)-dependent oxygenase superfamily protein
AT3G11160		transmembrane protein
AT3G11170	*FATTY ACID DESATURASE 7 (FAD7)*	Chloroplastic enzyme responsible for the synthesis of 16:3 and 18:3 fatty acids from galactolipids, sulpholipids and phosphatidylglycerol. Uses ferredoxin as electron donor. Gene expression is induced by wounding in shoot and root. The wound-response in shoot is independent of jasmonic acid mediated pathway whereas the root response is mediated by jasmonic acid. The mRNA is cell-to-cell mobile.
AT3G11180	*JASMONATE-INDUCED OXYGENASE1 (JOX1)*	One of 4 paralogs encoding a 2-oxoglutarate/Fe(II)-dependent oxygenases that hydroxylates JA to 12-OH-JA.
AT3G11210	*(GGL17)*	SGNH hydrolase-type esterase superfamily protein
AT3G11220	*ELONGATA 1 (ELO1)*	A subunit of Elongator, a histone acetyl transferase complex, consisting of six subunits (ELP1?ELP6), that copurifies with the elongating RNAPII in yeast and humans. Three Arabidopsis thaliana genes, encoding homologs of the yeast Elongator subunits ELP1, ELP3 (histone acetyl transferase), and ELP4 are responsible for the narrow leaf phenotype in elongata mutants and for reduced root growth that results from a decreased cell division rate.
AT3G11250		Ribosomal protein L10 family protein
AT3G11280		Putative transcription factors interacting with the gene product of VHA-B1 (vacuolar ATPase subunit B1; as shown through yeast two-hybrid assay).
AT3G11320		Nucleotide-sugar transporter family protein
AT3G11340	*UDP-DEPENDENT GLYCOSYLTRANSFERASE 76B1 (UGT76B1)*	Encodes a uridine diphosphate-dependent glucosyltransferase that conjugates isoleucic acid and modulates plant defense via glucosylation of N-hydroxypipecolic acid.
AT3G11350		Pentatricopeptide repeat (PPR) superfamily protein
AT3G11370		Cysteine/Histidine-rich C1 domain family protein
AT3G11380		Pentatricopeptide repeat (PPR) superfamily protein
AT3G11390		Cysteine/Histidine-rich C1 domain family protein
AT3G11410	*PROTEIN PHOSPHATASE 2CA (PP2CA)*	Encodes protein phosphatase 2C. Negative regulator of ABA signalling. Expressed in seeds during germination. mRNA up-regulated by drought and ABA.
AT3G11420		beta-1,3-N-acetylglucosaminyltransferase lunatic protein, putative (DUF604)
AT3G11430	*GLYCEROL-3-PHOSPHATE SN-2-ACYLTRANSFERASE 5 (GPAT5)*	sn-glycerol-3-phosphate 2-O-acyltransferas, involved in the biosynthesis of suberin polyester.
AT3G11450	*GONIDIALESS A/ZUOTIN RELATED FACTOR A1 (ATGLSA1)*	Encodes a ZRF1 chromatin regulator. Functions in regulating plant growth and development.
AT3G11460	*MITOCHONDRIAL RNA EDITING FACTOR 10 (MEF10)*	Encodes a DYW PPR protein that is involved in editing the mitochondrial NAD2 transcript at the site nad2-842.
AT3G11480	*(BSMT1)*	The gene encodes a SABATH methyltransferase that methylates both salicylic acid and benzoic acid. It is highly expressed in flowers, induced by biotic and abiotic stress and thought to be involved in direct defense mechanism.
AT3G11490	*ROP GUANOSINE TRIPHOSPHATASE (GTPASE)-ACTIVATING PROTEIN 4 (ROPGAP4)*	ROP (Rho of plant GTPases) family member Involved in cell wall patterning. Encodes ROP inactivator, regulates the formation of ROP-activated domains; these in turn determined the pattern of cell wall pits. Positively regulates pit formation, but negatively regulates pit size, required for periodic formation of secondary cell wall pits.
AT3G11520	*CYCLIN B1;3 (CYCB1;3)*	Encodes a B-type mitotic cyclin.

AT3G11540	*SPINDLY (SPY)*	Contains a tetratricopeptide repeat region, and a novel carboxy-terminal region. SPY acts as both a repressor of GA responses and as a positive regulation of cytokinin signalling. SPY may be involved in reducing ROS accumulation in response to stress. Regulates root hair patterning independently of 2 gibberellin signalling. Together with SEC functions to competitively regulate RGA1 (At2g01570).
AT3G11550	*CASPARIAN STRIP MEMBRANE DOMAIN PROTEIN 2 (CASP2)*	Uncharacterized protein family (UPF0497)
AT3G11560		LETM1-like protein
AT3G11580	*NGATHA-LIKE PROTEIN2 (NGAL2)*	SOD7 encodes nuclear localized B3 DNA binding domain and a transcriptional repression motif. Belongs to the RAV gene family. Functions in regulation of seed size and binds to and represses KLU. Transcription repressor involved in regulation of inflorescence architecture.
AT3G11590		golgin family A protein
AT3G11600	*(GIR2)*	One of two plant specific paralogs of unknown function. Interacts with GL2. GIR1/GIR2 loss of function resembles gl2 lof mutations.
AT3G11620		alpha/beta-Hydrolases superfamily protein
AT3G11630	*2-CYS PEROXIREDOXIN A (2CPA)*	Encodes a 2-Cys peroxiredoxin (2-Cys PrxA) that contains two catalytic Cys residues. Functions in redox cascade with TrxL2 via the ferredoxin-thioredoxin reductase (FTR)/thioredoxin (Trx) pathway to mediate the light-responsive reductive control of target proteins. Continuously transfers reducing power from TrxL2 to H2O2.
AT3G11640		transmembrane protein
AT3G11660	*NDR1/HIN1-LIKE 1 (NHL1)*	encodes a protein whose sequence is similar to tobacco hairpin-induced gene (HIN1) and Arabidopsis non-race specific disease resistance gene (NDR1). Expression of this gene is induced by cucumber mosaic virus. Localization of the gene product is similar to that of NHL3 (plasma membrane) but it is yet inconclusive.
AT3G11670	*DIGALACTOSYL DIACYLGLYCEROL DEFICIENT 1 (DGD1)*	Responsible for the final assembly of galactolipids in photosynthetic membranes. Provides stability to the PS I core complex (e.g. subunits PsaD, PsaE).
AT3G11690		hypothetical protein
AT3G11700	*FASCICLIN-LIKE ARABINOGALACTAN PROTEIN 18 PRECURSOR (FLA18)*	Fasciclin-like arabinogalactan protein. Possibly involved in embryogenesis and seed development.
AT3G11710	*LYSYL-TRNA SYNTHETASE 1 (ATKRS-1)*	lysyl-tRNA synthetase 1
AT3G11740		LURP-one-like protein (DUF567)
AT3G11750	*(FOLB1)*	Encodes an enzyme that can act as a aldolase or an epimerase for 7,8-dihydroneopterin and 7,8-dihydromonapterin in vitro. It is likely to act in tetrahydrofolate biosynthesis in vivo.
AT3G11760		structural maintenance of chromosomes flexible hinge domain protein
AT3G11770	*RISC-INTERACTING CLEARING 3?- 5? EXORIBONUCLEASE 1 (RICE1)*	RICE1 is a 23kDa protein with 3?- 5? exoribonuclease activity. It is expressed ubiquitously and localized to the cytoplasm. When RICE1 and its paralog RICE2 are knocked down, miRNA levels are decreased. RICE1 interacts with AGO1 and AGO10. It may affect miRNA accumulation by clearing RISC by degrading 5? products of AGO cleavage.
AT3G11790		
AT3G11820	*SYNTAXIN OF PLANTS 121 (SYP121)*	Encodes a syntaxin localized at the plasma membrane. SYR1/PEN1 is a member of the SNARE superfamily and functions in positioning anchoring of the KAT1 K+ channel protein at the plasma membrane. Transcription is upregulated by abscisic acid, suggesting a role in ABA signaling. Also functions in non-host resistance against barley powdery mildew. It is a nonessential component of the preinvasive resistance against Colletotrichum fungus. Required for mlo resistance. The syp121 point mutation results in stomatal phenotypes that reduce CO2 assimilation, slow vegetative growth and increase water use efficiency in the whole plant, conditional upon high light intensities and low relative humidity. The R20R21 motif of SYP121 are essential for SEC11 interaction. Mutation of the R20R21 motif blocks vesicle traffic without uncoupling the effects of SYP121 on solute and K+ uptake associated with the F9xRF motif; the mutation also mimicks the effects on traffic block observed on coexpression of the dominant negative SEC11?149 fragment.
AT3G11830	*CHAPERONIN CONTAINING T-COMPLEX POLYPEPTIDE-1 SUBUNIT 7 (CCT7)*	TCP-1/cpn60 chaperonin family protein
AT3G11840	*PLANT U-BOX 24 (PUB24)*	Encodes a U-box-domain-containing E3 ubiquitin ligase that acts as a negative regulator of PAMP-triggered immunity.
AT3G11860		sterile alpha motif (SAM) domain protein
AT3G11880		transmembrane protein, putative (Protein of unknown function DUF2359, transmembrane)
AT3G11920		glutaredoxin-like protein
AT3G11930		Adenine nucleotide alpha hydrolases-like superfamily protein
AT3G11950		publications Tian et al (2007) and Sadre et al (2006) refer to At3g11950. The prenyltransferase gene studied is actually At3g11945 which arises from a split of the previous At3g11950 gene model.
AT3G11964	*RIBOSOMAL RNA PROCESSING 5 (RRP5)*	Encodes a nucleolar protein that is a ribosome biogenesis co-factor. Mutants display aberrant RNA processing and female gametophyte development.
AT3G11980	*MALE STERILITY 2 (MS2)*	Similar to fatty acid reductases.
AT3G12000		S-locus related protein SLR1, putative (S1)
AT3G12010		C18orf8
AT3G12020	*KINESIN 7.3 (KIN7.3)*	P-loop containing nucleoside triphosphate hydrolases superfamily protein
AT3G12030		transmembrane/coiled-coil protein (Protein of unknown function DUF106, transmembrane)
AT3G12060	*TRICHOME BIREFRINGENCE-LIKE 1 (TBL1)*	Encodes a member of the TBL (TRICHOME BIREFRINGENCE-LIKE) gene family containing a plant-specific DUF231 (domain of unknown function) domain. TBL gene family has 46 members, two of which (TBR/AT5G06700 and TBL3/AT5G01360) have been shown to be involved in the synthesis and deposition of secondary wall cellulose, presumably by influencing the esterification state of pectic polymers. A nomenclature for this gene family has been proposed (Volker Bischoff & Wolf Scheible, 2010, personal communication).
AT3G12070	*RAB GERANYLGERANYL TRANSFERASE BETA SUBUNIT 2 (RGTB2)*	RAB geranylgeranyl transferase beta subunit 2
AT3G12090	*TETRASPANIN6 (TET6)*	TET6 encodes a member of the TETRASPANIN gene family that is expressed in the vascular system and is involved in organ growth redundantly with TET5.
AT3G12110	*ACTIN-11 (ACT11)*	Encodes an actin that is expressed predominantly during reproductive development.

AT3G12150		alpha/beta hydrolase family protein
AT3G12160	*RAB GTPASE HOMOLOG A4D (RABA4D)*	Encodes RABA4D, a member of the Arabidopsis RabA4 subfamily of Rab GTPase proteins. It is transported in exocytic vesicles to the apical tip of pollen tubes where it appears to promote tip growth. Proper localization of RabA4d depends on ROP1, RIC3, and RIC4 activity.
AT3G12190		golgin family A protein
AT3G12200	*NIMA-RELATED KINASE 7 (Nek7)*	Encodes AtNek7, a member of the NIMA-related serine/threonine kinases (Neks) that have been linked to cell-cycle regulation in fungi and mammals. Plant Neks might be involved in plant development processes.
AT3G12203	*SERINE CARBOXYPEPTIDASE-LIKE 17 (scpl17)*	serine carboxypeptidase-like 17
AT3G12210		DNA binding protein
AT3G12220	*SERINE CARBOXYPEPTIDASE-LIKE 16 (scpl16)*	serine carboxypeptidase-like 16
AT3G12230	*SERINE CARBOXYPEPTIDASE-LIKE 14 (scpl14)*	serine carboxypeptidase-like 14
AT3G12250	*TGACG MOTIF-BINDING FACTOR 6 (TGA6)*	basic leucine zipper transcription factor involved in the activation of SA-responsive genes.
AT3G12270	*PROTEIN ARGININE METHYLTRANSFERASE 3 (PRMT3)*	protein arginine methyltransferase 3
AT3G12320	*NIGHT LIGHT-INDUCIBLE AND CLOCK-REGULATED GENE 3 (LNK3)*	Member of a small gene family. Appears to be clock regulated.Somewhat redundant with LNK1/2 though more like LNK4 in having affects on biomass accumulation and phototrophism.
AT3G12340		FKBP-like peptidyl-prolyl cis-trans isomerase family protein
AT3G12370		Ribosomal protein L10 family protein
AT3G12420		Polynucleotidyl transferase, ribonuclease H-like superfamily protein
AT3G12440		Polynucleotidyl transferase, ribonuclease H-like superfamily protein
AT3G12460		Polynucleotidyl transferase, ribonuclease H-like superfamily protein
AT3G12470		Polynucleotidyl transferase, ribonuclease H-like superfamily protein
AT3G12490	*CYSTATIN B (CYSB)*	Encodes a protein with cysteine proteinase inhibitor activity. Overexpression increases tolerance to abiotic stressors (i.e.salt,osmotic, cold stress).
AT3G12500	*BASIC CHITINASE (HCHIB)*	encodes a basic chitinase involved in ethylene/jasmonic acid mediated signalling pathway during systemic acquired resistance based on expression analyses.
AT3G12510		MADS-box family protein
AT3G12520	*SULFATE TRANSPORTER 4;2 (SULTR4;2)*	Encodes a sulfate transporter that in induced under sulfate limitation.
AT3G12530	*(PSF2)*	PSF2
AT3G12540		ternary complex factor MIP1 leucine-zipper protein (Protein of unknown function, DUF547)
AT3G12550	*FACTOR OF DNA METHYLATION 3 (FDM3)*	Belongs to a subgroup of SGS3-like proteins that act redundantly in RNA-directed DNA methylation: AT1G15910 (FDM1), AT4G00380 (FDM2), AT3G12550 (FDM3), AT1G13790 (FDM4), AT1G80790 (FDM5).
AT3G12560	*TRF-LIKE 9 (TRFL9)*	Encodes a telomeric DNA-binding protein.
AT3G12570	*(FYD)*	FYD
AT3G12580	*HEAT SHOCK PROTEIN 70 (HSP70)*	heat shock protein 70
AT3G12600	*NUDIX HYDROLASE HOMOLOG 16 (NUDT16)*	nudix hydrolase homolog 16
AT3G12620	*D-CLADE TYPE 2C PROTEIN PHOSPHATASE 3 (PP2C.D3)*	Protein phosphatase 2C family protein
AT3G12660	*FASCICLIN-LIKE ARABINOGALACTAN PROTEIN 14 PRECURSOR (FLA14)*	fasciclin-like arabinogalactan-protein, family (FLA14). Possibly involved in embryogenesis and seed development.
AT3G12670	*EMBRYO DEFECTIVE 2742 (emb2742)*	Cytidine triphosphate synthase; essential for CTP supply in developing embryos.
AT3G12685		Acid phosphatase/vanadium-dependent haloperoxidase-related protein
AT3G12700	*NANA (NANA)*	Encodes an aspartic protease has an important regulatory function in chloroplasts that not only influences photosynthetic carbon metabolism but also plastid and nuclear gene expression.
AT3G12710		DNA glycosylase superfamily protein
AT3G12720	*MYB DOMAIN PROTEIN 67 (MYB67)*	Member of the R2R3 factor gene family.
AT3G12730		Homeodomain-like superfamily protein
AT3G12750	*ZINC TRANSPORTER 1 PRECURSOR (ZIP1)*	A member of Zrt- and Irt-related protein (ZIP) family. transcript is induced in response to zinc deficiency in the root.
AT3G12760		defective in cullin neddylation protein
AT3G12780	*PHOSPHOGLYCERATE KINASE 1 (PGK1)*	PGK1 was localized exclusively in the chloroplasts of photosynthetic tissues and is the photosynthetic isoform. The pgk1.1 knock-down mutant displayed reduced growth, lower photosynthetic capacity and starch content. Expression studies in PGK mutants showed that PGK1 and PGK3 were down-regulated in pgk3.2 and pgk1.1, respectively. These results indicate that the down-regulation of photosynthetic activity could be a plant strategy when glycolysis is impaired to achieve metabolic adjustment and optimize growth (DOI:10.1104/pp.17.01227).Functions redundantly with AT1G56190 in the chloroplast in the biosynthesis of thylakoid membrane galactolipids. Double mutants are photosynthetically incompetent, plants are albino and seedling lethal
AT3G12790		
AT3G12800	*SHORT-CHAIN DEHYDROGENASE-REDUCTASE B (SDRB)*	short-chain dehydrogenase-reductase B
AT3G12810	*PHOTOPERIOD-INDEPENDENT EARLY FLOWERING 1 (PIE1)*	Encodes a protein similar to ATP-dependent, chromatin-remodeling proteins of the ISWI and SWI2/SNF2 family. Genetic analyses suggest that this gene is involved in multiple flowering pathways. Mutations in PIE1 results in suppression of FLC-mediated delay of flowering and causes early flowering in noninductive photoperiods independently of FLC. PIE1 is required for expression of FLC in the shoot apex but not in the root.Along with ARP6 forms a complex to deposit modified histone H2A.Z at several loci within the genome. This modification alters the expression of the target genes (i.e. FLC, MAF4, MAF6). The mRNA is cell-to-cell mobile.
AT3G12820	*MYB DOMAIN PROTEIN 10 (MYB10)*	Member of the R2R3 factor gene family.
AT3G12830	*SMALL AUXIN UPREGULATED 72 (SAUR72)*	SAUR-like auxin-responsive protein family
AT3G12840		F-box/FBD-like domain protein

AT3G12850		COP9 signalosome complex-related / CSN complex-like protein
AT3G12870		transmembrane protein
AT3G12890	*ACTIVATOR OF SPOMIN::LUC2 (ASML2)*	Encodes a protein belonging to a class of CCT (CONSTANS, CONSTANS-like, TOC1) domain proteins. The protein contains a 43 amino acid-long sequence with high homology to the CCT domain but does not have any B-box or GATA-type zinc finger domains. Functions as a transcriptional activator and regulates the expression of at least a subset of sugar-inducible genes.
AT3G12900	*SCOPOLETIN 8- HYDROXYLASE (S8H)*	S8H hydroxylates scopoletin to generate fraxetin (8-hydroxyscopoletin). Fraxetin and its oxidized analog sideretin (5-hydroxyfraxetin) are catecholic coumarins secreted into the rhizosphere under conditions of low iron availability and help mobilize this nutrient from insoluble iron(III) pools in the soil.S8H hydroxylates scopoletin to generate fraxetin (8-hydroxyscopoletin). Fraxetin and its oxidized analog sideretin (5-hydroxyfraxetin) are catecholic coumarins secreted into the rhizosphere under conditions of low iron availability and help mobilize this nutrient from insoluble iron(III) pools in the soil.
AT3G12910		NAC (No Apical Meristem) domain transcriptional regulator superfamily protein
AT3G12920	*BOI-RELATED GENE 3 (BRG3)*	Encodes one of the BRGs (BOI-related gene) involved in resistance to Botrytis cinerea.
AT3G12930	*DELAYED GREENING 238 (DG238)*	Encodes a novel conserved chloroplast protein that interacts with components of the PEP complex. Mutants show delayed greening and reduced photosynthetic capcity.
AT3G12950		Trypsin family protein
AT3G12955	*SMALL AUXIN UPREGULATED RNA 74 (SAUR74)*	SAUR-like auxin-responsive protein family
AT3G12960	*SEED MATURATION PROTEIN 1 (SMP1)*	seed maturation protein
AT3G13000		ubiquinone biosynthesis protein (Protein of unknown function, DUF547)
AT3G13020		hAT transposon superfamily protein
AT3G13040	*(GAMMAMYB2)*	myb-like HTH transcriptional regulator family protein
AT3G13050	*NICOTINATE TRANSPORTER (NiaP)*	Encodes a plant nicotinate transporter than can also transport trigonelline (N-methylnicotinate).
AT3G13062		Polyketide cyclase/dehydrase and lipid transport superfamily protein
AT3G13065	*STRUBBELIG-RECEPTOR FAMILY 4 (SRF4)*	STRUBBELIG-receptor family 4
AT3G13070		CBS domain-containing protein / transporter associated domain-containing protein
AT3G13090	*ATP-BINDING CASSETTE C6 (ABCC6)*	member of MRP subfamily
AT3G13100	*ATP-BINDING CASSETTE C7 (ABCC7)*	member of MRP subfamily
AT3G13110	*SERINE ACETYLTRANSFERASE 2;2 (SERAT2;2)*	Encodes a mitochondrial serine O-acetyltransferase involved in sulfur assimilation and cysteine biosynthesis. Expressed in the vascular system.
AT3G13120	*PLASTID RIBOSOMAL PROTEIN OF THE 30S SUBUNIT 10 (PRPS10)*	Ribosomal protein S10p/S20e family protein
AT3G13130		transmembrane protein
AT3G13140		hydroxyproline-rich glycoprotein family protein
AT3G13150		Tetratricopeptide repeat (TPR)-like superfamily protein
AT3G13160	*RIBOSOMAL PENTATRICOPEPTIDE REPEAT PROTEIN 3B (RPPR3B)*	Ribosomal pentatricopeptide repeat protein
AT3G13175	*PSI-INTERACTING ROOT-CELL ENRICHED 1 (PRCE1)*	transmembrane protein
AT3G13190		WEB family protein (DUF827)
AT3G13210		crooked neck protein, putative / cell cycle protein
AT3G13220	*ATP-BINDING CASSETTE G26 (ABCG26)*	Encodes a ATP-binding cassette transporter G26 (ABCG26) involved in tapetal cell and pollen development. Required for male fertility and pollen exine formation.
AT3G13222	*GBF-INTERACTING PROTEIN 1 (GIP1)*	Encodes a protein that binds to G-box binding transcription factors and enhances their binding affinities to G-box in vitro. This protein localizes to the nucleus and is expressed predominantly in the root.
AT3G13225	*FORMIN BINDING PROTEIN 4 (FNBP4)*	WW domain-containing protein
AT3G13228		RING/U-box superfamily protein
AT3G13230		RNA-binding KH domain-containing protein
AT3G13240		hypothetical protein
AT3G13270		transposable_element_gene;similar to unknown protein [Arabidopsis thaliana] (TAIR:AT2G14450.1);(source:TAIR10)
AT3G13310	*DNA J PROTEIN C66 (DJC66)*	Chaperone DnaJ-domain superfamily protein
AT3G13320	*CATION EXCHANGER 2 (CAX2)*	low affinity calcium antiporter CAX2 The mRNA is cell-to-cell mobile.
AT3G13330	*PROTEASOME ACTIVATING PROTEIN 200 (PA200)*	Encodes a protein that interacts with the 26S proteasome. Mutants are phenotypically indistinguishable from wild type plants under a variety of growth conditions. Protein levels increase upon exposure of seedlings to MG132, a specific, potent, reversible, and cell-permeable proteasome inhibitor.
AT3G13340		Transducin/WD40 repeat-like superfamily protein
AT3G13360	*WPP DOMAIN INTERACTING PROTEIN 3 (WIP3)*	Encodes an outer nuclear membrane protein that anchors RanGAP1 to the nuclear envelope. It interacts with SUN proteins and is required for maintaining the elongated nuclear shape of epidermal cells.
AT3G13370		formin-like protein
AT3G13380	*BRI1-LIKE 3 (BRL3)*	Similar to BRI, brassinosteroid receptor protein.
AT3G13390	*SKU5 SIMILAR 11 (sks11)*	SKU5 similar 11
AT3G13400	*SKU5-SIMILAR 13 (SKS13)*	Encodes a pollen/pollen tube-specific gene that is essential for pollen tube growth in the transmitting tract by mediating the biosynthesis of JA that modifies the components of pollen tube cell walls. The SKS13 protein was distributed throughout the cytoplasm and pollen tube walls at the apical region.
AT3G13420		transmembrane protein
AT3G13430	*BCA2A ZINC FINGER ATL 7 (BTL07)*	RING/U-box superfamily protein

AT3G13440	*HEMK METHYLTRANSFERASE (NRF1)*	Encodes a HemK class glutamine‐methyltransferase that is involved in the termination of translation and essential for iron homeostasis.
AT3G13450	*DARK INDUCIBLE 4 (DIN4)*	branched chain alpha-keto acid dehydrogenase E1 beta
AT3G13460	*EVOLUTIONARILY CONSERVED C-TERMINAL REGION 2 (ECT2)*	Physically interacts with CIPK1. ECT2 regulates the mRNA levels of the roteasome regulator PTRE1 and of several 20S proteasome subunits, resulting in enhanced 26S proteasome activity. YTHDF protein which togeteher with ECT3 and ECT4 is involved in cell proliferation during plant organogenesis.
AT3G13480		nuclear polyadenylated RNA-binding protein
AT3G13510		carboxyl-terminal peptidase, putative (DUF239)
AT3G13540	*MYB DOMAIN PROTEIN 5 (MYB5)*	Encodes a member of the MYB family of transcriptional regulators. MYB5 act as a negative regulator of trichome branching and play a role in the correct formation of the seed coat and possibly the formation the underlying endosperm layers. Loss of function mutations have defects in seed coat mucilage and columella cells as well as trichome defects (smaller and reduced number of branches).
AT3G13560		O-Glycosyl hydrolases family 17 protein
AT3G13590		Cysteine/Histidine-rich C1 domain family protein
AT3G13610	*(F6'H1)*	Encodes a Fe(II)- and 2-oxoglutarate-dependent dioxygenase family gene F6'H1. Mutations in this gene compromise iron uptake and the production of fluorescent phenolics involved in Fe uptake. The mRNA is cell-to-cell mobile.
AT3G13620	*POLYAMINE UPTAKE TRANSPORTER 4 (PUT4)*	Encodes POLYAMINE UPTAKE TRANSPORTER 4, an amino acid permease family protein.
AT3G13630		hypothetical protein
AT3G13640	*ATP-BINDING CASSETTE E1 (ABCE1)*	member of RLI subfamily
AT3G13650		Disease resistance-responsive (dirigent-like protein) family protein
AT3G13660		Disease resistance-responsive (dirigent-like protein) family protein
AT3G13662		Disease resistance-responsive (dirigent-like protein) family protein
AT3G13672	*(SINA2)*	SINAT homolog with truncated RING finger and zinc finger domains.
AT3G13680		F-box and associated interaction domains-containing protein
AT3G13682	*LSD1-LIKE2 (LDL2)*	Encodes a homolog of human Lysine-Specific Demethylase1. Involved in H3K4 methylation of target genes including the flowering loci FLC and FWA.
AT3G13690		kinase with adenine nucleotide alpha hydrolases-like domain-containing protein
AT3G13700		RNA-binding (RRM/RBD/RNP motifs) family protein
AT3G13720	*(PRA8)*	PRA1 (Prenylated rab acceptor) family protein
AT3G13730	*CYTOCHROME P450, FAMILY 90, SUBFAMILY D, POLYPEPTIDE 1 (CYP90D1)*	Encodes a cytochrome P-450 gene that is involved in brassinosteroid biosynthesis, most likely in the conversion step of teasterone (TE) to 3-dehydroteasterone (3DT), and/or 6-deoxoteasterone (6-deoxoTE) to 6-deoxo-3-dehydroteasterone (6-deoxo3DT); or the conversion of cathasterone (CT) to TE, and/or 6-deoxocathasterone (6-deoxoCT) to 6-deoxoTE. Recently, CYP90D1 was shown to catalyse the C-23 hydroxylation of several brassinosteroids (the enzyme has a broad specificity for 22-hydroxylated substrates). Member of the CYP90C CYP450 family. Similar to Cytochrome P450 90C1 (ROT3).
AT3G13750	*BETA GALACTOSIDASE 1 (BGAL1)*	beta-galactosidase, glycosyl hydrolase family 35 The mRNA is cell-to-cell mobile.
AT3G13760		Cysteine/Histidine-rich C1 domain family protein
AT3G13770		Pentatricopeptide repeat (PPR) superfamily protein
AT3G13782	*NUCLEOSOME ASSEMBLY PROTEIN1;4 (NAP1;4)*	Plants mutated in three ubiquitously expressed NAP1 genes (NAP1;1~NAP1;3) and organ-specifically expressed NAP1;4 gene show hypersensitivity to genotoxic stresses including UV and DSB-inducing agent Bleomycin. The NAP1 genes act synergistically with NRP genes in promoting somatic homologous recombination.
AT3G13784	*CELL WALL INVERTASE 5 (CWINV5)*	cell wall invertase 5
AT3G13790	*(ATBFRUCT1)*	Encodes a protein with invertase activity.
AT3G13800		Metallo-hydrolase/oxidoreductase superfamily protein
AT3G13810	*INDETERMINATE(ID)-DOMAIN 11 (IDD11)*	indeterminate(ID)-domain 11
AT3G13820		F-box and associated interaction domains-containing protein
AT3G13830		F-box and associated interaction domains-containing protein
AT3G13840		GRAS family transcription factor
AT3G13860	*HEAT SHOCK PROTEIN 60-3A (HSP60-3A)*	heat shock protein 60-3A
AT3G13870	*ROOT HAIR DEFECTIVE 3 (RHD3)*	required for regulated cell expansion and normal root hair development. Encodes an evolutionarily conserved protein with putative GTP-binding motifs that is implicated in the control of vesicle trafficking between the endoplasmic reticulum and the Golgi compartments. Degraded by LNP1 and 2 to maintain a tubular ER network.
AT3G13880	*ORGANELLE TRANSCRIPT PROCESSING 72 (OTP72)*	Encodes a pentatricopeptide repeat (PPR) protein involved in RNA editing in mitochondria.
AT3G13890	*MYB DOMAIN PROTEIN 26 (MYB26)*	Encodes a putative transcription factor (MYB26). Mutants produces fertile pollen but plants are sterile because anthers do not dehisce. The cellulosic secondary wall thickenings are not formed in the endothecium as they are in non-mutant plants.
AT3G13900	*AMINOPHOSPHOLIPID ATPASE 7 (ALA7)*	Encodes a member of the P4 subfamily of P-type ATPases expressed in the pollen plasma membrane. Double mutants with ALA6 display pollen and pollen tube defects.
AT3G13910		hypothetical protein (DUF3511)
AT3G13930	*MITOCHONDRIAL PYRUVATE DEHYDROGENASE SUBUNIT 2-2 (MTE2-2)*	Encodes a subunit of the mitochondrial pyruvate dehydrogenase complex.
AT3G13940		DNA binding / DNA-directed RNA polymerase
AT3G13950		ankyrin
AT3G13960	*GROWTH-REGULATING FACTOR 5 (GRF5)*	Growth regulating factor encoding transcription activator. One of the nine members of a GRF gene family, containing nuclear targeting domain. Involved in leaf development and expressed in root, shoot and flower.

AT3G13970	*AUTOPHAGY 12 B (APG12B)*	Autophagy protein.
AT3G13980	*BIG GRAIN 4 (BG4)*	SKI/DACH domain protein
AT3G14000	*(ATBRXL2)*	Belongs to five-member BRX gene family. Arabidopsis BRX genes share high levels of similarity among each others, with several conserved domains. The most distinct is BRX domain - highly conserved in all BRX genes among distantly related species. This protein-protein interaction domain is required and sufficient for BRX activity.
AT3G14020	*NUCLEAR FACTOR Y, SUBUNIT A6 (NF-YA6)*	nuclear factor Y, subunit A6
AT3G14030		F-box associated ubiquitination effector family protein
AT3G14050	*RELA/SPOT HOMOLOG 2 (RSH2)*	Involved in the maintenance of the (p)ppGp level to accustom plastidial gene expression to darkness.
AT3G14060		hypothetical protein
AT3G14067	*SENESCENCE-ASSOCIATED SUBTILISIN PROTEASE (SASP)*	Encodes a protein with similarity to serine protease, subtilisin, that is upregulated during senescence and expressed in the arial portions of the plant.Loss of function mutations have increased branch number but normal silique length and seed set and therefore have increased fertility.
AT3G14070	*CATION EXCHANGER 9 (CAX9)*	Involved in cation (K, Na and Mn) homeostasis and transport
AT3G14160		2-oxoglutarate-dependent dioxygenase family protein
AT3G14180	*ARABIDOPSIS 6B-INTERACTING PROTEIN 1-LIKE 2 (ASIL2)*	sequence-specific DNA binding transcription factor
AT3G14190	*COPPER MODIFIED RESISTANCE 1 (CMR1)*	Encodes a 193 amino acid protein of unknown function. Contains a DEN-box (aa 14?16), a KEN-box, and the D-box (aa 46?54)and a third, unknown domain (aa 81?97). Loss of function alleles are defective in meiosis and have reduced fertility. pans1 mutants show premature loss of cohesion of sister chromatids during meioisis I and meiosis II resulting in abnormal chromosome segregation and unbalanced tetrads.
AT3G14200		Chaperone DnaJ-domain superfamily protein
AT3G14205	*SUPPRESSOR OF ACTIN 2 (SAC2)*	Phosphoinositide phosphatase family protein
AT3G14210	*EPITHIOSPECIFIER MODIFIER 1 (ESM1)*	A semidominant QTL which has an epistatic effect on the Epithiospecifier gene. Represses nitrile formation and favors isothiocyanate production during glucosinolate hydrolysis. The functional allele deters the insect herbivory T. ni.
AT3G14225	*GDSL-MOTIF LIPASE 4 (GLIP4)*	Contains lipase signature motif and GDSL domain.
AT3G14230	*RELATED TO AP2 2 (RAP2.2)*	encodes a member of the ERF (ethylene response factor) subfamily B-2 of ERF/AP2 transcription factor family (RAP2.2). The protein contains one AP2 domain. There are 5 members in this subfamily including RAP2.2 AND RAP2.12.
AT3G14240		Subtilase family protein
AT3G14250		RING/U-box superfamily protein
AT3G14280		LL-diaminopimelate aminotransferase
AT3G14300	*(ATPMEPCRC)*	pectinesterase family protein
AT3G14310	*PECTIN METHYLESTERASE 3 (PME3)*	encodes a pectin methylesterase, targeted by a cellulose binding protein (CBP) from the parasitic nematode Heterodera schachtii during parasitism.
AT3G14330	*CHLOROPLAST RNA EDITING FACTOR 3 (CREF3)*	Encodes a pentatricopeptide repeat protein involved in chloroplast mRNA editing. Mutants display defects in C-U editing of psbE.
AT3G14340		hypothetical protein
AT3G14350	*STRUBBELIG-RECEPTOR FAMILY 7 (SRF7)*	STRUBBELIG-receptor family 7
AT3G14360	*OIL BODY LIPASE 1 (ATOBL1)*	Lipid droplet-associated triacylglycerol lipase (TAG) involved in pollen tube growth. TAG is possibly a direct precursor for the synthesis of membrane lipids in pollen tubes.
AT3G14370	*(WAG2)*	The WAG2 and its homolog, WAG1 each encodes protein-serine/threonine kinase that are nearly 70% identical to PsPK3 protein. All three together with CsPK3 belong to PsPK3-type kinases. At the N-terminus, all four possess a serine/threonine-rich domain. They are closely related to Arabidopsis kinases PINOID. wag1/wag2 double mutants exhibit a pronounced wavy root phenotype when grown vertically on agar plates (while wild-type plants develop wavy roots only on plates inclined to angles less than 90 degrees), indicating an overlapping role for WAG1 and WAG2 as suppressors of root waving. Simultaneous disruption of PID(AT2G34650) and its 3 closest homologs (PID2/AT2G26700, WAG1/AT1G53700, and WAG2/AT3G14370) abolishes the formation of cotyledons.
AT3G14380	*CASP-LIKE PROTEIN 2A2 (CASPL2A2)*	Uncharacterized protein family (UPF0497)
AT3G14395	*MITE ATTACK TRIGGERED IMMUNITY 1 (MATI)*	Protein Involved in the Regulation of Herbivore-Associated Signaling Pathways, affecting the expression of genes involved in biosynthesis and signaling of the jasmonic acid and salicylic acid hormones.
AT3G14410		Nucleotide/sugar transporter family protein
AT3G14415	*GLYCOLATE OXIDASE 2 (GOX2)*	Encodes a glycolate oxidase that modulates reactive oxygen species-mediated signal transduction during nonhost resistance. The mRNA is cell-to-cell mobile.
AT3G14440	*NINE-CIS-EPOXYCAROTENOID DIOXYGENASE 3 (NCED3)*	Encodes 9-<i>cis</i>-epoxycarotenoid dioxygenase, a key enzyme in the biosynthesis of abscisic acid. Regulated in response to drought and salinity. Expressed in roots, flowers and seeds. Localized to the chloroplast stroma and thylakoid membrane.
AT3G14450	*CTC-INTERACTING DOMAIN 9 (CID9)*	RNA-binding protein, putative, contains Pfam profile: PF00076 RNA recognition motif. (a.k.a. RRM, RBD, or RNP domain) (2 copies). Contains PAM PABC binding domain.
AT3G14460	*LEUCINE-RICH REPEAT (LRR) PROTEIN 1 (LRRAC1)*	Leucine rich repeat protein that also contains an adenylate cyclase catalytic core motif. Capable of converting ATP to cAMP in vitro. Mutants show increased susceptibility to fungal pathogens.
AT3G14470		NB-ARC domain-containing disease resistance protein
AT3G14480		glycine/proline-rich protein
AT3G14490		Terpenoid cyclases/Protein prenyltransferases superfamily protein
AT3G14520	*TERPENE SYNTHASE 18 (TPS18)*	Encodes a sesterterpene synthase responsible for the biosynthesis of the tricyclic sesterterpene (+)-thalianatriene with a 11-6-5 fused ring system.
AT3G14530	*GERANYL-FARNESYL PYROPHOSPHATE SYNTHASE 1 (GFPPS1)*	Chloroplast localized GFDP synthase.
AT3G14560		Its transcript is targeted by miR824.
AT3G14570	*GLUCAN SYNTHASE-LIKE 4 (GSL04)*	encodes a protein similar to callose synthase
AT3G14580		Pentatricopeptide repeat (PPR) superfamily protein

AT3G14595		Ribosomal protein L18ae family
AT3G14610	*CYTOCHROME P450, FAMILY 72, SUBFAMILY A, POLYPEPTIDE 7 (CYP72A7)*	putative cytochrome P450
AT3G14620	*CYTOCHROME P450, FAMILY 72, SUBFAMILY A, POLYPEPTIDE 8 (CYP72A8)*	putative cytochrome P450 The mRNA is cell-to-cell mobile.
AT3G14630	*CYTOCHROME P450, FAMILY 72, SUBFAMILY A, POLYPEPTIDE 9 (CYP72A9)*	putative cytochrome P450
AT3G14640	*CYTOCHROME P450, FAMILY 72, SUBFAMILY A, POLYPEPTIDE 10 (CYP72A10)*	putative cytochrome P450
AT3G14650	*CYTOCHROME P450, FAMILY 72, SUBFAMILY A, POLYPEPTIDE 11 (CYP72A11)*	putative cytochrome P450 The mRNA is cell-to-cell mobile.
AT3G14660	*CYTOCHROME P450, FAMILY 72, SUBFAMILY A, POLYPEPTIDE 13 (CYP72A13)*	putative cytochrome P450 The mRNA is cell-to-cell mobile.
AT3G14670		hypothetical protein
AT3G14680	*CYTOCHROME P450, FAMILY 72, SUBFAMILY A, POLYPEPTIDE 14 (CYP72A14)*	putative cytochrome P450
AT3G14690	*CYTOCHROME P450, FAMILY 72, SUBFAMILY A, POLYPEPTIDE 15 (CYP72A15)*	putative cytochrome P450 The mRNA is cell-to-cell mobile.
AT3G14700		SART-1 family
AT3G14710		RNI-like superfamily protein
AT3G14730		Pentatricopeptide repeat (PPR) superfamily protein
AT3G14750	*FLX-LIKE 1 (FLL1)*	structural maintenance of chromosomes domain protein
AT3G14760		transmembrane protein
AT3G14770	*(SWEET2)*	Nodulin MtN3 family protein
AT3G14780		callose synthase
AT3G14790	*RHAMNOSE BIOSYNTHESIS 3 (RHM3)*	rhamnose biosynthesis 3
AT3G14820		GDSL-motif esterase/acyltransferase/lipase. Enzyme group with broad substrate specificity that may catalyze acyltransfer or hydrolase reactions with lipid and non-lipid substrates.
AT3G14840	*LYSM RLK1-INTERACTING KINASE 1 (LIK1)*	Encodes LRR-RLK protein that is localized to the plasma membrane and is involved in regulation of plant innate immunity to microbes. LIK1 is phosphorylated by CERK1, a kinase involved in chitin perception. The mRNA is cell-to-cell mobile.
AT3G14850	*TRICHOME BIREFRINGENCE-LIKE 41 (TBL41)*	Encodes a member of the TBL (TRICHOME BIREFRINGENCE-LIKE) gene family containing a plant-specific DUF231 (domain of unknown function) domain. TBL gene family has 46 members, two of which (TBR/AT5G06700 and TBL3/AT5G01360) have been shown to be involved in the synthesis and deposition of secondary wall cellulose, presumably by influencing the esterification state of pectic polymers. A nomenclature for this gene family has been proposed (Volker Bischoff & Wolf Scheible, 2010, personal communication).
AT3G14880		transcription factor-like protein
AT3G14930	*(HEME1)*	Uroporphyrinogen decarboxylase
AT3G14940	*PHOSPHOENOLPYRUVATE CARBOXYLASE 3 (PPC3)*	Encodes a cytosolic phosphoenolpyruvate carboxylase (PEPC) that has activity when expressed in E.coli. Its mRNA is most abundantly expressed in roots and siliques. PPC3 belongs to the plant-type PEPC family. It can form an enzymatically active complex with a castor bean ortholog of PPC4, which encodes a bacterial-type PEPC. The mRNA is cell-to-cell mobile.
AT3G14950	*TETRATRICOPETIDE-REPEAT THIOREDOXIN-LIKE 2 (TTL2)*	Encodes one of the 36 carboxylate clamp (CC)-tetratricopeptide repeat (TPR) proteins (Prasad 2010, Pubmed ID: 20856808) with potential to interact with Hsp90/Hsp70 as co-chaperones. The TTL family is required for osmotic stress tolerance and male sporogenesis.
AT3G14980	*REPRESSOR OF SILENCING 4 (ROS4)*	IDM1 is a histone H3 acetyltransferase that is capable of recognizing methylated DNA through its MBD domain and recognizing unmethylated histone H3K4 through its PHD domain. It negatively regulates DNA demethylation, preventing DNA hypermethylation of highly homologous multicopy genes and other repetitive sequences.
AT3G14990	*DJ-1 HOMOLOG A (DJ1A)*	Encodes a homolog of animal DJ-1 superfamily protein. In the A. thaliana genome, three genes encoding close homologs of human DJ-1 were identified AT3G14990 (DJ1A), AT1G53280 (DJ1B) and AT4G34020 (DJ1C). Among the three homologs, DJ1C is essential for chloroplast development and viability. It exhibits glyoxalase activity towards glyoxal and methylglyoxal. The mRNA is cell-to-cell mobile.
AT3G15000	*RNA-EDITING FACTOR INTERACTING PROTEIN 1 (RIP1)*	Encodes RIP1 (RNA-editing factor interacting protein 1). Involved in chloroplast and mitochondrial RNA editing. The mRNA is cell-to-cell mobile.
AT3G15010		RNA-binding (RRM/RBD/RNP motifs) family protein
AT3G15020	*MITOCHONDRIAL MALATE DEHYDROGENASE 2 (mMDH2)*	Lactate/malate dehydrogenase family protein
AT3G15030	*TCP FAMILY TRANSCRIPTION FACTOR 4 (TCP4)*	Arabidopsis thaliana TCP family transcription factor. Regulated by miR319. Involved in heterchronic regulation of leaf differentiation.
AT3G15060	*RAB GTPASE HOMOLOG A1G (RABA1g)*	RAB GTPase homolog A1G
AT3G15095	*HIGH CHLOROPHYLL FLUORESCENCE 243 (HCF243)*	Encodes HCF243 (high chlorophyll fluorescence), a chloroplast-localized protein involved in the D1 protein stability of the photosystem II complex1.
AT3G15110		transmembrane protein
AT3G15130		Tetratricopeptide repeat (TPR)-like superfamily protein
AT3G15140	*ENHANCER OF RNAI (ERI-1)*	ERI (At3g15140) encodes a protein of 337 amino acids of the ribonuclease H-like superfamily. The protein contains both DEDDh and SAP domains.The first exon contains a TCT-microsatellite structure (starting 226 bp after ATG) that, based on sequence complementarity, is a miR5021-cleavage target site. ERI is predicted to function as an siRNA exonuclease. Overexpression leads to increased post transcriptional gene silencing and reduced numbers of 21mers. Macroscopically, the growth rate is increased in overexpressors leading to increased biomass.
AT3G15150	*HIGH PLOIDY2 (HPY2)*	Encodes a SUMO E3 ligase that regulates endocycle onset and meristem maintenance.
AT3G15170	*CUP-SHAPED COTYLEDON1 (CUC1)*	Encodes a transcription factor involved in shoot apical meristem formation and cotyledon separation. Functions redundantly with CUC2 and CUC3. The cuc1 cuc2 double mutant phenotype is first detectable at the heart stage, as embryos lacking two distinct bulges of cotyledonary primordia.In post embryonic development it plays a role in axillary meristem formation, boundary separation, gynoecium and ovule development.Contains a MIR164 binding site.
AT3G15180		ARM repeat superfamily protein
AT3G15190	*PLASTID RIBOSOMAL PROTEIN S20 (PRPS20)*	chloroplast 30S ribosomal protein S20
AT3G15200		Tetratricopeptide repeat (TPR)-like superfamily protein

AT3G15210	*ETHYLENE RESPONSIVE ELEMENT BINDING FACTOR 4 (ERF4)*	Encodes a member of the ERF (ethylene response factor) subfamily B-1 of ERF/AP2 transcription factor family (ATERF-4). The protein contains one AP2 domain. Acts as a negative regulator of JA-responsive defense gene expression and resistance to the necrotrophic fungal pathogen Fusarium oxysporum and antagonizes JA inhibition of root elongation. The mRNA is cell-to-cell mobile.
AT3G15230		
AT3G15240		Serine/threonine-protein kinase WNK (With No Lysine)-like protein
AT3G15250		TPRXL
AT3G15260		Protein phosphatase 2C family protein
AT3G15270	*SQUAMOSA PROMOTER BINDING PROTEIN-LIKE 5 (SPL5)*	Encodes a member of the SPL (squamosa-promoter binding protein-like)gene family, a novel gene family encoding DNA binding proteins and putative transcription factors. Contains the SBP-box, which encodes the SBP-domain, required and sufficient for interaction with DNA. It is involved in regulation of flowering and vegetative phase change. Its temporal expression is regulated by the microRNA miR156. The target site for the microRNA is in the 3'UTR.
AT3G15280		hypothetical protein
AT3G15290		3-hydroxyacyl-CoA dehydrogenase family protein
AT3G15300	*MPK3/6-TARGETED VQP 4 (MVQ4)*	VQ motif-containing protein
AT3G15310		transposable_element_gene;similar to unknown protein [Arabidopsis thaliana] (TAIR:AT5G32621.1);(source:TAIR10)
AT3G15350		G14 enzyme
AT3G15352	*CYTOCHROME C OXIDASE 17 (COX17)*	Encodes protein similar to yeast COX17, a copper-binding protein that mediates the delivery of Cu to the mitochondria for the assembly of a functional cytochrome oxidase complex.
AT3G15353	*METALLOTHIONEIN 3 (MT3)*	metallothionein, binds to and detoxifies excess copper and other metals, limiting oxidative damage
AT3G15355	*UBIQUITIN-CONJUGATING ENZYME 25 (UBC25)*	ubiquitin-conjugating enzyme 25
AT3G15357		phosphopantothenoylcysteine decarboxylase subunit
AT3G15360	*THIOREDOXIN M-TYPE 4 (TRX-M4)*	encodes a prokaryotic thioredoxin The mRNA is cell-to-cell mobile.
AT3G15370	*EXPANSIN 12 (EXPA12)*	member of Alpha-Expansin Gene Family. Naming convention from the Expansin Working Group (Kende et al, 2004. Plant Mol Bio)
AT3G15400	*ANTHER 20 (ATA20)*	Encodes a protein with novel repeat sequences and a glycine-rich domain which has a 53% identity to GRP1, a petunia glycine-rich cell wall protein. DYT1 and bHLH089 specifically recognize the TCATGTGC box to activate expression.
AT3G15410		Leucine-rich repeat (LRR) family protein
AT3G15430		Regulator of chromosome condensation (RCC1) family protein
AT3G15440		RING/U-box protein
AT3G15450		aluminum induced protein with YGL and LRDR motifs
AT3G15460	*ARABIDOPSIS HOMOLOGUE OF YEAST BRX1 1 (ATBRX1-1)*	Encodes one of two Arabidopsis orthologs of yeast BRX1, a protein involved in maturation of the large ribosomal subunit. The proteins are mainly localized in nucleolus. Mutant plants are affected in pre-rRNA processing.
AT3G15470		Transducin/WD40 repeat-like superfamily protein
AT3G15480	*TRANVIA (TVA)*	Encodes a plant-specific protein that promotes trafficking of CSCs to the plasma membrane by facilitating exit from the TGN and/or interaction of CSC secretory vesicles with the plasma membrane.
AT3G15500	*NAC DOMAIN CONTAINING PROTEIN 3 (NAC3)*	Encodes an ATAF-like NAC-domain transcription factor that doesn't contain C-terminal sequences shared by CUC1, CUC2 and NAM. Note: this protein (AtNAC3) is not to be confused with the protein encoded by locus AT3G29035, which, on occasion, has also been referred to as AtNAC3. The mRNA is cell-to-cell mobile.
AT3G15510	*NAC DOMAIN CONTAINING PROTEIN 2 (NAC2)*	Note of caution: not to be confused with another protein (AtNAC6 locus AT5G39610) which on occasion has also been referred to as AtNAC2.
AT3G15520		Cyclophilin-like peptidyl-prolyl cis-trans isomerase family protein
AT3G15540	*INDOLE-3-ACETIC ACID INDUCIBLE 19 (IAA19)*	Primary auxin-responsive gene. Involved in the regulation stamen filaments development.
AT3G15550		trichohyalin
AT3G15560		
AT3G15570		Phototropic-responsive NPH3 family protein
AT3G15590		Tetratricopeptide repeat (TPR)-like superfamily protein
AT3G15600		transposable_element_gene;similar to unknown protein [Arabidopsis thaliana] (TAIR:AT2G07770.1);(source:TAIR10)
AT3G15605		nucleic acid binding protein
AT3G15610		Transducin/WD40 repeat-like superfamily protein
AT3G15620	*UV REPAIR DEFECTIVE 3 (UVR3)*	Required for photorepair of 6-4 photoproducts in Arabidopsis thaliana.
AT3G15630		plant/protein
AT3G15650		alpha/beta-Hydrolases superfamily protein
AT3G15660	*GLUTAREDOXIN 4 (GRX4)*	Mitochondrial glutaredoxin involved in Fe-S cluster assembly.
AT3G15670	*LATE EMBRYOGENESIS ACCUMULATING 76 (LEA76)*	Late embryogenesis abundant protein (LEA) family protein
AT3G15680		Ran BP2/NZF zinc finger-like superfamily protein
AT3G15690	*BCCP-LIKE PROTEIN 2 (BLP2)*	Single hybrid motif superfamily protein
AT3G15720		Pectin lyase-like superfamily protein
AT3G15730	*PHOSPHOLIPASE D ALPHA 1 (PLDALPHA1)*	Encodes phospholipase D alpha 1 (PLD alpha 1). Positive regulator of abscisic acid (ABA) mediated stomatal movements. PLD alpha 1 plays an important role in seed deterioration and aging in Arabidopsis. The mRNA is cell-to-cell mobile.
AT3G15740		RING/U-box superfamily protein
AT3G15760		cytochrome P450 family protein
AT3G15770		hypothetical protein

AT3G15780		transmembrane protein
AT3G15790	*METHYL-CPG-BINDING DOMAIN 11 (MBD11)*	Protein containing methyl-CpG-binding domain.Has sequence similarity to human MBD proteins.
AT3G15800		Glycosyl hydrolase superfamily protein
AT3G15810		LURP-one-like protein (DUF567)
AT3G15820	*REDUCED OLEATE DESATURATION 1 (ROD1)*	Functions as phosphatidylcholine:diacylglycerol cholinephosphotransferase, a major reaction for the transfer of 18:1 into phosphatidylcholine for desaturation and also for the reverse transfer of 18:2 and 18:3 into the triacylglycerols synthesis pathway
AT3G15840	*POST-ILLUMINATION CHLOROPHYLL FLUORESCENCE INCREASE (PIFI)*	Encodes a chloroplast-targeted protein localized in the stroma that is a novel component essential for NDH-mediated non-photochemical reduction of the plastoquinone pool in chlororespiratory electron transport.
AT3G15850	*FATTY ACID DESATURASE 5 (FAD5)*	Chloroplastic enzyme responsible for the synthesis of 16:1 fatty acids from galactolipids and sulpholipids. Uses ferredoxin as electron donor. The mRNA is cell-to-cell mobile.
AT3G15860		plant self-incompatibility protein S1 family protein
AT3G15870		Fatty acid desaturase family protein
AT3G15900		homoserine O-acetyltransferase
AT3G15910		hypothetical protein
AT3G15930		Pentatricopeptide repeat (PPR) superfamily protein
AT3G15940		UDP-Glycosyltransferase superfamily protein
AT3G15950	*(NAI2)*	Similar to TSK-associating protein 1 (TSA1), contains 10 EFE repeats, a novel repeat sequence unique to plants. Expressed preferentially in the roots.Protein is localized to ER bodies- an endoplasmic reticulum derived structure. Loss of function mutations lack ER bodies.
AT3G15990	*SULFATE TRANSPORTER 3;4 (SULTR3;4)*	Vascular cambium-localized sulfate transporter, mediates xylem-to-phloem transfer of phosphorus. 2 for its preferential distribution
AT3G16000	*MAR BINDING FILAMENT-LIKE PROTEIN 1 (MFP1)*	Encodes a DNA-binding protein that binds to plastid DNA non-specifically and is associated with nucleoids and thylakoid membranes. The expression of the gene is correlated with the development of thylakoid membranes.MFP1 is required for the normal initiation of starch granules in Arabidopsis chloroplasts. Mutants lacking MFP1 have fewer starch granules per chloroplast relative to wild-type plants. MFP1 interacts with PTST2 (At1g27070), which is also involved in starch granule initiation. MFP1 is required for proper PTST2 location within the chloroplast.
AT3G16010	*MITOCHONDRIAL INTRON SPLICING FACTOR 68 (MISF68)*	Pentatricopeptide Repeat Protein involved in splicing of nad4, nad 5 and nad2 introns which affects biogenesis of the respiratory complex I.
AT3G16020		F-box associated ubiquitination effector protein
AT3G16040		Translation machinery associated TMA7
AT3G16050	*PYRIDOXINE BIOSYNTHESIS 1.2 (PDX1.2)*	Encodes a protein with pyridoxal phosphate synthase activity whose transcripts were detected mostly in roots and accumulate during senescence. The protein was found in very low abundance, which prevented a specific localisation.
AT3G16060		ATP binding microtubule motor family protein
AT3G16100	*RAB GTPASE HOMOLOG G3C (RABG3c)*	RAB GTPase homolog G3C
AT3G16110	*PDI-LIKE 1-6 (PDIL1-6)*	Encodes a protein disulfide isomerase-like (PDIL) protein, a member of a multigene family within the thioredoxin (TRX) superfamily. Unlike several other PDI family members, transcript levels for this gene are not up-regulated in response to three different chemical inducers of ER stress (dithiothreitol, beta-mercaptoethanol, and tunicamycin).
AT3G16120		Dynein light chain type 1 family protein
AT3G16130	*ROP (RHO OF PLANTS) GUANINE NUCLEOTIDE EXCHANGE FACTOR 13 (ROPGEF13)*	Encodes a member of KPP-like gene family, homolog of KPP (kinase partner protein) gene in tomato. Also a member of the RopGEF (guanine nucleotide exchange factor) family, containing the novel PRONE domain (plant-specific Rop nucleotide exchanger), which is exclusively active towards members of the Rop subfamily.
AT3G16140	*PHOTOSYSTEM I SUBUNIT H-1 (PSAH-1)*	Encodes subunit H of photosystem I reaction center subunit VI.
AT3G16150	*ASPARAGINASE B1 (ASPGB1)*	Encodes an asparaginase that catalyzes the degradation of L-asparagine to L-aspartic acid and ammonia. The mRNA is cell-to-cell mobile.
AT3G16160	*(TCX8)*	TCX8 is a transcriptional regulatory protein. It binds the LOX2 promoter and represses its expression.
AT3G16170	*ACYL ACTIVATING ENZYME 13 (AAE13)*	Encodes a malonyl-CoA synthetase that is localized to the cytosol and mitochondrion. AAE13 produces two transcripts one of which includes an N terminal mitochondrial targeting motif. Loss of function of the mtAAE13 product results in growth arrest and lethality.
AT3G16175		Thioesterase superfamily protein
AT3G16190		Isochorismatase family protein
AT3G16220		Putative eukaryotic LigT
AT3G16240	*DELTA TONOPLAST INTEGRAL PROTEIN (DELTA-TIP)*	Delta tonoplast intrinsic protein, functions as a water channel and ammonium (NH3) transporter. Highly expressed in flower, shoot, and stem. Expression shows diurnal regulation and is induced by ammonium (NH3). Protein localized to vacuolar membrane. The mRNA is cell-to-cell mobile.
AT3G16250	*PHOTOSYNTHETIC NDH SUBCOMPLEX B 3 (PnsB3)*	encodes a novel subunit of the chloroplast NAD(P)H dehydrogenase complex, involved in cyclic electron flow around photosystem I to produce ATP. Contains a 4Fe-4S cluster.
AT3G16280	*ETHYLENE RESPONSE FACTOR 36 (ERF036)*	encodes a member of the DREB subfamily A-4 of ERF/AP2 transcription factor family. The protein contains one AP2 domain. There are 17 members in this subfamily including TINY.
AT3G16310		mitotic phosphoprotein N end (MPPN) family protein
AT3G16330		Avr9/Cf-9 rapidly elicited protein
AT3G16340	*ATP-BINDING CASSETTE G29 (ABCG29)*	Encodes a p-coumaryl alcohol exporter involved in lignin biosynthesis.
AT3G16350	*NITROGEN RESPONSE DEFICIENCY 1 (NID1)*	MYB-like transcription factor involved in nitrate signaling trough regulation of CHL1.
AT3G16360	*HPT PHOSPHOTRANSMITTER 4 (AHP4)*	Encodes AHP4, a histidine-containing phosphotransmitter involved in Histidine (His)-to-Aspartate (Asp) phosphorelay signal transduction. AHP4 is one of the six Arabidopsis thaliana histidine phosphotransfer proteins (AHPs). AHPs function as redundant positive regulators of cytokinin signaling. Members of the AHP gene family include: AT3G21510 (AHP1), AT3G29350 (AHP2), AT5G39340 (AHP3), AT3G16360 (AHP4), AT1G03430 (AHP5) and AT1G80100 (AHP6).

AT3G16370	*(GGL19)*	GDSL-motif esterase/acyltransferase/lipase. Enzyme group with broad substrate specificity that may catalyze acyltransfer or hydrolase reactions with lipid and non-lipid substrates. The mRNA is cell-to-cell mobile.
AT3G16380	*POLY(A) BINDING PROTEIN 6 (PAB6)*	polyadenylate-binding protein, putative / PABP, putative, similar to polyadenylate-binding protein (poly(A)-binding protein) from {Arabidopsis thaliana} SP:P42731, (Cucumis sativus) GI:7528270, {Homo sapiens} SP:Q13310, {Arabidopsis thaliana} SP:Q05196; contains InterPro entry IPR000504: RNA-binding region RNP-1 (RNA recognition motif) (RRM). Member of the class III family of PABP proteins.
AT3G16390	*NITRILE SPECIFIER PROTEIN 3 (NSP3)*	Encodes a nitrile-specifier protein NSP3. NSP3 is one out of five (At3g16400/NSP1, At2g33070/NSP2, At3g16390/NSP3, At3g16410/NSP4 and At5g48180/NSP5) A. thaliana epithiospecifier protein (ESP) homologues that promote simple nitrile, but not epithionitrile or thiocyanate formation. The mRNA is cell-to-cell mobile.
AT3G16430	*JACALIN-RELATED LECTIN 31 (JAL31)*	Encodes a protein that increases the beta-glucosidase activities of three scopolin glucosidases in vitro.
AT3G16440	*MYROSINASE-BINDING PROTEIN-LIKE PROTEIN-300B (MLP-300B)*	myrosinase-binding protein-like protein (AtMLP-300B) mRNA,
AT3G16450	*JACALIN-RELATED LECTIN 33 (JAL33)*	Mannose-binding lectin superfamily protein
AT3G16460	*JACALIN-RELATED LECTIN 34 (JAL34)*	Mannose-binding protein
AT3G16470	*JASMONATE RESPONSIVE 1 (JR1)*	Encodes a JA-responsive gene that coordinates with GRP7 in shaping plant development through the regulation of RNA processing in Arabidopsis. AtJAC1 interacts with RNA binding protein GRP7 specifically in the cytoplasm to regulate its nucleocytoplasmic distribution.
AT3G16480	*MITOCHONDRIAL PROCESSING PEPTIDASE ALPHA SUBUNIT (MPPalpha)*	mitochondrial processing peptidase alpha subunit
AT3G16490	*IQ-DOMAIN 26 (IQD26)*	Member of IQ67 (CaM binding) domain containing family.
AT3G16500	*PHYTOCHROME-ASSOCIATED PROTEIN 1 (PAP1)*	phytochrome-associated protein 1 (PAP1)
AT3G16520	*UDP-GLUCOSYL TRANSFERASE 88A1 (UGT88A1)*	UDP-glucosyl transferase 88A1
AT3G16530		Lectin like protein whose expression is induced upon treatment with chitin oligomers.
AT3G16540	*DEGRADATION OF PERIPLASMIC PROTEINS 11 (DEG11)*	Encodes a putative DegP protease.
AT3G16550	*DEGRADATION OF PERIPLASMIC PROTEINS 12 (DEG12)*	Encodes a putative DegP protease.
AT3G16560		Protein phosphatase 2C family protein
AT3G16580		F-box and associated interaction domains-containing protein
AT3G16600	*SNF2-RING-HELICASE?LIKE 3 (FRG3)*	SNF2 domain-containing protein / helicase domain-containing protein / zinc finger protein-like protein
AT3G16650	*PLEIOTROPIC REGULATORY LOCUS 2 (PRL2)*	Transducin/WD40 repeat-like superfamily protein
AT3G16670		Pollen Ole e 1 allergen and extensin family protein
AT3G16680		DNA binding / DNA-directed RNA polymerase
AT3G16690	*(SWEET16)*	Nodulin MtN3 family protein
AT3G16700	*FUMARYLACETOACETATE HYDROLASE DOMAIN CONTAINING PROTEIN 1B (FAHD1B)*	Fumarylacetoacetate hydrolase homolog.
AT3G16760		Tetratricopeptide repeat (TPR)-like superfamily protein
AT3G16770	*ETHYLENE-RESPONSIVE ELEMENT BINDING PROTEIN (EBP)*	Encodes a member of the ERF (ethylene response factor) subfamily B-2 of the plant specific ERF/AP2 transcription factor family (RAP2.3). The protein contains one AP2 domain. There are 5 members in this subfamily including RAP2.2 AND RAP2.12.It is localized to the nucleus and acts as a transcriptional activator through the GCC-box. It has been identified as a suppressor of Bax-induced cell death by functional screening in yeast and can also suppress Bax-induced cell death in tobacco plants. Overexpression of this gene in tobacco BY-2 cells confers resistance to H2O2 and heat stresses. Overexpression in Arabidopsis causes upregulation of PDF1.2 and GST6. It is part of the ethylene signaling pathway and is predicted to act downstream of EIN2 and CTR1, but not under EIN3. The mRNA is cell-to-cell mobile.
AT3G16790		
AT3G16800	*E GROWTH-REGULATING 3 (EGR3)*	EGR3 functions as a negative regulator of plant growth with prominent effect on plant growth during drought stress, EGR3 regulates microtubule organization and likely affects additional cytoskeleton and trafficking processes along the plasma membrane.
AT3G16810	*PUMILIO 24 (PUM24)*	Encodes a member of the Arabidopsis Pumilio (APUM) proteins containing PUF domain (eight repeats of approximately 36 amino acids each). PUF proteins regulate both mRNA stability and translation through sequence-specific binding to the 3' UTR of target mRNA transcripts.
AT3G16840		P-loop containing nucleoside triphosphate hydrolases superfamily protein
AT3G16855		
AT3G16860	*COBRA-LIKE PROTEIN 8 PRECURSOR (COBL8)*	COBRA-like protein 8 precursor
AT3G16870	*GATA TRANSCRIPTION FACTOR 17 (GATA17)*	Encodes a member of the GATA factor family of zinc finger transcription factors.
AT3G16880		F-box and associated interaction domains-containing protein
AT3G16890	*PENTATRICOPEPTIDE (PPR) DOMAIN PROTEIN 40 (PPR40)*	Encodes a mitochondrial pentatricopeptide repeat (PPR) domain protein, PPR40, which provides a signalling link between mitochondrial electron transport and regulation of stress and hormonal responses. Mutations in PPR40 result in enhanced sensitivity to salt, ABA and oxidative stress, as well as reduced electron transport through Complex III (cytochrome c reductase).
AT3G16920	*CHITINASE-LIKE PROTEIN 2 (CTL2)*	Encodes a chitinase-like protein expressed predominantly in stems. Mutants accumulate ligning in etiolated hypocotyls.
AT3G16930		hypothetical protein
AT3G16940	*CALMODULIN-BINDING TRANSCRIPTION ACTIVATOR 6 (CAMTA6)*	Calmodulin binding transcription factor. Mutants display increased salt tolerance during early germination. Involved in regulation of salt stress responsive genes.
AT3G16950	*LIPOAMIDE DEHYDROGENASE 1 (LPD1)*	encodes a plastid lipoamide dehydrogenase, subunit of the pyruvate dehydrogenase complex which provides acetyl-CoA for de novo fatty acid biosynthesis. The gene is highly expressed in developing seeds.
AT3G16960		plant self-incompatibility protein S1 family protein
AT3G16970		Plant self-incompatibility protein S1 family
AT3G16980	*(NRPB9A)*	One of two highly similar, non-catalytic subunits common to nuclear DNA-directed RNA polymerases II, IV and V; homologous to budding yeast RPB9. Appears to be redundant with At4g16265
AT3G16990		heme oxygenase-like, multi-helical

AT3G17010	*REPRODUCTIVE MERISTEM 22 (REM22)*	transcriptional factor B3 family protein, contains Pfam profile PF02362: B3 DNA binding domain. Activated by AGAMOUS ina a cal-1, ap1-1 background. Expressed in stamen primordia, the placental region of developing carpels and the ovary.
AT3G17020		Adenine nucleotide alpha hydrolases-like superfamily protein
AT3G17030		Nucleic acid-binding proteins superfamily
AT3G17040	*HIGH CHLOROPHYLL FLUORESCENT 107 (HCF107)*	It is a RNA tetratricopeptide repeat-containing protein required for normal processing of transcripts from the polycistronic chloroplast psbB-psbT-psbH-petB-petD operon coding for proteins of the photosystem II and cytochrome b6/f complexes. Localizes to the chloroplast membrane. Involved in regulating plastidial gene expression and biogenesis. It binds in the psbT?psbH intercistronic region and blocks the progression of 5′ → 3′ exoribonucleases, which defines the 5′ end of processed psbH transcripts and also stabilizes the downstream RNA segment. In addition, HCF107 binding remodels the structure of the psbH 5′ UTR in a way that can account for its ability to enhance psbH translation.
AT3G17050		transposable_element_gene;pseudogene, glycine-rich protein, similar to glycine-rich protein TIGR:At1g53620.1 (Arabidopsis thaliana);(source:TAIR10)
AT3G17060		Pectin lyase-like superfamily protein
AT3G17080		Plant self-incompatibility protein S1 family
AT3G17090	*D-CLADE TYPE 2C PROTEIN PHOSPHATASE 2 (PP2C.D2)*	Protein phosphatase 2C family protein
AT3G17100	*ATBS1 INTERACTING FACTOR 3 (AIF3)*	sequence-specific DNA binding transcription factor
AT3G17110		Probably not a pseudogene based on evidence for transcription (RNA-seq) and translation (Ribo-seq) described in PMID:27791167
AT3G17120		transmembrane protein
AT3G17130		Plant invertase/pectin methylesterase inhibitor superfamily protein
AT3G17140		Plant invertase/pectin methylesterase inhibitor superfamily protein
AT3G17150		Plant invertase/pectin methylesterase inhibitor superfamily protein
AT3G17160		hypothetical protein
AT3G17170	*REGULATOR OF FATTY-ACID COMPOSITION 3 (RFC3)*	Translation elongation factor EF1B/ribosomal protein S6 family protein
AT3G17180	*SERINE CARBOXYPEPTIDASE-LIKE 33 (scpl33)*	serine carboxypeptidase-like 33
AT3G17190		hypothetical protein
AT3G17210	*HEAT STABLE PROTEIN 1 (HS1)*	Encodes a heat stable protein with antimicrobial and antifungal activity.
AT3G17220	*PECTIN METHYLESTERASE INHIBITOR 2 (PMEI2)*	Pectin methylesterase inhibitor AtPMEI2. Inactivates AtPPME1 in vitro. Localized to Brefeldin A-induced compartments, and was found in FYVE-induced endosomal aggregates.
AT3G17230		plant invertase/pectin methylesterase inhibitor superfamily protein
AT3G17240	*LIPOAMIDE DEHYDROGENASE 2 (mtLPD2)*	lipoamide dehydrogenase precursor
AT3G17260		transposable_element_gene;hAT-like transposase family (hobo/Ac/Tam3), has a 2.6e-27 P-value blast match to GB:CAA29005 ORFa of Maize Ac (hAT-element) (Zea mays);(source:TAIR10)
AT3G17280		F-box and associated interaction domains-containing protein
AT3G17290		transposable_element_gene;hAT-like transposase family (hobo/Ac/Tam3), has a 3.1e-27 P-value blast match to GB:CAA29005 ORFa of Maize Ac (hAT-element) (Zea mays);(source:TAIR10)
AT3G17330	*EVOLUTIONARILY CONSERVED C-TERMINAL REGION 6 (ECT6)*	evolutionarily conserved C-terminal region 6
AT3G17340	*(PLANTKAP)*	Ran effector.
AT3G17350		wall-associated receptor kinase carboxy-terminal protein
AT3G17360	*PHRAGMOPLAST ORIENTING KINESIN 1 (POK1)*	PHRAGMOPLAST ORIENTING KINESIN 1 is one of the two Arabidopsis homologs isolated in yeast two-hybrid screen for interaction partners of maize gene TANGLED1 (TAN1). Based on sequence homology in their motor domains, POK1 and POK2 belong to the kinesin-12 class which also includes the well-characterized group of phragmoplast-associated kinesins AtPAKRPs. Both kinesins are composed of an N-terminal motor domain throughout the entire C terminus and putative cargo binding tail domains. The expression domains for POK1 constructs were more limited than those for POK2; both are expressed in tissues enriched for dividing cells. The phenotype of pok1/pok2 double mutants strongly resembles that of maize tan1 mutants, characterized by misoriented mitotic cytoskeletal arrays and misplaced cell walls.
AT3G17380		TRAF-like family protein
AT3G17390	*METHIONINE OVER-ACCUMULATOR 3 (MTO3)*	S-adenosylmethionine synthetase
AT3G17420	*GLYOXYSOMAL PROTEIN KINASE 1 (GPK1)*	Serine/threonine protein kinase-like protein expressed in etiolated cotyledons and found in glyoxysomes.
AT3G17465	*RIBOSOMAL PROTEIN L3 PLASTID (RPL3P)*	encodes a putative L3 ribosomal protein targeted to the plastid.
AT3G17470	*CA2+-ACTIVATED RELA-SPOT HOMOLOG (CRSH)*	CRSH-dependent ppGpp synthesis causes transient increase of intracellular ppGpp at night.
AT3G17490		F-box and associated interaction domains-containing protein
AT3G17510	*CBL-INTERACTING PROTEIN KINASE 1 (CIPK1)*	Encodes a CBL-interacting protein kinase. Specifically interacts with ECT1 and ECT2.
AT3G17520	*(SSLEA)*	Late embryogenesis abundant protein (LEA) family protein
AT3G17590	*BUSHY GROWTH (BSH)*	Encodes the Arabidopsis homologue of yeast SNF5 and represents a conserved subunit of plant SWI/SNF complexes.
AT3G17600	*INDOLE-3-ACETIC ACID INDUCIBLE 31 (IAA31)*	Encodes a member of the Aux/IAA family of proteins implicated in auxin signaling. IAA31 shares several residues with the conserved domain II region, believed to act as a degron in many of the rapidly degraded Aux/IAA family members. An IAA31 fusion protein is quite long-lived, but can be degraded more rapidly in the presence of auxin. Unlike many other family members, IAA31 transcript levels do not rise in response to auxin. Nevertheless, overexpression of IAA31 leads to defects in auxin-related processes such as gravitropism, root development, shoot development, and cotyledon vascular development.
AT3G17610		
AT3G17620		F-box and associated interaction domains-containing protein
AT3G17640		Leucine-rich repeat (LRR) family protein

AT3G17650	*YELLOW STRIPE LIKE 5 (YSL5)*	Arabidopsis thaliana metal-nicotianamine transporter YSL5
AT3G17680		Kinase interacting (KIP1-like) family protein
AT3G17690	*CYCLIC NUCLEOTIDE GATED CHANNEL 19 (CNGC19)*	member of Cyclic nucleotide gated channel family
AT3G17700	*CYCLIC NUCLEOTIDE-BINDING TRANSPORTER 1 (CNBT1)*	cyclic nucleotide-binding transporter 1, member of a family of cyclic nucleotide gated channels. The mRNA is cell-to-cell mobile.
AT3G17710		F-box and associated interaction domains-containing protein
AT3G17720		Pyridoxal phosphate (PLP)-dependent transferases superfamily protein
AT3G17730	*NAC DOMAIN CONTAINING PROTEIN 57 (NAC057)*	NAC domain containing protein 57
AT3G17740		hypothetical protein
AT3G17770		Dihydroxyacetone kinase
AT3G17790	*PURPLE ACID PHOSPHATASE 17 (PAP17)*	Expression is upregulated in the shoot of cax1/cax3 mutant and is responsive to phosphate (Pi) and not phosphite (Phi) in roots and shoots.
AT3G17800		mRNA level of the MEB5.2 gene (At3g17800) remains unchanged after cutting the inflorescence stem
AT3G17820	*GLUTAMINE SYNTHETASE 1.3 (GLN1.3)*	encodes a cytosolic glutamine synthetase, the enzyme has low affinity with substrate ammonium The mRNA is cell-to-cell mobile.
AT3G17830	*DNA J PROTEIN A4 (DJA4)*	Molecular chaperone Hsp40/DnaJ family protein
AT3G17840	*RECEPTOR-LIKE KINASE 902 (RLK902)*	Encodes a receptor-like kinase found at the cell surface of various tissues. Its function remains unknown.
AT3G17850	*NCOMPLETE ROOT HAIR ELONGATION 1 (IREH1)*	Protein kinase which together with IRE3 plays an important role in controlling root skewing and maintaining the microtubule network.
AT3G17860	*JASMONATE-ZIM-DOMAIN PROTEIN 3 (JAZ3)*	JAZs are direct targets of the SCFCOI1 E3 ubiquitin-ligase and JA treatment induces their proteasome-mediated degradation. Furthermore, JAI3 negatively regulates the key transcriptional activator of JA responses, AtMYC2. The C-terminal portion of JAZ3, including the Jas domain, appears to be important for JAZ3-COI1 binding in the presence of coronatine.
AT3G17890		hypothetical protein
AT3G17910	*SURFEIT 1A (SURF1A)*	Encodes one of two Arabidopsis mitochondrial proteins similar to human SURF1 which is known to be involved in cytochrome c oxidase assembly. Mutations result in embryo lethality.
AT3G17930	*DEFECTIVE ACCUMULATION OF CYTOCHROME B6/F COMPLEX (DAC)*	Encodes a thylakoid membrane protein involved in the accumulation of the cytochrome b6/f complex.
AT3G17950		transmembrane protein
AT3G17960		
AT3G17970	*TRANSLOCON AT THE OUTER MEMBRANE OF CHLOROPLASTS 64-III (TOC64-III)*	Integral chloroplast outer membrane protein. Belongs to one of the 36 carboxylate clamp (CC)-tetratricopeptide repeat (TPR) proteins (Prasad 2010, Pubmed ID: 20856808) with potential to interact with Hsp90/Hsp70 as co-chaperones.
AT3G17980	*C2 DOMAIN (C2)*	Calcium-dependent lipid-binding (CaLB domain) family protein
AT3G17990		
AT3G18000	*XIPOTL 1 (XPL1)*	Encodes a N-methyltransferase-like protein. Double mutants of NMT1 and NMT3 are defective in leaf, root, flower, seed, and pollen development.
AT3G18035	*(HON4)*	A linker histone like protein
AT3G18050		GPI-anchored protein
AT3G18060		transducin family protein / WD-40 repeat family protein
AT3G18070	*BETA GLUCOSIDASE 43 (BGLU43)*	beta glucosidase 43
AT3G18080	*B-S GLUCOSIDASE 44 (BGLU44)*	B-S glucosidase 44
AT3G18120		F-box associated ubiquitination effector family protein
AT3G18130	*RECEPTOR FOR ACTIVATED C KINASE 1C (RACK1C_AT)*	Encodes a protein with similarity to mammalian RACKs. RACKs function to shuttle activated protein kinase C to different subcellular sites and may also function as a scaffold through physical interactions with other proteins. RACK1C has no phenotype on its own and probably acts redundantly with RACK1A and RACK1B.
AT3G18160	*PEROXIN 3-1 (PEX3-1)*	Peroxin 3-1
AT3G18170		Glycosyltransferase family 61 protein
AT3G18180		Glycosyltransferase family 61 protein
AT3G18200	*USUALLY MULTIPLE ACIDS MOVE IN AND OUT TRANSPORTERS 4 (UMAMIT4)*	nodulin MtN21-like transporter family protein
AT3G18210	*CUPULIFORMIS2 (CP2)*	Belongs to the 2-oxoglutarate (2OG) and Fe(II)-dependent oxygenase superfamily proteins and contains an oxoglutarate/iron-dependent oxygenase domain (InterPro:IPR005123) of the prolyl 4-hydroxylase, alpha subunit subtype (P4Hc; InterPro:IPR006620), participates in epigenetic repression of flowering genes, works redundantly with ICU11 to repress several members of the MADS-box transcription factors family, during vegetative development via histone modification.
AT3G18230		Octicosapeptide/Phox/Bem1p family protein
AT3G18250		Putative membrane lipoprotein
AT3G18260	*RETICULON-LIKE B 9 (RTNLB9)*	Reticulon family protein
AT3G18270	*CYTOCHROME P450, FAMILY 77, SUBFAMILY A, POLYPEPTIDE 5 PSEUDOGENE (CYP77A5P)*	a cytochrome P450 pseudogene. the second half of the gene overlaps perfectly with the other gene model.
AT3G18280	*TRACHEARY ELEMENT DIFFERENTIATION-RELATED 4 (TED4)*	Bifunctional inhibitor/lipid-transfer protein/seed storage 2S albumin superfamily protein
AT3G18290	*BRUTUS (BTS)*	Encodes BRUTUS (BTS), a putative E3 ligase protein with metal ion binding and DNA binding domains, which negatively regulates the response to iron deficiency. The mRNA is cell-to-cell mobile.
AT3G18300		hypothetical protein
AT3G18360	*VQ MOTIF-CONTAINING PROTEIN 20 (VQ20)*	Member of VQ gene family. VQ proteins are named for the VQ motif (FxxxVQxxTG), a conserved amino acid region. Interacts with members of WRKY gene family, involved in pollen development.
AT3G18370	*(ATSYTF)*	C2 domain-containing protein
AT3G18390	*EMBRYO DEFECTIVE 1865 (EMB1865)*	CRS1 / YhbY (CRM) domain-containing protein
AT3G18400	*NAC DOMAIN CONTAINING PROTEIN 58 (NAC058)*	NAC domain containing protein 58
AT3G18450		PLAC8 family protein

AT3G18460		PLAC8 family protein
AT3G18490	*ASPARTIC PROTEASE IN GUARD CELL 1 (ASPG1)*	Encodes ASPG1 (ASPARTIC PROTEASE IN GUARD CELL 1). Functions in drought avoidance through abscisic acid (ABA) signalling in guard cells.
AT3G18500	*CATABOLITE REPRESSOR 4C (CCR4C)*	Deadenylase.
AT3G18510		ATP-dependent helicase/nuclease subunit
AT3G18524	*MUTS HOMOLOG 2 (MSH2)*	Encodes a DNA mismatch repair homolog of human MutS gene, MSH6. MSH2 is involved in maintaining genome stability and repressing recombination of mismatched heteroduplexes.There are four MutS genes in Arabidopsis, MSH2, MSH3, MSH6, and MSH7, which all act as heterodimers and bind to 51-mer duplexes. MSH2 has different binding specificity to different mismatches in combination with MSH3, MSH6, or MSH7.
AT3G18540		
AT3G18560		hypothetical protein
AT3G18570		Oleosin family protein
AT3G18600		P-loop containing nucleoside triphosphate hydrolases superfamily protein
AT3G18610	*NUCLEOLIN LIKE 2 (NUC-L2)*	Encodes ATNUC-L2 (NUCLEOLIN LIKE 2).
AT3G18630	*URACIL DNA GLYCOSYLASE (UNG)*	Encodes a uracil-DNA glycosylase (UDG) involved in a base excision DNA repair pathway in mitochondria.
AT3G18640		Zinc finger C-x8-C-x5-C-x3-H type family protein
AT3G18650	*AGAMOUS-LIKE 103 (agl103)*	AGAMOUS-like 103
AT3G18660	*PLANT GLYCOGENIN-LIKE STARCH INITIATION PROTEIN 1 (PGSIP1)*	Plants expressing an RNAi construct specifically targeting PGSIP1 was shown to have a dramatically reduced amount of starch. Encodes a glucuronyltransferase responsible for the addition of GlcA residues onto xylan and for secondary wall deposition.
AT3G18670		Ankyrin repeat family protein
AT3G18680	*PLASTID 55 UMP KINASE (PUMPKIN)*	Encodes a functional UMP Kinase located in the plastid that binds to group II intron plastid transcription products. Mutants show decreased accumulation of target transcripts/proteins.
AT3G18690	*MAP KINASE SUBSTRATE 1 (MKS1)*	Encodes a nuclear-localized member of a plant specific gene family involved in mediating responses to pathogens. Interacts with WRKY transcriptional regulators.
AT3G18700		transmembrane protein
AT3G18710	*PLANT U-BOX 29 (PUB29)*	Encodes a protein containing a U-box and an ARM domain. This protein has E3 ubiquitin ligase activity based on in vitro assays.
AT3G18720		F-box family protein
AT3G18750	*WITH NO LYSINE (K) KINASE 6 (WNK6)*	Encodes a member of the WNK family (9 members in all) of protein kinases, the structural design of which is clearly distinct from those of other known protein kinases, such as receptor-like kinases and mitogen-activated protein kinases. Its transcription is under the control of circadian rhythms.
AT3G18770	*AUTOPHAGY 13B (ATG13B)*	Autophagy protein.
AT3G18780	*ACTIN 2 (ACT2)*	Encodes an actin that is constitutively expressed in vegetative structures but not pollen. ACT2 is involved in tip growth of root hairs.
AT3G18800		transmembrane protein
AT3G18820	*RAB GTPASE HOMOLOG G3F (RAB7B)*	RAB7 homolog, forms retromer complex with VPS35; ES17 prevents the retromer complex to endosome anchoring, resulting in retention of RABG3f. The interaction of RABG3f?VPS35 functinons as a checkpoint in the control of traffic toward the vacuole.
AT3G18830	*POLYOL/MONOSACCHARIDE TRANSPORTER 5 (PMT5)*	This gene encodes a plasma membrane-localized polyol/cyclitol/monosaccharide-H+-symporter. The symporter is able to catalyze the energy-dependent membrane passage of a wide range of linear polyols (three to six carbon backbone), of cyclic polyols (<i>myo</i>-inositol), and of numerous monosaccharides, including pyranose ring-forming and furanose ring-forming hexoses and pentoses. This gene belongs to a monosaccharide transporter-like (MST-like) superfamily.
AT3G18840		LOW protein: PPR containing-like protein
AT3G18870	*(MTERF11)*	Mitochondrial transcription termination factor family member.
AT3G18890	*TRANSLOCON AT THE INNER ENVELOPE MEMBRANE OF CHLOROPLASTS 62 (Tic62)*	NAD(P)-binding Rossmann-fold superfamily protein
AT3G18900		ternary complex factor MIP1 leucine-zipper protein
AT3G18910	*EIN2 TARGETING PROTEIN2 (ETP2)*	EIN2 targeting protein2
AT3G18920		
AT3G18930	*ARABIDOPSIS T??XICOS EN LEVADURA 65 (ATL65)*	RING/U-box superfamily protein
AT3G18960	*REPRODUCTIVE MERISTEM 7 (REM7)*	Encodes a member of the REM (Reproductive Meristem) gene family, a part of the B3 DNA-binding domain superfamily. Together with ATHB25 induces the expression of genes controlling shoot stem characteristics by ectopic expression in roots.
AT3G19000		2-oxoglutarate (2OG) and Fe(II)-dependent oxygenase superfamily protein
AT3G19010	*2-OXOGLUTARATE OXYGENASE (2-OXOGLUTARATE OXYGENASE)*	2-oxoglutarate (2OG) and Fe(II)-dependent oxygenase superfamily protein
AT3G19020	*LEUCINE-RICH REPEAT/EXTENSIN 8 (LRX8)*	Pollen expressed protein required for pollen tube growth. Along with other members of the LRX family, interacts with RALF4 to control pollen tube growth and integrity. Loss of function results in premature pollen tube rupture and reduced fertility.
AT3G19030		transcription initiation factor TFIID subunit 1b-like protein
AT3G19040	*HISTONE ACETYLTRANSFERASE OF THE TAFII250 FAMILY 2 (HAF2)*	Encodes a protein similar to TATA-binding protein-associated factor TAF1 (a.k.a. TAFII250) with histone acetyltransferase activity. It is required in integrating light signals to regulate gene expression and growth.
AT3G19050	*PHRAGMOPLAST ORIENTING KINESIN 2 (POK2)*	PHRAGMOPLAST ORIENTING KINESIN 2 is one of the two Arabidopsis homologs isolated in yeast two-hybrid screen for interaction partners of maize gene TANGLED1 (TAN1). Based on sequence homology in their motor domains, POK1 and POK2 belong to the kinesin-12 class which also includes the well-characterized group of phragmoplast-associated kinesins AtPAKRPs. Both kinesins are composed of an N-terminal motor domain throughout the entire C terminus and putative cargo binding tail domains. The expression domains for POK2 constructs were broader than those for POK1; both are expressed in tissues enriched for dividing cells. The phenotype of pok1/pok2 double mutants strongly resembles that of maize tan1 mutants, characterized by misoriented mitotic cytoskeletal arrays and misplaced cell walls.

AT3G19070		Homeodomain-like superfamily protein
AT3G19080		SWIB complex BAF60b domain-containing protein
AT3G19085		F-box/RNI/FBD-like domain protein
AT3G19090	*LA RELATED PROTEIN 6C (LARP6C)*	RNA-binding protein
AT3G19100	*(TAGK2)*	Encodes a protein kinase that positively regulates gibberellic acid (GA) signaling by inactivating the E3 ubiquitin ligase GARU. GARU mediates ubiquitin-dependent degradation of GID1s, which are GA receptors.
AT3G19110		
AT3G19140	*DAY NEUTRAL FLOWERING (DNF)*	DAY NEUTRAL FLOWERING (DNF) is a membrane-bound E3 ligase involved in the regulation of flowering time in Arabidopsis. It negetively regulate the early flowering under Short Day condition.
AT3G19170	*PRESEQUENCE PROTEASE 1 (PREP1)*	Zinc metalloprotease pitrilysin subfamily A. Signal peptide degrading enzyme targeted to mitochondria and chloroplasts. Expressed only in siliques and flowers
AT3G19180	*PARALOG OF ARC6 (PARC6)*	Encodes a chloroplast division factor located in the plastid inner envelope with its N-terminus exposed to the stroma. PARC6 influences FtsZ assembly and is required for recruitment of PDV1 during chloroplast division.
AT3G19190	*AUTOPHAGY 2 (ATG2)*	Encodes autophagy-related 2 (ATG2). The mRNA is cell-to-cell mobile.
AT3G19200		hypothetical protein
AT3G19210	*HOMOLOG OF RAD54 (RAD54)*	Encodes RAD54, a member of the SWI2/SNF2 family of DNA-stimulated ATPases. Functions in DNA repair via homologous recombination.
AT3G19220	*SNOWY COTYLEDON 2 (SCO2)*	Encodes a zinc finger protein that is similar to a subgroup of DnaJ and is involved in cotyledon chloroplast biogenesis. Cyo1 is localized to the thylakoid membrane and has protein disulfide isomerase activity in vivo.Cyo1 is more highly expressed in light grown seedlings. Loss of function mutants have albino cotyledons and abnormal plastids.
AT3G19230	*(LLR4)*	Leucine-rich repeat (LRR) family protein
AT3G19240	*DEFECTIVE EMBRYO AND MERISTEMS 2 (DEM2)*	Together with DEM1 plays an essential role in cell division in plants, most likely through an interaction with RAN1.
AT3G19270	*CYTOCHROME P450, FAMILY 707, SUBFAMILY A, POLYPEPTIDE 4 (CYP707A4)*	Encodes a protein with ABA 8'-hydroxylase activity, involved in ABA catabolism. Member of the CYP707A gene family.
AT3G19280	*FUCOSYLTRANSFERASE 11 (FUT11)*	Encodes a protein with core α1,3-fucosyltransferase activity.
AT3G19290	*ABRE BINDING FACTOR 4 (ABF4)*	bZIP transcription factor with specificity for abscisic acid-responsive elements (ABRE). Mediates ABA-dependent stress responses.ABF4 acts through SnRK2 pathway and binds to ABA response elements of the promoters of NYE1 and regulates their expression to promote chlorophyll degradation.
AT3G19310		PLC-like phosphodiesterases superfamily protein
AT3G19350	*MATERNALLY EXPRESSED PAB C-TERMINAL (MPC)*	Encodes a the C-terminal domain of poly(A) binding proteins. MPC is imprinted such that only the maternal allele is expressed in the endosperm. MPC is silenced by the action of MET1 and its expression is promoted by DEM.
AT3G19360		Zinc finger (CCCH-type) family protein
AT3G19370		filament-like protein (DUF869)
AT3G19380	*PLANT U-BOX 25 (PUB25)*	PUB25 and PUB26 are closely related paralogs that encode functional E3 ligases. They function in immune response pathway by targeting BIK1 for degradation.
AT3G19390		Granulin repeat cysteine protease family protein
AT3G19400		Cysteine proteinases superfamily protein
AT3G19430		late embryogenesis abundant protein-related / LEA protein-like protein
AT3G19440		Pseudouridine synthase family protein
AT3G19450	*(ATCAD4)*	Encodes a catalytically active cinnamyl alcohol dehydrogenase which uses p-coumaryl aldehyde as a preferred substrate. It can also use caffeyl, coniferyl and d-hydroxyconiferyl aldehydes as substrates. The mRNA is cell-to-cell mobile.
AT3G19470		F-box and associated interaction domains-containing protein
AT3G19480	*(3-PGDH)*	Encodes a stromal phosphoglycerate dehydrogenase with a high NAD(H)-specificity that is active in photosynthesizing chloroplasts and draws its substrate 3-PGA directly from the Calvin-Benson-Bassham cycle.
AT3G19500		basic helix-loop-helix (bHLH) DNA-binding superfamily protein
AT3G19516		
AT3G19530		hypothetical protein
AT3G19550		glutamate racemase
AT3G19553	*POLYAMINE UPTAKE TRANSPORTER 5 (PUT5)*	Encodes POLYAMINE UPTAKE TRANSPORTER 5, an amino acid permease family protein.
AT3G19580	*ZINC-FINGER PROTEIN 2 (ZF2)*	Encodes zinc finger protein. mRNA levels are upregulated in response to ABA, high salt, and mild desiccation. The protein is localized to the nucleus and acts as a transcriptional repressor.
AT3G19610	*CORTICAL MICROTUBULE DISORDERING6 (CORD6)*	Member of a novel, plant specific family of microtubule associated proteins.
AT3G19620		Glycosyl hydrolase family protein
AT3G19630		Radical SAM superfamily protein
AT3G19640	*MAGNESIUM TRANSPORTER 4 (MGT4)*	Transmembrane magnesium transporter. One of nine family members.
AT3G19660		hypothetical protein
AT3G19680		hypothetical protein (DUF1005)
AT3G19690		CAP (Cysteine-rich secretory proteins, Antigen 5, and Pathogenesis-related 1 protein) superfamily protein
AT3G19700	*HAIKU2 (IKU2)*	Encodes leucine rich repeat (LRR) kinase. Iku2-3 identified in a screen for mutants with abnormal endosperm. Sporophytic recessive mutants have reduced embryo and endosperm size. Seed size is also reduced and the shape is abnormal suggesting an interaction between the endosperm and cell elongation in the integuments.

AT3G19710	*BRANCHED-CHAIN AMINOTRANSFERASE4 (BCAT4)*	Belongs to the branched-chain amino acid aminotransferase gene family. Encodes a methionine-oxo-acid transaminase. Involved in the methionine chain elongation pathway that leads to the ultimate biosynthesis of methionine-derived glucosinolates.
AT3G19730		
AT3G19750		
AT3G19770	*(VPS9A)*	Guanine nucleotide exchange factor VPS9a. Can activate all Rab5 members to GTP-bound forms in vitro. Required for embryogenesis. Regulates the localization of ARA7 and ARA6. Involved in postembryonic root development.
AT3G19780	*(DUF179)*	hypothetical protein
AT3G19790		hypothetical protein
AT3G19800	*DOMAIN OF UNKNOWN FUNCTION 177 B (DUF177B)*	Encodes the DUF177B version of the two DUF177 proteins in Arabidopsis. This version differs from DUF177A in containing a 23 aa insertion compared to the DUF177A sequence.
AT3G19820	*DWARF 1 (DWF1)*	Involved in the conversion of the early brassinosteroid precursor 24-methylenecholesterol to campesterol. Brassinosteroids affect cellular elongation. Mutants have dwarf phenotype. DWF1 is a Ca2+-dependent calmodulin-binding protein.
AT3G19860	*BASIC HELIX-LOOP-HELIX 121 (bHLH121)*	A basic helix?loop?helix (bHLH) transcription factor which acts as an essential part of the iron deficiency signaling pathway. The phosphorylated form of URI accumulates under Fe deficiency, forms heterodimers with subgroup IVc proteins, and induces transcription of bHLH38/39/100/101. These transcription factors in turn heterodimerize with FIT and drive the transcription of IRT1 and FRO2 to increase Fe uptake.
AT3G19880		F-box and associated interaction domains-containing protein
AT3G19890		F-box family protein
AT3G19910	*(CTL18)*	RING/U-box superfamily protein
AT3G19920		BTB/POZ domain protein
AT3G19930	*SUGAR TRANSPORTER 4 (STP4)*	Encodes a sucrose hydrogen symporter that is induced by wounding. The mRNA is cell-to-cell mobile.
AT3G19940	*SUGAR TRANSPORT PROTEIN 10 (STP10)*	Encodes a hexose-H(+) symporter that catalyzes the high-affinity uptake of glucose, galactose and mannose that is induced under low-glucose conditions in pollen tubes.
AT3G19960	*MYOSIN 1 (ATM1)*	member of Myosin-like proteins
AT3G19970		alpha/beta-Hydrolases superfamily protein
AT3G20000	*TRANSLOCASE OF THE OUTER MITOCHONDRIAL MEMBRANE 40 (TOM40)*	Encodes a component of the TOM receptor complex responsible for the recognition and translocation of cytosolically synthesized mitochondrial preproteins. With TOM22, functions as the transit peptide receptor at the surface of the mitochondrial outer membrane and facilitates the movement of preproteins into the translocation pore. The mRNA is cell-to-cell mobile.
AT3G20015		Eukaryotic aspartyl protease family protein
AT3G20030		F-box and associated interaction domains-containing protein
AT3G20080	*CYTOCHROME P450, FAMILY 705, SUBFAMILY A, POLYPEPTIDE 15 (CYP705A15)*	cytochrome P450, family 705, subfamily A, polypeptide 15
AT3G20090	*CYTOCHROME P450, FAMILY 705, SUBFAMILY A, POLYPEPTIDE 18 (CYP705A18)*	cytochrome P450, family 705, subfamily A, polypeptide 18
AT3G20100	*CYTOCHROME P450, FAMILY 705, SUBFAMILY A, POLYPEPTIDE 19 (CYP705A19)*	member of CYP705A The mRNA is cell-to-cell mobile.
AT3G20110	*CYTOCHROME P450, FAMILY 705, SUBFAMILY A, POLYPEPTIDE 20 (CYP705A20)*	member of CYP705A
AT3G20120	*CYTOCHROME P450, FAMILY 705, SUBFAMILY A, POLYPEPTIDE 21 (CYP705A21)*	cytochrome P450, family 705, subfamily A, polypeptide 21
AT3G20130	*CYTOCHROME P450, FAMILY 705, SUBFAMILY A, POLYPEPTIDE 22 (CYP705A22)*	Encodes a member of the CYP705A family of cytochrome P450 enzymes. Mutants show altered gravitropic responses.
AT3G20140	*CYTOCHROME P450, FAMILY 705, SUBFAMILY A, POLYPEPTIDE 23 (CYP705A23)*	member of CYP705A
AT3G20150		Kinesin motor family protein
AT3G20160	*POLYPRENYL PYROPHOSPHATE SYNTHASE 2 (PPPS2)*	Terpenoid synthases superfamily protein
AT3G20180		Copper transport protein family
AT3G20190	*POLLEN RECEPTOR LIKE KINASE 4 (PRK4)*	Leucine-rich repeat protein kinase family protein
AT3G20200		kinase with adenine nucleotide alpha hydrolases-like domain-containing protein
AT3G20210	*DELTA VACUOLAR PROCESSING ENZYME (DELTA-VPE)*	Encodes a vacuolar processing enzyme with caspase-1-like activity that is specifically expressed in inner integument of developing seeds. Mutants display abnormal seed coat development. It is speculated to be involved in cell death of limited cell layers, the purpose of which is to form a seed coat.
AT3G20220	*SMALL AUXIN UPREGULATED RNA 47 (SAUR47)*	SAUR-like auxin-responsive protein family
AT3G20240		Mitochondrial substrate carrier family protein
AT3G20260		DUF1666 family protein (DUF1666)
AT3G20270	*(LBR-2)*	Encodes one of the two LBP/BPI related proteins (AT1G04970/LBR-1, AT3G20270/LBR-2) that bind to LPS directly and regulate PR1 expression.
AT3G20280		RING/FYVE/PHD zinc finger superfamily protein
AT3G20300		extracellular ligand-gated ion channel protein (DUF3537)
AT3G20330	*PYRIMIDINE B (PYRB)*	encodes aspartate carbamoyltransferase catalyzing the second step in the de novo pyrimidine ribonucleotide biosynthesis
AT3G20340		Expression of the gene is downregulated in the presence of paraquat, an inducer of photoxidative stress.
AT3G20370		TRAF-like family protein
AT3G20400	*EMBRYO DEFECTIVE 2743 (EMB2743)*	F-box associated ubiquitination effector family protein
AT3G20420	*RNASE THREE-LIKE PROTEIN 2 (RTL2)*	double-stranded RNA binding / ribonuclease III. Required for 3' external transcribed spacer (ETS) cleavage of the pre-rRNA in vivo. Localizes in the nucleus and cytoplasm.
AT3G20430		phosphorylated adapter RNA export-like protein
AT3G20440	*EMBRYO DEFECTIVE 2729 (EMB2729)*	Encodes BE1, a putative glycoside hydrolase. Involved in organogenesis and somatic embryogenesis by regulating carbohydrate metabolism. Mutation in BE1 has pleotrophic effect on the whole plant development.
AT3G20450		B-cell receptor-associated protein 31-like protein

AT3G20460		Major facilitator superfamily protein
AT3G20470	*GLYCINE-RICH PROTEIN 5 (GRP5)*	encodes a glycine-rich protein that is expressed more abundantly in immature seed pods than in stems and leaves. Expression is not detected in roots or flowers.
AT3G20480	*LIPID X K (LPXK)*	tetraacyldisaccharide 4-kinase family protein
AT3G20490	*KNOTEN1 (KNO1)*	Involved in DNA repair. Mutants show accumulation of DNA lesions upon genotoxic stress
AT3G20500	*PURPLE ACID PHOSPHATASE 18 (PAP18)*	purple acid phosphatase 18
AT3G20510	*FATTY ACID EXPORT 6 (FAX6)*	Encodes a member of the Tmemb_14 family that is predicted to be localized to the membranes of the secretory pathway. The mRNA is cell-to-cell mobile.
AT3G20520	*SHV3-LIKE 3 (SVL3)*	Encodes a member of the glycerophosphodiester phosphodiesterase like (GDPD-like) family.
AT3G20530	*PBS1-LIKE 23 (PBL23)*	Protein kinase superfamily protein, expressed in the peroxisome.
AT3G20540	*POLYMERASE GAMMA 1 (POLGAMMA1)*	Encodes an organellar DNA polymerase I that is also involved in double strand break repair.
AT3G20560	*PDI-LIKE 5-3 (PDIL5-3)*	Encodes a protein disulfide isomerase-like (PDIL) protein, a member of a multigene family within the thioredoxin (TRX) superfamily. Unlike several other PDI family members, transcript levels for this gene are not up-regulated in response to three different chemical inducers of ER stress (dithiothreitol, beta-mercaptoethanol, and tunicamycin).
AT3G20570	*EARLY NODULIN-LIKE PROTEIN 9 (ENODL9)*	early nodulin-like protein 9
AT3G20580	*COBRA-LIKE PROTEIN 10 PRECURSOR (COBL10)*	COBRA-like protein 10 precursor
AT3G20590		Late embryogenesis abundant (LEA) hydroxyproline-rich glycoprotein family
AT3G20630	*UBIQUITIN-SPECIFIC PROTEASE 14 (UBP14)*	Encodes a ubiquitin-specific protease. Identical to TTN6. Loss of function mutations are embryo lethals, having development arrested at the preglobular/globular stage. Also involved in root responses to phosphate deficiency.
AT3G20660	*ORGANIC CATION/CARNITINE TRANSPORTER4 (OCT4)*	organic cation/carnitine transporter4
AT3G20670	*HISTONE H2A 13 (HTA13)*	Encodes HTA13, a histone H2A protein.
AT3G20680		plant/protein (DUF1995)
AT3G20690		F-box and associated interaction domains-containing protein
AT3G20710		F-box family protein
AT3G20740	*FERTILIZATION-INDEPENDENT ENDOSPERM (FIE)*	Encodes a protein similar to the transcriptional regular of the animal Polycomb group and is involved in regulation of establishment of anterior-posterior polar axis in the endosperm and repression of flowering during vegetative phase. Mutation leads endosperm to develop in the absence of fertilization and flowers to form in seedlings and non-reproductive organs. Also exhibits maternal effect gametophytic lethal phenotype, which is suppressed by hypomethylation. Forms part of a large protein complex that can include VRN2 (VERNALIZATION 2), VIN3 (VERNALIZATION INSENSITIVE 3) and polycomb group proteins FERTILIZATION INDEPENDENT ENDOSPERM (FIE), CURLY LEAF (CLF) and SWINGER (SWN or EZA1). The complex has a role in establishing FLC (FLOWERING LOCUS C) repression during vernalization. In the ovule, the FIE transcript levels increase transiently just after fertilization.
AT3G20760	*(NSE4B)*	Nse4, component of Smc5/6 DNA repair complex
AT3G20770	*ETHYLENE-INSENSITIVE3 (EIN3)*	Encodes EIN3 (ethylene-insensitive3), a nuclear transcription factor that initiates downstream transcriptional cascades for ethylene responses. EIN3 interacts with MYC2, MYC3 and MYC4 to inhibit jasmonate-induced expression of wound-responsive genes and herbivory-inducible genes, and plant defense against generalist herbivores.
AT3G20790		NAD(P)-binding Rossmann-fold superfamily protein
AT3G20810	*JUMONJI DOMAIN CONTAINING 5 (JMJD5)*	JMJD5 encodes a protein which contains a jumonji-C (jmjC) domain. jmjd5 mutant plants have a short-period circadian phenotype. JMJD5 has histone demethylase activity and interacts with EFM to repress FT.
AT3G20820		Leucine-rich repeat (LRR) family protein
AT3G20830	*UNICORN-LIKE (UCNL)*	AGC (cAMP-dependent, cGMP-dependent and protein kinase C) kinase family protein
AT3G20840	*PLETHORA 1 (PLT1)*	Encodes a member of the AINTEGUMENTA-like (AIL) subclass of the AP2/EREBP family of transcription factors and is essential for quiescent center (QC) specification and stem cell activity. It is a key effector for establishment of the stem cell niche during embryonic pattern formation. It is transcribed in response to auxin accumulation and is dependent on auxin response transcription factors.
AT3G20850		proline-rich family protein
AT3G20860	*NIMA-RELATED KINASE 5 (NEK5)*	Encodes a member of the NIMA-related serine/threonine kinases (Neks) that have been linked to cell-cycle regulation in fungi and mammals. Plant Neks might be involved in plant development processes.
AT3G20865	*ARABINOGALACTAN PROTEIN 40 (AGP40)*	Encodes a putative arabinogalactan-protein (AGP40) that is expressed in pollen.
AT3G20880	*WIP DOMAIN PROTEIN 4 (WIP4)*	WIP4 is a paralog of NTT and along with WIP5,acts redundantly in cell fate determination during primary root development. MP binds to AuxRE motifs within the WIP4 gene and likely regulates its expression.
AT3G20910	*NUCLEAR FACTOR Y, SUBUNIT A9 (NF-YA9)*	nuclear factor Y, subunit A9
AT3G20940	*CYTOCHROME P450, FAMILY 705, SUBFAMILY A, POLYPEPTIDE 30 (CYP705A30)*	a member of A-type cytochrome P450
AT3G20950	*CYTOCHROME P450, FAMILY 705, SUBFAMILY A, POLYPEPTIDE 32 (CYP705A32)*	member of CYP705A
AT3G20960	*CYTOCHROME P450, FAMILY 705, SUBFAMILY A, POLYPEPTIDE 33 (CYP705A33)*	cytochrome P450, family 705, subfamily A, polypeptide 33
AT3G20980		Gag-Pol-related retrotransposon family protein
AT3G20990		transposable_element_gene;copia-like retrotransposon family, has a 4.9e-07 P-value blast match to GB:CAA31653 polyprotein (Ty1_Copia-element) (Arabidopsis thaliana);(source:TAIR10)
AT3G21000		Gag-Pol-related retrotransposon family protein
AT3G21020		transposable_element_gene;copia-like retrotransposon family, has a 5.4e-14 P-value blast match to gb|AAO73529.1| gag-pol polyprotein (Glycine max) (SIRE1) (Ty1_Copia-family);(source:TAIR10)

AT3G21040		transposable_element_gene;copia-like retrotransposon family, has a 5.7e-20 P-value blast match to gb|AAO73527.1| gag-pol polyprotein (Glycine max) (SIRE1) (Ty1_Copia-family);(source:TAIR10)
AT3G21055	*PHOTOSYSTEM II SUBUNIT T (PSBTN)*	Encodes photosystem II 5 kD protein subunit PSII-T. This is a nuclear-encoded gene (PsbTn) which also has a plastid-encoded paralog (PsbTc).
AT3G21060	*RBBP5 LIKE (RBL)*	Encodes a structural core component of a COMPASS-like H3K4 histone methylation complex that is also involved in the timing of the floral transition.
AT3G21070	*NAD KINASE 1 (NADK1)*	Encodes a protein with NAD(H) kinase activity.
AT3G21080		ABC transporter-like protein
AT3G21090	*ATP-BINDING CASSETTE G15 (ABCG15)*	ABC-2 type transporter family protein
AT3G21100		RNA-binding (RRM/RBD/RNP motifs) family protein
AT3G21110	*PURIN 7 (PUR7)*	5'-phosphoribosyl-4-(N-succinocarboxamide)-5-aminoimidazole synthetase (PUR7, syn. SAICAR synthetase), catalyzes aspartate addition at the alpha-amino group to the growing purine backbone.
AT3G21140	*ONEIRIC1 (ONE1)*	Pyridoxamine 5-phosphate oxidase family protein
AT3G21160	*ALPHA-MANNOSIDASE 2 (MNS2)*	Encodes an alpha-mannosidase I enzyme responsible for N-glycan maturation.
AT3G21170		F-box/associated interaction domain protein
AT3G21180	*AUTOINHIBITED CA(2+)-ATPASE 9 (ACA9)*	one of the type IIB calcium pump isoforms. encodes an autoinhibited Ca(2+)-ATPase that contains an N-terminal calmodulin binding autoinhibitory domain.
AT3G21200	*PROTON GRADIENT REGULATION 7 (PGR7)*	Encodes a soluble glutamyl-tRNA reductase (GluTR) binding protein that forms a ternary complex with FLU and GluTR.
AT3G21210		zinc ion binding protein
AT3G21220	*MAP KINASE KINASE 5 (MKK5)*	Encodes a mitogen-activated kinase kinase, dual specific protein kinase that is expressed in vegetative tissues and floral buds. Involved in innate immunity. This protein activates MPK3/MPK6 and early-defense genes redundantly with MKK4. In plants with both MKK5 and MKK4 levels reduced by RNAi plants, floral organs do not abscise suggesting a role for both proteins in mediating floral organ abscission.MKK5 is part of a positive feedback loop that regulates HAE expression in floral receptacles.
AT3G21230	*4-COUMARATE:COA LIGASE 5 (4CL5)*	The gene encodes a 4-coumarate coenzyme A ligase being able to use sinapate as substrate. The catalytic efficiency was in the following (descending) order: p-coumaric acid, caffeic acid, 5-OH-ferulic acid, ferulic acid and sinapic acid. At4CL5 was unable to use cinnamic acid as substrate. Knockout of At4CL5 (4cl5) revealed no effect on syringyl lignin content indicating that the activity observed does probably not occur in vivo.
AT3G21240	*4-COUMARATE:COA LIGASE 2 (4CL2)*	encodes an isoform of 4-coumarate:CoA ligase (4CL), which is involved in the last step of the general phenylpropanoid pathway. The catalytic efficiency was in the following (descending) order: p-coumaric acid, caffeic acid, ferulic acid, 5-OH-ferulic acid and cinnamic acid. At4CL2 was unable to use sinapic acid as substrate.
AT3G21250	*ATP-BINDING CASSETTE C8 (ABCC8)*	member of MRP subfamily
AT3G21260	*GLYCOLIPID TRANSFER PROTEIN 3 (GLTP3)*	Glycolipid transfer protein (GLTP) family protein
AT3G21270	*DOF ZINC FINGER PROTEIN 2 (DOF2)*	Encodes Dof zinc finger protein adof2.
AT3G21290	*CONSTITUTIVE ALTERATIONS IN THE SMALL RNAS PATHWAYS9 (CARP9)*	Nuclear-localized intrinsically disordered protein involved in promoting miRNA activity.
AT3G21300	*TRNA METHYLTRANSFERASE 2A (TRM2A)*	RNA methyltransferase family protein
AT3G21310		Core-2/I-branching beta-1,6-N-acetylglucosaminyltransferase family protein
AT3G21340		Leucine-rich repeat protein kinase family protein
AT3G21350	*(MED6)*	RNA polymerase transcriptional regulation mediator-like protein
AT3G21370	*BETA GLUCOSIDASE 19 (BGLU19)*	beta glucosidase 19
AT3G21380		Mannose-binding lectin superfamily protein
AT3G21390		Encodes a mitochondrial thiamin diphosphate carrier.
AT3G21400		dynein beta chain, ciliary protein
AT3G21410		F-box and associated interaction domains-containing protein
AT3G21420	*LATERAL BRANCHING OXIDOREDUCTASE 1 (LBO1)*	LATERAL BRANCHING OXIDOREDUCTASE (LBO), encodes an oxidoreductase-like enzyme of the 2-oxoglutarate and Fe(II)-dependent dioxygenase superfamily. It is involved in the biosynthesis of strigolactones.
AT3G21430	*ALWAYS EARLY 3 (ALY3)*	DNA binding protein
AT3G21440		
AT3G21480		BRCT domain-containing DNA repair protein
AT3G21500	*1-DEOXY-D-XYLULOSE 5-PHOSPHATE SYNTHASE 1 (DXPS1)*	Encodes a protein postulated to have 1-deoxy-D-xylulose 5-phosphate synthase activity.
AT3G21510	*HISTIDINE-CONTAINING PHOSPHOTRANSMITTER 1 (AHP1)*	Encodes AHP1, one of the six Arabidopsis thaliana histidine phosphotransfer proteins (AHPs). AHPs function as redundant positive regulators of cytokinin signaling. Members of the AHP gene family include: AT3G21510 (AHP1), AT3G29350 (AHP2), AT5G39340 (AHP3), AT3G16360 (AHP4), AT1G03430 (AHP5) and AT1G80100 (AHP6).
AT3G21520	*DUF679 DOMAIN MEMBRANE PROTEIN 1 (DMP1)*	Encodes a protein is directly or indirectly involved in membrane fission during breakdown of the ER and the tonoplast during leaf senescence and in membrane fusion during vacuole biogenesis in roots. The mRNA is cell-to-cell mobile.
AT3G21540		transducin family protein / WD-40 repeat family protein
AT3G21550	*DUF679 DOMAIN MEMBRANE PROTEIN 2 (DMP2)*	transmembrane protein, putative (DUF679 domain membrane protein 2)
AT3G21560	*UDP-GLUCOSYL TRANSFERASE 84A2 (UGT84A2)*	Encodes a protein with sinapic acid:UDP-glucose glucosyltransferase activity. Mutants defective in this gene are hyper-fluorescent (which accumulate in their trichomes a compound that is likely to be 3',5'-dimethoxynaringenin chalcone or sinapoyltriacetic acid lactone, potential products of the concerted action of 4-coumarate CoA ligase and chalcone synthase on sinapic acid). Also shown to be required for Arabidopsis nonhost resistance to the Asian soybean rust pathogen Phakopsora pachyrhizi.
AT3G21570		proline-rich nuclear receptor coactivator
AT3G21580	*ATP-BINDING CASSETTE I12 (ABCI12)*	cobalt ion transmembrane transporter

AT3G21590		Senescence/dehydration-associated protein-like protein
AT3G21600		Senescence/dehydration-associated protein-like protein
AT3G21610		Acid phosphatase/vanadium-dependent haloperoxidase-related protein
AT3G21620		ERD (early-responsive to dehydration stress) family protein
AT3G21660		UBX domain-containing protein
AT3G21670	*NRT1/ PTR FAMILY 6.4 (NPF6.4)*	Major facilitator superfamily protein
AT3G21680		hypothetical protein
AT3G21700	*(SGP2)*	Monomeric G protein. Expressed in root epidermal cells that are destined to become atrichoblasts. Also expressed during pollen development and in the pollen tube tip.
AT3G21710	*VASCULAR-RELATED UNKNOWN PROTEIN 1 (VUP1)*	transmembrane protein
AT3G21720	*ISOCITRATE LYASE (ICL)*	Encodes a glyoxylate cycle enzyme isocitrate lyase (ICL) involved in salt tolerance.
AT3G21760	*HYPOSTATIN RESISTANCE 1 (HYR1)*	Encodes HYR1, a UDP glycosyltransferase (UGT). HYR1 glucosylates hypostatin, an inhibitor of cell expansion in vivo to form a bioactive glucoside.
AT3G21770		Peroxidase superfamily protein
AT3G21780	*UDP-GLUCOSYL TRANSFERASE 71B6 (UGT71B6)*	Encodes a protein with UDP-glucosyl transferase activity that was shown to preferentially glucosylates abscisic acid (ABA), and not its catabolites. Moreover, UGT71B6 was shown to have a strict preference for the naturally-occurring ABA enantiomer, (+)-ABA, and not its 'unnatural' relative, (-)-ABA. This is in contrast to the other identified UGT genes catalyzing the glucosylation of ABA which were shown to accept both stereoisomers as substrates.
AT3G21790		UDP-Glycosyltransferase superfamily protein
AT3G21800	*UDP-GLUCOSYL TRANSFERASE 71B8 (UGT71B8)*	UDP-glucosyl transferase 71B8
AT3G21830	*SKP1-LIKE 8 (SK8)*	SKP1-like 8
AT3G21840	*SKP1-LIKE 7 (SK7)*	SKP1-like 7
AT3G21850	*SKP1-LIKE 9 (SK9)*	one of Arabidopsis SKP1 homologues
AT3G21860	*SKP1-LIKE 10 (SK10)*	SKP1-like 10
AT3G21870	*CYCLIN P2;1 (CYCP2;1)*	cyclin p2
AT3G21890	*B-BOX DOMAIN PROTEIN 31 (BBX31)*	B-box type zinc finger family protein
AT3G21930		cysteine-rich repeat secretory protein
AT3G21950		SABATH family methyltransferase.
AT3G21960		Receptor-like protein kinase-related family protein
AT3G21970		cysteine-rich repeat secretory protein, putative (DUF26)
AT3G22000		cysteine-rich repeat secretory protein, putative (DUF26)
AT3G22020		Receptor-like protein kinase-related family protein
AT3G22040		cysteine-rich repeat secretory-like protein (DUF26)
AT3G22050		cysteine-rich repeat secretory protein, putative (DUF26)
AT3G22060		contains Pfam profile: PF01657 Domain of unknown function that is usually associated with protein kinase domain Pfam:PF00069, however this protein does not have the protein kinase domain
AT3G22070		proline-rich family protein
AT3G22090		hypothetical protein
AT3G22104		Phototropic-responsive NPH3 family protein
AT3G22120	*CELL WALL-PLASMA MEMBRANE LINKER PROTEIN (CWLP)*	Cell wall-plasma membrane linker protein homolog (CWLP)
AT3G22130		
AT3G22140		
AT3G22150	*ATPF EDITING FACTOR 1 (AEF1)*	Involved in RNA editing of plastid atpF and mitochondrial nad5.
AT3G22160	*JASMONATE-ASSOCIATED VQ MOTIF GENE 1 (JAV1)*	VQ motif-containing protein. JAV1 is a repressor of jasmonate-mediated defense responses.
AT3G22180		DHHC-type zinc finger family protein
AT3G22190	*IQ-DOMAIN 5 (IQD5)*	Member of IQ67 (CaM binding) domain containing family.
AT3G22210		transmembrane protein
AT3G22220		hAT transposon superfamily
AT3G22231	*PATHOGEN AND CIRCADIAN CONTROLLED 1 (PCC1)*	Encodes a member of a novel 6 member Arabidopsis gene family. Expression of PCC1 is regulated by the circadian clock and is upregulated in response to both virulent and avirulent strains of Pseudomonas syringae pv. tomato.
AT3G22240	*CYSTEINE-RICH TRANSMEMBRANE MODULE 9 (ATHCYSTM9)*	cysteine-rich/transmembrane domain PCC1-like protein
AT3G22250		UDP-Glycosyltransferase superfamily protein
AT3G22260		Cysteine proteinases superfamily protein
AT3G22280		
AT3G22300	*RIBOSOMAL PROTEIN S10 (RPS10)*	Nuclear-encoded gene for mitochondrial ribosomal small subunit protein S10
AT3G22310	*PUTATIVE MITOCHONDRIAL RNA HELICASE 1 (PMH1)*	Sequence similarity ot DEAD-box RNA helicases. Binds RNA and DNA. Involved in drought, salt and cold stress responses. The mRNA is cell-to-cell mobile.
AT3G22340		transposable_element_gene;copia-like retrotransposon family, has a 4.2e-60 P-value blast match to GB:AAC02666 polyprotein (Ty1_Copia-element) (Arabidopsis thaliana);(source:TAIR10)
AT3G22350		F-box and associated interaction domains-containing protein

AT3G22360	*ALTERNATIVE OXIDASE 1B (AOX1B)*	encodes an alternative oxidase whose expression is limited to flowers and floral buds.
AT3G22370	*ALTERNATIVE OXIDASE 1A (AOX1A)*	Encodes AOX1a, an isoform of alternative oxidase that is expressed in rosettes, flowers, and root. The alternative oxidase of plant mitochondria transfers electrons from the ubiquinone pool to oxygen without energy conservations. It is regulated through transcriptional control and by pyruvate. Plays a role in shoot acclimation to low temperature. Also is capable of ameliorating reactive oxygen species production when the cytochrome pathway is inhibited. AOX1a also functions as a marker for mitochondrial retrograde response. The mRNA is cell-to-cell mobile.
AT3G22400	*(LOX5)*	Encodes lipoxygenase5 (LOX5). LOX5 activity in roots facilitates green peach aphid colonization of Arabidopsis foliage by promoting green peach aphid feeding from sieve element and water consumption from xylem.
AT3G22420	*WITH NO LYSINE (K) KINASE 2 (WNK2)*	Encodes a member of the WNK family (9 members in all) of protein kinases, the structural design of which is clearly distinct from those of other known protein kinases, such as receptor-like kinases and mitogen-activated protein kinases. Its transcription is under the control of circadian rhythms.
AT3G22422		
AT3G22430		RNA recognition motif XS domain protein
AT3G22440		FRIGIDA-like protein
AT3G22460	*O-ACETYLSERINE (THIOL) LYASE (OAS-TL) ISOFORM A2 (OASA2)*	Encodes a member of a family of genes with O-acetylserine(thiol)lyase activity.
AT3G22490	*RESPONSIVE TO ABSCISIC ACID 28 (ATRAB28)*	Atrab28 plays a role in the ion cell balance during late embryogenesis and germination.
AT3G22500	*LATE EMBRYOGENESIS ABUNDANT PROTEIN ECP31 (ATECP31)*	late embryogenesis abundant (LEA) protein
AT3G22540		hypothetical protein (DUF1677)
AT3G22550		NAD(P)H-quinone oxidoreductase subunit, putative (DUF581)
AT3G22570	*GLYCOSYLPHOSPHATIDYLINOSITOL-ANCHORED LIPID PROTEIN TRANSFER 17 (LTPG17)*	Bifunctional inhibitor/lipid-transfer protein/seed storage 2S albumin superfamily protein
AT3G22600	*GLYCOSYLPHOSPHATIDYLINOSITOL-ANCHORED LIPID PROTEIN TRANSFER 5 (LTPG5)*	Glycosylphosphatidylinositol (GPI)-anchored LTPg protein, downregulated in syncytia induced by the beet cyst nematode Heterodera schachtii and root knot nematode Meloidogyne incognita. Infection with bacteria (Pseudomonas syringae) and fungi (Botrytis cinerea) leads to the induction of the gene in leaves.
AT3G22610		
AT3G22620	*GLYCOSYLPHOSPHATIDYLINOSITOL-ANCHORED LIPID PROTEIN TRANSFER 20 (LTPG20)*	Bifunctional inhibitor/lipid-transfer protein/seed storage 2S albumin superfamily protein
AT3G22630	*20S PROTEASOME BETA SUBUNIT D1 (PBD1)*	Encodes 20S proteasome beta subunit PBD1 (PBD1).
AT3G22640	*(PAP85)*	cupin family protein
AT3G22650	*CEGENDUO (CEG)*	F-box and associated interaction domains-containing protein
AT3G22660	*(EBP2)*	rRNA processing protein-like protein
AT3G22690	*YELLOW SEEDLING1 (YS1)*	YS1 is a PPR protein involved in RNA editing of plastid encoded genes. Natural variation in this locus is associated with increased photosynthetic acclimation.
AT3G22700		F-box and associated interaction domains-containing protein
AT3G22710		F-box family protein
AT3G22740	*HOMOCYSTEINE S-METHYLTRANSFERASE 3 (HMT3)*	homocysteine S-methyltransferase (HMT3)
AT3G22750		Protein kinase superfamily protein
AT3G22760	*(SOL1)*	CXC domain containing TSO1-like protein 1. The gene is expressed in stamens, pollen mother cells, and immature ovules. Regulates fate transition and cell Divisions in the stomatal lineage.
AT3G22770		F-box associated ubiquitination effector family protein
AT3G22790	*NETWORKED 1A (NET1A)*	Encodes a member of the NET superfamily of proteins that potentially couples different membranes to the actin cytoskeleton in plant cells. It binds filamentous actin and is localized to the plasma membrane and plasmodesmata.
AT3G22800		Leucine-rich repeat (LRR) family protein
AT3G22810	*FORKED-LIKE2 (FL2)*	FORKED-LIKE family member, part of Group 1 (FKD1, FL1-FL3; Group 2 consists of FL4 and FL8 and Group 3 consists of FL5- FL7). May coordinate leaf size with vein density, where Group 1 members and Group 3 members have opposing functions.
AT3G22830	*HEAT SHOCK TRANSCRIPTION FACTOR A6B (HSFA6B)*	member of Heat Stress Transcription Factor (Hsf) family
AT3G22840	*EARLY LIGHT-INDUCABLE PROTEIN (ELIP1)*	Encodes an early light-inducible protein.
AT3G22845		emp24/gp25L/p24 family/GOLD family protein
AT3G22850		aluminum induced protein with YGL and LRDR motifs
AT3G22860	*EUKARYOTIC TRANSLATION INITIATION FACTOR 3 SUBUNIT C2 (TIF3C2)*	member of eIF3c - eukaryotic initiation factor 3c
AT3G22880	*DISRUPTION OF MEIOTIC CONTROL 1 (DMC1)*	Expression of the AtDMC1 is restricted to pollen mother cells in anthers and to megaspore mother cells in ovules. Similar to meiosis-specific yeast DMC gene.
AT3G22890	*ATP SULFURYLASE 1 (APS1)*	encodes ATP sulfurylase, the first enzyme in the sulfate assimilation pathway of Arabidopsis. It may also participate in selenium metabolism. The mRNA is cell-to-cell mobile.
AT3G22910	*AUTO-INHIBITED CA2+ ATPASE 13 (ACA13)*	ATPase E1-E2 type family protein / haloacid dehalogenase-like hydrolase family protein
AT3G22920		Cyclophilin-like peptidyl-prolyl cis-trans isomerase family protein
AT3G22930	*CALMODULIN-LIKE 11 (CML11)*	Encodes a calmodulin-like protein.
AT3G22940		F-box associated ubiquitination effector family protein
AT3G22960	*(PKP-ALPHA)*	encodes a chloroplast pyruvate kinase alpha subunit. Important for seed oil biosynthesis. Ubiquitously expressed, with significantly increased expression in maturing seeds. The mRNA is cell-to-cell mobile.
AT3G22970		hypothetical protein (DUF506)
AT3G22980		Ribosomal protein S5/Elongation factor G/III/V family protein
AT3G23000	*CBL-INTERACTING PROTEIN KINASE 7 (CIPK7)*	Encodes a serine/threonine protein kinase with similarities to CBL-interacting protein kinases, SNF1 and SOS2. The mRNA is cell-to-cell mobile.
AT3G23010	*RECEPTOR LIKE PROTEIN 36 (RLP36)*	receptor like protein 36

AT3G23020	*PENTATRICOPEPTIDE REPEAT (PPR) CONTAINING PROTEIN 30 (PPR30)*	Encodes a chloroplast nucleoid-localized protein whose absence leads to broadly impaired plastid gene expression and chloroplast development.
AT3G23030	*INDOLE-3-ACETIC ACID INDUCIBLE 2 (IAA2)*	auxin inducible gene expressed in the nucleus
AT3G23040		
AT3G23050	*INDOLE-3-ACETIC ACID 7 (IAA7)*	Transcription regulator acting as repressor of auxin-inducible gene expression. Plays role in the control of gravitropic growth and development in light-grown seedlings. Auxin induces the degradation of the protein in a dosage-dependent manner in a process mediated by AtRac1. Auxin induced the relocalization of the protein within the nucleus from a diffused nucleoplasmic pattern to a discrete particulated pattern named nuclear protein bodies or NPB in a process also mediated by Rac1. Colocalizes with SCF, CSN and 26S proteasome components. Pseudomonas syringae type III effector AvrRpt2 stimulates AXR2 protein turnover.
AT3G23060	*(ATBM1C)*	RING/U-box superfamily protein
AT3G23080		Polyketide cyclase/dehydrase and lipid transport superfamily protein
AT3G23085		transposable_element_gene;hAT-like transposase family (hobo/Ac/Tam3), has a 1.1e-91 P-value blast match to GB:AAD24567 transposase Tag2 (hAT-element) (Arabidopsis thaliana);(source:TAIR10)
AT3G23090	*WAVE-DAMPENED 2-LIKE 3 (WDL3)*	Member of the microtubule regulatory protein WVD2/WDL family WDL3 stabilizes cortical microtubules and is involved in light induced hypocotyl elongation. WDL3 is ubiquinated by COP1, leading to its degadation in the dark,
AT3G23110	*RECEPTOR LIKE PROTEIN 37 (RLP37)*	receptor like protein 37
AT3G23130	*SUPERMAN (SUP)*	Flower-specific gene controlling the boundary of the stamen and carpel whorls. Similar to zinc finger transcription factors. Involved in shoot regenaration from root explants.
AT3G23150	*ETHYLENE RESPONSE 2 (ETR2)*	Involved in ethylene perception in Arabidopsis The mRNA is cell-to-cell mobile.
AT3G23160		plant/protein (DUF668)
AT3G23170	*PROLINE/SERINE-RICH PROTEIN (PRP)*	PRP is a proline/serine rich protein of unknown function. It interacts with defense related MAP kinase MPK6 and others. It's expression is induced by PAMP elicitors. May play a role in response to pathogens.
AT3G23190		HR-like lesion-inducing protein-like protein
AT3G23200	*CASP-LIKE PROTEIN 5B3 (CASPL5B3)*	Uncharacterized protein family (UPF0497)
AT3G23210	*BASIC HELIX-LOOP-HELIX 34 (bHLH34)*	bHLH34 is a basic helix loop helix transcription factor. It can bind GAGA and E-box cis elements.It is induced by abiotic stressors including ABA, salt and glucose. PGR, a plasma membrane glucose responsive regulator is a target of bHLH34. Involved in Fe regulation.
AT3G23220	*ETHYLENE AND SALT INDUCIBLE 1 (ESE1)*	encodes a member of the ERF (ethylene response factor) subfamily B-3 of ERF/AP2 transcription factor family. The protein contains one AP2 domain. There are 18 members in this subfamily including ATERF-1, ATERF-2, AND ATERF-5.
AT3G23230	*TRANSCRIPTIONAL REGULATOR OF DEFENSE RESPONSE 1 (TDR1)*	encodes a member of the ERF (ethylene response factor) subfamily B-3 of ERF/AP2 transcription factor family. The protein contains one AP2 domain. There are 18 members in this subfamily including ATERF-1, ATERF-2, AND ATERF-5.
AT3G23240	*ETHYLENE RESPONSE FACTOR 1 (ERF1)*	encodes a member of the ERF (ethylene response factor) subfamily B-3 of ERF/AP2 transcription factor family (ERF1). The protein contains one AP2 domain. There are 18 members in this subfamily including ATERF-1, ATERF-2, AND ATERF-5. EREBP like protein that binds GCC box of ethylene regulated promoters such as basic chitinases. Constitutive expression of ERF1 phenocopies ethylene over production. Involved in ethylene signaling cascade,downstream of EIN2 and EIN3.
AT3G23250	*MYB DOMAIN PROTEIN 15 (MYB15)*	Member of the R2R3 factor gene family. Key regulator of lignin biosynthesis in effector-triggered immunity
AT3G23260		F-box and associated interaction domains-containing protein
AT3G23270		Regulator of chromosome condensation (RCC1) family with FYVE zinc finger domain-containing protein
AT3G23290	*LIGHT SENSITIVE HYPOCOTYLS 4 (LSH4)*	LIGHT-DEPENDENT SHORT HYPOCOTYLS-like protein (DUF640)
AT3G23295		
AT3G23300		S-adenosyl-L-methionine-dependent methyltransferases superfamily protein
AT3G23310	*NUCLEAR DBF2-RELATED 3 (NDR3)*	AGC (cAMP-dependent, cGMP-dependent and protein kinase C) kinase family protein
AT3G23340	*CASEIN KINASE I-LIKE 10 (ckl10)*	Member of CKL gene family (CKL-C group).
AT3G23350		ENTH/VHS family protein
AT3G23400	*FIBRILLIN 4 (FIB4)*	Encodes FIBRILLIN 4 (FIB4). The fibrillins are a large family of chloroplast proteins that have been linked with stress tolerance and disease resistance. FIBRILLIN 4 is required for plastoglobule development and stress resistance.Iinvolved in plastoquinone transport.
AT3G23410	*FATTY ALCOHOL OXIDASE 3 (FAO3)*	Encodes a fatty alcohol oxidase.
AT3G23430	*PHOSPHATE 1 (PHO1)*	Encodes a protein with the mainly hydrophilic N-terminal and the C-terminal containing 6 potential membrane-spanning domains. The mutant is deficient in the transfer of phosphate from root epidermal and cortical cells to the xylem. Its expression is repressed by phosphate (Pi) in shoots, and transiently induced by phosphite (Phi) in roots and shoots. PHO is expressed in developing ovules and plays a role in the transfer of Ph from maternal tissues to filial tissues.
AT3G23450	*(KRATOS)*	Kratos restricts cell death during differentiation of tracheary elements.
AT3G23460		S-adenosyl-L-methionine-dependent methyltransferases superfamily protein
AT3G23470		Cyclopropane-fatty-acyl-phospholipid synthase
AT3G23490	*CYANASE (CYN)*	Encodes a cyanase that catalyzes the bicarbonate-dependent breakdown of cyanate to ammonia and bicarbonate. CYN forms a hexadecamer and is believed to be a cytosolic protein. Long-term exposure to NaCl increases CYN transcript levels. It is also expressed at higher levels in flowers relative to stems, roots, and seedlings.
AT3G23510		Cyclopropane-fatty-acyl-phospholipid synthase
AT3G23520		
AT3G23550	*DETOXIFICATION 18 (DTX18)*	MATE efflux family protein
AT3G23560	*ABERRANT LATERAL ROOT FORMATION 5 (ALF5)*	Member of the multidrug and toxic compound extrusion (MATE) family, protects roots from inhibitory compounds.
AT3G23570		alpha/beta-Hydrolases superfamily protein

AT3G23580	*RIBONUCLEOTIDE REDUCTASE 2A (RNR2A)*	Encodes one of the 3 ribonucleotide reductase (RNR) small subunit genes (RNR2A). Functionally redundant with the ribonucleotide reductase TSO2. mRNA was shown to specifically accumulate during the S-phase of the cell cycle in synchronized tobacco BY2 cells. Critical for cell cycle progression, DNA damage repair and plant development.
AT3G23600		alpha/beta-Hydrolases superfamily protein
AT3G23605		Ubiquitin-like superfamily protein
AT3G23620	*ARABIDOPSIS HOMOLOG OF YEAST RPF2 (ARPF2)*	BRIX domain containing protein, similar to RNA biogenesis factors in yeast. Binds rRNA and likely also functions in RNA biogenesis in Arabidopsis. Essential gene, mutants are embryo lethal and does not transmit well through the gametophyte.
AT3G23630	*ISOPENTENYLTRANSFERASE 7 (IPT7)*	Encodes an isopentenyl transferase involved in cytokinin biosynthesis.
AT3G23640	*HETEROGLYCAN GLUCOSIDASE 1 (HGL1)*	heteroglycan glucosidase 1
AT3G23650		kinase-like protein
AT3G23670	*(KINESIN-12B)*	Microtubule motor kinesin PAKRP1L/Kinesin-12B. Together with PAKRP1/Kinesin-12A, serve as linkers of the plus ends of antiparallel microtubules in the phragmoplast.
AT3G23680		F-box associated ubiquitination effector family protein
AT3G23690	*CIB1 LIKE PROTEIN 2 (CIL2)*	basic helix-loop-helix (bHLH) DNA-binding superfamily protein
AT3G23700	*S1 RNA-BINDING RIBOSOMAL PROTEIN 1 (SRRP1)*	Encodes a chloroplast-localized S1 domain-containing protein with RNA chaperone activity that affects the splicing and processing of chloroplast transcripts and plays a role in seedling growth in the presence of ABA. Binds the chloroplast psbA RNA and some other chloroplast RNAs. Required for the stability of the chloroplast ndhC RNA. Inhibits ribosome association with psbA RNA and ycf1 RNA. Not required for the splicing of chloroplast trnL, as had been reported previously.
AT3G23720		transposable_element_gene
AT3G23730	*XYLOGLUCAN ENDOTRANSGLUCOSYLASE/HYDROLASE 16 (XTH16)*	xyloglucan endotransglucosylase/hydrolase 16
AT3G23740		hypothetical protein
AT3G23760		transferring glycosyl group transferase
AT3G23770		O-Glycosyl hydrolases family 17 protein
AT3G23790	*ACYL ACTIVATING ENZYME 16 (AAE16)*	AMP-dependent synthetase and ligase family protein involved in fatty acid biosynthesis.
AT3G23800	*SELENIUM-BINDING PROTEIN 3 (SBP3)*	selenium-binding protein 3
AT3G23810	*S-ADENOSYL-L-HOMOCYSTEINE (SAH) HYDROLASE 2 (SAHH2)*	S-adenosyl-l-homocysteine (SAH) hydrolase 2
AT3G23820	*UDP-D-GLUCURONATE 4-EPIMERASE 6 (GAE6)*	Encodes a UDP-D-glucuronate 4-epimerase involved in pectin biosynthesis in the cell wall and affects cell wall integrity and immunity to fungi and bacteria. The mRNA is cell-to-cell mobile.
AT3G23830	*RNA-BINDING GLYCINE-RICH PROTEIN A4 (RBGA4)*	encodes a glycine-rich RNA binding protein. Gene expression is induced by cold and reduced by ionic (salt) and non-ionic (mannitol) osmotic stress. Lines overexpressing the gene are slightly more tolerant to osmotic stress during germination.
AT3G23840	*CER26-LIKE (CER26-LIKE)*	HXXXD-type acyl-transferase family protein
AT3G23850		
AT3G23870	*(ENOR3L2)*	magnesium transporter NIPA (DUF803)
AT3G23880		F-box and associated interaction domains-containing protein
AT3G23890	*TOPOISOMERASE II (TOPII)*	Encodes a topoisomerase II that is highly expressed in young seedlings. The protein is localized in the nucleus and gene expression levels are increased in proliferative tissues.
AT3G23900	*IMMUNOREGULATORY RNA-BINDING PROTEIN (IRR)*	Physically interacts with, and promotes canonical splicing of, transcripts encoding defense signaling proteins, including the key negative regulator of pattern recognition receptor signaling complexes, CALCIUM-DEPENDENT PROTEIN KINASE 28 (CPK28). Upon immune activation by Plant Elicitor Peptides (Peps), IRR is dephosphorylated, disrupting interaction with CPK28 transcripts and resulting in accumulation of an alternative splice variant encoding a truncated CPK28 protein with impaired kinase activity and diminished function as a negative regulator.
AT3G23910		reverse transcriptase-like protein
AT3G23920	*BETA-AMYLASE 1 (BAM1)*	Encodes a chloroplast beta-amylase. Is necessary for leaf starch breakdown in the absence of BAM3.Activity of BAM1 increases 4 days after osmotic stress. BAM1 has a higher temperature optimum than BAM3 (PMID:25293962).
AT3G23930		troponin T, skeletal protein
AT3G23940	*DIHYDROXYACID DEHYDRATASE (DHAD)*	Encodes a member of the dihydroxyacid dehydrates family of proteins that encode enzymes involved in branched chain amino acid biosynthesis. Loss of function mutations have significantly reduced transmission and fertility due to defects in male and female gametophyte development and embryo lethality. Mutants have increased sensitivity to abiotic stressors which may be partially compensated by addition of amino acids to the growth medium.
AT3G23960		F-box and associated interaction domains-containing protein
AT3G23970		F-box family protein
AT3G23990	*HEAT SHOCK PROTEIN 60 (HSP60)*	mitochondrial chaperonin HSP. assist in rapid assembly of the oligomeric protein structures in the mitochondria.
AT3G24000		Tetratricopeptide repeat (TPR)-like superfamily protein
AT3G24030		hydroxyethylthiazole kinase family protein
AT3G24060		Plant self-incompatibility protein S1 family
AT3G24070		Zinc knuckle (CCHC-type) family protein
AT3G24100	*(BIA)*	Encodes a secreted peptide that enhances stress indued cell death.
AT3G24110		Calcium-binding EF-hand family protein
AT3G24120	*PHR1-LIKE 2 (PHL2)*	MYB-CC protein involved in regulation of response to phosphate starvation.
AT3G24130		Pectin lyase-like superfamily protein

AT3G24140	*FAMA (FMA)*	Encodes a basic helix-loop-helix transcription factor whose activity is required to promote differentiation of stomatal guard cells and to halt proliferative divisions in their immediate precursors. It fulfills its role through recruitment of the Arabidopsis Retinoblastoma homologue, RETINOBLASTOMA-RELATED (RBR). Both transcript and protein are expressed in and are required for halting divisions at the end of the stomatal lineage. It also has a role in the promotion of guard cell fate and in controlling the transition from guard mother cell to guard cell. Its transcript levels change after inducing MUTE expression in a mute background.
AT3G24170	*GLUTATHIONE-DISULFIDE REDUCTASE (GR1)*	Encodes a cytosolic glutathione reductase.
AT3G24180		Beta-glucosidase, GBA2 type family protein
AT3G24210		Ankyrin repeat family protein
AT3G24220	*NINE-CIS-EPOXYCAROTENOID DIOXYGENASE 6 (NCED6)*	A member of gene NCED-related gene family, encodes 9-cis-epoxycarotenoid dioxygenase, a key enzyme in the biosynthesis of abscisic acid. The expression of this gene declines during the first 12h of imbibition.
AT3G24240	*(RGFR1)*	RGFR1 is a leucine--rich repeat receptor kinase that, together with RGFR2 and RGFR3, binds ROOT GROWTH FACTORS and is required for establishing the gradient of PLETHORA1 and PLETHORA2 essential for proper root growth and development.
AT3G24250		glycine-rich protein
AT3G24260		paired amphipathic helix Sin3-like protein
AT3G24270	*PUMILIO 25 (PUM25)*	Encodes a member of the Arabidopsis Pumilio (APUM) proteins containing PUF domain (eight repeats of approximately 36 amino acids each). PUF proteins regulate both mRNA stability and translation through sequence-specific binding to the 3' UTR of target mRNA transcripts.
AT3G24280	*SMALL ACIDIC PROTEIN 2 (SMAP2)*	small acidic protein 2
AT3G24290	*AMMONIUM TRANSPORTER 1;5 (AMT1;5)*	ammonium transporter 1
AT3G24310	*MYB DOMAIN PROTEIN 305 (MYB305)*	snapdragon myb protein 305 homolog (myb)
AT3G24330		O-Glycosyl hydrolases family 17 protein
AT3G24340	*CHROMATIN REMODELING 40 (chr40)*	Involved in gene silencing. Locus-specific regulator of 24nt-siRNA expression, works together with CLSY1-3 as the master regulators of essentially all Pol-IV-dependent 24nt-siRNAs.
AT3G24360		ATP-dependent caseinolytic (Clp) protease/crotonase family protein
AT3G24400	*PROLINE-RICH EXTENSIN-LIKE RECEPTOR KINASE 2 (PERK2)*	Encodes a member of the proline-rich extensin-like receptor kinase (PERK) family. This family consists of 15 predicted receptor kinases (PMID: 15653807).
AT3G24410		
AT3G24430	*HIGH-CHLOROPHYLL-FLUORESCENCE 101 (HCF101)*	Encodes chloroplast protein HCF101 (high chlorophyll fluorescence 101). Serves as a chloroplast scaffold protein that specifically assembles [4Fe-4S] clusters and transfers them to the chloroplast membrane and soluble target proteins.
AT3G24450	*HEAVY METAL ASSOCIATED PROTEIN 27 (ATHMP27)*	Heavy metal transport/detoxification superfamily protein
AT3G24470		
AT3G24490		Alcohol dehydrogenase transcription factor Myb/SANT-like family protein
AT3G24500	*MULTIPROTEIN BRIDGING FACTOR 1C (MBF1C)*	One of three genes in A. thaliana encoding multiprotein bridging factor 1, a highly conserved transcriptional coactivator. May serve as a bridging factor between a bZIP factor and TBP. Its expression is specifically elevated in response to pathogen infection, salinity, drought, heat, hydrogen peroxide, and application of abscisic acid or salicylic acid. Constitutive expression enhances the tolerance of transgenic plants to various biotic and abiotic stresses.
AT3G24506	*CONSERVED ONLY IN THE GREEN LINEAGE20B (CGL20B)*	Participates in the late stages of the biogenesis of 50S ribosomal subunits in plastids.
AT3G24510		Encodes a defensin-like (DEFL) family protein.
AT3G24520	*HEAT SHOCK TRANSCRIPTION FACTOR C1 (HSFC1)*	member of Heat Stress Transcription Factor (Hsf) family
AT3G24540	*PROLINE-RICH EXTENSIN-LIKE RECEPTOR KINASE 3 (PERK3)*	Encodes a member of the proline-rich extensin-like receptor kinase (PERK) family. This family consists of 15 predicted receptor kinases (PMID: 15653807).
AT3G24560	*RASPBERRY 3 (RSY3)*	novel gene involved in embryogenesis
AT3G24620	*ROP (RHO OF PLANTS) GUANINE NUCLEOTIDE EXCHANGE FACTOR 8 (ROPGEF8)*	Encodes a member of KPP-like gene family, homolog of KPP (kinase partner protein) gene in tomato. Also a member of the RopGEF (guanine nucleotide exchange factor) family, containing the novel PRONE domain (plant-specific Rop nucleotide exchanger), which is exclusively active towards members of the Rop subfamily.
AT3G24630	*TON1 RECRUITING MOTIF 34 (TRM34)*	hypothetical protein
AT3G24640		lyase
AT3G24650	*ABA INSENSITIVE 3 (ABI3)*	Homologous to the maize transcription factor Viviparous-1. Full length ABI3 protein binds to the highly conserved RY motif [DNA motif CATGCA(TG)], present in many seed-specific promoters, and the B3 domains of this transcription factor is necessary for the specific interaction with the RY element. Transcriptional activity of ABI3 requires the B3 DNA-binding domain and an activation domain. In addition to the known N-terminal-located activation domain, a second transcription activation domain was found in the B1 region of ABI3. ABI3 is essential for seed maturation. Regulator of the transition between embryo maturation and early seedling development. Putative seed-specific transcriptional activator. ABI3 is a central regulator in ABA signaling and is unstable in vivo. It interacts with and can by polyubiquitinated by AIP2 in vivo. Based on double mutant analyses, ABI3 interacts genetically with both FUS3 and LEC1 and is involved in controlling accumulation of chlorophyll and anthocyanins, sensitivity to abscisic acid, and expression of the members of the 12S storage protein gene family. In addition, both FUS3 and LEC1 regulate positively the abundance of the ABI3 protein in the seed. Alternative splicing of ABI3 is developmentally regulated by SUA (AT3G54230).
AT3G24660	*TRANSMEMBRANE KINASE-LIKE 1 (TMKL1)*	member of Receptor kinase-like protein family
AT3G24670		Pectin lyase-like superfamily protein
AT3G24680		transposable_element_gene;similar to zinc finger protein, putative [Arabidopsis thaliana] (TAIR:AT2G15520.1);(source:TAIR10)
AT3G24710		NADPH-dependent diflavin oxidoreductase
AT3G24720		

AT3G24750	*(LAZY5)*	Encodes a member of the LAZY gene family that is expressed in the hypocotyl and the root
AT3G24760		Galactose oxidase/kelch repeat superfamily protein
AT3G24770	*CLAVATA3/ESR-RELATED 41 (CLE41)*	Belongs to a large gene family, called CLE for CLAVATA3/ESR-related, encoding small peptides with conserved carboxyl termini. The C-terminal 12 amino acid sequence of CLE41 is identical to that of a dodeca peptide (TDIF, tracheary element differentiation inhibitory factor) isolated from Arabidopsis and functions as a suppressor of plant stem cell differentiation. TDIF sequence is also identical to the C-terminal 12 amino acids of CLE44 (At4g13195). The protein is expressed in the vascular system and is involved in axillary bud formation. The mRNA is cell-to-cell mobile.
AT3G24780		Uncharacterized conserved protein UCP015417, vWA
AT3G24790	*PBS1-LIKE 25 (PBL25)*	Protein kinase superfamily protein
AT3G24810	*(ICK3)*	Kip-related protein (KRP) gene, encodes CDK (cyclin-dependent kinase) inhibitor (CKI), negative regulator of cell division. A member of seven KRP genes found in Arabidopsis thaliana. Differential expression patterns for distinct KRPs were revealed by in situ hybridization.
AT3G24840		Sec14p-like phosphatidylinositol transfer family protein
AT3G24850		B3 domain protein (DUF313)
AT3G24900	*RECEPTOR LIKE PROTEIN 39 (RLP39)*	receptor like protein 39
AT3G24954		
AT3G24982	*RECEPTOR LIKE PROTEIN 40 (RLP40)*	receptor like protein 40
AT3G25010	*RECEPTOR LIKE PROTEIN 41 (RLP41)*	receptor like protein 41
AT3G25020	*RECEPTOR LIKE PROTEIN 42 (RLP42)*	receptor like protein 42
AT3G25050	*XYLOGLUCAN ENDOTRANSGLUCOSYLASE/HYDROLASE 3 (XTH3)*	Encodes an endotransglucosylase that cleaves the beta-1,4-glucosidic linkage in amorphous cellulose and ligates the nascent reducing end to a non-reducing terminus of either cellulosic or xyloglucan oligosaccharide. Higher expression in flowers and in response to IAA treatment.
AT3G25060	*MITOCHONDRIAL RNA EDITING FACTOR 25 (MEF25)*	Tetratricopeptide repeat (TPR)-like superfamily protein
AT3G25080		hypothetical protein
AT3G25090		F-box associated ubiquitination effector family protein
AT3G25100	*CELL DIVISION CYCLE 45 (CDC45)*	Required for normal meiosis, may act in the last round of DNA replication prior to meiosis, sequence similar to yeast CDC45
AT3G25110	*FATA ACYL-ACP THIOESTERASE (FaTA)*	Encodes a FatA acyl-ACP thioesterase
AT3G25130		acidic leucine-rich nuclear phosphoprotein 32 family B protein
AT3G25150		Nuclear transport factor 2 (NTF2) family protein with RNA binding (RRM-RBD-RNP motifs) domain-containing protein
AT3G25160		ER lumen protein retaining receptor family protein
AT3G25165	*RALF-LIKE 25 (RALFL25)*	Member of a diversely expressed predicted peptide family showing sequence similarity to tobacco Rapid Alkalinization Factor (RALF), and is believed to play an essential role in the physiology of Arabidopsis. Consists of a single exon and is characterized by a conserved C-terminal motif and N-terminal signal peptide.
AT3G25170	*RALF-LIKE 26 (RALFL26)*	Member of a diversely expressed predicted peptide family showing sequence similarity to tobacco Rapid Alkalinization Factor (RALF), and is believed to play an essential role in the physiology of Arabidopsis. Consists of a single exon and is characterized by a conserved C-terminal motif and N-terminal signal peptide.
AT3G25180	*CYTOCHROME P450, FAMILY 82, SUBFAMILY G, POLYPEPTIDE 1 (CYP82G1)*	Encodes a cytochrome P450 monooxygenase (CYP82G1) that catalyzes the production of two volatile homoterpenes, TMTT and DMNT, although it is only likely to produce TMTT in planta. TMTT can be involved in attracting predatory insects to protect Arabidopsis plants from herbivorous pests. Homoterpene synthesis is also stimulated by fungal elicitors which increase the transcript levels of CYP82G1.
AT3G25185		
AT3G25190	*VACUOLAR IRON TRANSPORTER-LIKE 5 (VTL5)*	The gene encodes nodulin-like21 whose transcript abundance was repressed under conditions of Fe-deficient growth.
AT3G25200	*DUF295 ORGANELLAR B 1 (ATDOB1)*	hypothetical protein
AT3G25210		Tetratricopeptide repeat (TPR)-like superfamily protein
AT3G25220	*FK506-BINDING PROTEIN 15 KD-1 (FKBP15-1)*	immunophilin (FKBP15-1)
AT3G25230	*ROTAMASE FKBP 1 (ROF1)*	Encodes a a high molecular weight member of the FK506 binding protein (FKBP) family. It has three FKBP12-like domains, tetratricopeptide repeats, and a putative calmodulin binding domain. Modulates thermotolerance by interacting with HSP90.1 and affecting the accumulation of HsfA2-regulated sHSPs. Belongs to one of the 36 carboxylate clamp (CC)-tetratricopeptide repeat (TPR) proteins (Prasad 2010, Pubmed ID: 20856808) with potential to interact with Hsp90/Hsp70 as co-chaperones.
AT3G25250	*AGC2 KINASE 1 (AGC2-1)*	Arabidopsis protein kinase The mRNA is cell-to-cell mobile.
AT3G25260	*NRT1/ PTR FAMILY 4.1 (NPF4.1)*	Major facilitator superfamily protein
AT3G25280	*NRT1/ PTR FAMILY 4.2 (NPF4.2)*	Major facilitator superfamily protein
AT3G25290		Auxin-responsive family protein
AT3G25400		dCTP pyrophosphatase-like protein
AT3G25410		Sodium Bile acid symporter family
AT3G25420	*SERINE CARBOXYPEPTIDASE-LIKE 21 (scpl21)*	serine carboxypeptidase-like 21
AT3G25440	*ACINUS (ACINUS)*	RNA-binding CRS1 / YhbY (CRM) domain protein
AT3G25450		transposable_element_gene;copia-like retrotransposon family, has a 6.7e-211 P-value blast match to gb|AAO73527.1| gag-pol polyprotein (Glycine max) (SIRE1) (Ty1_Copia-family);(source:TAIR10)
AT3G25480		Rhodanese/Cell cycle control phosphatase superfamily protein
AT3G25490		Protein kinase family protein
AT3G25510		disease resistance protein (TIR-NBS-LRR class) family protein
AT3G25530	*GLYOXYLATE REDUCTASE 1 (GLYR1)*	Encodes gamma-hydroxybutyrate dehydrogenase (AtGHBDH). Contains a NADP-binding domain. GHBDH is proposed to function in oxidative stress tolerance.

AT3G25540	*LAG ONE HOMOLOGUE 1 (LOH1)*	Encodes a ceramide synthase that together with LOH3 is essential for production of ceramides containing Very Long Chain Fatty acid VLCFA-Ceramides(mainly C 22 to 26).
AT3G25560	*NSP-INTERACTING KINASE 2 (NIK2)*	NSP-interacting kinase 2
AT3G25570	*S-ADENOSYLMETHIONINE DECARBOXYLASE 3 (SAMDC3)*	S-adenosylmethionine decarboxylase family member.
AT3G25580		Thioredoxin superfamily protein
AT3G25585	*AMINOALCOHOLPHOSPHOTRANSFERASE (AAPT2)*	aminoalcoholphosphotransferase (AAPT2)
AT3G25600	*CALMODULIN LIKE 16 (CML16)*	Calmodulin like protein. Paralog of CML15.
AT3G25610	*AMINOPHOSPHOLIPID ATPASE10 (ALA10)*	Encodes aminophospholipid ATPase10 (ALA10), a P4-type ATPase flippase that internalizes exogenous phospholipids across the plasma membrane.
AT3G25620	*ATP-BINDING CASSETTE G21 (ABCG21)*	ABC-2 type transporter family protein
AT3G25630		transposable_element_gene;gypsy-like retrotransposon family (Athila), has a 7.8e-20 P-value blast match to GB:CAA57397 Athila ORF 1 (Arabidopsis thaliana);(source:TAIR10)
AT3G25640		MIZU-KUSSEI-like protein (Protein of unknown function, DUF617)
AT3G25650	*SKP1-LIKE 15 (SK15)*	SKP1-like 15
AT3G25655	*INFLORESCENCE DEFICIENT IN ABSCISSION (IDA)-LIKE 1 (IDL1)*	Similar to Inflorescence Deficient in Abscission (IDA). Involved in floral organ abscission.
AT3G25660		Amidase family protein
AT3G25670		Leucine-rich repeat (LRR) family protein
AT3G25680		SLH domain protein
AT3G25690	*CHLOROPLAST UNUSUAL POSITIONING 1 (CHUP1)*	actin binding protein required for normal chloroplast positioning The mRNA is cell-to-cell mobile.
AT3G25710	*BASIC HELIX-LOOP-HELIX 32 (BHLH32)*	Encodes a basic helix-loop-helix transcription factor that is expressed in the hypophysis-adjacent embryo cells, and is required and partially sufficient for MP-dependent root initiation. Involved in response to phosphate starvation. Negative regulator of root hair development, anthocyanin formation and Pi content. Its expression is responsive to both phosphate (Pi) and phosphite (Phi) in shoots.
AT3G25720		RNA-directed DNA polymerase (reverse transcriptase)-related family protein
AT3G25730	*ETHYLENE RESPONSE DNA BINDING FACTOR 3 (EDF3)*	ethylene response DNA binding factor 3
AT3G25740	*METHIONINE AMINOPEPTIDASE 1C (MAP1B)*	Encodes a plastid localized methionine aminopeptidase. Formerly called MAP1C, now called MAP1B.
AT3G25750	*F-BOX/DUF295 ANCESTRAL 17 (ATFDA17)*	F-box SKIP23-like protein (DUF295)
AT3G25760	*ALLENE OXIDE CYCLASE 1 (AOC1)*	encodes allene oxide cyclase. One of four genes in Arabidopsis that encode this enzyme, which catalyzes an essential step in jasmonic acid biosynthesis. Gene expression is induced during senescence, a process that involves jasmonic acid signalling pathway. The mRNA is cell-to-cell mobile.
AT3G25780	*ALLENE OXIDE CYCLASE 3 (AOC3)*	Encodes allene oxide cyclase, one of the enzymes involved in jasmonic acid biosynthesis. One of four genes in Arabidopsis that encode this enzyme. mRNA expression is upregulated in senescing leaves. Note: Nomenclature for Arabidopsis allene oxide cyclase 3 (AOC3, AT3G25780) gene is based on Stenzel et al. 2003 Plant Molecular Biology 51:895-911. AOC3 (AT3G25780) is also referred to as AOC2 in He et al. 2002 Plant Physiology, 128:876-884. The mRNA is cell-to-cell mobile.
AT3G25790	*HRS1 HOMOLOGUE 1 (HHO1)*	Encodes a nuclear localized member of the GARP family of transcription factors. Along with AtNIGT1/HRS1 it is involved in nitrate and phosphate signaling in the root. Transcriptional repressors that functions with other NIGT genes as an important hub in the nutrient signaling network associated with the acquisition and use of nitrogen and phosphorus.
AT3G25805		transmembrane protein
AT3G25810		Terpenoid cyclases/Protein prenyltransferases superfamily protein
AT3G25820	*TERPENE SYNTHASE-LIKE SEQUENCE-1,8-CINEOLE (TPS-CIN)*	Encodes the monoterpene 1,8-cineole synthase, atTPS-Cin. This polypeptide was also shown to synthesize other monoterpenes albeit in minor quantities. The same polypeptide is encoded at two different loci, the result of gene duplication: at3g25820 and at3g25830.
AT3G25830	*TERPENE SYNTHASE-LIKE SEQUENCE-1,8-CINEOLE (TPS-CIN)*	Encodes the monoterpene 1,8-cineole synthase, atTPS-Cin. This polypeptide was also shown to synthesize other monoterpenes albeit in minor quantities. The same polypeptide is encoded at two different loci, the result of gene duplication: at3g25820 and at3g25830.
AT3G25850		Cysteine/Histidine-rich C1 domain family protein
AT3G25855	*HEAVY METAL ASSOCIATED PROTEIN 28 (ATHMP28)*	Copper transport protein family
AT3G25860	*(LTA2)*	Encodes the dihydrolipoamide S-acetyltransferase subunit of the plastid Pyruvate Dehydrogenase Complex (E2). Mutant has embryo defect.
AT3G25870		hypothetical protein
AT3G25880		NAD(P)-binding Rossmann-fold superfamily protein
AT3G25890	*CYTOKININ RESPONSE FACTOR 11 (CRF11)*	encodes a member of the ERF (ethylene response factor) subfamily B-6 of ERF/AP2 transcription factor family. The protein contains one AP2 domain. There are 12 members in this subfamily including RAP2.11. The mRNA is cell-to-cell mobile.
AT3G25900	*(HMT-1)*	Homocysteine S-methyltransferase family protein
AT3G25905	*CLAVATA3/ESR-RELATED 27 (CLE27)*	Member of a large family of putative ligands homologous to the Clavata3 gene. Consists of a single exon.
AT3G25910	*SALT INDUCED ZINC FINGER PROTEIN1 (SIZ1)*	C2H2-type zinc finger protein involved in salt tolerance. Induced by salt stress.
AT3G25920	*RIBOSOMAL PROTEIN L15 (RPL15)*	encodes a plastid ribosomal protein CL15, a constituent of the large subunit of the ribosomal complex
AT3G25930		Adenine nucleotide alpha hydrolases-like superfamily protein
AT3G25940		TFIIB zinc-binding protein
AT3G25950		TRAM, LAG1 and CLN8 (TLC) lipid-sensing domain containing protein
AT3G25960		Pyruvate kinase family protein
AT3G25970		Pentatricopeptide repeat (PPR) superfamily protein
AT3G25980	*MITOTIC ARREST-DEFICIENT 2 (MAD2)*	Encodes MAD2 (MITOTIC ARREST-DEFICIENT 2). May have the spindle assembly checkpoint protein functions conserved from yeast to humans.
AT3G26010		Galactose oxidase/kelch repeat superfamily protein

AT3G26030	*SERINE/THREONINE PROTEIN PHOSPHATASE 2A 55 KDA REGULATORY SUBUNIT B PRIME DELTA (ATB' DELTA)*	protein phosphatase 2A regulatory subunit isoform B' delta The mRNA is cell-to-cell mobile.
AT3G26040		HXXXD-type acyl-transferase family protein
AT3G26050		TPX2 (targeting protein for Xklp2) protein family
AT3G26060	*PEROXIREDOXIN Q (PRXQ)*	encodes periredoxin Q which decomposes peroxides and plays a role in the protection of the photosynthetic apparatus
AT3G26090	*REGULATOR OF G-PROTEIN SIGNALING 1 (RGS1)*	Encodes AtRGS1, a putative membrane receptor for D-glucose. Also functions as a regulator of G-protein signaling. Has GTPase-accelerating activity. Regulates the activity of AtGPA1. Lines over-expressing the gene are more tolerant to dehydration and root elongation. These phenotypes are dependent on ABA. Nuclear localization of the protein is dependent on ABA. RGS1 endocytosis is induced by JA which promotes its dissociation from GPA1.
AT3G26100		Regulator of chromosome condensation (RCC1) family protein
AT3G26110		Anther-specific protein agp1-like protein
AT3G26120	*TERMINAL EAR1-LIKE 1 (TEL1)*	Similar to terminal ear1 in Zea mays. A member of mei2-like gene family; phylogenetic analysis revealed that TEL1 belongs to the third clade of mei2-like proteins (TEL clade), with conserved two N-terminal RNA recognition motifs (RRM), in addition to the C-terminal RRM, shared among all mei2-like proteins.
AT3G26125	*CYTOCHROME P450, FAMILY 86, SUBFAMILY C, POLYPEPTIDE 2 (CYP86C2)*	encodes a protein with cytochrome P450 domain
AT3G26130		Cellulase (glycosyl hydrolase family 5) protein
AT3G26150	*CYTOCHROME P450, FAMILY 71, SUBFAMILY B, POLYPEPTIDE 16 (CYP71B16)*	putative cytochrome P450
AT3G26160	*CYTOCHROME P450, FAMILY 71, SUBFAMILY B, POLYPEPTIDE 17 (CYP71B17)*	putative cytochrome P450
AT3G26170	*CYTOCHROME P450, FAMILY 71, SUBFAMILY B, POLYPEPTIDE 19 (CYP71B19)*	putative cytochrome P450
AT3G26190	*CYTOCHROME P450, FAMILY 71, SUBFAMILY B, POLYPEPTIDE 21 (CYP71B21)*	putative cytochrome P450
AT3G26200	*CYTOCHROME P450, FAMILY 71, SUBFAMILY B, POLYPEPTIDE 22 (CYP71B22)*	putative cytochrome P450 The mRNA is cell-to-cell mobile.
AT3G26210	*CYTOCHROME P450, FAMILY 71, SUBFAMILY B, POLYPEPTIDE 23 (CYP71B23)*	putative cytochrome P450 The mRNA is cell-to-cell mobile.
AT3G26220	*CYTOCHROME P450, FAMILY 71, SUBFAMILY B, POLYPEPTIDE 3 (CYP71B3)*	cytochrome P450 monooxygenase
AT3G26230	*CYTOCHROME P450, FAMILY 71, SUBFAMILY B, POLYPEPTIDE 24 (CYP71B24)*	putative cytochrome P450
AT3G26240		Cysteine/Histidine-rich C1 domain family protein. Accumulation of this protein is regulated by a cis-Natural Antisense RNA (cis-NAT).
AT3G26270	*CYTOCHROME P450, FAMILY 71, SUBFAMILY B, POLYPEPTIDE 25 (CYP71B25)*	putative cytochrome P450
AT3G26280	*CYTOCHROME P450, FAMILY 71, SUBFAMILY B, POLYPEPTIDE 4 (CYP71B4)*	cytochrome P450 monooxygenase
AT3G26290	*CYTOCHROME P450, FAMILY 71, SUBFAMILY B, POLYPEPTIDE 26 (CYP71B26)*	putative cytochrome P450
AT3G26300	*CYTOCHROME P450, FAMILY 71, SUBFAMILY B, POLYPEPTIDE 34 (CYP71B34)*	putative cytochrome P450
AT3G26310	*CYTOCHROME P450, FAMILY 71, SUBFAMILY B, POLYPEPTIDE 35 (CYP71B35)*	putative cytochrome P450
AT3G26320	*CYTOCHROME P450, FAMILY 71, SUBFAMILY B, POLYPEPTIDE 36 (CYP71B36)*	putative cytochrome P450
AT3G26330	*CYTOCHROME P450, FAMILY 71, SUBFAMILY B, POLYPEPTIDE 37 (CYP71B37)*	putative cytochrome P450
AT3G26350		proline-rich receptor-like kinase
AT3G26360		Ribosomal protein S21 family protein
AT3G26380	*ARAPASE (APSE)*	APSE is a member of the Glycoside Hydrolase (GH27) family that functions as a β-l-arabinopyranosidase.
AT3G26390		hypothetical protein
AT3G26400	*EUKARYOTIC TRANSLATION INITIATION FACTOR 4B1 (EIF4B1)*	member of eIF4B - eukaryotic initiation factor 4B The mRNA is cell-to-cell mobile.
AT3G26410	*TRNA MODIFICATION 11 (TRM11)*	Encodes a protein involved in modification of nucleosides in tRNA. Mutants have only 7.3% 2-methylguanosine levels of wild type counterparts.
AT3G26420	*RNA-BINDING GLYCINE-RICH PROTEIN B2 (RBGB2)*	Zinc finger-containing glycine-rich RNA-binding protein. Cold-inducible. Contributes to the enhancement of freezing tolerance. Members of this protein family include AT3G26420 (ATRZ-1A), AT1G60650 (AtRZ-1b) and AT5G04280 (AtRZ-1c).
AT3G26430	*(GGL20)*	Encodes a functioning member of the GDS(L) lipase family with preference for long chain substrates that does not hydrolyze choline esters.
AT3G26440		transmembrane protein, putative (DUF707)
AT3G26450		Polyketide cyclase/dehydrase and lipid transport superfamily protein
AT3G26460		Polyketide cyclase/dehydrase and lipid transport superfamily protein
AT3G26470		Powdery mildew resistance protein, RPW8 domain-containing protein
AT3G26480		Transducin family protein / WD-40 repeat family protein
AT3G26490	*(NPY7)*	Encodes a gene homologous to the NPY family based on deep phylogeny.
AT3G26510		Octicosapeptide/Phox/Bem1p family protein
AT3G26520	*TONOPLAST INTRINSIC PROTEIN 2 (TIP2)*	gamma tonoplast intrinsic protein 2 (TIP2). expressed throughout the plant and transcript level is increased upon NaCl or ABA treatments. NaCl stress-sensitive yeast mutant strains exhibit more resistance to salt when expressing this protein.
AT3G26540		RGFR3 is a leucine--rich repeat receptor kinase that, together with RGFR1 and RGFR2, binds ROOT GROWTH FACTORS and is required for establishing the gradient of PLETHORA1 and PLETHORA2 essential for proper root growth and development.
AT3G26550		Cysteine/Histidine-rich C1 domain family protein
AT3G26570	*PHOSPHATE TRANSPORTER 2;1 (PHT2;1)*	low affinity phosphate transporter
AT3G26580		Tetratricopeptide repeat (TPR)-like superfamily protein
AT3G26590		MATE efflux family protein
AT3G26595		
AT3G26600	*ARMADILLO REPEAT ONLY 4 (ARO4)*	Armadillo repeat protein. One of a family of four in Arabidopsis. Expressed in vegetative tissues, anthers and ovules.
AT3G26610	*POLYGALACTURONASE INVOLVED IN EXPANSION 1 (PGX1)*	Encodes an apoplast-localized polygalacturonase involved in cell elongation and flower development.

AT3G26614		transposable_element_gene;non-LTR retrotransposon family (LINE), has a 3.0e-39 P-value blast match to GB:NP_038607 L1 repeat, Tf subfamily, member 9 (LINE-element) (Mus musculus);(source:TAIR10)
AT3G26618	*EUKARYOTIC RELEASE FACTOR 1-3 (ERF1-3)*	eukaryotic release factor 1-3
AT3G26620	*LOB DOMAIN-CONTAINING PROTEIN 23 (LBD23)*	LOB domain-containing protein 23
AT3G26650	*GLYCERALDEHYDE 3-PHOSPHATE DEHYDROGENASE A SUBUNIT (GAPA)*	Encodes one of the two subunits forming the photosynthetic glyceraldehyde-3-phosphate dehydrogenase (GAPDH) and as such a constituent of the supramolecular complex with phosphoribulokinase (PRK) thought to be linked by a small peptide encoded by CP12-2. GapA-1 is coordinately expressed by light with PRK and CP12-2. The enzyme activity, tested in leaf protein extracts dropped significantly after external sucrose treatment for the photosynthetic GAPDH (NADPH-dependent) but not for the cytosolic GAPDH (NADH-dependent).
AT3G26680	*SENSITIVE TO NITROGEN MUSTARD 1 (SNM1)*	involved in a SNM-dependent recombinational repair process of oxidatively induced DNA damage.
AT3G26690	*NUDIX HYDROLASE HOMOLOG 13 (NUDX13)*	Encodes AtNUDT13, a mitochondrial Nudix hydrolase specific for long-chain diadenosine polyphosphates.
AT3G26700		Protein kinase superfamily protein
AT3G26710	*COFACTOR ASSEMBLY OF COMPLEX C (CCB1)*	cofactor assembly of complex C
AT3G26720		Glycosyl hydrolase family 38 protein
AT3G26740	*CCR-LIKE (CCL)*	transcripts are differentially regulated at the level of mRNA stability at different times of day controlled by the circadian clock. mRNAs are targets of the mRNA degradation pathway mediated by the downstream (DST) instability determinant.
AT3G26744	*INDUCER OF CBF EXPRESSION 1 (ICE1)*	Encodes a MYC-like bHLH transcriptional activator that binds specifically to the MYC recognition sequences in the CBF3 promoter. It also binds to and inhibits the expression of ABI3. Mutants are defective in cold-regulated gene expression and ABA signaling druing seed germination.. Cold stress triggers protein degradation of nuclear GFPICE1 protein, and the RING finger protein HOS1 is required. Sumoylation of ICE1 controls CBF3/DREB1A expression and freezing tolerance. Together with ZOU, ICE1 determines primary seed dormancy depth independently of their joint role in endosperm development.ICE1 interacts with ABI5. Also members of the DELLA family, which repress ICE1 function.
AT3G26760		NAD(P)-binding Rossmann-fold superfamily protein
AT3G26770		NAD(P)-binding Rossmann-fold superfamily protein
AT3G26780	*MITOCHONDRIAL EDITING FACTOR 14 (MEF14)*	Encodes MEF14 (mitochondrial editing factor 14), a PPR (pentatricopeptide repeat proteins) protein required for RNA editing at site matR-1895 in mitochondria. The mRNA is cell-to-cell mobile.
AT3G26790	*FUSCA3 (FUS3)*	Transcriptional factor with high similarity to the B3 region of the VP1/ABI3-like proteins. Full length FUS3 protein binds to the highly conserved RY motif [DNA motif CATGCA(TG)], present in many seed-specific promoters, and the B3 domains of this transcription factor is necessary for the specific interaction with the RY element. Transcriptional activity of FUS3 requires the B3 DNA-binding domain and an activation domain. FUS3 specifies cotyledon identity. Regulator of gene expression during late embryogenesis. Involved in the control foliar organ identity in Arabidopsis by regulating the synthesis of two hormones, abscisic acid and gibberellin. FUS3 together with LEC1 positively regulate the abundance of the ABI3 protein in the seed.
AT3G26800		transmembrane protein
AT3G26830	*PHYTOALEXIN DEFICIENT 3 (PAD3)*	Mutations in pad3 are defective in biosynthesis of the indole derived phytoalexin camalexin. Encodes a cytochrome P450 enzyme that catalyzes the conversion of dihydrocamalexic acid to camalexin. The mRNA is cell-to-cell mobile.
AT3G26840	*PHYTYL ESTER SYNTHASE 2 (PES2)*	Encodes a protein with phytyl ester synthesis and diacylglycerol acyltransferase activities that is involved in the deposition of free phytol and free fatty acids in the form of phytyl esters in chloroplasts, a process involved in maintaining the integrity of the photosynthetic membrane during abiotic stress and senescence.
AT3G26850		histone-lysine N-methyltransferase
AT3G26860		Plant self-incompatibility protein S1 family
AT3G26870		Plant self-incompatibility protein S1 family
AT3G26880		Plant self-incompatibility protein S1 family
AT3G26910		hydroxyproline-rich glycoprotein family protein
AT3G26920		FBD / Leucine Rich Repeat domains containing protein
AT3G26932	*DSRNA-BINDING PROTEIN 3 (DRB3)*	dsRNA-binding protein 3
AT3G26940	*CONSTITUTIVE DIFFERENTIAL GROWTH 1 (CDG1)*	Receptor-like cytoplasmic kinase, RLCKVII subfamily. Overexpression causes abnormal differential and elongation growth after organ differentiation.
AT3G26960		Pollen Ole e 1 allergen and extensin family protein
AT3G26970		
AT3G26980	*MEMBRANE-ANCHORED UBIQUITIN-FOLD PROTEIN 4 PRECURSOR (MUB4)*	membrane-anchored ubiquitin-fold protein 4 precursor
AT3G27000	*ACTIN RELATED PROTEIN 2 (ARP2)*	encodes a protein whose sequence is similar to actin-related proteins (ARPs) in other organisms. its transcript level is down regulated by light and is expressed in very low levels in all organs examined.
AT3G27010	*TEOSINTE BRANCHED 1, CYCLOIDEA, PCF (TCP)-DOMAIN FAMILY PROTEIN 20 (TCP20)*	Belongs to a TCP protein transcription factor family. Members of this family contain a predicted basic-helix-loop-helix domain involved in DNA binding. Related to rice PCF1 and PCF2 genes. Binds to the GCCCR element of CYCB1;1. Involved in regulation of expression of cell cycle control and ribosomal protein genes.
AT3G27020	*YELLOW STRIPE LIKE 6 (YSL6)*	Arabidopsis thaliana metal-nicotianamine transporter YSL6
AT3G27060	*TSO MEANING 'UGLY' IN CHINESE 2 (TSO2)*	Encodes one of the 3 ribonucleotide reductase (RNR) small subunit genes. TSO2 transcription occurs predominantly at the S-phase of the cell cycle and its expression pattern is consistent with its role in dNDP biosynthesis during DNA replication in actively dividing cells. Critical for cell cycle progression, DNA damage repair and plant development.
AT3G27070	*TRANSLOCASE OUTER MEMBRANE 20-1 (TOM20-1)*	Form of TOM20, which is a component of the TOM complex, involved in transport of nuclear-encoded mitochondrial proteins
AT3G27080	*TRANSLOCASE OF OUTER MEMBRANE 20 KDA SUBUNIT 3 (TOM20-3)*	Component of the TOM complex involved in transport of nuclear-encoded mitochondrial proteins

AT3G27110	*(PGM48)*	PGM48 is a member of a plant specific clade of metallo-endopeptidase proteins. It is found in plastoglobules. Analysis of over-expression and loss of function phenotypes suggests PGM48 may have a role in positively regulating senescence.
AT3G27130		
AT3G27150		Target gene of MIR2111-5p.
AT3G27160	*GLUCOSE HYPERSENSITIVE 1 (GHS1)*	GHS1 encodes plastid ribosomal protein S21 The mRNA is cell-to-cell mobile.
AT3G27170	*CHLORIDE CHANNEL B (CLC-B)*	member of Anion channel protein family The mRNA is cell-to-cell mobile.
AT3G27190	*URIDINE KINASE-LIKE 2 (UKL2)*	One of the homologous genes predicted to encode proteins with UPRT domains (Uracil phosphoribosyltransferase). Five of these genes (At5g40870, At3g27190, At1g55810, At4g26510 and At3g27440) show a high level of identity, and are annotated as also containing a N-terminal uracil kinase (UK) domain. These genes are referred to as UKL1 (UK-like 1), UKL2, UKL3, UKL4 and UKL5, respectively.
AT3G27200		Cupredoxin superfamily protein
AT3G27210		hypothetical protein
AT3G27220	*HYPOXIA RESPONSE UNKNOWN PROTEIN 6 (HUP6)*	Galactose oxidase/kelch repeat superfamily protein
AT3G27230		S-adenosyl-L-methionine-dependent methyltransferases superfamily protein
AT3G27240	*(CYC1-1)*	Cytochrome C1 family
AT3G27260	*GLOBAL TRANSCRIPTION FACTOR GROUP E8 (GTE8)*	Kinase like protein with similarity to yeast BDF1 and human RING3 protein, which have two bromodomains GTE8 has a single bromodomain
AT3G27280	*PROHIBITIN 4 (PHB4)*	Part of protein complexes that are necessary for proficient mitochondrial function or biogenesis, thereby supporting cell division and differentiation in apical tissues
AT3G27300	*GLUCOSE-6-PHOSPHATE DEHYDROGENASE 5 (G6PD5)*	glucose-6-phosphate dehydrogenase 5
AT3G27325	*(PGAP1)*	ER localized GPI inositol-deacylase. Involved in GPI-anchor remodeling.
AT3G27330		zinc finger (C3HC4-type RING finger) family protein
AT3G27340		Myb domain protein
AT3G27350		transcriptional regulator ATRX-like protein
AT3G27370		
AT3G27400	*(PLL18)*	Encodes a pectate lyase involved in response to nematodes.
AT3G27410		transmembrane protein
AT3G27440	*URIDINE KINASE-LIKE 5 (UKL5)*	One of the homologous genes predicted to encode proteins with UPRT domains (Uracil phosphoribosyltransferase). Five of these genes (At5g40870, At3g27190, At1g55810, At4g26510 and At3g27440) show a high level of identity, and are annotated as also containing a N-terminal uracil kinase (UK) domain. These genes are referred to as UKL1 (UK-like 1), UKL2, UKL3, UKL4 and UKL5, respectively.
AT3G27450		pseudogene of Ribosomal protein S21e
AT3G27473		Cysteine/Histidine-rich C1 domain family protein
AT3G27490	*GERMOSTATIN RESISTANCE LOCUS 1 (GSR1)*	GSR1 is a tandem plant PhD homeodomain protein involved in auxin mediated seed dormancy and germination. It was identified in a screen for mutations resistant to the compound germostatin. It interacts with components of the auxin signaling pathway and may function as an auxin stimulated co-repressor.
AT3G27500		Cysteine/Histidine-rich C1 domain family protein
AT3G27510		Cysteine/Histidine-rich C1 domain family protein
AT3G27550	*(CFM9)*	Mitochondrial protein involved in RNA splicing. Required for normal mitochondrion biogenesis.
AT3G27580	*(ATPK7)*	D6PK family kinase involved in pulse-induced phototropism but also for time-dependent second positive phototropism, and continuous light-induced hypocotyl phototropism.D6PKL3 is polarly localized within the plasma membrane. It is involved in pollen aperture formation. The protein is localized within distinct regions of the pollen plasma membrane and mutants are also defective in pollen aperture formation.
AT3G27590		reverse transcriptase family protein
AT3G27620	*ALTERNATIVE OXIDASE 1C (AOX1C)*	encodes an isoform of alternate oxidase. expressed in all tissues examined and expression is not induced by antimycin A, an inhibitor of complex III in the mitochondrial respiratory chain.
AT3G27650	*LOB DOMAIN-CONTAINING PROTEIN 25 (LBD25)*	LOB domain-containing protein 25
AT3G27660	*OLEOSIN 4 (OLEO4)*	Encodes oleosin4 (Plant Cell, 2006, 18:1961), a protein found in oil bodies, involved in seed lipid accumulation. Functions in freezing tolerance of seeds. Note: also referred to as OLE3 in Plant Journal 2008, 55:798.
AT3G27670	*RESURRECTION1 (RST1)*	A novel protein, did not show high similarity to any protein of known function; reveals a novel genetic connection between lipid synthesis and embryo development. Expressed in all tissues examined including leaves, flowers, roots, stems, and siliques, but accumulation levels were not correlated with the degree to which different organs appeared affected by the mutation. Mutant plants showed alterations in the cuticular wax profiles and embryo development. The mRNA is cell-to-cell mobile.
AT3G27690	*PHOTOSYSTEM II LIGHT HARVESTING COMPLEX GENE 2.3 (LHCB2.3)*	Encodes Lhcb2.4. Belongs to the Lhc super-gene family encodes the light-harvesting chlorophyll a/b-binding (LHC) proteins that constitute the antenna system of the photosynthetic apparatus. The mRNA is cell-to-cell mobile.
AT3G27710	*ARIADNE 3 (ARI3)*	RING/U-box superfamily protein
AT3G27750	*EMBRYO DEFECTIVE 3123 (EMB3123)*	Encodes a pentatricopeptide repeat (PPR) protein required for the splicing of specific group II introns. Null alleles are embryo lethal.
AT3G27770	*HYPOXIA RESPONSE UNKNOWN PROTEIN 53 (HUP53)*	plant/protein
AT3G27780		
AT3G27785	*MYB DOMAIN PROTEIN 118 (MYB118)*	MYB118 encodes a myb transcription factor that represses endosperm maturation and, along with MYB115, regulates glucosinolate biosynthesis.
AT3G27800		
AT3G27810	*MYB DOMAIN PROTEIN 21 (MYB21)*	Encodes a member of the R2R3-MYB transcription factor gene family. Induced by jasmonate. Involved in jasmonate response during stamen development. MYB21 interacts with JAZ proteins, and functions redundantly with MYB24 and MYB57 to regulate stamen development. Promotes flavonol biosynthesis through regulation of FLS1 gene expression.

AT3G27812		
AT3G27820	*MONODEHYDROASCORBATE REDUCTASE 4 (MDAR4)*	Encodes a peroxisome membrane-bound monodehydroascorbate reductase, involved in the ascorbate-glutathione cycle which removes toxic H2O2
AT3G27840	*RIBOSOMAL PROTEIN L12-B (RPL12-B)*	50S ribosomal protein L12-B
AT3G27850	*RIBOSOMAL PROTEIN L12-C (RPL12-C)*	50S ribosomal protein L12-C The mRNA is cell-to-cell mobile.
AT3G27870	*AMINOPHOSPHOLIPID ATPASE 8 (ALA8)*	ATPase E1-E2 type family protein / haloacid dehalogenase-like hydrolase family protein
AT3G27880		hypothetical protein (DUF1645)
AT3G27890	*NADPH:QUINONE OXIDOREDUCTASE (NQR)*	Encodes NAD(P)H:quinone reductase which is an FMN binding protein that catalyzes the reduction of quinone substrates to hydroquinones.The enzyme activity was confirmed by in vitro assay.
AT3G27920	*GLABRA 1 (GL1)*	Encodes GL1, a Myb-like protein that is required for induction of trichome development. Interacts with JAZ and DELLA proteins to regulate trichome initiation. Natural hyperfunctional alleles producing trichome development in fruits and pedicels have been found.
AT3G27925	*DEGRADATION OF PERIPLASMIC PROTEINS 1 (DEG1)*	Encodes a DegP protease; nuclear gene encoding chloroplast-targeted protease that can degrade two lumenal proteins, plastocyanin and OE33, suggesting a role as a general-purpose protease in the thylakoid lumen. Involved in the degradation of D1 protein of PS II, hence participating in the repair of PS II damages caused by photoinhibition. The mRNA is cell-to-cell mobile.
AT3G27950		GDSL-motif esterase/acyltransferase/lipase. Enzyme group with broad substrate specificity that may catalyze acyltransfer or hydrolase reactions with lipid and non-lipid substrates.
AT3G27960	*KINESIN LIGHT CHAIN-RELATED 2 (KLCR2)*	CMU1 and CMU2 along with FRA1 contributes to lateral stability of cortical microtubules.
AT3G27980		Type II pectin methylesterase
AT3G27990		None
AT3G28000		
AT3G28007	*(SWEET4)*	Nodulin MtN3 family protein
AT3G28020		DNA-binding protein
AT3G28040		Leucine-rich receptor-like protein kinase family protein
AT3G28050	*USUALLY MULTIPLE ACIDS MOVE IN AND OUT TRANSPORTERS 41 (UMAMIT41)*	nodulin MtN21-like transporter family protein
AT3G28080	*USUALLY MULTIPLE ACIDS MOVE IN AND OUT TRANSPORTERS 47 (UMAMIT47)*	nodulin MtN21-like transporter family protein
AT3G28090		
AT3G28120		transmembrane protein
AT3G28130	*USUALLY MULTIPLE ACIDS MOVE IN AND OUT TRANSPORTERS 44 (UMAMIT44)*	nodulin MtN21-like transporter family protein
AT3G28150	*TRICHOME BIREFRINGENCE-LIKE 22 (TBL22)*	Encodes a member of the TBL (TRICHOME BIREFRINGENCE-LIKE) gene family containing a plant-specific DUF231 (domain of unknown function) domain. A putative xyloglucan O-acetyltransferase. TBL gene family has 46 members, two of which (TBR/AT5G06700 and TBL3/AT5G01360) have been shown to be involved in the synthesis and deposition of secondary wall cellulose, presumably by influencing the esterification state of pectic polymers. A nomenclature for this gene family has been proposed (Volker Bischoff & Wolf Scheible, 2010, personal communication).
AT3G28155		hypothetical protein
AT3G28160		transposable_element_gene;copia-like retrotransposon family, has a 1.4e-06 P-value blast match to GB:CAA31653 polyprotein (Ty1_Copia-element) (Arabidopsis thaliana);(source:TAIR10)
AT3G28170		hypothetical protein
AT3G28180	*CELLULOSE-SYNTHASE-LIKE C4 (CSLC04)*	encodes a gene similar to cellulose synthase The mRNA is cell-to-cell mobile.
AT3G28190		transmembrane protein
AT3G28200		Peroxidase superfamily protein
AT3G28210	*(PMZ)*	Encodes a putative zinc finger protein (PMZ).
AT3G28220		TRAF-like family protein
AT3G28230		something about silencing protein
AT3G28240		transposable_element_gene;similar to unknown protein [Arabidopsis thaliana] (TAIR:AT5G10670.1);(source:TAIR10)
AT3G28260		
AT3G28270	*AT14A-LIKE1 (AFL1)*	AFL1 was first identified by immunoscreening an Arabidopsis expression library with antisera recognizing mammalian β1-integrin. It is a peripheral membrane protein associated with endomembranes and plasmamembrane. Based on overexpression and knockdown phenotypes, AFL1 is postulated to function in regulation of growth and proline accumulation in response to drought. AFL1 protein co-localizes with clatharin coated vesicles and has been shown to interact with itself and several endomembrane associated proteins.
AT3G28280		F-box/associated interaction domain protein
AT3G28290	*(AT14A)*	Encodes a protein with sequence similarity to integrins. Localized to the cytoplasm and plasma membrane. Expressed in all tissues assayed.
AT3G28310		hypothetical protein (DUF677)
AT3G28320		UPF0496 protein; Commonly-enriched candidate LPS-interacting PM-associated proteins from the three affinity chromatography systems with LPS chemotype Xcc 8530 as ligand.
AT3G28330		F-box family protein-like protein
AT3G28340	*GALACTURONOSYLTRANSFERASE-LIKE 10 (GATL10)*	Encodes a protein with putative galacturonosyltransferase activity.
AT3G28345	*ATP-BINDING CASSETTE B15 (ABCB15)*	Encodes an ATP-binding cassette (ABC) transporter. Expressed in the vascular tissue of primary stem.
AT3G28350		Pseudogene of AT3G28350; unknown protein
AT3G28360	*ATP-BINDING CASSETTE B16 (ABCB16)*	P-glycoprotein 16
AT3G28380	*ATP-BINDING CASSETTE B17 (ABCB17)*	P-glycoprotein 17
AT3G28390	*ATP-BINDING CASSETTE B18 (ABCB18)*	P-glycoprotein 18

AT3G28400		transposable_element_gene;hAT-like transposase family (hobo/Ac/Tam3), has a 4.6e-39 P-value blast match to GB:CAA29005 ORFa of Maize Ac (hAT-element) (Zea mays);(source:TAIR10)
AT3G28420		Putative membrane lipoprotein
AT3G28430	*TRANSPARENT TESTA 9 (TT9)*	Encodes a peripheral membrane protein localized at the Golgi apparatus that is involved in membrane trafficking, vacuole development and in flavonoid accumulation in the seed coat. Mutant seed color is pale brown.
AT3G28440		
AT3G28470	*DEFECTIVE IN MERISTEM DEVELOPMENT AND FUNCTION 1 (TDF1)*	Member of the R2R3 factor gene family. Its E-box is critical for the DYT1- bHLH089 heterocomplex to bind to and activate its transcription.
AT3G28490		2-oxoglutarate-dependent dioxygenase
AT3G28500		60S acidic ribosomal protein family
AT3G28510		P-loop containing nucleoside triphosphate hydrolases superfamily protein
AT3G28520		P-loop containing nucleoside triphosphate hydrolases superfamily protein
AT3G28530		UDP-glucose 4-epimerase
AT3G28540		P-loop containing nucleoside triphosphate hydrolases superfamily protein
AT3G28550	*EXTENSIN 16 (EXT16)*	Proline-rich extensin-like family protein
AT3G28560		BCS1 AAA-type ATPase
AT3G28580		P-loop containing nucleoside triphosphate hydrolases superfamily protein
AT3G28590		transmembrane protein
AT3G28600		P-loop containing nucleoside triphosphate hydrolases superfamily protein
AT3G28610		P-loop containing nucleoside triphosphate hydrolases superfamily protein
AT3G28620		Zinc finger, C3HC4 type (RING finger) family protein
AT3G28630	*CROLIN 1 (CROLIN1)*	actin cross-linking protein, putative (DUF569)
AT3G28640		Tetratricopeptide repeat (TPR)-like superfamily protein
AT3G28680		Serine carboxypeptidase S28 family protein
AT3G28690	*PBS1-LIKE 36 (PBL36)*	Protein kinase superfamily protein
AT3G28700		NADH dehydrogenase ubiquinone complex I, assembly factor-like protein (DUF185)
AT3G28720		transmembrane protein
AT3G28740	*CYTOCHROME P450, FAMILY 81, SUBFAMILY D, POLYPEPTIDE 11 (CYP81D11)*	Encodes a member of the cytochrome p450 family. Expression is upregulated in response to cis-jasmonate treatment. Overexpression induces synthesis of volatile compounds that affect chemical ecology and insect interactions.
AT3G28750		hypothetical protein
AT3G28760		3-dehydroquinate synthase
AT3G28770		transmembrane protein, putative (DUF1216)
AT3G28780		transmembrane protein, putative (DUF1216)
AT3G28790		transmembrane protein, putative (DUF1216)
AT3G28810		mediator of RNA polymerase II transcription subunit-like protein, putative (DUF1216)
AT3G28830		mucin-like protein, putative (DUF1216)
AT3G28850		Glutaredoxin family protein
AT3G28860	*ATP-BINDING CASSETTE B19 (ABCB19)*	Encodes a member of the ATP-binding cassette (ABC) transporter family that is involved in auxin transport and is involved in postembryonic organ separation. Also known as AtMDR11 and PGP19. Possibly regulates auxin-dependent responses by influencing basipetal auxin transport in the root. Acts upstream of phyA in regulating hypocotyl elongation and gravitropic response. Exerts nonredundant, partially overlapping functions with the ABC transporter encoded by AtPGP1.
AT3G28870		paired amphipathic helix SIN3-like protein
AT3G28890	*RECEPTOR LIKE PROTEIN 43 (RLP43)*	receptor like protein 43
AT3G28910	*MYB DOMAIN PROTEIN 30 (MYB30)*	Encodes a MYB family transcriptional regulator.It is a a positive regulator of the pathogen-induced hypersensitive response and of brassinosteroid and abscisic acid signaling and a negative regulator of photomorphogenesis. Accumulation of MYB30 is light regulated and activity is modulated by SUMOlaytion. MYB30 can for complexes with different bHLH components to regulate expression of different pathways.
AT3G28920	*HOMEOBOX PROTEIN 34 (HB34)*	homeobox protein 34
AT3G28930	*AVRRPT2-INDUCED GENE 2 (AIG2)*	avrRpt2-induced gene that exhibits RPS2- and avrRpt2-dependent induction early after infection with Pseudomonas syringae pv maculicola strain ES4326 carrying avrRpt2
AT3G28940		AIG2-like (avirulence induced gene) family protein
AT3G28950		AIG2-like (avirulence induced gene) family protein
AT3G28960		Transmembrane amino acid transporter family protein
AT3G28970	*ANTIAUXIN-RESISTANT 3 (AAR3)*	Identified in a screen for mutants resistant to an anti-auxin. Encodes a protein with unknown function that shares homology with DCN protein family.
AT3G28980		mediator of RNA polymerase II transcription subunit-like protein, putative (DUF1216)
AT3G29020	*MYB DOMAIN PROTEIN 110 (MYB110)*	Encodes a putative transcription factor (MYB110).
AT3G29030	*EXPANSIN A5 (EXPA5)*	Encodes an expansin. Naming convention from the Expansin Working Group (Kende et al, 2004. Plant Mol Bio)
AT3G29035	*NAC DOMAIN CONTAINING PROTEIN 3 (NAC3)*	Encodes a protein with transcription factor activity. Note: this protein (AT3G29035) on occasion has also been referred to as AtNAC3, not to be confused with the AtNAC3 found at locus AT3G15500. The mRNA is cell-to-cell mobile.
AT3G29060		EXS (ERD1/XPR1/SYG1) family protein
AT3G29070		emp24/gp25L/p24 family/GOLD family protein
AT3G29080		hypothetical protein

AT3G29090	*PECTIN METHYLESTERASE 31 (PME31)*	Encodes an atypical pectin methylesterase that does not require salt for its activity and has a blockwise mode of pectin demethylesterification.
AT3G29110		Terpenoid cyclases/Protein prenyltransferases superfamily protein
AT3G29150		pseudogene of F-box family protein
AT3G29170		transmembrane protein (DUF872)
AT3G29185	*BIOGENESIS FACTOR REQUIRED FOR ATP SYNTHASE 1 (BFA1)*	Encodes a chloroplast protein that interacts with the CF1β, γ, and ε subunits of the chloroplast ATP synthase and is required for assembly of its F1 module. The protein is comprised primarily of two β-barrels and acts as a chaperone orchestrating the early steps of the CF1 assembly pathway via specific interaction with the CF1 β, γ, and ε subunits.
AT3G29190		Terpenoid cyclases/Protein prenyltransferases superfamily protein
AT3G29200	*CHORISMATE MUTASE 1 (CM1)*	L-ascorbate peroxidase
AT3G29210		transposable_element_gene;similar to Ulp1 protease family protein [Arabidopsis thaliana] (TAIR:AT3G47260.1);(source:TAIR10)
AT3G29220		transposable_element_gene;similar to unknown protein [Arabidopsis thaliana] (TAIR:AT3G30843.1);(source:TAIR10)
AT3G29240		PPR containing protein (DUF179)
AT3G29250	*SHORT-CHAIN DEHYDROGENASE REDUCTASE 4 (SDR4)*	NAD(P)-binding Rossmann-fold superfamily protein
AT3G29260		NAD(P)-binding Rossmann-fold superfamily protein
AT3G29265		transposable_element_gene;similar to zinc knuckle (CCHC-type) family protein [Arabidopsis thaliana] (TAIR:AT5G32482.1);(source:TAIR10)
AT3G29290	*EMBRYO DEFECTIVE 2076 (emb2076)*	Pentatricopeptide repeat (PPR) superfamily protein
AT3G29300		transmembrane protein
AT3G29310		calmodulin-binding protein-like protein
AT3G29330		zinc finger RNA-binding-like protein
AT3G29360	*UDP-GLUCOSE DEHYDROGENASE 2 (UGD2)*	Encodes one of four UDP-glucose dehydrogenase UGD) genes. Mutation of this gene in combination with UGD3 leads to swollen plant cell walls and severe developmental defects associated with changes in pectic polysaccharides.
AT3G29380	*PLANT-SPECIFIC TFIIB-RELATED PROTEIN 2 (pBRP2)*	Encodes a TFIIB-related protein expressed in the reproductive organs and seeds. Loss-of-function specifically affects the development of the syncytial endosperm. It is not required for RNA polymerase IV or V activities.
AT3G29410	*TERPENE SYNTHASE 25 (TPS25)*	Terpenoid cyclases/Protein prenyltransferases superfamily protein
AT3G29420		pseudogene of geranylgeranyl pyrophosphate synthase 3
AT3G29430	*GERANYL(GERANYL)DIPHOSPHATE SYNTHASE 9 (ATGGPS9)*	Chloroplast localized GFDP synthase.
AT3G29460		transposable_element_gene;copia-like retrotransposon family, has a 4.0e-34 P-value blast match to GB:BAA11674 ORF(AA 1-1338) (Ty1_Copia-element) (Nicotiana tabacum);(source:TAIR10)
AT3G29500		
AT3G29510		transposable_element_gene;copia-like retrotransposon family, has a 2.6e-122 P-value blast match to GB:CAA32025 ORF (Ty1_Copia-element) (Nicotiana tabacum)GB:CAA32025 ORF (Ty1_Copia-element) (Nicotiana tabacum);(source:TAIR10)
AT3G29570		hypothetical protein
AT3G29575	*ABI FIVE BINDING PROTEIN 3 (AFP3)*	ABI five binding protein 3
AT3G29590	*(AT5MAT)*	At3g29590 (At5MAT) encodes a malonyl-CoA:anthocyanidin 5-O-glucoside-6"-O-malonyltransferase that is coordinately expressed with a epistatic 5-O-anthocyanidin glucosyltransferase (At4g14090). The enzyme is involved in the malonylation of anthocyanins in Arabidopsis.
AT3G29600		transposable_element_gene;similar to unknown protein [Arabidopsis thaliana] (TAIR:AT1G54926.1);(source:TAIR10)
AT3G29610		transposable_element_gene;similar to unknown protein [Arabidopsis thaliana] (TAIR:AT3G31406.1);(source:TAIR10)
AT3G29630		UDP-Glycosyltransferase superfamily protein
AT3G29635		HXXXD-type acyl-transferase family protein
AT3G29640		transposable_element_gene;similar to unknown protein [Arabidopsis thaliana] (TAIR:AT3G29632.1);(source:TAIR10)
AT3G29650		transposable_element_gene;CACTA-like transposase family (Tnp1/En/Spm), has a 5.1e-81 P-value blast match to ref|NP_189784.1| TNP1-related protein (Arabidopsis thaliana) (CACTA-element);(source:TAIR10)
AT3G29660		transposable_element_gene;similar to unknown protein [Arabidopsis thaliana] (TAIR:AT5G37120.1);(source:TAIR10)
AT3G29670	*PHENOLIC GLUCOSIDE MALONYLTRANSFERASE 2 (PMAT2)*	Encodes a malonyltransferase that may play a role in phenolic xenobiotic detoxification. The mRNA is cell-to-cell mobile.
AT3G29690		HXXXD-type acyl-transferase family protein
AT3G29720		HXXXD-type acyl-transferase family protein
AT3G29732		transposable_element_gene;CACTA-like transposase family (Tnp2/En/Spm), has a 9.9e-152 P-value blast match to GB:CAA40555 TNP2 (CACTA-element) (Antirrhinum majus);(source:TAIR10)
AT3G29750		Eukaryotic aspartyl protease family protein
AT3G29760		Haloacid dehalogenase-like hydrolase (HAD) superfamily protein
AT3G29763		General transcription factor 2-related zinc finger protein
AT3G29770	*METHYL ESTERASE 11 (MES11)*	Encodes a protein predicted to act as a carboxylesterase. It has similarity to the SABP2 methyl salicylate esterase from tobacco. This protein does not act on methyl IAA, methyl JA, MeSA, MeGA4, or MEGA9 in vitro.
AT3G29775		transposable_element_gene;copia-like retrotransposon family, has a 2.8e-246 P-value blast match to gb|AAO73527.1| gag-pol polyprotein (Glycine max) (SIRE1) (Ty1_Copia-family);(source:TAIR10)
AT3G29780	*RALF-LIKE 27 (RALFL27)*	Member of a diversely expressed predicted peptide family showing sequence similarity to tobacco Rapid Alkalinization Factor (RALF), and is believed to play an essential role in the physiology of Arabidopsis. Consists of a single exon and is characterized by a conserved C-terminal motif and N-terminal signal peptide.
AT3G29787		transposable_element_gene;CACTA-like transposase family (Ptta/En/Spm), has a 4.8e-35 P-value blast match to At5g29026.1/8-244 CACTA-like transposase family (Ptta/En/Spm) (CACTA-element) (Arabidopsis thaliana);(source:TAIR10)

AT3G29791		hypothetical protein
AT3G29792		transposable_element_gene;copia-like retrotransposon family, has a 1.7e-243 P-value blast match to GB:CAA72989 open reading frame 1 (Ty1_Copia-element) (Brassica oleracea);(source:TAIR10)
AT3G29796		hypothetical protein
AT3G29800		P-loop containing nucleoside triphosphate hydrolases superfamily protein
AT3G29970		B12D protein
AT3G30140		
AT3G30150		transposable_element_gene;similar to unknown protein [Arabidopsis thaliana] (TAIR:AT3G31990.1);(source:TAIR10)
AT3G30160		transmembrane protein
AT3G30180	*BRASSINOSTEROID-6-OXIDASE 2 (BR6OX2)*	Encodes a cytochrome p450 enzyme that catalyzes the last reaction in the production of brassinolide. It is capable of converting 6-deoxocastasterone into castasterone, a C-6 oxidation, as well as the further conversion of castasterone into brassinolide by a Baeyer-Villinger oxidation reaction at C-6, resulting in the formation of an unusual seven-membered lactone ring. The enzyme possesses high affinity for both C28- and C27-Brassinosteroids. The expression of the gene using a CYP85A2 promoter:LUC fusion construct was shown to be under circadian and light control.
AT3G30190		transposable_element_gene;similar to unknown protein [Arabidopsis thaliana] (TAIR:AT1G24370.1);(source:TAIR10)
AT3G30200		Plant transposase (Ptta/En/Spm family)
AT3G30210	*MYB DOMAIN PROTEIN 121 (MYB121)*	Encodes a putative transcription factor, member of the R2R3 factor gene family (MYB121).
AT3G30220		hypothetical protein
AT3G30230		myosin heavy chain-like protein
AT3G30240		transposable_element_gene;contains InterPro domain Retrotransposon gag protein;(source:TAIR10)
AT3G30250		transposable_element_gene;similar to unknown protein [Arabidopsis thaliana] (TAIR:AT1G31150.1);(source:TAIR10)
AT3G30260	*AGAMOUS-LIKE 79 (AGL79)*	Agamous-like transcription factor. A target of SPL10, AGL79 knockdowns show defects in leaf shape, shoot branching, and flowering time.
AT3G30270		
AT3G30280		HXXXD-type acyl-transferase family protein
AT3G30290	*CYTOCHROME P450, FAMILY 702, SUBFAMILY A, POLYPEPTIDE 8 (CYP702A8)*	a member of cytochrome P450 gene family
AT3G30310		transposable_element_gene;similar to unknown protein [Arabidopsis thaliana] (TAIR:AT1G35920.1);(source:TAIR10)
AT3G30320		hypothetical protein
AT3G30330		transposable_element_gene;CACTA-like transposase family (Ptta/En/Spm), has a 1.7e-83 P-value blast match to At1g36190.1/92-340 CACTA-like transposase family (Ptta/En/Spm) (CACTA-element) (Arabidopsis thaliana);(source:TAIR10)
AT3G30340	*USUALLY MULTIPLE ACIDS MOVE IN AND OUT TRANSPORTERS 32 (UMAMIT32)*	nodulin MtN21-like transporter family protein
AT3G30350	*ROOT MERISTEM GROWTH FACTOR 4 (RGF4)*	Encodes a root meristem growth factor (RGF). Belongs to a family of functionally redundant homologous peptides that are secreted, tyrosine-sulfated, and expressed mainly in the stem cell area and the innermost layer of central columella cells. RGFs are required for maintenance of the root stem cell niche and transit amplifying cell proliferation. Members of this family include: At5g60810 (RGF1), At1g13620 (RGF2), At2g04025 (RGF3), At3g30350 (RGF4), At5g51451 (RGF5), At4g16515 (RGF6), At3g02240 (RGF7), At2g03830 (RGF8) and At5g64770 (RGF9).
AT3G30360		transposable_element_gene;similar to unknown protein [Arabidopsis thaliana] (TAIR:AT4G18420.1);(source:TAIR10)
AT3G30370		MuDR family transposase
AT3G30380		alpha/beta-Hydrolases superfamily protein
AT3G30390		Encodes a putative amino acid transporter.
AT3G30396		transposable_element_gene;CACTA-like transposase family (Ptta/En/Spm), has a 3.1e-39 P-value blast match to At5g29026.1/8-244 CACTA-like transposase family (Ptta/En/Spm) (CACTA-element) (Arabidopsis thaliana);(source:TAIR10)
AT3G30400		transposable_element_gene;gypsy-like retrotransposon family (Athila), has a 1.1e-46 P-value blast match to GB:CAA57397 Athila ORF 1 (Arabidopsis thaliana);(source:TAIR10)
AT3G30440		transposable_element_gene;similar to Ulp1 protease family protein [Arabidopsis thaliana] (TAIR:AT3G47260.1);(source:TAIR10)
AT3G30450		transposable_element_gene;similar to heat shock protein binding [Arabidopsis thaliana] (TAIR:AT3G47270.1);(source:TAIR10)
AT3G30460		RING/U-box superfamily protein
AT3G30480		transposable_element_gene;similar to unknown protein [Arabidopsis thaliana] (TAIR:AT2G24915.1);(source:TAIR10)
AT3G30490		transposable_element_gene;similar to unknown protein [Arabidopsis thaliana] (TAIR:AT2G24910.1);(source:TAIR10)
AT3G30500		transposable_element_gene;similar to unknown protein [Arabidopsis thaliana] (TAIR:AT4G07690.1);(source:TAIR10)
AT3G30510		transposable_element_gene
AT3G30530	*BASIC LEUCINE-ZIPPER 42 (bZIP42)*	basic leucine-zipper 42
AT3G30540		Glycosyl hydrolase superfamily protein
AT3G30560		transposable_element_gene;similar to AT hook motif-containing protein-related [Arabidopsis thaliana] (TAIR:AT1G35940.1);(source:TAIR10)
AT3G30570		transposable_element_gene;non-LTR retrotransposon family (LINE), has a 7.9e-38 P-value blast match to GB:AAB41224 ORF2 (LINE-element) (Rattus norvegicus);(source:TAIR10)
AT3G30585		transposable_element_gene;Mutator-like transposase family, has a 2.8e-33 P-value blast match to GB:AAA21566 mudrA of transposon=MuDR (MuDr-element) (Zea mays);(source:TAIR10)
AT3G30610		transposable_element_gene;similar to unknown protein [Arabidopsis thaliana] (TAIR:AT2G12685.1);(source:TAIR10)
AT3G30620		transposable_element_gene;gypsy-like retrotransposon family (Athila);(source:TAIR10)
AT3G30640		transposable_element_gene;similar to Ulp1 protease family protein [Arabidopsis thaliana] (TAIR:AT2G24930.1);(source:TAIR10)
AT3G30650		transposable_element_gene;similar to unknown protein [Arabidopsis thaliana] (TAIR:AT2G10175.1);(source:TAIR10)
AT3G30660		transposable_element_gene;similar to unknown protein [Arabidopsis thaliana] (TAIR:AT5G31753.1);(source:TAIR10)

AT3G30670		transposable_element_gene;similar to unknown protein [Arabidopsis thaliana] (TAIR:AT1G40125.1);(source:TAIR10)
AT3G30710		transposable_element_gene;similar to myosin heavy chain-related [Arabidopsis thaliana] (TAIR:AT4G08113.1);(source:TAIR10)
AT3G30720	*QUA-QUINE STARCH (QQS)*	QQS is an orphan gene that arose recently in the Arabidopsis thaliana lineage. It was first identified in a screen for genes with altered expression pattern in SS3 mutants which make an abundance of starch. Overexpression of QQS in Arabidopsis increases protein content and decreases total starch content. Thus it appears to function to modulate carbon/nitrogen allocation in Arabidopsis. Over expression of QQS in soybean, rice and maize also results in an increase in protein and decrease in starch levels suggesting that QQS affects similar pathways in a wide range of plants. QQS interacts with NF-YC4 in Arabidopsis and NF-YC4 homologs in rice, soybean and maize. In Arabidopsis QQS is localized the the cytoplasm and when complexed with NF-YC4 , it localizes to the nucleus. Putative OXS2-binding DEGs were constitutively activated by OXS2.
AT3G30730		hypothetical protein
AT3G30740		pseudogene of Ribosomal protein S25 family protein
AT3G30750		transposable_element_gene;similar to unknown protein [Arabidopsis thaliana] (TAIR:AT2G24910.1);(source:TAIR10)
AT3G30775	*EARLY RESPONSIVE TO DEHYDRATION 5 (ERD5)*	Encodes a proline oxidase that is predicted to localize to the inner mitochondrial membrane, its mRNA expression induced by high levels of Al and by osmotic stress. The promoter contains an L-proline-inducible element.
AT3G30800		transposable_element_gene;gypsy-like retrotransposon family, has a 3.6e-27 P-value blast match to GB:CAB39733 rotease, reverse transcriptase, ribonuclease H, integrase (Gypsy_Ty3-element) (Drosophila buzzatii);(source:TAIR10)
AT3G30810		transposable_element_gene;similar to unknown protein [Arabidopsis thaliana] (TAIR:AT4G04070.1);(source:TAIR10)
AT3G30820		retrotransposon ORF-1 protein
AT3G30830		transposable_element_gene;copia-like retrotransposon family, has a 6.8e-26 P-value blast match to gb|AAG52950.1| putative envelope protein (Endovir1-1) (Arabidopsis thaliana) (Ty1_Copia-family);(source:TAIR10)
AT3G30840		hypothetical protein
AT3G30843		transposable_element_gene;similar to unknown protein [Arabidopsis thaliana] (TAIR:AT4G08056.1);(source:TAIR10)
AT3G30845		hypothetical protein
AT3G30846		transposable_element_gene;gypsy-like retrotransposon family, has a 1.0e-303 P-value blast match to GB:AAD27547 polyprotein (Gypsy_Ty3-element) (Oryza sativa subsp. indica);(source:TAIR10)
AT3G31023		transposable_element_gene;copia-like retrotransposon family, has a 1.0e-152 P-value blast match to GB:CAA72989 open reading frame 1 (Ty1_Copia-element) (Brassica oleracea);(source:TAIR10)
AT3G31300		transposable_element_gene;similar to unknown protein [Arabidopsis thaliana] (TAIR:AT3G32903.1);(source:TAIR10)
AT3G31314		transposable_element_gene;CACTA-like transposase family (Ptta/En/Spm), has a 2.3e-23 P-value blast match to At5g59620.1/14-257 CACTA-like transposase family (Ptta/En/Spm) (CACTA-element) (Arabidopsis thaliana);(source:TAIR10)
AT3G31320		transposable_element_gene;similar to unknown protein [Arabidopsis thaliana] (TAIR:AT1G40133.1);(source:TAIR10)
AT3G31330		transposable_element_gene;similar to unknown protein [Arabidopsis thaliana] (TAIR:AT5G34855.1);(source:TAIR10)
AT3G31340		transposable_element_gene;gypsy-like retrotransposon family (Athila), has a 7.3e-111 P-value blast match to GB:CAA57397 Athila ORF 1 (Arabidopsis thaliana);(source:TAIR10)
AT3G31350		hypothetical protein
AT3G31360		transposable_element_gene;gypsy-like retrotransposon family (Athila), has a 4.2e-25 P-value blast match to GB:CAA57397 Athila ORF 1 (Arabidopsis thaliana);(source:TAIR10)
AT3G31380		transposable_element_gene;copia-like retrotransposon family, has a 4.2e-20 P-value blast match to gb|AAO73529.1| gag-pol polyprotein (Glycine max) (SIRE1) (Ty1_Copia-family);(source:TAIR10)
AT3G31390		transposable_element_gene;gypsy-like retrotransposon family (Athila), has a 1.6e-31 P-value blast match to GB:CAA57397 Athila ORF 1 (Arabidopsis thaliana);(source:TAIR10)
AT3G31403		transposable_element_gene;Mutator-like transposase family, has a 6.0e-61 P-value blast match to O80466 /172-336 Pfam PF03108 MuDR family transposase (MuDr-element domain);(source:TAIR10)
AT3G31410		transposable_element_gene;similar to unknown protein [Arabidopsis thaliana] (TAIR:AT3G32140.1);(source:TAIR10)
AT3G31415		
AT3G31430		zinc knuckle (CCHC-type) family protein
AT3G31460		transposable_element_gene;retroelement pol polyprotein -related;(source:TAIR10)
AT3G31540		transposable_element_gene;similar to unknown protein [Arabidopsis thaliana] (TAIR:AT4G06603.1);(source:TAIR10)
AT3G31900		ATP-dependent helicase family protein
AT3G31910		Ulp1 protease family protein (DUF1985)
AT3G31915		transposable_element_gene;similar to unknown protein [Arabidopsis thaliana] (TAIR:AT3G31370.1);(source:TAIR10)
AT3G31920		transposable_element_gene;CACTA-like transposase family (Tnp2/En/Spm), has a 3.0e-163 P-value blast match to gb|AAG52024.1|AC022456_5 Tam1-homologous transposon protein TNP2, putative;(source:TAIR10)
AT3G31930		transposable_element_gene;similar to unknown protein [Arabidopsis thaliana] (TAIR:AT5G28940.1);(source:TAIR10)
AT3G31940		transposable_element_gene;similar to unknown protein [Arabidopsis thaliana] (TAIR:AT4G07460.1);(source:TAIR10)
AT3G31955		transposable_element_gene;similar to unknown protein [Arabidopsis thaliana] (TAIR:AT2G04210.1);(source:TAIR10)
AT3G31970		transposable_element_gene;gypsy-like retrotransposon family, has a 5.3e-294 P-value blast match to GB:AAD27547 polyprotein (Gypsy_Ty3-element) (Oryza sativa subsp. indica);(source:TAIR10)
AT3G32000		transposable_element_gene;gypsy-like retrotransposon family (Athila), has a 5.4e-305 P-value blast match to GB:CAA57397 Athila ORF 1 (Arabidopsis thaliana);(source:TAIR10)
AT3G32020		transposable_element_gene;pseudogene, hypothetical protein;(source:TAIR10)

AT3G32040	*GERANYL(GERANYL)DIPHOSPHATE SYNTHASE 1 (ATGGPS10)*	Chloroplast localized GFDP synthase.
AT3G32050		hypothetical protein
AT3G32060		transposable_element_gene;Mutator-like transposase family, has a 4.0e-63 P-value blast match to O80466 /172-336 Pfam PF03108 MuDR family transposase (MuDr-element domain);(source:TAIR10)
AT3G32080		transposable_element_gene;similar to unknown protein [Arabidopsis thaliana] (TAIR:AT3G29710.1);(source:TAIR10)
AT3G32090		WRKY family transcription factor
AT3G32100		
AT3G32110		transposable_element_gene;non-LTR retrotransposon family (LINE), has a 1.9e-52 P-value blast match to GB:AAA39398 ORF2 (Mus musculus) (LINE-element);(source:TAIR10)
AT3G32120		
AT3G32130		non-LTR retroelement reverse transcriptase
AT3G32140		transposable_element_gene;similar to unknown protein [Arabidopsis thaliana] (TAIR:AT3G31410.1);(source:TAIR10)
AT3G32150		myosin heavy chain-like protein
AT3G32160		hypothetical protein
AT3G32180		hypothetical protein
AT3G32190		myosin heavy chain-like protein
AT3G32200		myosin heavy chain-like protein
AT3G32220		transposable_element_gene;gypsy-like retrotransposon family (Athila), has a 1.5e-59 P-value blast match to GB:CAA57397 Athila ORF 1 (Arabidopsis thaliana);(source:TAIR10)
AT3G32250		transposable_element_gene;gypsy-like retrotransposon family (Athila), has a 2.7e-106 P-value blast match to GB:CAA57397 Athila ORF 1 (Arabidopsis thaliana);(source:TAIR10)
AT3G32260		Nucleic acid-binding proteins superfamily
AT3G32270		transposable_element_gene;similar to unknown protein [Arabidopsis thaliana] (TAIR:AT4G07310.1);(source:TAIR10)
AT3G32280		ATP-dependent helicase family protein
AT3G32290		transposable_element_gene;gypsy-like retrotransposon family, has a 9.0e-22 P-value blast match to GB:AAD19359 polyprotein (gypsy_Ty3-element) (Sorghum bicolor);(source:TAIR10)
AT3G32350		
AT3G32900		transposable_element_gene;similar to Ulp1 protease family protein [Arabidopsis thaliana] (TAIR:AT5G34900.1);(source:TAIR10)
AT3G32910		transposable_element_gene;similar to unknown protein [Arabidopsis thaliana] (TAIR:AT4G03990.1);(source:TAIR10)
AT3G32917		transposable_element_gene;gypsy-like retrotransposon family, has a 1.0e-114 P-value blast match to GB:AAD27547 polyprotein (Gypsy_Ty3-element) (Oryza sativa subsp. indica);(source:TAIR10)
AT3G32920		P-loop containing nucleoside triphosphate hydrolases superfamily protein
AT3G33064		transposable_element_gene;similar to unknown protein [Arabidopsis thaliana] (TAIR:AT1G41770.1);(source:TAIR10)
AT3G33066		transposable_element_gene;gypsy-like retrotransposon family (Athila), has a 6.1e-125 P-value blast match to gb|AAL06421.1|AF378079_1 reverse transcriptase (Athila4) (Arabidopsis thaliana) (Gypsy_Ty3-family);(source:TAIR10)
AT3G33073		transposable_element_gene
AT3G33080		transposable_element_gene;similar to unknown protein [Arabidopsis thaliana] (TAIR:AT3G30650.1);(source:TAIR10)
AT3G42060		myosin heavy chain-like protein
AT3G42070		transposable_element_gene;similar to unknown protein [Arabidopsis thaliana] (TAIR:AT1G38380.1);(source:TAIR10)
AT3G42080		transposable_element_gene;contains InterPro domain t-snare;(source:TAIR10)
AT3G42090		transposable_element_gene;contains domain LIN-9 RELATED (PTHR21689);(source:TAIR10)
AT3G42100		transposable_element_gene;similar to AT hook motif-containing protein-related [Arabidopsis thaliana] (TAIR:AT1G35940.1);(source:TAIR10)
AT3G42110		transposable_element_gene;similar to unknown protein [Arabidopsis thaliana] (TAIR:AT1G35920.1);(source:TAIR10)
AT3G42120		transposable_element_gene;similar to unknown protein [Arabidopsis thaliana] (TAIR:AT2G12610.1);(source:TAIR10)
AT3G42130		glycine-rich protein
AT3G42140		zinc ion binding / nucleic acid binding protein
AT3G42160		Pectin lyase-like superfamily protein
AT3G42190		transposable_element_gene;similar to cysteine-type peptidase [Arabidopsis thaliana] (TAIR:AT3G42820.1);(source:TAIR10)
AT3G42210		
AT3G42220		transposable_element_gene;CACTA-like transposase family (Tnp2/En/Spm), has a 1.9e-166 P-value blast match to gb|AAG52024.1|AC022456_5 Tam1-homologous transposon protein TNP2, putative;(source:TAIR10)
AT3G42240		transposable_element_gene;similar to unknown protein [Arabidopsis thaliana] (TAIR:AT1G43940.1);(source:TAIR10)
AT3G42250		transposable_element_gene;similar to unknown protein [Arabidopsis thaliana] (TAIR:AT1G48720.1);(source:TAIR10)
AT3G42260		transposable_element_gene;gypsy-like retrotransposon family (Athila), has a 2.3e-60 P-value blast match to GB:CAA57397 Athila ORF 1 (Arabidopsis thaliana);(source:TAIR10)
AT3G42270		transposable_element_gene;gypsy-like retrotransposon family (Athila), has a 9.1e-62 P-value blast match to GB:CAA57397 Athila ORF 1 (Arabidopsis thaliana);(source:TAIR10)
AT3G42290		transposable_element_gene;retrotransposon family;(source:TAIR10)
AT3G42300		transposable_element_gene;similar to unknown protein [Arabidopsis thaliana] (TAIR:AT3G42120.1);(source:TAIR10)
AT3G42310		hypothetical protein

AT3G42320		transposable_element_gene;similar to unknown protein [Arabidopsis thaliana] (TAIR:AT1G64410.1);(source:TAIR10)
AT3G42330		transposable_element_gene;contains InterPro domain DNA polymerase III clamp loader subunit, C-terminal;(source:TAIR10)
AT3G42340		transposable_element_gene;similar to unknown protein [Arabidopsis thaliana] (TAIR:AT1G52960.1);(source:TAIR10)
AT3G42350		transposable_element_gene;similar to unknown protein [Arabidopsis thaliana] (TAIR:AT2G06095.1);(source:TAIR10)
AT3G42360		transposable_element_gene;pseudogene, similar to Putative 22 kDa kafirin cluster;(source:TAIR10)
AT3G42370		
AT3G42380		
AT3G42390		hypothetical protein
AT3G42400		transposable_element_gene;similar to unknown protein [Arabidopsis thaliana] (TAIR:AT5G33200.1);(source:TAIR10)
AT3G42420		transposable_element_gene;pseudogene, similar to putative helicase, various predicted proteins, including predicted Helicases.;(source:TAIR10)
AT3G42430		transposable_element_gene;similar to unknown protein [Arabidopsis thaliana] (TAIR:AT1G37045.1);(source:TAIR10)
AT3G42440		transposable_element_gene;similar to unknown protein [Arabidopsis thaliana] (TAIR:AT1G35090.1);(source:TAIR10)
AT3G42460		transposable_element_gene;similar to unknown protein [Arabidopsis thaliana] (TAIR:AT4G05570.1);(source:TAIR10)
AT3G42480		transposable_element_gene;similar to unknown protein [Arabidopsis thaliana] (TAIR:AT4G03979.1);(source:TAIR10)
AT3G42500		transposable_element_gene;similar to unknown protein [Arabidopsis thaliana] (TAIR:AT1G44860.1);(source:TAIR10)
AT3G42530		transposable_element_gene;similar to Ulp1 protease family protein [Arabidopsis thaliana] (TAIR:AT2G10350.1);(source:TAIR10)
AT3G42540		transposable_element_gene;similar to unknown protein [Arabidopsis thaliana] (TAIR:AT1G43940.1);(source:TAIR10)
AT3G42550		Eukaryotic aspartyl protease family protein
AT3G42580		transposable_element_gene;similar to Ulp1 protease family protein [Arabidopsis thaliana] (TAIR:AT2G12100.1);(source:TAIR10)
AT3G42590		transposable_element_gene;similar to unknown protein [Arabidopsis thaliana] (TAIR:AT2G12110.1);(source:TAIR10)
AT3G42600		transposable_element_gene;similar to unknown protein [Arabidopsis thaliana] (TAIR:AT2G12120.1);(source:TAIR10)
AT3G42640	*H(+)-ATPASE 8 (HA8)*	H[+]-ATPase 8
AT3G42680		transposable_element_gene;similar to unknown protein [Arabidopsis thaliana] (TAIR:AT2G05647.1);(source:TAIR10)
AT3G42690		transposable_element_gene;similar to Ulp1 protease family protein [Arabidopsis thaliana] (TAIR:AT4G04130.1);(source:TAIR10)
AT3G42700		transposable_element_gene;similar to Ulp1 protease family protein [Arabidopsis thaliana] (TAIR:AT4G04130.1);(source:TAIR10)
AT3G42710		transposable_element_gene;pseudogene, hypothetical protein;(source:TAIR10)
AT3G42725		Putative membrane lipoprotein
AT3G42730		transposable_element_gene;similar to Ulp1 protease family protein [Arabidopsis thaliana] (TAIR:AT1G27780.1);(source:TAIR10)
AT3G42770	*PHOSPHATE RESPONSE UBIQUITIN E3 LIGASE 1 (PRU1)*	E3 ligase involved in phosphate homeostasis. Under low Pi stress it targets WRKY6(AT1G62300) for degradation which in turn is a repressor of PHO1(AT3G23430).
AT3G42780		hypothetical protein
AT3G42800		AF-like protein
AT3G42820		transposable_element_gene;similar to Ulp1 protease family protein [Arabidopsis thaliana] (TAIR:AT1G35770.1);(source:TAIR10)
AT3G42830		RING/U-box superfamily protein
AT3G42860		zinc knuckle (CCHC-type) family protein
AT3G42880	*POLLEN RECEPTOR LIKE KINASE 3 (PRK3)*	Leucine-rich repeat protein kinase family protein
AT3G42900		transposable_element_gene;Mutator-like transposase family, has a 3.7e-44 P-value blast match to Q9SHN7 /450-633 Pfam PF03108 MuDR family transposase (MuDr-element domain);(source:TAIR10)
AT3G42910		transposable_element_gene;similar to Ulp1 protease family protein [Arabidopsis thaliana] (TAIR:AT5G28250.1);(source:TAIR10)
AT3G42920		transposable_element_gene;similar to Ulp1 protease family protein [Arabidopsis thaliana] (TAIR:AT4G04390.1);(source:TAIR10)
AT3G42930		transposable_element_gene;CACTA-like transposase family (Ptta/En/Spm), has a 1.0e-43 P-value blast match to At5g29026.1/8-244 CACTA-like transposase family (Ptta/En/Spm) (CACTA-element) (Arabidopsis thaliana);(source:TAIR10)
AT3G42940		C3HC4-type RING finger protein
AT3G42950	*POLYGALACTURONASE CLADE F 6 (PGF6)*	Pectin lyase-like superfamily protein
AT3G42960	*TAPETUM 1 (ATA1)*	Arabidopsis homolog of TASSELSEED2. Expressed specifically in tapetal cells.
AT3G42970		
AT3G42980		transposable_element_gene;similar to unknown protein [Arabidopsis thaliana] (TAIR:AT2G14450.1);(source:TAIR10)
AT3G42990		hypothetical protein
AT3G43010		
AT3G43020		transposable_element_gene;pseudogene, hypothetical protein, predicted proteins, Arabidopsis thaliana;(source:TAIR10)
AT3G43040		transposable_element_gene;pseudogene, hypothetical protein, includes At2g10370, At5g36050, At1g35090, At1g44860, At4g19300, At1g42400, At3g43040, At3g42500;(source:TAIR10)
AT3G43050		transposable_element_gene;Mutator-like transposase family, has a 5.0e-39 P-value blast match to Q9SUF8 /145-308 Pfam PF03108 MuDR family transposase (MuDr-element domain);(source:TAIR10)
AT3G43060		transposable_element_gene;pseudogene, hypothetical protein;(source:TAIR10)
AT3G43070		transposable_element_gene;pseudogene, hypothetical protein;(source:TAIR10)
AT3G43080		transposable_element_gene;Mutator-like transposase family, has a 2.8e-60 P-value blast match to Q9S9L6 /322-461 Pfam PF03108 MuDR family transposase (MuDr-element domain);(source:TAIR10)
AT3G43100		transposable_element_gene;similar to unknown protein [Arabidopsis thaliana] (TAIR:AT3G30550.1);(source:TAIR10)
AT3G43110		transmembrane protein

AT3G43120	*SMALL AUXIN UPREGULATED RNA 39 (SAUR39)*	SAUR-like auxin-responsive protein family
AT3G43130		transposable_element_gene;pseudogene, hypothetical protein, predicted proteins, Arabidopsis thaliana;(source:TAIR10)
AT3G43140		transposable_element_gene
AT3G43150		hypothetical protein
AT3G43160	*MATERNAL EFFECT EMBRYO ARREST 38 (MEE38)*	maternal effect embryo arrest 38
AT3G43170	*DUF295 ORGANELLAR B 2 (ATDOB2)*	hypothetical protein
AT3G43190	*SUCROSE SYNTHASE 4 (SUS4)*	Encodes a protein with sucrose synthase activity (SUS4).
AT3G43210	*TETRASPORE (TES)*	Encodes a kinesin TETRASPORE. Required for cytokinesis in pollen. In mutants, all four microspore nuclei remain within the same cytoplasm after meiosis.
AT3G43250		coiled-coil protein (DUF572)
AT3G43270		Plant invertase/pectin methylesterase inhibitor superfamily
AT3G43280		hypothetical protein
AT3G43310		pseudogene of myosin heavy chain-like protein
AT3G43320		transposable_element_gene;similar to unknown protein [Arabidopsis thaliana] (TAIR:AT1G37050.1);(source:TAIR10)
AT3G43330		transposable_element_gene;similar to unknown protein [Arabidopsis thaliana] (TAIR:AT3G30560.1);(source:TAIR10)
AT3G43350		transposable_element_gene;similar to unknown protein [Arabidopsis thaliana] (TAIR:AT1G52960.1);(source:TAIR10)
AT3G43390		transposable_element_gene;similar to Ulp1 protease family protein [Arabidopsis thaliana] (TAIR:AT1G27780.1);(source:TAIR10)
AT3G43400	*(ELMOD_D)*	Member of ELMOD family.
AT3G43420		hypothetical protein
AT3G43430		RING/U-box superfamily protein
AT3G43460		transposable_element_gene;similar to unknown protein [Arabidopsis thaliana] (TAIR:AT1G31150.1);(source:TAIR10)
AT3G43480		CCHC-type zinc knuckle protein
AT3G43500		hypothetical protein
AT3G43510		transposable_element_gene;copia-like retrotransposon family, has a 2.3e-11 P-value blast match to GB:BAA11674 ORF(AA 1-1338) (Ty1_Copia-element) (Nicotiana tabacum);(source:TAIR10)
AT3G43530		transposable_element_gene;similar to unknown protein [Arabidopsis thaliana] (TAIR:AT3G15600.1);(source:TAIR10)
AT3G43540		initiation factor 4F subunit (DUF1350)
AT3G43550		GDSL-motif esterase/acyltransferase/lipase. Enzyme group with broad substrate specificity that may catalyze acyltransfer or hydrolase reactions with lipid and non-lipid substrates.
AT3G43560		
AT3G43600	*ALDEHYDE OXIDASE 2 (AAO2)*	Encodes an aldehyde oxidase. AAO2 does not appear to act on abscisic aldehyde in vitro but it is possible that it may function in abscisic acid biosynthesis when the activity of At2g27150 (AAO3), the primary abscisic aldehyde oxidase, is lost.
AT3G43610		Spc97 / Spc98 family of spindle pole body (SBP) component
AT3G43630		The gene encodes nodulin-like3 whose transcript abundance was repressed under conditions of Fe-deficient growth.
AT3G43640		transposable_element_gene;copia-like retrotransposon family, has a 1.4e-25 P-value blast match to gb|AAG52950.1| putative envelope protein (Endovir1-1) (Arabidopsis thaliana) (Ty1_Copia-family);(source:TAIR10)
AT3G43650		transposable_element_gene;CACTA-like transposase family (Ptta/En/Spm), has a 3.0e-43 P-value blast match to At5g36655.1/81-333 CACTA-like transposase family (Ptta/En/Spm) (CACTA-element) (Arabidopsis thaliana);(source:TAIR10)
AT3G43660		The gene encodes a putative nodulin-like21 protein.
AT3G43670	*COPPER AMINE OXIDASE GAMMA 2 (CuAOgamma2)*	Copper amine oxidase family protein
AT3G43680		transposable_element_gene;similar to unknown protein [Arabidopsis thaliana] (TAIR:AT3G60930.1);(source:TAIR10)
AT3G43690		transposable_element_gene;copia-like retrotransposon family protein, has a 1.4e-29 P-value blast match to gb|AAG52950.1| putative envelope protein (Endovir1-1) (Arabidopsis thaliana) (Ty1_Copia-family);(source:TAIR10)
AT3G43700	*BTB-POZ AND MATH DOMAIN 6 (BPM6)*	Encodes a member of the MATH-BTB domain proteins (BPMs) that directly interact with and target for proteasomal degradation the class I homeobox-leucine zipper (HD-ZIP) transcription factor ATHB6. Known members include AT5G19000 (BPM1), AT3G06190 (BPM2), AT2G39760 (BPM3), AT3G03740 (BPM4), AT5G21010 (BPM5) and AT3G43700 (BPM6).
AT3G43720	*GLYCOSYLPHOSPHATIDYLINOSITOL-ANCHORED LIPID PROTEIN TRANSFER 2 (LTPG2)*	Bifunctional inhibitor/lipid-transfer protein/seed storage 2S albumin superfamily protein
AT3G43730		transposable_element_gene;pseudogene, similar to ring-infested erythrocyte surface antigen, contains Pfam profile PF03384: Drosophila protein of unknown function, DUF287;(source:TAIR10)
AT3G43750	*RING FINGER ABA-RELATED 1 (RFA1)*	E3 ubiquitin ligases, member of the RING between RING fingers (RBR)-type RSL1/RFA family, are key regulators of ABA receptor stability in root and leaf tissues, targeting ABA receptors for degradation in different subcellular locations.
AT3G43760		transposable_element_gene;similar to unknown protein [Arabidopsis thaliana] (TAIR:AT4G11710.1);(source:TAIR10)
AT3G43770		transposable_element_gene;similar to disease resistance protein (TIR-NBS-LRR class), putative [Arabidopsis thaliana] (TAIR:AT5G45230.1);(source:TAIR10)
AT3G43790	*ZINC INDUCED FACILITATOR-LIKE 2 (ZIFL2)*	zinc induced facilitator-like 2
AT3G43800	*GLUTATHIONE S-TRANSFERASE TAU 27 (GSTU27)*	Encodes glutathione transferase belonging to the tau class of GSTs. Naming convention according to Wagner et al. (2002). The mRNA is cell-to-cell mobile.
AT3G43820		pseudogene of Copper amine oxidase family protein
AT3G43840	*POLYPRENOL REDUCTASE 3 (PPRD3)*	3-oxo-5-alpha-steroid 4-dehydrogenase family protein
AT3G43860	*GLYCOSYL HYDROLASE 9A4 (GH9A4)*	glycosyl hydrolase 9A4

AT3G43880		hypothetical protein
AT3G43900		
AT3G43920	*DICER-LIKE 3 (DCL3)*	Encodes a ribonuclease III family protein that is required for endogenous RDR2-dependent siRNA (but not miRNA) formation.
AT3G43930		BRCT domain-containing DNA repair protein
AT3G43940		hypothetical protein
AT3G43950		Protein kinase superfamily protein
AT3G43960		Encodes a putative cysteine proteinase. Mutants exhibit shorter root hairs under phosphate-deficient conditions.
AT3G43970		hypothetical protein
AT3G43990		Bromo-adjacent homology (BAH) domain-containing protein
AT3G44000		transposable_element_gene;Mutator-like transposase family, has a 1.9e-07 P-value blast match to GB:AAA21566 mudrA of transposon=MuDR (MuDr-element) (Zea mays);(source:TAIR10)
AT3G44020		thylakoid lumenal P17.1 protein
AT3G44030		pseudogene of nuclease
AT3G44040		
AT3G44050	*(KINESIN-12E)*	Kinesin involved in spindle assembly.
AT3G44080		F-box family protein
AT3G44100		MD-2-related lipid recognition domain-containing protein
AT3G44140		transmembrane protein
AT3G44180		syntaxin-related family protein
AT3G44210		hypothetical protein
AT3G44230		transmembrane protein
AT3G44240	*CCR4-ASSOCIATED FACTOR 1F (CAF1F)*	Polynucleotidyl transferase, ribonuclease H-like superfamily protein
AT3G44250	*CYTOCHROME P450, FAMILY 71, SUBFAMILY B, POLYPEPTIDE 38 (CYP71B38)*	putative cytochrome P450
AT3G44260	*CCR4- ASSOCIATED FACTOR 1A (CAF1a)*	Encodes one of the homologs of the yeast CCR4-associated factor 1: AT3G44260 (CAF1a), AT5G22250 (CAF1b). Has mRNA deadenylation activity. Also plays a role in plant defense responses.
AT3G44270		transposable_element_gene;copia-like retrotransposon family, has a 1.1e-26 P-value blast match to gb|AAG52950.1| putative envelope protein (Endovir1-1) (Arabidopsis thaliana) (Ty1_Copia-family);(source:TAIR10)
AT3G44320	*NITRILASE 3 (NIT3)*	This enzyme catalyzes the hydrolysis of indole-3-acetonitrile (IAN) to indole-3-acetic acid (IAA) (EC 3.5.5.1) and IAN to indole-3-acetamide (IAM) at lower levels. It is the only one of the four Arabidopsis nitrilases whose mRNA levels are strongly induced when plants experience sulphur deprivation. This enzyme likely participates in other non-auxin-related metabolic pathways.
AT3G44330		M28 Zn-peptidase nicastrin
AT3G44350	*NAC DOMAIN CONTAINING PROTEIN 61 (NAC061)*	NAC domain containing protein 61
AT3G44370	*(OXA2B)*	Member of the Oxa1 super family protein insertases.It is structurally distinct having a tetratricopeptide repeat (TPR) domain at the C terminus. Paralog of OXA2a. Involved in biogenesis of mitochondrial respiratory chain complex IV, specifically via membrane insertion of COX2.
AT3G44420		transposable_element_gene;pseudogene, hypothetical protein;(source:TAIR10)
AT3G44430		transmembrane protein
AT3G44440		hypothetical protein
AT3G44450	*BLUE-LIGHT INHIBITOR OF CRYPTOCHROMES 2 (BIC2)*	Plant specific protein.BIC1 and BIC2 inhibit cryptochrome function by blocking blue light-dependent cryptochrome dimerization.Light activated transcription of BICs is mediated by cryptochromes.
AT3G44460	*(DPBF2)*	basic leucine zipper transcription factor (BZIP67), identical to basic leucine zipper transcription factor GI:18656053 from (Arabidopsis thaliana); identical to cDNA basic leucine zipper transcription factor (atbzip67 gene) GI:18656052. Located in the nucleus and expressed during seed maturation in the cotyledons.
AT3G44470		transposable_element_gene
AT3G44490	*HISTONE DEACETYLASE 17 (hda17)*	histone deacetylase 17
AT3G44500		transposable_element_gene;similar to Ulp1 protease family protein [Arabidopsis thaliana] (TAIR:AT3G47260.1);(source:TAIR10)
AT3G44510		alpha/beta-Hydrolases superfamily protein
AT3G44520		
AT3G44540	*FATTY ACID REDUCTASE 4 (FAR4)*	Encodes a member of the eight-member gene family encoding alcohol-forming fatty acyl-CoA reductases (FARs) identified in Arabidopsis thaliana. Three of the FARs, FAR1 (At5g22500), FAR4 (At3g44540) and FAR5 (At3g44550), are shown to generate the fatty alcohols found in root, seed coat, and wound-induced leaf tissue. The mRNA is cell-to-cell mobile.
AT3G44550	*FATTY ACID REDUCTASE 5 (FAR5)*	Encodes a member of the eight-member gene family encoding alcohol-forming fatty acyl-CoA reductases (FARs) identified in Arabidopsis thaliana. Three of the FARs, FAR1 (At5g22500), FAR4 (At3g44540) and FAR5 (At3g44550), are shown to generate the fatty alcohols found in root, seed coat, and wound-induced leaf tissue. The mRNA is cell-to-cell mobile.
AT3G44560	*FATTY ACID REDUCTASE 8 (FAR8)*	fatty acid reductase 8
AT3G44570		retrotransposon ORF-1 protein
AT3G44580		hypothetical protein
AT3G44590	*RIBOSOMAL PROTEIN P2D (RPP2D)*	cytosolic ribosomal protein gene, part of bL12 family
AT3G44610	*AGCVIII KINASE 1-12 (AGC1-12)*	Kinase involved in the first positive phototropism and gravitropism. Phosphorylates serine residues in the cytoplasmic loop of PIN1 and shares phosphosite preferences with D6PK. Critical component for both hypocotyl phototropism and gravitropism, control tropic responses mainly through regulation of PIN-mediated auxin transport by protein phosphorylation.

AT3G44630		Disease resistance protein (TIR-NBS-LRR class) family
AT3G44680	*HISTONE DEACETYLASE 9 (HDA9)*	Encodes HDA9 (a RPD3-like histone deacetylase). Functions in promoting the onset of leaf senescence.The hda9 mutant shows enhanced H3K9 acetylation levels,based on immunodetection using H3K9ac antibodies. Negatively controls gene expression in concert with interacting proteins POWERDRESS (PWR), HIGH EXPRESSION OF OSMOTICALLY RESPONSIVE GENES 15 (HOS15), WRKY53, ELONGATED HYPOCOTYL 5 (HY5), ABA INSENSITIVE 4 (ABI4) and EARLY FLOWERING 3 (ELF3). Involved in mutual negative feedback regulation with WRKY53. Mutations lead to a mild early flowering phenotype under SD.
AT3G44690		hypothetical protein
AT3G44730	*KINESIN-LIKE PROTEIN 1 (KP1)*	kinesin-like protein 1
AT3G44735	*PHYTOSULFOKINE 3 PRECURSOR (PSK3)*	Phytosulfokine 3 precursor, coding for a unique plant peptide growth factor.
AT3G44750	*HISTONE DEACETYLASE 3 (HDA3)*	Encodes a histone deacetylase. Controls the development of adaxial/abaxial leaf polarity. Two lines with RNAi-directed against this gene show reduced Agrobacterium-mediated DNA transformation of the roots. Involved in development of the vascular tissue of the stem by affecting cell proliferation and differentiation.
AT3G44760		transmembrane protein
AT3G44770		transmembrane protein, putative (DUF626)
AT3G44780		Cysteine proteinases superfamily protein
AT3G44800		Meprin and TRAF (MATH) homology domain-containing protein
AT3G44830		Lecithin:cholesterol acyltransferase family protein
AT3G44840		SABATH methyltransferase
AT3G44860	*FARNESOIC ACID CARBOXYL-O-METHYLTRANSFERASE (FAMT)*	Encodes a farnesoic acid carboxyl-O-methyltransferase. The mRNA is cell-to-cell mobile.
AT3G44880	*ACCELERATED CELL DEATH 1 (ACD1)*	Encodes a pheide a oxygenase (PAO). Accelerated cell death (acd1) mutants show rapid, spreading necrotic responses to both virulent and avirulent Pseudomonas syringae pv. maculicola or pv. tomato pathogens and to ethylene.
AT3G44890	*RIBOSOMAL PROTEIN L9 (RPL9)*	Plastid ribosomal protein CL9 The mRNA is cell-to-cell mobile.
AT3G44900	*CATION/H+ EXCHANGER 4 (CHX4)*	member of Putative Na+/H+ antiporter family
AT3G44910	*CATION/H+ EXCHANGER 12 (CHX12)*	member of Putative Na+/H+ antiporter family
AT3G44930	*CATION/H+ EXCHANGER 10 (CHX10)*	member of Putative Na+/H+ antiporter family
AT3G44940		enabled-like protein (DUF1635)
AT3G44950		glycine-rich protein
AT3G44960		shugoshin
AT3G44970		Cytochrome P450 superfamily protein
AT3G44980		hypothetical protein
AT3G44990	*XYLOGLUCAN ENDOTRANSGLUCOSYLASE/HYDROLASE 31 (XTH31)*	Encodes a xyloglucan endotransglycosylase/hydrolase. Protein sequence and phylogenetic analysis indicates that this enzyme resides in Group III-A of the XTH family, with high similarity to Tropaeolum majus (nasturtium) xyloglucanase 1 (TmNXG1).Enzyme kinetic analysis indicates predominant xyloglucan endo-hydrolase activity (EC 3.2.1.151) with only limited potential to act as a xyloglucan endo-transglycosylase (EC 2.4.1.207).
AT3G45000	*(VPS24.2)*	SNF7 family protein
AT3G45010	*SERINE CARBOXYPEPTIDASE-LIKE 48 (scpl48)*	serine carboxypeptidase-like 48
AT3G45060	*HIGH AFFINITY NITRATE TRANSPORTER 2.6 (NRT2.6)*	member of High affinity nitrate transporter family
AT3G45070	*SULFOTRANSFERASE 202B1 (SULT202B1)*	Encodes a sulfotransferase with sulfating activity toward flavonoids.
AT3G45090		P-loop containing nucleoside triphosphate hydrolases superfamily protein
AT3G45100	*(SETH2)*	encodes Arabidopsis homolog of a conserved protein involved in the first step of the GPI biosynthetic pathway.
AT3G45110		hypothetical protein
AT3G45120		transposable_element_gene;similar to unknown protein [Arabidopsis thaliana] (TAIR:AT1G17900.1);(source:TAIR10)
AT3G45130	*LANOSTEROL SYNTHASE 1 (LAS1)*	lanosterol synthase 1
AT3G45140	*LIPOXYGENASE 2 (LOX2)*	Chloroplast lipoxygenase required for wound-induced jasmonic acid accumulation in Arabidopsis.Mutants are resistant to Staphylococcus aureus and accumulate salicylic acid upon infection. The mRNA is cell-to-cell mobile.
AT3G45160		Putative membrane lipoprotein
AT3G45190		SIT4 phosphatase-associated family protein
AT3G45210		transcription initiation factor TFIID subunit (Protein of unknown function, DUF584)
AT3G45220		Serine protease inhibitor (SERPIN) family protein
AT3G45240	*GEMINIVIRUS REP INTERACTING KINASE 1 (GRIK1)*	Encodes a geminivirus Rep interacting kinase (GRIK; GRIK1/AT3G45240, GRIK2/AT5G60550). GRIKs are SnRK1 (SNF1-related kinases) activating kinases. Both GRIKs specifically bind to the SnRK1 catalytic subunit and phosphorylate the equivalent threonine residue in its activation loop in vitro. Involved in resistance to S. sclerotiorum, fungal sRNA target.
AT3G45250		transposable_element_gene;gypsy-like retrotransposon family, has a 9.2e-08 P-value blast match to GB:AAD22153 polyprotein (Gypsy_Ty3-element) (Sorghum bicolor);(source:TAIR10)
AT3G45260	*BALDIBIS (BIB)*	BIB is a member of the BIRD family of zinc finger proteins that includes JKD. BIB functions redundantly with JKD to retain SHR in the nucleus and thereby restrict SHR movement in root tissues.
AT3G45270		transposable_element_gene;hAT-like transposase family (hobo/Ac/Tam3), has a 5.7e-58 P-value blast match to GB:AAD24567 transposase Tag2 (hAT-element) (Arabidopsis thaliana);(source:TAIR10)
AT3G45280	*SYNTAXIN OF PLANTS 72 (SYP72)*	syntaxin of plants 72 (SYP72)

AT3G45290	*MILDEW RESISTANCE LOCUS O 3 (MLO3)*	A member of a large family of seven-transmembrane domain proteins specific to plants, homologs of the barley mildew resistance locus o (MLO) protein. The Arabidopsis genome contains 15 genes encoding MLO proteins, with localization in plasma membrane. Phylogenetic analysis revealed four clades of closely-related AtMLO genes. ATMLO3 belongs to the clade IV, with AtMLO2, AtMLO6 and AtMLO12. The gene is expressed during early seedling growth, in primary root and lateral root primordia, in fruit abscission zone, in vascular system of cotyledons and in trichomes of young leaves,; it was not expressed in mature rosette leaves, as shown by GUS activity patterns. The expression of several phylogenetically closely-related AtMLO genes showed similar or overlapping tissue specificity and analogous responsiveness to external stimuli, suggesting functional redundancy, co-function, or antagonistic function(s).
AT3G45310		Cysteine proteinases superfamily protein
AT3G45320		transmembrane protein
AT3G45330	*L-TYPE LECTIN RECEPTOR KINASE I.1 (LECRK-I.1)*	Concanavalin A-like lectin protein kinase family protein
AT3G45360		transposable_element_gene;similar to unknown protein [Arabidopsis thaliana] (TAIR:AT4G04394.1);(source:TAIR10)
AT3G45380		transposable_element_gene;similar to unknown protein [Arabidopsis thaliana] (TAIR:AT4G04400.1);(source:TAIR10)
AT3G45390	*L-TYPE LECTIN RECEPTOR KINASE I.2 (LECRK-I.2)*	LOW protein: L-type lectin-domain receptor kinase-like protein
AT3G45410	*L-TYPE LECTIN RECEPTOR KINASE I.3 (LECRK-I.3)*	encodes a receptor-like kinase that has serine/threonine kinase activity whose expression is induced by high salt stress. This induction is inhibited by tobacco ethylene receptor.
AT3G45430	*L-TYPE LECTIN RECEPTOR KINASE I.5 (LECRK-I.5)*	Extracellular ATP transmembrane receptor involved in innate immunity.
AT3G45450		Double Clp-N motif-containing P-loop nucleoside triphosphate hydrolases superfamily protein
AT3G45460	*RING FINGER ABA-RELATED 7 (RFA7)*	IBR domain containing protein
AT3G45480	*RING FINGER ABA-RELATED 6 (RFA6)*	RING/U-box protein with C6HC-type zinc finger
AT3G45490		reverse transcriptase-like protein
AT3G45500		hypothetical protein
AT3G45510		RING/U-box protein
AT3G45530		Cysteine/Histidine-rich C1 domain family protein
AT3G45540	*RING FINGER ABA-RELATED 8 (RFA8)*	RING/U-box protein with C6HC-type zinc finger
AT3G45560		zinc finger (C3HC4-type RING finger) family protein
AT3G45580	*RING FINGER ABA-RELATED 3 (RFA3)*	RING/U-box protein with C6HC-type zinc finger
AT3G45590	*SPLICING ENDONUCLEASE 1 (SEN1)*	Encodes a catalytic subunit of tRNA splicing endonuclease.
AT3G45600	*TETRASPANIN3 (TET3)*	Member of TETRASPANIN family
AT3G45610	*DOF TRANSCRIPTION FACTOR 6 (DOF6)*	PEAR protein involved in the formation of a short-range concentration gradient that peaks at protophloem sieve elements, and activates gene expression that promotes radial growth. Locally promotes transcription of inhibitory HD-ZIP III genes, and thereby establishes a negative-feedback loop that forms a robust boundary that demarks the zone of cell division.
AT3G45620		This gene is predicted to encode a protein with a DWD motif. It can bind to DDB1a in Y2H assays, and DDB1b in co-IP assays, and may be involved in the formation of a CUL4-based E3 ubiquitin ligase
AT3G45630	*(NOT4B)*	RNA binding (RRM/RBD/RNP motifs) family protein
AT3G45640	*MITOGEN-ACTIVATED PROTEIN KINASE 3 (MPK3)*	Encodes a mitogen-activated kinase whose mRNA levels increase in response to touch, cold, salinity stress and chitin oligomers.Also functions in ovule development. Heterozygous MPK3 mutants in a homozygous MPK6 background are female sterile due to defects in integument development. MPK3 can be dephosphorylated by MKP2 in vitro. The mRNA is cell-to-cell mobile.
AT3G45650	*NRT1/ PTR FAMILY 2.7 (NPF2.7)*	Encodes a nitrate efflux transporter NAXT1 (for NITRATE EXCRETION TRANSPORTER1). Localized to the plasma membrane. NAXT1 belongs to a subclass of seven NAXT members from the large NITRATE TRANSPORTER1/PEPTIDE TRANSPORTER family and is mainly expressed in the cortex of mature roots.
AT3G45670		Protein kinase superfamily protein
AT3G45680	*(NPF2.3)*	Major facilitator superfamily protein
AT3G45690		Encodes a member of the NAXT NPF subfamily.
AT3G45700	*NITRATE EXCRETION TRANSPORTER (NPF2.4)*	NPF2.4 is a member of the NAXT NPF subfamily. It encodes a plasmamembrane localized chloride transporter that is expressed in the root and is down regulated in response to ABA and salt treatment. NPF2.3 miRNA induced knockdowns have less Cl in the shoots when grown on low NaCl concentrations.
AT3G45710	*(NPF2.5)*	Encodes a chloride permeable transporter. Modulates chloride efflux from roots.
AT3G45720		Encodes a member of the NAXT NPF subfamily.
AT3G45730		hypothetical protein
AT3G45770		Encodes a mitochondrial enoyl-ACP reductase, a component of the fatty acid synthase complex.
AT3G45780	*PHOTOTROPIN 1 (PHOT1)*	Blue-light photoreceptor. Contains a light activated serine-threonine kinase domain and LOV1 and LOV2 repeats. Mutants are defective in blue-light response. Mediates blue light-induced growth enhancements. PHOT1 and PHOT2 mediate blue light-dependent activation of the plasma membrane H+-ATPase in guard cell protoplasts. PHOT1 undergoes blue-light-dependent autophosphorylation. At least eight phosphorylation sites have been identified in PHOT1. Phosphorylation of serine851 in the activation loop of PHOT1 appears to be required for stomatal opening, chloroplast accumulation, leaf flattening, and phototropism, and phosphorylation of serine849 may also contribute to the regulation of these responses. Phosphorylation-dependent binding of 14-3-3 proteins to the Hinge1 region of PHOT1 appears to require serine350 and serine376.
AT3G45800		Plant protein 1589 of unknown function
AT3G45810	*RESPIRATORY BURST OXIDASE HOMOLOG J (RBOHJ)*	ferric reductase-like transmembrane component family protein
AT3G45820		hypothetical protein
AT3G45840		Cysteine/Histidine-rich C1 domain family protein

AT3G45860	*CYSTEINE-RICH RLK (RECEPTOR-LIKE PROTEIN KINASE) 4 (CRK4)*	Encodes a cysteine-rich receptor-like protein kinase. Involved in programmed cell death and defense response to pathogen.
AT3G45870	*USUALLY MULTIPLE ACIDS MOVE IN AND OUT TRANSPORTERS 3 (UMAMIT3)*	nodulin MtN21-like transporter family protein
AT3G45880	*JUMONJI 32 (JMJ32)*	2-oxoglutarate (2OG) and Fe(II)-dependent oxygenase superfamily protein
AT3G45900		Ribonuclease P protein subunit P38-like protein
AT3G45910		hypothetical protein
AT3G45920		Protein kinase superfamily protein
AT3G45930		Histone superfamily protein
AT3G45940		Glycosyl hydrolases family 31 protein
AT3G45950		Pre-mRNA splicing Prp18-interacting factor
AT3G45960	*EXPANSIN-LIKE A3 (EXLA3)*	member of EXPANSIN-LIKE. Naming convention from the Expansin Working Group (Kende et al, 2004. Plant Mol Bio)
AT3G45970	*EXPANSIN-LIKE A1 (EXLA1)*	member of EXPANSIN-LIKE. Naming convention from the Expansin Working Group (Kende et al, 2004. Plant Mol Bio) The mRNA is cell-to-cell mobile.
AT3G45980	*(HTB9)*	Encodes a histone 2B (H2B) protein. This protein can be ubiquitinated in planta, and this modification depends on the HUB1 and HUB2 E3 ubiquitin ligases as well as the UBC1 and UBC2 E2 ubiquitin conjugating enzymes. Lysine 146 appears to be the site of the ubiquitin addition.
AT3G45990		Cofilin/tropomyosin-type actin-binding protein family
AT3G46000	*ACTIN DEPOLYMERIZING FACTOR 2 (ADF2)*	Encodes depolymerizing factor 2.
AT3G46030	*(HTB11)*	Histone superfamily protein
AT3G46070		C2H2-type zinc finger family protein
AT3G46100	*HISTIDYL-TRNA SYNTHETASE 1 (HRS1)*	histidyl-tRNA synthetase
AT3G46110	*SOSEKI4 (SOK4)*	DUF966 domain containing protein, expressed during embryogenesis.
AT3G46130	*MYB DOMAIN PROTEIN 48 (MYB48)*	Encodes a putative transcription factor (MYB48) that functions to regulate flavonol biosynthesis primarily in cotyledons.
AT3G46150		hypothetical protein
AT3G46170		NAD(P)-binding Rossmann-fold superfamily protein
AT3G46180	*UDP-GALACTOSE TRANSPORTER 5 (UTR5)*	UDP-galactose transporter 5
AT3G46210		Ribosomal protein S5 domain 2-like superfamily protein
AT3G46220	*(UFL1)*	E3 ligase that mediates ufmylation. Part of complex with C53 and the ER-resident adaptor protein DDRGK1. Involved in the pathway that links ribosome-associated quality control with selective autophagy at the ER.
AT3G46230	*HEAT SHOCK PROTEIN 17.4 (HSP17.4)*	Member of the class I small heat-shock protein (sHSP) family, which accounts for the majority of sHSPs in maturing seeds.Induced by heat, cold, salt, drought and high-light.
AT3G46240		ER protein carbohydrate-binding protein
AT3G46270		receptor protein kinase-like protein
AT3G46280		kinase-like protein
AT3G46340		Leucine-rich repeat protein kinase family protein
AT3G46350		LRR receptor-like Serine/Threonine-kinase
AT3G46360		transmembrane protein
AT3G46370		Leucine-rich repeat protein kinase family protein
AT3G46380		hypothetical protein
AT3G46390		NAC domain protein
AT3G46400		Leucine-rich repeat protein kinase family protein
AT3G46430	*(ATMTATP6)*	Mitochondrial F1F0-ATP synthase.
AT3G46440	*UDP-XYL SYNTHASE 5 (UXS5)*	Encodes a cytosolic isoform of UDP-glucuronic acid decarboxylase. UDP-glucuronic acid decarboxylase produces UDP-xylose, which is a substrate for many cell wall carbohydrates including hemicellulose and pectin. UDP-xylose is also known to feedback regulate several cell wall biosynthetic enzymes.
AT3G46480		2-oxoglutarate (2OG) and Fe(II)-dependent oxygenase superfamily protein
AT3G46500		2-oxoglutarate (2OG) and Fe(II)-dependent oxygenase superfamily protein
AT3G46510	*PLANT U-BOX 13 (PUB13)*	Encodes a protein containing a UND, a U-box, and an ARM domain. This protein has E3 ubiquitin ligase activity based on in vitro assays. Can be phosphorylated in vitro by MLPK, ARK1, and ARK2 but not by SD1-29. Involved in ubiquitination of pattern recognition receptor FLS2.
AT3G46520	*ACTIN-12 (ACT12)*	Member of actin subclass composed of ACT12 and ACT4. RNA is expressed at very low levels in vegetative organs, low levels in flowers and very high levels in pollen. Expression of an ACT12/GUS fusion was found in vascular tissues, tapetum, developing and mature pollen, the root cap and in a ring of pericycle tissues during lateral root initiation and early development.
AT3G46530	*RECOGNITION OF PERONOSPORA PARASITICA 13 (RPP13)*	Confers resistance to the biotrophic oomycete, Peronospora parasitica. Encodes an NBS-LRR type R protein with a putative amino-terminal leucine zipper. Fungal protein ATR13 induces RPP13 gene expression and disease resistance. The mRNA is cell-to-cell mobile.
AT3G46540		ENTH/VHS family protein
AT3G46560	*TRANSLOCASE OF THE INNER MEMBRANE 9 (TIM9)*	Encodes a small zinc finger-like protein that is a component of the mitochondrial protein import apparatus. Together with AtTIM10, AtTIM9 is non-redundantly essential for maintaining mitochondrial function of early embryo proper cells and endosperm free-nuclei.
AT3G46570		Glycosyl hydrolase superfamily protein
AT3G46590	*TRF-LIKE 1 (TRFL1)*	Encodes a protein that specifically binds plant telomeric DNA (TTTAGGG)n repeats. Involved in bending DNA. Expressed throughout the plant with highest levels in flowers.
AT3G46600		GRAS family transcription factor

AT3G46620	*RING AND DOMAIN OF UNKNOWN FUNCTION 1117 1 (RDUF1)*	Encodes an ABA- and drought-induced RING-DUF1117 gene whose mutation results in hyposensitive phenotypes toward ABA in terms of germination rate and stomatal closure and markedly reduced tolerance to drought stress relative to wild-type plants.
AT3G46630		DCL protein (DUF3223)
AT3G46640	*PHYTOCLOCK 1 (PCL1)*	Encodes a myb family transcription factor with a single Myb DNA-binding domain (type SHAQKYF) that is unique to plants and is essential for circadian rhythms, specifically for transcriptional regulation within the circadian clock. LUX is required for normal rhythmic expression of multiple clock outputs in both constant light and darkness. It is coregulated with TOC1 and seems to be repressed by CCA1 and LHY by direct binding of these proteins to the evening element in the LUX promoter. The mRNA is cell-to-cell mobile.
AT3G46650		UDP-Glycosyltransferase superfamily protein
AT3G46660	*UDP-GLUCOSYL TRANSFERASE 76E12 (UGT76E12)*	UDP-glucosyl transferase 76E12
AT3G46670	*UDP-GLUCOSYL TRANSFERASE 76E11 (UGT76E11)*	UDP-glucosyl transferase 76E11
AT3G46680		UDP-Glycosyltransferase superfamily protein
AT3G46690		UDP-Glycosyltransferase superfamily protein
AT3G46700		UDP-Glycosyltransferase superfamily protein
AT3G46710		NB-ARC domain-containing disease resistance protein
AT3G46720		UDP-Glycosyltransferase superfamily protein
AT3G46730		NB-ARC domain-containing disease resistance protein
AT3G46740	*TRANSLOCON AT THE OUTER ENVELOPE MEMBRANE OF CHLOROPLASTS 75-III (TOC75-III)*	Component of the translocon outer membrane (TOC) complex. Forms the outer envelope translocation channel (beta-barrel). Plays a role in preprotein conductance. Imported into chloroplast. Expressed in young dividing photosynthetic tissues. Knockout mutants are embryo lethal with arrested development at the two-cell stage. Knockout mutants have abnormal etioplasts.
AT3G46750		low-temperature-induced protein
AT3G46770	*(REM13)*	AP2/B3-like transcriptional factor family protein
AT3G46780	*PLASTID TRANSCRIPTIONALLY ACTIVE 16 (PTAC16)*	plastid transcriptionally active 16
AT3G46800		Cysteine/Histidine-rich C1 domain family protein
AT3G46810		Cysteine/Histidine-rich C1 domain family protein
AT3G46830	*RAB GTPASE HOMOLOG A2C (RABA2c)*	RAB GTPase homolog A2C
AT3G46840		Subtilase family protein
AT3G46860		Predicted to encode a PR (pathogenesis-related) peptide that belongs to the PR-6 proteinase inhibitor family. Six putative PR-6-type protein encoding genes are found in Arabidopsis: At2g38900, At2g38870, At5g43570, At5g43580, At3g50020 and At3g46860.
AT3G46880		hypothetical protein
AT3G46900	*COPPER TRANSPORTER 2 (COPT2)*	encodes a member of copper transporter family and functionally complements a high affinity copper transporter mutant in yeast
AT3G46910		Cullin family protein
AT3G46920		kinase superfamily with octicosapeptide/Phox/Bem1p domain-containing protein
AT3G46940	*DUTP-PYROPHOSPHATASE-LIKE 1 (DUT1)*	DUTP-PYROPHOSPHATASE-LIKE 1
AT3G46960	*(SKI2)*	The gene encodes a DExD⁄H box RNA helicase, involved in the regulation of K+ deprivation stress response.SKI2 encodes the RNA helicase subunit of the SKI complex. Mutation of SKI2 leads to production of secondary siRNAs from miRNA target transcripts
AT3G46970	*ALPHA-GLUCAN PHOSPHORYLASE 2 (PHS2)*	Encodes a cytosolic alpha-glucan phosphorylase. In vitro, the enzyme has a preference for branched polysaccharides, such as glycogen.
AT3G47000		Glycosyl hydrolase family protein
AT3G47010		Glycosyl hydrolase family protein
AT3G47040		Glycosyl hydrolase family protein
AT3G47050		Glycosyl hydrolase family protein
AT3G47060	*FTSH PROTEASE 7 (ftsh7)*	encodes an FtsH protease that is localized to the chloroplast
AT3G47070		thylakoid soluble phosphoprotein
AT3G47080		Tetratricopeptide repeat (TPR)-like superfamily protein
AT3G47090		Leucine-rich repeat protein kinase family protein
AT3G47100		hypothetical protein
AT3G47110		Leucine-rich repeat protein kinase family protein
AT3G47130		F-box associated ubiquitination effector family protein
AT3G47140		F-box associated ubiquitination effector family protein
AT3G47160		RING/U-box superfamily protein
AT3G47170		Encodes enzymes that can efficiently convert putrescine and caffeoyl-CoA to di-caffeoyl putrescine. Has a preference for caffeoyl CoA and putrescine.
AT3G47180	*(CTL16)*	RING/U-box superfamily protein
AT3G47210		hypothetical protein (DUF247)
AT3G47220	*PHOSPHATIDYLINOSITOL-SPECIFIC PHOSPHOLIPASE C9 (PLC9)*	Encodes a plasma membrane-localized phosphoinositide-specific phospholipase C with a role in thermotolerance.
AT3G47230		transposable_element_gene;similar to unknown protein [Arabidopsis thaliana] (TAIR:AT2G12505.1);(source:TAIR10)
AT3G47240		transposable_element_gene;similar to unknown protein [Arabidopsis thaliana] (TAIR:AT1G54926.1);(source:TAIR10)
AT3G47250		transmembrane protein, putative (DUF247)
AT3G47260		transposable_element_gene;similar to Ulp1 protease family protein [Arabidopsis thaliana] (TAIR:AT2G14130.1);(source:TAIR10)
AT3G47270		transposable_element_gene;similar to heat shock protein binding [Arabidopsis thaliana] (TAIR:AT3G30450.1);(source:TAIR10)
AT3G47295		hypothetical protein

AT3G47320		transposable_element_gene;similar to unknown protein [Arabidopsis thaliana] (TAIR:AT5G37385.1);(source:TAIR10)
AT3G47330		transposable_element_gene;similar to unknown protein [Arabidopsis thaliana] (TAIR:AT4G09370.1);(source:TAIR10)
AT3G47340	*GLUTAMINE-DEPENDENT ASPARAGINE SYNTHASE 1 (ASN1)*	encodes a glutamine-dependent asparagine synthetase, the predicted ASN1 peptide contains a purF-type glutamine-binding domain, and is expressed predominantly in shoot tissues, where light has a negative effect on its mRNA accumulation. Expression is induced within 3 hours of dark treatment, in senescing leaves and treatment with exogenous photosynthesis inhibitor. Induction of gene expression was suppressed in excised leaves supplied with sugar. The authors suggest that the gene's expression pattern is responding to the level of sugar in the cell.
AT3G47380	*(ATPMEI11)*	Pectin methylesterase inhibitor that is involved in resistance to Botrytis cinerea. Affects PME activity during infection to prevent disease.
AT3G47400		Plant invertase/pectin methylesterase inhibitor superfamily
AT3G47420	*GLYCEROL-3-PHOSPHATE PERMEASE 1 (G3Pp1)*	Encodes a Pi starvation-responsive protein AtPS3. A member of the phosphate starvation-induced glycerol-3-phosphate permease gene family: AT3G47420(G3Pp1), AT4G25220(G3Pp2), AT1G30560(G3Pp3), AT4G17550(G3Pp4) and AT2G13100(G3Pp5). Its expression is responsive to phosphate (Pi) and not phosphite (Phi) in roots and shoots.
AT3G47430	*PEROXIN 11B (PEX11B)*	member of the peroxin11 (PEX11) gene family, located on the peroxisome membrane, controls peroxisome proliferation. The mRNA is cell-to-cell mobile.
AT3G47440	*TONOPLAST INTRINSIC PROTEIN 5;1 (TIP5;1)*	Encodes AtTIP5;1, functions as water and urea channels in pollen. Target promoter of the male germline-specific transcription factor DUO1. Essential target of gibberellins, promotes hypocotyl cell elongation under excess boron stress.
AT3G47470	*LIGHT-HARVESTING CHLOROPHYLL-PROTEIN COMPLEX I SUBUNIT A4 (LHCA4)*	Encodes a chlorophyll a/b-binding protein that is more similar to the PSI Cab proteins than the PSII cab proteins. The predicted protein is about 20 amino acids shorter than most known Cab proteins.
AT3G47480	*Y CALMODULIN-LIKE 47 (CML47)*	Calcium-binding EF-hand family protein
AT3G47500	*CYCLING DOF FACTOR 3 (CDF3)*	Dof-type zinc finger domain-containing protein, identical to H-protein promoter binding factor-2a GI:3386546 from (Arabidopsis thaliana). Interacts with LKP2 and FKF1, but its overexpression does not change flowering time under short or long day conditions.
AT3G47510		transmembrane protein
AT3G47540		Chitinase family protein
AT3G47560		alpha/beta-Hydrolases superfamily protein
AT3G47570		Leucine-rich repeat protein kinase family protein
AT3G47600	*MYB DOMAIN PROTEIN 94 (MYB94)*	Encodes a putative transcription factor (MYB94).
AT3G47610		transcription regulator/ zinc ion binding protein
AT3G47620	*TEOSINTE BRANCHED, CYCLOIDEA AND PCF (TCP) 14 (TCP14)*	Encodes a transcription factor AtTCP14 that regulates seed germination. AtTCP14 shows elevated expression level just prior to germination. AtTCP14 is predominantly expressed in the vascular tissue of the embryo, and affects gene expression in radicles in a non-cell-autonomous manner. Modulates GA-dependent stamen filament elongation by direct activation of SAUR63 subfamily genes through conserved target sites in their promoters.
AT3G47640	*POPEYE (PYE)*	Encodes POPEYE (PYE), a bHLH transcription factor regulating response to iron deficiency in Arabidopsis roots.
AT3G47650	*BUNDLE SHEATH DEFECTIVE 2 (BSD2)*	DnaJ/Hsp40 cysteine-rich domain superfamily protein
AT3G47660		Regulator of chromosome condensation (RCC1) family protein
AT3G47700	*MAIGO2 (MAG2)*	Involved in transportation of seed storage proteins from the ER to the vacuole. Mutant seed cell accumulates the precursors of 12S globulin and 2S albumin instead of the vacuolar-located mature proteins. Member of MAG2 complex, involved in the development of vegetative organs.
AT3G47730	*ATP-BINDING CASSETTE A2 (ABCA2)*	member of ATH subfamily
AT3G47740	*ATP-BINDING CASSETTE A3 (ABCA3)*	member of ATH subfamily
AT3G47750	*ATP-BINDING CASSETTE A4 (ABCA4)*	member of ATH subfamily
AT3G47770	*ATP-BINDING CASSETTE A6 (ABCA6)*	ABC2 homolog 5
AT3G47780	*ATP-BINDING CASSETTE A7 (ABCA7)*	member of ATH subfamily The mRNA is cell-to-cell mobile.
AT3G47790	*ATP-BINDING CASSETTE A8 (ABCA8)*	ABC2 homolog 7
AT3G47800		Galactose mutarotase-like superfamily protein
AT3G47833	*SUCCINATE DEHYDROGENASE 7 (SDH7)*	predicted to encode subunit 7 of mitochondrial complex II and to participate in the respiratory chain
AT3G47840		Tetratricopeptide repeat (TPR)-like superfamily protein
AT3G47860	*CHLOROPLASTIC LIPOCALIN (CHL)*	Encodes a chloroplastic lipocalin AtCHL. Located in thylakoid lumen. Involved in the protection of thylakoidal membrane lipids against reactive oxygen species, especially singlet oxygen, produced upon excess light. LCNP is required for sustained photoprotective energy dissipation or NPQ (qH) to occur (PMID:29233855).
AT3G47890		Ubiquitin carboxyl-terminal hydrolase-related protein
AT3G47910		Ubiquitin carboxyl-terminal hydrolase-related protein
AT3G47930	*L-GALACTONO-1,4-LACTONE DEHYDROGENASE (GLDH)*	L-Galactono-1,4-lactone dehydrogenase, catalyzes the final step of ascorbate biosynthesis.
AT3G47950	*H(+)-ATPASE 4 (HA4)*	mutant has Slight reduction in root and shoot growth; Exaggerated defects in salt stress; Plasma Membrane H+ ATPase
AT3G47960	*NRT1/ PTR FAMILY 2.10 (NPF2.10)*	Encodes a high-affinity, proton-dependent glucosinolate-specific transporter that is crucial for the transport of both methionine- and tryptophan-derived glucosinolates to seeds.
AT3G47970		
AT3G47980	*(ATNITR2;2)*	Integral membrane HPP family protein. Putative nitrate transporter.
AT3G48000	*ALDEHYDE DEHYDROGENASE 2B4 (ALDH2B4)*	Encodes a putative (NAD+) aldehyde dehydrogenase.
AT3G48010	*CYCLIC NUCLEOTIDE-GATED CHANNEL 16 (CNGC16)*	member of Cyclic nucleotide gated channel family
AT3G48030	*HYPOXIA-INDUCED GENE DOMAIN 1 (ATHIGD1)*	Mitochondria localized, hypoxia induced gene similar to rice HIGD.
AT3G48040	*RHO-RELATED PROTEIN FROM PLANTS 10 (ROP10)*	Encodes a member of the Rop subfamily of Rho GTPases in Arabidopsis that contains a putative farnesylation motif. It is localized to the plasma membrane and involved in the negative regulation of ABA signalling.

AT3G48080		alpha/beta-Hydrolases superfamily protein
AT3G48090	*ENHANCED DISEASE SUSCEPTIBILITY 1 (EDS1)*	Component of R gene-mediated disease resistance in Arabidopsis thaliana with homology to eukaryotic lipases.
AT3G48100	*RESPONSE REGULATOR 5 (RR5)*	Encodes a transcription repressor that mediates a negative feedback loop in cytokinin signalling. ARR5 expression is upregulated by Class I KNOX genes. Arr5 protein is stabilized by cytokinin in a two-component phosphorelay.
AT3G48130	*(RSU1)*	Ribosomal protein L13 homolog.Evidence for transcription (RNA-seq) and translation (Ribo-seq) described in PMID:27791167
AT3G48150	*ANAPHASE-PROMOTING COMPLEX SUBUNIT 8 (APC8)*	anaphase-promoting complex or cyclosome subunit
AT3G48170	*ALDEHYDE DEHYDROGENASE 10A9 (ALDH10A9)*	ALDH10A9 encodes a protein that can function as a betaine aldehyde dehydrogenase in vitro. The C-terminal amino acids of this protein direct GFP to the peroxisome suggesting that ALDH10A9 accumulates in this organelle. ALDH10A9 transcript levels rise in response to ABA, NaCl, chilling, methyl viologen, and dehydration stress. The enzyme can catalyze the formation of glycine betaine in vitro, but there are still questions about whether Arabidopsis makes this protective compound under natural conditions. This enzyme may be involved in oxidizing aminoaldehydes formed through polyamine metabolism.
AT3G48185		transmembrane protein
AT3G48200		transmembrane protein
AT3G48250	*BUTHIONINE SULFOXIMINE-INSENSITIVE ROOTS 6 (BIR6)*	Encodes a pentatricopeptide repeat protein implicated in splicing of intron 1 of mitochondrial nad7 transcripts.
AT3G48350	*CYSTEINE ENDOPEPTIDASE 3 (CEP3)*	Involved in starvation-related responses that curtail primary root growth under severe nutrient limitation.
AT3G48360	*BTB AND TAZ DOMAIN PROTEIN 2 (bt2)*	Encodes a protein (BT2) that is an essential component of the TAC1-mediated telomerase activation pathway. Acts redundantly with BT3 and BT1 during female gametophyte development and with BT3 during male gametophyte development. BT2 also mediates multiple responses to nutrients, stresses, and hormones.
AT3G48370		transposable_element_gene;copia-like retrotransposon family, has a 7.2e-35 P-value blast match to GB:CAA72990 open reading frame 2 (Ty1_Copia-element) (Brassica oleracea);(source:TAIR10)
AT3G48410		alpha/beta-Hydrolases superfamily protein
AT3G48420		Haloacid dehalogenase-like hydrolase (HAD) superfamily protein
AT3G48450		RPM1-interacting protein 4 (RIN4) family protein
AT3G48460	*SEED FATTY ACID REDUCER 4 (SFAR4)*	GDSL-motif esterase/acyltransferase/lipase. Enzyme group with broad substrate specificity that may catalyze acyltransfer or hydrolase reactions with lipid and non-lipid substrates.
AT3G48490		hypothetical protein
AT3G48500	*PIGMENT DEFECTIVE 312 (PDE312)*	PEP complex component.
AT3G48510		ABA‐induced transcription repressor that acts as feedback regulator in ABA signalling.
AT3G48520	*CYTOCHROME P450, FAMILY 94, SUBFAMILY B, POLYPEPTIDE 3 (CYP94B3)*	CYP94B3 is a jasmonoyl-isoleucine-12-hydroxylase that catalyzes the formation of 12-OH-JA-Ile from JA-Ile. By reducing the levels of this the biologically active phytohormone, CYP94B3 attenuates the jasmonic acid signaling cascade. CYP94B3 transcript levels rise in response to wounding.
AT3G48530	*SNF1-RELATED PROTEIN KINASE REGULATORY SUBUNIT GAMMA 1 (KING1)*	SNF1-related protein kinase regulatory subunit gamma 1
AT3G48550		SHOOT GRAVITROPISM-like protein
AT3G48560	*CHLORSULFURON/IMIDAZOLINONE RESISTANT 1 (CSR1)*	Catalyzes the formation of acetolactate from pyruvate, the first step in valine and isoleucine biosynthesis. Requires FAD, thiamine pyrophosphate and Mg. Inhibited by the sulphonylurea herbicide, chlorsulphuron, and the imidazolinone herbicide, imazapyr. The obtained crystal structure of acetohydroxyacid synthase AHAS, EC 2.2.1.6)in complex with herbicides of the sulphonylurea and imidazolinone family reveals the molecular basis for substrate/inhibitor binding.
AT3G48565		
AT3G48580	*XYLOGLUCAN ENDOTRANSGLUCOSYLASE/HYDROLASE 11 (XTH11)*	xyloglucan endotransglucosylase/hydrolase 11
AT3G48610	*NON-SPECIFIC PHOSPHOLIPASE C6 (NPC6)*	Non-specific phospholipase C6 involved in gametophyte development.
AT3G48640		transmembrane protein
AT3G48650		pseudogene of pectinesterase
AT3G48680	*GAMMA CARBONIC ANHYDRASE-LIKE 2 (GAMMA CAL2)*	Encodes a mitochondrial gamma carbonic anhydrase-like protein. Component of the NADH dehydrogenase complex. The mRNA is cell-to-cell mobile.
AT3G48690	*(CXE12)*	Encodes a protein with carboxylesterase whose activity was tested using both pNA and 2,4-D-methyl.
AT3G48700	*CARBOXYESTERASE 13 (CXE13)*	carboxyesterase 13
AT3G48710		DEK domain-containing chromatin associated protein
AT3G48720	*DEFICIENT IN CUTIN FERULATE (DCF)*	Encodes a hydroxycinnamoyl-CoA: v-hydroxy fatty acid transferase involved in cutin synthesis. Mutants are almost devoid of ferulic acid.
AT3G48730	*GLUTAMATE-1-SEMIALDEHYDE 2,1-AMINOMUTASE 2 (GSA2)*	glutamate-1-semialdehyde 2,1-aminomutase 2
AT3G48740	*(SWEET11)*	Encodes a member of the SWEET sucrose efflux transporter family proteins.
AT3G48750	*CELL DIVISION CONTROL 2 (CDC2)*	A-type cyclin-dependent kinase. Together with its specific inhibitor, the Kip-related protein, KRP2 they regulate the mitosis-to-endocycle transition during leaf development. Dominant negative mutations abolish cell division. Loss of function phenotype has reduced fertility with failure to transmit via pollen. Pollen development is arrested at the second mitotic division. Expression is regulated by environmental and chemical signals. Part of the promoter is responsible for expression in trichomes. Functions as a positive regulator of cell proliferation during development of the male gametophyte, embryo and endosperm. Phosphorylation of threonine 161 is required for activation of its associated kinase.
AT3G48760	*(PAT5)*	DHHC-type zinc finger family protein
AT3G48770		ATP/DNA binding protein
AT3G48810	*ORGANELLE TRANSCRIPT PROCESSING 439 (OTP439)*	Pentatricopeptide repeat proteins involved in mitochondrial RNA processing.
AT3G48830		tRNA nucleotidyltransferase/polyA polymerase family protein
AT3G48850	*PHOSPHATE TRANSPORTER 3;2 (PHT3;2)*	Encodes a mitochondrial phosphate transporter. Modulates plant responses to salt stress.
AT3G48860	*STOMATAL CYTOKINESIS DEFECTIVE 2 (SCD2)*	coiled-coil protein

AT3G48880	*SNC1-INﬂUENCING PLANT E3 LIGASE REVERSE GENETIC SCREEN 4 (SNIPER4)*	Encodes an F-box protein, SNIPER4, that regulates the turnover of MUSE13 and MUSE14, redundant TRAF proteins serving as adaptors in the SCFCRP1 complex to facilitate the turnover of nucleotide-binding domain and leucine-rich repeats (NLR) immune receptors.
AT3G48910		
AT3G48920	*MYB DOMAIN PROTEIN 45 (MYB45)*	Member of the R2R3 factor gene family.
AT3G48940		Remorin family protein
AT3G48950	*POLYGALACTURONASE CLADE F 7 (PGF7)*	Pectin lyase-like superfamily protein
AT3G48960		Ribosomal protein L13e family protein
AT3G48970	*HEAVY METAL ASSOCIATED PROTEIN 29 (ATHMP29)*	Heavy metal transport/detoxification superfamily protein
AT3G48990	*ACYL-ACTIVATING ENZYME 3 (AAE3)*	Encodes an oxalyl-CoA synthetase and is required for oxalate degradation, for normal seed development, and for defense against an oxalate-producing fungal pathogen.
AT3G49020		FBD, F-box and Leucine Rich Repeat domains containing protein
AT3G49050		alpha/beta-Hydrolases superfamily protein
AT3G49060	*(PUB32)*	Plant U-box type E3 ubiquitin ligase (PUB).
AT3G49090		
AT3G49120	*PEROXIDASE CB (PRXCB)*	Class III peroxidase Perx34. Expressed in roots, leaves and stems. Located in the cell wall. Involved in cell elongation. Expression activated by light. May play a role in generating H2O2 during defense response. The mRNA is cell-to-cell mobile.
AT3G49130		SWAP (Suppressor-of-White-APricot)/surp RNA-binding domain-containing protein
AT3G49140		Pentatricopeptide repeat (PPR) superfamily protein
AT3G49150		F-box/FBD/LRR protein
AT3G49160		Expression of the gene is downregulated in the presence of paraquat, an inducer of photoxidative stress.
AT3G49170	*EMBRYO DEFECTIVE 2261 (EMB2261)*	Tetratricopeptide repeat (TPR)-like superfamily protein
AT3G49180	*ROOT INITIATION DEFECTIVE 3 (RID3)*	Transducin/WD40 repeat-like superfamily protein
AT3G49190		O-acyltransferase (WSD1-like) family protein
AT3G49210	*WS /DGAT 6 (WSD6)*	WSD6 can function in vitro as wax ester synthase but does not appear to be essential for cuticular wax biosynthesis.
AT3G49220	*PECTIN METHYLESTERASE 34 (PME34)*	Plant invertase/pectin methylesterase inhibitor superfamily
AT3G49230	*(DEG1)*	transmembrane protein
AT3G49250	*DEFECTIVE IN MERISTEM SILENCING 3 (DMS3)*	Similar to hinge-domain region of structural maintenance of chromosomes (SMC)proteins.Putative chromosome architecture protein that can potentialy link nucleic acids in facilitating an RNA1-mediated epigenetic modification involving secondary siRNA and spreading of DNA methylation.
AT3G49260	*IQ-DOMAIN 21 (iqd21)*	IQ-domain 21
AT3G49270		extensin-like protein
AT3G49280		transposable_element_gene;similar to unknown protein [Arabidopsis thaliana] (TAIR:AT5G13655.1);(source:TAIR10)
AT3G49300		proline-rich family protein
AT3G49310		Major facilitator superfamily protein
AT3G49320		Metal-dependent protein hydrolase
AT3G49340		Cysteine proteinases superfamily protein
AT3G49350		Ypt/Rab-GAP domain of gyp1p superfamily protein
AT3G49360	*6-PHOSPHOGLUCONOLACTONASE 2 (PGL2)*	Encodes a cytosolic 6-phosphogluconolactonase (PGL) thought to be involved in the oxidative pentose-phosphate pathway (OPPP).
AT3G49380	*IQ-DOMAIN 15 (iqd15)*	Member of IQ67 (CaM binding) domain containing family.
AT3G49400		Transducin/WD40 repeat-like superfamily protein
AT3G49420		Got1/Sft2-like vescicle transport protein family
AT3G49430	*SERINE/ARGININE-RICH PROTEIN SPLICING FACTOR 34A (SR34a)*	Barta et al (2010) have proposed a nomenclature for Serine/Arginine-Rich Protein Splicing Factors (SR proteins): Plant Cell. 2010, 22:2926.
AT3G49450	*F-BOX PROTEIN 113 (FBP113)*	F-box protein involved in protein binding and ubiquitination; involved in male fertility.
AT3G49460		60S acidic ribosomal-like protein
AT3G49470	*NASCENT POLYPEPTIDE-ASSOCIATED COMPLEX SUBUNIT ALPHA-LIKE PROTEIN 2 (NACA2)*	nascent polypeptide-associated complex subunit alpha-like protein 2
AT3G49480	*VIRD5 RESPONSE F-BOX PROTEIN (D5RF)*	F-Box Gene regulated by Agrobacterium virulence protein VirD5 and essential for Agrobacterium-mediated plant transformation.
AT3G49500	*RNA-DEPENDENT RNA POLYMERASE 6 (RDR6)*	Encodes RNA-dependent RNA polymerase. Involved in trans-acting siRNA and other siRNA biogenesis. Required for post-transcriptional gene silencing and natural virus resistance.Loss of function mutants produce ectopic megaspore mother cell and supernumary female gametophytes.
AT3G49510		F-box family protein
AT3G49520		F-box and associated interaction domains-containing protein
AT3G49580	*RESPONSE TO LOW SULFUR 1 (LSU1)*	response to low sulfur 1
AT3G49590	*AUTOPHAGY-RELATED 13A (ATG13a)*	Autophagy protein.
AT3G49600	*UBIQUITIN-SPECIFIC PROTEASE 26 (UBP26)*	Encodes a ubiquitin-specific protease which catalyzes deubiquitination of histone H2B and is required for heterochromatin silencing.Loss of function mutations display autonomous endosperm development and embryo arrest. Loss of function also results in an increase in expression of the PcG complex target gene PHE1.
AT3G49620	*DARK INDUCIBLE 11 (DIN11)*	encodes a protein similar to 2-oxoacid-dependent dioxygenase. Expression is induced after 24 hours of dark treatment, in senescing leaves and treatment with exogenous photosynthesis inhibitor. Induction of gene expression was suppressed in excised leaves supplied with sugar. The authors suggest that the gene's expression pattern is responding to the level of sugar in the cell.
AT3G49630		2-oxoglutarate (2OG) and Fe(II)-dependent oxygenase superfamily protein
AT3G49640		Aldolase-type TIM barrel family protein
AT3G49650		P-loop containing nucleoside triphosphate hydrolases superfamily protein

AT3G49660	*HUMAN WDR5 (WD40 REPEAT) HOMOLOG A (WDR5a)*	Encodes a structural core component of a COMPASS-like H3K4 histone methylation complex.
AT3G49670	*BARELY ANY MERISTEM 2 (BAM2)*	Encodes a CLAVATA1-related receptor kinase-like protein required for both shoot and flower meristem function. Very similar to BAM1,with more than 85% a.a. identity. It has a broad expression pattern and is involved in vascular strand development in the leaf, control of leaf shape, size and symmetry, male gametophyte development and ovule specification and function. Anthers of double mutants (bam1bam2) appeared abnormal at a very early stage and lack the endothecium, middle, and tapetum layers. Further analyses revealed that cells interior to the epidermis (in anther tissue) acquire some characteristics of pollen mother cells (PMCs), suggesting defects in cell fate specification. The pollen mother-like cells degenerate before the completion of meiosis, suggesting that these cells are defective. In addition, the BAM2 expression pattern supports both an early role in promoting somatic cell fates and a subsequent function in the PMCs. The mRNA is cell-to-cell mobile.
AT3G49690	*MYB DOMAIN PROTEIN 84 (MYB84)*	Putative homolog of the Blind gene in tomato. Together with RAX1 and RAX3 belong to the class R2R3 MYB genes; encoded by the Myb-like transcription factor MYB84, regulates axillary meristem formation.
AT3G49700	*1-AMINOCYCLOPROPANE-1-CARBOXYLATE SYNTHASE 9 (ACS9)*	encodes a a member of the 1-aminocyclopropane-1-carboxylate (ACC) synthase (S-adenosyl-L-methionine methylthioadenosine-lyase, EC 4.4.1.14) gene family. Mutants produce elevated levels of ethylene as etiolated seedlings.
AT3G49730		Tetratricopeptide repeat (TPR)-like superfamily protein
AT3G49750	*RECEPTOR LIKE PROTEIN 44 (RLP44)*	receptor like protein 44
AT3G49760	*BASIC LEUCINE-ZIPPER 5 (bZIP5)*	basic leucine-zipper 5
AT3G49770	*(PEG6)*	hypothetical protein
AT3G49780	*PHYTOSULFOKINE 4 PRECURSOR (PSK4)*	Phytosulfokine 3 precursor, coding for a unique plant peptide growth factor. Plants overexpressing this gene (under a 35S promoter), develop normal cotyledons and hypocotyls but their growth, in particular that of their roots, was faster than that of wildtype.
AT3G49790		Carbohydrate-binding protein
AT3G49810	*U-BOX PROTEIN 30 (ATPUB30)*	Encodes a protein with E3 ubiquitin ligase activity that is involved in negative regulation of salt stress tolerance during germination.
AT3G49820		hypothetical protein
AT3G49830		pseudogene
AT3G49840	*INNER NUCLEAR MEMBRANE PROTEIN C (NEMP_C)*	Encodes an orthlog of the Xenopus inner nuclear membrane (INM) protein Nemp1/TMEM194A.It is an inner nuclear membrane protein that binds with nucleoskeleton proteins.
AT3G49860	*ADP-RIBOSYLATION FACTOR-LIKE A1B (ARLA1B)*	A member of ARF-like GTPase family. A thaliana has 21 members, in two subfamilies, ARF and ARF-like (ARL) GTPases. Possible pseudogene because it lacks an N-terminal part that is conserved among the other ARL8 proteins.
AT3G49890		hypothetical protein
AT3G49900		Phototropic-responsive NPH3 family protein
AT3G49930		C2H2 and C2HC zinc fingers superfamily protein
AT3G49940	*LOB DOMAIN-CONTAINING PROTEIN 38 (LBD38)*	LOB domain-containing protein 38
AT3G49950		GRAS family transcription factor
AT3G49960		Its expression is enriched in root hair cells (compared to non-root hair cells) and this enrichment is associated with increase in the transcription-associated mark trimethylation of H3 lysine 4 (H3K4me3) and decrease in the Polycomb silencing-associated mark trimethylation of H3 lysine 27 (H3K27me3) in root hair cells relative to non-root hair cells.
AT3G50000	*CASEIN KINASE II, ALPHA CHAIN 2 (CKA2)*	Encodes the casein kinase II (CK2) catalytic subunit (alpha).
AT3G50010		Cysteine/Histidine-rich C1 domain family protein
AT3G50060	*MYB DOMAIN PROTEIN 77 (MYB77)*	Encodes a member of the R2R3 transcription factor gene family. Expressed in response to potassium deprivation and auxin. Involved in lateral root development. Interacts with ARF7 and regulates the expression of some auxin responsive genes.
AT3G50070	*CYCLIN D3;3 (CYCD3;3)*	Encode CYCD3;3, a CYCD3 D-type cyclin. Important for determining cell number in developing lateral organs. Mediating cytokinin effects in apical growth and development.
AT3G50080	*VIER F-BOX PROTEINE 2 (VFB2)*	Encodes an F-box protein. Based on genetic analysis appears to be functionally redundant with VFB1,3, and 4. When expression of all 4 genes is reduced plants show defects in growth and reduced expression of auxin response genes.
AT3G50120		transmembrane protein, putative (DUF247)
AT3G50130		transmembrane protein, putative (DUF247)
AT3G50140		transmembrane protein, putative (DUF247)
AT3G50150		transmembrane protein, putative (DUF247)
AT3G50160		transmembrane protein, putative (DUF247)
AT3G50170		transmembrane protein, putative (DUF247)
AT3G50190		transmembrane protein, putative (DUF247)
AT3G50200		hypothetical protein (DUF247)
AT3G50220	*IRREGULAR XYLEM 15 (IRX15)*	Encode a DUF579 (domain of unknown function 579) containing protein essential for normal xylan synthesis and deposition in the secondary cell wall.
AT3G50230		Leucine-rich repeat protein kinase family protein
AT3G50240	*(KICP-02)*	Encodes a kinesin-related protein.
AT3G50250		transmembrane protein
AT3G50260	*COOPERATIVELY REGULATED BY ETHYLENE AND JASMONATE 1 (CEJ1)*	Encodes a member of the DREB subfamily A-5 of ERF/AP2 transcription factor family. The protein contains one AP2 domain. Involved in defense and freezing stress responses. There are 16 members in this subfamily including RAP2.1, RAP2.9 and RAP2.10. The mRNA is cell-to-cell mobile.
AT3G50270		HXXXD-type acyl-transferase family protein
AT3G50280		HXXXD-type acyl-transferase family protein
AT3G50290		HXXXD-type acyl-transferase family protein

AT3G50300		HXXXD-type acyl-transferase family protein
AT3G50310	*MITOGEN-ACTIVATED PROTEIN KINASE KINASE KINASE 20 (MAPKKK20)*	Encodes a member of MEKK subfamily. Target promoter of the male germline-specific transcription factor DUO1. Involved in osmotic stress response via regulation of MPK6 activity. It also plays an important role in regulating cell division and cell elongation in the primary root meristematic and elongation areas. Mutants show defects in root microtubule organization.It phosphorylates MPK18 and MKK3.It is a positive regulator of ABA-induced stomatal closure that acts by phosphorylating MKK5.
AT3G50320		hypothetical protein
AT3G50340		hypothetical protein
AT3G50350		membrane insertase, putative (DUF1685)
AT3G50380		vacuolar protein sorting-associated protein, putative (DUF1162)
AT3G50400		GDSL-motif esterase/acyltransferase/lipase. Enzyme group with broad substrate specificity that may catalyze acyltransfer or hydrolase reactions with lipid and non-lipid substrates.
AT3G50410	*OBF BINDING PROTEIN 1 (OBP1)*	Arabidopsis Dof protein containing a single 51-amino acid zinc finger DNA-binding domain, which may play an important roles in plant growth and development.
AT3G50420		Pentatricopeptide repeat (PPR) superfamily protein
AT3G50440	*METHYL ESTERASE 10 (MES10)*	Encodes a protein shown to have methyl jasmonate esterase activity in vitro. This protein does not act on methyl IAA, MeSA, MeGA4, or MEGA9 in vitro.
AT3G50480	*HOMOLOG OF RPW8 4 (HR4)*	Homolog of RPW8
AT3G50500	*SNF1-RELATED PROTEIN KINASE 2.2 (SNRK2.2)*	encodes a member of SNF1-related protein kinases (SnRK2) whose activity is activated by ionic (salt) and non-ionic (mannitol) osmotic stress. Enzyme involved in the ABA signaling during seed germination, dormancy and seedling growth.
AT3G50540		hypothetical protein
AT3G50550		hypothetical protein
AT3G50560		NAD(P)-binding Rossmann-fold superfamily protein
AT3G50570		hydroxyproline-rich glycoprotein family protein
AT3G50580		transmembrane protein
AT3G50610	*C-TERMINALLY ENCODED PEPTIDE 9 (CEP9)*	DNA-directed RNA polymerase II subunit RPB1-like protein
AT3G50620		P-loop containing nucleoside triphosphate hydrolases superfamily protein
AT3G50630	*KIP-RELATED PROTEIN 2 (KRP2)*	Kip-related protein (KRP) gene, encodes CDK (cyclin-dependent kinase) inhibitor (CKI), negative regulator of cell division. A member of seven KRP genes found in Arabidopsis thaliana. Differential expression patterns for distinct KRPs were revealed by in situ hybridization. Gene was isolated from a yeast two hybrid screen as an interacting protein of CDC2A. Recombinant protein has a strong kinase inhibitor activity in vitro. Transcript is expressed in all tissues examined but is differentially distributed from ICK1. Controls the onset of the endoreduplication cycle through inhibition of CDKA;1. The KRP2 protein abundance is regulated by proteolysis through CDKB1;1 phosphorylation.
AT3G50640		hypothetical protein
AT3G50650		GRAS family transcription factor
AT3G50660	*DWARF 4 (DWF4)*	Encodes a 22α hydroxylase whose reaction is a rate-limiting step in brassinosteroid biosynthetic pathway. The protein is a member of CYP90B gene family. CLM is an epi-allele with small, compressed rosette, reduced internode length, and reduced fertility, appears in selfed ddm mutant plants possibly due to loss of cytosine methylation. Transcripts accumulate in actively growing tissues, and GUS expression is negatively regulated by brassinosteroids. Localized in the endoplasmic reticulum. The in vitro expressed protein can perform the C-22 hydroxylation of a variety of C27-, C28- and C29-sterols. Cholesterol was the best substrate, followed by campesterol. Sitosterol was a poor substrate.
AT3G50670	*U1 SMALL NUCLEAR RIBONUCLEOPROTEIN-70K (U1-70K)*	Encodes U1 snRNP 70K
AT3G50685		anti-muellerian hormone type-2 receptor
AT3G50690		Leucine-rich repeat (LRR) family protein
AT3G50700	*INDETERMINATE(ID)-DOMAIN 2 (IDD2)*	zinc finger protein, similar to maize Indeterminate1 (ID1)
AT3G50720	*(PEG7)*	Protein kinase superfamily protein
AT3G50730		Protein kinase superfamily protein
AT3G50740	*UDP-GLUCOSYL TRANSFERASE 72E1 (UGT72E1)*	UGT72E1 is an UDPG:coniferyl alcohol glucosyltransferase which specifically glucosylates sinapyl- and coniferyl aldehydes. The enzyme is thought to be involved in lignin metabolism.
AT3G50750	*BES1/BZR1 HOMOLOG 1 (BEH1)*	BES1/BZR1 homolog 1
AT3G50760	*GALACTURONOSYLTRANSFERASE-LIKE 2 (GATL2)*	Encodes a protein with putative galacturonosyltransferase activity. The mRNA is cell-to-cell mobile.
AT3G50770	*CALMODULIN-LIKE 41 (CML41)*	calmodulin-like 41
AT3G50790		esterase/lipase/thioesterase family protein
AT3G50810	*CASP-LIKE PROTEIN 5C2 (CASPL5C2)*	Uncharacterized protein family (UPF0497)
AT3G50820	*PHOTOSYSTEM II SUBUNIT O-2 (PSBO2)*	Encodes a protein which is an extrinsic subunit of photosystem II and which has been proposed to play a central role in stabilization of the catalytic manganese cluster. In <i>Arabidopsis thaliana</i> the PsbO proteins are encoded by two genes: <i>psbO1</i> and <i>psbO2</i>. PsbO2 is the minor isoform in the wild-type. Mutants defective in this gene have been shown to be affected in the dephosphorylation of the D1 protein of PSII.
AT3G50850		Putative methyltransferase family protein
AT3G50870	*MONOPOLE (MNP)*	Encodes a GATA transcriptional regulator required to position the proembryo boundary in the early embryo. Regulates shoot apical meristem and flower development.
AT3G50890	*HOMEOBOX PROTEIN 28 (HB28)*	homeobox protein 28
AT3G50900		hypothetical protein
AT3G50910		netrin receptor DCC

AT3G50930	*CYTOCHROME BC1 SYNTHESIS (BCS1)*	Encodes a protein that is present in a homo-multimeric protein complex on the outer mitochondrial membrane and plays a role in cell death and amplifying salicylic acid signalling. The mRNA is cell-to-cell mobile.
AT3G50940		P-loop containing nucleoside triphosphate hydrolases superfamily protein
AT3G50950	*HOPZ-ACTIVATED RESISTANCE 1 (ZAR1)*	Encodes a canonical CC-type NLR protein that is required for the recognition of the T3SE HopZ1a as well as several other Hop effectors from the pathogenic bacteria P. syringae.
AT3G50970	*LOW TEMPERATURE-INDUCED 30 (LTI30)*	Belongs to the dehydrin protein family, which contains highly conserved stretches of 7-17 residues that are repetitively scattered in their sequences, the K-, S-, Y- and lysine rich segments. LTI29 and LTI30 double overexpressors confer freeze tolerance. Located in membranes. mRNA upregulated by water deprivation and abscisic acid. The mRNA is cell-to-cell mobile.
AT3G50980	*DEHYDRIN XERO 1 (XERO1)*	dehydrin xero 1
AT3G50990	*PEROXIDASE 36 (PER36)*	Encodes a class III peroxidase family protein that functions as a mucilage extrusion factor. Its polarized and developmental stage-dependent secretion plays a role in cell wall modification of the cells in the second layer of the outer integument.
AT3G51000		alpha/beta-Hydrolases superfamily protein
AT3G51030	*THIOREDOXIN H-TYPE 1 (TRX1)*	encodes a cytosolic thioredoxin that reduces disulfide bridges of target proteins by the reversible formation of a disulfide bridge between two neighboring Cys residues present in the active site. Thioredoxins have been found to regulate a variety of biological reactions in prokaryotic and eukaryotic cells.
AT3G51040	*RTE1-HOMOLOG (RTH)*	Encodes a protein of 231 amino acids with 51% identity to RTE1 over 209 amino acids. Interacts with RTE1 in planta and appears to function in same pathway to positively regulate ethylene signaling.
AT3G51070		S-adenosyl-L-methionine-dependent methyltransferases superfamily protein
AT3G51100		altered inheritance of mitochondria protein
AT3G51110		Tetratricopeptide repeat (TPR)-like superfamily protein
AT3G51150		ATP binding microtubule motor family protein
AT3G51160	*MURUS 1 (MUR1)*	Catalyzes the first step in the de novo synthesis of GDP-L-fucose. Loss of function mutations result in reduced levels of fucosylation and decreased freezing tolerance.
AT3G51180		Zinc finger C-x8-C-x5-C-x3-H type family protein
AT3G51190		Ribosomal protein L2 family
AT3G51200	*SMALL AUXIN UPREGULATED RNA 18 (SAUR18)*	SAUR-like auxin-responsive protein family
AT3G51210		P-loop containing nucleoside triphosphate hydrolases superfamily protein
AT3G51240	*FLAVANONE 3-HYDROXYLASE (F3H)*	Encodes flavanone 3-hydroxylase that is coordinately expressed with chalcone synthase and chalcone isomerases and is involved in flavonoid biosynthesis. Not responsive to auxin or ethylene stimulus (qRT-PCR).
AT3G51270		protein serine/threonine kinase
AT3G51280		Tetratricopeptide repeat (TPR)-like superfamily protein
AT3G51290	*ALTERED PHOSPHATE STARVATION RESPONSE 1 (APSR1)*	pyridoxal-phosphate-dependent serine hydroxymethyltransferase, putative (DUF632)
AT3G51300	*RHO-RELATED PROTEIN FROM PLANTS 1 (ROP1)*	Encodes a pollen-specific Rop GTPase, member of the Rho family of small GTP binding proteins that interacts with RIC3 and RIC4 to control tip growth in pollen tubes. These three proteins promote the proper targeting of exocytic vesicles in the pollen tube tip. ROP1 activity is regulated by the REN1 GTPase activator protein.
AT3G51330		Eukaryotic aspartyl protease family protein
AT3G51350		Eukaryotic aspartyl protease family protein
AT3G51360		Eukaryotic aspartyl protease family protein
AT3G51400		hypothetical protein (DUF241)
AT3G51410		hypothetical protein (DUF241)
AT3G51420	*STRICTOSIDINE SYNTHASE-LIKE 4 (SSL4)*	Although this enzyme is predicted to encode a strictosidine synthase (SS), it lacks a conserved catalytic glutamate residue found in active SS enzymes and it is not expected to have SS activity.
AT3G51430	*YELLOW-LEAF-SPECIFIC GENE 2 (YLS2)*	Although this enzyme is predicted to encode a strictosidine synthase (SS), it lacks a conserved catalytic glutamate residue found in active SS enzymes and it is not expected to have SS activity.
AT3G51450		Calcium-dependent phosphotriesterase superfamily protein
AT3G51460	*ROOT HAIR DEFECTIVE 4 (RHD4)*	Encodes RHD4 (ROOT HAIR DEFECTIVE4), a phosphatidylinositol-4-phosphate phosphatase required for root hair development. The mRNA is cell-to-cell mobile.
AT3G51470		Protein phosphatase 2C family protein
AT3G51480	*GLUTAMATE RECEPTOR 3.6 (GLR3.6)*	member of Putative ligand-gated ion channel subunit family
AT3G51510		transmembrane protein
AT3G51520	*DIACYLGLYCEROL ACYLTRANSFERASE 2 (DGAT2)*	Encodes a functional acyl-CoA:diacylglycerol acyltransferase with different acyl-CoA substrate preferences and shows higher DAG to TAG conversion rate than AtDGAT1. It increases both C18:2 and C18:3 polyunsaturated fatty acids at the expense of C16:0.
AT3G51540	*BASIC PROLINE-RICH PROTEIN7 (BPP7)*	mucin-5AC-like protein
AT3G51560		Disease resistance protein (TIR-NBS-LRR class) family
AT3G51570		Disease resistance protein (TIR-NBS-LRR class) family
AT3G51580		transmembrane protein

AT3G51590	*LIPID TRANSFER PROTEIN 12 (LTP12)*	Encodes a member of the lipid transfer protein family. Proteins of this family are generally small (~9 kD), basic, expressed abundantly and contain eight Cys residues. The proteins can bind fatty acids and acylCoA esters and can transfer several different phospholipids. They are localized to the cell wall. The LTP12 promoter is active exclusively in the tapetum during the uninucleate microspore and bicellular pollen stages. Predicted to be a member of PR-14 pathogenesis-related protein family with the following members: At2g38540/LTP1, At2g38530/LTP2, At5g59320/LTP3, At5g59310/LTP4, At3g51600/LTP5, At3g08770/LTP6, At2g15050/LTP7, At2g18370/LTP8, At2g15325/LTP9, At5g01870/LTP10, At4g33355/LTP11, At3g51590/LTP12, At5g44265/LTP13, At5g62065/LTP14, At4g08530/LTP15.
AT3G51600	*LIPID TRANSFER PROTEIN 5 (LTP5)*	Predicted to encode a PR (pathogenesis-related) protein. Belongs to the lipid transfer protein (PR-14) family with the following members: At2g38540/LTP1, At2g38530/LTP2, At5g59320/LTP3, At5g59310/LTP4, At3g51600/LTP5, At3g08770/LTP6, At2g15050/LTP7, At2g18370/LTP8, At2g15325/LTP9, At5g01870/LTP10, At4g33355/LTP11, At3g51590/LTP12, At5g44265/LTP13, At5g62065/LTP14, At4g08530/LTP15.
AT3G51630	*WITH NO LYSINE (K) KINASE 5 (WNK5)*	Encodes a member of the WNK family (9 members in all) of protein kinases, the structural design of which is clearly distinct from those of other known protein kinases, such as receptor-like kinases and mitogen-activated protein kinases.
AT3G51660	*MIF/D-DT-LIKE 3 (MDL3)*	Chemokine-like MDL protein; modulate flowering time and innate immunity in plants.
AT3G51670	*(PATL6)*	PATLs belong to a family of proteins having a Golgi dynamics (GOLD) domain in tandem with the Sec14p-like domain. PATLs are auxin regulated. Quadruple mutants (patl2456) show altered PIN1 lateralization in root endodermis cells.
AT3G51680	*SHORT-CHAIN DEHYDROGENASE/REDUCTASE 2 (SDR2)*	NAD(P)-binding Rossmann-fold superfamily protein
AT3G51690		DNA helicase homolog PIF1.
AT3G51700		PIF1 helicase
AT3G51710		D-mannose binding lectin protein with Apple-like carbohydrate-binding domain-containing protein
AT3G51720		WEB family protein (DUF827)
AT3G51740	*INFLORESCENCE MERISTEM RECEPTOR-LIKE KINASE 2 (IMK2)*	encodes a leucine-repeat receptor kinase expressed in inflorescence meristem. Locus association was made from performing sequence analysis with IMK3 (MRLK) whose locus association was provided by the authors. The mRNA is cell-to-cell mobile.
AT3G51750		hypothetical protein
AT3G51760		hypothetical protein (DUF688)
AT3G51770	*ETHYLENE OVERPRODUCER 1 (ETO1)*	Encodes a negative regulator of 1-aminocyclopropane-1-carboxylic acid synthase5(ACS5), which catalyze the rate-limiting step in ethylene biosynthesis. ETO1 directly interacts with ACS5 and inhibits its enzyme activity and targets it for degradation via proteasome-dependent pathway. It also interacts with CUL3 (a component of ubiquitin ligase complexes). eto1 (and eto3) mutations elevate ethylene biosynthesis by affecting the posttranscriptional regulation of ACS
AT3G51780	*BCL-2-ASSOCIATED ATHANOGENE 4 (BAG4)*	A member of Arabidopsis BAG (Bcl-2-associated athanogene) proteins, plant homologs of mammalian regulators of apoptosis. BD domain of ATBAG4 had highest similarity to human DB domain of BAG protein. Plant BAG proteins are multi-functional and remarkably similar to their animal counterparts, as they regulate apoptotic-like processes ranging from pathogen attack, to abiotic stress, to plant development.
AT3G51790	*TRANSMEMBRANE PROTEIN G1P-RELATED 1 (G1)*	Encodes a heme-binding protein located in the mitochondrial inner membrane that is involved in cytochrome c maturation.
AT3G51810	*LATE EMBRYOGENESIS ABUNDANT 1 (EM1)*	Encodes a ABA-inducible protein that accumulates during seed maturation, in parallel with its corresponding mRNA but with a 3 d delay. During germination, AtEm1 protein undergoes two successive cleavages before being degraded. Both proteins are more stable than the corresponding mRNA. The gene can be activated by the basic leucine zipper transcription factor ABI5. Expressed predominantly in provascular tissues with the strongest expression in the root tip.
AT3G51820	*(G4)*	Encodes a protein with chlorophyll synthase activity. This enzyme has been shown to perform the esterification of chlorophyllide (a and b), the last step of chlorophyll biosynthesis. Although it can use either geranylgeranyl pyrophosphate (GGPP) or phytyl pyrophosphate (PhyPP) as substrates, the esterification reaction was faster with GGPP than with PhyPP.
AT3G51830	*SAC DOMAIN-CONTAINING PROTEIN 8 (SAC8)*	putative transmembrane protein G5p (AtG5) mRNA, complete. autophagy-related (ATG) gene
AT3G51850	*CALCIUM-DEPENDENT PROTEIN KINASE 13 (CPK13)*	member of Calcium Dependent Protein Kinase The mRNA is cell-to-cell mobile.
AT3G51860	*CATION EXCHANGER 3 (CAX3)*	cation exchanger 3
AT3G51890	*CLATHRIN LIGHT CHAIN 3 (CLC3)*	Clathrin light chain protein
AT3G51895	*SULFATE TRANSPORTER 3;1 (SULTR3;1)*	Encodes a chloroplast-localized sulfate transporter.
AT3G51910	*HEAT SHOCK TRANSCRIPTION FACTOR A7A (HSFA7A)*	member of Heat Stress Transcription Factor (Hsf) family The mRNA is cell-to-cell mobile.
AT3G51920	*CALMODULIN 9 (CAM9)*	encodes a divergent member of calmodulin, which is an EF-hand family of Ca2+-binding proteins. This gene is expressed in leaves, flowers and siliques. The gene functionally complements yeast calmodulin 1 (CAM1) but only when selected against the plasmid harboring wild-type yeast sequences. Also the protein does not form formed a complex with a basic amphiphilic helical peptide in the presence of Ca2+ in vitro. Authors suggest that this gene may represent a Ca2+-binding sensor protein that interacts with a more limited set of target proteins than do more conventional CaM isoforms. Mutations in this gene alter plant responses to abiotic stress and abscisic acid.
AT3G51940		oxidoreductase/transition metal ion-binding protein
AT3G51950		Contains single CCCH domain.
AT3G51960	*BASIC LEUCINE ZIPPER 24 (BZIP24)*	bZIP transcription factor induced by salt stress and promoted salt tolerance. Localized to the cytoplasm and nucleus under control conditions and targeted preferentially to the nucleus under salt stress
AT3G51970	*ACYL-COA STEROL ACYL TRANSFERASE 1 (ASAT1)*	acyl-CoA sterol acyl transferase 1
AT3G51990		Protein kinase superfamily protein
AT3G52000	*SERINE CARBOXYPEPTIDASE-LIKE 36 (scpl36)*	serine carboxypeptidase-like 36
AT3G52040		2,3-bisphosphoglycerate-dependent phosphoglycerate mutase
AT3G52060	*BETA-1,6-N-ACETYLGLUCOSAMINYL TRANSFERASE-LIKE (GNTL)*	Encodes a plasmodesmal glycosyltransferase-like protein. Mutation results in defects in seed germination and delayed plant growth.
AT3G52070		RNA/RNP complex-1-interacting phosphatase
AT3G52080	*CATION/HYDROGEN EXCHANGER 28 (chx28)*	encodes a cation:proton exchanger expressed in pollen

AT3G52090	*(NRPB11)*	Non-catalytic subunit common to nuclear DNA-dependent RNA polymerases II, IV and V; homologous to budding yeast RPB11 and the E. oli RNA polymerase alpha subunit.
AT3G52110		interferon-activable protein
AT3G52130		Bifunctional inhibitor/lipid-transfer protein/seed storage 2S albumin superfamily protein
AT3G52140	*NON RESPONDING TO OXYLIPINS 38 (NOXY38)*	Involved in regulating mitochondrial quality control. Regulates mitochondrial association time and thereby is involved in mitochondrial fusion. Mutants show unregulated autophagy and display transcriptomic markers of mitochondrial stress. Its activity can be modulated by Lys acetylation.
AT3G52150	*PLASTID-SPECIﬁC RIBOSOMAL PROTEIN 2 (PSRP2)*	RNA-binding (RRM/RBD/RNP motifs) family protein
AT3G52155		Phosphoglycerate mutase family protein
AT3G52160	*3-KETOACYL-COA SYNTHASE 15 (KCS15)*	Encodes KCS15, a member of the 3-ketoacyl-CoA synthase family involved in the biosynthesis of VLCFA (very long chain fatty acids).
AT3G52170		DNA binding protein
AT3G52180	*STARCH-EXCESS 4 (SEX4)*	Encodes a plant-specific glucan phosphatase that contains a noncatalytic carbohydrate-binding module as well as a dual specificity protein phosphatase domain. SEX4 can dephosphorylate C6- and C3-glucosyl residues on native starch grains and related maltodextrin compounds in vitro. This protein interacts with the plant SnRK AKIN11, binds starch, and is localized in the chloroplast. sex4 mutants have elevated levels of starch.
AT3G52200	*(LTA3)*	Encodes a dihydrolipoamide S-acetyltransferase, a subunit of the mitochondrial pyruvate dehydrogenase complex.
AT3G52210		S-adenosyl-L-methionine-dependent methyltransferases superfamily protein
AT3G52230		hypothetical protein
AT3G52240		transcriptional regulator ATRX
AT3G52260		Pseudouridine synthase family protein
AT3G52270		Transcription initiation factor IIF, beta subunit
AT3G52290	*IQ-DOMAIN 3 (IQD3)*	Member of IQ67 (CaM binding) domain containing family.
AT3G52340	*SUCROSE-6F-PHOSPHATE PHOSPHOHYDROLASE 2 (SPP2)*	sucrose-phosphatase (SPP2)
AT3G52370	*FASCICLIN-LIKE ARABINOGALACTAN PROTEIN 15 PRECURSOR (FLA15)*	Fasciclin-like arabinogalactan protein. Possibly involved in embryogenesis and seed development.
AT3G52380	*CHLOROPLAST RNA-BINDING PROTEIN 33 (CP33)*	Encodes a chloroplast RNA-binding protein that stabilizes chloroplast RNAs as evidenced by analyses of transcript accumulation in null mutants. Essential for seedling development (albino, strongly retarded growth even on sucrose-containing medium).
AT3G52390		TatD related DNase
AT3G52400	*SYNTAXIN OF PLANTS 122 (SYP122)*	syntaxin protein, involved in the negative regulation of defense pathways such as programmed cell death, salicylic acid signalling pathway, jasmonic acid signalling pathway
AT3G52430	*PHYTOALEXIN DEFICIENT 4 (PAD4)*	Encodes a lipase-like gene that is important for salicylic acid signaling and function in resistance (R) gene-mediated and basal plant disease resistance. PAD4 can interact directly with EDS1, another disease resistance signaling protein. Expressed at elevated level in response to green peach aphid (GPA) feeding, and modulates the GPA feeding-induced leaf senescence through a mechanism that doesn't require camalexin synthesis and salicylic acid (SA) signaling. Required for the ssi2-dependent heightened resistance to GPA. The mRNA is cell-to-cell mobile.
AT3G52450	*PLANT U-BOX 22 (PUB22)*	Encodes a cytoplasmically localized U-box domain E3 ubiquitin ligase protein that is involved in the response to water stress and acts as a negative regulator of PAMP-triggered immunity.
AT3G52460		hydroxyproline-rich glycoprotein family protein
AT3G52470		Late embryogenesis abundant (LEA) hydroxyproline-rich glycoprotein family
AT3G52480		transmembrane protein
AT3G52490	*SMAX1-LIKE 3 (SMXL3)*	Encodes a member of an eight-gene family (SMAX1 and SMAX1-like) that has weak similarity to AtHSP101, a ClpB chaperonin required for thermotolerance.
AT3G52500		Eukaryotic aspartyl protease family protein
AT3G52540	*OVATE FAMILY PROTEIN 18 (OFP18)*	ovate family protein 18
AT3G52550		transcription repressor OFP15-like protein
AT3G52570		alpha/beta-Hydrolases superfamily protein
AT3G52600	*CELL WALL INVERTASE 2 (CWINV2)*	Cell wall invertase expressed in flowers and ovary placental tissues. Reduced expression is correlated with decreased ovule production suggesting a link between sugar sensing and ovule initiation.
AT3G52620		transmembrane protein
AT3G52650		
AT3G52690		RNI-like superfamily protein
AT3G52700		hypothetical protein
AT3G52710		hypothetical protein
AT3G52720	*ALPHA CARBONIC ANHYDRASE 1 (ACA1)*	Encodes an alpha carbonic anhydrase (CAH1) located in the chloroplast stroma. Most chloroplast proteins are encoded by the nuclear genome and imported with the help of sorting signals that are intrinsic parts of the polypeptides. CAH1 takes an alternative route through the secretory pathway, and becomes N-glycosylated before entering the chloroplast. Glycosylation and intra-molecular disulfide bridge fromation are necessary for the correct folding, ER export, trafficking and activity of the protein.
AT3G52740	*BLUE-LIGHT INHIBITOR OF CRYPTOCHROMES 1 (BIC1)*	Plant specific protein.BIC1 and BIC2 inhibit cryptochrome function by blocking blue light-dependent cryptochrome dimerization.Light activated transcription of BICs is mediated by cryptochromes.
AT3G52750	*(FTSZ2-2)*	Nuclear gene that encodes a plastidial division protein (FtsZ2-2). FtsZ2-2 is involved in chloroplast morphology and internal organisation in addition to participating in chloroplast partition
AT3G52770	*LITTLE ZIPPER 3 (ZPR3)*	ZPR3 is a small-leucine zipper containing protein that is involved in the establishment of leaf polarity.
AT3G52780	*(PAP20)*	Purple acid phosphatases superfamily protein
AT3G52810	*PURPLE ACID PHOSPHATASE 21 (PAP21)*	purple acid phosphatase 21

AT3G52820	*PURPLE ACID PHOSPHATASE 22 (PAP22)*	purple acid phosphatase 22
AT3G52830		ankyrin repeat protein
AT3G52840	*BETA-GALACTOSIDASE 2 (BGAL2)*	beta-galactosidase 2
AT3G52870		IQ calmodulin-binding motif family protein
AT3G52890	*KCBP-INTERACTING PROTEIN KINASE (KIPK)*	KCBP-interacting protein kinase interacts specifically with the tail region of KCBP
AT3G52900		RAB6-interacting golgin (DUF662)
AT3G52920		transcriptional activator (DUF662)
AT3G52940	*FACKEL (FK)*	Encodes a sterol C-14 reductase required for cell division and expansion and is involved in proper organization of the embryo.
AT3G52950		CBS / octicosapeptide/Phox/Bemp1 (PB1) domains-containing protein
AT3G52960	*PEROXIREDOXIN-II-E (PRXIIE)*	Thioredoxin superfamily protein
AT3G52970	*CYTOCHROME P450, FAMILY 76, SUBFAMILY G, POLYPEPTIDE 1 (CYP76G1)*	member of CYP76G
AT3G53000	*PHLOEM PROTEIN 2-A15 (PP2-A15)*	phloem protein 2-A15
AT3G53040		late embryogenesis abundant protein, putative / LEA protein
AT3G53060	*SKP1-LIKE 6 (SK6)*	SKP1-like 6
AT3G53070		Putative membrane lipoprotein
AT3G53080		D-galactoside/L-rhamnose binding SUEL lectin protein
AT3G53100		GDSL-motif esterase/acyltransferase/lipase. Enzyme group with broad substrate specificity that may catalyze acyltransfer or hydrolase reactions with lipid and non-lipid substrates.
AT3G53110	*LOW EXPRESSION OF OSMOTICALLY RESPONSIVE GENES 4 (LOS4)*	Encodes a putative DEAD-Box RNA Helicase and has RNA-dependent ATPase activity. Mutant is Sensitive to chilling stress and heat stress. Germination of the mutant is inhibited by ABA. LOS4 may be involved in temperature sensing. Is enriched in the nuclear envelope and also located in the cytoplasm. LOS4 is involved in export of poly A RNA. The mRNA is cell-to-cell mobile.
AT3G53140	*NICOTINATE N-METHYLTRANSFERASE (NANMT)*	Nicotinate N-methyltransferase involved in N-methylnicotinate formation.
AT3G53150	*UDP-GLUCOSYL TRANSFERASE 73D1 (UGT73D1)*	UDP-glucosyl transferase 73D1
AT3G53160	*UDP-GLUCOSYL TRANSFERASE 73C7 (UGT73C7)*	UGT73C7 is induced by pathogen infection. It glycosylates p-coumaric acid and ferulic acid to modulate phenylpropanoid metabolism and induce innate immune response.
AT3G53170		LOW protein: PPR containing protein
AT3G53180	*NODULIN/GLUTAMINE SYNTHASE-LIKE PROTEIN (NodGS)*	Encodes a protein that is the product of a fusion gene with a C-terminal GSI like sequence and an N-terminal part sharing homology with nodulins. It self-assembles into oligomers and its expression is increased in response to flagellin treatment. The protein co-localizes with microtubules and binds gamma-tubulin. RNAi lines are affected in root morphogenesis.
AT3G53190		Pectin lyase-like superfamily protein
AT3G53200	*MYB DOMAIN PROTEIN 27 (MYB27)*	Member of the R2R3 factor gene family.
AT3G53210	*USUALLY MULTIPLE ACIDS MOVE IN AND OUT TRANSPORTERS 6 (UMAMIT6)*	nodulin MtN21-like transporter family protein
AT3G53220		Thioredoxin superfamily protein
AT3G53230	*CELL DIVISION CYCLE 48B (ATCDC48B)*	CDC48 is induced upon oilseed rape mosaic tobamovirus infection and appears to be involved in controlling virus movement.
AT3G53240	*RECEPTOR LIKE PROTEIN 45 (RLP45)*	receptor like protein 45
AT3G53250	*SMALL AUXIN UPREGULATED RNA 57 (SAUR57)*	SAUR-like auxin-responsive protein family
AT3G53260	*PHENYLALANINE AMMONIA-LYASE 2 (PAL2)*	Encodes phenylalanine lyase. Arabidopsis has four PALs: AT2G37040 (PAL1), AT3G53260 (PAL2), AT5G04230 (PAL3) and AT3G10340 (PAL4).
AT3G53270		Small nuclear RNA activating complex (SNAPc), subunit SNAP43 protein
AT3G53280	*CYTOCHROME P450 71B5 (CYP71B5)*	cytochrome P450 monooxygenase The mRNA is cell-to-cell mobile.
AT3G53290	*CYTOCHROME P450, FAMILY 71, SUBFAMILY B, POLYPEPTIDE 30 PSEUDOGENE (CYP71B30P)*	missing N-term 80 AA not found between end of 71B5 and start of this sequence probably a pseudogene, from http://drnelson.utmem.edu/biblioD.html
AT3G53300	*CYTOCHROME P450, FAMILY 71, SUBFAMILY B, POLYPEPTIDE 31 (CYP71B31)*	putative cytochrome P450
AT3G53310	*(REM16)*	B3 domain transcription factor that binds to and regulates the expression of SOC1 and FT.
AT3G53320	*GROWING PLUS-END TRACKING PROTEIN 2 (GPT2)*	Encodes a microtubule-associated protein track growing microtubule plus ends.
AT3G53350	*ROP INTERACTIVE PARTNER 3 (RIP3)*	Encodes RIP3 (ROP interactive partner 3), a microtubule-binding protein that is anchored to the plasma membrane domains and promotes local microtubule disassembly, forming as specific pattern of secondary walls in xylem vessel cells. Localized at microtubules and interacts with the plant-specific kinesin AtKinesin-13A.
AT3G53360		Tetratricopeptide repeat (TPR)-like superfamily protein
AT3G53370		S1FA-like DNA-binding protein
AT3G53380	*L-TYPE LECTIN RECEPTOR KINASE VIII.1 (LECRK-VIII.1)*	Concanavalin A-like lectin protein kinase family protein
AT3G53390		Transducin/WD40 repeat-like superfamily protein
AT3G53400		peptide upstream protein
AT3G53420	*PLASMA MEMBRANE INTRINSIC PROTEIN 2A (PIP2A)*	a member of the plasma membrane intrinsic protein subfamily PIP2. localizes to the plasma membrane and exhibits water transport activity in Xenopus oocyte. expressed specifically in the vascular bundles and protein level increases slightly during leaf dev. When expressed in yeast cells can conduct hydrogen peroxide into those cells.
AT3G53440		Homeodomain-like superfamily protein
AT3G53450	*LONELY GUY 4 (LOG4)*	Putative lysine decarboxylase family protein
AT3G53460	*CHLOROPLAST RNA-BINDING PROTEIN 29 (CP29)*	Encodes a nuclear gene with a consensus RNA-binding domain that is localized to the chloroplast.
AT3G53470		2,3-bisphosphoglycerate-independent phosphoglycerate mutase

AT3G53480	*ATP-BINDING CASSETTE G37 (ABCG37)*	Negative regulator of auxin polar transport inhibitors. ABCG37 regulates auxin distribution and homeostasis in roots by excluding IBA from the root apex, but does not act directly in basipetal transport. ABCG37 and ABCG36 act redundantly at outermost root plasma membranes and, transport IBA out of the cells. Also involved in root transmembrane secretion of fluorescent phenolics involved in Fe uptake. The mRNA is cell-to-cell mobile.
AT3G53510	*ATP-BINDING CASSETTE G20 (ABCG20)*	Belongs to a clade of five Arabidopsis thaliana ABCG half-transporters that are required for synthesis of an effective suberin barrier in roots and seed coats (ABCG2, ABCG6, and ABCG20) and for synthesis of an intact pollen wall (ABCG1 and ABCG16). Phloem-expressed and plasma membrane-localized jasmonate transporter which together with JAT3 and GLR3.3 involved in regulating long-distance translocation of JA, which is important for driving the loading, translocation of JA in the phloem pathway by a self-propagation mode, contributing to wound-induced systemic response/resistance.
AT3G53540	*TON1 RECRUITING MOTIF 19 (TRM19)*	afadin
AT3G53550		FBD-like domain family protein
AT3G53560		Tetratricopeptide repeat (TPR)-like superfamily protein
AT3G53570	*FUS3-COMPLEMENTING GENE 1 (FC1)*	a member of a CDC2-related kinase subfamily, the LAMMER kinases. activates STE12-dependent functions in yeast.
AT3G53580		diaminopimelate epimerase family protein
AT3G53590		LRR receptor-like Serine/Threonine-kinase
AT3G53600	*(ZAT18)*	Nuclear C2H2 zinc finger protein.Expression is induced by cold, osmotic, salt, and drought stress. Over expression confers some drought tolerance whereas mutants display some drought sensitivity.
AT3G53620	*PYROPHOSPHORYLASE 4 (PPa4)*	Encodes a soluble protein with inorganic pyrophosphatase activity that is highly specific for Mg-inorganic pyrophosphate. The mRNA is cell-to-cell mobile.
AT3G53630	*GUN1-INTERACTING PROTEIN 1 (GIP1)*	hypothetical protein
AT3G53660		
AT3G53670		hypothetical protein
AT3G53680		Acyl-CoA N-acyltransferase with RING/FYVE/PHD-type zinc finger domain-containing protein
AT3G53690		RING/U-box superfamily protein
AT3G53720	*CATION/H+ EXCHANGER 20 (CHX20)*	member of Putative Na+/H+ antiporter family. Involved in the osmoregulation through K(+) fluxes and possibly pH modulation of an active endomembrane system in guard cells.
AT3G53770		late embryogenesis abundant 3 (LEA3) family protein
AT3G53780	*RHOMBOID-LIKE PROTEIN 4 (RBL4)*	RHOMBOID-like protein 4
AT3G53790	*TRF-LIKE 4 (TRFL4)*	Arabidopsis thaliana telomere-binding protein, putative (At3g53790)
AT3G53800	*FES1B (Fes1B)*	Encodes one of the Arabidopsis orthologs of the human Hsp70-binding protein 1 (HspBP-1) and yeast Fes1p: Fes1A (AT3G09350), Fes1B (AT3G53800), Fes1C (AT5G02150).
AT3G53820		C2H2 and C2HC zinc fingers superfamily protein
AT3G53840		Protein kinase superfamily protein
AT3G53880	*ALDO-KETO REDUCTASE FAMILY 4 MEMBER C11 (AKR4C11)*	NAD(P)-linked oxidoreductase superfamily protein
AT3G53900	*URACIL PHOSPHORIBOSYLTRANSFERASE (UPP)*	Encodes UPP, a plastidial uracil phosphoribosyltransferase (UPRT) involved in uracil salvage. Loss-of-function mutation causes dramatic growth retardation, a pale-green to albino phenotype, abnormal root morphology and chloroplastic disorders.
AT3G53910		malate dehydrogenase-like protein
AT3G53920	*RNAPOLYMERASE SIGMA-SUBUNIT C (SIGC)*	Encodes a sigma-like transcription factor, Sigma 3 (SIG3 or SIGC). As a subunit of chloroplast RNA polymerase, SIG3 confers the ability to recognize promoter sequences on the core enzyme. SIG3 transcribes specifically the psbN gene in plastids.
AT3G53940		Mitochondrial substrate carrier family protein
AT3G53950	*GALACTOSE OXIDASE-LIKE 2 (GOXL2)*	Galactose oxydase; may function in tissues that require mechanical reinforcements in the absence of lignification.
AT3G53960		Major facilitator superfamily protein
AT3G53970	*PROTEASOME REGULATOR1 (PTRE1)*	PTRE1 was identified as homologous to human PI31. It has a conserved proline-rich domain at the C-terminus and a highly conserved FP (Fbxo7/PI31) dimerization domain at the N-terminus as well as some novel, conserved domains found only in plants. It regulates auxin signaling possibly via its proteosome suppressing activity.
AT3G53980		Bifunctional inhibitor/lipid-transfer protein/seed storage 2S albumin superfamily protein
AT3G53990	*UNIVERSAL STRESS PROTEIN (ATUSP)*	Encodes universal stress protein (USP). Functions as a molecular chaperone under heat shock and oxidative stress conditions. Chaperone activity and assembly into complexes is redox regulated.
AT3G54030	*BRASSINOSTEROID-SIGNALING KINASE 6 (BSK6)*	kinase with tetratricopeptide repeat domain-containing protein
AT3G54040		PAR1 protein
AT3G54050	*HIGH CYCLIC ELECTRON FLOW 1 (HCEF1)*	Encodes a chloroplastic fructose 1,6-bisphosphate phosphatase. also known as HCEF1 (High Cyclic Electron Flow 1). hcef1 mutants have constitutively elevated electron flow (CEFI) and plants with antisense suppression of this enzyme have higher levels of net leaf photosynthesis and increased sucrose biosynthesis. The mRNA is cell-to-cell mobile.
AT3G54060		myosin-M heavy protein
AT3G54070		LOW protein: ankyrin repeat protein
AT3G54080		Concanavalin A-like lectin family protein
AT3G54090	*FRUCTOKINASE-LIKE 1 (FLN1)*	Encodes a fructokinase-like protein (AT3G54090/FLN1, AT1G69200/FLN2), a member of the pfkB-carbohydrate kinase family. FLN1 and FLN2 are potential plastidial thioredoxin z (TRX z) targets. Mutants display mutant chloroplast development, general plant growth and development defects and defects in PEP-dependent transcription.
AT3G54100		O-fucosyltransferase family protein

AT3G54110	*PLANT UNCOUPLING MITOCHONDRIAL PROTEIN 1 (PUMP1)*	Member of Uncoupling protein PUMP2 family. Encodes a mitochondrial uncoupling protein AtUCP1 involved in maintain the redox poise of the mitochondrial electron transport chain to facilitate photosynthetic metabolism. Disruption of UCP1 results in a photosynthetic phenotype. Specifically there is a restriction in photorespiration with a decrease in the rate of oxidation of photorespiratory glycine in the mitochondrion. This change leads to an associated reduced photosynthetic carbon assimilation rate. The mRNA is cell-to-cell mobile.
AT3G54120		Reticulon family protein
AT3G54130		Josephin family protein
AT3G54150	*EXINE FORMATION DEFECTIVE (EFD)*	Encodes a DNA methyltransferase required for pollen exine formation and male fertility via the regulation of callose wall and primexine formation.
AT3G54200	*(NHL39)*	Late embryogenesis abundant (LEA) hydroxyproline-rich glycoprotein family
AT3G54210	*PLASTID RIBOSOMAL PROTEINS OF THE 50S SUBUNIT 17 (PRPL17)*	Ribosomal protein L17 family protein
AT3G54220	*SCARECROW (SCR)*	Encodes a member of a novel family having similarity to DNA binding proteins containing basic-leucine zipper regions; scr is expressed in cortex/endodermal initial cells and in the endodermal cell lineage. Regulates the radial organization of the root. Is required cell-autonomously for distal specification of the quiescent center, which in turn regulates stem cell fate of immediately surrounding cells. SCR appears to be a direct target of SHR. SCR and SCR-LIKE 23 act redundantly in bundle sheath cell fate specification. The scr mutant has a higher level of hydrogen peroxide in the elongation zone due to reduced expression of peroxidase genes.
AT3G54230	*SUPPRESSOR OF ABI3-5 (SUA)*	Encodes a splicing factor SUA (suppressor of abi3-5), homologous to the human protein RBM5. Controls alternative splicing of the developmental regulator ABI3.
AT3G54240		alpha/beta-Hydrolases superfamily protein
AT3G54260	*TRICHOME BIREFRINGENCE-LIKE 36 (TBL36)*	Encodes a member of the TBL (TRICHOME BIREFRINGENCE-LIKE) gene family containing a plant-specific DUF231 (domain of unknown function) domain. TBL gene family has 46 members, two of which (TBR/AT5G06700 and TBL3/AT5G01360) have been shown to be involved in the synthesis and deposition of secondary wall cellulose, presumably by influencing the esterification state of pectic polymers. A nomenclature for this gene family has been proposed (Volker Bischoff & Wolf Scheible, 2010, personal communication).
AT3G54270		sucrose-6F-phosphate phosphohydrolase family protein
AT3G54280	*ROOT GROWTH DEFECTIVE 3 (RGD3)*	ROOT GROWTH DEFECTIVE 3
AT3G54310		DNA-directed RNA polymerase subunit alpha
AT3G54320	*WRINKLED 1 (WRI1)*	WRINKLED1 encodes transcription factor of the AP2/ERWEBP class. Protein has two plant-specific (AP2/EREB) DNA-binding domains and is involved in the control of storage compound biosynthesis in Arabidopsis. Mutants have wrinkled seed phenotype, due to a defect in the incorporation of sucrose and glucose into triacylglycerols. Transgenic sGsL plants (21-day-old) grown on 6% sucrose for 24 hours had 2-fold increase in levels of expressions (sGsL line carries a single copy of T-DNA containing the Spomin::GUS-Spomin::LUC dual reporter genes in the upper arm of chromosome 5 of ecotype Col-0. The sporamin .minimal. promoter directs sugar-inducible expression of the LUC and GUS reporters in leaves). Regulation by LEC2 promotes fatty acid accumulation during seed maturation. Splice form 3 is the major form expressed in seedlings.Mutations in the C terminal intrinsically disordered region increase the stability of WRI1 and lead to increased oil production.
AT3G54330		
AT3G54340	*APETALA 3 (AP3)*	Floral homeotic gene encoding a MADS domain protein homologous to SRF transcription factors. Specifies petal and stamen identities. Associates with PISTILLATA.
AT3G54370		
AT3G54380	*YEAST SAC3 HOMOLOG C (SAC3C)*	SAC3/GANP/Nin1/mts3/eIF-3 p25 family
AT3G54390		sequence-specific DNA binding transcription factor
AT3G54400		Eukaryotic aspartyl protease family protein
AT3G54420	*HOMOLOG OF CARROT EP3-3 CHITINASE (EP3)*	encodes an EP3 chitinase that is expressed during somatic embryogenesis in 'nursing' cells surrounding the embryos but not in embryos themselves. The gene is also expressed in mature pollen and growing pollen tubes until they enter the receptive synergid, but not in endosperm and integuments as in carrot. Post-embryonically, expression is found in hydathodes, stipules, root epidermis and emerging root hairs.
AT3G54430	*SHI-RELATED SEQUENCE 6 (SRS6)*	A member of SHI gene family. Arabidopsis thaliana has ten members that encode proteins with a RING finger-like zinc finger motif. Despite being highly divergent in sequence, many of the SHI-related genes are partially redundant in function and synergistically promote gynoecium, stamen and leaf development in Arabidopsis.
AT3G54490	*RNA POLYMERASE II FIFTH LARGEST SUBUNIT, E (RPB5E)*	NRPE5-like protein of unknown function; homologous to budding yeast RPB5
AT3G54500	*NIGHT LIGHT-INDUCIBLE AND CLOCK-REGULATED GENE 2 (LNK2)*	Member of a small family (4 proteins) in Arabidopsis that have some overlap in function. LNK2 along with LNK1 functions in the integration of light signaling and circadian clock. It is regulated by the clock TOC1 complex. Functions as a transcriptional coactivator.
AT3G54510		Early-responsive to dehydration stress protein (ERD4)
AT3G54520		hypothetical protein
AT3G54560	*HISTONE H2A 11 (HTA11)*	Encodes HTA11, a histone H2A protein. Loss of all H2A.Z (triple mutant with HTA8 and HTA9) results in a reduction in DNA methylation of transposons but not that of genes. Loss of H2A.Z causes misregulation of many genes involved in the response to developmental and environmental cues, and that these genes tend to have high levels of gene-body H2A.Z.
AT3G54570		Plant calmodulin-binding protein-like protein
AT3G54580	*EXTENSIN 17 (EXT17)*	Proline-rich extensin-like family protein
AT3G54590	*HYDROXYPROLINE-RICH GLYCOPROTEIN (HRGP1)*	Encodes a hydroxyproline-rich glycoprotein. The mRNA is cell-to-cell mobile.
AT3G54620	*BASIC LEUCINE ZIPPER 25 (BZIP25)*	bZIP transcription factor-like protein mRNA
AT3G54630	*(NDC80)*	kinetochore protein
AT3G54640	*TRYPTOPHAN SYNTHASE ALPHA CHAIN (TSA1)*	Catalyzes the conversion of indole-3-glycerolphosphate to indole, the penultimate reaction in the biosynthesis of tryptophan. Functions as a heterocomplex with tryptophan synthase beta subunit (TSA2).
AT3G54650	*F BOX-LIKE17 (FBL17)*	F- box protein involved in regulation of cell cycle genes.

AT3G54660	*GLUTATHIONE REDUCTASE (GR)*	Encodes glutathione reductase that is most likely localized in the chloroplast. Flavoenzyme-encoding gene.
AT3G54670	*TITAN8 (TTN8)*	Encodes a member of the Arabidopsis cohesin complex that is essential for viability and sister chromatid alignment.
AT3G54680		proteophosphoglycan-like protein
AT3G54720	*ALTERED MERISTEM PROGRAM 1 (AMP1)*	Encodes glutamate carboxypeptidase. Various alleles show-increased cotyledon number and rate of leaf initiation, show transformation of leaves to cotyledons, altered flowering time and photomorphogenesis and an increased level of cytokinin biosynthesis. Involved in ethylene enhanced hypocotyl elongation in the light. Strong genetic interaction between TGH and AMP1.
AT3G54770	*ABA-REGULATED RNA-BINDING PROTEIN 1 (ARP1)*	Encodes a putative RNA binding protein that is localized in the nucleus and affects ABA-regulated seed germination of Arabidopsis.
AT3G54790	*PLANT U-BOX 3 (PUB3)*	ARM repeat superfamily protein
AT3G54800		Pleckstrin homology (PH) and lipid-binding START domains-containing protein
AT3G54810	*BLUE MICROPYLAR END 3 (BME3)*	Encodes a protein containing a GATA type zinc finger domain that is expressed in the embryo axis and involved in germination. Mutants have a reduced rate of germination even when stratified.
AT3G54820	*PLASMA MEMBRANE INTRINSIC PROTEIN 2;5 (PIP2;5)*	plasma membrane intrinsic protein 2
AT3G54850	*PLANT U-BOX 14 (PUB14)*	Encodes a protein with a typical U-box domain followed by an Armadillo repeat region, a domain organization that is frequently found in plant U-box proteins. Displays ubiquitin ligase activity in vitro. Regulator of flowering time.
AT3G54890	*PHOTOSYSTEM I LIGHT HARVESTING COMPLEX GENE 1 (LHCA1)*	Encodes a component of the light harvesting complex associated with photosystem I.
AT3G54900	*CAX INTERACTING PROTEIN 1 (CXIP1)*	A.thaliana PICOT protein.It activates CAX1 gene Calcium transport activity.In other organisms, PICOT proteins appear to play a negative regulatory role in cellular stress responses.
AT3G54920	*POWDERY MILDEW RESISTANT 6 (PMR6)*	Powdery mildew resistant mutant encodes a pectate lyase-like protein The mRNA is cell-to-cell mobile.
AT3G54940		Papain family cysteine protease
AT3G54950	*PATATIN-RELATED PHOSPHOLIPASE IIIBETA (pPLAIIIbeta)*	Encodes pPLAIIIbeta, a member of the Group 3 patatin-related phospholipases. pPLAIIIbeta hydrolyzes phospholipids and galactolipids and additionally has acyl-CoA thioesterase activity. Alterations of pPLAIIIβ result in changes in lipid levels and composition.
AT3G54980		Pentatricopeptide repeat (PPR) superfamily protein
AT3G55010	*(PUR5)*	encoding phosphoribosylformylglycinamidine cyclo-ligase (syn. AIR synthetase)that phosphorylates 5-phosphoribosyl-N-formylglycinamidine (FGAM) to form 5-aminoimidazole ribonucleotide (AIR)
AT3G55020		Ypt/Rab-GAP domain of gyp1p superfamily protein
AT3G55030	*PHOSPHATIDYLGLYCEROLPHOSPHATE SYNTHASE 2 (PGPS2)*	Encodes a phosphatidylglycerolphosphate synthase.
AT3G55060		CAP-gly domain linker
AT3G55090	*ATP-BINDING CASSETTE G16 (ABCG16)*	Belongs to a clade of five Arabidopsis thaliana ABCG half-transporters that are required for synthesis of an effective suberin barrier in roots and seed coats (ABCG2, ABCG6, and ABCG20) and for synthesis of an intact pollen wall (ABCG1 and ABCG16).
AT3G55100	*ATP-BINDING CASSETTE G17 (ABCG17)*	ABC-2 type transporter family protein
AT3G55120	*TRANSPARENT TESTA 5 (TT5)*	Catalyzes the conversion of chalcones into flavanones. Required for the accumulation of purple anthocyanins in leaves and stems. Co-expressed with CHS.
AT3G55130	*ATP-BINDING CASSETTE G19 (ABCG19)*	Encodes a vacuole localized protein of the ABC transporter White-Brown Complex (WBC) family. When overexpressed in planta, confers resistance to kanamycin.
AT3G55150	*EXOCYST SUBUNIT EXO70 FAMILY PROTEIN H1 (EXO70H1)*	A member of EXO70 gene family, putative exocyst subunits, conserved in land plants. Arabidopsis thaliana contains 23 putative EXO70 genes, which can be classified into eight clusters on the phylogenetic tree.
AT3G55170		Ribosomal L29 family protein
AT3G55180	*(MAGL10)*	alpha/beta-Hydrolases superfamily protein
AT3G55190	*(MAGL11)*	alpha/beta-Hydrolases superfamily protein
AT3G55250	*PIGMENT DEFECTIVE 329 (PDE329)*	Encodes a nucleus-encoded protein, Photosystem I Assembly 3 (PSA3), that is required for PSI accumulation.
AT3G55260	*BETA-HEXOSAMINIDASE 1 (HEXO1)*	Encodes a protein with β-hexosaminidase activity (the enzyme is active with p-nitrophenyl-β-N-acetylglucosaminide as substrate but displayed only a minor activity toward p-nitrophenyl-β-N-acetylgalactosaminide). The enzyme displays no distinct preference for a specific terminal GlcNAc residue and indeed cleaved the asialoagalactodabsylglycopeptide GnGn to a mixture of products.
AT3G55270	*MITOGEN-ACTIVATED PROTEIN KINASE PHOSPHATASE 1 (MKP1)*	Encodes MAP kinase phosphatase 1 (MKP1). Loss of MKP1 results in hypersensitivity to acute UV-B stress, but without impairing UV-B acclimation.
AT3G55300		transposable_element_gene;copia-like retrotransposon family, has a 4.5e-28 P-value blast match to GB:CAA37924 orf 2 (Ty1_Copia-element) (Arabidopsis thaliana);(source:TAIR10)
AT3G55310		NAD(P)-binding Rossmann-fold superfamily protein
AT3G55330	*PSBP-LIKE PROTEIN 1 (PPL1)*	Facilitates the assembly of the photosystem II supercomplexes and optimizes plant pitness under fluctuating light.
AT3G55350	*(HHP1)*	PIF / Ping-Pong family of plant transposase
AT3G55360	*ECERIFERUM 10 (CER10)*	Enoyl-CoA reductase is involved in all very long chain fatty acids (VLCFA) elongation reactions that are required for cuticular wax, storage lipid and sphingolipid metabolism. The protein is located in the ER, but in contrast to its yeast homolog TSC13 is not particularly enriched in the nuclear envelope-vacuole junction. Mutants in this gene show abnormal organ morphology and stem glossiness. Cells in all tissues are only about 1/3 of the size of wild type cells. The morphological changes are most likely to result from the reduction in the VLCFA content of sphingolipids. Mutants also show abnormalities in the endocytic membrane organization and transport as well as reduced trichome papillae.
AT3G55390	*CASP-LIKE PROTEIN 4C1 (CASPL4C1)*	Uncharacterized protein family (UPF0497)
AT3G55400	*OVULE ABORTION 1 (OVA1)*	methionyl-tRNA synthetase / methionine-tRNA ligase / MetRS (cpMetRS)
AT3G55410	*(E1-OGDH1)*	Encodes the E1 subunit of the 2-oxoglutarate dehydrogenase.
AT3G55420		hypothetical protein
AT3G55430		O-Glycosyl hydrolases family 17 protein

AT3G55470		Calcium-dependent lipid-binding (CaLB domain) family protein
AT3G55500	*EXPANSIN A16 (EXPA16)*	expansin-like protein. Naming convention from the Expansin Working Group (Kende et al, 2004. Plant Mol Bio). Involved in the formation of nematode-induced syncytia in roots of Arabidopsis thaliana.
AT3G55510	*REBELOTE (RBL)*	Encodes a regulator of floral determinacy in that interacts with both nucleolar and nucleoplasmic proteins.
AT3G55520		FKBP-like peptidyl-prolyl cis-trans isomerase family protein
AT3G55530	*SALT- AND DROUGHT-INDUCED RING FINGER1 (SDIR1)*	Encodes an intracellular membrane localized protein with E3 ligase activity, found in the ER with the C-terminus facing the cytoplasm. It is involved in regulation of ABA signaling. Loss of function alleles show decreased sensitivity to ABA. Overexpression results in increased sensitivity to ABA.
AT3G55550	*L-TYPE LECTIN RECEPTOR KINASE S.4 (LECRK-S.4)*	Concanavalin A-like lectin protein kinase family protein
AT3G55560	*AT-HOOK MOTIF NUCLEAR-LOCALIZED PROTEIN 15 (AHL15)*	AT-hook protein of GA feedback 2
AT3G55580	*TOLERANT TO CHILLING AND FREEZING1 (TCF1)*	TCF1 encodes a member of the RCC1 gene family and is required for chromatin based gene regulation of cold responsive genes in a CBF-independent manner. It is expressed in response to cold but not ABA.
AT3G55590		Glucose-1-phosphate adenylyltransferase family protein
AT3G55605		Mitochondrial glycoprotein family protein
AT3G55610	*DELTA 1-PYRROLINE-5-CARBOXYLATE SYNTHASE 2 (P5CS2)*	encodes delta 1-pyrroline-5-carboxylate synthetase B. Gene expression is induced by dehydration, high salt and ABA. Knock-out mutations in P5CS2 are embryo-lethal. P5CS2 appears to be present in different cells and/or different subcellular locations from P5CS1 in a tissue-dependent manner. Mutants are defective in pollen development.
AT3G55620	*EUKARYOTIC INITIATION FACOR 6A (eIF6A)*	Translation initiation factor IF6
AT3G55630	*DHFS-FPGS HOMOLOG D (DFD)*	Encodes one of the three folylpolyglutamate synthetase isoforms (FPGSs): FPGS1 (At5g05980, plastidic), FPGS2 (At3g10160, mitochondrial) and FPGS3 (At3g55630, cytosolic).
AT3G55640		Mitochondrial substrate carrier family protein
AT3G55650		Pyruvate kinase family protein
AT3G55660	*ROP (RHO OF PLANTS) GUANINE NUCLEOTIDE EXCHANGE FACTOR 6 (ROPGEF6)*	Encodes a member of KPP-like gene family, homolog of KPP (kinase partner protein) gene in tomato. Also a member of the RopGEF (guanine nucleotide exchange factor) family, containing the novel PRONE domain (plant-specific Rop nucleotide exchanger), which is exclusively active towards members of the Rop subfamily.
AT3G55670		F-box/FBD/LRR protein
AT3G55680		Plant invertase/pectin methylesterase inhibitor superfamily protein
AT3G55700	*UDP-GLYCOSYLTRANSFERASE 76F1 (UGT76F1)*	UDP-Glycosyltransferase superfamily protein
AT3G55710		UDP-Glycosyltransferase superfamily protein
AT3G55720	*BOUNDARY OF ROP DOMAIN7 (BDR7)*	replication factor C subunit, putative (DUF620)
AT3G55740	*PROLINE TRANSPORTER 2 (PROT2)*	Encodes a proline transporter with affinity for gly betaine, proline, and GABA. Protein is expressed most highly in the roots.
AT3G55760	*LIKE EARLY STARVATION (LESV)*	hypothetical protein
AT3G55780		Glycosyl hydrolase superfamily protein
AT3G55790		transmembrane protein
AT3G55800	*SEDOHEPTULOSE-BISPHOSPHATASE (SBPASE)*	Encodes the chloroplast enzyme sedoheptulose-1,7-bisphosphatase (SBPase), involved in the carbon reduction of the Calvin cycle. Increase in SBPase activity in transgenic lines accumulate up to 50% more sucrose and starch than wild-type. The mRNA is cell-to-cell mobile.
AT3G55820		Fasciclin-like arabinogalactan family protein
AT3G55850	*LONG AFTER FAR-RED 3 (LAF3)*	Encodes a product that might regulate nucleo-cytoplasmic trafficking of an intermediate(s) involved in phyA signal transduction. Differs from isoform 2 only in the first few N-terminal amino acids.
AT3G55870		ADC synthase superfamily protein
AT3G55880	*SULPHATE UTILIZATION EFFICIENCY 4 (SUE4)*	A gain-of-function mutant of SUE4 exhibited improved low sulphur tolerance.
AT3G55890		Yippee family putative zinc-binding protein
AT3G55910		ADP-ribosylation factor GTPase-activating protein
AT3G55930		pre-mRNA-splicing factor
AT3G55940	*PHOSPHOLIPASE C 7 (PLC7)*	Phospholipase C family member. Double mutants with PLC5 show defects in seed coat mucilage, leaf serration and over-expression improves drought tolerance.
AT3G55950	*CRINKLY4 RELATED 3 (CCR3)*	CRINKLY4 related 3
AT3G55970	*JASMONATE-REGULATED GENE 21 (JRG21)*	One of 4 paralogs encoding a 2-oxoglutarate/Fe(II)-dependent oxygenases that hydroxylates JA to 12-OH-JA.
AT3G55980	*SALT-INDUCIBLE ZINC FINGER 1 (SZF1)*	salt-inducible zinc finger 1
AT3G56000	*CELLULOSE SYNTHASE LIKE A14 (CSLA14)*	encodes a gene similar to cellulose synthase
AT3G56040	*UDP-GLUCOSE PYROPHOSPHORYLASE 3 (UGP3)*	UDP-glucose pyrophosphorylase 3
AT3G56050		Protein kinase family protein
AT3G56060		Glucose-methanol-choline (GMC) oxidoreductase family protein
AT3G56070	*ROTAMASE CYCLOPHILIN 2 (ROC2)*	rotamase cyclophilin 2 (ROC2) exhibiting peptidyl-prolyl cis-trans isomerase activity involved in signal transduction.
AT3G56080		S-adenosyl-L-methionine-dependent methyltransferases superfamily protein
AT3G56090	*FERRITIN 3 (FER3)*	Encodes FERRITIN 3, AtFER3. Ferritins are a class of 24-mer multi-meric proteins found in all kingdoms of life. Function as the main iron store in mammals. Evidence suggests that Arabidopsis ferritins are essential to protect cells against oxidative damage, but they do not constitute the major iron pool.
AT3G56100	*MERISTEMATIC RECEPTOR-LIKE KINASE (MRLK)*	Protein kinase expressed in meristematic cells. Phosphorylates AGL24.
AT3G56130	*BCCP-LIKE PROTEIN 3 (BLP3)*	biotin/lipoyl attachment domain-containing protein
AT3G56140	*RETICULATA-RELATED 6 (RER6)*	DUF399 family protein, putative (DUF399 and DUF3411)

AT3G56150	*EUKARYOTIC TRANSLATION INITIATION FACTOR 3C (EIF3C)*	member of eIF3c - eukaryotic initiation factor 3c
AT3G56160		Sodium Bile acid symporter family
AT3G56170	*CA-2+ DEPENDENT NUCLEASE (CAN)*	Encodes a calcium-dependent nuclease with similarity to staphylococcal nuclease.
AT3G56190	*ALPHA-SOLUBLE NSF ATTACHMENT PROTEIN 2 (ALPHA-SNAP2)*	Encodes one of two alpha-SNAPs (soluble NSF attachment protein) in Arabidopsis
AT3G56200		Encodes a putative amino acid transporter. The mRNA is cell-to-cell mobile.
AT3G56230		BTB/POZ domain-containing protein
AT3G56240	*COPPER CHAPERONE (CCH)*	CCH protein belongs to a family of eukaryotic proteins that participate in intracellular copper homeostasis by delivering this metal to the secretory pathway; mainly located along the vascular bundles of senescing leaves and petioles as well as in stem sieve elements; hypothesized to have a role in copper mobilization from decaying organs towards reproductive structures, as a result of metalloprotein breakdown. The plant-specific C-terminal domain of the CCH protein forms amyloid-like fibrils in vitro.
AT3G56250		hypothetical protein
AT3G56260		hypothetical protein
AT3G56270		WEB family protein (DUF827)
AT3G56280		pseudogene of Protein kinase superfamily protein
AT3G56290		potassium transporter
AT3G56350		Iron/manganese superoxide dismutase family protein
AT3G56360		hypothetical protein
AT3G56370	*INFLORESCENCE AND ROOT APICES RECEPTOR KINASE (IRK)*	LRR-RLK with distinct polar localization within the plasma membrane in different cell types of the root. Mutants show defects in cell divisions within the root ground tissue.
AT3G56380	*RESPONSE REGULATOR 17 (RR17)*	response regulator 17
AT3G56390	*TRICHOME RELATED PROTEIN (TRP)*	TRP is a novel transcription factor. The protein shows similarity to WRKY transcription factors but does not interact with W-box sequences, nor does it appear to activate transcription in one-hybrid assays.TRP binds to and acts antagonistically to ZPF5 in trichome initiation, possibly by blocking ZPF5 binding to its target ZPF8.
AT3G56400	*WRKY DNA-BINDING PROTEIN 70 (WRKY70)*	Member of WRKY Transcription Factor; Group III. Function as activator of SA-dependent defense genes and a repressor of JA-regulated genes. WRKY70-controlled suppression of JA-signaling is partly executed by NPR1.
AT3G56410		hypothetical protein (DUF3133)
AT3G56430	*ATTIM21-LIKE 2 (ATTIM21L-2)*	TIM domain protein. Associates with components of mitochondrial complex I and III. May be involved in biogenesis of respiratory chain components.
AT3G56480	*STOMATAL CLOSURE-RELATED ACTIN BINDING PROTEIN 3 (SCAB3)*	myosin heavy chain-like protein
AT3G56490	*HIS TRIAD FAMILY PROTEIN 3 (HIT3)*	Encodes a protein that has adenylylsulfate sulfohydrolase activity (E.C. 3.6.2.1) in vitro.
AT3G56500		serine-rich protein-like protein
AT3G56520		NAC (No Apical Meristem) domain transcriptional regulator superfamily protein
AT3G56540		alpha/beta-Hydrolases superfamily protein
AT3G56550		Pentatricopeptide repeat (PPR) superfamily protein
AT3G56560	*NAC DOMAIN CONTAINING PROTEIN 65 (NAC065)*	NAC domain containing protein 65
AT3G56580	*RING ZINC FINGER 1 (RZF1)*	Encodes a functional E3 ubiquitin ligase involved in the dehydration stress response and regulation of proline biosynthesis.
AT3G56590		hydroxyproline-rich glycoprotein family protein
AT3G56600		phosphatidylinositol 4-kinase gamma-like protein
AT3G56610		prolamin-like protein
AT3G56620	*USUALLY MULTIPLE ACIDS MOVE IN AND OUT TRANSPORTERS 10 (UMAMIT10)*	nodulin MtN21-like transporter family protein
AT3G56630	*CYTOCHROME P450, FAMILY 94, SUBFAMILY D, POLYPEPTIDE 2 (CYP94D2)*	member of CYP94D
AT3G56650	*PSBP-DOMAIN PROTEIN 6 (PPD6)*	thylakoid lumenal protein (Mog1/PsbP/DUF1795-like photosystem II reaction center PsbP family protein)
AT3G56660	*BASIC REGION/LEUCINE ZIPPER MOTIF PROTEIN 49 (BZIP49)*	basic region/leucine zipper motif protein 49
AT3G56670		F-box/associated interaction domain protein
AT3G56690	*CAM INTERACTING PROTEIN 111 (CIP111)*	encodes a protein similar to ATPases and binds to calmodulin in vitro. This is a single-copy gene and is expressed in all tissues examined.
AT3G56700	*FATTY ACID REDUCTASE 6 (FAR6)*	Encodes a fatty-acyl-CoA reductase that is expressed in response to wounding.
AT3G56710	*SIGMA FACTOR BINDING PROTEIN 1 (SIB1)*	Sig1 binding protein; interacts with Sig1R4. As well as Sig1, SibI is imported into chloroplasts and its expression is light-dependent in mature chloroplasts.
AT3G56730		Putative endonuclease or glycosyl hydrolase
AT3G56760		Protein kinase superfamily protein
AT3G56810		hypothetical protein
AT3G56850	*ABA-RESPONSIVE ELEMENT BINDING PROTEIN 3 (AREB3)*	Encodes an ABA-responsive element binding protein with a bZIP domain. Located in the nucleus and expressed in the embryo during seed maturation.
AT3G56870		hypothetical protein
AT3G56880		VQ motif-containing protein
AT3G56890		F-box associated ubiquitination effector family protein
AT3G56910	*PLASTID-SPECIFIC 50S RIBOSOMAL PROTEIN 5 (PSRP5)*	Encodes PSRP5 (PLASTID-SPECIFIC 50S RIBOSOMAL PROTEIN 5). Functions in plastid translation.
AT3G56930	*PROTEIN S-ACYL TRANSFERASE 4 (PAT4)*	Protein S-acyl transferase 4 (PAT4). Mutants display defects in root hair elongation. Along with SCN1 , it may be involved in targeting of ROP2 to the plasma membrane.
AT3G56940	*COPPER RESPONSE DEFECT 1 (CRD1)*	Encodes a putative ZIP protein with varying mRNA accumulation in leaves, stems and roots. Has a consensus carboxylate-bridged di-iron binding site. The mRNA is cell-to-cell mobile.

AT3G56950	*SMALL AND BASIC INTRINSIC PROTEIN 2;1 (SIP2;1)*	One of the Major Intrinsic Proteins(MIPs) which facilitate the passive transport of small molecules across membranes.Belongs to a family of plant aquaporins.Similar to yeast and radish aquaporins. Located on ER. Probably involved in the alleviation of ER stress; the lack of SIP2;1 reduces both pollen germination and pollen tube elongation.
AT3G56980	*BASIC HELIX-LOOP-HELIX 39 (BHLH39)*	Encodes a member of the basic helix-loop-helix transcription factor protein. bHLH IVc transcription factor repressed by bHLH11.
AT3G56990	*EMBRYO SAC DEVELOPMENT ARREST 7 (EDA7)*	embryo sac development arrest 7
AT3G57000		nucleolar essential protein-like protein
AT3G57010		Calcium-dependent phosphotriesterase superfamily protein
AT3G57020		Calcium-dependent phosphotriesterase superfamily protein
AT3G57030		Calcium-dependent phosphotriesterase superfamily protein
AT3G57040	*RESPONSE REGULATOR 9 (ARR9)*	response regulator ARR9, A two-component response regulator-like protein with a receiver domain with a conserved aspartate residue and a possible phosphorylation site and at the N-terminal half. Appears to interact with histidine kinase like genes ATHP3 and ATHP2
AT3G57050	*CYSTATHIONINE BETA-LYASE (CBL)*	Encodes cystathionine beta-lyase, the second enzyme in the methionine biosynthetic pathway. Mutants show defects in root development, reduced methylation and maintenance of the quiescent center.
AT3G57060	*(CAP-D2)*	Similar to mamalian condensin. Mutants have reduced fertility.
AT3G57070		Glutaredoxin family protein
AT3G57080	*(NRPE5)*	Non-catalytic subunit unique to Nuclear DNA-dependent RNA polymerase V; homologous to budding yeast RPB5.
AT3G57090	*(BIGYIN)*	Encodes a protein with similarity to yeast FIS proteins. These membrane anchored proteins bind DRP proteins and function during organelle division. FIS1B is expressed ubiquitously and appears to be involved in peroxisome division.
AT3G57100		transmembrane protein, putative (DUF677)
AT3G57110	*EXONUCLEASE V (EXOV)*	exonuclease V
AT3G57120		Protein kinase superfamily protein
AT3G57130	*BLADE ON PETIOLE 1 (BOP1)*	Encodes BOP1. Contains Pfam domain, PF00023: Ankyrin repeat and Pfam domain, PF00651: BTB/POZ domain. Lines carrying recessive mutations exhibit a number of visible defects, most pronounced being ectopic outgrowths of in leaf petioles of rosette leaves. Along with BOP2, BOP1 is required for nectary development and formation of normal abscission zones.Forms homodimers and heterodimers with BOP2. Nuclear localization is required for activity which includes positive regulation of AS2 in leaves. BOP1/2 promotes floral meristem fate and determinacy in a pathway targetting APETALA1 and AGAMOUS-LIKE24. PUCHI, BOP1 and BOP2 are redundantly required for expression of LFY and AP1. BOP1 is expressed in valve margin. Misexpression in stems causes short internodes and ectopic biosynthesis of lignin. BOP1 activity is antagonistic to BP (At4g08150) and PNY (At5g02030). BOP1 expression is restricted to pedicel axils by BP and PNY. BOP1 promotes KNAT6 (At1g23380) expression.BOP1 Interacts with BIL1/BZR1 and Inhibits BIL1/BZR1 transport into the nucleus.
AT3G57140	*SUGAR-DEPENDENT 1-LIKE (SDP1-LIKE)*	sugar-dependent 1-like protein
AT3G57150	*HOMOLOGUE OF NAP57 (NAP57)*	Encodes a putative pseudouridine synthase (NAP57).
AT3G57160		cysteine-rich TM module stress tolerance protein
AT3G57165		
AT3G57170		N-acetylglucosaminyl transferase component family protein / Gpi1 family protein
AT3G57180	*BRASSINAZOLE(BRZ) INSENSITIVE PALE GREEN 2 (BPG2)*	Encodes a chloroplast localized protein YL1/BPG2. Involved in seedling shoot response to salt stress through ABI4.
AT3G57190	*PEPTIDE CHAIN RELEASE FACTOR 3 (PrfB3)*	Encodes a chloroplast stroma-localized ribosomal peptide chain release factor that is involved in the light- and stress-dependent regulation of stability of 3' processed petB transcripts to adjust cytochrome b(6) levels. It appears to bind to the 3'-UTR of petB RNA, protecting it from 3'-5' exonucleolytic attack. At-prfB3 arose from a gene duplication of At-prfB1.
AT3G57210		hypothetical protein
AT3G57220		Glycosyl transferase family 4 protein
AT3G57230	*AGAMOUS-LIKE 16 (AGL16)*	MADS-box transcription factor. Expressed in leaf, root and stem, with higher RNA accumulation in guard cells and trichomes. AGL16 can directly interact with SVP and indirectly interact with FLC. Furthermore, the accumulation of AGL16 transcripts is modulated by miR824 (AT4G24415). The flowering time effect for the miR824/AGL16 module is more obvious in the Col-FRI background than in the Col-0 background. AGL16 controls flowering via a allelic dosage effect in long-day non-vernalized conditions.
AT3G57240	*BETA-1,3-GLUCANASE 3 (BG3)*	encodes a member of glycosyl hydrolase family 17
AT3G57250		Emsy N Terminus (ENT) domain-containing protein
AT3G57260	*PATHOGENESIS-RELATED PROTEIN 2 (PR2)*	beta 1,3-glucanase
AT3G57270	*BETA-1,3-GLUCANASE 1 (BG1)*	encodes a member of glycosyl hydrolase family 17
AT3G57290	*EUKARYOTIC TRANSLATION INITIATION FACTOR 3E (EIF3E)*	Encodes a protein that is found in not only the eif3 complex but also in association with subunits of the COP9 signalosome. eIF3e appears to be subjected to proteasome-dependent degradation that requires the PCI domain of eIF3e. The level of eIF3e present in cells appears to affect the rate of translation.
AT3G57300	*INO80 ORTHOLOG (INO80)*	Encodes the Arabidopsis INO80 ortholog of the SWI/SNF ATPase family that has been shown to interact with H2A.Z and facilitates the enrichment of H2A.Z at the ends of the flowering repressor genes FLC and MAF4/5. Functions as a positive regulator of DNA homologous recombination (HR) and plays a crucial role in genome stability maintenance. In INO80 mutants, the HR frequency is reduced to 15% of that in the wild-type. Plants mutated in INO80 display a pleiotropic phenotype including smaller plant and organ size, and late flowering but are not more sensitive to genotoxic agents or less efficient at T-DNA integration. INO80 has also been shown to regulate a subset of the Arabidopsis transcriptome.
AT3G57330	*AUTOINHIBITED CA2+-ATPASE 11 (ACA11)*	Lesion mimic phenotype when mutation in the gene is combined with a mutation in ACA4. Lesion mimic phenotype of double knockout can be suppressed by nutritional supplements that increase anion levels (e.g. 15 mM Nitrate, Chloride, or Phosphate)
AT3G57360		tRNA-splicing endonuclease subunit

AT3G57370	*(BRP4)*	Encodes a nuclear-localized member of the TFIIB-related protein family that is involved in regulation of the mitotic cell-cycle progression during male gametogenesis.
AT3G57380		Glycosyltransferase family 61 protein
AT3G57390	*AGAMOUS-LIKE 18 (AGL18)*	encodes a MADS-box containing protein likely to be a transcription factor that is expressed in endosperm and developing gametophytes. The protein sequence is most similar to that of AGL15, which is expressed in developing embryos.
AT3G57450		hypothetical protein
AT3G57460		catalytic/ metal ion binding / metalloendopeptidase/ zinc ion binding protein
AT3G57490		Ribosomal protein S5 family protein
AT3G57510	*ARABIDOPSIS DEHISCENCE ZONE POLYGALACTURONASE 1 (ADPG1)*	Encodes ADPG1, a polygalacturonase protein involved in silique and anther dihiscence. Loss of function mutations have reduced seed set, indehiscent fruit and reduced pollen shedding. Required for release of cell wall-derived PR elicitors.
AT3G57520	*SEED IMBIBITION 2 (SIP2)*	SIP2 encodes a raffinose-specific alpha-galactosidase that catalyzes the breakdown of raffinose into alpha-galatose and sucrose. This enzyme may function in unloading raffinose from the phloem as part of sink metabolism. Although it was originally predicted to act as a raffinose synthase (RS), that activity was not observed for recombinant SIP2.
AT3G57530	*CALCIUM-DEPENDENT PROTEIN KINASE 32 (CPK32)*	Calcium-dependent Protein Kinase. ABA signaling component that regulates the ABA-responsive gene expression via ABF4. AtCPK32 has autophosphorylation activity and can phosphorylate ABF4 in vitro
AT3G57540	*REMORIN GROUP 4 1 (REM4.1)*	Remorin family protein
AT3G57550	*GUANYLATE KINASE (AGK2)*	guanylate kinase
AT3G57560	*N-ACETYL-L-GLUTAMATE KINASE (NAGK)*	encodes a N-acetylglutamate kinase, involved in arginine biosynthesis
AT3G57600		encodes a member of the DREB subfamily A-2 of ERF/AP2 transcription factor family. The protein contains one AP2 domain. There are eight members in this subfamily including DREB2A AND DREB2B that are involved in response to drought.
AT3G57620	*GALACTOSE OXIDASE-LIKE 4 (GOXL4)*	Galactose oxydase; may function in tissues that require mechanical reinforcements in the absence of lignification.
AT3G57630	*EXTENSIN ARABINOSE DEFICIENT TRANSFERASE (EXAD)*	Encodes a glycoprotein glycosyl transferase ExAD. Knockout mutants show truncated root hair phenotype.
AT3G57650	*LYSOPHOSPHATIDYL ACYLTRANSFERASE 2 (LPAT2)*	Encodes an endoplasmic reticulum localized protein with lysophosphatidyl acyltransferase activity.
AT3G57660	*NUCLEAR RNA POLYMERASE A1 (NRPA1)*	Encodes a subunit of RNA polymerase I (aka RNA polymerase A). The mRNA is cell-to-cell mobile.
AT3G57670	*NO TRANSMITTING TRACT (NTT)*	Encodes a a C2H2/C2HC zinc finger transcription factor specifically expressed in the transmitting tract and involved in transmitting tract development and pollen tube growth.Acts redundantly with WIP4 and WIP5 to determine distal cell fate in the root. MP binds to regulatory elements within the NTT locus and likely regulates its expression.
AT3G57680	*(CTPB)*	C-terminal peptidase
AT3G57700	*(ZRK10)*	Protein kinase superfamily protein
AT3G57710	*RESISTANCE RELATED KINASE 1 (RKS1)*	Protein kinase superfamily protein
AT3G57720	*(ZRK2)*	Protein kinase superfamily protein
AT3G57760	*(ZRK6)*	Protein kinase superfamily protein
AT3G57770	*(ZRK7)*	Protein kinase superfamily protein
AT3G57780		nucleolar-like protein
AT3G57790	*POLYGALACTURONASE CLADE F 8 (PGF8)*	Pectin lyase-like superfamily protein
AT3G57795		
AT3G57830		Leucine-rich repeat protein kinase family protein
AT3G57840		Plant self-incompatibility protein S1 family
AT3G57850		transmembrane protein
AT3G57860	*UV-B-INSENSITIVE 4-LIKE (UVI4-LIKE)*	Encodes a protein that controls entry into the second meiotic division. Negatively regulates the Anaphase-Promoting Complex/Cyclosome.
AT3G57880	*MULTIPLE C2 DOMAIN AND TRANSMEMBRANE REGION PROTEIN 3 (MCTP3)*	Required for maintenance of inflorescence and shoot SAMs and normal development of the derived vascular cambium, functions in the SAM to promote continuous organogenesis, affects SAM development through STM, where it affects intracellular localization of STM in SAM cells in the peripheral region and prevents STM localization toward the cell wall of SAM cells in the peripheral region.
AT3G57900		import inner membrane translocase subunit Tim17/Tim22/Tim23 family protein
AT3G57920	*SQUAMOSA PROMOTER BINDING PROTEIN-LIKE 15 (SPL15)*	Encodes a putative transcriptional regulator that is involved in the vegetative to reproductive phase transition. Expression is regulated by MIR156b.
AT3G57930		rho GTPase-activating gacO-like protein
AT3G57940		GNAT acetyltransferase (DUF699)
AT3G57950		cotton fiber protein
AT3G57960		Emsy N Terminus (ENT) domain-containing protein
AT3G57970		Emsy N Terminus (ENT)/ plant Tudor-like domains-containing protein
AT3G57990	*(OEP40)*	Encodes a ?-barrel protein, named OEP40, locates in in the outer envelope of chloroplasts, and functions as a solute channel which is selectively permeable for glucose.
AT3G58000		VQ motif-containing protein
AT3G58020		Chaperone DnaJ-domain superfamily protein
AT3G58060	*(MTP8)*	TP8 is a tonoplast localized member of CDF family of cation transporters. It functions in roots as an Mn transporter.MTP8 transports manganese into root vacuoles of iron-deficient plants and thereby prevents inhibition of iron deficiency-induced ferric chelate reductase by manganese. In seed embryos, MTP8 is responsible for manganese and iron enrichment in the subepidermal cell layer (particularly in vit1 mutant background.)

AT3G58070	*GLABROUS INFLORESCENCE STEMS (GIS)*	Putative transcription factor, contains C2H2 domain, regulates aspects of shoot maturation in Arabidopsis thaliana. GIS loss-of-function mutations affect the epidermal differentiation of inflorescence organs, causing a premature decrease in trichome production on successive leaves, stem internodes, and branches. Overexpression has the opposite effect on trichome initiation and causes other heterochronic phenotypes, affecting flowering and juvenile?adult leaf transition and inducing the formation of rosette leaves on inflorescence stems.
AT3G58080		
AT3G58120	*(BZIP61)*	Encodes a member of the BZIP family of transcription factors. Forms heterodimers with the related protein AtbZIP34. Binds to G-boxes in vitro and is localized to the nucleus in onion epidermal cells.
AT3G58180		ARM repeat superfamily protein
AT3G58190	*LATERAL ORGAN BOUNDARIES-DOMAIN 29 (LBD29)*	This gene contains two auxin-responsive element (AuxRE). Required for triggering cell reprogramming during callus formation.
AT3G58200		TRAF-like family protein
AT3G58210		TRAF-like family protein
AT3G58230		Ubiquitin-specific protease family C19-related protein
AT3G58280		MATH domain/coiled-coil protein
AT3G58290		TRAF-like superfamily protein
AT3G58300		phospholipase-like protein (PEARLI 4) family protein
AT3G58330		phospholipase-like protein (PEARLI 4) family protein
AT3G58350	*RESTRICTED TEV MOVEMENT 3 (RTM3)*	Encodes RTM3 (Restricted Tobacco etch potyvirus Movement), a protein belonging to a protein family of 29 members which has a meprin and TRAF homology (MATH) domain in its N-terminal region and a coiled-coil (CC) domain at its C-terminal end. There are at least three RTMs in Arabidopsis. RTM proteins might form a multiprotein complex in the resistance mechanism to block the long distance movement of potyviruses.
AT3G58390	*PELOTA 2 (PEL2)*	Represses the RNA the non-stop decay (NSD) and no-go decay (NGD) quality control systems that act during translation. Impairs NSD likely by sequestering the HBS1 components of the NSD complex.
AT3G58450	*UNIVERSAL STRESS PROTEIN (USP)*	USP domain containing protein, member of the universal stress protein family, regulated by ABA and possibly regulated by the ABA-dependent transcription factor AREB/ABF. Involved in the regulation of seed germination.
AT3G58470		nucleic acid binding / methyltransferase
AT3G58510	*RNA HELICASE 11 (RH11)*	DEA(D/H)-box RNA helicase family protein
AT3G58520		Ubiquitin carboxyl-terminal hydrolase family protein
AT3G58530		RNI-like superfamily protein
AT3G58540		hypothetical protein
AT3G58550	*GLYCOSYLPHOSPHATIDYLINOSITOL-ANCHORED LIPID PROTEIN TRANSFER 22 (LTPG22)*	Bifunctional inhibitor/lipid-transfer protein/seed storage 2S albumin superfamily protein
AT3G58560	*(ATCCR4A)*	Encodes a protein that is involved in mRNA processing and localized to cytoplasmic p-bodies. Double mutants with CCR4b show decreased sensitivity to high concentrations of sucrose. Involved in starch and sucrose metabolism.
AT3G58570	*RNA HELICASE 52 (RH52)*	Immunoprecipitation with mRNA decapping complex members DCP1 and DCP2.
AT3G58590		Pentatricopeptide repeat (PPR) superfamily protein
AT3G58600		Adaptin ear-binding coat-associated protein 1 NECAP-1
AT3G58630		sequence-specific DNA binding transcription factor
AT3G58640		Mitogen activated protein kinase kinase kinase-like protein
AT3G58650	*TON1 RECRUITING MOTIF 7 (TRM7)*	Encodes a member of the TRM superfamily, that plays a role in preprophase band formation during plant cell division and controls the robustness of the orientation of that cell division. Its transcription is regulated by the cell cycle and peaks at the G2/M transition.
AT3G58660		Ribosomal protein L1p/L10e family
AT3G58670	*PLANT CYSTEINE OXIDASE 5 (PCO5)*	Plant Cysteine Oxidase (PCO). Involved in controlling the stability of Group VII ethylene response factors (ERF-VIIs) via N-Arg/degron pathway through catalyzing the oxidation of their N-Cys for subsequent Arginyl-tRNA--protein transferase 1 (ATE1) mediated arginine installation.
AT3G58710	*WRKY DNA-BINDING PROTEIN 69 (WRKY69)*	member of WRKY Transcription Factor; Group II-e
AT3G58740	*CITRATE SYNTHASE 1 (CSY1)*	Encodes a peroxisomal citrate synthase that is expressed in siliques and developing seeds.
AT3G58760		Integrin-linked protein kinase family
AT3G58770		hypothetical protein
AT3G58780	*SHATTERPROOF 1 (SHP1)*	One of two genes (SHP1 and SHP2) that are required for fruit dehiscence. The two genes control dehiscence zone differentiation and promote the lignification of adjacent cells.
AT3G58800		secretion-regulating guanine nucleotide exchange factor
AT3G58810	*METAL TOLERANCE PROTEIN A2 (MTPA2)*	Member of Zinc transporter (ZAT) family. Contributes to basic cellular Zn tolerance and controls Zn partitioning, particularly under conditions of high rates of Zn influx into the root symplasm. Localizes to the vacuolar membrane.
AT3G58840	*PEROXISOMAL AND MITOCHONDRIAL DIVISION FACTOR 1 (PMD1)*	Encodes PEROXISOMAL AND MITOCHONDRIAL DIVISION FACTOR1. Involved in the morphogenesis and proliferation of peroxisomes and mitochondria.
AT3G58860		F-box/RNI-like superfamily protein
AT3G58870		
AT3G58890		RNI-like superfamily protein
AT3G58990	*ISOPROPYLMALATE ISOMERASE 1 (IPMI1)*	Small subunit, which together with IPMI SSU1, IPMISSU2 and IPMI LSU1, is a member of heterodimeric isopropylmalate isomerase (IPMI). Together with IPMI SSU3 participates in the Met chain elongation pathway.
AT3G59000		F-box/RNI-like superfamily protein
AT3G59010	*PECTIN METHYLESTERASE 61 (PME61)*	Encodes PME35, a pectin methylesterase. PME35-mediated demethylesterification of the primary cell wall regulates the mechanical strength of the supporting tissue.

AT3G59020		ARM repeat superfamily protein
AT3G59030	*TRANSPARENT TESTA 12 (TT12)*	Encodes a proton antiporter. Involved in the transportation of proanthocyanidin precursors into the vacuole. In vitro transport experiments showed that cyanidin-3-O-glucoside (anthocyanin) was an effective substrate, whereas the proanthocyanidin precursor epicatechin was not transported. However catechin-3-O-glucoside inhibited anthocyanin transport in a dose-dependent manner suggesting that glycosylated epicatechin is the in vivo substrate. Recessive mutation has strong reduction of proanthocyanidin deposition in vacuoles and has reduced dormancy. Expressed in the endothelium of ovules and developing seeds.
AT3G59050	*POLYAMINE OXIDASE 3 (PAO3)*	Encodes a polyamine oxidase.
AT3G59060	*PHYTOCHROME INTERACTING FACTOR 3-LIKE 6 (PIL6)*	Encodes a novel Myc-related bHLH transcription factor, which physically associated with APRR1/TOC1 and is a member of PIF3 transcription factor family. Involved in shade avoidance. Functions as negative regulator of PhyB. Protein levels are modulated by phytochrome B. Controls the resistance to B. cinerea in a COI1- and EIN2-dependent manner.
AT3G59070		Cytochrome b561/ferric reductase transmembrane with DOMON related domain-containing protein
AT3G59080		Eukaryotic aspartyl protease family protein
AT3G59100	*GLUCAN SYNTHASE-LIKE 11 (GSL11)*	encodes a protein similar to callose synthase
AT3G59120		Cysteine/Histidine-rich C1 domain family protein
AT3G59130		Cysteine/Histidine-rich C1 domain family protein
AT3G59140	*ATP-BINDING CASSETTE C10 (ABCC10)*	member of MRP subfamily
AT3G59170		F-box/RNI-like superfamily protein
AT3G59180		Protein with RNI-like/FBD-like domain
AT3G59190		F-box/RNI-like superfamily protein
AT3G59220	*PIRIN (PRN)*	encodes a cupin-domain containing protein that is similar to pirins which interact with a CCAAT box binding transcription factor. The protein interacts with GPA1 (G protein alpha-subunit) in vitro. Mutants in the gene are affected in germination and early seedling development.
AT3G59260		pirin
AT3G59270		FBD-like domain family protein
AT3G59290	*EPSIN3 (EPS3)*	Involved in plant trans-Golgi network (TGN) transport.
AT3G59310		solute carrier family 35 protein (DUF914)
AT3G59340		solute carrier family 35 protein (DUF914)
AT3G59350	*MAZZA (MAZ)*	Pti-like protein. Interacts with CLV1 and functions in CLE peptide signaling pathway in root development. Membrane localization is dependent on palmytolation.
AT3G59370		Vacuolar calcium-binding protein-like protein
AT3G59400	*GENOMES UNCOUPLED 4 (GUN4)*	GUN, genomes uncoupled, is necessary for coupling the expression of some nuclear genes to the functional state of the chloroplast. Binds to the magnesium chelatase complex and promotes formation of the substrate,a tetrapyrrole signaling molecule. Porphyrin-binding protein that enhances the activity of Mg-chelatase. Although required for chlorophyll accumulation under normal growth conditions, GUN4 is not essential for chlorophyll synthesis.
AT3G59410	*GENERAL CONTROL NON-DEPRESSIBLE 2 (GCN2)*	Encodes an eIF2alpha kinase that can bind uncharged tRNA via its C-terminus and can phosphorylate both eIF2alpha homologues in Arabidopsis.
AT3G59420	*CRINKLY4 (CR4)*	Encodes a membrane localized protein with similarity to receptor kinases which is involved in epidermal cell differentiation. Flowers of mutants have disorganized ovule integument growth and abnormal sepal margins. In the roots, mutants initiate more lateral roots but fewer laterals actually emerge due to defects in lateral root formation. Mutants also display disorganized columella. The root phenotypes can be traced to abnormalities in asymmetric divisions in the pericycle and root apex. Conflicting data regarding the role of the kinase domain- which may or may not be required for function. Complementation studies indicate that the C-terminal domain is also not required for signaling function. May be regulated by protein turnover which is mediated by endocytic processes. ACR4 phosphorylates the PROTEIN PHOSPHATASE 2A-3 (PP2A-3) catalytic subunit of the PP2A phosphatase holoenzyme and PP2A
AT3G59430		hypothetical protein
AT3G59440	*(ATCML4)*	Encodes an endomembrane localized member of the CML subfamily VII. Contains a canonical CaM domain and unique N-terminal extension that distinguishes it from other members of the subfamily.
AT3G59480	*FRUCTOKINASE 7 (FRK7)*	Encodes a member of the fructokinase gene family. Nomenclature according to Riggs 2017 has been adopted for the family by the community (personal communication, Boernke, Callis, Granot, Boernke, and Smeekens).
AT3G59490		hypothetical protein
AT3G59530	*LESS ADHERENT POLLEN 3 (LAP3)*	Calcium-dependent phosphotriesterase superfamily protein
AT3G59550	*(SYN3)*	Encodes an alpha-kleisin protein that is localized primarily in the nucleolus and is essential for megagametogenesis and plays an important role in pollen development. alpha-kleisins are core components of meiotic and mitotic cohesin complexes.
AT3G59560		
AT3G59570		Ypt/Rab-GAP domain of gyp1p superfamily protein
AT3G59580	*NIN-LIKE PROTEIN 9 (NLP9)*	Plant regulator RWP-RK family protein
AT3G59610		F-box family protein / jacalin lectin family protein
AT3G59630		diphthamide synthesis DPH2 family protein
AT3G59670		elongation factor
AT3G59680		Serine/Threonine-kinase
AT3G59690	*IQ-DOMAIN 13 (IQD13)*	Member of IQ67 (CaM binding) domain containing family.
AT3G59700	*L-TYPE LECTIN RECEPTOR KINASE V.5 (LECRK-V.5)*	Member of Receptor kinase-like protein family. Represses stomatal immunity induced by Pseudomonas syringae pv. tomato DC3000.
AT3G59710		NAD(P)-binding Rossmann-fold superfamily protein

AT3G59730	*L-TYPE LECTIN RECEPTOR KINASE V.6 (LECRK-V.6)*	Concanavalin A-like lectin protein kinase family protein
AT3G59740	*L-TYPE LECTIN RECEPTOR KINASE V.7 (LECRK-V.7)*	Concanavalin A-like lectin protein kinase family protein
AT3G59760	*O-ACETYLSERINE (THIOL) LYASE ISOFORM C (OASC)*	Arabidopsis thaliana O-acetylserine (thiol) lyase (OAS-TL) isoform oasC. Required for pollen tube growth and/or fertilization.
AT3G59780		Rhodanese/Cell cycle control phosphatase superfamily protein
AT3G59790	*MAP KINASE 10 (MPK10)*	Encodes a member of the MAP Kinase family. Thought to be a pseuedogene, MAPK10 is expressed very transiently during germination and in the leaf tips/hydathodes. Loss of function mutations are late flowering in long days and exhibit abnormal patterning of cotyledon veins. MPK10 interacts with and may be regulated by MPKK2 another map kinase.
AT3G59820	*LEUCINE ZIPPER-EF-HAND-CONTAINING TRANSMEMBRANE PROTEIN 1 (LETM1)*	LETM1-like protein
AT3G59830		Integrin-linked protein kinase family
AT3G59845		Zinc-binding dehydrogenase family protein
AT3G59850		Pectin lyase-like superfamily protein
AT3G59860		transposable_element_gene;Mutator-like transposase family, has a 8.4e-21 P-value blast match to O65231 /281-442 Pfam PF03108 MuDR family transposase (MuDr-element domain);(source:TAIR10)
AT3G59900	*AUXIN-REGULATED GENE INVOLVED IN ORGAN SIZE (ARGOS)*	Encodes ARGOS (Auxin-Regulated Gene Involved in Organ Size). Inducible by auxin. Involved in lateral organ size control. Transgenic plants expressing sense or antisense ARGOS cDNA display enlarged or reduced aerial organs, respectively. The alteration in organ size is attributable mainly to changes in cell number and the duration of organ growth.
AT3G59930		Encodes a defensin-like (DEFL) family protein.
AT3G59940	*KISS ME DEADLY 4 (KMD4)*	Encodes a member of a family of F-box proteins, called the KISS ME DEADLY (KMD) family, that targets type-B ARR proteins for degradation and is involved in the negative regulation of the cytokinin response. Also named as KFB50, a member of a group of Kelch repeat F-box proteins that negatively regulate phenylpropanoid biosynthesis by targeting the phenypropanoid biosynthesis enzyme phenylalanine ammonia-lyase. The mRNA is cell-to-cell mobile.
AT3G59990	*METHIONINE AMINOPEPTIDASE 2B (MAP2B)*	Encodes a MAP2 like methionine aminopeptidase
AT3G60010	*SKP1-LIKE 13 (SK13)*	SKP1-like 13
AT3G60020	*SKP1-LIKE 5 (SK5)*	SKP1-like 5
AT3G60060		NAD(P)-binding Rossmann-fold superfamily protein
AT3G60100	*CITRATE SYNTHASE 5 (CSY5)*	citrate synthase 5
AT3G60110		DNA-binding bromodomain-containing protein
AT3G60120	*BETA GLUCOSIDASE 27 (BGLU27)*	beta glucosidase 27
AT3G60130	*BETA GLUCOSIDASE 16 (BGLU16)*	beta glucosidase 16
AT3G60140	*DARK INDUCIBLE 2 (DIN2)*	Encodes a protein similar to beta-glucosidase and is a member of glycoside hydrolase family 1. Expression is induced after 24 hours of dark treatment, in senescing leaves and treatment with exogenous photosynthesis inhibitor. Induction of gene expression was suppressed in excised leaves supplied with sugar. The authors suggest that the gene's expression pattern is responding to the level of sugar in the cell. The mRNA is cell-to-cell mobile.
AT3G60160	*ATP-BINDING CASSETTE C9 (ABCC9)*	member of MRP subfamily
AT3G60170		transposable_element_gene;copia-like retrotransposon family, has a 7.6e-229 P-value blast match to gb|AAO73527.1| gag-pol polyprotein (Glycine max) (SIRE1) (Ty1_Copia-family);(source:TAIR10)
AT3G60220	*TOXICOS EN LEVADURA 4 (ATL4)*	Encodes a putative RING-H2 zinc finger protein ATL4 (ATL4).
AT3G60250	*CASEIN KINASE II BETA CHAIN 3 (CKB3)*	Regulatory (beta) subunit of the protein kinase CK2. Involved in regulation of the circadian clock in Arabidopsis
AT3G60260	*(ELMOD_A)*	Acts upstream of aperture proteins, their expression levels influence the number of aperture domains that form on the surface of developing pollen grains.
AT3G60270		Cupredoxin superfamily protein
AT3G60280	*UCLACYANIN 3 (UCC3)*	Encodes blue copper-binding protein III.
AT3G60290		2-oxoglutarate (2OG) and Fe(II)-dependent oxygenase superfamily protein
AT3G60300		RWD domain-containing protein
AT3G60310		acyl-CoA synthetase family protein
AT3G60320	*NITRATE REGULATORY GENE 2 (NRG2)*	bZIP domain class transcription factor (DUF630 and DUF632)
AT3G60330	*H(+)-ATPASE 7 (HA7)*	H[+]-ATPase 7
AT3G60340		alpha/beta-Hydrolases superfamily protein
AT3G60360	*EMBRYO SAC DEVELOPMENT ARREST 14 (EDA14)*	embryo sac development arrest 14
AT3G60390	*HOMEOBOX-LEUCINE ZIPPER PROTEIN 3 (HAT3)*	Encodes homeobox protein HAT3.
AT3G60410		hypothetical protein (DUF1639)
AT3G60420		phosphoglycerate mutase family protein
AT3G60430		
AT3G60440		Phosphoglycerate mutase family protein
AT3G60470		transmembrane protein, putative (DUF247)
AT3G60490	*ETHYLENE-RESPONSIVE TRANSCRIPTION FACTOR 35 (ERF35)*	encodes a member of the DREB subfamily A-4 of ERF/AP2 transcription factor family. The protein contains one AP2 domain. There are 17 members in this subfamily including TINY.
AT3G60510		ATP-dependent caseinolytic (Clp) protease/crotonase family protein
AT3G60520		zinc ion-binding protein
AT3G60530	*GATA TRANSCRIPTION FACTOR 4 (GATA4)*	Encodes a member of the GATA factor family of zinc finger transcription factors.

AT3G60540		Preprotein translocase Sec, Sec61-beta subunit protein
AT3G60550	*CYCLIN P3;2 (CYCP3;2)*	cyclin p3
AT3G60560		hypothetical protein
AT3G60570	*EXPANSIN B5 (EXPB5)*	member of BETA-EXPANSINS. Naming convention from the Expansin Working Group (Kende et al, 2004. Plant Mol Bio)
AT3G60580	*(ZAT9)*	C2H2-like zinc finger protein
AT3G60590		cytochrome P450 family protein
AT3G60630	*HAIRY MERISTEM 2 (HAM2)*	Belongs to one of the LOM (LOST MERISTEMS) genes: AT2G45160 (LOM1), AT3G60630 (LOM2) and AT4G00150 (LOM3). LOM1 and LOM2 promote cell differentiation at the periphery of shoot meristems and help to maintain their polar organization.
AT3G60640	*AUTOPHAGY 8G (ATG8G)*	Autophagy protein.
AT3G60650		Encodes a peptide involved in the regulation of lateral root development and root growth pattern.
AT3G60660		spindle/kinetochore-associated-like protein
AT3G60700		hypothetical protein (DUF1163)
AT3G60720	*PLASMODESMATA-LOCATED PROTEIN 8 (PDLP8)*	Encodes a plasmodesmal protein that may be involved in the intercellular movement of molecules through the plasmodesmata. The protein has two DUF26 domains and a single transmembrane domain.
AT3G60730		Plant invertase/pectin methylesterase inhibitor superfamily
AT3G60750	*TRANSKETOLASE 1 (TKL1)*	Transketolase
AT3G60760		hypothetical protein
AT3G60780		hypothetical protein (DUF1442)
AT3G60790		F-box family protein
AT3G60800	*PROTEIN ACYLTRANSFERASE 14 (PAT14)*	Encodes a protein S-acyltransferase that, together with PAT13, cooperatively regulates leaf senescence.
AT3G60810		DUF1499 family protein
AT3G60840	*MICROTUBULE-ASSOCIATED PROTEIN 65-4 (MAP65-4)*	Encodes MAP65-4, a non-motor microtubule associated protein (MAP) that belongs to the evolutionarily conserved MAP65 family. MAP65-4 specifically associates with the forming mitotic spindle during prophase and with the kinetochore fibers from prometaphase to the end of anaphase. MAP65-4 cross-links microtubules and promotes microtubule bundle elongation.
AT3G60850		hypothetical protein
AT3G60880	*DIHYDRODIPICOLINATE SYNTHASE 1 (DHDPS1)*	Encodes a dihydropicolinate synthase involved in lysine biosynthesis. The enzyme is allosterically inhibited by lysine. It is predicted to localize to the cholorplast.
AT3G60900	*FASCICLIN-LIKE ARABINOGALACTAN-PROTEIN 10 (FLA10)*	Fasciclin-like arabinogalactan protein. Possibly involved in embryogenesis and seed development.
AT3G60910		S-adenosyl-L-methionine-dependent methyltransferases superfamily protein
AT3G60920	*BEACH-DOMAIN HOMOLOG C1 (BCHC1)*	beige/BEACH domain protein
AT3G60940		Putative endonuclease or glycosyl hydrolase
AT3G60980		Tetratricopeptide repeat (TPR)-like superfamily protein
AT3G61000		
AT3G61020		pseudogene of TRICHOME BIREFRINGENCE-LIKE 32
AT3G61040	*CYTOCHROME P450, FAMILY 76, SUBFAMILY C, POLYPEPTIDE 7 (CYP76C7)*	encodes a protein with cytochrome P450 domain
AT3G61050	*(NTMC2T4)*	Encodes a novel transcriptional regulator, a calcium-dependent lipid-binding protein containing a C2 domain, that binds specifically to the promoter of THAS1 (thalianol synthase 1). It can bind ceramide and is involved in drought and salt tolerance.
AT3G61060	*PHLOEM PROTEIN 2-A13 (PP2-A13)*	phloem protein 2-A13
AT3G61070	*PEROXIN 11E (PEX11E)*	member of the peroxin11 (PEX11) gene family, integral to peroxisome membrane, controls peroxisome proliferation.
AT3G61080		Protein kinase superfamily protein
AT3G61090		Putative endonuclease or glycosyl hydrolase
AT3G61100		Putative endonuclease or glycosyl hydrolase
AT3G61120	*AGAMOUS-LIKE 13 (AGL13)*	Encodes AGL13, a member of the AGL6 clade of the MADS-box gene family. Expressed in both pollen and ovules. Functions in male and female gametophyte morphogenesis.
AT3G61140	*FUSCA 6 (FUS6)*	Represses photomorphogenesis and induces skotomorphogenesis in the dark. Component of the nuclear-localized COP9 complex. Mutants display striking purple coloration due to anthocyanin accumulation in their cotyledons, first become defective during embryogenesis and exhibit limited seedling development.
AT3G61150	*HOMEODOMAIN GLABROUS 1 (HDG1)*	Encodes a homeobox-leucine zipper family protein belonging to the HD-ZIP IV family.
AT3G61160	*(SK31/ASKβ)*	GS3-like kinase, subgroup III.
AT3G61170		Tetratricopeptide repeat (TPR)-like superfamily protein
AT3G61190	*BON ASSOCIATION PROTEIN 1 (BAP1)*	Encodes a protein with a C2 domain that binds to BON1 in yeast two hybrid analyses. Its ability to bind to phospholipids is enhanced by calcium ions. Involved in maintaining cell homeostasis.
AT3G61200		Thioesterase superfamily protein
AT3G61210		S-adenosyl-L-methionine-dependent methyltransferases superfamily protein
AT3G61230	*POLLEN-SPECIFIC LIM PROTEIN 2C (PLIM2c)*	Encodes a member of the Arabidopsis LIM proteins: a family of actin bundlers with distinct expression patterns. WLIM1, WLIM2a, and WLIM2b are widely expressed, whereas PLIM2a, PLIM2b, and PLIM2c are predominantly expressed in pollen. Regulates actin cytoskeleton organization.
AT3G61250	*MYB DOMAIN PROTEIN 17 (MYB17)*	LATE MERISTEM IDENTITY2 (LMI2) is a target of the meristem identity regulator LEAFY (LFY). Has a role in the meristem identity transition from vegetative growth to flowering. Member of the R2R3 factor gene family.
AT3G61260	*REMORIN 1.2 (REM1.2)*	Lipid raft regulatory protein, crucial for plasma membrane nanodomain assembly to control plasmodesmata aperture and functionality. Negatively regulates the cell-to-cell movement of TuMV via competition with PCaP1 for binding actin filaments.

AT3G61300	*MULTIPLE C2 DOMAIN AND TRANSMEMBRANE REGION PROTEIN 1 (MCTP8)*	C2 calcium/lipid-binding plant phosphoribosyltransferase family protein
AT3G61310	*AT-HOOK MOTIF NUCLEAR LOCALIZED PROTEIN 11 (AHL11)*	AT hook motif DNA-binding family protein
AT3G61340		F-box and associated interaction domains-containing protein
AT3G61390	*(PUB36)*	Plant U-box type E3 ubiquitin ligase (PUB).
AT3G61400		1-aminocyclopropane-1-carboxylate oxidase-like protein
AT3G61410		U-box kinase family protein
AT3G61430	*PLASMA MEMBRANE INTRINSIC PROTEIN 1A (PIP1A)*	a member of the plasma membrane intrinsic protein subfamily PIP1. localizes to the plasma membrane and exhibits water transport activity in Xenopus oocyte. expressed ubiquitously and protein level decreases slightly during leaf development. The mRNA is cell-to-cell mobile.
AT3G61440	*CYSTEINE SYNTHASE C1 (CYSC1)*	Encodes a cysteine synthase isomer CysC1. The isomer is however less effective in cysteine biosynthesis. It is involved in beta-cyanoalanine biosynthesis, an intermediate of cyanide detoxification pathway. The mRNA is cell-to-cell mobile.
AT3G61450	*SYNTAXIN OF PLANTS 73 (SYP73)*	syntaxin of plants 73 (SYP73)
AT3G61460	*BRASSINOSTEROID-RESPONSIVE RING-H2 (BRH1)*	Encodes a novel ring finger protein and forms an N-terminal hydrophobic domain and a C-terminal RING-H2 signature. Expression is down regulated by brassinolide.
AT3G61470	*PHOTOSYSTEM I LIGHT HARVESTING COMPLEX GENE 2 (LHCA2)*	Encodes a component of the light harvesting antenna complex of photosystem I. The mRNA is cell-to-cell mobile.
AT3G61490	*POLYGALACTURONASE CLADE F 9 (PGF9)*	Pectin lyase-like superfamily protein
AT3G61530	*(PANB2)*	Encodes a ketopentoate hydroxymethyltransferase that appears to localize to the mitochondria. This protein is expected to play a role in pantothenate (vitamin B5) biosynthesis.
AT3G61550	*ARABIDOPSIS T??XICOS EN LEVADURA 68 (ATL68)*	RING/U-box superfamily protein
AT3G61610		Galactose mutarotase-like superfamily protein
AT3G61630	*CYTOKININ RESPONSE FACTOR 6 (CRF6)*	CRF6 encodes one of the six cytokinin response factors. CRF5 belongs to the AP2/ERF superfamily of the transcriptional factors. CRF proteins rapidly relocalize to the nucleus in response to cytokinin. Analysis of loos-of-function mutants revealed that the CRFs function redundantly to regulate the development of embryos, cotyledons and leaves.
AT3G61640	*ARABINOGALACTAN PROTEIN 20 (AGP20)*	arabinogalactan protein 20
AT3G61670		extra-large G-like protein, putative (DUF3133)
AT3G61700		helicase with zinc finger protein
AT3G61720	*MULTIPLE C2 DOMAIN AND TRANSMEMBRANE REGION PROTEIN 12 (MCTP12)*	Ca2+dependent plant phosphoribosyltransferase family protein
AT3G61770	*VACUOLAR PROTEIN SORTING 30 (VPS30)*	Acid phosphatase/vanadium-dependent haloperoxidase-related protein
AT3G61780	*EMBRYO DEFECTIVE 1703 (emb1703)*	embryo defective 1703
AT3G61810		Glycosyl hydrolase family 17 protein
AT3G61820		Eukaryotic aspartyl protease family protein
AT3G61830	*AUXIN RESPONSE FACTOR 18 (ARF18)*	auxin response factor 18
AT3G61840		auxin response factor, putative (DUF688)
AT3G61850	*DOF AFFECTING GERMINATION 1 (DAG1)*	Zinc finger transcription factor of the Dof family involved in the control of seed germination.
AT3G61870		plant/protein
AT3G61880	*CYTOCHROME P450 78A9 (CYP78A9)*	Encodes a cytochrome p450 monooxygenase. Overexpression of this gene allows fruit growth independently of fertilization. The gene is normally expressed only in floral organs(during the Arabidopsis stage 14 flower) and in the funiculus at anthesis.
AT3G61890	*HOMEOBOX 12 (HB-12)*	Encodes a homeodomain leucine zipper class I (HD-Zip I) protein. Loss of function mutant has abnormally shaped leaves and stems.
AT3G61900	*SMALL AUXIN UPREGULATED RNA 33 (SAUR33)*	SAUR-like auxin-responsive protein family
AT3G61910	*NAC DOMAIN PROTEIN 66 (NAC066)*	NAC transcription factor NST2. NST1 and NST2 are redundant in regulating secondary wall thickening in anther walls. NST2 promoter was particularly strong in anther tissue.
AT3G61920		PADRE protein.
AT3G61930		hypothetical protein
AT3G61940	*(MTPA1)*	Member of Zinc transporter (ZAT) family. Expressed in roots under low zinc conditions.
AT3G61950	*MYC-TYPE TRANSCRIPTION FACTOR 67 (MYC67)*	MYC-type transcription factor which interacts with ICE1 and negatively regulates cold-responsive genes and cold tolerance.
AT3G61970	*NGATHA2 (NGA2)*	AP2/B3-like transcriptional factor family protein
AT3G61990	*O-MTASE FAMILY 3 PROTEIN (OMTF3)*	Encodes a protein methyltransferase. Involved in the methylation of plant transmembrane proteins.
AT3G62000		S-adenosyl-L-methionine-dependent methyltransferases superfamily protein
AT3G62020	*GERMIN-LIKE PROTEIN 10 (GLP10)*	germin-like protein (GLP10)
AT3G62030	*ROTAMASE CYP 4 (ROC4)*	nuclear-encoded chloroplast stromal cyclophilin CYP20-3 (also known as ROC4). Protein is tyrosine-phosphorylated and its phosphorylation state is modulated in response to ABA in Arabidopsis thaliana seeds.
AT3G62040		Haloacid dehalogenase-like hydrolase (HAD) superfamily protein
AT3G62060	*PECTIN ACETYLESTERASE 6 (PAE6)*	Pectinacetylesterase family protein
AT3G62080	*CHARGED MULTI-VESICULAR BODY PROTEIN 7 (CHMP7)*	Encodes a charged multi-vesicular body protein (CHMP7) homolog, that is an ESCRT-III-related protein and functions in the endosomal sorting pathway in humans. The Brassica homolog has been shown to be involved in plant growth and leaf senescence.
AT3G62090	*PHYTOCHROME INTERACTING FACTOR 3-LIKE 2 (PIL2)*	encodes a novel Myc-related bHLH transcription factor, which physically associated with APRR1/TOC1 and is a member of PIF3 transcription factor family.
AT3G62100	*INDOLE-3-ACETIC ACID INDUCIBLE 30 (IAA30)*	Encodes a member of the Aux/IAA family of proteins implicated in auxin signaling. IAA30 lacks the conserved degron (domain II) found in many family members. IAA30 transcripts are induced by auxin treatment and accumulate preferentially in the quiescent center cells of the root meristem. Overexpression of IAA30 leads to defects in gravitropism, root development, root meristem maintenance, and cotyledon vascular development. Target of LEC2 and AGL15. Promotes somatyic embryogenesis.

AT3G62110	*POLYGALACTURONASE CLADE F 10 (PGF10)*	Pectin lyase-like superfamily protein
AT3G62130	*L-CYSTEINE DESULFHYDRASE (LCD)*	Encodes an enzyme that decomposes L-cysteine into pyruvate, H2S, and NH3.
AT3G62150	*ATP-BINDING CASSETTE B21 (ABCB21)*	Encodes a facultative transporter controlling auxin concentrations in plant cells.
AT3G62170	*VANGUARD 1 HOMOLOG 2 (VGDH2)*	VANGUARD-like protein
AT3G62180		Plant invertase/pectin methylesterase inhibitor superfamily protein
AT3G62230	*DUO1-ACTIVATED F-BOX 1 (DAF1)*	Target promoter of the male germline-specific transcription factor DUO1. Increases seed oil content by attenuating GL2 inhibition. Overexpression results in reduced trichome numbers.
AT3G62260	*TYPE 2C PROTEIN PHOSPHATASE 49 (PP2C49)*	Type 2C protein phosphatase (PP2C) which negatively regulates AtHKT1;1 activity and thus determines systemic Na+ allocation during salt stress.
AT3G62270	*REQUIRES HIGH BORON 2 (BOR2)*	BOR2 is involved in efficient borate crosslinking of rhamnogalacturonan II in cell walls under boron limitation.
AT3G62280		GDSL-motif esterase/acyltransferase/lipase. Enzyme group with broad substrate specificity that may catalyze acyltransfer or hydrolase reactions with lipid and non-lipid substrates.
AT3G62300	*DOMAIN OF UNKNOWN FUNCTION 724 7 (DUF7)*	Encodes a protein with Agenet/Tudor and DUF724 domains. It can interact with ABAP1, a negative regulator of DNA replication and transcription, with the plant histone modification 'reader' LHP1, and with non-modified histones. It may act as a link between DNA replication, transcription and chromatin remodeling during flower development. Loss of function mutant has a WT phenotype.
AT3G62310		RNA helicase family protein
AT3G62320		Polynucleotidyl transferase, ribonuclease H-like superfamily protein
AT3G62330		Zinc knuckle (CCHC-type) family protein
AT3G62350		F-box/associated interaction domain protein
AT3G62370		heme binding protein
AT3G62380		F-box/associated interaction domain protein
AT3G62390	*TRICHOME BIREFRINGENCE-LIKE 6 (TBL6)*	Encodes a member of the TBL (TRICHOME BIREFRINGENCE-LIKE) gene family containing a plant-specific DUF231 (domain of unknown function) domain. TBL gene family has 46 members, two of which (TBR/AT5G06700 and TBL3/AT5G01360) have been shown to be involved in the synthesis and deposition of secondary wall cellulose, presumably by influencing the esterification state of pectic polymers. A nomenclature for this gene family has been proposed (Volker Bischoff & Wolf Scheible, 2010, personal communication).
AT3G62410	*CP12 DOMAIN-CONTAINING PROTEIN 2 (CP12-2)*	CP12-2 encodes a small peptide found in the chloroplast stroma. It belongs to the CP12 gene family thought to be involved in the formation of a supramolecular complex with glyceraldehyde-3-phosphate dehydrogenase (GAPDH) and phosphoribulokinase (PRK) embedded in the Calvin cycle. CP12-2 is coordinately regulated by light with the photosynthetic GAPDH and PRK. The annotation of this gene is based on article 32494. The mRNA is cell-to-cell mobile.
AT3G62430		Protein with RNI-like/FBD-like domain
AT3G62440	*SECONDARY WALL THICKENING-ASSOCIATED F-BOX 1 (SAF1)*	Encodes an F-box protein which is predominantly expressed in flower tissues and interacts with ASK19 protein. Mutations in this gene suggest it acts as a negative regulator of endothecial secondary wall thickening in anthers.
AT3G62470		Pentatricopeptide repeat (PPR) superfamily protein
AT3G62480		transposable_element_gene;similar to unknown protein [Arabidopsis thaliana] (TAIR:AT2G01031.1);(source:TAIR10)
AT3G62490		transposable_element_gene;similar to ASY2, DNA binding [Arabidopsis thaliana] (TAIR:AT4G32200.1);(source:TAIR10)
AT3G62500		F-box protein RMF
AT3G62510		disulfide isomerase-like protein
AT3G62520		transposable_element_gene;hAT-like transposase family (hobo/Ac/Tam3), has a 1.1e-86 P-value blast match to GB:CAA29005 ORFa of Maize Ac (hAT-element) (Zea mays);(source:TAIR10)
AT3G62550		Adenine nucleotide alpha hydrolases-like superfamily protein
AT3G62590	*PLASTID LIPAS3 (PLIP3)*	PLIP3 is a glycerolipid A1 lipase with substrate specificity for phosphatidylglycerol. Expression is induced by ABA.
AT3G62610	*MYB DOMAIN PROTEIN 11 (MYB11)*	Member of the R2R3 factor gene family. Together with MYB12 and MYB111 redundantly regulates flavonol biosynthesis.
AT3G62630		stress response NST1-like protein (DUF1645)
AT3G62640		DUF3511 domain protein (DUF3511)
AT3G62650		hypothetical protein
AT3G62680	*PROLINE-RICH PROTEIN 3 (PRP3)*	Proline-rich protein The mRNA is cell-to-cell mobile.
AT3G62690	*ATL5 (ATL5)*	Encodes a RING-H2 zinc finger protein related to ATL2. The ATL gene family is represented by fifteen sequences that contain, in addition to the RING, a transmembrane domain which is located in most of them towards the N-terminal end.
AT3G62700	*ATP-BINDING CASSETTE C14 (ABCC14)*	member of MRP subfamily
AT3G62710		Glycosyl hydrolase family protein
AT3G62720	*XYLOSYLTRANSFERASE 1 (XT1)*	Encodes a protein with xylosyltransferase activity, which is specific for UDP-xylose as donor substrate and for oligosaccharides with a degree of polymerization >4. Although the enzyme utilizes either cellopentaose or cellohexaose, its activity is four-fold higher with cellohexaose as an acceptor compared to cellopentaose. The enzyme is able to add several xylosyl residues to the acceptor forming mono-, di- and trixylosylated polysaccharides.
AT3G62730		desiccation-like protein
AT3G62740	*BETA GLUCOSIDASE 7 (BGLU7)*	beta glucosidase 7
AT3G62750	*BETA GLUCOSIDASE 8 (BGLU8)*	Encodes a putative beta glucosidase, expressed in the peroxisome.
AT3G62760	*(ATGSTF13)*	Encodes glutathione transferase belonging to the phi class of GSTs. Naming convention according to Wagner et al. (2002).
AT3G62770	*AUTOPHAGY 18A (ATG18a)*	Required for autophagosome formation during nutrient deprivation and senescence, promotes pexophagy during seedling development.
AT3G62780		Calcium-dependent lipid-binding (CaLB domain) family protein
AT3G62810	*(LYRM7)*	complex 1 family protein / LVR family protein
AT3G62820		Plant invertase/pectin methylesterase inhibitor superfamily protein

AT3G62860	*(MAGL12)*	alpha/beta-Hydrolases superfamily protein
AT3G62910	*ALBINO AND PALE GREEN (APG3)*	Encodes a plastid-localized ribosome release factor 1 that is essential in chloroplast development. Pale green, albino mutant seedlings arrest early in seedling development.
AT3G62930	*(GRXS6)*	Encodes a member of the CC-type glutaredoxin (ROXY) family that has been shown to interact with the transcription factor TGA2 and suppress ORA59 promoter activity.
AT3G62940	*OVARIAN TUMOR DOMAIN (OTU)-CONTAINING DUB (DEUBIQUITILATING ENZYME) 5 (OTU5)*	Induces cross-talks among epigenomes that altogether impact the regulation of approximately 7060 genes of which 186 genes associated with root development.
AT3G62950	*(GRXC11)*	Encodes a member of the CC-type glutaredoxin (ROXY) family that has been shown to interact with the transcription factor TGA2 and suppress ORA59 promoter activity.
AT3G62960	*(ROXY8)*	Encodes a member of the CC-type glutaredoxin (ROXY) family that has been shown to interact with the transcription factor TGA2.
AT3G62980	*TRANSPORT INHIBITOR RESPONSE 1 (TIR1)*	Encodes an auxin receptor that mediates auxin-regulated transcription. It contains leucine-rich repeats and an F-box and interacts with ASK1, ASK2 and AtCUL1 to form SCF-TIR1, an SCF ubiquitin ligase complex. Related to yeast Grr1p and human SKP2 proteins, involved in ubiquitin-mediated processes. Required for normal response to auxin and repressed in response to flagellin. As part of the SCF complex and in the presence of auxin, TIR1 interacts with Aux/IAA transcriptional repressor proteins and mediates their degradation. Mutations in TIR1 block auxin stimulation of flavonoid synthesis.
AT3G63010	*GA INSENSITIVE DWARF1B (GID1B)*	Encodes a gibberellin (GA) receptor ortholog of the rice GA receptor gene (OsGID1). Has GA-binding activity, showing higher affinity to GA4. Interacts with DELLA proteins in vivo in the presence of GA4. The mRNA is cell-to-cell mobile.
AT3G63020		hypothetical protein (DUF3049)
AT3G63040		hypothetical protein
AT3G63050		hypothetical protein
AT3G63060	*EID1-LIKE 3 (EDL3)*	EDL3 is an F-box protein involved that mediated the regulation of abscisic acid signalling.
AT3G63090		Ubiquitin carboxyl-terminal hydrolase family protein
AT3G63100		
AT3G63110	*ISOPENTENYLTRANSFERASE 3 (IPT3)*	Encodes cytokinin synthase involved in cytokinin biosynthesis. IPT3 subcellular localization is modulated by farnesylation- when farnesylated it is localized to the nucleus, otherwise to the chloroplast.
AT3G63120	*CYCLIN P1;1 (CYCP1;1)*	cyclin p1
AT3G63130	*RAN GTPASE ACTIVATING PROTEIN 1 (RANGAP1)*	Encodes a RAN GTPase activating protein involved in nuclear import, cell plate formation and mitotic spindle formation. Associates with nuclear envelope membranes.
AT3G63140	*CHLOROPLAST STEM-LOOP BINDING PROTEIN OF 41 KDA (CSP41A)*	Encodes a protein with ribonuclease activity that is involved in plastid rRNA maturation.
AT3G63160	*OUTER ENVELOPE PROTEIN 6 (OEP6)*	Member of the Arabidopsis 7-kDa OEP family. Tail-anchored (TA) membrane protein which possesses a single C-terminal transmembrane domain targeting post-translationally to plastids.
AT3G63170	*FATTY-ACID-BINDING PROTEIN 1 (FAP1)*	Encodes a plastid stroma localized fatty acid binding protein involved in fatty acid metabolism.
AT3G63200	*PATATIN-LIKE PROTEIN 9 (PLP9)*	PATATIN-like protein 9
AT3G63210	*MEDIATOR OF ABA-REGULATED DORMANCY 1 (MARD1)*	encodes a novel zinc-finger protein with a proline-rich N-terminus, identical to senescence-associated protein SAG102
AT3G63230		senescence-associated-like protein (DUF581)
AT3G63260	*(ATMRK1)*	protein kinase, similar to mammal mixed-lineage kinase and Raf protein kinase
AT3G63290		2-oxoglutarate (2OG) and Fe(II)-dependent oxygenase superfamily protein
AT3G63300	*FORKED 1 (FKD1)*	Encodes a pleckstrin homology domain- and DUF828-containing protein. Mutants have defects in leaf vascular pattering, with vascular bundles that fail to meet distally in both the cotyledons and leaves. Necessary to the formation of the closed leaf vascular pattern characteristic of dicot leaves in response to auxin. Redundant with FKD2. FKD1 may influence PIN1 localization in an auxin dependent manner. proposed to be a key component of the auxin canalization pathway. FORKED-LIKE family member, part of Group 1 (FKD1, FL1-FL3; Group 2 consists of FL4 and FL8 and Group 3 consists of FL5-FL7). May coordinate leaf size with vein density, where Group 1 members and Group 3 members have opposing functions.
AT3G63310	*BRZ-INSENSITIVE-LONG HYPOCOTYLS 4 (BIL4)*	Mediates cell elongation in brassinosteroid signaling.
AT3G63320		Protein phosphatase 2C family protein
AT3G63330		
AT3G63350	*(AT-HSFA7B)*	member of Heat Stress Transcription Factor (Hsf) family
AT3G63360		Encodes a defensin-like (DEFL) family protein.
AT3G63370	*ORGANELLE TRANSCRIPT PROCESSING 86 (OTP86)*	Encodes a chloroplast RNA editing factor.
AT3G63380	*AUTO-INHIBITED CA2+ ATPASE 12 (ACA12)*	ATPase E1-E2 type family protein / haloacid dehalogenase-like hydrolase family protein
AT3G63390		hypothetical protein
AT3G63400		Cyclophilin-like peptidyl-prolyl cis-trans isomerase family protein
AT3G63410	*ALBINO OR PALE GREEN MUTANT 1 (APG1)*	Encodes a MPBQ/MSBQ methyltransferase located in the chloroplast inner envelope membrane. Mutant plants lack plastoquinone (PQ), suggesting that the APG1 protein is involved in the methylation step of PQ biosynthesis. The gene product is also involved in tocopherol (vitamin E) biosynthesis.
AT3G63440	*CYTOKININ OXIDASE/DEHYDROGENASE 6 (CKX6)*	This gene used to be called AtCKX7. It encodes a protein whose sequence is similar to cytokinin oxidase/dehydrogenase, which catalyzes the degradation of cytokinins.
AT3G63460	*(SEC31B)*	Together with SEC31A a component of the coat protein complex II (COPII) which promotes the formation of transport vesicles from the endoplasmic reticulum (ER).
AT3G63470	*SERINE CARBOXYPEPTIDASE-LIKE 40 (scpl40)*	serine carboxypeptidase-like 40
AT3G63490	*EMBRYO DEFECTIVE 3126 (EMB3126)*	Ribosomal protein L1p/L10e family

AT3G63520	*CAROTENOID CLEAVAGE DIOXYGENASE 1 (CCD1)*	Encodes a protein with 9-<i>cis</i>-epoxycarotenoid dioxygenase activity. The enzyme was shown to act on a variety of carotenoid including β-carotene, lutein, zeaxanthin, and all-<i>trans</i>-violaxanthin. When those compounds are used as substrates, the major reaction product detected is a C14 dialdehyde: 4,9-dimethyldodeca-2,4,6,8,10-pentaene-1,12-dial. The enzyme did not cleave as efficiently carotenoids containing 9-<i>cis</i>-double or allenic bonds. The mRNA is cell-to-cell mobile.
AT3G66652		fip1 motif-containing protein
AT3G66656	*AGAMOUS-LIKE 91 (AGL91)*	AGAMOUS-like 91
AT4G00010		
AT4G00020	*BREAST CANCER 2 LIKE 2A (BRCA2(IV))*	Ortholog of breast cancer susceptibility protein 2. Essential at meiosis. Interacts with either AtRad51 or AtDmc1 and ATDSS1(I). Involved in embryo sac development and defense gene transcription during plant immune responses.
AT4G00040		Chalcone and stilbene synthase family protein
AT4G00050	*UNFERTILIZED EMBRYO SAC 10 (UNE10)*	Encodes a phytochrome interacting factor that inhibits phytochrome A-mediated far-red light responses and binds to promoter regions of AT2G46970 and AT3G62090.
AT4G00070	*(CTL12)*	RING/U-box superfamily protein
AT4G00080	*UNFERTILIZED EMBRYO SAC 11 (UNE11)*	Plant invertase/pectin methylesterase inhibitor superfamily protein
AT4G00090		Transducin/WD40 repeat-like superfamily protein
AT4G00130		DNA-binding storekeeper protein-related transcriptional regulator
AT4G00150	*HAIRY MERISTEM 3 (HAM3)*	Belongs to one of the LOM (LOST MERISTEMS) genes: AT2G45160 (LOM1), AT3G60630 (LOM2) and AT4G00150 (LOM3). LOM1 and LOM2 promote cell differentiation at the periphery of shoot meristems and help to maintain their polar organization.
AT4G00160		F-box/RNI-like/FBD-like domains-containing protein
AT4G00180	*YABBY3 (YAB3)*	YABBY gene family member, likely has transcription factor activity, involved in specifying abaxial cell fate. Along with FIL, involved in patterning of the fruit. GUS reporter gene expression in seedlings is observed in the young leaves and as the leaf matures, expression is restricted to the abaxial tissues of leaves, expression is also observed on either side of the leaf margin in the younger tissues of leaf blades.
AT4G00190	*PECTIN METHYLESTERASE 38 (PME38)*	pectin methylesterase 38
AT4G00220	*JAGGED LATERAL ORGANS (JLO)*	Encodes a protein containing a LOB domain that is expressed in embryos, flower primordium and lateral floral organ boundaries. Overexpression is correlated with activation of STM and KNAT1 and down regulation of PIN1 and reduced auxin transport levels. Ectopic expression in plants results in premature termination of the shoot apical meristem and small, lobed leaves. A maternally expressed imprinted gene.
AT4G00230	*XYLEM SERINE PEPTIDASE 1 (XSP1)*	xylem serine peptidase 1
AT4G00231	*MATERNAL EFFECT EMBRYO ARREST 50 (MEE50)*	ARM repeat superfamily protein
AT4G00232		DNA-binding storekeeper protein-related transcriptional regulator
AT4G00234		mechanosensitive ion channel-like protein
AT4G00238	*(ATSTKL1)*	STKL1 is a DUF domain containing DNA binding protein that may be involved in mediating certain glucose responses. Binds to promoter of PGR, a putative plasma membrane glucose response regulator.
AT4G00240	*PHOSPHOLIPASE D BETA 2 (PLDBETA2)*	member of C2-PLD subfamily
AT4G00270	*GL1 ENHANCER BINDING PROTEIN (GeBP)*	GeBP is a non canonical, nuclear localized leucine zipper transcription factor that binds G-elements.It is strongly expressed in the vegetative meristem and leaf primordium. It regulates genes down stream of a number of signaling pathways including hormone and defense responses.
AT4G00310	*EMBRYO SAC DEVELOPMENT ARREST 8 (EDA8)*	Putative membrane lipoprotein
AT4G00320		F-box/RNI-like superfamily protein
AT4G00330	*CALMODULIN-BINDING RECEPTOR-LIKE CYTOPLASMIC KINASE 2 (CRCK2)*	high overall homology to CRCK1
AT4G00335	*RING-H2 FINGER B1A (RHB1A)*	RING-H2 finger B1A
AT4G00340	*RECEPTOR-LIKE PROTEIN KINASE 4 (RLK4)*	Encodes a receptor-like protein kinase that is expressed in roots.
AT4G00350		MATE efflux family protein
AT4G00360	*CYTOCHROME P450, FAMILY 86, SUBFAMILY A, POLYPEPTIDE 2 (CYP86A2)*	Encodes a member of the CYP86A subfamily of cytochrome p450 genes. Expressed at moderate levels in flowers, leaves, roots and stems.
AT4G00370	*(ANTR2)*	Encodes an inorganic phosphate transporter (PHT4;4) that can transport ascorbate and is located in the chloroplast envelope membrane. It has been shown to play a role in the xanthophyll cycle during photosynthesis and may be required for tolerance to strong light stress.
AT4G00390		DNA-binding storekeeper protein-related transcriptional regulator
AT4G00400	*GLYCEROL-3-PHOSPHATE SN-2-ACYLTRANSFERASE 8 (GPAT8)*	bifunctional sn-glycerol-3-phosphate 2-O-acyltransferase/phosphatase. Involved in cutin assembly and is functionally redundant with GPAT4.
AT4G00410		
AT4G00416	*METHYL-CPG-BINDING DOMAIN 3 (MBD3)*	Protein containing methyl-CpG-binding domain.Has sequence similarity to human MBD proteins.
AT4G00430	*PLASMA MEMBRANE INTRINSIC PROTEIN 1;4 (PIP1;4)*	a member of the plasma membrane intrinsic protein subfamily PIP1. involved redundantly with PIP1;1/2/3/5 in hydraulics and carbon fixation, regulates the expression of related genes that affect plant growth and development.
AT4G00440	*TON1 RECRUITING MOTIF 15 (TRM15)*	GPI-anchored adhesin-like protein, putative (DUF3741)
AT4G00460	*ROP (RHO OF PLANTS) GUANINE NUCLEOTIDE EXCHANGE FACTOR 3 (ROPGEF3)*	Encodes a member of KPP-like gene family, homolog of KPP (kinase partner protein) gene in tomato. Also a member of the RopGEF (guanine nucleotide exchange factor) family, containing the novel PRONE domain (plant-specific Rop nucleotide exchanger), which is exclusively active towards members of the Rop subfamily.
AT4G00480	*(ATMYC1)*	MYC-related protein with a basic helix-loop-helix motif at the C-terminus and a region similar to the maize B/R family at the N-terminus
AT4G00510		
AT4G00520		Acyl-CoA thioesterase family protein
AT4G00530		UvrABC system protein A
AT4G00550	*DIGALACTOSYL DIACYLGLYCEROL DEFICIENT 2 (DGD2)*	encodes a UDP-galactose-dependent digalactosyldiacylglycerol(DGDG) synthase. Located in chloroplast outer membrane.
AT4G00560		NAD(P)-binding Rossmann-fold superfamily protein

AT4G00570	*NAD-DEPENDENT MALIC ENZYME 2 (NAD-ME2)*	Encodes an NAD-dependent malic enzyme (NAD-ME) that does not act on oxaloacetate, indicating that it belongs to EC 1.1.1.39. It is a member of the beta family of NAD-MEs in plants. It appears to function as a homodimer or as a heterodimer with the alpha-type NAD-ME2 (At2g13560). NAD-ME2 transcript and protein levels are higher during the night than during the day.
AT4G00600		Amino acid dehydrogenase family protein
AT4G00640		
AT4G00670		Remorin family protein
AT4G00680	*ACTIN DEPOLYMERIZING FACTOR 8 (ADF8)*	actin depolymerizing factor 8
AT4G00690	*UB-LIKE PROTEASE 1B (ULP1B)*	UB-like protease 1B
AT4G00710	*BRASSINOSTEROID-SIGNALING KINASE 3 (BSK3)*	Encodes BR-signaling kinase 3 (BSK3), one of the three homologous BR-signaling kinases (BSK1, AT4G35230; BSK2, AT5G46570; BSK3, AT4G00710). Mediates signal transduction from receptor kinase BRI1 by functioning as the substrate of BRI1. Plasma membrane localized.
AT4G00720	*SHAGGY-LIKE PROTEIN KINASE 32 (SK32)*	Encodes ASKtheta, a group III Arabidopsis GSK3/shaggy-like kinase. Functions in the brassinosteroid signalling pathway.
AT4G00730	*ANTHOCYANINLESS 2 (ANL2)*	Encodes a homeodomain protein of the HD-GLABRA2 group. Involved in the accumulation of anthocyanin and in root development. Loss of function mutants have increased cell wall polysaccharide content.
AT4G00750		S-adenosyl-L-methionine-dependent methyltransferases superfamily protein
AT4G00770	*TON1 RECRUITING MOTIF 9 (TRM9)*	DUF4378 domain protein
AT4G00780		TRAF-like family protein
AT4G00790		
AT4G00800	*(SETH5)*	transducin family protein / WD-40 repeat family protein
AT4G00820	*IQ-DOMAIN 17 (iqd17)*	Member of IQ67 (CaM binding) domain containing family.
AT4G00850	*GRF1-INTERACTING FACTOR 3 (GIF3)*	Arabidopsis thaliana GRF1-interacting factor 3 (GIF3) mRNA
AT4G00860	*(ATOZI1)*	putative pathogenesis-related protein whose transcript level is induced in response to ozone and pathogenic Pseudomonas strains.
AT4G00870	*(BHLH14)*	bHLH14 interacts with JAZ proteins, and functions redundantly with bHLH3, bHLH13 and bHLH17 to negatively regulate jasmonate responses.
AT4G00880	*SMALL AUXIN UPREGULATED RNA 31 (SAUR31)*	SAUR-like auxin-responsive protein family
AT4G00890		Encodes a putative glycosyl hydrolase family 10 protein (xylanase).
AT4G00895		ATPase, F1 complex, OSCP/delta subunit protein
AT4G00900	*ER-TYPE CA2+-ATPASE 2 (ECA2)*	Type IIA (SERCA-type) Ca2+ ATPase, catalyzes the efflux of calcium from the cytoplasm.
AT4G00910		aluminum activated malate transporter family protein
AT4G00920		COP1-interacting protein-like protein
AT4G00940	*DNA BINDING WITH ONE FINGER 4.1 (DOF4.1)*	Dof-type zinc finger DNA-binding family protein
AT4G00950	*MATERNAL EFFECT EMBRYO ARREST 47 (MEE47)*	hypothetical protein (DUF688)
AT4G00955		wall-associated receptor kinase-like protein
AT4G00960		Protein kinase superfamily protein
AT4G00970	*CYSTEINE-RICH RLK (RECEPTOR-LIKE PROTEIN KINASE) 41 (CRK41)*	Encodes a cysteine-rich receptor-like protein kinase.
AT4G00990	*JMJC DOMAIN-CONTAINING PROTEIN 27 (JMJ27)*	jJumonji-domain-containing H3K9 histone demethylase. Loss of function mutants are susceptible to bacterial infection and early flowering.
AT4G01010	*CYCLIC NUCLEOTIDE-GATED CHANNEL 13 (CNGC13)*	member of Cyclic nucleotide gated channel family
AT4G01026	*PYR1-LIKE 7 (PYL7)*	Encodes a member of the PYR (pyrabactin resistance)/PYL(PYR1-like)/RCAR (regulatory components of ABA receptor) family proteins with 14 members. PYR/PYL/RCAR family proteins function as abscisic acid sensors. Mediate ABA-dependent regulation of protein phosphatase 2Cs ABI1 and ABI2. PYL/RCAR family proteins function as abscisic acid sensors. Mediate ABA-dependent regulation of ABI1 and ABI2.
AT4G01030		pentatricopeptide (PPR) repeat-containing protein
AT4G01040		Glycosyl hydrolase superfamily protein
AT4G01050	*THYLAKOID RHODANESE-LIKE (TROL)*	hydroxyproline-rich glycoprotein family protein, contains a rhodanese homology domain. Required for anchoring the FNR flavoenzyme to the thylakoid membranes and sustaining high efficiency photosynthetic linear electron flow. The mRNA is cell-to-cell mobile.
AT4G01070	*(GT72B1)*	the glycosyltransferase (UGT72B1) is involved in metabolizing xenobiotica (chloroaniline and chlorophenole). Comparison between wild type and knock-out mutant demonstrates the central role of this gene for metabolizing chloroaniline but significantly less for chlorophenole. The glucosyltransferase preferred UDP-xylose over UDP-glucose indicating its (additional) functioning as a xylosyltransferase in planta
AT4G01080	*TRICHOME BIREFRINGENCE-LIKE 26 (TBL26)*	Encodes a member of the TBL (TRICHOME BIREFRINGENCE-LIKE) gene family containing a plant-specific DUF231 (domain of unknown function) domain. TBL gene family has 46 members, two of which (TBR/AT5G06700 and TBL3/AT5G01360) have been shown to be involved in the synthesis and deposition of secondary wall cellulose, presumably by influencing the esterification state of pectic polymers. Functions as a mannan O-acetyltransferase, catalyzing the 2-O and 3-O-monoacetylation of mannosyl residues A nomenclature for this gene family has been proposed (Volker Bischoff & Wolf Scheible, 2010, personal communication).
AT4G01100	*ADENINE NUCLEOTIDE TRANSPORTER 1 (ADNT1)*	Adenine nucleotide transporter. Located in mitochondrion. Expressed in a broad range of tissues, but predominantly in root tips. Loss of function mutants exhibit reduced root growth and respiration.
AT4G01110		late embryogenesis abundant hydroxyproline-rich glycoprotein family protein
AT4G01120	*G-BOX BINDING FACTOR 2 (GBF2)*	bZIP (basic leucine zipper) transcription factor that binds to the G-box regulatory element found in many plant promoters. GBF2 nuclear localization is increased by blue light
AT4G01130		GDSL-motif esterase/acyltransferase/lipase. Enzyme group with broad substrate specificity that may catalyze acyltransfer or hydrolase reactions with lipid and non-lipid substrates.
AT4G01140		transmembrane protein, putative (DUF1191)
AT4G01150	*CURVATURE THYLAKOID 1A (CURT1A)*	Integral thylakoid membrane protein required for proper grana stack curvature.
AT4G01180		XH/XS domain-containing protein

AT4G01210		glycosyl transferase family 1 protein
AT4G01240		S-adenosyl-L-methionine-dependent methyltransferases superfamily protein
AT4G01250	*(WRKY22)*	AtWRKY22 is a member of WRKY Transcription Factor; Group II-e. It is involved in regulation of dark induced leaf senescence.
AT4G01270		RING/U-box superfamily protein
AT4G01280	*REVEILLE 5 (RVE5)*	RVE5 is one of eleven homologous MYB-like transcription factors in Arabidopsis and a member of the RVE8 clade. Plays a minor role in clock regulation.
AT4G01290	*CONSERVED BINDING OF EIF4E 1 (CBE1)*	Protein with evolutionarily conserved eIF4E-binding motif in its N-terminal domain that can form mRNA cap?binding complexes and has the potential for regulating gene expression as a translation factor associated plant-specific cell cycle regulator.
AT4G01300		
AT4G01310	*PLASTID RIBOSOMAL PROTEINS OF THE 50S SUBUNIT 5 (PRPL5)*	Ribosomal L5P family protein
AT4G01320	*(ATSTE24)*	CAAX protease with broad substrate specificity. Localized exclusively to the endoplasmic reticulum.
AT4G01330		Protein kinase superfamily protein
AT4G01340		CHP-rich zinc finger protein-like protein
AT4G01350		Cysteine/Histidine-rich C1 domain family protein
AT4G01380		plastocyanin-like domain-containing protein
AT4G01390		TRAF-like family protein
AT4G01410		Late embryogenesis abundant (LEA) hydroxyproline-rich glycoprotein family
AT4G01420	*CALCINEURIN B-LIKE PROTEIN 5 (CBL5)*	Encodes calcineurin B-like protein 5 (CBL5). Overexpression confers tolerance to drought and salt stress.
AT4G01430	*USUALLY MULTIPLE ACIDS MOVE IN AND OUT TRANSPORTERS 29 (UMAMIT29)*	Encodes a plasma membrane-localized amino acid transporter likely involved in amino acid export in the developing seed.
AT4G01440	*USUALLY MULTIPLE ACIDS MOVE IN AND OUT TRANSPORTERS 31 (UMAMIT31)*	nodulin MtN21-like transporter family protein
AT4G01450	*USUALLY MULTIPLE ACIDS MOVE IN AND OUT TRANSPORTERS 30 (UMAMIT30)*	nodulin MtN21-like transporter family protein
AT4G01460		basic helix-loop-helix (bHLH) DNA-binding superfamily protein
AT4G01470	*TONOPLAST INTRINSIC PROTEIN 1;3 (TIP1;3)*	Encodes AtTIP1;3, functions as water and urea channels in pollen.
AT4G01480	*PYROPHOSPHORYLASE 5 (PPa5)*	Encodes a protein that might have inorganic pyrophosphatase activity.
AT4G01490		transposable_element_gene;non-LTR retrotransposon family (LINE), has a 2.4e-44 P-value blast match to GB:AAB41224 ORF2 (LINE-element) (Rattus norvegicus);(source:TAIR10)
AT4G01500	*NGATHA4 (NGA4)*	AP2/B3-like transcriptional factor family protein
AT4G01510	*(ARV2)*	Arv1-like protein
AT4G01530		transposable_element_gene;similar to unknown protein [Arabidopsis thaliana] (TAIR:AT1G26860.1);(source:TAIR10)
AT4G01550	*NAC DOMAIN CONTAINING PROTEIN 69 (NAC069)*	Encodes a plasma-membrane bound NAC transcription factor, whose controlled proteolytic activation allows it to enter the nucleus.
AT4G01560	*MATERNAL EFFECT EMBRYO ARREST 49 (MEE49)*	Ribosomal RNA processing Brix domain protein
AT4G01590		DNA-directed RNA polymerase III subunit
AT4G01610	*(ATCATHB3)*	Encodes a capase involved in stress induced cell death. Activity detected in leaf and cell culture.
AT4G01630	*EXPANSIN A17 (EXPA17)*	member of Alpha-Expansin Gene Family. Naming convention from the Expansin Working Group (Kende et al, 2004. Plant Mol Bio)
AT4G01640		F-box associated ubiquitination effector family protein
AT4G01650		Polyketide cyclase / dehydrase and lipid transport protein
AT4G01660	*ABC TRANSPORTER 1 (ABC1)*	Encodes an ABC1-like protein, member of the ATH subfamily; putative ABC transporter; isolated by functional complementation of a yeast abc1 mutant The mRNA is cell-to-cell mobile.
AT4G01680	*MYB DOMAIN PROTEIN 55 (MYB55)*	Encodes a putative transcription factor (MYB55).
AT4G01690	*(PPOX)*	Encodes protoporphyrinogen oxidase (PPOX).
AT4G01700		Chitinase family protein
AT4G01720	*(WRKY47)*	member of WRKY Transcription Factor; Group II-b
AT4G01730		DHHC-type zinc finger family protein
AT4G01750	*RHAMNOGALACTURONAN XYLOSYLTRANSFERASE 2 (RGXT2)*	Encodes a protein with UDP-xylose-dependent xylosyltransferase activity, which transfers Xyl onto L-fucose and (albeit less efficiently) L-arabinose. The linkage to L-fucose was shown to be preferentially to the <i>O</i>-4 position. Analysis of mutant containing T-DNA insertion in this gene indicate that the RGXT2 protein might be involved in the synthesis of the α-D-Xyl-(1,3)-α-L-Fuc-(1,4)-L-Rha structure in pectic rhamnogalacturonan II. The mRNA is cell-to-cell mobile.
AT4G01760		Cysteine/Histidine-rich C1 domain family protein
AT4G01780		XH/XS domain-containing protein
AT4G01790		Ribosomal protein L7Ae/L30e/S12e/Gadd45 family protein
AT4G01800	*ALBINO OR GLASSY YELLOW 1 (AGY1)*	Encodes the ATPase subunit of the chloroplast Sec translocation machinery which plays an essential role in chloroplast biogenesis and the regulation of photosynthesis, the absence of which triggers a retrograde signal, eventually leading to a reprogramming of chloroplast and mitochondrial gene expression.
AT4G01820	*ATP-BINDING CASSETTE B3 (ABCB3)*	member of MDR subfamily
AT4G01840	*CA2+ ACTIVATED OUTWARD RECTIFYING K+ CHANNEL 5 (KCO5)*	Encodes AtTPK5, a member of the Arabidopsis thaliana K+ channel family of AtTPK/KCO proteins. AtTPK5 is targeted to the vacuolar membrane. May form homomeric ion channels in vivo.
AT4G01860		Transducin family protein / WD-40 repeat family protein
AT4G01870		tolB protein-like protein
AT4G01890		Pectin lyase-like superfamily protein
AT4G01895		systemic acquired resistance (SAR) regulator protein NIMIN-1-like protein

AT4G01900	*GLNB1 HOMOLOG (GLB1)*	encodes a PII protein that may function as part of a signal transduction network involved in perceiving the status of carbon and organic nitrogen. Forms a protein complex with N-acetylglutamate kinase and regulates the kinase activity by relieving the feedback inhibition of the kinase by arginine. Regulates acetyl-CoA carboxylase activity.
AT4G01910		Cysteine/Histidine-rich C1 domain family protein
AT4G01920		Cysteine/Histidine-rich C1 domain family protein
AT4G01930		Cysteine/Histidine-rich C1 domain family protein
AT4G01950	*GLYCEROL-3-PHOSPHATE SN-2-ACYLTRANSFERASE 3 (GPAT3)*	putative sn-glycerol-3-phosphate 2-O-acyltransferase
AT4G01960		transmembrane protein
AT4G01970	*STACHYOSE SYNTHASE (STS)*	Encodes a a raffinose and high affinity stachyose synthase as well as a stachyose and Gol specific galactosylhydrolase enzyme activity.AtRS4 is a sequential multifunctional RafS and StaS as well as a high affinity StaS, accepting only Raf and Gol for Sta product formation. AtRS4 possesses a Sta and Gol specific galactosylhydrolase enzyme activity.
AT4G01990		Tetratricopeptide repeat (TPR)-like superfamily protein
AT4G02020	*SWINGER (SWN)*	Encodes a polycomb group protein. Forms part of a large protein complex that can include VRN2 (VERNALIZATION 2), VIN3 (VERNALIZATION INSENSITIVE 3) and polycomb group proteins FERTILIZATION INDEPENDENT ENDOSPERM (FIE) and CURLY LEAF (CLF). The complex has a role in establishing FLC (FLOWERING LOCUS C) repression during vernalization. Performs a partially redundant role to MEA in controlling seed initiation by helping to suppress central cell nucleus endosperm proliferation within the FG.
AT4G02050	*SUGAR TRANSPORTER PROTEIN 7 (STP7)*	STP7 is a monosaccharide/H+ symporter that transports arabinose and xylose.
AT4G02075	*PITCHOUN 1 (PIT1)*	RING/FYVE/PHD zinc finger superfamily protein
AT4G02090		PADRE protein.
AT4G02120	*CTP SYNTHASE 3 (CTPS3)*	Cytidine triphosphate synthase.
AT4G02130	*GALACTURONOSYLTRANSFERASE-LIKE 6 (GATL6)*	Encodes a protein with putative galacturonosyltransferase activity.
AT4G02140		hypothetical protein
AT4G02160		cotton fiber protein
AT4G02170		cotton fiber protein
AT4G02180		DC1 domain-containing protein
AT4G02190		Cysteine/Histidine-rich C1 domain family protein
AT4G02220		zinc finger (MYND type) family protein / programmed cell death 2 C-terminal domain-containing protein
AT4G02230		Ribosomal protein L19e family protein
AT4G02240		
AT4G02250		Plant invertase/pectin methylesterase inhibitor superfamily protein
AT4G02260	*RELA/SPOT HOMOLOG 1 (RSH1)*	Displays guanosine-3',5'-bis(diphosphate) 3'-diphosphatase activity but not guanosine-3',5'-bis(diphosphate) 3'-diphosphate synthase activity. Involved in the maintenance of the (p)ppGp level to accustom plastidial gene expression to darkness.
AT4G02270	*ROOT HAIR SPECIFIC 13 (RHS13)*	root hair specific 13
AT4G02280	*SUCROSE SYNTHASE 3 (SUS3)*	Encodes a protein with sucrose synthase activity (SUS3). It appears to be important for sucrose metabolism in developing seeds, especially during the late maturation phase, about 18 days after flowering.
AT4G02290	*GLYCOSYL HYDROLASE 9B13 (GH9B13)*	glycosyl hydrolase 9B13
AT4G02300	*PECTIN METHYLESTERASE 39 (PME39)*	Plant invertase/pectin methylesterase inhibitor superfamily
AT4G02310		Galactose oxidase/kelch repeat superfamily protein
AT4G02320		Plant invertase/pectin methylesterase inhibitor superfamily
AT4G02330	*(ATPMEPCRB)*	Encodes a pectin methylesterase that is sensitive to chilling stress and brassinosteroid regulation, response to Botrytis cinerea.
AT4G02340		alpha/beta-Hydrolases superfamily protein
AT4G02360		transmembrane protein, putative (Protein of unknown function, DUF538)
AT4G02380	*SENESCENCE-ASSOCIATED GENE 21 (SAG21)*	Encodes AtLEA5 (late embryogenesis abundant like protein). Also known as SENESCENCE-ASSOCIATED GENE 21 (SAG21). Has a role on oxidative stress tolerance. mRNA levels are elevated in response to various stresses.
AT4G02390	*POLY(ADP-RIBOSE) POLYMERASE 2 (PARP2)*	Encodes a DNA dependent nuclear poly (ADP-ribose) polymerase (E.C.2.4.2.30), thought to be involved in post-translational modification .
AT4G02400		U3 ribonucleoprotein (Utp) family protein
AT4G02410	*L-TYPE LECTIN RECEPTOR KINASE IV.3 (LECRK-IV.3)*	Concanavalin A-like lectin protein kinase family protein
AT4G02420	*L-TYPE LECTIN RECEPTOR KINASE IV.4 (LECRK-IV.4)*	Concanavalin A-like lectin protein kinase family protein
AT4G02430	*SERINE/ARGININE-RICH PROTEIN SPLICING FACTOR 34B (SR34b)*	Barta et al (2010) have proposed a nomenclature for Serine/Arginine-Rich Protein Splicing Factors (SR proteins): Plant Cell. 2010, 22:2926.
AT4G02450	*(P23-1)*	Encodes one of two isoforms of a co-chaperone of HSP90 that is required for root growth, in particular in the maintenance of the root meristem.
AT4G02460	*POSTMEIOTIC SEGREGATION 1 (PMS1)*	Encodes a protein similar to PMS1 in yeast, a member of the family of eukaryotic MutL homologs. The protein appears to play a role in DNA mismatch repair and in the suppression of somatic homeologous recombination.
AT4G02480		AAA-type ATPase family protein
AT4G02490		transposable_element_gene;non-LTR retrotransposon family (LINE), has a 2.3e-36 P-value blast match to GB:AAA67727 reverse transcriptase (LINE-element) (Mus musculus);(source:TAIR10)
AT4G02500	*UDP-XYLOSYLTRANSFERASE 2 (XT2)*	Encodes a protein with xylosyltransferase activity, which is specific for UDP-xylose as donor substrate and for oligosaccharides with a degree of polymerization >4. Although the enzyme utilizes either cellopentaose or cellohexaose, its activity is four-fold higher with cellohexaose as an acceptor compared to cellopentaose. The enzyme is able to add several xylosyl residues to the acceptor forming mono-, di- and trixylosylated polysaccharides. The mRNA is cell-to-cell mobile.

AT4G02530	*MAINTENANCE OF PHOTOSYSTEM II UNDER HIGH LIGHT 2 (MPH2)*	MPH2 is a green lineage-specific thylakoid lumen protein required for photosynthetic acclimation of PSII to stressful light conditions (PMID:28874535).
AT4G02540		Cysteine/Histidine-rich C1 domain family protein
AT4G02560	*LUMINIDEPENDENS (LD)*	Encodes a nuclear localized protein with similarity to transcriptional regulators. Recessive mutants are late flowering. Expression of LFY is reduced in LD mutants. LD has been reported to exhibit prion like behavior in yeast but it remains to be determined if such activity exists during normal plant development.
AT4G02570	*CULLIN 1 (CUL1)*	Encodes a cullin that is a component of SCF ubiquitin ligase complexes involved in mediating responses to auxin and jasmonic acid. Homozygous auxin-resistant mutants arrest growth soon after germination, lacking a root and hypocotyl. Heterozygotes display a variety of phenotypes consistent with impaired auxin response.
AT4G02590	*UNFERTILIZED EMBRYO SAC 12 (UNE12)*	Basic helix loop helix class transcriptional regulator. Shows ecotype specific effects on temperature dependent salicylic acid accumulation and immunity. Governs the competence of pericycle cells to initiate lateral root primordium formation.
AT4G02600	*(MLO1)*	A member of a large family of seven-transmembrane domain proteins specific to plants, homologs of the barley mildew resistance locus o (MLO) protein. The Arabidopsis genome contains 15 genes encoding MLO proteins, with localization in plasma membrane. Phylogenetic analysis revealed four clades of closely-related AtMLO genes. ATMLO1 belongs to the clade II, with ATMLO13 and ATMLO15. The gene is expressed during early seedling growth, in root and cotyledon vascular system, in pollen and in papillae, as shown by GUS activity patterns. The expression of several phylogenetically closely-related AtMLO genes showed similar or overlapping tissue specificity and analogous responsiveness to external stimuli, suggesting functional redundancy, co-function, or antagonistic function(s).
AT4G02610		Aldolase-type TIM barrel family protein
AT4G02630		Protein kinase superfamily protein
AT4G02640	*(BZO2H1)*	Encodes a basic leucine zipper (bZIP) transcription factor AtbZIP10. AtbZIP10 shuttles between the nucleus and the cytoplasm. It binds consensus G- and C-box DNA sequences. AtbZIP10 acts antagonistically with LSD1 in both pathogen-induced hypersensitive response and basal defense responses.
AT4G02650	*PHOSPHATIDYLINOSITOL BINDING CLATHRIN ASSEMBLY PROTEIN 5B (PICALM5B)*	Phosphatidylinositol binding clathrin assembly protein 5A/B are recent paralogs with overlapping functions in recycling ANXUR proteins to the pollen tube membrane.
AT4G02670	*INDETERMINATE(ID)-DOMAIN 12 (IDD12)*	indeterminate(ID)-domain 12
AT4G02690	*LIFEGUARD 3 (LFG3)*	Stress induced membrane protein. Mutants show enhanced cell death under stress.
AT4G02700	*SULFATE TRANSPORTER 3;2 (SULTR3;2)*	sulfate transporter 3
AT4G02710	*NETWORKED 1C (NET1C)*	Kinase interacting (KIP1-like) family protein
AT4G02740		F-box/RNI-like superfamily protein
AT4G02770	*PHOTOSYSTEM I SUBUNIT D-1 (PSAD-1)*	Encodes a protein predicted by sequence similarity with spinach PsaD to be photosystem I reaction center subunit II (PsaD1)
AT4G02780	*GA REQUIRING 1 (GA1)*	Catalyzes the conversion of geranylgeranyl pyrophosphate (GGPP) to copalyl pyrophosphate (CPP) of gibberellin biosynthesis
AT4G02800	*(MADA1)*	GRIP/coiled-coil protein
AT4G02820		Pentatricopeptide repeat (PPR) superfamily protein
AT4G02850	*D-AMINO ACID RACEMASE1 (DAAR1)*	DAAR1 encodes a PLP-independent racemase that catalyzes the conversion from L-Ile to D-allo-Ile.
AT4G02880		ELKS/Rab6-interacting/CAST family protein
AT4G02890	*(UBQ14)*	Polyubiquitin gene containing 4 ubiquitin repeats.
AT4G02910		hypothetical protein
AT4G02930		GTP binding Elongation factor Tu family protein
AT4G02940	*ATALKBH10B (ALKBH10B)*	ALKBH10B is a functional RNA N6-methyladenosine demethylase. Reduction in ALKBH10B decreases m6A levels, and affects the stability of flowering time genes including FT, SPL3 and SPL9. Mutant plants are early flowering.
AT4G02960	*RETRO ELEMENT 2 (RE2)*	a copia-type retrotransposon element containing LTRs and encoding a polyprotein. This retro element exists in two loci in Landsberg erecta but only once in Columbia
AT4G02970	*7SL RNA1 (AT7SL-1)*	Signal recognition particle. Type 4 of RNA polymerase III dependent genes.
AT4G03010		RNI-like superfamily protein
AT4G03030		Galactose oxidase/kelch repeat superfamily protein
AT4G03040		hypothetical protein
AT4G03050	*(AOP3)*	The transcribed allele in ecotype Ler encodes a 2-oxoglutarate-dependent dioxygenase which is involved in glucosinolate biosynthesis. AOP3 is transcriptionally silent in leaf tissues of ecotype Col.The natural variation in this locus explains the diversification of hydroxyalkyl glucosinolates among different ecotypes of Arabidopsis.
AT4G03060	*ALKENYL HYDROXALKYL PRODUCING 2 (AOP2)*	Encodes a truncated and null function protein, due to a 5-bp deletion in cDNA. The functional allele in ecotype Cvi, AOP2, encodes a 2-oxoglutarate-dependent dioxygenase which is involved in glucosinolate biosynthesis. The natural variation in this locus explains the diversification of alkenyl glucosinolate among different ecotypes of Arabidopsis.
AT4G03070	*(AOP1)*	Encodes a possible 2-oxoglutarate-dependent dioxygenase that is involved in glucosinolate biosynthesis. The gene is expressed in all ecotypes examined but the enzymatic activity has not been determined experimentally. In Col, there is one copy of this gene (aka AOP1.1) but Ler contains two copies, AOP1.1 and a tightly linked AOP1.2.
AT4G03090	*NODULIN HOMEOBOX (NDX)*	AtNDX negatively regulates ABI4 expression during ABA signaling.
AT4G03100		Rho GTPase activating protein with PAK-box/P21-Rho-binding domain-containing protein
AT4G03110	*RNA-BINDING PROTEIN-DEFENSE RELATED 1 (RBP-DR1)*	Encodes a putative RNA-binding protein that is located in the cytoplasm and is involved in the hypersensitive response and positively regulates salicylic acid-mediated immunity.
AT4G03140		NAD(P)-binding Rossmann-fold superfamily protein

AT4G03150		plant/protein
AT4G03153	*NETWORKED 3B (NET3B)*	Kinase interacting (KIP1-like) family protein
AT4G03156		small GTPase-like protein
AT4G03160		B3 domain protein
AT4G03170		AP2/B3-like transcriptional factor family protein
AT4G03180		rRNA-processing protein
AT4G03190	*GRR1-LIKE PROTEIN 1 (GRH1)*	Encodes an F box protein belonging to the TIR1 subfamily. This protein forms SCF complexes with ASK1 and CUL1 and interacts with Aux/IAA proteins in an auxin-dependent manner. It also has sequence similarity to the yeast protein GRR1, which is involved in glucose repression.
AT4G03200		catalytics
AT4G03205	*(hemf2)*	Coproporphyrinogen III oxidase
AT4G03230		G-type lectin S-receptor-like Serine/Threonine-kinase
AT4G03240	*FRATAXIN HOMOLOG (FH)*	Encodes AtFH, a frataxin homolog. Frataxin is required for the biogenesis of mitochondria in different organisms. AtFH knock-out mutants are lethal. Required for full activity of mitochondrial Fe-S proteins. Deficiency of AtFH induces oxidative stress.
AT4G03260	*MICROTUBULE ASSOCIATED STRESS PROTEIN 1 (MASP1)*	MASP1 overexpression promotes growth during drought and promotes microtubule stability and recovery after stress as well as microtubule bundling.MASP1 growth promotion and microtubule stabilization activities are dependent upon serine 670 phosphorylation. MASP1 binds microtubules;this activity is not affected by MASP1 phosphorylation status.MASP1 protein levels are induced by drought post-transcriptionally.
AT4G03280	*PHOTOSYNTHETIC ELECTRON TRANSFER C (PETC)*	Encodes the Rieske FeS center of cytochrome b6f complex. Gene is expressed in shoot but not in root. Mutant has reduced electron transport at saturating light intensities and Q-cycle activity is hypersensitive to acidification of the thylakoid lumen. The mRNA is cell-to-cell mobile.
AT4G03290		EF hand calcium-binding protein family
AT4G03320	*TRANSLOCON AT THE INNER ENVELOPE MEMBRANE OF CHLOROPLASTS 20-IV (tic20-IV)*	Encodes a component of the TIC (translocon at the inner envelope membrane of chloroplasts) protein translocation machinery mediating the protein translocation across the inner envelope of plastids. The Arabidopsis genome encodes four Tic20 homologous proteins, AT1G04940(Tic20-I), AT2G47840(Tic20-II), AT4G03320(Tic20-IV) and AT5G55710(Tic20-V).
AT4G03330	*SYNTAXIN OF PLANTS 123 (SYP123)*	member of SYP12 Gene Family
AT4G03360		Ubiquitin family protein
AT4G03380		hypothetical protein
AT4G03390	*STRUBBELIG-RECEPTOR FAMILY 3 (SRF3)*	STRUBBELIG-receptor family 3
AT4G03400	*DWARF IN LIGHT 2 (DFL2)*	Encodes a GH3-related gene involved in red light-specific hypocotyl elongation. Analysis of sense and antisense transgenic plants suggests that DFL2 is located downstream of red light signal transduction and determines the degree of hypocotyl elongation.
AT4G03440		Ankyrin repeat family protein
AT4G03450		Ankyrin repeat family protein
AT4G03480		Ankyrin repeat family protein
AT4G03490		Ankyrin repeat family protein
AT4G03510	*RING MEMBRANE-ANCHOR 1 (RMA1)*	RMA1 encodes a novel 28 kDa protein with a RING finger motif and a C-terminal membrane-anchoring domain that is involved in the secretory pathway. Has E3 ubiquitin ligase activity.
AT4G03520	*(ATHM2)*	Encodes a redox activated co-chaperone, chloroplast localized thioredoxin, similar to prokaryotic types.
AT4G03530		transposable_element_gene;copia-like retrotransposon family, has a 2.4e-66 P-value blast match to GB:CAA72989 open reading frame 1 (Ty1_Copia-element) (Brassica oleracea);(source:TAIR10)
AT4G03570		Cystatin/monellin superfamily protein
AT4G03580		hypothetical protein
AT4G03600		pyrroline-5-carboxylate reductase
AT4G03610		Metallo-hydrolase/oxidoreductase superfamily protein
AT4G03620		myosin heavy chain-like protein
AT4G03630		RNI-like superfamily protein
AT4G03640		transposable_element_gene;similar to unknown protein [Arabidopsis thaliana] (TAIR:AT3G43100.1);(source:TAIR10)
AT4G03650	*(GP1)*	transposable_element_gene;gypsy-like retrotransposon family, has a 1.3e-314 P-value blast match to GB:AAD27547 polyprotein (Gypsy_Ty3-element) (Oryza sativa subsp. indica);(source:TAIR10)
AT4G03660		transposable_element_gene;similar to replication protein-related [Arabidopsis thaliana] (TAIR:AT4G07440.1);(source:TAIR10)
AT4G03670		transposable_element_gene;similar to unknown protein [Arabidopsis thaliana] (TAIR:AT2G10836.1);(source:TAIR10)
AT4G03680		transposable_element_gene;similar to unknown protein [Arabidopsis thaliana] (TAIR:AT3G30560.1);(source:TAIR10)
AT4G03690		transposable_element_gene;similar to unknown protein [Arabidopsis thaliana] (TAIR:AT3G30560.1);(source:TAIR10)
AT4G03700		transposable_element_gene;pseudogene, replication protein-related, similar to immunoglobulin heavy chain variable region (GI:4456528) (Homo sapiens);(source:TAIR10)
AT4G03710		transposable_element_gene;CACTA-like transposase family (En/Spm), has a 1.8e-111 P-value blast match to GB:AAD55677 putative transposase protein (CACTA-element) transposon=Shooter (Zea mays);(source:TAIR10)
AT4G03720		transposable_element_gene;pseudogene, similar to putative AP endonuclease/reverse transcriptase, blastp match of 44%25 identity and 1.5e-21 P-value to GP|21952510|gb|AAM82604.1|AF525305_2|AF525305 putative AP endonuclease/reverse transcriptase {Brassica napus};(source:TAIR10)
AT4G03730		transposable_element_gene;CACTA-like transposase family (En/Spm), has a 1.3e-102 P-value blast match to GB:AAD55677 putative transposase protein (CACTA-element) transposon=Shooter (Zea mays);(source:TAIR10)
AT4G03740		hypothetical protein

AT4G03750		transposable_element_gene;similar to disease resistance protein (TIR-NBS-LRR class), putative [Arabidopsis thaliana] (TAIR:AT4G16900.1);(source:TAIR10)
AT4G03760		transposable_element_gene;pseudogene, expressed protein;(source:TAIR10)
AT4G03770		transposable_element_gene;gypsy-like retrotransposon family, has a 2.8e-206 P-value blast match to GB:AAD11615 prpol (gypsy_Ty3-element) (Zea mays);(source:TAIR10)
AT4G03780		transposable_element_gene;gypsy-like retrotransposon family (Athila), has a 1.9e-59 P-value blast match to GB:CAA57397 Athila ORF 1 (Arabidopsis thaliana);(source:TAIR10)
AT4G03790		transposable_element_gene;gypsy-like retrotransposon family (Athila), has a 2.2e-286 P-value blast match to GB:CAA57397 Athila ORF 1 (Arabidopsis thaliana);(source:TAIR10)
AT4G03800		transposable_element_gene;gypsy-like retrotransposon family, has a 1.5e-125 P-value blast match to GB:AAD19359 polyprotein (gypsy_Ty3-element) (Sorghum bicolor);(source:TAIR10)
AT4G03810		transposable_element_gene;copia-like retrotransposon family, has a 0. P-value blast match to GB:BAA22288 pol polyprotein (Ty1_Copia-element) (Oryza australiensis)GB:BAA22288 polyprotein (Ty1_Copia-element) (Oryza australiensis)gi|2443320|dbj|BAA22288.1| polyprotein (RIRE1) (Oryza australiensis) (Ty1_Copia-element);(source:TAIR10)
AT4G03820		transmembrane protein, putative (DUF3537)
AT4G03830		hypothetical protein (Protein of unknown function, DUF601)
AT4G03840		transposable_element_gene;gypsy-like retrotransposon family, has a 2.4e-251 P-value blast match to GB:AAD27547 polyprotein (Gypsy_Ty3-element) (Oryza sativa subsp. indica);(source:TAIR10)
AT4G03850		transposable_element_gene;gypsy-like retrotransposon family (Athila), has a 1.8e-131 P-value blast match to GB:CAA57397 Athila ORF 1 (Arabidopsis thaliana);(source:TAIR10)
AT4G03860		transposable_element_gene;transposon protein -related, similar to athila retroelement;(source:TAIR10)
AT4G03870		transposable_element_gene;Mutator-like transposase family, has a 1.1e-61 P-value blast match to O81510 /24-166 Pfam PF03108 MuDR family transposase (MuDr-element domain);(source:TAIR10)
AT4G03880		transposable_element_gene;pseudogene, hypothetical protein;(source:TAIR10)
AT4G03900		transposable_element_gene;CACTA-like transposase family (Tnp2/En/Spm), has a 3.9e-150 P-value blast match to GB:CAA40555 TNP2 (CACTA-element) (Antirrhinum majus);(source:TAIR10)
AT4G03930	*(PME42)*	Plant invertase/pectin methylesterase inhibitor superfamily
AT4G03940		transmembrane protein
AT4G03960	*PLANT AND FUNGI ATYPICAL DUAL-SPECIﬁCITY PHOSPHATASE 4 (PFA-DSP4)*	Encodes an atypical dual-speciﬁcity phosphatase involved in the negative regulation of defense response to a bacterial pathogen, P. syringae pv. tomato.
AT4G03970		transposable_element_gene;similar to Ulp1 protease family protein [Arabidopsis thaliana] (TAIR:AT2G10350.1);(source:TAIR10)
AT4G03990		transposable_element_gene;similar to heat shock protein binding [Arabidopsis thaliana] (TAIR:AT1G32830.1);(source:TAIR10)
AT4G04000		transposable_element_gene;non-LTR retrotransposon family (LINE), has a 2.6e-39 P-value blast match to GB:AAB41224 ORF2 (LINE-element) (Rattus norvegicus);(source:TAIR10)
AT4G04010		transposable_element_gene;similar to Ulp1 protease family protein [Arabidopsis thaliana] (TAIR:AT3G47260.1);(source:TAIR10)
AT4G04020	*FIBRILLIN (FIB)*	Fibrillin precursor protein. The fibrillin preprotein, but not the mature protein interacts with ABI2. Regulated by abscisic acid response regulators. Involved in abscisic acid-mediated photoprotection. The mRNA is cell-to-cell mobile.
AT4G04040	*MATERNAL EFFECT EMBRYO ARREST 51 (MEE51)*	Encodes a pyrophosphate-dependent phosphofructokinase B subunit (PFPbeta2).
AT4G04050		transposable_element_gene;gypsy-like retrotransposon family, has a 2.2e-207 P-value blast match to GB:AAD11615 prpol (gypsy_Ty3-element) (Zea mays);(source:TAIR10)
AT4G04060		
AT4G04070		transposable_element_gene;similar to unknown protein [Arabidopsis thaliana] (TAIR:AT3G30810.1);(source:TAIR10)
AT4G04080	*ISCU-LIKE 3 (ISU3)*	Encodes a mitochondrial protein similar to E.coli IscU. In bacteria, IscU is a scaffold protein accepting sulfur and iron to build a transient Fe-S cluster,which is subsequently transferred to a target apoprotein.
AT4G04100		transposable_element_gene;CACTA-like transposase family (Ptta/En/Spm), has a 2.6e-16 P-value blast match to At5g29026.1/8-244 CACTA-like transposase family (Ptta/En/Spm) (CACTA-element) (Arabidopsis thaliana);(source:TAIR10)
AT4G04110	*TIR-NBS14 (TN14)*	Toll-Interleukin-Resistance (TIR) domain family protein
AT4G04120		transposable_element_gene;copia-like retrotransposon family, has a 3.8e-19 P-value blast match to GB:BAA78423 polyprotein (Ty1_Copia-element) (Arabidopsis thaliana)GB:BAA78423 polyprotein (Ty1_Copia-element) (Arabidopsis thaliana)GB:BAA78423 polyprotein (Ty1_Copia-element) (Arabidopsis thaliana)gi|4996361|dbj|BAA78423.1| polyprotein (Arabidopsis thaliana) (Ty1_Copia-element);(source:TAIR10)
AT4G04130		transposable_element_gene;similar to Ulp1 protease family protein [Arabidopsis thaliana] (TAIR:AT3G42690.1);(source:TAIR10)
AT4G04150		transposable_element_gene;gypsy-like retrotransposon family (Athila), has a 2.0e-27 P-value blast match to GB:CAA57397 Athila ORF 1 (Arabidopsis thaliana);(source:TAIR10)
AT4G04160		
AT4G04170		transposable_element_gene;CACTA-like transposase family (Ptta/En/Spm), has a 2.3e-87 P-value blast match to At5g36655.1/81-333 CACTA-like transposase family (Ptta/En/Spm) (CACTA-element) (Arabidopsis thaliana);(source:TAIR10)
AT4G04180		P-loop containing nucleoside triphosphate hydrolases superfamily protein
AT4G04220	*RECEPTOR LIKE PROTEIN 46 (RLP46)*	receptor like protein 46
AT4G04230		transposable_element_gene;gypsy-like retrotransposon family, has a 1.3e-95 P-value blast match to T27D20 reverse transcriptase (from Dan Voytas http://www.public.iastate.edu/~voytas) (Gypsy_Ty3-family);(source:TAIR10)

AT4G04260		Bromo-adjacent homology (BAH) domain-containing protein
AT4G04270		transposable_element_gene;CACTA-like transposase family (Ptta/En/Spm), has a 9.7e-78 P-value blast match to At5g29026.1/8-244 CACTA-like transposase family (Ptta/En/Spm) (CACTA-element) (Arabidopsis thaliana);(source:TAIR10)
AT4G04280		transposable_element_gene;copia-like retrotransposon family, has a 0. P-value blast match to GB:CAA31653 polyprotein (Ty1_Copia-element) (Arabidopsis thaliana);(source:TAIR10)
AT4G04290		transposable_element_gene;copia-like retrotransposon family, has a 1.8e-94 P-value blast match to GB:AAB82754 retrofit (TY1_Copia-element) (Oryza longistaminata);(source:TAIR10)
AT4G04300		transposable_element_gene;pseudogene, similar to putative helicase, blastp match of 38%25 identity and 2.6e-40 P-value to GP|21104536|dbj|BAB93131.1||AP003196 putative helicase {Oryza sativa (japonica cultivar-group)};(source:TAIR10)
AT4G04330	*HOMOLOGUE OF CYANOBACTERIAL RBCX 1 (RbcX1)*	Encodes a chloroplast thylakoid localized RbcX protein that acts as a chaperone in the folding of Rubisco.
AT4G04350	*EMBRYO DEFECTIVE 2369 (EMB2369)*	tRNA synthetase class I (I, L, M and V) family protein
AT4G04370		Tetratricopeptide repeat (TPR)-like superfamily protein
AT4G04380		transposable_element_gene;copia-like retrotransposon family, has a 7.5e-248 P-value blast match to GB:CAA31653 polyprotein (Ty1_Copia-element) (Arabidopsis thaliana);(source:TAIR10)
AT4G04410		transposable_element_gene;copia-like retrotransposon family, has a 1.7e-176 P-value blast match to dbj|BAA78426.1| polyprotein (AtRE2-1) (Arabidopsis thaliana) (Ty1_Copia-element);(source:TAIR10)
AT4G04420		transposable_element_gene;copia-like retrotransposon family, has a 0. P-value blast match to gb|AAG52949.1| gag/pol polyprotein (Endovir1-1) (Arabidopsis thaliana) (Ty1_Copia-family);(source:TAIR10)
AT4G04430		transposable_element_gene;CACTA-like transposase family (Ptta/En/Spm), has a 8.6e-122 P-value blast match to At5g36655.1/81-333 CACTA-like transposase family (Ptta/En/Spm) (CACTA-element) (Arabidopsis thaliana);(source:TAIR10)
AT4G04440		transposable_element_gene;copia-like retrotransposon family, has a 1.3e-215 P-value blast match to GB:CAA31653 polyprotein (Ty1_Copia-element) (Arabidopsis thaliana);(source:TAIR10)
AT4G04450	*(WRKY42)*	member of WRKY Transcription Factor; Group II-b. Interacts with lncRNA APOLO to trigger root hair cell expansion in response to cold.
AT4G04460	*PUTATIVE ASPARTIC PROTEINASE A3 (PASPA3)*	Saposin-like aspartyl protease family protein
AT4G04480		F-box protein with a domain protein
AT4G04490	*CYSTEINE-RICH RLK (RECEPTOR-LIKE PROTEIN KINASE) 36 (CRK36)*	Encodes a cysteine-rich receptor-like protein kinase.
AT4G04500	*CYSTEINE-RICH RLK (RECEPTOR-LIKE PROTEIN KINASE) 37 (CRK37)*	Encodes a cysteine-rich receptor-like protein kinase.
AT4G04510	*CYSTEINE-RICH RLK (RECEPTOR-LIKE PROTEIN KINASE) 38 (CRK38)*	Encodes a cysteine-rich receptor-like protein kinase.
AT4G04530		transposable_element_gene;similar to Ulp1 protease family protein [Arabidopsis thaliana] (TAIR:AT4G05280.1);(source:TAIR10)
AT4G04540	*CYSTEINE-RICH RLK (RECEPTOR-LIKE PROTEIN KINASE) 39 (CRK39)*	Encodes a cysteine-rich receptor-like protein kinase.
AT4G04550		transposable_element_gene;copia-like retrotransposon family, has a 1.9e-10 P-value blast match to gb|AAG52950.1| putative envelope protein (Endovir1-1) (Arabidopsis thaliana) (Ty1_Copia-family);(source:TAIR10)
AT4G04560		transposable_element_gene;copia-like retrotransposon family, has a 1.0e-94 P-value blast match to gb|AAG52949.1| gag/pol polyprotein (Endovir1-1) (Arabidopsis thaliana) (Ty1_Copia-family);(source:TAIR10)
AT4G04590		transposable_element_gene;CACTA-like transposase family (Tnp2/En/Spm), has a 5.3e-36 P-value blast match to gb|AAG52024.1|AC022456_5 Tam1-homologous transposon protein TNP2, putative;(source:TAIR10)
AT4G04600		transposable_element_gene;copia-like retrotransposon family, has a 0. P-value blast match to GB:CAA31653 polyprotein (Ty1_Copia-element) (Arabidopsis thaliana);(source:TAIR10)
AT4G04610	*APS REDUCTASE 1 (APR1)*	Encodes a protein disulfide isomerase-like (PDIL) protein, a member of a multigene family within the thioredoxin (TRX) superfamily. This protein also belongs to the adenosine 5'-phosphosulfate reductase-like (APRL) group. The mRNA is cell-to-cell mobile.
AT4G04620	*AUTOPHAGY 8B (ATG8B)*	Autophagy protein.
AT4G04630		senescence regulator (Protein of unknown function, DUF584)
AT4G04640	*(ATPC1)*	One of two genes (with ATPC2) encoding the gamma subunit of Arabidopsis chloroplast ATP synthase.
AT4G04650		RNA-directed DNA polymerase (reverse transcriptase)-related family protein
AT4G04660		pseudogene of Ta11-like non-LTR retrotransposon
AT4G04670	*TRNA METHYLTRANSFERASE 5C (TRM5C)*	Met-10+ like family protein / kelch repeat-containing protein
AT4G04700	*CALCIUM-DEPENDENT PROTEIN KINASE 27 (CPK27)*	member of Calcium Dependent Protein Kinase
AT4G04730		Ta11-like non-LTR retrotransposon
AT4G04740	*CALCIUM-DEPENDENT PROTEIN KINASE 23 (CPK23)*	member of Calcium Dependent Protein Kinase
AT4G04750	*MITOCHONDRIAL F1F0-ATP SYNTHASE INHIBITOR FACTOR 1 (IF1)*	Mitochondrial F1F0-ATPase inhibitor factor; has conserved function as an endogenous inhibitor affecting cellular energy status and plays an impor tant role in plant growth and reproduction, particularly in fertility.
AT4G04760	*(ESL3.03)*	Early response to dehydration six-like (ESL) family member.
AT4G04770	*ATP-BINDING CASSETTE I8 (ABCI8)*	Encodes an iron-stimulated ATPase. A member of the NAP subfamily of ABC transporters. Involved in Fe-S cluster assembly. Similar to SufB. Involved in the regulation of iron homeostasis. Able to form homodimers. Interacts with AtNAP7 inside the chloroplast.
AT4G04790		Tetratricopeptide repeat (TPR)-like superfamily protein
AT4G04820		transposable_element_gene;similar to Ulp1 protease family protein [Arabidopsis thaliana] (TAIR:AT2G24930.1);(source:TAIR10)
AT4G04830	*METHIONINE SULFOXIDE REDUCTASE B5 (MSRB5)*	methionine sulfoxide reductase B5
AT4G04840	*METHIONINE SULFOXIDE REDUCTASE B6 (MSRB6)*	methionine sulfoxide reductase B6

AT4G04850	*K+ EFFLUX ANTIPORTER 3 (KEA3)*	Encodes a potassium efflux antiporter; has three splice forms KEA3.1, KEA3.2, and KEA3.3, KEA3.2 is the most abundant splice form in all plant organs (silique, flower, leaf and root). KEA3.1 and KEA3.3 are minor variants that can be found in flowers and in leaves. KEA3 is localized to the thylakoid membrane and enriched in the stromal lamellae. It allows proton efflux from the thylakoid lumen by proton/potassium antiport.
AT4G04870	*CARDIOLIPIN SYNTHASE (CLS)*	Encodes a protein with cardiolipin synthase activity that is localized to the mitochondiria.
AT4G04890	*PROTODERMAL FACTOR 2 (PDF2)*	Encodes a homeodomain protein that is expressed in the LI layer of the vegetative, floral and inflorescence meristems. Binds to the L1 box promoter element which is required in some proteins for L1 specific expression.
AT4G04900	*ROP-INTERACTIVE CRIB MOTIF-CONTAINING PROTEIN 10 (RIC10)*	encodes a member of a novel protein family that contains contain a CRIB (for Cdc42/Rac-interactive binding) motif required for their specific interaction with GTP-bound Rop1 (plant-specific Rho GTPase). Most similar to RIC9 and RIC11 (subfamily group I). Gene is expressed predominantly in roots, leaves, and seedlings.
AT4G04920	*SENSITIVE TO FREEZING 6 (SFR6)*	Encodes a subunit of the MEDIATOR complex. Plays a role in the CBF pathway -downstream of CBF translation. Mutants have impaired cold responses, reduced levels of cold induced RNA transcripts, are sensitive to osmotic stress. Required for expression of CBF-controlled cold-upregulated genes and some, but not all, other cold up-regulated genes. Required for recruitment of the Mediator complex and RNA polymerase II to CBF-controlled cold-responsive genes. Required for expression of some dark-upregulated genes and auxin mediated gene expression. SFR6 was isolated as a suppressor of cell wall defects in cob6 mutant background.
AT4G04930	*(DES-1-LIKE)*	Encodes a sphingolipid delta4-desaturase, involved in sphingolipid biosynthesis. Specifically expressed in floral tissues. Knockout mutants were devoid of sphinga-4,8-dienine in floral tissues.
AT4G04940		transducin family protein / WD-40 repeat family protein
AT4G04950	*MONOTHIOL GLUTAREDOXIN 17 (GRXS17)*	Encodes a monothiol glutaredoxin that is a critical component involved in ROS accumulation, auxin signaling, and temperature-dependent postembryonic growth in plants. It has been shown to associate with the cytosolic Fe-S assembly (CIA) complex and contributes to, but is not essential for, the correct functioning of client Fe-S proteins in unchallenged conditions.
AT4G04955	*ALLANTOINASE (ALN)*	Encodes an allantoinase which is involved in allantoin degradation and assimilation. Gene expression was induced when allantoin was added to the medium. The insertion mutant, ataln m2-1, did not grow well on the MS medium where allantoin, instead of ammonium nitrate, was supplied.
AT4G04980		hypothetical protein
AT4G04990		serine/arginine repetitive matrix-like protein (DUF761)
AT4G05020	*NAD(P)H DEHYDROGENASE B2 (NDB2)*	Miitochondrial alternative NADH dehydrogenase.
AT4G05030		Copper transport protein family
AT4G05050	*UBIQUITIN 11 (UBQ11)*	polyubiquitin gene, belongs to a subtype group with UBQ10 and UBQ14. Various ecotypes of Arabidopsis have different numbers of ubiquitin repeats within this gene.
AT4G05070	*WOUND-INDUCED POLYPEPTIDE 2 (WIP2)*	Member of the wound-induced polypeptide (WIP) family. Positively regulates plant resistance against Pst DC3000 by enhancing PTI responses.
AT4G05080		F-box and associated interaction domains-containing protein
AT4G05100	*MYB DOMAIN PROTEIN 74 (MYB74)*	Member of the R2R3 factor gene family.
AT4G05110	*EQUILIBRATIVE NUCLEOSIDE TRANSPORTER 6 (ENT6)*	equilibrative nucleoside transporter 6
AT4G05140		Nucleoside transporter family protein
AT4G05150		Octicosapeptide/Phox/Bem1p family protein
AT4G05160		Encodes a peroxisomal protein involved in the activation of fatty acids through esterification with CoA. At4g05160 preferentially activates fatty acids with medium chain length (C6:0 and C7:0) as well as even-numbered long-chain fatty acids (C14:0, C16:0 and C18:0). At4g05160 was also able to catalyze the conversion of OPC-6:0 to its CoA ester and is therefore thought to be involved in the peroxisomal β-oxidation steps of jasmonic acid biosynthesis.
AT4G05170		basic helix-loop-helix (bHLH) DNA-binding superfamily protein
AT4G05180	*PHOTOSYSTEM II SUBUNIT Q-2 (PSBQ-2)*	Encodes the PsbQ subunit of the oxygen evolving complex of photosystem II.
AT4G05190	*KINESIN 5 (ATK5)*	ATK5 encodes a kinesin protein involved in microtubule spindle morphogenesis. It acts as a minus-end directed motor as well as a plus-end tracking protein (+TIP). Localizes to mitotic spindle midzones and regions rich in growing plus-ends within phragmoplasts.
AT4G05200	*CYSTEINE-RICH RLK (RECEPTOR-LIKE PROTEIN KINASE) 25 (CRK25)*	Encodes a cysteine-rich receptor-like protein kinase.
AT4G05210	*LIPID X D1 (LPXD1)*	Trimeric LpxA-like enzymes superfamily protein
AT4G05230		Ubiquitin-like superfamily protein
AT4G05240		Ubiquitin-like superfamily protein
AT4G05250		Ubiquitin-like superfamily protein
AT4G05260		Ubiquitin-like superfamily protein
AT4G05270		Ubiquitin-like superfamily protein
AT4G05280		transposable_element_gene;similar to Ulp1 protease family protein [Arabidopsis thaliana] (TAIR:AT1G27780.1);(source:TAIR10)
AT4G05300		transposable_element_gene;similar to unknown protein [Arabidopsis thaliana] (TAIR:AT3G43380.1);(source:TAIR10)
AT4G05310		Ubiquitin-like superfamily protein
AT4G05330	*ARF-GAP DOMAIN 13 (AGD13)*	A member of ARF GAP domain (AGD), A thaliana has 15 members, grouped into four classes.
AT4G05340		P-loop containing nucleoside triphosphate hydrolases superfamily protein
AT4G05370		
AT4G05390	*ROOT FNR 1 (RFNR1)*	Encodes a root-type ferredoxin:NADP(H) oxidoreductase.
AT4G05400		copper ion binding protein
AT4G05410	*YAOZHE (YAO)*	Encodes a nucleolar protein with seven WD40-repeats that plays a role in embryo sac development and is critical for the correct positioning of the division plane of zygote and the apical cell lineage in Arabidopsis. YAO may act by modulating nucleolar function, such as rRNA biogenesis, during early embryogenesis and gametogenesis.

AT4G05420	*DAMAGED DNA BINDING PROTEIN 1A (DDB1A)*	Structurally similar to damaged DNA binding proteins. DDB1a is part of a 350 KDa nuclear localized DET1 protein complex. This complex may physically interact with histone tails and while bound to chromatin- repress transcription of genes involved in photomorphogenesis. DDB1a is shown to be RUB-modified.
AT4G05440	*EMBRYO SAC DEVELOPMENT ARREST 35 (EDA35)*	Plays a role in pollen development and modulating DNA replication via interaction with MCM4 and MCM7 of the pre-replication complex.
AT4G05450	*MITOCHONDRIAL FERREDOXIN 1 (MFDX1)*	Iron-Sulfur (Fe-S) cluster gene, plays a role in host and nonhost disease resistance by accumulation of defense-related metabolites.
AT4G05470		
AT4G05490		RNI-like superfamily protein
AT4G05500		pseudogene of RNI-like superfamily protein
AT4G05510		transposable_element_gene;hAT-like transposase family (hobo/Ac/Tam3), has a 4.0e-144 P-value blast match to GB:AAD24567 transposase Tag2 (hAT-element) (Arabidopsis thaliana);(source:TAIR10)
AT4G05530	*INDOLE-3-BUTYRIC ACID RESPONSE 1 (IBR1)*	Encodes a peroxisomal member of the short-chain dehydrogenase/reductase (SDR) family of enzymes. Loss of IBR1 function causes increased resistance to indole-3-butyric acid without affecting plant responses to IAA, NAA, and 2,4-D. This enzyme may be responsible for catalyzing a dehydrogenation step in the beta-oxidation-like conversion of IBA to IAA. The mRNA is cell-to-cell mobile.
AT4G05550		transposable_element_gene;Mutator-like transposase family, has a 4.9e-80 P-value blast match to Q9S9W4 /247-408 Pfam PF03108 MuDR family transposase (MuDr-element domain);(source:TAIR10)
AT4G05570		transposable_element_gene;similar to unknown protein [Arabidopsis thaliana] (TAIR:AT3G42460.1);(source:TAIR10)
AT4G05580		transposable_element_gene;similar to zinc knuckle (CCHC-type) family protein [Arabidopsis thaliana] (TAIR:AT1G42630.1);(source:TAIR10)
AT4G05590	*NEGATIVE REGULATOR OF GUARD CELL ABA SIGNALING 1 (NRGA1)*	Encodes NRGA1, a putative mitochondrial pyruvate carrier that mediates ABA regulation of guard cell ion channels and drought stress responses.
AT4G05600		transposable_element_gene;gypsy-like retrotransposon family (Athila), has a 1.6e-10 P-value blast match to GB:CAA57397 Athila ORF 1 (Arabidopsis thaliana);(source:TAIR10)
AT4G05610		transposable_element_gene;pseudogene, hypothetical protein, putative retrotransposon-like orf - Arabidopsis thaliana,PID:g4309868;(source:TAIR10)
AT4G05620		Galactose oxidase/kelch repeat superfamily protein
AT4G05630		B3 domain protein
AT4G05640		transposable_element_gene;similar to unknown protein [Arabidopsis thaliana] (TAIR:AT1G41855.1);(source:TAIR10)
AT4G07330		transposable_element_gene;CACTA-like transposase family (Tnp2/En/Spm), has a 2.6e-13 P-value blast match to gb|AAG52024.1|AC022456_5 Tam1-homologous transposon protein TNP2, putative;(source:TAIR10)
AT4G07340		transposable_element_gene;similar to unknown protein [Arabidopsis thaliana] (TAIR:AT2G05090.1);(source:TAIR10)
AT4G07350		hypothetical protein
AT4G07360		transposable_element_gene;gypsy-like retrotransposon family (Athila), has a 1.7e-295 P-value blast match to GB:CAA57397 Athila ORF 1 (Arabidopsis thaliana);(source:TAIR10)
AT4G07370		transposable_element_gene;gypsy-like retrotransposon family (Athila), has a 1.7e-75 P-value blast match to GB:CAA57397 Athila ORF 1 (Arabidopsis thaliana);(source:TAIR10)
AT4G07380		hypothetical protein
AT4G07410	*POPCORN (PCN)*	Encodes a WD-40 protein expressed both during embryo development and postembryonically in the SAM and RAM that functions in the auxin pathway, integrating auxin signaling in the organization and maintenance of the SAM and RAM.
AT4G07420		transposable_element_gene;CACTA-like transposase family (Ptta/En/Spm), has a 1.5e-35 P-value blast match to At5g29026.1/8-244 CACTA-like transposase family (Ptta/En/Spm) (CACTA-element) (Arabidopsis thaliana);(source:TAIR10)
AT4G07430		transposable_element_gene;similar to unknown protein [Arabidopsis thaliana] (TAIR:AT4G07310.1);(source:TAIR10)
AT4G07450		transposable_element_gene;similar to unknown protein [Arabidopsis thaliana] (TAIR:AT3G30550.1);(source:TAIR10)
AT4G07460		transposable_element_gene;similar to unknown protein [Arabidopsis thaliana] (TAIR:AT3G42430.1);(source:TAIR10)
AT4G07480		transposable_element_gene;similar to unknown protein [Arabidopsis thaliana] (TAIR:AT4G19300.1);(source:TAIR10)
AT4G07490		transposable_element_gene;transposon protein -related;(source:TAIR10)
AT4G07500		transposable_element_gene;similar to unknown protein [Arabidopsis thaliana] (TAIR:AT2G09865.1);(source:TAIR10)
AT4G07510		transposable_element_gene;similar to unknown protein [Arabidopsis thaliana] (TAIR:AT3G42110.1);(source:TAIR10)
AT4G07515		ECA1 gametogenesis family protein (DUF784)
AT4G07530		transposable_element_gene;similar to unknown protein [Arabidopsis thaliana] (TAIR:AT3G30810.1);(source:TAIR10)
AT4G07540		transposable_element_gene;hAT-like transposase family (hobo/Ac/Tam3), has a 2.2e-153 P-value blast match to GB:AAD24567 transposase Tag2 (hAT-element) (Arabidopsis thaliana);(source:TAIR10)
AT4G07550		transposable_element_gene;gypsy-like retrotransposon family, has a 4.2e-07 P-value blast match to GB:AAD19359 polyprotein (gypsy_Ty3-element) (Sorghum bicolor);(source:TAIR10)
AT4G07580		transposable_element_gene;pseudogene, Ulp1 protease family, contains Pfam profile PF02902: Ulp1 protease family, C-terminal catalytic domain;(source:TAIR10)
AT4G07590		transposable_element_gene;gypsy-like retrotransposon family, has a 2.3e-17 P-value blast match to GB:AAD19359 polyprotein (gypsy_Ty3-element) (Sorghum bicolor);(source:TAIR10)
AT4G07600		transposable_element_gene;gypsy-like retrotransposon family (Athila), has a 1.9e-166 P-value blast match to GB:CAA57397 Athila ORF 1 (Arabidopsis thaliana);(source:TAIR10)
AT4G07610		
AT4G07620		transposable_element_gene;gypsy-like retrotransposon family (Athila), has a 7.1e-216 P-value blast match to GB:CAA57397 Athila ORF 1 (Arabidopsis thaliana);(source:TAIR10)

AT4G07640		transposable_element_gene;gypsy-like retrotransposon family (Athila), has a 1.1e-250 P-value blast match to GB:CAA57397 Athila ORF 1 (Arabidopsis thaliana);(source:TAIR10)
AT4G07650		transposable_element_gene;pseudogene, hypothetical protein;(source:TAIR10)
AT4G07660		transposable_element_gene;gypsy-like retrotransposon family (Athila), has a 6.9e-240 P-value blast match to GB:CAA57397 Athila ORF 1 (Arabidopsis thaliana);(source:TAIR10)
AT4G07670		protease-associated (PA) domain-containing protein
AT4G07680		transposable_element_gene;pseudogene, Ulp1 protease famiy, contains Pfam profile PF02902: Ulp1 protease family, C-terminal catalytic domain;(source:TAIR10)
AT4G07700		transposable_element_gene;gypsy-like retrotransposon family (Athila), has a 3.9e-13 P-value blast match to GB:CAA57397 Athila ORF 1 (Arabidopsis thaliana);(source:TAIR10)
AT4G07710		
AT4G07730		transposable_element_gene;gypsy-like retrotransposon family (Athila), has a 6.8e-198 P-value blast match to GB:CAA57397 Athila ORF 1 (Arabidopsis thaliana);(source:TAIR10)
AT4G07740		hypothetical protein (DUF3287)
AT4G07750		transposable_element_gene;CACTA-like transposase family (Tnp2/En/Spm), has a 2.9e-168 P-value blast match to gb|AAG52024.1|AC022456_5 Tam1-homologous transposon protein TNP2, putative;(source:TAIR10)
AT4G07760		transposable_element_gene;CACTA-like transposase family (Tnp1/En/Spm), has a 5.4e-220 P-value blast match to ref|NP_189784.1| TNP1-related protein (Arabidopsis thaliana) (CACTA-element);(source:TAIR10)
AT4G07780		transposable_element_gene;gypsy-like retrotransposon family (Athila), has a 3.1e-225 P-value blast match to GB:CAA57397 Athila ORF 1 (Arabidopsis thaliana);(source:TAIR10)
AT4G07790		transposable_element_gene;hypothetical protein;(source:TAIR10)
AT4G07800		transposable_element_gene;similar to unknown protein [Arabidopsis thaliana] (TAIR:AT3G13250.1);(source:TAIR10)
AT4G07830		transposable_element_gene;gypsy-like retrotransposon family, has a 6.3e-159 P-value blast match to GB:CAA73042 polyprotein (Gypsy_Ty3-element) (Ananas comosus);(source:TAIR10)
AT4G07840		transposable_element_gene;copia-like retrotransposon family, has a 0. P-value blast match to GB:BAA22288 pol polyprotein (Ty1_Copia-element) (Oryza australiensis)GB:BAA22288 polyprotein (Ty1_Copia-element) (Oryza australiensis)gi|2443320|dbj|BAA22288.1| polyprotein (RIRE1) (Oryza australiensis) (Ty1_Copia-element);(source:TAIR10)
AT4G07850		transposable_element_gene;gypsy-like retrotransposon family, has a 3.2e-135 P-value blast match to GB:AAD22153 polyprotein (Gypsy_Ty3-element) (Sorghum bicolor);(source:TAIR10)
AT4G07880		
AT4G07890		transposable_element_gene;gypsy-like retrotransposon family (Athila), has a 1.2e-14 P-value blast match to GB:CAA57397 Athila ORF 1 (Arabidopsis thaliana);(source:TAIR10)
AT4G07900		
AT4G07920		transposable_element_gene;pseudogene, hypothetical protein;(source:TAIR10)
AT4G07930		
AT4G07940		pre-mRNA-splicing factor CWC22-like protein, putative (DUF3245)
AT4G07950		DNA-directed RNA polymerase, subunit M, archaeal
AT4G07960	*CELLULOSE-SYNTHASE-LIKE C12 (CSLC12)*	encodes a XyG glucan synthase; gene similar to cellulose synthase
AT4G07970		transposable_element_gene;hypothetical protein, similar to A. thaliana hypothetical proteins;(source:TAIR10)
AT4G07990		Chaperone DnaJ-domain superfamily protein
AT4G08000		transposable_element_gene;CACTA-like transposase family (Tnp2/En/Spm), has a 1.0e-136 P-value blast match to GB:CAA40555 TNP2 (CACTA-element) (Antirrhinum majus);(source:TAIR10)
AT4G08020		transposable_element_gene;CACTA-like transposase family (En/Spm), has a 2.5e-93 P-value blast match to GB:BAA20532 ORF of transposon Tdc1 (CACTA-element) (Daucus carota);(source:TAIR10)
AT4G08030		transposable_element_gene;gypsy-like retrotransposon family (Athila), has a 1.5e-66 P-value blast match to GB:CAA57397 Athila ORF 1 (Arabidopsis thaliana);(source:TAIR10)
AT4G08040	*1-AMINOCYCLOPROPANE-1-CARBOXYLATE SYNTHASE 11 (ACS11)*	encodes an aminotransferase that belongs to ACC synthase gene family structurally
AT4G08050		transposable_element_gene;gypsy-like retrotransposon family (Athila), has a 0. P-value blast match to GB:CAA57397 Athila ORF 1 (Arabidopsis thaliana);(source:TAIR10)
AT4G08060		transposable_element_gene;CACTA-like transposase family (Tnp1/En/Spm), has a 0. P-value blast match to ref|NP_189784.1| TNP1-related protein (Arabidopsis thaliana) (CACTA-element);(source:TAIR10)
AT4G08090		transposable_element_gene;CACTA-like transposase family (Ptta/En/Spm), has a 1.4e-12 P-value blast match to At1g15560.1/58-302 CACTA-like transposase family (Ptta/En/Spm) (CACTA-element) (Arabidopsis thaliana);(source:TAIR10)
AT4G08100		transposable_element_gene;gypsy-like retrotransposon family, has a 1.2e-123 P-value blast match to GB:AAD22153 polyprotein (Gypsy_Ty3-element) (Sorghum bicolor);(source:TAIR10)
AT4G08110		transposable_element_gene;CACTA-like transposase family (Ptta/En/Spm), has a 4.2e-66 P-value blast match to At5g29026.1/8-244 CACTA-like transposase family (Ptta/En/Spm) (CACTA-element) (Arabidopsis thaliana);(source:TAIR10)
AT4G08130		transposable_element_gene;similar to unknown protein [Arabidopsis thaliana] (TAIR:AT1G37045.1);(source:TAIR10)
AT4G08140		26S proteasome non-ATPase regulatory subunit-like protein

AT4G08150	*KNOTTED-LIKE FROM ARABIDOPSIS THALIANA (KNAT1)*	A member of class I knotted1-like homeobox gene family (together with KNAT2). Similar to the knotted1 (kn1) homeobox gene of maize. Normally expressed in the peripheral and rib zone of shoot apical meristem but not in the leaf primordia. It is also expressed in the fourth floral whorl, in the region that would become style, particularly in the cell surrounding the transmitting tissue. No expression was detected in the first three floral whorls. Expression is repressed by auxin and AS1 which results in the promotion of leaf fate.
AT4G08160		Encodes a putative glycosyl hydrolase family 10 protein (xylanase).
AT4G08190		P-loop containing nucleoside triphosphate hydrolases superfamily protein
AT4G08210		Pentatricopeptide repeat (PPR-like) superfamily protein
AT4G08220		transposable_element_gene;Mutator-like transposase family, has a 5.3e-67 P-value blast match to Q9SUF8 /145-308 Pfam PF03108 MuDR family transposase (MuDr-element domain);(source:TAIR10)
AT4G08270		glutathione S-transferase T3-like protein
AT4G08290	*USUALLY MULTIPLE ACIDS MOVE IN AND OUT TRANSPORTERS 20 (UMAMIT20)*	nodulin MtN21-like transporter family protein
AT4G08300	*USUALLY MULTIPLE ACIDS MOVE IN AND OUT TRANSPORTERS 17 (UMAMIT17)*	nodulin MtN21-like transporter family protein
AT4G08310		DNA ligase
AT4G08330		hypothetical protein
AT4G08340		transposable_element_gene;similar to Ulp1 protease family protein [Arabidopsis thaliana] (TAIR:AT1G35770.1);(source:TAIR10)
AT4G08370	*EXTENSIN 20 (EXT20)*	Proline-rich extensin-like family protein
AT4G08380	*EXTENSIN 22 (EXT22)*	Proline-rich extensin-like family protein
AT4G08390	*STROMAL ASCORBATE PEROXIDASE (SAPX)*	Encodes a chloroplastic stromal ascorbate peroxidase sAPX. Ascorbate peroxidases are enzymes that scavenge hydrogen peroxide in plant cells. Eight types of APX have been described for Arabidopsis: three cytosolic (APX1, APX2, APX6), two chloroplastic types (stromal sAPX, thylakoid tAPX), and three microsomal (APX3, APX4, APX5) isoforms. The mRNA is cell-to-cell mobile.
AT4G08400	*EXTENSIN 7 (EXT7)*	Proline-rich extensin-like family protein
AT4G08420		transposable_element_gene;Mutator-like transposase family, has a 8.0e-59 P-value blast match to Q9S9L1 /206-367 Pfam PF03108 MuDR family transposase (MuDr-element domain);(source:TAIR10)
AT4G08430		Ulp1 protease family protein
AT4G08440		
AT4G08450		Disease resistance protein (TIR-NBS-LRR class) family
AT4G08460		hypothetical protein (DUF1644)
AT4G08470	*MAPK/ERK KINASE KINASE 3 (MEKK3)*	Encodes a member of the A1 subgroup of the MEKK (MAPK/ERK kinase kinase) family. MEKK is another name for Mitogen-Activated Protein Kinase Kinase Kinase (MAPKKK or MAP3K). This subgroup has four members: At4g08500 (MEKK1, also known as ARAKIN, MAP3Kb1, MAPKKK8), At4g08480 (MEKK2, also known as MAP3Kb4, MAPKKK9), At4g08470 (MEKK3, also known as MAP3Kb3, MAPKKK10) and At4g12020 (MEKK4, also known as MAP3Kb5, MAPKKK11, WRKY19). Nomenclatures for mitogen-activated protein kinases are described in Trends in Plant Science 2002, 7(7):301.
AT4G08480	*MITOGEN-ACTIVATED PROTEIN KINASE KINASE KINASE 9 (MAPKKK9)*	Encodes a member of the A1 subgroup of the MEKK (MAPK/ERK kinase kinase) family. MEKK is another name for Mitogen-Activated Protein Kinase Kinase Kinase (MAPKKK or MAP3K). This subgroup has four members: At4g08500 (MEKK1, also known as ARAKIN, MAP3Kb1, MAPKKK8), At4g08480 (MEKK2, also known as MAP3Kb4, MAPKKK9), At4g08470 (MEKK3, also known as MAP3Kb3, MAPKKK10) and At4g12020 (MEKK4, also known as MAP3Kb5, MAPKKK11, WRKY19). Nomenclatures for mitogen-activated protein kinases are described in Trends in Plant Science 2002, 7(7):301.
AT4G08490		transposable_element_gene;gypsy-like retrotransposon family (Athila), has a 4.7e-81 P-value blast match to GB:CAA57397 Athila ORF 1 (Arabidopsis thaliana);(source:TAIR10)
AT4G08530		Predicted to encode a PR (pathogenesis-related) protein. Belongs to the lipid transfer protein (PR-14) family with the following members: At2g38540/LTP1, At2g38530/LTP2, At5g59320/LTP3, At5g59310/LTP4, At3g51600/LTP5, At3g08770/LTP6, At2g15050/LTP7, At2g18370/LTP8, At2g15325/LTP9, At5g01870/LTP10, At4g33355/LTP11, At3g51590/LTP12, At5g44265/LTP13, At5g62065/LTP14, At4g08530/LTP15.
AT4G08560	*PUMILIO 15 (PUM15)*	Encodes a member of the Arabidopsis Pumilio (APUM) proteins containing PUF domain (eight repeats of approximately 36 amino acids each). PUF proteins regulate both mRNA stability and translation through sequence-specific binding to the 3' UTR of target mRNA transcripts.
AT4G08600		transposable_element_gene;hypothetical protein, contains Pfam profile PF03384: Drosophila protein of unknown function, DUF287;(source:TAIR10)
AT4G08610		transposable_element_gene;Mutator-like transposase family, has a 1.9e-87 P-value blast match to O22278 /203-375 Pfam PF03108 MuDR family transposase (MuDr-element domain);(source:TAIR10)
AT4G08620	*SULPHATE TRANSPORTER 1;1 (SULTR1;1)*	Encodes a high-af?nity sulfate transporter. Contains STAS domain. Expressed in roots and guard cells. Up-regulated by sulfur deficiency.
AT4G08640		ATP binding protein
AT4G08650		transposable_element_gene;hypothetical protein;(source:TAIR10)
AT4G08660		transposable_element_gene;Mutator-like transposase family, has a 3.0e-74 P-value blast match to O22273 /233-373 Pfam PF03108 MuDR family transposase (MuDr-element domain);(source:TAIR10)
AT4G08670	*GLYCOSYLPHOSPHATIDYLINOSITOL-ANCHORED LIPID PROTEIN TRANSFER 4 (LTPG4)*	Bifunctional inhibitor/lipid-transfer protein/seed storage 2S albumin superfamily protein
AT4G08680		transposable_element_gene;Mutator-like transposase family, has a 1.4e-46 P-value blast match to GB:AAA21566 mudrA of transposon=MuDR (MuDr-element) (Zea mays);(source:TAIR10)
AT4G08685	*(SAH7)*	Encodes a protein, expressed in leaves, with similarity to pollen allergens. The mRNA is cell-to-cell mobile.
AT4G08690		Sec14p-like phosphatidylinositol transfer family protein
AT4G08710		transposable_element_gene;similar to unknown protein [Arabidopsis thaliana] (TAIR:AT2G23480.1);(source:TAIR10)
AT4G08730		RNA-binding protein
AT4G08760		hypothetical protein

AT4G08770	*PEROXIDASE 37 (Prx37)*	Encodes a putative apoplastic peroxidase Prx37. Primarily expressed in the vascular bundles. Overexpression renders a dwarf phenotype with smaller plants and delayed development. Plants overexpressing Prx37 also shows an increase in the amount of esterified phenolic material associated with their walls.
AT4G08780		Peroxidase superfamily protein
AT4G08800		Protein kinase superfamily protein
AT4G08820		transposable_element_gene;similar to nucleic acid binding / zinc ion binding [Arabidopsis thaliana] (TAIR:AT2G01050.1);(source:TAIR10)
AT4G08830		transposable_element_gene;non-LTR retrotransposon family (LINE), has a 4.0e-49 P-value blast match to GB:NP_038603 L1 repeat, Tf subfamily, member 23 (LINE-element) (Mus musculus);(source:TAIR10)
AT4G08840	*PUMILIO 11 (PUM11)*	Encodes a member of the Arabidopsis Pumilio (APUM) proteins containing PUF domain (eight repeats of approximately 36 amino acids each). PUF proteins regulate both mRNA stability and translation through sequence-specific binding to the 3' UTR of target mRNA transcripts.
AT4G08850	*MDIS1-INTERACTING RECEPTOR LIKE KINASE2 (MIK2)*	MIK1 encodes a receptor kinase that forms a complex with MDIS1/MIK2 and binds LURE1, the female pollen guidance chemi-attractant. MIK1 phosphorylates MDIS1 and is autophosphorylated.
AT4G08870	*ARGININE AMIDOHYDROLASE 2 (ARGAH2)*	Encodes one of the two arginase in the genome. Gene expression is enhanced by methyl jasmonate treatment. It is involved in the defense response to B.cinerea.
AT4G08880		transposable_element_gene;similar to Ulp1 protease family protein [Arabidopsis thaliana] (TAIR:AT1G27780.1);(source:TAIR10)
AT4G08920	*CRYPTOCHROME 1 (CRY1)*	Encodes CRY1, a flavin-type blue-light photoreceptor with ATP binding and autophosphorylation activity. Functions in perception of blue / green ratio of light. The photoreceptor may be involved in electron transport. Mutant phenotype displays a blue light-dependent inhibition of hypocotyl elongation. Photoreceptor activity requires light-induced homodimerisation of the N-terminal CNT1 domains of CRY1. Involved in blue-light induced stomatal opening. The C-terminal domain of the protein undergoes a light dependent conformational change. Also involved in response to circadian rhythm. Mutants exhibit long hypocotyl under blue light and are out of phase in their response to circadian rhythm. CRY1 is present in the nucleus and cytoplasm. Different subcellular pools of CRY1 have different functions during photomorphogenesis of Arabidopsis seedlings. The mRNA is cell-to-cell mobile.
AT4G08930	*APR-LIKE 6 (APRL6)*	Encodes a protein disulfide isomerase-like (PDIL) protein, a member of a multigene family within the thioredoxin (TRX) superfamily. This protein also belongs to the adenosine 5'-phosphosulfate reductase-like (APRL) group. The mRNA is cell-to-cell mobile.
AT4G08950	*EXORDIUM (EXO)*	Phosphate-responsive 1 family protein
AT4G08960		phosphotyrosyl phosphatase activator (PTPA) family protein
AT4G08970		transposable_element_gene;Mutator-like transposase family, has a 8.1e-28 P-value blast match to Q9SL18 /349-510 Pfam PF03108 MuDR family transposase (MuDr-element domain);(source:TAIR10)
AT4G08990		DNA (cytosine-5-)-methyltransferase family protein
AT4G09010	*THYLAKOID LUMEN 29 (TL29)*	Encodes a thylakoid lumen protein that was initially believed to act as a microsomal ascorbate peroxidase APX4 but to date, no evidence of enzymatic activity has been found.
AT4G09020	*ISOAMYLASE 3 (ISA3)*	Encodes an isoamylase-like protein. Mutant studies show that the gene is strongly involved in starch breakdown. A GUS-protein fusion product was shown to localize to the surface of chloroplastic structures reminiscent of starch granules. In the mutants, the chloroplastic α-amylase AMY3 is upregulated. The mRNA is cell-to-cell mobile.
AT4G09030	*ARABINOGALACTAN PROTEIN 10 (AGP10)*	Encodes arabinogalactan protein (AGP10). The mRNA is cell-to-cell mobile.
AT4G09080	*TRANSLOCON AT THE OUTER ENVELOPE MEMBRANE OF CHLOROPLASTS 75-IV (TOC75-IV)*	Component of the translocon outer membrane (TOC) complex. Forms the outer envelope translocation channel (beta-barrel). Plays a role in preprotein conductance. Imported into chloroplast. Knockout mutants have abnormal etioplasts.
AT4G09090		Carbohydrate-binding X8 domain superfamily protein
AT4G09100	*ARABIDOPSIS T??XICOS EN LEVADURA 39 (ATL39)*	RING/U-box superfamily protein
AT4G09110	*ARABIDOPSIS T??XICOS EN LEVADURA 35 (ATL35)*	RING/U-box superfamily protein
AT4G09130	*ARABIDOPSIS T??XICOS EN LEVADURA 37 (ATL37)*	RING/U-box superfamily protein
AT4G09140	*MUTL-HOMOLOGUE 1 (MLH1)*	Encodes a protein with similarity to Mut1 DNA mismatch repair protein, from E.coli. The protein is expressed during prophase I of meiosis, colocalizes with MLH3 throughout pachytene and is dependent on MLH3 for proper localization.
AT4G09160	*(PATL5)*	PATLs belong to a family of proteins having a Golgi dynamics (GOLD) domain in tandem with the Sec14p-like domain. PATLs are auxin regulated. Quadruple mutants (patl2456) show altered PIN1 lateralization in root endodermis cells.
AT4G09230		transposable_element_gene;pseudogene, hypothetical protein;(source:TAIR10)
AT4G09270		
AT4G09290		transposable_element_gene;similar to Ulp1 protease family protein [Arabidopsis thaliana] (TAIR:AT3G30640.1);(source:TAIR10)
AT4G09300		LisH and RanBPM domains containing protein
AT4G09350	*NADH DEHYDROGENASE-LIKE COMPLEX T (NdhT)*	Chaperone DnaJ-domain superfamily protein
AT4G09360		NB-ARC domain-containing disease resistance protein
AT4G09390		transposable_element_gene;similar to unknown protein [Arabidopsis thaliana] (TAIR:AT4G04400.1);(source:TAIR10)
AT4G09400		transposable_element_gene;similar to unknown protein [Arabidopsis thaliana] (TAIR:AT3G33230.1);(source:TAIR10)
AT4G09410		transposable_element_gene;Mutator-like transposase family, has a 1.4e-40 P-value blast match to Q9SI25 /181-349 Pfam PF03108 MuDR family transposase (MuDr-element domain);(source:TAIR10)
AT4G09420	*TIR-NBS15 (TN15)*	Disease resistance protein (TIR-NBS class)
AT4G09430		disease resistance protein (TIR-NBS-LRR class)
AT4G09460	*MYB DOMAIN PROTEIN 6 (MYB6)*	Encodes myb6 DNA-binding protein. The mRNA is cell-to-cell mobile.
AT4G09480		transposable_element_gene;copia-like retrotransposon family, has a 4.8e-45 P-value blast match to GB:CAA72989 open reading frame 1 (Ty1_Copia-element) (Brassica oleracea);(source:TAIR10)

AT4G09490		Polynucleotidyl transferase, ribonuclease H-like superfamily protein
AT4G09500	*UDP-GLYCOSYLTRANSFERASE 79B7 (UGT79B7)*	Putative glycosyltransferase.
AT4G09510	*CYTOSOLIC INVERTASE 2 (CINV2)*	CINV2 appears to function as a neutral invertase based on the phenotype of a cinv1(AT1G35580)/cinv2 double mutant. It is predicted to be a cytosolic enzyme. CINV1, CINV2, and possibly other cytosolic invertases may play an important role in supplying carbon from sucrose to non-photosynthetic tissues.
AT4G09540		transposable_element_gene;copia-like retrotransposon family, has a 1.7e-43 P-value blast match to GB:AAC64917 gag-pol polyprotein (Ty1_Copia-element) (Glycine max);(source:TAIR10)
AT4G09570	*CALCIUM-DEPENDENT PROTEIN KINASE 4 (CPK4)*	Encodes a member of Calcium Dependent Protein Kinase (CDPK) gene family.Positive regulator of ABA signaling. Phosphorylates ABA responsive transcription factors ABF1 and ABF4.
AT4G09600	*GAST1 PROTEIN HOMOLOG 3 (GASA3)*	One of GASA gene family which is related to a GA-stimulated transcript (GAST) from tomato.
AT4G09610	*GAST1 PROTEIN HOMOLOG 2 (GASA2)*	GAST1 protein homolog 2
AT4G09640	*(ENOR3L6)*	magnesium transporter, putative (DUF803)
AT4G09650	*ATP SYNTHASE DELTA-SUBUNIT GENE (ATPD)*	Encodes the chloroplast ATPase delta-subunit. The mRNA is cell-to-cell mobile.
AT4G09670		Oxidoreductase family protein
AT4G09690		Cysteine/Histidine-rich C1 domain family protein
AT4G09700		transposable_element_gene;similar to unknown protein [Arabidopsis thaliana] (TAIR:AT2G06820.1);(source:TAIR10)
AT4G09730	*RH39 (RH39)*	Encodes RH39, a DEAD-box protein involved in the introduction of the hidden break into the 23S rRNA in the chloroplasts. Recombinant RH39 binds to the 23S rRNA in a segment adjacent to the stem-loop creating the hidden break target loop in a sequence-dependent manner. Has ATP-hydrolyzing activity at a Kcat of 5.3 /min in the presence of rRNA sequence. Mutants have drastically reduced level of level of ribulose 1,5-bisphosphate carboxylase/oxygenase. The mRNA is cell-to-cell mobile.
AT4G09740	*GLYCOSYL HYDROLASE 9B14 (GH9B14)*	glycosyl hydrolase 9B14
AT4G09760	*CHOLINE/ETHANOLAMINE KINASE 3 (CEK3)*	encodes a choline synthase whose gene expression is induced by high salt and mannitol.
AT4G09820	*TRANSPARENT TESTA 8 (TT8)*	TT8 is a regulation factor that acts in a concerted action with TT1, PAP1 and TTG1 on the regulation of flavonoid pathways, namely proanthocyanidin and anthocyanin biosynthesis. Affects dihydroflavonol 4-reductase gene expression. It is thought that a ternary complex composed of TT2, TT8 and TTG1 is necessary for correct expression of BAN in seed endothelium. Also important for important for marginal trichome development. It binds the promoter of both AT3G26790 and AT1G28300.TT8 interacts with JAZ proteins to regulate anthocyanin accumulation. TT8 acts maternally to affect seed FA biosynthesis and inhibits seed FA accumulation by down-regulating a group of genes either critical to embryonic development or important in the FA biosynthesis pathway. TT8 represses the activities of LEAFY COTYLEDON1, LEAFY COTYLEDON2, and FUSCA3, the critical transcriptional factors important for seed development.
AT4G09830		nuclear receptor family 2 group C protein
AT4G09840		hypothetical protein
AT4G09850		hypothetical protein
AT4G09860		hypothetical protein
AT4G09880		hypothetical protein
AT4G09890		mediator of RNA polymerase II transcription subunit, putative (DUF3511)
AT4G09900	*METHYL ESTERASE 12 (MES12)*	Encodes a protein predicted to act as a carboxylesterase. It has similarity to the SABP2 methyl salicylate esterase from tobacco. This protein does not act on methyl IAA, methyl JA, MeSA, MeGA4, or MEGA9 in vitro.
AT4G09910		
AT4G09930	*IMMUNE ASSOCIATED NUCLEOTIDE BINDING 11 (IAN11)*	Avirulence induced gene (AIG1) family protein
AT4G09940	*IMMUNE ASSOCIATED NUCLEOTIDE BINDING 12 (IAN12)*	P-loop containing nucleoside triphosphate hydrolases superfamily protein
AT4G09950	*IMMUNE ASSOCIATED NUCLEOTIDE BINDING 13 (IAN13)*	P-loop containing nucleoside triphosphate hydrolases superfamily protein
AT4G09960	*SEEDSTICK (STK)*	Encodes a MADS box transcription factor expressed in the carpel and ovules. Plays a maternal role in fertilization and seed development. Controls the structure and mechanical properties of the seed coat. Controls fruit size by regulating cytokinin levels and FRUITFULL.
AT4G09970		transmembrane protein
AT4G09990	*GLUCURONOXYLAN METHYLTRANSFERASE 2 (GXM2)*	glucuronoxylan 4-O-methyltransferase-like protein (DUF579)
AT4G10010		Protein kinase superfamily protein
AT4G10020	*HYDROXYSTEROID DEHYDROGENASE 5 (HSD5)*	Encodes a putative hydroxysteroid dehydrogenase (HSD). Genes that encode HSD include: At5g50600 and At5g50700 (HSD1), At3g47350(HSD2), At3g47360(HSD3), At5g50590 and At5g50690(HSD4), At5g50770(HSD6) (Plant Cell Physiology 50:1463). Two copies of HSD1 and HSD4 exist due to a gene duplication event. In Plant Physiology 145:87, At5g50690 is HSD7, At4g10020 is HSD5.
AT4G10030	*ALPHA/BETA HYDROLASE DOMAIN 11 (ABHD11)*	Alpha/beta hydrolase domain containing protein involved in lipid biosynthesis.
AT4G10040	*CYTOCHROME C-2 (CYTC-2)*	Encodes cytochrome c. Promoter directs preferential expression in vascular tissues of cotyledons, leaves, roots, and hypocotyls, and in anthers. Double mutants with CYTC-1 accumulate starch during the day, have delayed growth and development and reduced GA and DELLA proteins linking cellular metabolism and GA homeostasis.
AT4G10050	*PROTEIN PHOSPHATASE METHYLESTERASE (PMES)*	esterase/lipase/thioesterase family protein
AT4G10060	*GLCCER-DEGRADING ENZYME (GLUCOSYLCERAMIDASE) 3 (GCD3)*	Glucosylceramidase that preferentially hydrolyzes long acyl chain glucosylceramides.
AT4G10070		KH domain-containing protein
AT4G10100	*CO-FACTOR FOR NITRATE, REDUCTASE AND XANTHINE DEHYDROGENASE 7 (CNX7)*	molybdenum cofactor synthesis family protein, similar to Molybdenum cofactor synthesis protein 2 small subunit (Molybdopterin- synthase small subunit) (MOCS2A) (MOCO1-A) (Swiss-Prot:O96033) (Homo sapiens); contains TIGRFAM TIGR01682: molybdopterin converting factor, subunit 1; sir loss-of-function mutants are resistant to sirtinol, a modulator of auxin signaling.
AT4G10120	*SUCROSE PHOSPHATE SYNTHASE 4F (SPS4F)*	Encodes a sucrose-phosphate synthase.

AT4G10130		DNAJ heat shock N-terminal domain-containing protein
AT4G10140		transmembrane protein
AT4G10150	*ARABIDOPSIS T??XICOS EN LEVADURA 7 (ATL07)*	RING/U-box superfamily protein
AT4G10200		TTF-type zinc finger protein with HAT dimerization domain-containing protein
AT4G10210		transmembrane protein, putative (DUF239)
AT4G10220		NEP-interacting protein, putative (DUF239)
AT4G10240	*B-BOX DOMAIN PROTEIN 23 (BBX23)*	B-box zinc finger family protein
AT4G10250	*(ATHSP22.0)*	Columbia endomembrane-localized small heat shock protein
AT4G10260	*FRUCTOKINASE 4 (FRK4)*	Encodes a member of the fructokinase gene family. Nomenclature according to Riggs 2017 has been adopted for the family by the community (personal communication, Boernke, Callis, Granot, Boernke, and Smeekens).
AT4G10270	*WOUND-INDUCED POLYPEPTIDE 4 (WIP4)*	Member of the wound-induced polypeptide (WIP) family.
AT4G10280		RmlC-like cupins superfamily protein
AT4G10300	*(TRR14)*	RmlC-like cupins superfamily protein. Overexpression leads to trehalose resistance, drought and stress tolerance.
AT4G10320		tRNA synthetase class I (I, L, M and V) family protein
AT4G10340	*LIGHT HARVESTING COMPLEX OF PHOTOSYSTEM II 5 (LHCB5)*	photosystem II encoding the light-harvesting chlorophyll a/b binding protein CP26 of the antenna system of the photosynthetic apparatus The mRNA is cell-to-cell mobile.
AT4G10350	*NAC DOMAIN CONTAINING PROTEIN 70 (NAC070)*	NAC domain protein. SMB, BRN1, and BRN2 act to regulate root cap maturation, in a partially redundant fashion.BRN1 and BRN2, control the cell wall maturation processes that are required to detach root cap layers from the root.
AT4G10370		Cysteine/Histidine-rich C1 domain family protein
AT4G10380	*NOD26-LIKE INTRINSIC PROTEIN 5;1 (NIP5;1)*	Boric acid channel. Essential for efficient boron uptake and plant development under boron limitation. Also functions in arsenite transport and tolerance. Localized preferentially in outer membrane domains of root cells.
AT4G10390		Protein kinase superfamily protein
AT4G10400		F-box/RNI-like/FBD-like domains-containing protein
AT4G10410		Leucine-rich repeat (LRR) family protein
AT4G10420		FBD / Leucine Rich Repeat domains containing protein
AT4G10440		S-adenosyl-L-methionine-dependent methyltransferases superfamily protein
AT4G10450		Ribosomal protein L6 family
AT4G10460		transposable_element_gene;copia-like retrotransposon family, has a 3.8e-291 P-value blast match to GB:CAA31653 polyprotein (Ty1_Copia-element) (Arabidopsis thaliana);(source:TAIR10)
AT4G10490	*DMR6-LIKE OXYGENASE 2 (DLO2)*	2-oxoglutarate (2OG) and Fe(II)-dependent oxygenase superfamily protein
AT4G10500	*DMR6-LIKE OXYGENASE 1 (DLO1)*	2-oxoglutarate (2OG) and Fe(II)-dependent oxygenase superfamily protein
AT4G10510		Subtilase family protein
AT4G10520		Subtilase family protein
AT4G10540	*SUBTILASE 3.8 (SBT3.8)*	Proteolytic enzyme of that phytaspase family which at pH 5.5 is strictly Asp-specific. Strongly preferred cleavage motifs are YVAD and IETD.
AT4G10550		Subtilase family protein
AT4G10560	*MATERNAL EFFECT EMBRYO ARREST 53 (MEE53)*	Cysteine/Histidine-rich C1 domain family protein
AT4G10580		transposable_element_gene;gypsy-like retrotransposon family, has a 1.1e-306 P-value blast match to GB:AAD27547 polyprotein (Gypsy_Ty3-element) (Oryza sativa subsp. indica);(source:TAIR10)
AT4G10620		P-loop containing nucleoside triphosphate hydrolases superfamily protein
AT4G10640	*IQ-DOMAIN 16 (IQD16)*	Overexpression of IQD16 in transgenic Arabidopsis Pro35:IQD16 lines alters microtubule organization, cell shape, and plant growth. Phenotypes are reminiscent of lng1-1D plants, which overexpress LNG1/TRM2. IQD16 induces elongation of aerial tissues.
AT4G10670	*(GTC2)*	Homologous to yeast SPT16, a general chromatin factor required for transcription
AT4G10690		transposable_element_gene;copia-like retrotransposon family, has a 1.5e-256 P-value blast match to dbj|BAA78426.1| polyprotein (AtRE2-1) (Arabidopsis thaliana) (Ty1_Copia-element);(source:TAIR10)
AT4G10700		F-box/kelch-repeat protein
AT4G10710	*GLOBAL TRANSCRIPTION FACTOR C (SPT16)*	encodes a component of the FAcilitates Chromatin Transcription (FACT) complex, SPT16.Along with SSRP1 binds to the promoter of FLC.
AT4G10730		Protein kinase superfamily protein
AT4G10750		Phosphoenolpyruvate carboxylase family protein
AT4G10760	*MRNAADENOSINE METHYLASE (MTA)*	Encodes a member of a core set of mRNA m6A writer proteins and is required for N6-adenosine methylation of mRNA.
AT4G10770	*OLIGOPEPTIDE TRANSPORTER 7 (OPT7)*	oligopeptide transporter
AT4G10780		LRR and NB-ARC domains-containing disease resistance protein
AT4G10800		BTB/POZ domain protein
AT4G10810		hypothetical protein
AT4G10820	*F-BOX/DUF295 BRASSICEAE-SPECIFIC 22 (ATFDB22)*	F-box family protein
AT4G10830		transposable_element_gene;non-LTR retrotransposon family (LINE), has a 2.1e-39 P-value blast match to GB:NP_038605 L1 repeat, Tf subfamily, member 30 (LINE-element) (Mus musculus);(source:TAIR10)
AT4G10845		
AT4G10850	*(SWEET7)*	Nodulin MtN3 family protein
AT4G10880		hypothetical protein
AT4G10910		hypothetical protein

AT4G10930		RING/U-box protein
AT4G10940		
AT4G10950	*GDSL-TYPE ESTERASE/LIPASE 77 (GELP77)*	GDSL-type esterase/lipase. Required for pollen development.
AT4G10955	*(PRLIP9)*	alpha/beta-Hydrolases superfamily protein
AT4G10960	*UDP-D-GLUCOSE/UDP-D-GALACTOSE 4-EPIMERASE 5 (UGE5)*	Encodes a protein with UDP-D-glucose 4-epimerase activity.
AT4G10990		transposable_element_gene;copia-like retrotransposon family, has a 1.2e-190 P-value blast match to GB:CAA72989 open reading frame 1 (Ty1_Copia-element) (Brassica oleracea);(source:TAIR10)
AT4G11000		Ankyrin repeat family protein
AT4G11010	*NUCLEOSIDE DIPHOSPHATE KINASE 3 (NDPK3)*	nucleoside diphosphate kinase 3 (ndpk3), located to the inter-membrane space in mitochondria
AT4G11020		hypothetical protein
AT4G11030	*LONG-CHAIN ACYL-COENZYME A SYNTHETASE 5 (LACS5)*	AMP-dependent synthetase and ligase family protein
AT4G11050	*GLYCOSYL HYDROLASE 9C3 (GH9C3)*	glycosyl hydrolase 9C3
AT4G11060	*MITOCHONDRIALLY TARGETED SINGLE-STRANDED DNA BINDING PROTEIN (MTSSB)*	Participates in a minimal functioning DNA replisome in plant chloroplasts and mitochondria which consists of the DNA primase-helicase protein Twinkle along with DNA polymerase 1A or 1B.
AT4G11080	*3XHIGH MOBILITY GROUP-BOX1 (3xHMG-box1)*	Encodes a protein containing three copies of the HMG (high mobility group)-box domain. The two Arabidopsis 3xHMG-box proteins are: AT4G11080 (3xHMG-box1) and AT4G23800 (3xHMG-box2). Interacts with mitotic and meiotic chromosomes.
AT4G11100		gelsolin protein
AT4G11120		translation elongation factor Ts (EF-Ts)
AT4G11140	*CYTOKININ RESPONSE FACTOR 1 (CRF1)*	Encodes a member of the ERF (ethylene response factor) subfamily B-5 of ERF/AP2 transcription factor family. The protein contains one AP2 domain. There are 7 members in this subfamily. CRF proteins relocalize to the nucleus in response to cytokinin.
AT4G11160		Translation initiation factor 2, small GTP-binding protein
AT4G11170	*RESISTANCE METHYLATED GENE 1 (RMG1)*	Encodes RMG1 (Resistance Methylated Gene 1), a NB-LRR disease resistance protein with a Toll/interleukin-1 receptor (TIR) domain at its N terminus. RMG1 is expressed at high levels in response to flg22 and in naive met1/nrpd2 relative to wild-type plants. Expression of this gene is controlled by DNA methylation in its promoter region. The RMG1 promoter region is constitutively demethylated by active DNA demethylation mediated by the DNA glycosylase ROS1.
AT4G11175		Nucleic acid-binding, OB-fold-like protein
AT4G11180	*DIRIGENT PROTEIN 1 (DP1)*	Seed coat specific expressed protein. Together with LAC5 involved in neolignan biosynthesis via sinapoylcholine/feruloylcholine and is essential for the biosynthesis of 8-O-4′-type neolignans in seeds.
AT4G11190		Disease resistance-responsive (dirigent-like protein) family protein
AT4G11200		transposable_element_gene;similar to unknown protein [Arabidopsis thaliana] (TAIR:AT3G30370.1);(source:TAIR10)
AT4G11210		Disease resistance-responsive (dirigent-like protein) family protein
AT4G11230	*(RBOHI)*	NADPH-oxidase RbohI is expressed highly in seeds and roots. Mutants have inreased sensitivity to osmotic stress suggesting a role in mediating cellular response to stress in roots.
AT4G11240	*(TOPP7)*	encodes a type I serine/threonine protein phosphatase expressed in expressed in roots, rosettes and flowers.
AT4G11280	*1-AMINOCYCLOPROPANE-1-CARBOXYLIC ACID (ACC) SYNTHASE 6 (ACS6)*	encodes a a member of the 1-aminocyclopropane-1-carboxylate (ACC) synthase (S-adenosyl-L-methionine methylthioadenosine-lyase, EC 4.4.1.14) gene family The mRNA is cell-to-cell mobile.
AT4G11290	*PEROXIDASE 39 (PER39)*	Peroxidase required for casparian strip lignification as well as partially required for SGN-dependent compensatory lignification.
AT4G11300		ROH1, putative (DUF793)
AT4G11310	*CYSTEINE PROTEASE 1 (CP1)*	cysteine proteinase precursor-like protein
AT4G11340		Disease resistance protein (TIR-NBS-LRR class) family
AT4G11350		transferring glycosyl group transferase (DUF604)
AT4G11360	*RING-H2 FINGER A1B (RHA1B)*	Encodes a putative RING-H2 finger protein RHA1b. The mRNA is cell-to-cell mobile.
AT4G11370	*RING-H2 FINGER A1A (RHA1A)*	Encodes a putative RING-H2 finger protein RHA1a.
AT4G11400		ARID/BRIGHT DNA-binding , ELM2 domain and myb-like DNA-binding domain-containing protein
AT4G11410		NAD(P)-binding Rossmann-fold superfamily protein
AT4G11440		Mitochondrial substrate carrier family protein
AT4G11460	*CYSTEINE-RICH RLK (RECEPTOR-LIKE PROTEIN KINASE) 30 (CRK30)*	Encodes a cysteine-rich receptor-like protein kinase.
AT4G11470	*CYSTEINE-RICH RLK (RECEPTOR-LIKE PROTEIN KINASE) 31 (CRK31)*	Encodes a cysteine-rich receptor-like protein kinase.
AT4G11480	*CYSTEINE-RICH RLK (RECEPTOR-LIKE PROTEIN KINASE) 32 (CRK32)*	Encodes a cysteine-rich receptor-like protein kinase.
AT4G11510	*RALF-LIKE 28 (RALFL28)*	Member of a diversely expressed predicted peptide family showing sequence similarity to tobacco Rapid Alkalinization Factor (RALF), and is believed to play an essential role in the physiology of Arabidopsis. Consists of a single exon and is characterized by a conserved C-terminal motif and N-terminal signal peptide.
AT4G11530	*CYSTEINE-RICH RLK (RECEPTOR-LIKE PROTEIN KINASE) 34 (CRK34)*	Encodes a cysteine-rich receptor-like protein kinase. The mRNA is cell-to-cell mobile.
AT4G11550		Cysteine/Histidine-rich C1 domain family protein
AT4G11590		F-box associated ubiquitination effector family protein
AT4G11600	*GLUTATHIONE PEROXIDASE 6 (GPX6)*	Encodes glutathione peroxidase. Exhibits moderate binding affinity with dinotefuran.
AT4G11610	*MULTIPLE C2 DOMAIN AND TRANSMEMBRANE REGION PROTEIN 7 (MCTP7)*	C2 calcium/lipid-binding plant phosphoribosyltransferase family protein
AT4G11640	*SERINE RACEMASE (SR)*	Serine racemase, which is a bifunctional PLP-dependent enzyme catalyzing racemization of serine and dehydration of serine to pyruvate in the same way as mammalian serine racemases. similar to mammalian serine racemases.
AT4G11650	*OSMOTIN 34 (OSM34)*	Osmotin-like protein; functions as a positive regulator in the generation of ABA responses and is under post-translational control.

AT4G11660	*(AT-HSFB2B)*	member of Heat Stress Transcription Factor (Hsf) family
AT4G11700		hypothetical protein (DUF626)
AT4G11710		transposable_element_gene;similar to unknown protein [Arabidopsis thaliana] (TAIR:AT5G13655.1);(source:TAIR10)
AT4G11720	*HAPLESS 2 (HAP2)*	Encodes HAP2 with the following predicted motifs: an N-terminal secretion signal, a single transmembrane domain and a C-terminal histidine-rich domain. HAP2 is expressed only in the haploid sperm and is required for pollen tube guidance and fertilization. Predominantly localized to sperm endoplasmic reticulum membranes. May also reside in other endomembranes, including the plasma membrane. Target promoter of the male germline-specific transcription factor DUO1.
AT4G11730		Cation transporter/ E1-E2 ATPase family protein
AT4G11740	*(SAY1)*	Isolated as a suppressor of a dominant mutant in the Ara4 gene that was expressed in yeast ypt1 mutant strains. A novel protein with a small region of similarity to coil-coiled domain of yeast VSP27 protein.
AT4G11750		Galactose oxidase/kelch repeat superfamily protein
AT4G11760	*LOW-MOLECULAR-WEIGHT CYSTEINE-RICH 17 (LCR17)*	Encodes a member of a family of small,secreted, cysteine rich protein with sequence similarity to the PCP (pollen coat protein) gene family.
AT4G11770		Galactose oxidase/kelch repeat superfamily protein
AT4G11780	*TON1 RECRUITING MOTIF 10 (TRM10)*	GAR2-like protein
AT4G11820	*HYDROXYMETHYLGLUTARYL-COA SYNTHASE (HMGS)*	Encodes a protein with hydroxymethylglutaryl-CoA synthase activity which was characterized by phenotypical complementation of the S. cerevisiae mutant. Involved in glucosinolate biosynthesis.
AT4G11830	*PHOSPHOLIPASE D GAMMA 2 (PLDGAMMA2)*	Encodes one of three phospholipase D enzymes of the gamma class.
AT4G11840	*PHOSPHOLIPASE D GAMMA 3 (PLDGAMMA3)*	member of C2-PLD subfamily
AT4G11850	*PHOSPHOLIPASE D GAMMA 1 (PLDGAMMA1)*	Encodes a phospholipase D (gamma) that is involved in aluminum tolerance and plays a role in membrane lipid modulation under Al stress.
AT4G11870		hypothetical protein
AT4G11880	*AGAMOUS-LIKE 14 (AGL14)*	AGL12, AGL14, and AGL17 are all preferentially expressed in root tissues and therefore represent the only characterized MADS box genes expressed in roots. The mRNA is cell-to-cell mobile.
AT4G11890	*ABA- AND OSMOTIC-STRESS-INDUCIBLE RECEPTOR-LIKE CYTOSOLIC KINASE1 (ARCK1)*	Encodes a receptor-like cytosolic kinase ARCK1. Negatively controls abscisic acid and osmotic stress signal transduction.
AT4G11900		S-locus lectin protein kinase family protein
AT4G11930		hypothetical protein
AT4G11940	*ADMETOS (ADM)*	Encodes a nuclear localized dosage sensitive paternally expressed imprinted gene. It is a member of a family of molecular chaperones called J-domain. Loss of ADM function suppresses seed abortion of triploid embryos and also partially rescues the effect of mea mutations.
AT4G11950		transmembrane protein, putative (DUF1191)
AT4G11960	*PGR5-LIKE B (PGRL1B)*	Encodes PGRL1B, a transmembrane protein present in thylakoids. PGRL1B has a highly homologous isoform PGRL1A encoded by At4g22890. Plants lacking PGRL1 show perturbation of cyclic electron flow, similar to PGR5-deficient plants. PGRL1 and PGR5 interact physically and associate with PSI (photosystem I).
AT4G11980	*NUDIX HYDROLASE HOMOLOG 14 (NUDX14)*	nudix hydrolase homolog 14
AT4G12000		SNARE associated Golgi protein family
AT4G12010	*DOMINANT SUPRESSOR OF CAMTA3 NUMBER 1 (DSC1)*	Leucine-rich repeat domain (NLR) receptor. Dominant negative alleles suppress catma3 autoimmunity. Co-regulates with WRKY19 basal levels of immunity to root-knot nematodes.
AT4G12030	*BILE ACID TRANSPORTER 5 (BAT5)*	Required for the biosynthesis of methionine-derived glucosinolates. Involved in the transport of 2-keto acids between chloroplasts and the cytosol.
AT4G12060	*(CLPT2)*	Double Clp-N motif protein
AT4G12080	*AT-HOOK MOTIF NUCLEAR-LOCALIZED PROTEIN 1 (AHL1)*	AT-hook motif nuclear-localized protein 1
AT4G12110	*STEROL-4ALPHA-METHYL OXIDASE 1-1 (SMO1-1)*	Encodes a member of the SMO1 family of sterol 4alpha-methyl oxidases. More specifically functions as a 4,4-dimethyl-9beta,19-cyclopropylsterol-4alpha-methyl oxidase. Works together with SMO1-2 to maintain correct sterol composition and balance auxin and cytokinin activities during embryogenesis.
AT4G12120	*(SEC1B)*	member of KEULE Gene Family
AT4G12130	*(COG0354)*	Encodes a mitochondrial COG0354 protein that requires folate for its function in Fe/S cluster biogenesis.
AT4G12140		RING/U-box superfamily protein
AT4G12160		pseudogene of Ribosomal protein S4
AT4G12180		transposable_element_gene;non-LTR retrotransposon family (LINE), has a 2.2e-28 P-value blast match to GB:NP_038605 L1 repeat, Tf subfamily, member 30 (LINE-element) (Mus musculus);(source:TAIR10)
AT4G12190		RING/U-box superfamily protein
AT4G12220		hypothetical protein
AT4G12240		zinc finger (C2H2 type) family protein
AT4G12250	*UDP-D-GLUCURONATE 4-EPIMERASE 5 (GAE5)*	UDP-D-glucuronate 4-epimerase
AT4G12270		Copper amine oxidase family protein
AT4G12280		copper amine oxidase family protein
AT4G12300	*CYTOCHROME P450, FAMILY 706, SUBFAMILY A, POLYPEPTIDE 4 (CYP706A4)*	member of CYP706A
AT4G12310	*CYTOCHROME P450, FAMILY 706, SUBFAMILY A, POLYPEPTIDE 5 (CYP706A5)*	member of CYP706A
AT4G12330	*CYTOCHROME P450, FAMILY 706, SUBFAMILY A, POLYPEPTIDE 7 (CYP706A7)*	member of CYP706A
AT4G12340		copper ion binding protein
AT4G12350	*MYB DOMAIN PROTEIN 42 (MYB42)*	Encodes a transcriptional regulator that directly activates lignin biosynthesis genes and phenylalanine biosynthesis genes during secondary wall formation.
AT4G12360	*GLYCOSYLPHOSPHATIDYLINOSITOL-ANCHORED LIPID PROTEIN TRANSFER 24 (LTPG24)*	Bifunctional inhibitor/lipid-transfer protein/seed storage 2S albumin superfamily protein

AT4G12380		hypothetical protein
AT4G12390	*PECTIN METHYLESTERASE INHIBITOR 1 (PME1)*	pectin methylesterase inhibitor 1
AT4G12400	*HOP3 (Hop3)*	Encodes one of the 36 carboxylate clamp (CC)-tetratricopeptide repeat (TPR) proteins (Prasad 2010, Pubmed ID: 20856808) with potential to interact with Hsp90/Hsp70 as co-chaperones.
AT4G12410	*SMALL AUXIN UPREGULATED RNA 35 (SAUR35)*	SAUR-like auxin-responsive protein family
AT4G12420	*(SKU5)*	Encodes a protein of unknown function involved in directed root tip growth. It is a member of 19-member gene family and is distantly related structurally to the multiple-copper oxidases ascorbate oxidase and laccase, though it lacks the copper-binding domains. The protein is glycosylated and GPI-anchored. It is localized to the plasma membrane and the cell wall. The gene is expressed most strongly in expanding tissues.
AT4G12430	*TREHALOSE-6-PHOSPHATE PHOSPHATASE F (TPPF)*	Haloacid dehalogenase-like hydrolase (HAD) superfamily protein
AT4G12440	*ADENINE PHOSPHORIBOSYL TRANSFERASE 4 (APT4)*	adenine phosphoribosyl transferase 4
AT4G12460	*OSBP(OXYSTEROL BINDING PROTEIN)-RELATED PROTEIN 2B (ORP2B)*	OSBP(oxysterol binding protein)-related protein 2B
AT4G12470	*AZELAIC ACID INDUCED 1 (AZI1)*	Encodes AZI1 (AZELAIC ACID INDUCED 1). Involved in the priming of salicylic acid induction and systemic immunity triggered by pathogen or azelaic acid. Targeting if AZI1 to chloroplasts is increased during SAR induction and that localization requires the PRR domain.It is involved in the uptake and movement of the azelaic acid signal. AZI1 uses a previously undescribed variant of the signal anchor proteins mechanism to target plastids. AZI1 uses a bipartite N-terminal signature: a non-cleavable TMD that anchors the protein to membranes, followed by a proline rich region with features that are shared with bona fide chloroplastic transit peptides. flg22 MAMP treatment strongly induces AZI1/EARLI1 protein levels and increases their relative enrichment in the plastid fraction.
AT4G12480	*EARLY ARABIDOPSIS ALUMINUM INDUCED 1 (EARLI1)*	Encodes a putative lipid transfer protein, vernalization-responsive and cold-induced. It is involved in priming the SAR and ISR responses, specifically in propagating the cell-to-cell mobile signal. The kinases MPK3 (AT3G45640) and MPK6 (AT2G43790) promote the accumulation of AZI1/EARLI1 at plastids during defense priming induction. The kinases MPK3 (AT3G45640) and MPK6 (AT2G43790) promote the accumulation of AZI1/EARLI1 at plastids during defense priming induction.
AT4G12490	*(AZI3)*	Encodes a member of the AZI family of lipid transfer proteins. Contains a PRR domain that appears to be required for localization to the chloroplast.
AT4G12500		Bifunctional inhibitor/lipid-transfer protein/seed storage 2S albumin superfamily protein
AT4G12510	*(AZI5)*	Encodes a member of the AZI family of lipid transfer proteins.
AT4G12530	*(AZI7)*	Encodes a member of the AZI family of lipid transfer proteins.
AT4G12540		hypothetical protein
AT4G12550	*AUXIN-INDUCED IN ROOT CULTURES 1 (AIR1)*	isolated from differential screening of a cDNA library from auxin-treated root culture. encodes a protein that is related to a large family of proteins that consist of a proline-rich or glycine-rich N-terminus and a hydrophobic, possibly membrane spanning C-terminus.
AT4G12570	*UBIQUITIN PROTEIN LIGASE 5 (UPL5)*	Knock-out mutants showed accelerated senescence of leaves. The mRNA is cell-to-cell mobile.
AT4G12580		hypothetical protein
AT4G12600		Ribosomal protein L7Ae/L30e/S12e/Gadd45 family protein
AT4G12620	*ORIGIN OF REPLICATION COMPLEX 1B (ORC1B)*	Origin Recognition Complex subunit 1b. Involved in the initiation of DNA replication. Regulated transcriptionally during cell cycle, peaking at G1/S-phase. Target of E2F/DF family of transcription factors. Interacts with ORC2 and ORC5. Highly expressed in proliferating cells. Expression levels are independent of light regime.
AT4G12630		
AT4G12690		DUF868 family protein (DUF868)
AT4G12700		calcium ion-binding protein
AT4G12730	*FASCICLIN-LIKE ARABINOGALACTAN 2 (FLA2)*	AF333971 Arabidopsis thaliana fasciclin-like arabinogalactan-protein 2 (Fla2) mRNA, complete cds. Fasciclin-like arabinogalactan protein. Possibly involved in embryogenesis and seed development.
AT4G12750		Homeodomain-like transcriptional regulator
AT4G12770	*AUXILIN-LIKE2 (AUXILIN-LIKE2)*	Negative regulation of growth and endocytosis, most likely as a result of inhibition of the recruitment of clathrin to endocytic pits.
AT4G12800	*PHOTOSYSTEM I SUBUNIT L (PSAL)*	Encodes subunit L of photosystem I reaction center.
AT4G12830		alpha/beta-Hydrolases superfamily protein
AT4G12860	*UNFERTILIZED EMBRYO SAC 14 (UNE14)*	EF hand calcium-binding protein family
AT4G12880	*EARLY NODULIN-LIKE PROTEIN 19 (ENODL19)*	early nodulin-like protein 19
AT4G12890		Gamma interferon responsive lysosomal thiol (GILT) reductase family protein
AT4G12910	*SERINE CARBOXYPEPTIDASE-LIKE 20 (scpl20)*	serine carboxypeptidase-like 20
AT4G12920	*UNDEAD (UND)*	Eukaryotic aspartyl protease family protein
AT4G12930		hypothetical protein
AT4G12960	*GAMMA-INTERFERON-RESPONSIVE LYSOSOMAL THIOL REDUCTASE (GILT)*	Gamma interferon responsive lysosomal thiol (GILT) reductase family protein
AT4G12970	*STOMAGEN (STOMAGEN)*	Encodes a cysteine-rich peptide, a secretory factor that is produced in the mesophyll cells and acts on the epidermis to increase stomatal formation. Its mature form is a 45-aa peptide with three intramolecular disulfide bonds. It is proposed that STOMAGEN increases stomatal number by competing with two negative regulators of stomatal density, EPF1 and EPF2. STOMAGEN has been shown to compete with EPF2 for binding to the ER and TMM receptor kinases.Binding of STOMAGEN to ER prevents induction of the EPF2-ER MAPK cascade. It's transcript levels change after inducing MUTE expression in a mute background.
AT4G12980	*AUXIN-RESPONSIVE FAMILY PROTEIN GENE (DEG18)*	Activated by OXS2 under the treatment of salt.
AT4G13000		AGC (cAMP-dependent, cGMP-dependent and protein kinase C) kinase family protein
AT4G13010	*CHLOROPLAST ENVELOPE QUINONE OXIDOREDUCTASE HOMOLOG (CEQORH)*	Oxidoreductase, zinc-binding dehydrogenase family protein
AT4G13030		P-loop containing nucleoside triphosphate hydrolases superfamily protein
AT4G13050	*(FATA2)*	Acyl-ACP thioesterase

AT4G13060		F-box associated ubiquitination effector family protein
AT4G13070		RNA-binding CRS1 / YhbY (CRM) domain protein
AT4G13080	*XYLOGLUCAN ENDOTRANSGLUCOSYLASE/HYDROLASE 1 (XTH1)*	xyloglucan endotransglucosylase/hydrolase 1
AT4G13090	*XYLOGLUCAN ENDOTRANSGLUCOSYLASE/HYDROLASE 2 (XTH2)*	xyloglucan endotransglucosylase/hydrolase 2
AT4G13100		RING/U-box superfamily protein
AT4G13120		transposable_element_gene;hAT-like transposase family (hobo/Ac/Tam3), has a 2.6e-50 P-value blast match to GB:AAD24567 transposase Tag2 (hAT-element) (Arabidopsis thaliana);(source:TAIR10)
AT4G13130		Cysteine/Histidine-rich C1 domain family protein
AT4G13140		
AT4G13150		transmembrane protein
AT4G13160		zein-binding protein (Protein of unknown function, DUF593)
AT4G13180		NAD(P)-binding Rossmann-fold superfamily protein
AT4G13190	*PBS1-LIKE 24 (PBL24)*	Protein kinase superfamily protein
AT4G13195	*CLAVATA3/ESR-RELATED 44 (CLE44)*	Belongs to a large gene family, called CLE for CLAVATA3/ESR-related, encoding small peptides with conserved carboxyl termini. The C-terminal 12 amino acid sequence of CLE44 is identical to that of a dodeca peptide (TDIF, tracheary element differentiation inhibitory factor) isolated from Arabidopsis and functions as a suppressor of plant stem cell differentiation. TDIF sequence is also identical to the C-terminal 12 amino acids of CLE41 (At3g24770). The protein is expressed in the vascular system and is involved in axillary bud formation.
AT4G13210		Pectin lyase-like superfamily protein
AT4G13220		transmembrane protein
AT4G13230		Late embryogenesis abundant protein (LEA) family protein
AT4G13235	*EMBRYO SAC DEVELOPMENT ARREST 21 (EDA21)*	Encodes a defensin-like (DEFL) family protein.
AT4G13240	*ROP (RHO OF PLANTS) GUANINE NUCLEOTIDE EXCHANGE FACTOR 9 (ROPGEF9)*	Encodes a member of KPP-like gene family, homolog of KPP (kinase partner protein) gene in tomato. Also a member of the RopGEF (guanine nucleotide exchange factor) family, containing the novel PRONE domain (plant-specific Rop nucleotide exchanger), which is exclusively active towards members of the Rop subfamily.
AT4G13250	*NON-YELLOW COLORING 1 (NYC1)*	Encodes a chlorophyll b reductase involved in the degradation of chlorophyll b and LHCII (light harvesting complex II).
AT4G13260	*YUCCA2 (YUC2)*	Encodes YUC2. Catalyzes conversion of IPA (indole-3-pyruvic acid) to IAA (indole-3-acetic acid) in auxin biosynthesis pathway.
AT4G13280	*TERPENOID SYNTHASE 12 (TPS12)*	Catalyzes the conversion of farnesyl diphosphate to (Z)-gamma-bisabolene and the additional minor products E-nerolidol and alpha-bisabolol. Expressed in cortex and sub-epidermal layers of roots, leaf hydathodes and flower stigmata. Induced by wounding.
AT4G13290	*CYTOCHROME P450, FAMILY 71, SUBFAMILY A, POLYPEPTIDE 19 (CYP71A19)*	putative cytochrome P450
AT4G13320		hypothetical protein
AT4G13330		S-adenosyl-L-methionine-dependent methyltransferases superfamily protein
AT4G13340	*LEUCINE-RICH REPEAT/EXTENSIN 3 (LRX3)*	Leucine rich extensin protein involved in cell wall biogenesis and organization. Interacts with several members of the RALF family of ligand peptides.
AT4G13345	*MATERNAL EFFECT EMBRYO ARREST 55 (MEE55)*	Serinc-domain containing serine and sphingolipid biosynthesis protein
AT4G13380	*MATERNAL EFFECT EMBRYO ARREST 56 (MEE56)*	Heavy metal transport/detoxification superfamily protein
AT4G13390	*EXTENSIN 12 (EXT12)*	Proline-rich extensin-like family protein
AT4G13410	*(ATCSLA15)*	encodes a gene similar to cellulose synthase
AT4G13420	*HIGH AFFINITY K+ TRANSPORTER 5 (HAK5)*	Encodes a protein of the KUP/HAK/KT potassium channel class that is upregulated in the roots by K levels.
AT4G13440		Calcium-binding EF-hand family protein
AT4G13450		Adenine nucleotide alpha hydrolases-like superfamily protein
AT4G13480	*MYB DOMAIN PROTEIN 79 (MYB79)*	Member of the R2R3 factor gene family.
AT4G13510	*AMMONIUM TRANSPORTER 1;1 (AMT1;1)*	Encodes a plasma membrane localized ammonium transporter. Contains a cytosolic trans-activation domain essential for ammonium uptake. The mRNA is cell-to-cell mobile.
AT4G13520	*SMALL ACIDIC PROTEIN 1 (SMAP1)*	Encodes a small acid protein (SMAP1) that mediates responses Arabidopsis root to the synthetic auxin 2,4-Dichlorophenoxyacetic acid. The mRNA is cell-to-cell mobile.
AT4G13530		transmembrane protein
AT4G13560	*UNFERTILIZED EMBRYO SAC 15 (UNE15)*	Late embryogenesis abundant protein (LEA) family protein
AT4G13570	*HISTONE H2A 4 (HTA4)*	Encodes HTA4, a histone H2A protein. The mRNA is cell-to-cell mobile.
AT4G13580		Disease resistance-responsive (dirigent-like protein) family protein
AT4G13600		Carbohydrate-binding X8 domain superfamily protein
AT4G13610	*MATERNAL EFFECT EMBRYO ARREST 57 (MEE57)*	DNA (cytosine-5-)-methyltransferase family protein
AT4G13650		Pentatricopeptide repeat (PPR) superfamily protein
AT4G13660	*PINORESINOL REDUCTASE 2 (PRR2)*	Encodes a pinoresinol reductase involved in lignan biosynthesis. Expressed strongly in roots and less strongly in stems. Shows preference for pinoresinol and not lariciresinol. The mRNA is cell-to-cell mobile.
AT4G13670	*PLASTID TRANSCRIPTIONALLY ACTIVE 5 (PTAC5)*	plastid transcriptionally active 5
AT4G13690		RNA-binding protein
AT4G13700	*PURPLE ACID PHOSPHATASE 23 (PAP23)*	purple acid phosphatase 23
AT4G13710		Pectin lyase-like superfamily protein
AT4G13740		

AT4G13750	*NO VEIN (NOV)*	Encodes NO VEIN (NOV), a plant-specific nuclear factor required for leaf vascular development, cellular patterning and stem cell maintenance in the root meristem, as well as for cotyledon outgrowth and separation. nov mutations affect many aspects of auxin-dependent development without directly affecting auxin perception.
AT4G13760		Pectin lyase-like superfamily protein
AT4G13770	*CYTOCHROME P450, FAMILY 83, SUBFAMILY A, POLYPEPTIDE 1 (CYP83A1)*	Encodes a cytochrome p450 enzyme that catalyzes the initial conversion of aldoximes to thiohydroximates in the synthesis of glucosinolates not derived from tryptophan. Also has a role in auxin homeostasis.
AT4G13790	*SMALL AUXIN UPREGULATED RNA 25 (SAUR25)*	SAUR-like auxin-responsive protein family
AT4G13810	*RECEPTOR LIKE PROTEIN 47 (RLP47)*	receptor like protein 47
AT4G13820		Leucine-rich repeat (LRR) family protein
AT4G13830	*DNAJ-LIKE 20 (J20)*	DnaJ-like protein (J20); nuclear gene
AT4G13840	*ECERIFERUM 26 (CER26)*	HXXXD-type acyl-transferase family protein
AT4G13850	*RNA-BINDING GLYCINE-RICH PROTEIN A5 (RBGA5)*	Encodes a glycine-rich RNA-binding protein. Gene expression is induced by cold.
AT4G13860		RNA-binding (RRM/RBD/RNP motifs) family protein
AT4G13880	*RECEPTOR LIKE PROTEIN 48 (RLP48)*	receptor like protein 48
AT4G13900	*RECEPTOR LIKE PROTEIN 49 (RLP49)*	pseudogene of receptor like protein 47
AT4G13950	*RALF-LIKE 31 (RALFL31)*	Member of a diversely expressed predicted peptide family showing sequence similarity to tobacco Rapid Alkalinization Factor (RALF), and is believed to play an essential role in the physiology of Arabidopsis. Consists of a single exon and is characterized by a conserved C-terminal motif and N-terminal signal peptide.
AT4G13960		F-box/RNI-like superfamily protein
AT4G13985	*FBD-ASSOCIATED F-BOX PROTEIN (FBD1)*	FBD-associated F-box protein
AT4G13990		Exostosin family protein
AT4G14010	*RALF-LIKE 32 (RALFL32)*	Member of a diversely expressed predicted peptide family showing sequence similarity to tobacco Rapid Alkalinization Factor (RALF), and is believed to play an essential role in the physiology of Arabidopsis. Consists of a single exon and is characterized by a conserved C-terminal motif and N-terminal signal peptide.
AT4G14020		Rapid alkalinization factor (RALF) family protein
AT4G14050	*MITOCHONDRIAL EDITING FACTOR 35 (MEF35)*	Pentatricopeptide repeat (PPR) superfamily protein
AT4G14060		Polyketide cyclase/dehydrase and lipid transport superfamily protein
AT4G14080	*MATERNAL EFFECT EMBRYO ARREST 48 (MEE48)*	Involved in the formation of the pollen wall. DYT1 and bHLH089 specifically recognize the TCATGTGC box to activate expression.
AT4G14090		The At4g14090 encodes a anthocyanidin 5-O-glucosyltransferase specifically glucosylating the 5-position of the flavonoid A-ring.
AT4G14100		transferases, transferring glycosyl groups
AT4G14103		F-box/RNI-like superfamily protein
AT4G14130	*XYLOGLUCAN ENDOTRANSGLUCOSYLASE/HYDROLASE 15 (XTH15)*	xyloglucan endotransglycosylase-related protein (XTR7)
AT4G14150	*PHRAGMOPLAST-ASSOCIATED KINESIN-RELATED PROTEIN 1 (PAKRP1)*	Microtubule motor kinesin PAKRP1/Kinesin-12A. Together with PAKRP1L/Kinesin-12B, serve as linkers of the plus ends of antiparallel microtubules in the phragmoplast.
AT4G14170	*MITOCHONDRIAL EDITING FACTOR 32 (MEF32)*	Pentatricopeptide repeat (PPR) superfamily protein
AT4G14180	*PUTATIVE RECOMBINATION INITIATION DEFECT 1 (PRD1)*	Encodes a protein that is involved in meiotic recombination and is required for meiotic double strand break repair.
AT4G14200		Pentatricopeptide repeat (PPR) superfamily protein
AT4G14220	*RING-H2 GROUP F1A (RHF1A)*	encodes a RING-type E3 ubiquitin ligase implicated in gametogenesis. RHF1a can interact with the cell cycle inhibitor ICK4/KRP6 in vitro. It apppears to target ICK4KRP6 for degradation following meiosis in order to allow the mitoses associated with megagametogenesis and microgametogenesis to occur. RHF1a is expressed in the carpels throughout floral development. It is expressed in various tissues of the anthers during the early stages of anther development but not in stage 12 flowers and beyond. The mRNA is cell-to-cell mobile.
AT4G14270		Protein containing PAM2 motif which mediates interaction with the PABC domain of polyadenyl binding proteins.
AT4G14280		ARM repeat superfamily protein
AT4G14290		alpha/beta-Hydrolases superfamily protein
AT4G14300	*RNA-BINDING GLYCINE-RICH PROTEIN D4 (RBGD4)*	Belongs to a member of the RNA-binding glycine-rich (RBG) gene superfamily.
AT4G14310		Transducin/WD40 repeat-like superfamily protein
AT4G14330	*PHRAGMOPLAST-ASSOCIATED KINESIN-RELATED PROTEIN 2 (PAKRP2)*	Orphan kinesin with processive motility on single microtubules.
AT4G14340	*CASEIN KINASE I (CKI1)*	Phosphorylates serine or threonine residues that are near and C-terminal to acidic side chains on a variety of target proteins. Member of CKL gene family (CKL-C group).
AT4G14350	*NUCLEAR DBF2-RELATED 1 (NDR1)*	AGC (cAMP-dependent, cGMP-dependent and protein kinase C) kinase family protein
AT4G14365	*XB3 ORTHOLOG 4 IN ARABIDOPSIS THALIANA (XBAT34)*	hypothetical protein
AT4G14370		Disease resistance protein (TIR-NBS-LRR class) family
AT4G14380		cotton fiber protein
AT4G14390		Ankyrin repeat family protein
AT4G14400	*ACCELERATED CELL DEATH 6 (ACD6)*	encodes a novel protein with putative ankyrin and transmembrane regions. It is a member of one of the largest uncharacterized gene families in higher plants. The gene is involved in resistance to Pseudomonas syringae.
AT4G14410	*BASIC HELIX-LOOP-HELIX 104 (bHLH104)*	Basic helix loop helix transcription factor involved in stress mediated signaling during germination.
AT4G14430	*INDOLE-3-BUTYRIC ACID RESPONSE 10 (IBR10)*	Encodes a peroxisomal delta3, delta2-enoyl CoA isomerase, involved in unsaturated fatty acid degradation. This enzyme might also be involved in the conversion of indole-3-butyric acid to indole-3-acetic acid via a beta-oxidation-like pathway.
AT4G14440	*3-HYDROXYACYL-COA DEHYDRATASE 1 (HCD1)*	encodes a cytosolic delta3, delta2-enoyl CoA isomerase, involved in unsaturated fatty acid degradation

AT4G14450	*(PH1)*	A member of a small family of proline/serine rich proteins of unknown function. It interacts with defense related MAP kinase MPK6. It's expression is induced by PAMP elicitors. May play a role in response to pathogens.
AT4G14460		transposable_element_gene;copia-like retrotransposon family, has a 4.2e-253 P-value blast match to GB:CAA72989 open reading frame 1 (Ty1_Copia-element) (Brassica oleracea);(source:TAIR10)
AT4G14465	*AT-HOOK MOTIF NUCLEAR-LOCALIZED PROTEIN 20 (AHL20)*	AT-hook protein. Overexpression results in early flowering in short and long days.
AT4G14470		transposable_element_gene;non-LTR retrotransposon family (LINE), has a 2.1e-25 P-value blast match to GB:AAB41224 ORF2 (LINE-element) (Rattus norvegicus);(source:TAIR10)
AT4G14480	*BLUE LIGHT SIGNALING1 (BLUS1)*	Encodes a putative Ser/Thr protein kinase, BLUS1 (BLUE LIGHT SIGNALING1). BLUS1 functions as a phototropin substrate and primary regulator of stomatal control to enhance photosynthetic CO2 assimilation under natural light conditions.
AT4G14490		SMAD/FHA domain-containing protein
AT4G14510	*CRM FAMILY MEMBER 3B (CFM3B)*	Encodes a CRM domain protein CFM3b. Homolog of CFM3a (AT3G23070). CFM3a is shown to function in the splicing of group IIB introns in chloroplasts.
AT4G14530		agamous-like MADS-box protein
AT4G14540	*NUCLEAR FACTOR Y, SUBUNIT B3 (NF-YB3)*	Component of Nuclear factor Y transcription factor.
AT4G14550	*INDOLE-3-ACETIC ACID INDUCIBLE 14 (IAA14)*	IAA14 is a member of the Aux/IAA protein family. Involved in lateral root development. Gain of function mutation decreases auxin-inducible gene expression. Protein is localized to the nucleus. Expressed in stele and root tip epidermis. Functions as a negative regulator of ARF7/19.
AT4G14560	*INDOLE-3-ACETIC ACID INDUCIBLE 1 (IAA1)*	auxin (indole-3-acetic acid) induced gene (IAA1) encoding a short-lived nuclear-localized transcriptional regulator protein. The mRNA is cell-to-cell mobile.
AT4G14580	*CBL-INTERACTING PROTEIN KINASE 4 (CIPK4)*	CBL-interacting protein kinase
AT4G14590	*EMBRYO DEFECTIVE 2739 (emb2739)*	embryo defective 2739
AT4G14610		Probably not a pseudogene based on evidence for transcription (RNA-seq) and translation (Ribo-seq) described in PMID:27791167
AT4G14615		sporulation-specific protein
AT4G14630	*GERMIN-LIKE PROTEIN 9 (GLP9)*	germin-like protein with N-terminal signal sequence that may target it to the vacuole, plasma membrane and/or outside the cell. The mRNA is cell-to-cell mobile.
AT4G14640	*CALMODULIN 8 (CAM8)*	encodes a divergent member of calmodulin, which is an EF-hand family of Ca2+-binding proteins. This gene is expressed in leaves, flowers and siliques. The gene functionally complements yeast calmodulin 1 (CAM1). Also the protein does not form formed a complex with a basic amphiphilic helical peptide in the presence of Ca2+ in vitro. Authors suggest that this gene may represent a Ca2+-binding sensor protein that interacts with a more limited set of target proteins than do more conventional CaM isoforms.
AT4G14650		hypothetical protein
AT4G14670	*CASEIN LYTIC PROTEINASE B2 (CLPB2)*	This locus was originally annotated as encoding ClpB2 (also referred to as Hsp92.7), which belongs to the Casein lytic proteinase/heat shock protein 100 (Clp/Hsp100) family. However, according to Lee et al. (2007, Plant Journal, 49:115-127), there is no evidence for expression of an appropriate-sized mRNA from this locus. Re-annotation of the genome indicates that this locus potentially encodes a 68.8-kDa protein, containing only the N-terminal two thirds of the originally predicted open reading frame. This locus contains a 626-bp deletion in WS ecotype compared with the Col ecotype, which eliminates residues 1-86 of the predicted protein.
AT4G14680	*ATP-SULFURYLASE 3 (APS3)*	Encodes one of three A. thaliana ATP-sulfurylases. APS is the first enzyme of sulfate assimilation that catalyzes the formation of adenosine-5'-phosphosulfate from ATP and sulfate.
AT4G14690	*EARLY LIGHT-INDUCIBLE PROTEIN 2 (ELIP2)*	Encodes an early light-induced protein. ELIPs are thought not to be directly involved in the synthesis and assembly of specific photosynthetic complexes, but rather affect the biogenesis of all chlorophyll-binding complexes. A study (PMID 17553115) has shown that the chlorophyll synthesis pathway was downregulated as a result of constitutive ELIP2 expression, leading to decreased chlorophyll availability for the assembly of pigment-binding proteins for photosynthesis.
AT4G14695	*MITOCHONDRIAL PYRUVATE CARRIER 2 (MPC2)*	Uncharacterized protein family (UPF0041)
AT4G14716	*ACIREDUCTONE DIOXYGENASE 1 (ARD1)*	Encodes an acireductone dioxygenase that is activated by interaction with a heterotrimeric G protein beta subunit. It is a metalloenzyme and is likely involved in the methionine salvage pathway.
AT4G14720	*PEAPOD 2 (PPD2)*	PPD2 (and its paralog, PPD1) encode plant-specific putative DNA-binding proteins. Deletion of the PPD locus increases leaf lamina size and results in dome-shaped rather than flat leaves. Siliques are also altered in shape because of extra lamina growth.
AT4G14730	*LIFEGUARD 1 (LFG1)*	Stress induced membrane protein. Mutants show enhanced cell death under stress.
AT4G14750	*IQ-DOMAIN 19 (IQD19)*	Member of IQ67 (CaM binding) domain containing family.
AT4G14760	*NETWORKED 1B (NET1B)*	kinase interacting (KIP1-like) family protein
AT4G14770	*TESMIN/TSO1-LIKE CXC 2 (TCX2)*	Regulates fate transition and cell Divisions in the stomatal lineage.
AT4G14780		Protein kinase superfamily protein
AT4G14790	*(ATSUV3)*	encodes a nuclear-encoded DExH box RNA helicase, which is localized to mitochondria and whose in vitro ATPase activity is stimulated with mitochondrial RNA.
AT4G14810		hypothetical protein
AT4G14815	*GLYCOSYLPHOSPHATIDYLINOSITOL-ANCHORED LIPID PROTEIN TRANSFER 26 (LTPG26)*	Bifunctional inhibitor/lipid-transfer protein/seed storage 2S albumin superfamily protein
AT4G14850	*LOVASTATIN INSENSITIVE 1 (LOI1)*	Encodes a pentatricopeptide (PPR) protein that binds single-stranded RNA. The N-terminal portion of the protein can localize to the mitochondria. Mutations in this gene make plants less sensitive to inhibitors of the MEP and MVA pathways of isoprenoid biosynthesis and increase the activity of HMG CoA reductase.
AT4G14870	*(SECE1)*	Encodes a component of the thylakoid-localized Sec system involved in the translocation of cytoplasmic proteins into plastid. The mRNA is cell-to-cell mobile.
AT4G14890	*FERREDOXIN C 1 (FdC1)*	2Fe-2S ferredoxin-like superfamily protein

AT4G14930		Survival protein SurE-like phosphatase/nucleotidase
AT4G14940	*AMINE OXIDASE 1 (AO1)*	atao1 gene of Arabidopsis thaliana encodes an extracellular copper amine oxidase expressed during early stages of vascular tissue development.
AT4G14965	*MEMBRANE-ASSOCIATED PROGESTERONE BINDING PROTEIN 4 (MAPR4)*	membrane-associated progesterone binding protein 4
AT4G14980		Cysteine/Histidine-rich C1 domain family protein
AT4G14990	*PAT1 HOMOLOG 2 (PAT1H2)*	Topoisomerase II-associated protein PAT1
AT4G15010		Mitochondrial substrate carrier family protein
AT4G15020		hAT transposon superfamily
AT4G15030		folate-sensitive fragile site protein
AT4G15040		Subtilisin-like serine endopeptidase family protein
AT4G15050		NEP-interacting protein, putative (DUF239)
AT4G15060	*FOF2-LIKE 2 (FOL2)*	FBD, F-box/LRR protein
AT4G15093	*(LIGB)*	catalytic LigB subunit of aromatic ring-opening dioxygenase family
AT4G15100	*SERINE CARBOXYPEPTIDASE-LIKE 30 (scpl30)*	serine carboxypeptidase-like 30
AT4G15110	*CYTOCHROME P450, FAMILY 97, SUBFAMILY B, POLYPEPTIDE 3 (CYP97B3)*	member of CYP97B
AT4G15120		VQ motif-containing protein
AT4G15130	*PHOSPHORYLCHOLINE CYTIDYLYLTRANSFERASE2 (CCT2)*	phosphorylcholine cytidylyltransferase2
AT4G15140		hypothetical protein
AT4G15150		glycine-rich protein
AT4G15160	*(PAC2)*	According to PMID:14623884, PAC2 has not been correctly annotated; At4g15160 (protease inhibitor/seed storage/lipid transfer protein (LTP) family protein) containsAtPAC2(nucleotides 3269-3831).
AT4G15190		
AT4G15200	*FORMIN 3 (FH3)*	Actin nucleation factor that directs the formation of actin cables in pollen tubes. Involved in cytoplasmic streaming and polarized growth in pollen tubes.
AT4G15210	*BETA-AMYLASE 5 (BAM5)*	cytosolic beta-amylase expressed in rosette leaves and inducible by sugar. RAM1 mutants have reduced beta amylase in leaves and stems.
AT4G15230	*ATP-BINDING CASSETTE G30 (ABCG30)*	pleiotropic drug resistance 2
AT4G15240		glycosyltransferase (DUF604)
AT4G15250	*B-BOX DOMAIN PROTEIN 9 (BBX9)*	B-box type zinc finger protein with CCT domain-containing protein
AT4G15260		UDP-Glycosyltransferase superfamily protein
AT4G15280	*UDP-GLUCOSYL TRANSFERASE 71B5 (UGT71B5)*	UDP-glucosyl transferase 71B5
AT4G15290	*(ATCSLB05)*	Encodes a gene similar to cellulose synthase. Mutants exhibit shorter root hairs under phosphate-deficient conditions.
AT4G15300	*CYTOCHROME P450, FAMILY 702, SUBFAMILY A, POLYPEPTIDE 2 (CYP702A2)*	cytochrome P450, family 702, subfamily A, polypeptide 2
AT4G15310	*CYTOCHROME P450, FAMILY 702, SUBFAMILY A, POLYPEPTIDE 3 (CYP702A3)*	a member of the cytochrome P450 gene family. molecular function unknown.
AT4G15320	*CELLULOSE SYNTHASE-LIKE B6 (CSLB06)*	encodes a gene similar to cellulose synthase
AT4G15330	*CYTOCHROME P450, FAMILY 705, SUBFAMILY A, POLYPEPTIDE 1 (CYP705A1)*	cytochrome P450, family 705, subfamily A, polypeptide 1
AT4G15340	*PENTACYCLIC TRITERPENE SYNTHASE 1 (PEN1)*	Encodes a protein that catalyzes the production of the tricyclic triterpene arabidiol when expressed in yeast.
AT4G15350	*CYTOCHROME P450, FAMILY 705, SUBFAMILY A, POLYPEPTIDE 2 (CYP705A2)*	member of CYP705A
AT4G15360	*CYTOCHROME P450, FAMILY 705, SUBFAMILY A, POLYPEPTIDE 3 (CYP705A3)*	member of CYP705A
AT4G15370	*BARUOL SYNTHASE 1 (BARS1)*	Encodes an oxidosqualene cyclase that primarily produces the tetracyclic triterpene baruol in vitro and when expressed in yeast. It can also make 22 other minor triterpenoid products with varying numbers of rings.
AT4G15380	*CYTOCHROME P450, FAMILY 705, SUBFAMILY A, POLYPEPTIDE 4 (CYP705A4)*	member of CYP705A
AT4G15390		HXXXD-type acyl-transferase family protein
AT4G15400	*BRASSINOSTEROID INACTIVATOR1 (BIA1)*	Encodes BIA1, a member of the BAHD acyltransferase family. Plays a role in controlling brassinosteroids levels, particularly in the root and hypocotyl in darkness.
AT4G15420		Ubiquitin fusion degradation UFD1 family protein
AT4G15430		ERD (early-responsive to dehydration stress) family protein
AT4G15440	*HYDROPEROXIDE LYASE 1 (HPL1)*	Encodes a hydroperoxide lyase. Also a member of the CYP74B cytochrome p450 family. In the ecotype Columbia (Col) the gene contains a 10-nucleotide deletion in its first exon that causes it to code for a truncated protein that results in a non-functional hydroperoxide lyase.
AT4G15460		glycine-rich protein
AT4G15480	*(UGT84A1)*	Encodes a protein that might have sinapic acid:UDP-glucose glucosyltransferase activity.
AT4G15490	*(UGT84A3)*	Encodes a protein that might have sinapic acid:UDP-glucose glucosyltransferase activity.
AT4G15500	*(UGT84A4)*	Encodes a protein that might have sinapic acid:UDP-glucose glucosyltransferase activity.
AT4G15510	*PSBP-DOMAIN PROTEIN1 (PPD1)*	A nuclear-encoded thylakoid lumenal protein that is associated with photosystem I (PSI) but is not an integral subunit of PSI. It is an PSI assembly factor that assists the proper folding and integration of PsaB and PsaA into the thylakoid membrane. It is involved in the assembly of PSI reaction center heterodimer core.
AT4G15520		tRNA/rRNA methyltransferase (SpoU) family protein
AT4G15530	*PYRUVATE ORTHOPHOSPHATE DIKINASE (PPDK)*	Encodes a dual-targeted protein believed to act as a pyruvate, orthophosphate dikinase. These enzymes are normally associated with C4 photosynthesis which does not occur in Arabidopsis. However, PPDK may play a role in remobilizing nitrogen during leaf senescence in Arabidopsis. The product of the long transcript (.1 gene model) was shown to be targeted to the chloroplast, whereas the shorter transcript (no targeting sequence) accumulates in the cytosol. The two proteins were also found to be expressed in slightly different tissues.
AT4G15550	*INDOLE-3-ACETATE BETA-D-GLUCOSYLTRANSFERASE (IAGLU)*	UDP-glucose:indole-3-acetate beta-D-glucosyltransferase

AT4G15560	*CLOROPLASTOS ALTERADOS 1 (CLA1)*	Encodes a protein with 1-deoxyxylulose 5-phosphate synthase activity involved in the MEP pathway. It is essential for chloroplast development in Arabidopsis
AT4G15570	*MAGATAMA 3 (MAA3)*	Similar to yeast Sen1 (splicing endonuclease 1)helicase protein. Involved in female gametophyte development. The mRNA is cell-to-cell mobile.
AT4G15590		transposable_element_gene;non-LTR retrotransposon family (LINE), has a 1.5e-50 P-value blast match to GB:AAA67727 reverse transcriptase (LINE-element) (Mus musculus);(source:TAIR10)
AT4G15610	*CASP-LIKE PROTEIN 1D1 (CASPL1D1)*	Uncharacterized protein family (UPF0497)
AT4G15620	*CASP-LIKE PROTEIN 1E2 (CASPL1E2)*	Uncharacterized protein family (UPF0497)
AT4G15630	*CASP-LIKE PROTEIN 1E1 (CASPL1E1)*	Uncharacterized protein family (UPF0497)
AT4G15640		adenylyl cyclase
AT4G15650		kinase-like protein
AT4G15660	*(GRXS8)*	Encodes a member of the CC-type glutaredoxin (ROXY) family. Represses transcriptional and developmental responses to nitrate. Operates downstream of cytokinins in a signal transduction pathway that negatively regulates plant primary root growth in response to nitrate.Interacts with the TGA1 and TGA4 transcription factors, which are central regulators of early transcriptional responses to nitrate in root.
AT4G15680	*(GRXS4)*	Encodes a member of the CC-type glutaredoxin (ROXY) family. Operates downstream of cytokinins in a signal transduction pathway that negatively regulates plant primary root growth in response to nitrate.
AT4G15690	*(GRXS5)*	Encodes a member of the CC-type glutaredoxin (ROXY) family that has been shown to interact with the transcription factor TGA2 and suppress ORA59 promoter activity. Operates downstream of cytokinins in a signal transduction pathway that negatively regulates plant primary root growth in response to nitrate.
AT4G15700	*GLUTAREDOXIN 3 (GRXS3)*	Encodes a member of the CC-type glutaredoxin (ROXY) family. Operates downstream of cytokinins in a signal transduction pathway that negatively regulates plant primary root growth in response to nitrate.
AT4G15710		cystatin-like protein
AT4G15720	*REQUIRED FOR EFFICIENCY OF MITOCHONDRIAL EDITING 2 (REME2)*	Tetratricopeptide repeat (TPR)-like superfamily protein
AT4G15740		Calcium-dependent lipid-binding (CaLB domain) family protein
AT4G15750	*(PMEI13)*	Plant invertase/pectin methylesterase inhibitor superfamily protein
AT4G15760	*MONOOXYGENASE 1 (MO1)*	Encodes a protein with similarity to monooxygenases that are known to degrade salicylic acid (SA).
AT4G15770		RNA binding protein
AT4G15780	*VESICLE-ASSOCIATED MEMBRANE PROTEIN 724 (VAMP724)*	member of VAMP72 Gene Family
AT4G15810		P-loop containing nucleoside triphosphate hydrolases superfamily protein
AT4G15830		ARM repeat superfamily protein
AT4G15850	*RNA HELICASE 1 (RH1)*	plant DEAD box-like RNA helicase.
AT4G15860		transposable_element_gene;copia-like retrotransposon family, has a 2.3e-43 P-value blast match to dbj|BAA78425.1| polyprotein (Arabidopsis thaliana) (AtRE1) (Ty1_Copia-element);(source:TAIR10)
AT4G15870	*TERPENE SYNTHASE 1 (TS1)*	encodes a putative terpene synthase
AT4G15900	*PLEIOTROPIC REGULATORY LOCUS 1 (PRL1)*	Mutations confer hypersensitivity to glucose and sucrose and augments sensitivity to cytokinin, ethylene, ABA and auxin. Encodes a nuclear WD40 protein that is imported into the nucleus. Essential for plant innate immunity. Interacts with MOS4 and AtCDC5. It is also predicted to have two DWD motifs. It can bind to DDB1a in Y2H assays, and DDB1b in co-IP assays, and may be involved in the formation of a CUL4-based E3 ubiquitin ligase, and may affect the stability of AKIN10.
AT4G15910	*DROUGHT-INDUCED 21 (DI21)*	encodes a gene whose transcript level in root and leaves increases to progressive drought stress. The transcript level is also affected by changes of endogenous or exogenous abscisic acid level. It appears to be a member of plant-specific gene family that includes late embryo-abundant and zinc- IAA-induced proteins in other plants. The mRNA is cell-to-cell mobile.
AT4G15920	*(SWEET17)*	Encodes a vacuolar fructose transporter expressed in parenchyma and xylem that controls leaf fructose content. When its expression is reduced, fructose accumulates in leaves.
AT4G15930		Dynein light chain type 1 family protein
AT4G15940	*FUMARYLACETOACETATE HYDROLASE DOMAIN CONTAINING PROTEIN 1A (FAHD1A)*	Fumarylacetoacetate hydrolase homolog; involved in seed redox regulation and to affect seed quality traits such as seed thermo-dormancy and longevity.
AT4G15975	*ARABIDOPSIS T??XICOS EN LEVADURA 17 (ATL17)*	RING/U-box superfamily protein
AT4G15980		ProPME pectin methylesterase
AT4G16000		hypothetical protein
AT4G16010		transposable_element_gene;copia-like retrotransposon family, has a 3.9e-185 P-value blast match to GB:AAA57005 Hopscotch polyprotein (Ty1_Copia-element) (Zea mays);(source:TAIR10)
AT4G16030		Ribosomal protein L19e family protein
AT4G16040		transmembrane protein
AT4G16050		Aminotransferase-like, plant mobile domain family protein
AT4G16080	*DUF295 ORGANELLAR A 8 (ATDOA8)*	hypothetical protein (DUF295)
AT4G16090		hypothetical protein
AT4G16110	*RESPONSE REGULATOR 2 (RR2)*	Encodes a pollen-specific transcription factor involved in the expression of nuclear genes for components of mitochondrial complex I in Arabidopsis. Acts in concert with other type-B ARRs in the cytokinin signaling pathway. AHK3 mediates cytokinin-induced phosphorylation of ARR2 on the Asp-80 residue. This phosphorylation plays a positive role of ARR2 in cytokinin-mediated control of leaf longevity. Also involved in cytokinin-dependent inhibition of hypocotyl elongation.
AT4G16140		proline-rich family protein

AT4G16143	*IMPORTIN ALPHA ISOFORM 2 (IMPA-2)*	Protein interacts with Agrobacterium proteins VirD2 and VirE2. Is not individually essential for Agrobacterium-mediated root transformation, but when overexpressed can rescue the impa-4 decreased transformation susceptibility phenotype.
AT4G16146		cAMP-regulated phosphoprotein 19-related protein
AT4G16150	*CALMODULIN-BINDING TRANSCRIPTION ACTIVATOR 2 (CAMTA5)*	CATMA5 is a transcriptional activator. It acts in the cold response pathway, it can bind to and activate the expression of DREB1 genes.
AT4G16155		dihydrolipoamide dehydrogenase
AT4G16160	*(ATOEP16-2)*	Homologous to pea OEP16 and barley pPORA (OEP16), a member of Arabidopsis OEP16 family. Two OEP16 genes are closely related to each other and are conserved in all land plants, OEP16-2, also named OEP16-S, and OEP16-1 (renamed OEP16-L) are result of the gene duplication event that occurred prior to divergence of bryophytes and seed plants. Predominantly expressed in seed and is not inducible by cold treatment. atOEP16-S gained an additional exon. The promoter region of atOEP16-S (but not atOEP16-L) contains multiple G-box ABA-responsive elements. The atOEP16-S promoter conferred developmentally regulated seed- and pollen-specific GUS expression in tobacco.
AT4G16180		transmembrane protein
AT4G16190		Papain family cysteine protease
AT4G16210	*ENOYL-COA HYDRATASE/ISOMERASE A (ECHIA)*	enoyl-CoA hydratase/isomerase A
AT4G16230	*GDSL-TYPE ESTERASE/LIPASE 79 (GELP79)*	GDSL-motif esterase/acyltransferase/lipase. Enzyme group with broad substrate specificity that may catalyze acyltransfer or hydrolase reactions with lipid and non-lipid substrates.
AT4G16240		hypothetical protein
AT4G16250	*PHYTOCHROME D (PHYD)*	Encodes a phytochrome photoreceptor with a function similar to that of phyB that absorbs the red/far-red part of the light spectrum and is involved in light responses. It cannot compensate for phyB loss in Arabidopsis but can substitute for tobacco phyB in vivo.
AT4G16260		Encodes a putative beta-1,3-endoglucanase that interacts with the 30C02 cyst nematode effector. May play a role in host defense.
AT4G16270	*PEROXIDASE40 (PRX40)*	Encodes a class III peroxidase that is genetically redundant with PRX9, expressed in the tapetum, and essential for proper anther and pollen development.
AT4G16310	*LSD1-LIKE 3 (LDL3)*	FAD-dependent lysine-specific histone demethylase involved in the control of flowering time.
AT4G16340	*SPIKE1 (SPK1)*	Encodes SPIKE1 (SPK1), the lone DOCK family guanine nucleotide exchange factor (GEF) in Arabidopsis. SPK1 is a peripheral membrane protein that accumulates at, and promotes the formation of, a specialized domain of the endoplasmic reticulum (ER) termed the ER exit site (ERES). SPK1 promotes polarized growth and cell-cell adhesion in the leaf epidermis. Mutant has seedling lethal; cotyledon, leaf-shape, trichome defects.
AT4G16350	*CALCINEURIN B-LIKE PROTEIN 6 (CBL6)*	Calcium sensor protein. Binds CIPK14.
AT4G16370	*OLIGOPEPTIDE TRANSPORTER (OPT3)*	Encodes a phloem-specific iron transporter that is essential for systemic iron signaling and redistribution of iron and cadmium. It loads iron into the phloem, facilitates iron recirculation from the xylem to the phloem, and regulates both shoot-to-root iron signaling and iron redistribution from mature to developing tissues.
AT4G16380	*HEAVY METAL ASSOCIATED PROTEIN 35 (ATHMP35)*	Heavy metal transport/detoxification superfamily protein
AT4G16390	*SUPPRESSOR OF VARIEGATION 7 (SVR7)*	Encodes a pentatricopeptide repeat protein, SVR7 (SUPPRESSOR OF VARIEGATION7), required for FtsH-mediated chloroplast biogenesis. It is involved in accumulation and translation of chloroplast ATP synthase subunits.
AT4G16400		transmembrane protein
AT4G16410		transmembrane protein
AT4G16420	*HOMOLOG OF YEAST ADA2 2B (ADA2B)*	Transcriptional co-activator. Essential for the developmental switch from cell proliferation to cell differentiation in response to variations in auxin and cytokinin concentrations. Part of SAGA complex. Regulates gene expression by affecting histone H3 acetylation.
AT4G16442	*CASP-LIKE PROTEIN 2B1 (CASPL2B1)*	Uncharacterized protein family (UPF0497)
AT4G16447		hypothetical protein
AT4G16460		zinc finger CCCH domain protein
AT4G16470		Tetratricopeptide repeat (TPR)-like superfamily protein
AT4G16480	*INOSITOL TRANSPORTER 4 (INT4)*	Encodes a high affinity H+:<i>myo</i>-inositol symporter. The only other compound shown to be transported was pinitol, a methylated derivative of <i>myo</i>-inositol. The mRNA is cell-to-cell mobile.
AT4G16490		ARM repeat superfamily protein
AT4G16500	*PHYTOCYSTATIN 4 (CYS4)*	Cystatin/monellin superfamily protein
AT4G16515	*ROOT MERISTEM GROWTH FACTOR 6 (RGF6)*	Encodes a root meristem growth factor (RGF). Belongs to a family of functionally redundant homologous peptides that are secreted, tyrosine-sulfated, and expressed mainly in the stem cell area and the innermost layer of central columella cells. RGFs are required for maintenance of the root stem cell niche and transit amplifying cell proliferation. Members of this family include: At5g60810 (RGF1), At1g13620 (RGF2), At2g04025 (RGF3), At3g30350 (RGF4), At5g51451 (RGF5), At4g16515 (RGF6), At3g02240 (RGF7), At2g03830 (RGF8) and At5g64770 (RGF9).
AT4G16563		Eukaryotic aspartyl protease family protein
AT4G16566	*HISTIDINE TRIAD NUCLEOTIDE-BINDING 4 (HINT4)*	Encodes a protein that has an unexpected bifunctional capability in vitro. The purified enzyme has adenylylsulfate sulfohydrolase activity (E.C. 3.6.2.1) and ADP-sulfurylase activity (E.C. 2.7.7.5). The latter is activated at low pH. The enzyme can exert it phosphorylase activity on a range of related substrates in vitro, but it acts best with APS (adenosine 5'-phsophosulfate). This protein appears to function as a homodimer.
AT4G16590	*CELLULOSE SYNTHASE-LIKE A01 (CSLA01)*	encodes a gene similar to cellulose synthase
AT4G16600	*PLANT GLYCOGENIN-LIKE STARCH INITIATION PROTEIN 8 (PGSIP8)*	Nucleotide-diphospho-sugar transferases superfamily protein
AT4G16620	*USUALLY MULTIPLE ACIDS MOVE IN AND OUT TRANSPORTERS 8 (UMAMIT8)*	nodulin MtN21-like transporter family protein
AT4G16630		DEA(D/H)-box RNA helicase family protein
AT4G16640	*(AT1-MMP)*	Matrix metalloprotease.
AT4G16680		P-loop containing nucleoside triphosphate hydrolases superfamily protein

AT4G16690	*METHYL ESTERASE 16 (MES16)*	Encodes a protein shown to have carboxylesterase activity, methyl IAA esterase activity, and methyl jasmonate esterase activity in vitro. This protein does not act on MeSA, MeGA4, or MEGA9 in vitro. Although MES16 is similar to MES17, a MeIAA hydrolase, two mes16 mutant lines (SALK_151578) and (SALK_139756) do not show altered sensitivity to MeIAA in root growth assays. MES16 transcripts appear to be more than 10-fold less abundant than those of MES17 in roots.
AT4G16700	*PHOSPHATIDYLSERINE DECARBOXYLASE 1 (PSD1)*	Encodes a mitochondrial phosphatidylserine decarboxylase. Expressed mainly in roots and flowers.
AT4G16710		glycosyltransferase family protein 28
AT4G16730	*TERPENE SYNTHASE 02 (TPS02)*	In the Col ecotype, no functional protein is encoded at this locus due to the presence of a two-base (AT) insertion 184 nucleotides downstream of the start codon leading to a frame shift and premature translational termination. However, in the Ws ecotype, a functional terpene synthase that localizes to the chloroplast is encoded at this locus. It can catalyze the synthesis of (E)-beta-ocimene and (E,E)-alpha farnesene in vitro, but, it has more activity as an (E)-beta-ocimene synthase activity in vivo. This may reflect the greater availability of the GPP precursor of (E)-beta-ocimene than of the FPP precursor of (E,E)-alpha-farnesene in the chloroplasts where the Ws TPS02 is present.
AT4G16740	*TERPENE SYNTHASE 03 (TPS03)*	Encodes an (E,E)-alpha-farnesene synthase in the Col ecotype of Arabidopsis. This enzyme can also catalyze the formation of (E)-beta-ocimene as well as trace amounts of myrcene and other related compounds in vitro. The cytosolic localization of the protein may make it favor (E,E)-alpha-farnesene biosynthesis because the precursor of this product, FPP, is primarily cytosolic. Transcript levels for this gene increase in response to treatment with the jasmonic acid mimic coronalon or in response to the insect <i>Plutella xylostella</i>. TPS03 transcripts can also be detected in flowers. A similar protein from the C24 ecotype with one amino acid change (S267F) has a different substrate specificity.
AT4G16745		Exostosin family protein
AT4G16750	*ETHYLENE-RESPONSIVE TRANSCRIPTION FACTOR 39 (ERF39)*	encodes a member of the DREB subfamily A-4 of ERF/AP2 transcription factor family. The protein contains one AP2 domain. There are 17 members in this subfamily including TINY.
AT4G16760	*ACYL-COA OXIDASE 1 (ACX1)*	Encodes a medium to long-chain acyl-CoA oxidase. Catalyzes the first step of fatty acid beta-oxidation. Involved in jasmonate biosynthesis. Gene expression is induced by wounding, drought stress, abscisic acid, and jasmonate.
AT4G16770		2-oxoglutarate (2OG) and Fe(II)-dependent oxygenase superfamily protein
AT4G16790		hydroxyproline-rich glycoprotein family protein
AT4G16800		3-methylglutaconyl-CoA hydratase localized to mitochondria. Knockout displays accelerated senescence when subjected to extended dark conditions;knockout senescing leaves and knockout seeds accumulate leu, ile, and val.
AT4G16820	*PHOSPHOLIPASE A I BETA 2 (PLA-I[beta]2)*	Encodes a lipase that hydrolyzes phosphatidylcholine, glycolipids as well as triacylglycerols.
AT4G16830	*(ATRGGA)*	Encodes a perinuclear and cytoplasmically localized mRNA binding protein. AtRGGA is likely involved in stress responsivness. It is induced by salt and osmotic stress and loss of function mutations are more sensitive to stress. The mRNA is cell-to-cell mobile.
AT4G16840		transmembrane protein
AT4G16850	*(KSK)*	6-transmembrane (6TM) protein that underlies a QTL for petal size with increased expression correlating to increased petal size.
AT4G16860	*RECOGNITION OF PERONOSPORA PARASITICA 4 (RPP4)*	Confers resistance to Peronospora parasitica. RPP4 is coordinately regulated by transcriptional activation and RNA silencing.
AT4G16870		transposable_element_gene;copia-like retrotransposon family, has a 0. P-value blast match to dbj|BAA78426.1| polyprotein (AtRE2-1) (Arabidopsis thaliana) (Ty1_Copia-element);(source:TAIR10)
AT4G16880	*MONLP1-TRIGGERED CELL DEATH ON CHROMOSOME 4 (NTCD4)*	LRR-only protein, secreted into the apoplast; promotes NLP-triggered cell death and disease susceptibility by facilitating oligomerization of NLP.
AT4G16890	*SUPPRESSOR OF NPR1-1, CONSTITUTIVE 1 (SNC1)*	Encodes a Toll Interleukin1 receptor-nucleotide binding-Leu- rich repeat-type resistance gene (TIR-NB-LRR-type) involved in the salicylic acid-dependent defense response pathway. Mutant plants constitutively express pathogenesis-related (PR) genes and are pathogen resistant. Resistance signaling in snc1 requires EDS1, MOS3 and PAD4.
AT4G16900		Disease resistance protein (TIR-NBS-LRR class) family
AT4G16910		transposable_element_gene;gypsy-like retrotransposon family, has a 6.1e-185 P-value blast match to GB:AAD27547 polyprotein (Gypsy_Ty3-element) (Oryza sativa subsp. indica);(source:TAIR10)
AT4G16920		Disease resistance protein (TIR-NBS-LRR class) family
AT4G16930		Toll-Interleukin-Resistance (TIR) domain-containing protein
AT4G16950	*RECOGNITION OF PERONOSPORA PARASITICA 5 (RPP5)*	Contains a putative nucleotide binding site and leucine-rich repeats. Similar to the plant resistance genes N and L6, and to the toll and interleukin-1 receptors. Confers resistance to Peronospora parasitica.Redundant function together with SIKIC1 and 3 in SNC1-mediated autoimmunity. Protein levels controlled by MUSE1 and MUSE2.
AT4G16960	*SIDEKICK SNC1 3 (SIKIC3)*	Redundant function together with SIKIC1 and 2 in SNC1-mediated autoimmunity. Protein levels controlled by MUSE1 and MUSE2.
AT4G16980		arabinogalactan-protein family
AT4G16985		
AT4G16990	*RESISTANCE TO LEPTOSPHAERIA MACULANS 3 (RLM3)*	disease resistance protein (TIR-NBS class)
AT4G17000		neurofilament heavy protein
AT4G17020		transcription factor-like protein
AT4G17030	*EXPANSIN-LIKE B1 (EXLB1)*	Encodes EXLB1 (expansin-like B1), a member of the expansin family.
AT4G17050	*UREIDOGLYCINE AMINOHYDROLASE (UGLYAH)*	Encodes a protein with ureidoglycine aminohydrolase activity.
AT4G17070		peptidyl-prolyl cis-trans isomerase
AT4G17080		Histone H3 K4-specific methyltransferase SET7/9 family protein
AT4G17090	*CHLOROPLAST BETA-AMYLASE (CT-BMY)*	Encodes a beta-amylase targeted to the chloroplast. Transgenic BMY8 RNAi lines fail to accumulate maltose during cold shock suggesting that maltose accumulation coincides with BMY8 expression. Apart from maltose, the sugar content of the RNAi lines were similar to wildtype (glucose and sucrose unaffected).BAM3 activity declines 2 and 4 days after start of cold stress despite an increase in transcript levels. BAM3 activity has a lower temperature optimum than BAM1 (PMID:25293962).
AT4G17100		poly(U)-specific endoribonuclease-B protein

AT4G17120		
AT4G17130		
AT4G17150		alpha/beta-Hydrolases superfamily protein
AT4G17160	*RAB GTPASE HOMOLOG B1A (RABB1a)*	RAB GTPase homolog B1A
AT4G17190	*FARNESYL DIPHOSPHATE SYNTHASE 2 (FPS2)*	Encodes a protein with farnesyl diphosphate synthase activity, which catalyzes the rate limiting step in isoprenoid biosynthesis. Its mRNA is most abundantly expressed in flowers.
AT4G17200		F-box and associated interaction domains-containing protein
AT4G17215		Pollen Ole e 1 allergen and extensin family protein
AT4G17220	*MICROTUBULE-ASSOCIATED PROTEINS 70-5 (MAP70-5)*	Encodes a microtubule associated protein (MAP70-5). Regulates secondary wall patterning in wood cells. Expressed in all tissues.
AT4G17230	*SCARECROW-LIKE 13 (SCL13)*	Encodes a scarecrow-like protein (SCL13). Member of GRAS gene family. Regulated by heat shock.
AT4G17240		structural maintenance of chromosomes protein
AT4G17245	*ARABIDOPSIS T??XICOS EN LEVADURA 18 (ATL18)*	RING/U-box superfamily protein
AT4G17250		transmembrane protein
AT4G17260		Lactate/malate dehydrogenase family protein
AT4G17280		Auxin-responsive family protein
AT4G17310		hypothetical protein
AT4G17340	*TONOPLAST INTRINSIC PROTEIN 2;2 (TIP2;2)*	tonoplast intrinsic protein 2
AT4G17360		encodes one of the two putative formyltetrahydrofolate deformylase. Located in the mitochondrion. Involved in photorespiratory tetrahydrofolate cycle.
AT4G17380	*MUTS-LIKE PROTEIN 4 (MSH4)*	Encodes the Arabidopsis homolog of MSH4, a meiosis-specific member of the MutS-homolog family of genes. It is expressed only in floral tissues and only during early meiotic prophase I, preceding the synapsis of homologous chromosomes. It is involved in the early steps of recombination.
AT4G17410	*PARAQUAT TOLERANCE3 (PQT3)*	PQT3 is a nuclear localized E3 ligase involved in negative regulation of stress tolerance.PRMT4b is a substrate of PQT3.
AT4G17460	*(HAT1)*	Encodes a class II HD-ZIP protein that regulates meristematic activity in different tissues, and that it is necessary for the correct formation of the gynoecium.
AT4G17470	*CA2+-ACTIVATED RELA-SPOT HOMOLOG (CRSH)*	alpha/beta-Hydrolases superfamily protein
AT4G17480		alpha/beta-Hydrolases superfamily protein
AT4G17483		alpha/beta-Hydrolases superfamily protein
AT4G17490	*ETHYLENE RESPONSIVE ELEMENT BINDING FACTOR 6 (ERF6)*	Encodes a member of the ERF (ethylene response factor) subfamily B-3 of ERF/AP2 transcription factor family (ATERF-6). The protein contains one AP2 domain. There are 18 members in this subfamily including ATERF-1, ATERF-2, AND ATERF-5. It is involved in the response to reactive oxygen species and light stress. Involved in regulating root architecture and the response to cold stress.
AT4G17500	*ETHYLENE RESPONSIVE ELEMENT BINDING FACTOR 1 (ERF-1)*	Encodes a member of the ERF (ethylene response factor) subfamily B-3 of ERF/AP2 transcription factor family (ATERF-1). The protein contains one AP2 domain. There are 18 members in this subfamily including ATERF-1, ATERF-2, AND ATERF-5. The mRNA is cell-to-cell mobile.
AT4G17505	*DUF239 (DUF239)*	carboxyl-terminal proteinase-like protein (DUF239)
AT4G17550	*GLYCEROL-3-PHOSPHATE PERMEASE 4 (G3Pp4)*	Encodes a member of the phosphate starvation-induced glycerol-3-phosphate permease gene family: AT3G47420(G3Pp1), AT4G25220(G3Pp2), AT1G30560(G3Pp3), AT4G17550(G3Pp4) and AT2G13100(G3Pp5).
AT4G17560		Ribosomal protein L19 family protein
AT4G17570	*GATA TRANSCRIPTION FACTOR 26 (GATA26)*	Encodes a member of the GATA factor family of zinc finger transcription factors.
AT4G17585		aluminum activated malate transporter family protein
AT4G17590		NOL1/NOP2/sun family protein
AT4G17600	*LIGHT-HARVESTING-LIKE 3:1 (LIL3:1)*	Encodes Lil3:1 (light-harvesting-like) protein. Belongs to the Lhc super-gene family encodes the light-harvesting chlorophyll a/b-binding (LHC) proteins that constitute the antenna system of the photosynthetic apparatus. A generic LHC motif is present in Lil3:1. The mRNA is cell-to-cell mobile.
AT4G17610	*TRNA METHYLTRANSFERASE 3 (TRM3)*	tRNA/rRNA methyltransferase (SpoU) family protein
AT4G17650		Polyketide cyclase / dehydrase and lipid transport protein
AT4G17660	*PBS1-LIKE 20 (PBL20)*	Protein kinase superfamily protein
AT4G17670		senescence-associated family protein (DUF581)
AT4G17690		Peroxidase superfamily protein
AT4G17695	*KANADI 3 (KAN3)*	Homeodomain-like superfamily protein
AT4G17700		hypothetical protein
AT4G17710	*HOMEODOMAIN GLABROUS 4 (HDG4)*	Encodes a homeobox-leucine zipper family protein belonging to the HD-ZIP IV family.
AT4G17730	*SYNTAXIN OF PLANTS 23 (SYP23)*	member of SYP2 Gene Family. Together with SYP23 interacts with Tobacco mosaic virus 126 kDa protein; required for normal local virus accumulation and spread.
AT4G17740	*(CTPA)*	Cleaves the C-terminal extension of the D1 precursor (pD1) to form mature D1; initiation of the formation of the oxygenic D1/D2 type PSII.
AT4G17760		PCNA domain-containing protein
AT4G17770	*TREHALOSE PHOSPHATASE/SYNTHASE 5 (TPS5)*	Encodes an enzyme putatively involved in trehalose biosynthesis. The protein has a trehalose synthase (TPS)-like domain that may or may not be active as well as a trehalose phosphatase (TPP)-like domain. Phosphorylated TPS5 extracted from Arabidopsis cells binds directly to 14-3-3 isoforms. The mRNA is cell-to-cell mobile.
AT4G17785	*MYB DOMAIN PROTEIN 39 (MYB39)*	Encodes a putative transcription factor (MYB39) involved in the regulation of suberin biosynthetic genes.
AT4G17790		SNARE associated Golgi protein family
AT4G17800	*AT-HOOK MOTIF NUCLEAR LOCALIZED PROTEIN 23 (AHL23)*	Putative AT-hook DNA-binding family protein

AT4G17810	*ZINC FINGER PROTEIN 1 (ZP1)*	C2H2 domain regulatory protein. Functions downstream of GL2 during root hair development and regulates expression of targets RDH6, RSL2 and RSL4.
AT4G17840	*BALANCE OF CHLOROPHYLL METABOLISM 2 (BCM2)*	CAAX protease self-immunity protein
AT4G17860		carboxyl-terminal proteinase-like protein, putative (DUF239)
AT4G17870	*PYRABACTIN RESISTANCE 1 (PYR1)*	Encodes a member of the PYR (pyrabactin resistance)/PYL(PYR1-like)/RCAR (regulatory components of ABA receptor) family proteins with 14 members. PYR/PYL/RCAR family proteins function as abscisic acid sensors. Mediate ABA-dependent regulation of protein phosphatase 2Cs ABI1 and ABI2.
AT4G17880	*(MYC4)*	MYC4 is bHLH transcriptional regulator. It functions as a JAZ-interacting transcription factor that acts together with MYC2 and MYC3 to activate JA-responses. It also functions in blue light mediated secondary cell wall biogenesis via regulation of NST1 expression. MYC4 directly binds to NST1 promoter and activates its expression.
AT4G17900	*(PLATZ11)*	PLATZ transcription factor family protein
AT4G17910		transferases, transferring acyl groups
AT4G17930		
AT4G17940		Tetratricopeptide repeat (TPR)-like superfamily protein
AT4G17960		phospholipid hydroperoxide glutathione peroxidase
AT4G17970	*ALUMINUM-ACTIVATED, MALATE TRANSPORTER 12 (ALMT12)*	Anion transporter involved in stomatal closure. Gene has 3 splicing variants.
AT4G17980	*NAC DOMAIN CONTAINING PROTEIN 71 (NAC071)*	Encodes ANAC071, a transcription factor involved in cell proliferation in incised inflorescence stems.
AT4G17990		hypothetical protein
AT4G18000		
AT4G18010	*INOSITOL(1,4,5)P3 5-PHOSPHATASE II (IP5PII)*	Encodes an inositol polyphosphate 5-phosphatase that appears to have Type I activity. It can dephosphorylate IP3(inositol(1,4,5)P3) and IP4 (inositol(1,3,4,5)P4), but it does not appear to be active against phosphatidylinositol 4,5 bisphosphate. Overexpression of this gene renders plants insensitive to ABA in germination and growth assays.
AT4G18020	*(APRR2)*	Encodes pseudo-response regulator 2 (APRR2) that interacts with a calcium sensor (CML9).
AT4G18050	*ATP-BINDING CASSETTE B9 (ABCB9)*	P-glycoprotein 9
AT4G18070		suppressor
AT4G18080		transmembrane protein
AT4G18090		hypothetical protein
AT4G18120	*MEI2-LIKE 3 (ML3)*	A member of mei2-like gene family, predominantly plant-based family of genes encoding RNA binding proteins with characteristic presence of a highly conserved RNA binding motif first described in the mei2 gene of the fission yeast S. pombe. In silico analyses reveal nine mei2 -like genes in A. thaliana. They were grouped into four distinct clades, based on overall sequence similarity and subfamily-specific sequence elements. AML3 is a member of two sister clades of mei2-like gene family, AML1 through AML5, and belongs to the clade named ALM235. Among mei2-like genes, AML3 is the transcript with highest frequency of alternative splicing. Expression was detected during early embryo development (heart and torpedo stage); no accumulation was detected in vegetative and floral apices, as revealed by in situ hybridization.
AT4G18130	*PHYTOCHROME E (PHYE)*	member of Histidine Kinase
AT4G18140	*SCP1-LIKE SMALL PHOSPHATASE 4B (SSP4b)*	Encodes a SCP1-like small phosphatase (SSP). Three SSPs form a unique group with long N-terminal extensions: AT5G46410 (SSP4), AT5G11860 (SSP5), AT4G18140 (SSP4b). SSP4 and SSP4b were localized exclusively in the nuclei, whereas SSP5 accumulated in both nuclei and cytoplasm. All three SSPs encodes active CTD phosphatases like animal SCP1 family proteins, with distinct substrate specificities: SSP4 and SSP4b could dephosphorylate both Ser2-PO(4) and Ser5-PO(4) of CTD, whereas SSP5 dephosphorylated only Ser5-PO(4).
AT4G18170	*WRKY DNA-BINDING PROTEIN 28 (WRKY28)*	Member of WRKY Transcription Factor; Group II-c. Involved in the activation of salicylic acid biosynthesis genes ICS1 and PBS3. In the ovule, it is expressed in hypodermal somatic cells and appears to play a role in supression of megasporocyte cell fate. In the leaf if is upstream of FHY3 and regulates light-mediated leaf senescence.
AT4G18180		Pectin lyase-like superfamily protein
AT4G18210	*PURINE PERMEASE 10 (PUP10)*	Member of a family of proteins related to PUP1, a purine transporter. May be involved in the transport of purine and purine derivatives such as cytokinins, across the plasma membrane. The mRNA is cell-to-cell mobile.
AT4G18250		receptor Serine/Threonine kinase-like protein
AT4G18260		Cytochrome b561/ferric reductase transmembrane protein family
AT4G18270	*TRANSLOCASE 11 (TRANS11)*	Encodes protein similar to similar to bacterial translocase I (mra Y). Expressed during flower bud development.
AT4G18280	*GLYCINE RICH CELL WALL PROTEIN LIKE 1 (GRPL1)*	Encodes a gene whose transcription is induced in response to potassium-deficiency,drought, heat and salt stress. Overexpression increases tolerance to stressors.
AT4G18290	*POTASSIUM CHANNEL IN ARABIDOPSIS THALIANA 2 (KAT2)*	Encodes KAT2, a member of the Shaker family potassium ion (K+) channel. Critical to stomatal opening induced by blue light. Critical to circadian rhythm of stomatal opening. Involved in plant development in response to high light intensity. Under high light intensity, the mutant plant produced less biomass compared to the wild type. The Shaker family K+ ion channels include five groups based on phylogenetic analysis (FEBS Letters (2007) 581: 2357): I (inward rectifying channel): AKT1 (AT2G26650), AKT5 (AT4G32500) and SPIK (also known as AKT6, AT2G25600); II (inward rectifying channel): KAT1 (AT5G46240) and KAT2 (AT4G18290); III (weakly inward rectifying channel): AKT2 (AT4G22200); IV (regulatory subunit involved in inwardly rectifying conductance formation): KAT3 (also known as AtKC1, AT4G32650); V (outward rectifying channel): SKOR (AT3G02850) and GORK (AT5G37500).
AT4G18330		Translation elongation factor EF1A/initiation factor IF2gamma family protein
AT4G18340		Glycosyl hydrolase superfamily protein
AT4G18350	*NINE-CIS-EPOXYCAROTENOID DIOXYGENASE 2 (NCED2)*	Encodes 9-<i>cis</i>-epoxycarotenoid dioxygenase, a key enzyme in the biosynthesis of abscisic acid. The expression of this gene declines during the first 12h of imbibition.

AT4G18370	*DEGRADATION OF PERIPLASMIC PROTEINS 5 (DEG5)*	Encodes DEG5. Forms a hexamer with DEG8 in the thylakoid lumen. Involved in the cleavage of photodamaged D2 protein of photosystem II (PSII).
AT4G18390	*TEOSINTE BRANCHED 1, CYCLOIDEA AND PCF TRANSCRIPTION FACTOR 2 (TCP2)*	TEOSINTE BRANCHED 1, cycloidea and PCF transcription factor 2
AT4G18395		hypothetical protein
AT4G18410		transposable_element_gene;Mutator-like transposase family, has a 8.7e-30 P-value blast match to GB:AAA21566 mudrA of transposon=MuDR (MuDr-element) (Zea mays);(source:TAIR10)
AT4G18420		transposable_element_gene;similar to unknown protein [Arabidopsis thaliana] (TAIR:AT3G42810.1);(source:TAIR10)
AT4G18425	*DUF679 DOMAIN MEMBRANE PROTEIN 4 (DMP4)*	transmembrane protein, putative (DUF679)
AT4G18430	*RAB GTPASE HOMOLOG A1E (RABA1e)*	RAB GTPase homolog A1E
AT4G18440		L-Aspartase-like family protein
AT4G18450		encodes a member of the ERF (ethylene response factor) subfamily B-3 of ERF/AP2 transcription factor family. The protein contains one AP2 domain. There are 18 members in this subfamily including ATERF-1, ATERF-2, AND ATERF-5.
AT4G18460		D-Tyr-tRNA(Tyr) deacylase family protein
AT4G18470	*SUPPRESSOR OF NPR1-1, INDUCIBLE 1 (SNI1)*	Negative regulator of systemic acquired resistance (SAR), repressor of pathogenesis-related PR gene expression which is removed by NPR1 upon induction of SAR. Encodes leucine-rich nuclear protein. Conserved in plants, with putative orthologs found in several plant species. Many NPR1-dependent PR gene are specifically derepressed in the sni1 mutant. The structural similarity of SNI1 to Armadillo repeat proteins implies that SNI1 may form a scaffold for interaction with proteins that modulate transcription. Histone modification may be involved in SNI1-mediated repression of PR genes.SNI1 is the NSE6 subunit of the SMC5/6 complex. It can interact with and inhibit E2F transcription factors.
AT4G18480	*(CHLI1)*	Encodes the CHLI subunit of magnesium chelatase which is required for chlorophyll biosynthesis. All four cysteine residues of the protein form two disulfide bonds (Cys102-Cys193 and Cys354-Cys396) under oxidized conditions but are fully reduced by reduction. It was suggested that the redox state of CHLI is regulated in vivo by the change of the redox environment in the chloroplasts probably via the Trx system.
AT4G18500		hypothetical protein
AT4G18510	*CLAVATA3/ESR-RELATED 2 (CLE2)*	CLE2, putative ligand, member of large gene family homologous to Clavata3
AT4G18530	*STICKY GENERATIVE CELL (SGC)*	lysine ketoglutarate reductase trans-splicing-like protein, putative (DUF707)
AT4G18540		transmembrane protein
AT4G18550	*DAD1-LIKE SEEDING ESTABLISHMENT-RELATED LIPASE (DSEL)*	DSEL is cytosolic acylhydrolase that shows prefential lipase activity against the sn-1 position of several classes of lipids, including 1,3-diacylglycerols and 1-monoacylglycerols. Overexpression of DSEL leads to increased peroxisome and oil body levels in cotyledons and reduced beta-oxidation activity in seedlings.
AT4G18560		
AT4G18570	*INCREASED PETAL GROWTH ANISOTROPY 1 (IPGA1)*	Microtubule associated protein involved in cortical microtubule organization.
AT4G18580		hypothetical protein
AT4G18610	*LIGHT SENSITIVE HYPOCOTYLS 9 (LSH9)*	LIGHT-DEPENDENT SHORT HYPOCOTYLS-like protein (DUF640)
AT4G18630		hypothetical protein (DUF688)
AT4G18640	*MORPHOGENESIS OF ROOT HAIR 1 (MRH1)*	Required for root hair elongation during tip growth. The mRNA is cell-to-cell mobile.
AT4G18650	*DOG1-LIKE 4 (DOGL4)*	A maternally expressed imprinted gene in the endosperm. It's expression is positively regulated by ROS1.
AT4G18670	*LEUCINE-RICH REPEAT EXTENSIN 5 (LRX5)*	Leucine rich extensin protein involved in cell wall biogenesis and organization. Interacts with several members of the RALF family of ligand peptides.
AT4G18700	*CBL-INTERACTING PROTEIN KINASE 12 (CIPK12)*	Encodes CBL-interacting protein kinase 12 (CIPK12).
AT4G18710	*BRASSINOSTEROID-INSENSITIVE 2 (BIN2)*	Encodes BIN2, a member of the ATSK (shaggy-like kinase) family. BIN2 functions in the cross-talk between auxin and brassinosteroid signaling pathways. BIN2 regulates root epidermal cell fate specification by phosphorylating EGL3 and TTG1. BIN2-mediated phosphorylation appears to promote BZR1 export from the nucleus. KIB1 interacts with BIN2 blocking its interaction with substrates and promotes BIN2 degradation.
AT4G18720		Transcription factor IIS protein
AT4G18740		Rho termination factor
AT4G18750	*DEFECTIVELY ORGANIZED TRIBUTARIES 4 (DOT4)*	Encodes a pentatricopeptide (PPR) protein involved in leaf and root development. dot4 mutants have an aberrant midgap venation pattern in juvenile leaves and cotyledons.
AT4G18770	*MYB DOMAIN PROTEIN 98 (MYB98)*	MYB98 is a member of the R2R3-MYB gene family, the members of which likely encode transcription factors. Within an ovule, MYB98 is expressed exclusively in the synergid cells, and mutations in this gene affect the female gametophyte specifically. myb98 female gametophytes are affected in two unique features of the synergid cell, pollen tube guidance and the filiform apparatus, but are otherwise normal. This suggests that MYB98 controls the development of specific features within the synergid cell during female gametophyte development. MYB98 also is expressed in trichomes and endosperm. Homozygous myb98 mutants exhibit no sporophytic defects, including trichome and endosperm defects.
AT4G18780	*IRREGULAR XYLEM 1 (IRX1)*	Encodes a member of the cellulose synthase family involved in secondary cell wall biosynthesis. Mutants have abnormal xylem formation, reduced cellulose content, and enhanced drought and osmotic stress tolerance. Mediates resistance towards bacterial pathogens via ABA. Confers resistance towards bacterial and fungal pathogens, independent of salicylic acid, ethylene and jasmonate signaling.
AT4G18790	*(NRAMP5)*	member of Nramp2 family
AT4G18810		NAD(P)-binding Rossmann-fold superfamily protein
AT4G18820		AAA-type ATPase family protein
AT4G18850		
AT4G18860		hypothetical protein
AT4G18870		E2F/DP family winged-helix DNA-binding domain-containing protein
AT4G18880	*HEAT SHOCK TRANSCRIPTION FACTOR A4A (HSF A4A)*	Encodes a member of Heat Stress Transcription Factor(Hsf) family that is a substrate of the MPK3/MPK6 signaling and regulates stress responses.
AT4G18900		Transducin/WD40 repeat-like superfamily protein

AT4G18920		histone acetyltransferase (DUF1264)
AT4G18930		RNA ligase/cyclic nucleotide phosphodiesterase family protein
AT4G18960	*AGAMOUS (AG)*	Floral homeotic gene encoding a MADS domain transcription factor. Specifies floral meristem and carpel and stamen identity. Binds CArG box sequences. It is the only C function gene. It interacts genetically with the other homeotic genes to specify the floral organs.
AT4G18970	*(GGL22)*	GDSL-motif esterase/acyltransferase/lipase. Enzyme group with broad substrate specificity that may catalyze acyltransfer or hydrolase reactions with lipid and non-lipid substrates.
AT4G18980	*ATS40-3 (AtS40-3)*	Encodes a nuclear-targeted protein AtS40-3 that modulates senescence associated gene expression.
AT4G18990	*XYLOGLUCAN ENDOTRANSGLUCOSYLASE/HYDROLASE 29 (XTH29)*	xyloglucan endotransglucosylase/hydrolase 29
AT4G19000	*(IWS2)*	The C-terminal portion of this protein has homology to the C-termini of the IWS1 (Interacts With Spt6) proteins found in yeast and humans.
AT4G19010		Encodes for a 4-coumarate-CoA ligase involved in the biosynthesis of the benzenoid ring of ubiquinone from phenylalanine.
AT4G19020	*CHROMOMETHYLASE 2 (CMT2)*	Encodes a plant DNA methyltransferase that methylates mainly cytosines in CHH (H = any base but G) contexts. It is involved in heat tolerance.
AT4G19030	*NOD26-LIKE MAJOR INTRINSIC PROTEIN 1 (NLM1)*	an aquaporin whose expression level is reduced by ABA, NaCl, dark, and desiccation. is expressed at relatively low levels under normal conditions. Also functions in arsenite transport and tolerance.
AT4G19095		hypothetical protein
AT4G19100	*PHOTOSYNTHESIS AFFECTED MUTANT 68 (PAM68)*	Protein of unknown function (DUF3464) involved in photosystem II assembly.
AT4G19120	*EARLY-RESPONSIVE TO DEHYDRATION 3 (ERD3)*	S-adenosyl-L-methionine-dependent methyltransferases superfamily protein
AT4G19130	*REPLICATION PROTEIN A 1E (RPA1E)*	Replication factor-A protein 1-like protein
AT4G19160		transglutaminase family protein
AT4G19170	*NINE-CIS-EPOXYCAROTENOID DIOXYGENASE 4 (NCED4)*	Encodes a chloroplast-targeted member of a family of enzymes similar to nine-cis-epoxycarotenoid dioxygenase that acts as a major regulator of carotenoid degradation during dark-induced leaf senescence.. The mRNA is cell-to-cell mobile.
AT4G19185	*USUALLY MULTIPLE ACIDS MOVE IN AND OUT TRANSPORTERS 2 (UMAMIT2)*	nodulin MtN21-like transporter family protein
AT4G19190		zinc knuckle (CCHC-type) family protein
AT4G19200	*GLYCINE AND PROLINE RICH PROTEIN 3 (GPRP3)*	Glycine and proline rich protein.Mutants have increased size.
AT4G19230	*CYTOCHROME P450, FAMILY 707, SUBFAMILY A, POLYPEPTIDE 1 (CYP707A1)*	Encodes a protein with ABA 8'-hydroxylase activity, involved in ABA catabolism. Member of the CYP707A gene family. CYP707A1 appears to play an important role in determining the ABA levels in dry seeds. Gene involved in postgermination growth. Overexpression of CYP707A1 leads to a decrease in ABA levels and a reduction in after-ripening period to break dormancy.
AT4G19240		hypothetical protein
AT4G19260		Galactose oxidase/kelch repeat superfamily protein
AT4G19300		transposable_element_gene;similar to unknown protein [Arabidopsis thaliana] (TAIR:AT5G36050.1);(source:TAIR10)
AT4G19320		transposable_element_gene;similar to unknown protein [Arabidopsis thaliana] (TAIR:AT1G52087.1);(source:TAIR10)
AT4G19330	*COLD TEMPERATURE GERMINATING 10 (CTG10)*	Encodes a Kelch Repeat F-box protein involved in seed germination. In the current Araport11 annotation, At4g19330 is misannotated and is actually comprised of two genes with the more 3′ gene being intronless and producing a transcript encoding the F-box protein. The other encodes a ribonucleoprotein (10.1016/j.plgene.2017.05.012).
AT4G19370	*MODIFYING WALL LIGNIN-2 (MWL-2)*	chitin synthase, putative (DUF1218)
AT4G19380		Long-chain fatty alcohol dehydrogenase family protein
AT4G19390		Uncharacterized protein family (UPF0114)
AT4G19410	*PECTIN ACETYLESTERASE 7 (PAE7)*	Pectinacetylesterase family protein
AT4G19420	*PECTIN ACETYLESTERASE 8 (PAE8)*	Pectinacetylesterase family protein
AT4G19430		hypothetical protein
AT4G19440		Tetratricopeptide repeat (TPR)-like superfamily protein
AT4G19450		Major facilitator superfamily protein
AT4G19460		UDP-Glycosyltransferase superfamily protein
AT4G19470		Leucine-rich repeat (LRR) family protein
AT4G19480		hypothetical protein
AT4G19500		nucleoside-triphosphatase/transmembrane receptor/nucleotide binding/ATP binding protein
AT4G19510		Disease resistance protein (TIR-NBS-LRR class)
AT4G19520		disease resistance protein (TIR-NBS-LRR class) family
AT4G19530		Encodes a TIR-NB-LRR resistance protein. Transient expression in tobacco induces cell death.
AT4G19550		zinc ion binding / transcription regulator
AT4G19570		Chaperone DnaJ-domain superfamily protein
AT4G19580		DNAJ heat shock N-terminal domain-containing protein
AT4G19600	*(CYCT1;4)*	Encodes a cyclin T partner CYCT1;4. Plays important roles in infection with Cauliflower mosaic virus (CaMV). The mRNA is cell-to-cell mobile.
AT4G19620		hypothetical protein
AT4G19670		RING/U-box superfamily protein
AT4G19680	*IRON REGULATED TRANSPORTER 2 (IRT2)*	encodes an iron transporter whose expression is induced by iron and zinc deficiency. Gene is specifically expressed in the external cell layers of the root subapical zone.
AT4G19690	*IRON-REGULATED TRANSPORTER 1 (IRT1)*	The gene encodes Fe2+ transporter protein. It is a member of the Zrt/Irt-like protein (ZIP) family of transporters. AtIRT1 has broad specificity for divalent heavy metals, mediating the transport of zinc, manganese, cobalt and cadmium under Fe-deficient conditions. IRT1 is monoubiquitinated to promote endocytic trafficking. The mRNA is cell-to-cell mobile.
AT4G19720		Glycosyl hydrolase family protein with chitinase insertion domain-containing protein

AT4G19730		Glycosyl hydrolase superfamily protein
AT4G19740		Glycosyl hydrolase superfamily protein
AT4G19750		Glycosyl hydrolase family protein with chitinase insertion domain-containing protein
AT4G19770		Glycosyl hydrolase family protein with chitinase insertion domain-containing protein
AT4G19780		transposable_element_gene;copia-like retrotransposon family, has a 1.0e-72 P-value blast match to GB:CAA72990 open reading frame 2 (Ty1_Copia-element) (Brassica oleracea);(source:TAIR10)
AT4G19790		transposable_element_gene;copia-like retrotransposon family, has a 1.1e-45 P-value blast match to GB:CAA72989 open reading frame 1 (Ty1_Copia-element) (Brassica oleracea);(source:TAIR10)
AT4G19810	*CLASS V CHITINASE (ChiC)*	ChiC encodes a Class V chitinase that is a part of glycoside hydrolase family 18 based on CAZy groupings. It appears to primarily act as an exochitinase in vitro where it predominantly cleaves a chitobiose (GlcNAc)2 residue from the non-reducing end of a chitin oligosaccharide. However, it shows some minor endochitinase activity in vitro, as well. A putative 24 amino-acid signal peptide may direct this protein to the secretory system and it has been detected in cell wall apoplastic fluid. RT-PCR experiments demonstrate that ChiC transcript levels are increased in response to abscisisc acid, jasmonic acid, and NaCl stress. Microarray results also suggest that transcript levels rise in response to osmotic stress, two fungal pathogens, a bacterial pathogen, and the elicitor flagellin. The mRNA is cell-to-cell mobile.
AT4G19820		Glycosyl hydrolase family protein with chitinase insertion domain-containing protein
AT4G19840	*PHLOEM PROTEIN 2-A1 (PP2-A1)*	encodes a phloem lectin, similar to phloem lectin in cucumber and celery. Gene is expressed in the phloem, predominantly in the companion cells. The mRNA is cell-to-cell mobile.
AT4G19850	*PHLOEM PROTEIN 2-A2 (PP2-A2)*	encodes a protein similar to phloem protein 2 in cucumber. a member of a large gene family.
AT4G19860		Encodes a cytosolic calcium-independent phospholipase A.
AT4G19870		Galactose oxidase/kelch repeat superfamily protein
AT4G19880		Glutathione S-transferase family protein
AT4G19900		alpha 1,4-glycosyltransferase family protein
AT4G19920		Toll-Interleukin-Resistance (TIR) domain family protein
AT4G19940		F-box and associated interaction domains-containing protein
AT4G19960	*K+ UPTAKE PERMEASE 9 (KUP9)*	Encodes a potassium ion transmembrane transporter. Also mediates cesium uptake when expressed in E. coli. The mRNA is cell-to-cell mobile.
AT4G19980		hypothetical protein
AT4G19985	*GCN5‐RELATED N‐ACETYLTRANSFERASE 3 (GNAT3)*	Acyl-CoA N-acyltransferases (NAT) superfamily protein
AT4G19990	*FAR1-RELATED SEQUENCE 1 (FRS1)*	FAR1-related sequence 1
AT4G20000		VQ motif-containing protein
AT4G20010	*PLASTID TRANSCRIPTIONALLY ACTIVE 9 (PTAC9)*	Organellar Single-stranded DNA Binding protein. Decreases MMEJ on long ssDNA templates.
AT4G20020	*MULTIPLE ORGANELLAR RNA EDITING FACTOR 1 (MORF1)*	Encodes a protein involved in RNA editing in mitochondria. Member of MORF family consisting of of nine full-length proteins encoded in the nuclear genome. MORF proteins are required for all RNA editing events in plastids and for many, possibly also all, sites in mitochondria. Potential link between the RNA binding PPR protein and the protein contributing the enzymatic activity in RNA editing.
AT4G20050	*QUARTET 3 (QRT3)*	Encodes a polygalacturonase that plays a direct role in degrading the pollen mother cell wall during microspore development.
AT4G20070	*ALLANTOATE AMIDOHYDROLASE (AAH)*	The gene encoding Arabidopsis thaliana Allantoate Amidohydrolase (AtAAH)which catalyzes the allantoate deiminase reaction (EC 3.5.3.9)is expressed in all parts of the plant being consistent with a function in purine turnover in Arabidopsis. The mRNA is cell-to-cell mobile.
AT4G20080	*MULTIPLE C2 DOMAIN AND TRANSMEMBRANE REGION PROTEIN 11 (MCTP11)*	Calcium-dependent lipid-binding (CaLB domain) plant phosphoribosyltransferase family protein
AT4G20090	*EMBRYO DEFECTIVE 1025 (EMB1025)*	Pentatricopeptide repeat (PPR) superfamily protein
AT4G20100		PQ-loop repeat family protein / transmembrane family protein
AT4G20110	*VACUOLAR SORTING RECEPTOR 7 (VSR7)*	VACUOLAR SORTING RECEPTOR 7
AT4G20140	*GASSHO1 (GSO1)*	Encodes GASSHO1 (GSO1), a putative leucine-rich repeat transmembrane-type receptor kinase. GSO1 and a homolog GSO2 (At5g44700) are required for the formation of a normal epidermal surface during embryogenesis. Necessary for localizing CASPARIAN STRIP DOMAIN PROTEINS (CASPs) - major players of endodermal differentiation - into an uninterrupted, ring-like domain.
AT4G20160		golgin family A protein
AT4G20170	*GALACTAN SYNTHASE 3 (GALS3)*	glycosyltransferase family protein (DUF23)
AT4G20200		Terpenoid cyclases/Protein prenyltransferases superfamily protein
AT4G20210	*TERPENE SYNTHASE 8 (TPS08)*	Terpenoid cyclases/Protein prenyltransferases superfamily protein
AT4G20220		Reverse transcriptase (RNA-dependent DNA polymerase)
AT4G20230	*TERPENE SYNTHASE 30 (TPS30)*	terpenoid synthase superfamily protein
AT4G20240	*CYTOCHROME P450, FAMILY 71, SUBFAMILY A, POLYPEPTIDE 27 (CYP71A27)*	putative cytochrome P450
AT4G20250		hypothetical protein
AT4G20260	*PLASMA-MEMBRANE ASSOCIATED CATION-BINDING PROTEIN 1 (PCAP1)*	Encodes a Ca2+ and Cu2+ binding protein. N-terminal myristylation on glycine 2 appears to enable it to associate tightly with the plasma membrane. Recombinant PCaP1 interacts strongly with phosphatidylinositol 3,5-bisphosphate (PtdIns(3,5)P2) and PtdIns (3,4,5)P3, and weakly with PtdIns(3,5)P2 and PtdIns(4,5). It also interacts with calmodulin (CaM) in a calcium-dependent manner. CaM does not interfere with PCaP1 membrane localization but does weaken interactions between it and the PtdInsPs. PCaP1 has an apparent Kd of 10 uM for Cu2+ and can bind six ions per protein. Transcript levels for PCaP1 first fall and then rise following exposure to CuCl2. Mannitol, sorbitol, and the flg22 oligopeptide also increase expression levels. The mRNA is cell-to-cell mobile.
AT4G20270	*BARELY ANY MERISTEM 3 (BAM3)*	Encodes a CLAVATA1-related receptor kinase-like protein required for both shoot and flower meristem function. It has a broad expression pattern and is involved in vascular strand development in the leaf, control of leaf shape, size and symmetry, male gametophyte development and ovule specification and function. The mRNA is cell-to-cell mobile.

AT4G20290		transmembrane protein
AT4G20320	*CTP SYNTHASE 4 (CTPS4)*	Cytidine triphosphate synthase.
AT4G20330		Transcription initiation factor TFIIE, beta subunit
AT4G20350	*ALKB HOMOLOG 6 (ALKBH6)*	Putative RNA demethylase involved in seed germination, seedling growth and abiotic stress responses.
AT4G20360	*RAB GTPASE HOMOLOG E1B (ATRABE1B)*	Nuclear transcribed, plastid localized EF-Tu translation elongation factor. Referred to as AtRabE1b in DOI:10.1104/pp.013052. However, wider community usage and more publications assign the symbol RabE1b to At5g59840.
AT4G20370	*TWIN SISTER OF FT (TSF)*	Encodes a floral inducer that is a homolog of FT. Plants overexpressing this gene flower earlier than Col. Loss-of-function mutations flower later in short days. TSF and FT play overlapping roles in the promotion of flowering, with FT playing the dominant role and together playing an antagonistic role to TFL1 in the determination of inflorescence meristem identity. .TSF sequences show extensive variation in different accessions and may contribute to quantitative variation in flowering time in these accessions. TSF has a complex pattern of spatial expression; it is expressed mainly in phloem and expression is regulated by daylength and vernalization.
AT4G20390	*CASP-LIKE PROTEIN 1B2 (CASPL1B2)*	Uncharacterized protein family (UPF0497)
AT4G20420		Tapetum specific protein TAP35/TAP44
AT4G20430		Subtilase family protein
AT4G20460		NAD(P)-binding Rossmann-fold superfamily protein
AT4G20470		transmembrane protein
AT4G20490		transposable_element_gene;gypsy-like retrotransposon family, has a 1.5e-29 P-value blast match to GB:AAD11615 prpol (gypsy_Ty3-element) (Zea mays);(source:TAIR10)
AT4G20500		transposable_element_gene;similar to filament protein-related [Arabidopsis thaliana] (TAIR:AT4G20730.1);(source:TAIR10)
AT4G20510		transposable_element_gene;similar to filament protein-related [Arabidopsis thaliana] (TAIR:AT4G20730.1);(source:TAIR10)
AT4G20530		cysteine-rich repeat secretory-like protein
AT4G20770		Pentatricopeptide repeat (PPR) superfamily protein
AT4G20780	*CALMODULIN LIKE 42 (CML42)*	Calcium sensor involved in trichome branching.
AT4G20790		Leucine-rich repeat protein kinase family protein
AT4G20800	*(ATBBE17)*	FAD-binding Berberine family protein
AT4G20820	*(ATBBE18)*	FAD-binding Berberine family protein
AT4G20830	*(ATBBE19)*	Encodes an oligogalacturonide oxidase that inactivates the elicitor-active oligogalacturonides (OGs). It is involved in plant immunity. Overexpressing plants are more resistant to B. cinerea.
AT4G20840	*(ATBBE21)*	Encodes an oligogalacturonide oxidase that inactivates the elicitor-active oligogalacturonides (OGs).
AT4G20860	*(ATBBE22)*	involved in the generation of H2O2 from reduced compounds
AT4G20880		ethylene-responsive nuclear protein / ethylene-regulated nuclear protein (ERT2)
AT4G20890	*TUBULIN BETA-9 CHAIN (TUB9)*	tubulin 9 The mRNA is cell-to-cell mobile.
AT4G20900	*MALE-STERILE 5 (MS5)*	Encodes a tetratricopeptide repeat protein required for cell cycle exit after meiosis II.ms5 mutants are male sterile, pollen tetrads undergo an extra round of division after meiosis II without chromosome replication, resulting in chromosome abnormalities. Gene product has some similarity to SCP1, a rat synaptonemal complex protein.
AT4G20940	*GUARD CELL HYDROGEN PEROXIDE-RESISTANT 1 (GHR1)*	Encodes a plasma-membrane localized LRR receptor-like protein involved in both ABA and H202 mediated signaling involved in stomatal movement. TAIR10 annotation for this gene has a low confidence score (2-star). See Comments field for structural annotation by the community.
AT4G20950		
AT4G20960	*PYRIMIDINE DEAMINASE (PYRD)*	encodes diaminohydroxyphosphoribosylaminopyrimidine deaminase catalyzing the second step in the riboflavin biosynthesis
AT4G20990	*ALPHA CARBONIC ANHYDRASE 4 (ACA4)*	alpha carbonic anhydrase 4
AT4G21020		Late embryogenesis abundant protein (LEA) family protein
AT4G21050	*DNA-BINDING WITH ONE FINGER 4.4 (DOF4.4)*	Encodes a transcriptional activator involved in shoot branching and silique development. Involved in shoot regenaration from root explants.
AT4G21060	*AGP GALACTOSYLTRANSFERASE 2 (GALT2)*	Encodes an endomembrane system-localized, hydroxyproline-O-galactosyltransferase specific for arabinogalactan-protein biosynthesis.
AT4G21070	*BREAST CANCER SUSCEPTIBILITY1 (BRCA1)*	Encodes AtBRCA1, an ortholog of the human breast cancer susceptibility gene 1. Contains one N-terminal RING finger, two C-terminal BRCT and the p300/CBP interacting domain. Strongly induced by gamma rays, consistent with a putative role in DNA repair and in cell cycle control.
AT4G21080	*DNA-BINDING WITH ONE FINGER 4.5 (DOF4.5)*	Dof-type zinc finger domain-containing protein
AT4G21090	*MITOCHONDRIAL FERREDOXIN 2 (MFDX2)*	MITOCHONDRIAL FERREDOXIN 2
AT4G21120	*AMINO ACID TRANSPORTER 1 (AAT1)*	Encodes a member of the cationic amino acid transporter (CAT) subfamily of amino acid polyamine choline transporters. Mediates efficient uptake of Lys, Arg and Glu in a yeast system. The mRNA is cell-to-cell mobile.
AT4G21130	*EMBRYO DEFECTIVE 2271 (EMB2271)*	similar to man and yeast U3-55K genes, involved in processing of pre-ribosomal RNA.
AT4G21140		copper ion-binding protein
AT4G21170		Tetratricopeptide repeat (TPR)-like superfamily protein
AT4G21190	*EMBRYO DEFECTIVE 1417 (emb1417)*	Pentatricopeptide repeat (PPR) superfamily protein
AT4G21200	*GIBBERELLIN 2-OXIDASE 8 (GA2OX8)*	Encodes a protein with gibberellin 2-oxidase activity which acts specifically on C-20 gibberellins.
AT4G21210	*PPDK REGULATORY PROTEIN (RP1)*	Encodes a PPDK regulatory protein that has both protein kinase and protein phosphatase activities towards PPDK (pyruvate orthophosphate dikinase).
AT4G21230	*CYSTEINE-RICH RLK (RECEPTOR-LIKE PROTEIN KINASE) 27 (CRK27)*	Encodes a cysteine-rich receptor-like protein kinase.
AT4G21240		F-box and associated interaction domains-containing protein
AT4G21270	*KINESIN 1 (ATK1)*	Encodes a kinesin-like motor protein heavy chain. Loss of function mutations have reduced fertility and are defective in spindle formation in male meiosis.
AT4G21280	*PHOTOSYSTEM II SUBUNIT QA (PSBQA)*	Encodes the PsbQ subunit of the oxygen evolving complex of photosystem II.

AT4G21300	*(ATDEK36)*	Tetratricopeptide repeat (TPR)-like superfamily protein
AT4G21310	*DESIGUAL 2 (DEAL2)*	DUF1218 family member.
AT4G21320	*HEAT-STRESS-ASSOCIATED 32 (HSA32)*	Encodes heat-stress-associated 32-kD protein. Up-regulated by heat shock. Thermotolerance in a knockout mutant was compromised following a long recovery period (> 24 h) after acclimation heat shock treatment.
AT4G21323		Subtilase family protein
AT4G21340	*(B70)*	basic helix-loop-helix (bHLH) DNA-binding superfamily protein
AT4G21350	*PLANT U-BOX 8 (PUB8)*	Encodes a U-box/ARM repeat protein required fore self-incompatibility.
AT4G21360		transposable_element_gene;copia-like retrotransposon family, has a 0. P-value blast match to GB:CAA31653 polyprotein (Ty1_Copia-element) (Arabidopsis thaliana);(source:TAIR10)
AT4G21370	*PSEUDOGENE OF S-LOCUS RECEPTOR KINASE A (PSEUDOSRKA)*	The Col-0 pseudoSRKA allele contains a frameshift mutation that introduces a premature stop codon within the fourth of seven exons found in SRK genes. Its SCR sequences consist of several truncated pseudoSCR sequences, the longest of which is designated pseudoSCR1.
AT4G21380	*RECEPTOR KINASE 3 (RK3)*	encodes a putative receptor-like serine/threonine protein kinases that is similar to Brassica self-incompatibility (S) locus. Expressed in root. Shoot expression limited to limited to the root-hypocotyl transition zone and at the base of lateral roots as well as in axillary buds, and pedicels.
AT4G21390	*(B120)*	S-locus lectin protein kinase family protein
AT4G21400	*CYSTEINE-RICH RLK (RECEPTOR-LIKE PROTEIN KINASE) 28 (CRK28)*	Encodes a cysteine-rich receptor-like protein kinase.
AT4G21410	*CYSTEINE-RICH RLK (RECEPTOR-LIKE PROTEIN KINASE) 29 (CRK29)*	Encodes a cysteine-rich receptor-like protein kinase.
AT4G21420		transposable_element_gene;gypsy-like retrotransposon family, has a 6.9e-06 P-value blast match to GB:BAA84458 GAG-POL precursor (gypsy_Ty3-element) (Oryza sativa)gi|5902445|dbj|BAA84458.1| GAG-POL precursor (Oryza sativa (japonica cultivar-group)) (RIRE2) (Gypsy_Ty3-family);(source:TAIR10)
AT4G21430	*(JMJ28)*	protein B160
AT4G21440	*MYB-LIKE 102 (MYB102)*	Encodes a MYB transcription factor involved in wounding and osmotic stress response. Member of the R2R3 factor gene family.
AT4G21480	*SUGAR TRANSPORTER PROTEIN 12 (STP12)*	Putative sugar transporter. Expressed in nematode-induced root syncytia.
AT4G21490	*NAD(P)H DEHYDROGENASE B3 (NDB3)*	NAD(P)H dehydrogenase B3
AT4G21500		transmembrane protein
AT4G21510	*F-BOX STRESS INDUCED 2 (FBS2)*	F-box family protein; mutant is hypersensitive to salt stress.
AT4G21580		oxidoreductase, zinc-binding dehydrogenase family protein
AT4G21590	*ENDONUCLEASE 3 (ENDO3)*	Encodes a putative endonuclease but no demonstrable endonuclease activity, either towards single stranded DNA or mismatches, has been seen in vitro. Activated by AGAMOUS in a cal-1, ap1-1 background. Expressed in the floral meristem and during stamen development.
AT4G21600	*ENDONUCLEASE 5 (ENDO5)*	Encodes a protein with mismatch-specific endonuclease activity with a preference for T/G, A/G, and G/G of single base mismatches. It also has the ability to cleave indel types of mismatches (heteroduplexes with loops).
AT4G21610	*LSD ONE LIKE 2 (LOL2)*	Contains the same novel zinc finger motif with LSD1, a negative regulator of cell death and defense response. Due to differential splicing, it encodes two different proteins, one of which contains an additional, putative DNA binding motif. Northern analysis demonstrated that LOL2 transcripts containing the additional DNA binding motif are predominantly upregulated after treatment with both virulent and avirulent Pseudomonas syringae pv maculicola strains.
AT4G21620		glycine-rich protein
AT4G21630		Subtilase family protein
AT4G21640		Subtilase family protein
AT4G21650	*SUBTILASE 3.13 (SBT3.13)*	Subtilase family protein
AT4G21680	*NRT1/ PTR FAMILY 7.2 (NPF7.2)*	Encodes a nitrate transporter (NRT1.8). Functions in nitrate removal from the xylem sap. Mediates cadmium tolerance.
AT4G21690	*GIBBERELLIN 3-OXIDASE 3 (GA3OX3)*	gibberellin 3-oxidase 3
AT4G21710	*(NRPB2)*	Encodes the unique second-largest subunit of DNA-dependent RNA polymerase II; the ortholog of budding yeast RPB2 and a homolog of the E. coli RNA polymerase beta subunit.
AT4G21730		pseudogene of N-ethylmaleimide sensitive factor (NSF)
AT4G21750	*MERISTEM LAYER 1 (ATML1)*	Encodes a homeobox protein similar to GL2. It is expressed in both the apical and basal daughter cells of the zygote as well as their progeny. Expression is detected starting the two-celled stage of embryo development and is later restricted to the outermost, epidermal cell layer from its inception. Its promoter is highly modular with each region contributing to specific aspects of the gene's spatial and temporal expression. Double mutant analysis with PDF2, another L1-specific gene, suggests that their functions are partially redundant and the absence of both of the genes result in abnormal shoot development.
AT4G21780		hypothetical protein
AT4G21820		binding / calmodulin binding protein
AT4G21830	*METHIONINE SULFOXIDE REDUCTASE B7 (MSRB7)*	methionine sulfoxide reductase B7
AT4G21850	*METHIONINE SULFOXIDE REDUCTASE B9 (MSRB9)*	methionine sulfoxide reductase B9
AT4G21860	*METHIONINE SULFOXIDE REDUCTASE B 2 (MSRB2)*	2-Cys methionine sulfoxide reductase.
AT4G21865		hypothetical protein
AT4G21870		HSP20-like chaperone
AT4G21880		Pentatricopeptide repeat (PPR) superfamily protein
AT4G21910		MATE efflux family protein
AT4G21940	*CALCIUM-DEPENDENT PROTEIN KINASE 15 (CPK15)*	member of Calcium Dependent Protein Kinase
AT4G21950		hypothetical protein
AT4G21960	*(PRXR1)*	Encodes AT4g21960 (AT4g21960/T8O5_170). The mRNA is cell-to-cell mobile.

AT4G21980	*AUTOPHAGY 8A (APG8A)*	Encodes APG8, a component of autophagy conjugation pathway. Delivered to the lumens of vacuole under nitrogen-starvation condition. Highest expression in flowers. mRNA abundance increased during dark-induced carbon starvation. Predominantly cytoplasmic with or without N starvation. Upon concanamycin A the protein accumulates in the central vacuole as punctuate structures that resemble autophagic bodies. This localization is more abundant upon N starvation. The mRNA is cell-to-cell mobile.
AT4G21990	*APS REDUCTASE 3 (APR3)*	Encodes a protein disulfide isomerase-like (PDIL) protein, a member of a multigene family within the thioredoxin (TRX) superfamily. This protein also belongs to the adenosine 5'-phosphosulfate reductase-like (APRL) group.
AT4G22010	*SKU5 SIMILAR 4 (sks4)*	SKU5 similar 4
AT4G22020		Probably not a pseudogene based on evidence for transcription (RNA-seq) and translation (Ribo-seq) described in PMID:27791167
AT4G22030	*F-BOX/DUF295 BRASSICEAE-SPECIFIC 27 (ATFDB27)*	F-box protein with a domain protein
AT4G22040		transposable_element_gene;copia-like retrotransposon family, has a 5.3e-193 P-value blast match to GB:AAA57005 Hopscotch polyprotein (Ty1_Copia-element) (Zea mays);(source:TAIR10)
AT4G22050		Eukaryotic aspartyl protease family protein
AT4G22060	*F-BOX/DUF295 BRASSICEAE-SPECIFIC 29 (ATFDB29)*	F-box protein (DUF295)
AT4G22070	*WRKY DNA-BINDING PROTEIN 31 (WRKY31)*	member of WRKY Transcription Factor; Group II-b
AT4G22080	*ROOT HAIR SPECIFIC 14 (RHS14)*	root hair specific 14
AT4G22100	*BETA GLUCOSIDASE 2 (BGLU3)*	beta glucosidase 2
AT4G22110		GroES-like zinc-binding dehydrogenase family protein
AT4G22120	*CALCIUM PERMEABLE STRESS-GATED CATION CHANNEL 1 (CSC1)*	Calcium-permeable stretch activated cation channel.
AT4G22140	*EARLY BOLTING IN SHORT DAYS (EBS)*	Encoding a chromatin remodeling factor that regulates flowering time.
AT4G22150	*PLANT UBX DOMAIN-CONTAINING PROTEIN 3 (PUX3)*	Arabidopsis thaliana CDC48-interacting UBX-domain protein (PUX3)
AT4G22160		hypothetical protein
AT4G22200	*POTASSIUM TRANSPORT 2/3 (KT2/3)*	Encodes AKT2, a photosynthate- and light-dependent inward rectifying potassium channel with unique gating properties that are regulated by phosphorylation. Expressed in guard cell protoplasts and in the phloem and xylem of aerial portions of the plant. The channel can coassemble with another K+ channel, KAT1, in vitro. In guard cells, AKT2/3 is responsible for the Ca2+ sensitivity of the K+ uptake channel. In the phloem, it regulates the sucrose/H+ symporters via the phloem potential. AKT2 belongs to the Shaker family K+ channels which include the following groups based on phylogenetic analysis (FEBS Letters (2007) 581: 2357): I (inward rectifying channel): AKT1 (AT2G26650), AKT5 (AT4G32500) and SPIK (also known as AKT6, AT2G25600); II (inward rectifying channel): KAT1 (AT5G46240) and KAT2 (AT4G18290); III (weakly inward rectifying channel): AKT2 (AT4G22200); IV (regulatory subunit involved in inwardly rectifying conductance formation): KAT3 (also known as AtKC1, AT4G32650); V (outward rectifying channel): SKOR (AT3G02850) and GORK (AT5G37500).
AT4G22212		Encodes a defensin-like (DEFL) family protein.
AT4G22220	*(ISU1)*	Encodes a mitochondrial protein similar to E.coli IscU. In bacteria, IscU is a scaffold protein accepting sulfur and iron to build a transient Fe-S cluster,which is subsequently transferred to a target apoprotein.
AT4G22250		RING/U-box superfamily protein
AT4G22260	*IMMUTANS (IM)*	Similar to mitochondrial alternative oxidase. im mutants have a variegated phenotype and fail to differentiate chloroplasts in the majority of their cells under high light intensity continuous illumination. The white tissues of immutans accumulate phytoene, a non-colored C40 carotenoid intermediate. This suggests that immutans controls, either directly or indirectly, the activity of phytoene desaturase (PDS), the enzyme that converts phytoene to zeta-carotene in higher plants. However, im is not the structural gene for PDS. It is located in the lumenar face of the thylakoid membrane. IM is expressed ubiquitously in plant tissues.
AT4G22270	*MEMBRANE RELATED BIGGER1 (MRB1)*	Encodes a plasma membrane protein involved in the positive regulation of organ size development. Overexpression results in organ size enlargement.
AT4G22280		F-box/RNI-like superfamily protein
AT4G22320		golgin family A protein
AT4G22330	*(ATCES1)*	AtCES1 encodes a nuclear and endoplasmic reticulum localized Acyl-CoA independent ceramide synthase that is involved in sphingolipid metabolism, disease resistance, nutrient limitation, and response to salt stress. Facilitates adaptation to environmental stresses by regulating autophagy.
AT4G22370		transmembrane protein
AT4G22380		Ribosomal protein L7Ae/L30e/S12e/Gadd45 family protein
AT4G22400		hypothetical protein
AT4G22410		U4/U6.U5 tri-snRNP-associated-like protein
AT4G22420		
AT4G22430		LOW protein: F-box/kelch-repeat protein
AT4G22450		transposable_element_gene;similar to unknown protein [Arabidopsis thaliana] (TAIR:AT4G08200.1);(source:TAIR10)
AT4G22460		Bifunctional inhibitor/lipid-transfer protein/seed storage 2S albumin superfamily protein
AT4G22470	*DOUBLE HYBRID PROLINE-RICH PROTEIN 1 (DHYPRP1)*	Encodes a hybrid proline-rich protein that contains two tandem PRD-8CMs (proline-rich domain-eight cysteine motif). Its expression is induced by JA and certain pathogens. This pattern of induction, combined with overexpression and RNAi induced phenotypes, suggests involvement in defense response to pathogens.
AT4G22480		
AT4G22490		Bifunctional inhibitor/lipid-transfer protein/seed storage 2S albumin superfamily protein
AT4G22500		
AT4G22510		transmembrane protein
AT4G22530		S-adenosyl-L-methionine-dependent methyltransferases superfamily protein

AT4G22560		sulfated surface-like glycoprotein
AT4G22570	*ADENINE PHOSPHORIBOSYL TRANSFERASE 3 (APT3)*	Encodes an adenine phosphoribosyltransferase (APT; EC 2.4.2.7), which is a constitutively expressed enzyme involved in the one-step salvage of adenine to AMP. APT3 has higher affinity for zeatin, isopentenyladenine and benzyladenine than APT1 but lower Vmax than APT1.
AT4G22590	*TREHALOSE-6-PHOSPHATE PHOSPHATASE G (TPPG)*	Haloacid dehalogenase-like hydrolase (HAD) superfamily protein
AT4G22600	*INAPERTURATE POLLEN1 (INP1)*	Encodes a protein involved in involved in the formation of the pollen surface apertures. It acts late in aperture formation by excluding specific membrane domains from exine deposition.
AT4G22610		Bifunctional inhibitor/lipid-transfer protein/seed storage 2S albumin superfamily protein
AT4G22620	*SMALL AUXIN UPREGULATED RNA 34 (SAUR34)*	SAUR-like auxin-responsive protein family
AT4G22640		LTPG protein
AT4G22650	*GLYCOSYLPHOSPHATIDYLINOSITOL-ANCHORED LIPID PROTEIN TRANSFER 34 (LTPG34)*	lipid transfer protein
AT4G22680	*MYB DOMAIN PROTEIN 85 (MYB85)*	Encodes a transcriptional regulator that directly activates lignin biosynthesis genes and phenylalanine biosynthesis genes during secondary wall formation.
AT4G22700	*LOB DOMAIN-CONTAINING PROTEIN 32 (LBD32)*	LOB domain-containing protein 32
AT4G22710	*CYTOCHROME P450, FAMILY 706, SUBFAMILY A, POLYPEPTIDE 2 (CYP706A2)*	member of CYP706A
AT4G22720		Actin-like ATPase superfamily protein
AT4G22730		Leucine-rich repeat protein kinase family protein
AT4G22750	*PROTEIN ACYLTRANSFERASE 13 (PAT13)*	Encodes a protein S-acyltransferase that, together with PAT14, cooperatively regulates leaf senescence.
AT4G22753		
AT4G22756	*STEROL C4-METHYL OXIDASE 1-2 (SMO1-2)*	Encodes a member of the SMO1 family of sterol 4alpha-methyl oxidases. More specifically functions as a 4,4-dimethyl-9beta,19-cyclopropylsterol-4alpha-methyl oxidase. Works together with SMO1-1 to maintain correct sterol composition and balance auxin and cytokinin activities during embryogenesis.
AT4G22760		Tetratricopeptide repeat (TPR)-like superfamily protein
AT4G22780	*ACT DOMAIN REPEAT 7 (ACR7)*	Member of a family of ACT domain containing proteins . ACT domains are involved in amino acid binding .
AT4G22790	*RESISTANT TO HIGH CO2 (RHC1)*	Encodes a plasma membrane localized MATE type transporter that is involved in CO2 signaling during stomatal aperture regulation. RHC1 regulates HT1 which phosphorylates OST1, a kinase that regulates the SLAC1 anion channel and thus stomatal closing.
AT4G22800		
AT4G22840	*BILE ACID SODIUM SYMPORTER 6 (BASS6)*	Sodium Bile acid symporter family
AT4G22850		SNARE associated Golgi protein family
AT4G22870		2-oxoglutarate (2OG) and Fe(II)-dependent oxygenase superfamily protein
AT4G22890	*(PGR5-LIKE A)*	Encodes PGRL1A, a transmembrane protein present in thylakoids. PGRL1A has a highly homologous isoform PGRL1B encoded by At4g11960. Plants lacking PGRL1 show perturbation of cyclic electron flow, similar to PGR5-deficient plants. PGRL1 and PGR5 interact physically and associate with PSI (photosystem I). The mRNA is cell-to-cell mobile.
AT4G22910	*FIZZY-RELATED 2 (FZR2)*	FIZZY-related 2
AT4G22920	*NON-YELLOWING 1 (NYE1)*	Similar to the tomato senescence-inducible chloroplast stay-green protein 1. It is upregulated during maximal senescence in the Arabidopsis life cycle, especially in senescent leaves. Acts antagonistically with SGR2 to balance chlorophyll catabolism in chloroplasts with the dismantling and remobilizing of other cellular components in senescing leaf cells.
AT4G22930	*PYRIMIDIN 4 (PYR4)*	Encodes dihydroorotase (PYR4).
AT4G22940		Protein kinase superfamily protein
AT4G22950	*AGAMOUS-LIKE 19 (AGL19)*	MADS-box protein AGL19
AT4G22960		FAM63A-like protein (DUF544)
AT4G22980		molybdenum cofactor sulfurase-like protein
AT4G23000		Calcineurin-like metallo-phosphoesterase superfamily protein
AT4G23010	*UDP-GALACTOSE TRANSPORTER 2 (UTR2)*	UDP-galactose transporter 2
AT4G23030		MATE efflux family protein
AT4G23050	*(TES1)*	PAS domain-containing protein tyrosine kinase family protein
AT4G23060	*IQ-DOMAIN 22 (IQD22)*	Member of IQ67 (CaM binding) domain containing family.
AT4G23070	*RHOMBOID-LIKE PROTEIN 7 (RBL7)*	RHOMBOID-like protein 7
AT4G23080		transmembrane protein, putative (DUF239)
AT4G23090		transmembrane protein
AT4G23100	*GLUTAMATE-CYSTEINE LIGASE (GSH1)*	Encodes the enzyme glutamate-cysteine ligase catalyzing the first, and rate-limiting, step of glutathione biosynthesis. Required for cell proliferation at the root tip. Involved in susceptibility to the bacterial pathogen Pseudomonas syringae. Mutants are phytoalexin defective.
AT4G23110	*EARLY FLOWERING AND CURLY LEAVES (EFC)*	Encodes a protein that is expressed in nuclei of the central cell, endosperm, early embryo and seed coat. It is a phosphorylation target of protein kinase CK2. It also interacts with MSI1, one of the core members of the Polycomb Repressive Complex 2 (PRC2), a master epigenetic regulator. It is likely to play a role in seed development along with the FIS2-PRC2 complex.
AT4G23130	*CYSTEINE-RICH RLK (RECEPTOR-LIKE PROTEIN KINASE) 5 (CRK5)*	Encodes a receptor-like protein kinase. Naming convention from Chen et al 2003 (PMID 14756307)
AT4G23140	*CYSTEINE-RICH RLK (RECEPTOR-LIKE PROTEIN KINASE) 6 (CRK6)*	Arabidopsis thaliana receptor-like protein kinase. Naming convention from Chen et al 2003 (PMID 14756307)
AT4G23150	*CYSTEINE-RICH RLK (RECEPTOR-LIKE PROTEIN KINASE) 7 (CRK7)*	Encodes a cysteine-rich receptor-like protein kinase.
AT4G23180	*CYSTEINE-RICH RLK (RECEPTOR-LIKE PROTEIN KINASE) 10 (CRK10)*	Encodes a receptor-like protein kinase. Naming convention from Chen et al 2003 (PMID 14756307) The mRNA is cell-to-cell mobile.
AT4G23190	*CYSTEINE-RICH RLK (RECEPTOR-LIKE PROTEIN KINASE) 11 (CRK11)*	Encodes putative receptor-like protein kinase that is induced by the soil-borne vascular bacteria, Ralstonia solanacearum. Naming convention from Chen et al 2003 (PMID 14756307)

AT4G23200	*CYSTEINE-RICH RLK (RECEPTOR-LIKE PROTEIN KINASE) 12 (CRK12)*	Encodes a cysteine-rich receptor-like protein kinase.
AT4G23210	*CYSTEINE-RICH RLK (RECEPTOR-LIKE PROTEIN KINASE) 13 (CRK13)*	Encodes a Cysteine-rich receptor-like kinase (CRK13). Overexpression of CRK13 leads to hypersensitive response cell death, and induces defense against pathogens by causing increased accumulation of salicylic acid.
AT4G23220	*CYSTEINE-RICH RLK (RECEPTOR-LIKE PROTEIN KINASE) 14 (CRK14)*	Encodes a cysteine-rich receptor-like protein kinase.
AT4G23230	*CYSTEINE-RICH RLK (RECEPTOR-LIKE PROTEIN KINASE) 15 (CRK15)*	Encodes a cysteine-rich receptor-like protein kinase.
AT4G23240	*CYSTEINE-RICH RLK (RECEPTOR-LIKE PROTEIN KINASE) 16 (CRK16)*	Encodes a cysteine-rich receptor-like protein kinase.
AT4G23250	*EMBRYO DEFECTIVE 1290 (emb1290)*	cysteine-rich receptor-like protein kinase 17
AT4G23260	*CYSTEINE-RICH RLK (RECEPTOR-LIKE PROTEIN KINASE) 18 (CRK18)*	Encodes a cysteine-rich receptor-like protein kinase.
AT4G23270	*CYSTEINE-RICH RLK (RECEPTOR-LIKE PROTEIN KINASE) 19 (CRK19)*	Encodes a cysteine-rich receptor-like protein kinase. The mRNA is cell-to-cell mobile.
AT4G23280	*CYSTEINE-RICH RLK (RECEPTOR-LIKE PROTEIN KINASE) 20 (CRK20)*	Encodes a cysteine-rich receptor-like protein kinase.
AT4G23290	*CYSTEINE-RICH RLK (RECEPTOR-LIKE PROTEIN KINASE) 21 (CRK21)*	Encodes a cysteine-rich receptor-like protein kinase.
AT4G23300	*CYSTEINE-RICH RLK (RECEPTOR-LIKE PROTEIN KINASE) 22 (CRK22)*	Encodes a cysteine-rich receptor-like protein kinase.
AT4G23310	*CYSTEINE-RICH RLK (RECEPTOR-LIKE PROTEIN KINASE) 23 (CRK23)*	Encodes a cysteine-rich receptor-like protein kinase.
AT4G23320	*CYSTEINE-RICH RLK (RECEPTOR-LIKE PROTEIN KINASE) 24 (CRK24)*	Encodes a cysteine-rich receptor-like protein kinase.
AT4G23350		transmembrane protein, putative (DUF239)
AT4G23400	*PLASMA MEMBRANE INTRINSIC PROTEIN 1;5 (PIP1;5)*	Plasma membrane intrinsic protein, involved redundantly with PIP1;1/2/3/4 in hydraulics and carbon fixation, regulates the expression of related genes that affect plant growth and development.
AT4G23410	*TETRASPANIN5 (TET5)*	TET5 encodes a member of the TETRASPANIN gene family that is expressed in the embryo and vascular system and is involved in organ growth redundantly with TET6.
AT4G23420		NAD(P)-binding Rossmann-fold superfamily protein
AT4G23490		fringe-like protein (DUF604)
AT4G23493		hypothetical protein
AT4G23496	*SPIRAL1-LIKE5 (SP1L5)*	Belongs to a six-member gene family in Arabidopsis; all members share high sequence similarity in amino- and carboxy-terminal regions. Regulates cortical microtubule organization. Mutant plants exhibit altered patterns of root, leaf and petal growth as a result of defective anisotropic cell expansion.
AT4G23510		Disease resistance protein (TIR-NBS-LRR class) family
AT4G23520		Cysteine proteinases superfamily protein
AT4G23540		ARM repeat superfamily protein
AT4G23570	*(SGT1A)*	Closely related to SGT1B, may function in SCF(TIR1) mediated protein degradation. AtSGT1a and AtSGT1b are functionally redundant in the resistance to pathogenes. AtSGT1b was more highly expressed than AtSGT1. The N-terminal TPR domain of AtSGT1a reduces the steady-state level of Arabidopsis SGT1 proteins whereas the same domain from AtSGT1b enhances SGT1 accumulation. The TPR domain is dispensable for SGT1 resistance. AtSGT1a is induced upon pathogen infection and can function in R gene-mediated resistance.
AT4G23580		Galactose oxidase/kelch repeat superfamily protein
AT4G23590		Tyrosine transaminase family protein
AT4G23600	*CORONATINE INDUCED 1 (CORI3)*	Encodes cystine lyase which is expected to be involved in amino acid metabolism, providing the plant with cysteine and the generation of precursors of ethylene biosynthesis. mRNA levels are elevated in response to wounding.
AT4G23620		Ribosomal protein L25/Gln-tRNA synthetase, anti-codon-binding domain-containing protein
AT4G23650	*CALCIUM-DEPENDENT PROTEIN KINASE 6 (CDPK6)*	Encodes calcium dependent protein kinase 3 (CPK3), a member of the Arabidopsis CDPK gene family. CDPKs contain an intrinsic Ca2+-activation domain with four EF hand Ca2+-binding sites. CDPKs protein kinases have been proposed to function in multiple plant signal transduction pathways downstream of [Ca2+]cyt elevations, thus transducing various physiological responses. CPK3 is expressed in both guard cells and mesophyll cells. Functions in guard cell ion channel regulation. ABA and Ca(2+) activation of slow-type anion channels and, interestingly, ABA activation of plasma membrane Ca(2+)-permeable channels were impaired in independent alleles of single and double cpk3cpk6 mutant guard cells. Furthermore, ABA- and Ca(2+)-induced stomatal closing were partially impaired in these cpk3cpk6 mutant alleles. CPK6 is also a member of the Arabidopsis CDPK family.
AT4G23660	*POLYPRENYLTRANSFERASE 1 (PPT1)*	Encodes para-hydroxy benzoate polyprenyl diphosphate transferase. The enzyme was shown to be able to use a wide range of prenyl substrates : from GPP (C10) to decaprenyl diphosphate (C50).
AT4G23670		Polyketide cyclase/dehydrase and lipid transport superfamily protein
AT4G23680		Polyketide cyclase/dehydrase and lipid transport superfamily protein
AT4G23690	*DIRIGENT PROTEIN 6 (DIR6)*	Encodes a homodimeric all-beta dirigent protein in the superfamily of calycins. Dirigent proteins impart stereoselectivity on the phenoxy radical coupling reaction yielding optically active lignans from two molecules of coniferyl alcohol.
AT4G23700	*CATION/H+ EXCHANGER 17 (CHX17)*	member of Putative Na+/H+ antiporter family
AT4G23720		transmembrane protein, putative (DUF1191)
AT4G23730		Galactose mutarotase-like superfamily protein
AT4G23750	*CYTOKININ RESPONSE FACTOR 2 (CRF2)*	encodes a member of the ERF (ethylene response factor) subfamily B-5 of ERF/AP2 transcription factor family. The protein contains one AP2 domain. There are 7 members in this subfamily. Monopteros target gene. CRF proteins relocalize to the nucleus in response to cytokinin.
AT4G23760		Cox19-like CHCH family protein
AT4G23780		hypothetical protein
AT4G23800	*3XHIGH MOBILITY GROUP-BOX2 (3xHMG-box2)*	Encodes a protein containing three copies of the HMG (high mobility group)-box domain. The two Arabidopsis 3xHMG-box proteins are: AT4G11080 (3xHMG-box1) and AT4G23800 (3xHMG-box2). Interacts with mitotic and meiotic chromosomes.
AT4G23810	*(WRKY53)*	member of WRKY Transcription Factor; Group III

AT4G23820	*(PGF13)*	Pectin lyase-like superfamily protein
AT4G23830		
AT4G23840		Leucine-rich repeat (LRR) family protein
AT4G23850	*LONG-CHAIN ACYL-COA SYNTHETASE 4 (LACS4)*	Encodes a long chain acyl CoA synthetase that catalyzes the addition of CoA onto IBA, the first step in its β-oxidation.
AT4G23860		PHD finger protein-like protein
AT4G23870		hypothetical protein
AT4G23880		hypothetical protein
AT4G23885		hypothetical protein
AT4G23890	*NADH DEHYDROGENASE-LIKE COMPLEX S (NdhS)*	NAD(P)H-quinone oxidoreductase subunit S
AT4G23900		Nucleoside diphosphate kinase family protein
AT4G23920	*UDP-D-GLUCOSE/UDP-D-GALACTOSE 4-EPIMERASE 2 (UGE2)*	Encodes a protein with UDP-D-glucose 4-epimerase activity. Involved in growth and cell wall carbohydrate biosynthesis.
AT4G23930		Late embryogenesis abundant (LEA) hydroxyproline-rich glycoprotein family
AT4G23950	*SUN-DOMAIN PROTEIN 5 (SUN5)*	Encodes a member of the mid-SUN subfamily of SUN-domain proteins. It is involved in early seed development and nuclear morphology.
AT4G23960		F-box family protein
AT4G23970		hypothetical protein
AT4G23980	*AUXIN RESPONSE FACTOR 9 (ARF9)*	Encodes auxin response factor 9 (ARF9). The mRNA is cell-to-cell mobile.
AT4G23990	*CELLULOSE SYNTHASE LIKE G3 (CSLG3)*	encodes a protein similar to cellulose synthase
AT4G24000	*CELLULOSE SYNTHASE LIKE G2 (CSLG2)*	encodes a protein similar to cellulose synthase
AT4G24010	*CELLULOSE SYNTHASE LIKE G1 (CSLG1)*	encodes a protein similar to cellulose synthase
AT4G24020	*NIN LIKE PROTEIN 7 (NLP7)*	Encodes NIN Like Protein 7 (NLP7). Modulates nitrate sensing and metabolism. Mutants of NLP7 show features of nitrogen-starved plants and are tolerant to drought stress. Localized in the nucleus and functions as a putative transcription factor. The mRNA is cell-to-cell mobile.
AT4G24030		hypothetical protein
AT4G24040	*TREHALASE 1 (TRE1)*	Encodes a trehalase, member of Glycoside Hydrolase Family 37.
AT4G24050		NAD(P)-binding Rossmann-fold superfamily protein
AT4G24060	*(VDOF1)*	Plant-specific Dof transcription factor which regulates vascular cell di#erentiation and lignin biosynthesis.
AT4G24090		homer protein
AT4G24110	*HYPOXIA RESPONSE UNKNOWN PROTEIN 40 (HUP40)*	NADP-specific glutamate dehydrogenase
AT4G24120	*YELLOW STRIPE LIKE 1 (YSL1)*	Member of a small family of oligopeptide transporters similar to the yellow stripe locus of maize (ZmYS1).
AT4G24130	*(SVB5)*	ABA responsive SVB family gene.
AT4G24140		alpha/beta-Hydrolases superfamily protein
AT4G24150	*GROWTH-REGULATING FACTOR 8 (GRF8)*	Growth regulating factor encoding transcription activator. One of the nine members of a GRF gene family, containing nuclear targeting domain. Involved in leaf development and expressed in shoot and flower.
AT4G24160		Encodes a soluble lysophosphatidic acid acyltransferase with additional triacylglycerol lipase and phosphatidylcholine hydrolyzing enzymatic activities. Plays a pivotal role in maintaining the lipid homeostasis by regulating both phospholipid and neutral lipid levels.
AT4G24170		ATP binding microtubule motor family protein
AT4G24180	*THAUMATIN-LIKE PROTEIN 1 (TLP1)*	Root-specific expression activated in response to rhizobacteria and ACC. Role in induced systemic resistance.
AT4G24200		Transcription elongation factor (TFIIS) family protein
AT4G24220	*VEIN PATTERNING 1 (VEP1)*	encodes a progesterone-5beta-reductase-like protein. It has enone reductase activity against a wide range of substrates, including 3-oxo-Δ-4,5-steroids in vitro. The in vivo substrates and product of this enzyme have not yet been elucidated but it is likely to participate in steroid metabolism. The protein contains a mammalian death domain involved in programmed cell death. The gene is expressed in the vascular system and mutants carrying a dominant mutation in the gene have defective vascular patterning. VEP1 gene expression is induced specifically by wounding.
AT4G24240	*WRKY DNA-BINDING PROTEIN 7 (WRKY7)*	Encodes a Ca-dependent calmodulin binding protein. Sequence similarity to the WRKY transcription factor gene family.
AT4G24250	*MILDEW RESISTANCE LOCUS O 13 (MLO13)*	A member of a large family of seven-transmembrane domain proteins specific to plants, homologs of the barley mildew resistance locus o (MLO) protein. The Arabidopsis genome contains 15 genes encoding MLO proteins, with localization in plasma membrane. Phylogenetic analysis revealed four clades of closely-related AtMLO genes. ATMLO13 belongs to the clade II, with ATMLO1 and ATMLO15. The gene is expressed during early seedling growth, in root and cotyledon vascular system, in pollen and also in placenta of developing siliques, as shown by GUS activity patterns. The expression of several phylogenetically closely-related AtMLO genes showed similar or overlapping tissue specificity and analogous responsiveness to external stimuli, suggesting functional redundancy, co-function, or antagonistic function(s).
AT4G24260	*GLYCOSYL HYDROLASE 9A3 (GH9A3)*	Encodes a protein with similarity to endo-1,4-b-glucanases. KOR3 is induced by nemotodes and is expressed in syncitia induced by Heterodera schachtii.May be involved in the development and function of syncitia.
AT4G24290		MAC/Perforin domain-containing protein
AT4G24300		Peptidase C50, separase
AT4G24340		Phosphorylase superfamily protein
AT4G24350		Phosphorylase superfamily protein
AT4G24370	*POSITIVE REGULATOR OF SKD1 (PROS)*	hypothetical protein
AT4G24380		dihydrofolate reductase
AT4G24390	*AUXIN SIGNALING F-BOX 4 (AFB4)*	RNI-like superfamily protein
AT4G24410		hypothetical protein
AT4G24430		Rhamnogalacturonate lyase family protein
AT4G24450	*PHOSPHOGLUCAN, WATER DIKINASE (PWD)*	phosphoglucan, water dikinase

AT4G24480		Protein kinase superfamily protein
AT4G24510	*ECERIFERUM 2 (CER2)*	Encodes a component of the fatty acid elongation machinery required for C28 to C30 fatty acid elongation. It does not require the acyltransferase catalytic site for biological function.
AT4G24520	*P450 REDUCTASE 1 (ATR1)*	Encodes a cyp450 reductase likely to be involved in phenylpropanoid metabolism.
AT4G24540	*AGAMOUS-LIKE 24 (AGL24)*	Encodes a MADS-box protein involved in flowering. Regulates the expression of SOC1 and is also upregulated by SOC1. Binds with IMK3 kinase domain. Phosphorylated by IMK3; likely to be a target for IMK3 kinase domain.
AT4G24570	*DICARBOXYLATE CARRIER 2 (DIC2)*	Encodes one of the mitochondrial dicarboxylate carriers (DIC): DIC1 (AT2G22500), DIC2 (AT4G24570), DIC3 (AT5G09470). The mRNA is cell-to-cell mobile.
AT4G24580	*ROP1 ENHANCER 1 (REN1)*	Encodes a Rho GTPase-activating protein that interacts with ROP1 (a Rho GTPase) and regulates pollen tube development. This protein can be observed at the apical tip of growing pollen tubes and on endocytic vesicles traveling to this region of the pollen tube.
AT4G24600		hypothetical protein
AT4G24620	*PHOSPHOGLUCOSE ISOMERASE 1 (PGI1)*	The PGI1 gene encodes the plastid phospho-glucose (Glc) isomerase. While pgi1-1 mutant has a deficiency in leaf starch synthesis, it accumulates starch in root cap cells. Flowering time of the pgi1-1 mutant is significantly delayed under short-day conditions.
AT4G24630		DHHC-type zinc finger family protein
AT4G24640	*(APPB1)*	Encodes AppB protein (AppB1).
AT4G24650	*ISOPENTENYLTRANSFERASE 4 (IPT4)*	AB061402 Arabidopsis thaliana AtIPT4 mRNA for cytokinin synthase, complete cds
AT4G24660	*HOMEOBOX PROTEIN 22 (HB22)*	homeobox protein 22
AT4G24670	*TRYPTOPHAN AMINOTRANSFERASE RELATED 2 (TAR2)*	Encodes a protein with similarity to the TAA1 trytophan aminotransferase involved in IAA biosynthesis. Double mutant analyses suggest that this protein is involved in regulating many aspects of plant growth and development from embryogenesis to flower formation and plays a role in ethylene-mediated signaling.TAR2 is required for reprogramming root architecture in response to low nitrogen conditions.
AT4G24680	*MODIFIER OF SNC1 (MOS1)*	Encodes MOS1 (MODIFIER OF snc1). MOS1 contains a BAT2 domain that is conserved in plants and animals. MOS1 associates with the promoter of SNC1 and regulates its expression.
AT4G24700		hypothetical protein
AT4G24710	*(PCH2)*	Encodes an AAA+ ATPase that mediates meiotic chromosome remodeling and crossover maturation.
AT4G24750		Rhodanese/Cell cycle control phosphatase superfamily protein
AT4G24760		alpha/beta-Hydrolases superfamily protein
AT4G24780	*(PLL19)*	Encodes a pectate lyase involved in response to nematodes.
AT4G24790		AAA-type ATPase family protein
AT4G24810		similar to ABC1 family protein, contains InterPro domain ABC1 protein (InterPro:IPR004147)
AT4G24850		
AT4G24860		P-loop containing nucleoside triphosphate hydrolases superfamily protein
AT4G24880		snurportin-1 protein
AT4G24890	*PURPLE ACID PHOSPHATASE 24 (PAP24)*	purple acid phosphatase 24
AT4G24900	*TITAN-LIKE (TTL)*	Encodes a nuclear C2H2 domain-containing protein involved in embryo and endosperm development. Involved in brassinosteroid (BR)-mediated plant growth and catalyses the synthesis of S-allantoin, together with B1L participates in modulating plant growth and cold tolerance.
AT4G24920		secE/sec61-gamma protein transport protein
AT4G24930		thylakoid lumenal 17.9 kDa protein, chloroplast
AT4G24940	*SUMO-ACTIVATING ENZYME 1A (SAE1A)*	Encodes one of the two subunits of the SUMO activation enzyme required during sumolation. Sumolation is a post-translational protein modification process similar to ubiquitination during which a polypeptide (SUMO) is covalently attached to a target protein.
AT4G24950	*(SINE4)*	Encodes the plant KASH protein SINE4; SINE4 interacts with SUN1 and SUN2 and is localized at the nuclear envelope.
AT4G24960	*HVA22 HOMOLOGUE D (HVA22D)*	Homologous to a eukaryote specific ABA- and stress-inducible gene first isolated from barley. Groups in one subfamily with ATHVA22E. Along with other members of the ATHVA22 family, it may be involved in regulation of autophagy during development. The mRNA is cell-to-cell mobile.
AT4G24970	*(ATMORC7)*	MORC7 is a member of a family of GHKL ATPases. It is localized in the nuceloplasm and adjacent to chromocenters. Along with MORC4, it appears to repress the expression of genes involved in defense against pathogens.
AT4G24980	*USUALLY MULTIPLE ACIDS MOVE IN AND OUT TRANSPORTERS 16-PSEUDOGENE (UMAMIT16-PSI)*	nodulin MtN21-like transporter family protein
AT4G25000	*ALPHA-AMYLASE-LIKE (AMY1)*	Predicted to be secreted protein based on signalP prediction. Involved in starch mobilization. Mutants are defective in alpha-amylase activity. (Note: AMY1 has been found in the literature to be referred to as AMY3, which is not to be confused with AMY3/At1g69830).
AT4G25010	*(SWEET14)*	Encodes a member of the SWEET sucrose efflux transporter family proteins. Together with SWEET13, it is likely involved in modulating the GA response and is required for proper development of anthers, seeds and seedlings.
AT4G25020		D111/G-patch domain-containing protein
AT4G25030	*NON HOST RESISTANCE 2B (ATNHR2B)*	Plastid localized protein of unknown function. Mutants are more susceptible to P. syringae and produce less callose upon infection.
AT4G25040	*CASP-LIKE PROTEIN 1F1 (CASPL1F1)*	Uncharacterized protein family (UPF0497)
AT4G25050	*ACYL CARRIER PROTEIN 4 (ACP4)*	encodes an acyl carrier protein predominantly expressed in leaves. Gene expression is upregulated by light.
AT4G25060		
AT4G25080	*MAGNESIUM-PROTOPORPHYRIN IX METHYLTRANSFERASE (CHLM)*	Encodes a protein with methyltransferase activity responsible for the methylation of magnesium protoporphyrin IX. Mutants defective in this gene are affected in chlorophyll biosynthesis and show a reduction in the accumulation of a number of major thylakoid-associated proteins including components of PSI (LHCI), PSII (LHCII, D1, CP43) and the cytochrome b6f complex (Cytf). By contrast, no significant changes were detected for the proteins of the stroma and the chloroplast envelope.
AT4G25090	*(ATRBOHG)*	Riboflavin synthase-like superfamily protein

AT4G25100	*FE SUPEROXIDE DISMUTASE 1 (FSD1)*	Fe-superoxide dismutase
AT4G25110	*METACASPASE 2 (MC2)*	Encodes a type I metacaspase. Two Arabidopsis metacaspases, AT1G02170 (MC1) and AT4G25110 (MC2) antagonistically control programmed cell death in Arabidopsis. MC1 is a positive regulator of cell death and requires conserved caspase-like putative catalytic residues for its function. MC2 negatively regulates cell death. This function is independent of the putative catalytic residues. A third type I Arabidopsis metacaspase is MC3 (AT5g64240).
AT4G25120	*SUPPRESSOR OF RAD SIX-SCREEN MUTANT 2 (SRS2)*	Encodes a homolog of the yeast SRS2 (Suppressor of RAD Six-screen mutant 2) helicase. The Arabidopsis SRS2 is a functional 3?- to 5?-helicase. Biochemical studies show that SRS2 disrupts recombinogenic DNA intermediates and facilitates single strand annealing.
AT4G25130	*PEPTIDE MET SULFOXIDE REDUCTASE 4 (PMSR4)*	Encodes a chloroplast-localized methionine sulfoxide reductase that is a member of the MSRA family. Involved in protection of chloroplasts from oxidative stress.
AT4G25140	*OLEOSIN 1 (OLEO1)*	Encodes oleosin1, a protein found in oil bodies, involved in seed lipid accumulation. Suppression of OLEO1 (and OLEO2) resulted in an aberrant phenotype of embryo cells that contain unusually large oilbodies that are not normally observed in seeds. Changes in the size of oilbodies caused disruption of storage organelles, altering accumulation of lipids and proteins and causing delay in germination. Functions in freezing tolerance of seeds.
AT4G25150		HAD superfamily, subfamily IIIB acid phosphatase
AT4G25160	*(PUB35)*	Encodes a U-box domain-containing E3 ubiquitin ligase with central Ser/Thr protein kinase domain whose expression is responsive to both phosphate (Pi) and phosphite (Phi) in both roots and shoots.
AT4G25200	*MITOCHONDRION-LOCALIZED SMALL HEAT SHOCK PROTEIN 23.6 (HSP23.6-MITO)*	AtHSP23.6-mito mRNA, nuclear gene encoding mitochondrial
AT4G25220	*GLYCEROL-3-PHOSPHATE PERMEASE 2 (G3Pp2)*	Encodes a member of the phosphate starvation-induced glycerol-3-phosphate permease gene family: AT3G47420(G3Pp1), AT4G25220(G3Pp2), AT1G30560(G3Pp3), AT4G17550(G3Pp4) and AT2G13100(G3Pp5).
AT4G25230	*RPM1 INTERACTING PROTEIN 2 (RIN2)*	RPM1 interacting protein 2, has a CUE domain which is sufficient for the interaction with RPM1.Positive regulator of RPM1 and PRS2 mediated hypersensitive response.Functions as ubiquitin ligase and binds to RPM1.
AT4G25250	*PECTINMETHYLESTERASE INHIBITOR 4 (PMEI4)*	PMEI4 pectin methyleseterase inhibitor. Expressed in roots.
AT4G25260	*PECTIN METHYLESTERASE INIHIBITOR 7 (PMEI7)*	Pectin methylesterase inhibitor. Forms pH dependent complex with PME3.
AT4G25280		P-loop containing nucleoside triphosphate hydrolases superfamily protein
AT4G25300		2-oxoglutarate (2OG) and Fe(II)-dependent oxygenase superfamily protein
AT4G25320	*AT-HOOK MOTIF NUCLEAR LOCALIZED PROTEIN 3 (AHL3)*	AT hook motif DNA-binding family protein
AT4G25330		SAWADEE protein
AT4G25340	*FK506 BINDING PROTEIN 53 (FKBP53)*	Encodes a member of the FKBP-type immunophilin family that functions as a histone chaparone. Binds to 18S rDNA and represses its expression. The N-terminal nucleoplasmin domain interacts with H2A/H2B and H3/H4 histone oligomers, individually, as well as simultaneously, suggesting two different binding sites for H2A/H2B and H3/H4.
AT4G25350	*SHORT HYPOCOTYL UNDER BLUE1 (SHB1)*	SHB1 encodes a nuclear and cytosolic protein that has motifs homologous with SYG1 protein family members. Acts in cryptochrome signaling. Overexpression of SHB1 enhanced the expression of PHYTOCHROME-INTERACTING FACTOR4 (PIF4) under red light and promoted proteasome-mediated degradation of phytochrome A and hypocotyl elongation under far-red light. A knockout allele suppressed LONG HYPOCOTYL IN FAR-RED LIGHT1 (HFR1) expression and showed several deetiolation phenotypes. Acts upstream of HFR1. Regulates seed development.
AT4G25370	*(CLPT1)*	Double Clp-N motif protein
AT4G25380	*STRESS-ASSOCIATED PROTEIN 10 (SAP10)*	stress-associated protein 10
AT4G25410		basic helix-loop-helix (bHLH) DNA-binding superfamily protein
AT4G25420	*GIBBERELLIN 20 OXIDASE 1 (GA20OX1)*	Encodes gibberellin 20-oxidase that is involved in the later steps of the gibberellin biosynthetic pathway. Regulated by a circadian clock. Weak expression response to far red light.
AT4G25430	*TON1 RECRUITING MOTIF 23 (TRM23)*	hypothetical protein
AT4G25440	*ZINC FINGER WD40 REPEAT PROTEIN 1 (ZFWD1)*	zinc finger WD40 repeat protein 1
AT4G25450	*ATP-BINDING CASSETTE B28 (ABCB28)*	member of NAP subfamily
AT4G25460		
AT4G25470	*C-REPEAT/DRE BINDING FACTOR 2 (CBF2)*	Encodes a member of the DREB subfamily A-1 of ERF/AP2 transcription factor family (CBF2). The protein contains one AP2 domain. There are six members in this subfamily, including CBF1, CBF2, and CBF3. This gene is involved in response to low temperature, abscisic acid, and circadian rhythm. Overexpressing this gene leads to increased freeze tolerance and induces the expression level of 85 cold-induced genes and reduces the expression level of 8 cold-repressed genes, which constitute the CBF2 regulon. Mutations in CBF2 increases the expression level of CBF1 and CBF3, suggesting that this gene may be involved in a negative regulatory or feedback circuit of the CBF pathway.
AT4G25480	*DEHYDRATION RESPONSE ELEMENT B1A (DREB1A)*	Encodes a member of the DREB subfamily A-1 of ERF/AP2 transcription factor family (CBF3). The protein contains one AP2 domain. There are six members in this subfamily, including CBF1, CBF2, and CBF3. This gene is involved in response to low temperature and abscisic acid.
AT4G25490	*C-REPEAT/DRE BINDING FACTOR 1 (CBF1)*	Transcriptional activator that binds to the DRE/CRT regulatory element and induces COR (cold-regulated) gene expression increasing plant freezing tolerance. It encodes a member of the DREB subfamily A-1 of ERF/AP2 transcription factor family (CBF1). The protein contains one AP2 domain. There are six members in this subfamily, including CBF1, CBF2, and CBF3. This gene is involved in response to low temperature and abscisic acid.
AT4G25500	*ARGININE/SERINE-RICH SPLICING FACTOR 40 (RS40)*	Encodes an arginine/serine-rich splicing factor. The transcript is alternatively spliced and is differentially expressed in different tissues (flowers, roots, stems, and leaves) examined. Barta et al (2010) have proposed a nomenclature for Serine/Arginine-Rich Protein Splicing Factors (SR proteins): Plant Cell. 2010, 22:2926. RS40 binds to HYL1 and co-localizes to the nuclear dicing body. Along with RS41, it appears to be involved in pri-miRNA processing and miRNA biogenesis (DOI:10.1093/nar/gkv751).
AT4G25510		hypothetical protein

AT4G25530	*FLOWERING WAGENINGEN (FWA)*	Encodes a homeodomain-containing transcription factor that controls flowering. FWA is silenced in wild type plants and reverse of the imprinted silencing causes a late flowering phenotype. FWA gene contains two tandem repeats around the transcription start site that are necessary and sufficient for silencing via DNA methylation.
AT4G25540	*HOMOLOG OF DNA MISMATCH REPAIR PROTEIN MSH3 (MSH3)*	encodes a DNA mismatch repair homolog of human MutS gene, MSH6. There are four MutS genes in Arabidopsis, MSH2, MSH3, MSH6, and MSH7, which all act as heterodimers and bind to 51-mer duplexes. MSH2*MSH3 heterodimers bound 'insertion-deletion' DNA with three nucleotides (+AAG) or one nucleotide (+T) looped out much better than they bound DNA with a base/base mispair (T/G).
AT4G25560	*LONG AFTER FAR-RED LIGHT 1 (LAF1)*	LAF1 is a R2R3-MYB transcription factor and positive regulator of the phyA photoresponse. Interaction of LAF1 with HFR1 stabilize the proteins against ubiquitination by COP1(AT2G32950) and subsequent degrations. Mutants have an elongated hypocotyl specifically under far-red light but retain wild-type responses to other light wavelengths.
AT4G25570	*(ACYB-2)*	Encodes cytochrome b561.
AT4G25580		CAP160 protein
AT4G25590	*ACTIN DEPOLYMERIZING FACTOR 7 (ADF7)*	actin depolymerizing factor 7
AT4G25620		hydroxyproline-rich glycoprotein family protein
AT4G25630	*FIBRILLARIN 2 (FIB2)*	encodes a fibrillarin, a key nucleolar protein in eukaryotes which associates with box C/D small nucleolar RNAs (snoRNAs) directing 2'-O-ribose methylation of the rRNA. This gene also encodes a novel box C/D snoRNA, U60.2f in its fifth intron that accumulates in seedlings and that their targeted residue on the 25 S rRNA is methylated. The mRNA is cell-to-cell mobile.
AT4G25650	*ACD1-LIKE (ACD1-LIKE)*	Similar to ACD1. Leaves of antisense ACD1-like plants turn yellow in darkness like wild-type whereas antisense ACD1 plants remain dark after five days of dark treatment.
AT4G25670	*DOUBLE BENDING 1 (DOB1)*	stress response NST1-like protein
AT4G25700	*BETA-HYDROXYLASE 1 (BETA-OHASE 1)*	Converts beta-carotene to zeaxanthin via cryptoxanthin.
AT4G25710		Galactose oxidase/kelch repeat superfamily protein
AT4G25720	*GLUTAMINYL CYCLASE (QC)*	This locus encodes a protein with similarity to gamma-glutamylcyclotransferase that may be involved in catalyzing the formation of pyroglutate residue on proteins that have been post-translationally processed to reveal a glutamine at their N-terminus. Enzymatic assays to test the function of this protein were performed using a truncated form of the protein lacking a signal peptide that is most similar to the AT4G25720.1 protein model.
AT4G25730	*TRNA METHYLTRANSFERASE 7B (TRM7B)*	FtsJ-like methyltransferase family protein
AT4G25750	*ATP-BINDING CASSETTE G4 (ABCG4)*	ABC-2 type transporter family protein
AT4G25760	*GLUTAMINE DUMPER 2 (GDU2)*	Encodes a member of the GDU (glutamine dumper) family proteins involved in amino acid export: At4g31730 (GDU1), At4g25760 (GDU2), At5g57685 (GDU3), At2g24762 (GDU4), At5g24920 (GDU5), At3g30725 (GDU6) and At5g38770 (GDU7).
AT4G25770		alpha/beta-Hydrolases superfamily protein
AT4G25780	*(ATCAPE2)*	CAP (Cysteine-rich secretory proteins, Antigen 5, and Pathogenesis-related 1 protein) superfamily protein
AT4G25790	*(ATCAPE4)*	CAP (Cysteine-rich secretory proteins, Antigen 5, and Pathogenesis-related 1 protein) superfamily protein
AT4G25800	*(CBP60D)*	Calmodulin-binding protein
AT4G25810	*XYLOGLUCAN ENDOTRANSGLYCOSYLASE 6 (XTR6)*	xyloglucan endotransglycosylase-related protein (XTR6)
AT4G25820	*XYLOGLUCAN ENDOTRANSGLUCOSYLASE/HYDROLASE 14 (XTH14)*	Encodes a xyloglucan endotransglycosylase with a clear preference for non-fucosylated xyloglucan polymer. The mRNA is cell-to-cell mobile.
AT4G25830	*CASP-LIKE PROTEIN 2C1 (CASPL2C1)*	Uncharacterized protein family (UPF0497)
AT4G25835		P-loop containing nucleoside triphosphate hydrolases superfamily protein
AT4G25840	*GLYCEROL-3-PHOSPHATASE 1 (GPP1)*	glycerol-3-phosphatase 1
AT4G25860	*OSBP(OXYSTEROL BINDING PROTEIN)-RELATED PROTEIN 4A (ORP4A)*	OSBP(oxysterol binding protein)-related protein 4A
AT4G25870		Core-2/I-branching beta-1,6-N-acetylglucosaminyltransferase family protein
AT4G25880	*PUMILIO 6 (PUM6)*	Encodes a member of the Arabidopsis Pumilio (APUM) proteins containing PUF domain (eight repeats of approximately 36 amino acids each). PUF proteins regulate both mRNA stability and translation through sequence-specific binding to the 3' UTR of target mRNA transcripts.
AT4G25890		60S acidic ribosomal protein family
AT4G25900		Galactose mutarotase-like superfamily protein
AT4G25910	*NFU DOMAIN PROTEIN 3 (NFU3)*	Encodes a protein containing the NFU domain that may be involved in iron-sulfur cluster assembly. Part of a five member gene family, more closely related to NFU1 and 2 than to NFU4 and 5. Targeted to the chloroplast. The mRNA is cell-to-cell mobile.
AT4G25920	*DUF295 ORGANELLAR A 9 (ATDOA9)*	hypothetical protein (DUF295)
AT4G25940	*(PICALM2B)*	ENTH/ANTH/VHS superfamily protein
AT4G25950	*VACUOLAR ATP SYNTHASE G3 (VATG3)*	V-ATPase G-subunit like protein
AT4G25960	*ATP-BINDING CASSETTE B2 (ABCB2)*	P-glycoprotein 2
AT4G25970	*PHOSPHATIDYLSERINE DECARBOXYLASE 3 (PSD3)*	Encodes the major form of the two non-mitochondrail phosphatidylserine decarboxylase. Located at the ER. The mRNA is cell-to-cell mobile.
AT4G25980		peroxidase superfamily protein
AT4G25990	*CIA2-LIKE (CIL)*	chloroplast import apparatus CIA2-like. CIA2 is a transcription factor which upregulates chloroplast translocon genes
AT4G26010		Peroxidase superfamily protein
AT4G26020		4/1 protein short form protein
AT4G26040		hypothetical protein
AT4G26050	*PLANT INTRACELLULAR RAS GROUP-RELATED LRR 8 (PIRL8)*	Encodes PIRL8, a member of the Plant Intracellular Ras-group-related LRRs (Leucine rich repeat proteins). PIRLs are a distinct, plant-specific class of intracellular LRRs that likely mediate protein interactions, possibly in the context of signal transduction. The mRNA is cell-to-cell mobile.
AT4G26060		Ribosomal protein L18ae family
AT4G26070	*MAP KINASE/ ERK KINASE 1 (MEK1)*	Member of MAP Kinase Kinase. Likely functions in a stress-activated MAPK pathway. Can phosphorylate the MAPK AtMPK4, in response to stress. Gets phosphorylated by MEKK1 in response to wounding.

AT4G26080	*ABA INSENSITIVE 1 (ABI1)*	Involved in abscisic acid (ABA) signal transduction. Negative regulator of ABA promotion of stomatal closure.
AT4G26090	*RESISTANT TO P. SYRINGAE 2 (RPS2)*	Encodes a plasma membrane protein with leucine-rich repeat, leucine zipper, and P loop domains that confers resistance to Pseudomonas syringae infection by interacting with the avirulence gene avrRpt2. RPS2 protein interacts directly with plasma membrane associated protein RIN4 and this interaction is disrupted by avrRpt2. The mRNA is cell-to-cell mobile.
AT4G26090	*RESISTANT TO P. SYRINGAE 2 (RPS2)*	Encodes a plasma membrane protein with leucine-rich repeat, leucine zipper, and P loop domains that confers resistance to Pseudomonas syringae infection by interacting with the avirulence gene avrRpt2. RPS2 protein interacts directly with plasma membrane associated protein RIN4 and this interaction is disrupted by avrRpt2. The mRNA is cell-to-cell mobile.
AT4G26110	*NUCLEOSOME ASSEMBLY PROTEIN1;1 (NAP1;1)*	Encodes a member of a small gene family of proteins with similarity to nucleosome assembly proteins.May function in nucleotide excision repair. Loss of function mutations have no obvious visible phenotypes but do seem to affect transcription of NER related genes. The mRNA is cell-to-cell mobile. Plants mutated in three ubiquitously expressed NAP1 genes (NAP1;1~NAP1;3) and organ-specifically expressed NAP1;4 gene show hypersensitivity to genotoxic stresses including UV and DSB-inducing agent Bleomycin. The NAP1 genes act synergistically with NRP genes in promoting somatic homologous recombination.
AT4G26120		Ankyrin repeat family protein / BTB/POZ domain-containing protein
AT4G26140	*BETA-GALACTOSIDASE 12 (BGAL12)*	putative beta-galactosidase
AT4G26150	*CYTOKININ-RESPONSIVE GATA FACTOR 1 (CGA1)*	Encodes a member of the GATA factor family of zinc finger transcription factors. Modulate chlorophyll biosynthesis and glutamate synthase (GLU1/Fd-GOGAT) expression.
AT4G26160	*ATYPICAL CYS HIS RICH THIOREDOXIN 1 (ACHT1)*	Encodes a member of the thioredoxin family protein. Located in the chloroplast. Shows high activity towards the chloroplast 2-Cys peroxiredoxin A, and poor activity towards the chloroplast NADP-malate dehydrogenase.
AT4G26170	*EFFECTOR OF TRANSCRIPTION 1 (ET1)*	ET1 is a DNA and Zinc binding domain containing protein involved in DNA methylation.
AT4G26180	*COA CARRIER 2 (COAC2)*	Encodes a mitochondrial CoA transporter.
AT4G26190		Haloacid dehalogenase-like hydrolase (HAD) superfamily protein
AT4G26200	*1-AMINO-CYCLOPROPANE-1-CARBOXYLATE SYNTHASE 7 (ACS7)*	Member of a family of proteins in Arabidopsis that encode 1-Amino-cyclopropane-1-carboxylate synthase, an enzyme involved in ethylene biosynthesis. Not expressed in response to IAA.
AT4G26210		Mitochondrial ATP synthase subunit G protein
AT4G26220	*CAFFEOYL COENZYME A ESTER O-METHYLTRANSFERASE 7 (CCOAOMT7)*	Encodes a caffeoyl-coenzyme A O-methyltransferase (CCoAOMT)-like protein with a strong preference for methylating the para position of flavanones and dihydroflavonols, whereas flavones and flavonols are methylated in the meta-position.
AT4G26250	*GALACTINOL SYNTHASE 6 (GolS6)*	Predicted to encode a galactinol synthase.
AT4G26260	*MYO-INOSITOL OXYGENASE 4 (MIOX4)*	Encodes a myo-inositol oxygenase, which is the first enzyme in the inositol route to ascorbate (L‐ascorbic acid, AsA, vitamin C). Overexpression results in enhanced biomass and abiotic stress tolerance.
AT4G26270	*PHOSPHOFRUCTOKINASE 3 (PFK3)*	phosphofructokinase 3
AT4G26280		P-loop containing nucleoside triphosphate hydrolases superfamily protein
AT4G26290		hypothetical protein
AT4G26310		elongation factor P (EF-P) family protein
AT4G26320	*ARABINOGALACTAN PROTEIN 13 (AGP13)*	arabinogalactan protein 13
AT4G26330	*UNFERTILIZED EMBRYO SAC 17 (UNE17)*	Subtilisin-like serine endopeptidase family protein
AT4G26340		F-box/RNI-like/FBD-like domains-containing protein
AT4G26360		transposable_element_gene;non-LTR retrotransposon family (LINE), has a 4.9e-40 P-value blast match to GB:NP_038603 L1 repeat, Tf subfamily, member 23 (LINE-element) (Mus musculus);(source:TAIR10)
AT4G26370		antitermination NusB domain-containing protein
AT4G26380		Cysteine/Histidine-rich C1 domain family protein
AT4G26390		Pyruvate kinase family protein
AT4G26420	*GIBBERELLIN METHYLTRANSFERASE 1 (GAMT1)*	A member of the Arabidopsis SABATH methyltransferase gene family. Encodes GAMT1, a methyltransferase that uses S-adenosine-L-methionine (SAM) as a methyl donor to methylate the carboxyl group of GAs, resulting in the methyl esters of GAs (MeGAs). Expressed most highly in the siliques during seed development. SABATH family methyltransferase.
AT4G26430	*COP9 SIGNALOSOME SUBUNIT 6B (CSN6B)*	one of two genes encoding subunit 6 of COP9 signalosome complex
AT4G26440	*WRKY DNA-BINDING PROTEIN 34 (WRKY34)*	member of WRKY Transcription Factor; Group I
AT4G26460		S-adenosyl-L-methionine-dependent methyltransferases superfamily protein
AT4G26470		Calcium-binding EF-hand family protein
AT4G26480		RNA-binding KH domain-containing protein
AT4G26520	*FRUCTOSE-BISPHOSPHATE ALDOLASE 7 (FBA7)*	Aldolase superfamily protein
AT4G26530	*FRUCTOSE-BISPHOSPHATE ALDOLASE 5 (FBA5)*	Aldolase superfamily protein
AT4G26540	*(RGFR3)*	Leucine-rich repeat receptor-like protein kinase family protein
AT4G26555	*FK506-BINDING PROTEIN 16-1 (FKBP16-1)*	Encodes a chloroplast lumen-targeted immunophilin that plays a role in the acclimation of plants under photosynthetic stress conditions, probably by regulating PsaL stability.
AT4G26560	*CALCINEURIN B-LIKE PROTEIN 7 (CBL7)*	Encodes calcineurin B-like protein 7 (CBL7).Interacts with and modulates the activity of the PM ATPase AHA2.
AT4G26590	*OLIGOPEPTIDE TRANSPORTER 5 (OPT5)*	oligopeptide transporter
AT4G26600	*(NOP2B)*	S-adenosyl-L-methionine-dependent methyltransferases superfamily protein
AT4G26620		Sucrase/ferredoxin-like family protein
AT4G26630	*DEK-DOMAIN CONTAINING PROTEIN 3 (DEK3)*	Encodes a chromatin-associated protein that specifically binds histones H3 and H4 and contributes to modulation of Arabidopsis chromatin structure and function.

AT4G26650		RNA-binding (RRM/RBD/RNP motifs) family protein
AT4G26660		kinesin-like protein
AT4G26680		Tetratricopeptide repeat (TPR)-like superfamily protein
AT4G26700	*FIMBRIN 1 (FIM1)*	Encodes a member of the fimbrin family. Different members of the fimbrin/plastin family have diverged biochemically during evolution to generate either tight actin bundles or loose networks with distinct biochemical and biophysical properties. FIM4 generates both actin bundles and branched actin filaments whereas FIM5 only generates actin bundles.
AT4G26720	*PROTEIN PHOSPHATASE X 1 (PPX1)*	Encodes catalytic subunit of protein phosphatase X. Expressed at very low levels in A. thaliana flowers, leaves, stems and roots.
AT4G26730		S-adenosyl-L-methionine-dependent methyltransferases superfamily protein
AT4G26740	*SEED GENE 1 (ATS1)*	Encodes caleosin, a 27-kDa protein found within seed lipid bodies. Gene is expressed preferentially in the embryo, has similarity to a rice ABA-responsive gene, EFA27. Catalyze hydroperoxide-dependent mono-oxygenation reactions. Require calcium for peroxygenase activity. Probably deeply buried in lipid droplets or microsomes.
AT4G26770		Phosphatidate cytidylyltransferase family protein
AT4G26780	*(AR192)*	unknown function
AT4G26790	*(GGL23)*	GDSL-motif esterase/acyltransferase/lipase. Enzyme group with broad substrate specificity that may catalyze acyltransfer or hydrolase reactions with lipid and non-lipid substrates.
AT4G26800		Pentatricopeptide repeat (PPR) superfamily protein
AT4G26820		GrpE-like protein
AT4G26830		O-Glycosyl hydrolases family 17 protein
AT4G26850	*VITAMIN C DEFECTIVE 2 (VTC2)*	Encodes a novel protein involved in ascorbate biosynthesis, which was shown to catalyze the transfer of GMP from GDP-galactose to a variety of hexose-1-phosphate acceptors. Recessive mutation has a reduced amount of vitamin C, lower level of non-photochemical quenching, and reduced rate of conversion of violaxanthin to zeaxanthin in high light.
AT4G26880		Stigma-specific Stig1 family protein
AT4G26890	*MITOGEN-ACTIVATED PROTEIN KINASE KINASE KINASE 16 (MAPKKK16)*	Member of MEKK subfamily. Involved in wound response signaling. Interacts with At5g40440, and activates At1g59580.
AT4G26910	*(E2- OGDH1)*	Encodes the E2 subunit of the 2-oxoglutarate dehydrogenase.
AT4G26920		START (StAR-related lipid-transfer) lipid-binding domain-containing protein
AT4G26950		senescence regulator (Protein of unknown function, DUF584)
AT4G26970	*ACONITASE 2 (ACO2)*	Encodes an aconitase that can catalyze the conversion of citrate to isocitrate through a cis-aconitate intermediate, indicating that it may participate in the TCA cycle and other primary metabolic pathways. The protein is believed to accumulate in the mitochondria and the cytosol. It affects CSD2 (At2g28190 - a superoxide dismutase) transcript levels and may play a role in the response to oxidative stress. One member of the family (ACO1 - At35830) was shown to specifically bind to the 5' UTR of CSD2 in vitro. The mRNA is cell-to-cell mobile.
AT4G27000	*(ATRBP45C)*	RNA-binding (RRM/RBD/RNP motifs) family protein
AT4G27010	*EMBRYO DEFECTIVE 2788 (EMB2788)*	ribosome 60S biogenesis amino-terminal protein
AT4G27030	*FATTY ACID DESATURASE A (FADA)*	Encodes an unusual palmitate desaturase that is highly substrate specific. It introduces a delta-3 trans double bond at palmitate at the sn-2 position of phosphatidylglycerol. The mRNA is cell-to-cell mobile.
AT4G27040	*(VPS22)*	EAP30/Vps36 family protein
AT4G27070	*TRYPTOPHAN SYNTHASE BETA-SUBUNIT 2 (TSB2)*	Tryptophan synthase beta. Expressed at low levels in all tissues. The mRNA is cell-to-cell mobile.
AT4G27110	*COBRA-LIKE PROTEIN 11 PRECURSOR (COBL11)*	COBRA-like protein 11 precursor
AT4G27140	*SEED STORAGE ALBUMIN 1 (SESA1)*	seed storage albumin 1
AT4G27150	*SEED STORAGE ALBUMIN 2 (SESA2)*	seed storage albumin 2
AT4G27160	*SEED STORAGE ALBUMIN 3 (SESA3)*	seed storage albumin 3
AT4G27170	*SEED STORAGE ALBUMIN 4 (SESA4)*	seed storage albumin 4
AT4G27180	*KINESIN 2 (ATK2)*	kinesin heavy chain subunit
AT4G27190		NB-ARC domain-containing disease resistance protein
AT4G27200		transposable_element_gene;copia-like retrotransposon family, has a 1.7e-36 P-value blast match to GB:AAA57005 Hopscotch polyprotein (Ty1_Copia-element) (Zea mays);(source:TAIR10)
AT4G27220		NB-ARC domain-containing disease resistance protein
AT4G27230	*HISTONE H2A 2 (HTA2)*	Encodes HTA2, a histone H2A protein.
AT4G27240		zinc finger (C2H2 type) family protein
AT4G27250		NAD(P)-binding Rossmann-fold superfamily protein
AT4G27260	*(WES1)*	encodes an IAA-amido synthase that conjugates Asp and other amino acids to auxin in vitro. Lines carrying insertions in this gene are hypersensitive to auxin. It is involved in camalexin biosynthesis via conjugating indole-3-carboxylic acid (ICA) and cysteine (Cys). The mRNA is cell-to-cell mobile.
AT4G27280	*CA2+-DEPENDENT MODULATOR OF ICR1 (CMI1)*	EF-hand Ca2 + -binding protein, which is a Ca2+-dependent transducer of auxin-regulated gene expression and interacts with ICR1.
AT4G27290		S-locus lectin protein kinase family protein
AT4G27300		S-locus lectin protein kinase family protein
AT4G27320	*(PHOS34)*	Contains a universal stress protein domain. Protein is phosphorylated in response to Phytophthora infestans zoospores and xylanase.
AT4G27330	*SPOROCYTELESS (SPL)*	Encodes a putative transcription factor that is required for the initiation of both micro- and megagametogenesis and is expressed in the sporogenous tissue of the anther and the ovule. SPL is a chalaza identity gene that share overlapping functions in establishing the prospective chalaza of the ovule. It also plays a central role in patterning both the proximal-distal and the adaxial-abaxial axes in the ovule and generally interacts with YABBY proteins in vitro. Mutant is defective in the differentiation of primary sporogenous cells into microsporocytes, and does not properly form the anther wall. Regulator of anther cell differenctiation. Interacts with TPL and TCP proteins.

AT4G27350		membrane lipoprotein lipid attachment site-like protein, putative (DUF1223)
AT4G27370	*(VIIIB)*	member of Myosin-like proteins
AT4G27380		hypothetical protein
AT4G27390		transmembrane protein
AT4G27400		Late embryogenesis abundant (LEA) protein-like protein
AT4G27410	*RESPONSIVE TO DESICCATION 26 (RD26)*	Encodes a NAC transcription factor induced in response to desiccation. It is localized to the nucleus and acts as a transcriptional activator in ABA-mediated dehydration response.
AT4G27420	*ATP-BINDING CASSETTE G9 (ABCG9)*	ABC-2 type transporter family protein
AT4G27430	*COP1-INTERACTING PROTEIN 7 (CIP7)*	Positive regulator of light-regulated genes. Novel nuclear protein which requires light for its high level expression. The mRNA is cell-to-cell mobile.
AT4G27435		fiber (DUF1218)
AT4G27440	*PROTOCHLOROPHYLLIDE OXIDOREDUCTASE B (PORB)*	light-dependent NADPH:protochlorophyllide oxidoreductase B The mRNA is cell-to-cell mobile.
AT4G27450	*HYPOXIA RESPONSE UNKNOWN PROTEIN 54 (HUP54)*	aluminum induced protein with YGL and LRDR motifs
AT4G27460	*CBS DOMAIN CONTAINING PROTEIN 5 (CBSX5)*	Cystathionine beta-synthase (CBS) family protein
AT4G27470	*RING MEMBRANE-ANCHOR 3 (RMA3)*	Encodes a RING finger E3 ubiquitin ligase.
AT4G27490	*RRP41 LIKE (RRP41L)*	3-5-exoribonuclease family protein
AT4G27500	*PROTON PUMP INTERACTOR 1 (PPI1)*	interacts with H+-ATPase, and regulates its activity The mRNA is cell-to-cell mobile.
AT4G27510		2-isopropylmalate synthase
AT4G27520	*EARLY NODULIN-LIKE PROTEIN 2 (ENODL2)*	early nodulin-like protein 2
AT4G27530		hypothetical protein
AT4G27540	*PRENYLATED RAB ACCEPTOR 1.H (PRA1.H)*	prenylated RAB acceptor 1.H
AT4G27550	*TREHALOSE-6-PHOSPHATASE SYNTHASE S4 (TPS4)*	Encodes an enzyme putatively involved in trehalose biosynthesis. The protein has a trehalose synthase (TPS)-like domain that may or may not be active but no trehalose phosphatase (TPP)-like domain.
AT4G27570	*(UGT79B3)*	Encodes a UDP-glycosyltransferase that contributes to cold, salt and drought stress tolerance via modulating anthocyanin accumulation.
AT4G27580		phosphatidylinositol transfer SFH5-like protein
AT4G27590	*HEAVY METAL ASSOCIATED PROTEIN 37 (ATHMP37)*	Heavy metal transport/detoxification superfamily protein
AT4G27600	*GENES NECESSARY FOR THE ACHIEVEMENT OF RUBISCO ACCUMULATION 5 (NARA5)*	Encodes a phosphofructokinase B-type carbohydrate kinase family protein, NARA5. Regulates photosynthetic gene expression.
AT4G27620		intracellular protein transporter
AT4G27630	*GPCR-TYPE G PROTEIN 2 (GTG2)*	Encodes a GPCR-type G protein receptor with nine predicted transmembrane domains. The protein binds abscisic acid (ABA) and is predicted to function as an ABA receptor. It has GTP-binding and GTPase activity and binds to ABA more effectively in the presence of GDP. GTG2 binds to GPA1, the alpha subunit of the heterotrimeric G protein. GPA1 (in its GTP-bound state) affects the GTP binding and GTPase activity of GTG2 and may act to down-regulate GTG2 binding to ABA. GTG2 is widely expressed throughout the plant and appears to be involved in the regulation of several ABA-dependent responses including seed germination, plant development, and promotion of stomatal closure. GTG2 transcript levels do not appear to change in response to ABA or abiotic stresses.
AT4G27640	*IMPORTIN-BETA 4 (IMB4)*	Nuclear import receptor for GRF-interacting factors (GIFs),roles in ovule development.
AT4G27652		hypothetical protein
AT4G27654		transmembrane protein
AT4G27657		hypothetical protein
AT4G27660		hypothetical protein
AT4G27670	*HEAT SHOCK PROTEIN 21 (HSP21)*	Encodes Hsp21, a chloroplast located small heat shock protein. A structure model of Hsp21, obtained by homology modeling, single-particle electron microscopy, and lysine-specific chemical crosslinking, shows that the Hsp21 subunits are arranged in two hexameric discs, rotated by 25 degree in relation to each other.
AT4G27700		Rhodanese/Cell cycle control phosphatase superfamily protein
AT4G27710	*CYTOCHROME P450, FAMILY 709, SUBFAMILY B, POLYPEPTIDE 3 (CYP709B3)*	member of CYP709B The mRNA is cell-to-cell mobile.
AT4G27720		Major facilitator superfamily protein
AT4G27730	*OLIGOPEPTIDE TRANSPORTER 1 (OPT6)*	oligopeptide transporter
AT4G27760	*FOREVER YOUNG (FEY)*	Encodes an oxidoreductase required for vegetative shoot apex development. Mutants display disruptions in leaf positioning and meristem maintenance.
AT4G27780	*ACYL-COA BINDING PROTEIN 2 (ACBP2)*	Encodes acyl-CoA-binding protein with ankyrin repeats The mRNA is cell-to-cell mobile.
AT4G27790		Calcium-binding EF hand family protein
AT4G27800	*THYLAKOID-ASSOCIATED PHOSPHATASE 38 (TAP38)*	Choroplast protein phosphatase TAP38/PPH1 is required for efficient dephosphorylation of the LHCII anthena and state transition from state 2 to state 1.
AT4G27820	*BETA GLUCOSIDASE 9 (BGLU9)*	beta glucosidase 9
AT4G27830	*BETA GLUCOSIDASE 10 (BGLU10)*	Encodes a beta-glucosidase that may be responsible for acyl-glucose-dependent anthocyanin glucosyltransferase activity in Arabidopsis. In vitro efforts to demonstrate AAGT activity for BGLU10 have been unsuccessful but experiments with mutants in this gene suggest at least an indirect involvement in anthocyanin formation.
AT4G27850		Glycine-rich protein family
AT4G27860	*MEMBRANE OF ER BODY 1 (MEB1)*	vacuolar iron transporter (VIT) family protein
AT4G27890	*(BOB2)*	HSP20-like chaperones superfamily protein
AT4G27930		
AT4G27940	*MANGANESE TRACKING FACTOR FOR MITOCHONDRIAL SOD2 (MTM1)*	Involved in Mn and Fe homeostasis, root length, and flowering time.

AT4G27970	*SLAC1 HOMOLOGUE 2 (SLAH2)*	Encodes a protein with ten predicted transmembrane helices. The SLAH2 protein has similarity to the SLAC1 protein involved in ion homeostasis in guard cells. But, it is not expressed in guard cells and cannot complement a slac1-2 mutant suggesting that it performs a different function. SLAH2:GFP localizes to the plasma membrane.
AT4G27980		trichohyalin-like protein (DUF3444)
AT4G27990	*(YLMG1-2)*	One of four Arabidopsis homologs of bacterial ymlg proteins.
AT4G28000		P-loop containing nucleoside triphosphate hydrolases superfamily protein
AT4G28010	*RNA PROCESSING FACTOR 5 (RPF5)*	PPRP involved in processing of nad6,atp9 and 26S RNAs.
AT4G28020		tRNA-thr(GGU) m(6)t(6)A37 methyltransferase
AT4G28025		hypothetical protein
AT4G28030	*GCN5‐RELATED N‐ACETYLTRANSFERASE 7 (GNAT7)*	Acyl-CoA N-acyltransferases (NAT) superfamily protein
AT4G28040	*USUALLY MULTIPLE ACIDS MOVE IN AND OUT TRANSPORTERS 33 (UMAMIT33)*	nodulin MtN21-like transporter family protein
AT4G28050	*TETRASPANIN7 (TET7)*	Member of TETRASPANIN family
AT4G28080	*REDUCED CHLOROPLAST COVERAGE 2 (REC2)*	Encodes REDUCED CHLOROPLAST COVERAGE 2 (REC2). Along with REC1 and REC3 it contributes to establishing the size of the chloroplast compartment.
AT4G28085		transmembrane protein
AT4G28090	*SKU5 SIMILAR 10 (sks10)*	SKU5 similar 10
AT4G28100	*ENDODERMIS7 (EN7)*	transmembrane protein
AT4G28110	*MYB DOMAIN PROTEIN 41 (MYB41)*	Member of the R2R3 factor gene family. Expression is induced in response to desiccation, ABA and salt treatment. Overexpression of Myb41 results in abnormal cuticle development and decreased cell expansion.
AT4G28120		
AT4G28130	*DIACYLGLYCEROL KINASE 6 (DGK6)*	diacylglycerol kinase 6
AT4G28140	*ETHYLENE RESPONSIVE FACTOR54 (ERF54)*	encodes a member of the DREB subfamily A-6 of ERF/AP2 transcription factor family. The protein contains one AP2 domain. There are 8 members in this subfamily including RAP2.4. Regulated by heat shock.
AT4G28150		hypothetical protein (DUF789)
AT4G28160		hydroxyproline-rich glycoprotein family protein
AT4G28170		transmembrane protein
AT4G28190	*ULTRAPETALA1 (ULT1)*	Encodes a novel Cys-rich protein with a B-box like domain that acts as a negative regulator of meristem cell accumulation in inflorescence and floral meristems as loss-of-function ult1 mutations cause inflorescence meristem enlargement, the production of extra flowers and floral organs, and a decrease in floral meristem determinacy. Acts opposite to CLF which represses AG, but preventing deposition of CLF repressive methylation marks.ULT1 acts as an anti-repressor that counteracts EMF1 action through modulation of histone marks on target genes. Regulates developmental as well as biotic and abiotic stress response genes.
AT4G28200		U3 small nucleolar RNA-associated-like protein
AT4G28230		hypothetical protein
AT4G28240	*WOUND-INDUCED POLYPEPTIDE 1 (WIP1)*	Member of the wound-induced polypeptide (WIP) family. Positively regulates plant resistance against Pst DC3000 by enhancing PTI responses.
AT4G28250	*EXPANSIN B3 (EXPB3)*	putative beta-expansin/allergen protein. Naming convention from the Expansin Working Group (Kende et al, 2004. Plant Mol Bio). Involved in the formation of nematode-induced syncytia in roots of Arabidopsis thaliana.
AT4G28260		acyl-UDP-N-acetylglucosamine O-acyltransferase
AT4G28270	*RING MEMBRANE-ANCHOR 2 (RMA2)*	Encodes a RING finger E3 ubiquitin ligase. Binds and ubiquitinates ABP1 in vivo and in vitro.
AT4G28280	*LORELEI-LIKE-GPI ANCHORED PROTEIN 3 (LLG3)*	LORELEI-LIKE-GPI ANCHORED PROTEIN 3
AT4G28290		hypothetical protein
AT4G28300	*FLOE1 (FLOE1)*	Encodes a protein with 13.6% proline amino acids that is predicted to localize to the cell wall. The mRNA is cell-to-cell mobile.
AT4G28310		microtubule-associated protein
AT4G28350	*L-TYPE LECTIN RECEPTOR KINASE VII.2 (LECRK-VII.2)*	Concanavalin A-like lectin protein kinase family protein
AT4G28370	*FLYING SAUCER 1 (FLY1)*	Encodes an E3 ubiquitin ligase that is involved in plant cell wall modification, seed mucilage extrusion, and controls the degree of pectin methylesterification in seed mucilage. fly1 mutant seeds release more compact mucilage capsules and detached outer tangential primary walls when hydrated in water. Fly1 is located in the endomembrane system, likely localized in late endosome/multivesicular bodies/prevacular compartment. It has been shown to ubiquitinate proteins in conjunction with UBA1 and UBC8.
AT4G28380		Leucine-rich repeat (LRR) family protein
AT4G28390	*ADP/ATP CARRIER 3 (AAC3)*	Encodes a mitochondrial ADP/ATP carrier protein. Shown in heterologous systems to be located in the plasma membrane. Has comparable affinity for ADP and ATP (in E.coli).
AT4G28395	*ANTHER 7 (A7)*	related to lipid transfer proteins
AT4G28400		Protein phosphatase 2C family protein
AT4G28410	*ROOT SYSTEM ARCHITECTURE 1 (RSA1)*	Tyrosine transaminase family protein
AT4G28420		Tyrosine transaminase family protein
AT4G28430	*RETICULON 18 (RTN18)*	Reticulon family protein
AT4G28450		This gene is predicted to encode a protein with a DWD motif. It can bind to DDB1a in Y2H assays and may be involved in the formation of a CUL4-based E3 ubiquitin ligase
AT4G28460	*PRECURSOR OF PAMP-INDUCED PEPTIDE 1 (PREPIP1)*	Activates immune responses through RECEPTOR-LIKE KINASE7 (RLK7). Induces stomatal closure is dependent on RLK7 and the transcription of genes involved in SA production and SA-dependent stomatal closure. SA promotes the flg22-induced expression of PIP1 preligand, prePIP1.
AT4G28490	*HAESA (HAE)*	Member of Receptor kinase-like protein family. Controls the separation step of floral organ abscission. The mRNA is cell-to-cell mobile.

AT4G28500	*NAC DOMAIN CONTAINING PROTEIN 73 (NAC073)*	NAC domain containing protein 73
AT4G28520	*CRUCIFERIN 3 (CRU3)*	Encodes a 12S seed storage protein that is tyrosine-phosphorylated and its phosphorylation state is modulated in response to ABA in Arabidopsis thaliana seeds.
AT4G28560	*ROP-INTERACTIVE CRIB MOTIF-CONTAINING PROTEIN 7 (RIC7)*	encodes a member of a novel protein family that contains contain a CRIB (for Cdc42/Rac-interactive binding) motif required for their specific interaction with GTP-bound Rop1 (plant-specific Rho GTPase). Most similar to RIC6 and RIC8 (subfamily group II). Gene is expressed in all tissues examined.
AT4G28570		Long-chain fatty alcohol dehydrogenase family protein
AT4G28580	*MAGNESIUM TRANSPORT 5 (MGT5)*	Transmembrane magnesium transporter that induces Mg transport from tapetum cell to locule. One of nine family members. Functions in pollen development.
AT4G28620	*ATP-BINDING CASSETTE B24 (ABCB24)*	Half-molecule ABC transporter ATM2. Arabidopsis thaliana has three ATM genes, namely ATM1, ATM2 and ATM3. Only ATM3 has an important function for plant growth.
AT4G28640	*INDOLE-3-ACETIC ACID INDUCIBLE 11 (IAA11)*	Auxin induced gene, IAA11 (IAA11). Check the Comments field on the locus page to view updated sequence annotation.
AT4G28650	*PXY-LIKE2 (PXL2)*	Encodes one of the two putative eLRR kinase closely related to PXY (At1g08590/PXL1 and At4g28650/PXL2). Insertion mutants in either pxl1 or pxl2 do not exhibit an obvious phenotype in the stem; double-mutant combinations of a Col allele, of pxy (pxy-3) with pxl1 and pxl2, generate a more severe vascular phenotype than pxy-3 alone, suggesting that these genes act synergistically with PXY in regulating vascular-tissue development in the stem.
AT4G28660	*PHOTOSYSTEM II REACTION CENTER PSB28 PROTEIN (PSB28)*	Similar to PsbW subunit of photosystem II.
AT4G28670		cysteine-rich RECEPTOR-like kinase, putative (DUF26)
AT4G28680	*L-TYROSINE DECARBOXYLASE (TYRDC)*	Encodes a stress-induced tyrosine decarboxylase (TyrDC). Recombinant (His)6-TyrDC expressed in E. coli catalyzes the conversion of L-tyrosine to tyramine. Recombinant TyrDC forms tetramers.
AT4G28700	*AMMONIUM TRANSPORTER 1;4 (AMT1;4)*	ammonium transporter 1
AT4G28703		RmlC-like cupins superfamily protein
AT4G28706		pfkB-like carbohydrate kinase family protein
AT4G28720	*YUCCA 8 (YUC8)*	Auxin biosynthetic gene regulated by RVE1. Overexpression leads to suppression of bri1 phenotype.
AT4G28740		LOW PSII ACCUMULATION-like protein
AT4G28750	*PSA E1 KNOCKOUT (PSAE-1)*	mutant has Decreased effective quantum yield of photosystem II; Pale green plants; Reduced growth rate; Subunit E of Photosystem I
AT4G28780		GDSL-motif esterase/acyltransferase/lipase. Enzyme group with broad substrate specificity that may catalyze acyltransfer or hydrolase reactions with lipid and non-lipid substrates.
AT4G28810		
AT4G28830		S-adenosyl-L-methionine-dependent methyltransferases superfamily protein
AT4G28850	*XYLOGLUCAN ENDOTRANSGLUCOSYLASE/HYDROLASE 26 (XTH26)*	xyloglucan endotransglucosylase/hydrolase 26
AT4G28860	*CASEIN KINASE I-LIKE 4 (ckl4)*	Member of CKL gene family (CKL-A group)
AT4G28890	*ARABIDOPSIS T??XICOS EN LEVADURA 42 (ATL42)*	RING/U-box superfamily protein
AT4G28900		transposable_element_gene;copia-like retrotransposon family, has a 7.7e-236 P-value blast match to GB:AAA57005 Hopscotch polyprotein (Ty1_Copia-element) (Zea mays);(source:TAIR10)
AT4G28940		Phosphorylase superfamily protein
AT4G28950	*RHO-RELATED PROTEIN FROM PLANTS 9 (ROP9)*	A member of ROP GTPase gene family.
AT4G28960		transposable_element_gene;similar to unknown protein [Arabidopsis thaliana] (TAIR:AT1G23930.1);(source:TAIR10)
AT4G28980	*CDK-ACTIVATING KINASE 1AT (CAK1AT)*	Encodes a CDK-activating kinase that regulates root initial cell differentiation. Phosphorylates CDKD2 and CDKD3, but not CDKD1. Controls CDK activities and basal transcription.
AT4G29020		glycine-rich protein
AT4G29030		Putative membrane lipoprotein
AT4G29050	*L-TYPE LECTIN RECEPTOR KINASE V.9 (LECRK-V.9)*	Concanavalin A-like lectin protein kinase family protein
AT4G29060	*EMBRYO DEFECTIVE 2726 (emb2726)*	Involved in chloroplast biogenesis and early embryo development. May function as an EF-Ts to regulate plastid translation.
AT4G29070		Phospholipase A2 family protein
AT4G29080	*PHYTOCHROME-ASSOCIATED PROTEIN 2 (PAP2)*	phytochrome-associated protein 2 (PAP2)
AT4G29090		Ribonuclease H-like superfamily protein
AT4G29100	*BASIC HELIX LOOP HELIX 68 (BHLH68)*	Member of basic helix loop helix protein family. Expressed primarily in vascular system. Overexpression causes ABA sensitivity. Together with PFA1 and PFA2 governs the competence of pericycle cells to initiate lateral root primordium formation. Governs the competence of pericycle cells to initiate lateral root primordium formation.
AT4G29120		6-phosphogluconate dehydrogenase family protein
AT4G29130	*HEXOKINASE 1 (HXK1)*	Encodes a hexokinase (HXK1) in the plant glucose-signaling network. Functions as a glucose sensor to interrelate nutrient, light, and hormone signaling networks for controlling growth and development in response to the changing environment.
AT4G29140	*ACTIVATED DISEASE SUSCEPTIBILITY 1 (ADS1)*	Encodes Activated Disease Susceptibility 1 (ADS1), a putative MATE (multidrug and toxic compound extrusion) transport protein that negatively regulates plant disease resistance.
AT4G29180	*ROOT HAIR SPECIFIC 16 (RHS16)*	root hair specific 16
AT4G29190	*OXIDATION-RELATED ZINC FINGER 2 (OZF2)*	Zinc finger C-x8-C-x5-C-x3-H type family protein
AT4G29200	*(DEG3)*	Over-expressed by salt stress.

AT4G29210	*GAMMA-GLUTAMYL TRANSPEPTIDASE 4 (GGT4)*	The gene encodes a gamma-glutamyltransferase (AKA gamma-glutamyl transpeptidase, EC 2.3.2.2) that is located in the vacuole and is most active in roots. The encoded enzyme is involved in the initial degradation of glutathione conjugates in this cell compartment. It is also induced by xenobiotics and contributes to xenobiotics metabolism. Note that conflicting nomenclature exists in the literature: At4g29210 is named as GGT3 in Plant J. 2007 Mar 49(5):878-88; At4g29210 is named as GGT4 and At1g69820 as GGT3 in Plant Physiol. 2007 Aug 144(4):1715-32.
AT4G29220	*PHOSPHOFRUCTOKINASE 1 (PFK1)*	phosphofructokinase 1
AT4G29230	*NAC DOMAIN CONTAINING PROTEIN 75 (NAC075)*	NAC domain protein involved in negative regulation of flowering.
AT4G29240		Leucine-rich repeat (LRR) family protein
AT4G29250		HXXXD-type acyl-transferase family protein
AT4G29260	*VEGETATIVE STORAGE PROTEIN 3 (VSP3)*	VSP3 is a secreted acid phosphatase.
AT4G29270		HAD superfamily, subfamily IIIB acid phosphatase
AT4G29285	*LOW-MOLECULAR-WEIGHT CYSTEINE-RICH 24 (LCR24)*	Encodes a member of a family of small,secreted, cysteine rich protein with sequence similarity to the PCP (pollen coat protein) gene family.
AT4G29320		
AT4G29340	*PROFILIN 4 (PRF4)*	Profilin is a low-molecular weight, actin monomer-binding protein that regulates the organization of actin cytoskeleton in eukaryotes, including higher plants. PRF4 and PRF5 are late pollen-specific and are not detectable in other cell types of the plant body including microspores and root hairs. Immunocytochemical studies at the subcellular level reveal that both the constitutive and pollen-specific profilins are abundant in the cytoplasm. In vegetative cell types, such as root apical cells, profilins showed localization to nuclei in addition to the cytoplasmic staining.
AT4G29360		O-Glycosyl hydrolases family 17 protein
AT4G29370		Galactose oxidase/kelch repeat superfamily protein
AT4G29380	*VACUOLAR PROTEIN SORTING 15 (VPS15)*	The gene encodes phosphatidylinositol 3- kinase involved in the development and germination of pollen through the biosynthesis of phosphatidylinositol 3-phosphate (PI3P). The mRNA is cell-to-cell mobile.
AT4G29440	*IST1-LIKE 4 (ISTL4)*	Regulator of Vps4 activity in the MVB pathway protein
AT4G29450		Leucine-rich repeat protein kinase family protein
AT4G29490		Metallopeptidase M24 family protein
AT4G29510	*ARGININE METHYLTRANSFERASE 11 (PRMT11)*	Has arginine N-methyltransferase activity. Modifies AtMBD7.
AT4G29520	*SENSITIVE TO SALT1 (SES1)*	SES1 is an ER localized chaperone involved in salt and heat stress response.
AT4G29540	*(LPXA)*	Encodes a UDP-N-acetylglucosamine acyltransferase.
AT4G29550		hypothetical protein (DUF626)
AT4G29560		fanconi anemia group E protein FANCE protein
AT4G29570		Cytidine/deoxycytidylate deaminase family protein
AT4G29580		Cytidine/deoxycytidylate deaminase family protein
AT4G29590		S-adenosyl-L-methionine-dependent methyltransferases superfamily protein
AT4G29600		Cytidine/deoxycytidylate deaminase family protein
AT4G29610		Cytidine/deoxycytidylate deaminase family protein
AT4G29620		Cytidine/deoxycytidylate deaminase family protein
AT4G29630		Cytidine/deoxycytidylate deaminase family protein
AT4G29640		Cytidine/deoxycytidylate deaminase family protein
AT4G29650		Cytidine/deoxycytidylate deaminase family protein
AT4G29660	*EMBRYO DEFECTIVE 2752 (EMB2752)*	embryo defective 2752
AT4G29690		Alkaline-phosphatase-like family protein
AT4G29700		Alkaline-phosphatase-like family protein
AT4G29710		Alkaline-phosphatase-like family protein
AT4G29720	*POLYAMINE OXIDASE 5 (PAO5)*	polyamine oxidase 5
AT4G29770	*HEAT-INDUCED TAS1 TARGET 1 (HTT1)*	Target of trans acting-siR480/255. Testing.
AT4G29780		Expression of the gene is affected by multiple stresses. Knockout and overexpression lines show no obvious phenotypes.
AT4G29800	*PATATIN-LIKE PROTEIN 8 (PLP8)*	PATATIN-like protein 8
AT4G29810	*MAP KINASE KINASE 2 (MKK2)*	Encodes a MAP kinase kinase 2 that regulates MPK6 and MPK4 in response to cold and salt stresses. Co-expression with MEKK1 in protoplasts activated MKK2 activity, suggesting that MEKK1 may be a regulator of MKK2.
AT4G29820	*HOMOLOG OF CFIM-25 (CFIM-25)*	Encodes a homolog of the protein CFI-25, a polyadenylation factor subunit.
AT4G29890		choline monooxygenase, putative (CMO-like)
AT4G29900	*AUTOINHIBITED CA(2+)-ATPASE 10 (ACA10)*	one of the type IIB calcium pump isoforms. encodes an autoinhibited Ca(2+)-ATPase that contains an N-terminal calmodulin binding autoinhibitory domain.
AT4G29940	*PATHOGENESIS RELATED HOMEODOMAIN PROTEIN A (PRHA)*	Homeodomain protein (PRHA). Expression of the gene differs in various vegetative and floral plant tissues and is positively influenced by the phytohormone auxin. It is often associated with regions of developing vascular tissue. The prha promoter is highly responsive to the synthetic auxin, naphthalene acetic acid, in transient assays using tobacco protoplasts. The PRHA protein has the capacity to bind to TAATTG core sequence elements but requires additional adjacent bases for high-affinity binding.
AT4G29950		Ypt/Rab-GAP domain of gyp1p superfamily protein
AT4G29980		fasciclin-like arabinogalactan protein
AT4G29990		Leucine-rich repeat transmembrane protein kinase protein
AT4G30010		ATP-dependent RNA helicase
AT4G30020		PA-domain containing subtilase family protein

AT4G30030		Eukaryotic aspartyl protease family protein
AT4G30040		Eukaryotic aspartyl protease family protein
AT4G30050		transmembrane protein
AT4G30060		Core-2/I-branching beta-1,6-N-acetylglucosaminyltransferase family protein
AT4G30080	*AUXIN RESPONSE FACTOR 16 (ARF16)*	Involved in root cap cell differentiation. Gene expression is regulated by mir160.Located in the nucleus.
AT4G30090		golgin family A protein
AT4G30110	*HEAVY METAL ATPASE 2 (HMA2)*	encodes a protein similar to Zn-ATPase, a P1B-type ATPases transport zinc
AT4G30120	*HEAVY METAL ATPASE 3 (HMA3)*	encodes a protein similar to Zn-ATPase, a P1B-type ATPases transport zinc
AT4G30130		DUF630 family protein (DUF630 and DUF632)
AT4G30140	*CUTICLE DESTRUCTING FACTOR 1 (CDEF1)*	Member of the GDSL lipase/esterase family of proteins that functions as cutinase. Expressed in pollen and at the zone of lateral root emergence.
AT4G30150		Urb2/Npa2 family protein
AT4G30170		Peroxidase family protein
AT4G30190	*H(+)-ATPASE 2 (HA2)*	Belongs to the P-type ATPase superfamily of cation-transporting ATPases, pumps protons out of the cell, generating a proton gradient that drives the active transport of nutrients by proton symport. has two autoinhibitory regions within the C-terminal domain. Its plasma membrane localization is light-dependent.
AT4G30200	*VERNALIZATION5/VIN3-LIKE 1 (VEL1)*	Encodes a protein with similarity to VRN5 and VIN3.Contains both a fibronectin III and PHD finger domain. VEL1 is a part of a polycomb repressive complex (PRC2) that is involved in epigenetic silencing of the FLC flowering locus.
AT4G30230		hypothetical protein
AT4G30270	*XYLOGLUCAN ENDOTRANSGLUCOSYLASE/HYDROLASE 24 (XTH24)*	encodes a protein similar to endo xyloglucan transferase in sequence. It is also very similar to BRU1 in soybean, which is involved in brassinosteroid response.
AT4G30280	*XYLOGLUCAN ENDOTRANSGLUCOSYLASE/HYDROLASE 18 (XTH18)*	Encodes a xyloglucan endotransglucosylase/hydrolase with only only the endotransglucosylase (XET; EC 2.4.1.207) activity towards xyloglucan and non-detectable endohydrolytic (XEH; EC 3.2.1.151) activity. Expressed in the mature or basal regions of both the main and lateral roots, but not in the tip of these roots where cell division occurs.
AT4G30290	*XYLOGLUCAN ENDOTRANSGLUCOSYLASE/HYDROLASE 19 (XTH19)*	Encodes a xyloglucan endotransglucosylase/hydrolase with only only the endotransglucosylase (XET; EC 2.4.1.207) activity towards xyloglucan and non-detectable endohydrolytic (XEH; EC 3.2.1.151) activity. Expressed throughout both the main and the lateral root, with intensive expression at the dividing and elongating regions. Is expressed in lateral root primordia but expression ceases after lateral root begins to grow. Involved in cell proliferation in incised inflorescence stems.
AT4G30300	*ATP-BINDING CASSETTE E3 (ABCE3)*	member of NAP subfamily
AT4G30320	*(ATCAPE6)*	CAP (Cysteine-rich secretory proteins, Antigen 5, and Pathogenesis-related 1 protein) superfamily protein
AT4G30340	*DIACYLGLYCEROL KINASE 7 (DGK7)*	encodes a diacylglycerol kinase. Applying a specific diacylglycerol kinase inhibitor to the growth media resulted in reduced root elongation and plant growth. Gene is expressed throughout the plant but is strongest in flowers and young seedlings.
AT4G30350	*SMAX1-LIKE 2 (SMXL2)*	Encodes a member of an eight-gene family (SMAX1 and SMAX1-like) that has weak similarity to AtHSP101, a ClpB chaperonin required for thermotolerance. Regulates root and root hair development downstream of KAI2-mediated signaling.
AT4G30360	*CYCLIC NUCLEOTIDE-GATED CHANNEL 17 (CNGC17)*	member of Cyclic nucleotide gated channel family
AT4G30380		Encodes a Plant Natriuretic Peptide (PNP). PNPs are a class of systemically mobile molecules distantly related to expansins; their biological role has remained elusive.
AT4G30390		UDP-arabinopyranose mutase
AT4G30400	*ARABIDOPSIS T??XICOS EN LEVADURA 13 (ATL13)*	RING/U-box superfamily protein
AT4G30410	*IBH1-LIKE 1 (IBL1)*	sequence-specific DNA binding transcription factor
AT4G30420	*USUALLY MULTIPLE ACIDS MOVE IN AND OUT TRANSPORTERS 34 (UMAMIT34)*	nodulin MtN21-like transporter family protein
AT4G30430	*TETRASPANIN9 (TET9)*	Member of TETRASPANIN family
AT4G30450		glycine-rich protein
AT4G30460		glycine-rich protein
AT4G30470		NAD(P)-binding Rossmann-fold superfamily protein
AT4G30490		AFG1-like ATPase family protein
AT4G30500		transmembrane protein (DUF788)
AT4G30520	*SENESCENCE-ASSOCIATED RECEPTOR-LIKE KINASE (SARK)*	Encodes SARK (SENESCENCE-ASSOCIATED RECEPTOR-LIKE KINASE). Regulates leaf senescence through synergistic actions of auxin and ethylene. It is one of a group of LRR-RLKs, designated as CLAVATA3 INSENSITIVE RECEPTOR KINASES (CIKs), that act as co-receptors and have essential roles in regulating CLV3-mediated stem cell homeostasis.
AT4G30530	*GAMMA-GLUTAMYL PEPTIDASE 1 (GGP1)*	Encodes a gamma-glutamyl peptidase, outside the GGT family, that can hydrolyze gamma-glutamyl peptide bonds. The mRNA is cell-to-cell mobile.
AT4G30540		Class I glutamine amidotransferase-like superfamily protein
AT4G30580	*(ATS2)*	Encodes a plastidic lysophosphatidic acid acyltransferase (LPAAT). Is critical for chloroplasts phosphatidic acid biosynthesis. The null allele is embryo lethal.
AT4G30590	*EARLY NODULIN-LIKE PROTEIN 12 (ENODL12)*	early nodulin-like protein 12
AT4G30610	*BRI1 SUPPRESSOR 1 (BRS1)*	Encodes a secreted glycosylated serine carboxypeptidase with broad substrate preference that is involved in brassinosteroid signalling via BRI1. It is proteolytically processed in vivo by a separate as yet unidentified protease.
AT4G30620	*STIC2 LIKE (STCL)*	Homolog of STIC2, recent duplication.
AT4G30630	*(INB2A)*	hypothetical protein
AT4G30640		RNI-like superfamily protein
AT4G30650		Low temperature and salt responsive protein family

AT4G30670		Putative membrane lipoprotein
AT4G30690	*(ATINFC-4)*	SVR9-LIKE1 (SVR9L1)
AT4G30750		hypothetical protein
AT4G30770		Putative membrane lipoprotein
AT4G30800		Nucleic acid-binding, OB-fold-like protein
AT4G30810	*SERINE CARBOXYPEPTIDASE-LIKE 29 (scpl29)*	serine carboxypeptidase-like 29
AT4G30825	*BIOGENESIS FACTOR REQUIRED FOR ATP SYNTHASE 2 (BFA2)*	P-class pentatricopeptide repeat (PPR) protein essential for accumulation of the dicistronic atpH/F transcript in chloroplasts. Acts as barrier to prevent the atpH/F transcript degradation by exoribonucleases by binding to the consensus sequence of the atpF-atpA intergenic region.
AT4G30830		myosin-like protein (Protein of unknown function, DUF593)
AT4G30840	*(NUP43)*	Transducin/WD40 repeat-like superfamily protein
AT4G30850	*HEPTAHELICAL TRANSMEMBRANE PROTEIN2 (HHP2)*	heptahelical transmembrane protein homologous to human adiponectin receptors and progestin receptors
AT4G30890	*UBIQUITIN-SPECIFIC PROTEASE 24 (UBP24)*	Encodes a ubiquitin-specific protease.
AT4G30910		Cytosol aminopeptidase family protein
AT4G30930	*NUCLEAR FUSION DEFECTIVE 1 (NFD1)*	Encodes a ribosomal RPL21M protein that is localized to the mitochondrion and is involved in karyogamy during female gametophyte development and fertilization. Mutants display defects in both male and female gametophyte development (i.e.collapsed pollen and female gametophytes with unfused central cells).
AT4G30960	*SOS3-INTERACTING PROTEIN 3 (SIP3)*	Encodes CBL-interacting protein kinase 6 (CIPK6). Required for development and salt tolerance. The mRNA is cell-to-cell mobile.
AT4G30970		hypothetical protein
AT4G30990		ARM repeat superfamily protein
AT4G31000	*(CBP60F)*	Calmodulin-binding protein
AT4G31020	*ABHD17 (ALPHA/BETA HYDROLASE DOMAIN-CONTAINING PROTEIN 17)-LIKE ACYL PROTEIN THIOESTERASE 8 (ABAPT8)*	Encodes a de-S-acylation enzyme which specifically reduces protein S-acylation levels of five immunity-related proteins.
AT4G31030		Putative membrane lipoprotein
AT4G31060		encodes a member of the DREB subfamily A-5 of ERF/AP2 transcription factor family. The protein contains one AP2 domain. There are 15 members in this subfamily including RAP2.1, RAP2.9 and RAP2.10. A maternally expressed imprinted gene.
AT4G31070		PPR superfamily protein
AT4G31080	*LUNAPARK2.2 (LNP2.2)*	Encodes one of two LUNAPARK proteins in Arabidopsis. Both LNPA and LNPB are predominantly distributed throughout the ER, but not preferentially localized at the three-way junctions. Mutation of both LNPA and LNPB together caused the cortical ER to develop poor ER cisternae and a less dense tubular network. E3 ligase involved in degradation of RHD3 to maintain a tubular ER network.
AT4G31090		
AT4G31100		wall-associated kinase
AT4G31120	*SHK1 BINDING PROTEIN 1 (SKB1)*	Involved in vernalization. Required for epigenetic silencing of FLC, and for vernalization-mediated histone modification.
AT4G31150		endonuclease V family protein
AT4G31210		DNA topoisomerase, type IA, core
AT4G31220		
AT4G31230		kinase with adenine nucleotide alpha hydrolases-like domain-containing protein
AT4G31240	*NUCLEOREDOXIN 2 (NRX2)*	protein kinase C-like zinc finger protein
AT4G31260		hypothetical protein
AT4G31270	*IDM1-ASSOCIATED PROTEIN 2 (IDAP2)*	Encodes IDAP2. Acts together with IDAP1 and IDM1 to regulate active DNA demethylation.
AT4G31280		hypothetical protein
AT4G31290	*GAMMA-GLUTAMYL CYCLOTRANSFERASE 2;2 (GGCT2;2)*	ChaC-like family protein
AT4G31310		AIG2-like (avirulence induced gene) family protein
AT4G31320	*SMALL AUXIN UPREGULATED RNA 37 (SAUR37)*	SAUR-like auxin-responsive protein family
AT4G31330		transmembrane protein, putative (Protein of unknown function, DUF599)
AT4G31360		selenium binding protein
AT4G31420	*REI1-LIKE 1 (REIL1)*	Cytosolic ribosomal 60S-biogenesis factor.
AT4G31430	*(JAPANESE FOR NUCLEUS) 4 (KAKU4)*	Encodes a plant-specific protein that physically interacts with CRWN1 and its homolog CRWN4 and localizes at the inner nuclear membrane. KAKU4 deforms the nuclear envelope in a dose-dependent manner, in association with nuclear membrane invagination and stack formation.
AT4G31450	*(CTL09)*	RING/U-box superfamily protein
AT4G31470		CAP (Cysteine-rich secretory proteins, Antigen 5, and Pathogenesis-related 1 protein) superfamily protein
AT4G31500	*CYTOCHROME P450, FAMILY 83, SUBFAMILY B, POLYPEPTIDE 1 (CYP83B1)*	Encodes an oxime-metabolizing enzyme in the biosynthetic pathway of glucosinolates. Is required for phytochrome signal transduction in red light. Mutation confers auxin overproduction.
AT4G31520		SDA1 family protein
AT4G31550	*WRKY DNA-BINDING PROTEIN 11 (WRKY11)*	member of WRKY Transcription Factor; Group II-d; negative regulator of basal resistance to Pseudomonas syringae.
AT4G31560	*HIGH CHLOROPHYLL FLUORESCENCE 153 (HCF153)*	Encodes HCF153, a 15-KDa protein involved in the biogenesis of the cytochrome b(6)f complex. Associated with the thylakoid membrane.
AT4G31570		nucleoporin
AT4G31580	*RS-CONTAINING ZINC FINGER PROTEIN 22 (RSZ22)*	Encodes a Serine/arginine-rich (SR) protein RSZp22. SR proteins are splicing regulators that share a modular structure consisting of one or two N-terminal RNA recognition motif domains and a C-terminal RS-rich domain. RSZp22 is located in the nucleolus. It is a nucleocytoplasmic shuttling protein and an interacting partner to the Arabidopsis U1-70K. Barta et al (2010) have proposed a nomenclature for Serine/Arginine-Rich Protein Splicing Factors (SR proteins): Plant Cell. 2010, 22:2926.

AT4G31600	*UDP-GALACTOSE TRANSPORTER 7 (UTR7)*	Encodes a Golgi-localized UDP?glucose/UDP?galactose transporter that affects lateral root emergence.
AT4G31610	*REPRODUCTIVE MERISTEM 34 (REM34)*	Encodes a member of the REM (Reproductive Meristem) gene family, a part of the B3 DNA-binding domain superfamily. Expressed specifically in reproductive meristems.
AT4G31620	*REPRODUCTIVE MERISTEM 36 (REM36)*	Encodes a member of the REM (Reproductive Meristem) gene family, a part of the B3 DNA-binding domain superfamily.
AT4G31630	*(REM37)*	Transcriptional factor B3 family protein
AT4G31670	*UBIQUITIN-SPECIFIC PROTEASE 18 (UBP18)*	ubiquitin-specific protease 18
AT4G31680		Transcriptional factor B3 family protein
AT4G31730	*GLUTAMINE DUMPER 1 (GDU1)*	Glutamine dumper1 is a putative transmembrane protein. It is involved in glutamine secretion The mRNA is cell-to-cell mobile.
AT4G31740		Sec1/munc18-like (SM) proteins superfamily
AT4G31750	*HOPW1-1-INTERACTING 2 (WIN2)*	Encodes HopW1-1-Interacting protein 2 (WIN2). Interacts with the P. syringae effector HopW1-1. WIN2 has protein phosphatase activity. Modulates plant defenses against bacteria. Three WIN proteins are identified so far (WIN1: AT1G80600; WIN2: AT4G31750; WIN3: AT5G13320).
AT4G31760		peroxidase superfamily protein
AT4G31790		Tetrapyrrole (Corrin/Porphyrin) Methylase
AT4G31800	*WRKY DNA-BINDING PROTEIN 18 (WRKY18)*	Pathogen-induced transcription factor. Binds W-box sequences in vitro. Forms protein complexes with itself and with WRKY40 and WRKY60. Constitutive expression of WRKY18 enhanced resistance to P. syringae, but its coexpression with WRKY40 or WRKY60 made plants more susceptible to both P. syringae and B. cinerea. WRKY18, WRKY40, and WRKY60 have partially redundant roles in response to the hemibiotrophic bacterial pathogen Pseudomonas syringae and the necrotrophic fungal pathogen Botrytis cinerea, with WRKY18 playing a more important role than the other two. The mRNA is cell-to-cell mobile.
AT4G31805	*POLAR LOCALIZATION DURING ASYMMETRIC DIVISION AND REDISTRIBUTION (POLAR)*	Encodes POLAR, a scaffold protein associated with cellular asymmetry of meristemoids. Its transcript levels change after inducing MUTE expression in a mute background.
AT4G31810	*(CHY4)*	ATP-dependent caseinolytic (Clp) protease/crotonase family protein
AT4G31820	*ENHANCER OF PINOID (ENP)*	A member of the NPY family genes (NPY1/AT4G31820, NPY2/AT2G14820, NPY3/AT5G67440, NPY4/AT2G23050, NPY5/AT4G37590). Encodes a protein with similarity to NHP3. Contains BTB/POZ domain. Promoter region has canonical auxin response element binding site and Wus binding site. Co-localizes to the late endosome with PID. Regulates cotyledon development through control of PIN1 polarity in concert with PID. Also involved in sepal and gynoecia development.
AT4G31830		transmembrane protein
AT4G31840	*EARLY NODULIN-LIKE PROTEIN 15 (ENODL15)*	early nodulin-like protein 15
AT4G31850	*PROTON GRADIENT REGULATION 3 (PGR3)*	Encodes a protein containing 27 pentatrico-peptide repeat (PPR) motifs. Functions in the stabilization of petL operon RNA and also in the translation of petL.
AT4G31860		Protein phosphatase 2C family protein
AT4G31870	*GLUTATHIONE PEROXIDASE 7 (GPX7)*	Encodes glutathione peroxidase. Role in the degradation of H2O2 to water using glutathione as electron donor.
AT4G31875		hypothetical protein
AT4G31880	*(PDS5C)*	One of 5 PO76/PDS5 cohesion cofactor orthologs of Arabidopsis.
AT4G31890		ARM repeat superfamily protein
AT4G31900	*PICKLE RELATED 2 (PKR2)*	chromatin remodeling factor
AT4G31910	*BR-RELATED ACYLTRANSFERASE1 (BAT1)*	Encodes an acyltransferase that can modify brassinosteroids (BRs) by acylation and may modulate endogenous BR levels.
AT4G31920	*RESPONSE REGULATOR 10 (RR10)*	Encodes an Arabidopsis response regulator (ARR) protein that acts in concert with other type-B ARRs in the cytokinin signaling pathway. Also involved in cytokinin-dependent inhibition of hypocotyl elongation and cytokinin-dependent greening and shooting in tissue culture. ARR1, ARR10, and ARR12 are redundant regulators of drought response, with ARR1 being the most critical. ARR1, ARR10 and ARR12 redundantly bind to the promoter of WUSCHEL (WUS), directly activate its transcription. In parallel, ARR1, ARR10 and ARR12 repress the expression of YUCCAs (YUCs), which encode a key enzyme for auxin biosynthesis, indirectly promoting WUS induction. The regulation of ARR1, ARR10 and ARR12 on WUS and YUCs is required for regeneration and maintenance of shoot meristem.
AT4G31940	*CYTOCHROME P450, FAMILY 82, SUBFAMILY C, POLYPEPTIDE 4 (CYP82C4)*	The gene encodes a cytochrome P450 enzyme, CYP82C. It is involved in the early Fe deficiency response.CYP82C4 hydroxylates fraxetin to generate sideretin (5-hydroxyfraxetin). Fraxetin and sideretin are catecholic coumarins secreted into the rhizosphere under conditions of low iron availability and help mobilize this nutrient from insoluble iron(III) pools in the soil.The mRNA is cell-to-cell mobile.
AT4G31950	*CYTOCHROME P450, FAMILY 82, SUBFAMILY C, POLYPEPTIDE 3 (CYP82C3)*	member of CYP82C
AT4G31960		hypothetical protein
AT4G31970	*CYTOCHROME P450, FAMILY 82, SUBFAMILY C, POLYPEPTIDE 2 (CYP82C2)*	Functions in the biosynthesis of 4-hydroxy indole-3-carbonyl nitrile (4-OH-ICN), a cyanogenic phytoalexin in Arabidopsis. CYP82C2 acts as a hydroxylase on indole-3-carbonyl nitrile to generate 4-OH-ICN.
AT4G31980		PPPDE thiol peptidase family protein
AT4G31990	*ASPARTATE AMINOTRANSFERASE 5 (ASP5)*	Encodes a plastid-localized aspartate aminotransferase. Does not display any PAT (glutamate/aspartate-prephenate aminotransferase) activity even in the presence of a high concentration of prephenate.
AT4G32000		Protein kinase superfamily protein
AT4G32030		hypothetical protein
AT4G32050		neurochondrin family protein
AT4G32060	*(MICU)*	Encodes an EF-hand protein with homology to constituents of the mitochondrial Ca2+ uniporter machinery in mammals. MICU binds Ca2+ and localizes to the mitochondria in Arabidopsis. It is a negative regulator of mitochondrial calcium uptake. Mutants display elevated matrix calcium at steady state and modified calcium transient kinetics in vivo.
AT4G32080		hypothetical protein
AT4G32105		Beta-1,3-N-Acetylglucosaminyltransferase family protein

AT4G32120	*HYDROXYPROLINE O-GALACTOSYLTRANSFERASE 2 (HPGT2)*	Encodes a hydroxyproline O-galactosyltransferase.
AT4G32130		ER membrane protein complex subunit-like protein (DUF2012)
AT4G32150	*VESICLE-ASSOCIATED MEMBRANE PROTEIN 711 (VAMP711)*	AtVAMP711 is a member of Synaptobrevin-like AtVAMP7C, v-SNARE (soluble N-ethyl-maleimide sensitive factor attachment protein receptors) protein family. SNAREs have been divided into four subgroups: Qa-, Qb-, Qc- and R-SNAREs. R-SNAREs are classified into three groups, the Sec22-, YKT6- and VAMP7-like R-SNAREs. One R-SNARE and three Q-SNAREs (one of each subgroup) form the trans-SNARE complex, which governs specific membrane fusions. VAMP7 proteins consist of three distinct domain, the N-terminal longin-domain (LD), the SNARE motif (SNM) and a transmembrane domain. In spite of the high similarities among the VAMP7 proteins, they show different subcellular localizations. VAMP7C is vacuolar-localized and its LD is essential for the correct localization. Generally, it is suggested that the complete LD is the determinant of subcellular sorting in both animal and plant R-SNAREs.
AT4G32170	*CYTOCHROME P450, FAMILY 96, SUBFAMILY A, POLYPEPTIDE 2 (CYP96A2)*	member of CYP96A
AT4G32190	*PROTEIN INVOLVED IN STARCH INITIATION (PII1)*	Encodes a plastid-located coiled coil-containing protein that is required for normal starch granule initiation in Arabidopsis chloroplasts. Mutants lacking MRC have fewer starch granules per chloroplast than the wild type. Interacts with PTST2 (At1g27070), which is also required for normal starch granule initiation (DOI:10.1105/tpc.18.00219).
AT4G32210	*SUCCINATE DEHYDROGENASE 3-2 (SDH3-2)*	Encodes one of the membrane anchor subunits of the mitochondrial respiratory complex II. The protein is encoded by the nuclear genome but is imported into the mitochondrion. There are two genes that encode this protein, the other is SDH3-1.
AT4G32220		transposable_element_gene;gypsy-like retrotransposon family, has a 4.8e-57 P-value blast match to GB:AAD19359 polyprotein (gypsy_Ty3-element) (Sorghum bicolor);(source:TAIR10)
AT4G32230		hypothetical protein
AT4G32260	*PIGMENT DEFECTIVE 334 (PDE334)*	ATPase, F0 complex, subunit B/B, bacterial/chloroplast
AT4G32270		Ubiquitin-like superfamily protein
AT4G32280	*INDOLE-3-ACETIC ACID INDUCIBLE 29 (IAA29)*	indole-3-acetic acid inducible 29
AT4G32290		Core-2/I-branching beta-1,6-N-acetylglucosaminyltransferase family protein
AT4G32320	*ASCORBATE PEROXIDASE 6 (APX6)*	Encodes a cytosolic ascorbate peroxidase APX6. Ascorbate peroxidases are enzymes that scavenge hydrogen peroxide in plant cells. Eight types of APX have been described for Arabidopsis: three cytosolic (APX1, APX2, APX6), two chloroplastic types (stromal sAPX, thylakoid tAPX), and three microsomal (APX3, APX4, APX5) isoforms.
AT4G32340		Tetratricopeptide repeat (TPR)-like superfamily protein
AT4G32350	*IST1-LIKE 7 (ISTL7)*	Regulator of Vps4 activity in the MVB pathway protein
AT4G32370		Pectin lyase-like superfamily protein
AT4G32400	*SODIUM HYPERSENSITIVE 1 (SHS1)*	Encodes a plastidial nucleotide uniport carrier protein required to export newly synthesized adenylates into the cytosol.
AT4G32460	*(BDX)*	BDX is a DUF642 cell wall protein primarily expressed in vascular tissues of roots, leaves and embryos. Mutants show defects in seed and embryo development.
AT4G32470		Cytochrome bd ubiquinol oxidase, 14kDa subunit
AT4G32490	*EARLY NODULIN-LIKE PROTEIN 4 (ENODL4)*	early nodulin-like protein 4
AT4G32500	*K+ TRANSPORTER 5 (KT5)*	Encodes AKT5, a member of the Shaker family potassium ion (K+) channel. This family includes five groups based on phylogenetic analysis (FEBS Letters (2007) 581: 2357): I (inward rectifying channel): AKT1 (AT2G26650), AKT5 (AT4G32500) and SPIK (also known as AKT6, AT2G25600); II (inward rectifying channel): KAT1 (AT5G46240) and KAT2 (AT4G18290); III (weakly inward rectifying channel): AKT2 (AT4G22200); IV (regulatory subunit involved in inwardly rectifying conductance formation): KAT3 (also known as AtKC1, AT4G32650); V (outward rectifying channel): SKOR (AT3G02850) and GORK (AT5G37500).
AT4G32520	*SERINE HYDROXYMETHYLTRANSFERASE 3 (SHM3)*	Encodes a serine hydroxymethyltransferase SHMT3 located in the plastid.
AT4G32540	*YUCCA 1 (YUC1)*	Mutant has elevated levels of free IAA in dominant mutant allele; Flavin Monooxygenase-Like Enzyme; Auxin Biosynthesis
AT4G32590		2Fe-2S ferredoxin-like superfamily protein
AT4G32610		copper ion binding protein
AT4G32620	*(EPCR1)*	Polycomb related protein that is part of a protein complex involved in histone deacetylation and heterochromatin silencing.
AT4G32630		ArfGap/RecO-like zinc finger domain-containing protein
AT4G32640		Sec23/Sec24 protein transport family protein
AT4G32650	*POTASSIUM CHANNEL IN ARABIDOPSIS THALIANA 3 (KAT3)*	Encodes KAT3, a member of the Shaker family of voltage-gated potassium channel subunits. Does not form functional potassium channel on its own. Involved in down-regulating AKT1 and KAT1 channel activity by forming heteromers with AKT1 or KAT1. The Shaker family K+ ion channels include five groups based on phylogenetic analysis (FEBS Letters (2007) 581: 2357): I (inwardly rectifying conductance): AKT1 (AT2G26650), AKT5 (AT4G32500) and SPIK (also known as AKT6, AT2G25600); II (inward rectifying channel): KAT1 (AT5G46240) and KAT2 (AT4G18290); III (weakly inward rectifying channel): AKT2 (AT4G22200); IV (regulatory subunit involved in inwardly rectifying conductance formation): KAT3 (also known as AtKC1, AT4G32650); V (outward rectifying channel): SKOR (AT3G02850) and GORK (AT5G37500).
AT4G32690	*HEMOGLOBIN 3 (GLB3)*	Encodes a hemoglobin (Hb) with a central domain similar to the 'truncated Hbs of bacteria, protozoa and fungi. The 3D structure of these types of Hbs is a 2-on-2 arrangement of alpha-helices as opposed to the 3-on-3 arrangement of the standard globin fold. This type of Hb is not found in animals or yeast.
AT4G32695		
AT4G32710	*PROLINE-RICH EXTENSIN-LIKE RECEPTOR KINASE 14 (PERK14)*	Encodes a member of the proline-rich extensin-like receptor kinase (PERK) family. This family consists of 15 predicted receptor kinases (PMID: 15653807).
AT4G32720	*LA PROTEIN 1 (La1)*	Encodes AtLa1, a member of the highly abundant phosphoprotein La proteins. Predominantly localized to the nucleoplasm and was also detected in the nucleolar cavity. Has RNA binding activity. Required for normal ribosome biogenesis and embryogenesis.
AT4G32730	*(PC-MYB1)*	Encodes a putative c-myb-like transcription factor with three MYB repeats.

AT4G32760	*TOM1-LIKE 9 (TOL9)*	Encodes a member of the Arabidopsis TOL (TOM1-LIKE) family of ubiquitin binding proteins that acts redundantly in the recognition and further endocytic sorting of a PIN-FORMED (PIN)-type auxin carrier protein at the plasma membrane, modulating dynamic auxin distribution and associated growth responses.
AT4G32770	*VITAMIN E DEFICIENT 1 (VTE1)*	Tocopherol cyclase involved in tocopherol (vitamin E)synthesis. VTE1 over-expressing plants have increased tocopherol indicating VTE1 is a major limiting factor in tocopherol synthesis. Mutants defective in this gene accumulate high amounts of zeaxanthin in conditions of high light or low temperature. Plays a role in the adaptation to low temperature stress, notably phloem loading.
AT4G32780	*FORKED-LIKE4 (FL4)*	FORKED-LIKE family member, part of Group 2 (Group 1 consists of FKD1, FL1-FL3; Group 2 consists of FL4 and FL8 and Group 3 consists of FL5- FL7). May coordinate leaf size with vein density, where Group 1 members and Group 3 members have opposing functions.
AT4G32800	*ETHYLENE RESPONSE FACTOR 43 (ERF043)*	encodes a member of the DREB subfamily A-4 of ERF/AP2 transcription factor family. The protein contains one AP2 domain. There are 17 members in this subfamily including TINY.
AT4G32810	*CAROTENOID CLEAVAGE DIOXYGENASE 8 (CCD8)*	Encodes a protein with similarity to carotenoid cleaving deoxygenases, the enzymes that cleave beta-carotene. Involved in the production of a graft transmissable signal to suppress axillary branching. Protein is localized to chloroplast stroma and expressed primarily in root tip. Mutants in the gene exhibit increased shoot branching, and light-dependent defects in hook opening and hypocotyl/root elongation. Only upregulated by auxin in the root and hypocotyl, and this is not required for the inhibition of shoot branching.
AT4G32830	*ATAURORA1 (AUR1)*	Encodes a member of a family of Ser/Thr kinases whose activities peak during cell division. Transcripts are abundant in tissues rich in dividing cells like roots and flowers but are low or absent in fully expanded leaves and stems. In interphase cells, the protein is predominantly nuclear. During mitosis, the protein associates with plant-specific cytoskeletal structures (preprophase band, phragmoplast, nascent cell plate) that are necessary for cytokinesis as well as with the microtubule spindle. It specifically phosphorylates Ser10 of histone H3 and colocalizes with phosphorylated histone H3 during mitosis.
AT4G32850	*NUCLEAR POLY(A) POLYMERASE (nPAP)*	Encodes a nuclear poly(A) polymerase. Located in the nucleus. The mRNA is cell-to-cell mobile.
AT4G32860		Avr9/Cf-9 rapidly elicited protein
AT4G32870		Polyketide cyclase/dehydrase and lipid transport superfamily protein
AT4G32880	*HOMEOBOX GENE 8 (HB-8)*	member of homeodomain-leucine zipper family, acting as a differentiation-promoting transcription factor of the vascular meristems.
AT4G32890	*GATA TRANSCRIPTION FACTOR 9 (GATA9)*	Encodes a member of the GATA factor family of zinc finger transcription factors.
AT4G32910	*SUPPRESSOR OF BAK1 BKK1 (SBB1)*	SBB1 is a putative nucleoporin that is localized to the nuclear envelope. SBB1 was identified in a screen for supressors of BAK1, BKK1 mediated cell death.
AT4G32920		glycine-rich protein
AT4G32940	*GAMMA VACUOLAR PROCESSING ENZYME (GAMMA-VPE)*	Encodes a vacuolar processing enzyme belonging to a novel group of cysteine proteinases that is expressed in vegetative organs and is upregulated in association with various types of cell death and under stressed conditions. They are essential in processing seed storage proteins and for mediating the susceptible response of toxin-induced cell death.
AT4G32950		Protein phosphatase 2C family protein
AT4G32970		BRISC/BRCA1-A complex protein
AT4G32980	*HOMEOBOX GENE 1 (ATH1)*	Encodes transcription factor involved in photomorphogenesis. Regulates gibberellin biosynthesis. Activated by AGAMOUS in a cal-1, ap1-1 background. Expressed at low levels in developing stamens. Increased levels of ATH1 severely delay flowering in the C24 accession. Most remarkably, ectopically expressed ATH1 hardly had an effect on flowering time in the Col-0 and Ler accessions. ATH1 physically interacts with STM, BP and KNAT6 and enhances the shoot apical meristem defect of some of these genes suggesting a role in SAM maintenance. It acts to integrate light and hormone signaling to regulate internode elongation. Nuclear localization is dependent upon interaction with STM.
AT4G33000	*CALCINEURIN B-LIKE PROTEIN 10 (CBL10)*	Encodes a member of the calcineurin B-like calcium sensor gene family. Mediates salt tolerance by regulating ion homeostasis in Arabidopsis.
AT4G33010	*GLYCINE DECARBOXYLASE P-PROTEIN 1 (GLDP1)*	glycine decarboxylase P-protein 1
AT4G33020	*(ZIP9)*	member of Fe(II) transporter isolog family
AT4G33040	*(ROXY21)*	Encodes a member of the CC-type glutaredoxin (ROXY) family that has been shown to interact with the transcription factor TGA2.
AT4G33050	*EMBRYO SAC DEVELOPMENT ARREST 39 (EDA39)*	Encodes a calmodulin-binding protein involved in stomatal movement.
AT4G33070	*PYRUVATE DECARBOXYLASE 1 (PDC1)*	Thiamine pyrophosphate dependent pyruvate decarboxylase family protein
AT4G33080	*NUCLEAR DBF2-RELATED 6 (NDR6)*	Ubiquitously expressed protein kinase.
AT4G33130		rho GTPase-activating protein
AT4G33140		Haloacid dehalogenase-like hydrolase (HAD) superfamily protein
AT4G33150	*LYSINE-KETOGLUTARATE REDUCTASE (LKR)*	This is a splice variant of the LKR/SDH locus. It encodes a bifunctional polypeptide lysine-ketoglutarate reductase and saccharopine dehydrogenase involved in lysine degradation. There is another splice variant that encodes a mono saccharopine dehydrogenase protein. Gene expression is induced by abscisic acid, jasmonate, and under sucrose starvation.
AT4G33160		F-box family protein
AT4G33170		Tetratricopeptide repeat (TPR)-like superfamily protein
AT4G33190		
AT4G33220	*PECTIN METHYLESTERASE 44 (PME44)*	pectin methylesterase 44
AT4G33230		Plant invertase/pectin methylesterase inhibitor superfamily
AT4G33260	*CELL DIVISION CYCLE 20.2 (CDC20.2)*	Encodes a CDC20 protein that interacts with APC subunits, components of the mitochondrial checkpoint complex and mitotic cyclin substrates and is indispensable for normal plant development and fertility.
AT4G33280		AP2/B3-like transcriptional factor family protein
AT4G33290		F-box and associated interaction domains-containing protein
AT4G33300	*ADR1-LIKE 1 (ADR1-L1)*	Encodes a member of the ADR1 family nucleotide-binding leucine-rich repeat (NB-LRR) immune receptors.
AT4G33310		hypothetical protein

AT4G33330	*PLANT GLYCOGENIN-LIKE STARCH INITIATION PROTEIN 3 (PGSIP3)*	Encodes a glucuronyltransferase responsible for the addition of GlcA residues onto xylan and for secondary wall deposition.
AT4G33340		
AT4G33350	*TRANSLOCON AT THE INNER ENVELOPE MEMBRANE OF CHLOROPLASTS 22-IV (Tic22-IV)*	Tic22-like family protein
AT4G33370		DEA(D/H)-box RNA helicase family protein
AT4G33380		dimethylallyl, adenosine tRNA methylthiotransferase
AT4G33390		WEAK CHLOROPLAST MOVEMENT UNDER BLUE LIGHT-like protein (DUF827)
AT4G33420	*PEROXIDASE 47 (PRX47)*	Peroxidase superfamily protein
AT4G33450	*MYB DOMAIN PROTEIN 69 (MYB69)*	Member of the R2R3 factor gene family.
AT4G33460	*ATP-BINDING CASSETTE I10 (ABCI10)*	Member of NAP subfamily. Putative component of chloroplast ECF/ABC-Transporter involved in metal homeostasis.
AT4G33500	*PROBABLE PROTEIN PHOSPHATASE (PP2C62)*	Protein phosphatase 2C family protein. Loss of function enhances immunity to bacterial pathogens.
AT4G33520	*P-TYPE ATP-ASE 1 (PAA1)*	Encodes a putative metal-transporting P-type ATPase PAA1. An alternative-splicing event of the PAA1 pre-mRNA produces a copper chaperon named PCH1. The mRNA is cell-to-cell mobile.
AT4G33525		
AT4G33530	*K+ UPTAKE PERMEASE 5 (KUP5)*	potassium transporter
AT4G33550		Bifunctional inhibitor/lipid-transfer protein/seed storage 2S albumin superfamily protein
AT4G33560	*WOUND-INDUCED POLYPEPTIDE 5 (WIP5)*	Member of the wound-induced polypeptide (WIP) family.
AT4G33570		
AT4G33580	*BETA CARBONIC ANHYDRASE 5 (BCA5)*	beta carbonic anhydrase 5
AT4G33600		transmembrane protein
AT4G33610		glycine-rich protein
AT4G33630	*EXECUTER1 (EX1)*	Encodes one of the two plastid proteins EXECUTER (EX1, AT4G33630) and EX2 (AT1G27510). Mediates singlet oxygen induced programmed cell death.
AT4G33650	*DYNAMIN-RELATED PROTEIN 3A (DRP3A)*	Encodes a protein with high sequence similarity to the dynamin superfamily. Among those members ADL2 was most closely related to Dnm1p of yeast and likely a member of the Vps1p subfamily. Widely expressed in various tissues with highest expression in flower tissues. Localizes to the chloroplast, mitochondrion and peroxisome. Involved in peroxisome and mitochondria fission in combination with DRP3B.
AT4G33660	*CYSTEINE-RICH TRANSMEMBRANE MODULE 11 (ATHCYSTM11)*	cysteine-rich TM module stress tolerance protein
AT4G33663		
AT4G33666		hypothetical protein
AT4G33670	*L-GALACTOSE DEHYDROGENASE (L-GALDH)*	Encodes a L-galactose dehydrogenase, involved in ascorbate biosynthesis
AT4G33700		CBS domain protein (DUF21)
AT4G33710		CAP (Cysteine-rich secretory proteins, Antigen 5, and Pathogenesis-related 1 protein) superfamily protein
AT4G33720	*(ATCAPE3)*	CAP (Cysteine-rich secretory proteins, Antigen 5, and Pathogenesis-related 1 protein) superfamily protein
AT4G33730	*CAP-DERIVED PEPTIDE 1 (ATCAPE1)*	Member of CAP protein superfamily. Encoding a small 11 AA peptide (PAGNYIGARPY) involved in negative regulation of salt tolerance.
AT4G33750		
AT4G33770	*INOSITOL 1,3,4-TRISPHOSPHATE 5/6 KINASE 2 (ITPK2)*	Inositol pyrophosphate kinase. Catalyzes the phosphorylation of phytic acid (InsP6) to the symmetric InsP7 isomer 5-InsP7.
AT4G33790	*ECERIFERUM 4 (CER4)*	Encodes an alcohol-forming fatty acyl-CoA reductase, involved in cuticular wax biosynthesis. Lines carrying recessive mutations are deficient in primary alcohol and have glossy stem surfaces.
AT4G33800		hypothetical protein
AT4G33810		Glycosyl hydrolase superfamily protein
AT4G33850		Pseudogene of AT4G33850; glycosyl hydrolase family 10 protein
AT4G33870		Peroxidase superfamily protein
AT4G33900		Galactose oxidase/kelch repeat superfamily protein
AT4G33905		Peroxisomal membrane 22 kDa (Mpv17/PMP22) family protein
AT4G33910		2-oxoglutarate (2OG) and Fe(II)-dependent oxygenase superfamily protein
AT4G33930		Encodes a protein with 14.6% glycine residues, similar to hyphally regulated protein from Candida albicans, PIR2:S58135
AT4G33950	*OPEN STOMATA 1 (OST1)*	Encodes calcium-independent ABA-activated protein kinase, a member of SNF1-related protein kinases (SnRK2) whose activity is activated by ionic (salt) and non-ionic (mannitol) osmotic stress. Mutations disrupted ABA induction of stomatal closure as well as ABA inhibition of light-induced stomatal opening. However, regulation of stomatal opening/closing by light or CO(2) is not affected in these mutants. May act in the interval between ABA perception and reactive oxygen species production in the ABA signalling network.
AT4G33960		hypothetical protein
AT4G33970	*LEUCINE-RICH REPEAT/EXTENSIN 11 (LRX11)*	Pollen expressed protein required for pollen tube growth.Along with other members of the LRX family, itnteracts with RALF4 to control pollen tube growth and integrity. Loss of function results in premature pollen tube rupture and reduced fertility.
AT4G33980	*COLD-REGULATED GENE 28 (COR28)*	Acts with COR27 as a key regulator in the COP1-HY5 regulatory hub by regulating HY5 activity to ensure proper skotomorphogenic growth in the dark and photomorphogenic development in the light.
AT4G33985		membrane insertase, putative (DUF1685)
AT4G33990	*EMBRYO DEFECTIVE 2758 (EMB2758)*	Tetratricopeptide repeat (TPR)-like superfamily protein
AT4G34000	*ABSCISIC ACID RESPONSIVE ELEMENTS-BINDING FACTOR 3 (ABF3)*	Encodes an ABA-responsive element-binding protein with similarity to transcription factors that is expressed in response to stress and abscisic acid.
AT4G34040	*MED25 BINDING RING-H2 PROTEIN 2 (MBR2)*	RING/U-box superfamily protein
AT4G34050	*CAFFEOYL COENZYME A O-METHYLTRANSFERASE 1 (CCoAOMT1)*	Methyltransferase in the lignin biosynthetic pathway.
AT4G34090	*PROTEIN IN CHLOROPLAST ATPASE BIOGENESIS (PAB)*	cyclin delta-3

AT4G34135	*UDP-GLUCOSYLTRANSFERASE 73B2 (UGT73B2)*	The At4g34135 gene encodes a flavonol 7-O-glucosyltransferase (EC 2.4.1.237) that glucosylates also with a 20 fold lower activity flavonols (kaempferol and quercetin) at the 3-O-position.
AT4G34138	*UDP-GLUCOSYL TRANSFERASE 73B1 (UGT73B1)*	UDP-glucosyl transferase 73B1
AT4G34140		D111/G-patch domain-containing protein
AT4G34150		Calcium-dependent lipid-binding (CaLB domain) family protein
AT4G34160	*CYCLIN D3;1 (CYCD3;1)*	encodes a cyclin D-type protein involved in the switch from cell proliferation to the final stages of differentiation. The gene is transcriptionally regulated by cytokinin and brassinosteroid. Protein interacts with cyclin-dependent kinase inhibitor ICK1.
AT4G34180	*CYCLASE1 (CYCLASE1)*	Encodes a cyclase-family protein that is a negative regulator of cell death that regulates pathogen-induced symptom development.
AT4G34190	*STRESS ENHANCED PROTEIN 1 (SEP1)*	Encodes a stress enhanced protein that localizes to the thylakoid membrane and whose mRNA is upregulated in response to high light intensity. It may be involved in chlorophyll binding.
AT4G34200	*EMBRYO SAC DEVELOPMENT ARREST 9 (EDA9)*	Encodes a 3-phosphoglycerate dehydrogenase that is essential for embryo and pollen development.
AT4G34210	*SKP1-LIKE 11 (SK11)*	one of 20 SKP1 homologs in Arabidopsis. Protein is most similar to ASK12 and RNAi lines show defects in stamen development.
AT4G34220	*RECEPTOR DEAD KINASE1 (RDK1)*	Encodes a receptor like kinase involved in ABA-mediated seedling development and drought tolerance.RDK1 is an atypical or pseudokinase and has no phosphorylation activity. Its expression is upregulated in response to ABA.interacts with ABI1 and other PP2C phosphatases.
AT4G34230	*CINNAMYL ALCOHOL DEHYDROGENASE 5 (CAD5)*	Encodes a catalytically active cinnamyl alcohol dehydrogenase which uses p-coumaryl aldehyde as a preferred substrate. It can also use sinapyl, caffeyl, coniferyl and d-hydroxyconiferyl aldehydes as substrates.
AT4G34240	*ALDEHYDE DEHYDROGENASE 3I1 (ALDH3I1)*	Encodes an aldehyde dehydrogenase induced by ABA and dehydration that can oxidize saturated aliphatic aldehydes. It is also able to oxidize beta-unsaturated aldehydes, but not aromatic aldehydes. ALDH3I1 was able to use both NAD+ and NADP+ as cofactors.
AT4G34250	*3-KETOACYL-COA SYNTHASE 16 (KCS16)*	Encodes KCS16, a member of the 3-ketoacyl-CoA synthase family involved in the biosynthesis of VLCFA (very long chain fatty acids).
AT4G34260	*(FUC95A)*	1,2-alpha-L-fucosidase
AT4G34265		hypothetical protein
AT4G34290	*(SWIB3)*	SWIB/MDM2 domain superfamily protein
AT4G34320		transmembrane protein, putative (DUF677)
AT4G34330		transmembrane protein, putative (DUF677)
AT4G34340	*TBP-ASSOCIATED FACTOR 8 (TAF8)*	Member of SAGA complex, SPT module, interacts with HAG1.
AT4G34350	*4-HYDROXY-3-METHYLBUT-2-ENYL DIPHOSPHATE REDUCTASE (HDR)*	Arabidopsis ISPH is involved in the plastid nonmevalonate pathway of isoprenoid biosynthesis. It was shown to complement the lethal phenotype of E. coli ispH mutant and is therefore most likely encodes a protein with 4-hydroxy-3-methylbut-2-en-1-yl diphosphate reductase activity involved in the last step of mevalonate-independent isopentenyl biosynthesis. Mutant has Albino seedling.
AT4G34360		S-adenosyl-L-methionine-dependent methyltransferases superfamily protein
AT4G34400	*TARGET OF FLC AND SVP1 (TFS1)*	B3-type transcription factor, which promotes floral transition and is repressed by FLC/SVP and promoted by SOC1.
AT4G34410	*REDOX RESPONSIVE TRANSCRIPTION FACTOR 1 (RRTF1)*	Encodes a member of the ERF (ethylene response factor) subfamily B-3 of ERF/AP2 transcription factor family. The protein contains one AP2 domain. There are 18 members in this subfamily including ATERF-1, ATERF-2, AND ATERF-5. Regulates programmed cell death (PCD) inhibitor genes. Involved in retarding programmed cell death under salt stress due to the regulation of processes participating in ROS inhibition. ERF-regulated transcripts belong to the tryptophan biosynthesis, tryptophan metabolism, and downstream plant hormone signal transduction pathways, where ERF109 potentially acts as a 'master switch' mediator of a cascade of consecutive events across the three pathways, promoting plant growth and re-adjustment to homeostasis due the direct participation in auxin biosynthesis leading to the plants ability to tolerate salt stress.
AT4G34440	*PROLINE-RICH EXTENSIN-LIKE RECEPTOR KINASE 5 (PERK5)*	Encodes a member of the proline-rich extensin-like receptor kinase (PERK) family. This family consists of 15 predicted receptor kinases (PMID: 15653807).
AT4G34450	*GAMMA2 COAT PROTEIN (gamma2-COP)*	Member of the Coat Protein I (COPI) complex is a seven-subunit coatomer complex consisting of the α, β, β′, γ, δ, ε, and ζ proteins. COPI is required for retrograde transport from the Golgi to the endoplasmic reticulum, Golgi maintenance, and cell plate formation.
AT4G34480		O-Glycosyl hydrolases family 17 protein
AT4G34500		Protein kinase superfamily protein
AT4G34510	*3-KETOACYL-COA SYNTHASE 17 (KCS17)*	Encodes KCS17, a member of the 3-ketoacyl-CoA synthase family involved in the biosynthesis of VLCFA (very long chain fatty acids).
AT4G34520	*3-KETOACYL-COA SYNTHASE 18 (KCS18)*	Encodes KCS18, a member of the 3-ketoacyl-CoA synthase family involved in the biosynthesis of VLCFA (very long chain fatty acids).
AT4G34560		transmembrane protein
AT4G34590	*G-BOX BINDING FACTOR 6 (GBF6)*	Encodes a basic domain leucine zipper (bZip) transcription factor bZIP11. Translation is repressed by sucrose. Directly regulates gene expression of ASN1 and ProDH2, which are enzyme-coding genes involved in amino acid metabolism. Susceptibility factor during Pseudomonas syringae infection.
AT4G34600	*CASPARIAN STRIP INTEGRITY FACTOR 2 (CIF2)*	CAF2 is a peptide hormone expressed in the root stele that specifically binds the endodermis-expressed leucine-rich repeat receptor kinase GASSHO1 (GSO1)/SCHENGEN3 and its homolog, GSO2. Together with CAF1 it is required for formation of the casparian band.
AT4G34610	*BEL1-LIKE HOMEODOMAIN 6 (BLH6)*	BEL1-like homeodomain 6
AT4G34620	*SMALL SUBUNIT RIBOSOMAL PROTEIN 16 (SSR16)*	Encodes ribosomal protein S16, has embryo-defective lethal mutant phenotype
AT4G34630		prostatic spermine-binding-like protein
AT4G34640	*SQUALENE SYNTHASE 1 (SQS1)*	Encodes squalene synthase, which converts two molecules of farnesyl diphosphate (FPP) into squalene via an intermediate: presqualene diphosphate (PSPP). It is generally thought to be one of the key enzymes of sterol biosynthesis, since it catalyzes the first pathway-specific reaction of the sterol branch of the isoprenoid pathway. The mRNA is cell-to-cell mobile.
AT4G34650	*SQUALENE SYNTHASE 2 (SQS2)*	Encodes a protein with similarity to squalene synthase which catalyzes the first committed step in sterol biosynthesis. To date no experimental evidence exists that SQS2 functions as a squalene synthase and some experiments indicate it does not have this function.
AT4G34660	*SH3 DOMAIN-CONTAINING PROTEIN 2 (SH3P2)*	SH3 domain-containing protein
AT4G34680	*GATA TRANSCRIPTION FACTOR 3 (GATA3)*	Encodes a member of the GATA factor family of zinc finger transcription factors.

AT4G34690		transmembrane protein
AT4G34710	*ARGININE DECARBOXYLASE 2 (ADC2)*	Encodes a arginine decarboxylase (ADC), a rate-limiting enzyme that catalyzes the first step of polyamine (PA) biosynthesis via ADC pathway in Arabidopsis thaliana. Arabidopsis genome has two ADC paralogs, ADC1 and ADC2. ADC2 is stress-inducible (osmotic stress). Double mutant analysis showed that ADC genes are essential for the production of PA, and are required for normal seed development. Overexpression causes phenotypes similar to GA-deficient plants and these plants show reduced levels of GA due to lower expression levels of AtGA20ox1, AtGA3ox3 and AtGA3ox1.
AT4G34740	*GLN PHOSPHORIBOSYL PYROPHOSPHATE AMIDOTRANSFERASE 2 (ASE2)*	Encodes glutamine 5-phosphoribosylpyrophosphate amidotransferase. Mutants are deficient in leaf, but not cotyledon, plastid and palisade cell development. Mutants exhibit defective chloroplast development under non-low light, suggesting that the defect in chloroplast development is caused by photo-oxidative damage. Plays role in differential development of vascular-associated cells. Demonstrates a cell-specific difference in chloroplast development.Mutant leaves are highly reticulate with a green vascular pattern.
AT4G34750	*SMALL AUXIN UPREGULATED RNA 49 (SAUR49)*	SAUR-like auxin-responsive protein family
AT4G34760	*SMALL AUXIN UPREGULATED RNA 50 (SAUR50)*	SAUR-like auxin-responsive protein family
AT4G34770	*SMALL AUXIN UPREGULATED RNA 1 (SAUR1)*	SAUR-like auxin-responsive protein family
AT4G34820		
AT4G34830	*MATURATION OF RBCL 1 (MRL1)*	Encodes MRL1, a conserved pentatricopeptide repeat protein, required for stabilization of rbcL mRNA.
AT4G34840	*METHYLTHIOADENOSINE NUCLEOSIDASE 2 (MTN2)*	Encodes one of the 5'-methylthioadenosine nucleosidases (AT4G38800/MTN1; AT4G34840/MTN2). Double mutant, mtn1-1mtn2-1, retains approximately 14% of the MTN enzyme activity present in the wild type and displays a pleiotropic phenotype that includes altered vasculature and impaired fertility.
AT4G34850	*LESS ADHESIVE POLLEN 5 (LAP5)*	Chalcone and stilbene synthase family protein
AT4G34880		Amidase family protein
AT4G34890	*XANTHINE DEHYDROGENASE 1 (XDH1)*	Encodes a xanthine dehydrogenase, involved in purine catabolism. Ubiquitously expressed, but the transcript level is altered during aging, senescence, salt and cold stress, ABA treatment, and dark treatment. RNAi lines that suppress both XDH1 and XDH2 produce small plants with reduced fertility and accelerated leaf senescence. Role in drought tolerance.
AT4G34910		P-loop containing nucleoside triphosphate hydrolases superfamily protein
AT4G34920		PLC-like phosphodiesterases superfamily protein
AT4G34940	*ARMADILLO REPEAT ONLY 1 (ARO1)*	Armadillo repeat protein. One of a family of four in Arabidopsis. Located in the nucleus and cytoplasm of pollen vegetative cells, and in the cytoplasm of egg cells. Involved in the signaling network controlling tip growth and actin organization in the pollen tube.
AT4G34950	*MAJOR FACILITATOR SUPERFAMILY 1 (MFS1)*	Major facilitator superfamily protein
AT4G34960	*CYCLOPHYLIN 21-1 (CYP21-1)*	Cyclophilin-like peptidyl-prolyl cis-trans isomerase family protein
AT4G34970	*ACTIN DEPOLYMERIZING FACTOR 9 (ADF9)*	A member of actin polymerizing factors (ADFs)family, ADF9 primarily functions as an actin bundling protein.
AT4G34980	*SUBTILISIN-LIKE SERINE PROTEASE 2 (SLP2)*	Serine protease similar to subtilisin.
AT4G34990	*MYB DOMAIN PROTEIN 32 (MYB32)*	Member of the R2R3 factor gene family.
AT4G35010	*BETA-GALACTOSIDASE 11 (BGAL11)*	putative beta-galactosidase (BGAL11 gene)
AT4G35050	*MULTICOPY SUPPRESSOR OF IRA1 3 (MSI3)*	Encodes a WD-40 repeat protein similar to yeast MSI1. The predicted protein has a DWD motif. It can bind to DDB1a in Y2H assays, and DDB1b in co-IP assays, and may be involved in the formation of a CUL4-based E3 ubiquitin ligase
AT4G35060	*HEAVY METAL ASSOCIATED ISOPRENYLATED PLANT PROTEIN 25 (HIPP25)*	Heavy metal transport/detoxification superfamily protein
AT4G35070		SBP (S-ribonuclease binding protein) family protein
AT4G35090	*CATALASE 2 (CAT2)*	Encodes a peroxisomal catalase, highly expressed in bolts and leaves. mRNA expression patterns show circadian regulation with mRNA levels being high in the subjective early morning. Loss of function mutations have increased H2O2 levels and increased H2O2 sensitivity. Mutants accumulate more toxic ions yet show decreased sensitivity to Li+. This decreased sensitivity is most likely due to an insensitivity to ethylene. Note that in Queval et al. (2007) Plant Journal, 52(4):640, SALK_057998 is named as cat2-1, SALK_076998 is named as cat2-2; in Bueso et al. (2007) Plant Journal, 52(6):1052, SALK_076998 is named as cat2-1. TAIR has adopted the nomenclature consistent with that in Bueso et al. (2007) after consultation with the authors: SALK_076998 (cat2-1), SALK_057998 (cat2-2).
AT4G35120		Galactose oxidase/kelch repeat superfamily protein
AT4G35130		Tetratricopeptide repeat (TPR)-like superfamily protein
AT4G35140		Transducin/WD40 repeat-like superfamily protein
AT4G35150		O-methyltransferase family protein
AT4G35170	*COMPANION OF CELLULOSE SYNTHASE 4 (CC4)*	Late embryogenesis abundant (LEA) hydroxyproline-rich glycoprotein family
AT4G35180	*LYS/HIS TRANSPORTER 7 (LHT7)*	LYS/HIS transporter 7
AT4G35190	*LONELY GUY 5 (LOG5)*	Putative lysine decarboxylase family protein
AT4G35200		hypothetical protein (DUF241)
AT4G35210		carrier protein (DUF241)
AT4G35240		DNA-directed RNA polymerase subunit beta, putative (DUF630 and DUF632)
AT4G35250	*HIGH CHLOROPHYLL FLUORESCENCE PHENOTYPE 244 (HCF244)*	HCF244 is a member of the atypical short-chain dehydrogenase/reductase superfamily, a modified group, which has lost enzyme activity.HCF244 interacts with unknown partners in a 200-400 kD membrane associated complex.
AT4G35260	*ISOCITRATE DEHYDROGENASE 1 (IDH1)*	Encodes a regulatory subunit of the mitochondrially-localized NAD+- dependent isocitrate dehydrogenase.
AT4G35270	*NIN-LIKE PROTEIN 2 (NLP2)*	Plant regulator RWP-RK family protein
AT4G35280	*DUO1-ACTIVATED ZINC FINGER 2 (DAZ2)*	Target promoter of the male germline-specific transcription factor DUO1.
AT4G35290	*GLUTAMATE RECEPTOR 2 (GLUR2)*	Encodes a putative glutamate receptor like-protein, member of Putative ligand-gated ion channel subunit family
AT4G35310	*CALMODULIN-DOMAIN PROTEIN KINASE 5 (CPK5)*	calmodulin-domain protein kinase CDPK isoform 5 (CPK5)

AT4G35350	*XYLEM CYSTEINE PEPTIDASE 1 (XCP1)*	tracheary element vacuolar protein. A clubroot pathogen effector targets cruciferous cysteine proteases to suppress plant immunity.
AT4G35360		pantothenate kinase
AT4G35370		Transducin/WD40 repeat-like superfamily protein
AT4G35380	*BIG4 (BIG4)*	Encodes one of the functionally redundant ARF guanine-nucleotide exchange factors (ARF-GEFs). Functions as regulators of post-Golgi trafficking.
AT4G35400		transposable_element_gene;similar to unknown protein [Arabidopsis thaliana] (TAIR:AT1G48250.1);(source:TAIR10)
AT4G35420	*DIHYDROFLAVONOL 4-REDUCTASE-LIKE1 (DRL1)*	Encodes DRL1 (Dihydroflavonol 4-reductase-like1), a closely related homolog of the rice anther-specific gene OsDFR2. DRL1 may be involved in a metabolic pathway essential for pollen wall development and male fertility. Mutant plants have impaired pollen formation and seed production.
AT4G35440	*CHLORIDE CHANNEL E (CLC-E)*	Enclodes a choride channel protein that is localized to the thlakoid membrane.
AT4G35480	*RING-H2 FINGER A3B (RHA3B)*	Encodes a putative RING-H2 finger protein RHA3b.
AT4G35520	*MUTL PROTEIN HOMOLOG 3 (MLH3)*	DNA mismatch repair protein similar to MutL. Required for normal levels of meiotic crossovers
AT4G35550	*WUSCHEL RELATED HOMEOBOX 13 (WOX13)*	Encodes a WUSCHEL-related homeobox gene family member with 65 amino acids in its homeodomain. WOX13 is the only family member that does not contain a sequence of eight residues (TLPLFPMH) downstream of the homeodomain called the WUS box.
AT4G35560	*DUO1-ACTIVATED WD40 1 (DAW1)*	Target promoter of the male germline-specific transcription factor DUO1. The mRNA is cell-to-cell mobile.
AT4G35580	*NAC TRANSCRIPTION FACTOR-LIKE 9 (NTL9)*	Encodes a calmodulin-binding NAC protein (CBNAC). Contains calmodulin-binding domain in the C-terminus of the protein. Functions as a calmodulin-regulated transcriptional repressor.
AT4G35590	*RWP-RK DOMAIN-CONTAINING 5 (RKD5)*	RWP-RK domain-containing protein
AT4G35600	*CAST AWAY (CST)*	Encodes a receptor-like cytoplasmic kinase that acts as a spatial inhibitor of cell separation. Analysis of the cDNA previously described in Meiners et al., 1991 revealed mistakes in the predicted open reading frame. The mRNA is cell-to-cell mobile.
AT4G35620	*CYCLIN B2;2 (CYCB2;2)*	Cyclin B2
AT4G35630	*PHOSPHOSERINE AMINOTRANSFERASE 1 (PSAT1)*	Encodes a phosphoserine aminotransferase which is involved in serine biosynthesis in the chloroplast which operates via the phosphorylated pathway. The mRNA is cell-to-cell mobile.
AT4G35650	*ISOCITRATE DEHYDROGENASE III (IDH-III)*	Encodes a regulatory subunit of the mitochondrially-localized NAD+- dependent isocitrate dehydrogenase. In contrast to the broadly expressed other regulatory (IDH-I and IDH-II) and catalytic (IDH-V and IDH-VI) subunits of this enzyme, IDH-III expression appears to be restricted largely to pollen.
AT4G35660		selection/upkeep of intraepithelial T-cells protein, putative (DUF241)
AT4G35670		Pectin lyase-like superfamily protein
AT4G35690		hypothetical protein (DUF241)
AT4G35700	*DUO1-ACTIVATED ZINC FINGER 3 (DAZ3)*	Target promoter of the male germline-specific transcription factor DUO1.
AT4G35710		DUF241 domain protein, putative (DUF241)
AT4G35720		DUF241 domain protein, putative (DUF241)
AT4G35725		transmembrane protein
AT4G35750		SEC14 cytosolic factor family protein / phosphoglyceride transfer family protein
AT4G35770	*SENESCENCE 1 (SEN1)*	Senescence-associated gene that is strongly induced by phosphate starvation. Transcripts are differentially regulated at the level of mRNA stability at different times of day. mRNAs are targets of the mRNA degradation pathway mediated by the downstream (DST) instability determinant.
AT4G35820		2-oxoglutarate-dependent dioxygenase
AT4G35840	*ARABIDOPSIS T??XICOS EN LEVADURA 26 (ATL26)*	RING/U-box superfamily protein
AT4G35850		Pentatricopeptide repeat (PPR) superfamily protein
AT4G35880		Eukaryotic aspartyl protease family protein
AT4G35900	*(FD)*	bZIP protein required for positive regulation of flowering. Mutants are late flowering. FD interacts with FT to promote flowering.Expressed in the shoot apex in floral anlagen, then declines in floral primordia.
AT4G35910	*CYTOPLASMIC THIOURIDYLASE 2 (CTU2)*	Encodes a cytoplasmic thiouridylase that is essential for tRNA thiolation. Its activity appears to be important in root development.
AT4G35950	*RAC-LIKE 6 (RAC6)*	A member of ROP GTPases gene family-like. GTP binding protein Arac6.
AT4G35970	*ASCORBATE PEROXIDASE 5 (APX5)*	Encodes a microsomal ascorbate peroxidase APX5. Ascorbate peroxidases are enzymes that scavenge hydrogen peroxide in plant cells. Eight types of APX have been described for Arabidopsis: three cytosolic (APX1, APX2, APX6), two chloroplastic types (stromal sAPX, thylakoid tAPX), and three microsomal (APX3, APX4, APX5) isoforms.
AT4G35985		Senescence/dehydration-associated protein-like protein
AT4G36000		Pathogenesis-related thaumatin superfamily protein
AT4G36010		Pathogenesis-related thaumatin superfamily protein
AT4G36020	*COLD SHOCK DOMAIN PROTEIN 1 (CSDP1)*	Encodes a cold shock domain protein. Involved in cold acclimation by blocking the secondary structure of mRNA which in turn facilitates translation at cold temperature.
AT4G36040	*DNAJ11 (J11)*	Chaperone DnaJ-domain superfamily protein
AT4G36050	*APURINIC/APYRIMIDINIC ENDONUCLEASE2 (APE2)*	Encodes a base excision repair protein with 3'-phosphatase activity and strong 3'-5' exonuclease activity. Together with ZDP, it plays overlapping roles in the maintenance of epigenome and genome stability in plants.
AT4G36060	*BASIC HELIX-LOOP-HELIX 11 (bHLH11)*	Negative TF that regulates Fe homeostasis; inhibits bHLH IVc proteins by recruiting the TOPLESS/TOPLESS-RELATED corepressors.
AT4G36070	*CALCIUM-DEPENDENT PROTEIN KINASE 18 (CPK18)*	member of Calcium Dependent Protein Kinase
AT4G36105		polyamine-modulated factor 1-binding protein
AT4G36110	*SMALL AUXIN UPREGULATED RNA 9 (SAUR9)*	SAUR-like auxin-responsive protein family
AT4G36140		disease resistance protein (TIR-NBS-LRR class)
AT4G36160	*NAC DOMAIN CONTAINING PROTEIN 76 (NAC076)*	Encodes a NAC-domain transcription factor that is expressed in developing xylem. Over expression of this protein causes ectopic secondary cell wall growth. Complements some of the cell wall defects seen in SND1/NST1 double mutants.

AT4G36170		hypothetical protein
AT4G36180	*MUSTACHES-LIKE (MUL)*	LRR-RLK which regulates lateral root development.
AT4G36190		Serine carboxypeptidase S28 family protein
AT4G36200		
AT4G36210		transmembrane/coiled-coil protein (DUF726)
AT4G36220	*FERULIC ACID 5-HYDROXYLASE 1 (FAH1)*	encodes ferulate 5-hydroxylase (F5H). Involved in lignin biosynthesis.
AT4G36230		transmembrane protein
AT4G36250	*ALDEHYDE DEHYDROGENASE 3F1 (ALDH3F1)*	Encodes a putative aldehyde dehydrogenase. The gene is not responsive to osmotic stress and is expressed constitutively at a low level in plantlets and root cultures.
AT4G36260	*STYLISH 2 (STY2)*	A member of SHI gene family. Arabidopsis thaliana has ten members that encode proteins with a RING finger-like zinc finger motif. Despite being highly divergent in sequence, many of the SHI-related genes are partially redundant in function and synergistically promote gynoecium, stamen and leaf development in Arabidopsis. Encodes protein with a single zinc finger motif and a members of a small gene family of putative transcription factors in which the SHORT INTERNODES (SHI) gene is found. STY2/STY1 double mutants showed defective style, stigma as well as serrated leaves.
AT4G36270	*MICRORCHIDIA3 (MORC3)*	Member of the microrchidia protein family which have been described as epigenetic regulators and plant immune mediators, contains a hallmark GHKL-type ATPase domain in N-terminus.
AT4G36350	*PURPLE ACID PHOSPHATASE 25 (PAP25)*	purple acid phosphatase 25
AT4G36360	*BETA-GALACTOSIDASE 3 (BGAL3)*	putative beta-galactosidase (BGAL3 gene)
AT4G36370		hypothetical protein
AT4G36380	*ROTUNDIFOLIA 3 (ROT3)*	Encodes a cytochrome P-450 gene that is involved in leaf blade expansion by controlling polar cell expansion in the leaf length direction. Member of the CYP90C CYP450 family. ROT3 was shown to be involved in brassinosteroid biosynthesis, most likely in the conversion step of typhasterol (TY) to castasterone (CS). As 6-deoxo-CS was unable to restore the phenotype of rot3-1, it has been postulated that ROT3 might be specifically involved in the conversion of TY to CS in the C6-oxidation pathway of brassinolide. Recently, CYP90C1 was shown to catalyse the C-23 hydroxylation of several brassinosteroids (the enzyme has a broad specificity for 22-hydroxylated substrates).
AT4G36400	*D-2-HYDROXYGLUTARATE DEHYDROGENASE (D2HGDH)*	Encodes a (D)-2-hydroxyglutarate dehydrogenase.
AT4G36410	*UBIQUITIN-CONJUGATING ENZYME 17 (UBC17)*	ubiquitin-conjugating enzyme
AT4G36420		Ribosomal protein L12 family protein
AT4G36430		Peroxidase superfamily protein
AT4G36440		G-protein coupled receptor
AT4G36450	*MITOGEN-ACTIVATED PROTEIN KINASE 14 (MPK14)*	member of MAP Kinase
AT4G36460		transmembrane protein
AT4G36470		Encodes an S-adenosyl-l-methionine-dependent methyltransferase belonging to the SABATH family that catalyzes the specific carboxymethylation of (11R)-carlactonoic acid.
AT4G36480	*LONG-CHAIN BASE1 (LCB1)*	Encodes the LCB1 subunit of serine palmitoyltransferase. Together with the LCB2 subunit, forms a functional serine palmitoyltransferase complex, which catalyzes the first reaction of sphingolipid biosynthesis. Knockout of LCB1 was embryo lethal. Partial suppression of LCB1 expression led to smaller plants due to reduced cell expansion.
AT4G36490	*SEC14-LIKE 12 (SFH12)*	SEC14-like 12
AT4G36500		hypothetical protein
AT4G36510		hypothetical protein
AT4G36520	*AUXILIN-LIKE4 (AUXILIN-LIKE4)*	Chaperone DnaJ-domain superfamily protein
AT4G36530		alpha/beta-Hydrolases superfamily protein
AT4G36540	*BR ENHANCED EXPRESSION 2 (BEE2)*	Encodes the brassinosteroid signaling component BEE2 (BR-ENHANCED EXPRESSION 2). Positively modulates the shade avoidance syndrome in Arabidopsis seedlings.
AT4G36550	*(PUB5)*	Plant U-box type E3 ubiquitin ligase (PUB).
AT4G36560		transmembrane protein
AT4G36580	*SHOT1 BINDING ATPASE 4 (SBA4)*	Homologue of animal ATPase Family AAA Domain-Containing Protein 3 (ATAD3), which is involved in mitochondrial nucleoid organization; interacts with SHOT1.
AT4G36590	*AGAMOUS-LIKE 40 (AGL40)*	MADS-box transcription factor family protein
AT4G36600		Late embryogenesis abundant (LEA) protein
AT4G36610		alpha/beta-Hydrolases superfamily protein
AT4G36620	*GATA TRANSCRIPTION FACTOR 19 (GATA19)*	Encodes a member of the GATA factor family of zinc finger transcription factors.
AT4G36640		Sec14p-like phosphatidylinositol transfer family protein
AT4G36655		
AT4G36670	*POLYOL/MONOSACCHARIDE TRANSPORTER 6 (PMT6)*	Major facilitator superfamily protein
AT4G36680	*RIBOSOMAL PENTATRICOPEPTIDE REPEAT PROTEIN 7 (RPPR7)*	Ribosomal pentatricopeptide repeat protein
AT4G36700		RmlC-like cupins superfamily protein
AT4G36710	*(HAM4)*	GRAS family transcription factor
AT4G36740	*HOMEOBOX PROTEIN 40 (HB40)*	Encodes a homeodomain leucine zipper class I (HD-Zip I) protein.
AT4G36750		Quinone reductase family protein
AT4G36770		UDP-Glycosyltransferase superfamily protein
AT4G36780	*BES1/BZR1 HOMOLOG 2 (BEH2)*	BES1/BZR1 homolog 2

AT4G36790		Major facilitator superfamily protein
AT4G36810	*GERANYLGERANYL PYROPHOSPHATE SYNTHASE 1 (GGPS1)*	Encodes a protein with geranylgeranyl pyrophosphate synthase activity involved in isoprenoid biosynthesis. The enzyme appears to be targeted to the chloroplast in epidermal cells and guard cells of leaves, and in etioplasts in roots. The mRNA is cell-to-cell mobile.
AT4G36830	*(HOS3-1)*	ELO family protein.
AT4G36870	*BEL1-LIKE HOMEODOMAIN 2 (BLH2)*	Encodes a member of the BEL family of homeodomain proteins. Plants doubly mutant for saw1/saw2 (blh2/blh4) have serrated leaves. BP is expressed in the serrated leaves, therefore saw1/saw2 may act redundantly to repress BP in leaves. Regulates together with BLH4 demethylesterification of homogalacturonan in seed mucilage.
AT4G36880	*CYSTEINE PROTEINASE1 (CP1)*	cysteine proteinase1
AT4G36900	*RELATED TO AP2 10 (RAP2.10)*	Encodes a member of the DREB subfamily A-5 of ERF/AP2 transcription factor family (RAP2.10). The protein contains one AP2 domain. There are 16 members in this subfamily including RAP2.9 and RAP2.1.
AT4G36910	*LOSS OF THE TIMING OF ET AND JA BIOSYNTHESIS 2 (LEJ2)*	Encodes a single cystathionine beta-synthase domain-containing protein. Modulates development by regulating the thioredoxin system.
AT4G36920	*APETALA 2 (AP2)*	Encodes a floral homeotic gene, a member of the AP2/EREBP (ethylene responsive element binding protein) class of transcription factors and is involved in the specification of floral organ identity, establishment of floral meristem identity, suppression of floral meristem indeterminancy, and development of the ovule and seed coat. AP2 also has a role in controlling seed mass. Dominant negative allele I28, revealed a function in meristem maintenance-mutant meristems are smaller than normal siblings. AP2 appears to act on the WUS-CLV pathway in an AG independent manner.
AT4G36930	*SPATULA (SPT)*	Encodes a transcription factor of the bHLH protein family. Mutants have abnormal, unfused carpels and reduced seed dormancy.
AT4G36970		Remorin family protein
AT4G36990	*HEAT SHOCK FACTOR 4 (HSF4)*	Encodes a protein whose sequence is similar to heat shock factors that regulate the expression of heat shock proteins. Transcript level is increased in response to heat shock. However, overexpression of this gene did not result in the increase of decrease of heat shock proteins.
AT4G37000	*ACCELERATED CELL DEATH 2 (ACD2)*	Mutants have spontaneous spreading cell death lesions and constitutive activation of defenses in the absence of pathogen infection. Its product was shown to display red chlorophyll catabolite reductase (RCCR), which catalyzes one step in the breakdown of the porphyrin component of chlorophyll. The enzyme was further assessed to be a Type-1 (pFCC-1-producing) RCCR.Upon P. syringae infection, ACD2 localization shifts from being largely in chloroplasts to partitioning to chloroplasts, mitochondria, and to a small extent, cytosol. Overexpression of ACD2 delayed cell death and the replication of P. syringae.
AT4G37010	*CENTRIN 2 (CEN2)*	Encodes a member of the Centrin family. Mutants are hypersensitive to UV and prone to UV induced DNA damage. Based on sequence similarity and mutant phenotype CEN2 is thought to be involved in nucelotide excision repair/DNA repair.
AT4G37050	*PATATIN-LIKE PROTEIN 4 (PLP4)*	Patatin-related phospholipase A. Expressed in the floral gynaecium and is induced by abscisic acid (ABA) or phosphate deficiency in roots.
AT4G37070	*(PLP1)*	Patatin-related phospholipase A. Expressed strongly and exclusively in roots. AtplaIVA-null mutants have reduced lateral root development. Phosphorylation by calcium-dependent protein kinases in vitro enhances its activity.
AT4G37080		ternary complex factor MIP1 leucine-zipper protein (Protein of unknown function, DUF547)
AT4G37090		UDP-N-acetylmuramoyl-L-alanyl-D-glutamate-2, 6-diaminopimelate ligase
AT4G37110		Zinc-finger domain of monoamine-oxidase A repressor R1
AT4G37120	*SWELLMAP 2 (SMP2)*	Encodes a zinc finger containing protein similar to step II splicing factors that is similar to SMP1. SMP2 is also reduced in SMP1 epigenetic alleles; plants make smaller organs having reduced cell numbers but increased cell size. The mRNA is cell-to-cell mobile.
AT4G37150	*METHYL ESTERASE 9 (MES9)*	Encodes a protein shown to have carboxylesterase activity, methyl salicylate esterase activity, methyl jasmonate esterase activity, and methyl IAA esterase activity in vitro. MES9 appears to be involved in MeSA hydrolysis in planta. Expression of MES9 can restore systemic acquired resistance in SAR-deficient tobacco plants. This protein does not act on MeGA4, or MEGA9 in vitro.
AT4G37160	*SKU5 SIMILAR 15 (sks15)*	SKU5 similar 15
AT4G37170		Pentatricopeptide repeat (PPR) superfamily protein
AT4G37180	*ULT1 INTERACTING FACTOR 1 (UIF1)*	UIF1 is a nuclear and cytoplasmically localized myb-domain containing member of the GARP G2-like subfamily of transcription factors. Interacts with ULT1 and binds to the WUS promoter. UIF1 binding domains are also found in CUC and AG promoters suggesting they are also direct targets. This locus was also identified as a putative cytoskeletal protein in a yeast screen.
AT4G37190		plasma membrane, autoregulation-binding site, misato segment II, myosin-like, tubulin/FtsZ protein
AT4G37200	*HIGH CHLOROPHYLL FLUORESCENCE 164 (HCF164)*	Encodes thioredoxin-like protein with disulfide reductase activity that is involved in the biogenesis of the plastid cytochrome b6f complex. Protein is located in the thylakoid membrane with the C-terminal hydrophilic portion, containing the thioredoxin like domain, extending into the thylakoid lumen.
AT4G37220		Cold acclimation protein WCOR413 family
AT4G37230		Photosystem II manganese-stabilising protein (PsbO) family
AT4G37240		PADRE protein down-regulated after infection by S. sclerotiorun.
AT4G37250		Leucine-rich repeat protein kinase family protein
AT4G37260	*MYB DOMAIN PROTEIN 73 (MYB73)*	Member of the R2R3 factor gene family. The mRNA is cell-to-cell mobile.
AT4G37290	*PRECURSOR OF PAMP-INDUCED PEPTIDE 2 (PREPIP2)*	Encoding a precursor protein of a secreted peptide that is responsive to flg22 stimulus.
AT4G37295	*MP-INDUCED SECRETED PEPTIDE-LIKE 3 (PIPL3)*	Encodes an 86 AA polypeptide sequence that produces an 11 AA secreted, bioactive peptide. It is induced by BD16. The peptide is bound by the RLK7 receptor kinase and inhibits the formation of lateral root founder cells. Homolog of prePIP1.
AT4G37310	*CYTOCHROME P450, FAMILY 81, SUBFAMILY H, POLYPEPTIDE 1 (CYP81H1)*	member of CYP81H
AT4G37320	*CYTOCHROME P450, FAMILY 81, SUBFAMILY D, POLYPEPTIDE 5 (CYP81D5)*	member of CYP81D
AT4G37360	*CYTOCHROME P450, FAMILY 81, SUBFAMILY D, POLYPEPTIDE 2 (CYP81D2)*	member of CYP81D
AT4G37370	*CYTOCHROME P450, FAMILY 81, SUBFAMILY D, POLYPEPTIDE 8 (CYP81D8)*	member of CYP81D
AT4G37380		Tetratricopeptide repeat (TPR)-like superfamily protein
AT4G37400	*CYTOCHROME P450, FAMILY 81, SUBFAMILY F, POLYPEPTIDE 3 (CYP81F3)*	member of CYP81F
AT4G37410	*CYTOCHROME P450, FAMILY 81, SUBFAMILY F, POLYPEPTIDE 4 (CYP81F4)*	member of CYP81F The mRNA is cell-to-cell mobile.

AT4G37430	*CYTOCHROME P450, FAMILY 91, SUBFAMILY A, POLYPEPTIDE 2 (CYP91A2)*	Encodes a member of the CYP81F cytochrome P450 monooxygenase subfamily.
AT4G37445		calcium ion-binding protein
AT4G37450	*ARABINOGALACTAN PROTEIN 18 (AGP18)*	AGP18 is a lysine-rich arabinogalactan-protein (AGP) and part of a multi-gene family of glycoproteins with approx. 50 members. It falls into one subclass with AGP17 and AGP19, other lysine-rich AGPs. It is expressed in young leaves, shoots, roots and flowers and is active in the regulation of the selection and survival of megaspores.
AT4G37470	*KARRIKIN INSENSITIVE 2 (KAI2)*	HTL belonging to the alpha/beta fold hydrolase superfamily. Mutant and over-expression studies indicates its involvement in seedling de-etiolation process. Involved in the perception of karrikins. Interacts with MAX2. Important for cotyledon expansion.
AT4G37480		Chaperone DnaJ-domain superfamily protein
AT4G37490	*CYCLIN B1;1 (CYCB1;1)*	Cyclin-dependent protein kinase CYCB1;1. Functions as an effector of growth control at G2/M. Regulated by TCP20.
AT4G37510		Ribonuclease III family protein
AT4G37530		Peroxidase superfamily protein
AT4G37540	*LOB DOMAIN-CONTAINING PROTEIN 39 (LBD39)*	LOB domain-containing protein 39
AT4G37550	*IAMHYDROLASE1 (IAMH1)*	Indole-3-acetamide (IAM) hydrolase gene required for the auxin effects of IAM.
AT4G37560	*IAMHYDROLASE12 (IAMH2)*	Indole-3-acetamide (IAM) hydrolase gene required for the auxin effects of IAM.
AT4G37580	*HOOKLESS 1 (HLS1)*	involved in apical hook development. putative N-acetyltransferase
AT4G37610	*BTB AND TAZ DOMAIN PROTEIN 5 (bt5)*	BTB and TAZ domain protein. Located in cytoplasm and expressed in fruit, flower and leaves.
AT4G37640	*CALCIUM ATPASE 2 (ACA2)*	Encodes a calmodulin-regulated Ca(2+)-pump located in the endoplasmic reticulum. Belongs to plant 2B ATPase's with an N-terminal autoinhibitor.
AT4G37650	*SHORT ROOT (SHR)*	Involved in radial organization of the root and shoot axial organs. Essential for normal shoot gravitropism. The protein moves in a highly specific manner from the cells of the stele in which it is synthesized outward. Movement requires sequences within the GRAS and VHIID domains. SHORT-ROOT forms a network in combination with JACKDAW, BLUEJAY AND SCARECROW to regulate tissue patterning through asymmetric cell division. The ground tissue lineage remains in shortroot mutant, while it is progressively lost in the triple mutant bluejay jackdaw scarecrow and double mutant jackdaw scarecrow. In addition, ground tissue basal identity remains in shortroot mutant while it is defective in the quadruple mutant bluejay jackdaw magpie nutcracker.
AT4G37660		Ribosomal protein L12/ ATP-dependent Clp protease adaptor protein ClpS family protein
AT4G37670	*N-ACETYL-L-GLUTAMATE SYNTHASE 2 (NAGS2)*	N-acetyl-l-glutamate synthase 2
AT4G37710	*VQ MOTIF-CONTAINING PROTEIN 29 (VQ29)*	VQ motif-containing protein
AT4G37730	*BASIC LEUCINE-ZIPPER 7 (bZIP7)*	basic leucine-zipper 7
AT4G37740	*GROWTH-REGULATING FACTOR 2 (GRF2)*	Growth regulating factor encoding transcription activator. One of the nine members of a GRF gene family, containing nuclear targeting domain. Mutants result in smaller leaves indicating the role of the gene in leaf development. Expressed in root, shoot and flower
AT4G37750	*AINTEGUMENTA (ANT)*	ANT is required for control of cell proliferation and encodes a putative transcriptional regulator similar to AP2. Loss of function alleles have reduced fertility, abnormal ovules and abnormal lateral organs. Expressed in the chalaza, floral organ primordia, and lateral shoot organ primordia. Regulates growth and cell numbers during organogenesis. Modulates auxin biosynthesis in the ovule via regulation of YUC4.
AT4G37760	*SQUALENE EPOXIDASE 3 (SQE3)*	squalene epoxidase 3
AT4G37770	*1-AMINO-CYCLOPROPANE-1-CARBOXYLATE SYNTHASE 8 (ACS8)*	Encodes an auxin inducible ACC synthase.
AT4G37780	*MYB DOMAIN PROTEIN 87 (MYB87)*	encoded by the Myb-like transcription factor MYB87, regulates axillary meristem formation, expressed throughout the plant. Member of the R2R3 factor gene family.
AT4G37790	*(HAT22)*	Encodes homeobox protein HAT22, member of the HD-Zip II family. The mRNA is cell-to-cell mobile.
AT4G37800	*XYLOGLUCAN ENDOTRANSGLUCOSYLASE/HYDROLASE 7 (XTH7)*	xyloglucan endotransglucosylase/hydrolase 7
AT4G37820		transmembrane protein
AT4G37840	*HEXOKINASE-LIKE 3 (HKL3)*	Encodes a putative hexokinase.
AT4G37850		basic helix-loop-helix (bHLH) DNA-binding superfamily protein
AT4G37870	*PHOSPHOENOLPYRUVATE CARBOXYKINASE 1 (PCK1)*	Encodes a phosphoenolpyruvate carboxykinase that localizes to the cytosol.
AT4G37890	*EMBRYO SAC DEVELOPMENT ARREST 40 (EDA40)*	Involved in shoot regenaration from root explants.
AT4G37900	*(ATGRDP2)*	Protein of unknown function that contains DUF1399 domain and putative RNA binding motif. Expressed in many plant tissues and is involved in many aspects of plant growth and development as well as response to salt stress. Regulates ovule development via the auxin pathway.
AT4G37910	*MITOCHONDRIAL HEAT SHOCK PROTEIN 70-1 (mtHsc70-1)*	mitochondrial heat shock protein 70-1
AT4G37930	*SERINE TRANSHYDROXYMETHYLTRANSFERASE 1 (SHM1)*	Encodes a protein with mitochondrial serine hydroxymethyltransferase activity, which functions in the photorespiratory pathway, catalyzes the conversion of serine and tetrahydrofolate to glycine and 5,10-methylene tetrahydrofolate. Involved in controlling cell damage caused by abiotic stress, such as high light and salt and the hypersensitive defense response of plants.
AT4G37940	*AGAMOUS-LIKE 21 (AGL21)*	encodes a MADS box protein, highly expressed in the root.
AT4G37950		Rhamnogalacturonate lyase family protein
AT4G37960		
AT4G37970	*CINNAMYL ALCOHOL DEHYDROGENASE 6 (CAD6)*	cinnamyl alcohol dehydrogenase 6
AT4G37980	*ELICITOR-ACTIVATED GENE 3-1 (ELI3-1)*	NADPH-dependent cinnamaldehyde and hexenal reductase involved in the production of green leaf volitile compounds.
AT4G37990	*ELICITOR-ACTIVATED GENE 3-2 (ELI3-2)*	Encodes an aromatic alcohol:NADP+ oxidoreductase whose mRNA levels are increased in response to treatment with a variety of phytopathogenic bacteria. Though similar to mannitol dehydrogenases, this enzyme does not have mannitol dehydrogenase activity.
AT4G38000	*DNA BINDING WITH ONE FINGER 4.7 (DOF4.7)*	DNA binding with one finger 4.7
AT4G38010		Pentatricopeptide repeat (PPR-like) superfamily protein
AT4G38060	*CLAVATA COMPLEX INTERACTOR 2 (CCI2)*	hypothetical protein
AT4G38070		transcription factor bHLH131-like protein

AT4G38080		hydroxyproline-rich glycoprotein family protein
AT4G38100	*CURVATURE THYLAKOID 1D (CURT1D)*	CURVATURE THYLAKOID 1D-like protein; involved in thylakoid membrane organization.
AT4G38110		
AT4G38120		ARM repeat superfamily protein
AT4G38130	*HISTONE DEACETYLASE 1 (HD1)*	Encodes a histone deacetylase that enhances AtERF7-mediated transcriptional repression. Binds SIM3 and ERF7. Expressed in the nucleus in most tissues examined and throughout the life of the plant. Involved in jasmonic acid and ethylene dependent pathogen resistance. The sequence in GenBank has 17 AG dinucleotide repeats missing, which is also missing in Ler shotgun sequence from Cereon. Although it is annotated to be in Columbia, the GB sequence is probably not of Columbia origin. Plays a role in embryogenesis as mutants grown at higher temperatures display abnormalities in the organization of the root and shoot. Plant lines expressing an RNAi construct targeted against HDA19 shows some resistance to agrobacterium-mediated root transformation. HDA19 acts in a WOX5 mediated pathway to maintain columella stem cell fate by repressing CDF4. Class I RPD3-like family HDAC member which controls positive responses to salinity stress.
AT4G38150		Pentatricopeptide repeat (PPR) superfamily protein
AT4G38160	*PIGMENT DEFECTIVE 191 (pde191)*	Encodes a member of the mitochondrial transcription termination factor family. MTERF6 is localized to both the mitochondria and chloroplast. It is required for ILE tRNA maturation during chloroplast development. Loss of function of MTERF6 results in plants with defective chloroplasts or reduced rate of photosynthesis when MTERF6 expression is low.
AT4G38190	*CELLULOSE SYNTHASE LIKE D4 (CSLD4)*	encodes a gene similar to cellulose synthase
AT4G38210	*EXPANSIN A20 (EXPA20)*	expansin -like protein. Naming convention from the Expansin Working Group (Kende et al, 2004. Plant Mol Bio). Involved in the formation of nematode-induced syncytia in roots of Arabidopsis thaliana.
AT4G38220	*AQUAPORIN INTERACTOR (AQI)*	Peptidase M20/M25/M40 family protein
AT4G38230	*CALCIUM-DEPENDENT PROTEIN KINASE 26 (CPK26)*	member of Calcium Dependent Protein Kinase
AT4G38270	*GALACTURONOSYLTRANSFERASE 3 (GAUT3)*	Encodes a protein with putative galacturonosyltransferase activity.
AT4G38300		glycosyl hydrolase family 10 protein
AT4G38330		hemolysin-III integral membrane-like protein
AT4G38340	*NIN-LIKE PROTEIN 3 (NLP3)*	Chip-seq data indicates bZIP1 binds to the NLP3 promoter.
AT4G38360	*LAZARUS 1 (LAZ1)*	LAZ1 is a DUF300 domain protein that appears to function in vacuolar transport effecting brassinosteroid and programmed cell dealth signaling pathways.
AT4G38370		Phosphoglycerate mutase family protein
AT4G38380		MATE efflux family protein
AT4G38390	*ROOT HAIR SPECIFIC 17 (RHS17)*	root hair specific 17
AT4G38400	*EXPANSIN-LIKE A2 (EXLA2)*	member of EXPANSIN-LIKE. Naming convention from the Expansin Working Group (Kende et al, 2004. Plant Mol Bio)
AT4G38410		Dehydrin family protein
AT4G38420	*SKU5 SIMILAR 9 (sks9)*	SKU5 similar 9
AT4G38430	*ROP (RHO OF PLANTS) GUANINE NUCLEOTIDE EXCHANGE FACTOR 1 (ROPGEF1)*	Member of the RopGEF (guanine nucleotide exchange factor) family, containing the novel PRONE domain (plant-specific Rop nucleotide exchanger), which is exclusively active towards members of the Rop subfamily, also known as DUF315). Interacts with ROP1 but the whole protein lacks Rho guanyl-nucleotide exchange factor activity in vitro. The DUF315/PRONE domain is sufficient to confer RopGEF catalytic activity. ropgef1 mutants have defects in auxin transport that result in abnormal development of embryos and growth defects.
AT4G38440	*MINIYO (IYO)*	Encodes MINIYO (IYO), a positive regulator of transcriptional elongation that is essential for cells to initiate differentiation.
AT4G38460	*GERANYLGERANYL REDUCTASE (GGR)*	Encodes a type II small subunit of the heteromeric geranyl(geranyl) diphosphate synthase that is localized to the chloroplast, expressed in petals and sepals and is involved in monoterpene biosynthesis. The mRNA is cell-to-cell mobile.
AT4G38470	*SERINE/THREONINE/TYROSINE KINASE 46 (STY46)*	Serine/threonine kinase that phosphorylate transit peptides of chloroplast and mitochondria targeted pre-proteins.
AT4G38480	*ABA-HYPERSENSITIVE DCAF 1 (ABD1)*	Transducin/WD40 repeat-like superfamily protein
AT4G38510	*V-ATPASE B SUBUNIT 2 (VAB2)*	One of three genes encoding the vacuolar ATP synthase subunit B1. The protein binds to and co-localizes with F-actin, bundles F-actin to form higher-order structure, and stabilizes actin filaments in vitro.
AT4G38520	*ARABIDOPSIS PP2C CLADE D 6 (APD6)*	Protein phosphatase 2C family protein
AT4G38530	*PHOSPHOLIPASE C1 (PLC1)*	Encodes a putative phosphoinositide-specific phospholipase C. There are two genes called ATPLC1, one corresponding to AT4g38530 (this one) and one corresponding to AT5g58670.
AT4G38540		FAD/NAD(P)-binding oxidoreductase family protein
AT4G38560		phospholipase-like protein (PEARLI 4) family protein
AT4G38570	*PROBABLE CDP-DIACYLGLYCEROL--INOSITOL 3-PHOSPHATIDYLTRANSFERASE 2 (PIS2)*	Putative CDP-diacylglycerol-inositol 3-phosphatidyltransferase 2
AT4G38580	*FARNESYLATED PROTEIN 6 (FP6)*	putative farnesylated protein (At4g38580) mRNA, complete
AT4G38590	*BETA-GALACTOSIDASE 14 (BGAL14)*	putative beta-galactosidase (BGAL14 gene)
AT4G38610		
AT4G38620	*MYB DOMAIN PROTEIN 4 (MYB4)*	Encodes a R2R3 MYB protein which is involved in the response to UV-B. It functions as a repressor of target gene expression. One of its target genes encodes cinnamate 4-hydroxylase; mutants accumulate sinapate esters in their leaves. MYB4 binds to its own promoter and represses its own expression. Nuclear localization of MYB4 depends on the action of the beta importin SAD2. The mRNA is cell-to-cell mobile.
AT4G38660		Pathogenesis-related thaumatin superfamily protein
AT4G38680	*GLYCINE RICH PROTEIN 2 (GRP2)*	Encodes a glycine-rich protein that binds nucleic acids and promotes DNA melting. Its transcript and protein levels are up-regulated in response to cold treatment with protein levels peaking earlier in shoots (~10-14 days) than in roots (~21 days). It is normally expressed in meristematic regions and developing tissues where cell division occurs. RNAi and antisense lines with lower levels of CSP2/GRP2 transcripts flower earlier than wild type plants and have some defects in anther and seed development.

AT4G38690		PLC-like phosphodiesterases superfamily protein
AT4G38700		Disease resistance-responsive (dirigent-like protein) family protein
AT4G38710		glycine-rich protein
AT4G38740	*ROTAMASE CYP 1 (ROC1)*	Encodes cytosolic cyclophilin ROC1.
AT4G38760		nucleoporin (DUF3414)
AT4G38770	*PROLINE-RICH PROTEIN 4 (PRP4)*	Encodes one of four proline-rich proteins in Arabidopsis which are predicted to localize to the cell wall. Transcripts are most abundant in aerial organs of the plant.
AT4G38780		pre-mRNA-processing-splicing factor-like protein
AT4G38800	*METHYLTHIOADENOSINE NUCLEOSIDASE 1 (MTN1)*	Encodes one of the 5'-methylthioadenosine nucleosidases (AT4G38800/MTN1; AT4G34840/MTN2). Double mutant, mtn1-1mtn2-1, retains approximately 14% of the MTN enzyme activity present in the wild type and displays a pleiotropic phenotype that includes altered vasculature and impaired fertility.
AT4G38810	*CALCIUM SENSOR (SCS)*	SnRK2-Interacting Calcium Sensor. Encodes two different isoforms that can both inhibit SnRK2. The longer form (AT4G38810.2) is calcium dependant, the other is not.
AT4G38820		hypothetical protein
AT4G38830	*CYSTEINE-RICH RLK (RECEPTOR-LIKE PROTEIN KINASE) 26 (CRK26)*	Encodes a cysteine-rich receptor-like protein kinase.
AT4G38840	*SMALL AUXIN UPREGULATED RNA 14 (SAUR14)*	SAUR-like auxin-responsive protein family
AT4G38850	*SMALL AUXIN UPREGULATED 15 (SAUR15)*	mRNA is rapidly induced by auxin and is very short-lived. Has been used as a reporter gene in studying auxin mutants.
AT4G38860	*SMALL AUXIN UPREGULATED RNA 16 (SAUR16)*	SAUR-like auxin-responsive protein family
AT4G38870		F-box and associated interaction domains-containing protein
AT4G38890		FMN-linked oxidoreductases superfamily protein
AT4G38930		Ubiquitin fusion degradation UFD1 family protein
AT4G38940		Galactose oxidase/kelch repeat superfamily protein
AT4G38950		ATP binding microtubule motor family protein
AT4G38960	*B-BOX DOMAIN PROTEIN 19 (BBX19)*	BBX19 is a B-box containing transcriptional regulator involved in photomorphogenesis and flowering.
AT4G38970	*FRUCTOSE-BISPHOSPHATE ALDOLASE 2 (FBA2)*	Protein is tyrosine-phosphorylated and its phosphorylation state is modulated in response to ABA in Arabidopsis thaliana seeds.
AT4G38990	*GLYCOSYL HYDROLASE 9B16 (GH9B16)*	glycosyl hydrolase 9B16
AT4G39000	*GLYCOSYL HYDROLASE 9B17 (GH9B17)*	glycosyl hydrolase 9B17
AT4G39010	*GLYCOSYL HYDROLASE 9B18 (GH9B18)*	Cellulase involved in cell wall modification during valve dehiscence.
AT4G39030	*ENHANCED DISEASE SUSCEPTIBILITY 5 (EDS5)*	Encodes an orphan multidrug and toxin extrusion transporter. Essential component of salicylic acid-dependent signaling for disease resistance. Member of the MATE-transporter family. Expression induced by salicylic acid. Mutants are salicylic acid-deficient.
AT4G39040	*CRM FAMILY MEMBER SUBFAMILY 4 (CFM4)*	RNA-binding CRS1 / YhbY (CRM) domain protein
AT4G39070	*BZS1 (BZS1)*	Encodes BZS1, a brassinosteroids-regulated BZR1 target (BRBT) gene. BZS1 is a putative zinc finger transcription factor. Expression of BZS1 was increased under BR-deficient condition and repressed by BR. Transgenic Arabidopsis plants overexpressing BZS1 showed a hypersensitivity to the BR biosynthetic inhibitor brassinazole (BRZ). In contrast, transgenic plants expressing reduced level of BZS1 had longer hypocotyls than wild type when grown on BRZ.
AT4G39080	*VACUOLAR PROTON ATPASE A3 (VHA-A3)*	Vacuolar proton ATPase subunit VHA-a isoform 3. Localized in the tonoplast. The mRNA is cell-to-cell mobile.
AT4G39120	*MYO-INOSITOL MONOPHOSPHATASE LIKE 2 (IMPL2)*	Encodes a chloroplast-localized member of the myo-inositol monophosphatase family, IMPL2 (myo-Inositol monophosphatase like 2) that seems to have multiple enzymatic activities. It contributes to histidine biosynthesis based on it histidinol-phosphate phosphatase activity. In addition, the protein can act as an inositol monophosphatase and an L-galactose-1-phosphate phosphatase in vitro.
AT4G39130		Dehydrin family protein
AT4G39150		DNAJ heat shock N-terminal domain-containing protein
AT4G39160		Homeodomain-like superfamily protein
AT4G39180	*SECRETION 14 (SEC14)*	encodes a protein that complements the function of a sec14(ts) mutant of S. cerevisiae
AT4G39190		nucleolar-like protein
AT4G39200		Ribosomal protein S25 family protein
AT4G39210	*(APL3)*	Encodes the large subunit of ADP-Glucose Pyrophosphorylase which catalyzes the first, rate limiting step in starch biosynthesis. The large subunit plays a regulatory role whereas the small subunit (ApS) is the catalytic isoform. Four isoforms (ApL1-4) have been identified. ApL3 is the major large subunit isoform present in inflorescences, fruits and roots.
AT4G39235		hypothetical protein
AT4G39260	*RNA-BINDING GLYCINE-RICH PROTEIN A6 (RBGA6)*	Encodes a glycine-rich protein with RNA binding domain at the N-terminus. Protein is structurally similar to proteins induced by stress in other plants. Gene expression is induced by cold. Transcript undergoes circadian oscillations that is depressed by overexpression of AtGRP7. A substrate of the type III effector HopU1 (mono-ADP-ribosyltransferase).
AT4G39270		Leucine-rich repeat protein kinase family protein
AT4G39300		envelope glycoprotein
AT4G39320		microtubule-associated protein-like protein
AT4G39330	*CINNAMYL ALCOHOL DEHYDROGENASE 9 (CAD9)*	cinnamyl alcohol dehydrogenase 9
AT4G39350	*CELLULOSE SYNTHASE A2 (CESA2)*	Encodes a cellulose synthase isomer, related to CESA6. As inferred from the null role of secondary wall-type CesAs, included in a set of five primary wall-type CesAs that may support trichome cell wall thickening. The mRNA is cell-to-cell mobile.
AT4G39380		TSL-kinase interacting-like protein
AT4G39390	*NUCLEOTIDE SUGAR TRANSPORTER-KT 1 (NST-K1)*	Encodes a golgi localized nucleotide sugar transporter.

AT4G39400	*BRASSINOSTEROID INSENSITIVE 1 (BRI1)*	Encodes a plasma membrane localized leucine-rich repeat receptor kinase involved in brassinosteroid signal transduction. BRI1 ligand is brassinolide which binds at the extracellular domain. Binding results in phosphorylation of the kinase domain which activates the BRI1 protein leading to BR responses. Residue T-1049 and either S-1044 or T-1045 were essential for kinase function in vitro and normal BRI1 signaling in planta. The structure of BRI1 ligand-binding domain has been determined at 2.5A resolution. Although BAK1 and BRI1 alone localize in the plasma membrane, when BAK1 and BRI1 are coexpressed, the heterodimer BAK1/BRI1 they form is localized in the endosome. BRI1 appears to be involved in the autonomous pathway that regulates the transition to flowering, primarily through its effects on FLC expression levels, as uncovered by double mutant analyses. This most likely occurs as a result of BRI1-dependent effects on histone acetylation, but not histone triMeH3K4 methylation, at the FLC locus. The mRNA is cell-to-cell mobile.
AT4G39410	*WRKY DNA-BINDING PROTEIN 13 (WRKY13)*	Encodes a member of the Group II-c WRKY Transcription Factor family that is involved in stem development and has been shown to directly bind to the promoter of NST2. WRKY13 binds to the promoter of DCD to upregulate its expression and hydrogen sulfide production to enhance plant cadmium tolerance. Mutants show a weak stem phenotype and show decreased expression of lignin-synthesis-related genes.
AT4G39420		spatacsin carboxy-terminus protein
AT4G39430		
AT4G39440		
AT4G39460	*S-ADENOSYLMETHIONINE CARRIER 1 (SAMC1)*	Encodes a plastid metabolite transporter required for the import of S-Adenosylmethionine from the cytosol. Impaired function of SAMT1 led to decreased accumulation of prenyllipids and mainly affected the chlorophyll pathway.
AT4G39480	*CYTOCHROME P450, FAMILY 96, SUBFAMILY A, POLYPEPTIDE 9 (CYP96A9)*	member of CYP96A
AT4G39490	*CYTOCHROME P450, FAMILY 96, SUBFAMILY A, POLYPEPTIDE 10 (CYP96A10)*	member of CYP96A
AT4G39500	*CYTOCHROME P450, FAMILY 96, SUBFAMILY A, POLYPEPTIDE 11 (CYP96A11)*	cytochrome P450, family 96, subfamily A, polypeptide 11
AT4G39510	*CYTOCHROME P450, FAMILY 96, SUBFAMILY A, POLYPEPTIDE 12 (CYP96A12)*	member of CYP96A
AT4G39520	*(DRG1-1)*	Encodes a member of the DRG (developmentally regulated G-protein) family. Has GTPase activity.
AT4G39540	*SHIKIMATE KINASE 2 (SK2)*	Encodes a shikimate kinase. Its transcripts appear to be expressed in vegetative tissues and developing embryos. SK2 transcript levels rise in response to Phytophthora infestans spores. SK2 is believed to be localized to the chloroplast.
AT4G39550		Galactose oxidase/kelch repeat superfamily protein
AT4G39590		Galactose oxidase/kelch repeat superfamily protein
AT4G39610		MIZU-KUSSEI-like protein (Protein of unknown function, DUF617)
AT4G39640	*GAMMA-GLUTAMYL TRANSPEPTIDASE 1 (GGT1)*	The gene encodes a gamma-glutamyltransferase (AKA gamma-glutamyl transpeptidase, EC 2.3.2.2) that is located in vascular tissues (predominantly phloem) of leaves and is involved in the degradation of glutathione. The encoded enzyme also mitigates oxidative stress by metabolizing GSSG (oxidized form of GSH - glutathione) in the apoplast.
AT4G39650	*GAMMA-GLUTAMYL TRANSPEPTIDASE 2 (GGT2)*	The gene encodes a gamma-glutamyltransferase (AKA gamma-glutamyl transpeptidase, EC 2.3.2.2) that is located in the apoplast of young siliques (within the ovules of the carpel) and is involved in the degradation of glutathione. The encoded enzyme also acts as part of a GSH pumping gamma-glutamyl cycle in this tissue and may also be involved in gamma-glutamyl amino acid formation.
AT4G39660	*ALANINE:GLYOXYLATE AMINOTRANSFERASE 2 (AGT2)*	alanine:glyoxylate aminotransferase 2 homolog (AGT2). The mRNA is cell-to-cell mobile.
AT4G39670	*PHOSPHOLIPASE-LIKE PROTEIN (GLTP)*	Member of the glycolipid transfer protein (GLTP) superfamily, shuttles ceramide-1-phosphate (C1P) between membranes.
AT4G39675		hypothetical protein
AT4G39690	*(MIC60)*	Encodes a homolog of the yeast mic60 protein that is localized in the inner membrane of the mitochondrion, interacts with Tom40 as part of a large lipid-enriched complex called the mitochondrial transmembrane lipoprotein complex (MTL) and is involved in mitochondrial lipid trafficking.
AT4G39710	*PHOTOSYNTHETIC NDH SUBCOMPLEX L 4 (PnsL4)*	FK506-binding protein 16-2
AT4G39720		VQ motif-containing protein
AT4G39730	*PLAT DOMAIN PROTEIN 1 (PLAT1)*	PLAT1 domain stress protein family member. Involved in mediating response to stresses such as pathogen infection. It is found in endoplasmic reticulum bodies. PLAT1 is induced by pathogenic fungi and induces the production of scopolin.
AT4G39740	*HOMOLOGUE OF COPPER CHAPERONE SCO1 2 (HCC2)*	Encodes HCC2, one of the two Arabidopsis genes (HCC1 and HCC2) resulting from a duplication with homology to the SCO proteins involved in copper insertion during cytochrome c oxidase (COX) assembly in other organisms. HCC2, which lacks the cysteines and histidine putatively involved in copper binding, functions in copper sensing and redox homeostasis.
AT4G39753		Galactose oxidase/kelch repeat superfamily protein
AT4G39756		Galactose oxidase/kelch repeat superfamily protein
AT4G39770	*TREHALOSE-6-PHOSPHATE PHOSPHATASE H (TPPH)*	Haloacid dehalogenase-like hydrolase (HAD) superfamily protein
AT4G39780		encodes a member of the DREB subfamily A-6 of ERF/AP2 transcription factor family. The protein contains one AP2 domain. There are 8 members in this subfamily including RAP2.4.
AT4G39800	*D-MYO-INOSITOL 3-PHOSPHATE SYNTHASE 1 (MIPS1)*	** Referred to as MIPS2 in Mitsuhashi et al 2008. myo-inositol-1-phosphate synthase isoform 1.Expressed in leaf, root and silique. Immunolocalization experiments with an antibody recognizing MIPS1, MIPS2, and MIPS3 showed endosperm localization.
AT4G39820	*(TRAPPC12)*	Part of multi-protein complex, acting as guanine nucleotide exchange factors (GEFs) and possibly as tethers, regulating intracellular trafficking.
AT4G39840		cell wall integrity/stress response component-like protein
AT4G39850	*ATP-BINDING CASSETTE D1 (ABCD1)*	Encodes a peroxisomal protein of the ATP binding cassette (ABC) transporter class (PMP subfamily) with significant identity to the human X-linked adrenoleukodystrophy protein (ALDP). The gene product promotes germination and represses embryo dormancy. ABI3, ABA1, FUS3 and LEC1 are epistatic to this gene. Mutants accumulate fatty acyl CoA suggesting a defect in uptake of fatty acyl CoA into the peroxisome.
AT4G39880		Ribosomal protein L23/L15e family protein
AT4G39890	*RAB GTPASE HOMOLOG H1C (RABH1c)*	RAB GTPase homolog H1C
AT4G39900		adenine deaminase
AT4G39930		hypothetical protein

AT4G39940	*APS-KINASE 2 (AKN2)*	adenosine-5'-phosphosulfate-kinase (akn2) mRNA, complete The mRNA is cell-to-cell mobile.
AT4G39950	*CYTOCHROME P450, FAMILY 79, SUBFAMILY B, POLYPEPTIDE 2 (CYP79B2)*	Belongs to cytochrome P450 and is involved in tryptophan metabolism. Converts Trp to indo-3-acetaldoxime (IAOx), a precursor to IAA and indole glucosinolates. The mRNA is cell-to-cell mobile.
AT4G39955		alpha/beta-Hydrolases superfamily protein
AT4G39970		Haloacid dehalogenase-like hydrolase (HAD) superfamily protein
AT4G40000	*(TRM4A)*	S-adenosyl-L-methionine-dependent methyltransferases superfamily protein
AT4G40010	*SNF1-RELATED PROTEIN KINASE 2.7 (SNRK2.7)*	encodes a member of SNF1-related protein kinases (SnRK2) whose activity is activated by ionic (salt) and non-ionic (mannitol) osmotic stress.
AT4G40020		Myosin heavy chain-related protein
AT4G40060	*HOMEOBOX PROTEIN 16 (HB16)*	Encodes a homeodomain leucine zipper class I (HD-Zip I) protein.
AT4G40080	*(PICALM10A)*	ENTH/ANTH/VHS superfamily protein
AT4G40090	*ARABINOGALACTAN PROTEIN 3 (AGP3)*	arabinogalactan protein 3
AT4G40095		
AT5G01010		retinal-binding protein
AT5G01015		transmembrane protein
AT5G01030		enolase, putative (DUF3527)
AT5G01040	*LACCASE 8 (LAC8)*	putative laccase, knockout mutant showed early flowering
AT5G01060	*BRASSINOSTEROID-SIGNALING KINASE 10 (BSK10)*	kinase with tetratricopeptide repeat domain-containing protein
AT5G01070		RING/FYVE/PHD zinc finger superfamily protein
AT5G01075	*TWISTED SEED1 (TWS1)*	Encodes a small ER-localized protein that is strongly expressed in seeds and regulates both embryo development and accumulation of storage compounds. At the cellular level, TWS1 is responsible for cuticle deposition on epidermal cells and organization of the endomembrane system.
AT5G01080		Beta-galactosidase related protein
AT5G01090		Concanavalin A-like lectin family protein
AT5G01110		Tetratricopeptide repeat (TPR)-like superfamily protein
AT5G01150		hypothetical protein (DUF674)
AT5G01180	*NRT1/ PTR FAMILY 8.2 (NPF8.2)*	Encodes a dipeptide transporter expressed in pollen and ovules during early seed development. GFP-tagged PTR5 localizes to the plasma membrane.
AT5G01190	*LACCASE 10 (LAC10)*	putative laccase, a member of laccase family of genes (17 members in Arabidopsis).
AT5G01200		Duplicated homeodomain-like superfamily protein
AT5G01210		HXXXD-type acyl-transferase family protein
AT5G01220	*SULFOQUINOVOSYLDIACYLGLYCEROL 2 (SQD2)*	Encodes a UDP-sulfoquinovose:DAG sulfoquinovosyltransferase that is involved in sulfolipid biosynthesis and whose expression is responsive to both phosphate (Pi) and phosphite (Phi) in both roots and shoots.
AT5G01240	*LIKE AUXIN RESISTANT 1 (LAX1)*	Encodes LAX1 (LIKE AUXIN RESISTANT), a member of the AUX1 LAX family of auxin influx carriers. Required for the establishment of embryonic root cell organization.
AT5G01260		Carbohydrate-binding-like fold
AT5G01280	*BASIC PROLINE-RICH PROTEIN3 (BPP3)*	Encodes a microtubule-associated protein.
AT5G01300		PEBP (phosphatidylethanolamine-binding protein) family protein
AT5G01310	*APRATAXIN-LIKE (APTX)*	Encodes a protein that has adenylylsulfate sulfohydrolase activity (E.C. 3.6.2.1) in vitro.
AT5G01320		Thiamine pyrophosphate dependent pyruvate decarboxylase family protein
AT5G01340	*MITOCHONDRIAL SUCCINATE-FUMARATE CARRIER 1 (mSFC1)*	Transports citrate, isocitrate and aconitate, succinate and fumarate. Catalyzes a fast counter-exchange transport as well as a low uniport of substrates, exhibits a higher transport affinity for tricarboxylates than dicarboxylates. Might be involved in storage oil mobilization 78 at early stages of seedling growth and in nitrogen assimilation in root tissue by 79 catalyzing citrate/isocitrate or citrate/succinate exchanges.
AT5G01360	*TRICHOME BIREFRINGENCE-LIKE 3 (TBL3)*	Encodes a member of the TBL (TRICHOME BIREFRINGENCE-LIKE) gene family containing a plant-specific DUF231 (domain of unknown function) domain. TBL gene family has 46 members, two of which (TBR/AT5G06700 and TBL3/AT5G01360) have been shown to be involved in the synthesis and deposition of secondary wall cellulose, presumably by influencing the esterification state of pectic polymers. A nomenclature for this gene family has been proposed (Volker Bischoff & Wolf Scheible, 2010, personal communication).The dwarf phenotype can only be seen in tbl3 tbl31 esk1 triple mutant. tbl3 and tbl31 are specifically involved in 3-O-monoacetylation of xylan.
AT5G01370	*ALC-INTERACTING PROTEIN 1 (ACI1)*	Nuclear protein with a lysine-rich domain and a C-terminal serine-rich domain. Interacts with Alcatraz (ALC). ACI1 is mainly expressed in the vascular system. Involved in cell separation during fruit dehiscence.
AT5G01380		Homeodomain-like superfamily protein
AT5G01410	*REDUCED SUGAR RESPONSE 4 (RSR4)*	Encodes a protein predicted to function in tandem with PDX2 to form glutamine amidotransferase complex with involved in vitamin B6 biosynthesis.
AT5G01440		hypothetical protein
AT5G01450	*ABERRANT POLLEN DEVELOPMENT 2 (APD2)*	RING/U-box superfamily protein
AT5G01480		Cysteine/Histidine-rich C1 domain family protein
AT5G01490	*CATION EXCHANGER 4 (CAX4)*	Encodes a cation/proton antiporter, a member of low affinity calcium antiporter CAX2 family. Involved in root development under metal stress.
AT5G01520	*ABA INSENSITIVE RING PROTEIN 2 (AIRP2)*	Encodes a cytosolic RING-type E3 ubiquitin (Ub) ligase that is critical for ABA and high salinity responses during germination. AtAIRP2 and SDIR1 likely play a combinatory role in ABA signaling and the response to high salt in Arabidopsis.
AT5G01530	*LIGHT HARVESTING COMPLEX PHOTOSYSTEM II (LHCB4.1)*	light harvesting complex photosystem II
AT5G01540	*L-TYPE LECTIN RECEPTOR KINASE-VI.2 (LECRK-VI.2)*	Encodes LecRKA4.1, a member of the lectin receptor kinase subfamily A4 (LecRKA4.1 At5g01540; LecRKA4.2 At5g01550; LecRKA4.3 At5g01560). Together with other members of the subfamily, functions redundantly in the negative regulation of ABA response in seed germination. Positively regulates pattern-triggered immunity.

AT5G01550	*L-TYPE LECTIN RECEPTOR KINASE VI.3 (LECRK-VI.3)*	Encodes LecRKA4.2, a member of the lectin receptor kinase subfamily A4 (LecRKA4.1 At5g01540; LecRKA4.2 At5g01550; LecRKA4.3 At5g01560). Together with other members of the subfamily, functions redundantly in the negative regulation of ABA response in seed germination.
AT5G01560	*LECTIN RECEPTOR KINASE A4.3 (LECRKA4.3)*	Encodes LecRKA4.3, a member of the lectin receptor kinase subfamily A4 (LecRKA4.1 At5g01540; LecRKA4.2 At5g01550; LecRKA4.3 At5g01560). Together with other members of the subfamily, functions redundantly in the negative regulation of ABA response in seed germination.
AT5G01590	*TRANSLOCON AT THE INNER ENVELOPE MEMBRANE OF CHLOROPLASTS 56 (TIC56)*	histone-lysine N-methyltransferase ATXR3-like protein
AT5G01600	*FERRETIN 1 (FER1)*	Encodes a ferretin protein that is targeted to the chloroplast. Member of a Ferritin gene family. Gene expression is induced in response to iron overload and by nitric oxide. Expression of the gene is downregulated in the presence of paraquat, an inducer of photoxidative stress.
AT5G01610		hypothetical protein (Protein of unknown function, DUF538)
AT5G01620	*TRICHOME BIREFRINGENCE-LIKE 35 (TBL35)*	Encodes a member of the TBL (TRICHOME BIREFRINGENCE-LIKE) gene family containing a plant-specific DUF231 (domain of unknown function) domain. TBL gene family has 46 members, two of which (TBR/AT5G06700 and TBL3/AT5G01360) have been shown to be involved in the synthesis and deposition of secondary wall cellulose, presumably by influencing the esterification state of pectic polymers. A nomenclature for this gene family has been proposed (Volker Bischoff & Wolf Scheible, 2010, personal communication).TBL35 are required only for xylan 3-O-monoacetylation and 2,3-di-O-acetylation. The biochemical phenotype can be observed in tbl35 esk1, double mutant and tbl34 tbl35 esk1 triple mutants.
AT5G01650	*MIF/D-DT-LIKE 2 (MDL2)*	Chemokine-like MDL protein; modulate flowering time and innate immunity in plants.
AT5G01660		influenza virus NS1A-binding protein
AT5G01670		NAD(P)-linked oxidoreductase superfamily protein
AT5G01680	*CATION/H+ EXCHANGER 26 (CHX26)*	member of Putative Na+/H+ antiporter family
AT5G01690	*CATION/H+ EXCHANGER 27 (CHX27)*	member of Putative Na+/H+ antiporter family
AT5G01700		Protein phosphatase 2C family protein
AT5G01720	*REGULATION OF ATALMT1 EXPRESSION 1 (RAE1)*	RAE1 is an F-box protein component of a SCF-type E3 ligase complex. It is part of an alumium induced regulatory loop: its activity is induced by STOP1 and it in turn ubiquitinates STOP1 which is then targeted for degradation.
AT5G01730	*SCAR FAMILY PROTEIN 4 (SCAR4)*	Encodes a member of the SCAR family.These proteins are part of a complex (WAVE) complex.The SCAR subunit activates the ARP2/3 complex which in turn act as a nucleator for actin filaments.
AT5G01740		Unknown gene, induced by abiotic stress treatments.
AT5G01750		LURP-one-like protein (DUF567)
AT5G01780		2-oxoglutarate-dependent dioxygenase family protein
AT5G01790		hypothetical protein
AT5G01800		saposin B domain-containing protein
AT5G01810	*CBL-INTERACTING PROTEIN KINASE 15 (CIPK15)*	Encodes a CBL-interacting serine/threonine protein kinase, also has similarities to SOS2 kinase.
AT5G01820	*SERINE/THREONINE PROTEIN KINASE 1 (SR1)*	Encodes a CBL-interacting serine/threonine protein kinase.
AT5G01830	*(PUB16)*	Plant U-box type E3 ubiquitin ligase (PUB).
AT5G01840	*OVATE FAMILY PROTEIN 1 (OFP1)*	Encodes a member of the plant specific ovate protein family. Members of this family have been shown to bind to KNOX and BELL- like TALE class homeodomain proteins. This interaction may mediate relocalization of the TALE homeodomain from the nucleus to the cytoplasm. Functions as a transcriptional repressor that suppresses cell elongation. May also directly affect microtubule organization via interactions with TON2.
AT5G01850		Protein kinase superfamily protein
AT5G01860		C2H2 and C2HC zinc fingers superfamily protein
AT5G01870		Predicted to encode a PR (pathogenesis-related) protein. Belongs to the lipid transfer protein (PR-14) family with the following members: At2g38540/LTP1, At2g38530/LTP2, At5g59320/LTP3, At5g59310/LTP4, At3g51600/LTP5, At3g08770/LTP6, At2g15050/LTP7, At2g18370/LTP8, At2g15325/LTP9, At5g01870/LTP10, At4g33355/LTP11, At3g51590/LTP12, At5g44265/LTP13, At5g62065/LTP14, At4g08530/LTP15.
AT5G01880	*DAF-LIKE GENE 2 (DAFL2)*	RING/U-box superfamily protein
AT5G01890	*PXY/TDR-CORRELATED 2 (PXC2)*	Leucine-rich receptor-like protein kinase family protein
AT5G01920	*STATE TRANSITION 8 (STN8)*	Chloroplast thylakoid protein kinase STN8 is specific in phosphorylation of N-terminal threonine residues in D1, D2 and CP43 proteins, and Thr-4 in PsbH protein of photosystem II. Phosphorylation of Thr-4 in the wild type required both light and prior phosphorylation at Thr-2.
AT5G01930	*ENDO-BETA-MANNASE 6 (MAN6)*	Encodes a endo-beta-mannanase involved in seed germination.
AT5G01940		eukaryotic translation initiation factor 2B family protein / eIF-2B family protein
AT5G01950		Leucine-rich repeat protein kinase family protein
AT5G01970	*(MADA2)*	unknown protein;(source:TAIR10)
AT5G02000		hypothetical protein
AT5G02020	*SALT INDUCED SERINE RICH (SIS)*	Encodes a protein involved in salt tolerance, names SIS (Salt Induced Serine rich).
AT5G02030	*REPLUMLESS (RPL)*	Mutant has additional lateral organs and phyllotaxy defect. Encodes a homeodomain transcription factor. Has sequence similarity to the Arabidopsis ovule development regulator Bell1. Binds directly to the AGAMOUS cis-regulatory element. Its localization to the nucleus is dependent on the coexpression of either STM or BP.
AT5G02050		Mitochondrial glycoprotein family protein
AT5G02070		Protein kinase family protein
AT5G02080		phosphopantothenate-cysteine ligase-like protein
AT5G02120	*ONE-HELIX LHC-LIKE PROTEIN 1 (OHP1)*	Encodes a one helix protein homologous to cyanobacterial high-light inducible proteins. The protein is localized to the thylakoid membrane and its transcript is transiently induced by exposure to high light conditions. The mRNA is cell-to-cell mobile.
AT5G02140		Pathogenesis-related thaumatin superfamily protein

AT5G02150	*FES1C (Fes1C)*	Encodes one of the Arabidopsis orthologs of the human Hsp70-binding protein 1 (HspBP-1) and yeast Fes1p: Fes1A (AT3G09350), Fes1B (AT3G53800), Fes1C (AT5G02150).
AT5G02160	*FTSH5 INTERACTING PROTEIN (FIP)*	Zinc-finger domain containing protein involved in abiotic stress response. Possesses an N-terminal transit peptide followed by a hydrophobic domain and a zinc-finger domain. Despite the presence of a zinc-finger domain (C4-type) with two CXXCXGXG conserved repeats, characteristic of DNAJ protein, the conserved J domain is absent in FIP. Interacts with FtsH5. Gene expression levels are reduced and negatively regulates stress response genes during stress conditions.
AT5G02170		Transmembrane amino acid transporter family protein
AT5G02180		Transmembrane amino acid transporter family protein
AT5G02190	*PROMOTION OF CELL SURVIVAL 1 (PCS1)*	encodes an aspartic protease, has an important role in determining cell fate during embryonic development and in reproduction processes. The loss-of-function mutation of PCS1 causes degeneration of both male and female gametophytes and excessive cell death of developing embryos during torpedo stage.
AT5G02200	*FAR-RED-ELONGATED HYPOCOTYL1-LIKE (FHL)*	Encodes a small plant-specific protein with both nuclear localization and nuclear export signals that is specifically required, together with FHY1, for the light-regulated nuclear accumulation of phyA.
AT5G02210		GCK domain-containing protein
AT5G02220	*SIAMESE-RELATED 4 (SMR4)*	cyclin-dependent kinase inhibitor
AT5G02230		Haloacid dehalogenase-like hydrolase (HAD) superfamily protein
AT5G02240		Protein is tyrosine-phosphorylated and its phosphorylation state is modulated in response to ABA in Arabidopsis thaliana seeds. The mRNA is cell-to-cell mobile.
AT5G02250	*EMBRYO DEFECTIVE 2730 (EMB2730)*	Encodes a exoribonuclease involved in rRNA processing in mitochondria and chloroplasts.Loss of function mutations are pale green and require supplementation with sucrose for germination and early development. Plants are pale green due to defects in chloroplast biogenesis.
AT5G02260	*EXPANSIN A9 (EXPA9)*	member of Alpha-Expansin Gene Family. Naming convention from the Expansin Working Group (Kende et al, 2004. Plant Mol Bio)
AT5G02270	*ATP-BINDING CASSETTE I20 (ABCI20)*	member of NAP subfamily
AT5G02280	*(TRAPPC4)*	Part of multi-protein complex, acting as guanine nucleotide exchange factors (GEFs) and possibly as tethers, regulating intracellular trafficking.
AT5G02290	*(NAK)*	Encodes a candidate protein kinase NAK that is similar to the oncogenes met and abl.
AT5G02300		
AT5G02330		Cysteine/Histidine-rich C1 domain family protein
AT5G02350		Cysteine/Histidine-rich C1 domain family protein
AT5G02360		DC1 domain-containing protein
AT5G02370		ATP binding microtubule motor family protein
AT5G02390	*DUO1-ACTIVATED UNKNOWN 1 (DAU1)*	Target promoter of the male germline-specific transcription factor DUO1.
AT5G02430		Transducin/WD40 repeat-like superfamily protein
AT5G02440		60S ribosomal protein L36
AT5G02460	*PHLOEM EARLY DOF 2 (PEAR2)*	PEAR protein involved in the formation of a short-range concentration gradient that peaks at protophloem sieve elements, and activates gene expression that promotes radial growth. Locally promotes transcription of inhibitory HD-ZIP III genes, and thereby establishes a negative-feedback loop that forms a robust boundary that demarks the zone of cell division.
AT5G02470	*(DPA)*	core cell cycle genes
AT5G02480		HSP20-like chaperones superfamily protein
AT5G02490	*(Hsp70-2)*	Heat shock protein 70 (Hsp 70) family protein
AT5G02530	*(ALY2)*	RNA-binding (RRM/RBD/RNP motifs) family protein
AT5G02540		NAD(P)-binding Rossmann-fold superfamily protein
AT5G02550		hypothetical protein
AT5G02560	*HISTONE H2A 12 (HTA12)*	Encodes HTA12, a histone H2A protein.
AT5G02570	*(HTB10)*	Histone superfamily protein
AT5G02580		argininosuccinate lyase
AT5G02590		Tetratricopeptide repeat (TPR)-like superfamily protein
AT5G02600	*SODIUM POTASSIUM ROOT DEFECTIVE 1 (NAKR1)*	Encodes a phloem mobile metal binding protein necessary for phloem function and root meristem maintenance.
AT5G02630	*CANDIDATE G-PROTEIN COUPLED RECEPTOR 6 (CAND6)*	Lung seven transmembrane receptor family protein
AT5G02640		hypothetical protein
AT5G02650		hypothetical protein
AT5G02680		methionine-tRNA ligase
AT5G02690		hypothetical protein
AT5G02710		zinc/iron-chelating domain protein
AT5G02720		valine-tRNA ligase
AT5G02730		CAP (Cysteine-rich secretory proteins, Antigen 5, and Pathogenesis-related 1 protein) superfamily protein
AT5G02760	*ARABIDOPSIS PP2C CLADE D 7 (APD7)*	Encodes a phosphatase that functions in sustaining proper leaf longevity and preventing early senescence by suppressing or perturbing SARK-mediated senescence signal transduction.
AT5G02770	*MODIFIER OF SNC1, 11 (MOS11)*	Encodes a conserved eukaryotic protein with homology to the human RNA binding protein CIP29 that localizes to the nucleus. Mutants accumulate more poly(A) mRNAs in the nucleus, likely resulting from reduced mRNA export activity.
AT5G02780	*GLUTATHIONE TRANSFERASE LAMBDA 1 (GSTL1)*	Encodes a member of the lambda family of glutathione transferases. It has thiol transferase activity and self S-glutathionylation activity in vitro.
AT5G02790	*GLUTATHIONE TRANSFERASE L3 (GSTL3)*	GST functions in reductive deglutathionylation of glutathione conjugates of quercetin.

AT5G02810	*PSEUDO-RESPONSE REGULATOR 7 (PRR7)*	PRR7 and PRR9 are partially redundant essential components of a temperature-sensitive circadian system. CCA1 and LHY had a positive effect on PRR7 expression levels. Acts as transcriptional repressor of CCA1 and LHY. Acts additively with EC, PRR5 and PRR9 to regulate hypocotyl growth under photoperiodic conditions.
AT5G02820	*ROOT HAIRLESS 2 (RHL2)*	Involved in the patterning and shape of leaf trichomes. Encodes the DNA topoisomerase VI SPO11-3, involved in endoreduplication
AT5G02830		Tetratricopeptide repeat (TPR)-like superfamily protein
AT5G02890		Encodes a protein with similarity to transferases in plants and fungi.
AT5G02900	*CYTOCHROME P450, FAMILY 96, SUBFAMILY A, POLYPEPTIDE 13 (CYP96A13)*	member of CYP96A
AT5G02920		F-box/RNI-like superfamily protein
AT5G02940	*PLASTID ENVELOPE ION CHANNELS 1 (PEC1)*	Chloroplast envelope ion channel protein, forms oligomers with PEC2 with regulator of K+ conductance domains protruding into the intermembrane space.
AT5G02980		Galactose oxidase/kelch repeat superfamily protein
AT5G02990		galactose oxidase/kelch repeat protein
AT5G03000		Galactose oxidase/kelch repeat superfamily protein
AT5G03010		Galactose oxidase/kelch repeat superfamily protein
AT5G03020		Galactose oxidase/kelch repeat superfamily protein
AT5G03030	*(PAM18-3)*	Chaperone DnaJ-domain superfamily protein
AT5G03040	*IQ-DOMAIN 2 (iqd2)*	Member of IQ67 (CaM binding) domain containing family.
AT5G03050		knotted 1-binding protein
AT5G03060		striatin-like protein
AT5G03090		Mto 1 responding down protein
AT5G03120		transmembrane protein
AT5G03130		hypothetical protein
AT5G03140	*L-TYPE LECTIN RECEPTOR KINASE VIII.2 (LECRK-VIII.2)*	Concanavalin A-like lectin protein kinase family protein
AT5G03150	*JACKDAW (JKD)*	JKD is a nuclear-localized putative transcription factor of the BIRDS/IDD C2H2 zinc finger family. JKD and its homologue BIB, restrict SHR movement to a single layer, the endodermis, and delimit tissue boundaries in the root meristem through a process that involves nuclear retention through protein complex formation. JKD mutation leads to periclinal divisions in the cortex, increased cell numbers in the circumference of the cortical and epidermal layers, a disrupted QC marker expression pattern, and disorganized QC and columella cells. This effect is enhanced in jkd bib double mutants where tissue boundaries cannot be maintained due to excessive SHR movement. JKD and BIB restrict CYCIND6 expression to cortex and endodermis stem cells to prevent formative divisions in the ground tissue. JKD physically interacts with cell fate determinants SCR and SHR in a cell type specific manner. Native FRET-FLIM analysis showed higher JKD-SCR complex in the endodermis and predominant JKD-SHR in the QC and cortex/endodermis stem cells. In addition, JKD, SCR and SHR form a ternary complex whose conformation is cell type dependent, conformational changes of this complex differentially regulate SCR and WOX5 expression to specify endodermal cell fate and QC function respectively. Its mRNA is cell-to-cell mobile.
AT5G03170	*FASCICLIN-LIKE ARABINOGALACTAN-PROTEIN 11 (FLA11)*	Encodes FLA11, a member of fasciclin-like arabinogalactan proteins (FLAs) containing a cell adhesion fasciclin (FAS) domain. Mutations result in altered stem biomechanics with reduced tensile strength and reduced tensile modulus of elasticity, as well as altered cell wall architecture and composition, with increased cellulose microfibril angle, reduced arabinose, galactose and cellulose content.It is a GPI anchored protein.
AT5G03190	*CONSERVED PEPTIDE UPSTREAM OPEN READING FRAME 47 (CPuORF47)*	peptide upstream protein
AT5G03200	*LOG2-LIKE UBIQUITIN LIGASE1 (LUL1)*	Paralog of LOG2 (At3g09770), a ubiquitin ligase that regulates amino acid export.
AT5G03210	*DBP-INTERACTING PROTEIN 2 (DIP2)*	Encodes a small polypeptide contributing to resistance to potyvirus.
AT5G03230		senescence regulator (Protein of unknown function, DUF584)
AT5G03240	*POLYUBIQUITIN 3 (UBQ3)*	encodes ubiquitin that is attached to proteins destined for degradation. UBQ3 is most homologous with UBQ4, and is expressed in higher levels in vegetative tissue but lower levels in flowers than UBQ4. UBQ3 encodes different number of ubiquitins in different ecotypes. UBQ3 transcript level is modulated by UV-B and light/dark treatments.
AT5G03260	*LACCASE 11 (LAC11)*	LAC11 is a putative laccase, a member of laccase family of genes (17 members in Arabidopsis).
AT5G03270	*LONELY GUY 6 (LOG6)*	lysine decarboxylase family protein
AT5G03290	*ISOCITRATE DEHYDROGENASE V (IDH-V)*	Encodes a catalytic subunit of the mitochondrially-localized NAD+- dependent isocitrate dehydrogenase. The mRNA is cell-to-cell mobile.
AT5G03310	*SMALL AUXIN UPREGULATED RNA 44 (SAUR44)*	SAUR-like auxin-responsive protein family
AT5G03320	*PATTERN-TRIGGERED IMMUNITY COMPROMISED RECEPTOR-LIKE CYTOPLASMIC KINASE 2 (PCRK2)*	Protein kinase superfamily protein
AT5G03350	*SA-INDUCED LEGUME LECTIN-LIKE PROTEIN 1 (SAI-LLP1)*	Belongs to the group of early SA-activated genes. Involved in resistance to Pst Avr-Rpm1 as a component of the SA35 mediated defense processes associated to the ETI response. Involved in resistance to P.syringae pv. tomato Avr-Rpm1 in Arabidopsis, as a component of the SA-mediated defense processes associated with the effector-triggered immunity response.
AT5G03360		cysteine/histidine-rich C1 domain protein
AT5G03380	*HEAVY METAL ASSOCIATED PROTEIN 43 (ATHMP43)*	Heavy metal transport/detoxification superfamily protein
AT5G03390	*DUF295 ORGANELLAR A 11 (ATDOA11)*	hypothetical protein (DUF295)
AT5G03400		hypothetical protein
AT5G03430		phosphoadenosine phosphosulfate (PAPS) reductase family protein

AT5G03455	*(CDC25)*	Encodes a homolog of yeast cell cycle regulator CDC25. It has a sole catalytic domain and devoid of the N-terminal regulatory region found in the human CDC25 and is capable of reducing the mitotic cell length of transformed fission yeast. Non-plant CDC25 proteins have been shown to do this. However, the gene is more or less constant, regardless of whether the tissue examined contained proliferative cells. Also described as having arsenate reductase activity involved in arsenate resistance.
AT5G03470	*(ATB' ALPHA)*	Encodes B' regulatory subunit of PP2A (AtB'alpha), putative size of 57 kDa.Functions redundantly with the beta subunit do maintain sister chromatid cohesion during meiosis.
AT5G03490	*UDP GLYCOSYLTRANSFERASE 89A2 (UGT89A2)*	Encodes a dihydroxybenzoic acid (DHBA) glycosyltransferase. The Col-0 enzyme is responsible for biosynthesis of 2,3-DHBA xyloside and 2,5-DHBA xyloside. The Col-0 enzyme is specific for UDP-xylose and the C24 enzyme uses both UDP-glucose and UDP-xylose. This difference in sugar donor specificity was shown to be largely due to a single amino acid change between the two isoforms.
AT5G03510		C2H2-type zinc finger family protein
AT5G03530	*RAB GTPASE HOMOLOG C2A (RABC2A)*	Encodes a member of the Rab GTPase family of proteins. This protein interacts with the tail region of a myosin XI protein (AT5G43900) in a GTP-dependent manner. CFP:RabC2a appears to co-localize with peroxisomes.
AT5G03540	*EXOCYST SUBUNIT EXO70 FAMILY PROTEIN A1 (EXO70A1)*	AtEXO70A1 is a member of EXO70 gene family, putative exocyst subunits, conserved in land plants. It plays a central role in Casparian strip formation, generating a transient positional information that will be translated into a precisely localized cell wall modification.
AT5G03550		MATH domain/coiled-coil protein
AT5G03555	*NUCLEOBASE CATION SYMPORTER 1 (NCS1)*	Encodes PLUTO (plastidic nucleobase transporter), a member of the Nucleobase:Cation-Symporter1 protein family, capable of transporting purine and pyrimidine nucleobases.
AT5G03560		Tetratricopeptide repeat (TPR)-like superfamily protein
AT5G03570	*IRON REGULATED 2 (IREG2)*	Encodes FPN2, a tonoplast localized nickel transport protein. FPN2 is one of the Arabidopsis orthologs (AT2G38460/IREG1/FPN1 and AT5G03570/IREG2/FPN2) the iron efflux transporter ferroportin (FPN) identified in animals.
AT5G03590		GDSL-motif esterase/acyltransferase/lipase. Enzyme group with broad substrate specificity that may catalyze acyltransfer or hydrolase reactions with lipid and non-lipid substrates.
AT5G03600		SGNH hydrolase-type esterase superfamily protein
AT5G03610	*(GGL25)*	GDSL-motif esterase/acyltransferase/lipase. Enzyme group with broad substrate specificity that may catalyze acyltransfer or hydrolase reactions with lipid and non-lipid substrates.
AT5G03620		Subtilisin-like serine endopeptidase family protein
AT5G03630	*(MDAR2)*	Pyridine nucleotide-disulfide oxidoreductase family protein
AT5G03640	*AGCVIIIA KINASE 1-8 (AGC1-8)*	AGCVIII kinase involved in the pulse-induced first positive phototropism.
AT5G03670	*TON1 RECRUITING MOTIF 28 (TRM28)*	histone-lysine N-methyltransferase SETD1B-like protein
AT5G03680	*PETAL LOSS (PTL)*	Recessive mutations are defective in organ initiation and orientation in the second whorl. This gene encodes a trihelix transcription factor whose expression is limited to margins of floral and vegetative organs. Overexpression and double mutant analyses suggest that this gene is involved in limiting lateral growth of organs.
AT5G03700		D-mannose binding lectin protein with Apple-like carbohydrate-binding domain-containing protein
AT5G03720	*HEAT SHOCK TRANSCRIPTION FACTOR A3 (HSFA3)*	Member of Heat Stress Transcription Factor (Hsf) family. Expression is regulated by DREB2A and in turn HSFA3 regulates the expression of hsps Hsp18.1-CI and Hsp26.5-MII35S. Involved in establishing thermotolerence.
AT5G03760	*(ATCSLA09)*	encodes a beta-mannan synthase that is required for agrobacterium-mediated plant genetic transformation involves a complex interaction between the bacterium and the host plant. 3' UTR is involved in transcriptional regulation and the gene is expressed in the elongation zone of the root.
AT5G03770	*KDO TRANSFERASE A (KDTA)*	Encodes a putative KDO (3-deoxy-D-manno-octulosonate) transferase
AT5G03780	*TRF-LIKE 10 (TRFL10)*	Encodes a protein whose sequence is similar to human telomere proteins. This belongs to TRFL family 2, which do not show DNA binding in vitro.
AT5G03790	*HOMEOBOX 51 (HB51)*	Encodes a homeodomain leucine zipper class I (HD-Zip I) meristem identity regulator that acts together with LFY to induce CAL expression. It binds to the CAL promoter proximal CAATNATTG element. LMI1 acts primarily downstream of LFY in meristem identity regulation. The interaction between LFY, LMI1 and CAL resembles a feed-forward loop transcriptional network motif. The gene also had additional LFY-independent roles in leaf morphogenesis and bract formation.
AT5G03800	*EMBRYO DEFECTIVE 175 (EMB175)*	Encodes a protein with a large central domain of 14 internal pentatricopeptide motifs (some degenerate) arranged in tandem. Mutations in this locus result in embryo lethality.
AT5G03810		GDSL-motif esterase/acyltransferase/lipase. Enzyme group with broad substrate specificity that may catalyze acyltransfer or hydrolase reactions with lipid and non-lipid substrates.
AT5G03840	*TERMINAL FLOWER 1 (TFL1)*	Controls inflorescence meristem identity. Involved in the floral initiation process. Ortholog of the Antirrhinum gene CENTRORADIALIS (CEN). Involved in protein trafficking to the protein storage vacuole. TFL1 plays an antagonistic role to FT/TSF in the determination of inflorescence meristem identity.
AT5G03860	*MALATE SYNTHASE (MLS)*	Encodes a protein with malate synthase activity.
AT5G03870		Glutaredoxin family protein
AT5G03920		F-box protein
AT5G03930		F-box protein
AT5G03940	*CHLOROPLAST SIGNAL RECOGNITION PARTICLE 54 KDA SUBUNIT (CPSRP54)*	mutant has Yellow first leaves; Chloroplast Signal Recognition Particle Subunit
AT5G03960	*IQ-DOMAIN 12 (IQD12)*	Member of IQ67 (CaM binding) domain containing family.
AT5G03980		SGNH hydrolase-type esterase superfamily protein
AT5G04000		hypothetical protein
AT5G04010		F-box family protein
AT5G04020		calmodulin binding protein

AT5G04030		transmembrane protein
AT5G04040	*SUGAR-DEPENDENT1 (SDP1)*	Encodes a triacylglycerol lipase that is involved in storage lipid breakdown during seed germination. The mutant plant exhibits a much slower rate of postgerminative growth than the wild type.
AT5G04060		S-adenosyl-L-methionine-dependent methyltransferases superfamily protein
AT5G04080	*CYSTEINE-RICH TRANSMEMBRANE MODULE 12 (ATHCYSTM12)*	cysteine-rich TM module stress tolerance protein
AT5G04110	*DNA GYRASE B3 (GYRB3)*	DNA GYRASE B3
AT5G04120		Encodes a cofactor-dependent phosphoglycerate mutase (dPGM) - like protein with phosphoserine phosphatase activity that may be responsible for serine anabolism.
AT5G04140	*GLUTAMATE SYNTHASE 1 (GLU1)*	Encodes a gene whose sequence is similar to ferredoxin dependent glutamate synthase (Fd-GOGAT). Expression in leaves is induced by light and sucrose. Proposed to be involved in photorespiration and nitrogen assimilation. The mRNA is cell-to-cell mobile.
AT5G04150	*(BHLH101)*	Encodes a member of the basic helix-loop-helix transcription factor family protein. Functions as a key regulator of iron-deficiency responses independent of the master regulator FIT. Likely regulates genes involved in the distribution of iron within the plant.
AT5G04160	*(UUAT1)*	UUAT1 is a UDP-Uronic acid transporter that is localized to the Golgi. It is expressed in the seed coat epidermis and is involved in the development of seed coat mucilage.
AT5G04180	*ALPHA CARBONIC ANHYDRASE 3 (ACA3)*	alpha carbonic anhydrase 3
AT5G04190	*PHYTOCHROME KINASE SUBSTRATE 4 (PKS4)*	Encodes phytochrome kinase substrate 4, a phytochrome signaling component involved in phototropism. It is phosphorylated in a phot1-dependent manner in vitro. Phosphorylation is transient and regulated by a type 2- protein phosphatase.
AT5G04200	*METACASPASE 9 (MC9)*	Encodes a putative metacaspase. Arabidopsis contains three type I MCP genes (MCP1a-c) and six type II MCP genes (MCP2a?f): AtMCP1a/At5g64240, AtMCP1b/At1g02170, AtMCP1c/At4g25110, AtMCP2a/At1g79310, AtMCP2b/At1g79330, AtMCP2c/At1g79320, AtMCP2d/At1g79340, AtMCP2e/At1g16420, AtMCP2f/At5g04200.
AT5G04210		CCCH-type zinc fingerfamily protein with RNA-binding domain-containing protein
AT5G04220	*(SYTC)*	Calcium-dependent lipid-binding (CaLB domain) family protein
AT5G04230	*PHENYL ALANINE AMMONIA-LYASE 3 (PAL3)*	Member of Phenylalanine ammonialyase (PAL) gene family. Differs significantly from PAL1 and PAL2 and other sequenced plant PAL genes. Arabidopsis has four PALs: AT2G37040 (PAL1), AT3G53260 (PAL2), AT5G04230 (PAL3) and AT3G10340 (PAL4).
AT5G04240	*EARLY FLOWERING 6 (ELF6)*	Early Flowering 6 (ELF6) encodes a Jumonji N/C and zinc finger domain-containing protein that acts as a repressor in the photoperiod pathway. ELF6 interacts with BES1 in a Y2H assay, in vitro, and in Arabidosis protoplasts (based on BiFC). ELF6 may play a role in brassinosteroid signaling by affecting histone methylation in the promoters of BR-responsive genes.
AT5G04250		Cysteine proteinases superfamily protein
AT5G04290	*KOW DOMAIN-CONTAINING TRANSCRIPTION FACTOR 1 (KTF1)*	Encodes SPT5-Like, a member of the nuclear SPT5 (Suppressor of Ty insertion 5) RNA polymerase (RNAP) elongation factor family that is characterized by the presence of a carboxy-terminal extension with more than 40 WG/GW motifs. Interacts with AGO4. Required for RNA-directed DNA methylation. The mRNA is cell-to-cell mobile.
AT5G04300		
AT5G04310	*PECTATE LYASE LIKE12 (PLL12)*	Pectin-modifying gene involved in guard cell wall modifications that are important for proper maintenance of turgor pressure and stomatal movement.
AT5G04330	*CYTOCHROME P450 84A4 (CYP84A4)*	Cytochrome P450 superfamily protein
AT5G04340	*ZINC FINGER OF ARABIDOPSIS THALIANA 6 (ZAT6)*	Encodes a C2H2 zinc finger transcription factor that coordinately activates phytochelatin-synthesis related gene expression and directly targets GSH1 by binding to its promoter to positively regulate Cd accumulation and tolerance.
AT5G04360	*LIMIT DEXTRINASE (LDA)*	Encodes an enzyme thought to be involved in the hydrolysis of the α-1,6 linkages during starch degradation in seed endosperm. However, a knockout mutant of Arabidopsis lacking limit dextrinase has normal rates of starch degradation in the leaf at night, indicating that more than one isoamylases might be involved in this process.
AT5G04370	*(NAMT1)*	A member of the Arabidopsis SABATH methyltransferase gene family. Encodes NAMT1, a methyltransferase that methylates nicotinic acid to yield methyl nicotinate.
AT5G04380		S-adenosyl-L-methionine-dependent methyltransferases superfamily protein
AT5G04390		C2H2-type zinc finger family protein
AT5G04400	*NAC DOMAIN CONTAINING PROTEIN 77 (NAC077)*	NAC domain protein
AT5G04440		RAP release 2, galactose-binding-like domain protein, putative (DUF1997)
AT5G04450		
AT5G04460	*SUMO-TARGETED UBIQUITIN E3 LIGASE 5 (STUBL5)*	RING/U-box superfamily protein. SUMO- targeted ubiquitin ligase.
AT5G04470	*SIAMESE (SIM)*	Encodes a novel nuclear 14-kD protein containing a cyclin binding motif and a motif found in ICK/KRP cell cycle inhibitor proteins. It is required for coordinating cell division and cell differentiation during the development of Arabidopsis trichomes, playing a key role in the mitosis-to-endoreduplication transition. It interacts with D-type cyclins in vivo.
AT5G04490	*VITAMIN E PATHWAY GENE 5 (VTE5)*	Encodes a protein with phytol kinase activity involved in tocopherol biosynthesis.
AT5G04500	*GLUCOSAMINE INOSITOLPHOSPHORYLCERAMIDE TRANSFERASE 1 (GINT1)*	Encodes a member of the CAZy Glycosyltransferase Family 64 that is involved in glycosylinositolphosphorylceramide and sphingolipid glycosylation. In mutants, seed germination was less sensitive to salt stress than in wild-type plants. [The protein was expected to be Golgi-localized based on function as well as the Golgi localization of its homolog GMT1. However, GFP-fusion proteins localized both to the ER and Golgi, and especially to ER when co-expressed with Golgi markers. Therefore, localization cannot confidently be defined. (pers. communication, J. Mortimer)]
AT5G04530	*3-KETOACYL-COA SYNTHASE 19 (KCS19)*	Encodes KCS19, a member of the 3-ketoacyl-CoA synthase family involved in the biosynthesis of VLCFA (very long chain fatty acids).
AT5G04560	*DEMETER (DME)*	Encodes a DNA glycosylase DEMETER (DME). Responsible for endosperm maternal-allele-specific hypomethylation at the MEDEA (MEA) gene. DME can excise 5-methylcytosine in vitro and when expressed in E. coli. DME establishes MEA imprinting by removing 5-methylcytosine to activate the maternal allele.
AT5G04570		

AT5G04590	*SULFITE REDUCTASE (SIR)*	A.thaliana gene encoding sulfite reductase.
AT5G04600		RNA-binding (RRM/RBD/RNP motifs) family protein
AT5G04620	*BIOTIN F (BIOF)*	The cDNA encoding 7-keto-8-aminopelargonic acid (KAPA) synthase, the first committed enzyme of the biotin synthesis pathway has been cloned and its molecular function confirmed (functional complementation of an E. coli mutant). The subcellular localization of the enzyme (cytosol) proves that the biotin biosynthesis in plants takes place in different compartments which differs from the biosynthetic route found in microorganisms.
AT5G04630	*CYTOCHROME P450, FAMILY 77, SUBFAMILY A, POLYPEPTIDE 9 (CYP77A9)*	member of CYP77A
AT5G04640	*AGAMOUS-LIKE 99 (AGL99)*	AGAMOUS-like 99
AT5G04650		transposable_element_gene
AT5G04660	*CYTOCHROME P450, FAMILY 77, SUBFAMILY A, POLYPEPTIDE 4 (CYP77A4)*	encodes a protein with cytochrome P450 domain
AT5G04670	*(EPCR2)*	Polycomb related protein that is part of a protein complex involved in histone deacetylation and heterochromatin silencing.
AT5G04680		Ankyrin repeat family protein
AT5G04720	*ADR1-LIKE 2 (ADR1-L2)*	Encodes a member of the ADR1 family nucleotide-binding leucine-rich repeat (NB-LRR) immune receptors. The mRNA is cell-to-cell mobile.
AT5G04730		Ankyrin-repeat containing protein
AT5G04770	*CATIONIC AMINO ACID TRANSPORTER 6 (CAT6)*	Encodes a member of the cationic amino acid transporter (CAT) subfamily of amino acid polyamine choline transporters. Does not mediate efficient uptake of basic amino acids in yeast or Xenopus systems but can transport neutral and acidic amino acid analogs. Expressed in sink tissues. Induced during infestation of roots by the plant parasitic root-knot nematode, Meloidogyne incognita. Localized in the plasma membrane.
AT5G04790		transmembrane protein
AT5G04810	*PENTATRICOPEPTIDE REPEAT 4 (PPR4)*	Pentatricopeptide which is essential during the early stages of embryo development and acts in the plastid nucleoids as the factor responsible of rps12 intron 1 trans-splicing and, indirectly, in the assembly of 70S ribosomes and plastid translation.
AT5G04820	*OVATE FAMILY PROTEIN 13 (OFP13)*	ovate family protein 13
AT5G04830		Nuclear transport factor 2 (NTF2) family protein
AT5G04850	*(VPS60.2)*	SNF7 family protein
AT5G04860		splicing factor 3A subunit
AT5G04870	*CALCIUM DEPENDENT PROTEIN KINASE 1 (CPK1)*	A calcium-dependent protein kinase that can phosphorylate phenylalanine ammonia lyase (PAL), a key enzyme in pathogen defense.Phosphorylates, in vivo, the transcription factor ORE1, a master regulator of senescence.
AT5G04880		pseudogene of ABC transporter family protein
AT5G04890	*RESTRICTED TEV MOVEMENT 2 (RTM2)*	Specifically restricts the long-distance movement of tobacco etch potyvirus (TEV) without involving either hypersensitive cell death or systemic acquired resistance. Multidomain protein containing an N-terminal region with high similarity to plant small heat shock proteins (HSPs).
AT5G04900	*NYC1-LIKE (NOL)*	Encodes a chlorophyll b reducatase involved in the degradation of chlorophyll b and LHCII (light harvesting complex II).
AT5G04910		DNA repair REX1-B protein
AT5G04930	*AMINOPHOSPHOLIPID ATPASE 1 (ALA1)*	Encodes a putative aminophospholipid translocase (p-type ATPase) involved in chilling response. It is targeted to the plasma membrane following association in the endoplasmic reticulum with an ALIS protein beta-subunit. The mRNA is cell-to-cell mobile.
AT5G04940	*SU(VAR)3-9 HOMOLOG 1 (SUVH1)*	Encodes a SU(VAR)3-9 homolog, a SET domain protein. Known SET domain proteins are involved in epigenetic control of gene expression and act as histone methyltransferases. There are 10 SUVH genes in Arabidopsis and members of this subfamily of the SET proteins have an additional conserved SRA domain. SUVH1 has been shown to have a preference for binding methylated DNA.
AT5G04950	*NICOTIANAMINE SYNTHASE 1 (NAS1)*	Encodes a nicotianamide synthase.
AT5G04970		Plant invertase/pectin methylesterase inhibitor superfamily
AT5G04980		DNAse I-like superfamily protein
AT5G05030		NEP-interacting protein, putative (DUF239)
AT5G05050		Cysteine proteinases superfamily protein
AT5G05070		DHHC-type zinc finger family protein
AT5G05090		Homeodomain-like superfamily protein
AT5G05130		DNA/RNA helicase protein
AT5G05140		Transcription elongation factor (TFIIS) family protein
AT5G05150	*AUTOPHAGY-RELATED GENE 18E (G18E)*	autophagy-related protein 18E
AT5G05160	*REDUCED IN LATERAL GROWTH1 (RUL1)*	Encodes a receptor-like kinase that activates secondary growth, the production of secondary vascular tissues.
AT5G05180		myosin heavy chain, striated protein
AT5G05190	*ENHANCED DISEASE RESISTANCE4 (EDR4)*	hypothetical protein (DUF3133)
AT5G05200		Protein kinase superfamily protein
AT5G05220		hypothetical protein
AT5G05230		RING/U-box superfamily protein
AT5G05250		hypothetical protein
AT5G05260	*CYTOCHROME P450 79A2 (CYP79A2)*	Encodes cytochrome P450 CYP79A2; involved in turnover of benzyl glucosinolate and an additive effect of different aldoximes on phenylpropanoid repression.
AT5G05270	*CHALCONE ISOMERASE LIKE (CHIL)*	Chalcone-flavanone isomerase family protein
AT5G05280	*DEFECTIVE IN ANTHER DEHISCENCE1- (DAD1-) ACTIVATING FACTOR (DAF)*	Encodes a RING-finger E3 ligase protein that controls anther dehiscence by positively regulating the expression of DAD1 in the jasmonic acid biosynthesis pathway.
AT5G05290	*EXPANSIN A2 (EXPA2)*	Encodes an expansin. Naming convention from the Expansin Working Group (Kende et al, 2004. Plant Mol Bio)
AT5G05300	*(IDL6)*	IDL6 peptide is induced in response to Pathogen-Associated Molecular Patterns (PAMPs). Overexpression of IDL6 results in increased susceptibility to pathogens.

AT5G05320		FAD/NAD(P)-binding oxidoreductase family protein
AT5G05340	*PEROXIDASE 52 (PRX52)*	Encodes a protein with sequence similarity to peroxidases that is involved in lignin biosynthesis. Loss of function mutations show abnormal development of xylem fibers and reduced levels of lignin biosynthetic enxymes.
AT5G05390	*LACCASE 12 (LAC12)*	putative laccase, a member of laccase family of genes (17 members in Arabidopsis); involved in copper-iron crosstalk, root-to-shoot iron partitioning.
AT5G05400		LRR and NB-ARC domains-containing disease resistance protein
AT5G05410	*DRE-BINDING PROTEIN 2A (DREB2A)*	Encodes a transcription factor that specifically binds to DRE/CRT cis elements (responsive to drought and low-temperature stress). Belongs to the DREB subfamily A-2 of ERF/AP2 transcription factor family (DREB2A). There are eight members in this subfamily including DREB2B. The protein contains one AP2 domain. Overexpression of transcriptional activation domain of DREB2A resulted in significant drought stress tolerance but only slight freezing tolerance in transgenic Arabidopsis plants. Microarray and RNA gel blot analyses revealed that DREB2A regulates expression of many water stress?inducible genes. The mRNA is cell-to-cell mobile.
AT5G05420		FKBP-like peptidyl-prolyl cis-trans isomerase family protein
AT5G05430		RNA-binding protein
AT5G05440	*PYRABACTIN RESISTANCE 1-LIKE 5 (PYL5)*	Encodes a member of the PYR (pyrabactin resistance)/PYL(PYR1-like)/RCAR (regulatory components of ABA receptor) family proteins with 14 members. PYR/PYL/RCAR family proteins function as abscisic acid sensors. Mediate ABA-dependent regulation of protein phosphatase 2Cs ABI1 and ABI2.
AT5G05460	*ENDO-BETA-N-ACETYGLUCOSAMINIDASE 85A (ENGase85A)*	Encodes a cytosolic beta-endo-N-acetyglucosaminidase (ENGase). ENGases N-glycans cleave the O-glycosidic linkage between the two GlcNAc residues of the N-glycan core structure and thus generate a protein with a single GlcNAc attached to asparagine.
AT5G05480		Peptide-N4-(N-acetyl-beta-glucosaminyl)asparagine amidase A protein
AT5G05490	*SYNAPTIC 1 (SYN1)*	Encodes a RAD21-like gene essential for meiosis. Encodes a 627 a.a. protein that is slightly longer in the N-terminus than SYN1 BP5.
AT5G05500	*(MOP10)*	Encodes Proline-rich protein-like PRPL1, controls elongation of root hairs.
AT5G05520		Outer membrane OMP85 family protein
AT5G05540	*SMALL RNA DEGRADING NUCLEASE 2 (SDN2)*	small RNA degrading nuclease 2
AT5G05550	*VFP5 (VFP5)*	Encodes trihelix-domain transcription factor VFP5. Interacts with agrobacterium virulence protein VirF.
AT5G05560	*EMBRYO DEFECTIVE 2771 (EMB2771)*	Encodes a subunit of the Arabidopsis thaliana E3 ubiquitin ligase complex that plays a synergistic role with APC4 both in female gametogenesis and in embryogenesis.
AT5G05570	*TOMOSYN (TMS)*	transducin family protein / WD-40 repeat family protein
AT5G05600	*JASMONIC ACID OXIDASE 2 (JOX2)*	Encodes a protein with similarity to flavonol synthases that is involved in the detoxifcation polycyclic aromatic hydrocarbons.One of 4 paralogs encoding a 2-oxoglutarate/Fe(II)-dependent oxygenases that hydroxylates JA to 12-OH-JA.
AT5G05630	*RESISTANT TO METHYL VIOLOGEN 1 (RMV1)*	Encodes POLYAMINE UPTAKE TRANSPORTER 3, an amino acid permease family protein.
AT5G05640		nucleoprotein-like protein
AT5G05680	*MODIFIER OF SNC1,7 (MOS7)*	Encodes MOS7 (Modifier of snc1,7), homologous to human and Drosophila melanogaster nucleoporin Nup88. Resides at the nuclear envelope. Modulates the nuclear concentrations of certain defense proteins regulates defense outputs.
AT5G05690	*CONSTITUTIVE PHOTOMORPHOGENIC DWARF (CPD)*	Encodes a member of the CP90A family, a cytochrome P450 monooxygenase which converts 6-deoxocathasterone to 6-deoxoteasterone in the late C6 oxidation pathway and cathasterone to teasterone in the early C6 oxidation pathway of brassinolide biosynthesis. Expressed in cotyledons and leaves. Mutants display de-etiolation and derepression of light-induced genes in the dark, dwarfism, male sterility and activation of stress-regulated genes in the light. The expression of the gene using a CPD promoter:LUC fusion construct was shown to be under circadian and light control. Additionally, the circadian regulation was shown to be independent of BR levels as it remains unchanged in <i>bri1</i> mutant lines. CPD appears to be involved in the autonomous pathway that regulates the transition to flowering, primarily through a BRI1-mediated signaling pathway that affects FLC expression levels, as uncovered by double mutant analyses.
AT5G05730	*ANTHRANILATE SYNTHASE ALPHA SUBUNIT 1 (ASA1)*	ASA1 encodes the alpha subunit of anthranilate synthase, which catalyzes the rate-limiting step of tryptophan synthesis. ASA1 is induced by ethylene, and forms a link between ethylene signalling and auxin synthesis in roots.
AT5G05740	*ETHYLENE-DEPENDENT GRAVITROPISM-DEFICIENT AND YELLOW-GREEN-LIKE 2 (EGY2)*	S2P-like putative metalloprotease, also contain transmembrane helices near their C-termini and many of them, five of seven, contain a conserved zinc-binding motif HEXXH. Homolog of EGY1. Each of the EGY1 and EGY-like proteins share two additional highly conserved motifs, the previously reported NPDG motif (aa 442?454 in EGY1, Rudner et al., 1999) and a newly defined GNLR motif (aa 171?179 in EGY1). The GNLR motif is a novel signature motif unique to EGY1 and EGY-like proteins as well as other EGY1 orthologs found in cyanobacteria.
AT5G05750		DNAJ heat shock N-terminal domain-containing protein
AT5G05790		Duplicated homeodomain-like superfamily protein
AT5G05840	*BOUNDARY OF ROP DOMAIN6 (BDR6)*	replication factor C subunit, putative (DUF620)
AT5G05850	*PLANT INTRACELLULAR RAS GROUP-RELATED LRR 1 (PIRL1)*	Encodes PIRL1, a member of the Plant Intracellular Ras-group-related LRRs (Leucine rich repeat proteins). PIRLs are a distinct, plant-specific class of intracellular LRRs that likely mediate protein interactions, possibly in the context of signal transduction. PIRL1 (AT5G05850) and PIRL9 (AT3G11330) are genetically redundant and are required for differentiation of microspores into pollen.
AT5G05860	*UDP-GLUCOSYL TRANSFERASE 76C2 (UGT76C2)*	Encodes a cytokinin N-glucosyltransferase that is involved in cytokinin homeostasis and cytokinin response in planta through cytokinin N-glucosylation. Expression is induced by ABA, mannitol and drought stress. Analysis of overexpressors and loss of function mutants indicate a role in response to osmotic and drought stress.
AT5G05880	*UDP-GLUCOSYL TRANSFERASE 76C4 (UGT76C4)*	Encodes a nicotinate-N-glycosyltransferase.
AT5G05890	*UDP-GLUCOSYL TRANSFERASE 76C5 (UGT76C5)*	Encodes a nicotinate-N-glycosyltransferase.
AT5G05900		UDP-Glycosyltransferase superfamily protein
AT5G05920	*DEOXYHYPUSINE SYNTHASE (DHS)*	Encodes a deoxyhypusine synthase.
AT5G05930	*GUANYLYL CYCLASE 1 (GC1)*	guanylyl cyclase 1

AT5G05940	*ROP (RHO OF PLANTS) GUANINE NUCLEOTIDE EXCHANGE FACTOR 5 (ROPGEF5)*	Encodes a member of KPP-like gene family, homolog of KPP (kinase partner protein) gene in tomato. Also a member of the RopGEF (guanine nucleotide exchange factor) family, containing the novel PRONE domain (plant-specific Rop nucleotide exchanger), which is exclusively active towards members of the Rop subfamily.
AT5G05960		Bifunctional inhibitor/lipid-transfer protein/seed storage 2S albumin superfamily protein
AT5G05980	*DHFS-FPGS HOMOLOG B (DFB)*	Encodes one of the three folylpolyglutamate synthetase isoforms (FPGSs): FPGS1 (At5g05980, plastidic), FPGS2 (At3g10160, mitochondrial) and FPGS3 (At3g55630, cytosolic).
AT5G05990		Mitochondrial glycoprotein family protein
AT5G06020		Plant self-incompatibility protein S1 family
AT5G06040		self-incompatibility protein-like protein
AT5G06060		NAD(P)-binding Rossmann-fold superfamily protein
AT5G06070	*RABBIT EARS (RBE)*	Isolated as a mutation defective in petal development with specific effects on adaxial petals which are filamentous or absent. Encodes a Superman (SUP) like protein with zinc finger motifs. Transcript is detected in petal primordia and protein is localized to the nucleus.
AT5G06080	*LOB DOMAIN-CONTAINING PROTEIN 33 (LBD33)*	LOB domain-containing protein 33
AT5G06100	*MYB DOMAIN PROTEIN 33 (MYB33)*	Encodes a member of the myb family of transcription factors (MYB33), contains Pfam profile: PF00249 myb DNA-binding domain. Double mutants with MYB65 are male sterile- anthers are small, pollen development is defective. Spatial expression appears to be under the control of miR159, contains a target site for this micro RNA. A highly conserved RNA secondary structure abuts the miR159 binding site which facilitates its regulation by miR159. When the target site is mutated, expression is detected in leaves, roots, anther filament, pistil. The expression of a translational fusion is specific to anther locules in contrast to constructs lacking the miR159 target site. Phenotype is conditional and can be restored by lower temperature or higher light intensity.
AT5G06110	*GONIDIALESS A/ZUOTIN RELATED FACTOR A2 (ATGLSA2)*	Encodes a ZRF1 chromatin regulator. Functions in regulating plant growth and development.
AT5G06140	*SORTING NEXIN 1 (SNX1)*	Homolog of yeast retromer subunit VPS5. Part of a retromer-like protein complex involved in endosome to lysosome protein transport. In roots it co-localizes with the PIN2 auxin efflux carrier. Involved in endocytic sorting of membrane proteins including PIN2, BOR1 and BRI1.
AT5G06170	*SUCROSE-PROTON SYMPORTER 9 (SUC9)*	sucrose symporter with hight affinity for sucrose (K0.5=0.066 +/- 0.025mM), that can also transport a wide range of glucosides.
AT5G06180		fission ELM1-like protein (DUF1022)
AT5G06190		transmembrane protein
AT5G06200	*CASPARIAN STRIP MEMBRANE DOMAIN PROTEIN 4 (CASP4)*	Uncharacterized protein family (UPF0497)
AT5G06210	*SMALL RNA-BINDING PROTEIN 11 (S-RBP11)*	Encodes a chloroplast protein involved in the responses to salt and oxidative stresses.
AT5G06250	*DEVELOPMENT-RELATED PCG TARGET IN THE APEX 4 (DPA4)*	Transcription repressor involved in regulation of inflorescence architecture.
AT5G06270	*(GIR1)*	One of two plant specific paralogs of unknown function. Interacts with GL2. GIR1/GIR2 loss of function resembles gl2 lof mutations
AT5G06280		hypothetical protein
AT5G06290	*2-CYSTEINE PEROXIREDOXIN B (2-Cys Prx B)*	Encodes a 2-Cys peroxiredoxin (2-Cys PrxB) that contains two catalytic Cys residues. The mRNA is cell-to-cell mobile.
AT5G06300	*LONELY GUY 7 (LOG7)*	Putative lysine decarboxylase family protein
AT5G06320	*NDR1/HIN1-LIKE 3 (NHL3)*	encodes a protein whose sequence is similar to tobacco hairpin-induced gene (HIN1) and Arabidopsis non-race specific disease resistance gene (NDR1). Expression of this gene is induced by cucumber mosaic virus, spermine and Pseudomonas syringae pv. tomato DC3000. The gene product is localized to the plasma membrane.
AT5G06330		Late embryogenesis abundant (LEA) hydroxyproline-rich glycoprotein family
AT5G06340	*NUDIX HYDROLASE HOMOLOG 27 (NUDX27)*	nudix hydrolase homolog 27
AT5G06360		Ribosomal protein S8e family protein
AT5G06400		Pentatricopeptide repeat (PPR) superfamily protein
AT5G06410	*(HSCB)*	HscB is a mitochondrial cochaperone involved in [Fe-S] cluster biosynthesis and iron homeostasis.
AT5G06480		Immunoglobulin E-set superfamily protein
AT5G06490	*ARABIDOPSIS T??XICOS EN LEVADURA 71 (ATL71)*	RING/U-box superfamily protein
AT5G06500	*AGAMOUS-LIKE 96 (AGL96)*	AGAMOUS-like 96
AT5G06510	*NUCLEAR FACTOR Y, SUBUNIT A10 (NF-YA10)*	nuclear factor Y, subunit A10
AT5G06520		SWAP (Suppressor-of-White-APricot)/surp domain-containing protein
AT5G06530	*ATP-BINDING CASSETTE G22 (ABCG22)*	Encodes ABCG22, an ABC transporter gene. Mutation results in increased water transpiration and drought susceptibility.
AT5G06550	*JUMONJI DOMAIN-CONTAINING PROTEIN 22 (JMJ22)*	Encodes a HR demethylase that acts as a positive regulator of seed germination in the PHYB-PIL5-SOM pathway.
AT5G06570		alpha/beta-Hydrolases superfamily protein
AT5G06590		hypothetical protein
AT5G06610	*BOUNDARY OF ROP DOMAIN1 (BDR1)*	DUF620 domain protein. In xylem cells BRD1 is expressed in the secondary wall pit boundaries. BRD1 interacts with and appears to be necessary to recruit WALLIN to the plasma membrane.
AT5G06630	*EXTENSIN 9 (EXT9)*	proline-rich extensin-like family protein
AT5G06640	*EXTENSIN 10 (EXT10)*	Proline-rich extensin-like family protein
AT5G06650	*GLABROUS INFLORESCENCE STEMS 2 (GIS2)*	C2H2 and C2HC zinc fingers superfamily protein
AT5G06670	*KINESIN 7.5 (KIN7.5)*	P-loop containing nucleoside triphosphate hydrolases superfamily protein
AT5G06680	*SPINDLE POLE BODY COMPONENT 98 (SPC98)*	Encodes protein similar to yeast SCP98. Yeast SCP98 is essential for the microtubule nucleation activity of the gamma-tubulin ring complexes. Enriched at the post-cytokinetic cell edges in leaves and roots. The mRNA is cell-to-cell mobile.
AT5G06690	*WCRKC THIOREDOXIN 1 (WCRKC1)*	Encodes a thioredoxin (WCRKC1) localized in chloroplast stroma. Contains a WCRKC motif. Functions in redox cascade with 2CPA and 2CPB via the ferredoxin-thioredoxin reductase (FTR)/thioredoxin (Trx) pathway to mediate the light-responsive reductive control of target proteins. Oxidizes redox-regulated proteins.

AT5G06700	*TRICHOME BIREFRINGENCE (TBR)*	Encodes a member of the TBL (TRICHOME BIREFRINGENCE-LIKE) gene family containing a plant-specific DUF231 (domain of unknown function) domain. TBL gene family has 46 members, two of which (TBR/AT5G06700 and TBL3/AT5G01360) have been shown to be involved in the synthesis and deposition of secondary wall cellulose, presumably by influencing the esterification state of pectic polymers. A nomenclature for this gene family has been proposed (Volker Bischoff & Wolf Scheible, 2010, personal communication). A tbr mutant is impaired in its ability to deposit secondary wall cellulose in specific cell types, most notably in trichomes.
AT5G06710	*HOMEOBOX FROM ARABIDOPSIS THALIANA (HAT14)*	Homeobox-leucine zipper protein.
AT5G06720	*PEROXIDASE 2 (PA2)*	Encodes a peroxidase with diverse roles in the wound response, flower development, and syncytium formation.
AT5G06730		Peroxidase superfamily protein
AT5G06740	*L-TYPE LECTIN RECEPTOR KINASE S.5 (LECRK-S.5)*	Concanavalin A-like lectin protein kinase family protein
AT5G06750	*ARABIDOPSIS PP2C CLADE D 8 (APD8)*	Protein phosphatase 2C family protein
AT5G06760	*LATE EMBRYOGENESIS ABUNDANT 4-5 (LEA4-5)*	Encodes LEA4-5, a member of the Late Embryogenesis Abundant (LEA) proteins which typically accumulate in response to low water availability conditions imposed during development or by the environment. Most of the diverse set of LEA proteins can be grouped according to properties such as high hydrophilicity and high content of glycine or other small amino acids in what has been termed hydrophilins. LEA4-5 protects enzyme activities from the adverse effects induced by freeze-thaw cycles in vitro.
AT5G06790		cotton fiber protein
AT5G06800		myb-like HTH transcriptional regulator family protein
AT5G06820	*STRUBBELIG-RECEPTOR FAMILY 2 (SRF2)*	STRUBBELIG-receptor family 2
AT5G06840		
AT5G06850	*FT-INTERACTING PROTEIN 1 (FTIP1)*	Encodes an endoplasmic reticulum protein that is involved in the transport of the florigen FT from companion cells to sieve elements, thus affecting FT transport through the phloem to the SAM.
AT5G06860	*POLYGALACTURONASE INHIBITING PROTEIN 1 (PGIP1)*	Encodes a polygalacturonase inhibiting protein involved in defense response. PGIPs inhibit the function of cell wall pectin degrading enzymes such as those produced by fungal pathogens. PGIP1 is induced by fungal infection. Suppressed in the proton sensitive stop1-mutant, but the transcription level was recovered by transformation of STOP2. Knockout mutant showed severe damage in the root tip in low Ca and low pH medium.
AT5G06870	*POLYGALACTURONASE INHIBITING PROTEIN 2 (PGIP2)*	Encodes a polygalacturonase inhibiting protein involved in plant defense response. PGIPs inhibit the activity of pectin degrading enzymes such as those produced by fungal pathogens. PGIP2 is induced by fungal infection and methyl jasmonate.Suppressed in the proton sensitive stop1-mutant, but the transcription level was recovered by transformation of STOP2. Knockout mutant showed severe damage in the root tip in low Ca and low pH medium.
AT5G06900	*CYTOCHROME P450, FAMILY 93, SUBFAMILY D, POLYPEPTIDE 1 (CYP93D1)*	member of CYP93D
AT5G06920	*FASCICLIN-LIKE ARABINOGALACTAN PROTEIN 21 PRECURSOR (FLA21)*	Fasciclin-like arabinogalactan protein. Possibly involved in embryogenesis and seed development.
AT5G06930		nucleolar-like protein
AT5G06960	*OCS-ELEMENT BINDING FACTOR 5 (OBF5)*	Encodes a basic leucine zipper (B-ZIP) containing protein that interacts with NPR1 to promote expression of salicylic acid induced genes. Binds the ocs-element.
AT5G06980	*NIGHT LIGHT-INDUCIBLE AND CLOCK-REGULATED GENE 4 (LNK4)*	Member of a small gene family. Appears to be clock regulated.Somewhat redundant with LNK1/2 though more like LNK3 in having affects on biomass accumulation and phototrophism.
AT5G07000	*SULFOTRANSFERASE 2B (ST2B)*	Encodes a member of the sulfotransferase family of proteins. Although it has 85% amino acid identity with ST2A (At5g07010), this protein is not able to transfer a sulfate group to 11- or 12-hydroxyjasmonic acid in vitro. It may be able to act on structurally related jasmonates.
AT5G07010	*SULFOTRANSFERASE 2A (ST2A)*	Encodes a sulfotransferase that acts specifically on 11- and 12-hydroxyjasmonic acid. Transcript levels for this enzyme are increased by treatments with jasmonic acid (JA), 12-hydroxyJA, JA-isoleucine, and 12-oxyphytodienoic acid (a JA precursor).
AT5G07020	*MAINTENANCE OF PSII UNDER HIGH LIGHT 1 (MPH1)*	Encodes an integral thylakoid membrane protein that interacts with PSII core complexes and contributes to the maintenance of PSII homeostasis upon exposure to photoinhibitory light conditions by participating in the protection and stabilization of PSII under photoinhibitory stress.
AT5G07030		Eukaryotic aspartyl protease family protein
AT5G07040	*ARABIDOPSIS T??XICOS EN LEVADURA 69 (ATL69)*	RING/U-box superfamily protein
AT5G07050	*USUALLY MULTIPLE ACIDS MOVE IN AND OUT TRANSPORTERS 9 (UMAMIT9)*	nodulin MtN21-like transporter family protein
AT5G07060	*MOS4-ASSOCIATED COMPLEX SUBUNIT 5C (MAC5C)*	Encodes MAC5C, homologous to MAC5A. MAC5A is a component of the MOS4-associated complex (MAC) that contributes to snc1- mediated autoimmunity. Homologues include AT1G07360 (MAC5A), AT2G29580 (MAC5B) and AT5G07060 (MAC5C). MAC5A and MAC5B are more closely related to each other than to MAC5C.
AT5G07080		Encodes enzymes that can efficiently convert putrescine and caffeoyl-CoA to di-caffeoyl putrescine. Can convert spermidine/spermine and feruloyl CoA to mono-feruloyl spermidine/spermine. Has a preference for feruloyl-CoA binding, but little acyl-acceptor specificity.
AT5G07100	*WRKY DNA-BINDING PROTEIN 26 (WRKY26)*	Encodes WRKY DNA-binding protein 26 (WRKY26).
AT5G07110	*PRENYLATED RAB ACCEPTOR 1.B6 (PRA1.B6)*	Encodes PRA1.B6, an isoform of the PRA1 (Prenylated Rab acceptors) family. PRAs bind to prenylated Rab proteins and possibly aids in targeting Rabs to their respective compartments. PRA1.B6 localizes to the Golgi apparatus and its ER-to-Golgi trafficking and localization to the Golgi apparatus are regulated by multiple sequence motifs in both the C- and N-terminal cytoplasmic domains.
AT5G07150		Leucine-rich repeat protein kinase family protein
AT5G07160		Basic-leucine zipper (bZIP) transcription factor family protein
AT5G07180	*ERECTA-LIKE 2 (ERL2)*	Encodes a receptor-like kinase that, together with ER and ERL1 governs the initial decision of protodermal cells to either divide proliferatively to produce pavement cells or divide asymmetrically to generate stomatal complexes. It is also important for maintaining stomatal stem cell activity and preventing terminal differentiation of the meristemoid into the guard mother cell. When heterozygous in an er/erl1 null background, plants are female sterile due to cell division defect in the integuments.
AT5G07190	*SEED GENE 3 (ATS3)*	Gene is expressed preferentially in the embryo and encodes a unique protein of unknown function.
AT5G07200	*GIBBERELLIN 20-OXIDASE 3 (GA20OX3)*	encodes a gibberellin 20-oxidase.

AT5G07210	*RESPONSE REGULATOR 21 (RR21)*	member of Response Regulator: B- Type
AT5G07230	*Bifunctional inhibitor/lipid-transfer protein/seed storage 2S albumin superfamily protein (araport11).*	Tapetum specific gene.
AT5G07240	*IQ-DOMAIN 24 (IQD24)*	Member of IQ67 (CaM binding) domain containing family.
AT5G07250	*RHOMBOID-LIKE PROTEIN 3 (RBL3)*	RHOMBOID-like protein 3
AT5G07260		START (StAR-related lipid-transfer) lipid-binding domain-containing protein
AT5G07280	*EXCESS MICROSPOROCYTES1 (EMS1)*	Encodes EMS1 (EXCESS MICROSPOROCYTES1), a putative leucine-rich repeat receptor protein kinase that controls somatic and reproductive cell fates in Arabidopsis anther.
AT5G07300	*BONZAI 2 (BON2)*	Encodes a copine-like protein, which is a member of a newly identified class of calcium-dependent, phospholipid binding proteins that are present in a wide range of organisms.
AT5G07310	*ETHYLENE RESPONSE FACTOR 115 (ERF115)*	encodes a member of the ERF (ethylene response factor) subfamily B-4 of ERF/AP2 transcription factor family. The protein contains one AP2 domain. There are 7 members in this subfamily. Cytokinin production induced by jasmonate represses adventitious rooting.
AT5G07320	*ATP/PHOSPHATE CARRIER 3 (APC3)*	Encodes an APC isoform in Arabidopsis, a calcium-dependent mitochondrial ATP-Mg/Pi transporter.
AT5G07330		NFU1 iron-sulfur cluster protein
AT5G07350	*TUDOR-SN PROTEIN 1 (TUDOR1)*	RNA binding protein with nuclease activity essential for stress response. Involved in mechanisms acting on mRNAs entering the secretory pathway. Functionally redundant with TSN2.
AT5G07360		Amidase family protein
AT5G07380		hypothetical protein
AT5G07390	*RESPIRATORY BURST OXIDASE HOMOLOG A (RBOHA)*	respiratory burst oxidase homolog A
AT5G07410	*PECTIN METHYLESTERASE 48 (PME48)*	Encodes a pectin methylesterase implicated in the remodeling of pectins in pollen. Its expression is restricted to the male gametophyte (dry pollen, imbibed pollen and pollen tube). Mutants do not display any phenotype in the vegetative tissues. Pollen germination of KO mutant is strongly delayed in vivo and in vitro. Extra calcium restores the wild type phenotype in vitro.
AT5G07420		Pectin lyase-like superfamily protein
AT5G07430		Pectin lyase-like superfamily protein
AT5G07440	*GLUTAMATE DEHYDROGENASE 2 (GDH2)*	Encodes the beta-subunit of the glutamate dehydrogenase. The enzyme is almost exclusively found in the mitochondria of stem and leaf companion cells.
AT5G07450	*CYCLIN P4;3 (CYCP4;3)*	cyclin p4
AT5G07460	*PEPTIDEMETHIONINE SULFOXIDE REDUCTASE 2 (PMSR2)*	ubiquitous enzyme that repairs oxidatively damaged proteins. Methionine sulfoxide reductase activity. Mutant lacking reductase activity showed increased protein oxidation, nitration and glycation of specific amino acid residues during darkness.
AT5G07480	*KAR-UP OXIDOREDUCTASE 1 (KUOX1)*	KAR-UP oxidoreductase 1
AT5G07500	*(PEI1)*	Encodes an embryo-specific zinc finger transcription factor required for heart-stage embryo formation.
AT5G07510	*GLYCINE-RICH PROTEIN 14 (GRP14)*	encodes a glycine-rich protein that is expressed in low abundance in stems and leaves, and very low abundance in flowers.
AT5G07520	*GLYCINE-RICH PROTEIN 18 (GRP18)*	encodes a glycine-rich protein that is expressed only in flowers during a specific developmental stage (flower stage 12).
AT5G07530	*GLYCINE RICH PROTEIN 17 (GRP17)*	encodes a glycine-rich protein that has oleosin domain and is expressed specifically during flower stages 10 to 12. Protein is found on mature pollen coat.
AT5G07540	*GLYCINE-RICH PROTEIN 16 (GRP16)*	encodes a glycine-rich protein that is expressed only in flowers during a specific developmental stage (flower stages 11 and 12).
AT5G07550	*GLYCINE-RICH PROTEIN 19 (GRP19)*	member of Oleosin-like protein family
AT5G07560	*GLYCINE-RICH PROTEIN 20 (GRP20)*	Lipid-binding oleosins, glycine-rich protein.
AT5G07570		glycine/proline-rich protein
AT5G07580	*(ERF106)*	encodes a member of the ERF (ethylene response factor) subfamily B-3 of ERF/AP2 transcription factor family. The protein contains one AP2 domain. There are 18 members in this subfamily including ATERF-1, ATERF-2, AND ATERF-5.
AT5G07620		Protein kinase superfamily protein
AT5G07630		lipid transporter
AT5G07640		RING/U-box superfamily protein
AT5G07660	*STRUCTURAL MAINTENANCE OF CHROMOSOMES 6A (SMC6A)*	Encodes SMC6A (STRUCTURAL MAINTENANCE OF CHROMOSOMES 6A), a component of the SMC5/6 complex. SMC5/6 complex promotes sister chromatid alignment and homologous recombination after DNA damage.
AT5G07680	*NAC DOMAIN CONTAINING PROTEIN 80 (NAC080)*	NAC domain containing protein 80
AT5G07690	*MYB DOMAIN PROTEIN 29 (MYB29)*	Encodes a putative transcription factor (MYB29) that acts as a negative regulator of mitochondrial stress responses.
AT5G07700	*MYB DOMAIN PROTEIN 76 (MYB76)*	Encodes a putative transcription factor (MYB76), which inhibits the accumulation of seed oil.
AT5G07720	*XYLOGLUCAN XYLOSYLTRANSFERASE 3 (XXT3)*	Galactosyl transferase GMA12/MNN10 family protein
AT5G07730		transmembrane protein
AT5G07740		actin binding protein
AT5G07750		
AT5G07760		formin homology 2 domain-containing protein / FH2 domain-containing protein
AT5G07770	*FORMIN HOMOLOG 16 (FH16)*	Actin-binding FH2 protein
AT5G07780	*FORMIN HOMOLOG 19 (FH19)*	Encodes a class II formin that nucleates actin assembly, binds to the barbed-end of actin filaments and antagonizes the effect of FH1 on actin dynamics. The mRNA is cell-to-cell mobile.
AT5G07800		Flavin-binding monooxygenase family protein
AT5G07830	*GLUCURONIDASE 2 (GUS2)*	Belongs to the plant glycoside hydrolase family 79. Encodes a protein with several posttranslational modification sites including O-β-GlcNAc attachment sites and serine-, threonine- and tyrosine-phosphorylation sites, suggesting that this protein is extensively modified posttranslationally. The protein is predicted (WoLF PSORT program) to be membrane-associated. It is involved in cell elongation. The mRNA is cell-to-cell mobile.

AT5G07840	*PHYTOCHROME INTERACTING ANKYRIN-REPEAT PROTEIN 1 (PIA1)*	Ankyrin repeat family protein
AT5G07850		HXXXD-type acyl-transferase family protein
AT5G07880	*SYNAPTOSOMAL-ASSOCIATED PROTEIN SNAP25-LIKE 29 (SNAP29)*	member of mammalian SNAP25 Gene Family, a type of SNARE proteins with two chains. There are three members in Arabidopsis: SNAP30, SNAP29, and SNAP33.
AT5G07920	*DIACYLGLYCEROL KINASE1 (DGK1)*	Encodes a putative diacylglycerol kinase that is mainly expressed in roots, shoots and leaves, but its enzyme product was not active in vitro.
AT5G07930	*MEI2 C-TERMINAL RRM ONLY LIKE 2 (MCT2)*	A member of mei2-like gene family; phylogenetic analysis revealed that it belongs to the fourth clade of mei2-like proteins, with conserved C-terminal RNA recognition motif (RRM) only.
AT5G07950		hypothetical protein
AT5G07990	*TRANSPARENT TESTA 7 (TT7)*	Required for flavonoid 3' hydroxylase activity. Enzyme abundance relative to CHS determines Quercetin/Kaempferol metabolite ratio. The mRNA is cell-to-cell mobile.
AT5G08000	*GLUCAN ENDO-1,3-BETA-GLUCOSIDASE-LIKE PROTEIN 3 (E13L3)*	Encodes a member of the X8-GPI family of proteins. It localizes to the plasmodesmata and binds callose.
AT5G08030	*GLYCEROPHOSPHODIESTER PHOSPHODIESTERASE 6 (GDPD6)*	Encodes a member of the glycerophosphodiester phosphodiesterase (GDPD) family.
AT5G08050	*(RIQ1)*	Encodes a grana core localized protein. Mutant plants have reduced NPQ, affected organization of light-havesting complex II and an enhanced grana stacking.
AT5G08070	*TCP DOMAIN PROTEIN 17 (tcp17)*	TCP gene involved in heterochronic control of leaf differentiation.
AT5G08090		transmembrane protein
AT5G08110	*HOMOLOGOUS TO RECQ HELICASE 1 (HRQ1)*	Plays a role in the maintenance of genome stability and the repair of aberrant replication intermediates in the root meristem. Is involved with RAD1, FAN1, and RECQ4A in the repair of DNA CLs.
AT5G08130	*BES1-INTERACTING MYC-LIKE1 (BIM1)*	Encodes a basic helix-loop-helix (bHLH) family protein BIM1 (BES1-INTERACTING MYC-LIKE 1), involved in brassinosteroid signaling. It synergistically interacts with BES1 to bind to E box sequences (CANNTG). Positively modulates the shade avoidance syndrome in Arabidopsis seedlings.
AT5G08180		Ribosomal protein L7Ae/L30e/S12e/Gadd45 family protein
AT5G08200		peptidoglycan-binding LysM domain-containing protein
AT5G08210	*MICRORNA834A (MIR834A)*	Encodes a microRNA of unknown function. MicroRNAs are regulatory RNAs with a mature length of ~21-nucleotides that are processed from hairpin precursors by Dicer-like enzymes. MicroRNAs can negatively regulate gene expression by attenuating translation or by directing mRNA cleavage. Mature sequence: UGGUAGCAGUAGCGGUGGUAA
AT5G08220		
AT5G08240		transmembrane protein
AT5G08250		Cytochrome P450 superfamily protein
AT5G08260	*SERINE CARBOXYPEPTIDASE-LIKE 35 (scpl35)*	serine carboxypeptidase-like 35
AT5G08270		C5orf35
AT5G08280	*HYDROXYMETHYLBILANE SYNTHASE (HEMC)*	Encodes a protein with porphobilinogen deaminase activity. This protein is targeted to the chloroplast. Mutants spontaneously develop chlorotic leaf lesions in the absence of pathogen attack, resembling the phenotype of lesion-mimic mutants. It has been shown to interact with the PPR protein AtECB2 for chloroplast RNA editing.
AT5G08300		Succinyl-CoA ligase, alpha subunit
AT5G08310		Tetratricopeptide repeat (TPR)-like superfamily protein
AT5G08330	*TCP DOMAIN PROTEIN 21 (TCP21)*	Circadian oscillator protein which interacts with bZIP63 and regulates a response of the circadian oscillator to sugar. Is not required for the sugar-induced circadian phase advance in the morning; regulates a response of CCA1 to sugars.
AT5G08350		Mutants have decreased tolerance to cold and oxidative stress. Gene expression induced by drought and ABA.
AT5G08360		Stu1, putative (DUF789)
AT5G08370	*ALPHA-GALACTOSIDASE 2 (AGAL2)*	Member of Glycoside Hydrolase Family 27 (GH27)that functions as an α-galactosidase.
AT5G08380	*ALPHA-GALACTOSIDASE 1 (AGAL1)*	alpha-galactosidase 1
AT5G08400		structural maintenance of chromosomes-like protein, putative (DUF3531)
AT5G08410	*FERREDOXIN/THIOREDOXIN REDUCTASE SUBUNIT A (VARIABLE SUBUNIT) 2 (FTRA2)*	ferredoxin/thioredoxin reductase subunit A (variable subunit) 2
AT5G08415	*LIPOYL SYNTHASE 1 (LIP1)*	Radical SAM superfamily protein
AT5G08430		SWIB/MDM2 and Plus-3 and GYF domain-containing protein
AT5G08460		GDSL-motif esterase/acyltransferase/lipase. Enzyme group with broad substrate specificity that may catalyze acyltransfer or hydrolase reactions with lipid and non-lipid substrates.
AT5G08480		VQ motif-containing protein
AT5G08490	*SLOW GROWTH 1 (SLG1)*	Tetratricopeptide repeat (TPR)-like superfamily protein
AT5G08510		Pentatricopeptide repeat (PPR) superfamily protein
AT5G08565	*(SPT4-1)*	Transcription initiation Spt4-like protein
AT5G08570		Pyruvate kinase family protein
AT5G08600		U3 ribonucleoprotein (Utp) family protein
AT5G08610	*PIGMENT DEFECTIVE 340 (PDE340)*	P-loop containing nucleoside triphosphate hydrolases superfamily protein
AT5G08620	*STRESS RESPONSE SUPPRESSOR 2 (STRS2)*	Similar in sequence to DEAD-box RNA helicases. Binds RNA. Involved in drought, salt and cold stress responses.
AT5G08630	*DDT-WAC PROTEIN1 (DDW1)*	DDT domain-containing protein
AT5G08640	*FLAVONOL SYNTHASE 1 (FLS1)*	Encodes a flavonol synthase that catalyzes formation of flavonols from dihydroflavonols. Co-expressed with CHI and CHS (qRT-PCR).
AT5G08690		Encodes the mitochondrial ATP synthase beta-subunit. This subunit is encoded by a multigene family of three members (At5g08670, At5g08680, At5g08690) that shared 98% sequence identity at the amino acid level. The mRNA is cell-to-cell mobile.

AT5G09220	*AMINO ACID PERMEASE 2 (AAP2)*	member of AAAP family The mRNA is cell-to-cell mobile.
AT5G09230	*SIRTUIN 2 (SRT2)*	Encodes SRT2, a member of the SIR2 (sirtuin) family HDAC (histone deacetylase) (SRT1/AT5g55760, SRT2/AT5G09230).
AT5G09240		ssDNA-binding transcriptional regulator
AT5G09270		transmembrane protein
AT5G09280		Pectin lyase-like superfamily protein
AT5G09290		Inositol monophosphatase family protein
AT5G09300	*(E1A2)*	e1a2 subunit of branched chain ketoacid dehydrogenase (BCKDH) complex.
AT5G09320	*(VPS9B)*	vacuolar protein sorting-associated 9A-like protein
AT5G09340		Ubiquitin family protein
AT5G09360	*LACCASE 14 (LAC14)*	putative laccase, a member of laccase family of genes (17 members in Arabidopsis).
AT5G09370	*GLYCOSYLPHOSPHATIDYLINOSITOL-ANCHORED LIPID PROTEIN TRANSFER 29 (LTPG29)*	Bifunctional inhibitor/lipid-transfer protein/seed storage 2S albumin superfamily protein
AT5G09380		RNA polymerase III RPC4
AT5G09400	*K+ UPTAKE PERMEASE 7 (KUP7)*	Encodes a potassium uptake permease with a functional adenylate cyclase (AC) center. The first 100 aa of this protein can complement AC-deficient E. coli and display AC activity in vitro. KUP7 is localized to the plasma membrane where it functions in potassium uptake and translocation.
AT5G09420	*TRANSLOCON AT THE OUTER MEMBRANE OF CHLOROPLASTS 64-V (TOC64-V)*	Encodes one of the 36 carboxylate clamp (CC)-tetratricopeptide repeat (TPR) proteins (Prasad 2010, Pubmed ID: 20856808) with potential to interact with Hsp90/Hsp70 as co-chaperones.
AT5G09430		alpha/beta-Hydrolases superfamily protein
AT5G09440	*EXORDIUM LIKE 4 (EXL4)*	EXORDIUM like 4
AT5G09450		Tetratricopeptide repeat (TPR)-like superfamily protein
AT5G09460	*(SACL1)*	transcription factor bHLH143
AT5G09470	*DICARBOXYLATE CARRIER 3 (DIC3)*	Encodes one of the mitochondrial dicarboxylate carriers (DIC): DIC1 (AT2G22500), DIC2 (AT4G24570), DIC3 (AT5G09470).
AT5G09480		hydroxyproline-rich glycoprotein family protein
AT5G09490		Ribosomal protein S19 family protein
AT5G09500		Ribosomal protein S19 family protein
AT5G09520	*PRO-GLU-LEU|ILE|VAL-PRO-LYS 2 (PELPK2)*	hydroxyproline-rich glycoprotein family protein
AT5G09530	*PRO-GLU-LEU|ILE|VAL-PRO-LYS 1 (PELPK1)*	The gene encodes a unique protein which contains 36 repeats of a unique pentapeptide (Pro-Glu-Leu|Ile|Val-Pro-Lys). It has been shown tobe involved in growth and development.
AT5G09550	*RAB GDP-DISSOCIATION INHIBITOR (GDI)*	GDP dissociation inhibitor family protein / Rab GTPase activator family protein
AT5G09570	*(AT12CYS-2)*	Twin CX9C domain protein. Induced by low phosphate or iron, drought and heat stress. Loss of both At12cys-1 and At12cys-2 lead to enhanced tolerance to drought and light stress and increased anti-oxidant capacity.
AT5G09590	*MITOCHONDRIAL HSO70 2 (MTHSC70-2)*	heat shock protein 70 (Hsc70-5); nuclear
AT5G09630		LisH/CRA/RING-U-box domains-containing protein
AT5G09640	*SERINE CARBOXYPEPTIDASE-LIKE 19 (SCPL19)*	encodes a serine carboxypeptidase-like (SCPL) protein. Mutants accumulate sinapoylglucose instead of sinapoylcholine, and have increased levels of choline and decreased activity of the enzyme sinapoylglucose:choline sinapoyltransferase.
AT5G09650	*PYROPHOSPHORYLASE 6 (PPa6)*	Encodes a protein with inorganic pyrophosphatase activity.
AT5G09660	*PEROXISOMAL NAD-MALATE DEHYDROGENASE 2 (PMDH2)*	encodes a microbody NAD-dependent malate dehydrogenase encodes an peroxisomal NAD-malate dehydrogenase that is involved in fatty acid beta-oxidation through providing NAD to the process of converting fatty acyl CoA to acetyl CoA.
AT5G09670		loricrin-like protein
AT5G09700		pseudogene of glycosyl hydrolase family 3 protein
AT5G09710		Magnesium transporter CorA-like family protein
AT5G09730	*BETA-XYLOSIDASE 3 (BXL3)*	Encodes a protein similar to a beta-xylosidase located in the extracellular matrix. It is able to degrade terminal arabinosyl residues and likely participates in the in-vivo hydrolysis of arabinan. This is a member of glycosyl hydrolase family 3 and has six other closely related members.
AT5G09760		Plant invertase/pectin methylesterase inhibitor superfamily
AT5G09770		Ribosomal protein L17 family protein
AT5G09780	*REPRODUCTIVE MERISTEM 25 (REM25)*	Encodes a member of the REM (Reproductive Meristem) gene family, a part of the B3 DNA-binding domain superfamily.
AT5G09800	*(PUB28)*	Plant U-box type E3 ubiquitin ligase (PUB).
AT5G09820	*FIBRILLIN5 (FBN5)*	Encodes fibrillin 5 (FBN5). Located in chloroplast stroma. Essential for plastoquinone-9 biosynthesis. Stimulates enzymatic activity of solanesyl diphosphate synthases (SPS) 1 and 2 through binding to solanesyl moiety. Two splicing variants, named FBN5-A shorter one and FBN5-B longer one. FBN5-B is the protein detected in chloroplast stroma. Involved in plastoquinone biosynthesis.
AT5G09840	*MITOCHONDRIAL NUCLEASE2 (MNU2)*	Putative endonuclease or glycosyl hydrolase
AT5G09850	*MEDIATOR 26C (MED26C)*	Transcription elongation factor (TFIIS) family protein
AT5G09870	*CELLULOSE SYNTHASE 5 (CESA5)*	Encodes a cellulose synthase CESA5 that produces seed mucilage cellulose.Mutants are defective in seed coat mucilage.Involved in the regulation of mucilage composition and/or mucilage synthesis.
AT5G09890	*NUCLEAR DBF2-RELATED 8 (NDR8)*	Ubiquitously expressed protein kinase.
AT5G09920	*(NRPB4)*	Non-catalytic subunit specific to DNA-dependent RNA polymerase II; the ortholog of budding yeast RPB4)
AT5G09930	*ATP-BINDING CASSETTE F2 (ABCF2)*	ABCF2 is one of five members of the ABCF gene family in Arabidopsis, which are homologs of the yeast ABCF protein, GCN20.
AT5G09940		hypothetical protein (DUF1635)
AT5G09950	*MITOCHONDRIAL EDITING FACTOR 7 (MEF7)*	Encodes a DYW-class PPR protein required for RNA editing at four sites in mitochondria of A. thaliana.
AT5G09960		sorbin/SH3 domain protein

AT5G09970	*CYTOCHROME P450, FAMILY 78, SUBFAMILY A, POLYPEPTIDE 7 (CYP78A7)*	Member of CYP78A family. Paralog of CYP78A5 and appears to function in a shoot meristem maintainence pathway with LAMP1 that parallels AMP1/CYP87A5.
AT5G09980	*ELICITOR PEPTIDE 4 PRECURSOR (PROPEP4)*	elicitor peptide 4 precursor
AT5G09990	*ELICITOR PEPTIDE 5 PRECURSOR (PROPEP5)*	elicitor peptide 5 precursor
AT5G09995		transmembrane protein
AT5G10010	*HEAT INTOLERANT 4 (HIT4)*	myosin-H heavy protein
AT5G10030	*TGACG MOTIF-BINDING FACTOR 4 (TGA4)*	Encodes a member of basic leucine zipper transcription gene family. Nomenclature according to Xiang, et al. (1997).
AT5G10040	*HYPOXIA RESPONSE UNKNOWN PROTEIN 9 (HUP9)*	transmembrane protein
AT5G10070		RNase L inhibitor protein-like protein
AT5G10090	*TETRATRICOPEPTIDE REPEAT 13 (TPR13)*	Encodes one of the 36 carboxylate clamp (CC)-tetratricopeptide repeat (TPR) proteins (Prasad 2010, Pubmed ID: 20856808) with potential to interact with Hsp90/Hsp70 as co-chaperones.
AT5G10100	*TREHALOSE-6-PHOSPHATE PHOSPHATASE I (TPPI)*	Trehalose-6-phosphate phosphatase which enhances drought tolerance by regulating stomatal apertures.
AT5G10120	*(EIL4)*	Ethylene insensitive 3 family protein
AT5G10130		Pollen Ole e 1 allergen and extensin family protein
AT5G10140	*FLOWERING LOCUS C (FLC)*	MADS-box protein encoded by FLOWERING LOCUS C - transcription factor that functions as a repressor of floral transition and contributes to temperature compensation of the circadian clock. Expression is downregulated during cold treatment. Vernalization, FRI and the autonomous pathway all influence the state of FLC chromatin. Both maternal and paternal alleles are reset by vernalization, but their earliest activation differs in timing and location. Histone H3 trimethylation at lysine 4 and histone acetylation are associated with active FLC expression, whereas histone deacetylation and histone H3 dimethylation at lysines 9 and 27 are involved in FLC repression. Expression is also repressed by two small RNAs (30- and 24-nt) complementary to the FLC sense strand 3? to the polyA site. The small RNAs are most likely derived from an antisense transcript of FLC. Interacts with SOC1 and FT chromatin in vivo. Member of a protein complex.
AT5G10160		Thioesterase superfamily protein
AT5G10170	*MYO-INOSITOL-1-PHOSPHATE SYNTHASE 3 (MIPS3)*	myo-inositol-1-phosphate synthase isoform 3.Expressed in leaf, root and silique. Immunolocaliazation experiments with an antibody recognizing MIPS1, MIPS2, and MIPS3 showed endosperm localization.
AT5G10180	*SULFATE TRANSPORTER 2;1 (SULTR2;1)*	Encodes a low-affinity sulfate transporter expressed in the root cap and central cylinder, where it is induced by sulfur starvation. Expression in the shoot vascular system is not induced by sulfur starvation.
AT5G10190		Major facilitator superfamily protein
AT5G10210		nitric oxide synthase-interacting protein
AT5G10220	*ANNEXIN 6 (ANN6)*	Encodes a calcium-binding protein annexin (AnnAt6).
AT5G10230	*ANNEXIN 7 (ANNAT7)*	Encodes a calcium-binding protein annexin (AnnAt7).
AT5G10240	*ASPARAGINE SYNTHETASE 3 (ASN3)*	Encodes asparagine synthetase (ASN3).
AT5G10250	*DEFECTIVELY ORGANIZED TRIBUTARIES 3 (DOT3)*	Encodes a protein with an N-terminal BTB/POZ domain and a C-terminal NPH3 family domain. dot3 mutants have defects in shoot and primary root growth and produce an aberrant parallel venation pattern in juvenile leaves.
AT5G10260	*RAB GTPASE HOMOLOG H1E (RABH1e)*	RAB GTPase homolog H1E
AT5G10280	*MYB DOMAIN PROTEIN 92 (MYB92)*	Encodes a putative transcription factor (MYB92).
AT5G10290		leucine-rich repeat transmembrane protein kinase family protein
AT5G10300	*METHYL ESTERASE 5 (MES5)*	Encodes a protein with R-selective hydroxynitrile lyase activity. It has similarity to the SABP2 methyl salicylate esterase from tobacco. This protein does not act on methyl IAA, methyl JA, MeSA, MeGA4, or MEGA9 in vitro.
AT5G10310	*(ATEPFL1)*	Memmber of the EPF/EPFL (epidermal patterning factor/EPF-like) gene family, which genes encode plant-specific secretory peptides, several of which play a role in controlling stomatal density and patterning in the plant epidermis.
AT5G10370		helicase domain-containing protein / IBR domain-containing protein / zinc finger protein-like protein
AT5G10390	*(HTR13)*	Histone superfamily protein
AT5G10400	*HISTONE 3.1 (H3.1)*	Histone superfamily protein
AT5G10420		MATE efflux family protein
AT5G10430	*ARABINOGALACTAN PROTEIN 4 (AGP4)*	Encodes arabinogalactan-protein (AGP4) that is expressed in female reproductive tissues. It is involved in promoting degeneration of the persistent synergid after fertilization. In mutant ovules, the persistent synergid does not degrade resulting in polytuby.
AT5G10440	*CYCLIN D4;2 (CYCD4;2)*	Encodes a cyclin involved in cell proliferation during stomatal cell lineage development.
AT5G10460		Haloacid dehalogenase-like hydrolase (HAD) superfamily protein
AT5G10480	*PASTICCINO 2 (PAS2)*	Protein tyrosine phosphatase-like involved in cell division and differentiation. Interacts with CDKA;1 only in its phosphorylated form, preventing dephosphorylation. Overexpression slowed down cell division in suspension cell cultures at the G2-to-M transition and early mitosis and inhibited Arabidopsis seedling growth. Localized in the cytoplasm of dividing cells but moved into the nucleus upon cell differentiation. Based on complementation of yeast mutant PAS2 has acyl-CoA dehydratase activity. It interacts with CER10, a component of the microsomal fatty acid elongase complex, suggesting a role in synthesis of VLCFAs (very long chain fatty acids).
AT5G10500	*NETWORKED 2C (NET2C)*	Kinase interacting (KIP1-like) family protein
AT5G10510	*AINTEGUMENTA-LIKE 6 (AIL6)*	Encodes an AP2-domain transcription factor involved in root stem cell identity and root development. It is also required to maintain high levels of PIN1 expression at the periphery of the meristem and modulate local auxin production in the central region of the SAM which underlies phyllotactic transitions. Intronic sequences are required for its expression in flowers.Acts redundantly with PLT5 and 7 in lateral root pattern formation.
AT5G10520	*ROP BINDING PROTEIN KINASES 1 (RBK1)*	ROP binding protein kinases 1
AT5G10530	*L-TYPE LECTIN RECEPTOR KINASE IX.1 (LECRK-IX.1)*	Concanavalin A-like lectin protein kinase family protein

AT5G10550	*GLOBAL TRANSCRIPTION FACTOR GROUP E2 (GTE2)*	This gene is predicted to encode a bromodomain-containing protein. A plant line expressing RNAi constructs targeted against GTE7 shows some resistance to agrobacterium-mediated root transformation.
AT5G10560		Glycosyl hydrolase family protein
AT5G10580		plant/protein (Protein of unknown function, DUF599)
AT5G10620		methyltransferase
AT5G10650	*JAV1- 37 ASSOCIATED UBIQUITIN LIGASE 1 (JUL1)*	JUL1 encode a RING-type E3 ubiquitin ligase that is involved in JA responses. It ubiquitinates the JAV1 jasmonic acid response repressor which is then degraded by the proteosome. Participates in ABA-mediated microtubule depolymerization, stomatal closure, and tolerance response to drought stress.
AT5G10660		calmodulin-binding protein-like protein
AT5G10680		calmodulin-binding protein-like protein
AT5G10690		pentatricopeptide (PPR) repeat-containing protein / CBS domain-containing protein
AT5G10695		methionyl-tRNA synthetase
AT5G10720	*HISTIDINE KINASE 5 (HK5)*	member of Histidine Kinase
AT5G10730		NAD(P)-binding Rossmann-fold superfamily protein
AT5G10760	*APOPLASTIC, EDS1-DEPENDENT 1 (AED1)*	Eukaryotic aspartyl protease family protein
AT5G10770		Eukaryotic aspartyl protease family protein
AT5G10780		ER membrane protein complex subunit-like protein
AT5G10830		S-adenosyl-L-methionine-dependent methyltransferases superfamily protein
AT5G10850		transposable_element_gene;similar to nucleic acid binding / zinc ion binding [Arabidopsis thaliana] (TAIR:AT2G01050.1);(source:TAIR10)
AT5G10860	*CBS DOMAIN CONTAINING PROTEIN 3 (CBSX3)*	Encodes a single cystathionine beta-Synthase domain-containing protein. Modulates development by regulating the thioredoxin system.
AT5G10880		tRNA synthetase-related / tRNA ligase-like protein
AT5G10910	*CHLOROPLAST MRAW-LIKE (CMAL)*	Plastid rRNA methyltransferase involved in ribosome biogenesis and plant development. Accounts to the N4-methylation of C1352 in chloroplast 16S rRNA.
AT5G10920		L-Aspartase-like family protein
AT5G10930	*CBL-INTERACTING PROTEIN KINASE 5 (CIPK5)*	Encodes CBL-interacting protein kinase 5 (CIPK5).
AT5G10950		Tudor/PWWP/MBT superfamily protein
AT5G10960	*CCR4-ASSOCIATED FACTOR 1I (CAF1I)*	Polynucleotidyl transferase, ribonuclease H-like superfamily protein
AT5G10970		C2H2 and C2HC zinc fingers superfamily protein
AT5G11000		hypothetical protein (DUF868)
AT5G11010	*(GRC3)*	Nuclear-localizing protein.
AT5G11015		
AT5G11060	*KNOTTED1-LIKE HOMEOBOX GENE 4 (KNAT4)*	A member of Class II KN1-like homeodomain transcription factors (together with KNAT3 and KNAT5), with greatest homology to the maize knox1 homeobox protein. Expression regulated by light. Detected in all tissues examined, but most prominent in leaves and young siliques. Transient expression of GFP translational fusion protein suggests bipartite localization in nucleus and cytoplasm. KNAT4 promoter activity showed cell-type specific pattern along longitudinal root axis; GUS expression pattern started at the elongation zone, predominantly in the phloem and pericycle cells, extending to endodermis toward the base of the root.
AT5G11070		hypothetical protein
AT5G11080		Ubiquitin-like superfamily protein
AT5G11090		serine-rich protein-like protein
AT5G11100	*(SYTD)*	Calcium-dependent lipid-binding (CaLB domain) family protein
AT5G11110	*SUCROSE PHOSPHATE SYNTHASE 2F (SPS2F)*	Encodes a sucrose-phosphate synthase involved in pollen exine formation. This is the dominant SPS isoform in leaves with respect to protein levels.
AT5G11120		
AT5G11150	*VESICLE-ASSOCIATED MEMBRANE PROTEIN 713 (VAMP713)*	Member of Synaptobrevin-like AtVAMP7C, v-SNARE protein family.
AT5G11160	*ADENINE PHOSPHORIBOSYLTRANSFERASE 5 (APT5)*	adenine phosphoribosyltransferase 5
AT5G11180	*GLUTAMATE RECEPTOR 2.6 (GLR2.6)*	member of Putative ligand-gated ion channel subunit family
AT5G11210	*GLUTAMATE RECEPTOR 2.5 (GLR2.5)*	member of Putative ligand-gated ion channel subunit family
AT5G11220		hypothetical protein
AT5G11230	*UDPARABINOFURANOSE TRANSPORTER 2 (UAFT2)*	Nucleotide-sugar transporter family protein
AT5G11240	*NUCLEAR GLUCOSE-RESPONSIVE WD40 PROTEIN1 (NUGWD1)*	GHS40 encodes a WD40 protein, that is localized in the nucleus and nucleolus. In the presence of high glucose it negatively regulates the expression of abscisic acid degradation and signaling genes.
AT5G11250	*BNT1 (BURNOUT1)*	Encodes an atypical TIR-NBS-LRR protein that is involved in stress responses. Loss of function alleles overproduce stress hormones JA,SA, ABA, and ET.
AT5G11270	*OVEREXPRESSOR OF CATIONIC PEROXIDASE 3 (OCP3)*	Encodes a homeodomain transcription factor involved in mediating resistance to infection by necrotrophic pathogens dependent on perception of jasmonic acid through COI1. Expressed in the nucleus. Downregulated upon fungal infection. Also involved in drought tolerance.
AT5G11290		transmembrane protein, putative (DUF247)
AT5G11300	*MITOTIC-LIKE CYCLIN 3B FROM ARABIDOPSIS (CYC3B)*	mitotic-like cyclin, core cell cycle gene that is expressed only in roots (RT_PCR), portions with mitotic activity only (whole mount in situ).

AT5G11310	*SUPPRESSOR OF THE ABAR OVEREXPRESSOR 1 (SOAR1)*	The SOAR1 gene encodes a pentatricopeptide repeat (PPR) protein which localizes to both the cytosol and nucleus. Down-regulation of SOAR1 strongly enhances, but up-regulation of SOAR1 almost completely impairs, ABA responses, revealing that SOAR1 is a critical, negative, regulator of ABA signalling. Further genetic evidence supports that SOAR1 functions downstream of ABAR and probably upstream of an ABA-responsive transcription factor ABI5. Changes in the SOAR1 expression alter expression of a subset of ABA-responsive genes including ABI5. These findings provide important information to elucidate further the functional mechanism of PPR proteins and the complicated ABA signalling network.
AT5G11320	*YUCCA4 (YUC4)*	Belongs to the YUC gene family. Encodes a predicted flavin monooxygenase. YUC4 is part of a pathway linking auxin biosynthesis and gynoecium development. It is expressed in the stigma and the apical meristem and is ethylene inducible.
AT5G11340	*N-TERMINAL ACETYLTRANSFERASE 50 (NAA50)*	Encodes an N-terminal acetyltransferase involved in plant development and the suppression of stress responses, potentially through the regulation of ER stress.
AT5G11360	*BRASSIKIN3 (BKN3)*	Interleukin-1 receptor-associated kinase 4 protein
AT5G11400	*BRASSIKIN1 (BKN1)*	Psuedokinase that appears to produce a truncated (42AA protein) in Col-0 reference genome. Full length transcripts have been identified in Hh-0, Västervik and Dju-1 ecotypes.
AT5G11410	*SUPPRESSOR OF ZED1-D2 (SZE2)*	Similar to receptor like kinase but does not appear to have kinase activity (psuedokinase). It is involved in HopZ1a effector triggered immunity. Interacts with ZAR1 and ZED1.Localization to membrane is dependent on N-terminal myristoylation domain
AT5G11420		Encodes a DUF642 cell wall protein.
AT5G11430		SPOC domain / Transcription elongation factor S-II protein
AT5G11450	*PSBP DOMAIN PROTEIN 5 (PPD5)*	PsbP domain protein (Mog1/PsbP/DUF1795-like photosystem II reaction center PsbP family protein)
AT5G11460	*FCS LIKE ZINC FINGER 10 (FLZ10)*	FCS like zinc finger 10 is induced during energy starvation through SnRK1 signaling. Mutants accumulate more SnRK1alpha1 which results in the inhibition of seedling growth under favorable growth conditions. Increased SnRK1 activity in the mutant also results in the downregulation of TOR signaling (DOI:10.1111/tpj.13854).
AT5G11510	*MYB DOMAIN PROTEIN 3R-4 (MYB3R-4)*	Arabidopsis thaliana putative c-myb-like transcription factor MYB3R-4. Functions in powdery mildew induced host endoreduplication at the site of infection. Activates mitotic gene expression, cytokinin response.
AT5G11520	*ASPARTATE AMINOTRANSFERASE 3 (ASP3)*	Encodes the chloroplastic isozyme of aspartate aminotransferase. Involved in aspartate biosynthesis and nitrogen metabolism. mRNA is expressed in senescing leaves.
AT5G11540	*L -GULONO-1,4-LACTONE (L -GULL) OXIDASE 3 (GULLO3)*	Encodes a homolog of rat L-gulono-1,4-lactone (L-GulL) oxidase that is involved in the biosynthesis of L-ascorbic acid.
AT5G11550		ARM repeat superfamily protein
AT5G11570		Major facilitator superfamily protein
AT5G11580		Regulator of chromosome condensation (RCC1) family protein
AT5G11590	*TINY2 (TINY2)*	encodes a member of the DREB subfamily A-4 of ERF/AP2 transcription factor family. The protein contains one AP2 domain. There are 17 members in this subfamily including TINY.
AT5G11600		hypothetical protein
AT5G11610		Exostosin family protein
AT5G11640		Thioredoxin superfamily protein
AT5G11650	*(MAGL13)*	alpha/beta-Hydrolases superfamily protein
AT5G11670	*NADP-MALIC ENZYME 2 (NADP-ME2)*	The malic enzyme (EC 1.1.1.40) encoded by AtNADP-ME2 is presumably a cytosolic enzyme involved in malate metabolism and possibly assisting the oxidative pentose phosphate pathway. AtNADP-ME2 counts for the major part of NADP-ME activity in mature tissues of Arabidopsis.
AT5G11680		classical AGP protein
AT5G11720	*ALPHA-GLUCOSIDASE (AGLU1)*	Glycosyl hydrolases family 31 protein
AT5G11780		SNF2 domain protein
AT5G11790	*N-MYC DOWNREGULATED-LIKE 2 (NDL2)*	Plays a role in dehydration stress response.
AT5G11810		rhomboid family protein
AT5G11820		Plant self-incompatibility protein S1 family
AT5G11850	*MAPKKK DELTA-1 (MKD1)*	MAP3 kinase involved phosphorylation of a critical Ser171 for OST1/SnRK2.6 activation.
AT5G11870		Alkaline phytoceramidase (aPHC)
AT5G11920	*6-&1-FRUCTAN EXOHYDROLASE (cwINV6)*	Encodes a protein with fructan exohydrolase (FEH) activity acting on both inulin and levan-type fructans (1- and 6-FEH). The enzyme does not have invertase activity.
AT5G11930	*(ROXY20)*	Encodes a member of the CC-type glutaredoxin (ROXY) family that has been shown to interact with the transcription factor TGA2.
AT5G11940		Subtilase family protein
AT5G11950	*LONELY GUY 8 (LOG8)*	Encodes a protein of unknown function. It has been crystallized and shown to be structurally almost identical to the protein encoded by At2G37210.
AT5G11970		ABC family ABC transporter, putative (DUF3511)
AT5G11980	*CONSERVED OLIGOMERIC GOLGI COMPLEX 8 (COG8)*	COG8 is a component of a putative conserved oligomeric Golgi (COG) complex that is thought to be involved in tethering of retrograde intra Golgi vesicles. It is required for proper deposition of cell wall materials in pollen tube growth. In mutant pollen,golgi appear abnormal.When homozygotes can be produced (by complementing the defect in pollen), the plants are embryo lethal suggesting an essential function. COG8 interacts with several other putative COG components.
AT5G11990		proline-rich family protein
AT5G12000		kinase with adenine nucleotide alpha hydrolases-like domain-containing protein
AT5G12010		nuclease
AT5G12020	*17.6 KDA CLASS II HEAT SHOCK PROTEIN (HSP17.6II)*	17.6 kDa class II heat shock protein
AT5G12030	*HEAT SHOCK PROTEIN 17.6A (HSP17.6A)*	Encodes a cytosolic small heat shock protein with chaperone activity that is induced by heat and osmotic stress and is also expressed late in seed development.

AT5G12050	*BIG GRAIN 1 (BG1)*	rho GTPase-activating protein
AT5G12060		Plant self-incompatibility protein S1 family
AT5G12100		pentatricopeptide (PPR) repeat-containing protein
AT5G12110		elongation factor 1-beta 1
AT5G12120		Ubiquitin-associated/translation elongation factor EF1B protein
AT5G12140	*CYSTATIN-1 (CYS1)*	Encodes a cystatin.
AT5G12170	*CRT (CHLOROQUINE-RESISTANCE TRANSPORTER)-LIKE TRANSPORTER 3 (CLT3)*	Encodes one of the CRT-Like transporters (CLT1/AT5G19380, CLT2/AT4G24460, CLT3/AT5G12170). Required for glutathione homeostasis and stress responses. Mutants lacking these transporters are heavy metal-sensitive, glutathione(GSH)-deficient, and hypersensitive to Phytophthora infection. The mRNA is cell-to-cell mobile.
AT5G12180	*CALCIUM-DEPENDENT PROTEIN KINASE 17 (CPK17)*	member of Calcium Dependent Protein Kinase
AT5G12200	*PYRIMIDINE 2 (PYD2)*	Encodes a protein with dihydropyrimidine amidohydrolase activity. It localizes to the secretory system and plays a role in uracil metabolism.
AT5G12210	*RAB GERANYLGERANYL TRANSFERASE BETA SUBUNIT 1 (RGTB1)*	Encodes the Rab geranylgeranyl transferase beta subunit that is essential for embryo and seed development.
AT5G12220		las1-like family protein
AT5G12270		2-oxoglutarate (2OG) and Fe(II)-dependent oxygenase superfamily protein
AT5G12280		SWAP (Suppressor-of-White-APricot)/surp RNA-binding domain-containing protein
AT5G12290	*DGD1 SUPPRESSOR 1 (DGS1)*	Encodes a mitochondrial outer membrane protein that is found in a complex with MIC60, TOM40, RISP and TOM20. Involved in galactoglycerolipid biosynthesis/lipid homeostasis. The dgd1 mutant phenotype is suppressed in the dgs1 mutant background.
AT5G12330	*LATERAL ROOT PRIMORDIUM 1 (LRP1)*	A member of SHI gene family. Arabidopsis thaliana has ten members that encode proteins with a RING finger-like zinc finger motif. Despite being highly divergent in sequence, many of the SHI-related genes are partially redundant in function and synergistically promote gynoecium, stamen and leaf development in Arabidopsis. Expressed in lateral root primordia and induced by auxin. SWP1 is involved in the repression of LRP1 via histone deacetylation.
AT5G12340		PADRE protein up-regulated after infection by S. sclerotiorum.
AT5G12350		Regulator of chromosome condensation (RCC1) family with FYVE zinc finger domain-containing protein
AT5G12380	*ANNEXIN 8 (ANNAT8)*	Involved in multiple stress signaling pathways; negatively regulates RPW8.1-mediated cell death and disease resistance.
AT5G12400	*DDT-PHD PROTEIN3 (DDP3)*	PHD-finger and DNA binding domain-containing protein
AT5G12420	*WS /DGAT 7 (WSD7)*	WSD7 can function in vitro as wax ester synthase but does not appear to be essential for cuticular wax biosynthesis.
AT5G12430	*TETRATRICOPEPTIDE REPEAT 16 (TPR16)*	Encodes one of the 36 carboxylate clamp (CC)-tetratricopeptide repeat (TPR) proteins (Prasad 2010, Pubmed ID: 20856808) with potential to interact with Hsp90/Hsp70 as co-chaperones.
AT5G12440		CCCH-type zinc fingerfamily protein with RNA-binding domain-containing protein
AT5G12450		FBD-like domain family protein
AT5G12460		fringe-like protein (DUF604)
AT5G12480	*CALMODULIN-DOMAIN PROTEIN KINASE 7 (CPK7)*	calmodulin-domain protein kinase CDPK isoform 7 (CPK7)
AT5G12840	*NUCLEAR FACTOR Y, SUBUNIT A1 (NF-YA1)*	Encodes a subunit of CCAAT-binding complex, binds to CCAAT box motif present in some plant promoter sequences. One of three members of this class (HAP2A, HAP2B, HAP2C), it is expressed in vegetative and reproductive tissues.
AT5G12870	*MYB DOMAIN PROTEIN 46 (MYB46)*	Encodes MYB46, member of the R2R3 factor gene family. Modulates Disease Susceptibility to Botrytis cinerea.
AT5G12880		proline-rich family protein
AT5G12890		UDP-Glycosyltransferase superfamily protein
AT5G12900		DNA double-strand break repair RAD50 ATPase
AT5G12910	*HISTONE THREE RELATED 15 (H3.15)*	Plays role in cell fate reprogramming during plant regeneration; expression is rapidly induced upon wounding. Involved in release from PRC2-mediated gene repression by its deposition into chromatin, which is involved in reprogramming cell fate to produce pluripotent callus cells.
AT5G12930		inactive rhomboid protein
AT5G12940		Leucine-rich repeat (LRR) family protein
AT5G12950		proline-tRNA ligase (DUF1680)
AT5G12970	*MULTIPLE C2 DOMAIN AND TRANSMEMBRANE REGION PROTEIN 5 (MCTP5)*	Calcium-dependent lipid-binding (CaLB domain) plant phosphoribosyltransferase family protein
AT5G12980	*(NOT9B)*	Component of the CCR4-NOT complex; acts as negative regulator of phyA-specific light signalling when bound to NOT1, the scaffold protein of the complex. Photoactivated phyA can displace NOT9B from the CCR4-NOT complex.
AT5G12990	*CLAVATA3/ESR-RELATED 40 (CLE40)*	Member of a large family of putative ligands homologous to the Clavata3 gene. Consists of a single exon.
AT5G13020	*EMSY-LIKE 3 (EML3)*	Agenet domain containing nucleosome binding protein. Binds H3K36 sites.
AT5G13030	*SELENOPROTEIN O (SELO)*	Chloroplast localized homolog of SELO. Loss of function mutants have reduced production of reactive oxygen species (ROS) and higher ROS scavenging.
AT5G13070		MSF1-like family protein
AT5G13080	*WRKY DNA-BINDING PROTEIN 75 (WRKY75)*	WRKY75 is one of several transcription factors induced during Pi deprivation. It is nuclear localized and regulated differentially during Pi starvation. RNAi mediated suppression of WRKY75 made the plants more susceptible to Pi stress as indicated by the higher accumulation of anthocyanin during Pi starvation.
AT5G13090		hypothetical protein
AT5G13100		Gap junction beta-4 protein
AT5G13120	*PHOTOSYNTHETIC NDH SUBCOMPLEX L 5 (Pnsl5)*	Encodes a lumenal cyclophilin with peptidyl-prolyl isomerase activity that is associated with the NAD(P)H dehydrogenase complex in stromal regions of the thylakoid membrane. It is likely to be important for the accumulation of the hydrophobic domain of the NAD(P)H dehydrogenase complex. This complex is associated with PSI and is responsible for the reduction of plastoquinone.

AT5G13130	*MICRORCHIDIA 5 (MORC5)*	Member of the microrchidia protein family which have been described as epigenetic regulators and plant immune mediators, contains a hallmark GHKL-type ATPase domain in N-terminus. Possible role in the development of reproductive tissues.
AT5G13140		Pollen Ole e 1 allergen and extensin family protein
AT5G13150	*EXOCYST SUBUNIT EXO70 FAMILY PROTEIN C1 (EXO70C1)*	A member of EXO70 gene family, putative exocyst subunits, conserved in land plants. Arabidopsis thaliana contains 23 putative EXO70 genes, which can be classified into eight clusters on the phylogenetic tree. This particular member is expressed in pollen and, together with EXO70C2, is involved in pollen tube elongation. Found in the cytoplasm and surprisingly, not found in the plasma membrane.
AT5G13170	*SENESCENCE-ASSOCIATED GENE 29 (SAG29)*	Encodes a member of the SWEET sucrose efflux transporter family proteins.
AT5G13180	*NAC DOMAIN CONTAINING PROTEIN 83 (NAC083)*	Encodes a NAC domain transcription factor that interacts with VND7 and negatively regulates xylem vessel formation.
AT5G13190	*GSH-INDUCED LITAF DOMAIN PROTEIN (GILP)*	Encodes a plasma membrane localized LITAF domain protein that interacts with LSD1 and acts as a negative regulation of hypersensitive cell death.
AT5G13200	*GEM-RELATED 5 (GER5)*	Encodes a protein with unknown function that is involved in hormone mediated regulation of seed germination/dormancy.
AT5G13220	*JASMONATE-ZIM-DOMAIN PROTEIN 10 (JAZ10)*	Plants overexpressing At5g13220.3, but not At5g13220.1 showed enhanced insensitivity to MeJa.
AT5G13230		Tetratricopeptide repeat (TPR)-like superfamily protein
AT5G13260		myosin
AT5G13290	*CORYNE (CRN)*	Encodes a protein with predicted Ser/Thr kinase activity and membrane localization that is involved in the CLV3 signaling pathway that represses WUS expression in the meristem. Loss of function of CRN can suppress the phenotype caused by overexpression of CLV3. SOL2 isolated as a suppressor of root-specific overexpression of CLE19, a clavata3 like gene. sol2 partially suppresses the short root phenotype caused by CLE19 overexpression. Mutant flowers have extra carpels.
AT5G13310		hypothetical protein
AT5G13320	*AVRPPHB SUSCEPTIBLE 3 (PBS3)*	Encodes an enzyme capable of conjugating amino acids to 4-substituted benzoates. 4-HBA (4-hydroxybenzoic acid) and pABA (4-aminobenzoate) may be targets of the enzyme in Arabidopsis, leading to the production of pABA-Glu, 4HBA-Glu, or other related compounds. This enzyme is involved in disease-resistance signaling. It is required for the accumulation of salicylic acid, activation of defense responses, and resistance to Pseudomonas syringae. Salicylic acid can decrease this enzyme's activity in vitro and may act as a competitive inhibitor. Expression of PBS3/GH3.12 can be detected in cotyledons, true leaves, hypocotyls, and occasionally in some parts of roots from 10-day-old seedlings. No expression has been detected in root, stem, rosette or cauline leaves of mature 4- to 5-week-old plants.
AT5G13330	*RELATED TO AP2 6L (Rap2.6L)*	encodes a member of the ERF (ethylene response factor) subfamily B-4 of ERF/AP2 transcription factor family. The protein contains one AP2 domain. There are 7 members in this subfamily.
AT5G13360		Auxin-responsive GH3 family protein
AT5G13380		Auxin-responsive GH3 family protein
AT5G13400		Major facilitator superfamily protein
AT5G13410		FKBP-like peptidyl-prolyl cis-trans isomerase family protein
AT5G13420	*TRANSALDOLASE 2 (TRA2)*	Transaldolase which contributes to reactive oxygen species homeostasis in response to Glc during early seedling growth.
AT5G13430	*RIESKE FES PROTEIN (RISP)*	Rieske FeS protein.Ubiquinol-cytochrome C reductase iron-sulfur subunit
AT5G13470		hypothetical protein
AT5G13490	*ADP/ATP CARRIER 2 (AAC2)*	Encodes mitochondrial ADP/ATP carrier
AT5G13500	*HYDROXYPROLINE O-ARABINOSYLATRANSFERASE 3 (HPAT3)*	Hyp O-arabinosyltransferase-like protein
AT5G13510	*EMBRYO DEFECTIVE 3136 (EMB3136)*	Ribosomal protein L10 family protein
AT5G13530	*KEEP ON GOING (KEG)*	Encodes KEEP ON GOING (KEG), a RING E3 ligase involved in abscisic acid signaling. KEG is essential for Arabidopsis growth and development. ABA promotes KEG degradation via the ubiquitin dependent 26S proteasome pathway. Associates with and ubiquitinates MKK4 and MKK5 to regulate plant immunity.
AT5G13540		
AT5G13550	*SULFATE TRANSPORTER 4.1 (SULTR4;1)*	Encodes a sulfate transporter.
AT5G13580	*ATP-BINDING CASSETTE G6 (ABCG6)*	Belongs to a clade of five Arabidopsis thaliana ABCG half-transporters that are required for synthesis of an effective suberin barrier in roots and seed coats (ABCG2, ABCG6, and ABCG20) and for synthesis of an intact pollen wall (ABCG1 and ABCG16). Phloem-expressed and plasma membrane-localized jasmonate transporter which together with JAT4 and GLR3.3 involved in regulating long-distance translocation of JA, which is important for driving the loading, translocation of JA in the phloem pathway by a self-propagation mode, contributing to wound-induced systemic response/resistance.
AT5G13590		hypothetical protein
AT5G13600		Phototropic-responsive NPH3 family protein
AT5G13610	*RETARDED ROOT GROWTH-LIKE (RRL)*	Encodes a mitochondria-localized protein involved in ABI4-mediated mitochondrial retrograde signalling.
AT5G13620		hypothetical protein
AT5G13630	*GENOMES UNCOUPLED 5 (GUN5)*	Encodes magnesium chelatase involved in plastid-to-nucleus signal transduction.
AT5G13640	*PHOSPHOLIPID:DIACYLGLYCEROL ACYLTRANSFERASE (PDAT)*	arabidopsis phospholipid:diacylglycerol acyltransferase (PDAT)
AT5G13670	*USUALLY MULTIPLE ACIDS MOVE IN AND OUT TRANSPORTERS 15 (UMAMIT15)*	nodulin MtN21-like transporter family protein
AT5G13690	*CYCLOPS 1 (CYL1)*	Encodes an enzyme that is predicted to act as an alpha-N-acetylglucosaminidase (NAGLU). An naglu mutant arrests early in seed development but does not appear to have male or female gametophytic defects. Transcript levels for this gene are increased during reproductive development.
AT5G13700	*POLYAMINE OXIDASE 1 (PAO1)*	Encodes a protein with polyamine oxidase activity. The mRNA of this gene is only expressed in very low amounts in the organs where it was detected (light-grown plants).
AT5G13710	*STEROL METHYLTRANSFERASE 1 (SMT1)*	SMT1 controls the level of cholesterol in plants
AT5G13720		Uncharacterized protein family (UPF0114)

AT5G13730	*SIGMA FACTOR 4 (SIG4)*	Encodes sigma 4 factor, involved in regulating the activity of the plastid-encoded RNA polymerase PEP. Regulates the overall quantity of NDH complexes and thus influences NDH activity.
AT5G13740	*ZINC INDUCED FACILITATOR 1 (ZIF1)*	Encodes ZIF1 (ZINC-INDUCED FACILITATOR1), a member of the Major Facilitator Superfamily (MFS) of membrane proteins which are found in all organisms and transport a wide range of small, organic molecules. Involved in a mechanism of Zn sequestration, possibly by transport of a Zn ligand or Zn-ligand complex into vacuoles. The mRNA is cell-to-cell mobile.
AT5G13750	*ZINC INDUCED FACILITATOR-LIKE 1 (ZIFL1)*	zinc induced facilitator-like 1
AT5G13760		Plasma-membrane choline transporter family protein
AT5G13770		Pentatricopeptide repeat (PPR-like) superfamily protein
AT5G13780	*N-TERMINAL ACETYLTRANSFERASE 10 (NAA10)*	Encodes the catalytic subunit of a N-terminal acetyltransferase.
AT5G13790	*AGAMOUS-LIKE 15 (AGL15)*	AGL15 (AGAMOUS-Like 15) is a member of the MADS domain family of regulatory factors. Although AGL15 is preferentially expressed during embryogenesis, AGL15 is also expressed in leaf primordia, shoot apical meristems and young floral buds, suggesting that AGL15 may play a role during post-germinative development. Transgenic plants that ectopically express AGL15 show delays in the transition to flowering, perianth abscission and senescence and fruit and seed maturation. Role in embryogenesis and gibberellic acid catabolism. Targets B3 domain transcription factors that are key regulators of embryogenesis.AGL15 binds the HAE promoter in floral receptacles and represses HAE expression. AGL15 is phosphorylated in a MKK4/5 dependent manner in floral receptacles. Serines 231 and 257 are phosphorylated in floral receptacles. AGL15 also directly regulates the expression of the peroxidase PRX17, linking it to lignified tissue expression.
AT5G13830	*TRNA METHYLTRANSFERASE 7C (TRM7C)*	FtsJ-like methyltransferase family protein
AT5G13840	*FIZZY-RELATED 3 (FZR3)*	FIZZY-related 3
AT5G13850	*NASCENT POLYPEPTIDE-ASSOCIATED COMPLEX SUBUNIT ALPHA-LIKE PROTEIN 3 (NACA3)*	nascent polypeptide-associated complex subunit alpha-like protein 3
AT5G13870	*XYLOGLUCAN ENDOTRANSGLUCOSYLASE/HYDROLASE 5 (XTH5)*	EXGT-A4, endoxyloglucan transferase,
AT5G13880		cotton fiber protein
AT5G13900	*GLYCOSYLPHOSPHATIDYLINOSITOL-ANCHORED LIPID PROTEIN TRANSFER 30 (LTPG30)*	Bifunctional inhibitor/lipid-transfer protein/seed storage 2S albumin superfamily protein
AT5G13910	*LEAFY PETIOLE (LEP)*	Encodes a member of the ERF (ethylene response factor) subfamily B-1 of ERF/AP2 transcription factor family (LEAFY PETIOLE). The protein contains one AP2 domain. There are 15 members in this subfamily including ATERF-3, ATERF-4, ATERF-7, and LEAFY PETIOLE. Acts as a positive regulator of gibberellic acid-induced germination.
AT5G13930	*TRANSPARENT TESTA 4 (TT4)*	Encodes chalcone synthase (CHS), a key enzyme involved in the biosynthesis of flavonoids. Required for the accumulation of purple anthocyanins in leaves and stems. Also involved in the regulation of auxin transport and the modulation of root gravitropism. The mRNA is cell-to-cell mobile.
AT5G13960	*SU(VAR)3-9 HOMOLOG 4 (SUVH4)*	Encodes a histone 3 lysine 9 specific methyltransferase involved in the maintenance of DNA methylation. SUVH4/KYP is a SU(VAR)3-9 homolog, a SET domain protein. Known SET domain proteins are involved in epigenetic control of gene expression. There are 10 SUVH genes in Arabidopsis and members of this subfamily of the SET proteins have an additional conserved SRA domain. In kyp mutants, there is a loss of CpNpG methylation. The protein was shown to bind to methylated cytosines of CG, CNG and CNN motifs via its SRA domain but has a preference for the latter two. There is also evidence that KYP/SUVH4 might be involved in the telomerase-independent process known as Alternative Lengthening of Telomeres.
AT5G13980		Glycosyl hydrolase family 38 protein
AT5G13990	*EXOCYST SUBUNIT EXO70 FAMILY PROTEIN C2 (EXO70C2)*	A member of EXO70 gene family, putative exocyst subunits, conserved in land plants. Arabidopsis thaliana contains 23 putative EXO70 genes, which can be classified into eight clusters on the phylogenetic tree. This particular member is expressed in pollen and is involved in pollen tube elongation. Found in the cytoplasm and surprisingly, not found in the plasma membrane and is not found to colocalize with or interact with core exocyst subunits.
AT5G14000	*NAC DOMAIN CONTAINING PROTEIN 84 (NAC084)*	NAC domain containing protein 84
AT5G14020		Endosomal targeting BRO1-like domain-containing protein
AT5G14070	*(ROXY2)*	Encodes a member of the CC-type glutaredoxin (ROXY) family that has been shown to interact with the transcription factor TGA2 and suppress ORA59 promoter activity. ROXY2, together with ROXY1 (AT3G02000), controls anther development. roxy1 roxy2 double mutants are sterile and do not produce pollen.
AT5G14080		Tetratricopeptide repeat (TPR)-like superfamily protein
AT5G14090	*LAZY 1 (LAZY1)*	LAZY1 is required for gravitropic response. Mutants have abnormal shoot angles and abnormal root gravitropism. LZY1 affects the redistribution of auxin in response to gravity in shoots and roots via an unknown mechanism.
AT5G14105		hypothetical protein
AT5G14130		Peroxidase superfamily protein
AT5G14150	*(ATHD-1)*	Emb:.1 protein, putative (Protein of unknown function, DUF642)
AT5G14180	*MYZUS PERSICAE-INDUCED LIPASE 1 (MPL1)*	Myzus persicae-induced lipase 1
AT5G14190		
AT5G14210		Leucine-rich repeat protein kinase family protein
AT5G14230		ankyrin
AT5G14260	*SAFEGUARD1 (SAFE1)*	Suppresses singlet oxygen-induced stress responses by protecting grana margins.
AT5G14300	*PROHIBITIN 5 (PHB5)*	prohibitin 5
AT5G14310	*CARBOXYESTERASE 16 (CXE16)*	carboxyesterase 16
AT5G14320	*EMBRYO DEFECTIVE 3137 (EMB3137)*	Ribosomal protein S13/S18 family
AT5G14330		transmembrane protein
AT5G14340	*MYB DOMAIN PROTEIN 40 (MYB40)*	Member of the R2R3 factor gene family. Central regulator in arsenic resistance.
AT5G14370		CCT motif family protein
AT5G14380	*ARABINOGALACTAN PROTEIN 6 (AGP6)*	Encodes an arabinogalactan protein that is expressed in pollen, pollen sac and pollen tube. Loss of AGP6 function results in decreased fertility due to defects in pollen tube growth.

AT5G14390		alpha/beta-Hydrolases superfamily protein
AT5G14400	*CYTOCHROME P450, FAMILY 724, SUBFAMILY A, POLYPEPTIDE 1 (CYP724A1)*	Encodes a brassinosteroid C-22 hydroxylase.
AT5G14410		hypothetical protein
AT5G14440		Surfeit locus protein 2 (SURF2)
AT5G14450		GDSL-motif esterase/acyltransferase/lipase. Enzyme group with broad substrate specificity that may catalyze acyltransfer or hydrolase reactions with lipid and non-lipid substrates.
AT5G14500		aldose 1-epimerase family protein
AT5G14510	*(VPNB1)*	Armadillo (ARM) repeat containing protein involved in vascular development.
AT5G14520	*PESCADILLO (PES)*	Encodes a nucleolar protein that plays an essential role in cell growth and survival through its regulation of ribosome biogenesis and mitotic progression.
AT5G14530	*ANTHESIS PROMOTING FACTOR 1 (APRF1)*	Encodes a low molecular weight nuclear WDR protein which displays functional homology to the Swd2 protein, an essential subunit of the yeast histone methylation COMPASS complex. APRF1 acts upstream of FLC and promotes flowering under long day conditions.
AT5G14550		Core-2/I-branching beta-1,6-N-acetylglucosaminyltransferase family protein
AT5G14560		hypothetical protein
AT5G14570	*HIGH AFFINITY NITRATE TRANSPORTER 2.7 (NRT2.7)*	Encodes ATNRT2.7, a nitrate transporter that controls nitrate content in seeds. Expression is detected in reproductive organs and peaks in seeds. Localized to the vacuolar membrane.
AT5G14580		polyribonucleotide nucleotidyltransferase
AT5G14590		Isocitrate/isopropylmalate dehydrogenase family protein
AT5G14600	*TRNA METHYLTRANSFERASE 61 (TRM61)*	Part of complex with TRM6, plays a critical role in maintaining the stability of initiator methionyl-tRNA, embryo and endosperm development. In the complex TRM61 functions as the catalytic subunit, and TRM6 performs the role of the binding subunit
AT5G14610	*RNA HELICASE 46 (RH46)*	DEAD box RNA helicase family protein
AT5G14620	*DOMAINS REARRANGED METHYLTRANSFERASE 2 (DRM2)*	A putative DNA methyltransferase with rearranged catalytic domains; similar to mammalian DNMT3 methyltransferases; contains UBA domains. The 3'-end proximal part of the gene coding region is highly methylated at both adenine and cytosine residues.
AT5G14630		
AT5G14640	*SHAGGY-LIKE KINASE 13 (SK13)*	GSK3-like kinase, subgroup II.
AT5G14660	*PEPTIDE DEFORMYLASE 1B (PDF1B)*	Encodes a peptide deformylase PDF1B. The crystal structure has been determined at a resolution of 0.24 nm (Biochem J, 2008, vol 413:417-427).
AT5G14680		Adenine nucleotide alpha hydrolases-like superfamily protein
AT5G14690		transmembrane protein
AT5G14700		NAD(P)-binding Rossmann-fold superfamily protein
AT5G14720	*TARGET OF TEMPERATURE3 (TOT3)*	Membrane-localized protein kinase which regulates thermomorphogenesis.
AT5G14730		Unknown protein, expression induced by IDL7 and stress.
AT5G14740	*CARBONIC ANHYDRASE 2 (CA2)*	Encodes a beta carbonic anhydrase likely to be localized in the cytoplasm. Expression of its mRNA is seen in etiolated seedlings and points to a possible nonphotosynthetic role for this isoform.
AT5G14750	*MYB DOMAIN PROTEIN 66 (MYB66)*	Encodes a MyB-related protein containing R2 and R3 repeats, involved in root and hypocotyl epidermal cell fate determination. Loss of function mutations make extra root hairs. Nuclear localized protein is a positive regulator for expression of CAPRICE (CPC).
AT5G14760	*L-ASPARTATE OXIDASE (AO)*	At5g14760 encodes for L-aspartate oxidase involved in the early steps of NAD biosynthesis. In contrary to the EC 1.4.3.16 (l-aspartate oxidase - deaminating) the enzyme catalyzes the reaction L-aspartate + O2 = iminoaspartate (alpha-iminosuccinate) + H2O2. Flavoenzyme-encoding gene.
AT5G14770		PPR repeat protein
AT5G14780	*FORMATE DEHYDROGENASE (FDH)*	Encodes a NAD-dependent formate dehydrogenase.
AT5G14800	*PYRROLINE-5- CARBOXYLATE (P5C) REDUCTASE (P5CR)*	Delta 1-pyrroline-5-carboxylate reductase, catalyzes the final step in proline biosynthesis from glutamate and ornithine.In situ hybridization indicated that under normal growth conditions, the highest concentration of P5CR transcripts occurs in the cortical parenchyma, phloem, vascular cambium and pith parenchyma in the vicinity of the protoxylem. Single gene in Arabidopsis.
AT5G14830		transposable_element_gene;retrotransposon family;(source:TAIR10)
AT5G14840		pseudogene of hypothetical protein
AT5G14860		UDP-Glycosyltransferase superfamily protein
AT5G14870	*CYCLIC NUCLEOTIDE-GATED CHANNEL 18 (CNGC18)*	Encodes a member of the cyclic nucleotide gated channel family that is asymmetrically localized to the plasma membrane at the growing tip of the pollen tube and is involved in pollen tube growth and pollen tube guidance to ovules. It likely directly transduces a cNMP signal into an ion flux that can produce a localized signal capable of regulating the pollen tip-growth machinery. Also functions as a Ca2+ permeable channel.
AT5G14880	*POTASSIUM UPTAKE 8 (KUP8)*	Potassium transporter family protein
AT5G14890		potassium transporter
AT5G14900		helicase associated (HA2) domain-containing protein
AT5G14910		Heavy metal transport/detoxification superfamily protein
AT5G14920	*A-STIMULATED IN ARABIDOPSIS 14 (GASA14)*	Encodes a GASA domain containing protein that regulates increases in plant growth through GA-induced and DELLA-dependent signal transduction and that can increase abiotic stress resistance by reducing ROS accumulation.
AT5G14930	*SENESCENCE-ASSOCIATED GENE 101 (SAG101)*	encodes an acyl hydrolase involved in senescence .
AT5G14940		Major facilitator superfamily protein
AT5G14960	*DP-E2F-LIKE 2 (DEL2)*	DP-E2F-like 2
AT5G14970		seed maturation-like protein
AT5G14980	*(MAGL14)*	alpha/beta-Hydrolases superfamily protein
AT5G14990		WPP domain associated protein

AT5G15000		Encodes a ECA1 gametogenesis related family protein
AT5G15010		Tetratricopeptide repeat (TPR)-like superfamily protein
AT5G15060		Lateral organ boundaries (LOB) domain family protein
AT5G15080	*PBS1-LIKE 34 (PBL34)*	Protein kinase superfamily protein
AT5G15090	*VOLTAGE DEPENDENT ANION CHANNEL 3 (VDAC3)*	Encodes a voltage-dependent anion channel (VDAC: AT3G01280/VDAC1, AT5G67500/VDAC2, AT5G15090/VDAC3, AT5G57490/VDAC4, AT5G15090/VDAC5). VDACs are reported to be porin-type, beta-barrel diffusion pores. They are prominently localized in the outer mitochondrial membrane and are involved in metabolite exchange between the organelle and the cytosol. Purified VDAC3 is shown to have voltage-dependent anion channel activity.
AT5G15100	*PIN-FORMED 8 (PIN8)*	Encodes an auxin transporter with a strong expression in a male gametophyte. Mutant studies reveal a role for auxin transport in regulating pollen development and function. It acts together with PIN5.
AT5G15110		Pectate lyase family protein
AT5G15130	*WRKY DNA-BINDING PROTEIN 72 (WRKY72)*	member of WRKY Transcription Factor; Group II-b; contribute to basal immunity. The mRNA is cell-to-cell mobile.
AT5G15140	*PATERNALLY EXPRESSED GENE 9 (PEG9)*	Galactose mutarotase-like superfamily protein
AT5G15150	*HOMEOBOX 3 (HB-3)*	homeobox-containing gene with an unusual feature: a leucine zipper motif adjacent to the carboxyl-terminal of the homeodomain structure. This gene is expressed primarily in the cortex of the root and the stem.
AT5G15160	*BANQUO 2 (BNQ2)*	BNQ2 belongs to a family of atypical non-DNA binding basic helix-loop-helix (bHLH) proteins that heterodimerize with and negatively regulate bHLH transcription factors. Directly and negatively regulated by AP3 and PI in petals.Required for appropriate regulation of flowering time.
AT5G15180	*PEROXIDASE 56 (PRX56)*	Peroxidase superfamily protein
AT5G15190		hypothetical protein
AT5G15210	*HOMEOBOX PROTEIN 30 (HB30)*	Encodes ZFHD3, a member of the zinc finger homeodomain transcriptional factor family.
AT5G15230	*GAST1 PROTEIN HOMOLOG 4 (GASA4)*	Encodes gibberellin-regulated protein GASA4. Promotes GA responses and exhibits redox activity.
AT5G15240		Transmembrane amino acid transporter family protein
AT5G15250	*FTSH PROTEASE 6 (FTSH6)*	Encodes an FtsH protease that is localized to the chloroplast. AtFtsH6 is involved in the degradation of both Lhcb3 and Lhcb1 during senescence and high-light acclimation.
AT5G15270		RNA-binding KH domain-containing protein
AT5G15290	*CASPARIAN STRIP MEMBRANE DOMAIN PROTEIN 5 (CASP5)*	Uncharacterized protein family (UPF0497)
AT5G15300		Pentatricopeptide repeat (PPR) superfamily protein
AT5G15310	*MYB DOMAIN PROTEIN 16 (MYB16)*	Member of the R2R3 factor gene family; MIXTA-like transcription factor that controls trichome maturation and cuticle formation.
AT5G15330	*SPX DOMAIN GENE 4 (SPX4)*	Encodes a protein with a single SYG1/Pho81/XPR1 (SPX) domain that localizes to both the cytosol and nucleus, acting as a dose-dependent modulator of PHR1-dependent and PHR1-independent phosphate-starvation responses in shoots. SPX4 prevents translocation of PHR1 (AT4G28610) to the nucleus in a dose- and Pi-dependent manner. In contrast to SPX1, SPX4 modulates rather than suppresses phosphate-starvation response (PSR) gene expression.
AT5G15340		Pentatricopeptide repeat (PPR) superfamily protein
AT5G15360		transmembrane protein
AT5G15390		tRNA/rRNA methyltransferase (SpoU) family protein
AT5G15400	*MUTANT, SNC1 -ENHANCING 3 (MUSE3)*	Single copy gene encoding a putative ubiquitin conjugating E4 factor. Contains Ub elongating factor core domain and C-terminal U-box. Involved in ubiquitination of NLRs.
AT5G15410	*DEFENSE NO DEATH 1 (DND1)*	'defense, no death' gene (DND1) encodes a mutated cyclic nucleotide-gated cation channel; Same as CNGC2 (article ID 229): Cyclic nucleotide gated channel, activated by cAMP, conducts K+ and other monovalent cations but excludes Na+, does not contain the GYG amino acid sequence found in other channels with this conductivity profile. Conducts Ca2+ into cells which is linked to the generation of NO and the NO signaling pathway involved in the innate immune response to pathogens. CNGC2 could be the key step mediating bulk Ca2+ influx into leaf cells after unloading from the vascular and have no direct roles in the leaf development and HR.
AT5G15420		hypothetical protein
AT5G15440	*EID1-LIKE 1 (EDL1)*	EID1-like 1
AT5G15480	*MALE FERTILITY-ASSOCIATED ZINC FINGER PROTEIN 1 (MAZ1)*	Cys2/His2 zinc finger protein involved in pollen wall development.
AT5G15490	*UDP-GLUCOSE DEHYDROGENASE 3 (UGD3)*	Encodes one of four UDP-glucose dehydrogenase UGD) genes. Mutation of this gene in combination with UGD2 leads to swollen plant cell walls and severe developmental defects associated with changes in pectic polysaccharides.
AT5G15510	*(TPXL5)*	TPX2 (targeting protein for Xklp2) protein family
AT5G15530	*BIOTIN CARBOXYL CARRIER PROTEIN 2 (BCCP2)*	biotin carboxyl carrier protein isoform 2 (BCCP2) mRNA,
AT5G15550	*ARABIDOPSIS THALIANA PESCADILLO ORTHOLOG (ATPEP2)*	Transducin/WD40 repeat-like superfamily protein
AT5G15560		hypothetical protein
AT5G15570	*(TAF3)*	Bromodomain transcription factor
AT5G15580	*LONGIFOLIA1 (LNG1)*	Encodes LONGIFOLIA1 (LNG1). Regulates leaf morphology by promoting cell expansion in the leaf-length direction. The LNG1 homologue LNG2 (At3g02170) has similar function.
AT5G15590		
AT5G15600	*SPIRAL1-LIKE4 (SP1L4)*	SPIRAL1-LIKE4 belongs to a six-member gene family in Arabidopsis; all members share high sequence similarity in amino- and carboxy-terminal regions. Regulates cortical microtubule organization. Mutant plants exhibit altered patterns of root, leaf and petal growth as a result of defective anisotropic cell expansion.
AT5G15630	*IRREGULAR XYLEM 6 (IRX6)*	Encodes a member of the COBRA family, similar to phytochelatin synthetase. Involved in secondary cell wall biosynthesis. Mutants make smaller plants with reduced levels of cellulose and cell wall sugars.

AT5G15700		Nucleus encoded plastid RNA polymerase. Localized in mitochondria and chloroplast.
AT5G15710		Galactose oxidase/kelch repeat superfamily protein
AT5G15720	*GDSL-MOTIF LIPASE 7 (GLIP7)*	Contains lipase signature motif and GDSL domain.
AT5G15725	*GOLVEN 9 (GLV9)*	GOLVEN (GLV)/ROOT GROWTH FACTORS/CLE-Like small signaling peptide. Expressed in roots. Overexpression results in altered root gravitropism.
AT5G15730	*CALCIUM/CALMODULIN-REGULATED RECEPTOR-LIKE KINASE 2 (CRLK2)*	Protein kinase superfamily protein
AT5G15740	*RG-I RHAMNOSYLTRANSFERASE 1 (RRT1)*	RRT1 is a member of a novel glycosyltransferase famly in plants. It functions as a rhamnosyltransferase, elongating the RG-1 backbone. It functions during seed coat mucilage development.
AT5G15750		Alpha-L RNA-binding motif/Ribosomal protein S4 family protein
AT5G15760	*PLASTID-SPECIﬁC RIBOSOMAL PROTEIN 3/2 (PSRP3/2)*	Ribosomal protein PSRP-3/Ycf65
AT5G15770	*GLUCOSAMINE-6-PHOSPHATE N-ACETYLTRANSFERASE 1 (GNA1)*	Encodes a putative glucose-6-phosphate acetyltransferase that is likely involved in UDP-N-acetylglucosamine biosynthesis. A GFP:GNA1 fusion protein localizes to the endoplasmic reticulum.
AT5G15780		Pollen Ole e 1 allergen and extensin family protein
AT5G15790		RING/U-box superfamily protein
AT5G15800	*SEPALLATA1 (SEP1)*	Encodes a MADS box transcription factor involved flower and ovule development. Functionally redundant with SEP2 and SEP3.
AT5G15810	*TRNA METHYLTRANSFERASE 1B (TRM1B)*	Involved in posttranscriptional modification of tRNA.
AT5G15830	*BASIC LEUCINE-ZIPPER 3 (bZIP3)*	basic leucine-zipper 3
AT5G15840	*CONSTANS (CO)*	Encodes a protein showing similarities to zinc finger transcription factors, involved in regulation of flowering under long days. Acts upstream of FT and SOC1.
AT5G15850	*CONSTANS-LIKE 1 (COL1)*	Homologous to the flowering-time gene CONSTANS.
AT5G15890	*TRICHOME BIREFRINGENCE-LIKE 21 (TBL21)*	Encodes a member of the TBL (TRICHOME BIREFRINGENCE-LIKE) gene family containing a plant-specific DUF231 (domain of unknown function) domain. TBL gene family has 46 members, two of which (TBR/AT5G06700 and TBL3/AT5G01360) have been shown to be involved in the synthesis and deposition of secondary wall cellulose, presumably by influencing the esterification state of pectic polymers. A nomenclature for this gene family has been proposed (Volker Bischoff & Wolf Scheible, 2010, personal communication).
AT5G15900	*TRICHOME BIREFRINGENCE-LIKE 19 (TBL19)*	Encodes a member of the TBL (TRICHOME BIREFRINGENCE-LIKE) gene family containing a plant-specific DUF231 (domain of unknown function) domain. TBL gene family has 46 members, two of which (TBR/AT5G06700 and TBL3/AT5G01360) have been shown to be involved in the synthesis and deposition of secondary wall cellulose, presumably by influencing the esterification state of pectic polymers. A nomenclature for this gene family has been proposed (Volker Bischoff & Wolf Scheible, 2010, personal communication).
AT5G15910		NAD(P)-binding Rossmann-fold superfamily protein
AT5G15940		NAD(P)-binding Rossmann-fold superfamily protein
AT5G15950	*S-ADENOSYLMETHIONINE DECARBOXYLASE 2 (SAMDC2)*	Adenosylmethionine decarboxylase family protein
AT5G15960	*(KIN1)*	cold and ABA inducible protein kin1, possibly functions as an anti-freeze protein. Transcript level of this gene is induced by cold, ABA, dehydration and osmoticum (mannitol). However, protein activity of GUS fused to the promoter of this gene is inhibited by cold treatment, suggesting an inhibition of the protein by increased transcript level.
AT5G15980	*RIBOSOMAL PENTATRICOPEPTIDE REPEAT PROTEIN 8 (RPPR8)*	Ribosomal pentatricopeptide repeat protein
AT5G16010		3-oxo-5-alpha-steroid 4-dehydrogenase family protein
AT5G16030		mental retardation GTPase activating protein
AT5G16060		Cytochrome c oxidase biogenesis protein Cmc1-like protein
AT5G16070	*CHAPERONIN CONTAINING T-COMPLEX POLYPEPTIDE-1 SUBUNIT 6-1 (CCT6-1)*	TCP-1/cpn60 chaperonin family protein
AT5G16080	*CARBOXYESTERASE 17 (CXE17)*	carboxyesterase 17
AT5G16100		RWP-RK domain protein
AT5G16110		hypothetical protein
AT5G16150	*PLASTIDIC GLC TRANSLOCATOR (PGLCT)*	Encodes a putative plastidic glucose transporter.
AT5G16160		hypothetical protein
AT5G16180	*ORTHOLOG OF MAIZE CHLOROPLAST SPLICING FACTOR CRS1 (CRS1)*	Promotes the splicing of chloroplast group II introns. Splices atpF introns.
AT5G16190	*CELLULOSE SYNTHASE LIKE A11 (CSLA11)*	encodes a gene similar to cellulose synthase
AT5G16220		Octicosapeptide/Phox/Bem1p family protein
AT5G16230	*ACYL-ACYL CARRIER PROTEIN DESATURASE3 (AAD3)*	Encodes one of two ∆9 palmitoyl-ACP desaturases responsible for the biosynthesis of ω-7 fatty acids in the maturing endosperm.
AT5G16240	*ACYL-ACYL CARRIER PROTEIN DESATURASE1 (AAD1)*	Redundant Δ9 stearoyl-ACP desaturase gene which together with FAB2 and AAD5 during embryo development provide precursors for the elaboration of embryo cuticle and therefore plays a specific role during the phase of invasive embryo growth through the endosperm. Together with FAB2, AAD5, and AAD6 redundantly participates in oil storage during the maturation phase.
AT5G16250		transmembrane protein
AT5G16270	*SISTER CHROMATID COHESION 1 PROTEIN 4 (SYN4)*	Encodes a SCC1/REC8 ortholog that may be involved in mitosis and may represent a mitotic cohesin. Plays a role in somatic DNA double strand break damage repair. The mRNA is cell-to-cell mobile.
AT5G16340		AMP-dependent synthetase and ligase family protein
AT5G16390	*CHLOROPLASTIC ACETYLCOENZYME A CARBOXYLASE 1 (CAC1)*	Encodes for the biotin carboxyl-carrier subunit of the multi-enzyme plastidial acetyl-coenzyme A carboxylase complex.
AT5G16400	*THIOREDOXIN F2 (TRXF2)*	Encodes an f-type thioredoxin (Trx-f2) localized in chloroplast stroma.
AT5G16420		Pentatricopeptide repeat (PPR-like) superfamily protein
AT5G16430		Cysteine/Histidine-rich C1 domain family protein

AT5G16440	*ISOPENTENYL DIPHOSPHATE ISOMERASE 1 (IPP1)*	Encodes a protein with isopentenyl diphosphate:dimethylallyl diphosphate isomerase activity. There is genetic evidence that it functions in the mevalonate, but not the MEP biosynthetic pathway.
AT5G16450		Ribonuclease E inhibitor RraA/Dimethylmenaquinone methyltransferase
AT5G16460	*SEIPIN1 (SEIPIN1)*	Membrane protein involved in lipid droplet biogenesis primarily in embryos.
AT5G16480	*PLANT AND FUNGI ATYPICAL DUAL-SPECIﬁCITY PHOSPHATASE 5 (PFA-DSP5)*	Encodes an atypical dual-speciﬁcity phosphatase.
AT5G16490	*ROP-INTERACTIVE CRIB MOTIF-CONTAINING PROTEIN 4 (RIC4)*	encodes a member of a novel protein family that contains contain a CRIB (for Cdc42/Rac-interactive binding) motif required for their specific interaction with GTP-bound Rop1 (plant-specific Rho GTPase). It interacts with Rop1 and is involved in pollen tube growth and function, and exocytosis in the pollen tube tip. Protein most similar to RIC2 (family subgroup V). Gene is expressed in all tissues examined.Interacts with ROP2 during pavement cell morphogenesis and with ROP1 to promote apical F-actin assembly.
AT5G16500	*LOST IN POLLEN TUBE GUIDANCE 1 (LIP1)*	Encodes a receptor-like cytoplasmic kinase localized in the membrane of pollen tube tip regions that controls micropylar pollen tube guidance in Arabidopsis.
AT5G16505	*MUSTANG 4 (MUG4)*	Encodes a member of a domesticated transposable element gene family MUSTANG. Members of this family are derived from transposable elements genes but gained function in plant fitness and flower development. Known members include: AT3G04605 (MUG1), AT2G30640 (MUG2), AT1G06740 (MUG3), AT5G16505 (MUG4), AT3G06940 (MUG5), AT5G48965 (MUG6), AT3G05850 (MUG7) and AT5G34853 (MUG8).
AT5G16530	*PIN-FORMED 5 (PIN5)*	Encodes PIN5, an atypical member of the PIN family. PIN5 is a functional auxin transporter that is required for auxin-mediated development. PIN5 does not have a direct role in cell-to-cell transport but regulates intracellular auxin homeostasis and metabolism. PIN5 localizes, unlike other characterized plasma membrane PIN proteins, to endoplasmic reticulum (ER), presumably mediating auxin flow from the cytosol to the lumen of the ER. It acts together with PIN8 in affecting pollen development and auxin homeostasis.
AT5G16540	*ZINC FINGER NUCLEASE 3 (ZFN3)*	Encodes a zinc finger protein.
AT5G16560	*KANADI (KAN)*	Encodes a KANADI protein (KAN) that regulates organ polarity in Arabidopsis. KAN is required for abaxial identity in both leaves and carpels, and encodes a nuclear-localized protein in the GARP family of putative transcription factors. Together with KAN2, this gene appears to be involved in the development of the carpel and the outer integument of the ovule.Along with KAN2 and KAN4, KAN1 appears to be required for proper regulation of PIN1 in early embryogenesis.
AT5G16570	*GLUTAMINE SYNTHETASE 1;4 (GLN1;4)*	Encodes a cytosolic glutamine synthetase, the enzyme has high affinity for the substrate ammonium.
AT5G16580	*BETA GLUCOSIDASE 2 (BGLU2)*	beta glucosidase 2
AT5G16590	*LEUCINE RICH REPEAT PROTEIN 1 (LRR1)*	Leucine-rich repeat protein kinase family protein
AT5G16600	*MYB DOMAIN PROTEIN 43 (MYB43)*	Encodes a transcriptional regulator that directly activates lignin biosynthesis genes and phenylalanine biosynthesis genes during secondary wall formation.
AT5G16660		Low-density receptor-like protein
AT5G16690	*ORIGIN RECOGNITION COMPLEX SUBUNIT 3 (ORC3)*	Origin Recognition Complex subunit 3. Involved in the initiation of DNA replication. Regulated transcriptionally during cell cycle, peaking at G1/S-phase. Target of E2F/DF family of transcription factors. Interacts with all ORC subunits except ORC1b.
AT5G16710	*DEHYDROASCORBATE REDUCTASE 1 (DHAR3)*	DHAR3 protein undergoes thiolation following treatment with the oxidant tert-butylhydroperoxide.Encodes 30-40% of extractable leaf GSH-dependent DHAR activity. Single knockout mutants show unaltered ascorbate and glutathione status in optimal and oxidative stress conditions.Makes a minor contribution to glutathione oxidation in response to increased intracellular hydrogen peroxide (catalase deficiency) (PMID:28381499).
AT5G16720	*MYOSIN BINDING PROTEIN 3 (MYOB3)*	caldesmon-like protein (Protein of unknown function, DUF593)
AT5G16730		Encodes a microtubule-associated protein. The mRNA is cell-to-cell mobile.
AT5G16750	*TORMOZEMBRYO DEFECTIVE (TOZ)*	Encodes a nucleolar localized WD-40 repeat protein that is preferentially expressed in dividing cells and is required for regulated division planes and embryo development.
AT5G16770	*MYB DOMAIN PROTEIN 9 (MYB9)*	Member of the R2R3 factor gene family.
AT5G16790	*(THO7)*	Encodes a component of the putative Arabidopsis THO/TREX complex: THO1 or HPR1 (At5g09860), THO2 (At1g24706), THO3 or TEX1 (At5g56130), THO5 (At5g42920, At1g45233), THO6 (At2g19430), and THO7 (At5g16790, At3g02950). THO/TREX complexes in animals have been implicated in the transport of mRNA precursors. Mutants of THO3/TEX1, THO1, THO6 accumulate reduced amount of small interfering (si)RNA, suggesting a role of the putative Arabidopsis THO/TREX in siRNA biosynthesis.
AT5G16810		Protein kinase superfamily protein
AT5G16840	*BINDING PARTNER OF ACD11 1 (BPA1)*	Binds to ACD11 and fungal elicitor RxLR207. Regulates ROS mediated defense response.
AT5G16850	*TELOMERASE REVERSE TRANSCRIPTASE (TERT)*	Encodes the catalytic subunit of telomerase reverse transcriptase. Involved in telomere homeostasis. Homozygous double mutants with ATR show gross morphological defects over a period of generations. TERT shows Class II telomerase activity in vitro, indicating that it can initiate de novo telomerase synthesis on non-telomeric DNA, often using a preferred position within the telomerase-bound RNA. Loss of function mutants have reduced telomere length in roots and over a period of generations, decreasing root meristem function.
AT5G16860		Tetratricopeptide repeat (TPR)-like superfamily protein
AT5G16880	*TOM1-LIKE 1 (TOL1)*	Encodes a member of the Arabidopsis TOL (TOM1-LIKE) family of ubiquitin binding proteins that acts redundantly in the recognition and further endocytic sorting of a PIN-FORMED (PIN)-type auxin carrier protein at the plasma membrane, modulating dynamic auxin distribution and associated growth responses.
AT5G16920		Fasciclin-like arabinogalactan family protein
AT5G16930	*SHOT1 BINDING ATPASE 2 (SBA2)*	Homologue of animal ATPase Family AAA Domain-Containing Protein 3 (ATAD3), which is involved in mitochondrial nucleoid organization; interacts with SHOT1.
AT5G16940		carbon-sulfur lyase
AT5G16960		Zinc-binding dehydrogenase family protein
AT5G16970	*ALKENAL REDUCTASE (AER)*	encodes a 2-alkenal reductase (EC 1.3.1.74), plays a key role in the detoxification of reactive carbonyls
AT5G16980		Zinc-binding dehydrogenase family protein

AT5G16990		molecular function has not been defined, was shown involved in oxidative stress tolerance. The mRNA is cell-to-cell mobile.
AT5G17000		Zinc-binding dehydrogenase family protein
AT5G17010		Major facilitator superfamily protein
AT5G17020	*EXPORTIN 1A (XPO1A)*	Encodes a member of the exportin protein family (XPO1A) which functions as receptors for nuclear export. Binds to a variety of proteins having leucine rich export signals.Along with XPO1B involved with development of the male and female gametophytes. Sensitive to heat and oxidative stress.
AT5G17030	*UDP-GLUCOSYL TRANSFERASE 78D3 (UGT78D3)*	UDP-glucosyl transferase 78D3
AT5G17050	*UDP-GLUCOSYL TRANSFERASE 78D2 (UGT78D2)*	The At5g17050 encodes a anthocyanidin 3-O-glucosyltransferase which specifically glucosylates the 3-position of the flavonoid C-ring. Anthocyanidins such as cyanidin and pelargonidin as well as flavonols such as kaempferol and quercetin are accepted substrates.
AT5G17080		Cysteine proteinases superfamily protein
AT5G17100		Cystatin/monellin superfamily protein
AT5G17130		cysteine-type peptidase
AT5G17140		Cysteine proteinases superfamily protein
AT5G17150		Cystatin/monellin superfamily protein
AT5G17160		aspartic/glutamic acid-rich protein
AT5G17170	*ENHANCER OF SOS3-1 (ENH1)*	rubredoxin family protein
AT5G17180		hypothetical protein
AT5G17200		Pectin lyase-like superfamily protein
AT5G17220	*GLUTATHIONE S-TRANSFERASE PHI 12 (GSTF12)*	Encodes glutathione transferase belonging to the phi class of GSTs. Naming convention according to Wagner et al. (2002). Mutants display no pigments on leaves and stems. Likely to function as a carrier to transport anthocyanin from the cytosol to tonoplasts.
AT5G17230	*PHYTOENE SYNTHASE (PSY)*	Encodes phytoene synthase that is the rate-limiting enzyme in the carotenoid biosynthetic pathway and that interacts with the ORANGE (OR) protein. PSY's expression is posttransciptionally regulated by OR.
AT5G17260	*NAC DOMAIN CONTAINING PROTEIN 86 (NAC086)*	NAC domain containing protein 86
AT5G17270		Protein prenylyltransferase superfamily protein
AT5G17280		oxidoreductase-like protein, amino-terminal protein
AT5G17300	*REVEILLE 1 (RVE1)*	Myb-like transcription factor that regulates hypocotyl growth by regulating free auxin levels in a time-of-day specific manner.
AT5G17310	*UDP-GLUCOSE PYROPHOSPHORYLASE 2 (UGP2)*	UDP-glucose pyrophosphorylase 2
AT5G17320	*HOMEODOMAIN GLABROUS 9 (HDG9)*	Encodes a homeobox-leucine zipper family protein belonging to the HD-ZIP IV family.
AT5G17330	*GLUTAMATE DECARBOXYLASE (GAD)*	Encodes one of two isoforms of glutamate decarboxylase. The mRNA is cell-to-cell mobile.
AT5G17340		Putative membrane lipoprotein
AT5G17350		PADRE protein up-regulated after infection by S. sclerotiorum.
AT5G17360		DNA ligase
AT5G17380		Thiamine pyrophosphate dependent pyruvate decarboxylase family protein
AT5G17410		Spc97 / Spc98 family of spindle pole body (SBP) component
AT5G17420	*IRX3 is required for secondary cell wall biosynthesis.*	Encodes a xylem-specific cellulose synthase that is phosphorylated on one or more serine residues (on either S185 or one of S180 or S181).
AT5G17440	*LETHAL UNLESS CBC 7 B (LUC7B)*	LUC7 related protein
AT5G17450	*HEAVY METAL ASSOCIATED ISOPRENYLATED PLANT PROTEIN 21 (HIPP21)*	Heavy metal transport/detoxification superfamily protein
AT5G17470		EF hand calcium-binding protein family
AT5G17480	*POLLEN CALCIUM-BINDING PROTEIN 1 (PC1)*	pollen calcium-binding protein 1
AT5G17490	*RGA-LIKE PROTEIN 3 (RGL3)*	Encodes a DELLA subfamily member that acts as a negative regulator of GA signaling and as a coactivator of ABI3 to promote seed storage protein biosynthesis during the seed maturation stage.
AT5G17540		HXXXD-type acyl-transferase family protein
AT5G17570		TatD related DNase
AT5G17580		Phototropic-responsive NPH3 family protein
AT5G17600	*ARABIDOPSIS T??XICOS EN LEVADURA 52 (ATL52)*	RING/U-box superfamily protein
AT5G17640	*ABIOTIC STRESS GENE 1 (ASG1)*	Expression of this gene is induced by abscisic acid and salt stress.
AT5G17650	*GLYCINE AND PROLINE RICH PROTEIN 4 (GPRP4)*	glycine/proline-rich protein
AT5G17660	*TRNA METHYLTRANSFERASE 8B (TRM8B)*	tRNA (guanine-N-7) methyltransferase
AT5G17670		alpha/beta-Hydrolases superfamily protein
AT5G17680		disease resistance protein (TIR-NBS-LRR class)
AT5G17700		MATE efflux family protein
AT5G17720		alpha/beta-Hydrolases superfamily protein
AT5G17730		P-loop containing nucleoside triphosphate hydrolases superfamily protein
AT5G17750		P-loop containing nucleoside triphosphate hydrolases superfamily protein
AT5G17760		P-loop containing nucleoside triphosphate hydrolases superfamily protein
AT5G17770	*NADH:CYTOCHROME B5 REDUCTASE 1 (CBR)*	Encodes NADH:cytochrome (Cyt) b5 reductase that displayed strict specificity to NADH for the reduction of a recombinant Cyt b5 (AtB5-A), whereas no Cyt b5 reduction was observed when NADPH was used as the electron donor.
AT5G17780		alpha/beta-Hydrolases superfamily protein
AT5G17800	*MYB DOMAIN PROTEIN 56 (MYB56)*	Member of the R2R3 factor gene family that acts as a cell-specific repressor of quiescent center (QC) divisions in the primary root, acting through the BR signaling pathway. Works with BES1 to regulate QC division in the root.

AT5G17820	*PEROXIDASE 57 (PER57)*	Peroxidase superfamily protein, overexpression increases ROS.
AT5G17830		Plasma-membrane choline transporter family protein
AT5G17840		DnaJ/Hsp40 cysteine-rich domain superfamily protein
AT5G17850	*CATION/CA2+EXCHANGER2 (CCX2)*	CCX2 is a putative cation/Ca2+ exchange protein. It is located in the endoplasmic reticulum. It plays a role in salt induced calcium signaling. Loss of function results in decreased cytosolic and increased ER Ca2+ concentrations.
AT5G17860	*CALCIUM EXCHANGER 7 (CAX7)*	Cation/Ca2+ exchanger family member. Double mutants with CCX4 show delayed greening and defects in ROS response.
AT5G17870	*PLASTID-SPECIFIC 50S RIBOSOMAL PROTEIN 6 (PSRP6)*	plastid-specific ribosomal protein 6 precursor (Psrp-6) - like
AT5G17880	*CONSTITUTIVE SHADE-AVOIDANCE1 (CSA1)*	Encodes a TIR-NBS-LRR protein CSA1 that functions in photomorphogenic development. csa1 mutants display a constitutive shade-avoidance (CSA) phenotype (long stem) under high red:far-red rations (i.e. in the absence of a shade signal). csa1 mutation can be complemented by RPS4, a TIR-NBS-LRR protein that confers resistance against bacterium Pseudomonas syringae.
AT5G17890	*DA1-RELATED PROTEIN 4 (DAR4)*	Encodes a protein that appears to be involved in defense responses. Contains TIR, NB-LRR and LIM domains. A gain of function allele exhibits cold dependent phenotypes including apparent activation of defense responses and an increased freezing tolerance. The mRNA is cell-to-cell mobile.
AT5G17910		cardiomyopathy-associated protein
AT5G17950		disease resistance protein
AT5G17960		Encodes a member of a Cys-rich protein family known as C1-clan proteins, that contains C1_2, C1_3 and ZZ/PHD type C1 domains. Its expression is responsive to phytohormones and is affected by biotic (chitin) and different abiotic (salinity, drought, cold and UV) treatments.
AT5G17970		Disease resistance protein (TIR-NBS-LRR class) family
AT5G17980	*MULTIPLE C2 DOMAIN AND TRANSMEMBRANE REGION PROTEIN 16 (MCTP16)*	C2 calcium/lipid-binding plant phosphoribosyltransferase family protein
AT5G18000	*VERDANDI (VDD)*	Encodes VERDANDI (VDD), a putative transcription factor belonging to the reproductive meristem (REM) family. VDD is a direct target of the MADS domain ovule identity complex. Mutation in VDD affects embryo sac differentiation.
AT5G18060	*SMALL AUXIN UP RNA 23 (SAUR23)*	SAUR-like auxin-responsive protein family
AT5G18090		AP2/B3-like transcriptional factor family protein
AT5G18110	*NOVEL CAP-BINDING PROTEIN (NCBP)*	Putative cap-binding protein
AT5G18130		transmembrane protein
AT5G18160		F-box and associated interaction domains-containing protein
AT5G18180		H/ACA ribonucleoprotein complex, subunit Gar1/Naf1 protein
AT5G18200		encodes an adenylyltransferase
AT5G18220		O-Glycosyl hydrolases family 17 protein
AT5G18240	*MYB-RELATED PROTEIN 1 (MYR1)*	Encodes MYR1 (MYR1).
AT5G18270	*ARABIDOPSIS NAC DOMAIN CONTAINING PROTEIN 87 (ANAC087)*	NAC domain containing protein 87
AT5G18290	*(SIP1;2)*	Belongs to a family of plant aquaporins.Similar to yeast and radish aquaporins. Located on ER
AT5G18310		ubiquitin hydrolase
AT5G18340	*PLANT U-BOX 48 (PUB48)*	One of three tandemly located, paralogous plant U-box proteins. Mutants show increased sensitivity to water stress. E3 ligase which acts as a regulator in the heat response signaling pathway. Over-expressing AtPUB48 could induce the expression of the heat-related genes (HSP101, HSP70, HSP25.3, HSFA2, and ZAT12). Enhances plant resistance to heat stress during seed germination and seedling growth.
AT5G18350		Disease resistance protein (TIR-NBS-LRR class) family
AT5G18360	*HOPB-ACTIVATEDRESISTANCE1 (BAR1)*	Host immune receptor which recognizes the conserved effector HopB1.
AT5G18390		Pentatricopeptide repeat (PPR) superfamily protein
AT5G18400	*(DRE2)*	Cytosolic Iron-sulfur cluster Assembly protein.
AT5G18430	*(GGL26)*	GDSL-motif esterase/acyltransferase/lipase. Enzyme group with broad substrate specificity that may catalyze acyltransfer or hydrolase reactions with lipid and non-lipid substrates.
AT5G18450		encodes a member of the DREB subfamily A-2 of ERF/AP2 transcription factor family. The protein contains one AP2 domain. There are eight members in this subfamily including DREB2A AND DREB2B that are involved in response to drought.
AT5G18460		carboxyl-terminal peptidase (DUF239)
AT5G18470		Curculin-like (mannose-binding) lectin family protein
AT5G18490		vacuolar sorting-associated protein (DUF946)
AT5G18510		Aminotransferase-like, plant mobile domain family protein
AT5G18540		E3 ubiquitin-protein ligase
AT5G18550		Zinc finger C-x8-C-x5-C-x3-H type family protein
AT5G18560	*(PUCHI)*	Encodes PUCHI, a member of the ERF (ethylene response factor) subfamily B-1 of ERF/AP2 transcription factor family. The protein contains one AP2 domain. There are 15 members in this subfamily including ATERF-3, ATERF-4, ATERF-7, and leafy petiole. PUCHI is required for morphogenesis in the early lateral root primordium of Arabidopsis. Expressed in early floral meristem (stage 1 to 2). Required for early floral meristem growth and for bract suppression. Triple mutant with bop1 and bop2 displays a strong defect in the determination of floral meristem identity with reduced LFY expression and the lack of AP1 expression.
AT5G18590		Galactose oxidase/kelch repeat superfamily protein
AT5G18600	*(ROXY10)*	Encodes a member of the CC-type glutaredoxin (ROXY) family that has been shown to interact with the transcription factor TGA2 and suppress ORA59 promoter activity.
AT5G18610	*(PBL27)*	Encodes a receptor-like cytoplasmic kinase that is an immediate downstream component of the chitin receptor CERK1 and contributes to the regulation of chitin-induced immunity.
AT5G18640		alpha/beta-Hydrolases superfamily protein

AT5G18650	*MYB30-INTERACTING E3 LIGASE 1 (MIEL1)*	Encodes a RING-type E3 ubiquitin ligase that interacts with and ubiquitinates MYB30, leads to MYB30 proteasomal degradation and downregulation of its transcriptional activity. Since MYB30 is a positive regulator of Arabidopsis HR and defence responses, MIEL1 is involved in the negative regulation of these processes. The mRNA is cell-to-cell mobile.
AT5G18660	*PALE-GREEN AND CHLOROPHYLL B REDUCED 2 (PCB2)*	Encodes a protein with 3,8-divinyl protochlorophyllide a 8-vinyl reductase activity. Mutants accumulate divinyl chlorophyll rather than monovinyl chlorophyll.
AT5G18670	*BETA-AMYLASE 3 (BMY3)*	putative beta-amylase BMY3 (BMY3)
AT5G18680	*TUBBY LIKE PROTEIN 11 (TLP11)*	Member of TLP family of tubby like proteins that also contain an F-Box. Localized to the plasma membrane.
AT5G18690	*ARABINOGALACTAN PROTEIN 25 (AGP25)*	arabinogalactan protein 25
AT5G18710		DNAJ heat shock amino-terminal domain protein, putative (DUF3444)
AT5G18750		DNAJ heat shock N-terminal domain-containing protein
AT5G18770		F-box/FBD-like domains containing protein
AT5G18780		F-box/RNI-like superfamily protein
AT5G18790		Ribosomal protein L33 family protein
AT5G18810	*SC35-LIKE SPLICING FACTOR 28 (SCL28)*	encodes an SC35-like splicing factor of 28 kD localized to the nuclear specks. Barta et al (2010) have proposed a nomenclature for Serine/Arginine-Rich Protein Splicing Factors (SR proteins): Plant Cell. 2010, 22:2926.
AT5G18820	*(CPNA2)*	Encodes a subunit of chloroplasts chaperonins that are involved in mediating the folding of newly synthesized, translocated, or stress-denatured proteins. Cpn60 subunits are: Cpn60alpha1 (At2g28000), AtCpn60alpha2 (At5g18820), AtCpn60beta1 (At1g55490), AtCpn60beta2 (At3g13470), AtCpn60beta3 (At5g56500), AtCpn60beta4 (At1g26230).
AT5G18830	*SQUAMOSA PROMOTER BINDING PROTEIN-LIKE 7 (SPL7)*	Encodes a member of the Squamosa Binding Protein family of transcriptional regulators. SPL7 is expressed highly in roots and appears to play a role in copper homeostasis. Mutants are hypersensitive to copper deficient conditions and display a retarded growth phenotype. SPL7 binds to the promoter of the copper responsive miRNAs miR398b and miR389c.
AT5G18840		Major facilitator superfamily protein
AT5G18850		Low-density receptor-like protein
AT5G18860	*NUCLEOSIDE HYDROLASE 3 (NSH3)*	Encodes a purine nucleoside hydrolase active in the apoplast. It might play a role in salvaging extracellular ATP. NSH3 transcript levels rise in response to jasmonic acid and wounding.
AT5G18870	*NUCLEOSIDE HYDROLASE 5 (NSH5)*	Similar to N terminal region of NSH1 nucleoside hydrolase.
AT5G18880		RNA-directed DNA polymerase (reverse transcriptase)-related family protein
AT5G18910		Protein kinase superfamily protein
AT5G18920		Cox19-like CHCH family protein
AT5G18930	*BUSHY AND DWARF 2 (BUD2)*	S-adenosylmethionine decarboxylase family member.
AT5G18960	*FAR1-RELATED SEQUENCE 12 (FRS12)*	Transcriptional repressor that accumulates in short-day conditions. Regulates together with FRS7 and NINJA glucosinolate biosynthesis.
AT5G18970		AWPM-19-like family protein
AT5G18990		Pectin lyase-like superfamily protein
AT5G19010	*MITOGEN-ACTIVATED PROTEIN KINASE 16 (MPK16)*	member of MAP Kinase
AT5G19030		RNA-binding (RRM/RBD/RNP motifs) family protein
AT5G19040	*ISOPENTENYLTRANSFERASE 5 (IPT5)*	Encodes cytokinin synthase.
AT5G19090	*HEAVY METAL ASSOCIATED PROTEIN 46 (ATHMP46)*	Heavy metal transport/detoxification superfamily protein
AT5G19100		Eukaryotic aspartyl protease family protein
AT5G19110		Eukaryotic aspartyl protease family protein
AT5G19120		Eukaryotic aspartyl protease family protein
AT5G19140	*(AILP1)*	aluminum induced protein with YGL and LRDR motifs
AT5G19170		NEP-interacting protein, putative (DUF239)
AT5G19180	*E1 C-TERMINAL RELATED 1 (ECR1)*	Encodes a subunit of a RUB-activating enzyme analogous to the E1 ubiquitin-activating enzyme. ECR1 functions as a heterodimer with AXR1 to activate RUB, a ubiquitin-related protein.
AT5G19220	*ADP GLUCOSE PYROPHOSPHORYLASE LARGE SUBUNIT 1 (APL1)*	Encodes the large subunit of ADP-glucose pyrophosphorylase which catalyzes the first, rate limiting step in starch biosynthesis. The large subunit plays a regulatory role whereas the small subunit (ApS) is the catalytic isoform. Four isoforms (ApL1-4) have been identified. ApL1 is the major large subunit isoform present in leaves. Mutational analysis of APS1 suggests that APL1 and APL2 can compensate for loss of APS1 catalytic activity,suggesting both have catalytic as well as regulatory functions.
AT5G19240		Glycoprotein membrane precursor GPI-anchored
AT5G19250		Glycoprotein membrane precursor GPI-anchored
AT5G19260	*FANTASTIC FOUR 3 (FAF3)*	A member of the FAF family proteins encoded by the FANTASTIC FOUR (FAF) genes: AT4G02810 (FAF1), AT1G03170 (FAF2), AT5G19260 (FAF3) and AT3G06020 (FAF4). FAFs have the potential to regulate shoot meristem size in Arabidopsis thaliana. FAFs can repress WUS, which ultimately leads to an arrest of meristem activity in FAF overexpressing lines.
AT5G19270		reverse transcriptase-like protein
AT5G19280	*KINASE ASSOCIATED PROTEIN PHOSPHATASE (KAPP)*	kinase associated protein phosphatase composed of three domains: an amino-terminal signal anchor, a kinase interaction (KI) domain, and a type 2C protein phosphatase catalytic region
AT5G19290	*(MAGL16)*	alpha/beta-Hydrolases superfamily protein
AT5G19300		methyltransferase C9orf114 protein
AT5G19310	*CHROMATIN REMODELING 23 (CHR23)*	Encodes CHR23. Overexpression results in increased variability of growth and gene expression.
AT5G19320	*RAN GTPASE ACTIVATING PROTEIN 2 (RANGAP2)*	Encodes RAN GTPase activating protein 2. The protein is localized to the nuclear envelope during interphase.

AT5G19330	*ARM REPEAT PROTEIN INTERACTING WITH ABF2 (ARIA)*	Encodes an armadillo repeat protein involved in the abscisic acid response. The protein interacts with a transcription factor, ABF2, which controls ABA-dependent gene expression via the G-box-type ABA-responsive elements.
AT5G19340		hypothetical protein
AT5G19360	*CALCIUM-DEPENDENT PROTEIN KINASE 34 (CPK34)*	member of Calcium Dependent Protein Kinase
AT5G19410	*ATP-BINDING CASSETTE G23 (ABCG23)*	ABC-2 type transporter family protein
AT5G19440		similar to Eucalyptus gunnii alcohol dehydrogenase of unknown physiological function (GI:1143445), apple tree, PIR:T16995; NOT a cinnamyl-alcohol dehydrogenase
AT5G19480	*MEDIATOR 19B (MED19B)*	mediator of RNA polymerase II transcription subunit
AT5G19490		Histone superfamily protein
AT5G19500		Encodes a putative amino acid transporter that localizes to the chloroplast inner envelope membrane.
AT5G19520	*MECHANOSENSITIVE CHANNEL OF SMALL CONDUCTANCE-LIKE 9 (MSL9)*	mechanosensitive channel of small conductance-like 9
AT5G19530	*ACAULIS 5 (ACL5)*	Encodes a spermine synthase. Required for internode elongation and vascular development, specifically in the mechanism that defines the boundaries between veins and nonvein regions. This mechanism may be mediated by polar auxin transport. Though ACL5 has been shown to function as a spermine synthase in E. coli, an ACL5 knockout has no effect on the endogenous levels of free and conjugated polyamines in Arabidopsis, suggesting that ACL5 may have a very specific or altogether different in vivo function.
AT5G19550	*ASPARTATE AMINOTRANSFERASE 2 (ASP2)*	Nitrogen metabolism. Major cytosolic isoenzyme controlling aspartate biosynthesis in the light. The mRNA is cell-to-cell mobile.
AT5G19560	*ROP (RHO OF PLANTS) GUANINE NUCLEOTIDE EXCHANGE FACTOR 10 (ROPGEF10)*	Encodes a member of KPP-like gene family, homolog of KPP (kinase partner protein) gene in tomato. Also a member of the RopGEF (guanine nucleotide exchange factor) family, containing the novel PRONE domain (plant-specific Rop nucleotide exchanger), which is exclusively active towards members of the Rop subfamily.
AT5G19580	*GALACTOSE OXIDASE-LIKE 6 (GOXL6)*	Galactose oxydase; may function in tissues that require mechanical reinforcements in the absence of lignification.
AT5G19600	*SULFATE TRANSPORTER 3;5 (SULTR3;5)*	Encodes sulfate transporter Sultr3;5.
AT5G19610	*GNOM-LIKE 2 (GNL2)*	GNOM-like 2
AT5G19640	*(NFP7.1)*	Influences leaf N export via sink-to-source feedback, perhaps via a role in sensing plant internal N-status. Necessary for normal leaf N export under low N.
AT5G19660	*SITE-1 PROTEASE (S1P)*	S1P appears to function as a Golgi-localized subtilase and to help protect seedlings against salt and osmotic stress. The roots of s1p-3 mutants are hypersensitive to NaCl, KCl, LiCl, and mannitol. Several salt-stress responsive genes show weaker induction in an s1P-3 mutant background. The proteolytic cleavage of the bZIP17 transcription factor depends on S1P in vitro. And there is evidence that S1P can cleave bZIP17 in vitro.
AT5G19680	*PROTEIN PHOSPATASE 1 REGULATORY SUBUNIT 3 (PP1R3)*	PP1 Regulatory Subunit3. Interacts with members of the Type One Protein Phosphatases (TOPP) family.Facilitates the nuclear localization of TOPP4 which is required for its activity in mediating ABA responses.
AT5G19700	*EARLY LEAF SENESCENCE 1 (ELS1)*	Encodes a MATE transporter involved in leaf senescence and iron homeostasis.
AT5G19720		Ribosomal protein L25/Gln-tRNA synthetase, anti-codon-binding domain-containing protein
AT5G19730		Pectin lyase-like superfamily protein
AT5G19750		Peroxisomal membrane 22 kDa (Mpv17/PMP22) family protein
AT5G19790	*RELATED TO AP2 11 (RAP2.11)*	encodes a member of the ERF (ethylene response factor) subfamily B-6 of ERF/AP2 transcription factor family (RAP2.11). The protein contains one AP2 domain. There are 12 members in this subfamily including RAP2.11.
AT5G19800	*HYDROXYPROLINE-RICH GLYCOPROTEIN 2 (HRGP2)*	hydroxyproline-rich glycoprotein family protein
AT5G19810	*EXTENSIN 23 (EXT23)*	Proline-rich extensin-like family protein
AT5G19820	*(KARYOPHERIN ENABLING THE TRANSPORT OF THE CYTOPLASMIC HYL1 (KETCH1)*	Encodes an importin that transports HYL1, a component of the microprocessor, from the cytoplasm to the nucleus to constitute functional microprocessor, thereby affecting miRNA processing. Knockdown amiR mutants significantly reduced nuclear portion of HYL1 protein and correspondingly compromised the pri-miRNA processing in the nucleus.KETCH1 may protect RPs from the 26S proteasome-mediated degradation.
AT5G19830		Peptidyl-tRNA hydrolase family protein
AT5G19855	*HOMOLOGUE OF CYANOBACTERIAL RBCX 2 (RbcX2)*	Encodes a chloroplast stromal localized RbcX protein that acts as a chaperone in the folding of Rubisco.
AT5G19870		transmembrane epididymal protein (DUF716)
AT5G19875		transmembrane protein
AT5G19880		Peroxidase superfamily protein
AT5G19890		Peroxidase superfamily protein
AT5G19900		PRLI-interacting factor
AT5G19910	*(MED31)*	SOH1 family protein
AT5G19920		Transducin/WD40 repeat-like superfamily protein
AT5G19930	*PLASMA MEMBRANE GLUCOSE-RESPONSIVE REGULATOR (PGR)*	PGR is putative plasma membrane glucose- responsive regulator that is expressed in response to glucose stimulation.RNAi knockdown mutant seeds have enhanced sensitivity to glucose and 2-deoxyglucose.
AT5G19940	*FIBRILLIN6 (FBN6)*	Enables plants to cope with moderate light stress and affects cadmium tolerance.
AT5G19950		tudor domain protein (DUF1767)
AT5G19970		GRAS family transcription factor family protein
AT5G20040	*ISOPENTENYLTRANSFERASE 9 (IPT9)*	Encodes tRNA isopentenyltransferase AtIPT9.
AT5G20050		Protein kinase superfamily protein
AT5G20060		alpha/beta-Hydrolases superfamily protein
AT5G20110		Dynein light chain type 1 family protein
AT5G20120		testis- and ovary-specific PAZ domain protein

AT5G20140	*HAEM-BINDING PROTEIN 5 (HBP5)*	Encodes a haem-binding protein, HBP5. HBP5 binds haem and interacts with the haem oxygenase, HY1. Disrupting the binding of HBP5 to HY1 leads to oxidative stress.
AT5G20150	*SPX DOMAIN GENE 1 (SPX1)*	Expression is upregulated in the shoot of cax1/cax3 mutant. Additionally, its expression is responsive to both phosphate (Pi) and phosphite (Phi) in both roots and shoots. The mRNA is cell-to-cell mobile.
AT5G20160		Ribosomal protein L7Ae/L30e/S12e/Gadd45 family protein
AT5G20180		Ribosomal protein L36
AT5G20210		
AT5G20220		zinc knuckle (CCHC-type) family protein
AT5G20230	*BLUE-COPPER-BINDING PROTEIN (BCB)*	Encodes a Al-stress-induced gene. Along with TCF, it promotes lignin biosynthesis in response to cold stress. The mRNA is cell-to-cell mobile.
AT5G20240	*PISTILLATA (PI)*	Floral homeotic gene encoding a MADS domain transcription factor. Required for the specification of petal and stamen identities.
AT5G20250	*DARK INDUCIBLE 10 (DIN10)*	encodes a member of glycosyl hydrolase family 36. Expression is induced within 3 hours of dark treatment, in senescing leaves and treatment with exogenous photosynthesis inhibitor. Induction of gene expression was suppressed in excised leaves supplied with sugar. The authors suggest that the gene's expression pattern is responding to the level of sugar in the cell. The mRNA is cell-to-cell mobile.
AT5G20270	*HEPTAHELICAL TRANSMEMBRANE PROTEIN1 (HHP1)*	heptahelical transmembrane protein homologous to human adiponectin receptors and progestin receptors
AT5G20280	*SUCROSE PHOSPHATE SYNTHASE 1F (SPS1F)*	Encodes a sucrose-phosphate synthase activity. This is the major leaf isoform.
AT5G20300	*TRANSLOCON AT THE OUTER ENVELOPE MEMBRANE OF CHLOROPLASTS 90 (Toc90)*	Encodes Toc90, part of the TOC (translocon at the outer chloroplast membrane) machinery involved in the import of nucleus-encoded proteins into the chloroplast.
AT5G20310		Adenine nucleotide alpha hydrolases-like superfamily protein
AT5G20330	*BETA-1,3-GLUCANASE 4 (BETAG4)*	beta-1,3-glucanase 4
AT5G20340	*BETA-1,3-GLUCANASE 5 (BG5)*	Encodes a putative beta 1,3-glucanase.
AT5G20370		serine-rich protein-like protein
AT5G20380	*PHOSPHATE TRANSPORTER 4;5 (PHT4;5)*	Encodes an inorganic phosphate transporter (PHT4;5).
AT5G20390		Glycosyl hydrolase superfamily protein
AT5G20400		encodes a protein whose sequence is similar to flavanone 3 hydroxylase from Malus. The mRNA is cell-to-cell mobile.
AT5G20410	*MONOGALACTOSYLDIACYLGLYCEROL SYNTHASE 2 (MGD2)*	Encodes a type B monogalactosyldiacylglycerol (MGDG) synthase. Strongly induced by phosphate deprivation, and in non-photosynthetic tissues. Does not contribute to galactolipid synthesis under Pi-sufficient conditions but does under Pi starvation.
AT5G20420	*CHROMATIN REMODELING 42 (CHR42)*	Involved in gene silencing. Locus-specific regulator of 24nt-siRNA expression, works together with CLSY1-4 as the master regulators of essentially all Pol-IV-dependent 24nt-siRNAs.
AT5G20460		hypothetical protein
AT5G20470	*HEADLESS DERIVATIVE OF MYOSIN XI-K (HDK)*	Encodes a headless derivative of myosin XI-K, which likely arose from a partial duplication of the XI-K gene and is developmentally regulated.
AT5G20480	*EF-TU RECEPTOR (EFR)*	Encodes a predicted leucine-rich repeat receptor kinase (LRR-RLK). Functions as the receptor for bacterial PAMP (pathogen associated molecular patterns) EF-Tu.
AT5G20520	*WAVY GROWTH 2 (WAV2)*	Encodes a de-S-acylation enzyme which specifically reduces protein S-acylation levels of five immunity-related proteins. WAV2 negatively regulates root bending when roots alter their growth direction. It's not involved in sensing environmental stimuli (e.g. gravity, light, water, touch).
AT5G20530		
AT5G20540	*BREVIS RADIX-LIKE 4 (BRXL4)*	Belongs to five-member BRX gene family. Arabidopsis BRX genes share high levels of similarity among each others, with several conserved domains. The most distinct is BRX domain - highly conserved in all BRX genes among distantly related species. This protein-protein interaction domain is required and sufficient for BRX activity.
AT5G20550		2-oxoglutarate (2OG) and Fe(II)-dependent oxygenase superfamily protein
AT5G20560		Glycosyl hydrolase superfamily protein
AT5G20580		TMEM192 family protein
AT5G20590	*TRICHOME BIREFRINGENCE-LIKE 5 (TBL5)*	Encodes a member of the TBL (TRICHOME BIREFRINGENCE-LIKE) gene family containing a plant-specific DUF231 (domain of unknown function) domain. TBL gene family has 46 members, two of which (TBR/AT5G06700 and TBL3/AT5G01360) have been shown to be involved in the synthesis and deposition of secondary wall cellulose, presumably by influencing the esterification state of pectic polymers. A nomenclature for this gene family has been proposed (Volker Bischoff & Wolf Scheible, 2010, personal communication).
AT5G20600		ribosomal RNA processing-like protein
AT5G20630	*GERMIN 3 (GER3)*	Encodes a germin-like protein. Its transcripts are more abundant in RNA from leaves collected in the evening, suggesting some kind of circadian regulation.
AT5G20640		LURP-one-like protein (DUF567)
AT5G20690	*POLLEN RECEPTOR LIKE KINASE 6 (PRK6)*	PRK6 is pollen specific receptor kinase that functions as a receptor for the pollen attractant LURE1 in pollen tube guidance. It is localized to the tip the pollen tube and becomes asymmetrically distributed towards the source of the LURE1 signal prior to pollen tube growth reorientation.
AT5G20700		senescence-associated family protein, putative (DUF581)
AT5G20710	*BETA-GALACTOSIDASE 7 (BGAL7)*	beta-galactosidase 7
AT5G20730	*NON-PHOTOTROPHIC HYPOCOTYL (NPH4)*	Encodes an auxin-regulated transcriptional activator. Activates expression of IAA1 and IAA9 in the presence of auxin. Mutants affect blue light and gravitropic and auxin mediated growth responses. Together with AUX19, it is involved in the response to ethylene. In the arf7 arf19 double mutant, several auxin-responsive genes (e.g. IAA5, LBD16, LBD29 and LBD33) are no longer upregulated by auxin.
AT5G20740		Plant invertase/pectin methylesterase inhibitor superfamily protein
AT5G20760		transposable_element_gene;similar to unknown protein [Arabidopsis thaliana] (TAIR:AT3G42700.1);(source:TAIR10)
AT5G20770		transposable_element_gene;similar to unknown protein [Arabidopsis thaliana] (TAIR:AT5G34820.1);(source:TAIR10)
AT5G20790		transmembrane protein

AT5G20800		transposable_element_gene;pseudogene, similar to putative reverse transcriptase, predicted non-LTR reverse ranscriptase sequence fragments;(source:TAIR10)
AT5G20810	*SMALL AUXIN UPREGULATED RNA 70 (SAUR70)*	SAUR-like auxin-responsive protein family
AT5G20820	*SMALL AUXIN UPREGULATED RNA 76 (SAUR76)*	SAUR-like auxin-responsive protein family
AT5G20830	*SUCROSE SYNTHASE 1 (SUS1)*	Encodes a protein with sucrose synthase activity (SUS1).
AT5G20860		Plant invertase/pectin methylesterase inhibitor superfamily
AT5G20870		O-Glycosyl hydrolases family 17 protein
AT5G20885		RING/U-box superfamily protein
AT5G20900	*JASMONATE-ZIM-DOMAIN PROTEIN 12 (JAZ12)*	jasmonate-zim-domain protein 12
AT5G20910	*ABI3-INTERACTING PROTEIN 2 (AIP2)*	Encodes an E3 ligase that can interact with and polyubiquitinate ABI3 in vitro. AIP2 likely negatively regulates ABA signaling by targeting ABI3 for post-translational destruction.
AT5G20935	*CHLORORESPIRATORY REDUCTION 42 (CRR42)*	Chloroplast NADH dehydrogenase assembly protein. Mutants are defective in the accumulation of subcomplex A.
AT5G20940		Glycosyl hydrolase family protein
AT5G20950	*(BGLC1)*	Encodes a beta-glucosidase involved in xyloglucan metabolism.
AT5G20960	*ALDEHYDE OXIDASE 1 (AO1)*	Encodes aldehyde oxidase AA01.
AT5G20980	*METHIONINE SYNTHASE 3 (MS3)*	Encodes a plastidic methionine synthase, involved in methionine de novo synthesis in the chloroplast
AT5G21020		transmembrane protein
AT5G21030	*(AGO8)*	PAZ domain-containing protein / piwi domain-containing protein
AT5G21060		Glyceraldehyde-3-phosphate dehydrogenase-like family protein
AT5G21100		Plant L-ascorbate oxidase
AT5G21105		Plant L-ascorbate oxidase
AT5G21110		
AT5G21120	*ETHYLENE-INSENSITIVE3-LIKE 2 (EIL2)*	ethylene-insensitive3-like2 (EIL2)
AT5G21130		Late embryogenesis abundant (LEA) hydroxyproline-rich glycoprotein family
AT5G21150	*ARGONAUTE 9 (AGO9)*	AGO9-dependent sRNA silencing is crucial to specify cell fate in the Arabidopsis ovule. AGO9 is expressed in reproductive companion cells but not in the associated male or female gametes or their precursors. Therefore, AGO9 acts non-cell autonomously to silencing the activity of TEs activity in the female gametophyte.Loss of function mutants produce ectopic megaspore mother cell and supernumary female gametophytes.
AT5G21160	*LA RELATED PROTEIN 1A (LARP1A)*	Encodes a protein with sequence similarity to mRNA binding proteins from humans. LARP1a is involved in mRNA degradation in response to heat stress. Upon heat stress LARP1a interacts with XRN4 and appears to be responsible for addressing XRN4 to the polysome. LARP1/XRN4 double mutants are impaired in thermotolerance and lower levels of heat induced RNA turnover.
AT5G22000	*RING-H2 GROUP F2A (RHF2A)*	encodes a RING-type E3 ubiquitin ligase implicated in gametogenesis. Double mutant analyses with RHF1a suggests that RHF2a may be involved in targetting ICK4KRP6 for degradation following meiosis in order to allow the mitoses associated with megagametogenesis and microgametogenesis to occur. RHF2a is expressed in all four floral whorls and is present at ~8-fold higher levels than RHF1a in inflorescences by RT-PCR analyses.
AT5G22030	*UBIQUITIN-SPECIFIC PROTEASE 8 (UBP8)*	ubiquitin-specific protease 8
AT5G22250	*CCR4- ASSOCIATED FACTOR 1B (CAF1b)*	Encodes one of the homologs of the yeast CCR4-associated factor 1: AT3G44260 (CAF1a), AT5G22250 (CAF1b). Has mRNA deadenylation activity. Also plays a role in plant defense responses.
AT5G22270	*SALT-INDUCED AND EIN3/EIL1-DEPENDENT 1 (SIED1)*	hypothetical protein
AT5G22280		peptidyl-prolyl cis-trans isomerase G
AT5G22290	*NAC DOMAIN CONTAINING PROTEIN 89 (NAC089)*	Encodes ANAC089, a membrane-tethered transcription factor that negatively regulates floral initiation. Also controls ER-stress-induced programmed cell death.
AT5G22300	*NITRILASE 4 (NIT4)*	encodes a nitrilase isomer. The purified enzyme shows a strong substrate specificity for beta-cyano-L-alanine, a intermediate product of the cyanide detoxification pathway. The mRNA is cell-to-cell mobile.
AT5G22310		trichohyalin-like protein
AT5G22320		Leucine-rich repeat (LRR) family protein
AT5G22380	*NAC DOMAIN CONTAINING PROTEIN 90 (NAC090)*	NAC domain containing protein 90
AT5G22390		FANTASTIC four-like protein (DUF3049)
AT5G22410	*ROOT HAIR SPECIFIC 18 (RHS18)*	root hair specific 18
AT5G22420	*FATTY ACID REDUCTASE 7 (FAR7)*	fatty acid reductase 7
AT5G22430		Pollen Ole e 1 allergen and extensin family protein
AT5G22440		Ribosomal protein L1p/L10e family
AT5G22460		alpha/beta-Hydrolases superfamily protein
AT5G22470	*POLY(ADP-RIBOSE) POLYMERASE 3 (PARP3)*	PARP3 is one of three canonical PARPs in Arabidopsis.
AT5G22480		ZPR1 zinc-finger domain protein
AT5G22500	*FATTY ACID REDUCTASE 1 (FAR1)*	Encodes a member of the eight-member gene family encoding alcohol-forming fatty acyl-CoA reductases (FARs) identified in Arabidopsis thaliana. Three of the FARs, FAR1 (At5g22500), FAR4 (At3g44540) and FAR5 (At3g44550), are shown to generate the fatty alcohols found in root, seed coat, and wound-induced leaf tissue.
AT5G22530		Unknown protein, knockout shows increased sensitivity to Al stress.
AT5G22540		Associated with a QTL for quantitative disease resistance.
AT5G22550		transmembrane protein, putative (DUF247)
AT5G22555		transmembrane protein

AT5G22570	*WRKY DNA-BINDING PROTEIN 38 (WRKY38)*	member of WRKY Transcription Factor; Group III
AT5G22580		Stress responsive A/B Barrel Domain-containing protein
AT5G22590		Leucine Rich Repeat protein family
AT5G22600		FBD / Leucine Rich Repeat domains containing protein
AT5G22620		encodes a putative 2-carboxy-D-arabinitol 1-phosphate phosphatase
AT5G22630	*AROGENATE DEHYDRATASE 5 (ADT5)*	Encodes a plastid-localized arogenate dehydratase involved in phenylalanine biosynthesis. Not less than six genes encoding ADT were identiﬁed in the Arabidopsis genome: ADT1 [At1g11790]; ADT2 [At3g07630]; ADT3 [At2g27820]; ADT4 [At3g44720]; ADT5 [At5g22630]; and ADT6 [At1g08250]. The mRNA is cell-to-cell mobile.
AT5G22640	*EMBRYO DEFECTIVE 1211 (emb1211)*	EMB1211 is a MORN (multiple membrane occupation and recognition nexus) motif containing protein involved in embryo development and chloroplast biogenesis. The mRNA is cell-to-cell mobile.
AT5G22650	*HISTONE DEACETYLASE 2B (HD2B)*	Encodes a member of a plant-specific class of histone deacetylases. Controls the development of adaxial/abaxial leaf polarity. Its mRNA is widely expressed in stems, leaves, flowers and young siliques. Plant lines expressing RNAi constructs directed against this gene showed a marked reduction in agrobacterium-mediated root transformation.
AT5G22680		F-box protein
AT5G22690		Disease resistance protein (TIR-NBS-LRR class) family
AT5G22700		LOW protein: F-box/FBD/LRR-like protein
AT5G22710		
AT5G22730		F-box/RNI-like/FBD-like domains-containing protein
AT5G22770	*ALPHA-ADAPTIN (alpha-ADR)*	AP-2 complex subunit alpha-1. Part of endomembrane trafficking system.
AT5G22790	*RETICULATA-RELATED 1 (RER1)*	reticulata-related 1
AT5G22810		GDSL-motif esterase/acyltransferase/lipase. Enzyme group with broad substrate specificity that may catalyze acyltransfer or hydrolase reactions with lipid and non-lipid substrates.
AT5G22830	*MAGNESIUM (MG) TRANSPORTER 10 (MGT10)*	Transmembrane magnesium transporter that is essential for chloroplast development and photosynthesis. One of nine family members.
AT5G22840		Protein kinase superfamily protein
AT5G22850		Eukaryotic aspartyl protease family protein
AT5G22860		Serine carboxypeptidase S28 family protein
AT5G22870		Late embryogenesis abundant (LEA) hydroxyproline-rich glycoprotein family
AT5G22880	*HISTONE B2 (HTB2)*	Encodes a histone 2B (H2B) protein. This protein can be ubiquitinated in planta, and this modification depends on the HUB1 and HUB2 E3 ubiquitin ligases.
AT5G22890	*SENSITIVE TO PROTON RHIZOTOXICITY 2 (STOP2)*	An unique homologue of STOP1 (AT1G34370) in Arabidopsis genome. Transformation to the stop1-mutant activated several genes that are regulated by STOP1, and conferred proton sensitive phenotype.
AT5G22900	*CATION/H+ EXCHANGER 3 (CHX3)*	member of Putative Na+/H+ antiporter family
AT5G22910	*CATION/H+ EXCHANGER 9 (CHX9)*	member of Putative Na+/H+ antiporter family
AT5G22920	*RING ZINC-FINGER PROTEIN 34 (RZPF34)*	Encodes a protein with sequence similarity to RING, zinc finger proteins. Loss of function mutations show reduced (15%) stomatal aperture under non stress conditions.
AT5G22940	*FRA8 HOMOLOG (F8H)*	Homolog of FRA8 (AT2G28110), a member of a member of glycosyltransferase family 47; exhibits high sequence similarity to tobacco (Nicotiana plumbaginifolia) pectin glucuronyltransferase.
AT5G22950	*(VPS24.1)*	SNF7 family protein
AT5G22970		hypothetical protein
AT5G22980	*SERINE CARBOXYPEPTIDASE-LIKE 47 (scpl47)*	serine carboxypeptidase-like 47
AT5G22990	*GAZ-LIKE 3 (GAL3)*	Member of a small family of zinc finger containing putative transcription factors.Similar to GAZ.
AT5G23000	*MYB DOMAIN PROTEIN 37 (MYB37)*	Putative homolog of the Blind gene in tomato. Together with RAX2 and RAX3 belong to the class R2R3 MYB genes; encoded by the Myb-like transcription factor MYB37, regulates axillary meristem formation. RAX1 is expressed in a small central domain within the boundary zone separating SAM and leaf primordia during early leaf primordium development and is currently the earliest spatial marker for future axillary meristems. Member of the R2R3 factor gene family.
AT5G23010	*METHYLTHIOALKYLMALATE SYNTHASE 1 (MAM1)*	Encodes a methylthioalkylmalate synthase, catalyzes the condensation reactions of the first two rounds of methionine chain elongation in the biosynthesis of methionine-derived glucosinolates. The mRNA is cell-to-cell mobile.
AT5G23020	*2-ISOPROPYLMALATE SYNTHASE 2 (IMS2)*	methylthioalkymalate synthase-like. Also known as 2-isopropylmalate synthase (IMS2). encodes a methylthioalkylmalate synthase involved in the biosynthesis of aliphatic glucosinolates which accepts all the omega-methylthio-2-oxoalkanoic acids needed to form the known C3 to C8 glucosinolates in Arabidopsis. The mRNA is cell-to-cell mobile.
AT5G23030	*TETRASPANIN12 (TET12)*	Member of TETRASPANIN family
AT5G23050	*ACYL-ACTIVATING ENZYME 17 (AAE17)*	acyl-activating enzyme 17
AT5G23060	*CALCIUM SENSING RECEPTOR (CaS)*	Encodes a chloroplast-localized protein that modulates cytoplasmic Ca2+ concentration and is crucial for proper stomatal regulation in response to elevations of external Ca2+. Phosphorylation of this protein is dependent on calcium.
AT5G23080	*TOUGH (TGH)*	Interacts with TATA-box binding protein 2. Contains domains with strong similarity to G-patch and SWAP domains, characteristic of RNA binding and processing proteins. Colocalizes with the splicing regulator SRp34 to subnuclear particles. It is an important component of miRNA and siRNA biogenesis. Mutants display developmental defects, including reduced plant height, polycotyly, and reduced vascularization. Strong genetic interaction between TGH and AMP1.

AT5G23140	*NUCLEAR-ENCODED CLP PROTEASE P7 (NCLPP7)*	One of several nuclear-encoded ClpPs (caseinolytic protease). Contains a highly conserved catalytic triad of Ser-type proteases (Ser-His-Asp). The name reflects nomenclature described in Adam et. al (2001). This mitochondrial CLPP2 assists coordination and homeostasis of respiratory complexes.
AT5G23170		Protein kinase superfamily protein
AT5G23180		mediator-associated-like protein
AT5G23190	*CYTOCHROME P450, FAMILY 86, SUBFAMILY B, POLYPEPTIDE 1 (CYP86B1)*	cytochrome P450 CYP86B1, nuclear gene for chloroplast product. CYP86B1 is a very long chain fatty acid hydroxylase specifically involved in polyester monomer biosynthesis during the course of plant development.
AT5G23210	*SERINE CARBOXYPEPTIDASE-LIKE 34 (SCPL34)*	serine carboxypeptidase-like 34
AT5G23220	*NICOTINAMIDASE 3 (NIC3)*	nicotinamidase 3
AT5G23230	*NICOTINAMIDASE 2 (NIC2)*	nicotinamidase 2
AT5G23240	*DNA J PROTEIN C76 (DJC76)*	DNAJ heat shock N-terminal domain-containing protein
AT5G23250		Succinyl-CoA ligase, alpha subunit
AT5G23260	*TRANSPARENT TESTA16 (TT16)*	Encodes a MADS box protein. Regulates proanthocyanidin biosynthesis in the inner-most cell layer of the seed coat. Also controls cell shape of the inner-most cell layer of the seed coat. Also shown to be necessary for determining the identity of the endothelial layer within the ovule. Paralogous to GOA. Plays a maternal role in fertilization and seed development.
AT5G23270	*SUGAR TRANSPORTER 11 (STP11)*	Membrane localized sucrose transporter.
AT5G23300	*PYRIMIDINE D (PYRD)*	dihydroorotate dehydrogenase, catalyses fourth step of pyrimidine biosynthesis
AT5G23310	*FE SUPEROXIDE DISMUTASE 3 (FSD3)*	Fe superoxide dismutase
AT5G23320	*HOMOLOG OF YEAST STE14 A (STE14A)*	Encodes a prenylcysteine alpha-carboxyl methyltransferase involved in methylation of isoprenylated proteins. This protein appears to have lower catalytic activity and a lower transcript expression level than the other ICMT present in Arabidopsis (At5g08335). Analysis of ICMT RNAi lines suggests that this protein may be involved in flower and stem development.
AT5G23350		GRAM domain protein/ABA-responsive-like protein
AT5G23380		hypothetical protein (DUF789)
AT5G23400		Leucine-rich repeat (LRR) family protein
AT5G23420	*HIGH-MOBILITY GROUP BOX 6 (HMGB6)*	Encodes HMGB6, a protein belonging to the subgroup of HMGB (high mobility group B) proteins. Localized in the nucleus. Binds to supercoiled DNA in vitro. HMGB6 is phosphorylated by protein kinase CK2alpha within its acidic C-terminal domain.
AT5G23430	*KATANIN P80 SUBUNIT 4 (KTN80.4)*	One of four katanin p80 subunits. Involved in targeting of katanin complex to crossover and branch points to properly sever microtubules.
AT5G23440	*FERREDOXIN/THIOREDOXIN REDUCTASE SUBUNIT A (VARIABLE SUBUNIT) 1 (FTRA1)*	ferredoxin/thioredoxin reductase subunit A (variable subunit) 1
AT5G23460		hypothetical protein
AT5G23480		SWIB/MDM2, Plus-3 and GYF domain-containing protein
AT5G23510		hypothetical protein
AT5G23530	*CARBOXYESTERASE 18 (CXE18)*	carboxyesterase 18
AT5G23580	*CALMODULIN-LIKE DOMAIN PROTEIN KINASE 9 (CDPK9)*	Member of a unique family of enzymes containing a single polypeptide chain with a kinase domain at the amino terminus and a putative calcium-binding EF hands structure at the carboxyl terminus; recombinant protein is fully active and induced by Ca2+
AT5G23640		
AT5G23650		Homeodomain-like transcriptional regulator
AT5G23660	*(SWEET12)*	Encodes a member of the SWEET sucrose efflux transporter family proteins.
AT5G23670	*LONG CHAIN BASE2 (LCB2)*	Encodes the LCB2 subunit of serine palmitoyltransferase, an enzyme involved in sphingosine biosynthesis. The protein is localized to the endoplasmic reticulum.
AT5G23680		Sterile alpha motif (SAM) domain-containing protein
AT5G23690		Polynucleotide adenylyltransferase family protein
AT5G23710		DNA binding / DNA-directed RNA polymerase
AT5G23730	*REPRESSOR OF UV-B PHOTOMORPHOGENESIS 2 (RUP2)*	Encodes REPRESSOR OF UV-B PHOTOMORPHOGENESIS 2 (RUP2). Functions as a repressor of UV-B signaling.
AT5G23750		Remorin family protein
AT5G23760		Copper transport protein family
AT5G23790	*GALACTINOL SYNTHASE 5 (GolS5)*	Predicted to encode a galactinol synthase.
AT5G23800	*DOMAIN OF UNKNOWN FUNCTION 724 10 (DUF 10)*	Member of the plant-specific DUF724 protein family. Arabidopsis has 10 DUF724 proteins. Loss of function mutant has a WT phenotype
AT5G23810	*AMINO ACID PERMEASE 7 (AAP7)*	Encodes nonfunctional amino acid transporter. AAP7 is the most distantly related member of the AAP family, a group of well characterized amino acid transporters within the ATF1 superfamily. Expression of this gene has not been detected with RNA gel blots or promoter GUS studies.
AT5G23820	*MD2-RELATED LIPID RECOGNITION 3 (ML3)*	ML3 can be modified by NEDD8 and ubiquitin. ML3 expression is regulated by NAI1. ML3 expression is regulated by MeJA, ethylene and wounding. ml3-3 is more susceptible against infections with Alternaria brassicicola and more resistant against infections with Pseudomonas syringae DC3000.
AT5G23830		MD-2-related lipid recognition domain-containing protein
AT5G23840		MD-2-related lipid recognition domain-containing protein
AT5G23850		O-glucosyltransferase rumi-like protein (DUF821)
AT5G23860	*TUBULIN BETA 8 (TUB8)*	beta-tubulin, preferentially expressed in endodermal and phloem cells of primary roots and in the vascular tissues of leaves, stems, and flowers. The mRNA is cell-to-cell mobile.
AT5G23870	*PECTIN ACETYLESTERASE 9 (PAE9)*	Encodes a pectin acetylesterase that removes cell wall acetate associated with pectin formation in Arabidopsis leaves.
AT5G23890		GPI-anchored adhesin-like protein
AT5G23910		ATP binding microtubule motor family protein
AT5G23920		transmembrane protein

AT5G23940	*PERMEABLE LEAVES3 (PEL3)*	Encodes PERMEABLE LEAVES3 (PEL3), a putative acyl-transferase. Mutation in this locus results in altered trichome phenotype (trcichomes become tangled during leaf expansion). Additional phenotype includes altered cuticle layer.
AT5G23960	*TERPENE SYNTHASE 21 (TPS21)*	Encodes a sesquiterpene synthase involved in generating all of the group A sesquiterpenes found in the Arabidopsis floral volatile blend. Strongly expressed in the stigma.
AT5G23970		HXXXD-type acyl-transferase family protein
AT5G24010		Protein kinase superfamily protein
AT5G24030	*SLAC1 HOMOLOGUE 3 (SLAH3)*	Encodes a protein with ten predicted transmembrane helices. The SLAH3 protein has similarity to the SLAC1 protein involved in ion homeostasis in guard cells. Although it is not expressed in guard cells, it can complement an slac1-2 mutant suggesting that it performs a similar function. SLAH3:GFP localizes to the plasma membrane.
AT5G24070		Peroxidase superfamily protein
AT5G24080		Protein kinase superfamily protein
AT5G24090	*CHITINASE A (CHIA)*	Chitinase A (class III) expressed exclusively under environmental stress conditions. Shown be a plant lysozyme involved in plant immunity.
AT5G24100		Leucine-rich repeat protein kinase family protein
AT5G24110	*WRKY DNA-BINDING PROTEIN 30 (WRKY30)*	member of WRKY Transcription Factor; Group III
AT5G24120	*SIGMA FACTOR E (SIGE)*	Encodes a specialized sigma factor that functions in regulation of plastid genes and is responsible for the light-dependent transcription at the psbD LRP. Activation of SIG5 is dependent upon blue light and mediated by cryptochromes.
AT5G24130		polypyrimidine tract-binding-like protein
AT5G24140	*SQUALENE MONOOXYGENASE 2 (SQP2)*	Encodes a protein with similarity to squalene monoxygenases.
AT5G24160	*SQUALENE MONOXYGENASE 6 (SQE6)*	squalene monooxygenase 6
AT5G24165		hypothetical protein
AT5G24170		Got1/Sft2-like vescicle transport protein family
AT5G24210	*(PRLIP1)*	alpha/beta-Hydrolases superfamily protein
AT5G24240	*(AtPI4Kgamma3)*	Encodes PI4Kc3, localizes to the nucleus and has autophosphorylation activity, but no lipid kinase activity. Overexpression mutants display late-flowering phenotype.
AT5G24250		
AT5G24270	*SALT OVERLY SENSITIVE 3 (SOS3)*	encodes a calcium sensor that is essential for K+ nutrition, K+/Na+ selectivity, and salt tolerance. The protein is similar to calcineurin B. Lines carrying recessive mutations are hypersensitive to Na+ and Li+ stresses and is unable to grow in low K+. The growth defect is rescued by extracellular calcium.
AT5G24280	*GAMMA-IRRADIATION AND MITOMYCIN C INDUCED 1 (GMI1)*	Encodes GMI1, a structural-maintenance-of-chromosomes-hinge domain-containing protein. Involved in somatic homologous recombination.
AT5G24290	*MEMBRANE OF ER BODY 2 (MEB2)*	Vacuolar iron transporter (VIT) family protein
AT5G24310	*ABL INTERACTOR-LIKE PROTEIN 3 (ABIL3)*	One of four ABI-like proteins.
AT5G24313		transmembrane protein
AT5G24316		proline-rich family protein
AT5G24320		Transducin/WD40 repeat-like superfamily protein
AT5G24330	*ARABIDOPSIS TRITHORAX-RELATED PROTEIN 6 (ATXR6)*	Encodes a SET-domain protein, a H3K27 monomethyltransferases required for chromatin structure and gene silencing. Regulates heterochromatic DNA replication. Contains a PCNA-binding domain. ATXR6 accumulates preferentially during the late G1 or S phase, suggesting that it plays a role in cell-cycle regulation or progression.
AT5G24380	*YELLOW STRIPE LIKE 2 (YSL2)*	closest Arabidopsis homolog of Zea maize metal-phytosiderophore/metal-nicotianamine transporter ZmYS1
AT5G24390		Ypt/Rab-GAP domain of gyp1p superfamily protein
AT5G24400	*EMBRYO DEFECTIVE 2024 (emb2024)*	Encodes a protein with 6-phosphoglucunolactonase activity that localizes to the chloroplasts and the peroxisome. However, mutant phenotypes observed in pgl3 mutant plants can be complemented with a chloroplast-targeted version of the protein. PGL3 likely functions in the oxidative branch of the pentose phosphate pathway. pgl3 mutant phenotypes suggest that it is important in pathogen defense and maintenance of cellular redox homeostasis.
AT5G24410	*6-PHOSPHOGLUCONOLACTONASE 4 (PGL4)*	Encodes a cytosolic 6-phosphogluconolactonase (PGL) thought to be involved in the oxidative pentose-phosphate pathway (OPPP).
AT5G24420	*6-PHOSPHOGLUCONOLACTONASE 5 (PGL5)*	Encodes a cytosolic 6-phosphogluconolactonase (PGL) thought to be involved in the oxidative pentose-phosphate pathway (OPPP).
AT5G24430		Calcium-dependent protein kinase (CDPK) family protein
AT5G24440	*CTC-INTERACTING DOMAIN 13 (CID13)*	RNA-binding protein, putative. Contains PAM2, PABC binding domain.
AT5G24450		Transcription factor IIIC, subunit 5
AT5G24460		RING-H2 zinc finger protein
AT5G24470	*PSEUDO-RESPONSE REGULATOR 5 (PRR5)*	Encodes a pseudo-response regulator whose mutation affects various circadian-associated biological events such as flowering time in the long-day photoperiod conditions, red light sensitivity of seedlings during early photomorphogenesis, and the period of free-running rhythms of certain clock-controlled genes including CCA1 and APRR1/TOC1 in constant white light. Acts as transcriptional repressor of CCA1 and LHY. Acts additively with EC, PRR7 and PRR9 to regulate hypocotyl growth under photoperiodic conditions.
AT5G24480		Beta-galactosidase related protein
AT5G24490		30S ribosomal protein
AT5G24500	*ANTAGONIST OF LIKE HETEROCHROMATIN PROTEIN 2 (ALP2)*	fantom protein
AT5G24510		60S acidic ribosomal protein family
AT5G24530	*DOWNY MILDEW RESISTANT 6 (DMR6)*	Encodes a putative 2OG-Fe(II) oxygenase that is defense-associated but required for susceptibility to downy mildew. The mRNA is cell-to-cell mobile.
AT5G24540	*BETA GLUCOSIDASE 31 (BGLU31)*	beta glucosidase 31

AT5G24550	*BETA GLUCOSIDASE 32 (BGLU32)*	beta glucosidase 32
AT5G24560	*PHLOEM PROTEIN 2-B12 (PP2-B12)*	phloem protein 2-B12
AT5G24570		hypothetical protein
AT5G24580	*(HIPP09)*	Heavy metal transport/detoxification superfamily protein
AT5G24590	*TCV-INTERACTING PROTEIN (TIP)*	Member of NAc protein family. Interacts with turnip crinkle virus (TCV) capsid protein. Transcription factor involved in regulating the defense response of Arabidopsis to TCV.
AT5G24600		TRP-like ion channel protein (Protein of unknown function, DUF599)
AT5G24620		Pathogenesis-related thaumatin superfamily protein
AT5G24655	*RESPONSE TO LOW SULFUR 4 (LSU4)*	response to low sulfur 4
AT5G24660	*RESPONSE TO LOW SULFUR 2 (LSU2)*	response to low sulfur 2
AT5G24670	*EMBRYO DEFECTIVE 2820 (EMB2820)*	A protein coding gene with unknown function. The 5?UTR of this gene overlaps with a RNA coding gene TER2. TER2 (GenBank accession no. HQ401285) encodes a putative template sequence corresponding to 1.5 copies of the Arabidopsis telomere repeat (PNAS 2011, 108:73-78). Natural epiallele in Nok-1, transmission of the epiallele over generations depends only on the selfreinforcing loop between CHROMOMETHYLASE 3 and KRYPTONITE, involving DNA methylated in the CHG context and histone H3 lysine 9 methylation.
AT5G24700		
AT5G24730		
AT5G24740	*SHRUBBY (SHBY)*	Encodes a vacuolar sorting protein that interacts with the plant-specific GRAS family transcription factor SHORT-ROOT and acts in a pathway that controls root growth and radial patterning. It provides a connections between gibberellic acid, SHR and PLT signaling in the root.
AT5G24750		UDP-Glycosyltransferase superfamily protein
AT5G24760		GroES-like zinc-binding dehydrogenase family protein
AT5G24780	*VEGETATIVE STORAGE PROTEIN 1 (VSP1)*	encodes an acid phosphatase similar to soybean vegetative storage proteins. Gene expression is induced by wounding and jasmonic acid.
AT5G24800	*BASIC LEUCINE ZIPPER 9 (BZIP9)*	Encodes bZIP protein BZO2H2.
AT5G24810	*(ABC1K11)*	ABC1 family protein
AT5G24820		Eukaryotic aspartyl protease family protein
AT5G24850	*CRYPTOCHROME 3 (CRY3)*	Binds flavin adenine dinucleotide and DNA. It does not have photolyase activity, and it is likely to act as photoreceptor. Closely related to Synechocystis cryptochrome.
AT5G24860	*FLOWERING PROMOTING FACTOR 1 (FPF1)*	encodes a small protein of 12.6 kDa that regulates flowering and is involved in gibberellin signalling pathway. It is expressed in apical meristems immediately after the photoperiodic induction of flowering. Genetic interactions with flowering time and floral organ identity genes suggest that this gene may be involved in modulating the competence to flower. There are two other genes similar to FPF1, FLP1 (At4g31380) and FLP2 (no locus name yet, on BAC F8F16 on chr 4). This is so far a plant-specific gene and is only found in long-day mustard, arabidopsis, and rice.
AT5G24880		chromo domain cec-like protein
AT5G24900	*CYTOCHROME P450, FAMILY 714, SUBFAMILY A, POLYPEPTIDE 2 (CYP714A2)*	Member of CYP714A. Encodes one of the two tandemly duplicated gene pair ELA1 (CYP714A1) and ELA2 (CYP714A2), homologs of the rice cytochrome P450 monooxygenase gene EUI1. Double mutation of ELA1 and ELA2 results in increased biomass and enlarged organs.
AT5G24910	*CYTOCHROME P450, FAMILY 714, SUBFAMILY A, POLYPEPTIDE 1 (CYP714A1)*	Member of CYP714A. Encodes one of the two tandemly duplicated gene pair ELA1 (CYP714A1) and ELA2 (CYP714A2), homologs of the rice cytochrome P450 monooxygenase gene EUI1. Double mutation of ELA1 and ELA2 results in increased biomass and enlarged organs.
AT5G24930	*CONSTANS-LIKE 4 (COL4)*	Flowering repressor in long days (LD) and short days (SD) and acts on the expression of FT and FT-like genes as well as on SUPPRESSOR OF OVEREXPRESSION OF CONSTANS 1 (SOC1).
AT5G24940		Protein phosphatase 2C family protein
AT5G24950	*CYTOCHROME P450, FAMILY 71, SUBFAMILY A, POLYPEPTIDE 15 (CYP71A15)*	putative cytochrome P450
AT5G24970		Protein kinase superfamily protein
AT5G25020		enhanced disease resistance-like protein (DUF1336)
AT5G25040		Major facilitator superfamily protein
AT5G25090	*EARLY NODULIN-LIKE PROTEIN 13 (ENODL13)*	early nodulin-like protein 13
AT5G25100	*(TMN9)*	Endomembrane protein 70 protein family
AT5G25110	*CBL-INTERACTING PROTEIN KINASE 25 (CIPK25)*	salt- and anoxia-induced member of AtCIPK family.
AT5G25120	*CYTOCHROME P450, FAMILY 71, SUBFAMILY B, POLYPEPTIDE 11 (CYP71B11)*	putative cytochrome P450 The mRNA is cell-to-cell mobile.
AT5G25130	*CYTOCHROME P450, FAMILY 71, SUBFAMILY B, POLYPEPTIDE 12 (CYP71B12)*	putative cytochrome P450 The mRNA is cell-to-cell mobile.
AT5G25140	*CYTOCHROME P450, FAMILY 71, SUBFAMILY B, POLYPEPTIDE 13 (CYP71B13)*	putative cytochrome P450
AT5G25160	*ZINC FINGER PROTEIN 3 (ZFP3)*	Encodes a zinc finger protein containing only a single zinc finger.
AT5G25170		PPPDE putative thiol peptidase family protein
AT5G25180	*CYTOCHROME P450, FAMILY 71, SUBFAMILY B, POLYPEPTIDE 14 (CYP71B14)*	putative cytochrome P450
AT5G25190	*ETHYLENE AND SALT INDUCIBLE 3 (ESE3)*	encodes a member of the ERF (ethylene response factor) subfamily B-6 of ERF/AP2 transcription factor family. The protein contains one AP2 domain. There are 12 members in this subfamily including RAP2.11.
AT5G25210		hypothetical protein
AT5G25220	*KNOTTED1-LIKE HOMEOBOX GENE 3 (KNAT3)*	A member of class II knotted1-like homeobox gene family (together with KNAT4 and KNAT5). Expressed in: hypocotyl-root boundary, anther-filament junction in flowers, ovule-funiculus and peduncle-silique boundaries, petioles and root. Light-regulated expression with differential response to red/far-red light. KNAT3 promoter activity showed cell-type specific pattern along longitudinal root axis, restricted mainly to the differentiation zone of the root, namely in the cortex and pericycle. Not detected in lateral root primordia
AT5G25240		stress induced protein

AT5G25260	*FLOTILIN2 (FLOT2)*	Belongs to the group of plant flotillins, which are plasma membrane proteins. Flot2 complexes are found in microdomains and may be involved in plant-pathogen interactions, water transport and intracellular trafficking.
AT5G25280		serine-rich protein-like protein
AT5G25290	*F-BOX/DUF295 BRASSICEAE-SPECIFIC 35 (ATFDB35)*	F-box protein (DUF295)
AT5G25340		Ubiquitin-like superfamily protein
AT5G25350	*EIN3-BINDING F BOX PROTEIN 2 (EBF2)*	Arabidopsis thaliana EIN3-binding F-box protein 2 (EBF2) mRNA. Part of the SCF complex, it is located in the nucleus and is involved in the ethylene-response pathway.
AT5G25360		hypothetical protein
AT5G25370	*PHOSPHOLIPASE D ALPHA 3 (PLDALPHA3)*	member of C2-PLD subfamily. Analyses on the gene structures/sequences, overall amino acid sequences, and domain structures indicate that PLDalpha3 is most closely related to other two PLDalphas than to other PLDs. Phylogenetic analysis has not identified a true ortholog for PLDalpha3. Involved in hyperosmotic response.
AT5G25380	*CYCLIN A2;1 (CYCA2;1)*	core cell cycle genes
AT5G25390	*SHINE3 (SHN3)*	encodes a member of the ERF (ethylene response factor) subfamily B-6 of ERF/AP2 transcription factor family. The protein contains one AP2 domain. There are 12 members in this subfamily including RAP2.11.
AT5G25400		Nucleotide-sugar transporter family protein
AT5G25410		transmembrane protein, putative (DUF239)
AT5G25415		hypothetical protein (DUF239)
AT5G25420		Xanthine/uracil/vitamin C permease
AT5G25430		HCO3- transporter family
AT5G25440	*SUPPRESSOR OF ZED1-D1 (SZE1)*	Receptor like kinase involved in HopZ1a effector triggered immunity. Interacts with ZAR1. Localization to membrane is dependent on N-terminal myristoylation domain.
AT5G25450		Cytochrome bd ubiquinol oxidase, 14kDa subunit
AT5G25460	*DUF642 L-GALL RESPONSIVE GENE 2 (DGR2)*	Encodes a DUF642 cell wall protein.
AT5G25470		AP2/B3-like transcriptional factor family protein
AT5G25475		AP2/B3-like transcriptional factor family protein
AT5G25490		Ran BP2/NZF zinc finger-like superfamily protein
AT5G25500		exosome complex exonuclease
AT5G25530		DNAJ heat shock family protein
AT5G25550		Leucine-rich repeat (LRR) family protein
AT5G25580	*DDT-RELATED PROTEIN5 (DDR5)*	hypothetical protein
AT5G25590		DNA ligase (DUF630 and DUF632)
AT5G25610	*RESPONSIVE TO DESICCATION 22 (RD22)*	responsive to dehydration 22 (RD22) mediated by ABA
AT5G25620	*YUCCA6 (YUC6)*	Encodes a member of a family of flavin monooxygenases with an important role in auxin biosynthesis. YUC6 possesses an additional thiol-reductase activity that confers drought resistance independently of auxin biosynthesis.
AT5G25630		Tetratricopeptide repeat (TPR)-like superfamily protein
AT5G25640		Rhomboid-related intramembrane serine protease family protein
AT5G25750		hypothetical protein
AT5G25770		alpha/beta-Hydrolases superfamily protein
AT5G25780	*EUKARYOTIC TRANSLATION INITIATION FACTOR 3B-2 (EIF3B-2)*	member of eIF3b - eukaryotic initiation factor 3b
AT5G25800		Polynucleotidyl transferase, ribonuclease H-like superfamily protein
AT5G25810	*TINY (tny)*	encodes a member of the DREB subfamily A-4 of ERF/AP2 transcription factor family (TINY). The protein contains one AP2 domain. There are 17 members in this subfamily including TINY. Ectopic or overexpression of this gene in a Ds tagged line has reduced cell expansion. The expression of this gene is induced by ethylene and light and appears to stimulate cytokinin biosynthesis.
AT5G25820		Exostosin family protein
AT5G25830	*GATA TRANSCRIPTION FACTOR 12 (GATA12)*	Encodes a member of the GATA factor family of zinc finger transcription factors.
AT5G25840		DUF1677 family protein (DUF1677)
AT5G25860		F-box/RNI-like superfamily protein
AT5G25880	*NADP-MALIC ENZYME 3 (NADP-ME3)*	The malic enzyme (EC 1.1.1.40) encoded by the ATNADP-ME3 is presumably cytosolic and restricted in its expression by both developmental and cell-specific signals.
AT5G25890	*INDOLE-3-ACETIC ACID INDUCIBLE 28 (IAA28)*	encodes a protein that may be a negative regulator of lateral root formation in response to auxin. It is a member of IAA/ARF gene family and is plant-specific. Gain of function mutations in this gene suppresses lateral root formation and is resistant to inhibition of root elongation by auxin, cytokinin, and ethylene.
AT5G25910	*RECEPTOR LIKE PROTEIN 52 (RLP52)*	putative disease resistance protein induced by chitin oligomers.
AT5G25920		hypothetical protein
AT5G25930		kinase family with leucine-rich repeat domain-containing protein
AT5G25950		AslB, putative (DUF239)
AT5G25960		AslB (DUF239)
AT5G25980	*GLUCOSIDE GLUCOHYDROLASE 2 (TGG2)*	Myrosinase (thioglucoside glucohydrolase) gene involved in glucosinoloate metabolism. The mRNA is cell-to-cell mobile.
AT5G25990		core-2/I-branching beta-1,6-N-acetylglucosaminyltransferase family protein
AT5G26010		Protein phosphatase 2C family protein

AT5G26020		transposable_element_gene;similar to Glutamic acid-rich protein precursor (GB:P13816);(source:TAIR10)
AT5G26060		Plant self-incompatibility protein S1 family
AT5G26070		Encodes a protein with 23.5% proline residues and proline-rich extensin domains, INTERPRO:IPR002965; similar to root nodule extensin (Pisum sativum) gi:15021750/gb:AAK77902; Common family members: At5g19800, At5g57070, At1g72790 (Arabidopsis thaliana)
AT5G26080		proline-rich family protein
AT5G26100		hypothetical protein
AT5G26110		Protein kinase superfamily protein
AT5G26120	*ALPHA-L-ARABINOFURANOSIDASE 2 (ASD2)*	alpha-L-arabinofuranosidase 2
AT5G26130	*(ATCAPE8)*	CAP (Cysteine-rich secretory proteins, Antigen 5, and Pathogenesis-related 1 protein) superfamily protein
AT5G26140	*LONELY GUY 9 (LOG9)*	Putative lysine decarboxylase family protein
AT5G26150		protein kinase family protein
AT5G26180	*(NSUN5)*	S-adenosyl-L-methionine-dependent methyltransferases superfamily protein
AT5G26190		Cysteine/Histidine-rich C1 domain family protein
AT5G26200		Mitochondrial substrate carrier family protein
AT5G26210	*ALFIN-LIKE 4 (AL4)*	Encodes a member of the Alfin1-like family of nuclear-localized PHD (plant homeodomain) domain containing proteins. All AL proteins except AL3 bind to di- or trimethylated histone H3 (H3K4me3/2). Members of this family include: AT5G05610 (AL1), AT3G11200 (AL2), AT3G42790 (AL3), AT5G26210 (AL4), AT5G20510 (AL5), AT2G02470 (AL6), AT1G14510 (AL7).
AT5G26220	*GAMMA-GLUTAMYL CYCLOTRANSFERASE 2;1 (GGCT2;1)*	ChaC-like family protein
AT5G26230	*MEMBRANE-ASSOCIATED KINASE REGULATOR 1 (MAKR1)*	Encodes a member of the MAKR (MEMBRANE-ASSOCIATED KINASE REGULATOR) gene family. MAKRs have putative kinase interacting motifs and membrane localization signals. Known members include: AT5G26230 (MAKR1), AT1G64080 (MAKR2), AT2G37380 (MAKR3), AT2G39370 (MAKR4), AT5G52870 (MAKR5) and AT5G52900 (MAKR6).
AT5G26240	*CHLORIDE CHANNEL D (CLC-D)*	Anion channel protein family member. Involved in negative regulation of pattern triggered immunity.
AT5G26250	*SUGAR TRANSPORT PROTEIN 8 (STP8)*	Sugar transporter expressed strongly in pollen and pollen tubes.
AT5G26260		TRAF-like family protein
AT5G26270		transmembrane protein
AT5G26280		TRAF-like family protein
AT5G26310	*(UGT72E3)*	UGT72E3 is an UDPG:coniferyl alcohol glucosyltransferase which glucosylates sinapyl- and coniferyl alcohol as well as sinapic acid. The enzyme is thought to be involved in lignin- and phenylpropanoid metabolism. A knockdown mutant line (72E3KD) was obtained using RNAi silencing. No reduction in coniferyl alcohol 4-<i>O</i>-glucoside and sinapyl alcohol 4-<i>O</i>-glucoside was detected in this line compared to wildtype, in contrast with the knockdown line constructed for UGT72E2 displayed a twofold reduction in the these phenylpropanoid 4-<i>O</i>-glucosides. Can influence the kinetics of lignin deposition by regulating monolignol flow to the cell wall as well as the potential of this compartment to incorporate monomers into the growing lignin polymer.
AT5G26330		Cupredoxin superfamily protein
AT5G26340	*(MSS1)*	Encodes a protein with high affinity, hexose-specific/H+ symporter activity. The activity of the transporter appears to be negatively regulated by phosphorylation. Importantly, microarray analysis, as well as the study of the expression of this gene in mutants involved in programmed cell death (PCD) demonstrated a tight correlation between this gene's expression and PCD.
AT5G26350		transposable_element_gene;similar to unknown protein [Arabidopsis thaliana] (TAIR:AT5G33393.1);(source:TAIR10)
AT5G26595		
AT5G26605		
AT5G26615		
AT5G26625		
AT5G26635		
AT5G26645		
AT5G26655		
AT5G26665		
AT5G26675		
AT5G26696		
AT5G26718		
AT5G26731		hypothetical protein
AT5G26760	*RPAP2 IYO MATE (RIMA)*	Encodes RPAP2 IYO Mate (RIMA), a homologue of yeast and human proteins linked to nuclear import of selective cargo. Knockdown of RIMA causes delayed onset of cell differentiation.
AT5G26770	*(ATNEAP2)*	NEAP2 is a member of a small family containing coiled-coil domains, a nuclear localization signal and a C-terminal predicted transmembrane domain. It localizes to the nuclear periphery. Mutants have altered nuclear morphology and chromatin structure.
AT5G26780	*SERINE HYDROXYMETHYLTRANSFERASE 2 (SHM2)*	Encodes a protein with serine hydroxymethyltransferase activity which is thought to be localized in the mitochondrial matrix. SHM2 expression fails to rescue the conditional lethal phenotype of the shm1-1 mutant, defective in SHM1.
AT5G26790		transmembrane protein
AT5G26840		transmembrane protein
AT5G26850	*EFR3 OF PLANT 4 (EFOP4)*	Part of a nanodomain complex that tethers PI4Kα1 to the plasma membrane.

AT5G26860	*LON PROTEASE 1 (LON1)*	Encodes a member of the Lon protease-like proteins (Lon1/At5g26860, Lon2/At5g47040, Lon3/At3g05780, Lon4/At3g05790). Lon is a multifunctional ATP-dependent protease which exists in bacteria, archaea and within organelles in eukaryotic cells. Lon proteases are responsible for the degradation of abnormal, damaged and unstable proteins. The mRNA is cell-to-cell mobile.
AT5G26870		
AT5G26880	*AGAMOUS-LIKE 26 (AGL26)*	Root Specific
AT5G26890		hypothetical protein
AT5G26900	*CELL DIVISION CYCLE 20.4 (CDC20.4)*	No expression of gene detected yet.
AT5G26910	*TON1 RECRUITING MOTIF 8 (TRM8)*	Encodes a member of the TRM superfamily, that plays a role in preprophase band formation during plant cell division and controls the robustness of the orientation of that cell division.
AT5G26920	*CAM-BINDING PROTEIN 60-LIKE G (CBP60G)*	Encodes a calmodulin-binding protein CBP60g (calmodulin binding protein 60-like.g). The calmodulin-binding domain is located near the N-terminus; calmodulin binding is dependent on Ca(2+). Inducible by both bacterial pathogen and MAMP (microbe-associated molecular pattern) treatments. Bacterial growth is enhanced in cbp60g mutants. cbp60g mutants also show defects in salicylic acid (SA) accumulation and SA signaling.
AT5G26930	*GATA TRANSCRIPTION FACTOR 23 (GATA23)*	Encodes a member of the GATA factor family of zinc finger transcription factors. Controls lateral root founder cell specification.
AT5G26960		Galactose oxidase/kelch repeat superfamily protein
AT5G26970		hypothetical protein
AT5G26980	*SYNTAXIN OF PLANTS 41 (SYP41)*	member of SYP4 Gene Family
AT5G27000	*KINESIN 4 (ATK4)*	Encodes a kinesin-like protein that binds microtubules in an ATP-dependent manner.
AT5G27020		hypothetical protein
AT5G27040		
AT5G27050	*AGAMOUS-LIKE 101 (AGL101)*	AGAMOUS-like 101
AT5G27080	*CELL DIVISION CYCLE 20.3 (CDC20.3)*	No expression of gene detected yet.
AT5G27100	*GLUTAMATE RECEPTOR 2.1 (GLR2.1)*	member of Putative ligand-gated ion channel subunit family
AT5G27140		NOP56-like pre RNA processing ribonucleoprotein
AT5G27160		transposable_element_gene;similar to unknown protein [Arabidopsis thaliana] (TAIR:AT4G07520.1);(source:TAIR10)
AT5G27170		hypothetical protein
AT5G27180		transposable_element_gene;similar to unknown protein [Arabidopsis thaliana] (TAIR:AT4G07523.1);(source:TAIR10)
AT5G27190		transposable_element_gene;Mutator-like transposase family, has a 1.3e-76 P-value blast match to O22278 /203-375 Pfam PF03108 MuDR family transposase (MuDr-element domain);(source:TAIR10)
AT5G27200	*ACYL CARRIER PROTEIN 5 (ACP5)*	Encodes an acyl carrier protein family member. Expression is induced by salt stress and overexpression leads to increased salt tolerance.
AT5G27220		Frigida-like protein
AT5G27230		Frigida-like protein
AT5G27280		Zim17-type zinc finger protein
AT5G27290		stress regulated protein
AT5G27310		Transcription factor IIS family protein
AT5G27320	*GA INSENSITIVE DWARF1C (GID1C)*	Encodes a gibberellin (GA) receptor ortholog of the rice GA receptor gene (OsGID1). Has GA-binding activity, showing higher affinity to GA4. Interacts with DELLA proteins in vivo in the presence of GA4.
AT5G27330		Prefoldin chaperone subunit family protein
AT5G27340		hypothetical protein
AT5G27350	*(SFP1)*	Encodes a sugar-porter family protein that is induced during leaf senescence. The increase in its gene expression during leaf senescence is paralleled by an accumulation of monosaccharides. The mRNA is cell-to-cell mobile.
AT5G27360	*(SFP2)*	Encodes a sugar-porter family protein that unlike the closely related gene, SFP1, is not induced during leaf senescence.
AT5G27380	*GLUTATHIONE SYNTHETASE 2 (GSH2)*	Encodes a protein with similarity to glutathione synthetases, which catalyzes one of the early steps in glutathione biosynthesis. Two transcripts have been detected; the longer transcript is less abundant and the protein is localized to the chloroplast. The smaller transcript, in which the transit peptide is truncated, is localized to the cytosol. Increased glutathione accumulation in response to cesium stress.
AT5G27390		tagatose-6-phosphate ketose/aldose isomerase, putative (Mog1/PsbP/DUF1795-like photosystem II reaction center PsbP family protein)
AT5G27410		D-aminoacid aminotransferase-like PLP-dependent enzymes superfamily protein; FUNCTIONS IN: catalytic activity; INVOLVED IN: metabolic process; LOCATED IN: cellular_component unknown; EXPRESSED IN: 20 plant structures; EXPRESSED DURING: 10 growth stages; CONTAINS InterPro DOMAIN/s: Aminotransferase, class IV (InterPro:IPR001544); BEST Arabidopsis thaliana protein match is: D-aminoacid aminotransferase-like PLP-dependent enzymes superfamily protein (TAIR:AT3G05190.1). Note that the At5g27410.2 gene model (TAIR10) has been obsoleted due to the lack of experimental support.
AT5G27420	*CARBON/NITROGEN INSENSITIVE 1 (CNI1)*	Encodes CNI1 (Carbon/Nitrogen Insensitive1) (also named as ATL31), a RING type ubiquitin ligase that functions in the Carbon/Nitrogen response for growth phase transition in Arabidopsis seedlings. The mRNA is cell-to-cell mobile.
AT5G27440		transmembrane protein
AT5G27480		transposable_element_gene;pseudogene, hypothetical protein, putative replication proteins - Arabidopsis thaliana;(source:TAIR10)
AT5G27510		Protein kinase superfamily protein
AT5G27520	*PEROXISOMAL ADENINE NUCLEOTIDE CARRIER 2 (PNC2)*	encodes a peroxisomal adenine nucleotide transporter, involved in fatty acid beta-oxidation during early stage of postgerminative growth.
AT5G27530		Member of pectin lyase gene family.
AT5G27540	*MIRO-RELATED GTP-ASE 1 (MIRO1)*	Encodes a protein with similarity to GTPases that is localized to the mitochondrion. Involved in embryogenesis, pollen tube growth and required for mitochondrial development.
AT5G27550		P-loop containing nucleoside triphosphate hydrolases superfamily protein

AT5G27560		DUF1995 domain protein, putative (DUF1995)
AT5G27570	*CELL DIVISION CYCLE 20.5 (CDC20.5)*	No expression of gene detected yet.
AT5G27580	*AGAMOUS-LIKE 89 (AGL89)*	AGAMOUS-like 89
AT5G27600	*LONG-CHAIN ACYL-COA SYNTHETASE 7 (LACS7)*	Encode peroxisomal long-chain acyl-CoA synthetase. Activates fatty acids for further metabolism. Interacts with PEX5.
AT5G27610	*ALWAYS EARLY 1 (ALY1)*	protein ALWAYS EARLY 1
AT5G27630	*ACYL-COA BINDING PROTEIN 5 (ACBP5)*	Acyl-CoA binding protein with high affinity for oleoyl-CoA. Expressed in all plant organs. Involved in fatty acid transport. Plays a role in determining seed oil content.
AT5G27660	*DEGRADATION OF PERIPLASMIC PROTEINS 14 (DEG14)*	Encodes a protein with similarity to human PARK13, a mitochondrial protease implicated in Parkinson disease. DEG14 is induced by heat stress and involved in degradation of misfolded proteins.
AT5G27730		heparan-alpha-glucosaminide N-acetyltransferase-like protein (DUF1624)
AT5G27740	*EMBRYO DEFECTIVE 2775 (EMB2775)*	A locus involved in embryogenesis. Mutations in this locus result in embryo lethality.
AT5G27750		F-box/FBD-like domains containing protein
AT5G27760	*HYPOXIA-INDUCED GENE DOMAIN 2 (ATHIGD2)*	Hypoxia-responsive family protein
AT5G27800		Class II aminoacyl-tRNA and biotin synthetases superfamily protein
AT5G27810		MADS-box transcription factor family protein
AT5G27830	*FOLATE BINDING PROTEIN (FBP)*	Folate receptor family protein.Expression correlates with increase in bound folate in planta.
AT5G27860		hypothetical protein
AT5G27870		Plant invertase/pectin methylesterase inhibitor superfamily
AT5G27890		hypothetical protein
AT5G27900		transposable_element_gene;non-LTR retrotransposon family (LINE), has a 2.1e-26 P-value blast match to GB:AAB41224 ORF2 (LINE-element) (Rattus norvegicus);(source:TAIR10)
AT5G27910	*NUCLEAR FACTOR Y, SUBUNIT C8 (NF-YC8)*	nuclear factor Y, subunit C8
AT5G27920	*RAE1 HOMOLOG 1 (RAH1)*	Encodes a nuclear F-box protein that can directly interact with the C2H2‐type zinc finger transcription factor STOP1 and promote its ubiquitination and degradation. STOP1 is crucial for aluminum (Al) resistance.
AT5G27930	*E GROWTH-REGULATING 2 (EGR2)*	EGR2 functions as a negative regulator of plant growth with prominent effect on plant growth during drought stress. EGR2 regulates microtubule organization and likely affects additional cytoskeleton and trafficking processes along the plasma membrane.
AT5G27940	*WPP DOMAIN PROTEIN 3 (WPP3)*	WPP domain protein 3
AT5G27950		P-loop containing nucleoside triphosphate hydrolases superfamily protein
AT5G27980		Seed maturation protein
AT5G27990		Pre-rRNA-processing protein TSR2, conserved region
AT5G28010		Polyketide cyclase/dehydrase and lipid transport superfamily protein
AT5G28020	*CYSTEINE SYNTHASE D2 (CYSD2)*	Encodes cysteine synthase CysD2.
AT5G28030	*L-CYSTEINE DESULFHYDRASE 1 (DES1)*	Encodes a novel l-cysteine desulfhydrase involved in cysteine homeostasis that catalyzes the desulfuration of Cys, instead of the synthesis of Cys like the OASTL, to produce sulfide plus ammonia and pyruvate.
AT5G28040	*VIRF INTERACTING PROTEIN 4 (VFP4)*	Member of the GeBP/GPL family of leucine zipper transcription factors. VPF4 interacts with the F-box proteins from A.tumefaciens VirF and VBF. Over expression results in decreased tumor formation upon Agrobacterium infection. Mutants show changes in the level of expression of defense response genes.
AT5G28060	*(RPS24B)*	Ribosomal protein S24e family protein
AT5G28070		
AT5G28080	*(WNK9)*	Encodes a member of the WNK family (9 members in all) of protein kinases, the structural design of which is clearly distinct from those of other known protein kinases, such as receptor-like kinases and mitogen-activated protein kinases.
AT5G28090		hypothetical protein
AT5G28110		transposable_element_gene;similar to unknown protein [Arabidopsis thaliana] (TAIR:AT5G28120.1);(source:TAIR10)
AT5G28130		transposable_element_gene;similar to unknown protein [Arabidopsis thaliana] (TAIR:AT4G04155.1);(source:TAIR10)
AT5G28140		transposable_element_gene
AT5G28150		hypothetical protein (DUF868)
AT5G28160		Galactose oxidase/kelch repeat superfamily protein
AT5G28170		transposable_element_gene;similar to Ulp1 protease family protein [Arabidopsis thaliana] (TAIR:AT1G35110.1);(source:TAIR10)
AT5G28190		transmembrane protein
AT5G28200		transposable_element_gene;pseudogene, hypothetical protein, various predicted proteins, Arabidopsis thaliana;(source:TAIR10)
AT5G28210		mRNA capping enzyme family protein
AT5G28230		pseudogene of glucose-6-phosphate/phosphate translocator 2
AT5G28240		transposable_element_gene;similar to Ulp1 protease family protein [Arabidopsis thaliana] (TAIR:AT1G35770.1);(source:TAIR10)
AT5G28270		transposable_element_gene;similar to unknown protein [Arabidopsis thaliana] (TAIR:AT5G28480.1);(source:TAIR10)
AT5G28280		pseudogene of sterol desaturase domain-containing protein
AT5G28300	*GT-2LIKE PROTEIN (GT2L)*	Encodes a Ca(2+)-dependent CaM-binding protein. AtGT2L specifically targets the nucleus and possesses both transcriptional activation and DNA-binding abilities, implicating its function as a nuclear transcription factor.
AT5G28310		NAD(P)-binding Rossmann-fold superfamily protein
AT5G28370		Pentatricopeptide repeat (PPR) superfamily protein
AT5G28380		Tetratricopeptide repeat (TPR)-like superfamily protein

AT5G28410		hypothetical protein
AT5G28420		Pentatricopeptide repeat (PPR) superfamily protein
AT5G28440		transposable_element_gene;unknown protein;(source:TAIR10)
AT5G28470	*FLAVONOL SOPHOROSIDE TRANSPORTER 1 (FST1)*	Encodes a member of the nitrate/peptide NTR/PTR family of transporters is required for accumulation and transport of pollen-specific flavonol 3-O-sophorosides, characterized by a glycosidic β-1,2-linkage, to the pollen surface of Arabidopsis.
AT5G28490	*LIGHT-DEPENDENT SHORT HYPOCOTYLS 1 (LSH1)*	Encodes a nuclear protein that mediates light regulation of seedling development in a phytochrome-dependent manner.
AT5G28500		rubisco accumulation factor-like protein
AT5G28510	*BETA GLUCOSIDASE 24 (BGLU24)*	beta glucosidase 24
AT5G28520		Encodes a predicted target jacalin lectin that is transcriptionally upregulated by ABA and is cleaved, possibly by a variant 'isoMIR' of miR846 in roots.
AT5G28550		separase
AT5G28560		hypothetical protein
AT5G28570		transposable_element_gene;similar to unknown protein [Arabidopsis thaliana] (TAIR:AT1G12725.1);(source:TAIR10)
AT5G28580		transposable_element_gene;Mutator-like transposase family, has a 7.3e-33 P-value blast match to GB:AAA21566 mudrA of transposon=MuDR (MuDr-element) (Zea mays);(source:TAIR10)
AT5G28590		DNA-binding family protein
AT5G28600		transposable_element_gene;similar to Ulp1 protease family protein [Arabidopsis thaliana] (TAIR:AT1G35770.1);(source:TAIR10)
AT5G28610		LOW protein: ATP-dependent RNA helicase DRS1-like protein
AT5G28620		kinase C-like protein
AT5G28630		glycine-rich protein
AT5G28640	*ANGUSTIFOLIA 3 (AN3)*	Encodes a protein with similarity to mammalian transcriptional coactivator that is involved in cell proliferation during leaf and flower development. Loss of function mutations have narrow, pointed leaves and narrow floral organs. AN3 interacts with members of the growth regulating factor (GRF) family of transcription factors.
AT5G28650	*WRKY DNA-BINDING PROTEIN 74 (WRKY74)*	member of WRKY Transcription Factor; Group II-d
AT5G28670		transposable_element_gene;pseudogene, similar to putative helicase, predicted helicase proteins, Arabidopsis thaliana;(source:TAIR10)
AT5G28680	*ANXUR2 (ANX2)*	Receptor-like kinase required for maintenance of pollen tube growth. Display polar localization at the plasma membrane of the pollen tube tip.
AT5G28700		transposable_element_gene;pseudogene, similar to OSJNBa0026J14.30, various predicted proteins, Arabidopsis thaliana and others;(source:TAIR10)
AT5G28750	*TWIN-ARGININE TRANSLOCATION A (TATA)*	Small, dynamic subunit of the Tat-system that is believed to be the active component during target protein translocation. The twin-arginine translocase (Tat)system mediates the transport of already-folded proteins across membranes.
AT5G28760		transposable_element_gene;similar to unknown protein [Arabidopsis thaliana] (TAIR:AT5G27160.1);(source:TAIR10)
AT5G28770	*(BZO2H3)*	BASIC LEUCINE ZIPPER protein which regulates the circadian oscillator gene PSEUDO RESPONSE REGULATOR7 (PRR7) to change the circadian phase in response to sugars. It upregulates PRR7 in response to low energy. bZIP63 and PRR7 are required for correct oscillator phase under light/dark cycles. bZIP protein BZO2H3 mRNA, partial cds
AT5G28780		PIF1 helicase
AT5G28790		transposable_element_gene
AT5G28800		
AT5G28810		Ulp1 protease family protein
AT5G28820		Myb/SANT-like DNA-binding domain protein
AT5G28840	*GDP-D-MANNOSE 3 (GME)*	Encodes a protein with GDP-D-mannose 3',5'-epimerase activity. The enzyme is involved in ascorbate biosynthesis. It catalyzes the conversion of GDP-D-mannose to GDP-L-galactose.
AT5G28860		transposable_element_gene;similar to unknown protein [Arabidopsis thaliana] (TAIR:AT2G01031.1);(source:TAIR10)
AT5G28870		transposable_element_gene;pseudogene, hypothetical protein;(source:TAIR10)
AT5G28880		transposable_element_gene;pseudogene, hypothetical protein;(source:TAIR10)
AT5G28890		transposable_element_gene;similar to DNA binding [Arabidopsis thaliana] (TAIR:AT4G01980.1);(source:TAIR10)
AT5G28910		alpha-(1,6)-fucosyltransferase
AT5G28920		hypothetical protein
AT5G28930		transposable_element_gene;similar to Ulp1 protease family protein [Arabidopsis thaliana] (TAIR:AT2G07240.1);(source:TAIR10)
AT5G28940		transposable_element_gene;similar to unknown protein [Arabidopsis thaliana] (TAIR:AT2G07630.1);(source:TAIR10)
AT5G28950		nuclease
AT5G29000	*PHR1-LIKE 1 (PHL1)*	MYB-CC family member. PHL1 acts redundantly with PHR1 to regulate responses to Pi starvation.
AT5G29010		
AT5G29020		transposable_element_gene;similar to unknown protein [Arabidopsis thaliana] (TAIR:AT3G60930.1);(source:TAIR10)
AT5G29030		pseudogene of myosin heavy chain-like protein
AT5G29040		pseudogene of myosin heavy chain-like protein
AT5G29050		hypothetical protein (DUF3287)
AT5G29060		transposable_element_gene;pseudogene, hypothetical protein;(source:TAIR10)
AT5G29070		transmembrane protein
AT5G29080		
AT5G29090		transposable_element_gene;similar to unknown protein [Arabidopsis thaliana] (TAIR:AT3G33131.1);(source:TAIR10)

AT5G29100		transposable_element_gene;gypsy-like retrotransposon family (Athila), has a 1.2e-12 P-value blast match to GB:CAA57397 Athila ORF 1 (Arabidopsis thaliana);(source:TAIR10)
AT5G29210		hypothetical protein
AT5G29397		
AT5G29584		
AT5G29771		
AT5G29958		
AT5G30145		
AT5G30332		
AT5G30426		
AT5G30820		
AT5G31017		
AT5G31411		
AT5G32202		
AT5G32400		transposable_element_gene;hypothetical protein;(source:TAIR10)
AT5G32410		transposable_element_gene;similar to unknown protein [Arabidopsis thaliana] (TAIR:AT2G07710.1);(source:TAIR10)
AT5G32430		transposable_element_gene;pseudogene, hypothetical protein;(source:TAIR10)
AT5G32460		Transcriptional factor B3 family protein
AT5G32480		
AT5G32490		transposable_element_gene;pseudogene, hypothetical protein;(source:TAIR10)
AT5G32510		transposable_element_gene;pseudogene, hypothetical protein;(source:TAIR10)
AT5G32530		
AT5G32540		transposable_element_gene;pseudogene, hypothetical protein, predicted proteins, Arabidopsis thaliana similar to hypothetical protein GI:4263830 from (Arabidopsis thaliana) (similar to MYOSIN HEAVY CHAIN (Encephalitozoon cuniculi) GI:19074177) similar to hypothetical protein GI:4263830 from (Arabidopsis thaliana) (similar to MYOSIN HEAVY CHAIN (Encephalitozoon cuniculi) GI:19074177) (similar to MYOSIN HEAVY CHAIN (Encephalitozoon cuniculi) GI:19074177);(source:TAIR10)
AT5G32550		
AT5G32560		
AT5G32570		
AT5G32580		transposable_element_gene;gypsy-like retrotransposon family, has a 8.2e-241 P-value blast match to GB:AAD19359 polyprotein (gypsy_Ty3-element) (Sorghum bicolor);(source:TAIR10)
AT5G32590		myosin heavy chain-like protein
AT5G32600		transposable_element_gene;similar to unknown protein [Arabidopsis thaliana] (TAIR:AT3G60930.1);(source:TAIR10)
AT5G32610		transposable_element_gene;similar to unknown protein [Arabidopsis thaliana] (TAIR:AT5G33715.1);(source:TAIR10)
AT5G32630		transposable_element_gene;pseudogene, similar to putative helicase, various predicted helicase proteins, Arabidopsis thaliana and others;(source:TAIR10)
AT5G33200		transposable_element_gene;similar to unknown protein [Arabidopsis thaliana] (TAIR:AT3G42400.1);(source:TAIR10)
AT5G33240		transposable_element_gene;similar to unknown protein [Arabidopsis thaliana] (TAIR:AT3G42980.1);(source:TAIR10)
AT5G33250		transposable_element_gene;pseudogene, hypothetical protein, predicted proteins - Arabidopsis thaliana;(source:TAIR10)
AT5G33260		transposable_element_gene;pseudogene, hypothetical protein, predicted proteins - Arabidopsis thaliana;(source:TAIR10)
AT5G33290	*XYLOGALACTURONAN DEFICIENT 1 (XGD1)*	Acts as a xylogalacturonan xylosyltransferase within the XGA biosynthesis pathway. Involved in pectin biosynthesis.
AT5G33300		chromosome-associated kinesin-like protein
AT5G33370	*CUTIN SYNTHASE2 (CUS2)*	GDSL-motif esterase/acyltransferase/lipase. Enzyme group with broad substrate specificity that may catalyze acyltransfer or hydrolase reactions with lipid and non-lipid substrates. Mutants are defective in cuticle formation with reduced sepal cuticle ridge formation.
AT5G33380		transposable_element_gene;similar to unknown protein [Arabidopsis thaliana] (TAIR:AT5G28430.1);(source:TAIR10)
AT5G34450		transposable_element_gene;similar to unknown protein [Arabidopsis thaliana] (TAIR:AT2G05087.1);(source:TAIR10)
AT5G34460		transposable_element_gene;similar to replication protein-related [Arabidopsis thaliana] (TAIR:AT5G34950.1);(source:TAIR10)
AT5G34770		transposable_element_gene;CACTA-like transposase family (En/Spm), has a 3.2e-131 P-value blast match to GB:AAD55677 putative transposase protein (CACTA-element) transposon=Shooter (Zea mays);(source:TAIR10)
AT5G34790		transposable_element_gene;CACTA-like transposase family (Ptta/En/Spm), has a 2.9e-11 P-value blast match to At5g36655.1/81-333 CACTA-like transposase family (Ptta/En/Spm) (CACTA-element) (Arabidopsis thaliana);(source:TAIR10)
AT5G34800		transposable_element_gene;pseudogene, hypothetical protein, predicted proteins - Arabidopsis thaliana;(source:TAIR10)
AT5G34820		transposable_element_gene;similar to unknown protein [Arabidopsis thaliana] (TAIR:AT5G20770.1);(source:TAIR10)
AT5G34830		hypothetical protein
AT5G34840		transposable_element_gene;Mutator-like transposase family, has a 2.1e-07 P-value blast match to GB:AAA21566 mudrA of transposon=MuDR (MuDr-element) (Zea mays);(source:TAIR10)
AT5G34850	*PURPLE ACID PHOSPHATASE 26 (PAP26)*	Encodes a root-secreted purple acid phosphatase precursor involved in extracellular phosphate-scavenging.
AT5G34870		zinc knuckle (CCHC-type) family protein
AT5G34880		transposable_element_gene;gypsy-like retrotransposon family, has a 2.2e-11 P-value blast match to GB:AAD19359 polyprotein (gypsy_Ty3-element) (Sorghum bicolor);(source:TAIR10)

AT5G34885		inhibitor/lipid-transfer protein/seed storage 2S albumin superfamily protein (DUF784)
AT5G34895		transposable_element_gene;similar to heat shock protein binding [Arabidopsis thaliana] (TAIR:AT1G32830.1);(source:TAIR10)
AT5G34900		transposable_element_gene;similar to Ulp1 protease family protein [Arabidopsis thaliana] (TAIR:AT3G47260.1);(source:TAIR10)
AT5G34920		transposable_element_gene;CACTA-like transposase family (Ptta/En/Spm), has a 3.2e-40 P-value blast match to At5g59620.1/14-257 CACTA-like transposase family (Ptta/En/Spm) (CACTA-element) (Arabidopsis thaliana);(source:TAIR10)
AT5G34940	*GLUCURONIDASE 3 (GUS3)*	The protein is predicted (WoLF PSORT program) to be membrane-associated.
AT5G34950		transposable_element_gene;similar to unknown protein [Arabidopsis thaliana] (TAIR:AT2G05084.1);(source:TAIR10)
AT5G34990		transposable_element_gene;pseudogene, Ulp1 protease family, contains Pfam profile PF02902: Ulp1 protease family, C-terminal catalytic domain;(source:TAIR10)
AT5G35010		transposable_element_gene;similar to unknown protein [Arabidopsis thaliana] (TAIR:AT2G14774.1);(source:TAIR10)
AT5G35050		hypothetical protein (DUF1985)
AT5G35060		
AT5G35070		pseudogene of hypothetical protein
AT5G35090		hypothetical protein
AT5G35100		Cyclophilin-like peptidyl-prolyl cis-trans isomerase family protein
AT5G35130		transposable_element_gene;pseudogene, similar to putative helicase, predicted proteins from different species;(source:TAIR10)
AT5G35140		transposable_element_gene;CACTA-like transposase family (Ptta/En/Spm), has a 1.4e-23 P-value blast match to At1g36190.1/92-340 CACTA-like transposase family (Ptta/En/Spm) (CACTA-element) (Arabidopsis thaliana);(source:TAIR10)
AT5G35150		transposable_element_gene;CACTA-like transposase family (Ptta/En/Spm), has a 1.6e-26 P-value blast match to At5g29026.1/8-244 CACTA-like transposase family (Ptta/En/Spm) (CACTA-element) (Arabidopsis thaliana);(source:TAIR10)
AT5G35160	*(TMN11)*	Endomembrane protein 70 protein family
AT5G35170		adenylate kinase family protein
AT5G35180		ENHANCED DISEASE RESISTANCE protein (DUF1336)
AT5G35190	*EXTENSIN 13 (EXT13)*	proline-rich extensin-like family protein
AT5G35200	*(PICALM3)*	ENTH/ANTH/VHS superfamily protein
AT5G35210	*PHD TYPE TRANSCRIPTION FACTOR WITH TRANSMEMBRANE DOMAINS (PTM)*	Encodes a chloroplast envelope-bound plant homeodomain (PHD) transcription factor with transmembrane domains that functions in multiple retrograde signal pathways. The proteolytic cleavage of PTM occurs in response to retrograde signals and amino-terminal PTM accumulates in the nucleus, where it activates ABI4 transcription in a PHD-dependent manner associated with histone modifications.
AT5G35230		hypothetical protein
AT5G35240		transposable_element_gene;pseudogene, similar to putative transposable element, blastp match of 47%25 identity and 9.3e-52 P-value to GP|13122426|dbj|BAB32907.1||AP003047 putative transposable element {Oryza sativa (japonica cultivar-group)};(source:TAIR10)
AT5G35250		transposable_element_gene;similar to unknown protein [Arabidopsis thaliana] (TAIR:AT4G07450.1);(source:TAIR10)
AT5G35260		transposable_element_gene;similar to replication protein-related [Arabidopsis thaliana] (TAIR:AT4G07440.1);(source:TAIR10)
AT5G35270		transposable_element_gene;similar to cytochrome P-450 aromatase-related [Arabidopsis thaliana] (TAIR:AT4G07435.1);(source:TAIR10)
AT5G35280		transposable_element_gene;similar to unknown protein [Arabidopsis thaliana] (TAIR:AT4G07310.1);(source:TAIR10)
AT5G35290		
AT5G35300		hypothetical protein
AT5G35310		transposable_element_gene;copia-like retrotransposon family, has a 2.0e-24 P-value blast match to GB:BAA78424 polyprotein (Ty1_Copia-element) (Arabidopsis thaliana)gi|4996363|dbj|BAA78424.1| polyprotein (AtRE2) (Arabidopsis thaliana) (Ty1_Copia-element);(source:TAIR10)
AT5G35340		transposable_element_gene;pseudogene, hypothetical protein;(source:TAIR10)
AT5G35350		
AT5G35360	*ACETYL CO-ENZYME A CARBOXYLASE BIOTIN CARBOXYLASE SUBUNIT (CAC2)*	Encodes biotin carboxylase subunit (CAC2).
AT5G35370		S-locus lectin protein kinase family protein
AT5G35380		kinase with adenine nucleotide alpha hydrolases-like domain-containing protein
AT5G35390	*POLLEN RECEPTOR LIKE KINASE 1 (PRK1)*	Encodes a member of the receptor-like kinase family of genes. In pollen tubes, it accumulates in the plasma membrane of the apical growing tip through the process of exocytosis.
AT5G35410	*SALT OVERLY SENSITIVE 2 (SOS2)*	encodes a member of the CBL-interacting protein kinase family, is a regulatory component controlling plant potassium nutrition
AT5G35420		transposable_element_gene;pseudogene, hypothetical protein, similar to unknown protein (gb|AAD56332.1);(source:TAIR10)
AT5G35430	*(NOT10)*	Tetratricopeptide repeat (TPR)-like superfamily protein
AT5G35470		
AT5G35480		hypothetical protein
AT5G35490	*MTO 1 RESPONDING UP 1 (MRU1)*	Encodes MRU1 (mto 1 responding up). Up-regulated in mto1-1 mutant that over-accumulates soluble methionine. A maternally expressed imprinted gene.
AT5G35510		TIR-NBS-LRR class disease resistance protein
AT5G35540		transmembrane protein
AT5G35550	*TRANSPARENT TESTA 2 (TT2)*	TT2 encodes a R2R3 MYB domain putative transcription factor that acts as a key determinant in the proanthocyanidin accumulation of developing seed. It is thought that a ternary complex composed of TT2, TT8 and TTG1 is necessary for correct expression of BAN in seed endothelium.
AT5G35560		DENN (AEX-3) domain-containing protein
AT5G35570		O-fucosyltransferase family protein
AT5G35580	*(PBL13)*	Protein kinase superfamily protein

AT5G35600	*HISTONE DEACETYLASE7 (HDA7)*	Encodes a histone deacetylase that is crucial for female gametophyte development and embryogenesis.
AT5G35620	*LOSS OF SUSCEPTIBILITY TO POTYVIRUS 1 (LSP1)*	Cap-binding protein, binds to the 5' cap structure of nuclear-encoded mRNAs. Mutant is resistant to potyvirus infection.
AT5G35630	*GLUTAMINE SYNTHETASE 2 (GS2)*	chloroplastic glutamine synthetase The mRNA is cell-to-cell mobile.
AT5G35640		Putative endonuclease or glycosyl hydrolase
AT5G35660		Glycine-rich protein family
AT5G35670	*IQ-DOMAIN 33 (iqd33)*	Member of IQ67 (CaM binding) domain containing family.
AT5G35690	*WSS1/SPRTN TYPE REPAIR PROTEASE B (WSS1B)*	WT-like growth phenotype mutants of WSS1B do not display hypersensitivities after treatment with DNA-Protein crosslink inducing agents like camptothecin or cis-platin.
AT5G35700	*FIMBRIN5 (FIM5)*	Encodes FIMBRIN5, an actin bundling factor required for pollen germination and pollen tube growth. Different members of the fimbrin/plastin family have diverged biochemically during evolution to generate either tight actin bundles or loose networks with distinct biochemical and biophysical properties. FIM4 generates both actin bundles and branched actin filaments whereas FIM5 only generates actin bundles.
AT5G35710		transposable_element_gene;copia-like retrotransposon family, has a 2.5e-48 P-value blast match to dbj|BAA78425.1| polyprotein (Arabidopsis thaliana) (AtRE1) (Ty1_Copia-element);(source:TAIR10)
AT5G35735		Auxin-responsive family protein
AT5G35740		Carbohydrate-binding X8 domain superfamily protein
AT5G35750	*HISTIDINE KINASE 2 (HK2)*	Encodes histidine kinase AHK2.
AT5G35760		Beta-galactosidase related protein
AT5G35770	*STERILE APETALA (SAP)*	A recessive mutation in the Arabidopsis STERILE APETALA (SAP) causes severe aberrations in inflorescence and flower and ovule development.
AT5G35790	*GLUCOSE-6-PHOSPHATE DEHYDROGENASE 1 (G6PD1)*	Encodes a plastidic glucose-6-phosphate dehydrogenase that is sensitive to reduction by DTT and whose mRNA is more prevalent in developing organs but absent in the root.
AT5G35800		transposable_element_gene;pseudogene, similar to simiar to ribosomal protein, similar to unknown protein (gb|AAD32760.1);(source:TAIR10)
AT5G35820		transposable_element_gene;copia-like retrotransposon family, has a 0. P-value blast match to GB:CAA31653 polyprotein (Ty1_Copia-element) (Arabidopsis thaliana);(source:TAIR10)
AT5G35830		Ankyrin repeat family protein
AT5G35850		
AT5G35870		transmembrane protein
AT5G35880		transposable_element_gene;similar to unknown protein [Arabidopsis thaliana] (TAIR:AT1G32680.1);(source:TAIR10)
AT5G35890		Beta-galactosidase related protein
AT5G35900	*LOB DOMAIN-CONTAINING PROTEIN 35 (LBD35)*	LOB domain-containing protein 35
AT5G35920	*CYTOCHROME P450, FAMILY 79, SUBFAMILY A, POLYPEPTIDE 4 PSEUDOGENE (CYP79A4P)*	a cytochrome P450 pseudogene
AT5G35940		Mannose-binding lectin superfamily protein
AT5G35960		Protein kinase family protein
AT5G35970		P-loop containing nucleoside triphosphate hydrolases superfamily protein
AT5G36000		F-box protein RMF
AT5G36070		transposable_element_gene;similar to unknown protein [Arabidopsis thaliana] (TAIR:AT1G35080.1);(source:TAIR10)
AT5G36080		Myb/SANT-like DNA-binding domain protein
AT5G36090		transposable_element_gene;similar to unknown protein [Arabidopsis thaliana] (TAIR:AT3G28010.1);(source:TAIR10)
AT5G36110	*CYTOCHROME P450, FAMILY 716, SUBFAMILY A, POLYPEPTIDE 1 (CYP716A1)*	Encodes a member of the CYP716A subfamily of cytochrome P450 monooxygenases with triterpene oxidizing activity catalyzing C-28 hydroxylation of alpha-amyrin, beta-amyrin, and lupeol, producing uvaol, erythrodiol, and betulin, respectively. Additionally, it shows carboxylation activity for the C-28 position of alpha- and beta-amyrin.
AT5G36120	*COFACTOR ASSEMBLY, COMPLEX C (B6F) (CCB3)*	One of four Arabidopsis homologs of bacterial ymlg proteins.
AT5G36140	*CYTOCHROME P450, FAMILY 716, SUBFAMILY A, POLYPEPTIDE 2 (CYP716A2)*	Encodes a member of the CYP716A subfamily of cytochrome P450 monooxygenases with triterpene oxidizing activity catalyzing C-28 hydroxylation of alpha-amyrin, beta-amyrin, and lupeol, producing uvaol, erythrodiol, and betulin, respectively.In particular, 22alpha-hydroxylation activity has been observed against alpha-amyrin. Should be merged with At5g36130.
AT5G36150	*PUTATIVE PENTACYCLIC TRITERPENE SYNTHASE 3 (PEN3)*	putative pentacyclic triterpene synthase 3
AT5G36160	*TYR AMINOTRANSFERASE 2 (TAT2)*	Encodes a cytosolic L-tyrosine aminotransferase. AtTAT2 exhibits much broader amino donor specificity than AtTAT1 and can use not only Tyr but also Phe, Trp, His, Met, Leu, Ala, Ser, Cys, Asp, Asn, Gln, and Arg as amino donors.
AT5G36170	*HIGH CHLOROPHYLL FLUORESCENT 109 (HCF109)*	Required for normal processing of polycistronic plastidial transcripts
AT5G36190		F-box protein interaction domain protein
AT5G36220	*CYTOCHROME P450, FAMILY 81, SUBFAMILY D, POLYPEPTIDE 1 (CYP81D1)*	member of CYP81D family of cytochrome p450s. This gene was originally called CYP91A1, but was later renamed to CYP81D1.
AT5G36260	*(A36)*	Eukaryotic aspartyl protease family protein
AT5G36270		Annotated as pseudogene of dehydroascorbate reductase. Probably not a pseudogene based on evidence for transcription (RNA-seq) and translation (Ribo-seq) described in PMID:27791167
AT5G36280		acyl carrier protein
AT5G36650		transposable_element_gene;similar to unknown protein [Arabidopsiol thaliana] (TAIR:AT2G13320.1);(source:TAIR10)
AT5G36670		RING/FYVE/PHD zinc finger superfamily protein
AT5G36700	*2-PHOSPHOGLYCOLATE PHOSPHATASE 1 (PGLP1)*	2-phosphoglycolate phosphatase 1
AT5G36710		transmembrane protein
AT5G36720		Thionin-like gene.
AT5G36737		transposable_element_gene;pseudogene, hypothetical protein;(source:TAIR10)

AT5G36770		Pol-like polyprotein/retrotransposon, putative (DUF239)
AT5G36810		U3 small nucleolar RNA-associated protein
AT5G36840		transposable_element_gene;similar to unknown protein [Arabidopsis thaliana] (TAIR:AT3G24380.1);(source:TAIR10)
AT5G36870	*GLUCAN SYNTHASE-LIKE 9 (GSL09)*	encodes a gene similar to callose synthase
AT5G36880	*ACETYL-COA SYNTHETASE (ACS)*	Encodes a plastidic acetyl-coA synthetase. This enzyme plays a role in converting acetate to acetyl-coA in the plastids. It does not appear to be a major contributor to fatty acid biosynthesis based on mutant phenotypes. The enzyme seems to act as a monomer and may play an important role in preventing the toxic accumulation of fermentation products including acetaldehyde, acetate, and ethanol. It participates in the pyruvate dehydrogenase bypass pathway
AT5G36890	*BETA GLUCOSIDASE 42 (BGLU42)*	beta glucosidase 42
AT5G36900		hypothetical protein
AT5G36910	*THIONIN 2.2 (THI2.2)*	Encodes a thionin that is expressed at a low basal level in seedlings and shows circadian variation. Predicted to encode a PR (pathogenesis-related) protein. Belongs to the plant thionin (PR-13) family with the following members: At1g66100, At5g36910, At1g72260, At2g15010, At1g12663, At1g12660.
AT5G36930		Disease resistance protein (TIR-NBS-LRR class) family
AT5G36940	*CATIONIC AMINO ACID TRANSPORTER 3 (CAT3)*	Encodes a member of the cationic amino acid transporter (CAT) subfamily of amino acid polyamine choline transporters. Does not mediate efficient uptake of basic amino acids in yeast or Xenopus systems but can transport neutral and acidic amino acid analogs.
AT5G36950	*DEGRADATION OF PERIPLASMIC PROTEINS 10 (DEG10)*	Encodes a putative DegP protease.
AT5G36960		hypothetical protein
AT5G36990		transposable_element_gene;hAT-like transposase family (hobo/Ac/Tam3), has a 8.5e-61 P-value blast match to GB:AAD24567 transposase Tag2 (hAT-element) (Arabidopsis thaliana);(source:TAIR10)
AT5G37010		rho GTPase-activating protein
AT5G37030		P-loop containing nucleoside triphosphate hydrolases superfamily protein
AT5G37040		F-box family protein
AT5G37050		sorting nexin
AT5G37060	*CATION/H+ EXCHANGER 24 (CHX24)*	member of Putative Na+/H+ antiporter family
AT5G37070		hypothetical protein (Protein of unknown function, DUF538)
AT5G37080		transposable_element_gene;similar to unknown protein [Arabidopsis thaliana] (TAIR:AT2G05090.1);(source:TAIR10)
AT5G37090		transposable_element_gene;similar to unknown protein [Arabidopsis thaliana] (TAIR:AT2G05087.1);(source:TAIR10)
AT5G37110		transposable_element_gene;similar to unknown protein [Arabidopsis thaliana] (TAIR:AT3G13250.1);(source:TAIR10)
AT5G37140		P-loop containing nucleoside triphosphate hydrolases superfamily protein
AT5G37150		P-loop containing nucleoside triphosphate hydrolases superfamily protein
AT5G37160		P-loop nucleoside triphosphate hydrolase superfamily protein
AT5G37170		O-methyltransferase family protein
AT5G37180	*SUCROSE SYNTHASE 5 (SUS5)*	Encodes a protein with sucrose synthase activity (SUS5).
AT5G37210		Cysteine/Histidine-rich C1 domain family protein
AT5G37230		RING/U-box superfamily protein
AT5G37240		hypothetical protein
AT5G37260	*REVEILLE 2 (RVE2)*	Encodes a MYB family transcription factor Circadian 1 (CIR1). Involved in circadian regulation in Arabidopsis.
AT5G37280		RING/U-box superfamily protein
AT5G37300	*(WSD1)*	Encodes a bifunctional enzyme, wax ester synthase (WS) and diacylglycerol acyltransferase (DGAT). In vitro assay indicated a ratio of 10.9 between its WS and DGAT activities. Both mutant and in vivo expression/analysis in yeast studies indicated a role in wax biosynthesis.
AT5G37330		transposable_element_gene;pseudogene, hypothetical protein;(source:TAIR10)
AT5G37360		LOW protein: ammonium transporter 1-like protein
AT5G37370	*(ATSRL1)*	encodes a putative splicing factor. Over-expression in yeast and Arabidopsis result in increased tolerance to high salt.
AT5G37420		Note that previous reports (Plant Cell 2003,15:1538; PNAS 2003, 100:13407) have incorrectly named AT5G37420 as AGL105. AT5G37415 has now been named as AGL105 based on Plant Cell 2003, 15:1538 where the GenBank accession number given for AGL105 is AY141227 (Supplemental Table 3), which corresponds to AT5G37415.
AT5G37440		Chaperone DnaJ-domain superfamily protein
AT5G37450		Leucine-rich repeat protein kinase family protein
AT5G37490	*(PUB21)*	Plant U-box type E3 ubiquitin ligase (PUB).
AT5G37500	*GATED OUTWARDLY-RECTIFYING K+ CHANNEL (GORK)*	Encodes a guard cell outward potassium channel. Belongs to the Shaker family K+ channel. This family includes five groups based on phylogenetic analysis (FEBS Letters (2007) 581: 2357): I (inward rectifying channel): AKT1 (AT2G26650), AKT5 (AT4G32500) and SPIK (also known as AKT6, AT2G25600); II (inward rectifying channel): KAT1 (AT5G46240) and KAT2 (AT4G18290); III (weakly inward rectifying channel): AKT2 (AT4G22200); IV (regulatory subunit involved in inwardly rectifying conductance formation): KAT3 (also known as AtKC1, AT4G32650); V (outward rectifying channel): SKOR (AT3G02850) and GORK (AT5G37500). Mutants have increased water consumption and limited stomatal closure in response to abscisic and jasmonic acids. It forms a heteromeric K(out) channels with SKOR. The gene is expressed ubiquitously in root and the vasculature and guard cells of leaves. Expression is suppressed during agrobacterium-induced tumor formation and increased in response to water deprivation and cold.
AT5G37510	*EMBRYO DEFECTIVE 1467 (EMB1467)*	Encodes a subunit of the 400 kDa subcomplex of the mitochondrial NADH dehydrogenase (complex I). The protein has been isolated in the male gametophyte. The mRNA is cell-to-cell mobile.

AT5G37520		NEP-interacting protein, putative (DUF239)
AT5G37540		Eukaryotic aspartyl protease family protein
AT5G37550		hypothetical protein
AT5G37560	*RING FINGER ABA-RELATED 5 (RFA5)*	RING/U-box superfamily protein
AT5G37570		Pentatricopeptide repeat (PPR-like) superfamily protein
AT5G37580		
AT5G37590		Tetratricopeptide repeat (TPR)-like superfamily protein
AT5G37600	*GLUTAMINE SYNTHASE CLONE R1 (GSR 1)*	encodes a cytosolic glutamine synthetase, the enzyme has high affinity with substrate ammonium
AT5G37610		Eukaryotic porin family protein
AT5G37620		Cysteine/Histidine-rich C1 domain family protein
AT5G37640	*UBIQUITIN 9 (UBQ9)*	polyubiquitin gene with 4 ubiquitin repeats. The first ubiquitin repeat has 16 amino acid replacements.
AT5G37660	*PLASMODESMATA-LOCATED PROTEIN 7 (PDLP7)*	Encodes a plasmodesmal protein that may be involved in the intercellular movement of molecules through the plasmodesmata. The protein has two DUF26 domains and a single transmembrane domain.
AT5G37670	*(HSP15.7)*	HSP20-like chaperones superfamily protein
AT5G37680	*ADP-RIBOSYLATION FACTOR-LIKE A1A (ARLA1A)*	A member of ARF-like GTPase family. A thaliana has 21 members, in two subfamilies, ARF and ARL GTPases.
AT5G37690		SGNH hydrolase-type esterase superfamily protein
AT5G37730		hypothetical protein
AT5G37740		Calcium-dependent lipid-binding (CaLB domain) family protein
AT5G37770	*TOUCH 2 (TCH2)*	Encodes a protein with 40% similarity to calmodulin. Binds Ca(2+) and, as a consequence, undergoes conformational changes. CML24 expression occurs in all major organs, and transcript levels are increased from 2- to 15-fold in plants subjected to touch, darkness, heat, cold, hydrogen peroxide, abscisic acid (ABA), and indole-3-acetic acid. However, CML24 protein accumulation changes were not detectable. The putative CML24 regulatory region confers reporter expression at sites of predicted mechanical stress; in regions undergoing growth; in vascular tissues and various floral organs; and in stomata, trichomes, and hydathodes. CML24-underexpressing transgenics are resistant to ABA inhibition of germination and seedling growth, are defective in long-day induction of flowering, and have enhanced tolerance to CoCl(2), molybdic acid, ZnSO(4), and MgCl(2). Also regulates nitric oxide levels.
AT5G37790		Protein kinase superfamily protein
AT5G37810	*NOD26-LIKE INTRINSIC PROTEIN 4;1 (NIP4;1)*	NOD26-like intrinsic protein 4
AT5G37840		PADRE protein, up-regulated after infection by S. sclerotiorum.
AT5G37860	*HEAVY METAL ASSOCIATED PROTEIN 50 (ATHMP50)*	Heavy metal transport/detoxification superfamily protein
AT5G37870		Protein with RING/U-box and TRAF-like domain
AT5G37880		transposable_element_gene;similar to unknown protein [Arabidopsis thaliana] (TAIR:AT4G18420.1);(source:TAIR10)
AT5G37900		TRAF-like superfamily protein
AT5G37910		Protein with RING/U-box and TRAF-like domain
AT5G37950		UDP-Glycosyltransferase superfamily protein
AT5G37960		GroES-like family protein
AT5G37970		SABATH family methyltransferase.
AT5G37980		Zinc-binding dehydrogenase family protein
AT5G37990	*(CIMT1)*	SABATH family methyltransferase.
AT5G38000		Zinc-binding dehydrogenase family protein
AT5G38020		encodes a protein whose sequence is similar to SAM:salicylic acid carboxyl methyltransferase (SAMT) (GI:6002712)(Clarkia breweri) and to SAM:benzoic acid carboxyl methyltransferase (BAMT)(GI:9789277)(Antirrhinum majus). SABATH family methyltransferase.
AT5G38030	*DETOXIFICATION30 (DTX30)*	MATE transporter involved in auxin homeostasis in roots.
AT5G38035		transposable_element_gene;copia-like retrotransposon family, has a 9.9e-71 P-value blast match to GB:CAA31653 polyprotein (Ty1_Copia-element) (Arabidopsis thaliana);(source:TAIR10)
AT5G38040		UDP-Glycosyltransferase superfamily protein
AT5G38080		transmembrane protein
AT5G38100		SABATH family methyltransferase.
AT5G38110	*ANTI- SILENCING FUNCTION 1B (ASF1B)*	This gene is predicted to encode a silencing group A protein. Plant lines expressing RNAi constructs directed against SGA1 have reduced levels of agrobacterium-mediated root transformation. Its expression is regulated during cell cycle progression through E2F transcription factors Functions redundantly with AT1G66740 during gametogenesis
AT5G38120	*(4CL8)*	AMP-dependent synthetase and ligase family protein
AT5G38130		HXXXD-type acyl-transferase family protein
AT5G38140	*NUCLEAR FACTOR Y, SUBUNIT C12 (NF-YC12)*	nuclear factor Y, subunit C12
AT5G38160		Bifunctional inhibitor/lipid-transfer protein/seed storage 2S albumin superfamily protein
AT5G38170		Bifunctional inhibitor/lipid-transfer protein/seed storage 2S albumin superfamily protein
AT5G38180		Bifunctional inhibitor/lipid-transfer protein/seed storage 2S albumin superfamily protein
AT5G38195		Bifunctional inhibitor/lipid-transfer protein/seed storage 2S albumin superfamily protein
AT5G38210	*LEAF RUST 10 DISEASE-RESISTANCE LOCUS RECEPTOR- LIKE PROTEIN KINASE-LIKE 3 (LRK10L3)*	Protein kinase family protein
AT5G38260		Protein kinase superfamily protein

AT5G38280	*PR5-LIKE RECEPTOR KINASE (PR5K)*	putative receptor serine/threonine kinase PR5K (PR5K) mRNA, PR5-like receptor kinase
AT5G38290		Peptidyl-tRNA hydrolase family protein
AT5G38300		homeobox Hox-B3-like protein
AT5G38310		hypothetical protein
AT5G38320		hypothetical protein
AT5G38330	*LOW-MOLECULAR-WEIGHT CYSTEINE-RICH 80 (LCR80)*	Encodes a member of a family of small,secreted, cysteine rich protein with sequence similarity to the PCP (pollen coat protein) gene family. Predicted to encode a PR (pathogenesis-related) protein. Belongs to the plant defensin (PDF) family with the following members: At1g75830/PDF1.1, At5g44420/PDF1.2a, At2g26020/PDF1.2b, At5g44430/PDF1.2c, At2g26010/PDF1.3, At1g19610/PDF1.4, At1g55010/PDF1.5, At2g02120/PDF2.1, At2g02100/PDF2.2, At2g02130/PDF2.3, At1g61070/PDF2.4, At5g63660/PDF2.5, At2g02140/PDF2.6, At5g38330/PDF3.1 and At4g30070/PDF3.2.
AT5G38350		Disease resistance protein (NBS-LRR class) family
AT5G38360		alpha/beta-Hydrolases superfamily protein
AT5G38370		
AT5G38390		F-box/RNI-like superfamily protein
AT5G38400		hypothetical protein
AT5G38440		Plant self-incompatibility protein S1 family
AT5G38450	*CYTOCHROME P450, FAMILY 735, SUBFAMILY A, POLYPEPTIDE 1 (CYP735A1)*	cytochrome P450, family 735, subfamily A, polypeptide 1
AT5G38500		B3 domain protein (DUF313)
AT5G38510		Rhomboid-related intramembrane serine protease family protein
AT5G38520	*CHLOROPHYLL DEPHYTYLASE1 (CLD1)*	CLD1 is involved in steady-state chlorophyll turnover; CLD1 dephytylates chlorophyll a, chlorophyll b, and pheophytin a in vitro; CLD1 and CHLG form a salvage cycle in recycling chlorophyll. Suppression of CLD1 expression results in reduced tolerance to moderately high temperature.
AT5G38530	*TRYPTOPHAN SYNTHASE BETA TYPE 2 (TSBtype2)*	TSBtype2 encodes a type 2 tryptophan synthase beta subunit that catalyzes a condensation reaction between serine and indole to generate tryptophan.It appears to form a homodimer. Its biological role has not yet been determined, but it has a very high affinity for indole which may be involved in allowing TSBtype2 to carefully limit free indole build-up. But, to date no overall change in plant morphology or seedling root growth have been observed in tsbtype2 mutants, indicating that this gene is not essential under optimum conditions. n most organs, TSBtype2 is transcripts are expressed at a lower level than TSB1 but in dry seeds they are expressed at comparable levels.
AT5G38550		Jacalin lectin family protein gene
AT5G38560	*PROLINE-RICH EXTENSIN-LIKE RECEPTOR KINASE 8 (PERK8)*	Encodes a member of the proline-rich extensin-like receptor kinase (PERK) family. This family consists of 15 predicted receptor kinases (PMID: 15653807).
AT5G38580		FBD-like domain family protein
AT5G38610		Plant invertase/pectin methylesterase inhibitor superfamily protein
AT5G38650	*(UMP1B)*	Proteasome maturation factor UMP1
AT5G38670		Galactose oxidase/kelch repeat superfamily protein
AT5G38700		cotton fiber protein
AT5G38710	*(PDH2)*	Methylenetetrahydrofolate reductase family protein
AT5G38720	*RIBOSOMAL RNA PROCESSING7 I (RRP7)*	RRP7 shares limited sequence similarity to human and yeast RRP7. In Arabidopsis RRP7 functions in 18S ribosomal RNA maturation.
AT5G38750		asparaginyl-tRNA synthetase family
AT5G38760		Late embryogenesis abundant protein (LEA) family protein
AT5G38770	*GLUTAMINE DUMPER 7 (GDU7)*	Encodes a member of the GDU (glutamine dumper) family proteins involved in amino acid export: At4g31730 (GDU1), At4g25760 (GDU2), At5g57685 (GDU3), At2g24762 (GDU4), At5g24920 (GDU5), At3g30725 (GDU6) and At5g38770 (GDU7).
AT5G38780		SABATH methyltransferase.
AT5G38790		hypothetical protein
AT5G38800	*BASIC LEUCINE-ZIPPER 43 (bZIP43)*	basic leucine-zipper 43
AT5G38850	*MICRORNA-SILENCED TNL1 (MIST1)*	TNL-encoding miR825-5p target.
AT5G38870		transposable_element_gene;pseudogene, hypothetical protein;(source:TAIR10)
AT5G38880	*AUGMIN SUBUNIT 5 (AUG5)*	HAUS augmin-like complex subunit
AT5G38890		Nucleic acid-binding, OB-fold-like protein
AT5G38895		RING/U-box superfamily protein
AT5G38900	*PROTEIN DISULFIDE ISOMERASE (PDI)*	Thioredoxin superfamily protein
AT5G38910		RmlC-like cupins superfamily protein
AT5G38930	*(DEG8)*	RmlC-like cupins superfamily protein
AT5G38950		RmlC-like cupins superfamily protein
AT5G38960		RmlC-like cupins superfamily protein
AT5G38970	*BRASSINOSTEROID-6-OXIDASE 1 (BR6OX1)*	Encodes a polypeptide involved in the C-6 oxidation of brassinosteroids. Heterologous expression of the protein in yeast conferred the ability to catalyze multiple reactions in which the C-6 position of 6-deoxocastasterone, 6-deoxotyphasterol, 3-dehydro-6-deoxoteasterone and 6-deoxoteasterone are oxidized.
AT5G38980		transmembrane protein
AT5G38990	*MEDOS 1 (MDS1)*	Involved in growth adaptation upon exposure to metal ions. Contributes together with the other MDS genes to the complex network of CrRLK1Ls that positively and negatively affect growth.

AT5G39020	*MEDOS 3 (MDS3)*	Involved in growth adaptation upon exposure to metal ions. Contributes together with the other MDS genes to the complex network of CrRLK1Ls that positively and negatively affect growth.
AT5G39030	*MEDOS 4 (MDS4)*	Involved in growth adaptation upon exposure to metal ions. Contributes together with the other MDS genes to the complex network of CrRLK1Ls that positively and negatively affect growth.
AT5G39050	*PHENOLIC GLUCOSIDE MALONYLTRANSFERASE 1 (PMAT1)*	Encodes a malonyltransferase that may play a role in phenolic xenobiotic detoxification.
AT5G39070		transposable_element_gene;hAT-like transposase family (hobo/Ac/Tam3), has a 3.9e-50 P-value blast match to GB:AAD24567 transposase Tag2 (hAT-element) (Arabidopsis thaliana);(source:TAIR10)
AT5G39080		HXXXD-type acyl-transferase family protein
AT5G39090		HXXXD-type acyl-transferase family protein
AT5G39100	*GERMIN-LIKE PROTEIN 6 (GLP6)*	germin-like protein (GLP6)
AT5G39110		RmlC-like cupins superfamily protein
AT5G39130		RmlC-like cupins superfamily protein
AT5G39160		RmlC-like cupins superfamily protein
AT5G39190	*GERMIN-LIKE PROTEIN 2 (GER2)*	germin-like protein (GLP2a)
AT5G39200		myb-like protein Q
AT5G39210	*CHLORORESPIRATORY REDUCTION 7 (CRR7)*	Encodes a protein of the chloroplastic NAD(P)H dehydrogenase complex (NDH Complex) involved in respiration, photosystem I (PSI) cyclic electron transport and CO2 uptake. The product of this gene appears to be essential for the stable formation of the NDH Complex. The mRNA is cell-to-cell mobile.
AT5G39220		alpha/beta-Hydrolases superfamily protein
AT5G39230		TFIIB zinc-binding protein
AT5G39250		F-box family protein
AT5G39260	*EXPANSIN A21 (EXPA21)*	expansin-like protein. Naming convention from the Expansin Working Group (Kende et al, 2004. Plant Mol Bio)
AT5G39270	*EXPANSIN A22 (EXPA22)*	expansin-like protein. Naming convention from the Expansin Working Group (Kende et al, 2004. Plant Mol Bio)
AT5G39280	*EXPANSIN A23 (EXPA23)*	member of Alpha-Expansin Gene Family. Naming convention from the Expansin Working Group (Kende et al, 2004. Plant Mol Bio)
AT5G39310	*EXPANSIN A24 (EXPA24)*	member of Alpha-Expansin Gene Family. Naming convention from the Expansin Working Group (Kende et al, 2004. Plant Mol Bio)
AT5G39320	*UDP-GLUCOSE DEHYDROGENASE 4 (UDG4)*	UDP-glucose 6-dehydrogenase family protein
AT5G39340	*HISTIDINE-CONTAINING PHOSPHOTRANSMITTER 3 (AHP3)*	Encodes AHP3, one of the six Arabidopsis thaliana histidine phosphotransfer proteins (AHPs) involved in Histidine (His)-to-Aspartate (Asp) phosphorelay signal transduction. AHPs function as redundant positive regulators of cytokinin signaling. Members of the AHP gene family include: AT3G21510 (AHP1), AT3G29350 (AHP2), AT5G39340 (AHP3), AT3G16360 (AHP4), AT1G03430 (AHP5) and AT1G80100 (AHP6).
AT5G39350		Tetratricopeptide repeat (TPR)-like superfamily protein
AT5G39360	*EID1-LIKE 2 (EDL2)*	EID1-like 2
AT5G39370		Curculin-like (mannose-binding) lectin family protein
AT5G39390		Leucine-rich repeat protein kinase family protein
AT5G39400	*PHOSPHATASE AND TENSIN HOMOLOG DELETED ON CHROMOSOME TEN 1 (PTEN1)*	Calcium/lipid-binding (CaLB) phosphatase
AT5G39420	*CDC2C (cdc2cAt)*	CDC2C
AT5G39430		DUF1336 family protein, putative (DUF1336)
AT5G39440	*SNF1-RELATED PROTEIN KINASE 1.3 (SnRK1.3)*	SNF1-related protein kinase 1.3
AT5G39490		F-box family protein
AT5G39520	*CHLOROPLAST-LOCALIZED SENESCENCE-ASSOCIATED PROTEIN (CSAP)*	Plastid localized transmembrane protein involved in ABA mediated leaf senescence and stomatal movement.
AT5G39540		F-box associated ubiquitination effector family protein
AT5G39550	*VARIANT IN METHYLATION 3 (VIM3)*	Encodes the VIM3/ORTH1 protein that is similar to VIM1. This protein has an N-terminal PHD domain and two RING domains surrounding an SRA domain. The protein has been shown to bind to methylated cytosines of CG, CNG and CNN motifs via its SRA domain but has a preference for the former. This protein functions as an E3 ubiquitin ligase in vitro with members of the UBC8 family E2s. Either of the two RING domains present in the protein can promote ubiquitylation in vitro, but, not the PHD domain. Over-expression of ORTH1/VIM3 leads to decreased levels of FWA methylation, increased levels of FWA transcripts, and delayed flowering. Cen180 repeats are also hypomethylated in plants overexpressing this protein.
AT5G39560		Galactose oxidase/kelch repeat superfamily protein
AT5G39580	*(PRX62)*	Class III peroxidase cell wall-targeted protein localized to the micropylar endosperm facing the radicle. Involved in seed germination.
AT5G39590	*OXIDATION RESISTANCE 5 (OXR5)*	TLD-domain containing nucleolar protein
AT5G39610	*NAC DOMAIN CONTAINING PROTEIN 6 (NAC6)*	Encodes a NAC-domain transcription factor. Positively regulates aging-induced cell death and senescence in leaves. This gene is upregulated in response to salt stress in wildtype as well as NTHK1 transgenic lines although in the latter case the induction was drastically reduced. It was also upregulated by ABA, ACC and NAA treatment, although in the latter two cases, the induction occurred relatively late when compared with NaCl or ABA treatments. Note: this protein (AtNAC6) on occasion has also been referred to as AtNAC2, not to be confused with the AtNAC2 found at locus AT3G15510.
AT5G39620	*RAB GTPASE HOMOLOG G1 (RABG1)*	RAB GTPase homolog G1
AT5G39630		Vesicle transport v-SNARE family protein
AT5G39640		Putative endonuclease or glycosyl hydrolase
AT5G39650	*DUO1-ACTIVATED UNKNOWN 2 (DAU2)*	Target promoter of the male germline-specific transcription factor DUO1. Knock down mutants result in an aborted seed phenotype that is transmitted through the male, together with loss-of-function mutation in DMP9 induces maternal haploids, with an average haploid induction rate of 2.1 ? 1.1%.

AT5G39660	*CYCLING DOF FACTOR 2 (CDF2)*	Dof-type zinc finger domain-containing protein, identical to H-protein promoter binding factor-2a GI:3386546 from (Arabidopsis thaliana). Interacts with LKP2 and FKF1, but its overexpression does not change flowering time under short or long day conditions.
AT5G39690	*NAC DOMAIN CONTAINING PROTEIN 93 (NAC093)*	NAC domain containing protein 93
AT5G39700	*MYB DOMAIN PROTEIN 89 (MYB89)*	Encodes a putative transcription factor (MYB89) which inhibits the accumulation of seed oil.
AT5G39720	*AVIRULENCE INDUCED GENE 2 LIKE PROTEIN (AIG2L)*	avirulence induced protein 2 like protein
AT5G39730		AIG2-like (avirulence induced gene) family protein
AT5G39760	*HOMEOBOX PROTEIN 23 (HB23)*	Functions together with TZP in co-regulation of the expression of blue-light dependent transcriptional regulators. Coassociates with and regulates the expression of light-regulated loci as well as transcriptional regulators to shape plant development in response to environmental stimuli with targets in RNA processing factors as well as proteins involved in salt stress and ABA signaling, in addition to embryo development. Acts downstream of TZP action with regard to blue-light-regulated hypocotyl elongation.
AT5G39780		
AT5G39790	*PROTEIN TARGETING TO STARCH (PTST)*	Encodes a chloroplast localized protein that is involved in protein translocation and starch metabolism. PTST helps localize GBSS to the starch granules where GBSS functions in amylose biosynthesis.
AT5G39810	*AGAMOUS-LIKE 98 (AGL98)*	AGAMOUS-like 98
AT5G39820	*NAC DOMAIN CONTAINING PROTEIN 94 (NAC094)*	NAC domain containing protein 94
AT5G39840		ATP-dependent RNA helicase
AT5G39850		Ribosomal protein S4
AT5G39860	*PACLOBUTRAZOL RESISTANCE1 (PRE1)*	Encodes PRE1 (PACLOBUTRAZOL RESISTANCE1). PRE1 and IBH1 form a pair of antagonistic HLH/bHLH transcription factors that function downstream of BZR1 to mediate brassinosteroid regulation of cell elongation. BNQ1 is directly and negatively regulated by AP3 and PI in petals.Required for appropriate regulation of flowering time.
AT5G39870		hypothetical protein (DUF1216)
AT5G39880		transmembrane protein
AT5G39890	*PLANT CYSTEINE OXIDASE 2 (PCO2)*	Plant Cysteine Oxidase (PCO). Involved in controlling the stability of Group VII ethylene response factors (ERF-VIIs) via N-Arg/degron pathway through catalyzing the oxidation of their N-Cys for subsequent Arginyl-tRNA--protein transferase 1 (ATE1) mediated arginine installation.
AT5G39900		Small GTP-binding protein
AT5G39910		Pectin lyase-like superfamily protein
AT5G39930	*CLP1-SIMILAR PROTEIN 5 (CLPS5)*	Encodes a protein with similarity to the CLP1 polyadenylation factor.
AT5G39940		FAD/NAD(P)-binding oxidoreductase family protein
AT5G39960	*(ENGA-2)*	GTP-binding protein
AT5G39970		catalytics
AT5G40010	*AAA-ATPASE 1 (AATP1)*	Encodes a mitochondrial ATPase involved in seed and silique development.
AT5G40020		Pathogenesis-related thaumatin superfamily protein
AT5G40030	*PAX-LIKE (PAXL)*	Member of AGC VIIIa Kinase gene family.
AT5G40040	*RIBOSOMAL PROTEIN P2E (RPP2E)*	cytosolic ribosomal protein gene, part of bL12 family
AT5G40050		F-box/FBD-like domains containing protein
AT5G40060		Disease resistance protein (NBS-LRR class) family
AT5G40070		MADS-box family protein
AT5G40090	*CHS1-LIKE 1 (CHL1)*	Disease resistance protein (TIR-NBS class)
AT5G40100		Disease resistance protein (TIR-NBS-LRR class) family
AT5G40130		pseudogene of ribosomal protein L5 B
AT5G40140	*PLANT U-BOX 40 (PUB40)*	Encodes a ubiquitin E3 ligase of the U-box type that mediates the proteasomal degradation of BZR1 in a root-specific manner.
AT5G40150		Peroxidase superfamily protein
AT5G40155		Encodes a defensin-like (DEFL) family protein.
AT5G40160	*EMBRYO DEFECTIVE 506 (EMB506)*	Encodes ankyrin repeat protein EMB506. Mutations in this locus result in embryo lethality.
AT5G40170	*RECEPTOR LIKE PROTEIN 54 (RLP54)*	receptor like protein 54
AT5G40180		Pmr5/Cas1p GDSL/SGNH-like acyl-esterase family protein
AT5G40200	*DEGRADATION OF PERIPLASMIC PROTEINS 9 (DEG9)*	Encodes a putative DegP protease. The mRNA is cell-to-cell mobile.
AT5G40230	*USUALLY MULTIPLE ACIDS MOVE IN AND OUT TRANSPORTERS 37 (UMAMIT37)*	nodulin MtN21-like transporter family protein
AT5G40260	*(SWEET8)*	Encodes RPG1 (RUPTURED POLLEN GRAIN1), a member of the MtN3/saliva gene family. Crucial for exine pattern formation and cell integrity of microspores.
AT5G40270	*VENOSA 4 (VEN4)*	VEN4 is homologous to human SAMHD1 and functions in chloroplast biogenesis.
AT5G40290		HD domain-containing metal-dependent phosphohydrolase family protein
AT5G40300	*CASP-LIKE PROTEIN 4A1 (CASPL4A1)*	Uncharacterized protein family (UPF0497)
AT5G40320		Cysteine/Histidine-rich C1 domain family protein
AT5G40330	*MYB DOMAIN PROTEIN 23 (MYB23)*	Encodes a MYB gene that, when overexpressed ectopically, can induce ectopic trichome formation. It is a member of subgroup 15, together with WER and GL1. Members of this subgroup share a conserved motif of 19 amino acids in the putative transcription activation domain at the C-terminal end. The gene is expressed in leaves, stems, flowers, seeds and roots and quite strongly in trichomes. There is partial functional redundancy between ATMYB23 and GL1. The two proteins are functionally equivalent with respect to the regulation of trichome initiation but not with respect to trichome branching - which is controlled by MYB23 and not GL1.
AT5G40340	*PWWP DOMAIN PROTEIN 3 (PDP3)*	PWWP domain protein involved in regulation of FLC and flowering time.

AT5G40350	*MYB DOMAIN PROTEIN 24 (MYB24)*	Myb24 transcription factor. Member of the R2R3 factor gene family. Induced by jasmonate. Involved in jasmonate response during stamen development. MYB24 interacts with JAZ proteins, and functions redundantly with MYB21 and MYB57 to regulate stamen development. Possible positive regulator of CKL2 and 7 expression under high temperature. Promotes flavonol biosynthesis through regulation of FLS1 gene expression.
AT5G40360	*MYB DOMAIN PROTEIN 115 (MYB115)*	Encodes a member of the MYB family of transcription factors and in involved in regulation of glucosinolate (GLS) biosynthesis. MYB115 binds to the promoters of a number of GLS biosynthetic enzymes and mutations show differences in accumulation of GLS compared to wild type.
AT5G40370	*GLUTAREDOXIN C2 (GRXC2)*	Glutaredoxin family protein
AT5G40380	*CYSTEINE-RICH RLK (RECEPTOR-LIKE PROTEIN KINASE) 42 (CRK42)*	Encodes a cysteine-rich receptor-like protein kinase.
AT5G40390	*SEED IMBIBITION 1-LIKE (SIP1)*	Encodes a protein which might be involved in the formation of verbascose. A T-DNA insertion mutant was shown to have a decreased amount of verbascose (as well as mannitol) whereas the levels of raffinose and stachyose remained unchanged. Enhances drought tolerance through raffinose synthesis or galactinol hydrolysis.
AT5G40410		Tetratricopeptide repeat (TPR)-like superfamily protein
AT5G40420	*OLEOSIN 2 (OLEO2)*	Encodes oleosin2, a protein found in oil bodies, involved in seed lipid accumulation. Suppression of OLEO1 (and OLEO2) resulted in an aberrant phenotype of embryo cells that contain unusually large oilbodies that are not normally observed in seeds. Changes in the size of oilbodies caused disruption of storage organelles, altering accumulation of lipids and proteins and causing delay in germination. Functions in freezing tolerance of seeds.
AT5G40430	*MYB DOMAIN PROTEIN 22 (MYB22)*	Encodes a putative transcription factor (MYB22).
AT5G40440	*MITOGEN-ACTIVATED PROTEIN KINASE KINASE 3 (MKK3)*	Encodes a mitogen-activated protein kinase kinase. Activates MPK8 and is a target of MPKKK20. Mutant root growth is sensitive oryzalin and suggestive of a role in signaling during microtubule organization.
AT5G40450	*REGULATOR OF BULB BIOGENESIS1 (RBB1)*	Encodes a member of a plant gene family, APK_ORTHOMCL5144,of unknown function. RBB1 is localized to the cytosol and involved in vacuolar biogenesis and organization. RBB1 mutants have increased number of vacuolar bulbs and fewer trans-vacuolar strands.
AT5G40460	*(SMR6)*	cyclin-dependent kinase inhibitor SMR3-like protein
AT5G40480	*EMBRYO DEFECTIVE 3012 (EMB3012)*	embryo defective 3012
AT5G40500		hypothetical protein
AT5G40510		Sucrase/ferredoxin-like family protein
AT5G40540		Protein kinase superfamily protein
AT5G40560	*DEGRADATION OF PERIPLASMIC PROTEINS 13 (DEG13)*	Encodes a putative DegP protease.
AT5G40590		Cysteine/Histidine-rich C1 domain family protein
AT5G40610	*GLYCEROL-3-PHOSPHATE DEHYDROGENASE PLASTIDIC (GPDHP)*	NAD-dependent glycerol-3-phosphate dehydrogenase family protein
AT5G40630		Ubiquitin-like superfamily protein
AT5G40640		transmembrane protein
AT5G40645		RPM1-interacting protein 4 (RIN4) family protein
AT5G40650	*SUCCINATE DEHYDROGENASE 2-2 (SDH2-2)*	One of three isoforms of the iron-sulfur component of the succinate dehydrogenase complex, a component of the mitochondrial respiratory chain complex II. The product of the nuclear encoded gene is imported into the mitochondrion. Expressed during germination and post-germinative growth.
AT5G40660	*(P12)*	Encodes an F-type ATP Synthase Assembly factor that binds to beta subunits of mitochondrial ATPase.
AT5G40670		PQ-loop repeat family protein / transmembrane family protein
AT5G40690		histone-lysine N-methyltransferase trithorax-like protein
AT5G40730	*ARABINOGALACTAN PROTEIN 24 (AGP24)*	Encodes an arabinogalactan-protein (AGP24).
AT5G40740	*AUGMIN SUBUNIT 6 (AUG6)*	Encodes a conserved AUGMIN subunit 6 (AUG6) which is known to be involved in microtuble nucleation. Mutants affect both male and female gametogenesis.
AT5G40750		FBD / Leucine Rich Repeat domains containing protein
AT5G40770	*PROHIBITIN 3 (PHB3)*	prohibitin 3
AT5G40780	*LYSINE HISTIDINE TRANSPORTER 1 (LHT1)*	Encodes LHT1 (lysine histidine transporter), a high-affinity transporter for cellular amino acid uptake in both root epidermis and leaf mesophyll.
AT5G40810		Cytochrome C1 family
AT5G40830	*INCREASED CAMBIAL ACTIVITY (ICA)*	Encodes an SAM‐dependent methyltransferase superfamily protein that has an N‐terminal transmembrane domain and a putative methyltransferase domain, DUF248, and is strongly expressed in the vasculature. Overexpression results in increased phloem and xylem in the plant.
AT5G40840	*(SYN2)*	Cohesion family protein SYN2 (SYN2). Plays a role in somatic DNA double strand break damage repair.
AT5G40850	*UROPHORPHYRIN METHYLASE 1 (UPM1)*	Encodes a urophorphyrin III methylase that catalyzes S-adenosyl-L-methionine-dependent transmethylation in a multistep process involving the formation of a covalently linked complex with S-adenosyl-L-methionine. The mRNA is cell-to-cell mobile.
AT5G40860		transmembrane protein
AT5G40890	*CHLORIDE CHANNEL A (CLC-A)*	Encodes a member of the voltage-dependent chloride channel. Also functions as a NO3-/H+ exchanger that serves to accumulate nitrate nutrient in vacuoles. Mutants homozygous for the T-DNA insertion mutation have reduced nitrate uptake capacity in high nitrate environment and exhibit hypersensitivity to chlorate. Role in cytosolic pH homeostasis.
AT5G40910		Disease resistance protein (TIR-NBS-LRR class) family
AT5G40920		pseudogene of Disease resistance protein (TIR-NBS-LRR class) family
AT5G40940	*PUTATIVE FASCICLIN-LIKE ARABINOGALACTAN PROTEIN 20 (FLA20)*	Fasciclin-like arabinogalactan protein. Possibly involved in embryogenesis and seed development.
AT5G40950	*RIBOSOMAL PROTEIN LARGE SUBUNIT 27 (RPL27)*	ribosomal protein large subunit 27
AT5G40960		transmembrane protein, putative (DUF 3339)
AT5G40980		AT.I.24-6 protein, putative (DUF 3339)

AT5G40990	*GDSL LIPASE 1 (GLIP1)*	Component of plant resistance. Contains lipase signature motif and GDSL domain. Directly interferes with the fungal infection process by acting on fungal cell walls through its action as a antimicrobial compound. Critical component for both local and systemic resistance responses in the incompatible interaction with Alternaria brassicicola in the ethylene-dependent pathway.
AT5G41000	*YELLOW STRIPE LIKE 4 (YSL4)*	Arabidopsis thaliana metal-nicotianamine transporter YSL4
AT5G41010	*(NRPB12)*	Non-catalytic subunit common to nuclear DNA-dependent RNA polymerases II, IV and V; homologous to budding yeast RPB12.
AT5G41040	*REDUCED LEVELS OF WALL-BOUND PHENOLICS 1 (RWP1)*	Encodes a feruloyl-CoA transferase required for suberin synthesis. Has feruloyl-CoA-dependent feruloyl transferase activity towards substrates with a primary alcohol.
AT5G41050		Pollen Ole e 1 allergen and extensin family protein
AT5G41060		DHHC-type zinc finger family protein
AT5G41070	*DSRNA-BINDING PROTEIN 5 (DRB5)*	Encodes a double-stranded RNA binding protein.
AT5G41080	*GLYCEROPHOSPHODIESTER PHOSPHODIESTERASE 2 (GDPD2)*	Encodes a member of the glycerophosphodiester phosphodiesterase (GDPD) family.
AT5G41090	*NAC DOMAIN CONTAINING PROTEIN 95 (NAC095)*	NAC domain containing protein 95
AT5G41140		Myosin heavy chain-related protein
AT5G41190	*(NOB1)*	Encodes a cytoplasmic protein with RNA endonuclease activity. Mutants display aberrant RNA processing and male and female gametophyte development.
AT5G41210	*GLUTATHIONE S-TRANSFERASE THETA 1 (GSTT1)*	Encodes glutathione transferase belonging to the theta class of GSTs. Naming convention according to Wagner et al. (2002).
AT5G41220	*GLUTATHIONE S-TRANSFERASE THETA 3 (GSTT3)*	Encodes glutathione transferase belonging to the theta class of GSTs. Naming convention according to Wagner et al. (2002).
AT5G41240	*GLUTATHIONE S-TRANSFERASE THETA 2 (GSTT2)*	Encodes glutathione transferase belonging to the theta class of GSTs. Naming convention according to Wagner et al. (2002).
AT5G41250		Exostosin family protein
AT5G41260	*BRASSINOSTEROID-SIGNALING KINASE 8 (BSK8)*	kinase with tetratricopeptide repeat domain-containing protein
AT5G41270		RNase P Rpr2/Rpp21 subunit domain protein
AT5G41280		Receptor-like protein kinase-related family protein
AT5G41310		P-loop nucleoside triphosphate hydrolases superfamily protein with CH (Calponin Homology) domain-containing protein
AT5G41330		BTB/POZ domain with WD40/YVTN repeat-like protein
AT5G41340	*UBIQUITIN CONJUGATING ENZYME 4 (UBC4)*	Belongs to Ubiquitin conjugating enzyme family. Gene expression is developmentally regulated.
AT5G41350		RING/U-box superfamily protein
AT5G41370	*HOMOLOG OF XERODERMA PIGMENTOSUM COMPLEMENTATION GROUP B 1 (XPB1)*	Encodes XPB1, a DNA repair protein and transcription factor. Arabidopsis thaliana has duplicated XPB gene (AtXPB1 and AtXPB2, with high similarity to each other). XPB proteins are involved in both DNA repair and transcription, they are component of the transcription factor IIH (TFIIH) and are responsible for DNA helicase activity during nucleotide (nt) excision repair (NER). Complementation assays in yeast rad25 mutant strains suggest the involvement of AtXPB2 in DNA repair. Although both genes are expressed in a constitutive manner during the plant life cycle, Northern blot analyses suggest that light modulates the expression level of both XPB copies. The mRNA is cell-to-cell mobile.
AT5G41380		CCT motif family protein
AT5G41400		RING/U-box superfamily protein
AT5G41410	*BELL 1 (BEL1)*	Homeodomain protein required for ovule identity.Loss of function mutations show homeotic conversion of integuments to carpels.Forms heterodimers with STM and KNAT1. Interacts with AG-SEP heterodimers is thought to restrict WUS expression. BEL interacts with MADS box dimers composed of SEP1(or SEP3) and AG, SHP1, SHP2 and STK. The interaction of BEL1 with AG-SEP3 is required for proper integument development and specification of integument identity.
AT5G41440	*ARABIDOPSIS T??XICOS EN LEVADURA 86 (ATL86)*	RING/U-box superfamily protein
AT5G41450	*ARABIDOPSIS T??XICOS EN LEVADURA 87 (ATL87)*	RING/U-box superfamily protein
AT5G41460		transferring glycosyl group transferase (DUF604)
AT5G41490		F-box associated ubiquitination effector family protein
AT5G41540		Disease resistance protein (TIR-NBS-LRR class) family
AT5G41600	*VIRB2-INTERACTING PROTEIN 3 (BTI3)*	VIRB2-interacting protein 3
AT5G41610	*CATION/H+ EXCHANGER 18 (CHX18)*	member of Putative Na+/H+ antiporter family
AT5G41620		intracellular protein transporter USO1-like protein
AT5G41640		Mutants have decreased tolerance to osmotic stress.
AT5G41650	*GLYOXALASE I-LIKE;10 (GLXI-LIKE;10)*	Vicinal oxygen chelate (VOC) superfamily member. Responds to NaCl stress.
AT5G41660		transmembrane protein
AT5G41670	*6-PHOSPHOGLUCONATE DEHYDROGENASE 3 (PGD3)*	6-phosphogluconate dehydrogenase family protein
AT5G41680		Protein kinase superfamily protein
AT5G41700	*UBIQUITIN CONJUGATING ENZYME 8 (UBC8)*	One of the polypeptides that constitute the ubiquitin-conjugating enzyme E2 The mRNA is cell-to-cell mobile.
AT5G41710		transposable_element_gene;Mutator-like transposase family, has a 6.2e-18 P-value blast match to GB:AAA21566 mudrA of transposon=MuDR (MuDr-element) (Zea mays);(source:TAIR10)
AT5G41720		F-box associated ubiquitination effector family protein
AT5G41730		Protein kinase family protein
AT5G41740		Disease resistance protein (TIR-NBS-LRR class) family
AT5G41780		myosin heavy chain-like protein
AT5G41790	*COP1-INTERACTIVE PROTEIN 1 (CIP1)*	encodes a protein that physically interacts specifically with the putative coiled-coil region of COP1 in vitro. In hypocotyl and cotyledon protoplasts, it is associated to the cytoskeleton, but not in the root. expression is not regulated by light. The mRNA is cell-to-cell mobile.
AT5G41800		Transmembrane amino acid transporter family protein

AT5G41810		Avr9/Cf-9 rapidly elicited protein
AT5G41820	*RAB GERANYLGERANYL TRANSFERASE ALPHA SUBUNIT 2 (RGTA2)*	RAB geranylgeranyl transferase alpha subunit 2
AT5G41870	*POLYGALACTURONASE CLADE F 15 (PGF15)*	Pectin lyase-like superfamily protein
AT5G41880	*(POLA3)*	DNA primase POLA3
AT5G41890		GDSL-motif esterase/acyltransferase/lipase. Enzyme group with broad substrate specificity that may catalyze acyltransfer or hydrolase reactions with lipid and non-lipid substrates.
AT5G41900		alpha/beta-Hydrolases superfamily protein
AT5G41920	*SCARECROW-LIKE 23 (SCL23)*	Encodes a GRAS family transcription factor that is involved in bundle sheath cell fate specification.
AT5G41930		
AT5G41970		Metal-dependent protein hydrolase
AT5G41990	*WITH NO LYSINE (K) KINASE 8 (WNK8)*	Encodes a member of the WNK family (9 members in all) of protein kinases, the structural design of which is clearly distinct from those of other known protein kinases, such as receptor-like kinases and mitogen-activated protein kinases. Interacts specifically with and phosphorylates AtVHA-C, subunit C of the vacuolar H+-ATPase.
AT5G42030	*ABL INTERACTOR-LIKE PROTEIN 4 (ABIL4)*	ABL interactor-like protein 4
AT5G42050	*ASPARAGINE-RICH PROTEIN (NRP)*	Stress responsive asparagine-rich protein. Binds to PevD (Verticillium dahliae) fungal effector protein. NRP interacts with CRY2, leading to increased cytoplasmic accumulation of CRY2 in a blue light-independent manner (PMID:28633330).NRP also binds FyPP3 and recruits it to endosomes and thus targets it for degradation.Under ER stress NRP1 is activated by bZIP60 and inhibits cell death.
AT5G42060		DEK, chromatin associated protein
AT5G42070		hypothetical protein
AT5G42080	*DYNAMIN-LIKE PROTEIN (DL1)*	Encodes a dynamin-like protein related to phragmoplastin. Mutations in this gene, in combination with mutation in ADL1E, result in defects in embryogenesis, cell plate formation and trichome branching. Also controls vascular patterning in combination with VAN3 and GNOM. DRP2B and DRP1A participate together in clathrin-coated vesicle formation during endocytosis.
AT5G42100	*BETA-1,3-GLUCANASE_PUTATIVE (BG_PPAP)*	encodes a plasmodesmal (Pd)-associated membrane protein involved in plasmodesmal callose degradation, i.e. beta-1,3-glucanase (EC 3.2.1.39), and functions in the gating of Pd
AT5G42110		hypothetical protein
AT5G42120	*L-TYPE LECTIN RECEPTOR KINASE S.6 (LECRK-S.6)*	Concanavalin A-like lectin protein kinase family protein
AT5G42150		Glutathione S-transferase family protein
AT5G42170		SGNH hydrolase-type esterase superfamily protein
AT5G42180	*PEROXIDASE 64 (PER64)*	Peroxidase required for casparian strip lignification as well as partially required for SGN-dependent compensatory lignification.
AT5G42200	*ARABIDOPSIS T??XICOS EN LEVADURA 23 (ATL23)*	RING/U-box superfamily protein
AT5G42230	*SERINE CARBOXYPEPTIDASE-LIKE 41 (scpl41)*	Serine carboxypeptidase‐like (SCPL) enzyme involved in membrane lipid metabolism.
AT5G42240	*SERINE CARBOXYPEPTIDASE-LIKE 42 (scpl42)*	serine carboxypeptidase-like 42
AT5G42250		Zinc-binding alcohol dehydrogenase family protein
AT5G42260	*BETA GLUCOSIDASE 12 (BGLU12)*	beta glucosidase 12
AT5G42270	*VARIEGATED 1 (VAR1)*	VAR1 contains a conserved motif for ATPase and a metalloprotease characteristic to FtsH proteins, and is targeted into chloroplasts. A VAR1-fusion protein synthesized in vitro exhibited ATPase activity and partial metalloprotease activity. This protein is located to the thylakoid membrane and forms a complex with VAR2. FtsH1 (VAR1) and FtsH5 are interchangeable in thylakoid membranes. Phosphorylation of this protein is dependent on calcium. The mRNA is cell-to-cell mobile.
AT5G42280		Cysteine/Histidine-rich C1 domain family protein
AT5G42290		transcription activator-like protein
AT5G42300	*UBIQUITIN-LIKE PROTEIN 5 (UBL5)*	Ubiquitin like protein that appears to play a role in pre-mRNA splicing.
AT5G42310	*CHLOROPLAST RNA PROCESSING 1 (CRP1)*	Encodes a member of the Arabidopsis PPR family, expressed in the chloroplast, that acts as a regulator of chloroplast protein synthesis, interacts with the petB-petD intergenic region, and is required for the generation of petB and petD monocistronic RNAs.
AT5G42320		Zn-dependent exopeptidases superfamily protein
AT5G42330		hypothetical protein
AT5G42340	*PLANT U-BOX 15 (PUB15)*	Plant U-box type E3 ubiquitin ligase (PUB).
AT5G42360	*COP9 SIGNALOSOME INTERACTING F-BOX KELCH 2 (CFK2)*	Galactose oxidase/kelch repeat superfamily protein
AT5G42380	*CALMODULIN LIKE 37 (CML37)*	calmodulin like 37
AT5G42440		Protein kinase superfamily protein
AT5G42450		Pentatricopeptide repeat (PPR) superfamily protein
AT5G42490		ATP binding microtubule motor family protein
AT5G42500		Disease resistance-responsive (dirigent-like protein) family protein
AT5G42540	*EXORIBONUCLEASE 2 (XRN2)*	Encodes a protein with similarity to yeast 5'-3'exonucleases and can functionally complement the yeast mutations. In Arabidopsis XRN2 acts as a suppressor of posttranscriptional gene silencing.
AT5G42580	*CYTOCHROME P450, FAMILY 705, SUBFAMILY A, POLYPEPTIDE 12 (CYP705A12)*	a member of the cytochrome P450 family
AT5G42590	*CYTOCHROME P450, FAMILY 71, SUBFAMILY A, POLYPEPTIDE 16 (CYP71A16)*	putative cytochrome P450
AT5G42600	*MARNERAL SYNTHASE 1 (MRN1)*	Encodes an oxidosqualene synthase that produces the monocyclic triterpene marneral. Crucial for growth and development.
AT5G42620		metalloendopeptidase / zinc ion binding protein

AT5G42630	*ABERRANT TESTA SHAPE (ATS)*	Encodes a member of the KANADI family of putative transcription factors. Involved in integument formation during ovule development and expressed at the boundary between the inner and outer integuments. It is essential for directing laminar growth of the inner integument.Along with KAN1 and KAN2, KAN4 is involved in proper localization of PIN1 in early embryogenesis.
AT5G42640	*GAZ-LIKE 2 (GAL2)*	Member of a small family of zinc finger containing putative transcription factors.Similar to GAZ.
AT5G42650	*ALLENE OXIDE SYNTHASE (AOS)*	Encodes a member of the cytochrome p450 CYP74 gene family that functions as an allene oxide synthase. This enzyme catalyzes dehydration of the hydroperoxide to an unstable allene oxide in the JA biosynthetic pathway. It shows a dual catalytic activity, the major one being a 13-AOS but also expressing a 9-AOS activity. CFA-Leu, CFA-Val, CFA-Met and CFA-Ala can induce the expression of AOS.
AT5G42680		MIZU-KUSSEI-like protein (Protein of unknown function, DUF617)
AT5G42690		transcription factor, putative (Protein of unknown function, DUF547)
AT5G42720		Glycosyl hydrolase family 17 protein
AT5G42750	*BRI1 KINASE INHIBITOR 1 (BKI1)*	Encodes a plasma-membrane associated phosphoprotein that interacts directly with the kinase domain of BRI1 through the evolutionarily conserved C-terminal BIM motif binding to the C-lobe of the BRI1 kinase domain. It interferes with the interaction between BRI1 with its signalling partner, the plasma membrane localised LRR-receptor kinase BAK1 by inhibiting the transphosphorylation to keep BRI1 at a basal level of activity. It is phosphorylated by BRI1 at Ser270 & Ser274 and at tyrosine site Tyr211 and dissociates from plasma membrane to end up in the cytosol after phosphorylation. Its loss-of-function mutant shows higher sensitivity to BR treatment.
AT5G42760		Leucine carboxyl methyltransferase
AT5G42780	*HOMEOBOX PROTEIN 27 (HB27)*	Zinc finger and homeobox domain protein which interacts with RMB1 and ROS1 acting in the base excision repair pathway through DNA methylation.
AT5G42800	*DIHYDROFLAVONOL 4-REDUCTASE (DFR)*	dihydroflavonol reductase. Catalyzes the conversion of dihydroquercetin to leucocyanidin in the biosynthesis of anthocyanins. Not expressed in roots (qRT-PCR). The mRNA is cell-to-cell mobile.
AT5G42810	*INOSITOL-PENTAKISPHOSPHATE 2-KINASE 1 (IPK1)*	Encodes an inositol tetra-/pentaphosphate 2-kinase, involved in the biosynthesis of phytic acid, a regulator of intracellular signaling, a highly abundant animal antinutrient, and a phosphate and mineral storage compound in plant seeds. Is also required for growth and modulates phosphate homeostasis at the transcriptional level. Together with ITPK1 modulate crosstalks between SA-dependent immunity and phosphate-starvation responses.
AT5G42820	*(U2AF35B)*	U2 auxiliary factor small subunit. The atU2AF35b protein and its homolog, atU2AF35a, contain most of the conserved domains of hsU2AF35, including the psiRRM, one RS domain, two zinc fingers, and the two regions for interacting with U2AF large subunit. Both proteins lack the stretch of glycines present in human U2AF35. The sequences are overall 83% identical, and each Arabidopsis homolog shows approximately 70% similarity to hsU2AF35. U2AF(35) homologs were also identified from maize, rice and other plants with large-scale EST projects. Both genes are expressed in all major tissues, with atU2AF(35)a expressed at a higher level than atU2AF(35)b in most tissues. The expression patterns were different in roots: atU2AF(35)b expressed strongly in whole young roots and root tips and atU2AF(35)a limited to root vascular regions.
AT5G42830		HXXXD-type acyl-transferase family protein
AT5G42840		Cysteine/Histidine-rich C1 domain family protein
AT5G42850		Thioredoxin superfamily protein
AT5G42860	*COMPANION OF CELLULOSE SYNTHASE 2 (CC2)*	CC2 is a plant specific gene that interacts with with the cellulose synthase complex and microtubules. It appears to play a role in localizing CESA to the membrane, microtuble dynamics , particularly during salt stress.
AT5G42880		WEAK CHLOROPLAST MOVEMENT UNDER BLUE LIGHT-like protein (DUF827)
AT5G42890	*STEROL CARRIER PROTEIN 2 (SCP2)*	sterol carrier protein 2
AT5G42900	*COLD REGULATED GENE 27 (COR27)*	Acts with COR28 as a key regulator in the COP1-HY5 regulatory hub by regulating HY5 activity to ensure proper skotomorphogenic growth in the dark and photomorphogenic development in the light.
AT5G42910		Basic-leucine zipper (bZIP) transcription factor family protein
AT5G42940	*(CTL06)*	RING/U-box superfamily protein
AT5G42950	*ESSENTIAL FOR POTEXVIRUS ACCUMULATION 1 (EXA1)*	EXA1 is a GYF domain-containing gene of the SMY2 subgroup. Mutants exhibit resistance to potexviruses.
AT5G42955		inhibitor/lipid-transfer protein/seed storage 2S albumin superfamily protein (DUF784)
AT5G42960		outer envelope pore 24B-like protein
AT5G42970	*CONSTITUTIVE PHOTOMORPHOGENIC 8 (COP8)*	encodes subunit 4 of COP9 signalosome complex. sequence is similar to a subunit of the 19S regulatory particle of the 26S proteasome. recessive mutation causes derepression of photomorphogenesis.
AT5G42990	*UBIQUITIN-CONJUGATING ENZYME 18 (UBC18)*	ubiquitin-conjugating enzyme 18
AT5G43000		hypothetical protein
AT5G43010	*REGULATORY PARTICLE TRIPLE-A ATPASE 4A (RPT4A)*	26S proteasome AAA-ATPase subunit RPT4a (RPT4a) mRNA,
AT5G43020		Leucine-rich repeat protein kinase family protein
AT5G43030		Cysteine/Histidine-rich C1 domain family protein
AT5G43040		Cysteine/Histidine-rich C1 domain family protein
AT5G43060	*ESPONSIVE TO DEHYDRATION 21B (RD21B)*	Peptidase, activity detected in extracts of root, leaf and cell culture.
AT5G43070	*WPP DOMAIN PROTEIN 1 (WPP1)*	WPP family members contains an NE targeting domain. This domain, called the WPP domain after a highly conserved Trp-Pro-Pro motif, is necessary and sufficient for NE targeting of WPP1. RNAi suppression of WPP1 resulted in reduced mitotic activity.
AT5G43110	*PUMILIO 14 (PUM14)*	Encodes a member of the Arabidopsis Pumilio (APUM) proteins containing PUF domain (eight repeats of approximately 36 amino acids each). PUF proteins regulate both mRNA stability and translation through sequence-specific binding to the 3' UTR of target mRNA transcripts.
AT5G43120		ARM-repeat/Tetratricopeptide repeat (TPR)-like protein
AT5G43130	*TBP-ASSOCIATED FACTOR 4 (TAF4)*	TBP-associated factor 4
AT5G43140		Peroxisomal membrane 22 kDa (Mpv17/PMP22) family protein
AT5G43150		elongation factor
AT5G43160	*QWRF DOMAIN CONTAINING 9 (QWRF9)*	QWRF motif protein (DUF566)

AT5G43180		transmembrane protein, putative (Protein of unknown function, DUF599)
AT5G43190		Galactose oxidase/kelch repeat superfamily protein
AT5G43200		Zinc finger, C3HC4 type (RING finger) family protein
AT5G43220		
AT5G43230		EEIG1/EHBP1 protein amino-terminal domain protein
AT5G43260		chaperone protein dnaJ-like protein
AT5G43270	*SQUAMOSA PROMOTER BINDING PROTEIN-LIKE 2 (SPL2)*	Member of the SPL (squamosa-promoter binding protein-like) gene family, a novel gene family encoding DNA binding proteins and putative transcription factors. In conjunction with SPL10 and SPL11, SPL2 redundantly controls proper development of lateral organs in association with shoot maturation in the reproductive phase. SPL2, SPL10, and SPL11, suppress root regeneration with age by inhibiting wound-induced auxin biosynthesis.
AT5G43310		COP1-interacting protein-like protein
AT5G43330	*CYTOSOLIC-NAD-DEPENDENT MALATE DEHYDROGENASE 2 (c-NAD-MDH2)*	predicted to encode a cytosolic malate dehydrogenase. The mRNA is cell-to-cell mobile.
AT5G43340	*PHOSPHATE TRANSPORTER 1;6 (PHT1;6)*	Encodes Pht1;6, a member of the Pht1 family of phosphate transporters which include: Pht1;1/At5g43350, Pht1;2/At5g43370, Pht1;3/At5g43360, Pht1;4/At2g38940, Pht1;5/At2g32830, Pht1;6/At5g43340, Pht1;7/At3g54700, Pht1;8/At1g20860, Pht1;9/At1g76430 (Plant Journal 2002, 31:341).
AT5G43360	*PHOSPHATE TRANSPORTER 1;3 (PHT1;3)*	Encodes Pht1;3, a member of the Pht1 family of phosphate transporters which include: Pht1;1/At5g43350, Pht1;2/At5g43370, Pht1;3/At5g43360, Pht1;4/At2g38940, Pht1;5/At2g32830, Pht1;6/At5g43340, Pht1;7/At3g54700, Pht1;8/At1g20860, Pht1;9/At1g76430 (Plant Journal 2002, 31:341).
AT5G43370	*PHOSPHATE TRANSPORTER 2 (PHT1;2)*	Encodes a phosphate transporter Pht1;2. Members of the Pht1 family of phosphate transporters include: Pht1;1/At5g43350, Pht1;2/At5g43370, Pht1;3/At5g43360, Pht1;4/At2g38940, Pht1;5/At2g32830, Pht1;6/At5g43340, Pht1;7/At3g54700, Pht1;8/At1g20860, Pht1;9/At1g76430 (Plant Journal 2002, 31:341) The mRNA is cell-to-cell mobile.
AT5G43380	*TYPE ONE SERINE/THREONINE PROTEIN PHOSPHATASE 6 (TOPP6)*	encodes a type I serine/threonine protein phosphatase expressed in expressed in roots, rosettes and flowers.
AT5G43390		plant/protein
AT5G43410	*ETHYLENE RESPONSE FACTOR 96 (ERF96)*	Encodes a member of the ERF (ethylene response factor) subfamily B-3 of ERF/AP2 transcription factor family. The protein contains one AP2 domain. There are 18 members in this subfamily including ATERF-1, ATERF-2, AND ATERF-5. Expression of ERF96 is induced by pathogens, JA and ethylene and over expression leads to increased resistance to resistance to necrotrophic pathogens. It is a nuclear localized, transcriptional activator that binds to GCC elements that is involved in positive regulation of ABA responses.
AT5G43440		encodes a protein whose sequence is similar to ACC oxidase
AT5G43450		encodes a protein whose sequence is similar to ACC oxidase
AT5G43480		hypothetical protein
AT5G43500	*ACTIN-RELATED PROTEIN 9 (ARP9)*	encodes a protein whose sequence is similar to actin-related proteins (ARPs) in other organisms. Member of nuclear ARP family of genes.
AT5G43510	*LURE 1.2 (LURE1.2)*	Encodes a cysteine-rich peptide that acts as a pollen tube attractant guiding pollen tubes to the ovular micropyle. It is expressed in the synergid cell and appears to be secreted toward the funicular surface through the micropyle.
AT5G43520		Cysteine/Histidine-rich C1 domain family protein
AT5G43530	*(RAD5B)*	Helicase protein with RING/U-box domain-containing protein
AT5G43550		F-box associated ubiquitination effector family protein
AT5G43580	*UNUSUAL SERINE PROTEASE INHIBITOR (UPI)*	Predicted to encode a PR (pathogenesis-related) peptide that belongs to the PR-6 proteinase inhibitor family. Functions in resistance to necrotrophic fungi and insect herbivory. Six putative PR-6-type protein encoding genes are found in Arabidopsis: At2g38900, At2g38870, At5g43570, At5g43580, At3g50020 and At3g46860.
AT5G43590		Acyl transferase/acyl hydrolase/lysophospholipase superfamily protein
AT5G43600	*UREIDOGLYCOLATE AMIDOHYDROLASE (UAH)*	Encodes a protein with ureidoglycolate amidohydrolase activity in vitro. It is 27% identical and 43% similar to the E. coli allantoate amidohydrolase (AAH), but, in vitro assays with purified protein and allantoate as a substrate do not show any increase in ammonium concentration, indicating that there this enzyme has no AAH activity. The mRNA is cell-to-cell mobile.
AT5G43610	*SUCROSE-PROTON SYMPORTER 6 (SUC6)*	sucrose-proton symporter 6
AT5G43640	*RIBOSOMAL PROTEIN S15E (RPS15E)*	cytosolic ribosomal protein gene, part of uS19 family.
AT5G43670	*(ATSEC23G)*	Sec23/Sec24 protein transport family protein
AT5G43690		P-loop containing nucleoside triphosphate hydrolases superfamily protein
AT5G43700	*AUXIN INDUCIBLE 2-11 (ATAUX2-11)*	Auxin inducible protein similar to transcription factors.
AT5G43750	*PHOTOSYNTHETIC NDH SUBCOMPLEX B 5 (PnsB5)*	NAD(P)H dehydrogenase 18
AT5G43770		proline-rich family protein
AT5G43780	*(APS4)*	sulfate adenylyltransferase, ATP sulfurylase
AT5G43800		transposable_element_gene;copia-like retrotransposon family, has a 0. P-value blast match to gb|AAG52949.1| gag/pol polyprotein (Endovir1-1) (Arabidopsis thaliana) (Ty1_Copia-family);(source:TAIR10)
AT5G43830		aluminum induced protein with YGL and LRDR motifs
AT5G43840	*HEAT SHOCK TRANSCRIPTION FACTOR A6A (HSFA6A)*	member of Heat Stress Transcription Factor (Hsf) family
AT5G43850	*(ARD4)*	RmlC-like cupins superfamily protein
AT5G43860	*CHLOROPHYLLASE 2 (CLH2)*	Encodes a chlorophyllase, the first enzyme in chlorophyll degradation. It catalyzes the hydrolysis of the ester bond to chlorophyllide and phytol. AtCLH2 has a typical signal sequence for the chloroplast. Gene expression does not respond to methyljasmonate, a known promoter of senescence and chlorophyll degradation.
AT5G43870	*FORKED-LIKE1 (FL1)*	FORKED-LIKE family member, part of Group 1 (FKD1, FL1-FL3; Group 2 consists of FL4 and FL8 and Group 3 consists of FL5- FL7). May coordinate leaf size with vein density, where Group 1 members and Group 3 members have opposing functions.
AT5G43880	*TON1 RECRUITING MOTIF 21 (TRM21)*	methyl-coenzyme M reductase II subunit gamma, putative (DUF3741)

AT5G43890	*YUCCA5 (YUC5)*	Encodes a YUCCA-like putative flavin monooxygenase, the activation tagging mutant has increased level of IAA, increased auxin response and phenotype of auxin overproduction, rescues erecta mutant phenotype
AT5G43910		pfkB-like carbohydrate kinase family protein
AT5G43920		transducin family protein / WD-40 repeat family protein
AT5G43930	*RESISTANT TO PHYTOPHTHORA 5 (RTP5)*	Encodes a WD40 repeat domain-containing protein which negatively regulates plant resistance to Phytophthoravpathogens by modulating the biosynthesis of endogenous jasmonic acid and salicylic acid.
AT5G43960		Nuclear transport factor 2 (NTF2) family protein with RNA binding (RRM-RBD-RNP motifs) domain-containing protein
AT5G43980	*PLASMODESMATA-LOCATED PROTEIN 1 (PDLP1)*	Encodes a plasmodesmal protein that affects the intercellular movement of molecules through the plasmodesmata. The cytoplasmic C-terminal portion of the protein is connected to the apoplastic N-terminal portion of the protein by a single transmembrane domain (TMD). It is transported to the plasmodesmata through the secretory pathway. PDLP1 has two DUF26 domains and a signal peptide, but the proper localization of the protein appears to depend on the TMD.
AT5G43990	*(SUVR2)*	Encodes SUVR2, one of the four closely related Arabidopsis SUVR proteins that belong to the SU(VAR)3-9 subgroup of SET-domain proteins. Proteins containing the evolutionarily conserved SET domain are involved in regulation of eukaryotic gene expression and chromatin structure through their histone lysine methyltransferase (HMTase) activity. SUVR1, SUVR2 and SUVR4 proteins contain a novel domain at their N-terminus, and a SUVR specific region preceding the SET domain. Localized to the nucleolus, maybe involved in regulation of rRNA expression.
AT5G44010		fanconi anemia group F protein (FANCF)
AT5G44020		HAD superfamily, subfamily IIIB acid phosphatase
AT5G44030	*CELLULOSE SYNTHASE A4 (CESA4)*	Encodes a cellulose synthase involved in secondary cell wall biosynthesis. Confers resistance towards bacterial and fungal pathogens, independent of salicylic acid, ethylene and jasmonate signaling. The mRNA is cell-to-cell mobile.
AT5G44050	*(ATDTX28)*	MATE efflux family protein
AT5G44060		embryo sac development arrest protein
AT5G44070	*CADMIUM SENSITIVE 1 (CAD1)*	Phytochelatin synthase gene confers tolerance to cadmium ions. Catalyzes phytochelatin (PC) synthesis from glutathione (GSH) in the presence of Cd2+, Zn2+, Cu2+ and Fe3+, but not by Co2+ or Ni2+. The mRNA is cell-to-cell mobile.
AT5G44080		Basic-leucine zipper (bZIP) transcription factor family protein
AT5G44110	*ATP-BINDING CASSETTE A21 (ABCI21)*	Encodes a member of the NAP subfamily of ABC transporters whose expression pattern is regulated by light and sucrose.
AT5G44120	*CRUCIFERINA (CRA1)*	Encodes a 12S seed storage protein. The Landsberg erecta genome contains another copy of 12S globulin gene, CRA2, which is located tandemly with CRA1. Protein is tyrosine-phosphorylated and its phosphorylation state is modulated in response to ABA in Arabidopsis thaliana seeds.
AT5G44130	*FASCICLIN-LIKE ARABINOGALACTAN PROTEIN 13 PRECURSOR (FLA13)*	Fasciclin-like arabinogalactan protein. Possibly involved in embryogenesis and seed development.
AT5G44140	*PROHIBITIN 7 (PHB7)*	prohibitin 7
AT5G44150	*R RST1 INTERACTING PROTEIN (RIPR)*	Connects together with RST1 the cytosolic RNA exosome to the Ski complex.
AT5G44160	*NUTCRACKER (NUC)*	NUC is a member of the BIRD group of transcriptional regulators and is required for the formative divisions that pattern the root. the ground tissue into cortex and endodermis.
AT5G44170		S-adenosyl-L-methionine-dependent methyltransferases superfamily protein
AT5G44180	*RINGLET 2 (RLT2)*	Interacts with CHR11, CHR17, and ARID5, several known subunits of ISWI. JA biosynthesisis is positively regulated by this chromatin remodeling complex, thereby promoting stamen filament elongation.
AT5G44190	*GOLDEN2-LIKE 2 (GLK2)*	Encodes GLK2, Golden2-like 2, one of a pair of partially redundant nuclear transcription factors that regulate chloroplast development in a cell-autonomous manner. GLK1, Golden2-like 1, is encoded by At2g20570. GLK1 and GLK2 regulate the expression of the photosynthetic apparatus.
AT5G44230		Pentatricopeptide repeat (PPR) superfamily protein
AT5G44250		peptidase, S9A/B/C family, catalytic domain protein (Protein of unknown function DUF829, transmembrane 53)
AT5G44260	*TANDEM CCCH ZINC FINGER PROTEIN 5 (TZF5)*	Encodes a Tandem CCCH Zinc Finger protein. Interacts and co-localizes with MARD1 and RD21A in processing bodies (PBs) and stress granules (SGs).
AT5G44270	*(TPXL7)*	TPX2 (targeting protein for Xklp2) protein family
AT5G44280	*RING 1A (RING1A)*	Encodes a nuclear localized protein with similarity to animal polycomb repressive core complex1 (PRC1) core component RING.Appears to function redundantly with ATRING1b, a close paralog. Both interact physically with CLF and LHP1 and appear to function together to repress class I KNOX gene expression.
AT5G44300		Dormancy/auxin associated family protein
AT5G44310		Late embryogenesis abundant protein (LEA) family protein
AT5G44330		Tetratricopeptide repeat (TPR)-like superfamily protein
AT5G44350		ethylene-responsive nuclear protein-like protein
AT5G44360	*(ATBBE23)*	FAD-binding Berberine family protein
AT5G44380	*(AtBBE24)*	FAD-binding Berberine family protein
AT5G44390	*(ATBBE25)*	FAD-binding Berberine family protein
AT5G44400	*(ATBBE26)*	FAD-binding Berberine family protein
AT5G44410	*(ATBBE27)*	FAD-binding Berberine family protein
AT5G44420	*PLANT DEFENSIN 1.2 (PDF1.2)*	Encodes an ethylene- and jasmonate-responsive plant defensin. mRNA levels are not responsive to salicylic acid treatment; although jasmonate and salicylic acid can act synergistically to enhance the expression of this gene. Belongs to the plant defensin (PDF) family with the following members: At1g75830/PDF1.1, At5g44420/PDF1.2a, At2g26020/PDF1.2b, At5g44430/PDF1.2c, At2g26010/PDF1.3, At1g19610/PDF1.4, At1g55010/PDF1.5, At2g02120/PDF2.1, At2g02100/PDF2.2, At2g02130/PDF2.3, At1g61070/PDF2.4, At5g63660/PDF2.5, At2g02140/PDF2.6, At5g38330/PDF3.1 and At4g30070/PDF3.2. CFA-Leu, CFA-Val, CFA-Met and CFA-Ala can induce the expression of PDF.
AT5G44440	*(ATBBE28)*	FAD-binding Berberine family protein

AT5G44450		alpha amino-terminal protein methyltransferase
AT5G44470		ribonuclease H superfamily polynucleotidyl transferase
AT5G44480	*DEFECTIVE UGE IN ROOT (DUR)*	mutant has Altered lateral root; UDP Glucose Epimerase The mRNA is cell-to-cell mobile.
AT5G44500		Small nuclear ribonucleoprotein family protein
AT5G44520		NagB/RpiA/CoA transferase-like superfamily protein
AT5G44530		Subtilase family protein
AT5G44550	*CASP-LIKE PROTEIN 1B1 (CASPL1B1)*	Uncharacterized protein family (UPF0497)
AT5G44560	*(VPS2.2)*	SNF7 family protein
AT5G44570	*(PROSCOOP5)*	Encodes a PPR protein involved in mitochondrial functioning. Mutants suppress cell wall defects caused by C17 chemical inhibitor. Mutants are defective in cytochrome c maturation and activation of mitochondrial retrograde signalling.
AT5G44580	*(PROSCOOP10)*	transmembrane protein
AT5G44610	*MICROTUBULE-ASSOCIATED PROTEIN 18 (MAP18)*	Encodes a protein with seven repeated VEEKK motifs. RNAi and overexpression experiments suggest that the gene is not involved in cell division but might be consequential for cell shape of epidermal and cortical cells. The protein encoded by this gene binds to cortical microtubules and inhibits tubulin polymerization. Associates to the plasma membrane and interacts with calmodulin and phosphatidylinositol phosphates, indicating an involvement in cellular signal transduction. Expression is enhanced by abiotic and hormonal factors. Induced during senescence.Interacts with Ca2+/calmodulin complex, phosphatidylinositol phosphates, and free Ca2+.
AT5G44620	*CYTOCHROME P450, FAMILY 706, SUBFAMILY A, POLYPEPTIDE 3 (CYP706A3)*	member of CYP706A
AT5G44630		Encodes a sesquiterpene synthase involved in generating all of the group B sesquiterpenes found in the Arabidopsis floral volatile blend. Strongly expressed in intrafloral nectaries.
AT5G44650	*CHLOROPLAST PROTEIN-ENHANCING STRESS TOLERANCE (CEST)*	Encodes a nucleus-encoded thylakoid protein, cooperates with the plastid-encoded Ycf3 protein in photosystem i assembly. Also induces tolerance to multiple environmental stresses and reduces photooxidative damage.
AT5G44660		hypothetical protein
AT5G44670	*GALACTAN SYNTHASE 2 (GALS2)*	glycosyltransferase family protein (DUF23)
AT5G44680		DNA glycosylase superfamily protein
AT5G44700	*GASSHO 2 (GSO2)*	Encodes GASSHO2 (GSO2), a putative leucine-rich repeat transmembrane-type receptor kinase. GSO2 and a homolog GSO1 (At4g20140) are required for the formation of a normal epidermal surface during embryogenesis.
AT5G44720		Molybdenum cofactor sulfurase family protein
AT5G44730		Haloacid dehalogenase-like hydrolase (HAD) superfamily protein
AT5G44750	*(REV1)*	Homologous to Y-family DNA polymerases, contains BRCT domain. Mutants are sensitive to UV-B radiation. Gene is involved in damage-tolerance mechanisms through translesion synthesis(TLS).
AT5G44760		C2 domain-containing protein
AT5G44770		Cysteine/Histidine-rich C1 domain family protein
AT5G44780	*MULTIPLE ORGANELLAR RNA EDITING FACTOR 4 (MORF4)*	Member of MORF family consisting of of nine full-length proteins encoded in the nuclear genome. MORF proteins are required for all RNA editing events in plastids and for many, possibly also all, sites in mitochondria. Potential link between the RNA binding PPR protein and the protein contributing the enzymatic activity in RNA editing.
AT5G44785	*ORGANELLAR SINGLE-STRANDED DNA BINDING PROTEIN 3 (OSB3)*	Organellar Single-stranded DNA Binding protein. Decreases MMEJ on long ssDNA templates.
AT5G44790	*RESPONSIVE-TO-ANTAGONIST 1 (RAN1)*	ATP dependent copper transporter vital for ethylene response pathway
AT5G44810		
AT5G44820		Nucleotide-diphospho-sugar transferase family protein
AT5G44830		Pectin lyase-like superfamily protein
AT5G44870	*LAZARUS 5 (LAZ5)*	Encodes LAZ5, a TIR-class NB-LRR R protein of unknown pathogen specificity with sequence similarity to RPS4, an R protein conferring resistance to Pseudomonas syringae expressing the effector AvrRPS4. Overexpression of LAZ5 results in hypersensitive cell death (plants did not survive to set seeds).
AT5G44880		transposable_element_gene;similar to unknown protein [Arabidopsis thaliana] (TAIR:AT2G14020.1);(source:TAIR10)
AT5G44900		Toll-Interleukin-Resistance (TIR) domain family protein
AT5G44910		Toll-Interleukin-Resistance (TIR) domain family protein
AT5G44920	*TIR-KASH PROTEIN (TIK)*	Encodes a KASH domain protein that localizes to the nuclear envelope and affects nuclear morphology.
AT5G44930	*ARABINAN DEFICIENT 2 (ARAD2)*	Encodes a putative arabinosyltransferase that is associated with arabinan biosynthesis and is not redundant with ARAD1. The two glycosyltransferases may function in complexes held together by disulfide bridges.
AT5G44970		Protein with RNI-like/FBD-like domain
AT5G44980		F-box/RNI-like/FBD-like domains-containing protein
AT5G44990		Glutathione S-transferase family protein
AT5G45000		Disease resistance protein (TIR-NBS-LRR class) family
AT5G45020		Glutathione S-transferase family protein
AT5G45040	*CYTOCHROME C6A (CYTC6A)*	Encodes a Class I cytochrome c family member possessing a high structural homology with photosynthetic cytochrome c(6) from cyanobacteria, but structurally and functionally distinct through the presence of a disulfide bond.
AT5G45050	*(WRKY16)*	Encodes a member of the WRKY Transcription Factor (Group II-e) family.
AT5G45060		Disease resistance protein (TIR-NBS-LRR class) family
AT5G45070	*PHLOEM PROTEIN 2-A8 (PP2-A8)*	phloem protein 2-A8
AT5G45080	*PHLOEM PROTEIN 2-A6 (PP2-A6)*	phloem protein 2-A6

AT5G45090	*PHLOEM PROTEIN 2-A7 (PP2-A7)*	phloem protein 2-A7
AT5G45110	*NPR1-LIKE PROTEIN 3 (NPR3)*	Encodes NPR3, a paralog of NPR1. Involved in negative regulation of defense responses against bacterial and oomycete pathogens. npr3 mutants has elevated level of PR1 expression. Interacts with TGA2, TGA3, TGA5 and TGA6 in yeast two hybrid assays. NPR3 and NPR4 are receptors for the immune signal salicylic acid. The mRNA is cell-to-cell mobile.
AT5G45120		Eukaryotic aspartyl protease family protein
AT5G45140	*NUCLEAR RNA POLYMERASE C2 (NRPC2)*	Encodes a subunit of RNA polymerase III (aka RNA polymerase C).
AT5G45160	*RHD-LIKE2 (RL2)*	Root hair defective 3 GTP-binding protein (RHD3)
AT5G45170		Haloacid dehalogenase-like hydrolase (HAD) superfamily protein
AT5G45180		Flavin-binding monooxygenase family protein
AT5G45190		Encodes a cyclin T partner CYCT1;5. Plays important roles in infection with Cauliflower mosaic virus (CaMV).
AT5G45200		Disease resistance protein (TIR-NBS-LRR class) family
AT5G45210		Disease resistance protein (TIR-NBS-LRR class) family
AT5G45220		Disease resistance protein (TIR-NBS-LRR class) family
AT5G45230		Disease resistance protein (TIR-NBS-LRR class) family
AT5G45240		Disease resistance protein (TIR-NBS-LRR class)
AT5G45250	*RESISTANT TO P. SYRINGAE 4 (RPS4)*	RPS4 belongs to the Toll/interleukin-1 receptor (TIR)-nucleotide binding site (NBS)-Leu-rich repeat (LRR) class of disease resistance (R) genes. Confers specific resistance to Pseudomonas syringae pv. tomato carrying the avirulence gene AvrRPS4. Produces alternative transcripts with truncated open reading frames.
AT5G45260	*RESISTANT TO RALSTONIA SOLANACEARUM 1 (RRS1)*	Confers resistance to Ralstonia solanacearum. Similar to NBLS-TIR resistance genes,and also contains similarity to transcription factors. Interacts with pathogen effector protein AvrPop2.
AT5G45280	*PECTIN ACETYLESTERASE 11 (PAE11)*	Pectin acetylesterase involved in pectin remodelling.
AT5G45310		coiled-coil protein
AT5G45320		late embryogenesis abundant protein
AT5G45340	*CYTOCHROME P450, FAMILY 707, SUBFAMILY A, POLYPEPTIDE 3 (CYP707A3)*	Encodes a protein with ABA 8'-hydroxylase activity; involved in ABA catabolism. Mutant analyses show that disruption in the gene results in more drought tolerance whereas overexpression results in increased transpiration rate and reduced drought tolerance. Gene involved in postgermination growth. Plant P450 CYP707A3, ABA 8'-hydroxylase, binds enantioselectively (+)-ABA but not (-)-ABA, whereas the enzyme binds both enantiomers of AHI1 (a structural ABA analogue used as ABA 8'-hydroxylase competitive inhibitor).
AT5G45350	*GLYCINE AND PROLINE RICH PROTEIN 2 (GPRP2)*	proline-rich family protein
AT5G45360	*SKP1-INTERACTING PARTNER 31 (SKIP31)*	Encodes a F-box subunit of the SCF E3 ubiquitin ligase complex that mediates the degradation of 14-3-3 proteins.
AT5G45380	*DEGRADATION OF UREA 3 (DUR3)*	urea-proton symporter DEGRADATION OF UREA 3 (DUR3)
AT5G45390	*CLP PROTEASE P4 (CLPP4)*	One of several nuclear-encoded ClpPs (caseinolytic protease). Contains a highly conserved catalytic triad of Ser-type proteases (Ser-His-Asp). The name reflects nomenclature described in Adam et. al (2001). The mRNA is cell-to-cell mobile.
AT5G45400	*(RPA70C)*	Replication factor-A protein 1-like protein
AT5G45410	*NON HOST RESISTANCE 2A (ATNHR2A)*	Plastid localized protein of unknown function. Mutants are more susceptible to P. syringae and produce less callose upon infection.
AT5G45460		transmembrane protein
AT5G45480		transmembrane protein, putative (DUF594)
AT5G45500		RNI-like superfamily protein
AT5G45510		Leucine-rich repeat (LRR) family protein
AT5G45530		transmembrane protein, putative (DUF594)
AT5G45540		transmembrane protein, putative (DUF594)
AT5G45550	*MOB1-LIKE (MOB1-like)*	Encodes a gene product involved in both sporogenesis and gametogenesis and is required for the normal progression of megasporogenesis and microsporogenesis. Additional alleles were isolated in a screen for enhancers of PID and genetic analysis indicates a role for MOB1A in auxin mediated signaling.
AT5G45560		Pleckstrin homology (PH) domain-containing protein / lipid-binding START domain-containing protein
AT5G45570		Ulp1 protease family protein
AT5G45590		Ribosomal protein L35
AT5G45630		senescence regulator (Protein of unknown function, DUF584)
AT5G45640		Subtilisin-like serine endopeptidase family protein
AT5G45670		GDSL-motif esterase/acyltransferase/lipase. Enzyme group with broad substrate specificity that may catalyze acyltransfer or hydrolase reactions with lipid and non-lipid substrates.
AT5G45680	*FK506-BINDING PROTEIN 13 (FKBP13)*	Peptidyl-Prolyl Isomerase located in chloroplast thylakoid lumen The mRNA is cell-to-cell mobile.
AT5G45690		histone acetyltransferase (DUF1264)
AT5G45700		Haloacid dehalogenase-like hydrolase (HAD) superfamily protein
AT5G45770	*RECEPTOR LIKE PROTEIN 55 (RLP55)*	receptor like protein 55
AT5G45780	*CLAVATA3 INSENSITIVE RECEPTOR KINASE 4 (CIK4)*	Encodes one of a group of LRR-RLKs, designated as CLAVATA3 INSENSITIVE RECEPTOR KINASES (CIKs), that act as co-receptors and have essential roles in regulating CLV3-mediated stem cell homeostasis.
AT5G45790		Ubiquitin carboxyl-terminal hydrolase family protein
AT5G45800	*MATERNAL EFFECT EMBRYO ARREST 62 (MEE62)*	Leucine-rich repeat protein kinase family protein
AT5G45810	*CBL-INTERACTING PROTEIN KINASE 19 (CIPK19)*	Encodes a member of the SNF1-related kinase (SnRK) gene family (SnRK3.5), which has also been reported as a member of the CBL-interacting protein kinases (CIPK19).

AT5G45820	*CBL-INTERACTING PROTEIN KINASE 20 (CIPK20)*	Encodes a CBL-interacting serine/threonine protein kinase comprised of an N-terminal kinase catalytic domain similar to SNF1/AMPK and a unique C-terminal regulatory domain.
AT5G45830	*DELAY OF GERMINATION 1 (DOG1)*	Encodes DOG1 (DELAY OF GERMINATION 1). A quantitative trait locus involved in the control of seed dormancy. Belongs to a novel plant-specific gene family whose members include: DOG1-like 1-4 (DOGL1-4, At4g18660, At4g18680, At4g18690, At4g18650 respectively) and DOG1. DOG1 expression is seed-specific.
AT5G45840	*MALE DISCOVERER1 (MDIS1)*	Encodes a leucine-rich-repeat RLK that is localized to the plasma membrane of pollen tubes and functions with MIK1/2 as the male receptor of the pollen tube chemo-attractant LURE1.MDIS1 forms a complex with MIK1/2 and binds LURE1.
AT5G45850		hypothetical protein (DUF688)
AT5G45880		Pollen Ole e 1 allergen and extensin family protein
AT5G45890	*SENESCENCE-ASSOCIATED GENE 12 (SAG12)*	Senescence-associated gene 12 (SAG12) encoding a cysteine protease influenced by cytokinin, auxin, and sugars.Localized to special vacuole found during senescence called senescence associated vacuoles which are different from central vacuole in the tonoplast composition and pH.
AT5G45910		GDSL-motif esterase/acyltransferase/lipase. Enzyme group with broad substrate specificity that may catalyze acyltransfer or hydrolase reactions with lipid and non-lipid substrates.
AT5G45920	*GUARD-CELL-ENRICHED GDSL LIPASE 28 (GGL28)*	Guard-cell-enriched GDSL Lipase family member.
AT5G45930	*MAGNESIUM CHELATASE I2 (CHLI2)*	encodes a second Chl I gene (CHLI2), a subunit of magnesium chelatase which is required for chlorophyll biosynthesis. Has ATPase activity, regulated by TRX-f. Involved in the assembly of the Mg chelatase complex.
AT5G45940	*NUDIX HYDROLASE HOMOLOG 11 (NUDT11)*	Encodes a CoA pyrophosphatase, also has ppGpp pyrophosphohydrolase and exhibits minor activity of NADH pyrophosphatase. Most strongly expressed in embryo cotyledon and hypocotyl, flower, and phloem of vascular tissue. Over-expression mutant had a bigger plant with wider rosette.
AT5G45950	*(GGL28)*	GDSL-motif esterase/acyltransferase/lipase. Enzyme group with broad substrate specificity that may catalyze acyltransfer or hydrolase reactions with lipid and non-lipid substrates.
AT5G45960		GDSL-motif esterase/acyltransferase/lipase. Enzyme group with broad substrate specificity that may catalyze acyltransfer or hydrolase reactions with lipid and non-lipid substrates.
AT5G45970	*RAC-LIKE 2 (RAC2)*	Encodes a Rac-like protein ARAC2. A member of ROP GTPase gene family.
AT5G45980	*WUSCHEL RELATED HOMEOBOX 8 (WOX8)*	Arabidopsis thaliana WOX8 protein. Contains similarity to homeodomain transcription factor. Positively regulates early embryonic growth. Together with CLE8 it forms a signaling module that promotes seed growth and overall seed size.
AT5G46000		Mannose-binding lectin superfamily protein
AT5G46010		Homeodomain-like superfamily protein
AT5G46040		Major facilitator superfamily protein
AT5G46050	*NRT1/ PTR FAMILY 5.2 (NPF5.2)*	Encodes a di- and tri-peptide transporter involved in responses to wounding, virulent bacterial pathogens, and high NaCl concentrations. The protein is predicted to have 12 transmembrane helicies.
AT5G46070	*GUANYLATE-BINDING PROTEIN-LIKE 3 (GBPL3)*	Assembles liquid?liquid phase separation (LLPS)-driven condensates within the nucleus to protect against infection and autoimmunity. Within membraneless organelles termed GBPL defence-activated condensates (GDACs), directly binds defence-gene promoters and recruited specifc transcriptional coactivators of the Mediator complex and RNA polymerase II machinery to massively reprogram host gene expression for disease resistance.
AT5G46080		Protein kinase superfamily protein
AT5G46100		Pentatricopeptide repeat (PPR) superfamily protein
AT5G46110	*ACCLIMATION OF PHOTOSYNTHESIS TO ENVIRONMENT 2 (APE2)*	Encodes a chloroplast triose phosphate / 3-phosphoglycerate translocator that transports triose phosphates derived from the Calvin cycle in the stroma to the cytosol for use in sucrose synthesis and other biosynthetic processes. A tpt mutant has altered acclimation responses. The mRNA is cell-to-cell mobile.
AT5G46115		hypothetical protein
AT5G46120		transposable_element_gene;similar to unknown protein [Arabidopsis thaliana] (TAIR:AT4G05145.1);(source:TAIR10)
AT5G46140	*DUF295 ORGANELLAR A 13 (ATDOA13)*	hypothetical protein (DUF295)
AT5G46150		LEM3 (ligand-effect modulator 3) family protein / CDC50 family protein
AT5G46180	*ORNITHINE-DELTA-AMINOTRANSFERASE (DELTA-OAT)*	Encodes an ornithine delta-aminotransferase that is transcriptionally up-regulated in young seedlings and in response to salt stress. It is unlikely to play a role in salt-stress-induced proline accumulation, however, it appears to participate in arginine and ornithine catabolism.
AT5G46200		carboxyl-terminal proteinase-like protein (DUF239)
AT5G46230	*(SVB3)*	ABA responsive SVB family gene.
AT5G46240	*POTASSIUM CHANNEL IN ARABIDOPSIS THALIANA 1 (KAT1)*	Encodes a potassium channel protein (KAT1). ABA triggers KAT1 endocytosis both in epidermal cells as well as guard cells. Upon removal of ABA, KAT1 is recycled back to the plasma membrane. KAT1 is localized within 0.5?0.6 μm diameter microdomains at the plasma membrane surface. KAT1 belongs to the Shaker family K+ channel. This family includes five groups based on phylogenetic analysis (FEBS Letters (2007) 581: 2357): I (inward rectifying channel): AKT1 (AT2G26650), AKT5 (AT4G32500) and SPIK (also known as AKT6, AT2G25600); II (inward rectifying channel): KAT1 (AT5G46240) and KAT2 (AT4G18290); III (weakly inward rectifying channel): AKT2 (AT4G22200); IV (regulatory subunit involved in inwardly rectifying conductance formation): KAT3 (also known as AtKC1, AT4G32650); V (outward rectifying channel): SKOR (AT3G02850) and GORK (AT5G37500).
AT5G46270		Disease resistance protein (TIR-NBS-LRR class) family
AT5G46290	*3-KETOACYL-ACYL CARRIER PROTEIN SYNTHASE I (KASI)*	Encodes beta-ketoacyl-[acyl carrier protein] synthase I (KASI). Crucial for fatty acid synthesis. Plays a role in chloroplast division and embryo development.
AT5G46295		transmembrane protein
AT5G46300		hypothetical protein
AT5G46310		WRKY family transcription factor

AT5G46320		Mutants accumulate proline in response to drought. Potential QTL for drought tolerance.
AT5G46330	*FLAGELLIN-SENSITIVE 2 (FLS2)*	Encodes a leucine-rich repeat serine/threonine protein kinase that is expressed ubiquitously. FLS2 is involved in MAP kinase signalling relay involved in innate immunity. Essential in the perception of flagellin, a potent elicitor of the defense response. FLS2 is directed for degradation by the bacterial ubiquitin ligase AvrPtoB. The mRNA is cell-to-cell mobile.
AT5G46350	*WRKY DNA-BINDING PROTEIN 8 (WRKY8)*	member of WRKY Transcription Factor; Group II-c
AT5G46370	*CA2+ ACTIVATED OUTWARD RECTIFYING K+ CHANNEL 2 (KCO2)*	Encodes AtTPK2 (KCO2), a member of the Arabidopsis thaliana K+ channel family of AtTPK/KCO proteins. AtTPK2 is targeted to the vacuolar membrane. May form homomeric ion channels in vivo.
AT5G46390	*(CTPC)*	C-terminal peptidase
AT5G46410	*SCP1-LIKE SMALL PHOSPHATASE 4 (SSP4)*	Encodes a SCP1-like small phosphatase (SSP). Three SSPs form a unique group with long N-terminal extensions: AT5G46410 (SSP4), AT5G11860 (SSP5), AT4G18140 (SSP4b). SSP4 and SSP4b were localized exclusively in the nuclei, whereas SSP5 accumulated in both nuclei and cytoplasm. All three SSPs encodes active CTD phosphatases like animal SCP1 family proteins, with distinct substrate specificities: SSP4 and SSP4b could dephosphorylate both Ser2-PO(4) and Ser5-PO(4) of CTD, whereas SSP5 dephosphorylated only Ser5-PO(4). The mRNA is cell-to-cell mobile.
AT5G46420		16S rRNA processing protein RimM family
AT5G46440		PPR containing-like protein
AT5G46450		Disease resistance protein (TIR-NBS-LRR class) family
AT5G46460		Pentatricopeptide repeat (PPR) superfamily protein
AT5G46480		
AT5G46490		Disease resistance protein (TIR-NBS-LRR class) family
AT5G46500		protein VARIATION IN COMPOUND TRIGGERED ROOT growth protein
AT5G46510	*VARIATION IN COMPOUND TRIGGERED ROOT GROWTH RESPONSE-LIKE (VICTL)*	Disease resistance protein (TIR-NBS-LRR class) family
AT5G46520	*VARIATION IN COMPOUND TRIGGERED ROOT GROWTH RESPONSE (VICTR)*	VICTR (VARIATION IN COMPOUND TRIGGERED ROOT growth response) encodes a TIR-NB-LRR (for Toll-Interleukin1 Receptor-nucleotide binding-Leucine-rich repeat) protein. VICTR is necessary for DFPM-induced root growth arrest and inhibition of abscisic acid-induced stomatal closing (DFPM is [5-(3,4-dichlorophenyl)furan-2-yl]-piperidine-1-ylmethanethione)(PMID:21620700). DFPM-mediated root growth arrest is accession-specific and depends on EDS1 and PAD4; Col-0 has a functional copy of VICTR. Induction of the VICTR gene by DFPM treatment requires functional VICTR (Col). A close homolog to VICTR, named VICTL (At5g46510) lies in tandem with VICTR. The mRNA is cell-to-cell mobile.
AT5G46540	*ATP-BINDING CASSETTE B7 (ABCB7)*	P-glycoprotein 7
AT5G46550		DNA-binding bromodomain-containing protein
AT5G46570	*BRASSINOSTEROID-SIGNALING KINASE 2 (BSK2)*	Encodes BR-signaling kinase 2 (BSK2), one of the three homologous BR-signaling kinases (BSK1, AT4G35230; BSK2, AT5G46570; BSK3, AT4G00710). Mediates signal transduction from receptor kinase BRI1 by functioning as the substrate of BRI1. Plasma membrane localized.
AT5G46580	*SUPPRESSOR OF THYLAKOID FORMATION 1 (SOT1)*	PPR protein with 5'exonuclease activity that is involved in chloroplast rRNA processing.
AT5G46590	*NAC DOMAIN CONTAINING PROTEIN 96 (NAC096)*	Transcription factor required for the initiation of cell division during wound healing. Redundantly involved with ANAC071 in the process of "cambialization".
AT5G46640	*AT-HOOK MOTIF NUCLEAR LOCALIZED PROTEIN 8 (AHL8)*	AT hook motif DNA-binding family protein
AT5G46660		protein kinase C-like zinc finger protein
AT5G46670		Cysteine/Histidine-rich C1 domain family protein
AT5G46690	*BETA HLH PROTEIN 71 (bHLH071)*	beta HLH protein 71
AT5G46730		glycine-rich protein
AT5G46740	*UBIQUITIN-SPECIFIC PROTEASE 21 (UBP21)*	Encodes a ubiquitin-specific protease.
AT5G46760	*(MYC3)*	MYC3 is a JAZ-interacting transcription factor that act together with MYC2 and MYC4 to activate JA-responses. The mRNA is cell-to-cell mobile.
AT5G46770		hypothetical protein
AT5G46780		VQ motif-containing protein
AT5G46790	*PYR1-LIKE 1 (PYL1)*	Encodes a member of the PYR (pyrabactin resistance)/PYL(PYR1-like)/RCAR (regulatory components of ABA receptor) family proteins with 14 members. PYR/PYL/RCAR family proteins function as abscisic acid sensors. Mediate ABA-dependent regulation of protein phosphatase 2Cs ABI1 and ABI2. Negative regulation of ABA response as a result of phosphorylation of S136 and S182 sites by AEL1/3/4.
AT5G46795	*MICROSPORE-SPECIFIC PROMOTER 2 (MSP2)*	microspore-specific promoter 2
AT5G46800	*A BOUT DE SOUFFLE (BOU)*	Seedling lethal mutation; Mitochondrial Carnitine Acyl Carrier-Like Protein
AT5G46810		carboxyl-terminal proteinase-like protein, putative (DUF239)
AT5G46830	*NACL-INDUCIBLE GENE 1 (NIG1)*	Calcium-binding transcription factor involved in salt stress signaling.
AT5G46840		RNA-binding (RRM/RBD/RNP motifs) family protein
AT5G46880	*HOMEOBOX-7 (HB-7)*	homeobox-7
AT5G46900		Bifunctional inhibitor/lipid-transfer protein/seed storage 2S albumin superfamily protein
AT5G46910	*JUMONJI 13 (JMJ13)*	H3K27me3 demethylase involved in temperature and photoperiod dependent repressing of flowering.
AT5G46920		Intron maturase, type II family protein
AT5G46940		pectin methylesterase inhibitor
AT5G46960	*(ATPMEI12)*	Pectin methylesterase inhibitor that is involved in resistance to Botrytis cinerea. Affects PME activity during infection to prevent disease. Closely related paralog of AT5G46950 (InvINH2).
AT5G47000		Peroxidase superfamily protein
AT5G47020		MraZ
AT5G47050		SBP (S-ribonuclease binding protein) family protein

AT5G47070	*PBS1-LIKE 19 (PBL19)*	Encodes a member of the RLCK VII-4 subfamily of receptor-like cytoplasmic kinases that has been shown to phosphorylate MAPKKK5 Ser-599 and MEKK1 Ser-603, both players in PRR-mediated resistance to bacterial and fungal pathogens.
AT5G47080	*CASEIN KINASE II BETA CHAIN 1 (CKB1)*	Regulatory subunit beta of casein kinase II (CK2). purified CKB1 resulted in up 100-fold stimulation of casein kinase activity compared with the CKA1 activity alone. Forms a tetrameric complex with CKA1 (CKA1(2)CKB1(2)). One known substrate of CK2 is Phytochrome Interacting Factor 1 (PIF1). CK2-mediated phosphorylation enhances the light-induced degradation of PIF1 to promote photomorphogenesis.
AT5G47090		coiled-coil protein
AT5G47110	*LIGHT-HARVESTING-LIKE 3:2 (LIL3:2)*	Encodes a light-harvesting-like protein that is involved in chlorophyll and tocopherol biosynthesis anchoring geranylgeranyl reductase in the thylakoid membrane.
AT5G47130		Bax inhibitor-1 family protein
AT5G47150		YDG/SRA domain-containing protein
AT5G47160		YDG/SRA domain-containing protein
AT5G47180		Plant VAMP (vesicle-associated membrane protein) family protein
AT5G47190	*PLASTID RIBOSOMAL PROTEINS OF THE 50S SUBUNIT 19 (PRPL19)*	Ribosomal protein L19 family protein
AT5G47220	*ETHYLENE RESPONSIVE ELEMENT BINDING FACTOR 2 (ERF2)*	Encodes a member of the ERF (ethylene response factor) subfamily B-3 of ERF/AP2 transcription factor family (ATERF-2). The protein contains one AP2 domain. Functions as activator of GCC box?dependent transcription. Positive regulator of JA-responsive defense genes and resistance to F. oxysporum and enhances JA inhibition of root elongation.
AT5G47230	*ETHYLENE RESPONSIVE ELEMENT BINDING FACTOR 5 (ERF5)*	encodes a member of the ERF (ethylene response factor) subfamily B-3 of ERF/AP2 transcription factor family (ATERF-5). The protein contains one AP2 domain. There are 18 members in this subfamily including ATERF-1, ATERF-2, AND ATERF-5. The mRNA is cell-to-cell mobile.
AT5G47240	*NUDIX HYDROLASE HOMOLOG 8 (NUDT8)*	nudix hydrolase homolog 8
AT5G47250		LRR and NB-ARC domains-containing disease resistance protein
AT5G47260		putative disease resistance protein
AT5G47270		
AT5G47280	*ADR1-LIKE 3 (ADR1-L3)*	ADR1-like 3
AT5G47320	*RIBOSOMAL PROTEIN S19 (RPS19)*	Nuclear encoded mitochondrial ribosome subunit. The mRNA is cell-to-cell mobile.
AT5G47330		alpha/beta-Hydrolases superfamily protein
AT5G47340		alpha/beta-Hydrolases superfamily protein
AT5G47350		alpha/beta-Hydrolases superfamily protein
AT5G47370	*(HAT2)*	homeobox-leucine zipper genes induced by auxin, but not by other phytohormones. Plays opposite roles in the shoot and root tissues in regulating auxin-mediated morphogenesis.
AT5G47380		electron transporter, putative (Protein of unknown function, DUF547)
AT5G47390	*MYB HYPOCOTYL ELONGATION-RELATED (MYBH)*	Encodes a circadian-regulated transcription factor which specifically controls cell expansion during leaf development by controlling ROS homeostasis. The mRNA is cell-to-cell mobile.
AT5G47400	*(PNET4)*	sphingomyelin phosphodiesterase
AT5G47430		DWNN domain, a CCHC-type zinc finger
AT5G47440	*FORKED-LIKE6 (FL6)*	FORKED-LIKE family member, part of Group 3 (Group 1 consists of FKD1, FL1-FL3; Group 2 consists of FL4 and FL8 and Group 3 consists of FL5- FL7). May coordinate leaf size with vein density, where Group 1 members and Group 3 members have opposing functions.
AT5G47450	*TONOPLAST INTRINSIC PROTEIN 2;3 (TIP2;3)*	Tonoplast intrinsic protein, transports ammonium (NH3) and methylammonium across the tonoplast membrane, gene expression shows diurnal regulation and is upregulated by ammonium (NH3).
AT5G47455		hypothetical protein
AT5G47470	*USUALLY MULTIPLE ACIDS MOVE IN AND OUT TRANSPORTERS 7 (UMAMIT7)*	nodulin MtN21-like transporter family protein
AT5G47500	*PECTIN METHYLESTERASE 5 (PME5)*	predicted to encode a pectin methylesterase
AT5G47510		Sec14p-like phosphatidylinositol transfer family protein
AT5G47530		Auxin-responsive family protein
AT5G47540		Mo25 family protein
AT5G47550	*CYSTEINE PROTEINASE INHIBITOR 5 (CYS5)*	Putative phytocystatin expressed in seedlings and induced by heat stress and abscisic acid. Overexpression increases germination rate and heat stress tolerance. CYS5 is a target of ABF1 and ABF3 transcriptional regulators which bind to its promoter.
AT5G47560	*TONOPLAST DICARBOXYLATE TRANSPORTER (TDT)*	Encodes a tonoplast malate/fumarate transporter.
AT5G47570		NADH dehydrogenase ubiquinone 1 beta subcomplex subunit
AT5G47580	*ALTERED SEED GERMINATION 7 (ASG7)*	transmembrane protein
AT5G47600		HSP20-like chaperones superfamily protein
AT5G47610	*ARABIDOPSIS T??XICOS EN LEVADURA 79 (ATL79)*	RING/U-box superfamily protein
AT5G47635		Pollen Ole e 1 allergen and extensin family protein
AT5G47640	*NUCLEAR FACTOR Y, SUBUNIT B2 (NF-YB2)*	Involved in the regulation of response to nutrient levels.
AT5G47670	*NUCLEAR FACTOR Y, SUBUNIT B6 (NF-YB6)*	Encodes LEC1-Like (L1L), closely related to LEC1 (Leafy Cotyledon1). Functions as a regulator of embryo development.
AT5G47680	*TRNA MODIFICATION 10 (TRM10)*	Encodes a protein involved in modification of nucleosides in tRNA. Mutants have 50% less 1-methylguanosine than wt counterparts.
AT5G47690	*(PDS5A)*	One of 5 PO76/PDS5 cohesion cofactor orthologs of Arabidopsis.
AT5G47710		Calcium-dependent lipid-binding (CaLB domain) family protein
AT5G47720	*ACETOACETYL-COA THIOLASE 1 (AACT1)*	Encodes a functional acetoacetyl-CoA thiolase that is functionally redundant with AACT2. Loss-of-function mutants show no apparent growth phenotypes.
AT5G47730		Sec14p-like phosphatidylinositol transfer family protein

AT5G47740		Adenine nucleotide alpha hydrolases-like superfamily protein
AT5G47760	*2-PHOSPHOGLYCOLATE PHOSPHATASE 2 (PGLP2)*	serine/threonine protein kinase
AT5G47770	*FARNESYL DIPHOSPHATE SYNTHASE 1 (FPS1)*	Encodes a protein with farnesyl diphosphate synthase activity.
AT5G47790		SMAD/FHA domain-containing protein
AT5G47800	*(NPY6)*	Encodes a gene homologous to the NPY family based on deep phylogeny.
AT5G47810	*PHOSPHOFRUCTOKINASE 2 (PFK2)*	phosphofructokinase 2
AT5G47820	*FRAGILE FIBER 1 (FRA1)*	Encodes a kinesin-like protein with an N-terminal microtubule binding motor domain. Protein is localized to the periphery of the cytoplasm and mutants in the gene exhibit altered orientation of cellulose microfibrils and reduced mechanical strength of fibers.
AT5G47830		hypothetical protein
AT5G47840	*ADENOSINE MONOPHOSPHATE KINASE (AMK2)*	adenosine monophosphate kinase
AT5G47850	*CRINKLY4 RELATED 4 (CCR4)*	CRINKLY4 related 4
AT5G47870	*RADIATION SENSITIVE 52-2 (RAD52-2)*	cobalt ion-binding protein
AT5G47880	*EUKARYOTIC RELEASE FACTOR 1-1 (ERF1-1)*	Encodes a eukaryotic release factor 1 homolog. Cosuppression of the gene's expression results affects cell elongation of the inflorescence stem, specifically the internodes, and radial cell division. Expression of the protein is primarily observed in the vascular system and in actively growing and elongating zones.
AT5G47900		heparan-alpha-glucosaminide N-acetyltransferase-like protein (DUF1624)
AT5G47910	*RESPIRATORY BURST OXIDASE HOMOLOGUE D (RBOHD)*	NADPH/respiratory burst oxidase protein D (RbohD).Interacts with AtrbohF gene to fine tune the spatial control of ROI production and hypersensitive response to cell in and around infection site. The mRNA is cell-to-cell mobile.
AT5G47920		transcription elongation factor
AT5G47950	*BRASSINOSTEROID INACTIVATOR2 (BIA2)*	BIA2 is a putative HXXXD-type BAHD acyltransferase. Overexpression results in a BR deficient phenotype and is dependent on a functional HXXXD motif. BIA2 may function in BR homeostasis by regulating the pool of bioactive BR.
AT5G47960	*RAB GTPASE HOMOLOG A4C (RABA4C)*	Encodes a small molecular weight g-protein.
AT5G47980		HXXXD-type acyl-transferase family protein
AT5G47990	*CYTOCHROME P450, FAMILY 705, SUBFAMILY A, POLYPEPTIDE 5 (CYP705A5)*	Encodes an endomembrane system-expressed member of the CYP705A family of cytochrome P450 enzymes. It appears to catalyze the addition of a double bond to thalian-diol at carbon 15. Reduced levels of THAD expression lead to a build up of thalian-diol in root extracts. thad1-1 mutants also have longer roots than wild type seedlings and show altered gravitropic responses.
AT5G48000	*CYTOCHROME P450, FAMILY 708, SUBFAMILY A, POLYPEPTIDE 2 (CYP708A2)*	Encodes a member of the CYP708A family of cytochrome P450 enzymes. THAH appears to add a hydroxyl group to the triterpene thalianol. thah1 mutants have an elevated accumulation of thalianol. thah1-1 mutants have longer roots than wild type plants. Thalian-diol and desaturated thalian-diol are lost from the root extracts of thah1-1 mutants. Overexpression of the sequence from At5g48000.1 rescues the thah1-1 mutant phenotype (Field 2008); it is unknown whether the shorter sequences associated with other gene models would provide functional complementation.
AT5G48010	*THALIANOL SYNTHASE 1 (THAS1)*	Encodes an oxidosqualene cyclase involved in the biosynthesis of thalianol, a tricyclic triterpenoid of unknown function. Overexpression of THAS leads to dwarfing in the aerial tissues of Arabidopsis plants, but increases their root length. THAS is part of a small operon-like cluster of genes (with At5g48000 (THAH) and At5g47990 (THAD)) involved in thalianol metabolism.
AT5G48030	*GAMETOPHYTIC FACTOR 2 (GFA2)*	encodes a mitochondrially targeted DNAJ protein involved in female gametophyte development.
AT5G48050		Copia-like polyprotein/retrotransposon
AT5G48060	*MULTIPLE C2 DOMAIN AND TRANSMEMBRANE REGION PROTEIN 2 (MCTP2)*	C2 calcium/lipid-binding plant phosphoribosyltransferase family protein
AT5G48070	*XYLOGLUCAN ENDOTRANSGLUCOSYLASE/HYDROLASE 20 (XTH20)*	putative xyloglucan endotransglycosylase/hydrolase, expressed primarily in the stele of mature non-elongating regions of both the main and the lateral root. Is expressed in lateral root primordia but expression ceases after lateral root begins to grow. Involved in cell proliferation in incised inflorescence stems.
AT5G48080		
AT5G48090	*EDM2-LIKE PROTEIN1 (ELP1)*	EDM2-like protein1
AT5G48100	*TRANSPARENT TESTA 10 (TT10)*	Encodes a protein that is similar to laccase-like polyphenol oxidases. Involved in lignin and flavonoids biosynthesis. It has four conserved copper binding domains. Expressed in developing testa, where it colocalizes with the flavonoid end products proanthocyanidins and flavonols. Mutant plants exhibited a delay in developmentally determined browning of the testa, characterized by the pale brown color of seed coat. The tt10 mutant seeds accumulate more epicatechin monomers and more soluble proanthocyanidins than wild-type seeds. Flavonol composition was also affected in tt10 seeds, which exhibited a higher ratio of quercetin rhamnoside monomers versus dimers than wild-type seeds.
AT5G48110	*TERPENE SYNTHASE 20 (TPS20)*	The Col variant has no enzyme activity due to various substitution and deletion mutations.
AT5G48120	*HOMOLOG OF YEAST MET18 (MET18)*	ARM repeat superfamily protein
AT5G48130		Phototropic-responsive NPH3 family protein
AT5G48140		Pectin lyase-like superfamily protein
AT5G48150	*PHYTOCHROME A SIGNAL TRANSDUCTION 1 (PAT1)*	Member of GRAS gene family. Semi-dominant mutant has a reduced response to far-red light and appears to act early in the phytochrome A signaling pathway.
AT5G48170	*SLEEPY2 (SLY2)*	encodes an F-box protein whose protein sequence is similar to SLY1, which belongs to SCF-SLY1 E3 ligase complex. SCF-SLY1 E3 ligase degrades DELLA proteins that are involved in promoting growth. Overexpression of SLY2 can partially compensate sly1-10 mutant phenotype of dwarfism.
AT5G48175		transmembrane protein
AT5G48180	*NITRILE SPECIFIER PROTEIN 5 (NSP5)*	Encodes a nitrile-specifier protein NSP5. NSP5 is one out of five (At3g16400/NSP1, At2g33070/NSP2, At3g16390/NSP3, At3g16410/NSP4 and At5g48180/NSP5) A. thaliana epithiospecifier protein (ESP) homologues that promote simple nitrile, but not epithionitrile or thiocyanate formation.
AT5G48200		hypothetical protein
AT5G48210		prolamin-like protein (DUF1278)

AT5G48230	*ACETOACETYL-COA THIOLASE 2 (ACAT2)*	Encodes an acetoacetyl-CoA thiolase that generates the bulk of the acetoacetyl-CoA precursor needed for the cytosolic localized, mevalonate-derived isoprenoids biosynthetic pathway. Loss-of-function mutants are embryo lethal.
AT5G48240		Rrp15p protein
AT5G48250	*B-BOX DOMAIN PROTEIN 8 (BBX8)*	B-box type zinc finger protein with CCT domain-containing protein
AT5G48270		DUF868 family protein (DUF868)
AT5G48280		transmembrane protein
AT5G48290		Heavy metal transport/detoxification superfamily protein
AT5G48300	*ADP GLUCOSE PYROPHOSPHORYLASE 1 (ADG1)*	Encodes the small subunit of ADP-glucose pyrophosphorylase. The small subunit is the catalytic isoform responsible for ADP-glucose pyrophosphorylase activity. The presence of the small subunit is required for large subunit stability. Two isoforms of the small subunit (ApS1 and ApS2) have been described. ApS1 is the major small subunit isoform present in all plant tissues tested. The mRNA is cell-to-cell mobile.
AT5G48310	*RESPONSIVENESS TO ABA SALT AND DROUGHT 1 (RASD1)*	Protein of unknown function that may be involved in stress response. Strongly expressed in vascular tissues.Mutants are ABA- insensitive.
AT5G48330	*RCC1/UVR8/GEF-LIKE 2 (RUG2)*	Regulator of chromosome condensation (RCC1) family protein
AT5G48350		Polynucleotidyl transferase, ribonuclease H-like superfamily protein
AT5G48360	*FORMIN 9 (FH9)*	Actin-binding FH2 (formin homology 2) family protein
AT5G48380	*BAK1-INTERACTING RECEPTOR-LIKE KINASE 1 (BIR1)*	Encodes a BAK1-interacting receptor-like kinase named BIR1. Negatively regulates multiple plant resistance signaling pathways, one of which is the SOBIR1(AT2G31880)-dependent pathway.
AT5G48390	*A. THALIANA HOMOLOGUE OF YEAST SPO22 (ATSPO22)*	Defective in meiotic chromosome segregation. It is involved in crossover formation and involved in both male and female meiosis.
AT5G48400	*(ATGLR1.2)*	member of Putative ligand-gated ion channel subunit family
AT5G48410	*GLUTAMATE RECEPTOR 1.3 (GLR1.3)*	member of Putative ligand-gated ion channel subunit family
AT5G48420		hypothetical protein
AT5G48430		Eukaryotic aspartyl protease family protein
AT5G48460	*(ATFIM2)*	Encodes a member of the fimbrin family. Different members of the fimbrin/plastin family have diverged biochemically during evolution to generate either tight actin bundles or loose networks with distinct biochemical and biophysical properties. FIM4 generates both actin bundles and branched actin filaments whereas FIM5 only generates actin bundles.
AT5G48485	*DEFECTIVE IN INDUCED RESISTANCE 1 (DIR1)*	Encodes a putative apoplastic lipid transfer protein that is involved in systemic acquired resistance. Mutants in this gene exhibit wild-type local resistance to avirulent and virulent Pseudomonas syringae, but pathogenesis-related gene expression is abolished in uninoculated distant leaves and fail to develop SAR to virulent Pseudomonas or Peronospora parasitica. DIR1 protein is cell-to-cell mobile and is transported via phloem sap.
AT5G48490	*DIR1-LIKE (DIR1-LIKE)*	Encodes a protein with similarity to a lipid transfer protein that may contribute to systemic acquired resistance (SAR).
AT5G48500		pathogenic type III effector avirulence factor Avr AvrRpt-cleavage: cleavage site protein
AT5G48540		receptor-like protein kinase-related family protein
AT5G48550		F-box associated ubiquitination effector family protein
AT5G48560	*CRY2-INTERACTING BHLH 2 (CIB2)*	basic helix-loop-helix (bHLH) DNA-binding superfamily protein
AT5G48570	*(ROF2)*	Encodes one of the 36 carboxylate clamp (CC)-tetratricopeptide repeat (TPR) proteins (Prasad 2010, Pubmed ID: 20856808) with potential to interact with Hsp90/Hsp70 as co-chaperones.
AT5G48590		phosphoserine aminotransferase, putative (DUF760)
AT5G48600	*STRUCTURAL MAINTENANCE OF CHROMOSOME 3 (SMC3)*	member of SMC subfamily
AT5G48650	*GTPASE?ACTIVATING PROTEIN SH3-DOMAIN?BINDING PROTEIN LIKE (G3BP-LIKE)*	Negative regulator of defense response to Pseudomonas syringae pv. tomato through altered stomatal and apoplastic immunity.
AT5G48670	*AGAMOUS-LIKE 80 (AGL80)*	AGL80 is a member of the MADS box family of genes. AGL80 functions as a transcription factor within the central cell gene regulatory network and controls the expression of downstream genes required for central cell development and function.
AT5G48690		ubiquitin-associated (UBA)/TS-N domain protein
AT5G48710	*(SUMO6)*	Ubiquitin-like superfamily protein
AT5G48730		Pentatricopeptide repeat (PPR) superfamily protein
AT5G48740		Leucine-rich repeat protein kinase family protein
AT5G48750		Cytochrome b561/ferric reductase transmembrane with DOMON related domain-containing protein
AT5G48770		Disease resistance protein (TIR-NBS-LRR class) family
AT5G48780	*TIR-NBS20 (TN20)*	disease resistance protein (TIR-NBS class)
AT5G48790		LOW PSII ACCUMULATION protein (DUF1995)
AT5G48800		Phototropic-responsive NPH3 family protein
AT5G48810	*CYTOCHROME B5 ISOFORM D (CB5-D)*	Encodes a cytochrome b5 isoform that localizes to the ER. The C-terminal portion of the protein appears to be capable of inserting into a plant microsomal membrane in vitro and the protein appears to be subject to glycosylation. The mRNA is cell-to-cell mobile.
AT5G48840	*HOMOLOG OF BACTERIAL PANC (PANC)*	Encodes a pantothenate synthetase that appears to be located in the cytosol. This protein is expected to play a role in pantothenate (vitamin B5) biosynthesis. Analysis of the catalytic properties of this enzyme indicate that it might be able to synthesize adequate amounts of pantothenate even in the presence of low levels of pantoate.
AT5G48850	*SULPHUR DEFICIENCY-INDUCED 1 (ATSDI1)*	Homologous to the wheat sulphate deficiency-induced gene sdi1. Expression in root and leaf is induced by sulfur starvation. Knockout mutants retained higher root and leaf sulfate concentrations, indicating a role in regulation of stored sulfate pools.
AT5G48860		hypothetical protein
AT5G48880	*3-KETO-ACYL-COENZYME A THIOLASE 5 (KAT5)*	Encodes a peroxisomal 3-keto-acyl-CoA thiolase 2 precursor. EC2.3.1.16 thiolases. AT5G48880.1 is named PKT1 and AT5G48880.2 is named PKT2.
AT5G48900		Pectin lyase-like superfamily protein

AT5G48930	*HYDROXYCINNAMOYL-COA SHIKIMATE/QUINATE HYDROXYCINNAMOYL TRANSFERASE (HCT)*	At5g48930 has been shown to encode for the hydroxycinnamoyl-Coenzyme A shikimate/quinate hydroxycinnamoyltransferase (HCT) both synthesizing and catabolizing the hydroxycinnamoylesters (coumaroyl/caffeoyl shikimate and quinate) involved in the phenylpropanoid pathway. Influence on the accumulation of flavonoids which in turn inhibit auxin transport and reduce plant growth. The mRNA is cell-to-cell mobile.
AT5G48940	*(RGFR2)*	RGFR2 is a leucine--rich repeat receptor kinase that, together with RGFR1 and RGFR3, binds ROOT GROWTH FACTORS and is required for establishing the gradient of PLETHORA1 and PLETHORA2 essential for proper root growth and development.
AT5G48950	*DHNA-COA THIOESTERASE 2 (DHNAT2)*	Encodes one of the two functional DHNA-CoA (1,4-dihydroxy-2-naphthoyl-CoA) thioesterases found in Arabidopsis.
AT5G48970		Encodes a mitochondrial thiamin diphosphate carrier.
AT5G49000	*(PHIF1)*	F-box protein, part of SCF complex.
AT5G49020	*PROTEIN ARGININE METHYLTRANSFERASE 4A (PRMT4A)*	Encodes a type I protein arginine methyltransferase. PRMT4a can catalyze the asymmetric dimethylation of arginines 2,17, and 26 on histone 3 and can also methylate myelin basic protein in vitro. Double mutants lacking PRMT4a and 4b have reduced levels of histone 3 methylated at R17. These double mutants flower late due to defects in the autonomous pathway and they have elevated levels of FLC transcripts.
AT5G49050		universal stress A-like protein
AT5G49070	*3-KETOACYL-COA SYNTHASE 21 (KCS21)*	Encodes KCS21, a member of the 3-ketoacyl-CoA synthase family involved in the biosynthesis of VLCFA (very long chain fatty acids).
AT5G49080	*EXTENSIN 11 (EXT11)*	transposable_element_gene;similar to proline-rich extensin-like family protein [Arabidopsis thaliana] (TAIR:AT5G06640.1);(source:TAIR10)
AT5G49100		vitellogenin-like protein
AT5G49110		fanconi anemia group I-like protein
AT5G49120		DUF581 family protein, putative (DUF581)
AT5G49140		Disease resistance protein (TIR-NBS-LRR class) family
AT5G49150	*GAMETE EXPRESSED 2 (GEX2)*	Encodes a transmembrane domain containing protein expressed in sperm cells. Mutants are defective in gamete fusion. Target promoter of the male germline-specific transcription factor DUO1.
AT5G49160	*METHYLTRANSFERASE 1 (MET1)*	Encodes a cytosine methyltransferase MET1. Required for silencing of FWA paternal allele in endosperm. Two lines with RNAi constructs directed against DMT1 have reduced agrobacterium-mediated tumor formation. The mRNA is cell-to-cell mobile.
AT5G49170		hypothetical protein
AT5G49180	*PECTIN METHYLESTERASE 58 (PME58)*	Encodes a putative pectin methylesterase. The gene is preferentially expressed in floral buds and more specifically in mucilage secretory cells of seeds. Mutants have smaller mucilage cells and abnormal mucilage profiles.
AT5G49190	*SUCROSE SYNTHASE 2 (SUS2)*	Encodes a sucrose synthase (SUS2). The activity of the enzyme could not be assayed as proved to be insoluble (PMID 17257168). However, analyses of an sus2 mutant revealed a deficiency in sucrose synthase activity 12 and 15 days after flowering. There are some reports that SUS2 transcript levels are increased in leaves specifically by O(2) deficiency whereas other reports indicate that SUS2 is expressed only in seeds. Immulocalization shows that SUS2 is present in the cytosol of developing seeds, but, it also associated with plastids, though not located within them.
AT5G49240	*PSEUDO-RESPONSE REGULATOR 4 (APRR4)*	member of Response Regulator: Pseudo
AT5G49250		Beta-galactosidase related protein
AT5G49260		hypothetical protein
AT5G49270	*SHAVEN 2 (SHV2)*	Involved in successfully establishing tip growth in root hairs.
AT5G49280		hydroxyproline-rich glycoprotein family protein
AT5G49290	*RECEPTOR LIKE PROTEIN 56 (RLP56)*	receptor like protein 56
AT5G49310	*IMPORTIN ALPHA ISOFORM 5 (IMPA-5)*	Putative importin alpha isoform. When overexpressed can rescue the impa-4 decreased transformation susceptibility phenotype.
AT5G49320		transmembrane protein, putative (DUF1218)
AT5G49330	*MYB DOMAIN PROTEIN 111 (MYB111)*	Member of the R2R3 factor gene family. Together with MYB11 and MYB111 redundantly regulates flavonol biosynthesis.
AT5G49350		Glycine-rich protein family
AT5G49360	*BETA-XYLOSIDASE 1 (BXL1)*	Encodes a bifunctional {beta}-D-xylosidase/{alpha}-L-arabinofuranosidase required for pectic arabinan modification. Located in the extracellular matrix. Gene is expressed specifically in tissues undergoing secondary wall thickening. This is a member of glycosyl hydrolase family 3 and has six other closely related members.
AT5G49370		
AT5G49380		
AT5G49390		serine/threonine-protein phosphatase 4 regulatory subunit-like protein
AT5G49400		zinc knuckle (CCHC-type) family protein
AT5G49410		thiamine-phosphate synthase
AT5G49420	*AGAMOUS-LIKE 84 (AGL84)*	MADS-box transcription factor family protein
AT5G49430		WD40/YVTN repeat and Bromo-WDR9-I-like domain-containing protein
AT5G49450	*BASIC LEUCINE-ZIPPER 1 (bZIP1)*	Encodes a transcription activator is a positive regulator of plant tolerance to salt, osmotic and drought stresses.
AT5G49460	*ATP CITRATE LYASE SUBUNIT B 2 (ACLB-2)*	One of the two genes encoding subunit B of the cytosolic enzyme ATP Citrate Lyase (ACL)
AT5G49470	*(RAF10)*	Encodes a protein with similarity to RAF MAP Kinase that is expressed in most plant tissues. Based on loss of function and gain of function phenotypes, RAF10 appears to be involved in ABA response.
AT5G49480	*CA2+-BINDING PROTEIN 1 (CP1)*	AtCP1 encodes a novel Ca2+-binding protein, which shares sequence similarities with calmodulins. The expression of AtCP1 is induced by NaCl. The mRNA is cell-to-cell mobile.
AT5G49500		Signal recognition particle, SRP54 subunit protein
AT5G49520	*WRKY DNA-BINDING PROTEIN 48 (WRKY48)*	Encodes WRKY48, a member of the WRKY Transcription Factor. WRKY48 is a stress- and pathogen-induced transcriptional activator that represses plant basal defense. The mRNA is cell-to-cell mobile.
AT5G49560		Putative methyltransferase family protein
AT5G49590		hypothetical protein

AT5G49620	*MYB DOMAIN PROTEIN 78 (MYB78)*	Member of the R2R3 factor gene family.
AT5G49630	*AMINO ACID PERMEASE 6 (AAP6)*	Is a high affinity amino acid transporter capable of transporting aspartate and tryptophan. May be involved in the amino acid uptake from xylem.
AT5G49640		hypothetical protein
AT5G49660	*XYLEM INTERMIXED WITH PHLOEM 1 (XIP1)*	The gene encodes receptorlike kinase (RLK). Involved in the maintenance organization of cell files or cell morphology in conductive elements. Functions as a receptor for CEP1 peptide. Mediates nitrate uptake signaling.
AT5G49670		
AT5G49690	*UDP-GLYCOSYLTRANSFERASE 91C1 (UGT91C1)*	UDP-glycosyltransferase that can act upon sulcotrione herbicide. Overexpression confers resistance to herbicide.
AT5G49730	*FERRIC REDUCTION OXIDASE 6 (FRO6)*	Encodes a plasma membrane-located ferric chelate reductase. Its mRNA is expressed in green aerial tissues (shoot, flower and cotyledon) in a light- and cell differentiation-specific manner.
AT5G49750		Leucine-rich repeat (LRR) family protein
AT5G49760	*(HPCA1)*	Leucine rich receptor kinase. Encodes a receptor of extracellular reactive oxygen species.
AT5G49770		Leucine rich receptor kinase.
AT5G49780		Leucine-rich repeat protein kinase family protein
AT5G49790		transposable_element_gene;similar to unknown protein [Arabidopsis thaliana] (TAIR:AT5G36010.1);(source:TAIR10)
AT5G49800		Polyketide cyclase/dehydrase and lipid transport superfamily protein
AT5G49810	*METHIONINE S-METHYLTRANSFERASE (MMT)*	Arabidopsis thaliana methionine S-methyltransferase, an enzyme that catalyzes S -methylmethionine formation. The mRNA is cell-to-cell mobile.
AT5G49820	*ROOT UV-B SENSITIVE 6 (RUS6)*	root UVB sensitive protein (Protein of unknown function, DUF647)
AT5G49840		ATP-dependent Clp protease
AT5G49870		Mannose-binding lectin superfamily protein
AT5G49900		Beta-glucosidase, GBA2 type family protein
AT5G49910	*CHLOROPLAST HEAT SHOCK PROTEIN 70-2 (cpHsc70-2)*	Stromal heat shock protein involved in protein import into chloroplast. The mRNA is cell-to-cell mobile.
AT5G49945		hypothetical protein (DUF1682)
AT5G49960	*(DMI1)*	POLLUX family member.
AT5G49970	*PYRIDOXIN (PYRODOXAMINE) 5'-PHOSPHATE OXIDASE (PPOX)*	encodes the bifunctional pyridoxine (pyridoxamine) 5?-phosphate oxidase (PPOX)(EC 1.4.3.5) that is involved in the formation of pyridoxal 5'-phosphate (member of the vitamin B6 group). NAD(P)HX epimerase (AT5G49970) interconverts the two epimers of NAD(P)HX.
AT5G49980	*AUXIN F-BOX PROTEIN 5 (AFB5)*	auxin F-box protein 5
AT5G49990		Xanthine/uracil permease family protein
AT5G50000	*CONVERGENCE OF BLUE LIGHT AND CO2 2 (CBC2)*	Belongs to the Raf-like kinase subfamily of the mitogen-activated protein kinase kinase kinase (MAPKKK) family. Negatively regulates stomatal opening by negatively regulating plasma membrane H+-ATPase phosphorylation.
AT5G50010	*(SACL2)*	transcription factor bHLH145
AT5G50020	*(PAT9)*	DHHC-type zinc finger family protein
AT5G50030		Plant invertase/pectin methylesterase inhibitor superfamily protein
AT5G50100	*DXXCXXC MOTIF 1 (DCC1)*	Encodes a putative thioredoxin DCC1 involved in determining shoot regeneration capacity. It interacts directly with CARBONIC ANHYDRASE 2 (CA2), an essential subunit of respiratory chain NADH dehydrogenase complex (Complex I) and regulates Complex I activity via redox modification of CA2.
AT5G50110		S-adenosyl-L-methionine-dependent methyltransferases superfamily protein
AT5G50120		Transducin/WD40 repeat-like superfamily protein
AT5G50130		NAD(P)-binding Rossmann-fold superfamily protein
AT5G50140		Ankyrin repeat family protein
AT5G50160	*FERRIC REDUCTION OXIDASE 8 (FRO8)*	Encodes a ferric chelate reductase that is expressed in shoots and flowers.
AT5G50170		C2 calcium/lipid-binding and GRAM domain containing protein
AT5G50190		other_RNA
AT5G50200	*WOUND-RESPONSIVE 3 (WR3)*	Wound-responsive gene 3 (WR3). Encodes a high-affinity nitrate transporter. Up-regulated by nitrate. Involved in jasmonic acid-independent wound signal transduction.
AT5G50210	*QUINOLINATE SYNTHASE (QS)*	Encodes an Fe-S binding protein with quinolinate synthase (QS) activity and cysteine desulfurase activator activity. The QS activity was demonstrated by functional complementation of corresponding E. coli mutants and complementation of embryo-lethal phenotypes of the QS homozygous null allele in Arabidopsis. The SufE domain of the protein also stimulates the cysteine desulfurase activity of CpNifS (AT1G08490) in vitro. This protein binds a (4Fe-Su)2+ cluster in its NadA domain and is localized in the chloroplast.
AT5G50240	*PROTEIN-L-ISOASPARTATE METHYLTRANSFERASE 2 (PIMT2)*	L-isoaspartyl methyltransferase 2 (PIMT2)gene, alternatively spliced.
AT5G50250	*CHLOROPLAST RNA-BINDING PROTEIN 31B (CP31B)*	Encodes a RNA binding protein. A substrate of the type III effector HopU1 (mono-ADP-ribosyltransferase). Supports editing of specific CP31A-dependent sites.
AT5G50260	*CYSTEINE ENDOPEPTIDASE 1 (CEP1)*	Encodes a papain-like cysteine protease involved in tapetal programmed cell death and pollen development.CEP1 is expressed specifically in the tapetum from stages 5 to 11 of anther development. The CEP1 protein first appears as a proenzyme in precursor protease vesicles, and is then transported to the vacuole and transformed into the mature enzyme before rupture of the vacuole. CEP1 was also released to the tapetal cell wall during late stage 6 and stage 7. After the tapetal cell wall degenerated, the CEP1 enzyme entered the callose wall from the degenerated tapetal cell wall and was probably involved in degeneration of the callose wall.
AT5G50270		F-box/RNI-like/FBD-like domains-containing protein
AT5G50300	*AZA-GUANINE RESISTANT2 (AZG2)*	Encodes a homolog of the adenine-guanine-hypoxanthine transporter AzgA of Aspergillus nidulans. Function as a plant adenine-guanine transporter. Two closely related genes exist in Arabidopsis: AT3G10960 (Azg1) and AT5G50300 (Azg2).
AT5G50310		Galactose oxidase/kelch repeat superfamily protein

AT5G50315		transposable_element_gene;Mutator-like transposase family, has a 5.0e-14 P-value blast match to Q9XE24 /118-277 Pfam PF03108 MuDR family transposase (MuDr-element domain);(source:TAIR10)
AT5G50320	*ELONGATA 3 (ELO3)*	A subunit of Elongator, a histone acetyl transferase complex, consisting of six subunits (ELP1?ELP6), that copurifies with the elongating RNAPII in yeast and humans. Three Arabidopsis thaliana genes, encoding homologs of the yeast Elongator subunits ELP1, ELP3 (histone acetyl transferase), and ELP4 are responsible for the narrow leaf phenotype in elongata mutants and for reduced root growth that results from a decreased cell division rate. Two lines with RNAi constructs directed against HAG3 grow normally and can produce root calli, but have defects in agrobacterium-mediated transformation.
AT5G50335		hypothetical protein
AT5G50340	*RADA-LIKE (ATRADA)*	DNA repair protein RadA-like protein
AT5G50360	*ABA-INDUCED TRANSCRIPTION REPRESSOR 5 (AITR5)*	ABA‐induced transcription repressor that acts as feedback regulator in ABA signalling.
AT5G50370		Adenylate kinase family protein
AT5G50375	*CYCLOPROPYL ISOMERASE (CPI1)*	Converts pentacyclic cyclopropyl sterols to conventional tetracyclic sterols. CPI1 function during and just after division and support gravitropism by establishing polar PIN2 localization. Required for endocytosis of PIN2
AT5G50390	*EMBRYO DEFECTIVE 3141 (EMB3141)*	Pentatricopeptide repeat (PPR-like) superfamily protein
AT5G50400	*PURPLE ACID PHOSPHATASE 27 (PAP27)*	purple acid phosphatase 27
AT5G50420	*DEFECTIVE IN SYSTEMIC DEFENSE INDUCED BY ABIETANE DITERPENOID 1 (DSA1)*	O-fucosyltransferase family protein
AT5G50450		HCP-like superfamily protein with MYND-type zinc finger
AT5G50470	*NUCLEAR FACTOR Y, SUBUNIT C7 (NF-YC7)*	nuclear factor Y, subunit C7
AT5G50480	*NUCLEAR FACTOR Y, SUBUNIT C6 (NF-YC6)*	nuclear factor Y, subunit C6
AT5G50500		hypothetical protein
AT5G50510		Molecular chaperone Hsp40/DnaJ family protein
AT5G50530		CBS / octicosapeptide/Phox/Bemp1 (PB1) domains-containing protein
AT5G50540		transmembrane protein
AT5G50550		Transducin/WD40 repeat-like superfamily protein
AT5G50570	*SQUAMOSA PROMOTER-BINDING PROTEIN LIKE 13A (SPL13A)*	Squamosa promoter-binding protein-like (SBP domain) transcription factor family protein
AT5G50590	*HYDROXYSTEROID DEHYDROGENASE 4 (HSD4)*	Encodes a putative hydroxysteroid dehydrogenase (HSD). Genes that encode HSD include: At5g50600 and At5g50700 (HSD1), At3g47350(HSD2), At3g47360(HSD3), At5g50590 and At5g50690(HSD4), At5g50770(HSD6) (Plant Cell Physiology 50:1463). Two copies of HSD1 and HSD4 exist due to a gene duplication event. In Plant Physiology 145:87, At5g50690 is HSD7, At4g10020 is HSD5.
AT5G50600	*HYDROXYSTEROID DEHYDROGENASE 1 (HSD1)*	Encodes a hydroxysteroid dehydrogenase shown to act as a NADP+-dependent 11β-,17β-hydroxysteroid dehydrogenase/17β-ketosteroid reductase called HSD1. Two copies of HSD1 (At5g50600 and At5g50700) exist in the Arabidopsis genome as a result of an exact 33-kb duplication on chromosome 5 encompassing seven genes. There are five homologs of HSD1 in Arabidopsis (HSD2-At3g47350, HSD3-At3g47360, HSD4-At5g50590, HSD4-At5g50690 and HSD6-At5g50770; HSD4 has two copies due to the same gene duplication event occurred to HSD1) (Plant Cell Physiology 50:1463). At5g50690 is also named as HSD7 (Plant Physiology 145:87). HSD1 is identified from the proteome of oil bodies from mature seeds. Transcription of HSD1 is specifically and highly induced in oil-accumulating tissues of mature seeds; transcript disappears during germination. To date, the endogenous substrates of this enzyme are not known.
AT5G50710		transmembrane protein
AT5G50720	*HVA22 HOMOLOGUE E (HVA22E)*	Encodes one of five HVA22 homologs in Arabidopsis. HVA22 is an ABA- and stress-inducible gene first isolated from barley. Members of this gene family have only been found in eukaryotes. AtHVA22e mRNA is upregulated to varying degrees in response to cold stress, salt stress, ABA treatment or dehydration.
AT5G50730		
AT5G50740	*HEAVY METAL ASSOCIATED PROTEIN 52 (ATHMP52)*	Heavy metal transport/detoxification superfamily protein
AT5G50750	*REVERSIBLY GLYCOSYLATED POLYPEPTIDE 4 (RGP4)*	RGP4 is a reversibly glycosylated polypeptide. Analyses using tagged RGP4 suggest that it is present in the cytosol and in association with the Golgi apparatus. Recombinant RGP4 does not have UDP-arabinose mutase activity based on an in vitro assay even though the related RGP1, RGP2, and RGP3 proteins do have activity in the same assay. RGP4 can form complexes with RGP1 and RGP2. RGP4 is expressed during seed development.
AT5G50760	*SMALL AUXIN UPREGULATED RNA 55 (SAUR55)*	SAUR-like auxin-responsive protein family
AT5G50770	*HYDROXYSTEROID DEHYDROGENASE 6 (HSD6)*	Encodes a putative hydroxysteroid dehydrogenase (HSD). Genes that encode HSD include: At5g50600 and At5g50700 (HSD1), At3g47350(HSD2), At3g47360(HSD3), At5g50590 and At5g50690(HSD4), At5g50770(HSD6) (Plant Cell Physiology 50:1463). Two copies of HSD1 and HSD4 exist due to a gene duplication event. In Plant Physiology 145:87, At5g50690 is HSD7, At4g10020 is HSD5.
AT5G50790	*(SWEET10)*	Encodes a member of the SWEET sucrose efflux transporter family proteins. Transcriptionally activated by long photoperiods; activation depends on FT and SOC1. The ectopic expression of SWEET10 causes early flowering and leads to higher levels of transcription of flowering-time related genes in the shoot apex.
AT5G50800	*(SWEET13)*	Encodes a member of the SWEET sucrose efflux transporter family proteins, together with RPG1, it is involved in pollen development. Together with SWEET14, it is likely involved in modulating the GA response and is required for proper development of anthers, seeds and seedlings.
AT5G50810	*TRANSLOCASE INNER MEMBRANE SUBUNIT 8 (TIM8)*	Encodes a small zinc finger-like protein that is a component of the mitochondrial protein import apparatus.
AT5G50820	*NAC DOMAIN CONTAINING PROTEIN 97 (NAC097)*	NAC domain containing protein 97
AT5G50830		nuclear polyadenylated RNA-binding protein
AT5G50850	*MACCI-BOU (MAB1)*	Transketolase family protein
AT5G50880		
AT5G50910		hypothetical protein
AT5G50915	*CYTOKININ-RESPONSIVE GROWTH REGULATOR (CKG)*	Member of the bHLH family of transcription factors. Acts within a cytokinin signaling pathway to promote cell proliferation and expansion.

AT5G50920	*CLPC HOMOLOGUE 1 (CLPC1)*	Encodes a protein that is similar to ATP-dependent Clp protease ATP-binding subunit / ClpC. Involved in protein import into the chloroplast. May provide ATP source that drives the TIC (Translocon at the Inner envelope membrane of Chloroplasts) translocation machinery. Association of Hsp93 with the inner envelope membrane through its N domain is important for the functions of Hsp93 in vivo.
AT5G50930	*HOMOLOG OF HUMAN MHF1 (MHF1)*	Encodes a protein with similarity to mammalian MHF1 that acts in the same pathway as FANCM to restrain class II meiotic crossing over, and acts with FANCM during meiosis and to repair cross-links. It also assumes an opposing role from FANCM in homologous recombination and only FANCM is essential for replicative repair in the absence of the endonuclease MUS81.
AT5G50940		RNA-binding KH domain-containing protein
AT5G50950	*FUMARASE 2 (FUM2)*	Encodes a fumarase enzyme initially shown to be in the mitochondria through proteomic studies but later shown to be present in the cytosol using an RFP fluorescent protein tag. It appears to be important for the accumulation of fumarate from malate in leaves in the light, and helps to promote nitrogen assimilation under high nitrogen conditions. It does not appear to be necessary for lipid metabolism and seedling growth. Inhibition of fumarate accumulation results in an overall shift in the cold response of leaves, with a complete inhibition of cold acclimation of photosynthesis.
AT5G50960	*NUCLEOTIDE BINDING PROTEIN 35 (NBP35)*	Highly similar to Saccharomyces cerevisiae NBP35, locus YGL091C. Cytosolic protein that homodimerizes and can assemble both 4Fe-4S - type and 2Fe-2S - type clusters on its amino terminal and carboxy therminal respectively. Null mutants are embryo lethal.
AT5G50990		Tetratricopeptide repeat (TPR)-like superfamily protein
AT5G51010		Rubredoxin-like superfamily protein
AT5G51030		NAD(P)-binding Rossmann-fold superfamily protein
AT5G51040	*SUCCINATE DEHYDROGENASE ASSEMBLY FACTOR 2 (SDHAF2)*	Encodes succinate dehydrogenase assembly factor 2 (SDHAF2), a low abundance mitochondrial protein needed for assembly and activity of mitochondrial complex II and for normal root elongation.Sdhaf2 knockdown line showed lowered SDH1 protein abundance, lowered maximal SDH activity and less protein-bound FAD at the molecular mass of SDH1.
AT5G51060	*ROOT HAIR DEFECTIVE 2 (RHD2)*	RHD2 (along with RHD3 and RHD4) is required for normal root hair elongation. Has NADPH oxidase activity. Gene is expressed in the elongation and differention zone in trichoblasts and elongating root hairs. RDH2 is localized to the growing tips of root hair cells. It is required for the production of reactive oxygen species in response to extracellular ATP stimulus. The increase in ROS production stimulates Ca2+ influx.
AT5G51080		RNase H family protein
AT5G51090		hypothetical protein
AT5G51100	*FE SUPEROXIDE DISMUTASE 2 (FSD2)*	Fe superoxide dismutase whose mRNA levels are increased in response to exposure to UV-B.
AT5G51110	*SDIR1-INTERACTING PROTEIN1 (SDIRIP1)*	Encodes a protein involved in Rubisco assembly that also mediates Abscisic acid-dependent stress response. It is a ubiquitination target of the intracellular E3 ligase SDIR1. It selectively regulates the expression of the downstream basic region/leucine zipper motif transcription factor gene ABA-INSENSITIVE5, rather than ABA-RESPONSIVE ELEMENTS BINDING FACTOR3 (ABF3) or ABF4, to regulate ABA-mediated seed germination and the plant salt response.
AT5G51130		S-adenosyl-L-methionine-dependent methyltransferases superfamily protein
AT5G51160		Ankyrin repeat family protein
AT5G51170	*(ATUSB1)*	U6 snRNA phosphodiesterase-like protein
AT5G51180		alpha/beta-Hydrolases superfamily protein
AT5G51190	*ETHYLENE RESPONSE FACTOR 105 (ERF105)*	encodes a member of the ERF (ethylene response factor) subfamily B-3 of ERF/AP2 transcription factor family. The protein contains one AP2 domain. There are 18 members in this subfamily including ATERF-1, ATERF-2, AND ATERF-5. Involved in regulating root architecture and the response to cold stress.
AT5G51200	*EMBRYO DEFECTIVE 3142 (EMB3142)*	Originally identified as EDS4, enhanced disease sensitive phenotype and subsequently cloned and identified as NUCLEOPORIN205. Affects circadian clock and downstream genes including those involved in defense response.
AT5G51210	*OLEOSIN3 (OLEO3)*	Encodes oleosin3, a protein found in oil bodies, involved in seed lipid accumulation.
AT5G51220		ubiquinol-cytochrome C chaperone family protein
AT5G51230	*EMBRYONIC FLOWER 2 (EMF2)*	Polycomb group protein with zinc finger domain involved in negative regulation of reproductive development. Forms a complex with FIE, CLF, and MSI1. This complex modulates the expression of target genes including AG, PI and AP3.
AT5G51250		Galactose oxidase/kelch repeat superfamily protein
AT5G51270	*(PUB53)*	Plant U-box type E3 ubiquitin ligase (PUB).
AT5G51290	*ACCELERATED CELL DEATH 5 (ACD5)*	Encodes a ceramide kinase that plays a role in modulating cell death.
AT5G51310		Mutants exhibit longer root hairs under phosphate-deficient conditions.
AT5G51320		transposable_element_gene;similar to unknown protein [Arabidopsis thaliana] (TAIR:AT3G42556.1);(source:TAIR10)
AT5G51350	*MORE LATERAL GROWTH1 (MOL1)*	Encodes a receptor-like kinase that represses secondary growth, the production of secondary vascular tissues.
AT5G51360		Transcription elongation factor (TFIIS) family protein
AT5G51380		RNI-like superfamily protein
AT5G51390		hypothetical protein
AT5G51420		long-chain-alcohol O-fatty-acyltransferase family protein / wax synthase family protein
AT5G51440	*(HSP23.5)*	HSP20-like chaperones superfamily protein
AT5G51470		Auxin-responsive GH3 family protein
AT5G51480	*SKU5 SIMILAR 2 (SKS2)*	GPI anchored protein, highly expressed in reproductive tissues.
AT5G51490		Plant invertase/pectin methylesterase inhibitor superfamily
AT5G51500		Plant invertase/pectin methylesterase inhibitor superfamily
AT5G51520		Plant invertase/pectin methylesterase inhibitor superfamily protein
AT5G51550	*EXORDIUM LIKE 3 (EXL3)*	EXORDIUM like 3
AT5G51560		Leucine-rich repeat protein kinase family protein

AT5G51570	*HYPERSENSITIVE INDUCED REACTION 3 (HIR3)*	SPFH/Band 7/PHB domain-containing membrane-associated protein family
AT5G51580		hypothetical protein
AT5G51600	*PLEIADE (PLE)*	Mutant has defective roots. Essential for giant cell ontogenesis. Role in organizing the mitotic microtubule array during both early and late mitosis in all plant organs.
AT5G51610		Ribosomal protein L11 family protein
AT5G51620		Uncharacterized protein family (UPF0172)
AT5G51640	*YELLOW-LEAF-SPECIFIC GENE 7 (YLS7)*	Encodes leaf-senescence-related protein. A member of the TBL (TRICHOME BIREFRINGENCE-LIKE) gene family containing a plant-specific DUF231 (domain of unknown function) domain. TBL gene family has 46 members, two of which (TBR/AT5G06700 and TBL3/AT5G01360) have been shown to be involved in the synthesis and deposition of secondary wall cellulose, presumably by influencing the esterification state of pectic polymers. A nomenclature for this gene family has been proposed (Volker Bischoff & Wolf Scheible, 2010, personal communication).
AT5G51650		hypothetical protein
AT5G51670		hypothetical protein (DUF668)
AT5G51680		hydroxyproline-rich glycoprotein family protein
AT5G51690	*1-AMINO-CYCLOPROPANE-1-CARBOXYLATE SYNTHASE 12 (ACS12)*	Encodes an aminotransferase with broad specificity for aspartate and aromatic amino aids such as tyrosine and phenylalanine. It does not act on branched chain amino acids and does not have ACC synthase activity.
AT5G51710	*K+ EFFLUX ANTIPORTER 5 (KEA5)*	member of Putative potassium proton antiporter family
AT5G51720	*NEET GROUP PROTEIN (NEET)*	Encodes a protein with biochemical, structural, and biophysical characteristics of a NEET protein. It plays a key role in plant development, senescence, reactive oxygen homeostasis, and Fe metabolism.
AT5G51750	*SUBTILASE 1.3 (SBT1.3)*	subtilase 1.3
AT5G51760	*ABA-HYPERSENSITIVE GERMINATION 1 (AHG1)*	Encodes AHG1 (ABA-hypersensitive germination 1), a putative protein phosphatase 2C (PP2C). Expressed in seeds. AHG1 functions in seed development and germination.
AT5G51800		Protein kinase superfamily protein
AT5G51810	*GIBBERELLIN 20 OXIDASE 2 (GA20OX2)*	Encodes gibberellin 20-oxidase. Involved in gibberellin biosynthesis. Up-regulated by far red light in elongating petioles. Not regulated by a circadian clock. Mutation of GA20ox2 delays flowering.
AT5G51830	*FRUCTOKINASE 1 (FRK1)*	Encodes one of the several Arabidopsis fructokinases. Nomenclature according to Riggs 2017 has been adopted for the family by the community (personal communication, Boernke, Callis, Granot, Boernke, and Smeekens). Important for seed oil accumulation and vascular development.
AT5G51840		junctophilin-like protein
AT5G51850	*TON1 RECRUITING MOTIF 24 (TRM24)*	hypothetical protein
AT5G51860	*AGAMOUS-LIKE 72 (AGL72)*	Encodes a MADS-box transcription factor involved in floral transition.
AT5G51870	*AGAMOUS-LIKE 71 (AGL71)*	Encodes a MADS-box transcription factor involved in floral transition.
AT5G51890	*PEROXIDASE 66 (PRX66)*	encodes peroxidase involved in the lignification of tracheary elements (TE) in roots
AT5G51900		Cytochrome P450 family protein
AT5G51910	*TCP DOMAIN PROTEIN 19 (TCP19)*	TCP family transcription factor
AT5G51930		Glucose-methanol-choline (GMC) oxidoreductase family protein
AT5G51950		Glucose-methanol-choline (GMC) oxidoreductase family protein
AT5G51990	*C-REPEAT-BINDING FACTOR 4 (CBF4)*	encodes a member of the DREB subfamily A-1 of ERF/AP2 transcription factor family (CBF4). The protein contains one AP2 domain. There are six members in this subfamily, including CBF1, CBF2, and CBF3. This gene is involved in response to drought stress and abscisic acid treatment, but not to low temperature.
AT5G52000	*IMPORTIN ALPHA ISOFORM 8 (IMPA-8)*	Putative importin alpha isoform. When overexpressed can rescue the impa-4 decreased transformation susceptibility phenotype. Target promoter of the male germline-specific transcription factor DUO1.
AT5G52020		encodes a member of the DREB subfamily A-4 of ERF/AP2 transcription factor family. The protein contains one AP2 domain. There are 17 members in this subfamily including TINY.
AT5G52030		TraB family protein
AT5G52050	*DETOXIFICATION EFFLUX CARRIER 50 (DTX50)*	MATE efflux family protein
AT5G52060	*BCL-2-ASSOCIATED ATHANOGENE 1 (BAG1)*	A member of Arabidopsis BAG (Bcl-2-associated athanogene) proteins, plant homologs of mammalian regulators of apoptosis. Plant BAG proteins are multi-functional and remarkably similar to their animal counterparts, as they regulate apoptotic-like processes ranging from pathogen attack, to abiotic stress, to plant development.
AT5G52100	*CHLORORESPIRATION REDUCTION 1 (crr1)*	Is essential for chloroplast NAD(P)H dehydrogenase activity, which is involved in electron transfer between PSII and PSI. Likely functions in biogenesis or stabilization of the NAD(P)H dehydrogenase complex. The mRNA is cell-to-cell mobile.
AT5G52120	*PHLOEM PROTEIN 2-A14 (PP2-A14)*	phloem protein 2-A14
AT5G52130		hypothetical protein
AT5G52140	*(CTL17)*	RING/U-box superfamily protein
AT5G52170	*HOMEODOMAIN GLABROUS 7 (HDG7)*	Encodes a homeobox-leucine zipper family protein belonging to the HD-ZIP IV family.
AT5G52190		Sugar isomerase (SIS) family protein
AT5G52200	*INHIBITOR-2 (I-2)*	Encodes an inhibitor of protein phosphatase one (PP1).
AT5G52230	*METHYL-CPG-BINDING DOMAIN PROTEIN 13 (MBD13)*	Protein containing methyl-CpG-binding domain.Has sequence similarity to human MBD proteins.
AT5G52250	*REPRESSOR OF UV-B PHOTOMORPHOGENESIS 1 (RUP1)*	Encodes a transducin protein whose gene expression is induced by UV-B. This induction is reduced in hy5 mutant and may be a target of HY5 during UV-B response. Functions as a repressor of UV-B signaling.
AT5G52280		Myosin heavy chain-related protein

AT5G52290	*SHORTAGE IN CHIASMATA 1 (SHOC1)*	Encodes a protein with similarity to XPF endonucleases. Loss of function mutations have defects in meiosis- specifically a reduction in the number of chiasmata. As a result both pollen and embryo sacs are abnormal and plants have severely reduced fertility.
AT5G52300	*LOW-TEMPERATURE-INDUCED 65 (LTI65)*	Encodes a protein that is induced in expression in response to water deprivation such as cold, high-salt, and desiccation. The response appears to be via abscisic acid. The promoter region contains two ABA-responsive elements (ABREs) that are required for the dehydration-responsive expression of rd29B as cis-acting elements. Protein is a member of a gene family with other members found plants, animals and fungi. Upregulation by P. polymyxa CR1 increases drought resistance.
AT5G52310	*LOW-TEMPERATURE-INDUCED 78 (LTI78)*	cold regulated gene, the 5' region of cor78 has cis-acting regulatory elements that can impart cold-regulated gene expression The mRNA is cell-to-cell mobile.
AT5G52320	*CYTOCHROME P450, FAMILY 96, SUBFAMILY A, POLYPEPTIDE 4 (CYP96A4)*	cytochrome P450, family 96, subfamily A, polypeptide 4
AT5G52330		TRAF-like superfamily protein
AT5G52340	*EXOCYST SUBUNIT EXO70 FAMILY PROTEIN A2 (EXO70A2)*	A member of EXO70 gene family, putative exocyst subunits, conserved in land plants. Arabidopsis thaliana contains 23 putative EXO70 genes, which can be classified into eight clusters on the phylogenetic tree.
AT5G52350	*EXOCYST SUBUNIT EXO70 FAMILY PROTEIN A3 (EXO70A3)*	A member of EXO70 gene family, putative exocyst subunits, conserved in land plants. Arabidopsis thaliana contains 23 putative EXO70 genes, which can be classified into eight clusters on the phylogenetic tree.
AT5G52360	*ACTIN DEPOLYMERIZING FACTOR 10 (ADF10)*	ADF10 is an actin-depolymerizing factor that preferentially binds ADP-G-actin and inhibits G-actin nucleotide exchange. ADF10 promotes actin turnover in pollen , regulating organization of actin filaments and vesicle trafficking during pollen tube growth.
AT5G52380		
AT5G52390		PAR1 protein
AT5G52400	*CYTOCHROME P450, FAMILY 715, SUBFAMILY A, POLYPEPTIDE 1 (CYP715A1)*	member of CYP715A
AT5G52410		oxidoreductase/transition metal ion-binding protein
AT5G52420	*PSI-INTERACTING ROOT-CELL ENRICHED 3 (PRCE3)*	transmembrane protein
AT5G52440	*HIGH CHLOROPHYLL FLUORESCENCE 106 (HCF106)*	HCF106; nuclear gene for chloroplast. Thylakoid membrane delta pH translocation pathway component protein; related to Escherichia coli TatA and TatB The mRNA is cell-to-cell mobile.
AT5G52450		MATE efflux family protein
AT5G52460	*EMBRYO SAC DEVELOPMENT ARREST 41 (EDA41)*	FBD, F-box and Leucine Rich Repeat domains containing protein
AT5G52480		RNI-like superfamily protein
AT5G52490		Fibrillarin family protein
AT5G52500		transmembrane protein
AT5G52510	*SCARECROW-LIKE 8 (SCL8)*	SCARECROW-like 8
AT5G52530		dentin sialophosphoprotein-like protein
AT5G52540		keratin-associated protein, putative (DUF819)
AT5G52570	*BETA-CAROTENE HYDROXYLASE 2 (BETA-OHASE 2)*	Converts β-carotene to zeaxanthin via cryptoxanthin.
AT5G52580		RabGAP/TBC domain-containing protein
AT5G52620		F-box associated ubiquitination effector family protein
AT5G52630	*MITOCHONDRIAL RNA EDITING FACTOR 1 (MEF1)*	Encodes a member of the DYW subfamily of pentatricopeptide repeat (PPR) proteins. Loss of MEF1 function affects RNA editing at specific sites in the mitochondrial genome but do not exhibit obvious phenotypes.
AT5G52640	*HEAT SHOCK PROTEIN 90.1 (HSP90.1)*	Encodes a cytosolic heat shock protein AtHSP90.1. AtHSP90.1 interacts with disease resistance signaling components SGT1b and RAR1 and is required for RPS2-mediated resistance. The mRNA is cell-to-cell mobile.
AT5G52670		Member of plant specific copper transport protein family.
AT5G52680		Copper transport protein family
AT5G52690		Copper transport protein family
AT5G52710		Copper transport protein family
AT5G52720		Copper transport protein family
AT5G52740		Copper transport protein family
AT5G52750		Heavy metal transport/detoxification superfamily protein
AT5G52760		Copper transport protein family
AT5G52770		Copper transport protein family
AT5G52780	*PAM68-LIKE (PAM68L)*	Chloroplast NAD(P)H dehydrogenase complex assembly factor.
AT5G52790		CBS domain protein with a domain protein (DUF21)
AT5G52820	*NOTCHLESS (NLE)*	Encodes a NOTCHLESS homolog, a non-ribosomal protein involved in the maturation and assembly of the 60S ribosomal subunit, that is required for female gametogenesis. The mRNA is cell-to-cell mobile.
AT5G52830	*WRKY DNA-BINDING PROTEIN 27 (WRKY27)*	Encodes a WRKY transcription factor WRKY27. Mutation in Arabidopsis WRKY27 results in delayed symptom development in response to the bacterial wilt pathogen Ralstonia solanacearum.
AT5G52860	*ATP-BINDING CASSETTE G8 (ABCG8)*	ABC-2 type transporter family protein
AT5G52890		AT hook motif-containing protein
AT5G52900	*MEMBRANE-ASSOCIATED KINASE REGULATOR 6 (MAKR6)*	Encodes a member of the MAKR (MEMBRANE-ASSOCIATED KINASE REGULATOR) gene family. MAKRs have putative kinase interacting motifs and membrane localization signals. Known members include: AT5G26230 (MAKR1), AT1G64080 (MAKR2), AT2G37380 (MAKR3), AT2G39370 (MAKR4), AT5G52870 (MAKR5) and AT5G52900 (MAKR6).
AT5G52910	*TIMELESS (ATIM)*	homolog of Drosophila timeless

AT5G52920	*PLASTIDIC PYRUVATE KINASE BETA SUBUNIT 1 (PKP-BETA1)*	encodes a dominant chloroplast pyruvate kinase beta subunit. Important for seed oil biosynthesis. Ubiquitously expressed, with significantly increased expression in maturing seeds. The mutant plant has wrinkled seeds, with a 50-70% reduction in seed fatty acid content. The mRNA is cell-to-cell mobile.
AT5G52950		LIM domain protein
AT5G52960		tRNA dimethylallyltransferase
AT5G52970		thylakoid lumen 15.0 kDa protein
AT5G52975		egg cell-secreted-like protein (DUF1278)
AT5G53010		calcium-transporting ATPase
AT5G53030		hypothetical protein
AT5G53070		Ribosomal protein L9/RNase H1
AT5G53080	*WHITE TO GREEN 1 (WTG1)*	WTG1 is a chloroplast localized TPR protein required for chloroplast biogenesis. Mutants are delayed in greening and defective in splicing petL and ndhG. WTG1 does not itself bind RNA but it does bind known editing factors MORF8 and MORF9.
AT5G53100		NAD(P)-binding Rossmann-fold superfamily protein
AT5G53110	*ARABIDOPSIS T??XICOS EN LEVADURA 96 (ATL96)*	RING/U-box superfamily protein
AT5G53150		DnaJ heat shock amino-terminal domain protein
AT5G53160	*REGULATORY COMPONENTS OF ABA RECEPTOR 3 (RCAR3)*	Encodes RCAR3, a regulatory component of ABA receptor. Interacts with protein phosphatase 2Cs ABI1 and ABI2. Stimulates ABA signaling. The mRNA is cell-to-cell mobile.
AT5G53190	*(SWEET3)*	Nodulin MtN3 family protein
AT5G53200	*TRIPTYCHON (TRY)*	Encodes a R3MYB transcription inhibitor that regulates trichome patterning. Mutants produce trichome clusters whereas other transcriptional inhibitors involved in this patterning are involved in trichome density regulation. Natural hypofunctional alleles producing trichome development in fruits have been found.
AT5G53210	*SPEECHLESS (SPCH)*	Encodes a basic helix-loop-helix (bHLH) transcription factor that is necessary and sufficient for the asymmetric divisions that establish the stomatal lineage in Arabidopsis thaliana. Expression of SPCH in young epidermal cells allows these cells to make asymmetric divisions. SPCH is a substrate of a kinase MPK3 and MPK6. Its transcript levels change after inducing MUTE expression in a mute background.
AT5G53220		hypothetical protein
AT5G53240	*DUF295 ORGANELLAR B 7 (ATDOB7)*	hypothetical protein (DUF295)
AT5G53250	*ARABINOGALACTAN PROTEIN 22 (AGP22)*	arabinogalactan protein 22
AT5G53280	*PLASTID DIVISION1 (PDV1)*	An integral outer envelope membrane protein (as its homolog PDV2), component of the plastid division machinery. Similar to ARC5, PDV1 localized to a discontinuous ring at the division site in wild-type plants. PDV1 and PDV2 are required for localization of ARC5 at the chloroplast division site. Topological analysis showed that the large N-terminal region of PDV1 upstream of the transmembrane helix bearing a putative coiled-coil domain is exposed to the cytosol. Mutation of the conserved PDV1 C-terminal Gly residue did not block PDV1 insertion into the outer envelope membrane but did abolish its localization to the division site. The mRNA is cell-to-cell mobile.
AT5G53290	*CYTOKININ RESPONSE FACTOR 3 (CRF3)*	encodes a member of the ERF (ethylene response factor) subfamily B-5 of ERF/AP2 transcription factor family. The protein contains one AP2 domain. There are 7 members in this subfamily. CRF proteins relocalize to the nucleus in response to cytokinin.
AT5G53320		Leucine-rich repeat protein kinase family protein
AT5G53360	*(SINAT5)*	RING‐finger E3 ubiquitin ligase (SINATT) member that lacks ubiquitin ligase activity due to the absence of the RING domain, functions as a protector protein which stabilizes FREE1. Involved in response to iron deficiency stress.
AT5G53370	*PECTIN METHYLESTERASE PCR FRAGMENT F (PMEPCRF)*	pectin methylesterase PCR fragment F
AT5G53400	*BOBBER1 (BOB1)*	Encodes BOBBER1 (BOB1), a non-canonical small heat shock protein required for both development and thermotolerance. BOB1 is cytoplasmic at basal temperatures but forms heat shock granules containing canonical small heat shock proteins at high temperatures. The mRNA is cell-to-cell mobile.
AT5G53410		F-box associated ubiquitination effector family protein
AT5G53420	*CONSTANS, CO-LIKE AND TOC1 MOTIF; (CCT101)*	Member of ASML2 family of CCT domain proteins.There is a preferential accumulation of RNA isoforms CCT101.1 and CCT101.2 in response to N-treatment, each isoform has different targets.
AT5G53430	*SET DOMAIN GROUP 29 (SDG29)*	Homology Subgroup III; Orthology Group 2 - A putative histone methyltransferase (predicted to methylate H3K4) related to the Drosophila trithorax group proteins TRX and TRR and the yeast gene SET1. A plant line expressing an RNAi construct directed against this gene has reduced agrobacterium-mediated tumor formation.
AT5G53450	*OBP3-RESPONSIVE GENE 1 (ORG1)*	FBN11 contains a lipid-binding FBN domain and a kinase domain. It is induced by osmotic stress and loss of function mutants show decreased germination when grown under osmotic stress conditions.
AT5G53460	*NADH-DEPENDENT GLUTAMATE SYNTHASE 1 (GLT1)*	NADH-dependent glutamate synthase The mRNA is cell-to-cell mobile.
AT5G53470	*ACYL-COA BINDING PROTEIN 1 (ACBP1)*	Encodes an acyl-CoA binding protein that is localized to vesicles,and plasma membrane especially in epidermal cells of heart, torpedo and cotyledon stage embryos, cell wall of the seed coat. Northern blot analysis showed that the 1.4 kb ACBP1 mRNA was expressed in silique, root, stem, leaf and flower.
AT5G53480	*HOMOLOG OF HUMAN KPNB1 (KPNB1)*	Sensitive to ABA, role in drought stress.
AT5G53490	*THYLAKOID LUMENAL 17.4 KDA PROTEIN (TL17)*	thylakoid lumenal 17.4 kDa protein, chloroplast, identical to SP:P81760 Thylakoid lumenal 17.4 kDa protein, chloroplast precursor (P17.4) {Arabidopsis thaliana}. Putative pentapeptide protein.
AT5G53510	*OLIGOPEPTIDE TRANSPORTER 9 (OPT9)*	oligopeptide transporter
AT5G53520	*OLIGOPEPTIDE TRANSPORTER 8 (OPT8)*	Encodes an oligopeptide transporter. Target promoter of the male germline-specific transcription factor DUO1.
AT5G53540	*(APP1)*	Encodes a P-loop NTPase APP1. The disruption of APP1 is accompanied by a reduction in ROS level, a rise in the rate of cell division in the quiescent center (QC) and the promotion of root distal stem cell (DSC) differentiation.

AT5G53580	*PYRIDOXAL REDUCTASE 1 (PLR1)*	NAD(P)-linked oxidoreductase superfamily protein
AT5G53590	*SMALL AUXIN UPREGULATED RNA 30 (SAUR30)*	SAUR-like auxin-responsive protein family
AT5G53640		F-box/FBD/LRR protein
AT5G53660	*GROWTH-REGULATING FACTOR 7 (GRF7)*	Growth regulating factor encoding transcription activator. One of the nine members of a GRF gene family, containing nuclear targeting domain. Involved in leaf development and expressed in shoot and flower.
AT5G53670		transmembrane protein
AT5G53680		RNA-binding (RRM/RBD/RNP motifs) family protein
AT5G53690		
AT5G53710		hypothetical protein
AT5G53740		hypothetical protein
AT5G53760	*MILDEW RESISTANCE LOCUS O 11 (MLO11)*	A member of a large family of seven-transmembrane domain proteins specific to plants, homologs of the barley mildew resistance locus o (MLO) protein. The Arabidopsis genome contains 15 genes encoding MLO proteins, with localization in plasma membrane. Phylogenetic analysis revealed four clades of closely-related AtMLO genes. ATMLO11 belongs to the clade I, with AtMLO4 and AtMLO14. The gene is expressed during early seedling growth (in primary root), in root tips and lateral root primordia, and in very young leaves, and in flowers and fruit abscission zone, as shown by GUS activity patterns. The expression of several phylogenetically closely-related AtMLO genes showed similar or overlapping tissue specificity and analogous responsiveness to external stimuli, suggesting functional redundancy, co-function, or antagonistic function(s).
AT5G53770	*TRF4/5-LIKE (TRL)*	Nucleotidyltransferase family protein
AT5G53810		O-methyltransferase family protein
AT5G53820		Late embryogenesis abundant protein (LEA) family protein
AT5G53830	*MPK3/6-TARGETED VQP 3 (MVQ3)*	VQ motif-containing protein
AT5G53840		F-box/RNI-like/FBD-like domains-containing protein
AT5G53870	*EARLY NODULIN-LIKE PROTEIN 1 (ENODL1)*	early nodulin-like protein 1
AT5G53880		hypothetical protein
AT5G53890	*PHYTOSYLFOKINE-ALPHA RECEPTOR 2 (PSKR2)*	Encodes a leucine-rich repeat receptor kinase (LRR-RK) involved in the perception of phytosulfokine (PSK), which is a 5-aa tyrosine-sulfated peptide that primarily promotes cellular proliferation.
AT5G53900		Serine/threonine-protein kinase WNK (With No Lysine)-like protein
AT5G53920	*PROTEIN METHYLTRANSFERASE A (PRMA)*	Protein methyltransferase. One target is PRPL11 which it methylates on Lys 109.
AT5G53950	*CUP-SHAPED COTYLEDON 2 (CUC2)*	Transcriptional activator of the NAC gene family, with CUC1 redundantly required for embryonic apical meristem formation, cotyledon separation and expression of STM. Proper timing of CUC2 expression is required to maintain the phyllotactic pattern initiated in the meristem. CUC2 expression in leaf sinus region is required for serration and the extent of serration is modulated by mir164A mediated repression of CUC2. Together with CUC3-DA1-UBP15 part of a regulatory module which controls the initiation of axillary meristems, thereby determining plant architecture. Regulates the axillary meristem initiation, directly binding to the DA1 promoter.
AT5G53960		Mid-1-related chloride channel domain-containing protein
AT5G53970	*TYROSINE AMINOTRANSFERASE 7 (TAT7)*	Encodes a cytosolic tyrosine aminotransferase which is strongly induced upon aging and coronatine treatment. AtTAT1 prefers Tyr as an amino donor but can also use Phe, Trp, His, Met, and Leu. The mRNA is cell-to-cell mobile.
AT5G53980	*HOMEOBOX PROTEIN 52 (HB52)*	Encodes a homeodomain leucine zipper class I (HD-Zip I) protein.
AT5G53990		UDP-Glycosyltransferase superfamily protein
AT5G54000		2-oxoglutarate (2OG) and Fe(II)-dependent oxygenase superfamily protein
AT5G54010	*UDP-GLYCOSYLTRANSFERASE 79B6 (UGT79B6)*	Encodes a flavonoid 3-O-glucoside:2″-O-glucosyltransferase that determines pollen-specific flavonol structure.
AT5G54020		Cysteine/Histidine-rich C1 domain family protein
AT5G54030		Cysteine/Histidine-rich C1 domain family protein
AT5G54040		Cysteine/Histidine-rich C1 domain family protein
AT5G54050		Cysteine/Histidine-rich C1 domain family protein
AT5G54060	*UDP-GLUCOSE:FLAVONOID 3-O-GLUCOSYLTRANSFERASE (UF3GT)*	Encodes a anthocyanin 3-O-glucoside: 2"-O-xylosyl-transferase involved in anthocyanin modification that converts cyanidin 3-O-glucoside to cyanidin 3-O-xylosyl(1->2)glucoside. Its preferred sugar donor is UDP-xylose.
AT5G54070	*HEAT SHOCK TRANSCRIPTION FACTOR A9 (HSFA9)*	A member of Heat Stress Transcription Factor (Hsf) family. Not responding to heat stress. Is regulated by the seed-specific transcription factor ABI3. In turn, it regulates other heat stress proteins including Hsp17.4-CI, Hsp17.7-CII and Hsp101 during seed maturation.
AT5G54090		DNA mismatch repair protein MutS, type 2
AT5G54095		proteoglycan-like protein
AT5G54100	*STOMATIN-LIKE PROTEIN 2 (SLP2)*	SPFH/Band 7/PHB domain-containing membrane-associated protein family
AT5G54120		
AT5G54130		Calcium-binding endonuclease/exonuclease/phosphatase family
AT5G54140	*IAA-LEUCINE-RESISTANT (ILR1)-LIKE 3 (ILL3)*	encodes a protein similar to IAA amino acid conjugate hydrolase; member of ILR1-like (ILL) seven-gene family.
AT5G54150		hypothetical protein
AT5G54160	*O-METHYLTRANSFERASE 1 (OMT1)*	A caffeic acid/5-hydroxyferulic acid O-methyltransferase. Interacts with 14-4-3 proteins in yeast 2 hybrid assay. AtOMT1 (At5g54160) encodes a flavonol 3?-O-methyltransferase that is highly active towards quercetin and myricetin. The substrate specificity identifies the enzyme as flavonol 3?-methyltransferase which replaces the former annotation of the gene to encode a caffeic acid/5-hydroxyferulic acid O-methyltransferase The mRNA is cell-to-cell mobile.
AT5G54190	*PROTOCHLOROPHYLLIDE OXIDOREDUCTASE A (PORA)*	light-dependent NADPH:protochlorophyllide oxidoreductase A
AT5G54200	*WD40-DOMAIN 1 (WDD1)*	Transducin/WD40 repeat-like superfamily protein

AT5G54220		Encodes a defensin-like (DEFL) family protein.
AT5G54230	*MYB DOMAIN PROTEIN 49 (MYB49)*	MYB49 transcription factor. Binds to and promotes expression of genes involved in cadmium accumulation. Interacts with ABI5 which acts as a repressor preventing MYB49 induced expression of target genes.
AT5G54250	*CYCLIC NUCLEOTIDE-GATED CATION CHANNEL 4 (CNGC4)*	member of Cyclic nucleotide gated channel family, downstream component of the signaling pathways leading to HR resistance. mutant plants exhibit gene-for-gene disease resistance against avirulent Pseudomonas syringae despite the near-complete absence of the hypersensitive response (HR). Salicylic acid accumulation in dnd2 mutants is completely PAD4-independent.
AT5G54270	*LIGHT-HARVESTING CHLOROPHYLL B-BINDING PROTEIN 3 (LHCB3)*	Lhcb3 protein is a component of the main light harvesting chlorophyll a/b-protein complex of Photosystem II (LHC II).
AT5G54280	*MYOSIN 2 (ATM2)*	Type VII myosin gene
AT5G54290	*(CcdA)*	Encodes CcdA, a thylakoid membrane protein required for the transfer of reducing equivalents from stroma to thylakoid lumen.
AT5G54300		cotton fiber-like protein (DUF761)
AT5G54350		C2H2-type zinc finger protein
AT5G54370		Late embryogenesis abundant (LEA) protein-like protein
AT5G54380	*THESEUS1 (THE1)*	Encodes THESEUS1 (THE1), a receptor kinase regulated by Brassinosteroids and required for cell elongation during vegetative growth.
AT5G54390	*HAL2-LIKE (HL)*	Encodes a 3'-phosphoadenosine-5'-phosphate (PAP) phosphatase that is sensitive to physiological concentrations of Na+. It does not also act as inositol polyphosphate 1-phosphatases, which other members of the HAL2-like family do. It is proposed that AHL acts in concert with sulphotransferases to prevent both the toxicity of PAP on RNA processing enzymes as well as the product inhibition of PAP on sulphate conjugation. The mRNA is cell-to-cell mobile.
AT5G54400		S-adenosyl-L-methionine-dependent methyltransferases superfamily protein
AT5G54410		hypothetical protein
AT5G54420	*DUF295 ORGANELLAR B 10 (ATDOB10)*	hypothetical protein (DUF295)
AT5G54460		wound-responsive protein-like protein
AT5G54470	*B-BOX DOMAIN PROTEIN 29 (BBX29)*	B-box type zinc finger family protein
AT5G54480		hypothetical protein (DUF630 and DUF632)
AT5G54490	*PINOID-BINDING PROTEIN 1 (PBP1)*	Encodes a PINOID (PID)-binding protein containing putative EF-hand calcium-binding motifs. The interaction is dependent on the presence of calcium. mRNA expression is up-regulated by auxin. Not a phosphorylation target of PID, likely acts upstream of PID to regulate the activity of this protein in response to changes in calcium levels.
AT5G54500	*FLAVODOXIN-LIKE QUINONE REDUCTASE 1 (FQR1)*	Encodes a flavin mononucleotide-binding flavodoxin-like quinone reductase that is a primary auxin-response gene.
AT5G54510	*DWARF IN LIGHT 1 (DFL1)*	Encodes an IAA-amido synthase that conjugates Ala, Asp, Phe, and Trp to auxin. Lines overexpressing this gene accumulate IAA-ASP and are hypersensitive to several auxins. Identified as a dominant mutation that displays shorter hypocotyls in light grown plants when compared to wild type siblings. Protein is similar to auxin inducible gene from pea (GH3).
AT5G54530		serine protease, putative (Protein of unknown function, DUF538)
AT5G54570	*BETA GLUCOSIDASE 41 (BGLU41)*	beta glucosidase 41
AT5G54580	*(ORRM2)*	Encodes an RNA recognition motif (RRM) and is involved in C-> U RNA editing in mitochondria.
AT5G54590	*CALCIUM/CALMODULIN-REGULATED RECEPTOR-LIKE KINASE 1 (CRLK1)*	Splice variant At5g54590.2 encodes CRLK1 (440-amino acid in length) calcium/calmodulin-regulated receptor-like kinase crucial for cold tolerance. CRLK1 is Primarily localized in the plasma membrane.
AT5G54600	*PLASTID RIBOSOMAL PROTEIN L24 (RPL24)*	Translation protein SH3-like family protein
AT5G54610	*ANKYRIN (ANK)*	Induced in response to Salicylic acid. Belongs to the ankyrin repeat protein family.
AT5G54620		Ankyrin repeat family protein
AT5G54630		zinc finger protein-like protein
AT5G54660		HSP20-like chaperones superfamily protein
AT5G54670	*KINESIN 3 (ATK3)*	Encodes a truncated KatC polypeptide (KatC(207-754)), which includes the carboxyl-terminal region of KatC. This was expressed in Escherichia coli and was shown to possess microtubule-stimulated ATPase activity.
AT5G54690	*GALACTURONOSYLTRANSFERASE 12 (GAUT12)*	Encodes a protein with putative galacturonosyltransferase activity. Mutants defective in this gene displayed a notable reduction in xylose (>50%) in the cell walls from stems and roots and a reduction in cellulose (~25%).
AT5G54700		Ankyrin repeat family protein
AT5G54720		Ankyrin repeat family protein
AT5G54740	*SEED STORAGE ALBUMIN 5 (SESA5)*	seed storage albumin 5
AT5G54770	*(THI1)*	Encodes a thiamine biosynthetic gene that has a dual function in thiamine biosynthesis and mitochondrial DNA damage tolerance. It appears to be involved in producing the thiazole portion of thiamine (vitamin B1). A crystal structure of the protein reveals that it forms a 2-ring homo-octamer. The mRNA is cell-to-cell mobile.
AT5G54780		Ypt/Rab-GAP domain of gyp1p superfamily protein
AT5G54790	*VASCULAR-RELATED UNKNOWN PROTEIN 4 (VUP4)*	CTD small phosphatase-like protein
AT5G54800	*GLUCOSE 6-PHOSPHATE/PHOSPHATE TRANSLOCATOR 1 (GPT1)*	Encodes glucose6-Phosphate/phosphate transporter 1. Essential for pollen maturation and embryo sac development. The mRNA is cell-to-cell mobile.
AT5G54830		DOMON domain-containing protein / dopamine beta-monooxygenase N-terminal domain-containing protein
AT5G54840	*(SGP1)*	Monomeric G protein. Expressed in the root quiescent center, flowers, and leaf guard cells and hydathodes.
AT5G54855		Pollen Ole e 1 allergen and extensin family protein
AT5G54860		Major facilitator superfamily protein
AT5G54910		DEA(D/H)-box RNA helicase family protein
AT5G54940		Translation initiation factor SUI1 family protein

AT5G54950		Aconitase family protein
AT5G54960	*PYRUVATE DECARBOXYLASE-2 (PDC2)*	pyruvate decarboxylase-2
AT5G54970		hypothetical protein
AT5G54980	*CASP-LIKE PROTEIN 2D1 (CASPL2D1)*	Uncharacterized protein family (UPF0497)
AT5G55010		hypothetical protein
AT5G55020	*MYB DOMAIN PROTEIN 120 (MYB120)*	Encodes a putative transcription factor, member of the R2R3 factor gene family (MYB120).
AT5G55040	*BROMODOMAIN-CONTAINING PROTEIN 13 (BRD13)*	DNA-binding bromodomain-containing protein, interacts with core SWI/SNF complex components.
AT5G55050		GDSL-motif esterase/acyltransferase/lipase. Enzyme group with broad substrate specificity that may catalyze acyltransfer or hydrolase reactions with lipid and non-lipid substrates. The mRNA is cell-to-cell mobile.
AT5G55070	*(E2-OGDH2)*	Encodes the E2 subunit of the 2-oxoglutarate dehydrogenase.
AT5G55080	*RAS-RELATED NUCLEAR PROTEIN 4 (RAN4)*	A member of RAN GTPase gene family.
AT5G55090	*MITOGEN-ACTIVATED PROTEIN KINASE KINASE KINASE 15 (MAPKKK15)*	member of MEKK subfamily
AT5G55120	*VITAMIN C DEFECTIVE 5 (VTC5)*	Encodes a GDP-L-galactose phosphorylase, with similar biochemical properties as VTC2.
AT5G55130	*CO-FACTOR FOR NITRATE, REDUCTASE AND XANTHINE DEHYDROGENASE 5 (CNX5)*	putative molybdopterin synthase sulphurylase (cnx5)
AT5G55140		ribosomal protein L30 family protein
AT5G55170	*SMALL UBIQUITIN-LIKE MODIFIER 3 (SUMO3)*	Encodes a small ubiquitin-like modifier (SUMO) polypeptide that becomes covalently attached to various intracellular protein targets, much like ubiquitination, leading to post-translational modification of those targets.
AT5G55180		O-Glycosyl hydrolases family 17 protein
AT5G55200	*MITOCHONDRIAL GRPE 1 (MGE1)*	Co-chaperone GrpE family protein
AT5G55220	*(TIG1)*	Contains with HP22 a protein that is related to the bacterial trigger factor chaperone. Plants depleted of either HP22 or HP65b or even both were increasingly delayed in leaf senescence and retained much longer stromal chloroplast constituents than wild-type plants.
AT5G55230	*MICROTUBULE-ASSOCIATED PROTEINS 65-1 (MAP65-1)*	Binds and bundles microtubules. Plays a role in stabilizing anti-parallel microtubules in the central spindle at anaphase to early cytokinesis but is not essential at the midline of the phragmoplast at later stages. The timing with which the MAP65-1 was targeted to the spindle appears to be regulated by a phosphorylation sensitive switch. Enhances microtubule polymerization, promotes nucleation and stabilizes microtubules against cold treatment and dilution.
AT5G55240	*ARABIDOPSIS THALIANA PEROXYGENASE 2 (ATPXG2)*	Catalyze hydroperoxide-dependent mono-oxygenation reactions. Require calcium for peroxygenase activity. Probably deeply buried in lipid droplets or microsomes.
AT5G55250	*IAA CARBOXYLMETHYLTRANSFERASE 1 (IAMT1)*	Encodes an enzyme which specifically converts IAA to its methyl ester form MeIIAA. This gene belongs to the family of carboxyl methyltransferases whose members catalyze the transfer of the methyl group from S-adenosyl-L-methionine to carboxylic acid-containing substrates to form small molecule methyl esters. Expression of TCP genes is downregulated in mutant iamt1-D. SABATH methyltransferase.
AT5G55260	*PROTEIN PHOSPHATASE X 2 (PPX2)*	Encodes a protein with similarity to the catalytic subunit of the mammalian PPX protein phospatase.
AT5G55310	*DNA TOPOISOMERASE 1 BETA (TOP1BETA)*	Encodes one of two Arabidopsis type-I DNA topoisomerase I genes. Reducing the level of expression of this gene in a top1alpha (At5g55300) mutant background causes seedling lethality.
AT5G55320		MBOAT (membrane bound O-acyl transferase) family protein
AT5G55330		MBOAT (membrane bound O-acyl transferase) family protein
AT5G55340		MBOAT (membrane bound O-acyl transferase) family protein
AT5G55350		MBOAT (membrane bound O-acyl transferase) family protein
AT5G55360		MBOAT (membrane bound O-acyl transferase) family protein
AT5G55370	*LONG-CHAIN-ALCOHOL O-FATTY-ACYLTRANSFERASE 2 (AT2)*	MBOAT (membrane bound O-acyl transferase) family protein
AT5G55380		MBOAT (membrane bound O-acyl transferase) family protein
AT5G55400	*(ATFIM4)*	Encodes a member of the fimbrin family. Different members of the fimbrin/plastin family have diverged biochemically during evolution to generate either tight actin bundles or loose networks with distinct biochemical and biophysical properties. FIM4 generates both actin bundles and branched actin filaments whereas FIM5 only generates actin bundles.
AT5G55420		Encodes a Protease inhibitor/seed storage/LTP family protein [pseudogene]
AT5G55450	*(ATLTP4.4)*	Bifunctional inhibitor/lipid-transfer protein/seed storage 2S albumin superfamily protein
AT5G55470	*NA+/H+ (SODIUM HYDROGEN) EXCHANGER 3 (NHX3)*	member of Sodium proton exchanger family
AT5G55480	*SHV3-LIKE 1 (SVL1)*	Glycerophosphoryl diester phosphodiesterase-like protein involved in cell wall cellulose accumulation and pectin linking. Impacts root hair, trichome and epidermal cell development. The mRNA is cell-to-cell mobile.
AT5G55490	*GAMETE EXPRESSED PROTEIN 1 (GEX1)*	Encodes a transmembrane domain containing protein that is expressed in pollen germ cells.
AT5G55520		kinesin-like protein
AT5G55530		Calcium-dependent lipid-binding (CaLB domain) family protein
AT5G55550		RNA-binding (RRM/RBD/RNP motifs) family protein
AT5G55560		Protein kinase superfamily protein
AT5G55570		transmembrane protein
AT5G55580	*(MTERF9)*	Encodes a mitochondrial transcription termination factor (mTERF) family protein. The gene product is targeted to the chloroplast nucleoid and mutants are affected in plastid gene expression and chloroplast development. Mutants, named as twirt1 (twr-1), display altered root meristem function resulting in short roots. Mutation also affects shoot meristem function.

AT5G55590	*QUARTET 1 (QRT1)*	Encodes a protein with pectin methylesterase activity. No change in activity were detected in mutants defective in this gene, which was interpreted as a result of redundancy of product function with other pectin methylesterases. The gene product is required for pollen separation during normal development. In qrt mutants, the outer walls of the four meiotic products of the pollen mother cell are fused, and pollen grains are released in tetrads.May be required for cell type-specific pectin degradation.
AT5G55610		isopentenyl-diphosphate delta-isomerase
AT5G55620		hypothetical protein
AT5G55630	*TWO PORE K CHANNEL (KCO1)*	Encodes AtTPK1 (KCO1), a member of the Arabidopsis thaliana K+ channel family of AtTPK/KCO proteins. AtTPK1 is targeted to the vacuolar membrane. Forms homomeric ion channels in vivo. Voltage-independent and Ca2+-activated K+ channel. Activated by 14-3-3 proteins. Vacuolar K+-conducting TPC1 and TPK1/TPK3 channels act in concert to provide for Ca2+- and voltageinduced electrical excitability to the central organelle of plant cells.
AT5G55640		Na-translocating NADH-quinone reductase subunit A
AT5G55660		DEK domain-containing chromatin associated protein
AT5G55670		RNA-binding (RRM/RBD/RNP motifs) family protein
AT5G55720		Pectin lyase-like superfamily protein
AT5G55730	*FASCICLIN-LIKE ARABINOGALACTAN 1 (FLA1)*	Encodes fasciclin-like arabinogalactan-protein 1 (Fla1). fla1 mutants show defects in shoot regeneration. Possibly involved in embryogenesis and seed development.
AT5G55740	*CHLORORESPIRATORY REDUCTION 21 (CRR21)*	Encodes a member of the E+ subgroup of the PPR protein family, containing the E and E+ motifs following a tandem array of PPR motifs. It also contains an unknown motif consisting of 15 aa, which is highly conserved in some PPR proteins, including CRR4. CRR21 is involved in RNA editing of the site 2 of ndhD (ndhD-2),which encodes a subunit of the NDH complex. The RNA editing changes aa 128 from Ser to Leu. Mutants have impaired NDH complex activity.
AT5G55750		hydroxyproline-rich glycoprotein family protein
AT5G55770		Cysteine/Histidine-rich C1 domain family protein
AT5G55780		Cysteine/Histidine-rich C1 domain family protein
AT5G55790		hypothetical protein
AT5G55800		Cysteine/Histidine-rich C1 domain family protein
AT5G55820	*WYRD (WYR)*	Encodes a plant ortholog of the inner centromere protein (INCENP), which is implicated in the control of chromosome segregation and cytokinesis in yeast and animals. Required for female gametophytic cell specification and seed development.
AT5G55830	*L-TYPE LECTIN RECEPTOR KINASE S.7 (LECRK-S.7)*	Concanavalin A-like lectin protein kinase family protein
AT5G55850	*(NOI)*	NOI protein
AT5G55860	*TOUCH-REGULATED PHOSPHOPROTEIN1 (TREPH1)*	WEB1/PMI2 related protein involved in mecahnotransduction.TREPH1 is phosphorylated at position S625 in response to touch, and this is required for mechanosensitive growth response.
AT5G55870	*DUF295 ORGANELLAR B 15 (ATDOB15)*	hypothetical protein
AT5G55915		
AT5G55930	*OLIGOPEPTIDE TRANSPORTER 1 (OPT1)*	oligopeptide transporter
AT5G55940	*EMBRYO DEFECTIVE 2731 (emb2731)*	Uncharacterized protein family (UPF0172)
AT5G55950		Nucleotide/sugar transporter family protein
AT5G55960		transmembrane protein C9orf5 protein
AT5G55970	*DROUGHT-RESPONSIVE RING PROTEIN 1 (DDR1)*	Drought-induced gene encoding an ER-localized RING-type E3 Ub ligase.
AT5G55980		serine-rich protein-like protein
AT5G56040	*STERILITY-REGULATING KINASE MEMBER 2 (SKM2)*	Leucine-rich receptor-like protein kinase family protein
AT5G56070		hypothetical protein
AT5G56080	*NICOTIANAMINE SYNTHASE 2 (NAS2)*	Encodes a protein with nicotianamine synthase activity. Its transcript levels rise in roots in response to zinc deficiency and rise in leaves in response to elevated levels of zinc.
AT5G56090	*CYTOCHROME C OXIDASE 15 (COX15)*	Encodes a homolog of COX15. Microarray analysis show a 3.2 fold increase in transcription after treatment with rotenone, an electron transport chain inhibitor.
AT5G56100		glycine-rich protein / oleosin
AT5G56110	*(MYB80)*	Encodes a member of the R2R3 MYB transcription factor gene family that is required for anther development by regulation tapetum development, callose dissolution and exine formation. It acts upstream of MS2.
AT5G56140		RNA-binding KH domain-containing protein
AT5G56150	*UBIQUITIN-CONJUGATING ENZYME 30 (UBC30)*	ubiquitin-conjugating enzyme 30
AT5G56160		Sec14p-like phosphatidylinositol transfer family protein
AT5G56185		
AT5G56220	*TIR-NBS21 (TN21)*	P-loop containing nucleoside triphosphate hydrolases superfamily protein
AT5G56230	*PRENYLATED RAB ACCEPTOR 1.G2 (PRA1.G2)*	prenylated RAB acceptor 1.G2
AT5G56260		Ribonuclease E inhibitor RraA/Dimethylmenaquinone methyltransferase
AT5G56270	*WRKY DNA-BINDING PROTEIN 2 (WRKY2)*	Encodes WRKY transcription factor 2, a zinc-finger protein. In wrky2 mutants, egg cells polarize normally but zygotes fail to reestablish polar organelle positioning from a transient symmetric state, resulting in equal cell division and distorted embryo development.
AT5G56280	*COP9 SIGNALOSOME SUBUNIT 6A (CSN6A)*	one of two genes encoding subunit 6 of COP9 signalosome complex. Protein contains a MPR1p and PAD1p N-terminal (MPN) domain at the N-terminal region and belongs to the Mov34 superfamily. Mutant and antisense expression result in a number of developmental defects and in ubiquitin/proteasome-mediated protein degradation.

AT5G56300	*GIBBERELLIC ACID METHYLTRANSFERASE 2 (GAMT2)*	A member of the Arabidopsis SABATH methyltransferase gene family. Encodes GAMT2, a methyltransferase that uses S-adenosine-L-methionine (SAM) as a methyl donor to methylate the carboxyl group of GAs, resulting in the methyl esters of GAs (MeGAs). Expressed most highly in the siliques during seed development.
AT5G56310		Pentatricopeptide repeat (PPR) superfamily protein
AT5G56320	*EXPANSIN A14 (EXPA14)*	member of Alpha-Expansin Gene Family. Naming convention from the Expansin Working Group (Kende et al, 2004. Plant Mol Bio)
AT5G56330	*ALPHA CARBONIC ANHYDRASE 8 (ACA8)*	alpha carbonic anhydrase 8
AT5G56350		Pyruvate kinase family protein
AT5G56360	*PRIORITY IN SWEET LIFE 4 (PSL4)*	Encodes PSL4, beta-subunit of endoplasmic reticulum-resident glucosidase II, which is essential for stable accumulation and quality control of the elf18 receptor EFR but not the flg22 receptor FLS2. The mRNA is cell-to-cell mobile.
AT5G56370		F-box/RNI-like/FBD-like domains-containing protein
AT5G56380		F-box/RNI-like/FBD-like domains-containing protein
AT5G56460	*PBS1-LIKE16 (PBL16)*	Protein kinase superfamily protein
AT5G56470	*L -GULONO-1,4-LACTONE (L -GULL) OXIDASE 1 (GULLO7)*	FAD-dependent oxidoreductase family protein
AT5G56480	*END1-LIKE 2 (END2)*	Non specific lipid transfer protein, with embryo sac specific expression.
AT5G56490	*L -GULONO-1,4-LACTONE (L -GULL) OXIDASE 4 (GULLO4)*	D-arabinono-1,4-lactone oxidase family protein
AT5G56510	*PUMILIO 12 (PUM12)*	Encodes a member of the Arabidopsis Pumilio (APUM) proteins containing PUF domain (eight repeats of approximately 36 amino acids each). PUF proteins regulate both mRNA stability and translation through sequence-specific binding to the 3' UTR of target mRNA transcripts.
AT5G56520		hypothetical protein
AT5G56540	*ARABINOGALACTAN PROTEIN 14 (AGP14)*	Encodes arabinogalactan protein (AGP14). Mutants exhibit longer root hairs. The mRNA is cell-to-cell mobile.
AT5G56570		Leucine-rich repeat (LRR) family protein
AT5G56580	*MAP KINASE KINASE 6 (MKK6)*	Encodes a member of the MAP Kinase Kinase family of proteins. It can phosphorylate MPK12 in vitro and it can be dephosphorylated by MKP2 in vitro.
AT5G56590		O-Glycosyl hydrolases family 17 protein
AT5G56600	*PROFILIN 3 (PRF3)*	Encodes profilin3, a low-molecular weight, actin monomer-binding protein that regulates the organization of actin cytoskeleton. Originally known as profilin5, and later named profilin3. Expressed in vegetative organs. Mutants have slightly elongated petioles.
AT5G56620	*NAC DOMAIN CONTAINING PROTEIN 99 (NAC099)*	NAC domain containing protein 99
AT5G56630	*PHOSPHOFRUCTOKINASE 7 (PFK7)*	phosphofructokinase 7
AT5G56640	*MYO-INOSITOL OXYGENASE 5 (MIOX5)*	Myo-Inositol Oxygenase gene family
AT5G56700		FBD / Leucine Rich Repeat domains containing protein
AT5G56720	*CYTOSOLIC-NAD-DEPENDENT MALATE DEHYDROGENASE 3 (c-NAD-MDH3)*	predicted to encode a cytosolic malate dehydrogenase.
AT5G56750	*N-MYC DOWNREGULATED-LIKE 1 (NDL1)*	AGB1/AGG dimmer interacting protein, response to water deficit.
AT5G56760	*SERINE ACETYLTRANSFERASE 1;1 (SERAT1;1)*	Encodes a cytosolic serine O-acetyltransferase involved in sulfur assimilation and cysteine biosynthesis. Expressed in the vascular system.
AT5G56770	*EFFECTOR OF TRANSCRIPTION 3 (ET3)*	transcription repressor-like protein
AT5G56780	*EFFECTOR OF TRANSCRIPTION2 (ET2)*	Encodes a transcriptional regulator that is required for the induction of dormancy during late seed development.ET2 contains DNA and Zinc binding domains and is involved in DNA methylation. ET2 may function in DNA repair.
AT5G56790		Protein kinase superfamily protein
AT5G56800		Protein with RNI-like/FBD-like domain
AT5G56830		transposable_element_gene;copia-like retrotransposon family, has a 8.7e-14 P-value blast match to gb|AAO73521.1| gag-pol polyprotein (Glycine max) (SIRE1) (Ty1_Copia-family);(source:TAIR10)
AT5G56850		hypothetical protein
AT5G56860	*GATA, NITRATE-INDUCIBLE, CARBON METABOLISM-INVOLVED (GNC)*	Encodes a member of the GATA factor family of zinc finger transcription factors. Modulate chlorophyll biosynthesis and glutamate synthase (GLU1/Fd-GOGAT) expression.
AT5G56870	*BETA-GALACTOSIDASE 4 (BGAL4)*	beta-galactosidase 4
AT5G56880		hypothetical protein
AT5G56920		Cystatin/monellin superfamily protein
AT5G56950	*NUCLEOSOME ASSEMBLY PROTEIN 1;3 (NAP1;3)*	Encodes a member of a small gene family of proteins with similarity to nucleosome assembly proteins.May function in nucleotide excision repair. Loss of function mutations have no obvious visible phenotypes but do seem to affect transcription of NER related genes. Plants mutated in three ubiquitously expressed NAP1 genes (NAP1;1~NAP1;3) and organ-specifically expressed NAP1;4 gene show hypersensitivity to genotoxic stresses including UV and DSB-inducing agent Bleomycin. The NAP1 genes act synergistically with NRP genes in promoting somatic homologous recombination.
AT5G56970	*CYTOKININ OXIDASE 3 (CKX3)*	It encodes a protein whose sequence is similar to cytokinin oxidase/dehydrogenase, which catalyzes the degradation of cytokinins.Acts on N6-(2-isopentenyl)adenine 9-riboside.
AT5G56980		Pathogen-associated molecular pattern-induced gene.Responsive to jasmonic acid and wounding.
AT5G57000		DEAD-box ATP-dependent RNA helicase
AT5G57030	*LUTEIN DEFICIENT 2 (LUT2)*	Lutein-deficient 2 (LUT2) required for lutein biosynthesis, member of the xanthophyll class of carotenoids. Encodes lycopene epsilon cyclase
AT5G57050	*ABA INSENSITIVE 2 (ABI2)*	Encodes a protein phosphatase 2C and is involved in ABA signal transduction. Binds fibrillin preprotein in vitro and in vivo.
AT5G57060		60S ribosomal L18a-like protein
AT5G57070		hydroxyproline-rich glycoprotein family protein
AT5G57080		transmembrane protein

AT5G57090	*ETHYLENE INSENSITIVE ROOT 1 (EIR1)*	Encodes an auxin efflux carrier that is similar to bacterial membrane transporters. Root-specific role in the transport of auxin. Acts downstream of CTR1 and ethylene biosynthesis, in the same pathway as EIN2 and AUX1, and independent from EIN3 and EIN5/AIN1 pathway. In the root, the protein localizes apically in epidermal and lateral root cap cells and predominantly basally in cortical cells. Functions may be regulated by phosphorylation status. EIR1 expression is induced by brassinolide treatment in the brassinosteroid-insensitive br1 mutant. Gravistimulation results in asymmetric PIN2 distribution, with more protein degraded at the upper side of the gravistimulated root. Membrane sterol composition is essential for the acquisition of PIN2 polarity. Its expression is downregulated at hypoxic conditions. RAP2.12 overexpression inhibits this downregulation.
AT5G57100		Nucleotide/sugar transporter family protein
AT5G57130	*SMAX1-LIKE 5 (SMXL5)*	Encodes a member of an eight-gene family (SMAX1 and SMAX1-like) that has weak similarity to AtHSP101, a ClpB chaperonin required for thermotolerance.
AT5G57140	*PURPLE ACID PHOSPHATASE 28 (PAP28)*	purple acid phosphatase 28
AT5G57150		basic helix-loop-helix (bHLH) DNA-binding superfamily protein
AT5G57160	*(ATLIG4)*	Encodes the Arabidopsis orthologue of the yeast and mammalian DNA ligase IV. Involved in the repair of DNA damage but, unlike in yeast, not required for T-DNA integration. Interacts with the Arabidopsis homologue of XRCC4.
AT5G57180	*CHLOROPLAST IMPORT APPARATUS 2 (CIA2)*	Transcription regulator responsible for specific upregulation of the translocon genes atToc33 and atToc75 in leaves. Involved in protein import into chloroplast.
AT5G57190	*PHOSPHATIDYLSERINE DECARBOXYLASE 2 (PSD2)*	Encodes the minor form of the two non-mitochondrail phosphatidylserine decarboxylase. The gene expression level is very low. Located at the tonoplast.
AT5G57200	*(PICALM2A)*	ENTH/ANTH/VHS superfamily protein
AT5G57220	*CYTOCHROME P450, FAMILY 81, SUBFAMILY F, POLYPEPTIDE 2 (CYP81F2)*	member of CYP81F, involved in glucosinolate metabolism. Mutants had impaired resistance to fungi. The mRNA is cell-to-cell mobile.
AT5G57230		Thioredoxin superfamily protein
AT5G57240	*OSBP(OXYSTEROL BINDING PROTEIN)-RELATED PROTEIN 4C (ORP4C)*	OSBP(oxysterol binding protein)-related protein 4C
AT5G57250		Pentatricopeptide repeat (PPR) superfamily protein
AT5G57260	*CYTOCHROME P450, FAMILY 71, SUBFAMILY B, POLYPEPTIDE 10 (CYP71B10)*	putative cytochrome P450
AT5G57280	*ROOT INITIATION DEFECTIVE 2 (RID2)*	Gene encodes a methyltransferase-like protein involved in pre-rRNA processing.
AT5G57290	*RIBOSOMAL P3 PROTEIN B (P3B)*	60S acidic ribosomal protein family
AT5G57310		
AT5G57320	*VILLIN 5 (VLN5)*	actin filament bundling protein P-115-ABP protein
AT5G57330		Galactose mutarotase-like superfamily protein
AT5G57340		ras guanine nucleotide exchange factor Q-like protein
AT5G57345	*(ATOXR)*	OXR is a single copy gene in Arabidopsis. It is localized to the ER. It is expressed throughout the plant and expression is induced in response to abiotic stress. While the function of OXR is unknown, overexpression results in increased abiotic stress tolerance and increased ascorbic acid content.
AT5G57350	*H(+)-ATPASE 3 (HA3)*	member of Plasma membrane H+-ATPase family
AT5G57390	*AINTEGUMENTA-LIKE 5 (AIL5)*	Encodes a member of the AP2 family of transcriptional regulators. May be involved in germination and seedling growth. Mutants are resistant to ABA analogs and are resistant to high nitrogen concentrations.essential for the developmental transition between the embryonic and vegetative phases in plants. Overexpression results in the formation of somatic embryos on cotyledons. It is also required to maintain high levels of PIN1 expression at the periphery of the meristem and modulate local auxin production in the central region of the SAM which underlies phyllotactic transitions. Acts redundantly with PLT3 and 7 in lateral root pattern formation.
AT5G57400		transmembrane protein
AT5G57410		Encodes a microtubule-associated protein.
AT5G57420	*INDOLE-3-ACETIC ACID INDUCIBLE 33 (IAA33)*	Belongs to auxin inducible gene family.
AT5G57440	*(GS1)*	A member of haloacid dehalogenase-like hydrolase family, HAD-type phosphosugar phosphatase.
AT5G57450	*HOMOLOG OF X-RAY REPAIR CROSS COMPLEMENTING 3 (XRCC3)*	Involved in homologous recombination and recombinational repair, mutants are sterile, hypersensitive to DNA crosslinking agents, show aberrant meiosis with extensive chromosome fragmentation
AT5G57480		P-loop containing nucleoside triphosphate hydrolases superfamily protein
AT5G57490	*VOLTAGE DEPENDENT ANION CHANNEL 4 (VDAC4)*	Encodes a voltage-dependent anion channel (VDAC: AT3G01280/VDAC1, AT5G67500/VDAC2, AT5G15090/VDAC3, AT5G57490/VDAC4, AT5G15090/VDAC5). VDACs are reported to be porin-type, beta-barrel diffusion pores. They are prominently localized in the outer mitochondrial membrane and are involved in metabolite exchange between the organelle and the cytosol.
AT5G57500		Galactosyltransferase family protein
AT5G57520	*ZINC FINGER PROTEIN 2 (ZFP2)*	Encodes a zinc finger protein containing only a single zinc finger.
AT5G57530	*XYLOGLUCAN ENDOTRANSGLUCOSYLASE/HYDROLASE 12 (XTH12)*	xyloglucan endotransglucosylase/hydrolase 12
AT5G57540	*XYLOGLUCAN ENDOTRANSGLUCOSYLASE/HYDROLASE 13 (XTH13)*	Encodes a xyloglucan endotransglucosylase/hydrolase with only only the endotransglucosylase (XET; EC 2.4.1.207) activity towards xyloglucan and non-detectable endohydrolytic (XEH; EC 3.2.1.151) activity.
AT5G57550	*XYLOGLUCAN ENDOTRANSGLUCOSYLASE/HYDROLASE 25 (XTH25)*	xyloglucan endotransglycosylase-related protein (XTR3)
AT5G57560	*TOUCH 4 (TCH4)*	Encodes a cell wall-modifying enzyme, rapidly upregulated in response to environmental stimuli.
AT5G57570		GCK domain-containing protein
AT5G57580	*(CBP60B)*	Required for the expression of a guardee/decoy or a negative regulator of immunity mediated by receptors carrying an N-terminal Toll-interleukin-1 receptor-like domain.
AT5G57590	*BIOTIN AUXOTROPH 1 (BIO1)*	Encodes a bifunctional enzyme with both dethiobiotin synthetase and diaminopelargonic acid aminotransferase activities that is involved in biotin synthesis.

AT5G57600		
AT5G57610		kinase superfamily with octicosapeptide/Phox/Bem1p domain-containing protein
AT5G57620	*MYB DOMAIN PROTEIN 36 (MYB36)*	MYB36 is a transcriptional regulator that acts to promote differentiation of the endodermis during root development. It promotes the development the Casparian band in part by regulating the expression of genes involved in localizing lignin biosynthetic machinery to the Casparian band. MYB36 binds to and regulates the expression of factors involved in producing the Casparian band including CASP1, PER64, and ESB1.
AT5G57630	*CBL-INTERACTING PROTEIN KINASE 21 (CIPK21)*	CBL-interacting protein kinase.When mutated plants are hypersensitive to salt and osmotic stress.
AT5G57650		eukaryotic translation initiation factor-like protein
AT5G57655		xylose isomerase family protein
AT5G57660	*CONSTANS-LIKE 5 (COL5)*	CONSTANS-like 5
AT5G57670		Protein kinase superfamily protein
AT5G57680		
AT5G57690	*DIACYLGLYCEROL KINASE 4 (DGK4)*	Involved in nitric oxide-dependent pollen tube guidance and fertilization.
AT5G57710	*SUPPRESSOR OF MAX2 1 (SMAX1)*	SMAX1 (SUPPRESSOR OF MAX2 1) is a member of an eight-gene family in Arabidopsis that has weak similarity to AtHSP101, a ClpB chaperonin required for thermotolerance. SMAX1 is an important component of KAR/SL signaling during seed germination and seedling growth, but is not necessary for all MAX2-dependent responses. The mRNA is cell-to-cell mobile.
AT5G57720	*(REM15)*	AP2/B3-like transcriptional factor family protein
AT5G57730		
AT5G57760		hypothetical protein
AT5G57770	*FORKED-LIKE8 (FL8)*	FORKED-LIKE family member, part of Group 2 (Group 1 consists of FKD1, FL1-FL3; Group 2 consists of FL4 and FL8 and Group 3 consists of FL5- FL7). May coordinate leaf size with vein density, where Group 1 members and Group 3 members have opposing functions.
AT5G57780	*P1R1 (P1R1)*	Encodes a atypical member of the bHLH (basic helix-loop-helix) family transcriptional factors.
AT5G57785		hypothetical protein
AT5G57790	*ABORTED GAMETOPHYTE 1 (AOG1)*	Encodes a nuclear localized protein of unknown function that is involved in pollen and embryo sac development.
AT5G57800	*ECERIFERUM 3 (CER3)*	encodes a transmembrane protein with similarity to the sterol desaturase family at the N-terminus and to the short-chain dehydrogenase/reductase family at the C-terminus. Mutant analyses indicate this protein is involved in cuticle membrane and wax biosynthesis. The mRNA is cell-to-cell mobile.
AT5G57810	*TETRASPANIN15 (TET15)*	Member of TETRASPANIN family
AT5G57830	*(MYOB12)*	zein-binding protein (Protein of unknown function, DUF593)
AT5G57840		encodes a protein whose sequence is similar to anthranilate N-hydroxycinnamoyl/benzoyltransferase from Dianthus caryophyllus (gi:2239091)
AT5G57870	*EUKARYOTIC TRANSLATION INITIATION FACTOR ISOFORM 4G1 (eIFiso4G1)*	Encodes a putative eukaryotic translation initiation factor The mRNA is cell-to-cell mobile.
AT5G57900	*SKP1 INTERACTING PARTNER 1 (SKIP1)*	F-box protein, interacts with SKP1/ASK1 subunit of SCF ubiquitin ligase in a glucose-dependent manner
AT5G57920	*EARLY NODULIN-LIKE PROTEIN 10 (ENODL10)*	early nodulin-like protein 10
AT5G57940	*CYCLIC NUCLEOTIDE GATED CHANNEL 5 (CNGC5)*	Encodes a cyclic GMP activated Ca2+-permeable cation channel in the plasma membrane of guard cells. Required for constitutive growth of root hairs as Ca2+-permeable channels.
AT5G57950		26S proteasome regulatory subunit
AT5G57980	*RNA POLYMERASE II FIFTH LARGEST SUBUNIT, C (RPB5C)*	NRPB5-like protein of unknown function; homologous to budding yeast RPB5
AT5G57990	*UBIQUITIN-SPECIFIC PROTEASE 23 (UBP23)*	Encodes a ubiquitin-specific protease.
AT5G58010	*LJRHL1-LIKE 3 (LRL3)*	Encodes a basic helix-loop-helix (bHLH) protein that regulates root hair development. One of the three Arabidopsis homologs of the Lotus japonicus ROOTHAIRLESS1 (LjRHL1) gene: At2g24260 (AtLRL1), At4g30980 (AtLRL2), and At5g58010 (AtLRL3).
AT5G58020	*RTF2 DOMAIN PROTEIN (RTF2)*	Encodes a replication termination factor 2 domain containing protein involved in the regulation of pre-mRNA splicing.
AT5G58050	*SHV3-LIKE 4 (SVL4)*	Encodes a member of the glycerophosphodiester phosphodiesterase like (GDPD-like) family.
AT5G58070	*TEMPERATURE-INDUCED LIPOCALIN (TIL)*	Encodes a temperature-induced lipocalin TIL1. Involved in thermotolerance. Peripherally associated with plasma membrane.
AT5G58080	*RESPONSE REGULATOR 18 (RR18)*	member of Response Regulator: B- Type
AT5G58120	*DANGEROUS MIX 10 (DM10)*	DM10 is a singleton TIR-NLR, and causal QTL responsible for severe hybrid necrosis.
AT5G58130	*REPRESSOR OF SILENCING 3 (ROS3)*	Encodes ROS3 (repressor of silencing 3), a RNA-binding protein required for DNA demethylation.
AT5G58140	*PHOTOTROPIN 2 (PHOT2)*	Membrane-bound protein serine/threonine kinase that functions as blue light photoreceptor in redundancy with PHO1. Involved in stomatal opening, chloroplast movement and phototropism. Mediates blue light-induced growth enhancements. PHOT1 and PHOT2 mediate blue light-dependent activation of the plasma membrane H+-ATPase in guard cell protoplasts. PHOT2 possesses two LOV (LOV1 and LOV2, for light-oxygen-voltage-sensing) domains involved in FMN-binding and a C-terminus forming a serine/threonine kinase domain. LOV2 acts as an inhibitor of phototropin kinase in the dark, and light cancels the inhibition through cysteine-FMN adduct formation. LOV1 in contrast acts as an attenuator of photoactivation. Localized to the Golgi apparatus under the induction of blue light. The mRNA is cell-to-cell mobile.
AT5G58150		Leucine-rich repeat protein kinase family protein
AT5G58160	*FORMIN HOMOLOGY 13 (FH13)*	Class II formin; modulator of pollen tube elongation.
AT5G58170	*SHV3-LIKE 5 (SVL5)*	Encodes a member of the glycerophosphodiester phosphodiesterase like (GDPD-like) family.
AT5G58180	*(ATYKT62)*	member of YKT6 Gene Family
AT5G58200		Calcineurin-like metallo-phosphoesterase superfamily protein
AT5G58210		hydroxyproline-rich glycoprotein family protein
AT5G58250	*EMBRYO DEFECTIVE 3143 (EMB3143)*	Involved in tetrapyrrole biosynthesis. May function as a scaffold protein to stabilize CHL27.
AT5G58260	*NADH DEHYDROGENASE-LIKE COMPLEX N (NdhN)*	Encodes subunit NDH-N of NAD(P)H:plastoquinone dehydrogenase complex (Ndh complex) present in the thylakoid membrane of chloroplasts. This subunit is thought to be required for Ndh complex assembly.

AT5G58280		AP2/B3-like transcriptional factor family protein
AT5G58290	*REGULATORY PARTICLE TRIPLE-A ATPASE 3 (RPT3)*	26S proteasome AAA-ATPase subunit RPT3 (RPT3) mRNA,
AT5G58300		Leucine-rich repeat protein kinase family protein
AT5G58310	*METHYL ESTERASE 18 (MES18)*	Encodes a protein shown to have methyl IAA esterase activity in vitro. This protein does not act on methyl JA, MeSA, MeGA4, or MEGA9 in vitro.
AT5G58330	*NADP-DEPENDENT MALATE DEHYDROGENASE (NADP-MDH)*	lactate/malate dehydrogenase family protein
AT5G58340		myb-like HTH transcriptional regulator family protein
AT5G58350	*WITH NO LYSINE (K) KINASE 4 (WNK4)*	Encodes a member of the WNK family (9 members in all) of protein kinases, the structural design of which is clearly distinct from those of other known protein kinases, such as receptor-like kinases and mitogen-activated protein kinases. Its transcription is under the control of circadian rhythms.
AT5G58360	*OVATE FAMILY PROTEIN 3 (OFP3)*	ovate family protein 3
AT5G58370	*(ENGB-3)*	P-loop containing nucleoside triphosphate hydrolases superfamily protein
AT5G58390		Peroxidase superfamily protein
AT5G58400		Peroxidase superfamily protein
AT5G58440	*SORTING NEXIN 2A (SNX2a)*	sorting nexin 2A
AT5G58460	*CATION/H+ EXCHANGER 25 (CHX25)*	member of Putative Na+/H+ antiporter family
AT5G58470	*TBP-ASSOCIATED FACTOR 15B (TAF15b)*	TBP-associated factor 15B
AT5G58480		O-Glycosyl hydrolases family 17 protein
AT5G58500	*LIGHT SENSITIVE HYPOCOTYLS 5 (LSH5)*	LIGHT-DEPENDENT SHORT HYPOCOTYLS-like protein (DUF640)
AT5G58510		Rab3 GTPase-activating protein catalytic protein
AT5G58520		Protein kinase superfamily protein
AT5G58530		Glutaredoxin family protein
AT5G58540		Protein kinase superfamily protein
AT5G58550	*ETO1-LIKE 2 (EOL2)*	Encodes a paralog of ETO1, which is a negative regulator of ACS5 (a key enzyme in ethylene biosynthesis pathway). EOL2 also interacts with and inhibits the activity of ACS5.
AT5G58560	*FARNESOL KINASE (FOLK)*	FOLK is a farnesol kinase that can phosphorylate farnesol using an NTP donor. It can also phosphorylate geraniol, or geranylgeraniol, but it prefers farnesol in experiments performed using yeast membranes. folk loss-of-function mutants show ABA hypersensitivity in a seed germination assay and the mutants also exhibit abnormal flower development, including extra carpel formation, when subjected to water stress. The mRNA is cell-to-cell mobile.
AT5G58570		transmembrane protein
AT5G58575	*(SGF11)*	Component of the deubiquitination module of the SAGA complex.
AT5G58580	*TOXICOS EN LEVADURA 63 (ATL63)*	Encodes a functional E3 ligase that is involved in membrane trafficking and regulation of salt stress responses. It is localized to membranes including the plasma membrane, pre-vacuolar compartments and Golgi.
AT5G58590	*RAN BINDING PROTEIN 1 (RANBP1)*	Encodes a Ran-binding protein 1 homolog (RanBP1).
AT5G58610		PHD finger transcription factor
AT5G58630	*TON1 RECRUITING MOTIF 31 (TRM31)*	hypothetical protein
AT5G58650	*PLANT PEPTIDE CONTAINING SULFATED TYROSINE 1 (PSY1)*	Encodes PSY1, an18-aa tyrosine-sulfated glycopeptide that promotes cellular proliferation and expansion. PSY1 is widely expressed in various tissues, including shoot apical meristem, and is highly up-regulated by wounding. Perception of PSY1 depends on At1g72300, a leucine-rich repeat receptor kinase (LRR-RK).
AT5G58660	*GIBBERELLIN 2-OXIDASE 9 (GA2OX9)*	Encodes a class III gibberellin 2-oxidase that oxidizes GA12 to GA110 and GA9 to GA40.
AT5G58670	*PHOSPHOLIPASE C1 (PLC1)*	phosphatidylinositol-specific phospholipase C is induced to a significant extent under various environmental stresses, such as dehydration, salinity, and low temperature. May play a role in secondary ABA response. There are two genes called ATPLC1, one corresponding to AT4g38530 and one corresponding ot AT5g58670 (this one).
AT5G58680		ARM repeat superfamily protein
AT5G58690	*PHOSPHATIDYLINOSITOL-SPECIWC PHOSPHOLIPASE C5 (PLC5)*	phosphatidylinositol-speciwc phospholipase C5
AT5G58700	*PHOSPHATIDYLINOSITOL-SPECIFIC PHOSPHOLIPASE C4 (PLC4)*	phosphatidylinositol-speciwc phospholipase C4
AT5G58720		smr (Small MutS Related) domain-containing protein
AT5G58730	*MYO-INOSITOL KINASE (MIK)*	pfkB-like carbohydrate kinase family protein
AT5G58750	*PROGESTERONE 5β-REDUCTASE (PRISE)*	Putative PRISE (progesterone 5β-reductase and/or iridoid synthase-like 1,4-enone reductases).
AT5G58760	*DAMAGED DNA BINDING 2 (DDB2)*	Encodes a DDB1a interacting protein DDB2 required for UV-B tolerance and genomic integrity.
AT5G58770	*CIS-PRENYLTRANSFERASE 4 (CPT4)*	AtCPT7 synthesizes medium-chain polyprenols of approximately 55 carbons in length. The enzyme utlizes geranylgeranyl pyrophosphate (GGPP) and isopentenyl pyrophosphate (IPP) as substrates. The enzymatic product accumulates into plastdial membranes (DOI:10.1105/tpc.16.00796).
AT5G58780	*CIS -PRENYLTRANSFERASE 5 (CPT5)*	Encodes a novel Z,E-mixed heptaprenyl diphosphate (Z,E-HepPP) synthase, which may be responsible for short-chain betulaprenols. It catalyzes the formation of C 35 short-chain polyisoprenoids in which the optimal allylic substrate was E,E-FPP. It may have a role in response to cold stress in root.
AT5G58782	*CIS-PRENYLTRANSFERASE 6 (CPT6)*	Undecaprenyl pyrophosphate synthetase family protein
AT5G58784		Undecaprenyl pyrophosphate synthetase family protein
AT5G58790		hypothetical protein
AT5G58810		pseudogene of Subtilase family protein
AT5G58820		Subtilisin-like serine endopeptidase family protein
AT5G58830		Subtilisin-like serine endopeptidase family protein
AT5G58840		Subtilase family protein

AT5G58850	*MYB DOMAIN PROTEIN 119 (MYB119)*	Encodes a putative transcription factor, member of the R2R3 factor gene family (MYB119).
AT5G58860	*CYTOCHROME P450, FAMILY 86, SUBFAMILY A, POLYPEPTIDE 1 (CYP86A1)*	Encodes a member of the CYP86A subfamily of cytochrome p450 genes. Expressed significantly only in root tissue.
AT5G58870	*FTSH PROTEASE 9 (ftsh9)*	encodes an FtsH protease that is localized to the chloroplast
AT5G58890	*AGAMOUS-LIKE 82 (AGL82)*	AGAMOUS-like 82
AT5G58900	*DIVARICATA1 (DIV1)*	R-R-type MYB protein
AT5G58910	*LACCASE 16 (LAC16)*	putative laccase, a member of laccase family of genes (17 members in Arabidopsis).
AT5G58920		homeobox prospero protein
AT5G58930	*OCTOPUS LIKE 4 (OPL4)*	hypothetical protein (DUF740)
AT5G58960	*GRAVITROPIC IN THE LIGHT (GIL1)*	Mutant plants display impaired light-regulation of the hypocotyl randomization response.
AT5G58970	*UNCOUPLING PROTEIN 2 (UCP2)*	UCP2 and its paralog UCP1 is a member of the PUMP2 family of uncoupling proteins. It functions as a mitochondrial transporter of spartate, glutamate and dicarboxylates.
AT5G58980	*NEUTRAL CERAMIDASE3 (ATNCER3)*	Neutral/alkaline non-lysosomal ceramidase
AT5G59000		RING/FYVE/PHD zinc finger superfamily protein
AT5G59030	*COPPER TRANSPORTER 1 (COPT1)*	encodes a putative copper transport protein that contains copper-binding motif and functionally complements in copper-transport defective yeast strains
AT5G59040	*COPPER TRANSPORTER 3 (COPT3)*	encodes a member of copper transporter family and functionally complements a high affinity copper transporter mutant in yeast
AT5G59050		G patch domain protein
AT5G59060		reverse transcriptase family protein
AT5G59070		UDP-Glycosyltransferase superfamily protein
AT5G59080		hypothetical protein
AT5G59090	*SUBTILASE 4.12 (SBT4.12)*	subtilase 4.12
AT5G59100		Subtilisin-like serine endopeptidase family protein
AT5G59110		subtilisin-like serine protease-like protein
AT5G59120	*SUBTILASE 4.13 (SBT4.13)*	SBT4.13 subtilase. Activity is inhibited by SPI-1.
AT5G59130		Subtilase family protein
AT5G59170		Proline-rich extensin-like family protein
AT5G59190		subtilase family protein
AT5G59200	*ORGANELLE TRANSCRIPT PROCESSING 80 (OTP80)*	Encodes a chloroplast RNA editing factor.
AT5G59220	*HIGHLY ABA-INDUCED PP2C GENE 1 (HAI1)*	Encodes a member of the PP2C family (Clade A protein phosphatases type 2C). Functions as a negative regulator of osmotic stress and ABA signaling.
AT5G59240		Ribosomal protein S8e family protein
AT5G59250	*(HP59)*	Encodes a chloroplast localized H+/glucose antiporter.
AT5G59260	*L-TYPE LECTIN RECEPTOR KINASE II.1 (LECRK-II.1)*	Concanavalin A-like lectin protein kinase family protein
AT5G59270	*L-TYPE LECTIN RECEPTOR KINASE II.2 (LECRK-II.2)*	Concanavalin A-like lectin protein kinase family protein
AT5G59290	*UDP-GLUCURONIC ACID DECARBOXYLASE 3 (UXS3)*	Encodes a cytosolic isoform of UDP-glucuronic acid decarboxylase. This enzyme produces UDP-xylose, which is a substrate for many cell wall carbohydrates including hemicellulose and pectin. UDP-xylose is also known to feedback regulate several cell wall biosynthetic enzymes.
AT5G59305		CLAVATA 3/ESR (CLE)-like protein
AT5G59310	*LIPID TRANSFER PROTEIN 4 (LTP4)*	Encodes a member of the lipid transfer protein family. Proteins of this family are generally small (~9 kD), basic, expressed abundantly and contain eight Cys residues. The proteins can bind fatty acids and acylCoA esters and can transfer several different phospholipids. They are localized to the cell wall. The mRNA is present in flowers and siliques, and is strongly up-regulated by abscisic acid. Predicted to be a member of PR-14 pathogenesis-related protein family with the following members: At2g38540/LTP1, At2g38530/LTP2, At5g59320/LTP3, At5g59310/LTP4, At3g51600/LTP5, At3g08770/LTP6, At2g15050/LTP7, At2g18370/LTP8, At2g15325/LTP9, At5g01870/LTP10, At4g33355/LTP11, At3g51590/LTP12, At5g44265/LTP13, At5g62065/LTP14, At4g08530/LTP15.
AT5G59320	*LIPID TRANSFER PROTEIN 3 (LTP3)*	Predicted to encode a PR (pathogenesis-related) protein. Belongs to the lipid transfer protein (PR-14) family with the following members: At2g38540/LTP1, At2g38530/LTP2, At5g59320/LTP3, At5g59310/LTP4, At3g51600/LTP5, At3g08770/LTP6, At2g15050/LTP7, At2g18370/LTP8, At2g15325/LTP9, At5g01870/LTP10, At4g33355/LTP11, At3g51590/LTP12, At5g44265/LTP13, At5g62065/LTP14, At4g08530/LTP15. The mRNA is cell-to-cell mobile.
AT5G59330		Bifunctional inhibitor/lipid-transfer protein/seed storage 2S albumin superfamily protein
AT5G59340	*WUSCHEL RELATED HOMEOBOX 2 (WOX2)*	Encodes a WUSCHEL-related homeobox gene family member with 65 amino acids in its homeodomain. Proteins in this family contain a sequence of eight residues (TLPLFPMH) downstream of the homeodomain called the WUS box. WOX2 has a putative Zinc finger domain downstream of the homeodomain. Transcripts are expressed in the egg cell, the zygote and the apical cell lineage and are reduced in met3-1 early embryos. This gene is necessary for cell divisions that form the apical embryo domain.
AT5G59350		transmembrane protein
AT5G59360	*(SMR113)*	hypothetical protein
AT5G59380	*METHYL-CPG-BINDING DOMAIN 6 (MBD6)*	Protein containing methyl-CpG-binding domain.Has sequence similarity to human MBD proteins. The mRNA is cell-to-cell mobile.
AT5G59400		PGR5-like A protein
AT5G59430	*TELOMERIC REPEAT BINDING PROTEIN 1 (TRP1)*	Encodes a telomeric repeat binding protein with a DNA binding domain at its C terminus. The DNA binding domain has a preference for GGTTTAG sequences and at least five of these repeats are required for recognition by a nearly full-length TRP1 protein.

AT5G59440	*ZEUS1 (ZEU1)*	Encodes thymidylate kinase which exists in two isoforms in plants. The longer variant of 263 amino acids with a N-terminal extension that is required for localization to the mitochondrion. The second isoform of 224 residues is localized to the cytoplasm and nucleoplasm. Peak of expression occurs during G1/S phase transition.
AT5G59450		GRAS family transcription factor
AT5G59490		Haloacid dehalogenase-like hydrolase (HAD) superfamily protein
AT5G59510	*ROTUNDIFOLIA LIKE 5 (RTFL5)*	ROTUNDIFOLIA like 5
AT5G59515		
AT5G59530		2-oxoglutarate (2OG) and Fe(II)-dependent oxygenase superfamily protein
AT5G59540		2-oxoglutarate (2OG) and Fe(II)-dependent oxygenase superfamily protein
AT5G59580	*UDP-GLUCOSYL TRANSFERASE 76E1 (UGT76E1)*	UDP-glucosyl transferase 76E1
AT5G59590	*UDP-GLUCOSYL TRANSFERASE 76E2 (UGT76E2)*	UDP-glucosyl transferase 76E2
AT5G59600		Tetratricopeptide repeat (TPR)-like superfamily protein
AT5G59610	*DNA J PROTEIN C73 (DJC73)*	Chaperone DnaJ-domain superfamily protein
AT5G59630		pseudogene of serine carboxypeptidase-like 51
AT5G59640		transposable_element_gene;CACTA-like transposase family (En/Spm), has a 5.1e-159 P-value blast match to GB:AAD55677 putative transposase protein (CACTA-element) transposon=Shooter (Zea mays);(source:TAIR10)
AT5G59650		Leucine-rich repeat protein kinase family protein
AT5G59660		Leucine-rich repeat protein kinase family protein
AT5G59670		Leucine-rich repeat protein kinase family protein
AT5G59680		Leucine-rich repeat protein kinase family protein
AT5G59690		Histone superfamily protein
AT5G59700		Protein kinase superfamily protein
AT5G59720	*HEAT SHOCK PROTEIN 18.2 (HSP18.2)*	encodes a low molecular weight heat shock protein that contains the heat shock element in the promoter region. Expression is induced in response to heat shock.
AT5G59730	*EXOCYST SUBUNIT EXO70 FAMILY PROTEIN H7 (EXO70H7)*	A member of EXO70 gene family, putative exocyst subunits, conserved in land plants. Arabidopsis thaliana contains 23 putative EXO70 genes, which can be classified into eight clusters on the phylogenetic tree. The mRNA is cell-to-cell mobile.
AT5G59740		UDP-N-acetylglucosamine (UAA) transporter family
AT5G59760		hypothetical protein (DUF1635)
AT5G59780	*MYB DOMAIN PROTEIN 59 (MYB59)*	Encodes a putative transcription factor (MYB59). In roots it is involved in K+/NO3- transport and expression of the NPF7.3 transporter.
AT5G59800	*METHYL-CPG-BINDING DOMAIN 7 (MBD7)*	Encodes a protein containing a methyl-CpG-binding domain that acts as an anti-silencing factor that prevent gene repression and DNA hypermethylation by tethering other anti-silencing factors to methylated DNA, which enables the function of DNA demethylases that in turn limit DNA methylation and prevent transcriptional gene silencing.
AT5G59810	*(SBT5.4)*	Subtilase family protein
AT5G59820	*RESPONSIVE TO HIGH LIGHT 41 (RHL41)*	Encodes a zinc finger protein involved in high light and cold acclimation. Overexpression of this putative transcription factor increases the expression level of 9 cold-responsive genes and represses the expression level of 15 cold-responsive genes, including CBF genes. Also, lines overexpressing this gene exhibits a small but reproducible increase in freeze tolerance. Because of the repression of the CBF genes by the overexpression of this gene, the authors speculate that this gene may be involved in negative regulatory circuit of the CBF pathway. The mRNA is cell-to-cell mobile.
AT5G59845	*GIBBERELLIC ACID STIMULATED ARABIDOPSIS 10 (GASA10)*	Member of a family of proteins named as being GA inducible but GASA10 does not appear to be GA induced. It is likely to be secreted as the protein is found in the cell wall.
AT5G59860		RNA-binding (RRM/RBD/RNP motifs) family protein
AT5G59940		Cysteine/Histidine-rich C1 domain family protein
AT5G59950	*(ALY1)*	RNA-binding (RRM/RBD/RNP motifs) family protein
AT5G59980	*GAMETOPHYTE DEFECTIVE 1 (GAF1)*	Encodes a predicted protein subunit of RNases P/MRP that plays a role in female gametophyte development and male competence.
AT5G59990		CCT motif family protein
AT5G60010	*RESPIRATORY BURST OXIDASE HOMOLOG H (RBOHH)*	ferric reductase-like transmembrane component family protein
AT5G60020	*LACCASE 17 (LAC17)*	LAC17 appears to have laccase activity based on enzyme assays performed using lac17 mutants. Notably, these mutants appear to have a reduced deposition of G lignin units. LAC17 is expressed in interfascicular fibers and likely contributes to lignin biosynthesis, and hence, cell wall biosynthesis, there.
AT5G60030		hypothetical protein
AT5G60040	*NUCLEAR RNA POLYMERASE C1 (NRPC1)*	Encodes a subunit of RNA polymerase III (aka RNA polymerase C).
AT5G60070		ankyrin repeat family protein
AT5G60080		Protein kinase superfamily protein
AT5G60090		Protein kinase superfamily protein
AT5G60100	*PSEUDO-RESPONSE REGULATOR 3 (PRR3)*	Encodes pseudo-response regulator 3 (APRR3/PRR3). PRR3 transcript levels vary in a circadian pattern with peak expression at dusk under long and short day conditions. PRR3 affects the period of the circadian clock and seedlings with reduced levels of PRR3 have shorter periods, based on transcriptional assays of clock-regulated genes. PRR3 is expressed in the vasculature of cotyledons and leaves where it may help stabilize the TOC1 protein by preventing interactions between TOC1 and the F-box protein ZTL.
AT5G60110	*PUMILIO 18 (PUM18)*	Encodes a member of the Arabidopsis Pumilio (APUM) proteins containing PUF domain (eight repeats of approximately 36 amino acids each). PUF proteins regulate both mRNA stability and translation through sequence-specific binding to the 3' UTR of target mRNA transcripts.

AT5G60120	*TARGET OF EARLY ACTIVATION TAGGED (EAT) 2 (TOE2)*	AP2 family transcription factor that is involved in regulation of flowering and innate immunity.Interacts with CRY1 and CRY2 during flowering as part of a regulatory circuit including FT and CO. TOE1/TOE2 are also targets of MiR172b repression and functions in regulation of innate immunity via repression of FLS.
AT5G60130		AP2/B3-like transcriptional factor family protein
AT5G60140	*(REM11)*	REM-AP2/B3-like transcriptional factor family protein. Functions downstream of STK/SEP3 and with VDD in fertilization to regulate synergid cell death.
AT5G60150		hypothetical protein
AT5G60190		Encodes a protein that can cleave residues from the C-terminus of RUB1 to prepare it for conjugation to target proteins.
AT5G60200	*TARGET OF MONOPTEROS 6 (TMO6)*	Encodes a Dof-type transcription factor. PEAR protein involved in the formation of a short-range concentration gradient that peaks at protophloem sieve elements, and activates gene expression that promotes radial growth. Locally promotes transcription of inhibitory HD-ZIP III genes, and thereby establishes a negative-feedback loop that forms a robust boundary that demarks the zone of cell division.
AT5G60220	*TETRASPANIN4 (TET4)*	Member of TETRASPANIN family
AT5G60230	*SPLICING ENDONUCLEASE 2 (SEN2)*	putative subunit of tRNA splicing endonuclease
AT5G60240		hypothetical protein
AT5G60250		zinc finger (C3HC4-type RING finger) family protein
AT5G60260		hypothetical protein
AT5G60270	*L-TYPE LECTIN RECEPTOR KINASE I.7 (LECRK-I.7)*	Concanavalin A-like lectin protein kinase family protein
AT5G60280	*L-TYPE LECTIN RECEPTOR KINASE I.8 (LECRK-I.8)*	Plasma membrane localized receptor kinase. Binds NAD+ and induces expression of disease resistance genes.
AT5G60290		hypothetical protein
AT5G60300	*L-TYPE LECTIN RECEPTOR KINASE I.9 (LecRK-I.9)*	Encodes a legume-type lectin receptor kinase that is structurally distinct from the mammalian extracellular ATP receptors and acts as an extracellular ATP receptor in Arabidopsis. Extracellular ATP acts as a damage-associated molecular pattern in plants, and its signaling through P2K1 is important for mounting an effective defense response against various pathogenic microorganisms. It also plays a role in cell wall-plasma membrane adhesion.
AT5G60310	*L-TYPE LECTIN RECEPTOR KINASE I.10 (LECRK-I.10)*	Concanavalin A-like lectin protein kinase family protein
AT5G60320	*L-TYPE LECTIN RECEPTOR KINASE I.11 (LECRK-I.11)*	Concanavalin A-like lectin protein kinase family protein
AT5G60340	*ARABIDOPSIS ADENYLATE KINASE 6 (AAK6)*	Encodes a nuclear adenylate kinase that interacts with a putative homolog of Rps14, AtRPS14-1 and affects the elongation of cells in the stem.
AT5G60350		hypothetical protein
AT5G60360	*ALEURAIN-LIKE PROTEASE (ALP)*	Encodes a senescence-associated thiol protease. The mRNA is cell-to-cell mobile.
AT5G60400		hypothetical protein
AT5G60410	*SAP AND MIZ1 DOMAIN- CONTAINING LIGASE1 (SIZ1)*	Encodes a plant small ubiquitin-like modifier (SUMO) E3 ligase that is a focal controller of Pi starvation-dependent responses. Also required for SA and PAD4-mediated R gene signalling, which in turn confers innate immunity in Arabidopsis. Also involved in the regulation of plant growth, drought responses and freezing tolerance. This latter effect is most likely due to SIZ1 dependent ABI5 sumoylation. Regulates leaf cell division and expansion through salicylic acid accumulation. signaling
AT5G60420		
AT5G60440	*AGAMOUS-LIKE 62 (AGL62)*	AGL62 encodes a Type I MADS domain protein that likely functions as a transcription factor. It is expressed AGL62 is expressed exclusively in the endosperm. AGL62 supresses suppresses cellularization during the syncytial phase of endosperm development.
AT5G60450	*AUXIN RESPONSE FACTOR 4 (ARF4)*	Encodes a member of the ARF family of transcription factors which mediate auxin responses. ARF4 appears to have redundant function with ETT(ARF3) in specifying abaxial cell identity.
AT5G60460		Preprotein translocase Sec, Sec61-beta subunit protein
AT5G60470	*EGRET (EGRET)*	C2H2 and C2HC zinc fingers superfamily protein
AT5G60490	*FASCICLIN-LIKE ARABINOGALACTAN-PROTEIN 12 (FLA12)*	Encodes a member of fasciclin-like arabinogalactan proteins (FLAs) containing a cell adhesion fasciclin (FAS) domain. Mutations result in altered stem biomechanics with reduced tensile strength and reduced tensile modulus of elasticity, as well as altered cell wall architecture and composition, with increased cellulose microfibril angle, reduced arabinose, galactose and cellulose content. Possibly involved in embryogenesis and seed development.
AT5G60500	*CIS-PRENYLTRANSFERASE 8 (CPT8)*	Undecaprenyl pyrophosphate synthetase family protein
AT5G60520		Late embryogenesis abundant (LEA) protein-like protein
AT5G60530		Root tip expressed LEA protein involved in ribosome biogenesis.
AT5G60540	*PYRIDOXINE BIOSYNTHESIS 2 (PDX2)*	Encodes a protein predicted to function in tandem with PDX1 to form glutamine amidotransferase complex with involved in vitamin B6 biosynthesis. PDX2 is predicted to function as glutaminase within the complex.
AT5G60570		Galactose oxidase/kelch repeat superfamily protein
AT5G60590		DHBP synthase RibB-like alpha/beta domain-containing protein
AT5G60600	*4-HYDROXY-3-METHYLBUT-2-ENYL DIPHOSPHATE SYNTHASE (HDS)*	Encodes a chloroplast-localized hydroxy-2-methyl-2-(E)-butenyl 4-diphosphate (HMBPP) synthase (HDS), catalyzes the formation of HMBPP from 2-C-methyl-D-erythrytol 2,4-cyclodiphosphate (MEcPP). The HDS enzyme controls the penultimate steps of the biosynthesis of IPP and dimethylallyl diphosphate (DMAPP) via the MEP pathway and may serve as a metabolic control point for SA-mediated disease resistance. In the light, the electrons required for the reaction catalyzed by HDS are directly provided by the electron flow from photosynthesis via ferredoxin. In the dark however, the enzyme requires an electron shuttle: ferredoxin-NADP⁺ reductase. The mRNA is cell-to-cell mobile.
AT5G60610		F-box/RNI-like superfamily protein
AT5G60630		transmembrane protein
AT5G60660	*PLASMA MEMBRANE INTRINSIC PROTEIN 2;4 (PIP2;4)*	A member of the plasma membrane intrinsic protein subfamily PIP2.When expressed in yeast cells can conduct hydrogen peroxide into those cells. Mutants exhibit longer root hairs.
AT5G60680		transcription initiation factor TFIID subunit (Protein of unknown function, DUF584)

AT5G60690	*REVOLUTA (REV)*	REVOLUTA regulates meristem initiation at lateral positions. a member of a small homeodomain-leucine zipper family. Has overlapping functions with PHAVOLUTA and PHABULOSA. The mRNA is cell-to-cell mobile.
AT5G60710		Zinc finger (C3HC4-type RING finger) family protein
AT5G60720		electron transporter, putative (Protein of unknown function, DUF547)
AT5G60730	*GUIDED ENTRY OF TAIL-ANCHORED PROTEINS 3B (GET3C)*	One of 3 GET paralogs in Arabidopsis. GET3c is a mitochondrion localized protein with no obvious role in Tail Anchored (TA) protein insertion.
AT5G60740	*ATP-BINDING CASSETTE G28 (ABCG28)*	ABC transporter family protein. Localizes to the growing tip of pollen tubes where it appears to be critical for localizing polyamines and reactive oxygen species.
AT5G60760		P-loop containing nucleoside triphosphate hydrolases superfamily protein
AT5G60770	*NITRATE TRANSPORTER 2.4 (NRT2.4)*	member of High affinity nitrate transporter family
AT5G60780	*NITRATE TRANSPORTER 2.3 (NRT2.3)*	member of High affinity nitrate transporter family
AT5G60790	*ATP-BINDING CASSETTE F1 (ABCF1)*	Member of GCN subfamily; essential for translation inhibition under cold stress through interacting with GCN2 to phosphorylate eukaryotic translation initiation factor 2. GCN1 regulated gens are involved in flower development, seed dormancy and seed development, response to osmotic stress, amino acid biosynthesis, photosynthesis, cell wall organization, protein transport and localization, lipid biosynthesis, transcription, macroautophagy, proteolysis and cell death.
AT5G60800	*EAVY METAL-ASSOCIATED ISOPRENYLATED PLANT PROTEIN3 (HIPP3)*	Heavy metal transport/detoxification superfamily protein
AT5G60840		hypothetical protein
AT5G60850	*OBF BINDING PROTEIN 4 (OBP4)*	Encodes a zinc finger protein.
AT5G60860	*RAB GTPASE HOMOLOG A1F (RABA1f)*	RAB GTPase homolog A1F
AT5G60870	*RCC1/UVR8/GEF-LIKE 3 (RUG3)*	Encodes a mitochondrial protein RUG3 that is required for accumulation of mitochondrial respiratory chain complex I. RUG3 is related to human REGULATOR OF CHROMOSOME CONDENSATION 1 (RCC1) and Arabidopsis UV-B RESISTANCE 8 (UVR8).
AT5G60880	*BREAKING OF ASYMMETRY IN THE STOMATAL LINEAGE (BASL)*	Encodes BASL (BREAKING OF ASYMMETRY IN THE STOMATAL LINEAGE), a regulator of asymmetric divisions. In asymmetrically dividing stomatal-lineage cells, BASL accumulates in a polarized crescent at the cell periphery before division, and then localizes differentially to the nucleus and a peripheral crescent in self-renewing cells and their sisters after division. Its transcript levels change after inducing MUTE expression in a mute background.
AT5G60890	*MYB DOMAIN PROTEIN 34 (MYB34)*	Myb-like transcription factor that modulates expression of ASA1, a key point of control in the tryptophan pathway; mutant has deregulated expression of ASA1 in dominant allele. Loss of function allele suggests ATR1 also functions at a control point for regulating indole glucosinolate homeostasis.
AT5G60900	*RECEPTOR-LIKE PROTEIN KINASE 1 (RLK1)*	Encodes a receptor-like protein kinase.
AT5G60910	*AGAMOUS-LIKE 8 (AGL8)*	MADS box gene negatively regulated by APETALA1
AT5G60930		P-loop containing nucleoside triphosphate hydrolases superfamily protein
AT5G60950	*COBRA-LIKE PROTEIN 5 PRECURSOR (COBL5)*	COBRA-like protein 5 precursor
AT5G60960	*PPR PROTEIN LOCALIZED TO THE NUCLEUS AND MITOCHONDRIA 1 (PNM1)*	Encodes PNM1 (for PPR protein localized to the nucleus and mitochondria 1), a PPR protein that is dual localized to mitochondria and nuclei. Loss of PNM1 function in mitochondria, but not in nuclei, is lethal for the embryo. In mitochondria, it is associated with polysomes and may play a role in translation.
AT5G60970	*TEOSINTE BRANCHED 1, CYCLOIDEA AND PCF TRANSCRIPTION FACTOR 5 (TCP5)*	TCP gene involved in heterochronic control of leaf differentiation. Transcription factor which controls thermomorphogenesis by positively regulating PIF4 activity.
AT5G60980	*(NTF2)*	Nuclear transport factor 2 (NTF2) family protein with RNA binding (RRM-RBD-RNP motifs) domain-containing protein
AT5G60990	*RNA HELICASE10 (RH10)*	DEA(D/H)-box RNA helicase family protein
AT5G61000	*(RPA70D)*	Replication factor-A protein 1-like protein
AT5G61010	*EXOCYST SUBUNIT EXO70 FAMILY PROTEIN E2 (EXO70E2)*	A member of EXO70 gene family, putative exocyst subunits, conserved in land plants. Arabidopsis thaliana contains 23 putative EXO70 genes, which can be classified into eight clusters on the phylogenetic tree.
AT5G61030	*RNA-BINDING GLYCINE-RICH PROTEIN A7 (RBGA7)*	Encodes a glycine-rich RNA binding protein that is involved in C-> U RNA editing in mitochondria. Gene expression is induced by cold. The mRNA is cell-to-cell mobile.
AT5G61040		hypothetical protein
AT5G61050		histone deacetylase-related / HD-like protein
AT5G61060	*HISTONE DEACETYLASE 5 (HDA05)*	Encodes a member of the histone deacetylase family. Class II RPD3-like family HDAC member which controls negative responses to salinity stress.
AT5G61070	*HISTONE DEACETYLASE OF THE RPD3/HDA1 SUPERFAMILY 18 (HDA18)*	Encodes a protein with similarity to histone deacetylases, a class of chromatin remodeling factors which act on H3/H4 histones. Class II RPD3-like family HDAC member which controls negative responses to salinity stress. Expressed in roots where it appears to regulate the expression of epidermal cell fate genes controlling hair cell differentiation.
AT5G61110	*(PHD4)*	PHD finger-containing protein. Interacts with BDT1, acts with other PHD proteins to associate with flowering genes and thereby suppress their transcription.
AT5G61120	*(PHD6)*	PHD finger-containing protein. Interacts with BDT1, acts with other PHD proteins to associate with flowering genes and thereby suppress their transcription.
AT5G61160	*ANTHOCYANIN 5-AROMATIC ACYLTRANSFERASE 1 (AACT1)*	anthocyanin 5-aromatic acyltransferase 1
AT5G61190		putative endonuclease or glycosyl hydrolase with C2H2-type zinc finger domain-containing protein
AT5G61220	*(ISD11)*	LYR family of Fe/S cluster biogenesis protein
AT5G61230	*ANKYRIN REPEAT PROTEIN 6 (ANK6)*	Encodes ANK6, a mitochondrial ankyrin repeat protein required for male-female gamete recognition.
AT5G61260		Plant calmodulin-binding protein-like protein
AT5G61280		Remorin family protein
AT5G61290		Flavin-binding monooxygenase family protein
AT5G61310		Cytochrome c oxidase subunit Vc family protein

AT5G61330		rRNA processing protein-like protein
AT5G61340		transmembrane protein
AT5G61350	*[CA2+]CYT-ASSOCIATED PROTEIN KINASE 1 (CAP1)*	Encodes a membrane-localized receptor-like kinase that regulates root hair tip growth by maintaining cytoplasmic Ca2+ gradients. Knockouts of CAP1 produced more cytoplasmic NH4+ and ceased growth of root hairs on MS medium except when NH4+ was depleted; NH4+ depletion reestablished the Ca2+ gradient necessary for normal growth. The lower net NH4+ influx across the vacuolar membrane and relatively alkaline cytosolic pH of root hairs in cap1-1 relative to wild type implied that mutation of CAP1 results in more NH4+ accumulation in the cytoplasm. Furthermore, CAP1 functionally complemented npr1 kinase yeast mutant defective in high-affinity NH4+ uptake via MEP2, distinguishing CAP1 as a cytosolic modulator of NH4+ level that participates in NH4+ homeostasis-regulated root hair growth by modulating tip-focused cytoplasmic Ca2+ gradients.
AT5G61360		hypothetical protein
AT5G61370		Pentatricopeptide repeat (PPR) superfamily protein
AT5G61380	*TIMING OF CAB EXPRESSION 1 (TOC1)*	Pseudo response regulator involved in the generation of circadian rhythms. TOC1 appears to shorten the period of circumnutation speed. TOC1 contributes to the plant fitness (carbon fixation, biomass) by influencing the circadian clock period. PRR3 may increase the stability of TOC1 by preventing interactions between TOC1 and the F-box protein ZTL. Expression of TOC1 is correlated with rhythmic changes in chromatin organization. The mRNA is cell-to-cell mobile.
AT5G61400		Pentatricopeptide repeat (PPR) superfamily protein
AT5G61410	*D-RIBULOSE-5-PHOSPHATE-3-EPIMERASE (RPE)*	Arabidopsis thaliana ribulose-5-phosphate-3-epimerase mRNA
AT5G61420	*MYB DOMAIN PROTEIN 28 (MYB28)*	Encodes a nuclear localized member of the MYB transcription factor family. Involved in positive regulation of aliphatic glucosinolate biosynthesis.Expression is induced by touch, wounding and glucose.
AT5G61430	*NAC DOMAIN CONTAINING PROTEIN 100 (NAC100)*	NAC domain containing protein 100
AT5G61440	*ATYPICAL CYS HIS RICH THIOREDOXIN 5 (ACHT5)*	Encodes a member of the thioredoxin family protein. Located in the chloroplast. The mRNA is cell-to-cell mobile.
AT5G61450		P-loop containing nucleoside triphosphate hydrolases superfamily protein
AT5G61460	*HYPERSENSITIVE TO MMS, IRRADIATION AND MMC (MIM)*	Encodes SMC6B (STRUCTURAL MAINTENANCE OF CHROMOSOMES 6B), a component of the SMC5/6 complex. SMC5/6 complex promotes sister chromatid alignment and homologous recombination after DNA damage.
AT5G61470		C2H2-like zinc finger protein
AT5G61480	*PHLOEM INTERCALATED WITH XYLEM (PXY)*	Encodes PXY, a receptor-like kinase essential for maintaining polarity during plant vascular-tissue development.
AT5G61520		Major facilitator superfamily protein
AT5G61530		small G protein family protein / RhoGAP family protein
AT5G61540		N-terminal nucleophile aminohydrolases (Ntn hydrolases) superfamily protein
AT5G61550	*(PUB52)*	U-box domain-containing protein kinase family protein
AT5G61560	*(PUB51)*	Plant U-box type E3 ubiquitin ligase (PUB).
AT5G61570		Protein kinase superfamily protein
AT5G61590	*DECREASE WAX BIOSYNTHESIS (DEWAX)*	Encodes an AP2/ERF-type transcription factor that is preferentially expressed in the epidermis and induced by darkness and negatively regulates cuticular wax biosynthesis.
AT5G61600	*ETHYLENE RESPONSE FACTOR 104 (ERF104)*	encodes a member of the ERF (ethylene response factor) subfamily B-3 of ERF/AP2 transcription factor family. The protein contains one AP2 domain. There are 18 members in this subfamily including ATERF-1, ATERF-2, AND ATERF-5. Involved in regulating root architecture.
AT5G61605	*POLLEN COAT PROTEIN B CLASS ALPHA (PCP-BALPHA)*	EMBRYO SURROUNDING FACTOR-like protein. Expressed in pollen and involved in pollen hydration on the stigma.
AT5G61620		myb-like transcription factor family protein
AT5G61640	*PEPTIDEMETHIONINE SULFOXIDE REDUCTASE 1 (PMSR1)*	ubiquitous enzyme that repairs oxidatively damaged proteins
AT5G61650	*CYCLIN P4;2 (CYCP4;2)*	The P-type cyclins (CYCPs) share a conserved central region of 100 amino acids ('cyclin box') displaying homology to the corresponding region of the PHO80 cyclin from Saccharomyces cerevisiae and the related G1 cyclins from Trypanosoma cruzi and T. brucei.
AT5G61660		glycine-rich protein
AT5G61670	*ARABIDOPSIS THALIANA ORANGE (ATOR)*	Encodes a close homolog of the Cauliflower OR (Orange) protein that is located in the chloroplast of light grown organs but in the nucleus of etiolated cotyledons. The function of OR is to induce the differentiation of proplastids or other noncolored plastids into chromoplasts for carotenoid accumulation. Both proteins contain a Cysteine-rich zinc finger domain that is highly specific to DnaJ-like molecular chaperons. The AtOR protein interacts directly with the PSY (phytoene synthase) protein and acts as a positive posttranscriptional regulator of its expression, thereby affecting carotenoid biosynthesis.
AT5G61700	*ATP-BINDING CASSETTE A12 (ABCA12)*	ABC2 homolog 16
AT5G61710		cotton fiber protein
AT5G61720		hypothetical protein (DUF1216)
AT5G61740	*ATP-BINDING CASSETTE A10 (ABCA10)*	ABC2 homolog 14
AT5G61750		RmlC-like cupins superfamily protein
AT5G61770	*PETER PAN-LIKE PROTEIN (PPAN)*	A single-copy gene encoding a 346 aa protein with a single Brix domain. Similar to yeast ribosome biogenesis proteins Ssf1/2.
AT5G61800		Pentatricopeptide repeat (PPR) superfamily protein
AT5G61820		stress up-regulated Nod 19 protein
AT5G61850	*LEAFY (LFY)*	Encodes transcriptional regulator that promotes the transition to flowering.Involved in floral meristem development. LFY is involved in the regulation of AP3 expression, and appears to bring the F-box protein UFO to the AP3 promoter. Amino acids 46-120 define a protein domain that mediates self-interaction.
AT5G61880	*(PAM16L)*	Encodes PAM16L, a paralog of PAM16 (AT3G59280).

AT5G61890	*(ERF114)*	encodes a member of the ERF (ethylene response factor) subfamily B-4 of ERF/AP2 transcription factor family. The protein contains one AP2 domain. There are 7 members in this subfamily.
AT5G61930	*ACCUMULATION OF PHOTOSYSTEM ONE 3 (APO3)*	ACCUMULATION OF PHOTOSYSTEM ONE 3
AT5G61940		Ubiquitin carboxyl-terminal hydrolase-related protein
AT5G61960	*MEI2-LIKE PROTEIN 1 (ML1)*	A member of mei2-like gene family, predominantly plant-based family of genes encoding RNA binding proteins with characteristic presence of a highly conserved RNA binding motif first described in the mei2 gene of the fission yeast S. pombe. In silico analyses reveal nine mei2 -like genes in A. thaliana. They were grouped into four distinct clades, based on overall sequence similarity and subfamily-specific sequence elements. AML1 is a member of two sister clades of mei2-like gene family, AML1 through AML5 and belongs to the clade named ALM14. AML1 is expressed during early embryo development, particularly along embryonic axis at torpedo stage, in shoot apex (weaker expression) and in the organogenic regions of floral apices.
AT5G61980	*ARF-GAP DOMAIN 1 (AGD1)*	A member of ARF GAP domain (AGD), A thaliana has 15 members, grouped into four classes. AGD1 belongs to the class 1, together with AGD2, AGD3 and AGD4. Not expressed in hypocotyls and cotyledons.
AT5G62000	*AUXIN RESPONSE FACTOR 2 (ARF2)*	Encodes an auxin response factor. Mutants have many defects including enlarged rosette leaves, reduced fertility, later senescence, hypocotyl elongation defects, enlarged seeds and enlarged cotyledons. May not mediate auxin effects. Increase in seed size due to increased cell proliferation. The mRNA is cell-to-cell mobile.
AT5G62010		
AT5G62020	*HEAT SHOCK TRANSCRIPTION FACTOR B2A (HSFB2A)*	member of Heat Stress Transcription Factor (Hsf) family The mRNA is cell-to-cell mobile.
AT5G62030		diphthamide synthesis DPH2 family protein
AT5G62050	*HOMOLOG OF YEAST OXIDASE ASSEMBLY 1 (OXA1) (OXA1)*	essential factor for protein sorting and assembly into membranes
AT5G62080		Putative lipid transfer protein that is expressed in pollen and transmitted to the exine.
AT5G62090	*SEUSS-LIKE 2 (SLK2)*	Encodes a protein that functions with LUH to promote Al binding to the root cell wall.
AT5G62100	*BCL-2-ASSOCIATED ATHANOGENE 2 (BAG2)*	A member of Arabidopsis BAG (Bcl-2-associated athanogene) proteins, plant homologs of mammalian regulators of apoptosis. Plant BAG proteins are multi-functional and remarkably similar to their animal counterparts, as they regulate apoptotic-like processes ranging from pathogen attack, to abiotic stress, to plant development.
AT5G62110		Homeodomain-like superfamily protein
AT5G62120	*RESPONSE REGULATOR 23 (RR23)*	member of Response Regulator: B- Type
AT5G62130		Per1-like family protein
AT5G62140		ATP-dependent Clp protease ATP-binding subunit
AT5G62150		peptidoglycan-binding LysM domain-containing protein
AT5G62160	*ZINC TRANSPORTER 12 PRECURSOR (ZIP12)*	member of Fe(II) transporter isolog family
AT5G62165	*AGAMOUS-LIKE 42 (AGL42)*	Encodes a MADS box transcription factor. Expressed in quiescent center. Involved in floral transition.
AT5G62170	*TON1 RECRUITING MOTIF 25 (TRM25)*	LOW protein: M-phase inducer phosphatase-like protein
AT5G62180	*CARBOXYESTERASE 20 (CXE20)*	Carboxyesterase that binds stringolactones.
AT5G62190	*PLANT RNA HELICASE75 (PRH75)*	Encodes a ATP-dependent RNA unwinding protein targeted to the nucleolus and presumably involved in translation by assisting ribosome maturation. DEAD/DEAH box RNA helicase PRH75
AT5G62210		Embryo-specific protein 3, (ATS3)
AT5G62230	*ERECTA-LIKE 1 (ERL1)*	Encodes a receptor-like kinase that, together with ER and ERL2 governs the initial decision of protodermal cells to either divide proliferatively to produce pavement cells or divide asymmetrically to generate stomatal complexes. It is important for maintaining stomatal stem cell activity and preventing terminal differentiation of the meristemoid into the guard mother cell. Along with erl2 functionally compensates for loss of erecta during integument development. Its transcript levels change after inducing MUTE expression in a mute background.
AT5G62250	*MICROTUBULE-ASSOCIATED PROTEIN 65-9 (MAP65-9)*	microtubule-associated protein 65-9
AT5G62280		DUF1442 family protein (DUF1442)
AT5G62290		nucleotide-sensitive chloride conductance regulator (ICln) family protein
AT5G62310	*INCOMPLETE ROOT HAIR ELONGATION (IRE)*	Encodes a protein with a serine/threonine kinase domain. There are two other closely related members in Arabidopsis. Knock-out mutation results in incomplete root hair elongation. Expression is found all organs examined but is especially strong in elongating root hairs.
AT5G62320	*MYB DOMAIN PROTEIN 99 (MYB99)*	Encodes a putative transcription factor (MYB99).
AT5G62340		Plant invertase/pectin methylesterase inhibitor superfamily protein
AT5G62350		Plant invertase/pectin methylesterase inhibitor superfamily protein
AT5G62360	*PECTIN METHYL-ESTERASE INHIBITOR 13 (PMEI13)*	Pectin methylesterase inhibitor expressed throughout the plant.
AT5G62380	*NAC-DOMAIN PROTEIN 101 (NAC101)*	Encodes a NAC-domain transcription factor involved in xylem formation. Induces transdifferentiation of various cells into metaxylem vessel elements. Located in the nucleus. Expression induced in the presence of auxin, cytokinin and brassinosteroids.
AT5G62390	*BCL-2-ASSOCIATED ATHANOGENE 7 (BAG7)*	A member of Arabidopsis BAG (Bcl-2-associated athanogene) proteins, plant homologs of mammalian regulators of apoptosis. Plant BAG proteins are multi-functional and remarkably similar to their animal counterparts, as they regulate apoptotic-like processes ranging from pathogen attack, to abiotic stress, to plant development. Localized to the ER. Necessary for the proper maintenance of the unfolded protein response during heat and cold tolerance.
AT5G62400		transmembrane protein
AT5G62410	*STRUCTURAL MAINTENANCE OF CHROMOSOMES 2 (SMC2)*	SMC2-1 (SMC2)
AT5G62420		NAD(P)-linked oxidoreductase superfamily protein

AT5G62430	*CYCLING DOF FACTOR 1 (CDF1)*	Dof-type zinc finger domain-containing protein, similar to H-protein promoter binding factor-2a GI:3386546 from (Arabidopsis thaliana). Represses expression of Constans (CO), a circadian regulator of flowering time. Interacts with LKP2 and FKF1. Expression oscillates under constant light conditions. Mainly expressed in the vasculature of cotyledons, leaves and hypocotyls, but also in stomata. Localized to the nucleus and acts as a repressor of CONSTANS through binding to the Dof binding sites in the CO promoter. Protein gets degraded by FKF1 in the afternoon. CDF1 binds to the TOPLESS co-repressor protein through an N-terminal motif which is conserved across CDF-like proteins throughout land-plants. This interaction is important for the repression of CO and FT genes during the morning. Loss of CDF1 dependent repression through omission of TPL coordinating residues or through the loss of TPL function in phloem companion cells results in early flowering due to an up regulation of FT.
AT5G62440	*(DOM1)*	Encodes a protein DOMINO1 that belongs to a plant-specific gene family sharing a common motif present in the tomato DEFECTIVE CHLOROPLASTS AND LEAVES (LeDCL) protein. DOMINO1 is located in the nucleus. Arabidopsis embryos carrying the domino1 mutation grow slowly in comparison with wild type embryos and reach only the globular stage at desiccation. The primary defect of the mutation at the cellular level is the large size of the nucleolus that can be observed soon after fertilization in the nuclei of both the embryo and the endosperm. DOMINO1 might have a role in ribosome biogenesis and in determining the rate of cell division.
AT5G62450		
AT5G62470	*MYB DOMAIN PROTEIN 96 (MYB96)*	Encodes a R2R3 type Myb transcription factor whose expression is strongly induced by abscisic acid. Mediates abscisic acid signaling during drought stress response. Promotes seed fatty acid accumulation.
AT5G62480	*GLUTATHIONE S-TRANSFERASE TAU 9 (GSTU9)*	Encodes glutathione transferase belonging to the tau class of GSTs. Naming convention according to Wagner et al. (2002).
AT5G62490	*HVA22 HOMOLOGUE B (HVA22B)*	Part of the AtHVA22 family. Protein expression is ABA- and stress-inducible.
AT5G62500	*END BINDING PROTEIN 1B (EB1B)*	Encodes a homolog of animal microtubule-end-binding protein. There are two other members of this family. EB1 forms foci at regions where the minus ends of microtubules are gathered during mitosis and early cytokinesis.
AT5G62520	*SIMILAR TO RCD ONE 5 (SRO5)*	Encodes a protein with similarity to RCD1 but without the WWE domain. The protein does have a PARP signature upstream of the C-terminal protein interaction domain. The PARP signature may bind NAD+ and attach the ADP-ribose-moiety from NAD+ to the target molecule. Its presence suggests a role for the protein in ADP ribosylation. Up-regulated by NaCl. SRO5 and P5CDH (an overlapping gene in the antisense orientation) generate 24-nt and 21-nt siRNAs, which together are components of a regulatory loop controlling reactive oxygen species (ROS) production and stress response.
AT5G62530	*ALDEHYDE DEHYDROGENASE 12A1 (ALDH12A1)*	Encodes mitochondrial Delta-pyrroline-5- carboxylate dehydrogenase. Involved in the catabolism of proline to glutamate. Involved in protection from proline toxicity. Induced at pathogen infection sites. P5CDH and SRO5 (an overlapping gene in the sense orientation) generate 24-nt and 21-nt siRNAs, which together are components of a regulatory loop controlling reactive oxygen species (ROS) production and stress response.
AT5G62550		microtubule-associated futsch-like protein
AT5G62580		ARM repeat superfamily protein
AT5G62620	*(GALT6)*	Encodes a Golgi-localized hydroxyproline-O-galactosyltransferase. Mutants display multiple phenotypes including reduced seed coat mucilage and accelerated leaf senescence.
AT5G62630	*HIPL2 PROTEIN PRECURSOR (HIPL2)*	hipl2 protein precursor
AT5G62650		Tic22-like family protein
AT5G62670	*H(+)-ATPASE 11 (HA11)*	H[+]-ATPase 11
AT5G62680	*NRT1/ PTR FAMILY 2.11 (NPF2.11)*	Encodes a high-affinity, proton-dependent glucosinolate-specific transporter that is crucial for the transport of both methionine- and tryptophan-derived glucosinolates to seeds.
AT5G62720	*(ATNITR2;1)*	Integral membrane HPP family protein. Putative nitrate transporter.
AT5G62730		Major facilitator superfamily protein
AT5G62750		hypothetical protein
AT5G62760		P-loop containing nucleoside triphosphate hydrolases superfamily protein
AT5G62770		membrane-associated kinase regulator, putative (DUF1645)
AT5G62790	*1-DEOXY-D-XYLULOSE 5-PHOSPHATE REDUCTOISOMERASE (DXR)*	1-Deoxy-d-xylulose 5-phosphate reductoisomerase (DXR) catalyzes the first committed step of the 2-C-methyl-d-erythritol 4-phosphate pathway for isoprenoid biosynthesis. In Arabidopsis, DXR is encoded by a single-copy gene. Arabidopsis DXR is targeted to plastids and localizes into chloroplasts of leaf cells. DXR knockout or strongly silenced lines have a seedling lethal, albino phenotype. Transgenic, partially silenced lines expressing 35S:DXR have a variegated phenotype.
AT5G62800		Protein with RING/U-box and TRAF-like domain
AT5G62830		F-box associated ubiquitination effector family protein
AT5G62840		Phosphoglycerate mutase family protein
AT5G62850	*VEGETATIVE CELL EXPRESSED1 (AtVEX1)*	Encodes a protein that is expressed in vegetative cells of pollen. A member of the SWEET sucrose efflux transporter family proteins.
AT5G62860		F-box associated ubiquitination effector family protein
AT5G62890		Xanthine/uracil permease family protein
AT5G62900		PADRE protein down-regulated after infection by S. sclerotiorum.
AT5G62920	*RESPONSE REGULATOR 6 (ARR6)*	Encodes a Type-A response regulator that is responsive to cytokinin treatment. Its C-ter domain is very short in comparison to other Arabidopsis ARRs (17 total). Arr6 protein is stabilized by cytokinin.
AT5G62930	*(GGL29)*	Guard-cell-enriched GDSL Lipase family member.
AT5G62940	*HIGH CAMBIAL ACTIVITY2 (HCA2)*	HCA2 induces the formation of interfascicular cambium and regulates vascular tissue development in the aerial parts of the plant. Evidence from both gain of function and dominant negative alleles. PEAR protein involved in the formation of a short-range concentration gradient that peaks at protophloem sieve elements, and activates gene expression that promotes radial growth. Locally promotes transcription of inhibitory HD-ZIP III genes, and thereby establishes a negative-feedback loop that forms a robust boundary that demarks the zone of cell division.
AT5G62960		UDP-N-acetylglucosamine-N-acetylmuramyl-pyrophosphoryl-undecaprenol N-acetylglucosamine protein

AT5G62980	*(FOLB2)*	Encodes an enzyme that can act as a aldolase or an epimerase for 7,8-dihydroneopterin and 7,8-dihydromonapterin in vitro. It is likely to act in folate biosynthesis as a homooctamer in vivo.
AT5G63010		Transducin/WD40 repeat-like superfamily protein
AT5G63020	*SUPPRESSORS OF TOPP4-1 (SUT1)*	Nucleotide binding leucine rich repeat protein of the C-NB-LRR (CNL) type. Involved in TOPP4 mediated immune response.
AT5G63030	*GLUTAREDOXIN C1 (GRXC1)*	Thioredoxin superfamily protein, redox sensor.
AT5G63060	*CHLOROPLAST-LOCALIZED SEC14- LIKE PROTEIN (CPSFL1)*	Sec14p like protein involved in chloroplast vesicle transport. Required for photoauxotrophic growth.
AT5G63070		Ribosomal protein S19 family protein
AT5G63080	*JUMONJI DOMAIN-CONTAINING PROTEIN 20 (JMJ20)*	Encodes a HR demethylase that acts as a positive regulator of seed germination in the PHYB-PIL5-SOM pathway.
AT5G63100		S-adenosyl-L-methionine-dependent methyltransferases superfamily protein
AT5G63130		Octicosapeptide/Phox/Bem1p family protein
AT5G63140	*PURPLE ACID PHOSPHATASE 29 (PAP29)*	purple acid phosphatase 29
AT5G63180		Pectin lyase-like superfamily protein
AT5G63200		tetratricopeptide repeat (TPR)-containing protein
AT5G63290	*(ATHEMN1)*	CPO3 (At5g63290) has not been characterized per se, but is a homolog of the CPDH/HEMN (see review by Layer et al, 2010). The homolog from cyanobacteria was recently characterized to be a CPDH (see Goto et al, 2010), but not yet from plants.
AT5G63310	*NUCLEOSIDE DIPHOSPHATE KINASE 2 (NDPK2)*	Maintains intracellular dNTP levels except ATP. Plays a role in response to oxidative stress and UV. Involved in phytochrome-mediated light signaling. Participates in auxin-regulated processes, partly through the modulation of auxin transport. H-bonding with His-197 inside the nucleotide-binding pocket is critical for NDPK2 functioning.
AT5G63320	*NUCLEAR PROTEIN X1 (NPX1)*	Encodes NPX1 (Nuclear Protein X1), a nuclear factor regulating abscisic acid responses.
AT5G63340		hypothetical protein
AT5G63360		
AT5G63370	*CYCLIN-DEPENDENT KINASE G1 (CDKG1)*	CDKG1 interacts with the splicing factor RSZ33 to regulate proper splicing of Cals5 Pre-mRNA.
AT5G63380		Encodes a peroxisomal protein involved in the activation of fatty acids through esterification with CoA. At5g63380 preferentially activates fatty acids with increased chain length (C9:0 to C8:0) and thus shares characteristics with long-chain fatty acyl-CoA synthases. Also able to catalyze the conversion of OPDA to its CoA ester and is therefore thought to be involved in the peroxisomal β-oxidation steps of jasmonic acid biosynthesis.
AT5G63390		O-fucosyltransferase family protein
AT5G63420	*EMBRYO DEFECTIVE 2746 (emb2746)*	Encodes a member of the metallo-beta-lactamase protein family that plays a vital role in embryo morphogenesis and apical-basal pattern formation by regulating chloroplast development. In bacteria, RNase J plays an important role in rRNA maturation and in the 5′ stability of mRNA.
AT5G63450	*CYTOCHROME P450, FAMILY 94, SUBFAMILY B, POLYPEPTIDE 1 (CYP94B1)*	AtWRKY33 regulates root apoplastic barrier formation by controlling AtCYP94B1 leading to increased salt tolerance of Arabidopsis plants. Regulation by WRKY33 to control apoplastic barrier formation in roots to confer salt tolerance.
AT5G63470	*NUCLEAR FACTOR Y, SUBUNIT C4 (NF-YC4)*	Encodes a member of class of transcription regulators that his highly conserved across many plant species.In Arabidopsis and rice, NF-YC4 interacts with other members of this class and CO to regulate flowering. In Arabidopsis, it interacts with QQS to regulate C/N partitioning.
AT5G63520		F-box/LRR protein
AT5G63530	*FARNESYLATED PROTEIN 3 (FP3)*	Farnesylated protein that binds metals.
AT5G63560	*FATTY ALCOHOL:CAFFEOYL-COA CAFFEOYL TRANSFERASE (FACT)*	HXXXD-type acyl-transferase family protein
AT5G63580	*FLAVONOL SYNTHASE 2 (FLS2)*	encodes a protein whose sequence is similar to flavonol synthase
AT5G63590	*FLAVONOL SYNTHASE 3 (FLS3)*	flavonol synthase 3
AT5G63600	*FLAVONOL SYNTHASE 5 (FLS5)*	encodes a protein whose sequence is similar to flavonol synthase
AT5G63620	*HEXENAL RESPONSE 2 (HER2)*	Encodes an oxidoreductase involved in transducing the perception of E-2-hexenal, which changes the redox status of the mitochondria.
AT5G63650	*SNF1-RELATED PROTEIN KINASE 2.5 (SNRK2.5)*	encodes a member of SNF1-related protein kinases (SnRK2) whose activity is activated by ionic (salt) and non-ionic (mannitol) osmotic stress.
AT5G63660	*(PDF2.5)*	Predicted to encode a PR (pathogenesis-related) protein. Belongs to the plant defensin (PDF) family with the following members: At1g75830/PDF1.1, At5g44420/PDF1.2a, At2g26020/PDF1.2b, At5g44430/PDF1.2c, At2g26010/PDF1.3, At1g19610/PDF1.4, At1g55010/PDF1.5, At2g02120/PDF2.1, At2g02100/PDF2.2, At2g02130/PDF2.3, At1g61070/PDF2.4, At5g63660/PDF2.5, At2g02140/PDF2.6, At5g38330/PDF3.1 and At4g30070/PDF3.2.
AT5G63680		Pyruvate kinase family protein
AT5G63700		zinc ion binding / DNA binding protein
AT5G63710		Leucine-rich repeat protein kinase family protein
AT5G63720	*KOKOPELLI (KPL)*	Encodes KOKOPELLI (KPL). kokopelli (kpl) mutants display frequent single-fertilization events indicating that KPL is involved in double fertilization. KPL and an inversely transcribed gene, ARIADNE14 (ARI14), which encodes a putative ubiquitin E3 ligase, generate a sperm-specific natural cis-antisense siRNA pair. In the absence of KPL, ARI14 RNA levels in sperm are increased and fertilization is impaired.
AT5G63740		A paternally expressed imprinted gene.
AT5G63750	*ARIADNE 13 (ARI13)*	RING/U-box superfamily protein
AT5G63760	*ARIADNE 15 (ARI15)*	RING/U-box superfamily protein
AT5G63770	*DIACYLGLYCEROL KINASE 2 (DGK2)*	a member of the diacylglycerol kinase gene family. Encodes a functional diacylglycerol kinase. Involved in root elongation and plant development. Gene expression is induced by wounding or cold.
AT5G63790	*NAC DOMAIN CONTAINING PROTEIN 102 (NAC102)*	Encodes a member of the NAC family of transcription factors. ANAC102 appears to have a role in mediating response to low oxygen stress (hypoxia) in germinating seedlings. Its expression can be induced by beta-cyclocitral, an oxidized by-product of beta-carotene generated in the chloroplasts, mediates a protective retrograde response that lowers the levels of toxic peroxides and carbonyls, limiting damage to intracellular components.

AT5G63800	*MUCILAGE-MODIFIED 2 (MUM2)*	Involved in mucilage formation. Mutants form columella and outer cell wall architecture of the mucilage cells resembles wild-type. However, mum2 seeds completely lack seed coat mucilage. This mutation appears to represent a later step in the development of this cell-type. Encodes a beta-galactosidase involved in seed coat mucilage biosynthesis. Member of Glycoside Hydrolase Family 35
AT5G63810	*BETA-GALACTOSIDASE 10 (BGAL10)*	member of Glycoside Hydrolase Family 35
AT5G63820		hypothetical protein (DUF626)
AT5G63830		HIT-type Zinc finger family protein
AT5G63850	*AMINO ACID PERMEASE 4 (AAP4)*	Amino acid transporter whose expression is downregulated by dehydration.
AT5G63860	*UVB-RESISTANCE 8 (UVR8)*	UV-B-specific signaling component that orchestrates expression of a range of genes with vital UV-protective functions. Located in the nucleus and the cytosol. Associates with chromatin via histones. UV-B light promotes URV8 protein accumulation in the nucleus. UVR8 interaction with COP1 is negatively regulated by RUP1 and RUP2.
AT5G63880	*(VPS20.1)*	SNF7 family protein
AT5G63900		Acyl-CoA N-acyltransferase with RING/FYVE/PHD-type zinc finger domain-containing protein
AT5G63905		transmembrane protein
AT5G63930		Leucine-rich repeat protein kinase family protein
AT5G63940		kinase with adenine nucleotide alpha hydrolases-like domain-containing protein
AT5G63950	*CHROMATIN REMODELING 24 (CHR24)*	chromatin remodeling 24
AT5G63970	*RING DOMAIN LIGASE 3 (RGLG3)*	Encodes a ubiquitin ligase that is an essential upstream modulator of JA signaling in response to various stimuli.
AT5G63990		Inositol monophosphatase family protein
AT5G64000	*(SAL2)*	3'(2'),5'-bisphosphate nucleotidase
AT5G64040	*(PSAN)*	Encodes the only subunit of photosystem I located entirely in the thylakoid lumen. May be involved in the interaction between plastocyanin and the photosystem I complex. Phosphorylation of this protein is dependent on calcium.
AT5G64050	*GLUTAMATE TRNA SYNTHETASE (ERS)*	Glutamate-tRNA ligase. Targeted to mitochondria and chloroplast. Its inactivation causes developmental arrest of chloroplasts and mitochondria in Nicotiana benthamiana.
AT5G64060	*NAC DOMAIN CONTAINING PROTEIN 103 (NAC103)*	NAC domain containing protein 103
AT5G64070	*PHOSPHATIDYLINOSITOL 4-OH KINASE BETA1 (PI-4KBETA1)*	Encodes a phosphatidylinositol 4-OH kinase, PI-4Kbeta1. Arabidopsis contains 12 PI-4Ks in three separate families: PI-4Kalphs, PI-4kbeta, and PI-4Kgamma. PI-4Kbeta1 is 83% identical to PI-4kbeta2 encoded by At5g09350. Interacts with the RabA4b GTPase. Important for polarized root hair growth as the loss of this gene and its close relative PI-4kbeta2, leads to the formation of abnormal root hairs.
AT5G64080	*XYLOGEN PROTEIN 1 (XYP1)*	Bifunctional inhibitor/lipid-transfer protein/seed storage 2S albumin superfamily protein
AT5G64100	*(PRX69)*	Class III peroxidase cell wall-targeted protein localized to the micropylar endosperm facing the radicle. Involved in seed germination.
AT5G64110		Peroxidase superfamily protein
AT5G64120	*PEROXIDASE 71 (PRX71)*	Encodes a cell wall bound peroxidase that is induced by hypo-osmolarity and is involved in the lignification of cell walls. Class III peroxidase cell wall-targeted protein localized to the micropylar endosperm facing the radicle. Involved in seed germination.
AT5G64160		plant/protein
AT5G64170	*NIGHT LIGHT-INDUCIBLE AND CLOCK-REGULATED GENE 1 (LNK1)*	LNK1 is a member of a small family (4 proteins) in Arabidopsis that have some overlap in function. LNK1 functions in the integration of light signaling and circadian clock. It is regulated by the clock TOC1 complex.Functions as a transcriptional coactivator.
AT5G64180		tropomyosin
AT5G64190		neuronal PAS domain protein
AT5G64210	*ALTERNATIVE OXIDASE 2 (AOX2)*	encodes an isoform of alternative oxidase, which is expressed in rosettes, stems, and roots. Transcript accumulates in dry seeds and decreased upon germination and is not affected by actinomycin A. Protein is localized to mitochondria.
AT5G64220	*CALMODULIN-BINDING TRANSCRIPTION ACTIVATOR 2 (CAMTA2)*	CAMTA2 proteins bind to the AtALMT1 promoter at in vitro. The gene itself is Al inducible, and AtALMT1 expression is partially repressed in camta2 mutant. The mRNA is cell-to-cell mobile.
AT5G64230		1,8-cineole synthase
AT5G64240	*METACASPASE 3 (MC3)*	Encodes a type I metacaspase. Two Arabidopsis metacaspases, AT1G02170 (MC1) and AT4G25110 (MC2) antagonistically control programmed cell death in Arabidopsis. MC1 is a positive regulator of cell death and requires conserved caspase-like putative catalytic residues for its function. MC2 negatively regulates cell death. This function is independent of the putative catalytic residues. A third type I Arabidopsis metacaspase is MC3 (AT5g64240).
AT5G64250		Aldolase-type TIM barrel family protein
AT5G64260	*EXORDIUM LIKE 2 (EXL2)*	EXORDIUM like 2
AT5G64290	*DICARBOXYLATE TRANSPORT 2.1 (DIT2.1)*	dicarboxylate transport 2.1
AT5G64300	*GTP CYCLOHYDROLASE II (GCH)*	encodes GTP cyclohydrolase II that can functionally complement E. coli mutant deficient in this gene. It also has 3,4-dihydroxy-2-butanone-4-phosphate synthase activity which makes it a bifunctional enzyme involved in the formation of the pyrimidine and of the carbohydrate from GTP and ribulose-5-phosphate, respectively The mRNA is cell-to-cell mobile.
AT5G64310	*ARABINOGALACTAN PROTEIN 1 (AGP1)*	Encodes arabinogalactan-protein (AGP1). The mRNA is cell-to-cell mobile.
AT5G64330	*NON-PHOTOTROPIC HYPOCOTYL 3 (NPH3)*	Involved in blue light response signaling pathway; interacts with the blue light photoreceptor NPH1. Null mutations abolish phototrophic responses of etiolated seedlings to low fluence blue light. Protein contains multiple protein-protein interaction domains.
AT5G64340	*SUPPRESSOR OF ACAULIS 51 (SAC51)*	Encodes a bHLH(basic helix-loop-helix)-type transcription factor SAC51 [suppressor of acaulis 51]. Upregulation of SAC51 reverses the dwarf phenotype caused by a loss-of-function mutation in ACL5 (Arabidopsis thaliana ACAULIS 5) gene, suggesting that activation of SAC51 may lead to the expression of a subset of genes required for stem elongation.
AT5G64360	*EMF1-INTERACTING PROTEIN 9 (EIP9)*	EIP9 interacts with EMF1 to regulate flowering. It functions partially redundantly with SDJ2 and SDJ3 and interacts with SUVH1 and SUVH3 to form a SUVH-SDJ complex. The complex binds promoters with DNA methylation and mediates transcriptional activation of promoter methylated genes.

AT5G64380		Inositol monophosphatase family protein
AT5G64410	*OLIGOPEPTIDE TRANSPORTER 4 (OPT4)*	oligopeptide transporter
AT5G64420		DNA polymerase V family
AT5G64440	*FATTY ACID AMIDE HYDROLASE (FAAH)*	AtFAAH (fatty acid amide hydrolase) modulates endogenous NAEs (N-Acylethanolamines) levels in plants by hydrolyzing NAEs to ethanolamine and their corresponding free fatty acids. NAE depletion likely participates in the regulation of plant growth. The mRNA is cell-to-cell mobile.
AT5G64450		NYN domain protein
AT5G64460		Phosphoglycerate mutase family protein
AT5G64490	*PUMILIO HOMOLOG 26 (APUM26)*	ARM repeat superfamily protein
AT5G64510	*TUNICAMYCIN INDUCED 1 (TIN1)*	Encodes Tunicamycin Induced 1(TIN1), a plant-specic ER stress-inducible protein. TIN1 mutation affects pollen surface morphology. Transcriptionally induced by treatment with the N-linked glyclsylation inhibitor tunicamycin.
AT5G64530	*XYLEM NAC DOMAIN 1 (XND1)*	xylem NAC domain 1
AT5G64550		loricrin-like protein
AT5G64560	*MAGNESIUM TRANSPORTER 9 (MGT9)*	Transmembrane magnesium transporter that is located in plasma membrane of microspores to take up Mg from the locule. One of 9 family members.
AT5G64570	*BETA-D-XYLOSIDASE 4 (XYL4)*	Encodes a beta-d-xylosidase that belongs to family 3 of glycoside hydrolases.
AT5G64590		NYN domain protein
AT5G64600		O-fucosyltransferase family protein
AT5G64620	*CELL WALL / VACUOLAR INHIBITOR OF FRUCTOSIDASE 2 (C/VIF2)*	Plant cell wall (CWI) and vacuolar invertases (VI) play important roles in carbohydrate metabolism, stress responses and sugar signaling.
AT5G64640		Plant invertase/pectin methylesterase inhibitor superfamily
AT5G64660	*CYS, MET, PRO, AND GLY PROTEIN 2 (CMPG2)*	CYS, MET, PRO, and GLY protein 2
AT5G64680		mediator-associated protein
AT5G64690		neurofilament triplet H protein-like protein
AT5G64700	*USUALLY MULTIPLE ACIDS MOVE IN AND OUT TRANSPORTERS 21 (UMAMIT21)*	nodulin MtN21-like transporter family protein
AT5G64710	*MITOCHONDRIAL NUCLEASE 1 (MNU1)*	Putative endonuclease or glycosyl hydrolase
AT5G64720	*EGG CELL 1.5 (EC1.5)*	Encodes a small cysteine-rich protein that is secreted by the egg cell during gamete interactions. The regulated secretion of EC1 by the egg cell upon sperm-egg interaction is proposed to ensure the appropriate localization of the cell-fusion machinery in distinct sperm membrane domains to accomplish gamete fusion.
AT5G64740	*CELLULOSE SYNTHASE 6 (CESA6)*	Encodes a cellulose synthase isomer. CESA6 mutants have cellulose defect in the primary cell wall. Multiple lines of evidence suggest that CESA6, along with CESA1 and CESA3 are present in the same plasma membrane complex for cellulose biosynthesis. CESA2 and CESA5 are related to CESA6, having partially redundant roles. As inferred from the null role of secondary wall-type CesAs, included in a set of five primary wall-type CesAs that may support trichome cell wall thickening. The mRNA is cell-to-cell mobile.
AT5G64760	*REGULATORY PARTICLE NON-ATPASE SUBUNIT 5B (RPN5B)*	Encodes one of two isoforms for the 26S proteasome regulatory protein (RN) subunit RPN5. For many functions it acts redundantly with the paralogous genes RPN5a.
AT5G64770	*ROOT MERISTEM GROWTH FACTOR 9 (RGF9)*	Encodes a root meristem growth factor (RGF). Belongs to a family of functionally redundant homologous peptides that are secreted, tyrosine-sulfated, and expressed mainly in the stem cell area and the innermost layer of central columella cells. RGFs are required for maintenance of the root stem cell niche and transit amplifying cell proliferation. Members of this family include: At5g60810 (RGF1), At1g13620 (RGF2), At2g04025 (RGF3), At3g30350 (RGF4), At5g51451 (RGF5), At4g16515 (RGF6), At3g02240 (RGF7), At2g03830 (RGF8) and At5g64770 (RGF9).
AT5G64780		holocarboxylase synthetase
AT5G64790		O-Glycosyl hydrolases family 17 protein
AT5G64800	*CLAVATA3/ESR-RELATED 21 (CLE21)*	Member of a large family of putative ligands homologous to the Clavata3 gene. Consists of a single exon.
AT5G64813	*LIGHT INSENSITIVE PERIOD1 (LIP1)*	The LIP1 gene encodes a small GTPase that influences the light input pathway of the plant circadian network. An MBP:LIP1 fusion protein has GTP hydrolyzing abilities in vitro. In plants, LIP1 seems to play a negative role in regulating circadian period that can be suppressed by light. LIP1 also seems to negatively affect light-pulse-dependent resetting of the clock, especially during the first portion of the subjective evening. LIP1 expression levels are not significantly affected by the circadian clock in seedlings grown under LL conditions. The levels of the YFP:LIP1 protein expressed under the control of the 35S promoter, shows a low amplitude variation, with protein levels peaking near the beginning of subjective night under LL conditions. In hypocotyl epidermal cells of dark and light-grown seedlings, a YFP:LIP1 fusion protein can be seen in the cytoplasm and the nucleus, and does not cluster in nuclear speckles. LIP1 may also be involved in photomorphogenesis. The mRNA is cell-to-cell mobile.
AT5G64850		sorbin/SH3 domain protein
AT5G64870	*FLOTILLIN3 (FLOT3)*	Belongs to the group of plant flotillins, which are plasma membrane proteins. Flot3 is found in membrane nanodomains.
AT5G64900	*PRECURSOR OF PEPTIDE 1 (PROPEP1)*	Encodes a putative 92-aa protein that is the precursor of AtPep1, a 23-aa peptide which activates transcription of the defensive gene defensin (PDF1.2) and activates the synthesis of H2O2, both being components of the innate immune response.
AT5G64905	*ELICITOR PEPTIDE 3 PRECURSOR (PROPEP3)*	elicitor peptide 3 precursor
AT5G64940	*ABC2 HOMOLOG 13 (ATH13)*	ABC1K8 is a member of an atypical protein kinase family that is induced by heavy metals. Loss of function mutations affect the metabolic profile of chloroplast lipids. It appears to function along with ABC1K7 in mediating lipid membrane changes in response to stress. The mRNA is cell-to-cell mobile.
AT5G64980		transcription factor
AT5G64990	*RAB GTPASE HOMOLOG H1A (RABH1a)*	RAB GTPase homolog H1A
AT5G65020	*ANNEXIN 2 (ANNAT2)*	Annexins are calcium binding proteins that are localized in the cytoplasm. When cytosolic Ca2+ increases, they relocate to the plasma membrane. They may be involved in the Golgi-mediated secretion of polysaccharides.
AT5G65040	*INCREASED RESISTANCE TO MYZUS PERSICAE 1 (IRM1)*	senescence-associated family protein (DUF581)

AT5G65050	*AGAMOUS-LIKE 31 (AGL31)*	Originally published as Agamous like MADS-box protein AGL31. One of a group of MADS box genes involved in control of flowering time. Four variant sequences have been identified for this locus but have not been characterized for differences in expression pattern and/or function.
AT5G65070	*MADS AFFECTING FLOWERING 4 (MAF4)*	Encodes MADS-box containing FLC paralog. Five splice variants have been identified but not characterized with respect to expression patterns and/or differing function. Overexpression of the gene in the Landsberg ecotype leads to a delay in flowering, transcript levels of MAF4 are reduced after a 6 week vernalization.
AT5G65080	*MADS AFFECTING FLOWERING 5 (MAF5)*	Is upregulated during vernalization and regulates flowering time. Encodes MADS-domain protein. Two variants encoding proteins of 198 and 184 amino acids have been reported.
AT5G65090	*BRISTLED 1 (BST1)*	Encodes a protein involved in root hair morphogenesis and tip growth. Required for restricting both the size of the root-hair initiation site and the width of the root hairs during the transition to tip growth, but, apparently, is not required for normal subsequent tip growth.
AT5G65100		Ethylene insensitive 3 family protein
AT5G65110	*ACYL-COA OXIDASE 2 (ACX2)*	Encodes an acyl-CoA oxidase presumably involved in long chain fatty acid biosynthesis.
AT5G65130	*(WIND4)*	encodes a member of the DREB subfamily A-6 of ERF/AP2 transcription factor family. The protein contains one AP2 domain. There are 8 members in this subfamily including RAP2.4.
AT5G65140	*TREHALOSE-6-PHOSPHATE PHOSPHATASE J (TPPJ)*	Haloacid dehalogenase-like hydrolase (HAD) superfamily protein
AT5G65160	*TETRATRICOPEPTIDE REPEAT 14 (TPR14)*	Encodes one of the 36 carboxylate clamp (CC)-tetratricopeptide repeat (TPR) proteins (Prasad 2010, Pubmed ID: 20856808) with potential to interact with Hsp90/Hsp70 as co-chaperones.
AT5G65170		VQ motif-containing protein
AT5G65210	*TGACG SEQUENCE-SPECIFIC BINDING PROTEIN 1 (TGA1)*	Encodes TGA1, a redox-controlled regulator of systemic acquired resistance. TGA1 targets the activation sequence-1 (as-1) element of the promoter region of defense proteins. TGA1 are S-nitrosylated.
AT5G65220	*PLASTID RIBOSOMAL PROTEINS OF THE 50S SUBUNIT 29 (PRPL29)*	Ribosomal L29 family protein
AT5G65230	*MYB DOMAIN PROTEIN 53 (MYB53)*	Member of the R2R3 factor gene family.
AT5G65240		Leucine-rich repeat protein kinase family protein
AT5G65260		RNA-binding (RRM/RBD/RNP motifs) family protein
AT5G65280	*GCR2-LIKE 1 (GCL1)*	Encodes a protein with reported similarity to GCR2 a putative G protein coupled receptor thought to be an ABA receptor. Loss of function mutations in GCL1 show no ABA response defects based on assays of seed germination and seedling development.GCL1 also has similarity to LANCL1 and LANCL2, human homologs of bacterial lanthionine synthetase.
AT5G65300	*(SUPA)*	Gene of unknown function. Expression is induced by a variety of biotic (P. syringae) and abiotic stresses (salt, ABA,IAA, and more.)Member of a small family that includes AT1G35210, AT1G72240, and AT1G22470.Mutants have no obvious loss of function phenotype but overexpressors are early flowering.
AT5G65310	*HOMEOBOX PROTEIN 5 (HB5)*	Encodes a class I HDZip (homeodomain-leucine zipper) protein that is a positive regulator of ABA-responsiveness, mediating the inhibitory effect of ABA on growth during seedling establishment.
AT5G65350	*HISTONE 3 11 (HTR11)*	histone 3 11
AT5G65360	*HISTONE 3.1 (H3.1)*	Histone superfamily protein
AT5G65370	*(PICALM10C)*	ENTH/ANTH/VHS superfamily protein
AT5G65390	*ARABINOGALACTAN PROTEIN 7 (AGP7)*	arabinogalactan protein 7
AT5G65410	*HOMEOBOX PROTEIN 25 (HB25)*	Encodes ZFHD2, a member of the zinc finger homeodomain transcriptional factor family.Gain of function of ATHB25 (35S and UBQ10 proomoters) and double loss of function of ATHB25 and ATHB22 increases and decreases, respectively, seed longevity. This phenotype is maternal and related to seed coat alterations. Gain of function increases expression of GA3OX2 and GA4 and GA1 levels. Together with REM7 induces the expression of genes controlling shoot stem characteristics by ectopic expression in roots.
AT5G65420	*CYCLIN D4;1 (CYCD4;1)*	Encodes a D-type cyclin CYCD4;1 that physically interacts with CDC2A and is expressed during vascular tissue development, embryogenesis, and formation of lateral root primordia. Its expression is upregulated early during germination.Involved in stomatal cell lineage proliferation in the hypocotyl.
AT5G65450	*UBIQUITIN-SPECIFIC PROTEASE 17 (UBP17)*	Encodes a ubiquitin-specific protease. The mRNA is cell-to-cell mobile.
AT5G65460	*KINESIN LIKE PROTEIN FOR ACTIN BASED CHLOROPLAST MOVEMENT 2 (KAC2)*	Kinesin that binds cyclin-dependent kinase CDKA;1 as homodimer or as heterodimer with KCA1
AT5G65470		O-fucosyltransferase family protein
AT5G65480	*CLAVATA COMPLEX INTERACTOR 1 (CCI1)*	CCL1 is induced by WUS and binds to the kinase domains of BAM1 and CLV1. Localizes to lipid rich plasma membrane rafts. Likely to be involved in WUS/CLV signaling pathway.
AT5G65500	*(PUB50)*	Plant U-box type E3 ubiquitin ligase (PUB).
AT5G65530	*ARABIDOPSIS RECEPTOR-LIKE CYTOPLASMIC KINASE ATRLCK VI_A3 (ATRLCK VI_A3)*	Encodes a protein kinase involved in mediating resistance to fungi and also trichome branch number. Kinase activity is increased by ROP6 which also affects its sub-cellular localization (becomes localized to the cell periphery_
AT5G65550		UDP-Glycosyltransferase superfamily protein
AT5G65580		transmembrane protein
AT5G65590	*STOMATAL CARPENTER 1 (SCAP1)*	Encodes a plant-specific Dof-type transcription factor expressed in maturing guard cells, but not in guard mother cells. It regulates essential processes of stomatal guard cell maturation and functions as a key transcription factor regulating the final stages of guard cell differentiation.
AT5G65600	*L-TYPE LECTIN RECEPTOR KINASE IX.2 (LECRK-IX.2)*	L-type lectin receptor kinase which modulates metabolites and abiotic stress responses. Phosphorylates AvrPtoB which in turn reduces its virulence.
AT5G65610		hypothetical protein
AT5G65630	*GLOBAL TRANSCRIPTION FACTOR GROUP E7 (GTE7)*	This gene is predicted to encode a bromodomain-containing protein. Plant lines expressing RNAi constructs targeted against GTE7 show some resistance to agrobacterium-mediated root transformation.
AT5G65660		hydroxyproline-rich glycoprotein family protein
AT5G65670	*INDOLE-3-ACETIC ACID INDUCIBLE 9 (IAA9)*	auxin (indole-3-acetic acid) induced gene The mRNA is cell-to-cell mobile.

AT5G65700	*BARELY ANY MERISTEM 1 (BAM1)*	Encodes a CLAVATA1-related receptor kinase-like protein required for both shoot and flower meristem function. Very similar to BAM2,with more than 85% a.a. identity. It has a broad expression pattern and is involved in vascular strand development in the leaf, control of leaf shape, size and symmetry, male gametophyte development and ovule specification and function. Anthers of double mutants (bam1bam2) appeared abnormal at a very early stage and lack the endothecium, middle, and tapetum layers. Further analyses revealed that cells interior to the epidermis (in anther tissue) acquire some characteristics of pollen mother cells (PMCs), suggesting defects in cell fate specification. The pollen mother-like cells degenerate before the completion of meiosis, suggesting that these cells are defective. In addition, the BAM1 expression pattern supports both an early role in promoting somatic cell fates and a subsequent function in the PMCs. The mRNA is cell-to-cell mobile.
AT5G65710	*HAESA-LIKE 2 (HSL2)*	Encodes a protein controlling the separation step of floral organ abscission.Necessary for pathogen-triggered leaf abscission.
AT5G65730	*XYLOGLUCAN ENDOTRANSGLUCOSYLASE/HYDROLASE 6 (XTH6)*	xyloglucan endotransglucosylase/hydrolase 6
AT5G65750	*(E1-OGDH2)*	Encodes the E1 subunit of the 2-oxoglutarate dehydrogenase.
AT5G65770	*LITTLE NUCLEI4 (LINC4)*	Encodes a protein that localizes to the nuclear periphery and affects nuclear morphology. Member of a small gene family in Arabidopsis containing 4 proteins (LNC1-4 or CRWN 1-4) with redundant functions in protection from oxidative damage, control of nuclear morphology and degradation of ABI5.
AT5G65790	*MYB DOMAIN PROTEIN 68 (MYB68)*	Encodes a MYB family protein with N-terminal R2R3 DNA-binding domains involved in root development.
AT5G65800	*ACC SYNTHASE 5 (ACS5)*	1-aminocyclopropane-1-carboxylate synthase (ACS) is encoded by a multigene family consisting of at least five members whose expression is induced by hormones, developmental signals, and protein synthesis inhibition.
AT5G65820		Pentatricopeptide repeat (PPR) superfamily protein
AT5G65830	*RECEPTOR LIKE PROTEIN 57 (RLP57)*	receptor like protein 57
AT5G65840		Thioredoxin superfamily protein
AT5G65850		F-box and associated interaction domains-containing protein
AT5G65860		ankyrin repeat family protein
AT5G65870	*PHYTOSULFOKINE 5 PRECURSOR (PSK5)*	Probable phytosulfokines 5 precursor, coding for a unique plant peptide growth factor.
AT5G65880		transmembrane protein
AT5G65890	*ACT DOMAIN REPEAT 1 (ACR1)*	Member of ACT domain containing protein family. ACT domains are amino acid binding domains. Shows strongest expression in flowers and siliques.
AT5G65900	*RNA DEAD-BOX HELICASE 27 (RH27)*	DEA(D/H)-box RNA helicase family protein
AT5G65940	*BETA-HYDROXYISOBUTYRYL-COA HYDROLASE 1 (chy1)*	hydrolyzes beta-hydroxyisobutyryl-CoA
AT5G65960		GTP binding protein
AT5G65970	*MILDEW RESISTANCE LOCUS O 10 (MLO10)*	A member of a large family of seven-transmembrane domain proteins specific to plants, homologs of the barley mildew resistance locus o (MLO) protein. The Arabidopsis genome contains 15 genes encoding MLO proteins, with localization in plasma membrane. Phylogenetic analysis revealed four clades of closely-related AtMLO genes. ATMLO10 belongs to the clade III, with AtMLO5, AtMLO7, AtMLO8, and AtMLO9. The gene is expressed in root and cotyledon vascular system, in root-shoot junction and lateral root primordia and in developing siliques, as shown by GUS activity patterns. The expression of several phylogenetically closely-related AtMLO genes showed similar or overlapping tissue specificity and analogous responsiveness to external stimuli, suggesting functional redundancy, co-function, or antagonistic function(s
AT5G65990	*(ATAVT3)*	Transmembrane amino acid transporter family protein
AT5G66010		RNA-binding (RRM/RBD/RNP motifs) family protein
AT5G66020	*SUPPRESSOR OF ACTIN 1B (ATSAC1B)*	Mutants in this gene are unable to express female sterility in response to beta-aminobutyric acid, as wild type plants do. non-consensus AT donor splice site at exon 7, TA donor splice site at exon 10, AT acceptor splice at exon 13.
AT5G66040	*SULFURTRANSFERASE PROTEIN 16 (STR16)*	Encodes a protein with thiosulfate sulfurtransferase/rhodanese activity in vitro, however, it is likely to use a substrate other than thiosulfate or 3-mercaptopyruvate in vivo. The mRNA is cell-to-cell mobile.
AT5G66050		Wound-responsive family protein
AT5G66060		2-oxoglutarate-dependent dioxygenase
AT5G66070	*ABA-RELATED RING-TYPE E3 LIGASE (ATARRE)*	E3 ubiquitin ligase that functions in negative regulation of ABA signaling.
AT5G66080	*ARABIDOPSIS PP2C CLADE D 9 (APD9)*	Type 2C protein phosphatase located in the plasma membrane. Functions in heat shock response memory mantainance.
AT5G66090		cell wall integrity/stress response component
AT5G66100	*LA RELATED PROTEIN 1B (LARP1B)*	Encodes a LAM domain containing protein that is involved in leaf senescence.
AT5G66110	*HEAVY METAL ASSOCIATED ISOPRENYLATED PLANT PROTEIN 27 (HIPP27)*	Heavy metal transport/detoxification superfamily protein
AT5G66130	*RADIATION SENSITIVE 17 (ATRAD17)*	Encodes a homolog to yeast RAD17. Involved in the regulation of DNA damage repair and homologous recombination. Mutant has increased sensitivity to MMS and increased telomere lengths.
AT5G66150		Glycosyl hydrolase family 38 protein
AT5G66170	*SULFURTRANSFERASE 18 (STR18)*	Encodes a thiosulfate sulfurtransferase/rhodanese.
AT5G66180	*TRNA METHYLTRANSFERASE 4G (TRM4G)*	S-adenosyl-L-methionine-dependent methyltransferases superfamily protein
AT5G66190	*FERREDOXIN-NADP(+)-OXIDOREDUCTASE 1 (FNR1)*	Encodes a leaf-type ferredoxin:NADP(H) oxidoreductase. It is present in both chloroplast stroma and thylakoid membranes but is more abundant in the thylakoid. The affinity of this enzyme for ferredoxin is slightly, but significantly, higher than AtLFNR2, an isoform of the same enzyme. AtLFNR1 forms a heterodimer with AtFNR2 and is also a prerequisite to attach AtFNR2 to the thylakoid membrane.
AT5G66200	*ARMADILLO REPEAT ONLY 2 (ARO2)*	Armadillo repeat protein. One of a family of four in Arabidopsis. Expressed in vegetative tissues, anthers and ovules.
AT5G66210	*CALCIUM-DEPENDENT PROTEIN KINASE 28 (CPK28)*	Calcium Dependent Protein Kinase. Functions in the BIK1 innate immune response pathway.
AT5G66220		Chalcone-flavanone isomerase family protein
AT5G66230		Chalcone-flavanone isomerase family protein

AT5G66240	*UBIQUITIN LIGASE COMPLEX SUBUNIT 1 (ULCS1)*	Encodes a WD40-repeat protein that interacts with the E3 Cullin Ring Ligase subunit DDB1a and is involved in secondary wall modification and thickening by regulating the degradation of specific proteins. RNAi-mediated silencing results in anther indehiscence and infertility.
AT5G66250		kinectin-like protein
AT5G66260	*SMALL AUXIN UPREGULATED RNA 11 (SAUR11)*	SAUR-like auxin-responsive protein family
AT5G66280	*GDP-D-MANNOSE 4,6-DEHYDRATASE 1 (GMD1)*	GDP-D-mannose 4,6-dehydratase
AT5G66300	*NAC DOMAIN CONTAINING PROTEIN 105 (NAC105)*	Encodes a NAC-domain transcription factor. Expressed in the vascular tissue.
AT5G66310		ATP binding microtubule motor family protein
AT5G66320	*GATA TRANSCRIPTION FACTOR 5 (GATA5)*	Encodes GATA transcription factor gene GNC, involved in regulating carbon and nitrogen metabolism. Expression occurs in aerial tissue at an early stage of development and is inducible by nitrate.
AT5G66330		Leucine-rich repeat (LRR) family protein
AT5G66340		hypothetical protein
AT5G66350	*SHORT INTERNODES (SHI)*	A member of SHI gene family. Arabidopsis thaliana has ten members that encode proteins with a RING finger-like zinc finger motif. Despite being highly divergent in sequence, many of the SHI-related genes are partially redundant in function and synergistically promote gynoecium, stamen and leaf development in Arabidopsis. Shi mutant is dominant, has dwarf phenotype. Loss of function mutations have no observable phenotype. Putative zinc finger protein. Involved in the response to gibberellic acid.
AT5G66370		metal ion-binding protein
AT5G66380	*FOLATE TRANSPORTER 1 (FOLT1)*	Encodes a folate transporter that is located in the chloroplast envelope and is able to mediate exogenous folate uptake when expressed in E. coli. However, this is not the sole folate transporter for chloroplasts as null mutants of this gene have no discernible phenotype when grown under folate-sufficient conditions and contained wild-type levels of folates in leaves.
AT5G66390	*PEROXIDASE 72 (PRX72)*	Encodes a peroxidase that is involved in lignin biosynthesis. Required for casparian strip lignification as well as partially required for SGN-dependent compensatory lignification.
AT5G66400	*RESPONSIVE TO ABA 18 (RAB18)*	Belongs to the dehydrin protein family, which contains highly conserved stretches of 7-17 residues that are repetitively scattered in their sequences, the K-, S-, Y- and lysine rich segments. ABA- and drought-induced glycine-rice dehydrin protein. The ABA-induced expression of RAB18 was reduced following ACC application, indicating that ethylene inhibits the ABA signaling pathway. RAB18 is also expressed in response to the formation of the phospholipid diacylglycerol pyrophosphate. COR47 and RAB18 double overexpressor plants are cold tolerant. Expressed in guard cells.
AT5G66420		TIM-barrel signal transduction protein
AT5G66430		S-adenosyl-L-methionine-dependent methyltransferases superfamily protein
AT5G66440		tRNA-methyltransferase non-catalytic subunit trm6MTase subunit
AT5G66460	*ENDO-BETA-MANNASE 7 (MAN7)*	Encodes a endo-beta-mannanase involved in seed germination and silique dehiscence.
AT5G66470	*(ERA-1)*	GTP-binding protein Era-like protein
AT5G66490		hypothetical protein
AT5G66510	*GAMMA CARBONIC ANHYDRASE 3 (GAMMA CA3)*	Encodes mitochondrial gamma carbonic anhydrase. Component of the NADH dehydrogenase complex.
AT5G66520	*CHLOROPLAST RNA EDITING FACTOR 7 (CREF7)*	Encodes a pentatricopeptide repeat protein involved in chloroplast mRNA editing. Mutants display defects in C-U editing of ndhB.
AT5G66530		Galactose mutarotase-like superfamily protein
AT5G66540		U3 small nucleolar ribonucleoprotein
AT5G66550		Maf-like protein
AT5G66560		Phototropic-responsive NPH3 family protein
AT5G66570	*PS II OXYGEN-EVOLVING COMPLEX 1 (PSBO1)*	Encodes a protein which is an extrinsic subunit of photosystem II and which has been proposed to play a central role in stabilization of the catalytic manganese cluster. In <i>Arabidopsis thaliana</i> the PsbO proteins are encoded by two genes: <i>psbO1</i> and <i>psbO2</i>. PsbO1 is the major isoform in the wild-type. In plsp1-1 mutant plastids, the nonmature form of the protein localizes in the membrane. The mRNA is cell-to-cell mobile.
AT5G66590		CAP (Cysteine-rich secretory proteins, Antigen 5, and Pathogenesis-related 1 protein) superfamily protein
AT5G66610	*DA1-RELATED PROTEIN 7 (DAR7)*	DA1-related protein 7
AT5G66620	*DA1-RELATED PROTEIN 6 (DAR6)*	DA1-related protein 6
AT5G66630	*DA1-RELATED PROTEIN 5 (DAR5)*	DA1-related protein 5
AT5G66640	*DA1-RELATED PROTEIN 3 (DAR3)*	DA1-related protein 3
AT5G66660		pectinesterase, putative (DUF677)
AT5G66670		pectinesterase, putative (DUF677)
AT5G66690	*(UGT72E2)*	UGT72E2 is an UDPG:coniferyl alcohol glucosyltransferase which glucosylates sinapyl- and coniferyl aldehydes as well as sinapyl- and coniferyl alcohol. The enzyme is thought to be involved in lignin metabolism. A knockdown mutant line (72E2KD) was obtained using RNAi silencing. A twofold reduction in coniferyl alcohol 4-<i>O</i>-glucoside and sinapyl alcohol 4-<i>O</i>-glucoside was detected in this line compared to wildtype. In comparison, both knockout and knockdown lines of UGT72E1 and UGT72E3, respectively, failed to display the same reduction in phenylpropanoid 4-<i>O</i>-glucosides. The mRNA is cell-to-cell mobile.
AT5G66700	*HOMEOBOX 53 (HB53)*	Encodes a homeodomain protein. Member of HD-ZIP 1 family, most closely related to HB5. AtHB53 is auxin-inducible and its induction is inhibited by cytokinin, especially in roots therefore may be involved in root development.
AT5G66710		Protein kinase superfamily protein
AT5G66730	*INDETERMINATE DOMAIN 1 (IDD1)*	C2H2-like zinc finger protein
AT5G66740	*BOUNDARY OF ROP DOMAIN8 (BDR8)*	spindle assembly abnormal protein (DUF620)

AT5G66750	*CHROMATIN REMODELING 1 (CHR1)*	Protein is similar to SWI2/SNF2 chromatin remodeling proteins. DDM1 is appears to act as a chromatin-remodeling ATPase involved in cytosine methylation in CG and non-CG contexts. Involved in gene silencing and maintenance of DNA methylation and histone methylation. Hypomethylation of many genomic regions occurs in ddm1 mutants, and can cause several phenotypic abnormalities, but some loci, such as BONSAI (At1g73177) can be hypermethylated in ddm1 mutants after several generations, leading to different phenotypes. DDM1 might be involved in establishing a heterochromain boundary. A line expressing an RNAi targeted against DDM1 shows some resistance to agrobacterium-mediated root transformation.
AT5G66770		GRAS family transcription factor
AT5G66780		late embryogenesis abundant protein
AT5G66800		membrane-associated kinase regulator-like protein
AT5G66815	*C-TERMINALLY ENCODED PEPTIDE 5 (CEP5)*	Counteracts auxin effects by stabilizing AUX/IAA transcriptional repressors. Impact on abiotic stress processes.
AT5G66880	*SUCROSE NONFERMENTING 1(SNF1)-RELATED PROTEIN KINASE 2.3 (SNRK2.3)*	encodes a member of SNF1-related protein kinases (SnRK2) whose activity is activated by ionic (salt) and non-ionic (mannitol) osmotic stress. Enzyme involved in the ABA signaling during seed germination, dormancy and seedling growth. The mRNA is cell-to-cell mobile.
AT5G66890	*N REQUIREMENT GENE 1.3 (NRG1.3)*	RPW8 -CNL gene.
AT5G66900	*N REQUIREMENT GENE 1.1 (NRG1.1)*	RPW8 -CNL gene is required for signal transduction of TNLs; functionally redundant to NRG1.2. Exhibits autoimmunity.
AT5G66920	*SKU5 SIMILAR 17 (sks17)*	SKU5 similar 17
AT5G66940	*(ATDOF5.8)*	Encodes a nuclear localized DOF-domain binding transcription factor.
AT5G66960		Prolyl oligopeptidase family protein
AT5G66970		P-loop containing nucleoside triphosphate hydrolases superfamily protein
AT5G66980		AP2/B3-like transcriptional factor family protein
AT5G66985	*HYPOXIA RESPONSE UNKNOWN PROTEIN 44 (HUP44)*	hypothetical protein
AT5G67000		encodes a member of the ERF (ethylene response factor) subfamily B-6 of ERF/AP2 transcription factor family. The protein contains one AP2 domain. There are 12 members in this subfamily including RAP2.11.
AT5G67020		hypothetical protein
AT5G67040	*DUF295 ORGANELLAR A 17 (ATDOA17)*	F-box protein, putative (DUF295)
AT5G67050		alpha/beta-Hydrolases superfamily protein
AT5G67060	*HECATE 1 (HEC1)*	Encodes a bHLH transcription factor that is involved in transmitting tract and stigma development and acts as a local modulator of auxin and cytokinin responses to control gynoecium development. HEC1 affects auxin transport by acting as a transcriptional regulator of PIN1 and PIN3. Inhibits thermomorphogenesis.
AT5G67070	*RALF-LIKE 34 (RALFL34)*	Member of a diversely expressed predicted peptide family showing sequence similarity to tobacco Rapid Alkalinization Factor (RALF), and is believed to play an essential role in the physiology of Arabidopsis. Consists of a single exon and is characterized by a conserved C-terminal motif and N-terminal signal peptide. The mRNA is cell-to-cell mobile.
AT5G67080	*MITOGEN-ACTIVATED PROTEIN KINASE KINASE KINASE 19 (MAPKKK19)*	member of MEKK subfamily
AT5G67090	*SUBTILISIN-LIKE PROTEASE 1.9 (SBT1.9)*	Encodes a subtilisin-like serine protease with in vitro protease activity.
AT5G67150		HXXXD-type acyl-transferase family protein
AT5G67160	*ENHANCED PSEUDOMONAS SUSCEPTIBILTY 1 (EPS1)*	Encodes a member of the BAHD acyltransferase superfamily. Mutants have enhanced susceptibility to virulent and avirulent pathogens and are defective in pathogen induced SA biosynthesis. EPS1 may act upstream of SA biosynthesis as application of SA can rescue the mutant phenotype.
AT5G67180	*TARGET OF EARLY ACTIVATION TAGGED (EAT) 3 (TOE3)*	target of early activation tagged (EAT) 3
AT5G67190	*DREB AND EAR MOTIF PROTEIN 2 (DEAR2)*	encodes a member of the DREB subfamily A-5 of ERF/AP2 transcription factor family. The protein contains one AP2 domain. There are 16 members in this subfamily including RAP2.1, RAP2.9 and RAP2.10.
AT5G67210	*IRX15-LIKE (IRX15-L)*	Encode a DUF579 (domain of unknown function 579) containing protein essential for normal xylan synthesis and deposition in the secondary cell wall.
AT5G67220		FMN-linked oxidoreductases superfamily protein
AT5G67230	*IRREGULAR XYLEM 14-LIKE (IRX14-L)*	Encodes a member of the GT43 family glycosyltransferases involved in glucuronoxylan biosynthesis: AT2G37090 (IRX9) and AT1G27600 (IRX9-L or I9H, IRX9 homolog); AT4G36890 (IRX14) and AT5G67230 (IRX14-L or I14H, IRX14 homolog). They form two functionally non-redundant groups essential for the normal elongation of glucuronoxylan backbone. I9H functions redundantly with IRX9, I14H is redundant with IRX14. IRX9 or I9H do not complement IRX14, IRX14 or I14H do not complement IRX9.
AT5G67240	*SMALL RNA DEGRADING NUCLEASE 3 (SDN3)*	small RNA degrading nuclease 3
AT5G67250	*SKP1/ASK1-INTERACTING PROTEIN 2 (SKIP2)*	Encodes an SKP1 interacting partner (SKIP2).Encodes an F-box protein. Based on genetic analysis appears to be functionally redundant with VFB1,2, and 3. When expression of all 4 genes is reduced plants show defects in growth and reduced expression of auxin response genes.
AT5G67260	*CYCLIN D3;2 (CYCD3;2)*	Encode CYCD3;2, a CYCD3 D-type cyclin. Important for determining cell number in developing lateral organs and mediating cytokinin effects in apical growth and development. With PPD and NINJA, it plays a crucial role in leaf morphogenesis.
AT5G67270	*END BINDING PROTEIN 1C (EB1C)*	encodes a homolog of animal microtubule-end-binding protein. There are two other members of this family. EB1 forms foci at regions where the minus ends of microtubules are gathered during mitosis and early cytokinesis.
AT5G67280	*RECEPTOR-LIKE KINASE (RLK)*	receptor-like kinase
AT5G67300	*MYB DOMAIN PROTEIN R1 (MYBR1)*	Member of the R2R3 factor MYB gene family involved in mediating plant responses to a variety of abiotic stimiuli. The mRNA is cell-to-cell mobile.
AT5G67310	*CYTOCHROME P450, FAMILY 81, SUBFAMILY G, POLYPEPTIDE 1 (CYP81G1)*	member of CYP81G
AT5G67330	*NATURAL RESISTANCE ASSOCIATED MACROPHAGE PROTEIN 4 (NRAMP4)*	Encodes a member of the Nramp2 metal transporter family; like its homolog Atnramp3, localized in vacuolar membrane. Seedlings of double mutant, atnramp3-1 atnramp4-1, were arrested at early germination. The mRNA is cell-to-cell mobile.
AT5G67340	*(PUB2)*	Plant U-box type E3 ubiquitin ligase (PUB).
AT5G67350		hypothetical protein
AT5G67360	*(ARA12)*	Encodes a subtilisin-like serine protease essential for mucilage release from seed coats.

AT5G67370	*CONSERVED IN THE GREEN LINEAGE AND DIATOMS 27 (CGLD27)*	DUF1230 family protein (DUF1230)
AT5G67380	*CASEIN KINASE ALPHA 1 (CKA1)*	Casein kinase II (CK2) catalytic subunit (alpha 1). One known substrate of CK2 is Phytochrome Interacting Factor 1 (PIF1). CK2-mediated phosphorylation enhances the light-induced degradation of PIF1 to promote photomorphogenesis.
AT5G67390		glycosyltransferase-like protein
AT5G67400	*ROOT HAIR SPECIFIC 19 (RHS19)*	root hair specific 19
AT5G67420	*LOB DOMAIN-CONTAINING PROTEIN 37 (LBD37)*	Encodes a LOB-domain protein involved in nitrogen metabolism and affecting leaf morphogenesis.
AT5G67430		Acyl-CoA N-acyltransferases (NAT) superfamily protein
AT5G67440	*NAKED PINS IN YUC MUTANTS 3 (NPY3)*	A member of the NPY gene family (NPY1/AT4G31820, NPY2/AT2G14820, NPY3/AT5G67440, NPY4/AT2G23050, NPY5/AT4G37590). Involved in auxin-mediated organogenesis.
AT5G67450	*ZINC-FINGER PROTEIN 1 (ZF1)*	Encodes zinc-finger protein. mRNA levels are elevated in response to low temperature, cold temperatures and high salt. The protein is localized to the nucleus and acts as a transcriptional repressor.
AT5G67460		O-Glycosyl hydrolases family 17 protein
AT5G67480	*BTB AND TAZ DOMAIN PROTEIN 4 (BT4)*	BTB and TAZ domain protein. Located in cytoplasm and expressed in fruit, flower and leaves.
AT5G67500	*VOLTAGE DEPENDENT ANION CHANNEL 2 (VDAC2)*	Encodes a voltage-dependent anion channel (VDAC: AT3G01280/VDAC1, AT5G67500/VDAC2, AT5G15090/VDAC3, AT5G57490/VDAC4, AT5G15090/VDAC5). VDACs are reported to be porin-type, beta-barrel diffusion pores. They are prominently localized in the outer mitochondrial membrane and are involved in metabolite exchange between the organelle and the cytosol. The mRNA is cell-to-cell mobile.
AT5G67510		Translation protein SH3-like family protein
AT5G67520	*ADENOSINE-5'-PHOSPHOSULFATE (APS) KINASE 4 (APK4)*	Provides activated sulfate for the sulfation of secondary metabolites, including the glucosinolates. Redundant with APK3.
AT5G67550		transmembrane protein
AT5G67560	*ADP-RIBOSYLATION FACTOR-LIKE A1D (ARLA1D)*	A member of ARF-like GTPase family. A thaliana has 21 members, in two subfamilies, ARF and ARF-like (ARL) GTPases. Possible pseudogene because it lacks an N-terminal part that is conserved among the other ARL8 proteins. The mRNA is cell-to-cell mobile.
AT5G67570	*DELAYED GREENING 1 (DG1)*	Encodes a pentratricopeptide repeat containing protein that is targeted to the chloroplast. Mutants have pale young leave and reduced accumulation of plastid encoded transcripts suggesting a role for DG1 in regulation of plastid gene expression.
AT5G67580	*(TRB2)*	Encodes a telomeric DNA binding protein and Single Myb Histone (SMH) gene family member. In vitro, the protein preferentially binds double-stranded telomeric repeats, but it can also bind to the single G-rich telomeric strand.
AT5G67600	*WINDHOSE 1 (WIH1)*	cysteine-rich TM module stress tolerance protein
AT5G67630	*REPTIN, RVB2, RUVBL2, TIP49B (ISE4)*	P-loop containing nucleoside triphosphate hydrolases superfamily protein
ATCG00020	*PHOTOSYSTEM II REACTION CENTER PROTEIN A (PSBA)*	Encodes chlorophyll binding protein D1, a part of the photosystem II reaction center core
ATCG00040	*MATURASE K (MATK)*	Encodes a maturase located in the trnK intron in the chloroplast genome.
ATCG00050	*RIBOSOMAL PROTEIN S16 (RPS16)*	Homologous to the bacterial ribosomal protein S16
ATCG00070	*PHOTOSYSTEM II REACTION CENTER PROTEIN K PRECURSOR (PSBK)*	PSII K protein
ATCG00080	*PHOTOSYSTEM II REACTION CENTER PROTEIN I (PSBI)*	PSII I protein
ATCG00120	*ATP SYNTHASE SUBUNIT ALPHA (ATPA)*	Encodes the ATPase alpha subunit, which is a subunit of ATP synthase and part of the CF1 portion which catalyzes the conversion of ADP to ATP using the proton motive force. This complex is located in the thylakoid membrane of the chloroplast.
ATCG00130	*(ATPF)*	ATPase F subunit.
ATCG00140	*(ATPH)*	ATPase III subunit
ATCG00150	*(ATPI)*	Encodes a subunit of ATPase complex CF0, which is a proton channel that supplies the proton motive force to drive ATP synthesis by CF1 portion of the complex.
ATCG00160	*RIBOSOMAL PROTEIN S2 (RPS2)*	Chloroplast ribosomal protein S2
ATCG00170	*(RPOC2)*	RNA polymerase beta' subunit-2
ATCG00180	*(RPOC1)*	RNA polymerase beta' subunit-1
ATCG00190	*RNA POLYMERASE SUBUNIT BETA (RPOB)*	Chloroplast DNA-dependent RNA polymerase B subunit. The transcription of this gene is regulated by a nuclear encoded RNA polymerase. This gene has been transferred to mitochondrial genome during crucifer evolution.
ATCG00220	*PHOTOSYSTEM II REACTION CENTER PROTEIN M (PSBM)*	PSII low MW protein
ATCG00270	*PHOTOSYSTEM II REACTION CENTER PROTEIN D (PSBD)*	PSII D2 protein
ATCG00280	*PHOTOSYSTEM II REACTION CENTER PROTEIN C (PSBC)*	chloroplast gene encoding a CP43 subunit of the photosystem II reaction center. promoter contains a blue-light responsive element.
ATCG00330	*CHLOROPLAST RIBOSOMAL PROTEIN S14 (RPS14)*	30S chloroplast ribosomal protein S14
ATCG00340	*(PSAB)*	Encodes the D1 subunit of photosystem I reaction center.
ATCG00350	*(PSAA)*	Encodes psaA protein comprising the reaction center for photosystem I along with psaB protein; hydrophobic protein encoded by the chloroplast genome.
ATCG00360	*(YCF3)*	Encodes a protein required for photosystem I assembly and stability. In Chlamydomonas reinhardtii, this protein seems to act as a PSI specific chaperone facilitating the assembly of the complex by interacting with PsaA and PsaD. A loss of function mutation in tobacco leads to a loss of photosystem I.
ATCG00420	*NADH DEHYDROGENASE SUBUNIT J (NDHJ)*	Encodes NADH dehydrogenase subunit J. Its transcription is increased upon sulfur depletion.
ATCG00440	*(NDHC)*	Encodes NADH dehydrogenase D3 subunit of the chloroplast NAD(P)H dehydrogenase complex
ATCG00470	*ATP SYNTHASE EPSILON CHAIN (ATPE)*	ATPase epsilon subunit
ATCG00480	*ATP SYNTHASE SUBUNIT BETA (PB)*	chloroplast-encoded gene for beta subunit of ATP synthase
ATCG00490	*(RBCL)*	large subunit of RUBISCO. Protein is tyrosine-phosphorylated and its phosphorylation state is modulated in response to ABA in Arabidopsis thaliana seeds.

ATCG00500	*ACETYL-COA CARBOXYLASE CARBOXYL TRANSFERASE SUBUNIT BETA (ACCD)*	Encodes the carboxytransferase beta subunit of the Acetyl-CoA carboxylase (ACCase) complex in plastids. This complex catalyzes the carboxylation of acetyl-CoA to produce malonyl-CoA, the first committed step in fatty acid synthesis.
ATCG00510	*PHOTSYSTEM I SUBUNIT I (PSAI)*	Encodes subunit I of photosystem I.
ATCG00520	*(YCF4)*	Encodes a protein required for photosystem I assembly and stability. In cyanobacteria, loss of function mutation in this gene increases PSII/PSI ratio without any influence on photoautotrophic growth.
ATCG00540	*PHOTOSYNTHETIC ELECTRON TRANSFER A (PETA)*	Encodes cytochrome f apoprotein; involved in photosynthetic electron transport chain; encoded by the chloroplast genome and is transcriptionally repressed by a nuclear gene HCF2.
ATCG00550	*PHOTOSYSTEM II REACTION CENTER PROTEIN J (PSBJ)*	PSII component
ATCG00560	*PHOTOSYSTEM II REACTION CENTER PROTEIN L (PSBL)*	PSII L protein
ATCG00570	*PHOTOSYSTEM II REACTION CENTER PROTEIN F (PSBF)*	PSII cytochrome b559
ATCG00580	*PHOTOSYSTEM II REACTION CENTER PROTEIN E (PSBE)*	PSII cytochrome b559. There have been many speculations about the function of Cyt b559, but the most favored at present is that it plays a protective role by acting as an electron acceptor or electron donor under conditions when electron flow through PSII is not optimized.
ATCG00590	*(ORF31)*	electron carrier
ATCG00600	*(PETG)*	Cytochrome b6-f complex, subunit V. Disruption of homologous gene in Chlamydomonas results in disruption of cytochrome b6-f complex.
ATCG00630	*(PSAJ)*	Encodes subunit J of photosystem I.
ATCG00640	*RIBOSOMAL PROTEIN L33 (RPL33)*	encodes a chloroplast ribosomal protein L33, a constituent of the large subunit of the ribosomal complex
ATCG00650	*RIBOSOMAL PROTEIN S18 (RPS18)*	chloroplast-encoded ribosomal protein S18
ATCG00660	*RIBOSOMAL PROTEIN L20 (RPL20)*	encodes a chloroplast ribosomal protein L20, a constituent of the large subunit of the ribosomal complex
ATCG00680	*PHOTOSYSTEM II REACTION CENTER PROTEIN B (PSBB)*	encodes for CP47, subunit of the photosystem II reaction center.
ATCG00690	*PHOTOSYSTEM II REACTION CENTER PROTEIN T (PSBT)*	Encodes photosystem II 5 kD protein subunit PSII-T. This is a plastid-encoded gene (PsbTc) which also has a nuclear-encoded paralog (PsbTn).
ATCG00700	*PHOTOSYSTEM II REACTION CENTER PROTEIN N (PSBN)*	PSII low MW protein
ATCG00710	*PHOTOSYSTEM II REACTION CENTER PROTEIN H (PSBH)*	Encodes a 8 kD phosphoprotein that is a component of the photosystem II oxygen evolving core. Its exact molecular function has not been determined but it may play a role in mediating electron transfer between the secondary quinone acceptors, QA and QB, associated with the acceptor side of PSII.
ATCG00720	*PHOTOSYNTHETIC ELECTRON TRANSFER B (PETB)*	Encodes the cytochrome b(6) subunit of the cytochrome b6f complex.
ATCG00730	*PHOTOSYNTHETIC ELECTRON TRANSFER D (PETD)*	A chloroplast gene encoding subunit IV of the cytochrome b6/f complex
ATCG00740	*RNA POLYMERASE SUBUNIT ALPHA (RPOA)*	RNA polymerase alpha subunit
ATCG00750	*RIBOSOMAL PROTEIN S11 (RPS11)*	30S chloroplast ribosomal protein S11
ATCG00760	*RIBOSOMAL PROTEIN L36 (RPL36)*	encodes a chloroplast ribosomal protein L36, a constituent of the large subunit of the ribosomal complex
ATCG00770	*RIBOSOMAL PROTEIN S8 (RPS8)*	chloroplast 30S ribosomal protein S8
ATCG00780	*RIBOSOMAL PROTEIN L14 (RPL14)*	encodes a chloroplast ribosomal protein L14, a constituent of the large subunit of the ribosomal complex
ATCG00810	*RIBOSOMAL PROTEIN L22 (RPL22)*	encodes a chloroplast ribosomal protein L22, a constituent of the large subunit of the ribosomal complex
ATCG00820	*RIBOSOMAL PROTEIN S19 (RPS19)*	Encodes a 6.8-kDa protein of the small ribosomal subunit.
ATCG00890	*(NDHB.1)*	NADH dehydrogenase ND2
ATCG01000	*(YCF1.1)*	Ycf1 protein
ATCG01010	*(NDHF)*	Chloroplast encoded NADH dehydrogenase unit.
ATCG01020	*RIBOSOMAL PROTEIN L32 (RPL32)*	encodes a chloroplast ribosomal protein L32, a constituent of the large subunit of the ribosomal complex
ATCG01050	*(NDHD)*	Represents a plastid-encoded subunit of a NAD(P)H dehydrogenase complex. Its mRNA is edited at four positions. Translation data is not available for this gene.
ATCG01060	*(PSAC)*	Encodes the PsaC subunit of photosystem I.
ATCG01070	*(NDHE)*	NADH dehydrogenase ND4L
ATCG01080	*(NDHG)*	NADH dehydrogenase ND6
ATCG01090	*(NDHI)*	Encodes subunit of the chloroplast NAD(P)H dehydrogenase complex
ATCG01100	*(NDHA)*	NADH dehydrogenase ND1
ATCG01110	*NAD(P)H DEHYDROGENASE SUBUNIT H (NDHH)*	Encodes the 49KDa plastid NAD(P)H dehydrogenase subunit H protein. Its transcription is regulated by an ndhF-specific plastid sigma factor, SIG4.
ATCG01120	*CHLOROPLAST RIBOSOMAL PROTEIN S15 (RPS15)*	encodes a chloroplast ribosomal protein S15, a constituent of the small subunit of the ribosomal complex
ATMG00010	*(ORF153A)*	hypothetical protein
ATMG00050	*(ORF131)*	hypothetical protein
ATMG00070	*NADH DEHYDROGENASE SUBUNIT 9 (NAD9)*	NADH dehydrogenase subunit 9
ATMG00110	*ATP-BINDING CASSETTE I2 (ABCI2)*	Encodes a mitochondria-encoded cytochrome c biogenesis protein.
ATMG00120	*(ORF143)*	reverse transcriptase zinc-binding protein
ATMG00130	*(ORF121A)*	hypothetical protein
ATMG00140	*(ORF167)*	transmembrane protein
ATMG00150	*(ORF116)*	transmembrane protein
ATMG00160	*CYTOCHROME OXIDASE 2 (COX2)*	cytochrome c oxidase subunit 2
ATMG00180	*CYTOCHROME C BIOGENESIS 452 (CCB452)*	cytochrome c biogenesis orf452
ATMG00260	*(ORF101A)*	hypothetical protein
ATMG00270	*NADH DEHYDROGENASE 6 (NAD6)*	NADH dehydrogenase subunit 6
ATMG00280	*(ORF110A)*	Ribulose bisphosphate carboxylase large chain, catalytic domain-containing protein
ATMG00310	*(ORF154)*	RNA-directed DNA polymerase (reverse transcriptase)-related family protein
ATMG00320	*(ORF127)*	hypothetical protein

ATMG00400	*(ORF157)*	hypothetical protein
ATMG00450	*(ORF106B)*	hypothetical protein
ATMG00490	*(ORF107C)*	Mitovirus RNA-dependent RNA polymerase
ATMG00510	*NADH DEHYDROGENASE SUBUNIT 7 (NAD7)*	NADH dehydrogenase subunit 7
ATMG00520	*(MATR)*	Intron maturase, type II family protein
ATMG00530	*(ORF109)*	hypothetical protein
ATMG00570	*(ORFX)*	encodes a protein of unknown function. The transcript has extensive RNA editing at the 3' end. Protein has orthologous in other plants and sequence is similar to E. coli ORFs orf154 and orf131, both of unknown function. So far, similar proteins are found only in plants and prokaryotes.
ATMG00580	*NADH DEHYDROGENASE SUBUNIT 4 (NAD4)*	NADH dehydrogenase subunit 4
ATMG00600	*(ORF106C)*	hypothetical protein
ATMG00630	*(ORF110B)*	hypothetical protein
ATMG00640	*(ORF25)*	encodes a plant b subunit of mitochondrial ATP synthase based on structural similarity and the presence in the F(0) complex.
ATMG00650	*NADH DEHYDROGENASE SUBUNIT 4L (NAD4L)*	Encodes NADH dehydrogenase subunit 4L.
ATMG00660	*(ORF149)*	hypothetical protein
ATMG00670	*(ORF275)*	transmembrane protein
ATMG00680	*(ORF122C)*	transmembrane protein
ATMG00690	*(ORF240A)*	FO-ATPase subunit
ATMG00710	*(ORF120)*	Polynucleotidyl transferase, ribonuclease H-like superfamily protein
ATMG00720	*(ORF107D)*	hypothetical protein
ATMG00740	*(ORF100A)*	hypothetical protein
ATMG00750	*(ORF119)*	GAG/POL/ENV polyprotein
ATMG00820	*(ORF170)*	Reverse transcriptase (RNA-dependent DNA polymerase)
ATMG00830	*CYTOCHROME C BIOGENESIS 382 (CCB382)*	cytochrome c biogenesis orf382
ATMG00840	*(ORF121B)*	hypothetical protein
ATMG00850	*(ORF107E)*	DNA/RNA polymerases superfamily protein
ATMG00860	*(ORF158)*	DNA/RNA polymerases superfamily protein
ATMG00870	*(ORF184)*	hypothetical protein
ATMG00880	*(ORF187)*	hypothetical protein
ATMG00890	*(ORF106D)*	hypothetical protein
ATMG00960	*(CCB203)*	Encodes a protein of the mitochondrial membrane which has been shown to be present in a number of unidentified complexes including a 500-KDa complex postulated to have heme lyase activity, in which another protein (AtCCMH, AT1G15220) is also involved. cytochrome c biogenesis orf203.
ATMG00990	*NADH DEHYDROGENASE 3 (NAD3)*	NADH dehydrogenase subunit 3
ATMG01000	*(ORF114)*	hypothetical protein
ATMG01010	*(ORF118)*	Unknown conserved protein
ATMG01040	*(ORF107F)*	hypothetical protein
CLPP		
COX1		
MITOCHONDRIA.<br>1		
NAD5.1		
NAD5.2		
ORF100C		
ORF101B		
ORF102		
ORF105A		
ORF105B		
ORF106F		
ORF107G		
ORF107H		
ORF111B		
ORF111C		
ORF111D		
ORF113		
ORF135B		
ORF145B		
ORF145C		
ORF205		
ORF215A		
ORF215B		
ORF25		

ORF251
ORF262
ORF294
PSBG
RPL16.CHLOROPLAST
RPL16.MITOCHONDRIA
RPL2.1_CHLORO
RPL23.1
RPS12.1
YCF10_CEMA
YCF2.1
YCF5
YCF6
YCF9
