# Supplementary material for: Weighted Gene Correlation Network Analysis (WGCNA) of Arabidopsis Somatic Embryogenesis (SE) and Identification of Key Gene Modules to Uncover SE-Associated Hub Genes
Source: Int J Genomics. 2022 Jul 4;2022:7471063. doi: 10.1155/2022/7471063 (PMC9274236; doi:10.1155/2022/7471063)
Supplement: Supplementary 2 — Table S2. The list of differentially expressed genes. [file 7471063.f2.pdf]

Table S2. List of differentially expressed genes (|*log2 FC*| ≥ 2.0 and *p-value* < 0.05)

Gene identifier	Gene symbol	Gene description
AT1G01090	*PYRUVATE DEHYDROGENASE E1 ALPHA (PDH-E1 ALPHA)*	pyruvate dehydrogenase E1 alpha subunit
AT1G01200	*RAB GTPASE HOMOLOG A3 (RABA3)*	RAB GTPase homolog A3
AT1G01250		encodes a member of the DREB subfamily A-4 of ERF/AP2 transcription factor family. The protein contains one AP2 domain. There are 17 members in this subfamily including TINY.
AT1G01470	*LATE EMBRYOGENESIS ABUNDANT 14 (LEA14)*	Encodes late-embryogenesis abundant protein whose mRNA levels are induced in response to wounding and light stress. Might be involved in protection against desiccation.
AT1G01520	*ALTERED SEED GERMINATION 4 (ASG4)*	RVE3 is one of eleven homologous MYB-like transcription factors in Arabidopsis and a member of the RVE8 clade. Plays a minor role in clock regulation.
AT1G01600	*CYTOCHROME P450, FAMILY 86, SUBFAMILY A, POLYPEPTIDE 4 (CYP86A4)*	Encodes a member of the CYP86A subfamily of cytochrome p450 genes. Expressed significantly at highest level in mature stems and flowers.
AT1G01620	*PLASMA MEMBRANE INTRINSIC PROTEIN 1C (PIP1C)*	a member of the plasma membrane intrinsic protein subfamily PIP1. localizes to the plasma membrane and exhibits water transport activity in Xenopus oocyte. expressed ubiquitously and protein level decreases slightly during leaf development. Involved redundantly with PIP1;1/2/4/5 in hydraulics and carbon fixation, regulates the expression of related genes that affect plant growth and development.
AT1G01700	*ROP (RHO OF PLANTS) GUANINE NUCLEOTIDE EXCHANGE FACTOR 2 (ROPGEF2)*	Encodes a member of KPP-like gene family, homolog of KPP (kinase partner protein) gene in tomato. Also a member of the RopGEF (guanine nucleotide exchange factor) family, containing the novel PRONE domain (plant-specific Rop nucleotide exchanger), which is exclusively active towards members of the Rop subfamily.
AT1G02310	*ENDO-BETA-MANNANASE 1 (MAN1)*	Glycosyl hydrolase superfamily protein
AT1G02340	*LONG HYPOCOTYL IN FAR-RED (HFR1)*	Encodes a light-inducible, nuclear bHLH protein involved in phytochrome signaling. Mutants exhibit a long-hypocotyl phenotype only under far-red light but not under red light and are defective in other phytochrome A-related responses. Mutants also show blue light response defects. HFR1 interacts with COP1, co-localizes to the nuclear specks and is ubiquinated by COP1.
AT1G02390	*GLYCEROL-3-PHOSPHATE SN-2-ACYLTRANSFERASE 2 (GPAT2)*	putative sn-glycerol-3-phosphate 2-O-acyltransferase
AT1G02530	*ATP-BINDING CASSETTE B12 (ABCB12)*	P-glycoprotein 12
AT1G02720	*GALACTURONOSYLTRANSFERASE-LIKE 5 (GATL5)*	Encodes a protein with putative galacturonosyltransferase activity.
AT1G02790	*POLYGALACTURONASE 4 (PGA4)*	encodes a exopolygalacturonase.
AT1G02820	*LATE EMBRYOGENESIS ABUNDANT 3 (LEA3)*	Late embryogenesis abundant 3 (LEA3) family protein
AT1G02850	*BETA GLUCOSIDASE 11 (BGLU11)*	beta glucosidase 11
AT1G02900	*RAPID ALKALINIZATION FACTOR 1 (RALF1)*	Member of a diversely expressed predicted peptide family showing sequence similarity to tobacco Rapid Alkalinization Factor (RALF), and is believed to play an essential role in the physiology of Arabidopsis. Consists of a single exon and is characterized by a conserved C-terminal motif and N-terminal signal peptide. Mediates Ca2+-dependent signaling. Regulates the splicing of flowering genes and exerts an opposite effect on the flowering time compared with FER.
AT1G03010		Phototropic-responsive NPH3 family protein
AT1G03020	*(GRXS1)*	Encodes a member of the CC-type glutaredoxin (ROXY) family that has been shown to interact with the transcription factor TGA2.
AT1G03055	*DWARF27 (D27)*	Encodes the ortholog of rice D27. It is plastid-localized and is required for the inhibition of secondary bud outgrowth and operates on a nonmobile precursor upstream of MAX1 in the SL biosynthesis pathway.
AT1G03130	*PHOTOSYSTEM I SUBUNIT D-2 (PSAD-2)*	Encodes a protein predicted by sequence similarity with spinach PsaD to be photosystem I reaction center subunit II (PsaD2)
AT1G03550	*SECRETORY CARRIER MEMBRANE PROTEIN 4 (SCAMP4)*	Secretory carrier membrane protein (SCAMP) family protein
AT1G03600	*(PSB27)*	PSB27 is a chloroplast lumen localized protein that is involved in adaptation to changes in light intensity.
AT1G03620	*(ELMOD_F)*	Member of the ELMOD family.
AT1G03700	*CASP-LIKE PROTEIN 1C2 (CASPL1C2)*	Uncharacterized protein family (UPF0497)
AT1G03790	*SOMNUS (SOM)*	Encodes SOMNUS (SOM), a nucleus-localized CCCH-type zinc finger protein. SOM negatively regulates light-dependent seed germination downstream of PIL5 (AT2G20180).
AT1G03850	*GLUTAREDOXIN 13 (GRXS13)*	Encodes glutaredoxin ATGRXS13, required to facilitate Botrytis cinerea infection of Arabidopsis thaliana plants. Sylvain La Camera et al (2011, PMID:21756272) reported a third splice variant in addition to the two annotated in TAIR10. It is a member of the CC-type glutaredoxin (ROXY) family that has been shown to interact with the transcription factor TGA2 and suppress ORA59 promoter activity.
AT1G03870	*FASCICLIN-LIKE ARABINOOGALACTAN 9 (FLA9)*	fasciclin-like arabinogalactan-protein 9 (Fla9). Possibly involved in embryogenesis and seed development.
AT1G04040		HAD superfamily, subfamily IIIB acid phosphatase
AT1G04240	*SHORT HYPOCOTYL 2 (SHY2)*	SHY2/IAA3 regulates multiple auxin responses in roots. It is induced rapidly by IAA, and has been shown to be phosphorylated by oat phytochrome A in vitro.
AT1G04350		encodes a protein whose sequence is similar to 2-oxoglutarate-dependent dioxygenase
AT1G04470		hypothetical protein (DUF810)
AT1G05160	*CYTOCHROME P450, FAMILY 88, SUBFAMILY A, POLYPEPTIDE 3 (CYP88A3)*	Encodes an ent-kaurenoic acid hydroxylase, a member of the CYP88A cytochrome p450 family.
AT1G05190	*EMBRYO DEFECTIVE 2394 (emb2394)*	Encodes the plastid 50S ribosomal protein L6.
AT1G05280		ERV-F (C)1 provirus ancestral Env polyprotein, putative (DUF604)
AT1G05440		C-8 sterol isomerase
AT1G05460	*SILENCING DEFECTIVE (SDE3)*	Encodes a protein with similarity to RNA helicases. Mutants are defective in post-transcriptional gene silencing.
AT1G05480		
AT1G05580	*CATION/H+ EXCHANGER 23 (CHX23)*	member of Putative Na+/H+ antiporter family
AT1G05680	*URIDINE DIPHOSPHATE GLYCOSYLTRANSFERASE 74E2 (UGT74E2)*	Encodes a UDP-glucosyltransferase, UGT74E2, that acts on IBA (indole-3-butyric acid) and affects auxin homeostasis. The transcript and protein levels of this enzyme are strongly induced by H2O2 and may allow integration of ROS (reactive oxygen species) and auxin signaling. This enzyme can also transfer glycosyl groups to several compounds related to the explosive TNT when this synthetic compound is taken up from the environment.
AT1G05990	*ROOT HAIR SPECIFIC 2 (RHS2)*	EF hand calcium-binding protein family

AT1G06140	*MITOCHONDRIAL EDITING FACTOR 3 (MEF3)*	Encodes MEF3 (mitochondrial editing factor 3), a PPR (pentatricopeptide repeat) protein of the E domain subclass. Functions in mitochondrial RNA editing.
AT1G06420		DNA ligase-like protein
AT1G06520	*GLYCEROL-3-PHOSPHATE SN-2-ACYLTRANSFERASE 1 (GPAT1)*	sn-glycerol-3-phosphate 2-O-acyltransferase. Expressed in flower buds and siliques. Homozygous mutant plants are male sterile.
AT1G06620		encodes a protein whose sequence is similar to a 2-oxoglutarate-dependent dioxygenase The mRNA is cell-to-cell mobile.
AT1G06640		encodes a protein whose sequence is similar to a 2-oxoglutarate-dependent dioxygenase The mRNA is cell-to-cell mobile.
AT1G06680	*PHOTOSYSTEM II SUBUNIT P-1 (PSBP-1)*	Encodes a 23 kD extrinsic protein that is part of photosystem II and participates in the regulation of oxygen evolution. Phosphorylation of this protein is dependent on calcium. In plsp1-1 mutant plastids, the nonmature form of the protein localizes in the stroma. The mRNA is cell-to-cell mobile.
AT1G06980		PADRE protein
AT1G06990		GDSL-motif esterase/acyltransferase/lipase. Enzyme group with broad substrate specificity that may catalyze acyltransfer or hydrolase reactions with lipid and non-lipid substrates.
AT1G07160		Protein phosphatase 2C family protein
AT1G07290	*GOLGI NUCLEOTIDE SUGAR TRANSPORTER 2 (GONST2)*	Encodes a GDP-mannose transporter.
AT1G07320	*RIBOSOMAL PROTEIN L4 (RPL4)*	encodes a plastid ribosomal protein L4
AT1G07340	*SUGAR TRANSPORTER 2 (STP2)*	sugar transporter 2
AT1G07390	*RECEPTOR LIKE PROTEIN 1 (RLP1)*	receptor like protein 1
AT1G07430	*HIGHLY ABA-INDUCED PP2C GENE 2 (HAI2)*	Encodes a member of the group A protein phosphatase 2C (PP2C) family that is responsible for negatively regulating seed dormancy.
AT1G07450		NAD(P)-binding Rossmann-fold superfamily protein
AT1G07500	*SIAMESE-RELATED 5 (SMR5)*	SMR5 is a member of the SIAMESE-RELATED Cyclin-Dependent Kinase Inhibitor family. It is induced by ROS/oxidative stress.
AT1G07610	*METALLOTHIONEIN 1C (MT1C)*	one of the five metallothioneins (MTs) genes identified in Arabidopsis. MTs are cysteine-rich proteins required for heavy metal tolerance. The mRNA is cell-to-cell mobile.
AT1G07880	*(ATMPK13)*	member of MAP Kinase
AT1G08380	*PHOTOSYSTEM I SUBUNIT O (PSAO)*	Encodes subunit O of photosystem I.
AT1G08860	*BONZAI 3 (BON3)*	Encodes a copine-like protein, which is a member of a newly identified class of calcium-dependent, phospholipid binding proteins that are present in a wide range of organisms. Overexpression of this gene suppresses bon1-1 phenotypes. Double mutant analyses with bon1-1 suggest that BON1 and BON3 have overlapping functions in maintaining cellular homeostasis and inhibiting cell death.
AT1G08920	*ERD (EARLY RESPONSE TO DEHYDRATION) SIX-LIKE 1 (ESL1)*	Encodes ESL1, a transporter for monosaccharides.
AT1G08930	*EARLY RESPONSE TO DEHYDRATION 6 (ERD6)*	encodes a putative sucrose transporter whose gene expression is induced by dehydration and cold. The mRNA is cell-to-cell mobile.
AT1G09090	*RESPIRATORY BURST OXIDASE HOMOLOG B (RBOHB)*	NADPH-oxidase AtrbohB plays a role in seed after-ripening. Major producer of superoxide in germinating seeds. AtrbohB pre-mRNA is alternatively spliced in seeds in a hormonally and developmentally regulated manner. ABA caused accumulation of AtrbohB-? mRNA and prevented prevented AtrbohB-a mRNA expression in fresh seeds.
AT1G09155	*PHLOEM PROTEIN 2-B15 (PP2-B15)*	phloem protein 2-B15
AT1G09300	*(ATICP55)*	Encodes a mitochondrial protease ICP55. Alters the stability of proteins by removal of a single amino acid from their sequence.
AT1G09340	*CHLOROPLAST RNA BINDING (CRB)*	Encodes CHLOROPLAST RNA BINDING (CRB), a putative RNA-binding protein. CRB is important for the proper functioning of the chloroplast. Mutations in CRB also affects the circadian system, altering the expression of both oscillator and output genes. The mRNA is cell-to-cell mobile.
AT1G09390		GDSL-motif esterase/acyltransferase/lipase. Enzyme group with broad substrate specificity that may catalyze acyltransfer or hydrolase reactions with lipid and non-lipid substrates.
AT1G09440		Protein kinase superfamily protein
AT1G09560	*GERMIN-LIKE PROTEIN 5 (GLP5)*	Encodes a plasodesmata-located protein involved in regulating primary root growth by controlling phloem-mediated allocation of resources between the primary and lateral root meristems. The mRNA is cell-to-cell mobile.
AT1G09570	*PHYTOCHROME A (PHYA)*	Light-labile cytoplasmic red/far-red light photoreceptor involved in the regulation of photomorphogenesis. It exists in two inter-convertible forms: Pr and Pfr (active) and functions as a dimer.The N terminus carries a single tetrapyrrole chromophore, and the C terminus is involved in dimerization. It is the sole photoreceptor mediating the FR high irradiance response (HIR). Major regulator in red-light induction of phototropic enhancement. Involved in the regulation of de-etiolation. Involved in gravitropism and phototropism. Requires FHY1 for nuclear accumulation.
AT1G09750		Eukaryotic aspartyl protease family protein
AT1G09780	*2,3-BIPHOSPHOGLYCERATE-INDEPENDENT PHOSPHOGLYCERATE MUTASE 1 (iPGAM1)*	Encodes a 2,3-biphosphoglycerate-independent phosphoglycerate mutase that is involved in pollen development and stomatal movement.
AT1G09950	*RESPONSE TO ABA AND SALT 1 (RAS1)*	RESPONSE TO ABA AND SALT 1
AT1G10020		formin-like protein (DUF1005)
AT1G10140		Uncharacterized conserved protein UCP031279
AT1G10150		Carbohydrate-binding protein
AT1G10170	*NF-X-LIKE 1 (NFXL1)*	Encodes AtNFXL1, a homologue of the putative human transcription repressor NF-X1. Functions as a negative regulator of the trichothecene phytotoxin-induced defense response.
AT1G10270	*GLUTAMINE-RICH PROTEIN 23 (GRP23)*	glutamine-rich protein 23
AT1G10360	*GLUTATHIONE S-TRANSFERASE TAU 18 (GSTU18)*	Encodes glutathione transferase belonging to the tau class of GSTs. Naming convention according to Wagner et al. (2002).
AT1G10370	*EARLY-RESPONSIVE TO DEHYDRATION 9 (ERD9)*	Encodes GSTU17 (Glutathione S-Transferase U17). Functions as a negative component of stress-mediated signal transduction pathways in drought and salt stress responses.
AT1G10380		Putative membrane lipoprotein
AT1G10470	*RESPONSE REGULATOR 4 (ARR4)*	Encodes a two-component response regulator. Acts redundantly with ARR3 in the control of circadian period in a cytokinin-independent manner.
AT1G10530		PADRE protein
AT1G10960	*FERREDOXIN 1 (FD1)*	Phosphatidylinositol 4-phosphate 5-kinase (PIP5K) enzyme family member.

AT1G11000	*MILDEW RESISTANCE LOCUS O 4 (MLO4)*	A member of a large family of seven-transmembrane domain proteins specific to plants, homologs of the barley mildew resistance locus o (MLO) protein. The Arabidopsis genome contains 15 genes encoding MLO proteins, with localization in plasma membrane. Phylogenetic analysis revealed four clades of closely-related AtMLO genes. ATMLO4 belongs to the clade I, with AtMLO11 and AtMLO14. The gene is expressed during early seedling growth, in roots and lateral root primordia, in flower and fruit abscission zone, in vascular system of root, cotyledons and young leaves, it was not expressed in mature rosette leaves, as shown by GUS activity patterns. The expression of several phylogenetically closely-related AtMLO genes showed similar or overlapping tissue specificity and analogous responsiveness to external stimuli, suggesting functional redundancy, co-function, or antagonistic function(s).
AT1G11080	*SERINE CARBOXYPEPTIDASE-LIKE 31 (scpl31)*	serine carboxypeptidase-like 31
AT1G11190	*BIFUNCTIONAL NUCLEASE I (BFN1)*	Encodes a bifunctional nuclease that acts on both RNA and DNA involved in nucleic acid degradation to facilitate nucleotide and phosphate recovery during senescence. It has mismatch-specific endonuclease activity with wide recognition of single base mismatches as well as the ability to cleave indel types of mismatches (heteroduplexes with loops).
AT1G11440		hypothetical protein
AT1G11600	*CYTOCHROME P450, FAMILY 77, SUBFAMILY B, POLYPEPTIDE 1 (CYP77B1)*	Plant specific fatty acid epoxygenase.
AT1G11970		Ubiquitin-like superfamily protein
AT1G12030		phosphoenolpyruvate carboxylase, putative (DUF506)
AT1G12090	*EXTENSIN-LIKE PROTEIN (ELP)*	extensin-like protein (ELP)
AT1G12130	*(FMOGS-OX6)*	Encodes a flavin-containing monooxygenases involved in biosynthesis of aliphatic glucosinolates.
AT1G12160	*(FMOGS-OX7)*	Encodes a flavin-containing monooxygenases involved in biosynthesis of aliphatic glucosinolates.
AT1G12200	*FLAVIN MONOOXYGENASE (FMO)*	Putative flavin monooxygenase.
AT1G12250	*(TL20.3)*	Pentapeptide repeat-containing protein
AT1G12330		cyclin-dependent kinase-like protein
AT1G12740	*CYTOCHROME P450, FAMILY 87, SUBFAMILY A, POLYPEPTIDE 2 (CYP87A2)*	encodes a protein with cytochrome P450 domain
AT1G12845		transmembrane protein
AT1G12900	*GLYCERALDEHYDE 3-PHOSPHATE DEHYDROGENASE A SUBUNIT 2 (GAPA-2)*	glyceraldehyde 3-phosphate dehydrogenase A subunit 2
AT1G12940	*NITRATE TRANSPORTER2.5 (NRT2.5)*	member of High affinity nitrate transporter family
AT1G12950	*ROOT HAIR SPECIFIC 2 (RHS2)*	root hair specific 2
AT1G13590	*PHYTOSULFOKINE 1 PRECURSOR (PSK1)*	Encodes a phytosulfokine-alpha (PSK) precursor, a unique plant peptide growth factor first described in Asparagus.
AT1G13610		alpha/beta-Hydrolases superfamily protein
AT1G13700	*6-PHOSPHOGLUCONOLACTONASE 1 (PGL1)*	Encodes a cytosolic 6-phosphogluconolactonase (PGL) thought to be involved in the oxidative pentose-phosphate pathway (OPPP).
AT1G13710	*CYTOCHROME P450, FAMILY 78, SUBFAMILY A, POLYPEPTIDE 5 (CYP78A5)*	Encodes the cytochrome P450 CYP78A5 monooxygenase. Contributes to the generation of a growth-stimulating signal distinct from the classical phytohormones that prevents proliferation arrest, promoting organ growth. In ovules it is required for megagametogenesis, maternal control of seed size and restricting megaspore mother cell fate to a single cell.
AT1G13920		Remorin family protein
AT1G14080	*FUCOSYLTRANSFERASE 6 (FUT6)*	Encodes an alpha-(1,2)-fucosyltransferase.
AT1G14190		Glucose-methanol-choline (GMC) oxidoreductase family protein
AT1G14345		NAD(P)-linked oxidoreductase superfamily protein
AT1G14370	*PROTEIN KINASE 2A (APK2A)*	Encodes protein kinase APK2a. Protein is N-myristoylated.
AT1G14390		Leucine-rich repeat protein kinase family protein
AT1G14620	*DECOY (DECOY)*	decoy
AT1G14687	*HOMEOBOX PROTEIN 32 (HB32)*	homeobox protein 32
AT1G14700	*PURPLE ACID PHOSPHATASE 3 (PAP3)*	purple acid phosphatase 3
AT1G14720	*XYLOGLUCAN ENDOTRANSGLUCOSYLASE/HYDROLASE 28 (XTH28)*	member of Glycoside Hydrolase Family 16
AT1G14780		MAC/Perforin domain-containing protein
AT1G14840	*MICROTUBULE-ASSOCIATED PROTEINS 70-4 (MAP70-4)*	Encodes a microtubule associated protein (MAP70-4). Expressed in all tissues.
AT1G14940		Polyketide cyclase/dehydrase and lipid transport superfamily protein
AT1G15010		mediator of RNA polymerase II transcription subunit
AT1G15180		MATE efflux family protein
AT1G15210	*ATP-BINDING CASSETTE G35 (ABCG35)*	pleiotropic drug resistance 7
AT1G15290	*REDUCED CHLOROPLAST COVERAGE 3 (REC3)*	Encodes REDUCED CHLOROPLAST COVERAGE 3 (REC3). Contributes to establishing the size of the chloroplast compartment.
AT1G15340	*METHYL-CPG-BINDING DOMAIN 10 (MBD10)*	Protein containing methyl-CpG-binding domain.Has sequence similarity to human MBD proteins.
AT1G15440	*PERIODIC TRYPTOPHAN PROTEIN 2 (PWP2)*	Encodes a nucleolar protein that is a ribosome biogenesis co-factor. Mutants display aberrant RNA processing and female gametophyte development.
AT1G15550	*GIBBERELLIN 3-OXIDASE 1 (GA3OX1)*	Involved in later steps of the gibberellic acid biosynthetic pathway. Activated by AGAMOUS in a cal-1, ap1-1 background. Deletion of 208 bp from -1016 to -809 (Δ-808) resulted in loss of GA-negative feedback (this sequence, which contains a 43-bp sequence GNFEI, was shown to be sufficient for GA-negative feedback).
AT1G15580	*INDOLE-3-ACETIC ACID INDUCIBLE 5 (IAA5)*	auxin induced protein
AT1G15590		E3 ubiquitin-protein ligase

AT1G15670	*KISS ME DEADLY 2 (KMD2)*	Encodes a member of a family of F-box proteins, called the KISS ME DEADLY (KMD) family, that targets type-B ARR proteins for degradation and is involved in the negative regulation of the cytokinin response. Also named as KFB1, a member of a group of Kelch repeat F-box proteins that negatively regulate phenylpropanoid biosynthesis by targeting the phenypropanoid biosynthesis enzyme phenylalanine ammonia-lyase.
AT1G15790		mediator of RNA polymerase II transcription subunit 15a-like protein
AT1G15820	*LIGHT HARVESTING COMPLEX PHOTOSYSTEM II SUBUNIT 6 (LHCB6)*	Lhcb6 protein (Lhcb6), light harvesting complex of photosystem II.
AT1G15870		Mitochondrial glycoprotein family protein
AT1G16000	*OUTER ENVELOPE PROTEIN 9.1 (OEP9.1)*	Member of the Arabidopsis 7-kDa OEP family. Tail-anchored (TA) membrane protein which possesses a single C-terminal transmembrane domain targeting post-translationally to plastids.
AT1G16160	*WALL ASSOCIATED KINASE-LIKE 5 (WAKL5)*	WAK-like kinase The mRNA is cell-to-cell mobile.
AT1G16310	*METAL-TOLERANCE PROTEIN 10 (MTP10)*	Cation efflux family protein which affects ABA-JA crosstalk and susceptibility to Mamestra brassicae herbivory.
AT1G16410	*CYTOCHROME P450 79F1 (CYP79F1)*	member of CYP79F The mRNA is cell-to-cell mobile.
AT1G16490	*MYB DOMAIN PROTEIN 58 (MYB58)*	Member of the R2R3 factor gene family.
AT1G16510	*SMALL AUXIN UPREGULATED 41 (SAUR41)*	Encodes a clade III SAUR gene with a distinctive expression pattern in root meristems. It is normally expressed in the quiescent center and cortex/endodermis initials and upon auxin stimulation, the expression is found in the endodermal layer. Overexpression studies suggest roles in cell expansion and auxin transport.
AT1G16830		Pentatricopeptide repeat (PPR) superfamily protein
AT1G17010		2-oxoglutarate (2OG) and Fe(II)-dependent oxygenase superfamily protein
AT1G17020	*SENESCENCE-RELATED GENE 1 (SRG1)*	Encodes a novel member of the Fe(II)/ascorbate oxidase gene family; senescence-related gene.
AT1G17060	*CYTOCHROME P450 72C1 (CYP72C1)*	Encodes a protein with similarity to other cytochrome P450's and is a homolog of BAS1. Over expression causes a dwarf phenotype resembling brassinolide resistant mutants. Double mutant analysis of sob7/bas1 loss of function mutants suggests these genes have redundant functions in light responsiveness. SOB7 may function in metabolizing brassinolides. Expressed in leaf, root, stem and silique but expression highest in flower and cauline leaves. Dominant overexpressing plants have dwarf phenotype, short siliques/seeds, rounded dark green leaves and short hypocotyls in light and dark. Loss of function alleles result in plants with long hypocotyls.
AT1G17170	*GLUTATHIONE S-TRANSFERASE TAU 24 (GSTU24)*	Encodes glutathione transferase belonging to the tau class of GSTs. Naming convention according to Wagner et al. (2002). It is involved in the detoxification of the environmental pollutant 2,4,6-trinitrotoluene. Arabidopsis plants over-expressing At1g17170 were more resistant to TNT, removed more TNT from sterile and soil-based media, and had reduced levels of glutathione when grown in the presence of TNT.
AT1G17180	*GLUTATHIONE S-TRANSFERASE TAU 25 (GSTU25)*	Encodes glutathione transferase belonging to the tau class of GSTs. Naming convention according to Wagner et al. (2002). Detoxification of the environmental pollutant 2,4,6-trinitrotoluene. Arabidopsis plant over-expressing At1g17180 were more resistant to TNT, removed more TNT from sterile and soil-based media, and had reduced levels of glutathione when grown in the presence of TNT.
AT1G17190	*GLUTATHIONE S-TRANSFERASE TAU 26 (GSTU26)*	Encodes glutathione transferase belonging to the tau class of GSTs. Naming convention according to Wagner et al. (2002).
AT1G17200	*CASP-LIKE PROTEIN 2A1 (CASPL2A1)*	Uncharacterized protein family (UPF0497)
AT1G17290	*ALANINE AMINOTRANSFERASE (AlaAT1)*	Encodes for alanine aminotransferase (ALAAT1), involved in alanine catabolism during plants recovery from hypoxia The mRNA is cell-to-cell mobile.
AT1G17545		Protein phosphatase 2C family protein
AT1G17560	*HUELLENLOS (HLL)*	Encodes HUELLENLOS (HLL), a mitochondrial ribosome protein, similar to L14 ribosomal protein of eubacteria. HLL is essential for normal ovule development.
AT1G17710	*PHOSPHOETHANOLAMINE/PHOSPHOCHOLINE PHOSPHATASE 1 (PEPC1)*	Encodes a phosphoethanolamine/phosphocholine phosphatase. It is likely to be involved in the liberation of inorganic phosphate from intracellular sources. Expression is upregulated in the shoot of cax1/cax3 mutant.
AT1G17740		
AT1G17860	*ARABIDOPSIS THALIANA KUNITZ TRYPSIN INHIBITOR 5 (ATKTI5)*	Member of Kunitz trypsin inhibitor (KTI) family involved in plant defense response against spider mites.
AT1G18180		3-oxo-5-alpha-steroid 4-dehydrogenase (DUF1295)
AT1G18320	*(ATTIM22)*	Mitochondrial import inner membrane translocase subunit Tim17/Tim22/Tim23 family protein
AT1G18500	*METHYLTHIOALKYLMALATE SYNTHASE-LIKE 4 (MAML-4)*	Encodes an active Arabidopsis isopropylmalate synthase IPMS1. Involved in leucine biosynthesis. Do not participate in the chain elongation of glucosinolates. Expressed constitutively throughout the plant. Loss of IPMS1 can be compensated by a second isopropylmalate synthase gene IPMS2 (At1g74040). The mRNA is cell-to-cell mobile.
AT1G18730	*PHOTOSYNTHETIC NDH SUBCOMPLEX B 4 (PnsB4)*	likely a subunit of the chloroplast NAD(P)H dehydrogenase complex, involved in PSI cyclic electron transport. Located on the thylakoid membrane. Mutant has impaired NAD(P)H dehydrogenase activity. The mRNA is cell-to-cell mobile.
AT1G18810		phytochrome kinase substrate-like protein
AT1G18850	*LANT-SPECIFIC COMPONENT OF THE PRE- RRNA PROCESSING COMPLEX2 (PCP2)*	PCP2 encodes a novel plant specific protein that is co-expressed with components of pre-rRNA processing complex. Co-localizes with NuGWD1 and SWA1.
AT1G18980		RmlC-like cupins superfamily protein
AT1G19330	*SAP30 FUNCTION-RELATED 2 (AFR2)*	Evening-expressed key component of Sin3-HDAC complex, which bind directly to the CIRCADIAN CLOCK ASSOCIATED 1 (CCA1) and PSEUDO-RESPONSE REGULATOR 9 (PRR9) promoters and catalyze histone 3 (H3) deacetylation at the cognate regions to repress expression, allowing the declining phase of their expression at dusk.
AT1G19540		NmrA-like negative transcriptional regulator family protein
AT1G19630	*CYTOCHROME P450, FAMILY 722, SUBFAMILY A, POLYPEPTIDE 1 (CYP722A1)*	cytochrome P450, family 722, subfamily A, polypeptide 1
AT1G19700	*BEL1-LIKE HOMEODOMAIN 10 (BEL10)*	Encodes a member of the BEL family of homeodomain proteins. Its interaction with PLP (PAS/LOV PROTEIN) is diminished by blue light.
AT1G19970		ER lumen protein retaining receptor family protein
AT1G20020	*FERREDOXIN-NADP(+)-OXIDOREDUCTASE 2 (FNR2)*	Encodes a leaf-type ferredoxin:NADP(H) oxidoreductase. It is present in both chloroplast stroma and thylakoid membranes but is more abundant in the stroma The mRNA is cell-to-cell mobile.
AT1G20070		hypothetical protein
AT1G20510	*OPC-8:0 COA LIGASE1 (OPCL1)*	OPC-8:0 CoA ligase1

AT1G20823	*(ATATL80)*	Encodes a RING E3 ubiquitin ligase ATL80. Involved in phosphate mobilization and cold stress response in sufficient phosphate growth conditions. The mRNA is cell-to-cell mobile.
AT1G20850	*XYLEM CYSTEINE PEPTIDASE 2 (XCP2)*	Cysteine peptidase. Enzyme activity detected in leaf.
AT1G21000	*(PLATZ1)*	PLATZ transcription factor family protein
AT1G21360	*GLYCOLIPID TRANSFER PROTEIN 2 (GLTP2)*	glycolipid transfer protein 2
AT1G21400	*(E1A1)*	Branched chain alpha-keto acid dehydrogenase E1 alpha.
AT1G21440		Phosphoenolpyruvate carboxylase family protein
AT1G21500		hypothetical protein
AT1G21530		AMP-dependent synthetase and ligase family protein
AT1G21870	*GOLGI NUCLEOTIDE SUGAR TRANSPORTER 5 (GONST5)*	Encodes a Golgi-localized nucleotide-sugar transporter.
AT1G21910	*DEHYDRATION RESPONSE ELEMENT-BINDING PROTEIN 26 (DREB26)*	encodes a member of the DREB subfamily A-5 of ERF/AP2 transcription factor family. The protein contains one AP2 domain. There are 15 members in this subfamily including RAP2.1, RAP2.9 and RAP2.10.
AT1G22360	*UDP-GLUCOSYL TRANSFERASE 85A2 (UGT85A2)*	UDP-glucosyl transferase 85A2
AT1G22400	*(UGT85A1)*	UDP-Glycosyltransferase superfamily protein
AT1G22500	*ARABIDOPSIS TOXICOS EN LEVADURA 15 (ATL15)*	Gene encodes a putative C3HC4-type RING zinc finger factor. it is induced in response to light and ascorbate stimulus.
AT1G22570		Major facilitator superfamily protein
AT1G22590	*AGAMOUS-LIKE 87 (AGL87)*	AGAMOUS-like 87
AT1G22600		Late embryogenesis abundant protein (LEA) family protein
AT1G22710	*SUCROSE-PROTON SYMPORTER 2 (SUC2)*	Encodes for a high-affinity transporter essential for phloem loading and long-distance transport. A major sucrose transporter, AtSUC2 can also transport a wide range of physiological and synthetic glucose conjugates with both α- or β-linkage.
AT1G22890	*(STMP2)*	Secreted peptide which functions in plant growth and pathogen defense.
AT1G22980		Grap2/cyclin-D-interacting protein
AT1G22990	*HEAVY METAL ASSOCIATED ISOPRENYLATED PLANT PROTEIN 22 (HIPP22)*	Heavy metal transport/detoxification superfamily protein
AT1G23020	*FERRIC REDUCTION OXIDASE 3 (FRO3)*	Encodes a ferric chelate reductase whose transcription is regulated by FIT1. Expressed in the root, shoot, flower and cotyledon.
AT1G23040		hydroxyproline-rich glycoprotein family protein
AT1G23100		GroES-like family protein
AT1G23120		Polyketide cyclase/dehydrase and lipid transport superfamily protein
AT1G23130		Polyketide cyclase/dehydrase and lipid transport superfamily protein
AT1G23210	*GLYCOSYL HYDROLASE 9B6 (GH9B6)*	glycosyl hydrolase 9B6
AT1G23480	*CELLULOSE SYNTHASE-LIKE A3 (CSLA03)*	encodes a gene similar to cellulose synthase
AT1G23510		OBP32pep protein
AT1G23520		hypothetical protein (DUF220)
AT1G23580		transmembrane protein, putative (Domain of unknown function DUF220)
AT1G23590		OBP32pep protein, putative (Domain of unknown function DUF220)
AT1G23630		
AT1G23690		hypothetical protein (Domain of unknown function DUF220)
AT1G23740	*ALKENAL/ONE OXIDOREDUCTASE (AOR)*	AOR is an alkenal/one oxidoreductase that acts on compounds with unsaturated alpha,beta-carbonyls. The activity of this enzyme with a number of substrates, including acrolein and 3-buten-2-one, was demonstrated in vitro using a truncated form of the protein that lacked approximately 80 of the first amino acids. This protein appears to localize to the chloroplast where it likely helps to maintain the photosynthetic process by detoxifying reactive carbonyls formed during lipid peroxidation.
AT1G23760	*(JP630)*	Encodes aromatic rich glycoprotein JP630.
AT1G24030	*PBS1-LIKE 28 (PBL28)*	Protein kinase superfamily protein
AT1G24090	*(RNH1C)*	RNase H family protein
AT1G24470	*BETA-KETOACYL REDUCTASE 2 (KCR2)*	Encodes one of the two Arabidopsis homologues to YBR159w encoding a S. cerevisiae beta-ketoacyl reductase (KCR), which catalyzes the first reduction during VLCFA (very long chain fatty acids, >18 carbon) elongation: KCR1 (At1g67730), KCR2 (At1g24470). Complementation of the yeast ybr159Delta mutant demonstrated that the two KCR proteins are divergent and that only AtKCR1 can restore heterologous elongase activity similar to the native yeast KCR gene.
AT1G25230		Calcineurin-like metallo-phosphoesterase superfamily protein
AT1G25330	*CESTA (CES)*	Encodes CESTA, a positive regulator of brassinosteroid biosynthesis.
AT1G25560	*TEMPRANILLO 1 (TEM1)*	Encodes a member of the RAV transcription factor family that contains AP2 and B3 binding domains. Involved in the regulation of flowering under long days. Loss of function results in early flowering. Overexpression causes late flowering and repression of expression of FT. Novel transcriptional regulator involved in ethylene signaling. Promoter bound by EIN3. EDF1 in turn, binds to promoter elements in ethylene responsive genes.
AT1G26340	*CYTOCHROME B5 ISOFORM A (CB5-A)*	encodes a member of the cytochromes b5 family of proteins that localizes to the outer envelope of the chloroplast. The C-terminal portion of the protein appears to be capable of inserting into a plant microsomal membrane in vitro.
AT1G26530		PIN domain-like family protein
AT1G26680		transcriptional factor B3 family protein
AT1G26730		EXS (ERD1/XPR1/SYG1) family protein
AT1G26740		Ribosomal L32p protein family
AT1G26770	*EXPANSIN A10 (EXPA10)*	Encodes an expansin. Naming convention from the Expansin Working Group (Kende et al, Plant Mol Bio). Involved in the formation of nematode-induced syncytia in roots of Arabidopsis thaliana.

AT1G26870	*FEZ (FEZ)*	NAC-domain protein. Expressed in root cap stem cells, where it promotes periclinal root cap-forming divisions. Involved in a regulatory feedback loop with SMB. FEZ activates SMB in hte root cap daughter cells soon after division, and SMB in turn represses FEZ expression in these cells, thereby preventing further stem cell divisions.
AT1G27030		hypothetical protein
AT1G27120	*(GALT4)*	Encodes a Golgi-localized hydroxyproline-O-galactosyltransferase.
AT1G27480		alpha/beta-Hydrolases superfamily protein
AT1G27650	*(ATU2AF35A)*	U2 auxiliary factor small subunit. The atU2AF35a protein and its homolog, atU2AF35b, contain most of the conserved domains of hsU2AF35, including the psiRRM, one RS domain, two zinc fingers, and the two regions for interacting with U2AF large subunit. Both proteins lack the stretch of glycines present in human U2AF35. The sequences are overall 83% identical, and each Arabidopsis homolog shows approximately 70% similarity to hsU2AF35. U2AF(35) homologs were also identified from maize, rice and other plants with large-scale EST projects. Both genes are expressed in all major tissues, with atU2AF(35)a expressed at a higher level than atU2AF(35)b in most tissues. The expression patterns were different in roots: atU2AF(35)b expressed strongly in whole young roots and root tips and atU2AF(35)a limited to root vascular regions.
AT1G27740	*ROOT HAIR DEFECTIVE 6-LIKE 4 (RSL4)*	Basic helix-loop-helix (bHLH) transcription factor that is sufficient to promote postmitotic cell growth in root-hair cells. RSL4 is a direct transcriptional target of RHD6
AT1G27820	*CCR4-ASSOCIATED FACTOR 1C (CAF1C)*	Deadenylase.
AT1G28100		hypothetical protein
AT1G28130	*GRETCHEN HAGEN 3.17 (GH3.17)*	Encodes an IAA-amido synthase that conjugates Asp and other amino acids to auxin in vitro. Lines carrying insertions in this gene are hypersensitive to auxin.
AT1G28580		GDSL-motif esterase/acyltransferase/lipase. Enzyme group with broad substrate specificity that may catalyze acyltransfer or hydrolase reactions with lipid and non-lipid substrates.
AT1G29050	*TRICHOME BIREFRINGENCE-LIKE 38 (TBL38)*	Encodes a member of the TBL (TRICHOME BIREFRINGENCE-LIKE) gene family containing a plant-specific DUF231 (domain of unknown function) domain. TBL gene family has 46 members, two of which (TBR/AT5G06700 and TBL3/AT5G01360) have been shown to be involved in the synthesis and deposition of secondary wall cellulose, presumably by influencing the esterification state of pectic polymers. A nomenclature for this gene family has been proposed (Volker Bischoff & Wolf Scheible, 2010, personal communication).
AT1G29195		PADRE protein up-regulated after infection by S. sclerotiorum.
AT1G29280	*WRKY DNA-BINDING PROTEIN 65 (WRKY65)*	member of WRKY Transcription Factor; Group II-e The mRNA is cell-to-cell mobile.
AT1G29460	*SMALL AUXIN UPREGULATED RNA 65 (SAUR65)*	SAUR-like auxin-responsive protein family
AT1G29520		AWPM-19-like family protein
AT1G29630		5-3 exonuclease family protein
AT1G30100	*NINE-CIS-EPOXYCAROTENOID DIOXYGENASE 5 (NCED5)*	Encodes 9-cis-epoxycarotenoid dioxygenase, a key enzyme in the biosynthesis of abscisic acid. The expression of this gene increases during the first 6h of imbibition.
AT1G30120	*PYRUVATE DEHYDROGENASE E1 BETA (PDH-E1 BETA)*	Encodes a putative plastid pyruvate dehydrogenase E1 beta subunit that is distinct from the mitochondrial pyruvate dehydrogenase E1 beta subunit.
AT1G30380	*PHOTOSYSTEM I SUBUNIT K (PSAK)*	Encodes subunit K of photosystem I reaction center. The mRNA is cell-to-cell mobile.
AT1G30700	*(ATBBE8)*	FAD-binding Berberine family protein
AT1G30840	*PURINE PERMEASE 4 (PUP4)*	Member of a family of proteins related to PUP1, a purine transporter. May be involved in the transport of purine and purine derivatives such as cytokinins, across the plasma membrane.
AT1G30960	*(ERA-2)*	Ortholog of ERA (E. coli RAS-like protein)-related GTPase (ERG). Mitochondrial protein that associates with 18sRNA. Heterozygous mutants segregate for embryo lethality inherited as a sporphytic maternal effect. Increased ROS in the mutant ovule suggests a heritable mitochondrial defect results in lethality.
AT1G31050	*PERICYCLE FACTOR TYPE-A 1 (PFA1)*	Together with PFA2 and PFA3 governs the competence of pericycle cells to initiate lateral root primordium formation.
AT1G31290	*ARGONAUTE 3 (AGO3)*	ARGONAUTE 3
AT1G31310		hydroxyproline-rich glycoprotein family protein
AT1G31330	*PHOTOSYSTEM I SUBUNIT F (PSAF)*	Encodes subunit F of photosystem I.
AT1G31700		
AT1G31770	*ATP-BINDING CASSETTE G14 (ABCG14)*	ATP-binding cassette 14
AT1G31920		Tetratricopeptide repeat (TPR)-like superfamily protein
AT1G32060	*PHOSPHORIBULOKINASE (PRK)*	phosphoribulokinase
AT1G32080	*(LrgB)*	Encodes a plant LrgAB/CidAB protein localized to the chloroplast envelope that is involved in chloroplast development, carbon partitioning, ABA/drought response, and leaf senescence. The gene may have evolved from gene fusion of bacterial lrgA and lrgB.
AT1G32350	*ALTERNATIVE OXIDASE 1D (AOX1D)*	Alternative oxidase (AOX); terminal oxidases of electron transfer in mitochondria.
AT1G32450	*NRT1/ PTR FAMILY 7.3 (NPF7.3)*	Transmembrane nitrate transporter. Involved in xylem transport of nitrate from root to shoot. Induced in response to high and low concentrations of nitrate. Not involved in nitrate uptake. Expressed in root pericycle cells under the control of MYB59. Also functions as a proton-coupled H+/K+ antiporter for K+ loading into the xylem.
AT1G32470	*(GDC-H1)*	Single hybrid motif superfamily protein
AT1G32550	*FERREDOXIN C 2 (FdC2)*	Encodes FdC2, a ferredoxin protein capable of alternative electron partitioning. FdC1 level increases in conditions of acceptor limitation at PSI.
AT1G32740		SBP (S-ribonuclease binding protein) family protein
AT1G32760		Glutaredoxin family protein
AT1G32800		
AT1G32830		transposable_element_gene;similar to heat shock protein binding [Arabidopsis thaliana] (TAIR:AT3G30450.1);(source:TAIR10)
AT1G32870	*NAC DOMAIN PROTEIN 13 (NAC13)*	Expression in rosette leaves is activated by high concentration of boron.
AT1G32900	*GRANULE BOUND STARCH SYNTHASE 1 (GBSS1)*	UDP-Glycosyltransferase superfamily protein
AT1G33055	*HYPOXIA RESPONSE UNKNOWN PROTEIN 32 (HUP32)*	hypothetical protein
AT1G33110		MATE efflux family protein

AT1G33260		Protein kinase superfamily protein
AT1G33280	*NAC DOMAIN CONTAINING PROTEIN 15 (NAC015)*	NAC domain protein. SMB, BRN1, and BRN2 act to regulate root cap maturation, in a partially redundant fashion.BRN1 and BRN2, control the cell wall maturation processes that are required to detach root cap layers from the root.
AT1G33440	*NRT1/ PTR FAMILY 4.4 (NPF4.4)*	Major facilitator superfamily protein
AT1G33500		tropomyosin
AT1G33590		Leucine-rich repeat (LRR) family protein
AT1G33600		Leucine-rich repeat (LRR) family protein
AT1G33700		Beta-glucosidase, GBA2 type family protein
AT1G33750	*TERPENE SYNTHASE 22 (TPS22)*	Sesterterpene synthase which produces various sesterpne backbones bia type-A cyclization mechanism.
AT1G33811	*(GGL7)*	GDSL-motif esterase/acyltransferase/lipase. Enzyme group with broad substrate specificity that may catalyze acyltransfer or hydrolase reactions with lipid and non-lipid substrates.
AT1G33860		hypothetical protein
AT1G34180	*NAC DOMAIN CONTAINING PROTEIN 16 (NAC016)*	NAC domain containing protein 16
AT1G34430	*EMBRYO DEFECTIVE 3003 (EMB3003)*	2-oxoacid dehydrogenases acyltransferase family protein
AT1G34640		peptidase
AT1G34750	*CERK-1 INTERACTING PROTEIN PHOSPHATASE 1 (CIPP1)*	Protein phosphatase 2C family protein
AT1G35260	*MLP-LIKE PROTEIN 165 (MLP165)*	MLP-like protein 165
AT1G35290	*ACYL-LIPID THIOESTERASE 1 (ALT1)*	Thioesterase superfamily protein
AT1G35420		alpha/beta-Hydrolases superfamily protein
AT1G35910	*TREHALOSE-6-PHOSPHATE PHOSPHATASE D (TPPD)*	Haloacid dehalogenase-like hydrolase (HAD) superfamily protein
AT1G37110		transposable_element_gene;copia-like retrotransposon family, has a 0. P-value blast match to GB:CAA31653 polyprotein (Ty1_Copia-element) (Arabidopsis thaliana);(source:TAIR10)
AT1G41930		transposable_element_gene;CACTA-like transposase family (Tnp1/En/Spm), has a 3.8e-118 P-value blast match to ref|NP_189784.1| TNP1-related protein (Arabidopsis thaliana) (CACTA-element);(source:TAIR10)
AT1G42570		pseudogene of FAR1-related sequence 5
AT1G42970	*GLYCERALDEHYDE-3-PHOSPHATE DEHYDROGENASE B SUBUNIT (GAPB)*	Encodes chloroplast localized glyceraldehyde-3-phosphate dehydrogenase that can use both NADH and NADPH to reduce 1,3-diphosphate glycerate. It forms A2B2 heterotetramers with GapA forms of the GADPH enzyme. These complexes are active in the light under reducing conditions, but show reduced NADPH-dependent activity in response to oxidized thioredoxins and increased NAD(H)/NADP(H) ratios due to the formation of inactive A8B8 hexadecamers. The mRNA is cell-to-cell mobile.
AT1G43020		electron protein, putative (Protein of unknown function, DUF547)
AT1G43800	*FLORAL TRANSITION AT THE MERISTEM1 (FTM1)*	Δ9 stearoyl-ACP desaturase which together with FAB2, AAD1, and AAD5 redundantly participates in oil storage during the maturation phase.
AT1G43930		transposable_element_gene;Mutator-like transposase family, has a 1.2e-56 P-value blast match to GB:AAA21566 mudrA of transposon=MuDR (MuDr-element) (Zea mays);(source:TAIR10)
AT1G44000	*STAY-GREEN LIKE (SGRL)*	STAY-GREEN-like protein
AT1G44760		Adenine nucleotide alpha hydrolases-like superfamily protein
AT1G44830	*ERF TRANSCRIPTION FACTOR 14 (ERF014)*	Encodes a nuclear-localized member of the DREB subfamily A-5 of ERF/AP2 transcription factor family. The protein contains one AP2 domain. There are 15 members in this subfamily including RAP2.1, RAP2.9 and RAP2.10. Overexpression in cultured cells results in an increase in pectin deposition.ERF014 differentially regulates responses to bacterial and fungal pathogens.
AT1G45474	*PHOTOSYSTEM I LIGHT HARVESTING COMPLEX GENE 5 (LHCA5)*	Encodes a component of the light harvesting complex of photosystem I.
AT1G45616	*RECEPTOR LIKE PROTEIN 6 (RLP6)*	receptor like protein 6
AT1G47210	*CYCLIN-DEPENDENT PROTEIN KINASE 3;2 (CYCA3;2)*	cyclin-dependent protein kinase 3
AT1G47465		transposable_element_gene;gypsy-like retrotransposon family (Athila), has a 9.7e-17 P-value blast match to GB:CAA57397 Athila ORF 1 (Arabidopsis thaliana);(source:TAIR10)
AT1G47980		desiccation-like protein
AT1G48100	*POLYGALACTURONASE INVOLVED IN EXPANSION3 (PGX3)*	Pectin lyase-like superfamily protein
AT1G48260	*CBL-INTERACTING PROTEIN KINASE 17 (CIPK17)*	Encodes a member of the SNF1-related kinase (SnRK) gene family (SnRK3.21), which has also been reported as a member of the CBL-interacting protein kinases (CIPK17).
AT1G48350	*EMBRYO DEFECTIVE 3105 (EMB3105)*	Member of the uL18 RNA-binding protein family. uL18 proteins share a short structurally conserved domain that binds the 5S rRNA and allow its incorporation into ribosomes.
AT1G48510	*SURFEIT 1B (SURF1B)*	Encodes one of two Arabidopsis mitochondrial proteins similar to human SURF1 which is known to be involved in cytochrome c oxidase assembly. Mutations result in defects in hypocotyl elongation and changes in GA homeostasis.
AT1G48570		zinc finger (Ran-binding) family protein
AT1G48600	*PHOSPHOETHANOLAMINE N-METHYLTRANSFERASE (PMEAMT)*	Encodes a phosphoethanolamine N-methyltransferase that catalyses the last two methylation steps of the three sequential methylations of phosphoethanolamine (PEA) that are required for the synthesis of phosphocholine (PCho) in plants.
AT1G48660		Auxin-responsive GH3 family protein
AT1G49110		hypothetical protein
AT1G49430	*LONG-CHAIN ACYL-COA SYNTHETASE 2 (LACS2)*	Encodes a long chain acyl-CoA synthetase that catalyzes the synthesis of omega-hydroxy fatty acyl-CoA intermediates in the pathway to cutin synthesis. Required for repression of lateral root formation.
AT1G49470		transmembrane epididymal protein (DUF716)
AT1G49480	*RELATED TO VERNALIZATION1 1 (RTV1)*	Encodes a nuclear-localized DNA-binding protein that interacts with ITN1 at the PM and nuclei in vivo and may regulate ITN's subcellular localization.

AT1G49570		Peroxidase superfamily protein
AT1G49650		alpha/beta-Hydrolases superfamily protein
AT1G49830		basic helix-loop-helix (bHLH) DNA-binding superfamily protein
AT1G49840	*BOUNDARY OF ROP DOMAIN5 (BDR5)*	glutamyl-tRNA (Gln) amidotransferase subunit A (DUF620)
AT1G49860	*GLUTATHIONE S-TRANSFERASE (CLASS PHI) 14 (GSTF14)*	Encodes glutathione transferase belonging to the phi class of GSTs. Naming convention according to Wagner et al. (2002). The mRNA is cell-to-cell mobile.
AT1G49975		photosystem I reaction center subunit N
AT1G50060		CAP (Cysteine-rich secretory proteins, Antigen 5, and Pathogenesis-related 1 protein) superfamily protein
AT1G50080		ribonuclease
AT1G50110	*BRANCHED-CHAIN AMINOTRANSFERASE 6 (BCAT6)*	Encodes a cytosolic branched-chain aminotransferase that acts on Leu, Ile, Val and also on Met. Together with BCAT4 and BCAT3, it is involved in methionine salvage and glucosinolate biosynthesis.
AT1G50150		
AT1G50280	*BTB/POZ PROTEIN HYPERSENSITIVE TO ABA 1 (BPH1)*	BTB/POZ protein that forms a complex with CUL3a. Involved in repression of ABA responses.
AT1G50390		pfkB-like carbohydrate kinase family protein
AT1G50430	*DWARF 5 (DWF5)*	Mutants are defective in Brassinosteroid biosynthesis (delta7-sterol-C7 reduction step) and have a dwarf phenotype. EXO70 interactor and presumed negative secretion regulator.
AT1G50570		Calcium-dependent lipid-binding (CaLB domain) family protein
AT1G50630		extracellular ligand-gated ion channel protein (DUF3537)
AT1G50680		AP2/B3 transcription factor family protein
AT1G50720		Stigma-specific Stig1 family protein
AT1G50900	*GRANA DEFICIENT CHLOROPLAST 1 (GDC1)*	Encodes GDC1 (Grana Deficient Chloroplast 1), an ankyrin domain containing protein required fro chloroplast thylakoid grana formation. The mRNA is cell-to-cell mobile.
AT1G51080		golgin family A protein
AT1G51190	*PLETHORA 2 (PLT2)*	Encodes a member of the AINTEGUMENTA-like (AIL) subclass of the AP2/EREBP family of transcription factors and is essential for quiescent center (QC) specification and stem cell activity. It is a key effector for establishment of the stem cell niche during embryonic pattern formation. It is transcribed in response to auxin accumulation and is dependent on auxin response transcription factors.
AT1G51380		DEA(D/H)-box RNA helicase family protein
AT1G51400		Photosystem II 5 kD protein
AT1G51440	*DAD1-LIKE LIPASE 2 (DALL2)*	Encodes a lipase that hydrolyzes phosphatidylcholine, glycolipids as well as triacylglycerols.
AT1G51680	*4-COUMARATE:COA LIGASE 1 (4CL1)*	encodes an isoform of 4-coumarate:CoA ligase (4CL), which is involved in the last step of the general phenylpropanoid pathway. In addition to 4-coumarate, it also converts ferulate. The catalytic efficiency was in the following (descending) order: p-coumaric acid, ferulic acid, caffeic acid and 5-OH-ferulic acid. At4CL1 was unable to use sinapic acid as substrate.
AT1G51850	*STRESS INDUCED FACTOR 2 (SIF2)*	Malectin-like receptor-like kinase involved in MAMP mediated stomatal immunity. Interacts with BAK1/FLS2 signaling complex and subsequently phosphorylates and activates SLAC1.
AT1G51980		Insulinase (Peptidase family M16) protein
AT1G52070		Mannose-binding lectin superfamily protein
AT1G52100		Mannose-binding lectin superfamily protein
AT1G52200		PLAC8 family protein
AT1G52220	*CURVATURE THYLAKOID 1C (CURT1C)*	Thylakoid membrane localized protein that interacts with other CURT family proteins. Oligomerization is associated with grana thylakoid curavature.
AT1G52450		Ubiquitin carboxyl-terminal hydrolase-related protein
AT1G52510		alpha/beta-Hydrolases superfamily protein
AT1G52580	*RHOMBOID-LIKE PROTEIN 5 (RBL5)*	RHOMBOID-like protein 5
AT1G52680		late embryogenesis abundant protein-related / LEA protein-like protein
AT1G52710		Rubredoxin-like superfamily protein
AT1G52910		fiber (DUF1218)
AT1G52930	*ARABIDOPSIS HOMOLOGUE OF YEAST BRX1 2 (ATBRX1-2)*	Encodes one of two Arabidopsis orthologs of yeast BRX1, a protein involved in maturation of the large ribosomal subunit. The proteins are mainly localized in nucleolus. Mutant plants are affected in pre-rRNA processing.
AT1G53035		transmembrane protein
AT1G53170	*ETHYLENE RESPONSE FACTOR 8 (ERF8)*	encodes a member of the ERF (ethylene response factor) subfamily B-1 of ERF/AP2 transcription factor family (ATERF-8). The protein contains one AP2 domain. There are 15 members in this subfamily including ATERF-3, ATERF-4, ATERF-7, and leafy petiole.
AT1G53520	*FATTY-ACID-BINDING PROTEIN 3 (FAP3)*	Encodes a plastid stroma localized fatty acid binding protein involved in fatty acid metabolism.
AT1G53560		Ribosomal protein L18ae family
AT1G53645		hydroxyproline-rich glycoprotein family protein
AT1G53830	*PECTIN METHYLESTERASE 2 (PME2)*	encodes a pectin methylesterase
AT1G53885		linoleate 9S-lipoxygenase-4 protein, putative (DUF581). Senescence associated protein, expression regulated by cytokinin.
AT1G54040	*EPITHIOSPECIFIER PROTEIN (ESP)*	Epithiospecifier protein, interacts with WRKY53. Involved in pathogen resistance and leaf senescence.
AT1G54340	*ISOCITRATE DEHYDROGENASE (ICDH)*	NADP-specific isocitrate dehydrogenase (ICDH) The mRNA is cell-to-cell mobile.
AT1G54420		hypothetical protein
AT1G54500		RBD1 is a thylakoid membrane-bound iron-binding protein that is required for the proper assembly of photosystem II in Arabidopsis. It is found in all oxygenic photoautotrophic organisms (plants, algae and cyanobacteria).
AT1G54780	*THYLAKOID LUMEN PROTEIN 18.3 (TLP18.3)*	Encodes a thylakoid lumen protein regulating photosystem II repair cycle. Has acid phosphatase activity. The mRNA is cell-to-cell mobile.

AT1G54790		GDSL-motif esterase/acyltransferase/lipase. Enzyme group with broad substrate specificity that may catalyze acyltransfer or hydrolase reactions with lipid and non-lipid substrates.
AT1G54890		Late embryogenesis abundant (LEA) protein-like protein
AT1G55120	*BETA-FRUCTOFURANOSIDASE 5 (FRUCT5)*	Encodes a protein with fructan exohydrolase (FEH) activity acting on levan-type fructans (6-FEH, levanase). The enzyme does not have invertase activity.
AT1G55260	*GLYCOSYLPHOSPHATIDYLINOSITOL-ANCHORED LIPID PROTEIN TRANSFER 6 (LTPG6)*	Bifunctional inhibitor/lipid-transfer protein/seed storage 2S albumin superfamily protein
AT1G55265		DUF538 family protein, putative (Protein of unknown function, DUF538)
AT1G55440		Cysteine/Histidine-rich C1 domain family protein
AT1G55610	*BRI1 LIKE (BRL1)*	mutant has Altered vascular cell differentiation; LRR Receptor Kinase
AT1G55640	*PRENYLATED RAB ACCEPTOR 1.G1 (PRA1.G1)*	prenylated RAB acceptor 1.G1
AT1G55670	*PHOTOSYSTEM I SUBUNIT G (PSAG)*	Encodes subunit G of photosystem I, an 11-kDa membrane protein that plays an important role in electron transport between plastocyanin and PSI and is involved in the stability of the PSI complex. PSI-G subunit is bound to PSI-B and is in contact with Lhca1. The protein inserts into thylakoids by a direct or "spontaneous" pathway that does not involve the activities of any known chloroplast protein-targeting machinery. PSI-G appears to be directly or indirectly involved in the interaction between Photosystem I and plastocyanin.
AT1G55740	*SEED IMBIBITION 1 (SIP1)*	seed imbibition 1
AT1G55810	*URIDINE KINASE-LIKE 3 (UKL3)*	One of the homologous genes predicted to encode proteins with UPRT domains (Uracil phosphoribosyltransferase). Five of these genes (At5g40870, At3g27190, At1g55810, At4g26510 and At3g27440) show a high level of identity, and are annotated as also containing a N-terminal uracil kinase (UK) domain. These genes are referred to as UKL1 (UK-like 1), UKL2, UKL3, UKL4 and UKL5, respectively.
AT1G55850	*CELLULOSE SYNTHASE LIKE E1 (CSLE1)*	encodes a protein similar to cellulose synthase The mRNA is cell-to-cell mobile.
AT1G55920	*SERINE ACETYLTRANSFERASE 2;1 (SERAT2;1)*	Encodes a chloroplast/cytosol localized serine O-acetyltransferase involved in sulfur assimilation and cysteine biosynthesis. Expressed in the vascular system. The mRNA is cell-to-cell mobile.
AT1G56010	*NAC DOMAIN CONTAINING PROTEIN 1 (NAC1)*	Encodes a transcription factor involved auxin-mediated lateral root formation. Acts downstream of TIR1 and is regulated post-transcriptionally by miRNA164 and by SINAT5-dependent ubiquitination.
AT1G56060	*CYSTEINE-RICH TRANSMEMBRANE MODULE 3 (ATHCYSTM3)*	CYSTM3 is a mitochondrial protein that is induced by salt stress and is a negative regulator of salt stress.
AT1G56150	*SMALL AUXIN UPREGULATED 71 (SAUR71)*	SAUR-like auxin-responsive protein family
AT1G56170	*NUCLEAR FACTOR Y, SUBUNIT C2 (NF-YC2)*	Encodes a protein with similarity to a subunit of the CCAAT promoter motif binding complex of yeast.One of two members of this class (HAP5B) and expressed in vegetative and reproductive tissues. Involved in the regulation of response to nutrient levels.
AT1G56200	*EMBRYO DEFECTIVE 1303 (emb1303)*	Encodes a chloroplast localized protein that is essential for chloroplast development.
AT1G56680		Chitinase family protein
AT1G57790	*FBOX/DUF295-RELATED 1 (ATFDR1)*	F-box family protein
AT1G57990	*PURINE PERMEASE 18 (PUP18)*	Member of a family of proteins related to PUP1, a purine transporter. May be involved in the transport of purine and purine derivatives such as cytokinins, across the plasma membrane.
AT1G58150		phosphoglycerate kinase
AT1G58420		Uncharacterized conserved protein UCP031279
AT1G59124		Disease resistance protein (CC-NBS-LRR class) family
AT1G59500	*(GH3.4)*	encodes an IAA-amido synthase that conjugates Asp and other amino acids to auxin in vitro.
AT1G59740	*NRT1/ PTR FAMILY 4.3 (NPF4.3)*	Major facilitator superfamily protein
AT1G59860	*(HSP17.6A)*	HSP20-like chaperones superfamily protein
AT1G59870	*PENETRATION 3 (PEN3)*	ATP binding cassette transporter. Localized to the plasma membrane in uninfected cells. In infected leaves, the protein concentrated at infection sites. Contributes to nonhost resistance to inappropriate pathogens that enter by direct penetration in a salicylic acid?dependent manner. Required for mlo resistance. Has Cd transporter activity (Cd2+ extrusion pump) and contributes to heavy metal resistance. The mRNA is cell-to-cell mobile.
AT1G59940	*RESPONSE REGULATOR 3 (ARR3)*	Type A response regulator highly similar to bacterial two-component response regulators. Rapidly induced by cytokinin. Involved in red-light signaling. Acts redundantly with ARR3 in the control of circadian period in a cytokinin-independent manner.
AT1G60000		RNA-binding (RRM/RBD/RNP motifs) family protein
AT1G60060		Serine/threonine-protein kinase WNK (With No Lysine)-like protein
AT1G60090	*BETA GLUCOSIDASE 4 (BGLU4)*	beta glucosidase 4
AT1G60190	*PLANT U-BOX 19 (PUB19)*	Encodes PUB19, a plant U-box armadillo repeat protein. Involved in salt inhibition of germination together with PUB18. The mRNA is cell-to-cell mobile.
AT1G60270	*BETA GLUCOSIDASE 6 (BGLU6)*	beta glucosidase 6
AT1G60300		NAC (No Apical Meristem) domain transcriptional regulator superfamily protein
AT1G60730		NAD(P)-linked oxidoreductase superfamily protein
AT1G60810	*ATP-CITRATE LYASE A-2 (ACLA-2)*	One of the three genes encoding subunit A of the trimeric enzyme ATP Citrate lyase
AT1G60850	*(ATRPAC42)*	DNA-directed RNA polymerase family protein
AT1G60950	*(FED A)*	encodes a major leaf ferredoxin
AT1G60960	*IRON REGULATED TRANSPORTER 3 (IRT3)*	Encodes a plasma membrane localized zinc/iron transporter.
AT1G60970		SNARE-like superfamily protein
AT1G61070	*LOW-MOLECULAR-WEIGHT CYSTEINE-RICH 66 (LCR66)*	Predicted to encode a PR (pathogenesis-related) protein. Belongs to the plant defensin (PDF) family with the following members: At1g75830/PDF1.1, At5g44420/PDF1.2a, At2g26020/PDF1.2b, At5g44430/PDF1.2c, At2g26010/PDF1.3, At1g19610/PDF1.4, At1g55010/PDF1.5, At2g02120/PDF2.1, At2g02100/PDF2.2, At2g02130/PDF2.3, At1g61070/PDF2.4, At5g63660/PDF2.5, At2g02140/PDF2.6, At5g38330/PDF3.1 and At4g30070/PDF3.2.
AT1G61110	*NAC DOMAIN CONTAINING PROTEIN 25 (NAC025)*	NAC transcription regulator. Regulates endosperm cell expansion during germination.
AT1G61340	*F-BOX STRESS INDUCED 1 (FBS1)*	Encodes a F-box protein induced by various biotic or abiotic stress.

AT1G61380	*S-DOMAIN-1 29 (SD1-29)*	Encodes a membrane localized S-domain receptor kinase that is involved in lipopolysaccharide (LPS) sensing. SD1-29 detected LPS of Pseudomonas and Xanthomonas species for which it serves as a microbe associated molecular pattern triggering innate immunity. Loses of function mutants are hyper susceptible to P.syringae.
AT1G61590	*PBS1-LIKE 15 (PBL15)*	Protein kinase superfamily protein
AT1G61640		Protein kinase superfamily protein
AT1G61740		Sulfite exporter TauE/SafE family protein
AT1G61800	*GLUCOSE-6-PHOSPHATE/PHOSPHATE TRANSLOCATOR 2 (GPT2)*	glucose6-Phosphate/phosphate transporter 2. Expression is upregulated in the shoot of cax1/cax3 mutant. The mRNA is cell-to-cell mobile.
AT1G61930	*(S40-6)*	senescence regulator (Protein of unknown function, DUF584)
AT1G62030		Cysteine/Histidine-rich C1 domain family protein
AT1G62070		hypothetical protein
AT1G62300	*(WRKY6)*	Encodes a transcription factor WRKY6. Regulates Phosphate1 (Pho1) expression in response to low phosphate (Pi) stress.
AT1G62500	*(DEG27)*	Bifunctional inhibitor/lipid-transfer protein/seed storage 2S albumin superfamily protein
AT1G62510		Expressed in the root cortex.
AT1G62530		hypothetical protein (DUF863)
AT1G62620		Flavin-binding monooxygenase family protein
AT1G62730		Terpenoid synthases superfamily protein
AT1G62840		ankyrin repeat/KH domain protein (DUF1442)
AT1G62910	*RF-LIKE 9 (RFL9)*	Encodes PPR protein involved in mitochondrial 5' end processing. Ecotype variants show differences in processing.
AT1G62990	*KNOTTED-LIKE HOMEOBOX OF ARABIDOPSIS THALIANA 7 (KNAT7)*	Encodes a homeodomain transcription factor of the Knotted family. May be involved in secondary cell wall biosynthesis. Mutants have moderately irregular xylem development. Expression of this gene is upregulated by SND1 and MYB46.
AT1G63240	*ROS1-ASSOCIATED METHYL-DNA BINDING PROTEIN 1 (RMB1)*	Methyl-DNA binding protein which interacts with RMB1 and ROS1 acting in the base excision repair pathway through DNA methylation.
AT1G63440	*HEAVY METAL ATPASE 5 (HMA5)*	The Arabidopsis P-type ATPase HMA5 is involved in Cu detoxification. hma5 mutant plants exhibit Cu hypersensitivity, which is especially dramatic in roots where HMA5 is mostly expressed.
AT1G63550		Receptor-like protein kinase-related family protein
AT1G63650	*ENHANCER OF GLABRA 3 (EGL3)*	Mutant has reduced trichomes, anthocyanin, and seed coat mucilage and abnormally patterned stomates. Mutants are defective in jasmonate-induced anthocyanin accumulation. Encodes a bHLH Transcription Factor 1. The protein is functionally redundant with GL3 and TT8 and interacts with TTG1, the myb proteins GL1, PAP1 and 2, CPC and TRY, and it will form heterodimers with GL3. Expression in N (non-hair cell forming) cell layers is negatively regulated by WER. Expression in H cells (hair cell forming) is promoted by CPC/TRY.
AT1G63660		GMP synthase (glutamine-hydrolyzing), putative / glutamine amidotransferase
AT1G63750		miR825-5p target proposed as a phasiRNA producing locus.
AT1G63840		RING/U-box superfamily protein
AT1G64140		WRKY transcription factor
AT1G64170	*CATION/H+ EXCHANGER 16 (CHX16)*	member of Putative Na+/H+ antiporter family
AT1G64370	*PHLOEM ASSOCIATED RNA CHAPERONE-LIKE (PARCL)*	filaggrin-like protein
AT1G64400	*LONG-CHAIN ACYL-COA SYNTHETASE 3 (LACS3)*	AMP-dependent synthetase and ligase family protein
AT1G64470		
AT1G64600		copper ion binding / methyltransferase
AT1G64670	*BODYGUARD1 (BDG1)*	Encodes a epidermally expressed extracellular protein that likely functions as an alpha-beta hydrolase and is required for normal cuticle formation. Homozygous mutant plants are dwarfed and have abnormal leaves, collapsed cells, reduced numbers of trichomes. The specific role of BDG is unclear: it may function in cutin biosynthesis or as a cross-linking enzyme in the cell wall itself.
AT1G64680		beta-carotene isomerase D27
AT1G64720	*MEMBRANE RELATED PROTEIN CP5 (CP5)*	membrane related protein CP5
AT1G64950	*CYTOCHROME P450, FAMILY 89, SUBFAMILY A, POLYPEPTIDE 5 (CYP89A5)*	member of CYP89A The mRNA is cell-to-cell mobile.
AT1G65090		nucleolin
AT1G65180		Cysteine/Histidine-rich C1 domain family protein
AT1G65370		TRAF-like family protein
AT1G65510	*(STMP7)*	Secreted peptide which functions in plant growth and pathogen defense.
AT1G65570	*ROOT CAP POLYGALACTURONASE (RCPG)*	Encodes a glycosyl hydrolase 28 (GH28) family polygalacturonase (PG) protein. Involved in root cap development.
AT1G65620	*ASYMMETRIC LEAVES 2 (AS2)*	required for formation of a symmetric flat leaf lamina, encodes a member of a family of proteins characterized by cysteine repeats and a leucine zipper; involved in KNOX gene regulation. Acts together with ASL1 in proximal-distal symmetry determination. Forms a complex with AS1 that binds to the BP promoter and leads to silencing of BP.
AT1G65730	*YELLOW STRIPE LIKE 7 (YSL7)*	Arabidopsis thaliana metal-nicotianamine transporter YSL4
AT1G65880	*BENZOYLOXYGLUCOSINOLATE 1 (BZO1)*	Encodes a benzoate-CoA ligase. Involved in the biosynthesis of benzoyloxyglucosinolate in Arabidopsis seeds.
AT1G65900		plant/protein
AT1G66130		NAD(P)-binding Rossmann-fold superfamily protein
AT1G66430	*FRUCTOKINASE 3 (FRK3)*	Encodes one of the several Arabidopsis fructokinases. Nomenclature according to Riggs 2017 has been adopted for the family by the community (personal communication, Boernke, Callis, Granot, Boernke, and Smeekens). Important for seed oil accumulation and vascular development.
AT1G66470	*ROOT HAIR DEFECTIVE6 (RHD6)*	ROOT HAIR DEFECTIVE6
AT1G66830		Leucine-rich repeat protein kinase family protein

AT1G66840	*PLASTID MOVEMENT IMPAIRED 2 (PMI2)*	Encodes a coiled-coil protein WEB2 (weak chloroplast movement under blue light 2, also named PMI2/plastid movement impaired 2). Involved in chloroplast avoidance movement under intermediate and high light intensities. WEB2, together with another coiled-coil protein WEB1 (AT2G26570), maintains the chloroplast photorelocation movement velocity.
AT1G66920		Protein kinase superfamily protein
AT1G66930	*LEAF RUST 10 DISEASE-RESISTANCE LOCUS RECEPTOR- LIKE PROTEIN KINASE-LIKE2 (LRK10L2)*	Protein kinase superfamily protein
AT1G67560	*LIPOXYGENASE 6 (LOX6)*	PLAT/LH2 domain-containing lipoxygenase family protein
AT1G67590		Remorin family protein
AT1G67600		Acid phosphatase/vanadium-dependent haloperoxidase-related protein
AT1G67700	*HYPERSENSITIVE TO HIGH LIGHT 1 (HHL1)*	multidrug resistance protein
AT1G67740	*PHOTOSYSTEM II BY (PSBY)*	PsbY precursor (psbY) mRNA. This single nuclear gene is imported into the chloroplasts where it is processed into two integral membrane proteins with identical topology (PsbY-1 and PsbY-2). The protein appears to bind manganese. Important for the redox control of cytochrome b559.
AT1G67770	*TERMINAL EAR1-LIKE 2 (TEL2)*	Similar to terminal ear1 in Zea mays. A member of mei2-like gene family; phylogenetic analysis revealed that TEL2 belongs to the third clade of mei2-like proteins (TEL clade), with conserved two N-terminal RNA recognition motifs (RRM), in addition to the C-terminal RRM, shared among all mei2-like proteins. Expression patterns were similar to TEL1, with lower expression levels in most tissues examined.
AT1G67870		glycine-rich protein
AT1G68010	*HYDROXYPYRUVATE REDUCTASE (HPR)*	Encodes hydroxypyruvate reductase.
AT1G68330		membrane-associated kinase regulator
AT1G68400		leucine-rich repeat transmembrane protein kinase family protein
AT1G68440		Transmembrane protein. Expression induced by abiotic stressors such as ABA, drought, heat, light, NaCl, osmotic stress and wounding.
AT1G68520	*B-BOX DOMAIN PROTEIN 14 (BBX14)*	B-box type zinc finger protein with CCT domain-containing protein
AT1G68585		hypothetical protein
AT1G68590	*PLASTID-SPECIﬁC RIBOSOMAL PROTEIN 3/1 (PSRP3/1)*	Ribosomal protein PSRP-3/Ycf65
AT1G68650	*PHOTOSYNTHESIS-AFFECTED MUTANT 71 LIKE 5 (PML5)*	Member of the UPF0016 family of membrane proteins, belongs to the conserved group of Mn/Ca transporters. Might act to fine tune Mn allocation into the endoplasmic reticulum of specific cell types.
AT1G68740	*(PHO1;H1)*	Encodes PHO1;H1, a member of the PHO1 family. Involved in inorganic phosphate (Pi) transport and homeostasis. Complements pho1 mutation. Its expression is responsive to both phosphate (Pi) and phosphite (Phi) in shoots.
AT1G68765	*INFLORESCENCE DEFICIENT IN ABSCISSION (IDA)*	Encodes a small protein of 77 amino acids. Loss of function mutations are defective in the process of ethylene independent floral organ abscission. Although the mutants have a normal appearing abscission zone, the floral organs do not abscisce. The peptide appears to be secreted and may function as a ligand. Arabidopsis 35S:IDA lines constitutively overexpressing IDA exhibit earlier abscission of floral organs, showing that the abscission zones are responsive to IDA soon after the opening of the flowers. In addition, ectopic abscission was observed at the bases of the pedicel, branches of the inflorescence, and cauline leaves. The silique valves also dehisced prematurely.
AT1G68910	*WPP DOMAIN-INTERACTING PROTEIN 2 (WIT2)*	Encodes one of the WPP domain-interacting proteins (WIT1/AT5G11390, WIT2/AT1G68910) required for RanGAP nuclear envelope association in root tip cells. Ran GTPase plays essential roles in multiple cellular processes, including nucleocytoplasmic transport, spindle formation, and postmitotic nuclear envelope reassembly. The cytoplasmic Ran GTPase activating protein RanGAP is critical to establish a functional RanGTP/RanGDP gradient across the nuclear envelope and is associated with the outer surface of the nuclear envelope in metazoan and higher plant cells. Arabidopsis thaliana RanGAP association with the root tip nuclear envelope requires a family of likely plant-specific nucleoporins combining coiled-coil and transmembrane domains (CC-TMD) and WPP domain-interacting proteins (WIPs). WIT1 and WIT2 have been identified as a second family of CC-TMD proteins, structurally similar, yet clearly distinct from the WIP family, that is required for RanGAP nuclear envelop association in root tip cells.
AT1G69080		Adenine nucleotide alpha hydrolases-like superfamily protein
AT1G69230	*SPIRAL1-LIKE2 (SP1L2)*	SPIRAL1-LIKE2 belongs to a six-member gene family in Arabidopsis; all members share a high sequence similarity in amino- and carboxy-terminal regions. Regulates cortical microtubule organization. Mutant plants exhibit altered patterns of root and organ growth as a result of defective anisotropic cell expansion.
AT1G69260	*ABI FIVE BINDING PROTEIN (AFP1)*	ABI five binding protein
AT1G69480		EXS (ERD1/XPR1/SYG1) family protein
AT1G69600	*ZINC FINGER HOMEODOMAIN 1 (ZFHD1)*	Encodes ZFHD1, a member of the zinc finger homeodomain transcriptional factor family. Binds to the 62 bp promoter region of ERD1 (early responsive to dehydration stress 1). Expression of ZFHD1 is induced by drought, high salinity and abscisic acid.
AT1G69610		zinc finger FYVE domain protein, putative (DUF1666)
AT1G69750	*CYTOCHROME C OXIDASE 19-2 (COX19-2)*	cytochrome c oxidase 19-2
AT1G69850	*NRT1/ PTR FAMILY 4.6 (NPF4.6)*	Encodes an inducible component of low-affinity nitrate uptake. mRNA found primarily in root hairs and the epidermis of roots. It also acts as an ABA importer at the site of ABA biosynthesis and is important for the regulation of stomatal aperture in inflorescence stems.
AT1G69870	*NRT1/ PTR FAMILY 2.13 (NPF2.13)*	Encodes a low affinity nitrate transporter NRT1.7. Expressed in phloem. Responsible for source-to-sink remobilization of nitrate. The mRNA is cell-to-cell mobile.
AT1G69930	*GLUTATHIONE S-TRANSFERASE TAU 11 (GSTU11)*	Encodes glutathione transferase belonging to the tau class of GSTs. Naming convention according to Wagner et al. (2002).
AT1G70130	*L-TYPE LECTIN RECEPTOR KINASE V.2 (LECRK-V.2)*	Concanavalin A-like lectin protein kinase family protein
AT1G70210	*CYCLIN D1;1 (CYCD1;1)*	Encodes a D-type cyclin that physically interacts with CDC2A. Its expression is upregulated early during germination.
AT1G70290	*TREHALOSE-6-PHOSPHATASE SYNTHASE S8 (TPS8)*	Encodes an enzyme putatively involved in trehalose biosynthesis. Though the protein has both trehalose-6-phosphate synthase (TPS)-like and trehalose-6-phosphate phosphatase (TPP)-like domains, neither activity has been detected in enzymatic assays nor has the protein been able to complement yeast TPS or TPP mutants.
AT1G70410	*BETA CARBONIC ANHYDRASE 4 (BCA4)*	Encodes a putative beta-carbonic anhydrase betaCA4. Together with betaCA1 (At3g01500) regulates CO2-controlled stomatal movements in guard cells, as well as attenuates immunity. Differential CA gene expression in response to changing atmospheric CO2 conditions contribute to altered disease resistance levels.
AT1G70460	*PROLINE-RICH EXTENSIN-LIKE RECEPTOR KINASE 13 (PERK13)*	Encodes a member of the proline-rich extensin-like receptor kinase (PERK) family. This family consists of 15 predicted receptor kinases (PMID: 15653807).

AT1G70700	*(TIFY7)*	JAZ9 is a protein presumed to be involved in jasmonate signaling. JAZ9 transcript levels rise in response to a jasmonate stimulus. JAZ9 can interact with the COI1 F-box subunit of an SCF E3 ubiquitin ligase in a yeast-two-hybrid assay only in the presence of jasmonate-isoleucine (JA-ILE) or coronatine. The Jas domain appears to be important for JAZ9-COI1 interactions in the presence of coronatine. Two positive residues (R205 and R206) in the Jas domain shown to be important for coronatine -dependent COI1 binding are not required for binding AtMYC2. The mRNA is cell-to-cell mobile.
AT1G70850	*MLP-LIKE PROTEIN 34 (MLP34)*	MLP-like protein 34
AT1G70890	*MLP-LIKE PROTEIN 43 (MLP43)*	MLP-like protein 43
AT1G70990	*EXTENSIN 33 (EXT33)*	Short extensin family protein required during the first phase of dark-grown hypocotyl elongation, regulates the moment and extent of the growth acceleration by modulating cell wall extensibility.
AT1G71000		Chaperone DnaJ-domain superfamily protein
AT1G71080		RNA polymerase II transcription elongation factor
AT1G71140	*PROTEIN DETOXIFICATION 14 (DTX14)*	MATE transporter that can export the antibiotic norfloxacin.
AT1G71400	*RECEPTOR LIKE PROTEIN 12 (RLP12)*	Encodes a CLAVATA2 (CLV2)-related gene. Complements the clv2 mutant when expressed under the control of the CLV2 promoter. The mRNA is cell-to-cell mobile.
AT1G71690		glucuronoxylan 4-O-methyltransferase-like protein (DUF579)
AT1G71691		GDSL-motif esterase/acyltransferase/lipase. Enzyme group with broad substrate specificity that may catalyze acyltransfer or hydrolase reactions with lipid and non-lipid substrates.
AT1G71970		hypothetical protein
AT1G72030	*GCN5‐RELATED N‐ACETYLTRANSFERASE 10 (GNAT10)*	Acyl-CoA N-acyltransferases (NAT) superfamily protein
AT1G72100		late embryogenesis abundant domain-containing protein / LEA domain-containing protein
AT1G72180	*C-TERMINALLY ENCODED PEPTIDE RECEPTOR 2 (CEPR2)*	Encodes a leucine-rich repeat receptor kinase that functions as a receptor for CEP1 peptide. Mediates nitrate uptake signaling.
AT1G72220	*ARABIDOPSIS T??XICOS EN LEVADURA 54 (ATL54)*	RING/U-box superfamily protein
AT1G72450	*JASMONATE-ZIM-DOMAIN PROTEIN 6 (JAZ6)*	JAZ6 transcript levels rise in response to a jasmonate stimulus and a GFP:JAZ6 fusion protein localizes to the nucleus. Application of jasmonate methyl ester to Arabidopsis roots reduces the levels of a JAZ6:GUS fusion protein, presumably by stimulating ubiquitin-proteasome-mediated degradation.
AT1G72490	*DEEPER ROOTING 1 (DRO1)*	DRO1 is a member of the IGT gene family and has a unknown function . It is expressed in roots and involved in leaf root architecture, specifically the orientation of lateral root angles. Involved in determining lateral root branch angle.
AT1G72510		DUF1677 family protein (DUF1677)
AT1G72800		RNA-binding (RRM/RBD/RNP motifs) family protein
AT1G72870	*TIR-NBS5 (TN5)*	TIR-NBS gene.
AT1G72900	*TIR-NBS7 (TN7)*	Toll-Interleukin-Resistance (TIR) domain-containing protein
AT1G72940	*TIR-NBS11 (TN11)*	Nucleotide-binding, leucine-rich repeat (NLR) gene regulated by nonsense-mediated mRNA decay (NMD) genes UPF1 and UPF3.
AT1G73260	*KUNITZ TRYPSIN INHIBITOR 1 (KTI1)*	Encodes a trypsin inhibitor involved in modulating programmed cell death in plant-pathogen interactions.
AT1G73330	*DROUGHT-REPRESSED 4 (DR4)*	encodes a plant-specific protease inhibitor-like protein whose transcript level in root disappears in response to progressive drought stress. The decrease in transcript level is independent from abscisic acid level.
AT1G73340	*ABIETANE DITERPENE OXIDASE 1 (ADTO1)*	ADTO1 is required for the activation of systemic acquired resistance.
AT1G73370	*SUCROSE SYNTHASE 6 (SUS6)*	Encodes a protein with sucrose synthase activity (SUS6).
AT1G73600	*(NMT3)*	Encodes a S-adenosyl-L-methionine-dependent phosphoethanolamine N-methyltransferase whose expression is responsive to both phosphate (Pi) and phosphite (Phi) in roots. It catalyzes the three sequential P-base methylation of phosphoethanolamine to phosphocholine. Homologous biochemical function to NMT1 (At3g18000). Double mutants of NMT1 and NMT3 are defective in leaf, root, flower, seed, and pollen development.
AT1G73620		Pathogenesis-related thaumatin superfamily protein
AT1G74010		Calcium-dependent phosphotriesterase superfamily protein
AT1G74055		transmembrane protein
AT1G74070		Cyclophilin-like peptidyl-prolyl cis-trans isomerase family protein
AT1G74090	*DESULFO-GLUCOSINOLATE SULFOTRANSFERASE 18 (SOT18)*	encodes a desulfoglucosinolate sulfotransferase, involved in the final step of glucosinolate core structure biosynthesis. Has a broad-substrate specificity with preference with methionine-derived desulfoglucosinolates.
AT1G74420	*FUCOSYLTRANSFERASE 3 (FUT3)*	Predicted fucosyltransferase, based on similarity to FUT1, but not functionally redundantwith FUT1.
AT1G74470		Encodes for a multifunctional protein with geranylgeranyl reductase activity shown to catalyze the reduction of prenylated geranylgeranyl-chlorophyll a to phytyl-chlorophyll a (chlorophyll a) and free geranylgeranyl pyrophosphate to phytyl pyrophosphate. The mRNA is cell-to-cell mobile.
AT1G74490	*PBS1-LIKE 29 (PBL29)*	Protein kinase superfamily protein
AT1G74500	*ACTIVATION-TAGGED BRI1(BRASSINOSTEROID-INSENSITIVE 1)-SUPPRESSOR 1 (BS1)*	Encodes a basic helix/loop/helix transcription factor that acts downstream of MP in root initiation. TMO7 protein moves to the hypophysis and to vascular cells, contributing to MP-dependent root formation. Promotes the correct definition of the hypophysis cell division plane.
AT1G74580		Pentatricopeptide repeat (PPR) superfamily protein
AT1G74590	*GLUTATHIONE S-TRANSFERASE TAU 10 (GSTU10)*	Encodes glutathione transferase belonging to the tau class of GSTs. Naming convention according to Wagner et al. (2002).
AT1G74620		RING/U-box superfamily protein
AT1G74730	*(RIQ2)*	Encodes a grana core localized protein, is homologous to RIG1. Mutant plants have reduced NPQ, affected organization of light-havesting complex II and an enhanced grana stacking.
AT1G74940		cyclin-dependent kinase, putative (DUF581)
AT1G75160	*BOUNDARY OF ROP DOMAIN9 (BDR9)*	DUF620 family protein (DUF620)
AT1G75280		isoflavone reductase, putative, identical to SP:P52577 Isoflavone reductase homolog P3 (EC 1.3.1.-) {Arabidopsis thaliana}; contains Pfam profile PF02716: isoflavone reductase. Involved in response to oxidative stress. The mRNA is cell-to-cell mobile.
AT1G75300		encodes a protein whose sequence is similar to an isoflavone reductase

AT1G75450	*CYTOKININ OXIDASE 5 (CKX5)*	This gene used to be called AtCKX6. It encodes a protein whose sequence is similar to cytokinin oxidase/dehydrogenase, which catalyzes the degradation of cytokinins.
AT1G75460		ATP-dependent protease La (LON) domain protein
AT1G75670		DNA-directed RNA polymerase
AT1G75690	*LOW QUANTUM YIELD OF PHOTOSYSTEM II 1 (LQY1)*	Thylakoid Thiol/Disulfide-Modulating Protein.
AT1G75710		C2H2-like zinc finger protein
AT1G75720		WEB family protein (DUF827)
AT1G75750	*GAST1 PROTEIN HOMOLOG 1 (GASA1)*	GA-responsive GAST1 protein homolog regulated by BR and GA antagonistically. Possibly involved in cell elongation based on expression data The mRNA is cell-to-cell mobile.
AT1G75790	*SKU5 SIMILAR 18 (sks18)*	SKU5 similar 18
AT1G75830	*LOW-MOLECULAR-WEIGHT CYSTEINE-RICH 67 (LCR67)*	Predicted to encode a PR (pathogenesis-related) protein. Belongs to the plant defensin (PDF) family with the following members: At1g75830/PDF1.1, At5g44420/PDF1.2a, At2g26020/PDF1.2b, At5g44430/PDF1.2c, At2g26010/PDF1.3, At1g19610/PDF1.4, At1g55010/PDF1.5, At2g02120/PDF2.1, At2g02100/PDF2.2, At2g02130/PDF2.3, At1g61070/PDF2.4, At5g63660/PDF2.5, At2g02140/PDF2.6, At5g38330/PDF3.1 and At4g30070/PDF3.2.
AT1G76070		hypothetical protein
AT1G76100	*PLASTOCYANIN 1 (PETE1)*	One of two Arabidopsis plastocyanin genes. Expressed at 1/10th level of PETE2. Does not respond to increased copper levels and is thought to be the isoform that participates in electron transport under copper-limiting conditions. Mutation of this gene does not have obvious effect on photosynthesis.
AT1G76410	*(ATL8)*	RING/U-box superfamily protein
AT1G76470		NAD(P)-binding Rossmann-fold superfamily protein
AT1G76520	*PIN-LIKES 3 (PILS3)*	Auxin efflux carrier family protein
AT1G76590	*(PLATZ2)*	PLATZ transcription factor family protein
AT1G76600		PADRE protein up-regulated after infection by S. sclerotiorun.
AT1G76650	*CALMODULIN-LIKE 38 (CML38)*	calmodulin-like 38
AT1G76790	*INDOLE GLUCOSINOLATE O-METHYLTRANSFERASE 5 (IGMT5)*	Encodes a protein with similarity to N-acetylserotonin O-methyltransferase (ASMT) but it does not have ASMT activity in vitro.
AT1G76870		transcription factor
AT1G76990	*ACT DOMAIN REPEAT 3 (ACR3)*	ACT domain repeat 3
AT1G77000	*(SKP2B)*	AtSKP2;2 is a homolog of human SKP2, the human F-box protein that recruits E2F1. Contains an F-box motif at the N-terminal region and a C-terminal Leu-rich repeat domain. Forms part of an E3-ubiquitin-ligase SCF (Skp1, cullin, F-box) complex and recruits phosphorylated AtE2Fc, a transcriptional factor that might play a role in cell division and during the transition from skotomorphogenesis to photomorphogenesis. AtSKP2;1 (At1g21410) and AtSKP2;2 (At1g77000) may be duplicated genes. AtSKP2b may also be involved in the degradation of KRP1/ICK1, a CDK inhibitor.
AT1G77020		DNAJ heat shock N-terminal domain-containing protein
AT1G77060		Phosphoenolpyruvate carboxylase family protein
AT1G77200		encodes a member of the DREB subfamily A-4 of ERF/AP2 transcription factor family. The protein contains one AP2 domain. There are 17 members in this subfamily including TINY.
AT1G77380	*AMINO ACID PERMEASE 3 (AAP3)*	Amino acid permease which transports basic amino acids.
AT1G77530		O-methyltransferase family protein
AT1G77590	*LONG CHAIN ACYL-COA SYNTHETASE 9 (LACS9)*	Encodes major plastidic long chain acyl-CoA synthetase with a slight substrate preference of oleic acid over any of the other fatty acids.
AT1G77640		encodes a member of the DREB subfamily A-5 of ERF/AP2 transcription factor family. The protein contains one AP2 domain. There are 15 members in this subfamily including RAP2.1, RAP2.9 and RAP2.10.
AT1G77690	*LIKE AUX1 3 (LAX3)*	Encodes an auxin influx carrier LAX3 (Like Aux1) that promotes lateral root emergence. Auxin-induced expression of LAX3 in turn induces a selection of cell-wall-remodelling enzymes, which are likely to promote cell separation in advance of developing lateral root primordia.
AT1G77700	*UBIQUITIN FOLD MODIFIER 1 (UFM1)*	Osmotin-like protein.
AT1G77920	*TGACG SEQUENCE-SPECIFIC BINDING PROTEIN 7 (TGA7)*	bZIP transcription factor family protein
AT1G78100	*AUXIN UP-REGULATED F-BOX PROTEIN 1 (AUF1)*	F-box family protein
AT1G78120	*TETRATRICOPEPTIDE REPEAT 12 (TPR12)*	Encodes one of the 36 carboxylate clamp (CC)-tetratricopeptide repeat (TPR) proteins (Prasad 2010, Pubmed ID: 20856808) with potential to interact with Hsp90/Hsp70 as co-chaperones.
AT1G78170		E3 ubiquitin-protein ligase
AT1G78210		alpha/beta-Hydrolases superfamily protein
AT1G78290	*SNF1-RELATED PROTEIN KINASE 2-8 (SNRK2-8)*	encodes a member of SNF1-related protein kinase (SnRK2) family whose activity is activated by ionic (salt) and non-ionic (mannitol) osmotic stress and dehydration.
AT1G78370	*GLUTATHIONE S-TRANSFERASE TAU 20 (GSTU20)*	Encodes glutathione transferase belonging to the tau class of GSTs. Naming convention according to Wagner et al. (2002).
AT1G78430	*ROP INTERACTIVE PARTNER 2 (RIP2)*	Encodes RIP2 (ROP interactive partner 2), a putative Rho protein effector, interacting specifically with the active form of ROPs (Rho proteins of plants).
AT1G78670	*GAMMA-GLUTAMYL HYDROLASE 3 (GGH3)*	gamma-glutamyl hydrolase 3
AT1G78780		pathogenesis-related family protein
AT1G78815	*LIGHT SENSITIVE HYPOCOTYLS 7 (LSH7)*	LIGHT-DEPENDENT SHORT HYPOCOTYLS-like protein (DUF640)
AT1G78860	*APPLE DOMAIN LECTIN-2 (GAL2)*	curculin-like (mannose-binding) lectin family protein, low similarity to Ser/Thr protein kinase (Zea mays) GI:2598067; contains Pfam profile PF01453: Lectin (probable mannose binding) but not the protein kinase domain of the Z. mays protein
AT1G78940		kinase with adenine nucleotide alpha hydrolases-like domain-containing protein
AT1G78970	*LUPEOL SYNTHASE 1 (LUP1)*	Lupeol synthase. Converts oxidosqualene to multiple triterpene alcohols and a triterpene diols. This conversion proceeds through the formation of a 17β-dammarenyl cation.

AT1G79040	*PHOTOSYSTEM II SUBUNIT R (PSBR)*	Encodes for the 10 kDa PsbR subunit of photosystem II (PSII). This subunit appears to be involved in the stable assembly of PSII, particularly that of the oxygen-evolving complex subunit PsbP. Mutants defective in this gene have reduced amounts of subunits PsbP and PsbQ in PSII. In turn, assembly of PsbR is dependent on the presence of PsbJ.
AT1G79110	*BOI-RELATED GENE 2 (BRG2)*	Encodes one of the BRGs (BOI-related gene) involved in resistance to Botrytis cinerea.
AT1G79140		
AT1G79360	*ORGANIC CATION/CARNITINE TRANSPORTER 2 (OCT2)*	organic cation/carnitine transporter 2
AT1G79520		Cation efflux family protein
AT1G79580	*SOMBRERO (SMB)*	NAC-domain protein. Involved in root cap development. Involved in a regulatory feedback loop with FEZ. FEZ activates SMB in hte root cap daughter cells soon after division, and SMB in turn represses FEZ expression in these cells, thereby preventing further stem cell divisions.
AT1G79620	*VASCULAR-RELATED RLK 1 (VRLK1)*	VRLK1 is a LRR kinase involved in switching between cell elongation and secondary cell wall thickening.VRLK1 is a member of a gene family that includes a small number of recently duplicated paralogs.
AT1G79850	*RIBOSOMAL PROTEIN S17 (RPS17)*	nuclear-encoded 30S chloroplast ribosomal protein S17
AT1G79990		coatomer subunit beta-2
AT1G80080	*TOO MANY MOUTHS (TMM)*	Encodes a transmembrane leucine-repeat containing receptor-like protein that is expressed in proliferative postprotodermal cells. Recessive mutation leads to disruption of asymmetric cell division during stomata development. Its transcript levels change after inducing MUTE expression in a mute background.
AT1G80160	*GLYOXYLASE I 7 (GLYI7)*	Vicinal oxygen chelate (VOC) superfamily member.
AT1G80170		Pectin lyase-like superfamily protein
AT1G80240	*DUF642 L-GALL RESPONSIVE GENE 1 (DGR1)*	DUF642 gene
AT1G80270	*PENTATRICOPEPTIDE REPEAT 596 (PPR596)*	PENTATRICOPEPTIDE REPEAT 596
AT1G80290		a member of the Glycosyltransferase Family 64 (according to CAZy Database)
AT1G80330	*GIBBERELLIN 3-OXIDASE 4 (GA3OX4)*	Encodes a protein with gibberellin 3-oxidase activity. The enzyme, expressed and purified in E.coli, was shown to catalyze the 3β-hydroxylation of GA20 into GA29.
AT1G80340	*GIBBERELLIN 3-OXIDASE 2 (GA3OX2)*	Encodes a protein with gibberellin 3 β-hydroxylase activity. The protein was heterologously expressed in E. coli and shown to catalyze the hydroxylation of both GA9 and GA20.
AT1G80550		Pentatricopeptide repeat (PPR) superfamily protein
AT1G80720		Mitochondrial glycoprotein family protein
AT1G80760	*NOD26-LIKE INTRINSIC PROTEIN 6;1 (NIP6;1)*	Encodes a protein with boron transporter activity. It helps to preferentially direct boron to young developing tissues in the shoot, such as immature leaves, under low boron conditions. This boron channel appears to be impermeable to water, unlike the closely related NIP5;1 boron transporter. This protein also allows the transport of glycerol, urea, and formimide but not larger uncharged solutes such as arabitol and sucrose when it is expressed heterologously.
AT1G80820	*CINNAMOYL COA REDUCTASE (CCR2)*	Encodes an cinnamoyl CoA reductase isoform. Involved in lignin biosynthesis.
AT1G80970		XH domain-containing protein
AT2G01120	*ORIGIN RECOGNITION COMPLEX SUBUNIT 4 (ORC4)*	Origin Recognition Complex subunit 4. Involved in the initiation of DNA replication. Regulated transcriptionally during cell cycle, peaking at G1/S-phase. Target of E2F/DF family of transcription factors. Interacts with all ORC subunits except ORC1b.
AT2G01210	*ZYGOTIC ARREST 1 (ZAR1)*	ZAR1 encodes a plasma membrane localized leucine-rich repeat receptor-like kinase (LRR-RLK) that contains a putative CaM-binding domain and a Gβ-binding motif within its intracellular kinase region. Homozygous of function mutations are embryo-lethal and fail to properly make the first asymmetric division of the zygote. ZAR1 interacts with both CaM and Gβ in vivo and that interaction activates ZAR1 kinase activity.
AT2G01420	*PIN-FORMED 4 (PIN4)*	Encodes a putative auxin efflux carrier that is localized in developing and mature root meristems. It is involved in the maintenance of embryonic auxin gradients. A role for AtPIN4 in generating a sink for auxin below the quiescent center of the root meristem that is essential for auxin distribution and patterning is proposed. In the root, PIN4 is detected around the quiescent center and cells surrounding it, and localizes basally in provascular cells. PIN4 expression is upregulated in brassinosteroid-insensitive mutant (PMID 16141452).
AT2G01430	*HOMEOBOX-LEUCINE ZIPPER PROTEIN 17 (HB17)*	ATHB17 is a member of the HD-Zip transcription factor family. It is expressed most strongly in roots at different stages of development and induced by ABA, paraquat, drought, and NaCl treatments. Loss of function mutants are more sensitive to salt and drought stress.The protein is nuclear localized and has been shown to bind to the promoter of SIG5 and other genes.
AT2G01770	*VACUOLAR IRON TRANSPORTER 1 (VIT1)*	Encodes an iron transporter required for iron sequestration into vacuoles. Expressed in developing embryo and seed. Localized in the vacuolar membrane.
AT2G01890	*PURPLE ACID PHOSPHATASE 8 (PAP8)*	Encodes a purple acid phosphatase (PAP) belonging to the low molecular weight plant PAP group.
AT2G02390	*GLUTATHIONE S-TRANSFERASE ZETA 1 (GSTZ1)*	Encodes glutathione transferase belonging to the zeta class of GSTs. Naming convention according to Wagner et al. (2002). The protein undergoes spontaneous thiolation following treatment with the oxidant tert-butylhydroperoxide. It functions in vitro as a maleylacetoacetate isomerase and is likely to be involved in tyrosine catabolism.
AT2G02400		NAD(P)-binding Rossmann-fold superfamily protein
AT2G02540	*HOMEOBOX PROTEIN 21 (HB21)*	Zinc finger homeobox protein. Expressed in vascular tissue. In a yeast one hybrid system was not able to transactivate a reporter gene.
AT2G02780		Leucine-rich repeat protein kinase family protein
AT2G02850	*PLANTACYANIN (ARPN)*	Encodes plantacyanin one of blue copper proteins. Involved in anther development and pollination. Expressed in the transmitting tract of the pistil.
AT2G02990	*RIBONUCLEASE 1 (RNS1)*	Encodes a member of the ribonuclease T2 family that responds to inorganic phosphate starvation, and inhibits production of anthocyanin. Also involved in wound-induced signaling independent of jasmonic acid. Its expression is responsive to both phosphate (Pi) and phosphite (Phi) in roots.
AT2G03060	*AGAMOUS-LIKE 30 (AGL30)*	Encodes a member of the MIKC (MADS box, Keratin binding domain, and C terminal domain containing)family of transcriptional regulators. AGL30 is expressed in pollen.It forms heterodimers with other MICK family members.
AT2G03090	*EXPANSIN A15 (EXPA15)*	member of Alpha-Expansin Gene Family. Naming convention from the Expansin Working Group (Kende et al, 2004. Plant Mol Bio). Involved in the formation of nematode-induced syncytia in roots of Arabidopsis thaliana.
AT2G03260		EXS (ERD1/XPR1/SYG1) family protein
AT2G03730	*ACT DOMAIN REPEAT 5 (ACR5)*	Member of a small family of ACT domain containing proteins. ACT domains are thought to be involved in amino acid binding.

AT2G03750		P-loop containing nucleoside triphosphate hydrolases superfamily protein
AT2G03760	*SULPHOTRANSFERASE 12 (SOT12)*	Encodes a brassinosteroid sulfotransferase. In vitro experiements show that this enzyme has a preference for 24-epibrassinosteroids, particularly 24-epicathasterone, but does not act on castasterone and brassinolide. It also shows sulfating activity toward flavonoids. It is differentially expressed during development, being more abundant in young seedlings and actively growing cell cultures. Expression is induced in response to salicylic acid and methyl jasmonate and bacterial pathogens.
AT2G03830	*ROOT MERISTEM GROWTH FACTOR 8 (RGF8)*	Encodes a root meristem growth factor (RGF). Belongs to a family of functionally redundant homologous peptides that are secreted, tyrosine-sulfated, and expressed mainly in the stem cell area and the innermost layer of central columella cells. RGFs are required for maintenance of the root stem cell niche and transit amplifying cell proliferation. Members of this family include: At5g60810 (RGF1), At1g13620 (RGF2), At2g04025 (RGF3), At3g30350 (RGF4), At5g51451 (RGF5), At4g16515 (RGF6), At3g02240 (RGF7), At2g03830 (RGF8) and At5g64770 (RGF9).
AT2G04025	*ROOT MERISTEM GROWTH FACTOR 3 (RGF3)*	Encodes a root meristem growth factor (RGF). Belongs to a family of functionally redundant homologous peptides that are secreted, tyrosine-sulfated, and expressed mainly in the stem cell area and the innermost layer of central columella cells. RGFs are required for maintenance of the root stem cell niche and transit amplifying cell proliferation. Members of this family include: At5g60810 (RGF1), At1g13620 (RGF2), At2g04025 (RGF3), At3g30350 (RGF4), At5g51451 (RGF5), At4g16515 (RGF6), At3g02240 (RGF7), At2g03830 (RGF8) and At5g64770 (RGF9).
AT2G04040	*DETOXIFICATION 1 (DTX1)*	AtDTX1 (At2g04040) has been identified as a detoxifying efflux carrier for plant-derived antibiotics and other toxic compounds, including Cd2+. Expression in rosette leaves is activated by high concentration of boron.Mistakenly referred to as At2g04070 in PMID:11739388.
AT2G04080		MATE efflux family protein
AT2G04160	*AUXIN-INDUCED IN ROOT CULTURES 3 (AIR3)*	isolated from differential screening of a cDNA library from auxin-treated root culture. encodes a protein similar to subtilisin-like serine protease which is believed to be active outside the plant cell.
AT2G04480		hypothetical protein
AT2G04795		hypothetical protein
AT2G04850		Auxin-responsive family protein
AT2G05380	*GLYCINE-RICH PROTEIN 3 SHORT ISOFORM (GRP3S)*	glycine-rich protein 3 short isoform (GRP3S) mRNA, complete The mRNA is cell-to-cell mobile.
AT2G05510		Glycine-rich protein family
AT2G05560		transposable_element_gene;pseudogene, Ulp1 protease family, contains Pfam profile PF02902: Ulp1 protease family, C-terminal catalytic domain;(source:TAIR10)
AT2G05950		transposable_element_gene;similar to unknown protein [Arabidopsis thaliana] (TAIR:AT3G32050.1);(source:TAIR10)
AT2G06050	*OXOPHYTODIENOATE-REDUCTASE 3 (OPR3)*	Encodes a 12-oxophytodienoate reductase that is required for jasmonate biosynthesis. Mutants are male sterile and defective in pollen dehiscence. Shows activity towards 2,4,6-trinitrotoluene. CFA-Ile, CFA-Leu, CFA-Val, CFA-Met and CFA-Ala can restore the fertility of opr3 plants by inducing filament elongation and anther dehiscence.
AT2G06490		transposable_element_gene;CACTA-like transposase family (Ptta/En/Spm), has a 7.5e-83 P-value blast match to At5g29026.1/8-244 CACTA-like transposase family (Ptta/En/Spm) (CACTA-element) (Arabidopsis thaliana);(source:TAIR10)
AT2G06850	*XYLOGLUCAN ENDOTRANSGLUCOSYLASE/HYDROLASE 4 (XTH4)*	endoxyloglucan transferase (EXGT-A1) gene
AT2G07620		transposable_element_gene;pseudogene, similar to putative helicase, very low similarity to SP|Q9UUA2 DNA repair and recombination protein pif1, mitochondrial precursor {Schizosaccharomyces pombe};(source:TAIR10)
AT2G07692		hypothetical protein
AT2G07698		ATPase, F1 complex, alpha subunit protein
AT2G07700		transposable_element_gene;gypsy-like retrotransposon family, has a 4.7e-21 P-value blast match to GB:226407 retrotransposon del1-46 (Gypsy_Ty3-element) (Lilium henryi);(source:TAIR10)
AT2G07701		hypothetical protein
AT2G07702		hypothetical protein
AT2G07722		transmembrane protein
AT2G07728		hypothetical protein
AT2G07750		DEA(D/H)-box RNA helicase family protein
AT2G10140		transposable_element_gene;CACTA-like transposase family (Tnp2/En/Spm), has a 1.7e-140 P-value blast match to GB:CAA40555 TNP2 (CACTA-element) (Antirrhinum majus);(source:TAIR10)
AT2G10940		Bifunctional inhibitor/lipid-transfer protein/seed storage 2S albumin superfamily protein
AT2G10970		Plant invertase/pectin methylesterase inhibitor superfamily protein
AT2G12170		hypothetical protein
AT2G12870		transposable_element_gene;gypsy-like retrotransposon family, has a 3.8e-07 P-value blast match to GB:AAD19359 polyprotein (gypsy_Ty3-element) (Sorghum bicolor);(source:TAIR10)
AT2G13610	*ATP-BINDING CASSETTE G5 (ABCG5)*	ABC-2 type transporter family protein
AT2G13690		PRLI-interacting factor
AT2G13780		
AT2G14520		CBS domain protein (DUF21)
AT2G14550		pseudogene of RNA-binding (RRM/RBD/RNP motifs) family protein
AT2G14560	*LATE UPREGULATED IN RESPONSE TO HYALOPERONOSPORA PARASITICA (LURP1)*	Encodes LURP1, a member of the LURP cluster (late upregulated in response to Hyaloperonospora parasitica) which exhibits a pronounced upregulation after recognition of the pathogenic oomycte H. parasitica. LURP1 is required for full basal defense to H. parasitica and resistance to this pathogen mediated by the R-proteins RPP4 and RPP5. The mRNA is cell-to-cell mobile.
AT2G14620	*XYLOGLUCAN ENDOTRANSGLUCOSYLASE/HYDROLASE 10 (XTH10)*	xyloglucan endotransglucosylase/hydrolase 10
AT2G14960	*(GH3.1)*	encodes a protein similar to IAA-amido synthases. Lines carrying an insertion in this gene are hypersensitive to auxin.
AT2G15000		caspase-6 protein
AT2G15020		hypothetical protein

AT2G15480	*UDP-GLUCOSYL TRANSFERASE 73B5 (UGT73B5)*	UDP-glucosyl transferase 73B5
AT2G15490	*UDP-GLYCOSYLTRANSFERASE 73B4 (UGT73B4)*	UDP-glycosyltransferase 73B4
AT2G15580		RING/U-box superfamily protein
AT2G15620	*NITRITE REDUCTASE 1 (NIR1)*	Involved in the second step of nitrate assimilation. Its expression is induced by nitrate. The mRNA is cell-to-cell mobile.
AT2G15630		Pentatricopeptide repeat (PPR) superfamily protein
AT2G15890	*MATERNAL EFFECT EMBRYO ARREST 14 (MEE14)*	Encodes CBP1, a regulator of transcription initiation in central cell-mediated pollen tube guidance.
AT2G16060	*HEMOGLOBIN 1 (HB1)*	Encodes a class 1 nonsymbiotic hemoglobin induced by low oxygen levels with very high oxygen affinity. It is not likely to be a hemoglobin transporter because of its extremely high affinity for oxygen. Overexpression impairs cold stress-induced nitric oxide (NO) production.
AT2G16190		hypothetical protein
AT2G16230		O-Glycosyl hydrolases family 17 protein
AT2G16570	*GLN PHOSPHORIBOSYL PYROPHOSPHATE AMIDOTRANSFERASE 1 (ASE1)*	Amidophosphoribosyltransferase (ATase: EC 2.4.2.14) is a key enzyme in the pathway of purine nucleotide biosynthesis
AT2G16740	*UBIQUITIN-CONJUGATING ENZYME 29 (UBC29)*	ubiquitin-conjugating enzyme 29
AT2G16750		kinase with adenine nucleotide alpha hydrolases-like domain-containing protein
AT2G16760		Calcium-dependent phosphotriesterase superfamily protein
AT2G16990		Major facilitator superfamily protein
AT2G17160		Interleukin-1 receptor-associated kinase 4 protein
AT2G17230	*EXORDIUM LIKE 5 (EXL5)*	EXORDIUM like 5
AT2G17280		Phosphoglycerate mutase family protein
AT2G17290	*CALCIUM DEPENDENT PROTEIN KINASE 6 (CPK6)*	Encodes calcium dependent protein kinase 6 (CPK6), a member of the Arabidopsis CDPK gene family. CDPKs contain an intrinsic Ca2+-activation domain with four EF hand Ca2+-binding sites. CDPKs protein kinases have been proposed to function in multiple plant signal transduction pathways downstream of [Ca2+]cyt elevations, thus transducing various physiological responses. CPK6 is expressed in both guard cells and mesophyll cells. Functions in guard cell ion channel regulation. ABA and Ca(2+) activation of slow-type anion channels and, interestingly, ABA activation of plasma membrane Ca(2+)-permeable channels were impaired in independent alleles of single and double cpk3cpk6 mutant guard cells. Furthermore, ABA- and Ca(2+)-induced stomatal closing were partially impaired in these cpk3cpk6 mutant alleles. The protein kinase CPK6 is shown in biochemical assays to be directly activated by elevations in calcium concentrations in the physiological range (Laanements et al., 2013 PlantPhys.; PMID: 23766366). These data correlate with the in vivo function of CPK6 in Ca2+ and ABA activation of S-type anion channels (Mori et al., 2006 PLoS Biol.; PMID: 17032064) and the ability of CPK6 to mediate ABA activation of SLAC1 (Brandt et al., 2012 PNAS; PMID: 22689970). The mRNA is cell-to-cell mobile.
AT2G17490		transposable_element_gene;copia-like retrotransposon family, has a 9.3e-199 P-value blast match to gb|AAO73521.1| gag-pol polyprotein (Glycine max) (SIRE1) (Ty1_Copia-family);(source:TAIR10)
AT2G17500	*PIN-LIKES 5 (PILS5)*	Auxin efflux carrier family protein
AT2G17610		transposable_element_gene;non-LTR retrotransposon family (LINE), has a 3.6e-21 P-value blast match to GB:AAA39398 ORF2 (Mus musculus) (LINE-element);(source:TAIR10)
AT2G17650		AMP-dependent synthetase and ligase family protein
AT2G17660		RPM1-interacting protein 4 (RIN4) family protein
AT2G17850		Rhodanese/Cell cycle control phosphatase superfamily protein
AT2G17880	*DNA J PROTEIN C24 (DJC24)*	Chaperone DnaJ-domain superfamily protein
AT2G18010	*SMALL AUXIN UPREGULATED RNA 10 (SAUR10)*	SAUR-like auxin-responsive protein family
AT2G18140		Peroxidase superfamily protein
AT2G18210		hypothetical protein
AT2G19110	*HEAVY METAL ATPASE 4 (HMA4)*	Encodes a protein with similarity to Zn ATPase. Can rescue Zn deficiency in yeast and Cd resistance, suggesting a role in Zn and Cd transport. The mRNA is cell-to-cell mobile.
AT2G19200		pseudogene of hypothetical protein (DUF626)
AT2G19500	*CYTOKININ OXIDASE 2 (CKX2)*	It encodes a protein whose sequence is similar to cytokinin oxidase/dehydrogenase, which catalyzes the degradation of cytokinins.
AT2G19630		F-box and associated interaction domains-containing protein
AT2G19650		Cysteine/Histidine-rich C1 domain family protein
AT2G19670	*PROTEIN ARGININE METHYLTRANSFERASE 1A (PRMT1A)*	protein arginine methyltransferase 1A
AT2G19800	*MYO-INOSITOL OXYGENASE 2 (MIOX2)*	Encodes a myo-inositol oxygenase family gene.
AT2G19850		transcription repressor
AT2G19890		hypothetical protein
AT2G19900	*NADP-MALIC ENZYME 1 (NADP-ME1)*	The malic enzyme (EC 1.1.1.40) encoded by AtNADP-ME1 is expressed in response to developmental and cell-specific signals. The enzyme is active in vitro and appears to function as a homohexamer or homooctamer. It is believed to be a cytosolic protein.
AT2G19990	*PATHOGENESIS-RELATED PROTEIN-1-LIKE (PR-1-LIKE)*	Encodes a PR-1-like protein homolog that is differentially expressed in resistant compared to susceptible cultivars by powdery mildew infection. The deduced amino acid sequence has 24 amino acids comprising the signal peptide and 140 amino acids of the mature peptide (15 kDa). Northern blot analysis showed accumulation of the corresponding mRNA 12 h after inoculation of resistant barley cultivars with Erysiphe graminis. Though the Genbank record for the cDNA associated to this gene model is called 'PR-1', the sequence actually corresponds to PR-1-like. Expression of this gene is not salicylic-acid responsive.
AT2G20030	*ARABIDOPSIS T??XICOS EN LEVADURA 12 (ATL12)*	RING/U-box superfamily protein
AT2G20260	*PHOTOSYSTEM I SUBUNIT E-2 (PSAE-2)*	Encodes subunit E of photosystem I. The mRNA is cell-to-cell mobile.

AT2G20570	*GBF'S PRO-RICH REGION-INTERACTING FACTOR 1 (GPRI1)*	Encodes GLK1, Golden2-like 1, one of a pair of partially redundant nuclear transcription factors that regulate chloroplast development in a cell-autonomous manner. GLK2, Golden2-like 2, is encoded by At5g44190. GLK1 and GLK2 regulate the expression of the photosynthetic apparatus. GLK1 is also a member of the GARP transcription factor family.
AT2G20670		sugar phosphate exchanger, putative (DUF506)
AT2G20780		Major facilitator superfamily protein
AT2G20800	*NAD(P)H DEHYDROGENASE B4 (NDB4)*	NAD(P)H dehydrogenase B4
AT2G20830		folic acid binding / transferase
AT2G20940		transmembrane protein, putative (DUF1279)
AT2G21130		Cyclophilin-like peptidyl-prolyl cis-trans isomerase family protein
AT2G21185		transmembrane protein
AT2G21300		ATP binding microtubule motor family protein
AT2G21330	*FRUCTOSE-BISPHOSPHATE ALDOLASE 1 (FBA1)*	fructose-bisphosphate aldolase 1
AT2G21530		SMAD/FHA domain-containing protein
AT2G21540	*SEC14-LIKE 3 (SFH3)*	SEC14-like 3
AT2G21560		nucleolar-like protein
AT2G21640		Encodes a protein of unknown function that is a marker for oxidative stress response.Expression in rosette leaves is activated by high concentration of boron.
AT2G21950	*SKP1 INTERACTING PARTNER 6 (SKIP6)*	Encodes an SKP1 interacting partner (SKIP6).
AT2G21960		transmembrane protein
AT2G22000	*ELICITOR PEPTIDE 6 PRECURSOR (PROPEP6)*	elicitor peptide 6 precursor
AT2G22230		Thioesterase superfamily protein
AT2G22240	*MYO-INOSITOL-1-PHOSPHATE SYNTHASE 2 (MIPS2)*	** Referred to as MIPS1 in Mitsuhashi et al 2008. Myo-inositol-1-phosphate synthase isoform 2. Expressed in leaf, root and silique. Immunolocalization experiments with an antibody recognizing MIPS1, MIPS2, and MIPS3 showed endosperm localization.
AT2G22430	*HOMEOBOX PROTEIN 6 (HB6)*	Encodes a homeodomain leucine zipper class I (HD-Zip I) protein that is a target of the protein phosphatase ABI1 and regulates hormone responses in Arabidopsis.
AT2G22440		Frigida like gene.
AT2G22470	*ARABINOGALACTAN PROTEIN 2 (AGP2)*	Encodes arabinogalactan-protein (AGP2).
AT2G22490	*CYCLIN D2;1 (CYCD2;1)*	encodes a D-type cyclin whose transcription level is regulated by sucrose but not phytohormones or nitrate. Protein physically interacts with CDC2A. CycD2 kinase activity is regulated by sequestration of CycD2 protein in a form inaccessible to immunoprecipitation and probably not complexed to CDC2A.
AT2G22550		
AT2G22610	*MALECTIN DOMAIN KINESIN 2 (MDKIN2)*	Malectin domain kinesin. Possible role in cell division, with a possible secondary function in the nuclei.
AT2G22620	*RHAMNOGALACTURONAN LYASE6 (RGIL6)*	Rhamnogalacturonate lyase family protein
AT2G22860	*PHYTOSULFOKINE 2 PRECURSOR (PSK2)*	Phytosulfokine 2 precursor, coding for a unique plant peptide growth factor. The mRNA is cell-to-cell mobile.
AT2G22880	*(VQ12)*	VQ motif-containing protein
AT2G22920	*SERINE CARBOXYPEPTIDASE-LIKE 12 (SCPL12)*	serine carboxypeptidase-like 12
AT2G22930		UDP-Glycosyltransferase superfamily protein
AT2G22940		hypothetical protein
AT2G22990	*SINAPOYLGLUCOSE 1 (SNG1)*	sinapoylglucose:malate sinapoyltransferase. Catalyzes the formation of sinapoylmalate from sinapoylglucose. Mutants accumulate excess sinapoylglucose.
AT2G23030	*SNF1-RELATED PROTEIN KINASE 2.9 (SNRK2.9)*	encodes a member of SNF1-related protein kinases (SnRK2)
AT2G23060		Acyl-CoA N-acyltransferases (NAT) superfamily protein
AT2G23110		Late embryogenesis abundant protein, group 6
AT2G23170	*(GH3.3)*	encodes an IAA-amido synthase that conjugates Asp and other amino acids to auxin in vitro.
AT2G23180	*CYTOCHROME P450, FAMILY 96, SUBFAMILY A, POLYPEPTIDE 1 (CYP96A1)*	member of CYP96A
AT2G23200		Protein kinase superfamily protein
AT2G23240	*ARABIDOPSIS THALIANA METALLOTHIONEIN 4B (ATMT4B)*	AtMT4b is a member of Type 4 metallothionein (MT) genes. It is involved in the early develoment of the embryo and in the accumulation of metal ions especially Zn in the seeds.
AT2G23270	*PRECURSOR OF PAMP-INDUCED PEPTIDE 3 (PREPIP3)*	Encoding a precursor protein of a secreted peptide that is responsive to flg22 stimulus. Finetuning role in modulation of immunity through the regulation of SA and JA biosynthesis and signalling pathways.
AT2G23410	*CIS-PRENYLTRANSFERASE (CPT)*	Encodes cis-prenyltransferase involved in dolichol biosynthesis.
AT2G23480		transposable_element_gene;similar to unknown protein [Arabidopsis thaliana] (TAIR:AT5G35794.1);(source:TAIR10)
AT2G23500		transposable_element_gene;Mutator-like transposase family, has a 2.4e-80 P-value blast match to O80466 /172-336 Pfam PF03108 MuDR family transposase (MuDr-element domain);(source:TAIR10)
AT2G23540		GDSL-motif esterase/acyltransferase/lipase. Enzyme group with broad substrate specificity that may catalyze acyltransfer or hydrolase reactions with lipid and non-lipid substrates.
AT2G23670	*HOMOLOG OF SYNECHOCYSTIS YCF37 (YCF37)*	Phosphorylated protein in the thylakoid lumen.
AT2G23720		transposable_element_gene;Mutator-like transposase family, has a 6.7e-76 P-value blast match to Q9SI25 /181-349 Pfam PF03108 MuDR family transposase (MuDr-element domain);(source:TAIR10)
AT2G23760	*BEL1-LIKE HOMEODOMAIN 4 (BLH4)*	Encodes a member of the BEL family of homeodomain proteins. Plants doubly mutant for saw1/saw2 (blh2/blh4) have serrated leaves. BP is expressed in the serrated leaves, therefore saw2 and saw1 may act redundantly to repress BP in leaves. Regulates together with BLH2 demethylesterification of homogalacturonan in seed mucilage.
AT2G23890		HAD-superfamily hydrolase, subfamily IG, 5-nucleotidase

AT2G24090	*PLASTID RIBOSOMAL PROTEIN L35 (PRPL35)*	Ribosomal protein L35
AT2G24230		Leucine-rich repeat protein kinase family protein
AT2G24270	*ALDEHYDE DEHYDROGENASE 11A3 (ALDH11A3)*	Encodes a protein with non-phosphorylating NADP-dependent glyceraldehyde-3-phosphate dehydrogenase activity. The activity of the enzyme was determined from leaf extracts; the enzyme has not been purified to confirm activity.
AT2G24400	*SMALL AUXIN UPREGULATED RNA 38 (SAUR38)*	SAUR-like auxin-responsive protein family
AT2G24650		B3 domain-containing protein REM13
AT2G24740	*SET DOMAIN GROUP 21 (SDG21)*	Encodes a SU(VAR)3-9 homolog, a SET domain protein (Homology Subgroup V; Orthology Group 1). Known SET domain proteins are involved in epigenetic control of gene expression. There are 10 SUVH genes in Arabidopsis and members of this subfamily of the SET proteins have an additional conserved SRA domain. This protein is a putative histone methyltransferase (predicted to methylate H3K9/20) related to the the Drosophila Su(var)3-9 and mammalian G9a proteins.
AT2G24800		Peroxidase superfamily protein
AT2G24980	*EXTENSIN 6 (EXT6)*	Proline-rich extensin-like family protein
AT2G25160	*CYTOCHROME P450, FAMILY 82, SUBFAMILY F, POLYPEPTIDE 1 (CYP82F1)*	cytochrome P450, family 82, subfamily F, polypeptide 1
AT2G25450	*GLUCOSINOLATE HYDROXYLASE (GSL-OH)*	Encodes a 2-oxoacid-dependent dioxygenase involved in the production of 2-hydroxybut-3-enyl glucosinolate.
AT2G25630	*BETA GLUCOSIDASE 14 (BGLU14)*	beta glucosidase 14
AT2G25680	*MOLYBDATE TRANSPORTER 1 (MOT1)*	Encodes a high-affinity molybdate transporter. Mutant has reduced concentrations of molybdate in roots and shoots, and reduced shoot and root length when growing on Mo-limited medium.
AT2G25810	*TONOPLAST INTRINSIC PROTEIN 4;1 (TIP4;1)*	tonoplast intrinsic protein 4
AT2G25860		
AT2G25890		Oleosin family protein
AT2G25900	*(ATCTH)*	Encodes a protein with two tandem-arrayed CCCH-type zinc fingers that binds RNA and is involved in RNA turnover. The mRNA is cell-to-cell mobile.
AT2G25940	*ALPHA-VACUOLAR PROCESSING ENZYME (ALPHA-VPE)*	Encodes a vacuolar processing enzyme belonging to a novel group of cysteine proteinases that is expressed in vegetative organs and is upregulated in association with various types of cell death and under stressed conditions.
AT2G26020	*PLANT DEFENSIN 1.2B (PDF1.2b)*	Predicted to encode a PR (pathogenesis-related) protein. Belongs to the plant defensin (PDF) family with the following members: At1g75830/PDF1.1, At5g44420/PDF1.2a, At2g26020/PDF1.2b, At5g44430/PDF1.2c, At2g26010/PDF1.3, At1g19610/PDF1.4, At1g55010/PDF1.5, At2g02120/PDF2.1, At2g02100/PDF2.2, At2g02130/PDF2.3, At1g61070/PDF2.4, At5g63660/PDF2.5, At2g02140/PDF2.6, At5g38330/PDF3.1 and At4g30070/PDF3.2.
AT2G26170	*CYTOCHROME P450, FAMILY 711, SUBFAMILY A, POLYPEPTIDE 1 (CYP711A1)*	Encodes a protein with similarity to thromboxane-A synthase, member of the CYP711A cytochrome P450 family. MAX1 is a specific repressor of vegetative axillary buds generated by the axillary meristem. Expressed in vascular traces in the rosette stem and axillary buds throughout plant development. Mutants have increased axillary branches. Along with MAX3,4 thought to mediate control of shoot branching via synthesis of a signal molecule which is transported over long distance mediated by MAX2. cDNA supports the existence of the longer transcript predicted for this locus, no cDNA isolated for shorter transcript. MAX1 downregulates 11 genes involved in flavonoid pathway (CHS, CHI, F3H, F3'H, FLS, DFR, ANS, UFGT, RT, AAC and GST).
AT2G26290	*ROOT-SPECIFIC KINASE 1 (ARSK1)*	root-specific kinase 1
AT2G26330	*ERECTA (ER)*	Homologous to receptor protein kinases. Involved in specification of organs originating from the shoot apical meristem. Contains a cytoplasmic protein kinase catalytic domain, a transmembrane region, and an extracellular leucine-rich repeat. ER has been identified as a quantitative trait locus for transpiration efficiency by influencing epidermal and mesophyll development, stomatal density and porosity of leaves. It has been implicated in resistance to the bacterium Ralstonia solanacearum and to the necrotrophic fungus Plectosphaerella cucumerina. Together with ERL1 and ERL2, ER governs the initial decision of protodermal cells to either divide proliferatively to produce pavement cells or divide asymmetrically to generate stomatal complexes. ER binds to the peptides STOMAGEN and EPF2 which compete for the same binding site. The ER-EFP2 complex activates MAPK signaling that inhibits stomatal development. ER-STOMAGEN does not activate MAPK signaling. Plants harboring loss of function alleles of er are more susceptible to heat stress than wild type. In Arabidopsis and other organisms, overexpression of ER confers thermotolerance via as yet undefined mechanisms.
AT2G26400	*ACIREDUCTONE DIOXYGENASE 3 (ARD3)*	Encodes a protein predicted to belong to the acireductone dioxygenase (ARD/ARD?)family.
AT2G26500	*(PETM)*	Essential for the stabilization and function of the cytochrome b6f complex.
AT2G26520		transmembrane protein
AT2G26650	*K+ TRANSPORTER 1 (KT1)*	Encodes AKT1, a member of the Shaker family inward rectifying potassium channel predominantly expressed in predominantly in root hairs and root endodermis. This family includes five groups based on phylogenetic analysis (FEBS Letters (2007) 581: 2357): I (inward rectifying channel): AKT1 (AT2G26650), AKT5 (AT4G32500) and SPIK (also known as AKT6, AT2G25600); II (inward rectifying channel): KAT1 (AT5G46240) and KAT2 (AT4G18290); III (weakly inward rectifying channel): AKT2 (AT4G22200); IV (regulatory subunit involved in inwardly rectifying conductance formation): KAT3 (also known as AtKC1, AT4G32650); V (outward rectifying channel): SKOR (AT3G02850) and GORK (AT5G37500).
AT2G26710	*PHYB ACTIVATION TAGGED SUPPRESSOR 1 (BAS1)*	Encodes a member of the cytochrome p450 family that serves as a control point between multiple photoreceptor systems and brassinosteroid signal transduction. Involved in brassinolide metabolism. Mediates response to a variety of light signals including hypocotyl elongation and cotyledon expansion.
AT2G26740	*SOLUBLE EPOXIDE HYDROLASE (SEH)*	Encodes a soluble epoxide hydrolase whose expression is induced by auxin and water stress.
AT2G26910	*ATP-BINDING CASSETTE G32 (ABCG32)*	Encodes a member of the PLEIOTROPIC DRUG RESISTANCE family of ATP binding cassette transporters. Required for the formation of a functional cuticle.
AT2G27000	*CYTOCHROME P450, FAMILY 705, SUBFAMILY A, POLYPEPTIDE 8 (CYP705A8)*	member of CYP705A
AT2G27180		hypothetical protein
AT2G27300	*NTM1-LIKE 8 (NTL8)*	NTL8 is a membrane-associated NAC transcription factor that binds both TRY and TCL1. Overexpression results in fewer trichomes.
AT2G27360		GDSL-motif esterase/acyltransferase/lipase. Enzyme group with broad substrate specificity that may catalyze acyltransfer or hydrolase reactions with lipid and non-lipid substrates.
AT2G27380	*EXTENSIN PROLINE-RICH 1 (EPR1)*	Encodes an extensin like gene involved in seed germination.
AT2G27700		eukaryotic translation initiation factor 2 family protein / eIF-2 family protein

AT2G27775		PPR containing protein
AT2G27810	*NUCLEOBASE-ASCORBATE TRANSPORTER 12 (NAT12)*	Encodes a plasma-membrane localized nucleobase transporter capable of transporting adenine, guanine, uracil and hypoxanthine. Likely to be a proton-nucleobase symporter.
AT2G27830		hypothetical protein
AT2G27880	*ARGONAUTE 5 (AGO5)*	AGO5.Required for antiviral RNA silencing.Confers resistance to Potato virus X.
AT2G28400		senescence regulator (Protein of unknown function, DUF584)
AT2G28410		transmembrane protein
AT2G28530		
AT2G28650	*EXOCYST SUBUNIT EXO70 FAMILY PROTEIN H8 (EXO70H8)*	A member of EXO70 gene family, putative exocyst subunits, conserved in land plants. Arabidopsis thaliana contains 23 putative EXO70 genes, which can be classified into eight clusters on the phylogenetic tree.
AT2G28670	*ENHANCED SUBERIN 1 (ESB1)*	Encodes a protein of unknown function that is involved in formation of the casparian strip. ESB1 is localized to the casparian strip and this localization is depended on CASP1 and 2. esb1 mutants have increased levels of suberin, disordered casparian strip and altered levels of several ions in their leaves.
AT2G28755		UDP-D-glucuronate carboxy-lyase-like protein
AT2G28820		
AT2G28850	*CYTOCHROME P450, FAMILY 710, SUBFAMILY A, POLYPEPTIDE 3 (CYP710A3)*	member of CYP710A
AT2G28870	*(SMR10)*	cyclin-dependent kinase inhibitor SMR1-like protein
AT2G29090	*CYTOCHROME P450, FAMILY 707, SUBFAMILY A, POLYPEPTIDE 2 (CYP707A2)*	Encodes a protein with ABA 8'-hydroxylase activity, involved in ABA catabolism. Member of the CYP707A gene family. This gene predominantly accumulates in dry seeds and is up-regulated immediately following imbibition. CYP707A2 appears to play a major role in the rapid decrease in ABA levels during early seed imbibition.
AT2G29310		NAD(P)-binding Rossmann-fold superfamily protein
AT2G29330	*TROPINONE REDUCTASE (TRI)*	tropinone reductase
AT2G29350	*SENESCENCE-ASSOCIATED GENE 13 (SAG13)*	Encodes a senescence associated protein required for resistance against fungal pathogens. Negative regulator of defense against bacterial pathogens. Induced by ROS. Required for defense against ROS and fungal pathogens most likely by activating anthocyanin biosynthesis.
AT2G29370		NAD(P)-binding Rossmann-fold superfamily protein
AT2G29420	*GLUTATHIONE S-TRANSFERASE TAU 7 (GSTU7)*	Encodes glutathione transferase belonging to the tau class of GSTs. Naming convention according to Wagner et al. (2002). Induced by Salicylic acid. Independent of NPR1 for their induction by salicylic acid.
AT2G29440	*GLUTATHIONE S-TRANSFERASE TAU 6 (GSTU6)*	Encodes glutathione transferase belonging to the tau class of GSTs. Naming convention according to Wagner et al. (2002).
AT2G29460	*GLUTATHIONE S-TRANSFERASE TAU 4 (GSTU4)*	Encodes glutathione transferase belonging to the tau class of GSTs. Naming convention according to Wagner et al. (2002). Role in the degradation of H2O2 to water using glutathione as electron donor
AT2G29480	*GLUTATHIONE S-TRANSFERASE TAU 2 (GSTU2)*	Encodes glutathione transferase belonging to the tau class of GSTs. Naming convention according to Wagner et al. (2002).
AT2G29490	*GLUTATHIONE S-TRANSFERASE TAU 1 (GSTU1)*	Encodes glutathione transferase belonging to the tau class of GSTs. Naming convention according to Wagner et al. (2002).
AT2G29530	*TRANSLOCASE OF THE INNER MEMBRANE 10 (TIM10)*	Encodes a small zinc finger-like protein that is a component of the mitochondrial protein import apparatus. Together with AtTIM9, AtTIM10 is non-redundantly essential for maintaining mitochondrial function of early embryo proper cells and endosperm free-nuclei.
AT2G29630	*THIAMINC (THIC)*	Encodes a protein involved in thiamin biosynthesis. The protein is an iron-sulfur cluster protein predicted to catalyze the conversion of 5-aminoimidazole ribonucleotide (AIR) to hydroxymethylpyrimidine (HMP) or hydroxymethylpyrimidine phosphate (HMP-P). A severe reduction of THIC levels in plants decreases vitamin B1 (thiamin diphosphate (TPP)) levels and also leads to changes in the levels of numerous other metabolites since so many primary metabolic enzymes require a TPP co-factor. thiC mutants are chlorotic and arrest in their development at the cotyledon stage. A N-terminal targeting sequence directs the THIC protein to the chloroplast stroma. A conserved TPP-binding site is located in the 3' UTR of the At2g29630.2 gene model, and is predicted to function as a riboswitch. The riboswitch controls the formation of transcripts with alternative 3' UTR lengths, which affect mRNA accumulation and protein production. THIC transcripts are observed in seedlings 5 or more days after germination, and light promotes the expression of this gene. Recessive mutant isolated by Redei. Leaves but not cotyledons white, lethal; restored to normal by thiamine or 2,5-dimethyl-4-aminopyrimidine.
AT2G29750	*UDP-GLUCOSYL TRANSFERASE 71C1 (UGT71C1)*	UDP-glucosyl transferase 71C1
AT2G29890	*VILLIN 1 (VLN1)*	Encodes a ubiquitously expressed villin-like protein, whose mRNA may be alternatively processed. Villin belongs to a superfamily of actin binding proteins called the villin/gelsolin family. Animal villins are involved in actin binding. VLN1 protein co-localizes with actin filaments in several assays. VLN1 binds and bundles F-actin in a calcium-independent manner. It does not nucleate, cap or sever actin filaments and it stabilizes actin filaments, protecting them from ADF-mediated depolymerization.
AT2G29940	*ATP-BINDING CASSETTE G31 (ABCG31)*	pleiotropic drug resistance 3
AT2G30140	*UDP-GLUCOSYL TRANSFERASE 87A2 (UGT87A2)*	Encodes a putative glycosyltransferase. Regulates flowering time via FLOWERING LOCUS C.
AT2G30200	*EMBRYO DEFECTIVE 3147 (EMB3147)*	Malonyl-ACP expressed in developing seeds. Loss of function mutants are embryo lethal and over expression in seeds leads to increased seed oil content.
AT2G30250	*WRKY DNA-BINDING PROTEIN 25 (WRKY25)*	member of WRKY Transcription Factor; Group I. Located in nucleus. Involved in response to various abiotic stresses - especially salt stress.

AT2G30470	*HIGH-LEVEL EXPRESSION OF SUGAR-INDUCIBLE GENE 2 (HSI2)*	HSI2 is a member of the ABI3 family of B3 domain proteins and functions as an active repressor of the Spo minimal promoter through the EAR motif. It contains a plant-specific B3 DNA-binding domain. It is expressed at similar levels in all organs. Treatment with 6% sucrose showed a slight increase in transcript levels after 24 h. No changes were observed after treatment with 50?M ABA. It is localized in the nucleus via a nuclear localization sequence located in the fourth conserved region of the C-terminal B3 domain. HSI2 is also an epigenetic repressor as it also contains functional plant homeodomain-like (PHD-L) and zinc-finger Cys- and Trp-containing (CW) domains associated with epigenetic regulation. The PHD-L domain of HSI2 is connected to promoting trimethylation of Lys-27 on histone 3 (H3K27me3), while the CW domain can bind directly to H3K4me3. Through these domains, HSI2 represses the seed maturation program during seed germination by repressing transcription of the core LAFL (LEC1, ABI3, FUS3, and LEC2) seed developmental transcriptional regulators. In developing A. thaliana embryos, HSI2 suppresses expression of a large number of genes, many identified as targets of FUS3. However, the absence of HSI2 had no effect on transcript levels of the LAFL regulators and the levels of measured metabolites and phytohormones (ABA, auxin, and JA derivatives) in developing Arabidopsis embryos. HSI2 likely fine-tunes seed maturation by repressing genes involved in early embryogenesis that are not required later for seed maturation and desiccation.
AT2G30510		
AT2G30550	*DAD1-LIKE LIPASE 3 (DALL3)*	Encodes a lipase that hydrolyzes phosphatidylcholine, glycolipids as well as triacylglycerols.
AT2G30570	*PHOTOSYSTEM II REACTION CENTER W (PSBW)*	Encodes PsbW, a protein similar to photosystem II reaction center subunit W. Loss of PsbW destabilizes the supramolecular organization of PSII.
AT2G30790	*PHOTOSYSTEM II SUBUNIT P-2 (PSBP-2)*	Encodes a 23 kD extrinsic protein that is part of photosystem II and participates in the regulation of oxygen evolution.
AT2G30810	*(GASA12)*	Gibberellin-regulated family protein
AT2G30820		aspartyl/glutamyl-tRNA(Asn/Gln) amidotransferase subunit
AT2G30830		encodes a protein whose sequence is similar to 2-oxoglutarate-dependent dioxygenase
AT2G30870	*GLUTATHIONE S-TRANSFERASE PHI 10 (GSTF10)*	early dehydration-induced gene ERD13 homologous to tobacco and maize glutathione S-transferases. Encodes glutathione transferase belonging to the phi class of GSTs. Naming convention according to Wagner et al. (2002)
AT2G30890		Cytochrome b561/ferric reductase transmembrane protein family
AT2G30930		hypothetical protein
AT2G31060	*EMBRYO DEFECTIVE 2785 (EMB2785)*	elongation factor family protein
AT2G31070	*TCP DOMAIN PROTEIN 10 (tcp10)*	TCP family protein involved in heterchronic regulation of leaf differentiation.
AT2G31780	*ARIADNE 11 (ARI11)*	RING/U-box superfamily protein
AT2G31900	*MYOSIN-LIKE PROTEIN XIF (XIF)*	Encodes an novel myosin isoform.
AT2G31955	*COFACTOR OF NITRATE REDUCTASE AND XANTHINE DEHYDROGENASE 2 (CNX2)*	COFACTOR OF NITRATE REDUCTASE AND XANTHINE DEHYDROGENASE 2. Encodes a protein involved in molybdenum cofactor biosynthesis. Homologous to E.coli moaA. Expression is abundant in all tissues examined, particularly in roots. Appears to have targeting signals for chloroplast or mitochondria.
AT2G32020		Acyl-CoA N-acyltransferases (NAT) superfamily protein
AT2G32190	*CYSTEINE-RICH TRANSMEMBRANE MODULE 4 (ATHCYSTM4)*	cysteine-rich/transmembrane domain A-like protein
AT2G32210	*CYSTEINE-RICH TRANSMEMBRANE MODULE 6 (ATHCYSTM6)*	cysteine-rich/transmembrane domain A-like protein
AT2G32610	*CELLULOSE SYNTHASE-LIKE B1 (CSLB01)*	encodes a gene similar to cellulose synthase
AT2G32640	*LYCOPENE BETA-CYLCASE (LCYB)*	Encodes a lycopene beta cyclase that catalyzes the addition of beta-ionone end groups to the end of lycopene molecules.
AT2G32660	*RECEPTOR LIKE PROTEIN 22 (RLP22)*	receptor like protein 22
AT2G32770	*PURPLE ACID PHOSPHATASE 13 (PAP13)*	purple acid phosphatase 13
AT2G32800	*L-TYPE LECTIN RECEPTOR KINASE S.2 (LECRK-S.2)*	protein kinase family protein
AT2G32850		Protein kinase superfamily protein
AT2G32930	*ZINC FINGER NUCLEASE 2 (ZFN2)*	Encodes a zinc finger protein.
AT2G32990	*GLYCOSYL HYDROLASE 9B8 (GH9B8)*	glycosyl hydrolase 9B8
AT2G33030	*RECEPTOR LIKE PROTEIN 25 (RLP25)*	receptor like protein 25
AT2G33230	*YUCCA 7 (YUC7)*	Encodes a flavin monooxygenase gene which belongs to the tryptophan-dependent auxin biosynthetic pathway and enhances drought resistance.
AT2G33710		encodes a member of the ERF (ethylene response factor) subfamily B-4 of ERF/AP2 transcription factor family. The protein contains one AP2 domain. There are 7 members in this subfamily.
AT2G33830	*DORMANCY ASSOCIATED GENE 2 (DRM2)*	Negative regulator of local and systemic acquired resistance; target of FLD for activation of SAR.
AT2G34020		Calcium-binding EF-hand family protein
AT2G34080		Cysteine proteinases superfamily protein
AT2G34130	*MATERNAL EFFECT EMBRYO ARREST 19 (MEE19)*	transposable_element_gene;CACTA-like transposase family (Ptta/En/Spm), has a 5.8e-123 P-value blast match to At5g36655.1/81-333 CACTA-like transposase family (Ptta/En/Spm) (CACTA-element) (Arabidopsis thaliana);(source:TAIR10)
AT2G34300		S-adenosyl-L-methionine-dependent methyltransferases superfamily protein
AT2G34315		avirulence induced family protein
AT2G34370	*DYW DOMAIN PROTEIN 3 (DYW3)*	Pentatricopeptide repeat (PPR) superfamily protein
AT2G34500	*CYTOCHROME P450, FAMILY 710, SUBFAMILY A, POLYPEPTIDE 1 (CYP710A1)*	Encodes a protein with C22-sterol desaturase activity. The enzyme was shown to catalyze in the presence of NADPH the conversion of β-sitosterol to stigmasterol, but not that of 24-<i>epi</i>-campesterol to brassicasterol (unlike CYP710A2).
AT2G34590		Transketolase family protein
AT2G34600	*JASMONATE-ZIM-DOMAIN PROTEIN 7 (JAZ7)*	Key regulator in alternative splicing in the jasmonate signaling pathway, alone and in collaboration with other regulators.
AT2G34770	*FATTY ACID HYDROXYLASE 1 (FAH1)*	encodes a fatty acid hydroxylase, required for the AtBI-1-mediated suppression of programmed cell death.
AT2G34850	*MATERNAL EFFECT EMBRYO ARREST 25 (MEE25)*	NAD(P)-binding Rossmann-fold superfamily protein
AT2G34930		disease resistance family protein / LRR family protein
AT2G34990	*ARABIDOPSIS T??XICOS EN LEVADURA 38 (ATL38)*	RING/U-box superfamily protein
AT2G35150	*EXORDIUM LIKE 7 (EXL7)*	Encodes EXORDIUM LIKE 7.
AT2G35260	*BALANCE OF CHLOROPHYLL METABOLISM 1 (BCM1)*	CAAX protease self-immunity protein

AT2G35370	*GLYCINE DECARBOXYLASE COMPLEX H (GDCH)*	Encodes glycine decarboxylase complex H protein. Involved in photorespiration. The mRNA is cell-to-cell mobile.
AT2G35470		ribosome maturation factor
AT2G35570		pseudogene of Serine protease inhibitor (SERPIN) family protein
AT2G35720	*ORIENTATION UNDER VERY LOW FLUENCES OF LIGHT 1 (OWL1)*	Encodes OWL1, a J-domain protein involved in perception of very low light fluences.
AT2G35730	*HEAVY METAL ASSOCIATED PROTEIN 19 (ATHMP19)*	Heavy metal transport/detoxification superfamily protein
AT2G35760	*CASP-LIKE PROTEIN 2B2 (CASPL2B2)*	Uncharacterized protein family (UPF0497)
AT2G35930	*PLANT U-BOX 23 (PUB23)*	Encodes a cytoplasmically localized U-box domain containing E3 ubiquitin ligase that is involved in the response to water stress and acts as a negative regulator of PAMP-triggered immunity.
AT2G35980	*YELLOW-LEAF-SPECIFIC GENE 9 (YLS9)*	Encodes a protein whose sequence is similar to tobacco hairpin-induced gene (HIN1) and Arabidopsis non-race specific disease resistance gene (NDR1). Expression of this gene is induced by cucumber mosaic virus, spermine and during senescence. The gene product is localized to the chloroplast. The mRNA is cell-to-cell mobile.
AT2G36030		hypothetical protein
AT2G36100	*CASPARIAN STRIP MEMBRANE DOMAIN PROTEIN 1 (CASP1)*	Encodes a membrane bound protein involved in formation of the casparian strip. Along with CASP 2 it is required for the localization of ESB1.
AT2G36145		hypothetical protein
AT2G36270	*ABA INSENSITIVE 5 (ABI5)*	Encodes a member of the basic leucine zipper transcription factor family, involved in ABA signalling during seed maturation and germination. The Arabidopsis abscisic acid (ABA)-insensitive abi5 mutants have pleiotropic defects in ABA response, including decreased sensitivity to ABA inhibition of germination and altered expression of some ABA-regulated genes. Comparison of seed and ABA-inducible vegetative gene expression in wild-type and abi5-1 plants indicates that ABI5 regulates a subset of late embryogenesis-abundant genes during both developmental stages. Responsible for reducing cadmium uptake, mediated by interaction with MYB49 .
AT2G36320		A20/AN1-like zinc finger family protein
AT2G36430		transmembrane protein, putative (DUF247)
AT2G36460	*FRUCTOSE-BISPHOSPHATE ALDOLASE 6 (FBA6)*	Aldolase superfamily protein
AT2G36660	*POLY(A) BINDING PROTEIN 7 (PAB7)*	polyadenylate-binding protein, putative / PABP, putative. Member of the class III family of PABP proteins.
AT2G36690	*GERMINATION INSENSITIVE TO ABA MUTANT 2 (GIM2)*	Protein belonging to the Fe-dependent 2-oxoglutarate dioxygenase superfamily, catalyzes the stereospecific hydration of GA12 to produce DHGA12, negatively regulates ABA sensitivity during germination, phototrophic establishment and seedling development.
AT2G36790	*UDP-GLUCOSYL TRANSFERASE 73C6 (UGT73C6)*	The At2g36790 gene encodes a UDP-glucose:flavonol-3-O-glycoside-7-O-glucosyltransferase (UGT73C6)attaching a glucosyl residue to the 7-O-position of the flavonols kaempferol, quercetin and their 3-O-glycoside derivatives.
AT2G36885		translation initiation factor
AT2G36940		
AT2G36970		UDP-Glycosyltransferase superfamily protein
AT2G37090	*IRREGULAR XYLEM 9 (IRX9)*	The IRX9 gene encodes a putative family 43 glycosyl transferase. It was coordinately expressed with the cellulose synthase subunits during secondary cell wall formation. Cell wall analysis revealed a decrease in the abundance of xylan in the irx9 mutant, indicating that IRX9 is required for xylan synthesis. Mutants have irregular xylem phenotype suggesting a role in secondary cell wall biosynthesis.IRX9 was identified as MUCI65 in a reverse genetic screen for MUCILAGE-RELATED genes. Despite producing only a few seeds, the irx9-1 mutant displays normal mucilage properties.
AT2G37130		Peroxidase superfamily protein
AT2G37180	*RESPONSIVE TO DESICCATION 28 (RD28)*	a member of the plasma membrane intrinsic protein PIP2. functions as aquaporin and is involved in desiccation.
AT2G37250	*ADENOSINE KINASE (ADK)*	encodes adenylate kinase that is located in the chloroplast involved in the coordination of metabolism and growth
AT2G37300	*ATP-BINDING CASSETTE I16 (ABCI16)*	transmembrane protein
AT2G37390	*SODIUM POTASSIUM ROOT DEFECTIVE 2 (NAKR2)*	Chloroplast-targeted copper chaperone protein
AT2G37510		RNA-binding (RRM/RBD/RNP motifs) family protein
AT2G37630	*ASYMMETRIC LEAVES 1 (AS1)*	Encodes a MYB-domain protein involved in specification of the leaf proximodistal axis. Mutation results in lobed and dissected leaves with a characteristic asymmetry. Homologous to the Antirrhinum PHANTASTICA (PHAN) and maize ROUGH SHEATH2 (RS2) genes Asymmetric placement of auxin response at the distal leaf tip precedes visible asymmetric leaf growth. Acts alongside AXR1 to exclude BP expression in leaves and with PIN1 to repress BP and promote lateral organ growth. Interacts physically with AS2 to form a complex that binds to the BP promoter and silences BP. Also functions as a regulator of the plant immune response.
AT2G37670		Transducin/WD40 repeat-like superfamily protein
AT2G37690		phosphoribosylaminoimidazole carboxylase, putative / AIR carboxylase
AT2G37750		hypothetical protein
AT2G37820		Cysteine/Histidine-rich C1 domain family protein
AT2G37900		Major facilitator superfamily protein
AT2G38040	*ACETYL CO-ENZYME A CARBOXYLASE CARBOXYLTRANSFERASE ALPHA SUBUNIT (CAC3)*	encodes the carboxyltransferase alpha subunit of acetyl-CoA carboxylase, involved in de novo fatty acid biosynthesis
AT2G38210	*PUTATIVE PDX1-LIKE PROTEIN 4 (PDX1L4)*	putative PDX1-like protein 4
AT2G38390		Peroxidase superfamily protein
AT2G38470	*WRKY DNA-BINDING PROTEIN 33 (WRKY33)*	Member of the plant WRKY transcription factor family. Regulates the antagonistic relationship between defense pathways mediating responses to P. syringae and necrotrophic fungal pathogens. Located in nucleus. Involved in response to various abiotic stresses - especially salt stress. Regulates cytochrome P450 gene CYP94B1 to control apoplastic barrier formation in roots to confer salt tolerance.
AT2G38750	*ANNEXIN 4 (ANNAT4)*	Annexins are a family of calcium dependent membrane binding proteins though to be involved in Golgi mediated secretion. This is one of four annexins identified in Arabidopsis.

AT2G38760	*ANNEXIN 3 (ANNAT3)*	Annexins are calcium binding proteins that are localized in the cytoplasm. When cytosolic Ca2+ increases, they relocate to the plasma membrane. The mRNA is cell-to-cell mobile.
AT2G38860	*(YLS5)*	Encodes protease I (pfpI)-like protein YLS5.
AT2G38920		SPX (SYG1/Pho81/XPR1) domain-containing protein / zinc finger (C3HC4-type RING finger) protein-like protein
AT2G39010	*PLASMA MEMBRANE INTRINSIC PROTEIN 2E (PIP2E)*	plasma membrane intrinsic protein 2E
AT2G39050	*EUONYMUS LECTIN S3 (EULS3)*	Encodes a nucleocytoplasmic lectin that is capable of binding carbohydrates. It is involved in ABA mediated stomatal movement and increased expression is correlated with increased resistance to Pseudomonas syringae.
AT2G39210	*PICLORAM RESISTANT30 (PIC30)*	Major facilitator superfamily transmembrane transporter responsible for the uptake of picolinate herbicides.
AT2G39310	*JACALIN-RELATED LECTIN 22 (JAL22)*	jacalin-related lectin 22
AT2G39350	*ATP-BINDING CASSETTE G1 (ABCG1)*	Belongs to a clade of five Arabidopsis thaliana ABCG half-transporters that are required for synthesis of an effective suberin barrier in roots and seed coats (ABCG2, ABCG6, and ABCG20) and for synthesis of an intact pollen wall (ABCG1 and ABCG16).
AT2G39370	*MEMBRANE-ASSOCIATED KINASE REGULATOR 4 (MAKR4)*	Encodes a member of the MAKR (MEMBRANE-ASSOCIATED KINASE REGULATOR) gene family. MAKRs have putative kinase interacting motifs and membrane localization signals. Known members include: AT5G26230 (MAKR1), AT1G64080 (MAKR2), AT2G37380 (MAKR3), AT2G39370 (MAKR4), AT5G52870 (MAKR5) and AT5G52900 (MAKR6).
AT2G39430		Disease resistance-responsive (dirigent-like protein) family protein
AT2G39470	*PHOTOSYNTHETIC NDH SUBCOMPLEX L 1 (PnsL1)*	PsbP-like protein 2
AT2G39700	*EXPANSIN A4 (EXPA4)*	putative expansin. Naming convention from the Expansin Working Group (Kende et al, 2004. Plant Mol Bio). Involved in the formation of nematode-induced syncytia in roots of Arabidopsis thaliana.
AT2G39710		Encodes a Cysteine-rich peptide (CRP) family protein
AT2G39980		HXXXD-type acyl-transferase family protein
AT2G40080	*EARLY FLOWERING 4 (ELF4)*	Encodes a novel nuclear 111 amino-acid phytochrome-regulated component of a negative feedback loop involving the circadian clock central oscillator components CCA1 and LHY. ELF4 is necessary for light-induced expression of both CCA1 and LHY, and conversely, CCA1 and LHY act negatively on light-induced ELF4 expression. ELF4 promotes clock accuracy and is required for sustained rhythms in the absence of daily light/dark cycles. It is involved in the phyB-mediated constant red light induced seedling de-etiolation process and may function to coregulate the expression of a subset of phyB-regulated genes.
AT2G40100	*LIGHT HARVESTING COMPLEX PHOTOSYSTEM II (LHCB4.3)*	Lhcb4:3 protein (Lhcb4.3, light harvesting complex of photosystem II The mRNA is cell-to-cell mobile.
AT2G40150	*TRICHOME BIREFRINGENCE-LIKE 28 (TBL28)*	Encodes a member of the TBL (TRICHOME BIREFRINGENCE-LIKE) gene family containing a plant-specific DUF231 (domain of unknown function) domain. TBL gene family has 46 members, two of which (TBR/AT5G06700 and TBL3/AT5G01360) have been shown to be involved in the synthesis and deposition of secondary wall cellulose, presumably by influencing the esterification state of pectic polymers. A nomenclature for this gene family has been proposed (Volker Bischoff & Wolf Scheible, 2010, personal communication).
AT2G40170	*LATE EMBRYOGENESIS ABUNDANT 6 (GEA6)*	Encodes a group 1 LEA gene that is activated by direct binding of ABI5 to its promoter and is involved in response to ABA. Is required for normal seed development. Involved in regulating the timing of desiccation tolerance and rate of water loss during seed maturation.
AT2G40220	*ABA INSENSITIVE 4 (ABI4)*	Encodes a member of the DREB subfamily A-3 of ERF/AP2 transcription factor family (ABI4). The protein contains one AP2 domain. There is only one member in this family. Involved in abscisic acid (ABA) signal transduction, ABA-mediated glucose response, and hexokinase-dependent sugar responses. Acts downstream of GUN1 in retrograde signaling. Expressed most abundantly in developing siliques and to a lesser degree in seedlings.
AT2G40230		HXXXD-type acyl-transferase family protein
AT2G40280		S-adenosyl-L-methionine-dependent methyltransferases superfamily protein
AT2G40300	*FERRITIN 4 (FER4)*	Encodes FERRITIN 4, AtFER4. Ferritins are a class of 24-mer multi-meric proteins found in all kingdoms of life. Function as the main iron store in mammals. Evidence suggests that Arabidopsis ferritins are essential to protect cells against oxidative damage, but they do not constitute the major iron pool. Localize to mitochondria. Knock out mutants are not sensitive to abiotic stress.
AT2G40350		encodes a member of the DREB subfamily A-2 of ERF/AP2 transcription factor family. The protein contains one AP2 domain. There are eight members in this subfamily including DREB2A AND DREB2B that are involved in response to drought.
AT2G40460		Major facilitator superfamily protein
AT2G40470	*LOB DOMAIN-CONTAINING PROTEIN 15 (LBD15)*	LOB-domain containing protein. Involved in regulation of xylem differentiation- acts as a regulator of VND7 which is a master regulator of xylem cell differentiation.
AT2G40475	*ALTERED SEED GERMINATION 8 (ASG8)*	hypothetical protein
AT2G40610	*EXPANSIN A8 (EXPA8)*	member of Alpha-Expansin Gene Family. Naming convention from the Expansin Working Group (Kende et al, 2004. Plant Mol Bio). Involved in the formation of nematode-induced syncytia in roots of Arabidopsis thaliana.
AT2G40670	*RESPONSE REGULATOR 16 (RR16)*	response regulator 16
AT2G40880	*CYSTATIN A (CYSA)*	Encodes a protein with cysteine proteinase inhibitor activity. Overexpression increases tolerance to abiotic stressors (i.e.salt,osmotic, cold stress). The mRNA is cell-to-cell mobile.
AT2G40900	*USUALLY MULTIPLE ACIDS MOVE IN AND OUT TRANSPORTERS 11 (UMAMIT11)*	Encodes a plasma membrane-localized amino acid transporter likely involved in amino acid export in the developing seed.
AT2G40970	*(MYBC1)*	Homeodomain-like superfamily protein
AT2G41260	*(M17)*	Late-embryogenesis-abundant gene. Involved in the acquisition of desiccation tolerance during late phase of embryogenesis.
AT2G41340	*RNA POLYMERASE II FIFTH LARGEST SUBUNIT, D (RPB5D)*	NRPE5-like protein of unknown function; homologous to budding yeast RPB5
AT2G41640		Glycosyltransferase family 61 protein
AT2G41660	*MIZU-KUSSEI 1 (MIZ1)*	Essential for hydrotropism in roots. Mutant roots are defective in hydrotropism, and have slightly reduced phototropism and modified wavy growth response. Has normal gravitropism and root elongation.
AT2G41730	*(HRG1)*	Expression in rosette leaves is activated by high concentration of boron.
AT2G41800	*TEEBE, LONG IN THE MAYO-YOREME LANGUAGE (TEB)*	Encodes a DUF642 cell wall protein that is highly induced during the M/G1 phases of the cell cycle and is involved in hypocotyl cell elongation.
AT2G41810		imidazolonepropionase (Protein of unknown function, DUF642)

AT2G41990	*COMPANION OF CELLULOSE SYNTHASE 3 (CC3)*	late embryogenesis abundant protein
AT2G42190		rho GTPase-activating gacO-like protein
AT2G42200	*SQUAMOSA PROMOTER BINDING PROTEIN-LIKE 9 (SPL9)*	Encodes a putative transcriptional regulator that is involved in the vegetative to reproductive phase transition. Expression is regulated by MIR156b. SPL activity nonautonomously inhibits initiation of new leaves at the shoot apical meristem.
AT2G42350	*ARABIDOPSIS T??XICOS EN LEVADURA 40 (ATL40)*	RING/U-box superfamily protein
AT2G42360	*ARABIDOPSIS T??XICOS EN LEVADURA 41 (ATL41)*	RING/U-box superfamily protein
AT2G42430	*LATERAL ORGAN BOUNDARIES-DOMAIN 16 (LBD16)*	LOB-domain protein gene LBD16. This gene contains one auxin-responsive element (AuxRE). Regluates lateral root formation.
AT2G42570	*TRICHOME BIREFRINGENCE-LIKE 39 (TBL39)*	Encodes a member of the TBL (TRICHOME BIREFRINGENCE-LIKE) gene family containing a plant-specific DUF231 (domain of unknown function) domain. TBL gene family has 46 members, two of which (TBR/AT5G06700 and TBL3/AT5G01360) have been shown to be involved in the synthesis and deposition of secondary wall cellulose, presumably by influencing the esterification state of pectic polymers. A nomenclature for this gene family has been proposed (Volker Bischoff & Wolf Scheible, 2010, personal communication).
AT2G42590	*GENERAL REGULATORY FACTOR 9 (GRF9)*	14-3-3 gene. Binds calcium and displays induced structural changes.
AT2G42660		Homeodomain-like superfamily protein
AT2G42790	*CITRATE SYNTHASE 3 (CSY3)*	Encodes a peroxisomal citrate synthase that is expressed throughout seedling and shoot development.
AT2G42830	*SHATTERPROOF 2 (SHP2)*	AGAMOUS [AG]-like MADS box protein (AGL5) involved in fruit development (valve margin and dehiscence zone differentiation). A putative direct target of AG. SHP2 has been shown to be a downstream gene of the complex formed by AG and SEP proteins (SEP4 alone does not form a functional complex with AG).
AT2G42840	*PROTODERMAL FACTOR 1 (PDF1)*	Encodes a putative extracellular proline-rich protein is exclusively expressed in the L1 layer of vegetative, inflorescence and floral meristems and the protoderm of organ primordia.
AT2G42860		hypothetical protein
AT2G42870	*PHY RAPIDLY REGULATED 1 (PAR1)*	Encodes PHYTOCHROME RAPIDLY REGULATED1 (PAR1), an atypical basic helix-loop-helix (bHLP) protein. Closely related to PAR2 (At3g58850). Up regulated after simulated shade perception. Acts in the nucleus to control plant development and as a negative regulator of shade avoidance response. Functions as transcriptional repressor of auxin-responsive genes SAUR15 (AT4G38850) and SAUR68 (AT1G29510).
AT2G43000	*NAC DOMAIN CONTAINING PROTEIN 42 (NAC042)*	Encodes a NAC transcription factor induced by hydrogen peroxide (H_2O_2). Involved in senescence. Over expression of the gene strongly delays senescence and enhances tolerance to various abiotic stresses.
AT2G43030	*PLASTID RIBOSOMAL PROTEINS OF THE 50S SUBUNIT (PRPL3)*	Ribosomal protein L3 family protein
AT2G43060	*ILI1 BINDING BHLH 1 (IBH1)*	ILI1 binding bHLH 1
AT2G43270		F-box and associated interaction domains-containing protein
AT2G43480		Peroxidase superfamily protein
AT2G43530		Encodes a defensin-like (DEFL) family protein. The mRNA is cell-to-cell mobile.
AT2G43560		FKBP-like peptidyl-prolyl cis-trans isomerase family protein
AT2G43590		Chitinase family protein
AT2G43620		Chitinase family protein
AT2G43710	*SUPPRESSOR OF SA INSENSITIVE 2 (SSI2)*	Encodes a stearoyl-ACP desaturase, involved in fatty acid desaturation. The ssi2 mutants have increased 18:0 and reduced 18:1 fatty acids. Exogenous application of glycerol to wild type plants mimics the ssi2 mutant phenotype. The altered 18:1 fatty acid content in the ssi2 mutants has an impact on SA- and JA-mediated defense signaling. ssi2 mutants resulted in hyper-resistance to green peach aphid and antibiosis activity in petiole exudates. Redundant Δ9 stearoyl-ACP desaturase gene which together with AAD1 and AAD5 during embryo development provide precursors for the elaboration of embryo cuticle and therefore plays a specific role during the phase of invasive embryo growth through the endosperm. Together with AAD1, AAD5, and AAD6 redundantly participates in oil storage during the maturation phase.
AT2G43840	*UDP-GLYCOSYLTRANSFERASE 74 F1 (UGT74F1)*	UGT74F1 transfers UDP:glucose to salicylic acid (forming a glucoside), benzoic acid, quercetin, and athranilate in vitro. UGT74F1 shows a weak ability to catalyze the formation of the p-aminobenzoate-glucose ester in vitro. But, UGT75B1 appears to be the dominant pABA acylglucosyltransferase in vivo based on assays in leaves, flowers, and siliques. The true biological substrate(s) of UGT74F1 are not known, but mutant plants lacking UGT74F1 have a decreased level of salicylate glucoside.
AT2G44130	*KISS ME DEADLY 3 (KMD3)*	Encodes a member of a family of F-box proteins, called the KISS ME DEADLY (KMD) family. Component of SCF ubiquitin protein ligase, interacts with phenylalanine ammonia-lyase. AtKFB39 is a homolog of previously identified AtKFB50 (At3g59940) and specifically interacts with Arabidopsis PAL3 and PAL4 in vitro. In planta, together with AtKFB01, KFB20 and KFB50, it regulates PAL protein stability thus controlling phenylpropanoid biosynthesis .
AT2G44370		Cysteine/Histidine-rich C1 domain family protein
AT2G44460	*BETA GLUCOSIDASE 28 (BGLU28)*	Beta-glucosidase, major myrosinase which initiates sulfur reallocation by hydrolyzing particular GL species, conferring sulfur deficiency tolerance, especially during early development.
AT2G44470	*BETA GLUCOSIDASE 29 (BGLU29)*	beta glucosidase 29
AT2G44490	*PENETRATION 2 (PEN2)*	Encodes a glycosyl hydrolase that localizes to peroxisomes and acts as a component of an inducible preinvasion resistance mechanism. Required for mlo resistance. The mRNA is cell-to-cell mobile.
AT2G44510		CDK inhibitor P21 binding protein
AT2G44790	*UCLACYANIN 2 (UCC2)*	Encodes a uclacyanin, a protein precursor that is closely related to precursors of stellacyanins and a blue copper protein from pea pods.
AT2G44810	*DEFECTIVE ANTHER DEHISCENCE 1 (DAD1)*	Mutant has defects in anther dehiscence, pollen maturation, and flower opening. The DAD1 protein is a chloroplastic phospholipase A1 that catalyzes the initial step of jasmonic acid biosynthesis.
AT2G45030		Translation elongation factor EFG/EF2 protein
AT2G45210	*SMALL AUXIN UPREGULATED 36 (SAUR36)*	SAUR-like auxin-responsive protein family
AT2G45290	*TRANSKETOLASE 2 (TKL2)*	Transketolase
AT2G45340		Leucine-rich repeat protein kinase family protein
AT2G45400	*BRI1-5 ENHANCED 1 (BEN1)*	involved in the regulation of brassinosteroid metabolic pathway

AT2G45420	*LOB DOMAIN-CONTAINING PROTEIN 18 (LBD18)*	LOB domain-containing protein 18
AT2G45560	*CYTOCHROME P450, FAMILY 76, SUBFAMILY C, POLYPEPTIDE 1 (CYP76C1)*	cytochrome P450 monooxygenase
AT2G45970	*CYTOCHROME P450, FAMILY 86, SUBFAMILY A, POLYPEPTIDE 8 (CYP86A8)*	Encodes a member of the CYP86A subfamily of cytochrome p450 genes. Expressed at moderate levels in flowers, leaves, roots and stems.Mutant seeds have reduced seed longevity, higher tetrazolium salt uptake and reduction, and reduced lipid polyester barriers (PMID:32519347).
AT2G46140	*LATE EMBRYOGENESIS ABUNDANT 27 (LEA27)*	Late embryogenesis abundant protein
AT2G46150		Late embryogenesis abundant (LEA) hydroxyproline-rich glycoprotein family
AT2G46210	*SPHINGOID LCB DESATURASE 2 (SLD2)*	Fatty acid/sphingolipid desaturase
AT2G46440	*CYCLIC NUCLEOTIDE-GATED CHANNEL 11 (CNGC11)*	Member of Cyclic nucleotide gated channel family. Positive regulator of resistance against avirulent fungal pathogen. The mRNA is cell-to-cell mobile.
AT2G46550		transmembrane protein
AT2G46600		Calcium-binding EF-hand family protein
AT2G46820	*PHOTOSYSTEM I P SUBUNIT (PSI-P)*	Encodes the P subunit of Photosystem I. About 25% of the TMP14 pool appeared to be phosphorylated, and this ratio is not affected by light. Contains seven phosphorylation sites on threonine residue and chloroplast targeting signal. Located in the proximity of PSI-L, -H and -O subunits. Forms oligomers with other members of CURT1 family to modulate grana structure.
AT2G46830	*CIRCADIAN CLOCK ASSOCIATED 1 (CCA1)*	Encodes a transcriptional repressor that performs overlapping functions with LHY in a regulatory feedback loop that is closely associated with the circadian oscillator of Arabidopsis. Binds to the evening element in the promoter of TOC1 and represses TOC1 transcription. CCA1 and LHY colocalize in the nucleus and form heterodimers in vivo. CCA1 and LHY function synergistically in regulating circadian rhythms of Arabidopsis. CCA1 binds the GI promoter.
AT2G46850		Protein kinase superfamily protein
AT2G46960	*CYTOCHROME P450, FAMILY 709, SUBFAMILY B, POLYPEPTIDE 1 (CYP709B1)*	member of CYP709B
AT2G46990	*INDOLE-3-ACETIC ACID INDUCIBLE 20 (IAA20)*	Encodes a member of the Aux/IAA family of proteins implicated in auxin signaling. IAA20 lacks the conserved degron (domain II) found in many family members, and IAA20 fusion proteins are stable in Arabidopsis seedlings. IAA20 transcripts are induced by auxin treatment, and overexpression of IAA20 leads to defects in gravitropism, root development, root meristem maintenance, etiolation, and cotyledon vascular development.
AT2G47070	*SQUAMOSA PROMOTER BINDING PROTEIN-LIKE 1 (SPL1)*	member of SPL gene family, encodes DNA binding proteins and putative transcription factors. All have the SBP-box, which encodes the SBP-domain, required for and sufficient for interaction with DNA.
AT2G47130	*SHORT-CHAIN DEHYDROGENASE/REDUCTASE 3 (SDR3)*	Encodes a short-chain dehydrogenase/reductase that is not involved in ABA biosynthesis but plays an important role in plant defense response to bacteria.
AT2G47140	*SHORT-CHAIN DEHYDROGENASE REDUCTASE 5 (SDR5)*	NAD(P)-binding Rossmann-fold superfamily protein
AT2G47200		hypothetical protein
AT2G47240	*LONG-CHAIN ACYL-COA SYNTHASE 1 (LACS1)*	Encodes an acyl-CoA synthetase that acts on long-chain and very-long-chain fatty acids, involved in cuticular wax and cutin biosynthesis The mRNA is cell-to-cell mobile.
AT2G47520	*ETHYLENE RESPONSE FACTOR 71 (ERF71)*	encodes a member of the ERF (ethylene response factor) subfamily B-2 of ERF/AP2 transcription factor family. The protein contains one AP2 domain. There are 5 members in this subfamily including RAP2.2 AND RAP2.12. It plays a role in hypoxia-induced root slanting.
AT2G47550		Plant invertase/pectin methylesterase inhibitor superfamily
AT2G47750	*PUTATIVE INDOLE-3-ACETIC ACID-AMIDO SYNTHETASE GH3.9 (GH3.9)*	Encodes GH3.9, a member of the GH3 family auxin-responsive genes. gh3.9-1 mutants had greater primary root length, increased sensitivity to indole-3-acetic acid (IAA)-mediated root growth inhibition, but no obvious effects on apical dominance or leaf morphology.
AT2G47780	*LD-ASSOCIATED PROTEIN 2 (LDAP2)*	Encodes a small rubber particle protein homolog. Plays dual roles as positive factors for tissue growth and development and in drought stress responses.
AT2G47910	*CHLORORESPIRATORY REDUCTION 6 (CRR6)*	Encodes a chloroplast thylakoid membrane protein. Required for the assembly/accumulation of the NAD(P)H dehydrogenase complex of the photosynthetic electron transport chain.
AT2G48020	*ZINC-INDUCED FACILITATOR 2 (ZIF2)*	Encodes a zinc transporter ZIF2. Expression of ZIF2 is regulated by alternative splicing.
AT2G48130	*GLYCOSYLPHOSPHATIDYLINOSITOL-ANCHORED LIPID PROTEIN TRANSFER 15 (LTPG15)*	Encodes a plasma membrane-localized glycosylphosphatidylinositol-anchored lipid transfer protein expressed in root endodermis and seed coats that is involved in very long chain fatty acid (and their derivatives) transport.
AT2G48150	*GLUTATHIONE PEROXIDASE 4 (GPX4)*	Encodes glutathione peroxidase.
AT3G01480	*CYCLOPHILIN 38 (CYP38)*	Encodes a chloroplast cyclophilin functioning in the assembly and maintenance of photosystem II (PSII) supercomplexes. The mRNA is cell-to-cell mobile.
AT3G01490	*CONVERGENCE OF BLUE LIGHT AND CO2 (CBC1)*	Belongs to the Raf-like kinase subfamily of the mitogen-activated protein kinase kinase kinase (MAPKKK) family. Negatively regulates stomatal opening by negatively regulating plasma membrane H+-ATPase phosphorylation.
AT3G01820		P-loop containing nucleoside triphosphate hydrolases superfamily protein
AT3G01970	*WRKY DNA-BINDING PROTEIN 45 (WRKY45)*	member of WRKY Transcription Factor; Group I
AT3G02000	*(ROXY1)*	Encodes a member of the CC-type glutaredoxin (ROXY) family that has been shown to interact with the transcription factor TGA2 and suppress ORA59 promoter activity. It is required for proper petal initiation and organogenesis. It is likely to function in the temporal and spatial expression regulation of AGAMOUS in the first and second whorl. Its function is dependent on the Cysteine 49 residue and its nuclear localization. ROXY1 interacts in vitro and in vivo with members of the TGA family of transcription factors (e.g. TGA2, TGA3, TGA7 and PAN).
AT3G02110	*SERINE CARBOXYPEPTIDASE-LIKE 25 (scpl25)*	serine carboxypeptidase-like 25
AT3G02380	*CONSTANS-LIKE 2 (COL2)*	homologous to the flowering-time gene CONSTANS (CO) encoding zinc-finger proteins
AT3G02550	*LOB DOMAIN-CONTAINING PROTEIN 41 (LBD41)*	LOB domain-containing protein 41
AT3G02590		Fatty acid hydroxylase superfamily protein
AT3G02730	*THIOREDOXIN F-TYPE 1 (TRXF1)*	Encodes a type-f thioredoxin. Has a role in the short-term activation of carbon metabolism. Loss affects growth under short-day conditions.
AT3G02930		Encodes a microtubule-associated protein.
AT3G03000		Calmodulin like protein localized in the plant vacuolar compartment with a function of binding and modifying the activity of a tonoplast transporter (AtNHX1) from within the vacuole in a Ca+2- and pH-dependent manner

AT3G03060	*SHOT1 BINDING ATPASE 1 (SBA1)*	Homologue of animal ATPase Family AAA Domain-Containing Protein 3 (ATAD3), which is involved in mitochondrial nucleoid organization; interacts with SHOT1.
AT3G03230		alpha/beta-Hydrolases superfamily protein
AT3G03240		alpha/beta-Hydrolases superfamily protein
AT3G03270	*HYPOXIA RESPONSIVE UNIVERSAL STRESS PROTEIN 1 (HRU1)*	HRU1 is a hypoxia induced universal stress protein. It exists as two splice variants with AT3G03270.2 , which contains a putative dimerization domain, the predominant transcript found under anoxia. It is induced by RAP2.12. Subcellular localization is dynamic; under anoxia the localization of HRU1 shifts from cytoplasm to the plasma membrane.
AT3G03470	*CYTOCHROME P450, FAMILY 87, SUBFAMILY A, POLYPEPTIDE 9 (CYP89A9)*	P450 monooxygenase CYP89A9. Involved in NDCC accumulation during Arabidopsis leaf senescence.
AT3G03520	*NON-SPECIFIC PHOSPHOLIPASE C3 (NPC3)*	Lysophosphatidic acid phosphatase highly expressed during phosphate starvation and abiotic stresses. Role in lipid synthesis.
AT3G03590	*(SWIB4)*	SWIB/MDM2 domain superfamily protein
AT3G03620		MATE efflux family protein
AT3G03770		Leucine-rich repeat protein kinase family protein
AT3G03950	*EVOLUTIONARILY CONSERVED C-TERMINAL REGION 1 (ECT1)*	Physically interacts with CIPK1. Located in the nucleus.
AT3G04000	*CHLOROPLAST ALDEHYDE REDUCTASE (CHLADR)*	ChlADR is an aldehyde reductase that catalyzes the reduction of the aldehyde carbonyl groups on saturated and alpha,beta-unsaturated aldehydes with more than 5 carbons in vitro. The N-terminal region of this protein directs GFP to the chloroplast where where ChlADR likely helps to maintain the photosynthetic process by detoxifying reactive carbonyls formed during lipid peroxidation. In addition, this enzyme can also reduce cis-3-hexenal, a major plant volatile compound that contributes to green leaf odor, as well as methylglyoxal in vitro.
AT3G04050		Pyruvate kinase family protein
AT3G04230		Ribosomal protein S5 domain 2-like superfamily protein
AT3G04320		Kunitz family trypsin and protease inhibitor protein
AT3G04570	*AT-HOOK MOTIF NUCLEAR-LOCALIZED PROTEIN 19 (AHL19)*	AT-hook motif nuclear-localized protein 19
AT3G04770	*40S RIBOSOMAL PROTEIN SA B (RPSAb)*	40s ribosomal protein SA B
AT3G04800	*TRANSLOCASE INNER MEMBRANE SUBUNIT 23-3 (TIM23-3)*	translocase inner membrane subunit 23-3
AT3G04960		trichohyalin, putative (DUF3444)
AT3G05180	*(GGL16)*	GDSL-motif esterase/acyltransferase/lipase. Enzyme group with broad substrate specificity that may catalyze acyltransfer or hydrolase reactions with lipid and non-lipid substrates.
AT3G05360	*RECEPTOR LIKE PROTEIN 30 (RLP30)*	receptor like protein 30
AT3G05470	*FORMIN 11 (FH11)*	Actin-binding FH2 (formin homology 2) family protein
AT3G05980		hypothetical protein
AT3G06070		hypothetical protein
AT3G06530		ARM repeat superfamily protein
AT3G06800		
AT3G06840		hypothetical protein
AT3G06850	*(BCE2)*	dihydrolipoamide branched chain acyltransferase
AT3G06930	*PROTEIN ARGININE METHYLTRANSFERASE 4B (PRMT4B)*	Encodes an type I protein arginine methyltransferase. PRMT4b can catalyze the asymmetric dimethylation of arginines 2,17, and 26 on histone 3 and can also methylate myelin basic protein in vitro. Double mutants lacking PRMT4a and 4b have reduced levels of histone 3 methylated at R17. These double mutants flower late due to defects in the autonomous pathway and they have elevated levels of FLC transcripts.
AT3G07000		Cysteine/Histidine-rich C1 domain family protein
AT3G07010		Pectin lyase-like superfamily protein
AT3G07050	*NUCLEOSTEMIN-LIKE 1 (NSN1)*	Arabidopsis NSN1 encodes a nucleolar GTP- binding protein and is required for maintenance of inflorescence meristem identity and floral organ development.
AT3G07200	*SUMO-TARGETED UBIQUITIN E3 LIGASE 3 (STUBL3)*	RING/U-box superfamily protein. SUMO- targeted ubiquitin ligase.
AT3G07260		SMAD/FHA domain-containing protein
AT3G07350		sulfate/thiosulfate import ATP-binding protein, putative (DUF506)
AT3G07390	*AUXIN-INDUCED IN ROOT CULTURES 12 (AIR12)*	isolated from differential screening of a cDNA library from auxin-treated root culture. sequence does not show homology to any known proteins and is predicted to be extracellular. The mRNA is cell-to-cell mobile.
AT3G07650	*CONSTANS-LIKE 9 (COL9)*	This gene belongs to the CO (CONSTANS) gene family. This gene family is divided in three subgroups: groups III, to which COL9 belongs, is characterised by one B-box (supposed to regulate protein-protein interactions) and a second diverged zinc finger. COL9 downregulates expression of CO (CONSTANS) as well as FT and SOC1 which are known regulatory targets of CO. The mRNA is cell-to-cell mobile.
AT3G07730		hypothetical protein
AT3G07770	*HEAT SHOCK PROTEIN 89.1 (Hsp89.1)*	HEAT SHOCK PROTEIN 89.1
AT3G07870	*F-BOX PROTEIN92 (FBX92)*	FBX92 is an F-box containing protein. Overexpression produces plants with smaller leaves while reduced expression is correlated with increased leaf size and increased rates of cell proliferation.
AT3G07990	*SERINE CARBOXYPEPTIDASE-LIKE 27 (SCPL27)*	serine carboxypeptidase-like 27
AT3G08860	*PYRIMIDINE 4 (PYD4)*	Encodes a protein that is predicted to have beta-alanine aminotransferase activity.
AT3G08920	*RHODANESE-LIKE PROTEIN (STR10)*	Rhodanese/Cell cycle control phosphatase superfamily protein
AT3G08940	*LIGHT HARVESTING COMPLEX PHOTOSYSTEM II (LHCB4.2)*	Lhcb4.2 protein (Lhcb4.2, protein involved in the light harvesting complex of photosystem II The mRNA is cell-to-cell mobile.
AT3G09270	*GLUTATHIONE S-TRANSFERASE TAU 8 (GSTU8)*	Encodes glutathione transferase belonging to the tau class of GSTs. Naming convention according to Wagner et al. (2002).
AT3G09580		FAD/NAD(P)-binding oxidoreductase family protein
AT3G09590		CAP (Cysteine-rich secretory proteins, Antigen 5, and Pathogenesis-related 1 protein) superfamily protein

AT3G09660	*MINICHROMOSOME MAINTENANCE 8 (MCM8)*	Encodes a minichromosome maintenance protein that is involved with RAD51 in a backup pathway that repairs meiotic double strand breaks without giving meiotic crossovers when the major pathway, which relies on DMC1, fails.
AT3G09870	*SMALL AUXIN UPREGULATED RNA 48 (SAUR48)*	SAUR-like auxin-responsive protein family
AT3G09980	*ACETYLATED INTERACTING PROTEIN 1 (ACIP1)*	Encodes ACIP1, a microtubules-associated protein required for bacterial immunity. The mRNA is cell-to-cell mobile.
AT3G10020	*HYPOXIA RESPONSE UNKNOWN PROTEIN 26 (HUP26)*	plant/protein
AT3G10080		RmlC-like cupins superfamily protein
AT3G10120		PADRE protein down-regulated after infection by S. sclerotiorum.
AT3G10320	*MUCILAGE-RELATED 21 (MUCI21)*	MUCI21 is a GT61 protein required for the production of highly branched xylan in seed coat mucilage. MUCI21 likely decorates xylan with xylose side chains that seem to be necessary for pectin attachment to the seed surface.
AT3G10450	*SERINE CARBOXYPEPTIDASE-LIKE 7 (SCPL7)*	serine carboxypeptidase-like 7
AT3G10520	*HAEMOGLOBIN 2 (HB2)*	Encodes a class 2 non-symbiotic hemoglobin. Over-expression of AHb2 in seeds led to a 40% increase in the total fatty acid content of developing and mature seeds in three subsequent generations. This was mainly due to an increase in the poly-unsaturated C18:2 (omega-6) linoleic and C18:3 (omega-3) alpha-linolenic acids.
AT3G10720		Plant invertase/pectin methylesterase inhibitor superfamily
AT3G10890	*ENDO-BETA-MANNANASE 3 (MAN3)*	Encodes an endo beta mannanase that is localized to the apoplast and involved in glutathione mediated cadmium tolerance.
AT3G10930	*IDA-LIKE7 (IDL7)*	Encodes a small secreted signaling peptide that processed both N- and C-terminally after translation and is rapidly induced in response to ROS and flg22-induced stress and may act as a negative modulator of stress-induced ROS signalling.
AT3G11050	*FERRITIN 2 (FER2)*	ferritin 2
AT3G11180	*JASMONATE-INDUCED OXYGENASE1 (JOX1)*	One of 4 paralogs encoding a 2-oxoglutarate/Fe(II)-dependent oxygenases that hydroxylates JA to 12-OH-JA.
AT3G11280		Putative transcription factors interacting with the gene product of VHA-B1 (vacuolar ATPase subunit B1; as shown through yeast two-hybrid assay).
AT3G11340	*UDP-DEPENDENT GLYCOSYLTRANSFERASE 76B1 (UGT76B1)*	Encodes a uridine diphosphate-dependent glucosyltransferase that conjugates isoleucic acid and modulates plant defense via glucosylation of N-hydroxypipecolic acid.
AT3G11550	*CASPARIAN STRIP MEMBRANE DOMAIN PROTEIN 2 (CASP2)*	Uncharacterized protein family (UPF0497)
AT3G11640		transmembrane protein
AT3G12020	*KINESIN 7.3 (KIN7.3)*	P-loop containing nucleoside triphosphate hydrolases superfamily protein
AT3G12090	*TETRASPANIN6 (TET6)*	TET6 encodes a member of the TETRASPANIN gene family that is expressed in the vascular system and is involved in organ growth redundantly with TET5.
AT3G12500	*BASIC CHITINASE (HCHIB)*	encodes a basic chitinase involved in ethylene/jasmonic acid mediated signalling pathway during systemic acquired resistance based on expression analyses.
AT3G12520	*SULFATE TRANSPORTER 4;2 (SULTR4;2)*	Encodes a sulfate transporter that in induced under sulfate limitation.
AT3G12550	*FACTOR OF DNA METHYLATION 3 (FDM3)*	Belongs to a subgroup of SGS3-like proteins that act redundantly in RNA-directed DNA methylation: AT1G15910 (FDM1), AT4G00380 (FDM2), AT3G12550 (FDM3), AT1G13790 (FDM4), AT1G80790 (FDM5).
AT3G12700	*NANA (NANA)*	Encodes an aspartic protease has an important regulatory function in chloroplasts that not only influences photosynthetic carbon metabolism but also plastid and nuclear gene expression.
AT3G12710		DNA glycosylase superfamily protein
AT3G13220	*ATP-BINDING CASSETTE G26 (ABCG26)*	Encodes a ATP-binding cassette transporter G26 (ABCG26) involved in tapetal cell and pollen development. Required for male fertility and pollen exine formation.
AT3G13310	*DNA J PROTEIN C66 (DJC66)*	Chaperone DnaJ-domain superfamily protein
AT3G13610	*(F6'H1)*	Encodes a Fe(II)- and 2-oxoglutarate-dependent dioxygenase family gene F6'H1. Mutations in this gene compromise iron uptake and the production of fluorescent phenolics involved in Fe uptake. The mRNA is cell-to-cell mobile.
AT3G13650		Disease resistance-responsive (dirigent-like protein) family protein
AT3G13662		Disease resistance-responsive (dirigent-like protein) family protein
AT3G13760		Cysteine/Histidine-rich C1 domain family protein
AT3G13790	*(ATBFRUCT1)*	Encodes a protein with invertase activity.
AT3G13810	*INDETERMINATE(ID)-DOMAIN 11 (IDD11)*	indeterminate(ID)-domain 11
AT3G13940		DNA binding / DNA-directed RNA polymerase
AT3G13960	*GROWTH-REGULATING FACTOR 5 (GRF5)*	Growth regulating factor encoding transcription activator. One of the nine members of a GRF gene family, containing nuclear targeting domain. Involved in leaf development and expressed in root, shoot and flower.
AT3G14060		hypothetical protein
AT3G14310	*PECTIN METHYLESTERASE 3 (PME3)*	encodes a pectin methylesterase, targeted by a cellulose binding protein (CBP) from the parasitic nematode Heterodera schachtii during parasitism.
AT3G14360	*OIL BODY LIPASE 1 (ATOBL1)*	Lipid droplet-associated triacylglycerol lipase (TAG) involved in pollen tube growth. TAG is possibly a direct precursor for the synthesis of membrane lipids in pollen tubes.
AT3G14450	*CTC-INTERACTING DOMAIN 9 (CID9)*	RNA-binding protein, putative, contains Pfam profile: PF00076 RNA recognition motif. (a.k.a. RRM, RBD, or RNP domain) (2 copies). Contains PAM PABC binding domain.
AT3G14570	*GLUCAN SYNTHASE-LIKE 4 (GSL04)*	encodes a protein similar to callose synthase
AT3G14610	*CYTOCHROME P450, FAMILY 72, SUBFAMILY A, POLYPEPTIDE 7 (CYP72A7)*	putative cytochrome P450
AT3G14660	*CYTOCHROME P450, FAMILY 72, SUBFAMILY A, POLYPEPTIDE 13 (CYP72A13)*	putative cytochrome P450 The mRNA is cell-to-cell mobile.
AT3G14940	*PHOSPHOENOLPYRUVATE CARBOXYLASE 3 (PPC3)*	Encodes a cytosolic phosphoenolpyruvate carboxylase (PEPC) that has activity when expressed in E.coli. Its mRNA is most abundantly expressed in roots and siliques. PPC3 belongs to the plant-type PEPC family. It can form an enzymatically active complex with a castor bean ortholog of PPC4, which encodes a bacterial-type PEPC. The mRNA is cell-to-cell mobile.
AT3G15240		Serine/threonine-protein kinase WNK (With No Lysine)-like protein
AT3G15250		TPRXL

AT3G15280		hypothetical protein
AT3G15300	*MPK3/6-TARGETED VQP 4 (MVQ4)*	VQ motif-containing protein
AT3G15353	*METALLOTHIONEIN 3 (MT3)*	metallothionein, binds to and detoxifies excess copper and other metals, limiting oxidative damage
AT3G15357		phosphopantothenoylcysteine decarboxylase subunit
AT3G15450		aluminum induced protein with YGL and LRDR motifs
AT3G15460	*ARABIDOPSIS HOMOLOGUE OF YEAST BRX1 1 (ATBRX1-1)*	Encodes one of two Arabidopsis orthologs of yeast BRX1, a protein involved in maturation of the large ribosomal subunit. The proteins are mainly localized in nucleolus. Mutant plants are affected in pre-rRNA processing.
AT3G15510	*NAC DOMAIN CONTAINING PROTEIN 2 (NAC2)*	Note of caution: not to be confused with another protein (AtNAC6 locus AT5G39610) which on occasion has also been referred to as AtNAC2.
AT3G15540	*INDOLE-3-ACETIC ACID INDUCIBLE 19 (IAA19)*	Primary auxin-responsive gene. Involved in the regulation stamen filaments development.
AT3G15570		Phototropic-responsive NPH3 family protein
AT3G15820	*REDUCED OLEATE DESATURATION 1 (ROD1)*	Functions as phosphatidylcholine:diacylglycerol cholinephosphotransferase, a major reaction for the transfer of 18:1 into phosphatidylcholine for desaturation and also for the reverse transfer of 18:2 and 18:3 into the triacylglycerols synthesis pathway
AT3G15840	*POST-ILLUMINATION CHLOROPHYLL FLUORESCENCE INCREASE (PIFI)*	Encodes a chloroplast-targeted protein localized in the stroma that is a novel component essential for NDH-mediated non-photochemical reduction of the plastoquinone pool in chlororespiratory electron transport.
AT3G15950	*(NAI2)*	Similar to TSK-associating protein 1 (TSA1), contains 10 EFE repeats, a novel repeat sequence unique to plants. Expressed preferentially in the roots.Protein is localized to ER bodies- an endoplasmic reticulum derived structure. Loss of function mutations lack ER bodies.
AT3G16150	*ASPARAGINASE B1 (ASPGB1)*	Encodes an asparaginase that catalyzes the degradation of L-asparagine to L-aspartic acid and ammonia. The mRNA is cell-to-cell mobile.
AT3G16220		Putative eukaryotic LigT
AT3G16240	*DELTA TONOPLAST INTEGRAL PROTEIN (DELTA-TIP)*	Delta tonoplast intrinsic protein, functions as a water channel and ammonium (NH3) transporter. Highly expressed in flower, shoot, and stem. Expression shows diurnal regulation and is induced by ammonium (NH3). Protein localized to vacuolar membrane. The mRNA is cell-to-cell mobile.
AT3G16250	*PHOTOSYNTHETIC NDH SUBCOMPLEX B 3 (PnsB3)*	encodes a novel subunit of the chloroplast NAD(P)H dehydrogenase complex, involved in cyclic electron flow around photosystem I to produce ATP. Contains a 4Fe-4S cluster.
AT3G16360	*HPT PHOSPHOTRANSMITTER 4 (AHP4)*	Encodes AHP4, a histidine-containing phosphotransmitter involved in Histidine (His)-to-Aspartate (Asp) phosphorelay signal transduction. AHP4 is one of the six Arabidopsis thaliana histidine phosphotransfer proteins (AHPs). AHPs function as redundant positive regulators of cytokinin signaling. Members of the AHP gene family include: AT3G21510 (AHP1), AT3G29350 (AHP2), AT5G39340 (AHP3), AT3G16360 (AHP4), AT1G03430 (AHP5) and AT1G80100 (AHP6).
AT3G16390	*NITRILE SPECIFIER PROTEIN 3 (NSP3)*	Encodes a nitrile-specifier protein NSP3. NSP3 is one out of five (At3g16400/NSP1, At2g33070/NSP2, At3g16390/NSP3, At3g16410/NSP4 and At5g48180/NSP5) A. thaliana epithiospecifier protein (ESP) homologues that promote simple nitrile, but not epithionitrile or thiocyanate formation. The mRNA is cell-to-cell mobile.
AT3G16430	*JACALIN-RELATED LECTIN 31 (JAL31)*	Encodes a protein that increases the beta-glucosidase activities of three scopolin glucosidases in vitro.
AT3G16450	*JACALIN-RELATED LECTIN 33 (JAL33)*	Mannose-binding lectin superfamily protein
AT3G16460	*JACALIN-RELATED LECTIN 34 (JAL34)*	Mannose-binding protein
AT3G16470	*JASMONATE RESPONSIVE 1 (JR1)*	Encodes a JA-responsive gene that coordinates with GRP7 in shaping plant development through the regulation of RNA processing in Arabidopsis. AtJAC1 interacts with RNA binding protein GRP7 specifically in the cytoplasm to regulate its nucleocytoplasmic distribution.
AT3G16520	*UDP-GLUCOSYL TRANSFERASE 88A1 (UGT88A1)*	UDP-glucosyl transferase 88A1
AT3G16690	*(SWEET16)*	Nodulin MtN3 family protein
AT3G16810	*PUMILIO 24 (PUM24)*	Encodes a member of the Arabidopsis Pumilio (APUM) proteins containing PUF domain (eight repeats of approximately 36 amino acids each). PUF proteins regulate both mRNA stability and translation through sequence-specific binding to the 3' UTR of target mRNA transcripts.
AT3G16855		
AT3G17040	*HIGH CHLOROPHYLL FLUORESCENT 107 (HCF107)*	It is a RNA tetratricopeptide repeat-containing protein required for normal processing of transcripts from the polycistronic chloroplast psbB-psbT-psbH-petB-petD operon coding for proteins of the photosystem II and cytochrome b6/f complexes. Localizes to the chloroplast membrane. Involved in regulating plastidial gene expression and biogenesis. It binds in the psbT?psbH intercistronic region and blocks the progression of 5′ → 3′ exoribonucleases, which defines the 5′ end of processed psbH transcripts and also stabilizes the downstream RNA segment. In addition, HCF107 binding remodels the structure of the psbH 5′ UTR in a way that can account for its ability to enhance psbH translation.
AT3G17465	*RIBOSOMAL PROTEIN L3 PLASTID (RPL3P)*	encodes a putative L3 ribosomal protein targeted to the plastid.
AT3G17620		F-box and associated interaction domains-containing protein
AT3G17640		Leucine-rich repeat (LRR) family protein
AT3G17690	*CYCLIC NUCLEOTIDE GATED CHANNEL 19 (CNGC19)*	member of Cyclic nucleotide gated channel family
AT3G17730	*NAC DOMAIN CONTAINING PROTEIN 57 (NAC057)*	NAC domain containing protein 57
AT3G17840	*RECEPTOR-LIKE KINASE 902 (RLK902)*	Encodes a receptor-like kinase found at the cell surface of various tissues. Its function remains unknown.
AT3G17990		
AT3G18000	*XIPOTL 1 (XPL1)*	Encodes a N-methyltransferase-like protein. Double mutants of NMT1 and NMT3 are defective in leaf, root, flower, seed, and pollen development.
AT3G18080	*B-S GLUCOSIDASE 44 (BGLU44)*	B-S glucosidase 44
AT3G18370	*(ATSYTF)*	C2 domain-containing protein
AT3G18390	*EMBRYO DEFECTIVE 1865 (EMB1865)*	CRS1 / YhbY (CRM) domain-containing protein
AT3G18560		hypothetical protein
AT3G18600		P-loop containing nucleoside triphosphate hydrolases superfamily protein
AT3G18690	*MAP KINASE SUBSTRATE 1 (MKS1)*	Encodes a nuclear-localized member of a plant specific gene family involved in mediating responses to pathogens. Interacts with WRKY transcriptional regulators.
AT3G19390		Granulin repeat cysteine protease family protein
AT3G19430		late embryogenesis abundant protein-related / LEA protein-like protein
AT3G19440		Pseudouridine synthase family protein

AT3G19710	*BRANCHED-CHAIN AMINOTRANSFERASE4 (BCAT4)*	Belongs to the branched-chain amino acid aminotransferase gene family. Encodes a methionine-oxo-acid transaminase. Involved in the methionine chain elongation pathway that leads to the ultimate biosynthesis of methionine-derived glucosinolates.
AT3G20030		F-box and associated interaction domains-containing protein
AT3G20180		Copper transport protein family
AT3G20270	*(LBR-2)*	Encodes one of the two LBP/BPI related proteins (AT1G04970/LBR-1, AT3G20270/LBR-2) that bind to LPS directly and regulate PR1 expression.
AT3G20370		TRAF-like family protein
AT3G20510	*FATTY ACID EXPORT 6 (FAX6)*	Encodes a member of the Tmemb_14 family that is predicted to be localized to the membranes of the secretory pathway. The mRNA is cell-to-cell mobile.
AT3G20520	*SHV3-LIKE 3 (SVL3)*	Encodes a member of the glycerophosphodiester phosphodiesterase like (GDPD-like) family.
AT3G20630	*UBIQUITIN-SPECIFIC PROTEASE 14 (UBP14)*	Encodes a ubiquitin-specific protease. Identical to TTN6. Loss of function mutations are embryo lethals, having development arrested at the preglobular/globular stage. Also involved in root responses to phosphate deficiency.
AT3G20680		plant/protein (DUF1995)
AT3G20880	*WIP DOMAIN PROTEIN 4 (WIP4)*	WIP4 is a paralog of NTT and along with WIP5,acts redundantly in cell fate determination during primary root development. MP binds to AuxRE motifs within the WIP4 gene and likely regulates its expression.
AT3G20940	*CYTOCHROME P450, FAMILY 705, SUBFAMILY A, POLYPEPTIDE 30 (CYP705A30)*	a member of A-type cytochrome P450
AT3G21055	*PHOTOSYSTEM II SUBUNIT T (PSBTN)*	Encodes photosystem II 5 kD protein subunit PSII-T. This is a nuclear-encoded gene (PsbTn) which also has a plastid-encoded paralog (PsbTc).
AT3G21090	*ATP-BINDING CASSETTE G15 (ABCG15)*	ABC-2 type transporter family protein
AT3G21240	*4-COUMARATE:COA LIGASE 2 (4CL2)*	encodes an isoform of 4-coumarate:CoA ligase (4CL), which is involved in the last step of the general phenylpropanoid pathway. The catalytic efficiency was in the following (descending) order: p-coumaric acid, caffeic acid, ferulic acid, 5-OH-ferulic acid and cinnamic acid. At4CL2 was unable to use sinapic acid as substrate.
AT3G21250	*ATP-BINDING CASSETTE C8 (ABCC8)*	member of MRP subfamily
AT3G21420	*LATERAL BRANCHING OXIDOREDUCTASE 1 (LBO1)*	LATERAL BRANCHING OXIDOREDUCTASE (LBO), encodes an oxidoreductase-like enzyme of the 2-oxoglutarate and Fe(II)-dependent dioxygenase superfamily. It is involved in the biosynthesis of strigolactones.
AT3G21540		transducin family protein / WD-40 repeat family protein
AT3G21600		Senescence/dehydration-associated protein-like protein
AT3G21670	*NRT1/ PTR FAMILY 6.4 (NPF6.4)*	Major facilitator superfamily protein
AT3G21760	*HYPOSTATIN RESISTANCE 1 (HYR1)*	Encodes HYR1, a UDP glycosyltransferase (UGT). HYR1 glucosylates hypostatin, an inhibitor of cell expansion in vivo to form a bioactive glucoside.
AT3G21770		Peroxidase superfamily protein
AT3G21800	*UDP-GLUCOSYL TRANSFERASE 71B8 (UGT71B8)*	UDP-glucosyl transferase 71B8
AT3G21860	*SKP1-LIKE 10 (SK10)*	SKP1-like 10
AT3G22060		contains Pfam profile: PF01657 Domain of unknown function that is usually associated with protein kinase domain Pfam:PF00069, however this protein does not have the protein kinase domain
AT3G22210		transmembrane protein
AT3G22370	*ALTERNATIVE OXIDASE 1A (AOX1A)*	Encodes AOX1a, an isoform of alternative oxidase that is expressed in rosettes, flowers, and root. The alternative oxidase of plant mitochondria transfers electrons from the ubiquinone pool to oxygen without energy conservations. It is regulated through transcriptional control and by pyruvate. Plays a role in shoot acclimation to low temperature. Also is capable of ameliorating reactive oxygen species production when the cytochrome pathway is inhibited. AOX1a also functions as a marker for mitochondrial retrograde response. The mRNA is cell-to-cell mobile.
AT3G22420	*WITH NO LYSINE (K) KINASE 2 (WNK2)*	Encodes a member of the WNK family (9 members in all) of protein kinases, the structural design of which is clearly distinct from those of other known protein kinases, such as receptor-like kinases and mitogen-activated protein kinases. Its transcription is under the control of circadian rhythms.
AT3G22500	*LATE EMBRYOGENESIS ABUNDANT PROTEIN ECP31 (ATECP31)*	late embryogenesis abundant (LEA) protein
AT3G22540		hypothetical protein (DUF1677)
AT3G22600	*GLYCOSYLPHOSPHATIDYLINOSITOL-ANCHORED LIPID PROTEIN TRANSFER 5 (LTPG5)*	Glycosylphosphatidylinositol (GPI)-anchored LTPg protein, downregulated in syncytia induced by the beet cyst nematode Heterodera schachtii and root knot nematode Meloidogyne incognita. Infection with bacteria (Pseudomonas syringae) and fungi (Botrytis cinerea) leads to the induction of the gene in leaves.
AT3G22620	*GLYCOSYLPHOSPHATIDYLINOSITOL-ANCHORED LIPID PROTEIN TRANSFER 20 (LTPG20)*	Bifunctional inhibitor/lipid-transfer protein/seed storage 2S albumin superfamily protein
AT3G22760	*(SOL1)*	CXC domain containing TSO1-like protein 1. The gene is expressed in stamens, pollen mother cells, and immature ovules. Regulates fate transition and cell Divisions in the stomatal lineage.
AT3G22850		aluminum induced protein with YGL and LRDR motifs
AT3G22930	*CALMODULIN-LIKE 11 (CML11)*	Encodes a calmodulin-like protein.
AT3G22960	*(PKP-ALPHA)*	encodes a chloroplast pyruvate kinase alpha subunit. Important for seed oil biosynthesis. Ubiquitously expressed, with significantly increased expression in maturing seeds. The mRNA is cell-to-cell mobile.
AT3G22970		hypothetical protein (DUF506)
AT3G23000	*CBL-INTERACTING PROTEIN KINASE 7 (CIPK7)*	Encodes a serine/threonine protein kinase with similarities to CBL-interacting protein kinases, SNF1 and SOS2. The mRNA is cell-to-cell mobile.
AT3G23050	*INDOLE-3-ACETIC ACID 7 (IAA7)*	Transcription regulator acting as repressor of auxin-inducible gene expression. Plays role in the control of gravitropic growth and development in light-grown seedlings. Auxin induces the degradation of the protein in a dosage-dependent manner in a process mediated by AtRac1. Auxin induced the relocalization of the protein within the nucleus from a diffused nucleoplasmic pattern to a discrete particulated pattern named nuclear protein bodies or NPB in a process also mediated by Rac1. Colocalizes with SCF, CSN and 26S proteasome components. Pseudomonas syringae type III effector AvrRpt2 stimulates AXR2 protein turnover.
AT3G23080		Polyketide cyclase/dehydrase and lipid transport superfamily protein
AT3G23170	*PROLINE/SERINE-RICH PROTEIN (PRP)*	PRP is a proline/serine rich protein of unknown function. It interacts with defense related MAP kinase MPK6 and others. It's expression is induced by PAMP elicitors. May play a role in response to pathogens.
AT3G23470		Cyclopropane-fatty-acyl-phospholipid synthase

AT3G23560	*ABERRANT LATERAL ROOT FORMATION 5 (ALF5)*	Member of the multidrug and toxic compound extrusion (MATE) family, protects roots from inhibitory compounds.
AT3G23630	*ISOPENTENYLTRANSFERASE 7 (IPT7)*	Encodes an isopentenyl transferase involved in cytokinin biosynthesis.
AT3G23730	*XYLOGLUCAN ENDOTRANSGLUCOSYLASE/HYDROLASE 16 (XTH16)*	xyloglucan endotransglucosylase/hydrolase 16
AT3G23890	*TOPOISOMERASE II (TOPII)*	Encodes a topoisomerase II that is highly expressed in young seedlings. The protein is localized in the nucleus and gene expression levels are increased in proliferative tissues.
AT3G24070		Zinc knuckle (CCHC-type) family protein
AT3G24110		Calcium-binding EF-hand family protein
AT3G24310	*MYB DOMAIN PROTEIN 305 (MYB305)*	snapdragon myb protein 305 homolog (myb)
AT3G24410		
AT3G24640		lyase
AT3G25150		Nuclear transport factor 2 (NTF2) family protein with RNA binding (RRM-RBD-RNP motifs) domain-containing protein
AT3G25160		ER lumen protein retaining receptor family protein
AT3G25210		Tetratricopeptide repeat (TPR)-like superfamily protein
AT3G25250	*AGC2 KINASE 1 (AGC2-1)*	Arabidopsis protein kinase The mRNA is cell-to-cell mobile.
AT3G25260	*NRT1/ PTR FAMILY 4.1 (NPF4.1)*	Major facilitator superfamily protein
AT3G25610	*AMINOPHOSPHOLIPID ATPASE10 (ALA10)*	Encodes aminophospholipid ATPase10 (ALA10), a P4-type ATPase flippase that internalizes exogenous phospholipids across the plasma membrane.
AT3G25690	*CHLOROPLAST UNUSUAL POSITIONING 1 (CHUP1)*	actin binding protein required for normal chloroplast positioning The mRNA is cell-to-cell mobile.
AT3G25760	*ALLENE OXIDE CYCLASE 1 (AOC1)*	encodes allene oxide cyclase. One of four genes in Arabidopsis that encode this enzyme, which catalyzes an essential step in jasmonic acid biosynthesis. Gene expression is induced during senescence, a process that involves jasmonic acid signalling pathway. The mRNA is cell-to-cell mobile.
AT3G25820	*TERPENE SYNTHASE-LIKE SEQUENCE-1,8-CINEOLE (TPS-CIN)*	Encodes the monoterpene 1,8-cineole synthase, atTPS-Cin. This polypeptide was also shown to synthesize other monoterpenes albeit in minor quantities. The same polypeptide is encoded at two different loci, the result of gene duplication: at3g25820 and at3g25830.
AT3G25830	*TERPENE SYNTHASE-LIKE SEQUENCE-1,8-CINEOLE (TPS-CIN)*	Encodes the monoterpene 1,8-cineole synthase, atTPS-Cin. This polypeptide was also shown to synthesize other monoterpenes albeit in minor quantities. The same polypeptide is encoded at two different loci, the result of gene duplication: at3g25820 and at3g25830.
AT3G25900	*(HMT-1)*	Homocysteine S-methyltransferase family protein
AT3G25930		Adenine nucleotide alpha hydrolases-like superfamily protein
AT3G26060	*PEROXIREDOXIN Q (PRXQ)*	encodes periredoxin Q which decomposes peroxides and plays a role in the protection of the photosynthetic apparatus
AT3G26170	*CYTOCHROME P450, FAMILY 71, SUBFAMILY B, POLYPEPTIDE 19 (CYP71B19)*	putative cytochrome P450
AT3G26390		hypothetical protein
AT3G26490	*(NPY7)*	Encodes a gene homologous to the NPY family based on deep phylogeny.
AT3G26520	*TONOPLAST INTRINSIC PROTEIN 2 (TIP2)*	gamma tonoplast intrinsic protein 2 (TIP2). expressed throughout the plant and transcript level is increased upon NaCl or ABA treatments. NaCl stress-sensitive yeast mutant strains exhibit more resistance to salt when expressing this protein.
AT3G26610	*POLYGALACTURONASE INVOLVED IN EXPANSION 1 (PGX1)*	Encodes an apoplast-localized polygalacturonase involved in cell elongation and flower development.
AT3G26650	*GLYCERALDEHYDE 3-PHOSPHATE DEHYDROGENASE A SUBUNIT (GAPA)*	Encodes one of the two subunits forming the photosynthetic glyceraldehyde-3-phosphate dehydrogenase (GAPDH) and as such a constituent of the supramolecular complex with phosphoribulokinase (PRK) thought to be linked by a small peptide encoded by CP12-2. GapA-1 is coordinately expressed by light with PRK and CP12-2. The enzyme activity, tested in leaf protein extracts dropped significantly after external sucrose treatment for the photosynthetic GAPDH (NADPH-dependent) but not for the cytosolic GAPDH (NADH-dependent).
AT3G26690	*NUDIX HYDROLASE HOMOLOG 13 (NUDX13)*	Encodes AtNUDT13, a mitochondrial Nudix hydrolase specific for long-chain diadenosine polyphosphates.
AT3G26720		Glycosyl hydrolase family 38 protein
AT3G26770		NAD(P)-binding Rossmann-fold superfamily protein
AT3G26790	*FUSCA3 (FUS3)*	Transcriptional factor with high similarity to the B3 region of the VP1/ABI3-like proteins. Full length FUS3 protein binds to the highly conserved RY motif [DNA motif CATGCA(TG)], present in many seed-specific promoters, and the B3 domains of this transcription factor is necessary for the specific interaction with the RY element. Transcriptional activity of FUS3 requires the B3 DNA-binding domain and an activation domain. FUS3 specifies cotyledon identity. Regulator of gene expression during late embryogenesis. Involved in the control foliar organ identity in Arabidopsis by regulating the synthesis of two hormones, abscisic acid and gibberellin. FUS3 together with LEC1 positively regulate the abundance of the ABI3 protein in the seed.
AT3G26870		Plant self-incompatibility protein S1 family
AT3G26932	*DSRNA-BINDING PROTEIN 3 (DRB3)*	dsRNA-binding protein 3
AT3G27500		Cysteine/Histidine-rich C1 domain family protein
AT3G27785	*MYB DOMAIN PROTEIN 118 (MYB118)*	MYB118 encodes a myb transcription factor that represses endosperm maturation and, along with MYB115, regulates glucosinolate biosynthesis.
AT3G28050	*USUALLY MULTIPLE ACIDS MOVE IN AND OUT TRANSPORTERS 41 (UMAMIT41)*	nodulin MtN21-like transporter family protein
AT3G28150	*TRICHOME BIREFRINGENCE-LIKE 22 (TBL22)*	Encodes a member of the TBL (TRICHOME BIREFRINGENCE-LIKE) gene family containing a plant-specific DUF231 (domain of unknown function) domain. A putative xyloglucan O-acetyltransferase. TBL gene family has 46 members, two of which (TBR/AT5G06700 and TBL3/AT5G01360) have been shown to be involved in the synthesis and deposition of secondary wall cellulose, presumably by influencing the esterification state of pectic polymers. A nomenclature for this gene family has been proposed (Volker Bischoff & Wolf Scheible, 2010, personal communication).
AT3G28220		TRAF-like family protein
AT3G28350		Pseudogene of AT3G28350; unknown protein
AT3G28360	*ATP-BINDING CASSETTE B16 (ABCB16)*	P-glycoprotein 16
AT3G28420		Putative membrane lipoprotein
AT3G28500		60S acidic ribosomal protein family

AT3G29030	*EXPANSIN A5 (EXPA5)*	Encodes an expansin. Naming convention from the Expansin Working Group (Kende et al, 2004. Plant Mol Bio)
AT3G29035	*NAC DOMAIN CONTAINING PROTEIN 3 (NAC3)*	Encodes a protein with transcription factor activity. Note: this protein (AT3G29035) on occasion has also been referred to as AtNAC3, not to be confused with the AtNAC3 found at locus AT3G15500. The mRNA is cell-to-cell mobile.
AT3G29240		PPR containing protein (DUF179)
AT3G29510		transposable_element_gene;copia-like retrotransposon family, has a 2.6e-122 P-value blast match to GB:CAA32025 ORF (Ty1_Copia-element) (Nicotiana tabacum)GB:CAA32025 ORF (Ty1_Copia-element) (Nicotiana tabacum);(source:TAIR10)
AT3G29575	*ABI FIVE BINDING PROTEIN 3 (AFP3)*	ABI five binding protein 3
AT3G29970		B12D protein
AT3G30350	*ROOT MERISTEM GROWTH FACTOR 4 (RGF4)*	Encodes a root meristem growth factor (RGF). Belongs to a family of functionally redundant homologous peptides that are secreted, tyrosine-sulfated, and expressed mainly in the stem cell area and the innermost layer of central columella cells. RGFs are required for maintenance of the root stem cell niche and transit amplifying cell proliferation. Members of this family include: At5g60810 (RGF1), At1g13620 (RGF2), At2g04025 (RGF3), At3g30350 (RGF4), At5g51451 (RGF5), At4g16515 (RGF6), At3g02240 (RGF7), At2g03830 (RGF8) and At5g64770 (RGF9).
AT3G30500		transposable_element_gene;similar to unknown protein [Arabidopsis thaliana] (TAIR:AT4G07690.1);(source:TAIR10)
AT3G30843		transposable_element_gene;similar to unknown protein [Arabidopsis thaliana] (TAIR:AT4G08056.1);(source:TAIR10)
AT3G31955		transposable_element_gene;similar to unknown protein [Arabidopsis thaliana] (TAIR:AT2G04210.1);(source:TAIR10)
AT3G32080		transposable_element_gene;similar to unknown protein [Arabidopsis thaliana] (TAIR:AT3G29710.1);(source:TAIR10)
AT3G32100		
AT3G32140		transposable_element_gene;similar to unknown protein [Arabidopsis thaliana] (TAIR:AT3G31410.1);(source:TAIR10)
AT3G42310		hypothetical protein
AT3G43190	*SUCROSE SYNTHASE 4 (SUS4)*	Encodes a protein with sucrose synthase activity (SUS4).
AT3G43510		transposable_element_gene;copia-like retrotransposon family, has a 2.3e-11 P-value blast match to GB:BAA11674 ORF(AA 1-1338) (Ty1_Copia-element) (Nicotiana tabacum);(source:TAIR10)
AT3G43670	*COPPER AMINE OXIDASE GAMMA 2 (CuAOgamma2)*	Copper amine oxidase family protein
AT3G43800	*GLUTATHIONE S-TRANSFERASE TAU 27 (GSTU27)*	Encodes glutathione transferase belonging to the tau class of GSTs. Naming convention according to Wagner et al. (2002). The mRNA is cell-to-cell mobile.
AT3G43880		hypothetical protein
AT3G43960		Encodes a putative cysteine proteinase. Mutants exhibit shorter root hairs under phosphate-deficient conditions.
AT3G44350	*NAC DOMAIN CONTAINING PROTEIN 61 (NAC061)*	NAC domain containing protein 61
AT3G44540	*FATTY ACID REDUCTASE 4 (FAR4)*	Encodes a member of the eight-member gene family encoding alcohol-forming fatty acyl-CoA reductases (FARs) identified in Arabidopsis thaliana. Three of the FARs, FAR1 (At5g22500), FAR4 (At3g44540) and FAR5 (At3g44550), are shown to generate the fatty alcohols found in root, seed coat, and wound-induced leaf tissue. The mRNA is cell-to-cell mobile.
AT3G45070	*SULFOTRANSFERASE 202B1 (SULT202B1)*	Encodes a sulfotransferase with sulfating activity toward flavonoids.
AT3G45160		Putative membrane lipoprotein
AT3G45450		Double Clp-N motif-containing P-loop nucleoside triphosphate hydrolases superfamily protein
AT3G45530		Cysteine/Histidine-rich C1 domain family protein
AT3G45610	*DOF TRANSCRIPTION FACTOR 6 (DOF6)*	PEAR protein involved in the formation of a short-range concentration gradient that peaks at protophloem sieve elements, and activates gene expression that promotes radial growth. Locally promotes transcription of inhibitory HD-ZIP III genes, and thereby establishes a negative-feedback loop that forms a robust boundary that demarks the zone of cell division.
AT3G45780	*PHOTOTROPIN 1 (PHOT1)*	Blue-light photoreceptor. Contains a light activated serine-threonine kinase domain and LOV1 and LOV2 repeats. Mutants are defective in blue-light response. Mediates blue light-induced growth enhancements. PHOT1 and PHOT2 mediate blue light-dependent activation of the plasma membrane H+-ATPase in guard cell protoplasts. PHOT1 undergoes blue-light-dependent autophosphorylation. At least eight phosphorylation sites have been identified in PHOT1. Phosphorylation of serine851 in the activation loop of PHOT1 appears to be required for stomatal opening, chloroplast accumulation, leaf flattening, and phototropism, and phosphorylation of serine849 may also contribute to the regulation of these responses. Phosphorylation-dependent binding of 14-3-3 proteins to the Hinge1 region of PHOT1 appears to require serine350 and serine376.
AT3G45840		Cysteine/Histidine-rich C1 domain family protein
AT3G46110	*SOSEKI4 (SOK4)*	DUF966 domain containing protein, expressed during embryogenesis.
AT3G46130	*MYB DOMAIN PROTEIN 48 (MYB48)*	Encodes a putative transcription factor (MYB48) that functions to regulate flavonol biosynthesis primarily in cotyledons.
AT3G46170		NAD(P)-binding Rossmann-fold superfamily protein
AT3G46280		kinase-like protein
AT3G46500		2-oxoglutarate (2OG) and Fe(II)-dependent oxygenase superfamily protein
AT3G47070		thylakoid soluble phosphoprotein
AT3G47180	*(CTL16)*	RING/U-box superfamily protein
AT3G47220	*PHOSPHATIDYLINOSITOL-SPECIFIC PHOSPHOLIPASE C9 (PLC9)*	Encodes a plasma membrane-localized phosphoinositide-specific phospholipase C with a role in thermotolerance.
AT3G47295		hypothetical protein
AT3G47400		Plant invertase/pectin methylesterase inhibitor superfamily
AT3G47510		transmembrane protein
AT3G47560		alpha/beta-Hydrolases superfamily protein
AT3G47620	*TEOSINTE BRANCHED, CYCLOIDEA AND PCF (TCP) 14 (TCP14)*	Encodes a transcription factor AtTCP14 that regulates seed germination. AtTCP14 shows elevated expression level just prior to germination. AtTCP14 is predominantly expressed in the vascular tissue of the embryo, and affects gene expression in radicles in a non-cell-autonomous manner. Modulates GA-dependent stamen filament elongation by direct activation of SAUR63 subfamily genes through conserved target sites in their promoters.
AT3G47800		Galactose mutarotase-like superfamily protein

AT3G47970		
AT3G47980	*(ATNITR2;2)*	Integral membrane HPP family protein. Putative nitrate transporter.
AT3G48100	*RESPONSE REGULATOR 5 (RR5)*	Encodes a transcription repressor that mediates a negative feedback loop in cytokinin signalling. ARR5 expression is upregulated by Class I KNOX genes. Arr5 protein is stabilized by cytokinin in a two-component phosphorelay.
AT3G48185		transmembrane protein
AT3G48200		transmembrane protein
AT3G48360	*BTB AND TAZ DOMAIN PROTEIN 2 (bt2)*	Encodes a protein (BT2) that is an essential component of the TAC1-mediated telomerase activation pathway. Acts redundantly with BT3 and BT1 during female gametophyte development and with BT3 during male gametophyte development. BT2 also mediates multiple responses to nutrients, stresses, and hormones.
AT3G48420		Haloacid dehalogenase-like hydrolase (HAD) superfamily protein
AT3G48450		RPM1-interacting protein 4 (RIN4) family protein
AT3G48460	*SEED FATTY ACID REDUCER 4 (SFAR4)*	GDSL-motif esterase/acyltransferase/lipase. Enzyme group with broad substrate specificity that may catalyze acyltransfer or hydrolase reactions with lipid and non-lipid substrates.
AT3G48510		ABA‐induced transcription repressor that acts as feedback regulator in ABA signalling.
AT3G48550		SHOOT GRAVITROPISM-like protein
AT3G48580	*XYLOGLUCAN ENDOTRANSGLUCOSYLASE/HYDROLASE 11 (XTH11)*	xyloglucan endotransglucosylase/hydrolase 11
AT3G48650		pseudogene of pectinesterase
AT3G48740	*(SWEET11)*	Encodes a member of the SWEET sucrose efflux transporter family proteins.
AT3G48950	*POLYGALACTURONASE CLADE F 7 (PGF7)*	Pectin lyase-like superfamily protein
AT3G48970	*HEAVY METAL ASSOCIATED PROTEIN 29 (ATHMP29)*	Heavy metal transport/detoxification superfamily protein
AT3G49120	*PEROXIDASE CB (PRXCB)*	Class III peroxidase Perx34. Expressed in roots, leaves and stems. Located in the cell wall. Involved in cell elongation. Expression activated by light. May play a role in generating H2O2 during defense response. The mRNA is cell-to-cell mobile.
AT3G49140		Pentatricopeptide repeat (PPR) superfamily protein
AT3G49160		Expression of the gene is downregulated in the presence of paraquat, an inducer of photoxidative stress.
AT3G49210	*WS /DGAT 6 (WSD6)*	WSD6 can function in vitro as wax ester synthase but does not appear to be essential for cuticular wax biosynthesis.
AT3G49230	*(DEG1)*	transmembrane protein
AT3G49260	*IQ-DOMAIN 21 (iqd21)*	IQ-domain 21
AT3G49360	*6-PHOSPHOGLUCONOLACTONASE 2 (PGL2)*	Encodes a cytosolic 6-phosphogluconolactonase (PGL) thought to be involved in the oxidative pentose-phosphate pathway (OPPP).
AT3G49760	*BASIC LEUCINE-ZIPPER 5 (bZIP5)*	basic leucine-zipper 5
AT3G49780	*PHYTOSULFOKINE 4 PRECURSOR (PSK4)*	Phytosulfokine 3 precursor, coding for a unique plant peptide growth factor. Plants overexpressing this gene (under a 35S promoter), develop normal cotyledons and hypocotyls but their growth, in particular that of their roots, was faster than that of wildtype.
AT3G49860	*ADP-RIBOSYLATION FACTOR-LIKE A1B (ARLA1B)*	A member of ARF-like GTPase family. A thaliana has 21 members, in two subfamilies, ARF and ARF-like (ARL) GTPases. Possible pseudogene because it lacks an N-terminal part that is conserved among the other ARL8 proteins.
AT3G49950		GRAS family transcription factor
AT3G50010		Cysteine/Histidine-rich C1 domain family protein
AT3G50060	*MYB DOMAIN PROTEIN 77 (MYB77)*	Encodes a member of the R2R3 transcription factor gene family. Expressed in response to potassium deprivation and auxin. Involved in lateral root development. Interacts with ARF7 and regulates the expression of some auxin responsive genes.
AT3G50230		Leucine-rich repeat protein kinase family protein
AT3G50300		HXXXD-type acyl-transferase family protein
AT3G50560		NAD(P)-binding Rossmann-fold superfamily protein
AT3G50640		hypothetical protein
AT3G50685		anti-muellerian hormone type-2 receptor
AT3G50740	*UDP-GLUCOSYL TRANSFERASE 72E1 (UGT72E1)*	UGT72E1 is an UDPG:coniferyl alcohol glucosyltransferase which specifically glucosylates sinapyl- and coniferyl aldehydes. The enzyme is thought to be involved in lignin metabolism.
AT3G50760	*GALACTURONOSYLTRANSFERASE-LIKE 2 (GATL2)*	Encodes a protein with putative galacturonosyltransferase activity. The mRNA is cell-to-cell mobile.
AT3G50820	*PHOTOSYSTEM II SUBUNIT O-2 (PSBO2)*	Encodes a protein which is an extrinsic subunit of photosystem II and which has been proposed to play a central role in stabilization of the catalytic manganese cluster. In <i>Arabidopsis thaliana</i> the PsbO proteins are encoded by two genes: <i>psbO1</i> and <i>psbO2</i>. PsbO2 is the minor isoform in the wild-type. Mutants defective in this gene have been shown to be affected in the dephosphorylation of the D1 protein of PSII.
AT3G50870	*MONOPOLE (MNP)*	Encodes a GATA transcriptional regulator required to position the proembryo boundary in the early embryo. Regulates shoot apical meristem and flower development.
AT3G50930	*CYTOCHROME BC1 SYNTHESIS (BCS1)*	Encodes a protein that is present in a homo-multimeric protein complex on the outer mitochondrial membrane and plays a role in cell death and amplifying salicylic acid signalling. The mRNA is cell-to-cell mobile.
AT3G51110		Tetratricopeptide repeat (TPR)-like superfamily protein
AT3G51420	*STRICTOSIDINE SYNTHASE-LIKE 4 (SSL4)*	Although this enzyme is predicted to encode a strictosidine synthase (SS), it lacks a conserved catalytic glutamate residue found in active SS enzymes and it is not expected to have SS activity.
AT3G51510		transmembrane protein
AT3G51680	*SHORT-CHAIN DEHYDROGENASE/REDUCTASE 2 (SDR2)*	NAD(P)-binding Rossmann-fold superfamily protein
AT3G51690		DNA helicase homolog PIF1.
AT3G51790	*TRANSMEMBRANE PROTEIN G1P-RELATED 1 (G1)*	Encodes a heme-binding protein located in the mitochondrial inner membrane that is involved in cytochrome c maturation.

AT3G51810	*LATE EMBRYOGENESIS ABUNDANT 1 (EM1)*	Encodes a ABA-inducible protein that accumulates during seed maturation, in parallel with its corresponding mRNA but with a 3 d delay. During germination, AtEm1 protein undergoes two successive cleavages before being degraded. Both proteins are more stable than the corresponding mRNA. The gene can be activated by the basic leucine zipper transcription factor ABI5. Expressed predominantly in provascular tissues with the strongest expression in the root tip.
AT3G51820	*(G4)*	Encodes a protein with chlorophyll synthase activity. This enzyme has been shown to perform the esterification of chlorophyllide (a and b), the last step of chlorophyll biosynthesis. Although it can use either geranylgeranyl pyrophosphate (GGPP) or phytyl pyrophosphate (PhyPP) as substrates, the esterification reaction was faster with GGPP than with PhyPP.
AT3G51890	*CLATHRIN LIGHT CHAIN 3 (CLC3)*	Clathrin light chain protein
AT3G51910	*HEAT SHOCK TRANSCRIPTION FACTOR A7A (HSFA7A)*	member of Heat Stress Transcription Factor (Hsf) family The mRNA is cell-to-cell mobile.
AT3G51920	*CALMODULIN 9 (CAM9)*	encodes a divergent member of calmodulin, which is an EF-hand family of Ca2+-binding proteins. This gene is expressed in leaves, flowers and siliques. The gene functionally complements yeast calmodulin 1 (CAM1) but only when selected against the plasmid harboring wild-type yeast sequences. Also the protein does not form formed a complex with a basic amphiphilic helical peptide in the presence of Ca2+ in vitro. Authors suggest that this gene may represent a Ca2+-binding sensor protein that interacts with a more limited set of target proteins than do more conventional CaM isoforms. Mutations in this gene alter plant responses to abiotic stress and abscisic acid.
AT3G52370	*FASCICLIN-LIKE ARABINOGALACTAN PROTEIN 15 PRECURSOR (FLA15)*	Fasciclin-like arabinogalactan protein. Possibly involved in embryogenesis and seed development.
AT3G52840	*BETA-GALACTOSIDASE 2 (BGAL2)*	beta-galactosidase 2
AT3G52870		IQ calmodulin-binding motif family protein
AT3G53040		late embryogenesis abundant protein, putative / LEA protein
AT3G53160	*UDP-GLUCOSYL TRANSFERASE 73C7 (UGT73C7)*	UGT73C7 is induced by pathogen infection. It glycosylates p-coumaric acid and ferulic acid to modulate phenylpropanoid metabolism and induce innate immune response.
AT3G53180	*NODULIN/GLUTAMINE SYNTHASE-LIKE PROTEIN (NodGS)*	Encodes a protein that is the product of a fusion gene with a C-terminal GSI like sequence and an N-terminal part sharing homology with nodulins. It self-assembles into oligomers and its expression is increased in response to flagellin treatment. The protein co-localizes with microtubules and binds gamma-tubulin. RNAi lines are affected in root morphogenesis.
AT3G53210	*USUALLY MULTIPLE ACIDS MOVE IN AND OUT TRANSPORTERS 6 (UMAMIT6)*	nodulin MtN21-like transporter family protein
AT3G53230	*CELL DIVISION CYCLE 48B (ATCDC48B)*	CDC48 is induced upon oilseed rape mosaic tobamovirus infection and appears to be involved in controlling virus movement.
AT3G53260	*PHENYLALANINE AMMONIA-LYASE 2 (PAL2)*	Encodes phenylalanine lyase. Arabidopsis has four PALs: AT2G37040 (PAL1), AT3G53260 (PAL2), AT5G04230 (PAL3) and AT3G10340 (PAL4).
AT3G53280	*CYTOCHROME P450 71B5 (CYP71B5)*	cytochrome P450 monooxygenase The mRNA is cell-to-cell mobile.
AT3G53420	*PLASMA MEMBRANE INTRINSIC PROTEIN 2A (PIP2A)*	a member of the plasma membrane intrinsic protein subfamily PIP2. localizes to the plasma membrane and exhibits water transport activity in Xenopus oocyte. expressed specifically in the vascular bundles and protein level increases slightly during leaf dev. When expressed in yeast cells can conduct hydrogen peroxide into those cells.
AT3G53470		2,3-bisphosphoglycerate-independent phosphoglycerate mutase
AT3G53480	*ATP-BINDING CASSETTE G37 (ABCG37)*	Negative regulator of auxin polar transport inhibitors. ABCG37 regulates auxin distribution and homeostasis in roots by excluding IBA from the root apex, but does not act directly in basipetal transport. ABCG37 and ABCG36 act redundantly at outermost root plasma membranes and, transport IBA out of the cells. Also involved in root transmembrane secretion of fluorescent phenolics involved in Fe uptake. The mRNA is cell-to-cell mobile.
AT3G53690		RING/U-box superfamily protein
AT3G53800	*FES1B (Fes1B)*	Encodes one of the Arabidopsis orthologs of the human Hsp70-binding protein 1 (HspBP-1) and yeast Fes1p: Fes1A (AT3G09350), Fes1B (AT3G53800), Fes1C (AT5G02150).
AT3G53960		Major facilitator superfamily protein
AT3G54050	*HIGH CYCLIC ELECTRON FLOW 1 (HCEF1)*	Encodes a chloroplastic fructose 1,6-bisphosphate phosphatase. also known as HCEF1 (High Cyclic Electron Flow 1). hcef1 mutants have constitutively elevated electron flow (CEFI) and plants with antisense suppression of this enzyme have higher levels of net leaf photosynthesis and increased sucrose biosynthesis. The mRNA is cell-to-cell mobile.
AT3G54150	*EXINE FORMATION DEFECTIVE (EFD)*	Encodes a DNA methyltransferase required for pollen exine formation and male fertility via the regulation of callose wall and primexine formation.
AT3G54310		DNA-directed RNA polymerase subunit alpha
AT3G54320	*WRINKLED 1 (WRI1)*	WRINKLED1 encodes transcription factor of the AP2/ERWEBP class. Protein has two plant-specific (AP2/EREB) DNA-binding domains and is involved in the control of storage compound biosynthesis in Arabidopsis. Mutants have wrinkled seed phenotype, due to a defect in the incorporation of sucrose and glucose into triacylglycerols. Transgenic sGsL plants (21-day-old) grown on 6% sucrose for 24 hours had 2-fold increase in levels of expressions (sGsL line carries a single copy of T-DNA containing the Spomin::GUS-Spomin::LUC dual reporter genes in the upper arm of chromosome 5 of ecotype Col-0. The sporamin .minimal. promoter directs sugar-inducible expression of the LUC and GUS reporters in leaves). Regulation by LEC2 promotes fatty acid accumulation during seed maturation. Splice form 3 is the major form expressed in seedlings.Mutations in the C terminal intrinsically disordered region increase the stability of WRI1 and lead to increased oil production.
AT3G54330		
AT3G54390		sequence-specific DNA binding transcription factor
AT3G54420	*HOMOLOG OF CARROT EP3-3 CHITINASE (EP3)*	encodes an EP3 chitinase that is expressed during somatic embryogenesis in 'nursing' cells surrounding the embryos but not in embryos themselves. The gene is also expressed in mature pollen and growing pollen tubes until they enter the receptive synergid, but not in endosperm and integuments as in carrot. Post-embryonically, expression is found in hydathodes, stipules, root epidermis and emerging root hairs.
AT3G54500	*NIGHT LIGHT-INDUCIBLE AND CLOCK-REGULATED GENE 2 (LNK2)*	Member of a small family (4 proteins) in Arabidopsis that have some overlap in function. LNK2 along with LNK1 functions in the integration of light signaling and circadian clock. It is regulated by the clock TOC1 complex. Functions as a transcriptional coactivator.

AT3G54720	*ALTERED MERISTEM PROGRAM 1 (AMP1)*	Encodes glutamate carboxypeptidase. Various alleles show-increased cotyledon number and rate of leaf initiation, show transformation of leaves to cotyledons, altered flowering time and photomorphogenesis and an increased level of cytokinin biosynthesis. Involved in ethylene enhanced hypocotyl elongation in the light. Strong genetic interaction between TGH and AMP1.
AT3G54770	*ABA-REGULATED RNA-BINDING PROTEIN 1 (ARP1)*	Encodes a putative RNA binding protein that is localized in the nucleus and affects ABA-regulated seed germination of Arabidopsis.
AT3G55310		NAD(P)-binding Rossmann-fold superfamily protein
AT3G55330	*PSBP-LIKE PROTEIN 1 (PPL1)*	Facilitates the assembly of the photosystem II supercomplexes and optimizes plant pitness under fluctuating light.
AT3G55510	*REBELOTE (RBL)*	Encodes a regulator of floral determinacy in that interacts with both nucleolar and nucleoplasmic proteins.
AT3G55740	*PROLINE TRANSPORTER 2 (PROT2)*	Encodes a proline transporter with affinity for gly betaine, proline, and GABA. Protein is expressed most highly in the roots.
AT3G55800	*SEDOHEPTULOSE-BISPHOSPHATASE (SBPASE)*	Encodes the chloroplast enzyme sedoheptulose-1,7-bisphosphatase (SBPase), involved in the carbon reduction of the Calvin cycle. Increase in SBPase activity in transgenic lines accumulate up to 50% more sucrose and starch than wild-type. The mRNA is cell-to-cell mobile.
AT3G55870		ADC synthase superfamily protein
AT3G56080		S-adenosyl-L-methionine-dependent methyltransferases superfamily protein
AT3G56150	*EUKARYOTIC TRANSLATION INITIATION FACTOR 3C (EIF3C)*	member of eIF3c - eukaryotic initiation factor 3c
AT3G56240	*COPPER CHAPERONE (CCH)*	CCH protein belongs to a family of eukaryotic proteins that participate in intracellular copper homeostasis by delivering this metal to the secretory pathway; mainly located along the vascular bundles of senescing leaves and petioles as well as in stem sieve elements; hypothesized to have a role in copper mobilization from decaying organs towards reproductive structures, as a result of metalloprotein breakdown. The plant-specific C-terminal domain of the CCH protein forms amyloid-like fibrils in vitro.
AT3G56260		hypothetical protein
AT3G56520		NAC (No Apical Meristem) domain transcriptional regulator superfamily protein
AT3G56600		phosphatidylinositol 4-kinase gamma-like protein
AT3G56650	*PSBP-DOMAIN PROTEIN 6 (PPD6)*	thylakoid lumenal protein (Mog1/PsbP/DUF1795-like photosystem II reaction center PsbP family protein)
AT3G56910	*PLASTID-SPECIFIC 50S RIBOSOMAL PROTEIN 5 (PSRP5)*	Encodes PSRP5 (PLASTID-SPECIFIC 50S RIBOSOMAL PROTEIN 5). Functions in plastid translation.
AT3G56940	*COPPER RESPONSE DEFECT 1 (CRD1)*	Encodes a putative ZIP protein with varying mRNA accumulation in leaves, stems and roots. Has a consensus carboxylate-bridged di-iron binding site. The mRNA is cell-to-cell mobile.
AT3G56980	*BASIC HELIX-LOOP-HELIX 39 (BHLH39)*	Encodes a member of the basic helix-loop-helix transcription factor protein. bHLH IVc transcription factor repressed by bHLH11.
AT3G57010		Calcium-dependent phosphotriesterase superfamily protein
AT3G57160		cysteine-rich TM module stress tolerance protein
AT3G57210		hypothetical protein
AT3G57230	*AGAMOUS-LIKE 16 (AGL16)*	MADS-box transcription factor. Expressed in leaf, root and stem, with higher RNA accumulation in guard cells and trichomes. AGL16 can directly interact with SVP and indirectly interact with FLC. Furthermore, the accumulation of AGL16 transcripts is modulated by miR824 (AT4G24415). The flowering time effect for the miR824/AGL16 module is more obvious in the Col-FRI background than in the Col-0 background. AGL16 controls flowering via a allelic dosage effect in long-day non-vernalized conditions.
AT3G57330	*AUTOINHIBITED CA2+-ATPASE 11 (ACA11)*	Lesion mimic phenotype when mutation in the gene is combined with a mutation in ACA4. Lesion mimic phenotype of double knockout can be suppressed by nutritional supplements that increase anion levels (e.g. 15 mM Nitrate, Chloride, or Phosphate)
AT3G57360		tRNA-splicing endonuclease subunit
AT3G57380		Glycosyltransferase family 61 protein
AT3G57510	*ARABIDOPSIS DEHISCENCE ZONE POLYGALACTURONASE 1 (ADPG1)*	Encodes ADPG1, a polygalacturonase protein involved in silique and anther dihiscence. Loss of function mutations have reduced seed set, indehiscent fruit and reduced pollen shedding. Required for release of cell wall-derived PR elicitors.
AT3G57520	*SEED IMBIBITION 2 (SIP2)*	SIP2 encodes a raffinose-specific alpha-galactosidase that catalyzes the breakdown of raffinose into alpha-galatose and sucrose. This enzyme may function in unloading raffinose from the phloem as part of sink metabolism. Although it was originally predicted to act as a raffinose synthase (RS), that activity was not observed for recombinant SIP2.
AT3G57550	*GUANYLATE KINASE (AGK2)*	guanylate kinase
AT3G57670	*NO TRANSMITTING TRACT (NTT)*	Encodes a a C2H2/C2HC zinc finger transcription factor specifically expressed in the transmitting tract and involved in transmitting tract development and pollen tube growth.Acts redundantly with WIP4 and WIP5 to determine distal cell fate in the root. MP binds to regulatory elements within the NTT locus and likely regulates its expression.
AT3G57920	*SQUAMOSA PROMOTER BINDING PROTEIN-LIKE 15 (SPL15)*	Encodes a putative transcriptional regulator that is involved in the vegetative to reproductive phase transition. Expression is regulated by MIR156b.
AT3G58180		ARM repeat superfamily protein
AT3G58200		TRAF-like family protein
AT3G58450	*UNIVERSAL STRESS PROTEIN (USP)*	USP domain containing protein, member of the universal stress protein family, regulated by ABA and possibly regulated by the ABA-dependent transcription factor AREB/ABF. Involved in the regulation of seed germination.
AT3G58550	*GLYCOSYLPHOSPHATIDYLINOSITOL-ANCHORED LIPID PROTEIN TRANSFER 22 (LTPG22)*	Bifunctional inhibitor/lipid-transfer protein/seed storage 2S albumin superfamily protein
AT3G58990	*ISOPROPYLMALATE ISOMERASE 1 (IPMI1)*	Small subunit, which together with IPMI SSU1, IPMISSU2 and IPMI LSU1, is a member of heterodimeric isopropylmalate isomerase (IPMI). Together with IPMI SSU3 participates in the Met chain elongation pathway.
AT3G59050	*POLYAMINE OXIDASE 3 (PAO3)*	Encodes a polyamine oxidase.
AT3G59140	*ATP-BINDING CASSETTE C10 (ABCC10)*	member of MRP subfamily
AT3G59400	*GENOMES UNCOUPLED 4 (GUN4)*	GUN, genomes uncoupled, is necessary for coupling the expression of some nuclear genes to the functional state of the chloroplast. Binds to the magnesium chelatase complex and promotes formation of the substrate,a tetrapyrrole signaling molecule. Porphyrin-binding protein that enhances the activity of Mg-chelatase. Although required for chlorophyll accumulation under normal growth conditions, GUN4 is not essential for chlorophyll synthesis.

AT3G59420	*CRINKLY4 (CR4)*	Encodes a membrane localized protein with similarity to receptor kinases which is involved in epidermal cell differentiation. Flowers of mutants have disorganized ovule integument growth and abnormal sepal margins. In the roots, mutants initiate more lateral roots but fewer laterals actually emerge due to defects in lateral root formation. Mutants also display disorganized columella. The root phenotypes can be traced to abnormalities in asymmetric divisions in the pericycle and root apex. Conflicting data regarding the role of the kinase domain- which may or may not be required for function. Complementation studies indicate that the C-terminal domain is also not required for signaling function. May be regulated by protein turnover which is mediated by endocytic processes. ACR4 phosphorylates the PROTEIN PHOSPHATASE 2A-3 (PP2A-3) catalytic subunit of the PP2A phosphatase holoenzyme and PP2A
AT3G59440	*(ATCML4)*	Encodes an endomembrane localized member of the CML subfamily VII. Contains a canonical CaM domain and unique N-terminal extension that distinguishes it from other members of the subfamily.
AT3G59480	*FRUCTOKINASE 7 (FRK7)*	Encodes a member of the fructokinase gene family. Nomenclature according to Riggs 2017 has been adopted for the family by the community (personal communication, Boernke, Callis, Granot, Boernke, and Smeekens).
AT3G59710		NAD(P)-binding Rossmann-fold superfamily protein
AT3G59820	*LEUCINE ZIPPER-EF-HAND-CONTAINING TRANSMEMBRANE PROTEIN 1 (LETM1)*	LETM1-like protein
AT3G60140	*DARK INDUCIBLE 2 (DIN2)*	Encodes a protein similar to beta-glucosidase and is a member of glycoside hydrolase family 1. Expression is induced after 24 hours of dark treatment, in senescing leaves and treatment with exogenous photosynthesis inhibitor. Induction of gene expression was suppressed in excised leaves supplied with sugar. The authors suggest that the gene's expression pattern is responding to the level of sugar in the cell. The mRNA is cell-to-cell mobile.
AT3G60390	*HOMEOBOX-LEUCINE ZIPPER PROTEIN 3 (HAT3)*	Encodes homeobox protein HAT3.
AT3G60420		phosphoglycerate mutase family protein
AT3G60490	*ETHYLENE-RESPONSIVE TRANSCRIPTION FACTOR 35 (ERF35)*	encodes a member of the DREB subfamily A-4 of ERF/AP2 transcription factor family. The protein contains one AP2 domain. There are 17 members in this subfamily including TINY.
AT3G60650		Encodes a peptide involved in the regulation of lateral root development and root growth pattern.
AT3G60980		Tetratricopeptide repeat (TPR)-like superfamily protein
AT3G61050	*(NTMC2T4)*	Encodes a novel transcriptional regulator, a calcium-dependent lipid-binding protein containing a C2 domain, that binds specifically to the promoter of THAS1 (thalianol synthase 1). It can bind ceramide and is involved in drought and salt tolerance.
AT3G61150	*HOMEODOMAIN GLABROUS 1 (HDG1)*	Encodes a homeobox-leucine zipper family protein belonging to the HD-ZIP IV family.
AT3G61260	*REMORIN 1.2 (REM1.2)*	Lipid raft regulatory protein, crucial for plasma membrane nanodomain assembly to control plasmodesmata aperture and functionality. Negatively regulates the cell-to-cell movement of TuMV via competition with PCaP1 for binding actin filaments.
AT3G61400		1-aminocyclopropane-1-carboxylate oxidase-like protein
AT3G61430	*PLASMA MEMBRANE INTRINSIC PROTEIN 1A (PIP1A)*	a member of the plasma membrane intrinsic protein subfamily PIP1. localizes to the plasma membrane and exhibits water transport activity in Xenopus oocyte. expressed ubiquitously and protein level decreases slightly during leaf development. The mRNA is cell-to-cell mobile.
AT3G61550	*ARABIDOPSIS T??XICOS EN LEVADURA 68 (ATL68)*	RING/U-box superfamily protein
AT3G61630	*CYTOKININ RESPONSE FACTOR 6 (CRF6)*	CRF6 encodes one of the six cytokinin response factors. CRF5 belongs to the AP2/ERF superfamily of the transcriptional factors. CRF proteins rapidly relocalize to the nucleus in response to cytokinin. Analysis of loos-of-function mutants revealed that the CRFs function redundantly to regulate the development of embryos, cotyledons and leaves.
AT3G61870		plant/protein
AT3G61890	*HOMEOBOX 12 (HB-12)*	Encodes a homeodomain leucine zipper class I (HD-Zip I) protein. Loss of function mutant has abnormally shaped leaves and stems.
AT3G61930		hypothetical protein
AT3G62020	*GERMIN-LIKE PROTEIN 10 (GLP10)*	germin-like protein (GLP10)
AT3G62040		Haloacid dehalogenase-like hydrolase (HAD) superfamily protein
AT3G62100	*INDOLE-3-ACETIC ACID INDUCIBLE 30 (IAA30)*	Encodes a member of the Aux/IAA family of proteins implicated in auxin signaling. IAA30 lacks the conserved degron (domain II) found in many family members. IAA30 transcripts are induced by auxin treatment and accumulate preferentially in the quiescent center cells of the root meristem. Overexpression of IAA30 leads to defects in gravitropism, root development, root meristem maintenance, and cotyledon vascular development. Target of LEC2 and AGL15. Promotes somatyic embryogenesis.
AT3G62270	*REQUIRES HIGH BORON 2 (BOR2)*	BOR2 is involved in efficient borate crosslinking of rhamnogalacturonan II in cell walls under boron limitation.
AT3G62320		Polynucleotidyl transferase, ribonuclease H-like superfamily protein
AT3G62480		transposable_element_gene;similar to unknown protein [Arabidopsis thaliana] (TAIR:AT2G01031.1);(source:TAIR10)
AT3G62860	*(MAGL12)*	alpha/beta-Hydrolases superfamily protein
AT3G62930	*(GRXS6)*	Encodes a member of the CC-type glutaredoxin (ROXY) family that has been shown to interact with the transcription factor TGA2 and suppress ORA59 promoter activity.
AT3G63010	*GA INSENSITIVE DWARF1B (GID1B)*	Encodes a gibberellin (GA) receptor ortholog of the rice GA receptor gene (OsGID1). Has GA-binding activity, showing higher affinity to GA4. Interacts with DELLA proteins in vivo in the presence of GA4. The mRNA is cell-to-cell mobile.
AT3G63060	*EID1-LIKE 3 (EDL3)*	EDL3 is an F-box protein involved that mediated the regulation of abscisic acid signalling.
AT3G63350	*(AT-HSFA7B)*	member of Heat Stress Transcription Factor (Hsf) family
AT3G63380	*AUTO-INHIBITED CA2+ ATPASE 12 (ACA12)*	ATPase E1-E2 type family protein / haloacid dehalogenase-like hydrolase family protein
AT4G00050	*UNFERTILIZED EMBRYO SAC 10 (UNE10)*	Encodes a phytochrome interacting factor that inhibits phytochrome A-mediated far-red light responses and binds to promoter regions of AT2G46970 and AT3G62090.
AT4G00080	*UNFERTILIZED EMBRYO SAC 11 (UNE11)*	Plant invertase/pectin methylesterase inhibitor superfamily protein
AT4G00180	*YABBY3 (YAB3)*	YABBY gene family member, likely has transcription factor activity, involved in specifying abaxial cell fate. Along with FIL, involved in patterning of the fruit. GUS reporter gene expression in seedlings is observed in the young leaves and as the leaf matures, expression is restricted to the abaxial tissues of leaves, expression is also observed on either side of the leaf margin in the younger tissues of leaf blades.

AT4G00220	*JAGGED LATERAL ORGANS (JLO)*	Encodes a protein containing a LOB domain that is expressed in embryos, flower primordium and lateral floral organ boundaries. Overexpression is correlated with activation of STM and KNAT1 and down regulation of PIN1 and reduced auxin transport levels. Ectopic expression in plants results in premature termination of the shoot apical meristem and small, lobed leaves. A maternally expressed imprinted gene.
AT4G00460	*ROP (RHO OF PLANTS) GUANINE NUCLEOTIDE EXCHANGE FACTOR 3 (ROPGEF3)*	Encodes a member of KPP-like gene family, homolog of KPP (kinase partner protein) gene in tomato. Also a member of the RopGEF (guanine nucleotide exchange factor) family, containing the novel PRONE domain (plant-specific Rop nucleotide exchanger), which is exclusively active towards members of the Rop subfamily.
AT4G00570	*NAD-DEPENDENT MALIC ENZYME 2 (NAD-ME2)*	Encodes an NAD-dependent malic enzyme (NAD-ME) that does not act on oxaloacetate, indicating that it belongs to EC 1.1.1.39. It is a member of the beta family of NAD-MEs in plants. It appears to function as a homodimer or as a heterodimer with the alpha-type NAD-ME2 (At2g13560). NAD-ME2 transcript and protein levels are higher during the night than during the day.
AT4G00600		Amino acid dehydrogenase family protein
AT4G00670		Remorin family protein
AT4G00750		S-adenosyl-L-methionine-dependent methyltransferases superfamily protein
AT4G00780		TRAF-like family protein
AT4G00880	*SMALL AUXIN UPREGULATED RNA 31 (SAUR31)*	SAUR-like auxin-responsive protein family
AT4G00895		ATPase, F1 complex, OSCP/delta subunit protein
AT4G01026	*PYR1-LIKE 7 (PYL7)*	Encodes a member of the PYR (pyrabactin resistance)/PYL(PYR1-like)/RCAR (regulatory components of ABA receptor) family proteins with 14 members. PYR/PYL/RCAR family proteins function as abscisic acid sensors. Mediate ABA-dependent regulation of protein phosphatase 2Cs ABI1 and ABI2. PYL/RCAR family proteins function as abscisic acid sensors. Mediate ABA-dependent regulation of ABI1 and ABI2.
AT4G01050	*THYLAKOID RHODANESE-LIKE (TROL)*	hydroxyproline-rich glycoprotein family protein, contains a rhodanese homology domain. Required for anchoring the FNR flavoenzyme to the thylakoid membranes and sustaining high efficiency photosynthetic linear electron flow. The mRNA is cell-to-cell mobile.
AT4G01150	*CURVATURE THYLAKOID 1A (CURT1A)*	Integral thylakoid membrane protein required for proper grana stack curvature.
AT4G01310	*PLASTID RIBOSOMAL PROTEINS OF THE 50S SUBUNIT 5 (PRPL5)*	Ribosomal L5P family protein
AT4G01430	*USUALLY MULTIPLE ACIDS MOVE IN AND OUT TRANSPORTERS 29 (UMAMIT29)*	Encodes a plasma membrane-localized amino acid transporter likely involved in amino acid export in the developing seed.
AT4G01660	*ABC TRANSPORTER 1 (ABC1)*	Encodes an ABC1-like protein, member of the ATH subfamily; putative ABC transporter; isolated by functional complementation of a yeast abc1 mutant The mRNA is cell-to-cell mobile.
AT4G01700		Chitinase family protein
AT4G01750	*RHAMNOGALACTURONAN XYLOSYLTRANSFERASE 2 (RGXT2)*	Encodes a protein with UDP-xylose-dependent xylosyltransferase activity, which transfers Xyl onto L-fucose and (albeit less efficiently) L-arabinose. The linkage to L-fucose was shown to be preferentially to the <i>O</i>-4 position. Analysis of mutant containing T-DNA insertion in this gene indicate that the RGXT2 protein might be involved in the synthesis of the α-D-Xyl-(1,3)-α-L-Fuc-(1,4)-L-Rha structure in pectic rhamnogalacturonan II. The mRNA is cell-to-cell mobile.
AT4G01790		Ribosomal protein L7Ae/L30e/S12e/Gadd45 family protein
AT4G02090		PADRE protein.
AT4G02280	*SUCROSE SYNTHASE 3 (SUS3)*	Encodes a protein with sucrose synthase activity (SUS3). It appears to be important for sucrose metabolism in developing seeds, especially during the late maturation phase, about 18 days after flowering.
AT4G02380	*SENESCENCE-ASSOCIATED GENE 21 (SAG21)*	Encodes AtLEA5 (late embryogenesis abundant like protein). Also known as SENESCENCE-ASSOCIATED GENE 21 (SAG21). Has a role on oxidative stress tolerance. mRNA levels are elevated in response to various stresses.
AT4G02590	*UNFERTILIZED EMBRYO SAC 12 (UNE12)*	Basic helix loop helix class transcriptional regulator. Shows ecotype specific effects on temperature dependent salicylic acid accumulation and immunity. Governs the competence of pericycle cells to initiate lateral root primordium formation.
AT4G02710	*NETWORKED 1C (NET1C)*	Kinase interacting (KIP1-like) family protein
AT4G02770	*PHOTOSYSTEM I SUBUNIT D-1 (PSAD-1)*	Encodes a protein predicted by sequence similarity with spinach PsaD to be photosystem I reaction center subunit II (PsaD1)
AT4G02930		GTP binding Elongation factor Tu family protein
AT4G02940	*ATALKBH10B (ALKBH10B)*	ALKBH10B is a functional RNA N6-methyladenosine demethylase. Reduction in ALKBH10B decreases m6A levels, and affects the stability of flowering time genes including FT, SPL3 and SPL9. Mutant plants are early flowering.
AT4G03010		RNI-like superfamily protein
AT4G03050	*(AOP3)*	The transcribed allele in ecotype Ler encodes a 2-oxoglutarate-dependent dioxygenase which is involved in glucosinolate biosynthesis. AOP3 is transcriptionally silent in leaf tissues of ecotype Col.The natural variation in this locus explains the diversification of hydroxyalkyl glucosinolates among different ecotypes of Arabidopsis.
AT4G03070	*(AOP1)*	Encodes a possible 2-oxoglutarate-dependent dioxygenase that is involved in glucosinolate biosynthesis. The gene is expressed in all ecotypes examined but the enzymatic activity has not been determined experimentally. In Col, there is one copy of this gene (aka AOP1.1) but Ler contains two copies, AOP1.1 and a tightly linked AOP1.2.
AT4G03140		NAD(P)-binding Rossmann-fold superfamily protein
AT4G03280	*PHOTOSYNTHETIC ELECTRON TRANSFER C (PETC)*	Encodes the Rieske FeS center of cytochrome b6f complex. Gene is expressed in shoot but not in root. Mutant has reduced electron transport at saturating light intensities and Q-cycle activity is hypersensitive to acidification of the thylakoid lumen. The mRNA is cell-to-cell mobile.
AT4G03510	*RING MEMBRANE-ANCHOR 1 (RMA1)*	RMA1 encodes a novel 28 kDa protein with a RING finger motif and a C-terminal membrane-anchoring domain that is involved in the secretory pathway. Has E3 ubiquitin ligase activity.
AT4G03620		myosin heavy chain-like protein
AT4G03650	*(GP1)*	transposable_element_gene;gypsy-like retrotransposon family, has a 1.3e-314 P-value blast match to GB:AAD27547 polyprotein (Gypsy_Ty3-element) (Oryza sativa subsp. indica);(source:TAIR10)

AT4G03810		transposable_element_gene;copia-like retrotransposon family, has a 0. P-value blast match to GB:BAA22288 pol polyprotein (Ty1_Copia-element) (Oryza australiensis)GB:BAA22288 polyprotein (Ty1_Copia-element) (Oryza australiensis)gi|2443320|dbj|BAA22288.1| polyprotein (RIRE1) (Oryza australiensis) (Ty1_Copia-element);(source:TAIR10)
AT4G04180		P-loop containing nucleoside triphosphate hydrolases superfamily protein
AT4G04330	*HOMOLOGUE OF CYANOBACTERIAL RBCX 1 (RbcX1)*	Encodes a chloroplast thylakoid localized RbcX protein that acts as a chaperone in the folding of Rubisco.
AT4G04460	*PUTATIVE ASPARTIC PROTEINASE A3 (PASPA3)*	Saposin-like aspartyl protease family protein
AT4G04630		senescence regulator (Protein of unknown function, DUF584)
AT4G04640	*(ATPC1)*	One of two genes (with ATPC2) encoding the gamma subunit of Arabidopsis chloroplast ATP synthase.
AT4G04870	*CARDIOLIPIN SYNTHASE (CLS)*	Encodes a protein with cardiolipin synthase activity that is localized to the mitochondiria.
AT4G04940		transducin family protein / WD-40 repeat family protein
AT4G04955	*ALLANTOINASE (ALN)*	Encodes an allantoinase which is involved in allantoin degradation and assimilation. Gene expression was induced when allantoin was added to the medium. The insertion mutant, ataln m2-1, did not grow well on the MS medium where allantoin, instead of ammonium nitrate, was supplied.
AT4G05020	*NAD(P)H DEHYDROGENASE B2 (NDB2)*	Miitochondrial alternative NADH dehydrogenase.
AT4G05150		Octicosapeptide/Phox/Bem1p family protein
AT4G05180	*PHOTOSYSTEM II SUBUNIT Q-2 (PSBQ-2)*	Encodes the PsbQ subunit of the oxygen evolving complex of photosystem II.
AT4G05390	*ROOT FNR 1 (RFNR1)*	Encodes a root-type ferredoxin:NADP(H) oxidoreductase.
AT4G05470		
AT4G08000		transposable_element_gene;CACTA-like transposase family (Tnp2/En/Spm), has a 1.0e-136 P-value blast match to GB:CAA40555 TNP2 (CACTA-element) (Antirrhinum majus);(source:TAIR10)
AT4G08030		transposable_element_gene;gypsy-like retrotransposon family (Athila), has a 1.5e-66 P-value blast match to GB:CAA57397 Athila ORF 1 (Arabidopsis thaliana);(source:TAIR10)
AT4G08850	*MDIS1-INTERACTING RECEPTOR LIKE KINASE2 (MIK2)*	MIK1 encodes a receptor kinase that forms a complex with MDIS1/MIK2 and binds LURE1, the female pollen guidance chemi-attractant. MIK1 phosphorylates MDIS1 and is autophosphorylated.
AT4G08870	*ARGININE AMIDOHYDROLASE 2 (ARGAH2)*	Encodes one of the two arginase in the genome. Gene expression is enhanced by methyl jasmonate treatment. It is involved in the defense response to B.cinerea.
AT4G08950	*EXORDIUM (EXO)*	Phosphate-responsive 1 family protein
AT4G09010	*THYLAKOID LUMEN 29 (TL29)*	Encodes a thylakoid lumen protein that was initially believed to act as a microsomal ascorbate peroxidase APX4 but to date, no evidence of enzymatic activity has been found.
AT4G09110	*ARABIDOPSIS T??XICOS EN LEVADURA 35 (ATL35)*	RING/U-box superfamily protein
AT4G09460	*MYB DOMAIN PROTEIN 6 (MYB6)*	Encodes myb6 DNA-binding protein. The mRNA is cell-to-cell mobile.
AT4G09500	*UDP-GLYCOSYLTRANSFERASE 79B7 (UGT79B7)*	Putative glycosyltransferase.
AT4G09820	*TRANSPARENT TESTA 8 (TT8)*	TT8 is a regulation factor that acts in a concerted action with TT1, PAP1 and TTG1 on the regulation of flavonoid pathways, namely proanthocyanidin and anthocyanin biosynthesis. Affects dihydroflavonol 4-reductase gene expression. It is thought that a ternary complex composed of TT2, TT8 and TTG1 is necessary for correct expression of BAN in seed endothelium. Also important for important for marginal trichome development. It binds the promoter of both AT3G26790 and AT1G28300.TT8 interacts with JAZ proteins to regulate anthocyanin accumulation. TT8 acts maternally to affect seed FA biosynthesis and inhibits seed FA accumulation by down-regulating a group of genes either critical to embryonic development or important in the FA biosynthesis pathway. TT8 represses the activities of LEAFY COTYLEDON1, LEAFY COTYLEDON2, and FUSCA3, the critical transcriptional factors important for seed development.
AT4G09900	*METHYL ESTERASE 12 (MES12)*	Encodes a protein predicted to act as a carboxylesterase. It has similarity to the SABP2 methyl salicylate esterase from tobacco. This protein does not act on methyl IAA, methyl JA, MeSA, MeGA4, or MEGA9 in vitro.
AT4G09940	*IMMUNE ASSOCIATED NUCLEOTIDE BINDING 12 (IAN12)*	P-loop containing nucleoside triphosphate hydrolases superfamily protein
AT4G10030	*ALPHA/BETA HYDROLASE DOMAIN 11 (ABHD11)*	Alpha/beta hydrolase domain containing protein involved in lipid biosynthesis.
AT4G10150	*ARABIDOPSIS T??XICOS EN LEVADURA 7 (ATL07)*	RING/U-box superfamily protein
AT4G10240	*B-BOX DOMAIN PROTEIN 23 (BBX23)*	B-box zinc finger family protein
AT4G10300	*(TRR14)*	RmlC-like cupins superfamily protein. Overexpression leads to trehalose resistance, drought and stress tolerance.
AT4G10350	*NAC DOMAIN CONTAINING PROTEIN 70 (NAC070)*	NAC domain protein. SMB, BRN1, and BRN2 act to regulate root cap maturation, in a partially redundant fashion.BRN1 and BRN2, control the cell wall maturation processes that are required to detach root cap layers from the root.
AT4G10490	*DMR6-LIKE OXYGENASE 2 (DLO2)*	2-oxoglutarate (2OG) and Fe(II)-dependent oxygenase superfamily protein
AT4G10560	*MATERNAL EFFECT EMBRYO ARREST 53 (MEE53)*	Cysteine/Histidine-rich C1 domain family protein
AT4G10770	*OLIGOPEPTIDE TRANSPORTER 7 (OPT7)*	oligopeptide transporter
AT4G10800		BTB/POZ domain protein
AT4G10950	*GDSL-TYPE ESTERASE/LIPASE 77 (GELP77)*	GDSL-type esterase/lipase. Required for pollen development.
AT4G11120		translation elongation factor Ts (EF-Ts)
AT4G11140	*CYTOKININ RESPONSE FACTOR 1 (CRF1)*	Encodes a member of the ERF (ethylene response factor) subfamily B-5 of ERF/AP2 transcription factor family. The protein contains one AP2 domain. There are 7 members in this subfamily. CRF proteins relocalize to the nucleus in response to cytokinin.
AT4G11280	*1-AMINOCYCLOPROPANE-1-CARBOXYLIC ACID (ACC) SYNTHASE 6 (ACS6)*	encodes a a member of the 1-aminocyclopropane-1-carboxylate (ACC) synthase (S-adenosyl-L-methionine methylthioadenosine-lyase, EC 4.4.1.14) gene family The mRNA is cell-to-cell mobile.
AT4G11610	*MULTIPLE C2 DOMAIN AND TRANSMEMBRANE REGION PROTEIN 7 (MCTP7)*	C2 calcium/lipid-binding plant phosphoribosyltransferase family protein
AT4G11650	*OSMOTIN 34 (OSM34)*	Osmotin-like protein; functions as a positive regulator in the generation of ABA responses and is under post-translational control.
AT4G11820	*HYDROXYMETHYLGLUTARYL-COA SYNTHASE (HMGS)*	Encodes a protein with hydroxymethylglutaryl-CoA synthase activity which was characterized by phenotypical complementation of the S. cerevisiae mutant. Involved in glucosinolate biosynthesis.

AT4G11850	*PHOSPHOLIPASE D GAMMA 1 (PLDGAMMA1)*	Encodes a phospholipase D (gamma) that is involved in aluminum tolerance and plays a role in membrane lipid modulation under Al stress.
AT4G12110	*STEROL-4ALPHA-METHYL OXIDASE 1-1 (SMO1-1)*	Encodes a member of the SMO1 family of sterol 4alpha-methyl oxidases. More specifically functions as a 4,4-dimethyl-9beta,19-cyclopropylsterol-4alpha-methyl oxidase. Works together with SMO1-2 to maintain correct sterol composition and balance auxin and cytokinin activities during embryogenesis.
AT4G12300	*CYTOCHROME P450, FAMILY 706, SUBFAMILY A, POLYPEPTIDE 4 (CYP706A4)*	member of CYP706A
AT4G12330	*CYTOCHROME P450, FAMILY 706, SUBFAMILY A, POLYPEPTIDE 7 (CYP706A7)*	member of CYP706A
AT4G12400	*HOP3 (Hop3)*	Encodes one of the 36 carboxylate clamp (CC)-tetratricopeptide repeat (TPR) proteins (Prasad 2010, Pubmed ID: 20856808) with potential to interact with Hsp90/Hsp70 as co-chaperones.
AT4G12410	*SMALL AUXIN UPREGULATED RNA 35 (SAUR35)*	SAUR-like auxin-responsive protein family
AT4G12800	*PHOTOSYSTEM I SUBUNIT L (PSAL)*	Encodes subunit L of photosystem I reaction center.
AT4G12880	*EARLY NODULIN-LIKE PROTEIN 19 (ENODL19)*	early nodulin-like protein 19
AT4G12970	*STOMAGEN (STOMAGEN)*	Encodes a cysteine-rich peptide, a secretory factor that is produced in the mesophyll cells and acts on the epidermis to increase stomatal formation. Its mature form is a 45-aa peptide with three intramolecular disulfide bonds. It is proposed that STOMAGEN increases stomatal number by competing with two negative regulators of stomatal density, EPF1 and EPF2. STOMAGEN has been shown to compete with EPF2 for binding to the ER and TMM receptor kinases.Binding of STOMAGEN to ER prevents induction of the EPF2-ER MAPK cascade. It's transcript levels change after inducing MUTE expression in a mute background.
AT4G12980	*AUXIN-RESPONSIVE FAMILY PROTEIN GENE (DEG18)*	Activated by OXS2 under the treatment of salt.
AT4G13180		NAD(P)-binding Rossmann-fold superfamily protein
AT4G13235	*EMBRYO SAC DEVELOPMENT ARREST 21 (EDA21)*	Encodes a defensin-like (DEFL) family protein.
AT4G13250	*NON-YELLOW COLORING 1 (NYC1)*	Encodes a chlorophyll b reductase involved in the degradation of chlorophyll b and LHCII (light harvesting complex II).
AT4G13530		transmembrane protein
AT4G13580		Disease resistance-responsive (dirigent-like protein) family protein
AT4G13650		Pentatricopeptide repeat (PPR) superfamily protein
AT4G13710		Pectin lyase-like superfamily protein
AT4G13750	*NO VEIN (NOV)*	Encodes NO VEIN (NOV), a plant-specific nuclear factor required for leaf vascular development, cellular patterning and stem cell maintenance in the root meristem, as well as for cotyledon outgrowth and separation. nov mutations affect many aspects of auxin-dependent development without directly affecting auxin perception.
AT4G13770	*CYTOCHROME P450, FAMILY 83, SUBFAMILY A, POLYPEPTIDE 1 (CYP83A1)*	Encodes a cytochrome p450 enzyme that catalyzes the initial conversion of aldoximes to thiohydroximates in the synthesis of glucosinolates not derived from tryptophan. Also has a role in auxin homeostasis.
AT4G13810	*RECEPTOR LIKE PROTEIN 47 (RLP47)*	receptor like protein 47
AT4G14060		Polyketide cyclase/dehydrase and lipid transport superfamily protein
AT4G14100		transferases, transferring glycosyl groups
AT4G14560	*INDOLE-3-ACETIC ACID INDUCIBLE 1 (IAA1)*	auxin (indole-3-acetic acid) induced gene (IAA1) encoding a short-lived nuclear-localized transcriptional regulator protein. The mRNA is cell-to-cell mobile.
AT4G14640	*CALMODULIN 8 (CAM8)*	encodes a divergent member of calmodulin, which is an EF-hand family of Ca2+-binding proteins. This gene is expressed in leaves, flowers and siliques. The gene functionally complements yeast calmodulin 1 (CAM1). Also the protein does not form formed a complex with a basic amphiphilic helical peptide in the presence of Ca2+ in vitro. Authors suggest that this gene may represent a Ca2+-binding sensor protein that interacts with a more limited set of target proteins than do more conventional CaM isoforms.
AT4G14650		hypothetical protein
AT4G14690	*EARLY LIGHT-INDUCIBLE PROTEIN 2 (ELIP2)*	Encodes an early light-induced protein. ELIPs are thought not to be directly involved in the synthesis and assembly of specific photosynthetic complexes, but rather affect the biogenesis of all chlorophyll-binding complexes. A study (PMID 17553115) has shown that the chlorophyll synthesis pathway was downregulated as a result of constitutive ELIP2 expression, leading to decreased chlorophyll availability for the assembly of pigment-binding proteins for photosynthesis.
AT4G14695	*MITOCHONDRIAL PYRUVATE CARRIER 2 (MPC2)*	Uncharacterized protein family (UPF0041)
AT4G14730	*LIFEGUARD 1 (LFG1)*	Stress induced membrane protein. Mutants show enhanced cell death under stress.
AT4G14760	*NETWORKED 1B (NET1B)*	kinase interacting (KIP1-like) family protein
AT4G14890	*FERREDOXIN C 1 (FdC1)*	2Fe-2S ferredoxin-like superfamily protein
AT4G15250	*B-BOX DOMAIN PROTEIN 9 (BBX9)*	B-box type zinc finger protein with CCT domain-containing protein
AT4G15390		HXXXD-type acyl-transferase family protein
AT4G15400	*BRASSINOSTEROID INACTIVATOR1 (BIA1)*	Encodes BIA1, a member of the BAHD acyltransferase family. Plays a role in controlling brassinosteroids levels, particularly in the root and hypocotyl in darkness.
AT4G15510	*PSBP-DOMAIN PROTEIN1 (PPD1)*	A nuclear-encoded thylakoid lumenal protein that is associated with photosystem I (PSI) but is not an integral subunit of PSI. It is an PSI assembly factor that assists the proper folding and integration of PsaB and PsaA into the thylakoid membrane. It is involved in the assembly of PSI reaction center heterodimer core.
AT4G15560	*CLOROPLASTOS ALTERADOS 1 (CLA1)*	Encodes a protein with 1-deoxyxylulose 5-phosphate synthase activity involved in the MEP pathway. It is essential for chloroplast development in Arabidopsis
AT4G15610	*CASP-LIKE PROTEIN 1D1 (CASPL1D1)*	Uncharacterized protein family (UPF0497)
AT4G15620	*CASP-LIKE PROTEIN 1E2 (CASPL1E2)*	Uncharacterized protein family (UPF0497)
AT4G15640		adenylyl cyclase
AT4G15770		RNA binding protein
AT4G16155		dihydrolipoamide dehydrogenase
AT4G16250	*PHYTOCHROME D (PHYD)*	Encodes a phytochrome photoreceptor with a function similar to that of phyB that absorbs the red/far-red part of the light spectrum and is involved in light responses. It cannot compensate for phyB loss in Arabidopsis but can substitute for tobacco phyB in vivo.
AT4G16410		transmembrane protein
AT4G16480	*INOSITOL TRANSPORTER 4 (INT4)*	Encodes a high affinity H+:<i>myo</i>-inositol symporter. The only other compound shown to be transported was pinitol, a methylated derivative of <i>myo</i>-inositol. The mRNA is cell-to-cell mobile.

AT4G16680		P-loop containing nucleoside triphosphate hydrolases superfamily protein
AT4G16770		2-oxoglutarate (2OG) and Fe(II)-dependent oxygenase superfamily protein
AT4G16900		Disease resistance protein (TIR-NBS-LRR class) family
AT4G17030	*EXPANSIN-LIKE B1 (EXLB1)*	Encodes EXLB1 (expansin-like B1), a member of the expansin family.
AT4G17200		F-box and associated interaction domains-containing protein
AT4G17220	*MICROTUBULE-ASSOCIATED PROTEINS 70-5 (MAP70-5)*	Encodes a microtubule associated protein (MAP70-5). Regulates secondary wall patterning in wood cells. Expressed in all tissues.
AT4G17250		transmembrane protein
AT4G17280		Auxin-responsive family protein
AT4G17380	*MUTS-LIKE PROTEIN 4 (MSH4)*	Encodes the Arabidopsis homolog of MSH4, a meiosis-specific member of the MutS-homolog family of genes. It is expressed only in floral tissues and only during early meiotic prophase I, preceding the synapsis of homologous chromosomes. It is involved in the early steps of recombination.
AT4G17500	*ETHYLENE RESPONSIVE ELEMENT BINDING FACTOR 1 (ERF-1)*	Encodes a member of the ERF (ethylene response factor) subfamily B-3 of ERF/AP2 transcription factor family (ATERF-1). The protein contains one AP2 domain. There are 18 members in this subfamily including ATERF-1, ATERF-2, AND ATERF-5. The mRNA is cell-to-cell mobile.
AT4G17550	*GLYCEROL-3-PHOSPHATE PERMEASE 4 (G3Pp4)*	Encodes a member of the phosphate starvation-induced glycerol-3-phosphate permease gene family: AT3G47420(G3Pp1), AT4G25220(G3Pp2), AT1G30560(G3Pp3), AT4G17550(G3Pp4) and AT2G13100(G3Pp5).
AT4G17585		aluminum activated malate transporter family protein
AT4G17600	*LIGHT-HARVESTING-LIKE 3:1 (LIL3:1)*	Encodes Lil3:1 (light-harvesting-like) protein. Belongs to the Lhc super-gene family encodes the light-harvesting chlorophyll a/b-binding (LHC) proteins that constitute the antenna system of the photosynthetic apparatus. A generic LHC motif is present in Lil3:1. The mRNA is cell-to-cell mobile.
AT4G17610	*TRNA METHYLTRANSFERASE 3 (TRM3)*	tRNA/rRNA methyltransferase (SpoU) family protein
AT4G17660	*PBS1-LIKE 20 (PBL20)*	Protein kinase superfamily protein
AT4G17670		senescence-associated family protein (DUF581)
AT4G17695	*KANADI 3 (KAN3)*	Homeodomain-like superfamily protein
AT4G17700		hypothetical protein
AT4G17910		transferases, transferring acyl groups
AT4G17940		Tetratricopeptide repeat (TPR)-like superfamily protein
AT4G18010	*INOSITOL(1,4,5)P3 5-PHOSPHATASE II (IP5PII)*	Encodes an inositol polyphosphate 5-phosphatase that appears to have Type I activity. It can dephosphorylate IP3(inositol(1,4,5)P3) and IP4 (inositol(1,3,4,5)P4), but it does not appear to be active against phosphatidylinositol 4,5 bisphosphate. Overexpression of this gene renders plants insensitive to ABA in germination and growth assays.
AT4G18480	*(CHLI1)*	Encodes the CHLI subunit of magnesium chelatase which is required for chlorophyll biosynthesis. All four cysteine residues of the protein form two disulfide bonds (Cys102-Cys193 and Cys354-Cys396) under oxidized conditions but are fully reduced by reduction. It was suggested that the redox state of CHLI is regulated in vivo by the change of the redox environment in the chloroplasts probably via the Trx system.
AT4G18510	*CLAVATA3/ESR-RELATED 2 (CLE2)*	CLE2, putative ligand, member of large gene family homologous to Clavata3
AT4G18560		
AT4G18770	*MYB DOMAIN PROTEIN 98 (MYB98)*	MYB98 is a member of the R2R3-MYB gene family, the members of which likely encode transcription factors. Within an ovule, MYB98 is expressed exclusively in the synergid cells, and mutations in this gene affect the female gametophyte specifically. myb98 female gametophytes are affected in two unique features of the synergid cell, pollen tube guidance and the filiform apparatus, but are otherwise normal. This suggests that MYB98 controls the development of specific features within the synergid cell during female gametophyte development. MYB98 also is expressed in trichomes and endosperm. Homozygous myb98 mutants exhibit no sporophytic defects, including trichome and endosperm defects.
AT4G18780	*IRREGULAR XYLEM 1 (IRX1)*	Encodes a member of the cellulose synthase family involved in secondary cell wall biosynthesis. Mutants have abnormal xylem formation, reduced cellulose content, and enhanced drought and osmotic stress tolerance. Mediates resistance towards bacterial pathogens via ABA. Confers resistance towards bacterial and fungal pathogens, independent of salicylic acid, ethylene and jasmonate signaling.
AT4G18870		E2F/DP family winged-helix DNA-binding domain-containing protein
AT4G18920		histone acetyltransferase (DUF1264)
AT4G19120	*EARLY-RESPONSIVE TO DEHYDRATION 3 (ERD3)*	S-adenosyl-L-methionine-dependent methyltransferases superfamily protein
AT4G19230	*CYTOCHROME P450, FAMILY 707, SUBFAMILY A, POLYPEPTIDE 1 (CYP707A1)*	Encodes a protein with ABA 8'-hydroxylase activity, involved in ABA catabolism. Member of the CYP707A gene family. CYP707A1 appears to play an important role in determining the ABA levels in dry seeds. Gene involved in postgermination growth. Overexpression of CYP707A1 leads to a decrease in ABA levels and a reduction in after-ripening period to break dormancy.
AT4G19380		Long-chain fatty alcohol dehydrogenase family protein
AT4G19460		UDP-Glycosyltransferase superfamily protein
AT4G19680	*IRON REGULATED TRANSPORTER 2 (IRT2)*	encodes an iron transporter whose expression is induced by iron and zinc deficiency. Gene is specifically expressed in the external cell layers of the root subapical zone.
AT4G19810	*CLASS V CHITINASE (ChiC)*	ChiC encodes a Class V chitinase that is a part of glycoside hydrolase family 18 based on CAZy groupings. It appears to primarily act as an exochitinase in vitro where it predominantly cleaves a chitobiose (GlcNAc)2 residue from the non-reducing end of a chitin oligosaccharide. However, it shows some minor endochitinase activity in vitro, as well. A putative 24 amino-acid signal peptide may direct this protein to the secretory system and it has been detected in cell wall apoplastic fluid. RT-PCR experiments demonstrate that ChiC transcript levels are increased in response to abscisisc acid, jasmonic acid, and NaCl stress. Microarray results also suggest that transcript levels rise in response to osmotic stress, two fungal pathogens, a bacterial pathogen, and the elicitor flagellin. The mRNA is cell-to-cell mobile.
AT4G19820		Glycosyl hydrolase family protein with chitinase insertion domain-containing protein
AT4G19870		Galactose oxidase/kelch repeat superfamily protein
AT4G20000		VQ motif-containing protein

AT4G20020	*MULTIPLE ORGANELLAR RNA EDITING FACTOR 1 (MORF1)*	Encodes a protein involved in RNA editing in mitochondria. Member of MORF family consisting of of nine full-length proteins encoded in the nuclear genome. MORF proteins are required for all RNA editing events in plastids and for many, possibly also all, sites in mitochondria. Potential link between the RNA binding PPR protein and the protein contributing the enzymatic activity in RNA editing.
AT4G20050	*QUARTET 3 (QRT3)*	Encodes a polygalacturonase that plays a direct role in degrading the pollen mother cell wall during microspore development.
AT4G20070	*ALLANTOATE AMIDOHYDROLASE (AAH)*	The gene encoding Arabidopsis thaliana Allantoate Amidohydrolase (AtAAH)which catalyzes the allantoate deiminase reaction (EC 3.5.3.9)is expressed in all parts of the plant being consistent with a function in purine turnover in Arabidopsis. The mRNA is cell-to-cell mobile.
AT4G20260	*PLASMA-MEMBRANE ASSOCIATED CATION-BINDING PROTEIN 1 (PCAP1)*	Encodes a Ca2+ and Cu2+ binding protein. N-terminal myristylation on glycine 2 appears to enable it to associate tightly with the plasma membrane. Recombinant PCaP1 interacts strongly with phosphatidylinositol 3,5-bisphosphate (PtdIns(3,5)P2) and PtdIns (3,4,5)P3, and weakly with PtdIns(3,5)P2 and PtdIns(4,5). It also interacts with calmodulin (CaM) in a calcium-dependent manner. CaM does not interfere with PCaP1 membrane localization but does weaken interactions between it and the PtdInsPs. PCaP1 has an apparent Kd of 10 uM for Cu2+ and can bind six ions per protein. Transcript levels for PCaP1 first fall and then rise following exposure to CuCl2. Mannitol, sorbitol, and the flg22 oligopeptide also increase expression levels. The mRNA is cell-to-cell mobile.
AT4G20270	*BARELY ANY MERISTEM 3 (BAM3)*	Encodes a CLAVATA1-related receptor kinase-like protein required for both shoot and flower meristem function. It has a broad expression pattern and is involved in vascular strand development in the leaf, control of leaf shape, size and symmetry, male gametophyte development and ovule specification and function. The mRNA is cell-to-cell mobile.
AT4G20360	*RAB GTPASE HOMOLOG E1B (ATRABE1B)*	Nuclear transcribed, plastid localized EF-Tu translation elongation factor. Referred to as AtRabE1b in DOI:10.1104/pp.013052. However, wider community usage and more publications assign the symbol RabE1b to At5g59840.
AT4G20500		transposable_element_gene;similar to filament protein-related [Arabidopsis thaliana] (TAIR:AT4G20730.1);(source:TAIR10)
AT4G20830	*(ATBBE19)*	Encodes an oligogalacturonide oxidase that inactivates the elicitor-active oligogalacturonides (OGs). It is involved in plant immunity. Overexpressing plants are more resistant to B. cinerea.
AT4G20840	*(ATBBE21)*	Encodes an oligogalacturonide oxidase that inactivates the elicitor-active oligogalacturonides (OGs).
AT4G20990	*ALPHA CARBONIC ANHYDRASE 4 (ACA4)*	alpha carbonic anhydrase 4
AT4G21280	*PHOTOSYSTEM II SUBUNIT QA (PSBQA)*	Encodes the PsbQ subunit of the oxygen evolving complex of photosystem II.
AT4G21310	*DESIGUAL 2 (DEAL2)*	DUF1218 family member.
AT4G21323		Subtilase family protein
AT4G21400	*CYSTEINE-RICH RLK (RECEPTOR-LIKE PROTEIN KINASE) 28 (CRK28)*	Encodes a cysteine-rich receptor-like protein kinase.
AT4G21410	*CYSTEINE-RICH RLK (RECEPTOR-LIKE PROTEIN KINASE) 29 (CRK29)*	Encodes a cysteine-rich receptor-like protein kinase.
AT4G21500		transmembrane protein
AT4G21590	*ENDONUCLEASE 3 (ENDO3)*	Encodes a putative endonuclease but no demonstrable endonuclease activity, either towards single stranded DNA or mismatches, has been seen in vitro. Activated by AGAMOUS in a cal-1, ap1-1 background. Expressed in the floral meristem and during stamen development.
AT4G21990	*APS REDUCTASE 3 (APR3)*	Encodes a protein disulfide isomerase-like (PDIL) protein, a member of a multigene family within the thioredoxin (TRX) superfamily. This protein also belongs to the adenosine 5'-phosphosulfate reductase-like (APRL) group.
AT4G22060	*F-BOX/DUF295 BRASSICEAE-SPECIFIC 29 (ATFDB29)*	F-box protein (DUF295)
AT4G22250		RING/U-box superfamily protein
AT4G22470	*DOUBLE HYBRID PROLINE-RICH PROTEIN 1 (DHYPRP1)*	Encodes a hybrid proline-rich protein that contains two tandem PRD-8CMs (proline-rich domain-eight cysteine motif). Its expression is induced by JA and certain pathogens. This pattern of induction, combined with overexpression and RNAi induced phenotypes, suggests involvement in defense response to pathogens.
AT4G22530		S-adenosyl-L-methionine-dependent methyltransferases superfamily protein
AT4G22570	*ADENINE PHOSPHORIBOSYL TRANSFERASE 3 (APT3)*	Encodes an adenine phosphoribosyltransferase (APT; EC 2.4.2.7), which is a constitutively expressed enzyme involved in the one-step salvage of adenine to AMP. APT3 has higher affinity for zeatin, isopentenyladenine and benzyladenine than APT1 but lower Vmax than APT1.
AT4G22590	*TREHALOSE-6-PHOSPHATE PHOSPHATASE G (TPPG)*	Haloacid dehalogenase-like hydrolase (HAD) superfamily protein
AT4G22710	*CYTOCHROME P450, FAMILY 706, SUBFAMILY A, POLYPEPTIDE 2 (CYP706A2)*	member of CYP706A
AT4G22790	*RESISTANT TO HIGH CO2 (RHC1)*	Encodes a plasma membrane localized MATE type transporter that is involved in CO2 signaling during stomatal aperture regulation. RHC1 regulates HT1 which phosphorylates OST1, a kinase that regulates the SLAC1 anion channel and thus stomatal closing.
AT4G23050	*(TES1)*	PAS domain-containing protein tyrosine kinase family protein
AT4G23270	*CYSTEINE-RICH RLK (RECEPTOR-LIKE PROTEIN KINASE) 19 (CRK19)*	Encodes a cysteine-rich receptor-like protein kinase. The mRNA is cell-to-cell mobile.
AT4G23510		Disease resistance protein (TIR-NBS-LRR class) family
AT4G23620		Ribosomal protein L25/Gln-tRNA synthetase, anti-codon-binding domain-containing protein
AT4G23680		Polyketide cyclase/dehydrase and lipid transport superfamily protein
AT4G23750	*CYTOKININ RESPONSE FACTOR 2 (CRF2)*	encodes a member of the ERF (ethylene response factor) subfamily B-5 of ERF/AP2 transcription factor family. The protein contains one AP2 domain. There are 7 members in this subfamily. Monopteros target gene. CRF proteins relocalize to the nucleus in response to cytokinin.
AT4G23810	*(WRKY53)*	member of WRKY Transcription Factor; Group III
AT4G23890	*NADH DEHYDROGENASE-LIKE COMPLEX S (NdhS)*	NAD(P)H-quinone oxidoreductase subunit S
AT4G24000	*CELLULOSE SYNTHASE LIKE G2 (CSLG2)*	encodes a protein similar to cellulose synthase
AT4G24110	*HYPOXIA RESPONSE UNKNOWN PROTEIN 40 (HUP40)*	NADP-specific glutamate dehydrogenase
AT4G24180	*THAUMATIN-LIKE PROTEIN 1 (TLP1)*	Root-specific expression activated in response to rhizobacteria and ACC. Role in induced systemic resistance.
AT4G24290		MAC/Perforin domain-containing protein
AT4G24340		Phosphorylase superfamily protein
AT4G24450	*PHOSPHOGLUCAN, WATER DIKINASE (PWD)*	phosphoglucan, water dikinase
AT4G24930		thylakoid lumenal 17.9 kDa protein, chloroplast
AT4G25100	*FE SUPEROXIDE DISMUTASE 1 (FSD1)*	Fe-superoxide dismutase

AT4G25160	*(PUB35)*	Encodes a U-box domain-containing E3 ubiquitin ligase with central Ser/Thr protein kinase domain whose expression is responsive to both phosphate (Pi) and phosphite (Phi) in both roots and shoots.
AT4G25580		CAP160 protein
AT4G25590	*ACTIN DEPOLYMERIZING FACTOR 7 (ADF7)*	actin depolymerizing factor 7
AT4G25630	*FIBRILLARIN 2 (FIB2)*	encodes a fibrillarin, a key nucleolar protein in eukaryotes which associates with box C/D small nucleolar RNAs (snoRNAs) directing 2'-O-ribose methylation of the rRNA. This gene also encodes a novel box C/D snoRNA, U60.2f in its fifth intron that accumulates in seedlings and that their targeted residue on the 25 S rRNA is methylated. The mRNA is cell-to-cell mobile.
AT4G25760	*GLUTAMINE DUMPER 2 (GDU2)*	Encodes a member of the GDU (glutamine dumper) family proteins involved in amino acid export: At4g31730 (GDU1), At4g25760 (GDU2), At5g57685 (GDU3), At2g24762 (GDU4), At5g24920 (GDU5), At3g30725 (GDU6) and At5g38770 (GDU7).
AT4G25835		P-loop containing nucleoside triphosphate hydrolases superfamily protein
AT4G25860	*OSBP(OXYSTEROL BINDING PROTEIN)-RELATED PROTEIN 4A (ORP4A)*	OSBP(oxysterol binding protein)-related protein 4A
AT4G25900		Galactose mutarotase-like superfamily protein
AT4G25940	*(PICALM2B)*	ENTH/ANTH/VHS superfamily protein
AT4G25960	*ATP-BINDING CASSETTE B2 (ABCB2)*	P-glycoprotein 2
AT4G26020		4/1 protein short form protein
AT4G26150	*CYTOKININ-RESPONSIVE GATA FACTOR 1 (CGA1)*	Encodes a member of the GATA factor family of zinc finger transcription factors. Modulate chlorophyll biosynthesis and glutamate synthase (GLU1/Fd-GOGAT) expression.
AT4G26470		Calcium-binding EF-hand family protein
AT4G26700	*FIMBRIN 1 (FIM1)*	Encodes a member of the fimbrin family. Different members of the fimbrin/plastin family have diverged biochemically during evolution to generate either tight actin bundles or loose networks with distinct biochemical and biophysical properties. FIM4 generates both actin bundles and branched actin filaments whereas FIM5 only generates actin bundles.
AT4G26770		Phosphatidate cytidylyltransferase family protein
AT4G26790	*(GGL23)*	GDSL-motif esterase/acyltransferase/lipase. Enzyme group with broad substrate specificity that may catalyze acyltransfer or hydrolase reactions with lipid and non-lipid substrates.
AT4G26880		Stigma-specific Stig1 family protein
AT4G26910	*(E2- OGDH1)*	Encodes the E2 subunit of the 2-oxoglutarate dehydrogenase.
AT4G27260	*(WES1)*	encodes an IAA-amido synthase that conjugates Asp and other amino acids to auxin in vitro. Lines carrying insertions in this gene are hypersensitive to auxin. It is involved in camalexin biosynthesis via conjugating indole-3-carboxylic acid (ICA) and cysteine (Cys). The mRNA is cell-to-cell mobile.
AT4G27400		Late embryogenesis abundant (LEA) protein-like protein
AT4G27450	*HYPOXIA RESPONSE UNKNOWN PROTEIN 54 (HUP54)*	aluminum induced protein with YGL and LRDR motifs
AT4G27570	*(UGT79B3)*	Encodes a UDP-glycosyltransferase that contributes to cold, salt and drought stress tolerance via modulating anthocyanin accumulation.
AT4G27700		Rhodanese/Cell cycle control phosphatase superfamily protein
AT4G27710	*CYTOCHROME P450, FAMILY 709, SUBFAMILY B, POLYPEPTIDE 3 (CYP709B3)*	member of CYP709B The mRNA is cell-to-cell mobile.
AT4G27730	*OLIGOPEPTIDE TRANSPORTER 1 (OPT6)*	oligopeptide transporter
AT4G27940	*MANGANESE TRACKING FACTOR FOR MITOCHONDRIAL SOD2 (MTM1)*	Involved in Mn and Fe homeostasis, root length, and flowering time.
AT4G27970	*SLAC1 HOMOLOGUE 2 (SLAH2)*	Encodes a protein with ten predicted transmembrane helices. The SLAH2 protein has similarity to the SLAC1 protein involved in ion homeostasis in guard cells. But, it is not expressed in guard cells and cannot complement a slac1-2 mutant suggesting that it performs a different function. SLAH2:GFP localizes to the plasma membrane.
AT4G28030	*GCN5‐RELATED N‐ACETYLTRANSFERASE 7 (GNAT7)*	Acyl-CoA N-acyltransferases (NAT) superfamily protein
AT4G28040	*USUALLY MULTIPLE ACIDS MOVE IN AND OUT TRANSPORTERS 33 (UMAMIT33)*	nodulin MtN21-like transporter family protein
AT4G28170		transmembrane protein
AT4G28190	*ULTRAPETALA1 (ULT1)*	Encodes a novel Cys-rich protein with a B-box like domain that acts as a negative regulator of meristem cell accumulation in inflorescence and floral meristems as loss-of-function ult1 mutations cause inflorescence meristem enlargement, the production of extra flowers and floral organs, and a decrease in floral meristem determinacy. Acts opposite to CLF which represses AG, but preventing deposition of CLF repressive methylation marks.ULT1 acts as an anti-repressor that counteracts EMF1 action through modulation of histone marks on target genes. Regulates developmental as well as biotic and abiotic stress response genes.
AT4G28280	*LORELEI-LIKE-GPI ANCHORED PROTEIN 3 (LLG3)*	LORELEI-LIKE-GPI ANCHORED PROTEIN 3
AT4G28350	*L-TYPE LECTIN RECEPTOR KINASE VII.2 (LECRK-VII.2)*	Concanavalin A-like lectin protein kinase family protein
AT4G28400		Protein phosphatase 2C family protein
AT4G28450		This gene is predicted to encode a protein with a DWD motif. It can bind to DDB1a in Y2H assays and may be involved in the formation of a CUL4-based E3 ubiquitin ligase
AT4G28650	*PXY-LIKE2 (PXL2)*	Encodes one of the two putative eLRR kinase closely related to PXY (At1g08590/PXL1 and At4g28650/PXL2). Insertion mutants in either pxl1 or pxl2 do not exhibit an obvious phenotype in the stem; double-mutant combinations of a Col allele, of pxy (pxy-3) with pxl1 and pxl2, generate a more severe vascular phenotype than pxy-3 alone, suggesting that these genes act synergistically with PXY in regulating vascular-tissue development in the stem.
AT4G28703		RmlC-like cupins superfamily protein
AT4G28890	*ARABIDOPSIS T??XICOS EN LEVADURA 42 (ATL42)*	RING/U-box superfamily protein
AT4G29020		glycine-rich protein
AT4G29030		Putative membrane lipoprotein

AT4G29050	*L-TYPE LECTIN RECEPTOR KINASE V.9 (LECRK-V.9)*	Concanavalin A-like lectin protein kinase family protein
AT4G29080	*PHYTOCHROME-ASSOCIATED PROTEIN 2 (PAP2)*	phytochrome-associated protein 2 (PAP2)
AT4G29220	*PHOSPHOFRUCTOKINASE 1 (PFK1)*	phosphofructokinase 1
AT4G29240		Leucine-rich repeat (LRR) family protein
AT4G29510	*ARGININE METHYLTRANSFERASE 11 (PRMT11)*	Has arginine N-methyltransferase activity. Modifies AtMBD7.
AT4G29700		Alkaline-phosphatase-like family protein
AT4G30110	*HEAVY METAL ATPASE 2 (HMA2)*	encodes a protein similar to Zn-ATPase, a P1B-type ATPases transport zinc
AT4G30280	*XYLOGLUCAN ENDOTRANSGLUCOSYLASE/HYDROLASE 18 (XTH18)*	Encodes a xyloglucan endotransglucosylase/hydrolase with only only the endotransglucosylase (XET; EC 2.4.1.207) activity towards xyloglucan and non-detectable endohydrolytic (XEH; EC 3.2.1.151) activity. Expressed in the mature or basal regions of both the main and lateral roots, but not in the tip of these roots where cell division occurs.
AT4G30290	*XYLOGLUCAN ENDOTRANSGLUCOSYLASE/HYDROLASE 19 (XTH19)*	Encodes a xyloglucan endotransglucosylase/hydrolase with only only the endotransglucosylase (XET; EC 2.4.1.207) activity towards xyloglucan and non-detectable endohydrolytic (XEH; EC 3.2.1.151) activity. Expressed throughout both the main and the lateral root, with intensive expression at the dividing and elongating regions. Is expressed in lateral root primordia but expression ceases after lateral root begins to grow. Involved in cell proliferation in incised inflorescence stems.
AT4G30410	*IBH1-LIKE 1 (IBL1)*	sequence-specific DNA binding transcription factor
AT4G30650		Low temperature and salt responsive protein family
AT4G30800		Nucleic acid-binding, OB-fold-like protein
AT4G31070		PPR superfamily protein
AT4G31210		DNA topoisomerase, type IA, core
AT4G31330		transmembrane protein, putative (Protein of unknown function, DUF599)
AT4G31470		CAP (Cysteine-rich secretory proteins, Antigen 5, and Pathogenesis-related 1 protein) superfamily protein
AT4G31680		Transcriptional factor B3 family protein
AT4G31805	*POLAR LOCALIZATION DURING ASYMMETRIC DIVISION AND REDISTRIBUTION (POLAR)*	Encodes POLAR, a scaffold protein associated with cellular asymmetry of meristemoids. Its transcript levels change after inducing MUTE expression in a mute background.
AT4G31810	*(CHY4)*	ATP-dependent caseinolytic (Clp) protease/crotonase family protein
AT4G31860		Protein phosphatase 2C family protein
AT4G32280	*INDOLE-3-ACETIC ACID INDUCIBLE 29 (IAA29)*	indole-3-acetic acid inducible 29
AT4G32340		Tetratricopeptide repeat (TPR)-like superfamily protein
AT4G32460	*(BDX)*	BDX is a DUF642 cell wall protein primarily expressed in vascular tissues of roots, leaves and embryos. Mutants show defects in seed and embryo development.
AT4G32500	*K+ TRANSPORTER 5 (KT5)*	Encodes AKT5, a member of the Shaker family potassium ion (K+) channel. This family includes five groups based on phylogenetic analysis (FEBS Letters (2007) 581: 2357): I (inward rectifying channel): AKT1 (AT2G26650), AKT5 (AT4G32500) and SPIK (also known as AKT6, AT2G25600); II (inward rectifying channel): KAT1 (AT5G46240) and KAT2 (AT4G18290); III (weakly inward rectifying channel): AKT2 (AT4G22200); IV (regulatory subunit involved in inwardly rectifying conductance formation): KAT3 (also known as AtKC1, AT4G32650); V (outward rectifying channel): SKOR (AT3G02850) and GORK (AT5G37500).
AT4G32520	*SERINE HYDROXYMETHYLTRANSFERASE 3 (SHM3)*	Encodes a serine hydroxymethyltransferase SHMT3 located in the plastid.
AT4G32590		2Fe-2S ferredoxin-like superfamily protein
AT4G32650	*POTASSIUM CHANNEL IN ARABIDOPSIS THALIANA 3 (KAT3)*	Encodes KAT3, a member of the Shaker family of voltage-gated potassium channel subunits. Does not form functional potassium channel on its own. Involved in down-regulating AKT1 and KAT1 channel activity by forming heteromers with AKT1 or KAT1. The Shaker family K+ ion channels include five groups based on phylogenetic analysis (FEBS Letters (2007) 581: 2357): I (inwardly rectifying conductance): AKT1 (AT2G26650), AKT5 (AT4G32500) and SPIK (also known as AKT6, AT2G25600); II (inward rectifying channel): KAT1 (AT5G46240) and KAT2 (AT4G18290); III (weakly inward rectifying channel): AKT2 (AT4G22200); IV (regulatory subunit involved in inwardly rectifying conductance formation): KAT3 (also known as AtKC1, AT4G32650); V (outward rectifying channel): SKOR (AT3G02850) and GORK (AT5G37500).
AT4G33010	*GLYCINE DECARBOXYLASE P-PROTEIN 1 (GLDP1)*	glycine decarboxylase P-protein 1
AT4G33220	*PECTIN METHYLESTERASE 44 (PME44)*	pectin methylesterase 44
AT4G33280		AP2/B3-like transcriptional factor family protein
AT4G33560	*WOUND-INDUCED POLYPEPTIDE 5 (WIP5)*	Member of the wound-induced polypeptide (WIP) family.
AT4G33666		hypothetical protein
AT4G33730	*CAP-DERIVED PEPTIDE 1 (ATCAPE1)*	Member of CAP protein superfamily. Encoding a small 11 AA peptide (PAGNYIGARPY) involved in negative regulation of salt tolerance.
AT4G33800		hypothetical protein
AT4G33905		Peroxisomal membrane 22 kDa (Mpv17/PMP22) family protein
AT4G33950	*OPEN STOMATA 1 (OST1)*	Encodes calcium-independent ABA-activated protein kinase, a member of SNF1-related protein kinases (SnRK2) whose activity is activated by ionic (salt) and non-ionic (mannitol) osmotic stress. Mutations disrupted ABA induction of stomatal closure as well as ABA inhibition of light-induced stomatal opening. However, regulation of stomatal opening/closing by light or CO(2) is not affected in these mutants. May act in the interval between ABA perception and reactive oxygen species production in the ABA signalling network.
AT4G33960		hypothetical protein
AT4G34050	*CAFFEOYL COENZYME A O-METHYLTRANSFERASE 1 (CCoAOMT1)*	Methyltransferase in the lignin biosynthetic pathway.
AT4G34135	*UDP-GLUCOSYLTRANSFERASE 73B2 (UGT73B2)*	The At4g34135 gene encodes a flavonol 7-O-glucosyltransferase (EC 2.4.1.237) that glucosylates also with a 20 fold lower activity flavonols (kaempferol and quercetin) at the 3-O-position.
AT4G34250	*3-KETOACYL-COA SYNTHASE 16 (KCS16)*	Encodes KCS16, a member of the 3-ketoacyl-CoA synthase family involved in the biosynthesis of VLCFA (very long chain fatty acids).
AT4G34350	*4-HYDROXY-3-METHYLBUT-2-ENYL DIPHOSPHATE REDUCTASE (HDR)*	Arabidopsis ISPH is involved in the plastid nonmevalonate pathway of isoprenoid biosynthesis. It was shown to complement the lethal phenotype of E. coli ispH mutant and is therefore most likely encodes a protein with 4-hydroxy-3-methylbut-2-en-1-yl diphosphate reductase activity involved in the last step of mevalonate-independent isopentenyl biosynthesis. Mutant has Albino seedling.

AT4G34410	*REDOX RESPONSIVE TRANSCRIPTION FACTOR 1 (RRTF1)*	Encodes a member of the ERF (ethylene response factor) subfamily B-3 of ERF/AP2 transcription factor family. The protein contains one AP2 domain. There are 18 members in this subfamily including ATERF-1, ATERF-2, AND ATERF-5. Regulates programmed cell death (PCD) inhibitor genes. Involved in retarding programmed cell death under salt stress due to the regulation of processes participating in ROS inhibition. ERF-regulated transcripts belong to the tryptophan biosynthesis, tryptophan metabolism, and downstream plant hormone signal transduction pathways, where ERF109 potentially acts as a 'master switch' mediator of a cascade of consecutive events across the three pathways, promoting plant growth and re-adjustment to homeostasis due the direct participation in auxin biosynthesis leading to the plants ability to tolerate salt stress.
AT4G34520	*3-KETOACYL-COA SYNTHASE 18 (KCS18)*	Encodes KCS18, a member of the 3-ketoacyl-CoA synthase family involved in the biosynthesis of VLCFA (very long chain fatty acids).
AT4G34560		transmembrane protein
AT4G34760	*SMALL AUXIN UPREGULATED RNA 50 (SAUR50)*	SAUR-like auxin-responsive protein family
AT4G34970	*ACTIN DEPOLYMERIZING FACTOR 9 (ADF9)*	A member of actin polymerizing factors (ADFs)family, ADF9 primarily functions as an actin bundling protein.
AT4G35060	*HEAVY METAL ASSOCIATED ISOPRENYLATED PLANT PROTEIN 25 (HIPP25)*	Heavy metal transport/detoxification superfamily protein
AT4G35350	*XYLEM CYSTEINE PEPTIDASE 1 (XCP1)*	tracheary element vacuolar protein. A clubroot pathogen effector targets cruciferous cysteine proteases to suppress plant immunity.
AT4G35440	*CHLORIDE CHANNEL E (CLC-E)*	Enclodes a choride channel protein that is localized to the thlakoid membrane.
AT4G35520	*MUTL PROTEIN HOMOLOG 3 (MLH3)*	DNA mismatch repair protein similar to MutL. Required for normal levels of meiotic crossovers
AT4G36000		Pathogenesis-related thaumatin superfamily protein
AT4G36040	*DNAJ11 (J11)*	Chaperone DnaJ-domain superfamily protein
AT4G36410	*UBIQUITIN-CONJUGATING ENZYME 17 (UBC17)*	ubiquitin-conjugating enzyme
AT4G36430		Peroxidase superfamily protein
AT4G36540	*BR ENHANCED EXPRESSION 2 (BEE2)*	Encodes the brassinosteroid signaling component BEE2 (BR-ENHANCED EXPRESSION 2). Positively modulates the shade avoidance syndrome in Arabidopsis seedlings.
AT4G36600		Late embryogenesis abundant (LEA) protein
AT4G36700		RmlC-like cupins superfamily protein
AT4G36740	*HOMEOBOX PROTEIN 40 (HB40)*	Encodes a homeodomain leucine zipper class I (HD-Zip I) protein.
AT4G36880	*CYSTEINE PROTEINASE1 (CP1)*	cysteine proteinase1
AT4G37150	*METHYL ESTERASE 9 (MES9)*	Encodes a protein shown to have carboxylesterase activity, methyl salicylate esterase activity, methyl jasmonate esterase activity, and methyl IAA esterase activity in vitro. MES9 appears to be involved in MeSA hydrolysis in planta. Expression of MES9 can restore systemic acquired resistance in SAR-deficient tobacco plants. This protein does not act on MeGA4, or MEGA9 in vitro.
AT4G37200	*HIGH CHLOROPHYLL FLUORESCENCE 164 (HCF164)*	Encodes thioredoxin-like protein with disulfide reductase activity that is involved in the biogenesis of the plastid cytochrome b6f complex. Protein is located in the thylakoid membrane with the C-terminal hydrophilic portion, containing the thioredoxin like domain, extending into the thylakoid lumen.
AT4G37295	*MP-INDUCED SECRETED PEPTIDE-LIKE 3 (PIPL3)*	Encodes an 86 AA polypeptide sequence that produces an 11 AA secreted, bioactive peptide. It is induced by BD16. The peptide is bound by the RLK7 receptor kinase and inhibits the formation of lateral root founder cells. Homolog of prePIP1.
AT4G37310	*CYTOCHROME P450, FAMILY 81, SUBFAMILY H, POLYPEPTIDE 1 (CYP81H1)*	member of CYP81H
AT4G37370	*CYTOCHROME P450, FAMILY 81, SUBFAMILY D, POLYPEPTIDE 8 (CYP81D8)*	member of CYP81D
AT4G37400	*CYTOCHROME P450, FAMILY 81, SUBFAMILY F, POLYPEPTIDE 3 (CYP81F3)*	member of CYP81F
AT4G37410	*CYTOCHROME P450, FAMILY 81, SUBFAMILY F, POLYPEPTIDE 4 (CYP81F4)*	member of CYP81F The mRNA is cell-to-cell mobile.
AT4G37430	*CYTOCHROME P450, FAMILY 91, SUBFAMILY A, POLYPEPTIDE 2 (CYP91A2)*	Encodes a member of the CYP81F cytochrome P450 monooxygenase subfamily.
AT4G37530		Peroxidase superfamily protein
AT4G37540	*LOB DOMAIN-CONTAINING PROTEIN 39 (LBD39)*	LOB domain-containing protein 39
AT4G37550	*IAMHYDROLASE1 (IAMH1)*	Indole-3-acetamide (IAM) hydrolase gene required for the auxin effects of IAM.
AT4G37750	*AINTEGUMENTA (ANT)*	ANT is required for control of cell proliferation and encodes a putative transcriptional regulator similar to AP2. Loss of function alleles have reduced fertility, abnormal ovules and abnormal lateral organs. Expressed in the chalaza, floral organ primordia, and lateral shoot organ primordia. Regulates growth and cell numbers during organogenesis. Modulates auxin biosynthesis in the ovule via regulation of YUC4.
AT4G37870	*PHOSPHOENOLPYRUVATE CARBOXYKINASE 1 (PCK1)*	Encodes a phosphoenolpyruvate carboxykinase that localizes to the cytosol.
AT4G37900	*(ATGRDP2)*	Protein of unknown function that contains DUF1399 domain and putative RNA binding motif. Expressed in many plant tissues and is involved in many aspects of plant growth and development as well as response to salt stress. Regulates ovule development via the auxin pathway.
AT4G37910	*MITOCHONDRIAL HEAT SHOCK PROTEIN 70-1 (mtHsc70-1)*	mitochondrial heat shock protein 70-1
AT4G37930	*SERINE TRANSHYDROXYMETHYLTRANSFERASE 1 (SHM1)*	Encodes a protein with mitochondrial serine hydroxymethyltransferase activity, which functions in the photorespiratory pathway, catalyzes the conversion of serine and tetrahydrofolate to glycine and 5,10-methylene tetrahydrofolate. Involved in controlling cell damage caused by abiotic stress, such as high light and salt and the hypersensitive defense response of plants.
AT4G38000	*DNA BINDING WITH ONE FINGER 4.7 (DOF4.7)*	DNA binding with one finger 4.7
AT4G38080		hydroxyproline-rich glycoprotein family protein
AT4G38300		glycosyl hydrolase family 10 protein
AT4G38400	*EXPANSIN-LIKE A2 (EXLA2)*	member of EXPANSIN-LIKE. Naming convention from the Expansin Working Group (Kende et al, 2004. Plant Mol Bio)
AT4G38420	*SKU5 SIMILAR 9 (sks9)*	SKU5 similar 9

AT4G38540		FAD/NAD(P)-binding oxidoreductase family protein
AT4G38680	*GLYCINE RICH PROTEIN 2 (GRP2)*	Encodes a glycine-rich protein that binds nucleic acids and promotes DNA melting. Its transcript and protein levels are up-regulated in response to cold treatment with protein levels peaking earlier in shoots (~10-14 days) than in roots (~21 days). It is normally expressed in meristematic regions and developing tissues where cell division occurs. RNAi and antisense lines with lower levels of CSP2/GRP2 transcripts flower earlier than wild type plants and have some defects in anther and seed development.
AT4G38690		PLC-like phosphodiesterases superfamily protein
AT4G38970	*FRUCTOSE-BISPHOSPHATE ALDOLASE 2 (FBA2)*	Protein is tyrosine-phosphorylated and its phosphorylation state is modulated in response to ABA in Arabidopsis thaliana seeds.
AT4G39010	*GLYCOSYL HYDROLASE 9B18 (GH9B18)*	Cellulase involved in cell wall modification during valve dehiscence.
AT4G39070	*BZS1 (BZS1)*	Encodes BZS1, a brassinosteroids-regulated BZR1 target (BRBT) gene. BZS1 is a putative zinc finger transcription factor. Expression of BZS1 was increased under BR-deficient condition and repressed by BR. Transgenic Arabidopsis plants overexpressing BZS1 showed a hypersensitivity to the BR biosynthetic inhibitor brassinazole (BRZ). In contrast, transgenic plants expressing reduced level of BZS1 had longer hypocotyls than wild type when grown on BRZ.
AT4G39180	*SECRETION 14 (SEC14)*	encodes a protein that complements the function of a sec14(ts) mutant of S. cerevisiae
AT4G39420		spatacsin carboxy-terminus protein
AT4G39540	*SHIKIMATE KINASE 2 (SK2)*	Encodes a shikimate kinase. Its transcripts appear to be expressed in vegetative tissues and developing embryos. SK2 transcript levels rise in response to Phytophthora infestans spores. SK2 is believed to be localized to the chloroplast.
AT4G39670	*PHOSPHOLIPASE-LIKE PROTEIN (GLTP)*	Member of the glycolipid transfer protein (GLTP) superfamily, shuttles ceramide-1-phosphate (C1P) between membranes.
AT4G39675		hypothetical protein
AT4G39710	*PHOTOSYNTHETIC NDH SUBCOMPLEX L 4 (PnsL4)*	FK506-binding protein 16-2
AT4G39770	*TREHALOSE-6-PHOSPHATE PHOSPHATASE H (TPPH)*	Haloacid dehalogenase-like hydrolase (HAD) superfamily protein
AT4G39800	*D-MYO-INOSITOL 3-PHOSPHATE SYNTHASE 1 (MIPS1)*	** Referred to as MIPS2 in Mitsuhashi et al 2008. myo-inositol-1-phosphate synthase isoform 1.Expressed in leaf, root and silique. Immunolocalization experiments with an antibody recognizing MIPS1, MIPS2, and MIPS3 showed endosperm localization.
AT4G39890	*RAB GTPASE HOMOLOG H1C (RABH1c)*	RAB GTPase homolog H1C
AT4G39900		adenine deaminase
AT4G39955		alpha/beta-Hydrolases superfamily protein
AT4G39970		Haloacid dehalogenase-like hydrolase (HAD) superfamily protein
AT4G40020		Myosin heavy chain-related protein
AT5G01030		enolase, putative (DUF3527)
AT5G01040	*LACCASE 8 (LAC8)*	putative laccase, knockout mutant showed early flowering
AT5G01210		HXXXD-type acyl-transferase family protein
AT5G01530	*LIGHT HARVESTING COMPLEX PHOTOSYSTEM II (LHCB4.1)*	light harvesting complex photosystem II
AT5G01610		hypothetical protein (Protein of unknown function, DUF538)
AT5G01790		hypothetical protein
AT5G01870		Predicted to encode a PR (pathogenesis-related) protein. Belongs to the lipid transfer protein (PR-14) family with the following members: At2g38540/LTP1, At2g38530/LTP2, At5g59320/LTP3, At5g59310/LTP4, At3g51600/LTP5, At3g08770/LTP6, At2g15050/LTP7, At2g18370/LTP8, At2g15325/LTP9, At5g01870/LTP10, At4g33355/LTP11, At3g51590/LTP12, At5g44265/LTP13, At5g62065/LTP14, At4g08530/LTP15.
AT5G02070		Protein kinase family protein
AT5G02120	*ONE-HELIX LHC-LIKE PROTEIN 1 (OHP1)*	Encodes a one helix protein homologous to cyanobacterial high-light inducible proteins. The protein is localized to the thylakoid membrane and its transcript is transiently induced by exposure to high light conditions. The mRNA is cell-to-cell mobile.
AT5G02160	*FTSH5 INTERACTING PROTEIN (FIP)*	Zinc-finger domain containing protein involved in abiotic stress response. Possesses an N-terminal transit peptide followed by a hydrophobic domain and a zinc-finger domain. Despite the presence of a zinc-finger domain (C4-type) with two CXXCXGXG conserved repeats, characteristic of DNAJ protein, the conserved J domain is absent in FIP. Interacts with FtsH5. Gene expression levels are reduced and negatively regulates stress response genes during stress conditions.
AT5G02210		GCK domain-containing protein
AT5G02260	*EXPANSIN A9 (EXPA9)*	member of Alpha-Expansin Gene Family. Naming convention from the Expansin Working Group (Kende et al, 2004. Plant Mol Bio)
AT5G02430		Transducin/WD40 repeat-like superfamily protein
AT5G02540		NAD(P)-binding Rossmann-fold superfamily protein
AT5G02550		hypothetical protein
AT5G02560	*HISTONE H2A 12 (HTA12)*	Encodes HTA12, a histone H2A protein.
AT5G02580		argininosuccinate lyase
AT5G02790	*GLUTATHIONE TRANSFERASE L3 (GSTL3)*	GST functions in reductive deglutathionylation of glutathione conjugates of quercetin.
AT5G03350	*SA-INDUCED LEGUME LECTIN-LIKE PROTEIN 1 (SAI-LLP1)*	Belongs to the group of early SA-activated genes. Involved in resistance to Pst Avr-Rpm1 as a component of the SA35 mediated defense processes associated to the ETI response. Involved in resistance to P.syringae pv. tomato Avr-Rpm1 in Arabidopsis, as a component of the SA-mediated defense processes associated with the effector-triggered immunity response.
AT5G03560		Tetratricopeptide repeat (TPR)-like superfamily protein
AT5G03610	*(GGL25)*	GDSL-motif esterase/acyltransferase/lipase. Enzyme group with broad substrate specificity that may catalyze acyltransfer or hydrolase reactions with lipid and non-lipid substrates.
AT5G03940	*CHLOROPLAST SIGNAL RECOGNITION PARTICLE 54 KDA SUBUNIT (CPSRP54)*	mutant has Yellow first leaves; Chloroplast Signal Recognition Particle Subunit
AT5G04000		hypothetical protein
AT5G04020		calmodulin binding protein
AT5G04080	*CYSTEINE-RICH TRANSMEMBRANE MODULE 12 (ATHCYSTM12)*	cysteine-rich TM module stress tolerance protein

AT5G04120		Encodes a cofactor-dependent phosphoglycerate mutase (dPGM) - like protein with phosphoserine phosphatase activity that may be responsible for serine anabolism.
AT5G04310	*PECTATE LYASE LIKE12 (PLL12)*	Pectin-modifying gene involved in guard cell wall modifications that are important for proper maintenance of turgor pressure and stomatal movement.
AT5G04380		S-adenosyl-L-methionine-dependent methyltransferases superfamily protein
AT5G04490	*VITAMIN E PATHWAY GENE 5 (VTE5)*	Encodes a protein with phytol kinase activity involved in tocopherol biosynthesis.
AT5G04620	*BIOTIN F (BIOF)*	The cDNA encoding 7-keto-8-aminopelargonic acid (KAPA) synthase, the first committed enzyme of the biotin synthesis pathway has been cloned and its molecular function confirmed (functional complementation of an E. coli mutant). The subcellular localization of the enzyme (cytosol) proves that the biotin biosynthesis in plants takes place in different compartments which differs from the biosynthetic route found in microorganisms.
AT5G04770	*CATIONIC AMINO ACID TRANSPORTER 6 (CAT6)*	Encodes a member of the cationic amino acid transporter (CAT) subfamily of amino acid polyamine choline transporters. Does not mediate efficient uptake of basic amino acids in yeast or Xenopus systems but can transport neutral and acidic amino acid analogs. Expressed in sink tissues. Induced during infestation of roots by the plant parasitic root-knot nematode, Meloidogyne incognita. Localized in the plasma membrane.
AT5G04810	*PENTATRICOPEPTIDE REPEAT 4 (PPR4)*	Pentatricopeptide which is essential during the early stages of embryo development and acts in the plastid nucleoids as the factor responsible of rps12 intron 1 trans-splicing and, indirectly, in the assembly of 70S ribosomes and plastid translation.
AT5G05460	*ENDO-BETA-N-ACETYGLUCOSAMINIDASE 85A (ENGase85A)*	Encodes a cytosolic beta-endo-N-acetyglucosaminidase (ENGase). ENGases N-glycans cleave the O-glycosidic linkage between the two GlcNAc residues of the N-glycan core structure and thus generate a protein with a single GlcNAc attached to asparagine.
AT5G05480		Peptide-N4-(N-acetyl-beta-glucosaminyl)asparagine amidase A protein
AT5G05550	*VFP5 (VFP5)*	Encodes trihelix-domain transcription factor VFP5. Interacts with agrobacterium virulence protein VirF.
AT5G05740	*ETHYLENE-DEPENDENT GRAVITROPISM-DEFICIENT AND YELLOW-GREEN-LIKE 2 (EGY2)*	S2P-like putative metalloprotease, also contain transmembrane helices near their C-termini and many of them, five of seven, contain a conserved zinc-binding motif HEXXH. Homolog of EGY1. Each of the EGY1 and EGY-like proteins share two additional highly conserved motifs, the previously reported NPDG motif (aa 442?454 in EGY1, Rudner et al., 1999) and a newly defined GNLR motif (aa 171?179 in EGY1). The GNLR motif is a novel signature motif unique to EGY1 and EGY-like proteins as well as other EGY1 orthologs found in cyanobacteria.
AT5G05900		UDP-Glycosyltransferase superfamily protein
AT5G05990		Mitochondrial glycoprotein family protein
AT5G06190		transmembrane protein
AT5G06270	*(GIR1)*	One of two plant specific paralogs of unknown function. Interacts with GL2. GIR1/GIR2 loss of function resembles gl2 lof mutations
AT5G06290	*2-CYSTEINE PEROXIREDOXIN B (2-Cys Prx B)*	Encodes a 2-Cys peroxiredoxin (2-Cys PrxB) that contains two catalytic Cys residues. The mRNA is cell-to-cell mobile.
AT5G06550	*JUMONJI DOMAIN-CONTAINING PROTEIN 22 (JMJ22)*	Encodes a HR demethylase that acts as a positive regulator of seed germination in the PHYB-PIL5-SOM pathway.
AT5G06710	*HOMEOBOX FROM ARABIDOPSIS THALIANA (HAT14)*	Homeobox-leucine zipper protein.
AT5G06800		myb-like HTH transcriptional regulator family protein
AT5G06840		
AT5G06870	*POLYGALACTURONASE INHIBITING PROTEIN 2 (PGIP2)*	Encodes a polygalacturonase inhibiting protein involved in plant defense response. PGIPs inhibit the activity of pectin degrading enzymes such as those produced by fungal pathogens. PGIP2 is induced by fungal infection and methyl jasmonate.Suppressed in the proton sensitive stop1-mutant, but the transcription level was recovered by transformation of STOP2. Knockout mutant showed severe damage in the root tip in low Ca and low pH medium.
AT5G07060	*MOS4-ASSOCIATED COMPLEX SUBUNIT 5C (MAC5C)*	Encodes MAC5C, homologous to MAC5A. MAC5A is a component of the MOS4-associated complex (MAC) that contributes to snc1- mediated autoimmunity. Homologues include AT1G07360 (MAC5A), AT2G29580 (MAC5B) and AT5G07060 (MAC5C). MAC5A and MAC5B are more closely related to each other than to MAC5C.
AT5G07100	*WRKY DNA-BINDING PROTEIN 26 (WRKY26)*	Encodes WRKY DNA-binding protein 26 (WRKY26).
AT5G07110	*PRENYLATED RAB ACCEPTOR 1.B6 (PRA1.B6)*	Encodes PRA1.B6, an isoform of the PRA1 (Prenylated Rab acceptors) family. PRAs bind to prenylated Rab proteins and possibly aids in targeting Rabs to their respective compartments. PRA1.B6 localizes to the Golgi apparatus and its ER-to-Golgi trafficking and localization to the Golgi apparatus are regulated by multiple sequence motifs in both the C- and N-terminal cytoplasmic domains.
AT5G07150		Leucine-rich repeat protein kinase family protein
AT5G07200	*GIBBERELLIN 20-OXIDASE 3 (GA20OX3)*	encodes a gibberellin 20-oxidase.
AT5G07310	*ETHYLENE RESPONSE FACTOR 115 (ERF115)*	encodes a member of the ERF (ethylene response factor) subfamily B-4 of ERF/AP2 transcription factor family. The protein contains one AP2 domain. There are 7 members in this subfamily. Cytokinin production induced by jasmonate represses adventitious rooting.
AT5G07500	*(PEI1)*	Encodes an embryo-specific zinc finger transcription factor required for heart-stage embryo formation.
AT5G07780	*FORMIN HOMOLOG 19 (FH19)*	Encodes a class II formin that nucleates actin assembly, binds to the barbed-end of actin filaments and antagonizes the effect of FH1 on actin dynamics. The mRNA is cell-to-cell mobile.
AT5G08000	*GLUCAN ENDO-1,3-BETA-GLUCOSIDASE-LIKE PROTEIN 3 (E13L3)*	Encodes a member of the X8-GPI family of proteins. It localizes to the plasmodesmata and binds callose.
AT5G08030	*GLYCEROPHOSPHODIESTER PHOSPHODIESTERASE 6 (GDPD6)*	Encodes a member of the glycerophosphodiester phosphodiesterase (GDPD) family.
AT5G08050	*(RIQ1)*	Encodes a grana core localized protein. Mutant plants have reduced NPQ, affected organization of light-havesting complex II and an enhanced grana stacking.
AT5G08415	*LIPOYL SYNTHASE 1 (LIP1)*	Radical SAM superfamily protein
AT5G08460		GDSL-motif esterase/acyltransferase/lipase. Enzyme group with broad substrate specificity that may catalyze acyltransfer or hydrolase reactions with lipid and non-lipid substrates.
AT5G08480		VQ motif-containing protein
AT5G09270		transmembrane protein
AT5G09400	*K+ UPTAKE PERMEASE 7 (KUP7)*	Encodes a potassium uptake permease with a functional adenylate cyclase (AC) center. The first 100 aa of this protein can complement AC-deficient E. coli and display AC activity in vitro. KUP7 is localized to the plasma membrane where it functions in potassium uptake and translocation.
AT5G09480		hydroxyproline-rich glycoprotein family protein
AT5G09530	*PRO-GLU-LEU|ILE|VAL-PRO-LYS 1 (PELPK1)*	The gene encodes a unique protein which contains 36 repeats of a unique pentapeptide (Pro-Glu-Leu|Ile|Val-Pro-Lys). It has been shown tobe involved in growth and development.

AT5G09570	*(AT12CYS-2)*	Twin CX9C domain protein. Induced by low phosphate or iron, drought and heat stress. Loss of both At12cys-1 and At12cys-2 lead to enhanced tolerance to drought and light stress and increased anti-oxidant capacity.
AT5G09660	*PEROXISOMAL NAD-MALATE DEHYDROGENASE 2 (PMDH2)*	encodes a microbody NAD-dependent malate dehydrogenase encodes an peroxisomal NAD-malate dehydrogenase that is involved in fatty acid beta-oxidation through providing NAD to the process of converting fatty acyl CoA to acetyl CoA.
AT5G09670		loricrin-like protein
AT5G09710		Magnesium transporter CorA-like family protein
AT5G09840	*MITOCHONDRIAL NUCLEASE2 (MNU2)*	Putative endonuclease or glycosyl hydrolase
AT5G09970	*CYTOCHROME P450, FAMILY 78, SUBFAMILY A, POLYPEPTIDE 7 (CYP78A7)*	Member of CYP78A family. Paralog of CYP78A5 and appears to function in a shoot meristem maintainence pathway with LAMP1 that parallels AMP1/CYP87A5.
AT5G09990	*ELICITOR PEPTIDE 5 PRECURSOR (PROPEP5)*	elicitor peptide 5 precursor
AT5G10030	*TGACG MOTIF-BINDING FACTOR 4 (TGA4)*	Encodes a member of basic leucine zipper transcription gene family. Nomenclature according to Xiang, et al. (1997).
AT5G10040	*HYPOXIA RESPONSE UNKNOWN PROTEIN 9 (HUP9)*	transmembrane protein
AT5G10130		Pollen Ole e 1 allergen and extensin family protein
AT5G10160		Thioesterase superfamily protein
AT5G10180	*SULFATE TRANSPORTER 2;1 (SULTR2;1)*	Encodes a low-affinity sulfate transporter expressed in the root cap and central cylinder, where it is induced by sulfur starvation. Expression in the shoot vascular system is not induced by sulfur starvation.
AT5G10210		nitric oxide synthase-interacting protein
AT5G10230	*ANNEXIN 7 (ANNAT7)*	Encodes a calcium-binding protein annexin (AnnAt7).
AT5G10520	*ROP BINDING PROTEIN KINASES 1 (RBK1)*	ROP binding protein kinases 1
AT5G10770		Eukaryotic aspartyl protease family protein
AT5G10970		C2H2 and C2HC zinc fingers superfamily protein
AT5G11110	*SUCROSE PHOSPHATE SYNTHASE 2F (SPS2F)*	Encodes a sucrose-phosphate synthase involved in pollen exine formation. This is the dominant SPS isoform in leaves with respect to protein levels.
AT5G11160	*ADENINE PHOSPHORIBOSYLTRANSFERASE 5 (APT5)*	adenine phosphoribosyltransferase 5
AT5G11550		ARM repeat superfamily protein
AT5G11670	*NADP-MALIC ENZYME 2 (NADP-ME2)*	The malic enzyme (EC 1.1.1.40) encoded by AtNADP-ME2 is presumably a cytosolic enzyme involved in malate metabolism and possibly assisting the oxidative pentose phosphate pathway. AtNADP-ME2 counts for the major part of NADP-ME activity in mature tissues of Arabidopsis.
AT5G12030	*HEAT SHOCK PROTEIN 17.6A (HSP17.6A)*	Encodes a cytosolic small heat shock protein with chaperone activity that is induced by heat and osmotic stress and is also expressed late in seed development.
AT5G12210	*RAB GERANYLGERANYL TRANSFERASE BETA SUBUNIT 1 (RGTB1)*	Encodes the Rab geranylgeranyl transferase beta subunit that is essential for embryo and seed development.
AT5G12340		PADRE protein up-regulated after infection by S. sclerotiorum.
AT5G13140		Pollen Ole e 1 allergen and extensin family protein
AT5G13490	*ADP/ATP CARRIER 2 (AAC2)*	Encodes mitochondrial ADP/ATP carrier
AT5G13630	*GENOMES UNCOUPLED 5 (GUN5)*	Encodes magnesium chelatase involved in plastid-to-nucleus signal transduction.
AT5G13740	*ZINC INDUCED FACILITATOR 1 (ZIF1)*	Encodes ZIF1 (ZINC-INDUCED FACILITATOR1), a member of the Major Facilitator Superfamily (MFS) of membrane proteins which are found in all organisms and transport a wide range of small, organic molecules. Involved in a mechanism of Zn sequestration, possibly by transport of a Zn ligand or Zn-ligand complex into vacuoles. The mRNA is cell-to-cell mobile.
AT5G13830	*TRNA METHYLTRANSFERASE 7C (TRM7C)*	FtsJ-like methyltransferase family protein
AT5G14000	*NAC DOMAIN CONTAINING PROTEIN 84 (NAC084)*	NAC domain containing protein 84
AT5G14070	*(ROXY2)*	Encodes a member of the CC-type glutaredoxin (ROXY) family that has been shown to interact with the transcription factor TGA2 and suppress ORA59 promoter activity. ROXY2, together with ROXY1 (AT3G02000), controls anther development. roxy1 roxy2 double mutants are sterile and do not produce pollen.
AT5G14190		
AT5G14370		CCT motif family protein
AT5G14570	*HIGH AFFINITY NITRATE TRANSPORTER 2.7 (NRT2.7)*	Encodes ATNRT2.7, a nitrate transporter that controls nitrate content in seeds. Expression is detected in reproductive organs and peaks in seeds. Localized to the vacuolar membrane.
AT5G14580		polyribonucleotide nucleotidyltransferase
AT5G14610	*RNA HELICASE 46 (RH46)*	DEAD box RNA helicase family protein
AT5G14750	*MYB DOMAIN PROTEIN 66 (MYB66)*	Encodes a MyB-related protein containing R2 and R3 repeats, involved in root and hypocotyl epidermal cell fate determination. Loss of function mutations make extra root hairs. Nuclear localized protein is a positive regulator for expression of CAPRICE (CPC).
AT5G14800	*PYRROLINE-5- CARBOXYLATE (P5C) REDUCTASE (P5CR)*	Delta 1-pyrroline-5-carboxylate reductase, catalyzes the final step in proline biosynthesis from glutamate and ornithine.In situ hybridization indicated that under normal growth conditions, the highest concentration of P5CR transcripts occurs in the cortical parenchyma, phloem, vascular cambium and pith parenchyma in the vicinity of the protoxylem. Single gene in Arabidopsis.
AT5G14880	*POTASSIUM UPTAKE 8 (KUP8)*	Potassium transporter family protein
AT5G14920	*A-STIMULATED IN ARABIDOPSIS 14 (GASA14)*	Encodes a GASA domain containing protein that regulates increases in plant growth through GA-induced and DELLA-dependent signal transduction and that can increase abiotic stress resistance by reducing ROS accumulation.
AT5G14940		Major facilitator superfamily protein
AT5G15160	*BANQUO 2 (BNQ2)*	BNQ2 belongs to a family of atypical non-DNA binding basic helix-loop-helix (bHLH) proteins that heterodimerize with and negatively regulate bHLH transcription factors. Directly and negatively regulated by AP3 and PI in petals.Required for appropriate regulation of flowering time.
AT5G15240		Transmembrane amino acid transporter family protein
AT5G15530	*BIOTIN CARBOXYL CARRIER PROTEIN 2 (BCCP2)*	biotin carboxyl carrier protein isoform 2 (BCCP2) mRNA,
AT5G15550	*ARABIDOPSIS THALIANA PESCADILLO ORTHOLOG (ATPEP2)*	Transducin/WD40 repeat-like superfamily protein

AT5G15600	*SPIRAL1-LIKE4 (SP1L4)*	SPIRAL1-LIKE4 belongs to a six-member gene family in Arabidopsis; all members share high sequence similarity in amino- and carboxy-terminal regions. Regulates cortical microtubule organization. Mutant plants exhibit altered patterns of root, leaf and petal growth as a result of defective anisotropic cell expansion.
AT5G15700		Nucleus encoded plastid RNA polymerase. Localized in mitochondria and chloroplast.
AT5G15725	*GOLVEN 9 (GLV9)*	GOLVEN (GLV)/ROOT GROWTH FACTORS/CLE-Like small signaling peptide. Expressed in roots. Overexpression results in altered root gravitropism.
AT5G15740	*RG-I RHAMNOSYLTRANSFERASE 1 (RRT1)*	RRT1 is a member of a novel glycosyltransferase famly in plants. It functions as a rhamnosyltransferase, elongating the RG-1 backbone. It functions during seed coat mucilage development.
AT5G15780		Pollen Ole e 1 allergen and extensin family protein
AT5G15830	*BASIC LEUCINE-ZIPPER 3 (bZIP3)*	basic leucine-zipper 3
AT5G15840	*CONSTANS (CO)*	Encodes a protein showing similarities to zinc finger transcription factors, involved in regulation of flowering under long days. Acts upstream of FT and SOC1.
AT5G15850	*CONSTANS-LIKE 1 (COL1)*	Homologous to the flowering-time gene CONSTANS.
AT5G15950	*S-ADENOSYLMETHIONINE DECARBOXYLASE 2 (SAMDC2)*	Adenosylmethionine decarboxylase family protein
AT5G16030		mental retardation GTPase activating protein
AT5G16150	*PLASTIDIC GLC TRANSLOCATOR (PGLCT)*	Encodes a putative plastidic glucose transporter.
AT5G16240	*ACYL-ACYL CARRIER PROTEIN DESATURASE1 (AAD1)*	Redundant Δ9 stearoyl-ACP desaturase gene which together with FAB2 and AAD5 during embryo development provide precursors for the elaboration of embryo cuticle and therefore plays a specific role during the phase of invasive embryo growth through the endosperm. Together with FAB2, AAD5, and AAD6 redundantly participates in oil storage during the maturation phase.
AT5G16340		AMP-dependent synthetase and ligase family protein
AT5G16400	*THIOREDOXIN F2 (TRXF2)*	Encodes an f-type thioredoxin (Trx-f2) localized in chloroplast stroma.
AT5G16450		Ribonuclease E inhibitor RraA/Dimethylmenaquinone methyltransferase
AT5G16560	*KANADI (KAN)*	Encodes a KANADI protein (KAN) that regulates organ polarity in Arabidopsis. KAN is required for abaxial identity in both leaves and carpels, and encodes a nuclear-localized protein in the GARP family of putative transcription factors. Together with KAN2, this gene appears to be involved in the development of the carpel and the outer integument of the ovule.Along with KAN2 and KAN4, KAN1 appears to be required for proper regulation of PIN1 in early embryogenesis.
AT5G16570	*GLUTAMINE SYNTHETASE 1;4 (GLN1;4)*	Encodes a cytosolic glutamine synthetase, the enzyme has high affinity for the substrate ammonium.
AT5G16600	*MYB DOMAIN PROTEIN 43 (MYB43)*	Encodes a transcriptional regulator that directly activates lignin biosynthesis genes and phenylalanine biosynthesis genes during secondary wall formation.
AT5G16970	*ALKENAL REDUCTASE (AER)*	encodes a 2-alkenal reductase (EC 1.3.1.74), plays a key role in the detoxification of reactive carbonyls
AT5G17000		Zinc-binding dehydrogenase family protein
AT5G17050	*UDP-GLUCOSYL TRANSFERASE 78D2 (UGT78D2)*	The At5g17050 encodes a anthocyanidin 3-O-glucosyltransferase which specifically glucosylates the 3-position of the flavonoid C-ring. Anthocyanidins such as cyanidin and pelargonidin as well as flavonols such as kaempferol and quercetin are accepted substrates.
AT5G17170	*ENHANCER OF SOS3-1 (ENH1)*	rubredoxin family protein
AT5G17220	*GLUTATHIONE S-TRANSFERASE PHI 12 (GSTF12)*	Encodes glutathione transferase belonging to the phi class of GSTs. Naming convention according to Wagner et al. (2002). Mutants display no pigments on leaves and stems. Likely to function as a carrier to transport anthocyanin from the cytosol to tonoplasts.
AT5G17330	*GLUTAMATE DECARBOXYLASE (GAD)*	Encodes one of two isoforms of glutamate decarboxylase. The mRNA is cell-to-cell mobile.
AT5G17760		P-loop containing nucleoside triphosphate hydrolases superfamily protein
AT5G17970		Disease resistance protein (TIR-NBS-LRR class) family
AT5G18340	*PLANT U-BOX 48 (PUB48)*	One of three tandemly located, paralogous plant U-box proteins. Mutants show increased sensitivity to water stress. E3 ligase which acts as a regulator in the heat response signaling pathway. Over-expressing AtPUB48 could induce the expression of the heat-related genes (HSP101, HSP70, HSP25.3, HSFA2, and ZAT12). Enhances plant resistance to heat stress during seed germination and seedling growth.
AT5G18560	*(PUCHI)*	Encodes PUCHI, a member of the ERF (ethylene response factor) subfamily B-1 of ERF/AP2 transcription factor family. The protein contains one AP2 domain. There are 15 members in this subfamily including ATERF-3, ATERF-4, ATERF-7, and leafy petiole. PUCHI is required for morphogenesis in the early lateral root primordium of Arabidopsis. Expressed in early floral meristem (stage 1 to 2). Required for early floral meristem growth and for bract suppression. Triple mutant with bop1 and bop2 displays a strong defect in the determination of floral meristem identity with reduced LFY expression and the lack of AP1 expression.
AT5G18660	*PALE-GREEN AND CHLOROPHYLL B REDUCED 2 (PCB2)*	Encodes a protein with 3,8-divinyl protochlorophyllide a 8-vinyl reductase activity. Mutants accumulate divinyl chlorophyll rather than monovinyl chlorophyll.
AT5G18670	*BETA-AMYLASE 3 (BMY3)*	putative beta-amylase BMY3 (BMY3)
AT5G19140	*(AILP1)*	aluminum induced protein with YGL and LRDR motifs
AT5G19220	*ADP GLUCOSE PYROPHOSPHORYLASE LARGE SUBUNIT 1 (APL1)*	Encodes the large subunit of ADP-glucose pyrophosphorylase which catalyzes the first, rate limiting step in starch biosynthesis. The large subunit plays a regulatory role whereas the small subunit (ApS) is the catalytic isoform. Four isoforms (ApL1-4) have been identified. ApL1 is the major large subunit isoform present in leaves. Mutational analysis of APS1 suggests that APL1 and APL2 can compensate for loss of APS1 catalytic activity,suggesting both have catalytic as well as regulatory functions.
AT5G19260	*FANTASTIC FOUR 3 (FAF3)*	A member of the FAF family proteins encoded by the FANTASTIC FOUR (FAF) genes: AT4G02810 (FAF1), AT1G03170 (FAF2), AT5G19260 (FAF3) and AT3G06020 (FAF4). FAFs have the potential to regulate shoot meristem size in Arabidopsis thaliana. FAFs can repress WUS, which ultimately leads to an arrest of meristem activity in FAF overexpressing lines.
AT5G19600	*SULFATE TRANSPORTER 3;5 (SULTR3;5)*	Encodes sulfate transporter Sultr3;5.
AT5G19700	*EARLY LEAF SENESCENCE 1 (ELS1)*	Encodes a MATE transporter involved in leaf senescence and iron homeostasis.
AT5G19930	*PLASMA MEMBRANE GLUCOSE-RESPONSIVE REGULATOR (PGR)*	PGR is putative plasma membrane glucose- responsive regulator that is expressed in response to glucose stimulation.RNAi knockdown mutant seeds have enhanced sensitivity to glucose and 2-deoxyglucose.
AT5G19950		tudor domain protein (DUF1767)
AT5G20230	*BLUE-COPPER-BINDING PROTEIN (BCB)*	Encodes a Al-stress-induced gene. Along with TCF, it promotes lignin biosynthesis in response to cold stress. The mRNA is cell-to-cell mobile.
AT5G20250	*DARK INDUCIBLE 10 (DIN10)*	encodes a member of glycosyl hydrolase family 36. Expression is induced within 3 hours of dark treatment, in senescing leaves and treatment with exogenous photosynthesis inhibitor. Induction of gene expression was suppressed in excised leaves supplied with sugar. The authors suggest that the gene's expression pattern is responding to the level of sugar in the cell. The mRNA is cell-to-cell mobile.

AT5G20400		encodes a protein whose sequence is similar to flavanone 3 hydroxylase from Malus. The mRNA is cell-to-cell mobile.
AT5G20480	*EF-TU RECEPTOR (EFR)*	Encodes a predicted leucine-rich repeat receptor kinase (LRR-RLK). Functions as the receptor for bacterial PAMP (pathogen associated molecular patterns) EF-Tu.
AT5G20700		senescence-associated family protein, putative (DUF581)
AT5G20800		transposable_element_gene;pseudogene, similar to putative reverse transcriptase, predicted non-LTR reverse ranscriptase sequence fragments;(source:TAIR10)
AT5G20830	*SUCROSE SYNTHASE 1 (SUS1)*	Encodes a protein with sucrose synthase activity (SUS1).
AT5G20940		Glycosyl hydrolase family protein
AT5G21150	*ARGONAUTE 9 (AGO9)*	AGO9-dependent sRNA silencing is crucial to specify cell fate in the Arabidopsis ovule. AGO9 is expressed in reproductive companion cells but not in the associated male or female gametes or their precursors. Therefore, AGO9 acts non-cell autonomously to silencing the activity of TEs activity in the female gametophyte.Loss of function mutants produce ectopic megaspore mother cell and supernumary female gametophytes.
AT5G22390		FANTASTIC four-like protein (DUF3049)
AT5G22460		alpha/beta-Hydrolases superfamily protein
AT5G22550		transmembrane protein, putative (DUF247)
AT5G22580		Stress responsive A/B Barrel Domain-containing protein
AT5G22870		Late embryogenesis abundant (LEA) hydroxyproline-rich glycoprotein family
AT5G22890	*SENSITIVE TO PROTON RHIZOTOXICITY 2 (STOP2)*	An unique homologue of STOP1 (AT1G34370) in Arabidopsis genome. Transformation to the stop1-mutant activated several genes that are regulated by STOP1, and conferred proton sensitive phenotype.
AT5G23010	*METHYLTHIOALKYLMALATE SYNTHASE 1 (MAM1)*	Encodes a methylthioalkylmalate synthase, catalyzes the condensation reactions of the first two rounds of methionine chain elongation in the biosynthesis of methionine-derived glucosinolates. The mRNA is cell-to-cell mobile.
AT5G23020	*2-ISOPROPYLMALATE SYNTHASE 2 (IMS2)*	methylthioalkymalate synthase-like. Also known as 2-isopropylmalate synthase (IMS2). encodes a methylthioalkylmalate synthase involved in the biosynthesis of aliphatic glucosinolates which accepts all the omega-methylthio-2-oxoalkanoic acids needed to form the known C3 to C8 glucosinolates in Arabidopsis. The mRNA is cell-to-cell mobile.
AT5G23220	*NICOTINAMIDASE 3 (NIC3)*	nicotinamidase 3
AT5G23380		hypothetical protein (DUF789)
AT5G23680		Sterile alpha motif (SAM) domain-containing protein
AT5G23730	*REPRESSOR OF UV-B PHOTOMORPHOGENESIS 2 (RUP2)*	Encodes REPRESSOR OF UV-B PHOTOMORPHOGENESIS 2 (RUP2). Functions as a repressor of UV-B signaling.
AT5G23820	*MD2-RELATED LIPID RECOGNITION 3 (ML3)*	ML3 can be modified by NEDD8 and ubiquitin. ML3 expression is regulated by NAI1. ML3 expression is regulated by MeJA, ethylene and wounding. ml3-3 is more susceptible against infections with Alternaria brassicicola and more resistant against infections with Pseudomonas syringae DC3000.
AT5G23890		GPI-anchored adhesin-like protein
AT5G23920		transmembrane protein
AT5G24030	*SLAC1 HOMOLOGUE 3 (SLAH3)*	Encodes a protein with ten predicted transmembrane helices. The SLAH3 protein has similarity to the SLAC1 protein involved in ion homeostasis in guard cells. Although it is not expressed in guard cells, it can complement an slac1-2 mutant suggesting that it performs a similar function. SLAH3:GFP localizes to the plasma membrane.
AT5G24070		Peroxidase superfamily protein
AT5G24160	*SQUALENE MONOXYGENASE 6 (SQE6)*	squalene monooxygenase 6
AT5G24165		hypothetical protein
AT5G24270	*SALT OVERLY SENSITIVE 3 (SOS3)*	encodes a calcium sensor that is essential for K+ nutrition, K+/Na+ selectivity, and salt tolerance. The protein is similar to calcineurin B. Lines carrying recessive mutations are hypersensitive to Na+ and Li+ stresses and is unable to grow in low K+. The growth defect is rescued by extracellular calcium.
AT5G24290	*MEMBRANE OF ER BODY 2 (MEB2)*	Vacuolar iron transporter (VIT) family protein
AT5G24316		proline-rich family protein
AT5G24490		30S ribosomal protein
AT5G24700		
AT5G24800	*BASIC LEUCINE ZIPPER 9 (BZIP9)*	Encodes bZIP protein BZO2H2.
AT5G24860	*FLOWERING PROMOTING FACTOR 1 (FPF1)*	encodes a small protein of 12.6 kDa that regulates flowering and is involved in gibberellin signalling pathway. It is expressed in apical meristems immediately after the photoperiodic induction of flowering. Genetic interactions with flowering time and floral organ identity genes suggest that this gene may be involved in modulating the competence to flower. There are two other genes similar to FPF1, FLP1 (At4g31380) and FLP2 (no locus name yet, on BAC F8F16 on chr 4). This is so far a plant-specific gene and is only found in long-day mustard, arabidopsis, and rice.
AT5G25450		Cytochrome bd ubiquinol oxidase, 14kDa subunit
AT5G25460	*DUF642 L-GALL RESPONSIVE GENE 2 (DGR2)*	Encodes a DUF642 cell wall protein.
AT5G25470		AP2/B3-like transcriptional factor family protein
AT5G25620	*YUCCA6 (YUC6)*	Encodes a member of a family of flavin monooxygenases with an important role in auxin biosynthesis. YUC6 possesses an additional thiol-reductase activity that confers drought resistance independently of auxin biosynthesis.
AT5G25810	*TINY (tny)*	encodes a member of the DREB subfamily A-4 of ERF/AP2 transcription factor family (TINY). The protein contains one AP2 domain. There are 17 members in this subfamily including TINY. Ectopic or overexpression of this gene in a Ds tagged line has reduced cell expansion. The expression of this gene is induced by ethylene and light and appears to stimulate cytokinin biosynthesis.
AT5G25820		Exostosin family protein
AT5G25890	*INDOLE-3-ACETIC ACID INDUCIBLE 28 (IAA28)*	encodes a protein that may be a negative regulator of lateral root formation in response to auxin. It is a member of IAA/ARF gene family and is plant-specific. Gain of function mutations in this gene suppresses lateral root formation and is resistant to inhibition of root elongation by auxin, cytokinin, and ethylene.
AT5G26200		Mitochondrial substrate carrier family protein
AT5G26220	*GAMMA-GLUTAMYL CYCLOTRANSFERASE 2;1 (GGCT2;1)*	ChaC-like family protein
AT5G26260		TRAF-like family protein

AT5G26280		TRAF-like family protein
AT5G26330		Cupredoxin superfamily protein
AT5G26340	*(MSS1)*	Encodes a protein with high affinity, hexose-specific/H+ symporter activity. The activity of the transporter appears to be negatively regulated by phosphorylation. Importantly, microarray analysis, as well as the study of the expression of this gene in mutants involved in programmed cell death (PCD) demonstrated a tight correlation between this gene's expression and PCD.
AT5G26655		
AT5G26790		transmembrane protein
AT5G26920	*CAM-BINDING PROTEIN 60-LIKE G (CBP60G)*	Encodes a calmodulin-binding protein CBP60g (calmodulin binding protein 60-like.g). The calmodulin-binding domain is located near the N-terminus; calmodulin binding is dependent on Ca(2+). Inducible by both bacterial pathogen and MAMP (microbe-associated molecular pattern) treatments. Bacterial growth is enhanced in cbp60g mutants. cbp60g mutants also show defects in salicylic acid (SA) accumulation and SA signaling.
AT5G26930	*GATA TRANSCRIPTION FACTOR 23 (GATA23)*	Encodes a member of the GATA factor family of zinc finger transcription factors. Controls lateral root founder cell specification.
AT5G26960		Galactose oxidase/kelch repeat superfamily protein
AT5G27000	*KINESIN 4 (ATK4)*	Encodes a kinesin-like protein that binds microtubules in an ATP-dependent manner.
AT5G27050	*AGAMOUS-LIKE 101 (AGL101)*	AGAMOUS-like 101
AT5G27360	*(SFP2)*	Encodes a sugar-porter family protein that unlike the closely related gene, SFP1, is not induced during leaf senescence.
AT5G27390		tagatose-6-phosphate ketose/aldose isomerase, putative (Mog1/PsbP/DUF1795-like photosystem II reaction center PsbP family protein)
AT5G27440		transmembrane protein
AT5G27660	*DEGRADATION OF PERIPLASMIC PROTEINS 14 (DEG14)*	Encodes a protein with similarity to human PARK13, a mitochondrial protease implicated in Parkinson disease. DEG14 is induced by heat stress and involved in degradation of misfolded proteins.
AT5G28080	*(WNK9)*	Encodes a member of the WNK family (9 members in all) of protein kinases, the structural design of which is clearly distinct from those of other known protein kinases, such as receptor-like kinases and mitogen-activated protein kinases.
AT5G28230		pseudogene of glucose-6-phosphate/phosphate translocator 2
AT5G28500		rubisco accumulation factor-like protein
AT5G28610		LOW protein: ATP-dependent RNA helicase DRS1-like protein
AT5G28640	*ANGUSTIFOLIA 3 (AN3)*	Encodes a protein with similarity to mammalian transcriptional coactivator that is involved in cell proliferation during leaf and flower development. Loss of function mutations have narrow, pointed leaves and narrow floral organs. AN3 interacts with members of the growth regulating factor (GRF) family of transcription factors.
AT5G28770	*(BZO2H3)*	BASIC LEUCINE ZIPPER protein which regulates the circadian oscillator gene PSEUDO RESPONSE REGULATOR7 (PRR7) to change the circadian phase in response to sugars. It upregulates PRR7 in response to low energy. bZIP63 and PRR7 are required for correct oscillator phase under light/dark cycles. bZIP protein BZO2H3 mRNA, partial cds
AT5G33370	*CUTIN SYNTHASE2 (CUS2)*	GDSL-motif esterase/acyltransferase/lipase. Enzyme group with broad substrate specificity that may catalyze acyltransfer or hydrolase reactions with lipid and non-lipid substrates. Mutants are defective in cuticle formation with reduced sepal cuticle ridge formation.
AT5G35360	*ACETYL CO-ENZYME A CARBOXYLASE BIOTIN CARBOXYLASE SUBUNIT (CAC2)*	Encodes biotin carboxylase subunit (CAC2).
AT5G35370		S-locus lectin protein kinase family protein
AT5G35380		kinase with adenine nucleotide alpha hydrolases-like domain-containing protein
AT5G35470		
AT5G35670	*IQ-DOMAIN 33 (iqd33)*	Member of IQ67 (CaM binding) domain containing family.
AT5G35735		Auxin-responsive family protein
AT5G35740		Carbohydrate-binding X8 domain superfamily protein
AT5G35790	*GLUCOSE-6-PHOSPHATE DEHYDROGENASE 1 (G6PD1)*	Encodes a plastidic glucose-6-phosphate dehydrogenase that is sensitive to reduction by DTT and whose mRNA is more prevalent in developing organs but absent in the root.
AT5G35940		Mannose-binding lectin superfamily protein
AT5G36160	*TYR AMINOTRANSFERASE 2 (TAT2)*	Encodes a cytosolic L-tyrosine aminotransferase. AtTAT2 exhibits much broader amino donor specificity than AtTAT1 and can use not only Tyr but also Phe, Trp, His, Met, Leu, Ala, Ser, Cys, Asp, Asn, Gln, and Arg as amino donors.
AT5G36910	*THIONIN 2.2 (THI2.2)*	Encodes a thionin that is expressed at a low basal level in seedlings and shows circadian variation. Predicted to encode a PR (pathogenesis-related) protein. Belongs to the plant thionin (PR-13) family with the following members: At1g66100, At5g36910, At1g72260, At2g15010, At1g12663, At1g12660.
AT5G36960		hypothetical protein
AT5G37440		Chaperone DnaJ-domain superfamily protein
AT5G37550		hypothetical protein
AT5G37600	*GLUTAMINE SYNTHASE CLONE R1 (GSR 1)*	encodes a cytosolic glutamine synthetase, the enzyme has high affinity with substrate ammonium
AT5G37690		SGNH hydrolase-type esterase superfamily protein
AT5G37740		Calcium-dependent lipid-binding (CaLB domain) family protein
AT5G37790		Protein kinase superfamily protein
AT5G37990	*(CIMT1)*	SABATH family methyltransferase.
AT5G38030	*DETOXIFICATION30 (DTX30)*	MATE transporter involved in auxin homeostasis in roots.
AT5G38140	*NUCLEAR FACTOR Y, SUBUNIT C12 (NF-YC12)*	nuclear factor Y, subunit C12
AT5G38390		F-box/RNI-like superfamily protein
AT5G38520	*CHLOROPHYLL DEPHYTYLASE1 (CLD1)*	CLD1 is involved in steady-state chlorophyll turnover; CLD1 dephytylates chlorophyll a, chlorophyll b, and pheophytin a in vitro; CLD1 and CHLG form a salvage cycle in recycling chlorophyll. Suppression of CLD1 expression results in reduced tolerance to moderately high temperature.
AT5G38700		cotton fiber protein

AT5G38770	*GLUTAMINE DUMPER 7 (GDU7)*	Encodes a member of the GDU (glutamine dumper) family proteins involved in amino acid export: At4g31730 (GDU1), At4g25760 (GDU2), At5g57685 (GDU3), At2g24762 (GDU4), At5g24920 (GDU5), At3g30725 (GDU6) and At5g38770 (GDU7).
AT5G38780		SABATH methyltransferase.
AT5G38890		Nucleic acid-binding, OB-fold-like protein
AT5G38970	*BRASSINOSTEROID-6-OXIDASE 1 (BR6OX1)*	Encodes a polypeptide involved in the C-6 oxidation of brassinosteroids. Heterologous expression of the protein in yeast conferred the ability to catalyze multiple reactions in which the C-6 position of 6-deoxocastasterone, 6-deoxotyphasterol, 3-dehydro-6-deoxoteasterone and 6-deoxoteasterone are oxidized.
AT5G39100	*GERMIN-LIKE PROTEIN 6 (GLP6)*	germin-like protein (GLP6)
AT5G39210	*CHLORORESPIRATORY REDUCTION 7 (CRR7)*	Encodes a protein of the chloroplastic NAD(P)H dehydrogenase complex (NDH Complex) involved in respiration, photosystem I (PSI) cyclic electron transport and CO2 uptake. The product of this gene appears to be essential for the stable formation of the NDH Complex. The mRNA is cell-to-cell mobile.
AT5G39270	*EXPANSIN A22 (EXPA22)*	expansin-like protein. Naming convention from the Expansin Working Group (Kende et al, 2004. Plant Mol Bio)
AT5G39310	*EXPANSIN A24 (EXPA24)*	member of Alpha-Expansin Gene Family. Naming convention from the Expansin Working Group (Kende et al, 2004. Plant Mol Bio)
AT5G39860	*PACLOBUTRAZOL RESISTANCE1 (PRE1)*	Encodes PRE1 (PACLOBUTRAZOL RESISTANCE1). PRE1 and IBH1 form a pair of antagonistic HLH/bHLH transcription factors that function downstream of BZR1 to mediate brassinosteroid regulation of cell elongation. BNQ1 is directly and negatively regulated by AP3 and PI in petals.Required for appropriate regulation of flowering time.
AT5G39930	*CLP1-SIMILAR PROTEIN 5 (CLPS5)*	Encodes a protein with similarity to the CLP1 polyadenylation factor.
AT5G40100		Disease resistance protein (TIR-NBS-LRR class) family
AT5G40320		Cysteine/Histidine-rich C1 domain family protein
AT5G40450	*REGULATOR OF BULB BIOGENESIS1 (RBB1)*	Encodes a member of a plant gene family, APK_ORTHOMCL5144,of unknown function. RBB1 is localized to the cytosol and involved in vacuolar biogenesis and organization. RBB1 mutants have increased number of vacuolar bulbs and fewer trans-vacuolar strands.
AT5G40610	*GLYCEROL-3-PHOSPHATE DEHYDROGENASE PLASTIDIC (GPDHP)*	NAD-dependent glycerol-3-phosphate dehydrogenase family protein
AT5G40660	*(P12)*	Encodes an F-type ATP Synthase Assembly factor that binds to beta subunits of mitochondrial ATPase.
AT5G40780	*LYSINE HISTIDINE TRANSPORTER 1 (LHT1)*	Encodes LHT1 (lysine histidine transporter), a high-affinity transporter for cellular amino acid uptake in both root epidermis and leaf mesophyll.
AT5G40850	*UROPHORPHYRIN METHYLASE 1 (UPM1)*	Encodes a urophorphyrin III methylase that catalyzes S-adenosyl-L-methionine-dependent transmethylation in a multistep process involving the formation of a covalently linked complex with S-adenosyl-L-methionine. The mRNA is cell-to-cell mobile.
AT5G40890	*CHLORIDE CHANNEL A (CLC-A)*	Encodes a member of the voltage-dependent chloride channel. Also functions as a NO3-/H+ exchanger that serves to accumulate nitrate nutrient in vacuoles. Mutants homozygous for the T-DNA insertion mutation have reduced nitrate uptake capacity in high nitrate environment and exhibit hypersensitivity to chlorate. Role in cytosolic pH homeostasis.
AT5G40950	*RIBOSOMAL PROTEIN LARGE SUBUNIT 27 (RPL27)*	ribosomal protein large subunit 27
AT5G41040	*REDUCED LEVELS OF WALL-BOUND PHENOLICS 1 (RWP1)*	Encodes a feruloyl-CoA transferase required for suberin synthesis. Has feruloyl-CoA-dependent feruloyl transferase activity towards substrates with a primary alcohol.
AT5G41400		RING/U-box superfamily protein
AT5G41410	*BELL 1 (BEL1)*	Homeodomain protein required for ovule identity.Loss of function mutations show homeotic conversion of integuments to carpels.Forms heterodimers with STM and KNAT1. Interacts with AG-SEP heterodimers is thought to restrict WUS expression. BEL interacts with MADS box dimers composed of SEP1(or SEP3) and AG, SHP1, SHP2 and STK. The interaction of BEL1 with AG-SEP3 is required for proper integument development and specification of integument identity.
AT5G41640		Mutants have decreased tolerance to osmotic stress.
AT5G41650	*GLYOXALASE I-LIKE;10 (GLXI-LIKE;10)*	Vicinal oxygen chelate (VOC) superfamily member. Responds to NaCl stress.
AT5G41810		Avr9/Cf-9 rapidly elicited protein
AT5G42030	*ABL INTERACTOR-LIKE PROTEIN 4 (ABIL4)*	ABL interactor-like protein 4
AT5G42070		hypothetical protein
AT5G42120	*L-TYPE LECTIN RECEPTOR KINASE S.6 (LECRK-S.6)*	Concanavalin A-like lectin protein kinase family protein
AT5G42150		Glutathione S-transferase family protein
AT5G42180	*PEROXIDASE 64 (PER64)*	Peroxidase required for casparian strip lignification as well as partially required for SGN-dependent compensatory lignification.
AT5G42250		Zinc-binding alcohol dehydrogenase family protein
AT5G42500		Disease resistance-responsive (dirigent-like protein) family protein
AT5G42590	*CYTOCHROME P450, FAMILY 71, SUBFAMILY A, POLYPEPTIDE 16 (CYP71A16)*	putative cytochrome P450
AT5G42630	*ABERRANT TESTA SHAPE (ATS)*	Encodes a member of the KANADI family of putative transcription factors. Involved in integument formation during ovule development and expressed at the boundary between the inner and outer integuments. It is essential for directing laminar growth of the inner integument.Along with KAN1 and KAN2, KAN4 is involved in proper localization of PIN1 in early embryogenesis.
AT5G43020		Leucine-rich repeat protein kinase family protein
AT5G43040		Cysteine/Histidine-rich C1 domain family protein
AT5G43330	*CYTOSOLIC-NAD-DEPENDENT MALATE DEHYDROGENASE 2 (c-NAD-MDH2)*	predicted to encode a cytosolic malate dehydrogenase. The mRNA is cell-to-cell mobile.
AT5G43580	*UNUSUAL SERINE PROTEASE INHIBITOR (UPI)*	Predicted to encode a PR (pathogenesis-related) peptide that belongs to the PR-6 proteinase inhibitor family. Functions in resistance to necrotrophic fungi and insect herbivory. Six putative PR-6-type protein encoding genes are found in Arabidopsis: At2g38900, At2g38870, At5g43570, At5g43580, At3g50020 and At3g46860.
AT5G43690		P-loop containing nucleoside triphosphate hydrolases superfamily protein
AT5G43700	*AUXIN INDUCIBLE 2-11 (ATAUX2-11)*	Auxin inducible protein similar to transcription factors.
AT5G43750	*PHOTOSYNTHETIC NDH SUBCOMPLEX B 5 (PnsB5)*	NAD(P)H dehydrogenase 18
AT5G43780	*(APS4)*	sulfate adenylyltransferase, ATP sulfurylase
AT5G44050	*(ATDTX28)*	MATE efflux family protein

AT5G44360	*(ATBBE23)*	FAD-binding Berberine family protein
AT5G44380	*(AtBBE24)*	FAD-binding Berberine family protein
AT5G44440	*(ATBBE28)*	FAD-binding Berberine family protein
AT5G44580	*(PROSCOOP10)*	transmembrane protein
AT5G44630		Encodes a sesquiterpene synthase involved in generating all of the group B sesquiterpenes found in the Arabidopsis floral volatile blend. Strongly expressed in intrafloral nectaries.
AT5G44700	*GASSHO 2 (GSO2)*	Encodes GASSHO2 (GSO2), a putative leucine-rich repeat transmembrane-type receptor kinase. GSO2 and a homolog GSO1 (At4g20140) are required for the formation of a normal epidermal surface during embryogenesis.
AT5G44870	*LAZARUS 5 (LAZ5)*	Encodes LAZ5, a TIR-class NB-LRR R protein of unknown pathogen specificity with sequence similarity to RPS4, an R protein conferring resistance to Pseudomonas syringae expressing the effector AvrRPS4. Overexpression of LAZ5 results in hypersensitive cell death (plants did not survive to set seeds).
AT5G45060		Disease resistance protein (TIR-NBS-LRR class) family
AT5G45280	*PECTIN ACETYLESTERASE 11 (PAE11)*	Pectin acetylesterase involved in pectin remodelling.
AT5G45410	*NON HOST RESISTANCE 2A (ATNHR2A)*	Plastid localized protein of unknown function. Mutants are more susceptible to P. syringae and produce less callose upon infection.
AT5G45800	*MATERNAL EFFECT EMBRYO ARREST 62 (MEE62)*	Leucine-rich repeat protein kinase family protein
AT5G45980	*WUSCHEL RELATED HOMEOBOX 8 (WOX8)*	Arabidopsis thaliana WOX8 protein. Contains similarity to homeodomain transcription factor. Positively regulates early embryonic growth. Together with CLE8 it forms a signaling module that promotes seed growth and overall seed size.
AT5G46110	*ACCLIMATION OF PHOTOSYNTHESIS TO ENVIRONMENT 2 (APE2)*	Encodes a chloroplast triose phosphate / 3-phosphoglycerate translocator that transports triose phosphates derived from the Calvin cycle in the stroma to the cytosol for use in sucrose synthesis and other biosynthetic processes. A tpt mutant has altered acclimation responses. The mRNA is cell-to-cell mobile.
AT5G46180	*ORNITHINE-DELTA-AMINOTRANSFERASE (DELTA-OAT)*	Encodes an ornithine delta-aminotransferase that is transcriptionally up-regulated in young seedlings and in response to salt stress. It is unlikely to play a role in salt-stress-induced proline accumulation, however, it appears to participate in arginine and ornithine catabolism.
AT5G46270		Disease resistance protein (TIR-NBS-LRR class) family
AT5G46290	*3-KETOACYL-ACYL CARRIER PROTEIN SYNTHASE I (KASI)*	Encodes beta-ketoacyl-[acyl carrier protein] synthase I (KASI). Crucial for fatty acid synthesis. Plays a role in chloroplast division and embryo development.
AT5G46310		WRKY family transcription factor
AT5G46350	*WRKY DNA-BINDING PROTEIN 8 (WRKY8)*	member of WRKY Transcription Factor; Group II-c
AT5G46590	*NAC DOMAIN CONTAINING PROTEIN 96 (NAC096)*	Transcription factor required for the initiation of cell division during wound healing. Redundantly involved with ANAC071 in the process of "cambialization".
AT5G46690	*BETA HLH PROTEIN 71 (bHLH071)*	beta HLH protein 71
AT5G46795	*MICROSPORE-SPECIFIC PROMOTER 2 (MSP2)*	microspore-specific promoter 2
AT5G47150		YDG/SRA domain-containing protein
AT5G47240	*NUDIX HYDROLASE HOMOLOG 8 (NUDT8)*	nudix hydrolase homolog 8
AT5G47370	*(HAT2)*	homeobox-leucine zipper genes induced by auxin, but not by other phytohormones. Plays opposite roles in the shoot and root tissues in regulating auxin-mediated morphogenesis.
AT5G47440	*FORKED-LIKE6 (FL6)*	FORKED-LIKE family member, part of Group 3 (Group 1 consists of FKD1, FL1-FL3; Group 2 consists of FL4 and FL8 and Group 3 consists of FL5- FL7). May coordinate leaf size with vein density, where Group 1 members and Group 3 members have opposing functions.
AT5G47455		hypothetical protein
AT5G47500	*PECTIN METHYLESTERASE 5 (PME5)*	predicted to encode a pectin methylesterase
AT5G47600		HSP20-like chaperones superfamily protein
AT5G47670	*NUCLEAR FACTOR Y, SUBUNIT B6 (NF-YB6)*	Encodes LEC1-Like (L1L), closely related to LEC1 (Leafy Cotyledon1). Functions as a regulator of embryo development.
AT5G47800	*(NPY6)*	Encodes a gene homologous to the NPY family based on deep phylogeny.
AT5G47960	*RAB GTPASE HOMOLOG A4C (RABA4C)*	Encodes a small molecular weight g-protein.
AT5G48000	*CYTOCHROME P450, FAMILY 708, SUBFAMILY A, POLYPEPTIDE 2 (CYP708A2)*	Encodes a member of the CYP708A family of cytochrome P450 enzymes. THAH appears to add a hydroxyl group to the triterpene thalianol. thah1 mutants have an elevated accumulation of thalianol. thah1-1 mutants have longer roots than wild type plants. Thalian-diol and desaturated thalian-diol are lost from the root extracts of thah1-1 mutants. Overexpression of the sequence from At5g48000.1 rescues the thah1-1 mutant phenotype (Field 2008); it is unknown whether the shorter sequences associated with other gene models would provide functional complementation.
AT5G48060	*MULTIPLE C2 DOMAIN AND TRANSMEMBRANE REGION PROTEIN 2 (MCTP2)*	C2 calcium/lipid-binding plant phosphoribosyltransferase family protein
AT5G48070	*XYLOGLUCAN ENDOTRANSGLUCOSYLASE/HYDROLASE 20 (XTH20)*	putative xyloglucan endotransglycosylase/hydrolase, expressed primarily in the stele of mature non-elongating regions of both the main and the lateral root. Is expressed in lateral root primordia but expression ceases after lateral root begins to grow. Involved in cell proliferation in incised inflorescence stems.
AT5G48100	*TRANSPARENT TESTA 10 (TT10)*	Encodes a protein that is similar to laccase-like polyphenol oxidases. Involved in lignin and flavonoids biosynthesis. It has four conserved copper binding domains. Expressed in developing testa, where it colocalizes with the flavonoid end products proanthocyanidins and flavonols. Mutant plants exhibited a delay in developmentally determined browning of the testa, characterized by the pale brown color of seed coat. The tt10 mutant seeds accumulate more epicatechin monomers and more soluble proanthocyanidins than wild-type seeds. Flavonol composition was also affected in tt10 seeds, which exhibited a higher ratio of quercetin rhamnoside monomers versus dimers than wild-type seeds.
AT5G48130		Phototropic-responsive NPH3 family protein
AT5G48175		transmembrane protein
AT5G48210		prolamin-like protein (DUF1278)
AT5G48485	*DEFECTIVE IN INDUCED RESISTANCE 1 (DIR1)*	Encodes a putative apoplastic lipid transfer protein that is involved in systemic acquired resistance. Mutants in this gene exhibit wild-type local resistance to avirulent and virulent Pseudomonas syringae, but pathogenesis-related gene expression is abolished in uninoculated distant leaves and fail to develop SAR to virulent Pseudomonas or Peronospora parasitica. DIR1 protein is cell-to-cell mobile and is transported via phloem sap.
AT5G48560	*CRY2-INTERACTING BHLH 2 (CIB2)*	basic helix-loop-helix (bHLH) DNA-binding superfamily protein

AT5G48570	*(ROF2)*	Encodes one of the 36 carboxylate clamp (CC)-tetratricopeptide repeat (TPR) proteins (Prasad 2010, Pubmed ID: 20856808) with potential to interact with Hsp90/Hsp70 as co-chaperones.
AT5G48650	*GTPASE?ACTIVATING PROTEIN SH3-DOMAIN?BINDING PROTEIN LIKE (G3BP-LIKE)*	Negative regulator of defense response to Pseudomonas syringae pv. tomato through altered stomatal and apoplastic immunity.
AT5G48690		ubiquitin-associated (UBA)/TS-N domain protein
AT5G48800		Phototropic-responsive NPH3 family protein
AT5G48850	*SULPHUR DEFICIENCY-INDUCED 1 (ATSDI1)*	Homologous to the wheat sulphate deficiency-induced gene sdi1. Expression in root and leaf is induced by sulfur starvation. Knockout mutants retained higher root and leaf sulfate concentrations, indicating a role in regulation of stored sulfate pools.
AT5G48900		Pectin lyase-like superfamily protein
AT5G49120		DUF581 family protein, putative (DUF581)
AT5G49190	*SUCROSE SYNTHASE 2 (SUS2)*	Encodes a sucrose synthase (SUS2). The activity of the enzyme could not be assayed as proved to be insoluble (PMID 17257168). However, analyses of an sus2 mutant revealed a deficiency in sucrose synthase activity 12 and 15 days after flowering. There are some reports that SUS2 transcript levels are increased in leaves specifically by O(2) deficiency whereas other reports indicate that SUS2 is expressed only in seeds. Immulocalization shows that SUS2 is present in the cytosol of developing seeds, but, it also associated with plastids, though not located within them.
AT5G49250		Beta-galactosidase related protein
AT5G49330	*MYB DOMAIN PROTEIN 111 (MYB111)*	Member of the R2R3 factor gene family. Together with MYB11 and MYB111 redundantly regulates flavonol biosynthesis.
AT5G49460	*ATP CITRATE LYASE SUBUNIT B 2 (ACLB-2)*	One of the two genes encoding subunit B of the cytosolic enzyme ATP Citrate Lyase (ACL)
AT5G49520	*WRKY DNA-BINDING PROTEIN 48 (WRKY48)*	Encodes WRKY48, a member of the WRKY Transcription Factor. WRKY48 is a stress- and pathogen-induced transcriptional activator that represses plant basal defense. The mRNA is cell-to-cell mobile.
AT5G49630	*AMINO ACID PERMEASE 6 (AAP6)*	Is a high affinity amino acid transporter capable of transporting aspartate and tryptophan. May be involved in the amino acid uptake from xylem.
AT5G49730	*FERRIC REDUCTION OXIDASE 6 (FRO6)*	Encodes a plasma membrane-located ferric chelate reductase. Its mRNA is expressed in green aerial tissues (shoot, flower and cotyledon) in a light- and cell differentiation-specific manner.
AT5G49780		Leucine-rich repeat protein kinase family protein
AT5G49820	*ROOT UV-B SENSITIVE 6 (RUS6)*	root UVB sensitive protein (Protein of unknown function, DUF647)
AT5G50200	*WOUND-RESPONSIVE 3 (WR3)*	Wound-responsive gene 3 (WR3). Encodes a high-affinity nitrate transporter. Up-regulated by nitrate. Involved in jasmonic acid-independent wound signal transduction.
AT5G50260	*CYSTEINE ENDOPEPTIDASE 1 (CEP1)*	Encodes a papain-like cysteine protease involved in tapetal programmed cell death and pollen development.CEP1 is expressed specifically in the tapetum from stages 5 to 11 of anther development. The CEP1 protein first appears as a proenzyme in precursor protease vesicles, and is then transported to the vacuole and transformed into the mature enzyme before rupture of the vacuole. CEP1 was also released to the tapetal cell wall during late stage 6 and stage 7. After the tapetal cell wall degenerated, the CEP1 enzyme entered the callose wall from the degenerated tapetal cell wall and was probably involved in degeneration of the callose wall.
AT5G50300	*AZA-GUANINE RESISTANT2 (AZG2)*	Encodes a homolog of the adenine-guanine-hypoxanthine transporter AzgA of Aspergillus nidulans. Function as a plant adenine-guanine transporter. Two closely related genes exist in Arabidopsis: AT3G10960 (Azg1) and AT5G50300 (Azg2).
AT5G50390	*EMBRYO DEFECTIVE 3141 (EMB3141)*	Pentatricopeptide repeat (PPR-like) superfamily protein
AT5G50570	*SQUAMOSA PROMOTER-BINDING PROTEIN LIKE 13A (SPL13A)*	Squamosa promoter-binding protein-like (SBP domain) transcription factor family protein
AT5G50740	*HEAVY METAL ASSOCIATED PROTEIN 52 (ATHMP52)*	Heavy metal transport/detoxification superfamily protein
AT5G50750	*REVERSIBLY GLYCOSYLATED POLYPEPTIDE 4 (RGP4)*	RGP4 is a reversibly glycosylated polypeptide. Analyses using tagged RGP4 suggest that it is present in the cytosol and in association with the Golgi apparatus. Recombinant RGP4 does not have UDP-arabinose mutase activity based on an in vitro assay even though the related RGP1, RGP2, and RGP3 proteins do have activity in the same assay. RGP4 can form complexes with RGP1 and RGP2. RGP4 is expressed during seed development.
AT5G50760	*SMALL AUXIN UPREGULATED RNA 55 (SAUR55)*	SAUR-like auxin-responsive protein family
AT5G50770	*HYDROXYSTEROID DEHYDROGENASE 6 (HSD6)*	Encodes a putative hydroxysteroid dehydrogenase (HSD). Genes that encode HSD include: At5g50600 and At5g50700 (HSD1), At3g47350(HSD2), At3g47360(HSD3), At5g50590 and At5g50690(HSD4), At5g50770(HSD6) (Plant Cell Physiology 50:1463). Two copies of HSD1 and HSD4 exist due to a gene duplication event. In Plant Physiology 145:87, At5g50690 is HSD7, At4g10020 is HSD5.
AT5G51010		Rubredoxin-like superfamily protein
AT5G51060	*ROOT HAIR DEFECTIVE 2 (RHD2)*	RHD2 (along with RHD3 and RHD4) is required for normal root hair elongation. Has NADPH oxidase activity. Gene is expressed in the elongation and differention zone in trichoblasts and elongating root hairs. RDH2 is localized to the growing tips of root hair cells. It is required for the production of reactive oxygen species in response to extracellular ATP stimulus. The increase in ROS production stimulates Ca2+ influx.
AT5G51220		ubiquinol-cytochrome C chaperone family protein
AT5G51440	*(HSP23.5)*	HSP20-like chaperones superfamily protein
AT5G51500		Plant invertase/pectin methylesterase inhibitor superfamily
AT5G51520		Plant invertase/pectin methylesterase inhibitor superfamily protein
AT5G51550	*EXORDIUM LIKE 3 (EXL3)*	EXORDIUM like 3
AT5G51810	*GIBBERELLIN 20 OXIDASE 2 (GA20OX2)*	Encodes gibberellin 20-oxidase. Involved in gibberellin biosynthesis. Up-regulated by far red light in elongating petioles. Not regulated by a circadian clock. Mutation of GA20ox2 delays flowering.
AT5G52030		TraB family protein
AT5G52120	*PHLOEM PROTEIN 2-A14 (PP2-A14)*	phloem protein 2-A14
AT5G52300	*LOW-TEMPERATURE-INDUCED 65 (LTI65)*	Encodes a protein that is induced in expression in response to water deprivation such as cold, high-salt, and desiccation. The response appears to be via abscisic acid. The promoter region contains two ABA-responsive elements (ABREs) that are required for the dehydration-responsive expression of rd29B as cis-acting elements. Protein is a member of a gene family with other members found plants, animals and fungi. Upregulation by P. polymyxa CR1 increases drought resistance.
AT5G52310	*LOW-TEMPERATURE-INDUCED 78 (LTI78)*	cold regulated gene, the 5' region of cor78 has cis-acting regulatory elements that can impart cold-regulated gene expression The mRNA is cell-to-cell mobile.

AT5G52390		PAR1 protein
AT5G52640	*HEAT SHOCK PROTEIN 90.1 (HSP90.1)*	Encodes a cytosolic heat shock protein AtHSP90.1. AtHSP90.1 interacts with disease resistance signaling components SGT1b and RAR1 and is required for RPS2-mediated resistance. The mRNA is cell-to-cell mobile.
AT5G52760		Copper transport protein family
AT5G52790		CBS domain protein with a domain protein (DUF21)
AT5G53030		hypothetical protein
AT5G53210	*SPEECHLESS (SPCH)*	Encodes a basic helix-loop-helix (bHLH) transcription factor that is necessary and sufficient for the asymmetric divisions that establish the stomatal lineage in Arabidopsis thaliana. Expression of SPCH in young epidermal cells allows these cells to make asymmetric divisions. SPCH is a substrate of a kinase MPK3 and MPK6. Its transcript levels change after inducing MUTE expression in a mute background.
AT5G53320		Leucine-rich repeat protein kinase family protein
AT5G53450	*OBP3-RESPONSIVE GENE 1 (ORG1)*	FBN11 contains a lipid-binding FBN domain and a kinase domain. It is induced by osmotic stress and loss of function mutants show decreased germination when grown under osmotic stress conditions.
AT5G53480	*HOMOLOG OF HUMAN KPNB1 (KPNB1)*	Sensitive to ABA, role in drought stress.
AT5G53590	*SMALL AUXIN UPREGULATED RNA 30 (SAUR30)*	SAUR-like auxin-responsive protein family
AT5G53870	*EARLY NODULIN-LIKE PROTEIN 1 (ENODL1)*	early nodulin-like protein 1
AT5G53950	*CUP-SHAPED COTYLEDON 2 (CUC2)*	Transcriptional activator of the NAC gene family, with CUC1 redundantly required for embryonic apical meristem formation, cotyledon separation and expression of STM. Proper timing of CUC2 expression is required to maintain the phyllotactic pattern initiated in the meristem. CUC2 expression in leaf sinus region is required for serration and the extent of serration is modulated by mir164A mediated repression of CUC2. Together with CUC3-DA1-UBP15 part of a regulatory module which controls the initiation of axillary meristems, thereby determining plant architecture. Regulates the axillary meristem initiation, directly binding to the DA1 promoter.
AT5G53980	*HOMEOBOX PROTEIN 52 (HB52)*	Encodes a homeodomain leucine zipper class I (HD-Zip I) protein.
AT5G54100	*STOMATIN-LIKE PROTEIN 2 (SLP2)*	SPFH/Band 7/PHB domain-containing membrane-associated protein family
AT5G54160	*O-METHYLTRANSFERASE 1 (OMT1)*	A caffeic acid/5-hydroxyferulic acid O-methyltransferase. Interacts with 14-4-3 proteins in yeast 2 hybrid assay. AtOMT1 (At5g54160) encodes a flavonol 3?-O-methyltransferase that is highly active towards quercetin and myricetin. The substrate specificity identifies the enzyme as flavonol 3?-methyltransferase which replaces the former annotation of the gene to encode a caffeic acid/5-hydroxyferulic acid O-methyltransferase The mRNA is cell-to-cell mobile.
AT5G54370		Late embryogenesis abundant (LEA) protein-like protein
AT5G54510	*DWARF IN LIGHT 1 (DFL1)*	Encodes an IAA-amido synthase that conjugates Ala, Asp, Phe, and Trp to auxin. Lines overexpressing this gene accumulate IAA-ASP and are hypersensitive to several auxins. Identified as a dominant mutation that displays shorter hypocotyls in light grown plants when compared to wild type siblings. Protein is similar to auxin inducible gene from pea (GH3).
AT5G54530		serine protease, putative (Protein of unknown function, DUF538)
AT5G54630		zinc finger protein-like protein
AT5G54960	*PYRUVATE DECARBOXYLASE-2 (PDC2)*	pyruvate decarboxylase-2
AT5G55070	*(E2-OGDH2)*	Encodes the E2 subunit of the 2-oxoglutarate dehydrogenase.
AT5G55200	*MITOCHONDRIAL GRPE 1 (MGE1)*	Co-chaperone GrpE family protein
AT5G55360		MBOAT (membrane bound O-acyl transferase) family protein
AT5G55560		Protein kinase superfamily protein
AT5G55620		hypothetical protein
AT5G55730	*FASCICLIN-LIKE ARABINOGALACTAN 1 (FLA1)*	Encodes fasciclin-like arabinogalactan-protein 1 (Fla1). fla1 mutants show defects in shoot regeneration. Possibly involved in embryogenesis and seed development.
AT5G56090	*CYTOCHROME C OXIDASE 15 (COX15)*	Encodes a homolog of COX15. Microarray analysis show a 3.2 fold increase in transcription after treatment with rotenone, an electron transport chain inhibitor.
AT5G56320	*EXPANSIN A14 (EXPA14)*	member of Alpha-Expansin Gene Family. Naming convention from the Expansin Working Group (Kende et al, 2004. Plant Mol Bio)
AT5G56350		Pyruvate kinase family protein
AT5G56540	*ARABINOGALACTAN PROTEIN 14 (AGP14)*	Encodes arabinogalactan protein (AGP14). Mutants exhibit longer root hairs. The mRNA is cell-to-cell mobile.
AT5G56590		O-Glycosyl hydrolases family 17 protein
AT5G56630	*PHOSPHOFRUCTOKINASE 7 (PFK7)*	phosphofructokinase 7
AT5G56850		hypothetical protein
AT5G56870	*BETA-GALACTOSIDASE 4 (BGAL4)*	beta-galactosidase 4
AT5G57090	*ETHYLENE INSENSITIVE ROOT 1 (EIR1)*	Encodes an auxin efflux carrier that is similar to bacterial membrane transporters. Root-specific role in the transport of auxin. Acts downstream of CTR1 and ethylene biosynthesis, in the same pathway as EIN2 and AUX1, and independent from EIN3 and EIN5/AIN1 pathway. In the root, the protein localizes apically in epidermal and lateral root cap cells and predominantly basally in cortical cells. Functions may be regulated by phosphorylation status. EIR1 expression is induced by brassinolide treatment in the brassinosteroid-insensitive br1 mutant. Gravistimulation results in asymmetric PIN2 distribution, with more protein degraded at the upper side of the gravistimulated root. Membrane sterol composition is essential for the acquisition of PIN2 polarity. Its expression is downregulated at hypoxic conditions. RAP2.12 overexpression inhibits this downregulation.
AT5G57220	*CYTOCHROME P450, FAMILY 81, SUBFAMILY F, POLYPEPTIDE 2 (CYP81F2)*	member of CYP81F, involved in glucosinolate metabolism. Mutants had impaired resistance to fungi. The mRNA is cell-to-cell mobile.
AT5G57240	*OSBP(OXYSTEROL BINDING PROTEIN)-RELATED PROTEIN 4C (ORP4C)*	OSBP(oxysterol binding protein)-related protein 4C
AT5G57260	*CYTOCHROME P450, FAMILY 71, SUBFAMILY B, POLYPEPTIDE 10 (CYP71B10)*	putative cytochrome P450
AT5G57345	*(ATOXR)*	OXR is a single copy gene in Arabidopsis. It is localized to the ER. It is expressed throughout the plant and expression is induced in response to abiotic stress. While the function of OXR is unknown, overexpression results in increased abiotic stress tolerance and increased ascorbic acid content.
AT5G57400		transmembrane protein

AT5G57420	*INDOLE-3-ACETIC ACID INDUCIBLE 33 (IAA33)*	Belongs to auxin inducible gene family.
AT5G57560	*TOUCH 4 (TCH4)*	Encodes a cell wall-modifying enzyme, rapidly upregulated in response to environmental stimuli.
AT5G57780	*P1R1 (P1R1)*	Encodes a atypical member of the bHLH (basic helix-loop-helix) family transcriptional factors.
AT5G57920	*EARLY NODULIN-LIKE PROTEIN 10 (ENODL10)*	early nodulin-like protein 10
AT5G57980	*RNA POLYMERASE II FIFTH LARGEST SUBUNIT, C (RPB5C)*	NRPB5-like protein of unknown function; homologous to budding yeast RPB5
AT5G58080	*RESPONSE REGULATOR 18 (RR18)*	member of Response Regulator: B- Type
AT5G58550	*ETO1-LIKE 2 (EOL2)*	Encodes a paralog of ETO1, which is a negative regulator of ACS5 (a key enzyme in ethylene biosynthesis pathway). EOL2 also interacts with and inhibits the activity of ACS5.
AT5G58580	*TOXICOS EN LEVADURA 63 (ATL63)*	Encodes a functional E3 ligase that is involved in membrane trafficking and regulation of salt stress responses. It is localized to membranes including the plasma membrane, pre-vacuolar compartments and Golgi.
AT5G58700	*PHOSPHATIDYLINOSITOL-SPECIFIC PHOSPHOLIPASE C4 (PLC4)*	phosphatidylinositol-speciwc phospholipase C4
AT5G58750	*PROGESTERONE 5β-REDUCTASE (PRISE)*	Putative PRISE (progesterone 5β-reductase and/or iridoid synthase-like 1,4-enone reductases).
AT5G59060		reverse transcriptase family protein
AT5G59070		UDP-Glycosyltransferase superfamily protein
AT5G59080		hypothetical protein
AT5G59100		Subtilisin-like serine endopeptidase family protein
AT5G59320	*LIPID TRANSFER PROTEIN 3 (LTP3)*	Predicted to encode a PR (pathogenesis-related) protein. Belongs to the lipid transfer protein (PR-14) family with the following members: At2g38540/LTP1, At2g38530/LTP2, At5g59320/LTP3, At5g59310/LTP4, At3g51600/LTP5, At3g08770/LTP6, At2g15050/LTP7, At2g18370/LTP8, At2g15325/LTP9, At5g01870/LTP10, At4g33355/LTP11, At3g51590/LTP12, At5g44265/LTP13, At5g62065/LTP14, At4g08530/LTP15. The mRNA is cell-to-cell mobile.
AT5G59400		PGR5-like A protein
AT5G59530		2-oxoglutarate (2OG) and Fe(II)-dependent oxygenase superfamily protein
AT5G59730	*EXOCYST SUBUNIT EXO70 FAMILY PROTEIN H7 (EXO70H7)*	A member of EXO70 gene family, putative exocyst subunits, conserved in land plants. Arabidopsis thaliana contains 23 putative EXO70 genes, which can be classified into eight clusters on the phylogenetic tree. The mRNA is cell-to-cell mobile.
AT5G59780	*MYB DOMAIN PROTEIN 59 (MYB59)*	Encodes a putative transcription factor (MYB59). In roots it is involved in K+/NO3- transport and expression of the NPF7.3 transporter.
AT5G60280	*L-TYPE LECTIN RECEPTOR KINASE I.8 (LECRK-I.8)*	Plasma membrane localized receptor kinase. Binds NAD+ and induces expression of disease resistance genes.
AT5G60410	*SAP AND MIZ1 DOMAIN- CONTAINING LIGASE1 (SIZ1)*	Encodes a plant small ubiquitin-like modifier (SUMO) E3 ligase that is a focal controller of Pi starvation-dependent responses. Also required for SA and PAD4-mediated R gene signalling, which in turn confers innate immunity in Arabidopsis. Also involved in the regulation of plant growth, drought responses and freezing tolerance. This latter effect is most likely due to SIZ1 dependent ABI5 sumoylation. Regulates leaf cell division and expansion through salicylic acid accumulation. signaling
AT5G60470	*EGRET (EGRET)*	C2H2 and C2HC zinc fingers superfamily protein
AT5G60530		Root tip expressed LEA protein involved in ribosome biogenesis.
AT5G60660	*PLASMA MEMBRANE INTRINSIC PROTEIN 2;4 (PIP2;4)*	A member of the plasma membrane intrinsic protein subfamily PIP2.When expressed in yeast cells can conduct hydrogen peroxide into those cells. Mutants exhibit longer root hairs.
AT5G60890	*MYB DOMAIN PROTEIN 34 (MYB34)*	Myb-like transcription factor that modulates expression of ASA1, a key point of control in the tryptophan pathway; mutant has deregulated expression of ASA1 in dominant allele. Loss of function allele suggests ATR1 also functions at a control point for regulating indole glucosinolate homeostasis.
AT5G60950	*COBRA-LIKE PROTEIN 5 PRECURSOR (COBL5)*	COBRA-like protein 5 precursor
AT5G61010	*EXOCYST SUBUNIT EXO70 FAMILY PROTEIN E2 (EXO70E2)*	A member of EXO70 gene family, putative exocyst subunits, conserved in land plants. Arabidopsis thaliana contains 23 putative EXO70 genes, which can be classified into eight clusters on the phylogenetic tree.
AT5G61290		Flavin-binding monooxygenase family protein
AT5G61430	*NAC DOMAIN CONTAINING PROTEIN 100 (NAC100)*	NAC domain containing protein 100
AT5G61660		glycine-rich protein
AT5G61740	*ATP-BINDING CASSETTE A10 (ABCA10)*	ABC2 homolog 14
AT5G61820		stress up-regulated Nod 19 protein
AT5G61850	*LEAFY (LFY)*	Encodes transcriptional regulator that promotes the transition to flowering.Involved in floral meristem development. LFY is involved in the regulation of AP3 expression, and appears to bring the F-box protein UFO to the AP3 promoter. Amino acids 46-120 define a protein domain that mediates self-interaction.
AT5G61890	*(ERF114)*	encodes a member of the ERF (ethylene response factor) subfamily B-4 of ERF/AP2 transcription factor family. The protein contains one AP2 domain. There are 7 members in this subfamily.
AT5G62000	*AUXIN RESPONSE FACTOR 2 (ARF2)*	Encodes an auxin response factor. Mutants have many defects including enlarged rosette leaves, reduced fertility, later senescence, hypocotyl elongation defects, enlarged seeds and enlarged cotyledons. May not mediate auxin effects. Increase in seed size due to increased cell proliferation. The mRNA is cell-to-cell mobile.
AT5G62140		ATP-dependent Clp protease ATP-binding subunit
AT5G62180	*CARBOXYESTERASE 20 (CXE20)*	Carboxyesterase that binds stringolactones.
AT5G62230	*ERECTA-LIKE 1 (ERL1)*	Encodes a receptor-like kinase that, together with ER and ERL2 governs the initial decision of protodermal cells to either divide proliferatively to produce pavement cells or divide asymmetrically to generate stomatal complexes. It is important for maintaining stomatal stem cell activity and preventing terminal differentiation of the meristemoid into the guard mother cell. Along with erl2 functionally compensates for loss of erecta during integument development. Its transcript levels change after inducing MUTE expression in a mute background.
AT5G62440	*(DOM1)*	Encodes a protein DOMINO1 that belongs to a plant-specific gene family sharing a common motif present in the tomato DEFECTIVE CHLOROPLASTS AND LEAVES (LeDCL) protein. DOMINO1 is located in the nucleus. Arabidopsis embryos carrying the domino1 mutation grow slowly in comparison with wild type embryos and reach only the globular stage at desiccation. The primary defect of the mutation at the cellular level is the large size of the nucleolus that can be observed soon after fertilization in the nuclei of both the embryo and the endosperm. DOMINO1 might have a role in ribosome biogenesis and in determining the rate of cell division.

AT5G62480	*GLUTATHIONE S-TRANSFERASE TAU 9 (GSTU9)*	Encodes glutathione transferase belonging to the tau class of GSTs. Naming convention according to Wagner et al. (2002).
AT5G62520	*SIMILAR TO RCD ONE 5 (SRO5)*	Encodes a protein with similarity to RCD1 but without the WWE domain. The protein does have a PARP signature upstream of the C-terminal protein interaction domain. The PARP signature may bind NAD+ and attach the ADP-ribose-moiety from NAD+ to the target molecule. Its presence suggests a role for the protein in ADP ribosylation. Up-regulated by NaCl. SRO5 and P5CDH (an overlapping gene in the antisense orientation) generate 24-nt and 21-nt siRNAs, which together are components of a regulatory loop controlling reactive oxygen species (ROS) production and stress response.
AT5G62630	*HIPL2 PROTEIN PRECURSOR (HIPL2)*	hipl2 protein precursor
AT5G62920	*RESPONSE REGULATOR 6 (ARR6)*	Encodes a Type-A response regulator that is responsive to cytokinin treatment. Its C-ter domain is very short in comparison to other Arabidopsis ARRs (17 total). Arr6 protein is stabilized by cytokinin.
AT5G63560	*FATTY ALCOHOL:CAFFEOYL-COA CAFFEOYL TRANSFERASE (FACT)*	HXXXD-type acyl-transferase family protein
AT5G63580	*FLAVONOL SYNTHASE 2 (FLS2)*	encodes a protein whose sequence is similar to flavonol synthase
AT5G63600	*FLAVONOL SYNTHASE 5 (FLS5)*	encodes a protein whose sequence is similar to flavonol synthase
AT5G63650	*SNF1-RELATED PROTEIN KINASE 2.5 (SNRK2.5)*	encodes a member of SNF1-related protein kinases (SnRK2) whose activity is activated by ionic (salt) and non-ionic (mannitol) osmotic stress.
AT5G63790	*NAC DOMAIN CONTAINING PROTEIN 102 (NAC102)*	Encodes a member of the NAC family of transcription factors. ANAC102 appears to have a role in mediating response to low oxygen stress (hypoxia) in germinating seedlings. Its expression can be induced by beta-cyclocitral, an oxidized by-product of beta-carotene generated in the chloroplasts, mediates a protective retrograde response that lowers the levels of toxic peroxides and carbonyls, limiting damage to intracellular components.
AT5G63990		Inositol monophosphatase family protein
AT5G64040	*(PSAN)*	Encodes the only subunit of photosystem I located entirely in the thylakoid lumen. May be involved in the interaction between plastocyanin and the photosystem I complex. Phosphorylation of this protein is dependent on calcium.
AT5G64060	*NAC DOMAIN CONTAINING PROTEIN 103 (NAC103)*	NAC domain containing protein 103
AT5G64120	*PEROXIDASE 71 (PRX71)*	Encodes a cell wall bound peroxidase that is induced by hypo-osmolarity and is involved in the lignification of cell walls. Class III peroxidase cell wall-targeted protein localized to the micropylar endosperm facing the radicle. Involved in seed germination.
AT5G64240	*METACASPASE 3 (MC3)*	Encodes a type I metacaspase. Two Arabidopsis metacaspases, AT1G02170 (MC1) and AT4G25110 (MC2) antagonistically control programmed cell death in Arabidopsis. MC1 is a positive regulator of cell death and requires conserved caspase-like putative catalytic residues for its function. MC2 negatively regulates cell death. This function is independent of the putative catalytic residues. A third type I Arabidopsis metacaspase is MC3 (AT5g64240).
AT5G64790		O-Glycosyl hydrolases family 17 protein
AT5G65020	*ANNEXIN 2 (ANNAT2)*	Annexins are calcium binding proteins that are localized in the cytoplasm. When cytosolic Ca2+ increases, they relocate to the plasma membrane. They may be involved in the Golgi-mediated secretion of polysaccharides.
AT5G65070	*MADS AFFECTING FLOWERING 4 (MAF4)*	Encodes MADS-box containing FLC paralog. Five splice variants have been identified but not characterized with respect to expression patterns and/or differing function. Overexpression of the gene in the Landsberg ecotype leads to a delay in flowering, transcript levels of MAF4 are reduced after a 6 week vernalization.
AT5G65110	*ACYL-COA OXIDASE 2 (ACX2)*	Encodes an acyl-CoA oxidase presumably involved in long chain fatty acid biosynthesis.
AT5G65230	*MYB DOMAIN PROTEIN 53 (MYB53)*	Member of the R2R3 factor gene family.
AT5G65410	*HOMEOBOX PROTEIN 25 (HB25)*	Encodes ZFHD2, a member of the zinc finger homeodomain transcriptional factor family.Gain of function of ATHB25 (35S and UBQ10 proomoters) and double loss of function of ATHB25 and ATHB22 increases and decreases, respectively, seed longevity. This phenotype is maternal and related to seed coat alterations. Gain of function increases expression of GA3OX2 and GA4 and GA1 levels. Together with REM7 induces the expression of genes controlling shoot stem characteristics by ectopic expression in roots.
AT5G65600	*L-TYPE LECTIN RECEPTOR KINASE IX.2 (LECRK-IX.2)*	L-type lectin receptor kinase which modulates metabolites and abiotic stress responses. Phosphorylates AvrPtoB which in turn reduces its virulence.
AT5G65630	*GLOBAL TRANSCRIPTION FACTOR GROUP E7 (GTE7)*	This gene is predicted to encode a bromodomain-containing protein. Plant lines expressing RNAi constructs targeted against GTE7 show some resistance to agrobacterium-mediated root transformation.
AT5G65660		hydroxyproline-rich glycoprotein family protein
AT5G65750	*(E1-OGDH2)*	Encodes the E1 subunit of the 2-oxoglutarate dehydrogenase.
AT5G65860		ankyrin repeat family protein
AT5G66190	*FERREDOXIN-NADP(+)-OXIDOREDUCTASE 1 (FNR1)*	Encodes a leaf-type ferredoxin:NADP(H) oxidoreductase. It is present in both chloroplast stroma and thylakoid membranes but is more abundant in the thylakoid. The affinity of this enzyme for ferredoxin is slightly, but significantly, higher than AtLFNR2, an isoform of the same enzyme. AtLFNR1 forms a heterodimer with AtFNR2 and is also a prerequisite to attach AtFNR2 to the thylakoid membrane.
AT5G66280	*GDP-D-MANNOSE 4,6-DEHYDRATASE 1 (GMD1)*	GDP-D-mannose 4,6-dehydratase
AT5G66330		Leucine-rich repeat (LRR) family protein
AT5G66390	*PEROXIDASE 72 (PRX72)*	Encodes a peroxidase that is involved in lignin biosynthesis. Required for casparian strip lignification as well as partially required for SGN-dependent compensatory lignification.
AT5G66520	*CHLOROPLAST RNA EDITING FACTOR 7 (CREF7)*	Encodes a pentatricopeptide repeat protein involved in chloroplast mRNA editing. Mutants display defects in C-U editing of ndhB.
AT5G66570	*PS II OXYGEN-EVOLVING COMPLEX 1 (PSBO1)*	Encodes a protein which is an extrinsic subunit of photosystem II and which has been proposed to play a central role in stabilization of the catalytic manganese cluster. In <i>Arabidopsis thaliana</i> the PsbO proteins are encoded by two genes: <i>psbO1</i> and <i>psbO2</i>. PsbO1 is the major isoform in the wild-type. In plsp1-1 mutant plastids, the nonmature form of the protein localizes in the membrane. The mRNA is cell-to-cell mobile.
AT5G66620	*DA1-RELATED PROTEIN 6 (DAR6)*	DA1-related protein 6
AT5G66700	*HOMEOBOX 53 (HB53)*	Encodes a homeodomain protein. Member of HD-ZIP 1 family, most closely related to HB5. AtHB53 is auxin-inducible and its induction is inhibited by cytokinin, especially in roots therefore may be involved in root development.
AT5G66780		late embryogenesis abundant protein
AT5G67080	*MITOGEN-ACTIVATED PROTEIN KINASE KINASE KINASE 19 (MAPKKK19)*	member of MEKK subfamily
AT5G67220		FMN-linked oxidoreductases superfamily protein

AT5G67250	*SKP1/ASK1-INTERACTING PROTEIN 2 (SKIP2)*	Encodes an SKP1 interacting partner (SKIP2).Encodes an F-box protein. Based on genetic analysis appears to be functionally redundant with VFB1,2, and 3. When expression of all 4 genes is reduced plants show defects in growth and reduced expression of auxin response genes.
AT5G67420	*LOB DOMAIN-CONTAINING PROTEIN 37 (LBD37)*	Encodes a LOB-domain protein involved in nitrogen metabolism and affecting leaf morphogenesis.
AT5G67550		transmembrane protein
ATCG00120	*ATP SYNTHASE SUBUNIT ALPHA (ATPA)*	Encodes the ATPase alpha subunit, which is a subunit of ATP synthase and part of the CF1 portion which catalyzes the conversion of ADP to ATP using the proton motive force. This complex is located in the thylakoid membrane of the chloroplast.
ATCG00140	*(ATPH)*	ATPase III subunit
ATCG00170	*(RPOC2)*	RNA polymerase beta' subunit-2
ATCG00500	*ACETYL-COA CARBOXYLASE CARBOXYL TRANSFERASE SUBUNIT BETA (ACCD)*	Encodes the carboxytransferase beta subunit of the Acetyl-CoA carboxylase (ACCase) complex in plastids. This complex catalyzes the carboxylation of acetyl-CoA to produce malonyl-CoA, the first committed step in fatty acid synthesis.
ATCG00510	*PHOTSYSTEM I SUBUNIT I (PSAI)*	Encodes subunit I of photosystem I.
ATCG00540	*PHOTOSYNTHETIC ELECTRON TRANSFER A (PETA)*	Encodes cytochrome f apoprotein; involved in photosynthetic electron transport chain; encoded by the chloroplast genome and is transcriptionally repressed by a nuclear gene HCF2.
ATCG00560	*PHOTOSYSTEM II REACTION CENTER PROTEIN L (PSBL)*	PSII L protein
ATCG00570	*PHOTOSYSTEM II REACTION CENTER PROTEIN F (PSBF)*	PSII cytochrome b559
ATCG00630	*(PSAJ)*	Encodes subunit J of photosystem I.
ATMG00130	*(ORF121A)*	hypothetical protein
ATMG00150	*(ORF116)*	transmembrane protein
ATMG00520	*(MATR)*	Intron maturase, type II family protein
ATMG00860	*(ORF158)*	DNA/RNA polymerases superfamily protein
ATMG00920	*(ORF215B)*	hypothetical protein
ATMG01010	*(ORF118)*	Unknown conserved protein
ATMG01090	*(ORF262)*	ATP synthase 9 mitochondrial
ATMG01110	*(ORF251)*	Mitovirus RNA-dependent RNA polymerase
ATMG01220	*(ORF113)*	hypothetical protein
ATMG01240	*(ORF100C)*	hypothetical protein
ATMG01370	*(ORF111D)*	transmembrane protein
CLPP		
NAD5.2		
RPL16.CHLOROPLAST		
RPS12.2		
