# Supplementary material for: Weighted Gene Correlation Network Analysis (WGCNA) of Arabidopsis Somatic Embryogenesis (SE) and Identification of Key Gene Modules to Uncover SE-Associated Hub Genes
Source: Int J Genomics. 2022 Jul 4;2022:7471063. doi: 10.1155/2022/7471063 (PMC9274236; doi:10.1155/2022/7471063)
Supplement: Supplementary 3 — Table S3. The top 10 hub genes of each network module. [file 7471063.f3.pdf]

Table S3. The top 10 hub genes of each network module

Module	Gene identifier	Gene symbol	Gene description	Gene type	TF family	Differentially expressed	Embryo defective
Black	AT1G04920	*SUCROSE PHOSPHATE SYNTHASE 3F (SPS3F)*	Encodes a sucrose-phosphate synthase whose activity is stimulated by Glc-6-P and inhibited by Pi	protein_coding	-		
Black	AT1G62710	*BETA VACUOLAR PROCESSING ENZYME (BETA-VPE)*	Encodes a vacuolar processing enzyme belonging to a novel group of cysteine proteases that is expressed specifically in seeds and is essential for the proper processing of storage proteins	protein_coding	-		
Black	AT2G21820	*AT2G21820*	seed maturation protein	protein_coding	-		
Black	AT2G33520	*CYSTEINE-RICH TRANSMEMBRANE MODULE 7 (ATHCYSTM7)*	cysteine-rich/transmembrane domain protein A	protein_coding	-		
Black	AT2G38900	*AT2G38900*	Predicted to encode a pathogenesis-related peptide that belongs to the PR-6 proteinase inhibitor family	protein_coding	-		
Black	AT2G45510	*CYTOCHROME P450, FAMILY 704, SUBFAMILY A, POLYPEPTIDE 2 (CYP704A2)*	member of CYP704A	protein_coding	-		
Black	AT2G45600	*AT2G45600*	alpha/beta-Hydrolases superfamily protein	protein_coding	-		
Black	AT4G31670	*UBIQUITIN-SPECIFIC PROTEASE 18 (UBP18)*	ubiquitin-specific protease 18	protein_coding	-		
Black	AT5G16010	*AT5G16010*	3-oxo-5-alpha-steroid 4-dehydrogenase family protein	protein_coding	-		
Black	AT5G43770	*AT5G43770*	proline-rich family protein	protein_coding	-		
Blue	AT1G03900	*ATP-BINDING CASSETTE I18 (ABCI18)*	member of NAP family, an heterogeneous subfamily of the ATP-binding Cassette (ABC) superfamily of membrane transporters	protein_coding	-		
Blue	AT1G51600	*ZIM-LIKE 2 (ZML2)*	member of a novel family of plant-specific GATA-type transcription factors.	protein_coding	C2C2-Gata		
Blue	AT1G77220	*LAZ1 HOMOLOG1 (LAZ1H1)*	LAZ1H1 is a DUF300 that is localized to the tonoplast; along with LAZ1 it appears to play a role in maintaining the structural integrity of vacuoles and regulating BR signaling by modulating downstream subcellular distribution of BAK	protein_coding	-		
Blue	AT2G15240	*AT2G15240*	UNC-50 family protein	protein_coding	-		
Blue	AT4G10760	*MRNAADENOSINE METHYLASE (MTA)*	Encodes a member of a core set of mRNA m6A writer proteins and is required for N6-adenosine methylation of mRNA.	protein_coding	-		✓
Blue	AT5G05520	*AT5G05520*	Outer membrane OMP85 family protein	protein_coding	-		
Blue	AT5G50000	*CONVERGENCE OF BLUE LIGHT AND CO2 2 (CBC2)*	Belongs to the Raf-like kinase subfamily of the mitogen-activated protein kinase kinase kinase (MAPKKK) family; negatively regulates stomatal opening by negatively regulating plasma membrane H+-ATPase phosphorylation	protein_coding	-		
Blue	AT5G52820	*NOTCHLESS (NLE)*	Encodes a NOTCHLESS homolog, a non-ribosomal protein involved in the maturation and assembly of the 60S ribosomal subunit, that is required for female gametogenesis	protein_coding	-		
Blue	AT5G63400	*ADENYLATE KINASE 1 (ADK1)*	encodes a protein similar to adenylate kinase	protein_coding	-		
Blue	AT5G65860	*AT5G65860*	ankyrin repeat family protein	protein_coding	-	✓	
Brown	AT1G57990	*PURINE PERMEASE 18 (PUP18)*	Member of a family of proteins related to PUP1, a purine transporter. May be involved in the transport of purine and purine derivatives such as cytokinins, across the plasma membrane.	protein_coding	-	✓	
Brown	AT2G24970	*AT2G24970*	spindle/kinetochore-associated protein	protein_coding	-		
Brown	AT3G14060	*AT3G14060*	hypothetical protein	protein_coding	-	✓	
Brown	AT3G53180	*NODULIN/GLUTAMINE SYNTHASE-LIKE PROTEIN (NodGS)*	Encodes a protein that is the product of a fusion gene with a C-terminal GSI like sequence and an N-terminal part sharing homology with nodulins. It self-assembles into oligomers and its expression is increased in response to flagellin treatment.	protein_coding	-	✓	
Brown	AT3G56980	*BASIC HELIX-LOOP-HELIX 39 (BHLH39)*	Encodes a member of the basic helix-loop-helix transcription factor protein.	protein_coding	bHLH	✓	
Brown	AT3G57090	*FISSION 1A (FIS1A); (BIGYIN)*	Encodes a protein with similarity to yeast FIS proteins; these membrane anchored proteins bind DRP proteins and function during organelle division	protein_coding	-		
Brown	AT5G13070	*AT5G13070*	MSF1-like family protein	protein_coding	-		
Brown	AT5G39850	*RPS9C*	Ribosomal protein S4	protein_coding	-		
Brown	AT5G44380	*AT5G44380 (AtBBE24)*	FAD-binding Berberine family protein	protein_coding	-	✓	
Brown	AT5G55200	*MITOCHONDRIAL GRPE 1 (MGE1)*	Co-chaperone GrpE family protein	protein_coding	-	✓	
Cyan	AT1G62520	*AT1G62520*	sulfated surface-like glycoprotein	protein_coding	-		
Cyan	AT1G74450	*AT1G74450*	Plants overexpressing At1g74450 are stunted in height and have reduced male fertility.	protein_coding	-		
Cyan	AT2G30960	*AT2G30960*	myosin-M heavy chain-like protein	protein_coding	-		
Cyan	AT2G35450	*AT2G35450*	catalytic/ hydrolase	protein_coding	-		
Cyan	AT3G15970	*AT3G15970*	NUP50 (Nucleoporin 50 kDa) protein	protein_coding	-		

Cyan	AT3G16270	*MODIFIED TRANSPORT TO THE VACUOLE1 (MTV1)*	Involved in the plant trans-Golgi network (TGN), where it is part of an adaptor protein (AP) complex to promote vesicle generation with different cargo specificity and destination	protein_coding	-		
Cyan	AT3G50250	*AT3G50250*	transmembrane protein	protein_coding	-		
Cyan	AT4G10750	*AT4G10750*	Phosphoenolpyruvate carboxylase family protein	protein_coding	-		
Cyan	AT4G16490	*AT4G16490*	ARM repeat superfamily protein	protein_coding	-		
Cyan	AT5G01340	*MITOCHONDRIAL SUCCINATE-FUMARATE CARRIER 1 (mSFC1)*	Transports citrate, isocitrate and aconitate, succinate and fumarate. Catalyzes a fast counter-exchange transport as well as a low uniport of substrates. Might be involved in storage oil mobilization at early stages of seedling growth and in nitrogen assimilation in root tissue by catalyzing citrate/isocitrate or citrate/succinate exchanges.	protein_coding	-		
Darkgreen	AT1G18440	*AT1G18440*	Peptidyl-tRNA hydrolase family protein	protein_coding	-		
Darkgreen	AT2G03150	*SHORT ROOT IN SALT MEDIUM 1 (RSA1)*	Encodes a nuclear-localized calcium-binding protein RSA1 required for salt tolerance	protein_coding	-		✓
Darkgreen	AT2G48120	*PALE CRESS (PAC)*	The *pac* mutant affects chloroplast and leaf development; mutants are ABA-deficient and accumulate lower levels of carotenoids and chlorophyll compared to wild type. PAC binds 23srRNA and appears to be required for 50s ribosome assembly. PAC is essential for photoautotrophic growth.	protein_coding	-		
Darkgreen	AT3G18480	*CCAAT-DISPLACEMENT PROTEIN ALTERNATIVELY SPLICED PRODUCT (CASP)*	Predicted to encode a protein that functions as a Golgi apparatus structural component, known as a golgin in mammals and yeast.	protein_coding	-		
Darkgreen	AT3G55050	*D-CLADE TYPE 2C PROTEIN PHOSPHATASE 4 (PP2C.D4)*	Protein phosphatase 2C family protein	protein_coding	-		
Darkgreen	AT4G17695	*KANADI 3 (KAN3)*	Homeodomain-like superfamily protein	protein_coding	G2-like	✓	
Darkgreen	AT5G26605	*AT5G26605*	D111/G-patch domain-containing protein	protein_coding	-		
Darkgreen	AT5G59750	*HOMOLOG OF RIBA 3 (RIBA3)*	monofunctional riboflavin biosynthesis protein RIBA 3	protein_coding	-		
Darkgreen	AT5G65930	*ZWICHEL (ZWI)*	encodes a novel member of the kinesin superfamily of motor proteins. Recessive mutations have reduced number of trichome branches.	protein_coding	-		
Darkgreen	AT5G67020	*AT5G67020*	hypothetical protein	protein_coding	-		
Darkgrey	AT1G10870	*ARF-GAP DOMAIN 4 (AGD4)*	A member of ARF GAP domain (AGD), A thaliana has 15 members, grouped into four classes. AGD4 belongs to the Class 1, together with AGD1, AGD2, and AGD3.	protein_coding	-		
Darkgrey	AT1G16330	*CYCLIN B3;1 (CYCB3;1)*	core cell cycle genes	protein_coding	-		
Darkgrey	AT1G28600	*GUARD-CELL-ENRICHED GDSL LIPASE 2 (GGL2)*	GDSL-motif esterase/acyltransferase/lipase. Enzyme group with broad substrate specificity that may catalyze acyltransfer or hydrolase reactions with lipid and non-lipid substrates.	protein_coding	-		
Darkgrey	AT1G30600	*AT1G30600*	Subtilase family protein	protein_coding	-		
Darkgrey	AT1G75090	*AT1G75090*	DNA glycosylase superfamily protein	protein_coding	-		
Darkgrey	AT1G75390	*BASIC LEUCINE-ZIPPER 44 (bZIP44)*	basic leucine-zipper 44	protein_coding	bZIP		
Darkgrey	AT2G01630	*AT2G01630*	O-Glycosyl hydrolases family 17 protein	protein_coding	-		
Darkgrey	AT2G41720	*EMBRYO DEFECTIVE 2654 (EMB2654)*	Encodes a pentatricopeptide repeat protein that is essential for trans-splicing of a chloroplast small ribosomal subunit transcript.	protein_coding	-		✓
Darkgrey	AT3G61130	*GALACTURONOSYLTRANSFERASE 1 (GAUT1)*	Encodes a protein with putative galacturonosyltransferase activity	protein_coding	-		
Darkgrey	AT4G26370	*AT4G26370*	antitermination NusB domain-containing protein	protein_coding	-		
Darkorange	AT1G27760	*SALT-TOLERANCE 32 (SAT32)*	Encodes a protein with similarity to human interferon-related developmental regulator (IFRD) that is involved in salt tolerance. Loss of function mutations are hypersensitive to salt stress and have reduced fertility. SAT32 is found in the cytoplasm but appears to translocate to the nucleus when plants are subject to salt stress.	protein_coding	-		
Darkorange	AT1G54220	*MITOCHONDRIAL PYRUVATE DEHYDROGENASE SUBUNIT 2-3 (MTE2-3)*	Encodes a subunit of the mitochondrial pyruvate dehydrogenase complex.	protein_coding	-		
Darkorange	AT1G75990	*AT1G75990*	PAM domain (PCI/PINT associated module) protein	protein_coding	-		
Darkorange	AT1G76810	*AT1G76810 (EIF5B1)*	eukaryotic translation initiation factor 2 (eIF-2) family protein	protein_coding	-		
Darkorange	AT3G01160	*AT3G01160*	pre-rRNA-processing ESF1-like protein	protein_coding	-		
Darkorange	AT3G53350	*ROP INTERACTIVE PARTNER 3 (RIP3)*	Encodes a microtubule-binding protein that is anchored to the plasma membrane domains and promotes local microtubule disassembly, forming as specific pattern of secondary walls in xylem vessel cells. Localized at microtubules and interacts with the plant-specific kinesin AtKinesin-13A.	protein_coding	-		
Darkorange	AT4G28310	*AT4G28310*	microtubule-associated protein	protein_coding	-		

Darkorange	AT5G19920	*AT5G19920*	Transducin/WD40 repeat-like superfamily protein	protein_coding	-	
Darkorange	AT5G20470	*HEADLESS DERIVATIVE OF MYOSIN XI-K (HDK)*	Encodes a headless derivative of myosin XI-K, which likely arose from a partial duplication of the XI-K gene and is developmentally regulated.	protein_coding	-	
Darkorange	AT5G43160	*QWRF DOMAIN CONTAINING 9 (QWRF9)*	QWRF motif protein (DUF566)	protein_coding	-	
Darkred	AT1G70780	*AT1G70780*	hypothetical protein	protein_coding	-	
Darkred	AT4G17710	*HOMEODOMAIN GLABROUS 4 (HDG4)*	Encodes a homeobox-leucine zipper family protein belonging to the HD-ZIP IV family.	protein_coding	Homeobox	
Darkred	AT4G30850	*HEPTAHELICAL TRANSMEMBRANE PROTEIN2 (HHP2)*	heptahelical transmembrane protein homologous to human adiponectin receptors and progestin receptors	protein_coding	-	
Darkred	AT5G25520	*AT5G25520*	SPOC domain / Transcription elongation factor S-II protein	protein_coding	-	
Darkred	ATCG00420	*NADH DEHYDROGENASE SUBUNIT J (NDHJ)*	Encodes NADH dehydrogenase subunit J. Its transcription is increased upon sulfur depletion.	protein_coding	-	
Darkred	ATCG00430	*PHOTOSYSTEM II REACTION CENTER PROTEIN G (PSBG)*	Encodes a protein which was originally thought to be part of photosystem II but its wheat homolog was later shown to encode for subunit K of NADH dehydrogenase.	protein_coding	-	
Darkred	ATCG00600	*ATCG00600 (PETG)*	Cytochrome b6-f complex, subunit V. Disruption of homologous gene in Chlamydomonas results in disruption of cytochrome b6-f complex.	protein_coding	-	
Darkred	ATCG00870	*ATCG00870 (ORF77.1)*	Protein precursor Ycf15, putative, chloroplast	protein_coding	-	
Darkred	ATCG00890	*NAD(P)H-QUINONE OXIDOREDUCTASE SUBUNIT 2 A (NDHB.1)*	NADH dehydrogenase ND2	protein_coding	-	
Darkred	ATCG01070	*NAD(P)H-QUINONE OXIDOREDUCTASE SUBUNIT 4L (NDHE)*	NADH dehydrogenase ND4L	protein_coding	-	
Darkturquoise	AT1G16780	*AVP1-LIKE PROTEIN 2 (VHP2;2)*	Encodes a type II H+-PPases that localizes to and function as a proton pump of the Golgi apparatus in most tissues except for mature leaves.	protein_coding	-	
Darkturquoise	AT1G20380	*PROLYL-OLIGOPEPTIDASE ASSOCIATED WITH QUANTITATIVE RESISTANCE (POQR)*	Putative prolyl oligopeptidase, associated with quantitive disease resistance to S. sclerotiorum.	protein_coding	-	
Darkturquoise	AT1G31730	*ADAPTOR PROTEIN COMPLEX 4E (AP4E)*	Encodes a component of the AP4 complex and is involved in vacuolar sorting of storage proteins.	protein_coding	-	
Darkturquoise	AT1G65410	*ATP-BINDING CASSETTE I13 (ABCI13)*	Encodes a member of NAP subfamily of transporters. Mutations in this gene suppress the low temperature-induced phenotype of Arabidopsis tocopherol-deficient mutant vte2.	protein_coding	-	
Darkturquoise	AT1G74040	*2-ISOPROPYLMALATE SYNTHASE 1 (IMS1)*	Encodes an active Arabidopsis isopropylmalate synthase IPMS2. Involved in leucine biosynthesis. Expressed constitutively throughout the plant. Loss of IPMS2 can be compensated by a second isopropylmalate synthase gene IPMS1 (At1g18500).	protein_coding	-	
Darkturquoise	AT2G20140	*REGULATORY PARTICLE AAA-ATPASE 2B (RPT2b)*	Encodes one of the two RPT2 (26S proteasome subunit RPT2) paralogs: RPT2a (At4g29040) and RPT2b (At2g20140). RPT2b can not complement the rpt2a mutant phenotype. rpt2a rpt2b double mutants are embryo lethal.	protein_coding	-	
Darkturquoise	AT2G30920	*COENZYME Q 3 (COQ3)*	Encodes the enzyme which catalyzes the methylation of nonaprenyldihydroxybenzoate as it is the prevalent polyprenoid in Arabidopsis. The enzyme is a mitochondrial-localized methyltransferase involved in ubiquinone biosynthesis.	protein_coding	-	✓
Darkturquoise	AT3G09570	*AT3G09570 (7TM2)*	Lung seven transmembrane receptor family protein	protein_coding	-	
Darkturquoise	AT5G51660	*CLEAVAGE AND POLYADENYLATION SPECIFICITY FACTOR 160 (CPSF160)*	cleavage and polyadenylation specificity factor 160	protein_coding	-	
Darkturquoise	AT5G58530	*AT5G58530*	Glutaredoxin family protein	protein_coding	-	
Green	AT1G17680	*AT1G17680*	tetratricopeptide repeat (TPR)-containing protein	protein_coding	-	
Green	AT1G27930	*AT1G27930 (AGM1)*	Arabinogalactan methylesterase, involved in arabinogalactan glucuronic acid methylation. Interacts with eIF3.	protein_coding	-	
Green	AT1G28120	*OTU DOMAIN CONTAINING DUB 1 (OTU1)*	Deubiquitinase with preference towards M1 and K48 linkages.	protein_coding	-	
Green	AT1G71010	*FORMS APLOID AND BINUCLEATE CELLS 1C (FAB1C)*	Encodes a protein that is predicted to act as a phosphatidylinositol-3P 5-kinase, but, because it lacks a FYVE domain, it is unlikely to be efficiently targeted to membranes containing the proposed phosphatidylinositol-3P substrate. Therefore, its molecular function remains unknown.	protein_coding	-	
Green	AT1G75130	*CYTOCHROME P450, FAMILY 721, SUBFAMILY A, POLYPEPTIDE 1 (CYP721A1)*	member of CYP721A	protein_coding	-	
Green	AT2G32280	*VASCULATURE COMPLEXITY AND CONNECTIVITY (VCC)*	Encodes a member of a plant-specific gene family that is required for embryo provasculature development. The gene product regulates vascular network complexity and connectivity in cotyledons.	protein_coding	-	✓

Green	AT2G42280	*FLOWERING BHLH 4 (FBH4)*	basic helix-loop-helix (bHLH) DNA-binding superfamily protein	protein_coding	bHLH	
Green	AT5G51710	*K+ EFFLUX ANTIPORTER 5 (KEA5)*	member of Putative potassium proton antiporter family	protein_coding	-	
Green	AT5G63990	*AT5G63990*	Inositol monophosphatase family protein	protein_coding	-	✓
Green	AT5G65390	*ARABINOGALACTAN PROTEIN 7 (AGP7)*	arabinogalactan protein 7	protein_coding	-	
Greenyellow	AT1G14380	*IQ-DOMAIN 28 (IQD28)*	Encodes a microtubule-associated protein. Member of IQ67 (CaM binding) domain containing family.	protein_coding	-	
Greenyellow	AT1G19010	*AT1G19010*	hypothetical protein	protein_coding	-	
Greenyellow	AT1G67080	*ABSCISIC ACID (ABA)-DEFICIENT 4 (ABA4)*	Encodes a protein involved in the photoprotection of PSII. An aba4-1 mutant completely lacks neoxanthin,a component of the chromophore of the peripheral antenna system in PSII. ABA4 is required for neoxanthin biosynthesis, an intermediary step in abscisic acid biosynthesis, but no catalytic activity has been detected for the ABA4 protein.	protein_coding	-	
Greenyellow	AT2G02710	*PAS/LOV PROTEIN B (PLPB)*	Encodes a putative blue light receptor protein.	protein_coding	-	
Greenyellow	AT2G40420	*AT2G40420*	Encodes a putative amino acid transporter	protein_coding	-	
Greenyellow	AT3G26330	*CYTOCHROME P450, FAMILY 71, SUBFAMILY B, POLYPEPTIDE 37 (CYP71B37)*	putative cytochrome P450	protein_coding	-	
Greenyellow	AT4G01895	*AT4G01895*	systemic acquired resistance (SAR) regulator protein NIMIN-1-like protein	protein_coding	-	
Greenyellow	AT4G29890	*AT4G29890*	choline monooxygenase, putative (CMO-like)	protein_coding	-	
Greenyellow	AT5G27560	*AT5G27560*	DUF1995 domain protein, putative (DUF1995)	protein_coding	-	
Greenyellow	AT5G54540	*AT5G54540*	Uncharacterized conserved protein (UCP012943)	protein_coding	-	
Grey60	AT1G06040	*SALT TOLERANCE (STO)*	Encodes salt tolerance protein (STO) which confers salt tolerance to yeast cells. STO co-localizes with COP1 and plays a role in light signaling. STO transcript levels are regulated by photoperiod and phtyohormones. STO competes with FLC in the regulation of floral transition genes SOC1 and FT.	protein_coding	Orphan	
Grey60	AT1G65870	*AT1G65870*	Disease resistance-responsive (dirigent-like protein) family protein	protein_coding	-	
Grey60	AT3G02360	*6-PHOSPHOGLUCONATE DEHYDROGENASE 2 (PGD2)*	6-phosphogluconate dehydrogenase family protein	protein_coding	-	
Grey60	AT4G20930	*HYDROXYISOBUTYRATE DEHYDROGENASE 1 (HDH1)*	Encodes a 3-hydroxyisobutyrate dehydrogenase	protein_coding	-	
Grey60	AT4G35040	*BASIC LEUCINE ZIPPER 19 (bZIP19)*	Basic-region leucine zipper (bZIP19) transcription factor involved in the adaptation to zinc deficiency. Binds ZDRE motifs	protein_coding	bZIP	
Grey60	AT5G02840	*LHY/CCA1-LIKE 1 (LCL1)*	Encodes RVE4, a homolog of the circadian rhythm regulator RVE8. rve4 rve6 rve8 triple mutants display an extremely long circadian period, with delayed and reduced expression of evening-phased clock genes. Involved in heat shock response.	protein_coding	-	
Grey60	AT5G08380	*ALPHA-GALACTOSIDASE 1 (AGAL1)*	alpha-galactosidase 1	protein_coding	-	
Grey60	AT5G32450	*BPA1-LIKE 4 (BPL4)*	ACD11 binding partner, negatively regulates ROS-mediated defense response	protein_coding	-	
Grey60	AT5G60820	*AT5G60820*	RING/U-box superfamily protein	protein_coding	C3H	
Grey60	AT5G64880	*AT5G64880*	transmembrane protein	protein_coding	-	
Lightcyan	AT1G30460	*CLEAVAGE AND POLYADENYLATION SPECIFICITY FACTOR 30 (CPSF30)*	Encodes AtCPSF30, the 30-KDa subunit of cleavage and polyadenylation specificity factor. AtCPSF30 is a probable processing endonuclease. Nucleus-localized RNA binding protein capable of interacting with itself and with calmodulin. Its RNA-binding activity is inhibited by calmodulin in a calcium-dependent fashion.	protein_coding	-	
Lightcyan	AT1G78650	*POLD3*	Similar to DNA polymerase delta (POLD3), which in other organism was shown to be involved in the elongation of DNA replication.	protein_coding	-	
Lightcyan	AT2G15400	*NRPE3B*	Non-catalytic subunit of Nuclear DNA-dependent RNA polymerase V; homologous to budding yeast RPB3 and the E. coli RNA polymerase alpha subunit. A closely related paralog, At2g15430 can substitute for At2g15400 in the context of Pol V and encodes the equivalent subunit of Pol II and Pol IV.	protein_coding	-	
Lightcyan	AT2G41520	*TETRATRICOPEPTIDE REPEAT 15 (TPR15)*	Encodes one of the 36 carboxylate clamp (CC)-tetratricopeptide repeat (TPR) proteins with potential to interact with Hsp90/Hsp70 as co-chaperones.	protein_coding	-	
Lightcyan	AT2G47980	*SISTER-CHROMATID COHESION PROTEIN 3 (SCC3)*	Essential to the monopolar orientation of the kinetochores during meiosis.	protein_coding	-	
Lightcyan	AT4G36690	*AT4G36690 (ATU2AF65A)*	Regulates flowering time and displays a redundant role in pollen tube growth together with AtU2AF65b.	protein_coding	-	
Lightcyan	AT4G39050	*KINESIN 7.4 (KIN7.4)*	Kinesin motor family protein	protein_coding	-	
Lightcyan	AT5G14720	*TARGET OF TEMPERATURE3 (TOT3)*	Membrane-localized protein kinase which regulates thermomorphogenesis.	protein_coding	-	
Lightcyan	AT5G26360	*CHAPERONIN CONTAINING T-COMPLEX POLYPEPTIDE-1 SUBUNIT 3 (CCT3)*	TCP-1/cpn60 chaperonin family protein	protein_coding	-	

Lightcyan	AT5G54910	*AT5G54910*	DEA(D/H)-box RNA helicase family protein	protein_coding	-	
Lightgreen	AT1G50160	*AT1G50160*	ribonuclease H superfamily polynucleotidyl transferase	protein_coding	-	
Lightgreen	AT2G11560	*AT2G11560*	Mutator-like transposase / similar to MURA transposase of maize	transposable_element_gene	-	
Lightgreen	AT2G34520	*MITOCHONDRIAL RIBOSOMAL PROTEIN S14 (RPS14)*	nuclear-encoded mitochondrial ribosomal protein S14	protein_coding	-	
Lightgreen	AT3G33066	*AT3G33066*	transposable_element_gene; belonging to gypsy-like retrotransposon family	transposable_element_gene	-	
Lightgreen	AT4G02480	*AT4G02480*	AAA-type ATPase family protein	protein_coding	-	
Lightgreen	AT4G20530	*AT4G20530*	cysteine-rich repeat secretory-like protein	protein_coding	-	
Lightgreen	AT5G32430	*AT5G32430*	transposable_element_gene;pseudogene, hypothetical protein	transposable_element_gene	-	
Lightgreen	ATCG01010	*NAD(P)H-QUINONE OXIDOREDUCTASE SUBUNIT 5 (NDHF)*	Chloroplast encoded NADH dehydrogenase unit	protein_coding	-	
Lightgreen	ATMG00120	*ATMG00120 (ORF143)*	reverse transcriptase zinc-binding protein	protein_coding	-	
Lightgreen	ATMG01290	*ATMG01290 (ORF111C)*	hypothetical protein	protein_coding	-	
Lightyellow	AT1G31870	*BUD SITESELECTION PROTEIN 13 (BUD13)*	Ortholog of yeast BUD13 RES complex protein. Functions in pre mRNA processing of RNAs expressed in embryos.	protein_coding	-	
Lightyellow	AT1G69770	*CHROMOMETHYLASE 3 (CMT3)*	Encodes a chromomethylase involved in methylating cytosine residues at non-CG sites. Involved in preferentially methylating transposon-related sequences, reducing their mobility. CMT3 interacts with an Arabidopsis homologue of HP1 (heterochromatin protein 1), which in turn interacts with methylated histones. Involved in gene silencing.	protein_coding	-	
Lightyellow	AT3G44530	*HOMOLOG OF HISTONE CHAPERONE HIRA (HIRA)*	Encodes a nuclear localized WD-repeat containing protein involved in negative regulation of knox gene expression via epigenetic mechanism of chromatin re-organization. It is a part of the HISTONE REGULATOR complex that deposits histones in a DNA synthesis-independent manner and affects both nucleosome occupancy and the maintenance of transcriptional silencing. Interacts physically and genetically with AS1. Expressed in meristem and leaf primordia. Homozygous mutants are embryo lethal.	protein_coding	-	
Lightyellow	AT4G16660	*HEAT SHOCK PROTEIN 70 (HSP70)*	heat shock protein 70 (Hsp 70) family protein	protein_coding	-	
Lightyellow	AT4G35990	*AT4G35990*	hypothetical protein	protein_coding	-	
Lightyellow	AT4G38040	*AT4G38040*	Exostosin family protein	protein_coding	-	
Lightyellow	AT4G39680	*AT4G39680*	SAP domain-containing protein	protein_coding	-	
Lightyellow	AT5G02310	*PROTEOLYSIS 6 (PRT6)*	Encodes PROTEOLYSIS6 (PRT6), a component of the N-end rule pathway that targets protein degradation through the identity of the amino-terminal residue of specific protein substrates. Another component of the N-end rule pathway is arginyl-tRNA:protein arginyltransferase (ATE). PRT6 and ATE were shown to regulate seed after-ripening, seedling sugar sensitivity, seedling lipid breakdown, and abscisic acid (ABA) sensitivity of germination.	protein_coding	-	
Lightyellow	AT5G25150	*TBP-ASSOCIATED FACTOR 5 (TAF5)*	Encodes a putative TATA-binding-protein associated factor TAF5. TAFs are subunits of the general transcription factor IID (TFIID).	protein_coding	-	
Lightyellow	AT5G46710	*AT5G46710*	PLATZ transcription factor family protein	protein_coding	-	
Magenta	AT1G09770	*CELL DIVISION CYCLE 5 (CDC5)*	Member of MYB3R- and R2R3- type MYB- encoding genes. Essential for plant innate immunity. Interacts with MOS4 and PRL1.	protein_coding	MYB-related	✓
Magenta	AT1G72400	*AT1G72400*	hypothetical protein	protein_coding	-	
Magenta	AT2G20690	*AT2G20690*	A synthetic gene encoding the catalytic domain of the Arabidopsis thaliana gene At2g20690 was recombinant expressed in E. coli demonstrating the molecular function of riboflavin synthase.	protein_coding	-	
Magenta	AT2G43370	*SNRNP35*	RNA-binding (RRM/RBD/RNP motifs) family protein	protein_coding	-	
Magenta	AT3G19570	*SNOWY COTYLEDON 3 (SCO3)*	Encodes SCO3 (snowy cotyledon3), a member of a largely uncharacterized protein family unique to the plant kingdom. The sco3-1 mutation alters chloroplast morphology and development, reduces chlorophyll accumulation, impairs thylakoid formation and photosynthesis in seedlings, and results in photoinhibition under extreme CO(2) concentrations in mature leaves. SCO3 is targeted to the periphery of peroxisomes. Together with QWRF2 redundantly modulates cortical microtubule arrangement in floral organ growth and fertility.	protein_coding	-	
Magenta	AT3G55460	*SC35-LIKE SPLICING FACTOR 30 (SCL30)*	encodes an SC35-like splicing factor that is localized to nuclear specks	protein_coding	-	

Magenta	AT5G22400	*AT5G22400*	Rho GTPase activating protein with PAK-box/P21-Rho-binding domain-containing protein	protein_coding	-		
Magenta	AT5G23340	*AT5G23340*	RNI-like superfamily protein	protein_coding	-		
Magenta	AT5G50300	*AZA-GUANINE RESISTANT2 (AZG2)*	Encodes a homolog of the adenine-guanine-hypoxanthine transporter AzgA of Aspergillus nidulans. Function as a plant adenine-guanine transporter. Two closely related genes exist in Arabidopsis: AT3G10960 (Azg1) and AT5G50300 (Azg2).	protein_coding	-	✓	
Magenta	AT5G58190	*EVOLUTIONARILY CONSERVED C-TERMINAL REGION 10 (ECT10)*	evolutionarily conserved C-terminal region 10	protein_coding	-		
Midnightblue	AT1G15960	*NRAMP METAL ION TRANSPORTER 6 (NRAMP6)*	member of Nramp2 family	protein_coding	-		
Midnightblue	AT1G21510	*AT1G21510*	TPRXL	protein_coding	-		
Midnightblue	AT1G23530	*AT1G23530*	transmembrane protein	protein_coding	-		
Midnightblue	AT2G01450	*MAP KINASE 17 (MPK17)*	MPK17 Map kinase family member. Mutants have increased numbers of peroxisomes a phenotype that can be suppressed by mutations in PMD1. This and other treatments, suggests a function in control of peroxisome proliferation in salt stress.	protein_coding	-		
Midnightblue	AT2G23530	*AT2G23530*	Zinc-finger domain of monoamine-oxidase A repressor R1	protein_coding	-		
Midnightblue	AT2G23940	*AT2G23940*	transmembrane protein (DUF788)	protein_coding	-		
Midnightblue	AT3G01210	*BPA1 LIKE 6 (BPL6)*	ACD11 binding partner, may be involved in negative regulation of ROS-mediated defense response.	protein_coding	-		
Midnightblue	AT3G19930	*SUGAR TRANSPORTER 4 (STP4)*	Encodes a sucrose hydrogen symporter that is induced by wounding	protein_coding	-		
Midnightblue	AT4G11370	*RING-H2 FINGER A1A (RHA1A)*	Encodes a putative RING-H2 finger protein RHA1a.	protein_coding	-		
Midnightblue	AT5G06080	*LOB DOMAIN-CONTAINING PROTEIN 33 (LBD33)*	LOB domain-containing protein 33	protein_coding	-		
Orange	AT1G05300	*ZINC TRANSPORTER 5 PRECURSOR (ZIP5)*	member of Fe(II) transporter isolog family	protein_coding	-		
Orange	AT1G24706	*THO COMPLEX SUBUNIT 2 (THO2); EMBRYO DEFECTIVE 2793 (EMB2793)*	Encodes a component of the putative Arabidopsis THO/TREX complex. Mutants of THO3/TEX1, THO1, THO6 accumulate reduced amount of small interfering (si)RNA, suggesting a role of the putative Arabidopsis THO/TREX in siRNA biosynthesis. Mutations in THO have severe developmental defects and affect the production of several different classes of small RNAs indicating a broader role in small RNA biosynthesis.	.	-		✓
Orange	AT1G54680	*AT1G54680*	translation initiation factor 3 subunit I	protein_coding	-		
Orange	AT3G18680	*PLASTID 55 UMP KINASE (PUMPKIN)*	Encodes a functional UMP Kinase located in the plastid that binds to group II intron plastid transcription products. Mutants show decreased accumulation of target transcripts/proteins.	protein_coding	-		
Orange	AT4G26750	*EXTENSIN-LIKE (EXT-like); LYST-INTERACTING PROTEIN5 (LIP5)*	Encodes a protein involved in mediating plant responses to pathogenesis. Loss of function mutations have increased susceptibility to bacterial pathogens and are defective in pathogen induced endocytosis and vesicle trafficking. LIP5 physically interacts with SKD1 and that interaction is required for its resistance phenotype. LIP5 is phosphorylated by MPK3 and MPK6, both of which are components of the disease resistance pathway.	protein_coding	-		
Orange	AT4G32900	*AT4G32900*	Peptidyl-tRNA hydrolase II (PTH2) family protein	protein_coding	-		
Orange	AT5G16140	*AT5G16140*	Peptidyl-tRNA hydrolase family protein	protein_coding	-		
Orange	AT5G37850	*SALT OVERLY SENSITIVE 4 (SOS4)*	Encodes a pyridoxal kinase required for root hair development. Mutants are hypersensitive to Na+, K+ and Li+.	protein_coding	-		
Orange	AT5G43490	*AT5G43490*	myb-like protein X	protein_coding	-		
Orange	AT5G59210	*AT5G59210*	myosin heavy chain-like protein	protein_coding	-		
Pink	AT1G05800	*DONGLE (DGL)*	Encodes a galactolipase. Located in the chloroplast. Involved in the initial step of jasmonic acid biosynthesis. Expressed in vegetative tissues and is necessary for the biosynthesis of basal-level JAs in vegetative tissues.	protein_coding	-		
Pink	AT1G10070	*BRANCHED-CHAIN AMINO ACID TRANSAMINASE 2 (BCAT-2)*	Encodes a chloroplast branched-chain amino acid aminotransferase. Complements the yeast leu/iso-leu/val auxotrophy mutant. Involved in cell wall development.	protein_coding	-		
Pink	AT1G19540	*AT1G19540*	NmrA-like negative transcriptional regulator family protein	protein_coding	-	✓	
Pink	AT1G26400	*BERBERINE BRIDGE ENZYME-LIKE 5 (ATBBE5)*	FAD-binding Berberine family protein	protein_coding	-		
Pink	AT2G29300	*AT2G29300*	NAD(P)-binding Rossmann-fold superfamily protein	protein_coding	-		
Pink	AT2G32540	*CELLULOSE SYNTHASE-LIKE B4 (CSLB04)*	encodes a gene similar to cellulose synthase	protein_coding	-		
Pink	AT3G42820	*AT3G42820*	transposable_element_gene; similar to Ulp1 protease family protein	transposable_element_gene	-		
Pink	AT3G45990	*AT3G45990*	Cofilin/tropomyosin-type actin-binding protein family	protein_coding	-		

Pink	AT3G53980	*AT3G53980*	Bifunctional inhibitor/lipid-transfer protein/seed storage 2S albumin superfamily protein	protein_coding	-		
Pink	AT5G12270	*AT5G12270*	2-oxoglutarate (2OG) and Fe(II)-dependent oxygenase superfamily protein	protein_coding	-		
Purple	AT1G58360	*AMINO ACID PERMEASE 1 (AAP1)*	Encodes AAP1 (amino acid permease 1), a neutral amino acid transporter expressed in seeds. Functions in amino acid uptake into embryos. The transporter also functions in acquisition of glutamate and neutral amino acids by the root.	protein_coding	-		
Purple	AT2G04530	*TRNASE Z 2 (TRZ2); (CPZ)*	Encodes a protein with RNAse Z activity suggesting a role in tRNA processing. Protein contains a signal sequence for import into the chloroplast.	protein_coding	-		✓
Purple	AT2G21450	*CHROMATIN REMODELING 34 (CHR34)*	chromatin remodeling 34	protein_coding	-		
Purple	AT2G39650	*AT2G39650*	cruciferin (DUF506)	protein_coding	-		
Purple	AT3G08970	*THERMOSENSITIVE MALE STERILE 1 (TMS1); (ATERDJ3A)*	J domain protein localized in ER lumen. Can compensate for the growth defect in *jem1 scj1* mutant yeast. Also shows similarity to HSP40 proteins and is induced by heat stress. At high temperatures, mutant alleles are not transmitted through the pollen due to defects in pollen tube growth.	protein_coding	-		
Purple	AT4G01270	*AT4G01270*	RING/U-box superfamily protein	protein_coding	C3H		
Purple	AT4G12650	*AT4G12650*	Endomembrane protein 70 protein family	protein_coding	-		
Purple	AT5G59910	*HISTONE H2B.11 (HTB4)*	Histone superfamily protein	protein_coding	-		
Purple	AT5G65350	*HISTONE 3 11 (HTR11)*	histone 3 11	protein_coding	-		
Purple	AT5G65610	*AT5G65610*	hypothetical protein	protein_coding	-		
Red	AT1G60800	*NSP-INTERACTING KINASE 3 (NIK3)*	Encodes one of a group of LRR-RLKs, designated as CLAVATA3 INSENSITIVE RECEPTOR KINASES (CIKs), that act as co-receptors and have essential roles in regulating CLV3-mediated stem cell homeostasis.	protein_coding	-		
Red	AT1G67230	*LITTLE NUCLEI1 (LINC1)*	Encodes a nuclear coiled-coil protein that is involved in the determination of plant nuclear structure. Member of a small gene family in Arabidopsis containing 4 proteins (LNC1-4 or CRWN 1-4) with redundant functions in protection from oxidative damage, control of nuclear morphology and degradation of ABI5.	protein_coding	-		
Red	AT1G79110	*BOI-RELATED GENE 2 (BRG2)*	Encodes one of the BRGs (BOI-related gene) involved in resistance to Botrytis cinerea.	protein_coding	-	✓	
Red	AT2G29890	*VILLIN 1 (VLN1)*	Encodes a ubiquitously expressed villin-like protein, whose mRNA may be alternatively processed. Villin belongs to a superfamily of actin binding proteins called the villin/gelsolin family. VLN1 protein co-localizes with actin filaments in several assays. VLN1 binds and bundles F-actin in a calcium-independent manner. It does not nucleate, cap or sever actin filaments and it stabilizes actin filaments, protecting them from ADF-mediated depolymerization.	protein_coding	-	✓	
Red	AT2G47490	*NAD+ TRANSPORTER 1 (NDT1)*	Encodes a chloroplast-localized NAD+ transporter that transports NAD+ in a counter exchange mode with ADP and AMP in vitro.	protein_coding	-		
Red	AT3G51480	*GLUTAMATE RECEPTOR 3.6 (GLR3.6)*	member of Putative ligand-gated ion channel subunit family	protein_coding	-		
Red	AT4G01330	*AT4G01330*	Protein kinase superfamily protein	protein_coding	-		
Red	AT4G23980	*AUXIN RESPONSE FACTOR 9 (ARF9)*	Encodes auxin response factor 9 (ARF9)	protein_coding	ARF		
Red	AT4G39710	*PHOTOSYNTHETIC NDH SUBCOMPLEX L 4 (PnsL4)*	FK506-binding protein 16-2	protein_coding	-	✓	
Red	AT5G27420	*CARBON/NITROGEN INSENSITIVE 1 (CNI1)*	Encodes CNI1 (Carbon/Nitrogen Insensitive1) (also named as ATL31), a RING type ubiquitin ligase that functions in the Carbon/Nitrogen response for growth phase transition in Arabidopsis seedlings.	protein_coding	-		
Royalblue	AT1G09510	*AT1G09510*	similar to Eucalyptus gunnii alcohol dehydrogenase of unknown physiological function	protein_coding	-		
Royalblue	AT1G69240	*METHYL ESTERASE 15 (MES15)*	Encodes a protein predicted to act as a carboxylesterase. It has similarity to the SABP2 methyl salicylate esterase from tobacco but no enzymatic activity has been identified for this protein.	protein_coding	-		
Royalblue	AT2G33790	*ARABINOGALACTAN PROTEIN 30 (AGP30)*	Pistil-specific extensin-like protein precursor; Pollen proteins Ole e I family	protein_coding	-		
Royalblue	AT2G35460	*AT2G35460*	Late embryogenesis abundant (LEA) hydroxyproline-rich glycoprotein family	protein_coding	-		
Royalblue	AT2G42250	*CYTOCHROME P450, FAMILY 712, SUBFAMILY A, POLYPEPTIDE 1 (CYP712A1)*	member of CYP712A	protein_coding	-		
Royalblue	AT2G42670	*PLANT CYSTEINE OXIDASE 4 (PCO4)*	Involved in controlling the stability of Group VII ethylene response factors (ERF-VIIs) via N-Arg/degron pathway through catalyzing the oxidation of their N-Cys for subsequent Arginyl-tRNA--protein transferase 1 (ATE1) mediated arginine installation.	protein_coding	-		
Royalblue	AT3G25950	*AT3G25950*	TRAM, LAG1 and CLN8 (TLC) lipid-sensing domain containing protein	protein_coding	-		
Royalblue	AT3G42830	*AT3G42830*	RING/U-box superfamily protein	protein_coding	-		

Royalblue	AT4G28900	*AT4G28900*	transposable_element_gene; copia-like retrotransposon family	transposable_element_gene	-		
Royalblue	AT5G39360	*EID1-LIKE 2 (EDL2)*	EID1-like 2	protein_coding	-		
Salmon	AT1G19120	*SM-LIKE 1A (LSM1A)*	Small nuclear ribonucleoprotein family protein	protein_coding	-		
Salmon	AT1G60190	*PLANT U-BOX 19 (PUB19)*	Encodes PUB19, a plant U-box armadillo repeat protein. Involved in salt inhibition of germination together with PUB18.	protein_coding	-	✓	
Salmon	AT1G75630	*VACUOLAR H+-PUMPING ATPASE 16 KDA PROTEOLIPID SUBUNIT 4 (AVA-P4)*	Vacuolar H+-pumping ATPase 16 kD proteolipid (ava-p) mRNA	protein_coding	-		
Salmon	AT2G04500	*AT2G04500*	Cysteine/Histidine-rich C1 domain family protein	protein_coding	-		
Salmon	AT2G28890	*POLTERGEIST LIKE 4 (PLL4)*	Encodes a protein phosphatase 2C like gene, similar to POL. Involved in leaf development. Knockout mutants have abnormally shaped leaves.	protein_coding	-		
Salmon	AT2G39980	*AT2G39980*	HXXXD-type acyl-transferase family protein	protein_coding	-	✓	
Salmon	AT2G46150	*AT2G46150*	Late embryogenesis abundant (LEA) hydroxyproline-rich glycoprotein family	protein_coding	-	✓	
Salmon	AT3G62100	*INDOLE-3-ACETIC ACID INDUCIBLE 30 (IAA30)*	Encodes a member of the Aux/IAA family of proteins implicated in auxin signaling. IAA30 lacks the conserved degron (domain II) found in many family members. IAA30 transcripts are induced by auxin treatment and accumulate preferentially in the quiescent center cells of the root meristem. Overexpression of IAA30 leads to defects in gravitropism, root development, root meristem maintenance, and cotyledon vascular development. Target of LEC2 and AGL15. Promotes somatyic embryogenesis.	protein_coding	-	✓	
Salmon	AT4G23060	*IQ-DOMAIN 22 (IQD22)*	Member of IQ67 (CaM binding) domain containing family.	protein_coding	-		
Salmon	AT4G26470	*AT4G26470*	Calcium-binding EF-hand family protein	protein_coding	-	✓	
Tan	AT2G01910	*AT2G01910 (ATMAP65-6)*	Binds microtubules. Induces a crisscross mesh of microtubules, not bundles. Not involved in microtubule polymerization nor nucleation. Localizes to mitochondria.	protein_coding	-		
Tan	AT2G22990	*SINAPOYLGLUCOSE 1 (SNG1)*	sinapoylglucose:malate sinapoyltransferase. Catalyzes the formation of sinapoylmalate from sinapoylglucose. Mutants accumulate excess sinapoylglucose.	protein_coding	-	✓	
Tan	AT3G06390	*CASP-LIKE PROTEIN 1D2 (CASPL1D2)*	Uncharacterized protein family (UPF0497)	protein_coding	-		
Tan	AT3G07970	*QUARTET 2 (QRT2)*	Required for pollen separation during normal development. In *qrt* mutants, the outer walls of the four meiotic products of the pollen mother cell are fused, and pollen grains are released in tetrads. May be required for cell type-specific pectin degradation.	protein_coding	-		
Tan	AT4G09760	*CHOLINE/ETHANOLAMINE KINASE 3 (CEK3)*	encodes a choline synthase whose gene expression is induced by high salt and mannitol.	protein_coding	-		
Tan	AT4G21865	*AT4G21865*	hypothetical protein	protein_coding	-		
Tan	AT4G24890	*PURPLE ACID PHOSPHATASE 24 (PAP24)*	purple acid phosphatase 24	protein_coding	-		
Tan	AT5G07680	*NAC DOMAIN CONTAINING PROTEIN 80 (NAC080)*	NAC domain containing protein 80	protein_coding	NAC		
Tan	AT5G54020	*AT5G54020*	Cysteine/Histidine-rich C1 domain family protein	protein_coding	-		
Tan	AT5G54855	*AT5G54855*	Pollen Ole e 1 allergen and extensin family protein	protein_coding	-		
Turquoise	AT1G27120	*AT1G27120 (GALT4)*	Encodes a Golgi-localized hydroxyproline-O-galactosyltransferase.	protein_coding	-	✓	
Turquoise	AT2G22000	*ELICITOR PEPTIDE 6 PRECURSOR (PROPEP6)*	elicitor peptide 6 precursor	protein_coding	-	✓	
Turquoise	AT3G15190	*PLASTID RIBOSOMAL PROTEIN S20 (PRPS20)*	chloroplast 30S ribosomal protein S20	protein_coding	-		✓
Turquoise	AT3G26060	*PEROXIREDOXIN Q (PRXQ)*	encodes peridoxin Q which decomposes peroxides and plays a role in the protection of the photosynthetic apparatus	protein_coding	-	✓	
Turquoise	AT3G50685	*AT3G50685*	anti-muellerian hormone type-2 receptor	protein_coding	-	✓	
Turquoise	AT4G01050	*THYLAKOID RHODANESE-LIKE (TROL)*	hydroxyproline-rich glycoprotein family protein, contains a rhodanese homology domain. Required for anchoring the FNR flavoenzyme to the thylakoid membranes and sustaining high efficiency photosynthetic linear electron flow.	protein_coding	-	✓	
Turquoise	AT4G33666	*AT4G33666*	hypothetical protein	protein_coding	-	✓	
Turquoise	AT5G36160	*TYR AMINOTRANSFERASE 2 (TAT2)*	Encodes a cytosolic L-tyrosine aminotransferase. AtTAT2 exhibits much broader amino donor specificity than AtTAT1 and can use not only Tyr but also Phe, Trp, His, Met, Leu, Ala, Ser, Cys, Asp, Asn, Gln, and Arg as amino donors.	protein_coding	-	✓	
Turquoise	AT5G44630	*AT5G44630*	Encodes a sesquiterpene synthase involved in generating all of the group B sesquiterpenes found in the Arabidopsis floral volatile blend. Strongly expressed in intrafloral nectaries.	protein_coding	-	✓	
Turquoise	AT5G49820	*ROOT UV-B SENSITIVE 6 (RUS6)*	root UVB sensitive protein (Protein of unknown function, DUF647)	protein_coding	-	✓	
Yellow	AT1G62800	*ASPARTATE AMINOTRANSFERASE 4 (ASP4)*	Encodes aspartate aminotransferase (Asp4).	protein_coding	-		
Yellow	AT1G65960	*GLUTAMATE DECARBOXYLASE 2 (GAD2)*	glutamate decarboxylase (GAD2)	protein_coding	-		

Yellow	AT2G01520	*MLP-LIKE PROTEIN 328 (MLP328)*	Encodes a cis-cinnamic acid responsive gene that is a member of the major latex protein-like gene family and plays a role in promoting vegetative growth and delaying flowering	protein_coding	-	
Yellow	AT2G22550	*AT2G22550*	Unknown protein	protein_coding	-	✓
Yellow	AT2G30540	*AT2G30540*	Encodes a member of the CC-type glutaredoxin (ROXY) family that has been shown to interact with the transcription factor TGA2.	protein_coding	-	
Yellow	AT2G43100	*ISOPROPYLMALATE ISOMERASE 2 (IPMI2)*	Small subunit, which together with IPMI SSU2, IPMI SSU3 and IPMI LSU1, is a member of heterodimeric isopropylmalate isomerase (IPMI). Together with IPMI SSU3 participates in the Met chain elongation pathway.	protein_coding	-	
Yellow	AT3G26520	*TONOPLAST INTRINSIC PROTEIN 2 (TIP2)*	gamma tonoplast intrinsic protein 2 (TIP2). expressed throughout the plant and transcript level is increased upon NaCl or ABA treatments. NaCl stress-sensitive yeast mutant strains exhibit more resistance to salt when expressing this protein.	protein_coding	-	✓
Yellow	AT3G27650	*LOB DOMAIN-CONTAINING PROTEIN 25 (LBD25)*	LOB domain-containing protein 25	protein_coding	-	
Yellow	AT4G22212	*AT4G22212*	Encodes a defensin-like (DEFL) family protein.	protein_coding	-	
Yellow	AT5G23840	*AT5G23840*	MD-2-related lipid recognition domain-containing protein	protein_coding	-	
