# Supplementary material for: Weighted Gene Correlation Network Analysis (WGCNA) of Arabidopsis Somatic Embryogenesis (SE) and Identification of Key Gene Modules to Uncover SE-Associated Hub Genes
Source: Int J Genomics. 2022 Jul 4;2022:7471063. doi: 10.1155/2022/7471063 (PMC9274236; doi:10.1155/2022/7471063)
Supplement: Supplementary 4 — Table S4. Important hub genes based on differential gene expression analysis. [file 7471063.f4.pdf]

Table S4. Important hub genes based on differential gene expression analysis.

Gene identifier	Gene symbol	DEGs (|*log2 FC*| ≥ 2.0 and *p-value* < 0.05)	DEGs - Wickramasuriya & Dunwell (|*log2 FC*| ≥ 2.0 and *p-value* < 0.05)	Expression in SE tissues compared to leaf tissue	Arabidopsis eFP browser	Gene description
AT1G06040	*SALT TOLERANCE (STO)*		✓	Down-regulated		Encodes salt tolerance protein (STO) which confers salt tolerance to yeast cells.
AT1G16330	*CYCLIN B3;1 (CYCB3;1)*		✓	Up-regulated		core cell cycle genes
AT1G19540*	*AT1G19540*	✓	✓	Up-regulated	High and specific to the embryonic stages	NmrA-like negative transcriptional regulator family protein
AT1G27120*	*AT1G27120 (GALT4)*	✓		-		Encodes a Golgi-localized hydroxyproline-O-galactosyltransferase.
AT1G27930*	*AT1G27930 (AGM1)*		✓	Down-regulated		Arabinogalactan methylesterase,involved in arabinogalactan glucuronic acid methylation. Interacts with eIF3.
AT1G28600	*GUARD-CELL-ENRICHED GDSL LIPASE 2 (GGL2)*		✓	Down-regulated		GDSL-motif esterase/acyltransferase/lipase. Enzyme group with broad substrate specificity that may catalyze acyltransfer or hydrolase reactions with lipid and non-lipid substrates.
AT1G30600*	*AT1G30600*		✓	Up-regulated		Subtilase family protein
AT1G57990	*PURINE PERMEASE 18 (PUP18)*	✓		-		Member of a family of proteins related to PUP1, a purine transporter. May be involved in the transport of purine and purine derivatives such as cytokinins, across the plasma membrane.
AT1G60190	*PLANT U-BOX 19 (PUB19)*	✓		-		Encodes PUB19, a plant U-box armadillo repeat protein. Involved in salt inhibition of germination together with PUB18.
AT1G62520*	*AT1G62520*		✓	Up-regulated		sulfated surface-like glycoprotein
AT1G65870*	*AT1G65870*		✓	Down-regulated		Disease resistance-responsive (dirigent-like protein) family protein
AT1G65960	*GLUTAMATE DECARBOXYLASE 2 (GAD2)*		✓	Down-regulated		glutamate decarboxylase (GAD2)
AT1G67080	*ABSCISIC ACID (ABA)-DEFICIENT 4 (ABA4)*		✓	Down-regulated		Encodes a protein involved in the photoprotection of PSII. An aba4-1 mutant completely lacks neoxanthin,a component of the chromophore of the peripheral antenna system in PSII. ABA4 is required for neoxanthin biosynthesis, an intermediary step in abscisic acid biosynthesis, but no catalytic activity has been detected for the ABA4 protein.
AT1G69240	*METHYL ESTERASE 15 (MES15)*		✓	Up-regulated		Encodes a protein predicted to act as a carboxylesterase. It has similarity to the SABP2 methyl salicylate esterase from tobacco but no enzymatic activity has been identified for this protein.
AT1G79110	*BOI-RELATED GENE 2 (BRG2)*	✓		-		Encodes one of the BRGs (BOI-related gene) involved in resistance to Botrytis cinerea.
AT2G04500*	*AT2G04500*		✓	Up-regulated		Cysteine/Histidine-rich C1 domain family protein
AT2G15400*	*AT2G15400 (NRPE3B)*		✓	Up-regulated		Non-catalytic subunit of Nuclear DNA-dependent RNA polymerase V; homologous to budding yeast RPB3 and the E. coli RNA polymerase alpha subunit.
AT2G21820*	*AT2G21820*		✓	Up-regulated	High and specific to the embryonic stages	seed maturation protein
AT2G22000	*ELICITOR PEPTIDE 6 PRECURSOR (PROPEP6)*	✓	✓	Down-regulated		elicitor peptide 6 precursor
AT2G22550*	*AT2G22550 (SVP)/AT2G22540*	✓	✓	Down-regulated		Encodes a nuclear protein that acts as a floral repressor and functions within the thermosensory pathway
AT2G22990	*SINAPOYLGLUCOSE 1 (SNG1)*	✓	✓	Down-regulated		sinapoylglucose:malate sinapoyltransferase. Catalyzes the formation of sinapoylmalate from sinapoylglucose. Mutants accumulate excess sinapoylglucose.
AT2G23530*	*AT2G23530*		✓	Up-regulated		Zinc-finger domain of monoamine-oxidase A repressor R1
AT2G24970*	*AT2G24970*		✓	Up-regulated		spindle/kinetochore-associated protein
AT2G29300*	*AT2G29300*		✓	Down-regulated	High and specific to the embryonic stages	NAD(P)-binding Rossmann-fold superfamily protein
AT2G29890	*VILLIN 1 (VLN1)*	✓		-		Encodes a ubiquitously expressed villin-like protein, whose mRNA may be alternatively processed. Villin belongs to a superfamily of actin binding proteins called the villin/gelsolin family. VLN1 protein co-localizes with actin filaments in several assays. VLN1 binds and bundles F-actin in a calcium-independent manner. It does not nucleate, cap or sever actin filaments and it stabilizes actin filaments, protecting them from ADF-mediated depolymerization.
AT2G30540*	*AT2G30540 (ROXY7)*		✓	Down-regulated		Encodes a member of the CC-type glutaredoxin (ROXY) family that has been shown to interact with the transcription factor TGA2.
AT2G32280	*VASCULATURE COMPLEXITY AND CONNECTIVITY (VCC)*		✓	Up-regulated		Encodes a member of a plant-specific gene family that is required for embryo provasculature development. The gene product regulates vascular network complexity and connectivity in cotyledons.
AT2G32540	*CELLULOSE SYNTHASE-LIKE B4 (CSLB04)*		✓	Down-regulated		encodes a gene similar to cellulose synthase
AT2G33520	*CYSTEINE-RICH TRANSMEMBRANE MODULE 7 (ATHCYSTM7)*		✓	Up-regulated	High and specific to the embryonic stages	cysteine-rich/transmembrane domain protein A

AT2G38900*	*AT2G38900*		✓	Up-regulated	High and specific to the embryonic stages	Predicted to encode a PR (pathogenesis-related) peptide that belongs to the PR-6 proteinase inhibitor family. Six putative PR-6-type protein encoding genes are found in Arabidopsis: At2g38900, At2g38870, At5g43570, At5g43580, At3g50020 and At3g46860.
AT2G39980*	*AT2G39980*	✓		-		HXXXD-type acyl-transferase family protein
AT2G40420*	*AT2G40420*		✓	Down-regulated		Encodes a putative amino acid transporter.
AT2G43100	*ISOPROPYLMALATE ISOMERASE 2 (IPMI2)*		✓	Down-regulated		Small subunit, which together with IPMI SSU2, IPMI SSU3 and IPMI LSU1, is a member of heterodimeric isopropylmalate isomerase (IPMI). Together with IPMI SSU3 participates in the Met chain elongation pathway.
AT2G45600*	*AT2G45600*		✓	Down-regulated		alpha/beta-Hydrolases superfamily protein
AT2G46150*	*AT2G46150*	✓		-		Late embryogenesis abundant (LEA) hydroxyproline-rich glycoprotein family
AT3G14060*	*AT3G14060*	✓		-		hypothetical protein
AT3G15190	*PLASTID RIBOSOMAL PROTEIN S20 (PRPS20)*		✓	Down-regulated		chloroplast 30S ribosomal protein S20
AT3G25950*	*AT3G25950*		✓	Down-regulated		TRAM, LAG1 and CLN8 (TLC) lipid-sensing domain containing protein
AT3G26060	*PEROXIREDOXIN Q (PRXQ)*	✓	✓	Down-regulated		encodes periredoxin Q which decomposes peroxides and plays a role in the protection of the photosynthetic apparatus
AT3G26330	*CYTOCHROME P450, FAMILY 71, SUBFAMILY B, POLYPEPTIDE 37 (CYP71B37)*		✓	Up-regulated		putative cytochrome P450
AT3G26520	*TONOPLAST INTRINSIC PROTEIN 2 (TIP2)*	✓	✓	Down-regulated		gamma tonoplast intrinsic protein 2 (TIP2). expressed throughout the plant and transcript level is increased upon NaCl or ABA treatments. NaCl stress-sensitive yeast mutant strains exhibit more resistance to salt when expressing this protein.
AT3G27650	*LOB DOMAIN-CONTAINING PROTEIN 25 (LBD25)*		✓	Down-regulated		LOB domain-containing protein 25
AT3G50685*	*AT3G50685*	✓	✓	Down-regulated		anti-muellerian hormone type-2 receptor
AT3G53180	*NODULIN/GLUTAMINE SYNTHASE-LIKE PROTEIN (NodGS)*	✓		-		Encodes a protein that is the product of a fusion gene with a C-terminal GSI like sequence and an N-terminal part sharing homology with nodulins. It self-assembles into oligomers and its expression is increased in response to flagellin treatment.
AT3G53980*	*AT3G53980*		✓	Down-regulated		Bifunctional inhibitor/lipid-transfer protein/seed storage 2S albumin superfamily protein
AT3G56980	*BASIC HELIX-LOOP-HELIX 39 (BHLH39)*	✓		-		Encodes a member of the basic helix-loop-helix transcription factor protein.
AT3G62100	*INDOLE-3-ACETIC ACID INDUCIBLE 30 (IAA30)*	✓	✓	Up-regulated		Encodes a member of the Aux/IAA family of proteins implicated in auxin signaling. IAA30 lacks the conserved degron (domain II) found in many family members. IAA30 transcripts are induced by auxin treatment and accumulate preferentially in the quiescent center cells of the root meristem. Overexpression of IAA30 leads to defects in gravitropism, root development, root meristem maintenance, and cotyledon vascular development. Target of LEC2 and AGL15. Promotes somatic embryogenesis.
AT4G01050	*THYLAKOID RHODANESE-LIKE (TROL)*	✓	✓	Down-regulated		hydroxyproline-rich glycoprotein family protein, contains a rhodanese homology domain. Required for anchoring the FNR flavoenzyme to the thylakoid membranes and sustaining high efficiency photosynthetic linear electron flow.
AT4G01270*	*AT4G01270*		✓	Up-regulated		RING/U-box superfamily protein
AT4G01330*	*AT4G01330*		✓	Down-regulated		Protein kinase superfamily protein
AT4G09760	*CHOLINE/ETHANOLAMINE KINASE 3 (CEK3)*		✓	Down-regulated		encodes a choline synthase whose gene expression is induced by high salt and mannitol.
AT4G17695	*KANADI 3 (KAN3)*	✓	✓	Down-regulated		Homeodomain-like superfamily protein
AT4G20530*	*AT4G20530*		✓	Up-regulated		cysteine-rich repeat secretory-like protein
AT4G22212*	*AT4G22212*		✓	Up-regulated		Encodes a defensin-like (DEFL) family protein.
AT4G26370*	*AT4G26370*		✓	Down-regulated		antitermination NusB domain-containing protein
AT4G26470*	*AT4G26470*	✓	✓	Up-regulated		Calcium-binding EF-hand family protein
AT4G28310*	*AT4G28310*		✓	Up-regulated		microtubule-associated protein
AT4G30850	*HEPTAHELICAL TRANSMEMBRANE PROTEIN2 (HHP2)*		✓	Up-regulated		heptahelical transmembrane protein homologous to human adiponectin receptors and progestin receptors
AT4G33666*	*AT4G33666*	✓	✓	Down-regulated		hypothetical protein
AT4G39710	*PHOTOSYNTHETIC NDH SUBCOMPLEX L 4 (PnsL4)*	✓	✓	Down-regulated		FK506-binding protein 16-2
AT5G16010*	*AT5G16010*		✓	Down-regulated		3-oxo-5-alpha-steroid 4-dehydrogenase family protein
AT5G16140*	*AT5G16140*		✓	Down-regulated		Peptidyl-tRNA hydrolase family protein
AT5G27420	*CARBON/NITROGEN INSENSITIVE 1 (CNI1)*		✓	Down-regulated		Encodes CNI1 (Carbon/Nitrogen Insensitive1) (also named as ATL31), a RING type ubiquitin ligase that functions in the Carbon/Nitrogen response for growth phase transition in Arabidopsis seedlings.
AT5G27560*	*AT5G27560*		✓	Down-regulated		DUF1995 domain protein, putative (DUF1995)
AT5G36160	*TYR AMINOTRANSFERASE 2 (TAT2)*	✓	✓	Down-regulated		Encodes a cytosolic L-tyrosine aminotransferase. AtTAT2 exhibits much broader amino donor specificity than AtTAT1 and can use not only Tyr but also Phe, Trp, His, Met, Leu, Ala, Ser, Cys, Asp, Asn, Gln, and Arg as amino donors.

AT5G43770*	*AT5G43770*		✓	Up-regulated	High and specific to the embryonic stages	proline-rich family protein
AT5G44380*	*AT5G44380 (AtBBE24)*	✓	✓	Up-regulated	High and specific to the embryonic stages	FAD-binding Berberine family protein
AT5G44630*	*AT5G44630*	✓		-		Encodes a sesquiterpene synthase involved in generating all of the group B sesquiterpenes found in the Arabidopsis floral volatile blend. Strongly expressed in intrafloral nectaries.
AT5G49820	*ROOT UV-B SENSITIVE 6 (RUS6)*	✓		-		root UVB sensitive protein (Protein of unknown function, DUF647)
AT5G50300	*AZA-GUANINE RESISTANT2 (AZG2)*	✓		-		Encodes a homolog of the adenine-guanine-hypoxanthine transporter AzgA of Aspergillus nidulans. Function as a plant adenine-guanine transporter. Two closely related genes exist in Arabidopsis: AT3G10960 (Azg1) and AT5G50300 (Azg2).
AT5G54540*	*AT5G54540*		✓	Down-regulated		Uncharacterized conserved protein (UCP012943)
AT5G55200	*MITOCHONDRIAL GRPE 1 (MGE1)*	✓		-		Co-chaperone GrpE family protein
AT5G59750	*HOMOLOG OF RIBA 3 (RIBA3)*		✓	Down-regulated		monofunctional riboflavin biosynthesis protein RIBA 3
AT5G63990*	*AT5G63990*	✓		-		Inositol monophosphatase family protein
AT5G65860*	*AT5G65860*	✓		-		ankyrin repeat family protein
ATCG00420	*NADH DEHYDROGENASE SUBUNIT J (NDHJ)*		✓	Down-regulated		Encodes NADH dehydrogenase subunit J. Its transcription is increased upon sulfur depletion.
ATCG00430	*PHOTOSYSTEM II REACTION CENTER PROTEIN G (PSBG)*		✓	Down-regulated		Encodes a protein which was originally thought to be part of photosystem II but its wheat homolog was later shown to encode for subunit K of NADH dehydrogenase.
ATCG00890*	*ATCG00890 (NDHB.1)*		✓	Down-regulated		NADH dehydrogenase ND2

** - uncharacterized genes*
