# Supplementary material for: Weighted Gene Correlation Network Analysis (WGCNA) of Arabidopsis Somatic Embryogenesis (SE) and Identification of Key Gene Modules to Uncover SE-Associated Hub Genes
Source: Int J Genomics. 2022 Jul 4;2022:7471063. doi: 10.1155/2022/7471063 (PMC9274236; doi:10.1155/2022/7471063)
Supplement: Supplementary 5 — Table S5. EMB genes in the coexpression network. [file 7471063.f5.pdf]

Table S5. EMB genes in the co-expression network

Module	Gene identifier	Gene Symbol	Predicted protein function	Additional comments on protein function
Black	AT5G14850	*APTG1*	Mannosyltransferase	ER-localized; Biosynthesis of GPI anchors
Black	AT5G59340	*WOX2*	Homeodomain Protein	Transcriptional regulator
Black	AT5G51020	*CRL*	Plastid Outer Envelope Membrane Protein	Required for plastid division
Black	AT3G48930	*EMB1080*	Ribosomal Protein S11	Translation in cytosol
Black	AT4G23940	*FtsHi1*	Chloroplast AAA-ATPase; Ycf2-FtsHi Complex Subunit	Required for chloroplast protein import
Black	AT4G12130	*COG0354*	Mitochondrial Fe-S Cluster Biogenesis Protein	Folate dependent
Black	AT3G04400	*EMB2171*	Ribosomal Protein L17/L23	Translation in mitochondria or cytosol
Black	AT1G49880	*EMB3106*	Mitochondrial Sulfhydryl Oxidase	Proposed to stabilize mitochondrial proteins by oxidative protein folding
Black	AT1G02780	*EMB2386*	Ribosomal Protein L19	Translation in cytosol
Black	AT1G78580	*TPS1*	Trehalose-6-Phosphate Synthase 1	Catalyzes first step in trehalose biosynthesis
Black	AT2G03870	*EMB2816*	Pre-mRNA Splicing Factor	RNA processing and decay
Black	AT2G18020	*EMB2296*	Ribosomal Protein L8/L2	Translation in mitochondria or cytosol
Black	AT2G34480	*RPL18aB*	Ribosomal Protein	Translation in cytosol
Blue	AT2G39770	*CYT1*	Mannose-1-Phosphate-Guanyltransferase; GDP-MannosePyrophosphorylase	GDP-mannose production; N-glycosylation; Vitamin C biosynthesis
Blue	AT2G47620	*SWI3A*	Subunit of SWI/SNF Chromatin Remodeling Complex	ATP-dependent chromatin remodeling; Transcriptional regulation
Blue	AT4G14590	*EMB2739*	snRNA Processing Factor	snRNA 3' end maturation; Pre-mRNA splicing
Blue	AT5G19820	*EMB2734*	Karyopherin (Importin) Protein	Import of proteins into nucleus; miRNA biogenesis
Blue	AT5G20920	*EMB1401*	Translation Initiation Factor	Attachment of ribosome small subunit in translation
Blue	AT4G36480	*EMB2779*	Serine Palmitoyltransferase Subunit	Catalyzes first step in sphingolipid biosynthesis
Blue	AT4G36630	*EMB2754*	Putative Vacuolar Protein (VPS39)	Proposed role in vacuolar protein sorting
Blue	AT1G31860	*HISN2*	Histidine Biosynthetic Enzyme	Catalyzes second step in histidine biosynthesis
Blue	AT5G16750	*TOZ*	Uncertain	Nucleolar WD-40 repeat protein; Possible role in 18S rRNA biogenesis
Blue	AT5G27740	*EMB2775*	DNA Replication Factor C Subunit	DNA replication; PCNA clamp loading
Blue	AT5G27540	*EMB2473*	Mitochondrial GTPase	Regulation of mitochondrial trafficking and morphology
Blue	AT5G26760	*RIMA*	Putative Role in Nuclear Import and Transcriptional Regulation	Interacts with IYO transcriptional regulator
Blue	AT5G67570	*EMB1408*	PPR Protein	RNA binding protein; Organellar gene function
Blue	AT5G66055	*EMB2036*	Uncertain	Chloroplast-localized ankyrin repeat protein
Blue	AT5G63420	*EMB2746*	Chloroplast Ribonuclease J	Chloroplast RNA metabolism
Blue	AT5G62990	*EMB1692*	PORR Domain Protein	Putative role in organellar RNA splicing or metabolism
Blue	AT5G60540	*EMB2407*	Pyridoxine Glutaminase	Pyroxidine (vitamin B6) biosynthesis
Blue	AT5G59440	*ZEUS1*	Thymidylate Kinase	Biosynthesis of dTDP; Regulation of replication
Blue	AT5G58230	*MSI1*	Polycomb Repressive Complex Subunit	Chromatin modification; Histone methylation
Blue	AT5G58020	*RTF2*	Replication Termination Factor Domain Protein	Pre-mRNA splicing
Blue	AT5G55940	*EMB2731*	Unknown	None
Blue	AT5G53400	*BOB1*	Non-Canonical Small Heat Shock Protein	Putative molecular chaperone
Blue	AT5G49720	*KOR*	Endo-Β-1,4-Glucanase; Glycosyl Hydrolase	Cellulose biosynthesis
Blue	AT3G49010	*EMB2812*	GIN5 Complex Subunit	DNA replication
Blue	AT5G48720	*XRI1*	Novel DNA Repair Factor	Functions in meiosis; male gametophyte development
Blue	AT5G48840	*PTS*	Pantothenate Synthetase	Vitamin B5 biosynthesis; Coenzyme A function
Blue	AT5G42080	*ADL1A*	Dynamin-Like Protein	Associated with cell plate; Similar to phragmoplastin; Proposed role in vesicular trafficking and cytokinesis
Blue	AT5G37510	*EMB1467*	Mitochondrial NADH Dehydrogenase Complex Subunit	Role in electron transport chain
Blue	AT5G23880	*EMB1265*	Cleavage and Polyadenylation Specificity Factor	Location of poly(A) sites; Modulation of transcription termination
Blue	AT5G18580	*FASS*	Protein Phosphatase (PP2A Complex Subunit)	Preprophase band formation; Orientation of cell division
Blue	AT5G17710	*EMB1241*	Chloroplast GrpE Protein	Interacts with Hsp70; Chloroplast protein maintenance
Blue	AT5G13780	*NAA10*	N-Terminal Acetyltransferase, Catalytic Subunit	Protein modification
Blue	AT5G13010	*EMB3011*	RNA Helicase	Pre-mRNA splicing factor; miRNA biogenesis
Blue	AT5G11890	*EMB3135*	Unknown	None
Blue	AT5G09900	*EMB2107*	26S Proteasome Regulatory Subunit	Component of 26S proteasome lid
Blue	AT5G06240	*EMB2735*	Unknown	None
Blue	AT5G05680	*EMB2789*	Nuclear Envelope Protein (Nup88)	Nuclear protein export; Spindle apparatus function
Blue	AT5G02190	*ASP38*	Aspartic Protease	ER-localized protein
Blue	AT5G02250	*EMB2730*	Ribonuclease II Family Protein	3' to 5' Exoribonuclease; Maturation of chloroplast RNAs
Blue	AT3G60740	*TTN1*	Tubulin Folding Cofactor D	Regulation of Β-tubulin folding and microtubule dynamics
Blue	AT3G55010	*EMB2818*	Phosphoribosylformylglycinamidine Cyclo-Ligase (PUR5)	AIR synthetase; Purine biosynthesis
Blue	AT3G54650	*FBL17*	F-Box Protein	Cell cycle regulation in male gametophyte development
Blue	AT3G50870	*HAN*	GATA Factor Transcriptional Regulator	Regulation of transcription
Blue	AT3G49240	*EMB1796*	PPR Protein	Editing of mitochondrial and chloroplast transcripts; Interacts with DYW2
Blue	AT3G48750	*CDC2A*	A-Type Cyclin-Dependent Kinase	Cell cycle control
Blue	AT4G38440	*IYO*	Transcriptional Regulator	RNA polymerase II interacting factor
Blue	AT4G33090	*APM1*	M1 Aminopeptidase	Proposed role in auxin transport
Blue	AT4G32720	*LA1*	RNA Binding Protein	RNA maturation and stability in nucleus
Blue	AT4G32410	*RSW1*	Cellulose Synthase	Cellulose biosynthesis; Primary cell wall formation
Blue	AT4G32400	*EMB104*	Transmembrane Nucleotide Transporter	Protein localized to plastids and mitochondria

Blue	AT4G31810	*CHY4*	3-Hydroxyisobutyryl-CoA Hydrolase	Valine degradation in mitochondria
Blue	AT4G27010	*EMB2788*	Uncertain	Possible role in ribosome biogenesis
Blue	AT4G26500	*EMB1374*	Fe-S Cluster Assembly Factor	Activator of Plastidic and Mitochondrial Desulfurases
Blue	AT4G23660	*PPT1*	4-Hydroxybenzoate Polyprenyl Diphosphate Transferase	Ubiquinone Biosynthesis
Blue	AT4G21710	*EMB1989*	RNA Polymerase II Subunit	Transcription
Blue	AT4G20740	*EMB3131*	PPR Protein	RNA binding protein; Organellar gene function
Blue	AT4G10760	*EMB1706*	m6A Methyltransferase Complex Subunit	N6-adenosine methylation of mRNA; mRNA modification, splicing, metabolism
Blue	AT4G09980	*EMB1691*	m6A Methyltransferase Complex Subunit	N6-adenosine methylation of mRNA; mRNA modification, splicing, metabolism
Blue	AT4G07410	*PCN*	WD-40 Repeat Protein	Proposed role in auxin signaling
Blue	AT4G02570	*AXR6*	Cullin 1 Protein; SCF Ubiquitin Ligase Complex Subunit	Protein degradation; Auxin response pathways
Blue	AT4G02060	*PRL*	DNA Replication Licensing Factor	Initiation of replication
Blue	AT4G00231	*ZIX*	Unknown	Armadillo Repeat Protein
Blue	AT1G56200	*EMB1303*	Unknown	Chloroplast-localized protein
Blue	AT3G20630	*TTN6*	Deubiquitinating Enzyme	Ubiquitin processing and recycling; Negative regulator of endoreduplication
Blue	AT3G13200	*EMB2769*	U5 Associated Protein	Proposed role in pre-mRNA splicing
Blue	AT1G24340	*EMB2421*	Polyketide Hydroxylase-Related Monooxygenase	None
Blue	AT3G20440	*EMB2729*	1,4-Alpha-Glucan Branching Enzyme	Similar to starch branching enzyme
Blue	AT3G19770	*VPS9A*	Guanine Exchange Factor	Regulator of Rab5 GTPase; Membrane trafficking
Blue	AT3G17910	*EMB3121*	Cytochrome C Oxidase Assembly (Surf1)	Mitochondrial electron transport
Blue	AT3G27670	*RST1*	Uncertain	Plasma membrane protein; Cuticular wax formation
Blue	AT3G04680	*CLPS3*	Polyadenylation Factor	mRNA 3' end processing
Blue	AT3G10670	*NAP7*	Plastidic SufC-Like ABC/ATPase	Fe-S cluster maintenance and repair
Blue	AT3G08950	*HCC1*	Cytochrome C Oxidase Assembly Factor	Homolog of yeast copper chaperone
Blue	AT3G10220	*EMB2804*	Tubulin Folding Cofactor B	Regulation of Β-tubulin folding and microtubule dynamics
Blue	AT3G01610	*EMB1354*	AAA ATPase	Putative cell division control protein; Possible role in protein unfolding
Blue	AT1G63160	*EMB2811*	DNA Replication Factor C Subunit	DNA replication; PCNA clamp loading
Blue	AT1G77470	*EMB2810*	DNA Replication Factor C Subunit	DNA replication; PCNA clamp loading; Chromatin assembly
Blue	AT1G67630	*EMB2814*	DNA Polymerase Alpha; Beta Subunit	Initiation of DNA replication
Blue	AT1G80410	*EMB2753*	N-Terminal Acetyltransferase	Protein modification
Blue	AT1G51450	*TRAUCO*	Trithorax Class Transcriptional Regulator; H3K4 Methyltransferase Complex Component	Regulation of transcription; Histone modification
Blue	AT1G55870	*PARN*	Poly(A) Ribonuclease	Regulation of poly(A) status of mitochondrial mRNA
Blue	AT1G62360	*STM*	Knotted Class Homeodomain Protein	Transcriptional regulation; Shoot meristem formation
Blue	AT1G07910	*ZYG3*	tRNA Ligase	tRNA splicing in cytosol
Blue	AT1G34550	*EMB2756*	Unknown	None
Blue	AT1G71820	*SEC6*	Subunit of Exocyst Complex	Tethering of secretory vesicles to plasma membrane
Blue	AT1G08260	*EMB2284*	Subunit of DNA Polymerase Epsilon	DNA replication and repair; Cell cycle control
Blue	AT1G49400	*EMB1129*	Ribosomal Protein S17	Translation in cytosol
Blue	AT1G21690	*EMB1968*	Replication Factor C Subunit	DNA replication; PCNA clamp loading
Blue	AT1G15220	*CCMH*	Mitochondrial Thiol-Disulfide Oxidoreductase	Mitochondrial cytochrome C maturation
Blue	AT1G13980	*EMB30*	ARF Guanine Exchange Factor	Large membrane-associated ARF GEF; Role in vesicle trafficking and cell polarity
Blue	AT1G76060	*EMB1793*	Uncertain	Complex 1 LYR motif protein
Blue	AT1G79490	*EMB2217*	PPR Protein	RNA binding protein; Organellar gene function
Blue	AT2G17510	*EMB2763*	3' to 5' Exoribonuclease	Exosome component; Proposed role in ribosomal RNA processing
Blue	AT1G05600	*EMB3101*	PPR Protein	RNA binding protein; Organellar gene function
Blue	AT2G17250	*EMB2762*	Ribosome Biogenesis Factor	Nucleolar protein; Proposed role in rRNA processing
Blue	AT2G16440	*MCM4*	Minichromosome Maintenance Complex Subunit	DNA replication machinery
Blue	AT2G21470	*EMB2764*	SUMO Activating Enzyme	Protein modification
Blue	AT1G60170	*EMB1220*	Splicing Factor	Pre-mRNA processing; Transcriptional gene silencing
Blue	AT1G03360	*RRP4*	Exosome Subunit; Exonuclease	rRNA processing; RNA metabolism
Blue	AT1G10270	*GRP23*	Novel PPR Protein	Putative transcriptional regulator; Interacts with RNA polymerase II
Blue	AT2G18390	*TTN5*	ARL2 GTPase	Regulation of Β-tubulin folding and microtubule dynamics
Blue	AT2G20000	*HBT*	Anaphase Promoting Complex Subunit	Protein degradation; Cell cycle control
Blue	AT2G01350	*QPT*	Quinolinate Phosphoribosyl Transferase	NAD biosynthesis from aspartate in plastids
Blue	AT2G01420	*PIN4*	Transmembrane Auxin Efflux Carrier	Maintenance of embryonic auxin gradients
Blue	AT2G38770	*EMB2765*	RNA Helicase	Pre-mRNA splicing; miRNA biogenesis
Blue	AT2G31060	*EMB2785*	E2Fa Transcriptional Activator	Cell proliferation; Cell cycle control
Blue	AT2G26060	*EMB1345*	Cytosolic Fe-S Cluster Assembly Factor	WD-40 repeat protein
Blue	AT2G07690	*MCM5*	Minichromosome Maintenance Complex Subunit	DNA replication machinery
Blue	AT2G34260	*WDR55*	WD Repeat Protein	Substrate recruitment for ubiquitin E3 ligase complexes
Blue	AT2G38280	*FAC1*	AMP Deaminase	Purine biosynthesis; Outer mitochondrial membrane protein
Blue	AT2G41500	*EMB2776*	Pre-mRNA Splicing Factor	Proposed role in pre-mRNA splicing
Blue	AT2G35030	*COD1*	Mitochondrial PPR Protein	Editing of cytochrome C oxidase, NADH dehydrogenase transcripts associated with respiratory complex
Blue	AT2G26350	*PEX10*	Peroxisomal Membrane Protein; Ubiquitin-Protein Ligase	Import of matrix proteins into peroxisomes
Brown	AT4G16370	*OPT3*	Plasma Membrane Iron Transporter	Iron homeostasis
Brown	AT4G16440	*NAR1*	Cytosolic Fe-S Cluster Assembly Component	None

Brown	AT5G13480	*FY*	mRNA Polyadenylation Factor	3' End processing of specific mRNAs
Brown	AT5G15920	*EMB2782*	SMC5/6 Complex Subunit	Sister chromatid cohesion; Homologous recombination between sister chromatids; Double-strand break repair
Brown	AT5G66680	*DGL1*	Oligosaccharyltransferase (OST) Complex Subunit	Proposed role in N-linked glycosylation of proteins
Brown	AT5G63290	*HEMN1*	Coproporphyrinogen III Oxidase; Mitochondrial Localized	Tetrapyrrole biosynthesis
Brown	AT5G56290	*EMB2790*	Peroxisome Targeting Protein	Receptor for peroxisomal protein import
Brown	AT5G55490	*GEX1*	Plasma Membrane Protein	Proposed role in signaling pathway in gametophyte and early embryo
Brown	AT5G54800	*GPT1*	Plastidial Glucose-6-Phosphate/Phosphate Antiporter	Metabolite import into plastids
Brown	AT5G50960	*NBP35*	Cytosolic Fe-S Cluster Assembly Factor	Interacts with DRE2 protein
Brown	AT5G46280	*MCM3*	Minichromosome Maintenance Complex Subunit	DNA replication machinery
Brown	AT5G42390	*SPP*	Stromal Processing Peptidase	Cleavage of chloroplast transit peptide upon protein import from cytosol
Brown	AT5G41480	*GLA1*	Dihydrofolate Synthetase	Folate biosynthesis
Brown	AT5G40160	*EMB506*	Uncertain	Ankyrin repeat protein; Plastid differentiation
Brown	AT5G37630	*EMB2656*	Chromosome Condensation Factor	Similar to condensin subunit (CAP-G)
Brown	AT5G18820	*EMB3007*	Chloroplast Chaperonin	Folding of specific chloroplast proteins
Brown	AT5G08470	*EMB2817*	AAA ATPase	Peroxisome biogenesis
Brown	AT3G60830	*ARP7*	Actin-Related, Nuclear-Localized Protein	Proposed role in chromatin remodeling
Brown	AT3G55620	*EMB1624*	Translation Initiation Factor	Regulation of translation and 80S ribosome assembly
Brown	AT3G46560	*EMB2474*	Mitochondrial Inner Membrane Protein	Mitochondrial protein import
Brown	AT4G29660	*EMB2752*	Unknown	None
Brown	AT4G26300	*EMB1027*	Arginine tRNA Synthetase	Translation in chloroplasts
Brown	AT4G24270	*EMB140*	Uncertain	RNA recognition motif protein; Possible role in pre-mRNA processing and modification
Brown	AT4G22970	*AESP*	Separase; Kinesin Activating Protein	Separation of sister chromatids; Regulation of microtubule dynamics
Brown	AT4G21800	*QQT2*	ATP/GTP Binding Protein	Proposed role in microtubule localization
Brown	AT4G21300	*AtDEK36*	PPR Protein	RNA editing in mitochondria
Brown	AT4G13750	*EMB2597*	Uncertain	Plant-specific nuclear protein; Proposed role in auxin response
Brown	AT4G11150	*EMB2448*	Vacuolar H+ ATPase	Vacuolar proton pump component; Required for Golgi organization and secretory pathways
Brown	AT4G03240	*FH*	Frataxin	Mitochondrial and Chloroplast Fe-S cluster metabolism
Brown	AT4G02990	*BSM*	Uncertain	Plastid-localized mTERF protein; Plastid gene expression
Brown	AT3G18290	*EMB2454*	E3 Ubiquitin Ligase	Iron response mediator; Interacts with transcriptional regulators
Brown	AT3G16810	*APUM24*	Nucleolar Pumilio Class RNA Binding Protein	Pre-rRNA processing
Brown	AT3G13930	*MTE2-2*	Mitochondrial Pyruvate Dehydrogenase Subunit	Basic metabolism; Entry into citric acid cycle
Brown	AT3G15660	*GRXS15*	Mitochondrial Glutaredoxin S15	Fe-S protein maturation
Brown	AT3G17300	*EMB2786*	Uncertain	Putative Complex I (LYR domain) protein; Possible role in Fe-S cluster assembly; Mitochondrial electron transport
Brown	AT3G04460	*PEX12*	Peroxisomal Ubiquitin-Protein Ligase	Required for peroxisome biogenesis
Brown	AT3G08800	*SIEL1*	Subunit of snRNA 3' End Maturation Complex	Required for endosome localization of SHORT ROOT transcription factor
Brown	AT3G07430	*EMB1990*	Uncertain	Integral membrane, chloroplast division protein; Functions in distribution of nucleoids in chloroplasts
Brown	AT3G07630	*ADT2*	Arogenate Dehydrogenase; Prephenate Dehydratase	Phenylalanine biosynthesis; Bifunctional enzyme
Brown	AT1G73590	*PIN1*	Auxin Efflux Carrier	Maintenance of embryonic auxin gradients
Brown	AT1G55900	*EMB1860*	Inner Mitochondrial Membrane Protein	Import of mitochondrial matrix proteins
Brown	AT1G20200	*EMB2719*	26S Proteasome Regulatory Subunit	Component of 26S proteasome; Protein degradation
Brown	AT1G44900	*MCM2*	DNA Helicase; Minichromosome Maintenance Complex Subunit	DNA replication machinery
Brown	AT1G79810	*PEX2*	Peroxisomal Membrane Protein; Ubiquitin-Protein Ligase	Import of matrix proteins into peroxisomes
Brown	AT1G80070	*SUS2*	PRP8 Splicing Factor	Pre-mRNA processing factor; Spliceosome component
Brown	AT1G21650	*SECA2*	Chloroplast Inner Envelope Translocase	Chloroplast protein import
Brown	AT1G13120	*GLE1*	Nucleoporin; Nuclear Pore Complex Subunit	RNA transport from nucleus to cytosol
Brown	AT2G04030	*EMB1956*	Heat Shock Protein	Chloroplast-localized molecular chaperone; Proposed role in chloroplast protein import and thylakoid membrane maintenance
Brown	AT1G59990	*EMB3108*	DEAD Box RNA Helicase	Proposed role in chloroplast ribosome biogenesis
Brown	AT1G79230	*STR1*	Mitochondrial Sulfurtransferase 1	Rhodanese; Sulfur transfer; Cysteine degradation
Brown	AT1G67490	*KNF*	Alpha Glucosidase 1	Early step in N-glycan trimming of glycoproteins
Brown	AT1G67320	*EMB2813*	DNA Polymerase Alpha; Primase Subunit	Initiation of DNA replication
Brown	AT2G15690	*DYW2*	PPR Protein	Editing of chloroplast and mitochondrial transcripts; Interacts with NUWA
Brown	AT2G18510	*EMB2444*	Spliceosome-Associated Protein	RNA recognition motif (RRM) protein; RNA processing
Brown	AT2G29530	*TIM10*	Mitochondrial Inner Membrane Protein	Mitochondrial protein import
Brown	AT2G37560	*ORC2*	Origin Recognition Complex Subunit	DNA replication; Chromatin structure; Gene silencing
Brown	AT2G44520	*COX10*	Mitochondrial Farnesyltransferase	Cytochrome C oxidase biogenesis
Brown	AT2G45690	*SSE*	Peroxisomal Biogenesis Factor	Required for protein and oil body biogenesis
Cyan	AT5G08170	*EMB1873*	Agmatine Iminohydrolase	Putrescine biosynthesis
Cyan	AT1G08510	*FATB*	Acyl-Acyl Carrier Protein Thioesterase	Saturated fatty acid biosynthesis
Cyan	AT2G27100	*SERRATE*	Zinc Finger Protein	Pre-mRNA splicing; miRNA biogenesis
Darkgreen	AT1G19770	*PUP14*	Purine Permease Transporter	Uptake of bioactive cytokinins to cytosol
Darkgreen	AT2G28620	*RSW7*	Kinesin-5 Protein	Microtubule organization in mitotic spindles
Darkgrey	AT2G41720	*EMB2654*	PPR Protein	Trans-splicing of plastid rps12 transcript
Darkred	AT1G15110	*PSS1*	Phosphatidylserine Synthase	Phosphatidylserine (membrane phospholipid) biosynthesis
Darkturquoise	AT3G05680	*EMB2016*	m6A Methyltransferase Complex Subunit	N6-adenosine methylation of mRNA; mRNA modification, splicing, metabolism
Darkturquoise	AT1G63700	*YDA*	MAP3K Protein Kinase	Protein phosphorylation, Signal transduction pathways

Darkturquoise	AT2G30920	*EMB3002*	Hexaprenyldihydroxybenzoate Methyltransferase	Ubiquinone biosynthesis in mitochondria
Green	AT2G45330	*EMB1067*	2' tRNA Phosphotransferase	Catalytic role in tRNA splicing
Green	AT2G45270	*GCP1*	Mitochondrial Glycoprotease	HSP70 superfamily protein
Green	AT5G11040	*TRS120*	TRAPPII Tethering Factor	Membrane trafficking; Cell plate assembly during cytokinesis
Green	AT5G14800	*EMB2772*	Pyrroline-5-Carboxylate (P5C) Reductase;Proline Biosynthetic Enzyme	Catalyzes final step in proline biosynthesis from glutamate and ornithine
Green	AT5G26030	*FC1*	Ferrochelatase	Final enzyme for haem formation in the tetrapyrrole biosynthetic pathway
Green	AT5G24670	*EMB2820*	tRNA Adenosine Deaminase Subunit	Modification of wobble position of cytosolic tRNAs
Green	AT5G62440	*DOM1*	Uncertain	Nuclear protein; Similar to tomato LeDCLPutative role in ribosome biogenesis
Green	AT5G51430	*EYE*	Subunit of Golgi COG Complex	Vesicle trafficking; Golgi structure and function
Green	AT5G51200	*EMB3142*	Nuclear Pore Complex Protein (Nup205)	Nuclear protein export
Green	AT5G50210	*QS*	NAD Biosynthesis	Catalyzes second step in NAD biosynthesis from aspartatein plastids
Green	AT5G49930	*EMB1441*	Uncertain	Putative RNA binding protein; Possible role in ribosome biogenesis
Green	AT5G40480	*EMB3012*	Nuclear Pore Complex Protein (Nup210)	Nuclear protein export
Green	AT5G39710	*EMB2745*	PPR Protein	RNA binding protein; Organellar gene function
Green	AT5G22370	*EMB1705*	GTP Binding Protein	Microtubule organization
Green	AT5G08130	*BIM1*	bHLH Protein Interactor of Transcription Factors	Brassinosteroid signaling
Green	AT5G05560	*EMB2771*	E3 Ubiquitin Ligase Subunit	Anaphase promoting complex subunit; Cell cycle regulation
Green	AT5G04920	*VPS36*	Endosomal Sorting Complex (ESCRT-II) Subunit	Vacuolar biogenesis; Plasma membrane protein turnover
Green	AT3G55610	*P5CS2*	Delta 1-Pyrroline-5-Carboxylate Synthetase	Proline biosynthesis; Rate-limiting step
Green	AT3G55510	*RBL*	Uncertain	Noc2 domain protein
Green	AT3G48110	*EDD*	Glycine tRNA Synthetase	Translation in chloroplasts
Green	AT4G39920	*POR*	Tubulin Folding Cofactor C	Regulation of tubulin folding and microtubule dynamics
Green	AT4G39120	*IMPL2*	Histidinol-Phosphate Phosphatase	Histidine biosynthesis
Green	AT4G34200	*PGDH1*	3-Phosphoglycerate Dehydrogenase	Phosphoserine pathway of serine biosynthesis in plastids
Green	AT4G26900	*HISN4*	Histidine Biosynthetic Enzyme	Histidine biosynthesis
Green	AT4G20060	*EMB1895*	snRNA Processing Factor	snRNA 3' end maturation; Pre-mRNA splicing
Green	AT4G20090	*EMB1025*	PPR Protein	RNA binding protein; Organellar gene function
Green	AT4G05410	*YAO*	Nucleolar WD-40 Repeat Protein; Putative U3 snoRNP Complex Subunit	Potential role in 18S pre-rRNA processing
Green	AT4G03430	*EMB2770*	Spliceosome-Interacting Protein	Pre-mRNA splicing factor
Green	AT4G00450	*CCT*	Transcriptional Regulator	Modulates function of Mediator complex
Green	AT1G19850	*MP*	B3 Domain Transcription Factor	Transcriptional regulation; Auxin response pathway
Green	AT3G24560	*RSY3*	Putative Lysidine-tRNA-Ile Synthetase	Chloroplast tRNA editing
Green	AT3G07060	*EMB1974*	Unknown	None
Green	AT3G06350	*EMB3004*	Dehydroquinate Dehydratase; Shikimate Dehydrogenase	Chorismate biosynthesis
Green	AT3G02280	*ATR3*	Cytochrome P450 Reductase Like	NR1 subfamily of diflavin reductases; Potential role in Fe-S cluster biogenesis
Green	AT3G09070	*OPS*	Uncertain	Polar-localized, plasma membrane-associated protein;Role in protophloem development; cotyledon vascular complexity
Green	AT1G17690	*NOF1*	Nucleolar Protein	None
Green	AT1G19080	*TTN10*	GINS Complex Subunit	Initiation of replication
Green	AT1G12360	*KEU*	Cytokinesis-Related Sec1 Protein	Regulator of vesicle trafficking; Binds to cytokinesis-specific KNOLLE protein
Green	AT1G68990	*MGP3*	Mitochondrial Phage-Type RNA Polymerase	Transcription of mitochondrial genes
Green	AT1G71440	*PFI*	Tubulin Folding Cofactor E	Regulation of tubulin folding and microtubule dynamics
Green	AT1G67960	*POD1*	Uncertain	Proposed role in ER protein retention
Green	AT1G14610	*TWN2*	Valine tRNA Synthetase	Translation in mitochondria and cytosol
Green	AT1G43710	*EMB1075*	Serine Decarboxylase	Ethanolamine biosynthesis
Green	AT1G20050	*HYD1*	Sterol Isomerase	Catalyzes sterol biosynthetic step downstream of FACKEL
Green	AT1G50030	*TOR*	Protein Kinase (Target of Rapamycin)	Signaling pathways
Green	AT1G49510	*EMB1273*	Unknown	Putative chloroplast protein
Green	AT1G20960	*EMB1507*	Spliceosome-Associated RNA Helicase	RNA processing; Splicing of FLC transcripts
Green	AT1G75660	*XRN3*	5'-3' Exoribonuclease	Nuclear degradation of aberrant RNAs; Suppressor of post-transcriptional gene silencing
Green	AT2G31530	*EMB2289*	Chloroplast Inner Envelope Translocase	Import and integration of inner envelope membrane proteins
Green	AT2G21710	*EMB2219*	Uncertain	Chloroplast-localized mTERF domain protein;Putative role in chloroplast gene expression
Green	AT2G31340	*EMB1381*	Unknown	None
Green	AT1G08840	*EMB2411*	DNA Replication Helicase; Nuclease	DNA replication and repair; Okazaki fragment metabolism
Green	AT2G38020	*VCL1*	Vacuolar Sorting Protein (Vps16)	Vacuole biogenesis; Protein trafficking to the vacuole
Green	AT2G28880	*EMB1997*	Bifunctional PABA Synthase; Glutamine Amidotransferase	Para-aminobenzoic acid / folate biosynthesis
Green	AT2G32280	*VCC*	Unknown	Plant-specific protein; Role in cotyledon vascular complexity
Green	AT2G22870	*EMB2001*	Uncertain	Organelle-targeted GTPase (EngB/YihA family);Putative ribosome biogenesis GTPase
Green	AT2G34780	*EMB1611*	Uncertain	Putative role in promoting cell division
GreenYellow	AT5G57930	*EMB1629*	Putative Role in Accumulation of Fe-S Complexes	Chloroplast-localized protein
GreenYellow	AT4G04350	*EMB2369*	Leucine tRNA Synthetase	Translation in chloroplasts
GreenYellow	AT4G02790	*EMB3129*	Chloroplast-Targeted RbgA GTPase	Chloroplast ribosome biogenesis
GreenYellow	AT3G01370	*CFM2*	Chloroplast Intron Splicing Factor	Splicing of plastid group II introns
GreenYellow	AT2G31305	*INH3*	Inhibitor of Protein Phosphatase 1 Catalytic Activity	Regulation of signaling pathways
GreenYellow	AT1G70070	*ISE2*	RNA Helicase	Chloroplast RNA processing
GreenYellow	AT2G25660	*EMB2410*	Unknown	None

GreenYellow	AT2G30410	*KIS*	Tubulin Folding Cofactor A	Regulation of tubulin folding and microtubule dynamics
Grey	AT3G57870	*EMB1637*	SUMO Conjugating Enzyme	Protein modification; Role in stress response; Cell cycle control
Grey	AT3G27750	*EMB3123*	PPR Protein	Splicing of specific group II introns in plastids
Grey60	AT5G13710	*SMT1*	Sterol Methyltransferase	Sterol biosynthesis; Modulation of cholesterol levels
Lightcyan	AT3G54170	*FIP37*	m6A Methyltransferase Complex Subunit	N6-adenosine methylation of mRNA; mRNA modification, splicing, metabolism
Lightcyan	AT2G43650	*EMB2777*	Small Subunit (SSU) Processome Complex Interacting Protein	Nucleolar protein; Proposed role in rRNA biogenesis
LightGreen	AT4G31160	*DCAF1*	Nuclear Ubiquitin E3 Ligase	Protein degradation
LightYellow	AT4G31770	*DBR1*	Lariat Debranching Enzyme	Removal of spliced introns; Degradation of lariat RNAs; Regulation of miRNA biogenesis
LightYellow	AT3G12080	*EMB2738*	Uncertain	Putative ribosome biogenesis GTPase
LightYellow	AT1G21310	*RSH*	Cell Wall Hydroxyproline-Rich Glycoprotein; Extensin	Cell wall protein required for normal cell shape and positioning of cell plate during cytokinesis
LightYellow	AT1G32930	*GALT31A*	Galactosyltransferase; Galactosylation of Arabinogalactan Protein (AGP) Side Chains in Golgi Apparatus	Elongation of B-1,6-galactan side chains on AGPs
LightYellow	AT2G26890	*GRV2*	DnaJ Domain Protein (RME-8)	Endocytosis; Vacuolar sorting; Protein trafficking
Magenta	AT3G54350	*EMB1967*	Forkhead-Associated (FHA) Domain Protein	Putative role in signal transduction pathways
Magenta	AT4G02720	*MAS2*	Perinucleolar Protein; Homolog of Human NKAP	45S rDNA silencing; Interacts with splicing and ribosome biogenesis proteins
Magenta	AT1G01370	*CENH3*	Centromere-Specific Histone	Component of centromeric nucleosome
Magenta	AT1G64570	*DUO3*	Homeodomain Protein	Putative transcriptional regulator
Magenta	AT1G48410	*AGO1*	Argonaute; Effector in Small RNA-Based Gene Silencing	Cleavage or translational inhibition of target RNAs;Methylation of target DNAs
Magenta	AT2G36230	*HISN3*	BBMII Isomerase; Histidine Biosynthetic Enzyme	Catalyzes third step in histidine biosynthesis
Magenta	AT1G65440	*SPT6L*	Transcript Elongation Factor	Proposed role in transcription initiation and chromatin structure
Magenta	AT1G09770	*CDC5*	MYB Domain Transcription Factor	Cell cycle control; Proposed role in miRNA biogenesis
Magenta	AT2G39080	*EMB2799*	Uncertain	Potential role in chloroplast nutrient transport
Midnightblue	AT4G19350	*EMB3006*	Unknown	None
Midnightblue	AT2G26830	*EMB1187*	Choline/Ethanolamine Kinase	Enzyme in phospholipid (phosphatidylcholine) biosynthesis
Orange	AT1G24706	*EMB2793*	THO/TREX Complex Subunit	RNA transport and splicing; miRNA biogenesis
Pink	AT4G29910	*EMB2798*	Origin of Replication Complex	Initiation of replication
Pink	AT2G02150	*EMB2794*	Putative PPR Protein	Putative RNA binding protein
Purple	AT3G52590	*EMB2167*	Ubiquitin Fused to Ribosomal Protein L40	Ubiquitin ribosomal fusion protein
Purple	AT1G19800	*TGD1*	Chloroplast Envelope Protein; ABC Transporter	Import of lipids into chloroplasts
Purple	AT1G08130	*LIG1*	DNA Ligase	DNA replication and repair; Component of DNA demethylation machinery
Purple	AT2G04530	*TRZ2*	tRNase Z Enzyme; Plastid tRNA Processing	Cleavage of 3' end of plastid tRNA precursor
Red	AT5G19620	*OEP80*	Chloroplast Outer Envelope Protein	Uncertain role in chloroplast protein import
Red	AT5G63050	*EMB2759*	Unknown	None
Red	AT5G62410	*TTN3*	SMC2 Condensin	Chromosome condensation
Red	AT5G57590	*BIO1*	7-KAP, DAP Aminotransferase; Dethiobiotin Synthetase	Biotin synthesis in mitochondria; Bifunctional (BIO3-BIO1) protein
Red	AT5G48600	*SMC4*	Condensin Subunit	Chromatin condensation; Chromosome segregation; Silencing of pericentromeric transposons
Red	AT5G18570	*EMB3138*	Chloroplast Obg-Like GTPase	Plastid ribosome biogenesis
Red	AT3G61780	*EMB1703*	Unknown	None
Red	AT3G54670	*TTN8*	SMC1 Cohesin	Chromosomal ATPase; Sister chromatid cohesion; Chromosome segregation
Red	AT3G54720	*AMP1*	Putative Glutamate Carboxypeptidase	Similar to N-acetyl a-linked acidic dipeptidase; Precise biochemical function uncertain
Red	AT4G39620	*EMB2453*	PPR Protein	Proposed role in chloroplast tRNA maturation; Ribosome biogenesis
Red	AT4G38430	*RopGEF1*	Guanine Nucleotide Exchange Factor	Activator of Rho GTPases of plants
Red	AT4G35540	*PTF2*	Plant-Specific TFIIB-Related Protein	General transcription factor
Red	AT4G33460	*EMB2751*	ABC Transporter	Proposed role in chloroplast membrane transport
Red	AT4G31780	*EMB2797*	MGDG (Galactolipid) Synthesis	Monogalactosyldiacylglycerol biosynthesis
Red	AT3G20740	*FIE*	Polycomb Repressive Complex Subunit	Chromatin modification; Histone methylation
Red	AT3G04340	*EMB2458*	Chloroplast AAA-ATPase; Ycf2-FtsHi Complex Subunit	Required for chloroplast protein import
Red	AT1G55350	*EMB1275*	Calpain-Type Cysteine Protease	Homolog of maize DEK1; Transmembrane protein requiredfor mechanosensitive calcium ion channel function
Red	AT1G53580	*ETHE1*	Mitochondrial Sulfur Dioxygenase	Sulfide detoxification
Red	AT1G80260	*EMB1427*	Gamma Tubulin; GCP5 Subunit	Microtubule nucleation
Red	AT1G10510	*EMB2004*	Uncertain	Leucine-rich repeat protein; Chloroplast localized;Similar to ribonuclease inhibitor proteins
Red	AT1G67440	*EMB1688*	Uncertain	Putative ribosome biogenesis GTPase
Red	AT2G17800	*ROP3*	ROP GTPase	Auxin transport and response
Red	AT2G41350	*EMB2819*	Augmin Complex Subunit	Microtubule dynamics; Mitotic spindle formation
Red	AT2G03150	*EMB1579*	Uncertain	Calcium binding protein
Red	AT2G34650	*PID*	Serine-Threonine Protein Kinase	Positive regulator of polar auxin transport
Red	AT2G44190	*EMB3116*	Novel Microtubule-Associated Protein	Endosperm nuclear division and cellularization
RoyalBlue	AT4G13940	*EMB1395*	S-Adenosyl Homocysteine Hydrolase	Amino acid and nucleotide metabolism;Indirect role in gene silencing
Salmon	AT5G46210	*CUL4*	E3 Ubiquitin Ligase Subunit	None
Salmon	AT1G69270	*RPK1*	Receptor-Like Protein Kinase	Signaling pathways
Salmon	AT1G65450	*GLAUCE*	BAHD Family Acyltransferase	Unknown metabolic role in female gametophyte central cell; Required for fusion with second sperm cell
Salmon	AT2G38670	*PECT1*	Phosphatidylethanolamine Biosynthetic Enzyme	Rate-limiting enzyme in CDP-Etn pathway;Mitochondrial localized protein
Tan	AT4G21190	*EMB1417*	PPR Protein	RNA binding protein; Organellar gene function
Tan	AT3G20070	*TTN9*	Unknown	None
Turquoise	AT1G67730	*KCR1*	B-Ketoacyl-Coenzyme A Reductase	Long-chain fatty acid biosynthesis
Turquoise	AT1G28300	*LEC2*	B3 Domain Transcription Factor	Transcriptional regulation

Turquoise	AT5G13510	*EMB3136*	Chloroplast Ribosomal Protein L10	Translation in chloroplasts
Turquoise	AT5G08415	*LIP1*	Lipoyl Synthase	Lipoamide biosynthesis; Plastid-localized lipoylation of metabolic enzymes
Turquoise	AT4G36810	*GGPPS11*	Geranylgeranyl Diphosphate Synthase	Isoprenoid biosynthesis in plastids and cytosol
Turquoise	AT1G77360	*APPR6*	Mitochondrial PPR Protein	5' maturation and translational initiation of mitochondrial ribosomal protein S3 mRNA
Turquoise	AT5G27720	*EMB1644*	U6 snRNA Associated Protein	Component of spliceosome; Role in pre-mRNA splicing
Turquoise	AT5G64580	*EMB3144*	Chloroplast AAA-ATPase; Ycf2-FtsHi Complex Subunit	Required for chloroplast protein import
Turquoise	AT5G63920	*TOP3a*	Topoisomerase	DNA synthesis and repair
Turquoise	AT5G61410	*EMB2728*	Ribulose-5-Phosphate-3-Epimerase	Role in Calvin cycle; Oxidative pentose phosphate pathway
Turquoise	AT5G58250	*EMB3143*	Unknown	Chloroplast-localized protein
Turquoise	AT5G53860	*EMB2737*	Uncertain	Chloroplast-localized, DnaJ-like protein; Putative chaperone
Turquoise	AT5G52920	*PKP1*	Plastidic Pyruvate Kinase B1 Subunit	Fatty acid and seed oil biosynthesis
Turquoise	AT5G51600	*PLE*	Microtubule-Associated Protein	Coordination of microtubule and membrane dynamics during cytokinesis
Turquoise	AT5G50390	*EMB3141*	PPR Protein	RNA binding protein; Organellar gene function
Turquoise	AT5G50280	*EMB1006*	PPR Protein	RNA binding protein; Organellar gene function
Turquoise	AT5G48230	*EMB1276*	Acetoacetyl CoA Thiolase	Cytosolic mevalonate pathway of isoprenoid biosynthesis
Turquoise	AT5G40950	*PRPL27*	Chloroplast Ribosomal Protein	Translation in chloroplasts
Turquoise	AT5G24400	*EMB2024*	6-Phosphogluconolactonase	Role in pentose phosphate pathway
Turquoise	AT5G22640	*EMB1211*	Chloroplast Protein Import Complex Subunit	Component of 1 MD chloroplast inner membrane protein import complex
Turquoise	AT5G16390	*CAC1A*	Biotin Carboxyl Carrier Protein	Heteromeric acetyl-CoA carboxylase subunit; Fatty acid biosynthesis in plastids
Turquoise	AT5G14320	*EMB3137*	Chloroplast Ribosomal Protein S13	Translation in chloroplasts
Turquoise	AT5G13690	*CYL1*	Alpha-N-Acetyl-Glucosaminidase	Arabinogalactan protein metabolism
Turquoise	AT5G10480	*PAS2*	3-Hydroxyl-Acyl-CoA Dehydratase; Elongase Complex Subunit	Very-long-chain fatty acid biosynthesis
Turquoise	AT5G07280	*EXS*	LRR Receptor Kinase	Signal transduction pathway
Turquoise	AT5G04560	*DME*	DNA Glycosylase	DNA demethylation in central cell of female gametophyte, vegetative cell of male gametophyte
Turquoise	AT5G04810	*PPR4*	PPR Protein	Trans-splicing of chloroplast rps12 transcript
Turquoise	AT5G04660	*CYP77A4*	Cytochrome P450	Epoxidase of unsaturated fatty acids; ER-localized protein
Turquoise	AT5G03800	*EMB1899*	PPR Protein	RNA binding protein; Organellar gene function
Turquoise	AT3G63490	*EMB3126*	Chloroplast Ribosomal Protein L1	Translation in chloroplasts
Turquoise	AT5G01075	*TWS1*	Unknown	Small protein localized to endoplasmic reticulum
Turquoise	AT3G63190	*HFP108*	Chloroplast Ribosome Recycling Factor	Required for efficient chloroplast translation
Turquoise	AT3G57560	*NAGK*	Acetylglutamate Kinase	Arginine biosynthesis
Turquoise	AT3G54660	*EMB2360*	Chloroplast Glutathione Reductase	Protection against oxidative stress
Turquoise	AT3G54320	*WRI1*	AP2/EREB Domain Transcription Factor	Regulation of storage compound biosynthesis
Turquoise	AT3G52940	*FK*	Sterol C-14 Reductase	Sterol biosynthesis during embryo development
Turquoise	AT3G47520	*pNAD-MDH*	Plastid NAD-Dependent Malate Dehydrogenase	Redox homeostasis; Heterotrophic metabolism;Component of plastid Ycf2 complex required for protein import
Turquoise	AT3G46740	*TOC75-III*	Chloroplast Import Protein	Protein translocation channel in plastid outer membrane
Turquoise	AT4G34620	*SSR16*	Mitochondrial Ribosomal Protein S16	Translation in mitochondria and chloroplasts
Turquoise	AT4G33680	*AGD2*	LL-Diaminopimelate Aminotransferase	Lysine biosynthesis
Turquoise	AT4G29060	*EMB2726*	Translation Elongation Factor	Translation in chloroplasts
Turquoise	AT4G28210	*EMB1923*	Unknown	None
Turquoise	AT4G00220	*JLO*	LOB Domain Transcription Factor	Auxin signaling pathways
Turquoise	AT3G29290	*EMB2076*	PPR Protein	RNA binding protein; Organellar gene function
Turquoise	AT3G15190	*PRPS20*	Chloroplast Ribosomal Protein	Translation in chloroplasts
Turquoise	AT3G12670	*EMB2742*	CTP Synthase; UTP-Ammonia Ligase	Ribonucleotide metabolism
Turquoise	AT3G18390	*EMB1865*	Chloroplast Splicing Factor	Splicing of plastid group II introns
Turquoise	AT3G19810	*DUF177A*	Uncertain	23S rRNA accumulation
Turquoise	AT3G16290	*EMB2083*	Chloroplast AAA-ATPase; Ycf2-FtsHi Complex Subunit	Required for chloroplast protein import
Turquoise	AT3G25920	*RPL15*	Chloroplast Ribosomal Protein	Translation in chloroplasts
Turquoise	AT3G25860	*PLE2*	Chloroplast Pyruvate Dehydrogenase Complex E2 Subunit	Acetyl-CoA formation in plastids
Turquoise	AT3G26790	*FUS3*	B3 Domain Transcription Factor	Regulator of gene expression during embryogenesis
Turquoise	AT3G26744	*ICE1*	bHLH Transcriptional Activator	Regulation of stomatal development, cold tolerance, and endosperm breakdown
Turquoise	AT3G02660	*EMB2768*	Tyrosine tRNA Synthetase	Translation in mitochondria and chloroplasts
Turquoise	AT3G04790	*EMB3119*	Ribulose-5-Phosphate Isomerase	Carbohydrate metabolism
Turquoise	AT1G12410	*EMB3146*	Chloroplast Clp Protease Subunit	None
Turquoise	AT2G43360	*BIO2*	Biotin Synthase	Catalyzes final step in biotin synthesis in mitochondria
Turquoise	AT1G62340	*ALE1*	Subtilisin-Like Serine Protease	Cell signaling pathways; Possible role in processing of peptide ligands
Turquoise	AT1G06950	*TIC110*	Chloroplast Inner Membrane Protein	Chloroplast protein import
Turquoise	AT1G21970	*LEC1*	HAP3 Subunit of CCAAT-Binding Transcription Factor	Transcriptional regulation
Turquoise	AT1G02560	*CLPP5*	Chloroplast ClpP Protease Subunit	None
Turquoise	AT1G02680	*TAF13*	Transcriptional Regulator	Functions with PRC2 complex; Transcriptional repressor
Turquoise	AT1G07320	*EMB2784*	Chloroplast Ribosomal Protein L4	Translation in chloroplasts
Turquoise	AT1G75350	*EMB2184*	Chloroplast Ribosomal Protein L31	Translation in chloroplasts
Turquoise	AT1G04950	*EMB2781*	TATA Box Associated Factor; TFIID Subunit	Regulation and initiation of transcription
Turquoise	AT1G34430	*EMB3003*	Dihydrolipoamide S-Acetyltransferase; Chloroplast PyruvateDecarboxylase Complex, Putative E3 Binding Protein	Acetyl-CoA formation
Turquoise	AT1G12770	*EMB1586*	Mitochondrial RNA Helicase	RNA binding protein; Proposed role in processing mitochondrial transcripts

Turquoise	AT1G18370	*HIK*	Kinesin Required for Cytokinesis	Localized to site of cell plate formation
Turquoise	AT1G50650	*KRS*	Endosperm-Specific, Cysteine-Rich Peptide	STIG1 family of small proteins; Required for normal embryo-endosperm adhesion
Turquoise	AT1G35680	*RPL21C*	Chloroplast Ribosomal Protein L21	Translation in chloroplasts
Turquoise	AT1G74970	*TWN3*	Chloroplast Ribosomal Protein S9	Translation in chloroplasts
Turquoise	AT1G74960	*FAB1*	Ketoacyl Carrier Protein Synthase	Elongation of 16:0-ACP to 18:0-ACP in plastids
Turquoise	AT1G48350	*EMB3105*	Chloroplast Ribosomal Protein L18	Translation in chloroplasts
Turquoise	AT1G17220	*FUG1*	Translation Initiation Factor	Translation in chloroplasts
Turquoise	AT1G62750	*SCO1*	Plastid Elongation Factor G	Translation in chloroplasts
Turquoise	AT1G11680	*EMB1738*	Obtusifoliol 14-Alpha Demethylase	Cytochrome P450 monooxygenase; Sterol biosynthetic enzyme
Turquoise	AT1G23400	*CAF2*	Chloroplast Intron Splicing Factor	Splicing of plastid group II introns
Turquoise	AT2G05170	*VPS11*	Vacuolar Tethering Complex Subunit	Vacuolar biogenesis
Turquoise	AT1G78630	*EMB1473*	Chloroplast Ribosomal Protein L13	Translation in chloroplasts
Turquoise	AT1G36160	*ACC1*	Acetyl-CoA Carboxylase	Very-long-chain fatty acid biosynthesis in cytosol
Turquoise	AT1G30610	*EMB2279*	PPR Protein	Plastid rpl2 and trnK intron splicing
Turquoise	AT2G14170	*MMSD*	Methylmalonate Semialdehyde Dehydrogenase	Valine degradation in mitochondria
Turquoise	AT2G27170	*TTN7*	SMC3 Cohesin	Sister chromatid cohesion; Chromosome segregation
Turquoise	AT2G01860	*EMB975*	PPR Protein	RNA binding protein; Organellar gene function
Turquoise	AT2G36000	*EMB3114*	Uncertain	Chloroplast-localized mTERF domain protein
Turquoise	AT2G28000	*SLP*	Chaperonin 60 alpha 1	Protein folding in chloroplasts
Turquoise	AT1G43170	*EMB2207*	Ribosomal Protein L3A	Translation in cytosol
Turquoise	AT1G05190	*EMB2394*	Chloroplast Ribosomal Protein L6	Translation in chloroplasts
Turquoise	AT1G08560	*KN*	Cytokinesis-Specific Syntaxin	Vesicle fusion in cell division plane
Turquoise	AT2G01210	*ZAR1*	Plasma Membrane LRR-RLK	Signaling pathways; Calmodulin binding
Turquoise	AT2G37920	*EMB1513*	Uncertain	Similar to copper transporter proteins
Turquoise	AT2G24090	*PRPL35*	Chloroplast Ribosomal Protein	Translation in chloroplasts
Turquoise	AT2G03050	*EMB93*	Plastid mTERF-Related Protein	Plastid signaling pathways
Turquoise	AT2G32590	*EMB2795*	Non-SMC Condensin Complex Subunit H	Sister chromatid segregation in mitosis
Turquoise	AT2G19450	*TAG1*	Diacylglycerol Transferase	Storage lipid biosynthesis; Catalyzes final step in triacylglycerol biosynthetic pathway
Turquoise	AT2G30200	*EMB3147*	ACP-S-Malonyltransferase	Fatty acid biosynthesis
Turquoise	AT2G33800	*EMB3113*	Chloroplast Ribosomal Protein S5	Translation in chloroplasts
Yellow	AT1G48850	*EMB1144*	Chorismate Synthase	Aromatic amino acid biosynthesis
Yellow	AT5G16715	*EMB2247*	Valine tRNA Synthetase	Translation in chloroplasts
Yellow	AT5G15540	*EMB2773*	Cohesin-Loading Complex Subunit	Cohesin-chromatin interactions; Sister chromatid cohesion
Yellow	AT5G14760	*AO*	L-Aspartate Oxidase; NAD Biosynthetic Enzyme	Catalyzes first step in NAD biosynthesis from aspartate in plastids
Yellow	AT5G56270	*WRKY2*	WRKY Transcription Factor	Zygote polarity after fertilization
Yellow	AT5G54440	*CLUB*	TRAPPII Tethering Factor	Membrane trafficking; Cell plate assembly during cytokinesis
Yellow	AT5G51340	*SCC4*	Cohesin Loading Complex, Kleisin Subunit	TPR protein; Chromatin structure maintenance
Yellow	AT5G49160	*MET1*	DNA Methyltransferase	DNA (CG) methylation
Yellow	AT5G43810	*ZLL*	Argonaute / EIF2C Family Protein	Small RNA binding protein; Cleavage or translationalinhibition of target RNAs
Yellow	AT5G08080	*SYP132*	Cytokinesis-Specific SNARE Protein	Membrane vesicle fusion in cytokinesis
Yellow	AT3G54010	*PAS1*	Immunophilin-Like FK506 Binding Protein	Interacts with fatty acid elongase complex; Required for very-long-chain fatty acid biosynthesis
Yellow	AT4G30580	*EMB1995*	Plastidic Lysophosphatidic Acid Acyltransferase	Fatty acid and phospholipid metabolism
Yellow	AT4G24900	*TTL*	Unknown	C2H2 domain protein; Nuclear localized
Yellow	AT4G23100	*RML1*	Gamma-Glutamylcysteine Synthetase	First enzyme in glutathione biosynthetic pathway;Initiation and maintenance of cell division
Yellow	AT1G18640	*PSP1*	Phosphoserine Phosphatase	Phosphoserine pathway of serine biosynthesis in plastids
Yellow	AT3G23110	*EMB2800*	LRR Receptor-Like Protein	Putative signaling function
Yellow	AT3G18110	*EMB1270*	PPR Protein	RNA binding protein; Organellar gene function
Yellow	AT1G10910	*EMB3103*	PPR Protein	RNA binding protein; Organellar gene function
Yellow	AT1G32490	*EMB2733*	RNA Helicase	Proposed role in pre-mRNA splicing
Yellow	AT1G01040	*SUS1*	RNA Helicase; Dicer-Like Enzyme	Micro RNA biogenesis; Pri-miRNA processing
Yellow	AT1G34770	*NSE3*	Non-SMC Component of SMC5/6 Complex	DNA repair and genome stability
Yellow	AT1G50240	*TIO*	Protein Kinase; Ortholog of FUSED in Animal Systems	Signaling pathways required for cytokinesis
Yellow	AT1G79560	*EMB1047*	Chloroplast AAA-ATPase; Ycf2-FtsHi Complex Subunit	Required for chloroplast protein import
Yellow	AT1G55540	*LNO1*	Nucleoporin; Nuclear Pore Complex Subunit	RNA transport from nucleus to cytosol
Yellow	AT2G20300	*ALE2*	Receptor-Like Protein Kinase	Promotion of protoderm differentiation and cotyledon formation
Yellow	AT2G35650	*CSLA7*	Cellulose-Synthase-Like Glycosyltransferase; Beta-Mannan Synthase	Synthesis of cell wall polysaccharides
Yellow	AT2G25710	*HCS1*	Holocarboxylase Synthetase	Biotin attachment to carboxylases and decarboxylases
