# Supplementary material for: Weighted Gene Correlation Network Analysis (WGCNA) of Arabidopsis Somatic Embryogenesis (SE) and Identification of Key Gene Modules to Uncover SE-Associated Hub Genes
Source: Int J Genomics. 2022 Jul 4;2022:7471063. doi: 10.1155/2022/7471063 (PMC9274236; doi:10.1155/2022/7471063)
Supplement: Supplementary 6 — Table S6. TFs in the coexpression network. [file 7471063.f6.pdf]

**Table S6. TFs in the coexpression network**

| Gene identifier | Module | Alias | Description | TF Family (PlantTFDB) | Target of miRNA |
|---|---|---|---|---|---|
| AT1G05690 | Black | BT3 | BT3 (BTB and TAZ domain protein 3); protein binding / transcription regulator | TAZ | |
| AT1G19210 | Black | | AP2 domain-containing transcription factor, putative | AP2-EREBP | |
| AT1G19860 | Black | | zinc finger (CCCH-type) family protein | C3H | |
| AT1G24610 | Black | | SET domain-containing protein | SET | |
| AT1G27730 | Black | ZAT10, STZ | STZ (SALT TOLERANCE ZINC FINGER); nucleic acid binding / transcription factor/ zinc ion binding | C2H2 | |
| AT1G30500 | Black | NF-YA7 | CCAAT-binding transcription factor (CBF-B/NF-YA) family protein | CCAAT | |
| AT1G66500 | Black | | zinc finger (C2H2-type) family protein | | |
| AT1G71030 | Black | MYBL2, ATMYBL2 | ATMYBL2 (Arabidopsis myb-like 2); DNA binding / transcription factor | MYB-related | |
| AT1G75490 | Black | | DNA binding / transcription factor | AP2-EREBP | |
| AT2G31460 | Black | | unknown protein | | |
| AT2G31510 | Black | | IBR domain-containing protein / ARIADNE-like protein ARI7 (ARI7) | | |
| AT2G32700 | Black | LUH | WD-40 repeat family protein | LUG | |
| AT2G36480 | Black | | zinc finger (C2H2-type) family protein | | |
| AT2G38880 | Black | ATNF-YB1, ATHAP3, HAP3A | HAP3A (Heme activator protein (yeast) homolog 3A), HAP3A (Heme activator protein (yeast) homolog 3A); transcription factor | CCAAT | |
| AT2G42150 | Black | | DNA-binding bromodomain-containing protein | MYB-related | |
| AT2G42350 | Black | | zinc finger (C3HC4-type RING finger) family protein | | |
| AT2G42940 | Black | | DNA-binding family protein | | |
| AT2G46400 | Black | ATWRKY46, WRKY46 | WRKY46 (WRKY DNA-binding protein 46); transcription factor | WRKY | |
| AT3G03750 | Black | SUVR3, SDG20 | SET domain-containing protein | SET | |
| AT3G06400 | Black | CHR11 | CHR11 (CHROMATIN-REMODELING PROTEIN 11); DNA-dependent ATPase | SNF2 | |
| AT3G10330 | Black | | transcription initiation factor IIB-2 / general transcription factor TFIIB-2 (TFIIB2) | | |
| AT3G10810 | Black | | zinc finger (C3HC4-type RING finger) family protein | | |
| AT3G11110 | Black | | zinc finger (C3HC4-type RING finger) family protein | | |
| AT3G19580 | Black | AZF2 | AZF2 (ARABIDOPSIS ZINC-FINGER PROTEIN 2); nucleic acid binding / transcription factor/ zinc ion binding, unknown protein | C2H2 | |
| AT3G20310 | Black | ATERF7, ATERF-7 | ERF7 (ETHYLINE RESPONSE FACTOR7); DNA binding / protein binding / transcription factor/ transcriptional repressor | AP2-EREBP | |
| AT3G49850 | Black | TBP2, TRB3, ATTRB3 | ATTRB3/TRB3 (TELOMERE REPEAT BINDING FACTOR 1); DNA binding / transcription factor | MYB-related | |
| AT3G59580 | Black | NLP9 | RWP-RK domain-containing protein | RWP-RK | |
| AT3G62610 | Black | PFG2, ATMYB11 | AtMYB11 (myb domain protein 11); DNA binding / transcription factor | MYB | |
| AT4G18650 | Black | | transcription factor-related | | |
| AT4G18670 | Black | | leucine-rich repeat family protein / extensin family protein | | |
| AT4G23450 | Black | | zinc finger (C3HC4-type RING finger) family protein | | |
| AT4G23810 | Black | ATWRKY53, WRKY53 | WRKY53 (WRKY DNA-binding protein 53); transcription factor | WRKY | |
| AT4G28110 | Black | ATMYB41 | AtMYB41 (myb domain protein 41); DNA binding / transcription factor | MYB | |
| AT5G03410 | Black | DPB, | | | |
| AT5G08750 | Black | | zinc finger (C3HC4-type RING finger) family protein | | |
| AT5G10120 | Black | | ethylene insensitive 3 family protein | EIL | |
| AT5G10140 | Black | FLF, AGL25, FLC | FLC (FLOWERING LOCUS C), FLC (FLOWERING LOCUS C); transcription factor | MADS | |
| AT5G23610 | Black | | unknown protein | | |
| AT5G27650 | Black | | PWWP domain-containing protein | | |
| AT5G45830 | Black | GSQ5, DOG1 | DOG1 (DELAY OF GERMINATION 1) | | |
| AT5G47610 | Black | | zinc finger (C3HC4-type RING finger) family protein | | |
| AT5G48670 | Black | FEM111, AGL80 | AGL80/FEM111 (AGAMOUS-LIKE80); DNA binding / transcription factor | MADS | |
| AT5G59340 | Black | WOX2 | WOX2 (WUSCHEL-related homeobox 2); transcription factor | HB | |
| AT5G62470 | Black | ATMYB96, mybcov1 | MYB96 (myb domain protein 96); DNA binding / transcription factor | MYB | |
| AT5G65070 | Black | FCL4, MAF4, AGL69 | MAF4 (MADS AFFECTING FLOWERING 4); transcription factor, unknown protein | MADS | |
| AT1G02220 | Blue | ANAC003 | ANAC003 (Arabidopsis NAC domain containing protein 3); transcription factor | NAC | |
| AT1G04050 | Blue | SDG13, SUVR1 | SUVR1 (Arabidopsis homolog of SU(VAR)3-9 1); histone-lysine N-methyltransferase/ zinc ion binding | SET | |
| AT1G04250 | Blue | IAA17, AXR3 | AXR3 (AUXIN RESISTANT 3); transcription factor | AUX/IAA | |
| AT1G04790 | Blue | | zinc finger (C3HC4-type RING finger) family protein | | |
| AT1G05120 | Blue | | SNF2 domain-containing protein / helicase domain-containing protein / RING finger domain-containing protein | SNF2 | |

| | | | | |
|---|---|---|---|---|
| AT1G05380 | Blue | | DNA binding, unknown protein | PHD |
| AT1G06070 | Blue | AtbZIP69 | bZIP transcription factor, putative (bZIP69) | bZIP |
| AT1G06150 | Blue | bHLH089, EMB1444 | transcription factor | |
| AT1G07520 | Blue | | scarecrow transcription factor family protein | GRAS |
| AT1G08320 | Blue | | DNA binding, bZIP family transcription factor | bZIP |
| AT1G08620 | Blue | | transcription factor jumonji (jmj) family protein / zinc finger (C5HC2 type) family protein | Jumonji |
| AT1G08900 | Blue | | carbohydrate transporter, unknown protein | |
| AT1G09250 | Blue | | transcription factor | |
| AT1G10470 | Blue | IBC7, ATRR1, ARR4, MEE7 | ARR4 (RESPONSE REGULATOR 4); transcription regulator/ two-component response regulator | Orphans |
| AT1G11950 | Blue | | transcription factor jumonji (jmjC) domain-containing protein | Jumonji |
| AT1G14510 | Blue | AL7 | PHD finger family protein | Alfin-like |
| AT1G14920 | Blue | RGA2, GAI | GAI (GA INSENSITIVE); transcription factor | GRAS |
| AT1G16710 | Blue | HAC12 | TAZ zinc finger family protein / zinc finger (ZZ type) family protein | TAZ |
| AT1G19050 | Blue | ARR7 | ARR7 (RESPONSE REGULATOR 7); transcription regulator/ two-component response regulator | Orphans |
| AT1G19310 | Blue | | zinc finger (C3HC4-type RING finger) family protein | |
| AT1G21570 | Blue | | zinc finger (CCCH-type) family protein | C3H |
| AT1G22985 | Blue | | AP2 domain-containing transcription factor, putative | AP2-EREBP |
| AT1G25470 | Blue | | AP2 domain-containing transcription factor, putative | AP2-EREBP |
| AT1G27050 | Blue | ATHB54 | ATHB54 (ARABIDOPSIS THALIANA HOMEOBOX PROTEIN 54); nucleic acid binding / transcription factor | HB |
| AT1G27650 | Blue | ATU2AF35A | ATU2AF35A, ATU2AF35A; RNA binding | C3H |
| AT1G28050 | Blue | COL15 | zinc finger (B-box type) family protein | C2C2-CO-like |
| AT1G28520 | Blue | ATVOZ1, VOZ1 | unknown protein | VOZ |
| AT1G29860 | Blue | ATWRKY71, WRKY71 | WRKY71 (WRKY DNA-binding protein 71); transcription factor | WRKY |
| AT1G30650 | Blue | ATWRKY14, AR411, WRKY14 | WRKY14 (WRKY DNA-binding protein 14); transcription factor | WRKY |
| AT1G30970 | Blue | SUF4 | SUF4 (SUPPRESSOR OF FRIGIDA4), SUF4 (SUPPRESSOR OF FRIGIDA4); transcription factor | C2H2 |
| AT1G32800 | Blue | | | |
| AT1G34180 | Blue | ANAC016 | ANAC016 (Arabidopsis NAC domain containing protein 16), ANAC016 (Arabidopsis NAC domain containing protein 16); transcription factor | NAC |
| AT1G34190 | Blue | ANAC017 | ANAC017 (Arabidopsis NAC domain containing protein 17); transcription factor | NAC |
| AT1G36060 | Blue | | AP2 domain-containing transcription factor, putative | AP2-EREBP |
| AT1G43700 | Blue | AtbZIP51, VIP1 | VIP1 (VIRE2-INTERACTING PROTEIN 1); transcription factor | bZIP |
| AT1G43850 | Blue | SEU | SEU (SEUSS); transcription cofactor, unknown protein | |
| AT1G43860 | Blue | | transcription factor | |
| AT1G48000 | Blue | ATMYB112 | MYB112 (myb domain protein 112); DNA binding / transcription factor | MYB |
| AT1G48310 | Blue | CHR18, CHA18 | SNF2 domain-containing protein / helicase domain-containing protein | SNF2 |
| AT1G49560 | Blue | | myb family transcription factor | G2-like |
| AT1G49720 | Blue | ABF1 | ABF1 (ABSCISIC ACID RESPONSIVE ELEMENT-BINDING FACTOR 1); DNA binding / transcription factor/ transcriptional activator | bZIP |
| AT1G50640 | Blue | ATERF3 | ATERF3/ERF3 (ETHYLENE RESPONSIVE ELEMENT BINDING FACTOR 3); DNA binding / protein binding / transcription factor/ transcriptional repressor | AP2-EREBP |
| AT1G51140 | Blue | bHLH122 | basic helix-loop-helix (bHLH) family protein | bHLH |
| AT1G51190 | Blue | PLT2 | PLT2 (PLETHORA 2); transcription factor | AP2-EREBP |
| AT1G51600 | Blue | TIFY2A, ZML2 | ZML2 (ZIM-LIKE 2); transcription factor | C2C2-GATA |
| AT1G51950 | Blue | IAA18 | IAA18 (indoleacetic acid-induced protein 18); transcription factor | AUX/IAA |
| AT1G53320 | Blue | AtTLP7 | AtTLP7 (TUBBY LIKE PROTEIN 7); phosphoric diester hydrolase/ transcription factor | TUB |
| AT1G55460 | Blue | | Kin17 DNA-binding protein-related | |
| AT1G55520 | Blue | ATTBP2, TBP2 | TBP2 (TATA binding protein 2); DNA binding / RNA polymerase II transcription factor | |
| AT1G55580 | Blue | SCL18, LAS | LAS (LATERAL SUPPRESSOR); transcription factor | GRAS |
| AT1G56200 | Blue | EMB1303 | EMB1303 (EMBRYO DEFECTIVE 1303) | |
| AT1G58100 | Blue | TCP8 | TCP family transcription factor, putative | TCP |
| AT1G58330 | Blue | ZW2 | ZW2 | |
| AT1G59940 | Blue | ARR3 | ARR3 (RESPONSE REGULATOR 3); transcription regulator/ two-component response regulator | Orphans |
| AT1G62360 | Blue | WAM1, WAM, SHL, BUM1, STM | STM (SHOOT MERISTEMLESS); transcription factor | HB |
| AT1G66340 | Blue | EIN1, ETR1 | ETR1 (ETHYLENE RESPONSE 1); two-component response regulator | Orphans |
| AT1G66350 | Blue | RGL1 | RGL1 (RGA-LIKE 1); transcription factor | GRAS |

| | | | | | |
|---|---|---|---|---|---|
| AT1G66470 | Blue | bHLH083 | basic helix-loop-helix (bHLH) family protein | bHLH | |
| AT1G66810 | Blue | | zinc finger (CCCH-type) family protein | C3H | |
| AT1G67970 | Blue | AT-HSFA8 | AT-HSFA8 (Arabidopsis thaliana heat shock transcription factor A8); DNA binding / transcription factor | HSF | |
| AT1G68150 | Blue | ATWRKY9, WRKY9 | WRKY9 (WRKY DNA-binding protein 9); transcription factor | WRKY | |
| AT1G68550 | Blue | | AP2 domain-containing transcription factor, putative | AP2-EREBP | |
| AT1G69600 | Blue | ATHB29, ZFHD1 | ATHB29/ZFHD1 (ZINC FINGER HOMEODOMAIN 1); DNA binding / transcription factor/ transcriptional activator | zf-HD | |
| AT1G71260 | Blue | WHY2, ATWHY2 | ATWHY2 (A. THALIANA WHIRLY 2); DNA binding | PBF-2-like | |
| AT1G73100 | Blue | SDG19, SUVH3 | SUVH3 (SU(VAR)3-9 HOMOLOG 3) | SET | |
| AT1G74410 | Blue | | zinc finger (C3HC4-type RING finger) family protein | | |
| AT1G74500 | Blue | | bHLH family protein | bHLH | |
| AT1G75510 | Blue | | transcription initiation factor IIF beta subunit (TFIIF-beta) family protein | | |
| AT1G75520 | Blue | SRS5 | SRS5 (SHI-RELATED SEQUENCE 5) | SRS | |
| AT1G76580 | Blue | SPL16 | transcription factor | | |
| AT1G76710 | Blue | ASHH1 ,SDG26 | SET domain-containing protein (ASHH1) | SET | |
| AT1G79000 | Blue | ATHPCAT2, PCAT2, HAC1 | HAC1 (P300/CBP acetyltransferase-related protein 2 gene); transcription cofactor | TAZ | |
| AT2G01430 | Blue | ATHB17, ATHB-17 | homeobox-leucine zipper protein 17 (HB-17) / HD-ZIP transcription factor 17 | HB | |
| AT2G01810 | Blue | | PHD finger family protein | PHD | |
| AT2G02160 | Blue | | zinc finger (CCCH-type) family protein | C3H | |
| AT2G02740 | Blue | WHY3, ATWHY3, PTAC11 | ATWHY3/PTAC11 (A. THALIANA WHIRLY 3), ATWHY3/PTAC11 (A. THALIANA WHIRLY 3); DNA binding | PBF-2-like | |
| AT2G04890 | Blue | SCL21 | SCL21 (SCARECROW-LIKE 21); transcription factor | GRAS | |
| AT2G17870 | Blue | | cold-shock DNA-binding family protein | CSD | |
| AT2G19610 | Blue | | zinc finger (C3HC4-type RING finger) family protein | | |
| AT2G19640 | Blue | SDG39, ASHR2 | SET domain-containing protein | | |
| AT2G20280 | Blue | | zinc finger (CCCH-type) family protein | C3H | |
| AT2G21060 | Blue | ATGRP2B | ATGRP2B (GLYCINE-RICH PROTEIN 2B); nucleic acid binding | CSD | |
| AT2G21230 | Blue | AtbZIP30 | bZIP family transcription factor, unknown protein | bZIP | |
| AT2G22300 | Blue | SR1, CAMTA3 | ethylene-responsive calmodulin-binding protein, putative (SR1), unknown protein | CAMTA | |
| AT2G22430 | Blue | ATHB6 | ATHB6 (ARABIDOPSIS THALIANA HOMEOBOX PROTEIN 6); transcription factor | HB | |
| AT2G22770 | Blue | NAI1, bHLH020 | NAI1; DNA binding / transcription factor | bHLH | |
| AT2G23740 | Blue | AtCZS | nucleic acid binding / transcription factor/ zinc ion binding | C2H2 | |
| AT2G24500 | Blue | FZF | FZF; transcription factor | | |
| AT2G25640 | Blue | | unknown protein | | |
| AT2G27050 | Blue | EIL1 | EIL1 (ETHYLENE-INSENSITIVE3-LIKE 1); transcription factor | EIL | |
| AT2G27300 | Blue | NTL8, ANAC040 | ANAC040 (Arabidopsis NAC domain containing protein 40); transcription factor | NAC | |
| AT2G28350 | Blue | ARF10 | ARF10 (AUXIN RESPONSE FACTOR 10); miRNA binding / transcription factor | ARF | miR160, |
| AT2G28450 | Blue | | zinc finger (CCCH-type) family protein | C3H | |
| AT2G28530 | Blue | | | | |
| AT2G30590 | Blue | WRKY21 | WRKY21 (WRKY DNA-binding protein 21); transcription factor | WRKY | |
| AT2G32600 | Blue | | hydroxyproline-rich glycoprotein family protein | | |
| AT2G33610 | Blue | CHB2, ATSWI3B | ATSWI3B (Arabidopsis thaliana switching protein 3B); DNA binding | MYB-related | |
| AT2G34710 | Blue | ATHB14, PHB-1D, ATHB-14, PHB | PHB (PHABULOSA); DNA binding / transcription factor | HB | miR166/miR165, |
| AT2G35430 | Blue | | zinc finger (CCCH-type) family protein | C3H | |
| AT2G36010 | Blue | ATE2FA, E2F3 | E2F3 (E2F TRANSCRIPTION FACTOR-3), E2F3 (E2F TRANSCRIPTION FACTOR-3); transcription factor | E2F-DP | |
| AT2G36270 | Blue | EEL, GIA1, ABI5 | ABI5 (ABA INSENSITIVE 5); DNA binding / transcription factor/ transcriptional activator | bZIP | |
| AT2G36930 | Blue | | zinc finger (C2H2 type) family protein | | |
| AT2G37150 | Blue | | zinc finger (C3HC4-type RING finger) family protein | | |
| AT2G38950 | Blue | | transcription factor jumonji (jmj) family protein / zinc finger (C5HC2 type) family protein | Jumonji | |
| AT2G39100 | Blue | | zinc finger (C3HC4-type RING finger) family protein | | |
| AT2G40220 | Blue | GIN6, SUN6, SIS5, SAN5, ISI3, ABI4 | ABI4 (ABA INSENSITIVE 4); DNA binding / transcription factor | AP2-EREBP | |
| AT2G40820 | Blue | | proline-rich family protein | bHSH | |
| AT2G41310 | Blue | ARR8, ATRR3 | ATRR3 (RESPONSE REGULATOR 3); transcription regulator | Orphans | |
| AT2G41630 | Blue | TFIIB | TFIIB (TRANSCRIPTION FACTOR II B); RNA polymerase II transcription factor | | |
| AT2G41900 | Blue | | zinc finger (CCCH-type) family protein | C3H | |
| AT2G41980 | Blue | | seven in absentia (SINA) family protein | | |

| | | | | |
|---|---|---|---|---|
| AT2G42300 | Blue | bHLH048 | DNA binding / transcription factor, basic helix-loop-helix (bHLH) family protein | bHLH |
| AT2G42400 | Blue | ATVOZ2, VOZ2 | unknown protein | VOZ |
| AT2G42660 | Blue | | myb family transcription factor | G2-like |
| AT2G46040 | Blue | | ARID/BRIGHT DNA-binding domain-containing protein / ELM2 domain-containing protein | ARID |
| AT2G46310 | Blue | CRF5 | CRF5 (CYTOKININ RESPONSE FACTOR 5); DNA binding / transcription factor | AP2-EREBP |
| AT2G46680 | Blue | ATHB7, ATHB-7 | ATHB-7 (ARABIDOPSIS THALIANA HOMEOBOX 7), ATHB-7 (ARABIDOPSIS THALIANA HOMEOBOX 7); transcription factor | HB |
| AT2G46730 | Blue | REM18 | pseudogene | |
| AT2G47070 | Blue | SPL1 | SPL1 (SQUAMOSA PROMOTER BINDING PROTEIN-LIKE 1); DNA binding / transcription factor | SBP |
| AT2G47190 | Blue | AtMYB2 | MYB2 (myb domain protein 2); DNA binding / transcription factor | MYB |
| AT2G47520 | Blue | | AP2 domain-containing transcription factor, putative | AP2-EREBP |
| AT2G47620 | Blue | CHB1, ATSWI3A | ATSWI3A (Arabidopsis thaliana switching protein 3A); DNA binding | MYB-related |
| AT2G47680 | Blue | | zinc finger (CCCH type) helicase family protein | C3H |
| AT2G48160 | Blue | | PWWP domain-containing protein | |
| AT3G01970 | Blue | ATWRKY45, WRKY45 | WRKY45 (WRKY DNA-binding protein 45); transcription factor | WRKY |
| AT3G02260 | Blue | CRM1, TIR3, LPR1, DOC1, ASA1, UMB1, BIG | BIG (DARK OVER-EXPRESSION OF CAB 1); binding / ubiquitin-protein ligase/ zinc ion binding | |
| AT3G02550 | Blue | LBD41 | LOB domain protein 41 / lateral organ boundaries domain protein 41 (LBD41) | |
| AT3G02680 | Blue | ATNBS1, NBS1 | NBS1 (NIJMEGEN BREAKAGE SYNDROME 1) | FHA |
| AT3G03450 | Blue | RGL2 | RGL2 (RGA-LIKE 2); transcription factor | GRAS |
| AT3G04580 | Blue | EIN4 | EIN4 (ETHYLENE INSENSITIVE 4), EIN4 (ETHYLENE INSENSITIVE 4); receptor | Orphans |
| AT3G04590 | Blue | | DNA-binding family protein | |
| AT3G05760 | Blue | | nucleic acid binding / zinc ion binding | |
| AT3G07260 | Blue | | forkhead-associated domain-containing protein / FHA domain-containing protein | FHA |
| AT3G07650 | Blue | COL9 | COL9 (CONSTANS-LIKE 9); transcription factor/ zinc ion binding, COL9 (CONSTANS-LIKE 9); zinc ion binding, unknown protein | C2C2-CO-like |
| AT3G10030 | Blue | | aspartate/glutamate/uridylate kinase family protein | Trihelix |
| AT3G10490 | Blue | ANAC051, ANAC052 | ANAC051/ANAC052 (Arabidopsis NAC domain containing protein 51, Arabidopsis NAC domain containing protein 52); transcription factor | NAC |
| AT3G12250 | Blue | BZIP45, TGA6 | TGA6 (TGA1a-related gene 6), TGA6 (TGA1a-related gene 6); DNA binding / transcription factor | bZIP |
| AT3G12270 | Blue | ATPRMT3 | methyltransferase | |
| AT3G14180 | Blue | | transcription factor | Trihelix |
| AT3G15540 | Blue | MSG2, IAA19 | IAA19 (indoleacetic acid-induced protein 19); transcription factor | AUX/IAA |
| AT3G16870 | Blue | | zinc finger (GATA type) family protein | C2C2-GATA |
| AT3G16940 | Blue | | calmodulin-binding protein | CAMTA |
| AT3G18400 | Blue | ANAC058 | ANAC058 (Arabidopsis NAC domain containing protein 58); transcription factor | NAC |
| AT3G19210 | Blue | ATRAD54, CHR25 | ATRAD54/CHR25 (ARABIDOPSIS HOMOLOG OF RAD54); ATP binding / DNA binding / helicase | SNF2 |
| AT3G19360 | Blue | | zinc finger (CCCH-type) family protein | C3H |
| AT3G19860 | Blue | bHLH121 | DNA binding, basic helix-loop-helix (bHLH) family protein | bHLH |
| AT3G19910 | Blue | | zinc finger (C3HC4-type RING finger) family protein | |
| AT3G20770 | Blue | EIN3 | EIN3 (ETHYLENE-INSENSITIVE3); transcription factor | EIL |
| AT3G20840 | Blue | PLT1 | PLT1 (PLETHORA 1); transcription factor | AP2-EREBP |
| AT3G21175 | Blue | TIFY2B, ZML1 | ZML1 (ZIM-LIKE 1); transcription factor, unknown protein | C2C2-GATA |
| AT3G21270 | Blue | ADOF2, AtDof3. 1 | ADOF2 (Arabidopsis dof zinc finger protein 2); DNA binding / transcription factor | C2C2-Dof |
| AT3G22780 | Blue | ATTSO1, TSO1 | TSO1 (CHINESE FOR 'UGLY'); transcription factor | CPP |
| AT3G23050 | Blue | AXR2, IAA7 | IAA7 (AUXIN RESISTANT 2), IAA7 (AUXIN RESISTANT 2); transcription factor | AUX/IAA |
| AT3G23060 | Blue | | zinc finger (C3HC4-type RING finger) family protein | |
| AT3G24520 | Blue | AT-HSFC1 | AT-HSFC1 (Arabidopsis thaliana heat shock transcription factor C1); DNA binding / transcription factor | HSF |
| AT3G26640 | Blue | LWD2 | transducin family protein / WD-40 repeat family protein | |
| AT3G27700 | Blue | | RNA recognition motif (RRM)-containing protein | C3H |
| AT3G42670 | Blue | CLSY, CHR38 | CHR38 (chromatin remodeling 38); ATP binding / DNA binding / helicase | SNF2 |
| AT3G42790 | Blue | AL3 | PHD finger family protein | Alfin-like |
| AT3G44750 | Blue | HDT1, HDA3, ATHD2A, HD2A | HD2A (HISTONE DEACETYLASE 2A); nucleic acid binding / zinc ion binding | |
| AT3G46640 | Blue | PCL1, LUX | PCL1 (PHYTOCLOCK 1); DNA binding, PCL1 (PHYTOCLOCK 1); DNA binding / transcription factor | G2-like |
| AT3G47120 | Blue | | RNA recognition motif (RRM)-containing protein | C3H |
| AT3G47890 | Blue | | ubiquitin carboxyl-terminal hydrolase-related | |

| | | | | |
|---|---|---|---|---|
| AT3G50750 | Blue | | brassinosteroid signalling positive regulator-related | BES1 |
| AT3G50870 | Blue | HAN, MNP | MNP (MONOPOLE); transcription factor | C2C2-GATA |
| AT3G54280 | Blue | RGD3, ATBTAF1, CHR16, CHA16 | ATP binding / DNA binding / helicase | SNF2 |
| AT3G54620 | Blue | BZO2H4, ATBZIP25 | BZO2H4 (basic leucine zipper O2 homolog 4); transcription factor, unknown protein | bZIP |
| AT3G54810 | Blue | BME3, BME3-ZF | BME3/BME3-ZF (BLUE MICROPLYLAR END3); transcription factor | C2C2-GATA |
| AT3G55730 | Blue | ATMYB109 | MYB109 (myb domain protein 109); DNA binding / transcription factor | MYB |
| AT3G55770 | Blue | | LIM domain-containing protein, zinc ion binding | LIM |
| AT3G57150 | Blue | ATNAP57, ATCBF5, NAP57 | NAP57 (ARABIDOPSIS THALIANA HOMOLOGUE OF NAP57) | |
| AT3G57390 | Blue | AGL18 | AGL18 (AGAMOUS-LIKE 18); transcription factor | MADS |
| AT3G58630 | Blue | | transcription factor | Trihelix |
| AT3G61630 | Blue | CRF6 | CRF6 (CYTOKININ RESPONSE FACTOR 6); DNA binding / transcription factor | AP2-EREBP |
| AT3G63070 | Blue | | PWWP domain-containing protein | |
| AT4G00200 | Blue | | DNA binding | |
| AT4G00940 | Blue | AtDof4,1 | Dof-type zinc finger domain-containing protein | C2C2-Dof |
| AT4G01120 | Blue | ATBZIP54, GBF2 | GBF2 (G-BOX BINDING FACTOR 2); DNA binding / transcription factor | bZIP |
| AT4G01280 | Blue | | DNA binding / transcription factor, myb family transcription factor | MYB-related |
| AT4G09460 | Blue | ATMYB6, ATMYB8 | ATMYB6 (myb domain protein 6, myb domain protein 8); DNA binding / transcription factor | MYB |
| AT4G10350 | Blue | NST4, ANAC070 | ANAC070 (Arabidopsis NAC domain containing protein 70); transcription factor | NAC |
| AT4G10940 | Blue | | PHD finger family protein | |
| AT4G14720 | Blue | TIFY4B, PPD2 | PPD2 (PEAPOD 2) | ZIM |
| AT4G15420 | Blue | | PRLI-interacting factor K | |
| AT4G16110 | Blue | ARR2 | ARR2 (ARABIDOPSIS RESPONSE REGULATOR 2); transcription factor/ two-component response regulator | ARR-B |
| AT4G16150 | Blue | | calmodulin-binding protein | CAMTA |
| AT4G16850 | Blue | | unknown protein | |
| AT4G17020 | Blue | | transcription factor-related | |
| AT4G17230 | Blue | SCL13 | SCL13 (SCARECROW-LIKE 13); transcription factor | GRAS |
| AT4G17570 | Blue | | zinc finger (GATA type) family protein | C2C2-GATA |
| AT4G17785 | Blue | ATMYB39 | myb family transcription factor (MYB39) | MYB |
| AT4G17950 | Blue | | DNA-binding family protein | |
| AT4G19990 | Blue | FRS1 | FRS1 (FAR1-related sequence 1); zinc ion binding | Orphans |
| AT4G22250 | Blue | | zinc finger (C3HC4-type RING finger) family protein | |
| AT4G23860 | Blue | | PHD finger protein-related, protein binding / ubiquitin-protein ligase/ zinc ion binding | |
| AT4G24440 | Blue | | transcription initiation factor IIA gamma chain / TFIIA-gamma (TFIIA-S) | |
| AT4G24470 | Blue | TIFY1, ZIM | ZIM (ZINC-FINGER PROTEIN EXPRESSED IN INFLORESCENCE MERISTEM); transcription factor | C2C2-GATA |
| AT4G25320 | Blue | | DNA-binding protein-related | |
| AT4G28610 | Blue | ATPHR1, PHR1 | PHR1 (PHOSPHATE STARVATION RESPONSE 1); transcription factor | G2-like |
| AT4G29000 | Blue | | tesmin/TSO1-like CXC domain-containing protein | CPP |
| AT4G30860 | Blue | SDG4, ASHR3 | SET domain-containing protein | SET |
| AT4G30930 | Blue | WRKY32, NFD1 | NFD1 (NUCLEAR FUSION DEFECTIVE 1); structural constituent of ribosome | |
| AT4G31420 | Blue | | zinc finger (C2H2 type) family protein | |
| AT4G32010 | Blue | HSL1, HSI2-L1, VAL2 | transcriptional factor B3 family protein | ABI3VP1 |
| AT4G32040 | Blue | KNAT5 | KNAT5 (KNOTTED1-LIKE HOMEOBOX GENE 5); transcription factor | HB |
| AT4G32570 | Blue | TIFY8 | unknown protein | ZIM |
| AT4G32730 | Blue | ATMYB3R-1, PC-MYB1, ATMYB3R1 | PC-MYB1 (myb domain protein 3R1); DNA binding / transcription factor | MYB |
| AT4G32890 | Blue | | zinc finger (GATA type) family protein | C2C2-GATA |
| AT4G36020 | Blue | CSDP1 | CSDP1 (COLD SHOCK DOMAIN PROTEIN 1); nucleic acid binding | CSD |
| AT4G36260 | Blue | SRS2, STY2 | STY2 (STYLISH 2) | SRS |
| AT4G36740 | Blue | HB-5, ATHB40 | ATHB40 (ARABIDOPSIS THALIANA HOMEOBOX PROTEIN 40); DNA binding / transcription factor | HB |
| AT4G36780 | Blue | | brassinosteroid signalling positive regulator-related | BES1 |
| AT4G36900 | Blue | RAP2.10 | RAP2.10 (related to AP2 10); DNA binding / transcription factor | AP2-EREBP |
| AT4G37180 | Blue | | myb family transcription factor | G2-like |
| AT4G38960 | Blue | | zinc finger (B-box type) family protein, zinc ion binding | Orphans |
| AT4G39070 | Blue | | zinc finger (B-box type) family protein | Orphans |
| AT4G39100 | Blue | SHL1 | SHL1 (SHORT LIFE) | PHD |
| AT4G39160 | Blue | | DNA binding / transcription factor | MYB-related |

| | | | | | |
|---|---|---|---|---|---|
| AT5G01160 | Blue | | e-cadherin binding protein-related | | |
| AT5G04100 | Blue | | | | |
| AT5G04110 | Blue | | DNA topoisomerase II family protein | MYB-related | |
| AT5G04760 | Blue | | myb family transcription factor | MYB | |
| AT5G05130 | Blue | | SNF2 domain-containing protein / helicase domain-containing protein / RING finger domain-containing protein | SNF2 | |
| AT5G06160 | Blue | ATO | splicing factor-related | | |
| AT5G06550 | Blue | | unknown protein | Jumonji | |
| AT5G07060 | Blue | | zinc finger (CCCH-type) family protein | C3H | |
| AT5G07310 | Blue | | AP2 domain-containing transcription factor, putative | AP2-EREBP | |
| AT5G10280 | Blue | ATMYB92, AtMYB64 | MYB92 (myb domain protein 92); DNA binding / transcription factor | MYB | |
| AT5G10380 | Blue | RING1 | zinc finger (C3HC4-type RING finger) family protein | | |
| AT5G10720 | Blue | CKI2, AHK5 | AHK5 (CYTOKININ INDEPENDENT 2) | Orphans | |
| AT5G12310 | Blue | | zinc finger (C3HC4-type RING finger) family protein | | |
| AT5G13820 | Blue | HPPBF-1, ATTBP1, ATBP-1, ATBP1, TBP1 | TBP1 (TELOMERIC DNA BINDING PROTEIN 1); DNA binding | | |
| AT5G16070 | Blue | | chaperonin, putative | | |
| AT5G16470 | Blue | | zinc finger (C2H2 type) family protein | | |
| AT5G17430 | Blue | BBM | ovule development protein, putative | AP2-EREBP | |
| AT5G17800 | Blue | ATMYB56 | AtMYB56 (myb domain protein 56); DNA binding / transcription factor | MYB | |
| AT5G18560 | Blue | PUCHI | AP2 domain-containing transcription factor, putative | AP2-EREBP | |
| AT5G19280 | Blue | RAG1, KAPP | KAPP (Kinase-associated protein phosphatase); protein phosphatase type 2C | FHA | |
| AT5G22290 | Blue | FAN, ANAC089 | ANAC089 (Arabidopsis NAC domain containing protein 89); transcription factor | NAC | |
| AT5G22480 | Blue | | zinc finger (ZPR1-type) family protein | | |
| AT5G22750 | Blue | RAD5 | RAD5; ATP binding / DNA binding / helicase/ protein binding / zinc ion binding | SNF2 | |
| AT5G23405 | Blue | | high mobility group (HMG1/2) family protein | HMG | |
| AT5G25475 | Blue | | DNA binding | | |
| AT5G25830 | Blue | | zinc finger (GATA type) family protein | C2C2-GATA | |
| AT5G35770 | Blue | SAP | SAP (STERILE APETALA); transcription factor | SAP | |
| AT5G37020 | Blue | ARF8 | ARF8 (AUXIN RESPONSE FACTOR 8), ARF8 (AUXIN RESPONSE FACTOR 8); transcription factor | ARF | miR167, |
| AT5G42820 | Blue | ATU2AF35B | ATU2AF35B; RNA binding | C3H | |
| AT5G43170 | Blue | AZF3 | AZF3 (ARABIDOPSIS ZINC-FINGER PROTEIN 3); nucleic acid binding / transcription factor/ zinc ion binding | C2H2 | |
| AT5G43700 | Blue | IAA4, ATAUX2-11 | ATAUX2-11 (indoleacetic acid-induced protein 4); transcription factor | AUX/IAA | |
| AT5G45420 | Blue | | myb family transcription factor | MYB | |
| AT5G46640 | Blue | | DNA-binding family protein | | |
| AT5G47370 | Blue | HAT2 | HAT2; transcription factor | HB | |
| AT5G48250 | Blue | COL10 | zinc finger (B-box type) family protein | C2C2-CO-like | |
| AT5G48655 | Blue | | zinc finger (C3HC4-type RING finger) family protein | | |
| AT5G49700 | Blue | | DNA-binding protein-related | | |
| AT5G51230 | Blue | CYR1, VEF2, EMF2 | EMF2 (EMBRYONIC FLOWER 2); transcription factor, unknown protein | | |
| AT5G51980 | Blue | | WD-40 repeat family protein / zfwd2 protein (ZFWD2), putative, nucleic acid binding | C3H | |
| AT5G52660 | Blue | | myb family transcription factor | MYB-related | |
| AT5G53950 | Blue | CUC2, ANAC098 | CUC2 (CUP-SHAPED COTYLEDON 2); transcription factor | NAC | miR164, |
| AT5G56900 | Blue | | CwfJ-like family protein / zinc finger (CCCH-type) family protein | C3H | |
| AT5G59950 | Blue | | RNA and export factor-binding protein, putative, unknown protein | | |
| AT5G60410 | Blue | ATSIZ1, SIZ1 | ATSIZ1/SIZ1; DNA binding, ATSIZ1/SIZ1; DNA binding / SUMO ligase, unknown protein | PHD | |
| AT5G60850 | Blue | OBP4, AtDof5. 4 | OBP4 (OBF BINDING PROTEIN 4); DNA binding / transcription factor | C2C2-Dof | |
| AT5G61190 | Blue | | zinc finger protein-related | | |
| AT5G61770 | Blue | PPAN | brix domain-containing protein | | |
| AT5G63280 | Blue | | zinc finger (C2H2 type) family protein | C2H2 | |
| AT5G63420 | Blue | EMB2746 | EMB2746 (EMBRYO DEFECTIVE 2746); catalytic | Trihelix | |
| AT5G66055 | Blue | EMB2036, AKRP | AKRP/EMB2036 (EMBRYO DEFECTIVE 2036); protein binding | | |
| AT5G66730 | Blue | | zinc finger (C2H2 type) family protein | C2H2 | |
| AT5G67190 | Blue | | AP2 domain-containing transcription factor, putative | AP2-EREBP | |
| AT5G67300 | Blue | ATMYB44, AtMYBr1 | AtMYB44/AtMYBr1 (myb domain protein 44, myb domain protein r1); DNA binding / transcription factor | MYB | |
| AT1G01520 | Brown | | myb family transcription factor | MYB-related | |
| AT1G01720 | Brown | ATAF1, ANAC002 | ATAF1 (Arabidopsis NAC domain containing protein 2); transcription factor | NAC | |

| | | | | | |
|---|---|---|---|---|---|
| AT1G03790 | Brown | SOM | zinc finger (CCCH-type) family protein | C3H | |
| AT1G04990 | Brown | | zinc finger (CCCH-type) family protein | C3H | |
| AT1G07360 | Brown | | zinc finger (CCCH-type) family protein / RNA recognition motif (RRM)-containing protein | C3H | |
| AT1G07980 | Brown | NF-YC10 | histone-like transcription factor (CBF/NF-Y) family protein | CCAAT | |
| AT1G09530 | Brown | POC1, PAP3, PIF3 | PIF3 (PHYTOCHROME INTERACTING FACTOR 3); DNA binding / transcription factor/ transcription regulator | bHLH | |
| AT1G09950 | Brown | | transcription factor-related | | |
| AT1G10170 | Brown | ATNFXL1 | NF-X1 type zinc finger family protein | | |
| AT1G11020 | Brown | | zinc finger (C3HC4-type RING finger) family protein | | |
| AT1G12040 | Brown | LRX1 | LRX1 (LEUCINE-RICH REPEAT/EXTENSIN 1); protein binding / structural constituent of cell wall | | |
| AT1G15580 | Brown | ATAUX2-27, IAA5 | IAA5 (indoleacetic acid-induced protein 5); transcription factor | AUX/IAA | |
| AT1G17460 | Brown | TRFL3 | TRFL3 (TRF-LIKE 3); DNA binding / transcription factor | MYB-related | |
| AT1G20980 | Brown | ATSPL14, SPL1R2, FBR6, SPL14 | SPL14 (SQUAMOSA PROMOTER BINDING PROTEIN-LIKE 14); DNA binding / transcription factor | SBP | |
| AT1G21000 | Brown | | unknown protein, zinc-binding family protein | PLATZ | |
| AT1G21340 | Brown | AtDof1,2 | Dof-type zinc finger domain-containing protein | C2C2-Dof | |
| AT1G21450 | Brown | SCL1 | SCL1 (SCARECROW-LIKE 1); transcription factor | GRAS | |
| AT1G21910 | Brown | | AP2 domain-containing transcription factor family protein | AP2-EREBP | |
| AT1G22190 | Brown | | AP2 domain-containing transcription factor, putative | AP2-EREBP | |
| AT1G23420 | Brown | INO, YAB4 | INO (INNER NO OUTER); transcription factor | C2C2-YABBY | |
| AT1G25280 | Brown | AtTLP10 | AtTLP10 (TUBBY LIKE PROTEIN 10); phosphoric diester hydrolase/ transcription factor, unknown protein | TUB | |
| AT1G26870 | Brown | FEZ, ANAC009 | ANAC009 (Arabidopsis NAC domain containing protein 9); transcription factor | NAC | |
| AT1G32870 | Brown | ANAC13, ANAC013 | ANAC013 (Arabidopsis NAC domain containing protein 13); transcription factor, unknown protein | NAC | |
| AT1G33280 | Brown | ANAC015, NST5 | ANAC015 (Arabidopsis NAC domain containing protein 15); transcription factor | NAC | |
| AT1G33420 | Brown | | PHD finger family protein | PHD | |
| AT1G34360 | Brown | | translation initiation factor 3 (IF-3) family protein | | |
| AT1G34370 | Brown | STOP1 | nucleic acid binding / zinc ion binding, zinc finger (C2H2 type) family protein | C2H2 | |
| AT1G35460 | Brown | bHLH080 | basic helix-loop-helix (bHLH) family protein | bHLH | |
| AT1G42990 | Brown | AtBZIP60 | ATBZIP60 (BASIC REGION/LEUCINE ZIPPER MOTIF 60); DNA binding / transcription factor | bZIP | |
| AT1G44830 | Brown | | AP2 domain-containing transcription factor TINY, putative | AP2-EREBP | |
| AT1G44900 | Brown | | ATP binding / DNA binding / DNA-dependent ATPase | | |
| AT1G46768 | Brown | RAP2.1 | RAP2.1 (related to AP2 1); DNA binding / transcription factor | AP2-EREBP | |
| AT1G47270 | Brown | AtTLP6 | AtTLP6 (TUBBY LIKE PROTEIN 6); phosphoric diester hydrolase/ transcription factor | TUB | |
| AT1G50670 | Brown | | OTU-like cysteine protease family protein | | |
| AT1G54330 | Brown | ANAC020 | ANAC020 (Arabidopsis NAC domain containing protein 20); transcription factor | NAC | |
| AT1G54690 | Brown | HTA3 ,G-H2AX ,GAMMA-H2AX ,H2AXB | histone H2A, putative | CCAAT | |
| AT1G59640 | Brown | ZCW32, bHLH031, BPE, BPEUB, BPEP | ZCW32 (BIGPETAL, BIGPETALUB); DNA binding / transcription factor | bHLH | |
| AT1G62300 | Brown | ATWRKY6, WRKY6 | WRKY6 (WRKY DNA-binding protein 6); transcription factor | WRKY | |
| AT1G63040 | Brown | | pseudogene | | |
| AT1G63840 | Brown | | zinc finger (C3HC4-type RING finger) family protein | | |
| AT1G64380 | Brown | | AP2 domain-containing transcription factor, putative | AP2-EREBP | |
| AT1G65040 | Brown | | protein binding / zinc ion binding, unknown protein | | |
| AT1G66390 | Brown | PAP2, AtMYB90 | PAP2 (PRODUCTION OF ANTHOCYANIN PIGMENT 2); DNA binding / transcription factor | MYB | |
| AT1G68320 | Brown | BW62C, BW62B, AtMYB62 | MYB62 (myb domain protein 62); DNA binding / transcription factor | MYB | |
| AT1G68690 | Brown | | pseudogene | | |
| AT1G69170 | Brown | SPL6 | squamosa promoter-binding protein-like 6 (SPL6) | SBP | miR156/miR157, |
| AT1G70460 | Brown | | protein kinase, putative | | |
| AT1G72175 | Brown | | zinc finger (C3HC4-type RING finger) family protein | | |
| AT1G72570 | Brown | | DNA binding / transcription factor | AP2-EREBP | |
| AT1G73230 | Brown | | nascent polypeptide-associated complex (NAC) domain-containing protein | | |
| AT1G74250 | Brown | | DNAJ heat shock N-terminal domain-containing protein | | |
| AT1G74370 | Brown | | zinc finger (C3HC4-type RING finger) family protein | | |
| AT1G75340 | Brown | | zinc finger (CCCH-type) family protein | C3H | |
| AT1G76590 | Brown | | zinc-binding family protein | PLATZ | |
| AT1G77450 | Brown | ANAC032 | ANAC032 (Arabidopsis NAC domain containing protein 32); transcription factor | NAC | |
| AT1G77570 | Brown | HSFA5-like | DNA binding / transcription factor | HSF | |

| | | | | |
|---|---|---|---|---|
| AT1G77640 | Brown | | AP2 domain-containing transcription factor, putative | AP2-EREBP |
| AT1G78080 | Brown | CAF1, RAP2.4 | RAP2.4 (related to AP2 4); DNA binding / transcription factor | AP2-EREBP |
| AT1G78600 | Brown | STH3, LZF1 | zinc finger (B-box type) family protein | Orphans |
| AT1G79580 | Brown | ANAC033, SMB, NST6 | ANAC033, ANAC033; transcription factor | NAC |
| AT1G80400 | Brown | | zinc finger (C3HC4-type RING finger) family protein | |
| AT1G80840 | Brown | ATWRKY40, WRKY40 | WRKY40 (WRKY DNA-binding protein 40); transcription factor | WRKY |
| AT2G01060 | Brown | | myb family transcription factor | G2-like |
| AT2G01200 | Brown | IAA32, MEE10 | IAA32 (indoleacetic acid-induced protein 32, maternal effect embryo arrest 10), IAA32 (indoleacetic acid-induced protein 32, maternal effect embryo arrest 10); transcription factor | |
| AT2G07677 | Brown | | pseudogene | |
| AT2G18760 | Brown | CHR8 | CHR8 (chromatin remodeling 8); ATP binding / DNA binding / helicase | SNF2 |
| AT2G21050 | Brown | | amino acid permease, putative | |
| AT2G23320 | Brown | WRKY15 | WRKY15 (WRKY DNA-binding protein 15); transcription factor | WRKY |
| AT2G24570 | Brown | ATWRKY17, WRKY17 | WRKY17 (WRKY DNA-binding protein 17); transcription factor | WRKY |
| AT2G25000 | Brown | ATWRKY60, WRKY60 | WRKY60 (WRKY DNA-binding protein 60); transcription factor | WRKY |
| AT2G28710 | Brown | | zinc finger (C2H2 type) family protein | C2H2 |
| AT2G30250 | Brown | ATWRKY25, WRKY25 | WRKY25 (WRKY DNA-binding protein 25); transcription factor | WRKY |
| AT2G31370 | Brown | AtbZIP59, PosF21 | DNA binding / transcription factor, bZIP transcription factor (POSF21) | bZIP |
| AT2G32930 | Brown | ZFN2 | ZFN2 (ZINC FINGER PROTEIN 2); nucleic acid binding | C3H |
| AT2G33710 | Brown | | AP2 domain-containing transcription factor family protein | AP2-EREBP |
| AT2G35550 | Brown | BPC7, BBR/BPC7, ATBPC7 | ATBPC7/BBR/BPC7/BPC7 (BASIC PENTACYSTEINE 7), ATBPC7/BBR/BPC7/BPC7 (BASIC PENTACYSTEINE 7); DNA binding / transcription factor | BBR/BPC |
| AT2G35910 | Brown | | zinc finger (C3HC4-type RING finger) family protein | |
| AT2G37430 | Brown | ZAT11 | zinc finger (C2H2 type) family protein (ZAT11) | C2H2 |
| AT2G38250 | Brown | | DNA-binding protein-related | Trihelix |
| AT2G38340 | Brown | | AP2 domain-containing transcription factor, putative (DRE2B) | AP2-EREBP |
| AT2G38470 | Brown | ATWRKY33, WRKY33 | WRKY33 (WRKY DNA-binding protein 33); transcription factor | WRKY |
| AT2G40350 | Brown | | DNA binding / transcription factor | AP2-EREBP |
| AT2G40470 | Brown | LBD15 | LOB domain protein 15 / lateral organ boundaries domain protein 15 (LBD15) | |
| AT2G40950 | Brown | BZIP17 | bZIP transcription factor family protein | bZIP |
| AT2G41710 | Brown | | ovule development protein, putative | AP2-EREBP |
| AT2G42360 | Brown | | zinc finger (C3HC4-type RING finger) family protein | |
| AT2G42430 | Brown | ASL18, LBD16 | LBD16 (ASYMMETRIC LEAVES2-LIKE18) | |
| AT2G42680 | Brown | MBF1A, ATMBF1A | ATMBF1A/MBF1A (MULTIPROTEIN BRIDGING FACTOR 1A); DNA binding / transcription coactivator | MBF1 |
| AT2G43000 | Brown | ANAC042 | ANAC042 (Arabidopsis NAC domain containing protein 42); transcription factor | NAC |
| AT2G47260 | Brown | ATWRKY23, WRKY23 | WRKY23 (WRKY DNA-binding protein 23); transcription factor | WRKY |
| AT2G47850 | Brown | | zinc finger (CCCH-type) family protein | C3H |
| AT2G47890 | Brown | COL13 | zinc finger (B-box type) family protein | C2C2-CO-like |
| AT3G01330 | Brown | E2FF, E2L2, DEL3 | DEL3 (DP-E2F-like 3); transcription factor | E2F-DP |
| AT3G01560 | Brown | | proline-rich family protein | |
| AT3G02990 | Brown | ATHSFA1E | ATHSFA1E (Arabidopsis thaliana heat shock transcription factor A1E); DNA binding / transcription factor | HSF |
| AT3G03660 | Brown | WOX11 | DNA binding / transcription factor, unknown protein | HB |
| AT3G05400 | Brown | | sugar transporter, putative | |
| AT3G06410 | Brown | | nucleic acid binding | C3H |
| AT3G07610 | Brown | IBM1 | transcription factor jumonji (jmjC) domain-containing protein | Jumonji |
| AT3G10500 | Brown | ANAC053 | ANAC053 (Arabidopsis NAC domain containing protein 53); transcription factor | NAC |
| AT3G11020 | Brown | DREB2B | DREB2B (DRE-binding protein 2B); DNA binding / transcription factor/ transcriptional activator | AP2-EREBP |
| AT3G11200 | Brown | AL2 | PHD finger family protein | Alfin-like |
| AT3G11580 | Brown | | DNA-binding protein, putative | ABI3VP1 |
| AT3G12280 | Brown | RBL1, RBR1 | RBR1 (RETINOBLASTOMA-RELATED 1) | RB |
| AT3G13350 | Brown | | high mobility group (HMG1/2) family protein / ARID/BRIGHT DNA-binding domain-containing protein | ARID |
| AT3G13445 | Brown | TFIID-1, TBP1 | TBP1 (TRANSCRIPTION FACTOR IID-1); DNA binding / RNA polymerase II transcription factor | |
| AT3G14880 | Brown | | unknown protein | |
| AT3G18290 | Brown | EMB2454 | EMB2454 (EMBRYO DEFECTIVE 2454); protein binding / zinc ion binding | |
| AT3G20880 | Brown | | zinc finger (C2H2 type) protein (WIP4) | C2H2 |

| | | | | | |
|---|---|---|---|---|---|
| AT3G23250 | Brown | ATMYB15, ATY19 | AtMYB15/AtY19/MYB15 (myb domain protein 15); DNA binding, AtMYB15/AtY19/MYB15 (myb domain protein 15); DNA binding / transcription factor | MYB | |
| AT3G28210 | Brown | PMZ | PMZ; zinc ion binding | | |
| AT3G30530 | Brown | ATBZIP42 | bZIP transcription factor family protein | bZIP | |
| AT3G44350 | Brown | ANAC061 | ANAC061 (Arabidopsis NAC domain containing protein 61); transcription factor, unknown protein | NAC | |
| AT3G47640 | Brown | bHLH047 | basic helix-loop-helix (bHLH) family protein | bHLH | |
| AT3G50410 | Brown | OBP1, AtDof3. 4 | OBP1 (OBF BINDING PROTEIN 1); DNA binding / transcription factor | C2C2-Dof | |
| AT3G55560 | Brown | AGF2 | DNA-binding protein-related | | |
| AT3G56980 | Brown | ORG3, bHLH039 | ORG3 (OBP3-responsive gene 3); DNA binding / transcription factor | bHLH | |
| AT3G57670 | Brown | NTT | zinc finger (C2H2 type) protein (WIP2) | C2H2 | |
| AT3G58680 | Brown | MBF1B, ATMBF1B | ATMBF1B/MBF1B (MULTIPROTEIN BRIDGING FACTOR 1B); DNA binding / transcription coactivator | MBF1 | |
| AT4G00335 | Brown | RHB1A | RHB1A (RING-H2 finger B1A); protein binding / zinc ion binding | | |
| AT4G00850 | Brown | GIF3 | GIF3 (GRF1-INTERACTING FACTOR 3) | | |
| AT4G01550 | Brown | ANAC069, NTM2 | ANAC069 (Arabidopsis NAC domain containing protein 69), ANAC069 (Arabidopsis NAC domain containing protein 69); transcription factor | NAC | |
| AT4G01720 | Brown | ATWRKY47, WRKY47 | WRKY47 (WRKY DNA-binding protein 47); transcription factor | WRKY | |
| AT4G02540 | Brown | | DC1 domain-containing protein | | |
| AT4G02560 | Brown | LD | LD (LUMINIDEPENDENS); transcription factor | HB | |
| AT4G05100 | Brown | ATMYB74 | AtMYB74 (myb domain protein 74); DNA binding / transcription factor | MYB | |
| AT4G12240 | Brown | | zinc finger (C2H2 type) family protein | C2H2 | |
| AT4G12620 | Brown | ORC1B, ATORC1B, UNE13 | ATORC1B/ORC1B/UNE13 (ORIGIN OF REPLICATION COMPLEX 1B, unfertilized embryo sac 13); DNA binding / protein binding | PHD | |
| AT4G13130 | Brown | | DC1 domain-containing protein | | |
| AT4G14560 | Brown | AXR5, IAA1 | IAA1 (INDOLE-3-ACETIC ACID INDUCIBLE); transcription factor | AUX/IAA | |
| AT4G17750 | Brown | ATHSFA1A, ATHSF1, HSF1 | HSF1 (ARABIDOPSIS HEAT SHOCK FACTOR 1); DNA binding / transcription factor | HSF | |
| AT4G17980 | Brown | ANAC071 | ANAC071 (Arabidopsis NAC domain containing protein 71); transcription factor | NAC | |
| AT4G18880 | Brown | AT-HSFA4A | AT-HSFA4A (Arabidopsis thaliana heat shock transcription factor A4A); DNA binding / transcription factor | HSF | |
| AT4G18890 | Brown | | brassinosteroid signalling positive regulator-related | BES1 | |
| AT4G19660 | Brown | NPR4 | NPR4 (NPR1-LIKE PROTEIN 4); protein binding | | |
| AT4G22770 | Brown | | DNA-binding family protein | | |
| AT4G23750 | Brown | CRF2 | CRF2 (CYTOKININ RESPONSE FACTOR 2); DNA binding / transcription factor | AP2-EREBP | |
| AT4G24150 | Brown | ATGRF8 | AtGRF8 (GROWTH-REGULATING FACTOR 8) | GRF | miR396, |
| AT4G25990 | Brown | CIL | CIL | Orphans | |
| AT4G26120 | Brown | | ankyrin repeat family protein / BTB/POZ domain-containing protein | | |
| AT4G30080 | Brown | ARF16 | ARF16 (AUXIN RESPONSE FACTOR 16); miRNA binding / transcription factor | ARF | miR160, |
| AT4G31000 | Brown | | calmodulin-binding protein | | |
| AT4G31550 | Brown | ATWRKY11, WRKY11 | WRKY11 (WRKY DNA-binding protein 11); transcription factor | WRKY | |
| AT4G31800 | Brown | ATWRKY18, WRKY18 | WRKY18 (WRKY DNA-binding protein 18); transcription factor | WRKY | |
| AT4G32280 | Brown | IAA29 | IAA29 (indoleacetic acid-induced protein 29); transcription factor | AUX/IAA | |
| AT4G34990 | Brown | ATMYB32 | AtMYB32 (myb domain protein 32); DNA binding / transcription factor | MYB | |
| AT4G37130 | Brown | | hydroxyproline-rich glycoprotein family protein | | |
| AT4G38900 | Brown | AtbZIP29 | DNA binding, bZIP protein | bZIP | |
| AT4G39780 | Brown | | AP2 domain-containing transcription factor, putative | AP2-EREBP | |
| AT5G01380 | Brown | | transcription factor | Trihelix | |
| AT5G01960 | Brown | | zinc finger (C3HC4-type RING finger) family protein | | |
| AT5G04410 | Brown | NAC2, ANAC078 | NAC2 (Arabidopsis NAC domain containing protein 78); transcription factor | NAC | |
| AT5G09240 | Brown | | transcriptional coactivator p15 (PC4) family protein | | |
| AT5G09330 | Brown | ANAC082 | ANAC082 (Arabidopsis NAC domain containing protein 82), ANAC082 (Arabidopsis NAC domain containing protein 82); transcription factor | NAC | |
| AT5G10510 | Brown | PLT3, AIL6 | AIL6 (AINTEGUMENTA-LIKE 6); DNA binding / transcription factor, unknown protein | AP2-EREBP | |
| AT5G14000 | Brown | ANAC084 | ANAC084 (Arabidopsis NAC domain containing protein 84); transcription factor | NAC | |
| AT5G14960 | Brown | DEL2, E2L1, E2FD | DEL2/E2FD/E2L1 (DP-E2F-LIKE 2); DNA binding / transcription factor | E2F-DP | |
| AT5G15160 | Brown | | bHLH family protein | | |
| AT5G18450 | Brown | | AP2 domain-containing transcription factor, putative | AP2-EREBP | |
| AT5G18550 | Brown | | nucleic acid binding | C3H | |

| | | | | | |
|---|---|---|---|---|---|
| AT5G20510 | Brown | AL5 | PHD finger family protein | Alfin-like | |
| AT5G20910 | Brown | | zinc finger (C3HC4-type RING finger) family protein | | |
| AT5G24330 | Brown | SDG34, ATXR6 | ATXR6 (Arabidopsis thaliana Trithorax- related protein 6); DNA binding | PHD | |
| AT5G24800 | Brown | BZO2H2, ATBZIP9 | BZO2H2 (basic leucine zipper O2 homolog 2); DNA binding / transcription factor | bZIP | |
| AT5G25190 | Brown | | ethylene-responsive element-binding protein, putative | AP2-EREBP | |
| AT5G26930 | Brown | | zinc finger (GATA type) family protein | C2C2-GATA | |
| AT5G40880 | Brown | | WD-40 repeat family protein / zfwd3 protein (ZFWD3) | C3H | |
| AT5G41350 | Brown | | zinc finger (C3HC4-type RING finger) family protein | | |
| AT5G44260 | Brown | | zinc finger (CCCH-type) family protein | C3H | |
| AT5G46350 | Brown | ATWRKY8, WRKY8 | WRKY8 (WRKY DNA-binding protein 8); transcription factor | WRKY | |
| AT5G48150 | Brown | PAT1 | PAT1 (PHYTOCHROME A SIGNAL TRANSDUCTION 1); transcription factor | GRAS | |
| AT5G49520 | Brown | ATWRKY48, WRKY48 | WRKY48 (WRKY DNA-binding protein 48); transcription factor | WRKY | |
| AT5G50570 | Brown | SPL13 | squamosa promoter-binding protein, putative | SBP | miR156/miR157, |
| AT5G51240 | Brown | | | | |
| AT5G51590 | Brown | | DNA-binding protein-related | | |
| AT5G52830 | Brown | ATWRKY27, WRKY27 | WRKY27 (WRKY DNA-binding protein 27); transcription factor | WRKY | |
| AT5G54070 | Brown | AT-HSFA9 | AT-HSFA9 (Arabidopsis thaliana heat shock transcription factor A9); DNA binding / transcription factor | HSF | |
| AT5G57420 | Brown | IAA33 | IAA33 (indoleacetic acid-induced protein 33); transcription factor | | |
| AT5G58610 | Brown | | PHD finger transcription factor, putative | PHD | |
| AT5G59000 | Brown | | zinc finger (C3HC4-type RING finger) family protein | | |
| AT5G60100 | Brown | APRR3 | APRR3 (PSEUDO-RESPONSE REGULATOR 3); transcription regulator | Pseudo ARR-B | |
| AT5G61890 | Brown | | AP2 domain-containing transcription factor family protein | AP2-EREBP | |
| AT5G63780 | Brown | SHA1 | zinc finger (C3HC4-type RING finger) family protein | | |
| AT5G63790 | Brown | ANAC102 | ANAC102 (Arabidopsis NAC domain containing protein 102); transcription factor | NAC | |
| AT5G64060 | Brown | ANAC103 | ANAC103 (Arabidopsis NAC domain containing protein 103); transcription factor | NAC | |
| AT5G64980 | Brown | | unknown protein | | |
| AT5G65080 | Brown | AGL68, MAF5.2, MAF5 | MAF5 (MADS AFFECTING FLOWERING 5), MAF5 (MADS AFFECTING FLOWERING 5); transcription factor | MADS | |
| AT5G66750 | Brown | CHR01, CHA1, SOM1, CHR1, SOM4, DDM1 | DDM1 (DECREASED DNA METHYLATION 1); helicase | SNF2 | |
| AT5G67480 | Brown | ATBT4, BT4 | BT4 (BTB AND TAZ DOMAIN PROTEIN 4); protein binding, BT4 (BTB AND TAZ DOMAIN PROTEIN 4); protein binding / transcription regulator | TAZ | |
| AT5G67580 | Brown | ATTBP3, TRB2, ATTRB2 | ATTRB2/TRB2 (TELOMERE REPEAT BINDING FACTOR 2); DNA binding / transcription factor | MYB-related | |
| AT1G17920 | Cyan | HDG12 | HDG12 (HOMEODOMAIN GLABROUS12); transcription factor | HB | |
| AT2G24740 | Cyan | SUVH8, SDG21 | SDG21 (SET DOMAIN GROUP 21) | SET | |
| AT2G27100 | Cyan | SE | SE (SERRATE); transcription factor | | |
| AT2G27470 | Cyan | NF-YB11 | CCAAT-box binding transcription factor subunit HAP3-related | CCAAT | |
| AT2G35700 | Cyan | ATERF38 | AP2 domain-containing transcription factor, putative | AP2-EREBP | |
| AT3G02790 | Cyan | | zinc finger (C2H2 type) family protein | | |
| AT3G20010 | Cyan | | SNF2 domain-containing protein / helicase domain-containing protein / RING finger domain-containing protein | SNF2 | |
| AT4G11660 | Cyan | AT-HSFB2B, HSFB2B | AT-HSFB2B (Arabidopsis thaliana heat shock transcription factor B2B); transcription factor | HSF | |
| AT4G21550 | Cyan | HSI2-L2, VAL3 | transcriptional factor B3 family protein | ABI3VP1 | |
| AT4G26920 | Cyan | | unknown protein | | |
| AT5G07040 | Cyan | | zinc finger (C3HC4-type RING finger) family protein | | |
| AT5G12840 | Cyan | NF-YA1, ATHAP2A, HAP2A, EMB2220 | HAP2A (EMBRYO DEFECTIVE 2220); transcription factor | CCAAT | miR169, |
| AT1G33240 | Darkgreen | AT-GTL2, AT-GTL1 | AT-GTL1 (Arabidopsis thaliana GT2-like 1); transcription factor | Trihelix | |
| AT3G21810 | Darkgreen | | zinc finger (CCCH-type) family protein | C3H | |
| AT4G17695 | Darkgreen | KAN3, KANADI3 | KAN3 (KANADI 3); DNA binding / transcription factor | G2-like | |
| AT5G38840 | Darkgreen | | forkhead-associated domain-containing protein / FHA domain-containing protein | FHA | |
| AT1G08880 | Darkgrey | HTA5 ,G-H2AX ,GAMMA-H2AX ,H2AXA | histone H2A, putative | CCAAT | |
| AT1G75390 | Darkgrey | ATBZIP44 | bZIP transcription factor family protein | bZIP | |
| AT1G79350 | Darkorange | EMB1135 | EMB1135 (EMBRYO DEFECTIVE 1135); DNA binding | PHD | |
| AT2G31630 | Darkorange | TRX1, | | | |
| AT3G01460 | Darkorange | MBD9 | MBD9 (METHYL-CPG-BINDING DOMAIN 9); DNA binding | PHD | |
| AT5G25150 | Darkorange | TAF5 | nucleotide binding | | |
| AT1G24260 | Darkred | 3-Sep, AGL9, SEP3 | SEP3 (SEPALLATA3); transcription factor | MADS | |

| | | | | | |
|---|---|---|---|---|---|
| AT3G10800 | Darkred | BZIP28 | bZIP transcription factor family protein | bZIP | |
| AT4G17710 | Darkred | HDG4 | homeobox-leucine zipper family protein / lipid-binding START domain-containing protein | HB | |
| AT1G30330 | Darkturquoise | ARF6 | ARF6 (AUXIN RESPONSE FACTOR 6), ARF6 (AUXIN RESPONSE FACTOR 6); transcription factor | ARF | miR167, |
| AT1G30810 | Darkturquoise | | transcription factor jumonji (jmj) family protein / zinc finger (C5HC2 type) family protein | Jumonji | |
| AT1G57820 | Darkturquoise | ORTH2, VIM1 | VIM1 (VARIANT IN METHYLATION 1); DNA binding | | |
| AT3G51470 | Darkturquoise | | protein phosphatase 2C, putative / PP2C, putative | DBP | |
| AT4G16430 | Darkturquoise | bHLH003 | basic helix-loop-helix (bHLH) family protein | bHLH | |
| AT5G06770 | Darkturquoise | | KH domain-containing protein / zinc finger (CCCH type) family protein | C3H | |
| AT1G01010 | Green | ANAC001 | ANAC001 (Arabidopsis NAC domain containing protein 1); transcription factor | NAC | |
| AT1G01920 | Green | | SET domain-containing protein | SET | |
| AT1G04240 | Green | IAA3, SHY2 | SHY2 (SHORT HYPOCOTYL 2); transcription factor | AUX/IAA | |
| AT1G05050 | Green | | | | |
| AT1G05710 | Green | bHLH153 | ethylene-responsive protein, putative | | |
| AT1G07470 | Green | | transcription factor IIA large subunit, putative / TFIIA large subunit, putative | | |
| AT1G10480 | Green | ZFP5 | ZFP5 (ZINC FINGER PROTEIN 5); nucleic acid binding / transcription factor/ zinc ion binding | C2H2 | |
| AT1G11100 | Green | | SNF2 domain-containing protein / helicase domain-containing protein / zinc finger protein-related | SNF2 | |
| AT1G12630 | Green | | DNA binding / transcription factor/ transcriptional activator | AP2-EREBP | |
| AT1G12880 | Green | ATNUDT12 | ATNUDT12 (Arabidopsis thaliana Nudix hydrolase homolog 12); hydrolase | | |
| AT1G13400 | Green | JGL, NUB | JGL/NUB (NUBBIN); nucleic acid binding / zinc ion binding | | |
| AT1G13450 | Green | | DNA binding protein GT-1, unknown protein | Trihelix | |
| AT1G13960 | Green | WRKY4 | WRKY4 (WRKY DNA-binding protein 4); transcription factor | WRKY | |
| AT1G15100 | Green | RHA2A | RHA2A (RING-H2 finger A2A); protein binding / zinc ion binding | | |
| AT1G16640 | Green | | transcriptional factor B3 family protein | ABI3VP1 | |
| AT1G18570 | Green | HIG1, BW51B, BW51A, AtMYB51 | MYB51 (myb domain protein 51); DNA binding / transcription factor | MYB | |
| AT1G19850 | Green | IAA24, ARF5, MP | MP (MONOPTEROS); transcription factor | ARF | |
| AT1G20693 | Green | HMGBETA1, NFD2, NFD02, HMGB2 | HMGB2 (HIGH MOBILITY GROUP B 2), HMGB2 (HIGH MOBILITY GROUP B 2); transcription factor | HMG | |
| AT1G20696 | Green | NFD3, NFD03, HMGB3 | HMGB3 (HIGH MOBILITY GROUP B 3), HMGB3 (HIGH MOBILITY GROUP B 3); transcription factor | HMG | |
| AT1G20910 | Green | | ARID/BRIGHT DNA-binding domain-containing protein | ARID | |
| AT1G21160 | Green | | translation initiation factor | | |
| AT1G22490 | Green | bHLH094 | basic helix-loop-helix (bHLH) family protein | bHLH | |
| AT1G25560 | Green | TEM1 | AP2 domain-containing transcription factor, putative | AP2-EREBP | |
| AT1G25580 | Green | ANAC008 | ANAC008 (Arabidopsis NAC domain containing protein 8); transcription factor | NAC | |
| AT1G26580 | Green | | unknown protein | | |
| AT1G26800 | Green | | zinc finger (C3HC4-type RING finger) family protein | | |
| AT1G27360 | Green | SPL11 | squamosa promoter-binding protein-like 11 (SPL11) | SBP | miR156/miR157, |
| AT1G27370 | Green | SPL10 | DNA binding, squamosa promoter-binding protein-like 10 (SPL10) | SBP | miR156/miR157, |
| AT1G29280 | Green | ATWRKY65, WRKY65 | WRKY65 (WRKY DNA-binding protein 65); transcription factor | WRKY | |
| AT1G29950 | Green | | transcription factor/ transcription regulator, unknown protein | | |
| AT1G30490 | Green | ATHB9, PHV | PHV (PHAVOLUTA); DNA binding / transcription factor | HB | miR166/miR165, |
| AT1G31320 | Green | LBD4 | LOB domain protein 4 / lateral organ boundaries domain protein 4 (LBD4) | | |
| AT1G32330 | Green | ATHSFA1D | ATHSFA1D (Arabidopsis thaliana heat shock transcription factor A1D); DNA binding / transcription factor | HSF | |
| AT1G43770 | Green | | PHD finger family protein, unknown protein | PHD | |
| AT1G47870 | Green | ATE2FC, ATE2F2, E2FC | E2FC (ARABIDOPSIS HOMOLOG OF E2F C); transcription factor | E2F-DP | |
| AT1G49850 | Green | | zinc finger (C3HC4-type RING finger) family protein | | |
| AT1G50410 | Green | | SNF2 domain-containing protein / helicase domain-containing protein / RING finger domain-containing protein | SNF2 | |
| AT1G50420 | Green | SCL-3, SCL3 | SCL3 (SCARECROW-LIKE 3); transcription factor | GRAS | |
| AT1G52150 | Green | ATHB15, ICU4, CNA, ATHB-15 | ATHB-15 (INCURVATA 4), ATHB-15 (INCURVATA 4); DNA binding / transcription factor | HB | miR166/miR165, |
| AT1G52450 | Green | | ubiquitin carboxyl-terminal hydrolase-related | | |
| AT1G61140 | Green | EDA16 | EDA16 (embryo sac development arrest 16); ATP binding / DNA binding / helicase/ protein binding / zinc ion binding, unknown protein | SNF2 | |
| AT1G63490 | Green | | transcription factor jumonji (jmjC) domain-containing protein | Jumonji | |
| AT1G64105 | Green | ANAC027 | ANAC027 (Arabidopsis NAC domain containing protein 27); transcription factor | NAC | |
| AT1G64620 | Green | AtDof1,8 | Dof-type zinc finger domain-containing protein | C2C2-Dof | |
| AT1G68240 | Green | bHLH109 | transcription factor, unknown protein | bHLH | |
| AT1G68360 | Green | | zinc finger protein-related | | |

| | | | | | |
|---|---|---|---|---|---|
| AT1G68920 | Green | bHLH049 | basic helix-loop-helix (bHLH) family protein | bHLH | |
| AT1G69310 | Green | ATWRKY57, WRKY57 | WRKY57 (WRKY DNA-binding protein 57); transcription factor | WRKY | |
| AT1G69330 | Green | | zinc finger (C3HC4-type RING finger) family protein | | |
| AT1G69810 | Green | ATWRKY36, WRKY36 | WRKY36 (WRKY DNA-binding protein 36); transcription factor | WRKY | |
| AT1G70000 | Green | | DNA-binding family protein | MYB-related | |
| AT1G72050 | Green | | zinc finger (C2H2 type) family protein | C2H2 | |
| AT1G72650 | Green | TRFL6 | TRFL6 (TRF-LIKE 6); DNA binding / transcription factor | MYB-related | |
| AT1G72740 | Green | | DNA-binding family protein / histone H1/H5 family protein | MYB-related | |
| AT1G73730 | Green | SLIM1, EIL3 | EIL3 (ETHYLENE-INSENSITIVE3-LIKE3); transcription factor | EIL | |
| AT1G73950 | Green | | zinc finger (C3HC4-type RING finger) family protein | | |
| AT1G74660 | Green | MIF1 | MIF1 (MINI ZINC FINGER 1); DNA binding / transcription factor | zf-HD | |
| AT1G75540 | Green | STH2 | zinc finger (B-box type) family protein | Orphans | |
| AT1G77800 | Green | | PHD finger family protein | PHD | |
| AT1G77920 | Green | | bZIP family transcription factor | bZIP | |
| AT1G78280 | Green | | transcription factor jumonji (jmjC) domain-containing protein | Jumonji | |
| AT1G79700 | Green | | ovule development protein, putative | AP2-EREBP | |
| AT2G01650 | Green | PUX2 | PUX2 (PLANT UBX DOMAIN-CONTAINING PROTEIN 2); nucleic acid binding / zinc ion binding | | |
| AT2G02090 | Green | CHA19, ETL1, CHR19 | CHR19/ETL1 (chromatin remodeling 19); ATP binding / DNA binding / helicase | SNF2 | |
| AT2G13370 | Green | CHR5 | CHR5 (chromatin remodeling 5); ATP binding / DNA binding / chromatin binding / helicase | SNF2 | |
| AT2G15580 | Green | | zinc finger (C3HC4-type RING finger) family protein | | |
| AT2G17150 | Green | NLP1 | RWP-RK domain-containing protein | RWP-RK | |
| AT2G17410 | Green | | ARID/BRIGHT DNA-binding domain-containing protein | ARID | |
| AT2G18060 | Green | ANAC037, VND1 | VND1 (VASCULAR RELATED NAC-DOMAIN PROTEIN 1); transcription factor | NAC | |
| AT2G18090 | Green | | PHD finger family protein / SWIB complex BAF60b domain-containing protein / GYF domain-containing protein | | |
| AT2G18120 | Green | SRS4 | SRS4 (SHI-RELATED SEQUENCE 4) | SRS | |
| AT2G18750 | Green | | calmodulin binding, calmodulin-binding protein | | |
| AT2G20400 | Green | | myb family transcription factor | G2-like | |
| AT2G27230 | Green | LHW, bHLH156 | transcription factor-related | | |
| AT2G27760 | Green | IPPT, ATIPT2 | ATIPT2 (TRNA ISOPENTENYLTRANSFERASE) | | |
| AT2G27980 | Green | | protein binding / zinc ion binding | | |
| AT2G28510 | Green | AtDof2,1 | Dof-type zinc finger domain-containing protein | C2C2-Dof | |
| AT2G29580 | Green | | zinc finger (CCCH-type) family protein / RNA recognition motif (RRM)-containing protein | C3H | |
| AT2G31730 | Green | | ethylene-responsive protein, putative | | |
| AT2G33310 | Green | IAA13 | IAA13 (indoleacetic acid-induced protein 13); transcription factor, unknown protein | AUX/IAA | |
| AT2G33620 | Green | | DNA-binding family protein / AT-hook protein 1 (AHP1), unknown protein | | |
| AT2G34140 | Green | AtDof2. 3 | Dof-type zinc finger domain-containing protein | C2C2-Dof | |
| AT2G34450 | Green | | high mobility group (HMG1/2) family protein | HMG | |
| AT2G36720 | Green | | PHD finger transcription factor, putative | PHD | |
| AT2G37520 | Green | | PHD finger family protein | PHD | |
| AT2G37590 | Green | AtDof2. 4 | Dof-type zinc finger domain-containing protein | C2C2-Dof | |
| AT2G38090 | Green | | myb family transcription factor | MYB | |
| AT2G39880 | Green | ATMYB25 | MYB25 (myb domain protein 25); DNA binding / transcription factor | MYB | |
| AT2G40140 | Green | ATSZF2, CZF1, ZFAR1 | CZF1/ZFAR1, CZF1/ZFAR1; transcription factor | C3H | |
| AT2G42280 | Green | bHLH130 | basic helix-loop-helix (bHLH) family protein | bHLH | |
| AT2G44980 | Green | | transcription regulatory protein SNF2, putative | SNF2 | |
| AT2G46870 | Green | NGA1 | NGA1 (NGATHA1); transcription factor | ABI3VP1 | |
| AT2G47080 | Green | | | | |
| AT2G47270 | Green | bHLH151 | transcription factor/ transcription regulator | | |
| AT2G47460 | Green | PFG1, ATMYB12 | MYB12 (myb domain protein 12); DNA binding / transcription factor/ transcriptional activator | MYB | miR858, |
| AT2G47900 | Green | AtTLP3 | AtTLP3 (TUBBY LIKE PROTEIN 3); phosphoric diester hydrolase/ transcription factor | TUB | |
| AT2G48100 | Green | | exonuclease, exonuclease family protein | C2H2 | |
| AT3G01470 | Green | HD-ZIP-1, HAT5, ATHB1, ATHB-1 | ATHB-1 (Homeobox-leucine zipper protein HAT5); transcription factor | HB | |
| AT3G03550 | Green | | zinc finger (C3HC4-type RING finger) family protein | | |
| AT3G04570 | Green | | DNA-binding protein-related | | |
| AT3G04670 | Green | ATWRKY39, WRKY39 | WRKY39 (WRKY DNA-binding protein 39); transcription factor | WRKY | |

| | | | | | |
|---|---|---|---|---|---|
| AT3G04730 | Green | IAA16 | IAA16 (indoleacetic acid-induced protein 16); transcription factor | AUX/IAA | |
| AT3G05670 | Green | | PHD finger family protein | PHD | |
| AT3G14230 | Green | RAP2.2 | RAP2.2; DNA binding / transcription factor | AP2-EREBP | |
| AT3G16720 | Green | ATL2 | ATL2 (Arabidopsis T?xicos en Levadura 2); protein binding / zinc ion binding | | |
| AT3G16857 | Green | ARR1 | ARR1 (ARABIDOPSIS RESPONSE REGULATOR 1); transcription factor/ two-component response regulator | ARR-B | |
| AT3G19510 | Green | HAT3.1 | homeobox protein (HAT 3.1) | HB | |
| AT3G20280 | Green | | PHD finger family protein | PHD | |
| AT3G23030 | Green | IAA2 | IAA2 (indoleacetic acid-induced protein 2); transcription factor | AUX/IAA | |
| AT3G23150 | Green | ETR2 | ETR2 (ETHYLENE RESPONSE 2); receptor | Orphans | |
| AT3G23690 | Green | bHLH077 | basic helix-loop-helix (bHLH) family protein | bHLH | miR393, |
| AT3G24120 | Green | | myb family transcription factor | G2-like | |
| AT3G25730 | Green | | AP2 domain-containing transcription factor, putative | AP2-EREBP | |
| AT3G25790 | Green | | myb family transcription factor | G2-like | |
| AT3G26730 | Green | | zinc finger (C3HC4-type RING finger) family protein | | |
| AT3G27010 | Green | PCF1, AT-TCP20 | AT-TCP20 ("Arabidopsis thaliana teosinte branched 1, cycloidea, PCF (TCP)-domain family protein 20"); transcription factor | TCP | |
| AT3G29330 | Green | | unknown protein | | |
| AT3G42860 | Green | | zinc knuckle (CCHC-type) family protein | Orphans | |
| AT3G43240 | Green | | ARID/BRIGHT DNA-binding domain-containing protein | ARID | |
| AT3G46600 | Green | | scarecrow transcription factor family protein | GRAS | |
| AT3G47990 | Green | | zinc finger (C3HC4-type RING finger) family protein | | |
| AT3G48100 | Green | ATRR2, IBC6, ARR5 | ARR5 (ARABIDOPSIS RESPONSE REGULATOR 5); transcription regulator/ two-component response regulator | Orphans | |
| AT3G50260 | Green | CEJ1, ATERF#011 | ATERF#011/CEJ1 (COOPERATIVELY REGULATED BY ETHYLENE AND JASMONATE 1); DNA binding / transcription factor | AP2-EREBP | |
| AT3G50700 | Green | ATIDD2 | ATIDD2 (ARABIDOPSIS THALIANA INDETERMINATE(ID)-DOMAIN 2); nucleic acid binding / transcription factor/ zinc ion binding | C2H2 | |
| AT3G55080 | Green | | SET domain-containing protein | SET | |
| AT3G56400 | Green | ATWRKY70, WRKY70 | WRKY70 (WRKY DNA-binding protein 70); transcription factor | WRKY | |
| AT3G57300 | Green | ATINO80 | transcriptional activator, putative | SNF2 | |
| AT3G58710 | Green | ATWRKY69, WRKY69 | WRKY69 (WRKY DNA-binding protein 69); transcription factor | WRKY | |
| AT3G60030 | Green | SPL12 | SPL12 (SQUAMOSA PROMOTER-BINDING PROTEIN-LIKE 12); transcription factor | SBP | |
| AT3G61850 | Green | DAG1, AtDof3,7 | DAG1 (DOF AFFECTING GERMINATION 1), DAG1 (DOF AFFECTING GERMINATION 1); DNA binding / transcription factor, unknown protein | C2C2-Dof | |
| AT3G61890 | Green | ATHB12, ATHB-12 | ATHB-12 (ARABIDOPSIS THALIANA HOMEOBOX PROTEIN 1); transcription factor | HB | |
| AT3G62090 | Green | PIF6, PIL2 | PIL2 (PHYTOCHROME INTERACTING FACTOR 3-LIKE 2), PIL2 (PHYTOCHROME INTERACTING FACTOR 3-LIKE 2); transcription factor | bHLH | |
| AT4G01680 | Green | ATMYB55 | MYB55 (myb domain protein 55); DNA binding, MYB55 (myb domain protein 55); DNA binding / transcription factor, unknown protein | MYB | |
| AT4G12020 | Green | MAPKKK11, ATWRKY19, WRKY19 | WRKY19 (WRKY DNA-binding protein 19); transcription factor, unknown protein | WRKY | |
| AT4G14465 | Green | | DNA-binding protein-related | | |
| AT4G14550 | Green | SLR, IAA14 | IAA14 (SOLITARY ROOT); transcription factor | AUX/IAA | |
| AT4G16845 | Green | VRN2 | VRN2 (REDUCED VERNALIZATION RESPONSE 2), VRN2 (REDUCED VERNALIZATION RESPONSE 2); transcription factor | | |
| AT4G17460 | Green | HAT1 | HAT1 (homeobox-leucine zipper protein 1); DNA binding / transcription factor | HB | |
| AT4G17900 | Green | | zinc-binding family protein | PLATZ | |
| AT4G24060 | Green | AtDof4,6 | Dof-type zinc finger domain-containing protein | C2C2-Dof | |
| AT4G30410 | Green | | transcription factor, unknown protein | | |
| AT4G31920 | Green | ARR10 | ARR10 (ARABIDOPSIS RESPONSE REGULATOR 10); transcription factor/ two-component response regulator | ARR-B | |
| AT4G32880 | Green | ATHB8, ATHB-8 | ATHB-8 (HOMEOBOX GENE 8); DNA binding / transcription factor | HB | miR166/miR165, |
| AT4G34000 | Green | ABF3, DPBF5 | ABF3/DPBF5 (ABSCISIC ACID RESPONSIVE ELEMENTS-BINDING FACTOR 3); DNA binding / protein binding / transcription factor/ transcriptional activator | bZIP | |
| AT4G35550 | Green | HB-4, WOX13 | homeobox-leucine zipper protein (HB-2) / HD-ZIP protein | HB | |
| AT4G35570 | Green | NFD5, NFD05, HMGD, HMGB5 | HMGB5 (HIGH MOBILITY GROUP B 5); transcription factor | HMG | |
| AT4G35900 | Green | ATBZIP14, FD-1, FD | FD (FD); DNA binding / transcription factor | | |

| | | | | | |
|---|---|---|---|---|---|
| AT4G36860 | Green | DAR1 | LIM domain-containing protein | Orphans | |
| AT4G36920 | Green | FL1, FLO2, AP2 | AP2 (APETALA 2); transcription factor | AP2-EREBP | miR172, |
| AT4G37540 | Green | LBD39 | LOB domain protein 39 / lateral organ boundaries domain protein 39 (LBD39) | | |
| AT4G37650 | Green | SGR7, SHR | SHR (SHORT ROOT); transcription factor | GRAS | |
| AT4G37790 | Green | HAT22 | HAT22 (homeobox-leucine zipper protein 22); transcription factor | HB | |
| AT4G37940 | Green | AGL21 | AGL21 (AGAMOUS-LIKE 21); transcription factor | MADS | |
| AT4G38680 | Green | ATCSP2, CSDP2, GRP2 | GRP2 (COLD SHOCK DOMAIN PROTEIN 2); nucleic acid binding | CSD | |
| AT5G02470 | Green | DPA | DPA; transcription factor | E2F-DP | |
| AT5G04150 | Green | bHLH101 | basic helix-loop-helix (bHLH) family protein | bHLH | |
| AT5G05610 | Green | AL1 | PHD finger family protein | Alfin-like | |
| AT5G05660 | Green | ATNFXL2 | transcription factor | | |
| AT5G06110 | Green | | DNAJ heat shock N-terminal domain-containing protein / cell division protein-related | MYB | |
| AT5G06420 | Green | | zinc finger (CCCH-type/C3HC4-type RING finger) family protein | C3H | |
| AT5G06950 | Green | TGA2, AHBP-1B | AHBP-1B (bZIP transcription factor HBP-1b homolog), AHBP-1B (bZIP transcription factor HBP-1b homolog); DNA binding / transcription factor | bZIP | |
| AT5G06960 | Green | TGA5, OBF5 | OBF5 (OCS-ELEMENT BINDING FACTOR 5); DNA binding / transcription factor | bZIP | |
| AT5G08130 | Green | BIM1, bHLH046 | BIM1 (BES1-interacting Myc-like protein 1); DNA binding / transcription factor, unknown protein | bHLH | |
| AT5G09410 | Green | EICBP.B | EICBP.B (ETHYLENE INDUCED CALMODULIN BINDING PROTEIN); calmodulin binding / transcription regulator, unknown protein | CAMTA | |
| AT5G12330 | Green | LRP1 | LRP1 (LATERAL ROOT PRIMORDIUM 1) | SRS | |
| AT5G13080 | Green | ATWRKY75, WRKY75 | WRKY75 (WRKY DNA-binding protein 75); transcription factor | WRKY | |
| AT5G13330 | Green | RAP2.6L | RAP2.6L (related to AP2 6L); DNA binding / transcription factor | AP2-EREBP | |
| AT5G13730 | Green | SIGD, SIG4 | SIG4 (SIGMA FACTOR 4); DNA binding / DNA-directed RNA polymerase/ transcription factor | Sigma70-like | |
| AT5G13960 | Green | SDG33, KYP, SUVH4 | SUVH4 (SU(VAR)3-9 HOMOLOG 4) | SET | |
| AT5G16680 | Green | | PHD finger family protein | PHD | |
| AT5G17240 | Green | SDG40 | SET domain-containing protein | SET | |
| AT5G17490 | Green | RGL3 | RGL3 (RGA-LIKE 3); transcription factor | GRAS | |
| AT5G17600 | Green | | zinc finger (C3HC4-type RING finger) family protein | | |
| AT5G18830 | Green | SPL7 | SPL7 (SQUAMOSA PROMOTER BINDING PROTEIN-LIKE 7); DNA binding / transcription factor | SBP | |
| AT5G19490 | Green | | repressor protein-related | CCAAT | |
| AT5G20730 | Green | MSG1, IAA21, BIP, ARF7, IAA25, IAA23, TIR: | NPH4 (NON-PHOTOTROPHIC HYPOCOTYL); transcription factor | ARF | |
| AT5G22700 | Green | | F-box family protein | | |
| AT5G22760 | Green | | PHD finger family protein | DDT | |
| AT5G23090 | Green | NF-YB13 | TATA-binding protein-associated phosphoprotein Dr1 protein, putative (DR1) | CCAAT | |
| AT5G23150 | Green | HUA2 | HUA2 (ENHANCER OF AG-4 2) | | |
| AT5G24470 | Green | APRR5 | APRR5 (PSEUDO-RESPONSE REGULATOR 5); transcription regulator | Pseudo ARR-B | |
| AT5G26920 | Green | | calmodulin binding | | |
| AT5G28650 | Green | ATWRKY74, WRKY74 | WRKY74 (WRKY DNA-binding protein 74); transcription factor | WRKY | |
| AT5G29000 | Green | | myb family transcription factor, unknown protein | G2-like | |
| AT5G35210 | Green | | peptidase M50 family protein / sterol-regulatory element binding protein (SREBP) site 2 protease family protein | DDT | |
| AT5G38560 | Green | | protein kinase family protein | | |
| AT5G38895 | Green | | zinc finger (C3HC4-type RING finger) family protein | | |
| AT5G40710 | Green | | zinc finger (C2H2 type) family protein | | |
| AT5G42520 | Green | BPC6, BBR/BPC6, ATBPC6 | ATBPC6/BBR/BPC6/BPC6 (BASIC PENTACYSTEINE 6), ATBPC6/BBR/BPC6/BPC6 (BASIC PENTACYSTEINE 6); DNA binding / transcription factor | BBR/BPC | |
| AT5G43270 | Green | SPL2 | SPL2 (SQUAMOSA PROMOTER BINDING PROTEIN-LIKE 2); DNA binding, SPL2 (SQUAMOSA PROMOTER BINDING PROTEIN-LIKE 2); DNA binding / transcription factor | SBP | miR156/miR157, |
| AT5G44160 | Green | NUC | zinc finger (C2H2 type) family protein | C2H2 | |
| AT5G45260 | Green | ATWRKY52, SLH1, RRS1 | RRS1 (RESISTANT TO RALSTONIA SOLANACEARUM 1), RRS1 (RESISTANT TO RALSTONIA SOLANACEARUM 1); transcription factor | WRKY | |
| AT5G45710 | Green | RHA1, AT-HSFA4C | AT-HSFA4C (Arabidopsis thaliana heat shock transcription factor A4C); DNA binding / transcription factor | HSF | |
| AT5G46590 | Green | ANAC096 | ANAC096 (Arabidopsis NAC domain containing protein 96); transcription factor | NAC | |
| AT5G49620 | Green | ATMYB78 | AtMYB78 (myb domain protein 78); DNA binding / transcription factor | MYB | |
| AT5G51910 | Green | TCP19 | TCP family transcription factor, putative | TCP | |
| AT5G52170 | Green | HDG7 | homeobox-leucine zipper family protein / lipid-binding START domain-containing protein | HB | |

| | | | | |
|---|---|---|---|---|
| AT5G53290 | Green | CRF3 | CRF3 (CYTOKININ RESPONSE FACTOR 3); DNA binding / transcription factor | AP2-EREBP |
| AT5G53420 | Green | | unknown protein | Orphans |
| AT5G60200 | Green | AtDof5,3 | Dof-type zinc finger domain-containing protein | C2C2-Dof |
| AT5G60890 | Green | AtMYB34, MYB34, ATR1 | ATR1 (ALTERED TRYPTOPHAN REGULATION); DNA binding / kinase/ transcription factor | MYB |
| AT5G61380 | Green | APRR1, TOC1 | TOC1 (TIMING OF CAB1 1); transcription regulator | Pseudo ARR-B |
| AT5G61930 | Green | APO3 | APO3 (ACCUMULATION OF PHOTOSYSTEM ONE 3), unknown protein | |
| AT5G62570 | Green | | calmodulin-binding protein | |
| AT5G62610 | Green | bHLH079 | basic helix-loop-helix (bHLH) family protein | bHLH |
| AT5G62920 | Green | ARR6 | ARR6 (RESPONSE REGULATOR 6); transcription regulator/ two-component response regulator | Orphans |
| AT5G62940 | Green | AtDof5,6 | Dof-type zinc finger domain-containing protein | C2C2-Dof |
| AT5G63080 | Green | | transcription factor jumonji (jmjC) domain-containing protein | Jumonji |
| AT5G64220 | Green | | calmodulin binding, calmodulin-binding protein | CAMTA |
| AT5G64610 | Green | HAM1 | histone acetyltransferase, putative | |
| AT5G65050 | Green | MAF2, AGL31 | AGL31 (AGAMOUS LIKE MADS-BOX PROTEIN 31), AGL31 (AGAMOUS LIKE MADS-BOX PROTEIN 31); transcription factor, unknown protein | MADS |
| AT5G65310 | Green | ATHB-5, ATHB5 | ATHB5 (ARABIDOPSIS THALIANA HOMEOBOX PROTEIN 5), ATHB5 (ARABIDOPSIS THALIANA HOMEOBOX PROTEIN 5); transcription factor | HB |
| AT5G65670 | Green | IAA9 | IAA9 (indoleacetic acid-induced protein 9); transcription factor | AUX/IAA |
| AT5G66070 | Green | | protein binding / zinc ion binding, zinc finger (C3HC4-type RING finger) family protein | |
| AT5G66350 | Green | SHI | SHI (SHORT INTERNODES); transcription factor | SRS |
| AT5G66700 | Green | ATHB53, HB53, HB-8 | HB53 (homeobox-8); DNA binding / transcription factor | HB |
| AT5G67110 | Green | ALC, bHLH073 | ALC (ALCATRAZ), ALC (ALCATRAZ); DNA binding / transcription factor | bHLH |
| AT5G67420 | Green | LBD37 | LOB domain protein 37 / lateral organ boundaries domain protein 37 (LBD37) | |
| AT1G19000 | Greenyellow | | myb family transcription factor | MYB-related |
| AT1G32150 | Greenyellow | | bZIP transcription factor family protein | bZIP |
| AT1G50440 | Greenyellow | | unknown protein, zinc finger (C3HC4-type RING finger) family protein | |
| AT1G64280 | Greenyellow | SAI1, NIM1, NPR1 | NPR1 (NONEXPRESSER OF PR GENES 1); protein binding | |
| AT1G76900 | Greenyellow | AtTLP1 | AtTLP1 (TUBBY LIKE PROTEIN 1); phosphoric diester hydrolase/ transcription factor | TUB |
| AT2G26940 | Greenyellow | | zinc finger (C2H2 type) family protein | C2H2 |
| AT2G28290 | Greenyellow | CHR3, SYD | SYD (SPLAYED) | SNF2 |
| AT2G30410 | Greenyellow | TFCA, KIS | KIS (KIESEL); unfolded protein binding | |
| AT3G06140 | Greenyellow | | zinc finger (C3HC4-type RING finger) family protein | |
| AT3G07940 | Greenyellow | | zinc finger and C2 domain protein, putative | |
| AT3G09600 | Greenyellow | | DNA binding / transcription factor, myb family transcription factor | MYB-related |
| AT3G18990 | Greenyellow | REM39, VRN1 | VRN1 (REDUCED VERNALIZATION RESPONSE 1) | ABI3VP1 |
| AT3G50650 | Greenyellow | SCL7 | scarecrow-like transcription factor 7 (SCL7) | GRAS |
| AT4G00760 | Greenyellow | APRR8 | APRR8 (PSEUDO-RESPONSE REGULATOR 8); transcription regulator | Orphans |
| AT4G25560 | Greenyellow | ATMYB18 | AtMYB18 (myb domain protein 18); DNA binding / transcription factor | MYB |
| AT4G34610 | Greenyellow | BLH6 | BLH6 (BELL1-LIKE HOMEODOMAIN 5); DNA binding / transcription factor, unknown protein | HB |
| AT4G37730 | Greenyellow | ATBZIP7 | bZIP transcription factor family protein | bZIP |
| AT5G02460 | Greenyellow | AtDof5. 1 | Dof-type zinc finger domain-containing protein | C2C2-Dof |
| AT5G05090 | Greenyellow | | myb family transcription factor | G2-like |
| AT3G30210 | Grey | ATMYB121 | MYB121 (myb domain protein 121); DNA binding / transcription factor | MYB |
| AT1G06040 | Grey60 | STO | STO (SALT TOLERANCE); transcription factor/ zinc ion binding | Orphans |
| AT1G17520 | Grey60 | | DNA-binding protein, putative | MYB-related |
| AT1G18330 | Grey60 | EPR1 | EPR1 (EARLY-PHYTOCHROME-RESPONSIVE1); DNA binding / transcription factor, unknown protein | MYB-related |
| AT1G33060 | Grey60 | ANAC014 | ANAC014, ANAC014; transcription factor | NAC |
| AT1G76350 | Grey60 | NLP5 | RWP-RK domain-containing protein | RWP-RK |
| AT2G23660 | Grey60 | LBD10 | LOB domain family protein / lateral organ boundaries domain family protein (LBD10) | |
| AT2G33500 | Grey60 | COL14 | zinc finger (B-box type) family protein | C2C2-CO-like |
| AT2G44150 | Grey60 | SDG7, ASHH3 | SET domain-containing protein (ASHH3) | SET |
| AT3G05430 | Grey60 | | PWWP domain-containing protein | |
| AT3G10910 | Grey60 | | zinc finger (C3HC4-type RING finger) family protein | |
| AT4G05330 | Grey60 | AGD13 | AGD13 (ARF-GAP DOMAIN 13) | |
| AT4G16420 | Grey60 | PRZ1, ADA2B | ADA2B (PROPORZ1); DNA binding / transcription factor | MYB-related |

| | | | | |
|---|---|---|---|---|
| AT4G35040 | Grey60 | | bZIP transcription factor family protein | bZIP |
| AT5G02840 | Grey60 | LCL1 | DNA binding / transcription factor, myb family transcription factor | MYB-related |
| AT5G60820 | Grey60 | | zinc finger (C3HC4-type RING finger) family protein | |
| AT1G01930 | Lightcyan | | zinc finger protein-related | |
| AT1G30460 | Lightcyan | CPSF30, ATCPSF30 | ATCPSF30/CPSF30, ATCPSF30/CPSF30; RNA binding / calmodulin binding / nucleic acid binding | C3H |
| AT3G12130 | Lightcyan | | KH domain-containing protein / zinc finger (CCCH type) family protein | C3H |
| AT1G16070 | Lightyellow | AtTLP8 | AtTLP8 (TUBBY LIKE PROTEIN 8), AtTLP8 (TUBBY LIKE PROTEIN 8); transcription factor | TUB |
| AT1G61730 | Lightyellow | | DNA-binding storekeeper protein-related | GeBP |
| AT1G76500 | Lightyellow | SOB3, AHL29 | DNA-binding family protein | |
| AT3G09360 | Lightyellow | | RNA polymerase II transcription factor | |
| AT3G62240 | Lightyellow | | zinc finger (C2H2 type) family protein | |
| AT5G46710 | Lightyellow | | zinc-binding family protein | PLATZ |
| AT1G03970 | Magenta | GBF4 | GBF4 (G-box binding factor 4); transcription factor | bZIP |
| AT1G09770 | Magenta | ATCDC5, AtMYBCDC5 | ATCDC5 (Arabidopsis thaliana homolog of cdc5); DNA binding / transcription factor | MYB |
| AT1G44810 | Magenta | | transcription regulator | GeBP |
| AT1G46264 | Magenta | AT-HSFB4 | AT-HSFB4 (Arabidopsis thaliana heat shock transcription factor B4); DNA binding / transcription factor | HSF |
| AT1G54060 | Magenta | ASIL1 | transcription factor | Trihelix |
| AT1G68640 | Magenta | PAN | PAN (PERIANTHIA); DNA binding / transcription factor | bZIP |
| AT1G71130 | Magenta | | AP2 domain-containing transcription factor, putative | AP2-EREBP |
| AT2G01570 | Magenta | RGA1 | RGA1 (REPRESSOR OF GA1-3 1); transcription factor | GRAS |
| AT2G03340 | Magenta | WRKY3 | WRKY3 (WRKY DNA-binding protein 3); transcription factor | WRKY |
| AT2G23340 | Magenta | | AP2 domain-containing transcription factor, putative | AP2-EREBP |
| AT2G24790 | Magenta | COL3 | COL3 (CONSTANS-LIKE 3); protein binding / transcription factor/ zinc ion binding, COL3 (CONSTANS-LIKE 3); zinc ion binding | C2C2-CO-like |
| AT2G34720 | Magenta | NF-YA4 | CCAAT-binding transcription factor (CBF-B/NF-YA) family protein | CCAAT |
| AT2G37060 | Magenta | NF-YB8 | CCAAT-box binding transcription factor, putative | CCAAT |
| AT2G42030 | Magenta | | zinc finger (C3HC4-type RING finger) family protein | |
| AT2G42040 | Magenta | | unknown protein | Orphans |
| AT2G44730 | Magenta | | transcription factor | Trihelix |
| AT3G06010 | Magenta | ATCHR12 | homeotic gene regulator, putative | SNF2 |
| AT3G09770 | Magenta | | zinc finger (C3HC4-type RING finger) family protein | |
| AT3G11100 | Magenta | | transcription factor | Trihelix |
| AT3G14740 | Magenta | | PHD finger family protein | PHD |
| AT3G16770 | Magenta | RAP2.03, ATEBP, RAP2.3 ,ERF72 | ATEBP/RAP2.3 (RELATED TO AP2 3); DNA binding / protein binding / transcription factor/ transcriptional activator | AP2-EREBP |
| AT3G18640 | Magenta | | zinc finger protein-related | C3H |
| AT3G25890 | Magenta | | AP2 domain-containing transcription factor, putative | AP2-EREBP |
| AT3G28730 | Magenta | NFD, SSRP1, ATHMG | ATHMG (HIGH MOBILITY GROUP, STRUCTURE-SPECIFIC RECOGNITION PROTEIN 1); transcription factor | HMG |
| AT3G52800 | Magenta | | zinc finger (AN1-like) family protein | |
| AT3G54350 | Magenta | EMB1967 | EMB1967 (EMBRYO DEFECTIVE 1967) | FHA |
| AT4G10710 | Magenta | SPT16 | SPT16 (GLOBAL TRANSCRIPTION FACTOR C); metalloexopeptidase | |
| AT4G13460 | Magenta | SET22, SDG22, SUVH9 | SUVH9 (SU(VAR)3-9 HOMOLOG 9); histone-lysine N-methyltransferase/ zinc ion binding | SET |
| AT4G25210 | Magenta | | transcription regulator | GeBP |
| AT4G29190 | Magenta | | zinc finger (CCCH-type) family protein | C3H |
| AT4G35580 | Magenta | NTL9, CBNAC | no apical meristem (NAM) family protein, unknown protein | NAC |
| AT4G36730 | Magenta | GBF1 | GBF1 (G-box binding factor 1); transcription factor | bZIP |
| AT4G37260 | Magenta | ATMYB73 | AtMYB73/MYB73 (myb domain protein 73); DNA binding / transcription factor | MYB |
| AT5G05550 | Magenta | | transcription factor | Trihelix |
| AT5G09790 | Magenta | SDG15, ATXR5 | ATXR5 (Trithorax- related protein 5), ATXR5 (Trithorax- related protein 5); DNA binding | PHD |
| AT5G12400 | Magenta | | PHD finger transcription factor, putative | DDT |
| AT5G14460 | Magenta | | pseudouridylate synthase TruB family protein | |
| AT5G18620 | Magenta | CHR17 | CHR17 (CHROMATIN REMODELING FACTOR17); DNA-dependent ATPase | SNF2 |
| AT5G23420 | Magenta | HMGB6 | HMGB6 (High mobility group B 6); transcription factor | HMG |

| | | | | | |
|---|---|---|---|---|---|
| AT5G25220 | Magenta | KNAT3 | KNAT3 (KNOTTED1-LIKE HOMEOBOX GENE 3), KNAT3 (KNOTTED1-LIKE HOMEOBOX GENE 3); transcription factor | HB | |
| AT5G41370 | Magenta | XPB1, ATXPB1 | XPB1 (ARABIDOPSIS HOMOLOG OF XERODERMA PIGMENTOSUM COMPLEMENTATION GROUP B 1); ATP-dependent helicase | | |
| AT5G47790 | Magenta | | forkhead-associated domain-containing protein / FHA domain-containing protein | FHA | |
| AT5G62000 | Magenta | HSS, ARF1-BP, ARF2 | ARF2 (AUXIN RESPONSE FACTOR 2), ARF2 (AUXIN RESPONSE FACTOR 2); transcription factor | ARF | |
| AT5G62260 | Magenta | | DNA binding | | |
| AT1G51700 | Midnightblue | ADOF1, AtDof1. 7 | ADOF1 (Arabidopsis dof zinc finger protein 1); DNA binding / transcription factor | C2C2-Dof | |
| AT4G26150 | Midnightblue | CGA1, GNL | zinc finger (GATA type) family protein | C2C2-GATA | |
| AT4G30935 | Midnightblue | ATWRKY32, WRKY32 | WRKY32 (WRKY DNA-binding protein 32); transcription factor | WRKY | |
| AT5G06080 | Midnightblue | LBD33 | LOB domain protein 33 / lateral organ boundaries domain protein 33 (LBD33) | | |
| AT1G01160 | Orange | GIF2 | GIF2 (GRF1-INTERACTING FACTOR 2) | | |
| AT1G70790 | Orange | | C2 domain-containing protein | | |
| AT5G66610 | Orange | DAR7 | zinc ion binding | Orphans | |
| AT1G04100 | Pink | IAA10 | IAA10 (indoleacetic acid-induced protein 10); transcription factor | AUX/IAA | |
| AT1G10610 | Pink | bHLH090 | DNA binding / transcription factor | bHLH | |
| AT1G15050 | Pink | IAA34 | IAA34 (indoleacetic acid-induced protein 34); transcription factor | AUX/IAA | |
| AT1G18470 | Pink | | zinc finger (C3HC4-type RING finger) family protein | | |
| AT1G49900 | Pink | | zinc finger (C2H2 type) family protein | C2H2 | |
| AT1G55700 | Pink | | DC1 domain-containing protein | | |
| AT1G56160 | Pink | ATMYB72 | AtMYB72 (myb domain protein 72); DNA binding / transcription factor | MYB | |
| AT1G77950 | Pink | AGL67 | transcription factor, unknown protein | MADS | |
| AT2G04240 | Pink | XERICO | XERICO; protein binding / zinc ion binding | | |
| AT2G17770 | Pink | FDP, ATBZIP27 | ATBZIP27/FDP (FD PARALOG); transcription factor | | |
| AT2G19380 | Pink | | RNA recognition motif (RRM)-containing protein | | |
| AT2G22630 | Pink | AGL17 | AGL17 (AGAMOUS-LIKE 17); transcription factor | MADS | |
| AT2G23780 | Pink | | zinc finger (C3HC4-type RING finger) family protein | | |
| AT2G40770 | Pink | | SNF2 domain-containing protein / helicase domain-containing protein / zinc finger (C3HC4 type RING finger) family protein | PHD | |
| AT2G41370 | Pink | BOP2 | BOP2 (BLADE ON PETIOLE2); protein binding | | |
| AT2G44580 | Pink | | protein binding / zinc ion binding | | |
| AT2G47810 | Pink | NF-YB5 | histone-like transcription factor (CBF/NF-Y) family protein | CCAAT | |
| AT3G06490 | Pink | BOS1, AtMYB108 | MYB108 (BOTRYTIS-SUSCEPTIBLE1, myb domain protein 108); DNA binding / transcription factor | MYB | |
| AT3G11440 | Pink | AtMYB65 | ATMYB65 (myb domain protein 65); DNA binding / transcription factor | MYB | miR159/miR319, |
| AT3G12730 | Pink | | myb family transcription factor | G2-like | |
| AT3G12820 | Pink | ATMYB10 | AtMYB10 (myb domain protein 10); DNA binding / transcription factor | MYB | |
| AT3G20550 | Pink | DDL | DDL (DAWDLE) | FHA | |
| AT3G23230 | Pink | | ethylene-responsive factor, putative | AP2-EREBP | |
| AT3G55530 | Pink | SDIR1 | unknown protein | | |
| AT3G56660 | Pink | BZIP49 | bZIP transcription factor family protein | bZIP | |
| AT3G57480 | Pink | | zinc finger (C2H2 type, AN1-like) family protein | C2H2 | |
| AT4G25440 | Pink | ZFWD1 | ZFWD1 (zinc finger WD40 repeat protein 1); nucleic acid binding / zinc ion binding | C3H | |
| AT4G27470 | Pink | | zinc finger (C3HC4-type RING finger) family protein | | |
| AT5G07700 | Pink | ATMYB76 | MYB76 (myb domain protein 76); DNA binding / transcription factor | MYB | |
| AT5G11590 | Pink | DREB3, TINY2 | TINY2 (TINY2); DNA binding / transcription factor | AP2-EREBP | |
| AT5G16820 | Pink | ATHSFA1B, HSF3 | HSF3 (HEAT SHOCK FACTOR 3); DNA binding / transcription factor | HSF | |
| AT5G27910 | Pink | NF-YC8 | CCAAT-box binding transcription factor Hap5a, putative | CCAAT | |
| AT5G50915 | Pink | bHLH137 | basic helix-loop-helix (bHLH) family protein | bHLH | |
| AT5G53660 | Pink | ATGRF7 | AtGRF7 (GROWTH-REGULATING FACTOR 7) | GRF | miR396, |
| AT5G54230 | Pink | ATMYB49 | MYB49 (myb domain protein 49); transcription factor | MYB | |
| AT5G58010 | Pink | bHLH082 | basic helix-loop-helix (bHLH) family protein | bHLH | |
| AT5G61590 | Pink | | AP2 domain-containing transcription factor family protein | AP2-EREBP | |
| AT5G62380 | Pink | ANAC101, VND6 | VND6 (VASCULAR-RELATED NAC-DOMAIN 6); transcription factor | NAC | |
| AT1G21700 | Purple | CHB4, ATSWI3C | ATSWI3C (Arabidopsis thaliana switching protein 3C); DNA binding | MYB-related | |
| AT1G22510 | Purple | | zinc finger (C3HC4-type RING finger) family protein | | |

| | | | | | |
|---|---|---|---|---|---|
| AT1G48040 | Purple | | protein phosphatase type 2C | DBP | |
| AT1G63900 | Purple | | zinc finger (C3HC4-type RING finger) family protein | | |
| AT2G18670 | Purple | | zinc finger (C3HC4-type RING finger) family protein | | |
| AT2G21450 | Purple | CHR34 | CHR34 (chromatin remodeling 34); ATP binding / DNA binding / helicase | SNF2 | |
| AT2G40740 | Purple | ATWRKY55, WRKY55 | WRKY55 (WRKY DNA-binding protein 55), WRKY55 (WRKY DNA-binding protein 55); transcription factor | WRKY | |
| AT2G43500 | Purple | NLP8 | RWP-RK domain-containing protein | RWP-RK | |
| AT3G28910 | Purple | AtMYB30, MYB30 | MYB30 (myb domain protein 30); DNA binding / transcription factor | MYB | |
| AT3G28920 | Purple | ATHB34 | ATHB34 (ARABIDOPSIS THALIANA HOMEOBOX PROTEIN 34); DNA binding / transcription factor | zf-HD | |
| AT4G01270 | Purple | | zinc finger (C3HC4-type RING finger) family protein | | |
| AT4G29940 | Purple | PRHA | PRHA (PATHOGENESIS RELATED HOMEODOMAIN PROTEIN A); transcription factor | HB | |
| AT4G36650 | Purple | ATPBRP | transcription factor IIB (TFIIB) family protein | | |
| AT4G37490 | Purple | CYC1, CYCB1;1, CYCB1 | CYC1 (CYCLIN 1); cyclin-dependent protein kinase regulator | | |
| AT5G03740 | Purple | HD2C, HDT3 | HD2C (HISTONE DEACETYLASE 2C); nucleic acid binding / zinc ion binding | C2H2 | |
| AT5G03780 | Purple | TRFL10 | TRFL10 (TRF-LIKE 10); DNA binding | | |
| AT5G03790 | Purple | LMI1, ATHB51 | ATHB51/LMI1 (LATE MERISTEM IDENTITY1); DNA binding / transcription factor | HB | |
| AT5G05410 | Purple | DREB2A | DREB2A (DRE-BINDING PROTEIN 2A); DNA binding / transcription factor/ transcriptional activator | AP2-EREBP | |
| AT5G18270 | Purple | ANAC087 | ANAC087, ANAC087; transcription factor | NAC | |
| AT5G45110 | Purple | NPR3 | NPR3 (NPR1-LIKE PROTEIN 3); protein binding | | |
| AT5G54640 | Purple | HTA1, RAT5 | RAT5 (RESISTANT TO AGROBACTERIUM TRANSFORMATION 5); DNA binding | CCAAT | |
| AT5G61850 | Purple | LFY3, LFY | LFY (LEAFY); transcription factor | LFY | |
| AT5G65510 | Purple | AIL7 | AIL7 (AINTEGUMENTA-LIKE 7); DNA binding / transcription factor | AP2-EREBP | |
| AT1G02340 | Red | FBI1, RSF1, REP1, HFR1 | HFR1 (LONG HYPOCOTYL IN FAR-RED); DNA binding / transcription factor | bHLH | |
| AT1G03040 | Red | bHLH007 | basic helix-loop-helix (bHLH) family protein | bHLH | |
| AT1G03840 | Red | MGP | nucleic acid binding / zinc ion binding, zinc finger (C2H2 type) family protein | C2H2 | |
| AT1G04550 | Red | BDL, IAA12 | IAA12 (AUXIN-INDUCED PROTEIN 12); transcription factor | AUX/IAA | |
| AT1G06180 | Red | ATMYB13, AtMYBlfgn | ATMYB13 (myb domain protein 13); DNA binding / transcription factor | MYB | |
| AT1G08600 | Red | ATRX, CHR20 | ATRX/CHR20; ATP binding / DNA binding / helicase | SNF2 | |
| AT1G10120 | Red | bHLH074 | DNA binding / transcription factor | bHLH | |
| AT1G13880 | Red | | ELM2 domain-containing protein | | |
| AT1G19700 | Red | BEL10, BLH10 | BEL10 (BEL1-LIKE HOMEODOMAIN 10); DNA binding / transcription factor | HB | |
| AT1G20640 | Red | NLP4 | RWP-RK domain-containing protein | RWP-RK | |
| AT1G32640 | Red | JIN1, JAI1, ZBF1, RD22BP1, ATMYC2 | ATMYC2 (JASMONATE INSENSITIVE 1); DNA binding / transcription factor | bHLH | |
| AT1G34355 | Red | | forkhead-associated domain-containing protein / FHA domain-containing protein | FHA | |
| AT1G35560 | Red | TCP23 | TCP family transcription factor, putative | TCP | |
| AT1G50620 | Red | | PHD finger family protein | PHD | |
| AT1G51060 | Red | HTA10 | histone H2A, putative | CCAAT | |
| AT1G52890 | Red | ANAC019 | ANAC019 (Arabidopsis NAC domain containing protein 19); transcription factor | NAC | |
| AT1G53910 | Red | RAP2.12 | RAP2.12, RAP2.12; DNA binding / transcription factor | AP2-EREBP | |
| AT1G63470 | Red | | DNA-binding family protein | | |
| AT1G69570 | Red | AtDof1,10 | Dof-type zinc finger domain-containing protein | C2C2-Dof | |
| AT1G73760 | Red | | zinc finger (C3HC4-type RING finger) family protein | | |
| AT1G76870 | Red | | unknown protein | | |
| AT1G76880 | Red | | trihelix DNA-binding protein, putative | Trihelix | |
| AT2G04880 | Red | ATWRKY1, ZAP1 | ZAP1 (WRKY FAMILY TRANSCRIPTION FACTOR 1); transcription factor/ transcriptional activator | WRKY | |
| AT2G06200 | Red | ATGRF6 | AtGRF6 (GROWTH-REGULATING FACTOR 6) | GRF | |
| AT2G17560 | Red | NFD4, NFD04, HMGB4 | HMGB4 (HIGH MOBILITY GROUP B 4), HMGB4 (HIGH MOBILITY GROUP B 4); transcription factor | HMG | |
| AT2G18350 | Red | ATHB24 | ATHB24 (ARABIDOPSIS THALIANA HOMEOBOX PROTEIN 24); DNA binding / transcription factor | zf-HD | |
| AT2G20180 | Red | PIL5, bHLH015, PIF1 | PIL5 (PHYTOCHROME INTERACTING FACTOR 3-LIKE 5); transcription factor | bHLH | |
| AT2G23290 | Red | ATMYB70 | AtMYB70 (myb domain protein 70); DNA binding / transcription factor | MYB | |
| AT2G24630 | Red | ATCSLC8, ATCSLC08 | ATCSLC08 (Cellulose synthase-like C8); transferase, transferring glycosyl groups | | |
| AT2G25180 | Red | ARR12 | ARR12 (ARABIDOPSIS RESPONSE REGULATOR 12); transcription factor/ two-component response regulator | ARR-B | |
| AT2G25900 | Red | ATCTH | ATCTH (Arabidopsis thaliana Cys3His zinc finger protein); transcription factor | C3H | |
| AT2G31070 | Red | TCP10 | TCP10 (TCP domain protein 10); transcription factor | TCP | miR159/miR319, |
| AT2G31180 | Red | ATMYB14, Myb14at | AtMYB14/Myb14at (myb domain protein 14); DNA binding / transcription factor | MYB | |

| | | | | | |
|---|---|---|---|---|---|
| AT2G32370 | Red | HDG3 | homeobox-leucine zipper family protein / lipid-binding START domain-containing protein | HB | |
| AT2G37950 | Red | | zinc finger (C3HC4-type RING finger) family protein | | |
| AT2G40260 | Red | | myb family transcription factor | G2-like | |
| AT2G45460 | Red | | forkhead-associated domain-containing protein / FHA domain-containing protein | FHA | |
| AT2G45880 | Red | BAM7, BMY4 | BMY4 (beta-amylase 4); beta-amylase | BES1 | |
| AT2G46510 | Red | ATAIB, bHLH107, bHLH017 | basic helix-loop-helix (bHLH) family protein | bHLH | |
| AT2G46790 | Red | TL1, APRR9 | APRR9 (PSEUDO-RESPONSE REGULATOR 9), APRR9 (PSEUDO-RESPONSE REGULATOR 9); transcription regulator | Pseudo ARR-B | |
| AT3G04060 | Red | ANAC046 | ANAC046 (Arabidopsis NAC domain containing protein 46); transcription factor | NAC | |
| AT3G06740 | Red | | zinc finger (GATA type) family protein | C2C2-GATA | |
| AT3G08020 | Red | | protein binding / zinc ion binding | PHD | |
| AT3G09370 | Red | ATMYB3R-3, ATMYB3R3 | MYB3R-3 (myb domain protein 3R-3), MYB3R-3 (myb domain protein 3R-3); DNA binding / transcription factor | MYB | |
| AT3G10760 | Red | | myb family transcription factor | G2-like | |
| AT3G12480 | Red | NF-YC11 | transcription factor, putative | CCAAT | |
| AT3G13810 | Red | ATIDD11 | ATIDD11 (ARABIDOPSIS THALIANA INDETERMINATE(ID)-DOMAIN 11); nucleic acid binding / transcription factor/ zinc ion binding | C2H2 | |
| AT3G16280 | Red | | DNA binding / transcription factor | AP2-EREBP | |
| AT3G20740 | Red | FIE1, FIS3, FIE | FIE (FERTILIZATION-INDEPENDENT ENDOSPERM 1); nucleotide binding / transcription factor | | |
| AT3G24050 | Red | | GATA transcription factor 1 (GATA-1) | C2C2-GATA | |
| AT3G48190 | Red | ATATM, ATM | ATM (ATAXIA-TELANGIECTASIA MUTATED) | | |
| AT3G51120 | Red | | zinc finger (CCCH-type) family protein | C3H | |
| AT3G51880 | Red | NFD1, HMGB1 | HMGB1 (HIGH MOBILITY GROUP B 1), HMGB1 (HIGH MOBILITY GROUP B 1); transcription factor | HMG | |
| AT3G51950 | Red | | RNA binding, zinc finger (CCCH-type) family protein / RNA recognition motif (RRM)-containing protein | C3H | |
| AT3G53920 | Red | SIG3, SIGC | SIGC (RNA polymerase sigma subunit C); DNA binding / DNA-directed RNA polymerase/ transcription factor | Sigma70-like | |
| AT3G54990 | Red | SMZ | SMZ (SCHLAFMUTZE), SMZ (SCHLAFMUTZE); DNA binding / transcription factor | AP2-EREBP | miR172, |
| AT3G58120 | Red | ATBZIP61 | bZIP transcription factor family protein | bZIP | |
| AT3G62420 | Red | ATBZIP53 | ATBZIP53 (BASIC REGION/LEUCINE ZIPPER MOTIF 53); DNA binding / sequence-specific DNA binding / transcription factor | bZIP | |
| AT4G08150 | Red | BP1, KNAT1 | KNAT1 (BREVIPEDICELLUS 1); transcription factor | HB | |
| AT4G15180 | Red | SDG2, ATXR3 | SET domain-containing protein | SET | |
| AT4G20400 | Red | | transcription factor jumonji (jmj) family protein / zinc finger (C5HC2 type) family protein | Jumonji | |
| AT4G21160 | Red | ZAC, AGD12 | ZAC (ARF-GAP DOMAIN 12); ARF GTPase activator | | |
| AT4G22140 | Red | EBS | DNA binding | PHD | |
| AT4G23980 | Red | ARF9 | ARF9 (AUXIN RESPONSE FACTOR 9), ARF9 (AUXIN RESPONSE FACTOR 9); transcription factor | ARF | |
| AT4G27240 | Red | | zinc finger (C2H2 type) family protein | | |
| AT4G28260 | Red | | unknown protein | | |
| AT4G32551 | Red | RON2, LUG | LUG (LEUNIG) | LUG | |
| AT4G37750 | Red | DRG, CKC1, ANT | ANT (AINTEGUMENTA); DNA binding / transcription factor | AP2-EREBP | |
| AT5G01310 | Red | bHLH140 | basic helix-loop-helix (bHLH) family protein | bHLH | |
| AT5G07810 | Red | | SNF2 domain-containing protein / helicase domain-containing protein / HNH endonuclease domain-containing protein | SNF2 | |
| AT5G08190 | Red | NF-YB12 | TATA-binding protein-associated phosphoprotein Dr1 protein, putative | CCAAT | |
| AT5G08630 | Red | | DDT domain-containing protein | DDT | |
| AT5G09230 | Red | SRT2 | SRT2, SRT2; DNA binding | | |
| AT5G09740 | Red | HAM2 | histone acetyltransferase, putative | C2H2 | |
| AT5G11510 | Red | ATMYB3R-4, AtMYB3R4 | MYB3R-4 (C-MYB-LIKE TRANSCRIPTION FACTOR 3R-4, myb domain protein 3R-4); DNA binding / transcription factor | MYB | |
| AT5G15020 | Red | SNL2 | unknown protein | | |
| AT5G15840 | Red | FG, CO | CO (CONSTANS); transcription factor/ zinc ion binding, CO (CONSTANS); zinc ion binding | C2C2-CO-like | |
| AT5G28640 | Red | GIF1, AN3 | AN3 (ANGUSITFOLIA3) | | |
| AT5G39550 | Red | ORTH1, VIM3 | zinc finger (C3HC4-type RING finger) family protein | | |
| AT5G41920 | Red | | scarecrow transcription factor family protein | GRAS | |
| AT5G42400 | Red | SDG25, ATXR7 | SET domain-containing protein (TXR7) | SET | |
| AT5G42630 | Red | KAN4, KANADI4, ATS | ATS/KAN4 (ABERRANT TESTA SHAPE); DNA binding / transcription factor, unknown protein | G2-like | |

| | | | | | |
|---|---|---|---|---|---|
| AT5G43990 | Red | SDG18, SUVR2 | SUVR2; histone-lysine N-methyltransferase/ zinc ion binding | SET | |
| AT5G44080 | Red | | bZIP transcription factor family protein | bZIP | |
| AT5G44180 | Red | | homeobox transcription factor, putative | HB | |
| AT5G44800 | Red | CHR4, MI-2-LIKE | CHR4/MI-2-LIKE (chromatin remodeling 4); ATP binding / DNA binding / chromatin binding / helicase | PHD | |
| AT5G45300 | Red | BAM8, BMY2 | BMY2; beta-amylase | BES1 | |
| AT5G47390 | Red | | myb family transcription factor | MYB-related | |
| AT5G51190 | Red | | AP2 domain-containing transcription factor, putative | AP2-EREBP | |
| AT5G52510 | Red | SCL8 | scarecrow-like transcription factor 8 (SCL8) | GRAS | |
| AT5G53430 | Red | SET29, ATX5, SDG29 | SDG29 (SET DOMAIN GROUP 29); DNA binding | PHD | |
| AT5G55760 | Red | SRT1 | SRT1 (SIRTUIN 1); DNA binding | | |
| AT5G57180 | Red | CIA2 | CIA2 (CHLOROPLAST IMPORT APPARATUS 2) | Orphans | |
| AT5G58620 | Red | | zinc finger (CCCH-type) family protein | C3H | |
| AT5G59430 | Red | ATTRP1 | ATTRP1 (TELOMERE REPEAT BINDING PROTEIN 1), ATTRP1 (TELOMERE REPEAT BINDING PROTEIN 1); DNA binding | | |
| AT5G59450 | Red | SCL11 | scarecrow-like transcription factor 11 (SCL11) | GRAS | |
| AT5G63950 | Red | CHR24 | CHR24 (chromatin remodeling 24); ATP binding / DNA binding / helicase | SNF2 | |
| AT5G65640 | Red | bHLH093 | BHLH093 (BETA HLH PROTEIN 93), BHLH093 (BETA HLH PROTEIN 93); DNA binding / transcription factor | bHLH | |
| AT5G65770 | Red | LINC4 | nuclear matrix constituent protein-related | | |
| AT5G66770 | Red | | scarecrow transcription factor family protein | GRAS | |
| AT1G43000 | Royalblue | | zinc-binding family protein | PLATZ | |
| AT1G04850 | Salmon | | ubiquitin-associated (UBA)/TS-N domain-containing protein | | |
| AT1G54830 | Salmon | NF-YC3 | CCAAT-box binding transcription factor Hap5a, putative | CCAAT | |
| AT1G65620 | Salmon | AS2 | AS2 (ASYMMETRIC LEAVES 2), unknown protein | | |
| AT2G19810 | Salmon | | zinc finger (CCCH-type) family protein | C3H | |
| AT2G30340 | Salmon | LBD13 | LOB domain protein 13 / lateral organ boundaries domain protein 13 (LBD13) | | |
| AT2G45420 | Salmon | LBD18, ASL20 | LOB domain protein 18 / lateral organ boundaries domain protein 18 (LBD18) | | |
| AT2G46270 | Salmon | GBF3 | GBF3 (G-BOX BINDING FACTOR 3); transcription factor | bZIP | |
| AT2G46990 | Salmon | IAA20 | IAA20 (indoleacetic acid-induced protein 20); transcription factor | AUX/IAA | |
| AT3G15210 | Salmon | ATERF4, RAP2.5, ATERF-4 | ATERF-4/ATERF4/ERF4/RAP2.5 (ETHYLENE RESPONSIVE ELEMENT BINDING FACTOR 4); DNA binding / protein binding / transcription factor/ transcriptional repressor | AP2-EREBP | |
| AT3G62100 | Salmon | IAA30 | IAA30 (indoleacetic acid-induced protein 30); transcription factor | AUX/IAA | |
| AT4G11140 | Salmon | CRF1 | CRF1 (CYTOKININ RESPONSE FACTOR 1); DNA binding / transcription factor | AP2-EREBP | |
| AT4G17490 | Salmon | ERF-6-6, ATERF6 | ATERF6 (ETHYLENE RESPONSIVE ELEMENT BINDING FACTOR 6); DNA binding / transcription factor | AP2-EREBP | |
| AT4G28640 | Salmon | IAA11 | IAA11 (indoleacetic acid-induced protein 11), IAA11 (indoleacetic acid-induced protein 11); transcription factor | AUX/IAA | |
| AT5G04840 | Salmon | | bZIP protein | | |
| AT5G39820 | Salmon | ANAC094 | ANAC094 (Arabidopsis NAC domain containing protein 94); transcription factor | NAC | |
| AT5G63750 | Salmon | | IBR domain-containing protein | | |
| AT1G03750 | Tan | SWI2, SNF2, CHR9 | CHR9/SNF2/SWI2 (chromatin remodeling 9); helicase | SNF2 | |
| AT1G28420 | Tan | HB-1 | HB-1 (homeobox-1); transcription factor | HB | |
| AT1G70700 | Tan | TIFY7, JAZ9 | unknown protein | ZIM | |
| AT2G33480 | Tan | ANAC041 | ANAC041 (Arabidopsis NAC domain containing protein 41); transcription factor, unknown protein | NAC | |
| AT2G42200 | Tan | SPL9 | squamosa promoter-binding protein-like 9 (SPL9) | SBP | miR156/miR157, |
| AT3G09735 | Tan | | DNA-binding S1FA family protein | S1Fa-like | |
| AT3G17610 | Tan | | | | |
| AT3G19290 | Tan | AREB2, ABF4 | ABF4 (ABRE BINDING FACTOR 4); DNA binding / transcription factor/ transcriptional activator | bZIP | |
| AT4G28140 | Tan | | AP2 domain-containing transcription factor, putative | AP2-EREBP | |
| AT4G33940 | Tan | | zinc finger (C3HC4-type RING finger) family protein | | |
| AT5G07680 | Tan | ANAC079, ATNAC4, ANAC080 | ANAC079/ANAC080/ATNAC4 (Arabidopsis NAC domain containing protein 79, Arabidopsis NAC domain containing protein 80); transcription factor | NAC | miR164, |
| AT5G16540 | Tan | ZFN3 | ZFN3 (ZINC FINGER PROTEIN 3); nucleic acid binding | C3H | |
| AT5G24120 | Tan | ATSIG5, SIG5, SIGE | SIGE (RNA polymerase sigma subunit E); DNA binding / DNA-directed RNA polymerase/ sigma factor/ transcription factor | Sigma70-like | |

| | | | | | |
|---|---|---|---|---|---|
| AT5G39610 | Tan | AtNAC2, ANAC092, ATNAC6 | ANAC092/ATNAC2/ATNAC6 (Arabidopsis NAC domain containing protein 92); protein heterodimerization/ protein homodimerization/ transcription factor | NAC | miR164, |
| AT5G52010 | Tan | | zinc finger (C2H2 type) family protein | C2H2 | |
| AT1G01060 | Turquoise | LHY1, LHY | LHY (LATE ELONGATED HYPOCOTYL), LHY (LATE ELONGATED HYPOCOTYL); DNA binding, LHY (LATE ELONGATED HYPOCOTYL); DNA binding / transcription factor | MYB-related | |
| AT1G01250 | Turquoise | | AP2 domain-containing transcription factor, putative | AP2-EREBP | |
| AT1G01260 | Turquoise | bHLH013 | basic helix-loop-helix (bHLH) family protein | bHLH | |
| AT1G01380 | Turquoise | ETC1 | ETC1 (ENHANCER OF TRY AND CPC 1); DNA binding / transcription factor | MYB-related | |
| AT1G02680 | Turquoise | TAF13 | transcription initiation factor IID (TFIID) 18 kDa subunit (TAFII-18) family protein | | |
| AT1G04360 | Turquoise | | zinc finger (C3HC4-type RING finger) family protein | | |
| AT1G05230 | Turquoise | HDG2 | homeobox-leucine zipper family protein / lipid-binding START domain-containing protein, unknown protein | HB | |
| AT1G08000 | Turquoise | | zinc finger (GATA type) family protein | C2C2-GATA | |
| AT1G08540 | Turquoise | ATSIG1, SIG1, SIGA, SIG2, SIGB | SIGB (SIGMA FACTOR B); DNA binding / DNA-directed RNA polymerase/ transcription factor | Sigma70-like | |
| AT1G08810 | Turquoise | ATMYB60 | MYB60 (myb domain protein 60); DNA binding, MYB60 (myb domain protein 60); DNA binding / transcription factor | MYB | |
| AT1G08970 | Turquoise | NF-YC9, HAP5C | HAP5C (HEME ACTIVATED PROTEIN 5C); DNA binding, HAP5C (HEME ACTIVATED PROTEIN 5C); DNA binding / transcription factor | CCAAT | |
| AT1G09540 | Turquoise | AtMYB61 | MYB61 (myb domain protein 61); DNA binding / transcription factor | MYB | |
| AT1G12610 | Turquoise | DDF1 | DDF1 (DWARF AND DELAYED FLOWERING 1); DNA binding / transcription factor | AP2-EREBP | |
| AT1G12860 | Turquoise | SCRM2, ICE2, bHLH033 | basic helix-loop-helix (bHLH) family protein / F-box family protein | bHLH | |
| AT1G14030 | Turquoise | | ribulose-1,5 bisphosphate carboxylase oxygenase large subunit N-methyltransferase, putative | SET | |
| AT1G14200 | Turquoise | | zinc finger (C3HC4-type RING finger) family protein | | |
| AT1G14410 | Turquoise | WHY1, ATWHY1, PTAC1 | ATWHY1/PTAC1 (A. THALIANA WHIRLY 1, PLASTID TRANSCRIPTIONALLY ACTIVE1); DNA binding | PBF-2-like | |
| AT1G14440 | Turquoise | ATHB31 | ATHB31; DNA binding / transcription factor, ATHB31; transcription factor | zf-HD | |
| AT1G14490 | Turquoise | | DNA-binding protein-related | | |
| AT1G14580 | Turquoise | | zinc finger (C2H2 type) family protein | C2H2 | |
| AT1G14685 | Turquoise | BBR/BPC2, ATBPC2, BPC2 | BPC2 (BASIC PENTACYSTEINE 2) | BBR/BPC | |
| AT1G14687 | Turquoise | ATHB32 | ATHB32 (ARABIDOPSIS THALIANA HOMEOBOX PROTEIN 32); DNA binding / transcription factor | zf-HD | |
| AT1G15720 | Turquoise | TRFL5 | TRFL5 (TRF-LIKE 5); DNA binding / transcription factor | MYB-related | |
| AT1G16490 | Turquoise | ATMYB58 | AtMYB58 (myb domain protein 58); DNA binding / transcription factor | MYB | |
| AT1G19180 | Turquoise | TIFY10A, JAZ1 | unknown protein | ZIM | |
| AT1G20823 | Turquoise | | zinc finger (C3HC4-type RING finger) family protein | | |
| AT1G21740 | Turquoise | | unknown protein | | |
| AT1G21970 | Turquoise | NF-YB9, EMB212, LEC1 | LEC1 (LEAFY COTYLEDON 1); transcription factor | CCAAT | |
| AT1G22590 | Turquoise | AGL87 | MADS-box family protein | | |
| AT1G26260 | Turquoise | CIB5, bHLH076 | DNA binding / transcription factor, basic helix-loop-helix (bHLH) family protein | bHLH | |
| AT1G26680 | Turquoise | REM17 | transcriptional factor B3 family protein | ABI3VP1 | |
| AT1G26780 | Turquoise | ATMYB117 | MYB117 (myb domain protein 117); transcription factor | MYB | |
| AT1G28300 | Turquoise | LEC2 | LEC2 (LEAFY COTYLEDON 2); transcription factor | ABI3VP1 | |
| AT1G28360 | Turquoise | AtERF12 | ATERF12/ERF12 (ERF domain protein 12); DNA binding / transcription factor/ transcriptional repressor | AP2-EREBP | |
| AT1G28470 | Turquoise | ANA010, ANAC010, SND3, NST8 | ANAC010 (Arabidopsis NAC domain containing protein 10); transcription factor | NAC | |
| AT1G30210 | Turquoise | ATTCP24, TCP24 | TCP24; transcription factor | TCP | miR159/miR319, |
| AT1G31310 | Turquoise | | hydroxyproline-rich glycoprotein family protein | | |
| AT1G32240 | Turquoise | KAN2, KANADI2 | KAN2 (KANADI 2); DNA binding / transcription factor | G2-like | |
| AT1G32360 | Turquoise | | zinc finger (CCCH-type) family protein | C3H | |
| AT1G33760 | Turquoise | | AP2 domain-containing transcription factor, putative | AP2-EREBP | |
| AT1G34310 | Turquoise | ARF12 | ARF12 (AUXIN RESPONSE FACTOR 12); transcription factor | ARF | |
| AT1G43640 | Turquoise | AtTLP5 | AtTLP5 (TUBBY LIKE PROTEIN 5); phosphoric diester hydrolase/ transcription factor | TUB | |
| AT1G49010 | Turquoise | | myb family transcription factor | MYB | |
| AT1G49480 | Turquoise | REM19, RTV1 | RTV1 (RELATED TO VERNALIZATION1 1); DNA binding / transcription factor | ABI3VP1 | |
| AT1G49950 | Turquoise | ATTRB1, TRB1 | ATTRB1/TRB1 (TELOMERE REPEAT BINDING FACTOR 1); DNA binding, ATTRB1/TRB1 (TELOMERE REPEAT BINDING FACTOR 1); DNA binding / transcription factor | MYB-related | |
| AT1G50600 | Turquoise | SCL5 | SCL5; transcription factor | GRAS | |
| AT1G52880 | Turquoise | ATNAM, NAM, ANAC018, NARS2 | NAM (Arabidopsis NAC domain containing protein 18); transcription factor | NAC | |

| | | | | | |
|---|---|---|---|---|---|
| AT1G53170 | Turquoise | ATERF8, ATERF-8 | ATERF-8/ATERF8 (ETHYLENE RESPONSE ELEMENT BINDING FACTOR 4); DNA binding / transcription factor/ transcriptional repressor | AP2-EREBP | |
| AT1G53230 | Turquoise | TCP3 | TCP3 (TCP family transcription factor 3); transcription factor | TCP | miR159/miR319, |
| AT1G53670 | Turquoise | MSRB1 | transcription factor-related, unknown protein | | |
| AT1G54160 | Turquoise | NFYA5, NF-YA5 | CCAAT-binding transcription factor (CBF-B/NF-YA) family protein | CCAAT | miR169, |
| AT1G55110 | Turquoise | ARABIDOPSISTHALIANAINDETERMINATE | ARABIDOPSIS THALIANA INDETERMINATE(ID)-DOMAIN 7/ATIDD7; nucleic acid binding / transcription factor/ zinc ion binding | C2H2 | |
| AT1G55970 | Turquoise | HAG4, HAG04, HAC6, HAC04, HAC4 ,ATHPC | histone acetyltransferase 4 (HAC4) | TAZ | |
| AT1G56170 | Turquoise | NF-YC2, ATHAP5B, HAP5B | HAP5B (Heme activator protein (yeast) homolog 5B), HAP5B (Heme activator protein (yeast) homolog 5B); DNA binding / transcription factor | CCAAT | |
| AT1G59750 | Turquoise | ARF1 | ARF1 (AUXIN RESPONSE FACTOR 1); transcription factor | ARF | |
| AT1G62310 | Turquoise | | transcription factor jumonji (jmjC) domain-containing protein | Jumonji | |
| AT1G62990 | Turquoise | IXR11, KNAT7 | KNAT7 (Knotted-like Arabidopsis thaliana); DNA binding / transcription factor | HB | |
| AT1G63100 | Turquoise | | scarecrow transcription factor family protein | GRAS | |
| AT1G63480 | Turquoise | | DNA-binding family protein | | |
| AT1G63650 | Turquoise | EGL1, ATMYC-2, EGL3 | EGL3 (ENHANCER OF GLABRA3); DNA binding, EGL3 (ENHANCER OF GLABRA3); DNA binding / transcription factor | bHLH | |
| AT1G63820 | Turquoise | | unknown protein | Orphans | |
| AT1G64000 | Turquoise | ATWRKY56, WRKY56 | WRKY56 (WRKY DNA-binding protein 56); transcription factor | WRKY | |
| AT1G64860 | Turquoise | SIGB, SIG2, SIG1, RPOD1, SIGA | SIGA (SIGMA FACTOR A); DNA binding / DNA-directed RNA polymerase/ transcription factor | Sigma70-like | |
| AT1G65180 | Turquoise | | DC1 domain-containing protein | | |
| AT1G66370 | Turquoise | ATMYB113 | MYB113 (myb domain protein 113); DNA binding / transcription factor | MYB | miR828, |
| AT1G67100 | Turquoise | LBD40 | LOB domain protein 40 / lateral organ boundaries domain protein 40 (LBD40) | | |
| AT1G68070 | Turquoise | | zinc finger (C3HC4-type RING finger) family protein | | |
| AT1G68120 | Turquoise | BPC3, BBR/BPC3, ATBPC3 | ATBPC3/BBR/BPC3/BPC3 (BASIC PENTACYSTEINE 3); DNA binding / transcription factor | BBR/BPC | |
| AT1G68190 | Turquoise | | zinc finger (B-box type) family protein | Orphans | |
| AT1G68480 | Turquoise | JAG | JAG (JAGGED); nucleic acid binding / zinc ion binding | | |
| AT1G68510 | Turquoise | LBD42 | LOB domain protein 42 / lateral organ boundaries domain protein 42 (LBD42) | | |
| AT1G70510 | Turquoise | ATK1, KNAT2 | KNAT2 (KNOTTED-LIKE FROM ARABIDOPSIS THALIANA 2); transcription factor | HB | |
| AT1G72220 | Turquoise | | zinc finger (C3HC4-type RING finger) family protein | | |
| AT1G72830 | Turquoise | NF-YA3, ATHAP2C, HAP2C | HAP2C (Heme activator protein (yeast) homolog 2C); transcription factor | CCAAT | miR169, |
| AT1G73360 | Turquoise | ATHDG11, HDG11, EDT1 | homeobox-leucine zipper family protein / lipid-binding START domain-containing protein | HB | |
| AT1G73870 | Turquoise | COL7 | zinc finger (B-box type) family protein | C2C2-CO-like | |
| AT1G74890 | Turquoise | ARR15 | ARR15 (RESPONSE REGULATOR 15); transcription regulator | Orphans | |
| AT1G74930 | Turquoise | ORA47 | AP2 domain-containing transcription factor, putative | AP2-EREBP | |
| AT1G74950 | Turquoise | TIFY10B, JAZ2 | unknown protein | ZIM | |
| AT1G75240 | Turquoise | ATHB33 | ATHB33 (ARABIDOPSIS THALIANA HOMEOBOX PROTEIN 33); DNA binding / transcription factor | zf-HD | |
| AT1G75710 | Turquoise | | zinc finger (C2H2 type) family protein | C2H2 | |
| AT1G76110 | Turquoise | | high mobility group (HMG1/2) family protein / ARID/BRIGHT DNA-binding domain-containing protein | ARID | |
| AT1G76420 | Turquoise | NAC368, CUC3, ANAC031 | CUC3 (CUP SHAPED COTYLEDON3); transcription factor | NAC | |
| AT1G76510 | Turquoise | | ARID/BRIGHT DNA-binding domain-containing protein | ARID | |
| AT1G77200 | Turquoise | | AP2 domain-containing transcription factor TINY, putative | AP2-EREBP | |
| AT1G79840 | Turquoise | GL2 | GL2 (GLABRA 2); DNA binding / transcription factor | HB | |
| AT2G01930 | Turquoise | BPC1, BBR/BPC1, ATBPC1 | ATBPC1/BBR/BPC1/BPC1 (BASIC PENTACYSTEINE1), ATBPC1/BBR/BPC1/BPC1 (BASIC PENTACYSTEINE1); DNA binding / specific transcriptional repressor/ transcription factor | BBR/BPC | |
| AT2G01940 | Turquoise | SGR5, ATIDD15 | SGR5 (SHOOT GRAVITROPISM 5), SGR5 (SHOOT GRAVITROPISM 5); nucleic acid binding / transcription factor/ zinc ion binding, SGR5 (SHOOT GRAVITROPISM 5); nucleic acid binding / zinc ion binding | C2H2 | |
| AT2G02060 | Turquoise | | calcium-dependent protein kinase-related / CDPK-related | G2-like | |
| AT2G02070 | Turquoise | ATIDD5 | ATIDD5 (ARABIDOPSIS THALIANA INDETERMINATE(ID)-DOMAIN 5); nucleic acid binding / transcription factor/ zinc ion binding | C2H2 | |
| AT2G02080 | Turquoise | ATIDD4 | ATIDD4 (ARABIDOPSIS THALIANA INDETERMINATE(ID)-DOMAIN 4); transcription factor, unknown protein | C2H2 | |
| AT2G02470 | Turquoise | AL6 | PHD finger family protein | Alfin-like | |
| AT2G02540 | Turquoise | ATHB21, ZFHD4 | ATHB21/ZFHD4 (ZINC FINGER HOMEODOMAIN 4); DNA binding / transcription factor | zf-HD | |
| AT2G03470 | Turquoise | | myb family transcription factor / ELM2 domain-containing protein | MYB-related | |
| AT2G05160 | Turquoise | | zinc finger (CCCH-type) family protein / RNA recognition motif (RRM)-containing protein | C3H | |

| | | | | | |
|---|---|---|---|---|---|
| AT2G15530 | Turquoise | | unknown protein, zinc finger (C3HC4-type RING finger) family protein | | |
| AT2G15880 | Turquoise | | leucine-rich repeat family protein / extensin family protein | | |
| AT2G16390 | Turquoise | DMS1, CHR35, DRD1 | DRD1 (DEFECTIVE IN RNA-DIRECTED DNA METHYLATION 1); ATP binding / DNA binding / helicase/ nucleic acid binding | SNF2 | |
| AT2G17450 | Turquoise | RHA3A | RHA3A (RING-H2 finger A3A); protein binding / zinc ion binding | | |
| AT2G18650 | Turquoise | MEE16 | MEE16 (maternal effect embryo arrest 16); protein binding / zinc ion binding | | |
| AT2G18850 | Turquoise | | unknown protein | SET | |
| AT2G20570 | Turquoise | GLK1, GPRI1 | GPRI1 (GOLDEN2-LIKE 1); transcription factor | G2-like | |
| AT2G21240 | Turquoise | BPC4, BBR/BPC4, ATBPC4 | ATBPC4/BBR/BPC4/BPC4 (BASIC PENTACYSTEINE 4), ATBPC4/BBR/BPC4/BPC4 (BASIC PENTACYSTEINE 4); DNA binding / transcription factor | BBR/BPC | |
| AT2G21320 | Turquoise | | zinc finger (B-box type) family protein | Orphans | |
| AT2G21530 | Turquoise | | forkhead-associated domain-containing protein / FHA domain-containing protein | FHA | |
| AT2G21650 | Turquoise | ATRL2, MEE3 | MEE3 (maternal effect embryo arrest 3); DNA binding / transcription factor | | |
| AT2G22670 | Turquoise | IAA8 | IAA8 (indoleacetic acid-induced protein 8), IAA8 (indoleacetic acid-induced protein 8); transcription factor | AUX/IAA | |
| AT2G22740 | Turquoise | SDG23, SUVH6 | SUVH6 (SU(VAR)3-9 homolog 6) | SET | miR778, |
| AT2G22800 | Turquoise | HAT9 | HAT9 (homeobox-leucine zipper protein 9); DNA binding / transcription factor | HB | |
| AT2G22840 | Turquoise | ATGRF1 | AtGRF1 (GROWTH-REGULATING FACTOR 1) | GRF | miR396, |
| AT2G22900 | Turquoise | | galactosyl transferase GMA12/MNN10 family protein | | |
| AT2G23750 | Turquoise | | | | |
| AT2G23760 | Turquoise | BLH4, SAW2 | BLH4 (BLH4); DNA binding, BLH4 (BLH4); DNA binding / transcription factor | HB | |
| AT2G24430 | Turquoise | ANAC039, ANAC038 | no apical meristem (NAM) family protein | NAC | |
| AT2G24650 | Turquoise | REM13 | transcriptional factor B3 family protein | ABI3VP1 | |
| AT2G25620 | Turquoise | | protein phosphatase 2C, putative / PP2C, putative | DBP | |
| AT2G25650 | Turquoise | | DNA-binding storekeeper protein-related | GeBP | |
| AT2G26150 | Turquoise | ATHSFA2 | ATHSFA2 (Arabidopsis thaliana heat shock transcription factor A2); DNA binding / transcription factor, unknown protein | HSF | |
| AT2G26580 | Turquoise | YAB5 | plant-specific transcription factor YABBY family protein | C2C2-YABBY | |
| AT2G27940 | Turquoise | | zinc finger (C3HC4-type RING finger) family protein | | |
| AT2G27990 | Turquoise | PNF, BLH8 | BLH8 (BEL1-LIKE HOMEODOMAIN 8); DNA binding / transcription factor | HB | |
| AT2G30130 | Turquoise | PCK1, LBD12, ASL5 | ASL5 (phosphoenolpyruvate carboxykinase1) | | |
| AT2G30420 | Turquoise | ETC2 | myb family transcription factor | MYB-related | |
| AT2G30470 | Turquoise | HSI2, VAL1 | HSI2 (High-level expression of Sugar-Inducible gene 2); transcription factor/ transcriptional repressor | ABI3VP1 | |
| AT2G30580 | Turquoise | DRIP2 | zinc finger (C3HC4-type RING finger) family protein | | |
| AT2G32460 | Turquoise | ATMYB101, AtM1 | AtM1/AtMYB101/MYB101 (myb domain protein 101), AtM1/AtMYB101/MYB101 (myb domain protein 101); DNA binding / transcription factor | MYB | miR159/miR319, |
| AT2G33880 | Turquoise | STIP, WOX9, HB-3 | WOX9 (STIMPY); transcription factor | HB | |
| AT2G35160 | Turquoise | SGD9, SUVH5 | SUVH5 (SU(VAR)3-9 HOMOLOG 5) | SET | miR778, |
| AT2G35310 | Turquoise | | transcriptional factor B3 family protein | ABI3VP1 | |
| AT2G35530 | Turquoise | | bZIP transcription factor family protein | bZIP | |
| AT2G36400 | Turquoise | ATGRF3 | AtGRF3 (GROWTH-REGULATING FACTOR 3) | GRF | miR396, |
| AT2G36960 | Turquoise | TKI1 | TKI1 (TSL-KINASE INTERACTING PROTEIN 1); DNA binding / transcription factor | MYB-related | |
| AT2G36990 | Turquoise | SIG6, SIGF | SIGF (RNA POLYMERASE SIGMA-70 FACTOR); DNA binding / DNA-directed RNA polymerase/ transcription factor | Sigma70-like | |
| AT2G37120 | Turquoise | | DNA-binding S1FA family protein | S1Fa-like | |
| AT2G37260 | Turquoise | DSL1, ATWRKY44, TTG2 | TTG2 (TRANSPARENT TESTA GLABRA 2), TTG2 (TRANSPARENT TESTA GLABRA 2); transcription factor | WRKY | |
| AT2G37580 | Turquoise | | zinc finger (C3HC4-type RING finger) family protein | | |
| AT2G37630 | Turquoise | AtPHAN, AtMYB91, MYB91, AS1 | AS1 (ASYMMETRIC LEAVES 1); DNA binding / transcription factor | MYB | |
| AT2G37650 | Turquoise | SCL9 | scarecrow-like transcription factor 9 (SCL9) | GRAS | |
| AT2G39250 | Turquoise | SNZ | SNZ (SCHNARCHZAPFEN); DNA binding / transcription factor | AP2-EREBP | miR172, |
| AT2G40435 | Turquoise | | unknown protein | | |
| AT2G40620 | Turquoise | AtbZIP18 | bZIP transcription factor family protein | bZIP | |
| AT2G40670 | Turquoise | ARR16 | ARR16 (response regulator 16); transcription regulator/ two-component response regulator | Orphans | |
| AT2G41070 | Turquoise | DPBF4, ATBZIP12, EEL | EEL (ENHANCED EM LEVEL); DNA binding / transcription factor | bZIP | |
| AT2G41940 | Turquoise | ZFP8 | ZFP8 (ZINC FINGER PROTEIN 8); nucleic acid binding / transcription factor/ zinc ion binding | C2H2 | |

| | | | | | |
|---|---|---|---|---|---|
| AT2G42380 | Turquoise | ATBZIP34 | bZIP transcription factor family protein | bZIP | |
| AT2G42830 | Turquoise | AGL5, SHP2 | SHP2 (SHATTERPROOF 2); transcription factor | MADS | |
| AT2G43010 | Turquoise | SRL2, PIF4 | PIF4 (PHYTOCHROME INTERACTING FACTOR 4); DNA binding / transcription factor | bHLH | |
| AT2G43060 | Turquoise | bHLH158 | transcription factor | | |
| AT2G44840 | Turquoise | ATERF13, EREBP | ATERF13/EREBP (ETHYLENE-RESPONSIVE ELEMENT BINDING FACTOR 13); DNA binding / transcription factor | AP2-EREBP | |
| AT2G44910 | Turquoise | ATHB4, ATHB-4 | homeobox-leucine zipper protein 4 (HB-4) / HD-ZIP protein 4 | HB | |
| AT2G45100 | Turquoise | | RNA polymerase II transcription factor | | |
| AT2G45190 | Turquoise | FIL, YAB1, AFO | AFO (ABNORMAL FLORAL ORGANS); transcription factor | C2C2-YABBY | |
| AT2G45680 | Turquoise | TCP9 | TCP family transcription factor, putative | TCP | |
| AT2G45850 | Turquoise | | DNA-binding family protein | | |
| AT2G46020 | Turquoise | CHA2, ATBRM, CHR2, BRM | ATBRM/CHR2 (ARABIDOPSIS THALIANA BRAHMA); ATP binding / DNA binding / helicase/ transcription regulator | SNF2 | |
| AT2G46130 | Turquoise | ATWRKY43, WRKY43 | WRKY43 (WRKY DNA-binding protein 43); transcription factor | WRKY | |
| AT2G46530 | Turquoise | ARF11 | ARF11 (AUXIN RESPONSE FACTOR 11); transcription factor | ARF | |
| AT2G46830 | Turquoise | CCA1 | CCA1 (CIRCADIAN CLOCK ASSOCIATED 1); transcription factor | MYB-related | |
| AT3G02150 | Turquoise | TFPD, PTF1, TCP13 | PTF1 (PLASTID TRANSCRIPTION FACTOR 1); transcription factor | TCP | |
| AT3G02290 | Turquoise | | zinc finger (C3HC4-type RING finger) family protein | | |
| AT3G02310 | Turquoise | 2-Sep, AGL4, SEP2 | SEP2 (SEPALLATA2); DNA binding / transcription factor | MADS | |
| AT3G02380 | Turquoise | COL2 | COL2 (CONSTANS-LIKE 2); transcription factor/ zinc ion binding | C2C2-CO-like | |
| AT3G02400 | Turquoise | | forkhead-associated domain-containing protein / FHA domain-containing protein / AT hook motif-containing protein | FHA | |
| AT3G02890 | Turquoise | | PHD finger protein-related | | |
| AT3G02940 | Turquoise | ATMYB107 | MYB107 (myb domain protein 107); DNA binding / transcription factor | MYB | |
| AT3G04280 | Turquoise | ARR22 | ARR22 (ARABIDOPSIS RESPONSE REGULATOR 22), ARR22 (ARABIDOPSIS RESPONSE REGULATOR 22); transcription regulator/ two-component response regulator, ARR22 (ARABIDOPSIS RESPONSE REGULATOR 22); two-component response regulator | Orphans | |
| AT3G05150 | Turquoise | | sugar transporter family protein | | |
| AT3G06160 | Turquoise | | transcriptional factor B3 family protein | ABI3VP1 | |
| AT3G06220 | Turquoise | REM21 | DNA binding / transcription factor | ABI3VP1 | |
| AT3G07200 | Turquoise | | protein binding / zinc ion binding, zinc finger (C3HC4-type RING finger) family protein | | |
| AT3G09670 | Turquoise | | PWWP domain-containing protein | | |
| AT3G10040 | Turquoise | | transcription factor | | |
| AT3G10590 | Turquoise | | myb family transcription factor | MYB | |
| AT3G12560 | Turquoise | ATTBP2, TRFL9 | TRFL9 (TRF-LIKE 9); DNA binding | | |
| AT3G13540 | Turquoise | ATMYB5, ATM2 | ATMYB5 (myb domain protein 5); DNA binding / transcription factor | MYB | |
| AT3G13960 | Turquoise | ATGRF5 | AtGRF5 (GROWTH-REGULATING FACTOR 5) | GRF | |
| AT3G14020 | Turquoise | NF-YA6 | CCAAT-binding transcription factor (CBF-B/NF-YA) family protein | CCAAT | |
| AT3G14980 | Turquoise | | PHD finger transcription factor, putative | PHD | |
| AT3G15030 | Turquoise | TCP4, MEE35 | TCP4 (TCP family transcription factor 4, maternal effect embryo arrest 35); transcription factor | TCP | miR159/miR319, |
| AT3G15500 | Turquoise | ATNAC3, ANAC055 | ATNAC3 (ARABIDOPSIS NAC DOMAIN CONTAINING PROTEIN 55); transcription factor | NAC | |
| AT3G15510 | Turquoise | ATNAC2, ANAC056, NARS1 | ATNAC2 (Arabidopsis thaliana NAC domain containing protein 2); transcription factor | NAC | |
| AT3G16090 | Turquoise | | zinc finger (C3HC4-type RING finger) family protein | | |
| AT3G17600 | Turquoise | IAA31 | IAA31 (indoleacetic acid-induced protein 31); transcription factor | AUX/IAA | |
| AT3G18100 | Turquoise | ATMYB4R1 | MYB4R1 (myb domain protein 4R1); transcription factor | MYB | |
| AT3G18930 | Turquoise | | zinc finger (C3HC4-type RING finger) family protein | | |
| AT3G18960 | Turquoise | | transcriptional factor B3 family protein | ABI3VP1 | |
| AT3G19500 | Turquoise | bHLH113 | ethylene-responsive protein -related | | |
| AT3G20670 | Turquoise | HTA13 | histone H2A, putative | CCAAT | |
| AT3G20910 | Turquoise | NF-YA9 | CCAAT-binding transcription factor (CBF-B/NF-YA) family protein | CCAAT | miR169, |
| AT3G21890 | Turquoise | | zinc finger (B-box type) family protein | Orphans | |
| AT3G22760 | Turquoise | SOL1 | SOL1 (TSO1-Like); transcription factor | CPP | |
| AT3G24340 | Turquoise | CHR40 | CHR40 (chromatin remodeling 40); ATP binding / DNA binding / helicase | SNF2 | |
| AT3G24490 | Turquoise | | transcription factor | Trihelix | |

| | | | | | |
|---|---|---|---|---|---|
| AT3G24500 | Turquoise | MBF1C, ATMBF1C | ATMBF1C/MBF1C (MULTIPROTEIN BRIDGING FACTOR 1C); DNA binding / transcription coactivator/ transcription factor | MBF1 | |
| AT3G24650 | Turquoise | SIS10, ABI3 | ABI3 (ABA INSENSITIVE 3); DNA binding / transcription factor/ transcriptional activator | ABI3VP1 | |
| AT3G26744 | Turquoise | SCRM, ATICE1, ICE1, bHLH116 | ICE1 (INDUCER OF CBF EXPRESSION 1); DNA binding, ICE1 (INDUCER OF CBF EXPRESSION 1); DNA binding / transcription factor | bHLH | |
| AT3G26790 | Turquoise | FUS3 | FUS3 (FUSCA 3); DNA binding / transcription factor | ABI3VP1 | |
| AT3G27780 | Turquoise | MYB118, | | | |
| AT3G27785 | Turquoise | ATMYB118 | MYB118 (myb domain protein 118); DNA binding / transcription factor | MYB | |
| AT3G30460 | Turquoise | | zinc finger (C3HC4-type RING finger) family protein | | |
| AT3G44460 | Turquoise | DPBF2, ATBZIP67 | DPBF2 (BASIC LEUCINE ZIPPER TRANSCRIPTION FACTOR 67); DNA binding / transcription factor | bZIP | |
| AT3G45260 | Turquoise | | zinc finger (C2H2 type) family protein | C2H2 | |
| AT3G45610 | Turquoise | AtDof3,2 | Dof-type zinc finger domain-containing protein | C2C2-Dof | |
| AT3G47600 | Turquoise | ATMYB94, AtMYBCP70 | MYB94 (myb domain protein 94); DNA binding / transcription factor | MYB | |
| AT3G48440 | Turquoise | | zinc finger (CCCH-type) family protein | C3H | |
| AT3G49530 | Turquoise | ANAC062 | ANAC062 (Arabidopsis NAC domain containing protein 62); transcription factor | NAC | |
| AT3G49930 | Turquoise | | zinc finger (C2H2 type) family protein | C2H2 | |
| AT3G49950 | Turquoise | | scarecrow transcription factor family protein | GRAS | |
| AT3G50060 | Turquoise | ATMYB77 | myb family transcription factor | MYB | |
| AT3G50890 | Turquoise | ATHB28 | ATHB28 (ARABIDOPSIS THALIANA HOMEOBOX PROTEIN 28); DNA binding / transcription factor | zf-HD | |
| AT3G52910 | Turquoise | ATGRF4 | AtGRF4 (GROWTH-REGULATING FACTOR 4) | GRF | miR396, |
| AT3G53310 | Turquoise | REM20 | transcriptional factor B3 family protein | ABI3VP1 | |
| AT3G53370 | Turquoise | | DNA-binding S1FA family protein | S1Fa-like | |
| AT3G53680 | Turquoise | | PHD finger transcription factor, putative | PHD | |
| AT3G54220 | Turquoise | SGR1, SCR | SCR (SCARECROW); transcription factor | GRAS | |
| AT3G54320 | Turquoise | ASML1, WRI1 | WRI1 (WRINKLED 1); DNA binding / transcription factor | AP2-EREBP | |
| AT3G54340 | Turquoise | AP3 | AP3 (APETALA 3); DNA binding / transcription factor | MADS | |
| AT3G54390 | Turquoise | | transcription factor | Trihelix | |
| AT3G55980 | Turquoise | ATSZF1 | zinc finger (CCCH-type) family protein | C3H | |
| AT3G56850 | Turquoise | DPBF3, AREB3 | AREB3 (ABA-RESPONSIVE ELEMENT BINDING PROTEIN 3); DNA binding / transcription factor/ transcriptional activator | bZIP | |
| AT3G57600 | Turquoise | | AP2 domain-containing transcription factor, putative | AP2-EREBP | |
| AT3G57920 | Turquoise | SPL15, MSC1 | squamosa promoter-binding protein, putative | SBP | miR156/miR157, |
| AT3G58070 | Turquoise | GIS | GIS (GLABROUS INFLORESCENCE STEMS); nucleic acid binding / transcription factor/ zinc ion binding | C2H2 | |
| AT3G58780 | Turquoise | AGL1, SHP1 | SHP1 (SHATTERPROOF 1), SHP1 (SHATTERPROOF 1); DNA binding / transcription factor | MADS | |
| AT3G60390 | Turquoise | HAT3 | HAT3 (homeobox-leucine zipper protein 3); transcription factor | HB | |
| AT3G61150 | Turquoise | HD-GL2-1, HDG1 | HDG1 (HOMEODOMAIN GLABROUS1); DNA binding / transcription factor | HB | |
| AT3G61250 | Turquoise | ATMYB17 | AtMYB17 (myb domain protein 17); DNA binding / transcription factor | MYB | |
| AT3G61310 | Turquoise | | DNA-binding family protein | | |
| AT3G61830 | Turquoise | ARF18 | ARF18 (AUXIN RESPONSE FACTOR 18); transcription factor | ARF | |
| AT3G61970 | Turquoise | NGA2 | NGA2 (NGATHA2); transcription factor | ABI3VP1 | |
| AT3G62260 | Turquoise | | protein phosphatase 2C, putative / PP2C, putative | DBP | |
| AT3G63350 | Turquoise | AT-HSFA7B | AT-HSFA7B (Arabidopsis thaliana heat shock transcription factor A7B); DNA binding / transcription factor | HSF | |
| AT4G00180 | Turquoise | YAB3 | YAB3 (YABBY3), YAB3 (YABBY3); transcription factor | C2C2-YABBY | |
| AT4G00220 | Turquoise | JLO, LBD30, ASL19 | LOB domain protein 30 / lateral organ boundaries domain protein 30 (LBD30) | | |
| AT4G00238 | Turquoise | | DNA-binding storekeeper protein-related | GeBP | |
| AT4G00270 | Turquoise | | DNA-binding storekeeper protein-related | GeBP | |
| AT4G00480 | Turquoise | ATMYC1 | ATMYC1 (Arabidopsis thaliana myc-related transcription factor 1); DNA binding / transcription factor | bHLH | |
| AT4G00730 | Turquoise | AHDP, ANL2 | ANL2 (ANTHOCYANINLESS 2); transcription factor, unknown protein | HB | |
| AT4G01250 | Turquoise | ATWRKY22, WRKY22 | WRKY22 (WRKY DNA-binding protein 22); transcription factor | WRKY | |
| AT4G01460 | Turquoise | bHLH057 | basic helix-loop-helix (bHLH) family protein | bHLH | |
| AT4G04890 | Turquoise | PDF2 | PDF2 (PROTODERMAL FACTOR2); DNA binding / transcription factor | HB | |
| AT4G09820 | Turquoise | TT8, bHLH042 | TT8 (TRANSPARENT TESTA 8); DNA binding / transcription factor | bHLH | |
| AT4G09960 | Turquoise | AGL11, STK | STK (SEEDSTICK), STK (SEEDSTICK); transcription factor | MADS | |
| AT4G10150 | Turquoise | | zinc finger (C3HC4-type RING finger) family protein | | |
| AT4G11080 | Turquoise | | high mobility group (HMG1/2) family protein | HMG | |

| | | | | | |
|---|---|---|---|---|---|
| AT4G11400 | Turquoise | | ARID/BRIGHT DNA-binding domain-containing protein / ELM2 domain-containing protein / Myb-like DNA-binding domain-containing protein | ARID | |
| AT4G14540 | Turquoise | NF-YB3 | CCAAT-box binding transcription factor subunit B (NF-YB) (HAP3 ) (AHAP3) family | CCAAT | |
| AT4G14770 | Turquoise | ATTCX2 | tesmin/TSO1-like CXC domain-containing protein | CPP | |
| AT4G16610 | Turquoise | | zinc finger (C2H2 type) family protein | | |
| AT4G17600 | Turquoise | LIL3:1 | LIL3:1; transcription factor | | |
| AT4G17810 | Turquoise | ZFP12 | nucleic acid binding / transcription factor/ zinc ion binding | C2H2 | |
| AT4G18020 | Turquoise | APRR2 | APRR2 (PSEUDO-RESPONSE REGULATOR 2), APRR2 (PSEUDO-RESPONSE REGULATOR 2); transcription factor | ARR-B | |
| AT4G18390 | Turquoise | TCP2 | TCP family transcription factor, putative | TCP | miR159/miR319, |
| AT4G18770 | Turquoise | ATMYB98 | MYB98 (myb domain protein 98); DNA binding / transcription factor | MYB | |
| AT4G19510 | Turquoise | | disease resistance protein (TIR-NBS-LRR class), putative | | |
| AT4G21050 | Turquoise | AtDof4,4 | Dof-type zinc finger domain-containing protein | C2C2-Dof | |
| AT4G21430 | Turquoise | B160 | B160; transcription factor | Orphans | |
| AT4G21750 | Turquoise | ATML1 | ATML1 (MERISTEM LAYER 1); DNA binding, ATML1 (MERISTEM LAYER 1); DNA binding / transcription factor | HB | |
| AT4G23800 | Turquoise | AT4G23800 | high mobility group (HMG1/2) family protein | HMG | |
| AT4G24660 | Turquoise | ATHB22, MEE68 | ATHB22/MEE68 (ARABIDOPSIS THALIANA HOMEOBOX PROTEIN 22, maternal effect embryo arrest 68); DNA binding / transcription factor | zf-HD | |
| AT4G25470 | Turquoise | DREB1C, FTQ4, CBF2 | CBF2 (FREEZING TOLERANCE QTL 4); DNA binding / transcription factor/ transcriptional activator | AP2-EREBP | |
| AT4G25490 | Turquoise | DREB1B, CBF1 | CBF1 (C-REPEAT/DRE BINDING FACTOR 1); DNA binding / transcription factor/ transcriptional activator | AP2-EREBP | |
| AT4G27230 | Turquoise | HTA2 | histone H2A, putative | CCAAT | |
| AT4G27410 | Turquoise | RD26, ANAC072 | RD26 (RESPONSIVE TO DESSICATION 26), RD26 (RESPONSIVE TO DESSICATION 26); transcription factor | | |
| AT4G27900 | Turquoise | | unknown protein | Orphans | |
| AT4G28190 | Turquoise | ULT1 | ULT1 (ULTRAPETALA1); DNA binding | ULT | |
| AT4G29030 | Turquoise | | glycine-rich protein | | |
| AT4G29230 | Turquoise | ANAC075, NST9 | ANAC075 (Arabidopsis NAC domain containing protein 75); transcription factor | NAC | |
| AT4G31060 | Turquoise | | AP2 domain-containing transcription factor, putative | AP2-EREBP | |
| AT4G31270 | Turquoise | | transcription factor | | |
| AT4G31620 | Turquoise | REM2 | transcriptional factor B3 family protein | ABI3VP1 | |
| AT4G31680 | Turquoise | REM8 | transcriptional factor B3 family protein | ABI3VP1 | |
| AT4G32710 | Turquoise | | kinase | | |
| AT4G32800 | Turquoise | | AP2 domain-containing transcription factor TINY, putative | AP2-EREBP | |
| AT4G32980 | Turquoise | ATH1 | ATH1 (ARABIDOPSIS THALIANA HOMEOBOX GENE 1); transcription factor | HB | |
| AT4G33280 | Turquoise | REM16 | DNA binding / transcription factor | ABI3VP1 | |
| AT4G34410 | Turquoise | RRTF1 (Redox Responsive Transcription Factor 1 | AP2 domain-containing transcription factor, putative | AP2-EREBP | |
| AT4G34430 | Turquoise | ATSWI3D, CHB3 | CHB3 (Arabidopsis thaliana switch 3D); DNA binding / transcription factor | MYB-related | |
| AT4G34590 | Turquoise | ATB2, ATBZIP11, BZIP11, GBF6 | GBF6 (G-box binding factor 6); DNA binding / transcription factor | bZIP | |
| AT4G34680 | Turquoise | | GATA transcription factor 3, putative (GATA-3) | C2C2-GATA | |
| AT4G35590 | Turquoise | RKD5 | RWP-RK domain-containing protein | RWP-RK | |
| AT4G36870 | Turquoise | BLH2, SAW1 | BLH2 (BEL1-LIKE HOMEODOMAIN 2); DNA binding, BLH2 (BEL1-LIKE HOMEODOMAIN 2); DNA binding / transcription factor | HB | |
| AT4G36930 | Turquoise | SPT, bHLH024 | SPT (SPATULA); DNA binding / transcription factor | bHLH | |
| AT4G37740 | Turquoise | ATGRF2 | AtGRF2 (GROWTHREGULATING FACTOR 2) | GRF | miR396, |
| AT4G38000 | Turquoise | AtDof4,7 | Dof-type zinc finger domain-containing protein | C2C2-Dof | |
| AT5G01200 | Turquoise | | myb family transcription factor | MYB | |
| AT5G01980 | Turquoise | | zinc finger (C3HC4-type RING finger) family protein | | |
| AT5G02810 | Turquoise | APRR7, PRR7 | PRR7 (PSEUDO-RESPONSE REGULATOR 7); transcription regulator | Pseudo ARR-B | |
| AT5G03150 | Turquoise | JKD | zinc finger (C2H2 type) family protein | C2H2 | |
| AT5G04240 | Turquoise | ELF6 | ELF6 (EARLY FLOWERING 6); transcription factor | Jumonji | |
| AT5G04340 | Turquoise | ZAT6, CZF2, C2H2 | C2H2; nucleic acid binding / transcription factor/ zinc ion binding | C2H2 | |
| AT5G04940 | Turquoise | SUVH1 | SUVH1 (SU(VAR)3-9 HOMOLOG 1) | SET | |
| AT5G07210 | Turquoise | ARR21 | ARR21 (ARABIDOPSIS RESPONSE REGULATOR 21); transcription factor/ two-component response regulator | ARR-B | |

| | | | | |
|---|---|---|---|---|
| AT5G07260 | Turquoise | | homeobox protein-related | |
| AT5G07500 | Turquoise | PEI1 | PEI1; nucleic acid binding / transcription factor | C3H |
| AT5G08070 | Turquoise | TCP17 | TCP17 (TCP domain protein 17); transcription factor | TCP |
| AT5G08330 | Turquoise | TCP21 | TCP family transcription factor, putative | TCP |
| AT5G08520 | Turquoise | | myb family transcription factor | MYB |
| AT5G09250 | Turquoise | KIWI | KIWI; DNA binding / transcription coactivator, unknown protein | |
| AT5G13790 | Turquoise | AGL15 | AGL15 (AGAMOUS-LIKE 15); DNA binding / transcription factor | MADS |
| AT5G14260 | Turquoise | | SET domain-containing protein | SET |
| AT5G14370 | Turquoise | | unknown protein | Orphans |
| AT5G14540 | Turquoise | | proline-rich family protein | |
| AT5G14750 | Turquoise | WER1, WER, AtMYB66 | WER (WEREWOLF 1); DNA binding / transcription factor | MYB |
| AT5G15150 | Turquoise | ATHB3, HAT7, ATHB-3 | ATHB-3 (ARABIDOPSIS THALIANA HOMEOBOX 3); DNA binding / transcription factor | HB |
| AT5G15210 | Turquoise | ATHB30, ZFHD3 | ATHB30/ZFHD3 (ZINC FINGER HOMEODOMAIN 3); DNA binding / transcription factor | zf-HD |
| AT5G15800 | Turquoise | 1-Sep, AGL2, SEP1 | SEP1 (SEPALLATA1); DNA binding / transcription factor, unknown protein | MADS |
| AT5G15850 | Turquoise | COL1 | COL1 (CONSTANS-LIKE 1); transcription factor/ zinc ion binding | C2C2-CO-like |
| AT5G16560 | Turquoise | KAN1, KAN | KAN (KANADI); transcription factor | G2-like |
| AT5G16770 | Turquoise | ATMYB9 | AtMYB9 (myb domain protein 9); DNA binding, AtMYB9 (myb domain protein 9); DNA binding / transcription factor | MYB |
| AT5G17300 | Turquoise | | myb family transcription factor | MYB-related |
| AT5G19310 | Turquoise | | homeotic gene regulator, putative | SNF2 |
| AT5G19430 | Turquoise | | zinc finger (C3HC4-type RING finger) family protein | |
| AT5G20420 | Turquoise | CHR42 | CHR42 (chromatin remodeling 42); ATP binding / DNA binding / helicase | SNF2 |
| AT5G22220 | Turquoise | ATE2FB, E2F1 | E2F1; transcription factor | |
| AT5G24590 | Turquoise | TIP, ANAC091 | TIP (TCV-INTERACTING PROTEIN); transcription factor | |
| AT5G24930 | Turquoise | COL4 | zinc finger (B-box type) family protein | C2C2-CO-like |
| AT5G25060 | Turquoise | | RNA recognition motif (RRM)-containing protein | |
| AT5G25470 | Turquoise | | DNA binding | |
| AT5G26210 | Turquoise | AL4 | PHD finger family protein | Alfin-like |
| AT5G28040 | Turquoise | | transcription regulator | GeBP |
| AT5G28300 | Turquoise | | trihelix DNA-binding protein, putative | Trihelix |
| AT5G38140 | Turquoise | NF-YC12 | histone-like transcription factor (CBF/NF-Y) family protein | CCAAT |
| AT5G39860 | Turquoise | PRE1 | PRE1 (PACLOBUTRAZOL RESISTANCE1); DNA binding / transcription factor | |
| AT5G40340 | Turquoise | | PWWP domain-containing protein | |
| AT5G40360 | Turquoise | ATMYB115 | MYB115 (myb domain protein 115); DNA binding / transcription factor | MYB |
| AT5G41410 | Turquoise | BEL1 | BEL1 (BELL 1); DNA binding / transcription factor | HB |
| AT5G41580 | Turquoise | | zinc ion binding | |
| AT5G42200 | Turquoise | | zinc finger (C3HC4-type RING finger) family protein | |
| AT5G42780 | Turquoise | ATHB27 | ATHB27 (ARABIDOPSIS THALIANA HOMEOBOX PROTEIN 27); DNA binding / transcription factor | zf-HD |
| AT5G44190 | Turquoise | ATGLK2, GPRI2, GLK2 | GLK2 (GOLDEN2-LIKE 2); DNA binding / transcription factor | G2-like |
| AT5G44210 | Turquoise | AtERF9, ATERF-9 | ATERF-9/ATERF9/ERF9 (ERF domain protein 9); DNA binding / transcription factor/ transcriptional repressor | AP2-EREBP |
| AT5G45980 | Turquoise | STPL, WOX8 | WOX8 (WUSCHEL-related homeobox 8); DNA binding / transcription factor | HB |
| AT5G46690 | Turquoise | bHLH071 | BHLH071 (BETA HLH PROTEIN 71); DNA binding / transcription factor | bHLH |
| AT5G46880 | Turquoise | HDG5, HB-7 | HB-7 (homeobox-7); DNA binding / transcription factor | HB |
| AT5G46910 | Turquoise | | transcription factor jumonji (jmj) family protein | Jumonji |
| AT5G47670 | Turquoise | NF-YB6, L1L | CCAAT-box binding transcription factor family protein / leafy cotyledon 1-related (L1L) | CCAAT |
| AT5G49330 | Turquoise | ATMYB111 | AtMYB111 (myb domain protein 111); DNA binding / transcription factor | MYB |
| AT5G50480 | Turquoise | NF-YC6 | CCAAT-box binding transcription factor Hap5a, putative | CCAAT |
| AT5G51990 | Turquoise | CBF4, DREB1D | CBF4/DREB1D (C- REPEAT-BINDING FACTOR 4); DNA binding / transcription factor/ transcriptional activator | AP2-EREBP |
| AT5G53200 | Turquoise | TRY | TRY (TRIPTYCHON); DNA binding / transcription factor | MYB-related |
| AT5G53210 | Turquoise | SPCH, bHLH098 | DNA binding / transcription factor | bHLH |
| AT5G54470 | Turquoise | | zinc finger (B-box type) family protein | Orphans |
| AT5G54630 | Turquoise | | zinc finger protein-related | |
| AT5G54680 | Turquoise | bHLH105, ILR3 | ILR3 (IAA-LEUCINE RESISTANT3); DNA binding / transcription factor | bHLH |
| AT5G55970 | Turquoise | | zinc finger (C3HC4-type RING finger) family protein | |

| | | | | | |
|---|---|---|---|---|---|
| AT5G56780 | Turquoise | | unknown protein | HRT | |
| AT5G56860 | Turquoise | GNC | GNC (GATA, NITRATE-INDUCIBLE, CARBON METABOLISM-INVOLVED); transcription factor | C2C2-GATA | |
| AT5G57390 | Turquoise | CHO1, AIL5 | AIL5 (AINTEGUMENTA-LIKE 5); DNA binding / transcription factor | AP2-EREBP | |
| AT5G58280 | Turquoise | | transcriptional factor B3 family protein | ABI3VP1 | |
| AT5G58340 | Turquoise | | DNA binding | MYB-related | |
| AT5G59820 | Turquoise | ZAT12, RHL41 | RHL41 (RESPONSIVE TO HIGH LIGHT 41); nucleic acid binding / transcription factor/ zinc ion binding | C2H2 | |
| AT5G60140 | Turquoise | | transcriptional factor B3 family protein | ABI3VP1 | |
| AT5G61430 | Turquoise | ANAC100, ATNAC5 | ANAC100/ATNAC5 (Arabidopsis NAC domain containing protein 100); transcription factor | NAC | miR164, |
| AT5G61600 | Turquoise | | ethylene-responsive element-binding family protein | AP2-EREBP | |
| AT5G62010 | Turquoise | | | | |
| AT5G62020 | Turquoise | AT-HSFB2A | AT-HSFB2A (Arabidopsis thaliana heat shock transcription factor B2A); DNA binding / transcription factor | HSF | |
| AT5G63260 | Turquoise | | zinc finger (CCCH-type) family protein | C3H | |
| AT5G63430 | Turquoise | | | | |
| AT5G63920 | Turquoise | | DNA topoisomerase III alpha, putative | Orphans | |
| AT5G64530 | Turquoise | ANAC104, XND1 | ANAC104/XND1 (Arabidopsis NAC domain containing protein 104); transcription factor | NAC | |
| AT5G65230 | Turquoise | ATMYB53 | AtMYB53 (myb domain protein 53); DNA binding / transcription factor | MYB | |
| AT5G65410 | Turquoise | ATHB25, ZFHD2 | ATHB25/ZFHD2 (ZINC FINGER HOMEODOMAIN 2); DNA binding / transcription factor | zf-HD | |
| AT5G65590 | Turquoise | AtDof5,7 | Dof-type zinc finger domain-containing protein | C2C2-Dof | |
| AT5G66620 | Turquoise | DAR6 | LIM domain-containing protein | | |
| AT5G66940 | Turquoise | AtDof5. 8 | Dof-type zinc finger domain-containing protein | C2C2-Dof | |
| AT5G67030 | Turquoise | NPQ2, LOS6, IBS3, ATZEP, ATABA1, ABA1 | ABA1 (ABA DEFICIENT 1); zeaxanthin epoxidase | FHA | |
| AT1G01030 | Yellow | NGA3 | NGA3 (NGATHA3); transcription factor | ABI3VP1 | |
| AT1G01780 | Yellow | | LIM domain-containing protein | LIM | |
| AT1G05805 | Yellow | bHLH128 | basic helix-loop-helix (bHLH) family protein | bHLH | |
| AT1G06850 | Yellow | ATBZIP52 | DNA binding, bZIP transcription factor, putative | bZIP | |
| AT1G07530 | Yellow | SCL14, ATGRAS2 | scarecrow-like transcription factor 14 (SCL14) | GRAS | |
| AT1G07640 | Yellow | OBP2, AtDof1. 1 | OBP2 (OBF BINDING PROTEIN 2); DNA binding, OBP2 (OBF BINDING PROTEIN 2); DNA binding / transcription factor | C2C2-Dof | |
| AT1G08780 | Yellow | PDF4, AIP3, ABI3, API3 | prefoldin, putative | | |
| AT1G09060 | Yellow | | transcription factor, transcription factor jumonji (jmjC) domain-containing protein | Jumonji | |
| AT1G10200 | Yellow | WLIM1, SF3 | transcription factor LIM, putative | LIM | |
| AT1G10320 | Yellow | | U2 snRNP auxiliary factor-related | C3H | |
| AT1G10585 | Yellow | | transcription factor | bHLH | |
| AT1G13260 | Yellow | RAV1 | RAV1 (Related to ABI3/VP1 1); DNA binding / transcription factor | AP2-EREBP | |
| AT1G13300 | Yellow | | myb family transcription factor | G2-like | |
| AT1G13600 | Yellow | ATBZIP58 | bZIP transcription factor family protein | bZIP | |
| AT1G14350 | Yellow | FLP, AtMYB124 | FLP (FOUR LIPS), FLP (FOUR LIPS); DNA binding / transcription factor | MYB | |
| AT1G16060 | Yellow | | ovule development protein, putative | AP2-EREBP | |
| AT1G16530 | Yellow | ASL9, LBD3 | LOB domain protein 3 / lateral organ boundaries domain protein 3 (LBD3) | | |
| AT1G17380 | Yellow | TIFY11A, JAZ5 | unknown protein | ZIM | |
| AT1G17590 | Yellow | NF-YA8 | CCAAT-binding transcription factor (CBF-B/NF-YA) family protein | CCAAT | miR169, |
| AT1G19220 | Yellow | IAA22, ARF11, ARF19 | ARF19 (AUXIN RESPONSE FACTOR 11); transcription factor | ARF | |
| AT1G19270 | Yellow | DA1 | ubiquitin interaction motif-containing protein / LIM domain-containing protein | Orphans | |
| AT1G20700 | Yellow | ATWOX14, WOX14 | homeobox-leucine zipper family protein | HB | |
| AT1G20900 | Yellow | ORE7, AHL27, ESC | ESC (ESCAROLA) | | |
| AT1G21200 | Yellow | | transcription factor | | |
| AT1G22070 | Yellow | TGA3 | TGA3 (TGA1a-related gene 3); DNA binding / calmodulin binding / transcription factor | bZIP | |
| AT1G22640 | Yellow | AtMYB3 | MYB3 (myb domain protein 3); DNA binding / transcription factor | MYB | |
| AT1G23370 | Yellow | | | | |
| AT1G23380 | Yellow | KNAT6S, KNAT6L, KNAT6 | KNAT6 (Knotted-like Arabidopsis thaliana 6); DNA binding / transcription factor | HB | |
| AT1G24625 | Yellow | ZFP7 | ZFP7 (ZINC FINGER PROTEIN 7); nucleic acid binding / transcription factor/ zinc ion binding | C2H2 | |
| AT1G25550 | Yellow | AT1G25550 | myb family transcription factor | G2-like | |
| AT1G26150 | Yellow | | protein kinase | | |
| AT1G27320 | Yellow | AHK3 | AHK3 (ARABIDOPSIS HISTIDINE KINASE 3) | Orphans | |
| AT1G28370 | Yellow | AtERF11 | ATERF11/ERF11 (ERF domain protein 11); DNA binding / transcription factor/ transcriptional repressor | AP2-EREBP | |

| | | | | | |
|---|---|---|---|---|---|
| AT1G29160 | Yellow | AtDof1. 5 | Dof-type zinc finger domain-containing protein | C2C2-Dof | |
| AT1G31050 | Yellow | bHLH111 | transcription factor | | |
| AT1G32700 | Yellow | | zinc-binding family protein | PLATZ | |
| AT1G33480 | Yellow | | protein binding / zinc ion binding | | |
| AT1G34670 | Yellow | ATMYB93 | AtMYB93 (myb domain protein 93); DNA binding / transcription factor | MYB | |
| AT1G43160 | Yellow | RAP2.6, RAP2.06 | RAP2.6 (related to AP2 6); DNA binding / transcription factor | AP2-EREBP | |
| AT1G47655 | Yellow | | Dof-type zinc finger domain-containing protein | C2C2-Dof | |
| AT1G51070 | Yellow | bHLH115 | basic helix-loop-helix (bHLH) family protein | bHLH | |
| AT1G56010 | Yellow | NAC1, ANAC021, ANAC022 | NAC1 (Arabidopsis NAC domain containing protein 21, Arabidopsis NAC domain containing protein 22); transcription factor | NAC | miR164, |
| AT1G58110 | Yellow | | DNA binding / transcription factor, bZIP family transcription factor | | |
| AT1G58220 | Yellow | | myb family transcription factor | MYB-related | |
| AT1G61660 | Yellow | bHLH112 | basic helix-loop-helix (bHLH) family protein | bHLH | |
| AT1G64530 | Yellow | NLP6 | RWP-RK domain-containing protein | RWP-RK | |
| AT1G65910 | Yellow | ANAC028 | ANAC028 (Arabidopsis NAC domain containing protein 28); transcription factor | NAC | |
| AT1G66140 | Yellow | ZFP4 | ZFP4 (ZINC FINGER PROTEIN 4); nucleic acid binding / transcription factor/ zinc ion binding | C2H2 | |
| AT1G67030 | Yellow | ZFP6 | ZFP6 (ZINC FINGER PROTEIN 6); nucleic acid binding / transcription factor/ zinc ion binding | | |
| AT1G67310 | Yellow | | calmodulin binding / transcription regulator | CAMTA | |
| AT1G67710 | Yellow | ARR11 | ARR11 (RESPONSE REGULATOR 11); transcription factor/ two-component response regulator | ARR-B | |
| AT1G67910 | Yellow | | unknown protein | | |
| AT1G68130 | Yellow | ATIDD14 | ATIDD14 (ARABIDOPSIS THALIANA INDETERMINATE(ID)-DOMAIN 14), ATIDD14 (ARABIDOPSIS THALIANA INDETERMINATE(ID)-DOMAIN 14); nucleic acid binding / transcription factor/ zinc ion binding | C2H2 | |
| AT1G68520 | Yellow | COL6 | zinc finger (B-box type) family protein | C2C2-CO-like | |
| AT1G68670 | Yellow | | myb family transcription factor | G2-like | |
| AT1G68810 | Yellow | bHLH030 | basic helix-loop-helix (bHLH) family protein | bHLH | |
| AT1G68840 | Yellow | RAV2, RAP2.8, TEM2 | RAV2 (REGULATOR OF THE ATPASE OF THE VACUOLAR MEMBRANE); DNA binding / transcription factor | AP2-EREBP | |
| AT1G68880 | Yellow | ATBZIP | bZIP transcription factor family protein | bZIP | |
| AT1G69010 | Yellow | bHLH102, BIM2 | BIM2 (BES1-INTERACTING MYC-LIKE PROTEIN 2); DNA binding / transcription factor | bHLH | |
| AT1G69490 | Yellow | NAP, ANAC029, ATNAP | NAP (NAC-LIKE, ACTIVATED BY AP3/PI); transcription factor | NAC | |
| AT1G69580 | Yellow | | transcription factor, unknown protein | G2-like | |
| AT1G69780 | Yellow | ATHB13 | ATHB13 (ARABIDOPSIS THALIANA HOMEOBOX PROTEIN 12); DNA binding / transcription factor | HB | |
| AT1G70930 | Yellow | | | | |
| AT1G71930 | Yellow | VND7, ANAC030 | VND7 (VASCULAR RELATED NAC-DOMAIN PROTEIN 7); transcription factor | NAC | |
| AT1G72360 | Yellow | | ethylene-responsive element-binding protein, putative, unknown protein | AP2-EREBP | |
| AT1G72450 | Yellow | TIFY11B, JAZ6 | unknown protein | ZIM | |
| AT1G73830 | Yellow | bHLH050, BEE3 | BEE3 (BR ENHANCED EXPRESSION 3); DNA binding / transcription factor | bHLH | |
| AT1G74430 | Yellow | ATMYB95, AtMYBCP66 | MYB95 (myb domain protein 95); DNA binding / transcription factor | MYB | |
| AT1G74840 | Yellow | | myb family transcription factor | MYB-related | |
| AT1G75080 | Yellow | BZR1 | BZR1 (BRASSINAZOLE-RESISTANT 1), BZR1 (BRASSINAZOLE-RESISTANT 1); transcription regulator | BES1 | |
| AT1G75410 | Yellow | BLH3 | BLH3 (BLH3), BLH3 (BLH3); DNA binding / transcription factor | HB | |
| AT1G76410 | Yellow | ATL8 | ATL8; protein binding / zinc ion binding | | |
| AT1G76890 | Yellow | AT-GT2, GT2 | GT2 (GT2); transcription factor | Trihelix | |
| AT1G77080 | Yellow | FLM, AGL27, MAF1 | MAF1 (MADS AFFECTING FLOWERING 1); transcription factor | | |
| AT1G77250 | Yellow | | PHD finger family protein | PHD | |
| AT1G77850 | Yellow | ARF17 | ARF17 (AUXIN RESPONSE FACTOR 17); transcription factor | ARF | miR160, |
| AT1G78700 | Yellow | | brassinosteroid signalling positive regulator-related | BES1 | |
| AT1G79180 | Yellow | ATMYB63 | AtMYB63 (myb domain protein 63); DNA binding / transcription factor | MYB | |
| AT1G79430 | Yellow | WDY, APL | APL (ALTERED PHLOEM DEVELOPMENT); transcription factor | | |
| AT2G01760 | Yellow | ARR14 | ARR14 (ARABIDOPSIS RESPONSE REGULATOR 14); transcription factor/ two-component response regulator | ARR-B | |
| AT2G01830 | Yellow | CRE1, AHK4, WOL1, WOL | WOL (CYTOKININ RESPONSE 1) | Orphans | |
| AT2G03060 | Yellow | AGL30 | DNA binding / transcription factor, unknown protein | MADS | |
| AT2G03500 | Yellow | | myb family transcription factor | G2-like | |
| AT2G04038 | Yellow | ATBZIP48 | bZIP transcription factor family protein | bZIP | |

| | | | | | |
|---|---|---|---|---|---|
| AT2G16400 | Yellow | BLH7 | BLH7 (BELL1-LIKE HOMEODOMAIN 7); DNA binding / transcription factor | HB | |
| AT2G16720 | Yellow | AtY49, AtMYB7 | MYB7 (myb domain protein 7); DNA binding / transcription factor | MYB | |
| AT2G17040 | Yellow | ANAC036 | ANAC036 (Arabidopsis NAC domain containing protein 36); transcription factor | NAC | |
| AT2G17820 | Yellow | AHK1, ATHK1 | ATHK1 (HISTIDINE KINASE 1) | Orphans | |
| AT2G17900 | Yellow | SDG37 | SDG37 (SET DOMAIN GROUP 37); zinc ion binding | | |
| AT2G18160 | Yellow | GBF5, ATBZIP2 | GBF5 (G-box binding factor 5); DNA binding / transcription factor | bZIP | |
| AT2G18280 | Yellow | AtTLP2 | AtTLP2 (TUBBY LIKE PROTEIN 2); phosphoric diester hydrolase/ transcription factor | TUB | |
| AT2G18300 | Yellow | bHLH064 | basic helix-loop-helix (bHLH) family protein | bHLH | |
| AT2G18380 | Yellow | HANL1 | zinc finger (GATA type) family protein | C2C2-GATA | |
| AT2G18550 | Yellow | HB-2, ATHB21 | ATHB21/HB-2 (homeobox-2); DNA binding / transcription factor | HB | |
| AT2G22120 | Yellow | | protein binding / zinc ion binding | | |
| AT2G22540 | Yellow | AGL22, SVP | SVP (SHORT VEGETATIVE PHASE); transcription factor | MADS | |
| AT2G22750 | Yellow | bHLH018 | basic helix-loop-helix (bHLH) family protein | bHLH | |
| AT2G22850 | Yellow | ATBZIP6 | bZIP transcription factor family protein | bZIP | |
| AT2G23380 | Yellow | SET1, SDG1, ICU1, CLF | CLF (CURLY LEAF); transcription factor | SET | |
| AT2G24260 | Yellow | bHLH066 | basic helix-loop-helix (bHLH) family protein | bHLH | |
| AT2G24300 | Yellow | | calmodulin-binding protein | | |
| AT2G24830 | Yellow | | zinc finger (CCCH-type) family protein / D111/G-patch domain-containing protein | C3H | |
| AT2G25170 | Yellow | SSL2, GYM, CHD3, PKL, CHR6 | PKL/SSL2 (PICKLE, SUPPRESSOR OF SLR2) | PHD | |
| AT2G28200 | Yellow | | nucleic acid binding / transcription factor/ zinc ion binding | C2H2 | |
| AT2G28550 | Yellow | TOE1, RAP2.7 | TOE1 (TARGET OF EAT1 1); DNA binding / transcription factor | AP2-EREBP | miR172, |
| AT2G28810 | Yellow | AtDof2. 2 | Dof-type zinc finger domain-containing protein | C2C2-Dof | |
| AT2G29660 | Yellow | | zinc finger (C2H2 type) family protein | C2H2 | |
| AT2G31380 | Yellow | STH | STH (salt tolerance homologue); transcription factor/ zinc ion binding | Orphans | |
| AT2G33290 | Yellow | SDG3, ATSUVH2, SUVH2 | SUVH2 (SU(VAR)3-9 HOMOLOG 2) | SET | |
| AT2G33860 | Yellow | ARF3, ETT | ETT (ETTIN); transcription factor | ARF | |
| AT2G34210 | Yellow | | KOW domain-containing transcription factor family protein | | |
| AT2G34600 | Yellow | TIFY5B, JAZ7 | unknown protein | ZIM | |
| AT2G34830 | Yellow | ATWRKY35, WRKY35, MEE24 | WRKY35 (WRKY DNA-binding protein 35, maternal effect embryo arrest 24); transcription factor | WRKY | |
| AT2G35000 | Yellow | | zinc finger (C3HC4-type RING finger) family protein | | |
| AT2G35270 | Yellow | | DNA-binding protein-related | | |
| AT2G35940 | Yellow | BLH1, EDA29 | BLH1 (BLH1), BLH1 (BLH1); DNA binding / transcription factor, BLH1 (BLH1); transcription factor | HB | |
| AT2G36080 | Yellow | | DNA-binding protein, putative | ABI3VP1 | |
| AT2G36890 | Yellow | BIT1, ATMYB38, RAX2 | ATMYB38/MYB38/RAX2 (myb domain protein 38); DNA binding / transcription factor | MYB | |
| AT2G39830 | Yellow | DAR2 | zinc ion binding | Orphans | |
| AT2G39900 | Yellow | | LIM domain-containing protein | LIM | |
| AT2G40200 | Yellow | bHLH051 | basic helix-loop-helix (bHLH) family protein | bHLH | |
| AT2G40970 | Yellow | | myb family transcription factor | G2-like | |
| AT2G43140 | Yellow | bHLH129 | DNA binding / transcription factor | bHLH | |
| AT2G44940 | Yellow | | AP2 domain-containing transcription factor TINY, putative | AP2-EREBP | |
| AT2G45050 | Yellow | | zinc finger (GATA type) family protein | C2C2-GATA | |
| AT2G45160 | Yellow | | scarecrow transcription factor family protein | GRAS | miR170/miR171, |
| AT2G45430 | Yellow | | DNA-binding protein-related | | |
| AT2G45660 | Yellow | SOC1, AGL20 | AGL20 (AGAMOUS-LIKE 20); transcription factor | MADS | |
| AT2G46410 | Yellow | CPC | CPC (CAPRICE); DNA binding / transcription factor | MYB-related | |
| AT2G46590 | Yellow | DAG2, AtDof2,5 | DAG2 (DOF AFFECTING GERMINATION 2); DNA binding, DAG2 (DOF AFFECTING GERMINATION 2); DNA binding / transcription factor | C2C2-Dof | |
| AT3G01220 | Yellow | ATHB20 | ATHB20 (ARABIDOPSIS THALIANA HOMEOBOX PROTEIN 20); DNA binding / transcription factor | HB | |
| AT3G02340 | Yellow | | zinc finger (C3HC4-type RING finger) family protein | | |
| AT3G02830 | Yellow | ZFN1 | ZFN1 (ZINC FINGER PROTEIN 1); nucleic acid binding | C3H | |
| AT3G02860 | Yellow | | unknown protein | | |
| AT3G04030 | Yellow | | myb family transcription factor, unknown protein | G2-like | |
| AT3G04070 | Yellow | ANAC047 | ANAC047 (Arabidopsis NAC domain containing protein 47); transcription factor, unknown protein | NAC | |
| AT3G05690 | Yellow | NF-YA2, ATHAP2B, HAP2B, UNE8 | HAP2B (Heme activator protein (yeast) homolog 2B, unfertilized embryo sac 8); transcription factor | CCAAT | miR169, |
| AT3G05800 | Yellow | bHLH150 | transcription factor | | |

| | | | | | |
|---|---|---|---|---|---|
| AT3G06380 | Yellow | ATTLP9 | ATTLP9/AtTLP9 (TUBBY LIKE PROTEIN 9, TUBBY-LIKE PROTEIN 9); phosphoric diester hydrolase/ protein binding / transcription factor | TUB | |
| AT3G06590 | Yellow | bHLH148 | transcription factor, unknown protein | | |
| AT3G07340 | Yellow | bHLH062 | basic helix-loop-helix (bHLH) family protein | bHLH | |
| AT3G07670 | Yellow | | SET domain-containing protein | SET | |
| AT3G07740 | Yellow | HXA2, HXA02, HAC10, ATADA2A, ADA2A | ADA2A (Arabidopsis adaptor 2A homolog), ADA2A (Arabidopsis adaptor 2A homolog); DNA binding / transcription factor | MYB-related | |
| AT3G11090 | Yellow | LBD21 | LOB domain family protein / lateral organ boundaries domain family protein (LBD21) | | |
| AT3G11280 | Yellow | | myb family transcription factor | MYB | |
| AT3G11450 | Yellow | | DNAJ heat shock N-terminal domain-containing protein / cell division protein-related | MYB | |
| AT3G12680 | Yellow | HUA1 | HUA1 (ENHANCER OF AG-4 1) | C3H | |
| AT3G12980 | Yellow | HAC5 ,ATHPCAT4 | histone acetyltransferase 5 (HAC5) | TAZ | |
| AT3G13040 | Yellow | | myb family transcription factor | G2-like | |
| AT3G16350 | Yellow | | myb family transcription factor | MYB-related | |
| AT3G16500 | Yellow | IAA26, PAP1 | PAP1 (PHYTOCHROME-ASSOCIATED PROTEIN 1); transcription factor | AUX/IAA | |
| AT3G17100 | Yellow | bHLH147 | transcription factor | | |
| AT3G17730 | Yellow | ANAC057 | ANAC057 (Arabidopsis NAC domain containing protein 57); transcription factor | NAC | |
| AT3G17860 | Yellow | TIFY6B, JAI3, JAZ3 | unknown protein | ZIM | |
| AT3G23210 | Yellow | bHLH034 | basic helix-loop-helix (bHLH) family protein | bHLH | |
| AT3G23240 | Yellow | ERF1, ATERF1 | ATERF1/ERF1 (ETHYLENE RESPONSE FACTOR 1); DNA binding / transcription factor/ transcriptional activator | AP2-EREBP | |
| AT3G24010 | Yellow | ATING1 | PHD finger family protein | PHD | |
| AT3G24310 | Yellow | MYB305, ATMYB71 | MYB305 (myb domain protein 305); DNA binding / transcription factor | MYB | |
| AT3G25710 | Yellow | BHLH32, bHLH032 | basic helix-loop-helix (bHLH) family protein | bHLH | |
| AT3G27650 | Yellow | LBD25 | LOB domain protein 25 / lateral organ boundaries domain protein 25 (LBD25) | | |
| AT3G29035 | Yellow | AtNAC3, ANAC059 | ANAC059/ATNAC3 (Arabidopsis NAC domain containing protein 59); protein heterodimerization/ transcription factor | NAC | |
| AT3G43430 | Yellow | | zinc finger (C3HC4-type RING finger) family protein | | |
| AT3G46130 | Yellow | PFG3, ATMYB111 ATMYB48 | MYB111 (myb domain protein 111), MYB111 (myb domain protein 111); DNA binding, MYB111 (myb domain protein 111); DNA binding / transcription factor | MYB | |
| AT3G46590 | Yellow | ATTRP2, TRFL1 | TRFL1 (TRF-LIKE 1); DNA binding | | |
| AT3G47500 | Yellow | CDF3, AtDof3,3 | CDF3 (CYCLING DOF FACTOR 3); DNA binding / protein binding / transcription factor | C2C2-Dof | |
| AT3G47620 | Yellow | AtTCP14, TCP14 | TCP family transcription factor, putative | TCP | |
| AT3G48920 | Yellow | ATMYB45 | AtMYB45 (myb domain protein 45); DNA binding / transcription factor | MYB | |
| AT3G49690 | Yellow | ATMYB84, RAX3 | ATMYB84/MYB84/RAX3 (myb domain protein 84); DNA binding / transcription factor | MYB | |
| AT3G49760 | Yellow | ATBZIP5 | bZIP transcription factor family protein | bZIP | |
| AT3G49940 | Yellow | LBD38 | LOB domain protein 38 / lateral organ boundaries domain protein 38 (LBD38) | | |
| AT3G51910 | Yellow | AT-HSFA7A | AT-HSFA7A (Arabidopsis thaliana heat shock transcription factor A7A); DNA binding / transcription factor | HSF | |
| AT3G52100 | Yellow | | PHD finger family protein | | |
| AT3G53200 | Yellow | ATMYB27 | AtMYB27 (myb domain protein 27); DNA binding / transcription factor | MYB | |
| AT3G53340 | Yellow | NF-YB10 | CCAAT-box binding transcription factor, putative | CCAAT | |
| AT3G54430 | Yellow | SRS6 | SRS6 (SHI-RELATED SEQUENCE 6) | SRS | |
| AT3G57040 | Yellow | ATRR4, ARR9 | ARR9 (RESPONSE REACTOR 4); transcription regulator | Orphans | |
| AT3G57230 | Yellow | AGL16 | AGL16 (AGAMOUS-LIKE 16), AGL16 (AGAMOUS-LIKE 16); transcription factor | MADS | miR824, |
| AT3G59060 | Yellow | PIF5, PIL6 | PIL6 (PHYTOCHROME-INTERACTING FACTOR 5); DNA binding / transcription factor | bHLH | |
| AT3G60080 | Yellow | | zinc finger (C3HC4-type RING finger) family protein | | |
| AT3G60490 | Yellow | | AP2 domain-containing transcription factor TINY, putative | AP2-EREBP | |
| AT3G60530 | Yellow | | zinc finger (GATA type) family protein | C2C2-GATA | |
| AT3G60630 | Yellow | | scarecrow transcription factor family protein | GRAS | miR170/miR171, |
| AT4G00050 | Yellow | bHLH016, UNE10 | UNE10 (unfertilized embryo sac 10); DNA binding / transcription factor | bHLH | |
| AT4G00150 | Yellow | SCL6 | scarecrow-like transcription factor 6 (SCL6) | GRAS | miR170/miR171, |
| AT4G00990 | Yellow | | transcription factor jumonji (jmjC) domain-containing protein | Jumonji | |
| AT4G02020 | Yellow | SDG10, SWINGER, SWN, EZA1 | EZA1 (SWINGER); transcription factor | SET | |
| AT4G02590 | Yellow | bHLH059, UNE12 | UNE12 (unfertilized embryo sac 12); DNA binding, UNE12 (unfertilized embryo sac 12); DNA binding / transcription factor, unknown protein | bHLH | |

| | | | | | |
|---|---|---|---|---|---|
| AT4G02640 | Yellow | BZO2H1, AT4G02640, ATBZIP10 | BZO2H1 (basic leucine zipper O2 homolog 1); DNA binding / sequence-specific DNA binding / transcription factor, BZO2H1 (basic leucine zipper O2 homolog 1); DNA binding / transcription factor | bZIP | |
| AT4G02670 | Yellow | ATIDD12 | ATIDD12 (ARABIDOPSIS THALIANA INDETERMINATE(ID)-DOMAIN 12); nucleic acid binding / transcription factor/ zinc ion binding | C2H2 | |
| AT4G05170 | Yellow | bHLH114 | DNA binding / transcription factor | bHLH | |
| AT4G11880 | Yellow | AGL14 | AGL14 (AGAMOUS-LIKE 14); DNA binding / transcription factor | MADS | |
| AT4G12080 | Yellow | | DNA-binding family protein | | |
| AT4G13100 | Yellow | | zinc finger (C3HC4-type RING finger) family protein | | |
| AT4G13640 | Yellow | UNE16 | UNE16 (unfertilized embryo sac 16), UNE16 (unfertilized embryo sac 16); transcription factor | G2-like | |
| AT4G13980 | Yellow | AT-HSFA5 | AT-HSFA5 (Arabidopsis thaliana heat shock transcription factor A5); DNA binding / transcription factor | HSF | |
| AT4G14410 | Yellow | bHLH104 | basic helix-loop-helix (bHLH) family protein | bHLH | |
| AT4G14920 | Yellow | | PHD finger transcription factor, putative | PHD | |
| AT4G16750 | Yellow | | DRE-binding transcription factor, putative | AP2-EREBP | |
| AT4G16780 | Yellow | ATHB2, HAT4, ATHB-2 | ATHB-2 (Homeobox-leucine zipper protein HAT4); DNA binding / transcription factor | HB | |
| AT4G16870 | Yellow | | transposable element gene | | |
| AT4G17500 | Yellow | ATERF-1 | ATERF-1 (ETHYLENE RESPONSIVE ELEMENT BINDING FACTOR 1); DNA binding / transcription factor/ transcriptional activator | AP2-EREBP | |
| AT4G17800 | Yellow | | DNA-binding protein-related | | |
| AT4G17880 | Yellow | bHLH004 | basic helix-loop-helix (bHLH) family protein | bHLH | |
| AT4G18170 | Yellow | ATWRKY28, WRKY28 | WRKY28 (WRKY DNA-binding protein 28); transcription factor | WRKY | |
| AT4G24020 | Yellow | NLP7 | RWP-RK domain-containing protein | RWP-RK | |
| AT4G24240 | Yellow | ATWRKY7, WRKY7 | WRKY7 (WRKY DNA-binding protein 7); transcription factor | WRKY | |
| AT4G24540 | Yellow | AGL24 | AGL24 (AGAMOUS-LIKE 24); transcription factor | MADS | |
| AT4G26640 | Yellow | ATWRKY20, WRKY20 | WRKY20 (WRKY DNA-binding protein 20); transcription factor | WRKY | |
| AT4G27910 | Yellow | SDG16, ATX4 | PHD finger protein-related / SET domain-containing protein (TX4) | PHD | |
| AT4G28270 | Yellow | | zinc finger (C3HC4-type RING finger) family protein | | |
| AT4G28890 | Yellow | | zinc finger (C3HC4-type RING finger) family protein | | |
| AT4G29080 | Yellow | IAA27, PAP2 | PAP2 (PHYTOCHROME-ASSOCIATED PROTEIN 2); transcription factor | AUX/IAA | |
| AT4G29100 | Yellow | bHLH068 | ethylene-responsive family protein | | |
| AT4G35270 | Yellow | NLP2 | RWP-RK domain-containing protein | RWP-RK | |
| AT4G36050 | Yellow | | endonuclease/exonuclease/phosphatase family protein | Orphans | |
| AT4G36160 | Yellow | ANAC076, VND2 | ANAC076/VND2 (Arabidopsis NAC domain containing protein 76, VASCULAR-RELATED NAC-DOMAIN 2); transcription factor | NAC | |
| AT4G36540 | Yellow | bHLH058, BEE2 | BEE2 (BR ENHANCED EXPRESSION 2); DNA binding / transcription factor | bHLH | |
| AT4G36620 | Yellow | HANL2 | zinc finger (GATA type) family protein | C2C2-GATA | |
| AT4G36710 | Yellow | | scarecrow transcription factor family protein | GRAS | |
| AT4G36990 | Yellow | AT-HSFB1, ATHSF4, HSF4, HSFB1 | HSF4 (HEAT SHOCK FACTOR 4); DNA binding / transcription factor | HSF | |
| AT4G37110 | Yellow | | protein binding / zinc ion binding | | |
| AT4G37610 | Yellow | BT5 | BT5 (BTB and TAZ domain protein 5); protein binding / transcription regulator | TAZ | |
| AT4G38620 | Yellow | AtMYB4 | MYB4 (myb domain protein 4); transcription factor | MYB | |
| AT4G39410 | Yellow | ATWRKY13, WRKY13 | WRKY13 (WRKY DNA-binding protein 13); transcription factor | WRKY | |
| AT4G40060 | Yellow | ATHB16 ,ATHB-16 | ATHB16 (ARABIDOPSIS THALIANA HOMEOBOX PROTEIN 16); transcription factor | HB | |
| AT5G02030 | Yellow | PNY, BLR, BLH9, RPL, HB-6, VAN, LSN | LSN (LARSON, VAAMANA); DNA binding / transcription factor | HB | |
| AT5G03510 | Yellow | | zinc finger (C2H2 type) family protein | C2H2 | |
| AT5G03680 | Yellow | PTL | PTL (PETAL LOSS); transcription factor | Trihelix | |
| AT5G05790 | Yellow | | myb family transcription factor | MYB | |
| AT5G06100 | Yellow | ATMYB33 | ATMYB33/MYB33 (myb domain protein 33), ATMYB33/MYB33 (myb domain protein 33); DNA binding / transcription factor | MYB | miR159/miR319, |
| AT5G06510 | Yellow | NF-YA10 | CCAAT-binding transcription factor (CBF-B/NF-YA) family protein | CCAAT | miR169, |
| AT5G06710 | Yellow | HAT14 | HAT14 (homeobox-leucine zipper protein 14); DNA binding, HAT14 (homeobox-leucine zipper protein 14); DNA binding / transcription factor | HB | |
| AT5G06800 | Yellow | | myb family transcription factor | G2-like | |
| AT5G07100 | Yellow | WRKY26 | WRKY26 (WRKY DNA-binding protein 26); transcription factor | WRKY | |
| AT5G07580 | Yellow | | DNA binding / transcription factor | AP2-EREBP | |
| AT5G09460 | Yellow | bHLH143 | transcription factor/ transcription regulator | | |

| | | | | | |
|---|---|---|---|---|---|
| AT5G10030 | Yellow | OBF4, TGA4 | TGA4 (TGACG MOTIF-BINDING FACTOR 4); DNA binding / calmodulin binding / transcription factor | bZIP | |
| AT5G10970 | Yellow | | zinc finger (C2H2 type) family protein | C2H2 | |
| AT5G11060 | Yellow | KNAT4 | KNAT4 (KNOTTED1-LIKE HOMEOBOX GENE 4); transcription factor | HB | |
| AT5G11260 | Yellow | TED5, HY5 | HY5 (ELONGATED HYPOCOTYL 5); DNA binding / transcription factor | bZIP | |
| AT5G12440 | Yellow | | zinc finger (CCCH-type) family protein | C3H | |
| AT5G12850 | Yellow | PEI1 | zinc finger (CCCH-type) family protein | C3H | |
| AT5G13180 | Yellow | VNDIP2, ANAC083, VNI2 | ANAC083 (Arabidopsis NAC domain containing protein 83); transcription factor | NAC | |
| AT5G13220 | Yellow | JAS1, AT5G13220, TIFY9, JAZ10 | unknown protein | ZIM | |
| AT5G13910 | Yellow | LEAFY PETIOLE (LEP) | LEP (LEAFY PETIOLE); DNA binding / transcription factor | AP2-EREBP | |
| AT5G15790 | Yellow | | zinc finger (C3HC4-type RING finger) family protein | | |
| AT5G15830 | Yellow | ATBZIP3 | bZIP transcription factor family protein | bZIP | |
| AT5G16600 | Yellow | ATMYB43 | MYB43 (myb domain protein 43); DNA binding / transcription factor | MYB | |
| AT5G17890 | Yellow | DAR4 | LIM domain-containing protein / disease resistance protein-related | Orphans | |
| AT5G18240 | Yellow | MYR1 | MYR1 (MYB-RELATED PROTEIN 1), MYR1 (MYB-RELATED PROTEIN 1); transcription factor | G2-like | |
| AT5G18680 | Yellow | AtTLP11 | AtTLP11 (TUBBY LIKE PROTEIN 11); phosphoric diester hydrolase/ transcription factor | TUB | |
| AT5G20220 | Yellow | | zinc knuckle (CCHC-type) family protein | | |
| AT5G20240 | Yellow | PI | PI (PISTILLATA); DNA binding / transcription factor | MADS | |
| AT5G20885 | Yellow | | zinc finger (C3HC4-type RING finger) family protein | | |
| AT5G20900 | Yellow | TIFY3B, JAZ12 | unknown protein | ZIM | |
| AT5G22890 | Yellow | | zinc finger (C2H2 type) family protein | C2H2 | |
| AT5G23000 | Yellow | ATMYB37, RAX1 | ATMYB37/MYB37/RAX1 (myb domain protein 37); DNA binding / transcription factor | MYB | |
| AT5G25160 | Yellow | ZFP3 | ZFP3 (ZINC FINGER PROTEIN 3); nucleic acid binding / transcription factor/ zinc ion binding | C2H2 | |
| AT5G25810 | Yellow | TNY, TINY | TNY (TINY); DNA binding / transcription factor | AP2-EREBP | |
| AT5G25890 | Yellow | IAR2, IAA28 | IAA28 (IAA-ALANINE RESISTANT 2); transcription factor | AUX/IAA | |
| AT5G28590 | Yellow | | DNA-binding protein-related | | |
| AT5G28770 | Yellow | BZO2H3, ATBZIP63 | BZO2H3 (basic leucine zipper O2 homolog 3); DNA binding / transcription factor | bZIP | |
| AT5G35750 | Yellow | AHK2 | AHK2 (ARABIDOPSIS HISTIDINE KINASE 2) | Orphans | |
| AT5G36670 | Yellow | | PHD finger family protein | PHD | |
| AT5G37260 | Yellow | RVE2, CIR1 | myb family transcription factor | MYB-related | |
| AT5G38690 | Yellow | | unknown protein | | |
| AT5G39660 | Yellow | CDF2, AtDof5. 2 | CDF2 (CYCLING DOF FACTOR 2); DNA binding / protein binding / transcription factor, CDF2 (CYCLING DOF FACTOR 2); DNA binding / transcription factor | C2C2-Dof | |
| AT5G39760 | Yellow | ATHB23 | ATHB23 (ARABIDOPSIS THALIANA HOMEOBOX PROTEIN 23); DNA binding / transcription factor | zf-HD | |
| AT5G45050 | Yellow | ATWRKY16, TTR1 | TTR1 (WRKY domain family protein 16); transcription factor | WRKY | |
| AT5G45270 | Yellow | RRS1 | | | |
| AT5G46760 | Yellow | bHLH005, ATR2 | basic helix-loop-helix (bHLH) family protein | bHLH | |
| AT5G47220 | Yellow | ATERF-2, ERF2, ATERF2 | ATERF-2/ATERF2/ERF2 (ETHYLENE RESPONSE FACTOR 2); DNA binding / transcription factor/ transcriptional activator | AP2-EREBP | |
| AT5G47230 | Yellow | ATERF5, ATERF-5, ERF5 | ERF5 (ETHYLENE RESPONSIVE ELEMENT BINDING FACTOR 5); DNA binding / transcription factor/ transcriptional activator | AP2-EREBP | |
| AT5G47640 | Yellow | NF-YB2, HAP3b | CCAAT-box binding transcription factor subunit B (NF-YB) (HAP3 ) (AHAP3) family (Hap3b) | CCAAT | |
| AT5G48560 | Yellow | bHLH078 | basic helix-loop-helix (bHLH) family protein | bHLH | |
| AT5G49450 | Yellow | ATBZIP1 | bZIP family transcription factor | bZIP | |
| AT5G50010 | Yellow | | transcription factor/ transcription regulator | | |
| AT5G50820 | Yellow | ANAC097 | ANAC097 (Arabidopsis NAC domain containing protein 97); transcription factor | NAC | |
| AT5G53980 | Yellow | ATHB52 | ATHB52 (ARABIDOPSIS THALIANA HOMEOBOX PROTEIN 52); transcription factor | HB | |
| AT5G56270 | Yellow | ATWRKY2, WRKY2, WRKY23 | WRKY2 (WRKY DNA-binding protein 2); transcription factor | WRKY | |
| AT5G57150 | Yellow | bHLH035 | basic helix-loop-helix (bHLH) family protein | bHLH | |
| AT5G57410 | Yellow | | unknown protein | | |
| AT5G57580 | Yellow | | calmodulin-binding protein | | |
| AT5G57620 | Yellow | ATMYB36 | MYB36 (myb domain protein 36); DNA binding / transcription factor | MYB | |
| AT5G57660 | Yellow | COL5 | zinc finger (B-box type) family protein | C2C2-CO-like | |
| AT5G58900 | Yellow | | myb family transcription factor | MYB | |
| AT5G59780 | Yellow | ATMYB59 | MYB59 (myb domain protein 59); DNA binding / transcription factor | MYB-related | |
| AT5G60120 | Yellow | TOE2 | TOE2 (TARGET OF EAT1 2); DNA binding / transcription factor | AP2-EREBP | miR172, |

| | | | | | |
|---|---|---|---|---|---|
| AT5G60450 | Yellow | ARF4 | ARF4 (AUXIN RESPONSE FACTOR 4); transcription factor | ARF | |
| AT5G60690 | Yellow | IFL1, REV | REV (REVOLUTA); DNA binding / lipid binding / transcription factor | HB | miR166/miR165, |
| AT5G60910 | Yellow | FUL, AGL8 | AGL8 (AGAMOUS-LIKE 8), AGL8 (AGAMOUS-LIKE 8); transcription factor | MADS | |
| AT5G60970 | Yellow | TCP5 | TCP family transcription factor, putative | TCP | |
| AT5G61420 | Yellow | HAG1, PMG1, ATMYB28 | MYB28 (myb domain protein 28); DNA binding / transcription factor | | |
| AT5G62165 | Yellow | AGL42 | AGL42 (AGAMOUS LIKE 42); transcription factor | MADS | |
| AT5G62430 | Yellow | CDF1, AtDof5,5 | CDF1 (CYCLING DOF FACTOR 1); DNA binding / protein binding / transcription factor | C2C2-Dof | |
| AT5G64340 | Yellow | bHLH142, SAC51 | SAC51 (SUPPRESSOR OF ACAULIS 51); transcription factor/ transcription regulator | | |
| AT5G65210 | Yellow | TGA1 | DNA binding / calmodulin binding / transcription factor, bZIP family transcription factor (TGA1) | bZIP | |
| AT5G65790 | Yellow | AtMYB68 | MYB68 (myb domain protein 68); DNA binding / transcription factor | MYB | |
| AT5G66300 | Yellow | ANAC105, VND3 | ANAC105/VND3 (Arabidopsis NAC domain containing protein 105, VASCULAR-RELATED NAC-DOMAIN 3); transcription factor | NAC | |
| AT5G66320 | Yellow | | zinc finger (GATA type) family protein | C2C2-GATA | |
| AT5G67180 | Yellow | TOE3 | AP2 domain-containing transcription factor, putative | AP2-EREBP | miR172, |
