# Supplementary material for: Weighted Gene Correlation Network Analysis (WGCNA) of Arabidopsis Somatic Embryogenesis (SE) and Identification of Key Gene Modules to Uncover SE-Associated Hub Genes
Source: Int J Genomics. 2022 Jul 4;2022:7471063. doi: 10.1155/2022/7471063 (PMC9274236; doi:10.1155/2022/7471063)
Supplement: Supplementary 7 — Table S7. The list of microRNA-targeted TFs. [file 7471063.f7.pdf]

Table S7. The list of microRNA-targeted TFs

Gene identifier	Module	Alias	Description	TF Family (PlantTFDB)	Target of miRNA
AT2G28550	Yellow	TOE1, RAP2.7	TOE1 (TARGET OF EAT1 1); DNA binding / transcription factor	AP2-EREBP	miR172
AT2G39250	Turquoise	SNZ	SNZ (SCHNARCHZAPFEN); DNA binding / transcription factor	AP2-EREBP	miR172
AT3G54990	Red	SMZ	SMZ (SCHLAFMUTZE), SMZ (SCHLAFMUTZE); DNA binding / transcription factor	AP2-EREBP	miR172
AT4G36920	Green	FL1, FLO2, AP2	AP2 (APETALA 2); transcription factor	AP2-EREBP	miR172
AT5G60120	Yellow	TOE2	TOE2 (TARGET OF EAT1 2); DNA binding / transcription factor	AP2-EREBP	miR172
AT5G67180	Yellow	TOE3	AP2 domain-containing transcription factor, putative	AP2-EREBP	miR172
AT1G30330	Darkturquoise	ARF6	ARF6 (AUXIN RESPONSE FACTOR 6), ARF6 (AUXIN RESPONSE FACTOR 6); transcription factor	ARF	miR167
AT1G77850	Yellow	ARF17	ARF17 (AUXIN RESPONSE FACTOR 17); transcription factor	ARF	miR160
AT2G28350	Blue	ARF10	ARF10 (AUXIN RESPONSE FACTOR 10); miRNA binding / transcription factor	ARF	miR160
AT4G30080	Brown	ARF16	ARF16 (AUXIN RESPONSE FACTOR 16); miRNA binding / transcription factor	ARF	miR160
AT5G37020	Blue	ARF8	ARF8 (AUXIN RESPONSE FACTOR 8), ARF8 (AUXIN RESPONSE FACTOR 8); transcription factor	ARF	miR167
AT3G23690	Green	bHLH077	basic helix-loop-helix (bHLH) family protein	bHLH	miR393
AT1G17590	Yellow	NF-YA8	CCAAT-binding transcription factor (CBF-B/NF-YA) family protein	CCAAT	miR169
AT1G54160	Turquoise	NFYA5, NF-YA5	CCAAT-binding transcription factor (CBF-B/NF-YA) family protein	CCAAT	miR169
AT1G72830	Turquoise	NF-YA3, ATHAP2C, HAP2C	HAP2C (Heme activator protein (yeast) homolog 2C); transcription factor	CCAAT	miR169
AT3G05690	Yellow	NF-YA2, ATHAP2B, HAP2B, UNE8	HAP2B (Heme activator protein (yeast) homolog 2B, unfertilized embryo sac 8); transcription factor	CCAAT	miR169
AT3G20910	Turquoise	NF-YA9	CCAAT-binding transcription factor (CBF-B/NF-YA) family protein	CCAAT	miR169
AT5G06510	Yellow	NF-YA10	CCAAT-binding transcription factor (CBF-B/NF-YA) family protein	CCAAT	miR169
AT5G12840	Cyan	NF-YA1, ATHAP2A, HAP2A, EMB2220	HAP2A (EMBRYO DEFECTIVE 2220); transcription factor	CCAAT	miR169
AT2G45160	Yellow		scarecrow transcription factor family protein	GRAS	miR170/miR171
AT3G60630	Yellow		scarecrow transcription factor family protein	GRAS	miR170/miR171
AT4G00150	Yellow	SCL6	scarecrow-like transcription factor 6 (SCL6)	GRAS	miR170/miR171
AT2G22840	Turquoise	ATGRF1	AtGRF1 (GROWTH-REGULATING FACTOR 1)	GRF	miR396
AT2G36400	Turquoise	ATGRF3	AtGRF3 (GROWTH-REGULATING FACTOR 3)	GRF	miR396
AT3G52910	Turquoise	ATGRF4	AtGRF4 (GROWTH-REGULATING FACTOR 4)	GRF	miR396
AT4G24150	Brown	ATGRF8	AtGRF8 (GROWTH-REGULATING FACTOR 8)	GRF	miR396
AT4G37740	Turquoise	ATGRF2	AtGRF2 (GROWTHREGULATING FACTOR 2)	GRF	miR396
AT5G53660	Pink	ATGRF7	AtGRF7 (GROWTH-REGULATING FACTOR 7)	GRF	miR396
AT1G30490	Green	ATHB9, PHV	PHV (PHAVOLUTA); DNA binding / transcription factor	HB	miR166/miR165
AT1G52150	Green	ATHB15, ICU4, CNA, ATHB-15	ATHB-15 (INCURVATA 4), ATHB-15 (INCURVATA 4); DNA binding / transcription factor	HB	miR166/miR165
AT2G34710	Blue	ATHB14, PHB-1D, ATHB-14, PHB	PHB (PHABULOSA); DNA binding / transcription factor	HB	miR166/miR165
AT4G32880	Green	ATHB8, ATHB-8	ATHB-8 (HOMEOBOX GENE 8); DNA binding / transcription factor	HB	miR166/miR165
AT5G60690	Yellow	IFL1, REV	REV (REVOLUTA); DNA binding / lipid binding / transcription factor	HB	miR166/miR165
AT3G57230	Yellow	AGL16	AGL16 (AGAMOUS-LIKE 16), AGL16 (AGAMOUS-LIKE 16); transcription factor	MADS	miR824
AT1G66370	Turquoise	ATMYB113	MYB113 (myb domain protein 113); DNA binding / transcription factor	MYB	miR828
AT2G32460	Turquoise	ATMYB101, AtM1	AtM1/AtMYB101/MYB101 (myb domain protein 101), AtM1/AtMYB101/MYB101 (myb domain protein 101); DNA binding / transcription factor	MYB	miR159/miR319
AT2G47460	Green	PFG1, ATMYB12	MYB12 (myb domain protein 12); DNA binding / transcription factor/ transcriptional activator	MYB	miR858
AT3G11440	Pink	AtMYB65	ATMYB65 (myb domain protein 65); DNA binding / transcription factor	MYB	miR159/miR319
AT5G06100	Yellow	ATMYB33	ATMYB33/MYB33 (myb domain protein 33), ATMYB33/MYB33 (myb domain protein 33); DNA binding / transcription factor	MYB	miR159/miR319
AT1G56010	Yellow	NAC1, ANAC021, ANAC022	NAC1 (Arabidopsis NAC domain containing protein 21, Arabidopsis NAC domain containing protein 22); transcription factor	NAC	miR164
AT5G07680	Tan	ANAC079, ATNAC4, ANAC080	ANAC079/ANAC080/ATNAC4 (Arabidopsis NAC domain containing protein 79, Arabidopsis NAC domain containing protein 80); transcription factor	NAC	miR164
AT5G39610	Tan	AtNAC2, ANAC092, ATNAC6	ANAC092/ATNAC2/ATNAC6 (Arabidopsis NAC domain containing protein 92); protein heterodimerization/ protein homodimerization/ transcription factor	NAC	miR164
AT5G53950	Blue	CUC2, ANAC098	CUC2 (CUP-SHAPED COTYLEDON 2); transcription factor	NAC	miR164
AT5G61430	Turquoise	ANAC100, ATNAC5	ANAC100/ATNAC5 (Arabidopsis NAC domain containing protein 100); transcription factor	NAC	miR164
AT1G27360	Green	SPL11	squamosa promoter-binding protein-like 11 (SPL11)	SBP	miR156/miR157
AT1G27370	Green	SPL10	DNA binding, squamosa promoter-binding protein-like 10 (SPL10)	SBP	miR156/miR157
AT1G69170	Brown	SPL6	squamosa promoter-binding protein-like 6 (SPL6)	SBP	miR156/miR157
AT2G42200	Tan	SPL9	squamosa promoter-binding protein-like 9 (SPL9)	SBP	miR156/miR157
AT3G57920	Turquoise	SPL15, MSC1	squamosa promoter-binding protein, putative	SBP	miR156/miR157

AT5G43270	Green	SPL2	SPL2 (SQUAMOSA PROMOTER BINDING PROTEIN-LIKE 2); DNA binding, SPL2 (SQUAMOSA PROMOTER BINDING PROTEIN-LIKE 2); DNA binding / transcription factor	SBP	miR156/miR157
AT5G50570	Brown	SPL13	squamosa promoter-binding protein, putative	SBP	miR156/miR157
AT2G22740	Turquoise	SDG23, SUVH6	SUVH6 (SU(VAR)3-9 homolog 6)	SET	miR778
AT2G35160	Turquoise	SGD9, SUVH5	SUVH5 (SU(VAR)3-9 HOMOLOG 5)	SET	miR778
AT1G30210	Turquoise	ATTCP24, TCP24	TCP24; transcription factor	TCP	miR159/miR319
AT1G53230	Turquoise	TCP3	TCP3 (TCP family transcription factor 3); transcription factor	TCP	miR159/miR319
AT2G31070	Red	TCP10	TCP10 (TCP domain protein 10); transcription factor	TCP	miR159/miR319
AT3G15030	Turquoise	TCP4, MEE35	TCP4 (TCP family transcription factor 4, maternal effect embryo arrest 35); transcription factor	TCP	miR159/miR319
AT4G18390	Turquoise	TCP2	TCP family transcription factor, putative	TCP	miR159/miR319
