# Supplementary material for: Weighted Gene Correlation Network Analysis (WGCNA) of Arabidopsis Somatic Embryogenesis (SE) and Identification of Key Gene Modules to Uncover SE-Associated Hub Genes
Source: Int J Genomics. 2022 Jul 4;2022:7471063. doi: 10.1155/2022/7471063 (PMC9274236; doi:10.1155/2022/7471063)
Supplement: Supplementary 8 — Table S8. Distribution of gene encoding epigenetic regulators across network modules. [file 7471063.f8.pdf]

Table S8. Distribution of genes encoding epigenetic regulators across network modules

Function category	Gene identifier	Module	Gene acronym	Gene name	Confirmed or putative function of protein
RNA silencing	AT1G01040	Yellow	*DCL1/CAF1/SIN1/EMB76/SUS1*	Dicer-like, Carpel factory, short integuments, embryo-defective, suspensor	microRNAs (miRNA) processing
Chromatin formation or chromatin remodeling	AT1G03790	Brown	*SOM*	Decreased DNA methylation, somniferous	PAZ-PIWI domain protein, translational repression
RNA silencing	AT1G05460	Turquoise	*SDE3*	Silencing-defective	PAZ-PIWI domain protein, translational repression
Polycomb-group proteins and interacting components	AT1G06770	Red	*BMI1b*	B cell-specific Mo-MLV integration site 1	PAZ-PIWI domain protein, siRNA-binding
Chromatin formation or chromatin remodeling	AT1G08060	Red	*MOM1*	Morpheus' molecule	PAZ-PIWI domain protein, siRNA-binding
RNA silencing	AT1G08260	Blue	*ESD7*	Early in short days	PAZ-PIWI domain protein
Histone modification	AT1G08460	Pink	*HDA08*	Histone deacetylase	PAZ-PIWI domain protein, female gametophyte formation, siRNA binding
Histone modification	AT1G08620	Blue	*JMJ16*	Arabidopsis thaliana jumonji	Subunits of INO80 complex
RNA silencing	AT1G13790	Red	*FDM4*	Factor of DNA methylation	Subunits of INO80 complex
Histone modification	AT1G14400	Blue	*UBC1*	E2 ubiquitin-conjugating enzyme	Histone chaperone H3/H4
RNA silencing	AT1G14790	Yellow	*RDR1*	RNA-dependent RNA polymerase	Histone methyltransferase
DNA modification	AT1G15340	Turquoise	*MBD10*	Methylcytosine-binding domain protein	Histone methyltransferase (ATXR5,-6: H3K27me1)
RNA silencing	AT1G15910	Turquoise	*FDM1*	Factor of DNA methylation	Histone methyltransferase (ATXR5,-6: H3K27me1)
RNA silencing	AT1G16610	Black	*SR45*	Arginine/serine-rich	PRC1 subunit
Histone modification	AT1G16710	Blue	*HAC12*	Histone acetyltransferase CBP-like	PRC1 subunit
Chromatin formation or chromatin remodeling	AT1G18450	Blue	*ARP4*	Actin-related protein	SWI2/SNF2 ATPase family protein
Chromatin formation or chromatin remodeling	AT1G18800	Green	*NRP2*	NAP (nucleosome assembly protein)-related protein	Subunit of SWI/SNF-remodeling complexes
Chromatin formation or chromatin remodeling	AT1G19100	Turquoise	*DMS11*	Defective in meristem silencing	Chromodomain remodeling complex
Chromatin formation or chromatin remodeling	AT1G21700	Purple	*SWI3C*	Arabidopsis homolog of SWI3	SWI2/SNF2 family ATPase
Histone modification	AT1G32750	Turquoise	*HAF1*	Histone acetyltransferase CBP-like	ISWI-like chromatin remodeling protein
RNA silencing	AT1G48410	Magenta	*AGO1*	Argonaute	SNF2/Brahma-type protein
RNA silencing	AT1G48920	Blue	*NUC-l1*	Nucleolin-like	Polycomb-group protein (E(z))
RNA silencing	AT1G54490	Blue	*XRN4/EIN5*	XRN homolog, ethylene insensitive	DNA methyltransferase (mainly CHG and CHH)
Histone modification	AT1G55970	Turquoise	*HAC4*	Histone acetyltransferase CBP-like	Subunit of E3 ligase complex, MSI1 interactor
DNA modification	AT1G57820	Darkturquoise	*VIM1*	Variant in methylation	LHP1 and H3 interaction
Histone modification	AT1G62310	Turquoise	*JMJ29*	Arabidopsis thaliana jumonji	RNase III (dsRNase), miRNA and siRNA generation
RNA silencing	AT1G63020	Brown	*NRPD1/SDE4*	Nuclear RNA polymerase IV, silencing defective 4	RNase III (dsRNase), siRNA generation
Chromatin formation or chromatin remodeling	AT1G65470	Lightcyan	*FAS1*	Fasciated	RNase III (dsRNase), siRNA generation
Chromatin formation or chromatin remodeling	AT1G66740	Blue	*ASF1A*	Arabidopsis antisilencing factor	RNase III (dsRNase), siRNA generation
RNA silencing	AT1G69440	Turquoise	*AGO7/ZIP*	Argonaute, Zippy	Subunit of E3 ligase complex, MSI1 interactor
DNA modification	AT1G69770	Lightyellow	*CMT3*	Chromomethyltransferase	Subunit of E3 ligase complex, MSI1 interactor
Histone modification	AT1G73100	Blue	*SUVH3*	SET domain group 19	FHA domain protein, miRNA processing
Chromatin formation or chromatin remodeling	AT1G74560	Blue	*NRP1*	NAP (nucleosome assembly protein)-related protein	DNA glycosylase-domain protein, cytosine demethylation
RNA silencing	AT1G75660	Green	*XRN3*	XRN homolog	DNA glycosylase-domain protein, cytosine demethylation
Histone modification	AT1G79000	Blue	*HAC1*	Histone acetyltransferase CBP-like	DNA glycosylase-domain protein, cytosine demethylation
RNA silencing	AT1G80790	Magenta	*FDM5*	Factor of DNA methylation	GHKL ATPase, interaction with DMS3
Histone modification	AT2G02760	Blue	*UBC2*	E2 ubiquitin-conjugating enzyme	Structural-maintenance-of-chromosomes protein; required for RNA Pol V transcription
RNA silencing	AT2G13540	Brown	*ABH1/CBP80*	ABA-hypersensitive, cap-binding complex	Interaction with HD2
RNA silencing	AT2G15400	Lightcyan	*NRPE3b*	Nuclear RNA polymerases II, IV, and V	Dicer-interacting proteins, siRNA processing
Chromatin formation or chromatin remodeling	AT2G16390	Turquoise	*DRD1*	Defective in RNA-directed DNA methylation	Dicer-interacting proteins, siRNA processing
Histone modification	AT2G17900	Yellow	*SDG37*	SET domain group 37	Dicer-interacting proteins, siRNA processing
Chromatin formation or chromatin remodeling	AT2G19480	Blue	*NAP1;2*	Arabidopsis nucleosome assembly protein	SWI2/SNF2 ATPase; required for RNA Pol V transcription
Polycomb-group proteins and interacting components	AT2G19520	Black	*MSI4/FVE*	Multicopy suppressor of IRA homolog	Major de novo DNA methyltransferase (CG, CHG, and CHH)
Histone modification	AT2G22740	Turquoise	*SUVH6*	Su(Var)3-9 homolog	Histone demethylase (H3K4me1,-2,-3)
Polycomb-group proteins and interacting components	AT2G23380	Yellow	*CLF*	Curly leaf	Hhistone acetyl transferase complex
Chromatin formation or chromatin remodeling	AT2G24490	Green	*RPA2*	Replication protein A	Polycomb-group protein (Su(z)12)
Chromatin formation or chromatin remodeling	AT2G25170	Yellow	*PKL*	Pickle	Subunit of DNA polymerase epsilon
RNA silencing	AT2G27040	Turquoise	*AGO4*	Argonaute	Chromatin assembly factor subunit H3/H4
RNA silencing	AT2G27100	Cyan	*SE*	Serrate	Chromatin assembly factor subunit H3/H4
Histone modification	AT2G27350	Green	*OTLD1*	Otubain-like deubiquitinase	RRM-domain protein
Histone modification	AT2G27840	Green	*HD2d/HDT4*	Histone deacetylase	dsRNA-binding protein (FDM1)
Chromatin formation or chromatin remodeling	AT2G28290	Greenyellow	*SYD*	Splayed	dsRNA-binding protein (FDM1)
RNA silencing	AT2G28380	Turquoise	*DRB2*	Double-stranded RNA-binding protein	dsRNA-binding protein (FDM1)
RNA silencing	AT2G30280	Blue	*RDM4/DMS4*	RNA-directed DNA methylation, defective in meristem silencing	dsRNA-binding protein (FDM1)
Polycomb-group proteins and interacting components	AT2G30580	Turquoise	*BMI1a*	B cell-specific Mo-MLV integration site 1	Polycomb-group protein (Esc)

RNA silencing	AT2G32940	Black	*AGO6*	Argonaute	Histone demethylases
Histone modification	AT2G33290	Yellow	*SUVH2*	Su(Var)3-9 homolog	Histone acetyltransferase
Chromatin formation or chromatin remodeling	AT2G33610	Blue	*SWI3B*	Arabidopsis homolog of SWI3	Histone acetyltransferase
Histone modification	AT2G35160	Turquoise	*SUVH5*	Su(Var)3-9 homolog	Histone acetyltransferase
DNA modification	AT2G36490	Turquoise	*DML1/ROS1*	Repressor of silencing	Histone acetyltransferase
RNA silencing	AT2G40030	Turquoise	*NRPE1/DRD3*	Nuclear RNA Pol V, defective in RNA-directed DNA methylation	Histone deacetylase
Histone modification	AT2G44950	Lightcyan	*HUB1*	E3 ubiquitin ligase	Histone acetyltransferase
Chromatin formation or chromatin remodeling	AT2G46020	Turquoise	*BRM*	Brahma	Histone acetyltransferase
Chromatin formation or chromatin remodeling	AT2G47620	Blue	*SWI3A*	Arabidopsis homolog of SWI3	Histone acetyltransferase (H3K14ac)
RNA silencing	AT3G03300	Green	*DCL2*	Dicer-like	Histone acetyltransferase (H3K14ac)
RNA silencing	AT3G05040	Purple	*HST*	Hasty	Histone acetyltransferase
Chromatin formation or chromatin remodeling	AT3G06010	Magenta	*CHR12*	Arabidopsis chromatin remodeling	Histone acetyltransferase
Chromatin formation or chromatin remodeling	AT3G06400	Black	*CHR11*	Chromatin-remodeling protein	Atypical histone deacetylase
Histone modification	AT3G07610	Brown	*IBM1*	Increase in bonsai methylation	Atypical histone deacetylase
DNA modification	AT3G10010	Turquoise	*DML2*	Demeter-like proteins	Atypical histone deacetylase
Histone modification	AT3G10390	Cyan	*FLD*	Flowering locus D, LDS1-like	Histone deacetylase
Chromatin formation or chromatin remodeling	AT3G12380	Red	*ARP5*	Actin-related protein	Histone deacetylase
RNA silencing	AT3G12550	Turquoise	*FDM3*	Factor of DNA methylation	Histone deacetylase
Histone modification	AT3G12980	Yellow	*HAC5*	Histone acetyltransferase CBP-like	Histone deacetylase
Histone modification	AT3G13682	Pink	*LDL2*	Flowering locus D, LDS1-like	Histone deacetylase
DNA modification	AT3G14890	Turquoise	*ZDP*	Zinc finger DNA 3'-phosphoesterase	Histone deacetylase
RNA silencing	AT3G16980	Turquoise	*NRPB9a*	Nuclear RNA polymerases II, IV, and V	Histone deacetylase
Chromatin formation or chromatin remodeling	AT3G17590	Green	*BSH*	Bushy	Histone deacetylase
Histone modification	AT3G18520	Lightcyan	*HDA15*	Histone deacetylase	Histone deacetylase
Histone modification	AT3G19040	Pink	*HAF2*	Histone acetyltransferase CBP-like	dsRNA-binding, RNA methyltransferase
RNA silencing	AT3G20550	Pink	*DDL*	Dawdle	Histone chaperone H3/H4
Polycomb-group proteins and interacting components	AT3G20740	Red	*FIE, FIS3*	Fertilization-independent endosperm, fertilization-independent seeds	DNA methylation-dependent gene silencing
RNA silencing	AT3G22680	Blue	*RDM1*	RNA-directed DNA methylation	miRNA export receptor
RNA silencing	AT3G22900	Turquoise	*NRPD7a*	Nuclear RNA Pol IV	H2B monoubiquitination
Polycomb-group proteins and interacting components	AT3G24440	Blue	*VRN5*	Vernalization	Histone demethylase
RNA silencing	AT3G26932	Turquoise	*DRB3*	Double-stranded RNA-binding protein	Subunits of INO80 complex
Chromatin formation or chromatin remodeling	AT3G28730	Magenta	*SSRP1*	Structure-specific recognition protein	Histone demethylase (H3K4me1,-2,-3)
RNA silencing	AT3G43920	Red	*DCL3*	Dicer-like	Histone demethylase
Histone modification	AT3G44490	Blue	*HDA17*	Histone deacetylase	Histone demethylase
Chromatin formation or chromatin remodeling	AT3G44530	Green	*HIRA*	Histone regulator A	Histone demethylase
Polycomb-group proteins and interacting components	AT3G44600	Brown	*CYP71*	Cyclophilin	Putative Histone demethylase (H3K9me2)
Histone modification	AT3G44680	Blue	*HDA9*	Histone deacetylase	Part of RdDM effector complex
Histone modification	AT3G44750	Blue	*HDA3*	Histone deacetylase	Histone demethylases
Chromatin formation or chromatin remodeling	AT3G49250	Turquoise	*DMS3/IDN1*	Defective in meristem silencing, involved in de novo	RNA processing, LHP1 interaction
RNA silencing	AT3G49500	Green	*RDR6/SDE1/SGS2*	RNA-dependent RNA polymerase, silencing defective, suppressor of gene silencing	Methylcytosine-binding protein
Histone modification	AT3G54610	Blue	*HAG1*	Histone acetyltransferase GCN5-like	Methylcytosine-binding protein
RNA silencing	AT3G57080	Turquoise	*NRPE5*	Nuclear RNA Pol V	Cytosine methyltransferase
Chromatin formation or chromatin remodeling	AT3G57300	Green	*INO80*	Inositol-requiring	DNA topoisomerase
RNA silencing	AT3G62800	Pink	*DRB4*	Double-stranded RNA-binding protein	Genetic interaction with RNA Pol V
Polycomb-group proteins and interacting components	AT4G00830	Blue	*LIF2*	LHP1-interacting factor	Chromatin assembly factor subunit, H3/H4
Histone modification	AT4G00990	Yellow	*JMJ27*	Arabidopsis thaliana jumonji	Polycomb-group protein (p55), Cul4-DDB1 and PCR2 interactor
Polycomb-group proteins and interacting components	AT4G05420	Blue	*DDB1A*	Damaged DNA-binding	Histone chaperone H2A/H2B
Chromatin formation or chromatin remodeling	AT4G10710	Magenta	*SPT16*	Suppressor of Ty insertion-like	Histone chaperone H2A/H2B
RNA silencing	AT4G11130	Turquoise	*RDR2*	RNA-dependent RNA polymerase	Histone chaperone H2A/H2B
DNA modification	AT4G13940	Royalblue	*HOG1*	Homology-dependent gene silencing	Histone chaperone H2A/H2B
RNA silencing	AT4G14660	Blue	*NRPE7/NRPD7b*	Nuclear RNA Pol V	Histone chaperone H2A/H2B
Histone modification	AT4G15180	Red	*ATXR3/SDG2*	Arabidopsis homolog of trithorax, SET domain group	Alternative 9th subunits of Pols II, IV, and V
RNA silencing	AT4G16280	Blue	*FCA*	Flowering time	Pol IV largest subunit
Polycomb-group proteins and interacting components	AT4G16845	Green	*VRN2*	Vernalization	Major 7th subunit of RNA Pol IV
Histone modification	AT4G20400	Red	*JMJ14*	Arabidopsis thaliana jumonji	RNA Pol V largest subunit
RNA silencing	AT4G20910	Blue	*HEN1*	HUA enhancer	Alternative 3rd subunits of Pols II, IV, or V
Polycomb-group proteins and interacting components	AT4G21100	Blue	*DDB1B*	Damaged DNA-binding	5th subunit of RNA Pol V

Chromatin formation or chromatin remodeling	AT4G26110	Blue	*NAP1;1*	Arabidopsis nucleosome assembly protein	Major 7th subunit of RNA Pol V; alternative seventh subunit of RNA Pol IV
Histone modification	AT4G28190	Turquoise	*ULT1*	Ultrapetala	rRNA gene regulation
Polycomb-group proteins and interacting components	AT4G30200	Green	*VEL1*	Vernalization-like	H2B deubiquitination
Histone modification	AT4G33470	Red	*HDA14*	Histone deacetylase	CHD3 chromatin remodeling factor
Histone modification	AT4G38130	Blue	*HDA1*	Histone deacetylase	Component of DDR complex required for RNA Pol V transcription
Histone modification	AT5G03740	Purple	*HD2c/HDT3*	Histone deacetylase	IWR1-like regulator of multisubunit RNA polymerase assembly
Histone modification	AT5G04240	Turquoise	*ELF6*	Early flowering	RNA-dependent RNA polymerase
RNA silencing	AT5G04290	Turquoise	*KTF1/RDM3/SPT5-l*	KOW domain-containing transcription factor, suppressor of Ty insertion-like	RNA-dependent RNA polymerase
DNA modification	AT5G04560	Turquoise	*DME*	Demeter	RNA-dependent RNA polymerase
Histone modification	AT5G06550	Blue	*JMJ22*	Arabidopsis thaliana jumonji	PRC1 subunit
Histone modification	AT5G09230	Red	*SRT2*	Histone deacetylase SIR2-like	DNA glycosylase-domain protein, cytosine demethylation
Histone modification	AT5G09740	Red	*HAM2*	Histone acetyltransferase Myst-like	Single-stranded DNA-binding protein
Histone modification	AT5G09790	Magenta	*ATXR5*	Arabidopsis trithorax-related proteins	RNA helicase
Histone modification	AT5G13960	Green	*SUVH4/KYP1*	Su(Var)3-9 homolog, kryptonite	Histone methylation
Chromatin formation or chromatin remodeling	AT5G14170	Magenta	*CHC1*	Clathrin heavy chain 1	Putative histone methyltranferase
DNA modification	AT5G14620	Turquoise	*DRM2*	Domains-rearranged methyltransferase	Histone methyltranferase
RNA silencing	AT5G20320	Turquoise	*DCL4/SMD*	Dicer-like	Zn finger protein, miRNA processing
RNA silencing	AT5G21150	Turquoise	*AGO9*	Argonaute	Subunit of SWR1
Histone modification	AT5G22650	Blue	*HD2b/HDT2*	Histone deacetylase	Coiled-coil protein
RNA silencing	AT5G23570	Cyan	*SGS3*	Suppressor of gene silencing	SWI2/SNF2 family ATPase
Histone modification	AT5G24330	Brown	*ATXR6*	Arabidopsis trithorax-related proteins	Histone chaperone H2A/H2B
DNA modification	AT5G25480	Blue	*DNMT2*	DNA methyltransferase	Splicing factor
Histone modification	AT5G26040	Brown	*HDA2*	Histone deacetylase	Histone deacetylase
Chromatin formation or chromatin remodeling	AT5G37055	Yellow	*SEF/SWC6*	Serrated and early flowering, SWR1 complex	Histone deacetylase
DNA modification	AT5G39550	Red	*VIM3*	Variant in methylation	High mobility group (HMG) protein, histone chaperone subunit
Histone modification	AT5G42400	Red	*SDG25*	Arabidopsis homolog of trithorax, SET domain group	H3K9 methyltransferase
RNA silencing	AT5G42540	Lightcyan	*XRN2*	XRN homolog	Histone methyltranferase
RNA silencing	AT5G43810	Yellow	*AGO10/PNH/ZLL*	Argonaute, Pinhead, Zwille	H3K9 methyltransferase
Polycomb-group proteins and interacting components	AT5G44280	Turquoise	*RING1a*	RING finger protein	Recognition of DNA methylation (SUVH5)
Polycomb-group proteins and interacting components	AT5G46210	Salmon	*CUL4*	Cullin protein	Recognition of DNA methylation (SUVH5)
DNA modification	AT5G49160	Yellow	*MET1*	Methyltransferase	Subunits of SWITCH/SUCROSE NONFERMENTABLE (SWI/SNF)-remodeling complexes
Histone modification	AT5G50320	Green	*ELO3*	Histone acetyltransferase GNAT-like	Subunits of SWITCH/SUCROSE NONFERMENTABLE (SWI/SNF)-remodeling complexes
Polycomb-group proteins and interacting components	AT5G51230	Blue	*EMF2*	Embryonic flower	Subunits of SWITCH/SUCROSE NONFERMENTABLE (SWI/SNF)-remodeling complexes
Histone modification	AT5G53430	Red	*SDG29*	SET domain group 29	SWI2/SNF2 family ATPase
Chromatin formation or chromatin remodeling	AT5G55300	Red	*MGO1*	Mgoun	H2B monoubiquitination
Histone modification	AT5G55760	Red	*SRT1*	Histone deacetylase SIR2-like	H2B monoubiquitination
Histone modification	AT5G56740	Blue	*HAG2*	Histone acetyltransferase GCN5-like	Regulator of histone methylation, ATX1 interactor
Chromatin formation or chromatin remodeling	AT5G56950	Blue	*NAP1;3*	Arabidopsis nucleosome assembly protein	Homeodomain protein
DNA modification	AT5G58130	Blue	*ROS3*	Repressor of silencing	Methylcytosine-binding proteins
Chromatin formation or chromatin remodeling	AT5G58230	Blue	*MSI1*	Multicopy suppressor of IRA homolog	DNA methylation
DNA modification	AT5G59380	Blue	*MBD6*	Methylcytosine-binding domain protein	Polycomb-group protein (Su(z)12)
Histone modification	AT5G61060	Green	*HDA05*	Histone deacetylase	Homeodomain protein
Histone modification	AT5G63110	Blue	*HDA6/SIL1/AXE1/RTS1*	Histone deacetylase, modifier of silencing, auxin-gene repression, RNA-mediated transcriptional silencing	Exoribonuclease
Histone modification	AT5G64610	Green	*HAM1*	Histone acetyltransferase Myst-like	Exoribonuclease
Chromatin formation or chromatin remodeling	AT5G64630	Green	*FAS2*	Fasciated	Exoribonuclease, small RNA processing
Chromatin formation or chromatin remodeling	AT5G66750	Brown	*CHR1/DDM1*	Decreased DNA methylation, somniferous	3'-Phosphatase
